# RFECS: A Random-Forest Based Algorithm for Enhancer Identification from Chromatin State

**DOI:** 10.1371/journal.pcbi.1002968

**Published:** 2013-03-14

**Authors:** Nisha Rajagopal, Wei Xie, Yan Li, Uli Wagner, Wei Wang, John Stamatoyannopoulos, Jason Ernst, Manolis Kellis, Bing Ren

**Affiliations:** 1 Ludwig Institute for Cancer Research, University of California at San Diego, La Jolla, California, United States of America; 2 Bioinformatics and Systems Biology program, University of California at San Diego, La Jolla, California, United States of America; 3 Department of Chemistry and Biochemistry, University of California at San Diego, La Jolla, California, United States of America; 4 Department of Genome Sciences, University of Washington, Seattle, Washington, United States of America; 5 Department of Biological Chemistry, University of California Los Angeles, Los Angeles, California, United States of America; 6 Computer Science and Artificial Intelligence Laboratory, Massachusetts Institute of Technology, Cambridge, Massachusetts, United States of America; 7 Department of Cellular and Molecular Medicine, Institute of Genomic Medicine, and Moores Cancer Center, University of California at San Diego, La Jolla, California, United States of America; Princeton University, United States of America

## Abstract

Transcriptional enhancers play critical roles in regulation of gene expression, but their identification in the eukaryotic genome has been challenging. Recently, it was shown that enhancers in the mammalian genome are associated with characteristic histone modification patterns, which have been increasingly exploited for enhancer identification. However, only a limited number of cell types or chromatin marks have previously been investigated for this purpose, leaving the question unanswered whether there exists an optimal set of histone modifications for enhancer prediction in different cell types. Here, we address this issue by exploring genome-wide profiles of 24 histone modifications in two distinct human cell types, embryonic stem cells and lung fibroblasts. We developed a Random-Forest based algorithm, RFECS (Random Forest based Enhancer identification from Chromatin States) to integrate histone modification profiles for identification of enhancers, and used it to identify enhancers in a number of cell-types. We show that RFECS not only leads to more accurate and precise prediction of enhancers than previous methods, but also helps identify the most informative and robust set of three chromatin marks for enhancer prediction.

## Author Summary

Enhancers are regions in the genome that can activate the expression of a gene irrespective of their location with respect to the gene. Identifying these elements is critical in understanding regulatory differences between different cell-types. Since enhancers lack characteristic sequence features and can be far away from the gene they regulate, their identification is not trivial. Experimentally determining the genome-wide binding sites of transcriptional co-activator p300 is one way of finding enhancers but it can only identify a subset of enhancers. A few years ago, it was observed that the binding sites of p300 are marked by distinctive, post-translational histone modifications. Several groups have exploited this discovery to predict genome-wide enhancers based on their similarity to the histone modification profiles of p300 binding sites. We here report a novel algorithm for this purpose and show that it has much greater accuracy than existing methods. Another unique feature of our algorithm is the ability to automatically deduce the most informative set of histone modifications required for enhancer prediction. We expect that this method will become increasingly useful with the expanding number of known histone modifications and rapid accumulation of epigenomic datasets for various cell types and species.

## Introduction

Enhancers are distal regulatory elements with key roles in the regulation of gene expression. In higher eukaryotes, a diverse repertoire of transcription factors bind to enhancers to orchestrate critical cellular events including differentiation [1], [2], maintenance of cell-identity [3], [4] and response to stimuli [5]–[7]. While enhancers have long been recognized for their regulatory importance, the fact that they lack common sequence features and often reside far away from their target genes has made them difficult to identify. Computational techniques relying on transcription factor motif clustering or comparative analyses have had some success in identifying enhancers, but these predictions are neither comprehensive nor tissue-specific [8]–[13].

Recently, several high-throughput experimental approaches have been developed to identify enhancers in an unbiased, genome-wide manner. The first is mapping the binding sites of specific transcription factors by ChIP-seq [14]. Because this approach requires the knowledge of a subset of transcription factors (TFs) that are not only expressed but also occupy all active enhancer regions in the cell-type of interest, identification of all enhancers using this approach is not a trivial task. The second approach involves mapping the binding sites of transcriptional co-activators such as p300 and CBP [4], [5], [15], which are recruited by sequence-specific transcription factors to a large number of enhancers [6], [16], [17]. Since not all enhancers are marked by a given set of co-activators [18], [19], and ChIP-grade antibodies against these proteins may not always be available, systematic identification of enhancers by mapping the locations of co-activators is not generally feasible. A third approach relies on identifying open chromatin with techniques such as DNase I hypersensitivity mapping [20]. However, since open chromatin regions can correspond to not only enhancers, but also silencers/repressors, insulators, promoters [21], [22] or other functionally unknown sequences occupied by nuclear proteins, this approach lacks specificity in enhancer identification. Finally, a fourth approach interrogates covalent modifications of histones [5], [23]–[26] as it was observed that certain histone modifications form a consistent signature of enhancers. It is on this approach that the present work is focused.

Previously, we and others observed that distinct chromatin modification patterns were associated with transcriptional enhancers [5], [22], [27]. Specifically, active promoters are marked by trimethylation of Lys4 of histone H3 (H3K4me3), whereas enhancers are marked by monomethylation, but not trimethylation, of H3K4 (H3K4me1). This chromatin signature has been used to develop a profile-based method for enhancer discovery [5]. Both unsupervised [25], [28] and supervised learning approaches have also been employed to exploit chromatin modification-based differences to identify enhancers. The supervised machine learning techniques include HMM [8], [23], neural networks [24] and genetic algorithm-optimized SVM [26] based approaches, and have proved to be improvements over the profile-based method. While these methods have led to identification of a great number of enhancers in the human and mouse genomes [3], [25], [29], the current computational techniques have thus far been limited by the small number of the training set samples and limited number of chromatin modifications examined. Thus, it is possible that these approaches may not fully capture the entire range of chromatin modification patterns at enhancer elements. With the discovery of ever more histone modifications, it is likely that additional chromatin modifications may distinguish enhancers from other functional elements in the genome. This additional data should in principle allow us to answer the key question: what is the optimal set of modifications required for enhancer prediction?

Some researchers have tried to tackle this issue by using algorithms such as simulated annealing [23] or genetic-algorithm optimization [26]. We sought to develop a method in which the selection of the optimal set is automatically built into the training-process and is easily adapted to a large number of features.

As part of the NIH Epigenome Roadmap project, we have generated genome-wide profiles for 24 chromatin modifications and DNase-I hypersensitivity sites in 2 distinct cell types- human embryonic stem cell (H1) and a primary lung fibroblast cell line (IMR90) [30]. Additionally, we have experimentally determined a large number of promoter-distal p300 binding sites in each cell type, providing a rich training set for development of accurate and robust enhancer prediction algorithms. We now describe a random-forest [31] based method for integrative analysis of diverse histone modifications to predict enhancers. We show that this new algorithm outperforms the existing methods and leads to the automatic discovery of an optimal set of chromatin modifications for enhancer predictions.

## Results

### Prediction of enhancers using random forest and multiple chromatin marks

Random forests have recently become a popular machine learning technique in biology [32] due to their ability to run efficiently on large datasets without over-fitting, and their inherently non-parametric structure. Since random forests use a single variable at a time, they can give an automatic measure of feature importance [33]. Hence, we developed an algorithm based on this random forest technique for the purpose of enhancer prediction. Conventional random forests utilize a single scalar value associated with each feature at each node of the tree. In order to train a random-forest for enhancer prediction we wanted to use histone modification profiles at p300 binding sites. Because the spatial organization of histone modifications along a linear chromosome can be as informative as their actual levels, they are better represented as vectors of binned reads. Inspired by recent modifications to the random-forest approach such as discriminant random forests [34] or oblique random forests [35] that utilize a linear classifier at each node, we developed a new vector-based random forest algorithm RFECS or Random Forest for Enhancer Identification using Chromatin States (see Methods).

**Figure 1:**
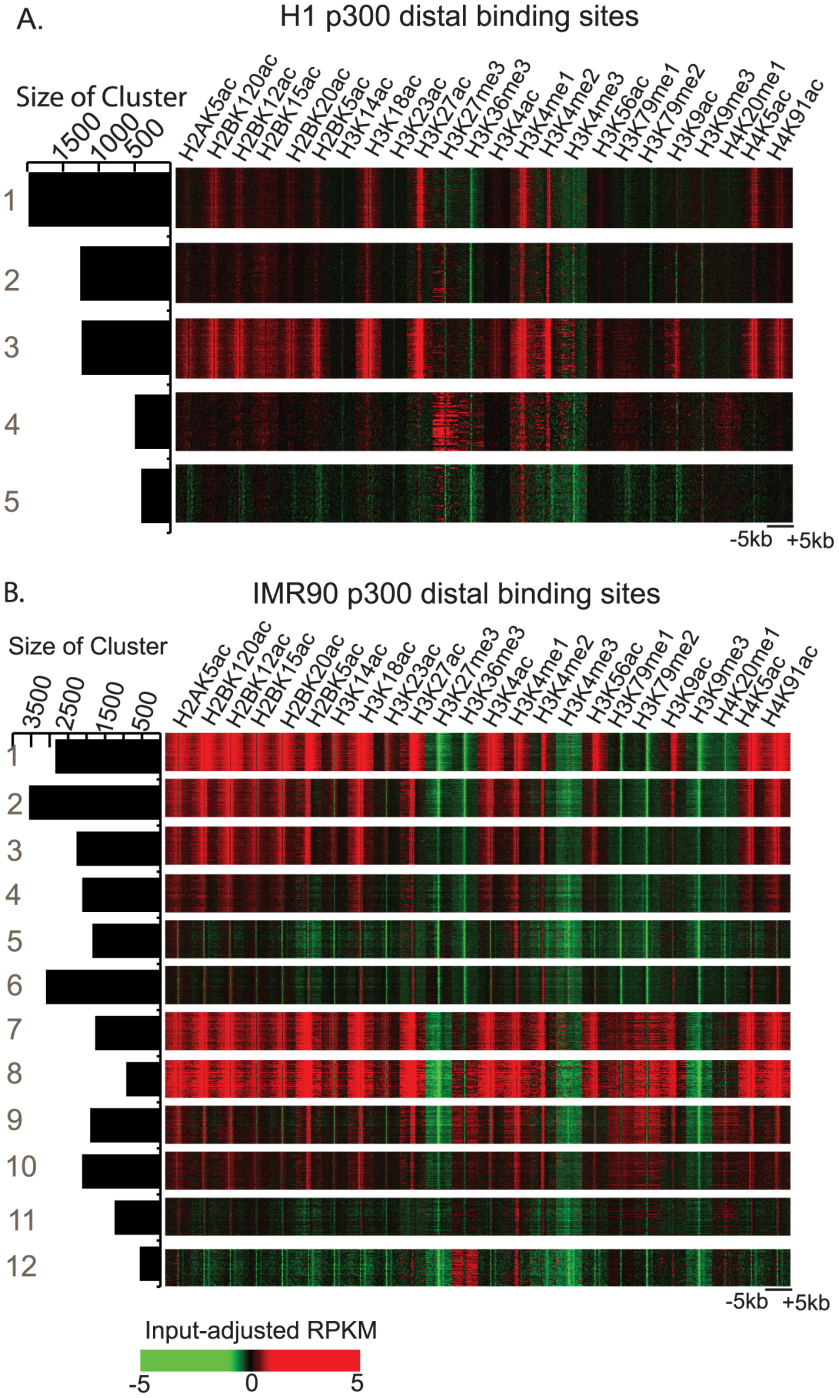
Histone modification patterns at distal p300 binding sites in H1 and IMR90. A.)Chromatin states for p300 binding sites in H1 cells. B.)Chromatin states for p300 binding sties identified in IMR90 cells, identified by clustering using ChromaSig [48]. The heatmap shows RPKM-normalized histone modification levels in 100 bp bins from −5 to +5 kb along p300 binding sites overlapping DHS and distal to known TSS.

Genome-wide distal p300 binding sites were found using ChIP-seq in H1 and IMR90 cell-lines. We selected p300 binding sites overlapping DNase-I hypersensitive sites and distal to annotated TSS as active p300 binding sites representative of enhancers. We found 5899 such p300 binding sites in H1 and 25109 such sites in IMR90 (Table S1,S2), and observed several distinct and diverse chromatin states using an unsupervised clustering technique, ChromaSig (fig. 1A,B). All clusters showed enrichment of H3K4me1 and depletion of H3K4me3 as previously observed [5]. However, different clusters were characterized by varying levels of histone acetylation, H3K4me2 or H3K27me3. Clusters with presence or absence of H3K36me3 may represent genic and intergenic enhancers respectively. In order to ensure we represented all these different chromatin states at active p300 binding sites, we selected a relatively large number of these sites (>5000) for training as compared to previous methods.

To train the forest, active and distal p300-binding sites (BS) were selected as representative of the enhancer class. As non-enhancer classes, we considered annotated transcription start sites (TSS) that overlap DNase-I, and random 100 bp bins that are distal to known p300 or TSS (see Methods). The confidence of each enhancer prediction is given by the percentage of trees that predict this site to be an enhancer. In general, a genomic region is predicted as an enhancer if it has a background cutoff greater than 0.5 (>50% trees vote in it's favor). At higher cutoffs, confidence of prediction is higher, but fewer enhancers are predicted.

We used Receiver Operating Characteristic (ROC) curves to determine optimal parameters for our classification algorithm [36]. In the case of enhancer predictions, we can only obtain an approximate measure of specificity since we can never be certain that the randomly selected elements of the non-p300 class are all true negatives. Hence, in addition to the ROC curves generated using 5-fold cross-validation, we also verified parameter selection by comparing the percentage of predicted enhancers at each cutoff that overlap markers of active enhancers (validation rate) or TSS (misclassification rate). The markers of active enhancers include distal DNase-I hypersensitivity sites (HS), p300 binding sites (excluding those used in training), occupancy by CBP or sequence-specific transcription factors known to act at embryonic stem cell enhancers such as NANOG, OCT4 and SOX2.

In the case of Random forests, the main parameter to be determined is the number of trees. Since the non-enhancer class is assumed to be several times enriched compared to the enhancer class in the genome, we select a greater number of non-p300 training sites as compared to p300 sites and this proportion is also adjusted using the above-described methods. Previous algorithms [23] as well as empirical observations showed a width of −1 kb to +1 kb around the p300 binding site as optimal but we further verified this selection by cross-validation in the H1 cell-type (fig. S1A). The difference in cross-validation curves using a width of 0.5 kb or 1 kb is not obvious on the cross-validation curve while a width of 1.5 kb clearly shows a sharp drop in the area under the ROC curve (fig. S1A). When we further made enhancer predictions using all three widths (fig. S1B,C), it can be seen that a width of 1 kb on either side shows best validation and misclassification rates as compared to 0.5 or 1.5 kb widths.

### Enhancer predictions in H1 and IMR90 cells

**Figure 2:**
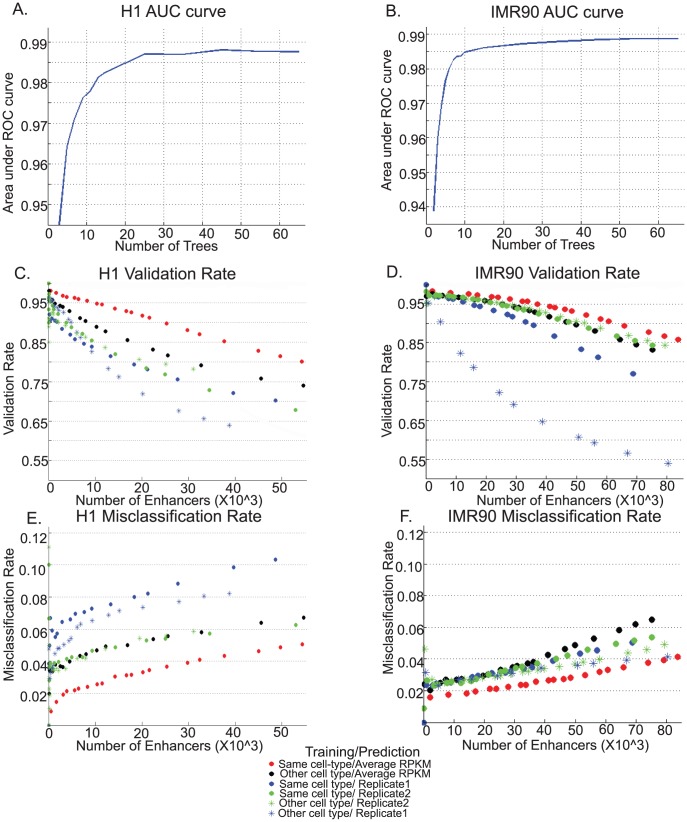
Performance of RFECS for enhancer predictions in H1 and IMR90 cells. Area under the 5-fold cross-validated ROC curve decreases with increase in number of trees stabilizing gradually in A.)H1 and B.)IMR90 cells. C.)Validation Rate of enhancer predicted in H1 cells, as measured by overlap with DNase-I HS and binding sites of p300, NANOG, OCT4 and SOX2. D.)Misclassification Rate of enhancer predicted using RFECS in H1 as measured as overlap of UCSC TSS, E.)Validation Rate of enhancers predicted by RFECS in IMR90 as measured by overlap with DNase-I HS or p300 binding sites in the same cells. F.)Misclassification Rate of enhancers predicted by RFECS in IMR90 as measured by overlap with UCSC TSS, versus total number of enhancers (upto 40000 enhancers) determined by taking different enrichment cutoffs, are shown for forest trained in the same cell type (⋅red), forest trained in other cell type and predictions made on modifications with averaged RPKM (⋅black), replicate 1 only (⋅blue), and replicate 2 only (⋅green). Training on one replicate and prediction on the other replicate of the same cell-type are indicated by asterisks.

To determine the optimal number of trees for the random-forest, we examined the area under the ROC curve in H1 and IMR90 and found both to be stable beyond 45 trees (fig. 2A,B). In order to verify this further, we made enhancer predictions using various number of trees such as 45, 65 and 85 and compared the validation and misclassification rates (fig. S2A–D). While H1 appeared to show no change at all (fig. S2A,,C) IMR90 showed a slight improvement from 45 to 65 trees (fig. S2B,D). In the end, we selected 65 trees for training the random forest as it appeared to be optimal for both cases. The training-set ratio of p300 to non-p300 was set at 1∶7 since the ROC curve did not appear to change much beyond this ratio. (fig. S2E,F)

In order to estimate the accuracy of the enhancer prediction by RFECS, we applied this algorithm to chromatin profiles of 24 marks obtained in H1 and IMR90. We then calculated the validation rate as the percentage of predicted enhancers overlapping with DNase-I hypersensitivity sites and binding sites of p300 and a few sequence specific transcription factors known to function in each cell type (true positive markers). We also computed the misclassification rate as the percentage of predicted enhancers overlapping with known promoters. These overlaps were computed using a window of −2.5 to +2.5 kb. Incase, both a true positive marker as well as promoter lay within this window, the criteria used to decide if the enhancer was “validated” or “misclassified” is discussed in detail in the Methods section. In H1 cells, we obtained a total of 55382 predicted enhancers at the lowest voting cutoff of 0.5. Over 80% of these predicted enhancers overlap with distal DNase-I hypersensitive sites and the binding sites of p300, NANOG, OCT4 and SOX2. Upon randomly generating enhancer predictions in the H1 genome 100 times, we found the average validation rate to be 18.43% and the actual validation rate of 80% to be highly significant with a one-sided t-test p-value of 10∧-256. Additionally, we found that 5% of them overlap with UCSC TSS, indicating a low misclassification rate of 5% (fig. 2C,E, in red). A similar high level of validation rate and low misclassification rate were observed when RFECS was applied to IMR90 cells, where 83581 enhancers were predicted with a validation rate of 85%(average random validation rate = 16.13%, pvalue = 2×10∧-279), and misclassification rate of 4% (fig. 2D,F). Thus, RFECS appears to accurately predict putative enhancer sequences based on chromatin modification state of the genome.

We next tried to assess the linear resolution of RFECS predictions. We calculated the distance between the predicted enhancers and locations of enhancer markers such as DNase-I hypersensitive sites, or p300 binding sites in each cell type, and found that the majority of predicted enhancers are within 200 bp of these sites (fig. S3A,B). In H1, nearly 62% of enhancers lie within 200 bp of an enhancer marker site (fig. S3A), while in IMR90 this value is around 70% (fig. S3B). Thus, the majority of enhancer predictions also show a high distance resolution in terms of proximity to the validation marker.

We also confirmed that our enhancer predictions showed an activation of gene expression in the proximal TSS. In order to do this, we compared RNA-seq datasets (Wei Xie et al., manuscript under revision) in H1 and IMR90 using edgeR [37] to identify H1-specific and IMR90-specific TSS. Then we identified enhancer predictions specific to either H1 or IMR90 using a filter distance of 2.5 kb. When we look at the average distribution of H1-specific enhancers they are clearly enriched in the vicinity of H1-specific TSS as compared to either non-specific TSS or IMR90-specific TSS (fig. S3C) and this enrichment is found to significant at distances up to at least 500 kb using a Wilcoxon test (p-value<10∧-6). Similarly, in the case of IMR90-specific enhancers, we observe them to be more enriched in the proximity of IMR90-specific TSS as compared to H1-specific TSS (fig. S3D, p-value<10∧-23).

As further evidence that RFECS accurately predicts enhancers, chromatin modifications at the predicted enhancers showed presence of all chromatin states observed in the training sets comprised of a subset of distal p300 binding sites (fig. 1). In H1, clusters 1,2 and 8 of enhancer predictions (fig. S4) are similar to clusters 1–3 of the p300 binding sites (fig. 1A), clusters 3–4 appear to correspond to cluster 5 of p300 BS, while clusters 5–6 look like cluster 4 of p300 BS. In IMR90, similar trends could be observed when comparing chromatin states at enhancer predictions (fig. S5) to those of p300 binding sites (fig. 1B). Further, it can be observed that clusters 3–6 of the enhancer predictions in H1 (fig. S4) that have weaker acetylation and/or enrichment of H3K27me3 also tend to have lower voting percentage of trees.

In summary, we showed that RFECS accurately predicted enhancers in the two cell lines H1 and IMR90 using a set of 24 chromatin modifications. These enhancers showed high validation rates, low misclassification rates and sharp linear resolution.

### Random forest trained on one cell-type can accurately predict enhancers in other cell-types

To make enhancer predictions, our approach requires a construction of a random forest trained on promoter-distal p300 binding sites. It is time-consuming and expensive to create a new training set for enhancer prediction in each new cell type, so it is desirable to use a random forest developed in one cell type to predict enhancers in another. To evaluate the feasibility of such approach, we first trained a random-forest using chromatin modification profiles obtained in H1, and then applied it to the IMR90 cells. Compared to RFECS predictions using IMR90 chromatin profiles as training set, RFECS predictions using H1 training dataset reduces the validation rate by ∼5–8% and increases the misclassification rate by ∼2% (fig. 2C,E black vs red). Similarly, we also developed a random forest using the IMR90 data as the training set and then applied it to H1. This led to an average reduction of 2–3% in validation rate (fig. 2D, black vs red). Therefore, RFECS trained using one cell type may be applied to a different cell type, albeit with slightly lower accuracy.

We sought to examine if this moderate decrease in performance was largely due to cell-type specific differences or was within the limits of technical or biological variability between replicates. To this end, we trained a random forest on one replicate of a cell-type, and made predictions on the other replicate of the same cell type. RFECS trained on IMR90 and then applied to the replicate 1 of the H1 profiles (blue dot vs asterisk) actually showed a higher validation rate and lower misclassification rate than RFECS trained using replicate 2 of H1 (fig. 2C,E), while similar performance was observed with enhancer predictions on replicate 2 of H1 independent of whether the random-forest was trained on H1 replicate 1 or IMR90 (green dot vs asterisk). Similar trends were observed when comparing predictions made on individual replicates of IMR90 using either H1-training or training on the other replicate (fig. 2D,F). In conclusion, predicting enhancers using the random forest built from a different cell type exhibits a modest decrease in performance compared to a same-cell training set. However, this decrease in performance is comparable to the decrease that can arise due to variability between two replicates of the same cell-type.

### Optimal set of chromatin marks required for enhancer prediction

**Figure 3:**
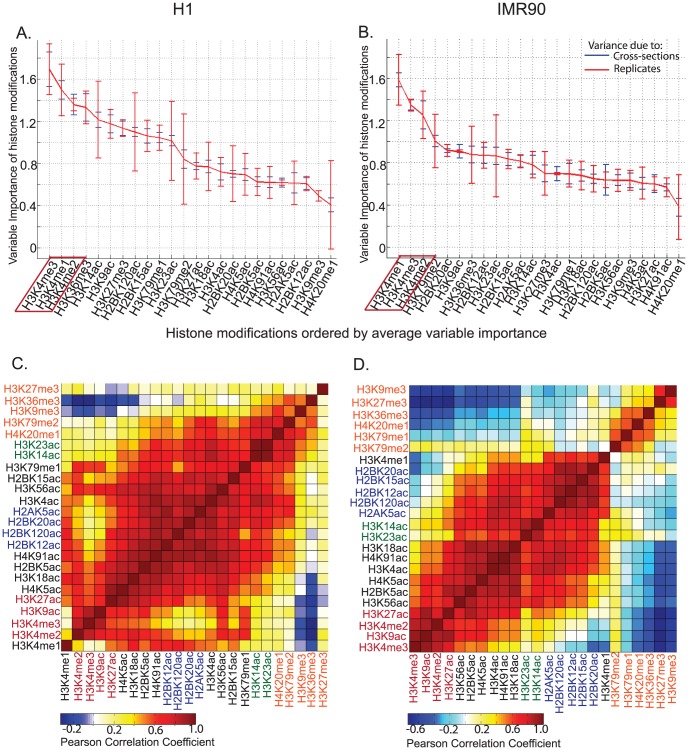
Out-of-bag variable importance of histone modifications in enhancer prediction. The average variable of histone modifications across 5 cross-sections of data in 2 sets of replicates as well as averaged replicates using all 24 modifications in A.)H1 and B.)IMR90 cells. Out-of-bag variable importance was calculated from the random-forest based classification of p300 binding sites against TSS+genomic background. Robust appearance of H3K4me1, H3K4me3 and H3K4me2 among the most important marks across replicates and cell types, indicates these may form a minimal set for prediction of enhancers. Differences observed in correlation clustering of the same 24 modifications in C.)H1 and D.)IMR90 explain some of the differences in ordering of variables in the two cell types. Same non-black colors of modifications indicate clusters that co-occur in both cell-types.

With the increasing number of histone modifications being discovered and mapped, determination of the relative importance of each mark in defining genomic elements is important. An out-of-bag measure of variable importance is a natural by-product of random forest classification scheme [33] wherein the relative importance of each feature is assessed as the increase in classification error upon permutation of feature values across classes. In both H1 and IMR90, the variable importance was assessed for random forests trained on 5 cross-sections of data for each of the 2 sets of replicates individually as well as the set of averaged replicates. Upon ranking histone modifications by variable importance, it is apparent that H3K4me1 and H3K4me3 are the top 2 most robust modifications across replicates and cross-sectional samples in both cell types, followed by H3K4me2 (fig. 3A, B). This indicates that these 3 modifications maybe the most informative in the prediction of enhancers in any unknown cell type as well.

Beyond the top 3 modifications, there is variability among the cell types. In IMR90, the other modifications appear to contribute almost equally, while in H1 there is a much clearer difference in variable importance. These differences are supported by correlation analyses in H1 and IMR90 (fig. 3C,D). In H1, several modifications are highly correlated, which could explain the larger differences in variable importance, as only a few variables maybe needed to form a non-redundant set. In IMR90, the correlations are lower and hence each of the modifications may contribute non-redundant information and thus contribute equally to the variable importance. Modifications that cluster together in both H1 and IMR90 (shown in the same non-black colors, fig. 3C,D) suggest cell-type independent redundancy.

**Figure 4:**
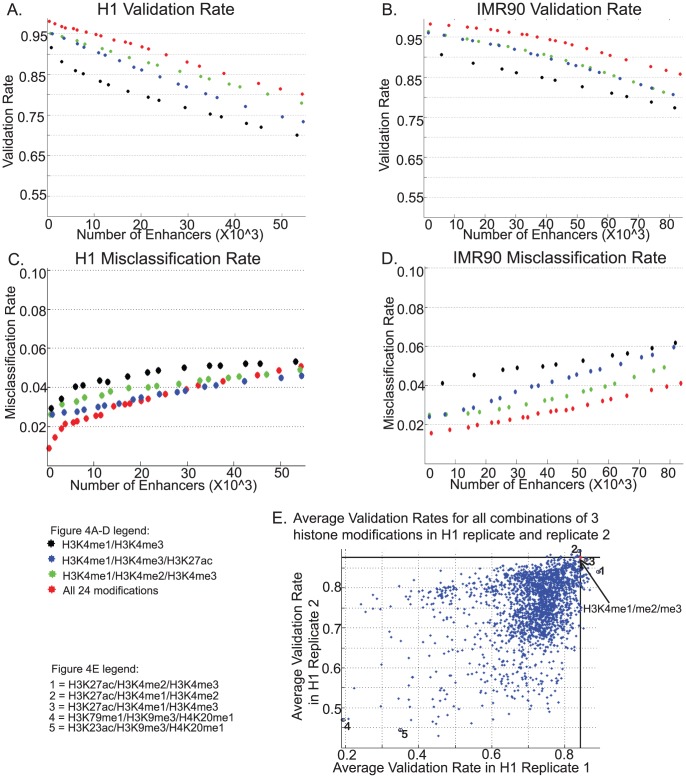
Validation rate and Misclassification rate of enhancers predicted using RFECS in H1 and IMR90. A.) Validation Rate in H1 measured by overlap with DNase-I HS, p300, NANOG, OCT4 or SOX2, B.) Misclassification Rate in H1 measured as overlap of UCSC TSS, C.) Validation Rate in IMR90 measured by overlap with DNase-I HS or p300, D.) Misclassification Rate in IMR90 measured as overlap of UCSC TSS, versus total number of enhancers determined by taking different enrichment cutoffs, are shown for all 24 modifications (red), predicted minimal set of H3K4me1/H3K4me2/H3K4me3 (green) and conventionally used marks H3K4me1/H3K4me3 (black) or H3K4me1/H3K4me3/H3K27ac (blue). E.) Comparison of average validation rates for enhancer predictions using all combinations of 3 histone modifications for 2 replicates of H1.

Having established the relative importance of each histone modification in predicting enhancers, we next examined the accuracy of predictions using different sets of modifications. Validation rates obtained by using the minimal set of H3K4me1-3 is within 2% of that for all 24 modifications in H1 (fig. 4A). Furthermore, this minimal set performs considerably better than the more conventionally selected set of H3K4me1 and H3K4me3 [3], [5] and at times, H3K27ac [38], [39] (fig. 4A,B, in black and blue). The set of H3K4me1-2-3 is more comparable to H3K4me1-H3K4me3-H3K27ac in IMR90 but does have a slightly lower misclassification rate (fig. 4D). In both cases the use of the minimal set of 3 modifications shows a much closer resemblance in performance to all 24 modifications than to the set of 2 marks H3K4me1 and H3K4me3 (fig. 4A–D).

It can also be observed that in conjunction with H3K4me1 and H3K4me3, using H3K4me2 picks up a larger proportion of enhancers with weaker acetylation enrichment as compared to H3K27ac (fig. S4,S5), supporting our prediction of the minimal set.

We also made enhancer predictions using all possible combinations of 3 modifications in chromosome 1 for replicate 1 and replicate 2 of H1. The average validation rate for a fixed range of enhancers was compared across replicates and it can be seen the set corresponding to H3K4me1, H3K4me2 and H3K4me3 (marked in *), is the highest performing combination common to both replicates (fig. 4E). We also found the performance of the combination of H3K27ac with H3K4me1 and H3K4me3 appears to be comparable in this case (3, fig. 4E), validating the use of H3K27ac as a feature for enhancer prediction when H3K4me2 is not available. Some of the worst performing combinations include H3K9me3 and H4K20me1 (4 and 5, fig. 4E), which also show up as variables with least importance in fig. 3A.

In many currently existing datasets, H3K27ac is the more commonly sequenced histone modification as compared to H3K4me2 due to it's perception as a marker of active enhancers. While using H3K4me2 may improve enhancer prediction in some cell-types, use of H3K27ac in addition to H3K4me1 and H3K4me3 marks does show considerable improvement over using just the top 2 marks H3K4me1 and H3K4me3 (fig. 4A–D). Hence, for many of the currently existing datasets, we could use H3K4me1, H3K4me3 and H3K27ac as the features in our random-forest with satisfactory performance.

Overall, these comparisons indicate the suitability of selecting H3K4me1, H3K4me2 and H3K4me3 as three minimal chromatin marks for purposes of enhancer prediction. Additional chromatin modifications required for improving upon enhancer predictions may depend on cell-type specific characteristics, as indicated by the differences in variable importance between H1 and IMR90 (fig. 3A,B).

### Comparison of RFECS with other enhancer prediction methods

**Figure 5:**
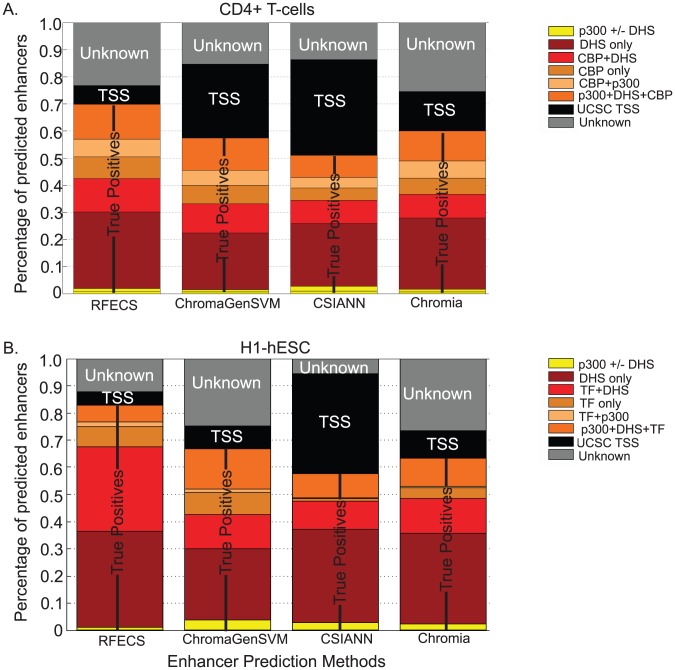
Comparison of enhancer predictions using RFECS, ChromaGenSVM, CSIANN and Chromia. A.) In CD4. True positive rates were measured as overlap with either DNase-I hypersensitive sites (DHS), p300 or CBP binding sites, while false positives were measured as overlap with UCSC TSS. B.) In H1. True positive rates were measured as overlap with either DNase-I hypersensitive sites (DHS), p300 or transcription factor binding sites such as NANOG, OCT4 and SOX2, while false positives were measured as overlap with UCSC TSS.

We next asked if our enhancer prediction algorithm performed better than several other current techniques for enhancer prediction – CSIANN, ChromaGenSVM and Chromia [23], [24], [26], [39]. In previous studies, CSIANN and ChromaGenSVM were applied on the histone modification dataset in CD4 T-cells [24], [26], [39]. In order to make a comparison of performance of our method with previous approaches, we applied RFECS to the CD4+ T cell dataset as well and determined parameters using cross-validation (fig. S6). Using H3K4me1, H3K4me3, and H3K27ac, CSIANN made 21832 predictions [39] and ChromaGenSVM method made 23574 predictions [26]. We made enhancer predictions using H3K4me1, H3K4me3 and H3K27ac with RFECS as well as Chromia [23]. Cutoffs were selected that yielded a similar number of enhancer predictions for both Chromia (21895) and RFECS (22947) (fig. 5A), so as to make a fair comparison across methods.

To compare these different sets of enhancer predictions, we computed validation rates by comparing them to TSS-distal DNase-I hypersensitive sites, p300 binding sites, and CBP binding sites and misclassification rates by comparing to known UCSC TSS using a window of −2.5 kb to +2.5 kb as described in the methods. (fig. 5A). The validation rate of RFECS predictions is around 70%, which is considerably higher than the other three methods (57% ChromaGenSVM, 51% CSIANN, 60% Chromia). Further, the misclassification rates of RFECS is less than 7%, much lower than the 27%, 35% and 15% rates of ChromaGenSVM, CSIANN and Chromia, respectively. These results suggested that overall procedure for RFECS, including selection of training set as well as training and prediction using the vector-random-forest, performs better than currently available techniques for enhancer prediction.

In the above comparison, we selected our enhancer-representative training set as p300 peaks called using MACS [40] that were distal to known UCSC TSS and overlapped DNase-I locations while CSIANN and ChromaGenSVM used a training-set of p300 peaks called using SICER previously [41]. We also wanted to compare the performance of the different algorithms on our own datasets using the same training-set to evaluate the performance of the random-forest based part of the algorithm. To achieve this, we ran the various enhancer prediction methods on H3K4me1, H3K4me2 and H3K4me3 datasets of H1, with help from the author of ChromaGenSVM [26] (fig. 5B). We tried to make the pre-processing stages of the various algorithms as consistent as possible by merging several replicates of each histone modification files and input files into single bed files and randomly selecting a smaller subset of p300 peaks for training, since these were the requirements of the other algorithms such as CSIANN and ChromaGenSVM. Incase of CSIANN, the selection of background was hard-coded in the software but in all other cases we used our own background training set as well. In fig. 5B, it can be observed that RFECS shows a maximum validation rate of around 82.8% as compared to 66.8%, 57.7% and 63.3% for ChromaGenSVM, CSIANN and chromia respectively. Further, RFECS showed the lowest misclassification rate of 4.9% as compared to 8.3%, 36,7% and 10.1% rates for the above-mentioned cases. Hence, the improvement in performance due to RFECS cannot be solely attributed to method of selecting the training-set. In summary, RFECS shows considerably improved performance over existing enhancer-prediction algorithms in two very different datasets and hence can be considered an advance in the field.

### Prediction of enhancers in multiple human cell-types

Comparing enhancer predictions across diverse cell-types can contribute to understanding differences in regulatory mechanisms between cell-types. The ENCODE dataset is an example of a collection of high-throughput datasets such as histone modifications and transcription factor binding data that are available for multiple cell-types [42]. Having a set of high-confidence enhancer predictions in these cell-types would be a valuable resource.

**Figure 6:**
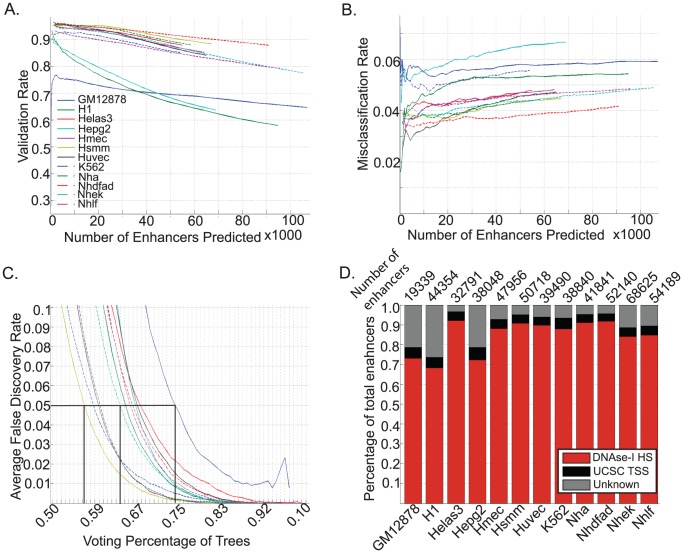
Enhancer predictions in ENCODE cell-lines using RFECS. A.)Validation Rate in the 12 cell-types measured by overlap with DNase-I HS, B.)Misclassification Rate in the cell-types measured as overlap of UCSC TSS, C.)Average false discovery rate (FDR) over the 22 autosomal chromosomes for each cell-type plotted as a function of voting percentage of trees, D.)Validation rate and misclassification rate for each cell-type at a FDR of 5% with number of enhancer predictions shown above the bar.

We trained our random forest on the p300 ENCODE data in H1 and made enhancer predictions in 12 ENCODE cell-types using the three marks H3K4me1, H3K4me3 and H3K27ac since these were available for all the cell-types. Validation rates were assessed based on overlap with existing DNAse-I hypersensitivity data while misclassification rates were calculated based on overlap with UCSC TSS. It can be seen that the majority of cell-types show high validation rates between 80 and 95%, while the misclassification rates lie within acceptable levels of 2–7% (fig. 6A,B).

In order to compare enhancers across cell-types, it is preferable to have enhancer predictions with the same level of confidence. To determine the appropriate cutoff for multiple number of cell-types, we calculate a False Discovery rate by randomly permuting 100 bp bins across the genome and computing the ratio of enhancers predicted in permuted data/enhancers predicted in real data for various cutoffs of voting percentages. In fig. 6C, it can be seen that different cell-types show a different relationship with FDR. For example, at an FDR of 5%, the voting percentage for GM12878 (solid dark blue) is 0.74, for Nhek (dashed cyan) 0.64 and for Hsmm (solid yellow) it is 0.56.

Using an FDR of 5%, we obtained a consistent set of high-confidence enhancer predictions in the 12 ENCODE cell-types. In fig. 6D, the numbers of enhancer predictions in each cell type is shown above the bar. The validation rates (in red) are above 90% for all cell-types except H1, Hepg2 and GM12878. In H1 and Hepg2, the numbers of DNase-I hypersensitivity sites are relatively less, i.e. ∼150 to 177K as compared to ∼230 to 380K in the other cell-lines. This may explain the somewhat lower validation rate in these two cell-types. GM12878 appears to be an outlier and we suspect that enhancer predictions may potentially be improved in this cell line by using a different training set.

In summary, we obtained a high-confidence set of enhancer predictions in multiple ENCODE cell-lines with the same level of confidence. This will enable more rigorous comparisons of regulatory characteristics of these cell-types in the future.

## Discussion

We describe here a novel machine-learning algorithm to accurately predict enhancers in a genome-wide manner based on chromatin modifications. We trained this algorithm using novel p300 training sets in H1 and IMR90 and 24 chromatin modifications in each cell-type. We showed that models trained on one cell-type could be effectively applied on another cell-type. Random forests enable detection of the most informative features required for a classification task. In the case of enhancer prediction, we identified a set of 3 histone modifications that appeared to be the most informative and robust across cell-types and replicates. Such an approach can once again be applied when the number of genome-wide modification maps is expanded in various different cell types and the most informative set of modifications can be further refined. We show that RFECS outperforms other machine-learning based prediction tools in CD4+ T cells, and can be applied in the future to multiple cell types. We successfully applied our enhancer prediction tool to 12 cell-lines in the publicly available ENCODE database and obtained a set of enhancers with a consistently high level of confidence across the cell-types.

In the future, we could potentially adapt the RFECS method to detect other regulatory genomic elements that can be observed to have a distinct chromatin signature and find the minimal set of chromatin marks for this purpose. The ability to detect diverse patterns of features within the training set indicates that the RFECS approach could be used to train on a composite training set comprised of different transcription factors. Combining information from different enhancer-binding proteins may improve prediction of regulatory elements. Random forests are non-parametric and have been shown to integrate a large number of diverse features. This could suggest the addition of other discrete and continuous data types such as sequence or motif based information or DNA methylation to the prediction of genomic elements.

## Methods

### Datasets used

The H1 and IMR90 datasets used in this study were generated as part of the NIH Roadmap Epigenome Project and have been released to the public prior to publication (http://www.genboree.org/epigenomeatlas/multiGridViewerPublic.rhtml). Briefly, 24 chromatin modifications in human embryonic stem cell (H1) and primary lung fibroblast cells (IMR90) were generated by the Ren lab and deposited under the NCBI Geo accession number GSE16256. Additionally, two replicates of H3K9me3 datasets deposited under Geo accession numbers GSM818057 and GSM42829 were used. Genome-wide binding data for p300 in H1 and IMR90, and transcription factors NANOG, SOX2 and OCT4 in H1 were generated in the Ren lab using ChIP-seq and deposited under accession numbers GSE37858, GSE18292 and GSE17917 respectively. Any data mapped to hg18 was converted to hg19 using liftover tools [43]. The DNase-I hypersensitivity datasets for H1 and IMR90 were produced by the Stammatoyanopoulos group at UW [44]. IMR90 DNase-I raw data may be accessed using GSM468792 and narrow peak calls are attached as supplemental information. Narrow DNase-I peaks in H1 were downloaded from UCSC ENCODE page (http://hgdownload.cse.ucsc.edu/goldenPath/hg19/encodeDCC/wgEncodeUwDnase/)

For CD4, previously generated datasets for p300 [41], CBP [41] and DNase-I [21] data as well as histone modifications [45], [46] were used. Histone modification data and DNase-I hypersensitivity data for the 12 ENCODE cell-lines was downloaded from http://genome.ucsc.edu/ENCODE/downloads.html.

### Data normalization for histone modifications

The ChIP-seq reads for the histone modification as well as corresponding input were binned into 100 bp intervals. The binned modification file was normalized against the binned input file using an RPKM (Reads per kilobase per million) measure [47]. In the case of 2 or more replicates, the RPKM- level for each bin is averaged to get a single histone modification file, in order to minimize batch-related differences.

### Determination of binding sites for p300 and other transcription factors

MACS [40] software was used to call peaks for p300, CBP and any other TF such as NANOG, SOX2 and OCT4. ChIP-seq input files were used as background and parameters of mfold = 20 and default p-value cutoffs were used. Peak calls are available as supplemental files. In case of the p300 and CBP binding sites used to validate enhancer predictions in CD4, we included the regions of enrichment that were previously published as well [41]

### Construction of random forest

We constructed the forest using the concept of binary classification trees, with each feature being a 20-dimensional vector of 100 bp bins from −1 to +1 kb along the genomic element. At each node in the tree, a linear classifier was constructed using the Fischer Discriminant approach using the histone modification vector, allowing for utilization of shape as well as abundance information (fig. S7A). The utilization of the linear discriminant at each node was inspired by the recent development of methods such as the discriminant random-forests [34] and oblique random forests [35]. The Vector-Random forest algorithm was implemented in MATLAB (MATLAB 7.14.0.739, The Mathworks Inc., Natick, MA, 2012a) as the function “multiclasstree” and utilizes functions from the “classregtree” and “classify” functions of MATLAB, implementing decision trees and linear discriminants respectively. The code used for RFECS can be downloaded from: http://enhancer.ucsd.edu/renlab/RFECS_enhancer_prediction/

### Training the random forest for enhancer predictions

Enhancer prediction involved two stages, which are classification of p300 vs non-p300 and peak-calling.

1. Classification of p300 vs non-p300 for enhancer prediction purposes
  i. Training
  In the first stage, a forest was constructed with two classes – a class containing p300 binding sites and a second class with an equal number of TSS and x times the number in random background sequences, where x = 9 for CD4 and x = 7 for H1 and IMR90.
  ii. Prediction
  In order to make predictions, each 100 bp bin along a chromosome is assigned either enhancer or non-enhancer status. The output from the forest is in the form of percentage of trees predicting a 100 bp bin to be one element or another. Only bins that have >50% trees voting for the enhancer class, are considered for further analysis.
2. Peak-calling
  Using the random forest previously trained to predict whether a 100 bp bin along a chromosome is an enhancer or not often yields values >50% for regions on either side of the exact location of a p300-binding site. However, the percentage of trees voting in favor of p300 decreases symmetrically on either side of the actual peak (fig. S7.B). This property is used to select the bin with maximum voting percentage within a certain peak-filtering distance as the enhancer peak based on the assumption that the flanking regions are part of this same enhancer.

### Computation of variable importance

A major advantage of the random forest is the inherent ability to select more important variables versus less important ones. In order to compute the order of variable importance, in this case, the importance of individual histone modifications for making enhancer predictions, we use an out-of-bag measure of variable importance [33] implemented in Matlab as the function oobVarImp.

### Application of variable importance to determine the minimal set of modifications required to predict enhancers

Based on the ordering of the variable importance across 5 different cross-sections of the training dataset of multiple replicates and cell types, certain modifications may always be observed to have priority. Due to the non-redundant nature of the ordering of variables as well as their robustness across replicates and samples, these modifications maybe selected as the most informative ones that are required to make enhancer predictions.

### Validation of enhancer predictions

Cross-validated ROC curves were used to estimate parameters for use within the same algorithm. However, comparisons across different algorithms may be biased depending upon the composition of the training set, so we validated enhancer predictions as described below.

Enhancer Predictions outputted from the random forest predictor have background enrichment scores of “voting percentage” ranging from 0.5 to 1 to enable detection of enhancers at different levels of confidence. At higher cutoffs, confidence of prediction is higher, but fewer enhancers are detected. The availability of large-scale datasets such as DNase-I hypersensitive sites, p300 binding sites, CBP binding sites and transcription factor binding sites enabled an estimate of the number of true positives at every cutoff. Further, the number of enhancers misclassified as TSS at each cutoff was also determined. Within the same cell type, an enhancer prediction method that performs better, should pick up more true positive validation markers and fewer TSS, given the number of predictions are the same.

Predicted enhancers are classified as “validated”, “misclassified” or “unknown” based on the criteria below. True Positive Markers (TPM) refer to DNase-I hypsersensitivity site, p300, CBP and Transcription factor binding sites.

1. If the nearest TPM lies within 2.5 kb of the enhancer and the nearest TSS is greater than 1 kb away from the TPM, the enhancer is “validated”
2. If a TSS lies within 2.5 kb of the enhancer, and the nearest TPM is either greater than 2.5 kb away from the enhancer or within 1 kb of the TSS, the enhancer is “misclassified”
3. If there is no TPM or TSS within 2.5 kb of the enhancer, it is “unknown”.

### Correlation graphs

The Pearson correlation coefficient between any two modifications was computed for RPKM-normalized histone modification reads between −1 to +1 kb for all elements within the selected training set. The correlation patterns of each histone modification was used to cluster the modifications and order them using MATLAB tools.

This enabled visualization of which modifications are the most similar in their correlation patterns. In the ordering of variable importance, if certain variables showed up as important in two different cell types, the redundancy based on their correlation plots could be used to explain away this variability.

### Visualization of chromatin modification patterns

ChromaSig [48] was used to cluster histone modification patterns along p300 binding sites and predicted enhancers using modification width as 4 kb. The resulting clusters were then visualized using Java TreeView [49].

## Supporting Information

Figure S1 **Determination of optimal peak width for training of RFECS predictor in H1 cells.** A.)ROC curves for 5-fold cross-validation at different proportions of peak widths of −0.5 to +0.5 kb, −1 to +1 kb and −1.5 to 1.5 kb around training set sites. B.)Percentage of enhancers validated by true positive markers at different numbers of enhancers determined by various cutoffs (Validation rate or VR curve). C.)Percentage of enhancers misclassified as TSS at different numbers of enhancers determined by various cutoffs. (Misclassification rate or MR curve). Overall, the width of −1 to +1 kb appears to show the best performance as expected based on previous observations.
(EPS)

Figure S2 **Determination of parameters for training of RFECS predictor in H1 and IMR90 cells.** A,B.)Percentage of enhancers validated by true positive markers at different numbers of enhancers determined by various cutoffs (Validation rate or VR curve) in A.)H1 and B.)IMR90, for different number of trees. C,D.)Percentage of enhancers misclassified as TSS at different numbers of enhancers determined by various cutoffs. (Misclassification rate or MR curve) in C.)H1 and D.) IMR90, for different number of trees. . VR and MR curves do not appear to change much beyond 45 trees, confirming the selection of 65 trees as valid. E,F.)ROC curves for 5-fold cross-validation at different proportions of training set ratios of p300∶non-p300 in E.) H1 and F.) IMR90. ROC curves appear to be most stable beyond the ratio of 1∶7.
(EPS)

Figure S3 **Linear resolution and association with expression of genes for enhancer predictions in H1 and IMR90.** Distribution of distances between predicted enhancers and known markers of active enhancers such as DNase-I hypersensitivity sites, p300 and transcription factor binding sites in A.)H1 and B.)IMR90. Distribution of average number of cell-type specific enhancers around the TSS specific to either H1 (blue), IMR90 (red) or non-specific (black) where the cell-type is C.)H1 or D.)IMR90.
(EPS)

Figure S4 **Histone modification patterns at enhancer predictions in H1.** Clustering was performed using ChromaSig. Java treeview-generated Heatmap shows RPKM-normalized histone modification levels in 100 bp bins from −5 to +5 kb along genomic elements overlapping enhancers in Chromosome1 predicted using all 24 modifications. On the left panel, the state number and sizes are indicated. On the right panel, percentage of each state detected by different combinations of histone modifications or H1-trained forest are shown. Also shown are the distribution of background cutoffs associated with each chromatin state.
(TIF)

Figure S5 **Histone modification patterns at enhancer predictions in IMR90.** Clustering was performed using ChromaSig. Java treeview-generated Heatmap shows RPKM-normalized histone modification levels in 100 bp bins from −5 to +5 kb along genomic elements overlapping enhancers in Chromosome1 predicted using all 24 modifications. On the left panel, the state number and sizes are indicated. On the right panel, percentage of each state detected by different combinations of histone modifications or H1-trained forest are shown. Also shown, are the distribution of background cutoffs associated with each chromatin state.
(TIF)

Figure S6 **Determination of parameters for training of RFECS predictor in CD4 T-cells.** A.)Area under the 5-fold cross-validated ROC curve decreases with increase in number of trees stabilising gradually B.) Percentage of enhancers validated by true positive markers at different numbers of enhancers determined by various cutoffs (Validation rate or VR curve) and C.) Percentage of enhancers misclassified as TSS at different numbers of enhancers determined by various cutoffs. (Misclassification rate or MR curve), for 41, 61 and 81 trees. VR and MR curves do not appear to change much beyond 61 trees, confirming the selection of 81 trees as valid. D.) ROC curves for 5-fold cross-validation at different proportions of training set ratios of p300∶non-p300. ROC curve does not appear to change much beyond a ratio of 1∶9 E.) Validation Rate curve for training set ratios of 1∶9 and 1∶11. F.) Misclassification Rate curve for training set ratios of 1∶9 and 1∶11. The VR and MR curves validate the choice of 1∶9 as an appropriate training set ratio.
(EPS)

Figure S7 **Training of RFECS for enhancer prediction.** A.)Example of the vector-based random-forest classifying p300 binding sites and TSS using histone modifications. B.)Average percentage of trees voting in favor of the enhancer class around a p300-binding site. Percentage of trees in the random forest predictor that vote in favor of the enhancer class decrease symmetrically with increasing distance from the p300-binding peak. This property is used to develop a peak-calling method that can predict the most probable location of the enhancer.
(EPS)

Table S1 **All p300 binding-sites called using MACS in H1 cells (hg19).**
(XLS)

Table S2 **p300 binding sites in H1 overlapping DNase-I hypersensitivity sites and distal to known UCSC and Gencode TSS.**
(XLS)

Table S3 **Transcription factor binding sites in H1.**
(XLS)

Table S4 **H1 enhancers predicted using all 24 modifications.**
(XLS)

Table S5 **All p300 binding-sites called using MACS in IMR90 cells (hg19).**
(XLS)

Table S6 **p300 binding sites in IMR90 overlapping DNase-I hypersensitivity sites and distal to known UCSC and Gencode TSS.**
(XLS)

Table S7 **IMR90 enhancers predicted using all 24 modifications.**
(XLS)

Table S8 **All p300 binding-sites called using MACS in CD4+ T-cells (hg18).**
(XLS)

Table S9 **p300 binding sites in CD4 T-cells overlapping DNase-I hypersensitivity sites and distal to known UCSC and Gencode TSS.**
(XLS)

Table S10 **CBP binding sites determined by MACS.**
(XLS)

Table S11 **CD4 enhancer predictions using RFECS with H3K27ac, H3K4me1 and H3K4me3 and peak filtering distance as 1 kb.**
(XLS)

Table S12 **CD4 enhancer predictions by Chromia using H3K27ac, H3K4me1 and H3K4me3.**
(XLS)

Table S13 **DNase-I hotspots in IMR90 (hg19).**
(TXT)

Table S14 **Enhancer predictions in the ENCODE cell-types GM12878, H1, Helas3, Hepg2, Hmec and Hsmm (hg18).**
(PDF)

Table S15 **Enhancer predictions in the ENCODE cell-types Huvec, K562, Nha, Nhdfad, Nhek and Nhlf (hg18).**
(PDF)
