# Supplementary figures and images for: RFECS: A Random-Forest Based Algorithm for Enhancer Identification from Chromatin State
Source: PLoS Comput Biol. 2013 Mar 14;9(3):e1002968. doi: 10.1371/journal.pcbi.1002968 (PMC3597546; doi:10.1371/journal.pcbi.1002968)

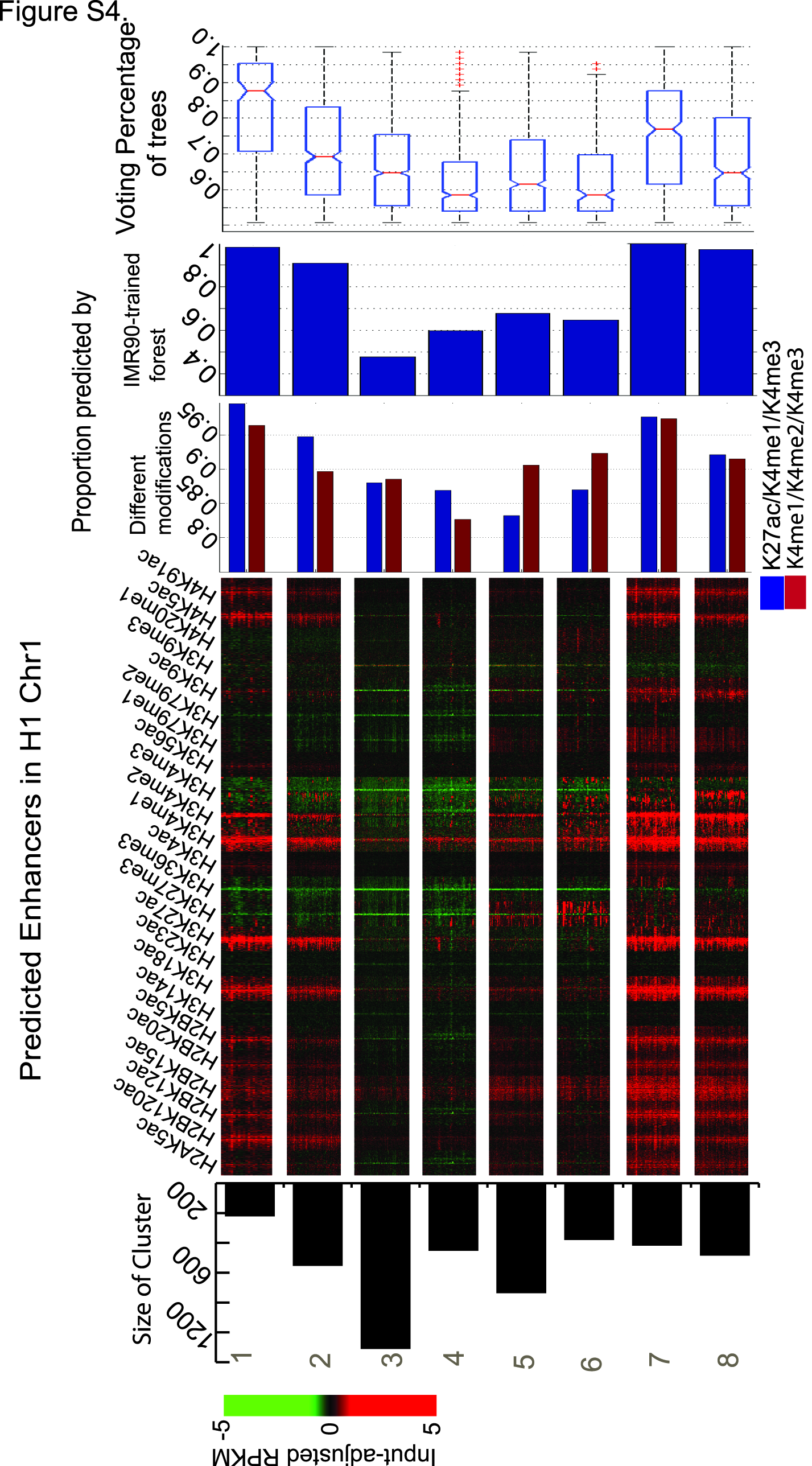

Supplement: Figure S4 — Histone modification patterns at enhancer predictions in H1. Clustering was performed using ChromaSig. Java treeview-generated Heatmap shows RPKM-normalized histone modification levels in 100 bp bins from −5 to +5 kb along genomic elements overlapping enhancers in Chromosome1 predicted using all 24 modifications. On the left panel, the state number and sizes are indicated. On the right panel, percentage of each state detected by different combinations of histone modifications or H1-trained forest are shown. Also shown are the distribution of background cutoffs associated with each chromatin state. (TIF) [file pcbi.1002968.s004.tif]

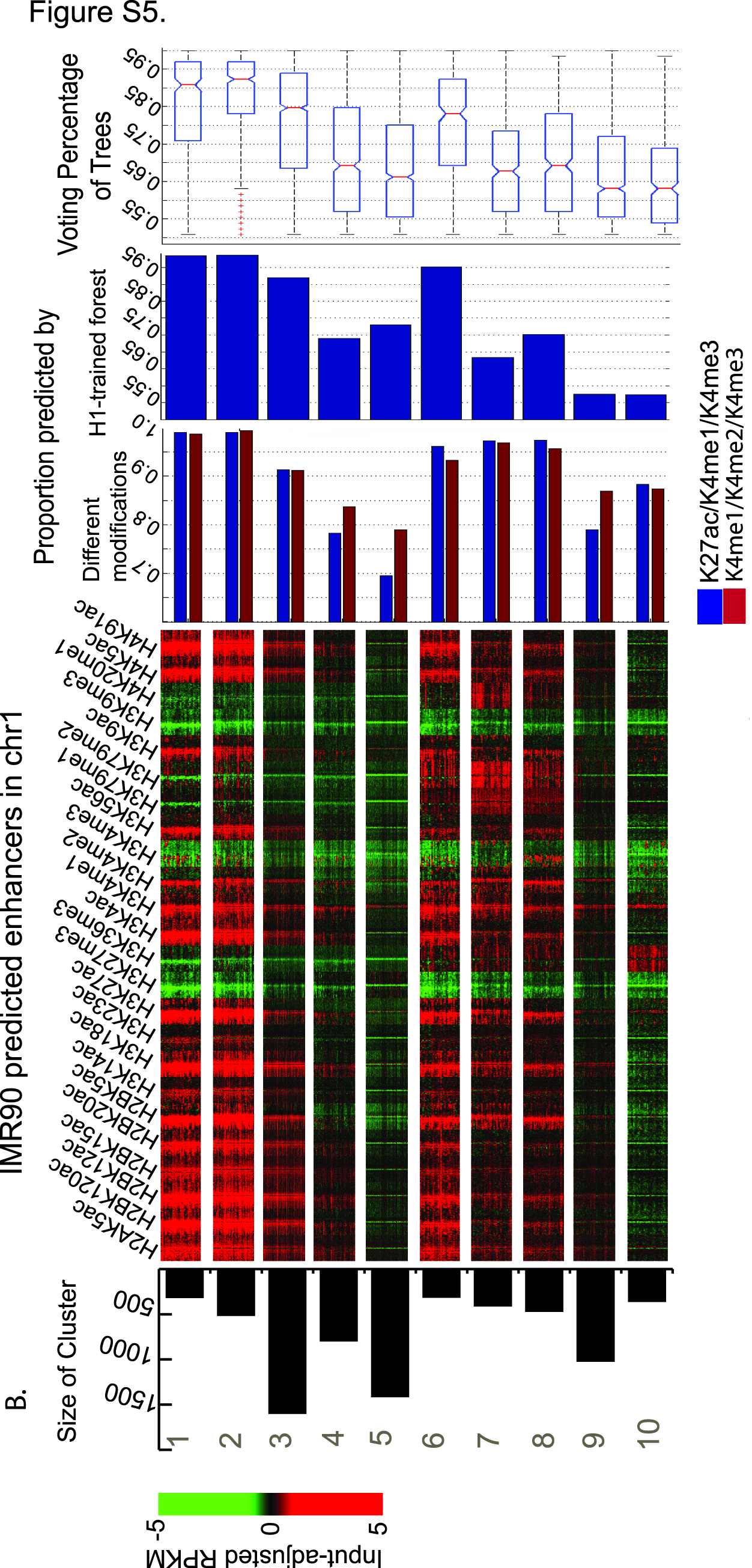

Supplement: Figure S5 — Histone modification patterns at enhancer predictions in IMR90. Clustering was performed using ChromaSig. Java treeview-generated Heatmap shows RPKM-normalized histone modification levels in 100 bp bins from −5 to +5 kb along genomic elements overlapping enhancers in Chromosome1 predicted using all 24 modifications. On the left panel, the state number and sizes are indicated. On the right panel, percentage of each state detected by different combinations of histone modifications or H1-trained forest are shown. Also shown, are the distribution of background cutoffs associated with each chromatin state. (TIF) [file pcbi.1002968.s005.tif]
