# Supplementary material for: RFECS: A Random-Forest Based Algorithm for Enhancer Identification from Chromatin State
Source: PLoS Comput Biol. 2013 Mar 14;9(3):e1002968. doi: 10.1371/journal.pcbi.1002968 (PMC3597546; doi:10.1371/journal.pcbi.1002968)
Supplement: Table S14 — Enhancer predictions in the ENCODE cell-types GM12878, H1, Helas3, Hepg2, Hmec and Hsmm (hg18). (PDF) [file pcbi.1002968.s021.pdf]

GM12878 enhancers

Chromosome	Location	Voting_cutoff
chr10	311000	0.834711
chr10	313500	0.884298
chr10	329100	0.776860
chr10	346400	0.752066
chr10	358100	0.735537
chr10	361200	0.867769
chr10	376200	0.900826
chr10	379700	0.752066
chr10	560100	0.735537
chr10	590800	0.768595
chr10	603800	0.752066
chr10	654100	0.917355
chr10	706400	0.900826
chr10	983100	0.776860
chr10	995800	0.760331
chr10	1034700	0.859504
chr10	1090200	0.818182
chr10	1296600	0.958678
chr10	1466600	0.909091
chr10	1469000	0.793388
chr10	1508300	0.876033
chr10	1582300	0.826446
chr10	1685500	0.752066
chr10	1755300	0.752066
chr10	1758400	0.966942
chr10	1760900	0.826446
chr10	1916900	0.793388
chr10	2993800	0.801653
chr10	3029300	0.752066
chr10	3051200	0.909091
chr10	3078800	0.909091
chr10	3107800	0.876033
chr10	3212300	0.735537
chr10	3216800	0.752066
chr10	3220800	0.809917
chr10	3240100	0.950413
chr10	3270500	0.925620
chr10	3396300	0.793388
chr10	3498200	0.735537
chr10	3520600	0.785124
chr10	3666800	0.834711
chr10	3689600	0.842975
chr10	3697900	0.809917
chr10	3709400	0.983471
chr10	3808100	0.809917
chr10	3908800	0.809917
chr10	3936100	0.735537
chr10	3945200	0.842975
chr10	3977400	0.809917
chr10	4023200	0.776860
chr10	4088800	0.851240

chr10	4109900	0.909091
chr10	4144700	0.760331
chr10	4164200	0.801653
chr10	4174800	0.760331
chr10	4178900	0.818182
chr10	4213400	0.752066
chr10	4219200	0.834711
chr10	4224200	0.776860
chr10	4557300	0.859504
chr10	4565600	0.851240
chr10	4694600	0.867769
chr10	5068100	0.768595
chr10	5398000	0.851240
chr10	5487200	0.760331
chr10	5521900	0.760331
chr10	5594100	0.892562
chr10	5628300	0.785124
chr10	5671700	0.818182
chr10	5699600	0.801653
chr10	5702000	0.776860
chr10	5882600	0.892562
chr10	5902500	0.892562
chr10	5938300	0.933884
chr10	6045700	0.826446
chr10	6079000	0.735537
chr10	6099400	0.735537
chr10	6108200	0.876033
chr10	6114500	0.933884
chr10	6128900	0.884298
chr10	6131300	0.752066
chr10	6163200	0.752066
chr10	6193800	0.826446
chr10	6206000	0.859504
chr10	6228800	0.826446
chr10	6317300	0.826446
chr10	6342800	0.876033
chr10	6371100	0.785124
chr10	6443800	0.776860
chr10	6812600	0.760331
chr10	6824300	0.793388
chr10	6927200	0.900826
chr10	6955200	0.867769
chr10	6958800	0.900826
chr10	7202600	0.768595
chr10	7227000	0.851240
chr10	7282300	0.809917
chr10	7362400	0.776860
chr10	7379800	0.942149
chr10	7436200	0.760331
chr10	7459800	0.760331
chr10	7462900	0.735537
chr10	7478900	0.743802
chr10	7488000	0.793388
chr10	7543700	0.917355

chr10	7562900	0.768595
chr10	7567800	0.892562
chr10	7661300	0.834711
chr10	8107600	0.760331
chr10	8111300	0.785124
chr10	8146400	0.818182
chr10	8179700	0.876033
chr10	8374600	0.801653
chr10	8403400	0.801653
chr10	9337000	0.760331
chr10	10526200	0.793388
chr10	10897300	0.851240
chr10	10912700	0.876033
chr10	11002200	0.884298
chr10	11072300	0.752066
chr10	11117900	0.743802
chr10	11147200	0.867769
chr10	11193000	0.818182
chr10	11196800	0.818182
chr10	11203300	0.785124
chr10	11271800	0.752066
chr10	11312200	0.743802
chr10	11326200	0.743802
chr10	11427400	0.826446
chr10	11489100	0.743802
chr10	11581900	0.760331
chr10	11637200	0.942149
chr10	11679200	0.768595
chr10	11684900	0.793388
chr10	11707000	0.743802
chr10	11711800	0.752066
chr10	11752600	0.876033
chr10	11762700	0.768595
chr10	11825900	0.859504
chr10	11880200	0.818182
chr10	11905900	0.826446
chr10	11917200	0.851240
chr10	11949100	0.735537
chr10	12069900	0.851240
chr10	12096100	0.842975
chr10	12111300	0.735537
chr10	12349900	0.842975
chr10	12379700	0.735537
chr10	12461800	0.743802
chr10	12490700	0.909091
chr10	12505000	0.768595
chr10	12508900	0.743802
chr10	12527500	0.867769
chr10	12542900	0.793388
chr10	12610100	0.735537
chr10	12648000	0.925620
chr10	12662400	0.776860
chr10	13330300	0.743802
chr10	13344200	0.958678

chr10	13394300	0.785124
chr10	13459600	0.818182
chr10	13465400	0.809917
chr10	13526500	0.826446
chr10	13754800	0.776860
chr10	13838500	0.892562
chr10	13873800	0.876033
chr10	13929500	0.801653
chr10	14083800	0.743802
chr10	14228400	0.851240
chr10	14428700	0.818182
chr10	14432600	0.842975
chr10	14499100	0.859504
chr10	14623000	0.867769
chr10	14642000	0.842975
chr10	14651200	0.785124
chr10	14659600	0.768595
chr10	14728900	0.958678
chr10	14740800	0.752066
chr10	14779100	0.785124
chr10	14842600	0.768595
chr10	14854700	0.752066
chr10	14924300	0.752066
chr10	14931600	0.818182
chr10	15001800	0.925620
chr10	15251100	0.785124
chr10	15297200	0.776860
chr10	15416400	0.867769
chr10	16479300	0.851240
chr10	16686500	0.735537
chr10	16813200	0.884298
chr10	17066700	0.760331
chr10	17248800	0.785124
chr10	17274900	0.884298
chr10	17471200	0.809917
chr10	17550000	0.809917
chr10	17698000	0.752066
chr10	18745900	0.818182
chr10	18790500	0.925620
chr10	21668800	0.809917
chr10	21789500	0.834711
chr10	21810900	0.760331
chr10	21959100	0.752066
chr10	21992200	0.785124
chr10	22178800	0.793388
chr10	22243900	0.818182
chr10	22283100	0.809917
chr10	22312200	0.735537
chr10	22511600	0.859504
chr10	22605700	0.760331
chr10	22634400	0.752066
chr10	22655000	0.859504
chr10	22679600	0.752066
chr10	22719500	0.859504

chr10	22733100	0.826446
chr10	22781200	0.876033
chr10	22795800	0.818182
chr10	22890200	0.735537
chr10	22995900	0.809917
chr10	23221600	0.876033
chr10	23235500	0.743802
chr10	23447400	0.884298
chr10	23657800	0.801653
chr10	23703500	0.785124
chr10	23811000	0.735537
chr10	24667400	0.768595
chr10	24720600	0.735537
chr10	24730200	0.900826
chr10	24738700	0.892562
chr10	24755300	0.933884
chr10	25112100	0.735537
chr10	25171400	0.752066
chr10	25271600	0.818182
chr10	25306100	0.851240
chr10	26191000	0.743802
chr10	26681500	0.768595
chr10	26738200	0.735537
chr10	26750000	0.743802
chr10	26759900	0.809917
chr10	26833700	0.752066
chr10	27119600	0.785124
chr10	27149600	0.818182
chr10	27389200	0.867769
chr10	27541800	0.942149
chr10	27545700	0.950413
chr10	27655100	0.842975
chr10	28550300	0.743802
chr10	28748500	0.776860
chr10	28835300	0.760331
chr10	28969400	0.735537
chr10	29322900	0.776860
chr10	29769200	0.867769
chr10	29776300	0.892562
chr10	29852500	0.933884
chr10	30323800	0.867769
chr10	30482500	0.743802
chr10	30509400	0.752066
chr10	30649000	0.768595
chr10	30687900	0.892562
chr10	30732500	0.892562
chr10	30800200	0.818182
chr10	30818600	0.851240
chr10	30825700	0.867769
chr10	30872800	0.785124
chr10	30952900	0.818182
chr10	30965200	0.851240
chr10	30976300	0.785124
chr10	30987000	0.768595

chr10	31109400	0.842975
chr10	31287600	0.851240
chr10	31297300	0.809917
chr10	31308900	0.933884
chr10	31886000	0.834711
chr10	32040200	0.842975
chr10	32097400	0.776860
chr10	32165900	0.826446
chr10	32258700	0.975207
chr10	32345400	0.735537
chr10	32456700	0.752066
chr10	32489400	0.842975
chr10	33228600	0.809917
chr10	33233900	0.826446
chr10	33238200	0.801653
chr10	33240800	0.925620
chr10	33251500	0.785124
chr10	33275100	0.842975
chr10	33285100	0.809917
chr10	33388700	0.851240
chr10	33403900	0.859504
chr10	33534200	0.760331
chr10	33798800	0.735537
chr10	34664000	0.785124
chr10	35070900	0.743802
chr10	35379600	0.933884
chr10	35425200	0.809917
chr10	35589600	0.768595
chr10	35631800	0.768595
chr10	35641300	0.826446
chr10	35751800	0.884298
chr10	35785000	0.801653
chr10	35788300	0.834711
chr10	35798500	0.933884
chr10	35812600	0.743802
chr10	38146700	0.900826
chr10	38265100	0.859504
chr10	43134100	0.809917
chr10	43278000	0.793388
chr10	43625400	0.743802
chr10	43629800	0.859504
chr10	43716900	0.752066
chr10	43886000	0.760331
chr10	43913800	0.776860
chr10	44091400	0.892562
chr10	44303400	0.752066
chr10	44439100	0.834711
chr10	44460600	0.785124
chr10	44694400	0.809917
chr10	44791000	0.859504
chr10	44806900	0.842975
chr10	45218800	0.842975
chr10	45221100	0.785124
chr10	45421400	0.834711

chr10	45431700	0.793388
chr10	45496500	0.859504
chr10	46053400	0.743802
chr10	46206100	0.909091
chr10	46800300	0.752066
chr10	47104500	0.826446
chr10	47918100	0.809917
chr10	48481600	0.892562
chr10	48989900	0.826446
chr10	49491100	0.809917
chr10	49518500	0.752066
chr10	49548100	0.785124
chr10	49599600	0.859504
chr10	49609000	0.826446
chr10	49831700	0.752066
chr10	49861400	0.867769
chr10	49866800	0.826446
chr10	49913800	0.743802
chr10	50221500	0.752066
chr10	50285300	0.809917
chr10	50389200	0.818182
chr10	50716700	0.884298
chr10	50790700	0.743802
chr10	51503600	0.743802
chr10	51580600	0.760331
chr10	52063400	0.801653
chr10	52076500	0.785124
chr10	52087400	0.743802
chr10	52157300	0.735537
chr10	52258100	0.876033
chr10	52340800	0.785124
chr10	52360800	0.793388
chr10	52397000	0.785124
chr10	52413100	0.785124
chr10	52484000	0.809917
chr10	53459400	0.809917
chr10	53620400	0.735537
chr10	53976700	0.818182
chr10	53992800	0.785124
chr10	54017400	0.826446
chr10	54882900	0.752066
chr10	58079700	0.776860
chr10	58383900	0.743802
chr10	60042100	0.768595
chr10	60083000	0.818182
chr10	61592300	0.867769
chr10	61625100	0.859504
chr10	61632700	0.776860
chr10	61635800	0.752066
chr10	61802700	0.801653
chr10	61853500	0.876033
chr10	61857400	0.842975
chr10	62118400	0.768595
chr10	62671000	0.851240

chr10	62896200	0.834711
chr10	63046700	0.809917
chr10	63546800	0.760331
chr10	63638700	0.760331
chr10	63643300	0.776860
chr10	63648600	0.842975
chr10	63786000	0.743802
chr10	63944800	0.768595
chr10	64003400	0.760331
chr10	64193400	0.859504
chr10	64220700	0.933884
chr10	64271800	0.909091
chr10	64301000	0.909091
chr10	64324400	0.834711
chr10	64392300	0.851240
chr10	64422300	0.793388
chr10	64431000	0.752066
chr10	64488500	0.834711
chr10	64577100	0.743802
chr10	64591200	0.793388
chr10	64613500	0.900826
chr10	64617200	0.834711
chr10	64631000	0.867769
chr10	64668100	0.892562
chr10	64677600	0.917355
chr10	64765600	0.785124
chr10	65006400	0.760331
chr10	65028700	0.809917
chr10	65395100	0.892562
chr10	65431100	0.735537
chr10	65458700	0.842975
chr10	65471700	0.793388
chr10	65480600	0.752066
chr10	69373400	0.809917
chr10	69456300	0.752066
chr10	69524300	0.851240
chr10	69539100	0.876033
chr10	69609600	0.925620
chr10	69644800	0.809917
chr10	69835100	0.776860
chr10	70012100	0.785124
chr10	70048600	0.760331
chr10	70166900	0.859504
chr10	70380500	0.884298
chr10	70444700	0.826446
chr10	70481400	0.900826
chr10	70661200	0.842975
chr10	70751600	0.834711
chr10	70821400	0.884298
chr10	70825900	0.735537
chr10	70833700	0.876033
chr10	70859300	0.776860
chr10	70940000	0.834711
chr10	70988900	0.958678

chr10	71022600	0.785124
chr10	71082200	0.743802
chr10	71087900	0.752066
chr10	71198500	0.809917
chr10	71221300	0.735537
chr10	71239800	0.743802
chr10	71334400	0.859504
chr10	71374300	0.768595
chr10	71507700	0.768595
chr10	71518200	0.768595
chr10	71886200	0.834711
chr10	71892700	0.917355
chr10	71934300	0.776860
chr10	71983700	0.752066
chr10	72091900	0.809917
chr10	72236800	0.909091
chr10	72269000	0.867769
chr10	72318900	0.760331
chr10	72336200	0.842975
chr10	72354600	0.809917
chr10	72376100	0.818182
chr10	72443200	0.851240
chr10	73103300	0.876033
chr10	73255100	0.809917
chr10	73381900	0.884298
chr10	73398300	0.793388
chr10	73454100	0.842975
chr10	73537300	0.768595
chr10	73542900	0.760331
chr10	73551100	0.909091
chr10	73561200	0.752066
chr10	73600700	0.834711
chr10	73619200	0.793388
chr10	73669200	0.752066
chr10	73693800	0.909091
chr10	73732000	0.809917
chr10	73734300	0.834711
chr10	73742600	0.735537
chr10	73813400	0.818182
chr10	73836000	0.785124
chr10	73957800	0.867769
chr10	73978900	0.842975
chr10	74023500	0.776860
chr10	74046100	0.884298
chr10	74076700	0.735537
chr10	74114700	0.900826
chr10	74322200	0.834711
chr10	74489400	0.768595
chr10	74562700	0.834711
chr10	74665300	0.933884
chr10	74927900	0.776860
chr10	75351700	0.768595
chr10	75477600	0.752066
chr10	75571100	0.876033

chr10	75639600	0.801653
chr10	75659800	0.760331
chr10	75682700	0.834711
chr10	75734600	0.933884
chr10	75768400	0.983471
chr10	75771800	0.776860
chr10	75816300	0.950413
chr10	75836500	0.884298
chr10	75857400	0.809917
chr10	75950100	0.892562
chr10	76511100	0.900826
chr10	76596200	0.735537
chr10	76614900	0.776860
chr10	76738300	0.917355
chr10	76921700	0.735537
chr10	76984900	0.884298
chr10	77096100	0.793388
chr10	77266300	0.768595
chr10	77288700	0.842975
chr10	77524400	0.776860
chr10	78985100	0.743802
chr10	79024200	0.842975
chr10	79170400	0.768595
chr10	79252100	0.884298
chr10	79363300	0.867769
chr10	79401400	0.735537
chr10	79443100	0.752066
chr10	79633800	0.925620
chr10	79659000	0.743802
chr10	79731300	0.752066
chr10	79967800	0.768595
chr10	80010800	0.884298
chr10	80338400	0.884298
chr10	80418900	0.884298
chr10	80524100	0.768595
chr10	80741800	0.818182
chr10	80832600	0.892562
chr10	80835700	0.867769
chr10	80844200	0.826446
chr10	80850600	0.818182
chr10	80862600	0.743802
chr10	80875400	0.851240
chr10	80990200	0.867769
chr10	81081100	0.826446
chr10	81087100	0.834711
chr10	81119100	0.785124
chr10	81265700	0.859504
chr10	81285500	0.834711
chr10	81300900	0.876033
chr10	81362200	0.809917
chr10	81435100	0.826446
chr10	81671000	0.867769
chr10	81864600	0.826446
chr10	81904300	0.909091

chr10	81965500	0.851240
chr10	81979000	0.842975
chr10	81994800	0.834711
chr10	82014800	0.859504
chr10	82196900	0.752066
chr10	82205200	0.834711
chr10	82209000	0.834711
chr10	82296000	0.892562
chr10	82339100	0.752066
chr10	82447300	0.776860
chr10	82793300	0.752066
chr10	84703300	0.876033
chr10	85304000	0.809917
chr10	85306700	0.760331
chr10	85719600	0.760331
chr10	85740900	0.925620
chr10	86052000	0.958678
chr10	86069600	0.752066
chr10	86077000	0.884298
chr10	86091500	0.735537
chr10	86323700	0.834711
chr10	87306300	0.834711
chr10	87372500	0.859504
chr10	87808000	0.942149
chr10	87930900	0.876033
chr10	88137300	0.785124
chr10	88260100	0.735537
chr10	88281200	0.743802
chr10	88439400	0.785124
chr10	88471800	0.818182
chr10	88494200	0.834711
chr10	88535100	0.801653
chr10	88545000	0.752066
chr10	88606500	0.826446
chr10	88609600	0.826446
chr10	88720500	0.776860
chr10	88728900	0.966942
chr10	89102700	0.884298
chr10	89279500	0.826446
chr10	89294500	0.735537
chr10	89404400	0.760331
chr10	89416500	0.776860
chr10	89478800	0.768595
chr10	89483300	0.818182
chr10	89676300	0.826446
chr10	89844300	0.760331
chr10	89897000	0.801653
chr10	89955000	0.884298
chr10	90005000	0.834711
chr10	90049400	0.735537
chr10	90071500	0.909091
chr10	90152100	0.925620
chr10	90172900	0.768595
chr10	90249500	0.793388

chr10	90304100	0.876033
chr10	90342800	0.900826
chr10	90493600	0.859504
chr10	90554200	0.809917
chr10	90591300	0.752066
chr10	90757800	0.776860
chr10	90844300	0.793388
chr10	90847200	0.776860
chr10	90916600	0.809917
chr10	90925900	0.884298
chr10	90964600	0.809917
chr10	90969600	0.818182
chr10	91238400	0.809917
chr10	91566200	0.752066
chr10	91689500	0.776860
chr10	91752200	0.752066
chr10	91779400	0.776860
chr10	91928900	0.818182
chr10	92049200	0.760331
chr10	92537300	0.735537
chr10	92678200	0.735537
chr10	92737600	0.776860
chr10	92744000	0.809917
chr10	92747100	0.743802
chr10	92931400	0.735537
chr10	92981000	0.793388
chr10	93001700	0.760331
chr10	93056600	0.842975
chr10	93114500	0.760331
chr10	93169700	0.933884
chr10	93175900	0.818182
chr10	93301300	0.876033
chr10	93317700	0.876033
chr10	93472600	0.793388
chr10	93564400	0.859504
chr10	93567200	0.876033
chr10	93771700	0.966942
chr10	93808200	0.785124
chr10	93902700	0.933884
chr10	93973900	0.867769
chr10	93993500	0.768595
chr10	94165500	0.793388
chr10	94304100	0.735537
chr10	94333700	0.818182
chr10	94480600	0.867769
chr10	94550500	0.735537
chr10	94562400	0.809917
chr10	94571300	0.876033
chr10	94840500	0.909091
chr10	94846000	0.793388
chr10	94958800	0.851240
chr10	95029200	0.735537
chr10	95097100	0.793388
chr10	95147100	0.809917

chr10	95262800	0.925620
chr10	95376300	0.818182
chr10	95382900	0.752066
chr10	95479200	0.859504
chr10	95511700	0.760331
chr10	95611100	0.801653
chr10	96405400	0.859504
chr10	96931600	0.752066
chr10	97036900	0.909091
chr10	97070200	0.917355
chr10	97137400	0.743802
chr10	97144400	0.801653
chr10	97148400	0.826446
chr10	97151100	0.735537
chr10	97155800	0.867769
chr10	97158200	0.743802
chr10	97186600	0.743802
chr10	97190000	0.842975
chr10	97192200	0.752066
chr10	97201500	0.735537
chr10	97490800	0.809917
chr10	97663400	0.752066
chr10	97748200	0.909091
chr10	97794900	0.760331
chr10	97850000	0.925620
chr10	97971600	0.785124
chr10	97999600	0.760331
chr10	98059800	0.867769
chr10	98067000	0.760331
chr10	98101800	0.776860
chr10	98182900	0.818182
chr10	98208400	0.760331
chr10	98349400	0.735537
chr10	98500800	0.793388
chr10	98516400	0.818182
chr10	98525000	0.785124
chr10	98549600	0.826446
chr10	98589200	0.752066
chr10	98681700	0.768595
chr10	98738900	0.900826
chr10	98810600	0.809917
chr10	98829900	0.801653
chr10	98856700	0.867769
chr10	98959400	0.768595
chr10	98967800	0.809917
chr10	99038100	0.834711
chr10	99167400	0.760331
chr10	99183000	0.900826
chr10	99310300	0.785124
chr10	99408500	0.776860
chr10	99629500	0.793388
chr10	99894800	0.942149
chr10	99901400	0.743802
chr10	100109600	0.851240

chr10	100138900	0.776860
chr10	100171900	0.743802
chr10	100206600	0.867769
chr10	100397100	0.909091
chr10	101040500	0.900826
chr10	101131000	0.900826
chr10	101292600	0.842975
chr10	101381300	0.859504
chr10	101492100	0.760331
chr10	101605900	0.884298
chr10	101781000	0.735537
chr10	102099200	0.752066
chr10	102138000	0.826446
chr10	102291800	0.876033
chr10	102457300	0.859504
chr10	102761600	0.760331
chr10	102773300	0.776860
chr10	102778700	0.793388
chr10	102981900	0.752066
chr10	103326800	0.842975
chr10	103431800	0.909091
chr10	103595000	0.743802
chr10	103746300	0.859504
chr10	103815700	0.892562
chr10	104016100	0.768595
chr10	104040300	0.760331
chr10	104191800	0.743802
chr10	104319600	0.752066
chr10	104375900	0.776860
chr10	104434200	0.801653
chr10	104614100	0.958678
chr10	104758300	0.785124
chr10	104827500	0.768595
chr10	104954400	0.776860
chr10	105133800	0.842975
chr10	105260900	0.809917
chr10	105275400	0.743802
chr10	105447700	0.785124
chr10	105524100	0.826446
chr10	105541800	0.818182
chr10	105560700	0.826446
chr10	105606300	0.752066
chr10	105668200	0.942149
chr10	105670800	0.933884
chr10	106018100	0.735537
chr10	106065500	0.859504
chr10	106077800	0.834711
chr10	106084400	0.793388
chr10	106098200	0.826446
chr10	106100700	0.776860
chr10	106108300	0.735537
chr10	106110500	0.842975
chr10	106139700	0.867769
chr10	106185200	0.793388

chr10	106577100	0.793388
chr10	107716700	0.826446
chr10	108475200	0.909091
chr10	108575900	0.884298
chr10	108633400	0.785124
chr10	109194700	0.760331
chr10	109415500	0.818182
chr10	110305900	0.735537
chr10	110609300	0.743802
chr10	110612600	0.760331
chr10	111641300	0.793388
chr10	111715600	0.785124
chr10	111717800	0.826446
chr10	111763400	0.768595
chr10	111853800	0.834711
chr10	111936200	0.793388
chr10	111973500	0.776860
chr10	111982100	0.917355
chr10	112009400	0.818182
chr10	112011600	0.851240
chr10	112034100	0.818182
chr10	112039100	0.768595
chr10	112065200	0.776860
chr10	112095400	0.743802
chr10	112105200	0.900826
chr10	112189400	0.876033
chr10	112266200	0.776860
chr10	112300200	0.793388
chr10	112327600	0.743802
chr10	112410100	0.859504
chr10	112412500	0.842975
chr10	112460900	0.785124
chr10	112494000	0.851240
chr10	112525700	0.776860
chr10	112529000	0.760331
chr10	112575300	0.760331
chr10	112688700	0.966942
chr10	113100000	0.818182
chr10	113755400	0.826446
chr10	113973000	0.900826
chr10	113993300	0.793388
chr10	114279000	0.826446
chr10	114306300	0.876033
chr10	114602900	0.818182
chr10	114711200	0.809917
chr10	114741300	0.760331
chr10	114746300	0.884298
chr10	114853900	0.809917
chr10	114930100	0.818182
chr10	115142000	0.793388
chr10	115426800	0.785124
chr10	115449200	0.752066
chr10	115563400	0.809917
chr10	115610900	0.760331

chr10	115759400	0.776860
chr10	115831300	0.785124
chr10	115928500	0.743802
chr10	115948400	0.793388
chr10	116021100	0.793388
chr10	116133500	0.818182
chr10	116181200	0.867769
chr10	116231500	0.760331
chr10	116253100	0.801653
chr10	116265600	0.743802
chr10	116298700	0.793388
chr10	116579700	0.834711
chr10	117639400	0.776860
chr10	118605700	0.776860
chr10	119262800	0.785124
chr10	119297500	0.735537
chr10	119307200	0.966942
chr10	119380200	0.793388
chr10	119410500	0.752066
chr10	119416900	0.892562
chr10	120493500	0.867769
chr10	120748100	0.743802
chr10	120880500	0.743802
chr10	120891300	0.917355
chr10	120938300	0.909091
chr10	121035200	0.834711
chr10	121072800	0.743802
chr10	121215700	0.876033
chr10	121221800	0.925620
chr10	121249200	0.859504
chr10	121259500	0.743802
chr10	121326400	0.826446
chr10	121423500	0.743802
chr10	121447000	0.752066
chr10	121453300	0.834711
chr10	121544400	0.892562
chr10	122365200	0.735537
chr10	122585200	0.743802
chr10	122587300	0.793388
chr10	122829200	0.818182
chr10	123581900	0.785124
chr10	123734500	0.785124
chr10	124056800	0.826446
chr10	124086600	0.818182
chr10	124088900	0.776860
chr10	124275800	0.793388
chr10	124714800	0.785124
chr10	124734000	0.925620
chr10	124740000	0.867769
chr10	124759900	0.752066
chr10	125083600	0.876033
chr10	125241200	0.743802
chr10	125299500	0.785124
chr10	125595400	0.958678

chr10	125754700	0.752066
chr10	125799500	0.809917
chr10	125825600	0.958678
chr10	125831800	0.917355
chr10	125926500	0.735537
chr10	125996200	0.809917
chr10	126125100	0.851240
chr10	126154400	0.760331
chr10	126212200	0.884298
chr10	126218700	0.900826
chr10	126229000	0.818182
chr10	126239300	0.900826
chr10	126290600	0.859504
chr10	126303700	0.760331
chr10	126314100	0.768595
chr10	126422200	0.735537
chr10	126426500	0.768595
chr10	126437800	0.909091
chr10	126490700	0.909091
chr10	126665300	0.735537
chr10	126677800	0.735537
chr10	126713900	0.859504
chr10	126746200	0.917355
chr10	126750800	0.859504
chr10	126833300	0.867769
chr10	126889800	0.876033
chr10	127581200	0.776860
chr10	127590100	0.735537
chr10	127606000	0.867769
chr10	127616300	0.809917
chr10	127730900	0.876033
chr10	129652800	0.785124
chr10	129945800	0.867769
chr10	129959500	0.818182
chr10	130171800	0.933884
chr10	130856900	0.925620
chr10	131299900	0.834711
chr10	131316600	0.743802
chr10	131926000	0.785124
chr10	133110200	0.735537
chr10	133963300	1.000000
chr10	134137200	0.859504
chr10	134140700	0.768595
chr10	134236900	0.768595
chr10	134266900	0.785124
chr10	134358500	0.818182
chr10	134361000	0.818182
chr10	134365300	0.867769
chr10	134369400	0.884298
chr10	134386700	0.760331
chr10	134389200	0.793388
chr10	134522700	0.776860
chr10	134599800	0.768595
chr10	134718600	0.809917

chr10	135039900	0.735537
chr10	135071500	0.933884
chr10	135150600	0.768595
chr10	135208300	0.925620
chr10	135462200	0.760331
chr10	135468800	0.760331
chr11	281000	0.801653
chr11	350900	0.917355
chr11	355800	0.900826
chr11	369700	0.942149
chr11	415200	0.818182
chr11	448200	0.859504
chr11	457200	0.826446
chr11	482600	0.842975
chr11	630900	0.743802
chr11	645700	0.760331
chr11	723900	0.834711
chr11	727800	0.743802
chr11	842600	0.900826
chr11	928700	0.735537
chr11	1093400	0.851240
chr11	1333300	0.760331
chr11	1357700	0.876033
chr11	1405800	0.768595
chr11	1610800	0.859504
chr11	1859900	0.842975
chr11	1866500	0.917355
chr11	1961300	0.991736
chr11	2328200	0.785124
chr11	2465700	0.743802
chr11	2488800	0.809917
chr11	2555000	0.909091
chr11	2643700	0.743802
chr11	2722000	0.851240
chr11	2800300	0.735537
chr11	2920100	0.917355
chr11	2967800	0.851240
chr11	3075800	0.768595
chr11	3400100	0.743802
chr11	3581300	0.768595
chr11	3636500	0.892562
chr11	3819200	0.768595
chr11	3863200	0.809917
chr11	3889200	0.743802
chr11	4414600	0.818182
chr11	4632100	0.735537
chr11	4721400	0.925620
chr11	4784700	0.776860
chr11	5255700	0.793388
chr11	5653400	0.735537
chr11	6400000	0.834711
chr11	6436900	0.867769
chr11	6454700	0.876033
chr11	6459700	0.842975

chr11	6490200	0.776860
chr11	6634100	0.760331
chr11	6649200	0.809917
chr11	6669900	0.735537
chr11	6695100	0.818182
chr11	7329100	0.884298
chr11	7444500	0.826446
chr11	7458300	0.826446
chr11	7523700	0.776860
chr11	7532000	0.752066
chr11	7634600	0.859504
chr11	7808900	0.752066
chr11	7912200	0.801653
chr11	8245000	0.818182
chr11	8256900	0.859504
chr11	8408300	0.884298
chr11	8555500	0.768595
chr11	8589500	0.735537
chr11	8663500	0.801653
chr11	8800600	0.867769
chr11	8832300	0.933884
chr11	8935900	0.785124
chr11	8978900	0.752066
chr11	8986400	0.752066
chr11	9039400	0.834711
chr11	9060900	0.743802
chr11	9082600	0.785124
chr11	9091600	0.735537
chr11	9145700	0.743802
chr11	9273400	0.760331
chr11	9281800	0.735537
chr11	9286200	0.909091
chr11	9302600	0.785124
chr11	9336800	0.909091
chr11	9385900	0.975207
chr11	9406500	0.834711
chr11	9532800	0.834711
chr11	9622700	0.876033
chr11	9636400	0.776860
chr11	9661700	0.818182
chr11	9778200	0.851240
chr11	9861300	0.884298
chr11	9877800	0.859504
chr11	9884100	0.743802
chr11	9969600	0.785124
chr11	9994800	0.793388
chr11	10320100	0.768595
chr11	10332400	0.834711
chr11	10336700	0.793388
chr11	10388600	0.760331
chr11	10436200	0.752066
chr11	10466600	0.809917
chr11	10482600	0.801653
chr11	10549100	0.859504

chr11	10586300	0.735537
chr11	10628600	0.818182
chr11	10747700	0.884298
chr11	10765300	0.809917
chr11	10772800	0.809917
chr11	10814400	0.826446
chr11	11001000	0.966942
chr11	11272900	0.867769
chr11	11438600	0.752066
chr11	11630000	0.768595
chr11	11684100	0.743802
chr11	12155700	0.818182
chr11	12181800	0.768595
chr11	12210800	0.834711
chr11	12220800	0.776860
chr11	12739800	0.909091
chr11	13227300	0.859504
chr11	13230600	0.826446
chr11	13312500	0.859504
chr11	13354900	0.809917
chr11	14224600	0.842975
chr11	14259100	0.826446
chr11	14270800	0.735537
chr11	14328800	0.743802
chr11	14437400	0.735537
chr11	14542200	0.768595
chr11	14995800	0.793388
chr11	15096300	0.776860
chr11	15135600	0.743802
chr11	15495900	0.900826
chr11	15505200	0.867769
chr11	16635300	0.851240
chr11	16933400	0.892562
chr11	17266200	0.776860
chr11	17315400	0.809917
chr11	17319200	0.785124
chr11	17408700	0.752066
chr11	17413000	0.809917
chr11	17422600	0.942149
chr11	17427600	0.884298
chr11	17900000	0.826446
chr11	18334800	0.735537
chr11	18405900	0.776860
chr11	18548600	0.785124
chr11	18641200	0.735537
chr11	18651000	0.826446
chr11	18656300	0.752066
chr11	18843000	0.818182
chr11	19131300	0.793388
chr11	19351600	0.834711
chr11	19433500	0.793388
chr11	19464000	0.785124
chr11	19468900	0.793388
chr11	19495100	0.818182

chr11	19547100	0.785124
chr11	19681800	0.851240
chr11	19792500	0.735537
chr11	19929200	0.975207
chr11	19981200	0.933884
chr11	19985700	0.884298
chr11	19991100	0.801653
chr11	20000700	0.768595
chr11	20059100	0.752066
chr11	20101500	0.785124
chr11	20106000	0.809917
chr11	20111100	0.809917
chr11	20296500	0.785124
chr11	22223900	0.752066
chr11	22678900	0.776860
chr11	24536100	0.785124
chr11	26050700	0.760331
chr11	26109500	0.826446
chr11	27378200	0.818182
chr11	27528700	0.752066
chr11	27776200	0.743802
chr11	27929900	0.768595
chr11	28318300	0.842975
chr11	30332600	0.818182
chr11	30345000	0.842975
chr11	30388000	0.818182
chr11	32017700	0.776860
chr11	32060800	0.785124
chr11	32074600	0.768595
chr11	32092000	0.735537
chr11	33086100	0.809917
chr11	33424500	0.834711
chr11	33499700	0.859504
chr11	33537500	0.834711
chr11	33597200	0.818182
chr11	33644900	0.752066
chr11	33693000	0.768595
chr11	33717600	0.834711
chr11	33728800	0.735537
chr11	33746900	0.735537
chr11	33775500	0.809917
chr11	33851200	0.834711
chr11	33922200	0.859504
chr11	33929000	0.809917
chr11	33971300	0.735537
chr11	34019100	0.884298
chr11	34021500	0.801653
chr11	34196100	0.793388
chr11	34198600	0.776860
chr11	34272900	0.900826
chr11	34301000	0.743802
chr11	34332400	0.818182
chr11	34371700	0.743802
chr11	34426400	0.892562

chr11	34534700	0.743802
chr11	34607500	0.834711
chr11	34628100	0.884298
chr11	34635400	0.876033
chr11	34645600	0.818182
chr11	34707400	0.818182
chr11	34848700	0.776860
chr11	35010900	0.752066
chr11	35025500	0.801653
chr11	35028600	0.768595
chr11	35035400	0.884298
chr11	35052400	0.851240
chr11	35059900	0.909091
chr11	35066800	0.743802
chr11	35091300	0.743802
chr11	35107300	0.876033
chr11	35136800	0.834711
chr11	35143100	0.867769
chr11	35159500	0.809917
chr11	35169300	0.785124
chr11	35175100	0.785124
chr11	35180100	0.760331
chr11	35191600	0.867769
chr11	35260500	0.826446
chr11	35279400	0.876033
chr11	35294300	0.743802
chr11	35304300	0.752066
chr11	35327200	0.834711
chr11	35338600	0.793388
chr11	35344100	0.809917
chr11	35356100	0.859504
chr11	35367400	0.809917
chr11	35389000	0.809917
chr11	35393300	0.793388
chr11	35397400	0.760331
chr11	35684500	0.826446
chr11	35933200	0.752066
chr11	35939400	0.867769
chr11	36378400	0.851240
chr11	36385300	0.776860
chr11	36407400	0.760331
chr11	36412500	0.876033
chr11	36421600	0.793388
chr11	36432700	0.834711
chr11	36442900	0.809917
chr11	36463900	0.743802
chr11	36472200	0.859504
chr11	36510300	0.752066
chr11	36563100	0.735537
chr11	36627300	0.826446
chr11	36653300	0.760331
chr11	36746900	0.917355
chr11	36912100	0.776860
chr11	37472500	0.743802

chr11	43333800	0.842975
chr11	43545200	0.933884
chr11	43575600	0.818182
chr11	43668500	0.752066
chr11	43708900	0.801653
chr11	43891000	0.884298
chr11	43966200	0.834711
chr11	44027400	0.768595
chr11	44368800	0.793388
chr11	44373100	0.842975
chr11	44650900	0.735537
chr11	44893900	0.925620
chr11	44964700	0.776860
chr11	45001900	0.933884
chr11	45054300	0.826446
chr11	45095500	0.743802
chr11	45115600	0.768595
chr11	45272000	0.801653
chr11	45845600	0.876033
chr11	45865200	0.776860
chr11	45872300	0.785124
chr11	45874900	0.826446
chr11	45944900	0.768595
chr11	45956300	0.884298
chr11	45960600	0.867769
chr11	46135000	0.752066
chr11	46149100	0.785124
chr11	46184900	0.867769
chr11	46232200	0.735537
chr11	46237800	0.942149
chr11	46298300	0.735537
chr11	46321800	0.743802
chr11	46340700	0.776860
chr11	46364100	0.760331
chr11	46450700	0.743802
chr11	46639100	0.735537
chr11	46860800	0.735537
chr11	46941500	0.768595
chr11	46998200	0.851240
chr11	47026700	0.735537
chr11	47042900	0.785124
chr11	47105800	0.743802
chr11	47153400	0.752066
chr11	47413200	0.735537
chr11	47416700	0.909091
chr11	47422600	0.768595
chr11	47429300	0.876033
chr11	47447900	0.867769
chr11	47562200	0.818182
chr11	47566100	0.900826
chr11	47614000	0.809917
chr11	47685400	0.743802
chr11	47736500	0.884298
chr11	47867200	0.752066

chr11	47870100	0.776860
chr11	47877800	0.735537
chr11	47937000	0.892562
chr11	47945800	0.760331
chr11	47950500	0.785124
chr11	47996300	0.842975
chr11	48009600	0.785124
chr11	48012800	0.925620
chr11	48133800	0.884298
chr11	48145800	0.818182
chr11	51567100	0.768595
chr11	55027000	0.760331
chr11	57045500	0.760331
chr11	57079300	0.793388
chr11	57108900	0.776860
chr11	57200400	0.785124
chr11	57247800	0.818182
chr11	57352600	0.760331
chr11	57408200	0.768595
chr11	57416600	0.826446
chr11	57509500	0.785124
chr11	57543700	0.760331
chr11	58271600	0.876033
chr11	58422400	0.743802
chr11	58761800	0.760331
chr11	58986500	0.842975
chr11	59102900	0.818182
chr11	59148300	0.785124
chr11	59318200	0.809917
chr11	59333400	0.842975
chr11	59379200	0.776860
chr11	59384000	0.942149
chr11	59436700	0.785124
chr11	59979400	0.743802
chr11	60019800	0.826446
chr11	60135300	0.834711
chr11	60248100	0.735537
chr11	60250700	0.793388
chr11	60272400	0.818182
chr11	60296600	0.884298
chr11	60362600	0.760331
chr11	60377300	0.851240
chr11	60400500	0.752066
chr11	60451900	0.776860
chr11	60482900	0.743802
chr11	60486100	0.752066
chr11	60524700	0.818182
chr11	60598900	0.818182
chr11	60602900	0.776860
chr11	60729300	0.760331
chr11	60737900	0.752066
chr11	60746900	0.793388
chr11	60795000	0.826446
chr11	60860100	0.892562

chr11	60883000	0.776860
chr11	60926300	0.925620
chr11	60932200	0.909091
chr11	60935200	0.834711
chr11	61153200	0.818182
chr11	61197700	0.809917
chr11	61496200	0.776860
chr11	61639100	0.752066
chr11	61648400	0.785124
chr11	61723300	0.859504
chr11	61735700	0.760331
chr11	61783300	0.842975
chr11	61809900	0.801653
chr11	61849500	0.768595
chr11	61873200	0.826446
chr11	62140900	0.884298
chr11	62154600	0.818182
chr11	62212400	0.793388
chr11	62230700	0.793388
chr11	62318300	0.785124
chr11	62328200	0.842975
chr11	62481900	0.818182
chr11	62521600	0.933884
chr11	62545200	0.743802
chr11	62570000	0.818182
chr11	62670700	0.826446
chr11	62673600	0.760331
chr11	62690100	0.768595
chr11	62693600	0.752066
chr11	63299200	0.743802
chr11	63328200	0.752066
chr11	63335400	0.909091
chr11	63434300	0.859504
chr11	63635900	0.735537
chr11	63656200	0.876033
chr11	63911600	0.785124
chr11	63920800	0.785124
chr11	63949900	0.867769
chr11	64059900	0.735537
chr11	64107600	0.809917
chr11	64110400	0.826446
chr11	64294500	0.776860
chr11	64440800	0.785124
chr11	64444000	0.785124
chr11	64456400	0.801653
chr11	64482800	0.776860
chr11	64547100	0.900826
chr11	64655900	0.752066
chr11	64691700	0.768595
chr11	64735500	0.851240
chr11	64739800	0.776860
chr11	64742600	0.851240
chr11	64764700	0.859504
chr11	64781500	0.826446

chr11	64784200	0.785124
chr11	64819500	0.867769
chr11	64949800	0.826446
chr11	65033600	0.785124
chr11	65047700	0.900826
chr11	65154000	0.735537
chr11	65171700	0.917355
chr11	65227300	0.785124
chr11	65239800	0.818182
chr11	65420500	0.892562
chr11	65468200	0.785124
chr11	65479700	0.909091
chr11	65653000	0.735537
chr11	65661500	0.884298
chr11	65684500	0.760331
chr11	65724700	0.950413
chr11	65916500	0.760331
chr11	65989800	0.966942
chr11	66025900	0.776860
chr11	66096300	0.743802
chr11	66104800	0.809917
chr11	66206200	0.892562
chr11	66250300	0.842975
chr11	66346800	0.735537
chr11	66363200	0.933884
chr11	66368200	0.851240
chr11	66406300	0.743802
chr11	66445600	0.842975
chr11	66611100	0.743802
chr11	66673100	0.793388
chr11	66687400	0.834711
chr11	66749900	0.735537
chr11	66792500	0.760331
chr11	66801100	0.851240
chr11	66822300	0.735537
chr11	66841900	0.793388
chr11	66850400	0.818182
chr11	66867800	0.892562
chr11	66879300	0.776860
chr11	66883300	0.826446
chr11	67084600	0.752066
chr11	67114500	0.735537
chr11	67136500	0.884298
chr11	67177300	0.818182
chr11	67187200	0.842975
chr11	67255700	0.867769
chr11	67276200	0.859504
chr11	67281600	0.785124
chr11	67327000	0.809917
chr11	67340400	0.809917
chr11	67347500	0.876033
chr11	67368400	0.793388
chr11	67381900	0.752066
chr11	67435900	0.876033

chr11	67867100	0.900826
chr11	67889300	0.942149
chr11	67981000	0.867769
chr11	67990700	0.909091
chr11	68050000	0.752066
chr11	68065200	0.851240
chr11	68083500	0.851240
chr11	68148600	0.735537
chr11	68241000	0.900826
chr11	68337200	0.876033
chr11	68808700	0.909091
chr11	68812400	0.859504
chr11	68929900	0.785124
chr11	69060600	0.768595
chr11	69217000	0.876033
chr11	69256200	0.768595
chr11	69282600	0.826446
chr11	69318500	0.826446
chr11	69330900	0.768595
chr11	69345800	0.793388
chr11	69503900	0.768595
chr11	69816700	0.743802
chr11	70099500	0.876033
chr11	70112000	0.925620
chr11	70129500	0.743802
chr11	70211700	0.818182
chr11	70249200	0.809917
chr11	70284100	0.776860
chr11	70314100	0.818182
chr11	70317400	0.752066
chr11	70329700	0.735537
chr11	70367900	0.925620
chr11	70825500	0.909091
chr11	71088900	0.818182
chr11	71132300	0.834711
chr11	71134500	0.909091
chr11	71137500	0.851240
chr11	71200600	0.950413
chr11	71259700	0.826446
chr11	71275000	0.867769
chr11	71291800	0.809917
chr11	71364500	0.842975
chr11	71426200	0.826446
chr11	71498600	0.776860
chr11	71508900	0.776860
chr11	71781000	0.768595
chr11	71783800	0.793388
chr11	71814200	0.867769
chr11	71824000	0.735537
chr11	71966300	0.785124
chr11	72123100	0.942149
chr11	72125600	0.752066
chr11	72238000	0.867769
chr11	72246100	0.735537

chr11	72303900	0.776860
chr11	72325500	0.826446
chr11	72505100	0.909091
chr11	72954000	0.760331
chr11	72981900	0.834711
chr11	72986200	0.735537
chr11	73001500	0.793388
chr11	73490700	0.950413
chr11	73677000	0.809917
chr11	73716800	0.793388
chr11	73723500	0.752066
chr11	74292900	0.768595
chr11	74408400	0.735537
chr11	74467100	0.752066
chr11	74647300	0.884298
chr11	74700100	0.760331
chr11	74815400	0.743802
chr11	74851900	0.834711
chr11	75100200	0.892562
chr11	75186800	0.735537
chr11	75307000	0.867769
chr11	75351200	0.776860
chr11	75402500	0.768595
chr11	75414400	0.743802
chr11	75865400	0.826446
chr11	75878000	0.752066
chr11	75880900	0.801653
chr11	75925900	0.785124
chr11	75942500	0.818182
chr11	76136100	0.892562
chr11	76148200	0.735537
chr11	76341700	0.760331
chr11	76388300	0.760331
chr11	76397900	0.834711
chr11	76549000	0.760331
chr11	76588200	0.768595
chr11	76791700	0.752066
chr11	77143500	0.859504
chr11	77168700	0.876033
chr11	77213100	0.785124
chr11	77495500	0.760331
chr11	77706100	0.818182
chr11	77727200	0.933884
chr11	77955500	0.826446
chr11	78044000	0.834711
chr11	78048600	0.776860
chr11	78132000	0.776860
chr11	78177100	0.818182
chr11	78244300	0.743802
chr11	78622000	0.760331
chr11	78913500	0.859504
chr11	78923900	0.958678
chr11	79117900	0.925620
chr11	79149500	0.826446

chr11	79197400	0.809917
chr11	80554900	0.876033
chr11	80560700	0.752066
chr11	80782700	0.942149
chr11	82268900	0.768595
chr11	82468900	0.752066
chr11	82671800	0.793388
chr11	82713600	0.768595
chr11	82736300	0.925620
chr11	82760100	0.851240
chr11	82768000	0.909091
chr11	82862200	0.743802
chr11	82960900	0.842975
chr11	82997300	0.752066
chr11	83354200	0.876033
chr11	83413900	0.752066
chr11	83547100	0.876033
chr11	83649900	0.834711
chr11	83961300	0.842975
chr11	84148600	0.900826
chr11	84774100	0.925620
chr11	84838400	0.851240
chr11	85028500	0.752066
chr11	85337200	0.776860
chr11	85411500	0.867769
chr11	85661200	0.768595
chr11	85673100	0.768595
chr11	85781000	0.917355
chr11	85863000	0.809917
chr11	85871400	0.743802
chr11	85888300	0.768595
chr11	85895200	0.752066
chr11	85913600	0.809917
chr11	85951900	0.842975
chr11	85963900	0.785124
chr11	86002900	0.752066
chr11	86155000	0.851240
chr11	86222300	0.760331
chr11	87063500	0.876033
chr11	87430900	0.834711
chr11	87898000	0.776860
chr11	87926900	0.876033
chr11	87929500	0.768595
chr11	88079200	0.785124
chr11	88277300	0.776860
chr11	93266100	0.884298
chr11	93386200	0.752066
chr11	93395100	0.834711
chr11	93868500	0.909091
chr11	94006000	0.776860
chr11	94277200	0.909091
chr11	94317600	0.801653
chr11	94601000	0.867769
chr11	94618800	0.768595

chr11	95013800	0.859504
chr11	95030700	0.776860
chr11	95073200	0.743802
chr11	95079500	0.958678
chr11	95177200	0.909091
chr11	95220900	0.933884
chr11	95290100	0.768595
chr11	95431800	0.752066
chr11	95434000	0.752066
chr11	95436600	0.776860
chr11	95887500	0.743802
chr11	95918600	0.743802
chr11	95931700	0.842975
chr11	95941900	0.876033
chr11	95955300	0.760331
chr11	95958700	0.760331
chr11	95977000	0.801653
chr11	96024600	0.760331
chr11	100555900	0.834711
chr11	100577200	0.760331
chr11	100612100	0.760331
chr11	100779900	0.760331
chr11	100782700	0.826446
chr11	100797100	0.834711
chr11	100800700	0.892562
chr11	100902200	0.876033
chr11	101425400	0.851240
chr11	102112100	0.760331
chr11	102166300	0.776860
chr11	102178100	0.950413
chr11	102232500	0.809917
chr11	102267800	0.735537
chr11	102274100	0.834711
chr11	102336200	0.760331
chr11	102340700	0.859504
chr11	102360300	0.793388
chr11	102363700	0.826446
chr11	102366500	0.818182
chr11	102391400	0.785124
chr11	102401900	0.793388
chr11	102435500	0.776860
chr11	102444600	0.735537
chr11	102463900	0.801653
chr11	102504600	0.743802
chr11	102587300	0.809917
chr11	103615800	0.818182
chr11	103715900	0.760331
chr11	103778900	0.900826
chr11	103795500	0.752066
chr11	103802100	0.760331
chr11	103829800	0.818182
chr11	104769500	0.743802
chr11	104828900	0.735537
chr11	105948700	0.785124

chr11	107123700	0.768595
chr11	107434200	0.785124
chr11	107641400	0.768595
chr11	107832100	0.735537
chr11	108075200	0.752066
chr11	108108000	0.809917
chr11	108338200	0.818182
chr11	108660300	0.801653
chr11	108725200	0.743802
chr11	108829300	0.735537
chr11	109817300	0.950413
chr11	109901700	0.809917
chr11	109915200	0.752066
chr11	109940700	0.892562
chr11	110167100	0.809917
chr11	110248300	0.818182
chr11	110306700	0.834711
chr11	110326900	0.876033
chr11	110397400	0.743802
chr11	110742500	0.768595
chr11	110876800	0.768595
chr11	110964100	0.950413
chr11	110969200	0.743802
chr11	111154800	0.785124
chr11	111300900	0.743802
chr11	111310500	0.917355
chr11	111317500	0.917355
chr11	111321500	0.900826
chr11	111382200	0.743802
chr11	111412400	0.743802
chr11	111647600	0.917355
chr11	111750000	0.809917
chr11	111820500	0.760331
chr11	111944800	0.768595
chr11	112046100	0.842975
chr11	112050400	0.892562
chr11	112193100	0.776860
chr11	112225200	0.834711
chr11	112390000	0.743802
chr11	112395000	0.818182
chr11	112410900	0.752066
chr11	112440900	0.909091
chr11	112456100	0.768595
chr11	112468000	0.735537
chr11	112485000	0.785124
chr11	112631000	0.884298
chr11	113235000	0.743802
chr11	113288500	0.842975
chr11	113311000	0.760331
chr11	113702600	0.768595
chr11	113730900	0.785124
chr11	113765500	0.776860
chr11	114030800	0.892562
chr11	114271700	0.735537

chr11	115094100	0.752066
chr11	115101300	0.793388
chr11	115171600	0.801653
chr11	115342600	0.876033
chr11	115368700	0.826446
chr11	115372300	0.909091
chr11	115767200	0.809917
chr11	115807600	0.826446
chr11	115859700	0.842975
chr11	116019500	0.950413
chr11	116390500	0.851240
chr11	116796200	0.933884
chr11	116799900	0.776860
chr11	116871500	0.735537
chr11	116878000	0.884298
chr11	116895500	0.743802
chr11	116916600	0.867769
chr11	116929700	0.743802
chr11	116961300	0.867769
chr11	117056700	0.900826
chr11	117172500	0.842975
chr11	117307000	0.876033
chr11	117309500	0.809917
chr11	117678300	0.743802
chr11	117680400	0.785124
chr11	117821700	0.760331
chr11	117824100	0.884298
chr11	117830900	0.743802
chr11	117861200	0.760331
chr11	117864900	0.752066
chr11	117874900	0.851240
chr11	117879000	0.776860
chr11	117887100	0.809917
chr11	117904700	0.785124
chr11	117922800	0.809917
chr11	118073700	0.876033
chr11	118077800	0.785124
chr11	118095700	0.776860
chr11	118111000	0.851240
chr11	118152200	0.900826
chr11	118189700	0.785124
chr11	118193300	0.826446
chr11	118269900	0.851240
chr11	118443500	0.867769
chr11	118492300	0.785124
chr11	118561300	0.884298
chr11	118576700	0.909091
chr11	118694800	0.801653
chr11	118723400	0.760331
chr11	118728400	0.752066
chr11	118743800	0.776860
chr11	118763700	0.760331
chr11	118767700	0.842975
chr11	118809600	0.884298

chr11	118919800	0.752066
chr11	118924800	0.768595
chr11	118955800	0.776860
chr11	118972900	0.760331
chr11	119039800	0.917355
chr11	119194800	0.768595
chr11	119205200	0.760331
chr11	119312100	0.785124
chr11	119439300	0.801653
chr11	119554600	0.793388
chr11	119562300	0.884298
chr11	119581200	0.743802
chr11	119593100	0.876033
chr11	119627000	0.793388
chr11	119630600	0.900826
chr11	119937700	0.743802
chr11	120426900	0.785124
chr11	120466500	0.842975
chr11	120469600	0.826446
chr11	120506500	0.933884
chr11	120544700	0.735537
chr11	120586400	0.892562
chr11	120594900	0.752066
chr11	120707000	0.818182
chr11	120711200	0.760331
chr11	120764800	0.818182
chr11	120823800	0.785124
chr11	120828300	0.785124
chr11	121078600	0.842975
chr11	121173700	0.842975
chr11	121197300	0.867769
chr11	121230800	0.743802
chr11	121265500	0.809917
chr11	121634500	0.793388
chr11	121775200	0.793388
chr11	122464500	0.826446
chr11	122537400	0.909091
chr11	122540100	0.809917
chr11	122561900	0.834711
chr11	122568900	0.768595
chr11	122588900	0.735537
chr11	122620700	0.884298
chr11	122625400	0.743802
chr11	122675500	0.752066
chr11	122721400	0.793388
chr11	122728800	0.785124
chr11	122743600	0.743802
chr11	122894300	0.842975
chr11	123132700	0.768595
chr11	123139000	0.752066
chr11	123148100	0.809917
chr11	123171600	0.752066
chr11	123178800	0.793388
chr11	123187100	0.768595

chr11	123196800	0.793388
chr11	123305000	0.809917
chr11	123310400	0.818182
chr11	123325800	0.851240
chr11	123377000	0.826446
chr11	123393600	0.752066
chr11	123408700	0.735537
chr11	123433400	0.743802
chr11	123632100	0.884298
chr11	124155800	0.735537
chr11	124200100	0.892562
chr11	124515000	0.826446
chr11	124588200	0.793388
chr11	124670600	0.851240
chr11	125011900	0.752066
chr11	125036000	0.834711
chr11	125047000	0.900826
chr11	125246200	0.818182
chr11	125439500	0.809917
chr11	125459400	0.793388
chr11	125757800	0.842975
chr11	125933200	0.842975
chr11	125954300	0.752066
chr11	125980400	0.826446
chr11	126086900	0.785124
chr11	126090100	0.776860
chr11	126177000	0.785124
chr11	126262800	0.851240
chr11	126274000	0.818182
chr11	126297200	0.768595
chr11	126335000	0.743802
chr11	126338200	0.826446
chr11	126348200	0.785124
chr11	126354900	0.842975
chr11	126571200	0.785124
chr11	126574400	0.735537
chr11	126606300	0.876033
chr11	126612300	0.851240
chr11	126733300	0.842975
chr11	126789300	0.760331
chr11	126943700	0.743802
chr11	128162800	0.842975
chr11	128176100	0.809917
chr11	128276300	0.834711
chr11	128283200	0.826446
chr11	128292200	0.917355
chr11	128322500	0.743802
chr11	128438500	0.801653
chr11	128445800	0.785124
chr11	128570200	0.809917
chr11	128574800	0.826446
chr11	128672500	0.793388
chr11	128716100	0.760331
chr11	128764200	0.785124

chr11	128798900	0.809917
chr11	128833800	0.793388
chr11	129288000	0.785124
chr11	129566000	0.834711
chr11	129817300	0.900826
chr11	129843500	0.909091
chr11	130016600	0.917355
chr11	130019400	0.867769
chr11	130024200	0.752066
chr11	130036400	0.900826
chr11	130072300	0.735537
chr11	131093300	0.735537
chr11	131514800	0.752066
chr11	131526300	0.884298
chr11	131529500	0.793388
chr11	131700200	0.834711
chr11	131757500	0.760331
chr11	131770000	0.851240
chr11	131994300	0.768595
chr11	132181900	0.900826
chr11	132207300	0.842975
chr11	132213800	0.826446
chr11	133259500	0.933884
chr11	133376700	0.785124
chr11	133399800	0.876033
chr11	133411700	0.785124
chr11	133416300	0.842975
chr11	133529900	0.743802
chr11	133768800	0.801653
chr11	133817900	0.950413
chr11	133823700	0.884298
chr11	133854900	0.859504
chr11	133943200	0.892562
chr11	134089000	0.900826
chr11	134100800	0.909091
chr11	134140600	0.900826
chr11	134281800	0.851240
chr11	134454000	0.842975
chr11	134458100	0.809917
chr12	77400	0.826446
chr12	221500	0.785124
chr12	289900	0.752066
chr12	316100	0.776860
chr12	385900	0.801653
chr12	396400	0.760331
chr12	648400	0.809917
chr12	656500	0.785124
chr12	673600	0.768595
chr12	786100	0.884298
chr12	791700	0.818182
chr12	1062300	0.768595
chr12	1068300	0.867769
chr12	1090500	0.942149
chr12	1180600	0.809917

chr12	1208100	0.743802
chr12	1295800	0.735537
chr12	1315500	0.834711
chr12	1336000	0.925620
chr12	1645000	0.842975
chr12	1655200	0.859504
chr12	1657800	0.743802
chr12	1680700	0.842975
chr12	1692700	0.909091
chr12	1778300	0.900826
chr12	2034100	0.793388
chr12	2042300	0.776860
chr12	2111500	0.776860
chr12	2173500	0.768595
chr12	2405000	0.942149
chr12	2408300	0.752066
chr12	2416100	0.925620
chr12	2465800	0.884298
chr12	2468100	0.818182
chr12	2635900	0.760331
chr12	2653500	0.884298
chr12	2893500	0.809917
chr12	3184200	0.735537
chr12	3207100	0.867769
chr12	3239400	0.900826
chr12	3296100	0.925620
chr12	3324900	0.801653
chr12	3327600	0.752066
chr12	3732300	0.743802
chr12	3837700	0.801653
chr12	3982600	0.818182
chr12	4179300	0.892562
chr12	4208400	0.785124
chr12	4301300	0.834711
chr12	4319200	0.834711
chr12	4333500	0.909091
chr12	4393100	0.776860
chr12	4396500	0.884298
chr12	4421600	0.884298
chr12	4549100	0.818182
chr12	4762000	0.909091
chr12	4843800	0.809917
chr12	4917100	0.776860
chr12	4934900	0.785124
chr12	4967700	0.760331
chr12	5021600	0.743802
chr12	5064200	0.793388
chr12	5073000	0.826446
chr12	5089300	0.809917
chr12	5093500	0.933884
chr12	5125600	0.917355
chr12	5144400	0.743802
chr12	5150100	0.892562
chr12	5156700	0.793388

chr12	5177900	0.809917
chr12	5205600	0.818182
chr12	5550700	0.776860
chr12	6011600	0.834711
chr12	6277500	0.735537
chr12	6340300	0.900826
chr12	6382100	0.743802
chr12	6409600	0.793388
chr12	6433000	0.809917
chr12	6455400	0.760331
chr12	6465600	0.735537
chr12	6470000	0.859504
chr12	6673000	0.826446
chr12	6753500	0.842975
chr12	6855300	0.892562
chr12	6862700	0.917355
chr12	6868300	0.776860
chr12	6893900	0.884298
chr12	6902400	0.851240
chr12	7001000	0.851240
chr12	7154300	0.735537
chr12	7263900	0.793388
chr12	7799000	0.752066
chr12	7995900	0.776860
chr12	8013000	0.818182
chr12	8148200	0.826446
chr12	8179800	0.859504
chr12	8219300	0.876033
chr12	8307400	0.785124
chr12	8608700	0.826446
chr12	8694300	0.892562
chr12	8732000	0.735537
chr12	8743700	0.842975
chr12	8790000	0.752066
chr12	8797300	0.900826
chr12	8802900	0.743802
chr12	8862500	0.752066
chr12	9057500	0.842975
chr12	9140600	0.818182
chr12	9269300	0.842975
chr12	9271500	0.785124
chr12	9299300	0.785124
chr12	9397900	0.876033
chr12	9472600	0.768595
chr12	9565000	0.809917
chr12	9618000	0.752066
chr12	9795500	0.793388
chr12	9918100	0.818182
chr12	9947800	0.785124
chr12	10025200	0.793388
chr12	10104200	0.743802
chr12	10113700	0.743802
chr12	10223700	0.884298
chr12	10247300	0.776860

chr12	10254300	0.826446
chr12	10282900	0.735537
chr12	10313800	0.917355
chr12	10349400	0.851240
chr12	10380800	0.743802
chr12	10707400	0.735537
chr12	10710200	0.917355
chr12	10828200	0.801653
chr12	10854700	0.776860
chr12	11639900	0.801653
chr12	11651200	0.826446
chr12	11653600	0.752066
chr12	12145900	0.776860
chr12	12199500	0.785124
chr12	12224300	0.892562
chr12	12325500	0.842975
chr12	12330000	0.768595
chr12	12354800	0.768595
chr12	12364600	0.760331
chr12	12401100	0.743802
chr12	12403300	0.785124
chr12	12476000	0.752066
chr12	12487800	0.818182
chr12	12628700	0.884298
chr12	12726200	0.884298
chr12	12764800	0.785124
chr12	12789900	0.900826
chr12	12807500	0.760331
chr12	12829600	0.752066
chr12	12846300	0.760331
chr12	12856600	0.842975
chr12	12858800	0.760331
chr12	12890100	0.776860
chr12	12961700	0.743802
chr12	13062300	0.776860
chr12	13243200	0.867769
chr12	13307400	0.735537
chr12	13318800	0.892562
chr12	13332700	0.760331
chr12	13349700	0.818182
chr12	13742100	0.735537
chr12	14218400	0.743802
chr12	14302100	0.818182
chr12	14325600	0.884298
chr12	14426600	0.768595
chr12	14543700	0.743802
chr12	14730900	0.859504
chr12	14773100	0.743802
chr12	14844300	0.785124
chr12	15008400	0.768595
chr12	15038800	0.818182
chr12	15057200	0.743802
chr12	15408200	0.859504
chr12	15680000	0.884298

chr12	15724600	0.785124
chr12	15841800	0.760331
chr12	16017100	0.801653
chr12	19585100	0.925620
chr12	21663100	0.834711
chr12	22199300	0.735537
chr12	22563300	0.752066
chr12	22584500	0.760331
chr12	22697300	0.884298
chr12	22840300	0.760331
chr12	22879200	0.933884
chr12	23179000	0.735537
chr12	24509400	0.925620
chr12	24552200	0.801653
chr12	24753500	0.768595
chr12	24872100	0.768595
chr12	24883800	0.818182
chr12	24956900	0.735537
chr12	24968000	0.743802
chr12	25018400	0.785124
chr12	25081000	0.735537
chr12	25130200	0.760331
chr12	25146400	0.743802
chr12	25174500	0.785124
chr12	25216600	0.818182
chr12	25223500	0.793388
chr12	25281900	0.834711
chr12	25340100	0.752066
chr12	25417100	0.826446
chr12	25425000	0.900826
chr12	25541500	0.909091
chr12	25612800	0.809917
chr12	25887400	0.793388
chr12	26212100	0.876033
chr12	26242200	0.752066
chr12	26270000	0.900826
chr12	26288700	0.842975
chr12	26307100	0.809917
chr12	26326500	0.834711
chr12	26436100	0.818182
chr12	26451000	0.735537
chr12	26481300	0.776860
chr12	26485400	0.752066
chr12	26494200	0.867769
chr12	26674400	0.892562
chr12	26953800	0.809917
chr12	27009100	0.768595
chr12	27028200	0.809917
chr12	27031900	0.743802
chr12	27038200	0.842975
chr12	27151500	0.735537
chr12	27175500	0.900826
chr12	27286600	0.793388
chr12	27299200	0.950413

chr12	27314600	0.834711
chr12	27353800	0.752066
chr12	27462800	0.950413
chr12	27496500	0.876033
chr12	27613900	0.876033
chr12	27620900	0.876033
chr12	27685400	0.859504
chr12	27691700	0.818182
chr12	27706800	0.743802
chr12	27709200	0.884298
chr12	27714600	0.776860
chr12	27732100	0.818182
chr12	27762800	0.809917
chr12	27978500	0.768595
chr12	27994400	0.735537
chr12	28219400	0.867769
chr12	28239500	0.809917
chr12	28406300	0.818182
chr12	29240200	0.867769
chr12	29305000	0.842975
chr12	29308100	0.735537
chr12	29311300	0.900826
chr12	29529700	0.834711
chr12	29533800	0.735537
chr12	31358200	0.933884
chr12	31391900	0.909091
chr12	31725700	0.776860
chr12	31785400	0.809917
chr12	31812400	0.876033
chr12	31868500	0.900826
chr12	31877500	0.752066
chr12	31912800	0.801653
chr12	31964400	0.776860
chr12	31986000	0.760331
chr12	32028100	0.909091
chr12	32044100	0.876033
chr12	32050600	0.793388
chr12	32216000	0.793388
chr12	32295800	0.801653
chr12	32455000	0.752066
chr12	32676300	0.958678
chr12	32908800	0.876033
chr12	33787800	0.900826
chr12	34175400	0.859504
chr12	38493700	0.900826
chr12	38745200	0.950413
chr12	39763700	0.776860
chr12	40587200	0.942149
chr12	40708600	0.809917
chr12	40909100	0.859504
chr12	42418100	0.768595
chr12	42603000	0.760331
chr12	42678500	0.793388
chr12	42767700	0.793388

chr12	42873100	0.859504
chr12	43157400	0.851240
chr12	43604900	0.859504
chr12	44200200	0.876033
chr12	44415600	0.785124
chr12	44769900	0.818182
chr12	45599400	0.876033
chr12	45629200	0.743802
chr12	45972500	0.876033
chr12	46025300	0.867769
chr12	46028000	0.785124
chr12	46371800	0.752066
chr12	46385700	0.743802
chr12	46573400	0.752066
chr12	46617600	0.809917
chr12	46861200	0.735537
chr12	46867600	0.884298
chr12	46882200	0.892562
chr12	47182700	0.867769
chr12	47461100	0.809917
chr12	47588300	0.785124
chr12	47610100	0.776860
chr12	47671300	0.752066
chr12	47701500	0.867769
chr12	47750400	0.917355
chr12	47802300	0.826446
chr12	48100300	0.760331
chr12	48103500	0.818182
chr12	48120100	0.884298
chr12	48141100	0.743802
chr12	48205200	0.735537
chr12	48222900	0.834711
chr12	48287900	0.752066
chr12	48384200	0.892562
chr12	48503200	0.809917
chr12	48551700	0.818182
chr12	48592300	0.876033
chr12	48691200	0.793388
chr12	49319300	0.793388
chr12	49385200	0.826446
chr12	49458000	0.801653
chr12	49464300	0.942149
chr12	49482200	0.818182
chr12	49486100	0.743802
chr12	49659300	0.818182
chr12	49682800	0.809917
chr12	49964800	0.933884
chr12	50016600	0.876033
chr12	50071300	0.735537
chr12	50110400	0.752066
chr12	50135900	0.768595
chr12	50171200	0.884298
chr12	50214800	0.793388
chr12	50219400	0.760331

chr12	50236700	0.900826
chr12	50241300	0.776860
chr12	50339800	0.776860
chr12	50506100	0.776860
chr12	50519100	0.818182
chr12	50560800	0.876033
chr12	50641200	0.752066
chr12	50648400	0.809917
chr12	50867700	0.909091
chr12	50952700	0.743802
chr12	50966700	0.859504
chr12	50983400	0.785124
chr12	50987700	0.752066
chr12	51312700	0.735537
chr12	51385400	0.826446
chr12	51566800	0.900826
chr12	51612000	0.842975
chr12	51632800	0.809917
chr12	51717800	0.834711
chr12	51721200	0.867769
chr12	51737800	0.768595
chr12	51763400	0.900826
chr12	51783200	0.760331
chr12	51789500	0.917355
chr12	51868000	0.818182
chr12	51906500	0.867769
chr12	51909500	0.842975
chr12	51925800	0.842975
chr12	51941800	0.917355
chr12	52306000	0.826446
chr12	52317000	0.752066
chr12	52364500	0.768595
chr12	52366900	0.909091
chr12	52374900	0.950413
chr12	52615900	0.752066
chr12	52621700	0.933884
chr12	52638200	0.917355
chr12	52643700	0.818182
chr12	52699200	0.809917
chr12	52721600	0.851240
chr12	52752700	0.867769
chr12	52776800	0.743802
chr12	53264700	0.760331
chr12	53443000	0.826446
chr12	53603200	0.867769
chr12	53607400	0.752066
chr12	53611500	0.793388
chr12	53615200	0.859504
chr12	53638200	0.785124
chr12	53662300	0.826446
chr12	53715500	0.933884
chr12	53734500	0.809917
chr12	53747100	0.752066
chr12	53772800	0.826446

chr12	53857200	0.900826
chr12	53864800	0.785124
chr12	53869700	0.743802
chr12	54132700	0.760331
chr12	54371800	0.942149
chr12	54374800	0.818182
chr12	54383200	0.809917
chr12	54412200	0.876033
chr12	54415700	0.752066
chr12	54421000	0.818182
chr12	54444500	0.776860
chr12	54447600	0.785124
chr12	54476200	0.768595
chr12	54569500	0.785124
chr12	54592100	0.892562
chr12	54762700	0.834711
chr12	54860500	0.834711
chr12	54891700	0.917355
chr12	54944100	0.752066
chr12	55059500	0.851240
chr12	55329800	0.842975
chr12	55378800	0.776860
chr12	55385400	0.851240
chr12	55393000	0.809917
chr12	55861800	0.793388
chr12	56023500	0.842975
chr12	56103100	0.776860
chr12	56136700	0.743802
chr12	56368200	0.743802
chr12	56436000	0.859504
chr12	56546000	0.793388
chr12	56615900	0.768595
chr12	56732900	0.867769
chr12	56843100	0.876033
chr12	56874500	0.793388
chr12	57034200	0.776860
chr12	57040100	0.743802
chr12	57094800	0.743802
chr12	57160800	0.851240
chr12	57335600	0.834711
chr12	57473300	0.752066
chr12	57525100	0.818182
chr12	57607800	0.966942
chr12	57621500	0.818182
chr12	57623800	0.785124
chr12	57674400	0.735537
chr12	57849900	0.801653
chr12	57852700	0.884298
chr12	57861900	0.867769
chr12	58008500	0.851240
chr12	58082700	0.776860
chr12	58087900	0.760331
chr12	58139000	0.876033
chr12	58147300	0.768595

chr12	58235400	0.834711
chr12	58282700	0.760331
chr12	58286300	0.876033
chr12	58290600	0.776860
chr12	58293300	0.826446
chr12	58329800	0.892562
chr12	58765500	0.743802
chr12	58819700	0.900826
chr12	62968800	0.933884
chr12	63024000	0.867769
chr12	63123300	0.768595
chr12	63177600	0.768595
chr12	63193500	0.859504
chr12	63199400	0.892562
chr12	63207200	0.785124
chr12	64106800	0.842975
chr12	64111200	0.900826
chr12	64174100	0.900826
chr12	64312100	0.818182
chr12	64322700	0.851240
chr12	64379400	0.867769
chr12	64688900	0.900826
chr12	64853200	0.917355
chr12	64925400	0.743802
chr12	64937200	0.917355
chr12	65108800	0.909091
chr12	65148900	0.826446
chr12	65195500	0.793388
chr12	65201800	0.859504
chr12	65370700	0.735537
chr12	65556900	0.834711
chr12	65687000	0.900826
chr12	66013000	0.752066
chr12	66320400	0.793388
chr12	66350000	0.768595
chr12	66563700	0.818182
chr12	67055700	0.842975
chr12	67171000	0.834711
chr12	67836100	0.900826
chr12	67847400	0.752066
chr12	67909800	0.818182
chr12	67935600	0.801653
chr12	68149900	0.793388
chr12	68152400	0.859504
chr12	68198400	0.851240
chr12	68314100	0.743802
chr12	68360800	0.776860
chr12	68377600	0.801653
chr12	68406200	0.867769
chr12	68515700	0.760331
chr12	68532000	0.785124
chr12	68554100	0.776860
chr12	68575400	0.842975
chr12	68630700	0.834711

chr12	68635100	0.834711
chr12	68654800	0.834711
chr12	68759000	0.859504
chr12	68841100	0.752066
chr12	68845200	0.867769
chr12	68881900	0.760331
chr12	68900000	0.743802
chr12	68981600	0.735537
chr12	68986100	0.809917
chr12	69013000	0.859504
chr12	69015400	0.785124
chr12	69022000	0.876033
chr12	69025400	0.760331
chr12	69257800	0.768595
chr12	69353200	0.809917
chr12	69678100	0.776860
chr12	69753700	0.900826
chr12	69861500	0.760331
chr12	69864400	0.859504
chr12	69908600	0.752066
chr12	69985400	0.785124
chr12	70279700	0.743802
chr12	70302000	0.768595
chr12	70441300	0.842975
chr12	70634800	0.768595
chr12	70853800	0.793388
chr12	70876900	0.834711
chr12	70975200	0.760331
chr12	72535700	0.842975
chr12	75785200	0.933884
chr12	75920900	0.752066
chr12	76035000	0.743802
chr12	76132700	0.776860
chr12	76207000	0.818182
chr12	76311200	0.735537
chr12	76328500	0.900826
chr12	76353900	0.851240
chr12	76479700	0.752066
chr12	76538600	0.752066
chr12	76546100	0.859504
chr12	76567600	0.900826
chr12	76579300	0.909091
chr12	76946400	0.826446
chr12	76953600	0.793388
chr12	77464700	0.925620
chr12	77948300	0.826446
chr12	78210600	0.859504
chr12	78571200	0.867769
chr12	78852200	0.826446
chr12	79926700	0.776860
chr12	80391100	0.884298
chr12	80569400	0.735537
chr12	80581700	0.851240
chr12	80613300	0.975207

chr12	80616500	0.917355
chr12	81102800	0.801653
chr12	81160900	0.768595
chr12	81215500	0.752066
chr12	81232800	0.834711
chr12	82022500	0.752066
chr12	82752300	0.826446
chr12	83042700	0.842975
chr12	83045300	0.776860
chr12	83265100	0.743802
chr12	85667500	0.768595
chr12	85921300	0.942149
chr12	88456600	0.785124
chr12	89554700	0.735537
chr12	89822100	0.752066
chr12	89869400	0.900826
chr12	89934000	0.735537
chr12	89961100	0.760331
chr12	89992300	0.809917
chr12	89996900	0.851240
chr12	90104000	0.851240
chr12	90160200	0.900826
chr12	90253200	0.884298
chr12	90353700	0.760331
chr12	90459100	0.983471
chr12	90645000	0.776860
chr12	90693200	0.752066
chr12	91401600	0.809917
chr12	91403700	0.876033
chr12	92172700	0.942149
chr12	92432900	0.876033
chr12	92446100	0.752066
chr12	92456900	0.743802
chr12	92459300	0.735537
chr12	92470600	0.809917
chr12	92472800	0.818182
chr12	92553000	0.867769
chr12	92892600	0.776860
chr12	92938100	0.793388
chr12	93293200	0.859504
chr12	93323600	0.818182
chr12	93400200	0.785124
chr12	93425000	0.735537
chr12	93492200	0.785124
chr12	93578600	0.859504
chr12	93594200	0.867769
chr12	93602000	0.892562
chr12	93691100	0.752066
chr12	93696800	0.743802
chr12	93758500	0.826446
chr12	93818100	0.793388
chr12	93861500	0.859504
chr12	93930900	0.826446
chr12	93988300	0.826446

chr12	94067600	0.867769
chr12	94071600	0.884298
chr12	94121000	0.776860
chr12	94197600	0.809917
chr12	94240400	0.743802
chr12	94298100	0.752066
chr12	94441900	0.834711
chr12	94570100	0.752066
chr12	94634300	0.768595
chr12	94661300	0.752066
chr12	94799800	0.809917
chr12	94854100	0.842975
chr12	94920000	0.801653
chr12	94962400	0.793388
chr12	95039000	0.793388
chr12	95041800	0.793388
chr12	95200900	0.793388
chr12	95206000	0.851240
chr12	95338400	0.760331
chr12	95364400	0.834711
chr12	95397500	0.884298
chr12	95497100	0.768595
chr12	95510900	0.801653
chr12	95604000	0.743802
chr12	95784700	0.785124
chr12	95834000	0.826446
chr12	96093900	0.859504
chr12	96125600	0.834711
chr12	96431900	0.925620
chr12	96438600	0.752066
chr12	96456500	0.801653
chr12	96530700	0.851240
chr12	96537800	0.735537
chr12	96591600	0.834711
chr12	96601000	0.950413
chr12	96604700	0.842975
chr12	96618000	0.876033
chr12	96719900	0.743802
chr12	96737600	0.842975
chr12	96762800	0.768595
chr12	96815100	0.752066
chr12	96819300	0.867769
chr12	96840300	0.752066
chr12	96849300	0.785124
chr12	96851700	0.752066
chr12	97798500	0.760331
chr12	98765300	0.842975
chr12	98897700	0.793388
chr12	99006700	0.925620
chr12	99034800	0.743802
chr12	99675900	0.760331
chr12	99849400	0.834711
chr12	100536600	0.809917
chr12	100558100	0.793388

chr12	101128300	0.884298
chr12	101382900	0.826446
chr12	101389700	0.917355
chr12	101395900	0.909091
chr12	101469100	0.768595
chr12	101801800	0.917355
chr12	102065700	0.842975
chr12	102138200	0.826446
chr12	102214000	0.735537
chr12	102278600	0.760331
chr12	102285400	0.834711
chr12	102404700	0.884298
chr12	102455500	0.867769
chr12	102490700	0.809917
chr12	102800300	0.809917
chr12	102908300	0.752066
chr12	102937900	0.760331
chr12	103099400	0.735537
chr12	103129300	0.834711
chr12	103170100	0.743802
chr12	103348000	0.892562
chr12	103356500	0.793388
chr12	103496800	0.834711
chr12	103707600	0.917355
chr12	103825700	0.785124
chr12	103864200	0.809917
chr12	103934100	0.818182
chr12	104034900	0.793388
chr12	104090100	0.735537
chr12	104283900	0.826446
chr12	104348000	0.876033
chr12	104351000	0.842975
chr12	104395000	0.851240
chr12	104444100	0.752066
chr12	104526600	0.851240
chr12	104618000	0.826446
chr12	104766200	0.760331
chr12	104806500	0.884298
chr12	104861500	0.785124
chr12	104864200	0.743802
chr12	104867600	0.842975
chr12	104901400	0.743802
chr12	104960200	0.768595
chr12	104979900	0.892562
chr12	104989600	0.760331
chr12	105058900	0.760331
chr12	105070500	0.793388
chr12	105080800	0.760331
chr12	105114800	0.925620
chr12	105143500	0.760331
chr12	105146500	0.752066
chr12	105240400	0.743802
chr12	105259300	0.776860
chr12	105264600	0.818182

chr12	105284500	0.735537
chr12	105328800	0.776860
chr12	105363200	0.826446
chr12	105370400	0.876033
chr12	105425200	0.933884
chr12	105446700	0.851240
chr12	105467400	0.925620
chr12	105718500	0.743802
chr12	105870300	0.785124
chr12	106115500	0.752066
chr12	106178400	0.842975
chr12	106381100	0.801653
chr12	106621400	0.892562
chr12	106669300	0.743802
chr12	106705400	0.867769
chr12	107065800	0.760331
chr12	107153900	0.785124
chr12	107174100	0.768595
chr12	107290300	0.966942
chr12	107305300	0.752066
chr12	107309200	0.793388
chr12	107763300	0.776860
chr12	107870100	0.801653
chr12	107884100	0.760331
chr12	107887000	0.818182
chr12	108056100	0.842975
chr12	108131100	0.735537
chr12	108195300	0.884298
chr12	108234500	0.760331
chr12	108450600	0.818182
chr12	108636300	0.867769
chr12	108662600	0.809917
chr12	108676900	0.752066
chr12	108696400	0.793388
chr12	108702000	0.743802
chr12	108705500	0.735537
chr12	108719700	0.793388
chr12	108733500	0.801653
chr12	108758400	0.743802
chr12	108840800	0.776860
chr12	108850800	0.752066
chr12	108868700	0.834711
chr12	108874100	0.768595
chr12	108909200	0.735537
chr12	109109400	0.776860
chr12	109113400	0.867769
chr12	109128800	0.867769
chr12	109252100	0.776860
chr12	109299700	0.785124
chr12	109324300	0.752066
chr12	109328200	0.818182
chr12	109333600	0.752066
chr12	109344800	0.768595
chr12	109688500	0.743802

chr12	109915300	0.900826
chr12	110453000	0.793388
chr12	110489300	0.768595
chr12	110517500	0.818182
chr12	110712100	0.826446
chr12	110841900	0.917355
chr12	110888100	0.892562
chr12	110940100	0.793388
chr12	111005900	0.768595
chr12	111029200	0.892562
chr12	111044000	0.892562
chr12	111057500	0.933884
chr12	111078500	0.809917
chr12	111120800	0.776860
chr12	111127600	0.793388
chr12	111170000	0.793388
chr12	111214900	0.809917
chr12	111221100	0.818182
chr12	111261200	0.892562
chr12	111264900	0.793388
chr12	111344700	0.768595
chr12	111834900	0.884298
chr12	111884800	0.752066
chr12	112213500	0.809917
chr12	112433600	0.768595
chr12	112446400	0.942149
chr12	112534700	0.818182
chr12	112541800	0.752066
chr12	112546800	0.876033
chr12	112825400	0.735537
chr12	112847100	0.917355
chr12	113332300	0.809917
chr12	113424800	0.752066
chr12	113469000	0.834711
chr12	113501300	0.735537
chr12	113506300	0.818182
chr12	113521800	0.776860
chr12	113539200	0.743802
chr12	113574200	0.760331
chr12	113593000	0.834711
chr12	113643400	0.768595
chr12	113653500	0.834711
chr12	113670700	0.793388
chr12	113842200	0.776860
chr12	114450700	0.950413
chr12	115447000	0.752066
chr12	115760100	0.851240
chr12	115792300	0.942149
chr12	115813200	0.884298
chr12	115818900	0.826446
chr12	115929800	0.826446
chr12	115933300	0.768595
chr12	116525900	0.752066
chr12	116559800	0.768595

chr12	116636600	0.760331
chr12	116649000	0.942149
chr12	116686800	0.785124
chr12	116799300	0.760331
chr12	116818100	0.793388
chr12	116823500	0.818182
chr12	116956200	0.851240
chr12	116977900	0.842975
chr12	117074400	0.842975
chr12	117126100	0.900826
chr12	117134000	0.826446
chr12	117147100	0.933884
chr12	117320100	0.842975
chr12	117360200	0.735537
chr12	117400600	0.842975
chr12	117417800	0.768595
chr12	117470400	0.859504
chr12	117557500	0.760331
chr12	117717700	0.760331
chr12	118006100	0.768595
chr12	118283900	0.818182
chr12	118503400	0.884298
chr12	118558600	0.942149
chr12	118606100	0.743802
chr12	118644100	0.735537
chr12	118768300	0.826446
chr12	118777200	0.867769
chr12	118796800	0.793388
chr12	119121000	0.768595
chr12	119835600	0.842975
chr12	120624900	0.735537
chr12	120632900	0.801653
chr12	120666900	0.859504
chr12	120690200	0.867769
chr12	120731200	0.768595
chr12	120837300	0.801653
chr12	120912900	0.785124
chr12	120924600	0.793388
chr12	120967200	0.884298
chr12	121085300	0.809917
chr12	121091000	0.776860
chr12	121103900	0.793388
chr12	121279600	0.743802
chr12	121292300	0.776860
chr12	121436800	0.768595
chr12	121472200	0.859504
chr12	121474700	0.801653
chr12	121496000	0.776860
chr12	121498300	0.975207
chr12	121567500	0.785124
chr12	121613900	0.752066
chr12	121623400	0.752066
chr12	121635500	0.768595
chr12	121648000	0.826446

chr12	121665100	0.818182
chr12	121715200	0.785124
chr12	121738800	0.892562
chr12	121843000	0.752066
chr12	121890500	0.776860
chr12	121952000	0.826446
chr12	121980300	0.809917
chr12	121989900	0.760331
chr12	121998700	0.876033
chr12	122232800	0.892562
chr12	122275800	0.892562
chr12	122365600	0.760331
chr12	122394200	0.809917
chr12	122407300	0.785124
chr12	122442900	0.743802
chr12	122448000	0.785124
chr12	122515200	0.793388
chr12	122584700	0.743802
chr12	122652100	0.785124
chr12	122669200	0.809917
chr12	122773000	0.818182
chr12	122942200	0.743802
chr12	122959700	0.752066
chr12	123000100	0.859504
chr12	123176200	0.793388
chr12	123201100	0.834711
chr12	123215100	0.793388
chr12	123270600	0.743802
chr12	123354800	0.760331
chr12	123363700	0.735537
chr12	123366000	0.876033
chr12	123380300	0.818182
chr12	123417500	0.752066
chr12	123478400	0.818182
chr12	123528700	0.818182
chr12	123568700	0.900826
chr12	123600400	0.900826
chr12	123611700	0.809917
chr12	123617000	0.793388
chr12	123820800	0.776860
chr12	123926700	0.752066
chr12	123968700	0.859504
chr12	124016500	0.752066
chr12	124297100	0.801653
chr12	124632500	0.867769
chr12	124878200	0.768595
chr12	124951100	0.925620
chr12	124979000	0.735537
chr12	125047700	0.785124
chr12	125077900	0.818182
chr12	125118300	0.933884
chr12	125131400	0.834711
chr12	125169700	0.859504
chr12	125179600	0.760331

chr12	125198600	0.735537
chr12	125212200	0.743802
chr12	125247600	0.809917
chr12	125340900	0.834711
chr12	125348700	0.768595
chr12	125354000	0.809917
chr12	125358700	0.818182
chr12	127568100	0.925620
chr12	128622400	0.743802
chr12	128751800	0.884298
chr12	129200500	0.785124
chr12	129288100	0.768595
chr12	129454300	0.859504
chr12	130183200	0.867769
chr12	131456700	0.900826
chr12	131590800	0.735537
chr12	131601600	0.735537
chr12	131702000	0.793388
chr12	132089700	0.743802
chr12	132195600	0.776860
chr12	132417000	0.752066
chr12	132434700	0.884298
chr12	133024900	0.834711
chr12	133028600	0.752066
chr12	133039500	0.735537
chr12	133306300	0.785124
chr12	133339100	0.801653
chr12	133397200	0.950413
chr12	133405800	0.950413
chr12	133464800	0.975207
chr13	20161500	0.809917
chr13	20356800	0.859504
chr13	20438800	0.801653
chr13	20794300	0.785124
chr13	20912600	0.752066
chr13	21347700	0.942149
chr13	21400800	0.752066
chr13	21452400	0.818182
chr13	21585400	0.826446
chr13	21793800	0.809917
chr13	21830900	0.785124
chr13	22143100	0.768595
chr13	22651800	0.743802
chr13	23897300	0.768595
chr13	24419700	0.743802
chr13	24530500	0.884298
chr13	24541100	0.752066
chr13	24567400	0.743802
chr13	24627900	0.785124
chr13	24778200	0.809917
chr13	24789000	0.851240
chr13	24791900	0.735537
chr13	24855300	0.776860
chr13	25033800	0.752066

chr13	25095100	0.834711
chr13	25139500	0.752066
chr13	25185400	0.826446
chr13	25245700	0.818182
chr13	25259500	0.793388
chr13	25283700	0.834711
chr13	25397700	0.776860
chr13	25425800	0.735537
chr13	25443700	0.768595
chr13	25505900	0.851240
chr13	25518500	0.818182
chr13	25563100	0.735537
chr13	25659500	0.776860
chr13	25689000	0.743802
chr13	25728800	0.851240
chr13	25760800	0.785124
chr13	25876100	0.818182
chr13	26352900	0.925620
chr13	26381400	0.760331
chr13	26481200	0.776860
chr13	26796400	0.809917
chr13	26900600	0.776860
chr13	27176300	0.876033
chr13	27296000	0.826446
chr13	27802200	0.776860
chr13	27867800	0.900826
chr13	27922400	0.801653
chr13	27927900	0.760331
chr13	27936600	0.752066
chr13	27944900	0.834711
chr13	28052200	0.834711
chr13	28055800	0.776860
chr13	28169900	0.793388
chr13	28182200	0.760331
chr13	28185200	0.826446
chr13	28349300	0.776860
chr13	28400600	0.809917
chr13	28554500	0.776860
chr13	28608300	0.785124
chr13	28876400	0.818182
chr13	28922400	0.776860
chr13	28932700	0.809917
chr13	28975800	0.809917
chr13	28983200	0.785124
chr13	29024700	0.859504
chr13	29099700	0.834711
chr13	29113800	0.760331
chr13	29225000	0.859504
chr13	29279500	0.851240
chr13	29738100	0.826446
chr13	30009100	0.867769
chr13	30029500	0.768595
chr13	30169600	0.735537
chr13	30290800	0.876033

chr13	30307700	0.776860
chr13	30310200	0.818182
chr13	30318100	0.760331
chr13	30498400	0.859504
chr13	30687200	0.818182
chr13	30695900	0.884298
chr13	30707400	0.818182
chr13	30715700	0.760331
chr13	30718100	0.743802
chr13	30832200	0.768595
chr13	30888100	0.793388
chr13	30899900	0.785124
chr13	30907400	0.909091
chr13	30920800	0.867769
chr13	30948700	0.735537
chr13	31018100	0.735537
chr13	31050100	0.801653
chr13	31052500	0.834711
chr13	31073900	0.892562
chr13	31145100	0.867769
chr13	31147300	0.818182
chr13	31152100	0.809917
chr13	31182500	0.933884
chr13	31185100	0.818182
chr13	31187900	0.876033
chr13	31270900	0.892562
chr13	31278200	0.851240
chr13	31293500	0.743802
chr13	31296600	0.809917
chr13	31314900	0.793388
chr13	31342000	0.785124
chr13	31422300	0.909091
chr13	31426400	0.876033
chr13	31612100	0.876033
chr13	31620600	0.785124
chr13	31650900	0.809917
chr13	31655900	0.867769
chr13	31742500	0.834711
chr13	32455300	0.909091
chr13	32495200	0.909091
chr13	32505500	0.892562
chr13	32522800	0.933884
chr13	32539400	0.818182
chr13	32542700	0.925620
chr13	32824400	0.801653
chr13	32884000	0.776860
chr13	32982600	0.768595
chr13	32998300	0.752066
chr13	33046300	0.743802
chr13	33404400	0.752066
chr13	33420300	0.760331
chr13	33480400	0.768595
chr13	33544200	0.867769
chr13	33786900	0.768595

chr13	33927900	0.851240
chr13	33938200	0.752066
chr13	33942800	0.834711
chr13	34092800	0.892562
chr13	34112500	0.735537
chr13	34219600	0.826446
chr13	34253900	0.752066
chr13	34304500	0.851240
chr13	34372100	0.826446
chr13	34619900	0.760331
chr13	34731600	0.834711
chr13	35519600	0.842975
chr13	36305200	0.768595
chr13	36992200	0.818182
chr13	37380600	0.826446
chr13	37462700	0.876033
chr13	38924100	0.801653
chr13	39619500	0.801653
chr13	39953600	0.752066
chr13	40263100	0.752066
chr13	40353700	0.793388
chr13	40598400	0.909091
chr13	40623200	0.760331
chr13	40629900	0.760331
chr13	40637400	0.917355
chr13	40676600	0.942149
chr13	40765000	0.760331
chr13	40778500	0.859504
chr13	40820200	0.900826
chr13	40849900	0.818182
chr13	40892300	0.752066
chr13	40919500	0.834711
chr13	40958300	0.735537
chr13	40977200	0.801653
chr13	41009400	0.809917
chr13	41013400	0.735537
chr13	41017700	0.785124
chr13	41027300	0.818182
chr13	41038900	0.776860
chr13	41055200	0.809917
chr13	41058900	0.834711
chr13	41069300	0.876033
chr13	41087300	0.793388
chr13	41139800	0.809917
chr13	41148500	0.818182
chr13	41161000	0.752066
chr13	41175100	0.752066
chr13	41201200	0.917355
chr13	41209900	0.743802
chr13	41214800	0.760331
chr13	41247200	0.793388
chr13	41268500	0.842975
chr13	41339200	0.776860
chr13	41593100	0.785124

chr13	41631500	0.900826
chr13	41857000	0.752066
chr13	41874100	0.884298
chr13	41889800	0.752066
chr13	42067200	0.933884
chr13	42077500	0.743802
chr13	42129100	0.876033
chr13	42494100	0.735537
chr13	42527200	0.752066
chr13	42552600	0.809917
chr13	42633100	0.776860
chr13	42945000	0.776860
chr13	43107700	0.900826
chr13	43177700	0.900826
chr13	43231200	0.851240
chr13	43295200	0.950413
chr13	43378800	0.851240
chr13	43543900	0.776860
chr13	43546700	0.876033
chr13	43597800	0.884298
chr13	43702800	0.752066
chr13	43882500	0.793388
chr13	43965100	0.760331
chr13	44449900	0.752066
chr13	44499300	0.933884
chr13	44507800	0.917355
chr13	44663600	0.892562
chr13	44772000	0.793388
chr13	44812200	0.834711
chr13	44816400	0.859504
chr13	44878500	0.851240
chr13	45152100	0.900826
chr13	45242500	0.760331
chr13	45338700	0.793388
chr13	45443100	0.884298
chr13	45492500	0.876033
chr13	45623500	0.909091
chr13	45630000	0.966942
chr13	45707300	0.876033
chr13	45904400	0.743802
chr13	46013600	0.735537
chr13	46016200	0.752066
chr13	46043300	0.735537
chr13	46312000	0.859504
chr13	46422400	0.909091
chr13	46498600	0.760331
chr13	46774900	0.818182
chr13	46975500	0.743802
chr13	47064200	0.809917
chr13	47371200	0.867769
chr13	47424900	0.760331
chr13	47429900	0.859504
chr13	47436700	0.768595
chr13	47509000	0.735537

chr13	47607900	0.950413
chr13	47610900	0.826446
chr13	47640700	0.752066
chr13	47710000	0.768595
chr13	47721700	0.867769
chr13	47742900	0.818182
chr13	47764900	0.876033
chr13	48612200	0.743802
chr13	48726400	0.900826
chr13	48730900	0.760331
chr13	48759000	0.793388
chr13	48795800	0.834711
chr13	48839800	0.859504
chr13	48861400	0.743802
chr13	49005300	0.752066
chr13	49236400	0.735537
chr13	49255900	0.966942
chr13	49324300	0.900826
chr13	49403500	0.735537
chr13	49428200	0.809917
chr13	49568700	0.743802
chr13	49808700	0.801653
chr13	49875900	0.743802
chr13	50114800	0.876033
chr13	50155700	0.735537
chr13	50256800	0.859504
chr13	50343500	0.842975
chr13	50603100	0.801653
chr13	50628700	0.776860
chr13	50681000	0.867769
chr13	50706900	0.768595
chr13	50733300	0.760331
chr13	50783300	0.876033
chr13	50797100	0.826446
chr13	50810100	0.752066
chr13	50819500	0.892562
chr13	50823300	0.859504
chr13	50930000	0.867769
chr13	50933300	0.876033
chr13	50935900	0.752066
chr13	51173500	0.801653
chr13	51210500	0.743802
chr13	51225100	0.842975
chr13	51288400	0.785124
chr13	51337600	0.735537
chr13	51375900	0.876033
chr13	51462000	0.743802
chr13	51491500	0.842975
chr13	51566800	0.917355
chr13	51899700	0.793388
chr13	51906900	0.809917
chr13	51981900	0.867769
chr13	51991000	0.760331
chr13	51995600	0.735537

chr13	52028000	0.785124
chr13	52074500	0.842975
chr13	52126700	0.884298
chr13	52339300	0.760331
chr13	52378000	0.876033
chr13	52386400	0.851240
chr13	52396700	0.925620
chr13	52400500	0.851240
chr13	52419600	0.760331
chr13	52487400	0.867769
chr13	52519400	0.876033
chr13	52532200	0.859504
chr13	52542500	0.752066
chr13	52815100	0.818182
chr13	52899200	0.785124
chr13	53024800	0.735537
chr13	53072800	0.851240
chr13	53145000	0.776860
chr13	53168800	0.834711
chr13	53429900	0.917355
chr13	53442000	0.793388
chr13	53444500	0.867769
chr13	53478000	0.768595
chr13	53587300	0.818182
chr13	53726300	0.859504
chr13	57733700	0.768595
chr13	59966200	0.809917
chr13	60320800	0.818182
chr13	60329800	0.752066
chr13	60648900	0.768595
chr13	60804900	0.867769
chr13	60902800	0.760331
chr13	62784200	0.793388
chr13	63086900	0.793388
chr13	73798200	0.760331
chr13	74342200	0.785124
chr13	74395500	0.801653
chr13	74488200	0.735537
chr13	74499600	0.818182
chr13	74508300	0.826446
chr13	74523800	0.842975
chr13	74530000	0.834711
chr13	74532600	0.834711
chr13	74541200	0.793388
chr13	74573300	0.752066
chr13	74704300	0.851240
chr13	74862400	0.785124
chr13	75949800	0.760331
chr13	75966300	0.768595
chr13	75984500	0.768595
chr13	76016900	0.785124
chr13	76023800	0.818182
chr13	76061100	0.809917
chr13	76085100	0.752066

chr13	76089300	0.735537
chr13	76214100	0.867769
chr13	76445100	0.760331
chr13	76450800	0.793388
chr13	76535000	0.900826
chr13	76561300	0.851240
chr13	76985800	0.925620
chr13	77015300	0.950413
chr13	77211800	0.760331
chr13	77310400	0.793388
chr13	77566700	0.809917
chr13	77601200	0.826446
chr13	77928600	0.867769
chr13	77961500	0.793388
chr13	78064600	0.743802
chr13	78076400	0.809917
chr13	78078500	0.851240
chr13	78083500	0.942149
chr13	78091300	0.859504
chr13	78099000	0.818182
chr13	78108700	0.735537
chr13	78119800	0.735537
chr13	78133100	0.834711
chr13	78270100	0.776860
chr13	78303800	0.809917
chr13	78874500	0.776860
chr13	78884300	0.851240
chr13	78915800	0.884298
chr13	78967900	0.752066
chr13	78977900	0.752066
chr13	78982700	0.801653
chr13	79185100	0.801653
chr13	79212900	0.735537
chr13	79450900	0.768595
chr13	79683600	0.760331
chr13	80026100	0.818182
chr13	80031900	0.752066
chr13	80043100	0.768595
chr13	80071200	0.801653
chr13	80099600	0.760331
chr13	80354600	0.752066
chr13	80624000	0.818182
chr13	80828400	0.752066
chr13	80851100	0.793388
chr13	91996800	0.867769
chr13	92058200	0.867769
chr13	92070400	0.785124
chr13	92438000	0.776860
chr13	94965400	0.925620
chr13	94970000	0.892562
chr13	94976700	0.760331
chr13	94980800	0.743802
chr13	94997600	0.752066
chr13	95201300	0.809917

chr13	95309400	0.900826
chr13	95897400	0.834711
chr13	95953700	0.818182
chr13	96033600	0.776860
chr13	96041600	0.785124
chr13	96162100	0.842975
chr13	96188200	0.809917
chr13	96348600	0.752066
chr13	96360800	0.735537
chr13	96608200	0.842975
chr13	97813800	0.752066
chr13	97868000	0.785124
chr13	97914100	0.760331
chr13	97936000	0.735537
chr13	97991900	0.776860
chr13	98039600	0.743802
chr13	98112900	0.876033
chr13	98501700	0.793388
chr13	98869500	0.785124
chr13	98919700	0.752066
chr13	99239000	0.793388
chr13	99303200	0.909091
chr13	99506400	0.859504
chr13	99527800	0.818182
chr13	99695100	0.900826
chr13	99698800	0.793388
chr13	99775200	0.892562
chr13	99803700	0.826446
chr13	99829000	0.743802
chr13	99859400	0.809917
chr13	99881800	0.768595
chr13	99904500	0.900826
chr13	99931600	0.752066
chr13	100090300	0.801653
chr13	100129600	0.876033
chr13	100151300	0.818182
chr13	100167100	0.752066
chr13	100231000	0.884298
chr13	100270000	0.768595
chr13	100289200	0.876033
chr13	100318900	0.735537
chr13	100346000	0.917355
chr13	100480700	0.768595
chr13	100536300	0.842975
chr13	100545200	0.950413
chr13	100564600	0.859504
chr13	100654200	0.867769
chr13	101076000	0.752066
chr13	101170100	0.768595
chr13	101195400	0.867769
chr13	101262800	0.760331
chr13	101991600	0.793388
chr13	102277500	0.776860
chr13	103237400	0.768595

chr13	103471500	0.735537
chr13	103588700	0.876033
chr13	103730900	0.859504
chr13	103754200	0.785124
chr13	103930100	0.776860
chr13	103935500	0.752066
chr13	105792300	0.752066
chr13	107124500	0.867769
chr13	107146300	0.900826
chr13	107153000	0.917355
chr13	107220200	0.900826
chr13	108857700	0.876033
chr13	108878800	0.809917
chr13	108968600	0.801653
chr13	108978800	0.892562
chr13	109819300	0.735537
chr13	110683200	0.752066
chr13	111064900	0.834711
chr13	111099700	0.842975
chr13	111116000	0.743802
chr13	111127900	0.793388
chr13	111162500	0.826446
chr13	111168500	0.785124
chr13	111178800	0.818182
chr13	111297600	0.776860
chr13	111472300	0.776860
chr13	111510300	0.768595
chr13	111699900	0.743802
chr13	111782300	0.809917
chr13	111868300	0.752066
chr13	111957100	0.842975
chr13	113242800	0.735537
chr13	113422300	0.867769
chr13	113528900	0.818182
chr13	113705200	0.991736
chr13	113807200	0.735537
chr13	113867200	0.776860
chr13	113947000	0.785124
chr13	113952100	0.818182
chr13	114141100	0.760331
chr13	114539400	0.842975
chr13	114584800	0.760331
chr13	114824900	0.809917
chr13	114890800	0.752066
chr13	114900400	0.892562
chr13	114906400	0.859504
chr13	114909200	0.826446
chr13	114912200	0.892562
chr13	114915700	0.743802
chr13	114927700	0.743802
chr13	114988100	0.752066
chr13	114992900	0.735537
chr14	19548700	0.743802
chr14	20881800	0.859504

chr14	20893800	0.842975
chr14	21056100	0.834711
chr14	21075800	0.776860
chr14	21121900	0.735537
chr14	21125800	0.785124
chr14	21136900	0.793388
chr14	21232900	0.776860
chr14	21270300	0.760331
chr14	21504100	0.760331
chr14	21566400	0.909091
chr14	21572500	0.867769
chr14	21660500	0.801653
chr14	21738500	0.925620
chr14	21769200	0.752066
chr14	21777100	0.851240
chr14	21902300	0.834711
chr14	21924600	0.892562
chr14	22631500	0.834711
chr14	22641800	0.892562
chr14	22646200	0.867769
chr14	22733300	0.892562
chr14	22750300	0.735537
chr14	22783500	0.785124
chr14	22866400	0.851240
chr14	22902100	0.809917
chr14	22966300	0.859504
chr14	22987100	0.735537
chr14	23039100	0.760331
chr14	23070800	0.826446
chr14	23083500	0.809917
chr14	23285100	0.818182
chr14	23288900	0.867769
chr14	23307200	0.834711
chr14	23426300	0.892562
chr14	23465700	0.876033
chr14	23590900	0.809917
chr14	23618500	0.826446
chr14	23707300	0.776860
chr14	23771600	0.752066
chr14	23776000	0.793388
chr14	24017500	0.743802
chr14	24458700	0.826446
chr14	24483500	0.752066
chr14	24593300	0.958678
chr14	24682500	0.867769
chr14	24702000	0.818182
chr14	24716200	0.785124
chr14	24777300	0.909091
chr14	24783000	0.752066
chr14	24912100	0.818182
chr14	24977900	0.842975
chr14	25114700	0.743802
chr14	25134900	0.892562
chr14	25168500	0.743802

chr14	29254900	0.793388
chr14	29300700	0.735537
chr14	30327300	0.925620
chr14	31091800	0.735537
chr14	31322500	0.735537
chr14	31332600	0.768595
chr14	31439800	0.851240
chr14	31669400	0.834711
chr14	31699000	0.892562
chr14	31709100	0.743802
chr14	31731400	0.834711
chr14	31735500	0.768595
chr14	31962400	0.760331
chr14	32161800	0.735537
chr14	32407400	0.818182
chr14	32414500	0.768595
chr14	32551500	0.752066
chr14	32572200	0.884298
chr14	32636000	0.867769
chr14	32655900	0.834711
chr14	32659300	0.752066
chr14	32667800	0.851240
chr14	32675600	0.809917
chr14	32730700	0.801653
chr14	32902600	0.867769
chr14	33042200	0.818182
chr14	33047900	0.933884
chr14	33505500	0.752066
chr14	34034400	0.768595
chr14	34407900	0.818182
chr14	34428400	0.809917
chr14	34473300	0.867769
chr14	34483200	0.760331
chr14	34496600	0.785124
chr14	34507200	0.743802
chr14	34511000	0.859504
chr14	34514100	0.735537
chr14	34519500	0.760331
chr14	34525700	0.801653
chr14	34560000	0.735537
chr14	34650300	0.859504
chr14	34690600	0.909091
chr14	34801600	0.909091
chr14	34934900	0.851240
chr14	34944800	0.925620
chr14	35015700	0.925620
chr14	35025900	0.975207
chr14	35099300	0.892562
chr14	35337500	0.818182
chr14	35400600	0.884298
chr14	35810100	0.809917
chr14	35835500	0.760331
chr14	36148500	0.768595
chr14	36278300	0.884298

chr14	36348400	0.735537
chr14	36357000	0.760331
chr14	36384900	0.785124
chr14	36409900	0.925620
chr14	36455500	0.760331
chr14	36491400	0.842975
chr14	36663700	0.933884
chr14	39639600	0.884298
chr14	39644800	0.735537
chr14	39736600	0.743802
chr14	40297800	0.776860
chr14	45268200	0.991736
chr14	45760300	0.776860
chr14	49660800	0.743802
chr14	49835300	0.809917
chr14	49950700	0.735537
chr14	50061400	0.826446
chr14	50096400	0.809917
chr14	50104400	0.801653
chr14	50109200	0.859504
chr14	50238000	0.752066
chr14	50316600	0.752066
chr14	50319600	0.776860
chr14	50330200	0.760331
chr14	50475900	0.818182
chr14	50571500	0.752066
chr14	50583600	0.942149
chr14	50698600	0.735537
chr14	50779500	0.851240
chr14	50830400	0.900826
chr14	51008200	0.785124
chr14	51230000	0.818182
chr14	51298400	0.776860
chr14	51342700	0.801653
chr14	51368500	0.743802
chr14	51395400	0.851240
chr14	51899600	0.760331
chr14	52034400	0.826446
chr14	52069600	0.752066
chr14	52247100	0.768595
chr14	52318800	0.842975
chr14	52489000	0.867769
chr14	52794700	0.867769
chr14	52819100	0.735537
chr14	52867600	0.859504
chr14	53190500	0.826446
chr14	53209100	0.950413
chr14	53291900	0.768595
chr14	53632700	0.752066
chr14	53658400	0.752066
chr14	54031900	0.743802
chr14	54081000	0.826446
chr14	54122400	0.842975
chr14	54360700	0.876033

chr14	54392100	0.851240
chr14	54430500	0.735537
chr14	54784800	0.752066
chr14	54908200	0.768595
chr14	54955600	0.735537
chr14	54999700	0.909091
chr14	55133400	0.735537
chr14	55141200	0.809917
chr14	55152600	0.752066
chr14	55189500	0.851240
chr14	55243700	0.809917
chr14	55273000	0.900826
chr14	55362600	0.752066
chr14	55742400	0.760331
chr14	55764600	0.760331
chr14	55807900	0.826446
chr14	55883500	0.768595
chr14	55918200	0.892562
chr14	56168800	0.834711
chr14	56229400	0.826446
chr14	56274300	0.892562
chr14	56662900	0.818182
chr14	56669100	0.842975
chr14	57046600	0.859504
chr14	57735900	0.793388
chr14	57952700	0.834711
chr14	59039200	0.818182
chr14	59434500	0.743802
chr14	59615400	0.768595
chr14	59628900	0.892562
chr14	59645700	0.867769
chr14	59660400	0.900826
chr14	59888900	0.760331
chr14	60053300	0.793388
chr14	60246400	0.785124
chr14	60286300	0.867769
chr14	60518900	0.884298
chr14	60712800	0.809917
chr14	60740800	0.752066
chr14	60798200	0.966942
chr14	61099700	0.892562
chr14	61225700	0.917355
chr14	61548400	0.793388
chr14	61587800	0.776860
chr14	61669500	0.818182
chr14	61763900	0.900826
chr14	61793800	0.735537
chr14	61796800	0.743802
chr14	62059400	0.818182
chr14	62061800	0.735537
chr14	62103500	0.776860
chr14	62134900	0.826446
chr14	62138100	0.876033
chr14	62178900	0.818182

chr14	62220400	0.933884
chr14	62308000	0.776860
chr14	62354900	0.842975
chr14	62482900	0.900826
chr14	62591200	0.892562
chr14	63751700	0.735537
chr14	63903100	0.743802
chr14	63951300	0.801653
chr14	63967600	0.743802
chr14	64226600	0.942149
chr14	64401200	0.776860
chr14	64472700	0.867769
chr14	64733600	0.752066
chr14	64763500	0.776860
chr14	64941200	0.752066
chr14	64977500	0.768595
chr14	65016700	0.735537
chr14	65065700	0.760331
chr14	65077000	0.818182
chr14	65099700	0.834711
chr14	65121600	0.818182
chr14	65172600	0.760331
chr14	65175800	0.752066
chr14	65279500	0.752066
chr14	65315200	0.884298
chr14	65381500	0.842975
chr14	65454000	0.776860
chr14	65623400	0.809917
chr14	65654100	0.826446
chr14	65708100	0.892562
chr14	65742400	0.776860
chr14	65802000	0.785124
chr14	65833600	0.809917
chr14	65938200	0.809917
chr14	65996600	0.776860
chr14	66271500	0.785124
chr14	66314100	0.760331
chr14	66458400	0.743802
chr14	66566700	0.859504
chr14	66841300	0.801653
chr14	66857100	0.834711
chr14	66862600	0.842975
chr14	66963800	0.834711
chr14	67219200	0.909091
chr14	67485800	0.834711
chr14	67511800	0.884298
chr14	67708300	0.776860
chr14	67827100	0.760331
chr14	67955600	0.768595
chr14	68141600	0.917355
chr14	68348500	0.818182
chr14	68429300	0.735537
chr14	68488200	0.809917
chr14	68760200	0.776860

chr14	68851000	0.776860
chr14	68938500	0.892562
chr14	68943800	0.909091
chr14	69014400	0.776860
chr14	69127800	0.925620
chr14	69140400	0.917355
chr14	69163100	0.785124
chr14	69208100	0.760331
chr14	69302200	0.809917
chr14	69378000	0.760331
chr14	69560800	0.867769
chr14	69818700	0.867769
chr14	69948700	0.826446
chr14	70041400	0.768595
chr14	70060600	0.851240
chr14	70074300	0.900826
chr14	70130100	0.809917
chr14	70134800	0.793388
chr14	70167000	0.752066
chr14	70193900	0.752066
chr14	70375300	0.917355
chr14	70444000	0.851240
chr14	70465700	0.809917
chr14	70467900	0.826446
chr14	70473800	0.760331
chr14	70476200	0.735537
chr14	70480100	0.776860
chr14	70651000	0.834711
chr14	71069000	0.752066
chr14	71243100	0.801653
chr14	71249200	0.735537
chr14	71279000	0.809917
chr14	71281900	0.785124
chr14	71339000	0.818182
chr14	71387400	0.752066
chr14	71606100	0.785124
chr14	71679300	0.760331
chr14	71758000	0.735537
chr14	71800600	0.859504
chr14	71945600	0.776860
chr14	72410300	0.760331
chr14	72490500	0.735537
chr14	72906800	0.735537
chr14	73048500	0.768595
chr14	73057200	0.884298
chr14	73102700	0.809917
chr14	73140400	0.752066
chr14	73142700	0.851240
chr14	73181400	0.752066
chr14	73277100	0.834711
chr14	73356200	0.867769
chr14	73374300	0.743802
chr14	73603600	0.735537
chr14	73711500	0.818182

chr14	73737100	0.743802
chr14	73893700	0.743802
chr14	73933300	0.793388
chr14	73935700	0.785124
chr14	73961400	0.768595
chr14	74017000	0.826446
chr14	74038700	0.842975
chr14	74049400	0.743802
chr14	74073500	0.760331
chr14	74094300	0.735537
chr14	74163300	0.743802
chr14	74217900	0.768595
chr14	74318800	0.818182
chr14	74413400	0.859504
chr14	74708300	0.785124
chr14	74868000	0.743802
chr14	74956400	0.809917
chr14	74981500	0.834711
chr14	75038900	0.793388
chr14	75081600	0.768595
chr14	75173400	0.743802
chr14	75238300	0.793388
chr14	75343200	0.892562
chr14	75413300	0.793388
chr14	75643500	0.859504
chr14	75651700	0.867769
chr14	75741900	0.859504
chr14	75830900	0.834711
chr14	75905200	0.776860
chr14	75947100	0.851240
chr14	75966000	0.933884
chr14	76018800	0.785124
chr14	76027600	0.925620
chr14	76035600	0.826446
chr14	76140200	0.851240
chr14	76342400	0.735537
chr14	76344700	0.801653
chr14	76435400	0.818182
chr14	76460400	0.735537
chr14	76482800	0.909091
chr14	76512700	0.818182
chr14	76522400	0.884298
chr14	76728600	0.809917
chr14	76886200	0.818182
chr14	77387000	0.842975
chr14	77396400	0.752066
chr14	77513000	0.768595
chr14	77532600	0.826446
chr14	77552600	0.900826
chr14	77567900	0.809917
chr14	77736300	0.958678
chr14	77787700	0.818182
chr14	77843100	0.760331
chr14	77873700	0.818182

chr14	77945300	0.801653
chr14	78021200	0.768595
chr14	78118900	0.735537
chr14	78278800	0.966942
chr14	78668600	0.768595
chr14	78758600	0.851240
chr14	78878100	0.768595
chr14	78922700	0.892562
chr14	81423700	0.834711
chr14	81688000	0.909091
chr14	81769900	0.867769
chr14	81941200	0.743802
chr14	82002300	0.776860
chr14	88407100	0.809917
chr14	88419300	0.851240
chr14	88477800	0.876033
chr14	88654300	0.801653
chr14	88694300	0.793388
chr14	88766700	0.809917
chr14	89134600	0.834711
chr14	89467000	0.900826
chr14	89670400	0.760331
chr14	89734800	0.793388
chr14	89737200	0.859504
chr14	89743200	0.752066
chr14	89791800	0.760331
chr14	89795000	0.752066
chr14	89884300	0.776860
chr14	89899300	0.818182
chr14	89929400	0.768595
chr14	89960600	0.793388
chr14	89970000	0.809917
chr14	90081400	0.735537
chr14	90087800	0.917355
chr14	90148200	0.917355
chr14	90191500	0.776860
chr14	90809900	0.776860
chr14	90816400	0.809917
chr14	90850200	0.752066
chr14	91018500	0.735537
chr14	91372600	0.884298
chr14	91436600	0.785124
chr14	91452600	0.933884
chr14	91479700	0.760331
chr14	91529700	0.793388
chr14	91535100	0.809917
chr14	91556800	0.768595
chr14	91608800	0.925620
chr14	91713400	0.834711
chr14	91731000	0.776860
chr14	91770100	0.768595
chr14	91824300	0.735537
chr14	91855100	0.743802
chr14	91877600	0.760331

chr14	91886000	0.776860
chr14	91945000	0.776860
chr14	92314500	0.826446
chr14	92325300	0.876033
chr14	92333800	0.859504
chr14	92340700	0.743802
chr14	92588800	0.752066
chr14	92783100	0.851240
chr14	92808100	0.842975
chr14	92904500	0.834711
chr14	92909000	0.760331
chr14	92928300	0.826446
chr14	92961100	0.809917
chr14	92973700	0.851240
chr14	93018400	0.735537
chr14	93020500	0.768595
chr14	93023300	0.793388
chr14	93050500	0.776860
chr14	93147900	0.809917
chr14	93165100	0.801653
chr14	93169200	0.752066
chr14	93171900	0.826446
chr14	93195300	0.826446
chr14	93204100	0.809917
chr14	93219000	0.760331
chr14	93227500	0.925620
chr14	93230900	0.776860
chr14	93242500	0.743802
chr14	93260700	0.760331
chr14	93364900	0.768595
chr14	93390700	0.752066
chr14	93540800	0.809917
chr14	93570200	0.735537
chr14	93582700	0.975207
chr14	93678800	0.867769
chr14	93698000	0.752066
chr14	93751800	0.818182
chr14	93842500	0.876033
chr14	93920100	0.826446
chr14	94419600	0.793388
chr14	94468400	0.900826
chr14	94481300	0.884298
chr14	94813400	0.950413
chr14	94822200	0.743802
chr14	94943300	0.809917
chr14	95501500	0.842975
chr14	95624300	0.909091
chr14	95652300	0.851240
chr14	95717300	0.801653
chr14	95722200	0.826446
chr14	95731900	0.752066
chr14	95767300	0.768595
chr14	95830500	0.735537
chr14	95947200	0.851240

chr14	96024300	0.743802
chr14	96044200	0.752066
chr14	96112300	0.768595
chr14	96144200	0.752066
chr14	96146900	0.735537
chr14	96241000	0.735537
chr14	96593400	0.793388
chr14	96644800	0.842975
chr14	96959900	0.834711
chr14	97070200	0.876033
chr14	97127100	0.752066
chr14	97150500	0.834711
chr14	97176700	0.826446
chr14	97194000	0.818182
chr14	97453800	0.834711
chr14	97541600	0.826446
chr14	97544100	0.793388
chr14	97719700	0.735537
chr14	97757500	0.892562
chr14	97817800	0.876033
chr14	97843400	0.776860
chr14	97853800	0.768595
chr14	97922500	0.867769
chr14	98173900	0.900826
chr14	99744000	0.785124
chr14	99753700	0.859504
chr14	99887000	0.834711
chr14	100035700	0.834711
chr14	100099400	0.884298
chr14	100131500	0.933884
chr14	100134100	0.760331
chr14	100211100	0.818182
chr14	100225600	0.793388
chr14	100359000	0.867769
chr14	100404100	0.958678
chr14	100413500	0.768595
chr14	100417200	0.801653
chr14	100462100	0.876033
chr14	100489000	0.900826
chr14	100614300	0.768595
chr14	100680800	0.950413
chr14	100713100	0.801653
chr14	100724400	0.743802
chr14	100808200	0.752066
chr14	100874900	0.776860
chr14	100901200	0.884298
chr14	101107000	0.834711
chr14	101120800	0.900826
chr14	101364200	0.876033
chr14	102171600	0.776860
chr14	102198400	0.735537
chr14	102249300	0.768595
chr14	102315900	0.909091
chr14	102384700	0.950413

chr14	102434900	0.743802
chr14	102837700	0.818182
chr14	102843200	0.818182
chr14	103032100	0.859504
chr14	103040500	0.892562
chr14	103085300	0.768595
chr14	103092000	0.776860
chr14	103113200	0.809917
chr14	103221600	0.859504
chr14	103223900	0.801653
chr14	103235200	0.760331
chr14	103261000	0.818182
chr14	103513100	0.793388
chr14	103540700	0.743802
chr14	103567400	0.785124
chr14	103574900	0.735537
chr14	103784000	0.743802
chr14	103796700	0.768595
chr14	103805700	0.917355
chr14	103848800	0.760331
chr14	104029200	0.859504
chr14	104061500	0.776860
chr14	104271900	0.834711
chr14	104274600	0.876033
chr14	104346700	0.909091
chr14	104862300	0.809917
chr14	104927800	0.917355
chr14	105144600	0.892562
chr14	105151700	0.752066
chr14	105367000	0.768595
chr14	105371100	0.768595
chr14	105480500	0.735537
chr14	105488200	0.851240
chr14	105510900	0.760331
chr14	105548800	0.876033
chr14	105567000	0.834711
chr14	105569600	0.735537
chr14	105660500	0.735537
chr14	105722600	0.834711
chr14	105784200	0.909091
chr14	105830900	0.867769
chr14	105842700	0.735537
chr14	105865000	0.785124
chr14	105952500	0.785124
chr14	106072600	0.768595
chr14	106099900	0.942149
chr14	106114600	0.752066
chr14	106175700	0.909091
chr14	106214900	0.859504
chr14	106314000	0.876033
chr14	106395000	0.818182
chr14	106829600	0.785124
chr14	106950800	0.768595
chr14	106973900	0.851240

chr14	106986700	0.818182
chr14	106990000	0.743802
chr14	107047100	0.950413
chr14	107069900	0.876033
chr14	107095300	0.801653
chr14	107110900	0.752066
chr14	107114300	0.768595
chr14	107147100	0.950413
chr14	107163800	0.735537
chr14	107170600	0.909091
chr14	107218500	0.809917
chr14	107223300	0.752066
chr14	107281500	0.735537
chr15	20851500	0.735537
chr15	21915700	0.776860
chr15	21928300	0.818182
chr15	21978100	0.818182
chr15	22461700	0.884298
chr15	23086900	0.842975
chr15	23098200	0.768595
chr15	23390000	0.925620
chr15	23677400	0.826446
chr15	25093200	0.876033
chr15	25985900	0.801653
chr15	26038900	0.793388
chr15	26056800	0.900826
chr15	28344700	0.818182
chr15	28529000	0.768595
chr15	28579000	0.884298
chr15	28823800	0.900826
chr15	28973000	0.826446
chr15	29247800	0.785124
chr15	29256000	0.752066
chr15	30028700	0.776860
chr15	30469500	0.876033
chr15	30829800	0.768595
chr15	30834300	0.760331
chr15	31135600	0.760331
chr15	31187100	0.834711
chr15	31198400	0.859504
chr15	31264300	0.776860
chr15	31284200	0.818182
chr15	31293900	0.776860
chr15	31298500	0.793388
chr15	31301900	0.826446
chr15	31306700	0.933884
chr15	31392000	0.818182
chr15	31421700	0.925620
chr15	31495800	0.793388
chr15	31531200	0.743802
chr15	31565500	0.776860
chr15	31600600	0.768595
chr15	31741200	0.752066
chr15	31763100	0.793388

chr15	31792800	0.809917
chr15	32871500	0.826446
chr15	32976300	0.735537
chr15	33023600	0.867769
chr15	33056100	0.876033
chr15	33126800	0.752066
chr15	33191700	0.900826
chr15	33452500	0.876033
chr15	33460600	0.851240
chr15	33482800	0.785124
chr15	33494100	0.809917
chr15	33616300	0.768595
chr15	33636600	0.752066
chr15	33931200	0.760331
chr15	33964200	0.809917
chr15	34008700	0.760331
chr15	34023400	0.818182
chr15	34072900	0.826446
chr15	34415900	0.785124
chr15	34606500	0.743802
chr15	34610700	0.884298
chr15	34958500	0.917355
chr15	35280800	0.818182
chr15	35311700	0.950413
chr15	35351200	0.925620
chr15	35838300	0.809917
chr15	36222500	0.942149
chr15	36871600	0.876033
chr15	37312400	0.735537
chr15	38335200	0.768595
chr15	38352300	0.785124
chr15	38361500	0.809917
chr15	38367800	0.818182
chr15	38372300	0.785124
chr15	38423700	0.743802
chr15	38441800	0.735537
chr15	38483400	0.859504
chr15	38530700	0.809917
chr15	38534700	0.966942
chr15	38601000	0.826446
chr15	38966800	0.735537
chr15	39110400	0.892562
chr15	39699000	0.834711
chr15	39703900	0.809917
chr15	39706100	0.826446
chr15	39784400	0.834711
chr15	39794300	0.735537
chr15	39845500	0.752066
chr15	39915800	0.735537
chr15	39921500	0.859504
chr15	39985500	0.743802
chr15	40166500	0.801653
chr15	40375200	0.818182
chr15	40405000	0.826446

chr15	40531300	0.809917
chr15	40569500	0.884298
chr15	40610200	0.818182
chr15	40690900	0.834711
chr15	40729200	0.818182
chr15	40785900	0.826446
chr15	40816300	0.785124
chr15	40889700	0.735537
chr15	41047800	0.950413
chr15	41053000	0.776860
chr15	41099300	0.776860
chr15	41176800	0.826446
chr15	41196300	0.760331
chr15	41218700	0.892562
chr15	41224700	0.834711
chr15	41716400	0.834711
chr15	41718700	0.876033
chr15	41721700	0.933884
chr15	41850400	0.859504
chr15	41896500	0.933884
chr15	41984800	0.785124
chr15	42181000	0.809917
chr15	42217000	0.801653
chr15	42224100	0.851240
chr15	42358300	0.752066
chr15	42783500	0.859504
chr15	42841100	0.785124
chr15	43398600	0.818182
chr15	43478000	0.834711
chr15	43546500	0.842975
chr15	43886300	0.818182
chr15	43931100	0.900826
chr15	44027600	0.942149
chr15	44069600	0.809917
chr15	44081600	0.892562
chr15	44116700	0.933884
chr15	44270200	0.768595
chr15	44279100	0.785124
chr15	44445700	0.793388
chr15	44450300	0.768595
chr15	44678000	0.942149
chr15	44838500	0.768595
chr15	45076700	0.826446
chr15	45098500	0.818182
chr15	45406800	0.760331
chr15	45410300	0.842975
chr15	45459200	0.925620
chr15	45462300	0.752066
chr15	45495100	0.768595
chr15	45684800	0.867769
chr15	45835700	0.752066
chr15	45848800	0.735537
chr15	45930000	0.859504
chr15	45937900	0.826446

chr15	46162800	0.785124
chr15	48107300	0.776860
chr15	48150500	0.768595
chr15	48279000	0.776860
chr15	48389300	0.752066
chr15	48618700	0.785124
chr15	48885000	0.801653
chr15	49087100	0.851240
chr15	49259000	0.768595
chr15	49439300	0.776860
chr15	49508900	0.776860
chr15	50443100	0.735537
chr15	50670800	0.958678
chr15	50736900	0.942149
chr15	50745600	0.735537
chr15	50777000	0.768595
chr15	50834900	0.809917
chr15	50979000	0.785124
chr15	51060300	0.826446
chr15	51342100	0.793388
chr15	51359100	0.925620
chr15	51533100	0.809917
chr15	51536700	0.876033
chr15	51595900	0.760331
chr15	51625400	0.950413
chr15	51628100	0.785124
chr15	51641300	0.818182
chr15	51661900	0.818182
chr15	51683200	0.760331
chr15	51893000	0.851240
chr15	51917900	0.743802
chr15	51984700	0.743802
chr15	52139100	0.785124
chr15	52274100	0.818182
chr15	52369800	0.735537
chr15	52404700	0.876033
chr15	52554800	0.909091
chr15	52772600	0.801653
chr15	52796300	0.867769
chr15	52857400	0.809917
chr15	52860800	0.859504
chr15	52952300	0.826446
chr15	52967200	0.917355
chr15	55489200	0.801653
chr15	55550300	0.884298
chr15	55560400	0.900826
chr15	55768200	0.801653
chr15	57019500	0.752066
chr15	57079500	0.859504
chr15	57091500	0.826446
chr15	57098400	0.851240
chr15	57130800	0.801653
chr15	57170200	0.826446
chr15	57338600	0.826446

chr15	57343900	0.876033
chr15	57426200	0.735537
chr15	57439900	0.776860
chr15	57482300	0.752066
chr15	57787700	0.826446
chr15	57802300	0.793388
chr15	57833300	0.743802
chr15	57999100	0.826446
chr15	58650900	0.818182
chr15	58814500	0.752066
chr15	58843700	0.768595
chr15	58920700	0.925620
chr15	59016700	0.867769
chr15	59575200	0.983471
chr15	59638300	0.867769
chr15	59648900	0.768595
chr15	59653400	0.793388
chr15	59659800	0.743802
chr15	59670000	0.892562
chr15	59711600	0.768595
chr15	59717200	0.752066
chr15	59734300	0.760331
chr15	59739800	0.892562
chr15	59809500	0.909091
chr15	59832800	0.760331
chr15	59882900	0.867769
chr15	59903200	0.966942
chr15	60090900	0.859504
chr15	60412100	0.818182
chr15	60417500	0.818182
chr15	60422400	0.768595
chr15	60521000	0.752066
chr15	60685300	0.834711
chr15	60836700	0.743802
chr15	60862900	0.760331
chr15	60988300	0.785124
chr15	61093200	0.752066
chr15	61134700	0.834711
chr15	61137200	0.743802
chr15	61151400	0.818182
chr15	61349900	0.760331
chr15	61363700	0.975207
chr15	61372100	0.876033
chr15	61377400	0.834711
chr15	61381100	0.760331
chr15	61405100	0.809917
chr15	61462400	0.793388
chr15	61619400	0.760331
chr15	61820800	0.801653
chr15	62376000	0.809917
chr15	62458300	0.842975
chr15	62533600	0.801653
chr15	62591200	0.859504
chr15	62950400	0.818182

chr15	63363700	0.851240
chr15	63454600	0.867769
chr15	63469900	0.834711
chr15	63496600	0.743802
chr15	63512300	0.752066
chr15	63565900	0.851240
chr15	63655200	0.876033
chr15	63779800	0.752066
chr15	63787600	0.768595
chr15	63811800	0.818182
chr15	63902800	0.826446
chr15	64126400	0.851240
chr15	64142500	0.776860
chr15	64231800	0.743802
chr15	64539800	0.851240
chr15	64673200	0.743802
chr15	64716600	0.776860
chr15	64737100	0.768595
chr15	65024600	0.785124
chr15	65118500	0.809917
chr15	65176300	0.801653
chr15	65282700	0.809917
chr15	65326400	0.760331
chr15	65504600	0.851240
chr15	65588300	0.975207
chr15	65591000	0.768595
chr15	65596800	0.933884
chr15	65629400	0.834711
chr15	65653000	0.785124
chr15	65809800	0.793388
chr15	65827300	0.809917
chr15	65847300	0.785124
chr15	65931400	0.785124
chr15	66065700	0.876033
chr15	66079800	0.925620
chr15	66106300	0.760331
chr15	66122000	0.768595
chr15	66125600	0.867769
chr15	66403800	0.752066
chr15	66648700	0.867769
chr15	66662600	0.925620
chr15	66687700	0.842975
chr15	66725800	0.760331
chr15	66866800	0.809917
chr15	66907100	0.752066
chr15	66930600	0.760331
chr15	67003000	0.942149
chr15	67053600	0.760331
chr15	67155500	0.884298
chr15	67158900	0.842975
chr15	67233300	0.809917
chr15	67318000	0.942149
chr15	67330000	0.801653
chr15	67343500	0.776860

chr15	67544000	0.752066
chr15	67552600	0.834711
chr15	67588800	0.826446
chr15	67753500	0.768595
chr15	67799400	0.842975
chr15	68133100	0.867769
chr15	68156100	0.735537
chr15	68173800	0.743802
chr15	68199600	0.760331
chr15	68239200	0.900826
chr15	68312100	0.793388
chr15	68372500	0.760331
chr15	68398900	0.867769
chr15	68521700	0.768595
chr15	68583800	0.809917
chr15	68818400	0.760331
chr15	68821900	0.785124
chr15	68967800	0.793388
chr15	69104300	0.884298
chr15	69478100	0.752066
chr15	69954300	0.785124
chr15	69967600	0.743802
chr15	69970300	0.826446
chr15	70012000	0.917355
chr15	70018500	0.801653
chr15	70042900	0.752066
chr15	70053600	0.776860
chr15	70173900	0.776860
chr15	70212600	0.776860
chr15	70282500	0.851240
chr15	70330300	0.785124
chr15	70333000	0.867769
chr15	70379900	0.793388
chr15	70435000	0.760331
chr15	70513000	0.743802
chr15	70534800	0.892562
chr15	70557200	0.851240
chr15	70559900	0.776860
chr15	70562500	0.752066
chr15	70615300	0.809917
chr15	70620800	0.867769
chr15	70629100	0.867769
chr15	70727200	0.735537
chr15	70753200	0.752066
chr15	70759800	0.793388
chr15	70767500	0.900826
chr15	70770000	0.785124
chr15	70799300	0.834711
chr15	70816900	0.859504
chr15	70820900	0.768595
chr15	70828100	0.818182
chr15	70851900	0.834711
chr15	70964800	0.834711
chr15	71068300	0.785124

chr15	71372900	0.743802
chr15	71769800	0.735537
chr15	72078400	0.768595
chr15	72512000	0.867769
chr15	72778700	0.876033
chr15	72812200	0.735537
chr15	72920500	0.818182
chr15	72963800	0.776860
chr15	73079700	0.735537
chr15	73551800	0.834711
chr15	73610900	0.884298
chr15	73656000	0.842975
chr15	73914200	0.851240
chr15	73919300	0.743802
chr15	73929100	0.768595
chr15	73931600	0.809917
chr15	74045700	0.785124
chr15	74200200	0.743802
chr15	74252100	0.826446
chr15	74268400	0.834711
chr15	74422100	0.752066
chr15	74480000	0.834711
chr15	74507900	0.884298
chr15	74528400	0.776860
chr15	74584800	0.851240
chr15	74669100	0.801653
chr15	74671900	0.876033
chr15	74728400	0.842975
chr15	75101700	0.818182
chr15	75114000	0.834711
chr15	75165600	0.809917
chr15	75230300	0.818182
chr15	75251700	0.793388
chr15	75253900	0.793388
chr15	75276500	0.826446
chr15	75283500	0.809917
chr15	75316300	0.942149
chr15	75387900	0.760331
chr15	75468700	0.892562
chr15	75471400	0.785124
chr15	75477700	0.801653
chr15	75487500	0.876033
chr15	75567100	0.768595
chr15	75626500	0.752066
chr15	75660700	0.867769
chr15	75918200	0.801653
chr15	75934600	0.752066
chr15	75952200	0.735537
chr15	76041800	0.793388
chr15	76051200	0.826446
chr15	76094800	0.818182
chr15	76109600	0.768595
chr15	76196300	0.884298
chr15	76354700	0.752066

chr15	76428500	0.760331
chr15	76438400	0.909091
chr15	76440900	0.752066
chr15	76443600	0.793388
chr15	76474400	0.785124
chr15	76510100	0.743802
chr15	76592500	0.752066
chr15	76603800	0.884298
chr15	77158500	0.818182
chr15	77187300	0.950413
chr15	77281300	0.760331
chr15	77308100	0.776860
chr15	77349900	0.768595
chr15	77352700	0.842975
chr15	77375800	0.801653
chr15	77378500	0.925620
chr15	77602300	0.809917
chr15	77664800	0.735537
chr15	77728600	0.785124
chr15	77733600	0.785124
chr15	77926300	0.785124
chr15	78246900	0.785124
chr15	78311700	0.785124
chr15	78347700	0.760331
chr15	78527100	0.818182
chr15	78667800	0.776860
chr15	78708000	0.917355
chr15	78745100	0.801653
chr15	78973800	0.735537
chr15	78978100	0.743802
chr15	78988100	0.793388
chr15	79002100	0.743802
chr15	79049700	0.834711
chr15	79186400	0.851240
chr15	79244400	0.826446
chr15	79297300	0.867769
chr15	79399700	0.900826
chr15	79959900	0.735537
chr15	79966300	0.950413
chr15	80127400	0.842975
chr15	80236400	0.793388
chr15	80254500	0.818182
chr15	80315500	0.859504
chr15	80345200	0.760331
chr15	80348900	0.752066
chr15	80361500	0.842975
chr15	80393800	0.818182
chr15	80966600	0.876033
chr15	81003900	0.801653
chr15	81085800	0.793388
chr15	81116500	0.785124
chr15	81213100	0.801653
chr15	81322700	0.826446
chr15	81332000	0.743802

chr15	81353800	0.851240
chr15	81404200	0.842975
chr15	81434700	0.851240
chr15	81454300	0.834711
chr15	81620200	0.867769
chr15	82136000	0.867769
chr15	82351200	0.892562
chr15	82375400	0.834711
chr15	82490000	0.743802
chr15	82579800	0.743802
chr15	82704700	0.752066
chr15	83081400	0.818182
chr15	83221100	0.933884
chr15	83497300	0.735537
chr15	83635500	0.735537
chr15	83683100	0.826446
chr15	83735700	0.867769
chr15	83778700	0.735537
chr15	83879400	0.768595
chr15	84049400	0.859504
chr15	84559300	0.776860
chr15	84611700	0.793388
chr15	84624900	0.752066
chr15	84769200	0.834711
chr15	85149600	0.818182
chr15	85259700	0.793388
chr15	85288600	0.809917
chr15	85297800	0.768595
chr15	85325800	0.735537
chr15	85431600	0.743802
chr15	85458800	0.776860
chr15	85570200	0.917355
chr15	85828400	0.925620
chr15	85851800	0.884298
chr15	85856100	0.900826
chr15	85859500	0.776860
chr15	85870000	0.892562
chr15	85932800	0.785124
chr15	85968600	0.768595
chr15	85988500	0.826446
chr15	86000800	0.942149
chr15	86033000	0.735537
chr15	86045600	0.776860
chr15	86083200	0.809917
chr15	86086400	0.867769
chr15	86384100	0.809917
chr15	86499300	0.760331
chr15	86929200	0.983471
chr15	88758900	0.785124
chr15	89052800	0.776860
chr15	89157300	0.776860
chr15	89195800	0.917355
chr15	89227400	0.793388
chr15	89388600	0.826446

chr15	89440500	0.818182
chr15	89462100	0.834711
chr15	89464300	0.752066
chr15	89493800	0.768595
chr15	89526200	0.752066
chr15	89546100	0.752066
chr15	89561300	0.768595
chr15	89618900	0.785124
chr15	89634500	0.735537
chr15	89711300	0.768595
chr15	89750500	0.826446
chr15	89955200	0.909091
chr15	89959900	0.867769
chr15	90039700	0.793388
chr15	90365700	0.793388
chr15	90383100	0.752066
chr15	90441500	0.809917
chr15	90574800	0.826446
chr15	90605200	0.826446
chr15	90673600	0.793388
chr15	90686400	0.801653
chr15	90737100	0.785124
chr15	90809200	0.760331
chr15	90880700	0.735537
chr15	90925900	0.900826
chr15	90931900	0.818182
chr15	91097800	0.752066
chr15	91101000	0.826446
chr15	91373900	0.851240
chr15	91429600	0.743802
chr15	91538000	0.801653
chr15	92401500	0.785124
chr15	92614800	0.892562
chr15	93388400	0.834711
chr15	93605500	0.785124
chr15	93722400	0.809917
chr15	94476200	0.785124
chr15	94803300	0.801653
chr15	95128400	0.752066
chr15	96764000	0.809917
chr15	96898700	0.735537
chr15	99322900	0.909091
chr15	99402600	0.851240
chr15	99417400	0.851240
chr15	99432500	0.826446
chr15	99525500	0.785124
chr15	99650600	0.785124
chr15	99670300	0.859504
chr15	99675800	0.834711
chr15	99818000	0.785124
chr15	99864600	0.768595
chr15	99907000	0.735537
chr15	99910000	0.793388
chr15	99974100	0.826446

chr15	100347000	0.933884
chr15	100583500	0.859504
chr15	100596200	0.826446
chr15	100731500	0.785124
chr15	100894500	0.752066
chr15	101001900	0.785124
chr15	101233900	0.793388
chr15	101326400	0.818182
chr15	101520900	0.842975
chr15	101629400	0.851240
chr15	101662500	0.801653
chr15	101692200	0.834711
chr15	101697200	0.851240
chr15	101743800	0.743802
chr15	101785500	0.818182
chr15	101835200	0.752066
chr15	101932600	0.876033
chr15	102041400	0.809917
chr15	102192800	0.793388
chr15	102285900	0.842975
chr15	102308000	0.760331
chr16	188600	0.735537
chr16	214700	0.991736
chr16	231700	0.867769
chr16	284800	0.785124
chr16	303000	0.900826
chr16	375200	0.793388
chr16	420900	0.809917
chr16	545100	0.776860
chr16	617100	0.942149
chr16	686600	0.752066
chr16	692700	0.752066
chr16	739000	0.735537
chr16	771000	0.785124
chr16	857600	0.933884
chr16	861000	0.752066
chr16	928000	0.752066
chr16	1342700	0.809917
chr16	1347300	0.760331
chr16	1351400	0.743802
chr16	1363600	0.801653
chr16	1394000	0.785124
chr16	1429200	0.950413
chr16	1471400	0.826446
chr16	1525400	0.917355
chr16	1543400	0.793388
chr16	1594100	0.834711
chr16	1731200	0.793388
chr16	1764300	0.933884
chr16	1927400	0.851240
chr16	2021700	0.776860
chr16	2031600	0.793388
chr16	2054600	0.826446
chr16	2201700	0.743802

chr16	2255500	0.809917
chr16	2318100	0.760331
chr16	2345500	0.809917
chr16	2537400	0.842975
chr16	2560100	0.942149
chr16	2583000	0.834711
chr16	2659300	0.785124
chr16	2721600	0.760331
chr16	2816800	0.735537
chr16	2827800	0.851240
chr16	2918200	0.983471
chr16	3006800	0.826446
chr16	3009600	0.826446
chr16	3030800	0.900826
chr16	3058500	0.900826
chr16	3083200	0.876033
chr16	3101400	0.867769
chr16	3104300	0.793388
chr16	3118700	0.793388
chr16	3222900	0.867769
chr16	3265700	0.818182
chr16	3285600	0.842975
chr16	3314300	0.867769
chr16	3333600	0.818182
chr16	3355700	0.950413
chr16	3687500	0.933884
chr16	4021900	0.884298
chr16	4069700	0.752066
chr16	4250500	0.743802
chr16	4303700	0.900826
chr16	4359800	0.834711
chr16	4375700	0.768595
chr16	4378300	0.818182
chr16	4415800	0.760331
chr16	4577500	0.785124
chr16	4617100	0.851240
chr16	4620500	0.776860
chr16	4666800	0.925620
chr16	4998000	0.834711
chr16	5026500	0.884298
chr16	5086900	0.752066
chr16	5092000	0.818182
chr16	5167600	0.892562
chr16	5203200	0.818182
chr16	6599300	0.966942
chr16	8774500	0.793388
chr16	9025700	0.851240
chr16	9046100	0.760331
chr16	9102800	0.768595
chr16	9171500	0.752066
chr16	9201100	0.752066
chr16	9222000	0.900826
chr16	9651900	0.760331
chr16	9924200	0.768595

chr16	10003900	0.776860
chr16	10008600	0.867769
chr16	10019900	0.834711
chr16	10023500	0.834711
chr16	10027400	0.752066
chr16	10030800	0.801653
chr16	10060400	0.809917
chr16	10121000	0.892562
chr16	10337400	0.752066
chr16	10351100	0.768595
chr16	10360800	0.809917
chr16	10369700	0.884298
chr16	10480200	0.867769
chr16	10609400	0.851240
chr16	10613200	0.809917
chr16	10646700	0.809917
chr16	10691200	0.925620
chr16	10837900	0.876033
chr16	10871200	0.752066
chr16	10881700	0.785124
chr16	10941100	0.752066
chr16	10977700	0.776860
chr16	11006800	0.743802
chr16	11224400	0.776860
chr16	11268000	0.876033
chr16	11307000	0.768595
chr16	11327200	0.867769
chr16	11332400	0.760331
chr16	11340600	0.842975
chr16	11410800	0.776860
chr16	11443700	0.876033
chr16	11454100	0.818182
chr16	11457700	0.793388
chr16	11464500	0.760331
chr16	11517800	0.925620
chr16	11691900	0.752066
chr16	11703300	0.933884
chr16	11716200	0.752066
chr16	11723600	0.793388
chr16	11735700	0.826446
chr16	11764200	0.809917
chr16	11785400	0.826446
chr16	11904100	0.801653
chr16	11945500	0.768595
chr16	11991500	0.743802
chr16	12044300	0.735537
chr16	12054500	0.834711
chr16	12126300	0.752066
chr16	12173900	0.876033
chr16	12181700	0.743802
chr16	12417000	0.842975
chr16	12562700	0.752066
chr16	13392900	0.760331
chr16	13410500	0.785124

chr16	13466100	0.752066
chr16	13929900	0.809917
chr16	14054100	0.752066
chr16	14064700	0.809917
chr16	14097000	0.809917
chr16	14101700	0.752066
chr16	14271200	0.925620
chr16	14300000	0.735537
chr16	14444800	0.925620
chr16	14472700	0.743802
chr16	14493700	0.909091
chr16	14581200	0.785124
chr16	14587100	0.859504
chr16	14597500	0.876033
chr16	14727100	0.768595
chr16	15021700	0.851240
chr16	15188200	0.909091
chr16	15278100	0.785124
chr16	15419400	0.776860
chr16	15684900	0.876033
chr16	15929900	0.785124
chr16	15949700	0.818182
chr16	15982700	0.752066
chr16	16092300	0.785124
chr16	16293700	0.776860
chr16	16301800	0.842975
chr16	16525200	0.776860
chr16	16816200	0.768595
chr16	16819800	0.900826
chr16	17023000	0.876033
chr16	17039000	0.752066
chr16	17083600	0.867769
chr16	17087500	0.834711
chr16	17096600	0.801653
chr16	17107600	0.909091
chr16	17148200	0.851240
chr16	17157400	0.876033
chr16	17165000	0.776860
chr16	17198700	0.743802
chr16	17267100	0.743802
chr16	17346500	0.818182
chr16	17353100	0.933884
chr16	17367100	0.876033
chr16	17377000	0.768595
chr16	17397400	0.809917
chr16	17400000	0.735537
chr16	17411100	0.785124
chr16	17421200	0.900826
chr16	17453200	0.818182
chr16	17459300	0.793388
chr16	17475100	0.876033
chr16	17545900	0.909091
chr16	17554500	0.809917
chr16	17570900	0.884298

chr16	17619000	0.834711
chr16	17621600	0.743802
chr16	17624300	0.867769
chr16	17626900	0.834711
chr16	17629200	0.768595
chr16	17649200	0.892562
chr16	18033400	0.917355
chr16	18190000	0.752066
chr16	18374700	0.826446
chr16	18603900	0.809917
chr16	18766600	0.826446
chr16	18771400	0.760331
chr16	18941900	0.818182
chr16	18958100	0.909091
chr16	19079200	0.925620
chr16	19118000	0.818182
chr16	19130400	0.809917
chr16	19139100	0.809917
chr16	19144800	0.776860
chr16	19222600	0.884298
chr16	19391800	0.900826
chr16	19422400	0.785124
chr16	19449100	0.818182
chr16	19567300	0.743802
chr16	20774800	0.826446
chr16	20877700	0.851240
chr16	20883800	0.768595
chr16	20912700	0.743802
chr16	20941000	0.925620
chr16	21200400	0.900826
chr16	21357200	0.909091
chr16	21500100	0.785124
chr16	21518700	0.809917
chr16	21521000	0.818182
chr16	21577600	0.826446
chr16	21616500	0.826446
chr16	21715300	0.884298
chr16	21946700	0.768595
chr16	21958900	0.842975
chr16	21962000	0.735537
chr16	22023200	0.818182
chr16	22229600	0.760331
chr16	22309100	0.867769
chr16	22312300	0.925620
chr16	22369300	0.859504
chr16	22527600	0.909091
chr16	22584900	0.859504
chr16	22676500	0.900826
chr16	23300400	0.760331
chr16	23362800	0.809917
chr16	23512800	0.834711
chr16	23521700	0.793388
chr16	23815100	0.942149
chr16	23818700	0.760331

chr16	23838200	0.892562
chr16	23882600	0.776860
chr16	23999000	0.809917
chr16	24013300	0.735537
chr16	24408700	0.884298
chr16	24537300	0.925620
chr16	25003900	0.818182
chr16	25006700	0.809917
chr16	25106800	0.834711
chr16	25269500	0.752066
chr16	25978200	0.801653
chr16	27181600	0.975207
chr16	27190900	0.818182
chr16	27246800	0.752066
chr16	27274400	0.900826
chr16	27279900	0.876033
chr16	27294800	0.876033
chr16	27311200	0.925620
chr16	27337900	0.743802
chr16	27379300	0.809917
chr16	27650900	0.785124
chr16	27864800	0.884298
chr16	28321800	0.801653
chr16	28371900	0.752066
chr16	28485700	0.876033
chr16	28506100	0.793388
chr16	28543800	0.785124
chr16	28554300	0.785124
chr16	28565200	0.793388
chr16	28611300	0.859504
chr16	28614700	0.876033
chr16	28624200	0.925620
chr16	28627000	0.818182
chr16	28652200	0.768595
chr16	28766300	0.752066
chr16	28937100	0.917355
chr16	29086300	0.867769
chr16	29296100	0.884298
chr16	29302800	0.826446
chr16	29461200	0.743802
chr16	29480100	0.826446
chr16	29514800	0.867769
chr16	29595900	0.785124
chr16	29611600	0.743802
chr16	29614200	0.818182
chr16	29653100	0.743802
chr16	29661700	0.743802
chr16	29722400	0.776860
chr16	29726400	0.735537
chr16	29765900	0.743802
chr16	29830200	0.743802
chr16	29874900	0.966942
chr16	29888800	0.752066
chr16	30087500	0.752066

chr16	30107800	0.826446
chr16	30134800	0.809917
chr16	30138300	0.884298
chr16	30143500	0.768595
chr16	30146800	0.842975
chr16	30178500	0.752066
chr16	30205200	0.743802
chr16	30346600	0.900826
chr16	30363400	0.785124
chr16	30366800	0.809917
chr16	30460000	0.752066
chr16	30486600	0.818182
chr16	30539300	0.876033
chr16	30569700	0.826446
chr16	30572900	0.851240
chr16	30583100	0.851240
chr16	30594200	0.752066
chr16	30621500	0.925620
chr16	30627100	0.743802
chr16	30640800	0.793388
chr16	30664700	0.743802
chr16	30759200	0.743802
chr16	30909100	0.752066
chr16	30969100	0.768595
chr16	31004800	0.752066
chr16	31008200	0.876033
chr16	31107300	0.760331
chr16	31229300	0.884298
chr16	31255900	0.768595
chr16	31277200	0.950413
chr16	31298900	0.735537
chr16	31312300	0.859504
chr16	31339900	0.942149
chr16	31345500	0.818182
chr16	31358800	0.818182
chr16	31369400	0.735537
chr16	31376300	0.760331
chr16	31378600	0.776860
chr16	31427000	0.834711
chr16	31432200	0.793388
chr16	31434500	0.900826
chr16	31442400	0.735537
chr16	32367400	0.735537
chr16	33404400	0.768595
chr16	33412600	0.917355
chr16	33429000	0.801653
chr16	33432200	0.768595
chr16	46663900	0.743802
chr16	46783500	0.735537
chr16	46788200	0.735537
chr16	46885200	0.826446
chr16	46920500	0.826446
chr16	46943400	0.760331
chr16	47010700	0.909091

chr16	47069300	0.743802
chr16	47352300	0.768595
chr16	47426300	0.809917
chr16	48089100	0.801653
chr16	48126000	0.884298
chr16	48192100	0.900826
chr16	48199300	0.743802
chr16	48223400	0.826446
chr16	48411700	0.760331
chr16	48419500	0.900826
chr16	48517700	0.859504
chr16	48632400	0.793388
chr16	48868600	0.735537
chr16	48935700	0.884298
chr16	49316700	0.842975
chr16	49434000	0.826446
chr16	49523700	0.735537
chr16	49537300	0.768595
chr16	49563600	0.884298
chr16	49625500	0.842975
chr16	49835100	0.776860
chr16	50156200	0.826446
chr16	50300700	0.768595
chr16	50467600	0.743802
chr16	50491200	0.785124
chr16	50497000	0.743802
chr16	50602200	0.834711
chr16	50618700	0.867769
chr16	50661300	0.785124
chr16	50724300	0.785124
chr16	50781300	0.966942
chr16	50843700	0.743802
chr16	50875500	0.793388
chr16	51118200	0.735537
chr16	53058100	0.735537
chr16	53128000	0.834711
chr16	53146300	0.776860
chr16	53468900	0.826446
chr16	53553300	0.892562
chr16	53699200	0.743802
chr16	53780100	0.826446
chr16	55479200	0.743802
chr16	55868400	0.859504
chr16	56198300	0.867769
chr16	56237000	0.933884
chr16	56628100	0.859504
chr16	56750700	0.876033
chr16	56892500	0.752066
chr16	56896700	0.867769
chr16	56905200	0.867769
chr16	56923000	0.776860
chr16	56954700	0.892562
chr16	57008800	0.826446
chr16	57013100	0.793388

chr16	57154400	0.900826
chr16	57162800	0.793388
chr16	57279100	0.760331
chr16	57334100	0.842975
chr16	57349000	0.851240
chr16	57377300	0.925620
chr16	57384000	0.842975
chr16	57435200	0.842975
chr16	57448300	0.826446
chr16	57453300	0.917355
chr16	57510300	0.768595
chr16	57575100	0.793388
chr16	57634500	0.760331
chr16	57729300	0.752066
chr16	57759700	0.760331
chr16	57842500	0.909091
chr16	57845200	0.752066
chr16	57910400	0.859504
chr16	58190000	0.809917
chr16	58371300	0.826446
chr16	58460300	0.834711
chr16	58529500	0.867769
chr16	58532100	0.801653
chr16	58554400	0.842975
chr16	58620200	0.950413
chr16	58664300	0.735537
chr16	58790200	0.752066
chr16	58916900	0.933884
chr16	62673700	0.776860
chr16	62676200	0.752066
chr16	66349600	0.785124
chr16	66452000	0.735537
chr16	66542500	0.900826
chr16	66615400	0.768595
chr16	66624700	0.752066
chr16	66629000	0.809917
chr16	66641000	0.768595
chr16	66685100	0.752066
chr16	66785700	0.900826
chr16	66912500	0.760331
chr16	66959800	0.785124
chr16	66970900	0.876033
chr16	66996000	0.752066
chr16	67190600	0.818182
chr16	67342500	0.859504
chr16	67355300	0.851240
chr16	67399400	0.892562
chr16	67447000	0.818182
chr16	67472400	0.826446
chr16	67523500	0.900826
chr16	67552600	0.842975
chr16	67555200	0.950413
chr16	67679200	0.958678
chr16	67876300	0.752066

chr16	67907100	0.743802
chr16	67973800	0.735537
chr16	68018700	0.909091
chr16	68034200	0.826446
chr16	68072100	0.876033
chr16	68115000	0.743802
chr16	68128400	0.859504
chr16	68154400	0.801653
chr16	68160000	0.760331
chr16	68310300	0.942149
chr16	68341700	0.743802
chr16	68362200	0.760331
chr16	68366400	0.785124
chr16	68399200	0.801653
chr16	68416400	0.785124
chr16	68419000	0.876033
chr16	68430700	0.752066
chr16	68525300	0.752066
chr16	68555000	0.826446
chr16	68679300	0.876033
chr16	68739000	0.743802
chr16	68775000	0.809917
chr16	68777400	0.785124
chr16	68795300	0.743802
chr16	69091700	0.925620
chr16	69123600	0.876033
chr16	69347700	0.925620
chr16	69364700	0.942149
chr16	69416800	0.768595
chr16	69514700	0.752066
chr16	69541200	0.743802
chr16	69555200	0.867769
chr16	69589200	0.735537
chr16	69647200	0.785124
chr16	69760700	0.859504
chr16	69801900	0.859504
chr16	69807500	0.900826
chr16	69858200	0.966942
chr16	70333100	0.859504
chr16	70338900	0.768595
chr16	70488300	0.909091
chr16	70688100	0.809917
chr16	70963700	0.884298
chr16	71177700	0.842975
chr16	71191600	0.876033
chr16	71323600	0.785124
chr16	71446900	0.793388
chr16	71511600	0.752066
chr16	71518200	0.900826
chr16	71645000	0.809917
chr16	71746500	0.884298
chr16	71915300	0.834711
chr16	71929800	0.735537
chr16	71940100	0.809917

chr16	71991000	0.809917
chr16	71993900	0.826446
chr16	72644200	0.892562
chr16	72684000	0.752066
chr16	72693400	0.776860
chr16	72868700	0.752066
chr16	72888100	0.752066
chr16	73075000	0.842975
chr16	74498600	0.752066
chr16	74599700	0.752066
chr16	74698600	0.768595
chr16	74734800	0.752066
chr16	74801700	0.826446
chr16	74880800	0.809917
chr16	75077300	0.876033
chr16	75099300	0.809917
chr16	75109300	0.760331
chr16	75182600	0.768595
chr16	75235800	0.958678
chr16	75240800	0.876033
chr16	75358700	0.743802
chr16	75368400	0.909091
chr16	75412000	0.834711
chr16	75414200	0.867769
chr16	75467700	0.809917
chr16	75494800	0.760331
chr16	75512200	0.760331
chr16	75526900	0.793388
chr16	75656900	0.793388
chr16	75808600	0.909091
chr16	75833900	0.818182
chr16	77166200	0.867769
chr16	77225400	0.768595
chr16	77362100	0.884298
chr16	77902700	0.909091
chr16	77997900	0.842975
chr16	78037600	0.735537
chr16	78223700	0.743802
chr16	78345100	0.809917
chr16	78509100	0.768595
chr16	78605100	0.818182
chr16	78763900	0.867769
chr16	78776800	0.909091
chr16	78784900	0.809917
chr16	78795800	0.801653
chr16	78991800	0.950413
chr16	79047500	0.834711
chr16	79116300	0.752066
chr16	79124500	0.826446
chr16	79127700	0.859504
chr16	79222500	0.785124
chr16	79297600	0.942149
chr16	79306700	0.760331
chr16	79350400	0.892562

chr16	79485600	0.818182
chr16	79640300	0.867769
chr16	79914800	0.900826
chr16	80061800	0.809917
chr16	80500800	0.809917
chr16	80561100	0.735537
chr16	80618700	0.768595
chr16	80626000	0.752066
chr16	80661500	0.818182
chr16	80691100	0.892562
chr16	80715100	0.776860
chr16	80729200	0.735537
chr16	81031200	0.834711
chr16	81130900	0.776860
chr16	81486800	0.768595
chr16	81539300	0.842975
chr16	81555200	0.752066
chr16	81792400	0.785124
chr16	81844700	0.752066
chr16	81898500	0.752066
chr16	82675100	0.842975
chr16	82769600	0.793388
chr16	82835500	0.834711
chr16	83534200	0.809917
chr16	83826500	0.752066
chr16	83987300	0.801653
chr16	84200800	0.735537
chr16	84209700	0.834711
chr16	84566500	0.826446
chr16	84645800	0.735537
chr16	84653300	0.826446
chr16	84662400	0.752066
chr16	84675100	0.876033
chr16	84682400	0.793388
chr16	84698500	0.743802
chr16	84706800	0.768595
chr16	84840400	0.801653
chr16	84845000	0.909091
chr16	84927700	0.876033
chr16	84983000	0.793388
chr16	85045500	0.859504
chr16	85051000	0.867769
chr16	85092400	0.793388
chr16	85139600	0.785124
chr16	85187500	0.851240
chr16	85191900	0.785124
chr16	85234800	0.752066
chr16	85264000	0.743802
chr16	85339700	0.743802
chr16	85482900	0.851240
chr16	85514700	0.793388
chr16	85532300	0.925620
chr16	85714800	0.942149
chr16	85752200	0.859504

chr16	85769700	0.842975
chr16	85781200	0.876033
chr16	85836000	0.768595
chr16	85863900	0.752066
chr16	85866800	0.826446
chr16	85887900	0.884298
chr16	85890600	0.834711
chr16	85904500	0.785124
chr16	85917100	0.752066
chr16	85945900	0.809917
chr16	85950400	0.760331
chr16	85972800	0.809917
chr16	86016600	0.735537
chr16	86035900	0.859504
chr16	86803100	0.752066
chr16	87094500	0.818182
chr16	87236800	0.735537
chr16	87270100	0.917355
chr16	87282800	0.785124
chr16	87441700	0.776860
chr16	87498800	0.793388
chr16	87636200	1.000000
chr16	87715900	0.752066
chr16	87800000	0.826446
chr16	87808600	0.785124
chr16	87855200	0.760331
chr16	87887300	0.909091
chr16	87889900	0.834711
chr16	87895100	0.768595
chr16	87908800	0.785124
chr16	87960600	0.867769
chr16	87982700	0.851240
chr16	88299300	0.867769
chr16	88509900	0.867769
chr16	88515800	0.743802
chr16	88600800	0.867769
chr16	88616900	0.752066
chr16	88658300	0.950413
chr16	88664200	0.735537
chr16	88706000	0.785124
chr16	88731900	0.760331
chr16	88758800	0.917355
chr16	88851700	0.793388
chr16	88856900	0.752066
chr16	88860500	0.842975
chr16	88866800	0.834711
chr16	88954700	0.966942
chr16	89003600	0.743802
chr16	89066200	0.983471
chr16	89071200	0.785124
chr16	89087400	0.925620
chr16	89098700	0.752066
chr16	89105100	0.743802
chr16	89131800	0.834711

chr16	89134700	0.743802
chr16	89166000	0.785124
chr16	89237500	0.876033
chr16	89307600	0.909091
chr16	89435200	0.809917
chr16	89441900	0.752066
chr16	89446500	0.752066
chr16	89502900	0.809917
chr16	89522900	0.851240
chr16	89557500	0.735537
chr16	89638900	0.809917
chr16	89753200	0.892562
chr16	89831800	0.743802
chr16	89883000	0.818182
chr16	89904300	0.842975
chr16	89911900	0.917355
chr16	89985200	0.735537
chr16	90011700	0.818182
chr16	90014100	0.776860
chr16	90039300	0.818182
chr16	90062000	0.900826
chr16	90080000	0.743802
chr17	266600	0.760331
chr17	565500	0.768595
chr17	783700	0.867769
chr17	900200	0.884298
chr17	1026800	0.801653
chr17	1120100	0.925620
chr17	1132800	0.776860
chr17	1267900	0.917355
chr17	1278100	0.834711
chr17	1292500	0.801653
chr17	1303400	0.752066
chr17	1378300	0.933884
chr17	1470200	0.942149
chr17	1481000	0.752066
chr17	1535000	0.842975
chr17	1554400	0.760331
chr17	1598800	0.892562
chr17	1655800	0.826446
chr17	1674000	0.818182
chr17	1724200	0.851240
chr17	1800300	0.776860
chr17	1812200	0.826446
chr17	1945500	0.884298
chr17	2016900	0.776860
chr17	2199500	0.752066
chr17	2206900	0.768595
chr17	2296900	0.735537
chr17	2301400	0.760331
chr17	2394600	0.809917
chr17	2412400	0.793388
chr17	2461800	0.752066
chr17	2527800	0.793388

chr17	2595100	0.768595
chr17	2697200	0.768595
chr17	2740800	0.768595
chr17	2834700	0.851240
chr17	2859000	0.909091
chr17	3540000	0.925620
chr17	3635900	0.834711
chr17	3675100	0.785124
chr17	3774400	0.818182
chr17	3790800	0.785124
chr17	3870900	0.768595
chr17	3947600	0.760331
chr17	4047300	0.859504
chr17	4111100	0.826446
chr17	4202800	0.785124
chr17	4249400	0.752066
chr17	4264300	0.900826
chr17	4285500	0.801653
chr17	4324500	0.859504
chr17	4340500	0.834711
chr17	4384900	0.768595
chr17	4396500	0.851240
chr17	4400900	0.735537
chr17	4413400	0.909091
chr17	4422400	0.752066
chr17	4464200	0.809917
chr17	4469300	0.884298
chr17	4477400	0.809917
chr17	4744500	0.760331
chr17	4807400	0.785124
chr17	5316200	0.809917
chr17	5390300	0.785124
chr17	5490400	0.818182
chr17	5515700	0.768595
chr17	5561500	0.793388
chr17	5679600	0.859504
chr17	6013800	0.867769
chr17	6353600	0.760331
chr17	6356500	0.818182
chr17	6364900	0.834711
chr17	6400000	0.876033
chr17	6449800	0.826446
chr17	6459700	0.818182
chr17	6544500	0.826446
chr17	6581300	0.785124
chr17	6593200	0.851240
chr17	7013400	0.818182
chr17	7081500	0.842975
chr17	7146200	0.851240
chr17	7155800	0.826446
chr17	7255700	0.884298
chr17	7461600	0.735537
chr17	7481100	0.818182
chr17	7483300	0.876033

chr17	7487300	0.826446
chr17	7559200	0.768595
chr17	7685100	0.793388
chr17	7743000	0.743802
chr17	7761100	0.785124
chr17	7785500	0.743802
chr17	7835800	0.826446
chr17	7901000	0.876033
chr17	7960400	0.876033
chr17	7976800	0.818182
chr17	7994500	0.735537
chr17	8042800	0.900826
chr17	8077300	0.942149
chr17	8099700	0.925620
chr17	8103400	0.785124
chr17	8111200	0.801653
chr17	8114100	0.752066
chr17	8192000	0.793388
chr17	8241800	0.876033
chr17	8313900	0.826446
chr17	8465800	0.851240
chr17	8829100	0.851240
chr17	8838500	0.834711
chr17	8889100	0.785124
chr17	8927500	0.752066
chr17	9108700	0.768595
chr17	9204600	0.793388
chr17	9293300	0.900826
chr17	9339300	0.752066
chr17	9957400	0.752066
chr17	9978000	0.917355
chr17	10018400	0.793388
chr17	10021400	0.776860
chr17	10023700	0.793388
chr17	10026200	0.917355
chr17	10075900	0.735537
chr17	10173600	0.851240
chr17	10196400	0.859504
chr17	10227600	0.809917
chr17	10537400	0.826446
chr17	10556800	0.892562
chr17	11924400	0.834711
chr17	12199900	0.809917
chr17	12763400	0.793388
chr17	12928200	0.735537
chr17	13948400	0.834711
chr17	14011000	0.760331
chr17	14096400	0.768595
chr17	14100500	0.760331
chr17	14222500	0.768595
chr17	14263000	0.851240
chr17	15406900	0.925620
chr17	15733300	0.776860
chr17	15756700	0.859504

chr17	15769800	0.752066
chr17	15863100	0.793388
chr17	15884700	0.752066
chr17	15980700	0.925620
chr17	16171300	0.867769
chr17	16284700	0.785124
chr17	16428400	0.859504
chr17	16613000	0.900826
chr17	16655100	0.933884
chr17	16790000	0.826446
chr17	16819700	0.876033
chr17	16884600	0.743802
chr17	16905200	0.809917
chr17	16914900	0.801653
chr17	16920300	0.851240
chr17	17028900	0.760331
chr17	17096400	0.793388
chr17	17140700	0.917355
chr17	17184300	0.776860
chr17	17209100	0.876033
chr17	17291400	0.752066
chr17	17295600	0.818182
chr17	17580000	0.884298
chr17	17638600	0.826446
chr17	17696200	0.917355
chr17	17712800	0.900826
chr17	17716000	0.818182
chr17	17743800	0.752066
chr17	17802400	0.735537
chr17	17936400	0.752066
chr17	17945900	0.851240
chr17	18077900	0.752066
chr17	18080100	0.834711
chr17	18124800	0.735537
chr17	18161800	0.876033
chr17	18218700	0.842975
chr17	18275400	0.818182
chr17	18301500	0.818182
chr17	18312600	0.785124
chr17	18424900	0.760331
chr17	18467800	0.801653
chr17	18473000	0.809917
chr17	18654100	0.958678
chr17	18684700	0.876033
chr17	18699100	0.760331
chr17	18743200	0.818182
chr17	18764300	0.735537
chr17	18897300	0.793388
chr17	18962000	0.793388
chr17	18967800	0.925620
chr17	19005000	0.760331
chr17	19016200	0.917355
chr17	19018800	0.735537
chr17	19090900	0.834711

chr17	19093900	0.925620
chr17	19130600	0.752066
chr17	19172700	0.760331
chr17	19187100	0.743802
chr17	19206600	0.768595
chr17	19211000	0.752066
chr17	19276300	0.801653
chr17	19399500	0.776860
chr17	19417700	0.735537
chr17	19441700	0.785124
chr17	19649100	0.776860
chr17	19659700	0.743802
chr17	19936600	0.809917
chr17	19969600	0.785124
chr17	19988200	0.818182
chr17	20285300	0.867769
chr17	20354700	0.826446
chr17	20445800	0.752066
chr17	20558000	0.818182
chr17	20569300	0.776860
chr17	20574200	0.851240
chr17	20579400	0.785124
chr17	20602100	0.818182
chr17	20870600	0.785124
chr17	20935800	0.752066
chr17	20947300	0.735537
chr17	20967900	0.867769
chr17	21191600	0.743802
chr17	21229100	0.735537
chr17	21247800	0.859504
chr17	21253400	0.785124
chr17	21342100	0.760331
chr17	21549800	0.776860
chr17	21902900	0.859504
chr17	25566400	0.801653
chr17	25572600	0.818182
chr17	25669000	0.826446
chr17	25673800	0.793388
chr17	25741300	0.925620
chr17	25790800	0.768595
chr17	25850800	0.735537
chr17	25858600	0.735537
chr17	25866900	0.859504
chr17	25869500	0.818182
chr17	25874700	0.743802
chr17	26073900	0.760331
chr17	26133000	0.834711
chr17	26145800	0.760331
chr17	26167400	0.785124
chr17	26173000	0.826446
chr17	26267100	0.818182
chr17	26302700	0.793388
chr17	26305000	0.842975
chr17	26313000	0.876033

chr17	26376200	0.752066
chr17	26380900	0.884298
chr17	26406400	0.752066
chr17	26554800	0.768595
chr17	26601900	0.826446
chr17	26605700	0.735537
chr17	26620600	0.752066
chr17	26742600	0.785124
chr17	26750500	0.752066
chr17	26794300	0.776860
chr17	26814100	0.818182
chr17	26828700	0.900826
chr17	26836900	0.760331
chr17	26904200	0.735537
chr17	26992300	0.809917
chr17	27062200	0.834711
chr17	27161500	0.859504
chr17	27198300	0.925620
chr17	27203500	0.735537
chr17	27261200	0.752066
chr17	27279200	0.925620
chr17	27310000	0.752066
chr17	27346600	0.776860
chr17	27477300	0.743802
chr17	27546800	0.743802
chr17	27621700	0.925620
chr17	27760800	0.942149
chr17	27887900	0.826446
chr17	27913600	0.867769
chr17	27945300	0.735537
chr17	28045800	0.834711
chr17	28054100	0.793388
chr17	28076300	0.768595
chr17	28088100	0.760331
chr17	28251700	0.760331
chr17	28259400	0.752066
chr17	28358800	0.735537
chr17	28428900	0.776860
chr17	28441000	0.859504
chr17	28546400	0.818182
chr17	28565700	0.760331
chr17	28628300	0.884298
chr17	28658300	0.892562
chr17	28672600	0.884298
chr17	28804600	0.851240
chr17	28878000	0.743802
chr17	28885600	0.743802
chr17	28983200	0.735537
chr17	29033600	0.809917
chr17	29059200	0.809917
chr17	29159300	0.876033
chr17	29254000	0.884298
chr17	29266300	0.917355
chr17	29280200	0.743802

chr17	29779300	0.876033
chr17	29838100	0.776860
chr17	29865900	0.842975
chr17	29909400	0.834711
chr17	29924300	0.834711
chr17	29988000	0.958678
chr17	30007500	0.942149
chr17	30162000	0.909091
chr17	30240700	0.851240
chr17	30251200	0.768595
chr17	30390100	0.752066
chr17	30398900	0.818182
chr17	30454700	0.859504
chr17	30584300	0.760331
chr17	30603500	0.851240
chr17	30620500	0.884298
chr17	30651200	0.776860
chr17	30984700	0.776860
chr17	31138400	0.735537
chr17	31146900	0.867769
chr17	31153300	0.760331
chr17	31198600	0.867769
chr17	31209800	0.752066
chr17	31226000	0.809917
chr17	31242400	0.768595
chr17	31786800	0.768595
chr17	31888800	0.818182
chr17	32299800	0.801653
chr17	32307400	0.900826
chr17	32367200	0.950413
chr17	32454000	0.818182
chr17	32529000	0.826446
chr17	32570100	0.776860
chr17	32579700	0.768595
chr17	32634500	0.842975
chr17	32698400	0.892562
chr17	32706900	0.851240
chr17	32709900	0.801653
chr17	33579800	0.859504
chr17	33616200	0.760331
chr17	33669800	0.809917
chr17	33695400	0.793388
chr17	33760100	0.760331
chr17	33820800	0.826446
chr17	33847100	0.900826
chr17	34036100	0.743802
chr17	34203900	0.768595
chr17	34329100	0.834711
chr17	34361400	0.851240
chr17	34400200	0.768595
chr17	34402500	0.793388
chr17	34409700	0.942149
chr17	34427500	0.892562
chr17	34476900	0.917355

chr17	34481800	0.842975
chr17	34537500	0.735537
chr17	34539900	0.809917
chr17	34641200	0.809917
chr17	34839100	0.876033
chr17	34905600	0.776860
chr17	35046300	0.925620
chr17	35120500	0.909091
chr17	35285900	0.867769
chr17	35291800	0.735537
chr17	35294600	0.768595
chr17	35306500	0.826446
chr17	35746600	0.735537
chr17	35843800	0.793388
chr17	36052400	0.793388
chr17	36078300	0.793388
chr17	36111400	0.867769
chr17	36121500	0.851240
chr17	36136000	0.809917
chr17	36151300	0.917355
chr17	36189700	0.760331
chr17	36194100	0.743802
chr17	36244500	0.768595
chr17	36453000	0.809917
chr17	36483500	0.743802
chr17	36600600	0.834711
chr17	36642200	0.917355
chr17	36713300	0.842975
chr17	36728300	0.842975
chr17	36748700	0.735537
chr17	36765200	0.818182
chr17	36769200	0.859504
chr17	36815400	0.785124
chr17	36828400	0.752066
chr17	36956300	0.925620
chr17	36982000	0.876033
chr17	37010300	0.776860
chr17	37082900	0.752066
chr17	37099400	0.867769
chr17	37275400	0.793388
chr17	37323700	0.884298
chr17	37366500	0.884298
chr17	37401000	0.793388
chr17	37762400	0.809917
chr17	37779200	0.818182
chr17	37793500	0.743802
chr17	37837300	0.752066
chr17	37886700	0.958678
chr17	37957100	0.842975
chr17	38221000	0.743802
chr17	38225500	0.867769
chr17	38310000	0.735537
chr17	38444200	0.743802
chr17	38472600	0.826446

chr17	38594100	0.842975
chr17	38636900	0.768595
chr17	38646500	0.768595
chr17	38689800	0.842975
chr17	38711600	0.842975
chr17	38723400	0.768595
chr17	38734200	0.826446
chr17	38739400	0.801653
chr17	38776400	0.735537
chr17	38779100	0.760331
chr17	38781600	0.793388
chr17	38804300	0.842975
chr17	39071300	0.735537
chr17	39333000	0.892562
chr17	39835000	0.909091
chr17	40075300	0.752066
chr17	40119100	0.876033
chr17	40268000	0.942149
chr17	40534800	0.760331
chr17	40555300	0.743802
chr17	40558700	0.876033
chr17	40567500	0.809917
chr17	40627300	0.752066
chr17	40676200	0.867769
chr17	40707200	0.834711
chr17	40719200	0.818182
chr17	40806300	0.768595
chr17	40809600	0.785124
chr17	40854900	0.743802
chr17	40894600	0.785124
chr17	40903300	0.752066
chr17	40988300	0.801653
chr17	41081100	0.785124
chr17	41159000	0.933884
chr17	41393300	0.966942
chr17	41439100	0.826446
chr17	41466200	0.834711
chr17	41488700	0.809917
chr17	41778900	0.776860
chr17	41846500	0.826446
chr17	42099700	0.760331
chr17	42215900	0.900826
chr17	42219500	0.809917
chr17	42278100	0.900826
chr17	42281100	0.768595
chr17	42324300	0.859504
chr17	42349800	0.743802
chr17	42358000	0.884298
chr17	42371300	0.851240
chr17	42409000	0.743802
chr17	42451600	0.834711
chr17	42601300	0.809917
chr17	42613900	0.735537
chr17	42625500	0.876033

chr17	42646200	0.785124
chr17	42845700	0.760331
chr17	42976800	0.917355
chr17	42997900	0.826446
chr17	43067500	0.793388
chr17	43128400	0.776860
chr17	43229900	0.752066
chr17	43233800	0.743802
chr17	43250300	0.933884
chr17	43297400	0.851240
chr17	43325900	0.834711
chr17	43369300	0.785124
chr17	43407300	0.834711
chr17	43449800	0.752066
chr17	43487800	0.768595
chr17	43507500	0.743802
chr17	43670000	0.801653
chr17	43674300	0.818182
chr17	44026600	0.752066
chr17	44217500	0.826446
chr17	44238000	0.760331
chr17	44251800	0.752066
chr17	44262300	0.842975
chr17	44342000	0.859504
chr17	44438800	0.785124
chr17	44512400	0.760331
chr17	44681000	0.735537
chr17	44730100	0.776860
chr17	44896700	0.760331
chr17	45040100	0.752066
chr17	45144600	0.950413
chr17	45277900	0.834711
chr17	45297100	0.785124
chr17	45317900	0.760331
chr17	45359100	0.809917
chr17	45735200	0.760331
chr17	45740000	0.776860
chr17	45781700	0.842975
chr17	45794800	0.776860
chr17	45809900	0.760331
chr17	45815300	0.818182
chr17	45942800	0.818182
chr17	45950600	0.743802
chr17	45960000	0.851240
chr17	45974000	0.809917
chr17	46067000	0.785124
chr17	46116000	0.735537
chr17	46221100	0.809917
chr17	46257800	0.743802
chr17	46372700	0.884298
chr17	46375400	0.760331
chr17	46507300	0.743802
chr17	46522400	0.809917
chr17	46531700	0.801653

chr17	46534800	0.776860
chr17	46555900	0.768595
chr17	46595500	0.768595
chr17	46667500	0.776860
chr17	46711100	0.785124
chr17	46719700	0.735537
chr17	46800900	0.826446
chr17	46834700	0.743802
chr17	46851900	0.933884
chr17	46869400	0.859504
chr17	46892900	0.785124
chr17	46919200	0.834711
chr17	46956100	0.826446
chr17	47109900	0.818182
chr17	47273900	0.776860
chr17	47406900	0.876033
chr17	47486200	0.752066
chr17	47564100	0.785124
chr17	47574900	0.809917
chr17	47610400	0.743802
chr17	47785400	0.909091
chr17	47808800	0.785124
chr17	48076300	0.793388
chr17	48205800	0.851240
chr17	48218300	0.884298
chr17	48222600	0.776860
chr17	48241500	0.768595
chr17	48248700	0.785124
chr17	48290600	0.743802
chr17	48299400	0.752066
chr17	48358100	0.842975
chr17	48382800	0.851240
chr17	48387000	0.909091
chr17	48450700	0.892562
chr17	48499200	0.958678
chr17	48530300	0.776860
chr17	48589900	0.752066
chr17	48715300	0.793388
chr17	48765600	0.793388
chr17	48786200	0.768595
chr17	48813900	0.818182
chr17	48841000	0.760331
chr17	48850000	0.752066
chr17	48929900	0.776860
chr17	48961800	0.834711
chr17	48997400	0.892562
chr17	49028200	0.851240
chr17	49107700	0.826446
chr17	49199000	0.818182
chr17	49210200	0.851240
chr17	49235000	0.735537
chr17	49244200	0.826446
chr17	53151000	0.776860
chr17	53250200	0.818182

chr17	53434000	0.818182
chr17	53522600	0.735537
chr17	53589700	0.834711
chr17	53602400	0.809917
chr17	53658400	0.818182
chr17	53820600	0.892562
chr17	53950100	0.801653
chr17	54098000	0.785124
chr17	54109500	0.876033
chr17	54502700	0.743802
chr17	54864900	0.925620
chr17	54958000	0.752066
chr17	54993600	0.818182
chr17	55038100	0.892562
chr17	55087400	0.867769
chr17	55131900	0.743802
chr17	55210900	0.950413
chr17	55213700	0.809917
chr17	55253900	0.735537
chr17	55402700	0.809917
chr17	55415900	0.909091
chr17	55485800	0.785124
chr17	55558200	0.743802
chr17	55770300	0.752066
chr17	55777400	0.776860
chr17	55822100	0.909091
chr17	55838600	0.867769
chr17	55866400	0.818182
chr17	55910900	0.760331
chr17	55938900	0.826446
chr17	55952300	0.818182
chr17	55955100	0.743802
chr17	55977700	0.768595
chr17	55980700	0.818182
chr17	55983500	0.760331
chr17	56019300	0.884298
chr17	56027200	0.958678
chr17	56403300	0.793388
chr17	56427300	0.785124
chr17	56494600	0.834711
chr17	56598400	0.785124
chr17	56655400	0.859504
chr17	56683200	0.752066
chr17	56709400	0.942149
chr17	56736700	0.950413
chr17	56744200	0.776860
chr17	56770200	0.785124
chr17	56838400	0.892562
chr17	57430300	0.834711
chr17	57441100	0.785124
chr17	57453700	0.735537
chr17	57490500	0.809917
chr17	57505400	0.760331
chr17	57516100	0.900826

chr17	57706300	0.776860
chr17	57710900	0.917355
chr17	57717900	0.752066
chr17	57721700	0.760331
chr17	57774400	0.867769
chr17	57807400	0.752066
chr17	57882800	0.859504
chr17	58114800	0.743802
chr17	58158300	0.760331
chr17	58184300	0.793388
chr17	58220700	0.809917
chr17	58228800	0.793388
chr17	58239200	0.818182
chr17	58393500	0.760331
chr17	58475600	0.826446
chr17	58808200	0.793388
chr17	59087900	0.933884
chr17	59216900	0.760331
chr17	59300000	0.768595
chr17	59308700	0.917355
chr17	59358000	0.884298
chr17	59396500	0.809917
chr17	59893800	0.752066
chr17	59940800	0.867769
chr17	59981900	0.768595
chr17	60143300	0.867769
chr17	60162800	0.752066
chr17	60169500	0.760331
chr17	60189800	0.768595
chr17	60197600	0.735537
chr17	60243100	0.735537
chr17	60630700	0.785124
chr17	60689200	0.834711
chr17	61045900	0.785124
chr17	61468100	0.900826
chr17	61542500	0.876033
chr17	61592900	0.917355
chr17	61780100	0.809917
chr17	61860100	0.735537
chr17	61921100	0.776860
chr17	61956100	0.826446
chr17	62103500	0.801653
chr17	62165300	0.785124
chr17	62181400	0.884298
chr17	62296100	0.809917
chr17	62350200	0.801653
chr17	62632300	0.768595
chr17	62710100	0.826446
chr17	62833400	0.867769
chr17	62915600	0.900826
chr17	62958300	0.884298
chr17	63096600	0.884298
chr17	63099900	0.768595
chr17	63139100	0.818182

chr17	63173000	0.801653
chr17	63225500	0.752066
chr17	63274200	0.809917
chr17	63359800	0.760331
chr17	63510600	0.735537
chr17	63554400	0.735537
chr17	63557600	0.909091
chr17	63561200	0.776860
chr17	63565500	0.768595
chr17	63832000	0.768595
chr17	64361100	0.735537
chr17	64438800	0.768595
chr17	65048400	0.818182
chr17	65079800	0.834711
chr17	65168300	0.760331
chr17	65254700	0.834711
chr17	65308100	0.884298
chr17	65355100	0.743802
chr17	65371100	0.752066
chr17	65379900	0.809917
chr17	65431300	0.768595
chr17	65457600	0.752066
chr17	65471400	0.760331
chr17	65489700	0.793388
chr17	65647800	0.818182
chr17	65714200	0.743802
chr17	65756500	0.768595
chr17	65781200	0.785124
chr17	65814400	0.801653
chr17	65834000	0.735537
chr17	66231500	0.876033
chr17	66255600	0.743802
chr17	66352900	0.884298
chr17	66905000	0.876033
chr17	67323700	0.900826
chr17	67363800	0.768595
chr17	67420800	0.876033
chr17	67454300	0.760331
chr17	67549800	0.917355
chr17	67571700	0.842975
chr17	68117000	0.876033
chr17	68132100	0.842975
chr17	68134900	0.826446
chr17	68527100	0.809917
chr17	69882500	0.859504
chr17	69966800	0.776860
chr17	69984200	0.801653
chr17	70030800	0.917355
chr17	70037700	0.809917
chr17	70041600	0.760331
chr17	70107500	0.884298
chr17	70166400	0.867769
chr17	70314900	0.809917
chr17	70320200	0.776860

chr17	70508900	0.818182
chr17	70594000	0.785124
chr17	70716500	0.752066
chr17	70863700	0.842975
chr17	71267800	0.867769
chr17	71287700	0.867769
chr17	71313700	0.818182
chr17	71453200	0.760331
chr17	71588400	0.818182
chr17	71707700	0.785124
chr17	71871300	0.809917
chr17	71979000	0.834711
chr17	72189800	0.834711
chr17	72200200	0.785124
chr17	72212500	0.809917
chr17	72365300	0.743802
chr17	72376300	0.867769
chr17	72414500	0.900826
chr17	72439700	0.743802
chr17	72599700	0.793388
chr17	72609300	0.752066
chr17	72628900	0.809917
chr17	72631800	0.743802
chr17	72638200	0.859504
chr17	72693800	0.785124
chr17	72735400	0.785124
chr17	72787500	0.768595
chr17	72869800	0.851240
chr17	73021400	0.801653
chr17	73064400	0.743802
chr17	73098600	0.859504
chr17	73108400	0.834711
chr17	73179000	0.809917
chr17	73201700	0.867769
chr17	73349300	0.842975
chr17	73359400	0.743802
chr17	73362800	0.768595
chr17	73369000	0.842975
chr17	73405100	0.867769
chr17	73483900	0.768595
chr17	73563300	0.768595
chr17	73567400	0.942149
chr17	73629900	0.785124
chr17	73642300	0.760331
chr17	73661000	0.867769
chr17	73775500	0.892562
chr17	73824500	0.735537
chr17	73852100	0.900826
chr17	73872600	0.859504
chr17	73976000	0.743802
chr17	73993400	0.933884
chr17	74131600	0.743802
chr17	74252700	0.818182
chr17	74350200	0.966942

chr17	74425500	0.785124
chr17	74437500	0.735537
chr17	74502800	0.735537
chr17	74511600	0.834711
chr17	74519700	0.760331
chr17	74668100	0.760331
chr17	74836000	0.950413
chr17	74954400	0.851240
chr17	74965500	0.735537
chr17	75033500	0.933884
chr17	75082700	0.859504
chr17	75085300	0.867769
chr17	75088200	0.801653
chr17	75124500	0.867769
chr17	75178800	0.743802
chr17	75270500	0.760331
chr17	75276500	0.760331
chr17	75343600	0.925620
chr17	75378500	0.826446
chr17	75407300	0.801653
chr17	75434200	0.752066
chr17	75462200	0.818182
chr17	75466200	0.743802
chr17	75486900	0.752066
chr17	75591500	0.768595
chr17	75606900	0.818182
chr17	75687300	0.884298
chr17	75721600	0.826446
chr17	75884100	0.818182
chr17	75952200	0.842975
chr17	76078900	0.752066
chr17	76187000	0.768595
chr17	76210500	0.809917
chr17	76226900	0.826446
chr17	76247800	0.842975
chr17	76269100	0.859504
chr17	76273200	0.842975
chr17	76286500	0.818182
chr17	76340300	0.826446
chr17	76345000	0.909091
chr17	76350400	0.785124
chr17	76372200	0.735537
chr17	76384500	0.818182
chr17	76390200	0.752066
chr17	76475500	0.851240
chr17	76780300	0.785124
chr17	76837300	0.801653
chr17	76840900	0.760331
chr17	76886200	0.859504
chr17	76930000	0.776860
chr17	76961800	0.743802
chr17	76976900	0.900826
chr17	76982900	0.942149
chr17	77249600	0.793388

chr17	77399800	0.743802
chr17	77671300	0.842975
chr17	77676700	0.851240
chr17	77720000	0.826446
chr17	77724500	0.876033
chr17	77772500	0.793388
chr17	77780600	0.760331
chr17	77789000	0.743802
chr17	77967800	0.776860
chr17	77971200	0.826446
chr17	78094300	0.900826
chr17	78121200	0.768595
chr17	78162300	0.752066
chr17	78235300	0.801653
chr17	78428500	0.735537
chr17	78638700	0.809917
chr17	78660300	0.785124
chr17	78735600	0.776860
chr17	78801700	0.768595
chr17	78807100	0.743802
chr17	78830900	0.735537
chr17	78872200	0.909091
chr17	78963200	0.760331
chr17	79069300	0.776860
chr17	79197800	0.768595
chr17	79216200	0.760331
chr17	79259500	0.925620
chr17	79339700	0.859504
chr17	79369300	0.768595
chr17	79458700	0.884298
chr17	79565400	0.851240
chr17	79620700	0.760331
chr17	79690500	0.917355
chr17	79791300	0.884298
chr17	79815300	0.842975
chr17	79832300	0.785124
chr17	79851900	0.735537
chr17	79881600	0.818182
chr17	79926100	0.809917
chr17	80057300	0.752066
chr17	80064700	0.884298
chr17	80167600	0.867769
chr17	80231400	0.917355
chr17	80233600	0.752066
chr17	80251100	0.933884
chr17	80255900	0.842975
chr17	80291000	0.785124
chr17	80317000	0.876033
chr17	80331400	0.851240
chr17	80372800	0.851240
chr17	80457000	0.801653
chr17	80498600	0.752066
chr17	80510400	0.809917
chr17	80802400	0.752066

chr17	80817700	0.760331
chr17	80926000	0.884298
chr17	80928700	0.768595
chr17	80931300	0.834711
chr17	81039900	0.752066
chr17	81147600	0.884298
chr17	81174400	0.760331
chr18	268500	0.859504
chr18	370900	0.768595
chr18	505900	0.826446
chr18	642000	0.776860
chr18	645000	0.884298
chr18	650600	0.851240
chr18	655300	0.809917
chr18	786800	0.809917
chr18	812800	0.776860
chr18	830900	0.809917
chr18	2559900	0.768595
chr18	2972700	0.809917
chr18	3016400	0.735537
chr18	3037700	0.991736
chr18	3046100	0.735537
chr18	3129600	0.834711
chr18	3175200	0.752066
chr18	3269500	0.801653
chr18	3320000	0.776860
chr18	3324000	0.876033
chr18	3403300	0.776860
chr18	3412300	0.884298
chr18	3473600	0.818182
chr18	3514400	0.942149
chr18	3625100	0.760331
chr18	3661800	0.867769
chr18	5289200	0.851240
chr18	5970700	0.735537
chr18	5973300	0.834711
chr18	5982600	0.842975
chr18	5985600	0.892562
chr18	5999700	0.785124
chr18	6006000	0.818182
chr18	6255100	0.834711
chr18	6271200	0.842975
chr18	6363700	0.735537
chr18	6407200	0.809917
chr18	6435300	0.768595
chr18	6510000	0.859504
chr18	8550100	0.900826
chr18	8604100	0.851240
chr18	8620000	0.760331
chr18	8756000	0.859504
chr18	8952000	0.933884
chr18	8967700	0.793388
chr18	8989200	0.752066
chr18	9017800	0.752066

chr18	9070200	0.793388
chr18	9083300	0.760331
chr18	9095700	0.925620
chr18	9185700	0.809917
chr18	9685200	0.917355
chr18	9705100	0.785124
chr18	9771900	0.900826
chr18	9862100	0.785124
chr18	9870500	0.818182
chr18	9966400	0.876033
chr18	10016800	0.776860
chr18	10262700	0.884298
chr18	10285600	0.867769
chr18	10387300	0.826446
chr18	10400600	0.950413
chr18	10476500	0.859504
chr18	10490900	0.768595
chr18	10560500	0.809917
chr18	10626600	0.851240
chr18	10787300	0.752066
chr18	11105100	0.842975
chr18	11141100	0.842975
chr18	11160300	0.925620
chr18	11326100	0.760331
chr18	11489900	0.876033
chr18	11500700	0.851240
chr18	11577800	0.867769
chr18	11637600	0.760331
chr18	11995900	0.859504
chr18	12042700	0.809917
chr18	12098900	0.925620
chr18	12237500	0.760331
chr18	12703000	0.743802
chr18	12750500	0.876033
chr18	12880300	0.809917
chr18	12921400	0.818182
chr18	12948400	0.933884
chr18	13150700	0.859504
chr18	13279900	0.785124
chr18	13289100	0.917355
chr18	13360000	0.801653
chr18	13372500	0.867769
chr18	13377900	0.842975
chr18	13382200	0.818182
chr18	13439000	0.867769
chr18	13451300	0.818182
chr18	13456600	0.876033
chr18	13502200	0.942149
chr18	13549900	0.809917
chr18	13558400	0.776860
chr18	13571100	0.801653
chr18	13607000	0.818182
chr18	13801500	0.818182
chr18	13804800	0.743802

chr18	18681300	0.735537
chr18	18718200	0.760331
chr18	19180600	0.834711
chr18	19192700	0.917355
chr18	19262700	0.909091
chr18	19294700	0.785124
chr18	19491600	0.752066
chr18	19612800	0.776860
chr18	19713400	0.867769
chr18	20427800	0.884298
chr18	20513700	0.768595
chr18	20722400	0.735537
chr18	20772100	0.752066
chr18	20913000	0.735537
chr18	20938700	0.942149
chr18	21068400	0.859504
chr18	21076300	0.768595
chr18	21296300	0.834711
chr18	21318500	0.834711
chr18	21452000	0.809917
chr18	21514400	0.892562
chr18	21518900	0.851240
chr18	21553200	0.818182
chr18	21567500	0.834711
chr18	21588000	0.743802
chr18	21603100	0.743802
chr18	21635400	0.785124
chr18	21717100	0.760331
chr18	21757200	0.818182
chr18	21767400	0.859504
chr18	21769900	0.842975
chr18	21826200	0.900826
chr18	21949400	0.900826
chr18	22024500	0.809917
chr18	22072400	0.768595
chr18	22502900	0.909091
chr18	22508900	0.752066
chr18	22570200	0.735537
chr18	22745300	0.735537
chr18	23395400	0.743802
chr18	23502100	0.752066
chr18	23508200	0.735537
chr18	23542800	0.801653
chr18	23557100	0.785124
chr18	23987300	0.793388
chr18	24005200	0.809917
chr18	24019500	0.867769
chr18	24034200	0.735537
chr18	24132300	0.776860
chr18	24230000	0.818182
chr18	24283500	0.785124
chr18	24329600	0.768595
chr18	24580200	0.867769
chr18	24621400	0.760331

chr18	28701100	0.809917
chr18	28786900	0.892562
chr18	28813700	0.752066
chr18	28855600	0.760331
chr18	29072600	0.760331
chr18	29091600	0.768595
chr18	29230200	0.752066
chr18	29665300	0.752066
chr18	29733400	0.768595
chr18	30310900	0.834711
chr18	30340900	0.876033
chr18	31302800	0.768595
chr18	31309700	0.842975
chr18	32339900	0.752066
chr18	32610200	0.975207
chr18	32619600	0.801653
chr18	32721900	0.768595
chr18	32821100	0.851240
chr18	32924300	0.950413
chr18	32988800	0.760331
chr18	33077700	0.966942
chr18	33087700	0.793388
chr18	33196800	0.760331
chr18	33199500	0.909091
chr18	33366700	0.743802
chr18	33596000	0.892562
chr18	33656500	0.826446
chr18	33706300	0.793388
chr18	33987300	0.793388
chr18	34028800	0.752066
chr18	34054100	0.801653
chr18	34076200	0.809917
chr18	34097000	0.752066
chr18	34114300	0.760331
chr18	34253400	0.768595
chr18	34973200	0.859504
chr18	35114900	0.776860
chr18	36521700	0.826446
chr18	39486600	0.859504
chr18	40772600	0.776860
chr18	41152400	0.859504
chr18	42088400	0.776860
chr18	42217100	0.768595
chr18	42292300	0.826446
chr18	42302300	0.735537
chr18	42589800	0.933884
chr18	42687800	0.776860
chr18	42717200	0.760331
chr18	43043700	0.743802
chr18	43518800	0.793388
chr18	43736400	0.793388
chr18	43801600	0.768595
chr18	43977200	0.760331
chr18	44154700	0.876033

chr18	44291700	0.768595
chr18	44350200	0.785124
chr18	44470800	0.768595
chr18	44648100	0.876033
chr18	44693100	0.752066
chr18	44699000	0.785124
chr18	44946600	0.776860
chr18	45428600	0.834711
chr18	45431000	0.793388
chr18	45445300	0.785124
chr18	45457700	0.876033
chr18	45472100	0.743802
chr18	45497600	0.768595
chr18	45651700	0.809917
chr18	45898200	0.809917
chr18	46149000	0.760331
chr18	46259700	0.776860
chr18	46464700	0.793388
chr18	46519400	0.917355
chr18	46696300	0.743802
chr18	46698900	0.876033
chr18	47013700	0.892562
chr18	47018500	0.917355
chr18	47058800	0.809917
chr18	47088200	0.760331
chr18	47139000	0.818182
chr18	47346700	0.776860
chr18	47401200	0.942149
chr18	47602700	0.834711
chr18	47626900	0.834711
chr18	47655600	0.809917
chr18	47658500	0.826446
chr18	47745100	0.760331
chr18	47795000	0.884298
chr18	47814600	0.768595
chr18	47865300	0.809917
chr18	47883000	0.768595
chr18	48223300	0.859504
chr18	48340800	0.834711
chr18	48348100	0.801653
chr18	48363200	0.776860
chr18	48389200	0.793388
chr18	48494600	0.892562
chr18	48580200	0.859504
chr18	48680600	0.876033
chr18	48724600	0.876033
chr18	48738200	0.884298
chr18	48778100	0.950413
chr18	49110800	0.776860
chr18	49238100	0.834711
chr18	49243000	0.809917
chr18	49476300	0.752066
chr18	49919300	0.925620
chr18	51613700	0.834711

chr18	52385500	0.735537
chr18	52789400	0.793388
chr18	52888500	0.752066
chr18	52972300	0.826446
chr18	53044100	0.867769
chr18	53072200	0.834711
chr18	53156900	0.933884
chr18	53665100	0.785124
chr18	53757400	0.743802
chr18	53779800	0.809917
chr18	53962600	0.884298
chr18	53984500	0.776860
chr18	54179300	0.842975
chr18	54306100	0.760331
chr18	54414800	0.801653
chr18	54589400	0.834711
chr18	54720900	0.793388
chr18	54725200	0.785124
chr18	54817900	0.851240
chr18	54877600	0.834711
chr18	55191100	0.768595
chr18	55213000	0.942149
chr18	55416400	0.909091
chr18	55503200	0.818182
chr18	55510100	0.735537
chr18	55598800	0.876033
chr18	55618900	0.735537
chr18	55803300	0.826446
chr18	55891300	0.785124
chr18	55898600	0.785124
chr18	55959400	0.743802
chr18	55978500	0.842975
chr18	55985000	0.752066
chr18	56161600	0.801653
chr18	56173100	0.768595
chr18	56214100	0.851240
chr18	56217100	0.793388
chr18	56307800	0.785124
chr18	56431600	0.909091
chr18	56435900	0.776860
chr18	56438500	0.760331
chr18	56468100	0.859504
chr18	56486800	0.768595
chr18	56515200	0.809917
chr18	56564400	0.884298
chr18	56614500	0.884298
chr18	56628700	0.851240
chr18	56641200	0.768595
chr18	56648600	0.760331
chr18	56666500	0.818182
chr18	56673300	0.884298
chr18	56706300	0.776860
chr18	56740400	0.859504
chr18	56751900	0.809917

chr18	56754400	0.768595
chr18	56758900	0.752066
chr18	56761700	0.768595
chr18	56813000	0.776860
chr18	56976200	0.760331
chr18	57026100	0.834711
chr18	57469600	0.818182
chr18	57511400	0.785124
chr18	57515100	0.933884
chr18	57543300	0.876033
chr18	57586100	0.743802
chr18	57618400	0.933884
chr18	57628900	0.752066
chr18	57907100	0.785124
chr18	57916100	0.826446
chr18	58230500	0.776860
chr18	58316800	0.859504
chr18	59028200	0.917355
chr18	59163200	0.876033
chr18	59514500	0.743802
chr18	59623600	0.785124
chr18	59963900	0.752066
chr18	59988600	0.859504
chr18	59997400	0.768595
chr18	59999800	0.752066
chr18	60024900	0.768595
chr18	60035900	0.826446
chr18	60039300	0.826446
chr18	60253700	0.851240
chr18	60259100	0.760331
chr18	60319100	0.933884
chr18	60397200	0.752066
chr18	60508500	0.735537
chr18	60537100	0.809917
chr18	60718700	0.776860
chr18	60742200	0.859504
chr18	60779800	0.809917
chr18	60803100	0.735537
chr18	60878700	0.809917
chr18	60941600	0.925620
chr18	60960600	0.851240
chr18	61090100	0.826446
chr18	61229500	0.826446
chr18	61285100	0.876033
chr18	61499400	0.867769
chr18	61637600	0.900826
chr18	62047900	0.776860
chr18	67726700	0.760331
chr18	67873100	0.892562
chr18	67959900	0.842975
chr18	68086600	0.851240
chr18	68879600	0.991736
chr18	71348500	0.752066
chr18	71358200	0.826446

chr18	71732200	0.752066
chr18	71753500	0.776860
chr18	71839900	0.760331
chr18	72002000	0.917355
chr18	72024600	0.785124
chr18	72075800	0.785124
chr18	72078600	0.776860
chr18	72152800	0.851240
chr18	72196100	0.859504
chr18	72214900	0.842975
chr18	72256500	0.743802
chr18	72261300	0.735537
chr18	72268000	0.752066
chr18	72486800	0.735537
chr18	72519200	0.785124
chr18	72538000	0.826446
chr18	72582500	0.735537
chr18	72628300	0.876033
chr18	72885700	0.768595
chr18	74061300	0.859504
chr18	74102400	0.876033
chr18	74130400	0.842975
chr18	74163000	0.785124
chr18	74170900	0.752066
chr18	74175900	0.818182
chr18	74208100	0.834711
chr18	74238600	0.801653
chr18	74332700	0.826446
chr18	74433400	0.776860
chr18	74516100	0.768595
chr18	74717200	0.809917
chr18	74922400	0.735537
chr18	75068000	0.743802
chr18	76818300	0.776860
chr18	77116300	0.818182
chr18	77139700	0.743802
chr18	77176400	0.801653
chr18	77182200	0.752066
chr18	77199200	0.776860
chr18	77203300	0.818182
chr18	77387100	0.809917
chr18	77404200	0.785124
chr18	77580400	0.933884
chr18	77616400	0.760331
chr18	77715000	0.743802
chr18	77720400	0.752066
chr18	77873200	0.735537
chr18	77912300	0.735537
chr18	77938000	0.818182
chr18	77949900	0.851240
chr18	77959700	0.743802
chr18	77962500	0.752066
chr19	291400	0.776860
chr19	488700	0.867769

chr19	507300	0.793388
chr19	620700	0.933884
chr19	845300	0.842975
chr19	893200	0.975207
chr19	909300	0.909091
chr19	937500	0.752066
chr19	984500	0.892562
chr19	1021300	0.933884
chr19	1036000	0.776860
chr19	1065300	0.892562
chr19	1077600	0.818182
chr19	1177700	0.776860
chr19	1192900	0.818182
chr19	1214000	0.876033
chr19	1269700	0.842975
chr19	1276200	0.834711
chr19	1383800	0.842975
chr19	1412000	0.785124
chr19	1445500	0.809917
chr19	1455800	0.942149
chr19	1471100	0.917355
chr19	1568100	0.760331
chr19	1605600	0.785124
chr19	1653000	0.933884
chr19	1655400	0.809917
chr19	1661100	0.826446
chr19	1848600	0.909091
chr19	1864800	0.925620
chr19	1897800	0.752066
chr19	1922100	0.752066
chr19	1930000	0.743802
chr19	1933800	0.793388
chr19	2188200	0.925620
chr19	2253400	0.785124
chr19	2302900	0.735537
chr19	2394600	0.851240
chr19	2427800	0.975207
chr19	2447200	0.818182
chr19	2467200	0.826446
chr19	2504300	0.818182
chr19	2524200	0.752066
chr19	2552900	0.958678
chr19	2562700	0.900826
chr19	2579700	0.743802
chr19	2599200	0.818182
chr19	2611100	0.933884
chr19	2614300	0.859504
chr19	2628500	0.834711
chr19	2634500	0.818182
chr19	2645600	0.743802
chr19	2681800	0.826446
chr19	2749000	0.876033
chr19	2783200	0.785124
chr19	2950400	0.818182

chr19	2984200	0.818182
chr19	3008200	0.793388
chr19	3029200	0.867769
chr19	3133500	0.809917
chr19	3155300	0.743802
chr19	3362500	0.785124
chr19	3435000	0.917355
chr19	3500600	0.958678
chr19	3573200	0.776860
chr19	3605500	0.818182
chr19	3627100	0.925620
chr19	3762800	0.859504
chr19	3808000	0.776860
chr19	3810800	0.793388
chr19	3989700	0.909091
chr19	4053500	0.884298
chr19	4078200	0.892562
chr19	4247300	0.975207
chr19	4338500	0.925620
chr19	4400000	0.801653
chr19	4432200	0.834711
chr19	4539500	0.876033
chr19	4636800	0.735537
chr19	4791800	0.884298
chr19	4831600	0.743802
chr19	5127200	0.752066
chr19	5138100	0.809917
chr19	5250700	0.809917
chr19	5288300	0.851240
chr19	5339000	0.834711
chr19	5627900	0.834711
chr19	5791000	0.950413
chr19	5827000	0.809917
chr19	5839500	0.752066
chr19	5903800	0.909091
chr19	5956100	0.743802
chr19	6087900	0.735537
chr19	6393600	0.776860
chr19	6425800	0.801653
chr19	6460600	0.735537
chr19	6464500	0.809917
chr19	6481800	0.876033
chr19	6489800	0.768595
chr19	6522600	0.842975
chr19	6544200	0.801653
chr19	6717900	0.743802
chr19	6773100	0.768595
chr19	6948500	0.876033
chr19	6988400	0.884298
chr19	7011400	0.818182
chr19	7099400	0.900826
chr19	7105200	0.809917
chr19	7192500	0.867769
chr19	7243200	0.752066

chr19	7273100	0.884298
chr19	7284300	0.785124
chr19	7405900	0.966942
chr19	7464200	0.785124
chr19	7813400	0.752066
chr19	7838300	0.768595
chr19	7888200	0.950413
chr19	7908700	0.743802
chr19	7927200	0.776860
chr19	7936700	0.776860
chr19	8018300	0.867769
chr19	8130800	0.785124
chr19	8373100	0.851240
chr19	8408300	0.793388
chr19	8455300	0.884298
chr19	8522100	0.793388
chr19	8578800	0.950413
chr19	8636400	0.834711
chr19	8639300	0.818182
chr19	8855700	0.909091
chr19	8872600	0.818182
chr19	8883900	0.760331
chr19	9420400	0.884298
chr19	9432200	0.983471
chr19	9695000	0.925620
chr19	9732200	0.851240
chr19	9785700	0.933884
chr19	9879400	0.942149
chr19	9929500	0.876033
chr19	9989400	0.900826
chr19	10028100	0.834711
chr19	10197600	0.884298
chr19	10207200	0.933884
chr19	10220500	0.826446
chr19	10341700	0.793388
chr19	10343800	0.776860
chr19	10347100	0.942149
chr19	10363000	0.884298
chr19	10392300	0.776860
chr19	10395700	0.826446
chr19	10404700	0.818182
chr19	10450600	0.760331
chr19	10491800	0.892562
chr19	10520000	0.752066
chr19	10610500	0.776860
chr19	10616600	0.834711
chr19	10620100	0.859504
chr19	10629500	0.818182
chr19	10654700	0.818182
chr19	10680300	0.975207
chr19	10707900	0.876033
chr19	10806800	0.818182
chr19	10947000	0.917355
chr19	10978900	0.809917

chr19	11079700	0.809917
chr19	11157300	0.826446
chr19	11290700	0.859504
chr19	11298300	0.785124
chr19	11354400	0.735537
chr19	11375600	0.760331
chr19	11486300	0.801653
chr19	11494200	0.826446
chr19	11647300	0.842975
chr19	11679200	0.942149
chr19	11708300	0.826446
chr19	11711500	0.851240
chr19	11723700	0.834711
chr19	11878000	0.768595
chr19	12041600	0.809917
chr19	12098800	0.760331
chr19	12175800	0.933884
chr19	12274000	0.834711
chr19	12405500	0.867769
chr19	12444200	0.900826
chr19	12662700	0.752066
chr19	12780300	0.900826
chr19	12792500	0.892562
chr19	12841600	0.925620
chr19	12848100	0.884298
chr19	12877300	0.818182
chr19	12958800	0.735537
chr19	13054200	0.776860
chr19	13068200	0.876033
chr19	13172300	0.834711
chr19	13205600	0.818182
chr19	13208200	0.900826
chr19	13216200	0.752066
chr19	13233200	0.776860
chr19	13278600	0.859504
chr19	13298600	0.785124
chr19	13320100	0.793388
chr19	13842600	0.768595
chr19	13885400	0.818182
chr19	13947900	0.776860
chr19	13961300	0.735537
chr19	13972600	0.809917
chr19	14117900	0.909091
chr19	14139800	0.776860
chr19	14142900	0.851240
chr19	14186700	0.760331
chr19	14193000	0.801653
chr19	14248200	0.925620
chr19	14277700	0.768595
chr19	14360300	0.851240
chr19	14460200	0.876033
chr19	14467100	0.925620
chr19	14486500	0.925620
chr19	14500300	0.735537

chr19	14523600	0.735537
chr19	14551200	0.925620
chr19	14629800	0.776860
chr19	14672500	0.735537
chr19	14703200	0.876033
chr19	14706700	0.826446
chr19	14772600	0.785124
chr19	14800600	0.818182
chr19	14892000	0.743802
chr19	14895700	0.867769
chr19	14897900	0.793388
chr19	15236500	0.842975
chr19	15343200	0.834711
chr19	15390900	0.735537
chr19	15472000	0.776860
chr19	15493400	0.760331
chr19	15578000	0.925620
chr19	15580800	0.760331
chr19	15591200	0.735537
chr19	15650900	0.925620
chr19	15662200	0.876033
chr19	15947600	0.900826
chr19	16111500	0.818182
chr19	16193400	0.826446
chr19	16196800	0.743802
chr19	16264700	0.776860
chr19	16364300	0.884298
chr19	16369200	0.842975
chr19	16394800	0.876033
chr19	16397200	0.735537
chr19	16413400	0.801653
chr19	16439400	0.743802
chr19	16451500	0.785124
chr19	16453600	0.909091
chr19	16466700	0.834711
chr19	16480500	0.785124
chr19	16485700	0.842975
chr19	16726600	0.760331
chr19	16730700	0.842975
chr19	16830500	0.752066
chr19	16945100	0.867769
chr19	16986000	0.842975
chr19	16999900	0.900826
chr19	17010800	0.809917
chr19	17175100	0.884298
chr19	17282000	0.942149
chr19	17321000	0.785124
chr19	17326100	0.950413
chr19	17420700	0.917355
chr19	17481200	0.958678
chr19	17485200	0.809917
chr19	17564700	0.776860
chr19	17599800	0.735537
chr19	17607900	0.867769

chr19	17622800	0.867769
chr19	17634400	0.785124
chr19	17640500	0.760331
chr19	17655500	0.743802
chr19	17666800	0.760331
chr19	17702200	0.785124
chr19	17713200	0.768595
chr19	17730400	0.752066
chr19	17887100	0.760331
chr19	17955700	0.743802
chr19	17961400	0.859504
chr19	17992400	0.768595
chr19	17998000	0.785124
chr19	18043900	0.876033
chr19	18154800	0.768595
chr19	18169500	0.826446
chr19	18197600	0.867769
chr19	18202200	0.958678
chr19	18338600	0.826446
chr19	18393200	0.892562
chr19	18504500	0.876033
chr19	18512800	0.760331
chr19	18544100	0.892562
chr19	18589700	0.892562
chr19	18705200	0.851240
chr19	18755800	0.768595
chr19	18760400	0.801653
chr19	18876200	0.752066
chr19	18952600	0.743802
chr19	18957000	0.892562
chr19	19146700	0.768595
chr19	19180300	0.950413
chr19	19271700	0.925620
chr19	19318400	0.892562
chr19	19325000	0.809917
chr19	19429400	0.743802
chr19	19475600	0.983471
chr19	19483600	0.925620
chr19	19497100	0.834711
chr19	19501700	0.768595
chr19	19520500	0.809917
chr19	19567900	0.752066
chr19	19652100	0.776860
chr19	19702300	0.925620
chr19	19811700	0.851240
chr19	19932000	0.834711
chr19	19977100	0.867769
chr19	20748200	0.933884
chr19	20750700	0.826446
chr19	20959100	0.752066
chr19	21106200	0.909091
chr19	21203600	0.834711
chr19	21324900	0.735537
chr19	21512000	0.900826

chr19	21541800	0.809917
chr19	21688400	0.834711
chr19	21950100	0.942149
chr19	23432900	0.917355
chr19	23456800	0.735537
chr19	23577900	0.942149
chr19	23752700	0.876033
chr19	23869700	0.958678
chr19	24216500	0.876033
chr19	24270200	0.975207
chr19	27764600	0.735537
chr19	30112500	0.859504
chr19	30171700	0.884298
chr19	30206600	0.809917
chr19	30262200	0.826446
chr19	30302100	0.900826
chr19	30322700	0.867769
chr19	30325700	0.793388
chr19	30333000	0.884298
chr19	30508700	0.801653
chr19	30557600	0.768595
chr19	30597500	0.809917
chr19	30645100	0.826446
chr19	32896500	0.818182
chr19	33239500	0.768595
chr19	33300800	0.735537
chr19	33311000	0.851240
chr19	33569100	0.785124
chr19	33667900	0.743802
chr19	33688800	0.752066
chr19	33726800	0.966942
chr19	33732900	0.842975
chr19	33752200	0.801653
chr19	34151800	0.760331
chr19	34311400	0.867769
chr19	34394200	0.917355
chr19	34761400	0.818182
chr19	34767700	0.735537
chr19	34909500	0.826446
chr19	34919500	0.735537
chr19	35168700	0.917355
chr19	35225000	0.900826
chr19	35464800	0.826446
chr19	35494500	0.760331
chr19	35531700	0.826446
chr19	35708400	0.785124
chr19	35716300	0.776860
chr19	35827700	0.735537
chr19	35868200	0.785124
chr19	35905400	0.876033
chr19	35940500	0.776860
chr19	35961000	0.752066
chr19	35987000	0.752066
chr19	36025100	0.809917

chr19	36101100	0.735537
chr19	36103700	0.933884
chr19	36193400	0.933884
chr19	36233400	0.809917
chr19	36249000	0.900826
chr19	36400500	0.867769
chr19	36429400	0.801653
chr19	36444600	0.925620
chr19	36619200	0.892562
chr19	36628200	0.818182
chr19	36631400	0.785124
chr19	36980900	0.752066
chr19	37019600	0.743802
chr19	37064600	0.859504
chr19	37329100	0.768595
chr19	37407000	0.859504
chr19	37709000	0.834711
chr19	38411500	0.809917
chr19	38442700	0.834711
chr19	38540300	0.760331
chr19	38700700	0.809917
chr19	38704900	0.760331
chr19	38827000	0.876033
chr19	38865700	0.851240
chr19	38920600	0.760331
chr19	39050700	0.933884
chr19	39053000	0.892562
chr19	39056300	0.818182
chr19	39059900	0.793388
chr19	39177200	0.735537
chr19	39322200	0.933884
chr19	39336800	0.851240
chr19	39369200	0.975207
chr19	39421700	0.884298
chr19	39423900	0.743802
chr19	39467300	0.818182
chr19	39728800	0.768595
chr19	39826200	0.876033
chr19	39881800	0.834711
chr19	39889000	0.801653
chr19	39921600	0.760331
chr19	39923900	0.760331
chr19	39926600	0.818182
chr19	40043400	0.785124
chr19	40046700	0.842975
chr19	40151100	0.809917
chr19	40187500	0.917355
chr19	40212100	0.768595
chr19	40226900	0.768595
chr19	40332600	0.768595
chr19	40596800	0.867769
chr19	40697200	0.851240
chr19	40786800	0.785124
chr19	40904900	0.776860

chr19	40923200	0.752066
chr19	40927100	0.925620
chr19	41035500	0.826446
chr19	41107800	0.760331
chr19	41194200	0.743802
chr19	41482500	0.842975
chr19	41727100	0.809917
chr19	41891200	0.809917
chr19	41903800	0.801653
chr19	41955700	0.884298
chr19	41960300	0.925620
chr19	41963400	0.909091
chr19	42028400	0.900826
chr19	42044100	0.876033
chr19	42299600	0.834711
chr19	42306100	0.760331
chr19	42373200	0.743802
chr19	42440400	0.900826
chr19	42456100	0.842975
chr19	42659400	0.867769
chr19	42722300	0.917355
chr19	42751300	0.735537
chr19	42874800	0.892562
chr19	42883200	0.793388
chr19	42974000	0.909091
chr19	43013800	0.752066
chr19	43019400	0.760331
chr19	43028600	0.818182
chr19	43943700	0.859504
chr19	44024000	0.743802
chr19	44123800	0.851240
chr19	44151200	0.752066
chr19	44263600	0.933884
chr19	44286400	0.785124
chr19	44295200	0.826446
chr19	44488200	0.735537
chr19	44711600	0.735537
chr19	44716500	0.776860
chr19	45189900	0.851240
chr19	45232400	0.826446
chr19	45303500	0.809917
chr19	45504900	0.892562
chr19	45542300	0.917355
chr19	45585700	0.876033
chr19	45623600	0.752066
chr19	45874300	0.809917
chr19	45909500	0.793388
chr19	45927700	0.809917
chr19	45972700	0.909091
chr19	45982200	0.975207
chr19	46110700	0.752066
chr19	46181700	0.785124
chr19	46192500	0.793388
chr19	46221400	0.752066

chr19	46233800	0.801653
chr19	46366000	0.867769
chr19	46390300	0.834711
chr19	46619100	0.900826
chr19	46766700	0.892562
chr19	46970900	0.760331
chr19	47017600	0.785124
chr19	47033600	0.735537
chr19	47140100	0.793388
chr19	47152000	0.917355
chr19	47420700	0.809917
chr19	47516600	0.867769
chr19	47634400	0.851240
chr19	47680200	0.768595
chr19	47777200	0.909091
chr19	47803200	0.752066
chr19	48081400	0.851240
chr19	48102200	0.809917
chr19	48119200	0.776860
chr19	48210300	0.776860
chr19	48248700	0.950413
chr19	48253100	0.760331
chr19	48281700	0.925620
chr19	48323300	0.760331
chr19	48611300	0.768595
chr19	48747600	0.900826
chr19	48759100	0.892562
chr19	48774600	0.892562
chr19	48819600	0.752066
chr19	48823500	0.785124
chr19	48829000	0.752066
chr19	48833700	0.768595
chr19	48868100	0.801653
chr19	48894400	0.909091
chr19	48928300	0.801653
chr19	48972700	0.950413
chr19	48993800	0.851240
chr19	49141500	0.884298
chr19	49164300	0.975207
chr19	49179000	0.760331
chr19	49184700	0.785124
chr19	49224200	0.752066
chr19	49389700	0.958678
chr19	49395600	0.768595
chr19	49458700	0.884298
chr19	49471600	0.743802
chr19	49594500	0.892562
chr19	49842200	0.743802
chr19	49846200	0.826446
chr19	49891700	0.752066
chr19	49957300	0.776860
chr19	50000100	0.809917
chr19	50015500	0.801653
chr19	50031500	0.818182

chr19	50166500	0.752066
chr19	50194000	0.801653
chr19	50283800	0.735537
chr19	50321700	0.818182
chr19	50327200	0.735537
chr19	50354200	0.818182
chr19	50373200	0.925620
chr19	50441500	0.752066
chr19	50457600	0.760331
chr19	50580200	0.776860
chr19	50811200	0.809917
chr19	50819500	0.785124
chr19	50853900	0.818182
chr19	50860800	0.785124
chr19	50866200	0.809917
chr19	50869000	0.842975
chr19	50872300	0.760331
chr19	50879700	0.958678
chr19	50918500	0.785124
chr19	50937900	0.809917
chr19	51071800	0.818182
chr19	51124800	0.826446
chr19	51374000	0.735537
chr19	51425500	0.842975
chr19	51627200	0.818182
chr19	51667400	0.752066
chr19	51671100	0.909091
chr19	51698200	0.867769
chr19	51876000	0.785124
chr19	51920800	0.933884
chr19	51978400	0.760331
chr19	52005300	0.818182
chr19	52035600	0.809917
chr19	52050400	0.818182
chr19	52068500	0.752066
chr19	52114100	0.809917
chr19	52150900	0.752066
chr19	52214900	0.752066
chr19	52531500	0.809917
chr19	52552400	0.842975
chr19	52773300	0.933884
chr19	52800600	0.892562
chr19	52873300	0.909091
chr19	53031300	0.876033
chr19	53039600	0.884298
chr19	53141200	0.942149
chr19	53360400	0.900826
chr19	53606700	0.876033
chr19	53935400	0.818182
chr19	54641700	0.818182
chr19	54666500	0.818182
chr19	54776400	0.776860
chr19	54837800	0.793388
chr19	54871500	0.752066

chr19	54873700	0.743802
chr19	54884300	0.892562
chr19	54886500	0.851240
chr19	54890600	0.760331
chr19	54982700	0.793388
chr19	54994700	0.801653
chr19	54997700	0.876033
chr19	55020900	0.884298
chr19	55040700	0.735537
chr19	55064100	0.785124
chr19	55067800	0.851240
chr19	55105200	0.876033
chr19	55141000	0.760331
chr19	55155000	0.760331
chr19	55177400	0.785124
chr19	55305900	0.768595
chr19	55445600	0.785124
chr19	55450200	0.760331
chr19	55581400	0.933884
chr19	55599200	0.785124
chr19	55632900	0.842975
chr19	55645000	0.925620
chr19	55678300	0.776860
chr19	55771200	0.859504
chr19	55788500	0.900826
chr19	55792900	0.743802
chr19	55891700	0.975207
chr19	55910300	0.851240
chr19	55916000	0.768595
chr19	55952700	0.900826
chr19	55979600	0.867769
chr19	56061600	0.743802
chr19	56117400	0.785124
chr19	56138300	0.818182
chr19	56143600	0.809917
chr19	56146700	0.818182
chr19	56504700	0.983471
chr19	56617600	0.752066
chr19	56633000	0.826446
chr19	56826300	0.876033
chr19	57752000	0.776860
chr19	57779100	0.776860
chr19	58011300	0.760331
chr19	58258200	0.735537
chr19	58281000	0.743802
chr19	58283500	0.809917
chr19	58315300	0.909091
chr19	58331200	0.892562
chr19	58361400	0.859504
chr19	58695000	0.768595
chr19	58740000	0.785124
chr19	58868200	0.752066
chr19	58873500	0.793388
chr19	58902200	0.851240

chr19	59001100	0.793388
chr19	59042900	0.809917
chr19	59066500	0.743802
chr19	59084500	0.884298
chr19	59087100	0.900826
chr19	59090400	0.818182
chr1	236600	0.752066
chr1	463700	0.793388
chr1	568100	0.884298
chr1	762700	0.925620
chr1	773800	0.859504
chr1	834300	0.776860
chr1	927100	0.892562
chr1	955100	0.851240
chr1	969800	0.743802
chr1	973100	0.793388
chr1	975600	0.801653
chr1	1015700	0.884298
chr1	1028700	0.785124
chr1	1093600	0.950413
chr1	1105200	0.768595
chr1	1219700	0.851240
chr1	1294600	0.834711
chr1	1307800	0.826446
chr1	1310800	0.752066
chr1	1354100	0.892562
chr1	1358600	0.818182
chr1	1376900	0.801653
chr1	1475700	0.900826
chr1	1551000	0.867769
chr1	1781700	0.884298
chr1	1790800	0.884298
chr1	1827100	0.801653
chr1	2058300	0.867769
chr1	2126700	0.933884
chr1	2132800	0.735537
chr1	2144500	0.867769
chr1	2160000	0.743802
chr1	2187600	0.884298
chr1	2223500	0.760331
chr1	2243900	0.785124
chr1	2311500	0.785124
chr1	2319300	0.909091
chr1	2326600	0.867769
chr1	2346500	0.752066
chr1	2512300	0.801653
chr1	2574200	0.958678
chr1	3448200	0.743802
chr1	3514500	0.851240
chr1	3597300	0.768595
chr1	3636300	0.818182
chr1	3692500	0.776860
chr1	3717800	0.925620
chr1	3817400	0.801653

chr1	3839300	0.752066
chr1	4855000	0.752066
chr1	6015300	0.801653
chr1	6093900	0.809917
chr1	6241400	0.859504
chr1	6266000	0.818182
chr1	6454100	0.876033
chr1	6488400	0.851240
chr1	6520800	0.884298
chr1	6663500	0.876033
chr1	6749800	0.743802
chr1	6831800	0.801653
chr1	6837600	0.743802
chr1	6849400	0.776860
chr1	6865400	0.793388
chr1	6949700	0.743802
chr1	6967100	0.760331
chr1	7056800	0.842975
chr1	7531200	0.826446
chr1	7539400	0.809917
chr1	7712800	0.776860
chr1	7945700	0.801653
chr1	7974100	0.735537
chr1	7991400	0.859504
chr1	8002700	0.826446
chr1	8014400	0.867769
chr1	8025000	0.809917
chr1	8054100	0.876033
chr1	8080200	0.776860
chr1	8230000	0.909091
chr1	8273300	0.735537
chr1	8378500	0.876033
chr1	8499800	0.752066
chr1	8518500	0.933884
chr1	8686900	0.776860
chr1	8689700	0.834711
chr1	8824700	0.867769
chr1	8890300	0.818182
chr1	8921200	0.768595
chr1	9047800	0.793388
chr1	9172000	0.892562
chr1	9235400	0.818182
chr1	9259800	0.809917
chr1	9295100	0.892562
chr1	9300400	0.876033
chr1	9335900	0.867769
chr1	9366300	0.768595
chr1	9423000	0.851240
chr1	9460700	0.760331
chr1	9483300	0.909091
chr1	9487400	0.801653
chr1	9509000	0.760331
chr1	9551300	0.876033
chr1	9555900	0.809917

chr1	9778200	0.752066
chr1	9796200	0.801653
chr1	9840800	0.884298
chr1	9848200	0.785124
chr1	9924500	0.752066
chr1	9961200	0.776860
chr1	9971100	0.768595
chr1	10011100	0.867769
chr1	10021200	0.785124
chr1	10490800	0.842975
chr1	10494600	0.859504
chr1	10821600	0.867769
chr1	10983400	0.842975
chr1	11000100	0.842975
chr1	11002500	0.842975
chr1	11072800	0.842975
chr1	11434600	0.785124
chr1	11710500	0.859504
chr1	11727700	0.752066
chr1	11845800	0.735537
chr1	11994500	0.752066
chr1	12079800	0.752066
chr1	12120100	0.859504
chr1	12235600	0.785124
chr1	12238500	0.776860
chr1	12348300	0.925620
chr1	12357200	0.859504
chr1	12494200	0.785124
chr1	12496800	0.760331
chr1	12499700	0.842975
chr1	12537400	0.793388
chr1	13821100	0.735537
chr1	13991300	0.785124
chr1	14083900	0.793388
chr1	14111900	0.768595
chr1	14580900	0.768595
chr1	14999200	0.859504
chr1	15066500	0.743802
chr1	15076900	0.768595
chr1	15208900	0.942149
chr1	15240900	0.917355
chr1	15502900	0.735537
chr1	15505400	0.818182
chr1	15508600	0.801653
chr1	15545700	0.809917
chr1	15570900	0.785124
chr1	15576000	0.900826
chr1	15579900	0.818182
chr1	15631500	0.735537
chr1	15679400	0.752066
chr1	15712700	0.735537
chr1	15849200	0.752066
chr1	15855800	0.785124
chr1	16115900	0.900826

chr1	16157100	0.793388
chr1	16212700	0.900826
chr1	16278100	0.760331
chr1	16332000	0.793388
chr1	16334300	0.793388
chr1	16449700	0.851240
chr1	16473900	0.909091
chr1	16478200	0.776860
chr1	16489300	0.768595
chr1	16517400	0.760331
chr1	16747600	0.760331
chr1	16835700	0.834711
chr1	16838600	0.851240
chr1	16858900	0.859504
chr1	16867100	0.785124
chr1	16870200	0.760331
chr1	16879400	0.818182
chr1	16887000	0.851240
chr1	16920400	0.958678
chr1	16927500	0.859504
chr1	16930600	0.975207
chr1	16934700	0.876033
chr1	16942000	0.818182
chr1	16993700	0.933884
chr1	16999000	0.909091
chr1	17002100	0.760331
chr1	17009400	0.760331
chr1	17013800	0.735537
chr1	17046900	0.809917
chr1	17052800	0.785124
chr1	17059200	0.752066
chr1	17061400	0.768595
chr1	17066800	0.942149
chr1	17072800	0.776860
chr1	17086900	0.859504
chr1	17099400	0.809917
chr1	17107800	0.809917
chr1	17191100	0.752066
chr1	17194100	0.842975
chr1	17200100	0.735537
chr1	17224800	0.818182
chr1	17231600	0.776860
chr1	17387000	0.752066
chr1	17446200	0.776860
chr1	17452600	0.768595
chr1	17488300	0.752066
chr1	17573300	0.909091
chr1	17779100	0.785124
chr1	17867400	0.793388
chr1	17962700	0.760331
chr1	18298000	0.826446
chr1	18326700	0.801653
chr1	18606600	0.752066
chr1	18807800	0.876033

chr1	19229200	0.917355
chr1	19256400	0.743802
chr1	19258800	0.809917
chr1	19275000	0.776860
chr1	19338000	0.809917
chr1	19537300	0.760331
chr1	19578300	0.876033
chr1	19644500	0.760331
chr1	19790300	0.884298
chr1	19812700	0.785124
chr1	19973000	0.776860
chr1	19980900	0.884298
chr1	20120800	0.768595
chr1	20385800	0.884298
chr1	20403800	0.793388
chr1	20406200	0.735537
chr1	20446200	0.793388
chr1	20481200	0.776860
chr1	20539700	0.859504
chr1	20686300	0.909091
chr1	20838900	0.752066
chr1	21005900	0.842975
chr1	21114300	0.909091
chr1	21333500	0.752066
chr1	21349300	0.942149
chr1	21503500	0.752066
chr1	21626400	0.768595
chr1	21661200	0.859504
chr1	21663800	0.760331
chr1	21668500	0.834711
chr1	21691800	0.785124
chr1	21711500	0.809917
chr1	21815500	0.801653
chr1	21841100	0.859504
chr1	21848000	0.867769
chr1	21996800	0.752066
chr1	22118600	0.876033
chr1	22125300	0.826446
chr1	22240900	0.735537
chr1	22358300	0.785124
chr1	22366900	0.851240
chr1	22369500	0.851240
chr1	22394800	0.760331
chr1	22416600	0.735537
chr1	22469800	0.867769
chr1	22505900	0.809917
chr1	22623700	0.834711
chr1	22746300	0.768595
chr1	22788700	0.826446
chr1	23035900	0.859504
chr1	23040500	0.793388
chr1	23090500	0.809917
chr1	23138000	0.892562
chr1	23181800	0.851240

chr1	23212100	0.867769
chr1	23289700	0.768595
chr1	23303000	0.851240
chr1	23338000	0.826446
chr1	23340900	0.735537
chr1	23446400	0.735537
chr1	23449400	0.876033
chr1	23482800	0.884298
chr1	23501500	0.942149
chr1	23506100	0.917355
chr1	23529000	0.851240
chr1	23580700	0.735537
chr1	23671300	0.801653
chr1	23730400	0.892562
chr1	23860500	0.768595
chr1	23887800	0.768595
chr1	23974900	0.752066
chr1	23991700	0.735537
chr1	24055700	0.752066
chr1	24134800	0.760331
chr1	24225800	0.834711
chr1	24243100	0.917355
chr1	24246900	0.752066
chr1	24258200	0.842975
chr1	24325700	0.917355
chr1	24330400	0.834711
chr1	24373600	0.768595
chr1	24477300	0.785124
chr1	24503900	0.743802
chr1	24520100	0.867769
chr1	24555900	0.876033
chr1	24563400	0.892562
chr1	24692100	0.867769
chr1	24863500	0.760331
chr1	24967000	0.818182
chr1	25032500	0.768595
chr1	25056600	0.785124
chr1	25063400	0.743802
chr1	25081000	0.776860
chr1	25088600	0.809917
chr1	25182700	0.793388
chr1	25193700	0.842975
chr1	25198000	0.834711
chr1	25257200	0.834711
chr1	25300700	0.834711
chr1	25305100	0.760331
chr1	25317000	0.735537
chr1	25327800	0.768595
chr1	25346300	0.768595
chr1	25407300	0.851240
chr1	25420900	0.809917
chr1	25423900	0.818182
chr1	25433300	0.752066
chr1	25447500	0.801653

chr1	25475500	0.768595
chr1	25525500	0.900826
chr1	25543400	0.752066
chr1	25559300	0.892562
chr1	25563800	0.752066
chr1	25566600	0.909091
chr1	25573900	0.768595
chr1	25594100	0.933884
chr1	25878600	0.743802
chr1	25911700	0.735537
chr1	25927000	0.818182
chr1	25950500	0.768595
chr1	25956000	0.768595
chr1	25969600	0.818182
chr1	26098100	0.900826
chr1	26104900	0.760331
chr1	26129200	0.768595
chr1	26136900	0.867769
chr1	26260600	0.834711
chr1	26346900	0.950413
chr1	26472000	0.892562
chr1	26602800	0.760331
chr1	26633600	0.842975
chr1	26687100	0.793388
chr1	26700100	0.867769
chr1	26796900	0.743802
chr1	26820000	0.801653
chr1	26827800	0.942149
chr1	26872500	0.743802
chr1	27004300	0.826446
chr1	27041100	0.752066
chr1	27045800	0.842975
chr1	27071300	0.776860
chr1	27147700	0.768595
chr1	27251100	0.768595
chr1	27339500	0.776860
chr1	27342200	0.743802
chr1	27371800	0.785124
chr1	27577700	0.760331
chr1	27648900	0.826446
chr1	27791800	0.867769
chr1	27819100	0.900826
chr1	27848500	0.809917
chr1	27852500	0.826446
chr1	27903000	0.884298
chr1	27926100	0.809917
chr1	27945900	0.735537
chr1	27970700	0.776860
chr1	27989600	0.909091
chr1	28016300	0.743802
chr1	28157500	0.809917
chr1	28196100	0.909091
chr1	28240800	0.818182
chr1	28261800	0.752066

chr1	28265300	0.851240
chr1	28414800	0.851240
chr1	28489500	0.876033
chr1	28504200	0.752066
chr1	28507500	0.768595
chr1	28583000	0.785124
chr1	28624500	0.867769
chr1	28656100	0.884298
chr1	28696400	0.752066
chr1	28845300	0.743802
chr1	28975000	0.876033
chr1	29313200	0.785124
chr1	30185300	0.859504
chr1	31192000	0.900826
chr1	31238700	0.760331
chr1	31247200	0.785124
chr1	31253800	0.743802
chr1	31256200	0.752066
chr1	31297300	0.809917
chr1	31342300	0.818182
chr1	31385000	0.760331
chr1	31532400	0.884298
chr1	31538200	0.892562
chr1	31905000	0.776860
chr1	32037600	0.793388
chr1	32062700	0.752066
chr1	32066800	0.884298
chr1	32110300	0.743802
chr1	32173900	0.776860
chr1	32216800	0.851240
chr1	32235700	0.834711
chr1	32314400	0.900826
chr1	32331900	0.842975
chr1	32347100	0.933884
chr1	32355200	0.958678
chr1	32357600	0.818182
chr1	32380500	0.735537
chr1	32405300	0.793388
chr1	32418200	0.743802
chr1	32427800	0.834711
chr1	32471100	0.842975
chr1	32532200	0.809917
chr1	32538600	0.793388
chr1	32582100	0.842975
chr1	32610800	0.793388
chr1	32688200	0.925620
chr1	32708700	0.752066
chr1	32722700	0.842975
chr1	32808100	0.793388
chr1	32824300	0.876033
chr1	32867100	0.851240
chr1	32930900	0.776860
chr1	32960600	0.752066
chr1	33182700	0.752066

chr1	33191000	0.785124
chr1	33199800	0.842975
chr1	33226000	0.735537
chr1	33386300	0.793388
chr1	33416500	0.776860
chr1	33422700	0.884298
chr1	33494200	0.809917
chr1	33595800	0.793388
chr1	33700000	0.785124
chr1	33739900	0.776860
chr1	33847300	0.958678
chr1	33863800	0.991736
chr1	34606000	0.892562
chr1	34832800	0.760331
chr1	35262800	0.867769
chr1	35385400	0.793388
chr1	35451000	0.842975
chr1	35497100	0.785124
chr1	35528800	0.842975
chr1	35545000	0.909091
chr1	35648200	0.818182
chr1	35939600	0.743802
chr1	36023300	0.842975
chr1	36047000	0.876033
chr1	36174400	0.776860
chr1	36184700	0.925620
chr1	36235200	0.909091
chr1	36243000	0.776860
chr1	36339400	0.760331
chr1	36391000	0.909091
chr1	36540100	0.743802
chr1	36566100	0.743802
chr1	36589400	0.900826
chr1	36673700	0.809917
chr1	36775100	0.818182
chr1	36779800	0.801653
chr1	36787100	0.834711
chr1	36840300	0.776860
chr1	36924800	0.752066
chr1	36930200	0.909091
chr1	37350300	0.809917
chr1	37377300	0.809917
chr1	37560100	0.876033
chr1	37755100	0.876033
chr1	37759800	0.793388
chr1	38041300	0.768595
chr1	38141700	0.768595
chr1	38232000	0.867769
chr1	38467800	0.867769
chr1	38478500	0.842975
chr1	38517900	0.793388
chr1	38549200	0.809917
chr1	38675200	0.834711
chr1	38679700	0.826446

chr1	38910400	0.760331
chr1	38965200	0.826446
chr1	39023000	0.842975
chr1	39492100	0.768595
chr1	39553300	0.801653
chr1	39582300	0.801653
chr1	39655600	0.735537
chr1	39978400	0.809917
chr1	40042200	0.909091
chr1	40119500	0.942149
chr1	40202300	0.851240
chr1	40265200	0.826446
chr1	40283100	0.925620
chr1	40358400	0.760331
chr1	40364900	0.735537
chr1	40412500	0.876033
chr1	40426700	0.958678
chr1	40452000	0.743802
chr1	40486500	0.760331
chr1	40703600	0.752066
chr1	40805000	0.793388
chr1	40810900	0.768595
chr1	41099600	0.818182
chr1	41268200	0.785124
chr1	41306300	0.752066
chr1	41317400	0.859504
chr1	41704800	0.818182
chr1	41708300	0.818182
chr1	41970100	0.776860
chr1	41982600	0.818182
chr1	42086700	0.752066
chr1	42122800	0.818182
chr1	42155800	0.735537
chr1	42190100	0.768595
chr1	42218200	0.735537
chr1	42269000	0.809917
chr1	42280500	0.785124
chr1	42284800	0.793388
chr1	42357200	0.909091
chr1	42474300	0.743802
chr1	42825100	0.801653
chr1	42935600	0.818182
chr1	43203200	0.785124
chr1	43282600	0.884298
chr1	43427300	0.776860
chr1	43468300	0.809917
chr1	43524900	0.818182
chr1	43573100	0.834711
chr1	43672800	0.760331
chr1	43687600	0.793388
chr1	43704200	0.743802
chr1	43761300	0.842975
chr1	44459600	0.785124
chr1	44513700	0.801653

chr1	44579600	0.884298
chr1	44828700	0.851240
chr1	44874800	0.785124
chr1	45159500	0.752066
chr1	45190400	0.752066
chr1	45285900	0.950413
chr1	45439400	0.785124
chr1	45449600	0.735537
chr1	45681900	0.867769
chr1	45772200	0.785124
chr1	45860200	0.859504
chr1	45920200	0.818182
chr1	45939000	0.859504
chr1	45958600	0.760331
chr1	46024500	0.793388
chr1	46050000	0.735537
chr1	46144700	0.859504
chr1	46395400	0.809917
chr1	46401400	0.760331
chr1	46563100	0.776860
chr1	46584900	0.809917
chr1	46626100	0.834711
chr1	46639700	0.826446
chr1	46711300	0.785124
chr1	46886000	0.859504
chr1	46932800	0.776860
chr1	47067600	0.743802
chr1	47082200	0.809917
chr1	47126500	0.867769
chr1	47780200	0.785124
chr1	47800500	0.735537
chr1	47911200	0.743802
chr1	48430700	0.942149
chr1	48648600	0.809917
chr1	49145800	0.768595
chr1	49196200	0.743802
chr1	49216400	0.950413
chr1	49227200	0.900826
chr1	49250900	0.752066
chr1	49256400	0.785124
chr1	49260000	0.867769
chr1	50576400	0.743802
chr1	50962600	0.752066
chr1	51190800	0.909091
chr1	51278800	0.768595
chr1	51396400	0.900826
chr1	51413700	0.743802
chr1	51634200	0.917355
chr1	51669700	0.743802
chr1	51680900	0.917355
chr1	51768500	0.743802
chr1	51770900	0.743802
chr1	51811000	0.842975
chr1	51896200	0.752066

chr1	51905300	0.743802
chr1	52145700	0.851240
chr1	52155300	0.760331
chr1	52208000	0.735537
chr1	52329100	0.809917
chr1	52440900	0.859504
chr1	52605900	0.834711
chr1	52729100	0.809917
chr1	53070200	0.785124
chr1	53100700	0.801653
chr1	53125900	0.776860
chr1	53309700	0.760331
chr1	53686300	0.884298
chr1	53856300	0.752066
chr1	53874500	0.743802
chr1	53880300	0.760331
chr1	53882900	0.752066
chr1	54304100	0.842975
chr1	54356000	0.793388
chr1	54360100	0.842975
chr1	54822600	0.909091
chr1	54963300	0.818182
chr1	55270700	0.793388
chr1	55280800	0.785124
chr1	55411200	0.909091
chr1	55431900	0.752066
chr1	55476700	0.801653
chr1	55526700	0.842975
chr1	55596000	0.752066
chr1	55681200	0.826446
chr1	55743600	0.834711
chr1	55754100	0.859504
chr1	56511000	0.867769
chr1	56527800	0.785124
chr1	56688900	0.743802
chr1	56762800	0.785124
chr1	56899100	0.884298
chr1	56939100	0.842975
chr1	56993600	0.735537
chr1	57634000	0.809917
chr1	58622400	0.793388
chr1	59034800	0.801653
chr1	59043700	0.743802
chr1	59051300	0.760331
chr1	59084900	0.785124
chr1	59193200	0.842975
chr1	59250200	0.735537
chr1	59325700	0.743802
chr1	59369500	0.900826
chr1	59434400	0.818182
chr1	59436900	0.752066
chr1	59465800	0.735537
chr1	59479600	0.842975
chr1	59486200	0.950413

chr1	59512900	0.752066
chr1	59543500	0.776860
chr1	59762700	0.859504
chr1	59850400	0.735537
chr1	59949800	0.826446
chr1	59998200	0.818182
chr1	60036300	0.826446
chr1	60216700	0.942149
chr1	60218800	0.760331
chr1	60437000	0.785124
chr1	61365300	0.760331
chr1	61713100	0.776860
chr1	61785000	0.876033
chr1	61798300	0.768595
chr1	61985600	0.842975
chr1	62190800	0.909091
chr1	62582300	0.752066
chr1	62738000	0.818182
chr1	62761800	0.776860
chr1	62913700	0.743802
chr1	63154000	0.859504
chr1	63156400	0.752066
chr1	63259800	0.760331
chr1	63430000	0.851240
chr1	63591400	0.876033
chr1	64059800	0.859504
chr1	64075500	0.735537
chr1	64159800	0.735537
chr1	64180000	0.933884
chr1	64356600	0.785124
chr1	64359100	0.826446
chr1	64660900	0.818182
chr1	64709700	0.776860
chr1	65295100	0.809917
chr1	65368500	0.942149
chr1	65374600	0.884298
chr1	65383300	0.768595
chr1	65412700	0.785124
chr1	65421000	0.801653
chr1	65424600	0.842975
chr1	65434100	0.859504
chr1	65453500	0.834711
chr1	65642200	0.867769
chr1	65662000	0.818182
chr1	66634400	0.752066
chr1	66767000	0.859504
chr1	66772300	0.826446
chr1	66911700	0.867769
chr1	67115500	0.925620
chr1	67218100	0.884298
chr1	67390800	0.776860
chr1	67578900	0.933884
chr1	67651500	0.768595
chr1	67730700	0.801653

chr1	67751800	0.917355
chr1	67816900	0.793388
chr1	68027700	0.818182
chr1	68188400	0.958678
chr1	68202100	0.925620
chr1	68209400	0.776860
chr1	68216300	0.752066
chr1	68221900	0.834711
chr1	68226100	0.892562
chr1	68232100	0.776860
chr1	68235900	0.735537
chr1	68297900	0.768595
chr1	68627700	0.851240
chr1	68647900	0.842975
chr1	68813000	0.735537
chr1	70549600	0.776860
chr1	70671700	0.851240
chr1	70687600	0.892562
chr1	70820300	0.793388
chr1	70825600	0.785124
chr1	70983800	0.785124
chr1	71546800	0.917355
chr1	72750800	0.776860
chr1	74983700	0.834711
chr1	75045600	0.743802
chr1	75168900	0.834711
chr1	75290900	0.793388
chr1	75601300	0.793388
chr1	75804400	0.826446
chr1	76549200	0.752066
chr1	76736100	0.842975
chr1	76891300	0.826446
chr1	77337600	0.900826
chr1	77405600	0.793388
chr1	77882100	0.925620
chr1	77887400	0.735537
chr1	78006000	1.000000
chr1	78514900	0.785124
chr1	78578300	0.809917
chr1	78609000	0.818182
chr1	79659400	0.743802
chr1	79923900	0.752066
chr1	79961900	0.809917
chr1	80177200	0.776860
chr1	80244200	0.768595
chr1	81897400	0.752066
chr1	81971900	0.735537
chr1	82064700	0.793388
chr1	83445800	0.735537
chr1	83937300	0.834711
chr1	84184200	0.785124
chr1	84212100	0.743802
chr1	84316200	0.876033
chr1	84618900	0.818182

chr1	84622100	0.876033
chr1	84633000	0.752066
chr1	84908700	0.768595
chr1	85004800	0.785124
chr1	85066100	0.743802
chr1	85224900	0.909091
chr1	85273700	0.859504
chr1	85286200	0.884298
chr1	85329900	0.743802
chr1	85742100	0.768595
chr1	85745000	0.760331
chr1	85764300	0.900826
chr1	85774700	0.760331
chr1	85786900	0.809917
chr1	85797000	0.735537
chr1	86848400	0.826446
chr1	86936700	0.842975
chr1	86939000	0.785124
chr1	87082700	0.752066
chr1	87375800	0.752066
chr1	87574500	0.752066
chr1	87588700	0.785124
chr1	87609400	0.859504
chr1	87621200	0.876033
chr1	87642200	0.842975
chr1	87674600	0.776860
chr1	87691700	0.801653
chr1	87697800	0.826446
chr1	87784100	0.818182
chr1	87901700	0.785124
chr1	87930000	0.818182
chr1	88056100	0.884298
chr1	88058200	0.884298
chr1	88658300	0.809917
chr1	89438400	0.752066
chr1	89447600	0.785124
chr1	89458300	0.752066
chr1	89503300	0.834711
chr1	89516200	0.752066
chr1	89531100	0.892562
chr1	89591700	0.743802
chr1	89595400	0.818182
chr1	89667000	0.768595
chr1	89740400	0.735537
chr1	89765500	0.917355
chr1	89895100	0.867769
chr1	89930000	0.793388
chr1	90012000	0.752066
chr1	90014800	0.851240
chr1	90018300	0.834711
chr1	90054800	0.785124
chr1	90069000	0.884298
chr1	90072400	0.735537
chr1	90096200	0.801653

chr1	90118100	0.818182
chr1	90223800	0.909091
chr1	90265900	0.884298
chr1	90276500	0.842975
chr1	90287600	0.735537
chr1	90298900	0.809917
chr1	90406900	0.809917
chr1	90413600	0.735537
chr1	90419200	0.743802
chr1	90422900	0.752066
chr1	90428400	0.876033
chr1	90432800	0.801653
chr1	90437400	0.859504
chr1	90513000	0.768595
chr1	91456800	0.743802
chr1	91478000	0.768595
chr1	91492400	0.842975
chr1	92042500	0.809917
chr1	92057500	0.842975
chr1	92116000	0.842975
chr1	92176200	0.760331
chr1	92250300	0.859504
chr1	92257200	0.768595
chr1	92287500	0.760331
chr1	92296000	0.809917
chr1	92330300	0.809917
chr1	92357800	0.818182
chr1	92762300	0.793388
chr1	92833100	0.735537
chr1	92940700	0.785124
chr1	92943200	0.785124
chr1	92945300	0.834711
chr1	93004300	0.867769
chr1	93325100	0.768595
chr1	93427600	0.801653
chr1	93434400	0.834711
chr1	93466700	0.884298
chr1	93470900	0.809917
chr1	93480900	0.867769
chr1	93499200	0.876033
chr1	93504500	0.859504
chr1	93508200	0.909091
chr1	93552500	0.776860
chr1	93576600	0.752066
chr1	94079600	0.909091
chr1	94083400	0.826446
chr1	94108100	0.867769
chr1	94152100	0.809917
chr1	94155700	0.909091
chr1	94159800	0.793388
chr1	94177900	0.851240
chr1	94344700	0.752066
chr1	94511300	0.752066
chr1	94558300	0.851240

chr1	94572200	0.900826
chr1	94577400	0.900826
chr1	94750700	0.867769
chr1	94798900	0.760331
chr1	94877000	0.735537
chr1	94890300	0.776860
chr1	95428400	0.925620
chr1	95532600	0.760331
chr1	95704200	0.834711
chr1	97629900	0.958678
chr1	97663100	0.859504
chr1	97893400	0.735537
chr1	98257900	0.867769
chr1	99694000	0.809917
chr1	100036200	0.809917
chr1	100232000	0.851240
chr1	100286600	0.760331
chr1	100501300	0.809917
chr1	100654900	0.950413
chr1	100843500	0.818182
chr1	100880800	0.859504
chr1	100890000	0.859504
chr1	100918700	0.818182
chr1	100989000	0.809917
chr1	100999100	0.892562
chr1	101336200	0.801653
chr1	101566700	0.859504
chr1	101588600	0.818182
chr1	101603100	0.884298
chr1	101666100	0.793388
chr1	101682400	0.809917
chr1	101687800	0.933884
chr1	101716400	0.826446
chr1	101728700	0.842975
chr1	101730800	0.826446
chr1	101741900	0.884298
chr1	101768800	0.793388
chr1	101836900	0.735537
chr1	101839200	0.735537
chr1	101910700	0.851240
chr1	102189600	0.785124
chr1	102194000	0.793388
chr1	104068500	0.752066
chr1	104323000	0.735537
chr1	105300600	0.776860
chr1	105308300	0.818182
chr1	106417900	0.826446
chr1	107450900	0.884298
chr1	107503100	0.867769
chr1	107973800	0.818182
chr1	108007900	0.851240
chr1	108015800	0.809917
chr1	108018200	0.958678
chr1	108078800	0.793388

chr1	108166100	0.735537
chr1	108319700	0.743802
chr1	108328100	0.917355
chr1	108376900	0.867769
chr1	108383500	0.809917
chr1	108387300	0.818182
chr1	108419600	0.859504
chr1	108443500	0.818182
chr1	108479900	0.776860
chr1	108498600	0.793388
chr1	108500700	0.859504
chr1	108584900	0.735537
chr1	108981600	0.809917
chr1	109047900	0.809917
chr1	109113000	0.735537
chr1	109204000	0.900826
chr1	109289500	0.892562
chr1	109469400	0.809917
chr1	109500300	0.793388
chr1	109618800	0.942149
chr1	109677400	0.743802
chr1	109697100	0.900826
chr1	109710600	0.785124
chr1	109729400	0.851240
chr1	109736400	0.909091
chr1	109820500	0.842975
chr1	109837700	0.876033
chr1	109911900	0.892562
chr1	109948300	0.826446
chr1	110026800	0.776860
chr1	110183000	0.793388
chr1	110186300	0.867769
chr1	110198400	0.851240
chr1	110203200	0.818182
chr1	110262500	0.752066
chr1	110352000	0.743802
chr1	110416600	0.735537
chr1	110430800	0.818182
chr1	110439000	0.801653
chr1	110490700	0.859504
chr1	110527700	0.876033
chr1	110571200	0.760331
chr1	110611400	0.809917
chr1	110630200	0.818182
chr1	110759900	0.909091
chr1	110779200	0.892562
chr1	110786700	0.801653
chr1	110825600	0.834711
chr1	110955800	0.851240
chr1	111045800	0.743802
chr1	111152800	0.785124
chr1	111170000	0.735537
chr1	111184100	0.768595
chr1	111285900	0.842975

chr1	111323800	0.867769
chr1	111435400	0.752066
chr1	111438100	0.752066
chr1	111459400	0.768595
chr1	111813700	0.768595
chr1	111949600	0.834711
chr1	112046700	0.785124
chr1	112050800	0.809917
chr1	112061300	0.785124
chr1	112113800	0.768595
chr1	112178500	0.735537
chr1	112248500	0.752066
chr1	112811400	0.735537
chr1	112815200	0.743802
chr1	112880100	0.752066
chr1	113005300	0.752066
chr1	113165200	0.801653
chr1	113180800	0.760331
chr1	113217500	0.760331
chr1	113313200	0.842975
chr1	113383800	0.809917
chr1	113585000	0.760331
chr1	113693200	0.743802
chr1	113730600	0.842975
chr1	113752800	0.859504
chr1	113769100	0.826446
chr1	114302500	0.834711
chr1	114355600	0.760331
chr1	114670200	0.867769
chr1	114896600	0.768595
chr1	115054300	0.876033
chr1	115102300	0.834711
chr1	115259400	0.859504
chr1	115300600	0.859504
chr1	115854200	0.809917
chr1	116220500	0.900826
chr1	116314700	0.735537
chr1	116798400	0.826446
chr1	117136300	0.760331
chr1	117138800	0.851240
chr1	117189100	0.735537
chr1	117351100	0.818182
chr1	117560300	0.801653
chr1	117902500	0.818182
chr1	117905400	0.826446
chr1	118158800	0.892562
chr1	118161300	0.735537
chr1	118165400	0.809917
chr1	118168300	0.785124
chr1	118170600	0.884298
chr1	118177000	0.768595
chr1	118185800	0.834711
chr1	118204200	0.950413
chr1	118213800	0.801653

chr1	118278200	0.768595
chr1	118301600	0.900826
chr1	118321200	0.760331
chr1	118528600	0.876033
chr1	118624500	0.768595
chr1	118679400	0.876033
chr1	118876500	0.768595
chr1	119503500	0.743802
chr1	119765600	0.760331
chr1	119770200	0.752066
chr1	119867000	0.818182
chr1	119902400	0.768595
chr1	119910000	0.826446
chr1	120006700	0.735537
chr1	120149800	0.735537
chr1	120160600	0.818182
chr1	120186500	0.768595
chr1	120199300	0.776860
chr1	120218400	0.851240
chr1	120390500	0.809917
chr1	120532600	0.975207
chr1	120536100	0.884298
chr1	120552800	0.735537
chr1	120555700	0.909091
chr1	120559300	0.752066
chr1	120574000	0.876033
chr1	120589800	0.801653
chr1	120594600	0.752066
chr1	120598100	0.966942
chr1	120604300	0.983471
chr1	120607600	0.826446
chr1	120620600	0.842975
chr1	120630100	0.966942
chr1	120635900	0.876033
chr1	120643200	0.876033
chr1	120647900	0.909091
chr1	120654300	0.892562
chr1	120656500	0.884298
chr1	120662200	0.859504
chr1	120671600	0.842975
chr1	120675200	0.818182
chr1	120750500	0.735537
chr1	120761500	0.925620
chr1	120787300	0.818182
chr1	120791900	0.809917
chr1	120807600	0.743802
chr1	120822700	0.851240
chr1	121132100	0.809917
chr1	142576000	0.785124
chr1	142589300	0.768595
chr1	142794500	0.818182
chr1	142867100	0.752066
chr1	143280300	0.776860
chr1	143481600	0.793388

chr1	143509800	0.859504
chr1	143737600	0.801653
chr1	143921800	0.867769
chr1	143929700	0.809917
chr1	143944500	0.917355
chr1	143951600	0.793388
chr1	143963000	0.900826
chr1	143965700	0.768595
chr1	143991100	0.826446
chr1	143997300	0.776860
chr1	144000400	0.925620
chr1	144002700	0.826446
chr1	144008600	0.776860
chr1	144011800	0.884298
chr1	144061800	0.735537
chr1	144066900	0.768595
chr1	144069700	0.809917
chr1	144091600	0.752066
chr1	144095100	0.809917
chr1	144314500	0.884298
chr1	144316800	0.884298
chr1	144384500	0.909091
chr1	144513900	0.834711
chr1	144530000	0.768595
chr1	144538200	0.818182
chr1	144540300	0.851240
chr1	144547100	0.768595
chr1	144679900	0.801653
chr1	144690900	0.776860
chr1	144693300	0.826446
chr1	144871500	0.859504
chr1	144937700	0.785124
chr1	144955400	0.809917
chr1	144983900	0.826446
chr1	145004700	0.842975
chr1	145030000	0.942149
chr1	145033300	0.876033
chr1	145044900	0.900826
chr1	145058900	0.859504
chr1	145092400	0.760331
chr1	145096600	0.851240
chr1	145144900	0.785124
chr1	145148300	0.818182
chr1	145164100	0.785124
chr1	145167100	0.776860
chr1	145169200	0.785124
chr1	145173400	0.933884
chr1	145177800	0.793388
chr1	145183900	0.917355
chr1	145190200	0.842975
chr1	145196000	0.752066
chr1	145199700	0.818182
chr1	145202000	0.752066
chr1	145214200	0.900826

chr1	145219700	0.752066
chr1	145225400	0.776860
chr1	145227600	0.917355
chr1	145235800	0.743802
chr1	145248200	0.809917
chr1	145255500	0.793388
chr1	145259800	0.785124
chr1	145283300	0.917355
chr1	145288600	0.842975
chr1	145563000	0.776860
chr1	145589100	0.867769
chr1	145610800	0.851240
chr1	145715600	0.793388
chr1	146374100	0.859504
chr1	147052200	0.925620
chr1	147511300	0.867769
chr1	147727000	0.859504
chr1	147782900	0.735537
chr1	147833800	0.752066
chr1	147918700	0.876033
chr1	147975700	0.867769
chr1	148176500	0.834711
chr1	148222300	0.776860
chr1	148241100	0.884298
chr1	148551500	0.909091
chr1	148605300	0.942149
chr1	148624700	0.735537
chr1	148767400	0.900826
chr1	148786900	0.768595
chr1	148945800	0.966942
chr1	149205100	0.776860
chr1	149224300	0.743802
chr1	149289300	0.966942
chr1	149310500	0.760331
chr1	149356600	0.743802
chr1	149514700	0.933884
chr1	149533300	0.884298
chr1	149581500	0.776860
chr1	149603200	0.801653
chr1	149606000	0.793388
chr1	149674400	0.818182
chr1	149686600	0.743802
chr1	149696700	0.826446
chr1	149744200	0.760331
chr1	149900200	0.925620
chr1	149909800	0.818182
chr1	149911900	0.735537
chr1	149974100	0.785124
chr1	150241300	0.818182
chr1	150294200	0.892562
chr1	150359700	0.826446
chr1	150676400	0.876033
chr1	150681300	0.900826
chr1	150738100	0.900826

chr1	150750900	0.809917
chr1	150863000	0.851240
chr1	150898900	0.859504
chr1	150952200	0.809917
chr1	150976300	0.818182
chr1	151043300	0.859504
chr1	151129700	0.909091
chr1	151372200	0.851240
chr1	151461600	0.801653
chr1	151467900	0.809917
chr1	151794000	0.735537
chr1	151801500	0.785124
chr1	151833100	0.867769
chr1	151918500	0.793388
chr1	151957500	0.826446
chr1	152011100	0.776860
chr1	152020600	0.876033
chr1	152143700	0.760331
chr1	152552300	0.793388
chr1	152853200	0.975207
chr1	153196300	0.909091
chr1	153224400	0.842975
chr1	153233600	0.743802
chr1	153322100	0.785124
chr1	153327600	0.760331
chr1	153330700	0.760331
chr1	153518400	0.793388
chr1	153541700	0.834711
chr1	153571500	0.818182
chr1	153599600	0.768595
chr1	153606700	0.752066
chr1	153631200	0.851240
chr1	153671600	0.818182
chr1	153700700	0.760331
chr1	153740800	0.743802
chr1	153755900	0.975207
chr1	153825000	0.776860
chr1	153876200	0.760331
chr1	153896300	0.950413
chr1	153930700	0.785124
chr1	154130700	0.859504
chr1	154155300	0.768595
chr1	154171900	0.768595
chr1	154245300	0.809917
chr1	154374700	0.760331
chr1	154413400	0.917355
chr1	154415500	0.768595
chr1	154463600	0.776860
chr1	154493000	0.876033
chr1	154525500	0.809917
chr1	154600500	0.785124
chr1	154660000	0.743802
chr1	154718000	0.942149
chr1	154820000	0.785124

chr1	154823500	0.743802
chr1	154859300	0.760331
chr1	154934700	0.743802
chr1	154990200	0.909091
chr1	155034300	0.785124
chr1	155043700	0.867769
chr1	155046600	0.925620
chr1	155055200	0.768595
chr1	155064400	0.876033
chr1	155140600	0.760331
chr1	155177200	0.909091
chr1	155197800	0.867769
chr1	155220300	0.809917
chr1	155518200	0.867769
chr1	155686900	0.859504
chr1	155822100	0.752066
chr1	155916800	0.735537
chr1	155938900	0.735537
chr1	155942800	0.743802
chr1	155987900	0.752066
chr1	156147800	0.842975
chr1	156169100	0.892562
chr1	156187700	0.876033
chr1	156196700	0.975207
chr1	156403300	0.834711
chr1	156408400	0.834711
chr1	156426800	0.975207
chr1	156478200	0.909091
chr1	156571100	0.752066
chr1	156578600	0.942149
chr1	156613900	0.752066
chr1	156626700	0.859504
chr1	156654000	0.785124
chr1	156659700	0.768595
chr1	156833600	0.785124
chr1	157082600	0.809917
chr1	157118500	0.809917
chr1	157163800	0.776860
chr1	157166700	0.818182
chr1	157239500	0.809917
chr1	157241600	0.752066
chr1	157281400	0.859504
chr1	157399900	0.892562
chr1	157467100	0.900826
chr1	157479500	0.735537
chr1	157495000	0.785124
chr1	157536000	0.809917
chr1	157545500	0.859504
chr1	157568800	0.785124
chr1	157623500	0.818182
chr1	157675200	0.785124
chr1	157697300	0.851240
chr1	157742700	0.735537
chr1	157747100	0.842975

chr1	157751800	0.735537
chr1	157773400	0.768595
chr1	157809200	0.743802
chr1	157820100	0.743802
chr1	157826400	0.859504
chr1	158096400	0.801653
chr1	158124100	0.884298
chr1	158146600	0.826446
chr1	158218800	0.760331
chr1	158245400	0.826446
chr1	158258200	0.743802
chr1	158280900	0.752066
chr1	158582200	0.884298
chr1	158687100	0.876033
chr1	158976100	0.842975
chr1	159040600	0.735537
chr1	159047700	0.735537
chr1	159059300	0.942149
chr1	159131600	0.851240
chr1	159209900	0.900826
chr1	159613200	0.892562
chr1	159806100	0.826446
chr1	159857600	0.834711
chr1	159894800	0.793388
chr1	159897700	0.867769
chr1	159908800	0.851240
chr1	160017000	0.834711
chr1	160040600	0.760331
chr1	160054300	0.743802
chr1	160143100	0.785124
chr1	160158900	0.785124
chr1	160164300	0.917355
chr1	160346400	0.826446
chr1	160408300	0.768595
chr1	160414400	0.900826
chr1	160430600	0.867769
chr1	160444700	0.942149
chr1	160540300	0.743802
chr1	160549000	0.809917
chr1	160647500	0.793388
chr1	160660500	0.826446
chr1	160692800	0.842975
chr1	160695000	0.743802
chr1	160723700	0.785124
chr1	160737800	0.925620
chr1	160941900	0.834711
chr1	160945100	0.925620
chr1	160952000	0.834711
chr1	161102900	0.760331
chr1	161123600	0.735537
chr1	161237900	0.768595
chr1	161268000	0.743802
chr1	161278100	0.818182
chr1	161284100	0.743802

chr1	161363200	0.768595
chr1	161390600	0.743802
chr1	161462200	0.909091
chr1	161501000	0.900826
chr1	161531000	0.743802
chr1	161533300	0.942149
chr1	161537000	0.958678
chr1	161582700	0.842975
chr1	161613000	0.876033
chr1	161618200	0.859504
chr1	161681900	0.785124
chr1	161700100	0.752066
chr1	161736300	0.743802
chr1	161743900	0.776860
chr1	162330300	0.925620
chr1	162359500	0.760331
chr1	162392100	0.851240
chr1	162603300	0.892562
chr1	162610100	0.793388
chr1	162671600	0.785124
chr1	162804300	0.785124
chr1	162922000	0.826446
chr1	163380400	0.776860
chr1	165362500	0.884298
chr1	165365400	0.785124
chr1	165394200	0.776860
chr1	165465100	0.809917
chr1	165563000	0.760331
chr1	165570800	0.884298
chr1	165600000	0.752066
chr1	165605100	0.752066
chr1	165609000	0.793388
chr1	165611900	0.818182
chr1	165667600	0.884298
chr1	165723300	0.752066
chr1	165725400	0.818182
chr1	165738500	0.809917
chr1	165819100	0.735537
chr1	165821300	0.743802
chr1	165840700	0.818182
chr1	165851800	0.735537
chr1	166059600	0.768595
chr1	166150300	0.826446
chr1	166242100	0.752066
chr1	166292500	0.826446
chr1	166312200	0.900826
chr1	166459300	0.958678
chr1	166845600	0.876033
chr1	166854200	0.768595
chr1	166917300	0.826446
chr1	166940100	0.768595
chr1	166948400	0.950413
chr1	167073700	0.884298
chr1	167201500	0.735537

chr1	167218600	0.735537
chr1	167326700	0.826446
chr1	167417400	0.752066
chr1	167525900	0.909091
chr1	167535900	0.909091
chr1	167572200	0.768595
chr1	167589700	0.826446
chr1	167680000	0.818182
chr1	167722400	0.925620
chr1	167868700	0.900826
chr1	168001500	0.925620
chr1	168243600	0.793388
chr1	168490600	0.743802
chr1	168940800	0.743802
chr1	168989300	0.809917
chr1	168997900	0.776860
chr1	169018800	0.768595
chr1	169022100	0.760331
chr1	169061800	0.793388
chr1	169085600	0.809917
chr1	169103000	0.801653
chr1	169108500	0.867769
chr1	169132700	0.793388
chr1	169333200	0.909091
chr1	169461300	0.834711
chr1	169498300	0.867769
chr1	169556000	0.768595
chr1	169563900	0.925620
chr1	169574500	0.752066
chr1	169863300	0.834711
chr1	169878200	0.867769
chr1	170183900	0.776860
chr1	170901600	0.735537
chr1	171256100	0.818182
chr1	171477200	0.752066
chr1	171480700	0.743802
chr1	171580300	0.876033
chr1	171594700	0.735537
chr1	171614300	0.884298
chr1	171625000	0.884298
chr1	171711600	0.876033
chr1	171775300	0.776860
chr1	171787400	0.743802
chr1	171853700	0.892562
chr1	172224900	0.760331
chr1	172243900	0.752066
chr1	172330600	0.760331
chr1	172396700	0.809917
chr1	172419500	0.735537
chr1	172515300	0.809917
chr1	172613400	0.801653
chr1	172632200	0.818182
chr1	172640000	0.900826
chr1	172667200	0.785124

chr1	172669900	0.818182
chr1	172715900	0.842975
chr1	172725800	0.752066
chr1	172759900	0.933884
chr1	172795900	0.909091
chr1	172808000	0.768595
chr1	172814900	0.752066
chr1	172876500	0.925620
chr1	173008700	0.867769
chr1	173012600	0.768595
chr1	173082300	0.900826
chr1	173159800	0.768595
chr1	173227900	0.801653
chr1	173269800	0.752066
chr1	173372100	0.760331
chr1	173571100	0.867769
chr1	174079400	0.826446
chr1	174161800	0.801653
chr1	174293700	0.752066
chr1	174710000	0.867769
chr1	174756600	0.909091
chr1	174787500	0.867769
chr1	174938200	0.735537
chr1	174959700	0.958678
chr1	175041900	0.892562
chr1	175087900	0.859504
chr1	175167100	0.842975
chr1	175178800	0.760331
chr1	175188200	0.917355
chr1	175199800	0.785124
chr1	175221800	0.834711
chr1	175243700	0.809917
chr1	175276400	0.760331
chr1	175282700	0.809917
chr1	175359600	0.818182
chr1	175382500	0.776860
chr1	175413300	0.752066
chr1	175480800	0.743802
chr1	175643300	0.859504
chr1	176162400	0.801653
chr1	177787900	0.735537
chr1	178542700	0.859504
chr1	178546000	0.818182
chr1	178569000	0.809917
chr1	178619400	0.743802
chr1	178634700	0.793388
chr1	178654700	0.876033
chr1	178895200	0.776860
chr1	178898200	0.867769
chr1	178903700	0.743802
chr1	178943400	0.859504
chr1	179099200	0.752066
chr1	179196400	0.785124
chr1	179334900	0.933884

chr1	179479300	0.809917
chr1	179771800	0.752066
chr1	179965800	0.842975
chr1	180183100	0.776860
chr1	180314000	0.826446
chr1	180472200	0.768595
chr1	180821600	0.818182
chr1	180858500	0.735537
chr1	180879400	0.834711
chr1	180888000	0.793388
chr1	180895900	0.809917
chr1	180904400	0.826446
chr1	180998200	0.842975
chr1	181014000	0.950413
chr1	181048700	0.743802
chr1	181072200	0.752066
chr1	181082200	0.752066
chr1	181112000	0.793388
chr1	181120300	0.776860
chr1	181123400	0.867769
chr1	181149400	0.752066
chr1	181226500	0.785124
chr1	181436900	0.785124
chr1	181464500	0.785124
chr1	181467600	0.743802
chr1	182115300	0.776860
chr1	182140800	0.752066
chr1	182218200	0.842975
chr1	182228800	0.818182
chr1	182273500	0.834711
chr1	182365900	0.826446
chr1	182411100	0.801653
chr1	182535300	0.909091
chr1	182539500	0.785124
chr1	182603000	0.826446
chr1	182620100	0.793388
chr1	182622300	0.752066
chr1	182641700	0.900826
chr1	182649700	0.768595
chr1	182686600	0.793388
chr1	182751400	0.785124
chr1	182796400	0.776860
chr1	183003100	0.826446
chr1	183014400	0.818182
chr1	183030100	0.793388
chr1	183149900	0.909091
chr1	183164800	0.842975
chr1	183274300	0.909091
chr1	183458100	0.768595
chr1	183554800	0.801653
chr1	183912800	0.818182
chr1	183958200	0.842975
chr1	184134900	0.809917
chr1	184182500	0.743802

chr1	184329400	0.925620
chr1	184465200	0.776860
chr1	184531300	0.826446
chr1	184537000	0.834711
chr1	184570700	0.785124
chr1	184574400	0.760331
chr1	184616500	0.909091
chr1	184654500	0.801653
chr1	184716600	0.735537
chr1	184772600	0.752066
chr1	184837000	0.884298
chr1	184933800	0.793388
chr1	184951500	0.818182
chr1	185287000	0.785124
chr1	185315900	0.768595
chr1	185378300	0.917355
chr1	185496500	0.752066
chr1	185530500	0.834711
chr1	186048100	0.768595
chr1	186051700	0.743802
chr1	186163900	0.834711
chr1	186345100	0.752066
chr1	186431800	0.785124
chr1	186584100	0.933884
chr1	186709400	0.735537
chr1	186733000	0.842975
chr1	186741500	0.768595
chr1	186869100	0.743802
chr1	191144900	0.760331
chr1	192493900	0.760331
chr1	192570600	0.818182
chr1	192578800	0.900826
chr1	192596000	0.892562
chr1	192609300	0.867769
chr1	192673400	0.743802
chr1	193091500	0.743802
chr1	193225000	0.859504
chr1	193240300	0.859504
chr1	193408100	0.768595
chr1	193426900	0.826446
chr1	193448900	0.760331
chr1	194054500	0.743802
chr1	195711300	0.842975
chr1	197115700	0.760331
chr1	197323700	0.958678
chr1	198101800	0.752066
chr1	198128600	0.768595
chr1	198168300	0.892562
chr1	198222700	0.884298
chr1	198227700	0.735537
chr1	198323400	0.818182
chr1	198333400	0.776860
chr1	198519600	0.785124
chr1	198545600	0.917355

chr1	198588500	0.752066
chr1	198601200	0.859504
chr1	198665700	0.818182
chr1	198889200	0.735537
chr1	199052500	0.793388
chr1	199126800	0.743802
chr1	199138800	0.859504
chr1	199278000	0.776860
chr1	199468200	0.859504
chr1	199480000	0.785124
chr1	199998500	0.735537
chr1	200045300	0.776860
chr1	200187400	0.809917
chr1	200196200	0.834711
chr1	200255800	0.752066
chr1	200333100	0.950413
chr1	200341400	0.768595
chr1	200422600	0.776860
chr1	200424700	0.801653
chr1	200443100	0.876033
chr1	200496700	0.809917
chr1	200594100	0.735537
chr1	200609400	0.735537
chr1	200750300	0.826446
chr1	200772200	0.776860
chr1	200836500	0.735537
chr1	200862500	0.793388
chr1	200997000	0.826446
chr1	201405400	0.851240
chr1	201417600	0.801653
chr1	201420900	0.867769
chr1	201452800	0.801653
chr1	201476700	0.809917
chr1	201478800	0.785124
chr1	201482000	0.818182
chr1	201509700	0.785124
chr1	201706300	0.851240
chr1	201798600	0.867769
chr1	201853200	0.859504
chr1	201874900	0.735537
chr1	201924800	0.760331
chr1	201941700	0.809917
chr1	202007900	0.892562
chr1	202083600	0.760331
chr1	202149100	0.752066
chr1	202197700	0.760331
chr1	202206400	0.809917
chr1	202265400	0.793388
chr1	202344400	0.867769
chr1	202417800	0.826446
chr1	202483500	0.950413
chr1	202486000	0.760331
chr1	202497200	0.925620
chr1	202508600	0.834711

chr1	202516900	0.818182
chr1	202523200	0.834711
chr1	202536700	0.859504
chr1	202553800	0.793388
chr1	202559100	0.776860
chr1	202566700	0.834711
chr1	202569800	0.867769
chr1	202594300	0.876033
chr1	202599000	0.793388
chr1	202805300	0.809917
chr1	202858200	0.892562
chr1	202896300	0.809917
chr1	202938900	0.785124
chr1	203052300	0.785124
chr1	203146900	0.735537
chr1	203150000	0.760331
chr1	203240800	0.892562
chr1	203310300	0.859504
chr1	203395600	0.925620
chr1	203423200	0.826446
chr1	203457100	0.818182
chr1	203652100	0.834711
chr1	203708600	0.867769
chr1	203723800	0.950413
chr1	203734600	0.785124
chr1	203765200	0.768595
chr1	204074200	0.793388
chr1	204158700	0.933884
chr1	204183300	0.809917
chr1	204352000	0.793388
chr1	204460000	0.826446
chr1	204477000	0.752066
chr1	204482700	0.809917
chr1	204548300	0.884298
chr1	204651600	0.735537
chr1	204682700	0.909091
chr1	204685000	0.735537
chr1	204733200	0.760331
chr1	204932300	0.793388
chr1	205031100	0.752066
chr1	205091300	0.917355
chr1	205093900	0.776860
chr1	205111600	0.793388
chr1	205201500	0.785124
chr1	205208000	0.809917
chr1	205216300	0.768595
chr1	205243600	0.834711
chr1	205249400	0.842975
chr1	205264100	0.876033
chr1	205286400	0.842975
chr1	205288800	0.752066
chr1	205294500	0.892562
chr1	205309100	0.801653
chr1	205404700	0.909091

chr1	205474300	0.859504
chr1	205551900	0.809917
chr1	205557800	0.743802
chr1	205607900	0.768595
chr1	205636200	0.793388
chr1	205662500	0.826446
chr1	205719700	0.933884
chr1	205763900	0.818182
chr1	206084200	0.785124
chr1	206088000	0.900826
chr1	206116800	0.809917
chr1	206122700	0.768595
chr1	206130200	0.768595
chr1	206308500	0.760331
chr1	206329200	0.842975
chr1	206500200	0.834711
chr1	206531500	0.818182
chr1	206554300	0.760331
chr1	206558000	0.809917
chr1	206582300	0.842975
chr1	206604100	0.826446
chr1	206812500	0.785124
chr1	206832300	0.892562
chr1	206836300	0.867769
chr1	206881700	0.801653
chr1	206884200	0.818182
chr1	206940400	0.793388
chr1	206951400	0.851240
chr1	207015800	0.760331
chr1	207070700	0.785124
chr1	207080000	0.776860
chr1	207084000	0.851240
chr1	207140800	0.768595
chr1	207144800	0.760331
chr1	207204300	0.743802
chr1	207256300	0.809917
chr1	207264700	0.760331
chr1	207392400	0.851240
chr1	207581500	0.752066
chr1	207633000	0.743802
chr1	207667500	0.760331
chr1	207793800	0.752066
chr1	207922700	0.785124
chr1	207973200	0.818182
chr1	207981800	0.752066
chr1	208042200	0.768595
chr1	208046800	0.785124
chr1	208067400	0.785124
chr1	208127800	0.851240
chr1	208358500	0.735537
chr1	208378500	0.743802
chr1	208413000	0.909091
chr1	208761000	0.892562
chr1	209029000	0.859504

chr1	209211500	0.809917
chr1	209255900	0.859504
chr1	209277400	0.809917
chr1	209307900	0.793388
chr1	209381200	0.834711
chr1	209833500	0.818182
chr1	209849000	0.785124
chr1	209859800	0.876033
chr1	209922900	0.809917
chr1	209925700	0.900826
chr1	209930100	0.785124
chr1	210506700	0.826446
chr1	210514700	0.793388
chr1	210771900	0.793388
chr1	210837300	0.793388
chr1	211509700	0.892562
chr1	211556500	0.900826
chr1	211687900	0.818182
chr1	211722000	0.909091
chr1	211828700	0.842975
chr1	211851300	0.743802
chr1	211861500	0.942149
chr1	211872300	0.909091
chr1	211905500	0.785124
chr1	211914100	0.925620
chr1	211997800	0.752066
chr1	212004000	0.785124
chr1	212007500	0.892562
chr1	212012900	0.909091
chr1	212399400	0.826446
chr1	212411600	0.950413
chr1	212441200	0.892562
chr1	212469300	0.776860
chr1	212588800	0.942149
chr1	212604600	0.768595
chr1	212629600	0.851240
chr1	212651300	0.785124
chr1	212669700	0.818182
chr1	212672200	0.752066
chr1	212770100	0.876033
chr1	212788100	0.842975
chr1	212795200	0.785124
chr1	212845300	0.884298
chr1	212866200	0.743802
chr1	212895400	0.752066
chr1	212923400	0.752066
chr1	212955000	0.768595
chr1	212968300	0.966942
chr1	213102300	0.876033
chr1	213120800	0.752066
chr1	213133500	0.809917
chr1	213284800	0.900826
chr1	213796000	0.776860
chr1	213798100	0.826446

chr1	214398400	0.768595
chr1	214433000	0.752066
chr1	214495600	0.809917
chr1	214699200	0.735537
chr1	214731800	0.809917
chr1	214776800	0.859504
chr1	215717000	0.809917
chr1	216702500	0.892562
chr1	217200900	0.859504
chr1	217496400	0.809917
chr1	217669600	0.785124
chr1	217698100	0.925620
chr1	217796200	0.834711
chr1	217804700	0.933884
chr1	217847500	0.785124
chr1	218550800	0.867769
chr1	218633400	0.760331
chr1	219256000	0.809917
chr1	219273100	0.900826
chr1	219345200	0.785124
chr1	219650400	0.900826
chr1	219748400	0.785124
chr1	219755900	0.826446
chr1	219947700	0.785124
chr1	220445700	0.851240
chr1	220511200	0.859504
chr1	220829100	0.884298
chr1	220876100	0.876033
chr1	221613200	0.752066
chr1	221683200	0.834711
chr1	221857800	0.826446
chr1	221954700	0.801653
chr1	221957500	0.768595
chr1	222011600	0.752066
chr1	222047400	0.752066
chr1	222052500	0.776860
chr1	222135400	0.743802
chr1	222629200	0.826446
chr1	222746600	0.785124
chr1	222817500	0.743802
chr1	222904200	0.834711
chr1	222924300	0.876033
chr1	222947300	0.793388
chr1	222962000	0.909091
chr1	222991000	0.743802
chr1	223231100	0.760331
chr1	223363600	0.793388
chr1	223656400	0.768595
chr1	223827900	0.735537
chr1	223893200	0.735537
chr1	224037500	0.851240
chr1	224091600	0.768595
chr1	224187200	0.743802
chr1	224294500	0.842975

chr1	224383600	0.867769
chr1	224391500	0.809917
chr1	224398000	0.809917
chr1	224456100	0.735537
chr1	224518100	0.900826
chr1	224552200	0.785124
chr1	224570900	0.776860
chr1	224698500	0.743802
chr1	224829200	0.859504
chr1	224926300	0.743802
chr1	224938700	0.735537
chr1	225438300	0.809917
chr1	225616300	0.793388
chr1	225624400	0.768595
chr1	225655200	0.809917
chr1	225665500	0.735537
chr1	225668700	0.776860
chr1	225952300	0.735537
chr1	225966000	0.884298
chr1	226109300	0.776860
chr1	226112000	0.867769
chr1	226132900	0.760331
chr1	226198900	0.752066
chr1	226300400	0.793388
chr1	226374400	0.859504
chr1	226496800	0.809917
chr1	226532200	0.760331
chr1	226537900	0.851240
chr1	226600000	0.809917
chr1	226634100	0.793388
chr1	226738000	0.735537
chr1	226820400	0.735537
chr1	226871400	0.801653
chr1	226879500	0.793388
chr1	226891200	0.925620
chr1	226913300	0.842975
chr1	227015600	0.983471
chr1	227103500	0.851240
chr1	227127400	0.768595
chr1	227178300	0.768595
chr1	227183500	0.768595
chr1	227355300	0.785124
chr1	227379900	0.851240
chr1	227751500	0.876033
chr1	228226000	0.760331
chr1	228279100	0.743802
chr1	228353900	0.826446
chr1	228457600	0.793388
chr1	228594200	0.776860
chr1	228670400	0.735537
chr1	228674700	0.768595
chr1	228688500	0.760331
chr1	228930100	0.768595
chr1	228947200	0.752066

chr1	229163700	0.884298
chr1	229171600	0.809917
chr1	229265400	0.842975
chr1	229272900	0.842975
chr1	229275800	0.809917
chr1	229335900	0.851240
chr1	229365200	0.801653
chr1	229367300	0.826446
chr1	229389500	0.859504
chr1	229394500	0.884298
chr1	229506400	0.834711
chr1	229707900	0.900826
chr1	229715300	0.809917
chr1	229832000	0.859504
chr1	229977600	0.909091
chr1	229992200	0.983471
chr1	230124200	0.851240
chr1	230173400	0.909091
chr1	230254000	0.909091
chr1	230276900	0.776860
chr1	230286200	0.851240
chr1	230350200	0.859504
chr1	230464200	0.752066
chr1	230745400	0.892562
chr1	231513100	0.793388
chr1	231515200	0.834711
chr1	231546300	0.768595
chr1	231558200	0.826446
chr1	231652000	0.851240
chr1	232055300	0.876033
chr1	232791400	0.768595
chr1	232803600	0.743802
chr1	232861300	0.983471
chr1	233080400	0.760331
chr1	233088500	0.818182
chr1	233276500	0.760331
chr1	233378900	0.768595
chr1	233397600	0.826446
chr1	233405200	0.925620
chr1	233686100	0.842975
chr1	233775300	0.768595
chr1	233778900	0.735537
chr1	234033600	0.760331
chr1	234299500	0.809917
chr1	234306900	0.768595
chr1	234406700	0.876033
chr1	234446000	0.834711
chr1	234458500	0.776860
chr1	234626100	0.793388
chr1	234648700	0.793388
chr1	234653400	0.900826
chr1	234665000	0.809917
chr1	234913600	0.975207
chr1	234950500	0.867769

chr1	234953600	0.809917
chr1	234986200	0.768595
chr1	234996200	0.826446
chr1	235127400	0.768595
chr1	235170800	0.933884
chr1	235177200	0.776860
chr1	235188300	0.735537
chr1	235242800	0.909091
chr1	235247000	0.933884
chr1	235253500	0.876033
chr1	235257500	0.884298
chr1	235292100	0.735537
chr1	235324500	0.809917
chr1	235422700	0.842975
chr1	235481600	0.776860
chr1	235664800	0.735537
chr1	235668600	0.851240
chr1	235796600	0.760331
chr1	235805700	0.958678
chr1	235931300	0.958678
chr1	236018100	0.768595
chr1	236027900	0.785124
chr1	236046600	0.867769
chr1	236051600	0.867769
chr1	236082300	0.942149
chr1	236135600	0.950413
chr1	236260600	0.793388
chr1	236288000	0.760331
chr1	236301900	0.892562
chr1	236568800	0.776860
chr1	236585800	0.818182
chr1	236589100	0.785124
chr1	236625400	0.785124
chr1	236672700	0.752066
chr1	237195600	0.900826
chr1	237670800	0.900826
chr1	238046800	0.975207
chr1	241649200	0.743802
chr1	241715300	0.925620
chr1	242003100	0.776860
chr1	242011400	0.752066
chr1	242310900	0.760331
chr1	242977100	0.859504
chr1	243264600	0.900826
chr1	243479800	0.743802
chr1	243646200	0.743802
chr1	243887700	0.793388
chr1	243891700	0.809917
chr1	243900500	0.818182
chr1	244081200	0.818182
chr1	244140800	0.859504
chr1	244274800	0.768595
chr1	244418200	0.892562
chr1	244463000	0.909091

chr1	244483300	0.785124
chr1	244535600	0.876033
chr1	244614700	0.884298
chr1	244829100	0.876033
chr1	245918400	0.785124
chr1	245920700	0.760331
chr1	245974700	0.892562
chr1	246117600	0.834711
chr1	246195700	0.826446
chr1	246497500	0.793388
chr1	246984600	0.735537
chr1	247095300	0.776860
chr1	247335300	0.768595
chr1	247373700	0.900826
chr1	247376700	0.785124
chr1	247521600	0.809917
chr1	247548900	0.801653
chr1	247552600	0.785124
chr1	247554700	0.752066
chr1	248626600	0.735537
chr1	248827200	0.884298
chr1	249095400	0.801653
chr1	249129000	0.818182
chr1	249167500	0.793388
chr1	249200500	0.809917
chr1	249203900	0.867769
chr20	328600	0.818182
chr20	340000	0.818182
chr20	342800	0.892562
chr20	346300	0.801653
chr20	524500	0.933884
chr20	573000	0.743802
chr20	597100	0.842975
chr20	644700	0.884298
chr20	814600	0.842975
chr20	837300	0.851240
chr20	873100	0.818182
chr20	991600	0.818182
chr20	1193600	0.768595
chr20	1196600	0.818182
chr20	1213300	0.735537
chr20	1268400	0.752066
chr20	1298700	0.842975
chr20	1306500	0.851240
chr20	1415700	0.834711
chr20	1451400	0.867769
chr20	1473100	0.826446
chr20	1475300	0.768595
chr20	1502100	0.834711
chr20	1505000	0.752066
chr20	1507700	0.768595
chr20	1520000	0.892562
chr20	1613400	0.900826
chr20	1634300	0.884298

chr20	1704500	0.793388
chr20	1745400	0.776860
chr20	1801100	0.826446
chr20	1812200	0.933884
chr20	1827400	0.760331
chr20	1839800	0.818182
chr20	1852900	0.801653
chr20	1877600	0.768595
chr20	1934200	0.867769
chr20	2091600	0.900826
chr20	2270200	0.900826
chr20	2282700	0.785124
chr20	2333100	0.743802
chr20	2372200	0.793388
chr20	2451600	0.768595
chr20	2710500	0.793388
chr20	2752500	0.826446
chr20	2803100	0.851240
chr20	2807600	0.867769
chr20	2820000	0.785124
chr20	3058500	0.752066
chr20	3083300	0.842975
chr20	3184700	0.909091
chr20	3401100	0.950413
chr20	3411600	0.884298
chr20	3690500	0.826446
chr20	3733100	0.776860
chr20	3742200	0.743802
chr20	3776700	0.735537
chr20	3783700	0.735537
chr20	3792700	0.859504
chr20	3801600	0.842975
chr20	3827800	0.735537
chr20	3880800	0.752066
chr20	3973900	0.735537
chr20	3998500	0.859504
chr20	4063700	0.909091
chr20	4066400	0.809917
chr20	4094800	0.809917
chr20	4132700	0.917355
chr20	4142000	0.867769
chr20	4232000	0.801653
chr20	4589000	0.900826
chr20	4594700	0.859504
chr20	4692800	0.826446
chr20	4701000	0.933884
chr20	4708000	0.793388
chr20	4747600	0.752066
chr20	4789000	0.842975
chr20	4876300	0.917355
chr20	4922500	0.743802
chr20	4928000	0.752066
chr20	5037900	0.760331
chr20	5117300	0.834711

chr20	5122400	0.776860
chr20	5126500	0.809917
chr20	5248200	0.735537
chr20	5471900	0.867769
chr20	5604600	0.793388
chr20	5646600	0.743802
chr20	5649000	0.867769
chr20	5656400	0.801653
chr20	5718900	0.752066
chr20	5778500	0.925620
chr20	5817000	0.776860
chr20	5822900	0.785124
chr20	5829900	0.876033
chr20	8197400	0.909091
chr20	8203000	0.776860
chr20	8721700	0.785124
chr20	8738100	0.785124
chr20	9372300	0.801653
chr20	9374500	0.892562
chr20	9379100	0.851240
chr20	9393300	0.876033
chr20	9410200	0.793388
chr20	9423000	0.884298
chr20	9463400	0.884298
chr20	9486300	0.975207
chr20	9554500	0.818182
chr20	9607800	0.851240
chr20	10020800	0.809917
chr20	10041000	0.925620
chr20	10331600	0.776860
chr20	10408700	0.842975
chr20	10413000	0.752066
chr20	10449600	0.735537
chr20	10492300	0.900826
chr20	10632400	0.743802
chr20	10644100	0.834711
chr20	11186100	0.735537
chr20	12990800	0.752066
chr20	13228900	0.785124
chr20	13304300	0.793388
chr20	13378400	0.752066
chr20	13636100	0.785124
chr20	13640200	0.842975
chr20	13665900	0.909091
chr20	14519200	0.752066
chr20	14782900	0.752066
chr20	14932300	0.867769
chr20	15247300	0.752066
chr20	15313800	0.851240
chr20	15366600	0.760331
chr20	15634800	0.776860
chr20	15838500	0.900826
chr20	15904700	0.842975
chr20	15988400	0.925620

chr20	16005400	0.884298
chr20	16139800	0.933884
chr20	16298700	0.793388
chr20	16373200	0.793388
chr20	16691900	0.809917
chr20	16710700	0.851240
chr20	16739900	0.842975
chr20	17487800	0.752066
chr20	17519200	0.809917
chr20	17547600	0.768595
chr20	17777300	0.752066
chr20	17828700	0.801653
chr20	17831300	0.842975
chr20	17840900	0.776860
chr20	17881700	0.785124
chr20	17924800	0.834711
chr20	18184400	0.785124
chr20	18257800	0.801653
chr20	18263500	0.760331
chr20	18393600	0.925620
chr20	18735400	0.809917
chr20	18783200	0.892562
chr20	19006900	0.735537
chr20	19218500	0.776860
chr20	19244500	0.793388
chr20	19308400	0.785124
chr20	19329000	0.785124
chr20	19409600	0.743802
chr20	19649800	0.818182
chr20	19687600	0.785124
chr20	19760900	0.743802
chr20	19802300	0.760331
chr20	19884500	0.834711
chr20	19922000	0.933884
chr20	19984400	0.867769
chr20	20210500	0.909091
chr20	20433000	0.743802
chr20	20662000	0.859504
chr20	20693600	0.818182
chr20	20724800	0.859504
chr20	20761300	0.826446
chr20	20934800	0.760331
chr20	21056000	0.859504
chr20	21261700	0.842975
chr20	21292600	0.768595
chr20	21484200	0.818182
chr20	21540400	0.876033
chr20	22327600	0.917355
chr20	22352800	0.809917
chr20	22368200	0.826446
chr20	22456800	0.776860
chr20	22709800	0.768595
chr20	23046400	0.851240
chr20	23055400	0.776860

chr20	23077900	0.801653
chr20	23119200	0.818182
chr20	23123100	0.768595
chr20	23125700	0.735537
chr20	23170800	0.809917
chr20	23181400	0.909091
chr20	23227400	0.859504
chr20	23315800	0.793388
chr20	23401800	0.834711
chr20	23423600	0.842975
chr20	23430500	0.776860
chr20	23473900	0.768595
chr20	23496100	0.760331
chr20	23512600	0.760331
chr20	23588000	0.818182
chr20	23616200	0.834711
chr20	23624400	0.809917
chr20	23636500	0.752066
chr20	24129400	0.752066
chr20	24590300	0.884298
chr20	24629900	0.785124
chr20	24720500	0.876033
chr20	24776800	0.735537
chr20	24821700	0.768595
chr20	24825800	0.743802
chr20	24832500	0.842975
chr20	24899400	0.925620
chr20	25035800	0.892562
chr20	25046700	0.892562
chr20	25071900	0.776860
chr20	25141500	0.752066
chr20	25233700	0.743802
chr20	25291600	0.892562
chr20	25300000	0.743802
chr20	25341700	0.743802
chr20	25360400	0.876033
chr20	25379800	0.801653
chr20	25388600	0.826446
chr20	25420000	0.735537
chr20	25604900	0.760331
chr20	25615000	0.876033
chr20	25677100	0.884298
chr20	29587200	0.776860
chr20	29593000	0.933884
chr20	29634800	0.909091
chr20	29638100	0.785124
chr20	30063100	0.809917
chr20	30190800	0.743802
chr20	30210200	0.768595
chr20	30229700	0.851240
chr20	30311200	0.859504
chr20	30424300	0.768595
chr20	30434300	0.867769
chr20	30446700	0.785124

chr20	30456600	0.876033
chr20	30506900	0.909091
chr20	30511900	0.735537
chr20	30545100	0.785124
chr20	30605700	0.842975
chr20	30677700	0.735537
chr20	30697500	0.801653
chr20	30710700	0.876033
chr20	30798300	0.776860
chr20	30957200	0.785124
chr20	31063500	0.776860
chr20	31098100	0.735537
chr20	31215900	0.826446
chr20	31253200	0.735537
chr20	31261100	0.834711
chr20	31326400	0.752066
chr20	31378300	0.768595
chr20	31382500	0.776860
chr20	31556000	0.876033
chr20	31585700	0.743802
chr20	32054200	0.859504
chr20	32143200	0.900826
chr20	32191200	0.834711
chr20	32195300	0.809917
chr20	32197800	0.768595
chr20	32236200	0.752066
chr20	32241800	0.851240
chr20	32260700	0.735537
chr20	32275300	0.892562
chr20	32321900	0.884298
chr20	32406100	0.760331
chr20	32472300	0.785124
chr20	32506500	0.917355
chr20	32657500	0.768595
chr20	32699800	0.735537
chr20	32804200	0.851240
chr20	32837400	0.867769
chr20	32891200	0.851240
chr20	32926100	0.950413
chr20	32951200	0.785124
chr20	32954300	0.752066
chr20	32989200	0.735537
chr20	33189400	0.752066
chr20	33207700	0.793388
chr20	33297200	0.743802
chr20	33335700	0.950413
chr20	33495900	0.760331
chr20	33543300	0.785124
chr20	33644000	0.884298
chr20	34042700	0.884298
chr20	34078800	0.842975
chr20	34242400	0.776860
chr20	34252700	0.900826
chr20	34300400	0.752066

chr20	34303000	0.867769
chr20	34407300	0.752066
chr20	34483900	0.900826
chr20	34616900	0.801653
chr20	34639000	0.859504
chr20	34702700	0.752066
chr20	34731300	0.867769
chr20	34734500	0.735537
chr20	34754000	0.834711
chr20	35098800	0.884298
chr20	35258700	0.735537
chr20	35386600	0.933884
chr20	35465600	0.909091
chr20	35575800	0.826446
chr20	35724700	0.768595
chr20	35819300	0.826446
chr20	35894000	0.809917
chr20	35899500	0.884298
chr20	35905600	0.793388
chr20	36037900	0.735537
chr20	36156200	0.809917
chr20	36327700	0.842975
chr20	36480000	0.768595
chr20	36810000	0.785124
chr20	36933100	0.735537
chr20	37056600	0.826446
chr20	37082600	0.735537
chr20	37230500	0.785124
chr20	38575100	0.752066
chr20	39305300	0.851240
chr20	39309600	0.785124
chr20	39333500	0.818182
chr20	39551900	0.859504
chr20	39599600	0.743802
chr20	39611400	0.743802
chr20	39783900	0.793388
chr20	39824400	0.752066
chr20	39861500	0.826446
chr20	39878400	0.768595
chr20	39933300	0.735537
chr20	39936400	0.851240
chr20	39958700	0.809917
chr20	39999400	0.851240
chr20	40057500	0.768595
chr20	40200800	0.826446
chr20	40238200	0.851240
chr20	40250400	0.743802
chr20	40257100	0.785124
chr20	40272200	0.809917
chr20	40302600	0.884298
chr20	40995900	0.892562
chr20	41064200	0.826446
chr20	41090800	0.809917
chr20	41121700	0.735537

chr20	41160500	0.950413
chr20	42067700	0.826446
chr20	42086800	0.826446
chr20	42286200	0.884298
chr20	42357500	0.793388
chr20	42550400	0.851240
chr20	42640500	0.851240
chr20	42764100	0.735537
chr20	42902300	0.752066
chr20	43189200	0.851240
chr20	43220000	0.859504
chr20	43361700	0.752066
chr20	43590100	0.735537
chr20	43602600	0.760331
chr20	43782700	0.801653
chr20	43883800	0.867769
chr20	43919600	0.876033
chr20	43922600	0.776860
chr20	43948000	0.842975
chr20	43984100	0.851240
chr20	43998600	0.752066
chr20	44002400	0.768595
chr20	44136400	0.752066
chr20	44381400	0.851240
chr20	44400000	0.842975
chr20	44413500	0.892562
chr20	44441500	0.735537
chr20	44563700	0.743802
chr20	44600700	0.793388
chr20	44622000	0.917355
chr20	44627700	0.851240
chr20	44718600	0.834711
chr20	44755100	0.809917
chr20	44835300	0.842975
chr20	44992800	0.966942
chr20	45176100	0.785124
chr20	45191500	0.851240
chr20	45313200	0.851240
chr20	45623300	0.776860
chr20	45825800	0.892562
chr20	45903800	0.809917
chr20	45949500	0.752066
chr20	45989200	0.892562
chr20	46191000	0.826446
chr20	46334100	0.925620
chr20	46354100	0.876033
chr20	46356900	0.793388
chr20	46457000	0.892562
chr20	46524200	0.876033
chr20	46531600	0.785124
chr20	46534400	0.776860
chr20	46604200	0.752066
chr20	46607200	0.768595
chr20	46998300	0.892562

chr20	47051300	0.768595
chr20	47085700	0.867769
chr20	47108500	0.793388
chr20	47119500	0.876033
chr20	47132500	0.933884
chr20	47161500	0.859504
chr20	47300100	0.735537
chr20	47353000	0.735537
chr20	47421400	0.818182
chr20	47437700	0.760331
chr20	47455800	0.950413
chr20	47460200	0.801653
chr20	47481800	0.809917
chr20	47489700	0.983471
chr20	47527200	0.776860
chr20	47833800	0.768595
chr20	47836100	0.867769
chr20	47889900	0.933884
chr20	48151700	0.743802
chr20	48190100	0.760331
chr20	48195700	0.834711
chr20	48214800	0.826446
chr20	48225700	0.884298
chr20	48228000	0.801653
chr20	48311200	0.809917
chr20	48313500	0.768595
chr20	48354200	0.867769
chr20	48385600	0.752066
chr20	48398800	0.776860
chr20	48406200	0.760331
chr20	48484700	0.859504
chr20	48596000	0.793388
chr20	48612700	0.752066
chr20	48615700	0.752066
chr20	48640000	0.760331
chr20	48719400	0.867769
chr20	48744200	0.760331
chr20	48763700	0.917355
chr20	48852400	0.809917
chr20	48914300	0.735537
chr20	48956300	0.793388
chr20	48963600	0.892562
chr20	49003300	0.818182
chr20	49044500	0.776860
chr20	49062800	0.793388
chr20	49069900	0.785124
chr20	49073500	0.909091
chr20	49081400	0.826446
chr20	49095100	0.892562
chr20	49198600	0.768595
chr20	49212800	0.809917
chr20	49216800	0.842975
chr20	49344000	0.958678
chr20	49407600	0.958678

chr20	49416300	0.776860
chr20	49548200	0.900826
chr20	49575000	0.776860
chr20	49745200	0.859504
chr20	49980200	0.793388
chr20	50019500	0.785124
chr20	50171700	0.818182
chr20	50214500	0.752066
chr20	50237200	0.867769
chr20	50248500	0.842975
chr20	50417500	0.809917
chr20	50868100	0.801653
chr20	51020000	0.818182
chr20	51022600	0.768595
chr20	51591900	0.859504
chr20	51618600	0.942149
chr20	51763200	0.842975
chr20	51772200	0.884298
chr20	51804300	0.752066
chr20	51843400	0.785124
chr20	51944800	0.809917
chr20	52030100	0.785124
chr20	52108300	0.834711
chr20	52187800	0.818182
chr20	52298600	0.785124
chr20	52302400	0.826446
chr20	52371200	0.760331
chr20	52384600	0.743802
chr20	52403400	0.876033
chr20	52479900	0.768595
chr20	52496200	0.851240
chr20	52534300	0.785124
chr20	52680700	0.760331
chr20	52684300	0.859504
chr20	52696400	0.892562
chr20	52707100	0.785124
chr20	52824700	0.909091
chr20	52844200	0.793388
chr20	53223700	0.776860
chr20	54928000	0.842975
chr20	54997600	0.859504
chr20	55004200	0.768595
chr20	55297600	0.876033
chr20	55750700	0.743802
chr20	55857600	0.743802
chr20	56008200	0.876033
chr20	56010600	0.818182
chr20	56024900	0.752066
chr20	56052700	0.768595
chr20	56074000	0.892562
chr20	56078500	0.768595
chr20	56098300	0.809917
chr20	56169000	0.809917
chr20	56172200	0.801653

chr20	56185000	0.752066
chr20	56251800	0.909091
chr20	56274500	0.892562
chr20	56277600	0.785124
chr20	56324100	0.909091
chr20	56345300	0.917355
chr20	56477700	0.768595
chr20	56964600	0.851240
chr20	56976700	0.760331
chr20	57132800	0.859504
chr20	57210100	0.760331
chr20	57244900	0.801653
chr20	57361300	0.793388
chr20	57445600	0.768595
chr20	57506100	0.776860
chr20	57521400	0.859504
chr20	57537100	0.851240
chr20	57545500	0.834711
chr20	57607600	0.826446
chr20	57646700	0.743802
chr20	57695400	0.809917
chr20	57753200	0.735537
chr20	57758000	0.801653
chr20	58147400	0.735537
chr20	58298400	0.950413
chr20	58342700	0.859504
chr20	58406100	0.743802
chr20	58413800	0.851240
chr20	58515900	0.917355
chr20	60488700	0.793388
chr20	60511200	0.909091
chr20	60614900	0.842975
chr20	60701500	0.752066
chr20	60758300	0.743802
chr20	60915200	0.867769
chr20	60982800	0.925620
chr20	60985300	0.826446
chr20	61200300	0.859504
chr20	61276800	0.743802
chr20	61439600	0.768595
chr20	61506400	0.735537
chr20	61533000	0.752066
chr20	61547200	0.793388
chr20	61557600	0.752066
chr20	61562500	0.867769
chr20	61572800	0.760331
chr20	61603800	0.793388
chr20	61620900	0.776860
chr20	62065700	0.785124
chr20	62104200	0.975207
chr20	62152300	0.859504
chr20	62160300	0.900826
chr20	62318600	0.735537
chr20	62413500	0.801653

chr20	62458100	0.793388
chr20	62535900	0.809917
chr20	62689900	0.752066
chr20	62731500	0.809917
chr20	62739500	0.760331
chr21	9685200	0.842975
chr21	9830800	0.818182
chr21	9848300	0.867769
chr21	9873400	0.776860
chr21	9916000	0.867769
chr21	9939200	0.826446
chr21	9995100	0.752066
chr21	10149200	0.909091
chr21	10164200	0.966942
chr21	10188200	0.760331
chr21	10455400	0.768595
chr21	11065200	0.809917
chr21	11071900	0.809917
chr21	11085200	0.867769
chr21	11112800	0.768595
chr21	11122400	0.760331
chr21	11165800	0.884298
chr21	15893200	0.818182
chr21	15975300	0.752066
chr21	16048800	0.793388
chr21	16244200	0.909091
chr21	16578800	0.735537
chr21	16780700	0.851240
chr21	18861300	0.785124
chr21	18864700	0.793388
chr21	18912000	0.859504
chr21	19072300	0.859504
chr21	19082000	0.842975
chr21	19274800	0.818182
chr21	27007100	0.752066
chr21	27163200	0.768595
chr21	27276900	0.760331
chr21	27340200	0.933884
chr21	27490300	0.900826
chr21	30257400	0.884298
chr21	30375000	0.818182
chr21	30391700	0.809917
chr21	30397100	0.768595
chr21	30445900	0.776860
chr21	30503200	0.826446
chr21	30569400	0.917355
chr21	30611300	0.768595
chr21	31069700	0.768595
chr21	32476000	0.859504
chr21	32504000	0.917355
chr21	32509900	0.826446
chr21	32926700	0.826446
chr21	33028200	0.785124
chr21	33038600	0.735537

chr21	33108000	0.826446
chr21	33388500	0.826446
chr21	33393500	0.752066
chr21	33509700	0.867769
chr21	33943500	0.842975
chr21	33997700	0.859504
chr21	34450500	0.785124
chr21	34569600	0.785124
chr21	34588800	0.735537
chr21	34634100	0.818182
chr21	34668700	0.900826
chr21	34773100	0.785124
chr21	34807600	0.785124
chr21	35220900	0.793388
chr21	35419200	0.743802
chr21	35455800	0.818182
chr21	35571300	0.793388
chr21	35574600	0.884298
chr21	35578700	0.876033
chr21	35734500	0.752066
chr21	35756800	0.809917
chr21	35770200	0.809917
chr21	35789900	0.776860
chr21	35855800	0.776860
chr21	35907100	0.743802
chr21	35954700	0.752066
chr21	36005300	0.818182
chr21	36174800	0.776860
chr21	36219400	0.818182
chr21	36247300	0.876033
chr21	36285500	0.933884
chr21	36342500	0.851240
chr21	36362800	0.809917
chr21	36408200	0.851240
chr21	36412900	0.776860
chr21	36430700	0.909091
chr21	36563000	0.809917
chr21	36571500	0.768595
chr21	36595100	0.785124
chr21	36607500	0.876033
chr21	36625300	0.917355
chr21	36694800	0.776860
chr21	36811500	0.900826
chr21	37268700	0.801653
chr21	37377200	0.975207
chr21	37393800	0.884298
chr21	37400900	0.760331
chr21	37499100	0.801653
chr21	37514400	0.743802
chr21	37662200	0.884298
chr21	37667900	0.776860
chr21	37675300	0.917355
chr21	37851100	0.785124
chr21	38226000	0.809917

chr21	38350000	0.867769
chr21	38626900	0.760331
chr21	38640600	0.925620
chr21	38646600	0.743802
chr21	38664500	0.735537
chr21	38746200	0.776860
chr21	38751200	0.793388
chr21	38906000	0.735537
chr21	38920100	0.743802
chr21	39621300	0.793388
chr21	39659300	0.842975
chr21	40145500	0.851240
chr21	40149700	0.966942
chr21	40160000	0.760331
chr21	40203900	0.826446
chr21	40358000	0.909091
chr21	40685900	0.876033
chr21	40721800	0.867769
chr21	40746200	0.760331
chr21	42602000	0.876033
chr21	42808300	0.735537
chr21	42855100	0.818182
chr21	42933900	0.809917
chr21	42943800	0.752066
chr21	43258500	0.743802
chr21	43294300	0.892562
chr21	43299800	0.942149
chr21	43375000	0.752066
chr21	43378900	0.768595
chr21	43423300	0.768595
chr21	43486000	0.760331
chr21	43522800	0.867769
chr21	43528400	0.743802
chr21	43534500	0.809917
chr21	43550200	0.867769
chr21	43608200	0.876033
chr21	43619800	0.768595
chr21	43655300	0.834711
chr21	43659400	0.785124
chr21	43670700	0.809917
chr21	43709500	0.884298
chr21	43758800	0.826446
chr21	43783200	0.834711
chr21	43800400	0.809917
chr21	43823100	0.760331
chr21	43866200	0.826446
chr21	43883000	0.785124
chr21	43899400	0.768595
chr21	43932400	0.735537
chr21	43950700	0.851240
chr21	44015400	0.900826
chr21	44198200	0.900826
chr21	44369700	0.842975
chr21	44395500	0.851240

chr21	44400000	0.867769
chr21	44466400	0.867769
chr21	44506900	0.867769
chr21	44574200	0.917355
chr21	44593800	0.768595
chr21	44597400	0.925620
chr21	44614900	0.768595
chr21	44700500	0.826446
chr21	44710600	0.851240
chr21	44739300	0.826446
chr21	44790200	0.809917
chr21	44830400	0.884298
chr21	44847400	0.867769
chr21	44859800	0.809917
chr21	44874100	0.876033
chr21	44876900	0.826446
chr21	44900600	0.752066
chr21	44985300	0.942149
chr21	45066900	0.909091
chr21	45123600	0.818182
chr21	45139600	0.801653
chr21	45153300	0.818182
chr21	45198700	0.826446
chr21	45203400	0.818182
chr21	45570600	0.785124
chr21	45593200	0.867769
chr21	45616600	0.735537
chr21	45624200	0.752066
chr21	45666400	0.743802
chr21	45703000	0.859504
chr21	45759700	0.950413
chr21	45925200	0.900826
chr21	46235300	0.735537
chr21	46238200	0.909091
chr21	46286600	0.785124
chr21	46385400	0.735537
chr21	46525700	0.785124
chr21	46533300	0.776860
chr21	46542700	0.818182
chr21	46547800	0.917355
chr21	46550800	0.884298
chr21	46687400	0.776860
chr21	46746300	0.793388
chr21	46921300	0.892562
chr21	47091800	0.958678
chr21	47247900	0.768595
chr21	47268300	0.785124
chr21	47306300	0.776860
chr21	47401700	0.991736
chr21	47478800	0.851240
chr21	47878200	0.776860
chr21	48055200	0.900826
chr22	17495300	0.909091
chr22	17514300	0.909091

chr22	17566200	0.793388
chr22	17599600	0.809917
chr22	17718300	0.809917
chr22	17724800	0.950413
chr22	17785300	0.842975
chr22	17916000	0.842975
chr22	17959700	0.776860
chr22	17981100	0.834711
chr22	18248100	0.876033
chr22	18269300	0.752066
chr22	18414700	0.834711
chr22	18424900	0.743802
chr22	18475800	0.752066
chr22	18478000	0.859504
chr22	18484200	0.909091
chr22	18493900	0.801653
chr22	18512600	0.892562
chr22	18551800	0.851240
chr22	18554400	0.867769
chr22	18574800	0.958678
chr22	18694800	0.851240
chr22	18795500	0.818182
chr22	18847600	0.842975
chr22	18881600	0.809917
chr22	19142200	0.785124
chr22	19163400	0.752066
chr22	19170400	0.768595
chr22	19407300	0.826446
chr22	19467200	0.818182
chr22	19564700	0.818182
chr22	19567500	0.752066
chr22	19596000	0.776860
chr22	19598800	0.884298
chr22	19602900	0.876033
chr22	19702700	0.752066
chr22	19761000	0.867769
chr22	19764200	0.743802
chr22	20105700	0.752066
chr22	20194000	0.776860
chr22	20235200	0.909091
chr22	20785200	0.801653
chr22	20878100	0.809917
chr22	20923400	0.809917
chr22	21280800	0.834711
chr22	21291800	0.900826
chr22	21410500	0.735537
chr22	21530300	0.801653
chr22	21598100	0.760331
chr22	21600300	0.752066
chr22	21612700	0.768595
chr22	21650600	0.760331
chr22	21961200	0.809917
chr22	21984200	0.925620
chr22	22062400	0.925620

chr22	22068200	0.876033
chr22	22109600	0.785124
chr22	22165600	0.752066
chr22	22171300	0.768595
chr22	22210700	0.768595
chr22	22222500	0.884298
chr22	22231000	0.801653
chr22	22257200	0.768595
chr22	22391700	0.743802
chr22	22413400	0.834711
chr22	22453200	0.900826
chr22	22511800	0.900826
chr22	22522700	0.933884
chr22	22560800	0.776860
chr22	22698500	0.842975
chr22	22724400	0.809917
chr22	22749600	0.768595
chr22	22764700	0.760331
chr22	22947200	0.818182
chr22	23015000	0.735537
chr22	23021000	0.760331
chr22	23024100	0.809917
chr22	23035600	0.785124
chr22	23040600	0.768595
chr22	23055700	0.752066
chr22	23058000	0.743802
chr22	23154600	0.735537
chr22	23343200	0.735537
chr22	23354500	0.760331
chr22	23485000	0.760331
chr22	23558900	0.760331
chr22	23658700	0.768595
chr22	23937900	0.851240
chr22	24038800	0.909091
chr22	24107300	0.776860
chr22	24110000	0.793388
chr22	24129300	0.892562
chr22	24193000	0.752066
chr22	24360100	0.760331
chr22	24373500	0.768595
chr22	24542200	0.876033
chr22	24594500	0.768595
chr22	24658600	0.752066
chr22	24674300	0.768595
chr22	24676800	0.743802
chr22	24695300	0.801653
chr22	24909300	0.793388
chr22	24918000	0.743802
chr22	24921700	0.752066
chr22	24926100	0.760331
chr22	24952000	0.743802
chr22	25042500	0.851240
chr22	25047800	0.818182
chr22	25072400	0.809917

chr22	25150800	0.785124
chr22	25482200	0.776860
chr22	25785800	0.760331
chr22	25871900	0.876033
chr22	25891000	0.760331
chr22	26016100	0.809917
chr22	26105700	0.743802
chr22	26228600	0.735537
chr22	26231200	0.752066
chr22	26341800	0.760331
chr22	26756500	0.768595
chr22	26800600	0.834711
chr22	26931600	0.785124
chr22	26976000	0.851240
chr22	26986300	0.785124
chr22	26988700	0.900826
chr22	27006700	0.909091
chr22	27041900	0.809917
chr22	27047800	0.785124
chr22	27387300	0.851240
chr22	28315800	0.909091
chr22	28693900	0.793388
chr22	29103500	0.776860
chr22	29188700	0.909091
chr22	29285800	0.818182
chr22	29293100	0.743802
chr22	29328300	0.851240
chr22	29352500	0.801653
chr22	29356200	0.826446
chr22	29373900	0.809917
chr22	29550600	0.793388
chr22	29592300	0.735537
chr22	29779100	0.735537
chr22	29866500	0.884298
chr22	29872100	0.876033
chr22	29875200	0.752066
chr22	30014600	0.776860
chr22	30268800	0.867769
chr22	30595100	0.735537
chr22	30618000	0.809917
chr22	30637200	0.826446
chr22	30744400	0.735537
chr22	30838500	0.851240
chr22	30841400	0.818182
chr22	31040300	0.818182
chr22	31102800	0.768595
chr22	31223200	0.909091
chr22	31318300	0.776860
chr22	31365600	0.867769
chr22	31391700	0.834711
chr22	31481400	0.760331
chr22	31634100	0.809917
chr22	31705000	0.809917
chr22	31743200	0.909091

chr22	31920600	0.859504
chr22	32128900	0.842975
chr22	32146100	0.851240
chr22	32374000	0.735537
chr22	32447200	0.735537
chr22	32540900	0.735537
chr22	33027700	0.809917
chr22	33119900	0.851240
chr22	33633600	0.933884
chr22	33644400	0.818182
chr22	33864100	0.776860
chr22	33972400	0.760331
chr22	34063000	0.826446
chr22	34250000	0.743802
chr22	34280300	0.801653
chr22	34285800	0.826446
chr22	34312900	0.752066
chr22	35343100	0.793388
chr22	35394400	0.801653
chr22	35653700	0.735537
chr22	35747400	0.876033
chr22	35756400	0.760331
chr22	35763700	0.793388
chr22	35768000	0.958678
chr22	35877000	0.785124
chr22	35902300	0.851240
chr22	36056700	0.842975
chr22	36148900	0.768595
chr22	36269800	0.785124
chr22	36299900	0.735537
chr22	36414000	0.768595
chr22	36499500	0.925620
chr22	36520300	0.776860
chr22	36559200	0.743802
chr22	36561500	0.867769
chr22	36618200	0.793388
chr22	36778400	0.809917
chr22	36798400	0.768595
chr22	36812200	0.851240
chr22	36832800	0.801653
chr22	36851600	0.851240
chr22	36945900	0.760331
chr22	36948800	0.809917
chr22	37173500	0.785124
chr22	37178400	0.892562
chr22	37249900	0.925620
chr22	37252900	0.892562
chr22	37350800	0.851240
chr22	37418400	0.834711
chr22	37449800	0.851240
chr22	37457200	0.776860
chr22	37484400	0.917355
chr22	37516500	0.801653
chr22	37536400	0.884298

chr22	37582600	0.793388
chr22	37656000	0.760331
chr22	37680600	0.735537
chr22	37727700	0.909091
chr22	37738000	0.826446
chr22	37926100	0.752066
chr22	37930400	0.818182
chr22	37958000	0.801653
chr22	37997700	0.867769
chr22	38001400	0.793388
chr22	38039600	0.892562
chr22	38074700	0.867769
chr22	38077800	0.760331
chr22	38082400	0.760331
chr22	38092900	0.793388
chr22	38142500	0.735537
chr22	38292500	0.933884
chr22	38544300	0.752066
chr22	38569200	0.735537
chr22	38595900	0.834711
chr22	38761100	0.760331
chr22	39009800	0.760331
chr22	39097100	0.900826
chr22	39154800	0.826446
chr22	39297600	0.785124
chr22	39339400	0.760331
chr22	39344300	0.884298
chr22	39378100	0.760331
chr22	39386000	0.752066
chr22	39389600	0.842975
chr22	39432200	0.851240
chr22	39473400	0.752066
chr22	39514000	0.859504
chr22	39567200	0.801653
chr22	39632800	0.909091
chr22	39637500	0.768595
chr22	39651300	0.859504
chr22	39673400	0.925620
chr22	39708700	0.900826
chr22	39716100	0.801653
chr22	39834200	0.818182
chr22	39854400	0.809917
chr22	39905400	0.735537
chr22	39928300	0.851240
chr22	39953700	0.826446
chr22	39997500	0.735537
chr22	40163500	0.776860
chr22	40170500	0.735537
chr22	40229300	0.735537
chr22	40236200	0.785124
chr22	40241500	0.743802
chr22	40271200	0.942149
chr22	40285600	0.900826
chr22	40324800	0.834711

chr22	40343800	0.809917
chr22	40346100	0.785124
chr22	40348400	0.752066
chr22	40351700	0.768595
chr22	40355000	0.851240
chr22	40359900	0.735537
chr22	40382000	0.917355
chr22	40452000	0.826446
chr22	40609600	0.752066
chr22	40614500	0.859504
chr22	40672900	0.801653
chr22	40728300	0.884298
chr22	40731200	0.776860
chr22	40734200	0.768595
chr22	40953200	0.735537
chr22	41047800	0.892562
chr22	41128800	0.735537
chr22	41339700	0.768595
chr22	41348300	0.818182
chr22	41465200	0.975207
chr22	41800100	0.760331
chr22	41990200	0.975207
chr22	41999900	0.809917
chr22	42063200	0.917355
chr22	42095200	0.859504
chr22	42239900	0.760331
chr22	42337100	0.884298
chr22	42345600	0.876033
chr22	42356800	0.793388
chr22	42573700	0.776860
chr22	42656600	0.851240
chr22	42659600	0.776860
chr22	42674700	0.834711
chr22	42680200	0.793388
chr22	42712900	0.867769
chr22	42739700	0.917355
chr22	42817700	0.735537
chr22	42931300	0.768595
chr22	43045700	0.917355
chr22	43093100	0.826446
chr22	43333600	0.743802
chr22	43366700	0.776860
chr22	43401300	0.743802
chr22	43510700	0.760331
chr22	43512800	0.768595
chr22	43544600	0.785124
chr22	43583600	0.917355
chr22	43653600	0.785124
chr22	43667100	0.752066
chr22	44041900	0.752066
chr22	44208400	0.776860
chr22	44280600	0.842975
chr22	44392200	0.950413
chr22	44435400	0.743802

chr22	44588600	0.884298
chr22	44601000	0.826446
chr22	44604800	0.876033
chr22	44653100	0.743802
chr22	44725600	0.809917
chr22	44851700	0.818182
chr22	44939800	0.768595
chr22	45060400	0.900826
chr22	45527900	0.859504
chr22	45687900	0.818182
chr22	45764100	0.785124
chr22	45837100	0.826446
chr22	45980000	0.801653
chr22	46035400	0.743802
chr22	46044100	0.785124
chr22	46463300	0.743802
chr22	46526000	0.892562
chr22	46537700	0.752066
chr22	46636000	0.743802
chr22	46684100	0.826446
chr22	46695500	0.735537
chr22	46770400	0.752066
chr22	46817800	0.884298
chr22	46829900	0.809917
chr22	46910800	0.867769
chr22	47070200	0.801653
chr22	47145900	0.867769
chr22	47166900	0.776860
chr22	48237400	0.909091
chr22	48441900	0.818182
chr22	48486800	0.884298
chr22	48840800	0.768595
chr22	50067300	0.884298
chr22	50205500	0.793388
chr22	50217400	0.776860
chr22	50319600	0.834711
chr22	50450900	0.892562
chr22	50473600	0.785124
chr22	50495300	0.851240
chr22	50541300	0.801653
chr22	50625300	0.760331
chr22	50706900	0.785124
chr22	50765600	0.842975
chr22	50769800	0.826446
chr22	50778600	0.768595
chr22	50929800	0.826446
chr22	50951400	0.752066
chr22	50965300	0.818182
chr22	51021300	0.867769
chr22	51136300	0.826446
chr2	275600	0.743802
chr2	290100	0.818182
chr2	394400	0.752066
chr2	585500	0.925620

chr2	637000	0.752066
chr2	677600	0.909091
chr2	706300	0.859504
chr2	715000	0.735537
chr2	975200	0.793388
chr2	1754200	0.983471
chr2	2875700	0.776860
chr2	2883900	0.851240
chr2	2906400	0.801653
chr2	3104200	0.859504
chr2	3383800	0.842975
chr2	3465800	0.966942
chr2	3643700	0.785124
chr2	3737700	0.983471
chr2	3752100	0.834711
chr2	6357000	0.752066
chr2	6913700	0.826446
chr2	7013100	0.776860
chr2	7075400	0.933884
chr2	7148100	0.818182
chr2	7192700	0.735537
chr2	7268300	1.000000
chr2	7383000	0.752066
chr2	7489000	0.834711
chr2	7571400	0.768595
chr2	7591100	0.925620
chr2	7658200	0.809917
chr2	7708400	0.735537
chr2	7761800	0.793388
chr2	7806900	0.867769
chr2	7843700	0.785124
chr2	7988500	0.826446
chr2	8039600	0.826446
chr2	8125300	0.735537
chr2	8137300	0.735537
chr2	8193400	0.785124
chr2	8220600	0.752066
chr2	8300900	0.818182
chr2	8342700	0.752066
chr2	8379800	0.892562
chr2	8389100	0.776860
chr2	8445700	0.752066
chr2	8508700	0.826446
chr2	8518700	0.909091
chr2	8529600	0.842975
chr2	8628900	0.735537
chr2	8652700	0.743802
chr2	8692900	0.793388
chr2	8728500	0.884298
chr2	8769400	0.909091
chr2	9013800	0.793388
chr2	9122600	0.834711
chr2	9615200	0.776860
chr2	9618800	0.809917

chr2	9781800	0.793388
chr2	9784400	0.842975
chr2	9798300	0.743802
chr2	9804100	0.776860
chr2	9807100	0.793388
chr2	9833300	0.793388
chr2	9840100	0.876033
chr2	9852800	0.826446
chr2	9861800	0.826446
chr2	9891900	0.851240
chr2	9895000	0.760331
chr2	9898100	0.809917
chr2	9908500	0.859504
chr2	9940100	0.768595
chr2	10030400	0.818182
chr2	10106000	0.842975
chr2	10183300	0.826446
chr2	10216200	0.851240
chr2	10242000	0.793388
chr2	10465200	0.892562
chr2	10516000	0.834711
chr2	10518400	0.818182
chr2	10554700	0.867769
chr2	10617800	0.760331
chr2	10634100	0.900826
chr2	10847900	0.884298
chr2	10934100	0.743802
chr2	11513400	0.776860
chr2	11528500	0.760331
chr2	11599300	0.867769
chr2	11647500	0.851240
chr2	11694500	0.884298
chr2	11829000	0.801653
chr2	11857200	0.752066
chr2	11884700	0.826446
chr2	11894400	0.925620
chr2	11919200	0.842975
chr2	11967300	0.752066
chr2	11970200	0.909091
chr2	11993400	0.743802
chr2	12020100	0.809917
chr2	12044200	0.760331
chr2	12050900	0.760331
chr2	12150900	0.776860
chr2	12176800	0.809917
chr2	12190900	0.743802
chr2	12216800	0.735537
chr2	12251300	0.834711
chr2	12403500	0.785124
chr2	12434800	0.752066
chr2	12553300	0.735537
chr2	12571500	0.735537
chr2	12627200	0.818182
chr2	12648000	0.760331

chr2	12695800	0.842975
chr2	12701400	0.752066
chr2	12708000	0.834711
chr2	12730500	0.925620
chr2	12862300	0.909091
chr2	12868700	0.818182
chr2	13684500	0.834711
chr2	13793200	0.876033
chr2	15171700	0.768595
chr2	15223100	0.809917
chr2	15821200	0.933884
chr2	15871100	0.876033
chr2	16790600	0.735537
chr2	16807100	0.785124
chr2	16832200	0.876033
chr2	16844700	0.785124
chr2	16894800	0.826446
chr2	17142200	0.834711
chr2	17697400	0.801653
chr2	17760100	0.743802
chr2	17997200	0.909091
chr2	18093600	0.859504
chr2	18741800	0.909091
chr2	19085600	0.834711
chr2	19122100	0.859504
chr2	19157900	0.785124
chr2	19547400	0.785124
chr2	19674100	0.876033
chr2	19843600	0.884298
chr2	19986100	0.743802
chr2	20063700	0.760331
chr2	20251800	0.818182
chr2	20358300	0.801653
chr2	20530600	0.768595
chr2	20553100	0.842975
chr2	20616700	0.942149
chr2	20630900	0.842975
chr2	20751600	0.768595
chr2	20755800	0.834711
chr2	20763900	0.801653
chr2	20848300	0.842975
chr2	21022500	0.834711
chr2	21068300	0.793388
chr2	23569300	0.818182
chr2	23571900	0.776860
chr2	23574800	0.752066
chr2	23577700	0.809917
chr2	23598800	0.842975
chr2	23612800	0.760331
chr2	23617400	0.859504
chr2	23623100	0.743802
chr2	23627200	0.785124
chr2	24074000	0.826446
chr2	24163600	0.892562

chr2	24284300	0.892562
chr2	24327800	0.735537
chr2	24346700	0.867769
chr2	24536800	0.818182
chr2	24583700	0.752066
chr2	24626300	0.958678
chr2	25150800	0.818182
chr2	25245000	0.842975
chr2	25298400	0.801653
chr2	25384300	0.966942
chr2	25418500	0.735537
chr2	25475000	0.900826
chr2	25518100	0.859504
chr2	25537700	0.900826
chr2	25557600	0.743802
chr2	25559900	0.826446
chr2	25565100	0.743802
chr2	25575900	0.909091
chr2	25585400	0.851240
chr2	25600400	0.826446
chr2	25627000	0.842975
chr2	25629500	0.892562
chr2	25676600	0.909091
chr2	25771600	0.752066
chr2	25781700	0.892562
chr2	25931800	0.842975
chr2	26029400	0.768595
chr2	26048200	0.818182
chr2	26076800	0.900826
chr2	26382900	0.735537
chr2	26623200	0.735537
chr2	26635000	0.776860
chr2	26648200	0.760331
chr2	26915700	0.867769
chr2	26974500	0.809917
chr2	27110300	0.760331
chr2	27227000	0.884298
chr2	27298100	0.735537
chr2	27304700	0.966942
chr2	27376300	0.834711
chr2	27388100	0.867769
chr2	27393400	0.867769
chr2	27494400	0.867769
chr2	27940000	0.884298
chr2	27943600	0.760331
chr2	28113600	0.859504
chr2	28475300	0.876033
chr2	28551300	0.826446
chr2	28582700	0.876033
chr2	28602000	0.735537
chr2	28619100	0.975207
chr2	28672700	0.801653
chr2	28810600	0.867769
chr2	28817700	0.776860

chr2	28848400	0.818182
chr2	28871200	0.743802
chr2	28873400	0.859504
chr2	29235100	0.735537
chr2	29263300	0.834711
chr2	29465000	0.801653
chr2	29484300	0.851240
chr2	29535500	0.809917
chr2	29538000	0.752066
chr2	29541200	0.743802
chr2	29545400	0.743802
chr2	29559400	0.785124
chr2	29671300	0.818182
chr2	29968300	0.842975
chr2	30089200	0.743802
chr2	30441100	0.776860
chr2	30460000	0.867769
chr2	30490000	0.818182
chr2	30500500	0.842975
chr2	30511500	0.834711
chr2	30547200	0.785124
chr2	30549900	0.735537
chr2	30645600	0.776860
chr2	30834800	0.867769
chr2	30889300	0.785124
chr2	30938000	0.851240
chr2	31090200	0.809917
chr2	31236000	0.851240
chr2	31394700	0.776860
chr2	31445500	0.909091
chr2	31474600	0.735537
chr2	31477200	0.743802
chr2	31481700	0.859504
chr2	31541600	0.801653
chr2	31547200	0.735537
chr2	31607700	0.785124
chr2	31680000	0.809917
chr2	32206300	0.958678
chr2	32236300	0.867769
chr2	32390800	0.735537
chr2	32489800	0.950413
chr2	32503100	0.809917
chr2	33136500	0.892562
chr2	33141400	0.760331
chr2	33309100	0.818182
chr2	33497400	0.917355
chr2	33547800	0.818182
chr2	33683100	0.768595
chr2	33844500	0.809917
chr2	34011800	0.818182
chr2	36374900	0.876033
chr2	36471300	0.826446
chr2	36474900	0.818182
chr2	36632200	0.917355

chr2	36756000	0.900826
chr2	36758600	0.743802
chr2	37016800	0.867769
chr2	37033300	0.801653
chr2	37187500	0.859504
chr2	37194200	0.867769
chr2	37233000	0.826446
chr2	37238700	0.752066
chr2	37577300	0.859504
chr2	37593300	0.793388
chr2	37607800	0.785124
chr2	37615600	0.809917
chr2	37734200	0.752066
chr2	37763500	0.768595
chr2	37934900	0.834711
chr2	37938900	0.851240
chr2	37995700	0.884298
chr2	38025900	0.884298
chr2	38050600	0.826446
chr2	38115900	0.768595
chr2	38156400	0.851240
chr2	38210000	0.826446
chr2	38334000	0.950413
chr2	38391100	0.768595
chr2	38604200	0.892562
chr2	38616000	0.768595
chr2	38645300	0.900826
chr2	38674900	0.851240
chr2	38761400	0.892562
chr2	38770100	0.809917
chr2	38832700	0.735537
chr2	38847300	0.909091
chr2	38850000	0.892562
chr2	38886700	0.793388
chr2	38891300	0.743802
chr2	38937300	0.876033
chr2	38947900	0.793388
chr2	38950500	0.826446
chr2	39133100	0.785124
chr2	39199300	0.760331
chr2	39316200	0.768595
chr2	39322800	0.752066
chr2	39337700	0.909091
chr2	39348400	0.752066
chr2	39351200	0.909091
chr2	39357600	0.818182
chr2	39368600	0.851240
chr2	39455800	0.867769
chr2	39630500	0.768595
chr2	39654900	0.851240
chr2	39659100	0.785124
chr2	39704400	0.735537
chr2	39722000	0.900826
chr2	39741600	0.760331

chr2	39753100	0.760331
chr2	39991600	0.752066
chr2	40008200	0.760331
chr2	40079200	0.900826
chr2	40256800	0.925620
chr2	41945400	0.793388
chr2	41963600	0.793388
chr2	42100600	0.867769
chr2	42124300	0.966942
chr2	42136000	0.785124
chr2	42180700	0.867769
chr2	42184400	0.809917
chr2	42193900	0.884298
chr2	42335400	0.851240
chr2	42346200	0.743802
chr2	42357500	0.752066
chr2	42360800	0.834711
chr2	42363300	0.933884
chr2	42384300	0.785124
chr2	42420400	0.768595
chr2	42588500	0.842975
chr2	42774300	0.785124
chr2	42949600	0.735537
chr2	43015400	0.876033
chr2	43055500	0.735537
chr2	43145100	0.818182
chr2	43190300	0.950413
chr2	43364200	0.743802
chr2	43367600	0.818182
chr2	43396700	0.900826
chr2	43521800	0.925620
chr2	43530600	0.743802
chr2	43603200	0.752066
chr2	43654400	0.793388
chr2	44325200	0.900826
chr2	44327700	0.785124
chr2	44330900	0.743802
chr2	44335100	0.793388
chr2	44418500	0.801653
chr2	44464700	0.776860
chr2	44774300	0.884298
chr2	44880000	0.842975
chr2	44936400	0.851240
chr2	45203300	0.867769
chr2	45211500	0.752066
chr2	45369400	0.793388
chr2	45725000	0.752066
chr2	45795700	0.892562
chr2	45865100	0.735537
chr2	45892100	0.826446
chr2	45958900	0.818182
chr2	46067900	0.768595
chr2	46088200	0.793388
chr2	46146800	0.793388

chr2	46236500	0.785124
chr2	46579600	0.867769
chr2	46709700	0.842975
chr2	46725400	0.900826
chr2	46749100	0.801653
chr2	46765900	0.834711
chr2	46776800	0.809917
chr2	46787500	0.735537
chr2	46790200	0.884298
chr2	46799600	0.851240
chr2	46898300	0.793388
chr2	46910500	0.909091
chr2	47465400	0.752066
chr2	47524600	0.867769
chr2	47528400	0.826446
chr2	47550100	0.917355
chr2	47553500	0.760331
chr2	47724900	0.851240
chr2	47883900	0.785124
chr2	47922400	0.859504
chr2	47925100	0.917355
chr2	47989600	0.851240
chr2	48010200	0.752066
chr2	48125200	0.735537
chr2	48211800	0.735537
chr2	48433300	0.859504
chr2	48500100	0.768595
chr2	48580500	0.752066
chr2	48648100	0.809917
chr2	48978300	0.752066
chr2	49218400	0.785124
chr2	53995300	0.735537
chr2	54067700	0.983471
chr2	54148500	0.809917
chr2	54304900	0.818182
chr2	54342900	0.925620
chr2	54698800	0.752066
chr2	54740600	0.735537
chr2	54783800	0.768595
chr2	55146500	0.776860
chr2	55180100	0.818182
chr2	55283400	0.735537
chr2	55364200	0.752066
chr2	55509300	0.867769
chr2	55680400	0.768595
chr2	55706300	0.743802
chr2	55849100	0.752066
chr2	56007700	0.892562
chr2	56103300	0.859504
chr2	56150100	0.785124
chr2	56212900	0.785124
chr2	56575700	0.859504
chr2	57953500	0.760331
chr2	58109900	0.752066

chr2	58251100	0.876033
chr2	58578400	0.801653
chr2	60286100	0.892562
chr2	60296200	0.834711
chr2	60367700	0.752066
chr2	60386100	0.942149
chr2	60639100	0.785124
chr2	60660900	0.826446
chr2	60765900	0.818182
chr2	60792500	0.884298
chr2	60888800	0.859504
chr2	60906700	0.801653
chr2	60973100	0.826446
chr2	61049500	0.743802
chr2	61057900	0.760331
chr2	61405000	0.743802
chr2	61598000	0.867769
chr2	61649400	0.752066
chr2	61698900	0.867769
chr2	61898400	0.867769
chr2	61932900	0.785124
chr2	61935700	0.958678
chr2	61991600	0.884298
chr2	62035300	0.785124
chr2	62082200	0.900826
chr2	62293300	0.768595
chr2	62435200	0.818182
chr2	62448100	0.842975
chr2	62450400	0.834711
chr2	62485600	0.760331
chr2	62572200	0.785124
chr2	62639200	0.950413
chr2	62663800	0.752066
chr2	62889700	0.867769
chr2	63039100	0.785124
chr2	63855300	0.752066
chr2	64254300	0.785124
chr2	64291500	0.735537
chr2	64394800	0.859504
chr2	64414100	0.768595
chr2	64487900	0.867769
chr2	64490700	0.842975
chr2	64510700	0.933884
chr2	64546000	0.818182
chr2	64566500	0.735537
chr2	64587400	0.752066
chr2	64699100	0.768595
chr2	64810700	0.735537
chr2	64883400	0.760331
chr2	64906900	0.735537
chr2	64951300	0.966942
chr2	64965600	0.760331
chr2	64978800	0.752066
chr2	64995200	0.876033

chr2	65053200	0.752066
chr2	65206900	0.760331
chr2	65209800	0.826446
chr2	65213200	0.760331
chr2	65455600	0.793388
chr2	65459500	0.776860
chr2	65524100	0.826446
chr2	65593000	0.842975
chr2	65598700	0.793388
chr2	65607500	0.743802
chr2	65617800	0.776860
chr2	65650400	0.867769
chr2	65742000	0.826446
chr2	65744100	0.909091
chr2	65763100	0.785124
chr2	65781800	0.876033
chr2	65808600	0.942149
chr2	65927100	0.752066
chr2	67387700	0.760331
chr2	67624700	0.892562
chr2	67899300	0.859504
chr2	68385100	0.752066
chr2	68428300	0.859504
chr2	68459200	0.851240
chr2	68480200	0.768595
chr2	68522800	0.768595
chr2	68665800	0.867769
chr2	68668700	0.950413
chr2	68881400	0.776860
chr2	68969700	0.809917
chr2	68972500	0.801653
chr2	68990100	0.743802
chr2	69056800	0.776860
chr2	69059900	0.884298
chr2	69063200	0.826446
chr2	69310000	0.842975
chr2	69391600	0.785124
chr2	69664700	0.859504
chr2	69703600	0.768595
chr2	69711200	0.793388
chr2	69763400	0.752066
chr2	69782200	0.958678
chr2	69793100	0.735537
chr2	69825100	0.900826
chr2	69880000	0.752066
chr2	69959100	0.735537
chr2	69963400	0.851240
chr2	69998300	0.867769
chr2	70296700	0.884298
chr2	70418100	0.752066
chr2	70475600	0.768595
chr2	70520500	0.768595
chr2	70528600	0.884298
chr2	70707600	0.809917

chr2	70743600	0.793388
chr2	71176100	0.735537
chr2	71212100	0.876033
chr2	71277500	0.743802
chr2	71605600	0.785124
chr2	71773100	0.752066
chr2	72560100	0.785124
chr2	73074000	0.735537
chr2	73118800	0.735537
chr2	73298800	0.776860
chr2	73517600	0.768595
chr2	73565300	0.925620
chr2	73851400	0.793388
chr2	73878600	0.793388
chr2	73881900	0.842975
chr2	74105200	0.776860
chr2	74148100	0.760331
chr2	74154200	0.900826
chr2	74195300	0.785124
chr2	74227100	0.785124
chr2	74245000	0.917355
chr2	74268000	0.776860
chr2	74347500	0.933884
chr2	74409000	0.826446
chr2	74413300	0.859504
chr2	74514400	0.859504
chr2	74619400	0.776860
chr2	74692700	0.818182
chr2	74700100	0.776860
chr2	74710200	0.909091
chr2	74734900	0.925620
chr2	74766700	0.735537
chr2	74790000	0.760331
chr2	74847400	0.743802
chr2	74992300	0.809917
chr2	75185700	0.942149
chr2	75258500	0.768595
chr2	75439000	0.752066
chr2	75836600	0.859504
chr2	75874400	0.752066
chr2	75938100	0.892562
chr2	76048800	0.743802
chr2	76674500	0.768595
chr2	76677800	0.826446
chr2	84440500	0.818182
chr2	84670800	0.842975
chr2	84686700	0.909091
chr2	85042800	0.876033
chr2	85074000	0.735537
chr2	85116400	0.834711
chr2	85147400	0.884298
chr2	85158800	0.809917
chr2	85174000	0.785124
chr2	85193500	0.900826

chr2	85444600	0.801653
chr2	85555300	0.793388
chr2	85558800	0.933884
chr2	85637700	0.867769
chr2	85645700	0.917355
chr2	85843400	0.785124
chr2	85876000	0.785124
chr2	85896800	0.743802
chr2	85932600	0.933884
chr2	85934800	0.809917
chr2	85973000	0.834711
chr2	85976300	0.900826
chr2	86055300	0.760331
chr2	86058400	0.917355
chr2	86067300	0.834711
chr2	86106500	0.735537
chr2	86221800	0.776860
chr2	86414700	0.801653
chr2	86426700	0.851240
chr2	86431400	0.917355
chr2	86456900	0.909091
chr2	86520900	0.842975
chr2	86596000	0.842975
chr2	86790400	0.842975
chr2	86811900	0.818182
chr2	86840900	0.776860
chr2	86855500	0.818182
chr2	86960300	0.752066
chr2	87304300	0.834711
chr2	87356300	0.760331
chr2	87483000	0.785124
chr2	87542600	0.752066
chr2	87552400	0.818182
chr2	87746400	0.842975
chr2	87756500	0.785124
chr2	87776100	0.752066
chr2	87801600	0.801653
chr2	87809700	0.793388
chr2	87821800	0.743802
chr2	87825600	0.752066
chr2	87848600	0.776860
chr2	87882000	0.801653
chr2	87970600	0.876033
chr2	87978100	0.735537
chr2	88316200	0.834711
chr2	88355300	0.909091
chr2	88364700	0.793388
chr2	88565300	0.735537
chr2	88864300	0.818182
chr2	88927800	0.809917
chr2	88938700	0.801653
chr2	89545700	0.793388
chr2	89573300	0.818182
chr2	89977800	0.776860

chr2	89987300	0.884298
chr2	91693300	0.851240
chr2	91764300	0.768595
chr2	91771900	0.801653
chr2	91782500	0.900826
chr2	91787100	0.793388
chr2	91795400	0.900826
chr2	91805900	0.983471
chr2	91814400	0.752066
chr2	91816600	0.867769
chr2	91818800	0.925620
chr2	91823000	0.743802
chr2	91830800	0.925620
chr2	95512300	0.760331
chr2	95681000	0.801653
chr2	95689700	0.867769
chr2	95787500	0.909091
chr2	96192600	0.793388
chr2	96253800	0.909091
chr2	96260700	0.735537
chr2	96441000	0.851240
chr2	96658100	0.760331
chr2	96769700	0.851240
chr2	96786000	0.735537
chr2	96865700	0.851240
chr2	96877600	0.735537
chr2	96891300	0.950413
chr2	96908100	0.867769
chr2	96971800	0.851240
chr2	97073700	0.768595
chr2	97176400	0.884298
chr2	97220100	0.818182
chr2	97436100	0.785124
chr2	97547500	0.785124
chr2	97554000	0.909091
chr2	97620400	0.743802
chr2	97627200	0.776860
chr2	97697200	0.801653
chr2	97703600	0.735537
chr2	97712700	0.776860
chr2	98041300	0.884298
chr2	98222200	0.809917
chr2	98262400	0.785124
chr2	98305000	0.760331
chr2	98335700	0.768595
chr2	98362600	0.834711
chr2	98499200	0.785124
chr2	98565600	0.768595
chr2	98578400	0.735537
chr2	98584100	0.760331
chr2	98587600	0.809917
chr2	98605300	0.785124
chr2	98637500	0.793388
chr2	98646500	0.818182

chr2	98701600	0.768595
chr2	98740000	0.876033
chr2	98751100	0.752066
chr2	99265500	0.768595
chr2	99345300	0.793388
chr2	99379100	0.876033
chr2	99381300	0.834711
chr2	99385300	0.735537
chr2	99486600	0.743802
chr2	99492000	0.809917
chr2	99683100	0.909091
chr2	99772000	0.785124
chr2	100380500	0.776860
chr2	100618300	0.809917
chr2	100726500	0.752066
chr2	100748200	0.801653
chr2	100755500	0.785124
chr2	100760400	0.867769
chr2	100862200	0.768595
chr2	100890900	0.851240
chr2	100997900	0.826446
chr2	101179800	0.801653
chr2	101197400	0.892562
chr2	101500500	0.768595
chr2	101558200	0.801653
chr2	101593700	0.826446
chr2	101698700	0.743802
chr2	101889700	0.776860
chr2	101916900	0.809917
chr2	101945400	0.917355
chr2	101978300	0.925620
chr2	102239200	0.760331
chr2	102292400	0.900826
chr2	102323500	0.859504
chr2	102327100	0.909091
chr2	102333800	0.892562
chr2	102368300	0.735537
chr2	102370800	0.809917
chr2	102374800	0.801653
chr2	102423200	0.809917
chr2	102517500	0.867769
chr2	102529300	0.851240
chr2	102607000	0.735537
chr2	102697400	0.826446
chr2	102844800	0.785124
chr2	102860000	0.760331
chr2	102875300	0.909091
chr2	102986400	0.859504
chr2	103008200	0.785124
chr2	103060100	0.760331
chr2	103115600	0.785124
chr2	103126200	0.785124
chr2	103218600	0.752066
chr2	105465800	0.809917

chr2	105506000	0.785124
chr2	105555300	0.752066
chr2	105747300	0.842975
chr2	105800600	0.760331
chr2	105954000	0.776860
chr2	106356000	0.826446
chr2	106373900	0.842975
chr2	106501900	0.859504
chr2	106522100	0.809917
chr2	106532400	0.743802
chr2	106866100	0.851240
chr2	106975100	0.933884
chr2	108533600	0.809917
chr2	108922000	0.859504
chr2	109025800	0.776860
chr2	109077500	0.925620
chr2	109182300	0.900826
chr2	109192600	0.867769
chr2	109209500	0.851240
chr2	109226500	0.834711
chr2	109231600	0.752066
chr2	109257800	0.752066
chr2	109336000	0.785124
chr2	109762200	0.752066
chr2	109766200	0.809917
chr2	109811300	0.735537
chr2	109813800	0.842975
chr2	109827400	0.776860
chr2	109832100	0.809917
chr2	109921900	0.809917
chr2	110087100	0.818182
chr2	110092600	0.735537
chr2	110274600	0.760331
chr2	110287700	0.809917
chr2	110297600	0.793388
chr2	110396200	0.818182
chr2	110401200	0.760331
chr2	110438100	0.735537
chr2	110459600	0.752066
chr2	110645800	0.933884
chr2	110664800	0.785124
chr2	111157500	0.768595
chr2	111223400	0.851240
chr2	111436000	0.735537
chr2	111489000	0.867769
chr2	111502600	0.735537
chr2	111563900	0.801653
chr2	111578300	0.884298
chr2	111593000	0.809917
chr2	111617200	0.760331
chr2	111624800	0.876033
chr2	111668000	0.826446
chr2	111809800	0.818182
chr2	111813200	0.859504

chr2	111820400	0.917355
chr2	111829400	0.735537
chr2	111858900	0.809917
chr2	111902900	0.776860
chr2	111916400	0.867769
chr2	111926100	0.818182
chr2	111960000	0.818182
chr2	112191600	0.933884
chr2	112223500	0.760331
chr2	112231100	0.859504
chr2	112249300	0.859504
chr2	112265000	0.842975
chr2	112291100	0.768595
chr2	112307800	0.776860
chr2	112363900	0.867769
chr2	112420900	0.801653
chr2	112463900	0.884298
chr2	112642000	0.752066
chr2	112731100	0.793388
chr2	112741800	0.826446
chr2	112763700	0.892562
chr2	112770300	0.809917
chr2	112914000	0.760331
chr2	113017700	0.785124
chr2	113033500	0.760331
chr2	113358100	0.809917
chr2	113381900	0.776860
chr2	113391900	0.876033
chr2	113398900	0.925620
chr2	113403700	0.768595
chr2	113470600	0.867769
chr2	113512600	0.760331
chr2	113551700	0.842975
chr2	113597000	0.768595
chr2	113599500	0.818182
chr2	113646100	0.760331
chr2	113737800	0.867769
chr2	113993600	0.801653
chr2	114148000	0.793388
chr2	114164200	0.801653
chr2	114166700	0.735537
chr2	114340000	0.752066
chr2	114549800	0.867769
chr2	117475800	0.743802
chr2	118807700	0.785124
chr2	118905600	0.834711
chr2	119009100	0.743802
chr2	119026300	0.785124
chr2	119613600	0.735537
chr2	119960700	0.818182
chr2	119996100	0.809917
chr2	120135000	0.768595
chr2	120156500	0.760331
chr2	120332700	0.876033

chr2	120387900	0.752066
chr2	120405400	0.876033
chr2	120455500	0.942149
chr2	120729300	0.909091
chr2	120863600	0.760331
chr2	120937200	0.776860
chr2	120981000	0.793388
chr2	121069200	0.859504
chr2	121561400	0.826446
chr2	122316200	0.760331
chr2	122358200	0.933884
chr2	122457800	0.785124
chr2	122489100	0.768595
chr2	122562200	0.909091
chr2	123098500	0.735537
chr2	125562700	0.735537
chr2	126409800	0.892562
chr2	127225800	0.942149
chr2	127374500	0.818182
chr2	127506400	0.801653
chr2	127845200	0.776860
chr2	127917000	0.785124
chr2	128071400	0.785124
chr2	128158300	0.851240
chr2	128162900	0.809917
chr2	128284100	0.752066
chr2	128332900	0.776860
chr2	128507500	0.760331
chr2	128528400	0.735537
chr2	128558400	0.760331
chr2	128663100	0.785124
chr2	129063800	0.785124
chr2	129379600	0.743802
chr2	129382400	0.851240
chr2	129862000	0.859504
chr2	130137800	0.785124
chr2	130222300	0.768595
chr2	130241400	0.801653
chr2	130570400	0.760331
chr2	131061200	0.785124
chr2	131068900	0.842975
chr2	131105300	0.801653
chr2	131135300	0.743802
chr2	131851100	0.859504
chr2	131903100	0.793388
chr2	131912000	0.760331
chr2	131914400	0.876033
chr2	132408000	0.785124
chr2	132984700	0.743802
chr2	133036500	0.867769
chr2	134785300	0.752066
chr2	134789500	0.809917
chr2	134988000	0.801653
chr2	135322900	0.776860

chr2	135328700	0.735537
chr2	135400500	0.876033
chr2	135427300	0.876033
chr2	135441800	0.752066
chr2	135472000	0.867769
chr2	135495400	0.752066
chr2	135503400	0.752066
chr2	135516300	0.917355
chr2	135676800	0.743802
chr2	135688500	0.768595
chr2	136373900	0.760331
chr2	136580900	0.859504
chr2	136583600	0.768595
chr2	136690000	0.909091
chr2	136695000	0.743802
chr2	136763900	0.834711
chr2	136776100	0.826446
chr2	136783500	0.793388
chr2	136787200	0.760331
chr2	136807800	0.743802
chr2	136865000	0.793388
chr2	136892100	0.917355
chr2	136894600	0.793388
chr2	136897300	0.933884
chr2	136965300	0.826446
chr2	137077600	0.818182
chr2	137082400	0.768595
chr2	137085000	0.909091
chr2	137142100	0.793388
chr2	139081400	0.818182
chr2	139281100	0.793388
chr2	139470300	0.768595
chr2	139488300	0.752066
chr2	141362900	0.768595
chr2	141964800	0.851240
chr2	142280200	0.809917
chr2	142287400	0.834711
chr2	142292900	0.735537
chr2	142563800	0.752066
chr2	143344100	0.867769
chr2	143673100	0.859504
chr2	143909100	0.752066
chr2	144226900	0.884298
chr2	144234300	0.834711
chr2	144321700	0.933884
chr2	144489500	0.776860
chr2	144560800	0.876033
chr2	144972000	0.743802
chr2	144991800	0.785124
chr2	145068800	0.793388
chr2	145082800	0.801653
chr2	145128700	0.826446
chr2	145189000	0.851240
chr2	145206800	0.760331

chr2	145212900	0.900826
chr2	145228900	0.793388
chr2	145237900	0.785124
chr2	145248500	0.776860
chr2	145319600	0.809917
chr2	145353900	0.818182
chr2	145944200	0.752066
chr2	146169700	0.818182
chr2	146509800	0.743802
chr2	146529300	0.826446
chr2	147235400	0.768595
chr2	147972500	0.743802
chr2	148258400	0.743802
chr2	148358600	0.793388
chr2	148386400	0.752066
chr2	148402300	0.785124
chr2	148404500	0.735537
chr2	148416200	0.752066
chr2	148430900	0.785124
chr2	148447500	0.892562
chr2	148868500	0.892562
chr2	149286100	0.793388
chr2	149298300	0.900826
chr2	149341100	0.785124
chr2	149411400	0.826446
chr2	149416500	0.826446
chr2	149480700	0.842975
chr2	149572500	0.752066
chr2	149856800	0.933884
chr2	149884300	0.752066
chr2	149891600	0.752066
chr2	149913800	0.809917
chr2	150002400	0.793388
chr2	150017800	0.909091
chr2	150024300	0.892562
chr2	150444200	0.925620
chr2	150454600	0.859504
chr2	151399100	0.809917
chr2	152118200	0.859504
chr2	152587000	0.859504
chr2	152628800	0.735537
chr2	152703300	0.909091
chr2	152709800	0.768595
chr2	152748400	0.818182
chr2	152804000	0.735537
chr2	152828800	0.776860
chr2	152862300	0.859504
chr2	152885300	0.752066
chr2	152912000	0.768595
chr2	152935700	0.743802
chr2	153023600	0.735537
chr2	153264200	0.834711
chr2	153346500	0.735537
chr2	153364800	0.735537

chr2	154078700	0.966942
chr2	154092400	0.793388
chr2	155249500	0.743802
chr2	155818100	0.917355
chr2	155867200	0.743802
chr2	156003100	0.818182
chr2	156039000	0.760331
chr2	157255000	0.785124
chr2	157287700	0.876033
chr2	157291000	0.809917
chr2	157314300	0.876033
chr2	157327700	0.768595
chr2	157442300	0.818182
chr2	157753100	0.892562
chr2	157920200	0.867769
chr2	157934200	0.909091
chr2	158046400	0.851240
chr2	158049700	0.793388
chr2	158113400	0.859504
chr2	158184400	0.818182
chr2	158278800	0.735537
chr2	158310000	0.834711
chr2	158348900	0.801653
chr2	158600200	0.768595
chr2	158754200	0.785124
chr2	159046500	0.900826
chr2	159150200	0.809917
chr2	159852200	0.859504
chr2	159924400	0.743802
chr2	160001800	0.809917
chr2	160014200	0.752066
chr2	160021600	0.752066
chr2	160066700	0.925620
chr2	160139900	0.768595
chr2	160143800	0.785124
chr2	160147600	0.776860
chr2	160325000	0.867769
chr2	160372600	0.768595
chr2	160736600	0.818182
chr2	160784400	0.776860
chr2	160863300	0.842975
chr2	161063100	0.884298
chr2	161067400	0.925620
chr2	161080800	0.859504
chr2	161251100	0.867769
chr2	161284900	0.884298
chr2	161447100	0.768595
chr2	161850900	0.834711
chr2	161920500	0.851240
chr2	161925300	0.842975
chr2	161935200	0.735537
chr2	161980600	0.760331
chr2	162065200	0.776860
chr2	162245400	0.752066

chr2	162920700	0.801653
chr2	162983200	0.735537
chr2	164205200	0.793388
chr2	166006500	0.793388
chr2	167583900	0.842975
chr2	168328800	0.752066
chr2	168898800	0.760331
chr2	169050200	0.785124
chr2	169052300	0.743802
chr2	169281200	0.776860
chr2	169287300	0.842975
chr2	169322000	0.809917
chr2	169329200	0.776860
chr2	169548700	0.768595
chr2	169893800	0.917355
chr2	169897900	0.793388
chr2	169931500	0.859504
chr2	169937500	0.752066
chr2	169965100	0.735537
chr2	170252100	0.859504
chr2	170516200	0.785124
chr2	170595200	0.793388
chr2	170655700	0.752066
chr2	171002500	0.785124
chr2	171012200	0.826446
chr2	171014300	0.859504
chr2	171155200	0.735537
chr2	171196700	0.752066
chr2	171265800	0.826446
chr2	171277800	0.826446
chr2	171292400	0.842975
chr2	171377700	0.752066
chr2	171537300	0.793388
chr2	171830200	0.776860
chr2	171949500	0.785124
chr2	172030300	0.776860
chr2	172039000	0.768595
chr2	172067600	0.950413
chr2	172187300	0.776860
chr2	172199000	0.950413
chr2	172370800	0.818182
chr2	172397700	0.809917
chr2	172598000	0.768595
chr2	173100200	0.760331
chr2	173303100	0.752066
chr2	173328300	0.933884
chr2	173411800	0.818182
chr2	173774700	0.752066
chr2	173850700	0.876033
chr2	173939000	0.801653
chr2	173941400	0.743802
chr2	173992000	0.818182
chr2	174129600	0.760331
chr2	174144800	0.859504

chr2	174151300	0.876033
chr2	174161500	0.735537
chr2	174203800	0.735537
chr2	174225300	0.818182
chr2	174267200	0.892562
chr2	174297300	0.892562
chr2	174316400	0.834711
chr2	174724000	0.884298
chr2	174808700	0.752066
chr2	174901900	0.768595
chr2	175249300	0.760331
chr2	175352400	0.851240
chr2	175358600	0.785124
chr2	175381600	0.867769
chr2	175401500	0.851240
chr2	175464800	0.776860
chr2	175509900	0.818182
chr2	175582200	0.768595
chr2	175615700	0.818182
chr2	175618500	0.768595
chr2	175643600	0.768595
chr2	176017800	0.900826
chr2	176046200	0.768595
chr2	176440800	0.809917
chr2	177134200	0.884298
chr2	177182700	0.768595
chr2	177220300	0.785124
chr2	177338100	0.900826
chr2	177359100	0.735537
chr2	177405700	0.785124
chr2	177610800	0.917355
chr2	177629000	0.776860
chr2	177634100	0.826446
chr2	177801500	0.793388
chr2	177803800	0.760331
chr2	177806300	0.776860
chr2	177924500	0.975207
chr2	178157000	0.933884
chr2	178417800	0.942149
chr2	178471900	0.876033
chr2	178483600	0.917355
chr2	178491300	0.851240
chr2	179313300	0.743802
chr2	179316100	0.842975
chr2	179407200	0.876033
chr2	179497900	0.752066
chr2	179793600	0.785124
chr2	179864000	0.743802
chr2	179887200	0.900826
chr2	179897200	0.900826
chr2	179961000	0.735537
chr2	180046400	0.735537
chr2	180125900	0.776860
chr2	180871900	0.743802

chr2	181817400	0.752066
chr2	181851000	0.760331
chr2	182107000	0.892562
chr2	182117900	0.809917
chr2	182146500	0.809917
chr2	182179300	0.752066
chr2	182227800	0.801653
chr2	182264100	0.743802
chr2	182471200	0.958678
chr2	182537800	0.859504
chr2	182546300	0.768595
chr2	182773700	0.768595
chr2	182858200	0.876033
chr2	183472200	0.876033
chr2	183959800	0.735537
chr2	184132700	0.768595
chr2	184213900	0.826446
chr2	184360900	0.892562
chr2	185309700	0.801653
chr2	186651200	0.892562
chr2	187292000	0.752066
chr2	187506300	0.793388
chr2	187611700	0.842975
chr2	187833200	0.776860
chr2	187916300	0.876033
chr2	188848500	0.842975
chr2	190539600	0.900826
chr2	190628100	0.735537
chr2	190986800	0.801653
chr2	191180100	0.743802
chr2	191299400	0.743802
chr2	191305200	0.801653
chr2	191428300	0.793388
chr2	191635400	0.801653
chr2	191659000	0.785124
chr2	191673000	0.785124
chr2	191709200	0.818182
chr2	191719000	0.842975
chr2	191903800	0.776860
chr2	191915000	0.859504
chr2	191917200	0.801653
chr2	191943500	0.743802
chr2	191974800	0.842975
chr2	191980800	0.818182
chr2	192017800	0.842975
chr2	192036300	0.752066
chr2	192041400	0.909091
chr2	192059800	0.834711
chr2	192098900	0.876033
chr2	192139400	0.933884
chr2	192187000	0.917355
chr2	192430600	0.826446
chr2	192494500	0.826446
chr2	192500500	0.785124

chr2	192503700	0.743802
chr2	192624200	0.818182
chr2	192792000	0.752066
chr2	193142900	0.834711
chr2	195041200	0.743802
chr2	196333100	0.768595
chr2	196365900	0.876033
chr2	196461400	0.876033
chr2	196484500	0.776860
chr2	196515200	0.892562
chr2	196931800	0.743802
chr2	196942500	0.735537
chr2	196968300	0.793388
chr2	197324800	0.917355
chr2	197396100	0.743802
chr2	197399100	0.752066
chr2	197616200	0.776860
chr2	197827100	0.876033
chr2	198010200	0.925620
chr2	198023700	0.752066
chr2	198025800	0.834711
chr2	198243000	0.785124
chr2	198294500	0.735537
chr2	198300300	0.785124
chr2	198466300	0.785124
chr2	198493500	0.933884
chr2	198809500	0.801653
chr2	198873200	0.842975
chr2	199237500	0.752066
chr2	200776200	0.776860
chr2	200820600	0.735537
chr2	201064400	0.826446
chr2	201165300	0.809917
chr2	201187100	0.735537
chr2	201481300	0.818182
chr2	201549300	0.909091
chr2	201568100	0.760331
chr2	201578300	0.983471
chr2	201723000	0.785124
chr2	201729100	0.809917
chr2	202032700	0.818182
chr2	202052200	0.809917
chr2	202069800	0.876033
chr2	202073900	0.818182
chr2	202088500	0.760331
chr2	202098700	0.785124
chr2	202126000	0.809917
chr2	202132300	0.752066
chr2	202209900	0.809917
chr2	202438200	0.793388
chr2	202747300	0.842975
chr2	202769800	0.851240
chr2	202871300	0.834711
chr2	202908600	0.760331

chr2	202934200	0.768595
chr2	202956800	0.768595
chr2	203006600	0.809917
chr2	203009900	0.859504
chr2	203124700	0.785124
chr2	203467400	0.826446
chr2	203538400	0.776860
chr2	203646000	0.776860
chr2	203696600	0.809917
chr2	203904300	0.809917
chr2	204104100	0.809917
chr2	204444600	0.851240
chr2	204448600	0.768595
chr2	204462800	0.801653
chr2	204465100	0.851240
chr2	204525000	0.900826
chr2	204539200	0.842975
chr2	204552400	0.760331
chr2	204559000	0.826446
chr2	204661900	0.834711
chr2	204666900	0.900826
chr2	204676800	0.826446
chr2	204736600	0.752066
chr2	204779700	0.842975
chr2	204863000	0.809917
chr2	204975100	0.826446
chr2	206511600	0.826446
chr2	206525700	0.884298
chr2	206579800	0.768595
chr2	207234500	0.818182
chr2	207337300	0.859504
chr2	207400200	0.785124
chr2	207630400	0.826446
chr2	207663300	0.735537
chr2	207802500	0.867769
chr2	207954100	0.884298
chr2	208015000	0.785124
chr2	208018100	0.735537
chr2	208020600	0.851240
chr2	208031500	0.801653
chr2	208037100	0.834711
chr2	208087400	0.834711
chr2	208092200	0.752066
chr2	208103900	0.776860
chr2	208273500	0.884298
chr2	208288700	0.793388
chr2	208325100	0.768595
chr2	208329100	0.743802
chr2	208371200	0.925620
chr2	208387000	0.760331
chr2	208391100	0.834711
chr2	208593800	0.785124
chr2	208676000	0.917355
chr2	208695400	0.851240

chr2	208817400	0.785124
chr2	208835400	0.851240
chr2	208896900	0.760331
chr2	208929800	0.752066
chr2	209131200	0.768595
chr2	209159800	0.793388
chr2	209231800	0.760331
chr2	209347500	0.859504
chr2	209987100	0.933884
chr2	212232800	0.735537
chr2	212733100	0.735537
chr2	212874700	0.809917
chr2	213803900	0.735537
chr2	213855900	0.925620
chr2	213991000	0.826446
chr2	216144900	0.809917
chr2	216476600	0.760331
chr2	216615200	0.752066
chr2	216661700	0.768595
chr2	216677500	0.785124
chr2	216711600	0.793388
chr2	216784800	0.826446
chr2	217007700	0.809917
chr2	217087900	0.809917
chr2	217139300	0.793388
chr2	217253800	0.942149
chr2	217277100	0.826446
chr2	217357200	0.818182
chr2	217787600	0.793388
chr2	217792400	0.884298
chr2	217967200	0.859504
chr2	218114000	0.752066
chr2	218280400	0.752066
chr2	218299700	0.768595
chr2	218310000	0.760331
chr2	218332000	0.743802
chr2	218480100	0.735537
chr2	218560300	0.752066
chr2	218911800	0.768595
chr2	218991200	0.867769
chr2	219015100	0.826446
chr2	219227700	0.760331
chr2	219259100	0.826446
chr2	219268700	0.768595
chr2	219336700	0.809917
chr2	219667800	0.743802
chr2	219670800	0.735537
chr2	219736600	0.876033
chr2	219828100	0.818182
chr2	219867300	0.876033
chr2	219946700	0.735537
chr2	220092200	0.752066
chr2	220110400	0.884298
chr2	220164100	0.859504

chr2	220209700	0.743802
chr2	220507900	0.867769
chr2	220716200	0.851240
chr2	221311200	0.768595
chr2	221998900	0.867769
chr2	222065500	0.785124
chr2	222068300	0.867769
chr2	222280900	0.760331
chr2	222328500	0.801653
chr2	222330800	0.793388
chr2	222385500	0.933884
chr2	222420300	0.876033
chr2	223177400	0.859504
chr2	223186900	0.760331
chr2	223292600	0.842975
chr2	223517400	0.876033
chr2	223521100	0.752066
chr2	223633000	0.768595
chr2	223678400	0.760331
chr2	223726000	0.735537
chr2	223731300	0.892562
chr2	223809100	0.809917
chr2	223836900	0.809917
chr2	223847300	0.760331
chr2	223941400	0.876033
chr2	223966800	0.818182
chr2	224512600	0.776860
chr2	224731400	0.966942
chr2	224747100	0.785124
chr2	224822200	0.900826
chr2	224891700	0.752066
chr2	225270500	0.834711
chr2	225390500	0.793388
chr2	225393500	0.892562
chr2	225435400	0.768595
chr2	225461200	0.942149
chr2	225635200	0.842975
chr2	225759700	0.735537
chr2	225814300	0.752066
chr2	225838900	0.826446
chr2	227354900	0.785124
chr2	227638600	0.760331
chr2	227656500	0.900826
chr2	227704200	0.876033
chr2	227706600	0.818182
chr2	228173900	0.793388
chr2	228216400	0.867769
chr2	228271800	0.735537
chr2	228399900	0.768595
chr2	228457900	0.842975
chr2	228473600	0.818182
chr2	228478700	0.743802
chr2	228881900	0.776860
chr2	230717800	0.842975

chr2	230990100	0.834711
chr2	230999900	0.793388
chr2	231072200	0.760331
chr2	231076100	0.776860
chr2	231081400	0.867769
chr2	231090700	0.735537
chr2	231099100	0.834711
chr2	231443200	0.826446
chr2	231476000	0.776860
chr2	231515700	0.834711
chr2	231559300	0.743802
chr2	231590000	0.867769
chr2	231623600	0.793388
chr2	231658800	0.867769
chr2	231689300	0.752066
chr2	231712800	0.760331
chr2	231727100	0.876033
chr2	231751100	0.785124
chr2	231921700	0.809917
chr2	232047100	0.867769
chr2	232227000	0.776860
chr2	232348200	0.892562
chr2	232389100	0.884298
chr2	232395600	0.826446
chr2	232419500	0.735537
chr2	232433800	0.760331
chr2	232552300	0.801653
chr2	232566900	0.752066
chr2	232878600	0.776860
chr2	232923400	0.826446
chr2	232967200	0.760331
chr2	233278100	0.785124
chr2	233415700	0.851240
chr2	233703200	0.859504
chr2	233820600	0.760331
chr2	233823900	0.776860
chr2	233989800	0.851240
chr2	234143100	0.776860
chr2	234221700	0.925620
chr2	234231000	0.892562
chr2	234247800	0.776860
chr2	234262500	0.752066
chr2	234325100	0.743802
chr2	234466200	0.826446
chr2	234475200	0.966942
chr2	234565100	0.851240
chr2	234710100	0.933884
chr2	234849600	0.876033
chr2	234899200	0.834711
chr2	234934600	0.793388
chr2	235355900	0.776860
chr2	235384600	0.768595
chr2	235860700	0.884298
chr2	235954700	0.818182

chr2	236180400	0.826446
chr2	236210600	0.859504
chr2	236479800	0.834711
chr2	237146200	0.826446
chr2	237275200	0.752066
chr2	237969800	0.760331
chr2	238337200	0.818182
chr2	238449700	0.793388
chr2	238490600	0.768595
chr2	238526200	0.818182
chr2	238571300	0.958678
chr2	238598400	0.809917
chr2	238724900	0.752066
chr2	238782500	0.776860
chr2	238840700	0.785124
chr2	238973000	0.743802
chr2	239048300	0.818182
chr2	239069400	0.851240
chr2	239215400	0.818182
chr2	239269300	0.793388
chr2	239292600	0.760331
chr2	239338800	0.760331
chr2	239870100	0.859504
chr2	240040900	0.818182
chr2	240138700	0.735537
chr2	240174600	0.743802
chr2	240180300	0.809917
chr2	240186800	0.826446
chr2	240197200	0.743802
chr2	240215900	0.793388
chr2	240220100	0.809917
chr2	240226700	0.735537
chr2	240241500	0.826446
chr2	240292200	0.818182
chr2	240323500	0.909091
chr2	240566700	0.743802
chr2	240835500	0.867769
chr2	241061000	0.818182
chr2	241118500	0.826446
chr2	241454000	0.743802
chr2	241567000	0.743802
chr2	241729700	0.826446
chr2	241850100	0.826446
chr2	241970800	0.851240
chr2	241979000	0.826446
chr2	241991200	0.735537
chr2	242028300	0.768595
chr2	242086500	0.776860
chr2	242157400	0.776860
chr2	242381100	0.752066
chr2	242451200	0.735537
chr2	242456900	0.818182
chr2	242577000	0.818182
chr2	242674400	0.892562

chr2	242679100	0.735537
chr2	242759200	0.809917
chr2	242788300	0.801653
chr2	242808000	0.785124
chr2	242824500	0.884298
chr3	2577600	0.760331
chr3	2697800	0.859504
chr3	2813100	0.752066
chr3	2832400	0.735537
chr3	2964700	0.942149
chr3	3010300	0.752066
chr3	3127900	0.859504
chr3	3131000	0.752066
chr3	3147500	0.842975
chr3	3157200	0.785124
chr3	3161800	0.776860
chr3	3323000	0.760331
chr3	3632000	0.752066
chr3	4103800	0.876033
chr3	4107400	0.842975
chr3	4345100	0.801653
chr3	4430100	0.818182
chr3	4550900	0.768595
chr3	4624000	0.785124
chr3	4800900	0.776860
chr3	4878400	0.776860
chr3	4883700	0.834711
chr3	4948000	0.909091
chr3	4985800	0.900826
chr3	5028600	0.826446
chr3	5044800	0.809917
chr3	5055900	0.743802
chr3	5058900	0.851240
chr3	5068700	0.768595
chr3	5077600	0.768595
chr3	5093800	0.752066
chr3	5099800	0.735537
chr3	5268900	0.801653
chr3	5432600	0.809917
chr3	5464800	0.760331
chr3	5527300	0.900826
chr3	6722100	0.793388
chr3	8558000	0.743802
chr3	8671300	0.834711
chr3	9074100	0.867769
chr3	9121800	0.834711
chr3	9188800	0.859504
chr3	9359500	0.776860
chr3	9384900	0.743802
chr3	9404800	0.768595
chr3	9546500	0.909091
chr3	9696300	0.842975
chr3	9851500	0.884298
chr3	9932600	0.900826

chr3	9958900	0.768595
chr3	9982200	0.735537
chr3	9993300	0.884298
chr3	10203800	0.793388
chr3	10212600	0.809917
chr3	10432100	0.785124
chr3	10479200	0.884298
chr3	10482600	0.760331
chr3	10499300	0.785124
chr3	10593900	0.776860
chr3	10645400	0.768595
chr3	10888100	0.842975
chr3	11182400	0.925620
chr3	11662000	0.760331
chr3	11697300	0.735537
chr3	11713900	0.768595
chr3	11812300	0.760331
chr3	11950900	0.768595
chr3	12183500	0.925620
chr3	12239500	0.801653
chr3	12329200	0.776860
chr3	12442500	0.826446
chr3	12607800	0.818182
chr3	12628600	0.867769
chr3	12668200	0.851240
chr3	12705700	0.735537
chr3	12748100	0.867769
chr3	12761800	0.818182
chr3	12795000	0.851240
chr3	12810000	0.884298
chr3	12860500	0.818182
chr3	12883200	0.842975
chr3	13015900	0.760331
chr3	13030100	0.735537
chr3	13133800	0.743802
chr3	13276900	0.900826
chr3	13304600	0.818182
chr3	13555100	0.818182
chr3	14123900	0.785124
chr3	14341900	0.752066
chr3	14419800	0.876033
chr3	14430800	0.743802
chr3	14481100	0.735537
chr3	14693500	0.884298
chr3	14727700	0.776860
chr3	14760500	0.809917
chr3	14842900	0.859504
chr3	14909900	0.760331
chr3	14967800	0.760331
chr3	15000900	0.752066
chr3	15106700	0.809917
chr3	15150000	0.776860
chr3	15162700	0.760331
chr3	15201500	0.735537

chr3	15257700	0.859504
chr3	15363300	0.884298
chr3	15509400	0.760331
chr3	15540100	0.900826
chr3	15577900	0.884298
chr3	15788500	0.760331
chr3	15843800	0.752066
chr3	16039500	0.818182
chr3	16290000	0.735537
chr3	16294800	0.793388
chr3	16444400	0.760331
chr3	16484900	0.793388
chr3	16632300	0.826446
chr3	16638600	0.735537
chr3	16648400	0.909091
chr3	16675800	0.842975
chr3	16735100	0.776860
chr3	16738900	0.818182
chr3	16842500	0.826446
chr3	16860800	0.958678
chr3	17089400	0.752066
chr3	17221700	0.809917
chr3	17274500	0.793388
chr3	17619800	0.793388
chr3	17674400	0.760331
chr3	17698100	0.768595
chr3	17797700	0.975207
chr3	17844700	0.851240
chr3	17848300	0.793388
chr3	17882200	0.785124
chr3	17894400	0.752066
chr3	18170300	0.776860
chr3	18781600	0.834711
chr3	19583300	0.768595
chr3	19602100	0.859504
chr3	19640700	0.826446
chr3	19753400	0.884298
chr3	20102700	0.768595
chr3	20122800	0.785124
chr3	20145700	0.801653
chr3	20167500	0.768595
chr3	20297700	0.735537
chr3	20367300	0.768595
chr3	23113700	0.876033
chr3	23250400	0.735537
chr3	23367400	0.735537
chr3	23409000	0.735537
chr3	23419800	0.801653
chr3	23429100	0.818182
chr3	23431400	0.884298
chr3	23444800	0.752066
chr3	23447300	0.776860
chr3	23462800	0.867769
chr3	23464900	0.793388

chr3	23609400	0.876033
chr3	23653800	0.760331
chr3	23754400	0.735537
chr3	23765900	0.776860
chr3	23768800	0.867769
chr3	23782100	0.900826
chr3	23809700	0.752066
chr3	23821500	0.818182
chr3	23839700	0.867769
chr3	23987800	0.743802
chr3	24196400	0.752066
chr3	24266900	0.834711
chr3	24545100	0.818182
chr3	25049800	0.809917
chr3	25503000	0.793388
chr3	25699200	0.842975
chr3	25858800	0.809917
chr3	25861100	0.867769
chr3	25872600	0.876033
chr3	25876500	0.842975
chr3	25905100	0.818182
chr3	27137600	0.809917
chr3	27566100	0.743802
chr3	27576100	0.793388
chr3	27656400	0.842975
chr3	28710000	0.818182
chr3	28883100	0.776860
chr3	28960000	0.842975
chr3	29031900	0.876033
chr3	29047100	0.801653
chr3	29222900	0.801653
chr3	29363400	0.760331
chr3	29366100	0.776860
chr3	29415400	0.900826
chr3	29452800	0.752066
chr3	29555200	0.760331
chr3	29728800	0.743802
chr3	29978300	0.776860
chr3	30129200	0.801653
chr3	30396000	0.884298
chr3	30519200	0.760331
chr3	30619900	0.834711
chr3	30671100	0.859504
chr3	30682000	0.867769
chr3	30686200	0.760331
chr3	30691200	0.826446
chr3	30696700	0.801653
chr3	30700900	0.785124
chr3	30703000	0.834711
chr3	30793800	0.735537
chr3	31138800	0.793388
chr3	31149000	0.884298
chr3	31434600	0.768595
chr3	31504200	0.785124

chr3	31614700	0.776860
chr3	31796600	0.785124
chr3	31966900	0.760331
chr3	31994100	0.859504
chr3	32020700	0.909091
chr3	32139600	0.826446
chr3	32178300	0.842975
chr3	32188500	0.735537
chr3	32351800	0.809917
chr3	32409100	0.867769
chr3	32435500	0.900826
chr3	32456700	0.776860
chr3	32462900	0.793388
chr3	32470500	0.809917
chr3	32478400	0.776860
chr3	32483300	0.743802
chr3	32497600	0.859504
chr3	32499800	0.776860
chr3	32502300	0.892562
chr3	32509800	0.793388
chr3	32535400	0.818182
chr3	32596400	0.826446
chr3	32619600	0.752066
chr3	32884300	0.743802
chr3	32892100	0.768595
chr3	32925500	0.834711
chr3	32931000	0.735537
chr3	33002300	0.785124
chr3	33015900	0.859504
chr3	33091900	0.851240
chr3	33153300	0.768595
chr3	33181900	0.826446
chr3	33359900	0.851240
chr3	33482800	0.958678
chr3	33772000	0.776860
chr3	33833300	0.809917
chr3	33925800	0.752066
chr3	34059900	0.809917
chr3	34063200	0.752066
chr3	34115900	0.785124
chr3	34119900	0.785124
chr3	34144100	0.743802
chr3	34290400	0.842975
chr3	34304400	0.818182
chr3	34365500	0.917355
chr3	34406100	0.909091
chr3	35564300	0.768595
chr3	35706800	0.876033
chr3	36717200	0.785124
chr3	36735300	0.752066
chr3	36885100	0.867769
chr3	36934400	0.809917
chr3	36937100	0.851240
chr3	36951300	0.752066

chr3	37000100	0.842975
chr3	37035500	0.809917
chr3	37500000	0.834711
chr3	37665100	0.859504
chr3	37778600	0.826446
chr3	37855200	0.917355
chr3	37941200	0.735537
chr3	37944300	0.743802
chr3	37949900	0.851240
chr3	38040400	0.826446
chr3	38157200	0.842975
chr3	38175600	0.752066
chr3	38183300	0.760331
chr3	38185900	0.768595
chr3	38217800	0.809917
chr3	39061500	0.809917
chr3	39091900	0.793388
chr3	39164500	0.867769
chr3	39267900	0.818182
chr3	39291800	0.768595
chr3	39295300	0.760331
chr3	39302600	0.900826
chr3	39326600	0.785124
chr3	39369200	0.867769
chr3	39425000	0.818182
chr3	39584500	0.826446
chr3	39979600	0.760331
chr3	40271300	0.752066
chr3	40346600	0.760331
chr3	40379300	0.826446
chr3	40566500	0.842975
chr3	40627000	0.834711
chr3	41011000	0.768595
chr3	41159300	0.867769
chr3	41232100	0.752066
chr3	41258600	0.917355
chr3	41565300	0.768595
chr3	41622300	0.818182
chr3	41650200	0.851240
chr3	41890900	0.776860
chr3	42082500	0.776860
chr3	42084700	0.735537
chr3	42093800	0.743802
chr3	42242000	0.884298
chr3	42289000	0.826446
chr3	42319600	0.752066
chr3	42332900	0.925620
chr3	42531800	0.892562
chr3	42632800	0.851240
chr3	42888700	0.760331
chr3	43080000	0.809917
chr3	43223200	0.776860
chr3	43369100	0.768595
chr3	43441100	0.752066

chr3	43490000	0.735537
chr3	43933900	0.842975
chr3	43949100	0.851240
chr3	43996100	0.768595
chr3	44001900	0.776860
chr3	44007600	0.793388
chr3	44108400	0.760331
chr3	44171900	0.818182
chr3	44481700	0.884298
chr3	45009100	0.909091
chr3	45152000	0.834711
chr3	45250400	0.909091
chr3	45423200	0.776860
chr3	45613900	0.892562
chr3	45616700	0.826446
chr3	45649500	0.925620
chr3	45657300	0.752066
chr3	45772600	0.752066
chr3	45926600	0.793388
chr3	45957600	0.818182
chr3	46014200	0.793388
chr3	46016400	0.892562
chr3	46020600	0.776860
chr3	46034400	0.801653
chr3	46037000	0.933884
chr3	46059800	0.826446
chr3	46068800	0.735537
chr3	46071900	0.785124
chr3	46074100	0.760331
chr3	46106500	0.900826
chr3	46233900	0.842975
chr3	46245400	0.785124
chr3	46255200	0.826446
chr3	46258900	0.867769
chr3	46265000	0.776860
chr3	46284100	0.818182
chr3	46294900	0.752066
chr3	46311600	0.760331
chr3	46318500	0.859504
chr3	46327600	0.950413
chr3	46331600	0.735537
chr3	46336000	0.776860
chr3	46339500	0.768595
chr3	46395400	0.950413
chr3	46404600	0.826446
chr3	46409700	0.743802
chr3	46412200	0.768595
chr3	46422300	0.818182
chr3	46435600	0.785124
chr3	46449300	0.818182
chr3	46482800	0.768595
chr3	46530800	0.760331
chr3	46545200	0.867769
chr3	46581200	0.917355

chr3	46588000	0.735537
chr3	46591900	0.892562
chr3	46671700	0.768595
chr3	46753600	0.785124
chr3	46955500	0.826446
chr3	46960500	0.752066
chr3	47018200	0.892562
chr3	47051300	0.942149
chr3	47156400	0.801653
chr3	47162600	0.776860
chr3	47267000	0.776860
chr3	47307600	0.842975
chr3	47462900	0.942149
chr3	47555300	0.801653
chr3	47773900	0.793388
chr3	48124400	0.809917
chr3	48216000	0.793388
chr3	48339800	0.818182
chr3	48552800	0.834711
chr3	48699200	0.760331
chr3	48777600	0.801653
chr3	48883300	0.743802
chr3	48936800	0.735537
chr3	49045200	0.859504
chr3	49314800	0.867769
chr3	49334700	0.834711
chr3	49341700	0.933884
chr3	49348800	0.743802
chr3	49351800	0.867769
chr3	49424200	0.760331
chr3	49438600	0.785124
chr3	49466800	0.909091
chr3	49726800	0.818182
chr3	49809200	0.818182
chr3	49851500	0.834711
chr3	49977800	0.818182
chr3	50232100	0.851240
chr3	50281000	0.752066
chr3	50310900	0.743802
chr3	50354700	0.752066
chr3	50359300	0.942149
chr3	50378200	0.735537
chr3	50606900	0.801653
chr3	50671100	0.760331
chr3	51333700	0.760331
chr3	51427700	0.735537
chr3	51432300	0.834711
chr3	51895500	0.867769
chr3	51977000	0.925620
chr3	52040500	0.809917
chr3	52049400	0.851240
chr3	52087100	0.867769
chr3	52230700	0.760331
chr3	52287800	0.884298

chr3	52334300	0.793388
chr3	52342700	0.752066
chr3	52352100	0.743802
chr3	52376700	0.743802
chr3	52481300	0.743802
chr3	52765400	0.826446
chr3	53073500	0.768595
chr3	53080200	0.793388
chr3	53164800	0.826446
chr3	53192900	0.826446
chr3	53215200	0.768595
chr3	53230600	0.867769
chr3	53234600	0.809917
chr3	53284500	0.867769
chr3	53647300	0.809917
chr3	53685200	0.785124
chr3	53744200	0.735537
chr3	53747700	0.760331
chr3	53778900	0.760331
chr3	53891100	0.735537
chr3	53915800	0.768595
chr3	53926200	0.801653
chr3	55556400	0.876033
chr3	55602700	0.768595
chr3	55606100	0.826446
chr3	55622500	0.867769
chr3	55627300	0.867769
chr3	55639400	0.776860
chr3	55663800	0.752066
chr3	55674300	0.834711
chr3	55723300	0.834711
chr3	55753200	0.752066
chr3	56717000	0.859504
chr3	56727800	0.809917
chr3	56814000	0.851240
chr3	56858900	0.933884
chr3	56898900	0.876033
chr3	56929800	0.876033
chr3	56934200	0.933884
chr3	56957900	0.776860
chr3	56977300	0.958678
chr3	56997300	0.867769
chr3	57014400	0.933884
chr3	57034300	0.735537
chr3	57080800	0.785124
chr3	57269300	0.958678
chr3	57657600	0.785124
chr3	57662000	0.834711
chr3	57678600	0.892562
chr3	57967100	0.809917
chr3	57970300	0.834711
chr3	58018800	0.735537
chr3	58023600	0.917355
chr3	58025800	0.834711

chr3	58030000	0.793388
chr3	58060800	0.760331
chr3	58072900	0.884298
chr3	58124900	0.743802
chr3	58292200	0.752066
chr3	58340600	0.809917
chr3	58415700	0.917355
chr3	58449900	0.933884
chr3	58460700	0.818182
chr3	58483100	0.735537
chr3	58593600	0.884298
chr3	58622000	0.785124
chr3	59326100	0.867769
chr3	59413400	0.958678
chr3	59513100	0.876033
chr3	59758900	0.743802
chr3	59770300	0.818182
chr3	59826200	0.752066
chr3	60067800	0.933884
chr3	60537200	0.768595
chr3	60542100	0.793388
chr3	61220300	0.884298
chr3	61239800	0.884298
chr3	61341600	0.917355
chr3	61400900	0.752066
chr3	61623200	0.859504
chr3	62213000	0.801653
chr3	62432300	0.876033
chr3	62610500	0.752066
chr3	62822100	0.752066
chr3	64026100	0.834711
chr3	64148200	0.793388
chr3	64448500	0.867769
chr3	65830000	0.851240
chr3	65950500	0.793388
chr3	66406400	0.876033
chr3	66504600	0.768595
chr3	66518100	0.768595
chr3	66523700	0.933884
chr3	66535800	0.867769
chr3	66541000	0.826446
chr3	66872800	0.752066
chr3	67126500	0.826446
chr3	67342200	0.867769
chr3	67400500	0.851240
chr3	67441000	0.884298
chr3	67701600	0.768595
chr3	67839800	0.735537
chr3	69107700	0.900826
chr3	69247400	0.892562
chr3	69291800	0.909091
chr3	69338000	0.834711
chr3	69414400	0.826446
chr3	69417900	0.834711

chr3	69813500	0.752066
chr3	70143900	0.752066
chr3	70146300	0.826446
chr3	70230200	0.884298
chr3	71137200	0.851240
chr3	71139700	0.785124
chr3	71410400	0.834711
chr3	71439800	0.818182
chr3	71452300	0.752066
chr3	71472300	0.842975
chr3	71925200	0.743802
chr3	71954900	0.743802
chr3	71957000	0.785124
chr3	71967300	0.933884
chr3	72016700	0.760331
chr3	72022000	0.735537
chr3	72038700	0.776860
chr3	72170700	0.842975
chr3	72183500	0.735537
chr3	72191900	0.876033
chr3	72231600	0.743802
chr3	72310900	0.768595
chr3	72360000	0.859504
chr3	72434300	0.842975
chr3	72442600	0.735537
chr3	72460600	0.768595
chr3	72474500	0.793388
chr3	72487500	0.876033
chr3	72490100	0.801653
chr3	72530500	0.743802
chr3	72537100	0.760331
chr3	72540600	0.760331
chr3	72583400	0.834711
chr3	72599800	0.809917
chr3	72631800	0.851240
chr3	72664000	0.892562
chr3	72675100	0.735537
chr3	72678900	0.743802
chr3	72681300	0.793388
chr3	72729600	0.826446
chr3	72806200	0.760331
chr3	73011000	0.859504
chr3	73026800	0.743802
chr3	73085500	0.851240
chr3	73093500	0.776860
chr3	73160000	0.851240
chr3	73498700	0.826446
chr3	75387500	0.900826
chr3	75733900	0.826446
chr3	81535100	0.917355
chr3	81540800	0.760331
chr3	81814900	0.785124
chr3	87724100	0.917355
chr3	88046900	0.760331

chr3	89154100	0.801653
chr3	89163900	0.760331
chr3	90448600	0.760331
chr3	93762200	0.950413
chr3	93791100	0.859504
chr3	95070300	0.834711
chr3	95076200	0.851240
chr3	97548800	0.834711
chr3	97701300	0.768595
chr3	98080500	0.809917
chr3	98164700	0.892562
chr3	98287200	0.867769
chr3	98317300	0.793388
chr3	98376500	0.859504
chr3	98505400	0.809917
chr3	98612700	0.752066
chr3	98731700	0.809917
chr3	99552900	0.826446
chr3	99563900	0.892562
chr3	99567900	0.917355
chr3	99615400	0.752066
chr3	99657800	0.826446
chr3	99762200	0.793388
chr3	99764500	0.867769
chr3	99791400	0.851240
chr3	99793500	0.834711
chr3	99806300	0.768595
chr3	99809500	0.925620
chr3	99929600	0.752066
chr3	100531400	0.867769
chr3	101099300	0.917355
chr3	101232300	0.958678
chr3	101289200	0.768595
chr3	101293400	0.785124
chr3	101441200	0.950413
chr3	101527700	0.785124
chr3	101530900	0.876033
chr3	101559000	0.760331
chr3	101562800	0.785124
chr3	101618300	0.851240
chr3	101621300	0.743802
chr3	101657700	0.876033
chr3	101712900	0.859504
chr3	101716100	0.760331
chr3	101719400	0.752066
chr3	101772900	0.735537
chr3	101854800	0.884298
chr3	101943100	0.842975
chr3	101953100	0.884298
chr3	101978000	0.776860
chr3	104246200	0.809917
chr3	104280600	0.735537
chr3	104436700	0.933884
chr3	104605200	0.851240

chr3	105053600	0.793388
chr3	105442700	0.826446
chr3	105484200	0.793388
chr3	105492800	0.743802
chr3	106784400	0.842975
chr3	106931800	0.760331
chr3	107133800	0.867769
chr3	107318200	0.909091
chr3	107346300	0.743802
chr3	107350200	0.752066
chr3	107399800	0.842975
chr3	107928300	0.818182
chr3	108015600	0.818182
chr3	108062200	0.884298
chr3	108065200	0.793388
chr3	108126500	0.818182
chr3	108254400	0.793388
chr3	108299400	0.760331
chr3	110534100	0.752066
chr3	111030700	0.892562
chr3	111258500	0.760331
chr3	111267400	0.859504
chr3	111402800	0.809917
chr3	111409800	0.818182
chr3	111442900	0.900826
chr3	111457300	0.793388
chr3	111602000	0.785124
chr3	111758300	0.867769
chr3	111794400	0.818182
chr3	111810200	0.867769
chr3	111855700	0.735537
chr3	111966300	0.768595
chr3	112003100	0.752066
chr3	112012700	0.900826
chr3	112016500	0.851240
chr3	112021600	0.735537
chr3	112223200	0.818182
chr3	112259700	0.884298
chr3	112311300	0.760331
chr3	112321200	0.776860
chr3	112324000	0.752066
chr3	112354300	0.917355
chr3	112389600	0.900826
chr3	112408900	0.942149
chr3	112459100	0.768595
chr3	112486100	0.876033
chr3	112494300	0.793388
chr3	112531000	0.793388
chr3	112533500	0.735537
chr3	112688400	0.768595
chr3	112695600	0.851240
chr3	112709900	0.801653
chr3	112738300	0.834711
chr3	112770700	0.834711

chr3	112777200	0.793388
chr3	112872900	0.768595
chr3	112943000	0.867769
chr3	112973400	0.884298
chr3	113000800	0.735537
chr3	113547700	0.826446
chr3	113704900	0.752066
chr3	113871800	0.818182
chr3	113927200	0.942149
chr3	113962200	0.900826
chr3	114010400	0.900826
chr3	114018600	0.776860
chr3	114027900	0.867769
chr3	114124600	0.776860
chr3	114137300	0.818182
chr3	114186200	0.768595
chr3	114219300	0.752066
chr3	114224300	0.752066
chr3	114238800	0.867769
chr3	114285700	0.752066
chr3	114302300	0.793388
chr3	114441500	0.818182
chr3	114475900	0.867769
chr3	114575100	0.851240
chr3	114739400	0.735537
chr3	114866900	0.950413
chr3	114905700	0.867769
chr3	115347100	0.785124
chr3	115375600	0.752066
chr3	115649900	0.818182
chr3	115652200	0.809917
chr3	115662700	0.818182
chr3	115666700	0.925620
chr3	115755100	0.818182
chr3	115786400	0.809917
chr3	115858900	0.900826
chr3	116605100	0.942149
chr3	116737700	0.768595
chr3	117322200	0.735537
chr3	117604300	0.876033
chr3	118199300	0.743802
chr3	118284000	0.809917
chr3	118529700	0.909091
chr3	118532400	0.892562
chr3	119051100	0.776860
chr3	119176900	0.785124
chr3	119217400	0.768595
chr3	119396600	0.760331
chr3	119465000	0.752066
chr3	119493600	0.785124
chr3	119631300	0.793388
chr3	119822500	0.818182
chr3	120118900	0.842975
chr3	120407300	0.818182

chr3	120438100	0.752066
chr3	120514600	0.735537
chr3	120521000	0.859504
chr3	120571800	0.768595
chr3	121268100	0.776860
chr3	121459400	0.900826
chr3	121541200	0.793388
chr3	121715500	0.735537
chr3	121783600	0.776860
chr3	121790000	0.818182
chr3	121804900	0.851240
chr3	121816000	0.760331
chr3	121965000	0.867769
chr3	122014600	0.743802
chr3	122041600	0.818182
chr3	122050400	0.776860
chr3	122135400	0.743802
chr3	122253100	0.776860
chr3	122329100	0.818182
chr3	122346400	0.760331
chr3	122362200	0.909091
chr3	122381400	0.793388
chr3	122409600	0.785124
chr3	122606100	0.743802
chr3	122635600	0.743802
chr3	122671900	0.743802
chr3	122694500	0.867769
chr3	122698300	0.809917
chr3	122792000	0.785124
chr3	122801700	0.785124
chr3	122803900	0.776860
chr3	123246000	0.801653
chr3	123255800	0.933884
chr3	123304500	0.793388
chr3	123414700	0.752066
chr3	123726400	0.760331
chr3	123968200	0.818182
chr3	124017900	0.859504
chr3	124215800	0.809917
chr3	124294700	0.834711
chr3	124302200	0.776860
chr3	124312200	0.735537
chr3	124339600	0.933884
chr3	124355200	0.876033
chr3	124492700	0.909091
chr3	124598100	0.801653
chr3	124646300	0.842975
chr3	124742900	0.793388
chr3	124919900	0.760331
chr3	125089600	0.743802
chr3	125094400	0.760331
chr3	125238800	0.818182
chr3	125327600	0.752066
chr3	125820200	0.867769

chr3	126061700	0.892562
chr3	126118700	0.735537
chr3	126398300	0.809917
chr3	126680300	0.743802
chr3	126701700	0.942149
chr3	126793900	0.876033
chr3	127124900	0.785124
chr3	127195400	0.801653
chr3	127203800	0.818182
chr3	127391500	0.884298
chr3	127521100	0.768595
chr3	127576200	0.752066
chr3	127609300	0.801653
chr3	127644600	0.826446
chr3	127833200	0.876033
chr3	128167200	0.942149
chr3	128292500	0.859504
chr3	128369600	0.793388
chr3	128393800	0.933884
chr3	128399800	0.909091
chr3	128736000	0.743802
chr3	128777800	0.892562
chr3	128880500	0.785124
chr3	128894100	0.859504
chr3	128964800	0.760331
chr3	128968800	0.867769
chr3	129002700	0.735537
chr3	129024900	0.768595
chr3	129037200	0.752066
chr3	129118500	0.909091
chr3	129133000	0.793388
chr3	129245100	0.785124
chr3	129312600	0.842975
chr3	129513600	0.925620
chr3	129546900	0.884298
chr3	129612100	0.801653
chr3	129814700	0.768595
chr3	130355700	1.000000
chr3	130495900	0.809917
chr3	130637000	0.867769
chr3	130794700	0.876033
chr3	131339400	0.826446
chr3	131364700	0.768595
chr3	131407700	0.876033
chr3	131515800	0.752066
chr3	131763600	0.983471
chr3	132134500	0.851240
chr3	132165200	0.735537
chr3	132372600	0.793388
chr3	132386100	0.752066
chr3	132829800	0.752066
chr3	132929200	0.842975
chr3	133199200	0.975207
chr3	133225700	0.752066

chr3	133241100	0.768595
chr3	133244400	0.743802
chr3	133267000	0.743802
chr3	133308300	0.793388
chr3	133395000	0.801653
chr3	133571500	0.776860
chr3	133576400	0.809917
chr3	133599300	0.743802
chr3	133601400	0.768595
chr3	133950900	0.834711
chr3	133956000	0.768595
chr3	133959000	0.743802
chr3	133975500	0.752066
chr3	134027200	0.793388
chr3	134150300	0.752066
chr3	134317900	0.752066
chr3	134528900	0.892562
chr3	134544100	0.818182
chr3	134563900	0.900826
chr3	134573300	0.760331
chr3	135828600	0.900826
chr3	135841000	0.809917
chr3	135916100	0.768595
chr3	136345600	0.768595
chr3	136384100	0.760331
chr3	136619200	0.752066
chr3	136652100	0.809917
chr3	136659700	0.743802
chr3	136752000	0.743802
chr3	136880300	0.743802
chr3	136893800	0.760331
chr3	137190300	0.809917
chr3	137780400	0.818182
chr3	138050300	0.735537
chr3	138087400	0.818182
chr3	138129900	0.842975
chr3	138135800	0.892562
chr3	138232200	0.834711
chr3	138297900	0.818182
chr3	138317400	0.743802
chr3	138497100	0.834711
chr3	138554000	0.752066
chr3	138800900	0.752066
chr3	138829400	0.859504
chr3	138921900	0.768595
chr3	139048400	0.793388
chr3	139108500	0.809917
chr3	139348100	0.760331
chr3	139890100	0.785124
chr3	140259700	0.867769
chr3	140475900	0.851240
chr3	140552000	0.793388
chr3	140564900	0.801653
chr3	140661500	0.776860

chr3	140764600	0.884298
chr3	141006300	0.785124
chr3	141022500	0.859504
chr3	141080300	0.752066
chr3	141088600	0.826446
chr3	141113900	0.809917
chr3	141160200	0.826446
chr3	141168800	0.760331
chr3	141218300	0.867769
chr3	141295700	0.785124
chr3	141711100	0.760331
chr3	142298100	0.801653
chr3	142847500	0.793388
chr3	142851400	0.752066
chr3	142879100	0.785124
chr3	142893800	0.743802
chr3	142911500	0.760331
chr3	142936100	0.735537
chr3	142950100	0.809917
chr3	143017500	0.834711
chr3	143068200	0.851240
chr3	143143400	0.867769
chr3	143234400	0.743802
chr3	143248200	0.859504
chr3	143301200	0.867769
chr3	143393100	0.834711
chr3	143495000	0.752066
chr3	143559900	0.793388
chr3	143644000	0.826446
chr3	143984100	0.752066
chr3	144276200	0.842975
chr3	145677900	0.785124
chr3	146222100	0.925620
chr3	146268800	0.768595
chr3	148037200	0.867769
chr3	148049800	0.735537
chr3	148558100	0.909091
chr3	148641300	0.743802
chr3	148722300	0.925620
chr3	148727900	0.743802
chr3	148815700	0.983471
chr3	148870300	0.818182
chr3	148892900	0.793388
chr3	148922000	0.867769
chr3	149226200	0.752066
chr3	149250600	0.917355
chr3	149322400	0.735537
chr3	149341600	0.776860
chr3	149369400	0.776860
chr3	149499300	0.851240
chr3	149792400	0.776860
chr3	149842300	0.735537
chr3	149850300	0.818182
chr3	149966400	0.867769

chr3	150103600	0.735537
chr3	150371400	0.768595
chr3	150399400	0.842975
chr3	150423600	0.818182
chr3	150463500	0.876033
chr3	150467000	0.793388
chr3	150516200	0.826446
chr3	150644400	0.785124
chr3	150679500	0.768595
chr3	150811800	0.826446
chr3	150889800	0.809917
chr3	150946200	0.867769
chr3	150949200	0.801653
chr3	150984100	0.818182
chr3	150987200	0.917355
chr3	150994800	0.768595
chr3	151106100	0.826446
chr3	151513700	0.909091
chr3	151740200	0.842975
chr3	151869500	0.768595
chr3	151971900	0.917355
chr3	152275100	0.818182
chr3	153340900	0.752066
chr3	153687800	0.867769
chr3	154116800	0.826446
chr3	155053900	0.776860
chr3	155212600	0.909091
chr3	155456100	0.743802
chr3	155573000	0.900826
chr3	155589100	0.752066
chr3	155769700	0.867769
chr3	156060300	0.735537
chr3	156106900	0.752066
chr3	156178800	0.942149
chr3	156212100	0.809917
chr3	156372800	0.752066
chr3	156544300	0.834711
chr3	156553300	0.776860
chr3	156692900	0.801653
chr3	156696500	0.909091
chr3	156790800	0.743802
chr3	156793600	0.752066
chr3	156884900	0.743802
chr3	158291300	0.793388
chr3	158577400	0.818182
chr3	158973900	0.785124
chr3	159502100	0.834711
chr3	159537500	0.776860
chr3	159579800	0.826446
chr3	159622900	0.859504
chr3	159629100	0.925620
chr3	159647500	0.801653
chr3	159724700	0.752066
chr3	159770600	0.776860

chr3	159845100	0.884298
chr3	159847600	0.826446
chr3	159854800	0.933884
chr3	159889900	0.826446
chr3	160380000	0.892562
chr3	160383600	0.925620
chr3	160415900	0.752066
chr3	160519900	0.925620
chr3	160785600	0.834711
chr3	160788400	0.752066
chr3	160800200	0.942149
chr3	161033600	0.842975
chr3	167445200	0.834711
chr3	167469300	0.735537
chr3	167480500	0.760331
chr3	167548600	0.867769
chr3	167771400	0.752066
chr3	168795700	0.801653
chr3	168876600	0.851240
chr3	169532300	0.876033
chr3	169547100	0.768595
chr3	169554500	0.917355
chr3	169581600	0.735537
chr3	169648600	0.743802
chr3	169872800	0.818182
chr3	169899300	0.776860
chr3	170045100	0.876033
chr3	170147100	0.851240
chr3	170291900	0.793388
chr3	170350300	0.752066
chr3	170353200	0.826446
chr3	170375700	0.909091
chr3	170431600	0.752066
chr3	170444900	0.801653
chr3	170587900	0.785124
chr3	171054800	0.834711
chr3	171095800	0.793388
chr3	171147700	0.785124
chr3	171186400	0.818182
chr3	171283200	0.752066
chr3	171556500	0.851240
chr3	171564700	0.818182
chr3	171637100	0.909091
chr3	171693700	0.842975
chr3	171806100	0.760331
chr3	171821700	0.743802
chr3	171860600	0.859504
chr3	171989100	0.776860
chr3	172196500	0.760331
chr3	172235500	0.793388
chr3	172248800	0.842975
chr3	172255300	0.851240
chr3	172265000	0.818182
chr3	172296300	0.760331

chr3	172300000	0.785124
chr3	172313700	0.925620
chr3	175195900	0.892562
chr3	176829900	0.809917
chr3	176863300	0.925620
chr3	176999800	0.752066
chr3	177018800	0.826446
chr3	177056600	0.752066
chr3	177071500	0.776860
chr3	177129000	0.826446
chr3	177135600	0.801653
chr3	177168100	0.793388
chr3	177184200	0.818182
chr3	177257100	0.892562
chr3	177365500	0.809917
chr3	177491400	0.768595
chr3	177606100	0.925620
chr3	178199900	0.776860
chr3	178240600	0.801653
chr3	178381700	0.768595
chr3	178388200	0.785124
chr3	178417700	0.859504
chr3	178483400	0.785124
chr3	178687500	0.900826
chr3	178851800	0.776860
chr3	178884600	0.776860
chr3	178906600	0.859504
chr3	178958500	0.793388
chr3	178984600	0.818182
chr3	179022600	0.876033
chr3	179027600	0.809917
chr3	179150200	0.760331
chr3	179166100	0.768595
chr3	179182900	0.818182
chr3	179322600	0.851240
chr3	179324900	0.743802
chr3	179331100	0.752066
chr3	179571200	0.867769
chr3	179604300	0.842975
chr3	179639600	0.925620
chr3	179667800	0.785124
chr3	179766800	0.752066
chr3	179985200	0.925620
chr3	180045000	0.768595
chr3	180120600	0.834711
chr3	180290400	0.768595
chr3	180345400	0.760331
chr3	181076800	0.826446
chr3	181614800	0.735537
chr3	181628600	0.826446
chr3	181673800	0.867769
chr3	182448300	0.851240
chr3	182451000	0.776860
chr3	182655900	0.826446

chr3	182690000	0.876033
chr3	182760800	0.768595
chr3	182842100	0.776860
chr3	182880000	0.818182
chr3	183025300	0.776860
chr3	183084700	0.776860
chr3	183093700	0.925620
chr3	183099700	0.892562
chr3	183154000	0.859504
chr3	183156200	0.743802
chr3	183231300	0.818182
chr3	183257600	0.760331
chr3	183291000	0.752066
chr3	183296500	0.851240
chr3	183310300	0.834711
chr3	183615300	0.842975
chr3	184033200	0.851240
chr3	184081200	0.776860
chr3	184474900	0.752066
chr3	184484000	0.818182
chr3	184503400	0.809917
chr3	185100000	0.884298
chr3	185216400	0.851240
chr3	185255700	0.884298
chr3	185304200	0.801653
chr3	185349100	0.983471
chr3	185364900	0.768595
chr3	185378600	0.892562
chr3	185464000	0.743802
chr3	185661600	0.793388
chr3	185695400	0.809917
chr3	185778500	0.892562
chr3	185823900	0.801653
chr3	185829700	0.933884
chr3	185944400	0.809917
chr3	185974300	0.752066
chr3	186100600	0.760331
chr3	186143500	0.834711
chr3	186176400	0.752066
chr3	186248300	0.735537
chr3	186424300	0.851240
chr3	186518700	0.752066
chr3	186543900	0.801653
chr3	186736400	0.743802
chr3	186747400	0.785124
chr3	186754500	0.851240
chr3	186820100	0.818182
chr3	186857200	0.958678
chr3	187086300	0.867769
chr3	187366100	0.760331
chr3	187459500	0.743802
chr3	187468500	0.834711
chr3	187495900	0.867769
chr3	187522400	0.909091

chr3	187547100	0.842975
chr3	187588200	0.842975
chr3	187636300	0.760331
chr3	187641300	0.752066
chr3	187644600	0.900826
chr3	187650800	0.735537
chr3	187653800	0.900826
chr3	187660500	0.826446
chr3	187681200	0.826446
chr3	187693000	0.834711
chr3	188002900	0.851240
chr3	188027800	0.867769
chr3	188108400	0.867769
chr3	188115600	0.900826
chr3	188119100	0.768595
chr3	188135800	0.801653
chr3	188157500	0.801653
chr3	188415600	0.743802
chr3	188471600	0.818182
chr3	188474600	0.785124
chr3	188635400	0.826446
chr3	189023700	0.776860
chr3	189043500	0.776860
chr3	189068200	0.867769
chr3	189074100	0.785124
chr3	189086800	0.785124
chr3	189208400	0.851240
chr3	189227700	0.735537
chr3	189231400	0.785124
chr3	189276300	0.876033
chr3	189280400	0.909091
chr3	189391600	0.834711
chr3	189433700	0.818182
chr3	189436800	0.958678
chr3	189475300	0.809917
chr3	189497100	0.768595
chr3	189517400	0.859504
chr3	189666500	0.842975
chr3	190335800	0.851240
chr3	190855800	0.834711
chr3	190955300	0.801653
chr3	190971900	0.752066
chr3	191008500	0.793388
chr3	191066500	0.884298
chr3	191760700	0.743802
chr3	191810600	0.826446
chr3	192394100	0.892562
chr3	192677000	0.735537
chr3	193495000	0.760331
chr3	193555500	0.842975
chr3	193586700	0.818182
chr3	193590400	0.735537
chr3	193681900	0.966942
chr3	193723800	0.776860

chr3	193763400	0.884298
chr3	193802200	0.933884
chr3	193857800	0.892562
chr3	193917200	0.818182
chr3	194202700	0.735537
chr3	194218700	0.735537
chr3	194262200	0.776860
chr3	194375600	0.942149
chr3	194393300	0.925620
chr3	194416400	0.842975
chr3	194452200	0.909091
chr3	194488200	0.776860
chr3	194586900	0.884298
chr3	194592700	0.785124
chr3	194723700	0.859504
chr3	194785400	0.801653
chr3	194871600	0.743802
chr3	194875600	0.793388
chr3	194980300	0.735537
chr3	194985500	0.752066
chr3	195419300	0.735537
chr3	195436800	0.958678
chr3	195443100	0.851240
chr3	195566300	0.743802
chr3	195578500	0.760331
chr3	195678600	0.818182
chr3	195683300	0.917355
chr3	195801300	0.785124
chr3	195820300	0.743802
chr3	195872800	0.785124
chr3	195887100	0.884298
chr3	195919600	0.760331
chr3	195930900	0.768595
chr3	196014900	0.776860
chr3	196019600	0.867769
chr3	196024000	0.743802
chr3	196184700	0.735537
chr3	196255700	0.785124
chr3	196595000	0.743802
chr3	196778600	0.793388
chr3	196913700	0.735537
chr3	196931700	0.752066
chr3	196967600	0.743802
chr3	197019000	0.809917
chr3	197033500	0.818182
chr3	197082000	0.859504
chr3	197155900	0.925620
chr3	197186800	0.776860
chr3	197226900	0.743802
chr3	197229500	0.768595
chr3	197237200	0.735537
chr3	197575100	0.900826
chr4	57100	0.735537
chr4	121700	0.818182

chr4	331800	0.743802
chr4	467800	0.958678
chr4	493100	0.801653
chr4	549400	0.842975
chr4	693700	0.735537
chr4	700000	0.818182
chr4	820300	0.991736
chr4	910500	0.801653
chr4	928600	0.768595
chr4	1016100	0.752066
chr4	1020100	0.785124
chr4	1056800	0.776860
chr4	1161000	0.851240
chr4	1166100	0.785124
chr4	1178300	0.892562
chr4	1192000	0.842975
chr4	1261400	0.892562
chr4	1299700	0.809917
chr4	1341500	0.909091
chr4	1346200	0.785124
chr4	1731100	0.768595
chr4	1769200	0.892562
chr4	1864700	0.876033
chr4	2010700	0.933884
chr4	2079800	0.785124
chr4	2293000	0.826446
chr4	2335600	0.834711
chr4	2395600	0.900826
chr4	2404200	0.983471
chr4	2471500	0.760331
chr4	2482500	0.818182
chr4	2597800	0.760331
chr4	2600600	0.793388
chr4	2607200	0.785124
chr4	2698900	0.752066
chr4	2803100	0.884298
chr4	2817500	0.826446
chr4	2956400	0.743802
chr4	3012000	0.743802
chr4	3016300	0.785124
chr4	3024400	0.867769
chr4	3036500	0.842975
chr4	3360900	0.917355
chr4	3486900	0.801653
chr4	4211500	0.785124
chr4	4289600	0.743802
chr4	4633600	0.842975
chr4	4691800	0.809917
chr4	4761100	0.859504
chr4	4946100	0.768595
chr4	5035700	0.892562
chr4	5721300	0.735537
chr4	5826000	0.925620
chr4	5943600	0.909091

chr4	5958000	0.842975
chr4	5984700	0.909091
chr4	5990300	0.760331
chr4	5998100	0.793388
chr4	6044000	0.925620
chr4	6281300	0.760331
chr4	6340200	0.801653
chr4	6427500	0.760331
chr4	6691200	0.884298
chr4	6698600	0.793388
chr4	6729700	0.991736
chr4	6780000	0.735537
chr4	6784900	0.809917
chr4	6796500	0.752066
chr4	6858300	0.785124
chr4	6894600	0.793388
chr4	6911200	0.851240
chr4	6928000	0.884298
chr4	6967700	0.752066
chr4	6986000	0.776860
chr4	7257000	0.801653
chr4	7296600	0.900826
chr4	7746000	0.801653
chr4	7764200	0.752066
chr4	7781000	0.785124
chr4	8037400	0.768595
chr4	8174200	0.867769
chr4	8177700	0.909091
chr4	8230600	0.793388
chr4	8442400	0.760331
chr4	8535800	0.801653
chr4	8563200	0.735537
chr4	8821400	0.760331
chr4	9814800	0.909091
chr4	9939400	0.892562
chr4	9992300	0.851240
chr4	10012700	0.917355
chr4	10025400	0.842975
chr4	10035200	0.884298
chr4	10107100	0.851240
chr4	10153200	0.768595
chr4	10579900	0.834711
chr4	10673400	0.743802
chr4	10675700	0.884298
chr4	10712800	0.735537
chr4	11100600	0.818182
chr4	11288800	0.834711
chr4	11343600	0.826446
chr4	11422100	0.826446
chr4	12725600	0.743802
chr4	13486300	0.958678
chr4	13624800	0.735537
chr4	13629800	0.793388
chr4	14151600	0.876033

chr4	14161900	0.743802
chr4	14171100	0.876033
chr4	14181900	0.842975
chr4	14785500	0.851240
chr4	14866400	0.768595
chr4	14879300	0.768595
chr4	14884400	0.818182
chr4	14907400	0.842975
chr4	15022600	0.776860
chr4	15412900	0.809917
chr4	15650100	0.776860
chr4	15657400	0.933884
chr4	15683600	0.743802
chr4	15759400	0.743802
chr4	15773300	0.776860
chr4	15776000	0.867769
chr4	15784500	0.743802
chr4	15796900	0.900826
chr4	16081300	0.900826
chr4	16207500	0.809917
chr4	17338000	0.867769
chr4	17387200	0.768595
chr4	17667100	0.809917
chr4	18027000	0.752066
chr4	18211800	0.793388
chr4	18595600	0.743802
chr4	19415200	0.900826
chr4	24547400	0.851240
chr4	24590100	0.793388
chr4	24659900	0.826446
chr4	24859100	0.735537
chr4	24887200	0.785124
chr4	25003500	0.785124
chr4	25162000	0.818182
chr4	25174900	0.735537
chr4	25299900	0.743802
chr4	25493400	0.785124
chr4	25844300	0.801653
chr4	25859400	0.735537
chr4	25869000	0.760331
chr4	25886400	0.809917
chr4	25971700	0.809917
chr4	25995600	0.851240
chr4	26052200	0.859504
chr4	26060800	0.917355
chr4	26073800	0.743802
chr4	26090700	0.776860
chr4	26135100	0.776860
chr4	26147000	0.793388
chr4	26199800	0.842975
chr4	26206200	0.760331
chr4	26275200	0.842975
chr4	26343200	0.876033
chr4	26367700	0.851240

chr4	26374700	0.826446
chr4	26379600	0.859504
chr4	26382700	0.826446
chr4	26403900	0.793388
chr4	26733500	0.884298
chr4	26748800	0.834711
chr4	26754100	0.760331
chr4	26789800	0.760331
chr4	26809400	0.925620
chr4	26820700	0.785124
chr4	26832600	0.809917
chr4	26868100	0.760331
chr4	26881400	0.809917
chr4	26914400	0.743802
chr4	27638200	0.752066
chr4	33106300	0.768595
chr4	36192100	0.884298
chr4	36246000	0.743802
chr4	36288100	0.785124
chr4	36290800	0.776860
chr4	36396500	0.768595
chr4	36553700	0.752066
chr4	36992800	0.743802
chr4	37604800	0.743802
chr4	37756300	0.818182
chr4	37898300	0.760331
chr4	37966900	0.785124
chr4	37983600	0.876033
chr4	38168500	0.743802
chr4	38188800	0.735537
chr4	38270600	0.867769
chr4	38308300	0.851240
chr4	38312700	0.867769
chr4	38368200	0.793388
chr4	38413200	0.826446
chr4	38494400	0.785124
chr4	38515400	0.809917
chr4	38531300	0.826446
chr4	38565700	0.793388
chr4	38569100	0.793388
chr4	38669100	0.785124
chr4	38706100	0.760331
chr4	38712400	0.933884
chr4	38755500	0.776860
chr4	38891200	0.892562
chr4	38942800	0.851240
chr4	39064300	0.752066
chr4	39356000	0.768595
chr4	39360800	0.785124
chr4	39363100	0.785124
chr4	39365200	0.760331
chr4	39570800	0.785124
chr4	39766300	0.818182
chr4	39935700	0.743802

chr4	39989200	0.768595
chr4	40061700	0.842975
chr4	40067300	0.743802
chr4	40225800	0.785124
chr4	40230500	0.818182
chr4	40267500	0.801653
chr4	40291800	0.801653
chr4	40311700	0.933884
chr4	40314300	0.892562
chr4	40319100	0.851240
chr4	40327000	0.867769
chr4	40576300	0.735537
chr4	40690400	0.785124
chr4	40692800	0.876033
chr4	40702200	0.826446
chr4	40827900	0.776860
chr4	40956600	0.735537
chr4	41174900	0.752066
chr4	41187900	0.909091
chr4	41191600	0.859504
chr4	41218600	0.942149
chr4	41823100	0.818182
chr4	41866400	0.785124
chr4	42170000	0.801653
chr4	42298900	0.876033
chr4	42304800	0.743802
chr4	42313100	0.818182
chr4	42346900	0.859504
chr4	42402600	0.752066
chr4	42463100	0.760331
chr4	42659700	0.876033
chr4	47145300	0.842975
chr4	47148100	0.983471
chr4	47162400	0.735537
chr4	47522200	0.809917
chr4	47567300	0.842975
chr4	47789200	0.900826
chr4	47829900	0.809917
chr4	47925600	0.785124
chr4	47929000	0.768595
chr4	47932100	0.735537
chr4	48130800	0.834711
chr4	48179900	0.752066
chr4	48229800	0.785124
chr4	48431700	0.752066
chr4	48568400	0.793388
chr4	48728800	0.735537
chr4	48794900	0.867769
chr4	48814200	0.851240
chr4	48833500	0.842975
chr4	49515000	0.851240
chr4	49617800	0.933884
chr4	52772900	0.760331
chr4	53525600	0.851240

chr4	53575800	0.826446
chr4	53588400	0.900826
chr4	53614700	0.801653
chr4	53879300	0.793388
chr4	53942200	0.776860
chr4	54244000	0.834711
chr4	54458200	0.760331
chr4	54468400	0.826446
chr4	54513300	0.785124
chr4	54614800	0.801653
chr4	54671100	0.917355
chr4	54960000	0.743802
chr4	54971400	0.793388
chr4	55576300	0.760331
chr4	56420100	0.801653
chr4	56428800	0.842975
chr4	56484100	0.752066
chr4	57292700	0.867769
chr4	57516900	0.909091
chr4	57547800	0.785124
chr4	57590000	0.735537
chr4	57629000	0.776860
chr4	57658000	0.785124
chr4	57695400	0.793388
chr4	57730600	0.735537
chr4	57845200	0.909091
chr4	58087700	0.743802
chr4	58732000	0.826446
chr4	60401900	0.735537
chr4	68082300	0.958678
chr4	68411100	0.909091
chr4	68727600	0.776860
chr4	69115600	0.785124
chr4	69138800	0.776860
chr4	69215600	0.917355
chr4	70697900	0.909091
chr4	70716000	0.752066
chr4	71456100	0.834711
chr4	71480000	0.776860
chr4	71535600	0.735537
chr4	71542200	0.809917
chr4	71575500	0.776860
chr4	71597500	0.834711
chr4	71603200	0.892562
chr4	71613700	0.760331
chr4	71616400	0.801653
chr4	71705200	0.876033
chr4	71710500	0.867769
chr4	71762100	0.743802
chr4	71781200	0.743802
chr4	71790400	0.851240
chr4	72172000	0.785124
chr4	72205100	0.809917
chr4	73902600	0.818182

chr4	74393700	0.752066
chr4	74442100	0.925620
chr4	74543100	0.752066
chr4	74759000	0.776860
chr4	75032700	0.809917
chr4	75101500	0.785124
chr4	75201600	0.809917
chr4	75231000	0.842975
chr4	75409600	0.876033
chr4	75799300	0.801653
chr4	75897300	0.768595
chr4	75901100	0.776860
chr4	75995100	0.752066
chr4	76098500	0.909091
chr4	76106800	0.760331
chr4	76162300	0.735537
chr4	76177400	0.909091
chr4	76354600	0.809917
chr4	76498800	0.735537
chr4	76621600	0.818182
chr4	76624100	0.933884
chr4	76650200	0.801653
chr4	76770000	0.925620
chr4	76950100	0.859504
chr4	76976500	0.793388
chr4	76980100	0.752066
chr4	76983100	0.793388
chr4	77117400	0.735537
chr4	77127600	0.851240
chr4	77223800	0.793388
chr4	77437600	0.785124
chr4	77466800	0.834711
chr4	77491200	0.743802
chr4	77528900	0.834711
chr4	77580000	0.818182
chr4	77658000	0.876033
chr4	77991600	0.818182
chr4	78048900	0.859504
chr4	78115100	0.925620
chr4	78507800	0.752066
chr4	78510400	0.752066
chr4	78523100	0.776860
chr4	78536600	0.743802
chr4	78551300	0.884298
chr4	78741400	0.735537
chr4	78783700	0.876033
chr4	78815200	0.876033
chr4	79221200	0.801653
chr4	79513900	0.785124
chr4	79537100	0.818182
chr4	79540000	0.809917
chr4	79552500	0.760331
chr4	79555300	0.801653
chr4	79560300	0.809917

chr4	79567900	0.776860
chr4	79575000	0.768595
chr4	79579100	0.735537
chr4	79584700	0.776860
chr4	79613000	0.892562
chr4	79627300	0.834711
chr4	79643600	0.776860
chr4	79677900	0.842975
chr4	79687300	0.826446
chr4	79690100	0.809917
chr4	79708600	0.752066
chr4	79719000	0.760331
chr4	79731400	0.900826
chr4	79778000	0.876033
chr4	79858300	0.752066
chr4	81047300	0.793388
chr4	81060700	0.776860
chr4	81462100	0.818182
chr4	81532700	0.867769
chr4	81961100	0.735537
chr4	81974500	0.818182
chr4	81982000	0.785124
chr4	82414000	0.842975
chr4	82487300	0.818182
chr4	82570500	0.933884
chr4	83215000	0.950413
chr4	83676700	0.900826
chr4	83941400	0.818182
chr4	84178200	0.818182
chr4	84219300	0.768595
chr4	84262600	0.760331
chr4	84278700	0.760331
chr4	84528200	0.785124
chr4	84953800	0.859504
chr4	85180700	0.801653
chr4	86320800	0.917355
chr4	86370700	0.785124
chr4	86394700	0.900826
chr4	86403800	0.760331
chr4	86442500	0.776860
chr4	86487900	0.851240
chr4	86525300	0.842975
chr4	86578700	0.958678
chr4	86593600	0.785124
chr4	86596100	0.752066
chr4	86601600	0.818182
chr4	86629500	0.785124
chr4	86647500	0.834711
chr4	86747600	0.735537
chr4	86936200	0.801653
chr4	87281600	0.826446
chr4	87319900	0.735537
chr4	87486600	0.768595
chr4	87508000	0.859504

chr4	87852500	0.842975
chr4	87861700	0.735537
chr4	87864300	0.834711
chr4	88141500	0.752066
chr4	88328300	0.834711
chr4	88599300	0.826446
chr4	89208400	0.826446
chr4	89242100	0.909091
chr4	89304100	0.801653
chr4	89447000	0.842975
chr4	89449500	0.768595
chr4	89526900	0.793388
chr4	89534600	0.933884
chr4	89658800	0.900826
chr4	89724600	0.760331
chr4	89780400	0.859504
chr4	89991000	0.735537
chr4	90002600	0.768595
chr4	90215900	0.842975
chr4	90580200	0.752066
chr4	90751200	0.785124
chr4	90820300	0.933884
chr4	90851100	0.785124
chr4	90993300	0.859504
chr4	91040000	0.760331
chr4	95129200	0.876033
chr4	99125100	0.933884
chr4	99369800	0.735537
chr4	99389200	0.768595
chr4	99440200	0.867769
chr4	99982900	0.752066
chr4	100368700	0.743802
chr4	100484900	0.867769
chr4	100730500	0.735537
chr4	100982100	0.801653
chr4	101017200	0.859504
chr4	101028300	0.950413
chr4	101130200	0.785124
chr4	101931300	0.743802
chr4	101943100	0.842975
chr4	102025900	0.752066
chr4	102063700	0.834711
chr4	102078600	0.776860
chr4	102204600	0.809917
chr4	102250300	0.826446
chr4	102340800	0.785124
chr4	102615500	0.851240
chr4	102706800	0.801653
chr4	102728500	0.809917
chr4	102929500	0.735537
chr4	103311900	0.743802
chr4	103648800	0.801653
chr4	103732300	0.867769
chr4	103940800	0.818182

chr4	103997700	0.842975
chr4	105664800	0.917355
chr4	105982800	0.818182
chr4	105996600	0.760331
chr4	106059600	0.776860
chr4	106137200	0.768595
chr4	106508200	0.826446
chr4	108514200	0.900826
chr4	109032400	0.900826
chr4	109081900	0.760331
chr4	109624200	0.818182
chr4	109771200	0.826446
chr4	110365700	0.801653
chr4	110509900	0.760331
chr4	110620400	0.768595
chr4	110799300	0.785124
chr4	110917500	0.867769
chr4	110977100	0.752066
chr4	111010300	0.818182
chr4	111189700	0.752066
chr4	111218800	0.768595
chr4	112188800	0.851240
chr4	112760100	0.752066
chr4	112852300	0.818182
chr4	113106800	0.735537
chr4	113121000	0.867769
chr4	113135100	0.735537
chr4	113234200	0.876033
chr4	113236800	0.818182
chr4	113240300	0.925620
chr4	113249800	0.752066
chr4	113558500	0.760331
chr4	113974200	0.785124
chr4	114275200	0.826446
chr4	114510700	0.743802
chr4	114528900	0.760331
chr4	114560700	0.752066
chr4	114566500	0.917355
chr4	114608900	0.834711
chr4	114628200	0.900826
chr4	114630300	0.809917
chr4	114674500	0.735537
chr4	115030300	0.834711
chr4	115522500	0.909091
chr4	115525100	0.842975
chr4	116256400	0.950413
chr4	117645700	0.909091
chr4	118188600	0.785124
chr4	119195600	0.818182
chr4	119205700	0.867769
chr4	119350000	0.785124
chr4	119513300	0.859504
chr4	119750100	0.809917
chr4	120159800	0.735537

chr4	120292100	0.859504
chr4	120971200	0.785124
chr4	121687100	0.793388
chr4	121695500	0.809917
chr4	121766100	0.933884
chr4	121787700	0.834711
chr4	121963200	0.834711
chr4	122059800	0.826446
chr4	122075600	0.859504
chr4	122111100	0.776860
chr4	122164900	0.884298
chr4	122178200	0.785124
chr4	122194200	0.809917
chr4	122200300	0.900826
chr4	122229300	0.785124
chr4	122431500	0.859504
chr4	122519400	0.743802
chr4	122576000	0.876033
chr4	122648500	0.851240
chr4	122710400	0.876033
chr4	123461900	0.768595
chr4	123500000	0.842975
chr4	123506100	0.834711
chr4	123556800	0.917355
chr4	123559300	0.735537
chr4	123612000	0.785124
chr4	123626400	0.743802
chr4	123683900	0.760331
chr4	123686400	0.900826
chr4	123719200	0.801653
chr4	123736300	0.785124
chr4	124265800	0.743802
chr4	124274300	0.826446
chr4	124284900	0.793388
chr4	124371600	0.809917
chr4	124570700	0.900826
chr4	125776500	0.892562
chr4	128620700	0.809917
chr4	128697500	0.909091
chr4	128778200	0.884298
chr4	129209600	0.884298
chr4	129471900	0.760331
chr4	129584800	0.851240
chr4	129596500	0.884298
chr4	129748700	0.752066
chr4	130017300	0.925620
chr4	131031300	0.735537
chr4	132211700	0.801653
chr4	132535500	0.735537
chr4	133568300	0.776860
chr4	138494500	0.809917
chr4	138924900	0.917355
chr4	138957100	0.785124
chr4	139112300	0.909091

chr4	139594300	0.743802
chr4	139899200	0.842975
chr4	140064000	0.809917
chr4	140216600	0.851240
chr4	140392500	0.752066
chr4	140450300	0.776860
chr4	140893900	0.801653
chr4	140975000	0.876033
chr4	141017100	0.735537
chr4	141035800	0.752066
chr4	141078200	0.867769
chr4	141489500	0.793388
chr4	141642100	0.801653
chr4	141723900	0.900826
chr4	141730900	0.842975
chr4	141784300	0.892562
chr4	141787200	0.743802
chr4	142129200	0.801653
chr4	142161900	0.809917
chr4	142298400	0.818182
chr4	142312800	0.735537
chr4	142570200	0.809917
chr4	143327900	0.809917
chr4	144277300	0.752066
chr4	144280800	0.950413
chr4	144324700	0.818182
chr4	144327000	0.818182
chr4	144460400	0.776860
chr4	144539300	0.752066
chr4	145277900	0.859504
chr4	145332100	0.917355
chr4	145636600	0.867769
chr4	146108400	0.892562
chr4	146116700	0.752066
chr4	146140000	0.785124
chr4	146411800	0.892562
chr4	146424900	0.793388
chr4	146547300	0.834711
chr4	146656300	0.859504
chr4	146696500	0.752066
chr4	146849700	0.735537
chr4	147034000	0.884298
chr4	147165700	0.760331
chr4	147200400	0.851240
chr4	147230700	0.809917
chr4	147257200	0.842975
chr4	147260500	0.768595
chr4	147264900	0.776860
chr4	147379800	0.867769
chr4	147916400	0.826446
chr4	148040400	0.826446
chr4	148061200	0.826446
chr4	148440800	0.809917
chr4	148508300	1.000000

chr4	148631900	0.876033
chr4	148891100	0.743802
chr4	148902900	0.768595
chr4	148936100	0.735537
chr4	148966500	0.785124
chr4	149756800	0.834711
chr4	150960700	0.768595
chr4	151000700	0.743802
chr4	151048500	0.818182
chr4	151134500	0.826446
chr4	151172500	0.785124
chr4	151247400	0.809917
chr4	151981800	0.776860
chr4	152226100	0.900826
chr4	152488600	0.859504
chr4	152720700	0.743802
chr4	152754800	0.826446
chr4	152809500	0.859504
chr4	152826900	0.842975
chr4	152974300	0.867769
chr4	153001800	0.809917
chr4	153037200	0.785124
chr4	153109900	0.743802
chr4	153141900	0.768595
chr4	153229700	0.933884
chr4	153494900	0.793388
chr4	153506400	0.909091
chr4	153512300	0.826446
chr4	153517000	0.859504
chr4	153602500	0.735537
chr4	153611300	0.752066
chr4	153655900	0.760331
chr4	153693800	0.785124
chr4	153771800	0.752066
chr4	153849600	0.834711
chr4	153930400	0.752066
chr4	153935000	0.793388
chr4	154011500	0.851240
chr4	154037500	0.867769
chr4	154055800	0.776860
chr4	154077700	0.884298
chr4	154138800	0.752066
chr4	154178600	0.785124
chr4	154263200	0.851240
chr4	154271100	0.760331
chr4	154340900	0.735537
chr4	154398700	0.776860
chr4	154426500	0.735537
chr4	154481600	0.743802
chr4	154491800	0.793388
chr4	154493900	0.785124
chr4	154563500	0.793388
chr4	154573100	0.752066
chr4	154627800	0.768595

chr4	154651100	0.876033
chr4	154702400	0.900826
chr4	154884000	0.925620
chr4	155347400	0.793388
chr4	155548500	0.768595
chr4	156388200	0.776860
chr4	156413200	0.876033
chr4	156829700	0.826446
chr4	156875000	0.950413
chr4	157116100	0.809917
chr4	159464200	0.818182
chr4	159532600	0.867769
chr4	159644500	0.785124
chr4	159699800	0.785124
chr4	159782700	0.735537
chr4	159939700	0.801653
chr4	159963300	0.818182
chr4	159967600	0.760331
chr4	159973100	0.859504
chr4	159977100	0.842975
chr4	160056000	0.884298
chr4	160090900	0.735537
chr4	160093300	0.900826
chr4	160154400	0.793388
chr4	160289300	0.966942
chr4	163772800	0.760331
chr4	163813900	0.776860
chr4	164165700	0.735537
chr4	164480500	0.776860
chr4	164490300	0.776860
chr4	164528600	0.876033
chr4	164595700	0.859504
chr4	164676100	0.785124
chr4	164736600	0.776860
chr4	165527900	0.818182
chr4	165656200	0.785124
chr4	165816400	0.867769
chr4	166033600	0.826446
chr4	166036700	0.900826
chr4	166040200	0.859504
chr4	166054700	0.851240
chr4	166177000	0.900826
chr4	166210000	0.743802
chr4	169506600	0.851240
chr4	169802500	0.743802
chr4	169976700	0.818182
chr4	170001700	0.867769
chr4	170090000	0.793388
chr4	170285100	0.801653
chr4	170533800	0.859504
chr4	170574000	0.793388
chr4	170874100	0.768595
chr4	170929700	0.818182
chr4	172486800	0.809917

chr4	174098300	0.760331
chr4	174164400	0.776860
chr4	174176400	0.851240
chr4	174186000	0.809917
chr4	174327800	0.809917
chr4	174367900	0.752066
chr4	174370900	0.768595
chr4	175205200	0.768595
chr4	175280300	0.768595
chr4	175459700	0.826446
chr4	176933600	0.752066
chr4	176958100	0.851240
chr4	177045500	0.876033
chr4	177230100	0.785124
chr4	177279300	0.909091
chr4	177284600	0.942149
chr4	177334700	0.752066
chr4	181201800	0.958678
chr4	181748800	0.809917
chr4	182751900	0.785124
chr4	183469400	0.735537
chr4	183640000	0.851240
chr4	183728400	0.735537
chr4	184521500	0.809917
chr4	184580600	0.735537
chr4	184950100	0.809917
chr4	185021200	0.909091
chr4	185371200	0.818182
chr4	185438400	0.776860
chr4	185578100	0.793388
chr4	185667600	0.826446
chr4	185683700	0.950413
chr4	185731800	0.768595
chr4	185741500	0.801653
chr4	185767000	0.735537
chr4	185769700	0.768595
chr4	185783400	0.785124
chr4	185820900	0.785124
chr4	186026500	0.743802
chr4	186087800	0.876033
chr4	186327200	0.933884
chr4	186347200	0.942149
chr4	186390500	0.900826
chr4	186393100	0.859504
chr4	186435000	0.809917
chr4	186645300	0.793388
chr4	186664700	0.735537
chr4	186709900	0.942149
chr4	186738900	0.743802
chr4	186819800	0.884298
chr4	186980200	0.900826
chr4	187027500	0.735537
chr4	187055900	0.851240
chr4	190201100	0.735537

chr4	190797600	0.768595
chr4	190807600	0.826446
chr4	190821800	0.768595
chr4	190828800	0.801653
chr4	190892000	0.818182
chr4	190970700	0.776860
chr4	190988600	0.900826
chr5	210100	0.743802
chr5	344600	0.933884
chr5	472800	0.768595
chr5	527600	0.809917
chr5	544900	0.760331
chr5	607900	0.752066
chr5	611200	0.752066
chr5	628800	0.867769
chr5	714800	0.933884
chr5	754700	0.884298
chr5	847200	0.867769
chr5	957800	0.801653
chr5	1131500	0.801653
chr5	1269200	0.793388
chr5	1366900	0.867769
chr5	1380800	0.842975
chr5	1536700	0.785124
chr5	1631400	0.859504
chr5	1765600	0.809917
chr5	1785700	0.760331
chr5	5494300	0.785124
chr5	5739700	0.809917
chr5	5872000	0.768595
chr5	6030300	0.752066
chr5	6195800	0.842975
chr5	6267000	0.743802
chr5	6360900	0.776860
chr5	6486100	0.801653
chr5	6488700	0.818182
chr5	6492600	0.834711
chr5	6633100	0.768595
chr5	6705500	0.752066
chr5	6709200	0.818182
chr5	6712800	0.743802
chr5	6828500	0.743802
chr5	6833200	0.801653
chr5	7779900	0.884298
chr5	7877200	0.842975
chr5	9363700	0.942149
chr5	9366000	0.785124
chr5	9384000	0.876033
chr5	9406000	0.785124
chr5	9432400	0.867769
chr5	9499600	0.826446
chr5	9597000	0.752066
chr5	10289000	0.842975
chr5	10374800	0.818182

chr5	10467700	0.752066
chr5	10493900	0.876033
chr5	10520100	0.834711
chr5	10525700	0.867769
chr5	10537500	0.892562
chr5	10601700	0.909091
chr5	10826600	0.760331
chr5	14151800	0.809917
chr5	14174300	0.842975
chr5	14190900	0.768595
chr5	14268300	0.752066
chr5	14347800	0.851240
chr5	14521100	0.876033
chr5	14535400	0.785124
chr5	14589400	0.909091
chr5	14591700	0.818182
chr5	14680400	0.776860
chr5	14763300	0.834711
chr5	14819200	0.809917
chr5	16784300	0.760331
chr5	17276900	0.884298
chr5	17279800	0.752066
chr5	17315700	0.768595
chr5	17374300	0.809917
chr5	17399600	0.975207
chr5	17427600	0.785124
chr5	31274700	0.760331
chr5	31382600	0.867769
chr5	31574100	0.859504
chr5	31599300	0.842975
chr5	31768100	0.785124
chr5	32207200	0.752066
chr5	32211300	0.966942
chr5	32281200	0.776860
chr5	32307000	0.743802
chr5	32444500	0.876033
chr5	32446900	0.834711
chr5	32490500	0.735537
chr5	32502400	0.851240
chr5	32505700	0.909091
chr5	32580600	0.768595
chr5	33236900	0.834711
chr5	33239900	0.842975
chr5	33242500	0.900826
chr5	33300600	0.776860
chr5	33773800	0.876033
chr5	33824400	0.752066
chr5	33831600	0.752066
chr5	33848400	0.892562
chr5	33850800	0.917355
chr5	33936600	0.743802
chr5	33974100	0.826446
chr5	34008700	0.735537
chr5	34157800	0.735537

chr5	34468100	0.876033
chr5	34556900	0.752066
chr5	34588100	0.760331
chr5	34638800	0.793388
chr5	34854900	0.809917
chr5	34930200	0.826446
chr5	35002700	0.834711
chr5	35017100	0.752066
chr5	35320300	0.867769
chr5	35821100	0.735537
chr5	35863300	0.809917
chr5	35878100	0.768595
chr5	35925700	0.735537
chr5	36075000	0.793388
chr5	36237300	0.776860
chr5	36690400	0.818182
chr5	36699900	0.834711
chr5	36882700	0.760331
chr5	37198200	0.892562
chr5	37738500	0.859504
chr5	37874300	0.859504
chr5	37881700	0.809917
chr5	37910500	0.876033
chr5	37915500	0.809917
chr5	39077700	0.752066
chr5	39188400	0.818182
chr5	39191600	0.826446
chr5	39202400	0.776860
chr5	39275000	0.942149
chr5	40224700	0.776860
chr5	40277400	0.909091
chr5	40378800	0.842975
chr5	40386600	0.809917
chr5	40402000	0.768595
chr5	40437800	0.826446
chr5	40442400	0.851240
chr5	40489100	0.735537
chr5	40495800	0.743802
chr5	40571100	0.785124
chr5	40591900	0.818182
chr5	40873000	0.735537
chr5	41276500	0.785124
chr5	41925500	0.809917
chr5	42719300	0.900826
chr5	43016200	0.851240
chr5	43137700	0.801653
chr5	43246600	0.735537
chr5	43314500	0.917355
chr5	43387500	0.760331
chr5	43559600	0.760331
chr5	45794600	0.793388
chr5	49726000	0.752066
chr5	49772700	0.942149
chr5	49860200	0.768595

chr5	49970800	0.876033
chr5	52064000	0.884298
chr5	52067900	0.909091
chr5	52337500	0.925620
chr5	52386100	0.892562
chr5	53117900	0.867769
chr5	53412200	0.760331
chr5	53527500	0.851240
chr5	53545800	0.884298
chr5	53698500	0.743802
chr5	53766000	0.818182
chr5	53788400	0.851240
chr5	53906800	0.785124
chr5	54032300	0.909091
chr5	54050400	0.768595
chr5	54052800	0.842975
chr5	54069200	0.809917
chr5	54082800	0.752066
chr5	54198800	0.900826
chr5	54472700	0.859504
chr5	54661200	0.809917
chr5	54897000	0.826446
chr5	55016500	0.826446
chr5	55060200	0.834711
chr5	55126100	0.752066
chr5	55190500	0.818182
chr5	55196400	0.776860
chr5	55202500	0.793388
chr5	55302200	0.851240
chr5	55315500	0.785124
chr5	55320900	0.801653
chr5	55356800	0.760331
chr5	55367300	0.851240
chr5	55369400	0.743802
chr5	55555500	0.760331
chr5	55566600	0.785124
chr5	55732100	0.776860
chr5	55778800	0.785124
chr5	55798200	0.785124
chr5	55877200	0.752066
chr5	55937300	0.859504
chr5	55955200	0.851240
chr5	55968700	0.735537
chr5	55988500	0.826446
chr5	56025800	0.743802
chr5	56100000	0.785124
chr5	56119000	0.818182
chr5	56137100	0.876033
chr5	56148100	0.768595
chr5	56196800	0.851240
chr5	56206300	0.776860
chr5	56279900	0.900826
chr5	56282700	0.735537
chr5	56327900	0.876033

chr5	56480900	0.834711
chr5	56484600	0.809917
chr5	56545700	0.818182
chr5	56826200	0.859504
chr5	58440500	0.809917
chr5	58474400	0.809917
chr5	58476700	0.842975
chr5	58916900	0.884298
chr5	59064700	0.752066
chr5	59096300	0.842975
chr5	59315600	0.743802
chr5	59635700	0.809917
chr5	59676500	0.752066
chr5	59790700	0.809917
chr5	59831100	0.818182
chr5	59881500	0.834711
chr5	60120100	0.752066
chr5	60126700	0.867769
chr5	60347600	0.826446
chr5	60458700	0.785124
chr5	60474200	0.876033
chr5	60525100	0.743802
chr5	60571900	0.743802
chr5	60624300	0.842975
chr5	60998500	0.917355
chr5	61390100	0.735537
chr5	61546900	0.776860
chr5	61552500	0.826446
chr5	62016500	0.834711
chr5	62079400	0.776860
chr5	64045700	0.760331
chr5	64055600	0.809917
chr5	64064800	0.826446
chr5	64110200	0.826446
chr5	64334000	0.743802
chr5	64368600	0.793388
chr5	64372400	0.760331
chr5	64375000	0.768595
chr5	64920900	0.818182
chr5	64995600	0.809917
chr5	65213300	0.760331
chr5	65736700	0.776860
chr5	65850400	0.925620
chr5	65935800	0.826446
chr5	66004700	0.801653
chr5	66136900	0.958678
chr5	66486000	0.752066
chr5	66495300	0.826446
chr5	66505100	0.793388
chr5	66519000	0.785124
chr5	66531100	0.925620
chr5	67103400	0.801653
chr5	67411800	0.809917
chr5	67584500	0.743802

chr5	67627600	0.760331
chr5	67664200	0.776860
chr5	67671200	0.801653
chr5	67761200	0.826446
chr5	68008500	0.834711
chr5	68036400	0.909091
chr5	68188000	0.842975
chr5	68463200	0.859504
chr5	68711200	0.793388
chr5	68728500	0.809917
chr5	69321300	0.826446
chr5	69760700	0.752066
chr5	70220900	0.793388
chr5	70314800	0.743802
chr5	70742500	0.752066
chr5	71053800	0.826446
chr5	71279300	0.760331
chr5	71435000	0.867769
chr5	71490200	0.876033
chr5	71540700	0.743802
chr5	71604000	0.809917
chr5	71683000	0.842975
chr5	71734300	0.752066
chr5	71747900	0.900826
chr5	72152400	0.743802
chr5	72406000	0.735537
chr5	72456700	0.785124
chr5	72509800	0.818182
chr5	72913800	0.743802
chr5	73092700	0.793388
chr5	73195200	0.735537
chr5	73209000	0.842975
chr5	73324700	0.842975
chr5	73537900	0.834711
chr5	73554100	0.876033
chr5	73623300	0.851240
chr5	73670800	0.834711
chr5	73699100	0.785124
chr5	73729900	0.859504
chr5	73749600	0.876033
chr5	73777000	0.768595
chr5	73856100	0.909091
chr5	73881000	0.842975
chr5	73924400	0.851240
chr5	73945000	0.834711
chr5	74185800	0.809917
chr5	74241800	0.793388
chr5	74340800	0.842975
chr5	74359300	0.760331
chr5	74663200	0.876033
chr5	74965100	0.942149
chr5	75013000	0.876033
chr5	75120400	0.776860
chr5	75503400	0.735537

chr5	75525000	0.743802
chr5	75552500	0.793388
chr5	75566000	0.752066
chr5	75589400	0.933884
chr5	75595100	0.842975
chr5	75600300	0.760331
chr5	75703700	0.793388
chr5	75817300	0.867769
chr5	75849100	0.743802
chr5	75858100	0.842975
chr5	75934900	0.818182
chr5	76017400	0.933884
chr5	76034800	0.809917
chr5	76234000	0.826446
chr5	76256000	0.818182
chr5	76326300	0.834711
chr5	76379600	0.818182
chr5	76432300	0.735537
chr5	76435700	0.818182
chr5	76646800	0.743802
chr5	76676800	0.776860
chr5	76692300	0.917355
chr5	76787900	0.834711
chr5	77071700	0.768595
chr5	77217000	0.735537
chr5	77663700	0.735537
chr5	77743700	0.925620
chr5	77822800	0.768595
chr5	78032400	0.752066
chr5	78289500	0.776860
chr5	78432100	0.933884
chr5	78753600	0.785124
chr5	78857200	0.826446
chr5	78867600	0.743802
chr5	79277500	0.851240
chr5	79286800	0.752066
chr5	79333400	0.859504
chr5	79366400	0.752066
chr5	79477500	0.785124
chr5	80052500	0.768595
chr5	80133400	0.776860
chr5	80213900	0.776860
chr5	80452500	0.818182
chr5	80457400	0.743802
chr5	80509800	0.735537
chr5	80583900	0.834711
chr5	80853400	0.735537
chr5	81047800	0.950413
chr5	81075200	0.975207
chr5	81153900	0.768595
chr5	81205200	0.884298
chr5	81219000	0.842975
chr5	81268300	0.793388
chr5	81401700	0.735537

chr5	81470700	0.859504
chr5	81570800	0.859504
chr5	82178200	0.752066
chr5	82181800	0.834711
chr5	82737600	0.909091
chr5	85913900	0.876033
chr5	86293300	0.776860
chr5	86377700	0.950413
chr5	86391000	0.768595
chr5	86409800	0.842975
chr5	86413300	0.859504
chr5	86507400	0.876033
chr5	86524100	0.818182
chr5	86650200	0.818182
chr5	87968800	0.950413
chr5	87976600	0.793388
chr5	88042200	0.752066
chr5	88275500	0.793388
chr5	88413400	0.842975
chr5	88605100	0.876033
chr5	88894500	0.760331
chr5	89230100	0.818182
chr5	89311400	0.785124
chr5	89561000	0.785124
chr5	89664800	0.801653
chr5	89731500	0.801653
chr5	89867000	0.785124
chr5	89909400	0.735537
chr5	90358400	0.851240
chr5	90375400	0.752066
chr5	90479400	0.900826
chr5	90562400	0.826446
chr5	90565600	0.760331
chr5	90568900	0.826446
chr5	90589000	0.909091
chr5	90607900	0.785124
chr5	90610200	0.793388
chr5	90651300	0.818182
chr5	90657800	0.793388
chr5	90694100	0.785124
chr5	93210400	0.793388
chr5	93506200	0.801653
chr5	94084000	0.752066
chr5	94186100	0.743802
chr5	94807800	0.768595
chr5	94982700	0.776860
chr5	95096300	0.876033
chr5	95152600	0.818182
chr5	95178800	0.735537
chr5	95197900	0.917355
chr5	95206500	0.801653
chr5	95499100	0.809917
chr5	95616000	0.818182
chr5	95662900	0.909091

chr5	95672100	0.867769
chr5	95676400	0.851240
chr5	95681200	0.884298
chr5	95770200	0.842975
chr5	95777100	0.743802
chr5	95983700	0.809917
chr5	95992900	0.768595
chr5	96042600	0.859504
chr5	96232600	0.760331
chr5	96431300	0.801653
chr5	96519300	0.958678
chr5	97174100	0.735537
chr5	97913000	0.826446
chr5	98269800	0.842975
chr5	98312100	0.735537
chr5	98373600	0.851240
chr5	99361200	0.826446
chr5	100116100	0.801653
chr5	100124200	0.752066
chr5	102049600	0.752066
chr5	102083700	0.834711
chr5	102151400	0.917355
chr5	102191800	0.735537
chr5	102599100	0.735537
chr5	102684600	0.776860
chr5	102824000	0.818182
chr5	102882900	0.884298
chr5	106600600	0.776860
chr5	106625500	0.867769
chr5	106630200	0.834711
chr5	106632300	0.776860
chr5	106710600	0.743802
chr5	106732400	0.818182
chr5	106904200	0.842975
chr5	106957800	0.785124
chr5	107255900	0.735537
chr5	107362200	0.776860
chr5	107720300	0.793388
chr5	108064100	0.842975
chr5	108066700	0.768595
chr5	108507300	0.793388
chr5	108746500	0.735537
chr5	108804800	0.768595
chr5	109032800	0.752066
chr5	109036600	0.776860
chr5	109079200	0.785124
chr5	109257800	0.735537
chr5	110188200	0.785124
chr5	110268900	0.743802
chr5	110573600	0.826446
chr5	111003000	0.851240
chr5	111093800	0.859504
chr5	111329400	0.793388
chr5	111447200	0.785124

chr5	111479300	0.900826
chr5	111539300	0.793388
chr5	111665900	0.942149
chr5	111684700	0.793388
chr5	111869100	0.776860
chr5	112028400	0.842975
chr5	112197100	0.809917
chr5	112205900	0.834711
chr5	112294600	0.760331
chr5	112305500	0.942149
chr5	112318100	0.809917
chr5	112451200	0.859504
chr5	112581900	0.925620
chr5	112656600	0.760331
chr5	114665100	0.793388
chr5	114668600	0.950413
chr5	114714000	0.768595
chr5	114816600	0.826446
chr5	114880300	0.818182
chr5	114887300	0.776860
chr5	114972000	0.743802
chr5	114975800	0.876033
chr5	115151800	0.785124
chr5	115316500	0.892562
chr5	115423300	0.743802
chr5	115471400	0.900826
chr5	115902400	0.801653
chr5	115963000	0.752066
chr5	115969000	0.818182
chr5	115982300	0.768595
chr5	116017600	0.859504
chr5	116032200	0.859504
chr5	116102400	0.859504
chr5	116216500	0.768595
chr5	116221900	0.933884
chr5	116245000	0.801653
chr5	116284000	0.752066
chr5	117017500	0.760331
chr5	117195200	0.842975
chr5	117336400	0.785124
chr5	117842900	0.826446
chr5	118400000	0.760331
chr5	118556600	0.768595
chr5	118741400	0.809917
chr5	118754600	0.859504
chr5	122030300	0.859504
chr5	122216700	0.760331
chr5	122303500	0.884298
chr5	122494100	0.768595
chr5	122759500	0.818182
chr5	122774600	0.793388
chr5	122786500	0.851240
chr5	122848600	0.776860
chr5	122859900	0.801653

chr5	122934200	0.768595
chr5	122942600	0.768595
chr5	123243200	0.933884
chr5	123312600	0.752066
chr5	123390900	0.793388
chr5	123499000	0.818182
chr5	123746900	0.793388
chr5	123780500	0.900826
chr5	123790700	0.785124
chr5	123840300	0.752066
chr5	123855600	0.768595
chr5	123860700	0.900826
chr5	123864300	0.826446
chr5	123880600	0.801653
chr5	123935800	0.793388
chr5	123983600	0.785124
chr5	123991800	0.842975
chr5	124060500	0.776860
chr5	124071100	0.809917
chr5	124129900	0.859504
chr5	124535000	0.776860
chr5	124744600	0.809917
chr5	125297300	0.876033
chr5	125696600	0.776860
chr5	125786200	0.776860
chr5	126016200	0.752066
chr5	126066800	0.925620
chr5	126083900	0.752066
chr5	126205000	0.793388
chr5	126330200	0.768595
chr5	126559100	0.826446
chr5	126736800	0.909091
chr5	126903900	0.834711
chr5	126914000	0.735537
chr5	126917100	0.752066
chr5	126926400	0.884298
chr5	127212200	0.842975
chr5	127263300	0.785124
chr5	127533900	0.743802
chr5	127537300	0.752066
chr5	128461900	0.801653
chr5	129448200	0.867769
chr5	130405800	0.876033
chr5	130500800	0.743802
chr5	130560800	0.801653
chr5	131084000	0.768595
chr5	131146800	0.793388
chr5	131154200	0.867769
chr5	131161500	0.752066
chr5	131190000	0.867769
chr5	131353100	0.793388
chr5	131362700	0.801653
chr5	131419500	0.900826
chr5	131441400	0.752066

chr5	131512800	0.917355
chr5	131515800	0.743802
chr5	131544300	0.834711
chr5	131558700	0.834711
chr5	131565500	0.867769
chr5	131865100	0.809917
chr5	131879800	0.735537
chr5	132088300	0.776860
chr5	132155900	0.876033
chr5	132393500	0.826446
chr5	132601000	0.818182
chr5	132661200	0.768595
chr5	133078900	0.842975
chr5	133196300	0.768595
chr5	133295900	0.793388
chr5	133390100	0.785124
chr5	133512800	0.909091
chr5	133611800	0.752066
chr5	133782400	0.818182
chr5	133931300	0.884298
chr5	134097500	0.785124
chr5	134389200	0.876033
chr5	134649700	0.793388
chr5	134735300	0.809917
chr5	134777600	0.826446
chr5	134784300	0.809917
chr5	134788600	0.793388
chr5	134794000	0.851240
chr5	134928200	0.760331
chr5	135223500	0.909091
chr5	135367400	0.793388
chr5	135418200	0.900826
chr5	135436200	0.876033
chr5	135499900	0.768595
chr5	135606200	0.892562
chr5	135820800	0.892562
chr5	136104900	0.785124
chr5	137178300	0.785124
chr5	137234600	0.809917
chr5	137275500	0.818182
chr5	137369000	0.776860
chr5	137432600	0.776860
chr5	137521500	0.909091
chr5	137589300	0.785124
chr5	137800500	0.818182
chr5	137911300	0.826446
chr5	137941400	0.760331
chr5	137947200	0.809917
chr5	137990300	0.834711
chr5	138004900	0.900826
chr5	138062900	0.809917
chr5	138075100	0.752066
chr5	138078300	0.950413
chr5	138089300	0.809917

chr5	138183600	0.785124
chr5	138404200	0.900826
chr5	138477300	0.818182
chr5	138484000	0.859504
chr5	138530200	0.818182
chr5	138582400	0.859504
chr5	138638000	0.776860
chr5	138640300	0.785124
chr5	138678000	0.760331
chr5	138735700	0.942149
chr5	138740100	0.942149
chr5	138775300	0.950413
chr5	138851100	0.760331
chr5	138862100	0.917355
chr5	138894200	0.743802
chr5	138946900	0.900826
chr5	138971900	0.760331
chr5	139077100	0.867769
chr5	139081400	0.752066
chr5	139085800	0.851240
chr5	139136000	0.752066
chr5	139155100	0.859504
chr5	139224300	0.768595
chr5	139242600	0.859504
chr5	139311100	0.809917
chr5	139327700	0.735537
chr5	139331100	0.768595
chr5	139379900	0.892562
chr5	139401900	0.743802
chr5	139429200	0.776860
chr5	139546900	0.801653
chr5	139549000	0.735537
chr5	139560400	0.768595
chr5	139646500	0.793388
chr5	139737400	0.801653
chr5	139927700	0.966942
chr5	139937200	0.876033
chr5	140090400	0.760331
chr5	140700200	0.909091
chr5	141003100	0.925620
chr5	141072200	0.743802
chr5	141096900	0.867769
chr5	141262300	0.793388
chr5	141296900	0.909091
chr5	141303400	0.760331
chr5	141386700	0.793388
chr5	141395900	0.867769
chr5	141398200	0.900826
chr5	141468500	0.851240
chr5	141603600	0.876033
chr5	141667900	0.768595
chr5	141800900	0.942149
chr5	142163700	0.785124
chr5	142187800	0.884298

chr5	142226100	0.735537
chr5	142305100	0.826446
chr5	142339300	0.785124
chr5	142410900	0.735537
chr5	142447000	0.876033
chr5	142725700	0.768595
chr5	142743900	0.809917
chr5	142890100	0.809917
chr5	142895600	0.752066
chr5	142944300	0.818182
chr5	142954000	0.776860
chr5	142963400	0.768595
chr5	142993800	0.859504
chr5	143022700	0.760331
chr5	143094600	0.809917
chr5	143172100	0.760331
chr5	143205300	0.760331
chr5	143208800	0.768595
chr5	143351900	0.768595
chr5	143522200	0.743802
chr5	144436900	0.917355
chr5	145459500	0.884298
chr5	145462600	0.884298
chr5	145827300	0.892562
chr5	145928400	0.793388
chr5	146445100	0.785124
chr5	146784400	0.768595
chr5	146859400	0.826446
chr5	147184600	0.884298
chr5	147763700	0.876033
chr5	148127500	0.834711
chr5	148140700	0.876033
chr5	148146500	0.867769
chr5	148174800	0.793388
chr5	148188700	0.876033
chr5	148196900	0.776860
chr5	148214800	0.801653
chr5	148542500	0.925620
chr5	148620500	0.900826
chr5	148675600	0.818182
chr5	148802500	0.752066
chr5	148908900	0.818182
chr5	149012600	0.818182
chr5	149057200	0.768595
chr5	149131900	0.735537
chr5	149447100	0.834711
chr5	149471900	0.785124
chr5	149679900	0.752066
chr5	149722700	0.925620
chr5	149737500	0.785124
chr5	149816100	0.809917
chr5	149829500	0.735537
chr5	149841100	0.884298
chr5	149860300	0.900826

chr5	149879000	0.785124
chr5	149966200	0.793388
chr5	150138700	0.867769
chr5	150512700	0.743802
chr5	150523900	0.743802
chr5	150576200	0.801653
chr5	150605400	0.809917
chr5	150618100	0.925620
chr5	150625700	0.735537
chr5	150832300	0.793388
chr5	150841600	0.809917
chr5	150877700	0.743802
chr5	150937000	0.842975
chr5	150957900	0.768595
chr5	150993300	0.776860
chr5	151129700	0.900826
chr5	151150300	0.735537
chr5	151842100	0.743802
chr5	153276500	0.776860
chr5	153354500	0.809917
chr5	153498800	0.876033
chr5	153563400	0.760331
chr5	153608600	0.793388
chr5	153730800	0.834711
chr5	153764800	0.851240
chr5	153850900	0.892562
chr5	153855100	0.735537
chr5	153857400	0.909091
chr5	153859800	0.867769
chr5	153863900	0.925620
chr5	153874500	0.752066
chr5	153890900	0.776860
chr5	153900800	0.793388
chr5	153923400	0.909091
chr5	153956000	0.809917
chr5	154094700	0.760331
chr5	154148100	0.776860
chr5	154336700	0.809917
chr5	154433000	0.776860
chr5	154444700	0.950413
chr5	154446900	0.776860
chr5	154814600	0.776860
chr5	155449300	0.809917
chr5	156253300	0.743802
chr5	156388400	0.785124
chr5	156403800	0.876033
chr5	156452900	0.917355
chr5	156456600	0.785124
chr5	156465700	0.842975
chr5	156504200	0.743802
chr5	156536700	0.900826
chr5	156558800	0.809917
chr5	156599700	0.925620
chr5	156605000	0.752066

chr5	156616500	0.776860
chr5	156643000	0.735537
chr5	156650300	0.950413
chr5	156691100	0.776860
chr5	156938100	0.933884
chr5	156957000	0.900826
chr5	156972800	0.867769
chr5	157158600	0.834711
chr5	157175600	0.809917
chr5	157255500	0.801653
chr5	157291100	0.801653
chr5	157302500	0.876033
chr5	157376200	0.768595
chr5	157626000	0.884298
chr5	157966000	0.752066
chr5	157992100	0.892562
chr5	158097200	0.884298
chr5	158106500	0.826446
chr5	158128600	0.735537
chr5	158177600	0.752066
chr5	158181100	0.859504
chr5	158269100	0.735537
chr5	158469200	0.768595
chr5	158560100	0.834711
chr5	158583900	0.826446
chr5	158738100	0.785124
chr5	158760300	0.776860
chr5	158833800	0.752066
chr5	158837000	0.851240
chr5	158851600	0.760331
chr5	158879100	0.743802
chr5	158881800	0.826446
chr5	158897000	0.834711
chr5	159253600	0.851240
chr5	159292800	0.909091
chr5	159523500	0.760331
chr5	159574300	0.735537
chr5	159580300	0.876033
chr5	159608800	0.909091
chr5	159648100	0.826446
chr5	159716800	0.834711
chr5	159722000	0.776860
chr5	159846900	0.743802
chr5	159927900	0.826446
chr5	160170700	0.909091
chr5	160429100	0.760331
chr5	160564700	0.859504
chr5	160706400	0.801653
chr5	160740800	0.760331
chr5	162533500	0.776860
chr5	162993400	0.735537
chr5	163343600	0.785124
chr5	167551800	0.818182
chr5	167563200	0.793388

chr5	167601100	0.917355
chr5	167655400	0.966942
chr5	167672500	0.801653
chr5	167735500	0.900826
chr5	167800000	0.867769
chr5	168007200	0.760331
chr5	168440400	0.743802
chr5	168844400	0.752066
chr5	169071300	0.793388
chr5	169595700	0.818182
chr5	169681600	0.809917
chr5	169740400	0.809917
chr5	169761300	0.776860
chr5	169821600	0.892562
chr5	170060700	0.851240
chr5	170203100	0.752066
chr5	170224900	0.785124
chr5	170271500	0.842975
chr5	170576900	0.760331
chr5	171061500	0.785124
chr5	171410400	0.785124
chr5	171577800	0.909091
chr5	171749200	0.909091
chr5	171840000	0.834711
chr5	171961100	0.842975
chr5	172118000	0.809917
chr5	172129000	0.752066
chr5	172140700	0.900826
chr5	172159200	0.760331
chr5	172205200	0.950413
chr5	172225400	0.851240
chr5	172234600	0.925620
chr5	172264600	0.900826
chr5	172437400	0.809917
chr5	172484700	0.909091
chr5	172571600	0.900826
chr5	172759400	0.851240
chr5	172966500	0.760331
chr5	173134800	0.768595
chr5	173205900	0.735537
chr5	173217400	0.743802
chr5	173221800	0.752066
chr5	173345200	0.892562
chr5	173524600	0.826446
chr5	173697700	0.867769
chr5	173700700	0.743802
chr5	173709800	0.801653
chr5	173751200	0.776860
chr5	173763800	0.752066
chr5	174966400	0.809917
chr5	175249700	0.809917
chr5	175567700	0.768595
chr5	175659800	0.760331
chr5	175864300	0.793388

chr5	176061000	0.876033
chr5	176080400	0.768595
chr5	176088200	0.801653
chr5	176433800	0.793388
chr5	176539300	0.768595
chr5	176587100	0.752066
chr5	176778800	0.933884
chr5	176924300	0.900826
chr5	176946900	0.776860
chr5	177022100	0.892562
chr5	177105100	0.752066
chr5	177239800	0.826446
chr5	177495600	0.818182
chr5	177533900	0.768595
chr5	177558300	0.776860
chr5	177591300	0.859504
chr5	177664500	0.743802
chr5	177729400	0.776860
chr5	178945900	0.752066
chr5	178986200	0.917355
chr5	179051400	0.785124
chr5	179107800	0.809917
chr5	179221000	0.818182
chr5	179248300	0.760331
chr5	179280000	0.933884
chr5	179967200	0.966942
chr5	180017600	0.760331
chr5	180146000	0.900826
chr5	180529500	0.768595
chr5	180532800	0.842975
chr5	180688800	0.768595
chr5	180711200	0.818182
chr6	158800	0.818182
chr6	200100	0.776860
chr6	211300	0.785124
chr6	260200	0.983471
chr6	263500	0.942149
chr6	345600	0.768595
chr6	367000	0.793388
chr6	373900	0.785124
chr6	423500	0.776860
chr6	432400	0.735537
chr6	447700	0.867769
chr6	468300	0.735537
chr6	475200	0.834711
chr6	479400	0.909091
chr6	506800	0.834711
chr6	685300	0.966942
chr6	711500	0.834711
chr6	722700	0.867769
chr6	788100	0.818182
chr6	875200	0.818182
chr6	996600	0.760331
chr6	1009200	0.826446

chr6	1317200	0.975207
chr6	1379900	0.776860
chr6	1486500	0.752066
chr6	1527600	0.842975
chr6	1578800	0.809917
chr6	1596900	0.752066
chr6	1613700	0.801653
chr6	1707900	0.867769
chr6	1737600	0.760331
chr6	1822500	0.743802
chr6	1993900	0.818182
chr6	2079900	0.884298
chr6	2083400	0.785124
chr6	2111100	0.793388
chr6	2168000	0.760331
chr6	2182600	0.892562
chr6	2188000	0.809917
chr6	2222600	0.818182
chr6	2543600	0.859504
chr6	2564300	0.876033
chr6	2659500	0.752066
chr6	2734100	0.752066
chr6	2842900	0.793388
chr6	2849500	0.735537
chr6	2863900	0.859504
chr6	2877000	0.768595
chr6	2911800	0.834711
chr6	2919600	0.752066
chr6	2941900	0.909091
chr6	3056300	0.785124
chr6	3069100	0.809917
chr6	3140000	0.735537
chr6	3338700	0.818182
chr6	3352600	0.752066
chr6	3368200	0.925620
chr6	3378200	0.900826
chr6	3450200	0.785124
chr6	3683100	0.867769
chr6	3727300	0.842975
chr6	3747100	0.834711
chr6	3903400	0.776860
chr6	4018800	0.867769
chr6	4138700	0.735537
chr6	4553800	0.826446
chr6	4638200	0.735537
chr6	4648200	0.776860
chr6	4791400	0.776860
chr6	4795000	0.760331
chr6	4823700	0.933884
chr6	4936200	0.793388
chr6	5035700	0.892562
chr6	5232600	0.752066
chr6	5235200	0.818182
chr6	5261800	0.776860

chr6	5516500	0.785124
chr6	5570600	0.743802
chr6	5696200	0.809917
chr6	5699400	0.743802
chr6	5724700	0.942149
chr6	5728200	0.933884
chr6	5765000	0.884298
chr6	5814400	0.958678
chr6	5847500	0.892562
chr6	5870400	0.752066
chr6	5969700	0.909091
chr6	6094200	0.743802
chr6	6201700	0.842975
chr6	6329000	0.752066
chr6	6396800	0.900826
chr6	6651700	0.925620
chr6	6685700	0.818182
chr6	6713700	0.809917
chr6	6759900	0.826446
chr6	6794100	0.752066
chr6	6806700	0.776860
chr6	6841900	0.793388
chr6	6916300	0.768595
chr6	6919500	0.785124
chr6	6933400	0.776860
chr6	7053700	0.752066
chr6	7074400	0.867769
chr6	7141300	0.917355
chr6	7203100	0.842975
chr6	7618900	0.834711
chr6	7648100	0.776860
chr6	7785200	0.768595
chr6	7907900	0.760331
chr6	7940300	0.801653
chr6	7976000	0.760331
chr6	7979400	0.900826
chr6	7992700	0.809917
chr6	8076200	0.785124
chr6	8435500	0.851240
chr6	10356000	0.743802
chr6	10511200	0.809917
chr6	10579000	0.768595
chr6	10643600	0.785124
chr6	10685800	0.768595
chr6	10695000	0.768595
chr6	10720500	0.801653
chr6	10727500	0.818182
chr6	10839400	0.776860
chr6	10890000	0.909091
chr6	10965400	0.933884
chr6	11009500	0.917355
chr6	11140100	0.768595
chr6	11165000	0.801653
chr6	11173300	0.834711

chr6	11223000	0.909091
chr6	11225600	0.768595
chr6	11259100	0.760331
chr6	11319800	0.752066
chr6	11354800	0.768595
chr6	11363600	0.743802
chr6	11389300	0.768595
chr6	11474200	0.760331
chr6	11576300	0.809917
chr6	11654000	0.768595
chr6	11705700	0.826446
chr6	11741700	0.760331
chr6	11801000	0.851240
chr6	11969200	0.735537
chr6	12262300	0.892562
chr6	12359800	0.826446
chr6	12436900	0.867769
chr6	12535000	0.801653
chr6	12609600	0.826446
chr6	12623200	0.826446
chr6	13230800	0.735537
chr6	13235300	0.859504
chr6	13264300	0.785124
chr6	13281700	0.785124
chr6	13287300	0.917355
chr6	13340900	0.760331
chr6	13381500	0.743802
chr6	13462600	0.785124
chr6	13467400	0.793388
chr6	13509800	0.743802
chr6	13527800	0.900826
chr6	13533600	0.743802
chr6	13575000	0.818182
chr6	13696900	0.768595
chr6	13712800	0.752066
chr6	13716200	0.752066
chr6	13748600	0.892562
chr6	13763400	0.834711
chr6	13834100	0.867769
chr6	13839300	0.892562
chr6	14025900	0.809917
chr6	14037600	0.793388
chr6	14075400	0.760331
chr6	14086000	0.909091
chr6	14145200	0.884298
chr6	14149300	0.785124
chr6	14241700	0.884298
chr6	14372500	0.818182
chr6	14374800	0.776860
chr6	14542900	0.884298
chr6	14637200	0.760331
chr6	14659600	0.735537
chr6	14664500	0.826446
chr6	14666700	0.876033

chr6	14720100	0.818182
chr6	14785600	0.900826
chr6	14947700	0.743802
chr6	15023400	0.950413
chr6	15074000	0.752066
chr6	15151900	0.884298
chr6	15171900	0.785124
chr6	15279200	0.752066
chr6	15401500	0.851240
chr6	15550100	0.743802
chr6	15621900	0.818182
chr6	15824700	0.776860
chr6	15844300	0.851240
chr6	15877100	0.743802
chr6	16006000	0.743802
chr6	16078700	0.851240
chr6	16125000	0.735537
chr6	16127500	0.735537
chr6	16163100	0.834711
chr6	16215800	0.876033
chr6	16302100	0.917355
chr6	16317100	0.851240
chr6	16375600	0.809917
chr6	16381400	0.735537
chr6	16498800	0.876033
chr6	16518500	0.950413
chr6	16533500	0.776860
chr6	16541700	0.785124
chr6	16586500	0.867769
chr6	16592600	0.818182
chr6	16604800	0.867769
chr6	16627400	0.867769
chr6	16631600	0.842975
chr6	16652700	0.900826
chr6	16669100	0.743802
chr6	16672200	0.818182
chr6	16686900	0.842975
chr6	16693100	0.900826
chr6	16695900	0.876033
chr6	16705200	0.851240
chr6	16708400	0.834711
chr6	16714000	0.735537
chr6	16744200	0.801653
chr6	16765500	0.842975
chr6	16771500	0.933884
chr6	16825800	0.859504
chr6	16856000	0.785124
chr6	17206600	0.776860
chr6	17629800	0.760331
chr6	17883600	0.776860
chr6	17886800	0.834711
chr6	17958200	0.892562
chr6	17994200	0.925620
chr6	18053900	0.768595

chr6	18341900	0.818182
chr6	18391400	0.933884
chr6	18401000	0.776860
chr6	18502000	0.859504
chr6	20024500	0.809917
chr6	20213300	0.801653
chr6	20320300	0.950413
chr6	20350000	0.768595
chr6	20366900	0.752066
chr6	20404600	0.884298
chr6	20534800	0.909091
chr6	20670400	0.826446
chr6	20689400	0.818182
chr6	20717500	0.834711
chr6	20723400	0.785124
chr6	20805400	0.752066
chr6	20920200	0.826446
chr6	20979100	0.785124
chr6	21004000	0.876033
chr6	21006300	0.793388
chr6	21191600	0.876033
chr6	21954500	0.842975
chr6	22021700	0.735537
chr6	23620200	0.933884
chr6	24256100	0.793388
chr6	24284000	0.768595
chr6	24392300	0.793388
chr6	24518200	0.909091
chr6	24694700	0.842975
chr6	24857100	0.752066
chr6	24860200	0.826446
chr6	24888300	0.867769
chr6	24891800	0.842975
chr6	24902200	0.743802
chr6	24923000	0.760331
chr6	24925500	0.884298
chr6	24927800	0.752066
chr6	24942300	0.785124
chr6	24975300	0.793388
chr6	25148000	0.859504
chr6	25224600	0.834711
chr6	25301800	0.785124
chr6	25333100	0.842975
chr6	25367800	0.917355
chr6	25378600	0.834711
chr6	25407300	0.809917
chr6	25411300	0.809917
chr6	25538600	0.809917
chr6	25843000	0.818182
chr6	26092600	0.809917
chr6	26104600	0.826446
chr6	26134400	0.793388
chr6	26224800	0.809917
chr6	26240900	0.776860

chr6	26258200	0.776860
chr6	26286400	0.735537
chr6	26296500	0.793388
chr6	26305400	0.818182
chr6	26307900	0.735537
chr6	26383500	0.752066
chr6	26398700	0.768595
chr6	26415100	0.884298
chr6	26424500	0.735537
chr6	26440800	0.859504
chr6	26458400	0.809917
chr6	26469200	0.776860
chr6	26512700	0.776860
chr6	26597100	0.785124
chr6	26600400	0.768595
chr6	26771700	0.768595
chr6	26987900	0.818182
chr6	27129800	0.743802
chr6	27619100	0.752066
chr6	27640600	0.876033
chr6	27739800	0.834711
chr6	27745300	0.785124
chr6	27976700	0.801653
chr6	28048900	0.776860
chr6	28072600	0.743802
chr6	28105300	0.760331
chr6	28340300	0.768595
chr6	28367600	0.776860
chr6	28394500	0.760331
chr6	28764500	0.768595
chr6	28770900	0.842975
chr6	28946800	0.752066
chr6	29194700	0.743802
chr6	29617400	0.867769
chr6	29645300	0.735537
chr6	29720600	0.818182
chr6	29728200	0.793388
chr6	29795600	0.735537
chr6	29855600	0.900826
chr6	29894700	0.958678
chr6	29927400	0.735537
chr6	29988500	0.834711
chr6	30079400	0.752066
chr6	30098600	0.768595
chr6	30104500	0.842975
chr6	30126200	0.876033
chr6	30181900	0.818182
chr6	30318400	0.752066
chr6	30418900	0.768595
chr6	30421600	0.809917
chr6	30431600	0.809917
chr6	30449100	0.809917
chr6	30461900	0.826446
chr6	30468800	0.818182

chr6	30484200	0.966942
chr6	30512000	0.809917
chr6	30594700	0.743802
chr6	30615100	0.826446
chr6	30699400	0.760331
chr6	30753800	0.785124
chr6	30761600	0.859504
chr6	30918500	0.859504
chr6	30920800	0.876033
chr6	30933400	0.818182
chr6	30947500	0.768595
chr6	30950600	0.743802
chr6	30958600	0.859504
chr6	31011100	0.752066
chr6	31048000	0.785124
chr6	31083500	0.785124
chr6	31130300	0.735537
chr6	31319100	0.785124
chr6	31515800	0.793388
chr6	31533300	0.743802
chr6	31626100	0.735537
chr6	31645000	0.743802
chr6	31671100	0.834711
chr6	31708600	0.842975
chr6	31763700	0.900826
chr6	31876900	0.884298
chr6	31940300	0.743802
chr6	32054200	0.818182
chr6	32116900	0.793388
chr6	32164200	0.834711
chr6	32209600	0.933884
chr6	32213000	0.735537
chr6	32220700	0.834711
chr6	32239400	0.818182
chr6	32295200	0.743802
chr6	32355600	0.776860
chr6	32365700	0.752066
chr6	32384100	0.743802
chr6	32441800	0.785124
chr6	32450700	0.876033
chr6	32522700	0.743802
chr6	32672700	0.752066
chr6	32767700	0.842975
chr6	32808800	0.743802
chr6	32818400	0.809917
chr6	32825900	0.826446
chr6	32850000	0.818182
chr6	32884600	0.760331
chr6	32955900	0.809917
chr6	32968400	0.785124
chr6	33030100	0.768595
chr6	33073400	0.801653
chr6	33096800	0.818182
chr6	33129600	0.867769

chr6	33137800	0.735537
chr6	33165800	0.842975
chr6	33176100	0.884298
chr6	33181500	0.735537
chr6	33245600	0.884298
chr6	33378800	0.752066
chr6	33382900	0.785124
chr6	33400500	0.785124
chr6	33600500	0.735537
chr6	33679600	0.892562
chr6	33689500	0.958678
chr6	33712200	0.752066
chr6	33824900	0.768595
chr6	33999700	0.876033
chr6	34010500	0.859504
chr6	34023800	0.752066
chr6	34117400	0.884298
chr6	34122500	0.909091
chr6	34151000	0.925620
chr6	34192200	0.958678
chr6	34197200	0.735537
chr6	34393500	0.842975
chr6	34437000	0.752066
chr6	34649300	0.801653
chr6	34760300	0.768595
chr6	34775200	0.776860
chr6	34881000	0.909091
chr6	34885700	0.768595
chr6	35002800	0.760331
chr6	35026700	0.966942
chr6	35109400	0.826446
chr6	35228100	0.851240
chr6	35279600	0.933884
chr6	35316700	0.809917
chr6	35344900	0.859504
chr6	35374600	0.818182
chr6	35477600	0.760331
chr6	35633700	0.752066
chr6	35693500	0.892562
chr6	35738900	0.752066
chr6	35748400	0.826446
chr6	35992700	0.867769
chr6	36033400	0.876033
chr6	36088900	0.735537
chr6	36104500	0.884298
chr6	36172900	0.785124
chr6	36209900	0.834711
chr6	36277400	0.842975
chr6	36279900	0.768595
chr6	36282100	0.851240
chr6	36306900	0.785124
chr6	36324500	0.793388
chr6	36333100	0.859504
chr6	36346400	0.743802

chr6	36368100	0.801653
chr6	36598200	0.735537
chr6	36624000	0.867769
chr6	36628900	0.876033
chr6	36652800	0.801653
chr6	36681900	0.826446
chr6	36688100	0.776860
chr6	36733900	0.892562
chr6	36750200	0.867769
chr6	36842900	0.743802
chr6	36922900	0.785124
chr6	37039900	0.785124
chr6	37066400	0.768595
chr6	37068800	0.859504
chr6	37073300	0.859504
chr6	37105900	0.768595
chr6	37128800	0.892562
chr6	37157700	0.760331
chr6	37173900	0.768595
chr6	37176200	0.735537
chr6	37208800	0.793388
chr6	37233600	0.809917
chr6	37241800	0.735537
chr6	37274700	0.752066
chr6	37467400	0.760331
chr6	37490700	0.752066
chr6	37587100	0.818182
chr6	37591000	0.801653
chr6	37599400	0.917355
chr6	37677900	0.760331
chr6	37700000	0.826446
chr6	37759600	0.851240
chr6	37874600	0.867769
chr6	37920400	0.735537
chr6	38002100	0.851240
chr6	38177700	0.826446
chr6	38184200	0.818182
chr6	38290200	0.752066
chr6	38316700	0.768595
chr6	38376400	0.760331
chr6	38400400	0.768595
chr6	38454400	0.735537
chr6	38604500	0.834711
chr6	38656700	0.809917
chr6	38694100	0.793388
chr6	38739500	0.818182
chr6	38752500	0.818182
chr6	38774200	0.933884
chr6	38844500	0.859504
chr6	39008600	0.785124
chr6	39054500	0.809917
chr6	39248700	0.785124
chr6	39298200	0.909091
chr6	39376100	0.809917

chr6	40555500	0.768595
chr6	40679100	0.801653
chr6	40757100	0.743802
chr6	40772200	0.785124
chr6	40880300	0.760331
chr6	41177400	0.793388
chr6	41224500	0.842975
chr6	41397100	0.851240
chr6	41431800	0.743802
chr6	41443000	0.768595
chr6	41458100	0.768595
chr6	41461900	0.809917
chr6	41476800	0.809917
chr6	41752600	0.752066
chr6	41814300	0.776860
chr6	41863700	0.892562
chr6	41889100	0.735537
chr6	41975900	0.785124
chr6	41996800	0.776860
chr6	42007300	0.834711
chr6	42018300	0.925620
chr6	42238000	0.752066
chr6	42309400	0.818182
chr6	42358600	0.809917
chr6	42375400	0.768595
chr6	42390900	0.768595
chr6	42394400	0.851240
chr6	42414000	0.735537
chr6	42694500	0.768595
chr6	42869900	0.826446
chr6	43021500	0.933884
chr6	43054600	0.743802
chr6	43062000	0.859504
chr6	43115900	0.809917
chr6	43209000	0.851240
chr6	43219200	0.909091
chr6	43239300	0.842975
chr6	43287300	0.768595
chr6	43290700	0.752066
chr6	43303400	0.884298
chr6	43540200	0.818182
chr6	43674300	0.859504
chr6	43678100	0.842975
chr6	43725300	0.735537
chr6	43823100	0.801653
chr6	44017200	0.809917
chr6	44027000	0.735537
chr6	44095000	0.818182
chr6	44140700	0.851240
chr6	44237000	0.834711
chr6	44355600	0.842975
chr6	44531700	0.743802
chr6	44623500	0.809917
chr6	44797700	0.909091

chr6	44801200	0.735537
chr6	44824000	0.735537
chr6	45384100	0.909091
chr6	45448000	0.900826
chr6	45516800	0.892562
chr6	45527100	0.818182
chr6	45627100	0.793388
chr6	45645700	0.809917
chr6	45655300	0.909091
chr6	45674400	0.884298
chr6	45710000	0.760331
chr6	45777000	0.743802
chr6	45803300	0.768595
chr6	45889000	0.818182
chr6	45892100	0.752066
chr6	46144000	0.809917
chr6	46228200	0.768595
chr6	46256600	0.760331
chr6	46259400	0.818182
chr6	46262100	0.809917
chr6	46305900	0.785124
chr6	46348500	0.809917
chr6	46470000	0.768595
chr6	47172100	0.776860
chr6	47328900	0.884298
chr6	47405800	0.768595
chr6	47409000	0.818182
chr6	47423900	0.900826
chr6	47469800	0.818182
chr6	47618800	0.933884
chr6	47669600	0.876033
chr6	47690900	0.809917
chr6	50931000	0.793388
chr6	51861700	0.884298
chr6	52009800	0.842975
chr6	52029700	0.851240
chr6	52042000	0.892562
chr6	52081800	0.785124
chr6	52086700	0.834711
chr6	52099400	0.851240
chr6	52116300	0.809917
chr6	52187300	0.785124
chr6	52191900	0.752066
chr6	52199000	0.925620
chr6	52217200	0.768595
chr6	52281000	0.859504
chr6	52291800	0.735537
chr6	52415700	0.801653
chr6	52431400	0.834711
chr6	52442300	0.884298
chr6	52590000	0.793388
chr6	52848100	0.760331
chr6	52855700	0.801653
chr6	52860200	0.958678

chr6	52913900	0.752066
chr6	52974800	0.834711
chr6	53037400	0.793388
chr6	53053200	0.909091
chr6	53064600	0.793388
chr6	53101500	0.826446
chr6	53105200	0.851240
chr6	53110200	0.834711
chr6	53203100	0.851240
chr6	53275700	0.793388
chr6	53305800	0.793388
chr6	53323300	0.785124
chr6	53349100	0.826446
chr6	53397700	0.809917
chr6	53496100	0.793388
chr6	53499600	0.834711
chr6	53512400	0.801653
chr6	53600500	0.867769
chr6	54156100	0.743802
chr6	56223000	0.752066
chr6	56277100	0.818182
chr6	56416000	0.842975
chr6	56614800	0.752066
chr6	56780200	0.768595
chr6	56782500	0.826446
chr6	57072900	0.851240
chr6	57216300	0.785124
chr6	64063100	0.752066
chr6	64130100	0.735537
chr6	64247700	0.851240
chr6	64395600	0.768595
chr6	64501100	0.834711
chr6	64593800	0.793388
chr6	70406900	0.752066
chr6	70502700	0.735537
chr6	70605300	0.785124
chr6	70980600	0.752066
chr6	71022200	0.826446
chr6	71025200	0.768595
chr6	71090200	0.884298
chr6	71361700	0.809917
chr6	71418400	0.892562
chr6	72206100	0.834711
chr6	73116900	0.760331
chr6	73379000	0.793388
chr6	73480400	0.900826
chr6	73740100	0.768595
chr6	73795900	0.909091
chr6	73840100	0.752066
chr6	74013900	0.801653
chr6	74039300	0.768595
chr6	74161600	0.834711
chr6	74171700	0.867769
chr6	74233500	0.735537

chr6	74266500	0.809917
chr6	74290500	0.735537
chr6	74457300	0.834711
chr6	75288000	0.768595
chr6	75918500	0.752066
chr6	75953500	0.826446
chr6	76200400	0.776860
chr6	79951100	0.867769
chr6	79960300	0.809917
chr6	80337200	0.900826
chr6	82691900	0.793388
chr6	82777400	0.735537
chr6	83107300	0.743802
chr6	83126200	0.743802
chr6	83278400	0.793388
chr6	83771200	0.867769
chr6	83796800	0.867769
chr6	83900900	0.785124
chr6	84144800	0.785124
chr6	84728200	0.884298
chr6	86115800	0.801653
chr6	86129200	0.826446
chr6	86173900	0.785124
chr6	86176000	0.834711
chr6	86354000	0.760331
chr6	86727000	0.801653
chr6	87769300	0.942149
chr6	87793300	0.826446
chr6	87874100	0.818182
chr6	87877400	0.909091
chr6	87882200	0.809917
chr6	87888200	0.752066
chr6	88043800	0.776860
chr6	88204900	0.818182
chr6	88284800	0.760331
chr6	88397600	0.809917
chr6	88441800	0.776860
chr6	88542300	0.909091
chr6	88625600	0.785124
chr6	88642700	0.776860
chr6	88851800	0.933884
chr6	88855200	0.768595
chr6	88884900	0.793388
chr6	88895100	0.760331
chr6	89210100	0.933884
chr6	89808600	0.793388
chr6	89854200	0.842975
chr6	89992000	0.785124
chr6	89997600	0.743802
chr6	90014700	0.743802
chr6	90049300	0.826446
chr6	90082100	0.785124
chr6	90185800	0.876033
chr6	90348300	0.917355

chr6	90573700	0.735537
chr6	90790700	0.876033
chr6	90813700	0.892562
chr6	90818200	0.760331
chr6	90854800	0.892562
chr6	90926200	0.809917
chr6	90984700	0.760331
chr6	91004900	0.809917
chr6	91079200	0.867769
chr6	91121600	0.735537
chr6	91169100	0.909091
chr6	91185400	0.834711
chr6	91188800	0.785124
chr6	91296800	0.884298
chr6	91651000	0.859504
chr6	92147900	0.743802
chr6	94164200	0.768595
chr6	96913900	0.909091
chr6	97070400	0.892562
chr6	97243000	0.809917
chr6	98771900	0.892562
chr6	99225900	0.760331
chr6	99423000	0.752066
chr6	99540100	0.900826
chr6	99542800	0.867769
chr6	99638400	0.925620
chr6	99782600	0.793388
chr6	99812400	0.909091
chr6	100553400	0.776860
chr6	104774200	0.760331
chr6	104908700	0.826446
chr6	105660800	0.859504
chr6	105879200	0.768595
chr6	105930900	0.884298
chr6	106005700	0.818182
chr6	106009800	0.801653
chr6	106036200	0.793388
chr6	106049700	0.917355
chr6	106097000	0.859504
chr6	106124500	0.743802
chr6	106189600	0.842975
chr6	106194300	0.752066
chr6	106197000	0.809917
chr6	106257600	0.809917
chr6	106328900	0.859504
chr6	106341500	0.785124
chr6	106348700	0.735537
chr6	106359400	0.760331
chr6	106555100	0.768595
chr6	106560600	0.826446
chr6	106567300	0.776860
chr6	106570000	0.818182
chr6	106586500	0.752066
chr6	106590500	0.801653

chr6	106597300	0.834711
chr6	106632700	0.760331
chr6	106643600	0.760331
chr6	106707200	0.809917
chr6	106719700	0.785124
chr6	106761400	0.776860
chr6	106765100	0.752066
chr6	106864600	0.752066
chr6	106893000	0.859504
chr6	106896200	0.785124
chr6	107056000	0.884298
chr6	107061900	0.851240
chr6	107161900	0.743802
chr6	107247500	0.735537
chr6	107351600	0.809917
chr6	107383100	0.785124
chr6	107386700	0.735537
chr6	107424200	0.776860
chr6	107450900	0.776860
chr6	107465800	0.793388
chr6	107857700	0.818182
chr6	107872100	0.743802
chr6	107940300	0.900826
chr6	107979700	0.809917
chr6	107996900	0.735537
chr6	108026100	0.743802
chr6	108062600	0.752066
chr6	108107300	0.785124
chr6	108435400	0.867769
chr6	108455300	0.801653
chr6	108464000	0.752066
chr6	108582600	0.785124
chr6	108616500	0.826446
chr6	108663200	0.809917
chr6	108867900	0.752066
chr6	108887200	0.826446
chr6	108900100	0.826446
chr6	108961300	0.793388
chr6	109006600	0.752066
chr6	109127200	0.884298
chr6	109217800	0.776860
chr6	109226200	0.851240
chr6	109277200	0.752066
chr6	109324100	0.752066
chr6	109373900	0.900826
chr6	109416700	0.859504
chr6	109443300	0.851240
chr6	109625300	0.851240
chr6	109706200	0.735537
chr6	109772000	0.768595
chr6	109901000	0.818182
chr6	109953700	0.760331
chr6	110386500	0.851240
chr6	110632500	0.768595

chr6	111187600	0.760331
chr6	111444100	0.760331
chr6	111493700	0.884298
chr6	111496100	0.826446
chr6	111572800	0.776860
chr6	111580800	0.892562
chr6	111927200	0.826446
chr6	112047800	0.785124
chr6	112130300	0.900826
chr6	112161600	0.776860
chr6	112173000	0.760331
chr6	112181300	0.752066
chr6	112364600	0.785124
chr6	112481100	0.809917
chr6	112525000	0.776860
chr6	112629200	0.776860
chr6	113684200	0.768595
chr6	113698900	0.826446
chr6	113883600	0.801653
chr6	114019100	0.884298
chr6	114386400	0.801653
chr6	116369000	0.793388
chr6	116424100	0.801653
chr6	116620400	0.793388
chr6	116768900	0.785124
chr6	116833700	0.752066
chr6	116852700	0.768595
chr6	116973400	0.735537
chr6	117800100	0.785124
chr6	117825500	0.842975
chr6	117869600	0.752066
chr6	117923700	0.785124
chr6	117929200	0.834711
chr6	118069600	0.743802
chr6	118090900	0.793388
chr6	118601500	0.900826
chr6	118676000	0.884298
chr6	119032700	0.752066
chr6	119069200	0.859504
chr6	119098500	0.867769
chr6	119186300	0.752066
chr6	119365300	0.793388
chr6	119381900	0.801653
chr6	119484000	0.892562
chr6	119527900	0.768595
chr6	119635800	0.752066
chr6	119775800	0.752066
chr6	119797900	0.826446
chr6	119823500	0.776860
chr6	119855300	0.826446
chr6	119908100	0.801653
chr6	120177900	0.793388
chr6	122086200	0.892562
chr6	122093600	0.809917

chr6	122792700	0.743802
chr6	125580200	0.768595
chr6	125667800	0.809917
chr6	125684500	0.752066
chr6	126039300	0.793388
chr6	126063000	0.793388
chr6	126102100	0.793388
chr6	127656000	0.859504
chr6	129059700	0.892562
chr6	129555100	0.743802
chr6	129770500	0.851240
chr6	129820600	0.917355
chr6	130008700	0.859504
chr6	130035500	0.752066
chr6	130204100	0.876033
chr6	130211900	0.752066
chr6	130229000	0.842975
chr6	130545100	0.851240
chr6	131230200	0.892562
chr6	131234400	0.768595
chr6	131351200	0.900826
chr6	131428300	0.818182
chr6	132005100	0.876033
chr6	132010100	0.876033
chr6	132589600	0.776860
chr6	132592300	0.752066
chr6	132688000	0.760331
chr6	132727800	0.818182
chr6	132788500	0.834711
chr6	132805800	0.768595
chr6	132970100	0.801653
chr6	132972400	0.793388
chr6	132991600	0.785124
chr6	133020300	0.760331
chr6	133047500	0.925620
chr6	133081800	0.752066
chr6	133119500	0.760331
chr6	133194400	0.785124
chr6	133286000	0.917355
chr6	134331100	0.735537
chr6	134401500	0.793388
chr6	134414200	0.735537
chr6	134521300	0.735537
chr6	134557600	0.826446
chr6	134570000	0.785124
chr6	134590500	0.900826
chr6	134594100	0.859504
chr6	134598900	0.776860
chr6	134720200	0.752066
chr6	134759000	0.743802
chr6	134768400	0.942149
chr6	134786100	0.892562
chr6	134941000	0.752066
chr6	134958900	0.818182

chr6	135192300	0.801653
chr6	135195400	0.834711
chr6	135366600	0.776860
chr6	135369400	0.826446
chr6	135376300	0.735537
chr6	135407300	0.867769
chr6	135412300	0.735537
chr6	135416100	0.735537
chr6	135470700	0.752066
chr6	135549700	0.867769
chr6	135553200	0.793388
chr6	135591800	0.735537
chr6	135644100	0.925620
chr6	135668300	0.809917
chr6	135877200	0.785124
chr6	136024700	0.925620
chr6	136201700	0.950413
chr6	136731700	0.735537
chr6	136785700	0.925620
chr6	136915700	0.809917
chr6	137046400	0.735537
chr6	137267300	0.768595
chr6	137580000	0.933884
chr6	137674900	0.743802
chr6	137703700	0.776860
chr6	137710600	0.851240
chr6	137747700	0.834711
chr6	137753500	0.743802
chr6	137969400	0.776860
chr6	138018700	0.818182
chr6	138021400	0.768595
chr6	138053700	0.768595
chr6	138062400	0.760331
chr6	138174200	0.735537
chr6	138182600	0.851240
chr6	138214100	0.760331
chr6	138286900	0.834711
chr6	138290500	0.859504
chr6	138467400	0.752066
chr6	138772100	0.826446
chr6	138976300	0.842975
chr6	139090800	0.752066
chr6	139094700	0.925620
chr6	139140400	0.851240
chr6	139309200	0.900826
chr6	139446800	0.818182
chr6	139468200	0.859504
chr6	139542500	0.760331
chr6	139573200	0.818182
chr6	139609700	0.785124
chr6	139638500	0.743802
chr6	139855900	0.818182
chr6	139861700	0.760331
chr6	139890600	0.768595

chr6	139902400	0.942149
chr6	139962500	0.859504
chr6	140204500	0.776860
chr6	140262500	0.834711
chr6	141095700	0.876033
chr6	141785400	0.793388
chr6	141805800	0.818182
chr6	142717000	0.842975
chr6	142891600	0.917355
chr6	142925800	0.785124
chr6	143021400	0.809917
chr6	143050700	0.785124
chr6	143105600	0.859504
chr6	143119200	0.966942
chr6	143228600	0.892562
chr6	143276600	0.809917
chr6	143328300	0.851240
chr6	143361000	0.760331
chr6	143620800	0.793388
chr6	143722500	0.743802
chr6	143775900	0.768595
chr6	143838300	0.851240
chr6	143969000	0.876033
chr6	144020000	0.768595
chr6	144048100	0.900826
chr6	144164700	0.760331
chr6	144218600	0.892562
chr6	144376700	0.950413
chr6	144396900	0.809917
chr6	144537600	0.900826
chr6	144660400	0.859504
chr6	144663000	0.876033
chr6	144686500	0.933884
chr6	144909300	0.809917
chr6	145018500	0.752066
chr6	145051200	0.752066
chr6	145074500	0.768595
chr6	146106200	0.793388
chr6	146474700	0.884298
chr6	146679400	0.834711
chr6	147643300	0.876033
chr6	148852100	0.785124
chr6	148881300	0.917355
chr6	148900300	0.892562
chr6	148951300	0.809917
chr6	148999700	0.842975
chr6	149112400	0.818182
chr6	149152400	0.859504
chr6	149280000	0.768595
chr6	149357300	0.785124
chr6	149360500	0.760331
chr6	149369600	0.768595
chr6	149376300	0.735537
chr6	149469200	0.776860

chr6	149490800	0.785124
chr6	149535200	0.735537
chr6	149556700	0.942149
chr6	149569800	0.809917
chr6	149592800	0.925620
chr6	149595300	0.859504
chr6	149606600	0.768595
chr6	149758600	0.768595
chr6	149950700	0.842975
chr6	150070900	0.752066
chr6	150174100	0.900826
chr6	150276200	0.801653
chr6	150321300	0.760331
chr6	150324000	0.900826
chr6	150336100	0.834711
chr6	150623800	0.851240
chr6	150812700	0.785124
chr6	150928200	0.743802
chr6	150964400	0.942149
chr6	151006000	0.851240
chr6	151012100	0.785124
chr6	151015100	0.735537
chr6	151020300	0.826446
chr6	151045900	0.859504
chr6	151054900	0.793388
chr6	151088400	0.925620
chr6	151209200	0.892562
chr6	151701700	0.909091
chr6	151762200	0.752066
chr6	151778500	0.768595
chr6	151949000	0.826446
chr6	151997500	0.785124
chr6	152379200	0.743802
chr6	152492800	0.768595
chr6	152593400	0.834711
chr6	152623300	0.909091
chr6	154279200	0.768595
chr6	154487500	0.768595
chr6	154573200	0.785124
chr6	154598700	0.834711
chr6	154619700	0.735537
chr6	154627800	0.826446
chr6	154925500	0.735537
chr6	155005400	0.785124
chr6	155011500	0.801653
chr6	155055100	0.826446
chr6	155086400	0.826446
chr6	155094800	0.752066
chr6	155302600	0.818182
chr6	155325200	0.809917
chr6	155401600	0.760331
chr6	155438100	0.768595
chr6	155443300	0.752066
chr6	155481100	0.950413

chr6	155487600	0.826446
chr6	155553300	0.752066
chr6	155569800	0.785124
chr6	155636600	0.909091
chr6	155693800	0.809917
chr6	155700200	0.851240
chr6	155761000	0.842975
chr6	155815100	0.917355
chr6	155841000	0.752066
chr6	156715200	0.884298
chr6	156719400	0.826446
chr6	156777100	0.917355
chr6	156959200	0.867769
chr6	157050700	0.851240
chr6	157074300	0.793388
chr6	157076800	0.735537
chr6	157095600	0.793388
chr6	157144000	0.801653
chr6	157183500	0.735537
chr6	157231200	0.867769
chr6	157305000	0.752066
chr6	157310400	0.793388
chr6	157365200	0.752066
chr6	157385200	0.793388
chr6	157388600	0.735537
chr6	157394800	0.752066
chr6	157415300	0.876033
chr6	157422300	0.818182
chr6	157544600	0.834711
chr6	157731600	0.834711
chr6	157735200	0.892562
chr6	157748400	0.867769
chr6	157790800	0.785124
chr6	157864700	0.760331
chr6	157878000	0.851240
chr6	157883200	0.776860
chr6	157920500	0.834711
chr6	157988300	0.809917
chr6	158035900	0.760331
chr6	158236400	0.867769
chr6	158262500	0.809917
chr6	158298400	0.826446
chr6	158314200	0.793388
chr6	158411900	0.743802
chr6	158628900	0.826446
chr6	158735200	0.752066
chr6	158974000	0.859504
chr6	159063000	0.867769
chr6	159081600	0.818182
chr6	159108200	0.776860
chr6	159125700	0.900826
chr6	159217700	0.793388
chr6	159227700	0.752066
chr6	159271400	0.768595

chr6	159291400	0.867769
chr6	159430200	0.743802
chr6	159462300	0.785124
chr6	159474300	0.743802
chr6	159477600	0.925620
chr6	159515900	0.785124
chr6	159639200	0.760331
chr6	160241700	0.826446
chr6	160250800	0.818182
chr6	160263500	0.818182
chr6	160326100	0.975207
chr6	160383000	0.760331
chr6	160395700	0.776860
chr6	160687700	0.818182
chr6	161059800	0.834711
chr6	161154800	0.776860
chr6	161341100	0.809917
chr6	161395200	0.834711
chr6	161591700	0.867769
chr6	161622900	0.776860
chr6	161657500	0.809917
chr6	161687800	0.892562
chr6	161795600	0.793388
chr6	161815800	0.859504
chr6	161964800	0.950413
chr6	161967000	0.876033
chr6	162603000	0.768595
chr6	163423000	0.809917
chr6	163820100	0.752066
chr6	163827200	0.834711
chr6	164106500	0.809917
chr6	164117000	0.818182
chr6	164142600	0.776860
chr6	164156500	0.760331
chr6	164159900	0.785124
chr6	164178600	0.752066
chr6	164210800	0.859504
chr6	164215000	0.752066
chr6	164309300	0.826446
chr6	164380500	0.752066
chr6	164405600	0.768595
chr6	166572100	0.760331
chr6	166665400	0.867769
chr6	166700700	0.975207
chr6	166817000	0.735537
chr6	166825600	0.801653
chr6	166901700	0.842975
chr6	167044600	0.801653
chr6	167070900	0.876033
chr6	167165000	0.900826
chr6	167244600	0.785124
chr6	167373100	0.892562
chr6	167463800	0.785124
chr6	167470100	0.793388

chr6	167487700	0.809917
chr6	167521600	0.785124
chr6	167579700	0.752066
chr6	167626400	0.900826
chr6	168100700	0.752066
chr6	168106900	0.842975
chr6	168688300	0.776860
chr6	169968900	0.776860
chr6	169990500	0.743802
chr6	170102700	0.752066
chr6	170109700	0.776860
chr6	170203500	0.925620
chr6	170368200	0.958678
chr6	170412300	0.909091
chr6	170463700	0.842975
chr6	170769400	0.859504
chr6	171005800	0.743802
chr6	171026400	0.760331
chr7	144700	0.809917
chr7	149800	0.925620
chr7	454000	0.826446
chr7	562600	0.876033
chr7	572300	0.801653
chr7	860500	0.851240
chr7	1004200	0.876033
chr7	1020100	0.876033
chr7	1200300	0.900826
chr7	1209200	0.735537
chr7	1216400	0.842975
chr7	1269700	0.950413
chr7	1373200	0.917355
chr7	1457700	0.933884
chr7	1546200	0.809917
chr7	1631900	0.793388
chr7	1646200	0.801653
chr7	1796400	0.859504
chr7	1960400	0.809917
chr7	2020500	0.801653
chr7	2077600	0.752066
chr7	2081000	0.884298
chr7	2152300	0.818182
chr7	2272600	0.859504
chr7	2282100	0.735537
chr7	2284600	0.818182
chr7	2353700	0.917355
chr7	2469500	0.735537
chr7	2491700	0.834711
chr7	2517900	0.760331
chr7	2552400	0.801653
chr7	2595200	0.975207
chr7	2649100	0.785124
chr7	2685200	0.958678
chr7	2761000	0.818182
chr7	2774800	0.859504

chr7	2813300	0.793388
chr7	2840900	0.801653
chr7	2848500	0.760331
chr7	2865100	0.752066
chr7	2877500	0.851240
chr7	2910400	0.851240
chr7	2936300	0.801653
chr7	3021600	0.909091
chr7	4681900	0.909091
chr7	4726100	0.760331
chr7	4729800	0.809917
chr7	4765200	0.950413
chr7	5022900	0.760331
chr7	5085700	0.900826
chr7	5322200	0.842975
chr7	5406400	0.818182
chr7	5437100	0.909091
chr7	5468700	0.851240
chr7	5475700	0.818182
chr7	5497800	0.768595
chr7	5578700	0.966942
chr7	5618600	0.743802
chr7	5817600	0.760331
chr7	6111200	0.743802
chr7	6202200	0.752066
chr7	6390400	0.826446
chr7	6407800	0.752066
chr7	6518800	0.859504
chr7	6523800	0.760331
chr7	6574100	0.752066
chr7	6585800	0.818182
chr7	6617200	0.909091
chr7	6655300	0.859504
chr7	6664400	0.785124
chr7	6769600	0.884298
chr7	7091300	0.809917
chr7	7199300	0.768595
chr7	7242400	0.760331
chr7	7244900	0.760331
chr7	7291700	0.925620
chr7	7295500	0.785124
chr7	7363700	0.809917
chr7	7604600	0.818182
chr7	7617100	0.735537
chr7	7746000	0.776860
chr7	7964200	0.851240
chr7	8027400	0.735537
chr7	8425100	0.834711
chr7	8427900	0.842975
chr7	11355500	0.776860
chr7	12521900	0.892562
chr7	12625000	0.801653
chr7	12641200	0.809917
chr7	14754400	0.818182

chr7	16973500	0.776860
chr7	17138000	0.801653
chr7	17148200	0.776860
chr7	17168300	0.809917
chr7	17174900	0.768595
chr7	17183500	0.785124
chr7	17189000	0.768595
chr7	17193400	0.884298
chr7	17195700	0.760331
chr7	17235300	0.760331
chr7	17442600	0.768595
chr7	17512200	0.735537
chr7	17532600	0.752066
chr7	17537300	0.834711
chr7	17546500	0.760331
chr7	17720600	0.743802
chr7	17883200	0.818182
chr7	17974500	0.760331
chr7	17986900	0.925620
chr7	17992300	0.768595
chr7	18264700	0.826446
chr7	18430200	0.884298
chr7	18546200	0.818182
chr7	18548700	0.900826
chr7	20062200	0.768595
chr7	20070500	0.768595
chr7	20105700	0.884298
chr7	20277900	0.735537
chr7	20324500	0.892562
chr7	20401300	0.752066
chr7	20488900	0.826446
chr7	21398000	0.752066
chr7	21476400	0.793388
chr7	21644100	0.834711
chr7	21712000	0.851240
chr7	21938200	0.842975
chr7	21979600	0.760331
chr7	22007800	0.785124
chr7	22372400	0.785124
chr7	22385000	0.785124
chr7	22391700	0.793388
chr7	22411500	0.760331
chr7	22478500	0.752066
chr7	22492300	0.917355
chr7	22521600	0.735537
chr7	22540100	0.735537
chr7	22589900	0.752066
chr7	22592000	0.851240
chr7	22605800	0.793388
chr7	22623700	0.975207
chr7	22651200	0.735537
chr7	22654900	0.842975
chr7	22662400	0.768595
chr7	22680100	0.752066

chr7	22734500	0.776860
chr7	22738300	0.809917
chr7	22740400	0.752066
chr7	22753000	0.785124
chr7	22766900	0.793388
chr7	22773900	0.842975
chr7	22805600	0.785124
chr7	22999100	0.793388
chr7	23045600	0.842975
chr7	23058300	0.842975
chr7	23274700	0.933884
chr7	23325800	0.826446
chr7	23462700	0.793388
chr7	23530200	0.933884
chr7	23579700	0.884298
chr7	24267000	0.818182
chr7	24285900	0.876033
chr7	24329900	0.900826
chr7	24365700	0.966942
chr7	24496900	0.752066
chr7	24585000	0.801653
chr7	24618700	0.735537
chr7	24700200	0.776860
chr7	24932200	0.752066
chr7	25071200	0.809917
chr7	25160700	0.768595
chr7	25359600	0.892562
chr7	25704600	0.892562
chr7	25863600	0.735537
chr7	25926800	0.867769
chr7	25948300	0.842975
chr7	25999800	0.867769
chr7	26118700	0.760331
chr7	26137400	0.743802
chr7	26141100	0.776860
chr7	26143700	0.793388
chr7	26157300	0.834711
chr7	26231000	0.752066
chr7	26319500	0.752066
chr7	26377300	0.818182
chr7	26427700	0.826446
chr7	26899100	0.818182
chr7	26931700	0.867769
chr7	27175100	0.776860
chr7	27356500	0.818182
chr7	27780300	0.834711
chr7	27930200	0.925620
chr7	27935500	0.743802
chr7	27942700	0.950413
chr7	27961200	0.752066
chr7	28011700	0.776860
chr7	28077100	0.768595
chr7	28189700	0.842975
chr7	28192500	0.826446

chr7	28208600	0.752066
chr7	28247600	0.892562
chr7	28332400	0.768595
chr7	28370100	0.735537
chr7	28579700	0.900826
chr7	28702200	0.752066
chr7	28739100	0.809917
chr7	28823000	0.925620
chr7	28832800	0.826446
chr7	28858600	0.826446
chr7	29125100	0.867769
chr7	29131200	0.900826
chr7	29236300	0.760331
chr7	29325200	0.876033
chr7	29360100	0.793388
chr7	29464900	0.752066
chr7	29604500	0.842975
chr7	30015400	0.752066
chr7	30060700	0.785124
chr7	30072800	0.859504
chr7	30126900	0.801653
chr7	30155000	0.752066
chr7	30188200	0.743802
chr7	30202000	0.842975
chr7	30219000	0.760331
chr7	30232900	0.735537
chr7	30242600	0.801653
chr7	30251200	0.801653
chr7	30255200	0.776860
chr7	30287700	0.768595
chr7	30321300	0.801653
chr7	30350300	0.859504
chr7	30358800	0.752066
chr7	30368100	0.801653
chr7	30441400	0.776860
chr7	30504900	0.752066
chr7	30591000	0.768595
chr7	30767100	0.752066
chr7	30804200	0.867769
chr7	31505500	0.735537
chr7	31518700	0.876033
chr7	31727300	0.793388
chr7	31729400	0.842975
chr7	31852200	0.735537
chr7	31908700	0.752066
chr7	31934500	0.818182
chr7	32076600	0.859504
chr7	32082600	0.760331
chr7	32295400	0.785124
chr7	32535500	0.809917
chr7	32782000	0.818182
chr7	32886700	0.851240
chr7	32932400	0.785124
chr7	33004500	0.842975

chr7	33028200	0.909091
chr7	33038700	0.752066
chr7	33231400	0.917355
chr7	33712400	0.752066
chr7	35255800	0.818182
chr7	35273100	0.768595
chr7	35611600	0.834711
chr7	35762300	0.785124
chr7	36155900	0.776860
chr7	36248500	0.735537
chr7	36331800	0.743802
chr7	36514400	0.884298
chr7	36566900	0.884298
chr7	36718200	0.785124
chr7	36730100	0.876033
chr7	36764300	0.842975
chr7	36766800	0.876033
chr7	36788200	0.801653
chr7	36825100	0.900826
chr7	36991600	0.743802
chr7	36995600	0.760331
chr7	37149700	0.768595
chr7	37252300	0.909091
chr7	37326900	0.752066
chr7	37395000	0.826446
chr7	37472700	0.818182
chr7	37507100	0.785124
chr7	37534700	0.842975
chr7	37581700	0.851240
chr7	37697400	0.826446
chr7	37715400	0.801653
chr7	37805800	0.892562
chr7	38033100	0.826446
chr7	38230000	0.834711
chr7	38290200	0.809917
chr7	38324800	0.876033
chr7	38347500	0.752066
chr7	38353100	0.809917
chr7	38393800	0.735537
chr7	38402800	0.834711
chr7	38434100	0.884298
chr7	38881100	0.785124
chr7	39649700	0.876033
chr7	39673700	0.884298
chr7	40515100	0.851240
chr7	41173700	0.826446
chr7	41772100	0.876033
chr7	41856800	0.809917
chr7	42057700	0.818182
chr7	43000700	0.752066
chr7	43343500	0.801653
chr7	43501900	0.884298
chr7	43510100	0.859504
chr7	43519200	0.834711

chr7	43528800	0.876033
chr7	43552400	0.785124
chr7	43742900	0.752066
chr7	43890200	0.735537
chr7	43966100	0.809917
chr7	44032700	0.735537
chr7	44119200	0.776860
chr7	44121700	0.793388
chr7	44297100	0.743802
chr7	44378700	0.760331
chr7	44471300	0.776860
chr7	44491200	0.760331
chr7	44530200	0.909091
chr7	44642400	0.867769
chr7	44662300	0.793388
chr7	44675700	0.752066
chr7	44840800	0.776860
chr7	44888200	0.809917
chr7	44925800	0.826446
chr7	44976100	0.892562
chr7	44999600	0.760331
chr7	45048400	0.735537
chr7	45076200	0.809917
chr7	45151600	0.801653
chr7	45200500	0.851240
chr7	45236300	0.826446
chr7	47294300	0.909091
chr7	47402300	0.867769
chr7	47440400	0.842975
chr7	47568500	0.735537
chr7	47592900	0.909091
chr7	47611200	0.743802
chr7	47616200	0.842975
chr7	47641400	0.917355
chr7	47667100	0.735537
chr7	47687100	0.793388
chr7	47755000	0.809917
chr7	47760900	0.892562
chr7	47772200	0.752066
chr7	47969300	0.876033
chr7	47991300	0.818182
chr7	48046500	0.768595
chr7	48128300	0.958678
chr7	48338900	0.834711
chr7	48430700	0.834711
chr7	49364500	0.785124
chr7	49803600	0.942149
chr7	49806000	0.826446
chr7	49808600	0.776860
chr7	50193000	0.809917
chr7	50207400	0.834711
chr7	50239400	0.818182
chr7	50314900	0.834711
chr7	50317100	0.851240

chr7	50475800	0.760331
chr7	50480900	0.752066
chr7	50535700	0.809917
chr7	50662700	0.909091
chr7	50719500	0.884298
chr7	50724700	0.884298
chr7	50728900	0.768595
chr7	54827300	0.743802
chr7	55156200	0.892562
chr7	55179000	0.809917
chr7	55199400	0.760331
chr7	55202800	0.768595
chr7	55374900	0.834711
chr7	55659200	0.752066
chr7	55661600	0.842975
chr7	55791200	0.801653
chr7	55955300	0.925620
chr7	56132200	0.809917
chr7	57699400	0.842975
chr7	61264400	0.867769
chr7	63087700	0.743802
chr7	63774700	0.900826
chr7	64023200	0.867769
chr7	64254900	0.743802
chr7	64363600	0.760331
chr7	64467100	0.892562
chr7	65081500	0.776860
chr7	65263600	0.793388
chr7	65580200	0.900826
chr7	65613700	0.876033
chr7	65958300	0.851240
chr7	66097400	0.884298
chr7	66146700	0.768595
chr7	66221800	0.768595
chr7	66343000	0.900826
chr7	66380400	0.809917
chr7	66594500	0.743802
chr7	68000300	0.925620
chr7	68631200	0.909091
chr7	68885000	0.809917
chr7	68899700	0.867769
chr7	69042600	0.909091
chr7	69083000	0.859504
chr7	69088100	0.760331
chr7	69136000	0.768595
chr7	69150300	0.776860
chr7	69159500	0.801653
chr7	69165500	0.925620
chr7	69168800	0.834711
chr7	69180800	0.851240
chr7	69202700	0.752066
chr7	69231100	0.818182
chr7	69331100	0.785124
chr7	69337300	0.752066

chr7	69355200	0.900826
chr7	69364300	0.793388
chr7	69373800	0.743802
chr7	69406700	0.785124
chr7	69596300	0.752066
chr7	69643200	0.743802
chr7	70144800	0.768595
chr7	70310700	0.809917
chr7	72436400	0.785124
chr7	72476700	0.818182
chr7	72505800	0.735537
chr7	72537500	0.768595
chr7	72571000	0.793388
chr7	72844800	0.900826
chr7	72889800	0.785124
chr7	72971800	0.925620
chr7	72975000	0.809917
chr7	72996800	0.876033
chr7	73386400	0.743802
chr7	73397400	0.752066
chr7	73725700	0.834711
chr7	74009800	0.760331
chr7	74053900	0.785124
chr7	74059400	0.867769
chr7	74065000	0.743802
chr7	74267800	0.966942
chr7	74347600	0.785124
chr7	74381000	0.826446
chr7	74541400	0.760331
chr7	74545600	0.785124
chr7	74587500	0.876033
chr7	75115700	0.909091
chr7	75341900	0.785124
chr7	75383200	0.752066
chr7	75406500	0.975207
chr7	75575500	0.743802
chr7	75618300	0.776860
chr7	75683500	0.809917
chr7	75831200	0.809917
chr7	76007200	0.851240
chr7	76009600	0.735537
chr7	76033500	0.867769
chr7	76054300	0.785124
chr7	76091300	0.752066
chr7	76146000	0.735537
chr7	76553300	0.743802
chr7	76557600	0.801653
chr7	76562200	0.793388
chr7	76589400	0.884298
chr7	76730800	0.743802
chr7	76758300	0.785124
chr7	76868800	0.768595
chr7	76899400	0.859504
chr7	77037600	0.809917

chr7	77055600	0.752066
chr7	77094300	0.826446
chr7	77136700	0.818182
chr7	77317600	0.826446
chr7	77397200	0.826446
chr7	79765700	0.776860
chr7	81254100	0.809917
chr7	81532800	0.776860
chr7	81814600	0.735537
chr7	83362000	0.884298
chr7	83428600	0.818182
chr7	84922900	0.768595
chr7	86378700	0.876033
chr7	86398400	0.892562
chr7	86418900	0.743802
chr7	86561300	0.752066
chr7	86595800	0.793388
chr7	86645700	0.900826
chr7	86757100	0.859504
chr7	86818500	0.785124
chr7	86832400	0.859504
chr7	86856300	0.876033
chr7	86946100	0.900826
chr7	87135800	0.876033
chr7	87574700	0.743802
chr7	89976200	0.851240
chr7	90292400	0.743802
chr7	90299400	0.785124
chr7	90330200	0.801653
chr7	90353600	0.793388
chr7	90367200	0.818182
chr7	90387100	0.859504
chr7	90577800	0.859504
chr7	90974900	0.760331
chr7	91473800	0.966942
chr7	91764200	0.785124
chr7	91788500	0.776860
chr7	91906200	0.933884
chr7	92079700	0.752066
chr7	92264700	0.876033
chr7	92271700	0.826446
chr7	92429700	0.768595
chr7	92470100	0.909091
chr7	92534200	0.958678
chr7	92667000	0.818182
chr7	92673300	0.776860
chr7	92747700	0.925620
chr7	93319900	0.851240
chr7	93587700	0.842975
chr7	93630800	0.826446
chr7	93682500	0.801653
chr7	93763100	0.735537
chr7	93790700	0.884298
chr7	93807500	0.826446

chr7	94135300	0.801653
chr7	94279400	0.743802
chr7	94954200	0.909091
chr7	94960500	0.743802
chr7	95100100	0.752066
chr7	95130700	0.909091
chr7	95147500	0.776860
chr7	95239200	0.760331
chr7	95930700	0.818182
chr7	95936200	0.743802
chr7	95951200	0.785124
chr7	96244000	0.743802
chr7	96467200	0.768595
chr7	97761300	0.884298
chr7	97764600	0.834711
chr7	97830000	0.793388
chr7	98311400	0.735537
chr7	98707400	0.743802
chr7	98788600	0.809917
chr7	98912200	0.768595
chr7	99070600	0.743802
chr7	99097700	0.925620
chr7	99102700	0.867769
chr7	99366000	0.752066
chr7	99385700	0.842975
chr7	99435800	0.826446
chr7	99438900	0.785124
chr7	99517200	0.834711
chr7	99541800	0.793388
chr7	99553500	0.801653
chr7	99578400	0.793388
chr7	99613500	0.867769
chr7	99725100	0.752066
chr7	99778500	0.735537
chr7	99901300	0.768595
chr7	99972200	0.917355
chr7	100005100	0.735537
chr7	100020800	0.867769
chr7	100034200	0.826446
chr7	100048100	0.991736
chr7	100076700	0.892562
chr7	100148100	0.818182
chr7	100209700	0.950413
chr7	100240400	0.768595
chr7	100550800	0.925620
chr7	100729200	0.859504
chr7	100765500	0.884298
chr7	100781800	0.735537
chr7	100809100	0.842975
chr7	100847100	0.768595
chr7	100926400	0.809917
chr7	101109500	0.752066
chr7	101448900	0.900826
chr7	101497600	0.793388

chr7	101507800	0.859504
chr7	101576200	0.768595
chr7	101580100	0.851240
chr7	101644900	0.760331
chr7	101686900	0.867769
chr7	101705200	0.785124
chr7	101765700	0.950413
chr7	101855500	0.867769
chr7	101883800	0.768595
chr7	101935900	0.776860
chr7	102056200	0.859504
chr7	102131200	0.842975
chr7	102336000	0.842975
chr7	102475800	0.752066
chr7	102481400	0.892562
chr7	102790300	0.818182
chr7	103047200	0.818182
chr7	103073600	0.851240
chr7	104492900	0.793388
chr7	104530600	0.752066
chr7	104563700	0.752066
chr7	104573900	0.793388
chr7	104579600	0.785124
chr7	104636500	0.735537
chr7	104641800	0.785124
chr7	104999900	0.793388
chr7	105042200	0.785124
chr7	105228600	0.892562
chr7	105315000	0.834711
chr7	105384200	0.942149
chr7	105440900	0.752066
chr7	105565900	0.768595
chr7	105652700	0.909091
chr7	105794400	0.776860
chr7	105818000	0.900826
chr7	105846600	0.785124
chr7	105926300	0.867769
chr7	105988100	0.801653
chr7	106066000	0.925620
chr7	106121400	0.785124
chr7	106201900	0.785124
chr7	106362900	0.818182
chr7	106436300	0.826446
chr7	106508400	0.752066
chr7	106529200	0.834711
chr7	106556600	0.809917
chr7	106766800	0.793388
chr7	106986400	0.743802
chr7	107285200	0.909091
chr7	107311600	0.776860
chr7	107704200	0.900826
chr7	107774100	0.752066
chr7	107798300	0.834711
chr7	107844300	0.859504

chr7	107911100	0.851240
chr7	107925200	0.818182
chr7	107949700	0.776860
chr7	107952400	0.909091
chr7	107968800	0.801653
chr7	108016500	0.925620
chr7	108026600	0.859504
chr7	108064300	0.768595
chr7	108071600	0.809917
chr7	108144300	0.909091
chr7	108167200	0.785124
chr7	108202100	0.760331
chr7	108264300	0.809917
chr7	110722000	0.768595
chr7	111202200	0.909091
chr7	111209300	0.925620
chr7	111906800	0.834711
chr7	111923800	0.760331
chr7	112084500	0.867769
chr7	112138100	0.851240
chr7	112151100	0.834711
chr7	112427400	0.793388
chr7	112430700	0.776860
chr7	113692900	0.752066
chr7	114195200	0.752066
chr7	114476100	0.776860
chr7	114776100	0.776860
chr7	115682200	0.867769
chr7	115734300	0.735537
chr7	115782900	0.801653
chr7	115800400	0.768595
chr7	115807400	0.809917
chr7	115862200	0.752066
chr7	115902100	0.793388
chr7	116086000	0.752066
chr7	116169100	0.752066
chr7	116188800	0.735537
chr7	116192400	0.776860
chr7	116195200	0.785124
chr7	116218900	0.851240
chr7	116228800	0.818182
chr7	116427300	0.933884
chr7	116469700	0.859504
chr7	116511900	0.776860
chr7	116603900	0.760331
chr7	116681500	0.776860
chr7	117409900	0.793388
chr7	117414000	0.900826
chr7	117417300	0.743802
chr7	117440800	0.743802
chr7	117479700	0.743802
chr7	117928500	0.842975
chr7	119701400	0.834711
chr7	120052700	0.942149

chr7	120082200	0.892562
chr7	120116200	0.776860
chr7	120255900	0.776860
chr7	120631500	0.884298
chr7	120637100	0.842975
chr7	120912200	0.900826
chr7	120946100	0.752066
chr7	121013200	0.859504
chr7	121015500	0.785124
chr7	121020800	0.752066
chr7	121033000	0.768595
chr7	121036100	0.859504
chr7	121072200	0.776860
chr7	121074800	0.834711
chr7	121081200	0.818182
chr7	122185300	0.809917
chr7	123172800	0.743802
chr7	123280600	0.884298
chr7	123615100	0.826446
chr7	126540300	0.776860
chr7	127044800	0.842975
chr7	127054600	0.760331
chr7	127496400	0.760331
chr7	127538100	0.743802
chr7	127642600	0.768595
chr7	127673800	0.760331
chr7	127752900	0.768595
chr7	128061500	0.785124
chr7	128117100	0.859504
chr7	128178000	0.768595
chr7	128595600	0.892562
chr7	128603200	0.768595
chr7	128682200	0.793388
chr7	128695300	0.809917
chr7	128735800	0.752066
chr7	128809500	0.826446
chr7	128858700	0.735537
chr7	128878400	0.743802
chr7	128902700	0.909091
chr7	128910800	0.768595
chr7	128991600	0.768595
chr7	128996100	0.809917
chr7	129134300	0.801653
chr7	129140500	0.768595
chr7	129565300	0.752066
chr7	129790300	0.859504
chr7	129792700	0.859504
chr7	129845100	0.900826
chr7	129993300	0.735537
chr7	130034400	0.826446
chr7	130080800	0.892562
chr7	130114100	0.768595
chr7	130116400	0.801653
chr7	130334100	0.892562

chr7	130426600	0.801653
chr7	130617500	0.809917
chr7	130630700	0.743802
chr7	130655800	0.743802
chr7	130786300	0.876033
chr7	130868700	0.743802
chr7	131052000	0.776860
chr7	131055400	0.834711
chr7	131305100	0.752066
chr7	131368800	0.842975
chr7	132342600	0.809917
chr7	132850200	0.900826
chr7	133489000	0.760331
chr7	133756600	0.735537
chr7	133840200	0.785124
chr7	133931200	0.801653
chr7	134239500	0.917355
chr7	134343000	0.884298
chr7	134354800	0.809917
chr7	134423300	0.776860
chr7	134467600	0.942149
chr7	134550600	0.818182
chr7	134596100	0.859504
chr7	134639700	0.785124
chr7	134787600	0.793388
chr7	134899000	0.867769
chr7	135152400	0.818182
chr7	135486100	0.876033
chr7	135528200	0.818182
chr7	135661700	0.851240
chr7	135679200	0.859504
chr7	136038500	0.760331
chr7	137442100	0.743802
chr7	137452100	0.752066
chr7	137495800	0.752066
chr7	137584300	0.826446
chr7	137602200	0.900826
chr7	137677700	0.752066
chr7	137776400	0.809917
chr7	137810500	0.834711
chr7	137854600	0.867769
chr7	137856800	0.776860
chr7	138784800	0.743802
chr7	138797000	0.776860
chr7	139006700	0.768595
chr7	139184900	0.809917
chr7	139358000	0.909091
chr7	139414600	0.933884
chr7	139463300	0.785124
chr7	139484300	0.851240
chr7	139542800	0.859504
chr7	139586900	0.826446
chr7	139594400	0.925620
chr7	139599500	0.809917

chr7	139736600	0.809917
chr7	139861200	0.743802
chr7	139915300	0.826446
chr7	139926200	0.801653
chr7	139933300	0.785124
chr7	140059200	0.752066
chr7	140092200	0.842975
chr7	140139500	0.785124
chr7	140356700	0.818182
chr7	140396800	0.776860
chr7	140576900	0.809917
chr7	140583800	0.735537
chr7	140624300	0.785124
chr7	141223200	0.752066
chr7	142144200	0.826446
chr7	142149200	0.834711
chr7	142153700	0.793388
chr7	142192400	0.925620
chr7	142247400	0.909091
chr7	142419700	0.752066
chr7	142447800	0.818182
chr7	142531300	0.809917
chr7	142712700	0.909091
chr7	142902900	0.735537
chr7	142954400	0.743802
chr7	142981400	0.826446
chr7	143023000	0.809917
chr7	143072600	0.768595
chr7	143134600	0.735537
chr7	143538000	0.818182
chr7	143985300	0.776860
chr7	144206100	0.785124
chr7	144227000	0.851240
chr7	144515700	0.735537
chr7	144539700	0.768595
chr7	144605600	0.900826
chr7	145700300	0.818182
chr7	145982700	0.859504
chr7	146722700	0.925620
chr7	147758400	0.752066
chr7	147951200	0.892562
chr7	147987000	0.768595
chr7	148144600	0.876033
chr7	148197700	0.859504
chr7	148232900	0.867769
chr7	148262500	0.884298
chr7	148281900	0.876033
chr7	148318300	0.842975
chr7	148325800	0.925620
chr7	148405400	0.776860
chr7	148410800	0.776860
chr7	148582100	0.917355
chr7	148606900	0.826446
chr7	148609500	0.793388

chr7	148617700	0.809917
chr7	148762600	0.942149
chr7	148788500	0.933884
chr7	149439500	0.884298
chr7	149887400	0.785124
chr7	150103700	0.884298
chr7	150118300	0.752066
chr7	150284300	0.768595
chr7	150346600	0.760331
chr7	150383200	0.801653
chr7	150482200	0.909091
chr7	150940900	0.809917
chr7	151049300	0.867769
chr7	151097900	0.942149
chr7	151106300	0.776860
chr7	151108600	0.776860
chr7	151203300	0.793388
chr7	151410000	0.752066
chr7	151536500	0.859504
chr7	151633100	0.768595
chr7	152053100	0.818182
chr7	152060700	0.735537
chr7	152081600	0.785124
chr7	152102400	0.917355
chr7	152106100	0.809917
chr7	152109500	0.867769
chr7	152409700	0.900826
chr7	152449200	0.884298
chr7	154653900	0.876033
chr7	155074300	0.884298
chr7	155077700	0.909091
chr7	155190700	0.760331
chr7	155282300	0.834711
chr7	155391700	0.793388
chr7	155518100	0.743802
chr7	155989400	0.851240
chr7	156405900	0.809917
chr7	156433700	0.900826
chr7	156497000	0.785124
chr7	156675500	0.793388
chr7	156686200	0.917355
chr7	156689900	0.809917
chr7	156715100	0.826446
chr7	156807000	0.818182
chr7	156929500	0.760331
chr7	157092700	0.760331
chr7	157095200	0.966942
chr7	157127500	0.851240
chr7	157179700	0.892562
chr7	157225800	0.760331
chr7	158611500	0.876033
chr8	182600	0.867769
chr8	359800	0.752066
chr8	714300	0.768595

chr8	1684700	0.818182
chr8	1970100	0.925620
chr8	2557600	0.760331
chr8	6149400	0.735537
chr8	6296100	0.818182
chr8	6539900	0.752066
chr8	6548700	0.752066
chr8	6633000	0.752066
chr8	6645800	0.809917
chr8	6655900	0.768595
chr8	6658600	0.892562
chr8	6698700	0.917355
chr8	6777900	0.884298
chr8	6780200	0.743802
chr8	6795700	0.809917
chr8	8001100	0.752066
chr8	8088300	0.760331
chr8	8100800	0.892562
chr8	8127100	0.809917
chr8	8137000	0.917355
chr8	8142500	0.785124
chr8	8147400	0.768595
chr8	8160600	0.818182
chr8	8182500	0.743802
chr8	8229700	0.867769
chr8	8234400	0.801653
chr8	8246000	0.785124
chr8	8297200	0.867769
chr8	8341300	0.809917
chr8	8367400	0.818182
chr8	8423900	0.743802
chr8	8427800	0.752066
chr8	8555200	0.743802
chr8	8567400	0.892562
chr8	8594900	0.834711
chr8	8603700	0.842975
chr8	8609000	0.809917
chr8	8654900	0.826446
chr8	8821200	0.793388
chr8	8853100	0.760331
chr8	8924400	0.785124
chr8	9006200	0.826446
chr8	9106900	0.859504
chr8	9268800	0.760331
chr8	9410000	0.818182
chr8	9413600	0.793388
chr8	9472800	0.884298
chr8	9503500	0.809917
chr8	9578200	0.884298
chr8	10191700	0.793388
chr8	10458200	0.760331
chr8	10488300	0.793388
chr8	10764100	0.801653
chr8	10921600	0.785124

chr8	10929400	0.752066
chr8	10988400	0.785124
chr8	11016900	0.809917
chr8	11035400	0.776860
chr8	11046600	0.768595
chr8	11106000	0.793388
chr8	11138700	0.760331
chr8	11271900	0.867769
chr8	11319300	0.801653
chr8	11322700	0.809917
chr8	11447300	0.859504
chr8	11716600	0.760331
chr8	11719600	0.876033
chr8	11733000	0.876033
chr8	11735800	0.842975
chr8	11801500	0.826446
chr8	12923100	0.900826
chr8	14866200	0.834711
chr8	17555000	0.785124
chr8	17598400	0.809917
chr8	17613300	0.958678
chr8	17942000	0.818182
chr8	18589300	0.735537
chr8	18697700	0.768595
chr8	18719700	0.752066
chr8	18769300	0.876033
chr8	18985800	0.867769
chr8	19205600	0.818182
chr8	19454200	0.752066
chr8	19495500	0.867769
chr8	19521000	0.776860
chr8	19527500	0.834711
chr8	19540400	0.752066
chr8	19820100	0.809917
chr8	20126800	0.768595
chr8	20499800	0.876033
chr8	20881100	0.735537
chr8	20900900	0.776860
chr8	20960900	0.785124
chr8	21622900	0.917355
chr8	21755400	0.942149
chr8	21913600	0.925620
chr8	21966900	0.842975
chr8	21996700	0.851240
chr8	22145300	0.785124
chr8	22395900	0.743802
chr8	22411500	0.809917
chr8	22419700	0.743802
chr8	22553200	0.743802
chr8	22602000	0.917355
chr8	22634400	0.909091
chr8	22639200	0.743802
chr8	22647400	0.892562
chr8	22662800	0.752066

chr8	22673800	0.752066
chr8	22718200	0.743802
chr8	22722500	0.892562
chr8	22726900	0.768595
chr8	22776400	0.859504
chr8	22876600	0.834711
chr8	22930500	0.785124
chr8	22943900	0.760331
chr8	22961800	0.785124
chr8	22987400	0.809917
chr8	23029700	0.842975
chr8	23038700	0.735537
chr8	23075200	0.826446
chr8	23079800	0.793388
chr8	23104300	0.917355
chr8	23305200	0.809917
chr8	23307800	0.834711
chr8	23348000	0.768595
chr8	23367900	0.752066
chr8	23396800	0.867769
chr8	23402500	0.743802
chr8	23410500	0.760331
chr8	23416900	0.834711
chr8	23619500	0.867769
chr8	24103100	0.842975
chr8	24129400	0.876033
chr8	24151800	0.842975
chr8	24225100	0.785124
chr8	24229000	0.809917
chr8	24242100	0.743802
chr8	24426300	0.826446
chr8	25227800	0.826446
chr8	25316700	0.867769
chr8	25963300	0.958678
chr8	25991500	0.884298
chr8	26059700	0.859504
chr8	26073000	0.958678
chr8	26243900	0.752066
chr8	26325400	0.752066
chr8	26453700	0.785124
chr8	26467300	0.809917
chr8	26487400	0.892562
chr8	26525000	0.859504
chr8	27201500	0.768595
chr8	27205900	0.785124
chr8	27260000	0.776860
chr8	27437700	0.818182
chr8	27472100	0.793388
chr8	27508700	0.900826
chr8	27632300	0.818182
chr8	27930800	0.867769
chr8	27933700	0.785124
chr8	27950600	0.842975
chr8	28027500	0.752066

chr8	28115200	0.743802
chr8	28182300	0.867769
chr8	28246400	0.752066
chr8	28264000	0.776860
chr8	28270100	0.785124
chr8	28569500	0.793388
chr8	28728100	0.752066
chr8	28873300	0.743802
chr8	29062400	0.793388
chr8	29097000	0.785124
chr8	29348700	0.743802
chr8	29490000	0.776860
chr8	29493000	0.842975
chr8	29499800	0.851240
chr8	29601000	0.785124
chr8	29610700	0.785124
chr8	29655200	0.793388
chr8	29848200	0.760331
chr8	29991500	0.735537
chr8	30325700	0.793388
chr8	30355500	0.809917
chr8	30414200	0.809917
chr8	30519600	0.900826
chr8	30827500	0.851240
chr8	31023800	0.809917
chr8	32078000	0.801653
chr8	32433000	0.768595
chr8	33125700	0.876033
chr8	33276300	0.842975
chr8	33370900	0.851240
chr8	33373900	0.818182
chr8	33385000	0.785124
chr8	33421500	0.760331
chr8	35755500	0.743802
chr8	37397600	0.876033
chr8	37431300	0.752066
chr8	37439300	0.768595
chr8	37613300	0.776860
chr8	37662600	0.760331
chr8	37744300	0.785124
chr8	37925100	0.776860
chr8	37963100	0.785124
chr8	37991900	0.909091
chr8	38044700	0.752066
chr8	38126700	0.909091
chr8	38289400	0.818182
chr8	38407100	0.768595
chr8	38411600	0.925620
chr8	38461400	0.826446
chr8	38492100	0.809917
chr8	38498800	0.743802
chr8	38504500	0.884298
chr8	38555300	0.801653
chr8	38584700	0.867769

chr8	38590400	0.884298
chr8	38723700	0.760331
chr8	38741900	0.876033
chr8	38862700	0.776860
chr8	39297300	0.842975
chr8	39771400	0.834711
chr8	39812200	0.785124
chr8	39819200	0.851240
chr8	39824000	0.776860
chr8	39850600	0.776860
chr8	41122300	0.743802
chr8	41329500	0.867769
chr8	41386900	0.785124
chr8	41470400	0.768595
chr8	41485700	0.743802
chr8	41502800	0.785124
chr8	41559500	0.818182
chr8	41686300	0.909091
chr8	41819600	0.776860
chr8	41896400	0.818182
chr8	41910100	0.925620
chr8	42040600	0.867769
chr8	42054100	0.909091
chr8	42066900	0.842975
chr8	42075000	0.851240
chr8	42113500	0.809917
chr8	42213500	0.768595
chr8	42356400	0.760331
chr8	42397200	0.826446
chr8	42494400	0.785124
chr8	42521400	0.785124
chr8	42549000	0.785124
chr8	42553700	0.909091
chr8	42592800	0.768595
chr8	42620700	0.917355
chr8	42629200	0.892562
chr8	42649300	0.892562
chr8	42698500	0.785124
chr8	42740800	0.752066
chr8	42749500	0.809917
chr8	42999000	0.776860
chr8	43002000	0.801653
chr8	43010700	0.785124
chr8	47829600	0.826446
chr8	48833700	0.768595
chr8	48921200	0.933884
chr8	49463600	0.851240
chr8	49577600	0.834711
chr8	49834500	0.776860
chr8	49842700	0.826446
chr8	49956000	0.752066
chr8	52530700	0.859504
chr8	52546400	0.752066
chr8	52798300	0.867769

chr8	52814800	0.909091
chr8	52853700	0.793388
chr8	53213100	0.809917
chr8	53246400	0.735537
chr8	53701700	0.760331
chr8	53706400	0.735537
chr8	54279000	0.818182
chr8	54567000	0.768595
chr8	54927100	0.735537
chr8	56116100	0.743802
chr8	56747400	0.884298
chr8	56753100	0.809917
chr8	56774300	0.834711
chr8	56928300	0.752066
chr8	57069500	0.776860
chr8	57426900	0.826446
chr8	57594900	0.785124
chr8	58163500	0.842975
chr8	58333900	0.793388
chr8	58492400	0.801653
chr8	59054700	0.752066
chr8	59110300	0.809917
chr8	59133900	0.752066
chr8	59178500	0.768595
chr8	59223300	0.834711
chr8	59234600	0.909091
chr8	59583000	0.892562
chr8	59712200	0.859504
chr8	59794800	0.818182
chr8	59864500	0.793388
chr8	59956700	0.842975
chr8	60176300	0.892562
chr8	60832600	0.851240
chr8	60876500	0.743802
chr8	61325500	0.776860
chr8	61438300	0.760331
chr8	61564500	0.768595
chr8	61613500	0.752066
chr8	61626700	0.809917
chr8	61639200	0.752066
chr8	61648800	0.776860
chr8	61672000	0.842975
chr8	61684000	0.793388
chr8	61694900	0.867769
chr8	61798700	0.735537
chr8	61812700	0.900826
chr8	61842300	0.917355
chr8	61846700	0.793388
chr8	61856900	0.743802
chr8	61889600	0.785124
chr8	61894200	0.876033
chr8	61928100	0.743802
chr8	61931400	0.735537
chr8	61968900	0.785124

chr8	61975800	0.834711
chr8	62334500	0.793388
chr8	62548700	0.818182
chr8	62613300	0.809917
chr8	63807200	0.950413
chr8	64135700	0.776860
chr8	64213200	0.776860
chr8	64243300	0.760331
chr8	64677300	0.768595
chr8	64680700	0.752066
chr8	64752100	0.735537
chr8	64812200	0.859504
chr8	65208900	0.743802
chr8	65542700	0.752066
chr8	65545100	0.776860
chr8	65548200	0.809917
chr8	65813500	0.859504
chr8	66595100	0.851240
chr8	66754700	0.768595
chr8	66901500	0.785124
chr8	66918900	0.752066
chr8	66922900	0.743802
chr8	66946200	0.818182
chr8	67033700	0.743802
chr8	67039500	0.785124
chr8	67045500	0.851240
chr8	67072100	0.785124
chr8	67303800	0.842975
chr8	67359000	0.859504
chr8	67380200	0.842975
chr8	67414400	0.867769
chr8	67426300	0.768595
chr8	67435800	0.785124
chr8	67444400	0.760331
chr8	67448800	0.793388
chr8	67454500	0.917355
chr8	67457500	0.785124
chr8	67605300	0.776860
chr8	67664800	0.785124
chr8	67837500	0.826446
chr8	68255800	0.785124
chr8	68306100	0.793388
chr8	68345400	0.933884
chr8	68402800	0.735537
chr8	68600900	0.876033
chr8	68654400	0.752066
chr8	68829900	0.884298
chr8	68881500	0.785124
chr8	69173800	0.826446
chr8	70510500	0.900826
chr8	70627500	0.793388
chr8	70746800	0.743802
chr8	70845200	0.809917
chr8	70879800	0.900826

chr8	70891700	0.834711
chr8	70947500	0.834711
chr8	70966700	0.867769
chr8	71217400	0.760331
chr8	71355000	0.809917
chr8	71369800	0.818182
chr8	71447100	0.884298
chr8	71456800	0.752066
chr8	71515400	0.752066
chr8	71520300	0.917355
chr8	71529800	0.859504
chr8	71595400	0.743802
chr8	71778500	0.842975
chr8	71965800	0.851240
chr8	72010000	0.826446
chr8	72778300	0.776860
chr8	72913500	0.892562
chr8	73020600	0.768595
chr8	73067800	0.859504
chr8	73199800	0.834711
chr8	73899300	0.909091
chr8	74024700	0.834711
chr8	74028500	0.826446
chr8	74037400	0.900826
chr8	74057700	0.785124
chr8	74064200	0.826446
chr8	74081900	0.793388
chr8	74103600	0.776860
chr8	74419600	0.760331
chr8	74751300	0.743802
chr8	74904200	0.851240
chr8	74961200	0.785124
chr8	75042300	0.867769
chr8	75207900	0.793388
chr8	77458200	0.760331
chr8	77529800	0.768595
chr8	77825000	0.752066
chr8	77867800	0.752066
chr8	78845100	0.801653
chr8	79428400	0.859504
chr8	79760500	0.752066
chr8	80563800	0.793388
chr8	80606000	0.942149
chr8	80636000	0.760331
chr8	80638300	0.760331
chr8	80704500	0.760331
chr8	80792700	0.884298
chr8	80846600	0.735537
chr8	80856200	0.834711
chr8	80870500	0.818182
chr8	80930500	0.735537
chr8	81183400	0.785124
chr8	81259500	0.900826
chr8	81276600	0.884298

chr8	81291000	0.768595
chr8	81304200	0.950413
chr8	81422900	0.785124
chr8	81831200	0.776860
chr8	81870100	0.793388
chr8	81896600	0.776860
chr8	81901000	0.851240
chr8	82013800	0.867769
chr8	82086800	0.768595
chr8	82143200	0.776860
chr8	82206800	0.809917
chr8	82231400	0.752066
chr8	82369400	0.884298
chr8	82372400	0.842975
chr8	82396500	0.752066
chr8	82598300	0.933884
chr8	82693100	0.809917
chr8	82708300	0.900826
chr8	86045900	0.760331
chr8	86084900	0.892562
chr8	86178200	0.867769
chr8	86284300	0.933884
chr8	86425900	0.768595
chr8	86561700	0.859504
chr8	87520700	0.942149
chr8	87720100	0.793388
chr8	90573600	0.752066
chr8	90776900	0.876033
chr8	90794600	0.842975
chr8	90827900	0.743802
chr8	90996700	0.743802
chr8	91019000	0.867769
chr8	91784200	0.776860
chr8	91997200	0.900826
chr8	92044600	0.768595
chr8	92061000	0.743802
chr8	92453100	0.801653
chr8	94753100	0.809917
chr8	94761300	0.760331
chr8	94896800	0.793388
chr8	94916900	0.909091
chr8	94945300	0.826446
chr8	94947800	0.884298
chr8	95000800	0.826446
chr8	95078400	0.818182
chr8	95179700	0.867769
chr8	95233000	0.743802
chr8	95712500	0.735537
chr8	95887400	0.752066
chr8	95904400	0.892562
chr8	95962100	0.851240
chr8	95975700	0.793388
chr8	96100500	0.752066
chr8	96110900	0.785124

chr8	96125300	0.834711
chr8	96161100	0.876033
chr8	96165700	0.851240
chr8	96194100	0.842975
chr8	96213000	0.752066
chr8	96226700	0.909091
chr8	96233600	0.752066
chr8	96239500	0.776860
chr8	96248300	0.752066
chr8	96269300	0.768595
chr8	97057300	0.809917
chr8	97138400	0.785124
chr8	97158300	0.867769
chr8	97175600	0.752066
chr8	97282100	0.809917
chr8	97874200	0.867769
chr8	98430300	0.776860
chr8	98582700	0.785124
chr8	98610700	0.900826
chr8	98670600	0.801653
chr8	98748700	0.785124
chr8	98773700	0.752066
chr8	98796200	0.834711
chr8	98857100	0.793388
chr8	98861300	0.776860
chr8	98941800	0.826446
chr8	98983800	0.768595
chr8	98992700	0.776860
chr8	99199400	0.743802
chr8	99313500	0.743802
chr8	99318000	0.743802
chr8	99370800	0.818182
chr8	99838200	0.925620
chr8	99866000	0.776860
chr8	99963700	0.768595
chr8	100093100	0.933884
chr8	100101200	0.768595
chr8	100511300	0.933884
chr8	100668300	0.768595
chr8	100787200	0.760331
chr8	101273800	0.760331
chr8	101308600	0.752066
chr8	101349500	0.793388
chr8	101367500	0.743802
chr8	101399000	0.760331
chr8	101426700	0.785124
chr8	101527600	0.809917
chr8	101576700	0.809917
chr8	101734800	0.760331
chr8	101775000	0.909091
chr8	101822300	0.735537
chr8	101845500	0.768595
chr8	101860000	0.768595
chr8	101950100	0.801653

chr8	102011200	0.776860
chr8	102064400	0.760331
chr8	102109100	0.884298
chr8	102149800	0.909091
chr8	102168200	0.826446
chr8	102179400	0.892562
chr8	102218500	0.760331
chr8	102345300	0.826446
chr8	102381400	0.842975
chr8	102504500	0.809917
chr8	102506800	0.752066
chr8	102514100	0.752066
chr8	102528100	0.809917
chr8	102619100	0.743802
chr8	102622200	0.801653
chr8	102644500	0.785124
chr8	102649200	0.851240
chr8	102656700	0.876033
chr8	102715900	0.785124
chr8	102738900	0.760331
chr8	102870700	0.743802
chr8	102992900	0.760331
chr8	103092900	0.735537
chr8	103117600	0.867769
chr8	103252000	0.776860
chr8	103398300	0.826446
chr8	103409900	0.809917
chr8	103413900	0.735537
chr8	103427900	0.735537
chr8	103469200	0.900826
chr8	103473400	0.760331
chr8	103486500	0.785124
chr8	103521700	0.826446
chr8	103584800	0.743802
chr8	103634100	0.735537
chr8	103647600	0.958678
chr8	103675200	0.966942
chr8	103695500	0.752066
chr8	103751600	0.809917
chr8	103786500	0.768595
chr8	103807600	0.933884
chr8	103810300	0.851240
chr8	103902300	0.917355
chr8	103911400	0.834711
chr8	103915900	0.867769
chr8	103937500	0.818182
chr8	104039700	0.809917
chr8	104151100	0.768595
chr8	104164600	0.801653
chr8	104186000	0.892562
chr8	104320300	0.785124
chr8	104349300	0.768595
chr8	104361800	0.793388
chr8	104366500	0.851240

chr8	104385100	0.785124
chr8	104474200	0.818182
chr8	104778200	0.842975
chr8	104844300	0.743802
chr8	105189100	0.826446
chr8	105286300	0.735537
chr8	105386300	0.892562
chr8	105459300	0.859504
chr8	107506700	0.768595
chr8	107513800	0.760331
chr8	107625600	0.768595
chr8	107630500	0.776860
chr8	107870600	0.851240
chr8	109268900	0.793388
chr8	109456300	0.826446
chr8	109481200	0.909091
chr8	110309700	0.768595
chr8	110463300	0.909091
chr8	114450600	0.809917
chr8	116199500	0.752066
chr8	116231500	0.752066
chr8	116441900	0.842975
chr8	116464100	0.818182
chr8	117485000	0.793388
chr8	117722800	0.785124
chr8	117886800	0.834711
chr8	118009500	0.776860
chr8	118525000	0.818182
chr8	118894000	0.809917
chr8	118907400	0.760331
chr8	118947200	0.785124
chr8	118982800	0.867769
chr8	119013300	0.876033
chr8	119017500	0.933884
chr8	119099300	0.776860
chr8	119129300	0.776860
chr8	119294800	0.958678
chr8	119398900	0.801653
chr8	119542700	0.760331
chr8	120090300	0.851240
chr8	120219700	0.809917
chr8	120429100	0.793388
chr8	120627500	0.851240
chr8	120889900	0.768595
chr8	120964500	0.760331
chr8	120995000	0.785124
chr8	121457400	0.826446
chr8	123467400	0.743802
chr8	123574500	0.834711
chr8	123983000	0.826446
chr8	124078400	0.942149
chr8	124173200	0.842975
chr8	124191500	0.760331
chr8	124215600	0.752066

chr8	124263700	0.760331
chr8	124408800	0.851240
chr8	124429200	0.859504
chr8	124542500	0.859504
chr8	124778800	0.735537
chr8	124829100	0.801653
chr8	124850600	0.743802
chr8	125047100	0.909091
chr8	125155600	0.760331
chr8	125260000	0.809917
chr8	125275900	0.793388
chr8	125324000	0.785124
chr8	125377400	0.942149
chr8	125438500	0.743802
chr8	125551600	0.826446
chr8	125577500	0.818182
chr8	125606100	0.760331
chr8	125608300	0.892562
chr8	125625100	0.851240
chr8	125631300	0.793388
chr8	125640700	0.776860
chr8	125654500	0.752066
chr8	125673100	0.818182
chr8	125685700	0.909091
chr8	125699400	0.743802
chr8	125702700	0.834711
chr8	125721900	0.867769
chr8	125764500	0.826446
chr8	125770700	0.826446
chr8	126185900	0.826446
chr8	126188200	0.867769
chr8	126233200	0.760331
chr8	126239900	0.826446
chr8	126298100	0.818182
chr8	126307700	0.809917
chr8	126312400	0.752066
chr8	126341500	0.809917
chr8	126365700	0.743802
chr8	126368100	0.818182
chr8	126404800	0.785124
chr8	126448700	0.768595
chr8	126471900	0.785124
chr8	126520700	0.735537
chr8	126525800	0.809917
chr8	126558400	0.776860
chr8	126592400	0.743802
chr8	126603000	0.950413
chr8	126654900	0.917355
chr8	126657300	0.776860
chr8	126709500	0.735537
chr8	126714100	0.859504
chr8	126719200	0.925620
chr8	126726500	0.826446
chr8	126788300	0.752066

chr8	127385400	0.867769
chr8	127414000	0.735537
chr8	127870300	0.933884
chr8	128149700	0.801653
chr8	128264900	0.818182
chr8	128591000	0.735537
chr8	128621900	0.776860
chr8	128630900	0.884298
chr8	128638100	0.809917
chr8	128646100	0.776860
chr8	128651500	0.809917
chr8	128654200	0.793388
chr8	128666500	0.826446
chr8	128763700	0.851240
chr8	128772300	0.884298
chr8	128796100	0.752066
chr8	128801700	0.867769
chr8	128844200	0.752066
chr8	128895000	0.785124
chr8	128933300	0.735537
chr8	129031200	0.760331
chr8	129121900	0.818182
chr8	129163700	0.917355
chr8	129185300	0.776860
chr8	129188200	0.842975
chr8	129200000	0.826446
chr8	129204000	0.842975
chr8	129219500	0.834711
chr8	129252000	0.818182
chr8	129254700	0.776860
chr8	129279800	0.793388
chr8	129351400	0.942149
chr8	129378900	0.826446
chr8	129694500	0.876033
chr8	130952100	0.842975
chr8	131019600	0.776860
chr8	131049900	0.842975
chr8	131055000	0.933884
chr8	131092200	0.801653
chr8	131156000	0.743802
chr8	131206900	0.876033
chr8	131214000	0.752066
chr8	131282100	0.735537
chr8	131296900	0.900826
chr8	131313900	0.793388
chr8	131327500	0.809917
chr8	131342400	0.776860
chr8	131348500	0.851240
chr8	131353200	0.793388
chr8	131356400	0.752066
chr8	131361400	0.876033
chr8	131374100	0.826446
chr8	131423600	0.768595
chr8	131432400	0.809917

chr8	131450000	0.851240
chr8	131458700	0.859504
chr8	131465500	0.826446
chr8	131470300	0.859504
chr8	131478700	0.942149
chr8	132840300	0.735537
chr8	132870500	0.809917
chr8	132874000	0.859504
chr8	132916500	0.760331
chr8	132926300	0.900826
chr8	132929200	0.826446
chr8	132934900	0.760331
chr8	132937900	0.735537
chr8	132940300	0.809917
chr8	133781000	0.851240
chr8	133799500	0.909091
chr8	133832300	0.801653
chr8	133849000	0.785124
chr8	133854900	0.834711
chr8	133876100	0.768595
chr8	133908100	0.793388
chr8	133930800	0.826446
chr8	134023700	0.743802
chr8	134029800	0.834711
chr8	134055800	0.809917
chr8	134060100	0.917355
chr8	134070200	0.818182
chr8	134105200	0.867769
chr8	134269800	0.793388
chr8	134291900	0.809917
chr8	134403800	0.851240
chr8	134492500	0.859504
chr8	134495400	0.801653
chr8	134517500	0.892562
chr8	134522800	0.876033
chr8	134535000	0.768595
chr8	134544200	0.752066
chr8	134565300	0.867769
chr8	134577000	0.826446
chr8	134658500	0.743802
chr8	134664400	0.876033
chr8	134695900	0.768595
chr8	134701800	0.859504
chr8	134819500	0.760331
chr8	134829200	0.851240
chr8	134906100	0.884298
chr8	134912000	0.884298
chr8	135056200	0.859504
chr8	135278600	0.760331
chr8	135337300	0.760331
chr8	135629300	0.735537
chr8	135634300	0.776860
chr8	135699500	0.735537
chr8	135725800	0.801653

chr8	135804300	0.834711
chr8	135918700	0.743802
chr8	136688900	0.735537
chr8	136769500	0.793388
chr8	137200200	0.743802
chr8	138947200	0.793388
chr8	139175400	0.884298
chr8	140227800	0.892562
chr8	140614200	0.900826
chr8	140718400	0.958678
chr8	141068200	0.818182
chr8	141125500	0.768595
chr8	141161700	0.867769
chr8	141179400	0.760331
chr8	141474900	0.867769
chr8	141477200	0.809917
chr8	141515600	0.900826
chr8	141518800	0.776860
chr8	141594400	0.743802
chr8	141600300	0.768595
chr8	141657000	0.867769
chr8	141996700	0.909091
chr8	142084000	0.752066
chr8	142095300	0.842975
chr8	142109700	0.818182
chr8	142151000	0.818182
chr8	142175100	0.768595
chr8	142185400	0.809917
chr8	142250000	0.752066
chr8	142255700	0.950413
chr8	142551200	0.793388
chr8	143024200	0.859504
chr8	143123000	0.842975
chr8	143408200	0.793388
chr8	143554100	0.851240
chr8	143811600	0.743802
chr8	143820700	0.933884
chr8	143952600	0.752066
chr8	144194600	0.785124
chr8	144218000	0.892562
chr8	144256100	0.743802
chr8	144273000	0.743802
chr8	144293800	0.760331
chr8	144303100	0.909091
chr8	144358600	0.752066
chr8	144484700	0.867769
chr8	144489800	0.834711
chr8	144511000	0.851240
chr8	144635600	0.826446
chr8	144718200	0.768595
chr8	144798900	0.793388
chr8	145012100	0.785124
chr8	145026700	0.950413
chr8	145065300	0.743802

chr8	145068900	0.752066
chr8	145104600	0.785124
chr8	145126200	0.909091
chr8	145150300	0.851240
chr8	145181300	0.867769
chr8	145273700	0.826446
chr8	145537200	0.826446
chr8	145628500	0.735537
chr8	145638700	0.818182
chr8	145973200	0.818182
chr8	146053100	0.884298
chr8	146277900	0.768595
chr9	11300	0.884298
chr9	201000	0.851240
chr9	223100	0.818182
chr9	567800	0.851240
chr9	626700	0.793388
chr9	653800	0.776860
chr9	848000	0.917355
chr9	900200	0.818182
chr9	949800	0.735537
chr9	994500	0.793388
chr9	999300	0.752066
chr9	1005400	0.842975
chr9	1522700	0.801653
chr9	1535400	0.776860
chr9	1760000	0.876033
chr9	1762400	0.776860
chr9	1930500	0.884298
chr9	1948100	0.785124
chr9	1993800	0.743802
chr9	2028100	0.842975
chr9	2030800	0.842975
chr9	2164300	0.760331
chr9	2210000	0.752066
chr9	2229900	0.851240
chr9	2710200	0.743802
chr9	3398600	0.884298
chr9	3731100	0.826446
chr9	3759400	0.801653
chr9	4339600	0.768595
chr9	4422700	0.735537
chr9	4560200	0.776860
chr9	4668600	0.735537
chr9	4887500	0.735537
chr9	4894900	0.818182
chr9	4927700	0.867769
chr9	4959300	0.735537
chr9	4971100	0.851240
chr9	5205500	0.735537
chr9	5278500	0.785124
chr9	5337300	0.801653
chr9	5494300	0.867769
chr9	5554600	0.801653

chr9	5566300	0.760331
chr9	5618400	0.842975
chr9	5684100	0.743802
chr9	5886700	0.752066
chr9	5977200	0.876033
chr9	6024700	0.735537
chr9	6134300	0.809917
chr9	6200400	0.768595
chr9	6239300	0.735537
chr9	6356900	0.768595
chr9	6441400	0.776860
chr9	6595200	0.752066
chr9	6605300	0.809917
chr9	6618000	0.793388
chr9	6628900	0.768595
chr9	6636500	0.809917
chr9	6704200	0.917355
chr9	6716500	0.950413
chr9	6765300	0.859504
chr9	6783500	0.768595
chr9	6869100	0.735537
chr9	6960600	0.776860
chr9	7289900	0.793388
chr9	7293700	0.776860
chr9	7298000	0.842975
chr9	7300500	0.818182
chr9	7342600	0.851240
chr9	7442700	0.900826
chr9	9793100	0.842975
chr9	14156200	0.785124
chr9	14452300	0.768595
chr9	14823500	0.917355
chr9	15180500	0.834711
chr9	15295900	0.743802
chr9	15404900	0.768595
chr9	16510400	0.793388
chr9	17294700	0.975207
chr9	18057400	0.867769
chr9	18757400	0.909091
chr9	18769500	0.900826
chr9	18879000	0.826446
chr9	18890500	0.760331
chr9	19026800	0.793388
chr9	19138900	0.859504
chr9	19156400	0.776860
chr9	19238700	0.983471
chr9	19424800	0.743802
chr9	19451800	0.876033
chr9	19493400	0.818182
chr9	19665700	0.801653
chr9	20280800	0.867769
chr9	20398200	0.842975
chr9	20418400	0.776860
chr9	20464400	0.785124

chr9	20508600	0.818182
chr9	20527300	0.859504
chr9	20530800	0.785124
chr9	20555300	0.752066
chr9	20571100	0.735537
chr9	20975300	0.818182
chr9	20981400	0.917355
chr9	21122400	0.768595
chr9	21142100	0.900826
chr9	21160300	0.834711
chr9	21183800	0.785124
chr9	21195800	0.842975
chr9	21214200	0.826446
chr9	21236400	0.818182
chr9	21295400	0.950413
chr9	21335300	0.892562
chr9	21428700	0.818182
chr9	21433400	0.892562
chr9	21444700	0.859504
chr9	21577400	0.818182
chr9	21808500	0.909091
chr9	21813900	0.876033
chr9	21852900	0.743802
chr9	21943600	0.785124
chr9	21967500	0.743802
chr9	22026700	0.842975
chr9	22029700	0.785124
chr9	22102800	0.867769
chr9	22171300	0.942149
chr9	22240000	0.818182
chr9	26727600	0.776860
chr9	26892800	0.884298
chr9	26947600	0.867769
chr9	27371200	0.900826
chr9	27384200	0.768595
chr9	27387300	0.900826
chr9	27442100	0.851240
chr9	27461000	0.876033
chr9	27500000	0.735537
chr9	31347200	0.834711
chr9	31446300	0.785124
chr9	32604300	0.909091
chr9	32906500	0.867769
chr9	33065700	0.785124
chr9	33221900	0.752066
chr9	33265400	0.826446
chr9	33304300	0.743802
chr9	33415600	0.826446
chr9	33862800	0.743802
chr9	33939600	0.834711
chr9	33955100	0.785124
chr9	34049500	0.809917
chr9	34128600	0.760331
chr9	34198300	0.752066

chr9	34220200	0.925620
chr9	34329700	0.826446
chr9	34452200	0.842975
chr9	34454900	0.752066
chr9	34629200	0.892562
chr9	34652700	0.785124
chr9	34687700	0.793388
chr9	34784400	0.876033
chr9	34862800	0.760331
chr9	34885400	0.793388
chr9	34957200	0.900826
chr9	34962700	0.760331
chr9	34987100	0.933884
chr9	35096400	0.950413
chr9	35322400	0.867769
chr9	35341500	0.842975
chr9	35621800	0.743802
chr9	35652400	0.776860
chr9	35680900	0.842975
chr9	35757300	0.752066
chr9	35806900	0.752066
chr9	35815000	0.867769
chr9	35826400	0.801653
chr9	35831300	0.826446
chr9	35933600	0.826446
chr9	35946100	0.851240
chr9	35977100	0.793388
chr9	36093200	0.752066
chr9	36140800	0.892562
chr9	36145800	0.743802
chr9	36154100	0.768595
chr9	36191300	0.768595
chr9	36258300	0.752066
chr9	36470800	0.785124
chr9	36488500	0.925620
chr9	36603200	0.834711
chr9	36856800	0.801653
chr9	36932300	0.793388
chr9	37075100	0.785124
chr9	37080400	0.776860
chr9	37374900	0.752066
chr9	37531800	0.942149
chr9	37574100	0.826446
chr9	37694600	0.801653
chr9	37951800	0.801653
chr9	38030800	0.826446
chr9	38062100	0.760331
chr9	38178100	0.776860
chr9	38339200	0.809917
chr9	38388400	0.867769
chr9	38409300	0.859504
chr9	38690500	0.818182
chr9	39475100	0.776860
chr9	40802400	0.842975

chr9	41619900	0.867769
chr9	41907600	0.776860
chr9	42019200	0.768595
chr9	42108500	0.834711
chr9	42242500	0.768595
chr9	42379300	0.752066
chr9	42416000	0.859504
chr9	42587400	0.842975
chr9	42847700	0.743802
chr9	42849800	0.859504
chr9	42856100	0.776860
chr9	42915000	0.925620
chr9	43011300	0.743802
chr9	44070500	0.752066
chr9	44245500	0.818182
chr9	44550700	0.752066
chr9	45351100	0.867769
chr9	45370000	0.884298
chr9	45451500	0.834711
chr9	45583600	0.768595
chr9	45740700	0.909091
chr9	45769300	0.859504
chr9	46110900	0.809917
chr9	46599000	0.826446
chr9	46687400	0.735537
chr9	46989800	0.743802
chr9	66050500	0.752066
chr9	66250300	0.768595
chr9	66463400	0.876033
chr9	66466500	0.760331
chr9	66469100	0.909091
chr9	66471900	0.776860
chr9	66507200	0.942149
chr9	66842400	0.793388
chr9	67029400	0.735537
chr9	67233000	0.809917
chr9	67294200	0.917355
chr9	67297800	0.801653
chr9	67318600	0.826446
chr9	67796800	0.859504
chr9	68157400	0.826446
chr9	68380600	0.834711
chr9	68395000	0.809917
chr9	68403100	0.842975
chr9	68409300	0.867769
chr9	68428000	0.826446
chr9	68484500	0.958678
chr9	68504300	0.743802
chr9	68678800	0.760331
chr9	68688500	0.867769
chr9	68691800	0.834711
chr9	68706200	0.743802
chr9	68735900	0.818182
chr9	69017100	0.801653

chr9	69030300	0.917355
chr9	69035400	0.909091
chr9	69074500	0.851240
chr9	69196700	0.776860
chr9	69200700	0.735537
chr9	69594700	0.760331
chr9	69653400	0.851240
chr9	70878600	0.760331
chr9	71199600	0.842975
chr9	71256900	0.867769
chr9	71261000	0.834711
chr9	71269400	0.785124
chr9	71293800	0.760331
chr9	71378300	0.793388
chr9	71457100	0.785124
chr9	71511300	0.826446
chr9	71549000	0.826446
chr9	71558900	0.925620
chr9	71600600	0.752066
chr9	71628200	0.867769
chr9	71645600	0.752066
chr9	71770900	0.818182
chr9	71797000	0.859504
chr9	71802000	0.743802
chr9	71808300	0.867769
chr9	71887900	0.818182
chr9	72504500	0.743802
chr9	73179200	0.884298
chr9	74159900	0.776860
chr9	74164500	0.842975
chr9	74262300	0.876033
chr9	74381700	0.760331
chr9	74474500	0.785124
chr9	74552400	0.743802
chr9	74557000	0.735537
chr9	74654700	0.752066
chr9	74888900	0.743802
chr9	74894400	0.892562
chr9	75124700	0.925620
chr9	75250500	0.768595
chr9	75264000	0.793388
chr9	75641800	0.834711
chr9	75747600	0.876033
chr9	75772100	0.785124
chr9	75795000	0.752066
chr9	77115300	0.801653
chr9	77204700	0.793388
chr9	77387400	0.859504
chr9	77392400	0.735537
chr9	77463400	0.876033
chr9	77502300	0.752066
chr9	77567800	0.793388
chr9	77616500	0.917355
chr9	77669500	0.801653

chr9	78054100	0.776860
chr9	78070800	0.760331
chr9	78086700	0.760331
chr9	78620000	0.735537
chr9	78985200	0.785124
chr9	78988700	0.867769
chr9	79048800	0.785124
chr9	79057200	0.933884
chr9	79071800	0.785124
chr9	79082300	0.826446
chr9	79132300	0.859504
chr9	79174000	0.876033
chr9	79221000	0.867769
chr9	79242400	0.818182
chr9	79244900	0.752066
chr9	79258700	0.876033
chr9	79296200	0.801653
chr9	79627500	0.785124
chr9	79712200	0.752066
chr9	80177700	0.735537
chr9	80347100	0.842975
chr9	80457100	0.760331
chr9	80719300	0.826446
chr9	80826000	0.925620
chr9	81323000	0.818182
chr9	81430700	0.917355
chr9	82180800	0.867769
chr9	82199400	0.760331
chr9	82239300	0.793388
chr9	82288700	0.785124
chr9	82310500	0.768595
chr9	82346600	0.752066
chr9	82449800	0.842975
chr9	84217100	0.793388
chr9	86087000	0.867769
chr9	86238700	0.826446
chr9	86263600	0.735537
chr9	86603400	0.859504
chr9	86754400	0.735537
chr9	86881400	0.743802
chr9	86905700	0.752066
chr9	86911600	0.743802
chr9	87385300	0.768595
chr9	87436600	0.785124
chr9	87582400	0.859504
chr9	87620400	0.760331
chr9	87680600	0.876033
chr9	87682900	0.933884
chr9	87904700	0.793388
chr9	88048200	0.785124
chr9	88058700	0.933884
chr9	88345900	0.909091
chr9	88511000	0.743802
chr9	88956900	0.768595

chr9	88969000	0.900826
chr9	89892300	0.933884
chr9	89989500	0.793388
chr9	90079700	0.809917
chr9	90095100	0.834711
chr9	90106300	0.760331
chr9	90110200	0.768595
chr9	90133300	0.892562
chr9	90183600	0.801653
chr9	90341300	0.776860
chr9	90408300	0.776860
chr9	90589600	0.776860
chr9	91193600	0.793388
chr9	91537100	0.842975
chr9	91681500	0.785124
chr9	91850300	0.809917
chr9	91892600	0.900826
chr9	91937400	0.818182
chr9	91955700	0.900826
chr9	92053200	0.793388
chr9	92064400	0.735537
chr9	92087000	0.785124
chr9	92123200	0.892562
chr9	92272700	0.776860
chr9	92296100	0.942149
chr9	92301800	0.743802
chr9	92306000	0.826446
chr9	92325900	0.735537
chr9	92331400	0.900826
chr9	92450700	0.859504
chr9	92455500	0.851240
chr9	92484000	0.785124
chr9	92747800	0.785124
chr9	92762000	0.785124
chr9	92841800	0.867769
chr9	92858600	0.735537
chr9	93022200	0.826446
chr9	93267900	0.809917
chr9	93278200	0.760331
chr9	93315100	0.752066
chr9	93562100	0.735537
chr9	93598100	0.785124
chr9	93604800	0.892562
chr9	93672200	0.884298
chr9	93697100	0.785124
chr9	93727200	0.909091
chr9	93796600	0.760331
chr9	93799400	0.752066
chr9	93862400	0.793388
chr9	93927400	0.818182
chr9	93940500	0.785124
chr9	93957200	0.752066
chr9	93962400	0.818182
chr9	93970900	0.826446

chr9	93975100	0.818182
chr9	94037500	0.752066
chr9	94158100	0.735537
chr9	94449000	0.752066
chr9	94483400	0.785124
chr9	94561500	0.760331
chr9	94877700	0.859504
chr9	94896600	0.809917
chr9	94927900	0.752066
chr9	94935800	0.768595
chr9	95088100	0.842975
chr9	95203400	0.925620
chr9	95273200	0.785124
chr9	95484800	0.760331
chr9	95527600	0.917355
chr9	95553400	0.867769
chr9	95583800	0.793388
chr9	95632800	0.876033
chr9	95680100	0.752066
chr9	95717000	0.768595
chr9	95857400	0.752066
chr9	95890300	0.793388
chr9	95894000	0.785124
chr9	95906700	0.818182
chr9	95911700	0.867769
chr9	95919300	0.884298
chr9	95988400	0.925620
chr9	96120300	0.776860
chr9	96226800	0.818182
chr9	96338600	0.752066
chr9	96936900	0.876033
chr9	96942000	0.851240
chr9	96944900	0.809917
chr9	97094900	0.743802
chr9	97109700	0.818182
chr9	97165300	0.752066
chr9	97339300	0.909091
chr9	97638100	0.785124
chr9	97952500	0.826446
chr9	97956400	0.760331
chr9	98079800	0.950413
chr9	98182600	0.735537
chr9	98203700	0.735537
chr9	98324200	0.867769
chr9	98487400	0.776860
chr9	98513100	0.834711
chr9	98520100	0.876033
chr9	98525000	0.917355
chr9	98564500	0.735537
chr9	98618500	0.735537
chr9	98622100	0.867769
chr9	98779400	0.785124
chr9	98785400	0.743802
chr9	98909800	0.752066

chr9	98919600	0.818182
chr9	99003800	0.768595
chr9	99015600	0.809917
chr9	99247700	0.785124
chr9	99753500	0.752066
chr9	100062200	0.925620
chr9	100121600	0.743802
chr9	100130100	0.826446
chr9	100147900	0.851240
chr9	100155800	0.851240
chr9	100169500	0.752066
chr9	100179500	0.917355
chr9	100194500	0.735537
chr9	100231600	0.842975
chr9	100269100	0.809917
chr9	100277800	0.826446
chr9	100684800	0.892562
chr9	100760300	0.826446
chr9	100797600	0.884298
chr9	100819300	0.876033
chr9	100931700	0.818182
chr9	100968300	0.801653
chr9	101158500	0.768595
chr9	101253800	0.867769
chr9	101335000	0.859504
chr9	101737500	0.760331
chr9	101820600	0.842975
chr9	101839800	0.826446
chr9	101848800	0.793388
chr9	101887000	0.801653
chr9	102016100	0.884298
chr9	102254800	0.818182
chr9	102272600	0.818182
chr9	102279700	0.785124
chr9	102324200	0.743802
chr9	102368600	0.818182
chr9	102482900	0.950413
chr9	102485600	0.785124
chr9	102525300	0.842975
chr9	102631300	0.859504
chr9	102829300	0.818182
chr9	102850700	0.818182
chr9	103212800	0.752066
chr9	103318400	0.851240
chr9	103930400	0.785124
chr9	107513300	0.842975
chr9	107543000	0.743802
chr9	107546400	0.834711
chr9	107626300	0.809917
chr9	107631500	0.793388
chr9	107684100	0.785124
chr9	107877700	0.743802
chr9	107890400	0.851240
chr9	107954900	0.892562

chr9	108005300	0.851240
chr9	108013700	0.776860
chr9	108071400	0.809917
chr9	108104800	0.752066
chr9	108120700	0.909091
chr9	108168800	0.768595
chr9	108282100	0.917355
chr9	108402200	0.735537
chr9	108424900	0.785124
chr9	108436000	0.793388
chr9	108646500	0.867769
chr9	109184400	0.884298
chr9	109266100	0.760331
chr9	109782400	0.818182
chr9	109786800	0.752066
chr9	109802500	0.867769
chr9	109809600	0.867769
chr9	109877400	0.859504
chr9	110946300	0.818182
chr9	111518700	0.818182
chr9	111757300	0.743802
chr9	111849000	0.735537
chr9	111861100	0.752066
chr9	111871500	0.900826
chr9	111906700	0.826446
chr9	111926900	0.760331
chr9	111944800	0.900826
chr9	111981300	0.958678
chr9	112074800	0.892562
chr9	112113800	0.876033
chr9	112118700	0.785124
chr9	112131600	0.884298
chr9	112140900	0.785124
chr9	112147800	0.760331
chr9	112190000	0.801653
chr9	112203900	0.900826
chr9	112210300	0.776860
chr9	112226500	0.809917
chr9	112670200	0.785124
chr9	112739400	0.900826
chr9	112818300	0.752066
chr9	112825900	0.776860
chr9	112867500	0.851240
chr9	112872800	0.785124
chr9	112890100	0.760331
chr9	112922100	0.752066
chr9	113389400	0.785124
chr9	113637800	0.760331
chr9	113696100	0.859504
chr9	113717400	0.867769
chr9	113779200	0.760331
chr9	113827800	0.818182
chr9	113942100	0.867769
chr9	114240400	0.752066

chr9	114246700	0.809917
chr9	114633500	0.867769
chr9	114649800	0.892562
chr9	114700700	0.851240
chr9	115085400	0.826446
chr9	115124200	0.818182
chr9	115282900	0.818182
chr9	115368200	0.884298
chr9	115506400	0.867769
chr9	115516900	0.842975
chr9	115523800	0.760331
chr9	115534700	0.809917
chr9	115580200	0.785124
chr9	115607900	0.842975
chr9	115624000	0.760331
chr9	115665700	0.826446
chr9	115969300	0.760331
chr9	115974300	0.760331
chr9	115995300	0.801653
chr9	116038300	0.752066
chr9	116173300	0.776860
chr9	116178900	0.768595
chr9	116247200	0.785124
chr9	116276700	0.785124
chr9	116378000	0.892562
chr9	116420000	0.735537
chr9	116452900	0.776860
chr9	116620800	0.818182
chr9	116638300	0.826446
chr9	117155800	0.768595
chr9	117167400	0.752066
chr9	117172300	0.768595
chr9	117211300	0.735537
chr9	117214300	0.818182
chr9	117280800	0.776860
chr9	117453800	0.743802
chr9	117457500	0.809917
chr9	117460800	0.842975
chr9	117481500	0.776860
chr9	117568800	0.876033
chr9	117670800	0.752066
chr9	117672900	0.925620
chr9	117692600	0.785124
chr9	117762700	0.785124
chr9	117799100	0.851240
chr9	117805800	0.900826
chr9	119143600	0.760331
chr9	119716900	0.867769
chr9	119721800	0.884298
chr9	120440500	0.735537
chr9	120467000	0.867769
chr9	120481000	0.900826
chr9	120540800	0.801653
chr9	122061000	0.760331

chr9	122499300	0.809917
chr9	123342700	0.842975
chr9	123605500	0.760331
chr9	123645300	0.752066
chr9	123659400	0.909091
chr9	124034300	0.834711
chr9	124116400	0.768595
chr9	124218500	0.793388
chr9	124248600	0.801653
chr9	124256500	0.884298
chr9	124260900	0.752066
chr9	124397100	0.735537
chr9	124501100	0.752066
chr9	124605100	0.735537
chr9	124692900	0.842975
chr9	124735300	0.884298
chr9	124814400	0.842975
chr9	124857900	0.834711
chr9	124976500	0.768595
chr9	125048900	0.776860
chr9	125089900	0.776860
chr9	125099800	0.809917
chr9	125130000	0.842975
chr9	125134700	0.735537
chr9	125152700	0.876033
chr9	125598000	0.760331
chr9	125663300	0.859504
chr9	125694100	0.752066
chr9	125980100	0.876033
chr9	126038000	0.834711
chr9	126045100	0.842975
chr9	126053800	0.735537
chr9	126067300	0.909091
chr9	126082300	0.867769
chr9	126099000	0.743802
chr9	126116800	0.991736
chr9	126121900	0.900826
chr9	126311700	0.760331
chr9	126325600	0.785124
chr9	126439900	0.743802
chr9	126450500	0.818182
chr9	126460300	0.917355
chr9	126597800	0.793388
chr9	126634400	0.752066
chr9	126693800	0.752066
chr9	126716600	0.867769
chr9	126722500	0.859504
chr9	126783600	0.851240
chr9	126803400	0.859504
chr9	126849000	0.793388
chr9	126908700	0.752066
chr9	126969300	0.752066
chr9	127182600	0.776860
chr9	127384600	0.867769

chr9	127402500	0.760331
chr9	127450900	0.818182
chr9	127533500	0.834711
chr9	127637700	0.735537
chr9	127962700	0.752066
chr9	128004400	0.826446
chr9	128024200	0.760331
chr9	128172400	0.876033
chr9	128190900	0.809917
chr9	128447000	0.851240
chr9	128586600	0.859504
chr9	129160300	0.859504
chr9	129262200	0.735537
chr9	129429600	0.743802
chr9	129467900	0.776860
chr9	129482300	0.752066
chr9	129508600	0.752066
chr9	129606300	0.867769
chr9	129630500	0.743802
chr9	129762700	0.752066
chr9	129768400	0.826446
chr9	129782600	0.900826
chr9	129811600	0.842975
chr9	129822200	0.884298
chr9	129830900	0.809917
chr9	129947000	0.818182
chr9	129983300	0.752066
chr9	130039700	0.842975
chr9	130341800	0.743802
chr9	130371000	0.834711
chr9	130462100	0.834711
chr9	130565700	0.859504
chr9	130628500	0.768595
chr9	130641000	0.743802
chr9	130660000	0.826446
chr9	130681500	0.760331
chr9	130684200	0.884298
chr9	130735300	0.793388
chr9	130743600	0.768595
chr9	130754000	0.809917
chr9	130890400	0.785124
chr9	130922400	0.793388
chr9	130952100	0.809917
chr9	131124700	0.851240
chr9	131127800	0.818182
chr9	131318900	0.735537
chr9	131406200	0.966942
chr9	131419100	0.892562
chr9	131685000	0.818182
chr9	131710400	0.909091
chr9	131783400	0.909091
chr9	131933200	0.818182
chr9	131947600	0.842975
chr9	131977500	0.834711

chr9	131984000	0.760331
chr9	131992500	0.743802
chr9	131998400	0.768595
chr9	132021100	0.801653
chr9	132024300	0.768595
chr9	132083300	0.925620
chr9	132091000	0.768595
chr9	132148100	0.851240
chr9	132176200	0.818182
chr9	132246500	0.735537
chr9	132360900	0.818182
chr9	132387400	0.752066
chr9	132515600	0.785124
chr9	132699400	0.826446
chr9	132728200	0.743802
chr9	132807700	0.818182
chr9	132816300	0.851240
chr9	132835800	0.884298
chr9	132998800	0.785124
chr9	133298900	0.851240
chr9	133572800	0.752066
chr9	133575200	0.752066
chr9	133588500	0.834711
chr9	133715400	0.743802
chr9	133801500	0.793388
chr9	134113800	0.834711
chr9	134248700	0.842975
chr9	134259300	0.776860
chr9	134280000	0.942149
chr9	134291400	0.760331
chr9	134507900	0.785124
chr9	134562700	0.743802
chr9	134577400	0.826446
chr9	134634500	0.925620
chr9	134853400	0.942149
chr9	134857200	0.842975
chr9	134859300	0.859504
chr9	134877400	0.834711
chr9	134883700	0.776860
chr9	134910700	0.925620
chr9	135145200	0.809917
chr9	135248700	0.752066
chr9	135348000	0.801653
chr9	135724600	0.859504
chr9	135764400	0.743802
chr9	135820300	0.917355
chr9	135870500	0.801653
chr9	135898600	0.818182
chr9	135909400	0.768595
chr9	135994700	0.818182
chr9	135997200	0.876033
chr9	136021000	0.776860
chr9	136240400	0.834711
chr9	136242600	0.859504

chr9	136283200	0.925620
chr9	136343700	0.917355
chr9	136405700	0.809917
chr9	136485400	0.768595
chr9	136574900	0.752066
chr9	136648200	0.760331
chr9	136766300	0.760331
chr9	136827200	0.917355
chr9	136925500	0.801653
chr9	137029900	0.785124
chr9	137136300	0.884298
chr9	137160600	0.793388
chr9	137218500	0.809917
chr9	137658400	0.735537
chr9	137663700	0.760331
chr9	137671200	0.884298
chr9	137718600	0.768595
chr9	137745600	0.801653
chr9	137750800	0.851240
chr9	137788400	0.801653
chr9	137823200	0.752066
chr9	137942800	0.801653
chr9	138155700	0.867769
chr9	138392700	0.801653
chr9	138803000	0.785124
chr9	138996800	0.776860
chr9	138999200	0.818182
chr9	139011200	0.842975
chr9	139089300	0.768595
chr9	139117800	0.851240
chr9	139258000	0.933884
chr9	139378200	0.884298
chr9	139424800	0.801653
chr9	139441200	0.925620
chr9	139459900	0.859504
chr9	139483400	0.900826
chr9	139513900	0.809917
chr9	139525700	0.768595
chr9	139539800	0.760331
chr9	139591400	0.842975
chr9	139598300	0.735537
chr9	139636400	0.818182
chr9	139781500	0.884298
chr9	139891400	0.933884
chr9	139927500	0.859504
chr9	139930300	0.900826
chr9	139990600	0.933884
chr9	140053900	0.834711
chr9	140065300	0.859504
chr9	140129800	0.785124
chr9	140176200	0.760331
chr9	140210900	0.859504
chr9	140306400	0.768595
chr9	140324800	0.760331

chr9	140377100	0.818182
chr9	140408400	0.735537
chr9	140429700	0.768595
chr9	140567800	0.809917
chr9	140610700	0.925620
chr9	140620800	0.892562
chr9	140761400	0.768595
chrX	289600	0.925620
chrX	300600	0.884298
chrX	347900	0.892562
chrX	569300	0.859504
chrX	628800	0.925620
chrX	951300	0.892562
chrX	1332300	0.950413
chrX	1401300	0.743802
chrX	1455800	0.892562
chrX	1474200	0.867769
chrX	1499200	0.925620
chrX	1567000	0.752066
chrX	1576600	0.793388
chrX	1590900	0.735537
chrX	1627200	0.933884
chrX	1650900	0.826446
chrX	1674300	0.752066
chrX	1684400	0.851240
chrX	1690200	0.884298
chrX	1695800	0.876033
chrX	1711300	0.834711
chrX	1776700	0.867769
chrX	1780700	0.925620
chrX	1854700	0.818182
chrX	2151700	0.809917
chrX	2248300	0.876033
chrX	2445200	0.752066
chrX	2460100	0.826446
chrX	2533700	0.909091
chrX	2616600	0.842975
chrX	2620000	0.801653
chrX	2627500	0.801653
chrX	2700200	0.826446
chrX	2730700	0.785124
chrX	2816500	0.842975
chrX	3618300	0.925620
chrX	5563000	0.933884
chrX	5565300	0.867769
chrX	5567900	0.760331
chrX	7123600	0.760331
chrX	7182900	0.818182
chrX	7895600	0.876033
chrX	7956400	0.768595
chrX	8006300	0.826446
chrX	8017000	0.752066
chrX	9321200	0.785124
chrX	9542300	0.743802

chrX	9707500	0.818182
chrX	10054300	0.809917
chrX	10068400	0.735537
chrX	10136400	0.834711
chrX	10145000	0.752066
chrX	11126900	0.760331
chrX	11129600	0.859504
chrX	11370400	0.768595
chrX	11843300	0.785124
chrX	12516200	0.842975
chrX	12629800	0.785124
chrX	12891900	0.752066
chrX	12901200	0.768595
chrX	13142100	0.818182
chrX	13506100	0.925620
chrX	13677200	0.925620
chrX	13703800	0.826446
chrX	13762800	0.793388
chrX	13872700	0.752066
chrX	13886500	0.851240
chrX	13909700	0.859504
chrX	14048100	0.768595
chrX	14690000	0.768595
chrX	15091500	0.859504
chrX	15169900	0.809917
chrX	15456900	0.776860
chrX	15584500	0.859504
chrX	15693500	0.826446
chrX	15800500	0.884298
chrX	15921800	0.743802
chrX	15990800	0.760331
chrX	16204600	0.793388
chrX	16730700	0.752066
chrX	16911400	0.809917
chrX	16989800	0.842975
chrX	17039600	0.909091
chrX	17755700	0.801653
chrX	18693500	0.933884
chrX	18874800	0.801653
chrX	18904300	0.760331
chrX	19003200	0.793388
chrX	19093900	0.876033
chrX	19216900	0.793388
chrX	19230700	0.776860
chrX	19496100	0.975207
chrX	19795700	0.900826
chrX	19826600	0.743802
chrX	19830200	0.884298
chrX	19851600	0.909091
chrX	19885400	0.793388
chrX	20079900	0.735537
chrX	20393900	0.735537
chrX	20397400	0.760331
chrX	20436800	0.809917

chrX	20464000	0.834711
chrX	20466400	0.735537
chrX	20471000	0.851240
chrX	22011800	0.809917
chrX	23686200	0.793388
chrX	24044200	0.842975
chrX	24177900	0.768595
chrX	24253400	0.909091
chrX	24498700	0.917355
chrX	24973400	0.785124
chrX	25036000	0.735537
chrX	27827700	0.884298
chrX	29000700	0.752066
chrX	29496500	0.900826
chrX	29503800	0.785124
chrX	29507400	0.752066
chrX	29546900	0.851240
chrX	29605900	0.884298
chrX	32462500	0.876033
chrX	37537400	0.760331
chrX	37636200	0.793388
chrX	38049100	0.876033
chrX	38509400	0.760331
chrX	38516700	0.776860
chrX	38777000	0.743802
chrX	38830100	0.809917
chrX	39008800	0.809917
chrX	39020400	0.760331
chrX	39027400	0.735537
chrX	39034100	0.801653
chrX	39196300	0.760331
chrX	39353500	0.793388
chrX	39437800	0.809917
chrX	39476000	0.859504
chrX	39688000	0.851240
chrX	39722600	0.826446
chrX	39730200	0.785124
chrX	39768200	0.785124
chrX	39882000	0.917355
chrX	39886500	0.785124
chrX	40008400	0.859504
chrX	40048700	0.785124
chrX	40332700	0.900826
chrX	40430500	0.760331
chrX	40618800	0.735537
chrX	41117500	0.917355
chrX	41302100	0.851240
chrX	41377800	0.900826
chrX	41456600	0.867769
chrX	41547200	0.735537
chrX	41566800	0.752066
chrX	41594600	0.743802
chrX	42475800	0.818182
chrX	42725600	0.785124

chrX	42856600	0.768595
chrX	42863200	0.801653
chrX	43513700	0.859504
chrX	44141700	0.925620
chrX	44402200	0.851240
chrX	44865400	0.752066
chrX	44931600	0.859504
chrX	45574800	0.801653
chrX	45620400	0.735537
chrX	46405300	0.909091
chrX	46456900	0.776860
chrX	46744400	0.809917
chrX	47420600	0.743802
chrX	47518500	0.909091
chrX	47863200	0.942149
chrX	47880800	0.975207
chrX	47931100	0.818182
chrX	48367000	0.826446
chrX	48456200	0.950413
chrX	48555600	0.826446
chrX	48595900	0.851240
chrX	48660900	0.925620
chrX	48755200	0.851240
chrX	48858800	0.942149
chrX	48900300	0.909091
chrX	48937700	0.950413
chrX	49023700	0.925620
chrX	49026400	0.752066
chrX	49610900	0.809917
chrX	49675200	0.892562
chrX	49933600	0.785124
chrX	51698700	0.834711
chrX	52953000	0.876033
chrX	52964200	0.876033
chrX	53025000	0.851240
chrX	53254500	0.826446
chrX	53317100	0.826446
chrX	53449300	0.900826
chrX	53745600	0.826446
chrX	54071500	0.818182
chrX	54209600	0.842975
chrX	54666400	0.826446
chrX	54774800	0.760331
chrX	55187700	0.809917
chrX	55744600	0.793388
chrX	56794500	0.892562
chrX	56797200	0.876033
chrX	56800100	0.892562
chrX	56805400	0.809917
chrX	56809800	0.933884
chrX	56829700	0.925620
chrX	56840100	0.818182
chrX	57618700	0.892562
chrX	57937000	0.785124

chrX	61745700	0.818182
chrX	62961500	0.884298
chrX	62977400	0.776860
chrX	63426400	0.834711
chrX	64815900	0.752066
chrX	64971700	0.735537
chrX	64989500	0.826446
chrX	65294800	0.793388
chrX	67718800	0.909091
chrX	70323700	0.743802
chrX	70503800	0.735537
chrX	70586700	0.826446
chrX	70849600	0.900826
chrX	70883700	0.809917
chrX	70922800	0.801653
chrX	70940500	0.793388
chrX	70981100	0.752066
chrX	71325900	0.859504
chrX	71341700	0.776860
chrX	71365600	0.900826
chrX	71401200	0.768595
chrX	71496800	0.892562
chrX	71529300	0.818182
chrX	71569800	0.809917
chrX	71748000	0.867769
chrX	71841400	0.818182
chrX	73071700	0.909091
chrX	73207300	0.735537
chrX	73512000	0.867769
chrX	73726500	0.752066
chrX	73754700	0.743802
chrX	74145100	0.809917
chrX	74376000	0.876033
chrX	75371600	0.867769
chrX	75393200	0.743802
chrX	77041500	0.842975
chrX	77321500	0.776860
chrX	77771300	0.752066
chrX	78009200	0.826446
chrX	78011900	0.834711
chrX	78363600	0.900826
chrX	78394700	0.884298
chrX	83757100	0.867769
chrX	92051300	0.909091
chrX	96581700	0.785124
chrX	96658200	0.752066
chrX	96750400	0.752066
chrX	96942700	0.735537
chrX	99880900	0.793388
chrX	99942200	0.809917
chrX	100085700	0.859504
chrX	100306900	0.851240
chrX	100663600	0.785124
chrX	100993100	0.768595

chrX	101636300	0.834711
chrX	102942500	0.925620
chrX	103401500	0.826446
chrX	106361800	0.851240
chrX	106709700	0.760331
chrX	106954600	0.752066
chrX	106965200	0.785124
chrX	106973000	0.735537
chrX	108515900	0.826446
chrX	108780600	0.867769
chrX	108976300	0.752066
chrX	109026700	0.809917
chrX	109168500	0.917355
chrX	109234200	0.768595
chrX	109561700	0.884298
chrX	109657700	0.752066
chrX	109674300	0.743802
chrX	109687200	0.809917
chrX	110924800	0.933884
chrX	112093100	0.851240
chrX	112118700	0.735537
chrX	112543300	0.917355
chrX	114972700	0.925620
chrX	114987600	0.801653
chrX	115004900	0.925620
chrX	117631100	0.809917
chrX	117662300	0.793388
chrX	117858700	0.743802
chrX	118149900	0.925620
chrX	118206100	0.859504
chrX	118602700	0.933884
chrX	118655800	0.785124
chrX	118683700	0.851240
chrX	118740600	0.917355
chrX	118813000	0.752066
chrX	118898300	0.743802
chrX	119170800	0.818182
chrX	119385300	0.818182
chrX	119602700	0.867769
chrX	119619800	0.809917
chrX	119738200	0.818182
chrX	119764200	0.785124
chrX	120181600	0.735537
chrX	120295800	0.793388
chrX	122547800	0.917355
chrX	122558700	0.809917
chrX	122834200	0.851240
chrX	122866800	0.842975
chrX	122933600	0.743802
chrX	123103000	0.760331
chrX	123623000	0.776860
chrX	123638700	0.867769
chrX	128771900	0.776860
chrX	128903300	0.752066

chrX	128911300	0.826446
chrX	129226200	0.785124
chrX	129244100	0.834711
chrX	129249500	0.859504
chrX	129456100	0.768595
chrX	129687200	0.826446
chrX	130863200	0.950413
chrX	130875700	0.942149
chrX	130881200	0.859504
chrX	130886900	0.958678
chrX	130913000	0.892562
chrX	130918300	0.735537
chrX	130927200	0.917355
chrX	130947200	0.842975
chrX	130951200	0.859504
chrX	130956400	0.909091
chrX	130962600	0.851240
chrX	130965200	0.942149
chrX	131121700	0.975207
chrX	133503000	0.768595
chrX	133972800	0.892562
chrX	135709500	0.760331
chrX	135717900	0.735537
chrX	135725600	0.776860
chrX	135792800	0.909091
chrX	135866200	0.785124
chrX	135962600	0.801653
chrX	138877600	0.851240
chrX	138934800	0.925620
chrX	139015100	0.752066
chrX	142070300	0.826446
chrX	146993900	0.834711
chrX	147541900	0.859504
chrX	147691700	0.876033
chrX	147694500	0.735537
chrX	147930600	0.876033
chrX	148558800	0.859504
chrX	148622800	0.876033
chrX	148889800	0.752066
chrX	148959200	0.925620
chrX	149374800	0.768595
chrX	149388100	0.776860
chrX	149647000	0.809917
chrX	149712100	0.809917
chrX	150731200	0.983471
chrX	152078800	0.876033
chrX	152238200	0.801653
chrX	152559900	0.768595
chrX	152740300	0.760331
chrX	152953500	0.818182
chrX	153001200	0.752066
chrX	153251100	0.876033
chrX	153342400	0.809917
chrX	153541800	0.826446

chrX	153549900	0.884298
chrX	153608300	0.760331
chrX	153665300	0.925620
chrX	153707700	0.950413
chrX	153755500	0.809917
chrX	153771800	0.768595
chrX	153776200	0.834711
chrX	153947400	0.809917
chrX	153955200	0.752066
chrX	154842200	0.917355

H1 enhancers

Chromosome	Location	Voting_cutoff
chr10	353200	0.727273
chr10	440900	0.793388
chr10	531200	0.933884
chr10	537000	0.917355
chr10	593600	0.876033
chr10	645000	0.669421
chr10	654600	0.652893
chr10	670800	0.900826
chr10	672900	0.727273
chr10	683000	0.776860
chr10	688600	0.702479
chr10	902900	0.760331
chr10	957000	0.760331
chr10	1019000	0.801653
chr10	1092300	0.818182
chr10	1147300	0.644628
chr10	1156900	0.826446
chr10	1241900	0.644628
chr10	1245300	0.892562
chr10	1335900	0.892562
chr10	1351300	0.942149
chr10	1354300	0.917355
chr10	1411700	0.933884
chr10	1526400	0.669421
chr10	1564900	0.727273
chr10	1654700	0.677686
chr10	1720000	0.859504
chr10	1758700	0.752066
chr10	1773800	0.776860
chr10	1797500	0.677686
chr10	1817200	0.752066
chr10	1828500	0.661157
chr10	2244400	0.884298
chr10	2519600	0.826446
chr10	2688500	0.719008
chr10	2841400	0.652893
chr10	2960700	0.900826
chr10	2976400	0.785124
chr10	3006600	0.933884
chr10	3088600	0.876033
chr10	3120700	0.884298
chr10	3133100	0.966942
chr10	3137400	0.694215
chr10	3216300	0.768595
chr10	3239900	0.644628
chr10	3313400	0.942149
chr10	3316500	0.727273
chr10	3495000	0.867769
chr10	3529300	0.818182
chr10	3539000	0.719008
chr10	3542900	0.661157

chr10	3545200	0.644628
chr10	3742400	0.677686
chr10	3788000	0.702479
chr10	3804500	0.768595
chr10	3818100	0.851240
chr10	3872100	0.677686
chr10	3915200	0.644628
chr10	3929800	0.925620
chr10	3962200	0.685950
chr10	3998100	0.834711
chr10	4003200	0.644628
chr10	4126300	0.644628
chr10	4284400	0.809917
chr10	4493600	0.661157
chr10	4986800	0.818182
chr10	5024500	0.727273
chr10	5137000	0.677686
chr10	5336500	0.793388
chr10	5459300	0.743802
chr10	5508700	0.793388
chr10	5519700	0.884298
chr10	5531300	0.760331
chr10	5629300	0.677686
chr10	5701500	0.793388
chr10	5706000	0.859504
chr10	5794300	0.867769
chr10	5938400	0.685950
chr10	5987300	0.702479
chr10	6047900	0.661157
chr10	6139800	0.950413
chr10	6147200	0.801653
chr10	6150900	0.702479
chr10	6182700	0.842975
chr10	6193300	0.702479
chr10	6254000	0.752066
chr10	6407900	0.710744
chr10	6585500	0.917355
chr10	6602400	0.694215
chr10	6624400	0.669421
chr10	6667200	0.710744
chr10	6764500	0.842975
chr10	6779600	0.925620
chr10	6805600	0.809917
chr10	6828400	0.752066
chr10	6837200	0.958678
chr10	6839400	0.917355
chr10	6845100	0.719008
chr10	6929200	0.710744
chr10	6942100	0.785124
chr10	7128200	0.685950
chr10	7253700	0.793388
chr10	7344100	0.842975
chr10	7376600	0.826446
chr10	7466000	0.694215

chr10	7471300	0.661157
chr10	7517900	0.661157
chr10	7536500	0.859504
chr10	7583300	0.950413
chr10	7603200	0.719008
chr10	7677400	0.909091
chr10	7896400	0.669421
chr10	8117600	0.793388
chr10	8128600	0.702479
chr10	8145700	0.677686
chr10	8331600	0.776860
chr10	8338400	0.752066
chr10	8349000	0.677686
chr10	8446100	0.644628
chr10	8545700	0.785124
chr10	8553200	0.776860
chr10	8670700	0.785124
chr10	8882900	0.760331
chr10	9552900	1.000000
chr10	9577700	0.677686
chr10	9587600	0.942149
chr10	9888200	0.710744
chr10	9953600	0.966942
chr10	10207400	0.809917
chr10	10336000	0.793388
chr10	10403800	0.975207
chr10	10518100	0.809917
chr10	10570500	0.859504
chr10	10834600	0.842975
chr10	10840300	0.859504
chr10	11077700	0.776860
chr10	11137700	0.785124
chr10	11186600	0.727273
chr10	11189300	0.925620
chr10	11196900	0.752066
chr10	11200200	0.669421
chr10	11232000	0.768595
chr10	11235000	0.694215
chr10	11239400	0.760331
chr10	11247000	0.975207
chr10	11249500	0.727273
chr10	11291600	0.892562
chr10	11308700	0.785124
chr10	11371100	0.842975
chr10	11379600	0.867769
chr10	11443800	0.876033
chr10	11477900	0.842975
chr10	11511900	0.942149
chr10	11515900	0.776860
chr10	11552800	0.652893
chr10	11637500	0.785124
chr10	11841400	0.768595
chr10	11852900	0.818182
chr10	11871200	0.727273

chr10	11873300	0.677686
chr10	11916900	0.975207
chr10	11944200	0.818182
chr10	11955500	0.834711
chr10	11979100	0.694215
chr10	12020400	0.669421
chr10	12222500	0.917355
chr10	12395600	0.809917
chr10	12401800	0.801653
chr10	12439200	0.983471
chr10	12477900	0.669421
chr10	12524800	0.950413
chr10	12527600	0.801653
chr10	12672000	0.743802
chr10	12680800	0.727273
chr10	12749900	0.909091
chr10	12753500	0.834711
chr10	12767000	0.702479
chr10	12807200	0.661157
chr10	12816600	0.867769
chr10	12861600	0.677686
chr10	13122200	0.884298
chr10	13276100	0.702479
chr10	13314100	0.801653
chr10	13371900	0.884298
chr10	13394900	0.727273
chr10	13433200	0.685950
chr10	13437100	0.719008
chr10	13447000	0.661157
chr10	13658400	0.950413
chr10	13670700	0.809917
chr10	13702000	0.727273
chr10	13715000	0.851240
chr10	13732800	0.925620
chr10	13752900	0.776860
chr10	13800900	0.925620
chr10	13830200	0.652893
chr10	13846100	0.727273
chr10	13851700	0.942149
chr10	13862800	0.826446
chr10	13865600	0.917355
chr10	13869600	0.851240
chr10	13873700	0.743802
chr10	13877000	0.669421
chr10	13908500	0.793388
chr10	13912400	0.760331
chr10	13923400	0.983471
chr10	13926000	0.760331
chr10	13936200	0.776860
chr10	13946500	0.669421
chr10	14013700	0.958678
chr10	14043500	0.652893
chr10	14052000	0.776860
chr10	14131200	0.661157

chr10	14136400	0.884298
chr10	14205900	0.652893
chr10	14227400	0.809917
chr10	14372400	0.933884
chr10	14443200	0.661157
chr10	14467700	0.752066
chr10	14471300	1.000000
chr10	14544600	0.752066
chr10	14561900	0.768595
chr10	14590300	0.702479
chr10	14598400	0.834711
chr10	14604000	0.644628
chr10	14627600	0.768595
chr10	14695400	0.760331
chr10	14716800	0.818182
chr10	15126700	0.942149
chr10	15252600	0.719008
chr10	15297100	0.900826
chr10	15358400	0.694215
chr10	15520900	0.694215
chr10	15921700	0.727273
chr10	16032800	0.809917
chr10	16238400	0.991736
chr10	16285400	1.000000
chr10	16391100	0.768595
chr10	16429200	0.958678
chr10	16464900	0.834711
chr10	16569500	0.809917
chr10	16608800	0.785124
chr10	16635300	0.785124
chr10	16910000	0.966942
chr10	16933600	0.776860
chr10	16971300	0.652893
chr10	16993500	0.958678
chr10	17028700	0.710744
chr10	17064500	0.909091
chr10	17165700	0.760331
chr10	17241200	0.859504
chr10	17257600	0.818182
chr10	17268600	0.801653
chr10	17422800	0.876033
chr10	17430300	0.644628
chr10	17604600	0.652893
chr10	17693000	0.991736
chr10	17713700	0.702479
chr10	17716400	0.644628
chr10	18386000	0.966942
chr10	18486400	0.710744
chr10	18523000	0.669421
chr10	18550100	0.702479
chr10	18787600	0.677686
chr10	19042700	0.727273
chr10	19663600	0.644628
chr10	19705700	0.702479

chr10	19710400	0.760331
chr10	19778200	0.809917
chr10	19918600	0.801653
chr10	20118200	0.925620
chr10	20122600	0.793388
chr10	20180500	0.710744
chr10	20196300	0.760331
chr10	20227200	0.760331
chr10	20253800	0.652893
chr10	20256300	0.760331
chr10	20271500	0.685950
chr10	20275500	0.942149
chr10	20293800	0.818182
chr10	20405200	0.942149
chr10	20407600	0.851240
chr10	20505300	0.991736
chr10	20546400	0.677686
chr10	20659300	0.867769
chr10	20662000	0.760331
chr10	20707200	0.743802
chr10	20887400	0.818182
chr10	20904300	0.917355
chr10	21238000	0.793388
chr10	21243100	0.950413
chr10	21435200	0.719008
chr10	21448800	0.694215
chr10	21451000	0.826446
chr10	21569700	0.925620
chr10	21572100	0.966942
chr10	21574500	0.975207
chr10	21579900	0.735537
chr10	21584000	0.735537
chr10	21587000	0.859504
chr10	21603400	0.942149
chr10	21605500	0.842975
chr10	21616900	0.677686
chr10	21625400	0.909091
chr10	21639300	0.677686
chr10	21653200	0.950413
chr10	21677800	0.950413
chr10	21680200	0.694215
chr10	21692600	0.966942
chr10	21706900	0.727273
chr10	21762900	0.710744
chr10	21791400	0.752066
chr10	21795000	0.818182
chr10	21926600	0.975207
chr10	22016500	0.661157
chr10	22021500	0.884298
chr10	22036900	0.867769
chr10	22040600	0.983471
chr10	22046700	0.752066
chr10	22096200	0.710744
chr10	22158100	0.644628

chr10	22265800	0.809917
chr10	22331900	0.884298
chr10	22494700	0.719008
chr10	22716800	0.851240
chr10	22771400	0.735537
chr10	22840800	0.710744
chr10	22893100	0.752066
chr10	22902600	0.685950
chr10	22905100	0.851240
chr10	22912700	0.752066
chr10	22917200	0.727273
chr10	22919800	0.694215
chr10	22944800	0.942149
chr10	22961800	0.685950
chr10	22969800	0.776860
chr10	22979800	0.892562
chr10	22989300	0.801653
chr10	23164000	0.834711
chr10	23267900	0.644628
chr10	23342300	0.727273
chr10	23345300	0.884298
chr10	23350100	0.809917
chr10	23447500	0.677686
chr10	23475900	0.809917
chr10	23478400	0.768595
chr10	23486500	0.785124
chr10	23490200	0.859504
chr10	23493700	0.809917
chr10	23569700	0.809917
chr10	23707600	0.818182
chr10	24298400	0.661157
chr10	24301400	0.644628
chr10	24407700	0.652893
chr10	24491400	0.867769
chr10	24496200	0.809917
chr10	24502200	0.694215
chr10	24527600	0.975207
chr10	24544800	0.851240
chr10	24548800	0.719008
chr10	24646800	0.917355
chr10	24658800	0.925620
chr10	24673200	0.900826
chr10	24730000	0.719008
chr10	24740100	0.809917
chr10	24772700	0.661157
chr10	24792900	0.743802
chr10	24841400	0.677686
chr10	24869100	0.677686
chr10	25005700	0.818182
chr10	25007900	0.685950
chr10	25357700	0.876033
chr10	25371900	0.768595
chr10	25405700	0.677686
chr10	25672600	0.727273

chr10	25757400	0.652893
chr10	25782100	0.694215
chr10	25797700	0.661157
chr10	25836600	0.652893
chr10	26015200	0.768595
chr10	26055400	0.661157
chr10	26065500	0.809917
chr10	26141200	0.661157
chr10	26188700	0.644628
chr10	26314700	0.694215
chr10	26375000	0.652893
chr10	26458900	0.702479
chr10	26461300	0.785124
chr10	27023400	0.735537
chr10	27045000	0.685950
chr10	27070300	0.735537
chr10	27092200	0.752066
chr10	27094900	0.752066
chr10	27824300	0.876033
chr10	28064000	0.752066
chr10	28078200	0.694215
chr10	28201000	0.975207
chr10	28284600	0.677686
chr10	28409100	0.735537
chr10	28504600	0.727273
chr10	28749000	0.867769
chr10	28760900	0.892562
chr10	28849200	0.925620
chr10	28974700	0.900826
chr10	29095800	0.876033
chr10	29099600	0.884298
chr10	29358500	0.685950
chr10	29368200	0.702479
chr10	29449600	0.983471
chr10	29493600	0.933884
chr10	29556200	0.702479
chr10	29669700	0.867769
chr10	29772100	0.768595
chr10	29785900	0.826446
chr10	29822700	0.801653
chr10	29859200	0.859504
chr10	29874600	0.710744
chr10	29879300	0.900826
chr10	29922400	0.669421
chr10	29925000	0.859504
chr10	29933100	0.727273
chr10	29956200	0.752066
chr10	29975100	0.752066
chr10	29977800	0.900826
chr10	29981300	0.925620
chr10	29998900	0.710744
chr10	30014600	0.652893
chr10	30017900	0.933884
chr10	30316700	0.677686

chr10	30323700	0.776860
chr10	30346000	0.694215
chr10	30649100	0.760331
chr10	30663000	0.942149
chr10	30667800	0.677686
chr10	30712500	0.876033
chr10	30720500	0.661157
chr10	30726800	0.801653
chr10	30791800	0.719008
chr10	30827500	0.719008
chr10	30842800	0.677686
chr10	30886400	0.867769
chr10	30932800	0.867769
chr10	30952700	0.702479
chr10	30994600	0.743802
chr10	30999200	0.743802
chr10	31100600	0.710744
chr10	31119700	0.801653
chr10	31175900	0.793388
chr10	31325500	0.677686
chr10	31362500	0.776860
chr10	31757500	0.727273
chr10	31872700	0.719008
chr10	31909500	0.818182
chr10	31955300	0.933884
chr10	31966900	0.801653
chr10	31983900	0.644628
chr10	31996800	0.752066
chr10	32017900	0.727273
chr10	32044100	0.991736
chr10	32046700	0.677686
chr10	32154200	0.760331
chr10	32202200	0.677686
chr10	32234000	0.785124
chr10	32290700	0.752066
chr10	32292800	0.876033
chr10	32341500	0.867769
chr10	32361700	0.727273
chr10	32428900	0.826446
chr10	32521300	0.867769
chr10	32551700	0.768595
chr10	32556200	0.950413
chr10	32617700	0.760331
chr10	32622300	0.677686
chr10	33230900	0.719008
chr10	33239900	0.661157
chr10	33244200	0.677686
chr10	33251600	0.793388
chr10	33261000	0.933884
chr10	33274900	0.719008
chr10	33299000	0.760331
chr10	33315600	0.776860
chr10	33331300	0.950413
chr10	33385400	0.735537

chr10	33396500	0.752066
chr10	33476500	0.884298
chr10	33773600	0.851240
chr10	33808700	0.892562
chr10	33825900	0.685950
chr10	33842900	0.710744
chr10	33898800	0.735537
chr10	34011600	0.652893
chr10	34062000	0.983471
chr10	34080500	0.776860
chr10	34402800	0.685950
chr10	34451100	0.760331
chr10	34467800	0.760331
chr10	34473200	0.933884
chr10	34476300	0.909091
chr10	34541300	0.785124
chr10	34583100	0.702479
chr10	34702600	0.793388
chr10	34720100	0.661157
chr10	34775200	0.743802
chr10	34794600	0.644628
chr10	34816200	0.991736
chr10	34827900	0.677686
chr10	34856900	0.694215
chr10	34865800	0.768595
chr10	34877100	0.644628
chr10	34926700	0.867769
chr10	34941700	0.644628
chr10	34945300	0.661157
chr10	35026600	0.884298
chr10	35029300	0.925620
chr10	35042400	0.826446
chr10	35056300	0.933884
chr10	35067400	0.752066
chr10	35070500	0.826446
chr10	35098100	0.942149
chr10	35100400	0.851240
chr10	35153500	0.735537
chr10	35194800	0.851240
chr10	35284400	0.892562
chr10	35301800	0.793388
chr10	35438500	0.785124
chr10	35500700	0.727273
chr10	35507700	0.727273
chr10	35618800	0.685950
chr10	35668200	0.719008
chr10	35683500	0.900826
chr10	35702500	0.677686
chr10	35710400	0.942149
chr10	35715800	0.694215
chr10	35738100	0.859504
chr10	35747600	0.867769
chr10	35751600	0.909091
chr10	35754500	0.793388

chr10	35760000	0.933884
chr10	35782000	0.652893
chr10	35784800	0.743802
chr10	35807000	0.652893
chr10	35809300	0.644628
chr10	35883500	0.966942
chr10	35890000	0.644628
chr10	35947400	0.735537
chr10	35958200	0.768595
chr10	35980700	0.925620
chr10	35986100	0.677686
chr10	36054300	0.719008
chr10	36060400	0.661157
chr10	36073000	0.644628
chr10	36161400	0.669421
chr10	36164600	0.900826
chr10	36223800	0.652893
chr10	36239200	0.785124
chr10	36248500	0.677686
chr10	36263500	0.867769
chr10	36459700	0.644628
chr10	36477100	0.867769
chr10	36483800	0.669421
chr10	36738800	0.917355
chr10	36978200	0.644628
chr10	38814900	0.752066
chr10	38872400	0.661157
chr10	38888600	0.867769
chr10	39084000	0.685950
chr10	39101800	0.801653
chr10	39106500	0.661157
chr10	39121600	0.768595
chr10	39123700	0.760331
chr10	39140400	0.661157
chr10	39147200	0.677686
chr10	42409400	0.685950
chr10	42525800	0.801653
chr10	42531700	0.661157
chr10	42539100	0.702479
chr10	42546300	0.867769
chr10	42602000	0.702479
chr10	42667300	0.702479
chr10	42791400	0.785124
chr10	42797100	0.685950
chr10	42801600	0.768595
chr10	42818300	0.925620
chr10	43308500	0.760331
chr10	43312800	1.000000
chr10	43323300	0.818182
chr10	43332200	0.785124
chr10	43362400	0.776860
chr10	43507100	0.884298
chr10	43509200	0.867769
chr10	43560500	0.785124

chr10	43868800	0.719008
chr10	43872400	0.719008
chr10	43954300	0.851240
chr10	44249900	0.900826
chr10	44538000	0.768595
chr10	44729400	0.851240
chr10	44754700	0.727273
chr10	44793600	0.876033
chr10	44806700	0.958678
chr10	44852200	0.884298
chr10	44866500	0.809917
chr10	44876700	0.785124
chr10	44894000	0.735537
chr10	44896600	0.818182
chr10	45353200	0.752066
chr10	45395400	0.776860
chr10	45403800	0.876033
chr10	45420900	0.727273
chr10	45457300	0.884298
chr10	45460100	0.950413
chr10	45468800	0.752066
chr10	45476700	0.661157
chr10	45481000	0.834711
chr10	45486300	0.892562
chr10	45540200	0.975207
chr10	45572100	0.768595
chr10	45591000	0.942149
chr10	45602100	0.710744
chr10	45904000	0.752066
chr10	45929400	0.884298
chr10	45967800	0.669421
chr10	46010700	0.776860
chr10	46053600	0.851240
chr10	46071300	0.793388
chr10	46603500	0.644628
chr10	46792600	0.842975
chr10	46903400	0.776860
chr10	46962500	0.776860
chr10	46976200	0.876033
chr10	46984100	0.851240
chr10	47006500	0.793388
chr10	47012500	0.867769
chr10	47044800	0.727273
chr10	47062900	0.669421
chr10	47108200	0.785124
chr10	47131200	0.826446
chr10	47152900	0.867769
chr10	47168700	0.892562
chr10	47273200	0.793388
chr10	47547000	0.677686
chr10	47599900	0.801653
chr10	47637700	0.661157
chr10	47686900	0.719008
chr10	47701200	0.702479

chr10	47752500	0.702479
chr10	47762100	0.735537
chr10	48261300	0.842975
chr10	48318600	0.702479
chr10	48336500	0.933884
chr10	48357600	0.685950
chr10	48366800	0.694215
chr10	48377700	0.652893
chr10	48396800	0.702479
chr10	48564300	0.710744
chr10	49093800	0.876033
chr10	49196400	0.892562
chr10	49380900	0.644628
chr10	49444200	0.677686
chr10	49460300	0.900826
chr10	49607900	0.975207
chr10	49653000	0.694215
chr10	49665100	0.727273
chr10	49678500	0.876033
chr10	49742300	0.652893
chr10	49745600	0.752066
chr10	49761100	0.768595
chr10	49803000	0.909091
chr10	49879900	0.760331
chr10	49941800	0.809917
chr10	50013700	0.842975
chr10	50088400	0.752066
chr10	50209900	0.644628
chr10	50351700	0.834711
chr10	50366800	0.842975
chr10	50451300	0.702479
chr10	50507300	0.876033
chr10	50511900	0.669421
chr10	50568400	0.851240
chr10	50613600	0.652893
chr10	50822900	0.826446
chr10	50976900	0.685950
chr10	51035500	0.644628
chr10	51038600	0.851240
chr10	51040800	0.942149
chr10	51356200	0.710744
chr10	51500200	0.743802
chr10	52086000	0.685950
chr10	52151200	0.644628
chr10	52167400	0.842975
chr10	52170600	0.661157
chr10	52231200	0.933884
chr10	52267000	0.851240
chr10	52315400	0.710744
chr10	52321500	0.735537
chr10	52324100	0.677686
chr10	52327600	0.743802
chr10	52332000	0.677686
chr10	52373100	0.867769

chr10	52379400	1.000000
chr10	52387700	0.669421
chr10	52390000	0.884298
chr10	52469600	0.685950
chr10	52548800	0.727273
chr10	52565900	0.760331
chr10	52766200	0.669421
chr10	52795600	0.809917
chr10	52840000	1.000000
chr10	52843500	0.933884
chr10	52855400	0.735537
chr10	52963600	0.834711
chr10	52977800	0.677686
chr10	52988500	0.685950
chr10	53005500	0.694215
chr10	53041900	0.702479
chr10	53127700	0.776860
chr10	53165900	0.752066
chr10	53334100	0.793388
chr10	53350500	0.809917
chr10	53379500	0.768595
chr10	53526900	0.768595
chr10	53593200	0.644628
chr10	53598800	0.669421
chr10	53670300	0.884298
chr10	53710700	0.735537
chr10	53779400	0.727273
chr10	53793000	0.925620
chr10	53854900	0.677686
chr10	53947700	0.694215
chr10	53967700	0.752066
chr10	53973800	0.983471
chr10	54025100	0.752066
chr10	54624700	0.694215
chr10	54644400	0.851240
chr10	54660900	0.669421
chr10	54663600	0.991736
chr10	54668800	1.000000
chr10	54725100	0.677686
chr10	54727200	0.900826
chr10	54730000	0.851240
chr10	54782700	0.991736
chr10	55104300	0.752066
chr10	55118200	0.876033
chr10	55236700	0.966942
chr10	55239700	0.752066
chr10	55268400	0.983471
chr10	55356900	0.966942
chr10	55360000	0.925620
chr10	56156100	0.652893
chr10	57020700	0.661157
chr10	57507300	0.752066
chr10	57582100	0.876033
chr10	57739400	0.793388

chr10	58346600	0.876033
chr10	58778600	0.958678
chr10	58862700	0.859504
chr10	58978700	0.694215
chr10	59388400	0.917355
chr10	59466600	0.669421
chr10	59546700	0.925620
chr10	59658500	0.710744
chr10	59809800	0.892562
chr10	59824700	0.760331
chr10	60025100	0.743802
chr10	60108800	0.677686
chr10	60401600	0.694215
chr10	60712800	0.694215
chr10	60723800	0.859504
chr10	60742200	0.818182
chr10	61050200	0.818182
chr10	61090500	0.760331
chr10	61274800	0.694215
chr10	61317500	0.876033
chr10	61386500	0.785124
chr10	61465600	0.710744
chr10	61564100	0.851240
chr10	61569600	0.677686
chr10	61603600	0.809917
chr10	61608100	0.669421
chr10	61624700	0.900826
chr10	61632400	0.752066
chr10	61660100	0.677686
chr10	61706000	0.834711
chr10	61726800	0.669421
chr10	61751600	0.966942
chr10	61763700	0.818182
chr10	61808300	0.694215
chr10	61890300	0.644628
chr10	62018600	0.884298
chr10	62060500	0.809917
chr10	62098700	0.809917
chr10	62110400	0.785124
chr10	62149300	0.644628
chr10	62282200	0.950413
chr10	62294800	0.768595
chr10	62333100	0.669421
chr10	62410100	0.719008
chr10	62495500	0.859504
chr10	62502500	0.867769
chr10	62522700	0.933884
chr10	62630600	0.685950
chr10	62895800	0.892562
chr10	62898700	0.694215
chr10	63174800	0.677686
chr10	63353100	0.702479
chr10	63515200	0.801653
chr10	63659100	0.644628

chr10	63724600	0.735537
chr10	63746600	0.892562
chr10	63796300	0.909091
chr10	63803800	0.677686
chr10	63809300	0.842975
chr10	64186900	0.702479
chr10	64370700	0.669421
chr10	64637900	0.694215
chr10	64722200	0.785124
chr10	64824600	0.685950
chr10	64866500	0.677686
chr10	64960400	0.669421
chr10	64966500	0.677686
chr10	65003900	0.776860
chr10	65057800	0.859504
chr10	65189900	0.727273
chr10	65195300	0.826446
chr10	65264500	0.917355
chr10	65275200	0.983471
chr10	65278900	0.834711
chr10	65333200	0.876033
chr10	65473300	0.983471
chr10	65526800	0.669421
chr10	65558900	0.685950
chr10	65626400	0.752066
chr10	65643900	0.685950
chr10	65647200	0.685950
chr10	65682500	0.867769
chr10	65772300	0.876033
chr10	65846100	0.867769
chr10	66145400	0.743802
chr10	67031700	0.661157
chr10	67086100	0.892562
chr10	68028700	0.669421
chr10	68138600	0.867769
chr10	68582900	0.942149
chr10	68901200	0.669421
chr10	69040300	0.867769
chr10	69162000	0.884298
chr10	69438100	0.669421
chr10	69621500	0.892562
chr10	69623700	0.983471
chr10	69695500	0.743802
chr10	69736800	0.826446
chr10	69848100	0.702479
chr10	69857200	0.818182
chr10	69914000	0.983471
chr10	70088900	0.834711
chr10	70256300	0.752066
chr10	70364400	0.719008
chr10	70631200	0.925620
chr10	70638800	0.760331
chr10	70992800	0.867769
chr10	71076500	0.661157

chr10	71081600	0.702479
chr10	71086100	0.966942
chr10	71089300	0.983471
chr10	71091500	0.644628
chr10	71093900	0.975207
chr10	71098900	0.859504
chr10	71105100	0.876033
chr10	71120200	0.669421
chr10	71135800	0.884298
chr10	71149300	0.818182
chr10	71166700	0.842975
chr10	71267100	0.760331
chr10	71405900	0.884298
chr10	71415500	0.727273
chr10	71470400	0.710744
chr10	71482200	0.752066
chr10	71491600	0.768595
chr10	71559600	0.719008
chr10	71570900	0.661157
chr10	71577000	0.710744
chr10	71587700	0.743802
chr10	71599300	0.669421
chr10	71605100	0.809917
chr10	71608100	0.834711
chr10	71610600	0.768595
chr10	71613000	0.950413
chr10	71620500	0.669421
chr10	71624900	0.685950
chr10	71628000	1.000000
chr10	71631200	0.719008
chr10	71633400	0.727273
chr10	71659500	0.892562
chr10	71674200	0.685950
chr10	71677900	0.735537
chr10	71685100	0.727273
chr10	71712900	0.892562
chr10	71802600	0.785124
chr10	71839100	0.752066
chr10	71867300	0.644628
chr10	72084400	0.694215
chr10	72097300	0.752066
chr10	72131700	0.900826
chr10	72203500	0.867769
chr10	72211000	0.752066
chr10	72220200	0.677686
chr10	72229400	0.661157
chr10	72249400	0.818182
chr10	72254100	0.983471
chr10	72259600	0.867769
chr10	72269100	0.826446
chr10	72273600	0.752066
chr10	72296400	0.917355
chr10	72319100	0.644628
chr10	72357400	0.727273

chr10	72382800	0.867769
chr10	72386100	0.966942
chr10	72430500	0.727273
chr10	72452200	0.702479
chr10	72476300	0.652893
chr10	72514100	0.661157
chr10	72520100	0.884298
chr10	72653200	0.793388
chr10	72727800	0.652893
chr10	72730800	0.859504
chr10	72766300	0.652893
chr10	72836800	0.685950
chr10	72887700	0.661157
chr10	72896100	0.752066
chr10	72936600	0.809917
chr10	72982700	0.884298
chr10	72991700	0.719008
chr10	73014100	0.900826
chr10	73020300	0.983471
chr10	73023800	0.752066
chr10	73026000	0.859504
chr10	73029200	0.892562
chr10	73038900	0.958678
chr10	73070000	0.661157
chr10	73074800	0.851240
chr10	73096900	0.685950
chr10	73104800	0.975207
chr10	73107000	0.702479
chr10	73169300	0.685950
chr10	73192200	0.776860
chr10	73203700	0.669421
chr10	73235100	0.702479
chr10	73257200	0.694215
chr10	73276900	0.652893
chr10	73288800	0.900826
chr10	73299600	0.743802
chr10	73303900	0.652893
chr10	73307600	0.727273
chr10	73310800	0.677686
chr10	73313200	0.719008
chr10	73334400	0.719008
chr10	73339000	0.710744
chr10	73366700	0.694215
chr10	73371000	0.685950
chr10	73373400	0.694215
chr10	73381800	0.735537
chr10	73384600	0.661157
chr10	73387700	0.768595
chr10	73392000	0.818182
chr10	73395400	0.785124
chr10	73398600	0.719008
chr10	73401200	0.644628
chr10	73408500	0.719008
chr10	73415000	0.876033

chr10	73419800	0.677686
chr10	73425600	0.851240
chr10	73445900	0.892562
chr10	73480000	0.867769
chr10	73496400	0.818182
chr10	73506100	0.702479
chr10	73517100	0.719008
chr10	73542800	0.826446
chr10	73566000	0.925620
chr10	73597300	1.000000
chr10	73600300	0.975207
chr10	73608500	0.752066
chr10	73620800	0.702479
chr10	73625500	0.785124
chr10	73740900	0.719008
chr10	73797700	0.644628
chr10	73808700	0.735537
chr10	73821300	0.793388
chr10	73839900	0.694215
chr10	73886400	0.801653
chr10	73906300	0.702479
chr10	74008500	0.809917
chr10	74014600	0.942149
chr10	74019400	0.785124
chr10	74021500	0.685950
chr10	74025100	0.925620
chr10	74056000	0.652893
chr10	74070100	0.768595
chr10	74075300	0.685950
chr10	74081800	0.826446
chr10	74085300	0.826446
chr10	74092000	0.917355
chr10	74095200	0.942149
chr10	74124400	0.801653
chr10	74181900	0.801653
chr10	74305600	0.669421
chr10	74506800	0.694215
chr10	74513900	0.776860
chr10	74517300	0.826446
chr10	74544500	0.917355
chr10	74552600	0.983471
chr10	74554800	0.669421
chr10	74586100	0.776860
chr10	74646200	0.983471
chr10	74713300	0.661157
chr10	74805100	0.785124
chr10	74878300	0.702479
chr10	75349400	0.661157
chr10	75361900	0.768595
chr10	75501600	0.785124
chr10	75535200	0.752066
chr10	75611100	0.809917
chr10	75616100	0.768595
chr10	75627700	0.719008

chr10	75648900	0.702479
chr10	75666800	0.826446
chr10	75674000	0.652893
chr10	75684500	0.702479
chr10	75692200	0.876033
chr10	75701100	0.661157
chr10	75710600	0.644628
chr10	75717000	0.710744
chr10	75725300	0.909091
chr10	75729100	0.933884
chr10	75771400	0.694215
chr10	75830800	0.702479
chr10	75839400	0.884298
chr10	75841600	0.685950
chr10	76015400	0.925620
chr10	76178300	0.925620
chr10	76181200	0.752066
chr10	76490400	0.966942
chr10	76513700	0.719008
chr10	76614700	0.760331
chr10	76623000	0.876033
chr10	76627600	0.644628
chr10	76684000	0.876033
chr10	76708000	0.702479
chr10	76860300	0.776860
chr10	76866100	0.983471
chr10	76868800	0.842975
chr10	76947800	0.942149
chr10	76967900	0.661157
chr10	76982900	0.950413
chr10	76986000	0.942149
chr10	77006100	0.661157
chr10	77035900	0.884298
chr10	77177700	0.719008
chr10	77212400	0.900826
chr10	77235400	0.793388
chr10	77298200	0.859504
chr10	77301900	0.727273
chr10	77354000	0.710744
chr10	77357600	0.884298
chr10	77360500	0.884298
chr10	77371700	0.917355
chr10	77542400	0.776860
chr10	77548500	0.942149
chr10	77605700	0.809917
chr10	77617700	0.917355
chr10	77637300	0.710744
chr10	77727600	0.834711
chr10	77769700	0.719008
chr10	77909800	0.991736
chr10	77938200	0.694215
chr10	78030100	0.859504
chr10	78123900	0.661157
chr10	78189600	0.661157

chr10	78192900	0.719008
chr10	78392600	0.809917
chr10	78409400	0.818182
chr10	78483800	0.644628
chr10	78538500	0.991736
chr10	78689000	0.702479
chr10	78798000	0.710744
chr10	78812000	0.669421
chr10	78840600	0.876033
chr10	78842700	0.685950
chr10	78869000	0.909091
chr10	78871300	0.710744
chr10	78874700	0.867769
chr10	78909400	0.661157
chr10	78912200	0.785124
chr10	78939500	0.661157
chr10	78946000	0.801653
chr10	79046600	0.669421
chr10	79085300	0.892562
chr10	79148900	0.710744
chr10	79166100	0.661157
chr10	79271200	0.735537
chr10	79287300	0.694215
chr10	79289600	0.768595
chr10	79325000	0.809917
chr10	79363700	0.661157
chr10	79389400	0.785124
chr10	79408400	0.677686
chr10	79410800	0.702479
chr10	79419400	0.867769
chr10	79459200	0.826446
chr10	79473400	0.702479
chr10	79516600	0.727273
chr10	79561500	0.876033
chr10	79598000	0.694215
chr10	79607600	0.966942
chr10	79611000	0.876033
chr10	79624800	0.702479
chr10	79627500	0.826446
chr10	79635700	0.809917
chr10	79638300	0.785124
chr10	79642700	0.702479
chr10	79645200	0.735537
chr10	79651700	0.710744
chr10	79658800	0.785124
chr10	79709200	0.876033
chr10	79731600	0.677686
chr10	79801000	0.859504
chr10	79899300	0.661157
chr10	79932000	0.702479
chr10	79937700	0.834711
chr10	80053400	0.826446
chr10	80117200	0.958678
chr10	80120000	0.644628

chr10	80162000	0.661157
chr10	80181700	0.942149
chr10	80232700	0.735537
chr10	80311400	0.983471
chr10	80514800	0.851240
chr10	80577900	0.917355
chr10	80635600	0.909091
chr10	80655400	0.925620
chr10	80673900	0.983471
chr10	80677000	0.768595
chr10	80684100	0.776860
chr10	80698100	0.727273
chr10	80721400	0.710744
chr10	80725300	0.975207
chr10	80741800	0.826446
chr10	80745700	0.760331
chr10	80785400	0.719008
chr10	80811200	0.809917
chr10	80817100	0.892562
chr10	80833300	0.719008
chr10	80836200	0.661157
chr10	80844200	0.909091
chr10	80859700	0.652893
chr10	80862300	0.768595
chr10	80867400	0.694215
chr10	80873000	0.719008
chr10	80875900	0.685950
chr10	80883800	0.661157
chr10	80886300	0.752066
chr10	80889000	0.793388
chr10	80902400	0.694215
chr10	80904800	0.933884
chr10	80911400	0.752066
chr10	80915900	0.826446
chr10	80919600	0.851240
chr10	80925600	0.933884
chr10	80928000	0.793388
chr10	80984200	0.669421
chr10	80990300	0.975207
chr10	80996700	0.818182
chr10	80998800	0.661157
chr10	81077600	0.925620
chr10	81096500	0.669421
chr10	81159500	0.867769
chr10	81164300	0.925620
chr10	81195500	0.702479
chr10	81460500	0.884298
chr10	81598600	0.644628
chr10	81709200	0.776860
chr10	81711700	0.743802
chr10	81776200	0.867769
chr10	81830600	0.710744
chr10	81842200	0.776860
chr10	81860900	0.743802

chr10	81865300	0.942149
chr10	81921500	0.752066
chr10	81954900	0.661157
chr10	82016700	0.661157
chr10	82025900	0.719008
chr10	82049700	0.768595
chr10	82177200	0.702479
chr10	82217900	0.727273
chr10	82220200	0.958678
chr10	82228000	0.851240
chr10	82245900	0.752066
chr10	82258200	0.785124
chr10	82264300	0.801653
chr10	82290900	0.669421
chr10	82312300	0.710744
chr10	82500300	0.677686
chr10	82649900	0.785124
chr10	82654700	0.892562
chr10	82657100	0.909091
chr10	82687300	0.900826
chr10	83011600	0.991736
chr10	83457100	0.867769
chr10	83607800	0.917355
chr10	83625700	0.785124
chr10	83638900	0.685950
chr10	83666400	0.859504
chr10	83677900	0.661157
chr10	83788400	0.743802
chr10	83801100	0.669421
chr10	83849600	0.760331
chr10	84011200	0.694215
chr10	84028100	0.694215
chr10	84079500	0.669421
chr10	84413400	0.826446
chr10	84502800	0.644628
chr10	84649100	0.851240
chr10	84724900	0.702479
chr10	84838300	0.991736
chr10	84971500	0.685950
chr10	84983300	0.669421
chr10	85049800	0.727273
chr10	85196200	0.727273
chr10	85577800	0.652893
chr10	85684800	0.710744
chr10	85692300	0.644628
chr10	85717300	0.900826
chr10	85920900	0.801653
chr10	85997300	0.694215
chr10	86056500	0.801653
chr10	86074100	0.743802
chr10	86102000	0.884298
chr10	86190100	0.776860
chr10	86273900	0.727273
chr10	86327100	0.925620

chr10	86343800	0.702479
chr10	86348000	0.851240
chr10	86523100	0.735537
chr10	86571400	0.652893
chr10	86751100	0.652893
chr10	86803100	0.752066
chr10	86908700	0.719008
chr10	87252900	0.867769
chr10	87273800	0.677686
chr10	87308000	0.743802
chr10	87428000	0.966942
chr10	87676200	0.644628
chr10	87838900	0.727273
chr10	87992600	0.809917
chr10	87994700	0.760331
chr10	88013400	0.818182
chr10	88022200	0.702479
chr10	88051700	0.785124
chr10	88053800	0.909091
chr10	88100900	0.768595
chr10	88147800	0.867769
chr10	88162800	0.710744
chr10	88183900	0.809917
chr10	88360400	0.942149
chr10	88428400	0.694215
chr10	88433800	0.760331
chr10	88444800	0.801653
chr10	88453800	0.859504
chr10	88465800	0.727273
chr10	88513100	0.694215
chr10	88527500	0.760331
chr10	88530400	0.719008
chr10	88546300	0.644628
chr10	88559500	0.991736
chr10	88572900	0.685950
chr10	88587900	0.752066
chr10	88590100	0.694215
chr10	88593800	0.743802
chr10	88597600	0.719008
chr10	88601700	0.809917
chr10	88612000	0.735537
chr10	88614300	0.685950
chr10	88617100	0.942149
chr10	88622100	0.958678
chr10	88626100	0.958678
chr10	88628500	0.958678
chr10	88631400	0.834711
chr10	88659000	0.809917
chr10	88699500	0.809917
chr10	88714400	0.909091
chr10	88716700	0.710744
chr10	88719600	0.793388
chr10	88726700	0.842975
chr10	88965500	0.661157

chr10	89151200	0.661157
chr10	89308300	0.652893
chr10	89319200	0.851240
chr10	89368900	0.644628
chr10	89385700	0.991736
chr10	89388300	0.735537
chr10	89411700	0.884298
chr10	89422400	0.743802
chr10	89494900	0.727273
chr10	89736600	0.652893
chr10	89802700	0.776860
chr10	89878300	0.669421
chr10	90042000	0.661157
chr10	90107800	0.834711
chr10	90235300	0.785124
chr10	90265600	0.669421
chr10	90463300	0.925620
chr10	90488700	0.661157
chr10	90491800	0.652893
chr10	90657300	0.652893
chr10	90792400	0.826446
chr10	90846900	0.826446
chr10	90946900	0.710744
chr10	90995200	0.719008
chr10	91020500	0.917355
chr10	91044200	0.776860
chr10	91087900	0.867769
chr10	91093700	0.884298
chr10	91106900	0.958678
chr10	91117400	0.710744
chr10	91146300	0.652893
chr10	91152500	0.876033
chr10	91188600	0.669421
chr10	91238800	0.818182
chr10	91251300	0.644628
chr10	91275800	0.669421
chr10	91407100	0.661157
chr10	91455100	0.801653
chr10	91555200	0.719008
chr10	92062200	0.958678
chr10	92079700	0.991736
chr10	92124000	0.933884
chr10	92244700	0.644628
chr10	92484100	0.677686
chr10	92530900	0.727273
chr10	92535400	0.661157
chr10	92546600	0.752066
chr10	92564600	0.859504
chr10	92573800	0.809917
chr10	92690400	0.735537
chr10	92795200	0.661157
chr10	92964600	0.652893
chr10	93045000	0.768595
chr10	93429200	0.768595

chr10	93432300	0.652893
chr10	93441700	0.876033
chr10	93444000	0.826446
chr10	93505000	0.760331
chr10	93542700	0.801653
chr10	93548400	0.719008
chr10	93626000	0.818182
chr10	93640100	0.776860
chr10	93643700	0.669421
chr10	93793100	0.752066
chr10	94063800	0.768595
chr10	94066700	0.785124
chr10	94142600	0.702479
chr10	94146900	0.685950
chr10	94487300	0.842975
chr10	94548400	0.727273
chr10	94596600	0.727273
chr10	94652800	0.743802
chr10	94676300	0.876033
chr10	94848200	0.851240
chr10	94958800	0.842975
chr10	95031200	0.661157
chr10	95033700	0.925620
chr10	95041900	0.966942
chr10	95048300	0.826446
chr10	95094200	0.710744
chr10	95136500	0.669421
chr10	95181700	0.776860
chr10	95196200	0.975207
chr10	95202000	0.661157
chr10	95205800	0.991736
chr10	95219000	0.785124
chr10	95351800	0.950413
chr10	95511500	0.826446
chr10	95517900	0.776860
chr10	95520600	0.760331
chr10	95550600	0.876033
chr10	95564100	0.661157
chr10	95575700	0.644628
chr10	95602500	0.785124
chr10	95609300	0.900826
chr10	95657200	0.834711
chr10	95974300	0.644628
chr10	96048700	0.884298
chr10	96299900	0.743802
chr10	96309200	0.710744
chr10	96383400	0.776860
chr10	96386500	0.752066
chr10	96758000	0.785124
chr10	96969400	0.900826
chr10	96988300	0.842975
chr10	96992000	0.867769
chr10	97024600	0.991736
chr10	97028800	0.644628

chr10	97033700	0.809917
chr10	97038300	0.719008
chr10	97064300	0.760331
chr10	97073200	0.867769
chr10	97131400	0.661157
chr10	97169600	0.950413
chr10	97219500	0.661157
chr10	97222000	0.752066
chr10	97227600	0.669421
chr10	97231900	0.727273
chr10	97234600	0.760331
chr10	97236800	0.876033
chr10	97240600	0.785124
chr10	97260700	0.776860
chr10	97271300	0.801653
chr10	97278700	0.867769
chr10	97291100	0.702479
chr10	97300600	0.677686
chr10	97304300	0.801653
chr10	97307500	0.892562
chr10	97311200	0.826446
chr10	97317600	0.743802
chr10	97681000	0.826446
chr10	97884700	0.776860
chr10	97965900	0.776860
chr10	97982300	0.884298
chr10	98068600	0.876033
chr10	98070900	0.933884
chr10	98101000	0.942149
chr10	98105200	0.644628
chr10	98130700	0.776860
chr10	98156500	0.867769
chr10	98201700	0.710744
chr10	98207500	0.776860
chr10	98210900	1.000000
chr10	98229500	0.851240
chr10	98269200	0.925620
chr10	98596100	0.876033
chr10	98604600	0.752066
chr10	98617400	0.661157
chr10	98744700	0.991736
chr10	98780900	0.735537
chr10	98792100	0.652893
chr10	98798100	0.661157
chr10	98840800	0.867769
chr10	98861200	0.677686
chr10	98900500	0.834711
chr10	98939900	0.743802
chr10	99115800	0.719008
chr10	99174000	0.652893
chr10	99178900	0.925620
chr10	99240100	0.942149
chr10	99255200	0.776860
chr10	99278200	0.685950

chr10	99281300	0.867769
chr10	99292400	0.942149
chr10	99302700	0.933884
chr10	99310200	0.694215
chr10	99314000	0.776860
chr10	99344000	0.677686
chr10	99372000	0.900826
chr10	99408900	0.826446
chr10	99469400	0.710744
chr10	99476900	0.644628
chr10	99479200	0.942149
chr10	99486800	0.652893
chr10	99522600	0.884298
chr10	99533400	0.719008
chr10	99538400	0.677686
chr10	99541800	0.752066
chr10	99565900	0.768595
chr10	99610800	0.785124
chr10	99621600	0.702479
chr10	99629700	0.876033
chr10	99649200	0.975207
chr10	99717800	0.826446
chr10	99786400	0.644628
chr10	99798300	0.851240
chr10	99822500	0.818182
chr10	99929000	0.752066
chr10	99933400	0.958678
chr10	100029600	0.785124
chr10	100033000	0.752066
chr10	100056500	0.785124
chr10	100127700	0.702479
chr10	100140800	0.876033
chr10	100216900	0.809917
chr10	100381200	0.909091
chr10	100407500	0.900826
chr10	100431800	0.727273
chr10	100676800	0.677686
chr10	100844400	0.942149
chr10	100930000	0.669421
chr10	100981700	0.652893
chr10	101118300	0.933884
chr10	101154300	0.760331
chr10	101211500	0.727273
chr10	101280000	0.702479
chr10	101285000	0.677686
chr10	101290600	0.735537
chr10	101293200	0.644628
chr10	101298600	0.801653
chr10	101301000	0.727273
chr10	101329500	0.694215
chr10	101544900	0.785124
chr10	101606300	0.677686
chr10	101610000	0.983471
chr10	101613900	0.694215

chr10	101675100	0.694215
chr10	101678100	0.785124
chr10	101690900	0.909091
chr10	101780300	0.727273
chr10	102060300	0.760331
chr10	102101100	0.884298
chr10	102115900	0.685950
chr10	102131100	0.809917
chr10	102193900	0.677686
chr10	102246100	0.958678
chr10	102268700	0.669421
chr10	102322200	0.685950
chr10	102385800	0.983471
chr10	102397900	0.842975
chr10	102402600	0.818182
chr10	102405800	0.752066
chr10	102419900	0.925620
chr10	102428200	0.826446
chr10	102441300	0.768595
chr10	102484700	0.867769
chr10	102491900	0.785124
chr10	102495500	0.661157
chr10	102497600	0.818182
chr10	102501400	0.768595
chr10	102503900	0.760331
chr10	102508700	0.669421
chr10	102536200	0.867769
chr10	102548000	0.884298
chr10	102553300	0.752066
chr10	102563500	0.710744
chr10	102576700	0.669421
chr10	102590000	0.801653
chr10	102634000	0.933884
chr10	102636800	0.694215
chr10	102670000	0.652893
chr10	102731900	0.909091
chr10	102773900	0.917355
chr10	102776900	0.677686
chr10	102789600	0.735537
chr10	102806600	0.694215
chr10	102810600	0.710744
chr10	102833500	0.669421
chr10	102836000	0.710744
chr10	102855300	0.719008
chr10	102871500	0.719008
chr10	102883500	0.842975
chr10	102891400	0.743802
chr10	102900100	0.801653
chr10	102949800	0.884298
chr10	102975200	0.768595
chr10	102977500	0.851240
chr10	102981900	0.743802
chr10	102985600	0.710744
chr10	102988900	0.876033

chr10	102998600	0.743802
chr10	103021000	0.677686
chr10	103027000	0.867769
chr10	103032600	0.768595
chr10	103048000	0.958678
chr10	103050100	0.661157
chr10	103058300	0.644628
chr10	103071200	0.867769
chr10	103079400	0.785124
chr10	103246000	0.801653
chr10	103249600	0.768595
chr10	103289500	0.842975
chr10	103318500	0.661157
chr10	103326700	0.909091
chr10	103340600	0.793388
chr10	103367500	0.809917
chr10	103374400	0.834711
chr10	103403700	0.983471
chr10	103406100	0.669421
chr10	103519500	0.867769
chr10	103530600	0.669421
chr10	103595600	0.652893
chr10	103634900	0.793388
chr10	103662500	0.900826
chr10	103995200	0.727273
chr10	104102700	0.876033
chr10	104213000	0.801653
chr10	104215700	0.933884
chr10	104342900	0.652893
chr10	104375400	0.776860
chr10	104386600	0.644628
chr10	104389800	0.950413
chr10	104394100	0.884298
chr10	104412100	0.727273
chr10	104421300	0.719008
chr10	104428200	0.743802
chr10	104430600	0.826446
chr10	104434500	0.834711
chr10	104439000	0.884298
chr10	104478600	0.710744
chr10	104536800	0.760331
chr10	104563400	0.958678
chr10	104575900	0.867769
chr10	104705500	0.685950
chr10	104813900	0.859504
chr10	104823300	0.942149
chr10	104829500	0.785124
chr10	104913500	0.826446
chr10	104944600	0.719008
chr10	104965000	0.975207
chr10	104976100	0.661157
chr10	104991300	0.933884
chr10	105001300	0.809917
chr10	105041900	0.719008

chr10	105218900	0.735537
chr10	105236500	0.735537
chr10	105303400	0.818182
chr10	105395100	0.793388
chr10	105398800	0.685950
chr10	105407900	0.859504
chr10	105411100	0.727273
chr10	105421300	0.925620
chr10	105429100	0.826446
chr10	105445900	0.735537
chr10	105490100	0.851240
chr10	105492900	0.661157
chr10	105499500	0.768595
chr10	105517200	0.942149
chr10	105594100	0.917355
chr10	105600600	0.917355
chr10	105609200	0.768595
chr10	105630800	0.801653
chr10	105644900	0.652893
chr10	105668500	0.933884
chr10	105679100	0.851240
chr10	105681600	0.661157
chr10	105729500	0.793388
chr10	105819900	0.909091
chr10	105827400	0.809917
chr10	105836900	0.991736
chr10	105887400	0.644628
chr10	106016800	0.743802
chr10	106051500	0.793388
chr10	106065900	0.710744
chr10	106076800	0.900826
chr10	106081800	0.809917
chr10	106089200	0.644628
chr10	106092200	0.991736
chr10	106095000	0.801653
chr10	106109800	0.669421
chr10	106128200	0.644628
chr10	106177000	0.644628
chr10	106190000	0.735537
chr10	106262600	0.727273
chr10	106303200	0.669421
chr10	106420200	0.760331
chr10	106468200	0.677686
chr10	106495400	0.669421
chr10	106537500	0.727273
chr10	106764500	0.719008
chr10	106862900	0.652893
chr10	106873300	0.652893
chr10	107298900	0.677686
chr10	107460400	0.809917
chr10	107604700	0.735537
chr10	107712500	0.958678
chr10	108068200	0.652893
chr10	108071000	0.743802

chr10	108468000	0.776860
chr10	108480900	0.826446
chr10	108526100	0.760331
chr10	108540800	0.661157
chr10	108567500	0.661157
chr10	108664200	0.677686
chr10	108669000	0.818182
chr10	108673900	0.793388
chr10	108689400	0.710744
chr10	108734900	0.702479
chr10	108785700	0.826446
chr10	108863700	0.859504
chr10	109308500	0.933884
chr10	109432200	0.752066
chr10	109619000	0.694215
chr10	109640400	0.826446
chr10	109712900	0.818182
chr10	109800000	0.851240
chr10	111035100	0.768595
chr10	111153600	0.917355
chr10	111621900	0.719008
chr10	111644500	0.876033
chr10	111653400	0.719008
chr10	111685100	0.652893
chr10	111695800	0.933884
chr10	111721500	0.842975
chr10	111763300	0.909091
chr10	111773600	0.760331
chr10	111793900	0.661157
chr10	111834700	0.661157
chr10	111841900	0.669421
chr10	111911500	0.677686
chr10	111923300	0.826446
chr10	111928700	0.876033
chr10	111931000	0.917355
chr10	111935700	0.909091
chr10	111981900	0.925620
chr10	112096200	0.859504
chr10	112118600	0.851240
chr10	112153000	0.950413
chr10	112158800	0.669421
chr10	112168000	0.785124
chr10	112248200	0.702479
chr10	112261500	0.842975
chr10	112268100	0.876033
chr10	112290300	0.776860
chr10	112314100	0.925620
chr10	112371300	0.727273
chr10	112389900	0.719008
chr10	112397100	0.876033
chr10	112401500	0.685950
chr10	112427800	0.702479
chr10	112445100	0.694215
chr10	112461700	0.801653

chr10	112582600	0.925620
chr10	112594300	0.661157
chr10	112630500	0.652893
chr10	112842800	0.735537
chr10	112888800	0.743802
chr10	112896900	0.958678
chr10	113267100	0.909091
chr10	113322100	0.644628
chr10	113484300	0.818182
chr10	113624000	0.661157
chr10	113759400	0.942149
chr10	113900300	0.793388
chr10	113995400	0.644628
chr10	114039300	0.851240
chr10	114323400	0.702479
chr10	114430600	0.917355
chr10	114438600	0.809917
chr10	114492700	0.900826
chr10	114527300	0.735537
chr10	114553100	0.760331
chr10	114564300	0.768595
chr10	114569300	0.958678
chr10	114592400	0.900826
chr10	114595300	0.867769
chr10	114646700	0.925620
chr10	114654300	0.834711
chr10	114676000	0.669421
chr10	114688000	0.652893
chr10	114698800	0.818182
chr10	114708800	0.727273
chr10	114721000	0.909091
chr10	114725200	0.694215
chr10	114749200	0.793388
chr10	114760700	0.644628
chr10	114765800	0.884298
chr10	114772100	0.661157
chr10	114787600	0.752066
chr10	114804800	0.702479
chr10	114808000	0.851240
chr10	114810200	0.735537
chr10	114813900	0.876033
chr10	114818900	0.876033
chr10	114823500	0.884298
chr10	114833800	0.669421
chr10	114840400	0.867769
chr10	114847000	0.669421
chr10	114888600	0.743802
chr10	114893000	0.776860
chr10	114896300	0.702479
chr10	114900300	0.752066
chr10	114924700	0.727273
chr10	114928700	0.933884
chr10	114938400	0.851240
chr10	115024700	0.876033

chr10	115033800	0.644628
chr10	115058900	0.661157
chr10	115086700	0.925620
chr10	115170500	0.876033
chr10	115318400	0.892562
chr10	115374100	0.669421
chr10	115380700	0.809917
chr10	115465900	0.727273
chr10	115469000	0.867769
chr10	115472100	0.768595
chr10	115581200	0.933884
chr10	115821600	0.719008
chr10	115926300	0.644628
chr10	115939800	0.859504
chr10	116045800	0.677686
chr10	116080600	0.719008
chr10	116097600	0.752066
chr10	116119900	0.727273
chr10	116134300	0.719008
chr10	116153800	0.768595
chr10	116169200	0.685950
chr10	116232700	0.925620
chr10	116249700	0.768595
chr10	116275400	0.876033
chr10	116279500	0.743802
chr10	116283400	0.809917
chr10	116298300	0.900826
chr10	116423100	0.752066
chr10	116641700	0.917355
chr10	116730600	0.826446
chr10	116759200	0.818182
chr10	117050500	0.694215
chr10	117561200	0.834711
chr10	117641500	0.743802
chr10	117735800	0.834711
chr10	117829300	0.809917
chr10	117881400	0.677686
chr10	117954900	0.702479
chr10	118084800	0.958678
chr10	118158000	0.644628
chr10	118442200	0.685950
chr10	118451100	0.760331
chr10	118454300	0.942149
chr10	118467100	0.809917
chr10	118527200	0.652893
chr10	118547500	0.719008
chr10	118560400	0.719008
chr10	118562700	0.685950
chr10	118593600	0.826446
chr10	118633600	0.768595
chr10	118646600	0.776860
chr10	118707700	0.876033
chr10	118749800	0.867769
chr10	118752100	0.719008

chr10	118855000	0.851240
chr10	118900200	0.644628
chr10	118947000	0.669421
chr10	118981100	0.644628
chr10	119087100	0.669421
chr10	119100000	0.884298
chr10	119104800	0.983471
chr10	119107900	0.727273
chr10	119113400	0.661157
chr10	119185500	0.884298
chr10	119202700	0.727273
chr10	119221900	0.776860
chr10	119291900	0.900826
chr10	119295300	0.818182
chr10	119298200	0.776860
chr10	119370000	0.735537
chr10	119590700	0.975207
chr10	119599000	0.685950
chr10	119659000	0.677686
chr10	119689200	0.900826
chr10	119695000	0.644628
chr10	119715400	0.644628
chr10	119733900	0.685950
chr10	119748700	0.793388
chr10	119830200	0.661157
chr10	119964300	0.809917
chr10	120031800	0.826446
chr10	120055000	0.710744
chr10	120167800	0.818182
chr10	120282800	0.776860
chr10	120461600	0.876033
chr10	120491900	0.710744
chr10	120782900	0.884298
chr10	120977800	0.685950
chr10	120982200	0.702479
chr10	120997800	0.719008
chr10	121001800	0.669421
chr10	121013300	0.652893
chr10	121075300	0.727273
chr10	121077900	0.867769
chr10	121172800	0.652893
chr10	121182100	0.933884
chr10	121239100	0.669421
chr10	121413600	0.702479
chr10	121427500	0.884298
chr10	121432600	0.685950
chr10	121449000	0.652893
chr10	121466700	0.743802
chr10	121510500	0.652893
chr10	121546400	0.966942
chr10	121577300	0.760331
chr10	121811500	0.851240
chr10	121837400	0.809917
chr10	121858800	0.727273

chr10	121891400	0.768595
chr10	122038300	0.892562
chr10	122358900	0.776860
chr10	122400900	0.652893
chr10	122404800	0.793388
chr10	122525100	0.760331
chr10	122552300	0.809917
chr10	122585200	0.851240
chr10	122700200	0.851240
chr10	122702700	0.710744
chr10	122711200	0.719008
chr10	122723000	0.752066
chr10	122741700	0.809917
chr10	122792800	0.702479
chr10	122864400	0.677686
chr10	123044400	0.677686
chr10	123049000	0.991736
chr10	123098500	0.694215
chr10	123100600	0.958678
chr10	123196800	0.785124
chr10	123219600	0.991736
chr10	123288100	0.719008
chr10	123296400	0.677686
chr10	123303000	0.983471
chr10	123306900	0.677686
chr10	123319100	0.768595
chr10	123326100	0.801653
chr10	123330200	0.909091
chr10	123337600	0.818182
chr10	123360500	0.909091
chr10	123368400	0.694215
chr10	123428400	0.719008
chr10	123468000	0.677686
chr10	123470200	0.752066
chr10	123483600	0.760331
chr10	123492200	0.669421
chr10	123507400	0.661157
chr10	123651200	1.000000
chr10	123660300	0.702479
chr10	123754600	0.842975
chr10	123779200	0.859504
chr10	123804400	0.950413
chr10	123880100	0.752066
chr10	123886800	0.958678
chr10	123892900	0.933884
chr10	123900600	0.859504
chr10	123914600	0.727273
chr10	123919600	0.925620
chr10	123931100	0.892562
chr10	123933200	0.818182
chr10	123938400	0.983471
chr10	123941800	0.975207
chr10	123944000	0.785124
chr10	123947200	0.818182

chr10	123952400	0.702479
chr10	124018400	0.917355
chr10	124058800	0.743802
chr10	124103000	0.685950
chr10	124128700	0.694215
chr10	124219000	0.727273
chr10	124238400	0.834711
chr10	124606200	0.950413
chr10	124610300	0.884298
chr10	124616500	0.644628
chr10	124636800	0.975207
chr10	124639900	0.785124
chr10	124775200	0.818182
chr10	124777800	0.933884
chr10	124780100	0.768595
chr10	124803200	0.677686
chr10	124828800	0.661157
chr10	124877800	0.909091
chr10	125117500	0.867769
chr10	125139300	0.685950
chr10	125189900	0.669421
chr10	125219400	0.917355
chr10	125223100	0.743802
chr10	125252000	0.851240
chr10	125257000	0.735537
chr10	125259900	0.685950
chr10	125273400	0.760331
chr10	125290400	0.719008
chr10	125298600	0.950413
chr10	125318800	0.652893
chr10	125344400	0.743802
chr10	125366400	0.909091
chr10	125399700	0.892562
chr10	125402600	0.702479
chr10	125415700	0.694215
chr10	125488900	0.834711
chr10	125515400	0.851240
chr10	125542000	0.768595
chr10	125664900	0.818182
chr10	125667200	0.644628
chr10	125730300	0.826446
chr10	125766000	0.760331
chr10	125770700	0.735537
chr10	125776400	0.661157
chr10	125781200	0.876033
chr10	125786700	0.933884
chr10	125793000	0.909091
chr10	125825400	0.859504
chr10	125835200	0.735537
chr10	125847900	0.661157
chr10	125929000	0.900826
chr10	126051400	0.884298
chr10	126063800	0.661157
chr10	126075100	0.743802

chr10	126104900	0.669421
chr10	126143100	0.966942
chr10	126145300	0.644628
chr10	126148100	0.876033
chr10	126159000	0.900826
chr10	126166600	0.760331
chr10	126170500	0.694215
chr10	126185700	0.719008
chr10	126211900	0.818182
chr10	126229200	0.809917
chr10	126254300	0.685950
chr10	126314900	0.909091
chr10	126339900	0.867769
chr10	126343900	0.661157
chr10	126387100	0.644628
chr10	126394300	0.719008
chr10	126413000	0.834711
chr10	126425400	0.826446
chr10	126429500	0.727273
chr10	126445400	0.776860
chr10	126488700	0.768595
chr10	126698600	0.776860
chr10	126701400	0.826446
chr10	126706300	0.752066
chr10	126718900	0.809917
chr10	126721100	0.834711
chr10	126725700	0.909091
chr10	126728100	0.925620
chr10	126731500	0.677686
chr10	126737800	0.685950
chr10	126744200	0.842975
chr10	126746600	0.851240
chr10	126750400	0.760331
chr10	126753600	0.983471
chr10	126782200	0.719008
chr10	126784300	0.842975
chr10	126789800	0.677686
chr10	126792400	0.876033
chr10	126802500	0.669421
chr10	126808100	0.652893
chr10	126810300	0.851240
chr10	126812600	0.685950
chr10	126815900	0.958678
chr10	126819900	0.884298
chr10	126824900	0.851240
chr10	126828000	0.859504
chr10	126831200	0.818182
chr10	126833500	0.727273
chr10	126837100	0.950413
chr10	126947400	0.694215
chr10	127059500	0.677686
chr10	127234900	0.859504
chr10	127244000	0.719008
chr10	127569700	0.735537

chr10	127623500	0.652893
chr10	127726700	0.710744
chr10	127804400	0.785124
chr10	127812600	0.942149
chr10	127836900	0.892562
chr10	127842900	0.983471
chr10	127846100	0.727273
chr10	127864300	0.818182
chr10	127866500	0.917355
chr10	127904700	0.719008
chr10	128087000	0.785124
chr10	128233800	0.735537
chr10	128250900	0.661157
chr10	128259700	0.719008
chr10	128274800	0.818182
chr10	128288800	0.752066
chr10	128339800	0.644628
chr10	128376100	0.793388
chr10	128466700	0.710744
chr10	128780400	0.652893
chr10	128948100	0.900826
chr10	128951000	0.735537
chr10	129014900	0.661157
chr10	129079100	0.685950
chr10	129095000	0.735537
chr10	129109400	0.644628
chr10	129222300	0.710744
chr10	129240300	0.892562
chr10	129283800	0.793388
chr10	129494000	0.818182
chr10	129562000	0.752066
chr10	129631200	0.719008
chr10	129766700	0.876033
chr10	129861800	0.743802
chr10	129884400	0.677686
chr10	129984300	0.900826
chr10	130010900	0.876033
chr10	130110500	0.785124
chr10	130152100	0.644628
chr10	130213300	0.677686
chr10	130238900	0.925620
chr10	130326200	0.851240
chr10	130336600	0.851240
chr10	130355800	0.661157
chr10	130425100	0.694215
chr10	130478600	0.685950
chr10	130525900	0.760331
chr10	130820400	0.909091
chr10	130831200	0.776860
chr10	131230800	0.644628
chr10	131320500	0.710744
chr10	131330100	0.884298
chr10	131378600	0.760331
chr10	131441600	0.677686

chr10	131460800	0.644628
chr10	131509800	0.867769
chr10	131662000	0.652893
chr10	131666800	0.661157
chr10	131688000	0.727273
chr10	131696800	0.644628
chr10	131700400	0.909091
chr10	131710800	0.842975
chr10	131748800	0.834711
chr10	131755500	0.867769
chr10	131904300	0.661157
chr10	131992600	0.652893
chr10	132176900	0.851240
chr10	132194300	0.677686
chr10	132199700	0.685950
chr10	132414600	0.694215
chr10	132433700	0.785124
chr10	132541600	0.826446
chr10	132544000	0.710744
chr10	132609200	0.702479
chr10	132645700	0.652893
chr10	132723000	0.669421
chr10	132733300	0.909091
chr10	132799700	0.809917
chr10	132847600	0.975207
chr10	132864500	0.785124
chr10	132893300	0.925620
chr10	132913600	0.661157
chr10	132925400	0.818182
chr10	132930700	0.702479
chr10	133030100	0.669421
chr10	133119800	0.719008
chr10	133148700	0.685950
chr10	133201800	0.842975
chr10	133315600	0.669421
chr10	133484000	0.867769
chr10	133509500	0.925620
chr10	133575800	0.892562
chr10	133581300	0.909091
chr10	133590900	0.760331
chr10	133593500	0.909091
chr10	133596100	0.694215
chr10	133621000	0.842975
chr10	133657600	0.809917
chr10	133735400	0.818182
chr10	133744400	0.727273
chr10	133792000	0.867769
chr10	133811500	0.884298
chr10	133820900	0.669421
chr10	133825300	0.727273
chr10	133837300	0.900826
chr10	133857800	0.942149
chr10	133874000	0.644628
chr10	133879300	0.851240

chr10	133933800	0.694215
chr10	133949700	0.727273
chr10	133956800	0.818182
chr10	133976800	0.776860
chr10	133989900	0.818182
chr10	134107300	0.776860
chr10	134178100	0.842975
chr10	134183300	0.644628
chr10	134214800	0.834711
chr10	134217600	0.760331
chr10	134220300	0.876033
chr10	134224200	0.785124
chr10	134232400	0.752066
chr10	134243900	0.644628
chr10	134258600	0.876033
chr10	134261200	0.719008
chr10	134264600	0.669421
chr10	134281700	0.834711
chr10	134330000	0.644628
chr10	134355500	0.900826
chr10	134385200	0.809917
chr10	134392800	0.710744
chr10	134439000	0.793388
chr10	134528200	0.917355
chr10	134556600	1.000000
chr10	134560300	0.793388
chr10	134588800	0.677686
chr10	134599300	0.661157
chr10	134650400	0.685950
chr10	134672200	0.760331
chr10	134693200	0.834711
chr10	134704500	0.760331
chr10	134707400	0.735537
chr10	134724900	0.727273
chr10	134727000	0.644628
chr10	134785500	0.809917
chr10	134838300	0.785124
chr10	134914900	0.818182
chr10	134965300	0.842975
chr10	134978400	0.809917
chr10	135038800	0.809917
chr10	135061400	0.644628
chr10	135143400	0.768595
chr11	268300	0.644628
chr11	284200	0.859504
chr11	300800	0.685950
chr11	306000	0.801653
chr11	326800	0.743802
chr11	334200	0.933884
chr11	336500	0.909091
chr11	341100	0.859504
chr11	356100	0.983471
chr11	368600	0.710744
chr11	410400	0.644628

chr11	446600	0.719008
chr11	469300	0.752066
chr11	571800	0.644628
chr11	626800	0.702479
chr11	640400	0.644628
chr11	642700	0.876033
chr11	693800	0.801653
chr11	708000	0.768595
chr11	710200	0.694215
chr11	720800	0.760331
chr11	728400	0.809917
chr11	744200	0.966942
chr11	763300	0.917355
chr11	772300	0.818182
chr11	779800	0.652893
chr11	789600	0.776860
chr11	852800	0.644628
chr11	855200	0.685950
chr11	860100	0.702479
chr11	863600	0.909091
chr11	934500	0.933884
chr11	941100	0.776860
chr11	945800	0.991736
chr11	953000	0.842975
chr11	955700	0.909091
chr11	1093300	0.685950
chr11	1246000	0.785124
chr11	1284100	0.785124
chr11	1324300	0.719008
chr11	1333000	0.644628
chr11	1370800	0.785124
chr11	1423400	0.652893
chr11	1463900	0.876033
chr11	1528900	0.975207
chr11	1534500	0.735537
chr11	1563700	0.685950
chr11	1578000	0.809917
chr11	1580500	0.900826
chr11	1586100	0.917355
chr11	1589700	0.917355
chr11	1596100	0.719008
chr11	1618400	0.685950
chr11	1650600	0.685950
chr11	1674500	0.752066
chr11	1722600	0.652893
chr11	1794300	0.925620
chr11	1885500	0.719008
chr11	1904100	0.669421
chr11	1948800	0.702479
chr11	1986400	0.644628
chr11	1991000	0.826446
chr11	2004600	0.785124
chr11	2026100	0.975207
chr11	2058200	0.685950

chr11	2119200	0.966942
chr11	2121400	0.669421
chr11	2162100	0.793388
chr11	2165500	0.760331
chr11	2172400	0.727273
chr11	2216500	0.677686
chr11	2235200	0.834711
chr11	2299400	0.694215
chr11	2311800	0.760331
chr11	2360800	0.677686
chr11	2411700	0.900826
chr11	2442100	0.719008
chr11	2555400	0.884298
chr11	2709400	0.809917
chr11	2712600	0.909091
chr11	2714800	0.876033
chr11	2717700	0.735537
chr11	2725100	0.834711
chr11	2740000	0.652893
chr11	2742600	0.859504
chr11	2810000	0.719008
chr11	2813500	0.694215
chr11	2827600	0.793388
chr11	2847700	0.793388
chr11	2859400	0.702479
chr11	2867800	0.661157
chr11	2884200	0.884298
chr11	2892500	0.702479
chr11	2903800	0.768595
chr11	2912600	0.760331
chr11	3022200	0.801653
chr11	3073700	0.842975
chr11	3128900	0.867769
chr11	3160400	0.644628
chr11	3175400	0.669421
chr11	3190900	0.859504
chr11	3210800	0.958678
chr11	3238100	0.669421
chr11	3242100	0.727273
chr11	3396400	0.884298
chr11	3731700	0.735537
chr11	3846600	0.702479
chr11	3849000	0.768595
chr11	3856400	0.942149
chr11	3859900	0.760331
chr11	3880900	0.652893
chr11	3883500	0.818182
chr11	3972800	0.661157
chr11	4485500	0.917355
chr11	4488100	0.917355
chr11	4725800	0.950413
chr11	4884600	0.818182
chr11	5279600	0.685950
chr11	5646400	0.694215

chr11	6325400	0.702479
chr11	6345100	1.000000
chr11	6384400	0.644628
chr11	6402200	0.694215
chr11	6406800	0.942149
chr11	6426400	0.966942
chr11	6490500	0.909091
chr11	6497500	0.685950
chr11	6519100	0.801653
chr11	6560100	0.727273
chr11	6628500	0.983471
chr11	6822500	0.727273
chr11	6831900	0.925620
chr11	6865300	0.760331
chr11	6869800	0.760331
chr11	6950700	0.785124
chr11	7261500	0.710744
chr11	7370200	0.710744
chr11	7436800	0.694215
chr11	7469700	0.859504
chr11	7513500	0.743802
chr11	7593300	0.768595
chr11	7596100	0.661157
chr11	7599600	0.818182
chr11	7616800	0.809917
chr11	7642700	0.743802
chr11	7927000	0.900826
chr11	7929200	0.652893
chr11	7931600	0.752066
chr11	7992200	0.826446
chr11	8038200	0.917355
chr11	8054700	0.818182
chr11	8094500	0.925620
chr11	8105300	0.685950
chr11	8108400	0.884298
chr11	8214700	0.834711
chr11	8264900	0.900826
chr11	8306200	0.677686
chr11	8351200	0.900826
chr11	8364500	0.884298
chr11	8662700	0.661157
chr11	8680100	0.760331
chr11	8693200	0.950413
chr11	8719900	0.867769
chr11	8724000	0.859504
chr11	8730600	0.892562
chr11	8740400	0.677686
chr11	8743800	0.801653
chr11	8833500	0.719008
chr11	8836100	0.950413
chr11	8848400	0.933884
chr11	8850500	0.710744
chr11	8853000	0.743802
chr11	8855600	0.892562

chr11	8861900	0.991736
chr11	8938900	0.826446
chr11	9037500	0.677686
chr11	9058900	0.809917
chr11	9143000	0.917355
chr11	9159900	0.785124
chr11	9251000	0.719008
chr11	9253400	0.884298
chr11	9333200	0.685950
chr11	9341300	0.801653
chr11	9384700	0.644628
chr11	9528900	0.983471
chr11	9551200	0.644628
chr11	9555900	0.719008
chr11	9588000	0.925620
chr11	9590800	0.859504
chr11	9616500	0.727273
chr11	9623100	0.702479
chr11	9629200	0.785124
chr11	9649200	0.942149
chr11	9697300	0.859504
chr11	9700400	0.752066
chr11	9710600	0.793388
chr11	9733500	0.768595
chr11	9787400	0.834711
chr11	9797100	0.652893
chr11	9824600	0.925620
chr11	9842400	0.785124
chr11	9861200	0.727273
chr11	9880200	0.933884
chr11	9927500	0.909091
chr11	9945300	0.752066
chr11	9995500	0.801653
chr11	10024800	0.719008
chr11	10043400	0.652893
chr11	10065900	0.900826
chr11	10089400	0.818182
chr11	10224800	0.710744
chr11	10311600	0.694215
chr11	10332000	0.884298
chr11	10339800	0.685950
chr11	10345000	0.793388
chr11	10371800	0.702479
chr11	10375200	0.975207
chr11	10388400	0.661157
chr11	10428100	0.719008
chr11	10477800	0.925620
chr11	10514600	0.661157
chr11	10531200	0.677686
chr11	10533300	0.818182
chr11	10628000	0.826446
chr11	10659300	0.735537
chr11	10757100	0.661157
chr11	10788300	0.694215

chr11	10806400	0.768595
chr11	10814500	0.685950
chr11	10930200	0.702479
chr11	10934100	0.826446
chr11	11032700	0.867769
chr11	11042500	0.685950
chr11	11087200	0.983471
chr11	11093800	0.677686
chr11	11103300	0.727273
chr11	11143100	0.743802
chr11	11259400	0.727273
chr11	11265700	0.884298
chr11	11302300	0.702479
chr11	11426100	0.851240
chr11	11443600	0.694215
chr11	11523100	0.702479
chr11	11536200	0.900826
chr11	11567700	0.818182
chr11	11611000	0.867769
chr11	11629700	0.702479
chr11	11650800	0.818182
chr11	11683500	0.752066
chr11	11713500	0.933884
chr11	11757500	0.661157
chr11	11811600	0.669421
chr11	11866700	0.818182
chr11	11871100	0.851240
chr11	11886400	0.652893
chr11	11893600	0.719008
chr11	12072400	0.743802
chr11	12089000	0.719008
chr11	12114000	0.966942
chr11	12125600	0.900826
chr11	12159900	0.644628
chr11	12264000	0.826446
chr11	12270700	0.652893
chr11	12274100	0.793388
chr11	12309800	0.710744
chr11	12352700	0.909091
chr11	12434100	0.900826
chr11	12495100	0.661157
chr11	12578700	0.925620
chr11	12609300	0.677686
chr11	12700200	0.669421
chr11	12702300	0.942149
chr11	12706600	0.677686
chr11	12717100	0.859504
chr11	12720500	0.652893
chr11	12724200	0.710744
chr11	12726400	0.661157
chr11	12729000	0.661157
chr11	12739200	0.694215
chr11	12745300	0.933884
chr11	12747500	0.834711

chr11	12767600	0.851240
chr11	12773800	0.677686
chr11	12777700	0.876033
chr11	12790800	0.801653
chr11	12811300	0.867769
chr11	12820300	0.702479
chr11	12825800	0.768595
chr11	12829400	0.818182
chr11	12852300	0.958678
chr11	12868300	0.909091
chr11	12887400	0.966942
chr11	12890300	0.900826
chr11	12894800	0.685950
chr11	12924400	0.818182
chr11	12936500	0.867769
chr11	13025800	0.661157
chr11	13034600	0.801653
chr11	13124100	0.851240
chr11	13136500	0.826446
chr11	13157400	0.677686
chr11	13229700	0.793388
chr11	13238300	0.652893
chr11	13305700	0.818182
chr11	13336400	0.685950
chr11	13456300	0.859504
chr11	13462100	0.719008
chr11	13494100	0.851240
chr11	13799400	0.818182
chr11	13895200	0.719008
chr11	13939100	0.760331
chr11	13988100	0.884298
chr11	14201500	0.702479
chr11	14246300	0.661157
chr11	14254300	0.834711
chr11	14270500	0.785124
chr11	14321700	0.892562
chr11	14330000	0.710744
chr11	14332100	0.785124
chr11	14335900	0.768595
chr11	14340000	0.710744
chr11	14351600	0.661157
chr11	14357500	0.925620
chr11	14361100	0.867769
chr11	14364500	0.826446
chr11	14372300	0.892562
chr11	14429500	0.909091
chr11	14444300	0.950413
chr11	14563400	0.702479
chr11	14748500	0.710744
chr11	14929600	0.702479
chr11	14991500	0.727273
chr11	15000700	0.710744
chr11	15063500	0.694215
chr11	15329200	0.859504

chr11	15350000	0.768595
chr11	15363700	0.867769
chr11	15493600	0.644628
chr11	15574000	0.743802
chr11	15580500	0.768595
chr11	15629300	0.685950
chr11	15672100	0.892562
chr11	15685800	0.694215
chr11	15733200	0.867769
chr11	15865200	0.975207
chr11	15867500	0.661157
chr11	15893800	0.760331
chr11	16068800	0.652893
chr11	16133700	0.818182
chr11	16193900	0.677686
chr11	16204100	0.661157
chr11	16216700	0.652893
chr11	16425000	0.710744
chr11	16632900	0.785124
chr11	16691500	0.867769
chr11	16834600	0.785124
chr11	16854700	0.677686
chr11	16885000	0.809917
chr11	16915800	0.851240
chr11	16919300	0.842975
chr11	16921700	0.727273
chr11	16935100	0.652893
chr11	16978300	0.876033
chr11	16982100	0.900826
chr11	16998100	0.677686
chr11	17004300	0.743802
chr11	17009200	0.818182
chr11	17479800	0.785124
chr11	17495100	0.685950
chr11	17501800	0.685950
chr11	17520200	0.809917
chr11	17564200	0.652893
chr11	17597400	0.685950
chr11	17602100	0.652893
chr11	17623500	0.644628
chr11	17760800	0.776860
chr11	17823700	0.644628
chr11	17885000	0.652893
chr11	17897700	0.760331
chr11	17909500	0.776860
chr11	17928100	0.677686
chr11	17940100	0.801653
chr11	17943700	0.661157
chr11	18000500	0.719008
chr11	18405900	0.727273
chr11	18748100	0.743802
chr11	18750200	0.743802
chr11	18887400	0.669421
chr11	18957800	0.652893

chr11	19095900	0.950413
chr11	19333600	0.735537
chr11	19417300	0.661157
chr11	19452900	0.677686
chr11	19455000	0.867769
chr11	19477900	0.876033
chr11	19497200	0.644628
chr11	19696100	0.735537
chr11	19698500	0.818182
chr11	19702100	0.842975
chr11	19761600	0.685950
chr11	19775700	0.958678
chr11	19782100	0.809917
chr11	19794100	0.933884
chr11	19803300	0.677686
chr11	19813400	0.867769
chr11	19816100	0.867769
chr11	19818900	0.826446
chr11	19822600	0.752066
chr11	19825600	0.809917
chr11	19837000	0.669421
chr11	19843300	0.793388
chr11	19848400	0.776860
chr11	19869900	0.768595
chr11	19886100	0.793388
chr11	19889500	0.719008
chr11	19895600	0.719008
chr11	19925100	0.644628
chr11	19956900	0.735537
chr11	19974200	0.727273
chr11	20010500	0.942149
chr11	20020600	0.735537
chr11	20023500	0.743802
chr11	20037900	0.685950
chr11	20053400	0.909091
chr11	20072900	0.876033
chr11	20087200	0.867769
chr11	20150800	0.743802
chr11	20176300	0.809917
chr11	20269600	0.909091
chr11	20364600	0.925620
chr11	20496000	0.719008
chr11	20519200	0.975207
chr11	20544600	0.900826
chr11	20566500	0.809917
chr11	20643800	0.661157
chr11	20680000	0.719008
chr11	20761500	0.801653
chr11	20855400	0.644628
chr11	20903200	0.884298
chr11	20935100	0.719008
chr11	20972300	0.669421
chr11	21081600	0.661157
chr11	21271600	0.669421

chr11	21317300	0.727273
chr11	21357000	0.694215
chr11	21469700	0.719008
chr11	21498700	0.842975
chr11	21883100	0.793388
chr11	21934300	0.652893
chr11	22256900	0.677686
chr11	22393000	0.900826
chr11	22657600	0.867769
chr11	22662600	0.818182
chr11	23013500	0.694215
chr11	23513100	0.884298
chr11	25005600	0.785124
chr11	25566600	0.702479
chr11	25601300	0.983471
chr11	26165900	0.702479
chr11	26293900	0.710744
chr11	26900900	0.694215
chr11	26937100	0.644628
chr11	27224000	0.710744
chr11	27237800	0.644628
chr11	27350400	0.917355
chr11	27369000	0.760331
chr11	27371700	0.842975
chr11	27409800	0.669421
chr11	27412200	0.710744
chr11	27417100	0.776860
chr11	27449300	0.702479
chr11	27453800	0.702479
chr11	27462600	0.818182
chr11	27465700	0.966942
chr11	27482000	0.685950
chr11	27486700	0.801653
chr11	27657100	0.925620
chr11	27659200	0.892562
chr11	27672000	0.710744
chr11	27709800	0.818182
chr11	27762300	0.859504
chr11	27818200	0.933884
chr11	28650400	0.867769
chr11	28719900	0.652893
chr11	28737600	0.809917
chr11	28815500	0.991736
chr11	28856100	0.669421
chr11	29247100	0.661157
chr11	29374100	0.727273
chr11	29644900	0.933884
chr11	29981600	0.793388
chr11	30047700	0.694215
chr11	30429000	0.694215
chr11	30444600	0.925620
chr11	30466800	0.677686
chr11	30469100	0.685950
chr11	30473400	0.694215

chr11	30477000	0.842975
chr11	30488400	0.685950
chr11	30503000	0.975207
chr11	30560300	0.743802
chr11	30568400	0.661157
chr11	30572000	0.694215
chr11	30575300	0.842975
chr11	30582000	0.975207
chr11	30586900	0.768595
chr11	30657300	0.776860
chr11	30771800	0.743802
chr11	30871800	0.652893
chr11	30875500	0.884298
chr11	30928000	0.661157
chr11	30936000	0.652893
chr11	31142700	0.710744
chr11	31260100	0.859504
chr11	31264200	0.809917
chr11	31439300	0.661157
chr11	31441700	0.876033
chr11	31465700	0.719008
chr11	31628700	0.677686
chr11	31672600	0.842975
chr11	31684500	0.652893
chr11	31744500	0.644628
chr11	31822300	0.677686
chr11	31824900	0.801653
chr11	31829500	0.735537
chr11	31885400	0.644628
chr11	31893100	0.776860
chr11	31898600	0.785124
chr11	31910200	0.892562
chr11	32095000	0.661157
chr11	32104800	0.842975
chr11	32284300	0.661157
chr11	32318000	0.652893
chr11	32334700	0.677686
chr11	32455400	0.917355
chr11	32461300	0.752066
chr11	32896200	0.644628
chr11	32912000	0.842975
chr11	32922100	0.661157
chr11	32924200	0.760331
chr11	33039500	0.669421
chr11	33143900	0.876033
chr11	33200000	0.826446
chr11	33209700	0.842975
chr11	33217400	0.669421
chr11	33516200	0.710744
chr11	33690500	0.917355
chr11	33705600	0.735537
chr11	33713700	0.685950
chr11	33800600	0.801653
chr11	33993000	0.652893

chr11	34018900	0.685950
chr11	34067100	0.644628
chr11	34208100	0.975207
chr11	34214600	0.884298
chr11	34226100	0.884298
chr11	34228600	0.818182
chr11	34236900	0.677686
chr11	34244700	0.917355
chr11	34256500	0.909091
chr11	34262000	0.760331
chr11	34266200	0.685950
chr11	34285000	0.834711
chr11	34332000	0.785124
chr11	34337700	0.661157
chr11	34535800	0.793388
chr11	34608200	0.727273
chr11	34653900	0.644628
chr11	34673300	0.669421
chr11	34676200	0.644628
chr11	34702700	0.900826
chr11	34858700	0.677686
chr11	35173700	0.776860
chr11	35201800	0.710744
chr11	35259900	0.859504
chr11	35500400	0.834711
chr11	35571500	0.677686
chr11	35630700	0.685950
chr11	35637500	0.669421
chr11	35676800	0.793388
chr11	35774100	0.669421
chr11	35968600	0.661157
chr11	35987400	0.685950
chr11	36000200	0.826446
chr11	36008500	0.669421
chr11	36013800	0.859504
chr11	36020300	0.677686
chr11	36024100	0.884298
chr11	36046100	0.727273
chr11	36055400	0.983471
chr11	36083100	0.677686
chr11	36091800	0.876033
chr11	36095500	0.818182
chr11	36108600	0.809917
chr11	36112900	0.933884
chr11	36115600	0.867769
chr11	36126400	0.785124
chr11	36128500	0.884298
chr11	36131200	0.719008
chr11	36134000	0.702479
chr11	36217800	0.677686
chr11	36341000	0.669421
chr11	36477000	0.925620
chr11	36489600	0.719008
chr11	36586600	0.669421

chr11	36722500	0.834711
chr11	36741000	0.752066
chr11	36769100	0.669421
chr11	36815000	0.818182
chr11	38123200	0.685950
chr11	38158900	0.892562
chr11	38174200	0.776860
chr11	38461600	0.760331
chr11	39367900	0.793388
chr11	39685300	0.735537
chr11	39729300	0.677686
chr11	40121800	0.719008
chr11	40224000	0.685950
chr11	40226200	0.933884
chr11	40249200	0.859504
chr11	40667500	0.727273
chr11	40701400	0.950413
chr11	40738900	0.710744
chr11	41040100	0.760331
chr11	41138000	0.710744
chr11	41140300	0.867769
chr11	41414200	0.727273
chr11	41416400	0.958678
chr11	41478200	0.925620
chr11	41481100	0.760331
chr11	41519600	0.925620
chr11	41544800	0.826446
chr11	41944100	0.809917
chr11	42256400	0.743802
chr11	42265800	0.669421
chr11	42429300	0.834711
chr11	42546400	0.661157
chr11	42617600	0.702479
chr11	42620300	0.834711
chr11	42863100	0.661157
chr11	43094000	0.818182
chr11	43234300	0.818182
chr11	43291200	0.694215
chr11	43571600	0.669421
chr11	43576200	0.768595
chr11	43581600	0.669421
chr11	43598900	0.727273
chr11	43653500	0.876033
chr11	43660400	0.793388
chr11	43758300	0.793388
chr11	43761000	0.669421
chr11	43935700	0.884298
chr11	43942300	0.834711
chr11	43946800	0.834711
chr11	43955800	0.677686
chr11	43976300	0.966942
chr11	44011000	0.752066
chr11	44027100	0.735537
chr11	44052500	0.851240

chr11	44200100	0.661157
chr11	44212900	0.685950
chr11	44221700	0.818182
chr11	44265500	1.000000
chr11	44308100	0.785124
chr11	44348700	0.743802
chr11	44411000	0.900826
chr11	44467900	0.859504
chr11	44489300	0.909091
chr11	44561300	0.677686
chr11	44581500	0.867769
chr11	44609300	0.644628
chr11	44637200	0.652893
chr11	44643400	0.743802
chr11	44693100	0.991736
chr11	44707200	0.644628
chr11	44755900	0.719008
chr11	44762800	0.884298
chr11	44765500	0.785124
chr11	44788300	0.752066
chr11	44796100	0.785124
chr11	44799400	0.776860
chr11	44802600	0.818182
chr11	44807900	0.884298
chr11	44819900	0.876033
chr11	44826900	1.000000
chr11	44846100	0.975207
chr11	44856300	0.842975
chr11	44864700	0.752066
chr11	44870500	0.710744
chr11	44873600	0.652893
chr11	44880600	0.933884
chr11	44884300	0.892562
chr11	44900000	0.710744
chr11	44907800	0.859504
chr11	44915100	0.876033
chr11	44933000	0.752066
chr11	44949600	0.801653
chr11	44953600	0.876033
chr11	44958800	0.851240
chr11	44968900	0.702479
chr11	44976300	0.702479
chr11	44978900	0.876033
chr11	44982100	0.917355
chr11	44984500	0.917355
chr11	44989600	0.702479
chr11	45001700	0.925620
chr11	45008800	0.661157
chr11	45078400	0.942149
chr11	45101600	0.859504
chr11	45104800	1.000000
chr11	45107300	0.909091
chr11	45109800	0.752066
chr11	45113900	0.917355

chr11	45124500	0.652893
chr11	45231700	0.958678
chr11	45320500	0.809917
chr11	45367300	0.652893
chr11	45373200	0.644628
chr11	45434100	0.743802
chr11	45453300	0.694215
chr11	45466800	0.793388
chr11	45599600	0.900826
chr11	45628500	0.917355
chr11	45680600	0.909091
chr11	45684400	0.867769
chr11	45699700	0.644628
chr11	45745500	0.669421
chr11	45838100	0.859504
chr11	45874700	0.867769
chr11	45880500	0.867769
chr11	45914900	0.900826
chr11	45918500	0.851240
chr11	45930000	0.818182
chr11	45941900	0.859504
chr11	46005100	0.975207
chr11	46016000	0.917355
chr11	46019300	0.735537
chr11	46022500	0.966942
chr11	46024600	0.900826
chr11	46027300	0.776860
chr11	46034300	0.917355
chr11	46067800	0.933884
chr11	46072500	0.942149
chr11	46077700	0.950413
chr11	46086200	0.702479
chr11	46121500	0.685950
chr11	46128000	0.669421
chr11	46136200	0.661157
chr11	46224600	0.950413
chr11	46270500	0.752066
chr11	46294000	0.793388
chr11	46302900	0.793388
chr11	46312300	0.785124
chr11	46352300	0.917355
chr11	46357200	0.661157
chr11	46360100	0.925620
chr11	46363800	0.933884
chr11	46367300	0.809917
chr11	46374600	0.909091
chr11	46379000	0.776860
chr11	46384300	0.933884
chr11	46388700	0.685950
chr11	46391500	0.719008
chr11	46440000	0.669421
chr11	46457600	0.917355
chr11	46467800	0.661157
chr11	46478300	0.785124

chr11	46486600	0.884298
chr11	46527500	0.909091
chr11	46534200	0.785124
chr11	46543200	0.710744
chr11	46550900	0.710744
chr11	46559100	0.975207
chr11	46576400	0.933884
chr11	46696400	0.983471
chr11	46760900	0.685950
chr11	46787300	0.677686
chr11	46857700	0.669421
chr11	46925800	0.826446
chr11	46928600	0.867769
chr11	46999000	0.900826
chr11	47042900	0.851240
chr11	47055400	0.743802
chr11	47083700	0.958678
chr11	47126600	0.925620
chr11	47152300	0.727273
chr11	47226300	0.859504
chr11	47279400	0.851240
chr11	47294200	0.677686
chr11	47373100	0.685950
chr11	47398600	0.652893
chr11	47489200	0.933884
chr11	47491900	0.818182
chr11	47529800	0.826446
chr11	47537400	0.950413
chr11	47545900	0.776860
chr11	47733000	0.735537
chr11	47966100	0.842975
chr11	47996300	0.884298
chr11	48013200	0.826446
chr11	48025900	0.809917
chr11	48040500	0.942149
chr11	48074200	0.900826
chr11	48077800	0.958678
chr11	48110100	0.694215
chr11	48118600	0.826446
chr11	48131400	0.859504
chr11	48156500	0.785124
chr11	48718300	0.735537
chr11	48805300	0.760331
chr11	48895400	0.702479
chr11	50760100	0.661157
chr11	51250500	0.694215
chr11	51575800	0.735537
chr11	51581100	0.669421
chr11	51587100	0.785124
chr11	51589200	0.719008
chr11	51593300	0.719008
chr11	54961900	0.735537
chr11	56105100	0.958678
chr11	56428000	0.917355

chr11	56892400	0.768595
chr11	56923900	0.867769
chr11	56972900	0.785124
chr11	56981300	0.851240
chr11	57031100	0.909091
chr11	57045700	0.942149
chr11	57057000	0.644628
chr11	57080900	0.892562
chr11	57084400	0.909091
chr11	57107500	0.710744
chr11	57111000	0.760331
chr11	57121000	0.694215
chr11	57133100	0.809917
chr11	57192700	0.661157
chr11	57231700	0.760331
chr11	57235900	0.793388
chr11	57241700	0.752066
chr11	57261500	0.859504
chr11	57268500	0.669421
chr11	57305100	0.909091
chr11	57311200	0.793388
chr11	57315100	0.685950
chr11	57341900	0.809917
chr11	57410100	0.859504
chr11	57496900	0.652893
chr11	57546500	0.685950
chr11	57550100	0.694215
chr11	57555800	0.842975
chr11	57636400	1.000000
chr11	57826200	0.925620
chr11	57829400	0.809917
chr11	58564300	0.826446
chr11	58669400	0.652893
chr11	58691400	0.942149
chr11	58723200	0.644628
chr11	58818600	0.917355
chr11	58861100	0.842975
chr11	58866000	0.834711
chr11	58943100	0.702479
chr11	58945200	0.685950
chr11	58953200	0.652893
chr11	59023000	0.719008
chr11	59194400	1.000000
chr11	59296300	0.785124
chr11	59525600	0.677686
chr11	59582200	0.652893
chr11	59614400	0.702479
chr11	59659600	0.925620
chr11	60052500	0.702479
chr11	60131700	0.909091
chr11	60135500	0.652893
chr11	60268500	0.950413
chr11	60469100	0.694215
chr11	60524700	0.752066

chr11	60543700	0.727273
chr11	60620000	0.760331
chr11	60686200	0.661157
chr11	60700200	0.900826
chr11	60715500	0.661157
chr11	60809900	0.752066
chr11	60843700	0.652893
chr11	60917600	0.793388
chr11	60920000	0.834711
chr11	60990200	0.685950
chr11	61009200	0.652893
chr11	61049100	0.727273
chr11	61103500	0.950413
chr11	61214100	0.661157
chr11	61245500	0.694215
chr11	61249300	0.851240
chr11	61256800	0.719008
chr11	61267800	0.793388
chr11	61270900	0.685950
chr11	61273800	0.793388
chr11	61280700	0.768595
chr11	61284600	0.818182
chr11	61307300	0.702479
chr11	61309700	0.661157
chr11	61337300	0.735537
chr11	61345700	0.669421
chr11	61363000	0.768595
chr11	61367200	0.851240
chr11	61390000	0.752066
chr11	61394800	0.900826
chr11	61397400	0.710744
chr11	61400400	0.661157
chr11	61404800	0.694215
chr11	61424700	0.809917
chr11	61426900	0.652893
chr11	61459200	0.694215
chr11	61462500	0.661157
chr11	61469200	0.669421
chr11	61477800	0.685950
chr11	61513300	0.644628
chr11	61517500	0.859504
chr11	61524500	0.661157
chr11	61529200	0.752066
chr11	61532800	0.685950
chr11	61539900	0.685950
chr11	61544800	0.677686
chr11	61602400	0.983471
chr11	61638800	0.677686
chr11	61648700	0.884298
chr11	61653100	0.776860
chr11	61665500	0.925620
chr11	61672600	0.702479
chr11	61676800	0.826446
chr11	61691300	0.876033

chr11	61739500	0.785124
chr11	61766700	0.966942
chr11	61769400	0.809917
chr11	62163300	0.669421
chr11	62169800	0.710744
chr11	62268100	0.867769
chr11	62276500	0.900826
chr11	62304000	0.685950
chr11	62319200	0.809917
chr11	62323800	0.834711
chr11	62327000	0.818182
chr11	62329300	0.694215
chr11	62338800	0.710744
chr11	62455600	0.652893
chr11	62457700	0.752066
chr11	62482600	0.925620
chr11	62651900	0.876033
chr11	62677000	0.975207
chr11	62783500	0.677686
chr11	62947400	0.793388
chr11	63195900	0.793388
chr11	63216600	0.917355
chr11	63246800	0.694215
chr11	63267700	0.776860
chr11	63270700	0.752066
chr11	63276200	0.710744
chr11	63278500	0.925620
chr11	63295500	0.702479
chr11	63299900	0.983471
chr11	63322100	0.652893
chr11	63334800	0.727273
chr11	63455500	0.760331
chr11	63467300	0.669421
chr11	63513500	0.661157
chr11	63599700	0.776860
chr11	63610400	0.760331
chr11	63612600	0.644628
chr11	63615100	0.991736
chr11	63636600	0.652893
chr11	63640200	0.661157
chr11	63647600	0.719008
chr11	63650600	0.793388
chr11	63655900	0.694215
chr11	63673700	0.958678
chr11	63676500	0.851240
chr11	63678600	0.793388
chr11	63746900	0.768595
chr11	63750100	0.669421
chr11	63786800	0.727273
chr11	63789600	0.867769
chr11	63792200	0.842975
chr11	63796600	0.685950
chr11	63802200	0.809917
chr11	63808900	0.719008

chr11	63835400	0.644628
chr11	63860200	0.785124
chr11	63868500	0.727273
chr11	63890000	0.809917
chr11	63895800	0.652893
chr11	63899600	0.743802
chr11	63916400	0.644628
chr11	63921400	0.909091
chr11	63949800	0.661157
chr11	64060400	0.685950
chr11	64065200	0.735537
chr11	64092400	0.735537
chr11	64099300	0.669421
chr11	64116400	0.644628
chr11	64119100	0.661157
chr11	64122900	0.950413
chr11	64141000	0.851240
chr11	64152600	0.710744
chr11	64163700	0.892562
chr11	64174100	0.727273
chr11	64245200	0.661157
chr11	64257700	0.768595
chr11	64307400	0.727273
chr11	64326200	0.809917
chr11	64334300	0.975207
chr11	64339700	0.785124
chr11	64376100	0.702479
chr11	64387900	0.661157
chr11	64397900	0.876033
chr11	64416600	0.776860
chr11	64435500	0.727273
chr11	64478400	0.661157
chr11	64484200	0.719008
chr11	64490900	0.677686
chr11	64493400	0.826446
chr11	64497900	0.752066
chr11	64527300	0.669421
chr11	64534600	0.991736
chr11	64609800	0.760331
chr11	64619300	0.719008
chr11	64631400	0.884298
chr11	64637900	0.834711
chr11	64655800	0.834711
chr11	64703300	0.933884
chr11	64764600	0.909091
chr11	64834700	0.867769
chr11	64953400	0.677686
chr11	64972600	0.933884
chr11	64979600	0.834711
chr11	65011100	0.727273
chr11	65069900	0.735537
chr11	65150100	0.867769
chr11	65158200	0.652893
chr11	65184000	0.834711

chr11	65188100	0.735537
chr11	65194200	0.818182
chr11	65227100	0.801653
chr11	65247100	0.842975
chr11	65253300	0.892562
chr11	65257600	0.710744
chr11	65264300	0.842975
chr11	65315400	0.702479
chr11	65323000	0.669421
chr11	65328600	0.735537
chr11	65344700	0.702479
chr11	65356100	0.685950
chr11	65361200	0.677686
chr11	65379700	0.644628
chr11	65404500	0.661157
chr11	65411700	0.735537
chr11	65671000	0.793388
chr11	65679900	0.760331
chr11	65714900	0.809917
chr11	65775300	0.644628
chr11	65813100	0.809917
chr11	65855000	0.735537
chr11	65880200	0.652893
chr11	65887400	0.867769
chr11	65894800	0.702479
chr11	65989900	0.809917
chr11	65996300	0.652893
chr11	66007900	0.644628
chr11	66050300	0.785124
chr11	66065400	0.760331
chr11	66085400	0.785124
chr11	66092600	0.859504
chr11	66096300	0.876033
chr11	66105200	0.818182
chr11	66136400	0.727273
chr11	66182300	0.652893
chr11	66185800	0.710744
chr11	66259700	0.958678
chr11	66307200	0.669421
chr11	66316900	0.768595
chr11	66337900	0.818182
chr11	66350800	0.842975
chr11	66411700	0.743802
chr11	66486200	0.809917
chr11	66488400	0.652893
chr11	66492600	0.710744
chr11	66578600	0.991736
chr11	66636900	0.652893
chr11	66647400	0.702479
chr11	66650400	0.909091
chr11	66673500	0.859504
chr11	66680300	0.876033
chr11	66683200	0.768595
chr11	66718700	0.702479

chr11	66755100	0.661157
chr11	66770500	0.867769
chr11	66774600	0.677686
chr11	66799500	0.851240
chr11	66822300	0.809917
chr11	66850000	0.760331
chr11	66881100	0.685950
chr11	66965100	0.760331
chr11	67030600	0.834711
chr11	67038000	0.884298
chr11	67045200	0.975207
chr11	67124600	0.760331
chr11	67147100	0.735537
chr11	67150400	0.785124
chr11	67157400	0.677686
chr11	67178000	0.892562
chr11	67184400	0.801653
chr11	67204000	0.925620
chr11	67310800	0.702479
chr11	67349500	0.966942
chr11	67353900	0.694215
chr11	67372400	0.793388
chr11	67382100	0.710744
chr11	67385700	0.685950
chr11	67395700	0.925620
chr11	67442400	0.785124
chr11	67560100	0.842975
chr11	67593400	0.743802
chr11	67652600	0.652893
chr11	67711100	0.710744
chr11	67746500	0.958678
chr11	67778000	0.793388
chr11	67808500	0.851240
chr11	67875100	0.727273
chr11	67885300	0.900826
chr11	67892900	0.752066
chr11	67896700	0.702479
chr11	67954200	0.826446
chr11	67960800	0.652893
chr11	67971600	0.685950
chr11	67977300	0.752066
chr11	68024200	0.685950
chr11	68083100	0.702479
chr11	68093200	0.677686
chr11	68096200	0.909091
chr11	68099800	0.859504
chr11	68105300	0.652893
chr11	68110600	0.917355
chr11	68122100	0.975207
chr11	68207600	0.752066
chr11	68210000	0.752066
chr11	68214900	0.685950
chr11	68237800	0.785124
chr11	68266800	0.801653

chr11	68276400	0.661157
chr11	68307400	0.760331
chr11	68312000	0.727273
chr11	68317200	0.768595
chr11	68377000	0.685950
chr11	68458400	0.752066
chr11	68461800	0.925620
chr11	68474000	0.727273
chr11	68566000	0.719008
chr11	68603500	0.768595
chr11	68633100	0.710744
chr11	68637800	0.760331
chr11	68766200	0.644628
chr11	68778800	0.719008
chr11	68803400	0.669421
chr11	68812300	0.925620
chr11	68819700	0.752066
chr11	68854000	0.719008
chr11	68862300	0.694215
chr11	68868000	0.933884
chr11	68891100	0.933884
chr11	68899400	0.925620
chr11	68920400	0.801653
chr11	68934500	0.677686
chr11	68968200	0.818182
chr11	69017900	0.661157
chr11	69026000	0.710744
chr11	69061000	0.652893
chr11	69235500	0.752066
chr11	69249900	0.892562
chr11	69258400	0.867769
chr11	69276500	0.776860
chr11	69286800	0.834711
chr11	69291800	0.909091
chr11	69299000	0.809917
chr11	69335800	0.793388
chr11	69418300	0.834711
chr11	69431000	0.727273
chr11	69473300	0.826446
chr11	69515600	0.925620
chr11	69521900	0.719008
chr11	69524200	0.801653
chr11	69527200	0.694215
chr11	69533300	0.834711
chr11	69586700	0.760331
chr11	69647800	0.842975
chr11	69657900	0.876033
chr11	69683400	0.760331
chr11	69704600	0.727273
chr11	69778800	0.809917
chr11	69781600	0.760331
chr11	69793400	0.760331
chr11	69795700	0.826446
chr11	69802300	0.677686

chr11	69812200	0.876033
chr11	69825700	0.760331
chr11	69828900	0.661157
chr11	69846400	0.900826
chr11	69875900	0.785124
chr11	69895000	0.702479
chr11	69919500	0.793388
chr11	69958100	0.760331
chr11	69969400	0.834711
chr11	69974600	0.768595
chr11	70021500	0.900826
chr11	70072800	0.719008
chr11	70094000	0.661157
chr11	70157500	0.942149
chr11	70159800	0.892562
chr11	70165400	0.785124
chr11	70228600	0.669421
chr11	70236400	0.900826
chr11	70270400	0.867769
chr11	70278900	0.685950
chr11	70310900	0.826446
chr11	70369500	0.710744
chr11	70373800	0.719008
chr11	70378500	0.933884
chr11	70391600	0.735537
chr11	70414800	0.859504
chr11	70431100	0.719008
chr11	70451100	0.867769
chr11	70458400	0.652893
chr11	70484500	0.694215
chr11	70488600	0.851240
chr11	70491000	0.694215
chr11	70494400	0.900826
chr11	70503700	0.818182
chr11	70534700	0.768595
chr11	70560000	0.752066
chr11	70589700	0.851240
chr11	70661400	0.719008
chr11	70672800	0.933884
chr11	70680800	0.983471
chr11	70708100	0.694215
chr11	70719400	0.727273
chr11	70760500	0.793388
chr11	70774500	0.710744
chr11	70823800	0.768595
chr11	70831900	0.727273
chr11	70864100	0.785124
chr11	70886700	0.900826
chr11	70952500	0.859504
chr11	70955800	0.669421
chr11	70959400	0.669421
chr11	70966900	0.776860
chr11	70977600	0.884298
chr11	71216100	0.785124

chr11	71278400	0.677686
chr11	71280800	0.719008
chr11	71290000	0.710744
chr11	71555100	0.719008
chr11	71750100	0.867769
chr11	71759900	0.851240
chr11	71781300	0.851240
chr11	71802900	0.859504
chr11	72026200	0.719008
chr11	72045000	0.826446
chr11	72107300	0.702479
chr11	72120500	0.892562
chr11	72123000	0.793388
chr11	72125800	0.694215
chr11	72129200	0.652893
chr11	72237400	0.719008
chr11	72246100	0.694215
chr11	72296600	0.652893
chr11	72303300	0.818182
chr11	72306200	0.801653
chr11	72311600	0.859504
chr11	72337500	0.661157
chr11	72347800	0.752066
chr11	72364500	0.834711
chr11	72374400	0.677686
chr11	72381200	0.652893
chr11	72388200	0.900826
chr11	72424200	0.801653
chr11	72447500	0.702479
chr11	72452600	0.909091
chr11	72455600	0.760331
chr11	72458600	0.677686
chr11	72466000	0.867769
chr11	72475600	0.842975
chr11	72479400	0.685950
chr11	72510000	0.702479
chr11	72543300	0.644628
chr11	72662300	0.719008
chr11	72723900	0.966942
chr11	72811700	0.652893
chr11	72859100	0.793388
chr11	72864300	0.743802
chr11	72880800	0.793388
chr11	72910000	0.685950
chr11	72912200	0.743802
chr11	72939800	0.694215
chr11	72954100	0.785124
chr11	72981100	0.818182
chr11	73000600	0.735537
chr11	73034000	0.694215
chr11	73046500	0.677686
chr11	73055000	0.710744
chr11	73114100	0.669421
chr11	73161300	0.776860

chr11	73205600	0.809917
chr11	73222000	0.876033
chr11	73240700	0.867769
chr11	73249700	0.950413
chr11	73340500	0.925620
chr11	73360400	0.644628
chr11	73363600	0.752066
chr11	73495600	0.776860
chr11	73530000	0.851240
chr11	73549800	0.867769
chr11	73811900	0.669421
chr11	73850500	0.859504
chr11	74099000	0.719008
chr11	74408400	0.884298
chr11	74432200	0.644628
chr11	74466700	0.909091
chr11	74561200	0.925620
chr11	74816900	0.644628
chr11	74902900	0.900826
chr11	74947800	0.933884
chr11	74988100	0.809917
chr11	75014800	0.652893
chr11	75045900	0.644628
chr11	75051800	0.876033
chr11	75055700	0.851240
chr11	75058400	0.661157
chr11	75060800	0.876033
chr11	75100200	0.685950
chr11	75114400	0.710744
chr11	75136700	0.685950
chr11	75150900	0.752066
chr11	75162600	0.661157
chr11	75166900	0.776860
chr11	75173500	0.884298
chr11	75177300	0.900826
chr11	75186600	0.818182
chr11	75201900	0.884298
chr11	75243200	0.669421
chr11	75259200	0.644628
chr11	75266100	1.000000
chr11	75270400	0.842975
chr11	75277300	0.809917
chr11	75308800	0.669421
chr11	75336200	0.834711
chr11	75339500	0.950413
chr11	75428400	0.752066
chr11	75478500	0.801653
chr11	75486400	0.818182
chr11	75523900	0.702479
chr11	75741900	0.669421
chr11	75775700	0.685950
chr11	75831500	0.785124
chr11	75887800	0.669421
chr11	75923900	0.702479

chr11	75954700	0.661157
chr11	75995300	0.685950
chr11	76019300	0.785124
chr11	76025500	0.743802
chr11	76041100	0.694215
chr11	76160600	0.925620
chr11	76164500	0.842975
chr11	76167600	0.842975
chr11	76221700	0.818182
chr11	76290100	0.859504
chr11	76321800	0.884298
chr11	76367200	0.644628
chr11	76375000	0.760331
chr11	76379000	0.719008
chr11	76397500	0.826446
chr11	76410500	0.842975
chr11	76415100	0.983471
chr11	76433700	0.677686
chr11	76446400	0.917355
chr11	76482500	0.942149
chr11	76497700	0.669421
chr11	76500100	0.752066
chr11	76505200	0.842975
chr11	76510400	0.644628
chr11	76518000	0.818182
chr11	76530400	0.785124
chr11	76549600	0.760331
chr11	76631100	0.669421
chr11	76751000	0.652893
chr11	76794100	0.776860
chr11	76851700	0.727273
chr11	76882000	0.859504
chr11	76887300	0.851240
chr11	77119400	0.892562
chr11	77129800	0.760331
chr11	77132300	0.719008
chr11	77160700	0.826446
chr11	77166300	0.669421
chr11	77168800	0.983471
chr11	77181200	0.942149
chr11	77259200	0.983471
chr11	77464500	0.942149
chr11	77587500	0.677686
chr11	77597800	0.785124
chr11	77711600	0.677686
chr11	77757500	0.809917
chr11	77762500	0.752066
chr11	77859300	0.685950
chr11	78030100	0.644628
chr11	78061900	0.652893
chr11	78076600	1.000000
chr11	78215500	0.702479
chr11	78261300	0.909091
chr11	78306600	0.768595

chr11	78362700	0.694215
chr11	78395500	0.719008
chr11	78399900	0.950413
chr11	78513600	0.818182
chr11	78524400	0.743802
chr11	78580600	0.867769
chr11	78639900	0.851240
chr11	78652300	0.834711
chr11	78654500	0.710744
chr11	78771600	0.752066
chr11	78774400	0.719008
chr11	78778700	0.933884
chr11	78784900	0.859504
chr11	78791600	0.702479
chr11	78799900	0.842975
chr11	78885600	0.752066
chr11	78939000	0.727273
chr11	78981200	0.702479
chr11	79042200	0.685950
chr11	79087100	0.909091
chr11	79104600	0.768595
chr11	79144200	0.727273
chr11	79182500	0.785124
chr11	79185200	0.719008
chr11	79197300	0.876033
chr11	79868900	0.867769
chr11	79907600	0.752066
chr11	80039500	0.727273
chr11	80493800	0.900826
chr11	80704700	0.793388
chr11	81132300	0.851240
chr11	81358900	0.694215
chr11	82586700	0.793388
chr11	82620000	0.809917
chr11	83004400	0.644628
chr11	83075800	0.661157
chr11	83079600	0.975207
chr11	83195500	0.694215
chr11	83360900	0.669421
chr11	83393100	0.793388
chr11	83404900	0.652893
chr11	83733600	0.950413
chr11	84101900	0.768595
chr11	84131300	0.942149
chr11	84137300	0.694215
chr11	84182800	0.966942
chr11	84254900	0.991736
chr11	84357600	0.776860
chr11	84361600	0.793388
chr11	84367200	0.785124
chr11	84404000	0.752066
chr11	84621000	0.735537
chr11	84866000	0.776860
chr11	84882500	0.884298

chr11	85058000	0.859504
chr11	85060400	0.727273
chr11	85182200	0.909091
chr11	85195300	1.000000
chr11	85237400	0.826446
chr11	85243000	0.685950
chr11	85252200	0.719008
chr11	85430700	0.925620
chr11	85460700	0.669421
chr11	85484300	0.776860
chr11	85630300	0.685950
chr11	85863100	0.685950
chr11	85961300	0.644628
chr11	86068100	0.702479
chr11	86189400	0.661157
chr11	86206800	0.768595
chr11	86300000	0.917355
chr11	86303700	0.909091
chr11	86306300	0.661157
chr11	86452000	0.933884
chr11	86581300	0.809917
chr11	86635800	0.842975
chr11	86651500	0.669421
chr11	86794200	0.876033
chr11	87003400	0.842975
chr11	87080300	0.776860
chr11	87205100	0.760331
chr11	87224400	0.702479
chr11	87285100	0.727273
chr11	87678900	0.694215
chr11	87682300	0.826446
chr11	87836900	0.801653
chr11	88034700	1.000000
chr11	88040800	0.793388
chr11	88042900	0.983471
chr11	88045700	0.752066
chr11	88060400	0.867769
chr11	88064600	0.900826
chr11	88190200	0.661157
chr11	88255500	0.752066
chr11	88303100	0.669421
chr11	88954500	0.818182
chr11	89235100	0.677686
chr11	89339800	0.710744
chr11	89973900	0.677686
chr11	90171700	1.000000
chr11	90187200	0.661157
chr11	90470900	0.809917
chr11	90508500	0.826446
chr11	90532000	0.644628
chr11	91261000	0.644628
chr11	91450800	0.685950
chr11	91537500	0.876033
chr11	91553000	0.719008

chr11	91948600	0.661157
chr11	91952400	0.677686
chr11	91962400	0.743802
chr11	91974000	0.785124
chr11	91987100	0.867769
chr11	92019800	0.743802
chr11	92041300	0.710744
chr11	92045400	0.892562
chr11	92049800	0.727273
chr11	92053200	0.950413
chr11	92063200	0.652893
chr11	92065300	0.669421
chr11	92080600	0.710744
chr11	92083100	0.752066
chr11	92134600	0.793388
chr11	92140400	0.809917
chr11	92174400	0.983471
chr11	92244700	0.925620
chr11	92273300	0.735537
chr11	92279000	0.752066
chr11	92526600	0.975207
chr11	92538400	0.809917
chr11	92675500	0.743802
chr11	92805800	0.991736
chr11	92808000	0.809917
chr11	92815000	0.826446
chr11	92846900	0.685950
chr11	92851300	0.677686
chr11	92925600	0.661157
chr11	93082500	0.752066
chr11	93178400	0.818182
chr11	93211300	0.859504
chr11	93216500	0.752066
chr11	93220300	0.710744
chr11	93224300	0.933884
chr11	93232000	0.950413
chr11	93250400	0.776860
chr11	93258300	0.661157
chr11	93269500	0.669421
chr11	93279300	0.644628
chr11	93294000	0.785124
chr11	93305000	0.702479
chr11	93339500	0.851240
chr11	93553600	0.685950
chr11	93612600	0.661157
chr11	93868300	0.975207
chr11	93876000	0.669421
chr11	93880600	0.743802
chr11	93886900	0.652893
chr11	93905500	0.669421
chr11	93971500	0.892562
chr11	94017500	0.776860
chr11	94036200	0.652893
chr11	94391400	0.768595

chr11	94395500	0.776860
chr11	94456300	0.644628
chr11	94468400	0.925620
chr11	94498000	0.842975
chr11	94504500	0.694215
chr11	94509800	0.900826
chr11	94514300	0.661157
chr11	94630800	0.652893
chr11	94674900	0.694215
chr11	94931400	0.652893
chr11	94966900	0.743802
chr11	94976000	0.661157
chr11	94982100	0.892562
chr11	94987100	0.983471
chr11	95013900	0.884298
chr11	95036300	0.727273
chr11	95069700	0.710744
chr11	95073300	0.859504
chr11	95093300	0.851240
chr11	95097400	0.735537
chr11	95112300	0.884298
chr11	95178200	0.661157
chr11	95232700	0.768595
chr11	95289600	0.669421
chr11	95325900	0.743802
chr11	95439000	0.702479
chr11	95723000	0.685950
chr11	95779800	0.719008
chr11	95789300	0.950413
chr11	95816300	0.719008
chr11	95894100	0.719008
chr11	95900300	0.752066
chr11	95902700	0.677686
chr11	95914300	0.842975
chr11	95928600	0.710744
chr11	96001100	0.677686
chr11	96006200	0.826446
chr11	96011800	0.776860
chr11	96040400	0.834711
chr11	96047200	0.669421
chr11	96065900	0.719008
chr11	96069600	0.768595
chr11	96159900	0.752066
chr11	96198600	0.752066
chr11	96479500	0.834711
chr11	97029600	0.760331
chr11	97593600	0.694215
chr11	97601300	0.685950
chr11	97636000	0.842975
chr11	97727600	0.685950
chr11	98940800	0.867769
chr11	99071800	0.743802
chr11	99287500	0.702479
chr11	99290000	0.842975

chr11	99300800	0.785124
chr11	99505500	0.809917
chr11	99797100	0.801653
chr11	99976800	0.702479
chr11	100275700	0.809917
chr11	100385900	0.727273
chr11	100423400	0.809917
chr11	100548000	0.801653
chr11	100575300	0.760331
chr11	100587000	0.644628
chr11	100590300	0.950413
chr11	100598500	0.966942
chr11	100606100	0.677686
chr11	100611200	0.909091
chr11	100614100	0.892562
chr11	100624600	0.752066
chr11	100656400	0.851240
chr11	100660300	0.975207
chr11	100662700	0.851240
chr11	100699300	0.834711
chr11	100702500	0.694215
chr11	100726800	0.826446
chr11	100731600	0.884298
chr11	100745800	0.752066
chr11	101385500	0.644628
chr11	101387800	0.652893
chr11	101438000	0.966942
chr11	101619500	0.834711
chr11	101664100	0.776860
chr11	101990600	0.727273
chr11	102010100	0.661157
chr11	102056000	0.644628
chr11	102069100	0.950413
chr11	102079900	0.900826
chr11	102151200	0.793388
chr11	102320800	0.652893
chr11	102366700	0.710744
chr11	102484900	0.694215
chr11	102651300	0.661157
chr11	102707400	0.809917
chr11	103197100	0.677686
chr11	103362500	0.801653
chr11	103553400	0.925620
chr11	103609700	0.694215
chr11	104274200	0.826446
chr11	104405500	0.669421
chr11	104494800	0.644628
chr11	104621100	0.892562
chr11	104840000	0.702479
chr11	104952500	0.685950
chr11	105693000	0.785124
chr11	105786000	0.743802
chr11	105831400	1.000000
chr11	106121400	0.752066

chr11	106148400	0.669421
chr11	106197800	0.859504
chr11	106806100	0.685950
chr11	106828000	0.884298
chr11	106840600	0.785124
chr11	107268100	0.851240
chr11	107465100	0.685950
chr11	107496500	0.727273
chr11	107521000	0.677686
chr11	107524200	0.702479
chr11	107541400	0.694215
chr11	107578900	0.859504
chr11	107704300	0.867769
chr11	107863000	0.909091
chr11	107986400	0.719008
chr11	108090400	0.867769
chr11	108466300	0.851240
chr11	108541100	0.677686
chr11	108662900	0.652893
chr11	108741500	0.669421
chr11	108757000	0.702479
chr11	108762000	0.818182
chr11	108831500	0.884298
chr11	108977600	0.900826
chr11	108983600	0.685950
chr11	108991800	0.669421
chr11	108995200	0.644628
chr11	109259900	0.735537
chr11	109947200	0.933884
chr11	110002400	0.760331
chr11	110044000	0.735537
chr11	110046800	0.685950
chr11	110057000	0.776860
chr11	110182400	0.892562
chr11	110234200	0.966942
chr11	110345400	0.760331
chr11	110363000	0.942149
chr11	110436000	0.826446
chr11	110634300	0.933884
chr11	110876500	0.826446
chr11	110892700	0.702479
chr11	111006400	0.727273
chr11	111101500	0.809917
chr11	111173900	0.702479
chr11	111325900	0.719008
chr11	111420500	0.677686
chr11	111432600	0.669421
chr11	111604300	0.826446
chr11	111609800	0.884298
chr11	111642100	0.719008
chr11	111694300	0.801653
chr11	111783200	0.859504
chr11	111789900	0.727273
chr11	111810300	0.727273

chr11	111832100	0.925620
chr11	111855300	0.776860
chr11	112192800	0.909091
chr11	112216800	0.644628
chr11	112233500	0.652893
chr11	112279600	0.760331
chr11	112426900	0.661157
chr11	112467500	0.743802
chr11	112483700	0.652893
chr11	112505900	0.719008
chr11	112668400	0.958678
chr11	112677700	0.702479
chr11	112693700	0.917355
chr11	112697100	0.925620
chr11	112793800	0.950413
chr11	112853300	0.743802
chr11	112958800	0.892562
chr11	113050100	0.793388
chr11	113063400	0.652893
chr11	113114100	0.652893
chr11	113258700	0.785124
chr11	113328700	0.925620
chr11	113411500	0.950413
chr11	113434600	0.867769
chr11	113439300	0.892562
chr11	113500300	0.842975
chr11	113515000	0.677686
chr11	113718700	0.884298
chr11	113721600	0.942149
chr11	113726000	0.884298
chr11	113730900	0.727273
chr11	113733700	0.942149
chr11	113766700	0.768595
chr11	113775900	0.677686
chr11	113803600	0.702479
chr11	113829100	0.669421
chr11	113846300	0.826446
chr11	113944800	0.644628
chr11	113948500	0.710744
chr11	113963100	0.694215
chr11	114031400	0.710744
chr11	114099900	0.685950
chr11	114111500	0.677686
chr11	114161200	0.710744
chr11	114186900	0.719008
chr11	114211600	0.677686
chr11	114248800	0.694215
chr11	114349400	0.975207
chr11	114353300	0.867769
chr11	114437500	0.909091
chr11	114644900	0.785124
chr11	114670500	0.785124
chr11	114688800	0.876033
chr11	114692300	0.694215

chr11	114703600	0.685950
chr11	114718400	0.818182
chr11	114760800	0.809917
chr11	114989600	0.859504
chr11	115036000	0.842975
chr11	115041100	0.644628
chr11	115069000	0.677686
chr11	115097900	0.694215
chr11	115127700	0.644628
chr11	115280000	0.801653
chr11	115294400	0.776860
chr11	115369200	0.768595
chr11	115398900	0.983471
chr11	115420300	0.793388
chr11	115452300	0.652893
chr11	115461500	0.669421
chr11	115483000	0.752066
chr11	115501100	0.710744
chr11	115517000	0.776860
chr11	115545300	0.644628
chr11	115567000	0.768595
chr11	115616000	0.702479
chr11	115689100	0.818182
chr11	115774100	0.909091
chr11	115832500	0.727273
chr11	115860300	0.719008
chr11	115907300	0.867769
chr11	115915900	0.694215
chr11	115924500	0.842975
chr11	115931100	0.661157
chr11	115943300	0.702479
chr11	115978900	0.842975
chr11	116033700	0.867769
chr11	116045200	0.735537
chr11	116048300	0.760331
chr11	116061500	0.719008
chr11	116096200	0.826446
chr11	116106200	0.669421
chr11	116115500	0.669421
chr11	116134800	0.710744
chr11	116237900	0.801653
chr11	116271300	0.735537
chr11	116294300	0.834711
chr11	116299500	0.677686
chr11	116316700	0.975207
chr11	116325200	0.710744
chr11	116332900	0.785124
chr11	116344400	0.818182
chr11	116377100	0.842975
chr11	116404600	0.925620
chr11	116522900	0.677686
chr11	116532000	0.644628
chr11	116558600	0.818182
chr11	116567500	0.694215

chr11	116574300	0.867769
chr11	116578400	0.710744
chr11	116581700	0.900826
chr11	116585800	0.876033
chr11	116602200	0.685950
chr11	116801600	0.694215
chr11	116827100	0.685950
chr11	116857900	0.752066
chr11	116861300	0.760331
chr11	116908600	0.727273
chr11	116948300	0.793388
chr11	117017800	0.793388
chr11	117072400	0.785124
chr11	117171000	0.785124
chr11	117175000	0.867769
chr11	117300900	0.644628
chr11	117344300	0.859504
chr11	117355100	0.669421
chr11	117363300	0.785124
chr11	117373500	0.884298
chr11	117381700	0.652893
chr11	117383800	0.743802
chr11	117399400	0.752066
chr11	117418000	0.925620
chr11	117454700	0.884298
chr11	117482600	0.652893
chr11	117534500	0.694215
chr11	117537400	0.644628
chr11	117541700	0.826446
chr11	117553200	0.685950
chr11	117560600	0.702479
chr11	117562800	0.719008
chr11	117567600	0.785124
chr11	117573300	0.694215
chr11	117582600	0.694215
chr11	117594600	0.776860
chr11	117605900	0.776860
chr11	117630500	0.834711
chr11	117634600	0.752066
chr11	117685800	0.644628
chr11	117688300	0.809917
chr11	117691100	0.743802
chr11	117695000	0.694215
chr11	117702300	0.652893
chr11	117722200	0.669421
chr11	117732600	0.702479
chr11	117740800	0.702479
chr11	117778600	0.900826
chr11	117800900	0.983471
chr11	117947900	0.776860
chr11	117970500	0.900826
chr11	118019000	0.760331
chr11	118026100	0.785124
chr11	118037200	0.826446

chr11	118051500	0.842975
chr11	118065900	0.743802
chr11	118069200	0.909091
chr11	118082200	0.991736
chr11	118084500	0.834711
chr11	118187900	0.652893
chr11	118286600	0.942149
chr11	118310900	0.809917
chr11	118313200	0.892562
chr11	118476700	0.859504
chr11	118483100	0.884298
chr11	118486100	0.925620
chr11	118490100	0.719008
chr11	118496400	0.719008
chr11	118530600	0.801653
chr11	118534000	0.851240
chr11	118550200	0.644628
chr11	118563800	0.644628
chr11	118576800	0.702479
chr11	118586400	0.925620
chr11	118741300	0.768595
chr11	118770400	0.752066
chr11	118778600	0.768595
chr11	118785500	0.900826
chr11	118787900	0.719008
chr11	118811100	0.876033
chr11	118851100	0.909091
chr11	118916200	0.661157
chr11	118967500	0.727273
chr11	119189800	0.702479
chr11	119208100	0.876033
chr11	119238900	0.809917
chr11	119242000	0.818182
chr11	119245600	0.884298
chr11	119249500	0.867769
chr11	119288300	0.752066
chr11	119290400	0.727273
chr11	119296600	0.917355
chr11	119303600	0.884298
chr11	119330500	0.727273
chr11	119345800	0.752066
chr11	119367900	0.933884
chr11	119383500	0.702479
chr11	119390900	0.685950
chr11	119405100	0.661157
chr11	119461800	0.851240
chr11	119479200	0.809917
chr11	119488900	0.743802
chr11	119495100	0.743802
chr11	119518000	0.719008
chr11	119522800	0.776860
chr11	119534600	0.900826
chr11	119537400	0.677686
chr11	119555400	0.785124

chr11	119558000	0.702479
chr11	119563400	0.652893
chr11	119568200	0.826446
chr11	119580600	0.793388
chr11	119584200	0.801653
chr11	119593000	0.909091
chr11	119626300	0.752066
chr11	119662400	0.768595
chr11	119665300	0.719008
chr11	119668900	0.719008
chr11	119698400	0.793388
chr11	119735100	0.652893
chr11	119751300	0.727273
chr11	119807000	0.892562
chr11	119812600	0.809917
chr11	119835300	0.900826
chr11	119862800	0.677686
chr11	119872400	0.752066
chr11	119894500	0.702479
chr11	119901500	0.685950
chr11	119926600	0.892562
chr11	119929100	0.991736
chr11	119967800	0.826446
chr11	119978200	0.677686
chr11	120018700	0.685950
chr11	120051400	0.727273
chr11	120067600	0.719008
chr11	120084100	0.842975
chr11	120106700	0.818182
chr11	120116300	0.760331
chr11	120119700	0.942149
chr11	120170600	0.702479
chr11	120177600	0.867769
chr11	120188000	0.884298
chr11	120269100	0.694215
chr11	120271900	0.702479
chr11	120275300	0.818182
chr11	120358600	0.743802
chr11	120374600	0.867769
chr11	120394200	0.669421
chr11	120398400	0.942149
chr11	120426800	0.669421
chr11	120441100	0.867769
chr11	120446200	0.669421
chr11	120463000	0.644628
chr11	120481900	0.677686
chr11	120516500	0.652893
chr11	120533700	0.685950
chr11	120552900	0.669421
chr11	120581700	0.776860
chr11	120590000	0.694215
chr11	120619400	0.710744
chr11	120630200	0.661157
chr11	120642900	0.760331

chr11	120646800	0.719008
chr11	120678600	0.900826
chr11	120800300	0.644628
chr11	120809400	0.768595
chr11	120823800	0.958678
chr11	120828200	0.867769
chr11	120875200	0.760331
chr11	120910600	0.735537
chr11	120942800	0.950413
chr11	121112600	0.743802
chr11	121117500	0.760331
chr11	121120200	0.644628
chr11	121126900	0.826446
chr11	121169100	0.826446
chr11	121243600	0.983471
chr11	121250200	0.884298
chr11	121260500	0.909091
chr11	121277200	0.851240
chr11	121279400	0.719008
chr11	121317300	0.685950
chr11	121330000	0.768595
chr11	121334900	0.719008
chr11	121339400	0.942149
chr11	121350200	0.942149
chr11	121353600	0.867769
chr11	121365600	0.826446
chr11	121368600	0.818182
chr11	121379400	0.942149
chr11	121435400	0.743802
chr11	121493800	0.644628
chr11	121514800	0.867769
chr11	121559000	0.776860
chr11	121626700	0.818182
chr11	121665800	0.652893
chr11	121674000	0.661157
chr11	121712900	0.776860
chr11	122030500	0.735537
chr11	122073900	0.809917
chr11	122086000	0.719008
chr11	122100700	0.925620
chr11	122124700	0.785124
chr11	122177400	0.694215
chr11	122180100	0.727273
chr11	122562700	0.793388
chr11	122595100	0.644628
chr11	122684400	0.818182
chr11	122750700	0.851240
chr11	122852800	0.950413
chr11	122874500	0.727273
chr11	122882700	0.644628
chr11	123008700	0.785124
chr11	123040600	0.661157
chr11	123056100	0.752066
chr11	123059500	0.776860

chr11	123063300	0.785124
chr11	123069000	0.900826
chr11	123073500	0.685950
chr11	123089800	0.652893
chr11	123184000	0.892562
chr11	123222300	0.652893
chr11	123237100	0.859504
chr11	123248300	0.900826
chr11	123279100	0.652893
chr11	123349900	0.644628
chr11	123365000	0.909091
chr11	123377100	0.768595
chr11	123381300	0.876033
chr11	123390500	0.851240
chr11	123397000	0.652893
chr11	123402000	0.661157
chr11	123408200	0.975207
chr11	123421100	0.669421
chr11	123425900	0.685950
chr11	123431600	0.909091
chr11	123452300	0.702479
chr11	123542500	0.652893
chr11	123581900	0.809917
chr11	123617400	0.685950
chr11	123645300	0.975207
chr11	123648700	0.669421
chr11	123652000	0.859504
chr11	123724200	0.685950
chr11	123730700	0.884298
chr11	124061200	0.785124
chr11	124352100	0.801653
chr11	124514800	0.702479
chr11	124527500	0.752066
chr11	124595700	0.842975
chr11	124627000	0.652893
chr11	124660900	0.818182
chr11	124663300	0.685950
chr11	124713400	0.958678
chr11	124718400	0.669421
chr11	124733500	0.909091
chr11	124768600	0.752066
chr11	124962500	0.677686
chr11	125007600	0.669421
chr11	125009700	0.851240
chr11	125011900	0.975207
chr11	125039700	0.867769
chr11	125041900	0.785124
chr11	125047000	0.710744
chr11	125051500	0.793388
chr11	125071600	0.809917
chr11	125074200	0.892562
chr11	125077200	0.892562
chr11	125085000	0.859504
chr11	125098800	0.834711

chr11	125106100	0.685950
chr11	125110300	0.983471
chr11	125133300	0.867769
chr11	125157100	0.719008
chr11	125185200	0.661157
chr11	125190800	0.694215
chr11	125201200	0.776860
chr11	125212900	0.685950
chr11	125218400	0.752066
chr11	125242400	0.719008
chr11	125246300	0.677686
chr11	125261500	0.925620
chr11	125270400	0.876033
chr11	125274800	0.727273
chr11	125280400	0.727273
chr11	125288800	0.809917
chr11	125299700	0.685950
chr11	125303700	0.752066
chr11	125314300	0.818182
chr11	125322100	0.834711
chr11	125328100	0.685950
chr11	125335300	0.702479
chr11	125352100	0.727273
chr11	125387100	0.768595
chr11	125744300	0.760331
chr11	125811900	0.884298
chr11	125863500	0.694215
chr11	125904400	0.710744
chr11	125929600	0.735537
chr11	125942700	0.925620
chr11	125950000	0.719008
chr11	125954100	0.867769
chr11	125971200	0.752066
chr11	125986300	0.677686
chr11	126010100	0.776860
chr11	126016900	0.917355
chr11	126025700	0.710744
chr11	126206900	0.834711
chr11	126215800	0.768595
chr11	126223300	0.669421
chr11	126228500	0.652893
chr11	126236300	0.785124
chr11	126238900	0.876033
chr11	126256900	0.842975
chr11	126273300	0.793388
chr11	126305100	0.644628
chr11	126311100	0.892562
chr11	126313500	0.760331
chr11	126350800	0.785124
chr11	126365600	0.760331
chr11	126369900	0.644628
chr11	126372900	0.685950
chr11	126414500	0.884298
chr11	126434900	0.793388

chr11	126452700	0.719008
chr11	126456200	0.966942
chr11	126459300	0.710744
chr11	126472300	0.958678
chr11	126477900	0.859504
chr11	126577900	0.677686
chr11	126686800	0.652893
chr11	126694300	0.900826
chr11	126760800	0.851240
chr11	126848300	0.842975
chr11	126860500	0.900826
chr11	126922700	0.685950
chr11	126928900	0.644628
chr11	126980200	0.958678
chr11	127063000	0.876033
chr11	127209000	0.776860
chr11	127263300	0.851240
chr11	127325000	0.719008
chr11	127437800	0.652893
chr11	127444800	0.752066
chr11	127764400	0.661157
chr11	128003600	0.669421
chr11	128045100	0.702479
chr11	128205900	0.727273
chr11	128212500	0.876033
chr11	128377000	0.652893
chr11	128396100	0.859504
chr11	128402900	0.892562
chr11	128420800	0.652893
chr11	128426200	0.694215
chr11	128450700	0.644628
chr11	128454100	0.644628
chr11	128463500	0.933884
chr11	128524100	0.661157
chr11	128588200	0.892562
chr11	128591000	0.652893
chr11	128603800	0.983471
chr11	128647500	0.900826
chr11	128654100	0.760331
chr11	128733200	0.661157
chr11	128742800	0.727273
chr11	128763400	0.652893
chr11	128799900	0.809917
chr11	128806900	0.975207
chr11	128834600	0.661157
chr11	129054400	0.768595
chr11	129066300	0.785124
chr11	129080400	0.900826
chr11	129085700	0.851240
chr11	129089000	0.752066
chr11	129091900	0.661157
chr11	129119300	0.661157
chr11	129122400	0.776860
chr11	129152800	0.867769

chr11	129161600	0.859504
chr11	129183200	0.752066
chr11	129186100	0.892562
chr11	129190600	0.818182
chr11	129202000	0.834711
chr11	129234400	0.669421
chr11	129323700	0.983471
chr11	129326300	0.826446
chr11	129459700	0.735537
chr11	129806000	0.884298
chr11	129816900	0.859504
chr11	129843000	0.950413
chr11	129862600	0.793388
chr11	129957500	0.710744
chr11	129985200	0.917355
chr11	130016300	0.859504
chr11	130027000	0.727273
chr11	130083300	0.735537
chr11	130087500	0.685950
chr11	130117000	0.702479
chr11	130166100	0.975207
chr11	130192900	0.842975
chr11	130200200	0.669421
chr11	130241300	0.818182
chr11	130247900	0.925620
chr11	130253500	0.975207
chr11	130260000	0.719008
chr11	130274300	0.983471
chr11	130386400	0.876033
chr11	130393100	0.710744
chr11	130470000	0.884298
chr11	130547800	0.719008
chr11	130653100	0.760331
chr11	130672700	0.669421
chr11	130735600	0.809917
chr11	130763200	0.942149
chr11	130933700	0.909091
chr11	130980000	0.644628
chr11	131084800	0.710744
chr11	131093600	0.867769
chr11	131105700	0.752066
chr11	131116700	0.793388
chr11	131160400	0.950413
chr11	131224500	0.801653
chr11	131241300	0.842975
chr11	131335800	0.644628
chr11	131341900	0.818182
chr11	131374500	0.818182
chr11	131416800	0.702479
chr11	131455500	0.867769
chr11	131461300	0.826446
chr11	131465500	0.818182
chr11	131468400	0.694215
chr11	131510500	0.884298

chr11	131515800	0.652893
chr11	131523300	0.760331
chr11	131527000	0.752066
chr11	131530400	0.842975
chr11	131533100	0.917355
chr11	131539500	0.677686
chr11	131544200	0.735537
chr11	131547800	0.768595
chr11	131558500	0.793388
chr11	131564800	0.735537
chr11	131632800	0.694215
chr11	131671100	0.760331
chr11	131673200	0.710744
chr11	131685200	0.677686
chr11	131734500	0.842975
chr11	131736700	0.950413
chr11	131740200	0.694215
chr11	131760200	1.000000
chr11	131762700	0.752066
chr11	131775000	0.685950
chr11	131788000	0.867769
chr11	131797800	0.661157
chr11	131825900	0.710744
chr11	131848400	0.917355
chr11	131852300	0.950413
chr11	131855100	0.644628
chr11	131873500	0.702479
chr11	131877000	0.719008
chr11	131879100	0.909091
chr11	131907200	0.644628
chr11	131942300	0.669421
chr11	131973100	0.710744
chr11	131983300	0.727273
chr11	131985800	0.685950
chr11	131993800	0.851240
chr11	132154700	0.834711
chr11	132202900	0.719008
chr11	132275900	0.876033
chr11	132299900	0.834711
chr11	132322000	0.900826
chr11	132327200	0.677686
chr11	132372700	0.685950
chr11	132650200	0.768595
chr11	132656600	0.793388
chr11	132691800	0.661157
chr11	132710500	0.685950
chr11	132728400	0.760331
chr11	132735000	0.735537
chr11	132772300	0.851240
chr11	132780200	0.743802
chr11	132832300	0.809917
chr11	132853400	0.644628
chr11	132869600	0.669421
chr11	132921700	0.776860

chr11	132940400	0.859504
chr11	132949100	0.710744
chr11	132966600	0.661157
chr11	132992900	0.834711
chr11	133006700	0.710744
chr11	133098400	0.702479
chr11	133109900	0.842975
chr11	133155300	0.768595
chr11	133188300	0.752066
chr11	133208300	0.809917
chr11	133295400	0.909091
chr11	133371800	0.677686
chr11	133376500	0.710744
chr11	133380400	0.892562
chr11	133411700	0.942149
chr11	133450000	0.834711
chr11	133469100	0.776860
chr11	133530200	0.975207
chr11	133582000	0.884298
chr11	133585100	0.884298
chr11	133602100	0.685950
chr11	133724700	0.867769
chr11	133765200	0.842975
chr11	133816400	0.876033
chr11	133818800	0.727273
chr11	133823800	0.677686
chr11	133837700	0.669421
chr11	133878800	0.685950
chr11	133903800	0.752066
chr11	133916900	0.677686
chr11	133920800	0.900826
chr11	133942900	0.644628
chr11	134140600	0.867769
chr11	134219900	0.917355
chr11	134247000	0.661157
chr11	134285300	0.752066
chr11	134336500	0.776860
chr11	134347700	0.859504
chr11	134373100	0.826446
chr11	134446000	0.685950
chr11	134482400	0.694215
chr11	134505700	0.652893
chr11	134524900	0.694215
chr11	134600800	0.892562
chr11	134620000	0.760331
chr11	134627000	0.644628
chr11	134671900	0.834711
chr11	134689700	0.702479
chr11	134721900	0.727273
chr11	134738800	0.809917
chr11	134753200	0.661157
chr11	134852200	0.685950
chr11	134860600	1.000000
chr11	134865100	0.950413

chr11	134869800	0.933884
chr11	134892700	0.826446
chr12	67000	0.752066
chr12	95600	0.677686
chr12	194800	0.752066
chr12	218300	0.661157
chr12	249500	0.644628
chr12	253700	0.652893
chr12	258700	0.776860
chr12	278100	0.834711
chr12	280900	0.661157
chr12	288600	0.892562
chr12	323800	0.842975
chr12	329800	0.892562
chr12	574500	0.644628
chr12	583400	0.694215
chr12	619800	0.925620
chr12	651300	0.702479
chr12	688900	0.644628
chr12	738000	0.652893
chr12	831900	0.925620
chr12	927900	0.785124
chr12	1113200	0.760331
chr12	1157600	0.809917
chr12	1161100	0.710744
chr12	1163800	0.785124
chr12	1166300	0.925620
chr12	1192800	0.677686
chr12	1313900	0.710744
chr12	1322000	0.752066
chr12	1359600	0.661157
chr12	1555400	0.925620
chr12	1584000	0.652893
chr12	1633100	0.842975
chr12	1697700	0.652893
chr12	1734500	0.710744
chr12	1760100	0.834711
chr12	1910200	0.785124
chr12	1953600	0.826446
chr12	2027600	0.719008
chr12	2056900	0.727273
chr12	2104300	0.958678
chr12	2111500	0.652893
chr12	2132700	0.694215
chr12	2144500	0.710744
chr12	2159000	0.669421
chr12	2245700	0.826446
chr12	2293500	0.809917
chr12	2297100	0.694215
chr12	2299900	0.793388
chr12	2307900	0.975207
chr12	2353800	0.694215
chr12	2365300	0.768595
chr12	2377500	0.752066

chr12	2392000	0.644628
chr12	2394800	0.685950
chr12	2398100	0.834711
chr12	2410900	0.644628
chr12	2416700	0.942149
chr12	2453900	0.752066
chr12	2520800	0.925620
chr12	2527900	0.743802
chr12	2692600	0.917355
chr12	2710100	0.694215
chr12	2734600	0.884298
chr12	2736700	0.677686
chr12	2750300	0.876033
chr12	2783600	0.710744
chr12	2793700	0.719008
chr12	2859500	0.752066
chr12	2863400	0.677686
chr12	2874900	0.669421
chr12	2888600	0.793388
chr12	2934500	1.000000
chr12	2944500	0.876033
chr12	2966600	0.851240
chr12	3022000	0.933884
chr12	3054100	0.677686
chr12	3072900	0.958678
chr12	3086800	0.983471
chr12	3112600	0.776860
chr12	3146400	0.702479
chr12	3193300	0.842975
chr12	3214200	0.859504
chr12	3217100	0.760331
chr12	3240300	0.727273
chr12	3258100	0.677686
chr12	3260700	0.892562
chr12	3274500	0.809917
chr12	3278000	0.752066
chr12	3281800	0.685950
chr12	3288500	0.652893
chr12	3305100	0.776860
chr12	3312700	0.685950
chr12	3317100	0.826446
chr12	3324100	0.909091
chr12	3336400	0.768595
chr12	3340700	0.834711
chr12	3346200	0.661157
chr12	3381000	0.644628
chr12	3409800	0.826446
chr12	3443100	0.776860
chr12	3446300	0.785124
chr12	3451800	0.727273
chr12	3531100	0.809917
chr12	3566300	0.776860
chr12	3673200	0.710744
chr12	3697400	0.677686

chr12	3709400	0.884298
chr12	3712400	0.776860
chr12	3771900	0.776860
chr12	3841900	0.801653
chr12	3885000	0.719008
chr12	3899400	0.677686
chr12	4054400	0.793388
chr12	4133900	0.966942
chr12	4140700	0.966942
chr12	4216900	0.677686
chr12	4225700	0.685950
chr12	4245000	0.826446
chr12	4256300	0.760331
chr12	4277800	0.851240
chr12	4303600	0.677686
chr12	4310800	0.851240
chr12	4350600	0.685950
chr12	4387000	0.834711
chr12	4416900	0.859504
chr12	4550300	0.694215
chr12	4560400	0.669421
chr12	4739900	0.851240
chr12	4832000	0.859504
chr12	4873100	0.727273
chr12	4967400	0.768595
chr12	5088100	0.669421
chr12	5101300	0.760331
chr12	5137000	0.669421
chr12	5148200	0.719008
chr12	5157000	0.785124
chr12	5310700	0.760331
chr12	5313900	0.727273
chr12	5355900	0.851240
chr12	5378000	0.694215
chr12	5438700	0.710744
chr12	5550800	0.669421
chr12	5587800	0.785124
chr12	5621800	0.818182
chr12	5697200	0.909091
chr12	5758200	0.818182
chr12	5854800	0.677686
chr12	5978900	0.661157
chr12	5982800	0.727273
chr12	5992000	0.842975
chr12	6074400	0.851240
chr12	6098200	0.859504
chr12	6180700	0.752066
chr12	6184500	0.652893
chr12	6194000	0.975207
chr12	6198600	0.851240
chr12	6291800	0.661157
chr12	6307400	0.694215
chr12	6312800	0.859504
chr12	6316800	0.760331

chr12	6320400	0.859504
chr12	6323700	0.991736
chr12	6327600	0.644628
chr12	6340200	0.727273
chr12	6373700	0.669421
chr12	6378300	0.933884
chr12	6383300	0.958678
chr12	6386100	0.710744
chr12	6389300	0.669421
chr12	6422200	0.867769
chr12	6432000	0.809917
chr12	6434600	0.867769
chr12	6436700	0.818182
chr12	6439000	0.809917
chr12	6443900	0.735537
chr12	6453000	0.793388
chr12	6455900	0.652893
chr12	6464800	0.727273
chr12	6493300	0.719008
chr12	6543800	0.785124
chr12	6562700	0.677686
chr12	6638200	0.809917
chr12	6649800	0.743802
chr12	6661700	0.702479
chr12	6717700	0.669421
chr12	6723400	0.834711
chr12	6729800	0.859504
chr12	6813100	0.884298
chr12	6873200	0.834711
chr12	6896400	0.834711
chr12	6927900	0.801653
chr12	6935000	0.752066
chr12	6949700	0.776860
chr12	6979800	0.859504
chr12	6994400	0.702479
chr12	7013200	0.801653
chr12	7033400	0.644628
chr12	7061600	0.826446
chr12	7074900	0.809917
chr12	7144100	0.652893
chr12	7243600	0.851240
chr12	7281200	0.677686
chr12	7315700	0.768595
chr12	7449800	0.942149
chr12	7455300	0.669421
chr12	7583500	0.661157
chr12	7795000	0.719008
chr12	7799100	0.793388
chr12	7830400	0.785124
chr12	7847600	0.975207
chr12	7863100	0.702479
chr12	7911100	0.834711
chr12	7940200	0.876033
chr12	7950500	0.884298

chr12	7952700	0.884298
chr12	7959800	0.669421
chr12	7985600	0.752066
chr12	7995800	0.768595
chr12	8000500	0.958678
chr12	8004100	0.842975
chr12	8013700	0.966942
chr12	8043500	1.000000
chr12	8058100	0.702479
chr12	8060200	0.933884
chr12	8069300	0.991736
chr12	8125400	0.867769
chr12	8130300	0.727273
chr12	8147600	0.851240
chr12	8188500	0.702479
chr12	8299200	0.785124
chr12	8549400	0.669421
chr12	8638200	0.826446
chr12	8661800	0.818182
chr12	8717200	0.752066
chr12	8789400	0.826446
chr12	8834400	0.801653
chr12	8842300	0.958678
chr12	8845600	0.991736
chr12	8864800	0.818182
chr12	8875000	0.735537
chr12	8877300	0.818182
chr12	8963200	0.727273
chr12	8967400	0.884298
chr12	8970100	0.785124
chr12	8972300	0.958678
chr12	8981500	0.809917
chr12	8984200	0.793388
chr12	8987400	0.859504
chr12	9030100	0.644628
chr12	9059800	0.958678
chr12	9063600	0.752066
chr12	9071900	0.719008
chr12	9075500	0.669421
chr12	9323200	0.876033
chr12	9506800	0.710744
chr12	9517500	0.950413
chr12	9803300	0.809917
chr12	9903400	0.669421
chr12	9937400	0.661157
chr12	9940200	0.785124
chr12	9967000	0.694215
chr12	10052100	0.702479
chr12	10055900	0.710744
chr12	10065100	0.942149
chr12	10170100	0.776860
chr12	10285900	0.661157
chr12	10358200	0.694215
chr12	10394700	0.652893

chr12	10513800	1.000000
chr12	10525600	0.685950
chr12	10789700	0.867769
chr12	10869800	0.876033
chr12	10872000	0.900826
chr12	10955300	0.925620
chr12	11127900	0.702479
chr12	11614000	0.719008
chr12	11662300	0.760331
chr12	11757900	0.900826
chr12	11761000	0.933884
chr12	11776400	0.809917
chr12	11805900	0.818182
chr12	11810900	0.719008
chr12	11815100	0.702479
chr12	11819500	0.776860
chr12	11854400	0.776860
chr12	11859700	0.760331
chr12	11894700	0.743802
chr12	11897000	0.859504
chr12	11899400	0.801653
chr12	11905900	0.851240
chr12	11948800	0.776860
chr12	11960300	0.826446
chr12	11965700	0.975207
chr12	11975100	0.851240
chr12	11980400	0.925620
chr12	12009200	0.983471
chr12	12015000	0.661157
chr12	12031400	0.776860
chr12	12038000	0.785124
chr12	12123100	0.966942
chr12	12162100	0.876033
chr12	12210200	0.909091
chr12	12240400	0.768595
chr12	12447900	0.743802
chr12	12473200	0.842975
chr12	12484700	0.834711
chr12	12519200	0.842975
chr12	12538000	0.950413
chr12	12552300	0.809917
chr12	12672300	0.685950
chr12	12680600	0.785124
chr12	12687000	0.702479
chr12	12710800	0.702479
chr12	12797100	0.677686
chr12	12811300	0.644628
chr12	12814600	0.842975
chr12	12829800	0.975207
chr12	12835000	0.677686
chr12	12839200	0.975207
chr12	12867800	0.867769
chr12	12938500	0.785124
chr12	12941100	0.669421

chr12	13025400	0.743802
chr12	13038600	0.685950
chr12	13046200	0.702479
chr12	13069800	0.776860
chr12	13133000	0.735537
chr12	13145200	0.842975
chr12	13150700	0.685950
chr12	13159200	0.661157
chr12	13181800	0.644628
chr12	13184500	0.776860
chr12	13245200	0.876033
chr12	13267700	0.669421
chr12	13277900	0.743802
chr12	13332700	0.859504
chr12	13352000	0.661157
chr12	13357400	0.867769
chr12	13364600	0.826446
chr12	13409100	0.975207
chr12	13431000	0.677686
chr12	13472900	0.719008
chr12	13588800	0.685950
chr12	13684500	0.661157
chr12	13687800	0.702479
chr12	13736700	0.661157
chr12	13741600	0.735537
chr12	13748600	0.768595
chr12	13881200	0.818182
chr12	13883600	0.694215
chr12	13899100	0.958678
chr12	13903100	0.661157
chr12	13953000	0.776860
chr12	14005200	0.710744
chr12	14117700	0.685950
chr12	14264700	0.743802
chr12	14347400	0.785124
chr12	14361100	0.842975
chr12	14365300	0.735537
chr12	14393000	0.942149
chr12	14409700	0.975207
chr12	14412900	0.892562
chr12	14415500	0.785124
chr12	14421000	0.933884
chr12	14437000	1.000000
chr12	14526000	0.851240
chr12	14537800	0.925620
chr12	14544300	0.661157
chr12	14547600	0.818182
chr12	14556600	0.652893
chr12	14571100	0.925620
chr12	14819300	0.801653
chr12	14858200	0.752066
chr12	14864500	0.909091
chr12	14867500	0.719008
chr12	14870100	0.694215

chr12	14909800	0.743802
chr12	14930200	0.694215
chr12	15155100	0.818182
chr12	15360100	0.727273
chr12	15924700	0.652893
chr12	16266400	0.710744
chr12	16478000	0.644628
chr12	16751600	0.644628
chr12	16941800	0.652893
chr12	17034300	0.710744
chr12	17173900	0.809917
chr12	17742200	0.677686
chr12	17972300	0.768595
chr12	18607700	0.900826
chr12	18630800	0.760331
chr12	18636800	0.685950
chr12	18835100	0.876033
chr12	18906600	0.752066
chr12	19017300	0.859504
chr12	19041400	0.809917
chr12	19048700	0.776860
chr12	19088700	0.652893
chr12	19104500	0.834711
chr12	19118100	0.809917
chr12	19124800	0.909091
chr12	19157000	0.991736
chr12	19159900	0.884298
chr12	19206100	0.661157
chr12	19287600	0.842975
chr12	19292200	0.677686
chr12	19335700	0.719008
chr12	19338600	0.991736
chr12	19341000	0.669421
chr12	19356800	0.727273
chr12	19367200	0.752066
chr12	19387500	0.661157
chr12	19417100	0.917355
chr12	19442000	0.735537
chr12	19535200	0.983471
chr12	19564600	0.925620
chr12	19585200	0.842975
chr12	19590700	0.900826
chr12	19600200	0.719008
chr12	19602900	0.644628
chr12	19610800	0.702479
chr12	19635800	0.694215
chr12	19750400	0.851240
chr12	20124800	0.867769
chr12	20250100	0.719008
chr12	20252800	0.818182
chr12	20418200	0.719008
chr12	20508600	0.801653
chr12	20524800	0.652893
chr12	20563300	0.793388

chr12	20704600	1.000000
chr12	20782400	0.851240
chr12	21477800	0.991736
chr12	21492700	0.727273
chr12	21748400	0.652893
chr12	21804400	0.661157
chr12	22201800	0.925620
chr12	22204400	0.685950
chr12	22235600	0.652893
chr12	22415800	0.677686
chr12	22441300	0.801653
chr12	23178500	0.826446
chr12	23208300	0.727273
chr12	23252700	0.851240
chr12	23658000	0.917355
chr12	23738800	0.958678
chr12	23785200	0.727273
chr12	23793800	0.909091
chr12	23858900	0.669421
chr12	23910300	0.876033
chr12	23920800	0.785124
chr12	23969800	0.661157
chr12	24048900	0.867769
chr12	24286700	0.983471
chr12	24350600	0.842975
chr12	24383400	0.818182
chr12	24406000	0.942149
chr12	24461900	0.942149
chr12	24533100	0.933884
chr12	24689100	0.710744
chr12	24711800	0.809917
chr12	24744500	0.644628
chr12	24852000	0.694215
chr12	24901900	0.793388
chr12	24914000	0.710744
chr12	25064500	0.727273
chr12	25082600	0.694215
chr12	25094500	0.892562
chr12	25098200	0.809917
chr12	25105000	0.909091
chr12	25305500	0.834711
chr12	25410500	0.958678
chr12	25418000	0.760331
chr12	25845300	0.851240
chr12	25864000	0.884298
chr12	25897700	0.702479
chr12	26132700	0.801653
chr12	26145900	0.801653
chr12	26150100	0.950413
chr12	26160200	0.710744
chr12	26163800	0.785124
chr12	26170900	0.677686
chr12	26174500	0.685950
chr12	26201200	0.933884

chr12	26222000	0.752066
chr12	26620300	0.710744
chr12	26663900	0.966942
chr12	26666400	0.842975
chr12	26726800	0.743802
chr12	26797400	0.760331
chr12	26831600	0.942149
chr12	26844500	0.776860
chr12	26975900	0.644628
chr12	27038200	0.809917
chr12	27311900	0.735537
chr12	27400300	0.685950
chr12	27428400	0.677686
chr12	27472900	0.851240
chr12	27498000	0.793388
chr12	27589900	0.785124
chr12	27713200	0.710744
chr12	28058800	0.917355
chr12	28105200	0.851240
chr12	28542500	0.743802
chr12	28613700	0.950413
chr12	28707300	0.644628
chr12	28912700	0.892562
chr12	29022000	0.694215
chr12	29026000	0.652893
chr12	29177300	0.669421
chr12	29223800	0.793388
chr12	29225900	0.652893
chr12	29299200	0.966942
chr12	29379100	0.867769
chr12	29381700	0.826446
chr12	29426600	0.669421
chr12	29470200	0.776860
chr12	29698100	0.809917
chr12	29744800	0.950413
chr12	29794200	0.652893
chr12	29820800	0.710744
chr12	29852000	0.719008
chr12	29916400	0.694215
chr12	29941100	0.702479
chr12	30024100	0.644628
chr12	30033000	0.809917
chr12	30049000	0.752066
chr12	30251700	0.925620
chr12	30404600	0.644628
chr12	30667800	0.652893
chr12	30904500	0.735537
chr12	30939000	0.826446
chr12	30946400	0.801653
chr12	30996900	0.743802
chr12	31058900	0.818182
chr12	31063000	0.669421
chr12	31069800	0.710744
chr12	31081600	0.661157

chr12	31127500	0.652893
chr12	31130500	0.876033
chr12	31141400	0.719008
chr12	31229500	0.801653
chr12	31233600	0.884298
chr12	31359500	0.669421
chr12	31370700	0.735537
chr12	31374000	0.719008
chr12	31378500	0.644628
chr12	31388100	0.702479
chr12	31441800	0.743802
chr12	31456800	0.793388
chr12	31463500	0.644628
chr12	31471100	0.933884
chr12	31473500	0.867769
chr12	31493500	0.677686
chr12	31515000	0.842975
chr12	31517100	0.710744
chr12	31521800	0.694215
chr12	31537200	0.785124
chr12	31541300	0.975207
chr12	31544300	0.809917
chr12	31632900	0.719008
chr12	31650700	0.909091
chr12	31660100	0.925620
chr12	31662800	0.933884
chr12	31672000	1.000000
chr12	31675900	0.876033
chr12	31872300	0.710744
chr12	31885200	0.727273
chr12	31902500	0.950413
chr12	32115400	0.644628
chr12	32119700	0.809917
chr12	32163900	0.661157
chr12	32166400	0.867769
chr12	32193400	0.950413
chr12	32213900	0.661157
chr12	32269000	0.719008
chr12	32272900	0.950413
chr12	32277700	0.884298
chr12	32294100	0.652893
chr12	32324700	0.694215
chr12	32331800	0.909091
chr12	32363900	0.842975
chr12	32402300	0.876033
chr12	32424100	0.661157
chr12	32426800	0.669421
chr12	32438800	0.991736
chr12	32518800	0.710744
chr12	32532800	0.652893
chr12	32572300	0.834711
chr12	32594400	0.735537
chr12	32599500	0.884298
chr12	32607500	0.917355

chr12	32610300	0.966942
chr12	32654900	0.809917
chr12	32657400	0.793388
chr12	32675800	0.644628
chr12	32685700	0.669421
chr12	32702000	0.719008
chr12	32720000	0.694215
chr12	32989500	0.735537
chr12	32996600	0.818182
chr12	33045300	0.983471
chr12	33143000	0.685950
chr12	33209600	0.760331
chr12	33265300	0.702479
chr12	33741200	0.983471
chr12	33824800	0.933884
chr12	34372800	0.925620
chr12	34427200	0.652893
chr12	34834700	0.644628
chr12	34841700	0.851240
chr12	38027800	0.727273
chr12	38038300	0.785124
chr12	38447000	0.826446
chr12	38449400	0.685950
chr12	38857000	0.644628
chr12	38880300	0.669421
chr12	39485800	0.694215
chr12	39516700	0.917355
chr12	39787000	0.842975
chr12	39800800	0.776860
chr12	39896700	0.719008
chr12	39969400	0.942149
chr12	40147000	0.942149
chr12	40158100	0.644628
chr12	40383200	0.785124
chr12	40414200	0.669421
chr12	40427500	0.925620
chr12	40868600	0.867769
chr12	40912600	0.785124
chr12	41129400	0.727273
chr12	41182300	0.661157
chr12	41187300	0.900826
chr12	41213700	0.909091
chr12	41222100	0.876033
chr12	41245800	0.652893
chr12	41597100	0.652893
chr12	41728200	0.933884
chr12	41757600	0.991736
chr12	41759700	0.669421
chr12	41813000	0.884298
chr12	41833200	0.644628
chr12	41870700	0.809917
chr12	41971200	0.966942
chr12	42000300	0.644628
chr12	42103500	0.727273

chr12	42114200	0.702479
chr12	42161700	0.727273
chr12	42362200	0.909091
chr12	42519500	0.876033
chr12	42776200	0.793388
chr12	42853900	0.801653
chr12	42869300	0.644628
chr12	42880300	0.809917
chr12	42905500	0.719008
chr12	42946800	0.727273
chr12	42997600	0.661157
chr12	43078100	0.710744
chr12	43151000	0.975207
chr12	43548900	0.785124
chr12	43666500	0.950413
chr12	43675600	0.677686
chr12	43732400	0.685950
chr12	44158900	0.752066
chr12	44308400	0.652893
chr12	44316200	0.867769
chr12	44799400	0.966942
chr12	44823300	0.652893
chr12	44965000	0.719008
chr12	44975900	0.909091
chr12	44978700	0.867769
chr12	45048900	0.917355
chr12	45156600	0.760331
chr12	45165200	0.710744
chr12	45354500	0.809917
chr12	45471200	0.909091
chr12	45614600	0.834711
chr12	45626300	0.958678
chr12	45629200	0.842975
chr12	45651300	0.644628
chr12	45654200	0.867769
chr12	45663200	0.694215
chr12	45678300	0.809917
chr12	45722400	0.669421
chr12	45819800	0.644628
chr12	45827400	0.876033
chr12	45970600	0.727273
chr12	46438000	0.801653
chr12	46615900	0.884298
chr12	46639200	0.644628
chr12	46654400	0.983471
chr12	46657900	0.694215
chr12	46665700	0.694215
chr12	46693600	0.760331
chr12	46781000	0.933884
chr12	46785500	0.925620
chr12	46844400	0.669421
chr12	46877900	0.694215
chr12	46888500	0.950413
chr12	46980000	0.958678

chr12	47019200	0.834711
chr12	47064100	0.983471
chr12	47359200	0.685950
chr12	47427600	0.694215
chr12	47462900	0.818182
chr12	47479800	0.694215
chr12	47584200	0.685950
chr12	47587600	0.768595
chr12	47603200	0.685950
chr12	47617600	0.942149
chr12	47619900	0.652893
chr12	47779300	0.669421
chr12	48030900	0.652893
chr12	48046400	0.702479
chr12	48112200	0.876033
chr12	48128900	0.818182
chr12	48135000	0.801653
chr12	48138400	0.735537
chr12	48147700	0.900826
chr12	48196000	0.661157
chr12	48202400	0.743802
chr12	48204900	0.785124
chr12	48208500	0.735537
chr12	48211000	0.801653
chr12	48223100	0.735537
chr12	48232700	0.785124
chr12	48237700	0.661157
chr12	48240800	0.818182
chr12	48245200	0.677686
chr12	48336300	0.909091
chr12	48382500	0.768595
chr12	48393000	0.652893
chr12	48417000	0.685950
chr12	48431600	1.000000
chr12	48537000	0.983471
chr12	48570200	0.876033
chr12	48580300	0.644628
chr12	48995200	0.991736
chr12	49073500	0.752066
chr12	49162500	0.818182
chr12	49171600	0.991736
chr12	49205200	0.876033
chr12	49256600	0.669421
chr12	49275900	0.661157
chr12	49278600	0.710744
chr12	49359300	0.950413
chr12	49367900	0.867769
chr12	49385100	0.719008
chr12	49450000	0.710744
chr12	49482700	0.752066
chr12	49522200	0.652893
chr12	49592900	0.801653
chr12	49595800	0.669421
chr12	49627200	0.785124

chr12	49674000	0.760331
chr12	49687100	0.826446
chr12	49733500	0.644628
chr12	49737900	0.652893
chr12	49783600	0.793388
chr12	49870100	0.694215
chr12	49877500	0.867769
chr12	49883800	0.694215
chr12	50067700	0.950413
chr12	50087100	0.661157
chr12	50091100	0.677686
chr12	50144600	0.826446
chr12	50172200	0.702479
chr12	50204600	0.917355
chr12	50215400	0.644628
chr12	50261500	0.661157
chr12	50276900	0.876033
chr12	50279500	0.685950
chr12	50288400	0.743802
chr12	50298100	0.685950
chr12	50304200	0.677686
chr12	50336700	0.661157
chr12	50339900	0.851240
chr12	50349800	0.917355
chr12	50353600	0.809917
chr12	50412700	0.719008
chr12	50464600	0.652893
chr12	50497500	0.842975
chr12	50504200	0.677686
chr12	50596300	0.942149
chr12	50782100	0.842975
chr12	50819900	0.702479
chr12	50932300	0.685950
chr12	50962600	0.702479
chr12	50968400	0.900826
chr12	50992500	0.644628
chr12	51007500	0.958678
chr12	51016000	0.917355
chr12	51399600	0.809917
chr12	51422000	0.809917
chr12	51641800	0.933884
chr12	51647000	0.669421
chr12	51713200	0.859504
chr12	51735400	0.685950
chr12	51745800	0.644628
chr12	51765700	0.867769
chr12	51776700	0.776860
chr12	51813600	0.710744
chr12	51842900	0.743802
chr12	51925800	0.661157
chr12	51928300	0.710744
chr12	51931300	0.776860
chr12	51939300	0.669421
chr12	52060600	0.719008

chr12	52071600	0.661157
chr12	52081400	0.685950
chr12	52211400	0.685950
chr12	52223200	0.867769
chr12	52226300	0.669421
chr12	52228900	0.702479
chr12	52231300	0.719008
chr12	52235600	0.644628
chr12	52239100	0.735537
chr12	52245000	0.727273
chr12	52260500	0.685950
chr12	52265700	0.735537
chr12	52315100	0.760331
chr12	52317800	0.851240
chr12	52359800	0.652893
chr12	52368500	0.859504
chr12	52404400	0.933884
chr12	52408900	0.661157
chr12	52423800	0.752066
chr12	52427100	0.752066
chr12	52476100	0.884298
chr12	52513900	0.694215
chr12	52516300	0.752066
chr12	52518800	0.719008
chr12	52521100	0.801653
chr12	52524700	0.859504
chr12	52539400	0.950413
chr12	52542500	0.950413
chr12	52546700	0.719008
chr12	52558300	0.727273
chr12	52570400	0.669421
chr12	52575700	0.776860
chr12	52577900	0.892562
chr12	52625200	0.818182
chr12	52632100	0.661157
chr12	52637700	0.685950
chr12	52641400	0.727273
chr12	52836300	0.900826
chr12	52890200	0.702479
chr12	53194400	0.694215
chr12	53274000	0.669421
chr12	53300900	0.760331
chr12	53303900	0.677686
chr12	53320900	0.983471
chr12	53340800	0.694215
chr12	53365700	0.859504
chr12	53375000	0.669421
chr12	53438400	0.818182
chr12	53463500	0.661157
chr12	53553800	0.752066
chr12	53582900	0.785124
chr12	53617500	0.694215
chr12	53619600	0.884298
chr12	53630700	0.719008

chr12	53635500	0.859504
chr12	53777100	0.925620
chr12	53876900	0.760331
chr12	53889800	0.983471
chr12	53938000	0.702479
chr12	54053100	0.975207
chr12	54073100	0.719008
chr12	54137300	0.685950
chr12	54141700	0.719008
chr12	54148500	0.768595
chr12	54175200	0.859504
chr12	54239400	0.644628
chr12	54322000	0.793388
chr12	54329600	0.661157
chr12	54344200	0.809917
chr12	54350000	0.776860
chr12	54367500	0.752066
chr12	54369600	0.785124
chr12	54380000	0.735537
chr12	54394600	0.785124
chr12	54403800	0.867769
chr12	54441400	0.768595
chr12	54447500	0.818182
chr12	54449800	0.735537
chr12	54452900	0.933884
chr12	54469500	0.677686
chr12	54471600	0.743802
chr12	54475900	0.735537
chr12	54523100	0.917355
chr12	54535300	0.760331
chr12	54550200	0.694215
chr12	54553800	0.727273
chr12	54558100	0.760331
chr12	54601400	0.652893
chr12	54651700	0.900826
chr12	54656100	0.867769
chr12	54688700	0.710744
chr12	54698300	0.801653
chr12	54701200	0.768595
chr12	54723500	0.644628
chr12	54744100	0.826446
chr12	54750000	0.768595
chr12	54754900	0.644628
chr12	54765300	0.644628
chr12	54778800	0.702479
chr12	54787600	0.925620
chr12	54815200	0.826446
chr12	54826200	0.801653
chr12	54867300	0.710744
chr12	54903800	0.661157
chr12	54921400	0.826446
chr12	54923600	0.760331
chr12	54936400	0.884298
chr12	54965900	0.644628

chr12	54977800	0.743802
chr12	54984500	0.909091
chr12	55052500	0.685950
chr12	55071600	0.644628
chr12	55076800	0.735537
chr12	55392300	0.991736
chr12	55438500	0.752066
chr12	55749000	0.842975
chr12	55823300	0.876033
chr12	56040500	0.801653
chr12	56112900	0.942149
chr12	56206300	0.917355
chr12	56208400	0.702479
chr12	56237000	0.818182
chr12	56334300	0.900826
chr12	56414200	0.677686
chr12	56476600	0.694215
chr12	56555900	0.900826
chr12	56558000	0.677686
chr12	56649700	0.661157
chr12	56695900	0.719008
chr12	56732700	0.925620
chr12	56838500	0.719008
chr12	56845000	0.644628
chr12	56857000	0.983471
chr12	56921200	0.776860
chr12	56959900	0.743802
chr12	56982200	0.694215
chr12	56985100	0.785124
chr12	56988400	0.685950
chr12	57026200	0.702479
chr12	57111100	0.677686
chr12	57115300	0.743802
chr12	57322600	0.785124
chr12	57382100	0.776860
chr12	57402400	0.785124
chr12	57410400	0.851240
chr12	57425400	0.752066
chr12	57485100	0.727273
chr12	57488100	1.000000
chr12	57525500	0.768595
chr12	57529800	0.975207
chr12	57546100	0.884298
chr12	57608000	0.834711
chr12	57613300	0.727273
chr12	57615500	0.900826
chr12	57638600	0.661157
chr12	57645100	0.768595
chr12	57655500	0.644628
chr12	57694000	0.735537
chr12	57736000	0.652893
chr12	57877400	0.669421
chr12	57946500	0.727273
chr12	58136100	0.694215

chr12	58226300	0.644628
chr12	58228400	0.900826
chr12	58231600	0.851240
chr12	58234600	0.743802
chr12	58236700	0.834711
chr12	58282800	0.867769
chr12	58287700	0.917355
chr12	58299500	0.760331
chr12	58680100	0.719008
chr12	58820100	0.958678
chr12	58844900	0.851240
chr12	58934700	0.719008
chr12	59064900	0.785124
chr12	59106400	0.702479
chr12	59139700	0.710744
chr12	59235300	0.719008
chr12	59255000	0.661157
chr12	59311100	0.644628
chr12	59478900	0.710744
chr12	59628600	0.677686
chr12	59677400	0.710744
chr12	59716600	0.933884
chr12	59723500	0.867769
chr12	59776500	0.727273
chr12	59784200	0.644628
chr12	59894800	0.677686
chr12	60269200	0.785124
chr12	61229400	0.669421
chr12	61375100	0.652893
chr12	61806300	0.793388
chr12	61898500	0.933884
chr12	61915200	0.768595
chr12	61960400	0.661157
chr12	62049100	0.652893
chr12	62172100	0.842975
chr12	62174900	0.743802
chr12	62334100	0.801653
chr12	62498100	0.735537
chr12	62507700	0.809917
chr12	63016100	0.834711
chr12	63023800	0.776860
chr12	63041100	0.702479
chr12	63046500	0.694215
chr12	63050600	0.884298
chr12	63054700	0.818182
chr12	63067300	0.710744
chr12	63078600	0.925620
chr12	63097500	0.983471
chr12	63108800	0.694215
chr12	63118900	0.958678
chr12	63123200	0.793388
chr12	63164900	0.834711
chr12	63178000	0.652893
chr12	63188000	0.669421

chr12	63211800	0.727273
chr12	63237900	0.892562
chr12	63253800	0.685950
chr12	63256100	0.876033
chr12	63260400	0.752066
chr12	63307900	0.950413
chr12	63322900	0.669421
chr12	63325100	0.677686
chr12	63415100	0.768595
chr12	63421600	0.727273
chr12	63479100	0.958678
chr12	64256700	0.685950
chr12	64264900	0.793388
chr12	64276800	0.752066
chr12	64287300	0.859504
chr12	64433600	0.735537
chr12	64482800	0.677686
chr12	64564400	0.785124
chr12	64613400	0.669421
chr12	64713300	0.702479
chr12	64794200	0.785124
chr12	64853200	0.661157
chr12	64916300	0.925620
chr12	64960700	0.925620
chr12	65019200	0.876033
chr12	65060300	0.694215
chr12	65063600	0.752066
chr12	65068100	0.669421
chr12	65077100	0.842975
chr12	65082200	0.851240
chr12	65085600	0.867769
chr12	65089400	0.867769
chr12	65182400	0.677686
chr12	65283800	0.785124
chr12	65386800	0.975207
chr12	65450000	0.677686
chr12	65589900	0.669421
chr12	65905100	0.669421
chr12	65989400	0.644628
chr12	65992600	0.661157
chr12	66148600	0.719008
chr12	66155700	0.942149
chr12	66192700	0.925620
chr12	66222400	0.851240
chr12	66240100	0.801653
chr12	66276000	0.710744
chr12	66289300	0.793388
chr12	66291600	0.950413
chr12	66320600	0.884298
chr12	66324500	0.834711
chr12	66456400	0.710744
chr12	66526700	0.710744
chr12	66821100	0.942149
chr12	66892000	0.983471

chr12	66996100	0.876033
chr12	67010800	0.719008
chr12	67022600	0.710744
chr12	67073000	0.719008
chr12	67128600	0.652893
chr12	67194000	0.694215
chr12	67198500	0.867769
chr12	67279200	0.694215
chr12	67409200	0.884298
chr12	67413000	0.776860
chr12	67428900	0.942149
chr12	67437000	0.793388
chr12	67439800	0.669421
chr12	67758400	0.694215
chr12	67782800	0.735537
chr12	67808500	0.859504
chr12	67814100	0.719008
chr12	67864000	0.900826
chr12	67914000	0.851240
chr12	67919800	0.760331
chr12	67929700	0.793388
chr12	68025400	0.661157
chr12	68167100	0.809917
chr12	68270800	0.909091
chr12	68310500	0.702479
chr12	68314400	0.719008
chr12	68475000	0.652893
chr12	68512500	0.768595
chr12	68534500	0.719008
chr12	68671900	0.644628
chr12	68738100	0.793388
chr12	68752600	0.752066
chr12	68759200	0.826446
chr12	68770400	0.710744
chr12	68833700	0.710744
chr12	68841100	0.735537
chr12	68849900	0.752066
chr12	68933200	0.900826
chr12	68954500	0.669421
chr12	69002100	0.727273
chr12	69011000	0.669421
chr12	69199000	0.818182
chr12	69357900	0.743802
chr12	69447100	0.809917
chr12	69493200	0.727273
chr12	69502300	0.876033
chr12	69562100	0.702479
chr12	69678300	0.677686
chr12	69716700	0.785124
chr12	69722800	0.809917
chr12	69725400	0.966942
chr12	69809900	0.909091
chr12	69861800	0.818182
chr12	69869900	0.760331

chr12	69928600	0.851240
chr12	70083200	0.834711
chr12	70273500	0.719008
chr12	70288800	0.851240
chr12	70292200	0.942149
chr12	70294700	0.710744
chr12	70327200	0.975207
chr12	70330300	0.925620
chr12	70342900	0.644628
chr12	70346800	0.876033
chr12	70424700	0.652893
chr12	70468800	0.776860
chr12	70495500	0.644628
chr12	71039800	0.933884
chr12	71058600	0.851240
chr12	71084000	0.661157
chr12	71150700	0.702479
chr12	71180300	0.652893
chr12	71200500	0.652893
chr12	71203200	0.669421
chr12	71249600	0.818182
chr12	71377700	0.743802
chr12	71608800	0.991736
chr12	71957200	0.735537
chr12	71981400	0.644628
chr12	72338700	0.661157
chr12	72340900	0.851240
chr12	72344000	0.801653
chr12	72366700	0.661157
chr12	72609600	0.685950
chr12	72663500	0.702479
chr12	72675300	0.752066
chr12	73266900	0.768595
chr12	73880100	0.826446
chr12	74598400	0.760331
chr12	74802900	0.760331
chr12	75760400	0.685950
chr12	75878600	0.909091
chr12	76026000	0.702479
chr12	76029300	0.917355
chr12	76093600	0.685950
chr12	76115000	0.859504
chr12	76203800	0.925620
chr12	76206500	0.859504
chr12	76338400	1.000000
chr12	76347500	0.702479
chr12	76352400	0.859504
chr12	76354500	0.685950
chr12	76413900	0.661157
chr12	76449100	0.818182
chr12	76554900	0.644628
chr12	76644200	0.727273
chr12	76706900	0.652893
chr12	77015900	0.677686

chr12	77027800	0.710744
chr12	77060100	0.851240
chr12	77093000	0.867769
chr12	77156100	0.710744
chr12	77175900	0.743802
chr12	77260400	0.776860
chr12	77266500	0.760331
chr12	77301800	0.669421
chr12	77315100	0.694215
chr12	77358900	0.892562
chr12	78040300	0.867769
chr12	78154400	0.719008
chr12	78623800	0.785124
chr12	78626800	0.776860
chr12	78637900	0.710744
chr12	79261000	0.644628
chr12	79274800	0.710744
chr12	79396500	0.669421
chr12	79456500	0.677686
chr12	79479600	0.661157
chr12	79715900	0.702479
chr12	79775800	0.876033
chr12	79797400	0.743802
chr12	79803000	0.966942
chr12	79809600	0.768595
chr12	79814100	0.785124
chr12	79876600	0.768595
chr12	79916700	0.942149
chr12	79940000	0.842975
chr12	80039900	0.702479
chr12	80049100	0.719008
chr12	80073500	0.694215
chr12	80079700	0.785124
chr12	80178700	1.000000
chr12	80242200	0.644628
chr12	80441400	0.669421
chr12	80513200	0.644628
chr12	80624200	0.801653
chr12	80864400	0.652893
chr12	80884900	0.710744
chr12	81026900	0.694215
chr12	81077800	0.909091
chr12	81163800	0.785124
chr12	81176400	0.942149
chr12	81215400	0.909091
chr12	81241000	0.859504
chr12	81286700	0.677686
chr12	81474700	0.768595
chr12	81477100	0.702479
chr12	81479300	0.892562
chr12	81601400	0.975207
chr12	81606100	0.644628
chr12	81640100	0.785124
chr12	81805400	0.652893

chr12	81845300	0.652893
chr12	82192000	0.702479
chr12	82195100	0.793388
chr12	82282500	0.694215
chr12	82723500	0.685950
chr12	83076800	0.710744
chr12	83102500	0.752066
chr12	83150700	0.942149
chr12	83175900	0.834711
chr12	83188100	0.735537
chr12	83243300	0.735537
chr12	83252000	0.661157
chr12	83437100	0.892562
chr12	83593000	0.958678
chr12	83760400	0.685950
chr12	84220300	0.652893
chr12	84349200	0.859504
chr12	84432300	0.834711
chr12	85118000	0.719008
chr12	85124900	0.933884
chr12	85199500	0.842975
chr12	85399300	0.644628
chr12	85913200	0.909091
chr12	85985500	0.826446
chr12	86169900	0.644628
chr12	86230000	0.975207
chr12	86268400	0.694215
chr12	86342600	0.933884
chr12	87231400	0.768595
chr12	87501400	0.652893
chr12	87931900	0.677686
chr12	88264800	0.669421
chr12	88539200	0.892562
chr12	88549600	0.809917
chr12	88638200	0.867769
chr12	88865900	0.933884
chr12	88953700	0.694215
chr12	88967600	0.652893
chr12	89257100	0.719008
chr12	89315800	0.752066
chr12	89340600	0.727273
chr12	89346000	0.818182
chr12	89352500	0.900826
chr12	89475800	0.710744
chr12	89479100	0.801653
chr12	89481800	0.776860
chr12	89492700	0.983471
chr12	89620000	0.694215
chr12	89639200	0.909091
chr12	89642000	0.884298
chr12	89701300	0.677686
chr12	89730000	0.685950
chr12	89740800	0.925620
chr12	89778100	0.735537

chr12	89845500	0.760331
chr12	89855600	0.785124
chr12	89891400	0.669421
chr12	89910100	0.685950
chr12	89986800	0.735537
chr12	90258200	0.727273
chr12	90278100	0.892562
chr12	90331700	0.644628
chr12	90360000	0.685950
chr12	90485500	0.719008
chr12	90748000	0.752066
chr12	91669600	0.719008
chr12	91764600	0.760331
chr12	91775100	0.752066
chr12	91817000	0.644628
chr12	91905800	0.859504
chr12	92305400	0.727273
chr12	92428800	0.801653
chr12	92432900	0.719008
chr12	92501400	0.942149
chr12	92528200	0.884298
chr12	92531000	0.892562
chr12	92666500	0.809917
chr12	92818800	0.867769
chr12	92860100	0.958678
chr12	92871100	0.652893
chr12	92934300	0.702479
chr12	93042900	0.752066
chr12	93132200	0.644628
chr12	93274200	0.851240
chr12	93327800	0.743802
chr12	93474800	0.966942
chr12	93614400	0.710744
chr12	93775800	0.644628
chr12	93819900	0.652893
chr12	93832500	0.710744
chr12	93978600	0.644628
chr12	94010700	0.950413
chr12	94093400	0.958678
chr12	94135000	0.776860
chr12	94137900	0.652893
chr12	94192900	0.801653
chr12	94197200	0.652893
chr12	94218400	0.776860
chr12	94404400	0.677686
chr12	94559100	0.809917
chr12	94598200	0.694215
chr12	94641100	0.735537
chr12	94655100	0.909091
chr12	94658700	0.694215
chr12	94698800	0.983471
chr12	94727900	0.834711
chr12	94783900	0.685950
chr12	94819000	0.801653

chr12	94823400	0.801653
chr12	94955200	0.900826
chr12	95189500	0.727273
chr12	95226800	0.851240
chr12	95316600	0.842975
chr12	95508900	0.876033
chr12	95582500	0.785124
chr12	95597900	0.909091
chr12	95600100	0.925620
chr12	95651500	0.818182
chr12	95730200	0.644628
chr12	95930700	0.768595
chr12	95969000	0.933884
chr12	96002100	0.834711
chr12	96018200	0.942149
chr12	96101000	0.694215
chr12	96162300	0.958678
chr12	96380900	0.669421
chr12	96392600	0.661157
chr12	96464700	0.727273
chr12	96488200	0.826446
chr12	96515000	0.644628
chr12	96610500	0.685950
chr12	96652700	0.801653
chr12	96789800	0.677686
chr12	96916400	0.933884
chr12	97144200	0.685950
chr12	97303700	0.719008
chr12	97491700	0.760331
chr12	97525300	0.876033
chr12	97528300	0.743802
chr12	97670200	0.925620
chr12	97706800	0.694215
chr12	97716800	0.933884
chr12	97820500	1.000000
chr12	97864200	0.859504
chr12	97868700	0.760331
chr12	97925100	0.760331
chr12	97946200	0.975207
chr12	97952400	0.966942
chr12	97955700	0.983471
chr12	98106900	0.793388
chr12	98179000	0.933884
chr12	98648200	0.710744
chr12	98738500	0.785124
chr12	98767100	0.702479
chr12	98793500	0.694215
chr12	98884200	0.685950
chr12	98887100	0.677686
chr12	98907200	0.661157
chr12	99090300	0.925620
chr12	99136200	0.876033
chr12	99214800	0.884298
chr12	99250800	0.760331

chr12	99256900	0.842975
chr12	99420800	0.677686
chr12	99525800	0.966942
chr12	99548600	0.694215
chr12	99644000	0.727273
chr12	100117000	0.652893
chr12	100545700	0.975207
chr12	100548300	0.652893
chr12	100557900	0.892562
chr12	100673800	0.876033
chr12	100751100	0.834711
chr12	100769100	0.644628
chr12	100841600	0.752066
chr12	100922700	0.752066
chr12	101173300	0.677686
chr12	101189000	0.884298
chr12	101346700	0.669421
chr12	101349000	0.818182
chr12	101365500	0.735537
chr12	101382000	0.900826
chr12	101960400	0.975207
chr12	101975400	0.735537
chr12	101998600	0.727273
chr12	102215300	0.851240
chr12	102219600	0.644628
chr12	102873000	0.859504
chr12	102943300	0.702479
chr12	103006000	0.851240
chr12	103311400	0.818182
chr12	103325900	0.661157
chr12	103356500	0.809917
chr12	103407100	0.917355
chr12	103438000	0.702479
chr12	103592000	0.644628
chr12	103696400	0.702479
chr12	103839700	0.710744
chr12	103969500	0.727273
chr12	104163300	0.842975
chr12	104272100	0.652893
chr12	104449000	0.809917
chr12	104501200	0.785124
chr12	104643100	0.661157
chr12	104744900	0.859504
chr12	104761700	0.685950
chr12	104766000	0.867769
chr12	104807100	0.867769
chr12	104867000	0.743802
chr12	104894400	0.900826
chr12	104898300	0.677686
chr12	104901100	0.801653
chr12	104920400	0.900826
chr12	105017100	0.743802
chr12	105048600	0.702479
chr12	105065800	0.884298

chr12	105089800	0.685950
chr12	105098300	0.867769
chr12	105103200	0.842975
chr12	105107400	0.719008
chr12	105115000	0.884298
chr12	105117100	0.876033
chr12	105132200	0.818182
chr12	105162300	0.677686
chr12	105184500	0.760331
chr12	105486400	0.900826
chr12	105728400	0.652893
chr12	105762500	0.644628
chr12	105777200	0.710744
chr12	105874500	0.818182
chr12	106098600	0.859504
chr12	106134400	0.652893
chr12	106215200	0.710744
chr12	106291200	0.652893
chr12	106475000	0.669421
chr12	106478200	0.975207
chr12	106492000	0.925620
chr12	106500500	0.669421
chr12	106502600	0.991736
chr12	106506300	0.867769
chr12	106509800	0.677686
chr12	106522000	0.677686
chr12	106529500	0.702479
chr12	106539600	0.768595
chr12	106582300	0.743802
chr12	106648600	0.826446
chr12	106723700	0.752066
chr12	106974900	0.669421
chr12	106995200	0.900826
chr12	107040000	0.644628
chr12	107066800	0.735537
chr12	107103900	0.867769
chr12	107134600	0.710744
chr12	107153700	0.909091
chr12	107158700	0.652893
chr12	107161000	0.677686
chr12	107163300	0.991736
chr12	107259500	0.735537
chr12	107307400	0.644628
chr12	107312900	0.942149
chr12	107516500	0.702479
chr12	107521400	0.661157
chr12	107619700	0.719008
chr12	107700200	0.685950
chr12	107725500	0.867769
chr12	107792100	0.735537
chr12	107820100	0.644628
chr12	107833500	0.644628
chr12	107844900	0.644628
chr12	107862000	0.809917

chr12	107890000	0.677686
chr12	107978700	0.661157
chr12	108004200	0.809917
chr12	108028900	0.793388
chr12	108040100	0.991736
chr12	108054600	0.644628
chr12	108065000	0.892562
chr12	108211200	0.719008
chr12	108427600	0.652893
chr12	108507000	0.867769
chr12	108525800	0.834711
chr12	108535400	0.809917
chr12	108540700	0.785124
chr12	108567300	0.867769
chr12	108569400	0.752066
chr12	108585600	0.776860
chr12	108596300	0.677686
chr12	108605000	0.727273
chr12	108629700	1.000000
chr12	108634500	0.669421
chr12	108647400	0.719008
chr12	108678400	0.694215
chr12	108696700	0.851240
chr12	108698800	0.752066
chr12	108710700	0.975207
chr12	108715100	0.909091
chr12	108733500	0.652893
chr12	108784900	0.834711
chr12	108804300	0.727273
chr12	108819900	0.793388
chr12	108836100	0.859504
chr12	108851800	0.785124
chr12	108987200	0.933884
chr12	109007400	0.685950
chr12	109019500	0.876033
chr12	109028900	0.876033
chr12	109032500	0.975207
chr12	109047700	0.727273
chr12	109058400	0.884298
chr12	109064100	0.776860
chr12	109083900	0.900826
chr12	109087100	0.851240
chr12	109096100	0.661157
chr12	109116300	0.818182
chr12	109178000	0.809917
chr12	109196300	0.983471
chr12	109220900	0.826446
chr12	109233000	0.892562
chr12	109274100	0.801653
chr12	109507800	0.900826
chr12	109531200	0.752066
chr12	109549200	0.768595
chr12	109553900	0.719008
chr12	109711600	0.842975

chr12	109729600	0.842975
chr12	109873900	0.876033
chr12	109898000	0.950413
chr12	109901600	0.710744
chr12	109968300	0.867769
chr12	110036400	0.809917
chr12	110044900	0.669421
chr12	110060200	0.652893
chr12	110091000	0.801653
chr12	110108900	0.719008
chr12	110117900	0.694215
chr12	110150700	0.735537
chr12	110157800	0.851240
chr12	110173100	0.661157
chr12	110196800	0.669421
chr12	110209300	0.669421
chr12	110260500	0.694215
chr12	110278600	0.768595
chr12	110283400	0.785124
chr12	110302600	0.677686
chr12	110383200	0.826446
chr12	110560800	0.793388
chr12	110603500	0.719008
chr12	110637700	0.694215
chr12	110682900	0.727273
chr12	111017300	0.768595
chr12	111029700	0.702479
chr12	111254000	0.867769
chr12	111286500	0.652893
chr12	111299300	0.644628
chr12	111332700	0.685950
chr12	111372300	0.942149
chr12	111379200	0.735537
chr12	111467600	0.743802
chr12	111510500	0.925620
chr12	111512700	0.677686
chr12	111522600	0.661157
chr12	111592000	0.760331
chr12	111631300	0.917355
chr12	111639000	0.661157
chr12	111722900	0.710744
chr12	111736700	0.644628
chr12	111777300	0.867769
chr12	111841300	0.776860
chr12	111850500	0.991736
chr12	111859800	0.809917
chr12	111863500	0.909091
chr12	111868200	0.818182
chr12	111875700	0.793388
chr12	111883800	0.859504
chr12	112074300	0.958678
chr12	112103400	0.876033
chr12	112196200	0.727273
chr12	112212900	0.685950

chr12	112273200	0.644628
chr12	112306800	0.925620
chr12	112550900	0.776860
chr12	112554600	0.909091
chr12	112621900	0.958678
chr12	112694700	0.694215
chr12	112760400	0.743802
chr12	112782000	0.760331
chr12	113016100	0.793388
chr12	113028500	0.884298
chr12	113040500	0.884298
chr12	113055000	0.793388
chr12	113215700	0.950413
chr12	113226400	0.644628
chr12	113236300	0.661157
chr12	113262900	0.652893
chr12	113285900	0.669421
chr12	113293700	0.826446
chr12	113370900	0.793388
chr12	113468700	0.727273
chr12	113503400	0.867769
chr12	113507700	0.727273
chr12	113525000	0.809917
chr12	113536000	0.826446
chr12	113587800	0.834711
chr12	113600400	0.760331
chr12	113669600	0.900826
chr12	113679600	0.677686
chr12	113682600	0.768595
chr12	113737400	0.826446
chr12	113787300	0.776860
chr12	113860300	0.760331
chr12	113864300	0.892562
chr12	113895000	0.669421
chr12	113923500	0.842975
chr12	113939100	0.785124
chr12	113950500	0.834711
chr12	114026800	0.842975
chr12	114047300	0.685950
chr12	114050700	0.785124
chr12	114070900	0.702479
chr12	114102100	0.768595
chr12	114136000	0.776860
chr12	114173500	0.694215
chr12	114217000	0.809917
chr12	114296900	0.661157
chr12	114301100	0.983471
chr12	114337900	0.685950
chr12	114367000	0.983471
chr12	114370800	0.909091
chr12	114484500	0.867769
chr12	114624600	0.933884
chr12	114664100	0.768595
chr12	114691100	0.685950

chr12	114709700	0.694215
chr12	114714100	0.958678
chr12	114724100	0.925620
chr12	114726400	0.735537
chr12	114773800	0.785124
chr12	114829600	0.776860
chr12	114833400	0.719008
chr12	114835500	0.727273
chr12	114838700	0.644628
chr12	114841500	0.685950
chr12	114845500	0.669421
chr12	114848800	0.669421
chr12	114878000	0.743802
chr12	114882500	0.826446
chr12	114886200	0.677686
chr12	114888600	0.727273
chr12	114985800	0.710744
chr12	115000900	0.719008
chr12	115074800	0.793388
chr12	115077300	0.719008
chr12	115095600	0.842975
chr12	115107700	0.710744
chr12	115114300	0.785124
chr12	115118000	0.793388
chr12	115125300	0.842975
chr12	115131300	0.818182
chr12	115138500	0.743802
chr12	115141600	0.710744
chr12	115149700	0.644628
chr12	115190100	0.685950
chr12	115251300	0.801653
chr12	115303300	0.834711
chr12	115456000	0.719008
chr12	115490500	0.735537
chr12	115510500	0.702479
chr12	115637500	0.652893
chr12	115651800	0.727273
chr12	115849200	0.677686
chr12	115875400	0.768595
chr12	115946000	0.776860
chr12	115949900	0.743802
chr12	115957500	0.727273
chr12	116062000	0.727273
chr12	116197000	0.752066
chr12	116311000	0.958678
chr12	116386300	0.752066
chr12	116390800	0.727273
chr12	116426000	0.768595
chr12	116561200	0.793388
chr12	116571300	0.661157
chr12	116592000	0.925620
chr12	116595600	0.752066
chr12	116640700	0.694215
chr12	116702000	0.727273

chr12	116704700	0.933884
chr12	116718600	0.652893
chr12	116778200	0.809917
chr12	116796600	0.892562
chr12	116814300	0.661157
chr12	116822800	0.719008
chr12	116854200	0.876033
chr12	116865100	0.966942
chr12	116872500	0.801653
chr12	116893300	0.793388
chr12	116944500	0.785124
chr12	116955700	0.743802
chr12	116963300	0.859504
chr12	116978100	0.917355
chr12	116997300	0.727273
chr12	117037500	0.719008
chr12	117043400	0.743802
chr12	117084300	0.652893
chr12	117096700	0.768595
chr12	117104200	0.801653
chr12	117126100	0.685950
chr12	117147100	0.727273
chr12	117364100	0.776860
chr12	117438700	0.677686
chr12	117447900	1.000000
chr12	117450200	0.892562
chr12	117483600	0.743802
chr12	117486500	0.768595
chr12	117490300	0.719008
chr12	117496700	0.685950
chr12	117498900	0.743802
chr12	117502000	0.752066
chr12	117525200	0.644628
chr12	117529700	0.793388
chr12	117540000	0.644628
chr12	117550000	0.702479
chr12	117556900	0.933884
chr12	117593500	0.727273
chr12	117673700	0.752066
chr12	117715700	0.685950
chr12	117717900	0.950413
chr12	117771900	0.719008
chr12	117846200	0.809917
chr12	117911100	0.694215
chr12	117952600	0.776860
chr12	118018700	0.661157
chr12	118022200	0.933884
chr12	118025500	0.652893
chr12	118110100	0.644628
chr12	118123300	0.677686
chr12	118141900	0.867769
chr12	118228200	0.644628
chr12	118275400	0.752066
chr12	118314000	0.735537

chr12	118558200	0.884298
chr12	118628000	0.809917
chr12	118971900	0.685950
chr12	118975700	0.950413
chr12	119120200	0.892562
chr12	119143200	0.652893
chr12	119208100	0.801653
chr12	119220200	0.661157
chr12	119252000	0.809917
chr12	119293300	0.694215
chr12	119313200	0.950413
chr12	119442100	0.768595
chr12	119446900	0.752066
chr12	119455800	0.801653
chr12	119458900	0.975207
chr12	119520400	0.652893
chr12	119525400	0.743802
chr12	119528800	0.909091
chr12	119583500	0.743802
chr12	119614400	0.735537
chr12	119616900	0.876033
chr12	119619300	0.942149
chr12	119639900	0.809917
chr12	119655700	0.735537
chr12	119658200	0.966942
chr12	119670500	0.710744
chr12	119674100	0.818182
chr12	119712400	0.719008
chr12	119715600	0.702479
chr12	119747200	0.768595
chr12	119776800	0.842975
chr12	119802000	0.867769
chr12	119807400	0.859504
chr12	119828800	0.842975
chr12	119851000	0.661157
chr12	119935400	0.867769
chr12	119949600	0.661157
chr12	120108300	0.801653
chr12	120139300	0.661157
chr12	120479500	0.661157
chr12	120525000	0.884298
chr12	120528800	0.809917
chr12	120532100	0.942149
chr12	120534300	0.983471
chr12	120540100	0.826446
chr12	120543200	0.677686
chr12	120546500	0.834711
chr12	120549000	0.760331
chr12	120663200	0.702479
chr12	120666300	0.652893
chr12	120670400	0.942149
chr12	120673300	0.710744
chr12	120679700	0.793388
chr12	120683200	0.727273

chr12	120695300	0.685950
chr12	120780300	0.644628
chr12	120796600	0.925620
chr12	120801900	0.702479
chr12	120809600	0.652893
chr12	120815000	0.661157
chr12	120827200	0.983471
chr12	120832700	0.900826
chr12	120840300	0.892562
chr12	120852100	0.958678
chr12	120943000	0.818182
chr12	121057500	0.809917
chr12	121090700	0.661157
chr12	121107200	0.884298
chr12	121131800	0.983471
chr12	121197500	0.685950
chr12	121261200	0.975207
chr12	121363800	0.983471
chr12	121367300	1.000000
chr12	121437600	0.917355
chr12	121534000	0.652893
chr12	121676800	0.950413
chr12	121679300	0.735537
chr12	121717600	0.669421
chr12	121719900	0.859504
chr12	121726700	0.669421
chr12	121882100	0.917355
chr12	121902800	0.644628
chr12	121914800	0.727273
chr12	121928800	0.842975
chr12	121938600	0.842975
chr12	121952000	0.884298
chr12	121969100	0.685950
chr12	122012900	0.892562
chr12	122028700	0.685950
chr12	122058400	0.851240
chr12	122062900	0.818182
chr12	122068000	0.644628
chr12	122097100	0.876033
chr12	122107000	0.892562
chr12	122110400	0.719008
chr12	122117300	0.644628
chr12	122297300	0.760331
chr12	122321600	0.710744
chr12	122332600	0.677686
chr12	122371900	0.892562
chr12	122385900	0.652893
chr12	122502600	0.702479
chr12	122608100	0.661157
chr12	122611400	0.677686
chr12	122622700	0.809917
chr12	122690500	0.685950
chr12	122712000	0.661157
chr12	122870400	0.702479

chr12	122884700	0.900826
chr12	123360700	0.743802
chr12	123373400	0.859504
chr12	123376600	0.826446
chr12	123388400	0.768595
chr12	123394900	0.851240
chr12	123560200	0.727273
chr12	123587300	0.818182
chr12	123591200	0.710744
chr12	123625100	0.685950
chr12	123638600	0.735537
chr12	123746800	0.892562
chr12	123750000	0.685950
chr12	123753100	0.677686
chr12	123887300	0.760331
chr12	123973600	0.727273
chr12	124385900	0.735537
chr12	124530000	0.719008
chr12	124601400	0.966942
chr12	124615900	0.909091
chr12	124625700	0.991736
chr12	124629500	0.661157
chr12	124633100	0.652893
chr12	124642400	0.669421
chr12	124644900	0.785124
chr12	124692900	0.694215
chr12	124726300	0.669421
chr12	124764000	0.785124
chr12	124778700	0.702479
chr12	124803800	0.710744
chr12	124878000	0.925620
chr12	124887400	0.859504
chr12	124895700	1.000000
chr12	124907800	0.876033
chr12	124917200	0.652893
chr12	124919500	0.900826
chr12	124938400	0.884298
chr12	124964800	0.925620
chr12	124984700	0.809917
chr12	124987300	0.842975
chr12	124989500	0.900826
chr12	124992300	0.644628
chr12	124996300	0.876033
chr12	124999400	0.975207
chr12	125003200	0.958678
chr12	125017600	0.752066
chr12	125024100	0.950413
chr12	125028300	0.983471
chr12	125030500	0.818182
chr12	125034400	0.793388
chr12	125036700	0.876033
chr12	125039200	0.644628
chr12	125041700	0.892562
chr12	125045600	0.925620

chr12	125048200	0.801653
chr12	125054800	0.669421
chr12	125058500	0.702479
chr12	125090600	0.768595
chr12	125106100	0.669421
chr12	125116600	0.785124
chr12	125140500	0.727273
chr12	125219400	0.818182
chr12	125227300	0.694215
chr12	125230500	0.867769
chr12	125235700	0.809917
chr12	125240100	0.702479
chr12	125245000	0.793388
chr12	125257000	0.785124
chr12	125271400	0.752066
chr12	125282300	0.966942
chr12	125308900	0.644628
chr12	125318600	0.966942
chr12	125323200	0.677686
chr12	125354000	0.652893
chr12	125369400	0.743802
chr12	125389600	0.752066
chr12	125402200	0.652893
chr12	125404400	0.685950
chr12	125408700	0.859504
chr12	125548000	0.867769
chr12	125629300	0.644628
chr12	125678700	0.702479
chr12	125694000	0.867769
chr12	125713300	0.677686
chr12	125800400	0.719008
chr12	125809400	0.652893
chr12	125814000	0.793388
chr12	125830300	0.867769
chr12	126181200	0.661157
chr12	126374700	0.677686
chr12	126412000	0.776860
chr12	126570900	0.661157
chr12	126642200	0.842975
chr12	126689100	0.661157
chr12	126699100	0.752066
chr12	126718400	0.768595
chr12	126904200	0.776860
chr12	126965200	0.743802
chr12	127039600	0.834711
chr12	127219200	0.818182
chr12	127509400	0.702479
chr12	127568400	0.809917
chr12	127598700	0.785124
chr12	127618900	0.710744
chr12	127627900	0.884298
chr12	127632600	0.950413
chr12	127651100	0.884298
chr12	127765300	0.785124

chr12	128072900	0.859504
chr12	128079400	0.983471
chr12	128092000	0.818182
chr12	128105900	0.900826
chr12	128223500	0.735537
chr12	128259100	0.685950
chr12	128388100	0.710744
chr12	128541400	0.925620
chr12	128563100	0.842975
chr12	128576700	0.661157
chr12	128597100	0.661157
chr12	128643300	0.950413
chr12	128653700	0.859504
chr12	128716700	0.669421
chr12	128744400	0.727273
chr12	128754500	0.743802
chr12	128776500	0.652893
chr12	128783200	0.644628
chr12	128822100	0.768595
chr12	128824300	0.677686
chr12	128841300	0.644628
chr12	128858600	0.661157
chr12	128887600	0.702479
chr12	128893100	0.677686
chr12	128928600	0.669421
chr12	128943700	0.809917
chr12	128959300	0.644628
chr12	128994000	0.677686
chr12	128998400	0.702479
chr12	129036000	0.876033
chr12	129066200	0.735537
chr12	129091200	0.644628
chr12	129104400	0.793388
chr12	129157800	0.719008
chr12	129214000	0.900826
chr12	129252400	0.710744
chr12	129266700	0.785124
chr12	129282300	0.859504
chr12	129302700	0.851240
chr12	129305300	0.752066
chr12	129456900	0.710744
chr12	129459400	0.752066
chr12	129466000	0.892562
chr12	129554800	0.793388
chr12	129634600	0.685950
chr12	129648600	0.884298
chr12	129711000	0.702479
chr12	129824100	0.966942
chr12	129870600	0.719008
chr12	130015800	0.669421
chr12	130018100	0.892562
chr12	130092500	0.719008
chr12	130116500	0.809917
chr12	130120300	0.859504

chr12	130142500	0.768595
chr12	130181700	0.727273
chr12	130256400	0.801653
chr12	130261500	0.719008
chr12	130276300	0.834711
chr12	130315600	0.785124
chr12	130322300	0.743802
chr12	130356900	0.966942
chr12	130422600	0.694215
chr12	130451200	0.884298
chr12	130469100	0.785124
chr12	130499300	0.842975
chr12	130529800	0.826446
chr12	130560500	0.793388
chr12	130641600	0.652893
chr12	130649600	0.768595
chr12	130660800	0.719008
chr12	130705200	0.776860
chr12	130710500	0.867769
chr12	130967200	0.917355
chr12	130989300	0.760331
chr12	130999000	0.867769
chr12	131118900	0.785124
chr12	131214700	0.809917
chr12	131267800	0.809917
chr12	131272800	0.727273
chr12	131297700	0.694215
chr12	131301000	0.826446
chr12	131303500	0.752066
chr12	131310700	0.669421
chr12	131314600	0.710744
chr12	131334900	0.975207
chr12	131338400	0.958678
chr12	131361600	0.685950
chr12	131433200	0.669421
chr12	131703300	0.826446
chr12	131708400	0.801653
chr12	131714700	0.884298
chr12	131719300	0.818182
chr12	131762400	0.694215
chr12	131796500	0.661157
chr12	131825800	0.661157
chr12	131833500	0.867769
chr12	131865900	0.727273
chr12	131914100	0.966942
chr12	131922100	0.685950
chr12	131996500	0.785124
chr12	132073100	0.735537
chr12	132106500	0.752066
chr12	132109800	0.851240
chr12	132118600	0.677686
chr12	132128900	0.900826
chr12	132131300	0.818182
chr12	132167300	0.900826

chr12	132205100	0.826446
chr12	132207200	0.859504
chr12	132209300	0.884298
chr12	132213400	0.925620
chr12	132215700	0.719008
chr12	132236000	0.809917
chr12	132257200	0.685950
chr12	132259500	0.793388
chr12	132271300	0.909091
chr12	132281300	0.900826
chr12	132296100	0.834711
chr12	132317300	0.743802
chr12	132337500	0.702479
chr12	132340900	0.818182
chr12	132345400	0.991736
chr12	132473300	0.983471
chr12	132477200	0.925620
chr12	132482100	0.677686
chr12	132531000	0.900826
chr12	132534100	0.884298
chr12	132588100	0.694215
chr12	132851400	0.785124
chr12	132862900	0.876033
chr12	132866400	0.677686
chr12	132904500	0.661157
chr12	132933700	0.933884
chr12	132951300	0.851240
chr12	132963400	0.826446
chr12	132974300	0.900826
chr12	132977300	0.685950
chr12	133006200	0.818182
chr12	133079100	0.768595
chr12	133103400	0.925620
chr12	133134800	0.735537
chr12	133177400	0.752066
chr12	133424700	0.867769
chr13	20260000	0.768595
chr13	20270000	0.818182
chr13	20369100	0.785124
chr13	20624200	0.669421
chr13	20628800	0.900826
chr13	20673500	0.776860
chr13	20681400	0.801653
chr13	20694800	0.876033
chr13	20936900	0.661157
chr13	20987200	0.958678
chr13	20989600	0.760331
chr13	21000000	0.727273
chr13	21086200	0.710744
chr13	21259200	0.743802
chr13	21287700	0.842975
chr13	21291500	0.809917
chr13	21399300	0.760331
chr13	21457100	0.851240

chr13	21513900	0.851240
chr13	21520800	0.710744
chr13	21572600	0.719008
chr13	21624500	0.867769
chr13	21689800	0.743802
chr13	21698400	0.768595
chr13	21701000	0.768595
chr13	21880800	0.644628
chr13	21903900	0.743802
chr13	21913300	0.834711
chr13	21985000	0.710744
chr13	22040400	0.694215
chr13	22157700	0.933884
chr13	22170300	0.760331
chr13	22273200	0.685950
chr13	22496600	0.727273
chr13	22499600	0.826446
chr13	22635900	0.785124
chr13	22640100	0.768595
chr13	22773100	0.669421
chr13	22810000	0.752066
chr13	22890700	0.661157
chr13	22979300	0.694215
chr13	23030300	0.925620
chr13	23125500	0.644628
chr13	23332600	0.760331
chr13	23388200	0.685950
chr13	23423500	0.793388
chr13	23455500	0.776860
chr13	23954300	0.719008
chr13	23974000	0.661157
chr13	23985900	0.652893
chr13	24002300	0.652893
chr13	24023400	0.892562
chr13	24025800	0.776860
chr13	24044700	0.743802
chr13	24126600	0.760331
chr13	24144900	0.644628
chr13	24173600	0.652893
chr13	24201400	0.710744
chr13	24206600	0.851240
chr13	24212100	0.694215
chr13	24218300	0.727273
chr13	24228300	0.677686
chr13	24270700	0.768595
chr13	24280000	0.644628
chr13	24323500	0.776860
chr13	24486700	0.760331
chr13	24496500	0.743802
chr13	24627800	0.694215
chr13	24641000	0.867769
chr13	24650900	0.661157
chr13	24658200	0.661157
chr13	24694400	0.900826

chr13	24701700	0.694215
chr13	24720300	0.661157
chr13	24766800	0.727273
chr13	24792700	0.818182
chr13	25019000	0.652893
chr13	25103400	0.975207
chr13	25202000	0.818182
chr13	25245800	0.876033
chr13	25258800	0.876033
chr13	25290700	0.752066
chr13	25462600	0.685950
chr13	25689000	0.685950
chr13	25706500	0.818182
chr13	25711300	0.933884
chr13	25724800	0.760331
chr13	25728100	0.661157
chr13	25739800	0.983471
chr13	25747800	0.669421
chr13	25755700	0.702479
chr13	25919200	0.652893
chr13	25959200	0.702479
chr13	25994600	0.661157
chr13	26052700	0.917355
chr13	26063900	0.809917
chr13	26127900	0.661157
chr13	26206800	0.909091
chr13	26227300	0.702479
chr13	26303400	0.652893
chr13	26345500	0.818182
chr13	26422700	0.719008
chr13	26456400	0.900826
chr13	26486300	0.925620
chr13	26497700	0.826446
chr13	26501900	0.710744
chr13	26504800	0.760331
chr13	26515100	0.727273
chr13	26545600	0.958678
chr13	26548200	0.669421
chr13	26551300	0.983471
chr13	26554700	0.776860
chr13	26599500	0.776860
chr13	26707600	0.702479
chr13	26923200	0.644628
chr13	27048300	0.842975
chr13	27092700	0.983471
chr13	27103400	0.818182
chr13	27136400	0.826446
chr13	27142500	0.768595
chr13	27254300	0.694215
chr13	27282100	0.694215
chr13	27317100	0.884298
chr13	27411000	0.644628
chr13	27447900	0.702479
chr13	27455500	0.719008

chr13	27469500	0.719008
chr13	27539100	0.727273
chr13	27579800	0.652893
chr13	27593100	0.685950
chr13	27654700	0.768595
chr13	27695200	0.669421
chr13	27712600	0.652893
chr13	27732200	0.702479
chr13	27734700	0.743802
chr13	27752800	0.818182
chr13	27817800	0.818182
chr13	27820300	0.867769
chr13	27834500	0.859504
chr13	27854600	0.669421
chr13	27910600	0.685950
chr13	27930300	0.760331
chr13	27933200	0.710744
chr13	27955200	0.776860
chr13	27961900	0.661157
chr13	27977800	0.801653
chr13	28033900	0.834711
chr13	28319400	0.876033
chr13	28345200	0.685950
chr13	28367000	0.793388
chr13	28381400	0.892562
chr13	28428000	0.752066
chr13	28434900	0.719008
chr13	28437000	0.669421
chr13	28492300	0.776860
chr13	28496500	0.719008
chr13	28499000	0.710744
chr13	28502800	0.909091
chr13	28537100	0.851240
chr13	28540400	0.834711
chr13	28545800	0.677686
chr13	28550100	0.776860
chr13	28552800	0.735537
chr13	28555100	0.719008
chr13	28575600	0.661157
chr13	28597100	0.760331
chr13	28624600	0.950413
chr13	28716600	0.768595
chr13	28736100	0.975207
chr13	28746300	0.818182
chr13	28812900	0.768595
chr13	28876500	0.975207
chr13	28883000	0.661157
chr13	28971300	0.694215
chr13	28982900	0.925620
chr13	29001600	0.727273
chr13	29005700	0.909091
chr13	29008500	0.685950
chr13	29012700	0.909091
chr13	29015200	0.958678

chr13	29025700	0.950413
chr13	29043500	0.826446
chr13	29072400	0.917355
chr13	29153700	0.702479
chr13	29161200	0.793388
chr13	29198600	0.644628
chr13	29259100	0.809917
chr13	29268100	0.942149
chr13	29318500	0.818182
chr13	29320900	0.669421
chr13	29648400	0.917355
chr13	29874900	0.710744
chr13	30058400	0.661157
chr13	30112900	0.826446
chr13	30115100	0.909091
chr13	30137600	0.702479
chr13	30156600	0.851240
chr13	30163900	0.710744
chr13	30297000	0.669421
chr13	30328400	0.727273
chr13	30415700	0.909091
chr13	30452500	0.834711
chr13	30459300	0.826446
chr13	30498800	0.652893
chr13	30517800	0.950413
chr13	30531700	0.669421
chr13	30538900	0.966942
chr13	30589500	0.702479
chr13	30592500	0.719008
chr13	30627300	0.909091
chr13	30754200	0.809917
chr13	30951200	0.768595
chr13	30969800	0.743802
chr13	30976200	0.677686
chr13	30983100	0.851240
chr13	31018400	0.933884
chr13	31051700	0.925620
chr13	31054000	0.652893
chr13	31061100	0.702479
chr13	31240200	0.735537
chr13	31353100	0.694215
chr13	31377700	0.727273
chr13	31407800	0.669421
chr13	31445000	0.900826
chr13	31447300	0.809917
chr13	31586100	0.900826
chr13	31669500	0.834711
chr13	31672000	0.644628
chr13	31783800	0.933884
chr13	31800100	0.925620
chr13	31802700	0.669421
chr13	32205800	0.719008
chr13	32239000	0.859504
chr13	32274100	0.661157

chr13	32497500	0.776860
chr13	32600100	0.661157
chr13	32620600	0.719008
chr13	32717100	0.652893
chr13	32859700	0.702479
chr13	33398700	0.694215
chr13	33404000	0.727273
chr13	33412600	0.785124
chr13	33599900	0.710744
chr13	33666500	0.760331
chr13	33697800	0.727273
chr13	33760700	0.735537
chr13	33768100	0.867769
chr13	33780500	0.760331
chr13	33788500	0.876033
chr13	33833700	0.909091
chr13	34064600	0.925620
chr13	34185200	0.809917
chr13	34188500	0.743802
chr13	34212200	0.727273
chr13	34214400	0.818182
chr13	34510800	0.818182
chr13	34702100	0.950413
chr13	34736700	0.842975
chr13	34932200	0.644628
chr13	34950800	0.818182
chr13	35323200	0.694215
chr13	35351200	0.677686
chr13	35390700	0.950413
chr13	35444900	0.909091
chr13	35474500	0.851240
chr13	35479300	0.694215
chr13	35488700	0.983471
chr13	35491900	0.727273
chr13	35510600	0.834711
chr13	35523200	0.884298
chr13	35526300	0.818182
chr13	35589000	0.834711
chr13	35605300	0.719008
chr13	36089300	0.735537
chr13	36147900	0.793388
chr13	36271500	0.669421
chr13	36397500	0.867769
chr13	36430700	0.851240
chr13	36490800	0.785124
chr13	36495600	0.834711
chr13	36505400	0.677686
chr13	36522100	0.793388
chr13	36559500	0.958678
chr13	36563200	0.851240
chr13	36567400	0.793388
chr13	36570300	0.719008
chr13	36642400	0.826446
chr13	36833100	0.867769

chr13	36874600	0.917355
chr13	37028800	0.851240
chr13	37093600	0.752066
chr13	37104900	0.644628
chr13	37186700	0.694215
chr13	37336500	0.900826
chr13	37348000	0.694215
chr13	37353000	0.991736
chr13	37361800	0.727273
chr13	37462500	0.884298
chr13	37640500	0.867769
chr13	37773000	0.909091
chr13	38169500	0.685950
chr13	38176200	0.685950
chr13	38370800	0.661157
chr13	38493700	0.727273
chr13	38508800	0.809917
chr13	38517400	0.685950
chr13	39093900	1.000000
chr13	39181500	0.644628
chr13	39199200	0.917355
chr13	39327900	0.694215
chr13	39377100	0.652893
chr13	39543800	0.752066
chr13	39555300	0.727273
chr13	39560900	0.785124
chr13	39596700	0.900826
chr13	39600000	0.900826
chr13	39622100	0.710744
chr13	39701500	0.669421
chr13	39704700	0.884298
chr13	39764600	0.793388
chr13	39903500	0.966942
chr13	39923000	0.826446
chr13	39960100	0.892562
chr13	39963600	0.661157
chr13	39975900	0.760331
chr13	39985100	0.702479
chr13	39992000	0.851240
chr13	40027000	0.702479
chr13	40058400	0.966942
chr13	40065000	0.809917
chr13	40068500	0.950413
chr13	40073000	0.975207
chr13	40140000	0.975207
chr13	40150100	0.710744
chr13	40152800	0.743802
chr13	40185800	0.644628
chr13	40361500	0.768595
chr13	40397500	0.694215
chr13	40410200	0.743802
chr13	40516200	0.669421
chr13	40846200	0.661157
chr13	40893100	0.694215

chr13	40915600	0.809917
chr13	40920200	0.710744
chr13	40954300	0.669421
chr13	40957500	0.851240
chr13	40965300	0.785124
chr13	41054800	0.644628
chr13	41114500	0.818182
chr13	41137100	0.735537
chr13	41139300	0.785124
chr13	41150500	0.727273
chr13	41153500	0.752066
chr13	41160500	0.909091
chr13	41171700	0.661157
chr13	41174300	0.719008
chr13	41177300	0.652893
chr13	41184600	0.661157
chr13	41189200	0.793388
chr13	41205100	0.719008
chr13	41214300	0.917355
chr13	41223500	0.710744
chr13	41227300	1.000000
chr13	41234600	0.933884
chr13	41246000	0.892562
chr13	41248200	0.900826
chr13	41256600	0.702479
chr13	41568700	0.702479
chr13	41589800	0.710744
chr13	41593300	0.669421
chr13	41731400	0.710744
chr13	41733800	0.694215
chr13	41861800	0.743802
chr13	41889000	0.685950
chr13	42271500	0.925620
chr13	42377100	0.685950
chr13	42575900	0.727273
chr13	42586300	0.719008
chr13	42703800	0.776860
chr13	42742100	0.694215
chr13	42790300	0.685950
chr13	42795600	0.867769
chr13	42833200	0.793388
chr13	42858200	0.677686
chr13	43142700	0.884298
chr13	43152000	0.743802
chr13	43166200	0.876033
chr13	43169300	0.983471
chr13	43191800	0.669421
chr13	43306300	0.685950
chr13	43447800	0.809917
chr13	43451500	0.702479
chr13	43543200	0.644628
chr13	43545600	0.719008
chr13	43556200	0.702479
chr13	43615700	0.677686

chr13	43647000	0.685950
chr13	43701600	0.719008
chr13	43707100	0.776860
chr13	43712200	0.677686
chr13	43715900	0.818182
chr13	43841500	0.685950
chr13	43890500	0.818182
chr13	43893900	0.983471
chr13	43904700	0.785124
chr13	43922500	0.669421
chr13	43934200	0.983471
chr13	43938800	0.752066
chr13	43999400	0.842975
chr13	44003400	0.710744
chr13	44016400	0.685950
chr13	44268400	0.735537
chr13	44339400	0.644628
chr13	44379800	0.909091
chr13	44627400	0.785124
chr13	44653500	1.000000
chr13	44691100	0.851240
chr13	44785600	0.685950
chr13	44806400	0.710744
chr13	44858300	0.719008
chr13	44953100	0.644628
chr13	44971700	0.809917
chr13	44981900	0.793388
chr13	44991000	0.925620
chr13	45067700	0.933884
chr13	45082500	0.785124
chr13	45223700	0.826446
chr13	45238900	0.793388
chr13	45391600	0.851240
chr13	45793500	0.644628
chr13	45883700	0.785124
chr13	45945800	0.669421
chr13	46042700	0.669421
chr13	46196500	0.661157
chr13	46201300	0.876033
chr13	46252300	0.909091
chr13	46255000	0.677686
chr13	46343600	0.892562
chr13	46402600	0.743802
chr13	46412700	0.677686
chr13	46457100	0.809917
chr13	46498600	0.842975
chr13	46709800	0.768595
chr13	46745400	0.710744
chr13	46964100	0.900826
chr13	47204900	0.851240
chr13	47237600	0.876033
chr13	47241100	0.867769
chr13	47312600	0.644628
chr13	47521800	0.801653

chr13	47610900	0.876033
chr13	47622300	0.793388
chr13	47669600	0.685950
chr13	47687400	0.958678
chr13	47947300	0.685950
chr13	47971500	0.809917
chr13	48046900	0.975207
chr13	48138500	0.752066
chr13	48206900	0.652893
chr13	48348700	0.644628
chr13	48388200	0.752066
chr13	48391300	0.958678
chr13	48671100	0.702479
chr13	48701800	0.694215
chr13	48810900	0.760331
chr13	48987100	0.752066
chr13	49071800	0.851240
chr13	49117100	0.661157
chr13	49168800	0.793388
chr13	49448400	0.834711
chr13	49553600	0.925620
chr13	49758600	0.867769
chr13	49853700	0.652893
chr13	49942600	0.876033
chr13	49975700	0.735537
chr13	50202900	0.652893
chr13	50244700	0.834711
chr13	50575000	0.702479
chr13	50652900	0.776860
chr13	50681900	0.842975
chr13	50715400	0.760331
chr13	50783100	0.909091
chr13	50851400	0.768595
chr13	50856900	0.917355
chr13	50952700	0.785124
chr13	50961100	0.652893
chr13	51210600	0.925620
chr13	51260100	0.677686
chr13	51361700	0.776860
chr13	51375400	0.669421
chr13	51419400	0.644628
chr13	51468100	0.876033
chr13	51486600	0.669421
chr13	51490700	0.884298
chr13	51565200	0.900826
chr13	51575200	0.793388
chr13	51579700	0.900826
chr13	51633400	0.702479
chr13	51672100	0.884298
chr13	51724400	0.950413
chr13	51727900	0.892562
chr13	51730900	0.644628
chr13	51733300	0.776860
chr13	51742300	0.818182

chr13	51749900	0.801653
chr13	51783300	0.652893
chr13	51786300	0.652893
chr13	51799700	0.809917
chr13	51803900	0.652893
chr13	51809800	0.818182
chr13	51814800	0.677686
chr13	51817300	0.859504
chr13	51820600	0.925620
chr13	51823200	0.677686
chr13	51830100	0.983471
chr13	51833500	0.752066
chr13	51854700	0.909091
chr13	51871700	0.743802
chr13	51877600	0.991736
chr13	51879700	0.768595
chr13	51899600	0.644628
chr13	51902900	0.669421
chr13	51921300	0.727273
chr13	51994900	0.735537
chr13	51997300	0.685950
chr13	52113100	0.677686
chr13	52162000	0.768595
chr13	52192200	0.776860
chr13	52195000	0.685950
chr13	52207500	0.826446
chr13	52210500	0.669421
chr13	52213000	0.785124
chr13	52218700	0.975207
chr13	52222000	0.752066
chr13	52257900	0.644628
chr13	52308100	0.859504
chr13	52343300	0.818182
chr13	52346000	0.644628
chr13	52372200	0.727273
chr13	52390000	0.743802
chr13	52393600	0.669421
chr13	52467400	0.702479
chr13	52480600	0.652893
chr13	52573100	0.801653
chr13	52786000	0.669421
chr13	52810300	0.727273
chr13	53152000	0.661157
chr13	53286900	0.735537
chr13	53303100	0.719008
chr13	53311700	0.644628
chr13	53383000	0.719008
chr13	53413000	0.826446
chr13	53442500	0.752066
chr13	53457000	0.892562
chr13	53479000	0.867769
chr13	53530000	0.876033
chr13	53538100	0.694215
chr13	53566300	0.818182

chr13	53595300	0.710744
chr13	53649800	0.884298
chr13	53695600	0.876033
chr13	53708700	0.644628
chr13	53716000	0.719008
chr13	53765200	0.719008
chr13	53812600	0.818182
chr13	53844100	0.661157
chr13	53850700	0.917355
chr13	53869600	0.694215
chr13	53917100	0.933884
chr13	53938600	0.768595
chr13	54082400	0.685950
chr13	54088000	0.661157
chr13	54094800	0.752066
chr13	54166100	0.702479
chr13	54516100	0.710744
chr13	54759500	0.710744
chr13	54768700	0.809917
chr13	54785300	0.785124
chr13	54838100	0.710744
chr13	54842000	0.884298
chr13	54875700	0.685950
chr13	54929800	0.842975
chr13	54932000	0.760331
chr13	54948300	0.768595
chr13	54956500	0.661157
chr13	54962100	0.842975
chr13	55449700	0.735537
chr13	55519700	0.644628
chr13	55575900	0.950413
chr13	55610700	0.975207
chr13	55661500	0.933884
chr13	56015100	0.950413
chr13	56158700	0.900826
chr13	56228500	0.727273
chr13	56235700	0.842975
chr13	56248500	0.900826
chr13	56279300	0.743802
chr13	56892200	0.644628
chr13	57733700	0.735537
chr13	57885500	0.694215
chr13	58257000	0.776860
chr13	58299200	0.776860
chr13	59050700	0.793388
chr13	59193700	0.768595
chr13	59459100	0.867769
chr13	59508000	0.958678
chr13	59833900	0.661157
chr13	59966100	0.735537
chr13	59984200	0.991736
chr13	60022700	1.000000
chr13	60054200	0.966942
chr13	60058100	0.892562

chr13	60106200	0.876033
chr13	60118900	0.842975
chr13	60161000	0.661157
chr13	60163100	0.818182
chr13	60197700	0.958678
chr13	60239100	0.727273
chr13	60263500	0.958678
chr13	60325200	0.876033
chr13	60367700	0.917355
chr13	60373100	0.710744
chr13	60718600	0.719008
chr13	60733100	0.801653
chr13	61147000	0.975207
chr13	61157900	0.859504
chr13	61160000	0.793388
chr13	61203700	0.652893
chr13	61221700	0.677686
chr13	61329700	0.900826
chr13	61669400	0.785124
chr13	61754200	0.925620
chr13	61776800	0.652893
chr13	61829600	0.702479
chr13	61891100	0.735537
chr13	62182800	0.685950
chr13	62475400	0.933884
chr13	62760300	0.710744
chr13	62884700	0.892562
chr13	63048100	0.661157
chr13	63086200	0.652893
chr13	63264600	0.826446
chr13	63742300	0.991736
chr13	63759100	0.669421
chr13	64398900	0.685950
chr13	65103500	0.876033
chr13	65108700	0.661157
chr13	65258900	0.917355
chr13	65321500	0.702479
chr13	65324300	0.661157
chr13	65332900	0.975207
chr13	66501200	0.859504
chr13	66799200	0.735537
chr13	67103300	0.975207
chr13	67500800	0.809917
chr13	67523600	0.669421
chr13	67833100	0.785124
chr13	68093200	0.768595
chr13	68310500	0.867769
chr13	68338100	0.685950
chr13	68429500	0.661157
chr13	68598500	0.834711
chr13	69545000	0.892562
chr13	69809100	0.933884
chr13	70154900	0.702479
chr13	70368800	0.685950

chr13	70663200	0.702479
chr13	70700000	0.669421
chr13	70966100	0.809917
chr13	70968600	0.809917
chr13	71614300	0.644628
chr13	71621200	0.900826
chr13	71794000	0.685950
chr13	72259800	0.776860
chr13	72334200	0.859504
chr13	72444100	0.644628
chr13	72451500	0.694215
chr13	72455200	0.644628
chr13	72637100	0.727273
chr13	72695700	0.727273
chr13	73101800	0.776860
chr13	73142100	0.669421
chr13	73213200	0.702479
chr13	73395200	0.776860
chr13	73535100	0.710744
chr13	73713100	0.685950
chr13	73743700	0.685950
chr13	73973800	0.661157
chr13	74193200	0.644628
chr13	74271000	0.727273
chr13	74276600	0.859504
chr13	74296900	0.669421
chr13	74304400	0.743802
chr13	74340200	0.851240
chr13	74445400	0.768595
chr13	74512800	0.776860
chr13	74547400	0.710744
chr13	74599300	0.743802
chr13	74601500	0.933884
chr13	74610900	0.991736
chr13	74750200	0.702479
chr13	74862400	0.818182
chr13	74864500	0.644628
chr13	74876300	0.892562
chr13	74921400	0.685950
chr13	75348800	0.661157
chr13	76033500	0.925620
chr13	76377500	0.925620
chr13	76393300	0.776860
chr13	76399600	0.809917
chr13	76463900	0.826446
chr13	77145000	0.785124
chr13	77259300	0.727273
chr13	77299900	0.677686
chr13	77622400	0.867769
chr13	77669400	0.760331
chr13	77862700	0.801653
chr13	77881000	0.669421
chr13	77885900	0.876033
chr13	77948600	0.661157

chr13	78023300	0.685950
chr13	78052700	0.925620
chr13	78054800	0.719008
chr13	78428000	0.958678
chr13	78430100	0.834711
chr13	79161900	0.743802
chr13	79179700	0.793388
chr13	79230300	0.900826
chr13	79352200	0.793388
chr13	79772500	0.768595
chr13	79856100	0.950413
chr13	79862300	0.867769
chr13	80026300	0.735537
chr13	80110000	0.776860
chr13	80357300	0.966942
chr13	80385000	0.677686
chr13	80444200	0.991736
chr13	80487900	0.702479
chr13	80541000	0.661157
chr13	80715900	0.710744
chr13	80792800	0.735537
chr13	80810000	0.702479
chr13	80934000	0.752066
chr13	81159200	0.776860
chr13	81291200	0.727273
chr13	81358200	0.809917
chr13	81537400	0.652893
chr13	81715300	0.752066
chr13	81968200	0.743802
chr13	82447200	0.950413
chr13	82450700	0.809917
chr13	82562100	0.826446
chr13	82565100	0.652893
chr13	82585700	0.743802
chr13	82659800	0.644628
chr13	83249000	0.669421
chr13	83332600	0.685950
chr13	83475200	0.644628
chr13	84255800	0.644628
chr13	84511500	0.876033
chr13	84516100	0.818182
chr13	84600300	0.793388
chr13	85229600	0.975207
chr13	85236700	0.851240
chr13	85391200	0.702479
chr13	86075700	0.851240
chr13	86142400	0.768595
chr13	87099000	0.859504
chr13	87535100	0.834711
chr13	87571300	0.661157
chr13	87777000	0.685950
chr13	88490500	0.652893
chr13	88776900	0.859504
chr13	89113900	0.867769

chr13	89178900	0.743802
chr13	89416800	0.677686
chr13	90167100	0.851240
chr13	90170600	0.785124
chr13	90661200	0.859504
chr13	91067600	0.685950
chr13	91426600	0.694215
chr13	91525100	0.652893
chr13	91627000	0.991736
chr13	91791900	0.644628
chr13	91869900	0.809917
chr13	93798100	0.776860
chr13	93869200	0.876033
chr13	93897800	0.644628
chr13	93901500	0.735537
chr13	93921100	0.752066
chr13	93932600	0.694215
chr13	93969500	0.884298
chr13	93971900	0.727273
chr13	93974000	0.669421
chr13	94067100	0.652893
chr13	94072900	0.768595
chr13	94172400	0.727273
chr13	94177100	0.652893
chr13	94210600	0.644628
chr13	94214200	0.727273
chr13	94491500	0.652893
chr13	94686500	0.743802
chr13	94796600	0.966942
chr13	94823900	0.809917
chr13	94841200	0.752066
chr13	94870500	0.710744
chr13	94956300	0.702479
chr13	94965100	0.859504
chr13	94996900	0.851240
chr13	95086100	0.727273
chr13	95146600	0.652893
chr13	95198600	0.818182
chr13	95313600	0.801653
chr13	95368300	0.735537
chr13	95413900	0.942149
chr13	95478200	0.942149
chr13	95481800	0.644628
chr13	95600100	0.743802
chr13	95649000	0.785124
chr13	95655800	0.851240
chr13	95719100	0.719008
chr13	96100300	0.752066
chr13	96199800	0.727273
chr13	96606800	0.876033
chr13	96652100	0.950413
chr13	96686700	0.727273
chr13	96693900	0.727273
chr13	96756800	0.694215

chr13	96783300	0.752066
chr13	96947800	0.983471
chr13	97362700	0.694215
chr13	97420900	0.909091
chr13	97551100	0.719008
chr13	97556700	0.644628
chr13	97636600	0.727273
chr13	97653200	0.710744
chr13	97696000	0.694215
chr13	97720700	0.834711
chr13	97794500	0.917355
chr13	97877000	0.793388
chr13	97893500	0.859504
chr13	97920100	0.859504
chr13	97981100	0.925620
chr13	98221300	0.760331
chr13	98274300	0.652893
chr13	98679500	0.677686
chr13	98714200	0.743802
chr13	98757600	0.785124
chr13	98799400	0.752066
chr13	98821200	0.727273
chr13	98825600	0.975207
chr13	98854200	0.983471
chr13	98892300	0.966942
chr13	98913700	0.644628
chr13	98926600	0.966942
chr13	98968500	0.710744
chr13	98978700	0.942149
chr13	98981000	0.694215
chr13	98985500	0.909091
chr13	99024000	0.652893
chr13	99102600	0.851240
chr13	99128300	0.719008
chr13	99136700	0.867769
chr13	99142100	0.933884
chr13	99145200	0.859504
chr13	99148600	0.768595
chr13	99159700	0.950413
chr13	99173200	0.719008
chr13	99176000	0.859504
chr13	99180900	0.760331
chr13	99189900	0.752066
chr13	99196700	0.818182
chr13	99215600	0.793388
chr13	99224400	0.818182
chr13	99328000	0.710744
chr13	99341300	0.859504
chr13	99460200	0.785124
chr13	99474000	0.958678
chr13	99479000	0.942149
chr13	99492800	0.884298
chr13	99527900	0.983471
chr13	99541100	0.644628

chr13	99545400	0.652893
chr13	99574600	0.809917
chr13	99578600	0.694215
chr13	99591000	0.950413
chr13	99606700	0.719008
chr13	99638300	0.727273
chr13	99828900	0.702479
chr13	99861400	0.768595
chr13	99867200	0.702479
chr13	100012300	0.694215
chr13	100031600	0.644628
chr13	100069900	0.851240
chr13	100092300	0.685950
chr13	100129200	0.719008
chr13	100148500	0.661157
chr13	100253600	0.752066
chr13	100277900	0.933884
chr13	100312300	0.900826
chr13	100315700	0.752066
chr13	100330500	0.867769
chr13	100334100	0.685950
chr13	100338300	0.867769
chr13	100341100	0.842975
chr13	100343400	0.900826
chr13	100346400	0.851240
chr13	100355800	0.983471
chr13	100358000	0.842975
chr13	100370900	0.851240
chr13	100374000	0.859504
chr13	100378600	0.975207
chr13	100397700	0.958678
chr13	100402200	0.933884
chr13	100411200	0.776860
chr13	100419300	0.859504
chr13	100421400	0.719008
chr13	100445200	0.694215
chr13	100453200	0.826446
chr13	100459400	0.760331
chr13	100472300	0.719008
chr13	100500100	0.652893
chr13	100506900	0.884298
chr13	100514600	0.785124
chr13	100517700	0.801653
chr13	100545000	0.842975
chr13	100557500	0.652893
chr13	100579100	0.694215
chr13	100744000	0.685950
chr13	100801400	0.950413
chr13	100807100	0.909091
chr13	100828300	0.917355
chr13	100835700	0.644628
chr13	100882900	0.801653
chr13	100887100	0.950413
chr13	100897400	0.719008

chr13	100958900	0.760331
chr13	101010000	0.719008
chr13	101091500	0.991736
chr13	101125900	0.826446
chr13	101151500	0.743802
chr13	101162800	0.991736
chr13	101166900	0.727273
chr13	101174200	0.892562
chr13	101305700	0.652893
chr13	101426600	0.661157
chr13	101468700	0.793388
chr13	101506800	0.743802
chr13	101640600	0.743802
chr13	101766900	0.884298
chr13	101769600	0.669421
chr13	101773100	0.661157
chr13	102413200	0.776860
chr13	102494500	0.859504
chr13	102520000	0.826446
chr13	102865900	0.826446
chr13	103153400	0.685950
chr13	103252400	0.851240
chr13	103340800	0.652893
chr13	103344800	0.743802
chr13	103446400	0.950413
chr13	103718500	0.768595
chr13	103721000	0.892562
chr13	104003700	0.818182
chr13	104087000	0.677686
chr13	104169100	0.702479
chr13	104796500	0.652893
chr13	104833800	0.917355
chr13	104905700	0.826446
chr13	105183500	0.884298
chr13	105238800	0.685950
chr13	105267200	0.752066
chr13	105288800	0.826446
chr13	105432200	0.760331
chr13	105931900	0.685950
chr13	105934500	0.900826
chr13	106128000	0.661157
chr13	106469900	0.785124
chr13	106572200	0.710744
chr13	106753400	0.966942
chr13	106865700	0.776860
chr13	106886000	0.785124
chr13	106954800	0.652893
chr13	107058400	0.900826
chr13	107076200	0.727273
chr13	107165800	0.966942
chr13	107168600	0.685950
chr13	107173900	0.685950
chr13	107183100	0.867769
chr13	107216900	0.685950

chr13	107333900	0.933884
chr13	107448800	0.958678
chr13	107458000	0.652893
chr13	107468100	0.867769
chr13	107511300	0.933884
chr13	107513700	0.727273
chr13	107559200	0.760331
chr13	107568200	0.793388
chr13	107572400	0.876033
chr13	108215500	0.644628
chr13	108227600	0.685950
chr13	108407500	0.677686
chr13	108451700	0.661157
chr13	108470300	0.652893
chr13	108491400	0.760331
chr13	108496000	0.743802
chr13	109439100	0.785124
chr13	109810100	0.702479
chr13	109849500	0.760331
chr13	109924200	0.884298
chr13	109927300	0.735537
chr13	110022000	0.644628
chr13	110076800	1.000000
chr13	110198500	0.785124
chr13	110321600	0.785124
chr13	110340600	0.818182
chr13	110434400	0.884298
chr13	110443700	0.644628
chr13	110469400	0.727273
chr13	110680400	0.694215
chr13	110689200	0.785124
chr13	110762000	0.785124
chr13	110779300	0.950413
chr13	110785200	0.876033
chr13	110788100	0.900826
chr13	110914400	0.735537
chr13	110917600	0.743802
chr13	110951500	0.694215
chr13	110954700	0.785124
chr13	110976300	0.735537
chr13	110979400	0.917355
chr13	111007800	0.900826
chr13	111032600	0.809917
chr13	111068300	0.776860
chr13	111108100	0.702479
chr13	111118900	0.801653
chr13	111137900	0.801653
chr13	111140500	0.793388
chr13	111179500	0.876033
chr13	111184200	0.876033
chr13	111228200	0.719008
chr13	111297600	0.884298
chr13	111409100	0.685950
chr13	111450500	0.685950

chr13	111539900	0.884298
chr13	111559200	0.950413
chr13	111561300	0.975207
chr13	111563400	0.644628
chr13	111646600	0.685950
chr13	111716100	0.834711
chr13	111823600	0.834711
chr13	111854500	0.884298
chr13	111864100	0.975207
chr13	111868300	0.925620
chr13	111871700	0.851240
chr13	111876000	0.644628
chr13	111893900	0.735537
chr13	111918100	0.719008
chr13	111965500	0.661157
chr13	111997400	0.776860
chr13	112012200	0.809917
chr13	112065800	0.826446
chr13	112169800	0.661157
chr13	112183000	0.842975
chr13	112185300	0.826446
chr13	112249900	0.719008
chr13	112285500	0.818182
chr13	112289000	0.826446
chr13	112318700	0.652893
chr13	112332700	0.818182
chr13	112514400	0.644628
chr13	112574300	0.644628
chr13	112609400	0.776860
chr13	112715800	0.685950
chr13	112718600	0.776860
chr13	112813900	0.785124
chr13	112816900	0.851240
chr13	112997100	0.685950
chr13	113006200	0.685950
chr13	113098100	0.884298
chr13	113215700	0.752066
chr13	113228300	0.685950
chr13	113352300	0.685950
chr13	113359300	0.768595
chr13	113365600	0.743802
chr13	113368000	0.669421
chr13	113370400	0.859504
chr13	113375800	0.702479
chr13	113379500	0.876033
chr13	113381800	0.710744
chr13	113383900	0.776860
chr13	113388700	0.966942
chr13	113392300	0.685950
chr13	113395600	0.834711
chr13	113398500	0.950413
chr13	113416800	0.677686
chr13	113420000	0.702479
chr13	113425300	0.867769

chr13	113435400	0.768595
chr13	113462600	0.652893
chr13	113540300	0.818182
chr13	113557400	0.669421
chr13	113560000	0.834711
chr13	113574300	0.652893
chr13	113585900	0.826446
chr13	113634900	0.644628
chr13	113639800	0.702479
chr13	113645300	0.851240
chr13	113677400	0.975207
chr13	113705500	0.694215
chr13	113724700	0.892562
chr13	113744600	0.685950
chr13	113777500	0.702479
chr13	113813300	0.851240
chr13	113869700	0.933884
chr13	113872000	0.694215
chr13	113898700	0.801653
chr13	113946700	0.842975
chr13	113986600	0.685950
chr13	113992400	0.793388
chr13	114031300	0.950413
chr13	114034900	0.677686
chr13	114045900	0.925620
chr13	114053600	0.727273
chr13	114123500	0.925620
chr13	114138300	0.710744
chr13	114150400	0.892562
chr13	114187000	0.933884
chr13	114215800	0.867769
chr13	114271500	0.876033
chr13	114469100	0.743802
chr13	114505500	0.776860
chr13	114541200	0.966942
chr13	114552500	0.710744
chr13	114564400	0.685950
chr13	114594300	0.900826
chr13	114638300	0.876033
chr13	114801500	0.727273
chr13	114805700	0.876033
chr13	114830900	0.917355
chr13	114844700	0.892562
chr13	114855200	0.776860
chr13	114864300	0.884298
chr13	114875500	0.760331
chr13	114878600	0.661157
chr13	114885100	0.809917
chr13	114996600	0.785124
chr13	114999400	0.677686
chr13	115022500	0.834711
chr14	19004300	0.702479
chr14	19038600	0.694215
chr14	20967600	0.776860

chr14	21046700	0.983471
chr14	21096900	0.677686
chr14	21175400	0.925620
chr14	21243500	0.933884
chr14	21252400	0.677686
chr14	21268500	0.644628
chr14	21289100	0.900826
chr14	21317600	0.776860
chr14	21372900	0.661157
chr14	21467200	0.909091
chr14	21507600	0.760331
chr14	21555900	0.859504
chr14	21560100	0.958678
chr14	21575200	0.867769
chr14	21738900	0.644628
chr14	21788100	0.661157
chr14	21909300	0.661157
chr14	21967200	0.950413
chr14	21989700	0.826446
chr14	21992100	0.752066
chr14	21997400	0.925620
chr14	22007800	0.975207
chr14	22011700	0.909091
chr14	22021900	0.925620
chr14	22025400	0.917355
chr14	22111900	0.702479
chr14	22121000	0.785124
chr14	22198300	0.801653
chr14	22249100	0.801653
chr14	22549800	0.661157
chr14	22640400	0.669421
chr14	22697400	0.818182
chr14	22802000	0.661157
chr14	22945800	0.677686
chr14	22949000	0.743802
chr14	23015900	0.842975
chr14	23019700	0.950413
chr14	23026200	0.966942
chr14	23039600	0.933884
chr14	23155600	0.652893
chr14	23266300	0.975207
chr14	23282200	0.892562
chr14	23284800	0.826446
chr14	23289000	0.818182
chr14	23318300	0.801653
chr14	23322600	0.727273
chr14	23352500	0.785124
chr14	23369300	0.776860
chr14	23447400	0.743802
chr14	23464400	0.801653
chr14	23495200	0.793388
chr14	23537900	0.727273
chr14	23575800	0.743802
chr14	23589000	0.826446

chr14	23624500	0.867769
chr14	23629900	0.991736
chr14	23644400	0.950413
chr14	23676600	0.826446
chr14	23722100	0.851240
chr14	23749600	0.752066
chr14	23763600	0.694215
chr14	23846300	0.942149
chr14	23849500	0.685950
chr14	23908000	0.785124
chr14	23912600	0.809917
chr14	23952100	0.900826
chr14	23956000	0.859504
chr14	23963600	0.867769
chr14	23998000	0.818182
chr14	24013500	0.925620
chr14	24017000	0.669421
chr14	24028400	0.950413
chr14	24031900	0.719008
chr14	24038800	0.925620
chr14	24058600	0.818182
chr14	24101500	0.760331
chr14	24110400	0.743802
chr14	24163500	0.719008
chr14	24380600	0.925620
chr14	24601600	0.727273
chr14	24630800	0.743802
chr14	24672400	0.760331
chr14	24862000	0.719008
chr14	24896800	0.727273
chr14	25275900	0.727273
chr14	25319900	0.768595
chr14	25322300	0.776860
chr14	25327800	0.925620
chr14	25411400	0.975207
chr14	25413600	0.735537
chr14	25422600	0.975207
chr14	25479000	0.785124
chr14	25521800	0.710744
chr14	25602100	0.661157
chr14	25707300	0.752066
chr14	26046200	0.735537
chr14	26226600	1.000000
chr14	26235700	0.677686
chr14	26338400	0.702479
chr14	26486800	0.685950
chr14	26731400	1.000000
chr14	26751200	0.958678
chr14	26862500	0.752066
chr14	27023500	0.685950
chr14	27034900	0.644628
chr14	27245800	0.735537
chr14	27341000	0.661157
chr14	27395700	0.760331

chr14	27411000	0.826446
chr14	28158600	0.867769
chr14	28555300	0.661157
chr14	29130900	0.966942
chr14	29231300	0.661157
chr14	29233900	0.776860
chr14	29247600	0.752066
chr14	29251100	0.727273
chr14	29345900	0.743802
chr14	29498700	0.884298
chr14	29508800	0.652893
chr14	30054500	0.677686
chr14	30208600	0.785124
chr14	30275300	0.685950
chr14	30280400	0.719008
chr14	30411400	0.727273
chr14	30792700	0.661157
chr14	30954300	0.760331
chr14	30998500	0.942149
chr14	31466200	0.694215
chr14	31470200	0.809917
chr14	31528700	0.644628
chr14	31592700	0.727273
chr14	31699300	0.760331
chr14	31763000	0.702479
chr14	32362600	0.743802
chr14	32420500	0.917355
chr14	32529100	0.644628
chr14	32556200	0.661157
chr14	32806300	0.661157
chr14	32828000	0.785124
chr14	32831400	0.677686
chr14	32836000	0.702479
chr14	32866100	0.661157
chr14	32942400	0.768595
chr14	32954200	0.975207
chr14	33604000	0.685950
chr14	33811600	0.719008
chr14	33820000	0.909091
chr14	33832400	0.900826
chr14	33936500	0.677686
chr14	33947200	0.859504
chr14	33955700	0.661157
chr14	34081500	0.834711
chr14	34111300	0.966942
chr14	34234700	0.652893
chr14	34306400	0.900826
chr14	34321500	0.685950
chr14	34386900	0.719008
chr14	34407300	0.818182
chr14	34410500	0.884298
chr14	34483100	0.752066
chr14	34487900	0.727273
chr14	34966700	0.958678

chr14	34972200	0.719008
chr14	35017200	0.694215
chr14	35155600	0.710744
chr14	35162800	0.702479
chr14	35365200	0.702479
chr14	35628900	0.669421
chr14	35802100	0.867769
chr14	35806500	0.685950
chr14	35811100	0.752066
chr14	35816900	0.710744
chr14	35822000	0.727273
chr14	35836900	0.727273
chr14	35867300	0.983471
chr14	35882500	0.793388
chr14	35885400	0.826446
chr14	36367600	0.710744
chr14	36467300	0.768595
chr14	36479200	0.801653
chr14	36631400	0.669421
chr14	36692400	0.661157
chr14	36699200	0.661157
chr14	36713100	0.818182
chr14	36739200	0.785124
chr14	36741800	0.809917
chr14	36749800	0.950413
chr14	36765500	0.694215
chr14	36899800	0.884298
chr14	36910300	0.809917
chr14	36912900	0.842975
chr14	36979400	0.818182
chr14	36983100	0.785124
chr14	36987100	0.834711
chr14	36992100	0.867769
chr14	37025900	0.644628
chr14	37053300	0.719008
chr14	37059600	0.644628
chr14	37127700	0.685950
chr14	37145900	0.694215
chr14	37193600	0.694215
chr14	37237400	0.892562
chr14	37274600	0.776860
chr14	37295600	0.735537
chr14	37530400	0.785124
chr14	37637200	0.933884
chr14	38067100	0.644628
chr14	38279600	0.768595
chr14	38358600	0.694215
chr14	38464700	0.942149
chr14	38548000	0.685950
chr14	38597000	0.719008
chr14	38613400	0.933884
chr14	38659300	0.909091
chr14	38666700	0.685950
chr14	38690800	0.702479

chr14	38779900	0.669421
chr14	38994800	0.859504
chr14	39081600	0.867769
chr14	39261300	0.776860
chr14	39656800	0.851240
chr14	39673300	0.652893
chr14	39836600	0.661157
chr14	40337900	0.694215
chr14	40347000	0.661157
chr14	40787800	0.669421
chr14	41085700	0.834711
chr14	41328600	0.710744
chr14	42069500	0.884298
chr14	42805500	0.743802
chr14	42983200	0.851240
chr14	43134100	0.685950
chr14	44056700	0.752066
chr14	44224500	0.909091
chr14	44250000	0.677686
chr14	44342100	0.966942
chr14	44419000	0.702479
chr14	44500400	0.669421
chr14	45290000	0.768595
chr14	45681700	0.793388
chr14	45840300	0.685950
chr14	46241800	0.859504
chr14	46361300	0.677686
chr14	46592900	0.669421
chr14	46791900	0.809917
chr14	46874600	0.917355
chr14	47573000	0.785124
chr14	47835300	0.867769
chr14	47907800	0.752066
chr14	47910100	0.702479
chr14	48527400	0.867769
chr14	48542300	0.760331
chr14	48720500	0.669421
chr14	48731200	0.685950
chr14	49031300	0.867769
chr14	49033400	0.876033
chr14	49360300	0.702479
chr14	49884900	0.652893
chr14	49931900	0.785124
chr14	49973900	0.809917
chr14	50017200	0.933884
chr14	50049500	0.818182
chr14	50158400	0.652893
chr14	50276200	0.727273
chr14	50433400	0.785124
chr14	50528300	0.975207
chr14	50802800	0.710744
chr14	51067600	0.727273
chr14	51125300	0.909091
chr14	51137400	0.966942

chr14	51280200	0.859504
chr14	51326800	0.884298
chr14	51365100	0.991736
chr14	51450500	0.685950
chr14	51452600	0.842975
chr14	51505800	0.743802
chr14	51587800	0.768595
chr14	51606100	0.727273
chr14	51634900	0.652893
chr14	51643600	0.652893
chr14	51862400	0.752066
chr14	51878400	0.644628
chr14	52042900	0.685950
chr14	52051900	0.644628
chr14	52172300	0.743802
chr14	52183100	0.669421
chr14	52286000	0.652893
chr14	52313000	0.677686
chr14	52333500	0.727273
chr14	52591900	0.884298
chr14	52720500	0.719008
chr14	53200300	0.652893
chr14	53259500	0.752066
chr14	53298700	0.768595
chr14	53302200	0.785124
chr14	53311700	0.884298
chr14	53339800	0.892562
chr14	53343000	0.694215
chr14	53390800	0.685950
chr14	53478300	0.900826
chr14	53487000	0.933884
chr14	53795200	0.644628
chr14	53807100	0.884298
chr14	53823300	0.942149
chr14	54038600	0.826446
chr14	54208900	0.876033
chr14	54388300	0.867769
chr14	54410200	0.975207
chr14	54432300	0.694215
chr14	54448600	0.776860
chr14	54689200	0.768595
chr14	54816200	0.702479
chr14	54829000	0.768595
chr14	54834000	0.669421
chr14	55073400	0.677686
chr14	55117400	0.900826
chr14	55120200	0.801653
chr14	55140600	0.685950
chr14	55144400	0.991736
chr14	55151300	0.801653
chr14	55158600	0.768595
chr14	55193200	0.661157
chr14	55240200	0.694215
chr14	55272200	0.826446

chr14	55355600	0.694215
chr14	55491000	0.958678
chr14	55570300	0.884298
chr14	55668200	0.818182
chr14	55774100	0.644628
chr14	55807000	0.685950
chr14	56073200	0.727273
chr14	56108700	0.719008
chr14	56216700	0.644628
chr14	56219900	0.685950
chr14	56358400	0.752066
chr14	56360500	0.652893
chr14	56377700	0.661157
chr14	56424900	0.966942
chr14	56472200	0.942149
chr14	56593000	0.661157
chr14	56596400	0.876033
chr14	56599800	0.694215
chr14	56605000	0.809917
chr14	56615300	0.669421
chr14	56654600	0.694215
chr14	56662500	0.694215
chr14	56771400	0.685950
chr14	56959000	0.834711
chr14	56992300	0.809917
chr14	57019200	0.677686
chr14	57127600	0.884298
chr14	57157800	0.719008
chr14	57197600	0.917355
chr14	57201000	0.677686
chr14	57311800	0.876033
chr14	57316300	0.694215
chr14	57321400	0.644628
chr14	57352400	0.842975
chr14	57355600	0.809917
chr14	57360000	0.900826
chr14	57364400	0.752066
chr14	57367000	0.933884
chr14	57369900	0.867769
chr14	57372500	0.851240
chr14	57386100	0.859504
chr14	57420800	0.669421
chr14	57496700	0.834711
chr14	57525400	0.735537
chr14	57547900	0.818182
chr14	57558000	0.719008
chr14	57603500	0.876033
chr14	57628900	0.793388
chr14	57693100	0.669421
chr14	57832500	0.652893
chr14	57968300	0.644628
chr14	58181600	0.983471
chr14	58196700	0.694215
chr14	58616200	0.909091

chr14	59087400	0.876033
chr14	59092400	0.859504
chr14	59095000	0.727273
chr14	59102100	0.801653
chr14	59123900	0.669421
chr14	59164800	0.661157
chr14	59222900	0.950413
chr14	59236200	0.661157
chr14	59240100	0.677686
chr14	59256100	0.752066
chr14	59438000	0.925620
chr14	59688700	0.644628
chr14	59736900	0.685950
chr14	59979100	0.644628
chr14	60278900	0.942149
chr14	60562000	0.768595
chr14	60628400	0.727273
chr14	60671500	0.909091
chr14	60687900	0.743802
chr14	60712900	0.900826
chr14	60745400	0.884298
chr14	60770400	0.669421
chr14	60784800	0.942149
chr14	60817300	0.776860
chr14	60824000	0.917355
chr14	60979700	0.669421
chr14	61107900	0.719008
chr14	61110900	0.900826
chr14	61113000	0.644628
chr14	61146700	0.851240
chr14	61177600	0.702479
chr14	61228100	0.900826
chr14	61247200	0.809917
chr14	61547500	0.818182
chr14	61557100	0.851240
chr14	61568200	0.652893
chr14	61571200	0.702479
chr14	61586800	0.694215
chr14	61620800	0.933884
chr14	61645000	0.735537
chr14	61654700	0.867769
chr14	61669600	0.826446
chr14	61705600	0.991736
chr14	61722100	0.702479
chr14	61764400	0.652893
chr14	61767700	0.826446
chr14	61823200	0.644628
chr14	61906200	0.752066
chr14	61929800	0.669421
chr14	61933500	0.859504
chr14	61937300	0.826446
chr14	61965700	0.644628
chr14	61969800	0.983471
chr14	61973200	0.661157

chr14	61991400	0.710744
chr14	62004600	0.884298
chr14	62020400	0.661157
chr14	62028800	0.661157
chr14	62063800	0.768595
chr14	62069200	0.760331
chr14	62074200	0.760331
chr14	62076500	0.991736
chr14	62145800	0.809917
chr14	62158300	0.809917
chr14	62180700	0.867769
chr14	62222300	0.727273
chr14	62232800	0.727273
chr14	62265600	0.818182
chr14	62303300	0.644628
chr14	62394500	0.768595
chr14	62397400	0.661157
chr14	62435600	0.661157
chr14	62876300	0.644628
chr14	62912600	0.785124
chr14	62916900	0.652893
chr14	63129800	0.644628
chr14	63163600	0.909091
chr14	63210600	0.644628
chr14	63356200	0.710744
chr14	63641800	0.719008
chr14	63671500	0.925620
chr14	63872000	0.652893
chr14	63959900	0.669421
chr14	63981100	0.702479
chr14	64331800	0.826446
chr14	64358400	0.983471
chr14	64430800	0.801653
chr14	64527800	0.975207
chr14	64542400	0.735537
chr14	64557400	0.892562
chr14	64563100	0.727273
chr14	64659800	0.677686
chr14	64663600	0.801653
chr14	64667600	0.685950
chr14	64745900	0.644628
chr14	64770500	0.719008
chr14	64807600	0.669421
chr14	64858400	0.884298
chr14	64861700	0.760331
chr14	64942700	0.644628
chr14	65011800	0.702479
chr14	65040100	0.909091
chr14	65048500	0.710744
chr14	65106900	0.752066
chr14	65112500	0.975207
chr14	65149500	0.710744
chr14	65152900	0.900826
chr14	65165900	0.685950

chr14	65168300	0.867769
chr14	65176700	0.669421
chr14	65185200	0.685950
chr14	65188000	0.735537
chr14	65193300	0.760331
chr14	65203500	0.892562
chr14	65229400	0.867769
chr14	65231700	0.710744
chr14	65235800	0.735537
chr14	65279500	0.677686
chr14	65290100	0.743802
chr14	65298200	0.719008
chr14	65308600	0.793388
chr14	65312700	0.669421
chr14	65323000	0.900826
chr14	65325400	0.942149
chr14	65332800	0.834711
chr14	65410300	0.727273
chr14	65422700	0.743802
chr14	65427700	0.826446
chr14	65443900	0.785124
chr14	65464700	0.661157
chr14	65638000	0.900826
chr14	65641300	0.785124
chr14	65654400	0.859504
chr14	65696800	0.793388
chr14	65705000	0.809917
chr14	65722800	0.818182
chr14	65749300	0.884298
chr14	65834200	0.752066
chr14	65937200	0.900826
chr14	65952800	0.727273
chr14	66061400	0.859504
chr14	66131500	0.752066
chr14	66152900	0.710744
chr14	66281000	0.900826
chr14	66288300	0.735537
chr14	66302600	0.727273
chr14	66393500	0.842975
chr14	66404300	0.661157
chr14	66408400	0.644628
chr14	66424800	0.867769
chr14	66461100	0.867769
chr14	66720900	0.727273
chr14	66826300	0.892562
chr14	66850300	0.842975
chr14	67499300	0.925620
chr14	67735600	0.933884
chr14	67894700	0.743802
chr14	67911400	0.933884
chr14	67958700	0.975207
chr14	68006000	0.975207
chr14	68077000	0.900826
chr14	68090000	0.818182

chr14	68189600	0.884298
chr14	68196100	0.909091
chr14	68401000	0.892562
chr14	68408800	0.760331
chr14	68507800	0.760331
chr14	68510700	0.685950
chr14	68542400	0.851240
chr14	68552800	0.669421
chr14	68556000	0.644628
chr14	68666700	0.884298
chr14	68690800	0.727273
chr14	68698100	0.991736
chr14	68718500	0.661157
chr14	68727200	0.876033
chr14	68750200	0.809917
chr14	68759000	0.867769
chr14	68762400	0.719008
chr14	68765300	0.702479
chr14	68807500	0.876033
chr14	68810500	0.652893
chr14	68832300	0.661157
chr14	68839400	0.702479
chr14	68861700	0.909091
chr14	68916600	1.000000
chr14	68936100	0.801653
chr14	68938600	0.958678
chr14	68943800	0.900826
chr14	68959800	0.925620
chr14	69015300	0.801653
chr14	69017500	0.661157
chr14	69021300	0.727273
chr14	69052000	0.809917
chr14	69055700	0.834711
chr14	69061400	0.851240
chr14	69074400	0.735537
chr14	69101400	0.652893
chr14	69105700	0.735537
chr14	69198200	0.785124
chr14	69230000	0.661157
chr14	69247500	0.719008
chr14	69264900	0.793388
chr14	69286900	0.909091
chr14	69291900	0.702479
chr14	69389300	0.842975
chr14	69392400	0.892562
chr14	69410000	0.727273
chr14	69419700	0.884298
chr14	69429500	0.719008
chr14	69431900	0.644628
chr14	69437800	1.000000
chr14	69443100	0.652893
chr14	69472600	0.809917
chr14	69481300	0.710744
chr14	69511500	0.851240

chr14	69517500	0.966942
chr14	69540900	0.652893
chr14	69565100	0.752066
chr14	69596500	0.867769
chr14	69650900	0.991736
chr14	69755200	0.677686
chr14	69790900	0.768595
chr14	69813800	0.876033
chr14	69817800	0.735537
chr14	69948900	0.702479
chr14	69986100	0.884298
chr14	70039100	0.702479
chr14	70099100	0.652893
chr14	70105300	0.842975
chr14	70117300	0.760331
chr14	70125100	0.942149
chr14	70141000	0.644628
chr14	70176200	0.842975
chr14	70178500	0.768595
chr14	70180800	0.793388
chr14	70219800	0.702479
chr14	70309900	0.685950
chr14	70327600	0.859504
chr14	70447700	0.669421
chr14	70454700	0.785124
chr14	70499700	0.685950
chr14	70679700	0.685950
chr14	70697200	0.942149
chr14	70706800	0.851240
chr14	70749200	0.793388
chr14	70759400	0.818182
chr14	70869700	0.851240
chr14	70938100	0.842975
chr14	71035000	0.710744
chr14	71113100	0.884298
chr14	71115500	0.793388
chr14	71120300	0.702479
chr14	71128400	0.892562
chr14	71133200	0.677686
chr14	71138400	0.719008
chr14	71152700	0.702479
chr14	71173700	0.859504
chr14	71177700	0.884298
chr14	71180400	0.818182
chr14	71377600	0.743802
chr14	71397600	0.727273
chr14	71606200	0.859504
chr14	71655000	0.694215
chr14	71660600	0.743802
chr14	71679600	0.661157
chr14	71792000	0.958678
chr14	71795000	0.677686
chr14	71822400	0.801653
chr14	71875700	0.727273

chr14	71898000	0.644628
chr14	71905300	0.710744
chr14	72053300	0.752066
chr14	72114300	0.991736
chr14	72126100	0.719008
chr14	72142300	0.867769
chr14	72148200	0.793388
chr14	72244000	0.876033
chr14	72250800	0.793388
chr14	72301800	0.685950
chr14	72358800	0.958678
chr14	72490900	0.743802
chr14	72718700	0.793388
chr14	72799900	0.892562
chr14	72802100	0.677686
chr14	73011500	0.685950
chr14	73067400	0.752066
chr14	73088800	0.809917
chr14	73168100	0.685950
chr14	73264100	0.818182
chr14	73271100	0.826446
chr14	73318300	0.677686
chr14	73344000	0.809917
chr14	73355200	0.661157
chr14	73357800	0.661157
chr14	73400400	0.727273
chr14	73447100	0.727273
chr14	73528800	0.710744
chr14	73592300	0.950413
chr14	73637500	0.652893
chr14	73665600	0.842975
chr14	73690000	0.842975
chr14	73695500	0.793388
chr14	73699600	0.694215
chr14	73701800	0.661157
chr14	73708700	0.851240
chr14	73751100	0.867769
chr14	73754300	0.917355
chr14	73757100	0.793388
chr14	73806900	0.652893
chr14	73926500	0.685950
chr14	73974000	0.859504
chr14	74006400	0.652893
chr14	74042100	0.661157
chr14	74188100	0.785124
chr14	74195500	0.661157
chr14	74208100	0.710744
chr14	74223400	0.644628
chr14	74238700	0.950413
chr14	74250800	0.661157
chr14	74300700	0.694215
chr14	74710700	0.851240
chr14	74713900	0.727273
chr14	74761900	0.917355

chr14	74795100	0.685950
chr14	74819900	0.818182
chr14	74824100	0.818182
chr14	74826300	0.702479
chr14	74829000	0.925620
chr14	74867400	0.727273
chr14	74869500	0.834711
chr14	74881500	0.909091
chr14	74896200	0.983471
chr14	74926300	0.793388
chr14	74967900	0.909091
chr14	74975300	0.859504
chr14	74983600	0.719008
chr14	74991800	0.677686
chr14	75018600	0.818182
chr14	75037100	0.727273
chr14	75084200	0.669421
chr14	75098600	0.644628
chr14	75115100	0.669421
chr14	75343000	0.719008
chr14	75353000	0.669421
chr14	75355400	0.760331
chr14	75370000	0.677686
chr14	75420700	0.834711
chr14	75441500	0.719008
chr14	75447700	0.743802
chr14	75559900	0.719008
chr14	75614900	0.768595
chr14	75690600	0.752066
chr14	75716700	0.652893
chr14	75736400	0.661157
chr14	75763500	0.644628
chr14	75777600	0.925620
chr14	75877600	0.884298
chr14	75905300	0.752066
chr14	75910000	0.752066
chr14	75920200	0.925620
chr14	75924000	1.000000
chr14	75946800	0.933884
chr14	76006500	0.826446
chr14	76027100	0.801653
chr14	76035300	0.727273
chr14	76099400	0.719008
chr14	76178200	0.785124
chr14	76278400	0.966942
chr14	76327700	0.644628
chr14	76384000	0.917355
chr14	76424200	0.702479
chr14	76518200	0.950413
chr14	76550600	0.793388
chr14	76556100	0.735537
chr14	76571400	0.644628
chr14	76580200	0.909091
chr14	76582800	0.669421

chr14	76603400	0.669421
chr14	76621200	0.694215
chr14	76734400	0.694215
chr14	76779800	0.727273
chr14	76797200	0.710744
chr14	76801200	0.743802
chr14	76819400	0.826446
chr14	76853800	0.652893
chr14	76874000	0.685950
chr14	76879500	0.694215
chr14	76932200	0.909091
chr14	77079300	0.743802
chr14	77110500	0.727273
chr14	77234800	0.876033
chr14	77239300	0.768595
chr14	77295900	0.652893
chr14	77330000	0.776860
chr14	77380400	0.669421
chr14	77384400	0.694215
chr14	77387000	0.818182
chr14	77391300	0.975207
chr14	77396200	0.826446
chr14	77399600	0.661157
chr14	77414100	0.983471
chr14	77428600	0.975207
chr14	77431700	0.809917
chr14	77443600	0.661157
chr14	77451500	0.727273
chr14	77461700	0.859504
chr14	77470700	0.867769
chr14	77474400	0.735537
chr14	77489500	0.867769
chr14	77504200	0.925620
chr14	77511900	0.851240
chr14	77519000	0.694215
chr14	77526000	0.727273
chr14	77542900	0.958678
chr14	77552500	0.933884
chr14	77555100	0.991736
chr14	77571800	0.909091
chr14	77576300	0.933884
chr14	77588000	0.851240
chr14	77611000	0.826446
chr14	77613100	0.694215
chr14	77618400	0.661157
chr14	77621000	0.702479
chr14	77625100	0.826446
chr14	77672700	0.719008
chr14	77675300	0.661157
chr14	77689300	0.900826
chr14	77691900	0.694215
chr14	77774000	0.752066
chr14	77781800	0.842975
chr14	77791100	0.694215

chr14	77805000	0.991736
chr14	77810300	0.727273
chr14	77891500	0.776860
chr14	77939200	0.785124
chr14	78121000	0.859504
chr14	78230300	0.900826
chr14	78379700	0.900826
chr14	78445900	0.677686
chr14	78496400	0.917355
chr14	78528700	0.677686
chr14	78548100	0.859504
chr14	78553100	0.768595
chr14	78758600	0.826446
chr14	78781000	0.677686
chr14	78834300	0.661157
chr14	78870500	0.867769
chr14	78952800	0.652893
chr14	79032800	0.809917
chr14	79105700	0.818182
chr14	79111700	0.809917
chr14	79192000	0.677686
chr14	79204200	0.685950
chr14	79316900	0.884298
chr14	79341700	0.727273
chr14	79359500	0.867769
chr14	79454200	0.719008
chr14	79561400	0.735537
chr14	79782500	0.760331
chr14	79799700	0.834711
chr14	79809100	0.652893
chr14	79821700	0.669421
chr14	79845400	0.991736
chr14	79933900	0.694215
chr14	79950500	0.677686
chr14	80010700	0.785124
chr14	80287600	0.669421
chr14	81735300	0.685950
chr14	81778300	0.818182
chr14	81783100	0.942149
chr14	81791100	0.685950
chr14	81832600	0.669421
chr14	81852500	0.652893
chr14	81916500	0.752066
chr14	82148800	0.752066
chr14	82437000	0.719008
chr14	82547100	0.768595
chr14	82898600	0.719008
chr14	83417900	0.801653
chr14	83696300	0.785124
chr14	84131600	0.719008
chr14	84173600	0.702479
chr14	84231900	0.652893
chr14	84360600	0.760331
chr14	84427500	0.942149

chr14	84526000	0.644628
chr14	84721400	0.735537
chr14	84870400	0.925620
chr14	85217400	0.644628
chr14	85645600	0.793388
chr14	85668700	0.710744
chr14	85689600	0.892562
chr14	86003200	0.710744
chr14	86013100	0.958678
chr14	86042000	0.826446
chr14	86704100	0.694215
chr14	87521700	0.644628
chr14	87599000	0.644628
chr14	87616200	0.661157
chr14	87620400	0.661157
chr14	87653700	0.719008
chr14	87995700	0.652893
chr14	88212200	0.685950
chr14	88232400	0.685950
chr14	88237700	0.677686
chr14	88265900	0.760331
chr14	88503500	0.685950
chr14	88828200	0.768595
chr14	88831800	0.884298
chr14	88949100	0.842975
chr14	88959100	0.735537
chr14	89084900	0.743802
chr14	89507400	0.793388
chr14	89588900	0.867769
chr14	89606400	0.760331
chr14	89653400	0.735537
chr14	89672200	0.735537
chr14	89710500	0.851240
chr14	89733000	0.702479
chr14	89736100	0.661157
chr14	89750600	0.760331
chr14	89753100	0.694215
chr14	89757200	0.652893
chr14	89760300	0.925620
chr14	89762900	0.958678
chr14	89772400	0.892562
chr14	89774600	0.900826
chr14	89794600	0.719008
chr14	89803500	0.677686
chr14	89806400	0.950413
chr14	89808800	0.652893
chr14	89815600	0.892562
chr14	89831900	0.743802
chr14	89838400	0.809917
chr14	89852600	0.661157
chr14	89855600	0.892562
chr14	89864800	0.719008
chr14	89867600	0.818182
chr14	89870200	0.644628

chr14	89874000	0.776860
chr14	89876400	0.942149
chr14	89890200	0.925620
chr14	89895400	0.909091
chr14	89898800	0.702479
chr14	89904600	0.702479
chr14	89908400	0.975207
chr14	89916400	0.735537
chr14	89926500	0.884298
chr14	89933400	0.652893
chr14	89960800	0.876033
chr14	89976700	0.685950
chr14	90010000	0.925620
chr14	90016700	0.900826
chr14	90030200	0.669421
chr14	90042000	0.826446
chr14	90052700	0.644628
chr14	90057300	0.760331
chr14	90082300	0.694215
chr14	90097000	0.752066
chr14	90118900	0.768595
chr14	90187700	0.983471
chr14	90240300	0.859504
chr14	90301200	0.743802
chr14	90378200	0.983471
chr14	90424700	0.652893
chr14	90757500	0.818182
chr14	90769300	0.991736
chr14	90855200	0.834711
chr14	90872300	0.760331
chr14	90891300	0.942149
chr14	90906600	0.851240
chr14	90910400	0.818182
chr14	90920800	0.892562
chr14	90958300	0.818182
chr14	90967200	0.719008
chr14	90980200	0.917355
chr14	90984900	0.727273
chr14	91013000	0.809917
chr14	91022900	0.785124
chr14	91034300	0.958678
chr14	91041000	0.925620
chr14	91066600	0.793388
chr14	91071200	0.900826
chr14	91075500	0.719008
chr14	91081700	0.760331
chr14	91091600	0.760331
chr14	91108800	0.958678
chr14	91112500	0.826446
chr14	91115800	0.785124
chr14	91128200	0.644628
chr14	91224800	0.859504
chr14	91294700	0.793388
chr14	91583400	0.884298

chr14	91695200	0.719008
chr14	91770000	0.950413
chr14	91773400	0.809917
chr14	91790700	0.677686
chr14	91815900	0.834711
chr14	91819300	0.950413
chr14	91821700	0.735537
chr14	91831000	0.818182
chr14	91834200	0.719008
chr14	91837200	0.677686
chr14	91841200	0.760331
chr14	91848600	0.933884
chr14	91850800	0.842975
chr14	91854600	0.867769
chr14	91859200	0.801653
chr14	91861600	0.652893
chr14	91863700	0.743802
chr14	91866700	0.900826
chr14	91880200	0.991736
chr14	92048200	0.719008
chr14	92220300	0.685950
chr14	92575300	0.867769
chr14	92666100	0.752066
chr14	92673700	0.702479
chr14	92852800	0.925620
chr14	92856500	0.669421
chr14	92864000	0.950413
chr14	92867900	0.892562
chr14	92937900	0.768595
chr14	92954200	0.719008
chr14	93040500	0.702479
chr14	93096100	0.785124
chr14	93327400	0.776860
chr14	93344500	0.867769
chr14	93347700	0.801653
chr14	93436800	0.644628
chr14	93439700	0.719008
chr14	93484500	0.735537
chr14	93486600	0.785124
chr14	93490900	0.743802
chr14	93500000	0.884298
chr14	93507700	0.826446
chr14	93517100	0.884298
chr14	93519600	0.950413
chr14	93523400	0.983471
chr14	93527400	0.983471
chr14	93553300	0.925620
chr14	93561200	0.719008
chr14	93567100	0.652893
chr14	93577000	0.677686
chr14	93768800	0.818182
chr14	93785400	0.677686
chr14	93810600	0.933884
chr14	93869700	0.685950

chr14	93992500	0.661157
chr14	94155800	0.661157
chr14	94160600	0.867769
chr14	94179700	0.644628
chr14	94212000	0.677686
chr14	94346500	0.884298
chr14	94361000	0.842975
chr14	94372100	0.644628
chr14	94408500	0.752066
chr14	94420300	0.851240
chr14	94422800	0.776860
chr14	94454000	0.710744
chr14	94468500	0.768595
chr14	94489000	0.793388
chr14	94529300	0.685950
chr14	94611700	0.900826
chr14	94631800	0.851240
chr14	94644300	0.909091
chr14	94660100	0.669421
chr14	94841900	0.958678
chr14	94857200	0.760331
chr14	94864700	0.958678
chr14	94941500	0.652893
chr14	94953100	0.925620
chr14	94984200	0.801653
chr14	95156500	0.768595
chr14	95242200	0.842975
chr14	95247400	0.900826
chr14	95300400	0.752066
chr14	95334900	0.710744
chr14	95428900	0.710744
chr14	95465500	0.785124
chr14	95545700	0.752066
chr14	95604700	0.727273
chr14	95609100	0.727273
chr14	95613100	0.834711
chr14	95664200	0.818182
chr14	95666600	0.768595
chr14	95685100	0.735537
chr14	95688200	0.727273
chr14	95691900	0.727273
chr14	95722900	0.876033
chr14	95726300	0.685950
chr14	95827100	0.834711
chr14	95837300	0.719008
chr14	95841000	0.752066
chr14	95927900	0.900826
chr14	95947900	0.727273
chr14	95952000	0.743802
chr14	95981300	0.851240
chr14	96023900	0.719008
chr14	96028500	0.710744
chr14	96064500	0.785124
chr14	96086600	0.768595

chr14	96133800	0.685950
chr14	96147000	0.909091
chr14	96153100	0.677686
chr14	96175600	0.776860
chr14	96219900	0.793388
chr14	96228200	0.702479
chr14	96285200	0.677686
chr14	96298600	0.752066
chr14	96317200	0.661157
chr14	96351900	0.851240
chr14	96363300	0.876033
chr14	96377500	0.809917
chr14	96382000	0.760331
chr14	96414000	0.909091
chr14	96423300	0.644628
chr14	96425500	0.669421
chr14	96445400	0.710744
chr14	96448400	0.834711
chr14	96460600	0.950413
chr14	96972000	0.834711
chr14	97040200	0.793388
chr14	97127300	0.909091
chr14	97160700	0.826446
chr14	97205800	0.752066
chr14	97329300	0.743802
chr14	97489800	0.694215
chr14	97492300	0.925620
chr14	97494600	0.710744
chr14	97502700	0.834711
chr14	97745000	0.694215
chr14	97747500	0.760331
chr14	97833000	0.876033
chr14	97845500	0.859504
chr14	97854500	0.694215
chr14	97874500	0.768595
chr14	97882800	0.917355
chr14	98039300	0.694215
chr14	98097800	0.909091
chr14	98101200	0.669421
chr14	98106700	0.851240
chr14	98121700	0.685950
chr14	98152800	0.644628
chr14	98224300	0.859504
chr14	98358500	0.685950
chr14	98489800	0.909091
chr14	98536700	0.917355
chr14	98576000	0.661157
chr14	98603000	0.859504
chr14	98639900	0.669421
chr14	98670900	0.710744
chr14	99118100	0.760331
chr14	99286300	1.000000
chr14	99320500	0.644628
chr14	99373900	0.719008

chr14	99560700	0.661157
chr14	99568200	0.793388
chr14	99599900	0.867769
chr14	99655400	0.801653
chr14	99658200	0.851240
chr14	99710200	0.685950
chr14	99715100	0.685950
chr14	99719900	0.702479
chr14	99724900	0.859504
chr14	99729400	0.719008
chr14	99733100	0.892562
chr14	99751200	0.669421
chr14	99766900	0.785124
chr14	99781000	0.644628
chr14	99830400	0.702479
chr14	99850900	0.702479
chr14	99861100	0.809917
chr14	99879200	0.942149
chr14	99888900	0.735537
chr14	99891100	0.892562
chr14	99929500	0.917355
chr14	99969500	0.975207
chr14	99976300	0.702479
chr14	99989600	0.958678
chr14	99992500	0.752066
chr14	99997400	0.876033
chr14	100004700	0.661157
chr14	100012800	0.818182
chr14	100016400	0.752066
chr14	100027600	0.818182
chr14	100031100	0.685950
chr14	100035000	0.710744
chr14	100038200	0.702479
chr14	100041200	0.793388
chr14	100045900	0.752066
chr14	100048200	0.892562
chr14	100059300	0.884298
chr14	100127000	0.760331
chr14	100130700	0.983471
chr14	100133900	0.644628
chr14	100138300	0.925620
chr14	100225700	0.735537
chr14	100234800	0.644628
chr14	100241500	0.859504
chr14	100289100	0.876033
chr14	100305700	0.876033
chr14	100309000	0.735537
chr14	100338600	0.834711
chr14	100354200	0.669421
chr14	100356400	0.925620
chr14	100359800	0.685950
chr14	100416900	0.876033
chr14	100435800	0.727273
chr14	100545800	0.826446

chr14	100597700	0.950413
chr14	100607200	0.942149
chr14	100621700	0.669421
chr14	100632600	0.669421
chr14	100659600	0.851240
chr14	100666100	0.710744
chr14	100713100	0.851240
chr14	100750400	0.752066
chr14	100757200	0.752066
chr14	100778300	0.776860
chr14	100838600	0.909091
chr14	100865100	0.809917
chr14	100876600	0.942149
chr14	100910200	0.768595
chr14	100913700	0.900826
chr14	100920000	0.793388
chr14	100932800	0.685950
chr14	100989400	0.644628
chr14	101013000	0.867769
chr14	101022000	0.818182
chr14	101111900	0.685950
chr14	101124700	0.785124
chr14	101140000	0.867769
chr14	101177900	0.776860
chr14	101190100	0.669421
chr14	101197500	0.950413
chr14	101271500	0.694215
chr14	101298200	0.933884
chr14	101305500	0.834711
chr14	101308000	0.983471
chr14	101365600	0.677686
chr14	101369000	0.851240
chr14	101539800	0.743802
chr14	101550500	0.652893
chr14	101617500	0.834711
chr14	101660700	0.768595
chr14	101689900	0.933884
chr14	101692700	0.719008
chr14	101755500	0.702479
chr14	101943700	0.851240
chr14	101963400	0.818182
chr14	102095400	0.776860
chr14	102174400	0.752066
chr14	102183700	0.793388
chr14	102186600	0.752066
chr14	102198100	0.892562
chr14	102226500	0.644628
chr14	102274700	0.785124
chr14	102312300	0.793388
chr14	102383500	0.760331
chr14	102427600	0.644628
chr14	102563800	0.925620
chr14	102621800	0.776860
chr14	102688000	0.842975

chr14	102714700	0.892562
chr14	102729700	0.685950
chr14	102860900	0.702479
chr14	102863400	0.694215
chr14	102878600	0.892562
chr14	102881700	0.727273
chr14	103051700	0.809917
chr14	103088800	0.859504
chr14	103097000	0.958678
chr14	103129800	1.000000
chr14	103160400	0.652893
chr14	103216000	0.892562
chr14	103227100	0.909091
chr14	103272500	0.710744
chr14	103286200	0.892562
chr14	103295500	0.694215
chr14	103300300	0.719008
chr14	103304800	0.727273
chr14	103392200	0.867769
chr14	103397600	0.719008
chr14	103488800	0.719008
chr14	103532900	0.719008
chr14	103550800	0.752066
chr14	103563900	0.677686
chr14	103566100	0.760331
chr14	103568900	0.694215
chr14	103571300	0.834711
chr14	103575200	0.876033
chr14	103587800	0.644628
chr14	103593600	0.785124
chr14	103623600	0.710744
chr14	103653500	0.925620
chr14	103710200	0.925620
chr14	103727200	0.776860
chr14	103748100	0.669421
chr14	103751100	0.925620
chr14	103783700	0.652893
chr14	103796500	0.661157
chr14	103813900	0.719008
chr14	103821700	0.917355
chr14	103860600	0.677686
chr14	103911900	0.710744
chr14	103985300	0.727273
chr14	104010500	0.719008
chr14	104013400	0.685950
chr14	104178200	0.884298
chr14	104202400	0.661157
chr14	104206900	0.644628
chr14	104229400	0.669421
chr14	104286600	0.818182
chr14	104322000	0.909091
chr14	104334400	0.719008
chr14	104346800	0.876033
chr14	104352700	0.735537

chr14	104544600	0.644628
chr14	104614200	0.710744
chr14	104617600	0.809917
chr14	104635000	0.644628
chr14	104637700	0.801653
chr14	104690400	0.809917
chr14	104706900	0.826446
chr14	104757900	0.884298
chr14	104809600	0.677686
chr14	104852200	0.661157
chr14	104870500	0.760331
chr14	104909900	0.743802
chr14	105030500	0.702479
chr14	105106600	0.661157
chr14	105132600	0.785124
chr14	105146200	0.652893
chr14	105148500	0.925620
chr14	105154400	0.669421
chr14	105161300	0.834711
chr14	105164400	0.677686
chr14	105167300	0.652893
chr14	105174400	0.685950
chr14	105235500	0.710744
chr14	105264200	0.652893
chr14	105318700	0.727273
chr14	105339500	0.702479
chr14	105342600	0.694215
chr14	105435700	0.776860
chr14	105442800	0.677686
chr14	105482400	0.909091
chr14	105525500	0.669421
chr14	105531600	0.661157
chr14	105542000	0.909091
chr14	105557300	0.760331
chr14	105622500	0.735537
chr14	105668600	0.727273
chr14	105773900	0.851240
chr14	105794000	0.760331
chr14	105796600	0.776860
chr14	105799100	0.685950
chr14	105804600	0.661157
chr14	105818900	0.735537
chr14	105823500	0.834711
chr14	105878100	0.826446
chr14	105889000	0.694215
chr14	105931900	0.719008
chr14	105945700	0.900826
chr14	105948300	0.776860
chr14	105952500	0.702479
chr15	20478200	0.801653
chr15	20639000	0.785124
chr15	20684100	0.834711
chr15	22904200	0.652893
chr15	22920000	0.694215

chr15	22999900	0.991736
chr15	23028000	0.694215
chr15	23202000	0.677686
chr15	23306400	0.644628
chr15	23936200	0.677686
chr15	24123400	0.818182
chr15	25017600	0.661157
chr15	25030000	0.876033
chr15	25069200	0.983471
chr15	25103500	0.867769
chr15	25108600	0.785124
chr15	25145200	0.900826
chr15	25165700	0.752066
chr15	25220000	0.793388
chr15	25588600	0.876033
chr15	25651500	0.785124
chr15	25726600	0.991736
chr15	25747500	0.793388
chr15	25750100	0.876033
chr15	25777500	0.818182
chr15	25801500	0.809917
chr15	25888200	0.884298
chr15	25901500	0.702479
chr15	25907000	0.884298
chr15	25915300	0.925620
chr15	25934900	0.768595
chr15	25942100	0.685950
chr15	26048100	0.669421
chr15	26056300	0.859504
chr15	26097900	0.644628
chr15	26179000	0.727273
chr15	26348200	0.710744
chr15	26379300	0.851240
chr15	26416900	0.917355
chr15	26447000	0.834711
chr15	26480900	1.000000
chr15	26483100	0.694215
chr15	26488300	0.950413
chr15	26540500	0.859504
chr15	26570400	0.785124
chr15	26610800	0.710744
chr15	26695000	0.719008
chr15	26861000	0.694215
chr15	26865900	0.685950
chr15	26868100	0.743802
chr15	26874400	0.727273
chr15	26876900	0.884298
chr15	26879300	0.768595
chr15	26894700	0.793388
chr15	26897400	0.892562
chr15	26904800	0.834711
chr15	26911700	0.685950
chr15	26928400	0.884298
chr15	26934500	0.842975

chr15	26937700	0.909091
chr15	26956900	0.702479
chr15	26961800	0.760331
chr15	26964600	0.793388
chr15	26968600	0.727273
chr15	26974800	0.735537
chr15	26977200	0.652893
chr15	26984400	0.752066
chr15	27003200	0.776860
chr15	27005900	0.826446
chr15	27010400	0.743802
chr15	27014800	0.818182
chr15	27087800	0.900826
chr15	27137300	0.768595
chr15	27316700	0.727273
chr15	27417800	0.785124
chr15	27420800	0.884298
chr15	27503600	0.867769
chr15	27588200	0.727273
chr15	27644500	0.677686
chr15	27657800	0.685950
chr15	27660100	0.876033
chr15	27728000	0.785124
chr15	27884600	0.727273
chr15	27891200	0.644628
chr15	27924800	0.826446
chr15	28009600	0.768595
chr15	28013800	0.685950
chr15	28145500	0.900826
chr15	28267700	0.685950
chr15	28539600	0.909091
chr15	28837500	0.752066
chr15	28861800	0.727273
chr15	28990100	0.842975
chr15	29099200	0.958678
chr15	29158400	0.661157
chr15	29211200	0.785124
chr15	29277400	0.669421
chr15	29349300	0.694215
chr15	29406700	0.644628
chr15	29489000	0.851240
chr15	29514300	0.652893
chr15	29519800	0.661157
chr15	29533000	0.694215
chr15	29552300	0.661157
chr15	29612300	0.933884
chr15	29638300	0.983471
chr15	29693500	0.644628
chr15	29696500	0.884298
chr15	29702300	0.760331
chr15	29712500	0.694215
chr15	29726400	0.694215
chr15	29763200	0.661157
chr15	29773300	0.669421

chr15	29826000	0.793388
chr15	29903700	0.876033
chr15	29924000	0.661157
chr15	29949000	0.710744
chr15	29963900	0.702479
chr15	30060800	0.685950
chr15	30163300	0.735537
chr15	30181800	0.892562
chr15	30196700	0.933884
chr15	30213400	0.909091
chr15	30217300	0.776860
chr15	30254600	0.884298
chr15	30278000	0.735537
chr15	30338100	0.785124
chr15	30442400	0.727273
chr15	30478200	0.826446
chr15	30513200	0.793388
chr15	30587800	0.719008
chr15	30668500	0.702479
chr15	30691900	0.661157
chr15	30770300	0.842975
chr15	30829800	0.743802
chr15	30989100	0.694215
chr15	31116000	0.760331
chr15	31187000	0.710744
chr15	31213800	0.842975
chr15	31216200	0.776860
chr15	31317000	0.942149
chr15	31370500	0.702479
chr15	31373800	0.826446
chr15	31440800	0.743802
chr15	31444100	0.925620
chr15	31493500	0.652893
chr15	31515200	0.859504
chr15	31589400	0.834711
chr15	31596100	0.685950
chr15	31599200	0.975207
chr15	31602000	0.809917
chr15	31612300	0.818182
chr15	31623900	0.743802
chr15	31631600	0.809917
chr15	31635100	0.644628
chr15	31638800	0.826446
chr15	31645700	0.702479
chr15	31653700	0.743802
chr15	31659200	0.752066
chr15	31665100	0.685950
chr15	31675400	0.669421
chr15	31690300	0.776860
chr15	31728000	0.661157
chr15	31883500	0.685950
chr15	31891000	0.768595
chr15	32197400	0.818182
chr15	32207000	0.727273

chr15	32267600	0.793388
chr15	32384600	0.867769
chr15	32390100	0.669421
chr15	32392600	0.677686
chr15	32400300	0.743802
chr15	32536000	0.644628
chr15	32610400	0.876033
chr15	32645500	0.702479
chr15	32680700	0.768595
chr15	32762600	0.661157
chr15	32822100	0.768595
chr15	32935700	0.727273
chr15	32962100	0.950413
chr15	33023600	0.884298
chr15	33211100	0.669421
chr15	33245600	0.851240
chr15	33315000	0.859504
chr15	33360900	0.950413
chr15	33376200	0.867769
chr15	33403800	0.710744
chr15	33534700	0.793388
chr15	33851700	0.785124
chr15	33854100	0.677686
chr15	33873300	0.958678
chr15	33885400	0.702479
chr15	33905700	0.735537
chr15	34059400	0.685950
chr15	34127500	0.859504
chr15	34136700	0.925620
chr15	34201400	0.669421
chr15	34208200	0.727273
chr15	34453500	0.834711
chr15	34458500	0.702479
chr15	34506500	0.942149
chr15	34533400	0.661157
chr15	34638700	0.876033
chr15	34646300	0.685950
chr15	34950300	0.719008
chr15	35018900	0.975207
chr15	35045300	0.644628
chr15	35061500	0.677686
chr15	35287600	0.925620
chr15	35312100	0.702479
chr15	35354700	1.000000
chr15	35357600	0.743802
chr15	35377400	0.644628
chr15	35474000	0.710744
chr15	35479200	0.652893
chr15	35482300	0.677686
chr15	35539100	0.826446
chr15	35546600	0.942149
chr15	35729700	0.702479
chr15	35840600	0.950413
chr15	35842700	0.917355

chr15	35961000	0.966942
chr15	35990400	0.809917
chr15	36131500	0.710744
chr15	36162700	0.752066
chr15	36291700	0.702479
chr15	36332600	0.809917
chr15	36383100	0.917355
chr15	36509000	0.685950
chr15	36524500	0.710744
chr15	36561500	0.652893
chr15	36699600	0.876033
chr15	36771900	0.694215
chr15	36820000	0.719008
chr15	36838200	0.867769
chr15	36936400	0.719008
chr15	37047800	0.958678
chr15	37112300	0.685950
chr15	37178400	0.809917
chr15	37197200	0.892562
chr15	37307800	0.702479
chr15	37325500	0.644628
chr15	37339700	0.702479
chr15	37396900	0.826446
chr15	37405700	0.719008
chr15	37438900	0.694215
chr15	37441900	0.743802
chr15	38033300	0.768595
chr15	38351200	1.000000
chr15	38369300	0.842975
chr15	38407500	0.694215
chr15	38456500	0.685950
chr15	38464800	0.760331
chr15	38482100	0.694215
chr15	38552700	0.851240
chr15	38809900	0.876033
chr15	38844700	0.909091
chr15	38998200	0.719008
chr15	39309200	0.652893
chr15	39388300	0.900826
chr15	39462100	0.892562
chr15	39464600	0.975207
chr15	39505900	0.975207
chr15	39637500	0.933884
chr15	39727700	0.867769
chr15	39823700	0.809917
chr15	39875500	0.661157
chr15	39985300	0.867769
chr15	39996900	0.851240
chr15	40070100	0.652893
chr15	40077800	0.719008
chr15	40080600	0.933884
chr15	40101500	0.884298
chr15	40113200	0.785124
chr15	40116100	0.661157

chr15	40123700	0.768595
chr15	40129400	0.768595
chr15	40177100	0.851240
chr15	40179500	0.966942
chr15	40181700	0.991736
chr15	40184100	0.834711
chr15	40191600	0.867769
chr15	40195000	0.933884
chr15	40199000	0.859504
chr15	40201900	0.644628
chr15	40215100	0.677686
chr15	40217400	0.702479
chr15	40337700	0.826446
chr15	40343500	0.851240
chr15	40347500	0.727273
chr15	40361700	0.719008
chr15	40374400	0.917355
chr15	40381400	0.793388
chr15	40384500	0.925620
chr15	40388500	0.719008
chr15	40390800	0.809917
chr15	40397900	0.719008
chr15	40404500	0.752066
chr15	40417900	0.644628
chr15	40550800	0.876033
chr15	40566900	0.669421
chr15	40577900	0.900826
chr15	40589100	0.768595
chr15	40594400	0.851240
chr15	40599400	0.818182
chr15	40601900	0.834711
chr15	40619500	0.735537
chr15	40626900	0.702479
chr15	40630000	0.669421
chr15	40633200	0.851240
chr15	40646800	0.900826
chr15	40714000	0.702479
chr15	40722100	0.661157
chr15	40736500	0.942149
chr15	40738800	0.809917
chr15	40745000	0.809917
chr15	40747500	0.809917
chr15	40749700	0.884298
chr15	40762500	0.661157
chr15	40799700	0.661157
chr15	40846800	0.925620
chr15	41031600	0.991736
chr15	41037100	0.785124
chr15	41059200	0.752066
chr15	41075300	0.793388
chr15	41082100	0.826446
chr15	41091500	0.834711
chr15	41139700	0.909091
chr15	41204000	0.966942

chr15	41207200	0.702479
chr15	41230200	0.694215
chr15	41241600	0.710744
chr15	41248100	0.900826
chr15	41253000	0.652893
chr15	41511800	0.801653
chr15	41556600	0.925620
chr15	41595800	0.933884
chr15	41854300	0.785124
chr15	41857600	0.842975
chr15	41861700	0.892562
chr15	41870900	0.793388
chr15	41896900	0.785124
chr15	41922300	0.859504
chr15	42225100	0.694215
chr15	42227200	0.743802
chr15	42234700	0.719008
chr15	42248300	0.685950
chr15	42327800	0.661157
chr15	42349600	0.685950
chr15	42372800	0.933884
chr15	42377500	0.669421
chr15	42385100	0.719008
chr15	42411400	0.727273
chr15	42656200	0.867769
chr15	42677500	0.685950
chr15	42750100	0.818182
chr15	42876500	0.958678
chr15	42912300	0.760331
chr15	43021700	0.735537
chr15	43051700	0.925620
chr15	43183400	0.876033
chr15	43511000	0.793388
chr15	43806500	0.710744
chr15	43808600	0.743802
chr15	43823500	0.677686
chr15	44037700	0.685950
chr15	44087400	0.917355
chr15	44089500	0.867769
chr15	44173100	0.793388
chr15	44196100	0.669421
chr15	44218100	0.785124
chr15	44220400	0.975207
chr15	44223500	0.669421
chr15	44268300	0.652893
chr15	44331400	0.884298
chr15	44399600	0.710744
chr15	44589300	0.933884
chr15	44733900	0.793388
chr15	44975700	0.801653
chr15	45019000	0.793388
chr15	45250200	0.652893
chr15	45393500	0.818182
chr15	45406800	0.892562

chr15	45455400	0.685950
chr15	45462000	0.652893
chr15	45472500	0.644628
chr15	45476700	0.892562
chr15	45618900	0.727273
chr15	45664600	0.719008
chr15	45866700	0.702479
chr15	46007400	0.661157
chr15	46035900	0.719008
chr15	46234400	0.652893
chr15	46240600	0.694215
chr15	46636600	0.685950
chr15	47481500	0.702479
chr15	47488700	0.644628
chr15	47618600	0.760331
chr15	47686700	0.809917
chr15	47733300	0.644628
chr15	48008200	0.702479
chr15	48013500	0.760331
chr15	48028900	0.826446
chr15	48035100	0.884298
chr15	48070600	0.933884
chr15	48110600	0.710744
chr15	48153800	0.859504
chr15	48178100	0.719008
chr15	48339800	0.950413
chr15	48442400	0.826446
chr15	48559900	0.818182
chr15	48578200	0.685950
chr15	48590700	0.966942
chr15	48592800	0.727273
chr15	48596600	0.909091
chr15	48607600	0.719008
chr15	48714100	0.661157
chr15	48733100	0.958678
chr15	49090700	0.809917
chr15	49148400	0.909091
chr15	49158200	0.685950
chr15	49189700	0.900826
chr15	49356100	0.760331
chr15	49403000	0.752066
chr15	49480800	0.702479
chr15	49563700	0.694215
chr15	49608300	0.727273
chr15	49621300	0.975207
chr15	49676700	0.661157
chr15	49936900	0.785124
chr15	50188100	0.975207
chr15	50296400	0.694215
chr15	50352400	0.661157
chr15	50623600	0.818182
chr15	50759200	0.851240
chr15	51330300	0.661157
chr15	51369900	0.917355

chr15	51376400	0.743802
chr15	51533300	0.876033
chr15	51683500	0.801653
chr15	51708700	0.818182
chr15	52019100	0.752066
chr15	52147000	0.768595
chr15	52149400	0.842975
chr15	52157200	0.876033
chr15	52332900	0.669421
chr15	52550000	0.909091
chr15	52553400	0.876033
chr15	52555500	0.942149
chr15	52581700	0.834711
chr15	52630300	0.876033
chr15	52632700	0.685950
chr15	52685000	0.917355
chr15	52740700	0.669421
chr15	52759000	0.892562
chr15	52778500	0.760331
chr15	52815800	0.760331
chr15	52944100	0.644628
chr15	53038000	0.909091
chr15	53043100	0.851240
chr15	53070900	0.669421
chr15	53073100	0.801653
chr15	53085600	0.834711
chr15	53093400	0.834711
chr15	53150900	0.834711
chr15	53373000	0.793388
chr15	53403500	0.884298
chr15	53740800	0.851240
chr15	53918500	0.900826
chr15	54618500	0.669421
chr15	55636400	0.851240
chr15	55640900	0.760331
chr15	55645700	0.644628
chr15	55945000	0.661157
chr15	55953900	0.826446
chr15	55968700	0.842975
chr15	55987800	0.826446
chr15	56078800	0.892562
chr15	56500900	0.685950
chr15	56508600	0.768595
chr15	56519100	0.702479
chr15	56538500	0.834711
chr15	56643900	0.933884
chr15	57011400	0.842975
chr15	57023200	0.669421
chr15	57029400	0.710744
chr15	57031800	0.809917
chr15	57277400	0.768595
chr15	57309100	0.644628
chr15	57332400	0.685950
chr15	57350000	0.859504

chr15	57363200	0.669421
chr15	57404300	0.677686
chr15	57411300	0.776860
chr15	57426900	0.669421
chr15	57492300	0.958678
chr15	57511600	0.644628
chr15	57528200	0.793388
chr15	57545100	0.925620
chr15	57574000	0.743802
chr15	57579100	0.652893
chr15	57595400	0.768595
chr15	57638300	0.644628
chr15	57645300	0.892562
chr15	57649000	0.900826
chr15	57664400	0.752066
chr15	57671500	0.694215
chr15	57675000	0.818182
chr15	57677400	0.669421
chr15	57695800	0.966942
chr15	57698300	0.685950
chr15	57704500	0.694215
chr15	57720300	0.925620
chr15	57724200	0.909091
chr15	57737800	0.826446
chr15	57770600	0.950413
chr15	57798200	0.685950
chr15	57844000	0.694215
chr15	57846800	0.719008
chr15	57908000	0.818182
chr15	57919400	0.661157
chr15	58039700	0.669421
chr15	58043700	0.809917
chr15	58303400	0.652893
chr15	58342400	0.884298
chr15	58431400	0.768595
chr15	58522600	0.702479
chr15	58553000	0.801653
chr15	58562400	0.669421
chr15	58590700	0.892562
chr15	58599900	0.743802
chr15	58622600	0.677686
chr15	58624900	0.785124
chr15	58650700	0.826446
chr15	58736800	0.735537
chr15	58813900	0.661157
chr15	58848000	0.793388
chr15	58891300	0.785124
chr15	58960500	0.834711
chr15	59126300	0.900826
chr15	59278000	0.727273
chr15	59440300	0.727273
chr15	59530100	0.826446
chr15	59585300	0.694215
chr15	59588200	0.818182

chr15	59606800	0.834711
chr15	59627600	0.727273
chr15	59640700	0.958678
chr15	59644000	0.661157
chr15	59646100	0.710744
chr15	59693500	0.760331
chr15	59695800	0.644628
chr15	59713500	0.694215
chr15	59785400	0.710744
chr15	60104800	0.677686
chr15	60154900	0.661157
chr15	60197300	0.884298
chr15	60226000	0.685950
chr15	60229400	0.917355
chr15	60234100	0.727273
chr15	60246000	0.669421
chr15	60300900	0.702479
chr15	60314700	0.793388
chr15	60330600	0.834711
chr15	60333900	0.685950
chr15	60337000	0.776860
chr15	60369400	0.958678
chr15	60409000	0.702479
chr15	60413800	0.735537
chr15	60421700	0.760331
chr15	60667300	0.743802
chr15	60686000	0.801653
chr15	60812500	0.793388
chr15	60837300	0.793388
chr15	60844500	0.694215
chr15	60849100	0.826446
chr15	60852300	0.859504
chr15	60861400	0.752066
chr15	60868800	0.900826
chr15	60873000	0.661157
chr15	60908400	0.809917
chr15	60919300	0.752066
chr15	60935900	0.685950
chr15	60942900	0.925620
chr15	60960300	0.851240
chr15	61012700	0.785124
chr15	61070900	0.719008
chr15	61073900	0.685950
chr15	61093100	0.735537
chr15	61097500	0.991736
chr15	61110700	0.768595
chr15	61115200	0.851240
chr15	61127300	0.727273
chr15	61168300	0.752066
chr15	61197800	0.694215
chr15	61208900	0.719008
chr15	61296300	0.925620
chr15	61304100	0.983471
chr15	61310300	0.719008

chr15	61343600	0.859504
chr15	61377800	0.776860
chr15	61409000	0.710744
chr15	61439500	0.818182
chr15	61461000	0.652893
chr15	61463800	0.900826
chr15	61466800	0.900826
chr15	61471600	0.661157
chr15	61657500	0.892562
chr15	61794100	0.727273
chr15	61820800	0.752066
chr15	61847700	0.661157
chr15	61932300	0.652893
chr15	61973500	0.876033
chr15	62011800	0.809917
chr15	62082300	0.710744
chr15	62109700	0.702479
chr15	62588400	0.942149
chr15	62744800	0.933884
chr15	62749300	0.776860
chr15	62757700	0.661157
chr15	62762700	0.768595
chr15	62765100	0.842975
chr15	62776000	0.768595
chr15	62796000	0.743802
chr15	62799000	0.743802
chr15	62814000	0.826446
chr15	62853900	0.876033
chr15	62929300	0.685950
chr15	62937300	0.652893
chr15	63027300	0.876033
chr15	63035000	0.694215
chr15	63041700	0.669421
chr15	63050700	0.842975
chr15	63075000	0.644628
chr15	63084900	0.867769
chr15	63100200	0.669421
chr15	63126100	0.710744
chr15	63138300	0.644628
chr15	63142500	0.966942
chr15	63167200	0.735537
chr15	63179300	0.884298
chr15	63188400	0.975207
chr15	63195000	0.694215
chr15	63205000	0.644628
chr15	63207800	0.752066
chr15	63250800	0.644628
chr15	63312200	0.801653
chr15	63343600	0.702479
chr15	63367200	0.685950
chr15	63369700	0.950413
chr15	63381700	0.661157
chr15	63385800	0.694215
chr15	63465700	0.677686

chr15	63490200	0.743802
chr15	63502700	0.752066
chr15	63548800	0.958678
chr15	63608800	0.669421
chr15	63615900	0.694215
chr15	63633700	0.776860
chr15	63661700	0.909091
chr15	63688600	0.727273
chr15	63727300	0.867769
chr15	63729700	0.867769
chr15	63739900	0.735537
chr15	63776300	0.661157
chr15	63783800	0.677686
chr15	63788200	0.818182
chr15	63800800	0.702479
chr15	63807500	0.735537
chr15	63810100	0.801653
chr15	63824200	0.710744
chr15	63829200	0.785124
chr15	63832500	0.834711
chr15	63845800	0.768595
chr15	63848900	0.652893
chr15	63853100	0.760331
chr15	63887400	0.719008
chr15	64057800	0.851240
chr15	64061200	0.818182
chr15	64108100	0.851240
chr15	64136200	0.785124
chr15	64183600	0.710744
chr15	64189200	0.702479
chr15	64210700	0.661157
chr15	64265000	0.710744
chr15	64302700	0.694215
chr15	64429900	0.785124
chr15	64446400	0.735537
chr15	64457000	0.685950
chr15	64539900	0.925620
chr15	64634400	0.801653
chr15	64722700	0.760331
chr15	64747000	0.652893
chr15	64756500	0.768595
chr15	64759200	0.867769
chr15	64795600	0.661157
chr15	64807600	0.710744
chr15	64813800	0.900826
chr15	64889500	0.743802
chr15	64910800	0.743802
chr15	64926300	0.933884
chr15	64928500	0.809917
chr15	64960400	0.851240
chr15	64977400	0.942149
chr15	64980500	0.925620
chr15	64984800	0.842975
chr15	65024700	0.917355

chr15	65041900	0.719008
chr15	65051200	0.925620
chr15	65055900	0.677686
chr15	65063500	0.669421
chr15	65071400	0.768595
chr15	65104700	0.834711
chr15	65108300	0.661157
chr15	65114900	0.752066
chr15	65128400	0.661157
chr15	65137300	0.834711
chr15	65140100	0.785124
chr15	65143900	0.834711
chr15	65149700	0.909091
chr15	65154400	0.884298
chr15	65158600	0.950413
chr15	65167100	0.917355
chr15	65185000	0.727273
chr15	65197200	0.661157
chr15	65208200	0.677686
chr15	65272200	0.958678
chr15	65342400	0.694215
chr15	65361400	0.669421
chr15	65364200	0.685950
chr15	65376000	0.917355
chr15	65563600	0.760331
chr15	65618900	0.760331
chr15	65627100	0.818182
chr15	65629800	0.652893
chr15	65632700	0.867769
chr15	65653900	0.752066
chr15	65657000	0.809917
chr15	65659300	0.785124
chr15	65665300	0.685950
chr15	65672500	0.801653
chr15	65682600	0.727273
chr15	65694000	0.851240
chr15	65696600	0.785124
chr15	65703600	0.685950
chr15	65711700	0.842975
chr15	65843200	0.776860
chr15	65911400	0.768595
chr15	66093700	0.710744
chr15	66125400	0.958678
chr15	66194600	0.677686
chr15	66209100	0.735537
chr15	66246900	0.876033
chr15	66285200	0.727273
chr15	66310300	0.809917
chr15	66315700	0.793388
chr15	66318700	0.925620
chr15	66335200	0.867769
chr15	66339500	0.694215
chr15	66351200	0.801653
chr15	66358800	0.809917

chr15	66365700	0.652893
chr15	66389000	0.644628
chr15	66394100	0.669421
chr15	66471300	0.933884
chr15	66473600	0.644628
chr15	66477500	0.652893
chr15	66542300	0.760331
chr15	66697000	0.975207
chr15	66703900	0.694215
chr15	66755400	0.669421
chr15	66763200	0.842975
chr15	66766400	0.975207
chr15	66769800	0.826446
chr15	66774800	0.752066
chr15	66858700	0.702479
chr15	66881600	0.735537
chr15	66915000	0.900826
chr15	66922600	0.694215
chr15	66925100	0.735537
chr15	66927600	0.867769
chr15	66946900	0.644628
chr15	66950100	0.884298
chr15	66965900	0.818182
chr15	66972300	0.776860
chr15	67000200	0.760331
chr15	67011300	0.710744
chr15	67022400	0.842975
chr15	67026800	0.644628
chr15	67039600	0.735537
chr15	67063500	0.677686
chr15	67067000	1.000000
chr15	67072500	0.809917
chr15	67095700	0.900826
chr15	67119900	0.925620
chr15	67127900	0.950413
chr15	67143700	0.685950
chr15	67176200	0.818182
chr15	67306400	0.776860
chr15	67316800	0.702479
chr15	67323000	0.892562
chr15	67326600	0.661157
chr15	67332100	0.983471
chr15	67336600	0.876033
chr15	67384100	0.933884
chr15	67398600	0.743802
chr15	67426900	0.859504
chr15	67431500	0.859504
chr15	67437700	0.917355
chr15	67458300	0.900826
chr15	67469100	0.793388
chr15	67560000	0.727273
chr15	67575300	0.776860
chr15	67589000	0.702479
chr15	67652200	0.694215

chr15	67701800	0.851240
chr15	67794700	0.710744
chr15	67841100	0.818182
chr15	67851300	0.826446
chr15	68023900	0.727273
chr15	68026400	0.661157
chr15	68043000	0.652893
chr15	68063500	0.925620
chr15	68075100	0.909091
chr15	68080300	0.735537
chr15	68083800	0.834711
chr15	68096900	0.867769
chr15	68104900	0.760331
chr15	68114100	0.702479
chr15	68119500	0.644628
chr15	68136100	0.876033
chr15	68145900	0.652893
chr15	68175800	0.644628
chr15	68207500	0.925620
chr15	68210800	0.702479
chr15	68238900	0.867769
chr15	68254100	0.826446
chr15	68300800	0.669421
chr15	68355700	0.677686
chr15	68399600	0.661157
chr15	68484100	0.685950
chr15	68494000	0.652893
chr15	68498700	0.809917
chr15	68504000	0.884298
chr15	68556400	0.760331
chr15	68564500	0.851240
chr15	68574600	0.710744
chr15	68599100	0.909091
chr15	68605800	0.826446
chr15	68608500	0.933884
chr15	68623500	0.867769
chr15	68659200	0.702479
chr15	68713600	0.743802
chr15	68810600	0.818182
chr15	68853900	0.818182
chr15	68859400	0.735537
chr15	68862900	0.892562
chr15	68866900	0.760331
chr15	68888300	0.652893
chr15	68892400	0.925620
chr15	68900700	0.776860
chr15	68907400	0.933884
chr15	68927500	0.669421
chr15	68959200	0.942149
chr15	68993100	0.752066
chr15	69023400	0.818182
chr15	69076000	0.644628
chr15	69081700	0.842975
chr15	69084800	0.933884

chr15	69088200	0.909091
chr15	69100400	0.727273
chr15	69283200	0.685950
chr15	69293400	0.719008
chr15	69368600	0.685950
chr15	69373800	0.735537
chr15	69376800	0.677686
chr15	69379000	0.975207
chr15	69383400	0.694215
chr15	69412600	0.702479
chr15	69486600	0.644628
chr15	69517200	0.752066
chr15	69564300	0.685950
chr15	69582000	0.760331
chr15	69610100	0.818182
chr15	69635900	0.925620
chr15	69687200	0.776860
chr15	69816500	0.760331
chr15	69818800	0.669421
chr15	69824100	0.685950
chr15	69849800	0.752066
chr15	69940300	0.793388
chr15	69960900	0.809917
chr15	70020100	0.677686
chr15	70028000	0.768595
chr15	70048400	0.785124
chr15	70105900	0.826446
chr15	70118800	0.768595
chr15	70203400	0.884298
chr15	70206700	0.685950
chr15	70209300	0.652893
chr15	70212600	0.900826
chr15	70232300	0.727273
chr15	70307800	0.900826
chr15	70340200	0.768595
chr15	70373700	0.768595
chr15	70376200	0.785124
chr15	70380400	0.826446
chr15	70384500	0.694215
chr15	70404000	0.859504
chr15	70433100	0.917355
chr15	70446700	0.851240
chr15	70458200	0.752066
chr15	70489100	0.933884
chr15	70505600	0.917355
chr15	70540700	0.727273
chr15	70578700	0.652893
chr15	70602000	0.652893
chr15	70607100	0.793388
chr15	70655200	0.776860
chr15	70671000	0.669421
chr15	70678900	0.644628
chr15	70699700	0.909091
chr15	70720200	0.669421

chr15	70727100	0.719008
chr15	70736500	0.842975
chr15	70798300	0.760331
chr15	70851500	0.719008
chr15	70921200	0.685950
chr15	70924300	0.917355
chr15	70929500	0.694215
chr15	70962000	0.661157
chr15	71126800	0.818182
chr15	71342500	0.685950
chr15	71378500	0.900826
chr15	71480100	0.694215
chr15	71496000	0.991736
chr15	71532100	0.743802
chr15	71573500	0.760331
chr15	71623900	0.710744
chr15	71629700	0.644628
chr15	71641700	0.694215
chr15	71649900	0.694215
chr15	71657400	0.975207
chr15	71668300	0.727273
chr15	71679200	0.966942
chr15	71681300	0.652893
chr15	71685100	0.735537
chr15	71735900	0.793388
chr15	71744500	0.702479
chr15	71758400	0.661157
chr15	71808300	0.785124
chr15	71885000	0.669421
chr15	71892600	0.669421
chr15	71904700	0.933884
chr15	71927200	0.661157
chr15	71940800	0.785124
chr15	72100200	0.851240
chr15	72119000	0.867769
chr15	72448400	0.793388
chr15	72519700	0.685950
chr15	72535600	0.669421
chr15	72897600	0.719008
chr15	72908300	0.752066
chr15	72935100	0.776860
chr15	72942900	0.768595
chr15	72958500	0.652893
chr15	72963100	0.710744
chr15	73073900	0.644628
chr15	73113300	0.669421
chr15	73127900	0.809917
chr15	73140800	0.826446
chr15	73232500	0.685950
chr15	73311700	0.685950
chr15	73314400	0.834711
chr15	73371000	0.851240
chr15	73467600	0.644628
chr15	73475900	0.876033

chr15	73518200	0.768595
chr15	73611200	0.760331
chr15	73640700	0.809917
chr15	73644600	0.719008
chr15	73646800	0.867769
chr15	73655700	0.867769
chr15	73657900	0.892562
chr15	73664100	0.710744
chr15	73668000	0.933884
chr15	73679800	0.851240
chr15	73682400	0.801653
chr15	73811000	0.785124
chr15	73891100	0.702479
chr15	73894500	0.842975
chr15	73899300	0.776860
chr15	73910400	0.950413
chr15	73989000	0.900826
chr15	73992900	0.743802
chr15	74040800	0.685950
chr15	74073600	0.727273
chr15	74077000	0.884298
chr15	74079100	0.958678
chr15	74081200	0.685950
chr15	74134600	0.685950
chr15	74137500	0.694215
chr15	74159500	0.859504
chr15	74190500	0.793388
chr15	74215100	0.876033
chr15	74217200	0.876033
chr15	74243800	0.818182
chr15	74258000	0.809917
chr15	74265700	0.925620
chr15	74289600	0.669421
chr15	74330000	0.743802
chr15	74374700	0.743802
chr15	74415400	0.834711
chr15	74433000	0.818182
chr15	74466000	0.752066
chr15	74501500	0.950413
chr15	74509200	0.719008
chr15	74513600	0.768595
chr15	74516500	0.809917
chr15	74520700	0.842975
chr15	74526800	0.702479
chr15	74532000	0.727273
chr15	74535100	0.669421
chr15	74551300	0.661157
chr15	74556700	0.925620
chr15	74584000	0.884298
chr15	74596200	0.793388
chr15	74601100	0.917355
chr15	74604400	0.652893
chr15	74607300	0.752066
chr15	74610500	0.735537

chr15	74612600	0.760331
chr15	74652300	0.669421
chr15	74678900	0.702479
chr15	74712500	0.652893
chr15	74715400	0.900826
chr15	74782800	0.818182
chr15	74838800	0.644628
chr15	74881600	0.859504
chr15	74891100	0.892562
chr15	74914300	0.644628
chr15	74935500	0.975207
chr15	75061100	0.735537
chr15	75066000	0.702479
chr15	75081300	0.694215
chr15	75085400	0.851240
chr15	75092600	0.727273
chr15	75114100	0.644628
chr15	75119100	0.809917
chr15	75243100	0.809917
chr15	75322000	0.760331
chr15	75339500	0.760331
chr15	75472500	0.768595
chr15	75497500	0.818182
chr15	75508100	0.669421
chr15	75546700	0.983471
chr15	75567100	0.892562
chr15	75571100	0.900826
chr15	75575700	0.685950
chr15	75578000	0.694215
chr15	75865900	0.867769
chr15	75943500	0.677686
chr15	75965200	0.768595
chr15	75976300	0.710744
chr15	75979000	0.735537
chr15	76028400	0.727273
chr15	76051000	0.867769
chr15	76064000	0.925620
chr15	76068500	0.859504
chr15	76094800	0.652893
chr15	76103000	0.669421
chr15	76116600	0.991736
chr15	76124000	0.727273
chr15	76160200	0.900826
chr15	76164700	0.652893
chr15	76194200	0.801653
chr15	76219300	0.694215
chr15	76349800	0.752066
chr15	76401000	0.867769
chr15	76408700	0.842975
chr15	76421100	0.793388
chr15	76432700	0.867769
chr15	76444000	0.900826
chr15	76492900	0.793388
chr15	76527500	0.909091

chr15	76546200	0.752066
chr15	76622900	0.652893
chr15	76630700	0.652893
chr15	76635900	0.644628
chr15	77095900	0.933884
chr15	77323200	0.685950
chr15	77376400	0.719008
chr15	77400300	0.818182
chr15	77423300	0.710744
chr15	77709000	0.760331
chr15	77775700	0.983471
chr15	77789100	0.719008
chr15	77796100	0.917355
chr15	77798800	0.661157
chr15	77803000	0.983471
chr15	77826500	0.983471
chr15	77830600	0.876033
chr15	77836000	0.818182
chr15	77850000	0.677686
chr15	77861700	0.685950
chr15	77897200	0.884298
chr15	77903900	0.652893
chr15	77909300	0.834711
chr15	77917400	0.851240
chr15	77921800	0.760331
chr15	77929200	0.752066
chr15	77943000	0.925620
chr15	78005000	0.644628
chr15	78015800	0.743802
chr15	78033000	0.801653
chr15	78035600	0.743802
chr15	78040000	0.900826
chr15	78077700	0.768595
chr15	78096900	0.785124
chr15	78104800	0.669421
chr15	78129000	0.735537
chr15	78133600	0.694215
chr15	78156900	0.685950
chr15	78175600	0.785124
chr15	78223200	0.958678
chr15	78252100	0.942149
chr15	78276600	0.900826
chr15	78283300	0.644628
chr15	78292800	0.652893
chr15	78296200	0.644628
chr15	78343800	0.702479
chr15	78358600	0.826446
chr15	78362200	0.677686
chr15	78413400	0.859504
chr15	78421900	0.677686
chr15	78527500	0.669421
chr15	78536100	0.760331
chr15	78604800	0.710744
chr15	78609200	0.900826

chr15	78614900	0.818182
chr15	78624100	0.892562
chr15	78661300	0.677686
chr15	78679700	0.652893
chr15	78687900	0.925620
chr15	78698000	0.652893
chr15	78707800	0.710744
chr15	78753900	0.917355
chr15	78964500	0.776860
chr15	78968900	0.752066
chr15	78974200	0.727273
chr15	78978100	0.785124
chr15	79033400	0.661157
chr15	79044000	0.644628
chr15	79097500	0.851240
chr15	79105000	0.702479
chr15	79249800	0.677686
chr15	79261300	0.727273
chr15	79264200	0.710744
chr15	79286400	0.859504
chr15	79288900	0.702479
chr15	79297100	0.710744
chr15	79321700	0.694215
chr15	79328500	0.892562
chr15	79353000	0.900826
chr15	79429200	0.925620
chr15	79447300	0.818182
chr15	79464600	0.892562
chr15	79471900	0.685950
chr15	79490200	0.694215
chr15	79502500	0.727273
chr15	79532600	0.801653
chr15	79666400	0.702479
chr15	79700900	0.661157
chr15	79711300	0.669421
chr15	79744200	0.735537
chr15	79966400	0.661157
chr15	80109800	0.719008
chr15	80213300	0.719008
chr15	80321700	0.793388
chr15	80325200	0.793388
chr15	80328000	0.652893
chr15	80334500	0.710744
chr15	80363600	0.669421
chr15	80368700	0.685950
chr15	80406400	0.760331
chr15	80492300	0.768595
chr15	80711500	0.735537
chr15	80738300	0.644628
chr15	80743600	0.702479
chr15	80760800	0.768595
chr15	80766400	0.942149
chr15	80773100	0.909091
chr15	80860700	0.661157

chr15	81040300	0.834711
chr15	81048600	0.702479
chr15	81097600	0.677686
chr15	81109000	0.669421
chr15	81116100	0.719008
chr15	81149500	0.644628
chr15	81156500	0.859504
chr15	81160000	0.867769
chr15	81246800	0.925620
chr15	81260100	0.983471
chr15	81263600	0.867769
chr15	81406300	0.661157
chr15	81509000	0.661157
chr15	81586600	0.793388
chr15	81596700	0.842975
chr15	81649500	0.677686
chr15	81711600	0.793388
chr15	81823900	0.727273
chr15	81841200	0.661157
chr15	81902600	0.644628
chr15	82051500	0.661157
chr15	82559400	0.694215
chr15	82562200	0.727273
chr15	82574700	0.652893
chr15	82586600	0.644628
chr15	82761900	0.685950
chr15	83026300	0.669421
chr15	83220500	0.685950
chr15	83254700	0.727273
chr15	83315400	0.710744
chr15	83519000	0.752066
chr15	83564200	0.710744
chr15	83726600	0.942149
chr15	83732900	0.719008
chr15	83917900	0.785124
chr15	83974800	0.826446
chr15	83979900	0.933884
chr15	84041600	0.851240
chr15	84049400	0.710744
chr15	84065100	0.677686
chr15	84168200	0.991736
chr15	84181300	0.793388
chr15	84202700	0.826446
chr15	84215500	0.867769
chr15	84363600	0.917355
chr15	85131300	0.652893
chr15	85138500	0.735537
chr15	85147000	0.694215
chr15	85149300	0.727273
chr15	85203400	0.743802
chr15	85278700	0.793388
chr15	85321800	0.677686
chr15	85327400	0.884298
chr15	85366000	0.735537

chr15	85498800	0.735537
chr15	85504300	0.818182
chr15	85546000	0.834711
chr15	85575400	0.719008
chr15	85856300	0.719008
chr15	85874600	0.793388
chr15	85937800	0.727273
chr15	85948200	0.661157
chr15	85973600	0.702479
chr15	85987500	0.834711
chr15	85997800	0.785124
chr15	86007000	0.719008
chr15	86042600	0.685950
chr15	86047000	0.661157
chr15	86073400	0.702479
chr15	86087100	0.661157
chr15	86233000	0.809917
chr15	86246100	0.669421
chr15	86295500	0.818182
chr15	86301600	0.677686
chr15	86307500	0.727273
chr15	86329800	0.785124
chr15	86332800	0.909091
chr15	86441100	0.884298
chr15	86469000	0.702479
chr15	86517600	0.809917
chr15	86584400	0.933884
chr15	86623200	0.661157
chr15	86626200	0.785124
chr15	86665900	0.909091
chr15	86776100	0.818182
chr15	86780500	0.801653
chr15	86951700	0.719008
chr15	87131400	0.884298
chr15	87436800	0.867769
chr15	87455900	0.834711
chr15	87460300	0.752066
chr15	87531500	0.776860
chr15	88021500	0.719008
chr15	88308200	0.925620
chr15	88559500	0.834711
chr15	88580200	0.909091
chr15	88616100	0.735537
chr15	88776500	0.727273
chr15	88790500	0.719008
chr15	88843200	0.909091
chr15	88918900	0.768595
chr15	88932200	0.644628
chr15	88940100	0.694215
chr15	88946900	0.826446
chr15	88977200	0.685950
chr15	89028500	0.859504
chr15	89048200	0.801653
chr15	89153000	0.793388

chr15	89158200	0.809917
chr15	89171400	0.785124
chr15	89179400	0.727273
chr15	89182300	0.950413
chr15	89196400	0.768595
chr15	89288000	0.752066
chr15	89336600	0.884298
chr15	89428400	0.834711
chr15	89441100	0.884298
chr15	89444600	0.661157
chr15	89447900	0.876033
chr15	89452300	0.909091
chr15	89482900	0.942149
chr15	89516500	0.644628
chr15	89529500	0.818182
chr15	89532100	0.661157
chr15	89535000	0.809917
chr15	89538100	0.933884
chr15	89542700	0.933884
chr15	89545600	0.735537
chr15	89547800	0.950413
chr15	89561300	0.958678
chr15	89601100	0.685950
chr15	89660100	0.859504
chr15	89666100	0.851240
chr15	89741000	0.826446
chr15	89743400	0.942149
chr15	89875500	0.644628
chr15	89890100	0.851240
chr15	89900800	0.710744
chr15	89906000	0.818182
chr15	89911300	0.752066
chr15	89915700	0.752066
chr15	89918200	0.669421
chr15	89920800	0.710744
chr15	89927000	0.644628
chr15	89932600	0.727273
chr15	89935200	0.785124
chr15	89938600	0.661157
chr15	89940700	0.727273
chr15	89950100	0.652893
chr15	89953600	0.685950
chr15	89967500	0.842975
chr15	89977600	0.867769
chr15	89988000	0.876033
chr15	89993300	0.694215
chr15	90016400	0.677686
chr15	90030200	0.752066
chr15	90035800	0.677686
chr15	90304400	0.768595
chr15	90327200	0.909091
chr15	90350100	0.644628
chr15	90367900	0.735537
chr15	90526100	0.685950

chr15	90548400	0.851240
chr15	90563500	0.760331
chr15	90574900	0.867769
chr15	90578300	0.859504
chr15	90583900	0.702479
chr15	90597800	0.710744
chr15	90602700	0.735537
chr15	90605500	0.966942
chr15	90619600	0.793388
chr15	90670300	0.892562
chr15	90695900	0.892562
chr15	90704500	0.925620
chr15	90718800	0.884298
chr15	90735500	0.752066
chr15	90755500	0.694215
chr15	90762900	0.710744
chr15	90830800	0.776860
chr15	90838400	0.661157
chr15	90845000	0.776860
chr15	90863800	0.719008
chr15	90934400	0.760331
chr15	90944600	0.983471
chr15	90949100	0.685950
chr15	90955300	0.851240
chr15	90957900	0.719008
chr15	90963500	0.743802
chr15	91105600	0.669421
chr15	91120500	0.793388
chr15	91124700	0.842975
chr15	91127500	0.975207
chr15	91151900	0.917355
chr15	91154300	0.752066
chr15	91167600	0.652893
chr15	91204100	0.760331
chr15	91305200	0.801653
chr15	91357400	0.867769
chr15	91380300	0.760331
chr15	91383800	0.909091
chr15	91412600	0.768595
chr15	91417600	0.809917
chr15	91427400	0.710744
chr15	91433600	0.743802
chr15	91459500	0.652893
chr15	91576100	0.694215
chr15	91671300	0.760331
chr15	91964600	0.735537
chr15	92399900	0.669421
chr15	92406900	0.661157
chr15	92410900	0.669421
chr15	92425500	0.694215
chr15	92438400	0.768595
chr15	92470900	0.760331
chr15	92484300	0.760331
chr15	92493900	0.917355

chr15	92544500	0.710744
chr15	92549300	0.818182
chr15	92557900	0.801653
chr15	92628700	0.644628
chr15	92634700	0.719008
chr15	92641100	0.785124
chr15	92645500	0.735537
chr15	92647700	0.652893
chr15	92656700	0.776860
chr15	92662600	0.942149
chr15	92667500	0.768595
chr15	92674000	0.652893
chr15	92701800	0.710744
chr15	92704000	0.983471
chr15	92709200	0.900826
chr15	92812600	0.735537
chr15	92849200	0.917355
chr15	92887900	0.942149
chr15	92957600	0.760331
chr15	93053800	0.991736
chr15	93065100	0.768595
chr15	93191300	0.983471
chr15	93211000	0.842975
chr15	93255200	0.685950
chr15	93286700	0.966942
chr15	93355100	0.743802
chr15	93361600	0.652893
chr15	93363800	0.876033
chr15	93366100	0.950413
chr15	93375400	0.991736
chr15	93424200	0.710744
chr15	93451200	0.760331
chr15	93457600	0.834711
chr15	93461200	0.776860
chr15	93465600	0.942149
chr15	93493200	0.801653
chr15	93605300	0.983471
chr15	93608600	0.867769
chr15	93612100	0.818182
chr15	93649000	0.925620
chr15	93679700	0.809917
chr15	93695900	0.661157
chr15	93723700	0.727273
chr15	93733500	0.876033
chr15	93779800	0.710744
chr15	93785600	0.834711
chr15	93814600	0.826446
chr15	93824200	0.867769
chr15	93870900	0.801653
chr15	93902000	0.727273
chr15	93905700	0.867769
chr15	93909600	0.925620
chr15	93925800	0.760331
chr15	93994900	0.743802

chr15	94015700	0.834711
chr15	94021100	0.644628
chr15	94041900	0.702479
chr15	94207600	0.760331
chr15	94462400	0.735537
chr15	94831300	0.818182
chr15	94867400	0.669421
chr15	94944100	0.760331
chr15	95181700	0.719008
chr15	95215600	0.851240
chr15	95287800	0.685950
chr15	95304200	0.652893
chr15	95374000	0.776860
chr15	95388800	0.677686
chr15	95562100	0.644628
chr15	95831100	0.884298
chr15	96170100	0.694215
chr15	96314000	0.801653
chr15	96456200	0.892562
chr15	96501300	0.685950
chr15	96632700	0.884298
chr15	96710000	0.702479
chr15	96723900	0.752066
chr15	96726300	0.694215
chr15	96838300	0.719008
chr15	96871800	0.752066
chr15	96878100	0.694215
chr15	96880300	0.818182
chr15	96892100	0.685950
chr15	96895600	0.983471
chr15	96898200	0.661157
chr15	96906400	0.793388
chr15	96913700	0.884298
chr15	96936000	0.958678
chr15	96960100	0.669421
chr15	96964900	0.652893
chr15	97129100	0.818182
chr15	97131400	0.669421
chr15	97133600	0.677686
chr15	97162700	0.776860
chr15	97243200	0.876033
chr15	97247300	0.809917
chr15	97434900	0.661157
chr15	97740000	0.669421
chr15	97879000	0.669421
chr15	97896500	0.818182
chr15	98198300	0.801653
chr15	98442800	0.661157
chr15	98632700	0.743802
chr15	98670900	0.661157
chr15	98711500	0.909091
chr15	98971900	0.669421
chr15	99095800	0.710744
chr15	99124100	0.644628

chr15	99141000	0.652893
chr15	99143800	0.851240
chr15	99153100	0.735537
chr15	99197100	0.685950
chr15	99206600	0.834711
chr15	99211300	0.851240
chr15	99215000	0.933884
chr15	99225100	0.661157
chr15	99231000	0.933884
chr15	99236000	0.809917
chr15	99239000	0.644628
chr15	99243500	0.661157
chr15	99246800	0.752066
chr15	99252300	0.669421
chr15	99256400	0.661157
chr15	99259300	0.785124
chr15	99263000	0.900826
chr15	99266100	0.661157
chr15	99271300	0.933884
chr15	99280700	0.801653
chr15	99287900	0.776860
chr15	99297800	0.983471
chr15	99307100	0.785124
chr15	99314200	0.859504
chr15	99316900	0.942149
chr15	99320400	0.851240
chr15	99329600	0.900826
chr15	99338000	0.917355
chr15	99340200	0.851240
chr15	99349300	0.694215
chr15	99356600	0.727273
chr15	99367400	0.950413
chr15	99370900	0.933884
chr15	99398700	0.685950
chr15	99409700	0.793388
chr15	99426300	0.661157
chr15	99443400	0.710744
chr15	99450800	0.966942
chr15	99631100	0.710744
chr15	99664500	0.768595
chr15	99683700	0.917355
chr15	99836200	0.644628
chr15	99847600	0.685950
chr15	99979100	0.776860
chr15	99987800	0.644628
chr15	100103800	0.818182
chr15	100109400	0.826446
chr15	100119500	0.776860
chr15	100169300	0.694215
chr15	100172500	0.685950
chr15	100518700	0.760331
chr15	100524000	0.909091
chr15	100536200	0.677686
chr15	100543200	0.702479

chr15	100560700	0.661157
chr15	100655100	0.818182
chr15	100731800	0.834711
chr15	100785900	0.694215
chr15	101203900	0.652893
chr15	101322400	0.785124
chr15	101450500	0.727273
chr15	101469500	0.702479
chr15	101515700	0.677686
chr15	101541300	0.752066
chr15	101555800	0.933884
chr15	101588100	0.909091
chr15	101592900	0.644628
chr15	101603800	0.834711
chr15	101611900	1.000000
chr15	101619100	0.818182
chr15	101629300	0.867769
chr15	101667500	0.735537
chr15	101725700	0.710744
chr15	101728900	0.818182
chr15	101734100	0.727273
chr15	101739300	0.710744
chr15	101742500	0.859504
chr15	101783400	0.950413
chr15	101799100	0.768595
chr15	101903500	0.669421
chr15	101913400	0.809917
chr15	101922700	0.776860
chr15	101951100	0.661157
chr15	101966200	0.892562
chr15	102021800	0.743802
chr15	102037400	0.685950
chr15	102156800	0.776860
chr16	119500	0.652893
chr16	125700	0.884298
chr16	219900	0.834711
chr16	224900	0.776860
chr16	369200	0.743802
chr16	375100	0.752066
chr16	382000	0.892562
chr16	421400	0.685950
chr16	446300	0.652893
chr16	449400	0.834711
chr16	508000	0.669421
chr16	521800	0.859504
chr16	533300	0.917355
chr16	535400	0.702479
chr16	617400	0.826446
chr16	633700	0.793388
chr16	658400	0.719008
chr16	679200	0.942149
chr16	689100	0.685950
chr16	710600	0.719008
chr16	727900	0.743802

chr16	767100	0.719008
chr16	796600	0.677686
chr16	802100	0.694215
chr16	819500	0.818182
chr16	835700	0.785124
chr16	937200	0.719008
chr16	979800	0.917355
chr16	1098800	0.892562
chr16	1120500	0.793388
chr16	1141100	0.661157
chr16	1195600	0.876033
chr16	1304100	0.685950
chr16	1309400	0.652893
chr16	1328600	0.851240
chr16	1333800	0.661157
chr16	1336700	0.644628
chr16	1341800	0.685950
chr16	1351200	0.900826
chr16	1354300	0.867769
chr16	1393900	0.719008
chr16	1730900	0.942149
chr16	1764200	0.818182
chr16	1814200	0.669421
chr16	1863100	0.851240
chr16	1964700	0.776860
chr16	1970600	0.661157
chr16	1974200	0.801653
chr16	2012000	0.685950
chr16	2031800	0.884298
chr16	2054200	0.950413
chr16	2175000	0.851240
chr16	2178300	0.933884
chr16	2210900	0.702479
chr16	2289100	0.677686
chr16	2436000	0.710744
chr16	2552000	0.884298
chr16	2561700	0.900826
chr16	2566800	0.752066
chr16	2597200	0.710744
chr16	2601700	0.719008
chr16	2610500	0.818182
chr16	2659000	0.884298
chr16	2743400	0.719008
chr16	2766000	0.677686
chr16	2769800	0.694215
chr16	2806200	0.652893
chr16	2886300	0.851240
chr16	2975800	0.933884
chr16	3006500	0.719008
chr16	3032800	0.652893
chr16	3039000	0.719008
chr16	3041200	0.785124
chr16	3047200	0.644628
chr16	3051700	0.727273

chr16	3056500	0.685950
chr16	3078800	0.719008
chr16	3088700	0.834711
chr16	3106300	0.876033
chr16	3114700	0.644628
chr16	3137800	0.942149
chr16	3139900	0.694215
chr16	3146100	0.719008
chr16	3152300	0.776860
chr16	3177600	0.859504
chr16	3179900	0.652893
chr16	3511400	0.809917
chr16	3515100	0.661157
chr16	3518400	0.851240
chr16	3531800	0.644628
chr16	3592300	0.652893
chr16	3599800	0.644628
chr16	3603300	0.917355
chr16	3629000	0.743802
chr16	3687600	0.752066
chr16	3692100	0.785124
chr16	3696000	0.909091
chr16	3702900	0.826446
chr16	3705400	0.900826
chr16	3848600	0.661157
chr16	3861200	0.892562
chr16	3867000	0.661157
chr16	3907700	0.785124
chr16	3913300	0.677686
chr16	3960600	0.661157
chr16	3989200	0.669421
chr16	4014200	0.685950
chr16	4033600	0.685950
chr16	4049500	0.933884
chr16	4058000	0.644628
chr16	4079700	0.793388
chr16	4131600	0.644628
chr16	4184200	0.834711
chr16	4264500	0.867769
chr16	4268300	0.818182
chr16	4314300	0.809917
chr16	4338800	0.851240
chr16	4344400	0.644628
chr16	4360200	0.785124
chr16	4371200	0.768595
chr16	4374200	0.809917
chr16	4378700	0.719008
chr16	4424600	0.842975
chr16	4429000	0.851240
chr16	4439100	0.652893
chr16	4441700	0.677686
chr16	4444000	0.925620
chr16	4511800	0.702479
chr16	4564900	0.727273

chr16	4573000	0.809917
chr16	4579500	0.950413
chr16	4585800	0.694215
chr16	4621000	1.000000
chr16	4625600	0.809917
chr16	4643000	0.801653
chr16	4652600	0.644628
chr16	4668700	0.983471
chr16	4700800	0.702479
chr16	4764400	0.867769
chr16	4768900	0.793388
chr16	4793600	0.801653
chr16	4969000	0.735537
chr16	4975200	0.644628
chr16	4991300	0.710744
chr16	4994400	0.991736
chr16	4998800	0.942149
chr16	5019300	0.983471
chr16	5038000	0.702479
chr16	5061500	0.661157
chr16	5075500	0.727273
chr16	5086700	0.727273
chr16	5109400	0.801653
chr16	5184500	0.710744
chr16	5216900	0.867769
chr16	5263300	0.776860
chr16	5276200	0.818182
chr16	5302200	0.669421
chr16	5373800	0.834711
chr16	5400300	0.859504
chr16	5435500	0.644628
chr16	5452200	0.826446
chr16	5479600	0.851240
chr16	5530100	0.702479
chr16	5640700	0.661157
chr16	5693000	0.710744
chr16	5700600	0.694215
chr16	5804100	0.768595
chr16	5806200	0.677686
chr16	5813700	0.834711
chr16	5843200	0.727273
chr16	5846300	0.652893
chr16	5911900	0.652893
chr16	5921500	0.859504
chr16	5966900	0.925620
chr16	5976300	0.892562
chr16	5979100	0.917355
chr16	5983000	0.809917
chr16	6030600	0.760331
chr16	6034500	0.851240
chr16	6094300	0.884298
chr16	6244200	0.661157
chr16	6246800	0.983471
chr16	6267300	0.793388

chr16	6300500	0.801653
chr16	6318600	0.644628
chr16	6335300	0.818182
chr16	6348700	0.661157
chr16	6368400	0.768595
chr16	6393400	0.966942
chr16	6396400	0.942149
chr16	6402900	0.909091
chr16	6407400	0.809917
chr16	6414200	0.677686
chr16	6426000	0.776860
chr16	6608400	1.000000
chr16	6621300	0.776860
chr16	6714100	0.818182
chr16	6911900	0.809917
chr16	6976900	0.876033
chr16	7076800	0.752066
chr16	7137700	0.669421
chr16	7140400	0.661157
chr16	7258300	0.652893
chr16	7269100	0.966942
chr16	7277500	0.983471
chr16	7374800	0.694215
chr16	7419600	0.644628
chr16	7502200	0.760331
chr16	7560300	0.727273
chr16	7675100	0.826446
chr16	7684800	0.719008
chr16	7702100	0.942149
chr16	7718500	0.685950
chr16	7796800	0.752066
chr16	7812500	0.661157
chr16	7814900	0.669421
chr16	7817500	0.826446
chr16	7878700	0.661157
chr16	7917500	0.661157
chr16	8578900	0.727273
chr16	8620000	0.842975
chr16	8623400	0.760331
chr16	8759800	0.743802
chr16	8780300	0.801653
chr16	8807300	0.685950
chr16	8815700	0.809917
chr16	8858500	0.719008
chr16	8923500	0.809917
chr16	8927300	0.793388
chr16	8944000	0.809917
chr16	8947100	0.975207
chr16	8956100	0.842975
chr16	8972700	0.685950
chr16	8980100	0.917355
chr16	9026100	0.644628
chr16	9030300	0.834711
chr16	9037100	0.669421

chr16	9044100	0.685950
chr16	9050600	0.702479
chr16	9054300	0.801653
chr16	9060600	0.859504
chr16	9084700	0.719008
chr16	9102500	0.661157
chr16	9107500	0.743802
chr16	9142000	0.867769
chr16	9202700	0.950413
chr16	9219200	0.694215
chr16	9233300	0.768595
chr16	9239600	0.669421
chr16	9243100	0.859504
chr16	9254300	0.710744
chr16	9352700	0.818182
chr16	9405400	0.776860
chr16	9437100	0.710744
chr16	9597700	0.710744
chr16	9616400	0.743802
chr16	9621300	0.892562
chr16	9706500	0.694215
chr16	9764100	0.884298
chr16	9771800	0.884298
chr16	9774400	0.958678
chr16	9798600	0.727273
chr16	9807300	0.760331
chr16	9857800	0.702479
chr16	9910500	0.644628
chr16	9916100	0.801653
chr16	9921600	0.768595
chr16	10040400	0.743802
chr16	10072500	0.669421
chr16	10170500	0.652893
chr16	10215900	0.834711
chr16	10350800	0.909091
chr16	10598500	0.958678
chr16	10700800	0.793388
chr16	10711200	0.677686
chr16	10716800	0.677686
chr16	10736500	0.942149
chr16	10755500	0.685950
chr16	10786400	0.644628
chr16	11041000	0.859504
chr16	11043600	0.793388
chr16	11145200	0.809917
chr16	11182200	0.719008
chr16	11235200	0.644628
chr16	11277300	0.842975
chr16	11296200	0.776860
chr16	11300300	0.776860
chr16	11327200	0.644628
chr16	11367800	0.743802
chr16	11402400	0.809917
chr16	11407200	0.925620

chr16	11410800	0.710744
chr16	11423300	0.818182
chr16	11435200	0.677686
chr16	11443500	0.909091
chr16	11449900	0.677686
chr16	11481100	0.652893
chr16	11485600	0.801653
chr16	11592000	0.933884
chr16	11595700	0.793388
chr16	11612100	0.652893
chr16	11624700	0.661157
chr16	11655300	0.933884
chr16	11665800	0.892562
chr16	11691600	0.801653
chr16	11709600	0.975207
chr16	11726600	0.719008
chr16	11730500	0.842975
chr16	11735000	0.876033
chr16	11753400	0.727273
chr16	11759600	0.801653
chr16	11765200	0.760331
chr16	11768200	0.768595
chr16	12186100	0.685950
chr16	12189300	0.661157
chr16	12242000	0.909091
chr16	12275400	0.818182
chr16	12308800	0.644628
chr16	12405600	0.785124
chr16	12414400	0.661157
chr16	12479900	0.752066
chr16	12686000	0.842975
chr16	12774600	0.851240
chr16	12993200	0.685950
chr16	13012100	0.958678
chr16	13034200	0.909091
chr16	13041400	0.694215
chr16	13065400	0.644628
chr16	13089700	0.768595
chr16	13107200	0.826446
chr16	13112400	0.776860
chr16	13118500	0.677686
chr16	13131400	0.719008
chr16	13135200	0.859504
chr16	13155500	0.652893
chr16	13164400	1.000000
chr16	13177700	0.644628
chr16	13193800	0.826446
chr16	13204300	0.776860
chr16	13215000	0.661157
chr16	13249000	0.818182
chr16	13251500	0.892562
chr16	13256000	0.727273
chr16	13262300	0.760331
chr16	13265600	0.677686

chr16	13325600	0.950413
chr16	13329200	0.785124
chr16	13393200	0.727273
chr16	13419300	0.809917
chr16	13427100	0.851240
chr16	13446300	0.793388
chr16	13530500	0.652893
chr16	13615200	0.801653
chr16	13646800	0.752066
chr16	13689300	0.867769
chr16	13715900	0.644628
chr16	13722500	0.719008
chr16	13803100	0.710744
chr16	14097300	0.727273
chr16	14107600	0.876033
chr16	14145000	0.942149
chr16	14204700	0.851240
chr16	14219500	0.719008
chr16	14280500	0.768595
chr16	14401700	0.942149
chr16	14404600	0.917355
chr16	14553000	0.859504
chr16	14581100	0.826446
chr16	14596100	0.876033
chr16	14604300	0.909091
chr16	14616500	0.884298
chr16	14643500	0.694215
chr16	14658800	0.884298
chr16	14730500	0.892562
chr16	14734300	0.735537
chr16	14884500	0.710744
chr16	14995900	0.652893
chr16	15072700	0.785124
chr16	15122500	0.818182
chr16	15235900	0.743802
chr16	15238700	0.652893
chr16	15241200	0.966942
chr16	15243700	0.719008
chr16	15256600	0.909091
chr16	15272600	0.892562
chr16	15350700	0.661157
chr16	15357100	0.884298
chr16	15360900	0.644628
chr16	15397800	0.752066
chr16	15496200	0.694215
chr16	15604100	0.867769
chr16	15902000	0.909091
chr16	16060200	0.760331
chr16	16065300	0.867769
chr16	16070100	0.669421
chr16	16083200	0.876033
chr16	16168800	0.818182
chr16	16187600	0.851240
chr16	16369100	0.694215

chr16	16394700	0.809917
chr16	16410800	0.793388
chr16	16546200	0.768595
chr16	16583700	0.818182
chr16	16589200	0.925620
chr16	16701700	0.909091
chr16	16882100	0.776860
chr16	16963000	0.917355
chr16	16975300	0.834711
chr16	17034300	0.809917
chr16	17176800	0.900826
chr16	17255400	0.909091
chr16	17299800	0.859504
chr16	17305600	0.661157
chr16	17361000	0.776860
chr16	17394000	0.884298
chr16	17399600	0.842975
chr16	17459500	0.677686
chr16	17462100	0.900826
chr16	17464300	0.966942
chr16	17472700	0.677686
chr16	17480200	0.925620
chr16	17482700	0.809917
chr16	17486800	0.785124
chr16	17506400	0.950413
chr16	17510900	0.867769
chr16	17517600	0.876033
chr16	17523100	0.735537
chr16	17525900	0.652893
chr16	17528200	0.652893
chr16	17561500	0.834711
chr16	17673400	0.727273
chr16	17751100	0.752066
chr16	17837300	0.661157
chr16	17839400	0.917355
chr16	17842500	0.809917
chr16	17852100	0.743802
chr16	17893000	0.652893
chr16	17931300	1.000000
chr16	18043400	0.776860
chr16	18136000	0.644628
chr16	18169000	0.743802
chr16	18184500	0.685950
chr16	18228900	0.719008
chr16	18311700	0.661157
chr16	18324500	0.710744
chr16	18353500	0.768595
chr16	18505000	0.776860
chr16	18531100	0.669421
chr16	18757400	0.801653
chr16	18815700	0.818182
chr16	19017300	0.892562
chr16	19142700	0.644628
chr16	19197100	0.826446

chr16	19239700	0.727273
chr16	19299300	0.966942
chr16	19303600	0.983471
chr16	19328300	0.842975
chr16	19360400	0.851240
chr16	19418800	0.818182
chr16	19499700	0.933884
chr16	19719200	0.801653
chr16	19722300	0.991736
chr16	19724500	0.859504
chr16	19816500	0.900826
chr16	19849700	0.975207
chr16	19862900	0.760331
chr16	19865900	0.760331
chr16	19888500	0.677686
chr16	19891700	0.975207
chr16	19910400	0.694215
chr16	19960200	0.909091
chr16	19962800	0.727273
chr16	20016400	0.677686
chr16	20053200	0.909091
chr16	20186400	0.975207
chr16	20255300	0.900826
chr16	20280300	0.760331
chr16	20421400	0.768595
chr16	20599000	0.694215
chr16	20689200	0.768595
chr16	20694300	0.768595
chr16	20901700	0.867769
chr16	20909400	0.661157
chr16	20914800	0.644628
chr16	20980400	0.801653
chr16	21116200	0.710744
chr16	21138400	0.677686
chr16	21235000	0.884298
chr16	21359100	0.685950
chr16	21401700	0.760331
chr16	21499700	0.652893
chr16	21524500	0.917355
chr16	21535200	0.760331
chr16	21538300	0.669421
chr16	21620300	0.727273
chr16	21623500	0.876033
chr16	21678200	0.900826
chr16	21768800	0.752066
chr16	21773800	0.834711
chr16	21811200	0.685950
chr16	21946800	0.719008
chr16	22053200	0.694215
chr16	22104100	0.950413
chr16	22115100	0.867769
chr16	22236900	0.892562
chr16	22290400	0.661157
chr16	22333400	0.909091

chr16	22365300	1.000000
chr16	22382500	0.826446
chr16	22589100	0.884298
chr16	22628300	0.768595
chr16	22644700	0.942149
chr16	22648000	0.942149
chr16	22650500	0.826446
chr16	22653300	0.809917
chr16	22682100	0.694215
chr16	22776400	0.652893
chr16	22860300	0.685950
chr16	22871500	0.809917
chr16	22910600	0.752066
chr16	23071300	0.826446
chr16	23132900	0.652893
chr16	23137000	0.685950
chr16	23145600	0.735537
chr16	23157300	0.702479
chr16	23208000	0.826446
chr16	23212500	0.925620
chr16	23291200	0.644628
chr16	23410800	0.950413
chr16	23421400	0.661157
chr16	23431800	0.669421
chr16	23504800	0.644628
chr16	23511900	0.900826
chr16	23515900	0.719008
chr16	23518400	0.694215
chr16	23610500	0.991736
chr16	23712500	0.900826
chr16	23798700	1.000000
chr16	23877600	0.793388
chr16	23913200	0.710744
chr16	23916600	0.743802
chr16	23963900	0.900826
chr16	24057500	0.793388
chr16	24066700	0.917355
chr16	24110100	0.909091
chr16	24129200	0.702479
chr16	24147600	0.867769
chr16	24150500	1.000000
chr16	24197200	0.702479
chr16	24220600	0.710744
chr16	24265200	0.735537
chr16	24358600	1.000000
chr16	24360800	0.719008
chr16	24394000	0.983471
chr16	24408500	0.644628
chr16	24494500	0.892562
chr16	24508800	0.900826
chr16	24682500	0.909091
chr16	24695100	0.743802
chr16	24697800	0.694215
chr16	24757200	0.950413

chr16	24781700	0.826446
chr16	24932100	0.644628
chr16	25060200	0.694215
chr16	25329700	0.727273
chr16	25577200	0.760331
chr16	25762900	0.652893
chr16	25850500	0.644628
chr16	25867700	0.975207
chr16	25896700	0.743802
chr16	25963400	0.975207
chr16	26031500	0.735537
chr16	26062400	0.785124
chr16	26067400	0.991736
chr16	26069800	0.809917
chr16	26082800	0.917355
chr16	26189400	0.900826
chr16	26228900	0.735537
chr16	26232300	1.000000
chr16	26329200	0.685950
chr16	26427400	0.776860
chr16	26487100	0.752066
chr16	26502500	0.727273
chr16	26610600	0.942149
chr16	26699600	0.719008
chr16	26762000	0.760331
chr16	26996600	0.867769
chr16	26998700	0.859504
chr16	27078900	0.834711
chr16	27095600	0.917355
chr16	27270800	0.694215
chr16	27327300	0.710744
chr16	27330100	0.677686
chr16	27335700	0.867769
chr16	27338600	1.000000
chr16	27342500	0.652893
chr16	27630100	0.644628
chr16	27648300	0.710744
chr16	27714300	0.644628
chr16	27732600	0.752066
chr16	27815600	0.710744
chr16	27865400	0.694215
chr16	27932000	0.661157
chr16	28104900	1.000000
chr16	28175800	0.768595
chr16	28267300	0.793388
chr16	28269800	0.793388
chr16	28272000	0.834711
chr16	28274600	0.685950
chr16	28309900	0.793388
chr16	28342000	0.652893
chr16	28585100	0.842975
chr16	28633700	0.702479
chr16	28700200	0.661157
chr16	28894400	0.809917

chr16	29040800	0.818182
chr16	29127000	0.719008
chr16	29132900	0.876033
chr16	29168900	0.966942
chr16	29213900	0.661157
chr16	29242400	0.652893
chr16	29263100	0.727273
chr16	29290600	0.776860
chr16	29296800	0.900826
chr16	29302500	0.909091
chr16	29327900	0.685950
chr16	29331400	0.752066
chr16	29381000	0.644628
chr16	29472600	0.991736
chr16	29480000	0.677686
chr16	29581400	0.694215
chr16	29612200	0.826446
chr16	29657500	0.644628
chr16	29674700	0.702479
chr16	29703400	0.776860
chr16	29724000	0.644628
chr16	29731000	0.900826
chr16	29837100	0.966942
chr16	29860200	0.710744
chr16	29955800	0.876033
chr16	30012600	0.876033
chr16	30040300	0.685950
chr16	30124700	0.661157
chr16	30198800	0.694215
chr16	30211700	0.685950
chr16	30219500	0.694215
chr16	30387700	0.826446
chr16	30409800	0.685950
chr16	30442600	0.652893
chr16	30553600	0.958678
chr16	30560500	0.867769
chr16	30567500	0.818182
chr16	30703900	0.876033
chr16	30752300	0.950413
chr16	30825900	0.876033
chr16	30922300	0.685950
chr16	30938700	0.950413
chr16	30954100	0.826446
chr16	30957900	0.669421
chr16	30965600	0.719008
chr16	30996700	0.859504
chr16	31019000	0.677686
chr16	31141800	0.876033
chr16	31143900	0.809917
chr16	31150700	0.925620
chr16	31154000	0.752066
chr16	31161700	0.842975
chr16	31257800	0.661157
chr16	31277400	0.851240

chr16	31439300	0.652893
chr16	31464600	0.909091
chr16	31496400	0.785124
chr16	31513600	0.752066
chr16	33902400	0.793388
chr16	33950000	0.785124
chr16	33954700	0.694215
chr16	33960100	0.652893
chr16	34022900	0.950413
chr16	34197100	0.735537
chr16	35239800	0.809917
chr16	35281500	0.644628
chr16	46414500	0.661157
chr16	46782600	0.867769
chr16	46791200	0.909091
chr16	46888200	0.818182
chr16	46921300	0.867769
chr16	46993800	0.942149
chr16	47114200	0.834711
chr16	47400500	0.776860
chr16	47526700	0.892562
chr16	47660700	0.818182
chr16	47733600	0.661157
chr16	47881900	0.702479
chr16	47918700	0.826446
chr16	47921500	0.801653
chr16	47999500	0.991736
chr16	48020200	0.677686
chr16	48089000	0.652893
chr16	48370700	0.743802
chr16	48407900	0.694215
chr16	48413700	0.652893
chr16	48513700	0.818182
chr16	48641300	0.793388
chr16	48647400	0.735537
chr16	48664400	0.768595
chr16	49056000	0.851240
chr16	49310900	0.644628
chr16	49346800	0.867769
chr16	49367000	0.851240
chr16	49401300	0.900826
chr16	49475700	0.867769
chr16	49479300	0.661157
chr16	49486900	0.760331
chr16	49525900	0.661157
chr16	49562800	0.809917
chr16	49567000	0.801653
chr16	49580900	0.652893
chr16	49615900	0.809917
chr16	49620500	0.719008
chr16	49624300	0.785124
chr16	49630900	0.752066
chr16	49636600	0.793388
chr16	49642700	0.752066

chr16	49673900	0.983471
chr16	49699800	0.876033
chr16	49733200	0.867769
chr16	49735700	0.677686
chr16	49738000	0.925620
chr16	49747400	0.652893
chr16	49778100	0.702479
chr16	49784300	0.859504
chr16	49808200	0.785124
chr16	49810600	0.917355
chr16	49833200	0.702479
chr16	49845900	0.702479
chr16	49855500	0.694215
chr16	49858600	0.661157
chr16	49866800	0.644628
chr16	49869900	0.809917
chr16	49875100	0.719008
chr16	49880900	0.710744
chr16	49886200	0.710744
chr16	49901100	0.917355
chr16	49911700	0.644628
chr16	49918000	0.727273
chr16	49955700	0.677686
chr16	50055700	0.694215
chr16	50102400	0.735537
chr16	50282400	0.677686
chr16	50293900	0.652893
chr16	50300600	0.834711
chr16	50408900	0.652893
chr16	50429200	0.743802
chr16	50480100	0.909091
chr16	50483100	0.917355
chr16	50493000	0.983471
chr16	50497600	0.917355
chr16	50501400	0.661157
chr16	50543800	0.727273
chr16	50576100	0.644628
chr16	50615600	0.809917
chr16	50627300	0.842975
chr16	50644700	0.867769
chr16	50679700	0.793388
chr16	50754200	0.851240
chr16	50778500	0.661157
chr16	51059100	0.776860
chr16	51069100	0.752066
chr16	51118700	0.900826
chr16	51129700	0.743802
chr16	51145600	0.644628
chr16	51156400	0.884298
chr16	51180700	0.652893
chr16	51191800	0.809917
chr16	51200200	0.661157
chr16	51216300	0.876033
chr16	51262600	0.942149

chr16	51271700	0.735537
chr16	51325200	0.942149
chr16	51341200	0.743802
chr16	51393500	0.834711
chr16	51418100	0.702479
chr16	51427200	0.677686
chr16	51460900	0.677686
chr16	51477000	0.842975
chr16	51573400	0.851240
chr16	51579300	1.000000
chr16	51664400	0.752066
chr16	51671500	0.801653
chr16	51788600	0.735537
chr16	51791000	0.851240
chr16	51793900	0.685950
chr16	51797200	0.884298
chr16	51807500	0.719008
chr16	51913900	0.834711
chr16	51973800	0.768595
chr16	51986100	0.884298
chr16	52151700	0.710744
chr16	52286300	0.917355
chr16	52300800	0.702479
chr16	52344300	0.719008
chr16	52417500	0.685950
chr16	52479700	0.694215
chr16	52503400	0.685950
chr16	52532900	0.859504
chr16	52547100	0.909091
chr16	52556100	0.669421
chr16	52560700	0.942149
chr16	52583900	0.694215
chr16	52586900	0.727273
chr16	52596400	0.685950
chr16	52632000	0.644628
chr16	52681500	0.644628
chr16	52775800	0.966942
chr16	52907800	0.818182
chr16	52998000	0.933884
chr16	53105800	0.966942
chr16	53120400	0.702479
chr16	53124200	0.867769
chr16	53128300	0.710744
chr16	53145200	0.710744
chr16	53191500	0.685950
chr16	53749100	0.826446
chr16	53899400	0.851240
chr16	54025900	0.867769
chr16	54084100	0.942149
chr16	54088400	0.867769
chr16	54210700	0.942149
chr16	54365100	0.909091
chr16	54397000	0.694215
chr16	54445600	0.661157

chr16	54465300	0.719008
chr16	54489500	0.652893
chr16	54539100	0.826446
chr16	54611600	0.917355
chr16	54619800	0.842975
chr16	54623300	0.776860
chr16	54626200	0.884298
chr16	54629100	0.685950
chr16	54685800	0.785124
chr16	54689600	0.933884
chr16	54852400	0.669421
chr16	55020700	0.876033
chr16	55167400	0.710744
chr16	55224500	0.760331
chr16	55284300	0.809917
chr16	55318600	0.735537
chr16	55326500	0.809917
chr16	55336900	0.661157
chr16	55405300	0.851240
chr16	55422600	0.694215
chr16	55509200	0.652893
chr16	55702500	0.685950
chr16	56000400	0.826446
chr16	56005400	0.752066
chr16	56040100	0.793388
chr16	56142800	0.710744
chr16	56147500	0.768595
chr16	56150300	0.892562
chr16	56231600	0.867769
chr16	56280100	0.694215
chr16	56298100	0.669421
chr16	56303800	0.743802
chr16	56311700	0.892562
chr16	56315700	0.867769
chr16	56330500	0.925620
chr16	56340300	0.694215
chr16	56352200	0.661157
chr16	56391000	0.727273
chr16	56456700	0.719008
chr16	56581700	0.925620
chr16	56644800	0.768595
chr16	56652900	0.776860
chr16	56661400	0.752066
chr16	56672700	0.826446
chr16	56682000	0.752066
chr16	56711600	0.694215
chr16	56718200	0.652893
chr16	56777800	0.719008
chr16	56782200	0.735537
chr16	56817900	0.743802
chr16	56821300	0.909091
chr16	56857700	0.785124
chr16	57038700	0.661157
chr16	57150300	0.892562

chr16	57168600	0.719008
chr16	57181800	0.983471
chr16	57185600	0.925620
chr16	57193600	0.702479
chr16	57294200	0.752066
chr16	57352400	0.851240
chr16	57453200	0.661157
chr16	57506100	0.975207
chr16	57509400	0.876033
chr16	57511500	0.867769
chr16	57541600	0.826446
chr16	57567100	0.867769
chr16	57610600	0.661157
chr16	57654600	0.702479
chr16	57657600	0.685950
chr16	57662000	0.900826
chr16	57673700	0.694215
chr16	57710300	0.884298
chr16	57713900	0.685950
chr16	57794400	0.710744
chr16	57810100	0.818182
chr16	57850600	0.909091
chr16	57871800	0.809917
chr16	57880500	0.859504
chr16	57897100	0.743802
chr16	57907500	0.710744
chr16	57946300	0.727273
chr16	57992100	0.842975
chr16	58045400	0.719008
chr16	58057000	0.776860
chr16	58063300	0.909091
chr16	58081600	0.768595
chr16	58097300	0.727273
chr16	58144900	0.677686
chr16	58239600	0.727273
chr16	58256700	0.669421
chr16	58278100	0.809917
chr16	58365700	0.975207
chr16	58371200	0.768595
chr16	58453500	0.834711
chr16	58506000	0.677686
chr16	58509000	0.710744
chr16	58527100	0.661157
chr16	58529400	0.991736
chr16	58790300	0.760331
chr16	58896900	0.975207
chr16	58973200	0.735537
chr16	59158000	0.694215
chr16	59204400	0.925620
chr16	59235900	0.909091
chr16	59245000	0.735537
chr16	59722300	0.652893
chr16	60248600	0.933884
chr16	60587500	0.776860

chr16	60670000	0.752066
chr16	60679800	0.768595
chr16	61165700	0.702479
chr16	61331600	0.661157
chr16	61667200	0.652893
chr16	61711800	0.793388
chr16	61765500	0.785124
chr16	61846600	0.793388
chr16	61906900	0.785124
chr16	61991400	0.644628
chr16	62002900	0.685950
chr16	62057000	0.685950
chr16	62072800	0.785124
chr16	62085100	0.768595
chr16	62089800	0.694215
chr16	62092700	0.710744
chr16	62095000	0.685950
chr16	62098200	0.793388
chr16	62141400	0.727273
chr16	62274800	0.925620
chr16	62310700	0.694215
chr16	62541600	0.661157
chr16	62699600	0.917355
chr16	62984500	0.801653
chr16	62988500	0.909091
chr16	63101500	0.677686
chr16	63407100	0.644628
chr16	63609400	0.685950
chr16	64012400	0.785124
chr16	64017200	0.809917
chr16	64479600	0.900826
chr16	64489100	0.735537
chr16	64568600	0.818182
chr16	64649900	0.876033
chr16	64944600	0.702479
chr16	65084300	0.776860
chr16	65152100	0.694215
chr16	65259200	0.752066
chr16	65270200	0.884298
chr16	65274900	0.752066
chr16	65284000	0.669421
chr16	65315400	0.933884
chr16	65328200	0.644628
chr16	65340800	0.694215
chr16	65555300	0.785124
chr16	65700600	0.702479
chr16	65747900	0.892562
chr16	65762900	0.909091
chr16	65771900	0.685950
chr16	65783500	0.760331
chr16	65806700	0.975207
chr16	65851300	0.785124
chr16	65859600	0.776860
chr16	65951900	0.760331

chr16	65993800	0.652893
chr16	66088300	0.685950
chr16	66099000	0.768595
chr16	66116100	0.644628
chr16	66161900	0.768595
chr16	66304800	0.719008
chr16	66312600	0.752066
chr16	66393700	0.735537
chr16	66402200	0.859504
chr16	66463600	0.727273
chr16	66468900	0.752066
chr16	66509300	0.661157
chr16	66546400	0.958678
chr16	66576400	0.669421
chr16	66613200	0.892562
chr16	66643000	0.685950
chr16	66651000	0.785124
chr16	66685400	0.710744
chr16	66728500	0.669421
chr16	66762900	0.892562
chr16	66774500	0.669421
chr16	66961200	0.644628
chr16	66995500	0.652893
chr16	67201000	0.677686
chr16	67275300	0.917355
chr16	67278700	0.669421
chr16	67421400	0.644628
chr16	67430600	0.851240
chr16	67438900	0.917355
chr16	67448600	0.818182
chr16	67458400	0.983471
chr16	67462400	0.760331
chr16	67468200	0.867769
chr16	67479000	0.826446
chr16	67523500	0.834711
chr16	67540700	0.760331
chr16	67581900	0.644628
chr16	67628700	0.892562
chr16	67702500	0.644628
chr16	67747800	0.735537
chr16	67783600	0.884298
chr16	67859700	0.826446
chr16	67879000	0.652893
chr16	67892200	0.983471
chr16	67897700	0.760331
chr16	67920000	0.702479
chr16	67949300	0.793388
chr16	67960400	0.933884
chr16	68419000	0.909091
chr16	68431200	0.842975
chr16	68443500	0.743802
chr16	68513400	0.694215
chr16	68525400	0.851240
chr16	68555000	0.785124

chr16	68623900	0.677686
chr16	68738600	0.644628
chr16	68741300	0.719008
chr16	68747400	0.661157
chr16	68766700	0.958678
chr16	68774800	0.677686
chr16	68776900	0.719008
chr16	68779700	0.925620
chr16	68795500	0.694215
chr16	68799300	0.859504
chr16	68812900	0.983471
chr16	68816400	0.801653
chr16	68824300	0.950413
chr16	68827800	0.892562
chr16	68832800	0.859504
chr16	68904000	0.644628
chr16	68942100	0.652893
chr16	68994900	0.785124
chr16	69007100	0.760331
chr16	69022600	0.752066
chr16	69030000	0.752066
chr16	69099000	0.793388
chr16	69106500	0.867769
chr16	69111300	0.719008
chr16	69123700	0.719008
chr16	69134400	0.842975
chr16	69136900	0.661157
chr16	69143700	0.694215
chr16	69564400	0.942149
chr16	69603200	0.900826
chr16	69762500	0.644628
chr16	69830700	0.884298
chr16	69853100	0.760331
chr16	69868000	0.652893
chr16	69951800	0.735537
chr16	69961600	0.661157
chr16	69975300	0.785124
chr16	69977400	0.975207
chr16	69987600	0.719008
chr16	70002000	0.826446
chr16	70230300	0.851240
chr16	70435200	0.719008
chr16	70442500	0.867769
chr16	70455500	0.884298
chr16	70462500	0.867769
chr16	70465000	0.909091
chr16	70467300	0.958678
chr16	70513200	0.685950
chr16	70669500	0.735537
chr16	70714300	0.809917
chr16	70727700	0.776860
chr16	70738000	0.661157
chr16	70742400	0.768595
chr16	70747100	0.842975

chr16	70770400	0.884298
chr16	70784300	0.743802
chr16	71002500	0.801653
chr16	71040800	0.768595
chr16	71043900	0.719008
chr16	71062400	0.991736
chr16	71068400	0.801653
chr16	71074300	0.685950
chr16	71080300	0.685950
chr16	71089800	0.685950
chr16	71098800	0.834711
chr16	71101100	0.876033
chr16	71144300	0.743802
chr16	71187500	0.776860
chr16	71289300	0.818182
chr16	71379600	0.644628
chr16	71421400	0.983471
chr16	71435800	0.867769
chr16	71549100	0.661157
chr16	71645300	0.884298
chr16	71663100	0.809917
chr16	71671800	0.942149
chr16	71741400	0.876033
chr16	71746200	0.793388
chr16	71972000	0.644628
chr16	72107300	0.942149
chr16	72196400	0.867769
chr16	72291200	0.900826
chr16	72319300	0.900826
chr16	72405700	0.809917
chr16	72603700	0.743802
chr16	72756900	0.892562
chr16	72803100	0.719008
chr16	72867300	0.694215
chr16	72882300	0.776860
chr16	72965800	0.768595
chr16	73024000	0.661157
chr16	73026600	0.685950
chr16	73067400	0.669421
chr16	73089400	0.867769
chr16	73094700	0.652893
chr16	73098700	0.859504
chr16	73179400	0.752066
chr16	73206400	0.793388
chr16	73248000	0.644628
chr16	73368900	0.743802
chr16	73390400	0.727273
chr16	73467500	0.793388
chr16	73517600	0.884298
chr16	73578100	0.768595
chr16	73581900	0.652893
chr16	73639400	0.785124
chr16	73697600	0.685950
chr16	73705800	0.661157

chr16	73910800	0.652893
chr16	73922300	0.809917
chr16	74060600	0.909091
chr16	74063400	0.892562
chr16	74135600	1.000000
chr16	74282100	0.652893
chr16	74432800	0.710744
chr16	74438600	0.719008
chr16	74656800	0.677686
chr16	74768500	0.909091
chr16	74771700	0.685950
chr16	74783600	0.884298
chr16	74846100	0.710744
chr16	74901000	0.900826
chr16	74905400	0.917355
chr16	75032100	0.669421
chr16	75087700	0.776860
chr16	75097000	0.867769
chr16	75099100	0.685950
chr16	75109500	0.644628
chr16	75115100	0.876033
chr16	75121700	0.694215
chr16	75130000	0.933884
chr16	75133300	0.859504
chr16	75143300	0.834711
chr16	75145600	0.867769
chr16	75150200	0.661157
chr16	75153200	0.909091
chr16	75273800	0.793388
chr16	75278000	0.694215
chr16	75282700	0.876033
chr16	75287600	0.834711
chr16	75290000	0.768595
chr16	75292500	0.735537
chr16	75449500	0.776860
chr16	75455200	0.801653
chr16	75490800	0.793388
chr16	75494900	0.743802
chr16	75506400	0.958678
chr16	75569200	0.743802
chr16	75584500	0.950413
chr16	75610600	0.702479
chr16	75625200	0.884298
chr16	75628200	0.950413
chr16	75679200	0.685950
chr16	75955400	0.892562
chr16	76018000	0.884298
chr16	76228600	0.768595
chr16	76242600	0.933884
chr16	76257000	0.644628
chr16	76553300	0.644628
chr16	76717400	0.719008
chr16	77098100	0.818182
chr16	77167800	0.719008

chr16	77240400	0.719008
chr16	77254600	0.710744
chr16	77273400	0.727273
chr16	77308300	0.644628
chr16	77366500	0.685950
chr16	77370600	0.933884
chr16	77421700	0.834711
chr16	77534300	0.851240
chr16	77575300	0.859504
chr16	77868300	0.958678
chr16	77891000	0.826446
chr16	77989100	0.719008
chr16	77997500	0.900826
chr16	78000000	0.950413
chr16	78002800	0.950413
chr16	78025600	0.933884
chr16	78106600	0.966942
chr16	78246200	0.743802
chr16	78309500	0.876033
chr16	78311900	0.925620
chr16	78342400	0.727273
chr16	78356000	0.801653
chr16	78359600	0.818182
chr16	78406900	0.892562
chr16	78483000	0.652893
chr16	78585700	0.743802
chr16	78618900	0.776860
chr16	78640300	0.867769
chr16	78675700	0.661157
chr16	78911800	0.776860
chr16	78940300	0.785124
chr16	79029100	0.876033
chr16	79035400	0.727273
chr16	79088600	0.892562
chr16	79111100	0.669421
chr16	79130200	0.677686
chr16	79203700	0.842975
chr16	79222400	0.652893
chr16	79236500	0.743802
chr16	79246800	0.950413
chr16	79321400	0.727273
chr16	79333500	1.000000
chr16	79335800	0.752066
chr16	79352400	0.917355
chr16	79362800	0.942149
chr16	79381200	0.801653
chr16	79384700	0.892562
chr16	79441700	0.735537
chr16	79446600	0.826446
chr16	79452800	0.669421
chr16	79472100	0.909091
chr16	79584600	0.669421
chr16	79598400	0.867769
chr16	79606200	0.909091

chr16	79614300	0.652893
chr16	79757900	0.752066
chr16	79810900	0.702479
chr16	79839700	0.760331
chr16	80500300	0.892562
chr16	81021100	0.776860
chr16	81214600	0.809917
chr16	81272300	0.851240
chr16	81418400	0.859504
chr16	81437600	0.727273
chr16	81466900	0.752066
chr16	81474600	0.694215
chr16	81482400	0.892562
chr16	81485700	0.793388
chr16	81492000	0.900826
chr16	81499000	0.826446
chr16	81512300	0.760331
chr16	81526600	0.917355
chr16	81533300	0.925620
chr16	81547600	0.876033
chr16	81553500	0.727273
chr16	81578900	0.644628
chr16	81588600	0.644628
chr16	81616500	0.752066
chr16	81619800	0.735537
chr16	81650000	0.661157
chr16	81663900	0.694215
chr16	81666700	0.727273
chr16	81668900	0.768595
chr16	81681300	0.801653
chr16	81686700	0.958678
chr16	81694800	0.867769
chr16	81721800	0.809917
chr16	81747700	0.859504
chr16	81750100	0.743802
chr16	81764000	0.859504
chr16	81849300	0.809917
chr16	81882400	0.809917
chr16	81908500	0.727273
chr16	81913400	0.677686
chr16	81972800	0.710744
chr16	81976500	0.900826
chr16	82007600	0.942149
chr16	82129100	0.958678
chr16	82160600	0.702479
chr16	82172900	0.892562
chr16	82640300	0.975207
chr16	82663700	0.719008
chr16	82781600	0.677686
chr16	82792700	0.694215
chr16	82816800	0.950413
chr16	82864600	0.859504
chr16	82867800	0.942149
chr16	82922300	0.677686

chr16	83148500	0.776860
chr16	83199300	0.801653
chr16	83273300	0.677686
chr16	83616400	0.685950
chr16	83636900	0.900826
chr16	83658400	0.669421
chr16	83682800	0.694215
chr16	83715800	0.685950
chr16	83803600	0.909091
chr16	83836600	0.876033
chr16	83847800	0.975207
chr16	83860100	0.685950
chr16	83864700	0.760331
chr16	83869800	0.644628
chr16	83873100	0.768595
chr16	83875200	0.834711
chr16	83882000	0.652893
chr16	83961100	0.842975
chr16	83974400	0.644628
chr16	84029400	0.867769
chr16	84034800	0.652893
chr16	84077500	0.975207
chr16	84096800	0.685950
chr16	84170700	0.727273
chr16	84172900	0.842975
chr16	84241700	0.694215
chr16	84262300	0.818182
chr16	84312600	0.710744
chr16	84315200	0.669421
chr16	84317900	0.760331
chr16	84333800	0.867769
chr16	84543500	0.867769
chr16	84548700	0.677686
chr16	84566300	0.933884
chr16	84569500	0.950413
chr16	84581700	0.859504
chr16	84593600	0.925620
chr16	84595700	0.933884
chr16	84600700	0.867769
chr16	84631400	0.900826
chr16	84634400	0.743802
chr16	84638700	0.826446
chr16	84653200	0.710744
chr16	84701600	0.735537
chr16	84711600	0.801653
chr16	84718100	0.809917
chr16	84743400	0.710744
chr16	84747000	0.694215
chr16	84759200	0.826446
chr16	84767200	0.735537
chr16	84771700	0.702479
chr16	84776200	0.661157
chr16	84876000	0.900826
chr16	84878400	0.842975

chr16	84882400	0.900826
chr16	84919300	0.719008
chr16	84959500	0.768595
chr16	85037700	0.867769
chr16	85117300	0.727273
chr16	85120700	0.834711
chr16	85158300	0.719008
chr16	85160600	0.809917
chr16	85170700	0.702479
chr16	85186000	0.661157
chr16	85192100	0.760331
chr16	85208800	0.652893
chr16	85221400	0.818182
chr16	85230100	0.652893
chr16	85236500	0.900826
chr16	85240700	0.900826
chr16	85243600	0.694215
chr16	85254400	0.768595
chr16	85261700	0.785124
chr16	85272700	0.851240
chr16	85275700	0.768595
chr16	85287500	0.661157
chr16	85293000	0.760331
chr16	85310600	0.859504
chr16	85325200	0.652893
chr16	85337000	0.735537
chr16	85344500	0.760331
chr16	85347400	0.669421
chr16	85364400	0.702479
chr16	85366600	0.702479
chr16	85378600	0.644628
chr16	85386300	0.661157
chr16	85399400	0.826446
chr16	85404300	0.752066
chr16	85428300	0.826446
chr16	85431000	0.818182
chr16	85456300	0.661157
chr16	85471100	0.975207
chr16	85482900	0.644628
chr16	85486400	0.694215
chr16	85490100	0.727273
chr16	85494200	0.876033
chr16	85526100	0.834711
chr16	85541000	0.752066
chr16	85544200	0.685950
chr16	85558800	0.950413
chr16	85571500	0.685950
chr16	85575900	0.842975
chr16	85585300	0.867769
chr16	85596200	0.652893
chr16	85603800	0.826446
chr16	85606100	0.809917
chr16	85608500	0.892562
chr16	85610900	0.735537

chr16	85613800	0.735537
chr16	85617700	0.760331
chr16	85622100	0.677686
chr16	85633100	0.752066
chr16	85637700	0.719008
chr16	85640900	0.983471
chr16	85650900	0.727273
chr16	85656300	0.702479
chr16	85660400	0.735537
chr16	85669900	0.644628
chr16	85673700	0.826446
chr16	85733300	0.760331
chr16	85750000	0.818182
chr16	85768100	0.801653
chr16	85780300	0.818182
chr16	85786800	0.677686
chr16	85802000	0.785124
chr16	85855400	0.644628
chr16	86068100	0.851240
chr16	86108800	0.909091
chr16	86122500	0.809917
chr16	86335000	0.776860
chr16	86370400	0.685950
chr16	86401500	0.842975
chr16	86458800	0.710744
chr16	86528300	0.818182
chr16	86546900	0.834711
chr16	86550100	0.752066
chr16	86602400	0.694215
chr16	86604800	0.809917
chr16	86607200	0.826446
chr16	86703100	0.909091
chr16	86767000	0.752066
chr16	86775300	0.801653
chr16	86793700	0.834711
chr16	86859500	0.785124
chr16	86917900	0.685950
chr16	86929700	0.702479
chr16	86954400	0.652893
chr16	87019000	0.702479
chr16	87081700	0.677686
chr16	87096600	0.710744
chr16	87128200	0.876033
chr16	87133700	0.793388
chr16	87211700	0.884298
chr16	87213800	0.776860
chr16	87220400	0.760331
chr16	87232600	0.644628
chr16	87237200	0.669421
chr16	87276900	0.950413
chr16	87329900	0.727273
chr16	87421900	0.842975
chr16	87457100	0.644628
chr16	87460600	0.834711

chr16	87521600	0.785124
chr16	87570500	0.776860
chr16	87574500	0.809917
chr16	87609800	0.884298
chr16	87616500	0.702479
chr16	87623200	0.694215
chr16	87692200	0.933884
chr16	87812200	0.975207
chr16	87852000	0.694215
chr16	87854200	0.884298
chr16	87856600	0.793388
chr16	87858900	0.884298
chr16	87862600	0.884298
chr16	87876800	0.661157
chr16	87881800	0.851240
chr16	87885400	0.801653
chr16	87887700	0.834711
chr16	87890100	0.669421
chr16	87896400	0.644628
chr16	87905900	0.900826
chr16	87916100	0.743802
chr16	87943000	0.735537
chr16	87961000	0.652893
chr16	87979200	0.809917
chr16	87981300	0.694215
chr16	87987700	0.768595
chr16	87990400	0.760331
chr16	87998800	0.743802
chr16	88043400	0.719008
chr16	88099000	0.752066
chr16	88102100	0.702479
chr16	88153000	0.950413
chr16	88228000	0.694215
chr16	88292600	0.727273
chr16	88295200	0.677686
chr16	88298800	0.909091
chr16	88504000	0.842975
chr16	88509400	0.677686
chr16	88528800	0.743802
chr16	88535400	0.876033
chr16	88549200	0.760331
chr16	88594200	0.661157
chr16	88667700	0.785124
chr16	88677800	0.842975
chr16	88695000	0.644628
chr16	88703000	0.859504
chr16	88706100	0.727273
chr16	88826000	0.851240
chr16	88831200	0.958678
chr16	88857600	0.669421
chr16	88859900	0.801653
chr16	88947700	0.867769
chr16	88972700	0.644628
chr16	88985500	0.909091

chr16	88990300	0.727273
chr16	88993100	0.892562
chr16	89059500	0.851240
chr16	89101600	0.909091
chr16	89163000	0.776860
chr16	89180800	0.801653
chr16	89184900	0.760331
chr16	89239900	0.809917
chr16	89355400	0.859504
chr16	89357600	0.900826
chr16	89360300	0.818182
chr16	89363100	0.851240
chr16	89384400	0.801653
chr16	89386900	0.900826
chr16	89389800	0.925620
chr16	89392800	0.851240
chr16	89402000	0.818182
chr16	89411500	0.661157
chr16	89424100	0.851240
chr16	89427300	0.710744
chr16	89429600	0.892562
chr16	89436800	0.743802
chr16	89450500	0.958678
chr16	89458600	1.000000
chr16	89462200	0.776860
chr16	89469300	0.917355
chr16	89472400	0.694215
chr16	89475600	0.851240
chr16	89480300	0.867769
chr16	89498100	0.909091
chr16	89500300	0.793388
chr16	89509200	0.752066
chr16	89532700	0.793388
chr16	89542700	0.768595
chr16	89549200	0.917355
chr16	89552700	0.710744
chr16	89669100	0.752066
chr16	89680100	0.710744
chr16	89687100	0.694215
chr16	89772900	0.768595
chr16	89776300	0.685950
chr16	89796600	0.727273
chr16	89889900	0.702479
chr16	89906400	0.685950
chr16	89915300	0.644628
chr16	89976600	0.826446
chr16	90102500	0.884298
chr17	32400	0.719008
chr17	87600	0.677686
chr17	110000	0.917355
chr17	136300	0.834711
chr17	160700	0.694215
chr17	171000	0.818182
chr17	186300	0.776860

chr17	202800	0.644628
chr17	232900	0.826446
chr17	277600	0.834711
chr17	424000	0.842975
chr17	426100	0.809917
chr17	441200	0.710744
chr17	448900	0.677686
chr17	564800	0.760331
chr17	629800	0.942149
chr17	694200	0.933884
chr17	698500	0.809917
chr17	708200	0.644628
chr17	739300	0.710744
chr17	750700	0.752066
chr17	805300	0.966942
chr17	819300	0.884298
chr17	828500	0.809917
chr17	831400	0.925620
chr17	836700	0.892562
chr17	840700	0.818182
chr17	853700	0.983471
chr17	866100	0.925620
chr17	868800	0.818182
chr17	873600	0.859504
chr17	908700	0.909091
chr17	942700	0.702479
chr17	977400	0.685950
chr17	981600	0.834711
chr17	1014200	0.801653
chr17	1018700	0.826446
chr17	1085300	0.933884
chr17	1090300	0.776860
chr17	1174300	0.669421
chr17	1202400	0.685950
chr17	1271200	0.801653
chr17	1278300	0.793388
chr17	1297500	0.702479
chr17	1472900	0.785124
chr17	1478600	0.826446
chr17	1489100	1.000000
chr17	1491200	0.661157
chr17	1508600	0.685950
chr17	1549600	0.694215
chr17	1639800	0.785124
chr17	1650400	0.685950
chr17	1656500	0.694215
chr17	1666900	0.652893
chr17	1835800	0.768595
chr17	1871000	0.669421
chr17	1903200	0.727273
chr17	1915500	0.892562
chr17	1961500	0.652893
chr17	1968400	0.710744
chr17	1974300	0.851240

chr17	1994500	0.785124
chr17	1998800	0.752066
chr17	2027100	0.917355
chr17	2041100	0.933884
chr17	2085100	0.776860
chr17	2098200	0.950413
chr17	2119000	0.694215
chr17	2249600	0.818182
chr17	2251800	0.776860
chr17	2289100	0.685950
chr17	2339100	0.975207
chr17	2388600	0.661157
chr17	2394800	0.809917
chr17	2455500	0.892562
chr17	2527700	0.892562
chr17	2610800	0.694215
chr17	2620200	0.743802
chr17	2702100	0.876033
chr17	2719200	0.826446
chr17	2732200	0.727273
chr17	2748100	0.892562
chr17	2752400	0.818182
chr17	2760600	0.801653
chr17	2763700	0.661157
chr17	2771300	0.900826
chr17	2775500	0.942149
chr17	2778700	0.975207
chr17	2787200	0.677686
chr17	2802100	0.867769
chr17	2807500	0.966942
chr17	2813500	0.644628
chr17	2835200	0.900826
chr17	2843700	0.719008
chr17	2861200	0.942149
chr17	3379300	0.669421
chr17	3386500	0.809917
chr17	3414100	0.776860
chr17	3416600	0.768595
chr17	3433500	0.694215
chr17	3581700	0.694215
chr17	3586600	0.661157
chr17	3641800	0.735537
chr17	3792100	0.801653
chr17	3807400	0.884298
chr17	3811300	0.735537
chr17	3860000	0.851240
chr17	3880400	0.842975
chr17	3884500	0.826446
chr17	3889800	0.669421
chr17	3907600	0.892562
chr17	4028700	0.958678
chr17	4091000	0.966942
chr17	4208100	0.694215
chr17	4211100	0.793388

chr17	4222000	0.677686
chr17	4292600	0.884298
chr17	4297600	0.925620
chr17	4301100	0.851240
chr17	4396800	0.661157
chr17	4417800	0.983471
chr17	4430900	0.661157
chr17	4440100	0.694215
chr17	4498600	0.809917
chr17	4503500	0.909091
chr17	4535900	0.752066
chr17	4539500	0.826446
chr17	4609800	0.942149
chr17	4752400	0.909091
chr17	4754600	0.760331
chr17	4757400	0.975207
chr17	4761000	0.975207
chr17	4776800	0.859504
chr17	4812200	0.702479
chr17	4838000	0.644628
chr17	4848500	0.818182
chr17	4864100	0.677686
chr17	5190300	0.809917
chr17	5192600	0.652893
chr17	5265800	0.727273
chr17	5339000	0.652893
chr17	5584600	0.669421
chr17	5603100	0.818182
chr17	5606600	0.933884
chr17	5673900	0.809917
chr17	5693400	0.785124
chr17	5701700	0.900826
chr17	5704400	0.702479
chr17	5716600	0.884298
chr17	5736500	0.909091
chr17	5787500	0.702479
chr17	5800900	0.909091
chr17	5805900	0.652893
chr17	5822900	0.876033
chr17	5926900	0.719008
chr17	6002600	0.710744
chr17	6005400	0.900826
chr17	6042400	0.867769
chr17	6066000	0.983471
chr17	6076200	0.925620
chr17	6135500	0.669421
chr17	6143300	0.801653
chr17	6145500	0.685950
chr17	6160800	0.793388
chr17	6163500	0.685950
chr17	6165600	0.685950
chr17	6323600	0.694215
chr17	6341100	0.752066
chr17	6343700	0.809917

chr17	6353900	0.876033
chr17	6362100	0.661157
chr17	6365400	0.876033
chr17	6376000	0.652893
chr17	6389900	0.735537
chr17	6401800	0.925620
chr17	6412800	0.644628
chr17	6434100	0.776860
chr17	6479100	0.900826
chr17	6569800	0.727273
chr17	6703500	0.768595
chr17	6818200	0.677686
chr17	6923600	0.958678
chr17	7032800	0.760331
chr17	7036700	0.942149
chr17	7043500	0.801653
chr17	7121800	0.669421
chr17	7139800	0.685950
chr17	7214400	0.867769
chr17	7240200	0.884298
chr17	7259900	0.694215
chr17	7321900	0.752066
chr17	7324200	0.677686
chr17	7365000	0.818182
chr17	7461800	0.884298
chr17	7483400	0.917355
chr17	7495100	0.760331
chr17	7560700	0.942149
chr17	7580400	0.735537
chr17	7606400	0.834711
chr17	7615800	0.842975
chr17	7626000	0.743802
chr17	7697800	0.702479
chr17	7727300	0.719008
chr17	7792800	0.867769
chr17	7807200	0.752066
chr17	7961500	0.818182
chr17	7994400	0.685950
chr17	8037300	0.876033
chr17	8057300	0.760331
chr17	8204300	0.685950
chr17	8245100	0.793388
chr17	8263700	0.801653
chr17	8295400	0.793388
chr17	8312000	0.685950
chr17	8314400	0.867769
chr17	8510100	0.694215
chr17	8512900	0.710744
chr17	8578900	0.876033
chr17	8600800	0.933884
chr17	8642900	0.735537
chr17	8693600	0.884298
chr17	8707200	0.661157
chr17	8731200	0.652893

chr17	8736600	0.644628
chr17	8760900	0.685950
chr17	8777400	0.892562
chr17	8779500	0.661157
chr17	8804300	0.859504
chr17	8920800	0.702479
chr17	8948700	0.710744
chr17	8965800	0.818182
chr17	9009500	0.661157
chr17	9013600	0.694215
chr17	9066600	0.867769
chr17	9074200	0.785124
chr17	9082600	0.702479
chr17	9103700	0.669421
chr17	9124400	0.925620
chr17	9129200	0.884298
chr17	9145200	0.669421
chr17	9149200	0.876033
chr17	9161800	0.661157
chr17	9189500	0.677686
chr17	9213100	0.842975
chr17	9226000	0.900826
chr17	9234900	0.884298
chr17	9328000	0.768595
chr17	9552100	0.752066
chr17	9835100	0.669421
chr17	9860700	0.950413
chr17	9885500	0.694215
chr17	9930000	0.685950
chr17	9963400	0.768595
chr17	9985700	0.702479
chr17	10021400	0.710744
chr17	10023500	0.909091
chr17	10030200	0.719008
chr17	10081300	0.917355
chr17	10088000	0.909091
chr17	10113600	0.925620
chr17	10119900	0.933884
chr17	10179900	0.677686
chr17	10195400	0.669421
chr17	10209300	0.694215
chr17	10452400	0.909091
chr17	10475400	0.842975
chr17	10635100	0.702479
chr17	10651900	0.661157
chr17	10935500	0.752066
chr17	10986300	0.859504
chr17	11103300	0.801653
chr17	11106900	0.719008
chr17	11160300	0.694215
chr17	11212000	0.776860
chr17	11234800	0.685950
chr17	11397800	0.876033
chr17	11422900	0.702479

chr17	11572600	0.884298
chr17	11759200	0.702479
chr17	11803500	0.694215
chr17	11894700	0.735537
chr17	11950300	0.958678
chr17	11953100	0.652893
chr17	12025500	0.892562
chr17	12126000	0.669421
chr17	12200000	0.859504
chr17	12316700	0.685950
chr17	12426400	0.644628
chr17	12469900	0.677686
chr17	12523500	0.661157
chr17	12527600	0.661157
chr17	12547600	0.702479
chr17	12621800	0.652893
chr17	12634700	0.950413
chr17	12801400	0.752066
chr17	12880800	1.000000
chr17	13018200	0.719008
chr17	13209500	0.694215
chr17	13240900	0.727273
chr17	13479400	0.925620
chr17	13547000	0.652893
chr17	13575300	0.661157
chr17	13654500	0.851240
chr17	13874600	0.677686
chr17	13882000	0.735537
chr17	13914900	0.710744
chr17	14029400	0.735537
chr17	14080400	0.809917
chr17	14210700	0.917355
chr17	14219200	0.752066
chr17	14224700	0.900826
chr17	14227400	0.917355
chr17	14259600	0.983471
chr17	14277100	0.876033
chr17	14417400	0.685950
chr17	14429600	0.884298
chr17	14463300	0.710744
chr17	14484200	0.752066
chr17	14603500	0.685950
chr17	14822900	0.669421
chr17	14936900	0.785124
chr17	14940000	0.702479
chr17	14952700	0.900826
chr17	14990200	0.826446
chr17	15083700	0.942149
chr17	15182100	0.826446
chr17	15287900	0.826446
chr17	15394500	0.900826
chr17	15396800	0.793388
chr17	15406300	0.826446
chr17	15472100	0.834711

chr17	15522900	0.694215
chr17	15549300	0.983471
chr17	15554800	0.818182
chr17	15862200	0.652893
chr17	15871600	0.785124
chr17	15874600	0.719008
chr17	16103100	0.719008
chr17	16159000	0.834711
chr17	16190000	0.710744
chr17	16201100	0.719008
chr17	16302100	0.809917
chr17	16311900	0.826446
chr17	16319300	0.677686
chr17	16331800	0.652893
chr17	16346100	0.859504
chr17	16349900	0.710744
chr17	16363700	0.925620
chr17	16373600	0.719008
chr17	16381400	0.677686
chr17	16387700	0.975207
chr17	16392600	0.793388
chr17	16415400	0.876033
chr17	16590800	1.000000
chr17	16625300	0.685950
chr17	16655700	0.685950
chr17	16739300	0.876033
chr17	16804800	0.669421
chr17	16955800	0.685950
chr17	16959800	0.669421
chr17	16995800	0.834711
chr17	16998600	0.884298
chr17	17035600	0.768595
chr17	17046200	0.694215
chr17	17258900	0.735537
chr17	17267600	0.702479
chr17	17307200	0.752066
chr17	17349200	0.644628
chr17	17396800	0.719008
chr17	17419000	0.876033
chr17	17426500	0.818182
chr17	17529900	0.966942
chr17	17566800	0.776860
chr17	17579600	0.933884
chr17	17589700	0.818182
chr17	17598600	0.669421
chr17	17601800	0.752066
chr17	17604800	0.652893
chr17	17610300	0.652893
chr17	17613300	0.867769
chr17	17618000	0.834711
chr17	17630500	0.677686
chr17	17633300	0.661157
chr17	17641600	0.652893
chr17	17645100	0.884298

chr17	17656000	0.942149
chr17	17670300	0.644628
chr17	17682700	0.859504
chr17	17696200	0.768595
chr17	17715800	0.768595
chr17	17726000	0.644628
chr17	17742400	0.842975
chr17	17772600	0.834711
chr17	17781300	0.842975
chr17	17785900	0.809917
chr17	17788400	0.818182
chr17	17792700	0.826446
chr17	17817700	0.685950
chr17	17825200	1.000000
chr17	17847100	0.785124
chr17	18039800	0.677686
chr17	18106700	0.652893
chr17	18127400	0.826446
chr17	18134500	0.727273
chr17	18159700	0.743802
chr17	18221200	0.661157
chr17	18286800	0.685950
chr17	18339700	0.710744
chr17	18389600	0.933884
chr17	18467200	0.644628
chr17	18483500	0.661157
chr17	18497200	0.669421
chr17	18622600	0.909091
chr17	18870700	0.719008
chr17	18872800	0.685950
chr17	18933500	0.900826
chr17	18936500	0.851240
chr17	18950400	0.702479
chr17	18964600	0.801653
chr17	18968200	0.652893
chr17	18991000	0.760331
chr17	18995200	0.644628
chr17	19016500	0.876033
chr17	19031100	0.702479
chr17	19039700	0.710744
chr17	19044900	0.694215
chr17	19048000	0.900826
chr17	19068200	0.677686
chr17	19085100	0.702479
chr17	19094200	0.884298
chr17	19116900	0.867769
chr17	19151400	0.826446
chr17	19189200	0.719008
chr17	19191700	0.925620
chr17	19220500	0.884298
chr17	19247500	0.727273
chr17	19284400	0.669421
chr17	19290800	0.884298
chr17	19295200	0.785124

chr17	19301700	0.793388
chr17	19314900	0.851240
chr17	19329900	0.776860
chr17	19337200	0.925620
chr17	19351400	0.975207
chr17	19360400	0.669421
chr17	19365100	0.793388
chr17	19393300	0.652893
chr17	19396100	0.818182
chr17	19441900	0.644628
chr17	19546100	0.694215
chr17	19549900	0.677686
chr17	19607400	0.917355
chr17	19610300	0.801653
chr17	19652100	0.652893
chr17	19656700	0.644628
chr17	19814700	0.702479
chr17	19891000	0.975207
chr17	19996600	0.652893
chr17	20037800	0.834711
chr17	20045000	0.983471
chr17	20075700	0.776860
chr17	20078900	0.735537
chr17	20093300	0.950413
chr17	20106400	0.685950
chr17	20109500	0.710744
chr17	20114700	0.776860
chr17	20121300	0.719008
chr17	20128600	0.834711
chr17	20134500	0.826446
chr17	20165800	0.760331
chr17	20255600	0.809917
chr17	20358600	0.884298
chr17	20361600	0.752066
chr17	20388800	0.694215
chr17	20405500	0.735537
chr17	20411200	0.834711
chr17	20585600	0.752066
chr17	20802500	1.000000
chr17	20811800	0.685950
chr17	20847800	0.760331
chr17	20865400	0.826446
chr17	20896800	0.710744
chr17	20935800	0.661157
chr17	20940200	0.834711
chr17	20955400	0.809917
chr17	21013100	0.925620
chr17	21102900	0.859504
chr17	21143900	0.752066
chr17	21244800	0.661157
chr17	21263800	0.694215
chr17	21274000	0.793388
chr17	21356400	0.685950
chr17	21361300	0.694215

chr17	21386000	0.760331
chr17	22024000	0.909091
chr17	25267600	0.652893
chr17	25572200	0.884298
chr17	25604300	0.950413
chr17	25666300	0.719008
chr17	25793800	0.983471
chr17	25796300	0.694215
chr17	25804000	0.809917
chr17	25816900	0.661157
chr17	25821600	0.826446
chr17	25824100	0.925620
chr17	25833000	0.991736
chr17	25841500	0.958678
chr17	25847600	0.942149
chr17	25852300	0.966942
chr17	25857900	0.842975
chr17	25862300	0.851240
chr17	25879200	0.710744
chr17	25887900	0.818182
chr17	25901300	0.776860
chr17	25904900	0.752066
chr17	25930000	0.694215
chr17	26104300	0.677686
chr17	26131600	0.694215
chr17	26157500	0.719008
chr17	26167200	0.652893
chr17	26216100	0.917355
chr17	26218400	0.760331
chr17	26243400	0.793388
chr17	26267300	0.785124
chr17	26287000	0.743802
chr17	26383200	0.652893
chr17	26400300	0.685950
chr17	26418600	0.735537
chr17	26421900	0.909091
chr17	26498300	0.884298
chr17	26501600	0.801653
chr17	26557800	0.752066
chr17	26561100	0.669421
chr17	26575600	0.652893
chr17	26729600	0.884298
chr17	26735300	0.909091
chr17	26739400	0.719008
chr17	26808600	0.743802
chr17	26828900	0.826446
chr17	26835100	0.710744
chr17	26848300	0.743802
chr17	27029600	0.809917
chr17	27075200	0.694215
chr17	27077700	0.685950
chr17	27088300	0.917355
chr17	27090500	0.652893
chr17	27189300	0.644628

chr17	27214700	0.702479
chr17	27239000	0.652893
chr17	27264200	0.966942
chr17	27284100	0.702479
chr17	27295400	0.694215
chr17	27299900	0.677686
chr17	27313500	0.900826
chr17	27323900	0.644628
chr17	27329600	0.661157
chr17	27334100	0.694215
chr17	27338000	0.925620
chr17	27342000	0.710744
chr17	27344500	0.900826
chr17	27347800	0.710744
chr17	27355200	0.694215
chr17	27359300	0.917355
chr17	27363600	0.785124
chr17	27366200	0.925620
chr17	27370000	0.966942
chr17	27393600	0.735537
chr17	27404500	0.842975
chr17	27412000	0.694215
chr17	27414600	0.793388
chr17	27418800	0.776860
chr17	27436900	0.644628
chr17	27460600	0.826446
chr17	27468200	0.842975
chr17	27476500	0.644628
chr17	27483500	0.892562
chr17	27546800	0.661157
chr17	27568200	0.867769
chr17	27696700	0.826446
chr17	27704000	0.652893
chr17	27720300	0.685950
chr17	27900400	0.735537
chr17	27912200	0.710744
chr17	27924700	0.917355
chr17	27952100	0.694215
chr17	27966900	0.702479
chr17	28046000	0.710744
chr17	28054100	0.661157
chr17	28114000	0.933884
chr17	28166300	0.661157
chr17	28550100	0.661157
chr17	28667700	0.735537
chr17	28715600	0.685950
chr17	28901200	0.669421
chr17	28972300	0.867769
chr17	28978300	0.760331
chr17	28990800	0.677686
chr17	29014100	0.818182
chr17	29043800	0.677686
chr17	29284000	0.851240
chr17	29359400	0.719008

chr17	29402400	0.694215
chr17	29458300	0.942149
chr17	29471900	0.851240
chr17	29597000	0.925620
chr17	29705800	0.719008
chr17	29716900	0.702479
chr17	29740100	0.661157
chr17	29743600	0.677686
chr17	29746500	0.719008
chr17	29752900	0.677686
chr17	29759100	0.677686
chr17	29779900	0.652893
chr17	29784200	0.694215
chr17	29795000	0.859504
chr17	29798400	0.909091
chr17	29818400	0.677686
chr17	29821700	0.884298
chr17	29827400	0.834711
chr17	29844400	0.752066
chr17	29889300	0.694215
chr17	29892700	0.909091
chr17	29923600	0.652893
chr17	29937600	0.983471
chr17	30387900	0.743802
chr17	30438700	0.719008
chr17	30555200	0.702479
chr17	30568900	0.793388
chr17	30585600	0.826446
chr17	30603600	0.876033
chr17	30608500	0.760331
chr17	30612200	0.743802
chr17	30615300	0.735537
chr17	30617500	0.809917
chr17	30630800	0.991736
chr17	30638600	0.826446
chr17	30651200	0.842975
chr17	30682800	0.685950
chr17	30805700	0.661157
chr17	30810900	0.909091
chr17	30813200	0.661157
chr17	30816800	0.702479
chr17	30825700	0.818182
chr17	30844100	0.950413
chr17	30848100	0.685950
chr17	30850600	0.752066
chr17	30869200	0.859504
chr17	30949000	0.892562
chr17	30954800	0.710744
chr17	31239700	0.702479
chr17	31245300	0.760331
chr17	31264200	0.892562
chr17	31349400	0.760331
chr17	31352200	0.677686
chr17	31479900	0.752066

chr17	31520700	0.942149
chr17	31529300	0.685950
chr17	31587800	0.685950
chr17	31671400	0.851240
chr17	31697600	0.743802
chr17	31780100	0.909091
chr17	31909900	0.710744
chr17	31967500	0.735537
chr17	32078200	0.652893
chr17	32121300	0.793388
chr17	32133600	0.892562
chr17	32185800	0.859504
chr17	32224000	0.801653
chr17	32228400	0.702479
chr17	32256500	0.859504
chr17	32463300	0.851240
chr17	32481900	0.694215
chr17	32485800	0.842975
chr17	32488600	0.702479
chr17	32505500	0.900826
chr17	32741700	0.859504
chr17	32859600	0.809917
chr17	32919500	0.694215
chr17	32925600	0.834711
chr17	32932200	0.694215
chr17	32936700	0.950413
chr17	32940600	0.867769
chr17	32943900	0.694215
chr17	32952700	0.876033
chr17	32960600	0.909091
chr17	32965500	0.694215
chr17	32992900	0.917355
chr17	33084000	0.685950
chr17	33126100	0.834711
chr17	33148300	0.735537
chr17	33164400	0.685950
chr17	33168600	0.826446
chr17	33199100	0.793388
chr17	33211000	0.876033
chr17	33390900	0.966942
chr17	33407600	0.842975
chr17	33478500	0.793388
chr17	33695600	0.933884
chr17	33756800	0.661157
chr17	33772800	0.735537
chr17	33817300	0.776860
chr17	33825400	0.652893
chr17	34011500	0.743802
chr17	34056800	0.710744
chr17	34077500	0.851240
chr17	34093200	0.661157
chr17	34099800	0.743802
chr17	34217900	0.743802
chr17	34451500	0.652893

chr17	34838700	0.694215
chr17	34970600	0.752066
chr17	34986900	0.743802
chr17	34990000	0.760331
chr17	35006800	0.842975
chr17	35037500	0.867769
chr17	35044100	0.917355
chr17	35088700	0.842975
chr17	35129300	0.677686
chr17	35149400	0.900826
chr17	35170900	0.752066
chr17	35181400	0.735537
chr17	35184500	0.801653
chr17	35194600	0.735537
chr17	35200200	0.818182
chr17	35211200	0.652893
chr17	35228400	0.702479
chr17	35233400	0.760331
chr17	35242600	0.719008
chr17	35246400	0.818182
chr17	35249500	0.661157
chr17	35275200	0.694215
chr17	35279200	0.752066
chr17	35289700	0.694215
chr17	35304400	0.735537
chr17	35425600	0.942149
chr17	35442100	0.768595
chr17	35458800	0.702479
chr17	35461300	0.909091
chr17	35464700	0.694215
chr17	35474700	0.851240
chr17	35488600	0.950413
chr17	35528100	0.785124
chr17	35530700	0.694215
chr17	35571900	0.809917
chr17	35576900	0.867769
chr17	35582700	0.958678
chr17	35652200	0.909091
chr17	35656800	0.975207
chr17	35810600	0.652893
chr17	35855300	0.867769
chr17	35922800	0.710744
chr17	36070900	0.983471
chr17	36184900	0.652893
chr17	36204500	0.834711
chr17	36433300	0.702479
chr17	36481900	0.818182
chr17	36497800	0.694215
chr17	36560500	0.727273
chr17	36578100	0.826446
chr17	36581900	0.760331
chr17	36595600	0.768595
chr17	36597700	0.942149
chr17	36600700	0.735537

chr17	36605900	0.727273
chr17	36610300	0.644628
chr17	36612400	0.917355
chr17	36614600	0.942149
chr17	36617500	0.710744
chr17	36640800	0.793388
chr17	36664900	0.727273
chr17	36668700	0.677686
chr17	36687800	0.661157
chr17	36717900	0.702479
chr17	36725100	0.785124
chr17	36731000	0.818182
chr17	36736500	0.909091
chr17	36741800	0.752066
chr17	36745000	0.859504
chr17	36752900	0.900826
chr17	36756000	0.685950
chr17	36766600	0.966942
chr17	36770300	0.677686
chr17	36773900	0.752066
chr17	36776700	0.694215
chr17	36792700	0.743802
chr17	36811100	0.694215
chr17	36814200	0.752066
chr17	36819800	0.677686
chr17	36864800	0.694215
chr17	36870100	0.925620
chr17	36872600	0.867769
chr17	36900100	0.826446
chr17	36946400	0.743802
chr17	36953700	0.685950
chr17	37031700	0.892562
chr17	37034200	0.892562
chr17	37038200	0.892562
chr17	37040400	0.950413
chr17	37057600	0.702479
chr17	37078900	0.809917
chr17	37082300	0.669421
chr17	37123800	0.917355
chr17	37202300	0.892562
chr17	37204400	0.851240
chr17	37220000	0.793388
chr17	37225800	0.644628
chr17	37237300	0.752066
chr17	37279200	0.652893
chr17	37330400	0.644628
chr17	37333800	0.925620
chr17	37359100	0.685950
chr17	37396300	0.661157
chr17	37720200	0.933884
chr17	37736400	0.884298
chr17	37748500	0.768595
chr17	37753300	0.867769
chr17	37755700	0.685950

chr17	37765300	0.685950
chr17	37781000	0.669421
chr17	37785900	0.702479
chr17	37796100	0.776860
chr17	37799100	0.710744
chr17	37801700	0.851240
chr17	37809100	0.685950
chr17	37814500	0.661157
chr17	37831900	0.677686
chr17	37847100	0.884298
chr17	37852800	0.958678
chr17	37859800	0.801653
chr17	37862600	0.892562
chr17	37880000	0.652893
chr17	37892700	0.702479
chr17	37898300	0.719008
chr17	37907300	0.925620
chr17	37957100	0.809917
chr17	38020700	0.685950
chr17	38080900	0.900826
chr17	38090500	0.719008
chr17	38171900	0.917355
chr17	38175200	0.892562
chr17	38191800	0.785124
chr17	38195700	0.661157
chr17	38222200	0.702479
chr17	38225600	0.743802
chr17	38228500	0.677686
chr17	38232200	0.884298
chr17	38243100	0.735537
chr17	38248600	0.669421
chr17	38259400	0.876033
chr17	38264000	0.669421
chr17	38266600	0.933884
chr17	38270600	0.818182
chr17	38324400	0.785124
chr17	38438400	1.000000
chr17	38452200	0.909091
chr17	38463500	0.867769
chr17	38470900	0.859504
chr17	38493800	0.644628
chr17	38500500	0.743802
chr17	38504600	0.702479
chr17	38507500	0.925620
chr17	38517700	0.661157
chr17	38567300	0.876033
chr17	38576400	0.801653
chr17	38578800	0.652893
chr17	38590100	0.743802
chr17	38602800	0.661157
chr17	38614100	0.694215
chr17	38619000	0.760331
chr17	38621300	0.900826
chr17	38627800	0.719008

chr17	38631900	0.776860
chr17	38637100	0.661157
chr17	38640900	0.785124
chr17	38647200	0.752066
chr17	38656800	0.760331
chr17	38666700	0.710744
chr17	38699400	0.719008
chr17	38716500	0.677686
chr17	38721300	0.685950
chr17	38764200	0.677686
chr17	39010500	0.785124
chr17	39030100	0.859504
chr17	39165700	0.644628
chr17	39222000	0.785124
chr17	39316700	0.727273
chr17	39394700	0.652893
chr17	39653300	0.694215
chr17	39656800	0.859504
chr17	39667100	0.834711
chr17	39678600	0.909091
chr17	39695500	0.644628
chr17	39701100	0.851240
chr17	39705200	0.958678
chr17	39811900	0.884298
chr17	39832800	1.000000
chr17	39848300	0.743802
chr17	39862100	0.719008
chr17	39928600	0.851240
chr17	39932000	0.859504
chr17	39934400	0.867769
chr17	39975900	0.917355
chr17	40017400	0.694215
chr17	40071700	0.851240
chr17	40112000	0.983471
chr17	40162500	0.727273
chr17	40164700	0.991736
chr17	40180300	0.818182
chr17	40190300	0.876033
chr17	40207900	0.867769
chr17	40288300	0.652893
chr17	40346600	0.785124
chr17	40435800	0.652893
chr17	40475300	0.661157
chr17	40480800	0.884298
chr17	40483300	0.809917
chr17	40514000	0.842975
chr17	40519600	0.652893
chr17	40531500	0.966942
chr17	40552100	0.842975
chr17	40554800	0.842975
chr17	40559400	0.801653
chr17	40567200	0.801653
chr17	40581100	0.735537
chr17	40584100	0.719008

chr17	40672100	0.933884
chr17	40679600	0.768595
chr17	40702600	0.685950
chr17	40708800	0.710744
chr17	40820300	0.694215
chr17	40915300	0.925620
chr17	40936000	0.644628
chr17	41030500	0.842975
chr17	41044000	0.925620
chr17	41167900	0.876033
chr17	41179900	0.900826
chr17	41183500	0.719008
chr17	41186300	0.983471
chr17	41188500	0.876033
chr17	41192400	0.933884
chr17	41195500	0.884298
chr17	41231800	0.776860
chr17	41382500	0.909091
chr17	41387500	0.710744
chr17	41415500	0.867769
chr17	41421000	0.809917
chr17	41471600	0.702479
chr17	41516500	0.702479
chr17	41603800	0.818182
chr17	41608600	0.719008
chr17	41614300	0.652893
chr17	41619100	0.900826
chr17	41627300	0.793388
chr17	41631800	0.710744
chr17	41640800	0.710744
chr17	41654300	0.752066
chr17	41656900	0.801653
chr17	41676600	0.694215
chr17	41721800	0.710744
chr17	41734200	0.892562
chr17	41740200	0.710744
chr17	41743600	0.685950
chr17	41747700	0.818182
chr17	41757900	0.768595
chr17	41778800	0.785124
chr17	41823500	0.867769
chr17	41837400	0.834711
chr17	41881700	0.942149
chr17	41924600	0.768595
chr17	41957000	0.801653
chr17	41970400	0.818182
chr17	41980100	0.793388
chr17	41987400	0.644628
chr17	41993300	0.644628
chr17	42013400	0.785124
chr17	42018300	0.661157
chr17	42021200	0.661157
chr17	42162100	0.909091
chr17	42165900	0.661157

chr17	42175300	0.909091
chr17	42186600	0.727273
chr17	42215900	0.917355
chr17	42245500	0.818182
chr17	42248600	0.842975
chr17	42270300	0.661157
chr17	42282000	0.752066
chr17	42288400	0.966942
chr17	42357500	0.851240
chr17	42388200	0.809917
chr17	42390400	0.776860
chr17	42398800	0.710744
chr17	42438500	0.727273
chr17	42453600	0.669421
chr17	42457800	0.652893
chr17	42589200	0.909091
chr17	42611500	0.669421
chr17	42614200	0.710744
chr17	42622100	0.793388
chr17	42625400	0.867769
chr17	42627500	0.702479
chr17	42677200	0.867769
chr17	42686100	0.909091
chr17	42706100	0.793388
chr17	42815700	0.661157
chr17	42846000	0.925620
chr17	42859900	0.851240
chr17	42910400	0.702479
chr17	42920400	0.834711
chr17	42998500	0.834711
chr17	43019000	0.652893
chr17	43040000	0.644628
chr17	43049800	0.694215
chr17	43055300	0.785124
chr17	43081800	0.826446
chr17	43118400	0.942149
chr17	43191800	0.793388
chr17	43205400	0.776860
chr17	43248200	0.685950
chr17	43250500	0.776860
chr17	43275100	0.851240
chr17	43303100	0.752066
chr17	43306500	0.909091
chr17	43308900	0.661157
chr17	43313700	0.917355
chr17	43334300	0.925620
chr17	43419700	0.652893
chr17	43442200	0.785124
chr17	43449200	0.950413
chr17	43451300	0.694215
chr17	43454000	0.727273
chr17	43462700	0.685950
chr17	43466900	0.669421
chr17	43487700	0.694215

chr17	43514900	0.719008
chr17	43517900	0.834711
chr17	43585700	0.834711
chr17	43636800	0.644628
chr17	43653600	0.785124
chr17	43657400	0.735537
chr17	43724200	0.760331
chr17	43781000	0.752066
chr17	43818600	0.677686
chr17	43840400	0.644628
chr17	43865400	0.867769
chr17	43868300	0.776860
chr17	43890800	0.677686
chr17	44020200	0.809917
chr17	44049200	0.669421
chr17	44061600	0.669421
chr17	44071100	0.876033
chr17	44104900	0.892562
chr17	44135400	0.892562
chr17	44179100	0.842975
chr17	44184800	0.925620
chr17	44187200	0.702479
chr17	44196100	0.685950
chr17	44205700	0.809917
chr17	44208200	0.702479
chr17	44216800	0.933884
chr17	44222700	0.785124
chr17	44231800	0.826446
chr17	44235000	0.661157
chr17	44237400	0.661157
chr17	44240800	0.933884
chr17	44250400	0.925620
chr17	44415700	0.735537
chr17	44419700	0.710744
chr17	44485200	0.702479
chr17	44512500	0.809917
chr17	44581400	0.760331
chr17	44632800	0.735537
chr17	44637000	0.727273
chr17	44703100	0.661157
chr17	44735600	0.669421
chr17	44750200	0.801653
chr17	44810600	0.900826
chr17	44909700	0.719008
chr17	44931700	0.876033
chr17	45021100	0.702479
chr17	45058800	0.677686
chr17	45132100	0.743802
chr17	45197900	0.826446
chr17	45214200	0.702479
chr17	45220000	0.685950
chr17	45223400	0.760331
chr17	45249700	0.760331
chr17	45259000	0.652893

chr17	45283000	0.677686
chr17	45298500	0.652893
chr17	45307800	0.801653
chr17	45329600	0.644628
chr17	45353200	0.958678
chr17	45589800	0.727273
chr17	45742700	0.933884
chr17	45765200	0.793388
chr17	45795200	0.785124
chr17	45798800	0.818182
chr17	45820500	0.644628
chr17	45920900	0.859504
chr17	45947800	0.644628
chr17	45952700	0.867769
chr17	45956300	0.809917
chr17	45962800	0.743802
chr17	45979400	0.768595
chr17	46027200	0.785124
chr17	46033300	0.669421
chr17	46036100	0.685950
chr17	46056100	0.685950
chr17	46065200	0.876033
chr17	46068900	0.768595
chr17	46073500	0.909091
chr17	46079800	0.842975
chr17	46082300	0.950413
chr17	46091800	0.768595
chr17	46128900	0.661157
chr17	46234100	0.677686
chr17	46256200	0.652893
chr17	46269700	0.719008
chr17	46297300	0.760331
chr17	46303000	0.785124
chr17	46494400	0.809917
chr17	46549800	0.892562
chr17	46583200	0.677686
chr17	46593900	0.834711
chr17	46598300	0.785124
chr17	46604800	0.702479
chr17	46621400	0.727273
chr17	46624400	0.752066
chr17	46627500	0.809917
chr17	46629600	0.793388
chr17	46632600	0.859504
chr17	46642000	0.776860
chr17	46648600	0.652893
chr17	46659900	0.644628
chr17	46663400	0.892562
chr17	46671600	0.818182
chr17	46675500	0.685950
chr17	46688200	0.661157
chr17	46714300	0.694215
chr17	46719600	0.785124
chr17	46749600	0.652893

chr17	46756100	0.694215
chr17	46806500	0.652893
chr17	46830200	0.719008
chr17	46832700	0.834711
chr17	46868800	0.661157
chr17	46897100	0.669421
chr17	47031900	0.702479
chr17	47038800	0.719008
chr17	47049600	0.867769
chr17	47068500	0.752066
chr17	47084500	0.826446
chr17	47093500	0.785124
chr17	47096300	0.867769
chr17	47099200	0.867769
chr17	47102400	0.669421
chr17	47113300	0.925620
chr17	47130000	0.702479
chr17	47132600	0.867769
chr17	47144800	0.644628
chr17	47164700	0.801653
chr17	47185900	0.900826
chr17	47288000	0.842975
chr17	47449000	0.958678
chr17	47454300	0.652893
chr17	47472700	0.661157
chr17	47480300	0.801653
chr17	47513200	0.768595
chr17	47536900	0.644628
chr17	47563800	0.719008
chr17	47586300	0.694215
chr17	47590200	0.760331
chr17	47592500	0.950413
chr17	47643600	0.727273
chr17	47651400	0.685950
chr17	47711600	0.677686
chr17	47833900	0.710744
chr17	47886900	0.776860
chr17	47891600	0.785124
chr17	47895400	0.909091
chr17	47898800	0.644628
chr17	47925800	0.710744
chr17	47939100	0.677686
chr17	47943000	0.652893
chr17	47953400	0.966942
chr17	47965000	0.752066
chr17	47968100	0.826446
chr17	47976800	0.867769
chr17	47985200	0.702479
chr17	47993500	0.669421
chr17	48012900	0.702479
chr17	48022200	0.859504
chr17	48053400	0.785124
chr17	48055500	0.752066
chr17	48058400	0.892562

chr17	48065700	0.900826
chr17	48074500	0.851240
chr17	48105400	0.818182
chr17	48125300	0.677686
chr17	48178800	0.826446
chr17	48192900	0.694215
chr17	48201500	0.809917
chr17	48224400	0.801653
chr17	48230400	0.669421
chr17	48245400	0.768595
chr17	48255900	0.876033
chr17	48284100	0.661157
chr17	48288300	0.752066
chr17	48291100	0.694215
chr17	48297800	0.785124
chr17	48348100	0.768595
chr17	48354600	0.694215
chr17	48357300	0.752066
chr17	48361700	0.694215
chr17	48381000	0.884298
chr17	48411300	0.867769
chr17	48440700	0.793388
chr17	48443000	0.685950
chr17	48553800	0.859504
chr17	48581300	0.834711
chr17	48642500	0.859504
chr17	48653000	0.760331
chr17	48661000	0.661157
chr17	48670800	0.942149
chr17	48686900	0.776860
chr17	48698600	0.694215
chr17	48701000	0.842975
chr17	48703700	0.685950
chr17	48705800	0.826446
chr17	48739200	0.702479
chr17	48745900	0.685950
chr17	48768300	0.892562
chr17	48800500	0.652893
chr17	48912700	0.710744
chr17	48981000	0.909091
chr17	48983100	0.644628
chr17	48987000	0.859504
chr17	48989700	0.760331
chr17	48992400	0.702479
chr17	48995100	0.719008
chr17	48998000	0.727273
chr17	49027200	0.884298
chr17	49032100	0.950413
chr17	49113700	0.776860
chr17	49135000	0.876033
chr17	49441000	0.743802
chr17	49450200	0.909091
chr17	49452300	0.818182
chr17	49676700	0.685950

chr17	49707800	0.685950
chr17	49762200	0.743802
chr17	49816900	0.727273
chr17	49822900	0.752066
chr17	49987600	0.685950
chr17	49990700	0.818182
chr17	50213800	0.644628
chr17	51111000	0.743802
chr17	51139500	0.702479
chr17	51183600	0.983471
chr17	51220600	0.818182
chr17	51532400	0.867769
chr17	51594300	0.652893
chr17	51685300	0.661157
chr17	51708300	0.826446
chr17	51826600	0.719008
chr17	52250200	0.685950
chr17	52333900	0.785124
chr17	52351100	0.652893
chr17	52438900	0.776860
chr17	52703900	1.000000
chr17	52980800	0.685950
chr17	52986000	0.859504
chr17	52997100	0.743802
chr17	53034700	0.842975
chr17	53167800	0.652893
chr17	53218600	0.685950
chr17	53221000	0.826446
chr17	53413100	0.644628
chr17	53428400	0.892562
chr17	53434300	0.685950
chr17	53544400	0.933884
chr17	53570700	0.702479
chr17	53679800	0.900826
chr17	53785100	0.719008
chr17	53809500	0.776860
chr17	53812100	0.818182
chr17	53872700	0.793388
chr17	54004300	0.685950
chr17	54016900	0.702479
chr17	54047500	0.685950
chr17	54068300	0.768595
chr17	54398300	0.826446
chr17	54655600	0.702479
chr17	54785700	0.661157
chr17	54832500	0.669421
chr17	54858000	0.685950
chr17	54876500	0.719008
chr17	54968000	0.719008
chr17	55004100	0.859504
chr17	55059800	0.685950
chr17	55094600	0.760331
chr17	55131900	0.785124
chr17	55150200	0.991736

chr17	55166100	0.851240
chr17	55168300	0.768595
chr17	55170600	0.677686
chr17	55174300	0.727273
chr17	55177300	0.710744
chr17	55186400	0.842975
chr17	55211000	0.842975
chr17	55214000	0.785124
chr17	55222500	0.851240
chr17	55244500	0.685950
chr17	55338400	0.818182
chr17	55348300	0.661157
chr17	55366500	0.719008
chr17	55372000	0.826446
chr17	55390800	0.909091
chr17	55398700	0.735537
chr17	55412000	0.669421
chr17	55416100	0.809917
chr17	55419400	0.909091
chr17	55434600	0.867769
chr17	55447600	0.669421
chr17	55453500	0.652893
chr17	55457000	0.975207
chr17	55469400	0.983471
chr17	55473900	0.809917
chr17	55484800	0.909091
chr17	55503100	0.644628
chr17	55510200	0.760331
chr17	55518700	0.909091
chr17	55532900	0.727273
chr17	55543900	0.975207
chr17	55547300	0.710744
chr17	55549800	0.867769
chr17	55552200	0.669421
chr17	55555000	0.669421
chr17	55563100	0.669421
chr17	55583800	0.851240
chr17	55589200	0.859504
chr17	55605300	0.958678
chr17	55615800	0.719008
chr17	55623200	0.743802
chr17	55626100	0.743802
chr17	55629200	0.983471
chr17	55632600	0.892562
chr17	55655400	0.809917
chr17	55665900	0.801653
chr17	55682900	0.768595
chr17	55686100	0.909091
chr17	55690000	0.842975
chr17	55714100	0.768595
chr17	55720500	0.884298
chr17	55727600	0.933884
chr17	55740800	0.793388
chr17	55743800	0.760331

chr17	55762200	0.644628
chr17	55785600	0.752066
chr17	55840000	0.942149
chr17	55859600	0.818182
chr17	55880700	0.685950
chr17	55933300	0.694215
chr17	55952100	0.710744
chr17	55956900	0.809917
chr17	55959100	0.900826
chr17	55976600	0.793388
chr17	55989000	0.652893
chr17	55994200	0.925620
chr17	55997500	0.644628
chr17	56004500	0.735537
chr17	56013100	0.966942
chr17	56017700	0.685950
chr17	56026500	0.768595
chr17	56062700	0.727273
chr17	56110700	0.685950
chr17	56124800	0.677686
chr17	56306500	0.801653
chr17	56320000	0.760331
chr17	56357000	0.842975
chr17	56362300	0.760331
chr17	56386000	0.752066
chr17	56406500	0.776860
chr17	56409700	0.727273
chr17	56416500	1.000000
chr17	56442100	0.793388
chr17	56454000	0.859504
chr17	56466600	0.900826
chr17	56476500	0.876033
chr17	56480900	0.892562
chr17	56524400	0.925620
chr17	56643000	0.818182
chr17	56645900	0.644628
chr17	56679700	0.793388
chr17	56736200	0.694215
chr17	56990100	0.933884
chr17	57046600	0.958678
chr17	57407200	0.768595
chr17	57431700	0.975207
chr17	57444100	0.983471
chr17	57448500	0.685950
chr17	57501100	0.727273
chr17	57518500	0.801653
chr17	57861500	0.677686
chr17	57864600	0.884298
chr17	57906600	0.677686
chr17	57923900	0.975207
chr17	57934900	0.818182
chr17	58115200	0.685950
chr17	58165300	0.892562
chr17	58174200	0.644628

chr17	58220700	0.661157
chr17	58223100	0.909091
chr17	58232800	0.694215
chr17	58236400	0.818182
chr17	58239300	0.876033
chr17	58405400	0.710744
chr17	58462100	0.917355
chr17	58655100	0.859504
chr17	58821800	0.818182
chr17	58825100	0.933884
chr17	59143200	0.876033
chr17	59233700	0.669421
chr17	59238400	0.644628
chr17	59331800	0.710744
chr17	59363600	0.776860
chr17	59372400	0.826446
chr17	59386100	0.702479
chr17	59431000	0.809917
chr17	59471600	0.743802
chr17	59479500	0.785124
chr17	59494100	0.743802
chr17	59532600	0.892562
chr17	59535400	0.909091
chr17	59541600	0.826446
chr17	59545500	0.892562
chr17	60539800	0.892562
chr17	60550400	0.652893
chr17	60620500	0.909091
chr17	60695200	0.892562
chr17	60710300	0.685950
chr17	60732000	0.752066
chr17	60747800	0.661157
chr17	60789600	0.685950
chr17	60813300	0.809917
chr17	60817500	0.785124
chr17	60828500	0.925620
chr17	60878300	0.669421
chr17	60902800	0.776860
chr17	60938500	0.752066
chr17	60948000	0.983471
chr17	60970400	0.818182
chr17	60980300	0.685950
chr17	61054000	0.876033
chr17	61119700	0.793388
chr17	61122400	0.719008
chr17	61214400	0.727273
chr17	61229200	0.925620
chr17	61368200	0.768595
chr17	61505600	0.669421
chr17	61621700	0.685950
chr17	61656800	0.851240
chr17	61711600	0.867769
chr17	61725500	0.768595
chr17	61753000	0.644628

chr17	61774900	0.867769
chr17	61916500	0.925620
chr17	61944000	0.661157
chr17	61959000	0.859504
chr17	61988500	0.669421
chr17	62009100	0.776860
chr17	62040100	0.818182
chr17	62098000	0.685950
chr17	62225900	0.793388
chr17	62278200	0.809917
chr17	62294500	0.975207
chr17	62318000	0.719008
chr17	62334800	0.785124
chr17	62498000	0.933884
chr17	62902100	0.842975
chr17	62962200	0.644628
chr17	63054600	0.735537
chr17	63057900	0.694215
chr17	63060800	0.826446
chr17	63069500	0.644628
chr17	63180300	0.983471
chr17	63228200	0.694215
chr17	63240400	0.834711
chr17	63267300	0.685950
chr17	63274600	0.661157
chr17	63287000	0.652893
chr17	63290500	0.776860
chr17	63456000	0.785124
chr17	63491800	0.842975
chr17	63512500	0.776860
chr17	63527200	0.818182
chr17	63535800	0.826446
chr17	63543000	0.892562
chr17	63547200	0.760331
chr17	63550800	0.859504
chr17	63570400	0.818182
chr17	63583600	0.727273
chr17	63590300	0.842975
chr17	63687400	0.752066
chr17	63738900	0.694215
chr17	63786800	0.661157
chr17	64306400	0.669421
chr17	64357000	0.719008
chr17	64362200	0.652893
chr17	64400700	0.876033
chr17	64444700	0.661157
chr17	64446800	0.826446
chr17	64465600	0.652893
chr17	64470500	0.933884
chr17	64472600	0.760331
chr17	64481900	0.719008
chr17	64527300	0.669421
chr17	64529900	0.917355
chr17	64536900	0.867769

chr17	64546100	1.000000
chr17	64562100	0.818182
chr17	64574300	0.760331
chr17	64599400	0.702479
chr17	64708600	0.859504
chr17	64711100	0.884298
chr17	64721100	0.685950
chr17	64726900	0.925620
chr17	64744700	0.669421
chr17	64760800	0.727273
chr17	64771500	0.644628
chr17	64773600	0.925620
chr17	64775700	0.834711
chr17	64779800	0.876033
chr17	64800200	0.809917
chr17	64826100	0.917355
chr17	64941200	0.793388
chr17	64948400	0.983471
chr17	64954400	0.876033
chr17	64957100	0.768595
chr17	65254300	0.685950
chr17	65277300	0.694215
chr17	65350600	0.892562
chr17	65371100	0.710744
chr17	65442100	0.694215
chr17	65471100	0.661157
chr17	65475300	0.685950
chr17	65487100	0.859504
chr17	65489300	0.669421
chr17	65525200	0.826446
chr17	65527900	0.842975
chr17	65574600	0.719008
chr17	65633000	0.652893
chr17	65646800	0.719008
chr17	65654800	0.917355
chr17	65730400	0.909091
chr17	65747700	0.826446
chr17	65749800	0.867769
chr17	65827400	0.826446
chr17	65955800	0.669421
chr17	65972500	0.842975
chr17	66030200	0.942149
chr17	66034700	0.677686
chr17	66180700	0.669421
chr17	66251400	0.818182
chr17	66290700	0.892562
chr17	66293200	0.768595
chr17	66374800	0.752066
chr17	66432900	0.710744
chr17	66511300	0.694215
chr17	66599400	0.685950
chr17	66728100	0.785124
chr17	66731200	0.892562
chr17	66746600	0.793388

chr17	66755800	0.809917
chr17	67317200	0.776860
chr17	67319500	0.661157
chr17	67355200	0.719008
chr17	67409000	0.719008
chr17	67414100	0.768595
chr17	67420100	0.834711
chr17	67422500	0.727273
chr17	67426400	0.661157
chr17	67441800	0.669421
chr17	67444900	1.000000
chr17	67447800	0.958678
chr17	67457900	0.859504
chr17	67463600	0.900826
chr17	67498400	0.851240
chr17	67530500	0.793388
chr17	67570900	0.752066
chr17	67958100	0.876033
chr17	68497800	0.851240
chr17	68535900	0.685950
chr17	68894100	0.776860
chr17	68984800	0.950413
chr17	69000200	0.677686
chr17	69016500	0.760331
chr17	69029900	0.851240
chr17	69283500	0.776860
chr17	69827400	0.842975
chr17	70026700	0.710744
chr17	70104200	0.768595
chr17	70107700	0.752066
chr17	70121600	0.644628
chr17	70147700	0.983471
chr17	70162100	0.834711
chr17	70172800	0.644628
chr17	70179200	0.735537
chr17	70201700	0.652893
chr17	70218900	0.735537
chr17	70233600	0.719008
chr17	70265200	0.785124
chr17	70323100	0.760331
chr17	70332400	0.719008
chr17	70378500	0.925620
chr17	70383200	0.776860
chr17	70390500	0.719008
chr17	70462100	0.785124
chr17	70464700	0.851240
chr17	70471800	0.909091
chr17	70498300	0.900826
chr17	70512600	0.727273
chr17	70515400	0.801653
chr17	70536100	0.826446
chr17	70572600	0.851240
chr17	70584400	0.652893
chr17	70599700	0.661157

chr17	70620100	0.826446
chr17	70663100	0.694215
chr17	70739400	0.900826
chr17	70771700	0.785124
chr17	70897200	0.644628
chr17	70949600	0.909091
chr17	70967300	0.793388
chr17	71010500	0.743802
chr17	71152600	0.809917
chr17	71222900	0.900826
chr17	71225400	0.760331
chr17	71249800	0.685950
chr17	71253000	0.652893
chr17	71258900	0.801653
chr17	71280500	0.809917
chr17	71301800	0.710744
chr17	71304700	0.702479
chr17	71321100	0.818182
chr17	71344800	0.685950
chr17	71355700	0.652893
chr17	71359700	0.702479
chr17	71463200	0.644628
chr17	71473600	0.743802
chr17	71486000	0.661157
chr17	71489300	0.818182
chr17	71512100	0.702479
chr17	71530200	0.743802
chr17	71533300	0.719008
chr17	71538500	0.851240
chr17	71543100	0.793388
chr17	71549400	0.752066
chr17	71556000	0.685950
chr17	71563300	0.991736
chr17	71609100	0.958678
chr17	71611200	0.859504
chr17	71614100	0.900826
chr17	71622900	0.752066
chr17	71643400	0.702479
chr17	71680300	0.859504
chr17	71682400	0.942149
chr17	71687400	0.826446
chr17	71732100	0.669421
chr17	71756300	0.661157
chr17	71783600	0.661157
chr17	71800700	0.909091
chr17	71810900	0.710744
chr17	71820300	0.735537
chr17	71824200	0.826446
chr17	71926600	0.661157
chr17	71953000	0.760331
chr17	71970600	0.661157
chr17	72004900	0.694215
chr17	72115200	0.842975
chr17	72146500	0.760331

chr17	72202500	0.727273
chr17	72242000	0.917355
chr17	72256700	0.776860
chr17	72269100	0.661157
chr17	72328900	0.677686
chr17	72338500	0.644628
chr17	72348300	0.876033
chr17	72350600	0.752066
chr17	72365500	0.652893
chr17	72400500	0.892562
chr17	72406300	0.652893
chr17	72423600	0.743802
chr17	72439700	0.851240
chr17	72448300	0.752066
chr17	72451900	0.859504
chr17	72595400	0.652893
chr17	72631800	0.867769
chr17	72667400	0.677686
chr17	72674200	0.842975
chr17	72742600	0.933884
chr17	72748100	0.752066
chr17	72754200	0.867769
chr17	72757000	0.834711
chr17	72836600	0.661157
chr17	72861600	0.760331
chr17	72879500	0.644628
chr17	72885500	0.644628
chr17	72900700	0.669421
chr17	72917600	0.876033
chr17	72964800	0.710744
chr17	73032800	0.735537
chr17	73038300	0.892562
chr17	73040700	0.950413
chr17	73079200	0.760331
chr17	73088000	0.933884
chr17	73122000	0.694215
chr17	73236400	0.859504
chr17	73264200	0.702479
chr17	73322200	0.966942
chr17	73338400	0.842975
chr17	73343400	0.851240
chr17	73409000	0.859504
chr17	73475000	0.867769
chr17	73477900	0.793388
chr17	73504700	0.710744
chr17	73527600	0.735537
chr17	73541500	0.661157
chr17	73554200	0.743802
chr17	73616400	0.884298
chr17	73630100	0.727273
chr17	73639400	0.834711
chr17	73690300	0.785124
chr17	73771100	0.776860
chr17	73824100	0.909091

chr17	73829200	0.818182
chr17	73841100	0.818182
chr17	73861300	0.710744
chr17	73956300	0.884298
chr17	73980500	0.694215
chr17	74010900	0.652893
chr17	74023600	0.768595
chr17	74026100	0.818182
chr17	74033400	0.760331
chr17	74036700	0.644628
chr17	74102500	0.851240
chr17	74118300	0.776860
chr17	74121600	0.677686
chr17	74126900	0.752066
chr17	74131700	0.809917
chr17	74141300	0.760331
chr17	74160800	0.793388
chr17	74210300	0.867769
chr17	74268400	0.942149
chr17	74376000	0.661157
chr17	74382800	0.884298
chr17	74386000	0.958678
chr17	74410600	0.710744
chr17	74417000	0.892562
chr17	74427800	0.661157
chr17	74435000	0.694215
chr17	74437500	0.892562
chr17	74444600	0.785124
chr17	74489300	0.925620
chr17	74494500	0.867769
chr17	74502800	0.776860
chr17	74519900	0.727273
chr17	74524300	0.760331
chr17	74540400	0.743802
chr17	74543300	0.826446
chr17	74563500	0.958678
chr17	74565900	0.818182
chr17	74577400	0.661157
chr17	74609500	0.677686
chr17	74680100	0.801653
chr17	74693900	0.867769
chr17	74697600	0.710744
chr17	74704500	0.661157
chr17	74841000	0.652893
chr17	74881700	0.735537
chr17	74937000	0.694215
chr17	74939400	0.760331
chr17	74953900	0.851240
chr17	74963200	0.677686
chr17	74968700	0.818182
chr17	74979300	0.694215
chr17	74991700	0.942149
chr17	74998800	0.661157
chr17	75052200	0.685950

chr17	75094300	0.743802
chr17	75106400	0.809917
chr17	75144000	0.776860
chr17	75161300	0.785124
chr17	75171400	0.752066
chr17	75251800	0.826446
chr17	75279600	0.876033
chr17	75283000	0.983471
chr17	75285100	0.710744
chr17	75312700	0.702479
chr17	75319400	0.809917
chr17	75347700	0.900826
chr17	75372700	0.735537
chr17	75379200	0.983471
chr17	75383200	0.719008
chr17	75387900	0.958678
chr17	75390600	0.842975
chr17	75396500	0.743802
chr17	75405600	0.975207
chr17	75417400	0.991736
chr17	75420800	0.760331
chr17	75426100	0.661157
chr17	75432600	0.975207
chr17	75435200	0.752066
chr17	75445700	0.884298
chr17	75450300	0.743802
chr17	75457400	0.842975
chr17	75461700	0.826446
chr17	75463800	0.867769
chr17	75466700	0.876033
chr17	75468900	0.983471
chr17	75472000	0.917355
chr17	75476800	0.826446
chr17	75500700	0.710744
chr17	75537500	0.892562
chr17	75545100	0.702479
chr17	75633500	0.818182
chr17	75651200	0.793388
chr17	75681500	0.719008
chr17	75705900	0.776860
chr17	75731300	0.661157
chr17	75738900	0.900826
chr17	75759900	0.917355
chr17	75769300	0.735537
chr17	75776500	0.859504
chr17	75778700	0.727273
chr17	75782400	0.859504
chr17	75786600	0.892562
chr17	75799200	0.809917
chr17	75824200	0.719008
chr17	75827500	0.727273
chr17	75861300	0.785124
chr17	75920100	0.768595
chr17	75925300	0.851240

chr17	75933000	0.694215
chr17	75958400	0.776860
chr17	75999900	0.710744
chr17	76015700	0.768595
chr17	76130600	0.694215
chr17	76215700	0.867769
chr17	76246900	0.702479
chr17	76254400	0.785124
chr17	76269300	0.851240
chr17	76291100	0.685950
chr17	76311900	0.685950
chr17	76314200	0.644628
chr17	76321400	0.785124
chr17	76339800	0.826446
chr17	76350500	0.702479
chr17	76352600	0.793388
chr17	76358900	0.818182
chr17	76362000	0.884298
chr17	76364100	0.826446
chr17	76366300	0.966942
chr17	76369600	0.933884
chr17	76377700	0.809917
chr17	76381000	0.694215
chr17	76384900	0.685950
chr17	76409900	0.677686
chr17	76418200	0.669421
chr17	76431200	0.760331
chr17	76494900	0.685950
chr17	76572000	0.644628
chr17	76593500	0.727273
chr17	76713800	0.876033
chr17	76721400	0.743802
chr17	76771300	0.669421
chr17	76880100	0.917355
chr17	76893300	0.644628
chr17	76912600	0.719008
chr17	76917000	0.826446
chr17	76930600	0.768595
chr17	76948700	0.644628
chr17	76976400	0.785124
chr17	77003200	0.644628
chr17	77094000	0.694215
chr17	77111000	0.727273
chr17	77146900	0.685950
chr17	77149100	0.801653
chr17	77184800	0.768595
chr17	77189600	0.925620
chr17	77198100	0.694215
chr17	77208400	0.900826
chr17	77211300	0.735537
chr17	77215200	0.677686
chr17	77266000	0.710744
chr17	77283000	0.652893
chr17	77339100	0.710744

chr17	77384500	0.727273
chr17	77404300	0.752066
chr17	77482100	0.661157
chr17	77632800	0.768595
chr17	77639100	0.719008
chr17	77665700	0.826446
chr17	77669800	0.661157
chr17	77673500	0.652893
chr17	77682200	0.851240
chr17	77702600	0.702479
chr17	77760400	0.809917
chr17	77776300	0.752066
chr17	77780600	0.851240
chr17	77815700	0.719008
chr17	77836700	0.727273
chr17	77890000	0.702479
chr17	77899700	0.752066
chr17	77906400	0.702479
chr17	77911900	0.661157
chr17	77930600	0.859504
chr17	77940700	0.702479
chr17	77949600	0.702479
chr17	77962800	0.842975
chr17	77980000	0.785124
chr17	77987300	0.669421
chr17	78004500	0.727273
chr17	78039700	0.942149
chr17	78086900	0.933884
chr17	78098500	0.867769
chr17	78126200	0.809917
chr17	78134600	0.719008
chr17	78149500	0.760331
chr17	78179200	0.785124
chr17	78199600	0.785124
chr17	78231400	0.752066
chr17	78237600	0.801653
chr17	78349000	0.710744
chr17	78418300	0.842975
chr17	78455900	0.694215
chr17	78461000	0.727273
chr17	78467800	0.702479
chr17	78495200	0.743802
chr17	78500600	0.727273
chr17	78606000	0.727273
chr17	78624400	0.743802
chr17	78638100	0.644628
chr17	78653400	0.842975
chr17	78668500	0.876033
chr17	78672100	0.743802
chr17	78680400	0.842975
chr17	78698800	0.735537
chr17	78725900	0.876033
chr17	78755000	0.652893
chr17	78760400	0.644628

chr17	78801900	0.933884
chr17	78820000	0.834711
chr17	78833800	0.909091
chr17	78847700	0.917355
chr17	78851400	0.677686
chr17	78901100	0.652893
chr17	79038700	0.900826
chr17	79065000	0.950413
chr17	79068800	0.900826
chr17	79071500	0.727273
chr17	79096400	0.793388
chr17	79105800	0.785124
chr17	79292000	0.818182
chr17	79303300	0.793388
chr17	79305600	0.867769
chr17	79310900	0.710744
chr17	79313600	0.719008
chr17	79380000	0.694215
chr17	79393400	0.702479
chr17	79407900	0.644628
chr17	79424200	0.925620
chr17	79459300	0.793388
chr17	79490700	0.669421
chr17	79615300	0.768595
chr17	79620700	0.760331
chr17	79635600	0.702479
chr17	79688300	0.719008
chr17	79699900	0.809917
chr17	79783700	0.760331
chr17	79825800	0.966942
chr17	79879700	0.735537
chr17	79882700	0.661157
chr17	79899800	0.785124
chr17	79925200	0.818182
chr17	79949000	0.834711
chr17	79952400	0.644628
chr17	80001800	0.900826
chr17	80059100	0.818182
chr17	80061600	0.892562
chr17	80164100	0.661157
chr17	80194700	0.743802
chr17	80226900	0.768595
chr17	80274000	0.776860
chr17	80285900	0.826446
chr17	80293300	0.818182
chr17	80297500	0.752066
chr17	80314900	0.719008
chr17	80398100	0.983471
chr17	80400200	0.851240
chr17	80480600	0.785124
chr17	80486600	0.793388
chr17	80494800	0.909091
chr17	80511300	0.710744
chr17	80524900	0.809917

chr17	80551500	0.851240
chr17	80626700	0.685950
chr17	80713300	0.876033
chr17	80733300	0.710744
chr17	80798400	0.702479
chr17	80817800	0.809917
chr17	80821700	0.876033
chr17	80834000	0.975207
chr17	80847700	0.991736
chr17	80944000	0.760331
chr17	80968600	0.876033
chr17	81041000	0.950413
chr17	81045100	0.876033
chr17	81057400	0.776860
chr18	304900	0.859504
chr18	307100	0.694215
chr18	362900	0.644628
chr18	366800	0.966942
chr18	377600	0.669421
chr18	475900	0.768595
chr18	573100	0.694215
chr18	575400	0.900826
chr18	635200	0.719008
chr18	687000	0.752066
chr18	839800	0.669421
chr18	939800	0.652893
chr18	986000	0.975207
chr18	1015000	0.677686
chr18	1018100	0.793388
chr18	1297100	0.661157
chr18	1515100	0.719008
chr18	2002600	0.851240
chr18	2105300	0.644628
chr18	2653500	0.719008
chr18	2842400	0.958678
chr18	2898700	0.669421
chr18	2916800	0.842975
chr18	2933100	0.818182
chr18	2998600	0.785124
chr18	3266800	0.652893
chr18	3279300	0.752066
chr18	3305900	0.710744
chr18	3324000	0.735537
chr18	3330800	0.685950
chr18	3333500	0.818182
chr18	3456900	0.743802
chr18	3459000	0.859504
chr18	3467000	0.917355
chr18	3469100	0.669421
chr18	3482100	0.818182
chr18	3494200	0.652893
chr18	3521000	0.917355
chr18	3526700	0.867769
chr18	3552800	0.735537

chr18	3556400	0.719008
chr18	3596300	0.826446
chr18	3602100	0.743802
chr18	3607900	0.644628
chr18	3616000	0.752066
chr18	3624600	0.942149
chr18	3649300	0.644628
chr18	3665800	0.719008
chr18	3773700	0.760331
chr18	3805100	0.785124
chr18	3822300	0.892562
chr18	3832800	0.768595
chr18	3868400	0.669421
chr18	3874400	0.644628
chr18	3965200	0.677686
chr18	3973800	0.677686
chr18	4085400	0.892562
chr18	4093800	0.685950
chr18	4106900	0.884298
chr18	4118200	0.909091
chr18	4121100	0.768595
chr18	4123200	0.776860
chr18	4126400	1.000000
chr18	4135300	0.719008
chr18	4144600	0.694215
chr18	4207500	0.719008
chr18	4254900	0.644628
chr18	4284300	0.685950
chr18	4328500	0.702479
chr18	4345800	0.644628
chr18	4354200	0.834711
chr18	4359100	0.867769
chr18	4407000	0.983471
chr18	4441600	0.644628
chr18	4854800	0.809917
chr18	4869300	0.801653
chr18	4988100	0.685950
chr18	5038900	0.652893
chr18	5104600	0.694215
chr18	5152700	0.834711
chr18	5335200	0.768595
chr18	5415100	0.760331
chr18	5457200	0.834711
chr18	5477800	0.685950
chr18	5512900	0.661157
chr18	5578400	0.677686
chr18	5582700	0.851240
chr18	5625400	0.793388
chr18	5679200	0.801653
chr18	5824600	0.727273
chr18	5885400	0.710744
chr18	5970800	0.942149
chr18	6023500	0.727273
chr18	6120200	0.644628

chr18	6272400	0.793388
chr18	6358000	0.909091
chr18	6482900	0.743802
chr18	6488200	0.975207
chr18	6502600	0.743802
chr18	6557900	0.867769
chr18	6567100	0.743802
chr18	6598900	0.644628
chr18	6607300	0.809917
chr18	6624800	0.760331
chr18	6661800	0.735537
chr18	6732000	0.826446
chr18	6752500	0.958678
chr18	6758800	0.669421
chr18	6775400	0.685950
chr18	6781400	0.867769
chr18	6785100	0.677686
chr18	6861200	0.917355
chr18	6880000	0.818182
chr18	6920500	0.685950
chr18	7207400	0.859504
chr18	7273100	0.661157
chr18	7381900	0.661157
chr18	7515400	0.983471
chr18	7524500	0.752066
chr18	7577600	0.793388
chr18	7604100	0.851240
chr18	7647000	0.735537
chr18	7666800	0.876033
chr18	7683600	0.669421
chr18	7723900	0.661157
chr18	7741300	0.652893
chr18	7750000	0.752066
chr18	7790600	0.809917
chr18	7823200	0.760331
chr18	7898600	0.727273
chr18	8008700	0.685950
chr18	8029600	0.826446
chr18	8066100	0.785124
chr18	8084700	0.867769
chr18	8122400	0.685950
chr18	8217300	0.933884
chr18	8253000	0.818182
chr18	8261300	0.669421
chr18	8313000	0.702479
chr18	8362400	0.694215
chr18	8425300	0.710744
chr18	8615900	0.867769
chr18	8619400	0.768595
chr18	8621500	0.801653
chr18	8623700	0.859504
chr18	8626000	0.818182
chr18	8629400	0.851240
chr18	8662200	0.884298

chr18	8709500	0.719008
chr18	8717600	0.785124
chr18	8724600	0.719008
chr18	8733100	0.867769
chr18	8778600	0.809917
chr18	8796500	0.785124
chr18	8801100	0.876033
chr18	8891100	0.760331
chr18	8912800	0.801653
chr18	8931800	0.776860
chr18	8937300	0.876033
chr18	8939400	0.661157
chr18	8945600	0.685950
chr18	8949400	0.669421
chr18	8956800	0.702479
chr18	8959900	0.785124
chr18	8981000	0.818182
chr18	9017500	0.826446
chr18	9022800	0.867769
chr18	9073900	0.702479
chr18	9096900	0.925620
chr18	9107300	0.776860
chr18	9109700	0.685950
chr18	9201400	0.900826
chr18	9243500	0.834711
chr18	9366300	0.694215
chr18	9381000	0.785124
chr18	9417500	0.652893
chr18	9434800	0.884298
chr18	9494900	0.983471
chr18	9497000	0.958678
chr18	9552100	0.809917
chr18	9575000	0.859504
chr18	9609600	0.677686
chr18	9612500	0.661157
chr18	9643900	0.983471
chr18	9688600	0.818182
chr18	9738000	0.677686
chr18	9742000	0.669421
chr18	9744100	0.933884
chr18	9804100	0.950413
chr18	9808800	1.000000
chr18	9835400	0.818182
chr18	9837600	0.776860
chr18	9839700	1.000000
chr18	9870500	0.719008
chr18	9900800	0.661157
chr18	9929200	0.677686
chr18	9960400	0.685950
chr18	10033000	0.826446
chr18	10068200	0.694215
chr18	10070500	0.776860
chr18	10101600	0.859504
chr18	10203000	0.685950

chr18	10318300	0.876033
chr18	10406600	0.785124
chr18	10477900	0.909091
chr18	10799100	0.809917
chr18	10860700	0.677686
chr18	10922500	0.768595
chr18	11088100	0.743802
chr18	11163500	0.719008
chr18	11667000	0.644628
chr18	11714500	0.900826
chr18	11743300	0.661157
chr18	11761700	0.702479
chr18	11766600	0.925620
chr18	11785900	0.900826
chr18	11791800	0.661157
chr18	11807600	0.925620
chr18	11810600	0.859504
chr18	11826800	0.735537
chr18	11829700	0.842975
chr18	11857400	0.983471
chr18	11886600	0.644628
chr18	11902700	0.801653
chr18	11952100	0.685950
chr18	11986500	0.801653
chr18	11995400	0.917355
chr18	12027100	0.834711
chr18	12034200	0.818182
chr18	12247400	0.677686
chr18	12279100	0.876033
chr18	12336900	0.991736
chr18	12480600	0.702479
chr18	12610600	0.793388
chr18	12646100	0.644628
chr18	12648200	0.776860
chr18	12652600	0.644628
chr18	12862900	0.809917
chr18	12880500	0.867769
chr18	13181800	0.685950
chr18	13239800	0.702479
chr18	13256900	0.818182
chr18	13268600	0.768595
chr18	13415000	0.661157
chr18	13447700	0.661157
chr18	13452400	0.909091
chr18	13487400	0.652893
chr18	13501000	0.760331
chr18	13567500	0.752066
chr18	13611600	0.669421
chr18	13630500	0.644628
chr18	13654400	0.719008
chr18	13713300	0.851240
chr18	13775700	0.776860
chr18	13809100	0.933884
chr18	13812400	0.719008

chr18	13932400	0.760331
chr18	14026000	0.958678
chr18	14127500	0.727273
chr18	18520400	0.644628
chr18	18697800	0.900826
chr18	18842700	0.768595
chr18	18907300	0.752066
chr18	18910600	0.661157
chr18	18923100	0.801653
chr18	18947000	0.876033
chr18	19315700	0.876033
chr18	19327600	0.685950
chr18	19514700	0.760331
chr18	19571800	0.842975
chr18	19752700	0.768595
chr18	19911300	0.710744
chr18	20050100	0.644628
chr18	20272300	0.652893
chr18	20291300	0.809917
chr18	20419300	0.752066
chr18	20494900	0.942149
chr18	20634300	0.652893
chr18	20674700	0.760331
chr18	20711300	0.785124
chr18	20721800	0.809917
chr18	20724600	0.760331
chr18	20736100	0.669421
chr18	20749400	1.000000
chr18	20769600	0.917355
chr18	20776700	0.876033
chr18	20812200	0.710744
chr18	20816000	0.859504
chr18	20824500	0.801653
chr18	20840200	0.900826
chr18	20862200	0.727273
chr18	20865500	0.942149
chr18	20895200	0.768595
chr18	20911500	0.644628
chr18	20953800	0.702479
chr18	20979600	0.801653
chr18	20981900	0.826446
chr18	21228400	0.801653
chr18	21393300	0.793388
chr18	21401500	0.735537
chr18	21424800	0.685950
chr18	21442100	0.933884
chr18	21453200	0.694215
chr18	21562300	0.644628
chr18	21602700	0.669421
chr18	21636000	0.809917
chr18	21716900	0.694215
chr18	21812800	0.809917
chr18	21846200	0.851240
chr18	21958200	0.768595

chr18	21960400	0.991736
chr18	21975000	0.768595
chr18	21980300	0.809917
chr18	22140800	0.760331
chr18	22165000	0.925620
chr18	22212400	0.834711
chr18	22372400	0.859504
chr18	22398600	0.809917
chr18	22407000	0.834711
chr18	22410000	0.851240
chr18	22442200	0.925620
chr18	22504400	0.727273
chr18	22511500	0.842975
chr18	22530300	0.727273
chr18	22559700	0.925620
chr18	22563800	0.842975
chr18	22568400	0.685950
chr18	22628000	0.727273
chr18	22654200	0.727273
chr18	22691600	0.727273
chr18	22772500	0.801653
chr18	22847100	0.743802
chr18	22862700	0.933884
chr18	22905600	0.652893
chr18	23256000	1.000000
chr18	23259500	0.793388
chr18	23314600	0.892562
chr18	23323800	0.743802
chr18	23572800	0.644628
chr18	23615800	0.842975
chr18	23813900	0.842975
chr18	23842400	0.735537
chr18	23995700	0.909091
chr18	24037700	0.776860
chr18	24059400	0.851240
chr18	24062600	0.661157
chr18	24096200	0.884298
chr18	24106500	0.950413
chr18	24112400	0.884298
chr18	24155600	0.793388
chr18	24194800	0.719008
chr18	24265900	0.876033
chr18	24290300	0.685950
chr18	24297400	0.760331
chr18	24326300	0.661157
chr18	24445500	0.842975
chr18	24537400	0.859504
chr18	24704500	0.942149
chr18	24710300	0.727273
chr18	24756100	0.991736
chr18	24794200	0.685950
chr18	24804200	0.760331
chr18	24808100	0.834711
chr18	24882100	0.661157

chr18	25027700	0.677686
chr18	25122800	0.661157
chr18	25142600	0.900826
chr18	25158000	0.685950
chr18	25185100	0.752066
chr18	25195500	0.652893
chr18	25242700	0.685950
chr18	25246000	0.884298
chr18	25293900	0.818182
chr18	25297600	0.760331
chr18	25304100	0.669421
chr18	25342100	0.685950
chr18	25359400	0.694215
chr18	25405900	0.710744
chr18	25545000	0.760331
chr18	25625900	0.752066
chr18	25643700	0.768595
chr18	25654000	0.669421
chr18	25667700	0.685950
chr18	25690200	0.677686
chr18	25731500	0.793388
chr18	25735400	0.975207
chr18	25760200	0.644628
chr18	25931200	0.694215
chr18	26272300	0.702479
chr18	26294500	0.661157
chr18	26408600	0.760331
chr18	26412100	0.851240
chr18	26585000	0.818182
chr18	28203500	0.661157
chr18	28212400	0.917355
chr18	28344000	0.785124
chr18	28675600	0.652893
chr18	28806100	0.867769
chr18	28824800	0.685950
chr18	28862000	0.702479
chr18	28881400	0.834711
chr18	29081000	0.727273
chr18	29093800	0.661157
chr18	29096900	0.661157
chr18	29129700	0.851240
chr18	29227600	0.752066
chr18	29253000	0.942149
chr18	29258300	0.851240
chr18	29261000	0.661157
chr18	29267500	0.752066
chr18	29391500	0.652893
chr18	29402200	0.644628
chr18	29406700	0.842975
chr18	29531000	0.669421
chr18	29557600	0.851240
chr18	29573600	0.727273
chr18	29602100	0.685950
chr18	29822600	0.669421

chr18	29852000	0.719008
chr18	30129100	0.950413
chr18	30131700	0.727273
chr18	30335200	0.925620
chr18	30341600	0.834711
chr18	30428000	0.661157
chr18	30470500	0.735537
chr18	31162100	0.661157
chr18	31176400	0.652893
chr18	31224900	0.644628
chr18	31227700	0.925620
chr18	31417500	0.884298
chr18	31697800	0.727273
chr18	32068700	0.661157
chr18	32129100	0.892562
chr18	32163800	0.752066
chr18	32173300	0.826446
chr18	32212200	0.991736
chr18	32220700	0.809917
chr18	32238700	0.867769
chr18	32277600	0.785124
chr18	32288400	0.727273
chr18	32387700	0.909091
chr18	32656400	0.677686
chr18	32703200	0.809917
chr18	32724300	0.719008
chr18	32771400	0.760331
chr18	32795800	0.710744
chr18	32935800	0.644628
chr18	32966800	0.785124
chr18	33039100	0.785124
chr18	33068500	0.917355
chr18	33071200	0.975207
chr18	33095900	0.669421
chr18	33169200	0.677686
chr18	33171900	0.727273
chr18	33199200	0.644628
chr18	33242200	0.652893
chr18	33334300	0.760331
chr18	33336900	0.743802
chr18	33350900	0.752066
chr18	33398200	0.652893
chr18	33406700	0.834711
chr18	33408900	0.900826
chr18	33432800	0.950413
chr18	33464500	0.867769
chr18	33492900	0.685950
chr18	33504000	0.785124
chr18	33519800	0.760331
chr18	33712700	0.661157
chr18	33769600	0.652893
chr18	33883400	0.661157
chr18	33889600	0.801653
chr18	33910200	0.776860

chr18	33912600	0.710744
chr18	33931600	0.892562
chr18	33992000	0.677686
chr18	34029200	0.809917
chr18	34041400	0.793388
chr18	34051100	0.644628
chr18	34060700	0.909091
chr18	34074100	0.909091
chr18	34108200	0.694215
chr18	34140000	0.661157
chr18	34147700	0.867769
chr18	34163600	0.975207
chr18	34214900	0.909091
chr18	34253500	0.652893
chr18	34467800	0.785124
chr18	34526900	0.917355
chr18	34530200	0.743802
chr18	34756500	0.892562
chr18	34915300	0.685950
chr18	34948700	0.785124
chr18	34964400	0.809917
chr18	34989900	0.677686
chr18	35002200	0.859504
chr18	35010400	0.652893
chr18	35030800	0.925620
chr18	35067600	0.727273
chr18	35070400	0.876033
chr18	35106700	0.793388
chr18	35114500	0.652893
chr18	35118700	0.719008
chr18	35151400	0.752066
chr18	35159300	0.685950
chr18	35166000	0.760331
chr18	35200200	0.661157
chr18	35220700	0.710744
chr18	35278900	0.752066
chr18	36001600	0.694215
chr18	36132400	0.983471
chr18	36278300	0.851240
chr18	36382100	0.727273
chr18	36386100	0.735537
chr18	36512200	0.694215
chr18	36542400	0.694215
chr18	36545100	0.752066
chr18	36749200	0.719008
chr18	36870600	0.652893
chr18	36940300	0.900826
chr18	36961000	0.983471
chr18	36970200	0.743802
chr18	37172800	0.884298
chr18	37187200	0.793388
chr18	37227800	0.669421
chr18	37265600	0.785124
chr18	37273200	0.685950

chr18	37312700	0.719008
chr18	37364400	0.752066
chr18	37405000	0.661157
chr18	37526900	0.644628
chr18	37550200	0.677686
chr18	37598300	0.644628
chr18	37601600	0.710744
chr18	38157100	0.727273
chr18	38478200	0.818182
chr18	38482900	0.661157
chr18	38892900	0.942149
chr18	38952800	0.834711
chr18	39045100	0.867769
chr18	39324600	0.818182
chr18	39688100	0.644628
chr18	39879300	0.685950
chr18	39919400	0.933884
chr18	40323000	0.834711
chr18	40402000	0.652893
chr18	40615100	0.925620
chr18	40686900	0.694215
chr18	41208100	0.644628
chr18	41286400	0.685950
chr18	41427500	0.826446
chr18	41622200	0.793388
chr18	41629200	0.809917
chr18	42133000	0.768595
chr18	42277400	0.876033
chr18	42281000	0.702479
chr18	42284000	0.727273
chr18	42292500	0.785124
chr18	42296400	0.768595
chr18	42304700	0.826446
chr18	42313200	0.743802
chr18	42383300	0.760331
chr18	42419400	0.735537
chr18	42447700	0.768595
chr18	42523800	0.743802
chr18	42818200	0.652893
chr18	42996200	0.884298
chr18	43147800	0.818182
chr18	43157200	0.652893
chr18	43170000	0.942149
chr18	43207200	0.727273
chr18	43233500	0.760331
chr18	43242400	0.851240
chr18	43266600	0.867769
chr18	43271600	0.719008
chr18	43320700	0.851240
chr18	43337300	0.768595
chr18	43340700	0.735537
chr18	43363400	0.661157
chr18	43387200	0.661157
chr18	43558400	0.727273

chr18	43733200	0.909091
chr18	43762800	0.644628
chr18	43830700	0.793388
chr18	43845200	0.917355
chr18	43858600	0.785124
chr18	43874200	0.842975
chr18	43902400	0.793388
chr18	43917900	0.719008
chr18	43924000	0.859504
chr18	43940700	0.644628
chr18	43994500	0.760331
chr18	44005100	0.809917
chr18	44013200	0.842975
chr18	44028500	0.917355
chr18	44038800	0.785124
chr18	44042700	0.677686
chr18	44046500	0.876033
chr18	44049500	0.669421
chr18	44065300	0.735537
chr18	44126600	0.768595
chr18	44143500	0.809917
chr18	44273100	0.677686
chr18	44348200	0.752066
chr18	44353200	0.743802
chr18	44447600	0.776860
chr18	44457800	0.842975
chr18	44479600	0.826446
chr18	44494400	0.661157
chr18	44545000	0.710744
chr18	44547900	0.793388
chr18	44550800	0.876033
chr18	44553300	0.677686
chr18	44556800	0.776860
chr18	44602100	0.900826
chr18	44635400	0.743802
chr18	44640800	0.661157
chr18	44687700	0.652893
chr18	44775500	0.760331
chr18	44780500	0.710744
chr18	44931600	0.727273
chr18	44945200	0.661157
chr18	45084600	0.826446
chr18	45087600	0.892562
chr18	45121600	0.735537
chr18	45342300	0.710744
chr18	45344800	0.975207
chr18	45379800	1.000000
chr18	45425700	0.909091
chr18	45430400	0.652893
chr18	45442300	0.652893
chr18	45445100	0.702479
chr18	45449600	0.694215
chr18	45453000	0.694215
chr18	45494900	0.958678

chr18	45499500	0.809917
chr18	45501900	0.818182
chr18	45505900	0.776860
chr18	45523100	0.669421
chr18	45557700	0.809917
chr18	45586300	0.727273
chr18	45606900	0.685950
chr18	45627700	0.677686
chr18	45636800	0.735537
chr18	45656500	0.685950
chr18	45665400	0.677686
chr18	45708100	0.727273
chr18	45821700	0.884298
chr18	45827400	0.785124
chr18	45841300	0.933884
chr18	45883700	0.826446
chr18	45973400	0.694215
chr18	46069900	0.842975
chr18	46072300	0.867769
chr18	46107300	0.677686
chr18	46140100	0.702479
chr18	46149800	0.743802
chr18	46354400	0.900826
chr18	46356600	0.884298
chr18	46369200	0.760331
chr18	46377500	0.834711
chr18	46386900	0.867769
chr18	46405500	0.669421
chr18	46441500	0.735537
chr18	46450900	0.727273
chr18	46455000	0.867769
chr18	46459300	0.867769
chr18	46461700	0.694215
chr18	46466100	0.925620
chr18	46468700	0.776860
chr18	46471100	0.685950
chr18	46481800	0.644628
chr18	46486600	0.933884
chr18	46495300	0.851240
chr18	46524800	0.859504
chr18	46543400	0.644628
chr18	46548100	0.727273
chr18	46572100	0.743802
chr18	46574500	0.801653
chr18	46639200	0.842975
chr18	46785600	0.669421
chr18	46846400	0.752066
chr18	46907500	0.859504
chr18	46992100	0.942149
chr18	47026100	0.776860
chr18	47037200	0.735537
chr18	47042600	0.669421
chr18	47045800	0.685950
chr18	47139800	0.933884

chr18	47195700	0.809917
chr18	47224700	0.677686
chr18	47252700	0.876033
chr18	47257600	0.768595
chr18	47373200	0.801653
chr18	47376700	0.661157
chr18	47385100	0.685950
chr18	47416400	0.644628
chr18	47438400	0.694215
chr18	47515900	0.785124
chr18	47617200	0.661157
chr18	47654800	0.702479
chr18	47689900	0.644628
chr18	47710400	0.685950
chr18	47928800	0.669421
chr18	47932600	0.975207
chr18	47985000	0.768595
chr18	48101400	0.776860
chr18	48223400	0.768595
chr18	48227200	0.702479
chr18	48232700	0.719008
chr18	48275500	0.867769
chr18	48311400	0.743802
chr18	48344200	0.644628
chr18	48518800	0.950413
chr18	48541400	0.677686
chr18	48544200	0.900826
chr18	48564800	0.809917
chr18	48691600	0.950413
chr18	48694800	0.950413
chr18	48699400	0.652893
chr18	48713700	0.669421
chr18	48719200	0.669421
chr18	48803900	0.768595
chr18	48848200	0.768595
chr18	48877200	0.652893
chr18	48929400	0.834711
chr18	48944600	0.884298
chr18	48947800	0.925620
chr18	48974200	0.727273
chr18	49001000	0.669421
chr18	49149000	0.818182
chr18	49181900	0.867769
chr18	49238700	0.834711
chr18	49587700	0.892562
chr18	49814000	0.776860
chr18	49850400	0.644628
chr18	50780800	0.694215
chr18	50943400	0.760331
chr18	50985600	0.785124
chr18	51377400	0.719008
chr18	51613200	0.735537
chr18	51650500	0.652893
chr18	51714400	0.669421

chr18	51773500	0.933884
chr18	51781000	0.694215
chr18	51784600	0.702479
chr18	51909600	0.652893
chr18	51915500	0.991736
chr18	52055400	0.752066
chr18	52275100	0.933884
chr18	52777400	0.752066
chr18	52882400	0.867769
chr18	52919300	0.876033
chr18	52964700	0.661157
chr18	52968100	0.958678
chr18	53014600	0.661157
chr18	53017300	0.884298
chr18	53019400	0.818182
chr18	53022000	0.685950
chr18	53069400	0.867769
chr18	53072900	0.669421
chr18	53077800	0.760331
chr18	53082800	0.661157
chr18	53087200	0.933884
chr18	53093200	0.735537
chr18	53107200	1.000000
chr18	53135000	0.661157
chr18	53140600	0.776860
chr18	53156200	0.983471
chr18	53159100	0.669421
chr18	53177900	0.958678
chr18	53197500	0.818182
chr18	53215700	0.776860
chr18	53240900	0.727273
chr18	53248900	0.859504
chr18	53435800	0.842975
chr18	53449000	0.685950
chr18	53524100	0.677686
chr18	53531800	0.801653
chr18	53665700	0.917355
chr18	53835500	0.719008
chr18	54075400	0.842975
chr18	54121100	0.867769
chr18	54230800	0.719008
chr18	54244100	0.677686
chr18	54541000	0.834711
chr18	54586700	0.694215
chr18	54590700	0.652893
chr18	54608000	0.644628
chr18	54634800	0.743802
chr18	54654500	0.768595
chr18	54684600	0.661157
chr18	54740300	0.719008
chr18	54743600	0.652893
chr18	54759700	0.834711
chr18	54763500	0.776860
chr18	54790700	0.727273

chr18	54817400	0.776860
chr18	54875100	0.752066
chr18	54878000	0.752066
chr18	54888400	0.752066
chr18	55001600	0.669421
chr18	55100300	0.859504
chr18	55114400	0.785124
chr18	55200900	0.801653
chr18	55228900	0.793388
chr18	55411000	0.644628
chr18	55416200	0.719008
chr18	55575000	0.702479
chr18	55610100	0.859504
chr18	55682700	0.743802
chr18	55694200	0.743802
chr18	55721900	0.876033
chr18	55737200	0.876033
chr18	55755700	0.909091
chr18	55759500	0.752066
chr18	55773700	0.801653
chr18	55783700	0.661157
chr18	55803500	0.743802
chr18	55841900	0.677686
chr18	55844600	0.743802
chr18	55847300	0.793388
chr18	55856400	0.950413
chr18	55865100	0.710744
chr18	55869700	0.677686
chr18	55884000	0.917355
chr18	55891500	0.884298
chr18	55896200	0.793388
chr18	55898500	0.809917
chr18	55915000	0.776860
chr18	55922900	0.735537
chr18	55955700	0.818182
chr18	55984000	0.694215
chr18	56002800	0.834711
chr18	56006900	0.991736
chr18	56012900	1.000000
chr18	56022800	0.702479
chr18	56059800	0.793388
chr18	56070700	0.950413
chr18	56111200	0.710744
chr18	56114800	0.851240
chr18	56153000	0.884298
chr18	56181300	0.677686
chr18	56213300	0.743802
chr18	56252200	0.876033
chr18	56270900	0.727273
chr18	56279800	0.818182
chr18	56286700	0.694215
chr18	56307600	0.983471
chr18	56422300	0.669421
chr18	56436200	0.677686

chr18	56439600	0.685950
chr18	56452500	0.710744
chr18	56456900	0.669421
chr18	56472800	0.669421
chr18	56475800	0.884298
chr18	56483700	0.743802
chr18	56499700	0.644628
chr18	56506100	0.644628
chr18	56514000	0.859504
chr18	56516500	0.991736
chr18	56518800	0.809917
chr18	56527700	0.958678
chr18	56535600	0.652893
chr18	56538400	0.818182
chr18	56559900	0.809917
chr18	56563300	0.644628
chr18	56579300	0.900826
chr18	56584000	0.900826
chr18	56590400	0.818182
chr18	56595700	0.917355
chr18	56675900	0.719008
chr18	56716900	0.776860
chr18	56738000	0.677686
chr18	56758700	0.677686
chr18	56943700	0.694215
chr18	57054600	0.710744
chr18	57070000	0.677686
chr18	57127400	0.702479
chr18	57215300	0.719008
chr18	57223900	0.710744
chr18	57238000	0.694215
chr18	57301600	0.669421
chr18	57349200	0.719008
chr18	57352900	0.752066
chr18	57356700	0.917355
chr18	57367900	0.727273
chr18	57501600	0.735537
chr18	57532100	0.851240
chr18	57534600	0.818182
chr18	57539600	0.760331
chr18	57549800	0.694215
chr18	57572500	0.727273
chr18	57576100	0.776860
chr18	57664000	0.652893
chr18	57671300	0.702479
chr18	57734700	0.958678
chr18	57987600	0.644628
chr18	58007000	0.793388
chr18	58040100	0.685950
chr18	58116200	0.950413
chr18	58472900	0.917355
chr18	58648100	0.669421
chr18	58732300	0.694215
chr18	58795600	0.867769

chr18	58942000	0.752066
chr18	59150800	0.644628
chr18	59273900	0.702479
chr18	59306200	0.727273
chr18	59329300	0.644628
chr18	59619900	0.685950
chr18	59990400	0.652893
chr18	60012500	0.776860
chr18	60041500	0.661157
chr18	60105500	0.652893
chr18	60118200	0.661157
chr18	60125800	0.710744
chr18	60273500	0.702479
chr18	60279100	0.669421
chr18	60315400	0.652893
chr18	60346600	0.710744
chr18	60350200	0.768595
chr18	60381400	0.685950
chr18	60391100	0.842975
chr18	60404500	0.933884
chr18	60408600	0.710744
chr18	60417100	0.735537
chr18	60422000	0.776860
chr18	60424700	0.958678
chr18	60428200	0.892562
chr18	60430900	0.743802
chr18	60446700	0.942149
chr18	60449100	0.661157
chr18	60452300	0.661157
chr18	60455300	0.760331
chr18	60465700	0.727273
chr18	60479600	0.685950
chr18	60482400	0.652893
chr18	60495000	0.776860
chr18	60498900	0.785124
chr18	60502200	0.785124
chr18	60506900	0.975207
chr18	60544800	0.983471
chr18	60755600	0.727273
chr18	60806100	0.983471
chr18	60905500	0.809917
chr18	60990200	0.710744
chr18	61007400	0.909091
chr18	61179000	0.710744
chr18	61181800	0.818182
chr18	61311900	0.818182
chr18	61351300	0.834711
chr18	61687300	0.694215
chr18	61784600	0.752066
chr18	61801900	0.975207
chr18	61833700	0.644628
chr18	61939400	0.983471
chr18	61943400	0.793388
chr18	62758500	0.669421

chr18	62874100	0.661157
chr18	63033200	0.917355
chr18	63082600	0.735537
chr18	63421600	0.752066
chr18	63938200	0.644628
chr18	63984500	0.785124
chr18	64314100	0.644628
chr18	64336700	0.669421
chr18	64344300	0.710744
chr18	64426500	0.752066
chr18	64598600	0.652893
chr18	64640500	0.702479
chr18	64702600	0.644628
chr18	64749400	0.801653
chr18	64900300	0.727273
chr18	65161700	0.727273
chr18	65192800	0.652893
chr18	65277500	0.685950
chr18	65302300	0.991736
chr18	65624700	0.719008
chr18	65888900	0.644628
chr18	66156700	0.809917
chr18	66633600	0.719008
chr18	67350000	0.710744
chr18	67425800	0.884298
chr18	67546500	0.966942
chr18	67649600	0.727273
chr18	67888800	0.818182
chr18	68033800	0.884298
chr18	68090800	0.776860
chr18	68121800	0.735537
chr18	68132100	0.710744
chr18	69759000	0.661157
chr18	70359900	0.851240
chr18	70515500	0.652893
chr18	70998800	0.685950
chr18	71356600	0.710744
chr18	71559200	0.842975
chr18	71563400	0.851240
chr18	71691000	0.809917
chr18	71781700	0.652893
chr18	71783900	0.644628
chr18	71851600	0.752066
chr18	71865000	0.694215
chr18	71924200	0.942149
chr18	71949000	0.735537
chr18	71951500	0.975207
chr18	71956100	0.975207
chr18	72079800	0.917355
chr18	72247100	0.793388
chr18	72334900	0.876033
chr18	72405000	0.694215
chr18	72418400	0.661157
chr18	72506200	0.661157

chr18	72532200	0.909091
chr18	72548500	0.818182
chr18	72634000	0.768595
chr18	72660200	0.760331
chr18	72663000	0.917355
chr18	72715000	0.661157
chr18	72802300	0.677686
chr18	73062500	0.842975
chr18	73119700	0.826446
chr18	73298600	0.743802
chr18	73351200	0.876033
chr18	73367300	0.677686
chr18	73495800	0.702479
chr18	73649700	0.776860
chr18	73776800	0.719008
chr18	73863100	0.743802
chr18	73970500	0.760331
chr18	74026500	0.776860
chr18	74061700	0.702479
chr18	74134700	0.727273
chr18	74138400	0.917355
chr18	74200300	0.652893
chr18	74216100	0.644628
chr18	74233800	0.669421
chr18	74241100	0.900826
chr18	74277600	0.677686
chr18	74301100	0.851240
chr18	74335500	0.719008
chr18	74399300	0.685950
chr18	74515700	0.661157
chr18	74679300	0.983471
chr18	74693500	0.909091
chr18	74695900	0.851240
chr18	74719300	0.859504
chr18	74723500	0.793388
chr18	74766600	0.818182
chr18	74768700	0.842975
chr18	74774600	0.644628
chr18	74781500	0.876033
chr18	74801600	0.801653
chr18	74809500	0.958678
chr18	74813900	0.900826
chr18	74872700	0.793388
chr18	74881000	0.727273
chr18	74983300	0.867769
chr18	75173700	0.669421
chr18	75183200	0.677686
chr18	75389400	0.743802
chr18	75445500	0.710744
chr18	75474900	0.677686
chr18	75624200	0.719008
chr18	75682800	0.652893
chr18	75718500	0.917355
chr18	75792500	0.669421

chr18	75855100	0.694215
chr18	75899400	0.776860
chr18	75989400	0.677686
chr18	76054200	0.776860
chr18	76266500	0.694215
chr18	76302500	0.669421
chr18	76431100	0.727273
chr18	76461800	0.719008
chr18	76481200	0.818182
chr18	76484800	0.909091
chr18	76574900	0.809917
chr18	76581400	0.760331
chr18	76708200	0.859504
chr18	76747900	0.669421
chr18	76751400	0.768595
chr18	76838000	0.710744
chr18	76856200	0.818182
chr18	76942300	0.958678
chr18	76947600	0.669421
chr18	76999700	0.669421
chr18	77035300	0.752066
chr18	77139400	0.867769
chr18	77221200	0.652893
chr18	77225900	0.768595
chr18	77245100	0.735537
chr18	77268300	0.809917
chr18	77286400	0.752066
chr18	77327700	0.793388
chr18	77348800	0.710744
chr18	77382400	0.892562
chr18	77418000	0.776860
chr18	77671600	0.702479
chr18	77675700	0.644628
chr18	77687400	0.842975
chr18	77705000	0.669421
chr18	77720300	0.900826
chr18	77753900	0.735537
chr18	77862700	0.876033
chr18	77878100	0.884298
chr18	77955300	0.876033
chr18	77959000	0.867769
chr19	78400	0.925620
chr19	288300	0.768595
chr19	384600	0.768595
chr19	389400	0.735537
chr19	412900	0.677686
chr19	427900	0.652893
chr19	486000	0.702479
chr19	511500	0.793388
chr19	575800	0.826446
chr19	584000	0.760331
chr19	589100	0.669421
chr19	711300	0.859504
chr19	718900	0.677686

chr19	773500	0.677686
chr19	782000	0.743802
chr19	789500	0.851240
chr19	796500	0.661157
chr19	802200	0.735537
chr19	867700	0.735537
chr19	900600	0.743802
chr19	928700	0.909091
chr19	933700	0.917355
chr19	953400	0.958678
chr19	956700	0.950413
chr19	960600	0.892562
chr19	980400	0.644628
chr19	996600	0.719008
chr19	1015700	0.925620
chr19	1018100	0.975207
chr19	1065300	0.785124
chr19	1082500	0.735537
chr19	1102000	0.735537
chr19	1113600	0.958678
chr19	1132300	0.644628
chr19	1146900	0.925620
chr19	1171100	0.950413
chr19	1181300	0.892562
chr19	1211000	0.884298
chr19	1214500	0.983471
chr19	1222800	0.719008
chr19	1244600	0.652893
chr19	1246700	0.776860
chr19	1259200	0.966942
chr19	1264800	0.900826
chr19	1272000	0.743802
chr19	1360700	0.760331
chr19	1381800	0.702479
chr19	1411600	0.950413
chr19	1415600	0.966942
chr19	1417900	0.735537
chr19	1446900	0.851240
chr19	1452800	0.818182
chr19	1457000	0.917355
chr19	1465800	0.842975
chr19	1503800	0.892562
chr19	1513100	0.677686
chr19	1620800	0.809917
chr19	1648800	0.768595
chr19	1672800	0.694215
chr19	1691400	0.685950
chr19	1720200	0.727273
chr19	1749800	0.859504
chr19	1753000	0.752066
chr19	1755900	0.735537
chr19	1758300	0.702479
chr19	1765600	0.785124
chr19	1767800	0.760331

chr19	1796200	0.669421
chr19	1827200	0.991736
chr19	1843800	0.785124
chr19	1852000	0.842975
chr19	1855400	0.892562
chr19	1882900	0.727273
chr19	1887800	0.892562
chr19	1944600	0.776860
chr19	1956700	0.652893
chr19	2028700	0.694215
chr19	2035400	0.892562
chr19	2040800	0.785124
chr19	2043200	0.834711
chr19	2052400	0.661157
chr19	2056700	0.809917
chr19	2090500	0.776860
chr19	2167400	0.661157
chr19	2171200	0.975207
chr19	2240900	0.760331
chr19	2273600	0.818182
chr19	2275700	0.793388
chr19	2303100	0.743802
chr19	2308300	0.818182
chr19	2313100	0.719008
chr19	2331800	0.710744
chr19	2337500	0.661157
chr19	2385400	0.743802
chr19	2416100	0.652893
chr19	2453800	0.644628
chr19	2540900	0.900826
chr19	2543700	0.925620
chr19	2547100	0.685950
chr19	2581600	0.809917
chr19	2587700	0.818182
chr19	2603000	0.818182
chr19	2613800	0.661157
chr19	2637700	0.719008
chr19	2675500	0.776860
chr19	2923600	0.710744
chr19	2978300	0.685950
chr19	2984100	0.851240
chr19	2987500	0.851240
chr19	3013400	0.710744
chr19	3025300	0.644628
chr19	3042500	0.760331
chr19	3053800	0.760331
chr19	3056400	0.818182
chr19	3076200	0.760331
chr19	3116500	0.859504
chr19	3189500	0.685950
chr19	3249100	0.677686
chr19	3270800	0.685950
chr19	3361700	0.917355
chr19	3390300	0.727273

chr19	3397800	0.760331
chr19	3444600	0.661157
chr19	3459400	0.776860
chr19	3481300	0.652893
chr19	3519800	0.851240
chr19	3539300	0.677686
chr19	3572100	0.677686
chr19	3670100	0.644628
chr19	3672600	0.785124
chr19	3681700	0.801653
chr19	3683900	0.785124
chr19	3688300	0.752066
chr19	3710000	0.900826
chr19	3726000	0.818182
chr19	3951200	0.644628
chr19	3968600	0.669421
chr19	3977500	0.710744
chr19	3981700	0.909091
chr19	4010300	0.768595
chr19	4015900	0.834711
chr19	4059300	0.867769
chr19	4061700	0.859504
chr19	4173300	0.752066
chr19	4204900	0.735537
chr19	4229500	0.933884
chr19	4284700	0.685950
chr19	4369300	0.991736
chr19	4372400	0.702479
chr19	4375800	0.909091
chr19	4377900	0.752066
chr19	4394600	0.694215
chr19	4405800	0.710744
chr19	4409400	0.925620
chr19	4415500	0.975207
chr19	4557000	0.710744
chr19	4575600	0.983471
chr19	4610700	0.909091
chr19	4679300	0.884298
chr19	4743200	0.685950
chr19	4878500	0.768595
chr19	4912100	0.785124
chr19	4977000	0.842975
chr19	4986300	0.677686
chr19	4988800	0.743802
chr19	5048800	0.685950
chr19	5059600	0.661157
chr19	5070900	0.652893
chr19	5074700	0.884298
chr19	5093300	0.694215
chr19	5115400	0.909091
chr19	5121800	0.727273
chr19	5124000	0.884298
chr19	5126800	0.900826
chr19	5129200	0.702479

chr19	5132000	0.884298
chr19	5151000	0.983471
chr19	5193100	1.000000
chr19	5248400	0.776860
chr19	5251700	0.917355
chr19	5295700	0.669421
chr19	5306800	0.859504
chr19	5337100	0.735537
chr19	5352600	0.892562
chr19	5365100	0.809917
chr19	5395000	0.702479
chr19	5398200	0.851240
chr19	5484700	0.644628
chr19	5493400	0.793388
chr19	5524400	0.661157
chr19	5539700	0.966942
chr19	5560800	0.834711
chr19	5580500	0.735537
chr19	5668000	0.966942
chr19	5806200	0.677686
chr19	5881300	0.867769
chr19	5892800	0.752066
chr19	5951300	0.809917
chr19	5953800	0.760331
chr19	5957600	0.809917
chr19	5969100	0.760331
chr19	6022500	0.694215
chr19	6048600	0.719008
chr19	6066900	0.958678
chr19	6092600	0.727273
chr19	6217100	0.917355
chr19	6228900	0.752066
chr19	6234300	0.644628
chr19	6243600	0.694215
chr19	6254700	0.661157
chr19	6271900	0.917355
chr19	6277400	0.776860
chr19	6395400	0.677686
chr19	6412400	0.867769
chr19	6552200	0.727273
chr19	6675200	0.834711
chr19	6678500	0.702479
chr19	6721000	0.702479
chr19	6730600	0.644628
chr19	6734500	0.809917
chr19	6744300	0.983471
chr19	6752100	0.669421
chr19	6773600	0.644628
chr19	6801700	0.900826
chr19	7110300	0.743802
chr19	7212400	0.834711
chr19	7214500	0.752066
chr19	7284800	0.644628
chr19	7326500	0.719008

chr19	7441400	0.752066
chr19	7451600	0.702479
chr19	7471400	0.644628
chr19	7548400	0.793388
chr19	7580200	0.727273
chr19	7616000	0.727273
chr19	7620600	0.735537
chr19	7813100	0.818182
chr19	7896800	0.900826
chr19	7918500	0.917355
chr19	7936700	0.942149
chr19	7960200	0.776860
chr19	7986600	0.652893
chr19	8033900	0.900826
chr19	8036400	0.694215
chr19	8059300	0.917355
chr19	8067000	0.669421
chr19	8094200	0.892562
chr19	8098800	0.933884
chr19	8118000	0.719008
chr19	8185900	0.933884
chr19	8259200	0.892562
chr19	8268400	0.900826
chr19	8270700	0.892562
chr19	8276800	0.669421
chr19	8305500	0.809917
chr19	8369500	0.685950
chr19	8417600	0.669421
chr19	8432400	0.785124
chr19	8462400	0.884298
chr19	8506700	0.909091
chr19	8513800	0.942149
chr19	8522800	0.661157
chr19	8552600	0.818182
chr19	8641000	0.677686
chr19	8677300	0.818182
chr19	9166000	0.983471
chr19	9181300	0.917355
chr19	9186500	0.975207
chr19	9958000	0.801653
chr19	9961500	0.876033
chr19	9994500	0.685950
chr19	10028100	0.884298
chr19	10056500	0.785124
chr19	10063700	0.851240
chr19	10070900	0.644628
chr19	10113000	0.652893
chr19	10120200	0.644628
chr19	10178500	0.760331
chr19	10331500	0.892562
chr19	10450600	0.652893
chr19	10565200	0.991736
chr19	10579300	0.933884
chr19	10610800	0.842975

chr19	10621300	0.661157
chr19	10692000	0.760331
chr19	10754500	0.966942
chr19	10773500	0.719008
chr19	10806300	0.702479
chr19	10859300	0.685950
chr19	10867400	0.669421
chr19	10873700	0.768595
chr19	10989900	0.892562
chr19	10992500	0.975207
chr19	10995900	0.876033
chr19	10999700	0.925620
chr19	11002600	0.735537
chr19	11005100	0.719008
chr19	11007700	0.900826
chr19	11203800	0.677686
chr19	11249300	0.702479
chr19	11253700	0.876033
chr19	11386300	0.826446
chr19	11405100	0.793388
chr19	11466600	0.710744
chr19	11517400	0.851240
chr19	11576700	0.719008
chr19	11593800	0.735537
chr19	11646600	0.702479
chr19	11651600	0.785124
chr19	11671700	0.752066
chr19	12775500	0.702479
chr19	12814100	0.818182
chr19	12881000	0.735537
chr19	12907600	0.727273
chr19	12939600	0.727273
chr19	12943800	0.685950
chr19	12947700	0.727273
chr19	12958800	0.834711
chr19	12976500	0.809917
chr19	13065500	0.677686
chr19	13096300	0.743802
chr19	13101400	0.669421
chr19	13107300	0.702479
chr19	13121300	0.776860
chr19	13124000	0.785124
chr19	13126100	0.834711
chr19	13128400	0.801653
chr19	13131500	0.644628
chr19	13133600	0.842975
chr19	13136500	0.719008
chr19	13162800	0.958678
chr19	13165300	0.727273
chr19	13188300	0.652893
chr19	13194000	0.900826
chr19	13203100	0.752066
chr19	13208600	0.727273
chr19	13215700	0.768595

chr19	13232500	0.661157
chr19	13279400	0.793388
chr19	13349000	0.710744
chr19	13507800	0.809917
chr19	13570500	0.834711
chr19	13572700	0.785124
chr19	13733600	0.801653
chr19	13744200	0.661157
chr19	13763800	0.884298
chr19	13950800	0.892562
chr19	13961500	0.975207
chr19	13991900	0.867769
chr19	13994600	0.702479
chr19	14045400	0.842975
chr19	14049200	0.760331
chr19	14094500	0.685950
chr19	14097900	0.702479
chr19	14224000	0.834711
chr19	14276100	0.685950
chr19	14329100	0.735537
chr19	14332600	0.727273
chr19	14339500	0.867769
chr19	14365100	0.727273
chr19	14379900	0.809917
chr19	14404200	0.785124
chr19	14406400	0.809917
chr19	14459400	0.719008
chr19	14492700	0.752066
chr19	14504800	0.735537
chr19	14517300	0.991736
chr19	14586300	0.768595
chr19	14591300	0.876033
chr19	14616800	0.652893
chr19	14637100	0.669421
chr19	14648200	0.735537
chr19	14665100	0.933884
chr19	14669700	0.685950
chr19	14672200	0.983471
chr19	14674300	0.925620
chr19	14869800	0.900826
chr19	15220300	0.652893
chr19	15289000	0.661157
chr19	15292000	0.710744
chr19	15361400	0.752066
chr19	15363700	0.818182
chr19	15397400	0.842975
chr19	15399700	0.685950
chr19	15404800	0.694215
chr19	15435400	0.760331
chr19	16177000	0.793388
chr19	16191200	0.743802
chr19	16397500	0.760331
chr19	16449900	0.818182
chr19	16477700	0.801653

chr19	16531500	0.859504
chr19	16556900	0.727273
chr19	16565500	0.867769
chr19	16569200	0.884298
chr19	16695400	0.793388
chr19	16698400	0.867769
chr19	16702900	0.793388
chr19	16844800	0.884298
chr19	16936800	0.669421
chr19	17190600	0.752066
chr19	17224700	0.892562
chr19	17227700	0.719008
chr19	17230100	0.702479
chr19	17246500	0.876033
chr19	17270900	0.876033
chr19	17292300	0.892562
chr19	17320600	0.867769
chr19	17348000	0.661157
chr19	17359300	0.842975
chr19	17375200	0.760331
chr19	17405300	0.752066
chr19	17415300	0.752066
chr19	17423200	0.859504
chr19	17451700	0.760331
chr19	17459800	0.702479
chr19	17517700	0.677686
chr19	17526200	0.909091
chr19	17536000	0.876033
chr19	17586900	0.685950
chr19	17598100	0.752066
chr19	17641800	0.826446
chr19	17688300	0.876033
chr19	17690800	0.950413
chr19	17713000	0.859504
chr19	17728000	0.694215
chr19	17766300	0.652893
chr19	17818000	0.752066
chr19	17866300	0.851240
chr19	17959000	0.677686
chr19	17973800	0.785124
chr19	17979800	0.727273
chr19	18020300	0.710744
chr19	18054100	0.933884
chr19	18057900	0.652893
chr19	18073100	0.719008
chr19	18156300	0.776860
chr19	18211700	0.710744
chr19	18230900	0.710744
chr19	18268600	0.702479
chr19	18272400	0.975207
chr19	18331900	0.834711
chr19	18366100	0.884298
chr19	18405600	0.760331
chr19	18415600	0.917355

chr19	18429600	0.842975
chr19	18435100	0.677686
chr19	18440000	0.644628
chr19	18457300	0.851240
chr19	18476200	0.851240
chr19	18485000	0.950413
chr19	18508400	0.859504
chr19	18526500	0.719008
chr19	18534900	0.884298
chr19	18551100	0.743802
chr19	18562300	0.710744
chr19	18577900	0.644628
chr19	18589200	0.694215
chr19	18606500	0.727273
chr19	18707000	0.809917
chr19	18716900	0.677686
chr19	18725300	0.669421
chr19	18755900	0.809917
chr19	18759100	0.710744
chr19	18763100	0.785124
chr19	18769000	0.925620
chr19	18774200	0.719008
chr19	18783000	0.710744
chr19	18789000	0.859504
chr19	18857400	0.801653
chr19	18861100	0.801653
chr19	18896500	0.776860
chr19	18977300	0.809917
chr19	18981600	0.876033
chr19	19043800	0.694215
chr19	19072900	0.801653
chr19	19201500	0.892562
chr19	19231700	0.842975
chr19	19256800	0.685950
chr19	19272000	0.719008
chr19	19282500	0.669421
chr19	19325700	0.661157
chr19	19521600	0.785124
chr19	19555300	0.685950
chr19	19576400	0.909091
chr19	19586600	0.958678
chr19	19593700	0.677686
chr19	19596700	0.793388
chr19	19601100	0.768595
chr19	19618900	0.661157
chr19	19636600	0.785124
chr19	19681400	0.669421
chr19	19686500	0.735537
chr19	19702400	0.859504
chr19	19748800	0.677686
chr19	20179200	0.909091
chr19	20350700	0.776860
chr19	20370500	0.966942
chr19	20471300	0.702479

chr19	20479000	0.785124
chr19	20482600	0.735537
chr19	20485000	0.719008
chr19	20848300	0.644628
chr19	21041600	0.900826
chr19	21404800	0.966942
chr19	21783400	0.685950
chr19	21806900	0.727273
chr19	21877400	0.652893
chr19	21888600	1.000000
chr19	21917100	0.826446
chr19	23350300	0.719008
chr19	24257900	0.760331
chr19	24261500	0.892562
chr19	24587200	0.652893
chr19	27740500	0.685950
chr19	27764800	0.694215
chr19	27779100	0.661157
chr19	27907800	0.694215
chr19	28659700	0.818182
chr19	28779900	0.818182
chr19	28782600	0.917355
chr19	28784700	0.942149
chr19	28805500	0.752066
chr19	28815500	0.661157
chr19	28885100	0.710744
chr19	28915400	0.983471
chr19	28918500	0.727273
chr19	28935200	0.685950
chr19	28938000	0.917355
chr19	28973200	0.818182
chr19	28981500	0.694215
chr19	28985800	0.834711
chr19	28994800	0.727273
chr19	29012400	0.710744
chr19	29072200	0.768595
chr19	29074600	0.669421
chr19	29124400	0.966942
chr19	29314500	0.892562
chr19	29323000	0.793388
chr19	29332000	0.876033
chr19	29374500	0.818182
chr19	29377700	0.702479
chr19	29412000	0.661157
chr19	29480800	0.727273
chr19	29537100	0.818182
chr19	29553800	0.818182
chr19	29575000	0.644628
chr19	29582400	0.752066
chr19	29653800	0.809917
chr19	29739100	0.685950
chr19	29800100	0.727273
chr19	29802600	0.826446
chr19	29855700	0.801653

chr19	29884800	0.702479
chr19	29896300	0.793388
chr19	29989500	0.661157
chr19	29998700	0.685950
chr19	30059500	0.661157
chr19	30067300	0.785124
chr19	30087500	0.925620
chr19	30101600	0.644628
chr19	30116200	0.685950
chr19	30136800	0.933884
chr19	30159900	0.933884
chr19	30215300	0.826446
chr19	30349700	0.743802
chr19	30373900	0.669421
chr19	30419500	0.826446
chr19	30537800	0.727273
chr19	30728000	0.776860
chr19	30747500	0.752066
chr19	30755700	0.685950
chr19	30760300	0.644628
chr19	30768500	0.909091
chr19	30840600	0.768595
chr19	30842700	0.909091
chr19	30851700	0.685950
chr19	30864000	1.000000
chr19	30894100	0.768595
chr19	30941400	0.652893
chr19	30984400	0.801653
chr19	31037400	0.892562
chr19	31047600	0.694215
chr19	31056000	0.842975
chr19	31085400	0.677686
chr19	31088200	0.933884
chr19	31153600	0.710744
chr19	31182600	0.809917
chr19	31296400	0.876033
chr19	31387700	0.925620
chr19	31470100	0.966942
chr19	31565500	0.842975
chr19	31593000	0.752066
chr19	31597200	0.644628
chr19	31807700	0.950413
chr19	31811000	0.834711
chr19	31825900	0.818182
chr19	31836400	0.884298
chr19	31899600	0.884298
chr19	31912100	0.925620
chr19	31916300	0.735537
chr19	32065600	0.677686
chr19	32080300	0.702479
chr19	32119700	0.727273
chr19	32252400	0.801653
chr19	32309800	0.727273
chr19	32527800	0.727273

chr19	32589700	0.669421
chr19	32664000	0.677686
chr19	32753900	0.785124
chr19	32778800	0.661157
chr19	32810500	0.859504
chr19	33164000	0.685950
chr19	33168900	0.694215
chr19	33308000	0.818182
chr19	33315800	0.809917
chr19	33319900	0.644628
chr19	33393600	0.677686
chr19	33409000	0.752066
chr19	33513300	0.669421
chr19	33523700	0.876033
chr19	33532500	0.933884
chr19	33678100	0.752066
chr19	33765600	0.760331
chr19	33769800	0.702479
chr19	33867500	0.685950
chr19	33888500	0.776860
chr19	33907600	0.661157
chr19	33910300	0.834711
chr19	34001900	0.809917
chr19	34020900	0.644628
chr19	34024900	0.785124
chr19	34057700	0.776860
chr19	34094400	0.735537
chr19	34120800	0.677686
chr19	34130000	0.818182
chr19	34140700	0.933884
chr19	34151600	0.719008
chr19	34175700	0.818182
chr19	34179600	0.809917
chr19	34227600	0.826446
chr19	34269900	0.743802
chr19	34360100	0.842975
chr19	34375400	0.950413
chr19	34419300	0.834711
chr19	34576600	0.719008
chr19	34716100	0.933884
chr19	34722400	0.818182
chr19	34728900	0.661157
chr19	34752200	0.710744
chr19	34756500	0.652893
chr19	34761300	0.727273
chr19	34796900	0.735537
chr19	34814700	0.826446
chr19	35335700	0.694215
chr19	35476800	0.826446
chr19	35494600	0.900826
chr19	35536500	0.710744
chr19	35540400	0.727273
chr19	35547500	0.785124
chr19	35557700	0.842975

chr19	35560200	0.909091
chr19	35581900	0.727273
chr19	35587400	0.719008
chr19	35592000	0.834711
chr19	35607100	0.652893
chr19	35610400	0.710744
chr19	35614500	0.768595
chr19	35626300	0.859504
chr19	35635900	0.818182
chr19	35643000	0.768595
chr19	35690700	0.826446
chr19	35694400	0.933884
chr19	35696700	0.867769
chr19	35738000	0.735537
chr19	35744900	0.818182
chr19	35776100	0.727273
chr19	35786800	0.685950
chr19	36015800	0.917355
chr19	36027900	0.752066
chr19	36040700	0.776860
chr19	36066800	0.958678
chr19	36130200	0.876033
chr19	36132900	0.909091
chr19	36185000	0.719008
chr19	36202100	0.669421
chr19	36258900	0.834711
chr19	36335000	0.694215
chr19	36342700	0.661157
chr19	36370600	0.785124
chr19	36400900	0.768595
chr19	36542700	0.776860
chr19	36599300	0.669421
chr19	36602400	0.752066
chr19	36611300	0.900826
chr19	36652500	0.884298
chr19	36673900	0.644628
chr19	37812000	0.909091
chr19	38207400	0.702479
chr19	38420200	0.876033
chr19	38444700	0.933884
chr19	38457500	0.677686
chr19	38480800	0.983471
chr19	38493700	0.727273
chr19	38496400	0.710744
chr19	38503800	0.859504
chr19	38516100	0.776860
chr19	38524600	0.818182
chr19	38534700	0.809917
chr19	38540300	0.661157
chr19	38546500	0.818182
chr19	38549100	0.801653
chr19	38626300	0.719008
chr19	38633400	0.644628
chr19	38698400	0.685950

chr19	38744200	0.900826
chr19	38758700	0.776860
chr19	38804600	0.727273
chr19	38882400	0.702479
chr19	38885600	0.727273
chr19	38916800	0.735537
chr19	38985200	0.702479
chr19	38997500	0.752066
chr19	39128000	0.710744
chr19	39146000	0.851240
chr19	39148900	0.760331
chr19	39154800	0.768595
chr19	39157700	0.760331
chr19	39165800	0.884298
chr19	39169200	0.851240
chr19	39172900	0.867769
chr19	39177800	0.884298
chr19	39183500	0.975207
chr19	39185700	0.793388
chr19	39188700	0.818182
chr19	39369200	0.694215
chr19	39480000	0.710744
chr19	39615100	0.694215
chr19	39622400	0.958678
chr19	39629700	0.900826
chr19	39631900	0.752066
chr19	39637900	0.685950
chr19	39645500	0.859504
chr19	39659000	0.867769
chr19	39694900	0.851240
chr19	39796100	0.933884
chr19	39801500	0.694215
chr19	39818400	0.685950
chr19	39906500	0.677686
chr19	39919100	0.826446
chr19	39921900	0.958678
chr19	39924600	0.752066
chr19	40033300	0.685950
chr19	40098300	0.719008
chr19	40115200	0.685950
chr19	40259800	0.727273
chr19	40287200	0.859504
chr19	40333600	0.661157
chr19	40361200	0.818182
chr19	40699500	0.685950
chr19	40729500	0.743802
chr19	40738100	0.826446
chr19	40758900	0.983471
chr19	40780700	0.669421
chr19	40786500	0.925620
chr19	40905200	0.867769
chr19	40916600	0.876033
chr19	40939000	0.735537
chr19	40952600	0.826446

chr19	40973700	0.677686
chr19	41019100	0.743802
chr19	41029600	0.719008
chr19	41060600	0.644628
chr19	41101000	0.694215
chr19	41140500	0.768595
chr19	41208700	0.909091
chr19	41251200	0.776860
chr19	41310900	0.925620
chr19	41313800	0.818182
chr19	41324900	0.909091
chr19	41386400	0.710744
chr19	41487000	0.702479
chr19	41489300	0.785124
chr19	41634300	0.702479
chr19	41678800	0.719008
chr19	41682000	0.801653
chr19	41693000	0.884298
chr19	41696200	0.958678
chr19	41701900	0.867769
chr19	41730100	0.719008
chr19	41773400	0.776860
chr19	41795300	0.809917
chr19	41814200	0.925620
chr19	41829700	0.702479
chr19	41837500	0.661157
chr19	41913200	0.818182
chr19	42071200	0.702479
chr19	42368200	0.983471
chr19	42392200	0.851240
chr19	42419200	0.818182
chr19	42503800	0.669421
chr19	42508600	0.752066
chr19	42536900	1.000000
chr19	42539000	0.851240
chr19	42603800	0.661157
chr19	42618400	0.760331
chr19	42625600	0.809917
chr19	42655700	0.768595
chr19	42659800	0.909091
chr19	42683800	0.735537
chr19	42688800	0.677686
chr19	42701500	0.818182
chr19	42751100	0.644628
chr19	42755100	0.727273
chr19	42775100	0.661157
chr19	42800200	0.652893
chr19	42811200	0.644628
chr19	42865400	0.809917
chr19	42874300	0.892562
chr19	42947100	0.776860
chr19	42975600	0.851240
chr19	42977900	1.000000
chr19	42980400	0.851240

chr19	43002100	0.818182
chr19	43033400	0.752066
chr19	43059800	0.867769
chr19	43063500	0.644628
chr19	43820800	0.719008
chr19	43835000	0.677686
chr19	43837100	0.851240
chr19	43861400	0.892562
chr19	43868700	0.818182
chr19	43882000	0.859504
chr19	43884800	0.735537
chr19	43970200	0.685950
chr19	43979600	0.710744
chr19	44074800	0.785124
chr19	44081200	0.884298
chr19	44141500	0.768595
chr19	44281900	0.685950
chr19	45193000	0.793388
chr19	45195900	0.735537
chr19	45222200	0.760331
chr19	45245500	0.661157
chr19	45300200	0.818182
chr19	45303700	0.776860
chr19	45354300	0.702479
chr19	45382600	0.677686
chr19	45563500	0.876033
chr19	45585500	0.669421
chr19	45594000	0.677686
chr19	45601600	0.818182
chr19	45607000	0.867769
chr19	45629700	0.917355
chr19	45634300	0.917355
chr19	45640400	0.966942
chr19	45660300	0.644628
chr19	45664400	0.685950
chr19	45691000	0.719008
chr19	45739200	0.776860
chr19	45889200	0.694215
chr19	45895300	0.669421
chr19	45906100	0.743802
chr19	45959500	0.842975
chr19	45977200	0.644628
chr19	46005100	0.710744
chr19	46057500	0.669421
chr19	46068100	0.752066
chr19	46080800	0.768595
chr19	46094100	0.644628
chr19	46133800	0.942149
chr19	46136500	0.801653
chr19	46185200	0.644628
chr19	46268100	0.652893
chr19	46284900	0.694215
chr19	46417300	0.768595
chr19	46464300	0.752066

chr19	46625400	0.644628
chr19	46635400	0.710744
chr19	46637800	0.942149
chr19	46715000	0.752066
chr19	46774700	0.859504
chr19	46804400	0.933884
chr19	46807300	0.876033
chr19	46855900	0.760331
chr19	46878600	0.909091
chr19	46910200	0.644628
chr19	46912400	0.661157
chr19	46984500	0.669421
chr19	47033400	0.900826
chr19	47049900	0.785124
chr19	47107600	0.727273
chr19	47124800	0.818182
chr19	47128700	0.694215
chr19	47134500	0.768595
chr19	47201000	0.818182
chr19	47245000	0.876033
chr19	47368600	0.702479
chr19	47370900	0.685950
chr19	47379000	0.685950
chr19	47429100	0.752066
chr19	47448200	0.735537
chr19	47479500	0.991736
chr19	47494900	0.727273
chr19	47516700	0.909091
chr19	47531700	0.743802
chr19	47535600	0.760331
chr19	47600700	0.867769
chr19	47603500	0.727273
chr19	47609500	0.867769
chr19	47655900	0.743802
chr19	47660300	0.685950
chr19	47713500	1.000000
chr19	47736300	0.809917
chr19	47744700	0.760331
chr19	47793200	0.644628
chr19	47855500	0.760331
chr19	47902500	0.966942
chr19	47953000	0.768595
chr19	47972400	0.702479
chr19	48003700	1.000000
chr19	48011200	0.925620
chr19	48116900	0.975207
chr19	48123500	0.975207
chr19	48127300	0.966942
chr19	48151500	0.834711
chr19	48196700	0.785124
chr19	48201900	0.752066
chr19	48250800	0.702479
chr19	48269500	0.685950
chr19	48273900	0.752066

chr19	48284000	0.669421
chr19	48323500	0.842975
chr19	48325700	0.851240
chr19	48352200	0.785124
chr19	48367500	0.743802
chr19	48395500	0.917355
chr19	48565400	0.685950
chr19	48567800	0.834711
chr19	48694000	0.842975
chr19	48699700	0.677686
chr19	48776800	0.652893
chr19	48812600	0.752066
chr19	48846200	0.743802
chr19	48868600	0.685950
chr19	48899800	0.760331
chr19	48904300	0.991736
chr19	48910400	0.694215
chr19	48918000	0.785124
chr19	48994900	0.735537
chr19	49046900	0.727273
chr19	49049200	0.644628
chr19	49057200	0.694215
chr19	49059800	0.752066
chr19	49072100	0.793388
chr19	49142900	0.776860
chr19	49176900	0.776860
chr19	49179100	0.958678
chr19	49219800	0.776860
chr19	49232500	0.793388
chr19	49245200	0.752066
chr19	49258400	0.768595
chr19	49303000	0.818182
chr19	49310600	0.942149
chr19	49316100	0.793388
chr19	49371900	0.760331
chr19	49385300	0.652893
chr19	49399600	0.644628
chr19	49465700	0.752066
chr19	49473900	0.785124
chr19	49503300	0.826446
chr19	49610800	0.694215
chr19	49628800	0.727273
chr19	49636700	0.727273
chr19	49639500	0.752066
chr19	49650000	0.719008
chr19	49659100	0.826446
chr19	49905000	0.652893
chr19	49907400	0.834711
chr19	49919600	0.644628
chr19	49929700	0.702479
chr19	49951300	0.702479
chr19	49972500	0.752066
chr19	49994200	0.768595
chr19	50003900	0.966942

chr19	50015500	0.677686
chr19	50061600	0.644628
chr19	50064100	0.719008
chr19	50074200	0.719008
chr19	50088300	0.694215
chr19	50141900	0.859504
chr19	50160600	0.735537
chr19	50175600	0.966942
chr19	50183700	0.719008
chr19	50191500	0.652893
chr19	50262100	0.760331
chr19	50305900	0.909091
chr19	50364200	0.892562
chr19	50366300	0.876033
chr19	50400800	0.669421
chr19	50411000	0.925620
chr19	50425100	0.983471
chr19	50427200	0.801653
chr19	50441500	0.768595
chr19	50457600	0.710744
chr19	50718600	0.950413
chr19	50737600	0.884298
chr19	50747900	0.826446
chr19	50828200	0.644628
chr19	50861100	0.892562
chr19	50931800	0.768595
chr19	50969700	0.727273
chr19	50995400	0.735537
chr19	51066600	0.710744
chr19	51073200	0.834711
chr19	51153000	0.719008
chr19	51157100	0.694215
chr19	51170200	0.669421
chr19	51181200	0.785124
chr19	51190000	0.644628
chr19	51195900	0.719008
chr19	51224300	0.876033
chr19	51231700	0.760331
chr19	51281500	0.826446
chr19	51298100	0.727273
chr19	51362200	0.685950
chr19	51380900	0.752066
chr19	51410600	0.652893
chr19	51425600	0.859504
chr19	51472500	0.809917
chr19	51479500	0.685950
chr19	51483000	0.834711
chr19	51487400	0.851240
chr19	51504800	0.867769
chr19	51550900	0.925620
chr19	51580300	0.694215
chr19	51600500	0.694215
chr19	51644400	0.760331
chr19	51796100	0.851240

chr19	51798300	0.719008
chr19	51845700	0.859504
chr19	51894200	0.785124
chr19	51911500	0.652893
chr19	51925300	0.652893
chr19	52056600	0.669421
chr19	52196300	0.909091
chr19	52281600	0.661157
chr19	52412700	0.809917
chr19	52698300	0.776860
chr19	52989200	0.809917
chr19	53021900	0.702479
chr19	53246500	0.851240
chr19	53448100	0.685950
chr19	53964700	0.661157
chr19	53966900	0.950413
chr19	53973600	0.685950
chr19	54007700	0.876033
chr19	54015500	0.702479
chr19	54052500	0.702479
chr19	54085200	0.925620
chr19	54097100	0.760331
chr19	54099800	0.842975
chr19	54116300	0.958678
chr19	54147200	0.983471
chr19	54164600	0.917355
chr19	54170000	0.950413
chr19	54212200	0.669421
chr19	54274800	0.909091
chr19	54276900	0.735537
chr19	54345700	0.677686
chr19	54361600	0.760331
chr19	54374400	0.834711
chr19	54455300	0.859504
chr19	54492900	0.809917
chr19	54501800	0.818182
chr19	54534300	0.727273
chr19	54539000	0.933884
chr19	54683900	0.768595
chr19	54708300	0.694215
chr19	54710900	0.917355
chr19	54713200	0.925620
chr19	54755700	0.710744
chr19	54758700	0.644628
chr19	54770500	0.644628
chr19	54903500	0.851240
chr19	54922800	0.661157
chr19	54933300	0.785124
chr19	54947500	0.702479
chr19	54950500	0.719008
chr19	54984700	0.876033
chr19	55404800	0.719008
chr19	55418100	0.818182
chr19	55582800	0.652893

chr19	55587800	0.752066
chr19	55600700	0.776860
chr19	55606200	0.933884
chr19	55611500	0.727273
chr19	55625300	0.975207
chr19	55670400	0.876033
chr19	55687600	0.876033
chr19	55718700	0.677686
chr19	55765900	0.867769
chr19	55792700	0.785124
chr19	55796200	0.694215
chr19	55813300	0.900826
chr19	55857800	0.958678
chr19	55860600	0.983471
chr19	55874400	0.752066
chr19	55877800	0.710744
chr19	55885300	0.785124
chr19	55900800	0.925620
chr19	55904300	0.900826
chr19	55911800	0.743802
chr19	55920700	0.925620
chr19	55976300	0.867769
chr19	55984900	0.677686
chr19	55998900	0.834711
chr19	56001200	0.826446
chr19	56008400	0.809917
chr19	56049300	0.950413
chr19	56058400	0.719008
chr19	56064600	0.752066
chr19	56105200	0.694215
chr19	56114400	0.719008
chr19	56170300	0.966942
chr19	56182200	0.710744
chr19	56597200	0.644628
chr19	56604000	0.809917
chr19	56616500	0.867769
chr19	56620700	0.793388
chr19	56623000	0.859504
chr19	56626000	0.867769
chr19	56648300	0.892562
chr19	56665200	0.760331
chr19	56821300	0.685950
chr19	56859800	0.644628
chr19	57814200	0.685950
chr19	58231600	0.677686
chr19	58298300	0.661157
chr19	58313100	0.876033
chr19	58317300	0.669421
chr19	58344800	0.958678
chr19	58393300	0.933884
chr19	58397600	0.652893
chr19	58550000	0.958678
chr19	58554600	0.785124
chr19	58567000	0.685950

chr19	59028100	0.760331
chr19	59059200	0.851240
chr1	36900	0.859504
chr1	570200	0.685950
chr1	781800	0.917355
chr1	808600	0.876033
chr1	856300	0.743802
chr1	871300	0.752066
chr1	875000	0.702479
chr1	906300	0.834711
chr1	911800	0.661157
chr1	933000	0.669421
chr1	940800	0.925620
chr1	962500	0.727273
chr1	997600	0.685950
chr1	999700	0.760331
chr1	1058100	0.801653
chr1	1072100	0.826446
chr1	1098100	0.644628
chr1	1100400	0.933884
chr1	1105800	0.851240
chr1	1109300	0.702479
chr1	1112200	0.867769
chr1	1182300	0.933884
chr1	1193100	0.859504
chr1	1206800	0.669421
chr1	1228200	0.752066
chr1	1240600	0.859504
chr1	1251200	0.900826
chr1	1281700	0.809917
chr1	1294000	0.785124
chr1	1307500	0.727273
chr1	1318400	0.652893
chr1	1337500	0.834711
chr1	1346000	0.776860
chr1	1357600	0.942149
chr1	1362000	0.809917
chr1	1366500	0.942149
chr1	1532900	0.661157
chr1	1568800	0.652893
chr1	1610300	0.876033
chr1	1615400	0.958678
chr1	1631800	0.909091
chr1	1780800	0.842975
chr1	1804500	0.661157
chr1	1826600	0.735537
chr1	1851900	0.727273
chr1	1892700	0.900826
chr1	1976500	0.834711
chr1	1979200	0.727273
chr1	2019700	0.760331
chr1	2024200	0.801653
chr1	2027400	0.719008
chr1	2057500	0.768595

chr1	2121500	0.776860
chr1	2130900	0.909091
chr1	2141600	0.727273
chr1	2165200	0.884298
chr1	2178500	0.917355
chr1	2180800	0.661157
chr1	2183000	0.867769
chr1	2188300	0.851240
chr1	2191200	0.719008
chr1	2199100	0.719008
chr1	2202700	0.851240
chr1	2205800	0.933884
chr1	2208100	0.776860
chr1	2211200	0.991736
chr1	2215500	0.652893
chr1	2220900	0.768595
chr1	2224700	0.826446
chr1	2231100	0.776860
chr1	2243200	0.776860
chr1	2246100	0.826446
chr1	2249000	0.834711
chr1	2253700	0.884298
chr1	2346400	0.677686
chr1	2378900	0.867769
chr1	2399300	0.702479
chr1	2467100	0.925620
chr1	2475800	0.826446
chr1	2509600	0.661157
chr1	2515400	0.785124
chr1	2543200	0.785124
chr1	2564500	0.702479
chr1	3021000	0.710744
chr1	3045000	0.710744
chr1	3121900	0.801653
chr1	3158300	0.644628
chr1	3202600	0.752066
chr1	3230100	0.809917
chr1	3236300	0.652893
chr1	3269400	0.917355
chr1	3280000	0.710744
chr1	3308700	0.892562
chr1	3313000	0.685950
chr1	3388400	0.975207
chr1	3413100	0.719008
chr1	3460500	0.661157
chr1	3507100	0.752066
chr1	3510200	0.719008
chr1	3515500	0.917355
chr1	3525600	0.793388
chr1	3530300	0.743802
chr1	3535900	0.768595
chr1	3548900	0.909091
chr1	3585800	0.743802
chr1	3588600	0.727273

chr1	3591800	0.958678
chr1	3595100	0.719008
chr1	3601200	0.735537
chr1	3605700	0.842975
chr1	3612600	0.669421
chr1	3626200	0.826446
chr1	3637100	0.677686
chr1	3649800	0.702479
chr1	3706700	0.727273
chr1	3710100	0.801653
chr1	3776200	0.669421
chr1	3820900	0.867769
chr1	3828000	0.867769
chr1	4087800	0.950413
chr1	4094200	0.933884
chr1	4129800	0.661157
chr1	4143600	0.710744
chr1	4175300	0.859504
chr1	4179800	0.644628
chr1	4233100	0.735537
chr1	4336900	0.809917
chr1	4363400	0.793388
chr1	4380000	0.685950
chr1	4711000	0.818182
chr1	4719500	0.933884
chr1	4744700	0.801653
chr1	4815700	0.942149
chr1	4993500	0.760331
chr1	5080500	0.661157
chr1	5179800	0.719008
chr1	5286600	0.644628
chr1	5328100	0.842975
chr1	5485300	0.760331
chr1	5491200	0.752066
chr1	5570400	0.776860
chr1	5646700	0.768595
chr1	5713200	0.702479
chr1	5751000	0.669421
chr1	5765500	0.652893
chr1	5777800	0.809917
chr1	5806800	0.892562
chr1	5812300	0.834711
chr1	5827900	0.909091
chr1	5858400	0.652893
chr1	5891700	0.685950
chr1	5904600	0.735537
chr1	5957000	0.975207
chr1	5962200	0.735537
chr1	5976200	0.669421
chr1	6041100	0.867769
chr1	6063700	0.644628
chr1	6086500	0.842975
chr1	6094700	0.719008
chr1	6113400	0.818182

chr1	6137800	0.652893
chr1	6188100	0.785124
chr1	6205200	0.752066
chr1	6209200	0.694215
chr1	6267900	0.661157
chr1	6298600	0.842975
chr1	6333900	0.768595
chr1	6399200	0.950413
chr1	6409400	0.644628
chr1	6412300	0.768595
chr1	6418700	0.933884
chr1	6421000	0.752066
chr1	6445900	0.776860
chr1	6448000	0.752066
chr1	6475200	0.900826
chr1	6490100	0.776860
chr1	6492400	0.702479
chr1	6498000	0.661157
chr1	6516400	0.785124
chr1	6520600	0.950413
chr1	6525900	0.669421
chr1	6531600	0.644628
chr1	6535000	0.752066
chr1	6580100	0.743802
chr1	6706900	0.702479
chr1	6754900	0.933884
chr1	6795800	0.867769
chr1	6831800	0.661157
chr1	6927100	0.652893
chr1	6967100	0.884298
chr1	6972400	0.859504
chr1	7017300	0.694215
chr1	7039800	0.958678
chr1	7056300	0.719008
chr1	7082100	0.768595
chr1	7103700	0.925620
chr1	7178200	0.909091
chr1	7209900	0.685950
chr1	7259400	0.694215
chr1	7317700	0.694215
chr1	7354400	0.677686
chr1	7358100	0.785124
chr1	7362500	0.735537
chr1	7439900	0.942149
chr1	7459400	0.834711
chr1	7462600	0.884298
chr1	7551700	0.851240
chr1	7561900	0.652893
chr1	7599500	0.685950
chr1	7610900	0.834711
chr1	7669100	0.842975
chr1	7727900	0.752066
chr1	7730200	0.818182
chr1	7762600	0.818182

chr1	7788600	0.661157
chr1	7813200	0.793388
chr1	8002400	0.702479
chr1	8052200	0.652893
chr1	8065000	0.975207
chr1	8079000	0.727273
chr1	8083000	0.644628
chr1	8088500	0.644628
chr1	8131000	0.776860
chr1	8194200	0.661157
chr1	8217300	0.702479
chr1	8227600	0.942149
chr1	8278000	0.661157
chr1	8299400	0.884298
chr1	8319900	0.661157
chr1	8327200	0.867769
chr1	8329300	0.669421
chr1	8375300	0.892562
chr1	8427800	0.776860
chr1	8431900	0.917355
chr1	8434300	0.826446
chr1	8456800	0.933884
chr1	8468200	0.975207
chr1	8472100	0.834711
chr1	8486000	0.661157
chr1	8491200	0.677686
chr1	8499200	0.917355
chr1	8504500	0.884298
chr1	8508300	0.760331
chr1	8510900	0.826446
chr1	8520500	0.983471
chr1	8533800	0.760331
chr1	8578200	0.661157
chr1	8586300	0.884298
chr1	8613500	0.785124
chr1	8652800	0.760331
chr1	8686500	0.917355
chr1	8721600	0.801653
chr1	8724600	0.735537
chr1	8731200	0.867769
chr1	8741100	0.644628
chr1	8750600	0.776860
chr1	8756100	0.694215
chr1	8765000	0.785124
chr1	8767500	0.644628
chr1	8772800	0.950413
chr1	8778800	0.859504
chr1	8845500	0.669421
chr1	8850100	0.975207
chr1	8882700	0.719008
chr1	8918500	0.710744
chr1	8924300	0.942149
chr1	8931500	0.876033
chr1	8940800	0.809917

chr1	8975600	0.793388
chr1	9048200	0.644628
chr1	9094100	0.669421
chr1	9129900	0.884298
chr1	9190300	0.661157
chr1	9292200	0.661157
chr1	9300400	0.661157
chr1	9302700	0.760331
chr1	9310700	0.719008
chr1	9313500	0.933884
chr1	9330700	0.710744
chr1	9336100	0.669421
chr1	9340500	0.884298
chr1	9380900	0.801653
chr1	9412200	0.669421
chr1	9509300	0.661157
chr1	9651600	0.760331
chr1	9654300	0.719008
chr1	9687900	0.851240
chr1	9717900	0.652893
chr1	9752100	0.685950
chr1	9779000	0.933884
chr1	9821600	0.983471
chr1	9845000	0.685950
chr1	9852900	0.685950
chr1	9865400	0.694215
chr1	9868700	0.867769
chr1	9896100	0.768595
chr1	9906800	0.834711
chr1	9910800	0.685950
chr1	9913500	0.743802
chr1	9920000	0.884298
chr1	9932400	0.785124
chr1	9935700	0.776860
chr1	9950500	0.958678
chr1	9983000	0.768595
chr1	9989800	0.966942
chr1	10011200	0.867769
chr1	10021500	0.776860
chr1	10125500	0.727273
chr1	10239400	0.942149
chr1	10380100	0.776860
chr1	10408600	0.669421
chr1	10448300	0.876033
chr1	10550500	0.793388
chr1	10555600	0.702479
chr1	10568600	0.933884
chr1	10570800	0.702479
chr1	10574600	0.677686
chr1	10579400	0.933884
chr1	10592400	0.702479
chr1	10601200	0.768595
chr1	10616100	0.867769
chr1	10638800	0.669421

chr1	10655100	0.685950
chr1	10666200	0.644628
chr1	10676100	0.685950
chr1	10695500	0.892562
chr1	10712100	0.677686
chr1	10724400	0.818182
chr1	10730800	0.661157
chr1	10737300	0.900826
chr1	10739900	0.652893
chr1	10742800	0.876033
chr1	10747100	0.702479
chr1	10759400	0.677686
chr1	10766200	0.644628
chr1	10769800	0.834711
chr1	10774900	0.735537
chr1	10789400	0.859504
chr1	10795200	0.752066
chr1	10851900	0.760331
chr1	10875500	0.867769
chr1	10885600	0.801653
chr1	10899300	0.818182
chr1	10920800	0.735537
chr1	10923500	0.834711
chr1	10961500	0.834711
chr1	10967900	0.727273
chr1	10995000	0.842975
chr1	10997800	0.644628
chr1	11002400	0.727273
chr1	11018400	0.842975
chr1	11021300	0.834711
chr1	11029700	0.851240
chr1	11036600	0.768595
chr1	11041500	0.793388
chr1	11105700	0.644628
chr1	11113600	0.826446
chr1	11345000	0.694215
chr1	11348000	0.991736
chr1	11359200	0.644628
chr1	11385100	0.669421
chr1	11400100	0.702479
chr1	11405900	0.867769
chr1	11434000	0.892562
chr1	11477400	0.652893
chr1	11547600	0.652893
chr1	11618700	0.776860
chr1	11624100	0.801653
chr1	11654700	0.876033
chr1	11662600	0.776860
chr1	11712900	0.768595
chr1	11748700	0.809917
chr1	11754500	0.644628
chr1	11758000	0.669421
chr1	11761300	0.719008
chr1	11764500	0.694215

chr1	11768100	0.669421
chr1	11786400	0.768595
chr1	11789800	0.826446
chr1	11792200	0.900826
chr1	11801100	0.859504
chr1	11822600	0.933884
chr1	11845200	0.884298
chr1	11849000	0.876033
chr1	11919400	0.719008
chr1	11943400	0.809917
chr1	11959300	0.826446
chr1	11967700	0.760331
chr1	12004700	0.900826
chr1	12112700	0.727273
chr1	12130200	0.801653
chr1	12139200	0.842975
chr1	12207100	0.842975
chr1	12218600	0.776860
chr1	12245100	0.661157
chr1	12249000	0.876033
chr1	12264500	0.727273
chr1	12302500	0.727273
chr1	12310000	0.818182
chr1	12400700	0.644628
chr1	12419200	0.644628
chr1	12514400	0.669421
chr1	12538800	0.818182
chr1	12605000	0.652893
chr1	12653000	0.809917
chr1	12662700	0.876033
chr1	12682400	0.652893
chr1	12687500	0.743802
chr1	12689900	0.975207
chr1	12692500	0.669421
chr1	12697000	0.942149
chr1	12719300	0.702479
chr1	12775000	0.768595
chr1	12777300	0.917355
chr1	13832300	0.727273
chr1	13836700	0.661157
chr1	13876400	0.743802
chr1	13914300	0.809917
chr1	13920200	0.925620
chr1	13931300	0.785124
chr1	14086000	0.892562
chr1	14088500	0.842975
chr1	14091400	0.793388
chr1	14337800	0.685950
chr1	14440800	0.685950
chr1	14463200	0.991736
chr1	14492200	0.735537
chr1	14504900	0.694215
chr1	14623800	0.677686
chr1	14672900	0.669421

chr1	14731400	0.892562
chr1	14839400	0.950413
chr1	14877400	0.694215
chr1	14917400	0.727273
chr1	14920200	0.917355
chr1	14928800	0.925620
chr1	14943400	0.669421
chr1	15005800	0.942149
chr1	15052700	0.876033
chr1	15059800	0.752066
chr1	15066700	0.768595
chr1	15069400	0.867769
chr1	15086100	0.867769
chr1	15094200	0.876033
chr1	15106600	0.661157
chr1	15202900	0.826446
chr1	15211500	0.809917
chr1	15222000	0.685950
chr1	15272400	0.735537
chr1	15277000	0.685950
chr1	15335600	0.710744
chr1	15380100	0.958678
chr1	15417100	0.859504
chr1	15426700	0.719008
chr1	15434800	0.785124
chr1	15439000	0.768595
chr1	15467100	0.752066
chr1	15483500	0.694215
chr1	15505500	0.677686
chr1	15513700	0.966942
chr1	15523600	0.793388
chr1	15528200	0.785124
chr1	15539600	0.760331
chr1	15599300	0.760331
chr1	15631600	0.661157
chr1	15642900	0.644628
chr1	15715600	0.818182
chr1	15743100	0.685950
chr1	15782500	0.719008
chr1	15836400	0.768595
chr1	15923700	0.702479
chr1	16062700	0.669421
chr1	16068700	0.752066
chr1	16076400	0.694215
chr1	16083000	0.793388
chr1	16088000	0.826446
chr1	16156900	0.669421
chr1	16209700	0.677686
chr1	16213100	0.991736
chr1	16227300	0.677686
chr1	16275400	0.809917
chr1	16285000	0.867769
chr1	16288200	0.669421
chr1	16359700	0.652893

chr1	16377300	0.809917
chr1	16382600	0.834711
chr1	16415800	0.793388
chr1	16442800	0.983471
chr1	16466500	0.975207
chr1	16471100	0.719008
chr1	16479000	0.950413
chr1	16489000	0.776860
chr1	16501000	0.859504
chr1	16508300	0.917355
chr1	16536500	0.694215
chr1	16555300	0.669421
chr1	16711900	0.661157
chr1	16719300	0.818182
chr1	16817700	0.818182
chr1	16820600	0.694215
chr1	16867200	0.652893
chr1	16887100	0.884298
chr1	16919600	0.859504
chr1	16946200	0.760331
chr1	16957400	0.710744
chr1	16983800	0.818182
chr1	16998400	0.644628
chr1	17002000	0.694215
chr1	17023000	0.727273
chr1	17030100	0.719008
chr1	17044600	0.644628
chr1	17055200	0.727273
chr1	17062300	0.685950
chr1	17075100	0.917355
chr1	17077200	0.793388
chr1	17095500	0.776860
chr1	17124700	0.834711
chr1	17193900	0.719008
chr1	17253500	0.933884
chr1	17256400	0.685950
chr1	17265800	0.909091
chr1	17279600	0.917355
chr1	17298700	0.702479
chr1	17302100	0.776860
chr1	17333900	0.834711
chr1	17340400	0.685950
chr1	17342600	0.942149
chr1	17386600	0.925620
chr1	17448300	0.727273
chr1	17457500	0.760331
chr1	17493900	0.727273
chr1	17541500	0.768595
chr1	17572000	0.727273
chr1	17631600	0.760331
chr1	17642000	0.851240
chr1	17704600	0.801653
chr1	17728100	0.644628
chr1	17731500	0.719008

chr1	17750100	0.677686
chr1	17752400	0.768595
chr1	17756000	0.867769
chr1	17759000	0.760331
chr1	17796600	0.900826
chr1	17804000	0.719008
chr1	17829200	0.834711
chr1	17843100	0.661157
chr1	17848000	0.958678
chr1	17854000	0.818182
chr1	17859300	0.677686
chr1	17872200	0.702479
chr1	17877300	0.966942
chr1	17881300	0.859504
chr1	17888000	0.842975
chr1	17892000	0.925620
chr1	17894300	0.867769
chr1	17896700	0.801653
chr1	17899200	0.752066
chr1	17902900	0.876033
chr1	17906500	0.842975
chr1	17909000	0.892562
chr1	17915100	0.834711
chr1	17998300	0.785124
chr1	18027200	0.818182
chr1	18063000	0.644628
chr1	18117500	0.669421
chr1	18188800	0.669421
chr1	18201700	0.826446
chr1	18237100	0.776860
chr1	18246800	0.685950
chr1	18275400	0.768595
chr1	18285000	0.834711
chr1	18300600	0.702479
chr1	18330000	0.652893
chr1	18417700	0.719008
chr1	18459700	0.842975
chr1	18503400	0.752066
chr1	18538900	0.694215
chr1	18560400	0.983471
chr1	18565500	0.793388
chr1	18589600	0.768595
chr1	18605900	0.661157
chr1	18609300	0.685950
chr1	18611700	0.743802
chr1	18615600	0.785124
chr1	18618400	0.685950
chr1	18621600	1.000000
chr1	18625700	0.776860
chr1	18659500	0.776860
chr1	18675600	0.652893
chr1	18678900	0.661157
chr1	18701000	0.652893
chr1	18712100	0.735537

chr1	18726400	0.760331
chr1	18743800	0.851240
chr1	18746500	0.900826
chr1	18767500	0.851240
chr1	18775000	0.801653
chr1	18797300	0.669421
chr1	18803300	0.685950
chr1	18807500	0.950413
chr1	18813700	0.818182
chr1	18869300	0.917355
chr1	18874300	0.826446
chr1	18902400	0.826446
chr1	18930200	0.661157
chr1	18954300	0.677686
chr1	18960000	0.793388
chr1	18965700	0.809917
chr1	18968600	0.669421
chr1	18972700	0.809917
chr1	18976500	0.826446
chr1	18978900	0.975207
chr1	18981300	0.677686
chr1	19001000	0.760331
chr1	19003100	0.909091
chr1	19016600	0.661157
chr1	19020400	0.694215
chr1	19052300	0.768595
chr1	19121500	0.644628
chr1	19147400	0.826446
chr1	19206500	0.685950
chr1	19250200	0.760331
chr1	19254200	0.694215
chr1	19262000	0.644628
chr1	19265300	0.793388
chr1	19268200	0.884298
chr1	19292700	0.727273
chr1	19337400	0.917355
chr1	19393800	0.661157
chr1	19581100	0.694215
chr1	19680200	0.958678
chr1	19720100	0.669421
chr1	19728800	1.000000
chr1	19732700	0.801653
chr1	19769600	0.661157
chr1	19772800	0.834711
chr1	19775200	0.818182
chr1	19780800	0.694215
chr1	19784000	0.867769
chr1	19790000	0.735537
chr1	19797100	0.727273
chr1	19808600	0.768595
chr1	19946400	0.942149
chr1	19967500	0.925620
chr1	19984200	0.760331
chr1	20009400	0.719008

chr1	20012500	0.867769
chr1	20015900	0.685950
chr1	20030900	0.818182
chr1	20042400	0.677686
chr1	20095800	0.669421
chr1	20143400	1.000000
chr1	20301900	0.669421
chr1	20308800	0.892562
chr1	20317900	0.884298
chr1	20441200	0.735537
chr1	20479200	0.859504
chr1	20521500	0.801653
chr1	20605600	0.950413
chr1	20617400	0.719008
chr1	20712900	0.669421
chr1	20726800	0.652893
chr1	20757800	0.876033
chr1	20771100	0.925620
chr1	20775600	0.694215
chr1	20800300	0.710744
chr1	20803900	0.702479
chr1	20808500	0.768595
chr1	20821100	0.942149
chr1	20916200	0.776860
chr1	20940100	0.768595
chr1	20968900	0.727273
chr1	21049300	0.685950
chr1	21054200	0.801653
chr1	21056600	0.809917
chr1	21233200	0.669421
chr1	21448800	0.776860
chr1	21544200	0.818182
chr1	21564900	0.842975
chr1	21589100	0.933884
chr1	21604500	0.702479
chr1	21620100	0.818182
chr1	21629500	0.685950
chr1	21641200	0.900826
chr1	21649200	0.727273
chr1	21653800	0.793388
chr1	21656200	0.834711
chr1	21659200	0.760331
chr1	21661700	0.652893
chr1	21794600	0.785124
chr1	21824500	0.826446
chr1	21838600	0.776860
chr1	21845500	1.000000
chr1	21851900	0.644628
chr1	21861300	0.719008
chr1	21864400	0.793388
chr1	21867300	0.652893
chr1	21874300	0.818182
chr1	21877700	0.669421
chr1	21880500	0.900826

chr1	21901300	0.694215
chr1	21934700	0.661157
chr1	21945700	0.776860
chr1	21956100	0.842975
chr1	21958500	0.710744
chr1	21993700	0.719008
chr1	21997800	0.652893
chr1	22033000	0.752066
chr1	22091700	0.677686
chr1	22192900	0.818182
chr1	22200400	0.826446
chr1	22228900	0.669421
chr1	22236600	0.727273
chr1	22238800	0.727273
chr1	22241100	0.776860
chr1	22248300	0.685950
chr1	22258300	0.702479
chr1	22260900	0.652893
chr1	22265000	0.644628
chr1	22270900	0.735537
chr1	22283300	0.900826
chr1	22290300	0.735537
chr1	22364400	0.735537
chr1	22416100	0.950413
chr1	22419500	0.950413
chr1	22441400	0.826446
chr1	22447500	0.768595
chr1	22449700	0.876033
chr1	22458500	0.652893
chr1	22460900	0.859504
chr1	22464000	0.900826
chr1	22534500	0.768595
chr1	22552600	0.719008
chr1	22556000	0.834711
chr1	22560800	0.851240
chr1	22563300	0.842975
chr1	22575500	0.685950
chr1	22581100	0.661157
chr1	22588800	0.760331
chr1	22591300	0.785124
chr1	22648100	0.900826
chr1	22677600	0.760331
chr1	22691800	0.694215
chr1	22696200	0.710744
chr1	22716000	0.752066
chr1	22722100	0.644628
chr1	22740200	0.743802
chr1	22770000	0.685950
chr1	22815400	0.900826
chr1	22819100	0.785124
chr1	22827000	0.710744
chr1	22893800	0.842975
chr1	22919900	0.677686
chr1	22945200	0.694215

chr1	22953100	0.900826
chr1	22957000	0.661157
chr1	23057700	0.710744
chr1	23063500	0.661157
chr1	23105300	0.661157
chr1	23119800	0.702479
chr1	23122300	0.752066
chr1	23128300	0.652893
chr1	23132000	0.785124
chr1	23153500	0.925620
chr1	23176900	0.702479
chr1	23202900	0.859504
chr1	23298200	0.685950
chr1	23449400	0.644628
chr1	23461900	0.760331
chr1	23590500	0.917355
chr1	23698100	0.785124
chr1	23721500	0.892562
chr1	23764000	0.867769
chr1	23801700	0.785124
chr1	23852000	0.826446
chr1	23866700	0.818182
chr1	23903100	0.652893
chr1	23925100	0.933884
chr1	23927700	0.694215
chr1	23946700	0.727273
chr1	23961800	0.809917
chr1	23971000	0.652893
chr1	24231100	0.735537
chr1	24263600	0.884298
chr1	24308200	0.743802
chr1	24330300	0.925620
chr1	24348900	0.685950
chr1	24398100	0.735537
chr1	24434800	0.644628
chr1	24477000	0.710744
chr1	24479300	0.743802
chr1	24529700	0.818182
chr1	24563600	0.727273
chr1	24613500	0.933884
chr1	24621700	0.801653
chr1	24635200	1.000000
chr1	24638600	0.677686
chr1	24656300	0.760331
chr1	24664300	0.685950
chr1	24667000	0.900826
chr1	24669300	0.876033
chr1	24719800	0.743802
chr1	24808200	0.752066
chr1	24834300	0.851240
chr1	24878800	0.702479
chr1	24889400	0.669421
chr1	25027000	0.826446
chr1	25032200	0.834711

chr1	25051700	0.958678
chr1	25057200	0.677686
chr1	25062700	0.909091
chr1	25066700	0.735537
chr1	25070000	0.752066
chr1	25091900	0.661157
chr1	25236400	0.809917
chr1	25242900	0.669421
chr1	25260700	0.677686
chr1	25262800	0.834711
chr1	25265600	0.669421
chr1	25276900	0.818182
chr1	25296800	0.735537
chr1	25299400	0.719008
chr1	25308200	0.685950
chr1	25338800	0.727273
chr1	25346200	0.677686
chr1	25349500	0.710744
chr1	25354100	0.735537
chr1	25381900	0.727273
chr1	25526500	0.735537
chr1	25591400	0.719008
chr1	25598600	0.785124
chr1	25653400	0.826446
chr1	25661700	0.768595
chr1	25691400	0.694215
chr1	25694900	0.785124
chr1	25747700	0.785124
chr1	25811100	0.752066
chr1	25813200	0.652893
chr1	25830200	0.760331
chr1	25833100	0.867769
chr1	25856700	0.694215
chr1	25891900	0.661157
chr1	25904300	0.702479
chr1	25908300	0.694215
chr1	25915300	0.760331
chr1	25946600	0.685950
chr1	25988400	0.760331
chr1	25993100	0.851240
chr1	26022000	0.685950
chr1	26024500	0.644628
chr1	26027400	0.818182
chr1	26031400	0.685950
chr1	26035000	0.909091
chr1	26051800	0.818182
chr1	26054900	0.859504
chr1	26075100	0.892562
chr1	26084400	0.702479
chr1	26107100	0.933884
chr1	26194500	0.793388
chr1	26219100	0.991736
chr1	26254100	0.909091
chr1	26298000	0.768595

chr1	26320500	0.710744
chr1	26337500	0.743802
chr1	26348800	0.694215
chr1	26405800	0.702479
chr1	26421400	0.876033
chr1	26485700	0.793388
chr1	26504200	0.702479
chr1	26517300	0.752066
chr1	26527800	0.669421
chr1	26530900	0.710744
chr1	26547100	0.768595
chr1	26602100	1.000000
chr1	26609200	0.661157
chr1	26612000	0.851240
chr1	26617600	0.933884
chr1	26644800	0.785124
chr1	26677800	0.867769
chr1	26690600	0.677686
chr1	26744900	0.834711
chr1	26747200	0.876033
chr1	26752400	0.727273
chr1	26754500	0.900826
chr1	26868700	0.793388
chr1	26872600	0.694215
chr1	26937500	0.719008
chr1	27029100	0.933884
chr1	27033000	0.661157
chr1	27044900	0.727273
chr1	27049800	0.892562
chr1	27054100	0.694215
chr1	27183800	0.818182
chr1	27189100	0.793388
chr1	27194500	0.652893
chr1	27318100	0.818182
chr1	27323900	0.917355
chr1	27326300	1.000000
chr1	27329100	0.925620
chr1	27333000	0.892562
chr1	27348400	0.809917
chr1	27353900	0.834711
chr1	27358400	0.867769
chr1	27372000	0.652893
chr1	27384300	0.809917
chr1	27397500	0.768595
chr1	27426800	0.826446
chr1	27465400	0.859504
chr1	27469000	0.925620
chr1	27472500	0.950413
chr1	27628200	0.859504
chr1	27634800	0.776860
chr1	27684300	0.876033
chr1	27725100	0.834711
chr1	27729900	0.975207
chr1	27733400	0.975207

chr1	27745200	0.859504
chr1	27751100	0.669421
chr1	27755700	0.950413
chr1	27757900	0.801653
chr1	27818900	0.801653
chr1	27823700	0.975207
chr1	27831500	0.809917
chr1	27846100	0.851240
chr1	27852300	0.685950
chr1	27856500	0.661157
chr1	27863700	0.925620
chr1	27867200	0.685950
chr1	27869800	0.892562
chr1	27883900	0.942149
chr1	27887400	0.942149
chr1	27890800	0.942149
chr1	27894300	0.644628
chr1	27897600	0.851240
chr1	27915500	0.867769
chr1	27918100	0.776860
chr1	27927100	0.818182
chr1	27955900	0.776860
chr1	27970700	0.867769
chr1	27977500	0.768595
chr1	27982600	0.851240
chr1	27989800	0.669421
chr1	27999300	0.644628
chr1	28016800	0.942149
chr1	28133900	0.826446
chr1	28185300	0.743802
chr1	28288500	0.710744
chr1	28432100	0.719008
chr1	28490200	0.710744
chr1	28523900	0.826446
chr1	28575400	0.991736
chr1	28594000	0.942149
chr1	28623700	0.942149
chr1	28726600	0.694215
chr1	28741600	0.743802
chr1	28837200	0.942149
chr1	28848100	0.644628
chr1	28921800	0.768595
chr1	28926200	0.942149
chr1	28929700	0.644628
chr1	28991000	0.677686
chr1	29081700	0.694215
chr1	29123300	0.661157
chr1	29211100	0.842975
chr1	29254600	0.851240
chr1	29280700	0.719008
chr1	29301100	0.669421
chr1	29310300	0.859504
chr1	29439600	0.760331
chr1	29491300	0.983471

chr1	29493700	0.677686
chr1	29513400	0.983471
chr1	29567300	0.859504
chr1	29577200	0.710744
chr1	29584000	0.834711
chr1	29598600	0.933884
chr1	29652000	0.801653
chr1	29671000	0.809917
chr1	29742600	0.760331
chr1	29751300	0.752066
chr1	29770800	0.719008
chr1	29827500	0.735537
chr1	29830300	0.876033
chr1	30179800	0.644628
chr1	30370200	0.694215
chr1	30374800	0.677686
chr1	30397300	0.917355
chr1	30399800	0.710744
chr1	30404700	0.702479
chr1	30447400	0.958678
chr1	30455000	0.644628
chr1	30475600	0.743802
chr1	30479300	0.801653
chr1	30527300	0.669421
chr1	30574700	0.661157
chr1	30630700	0.644628
chr1	30836000	0.752066
chr1	30853100	0.669421
chr1	30985000	0.842975
chr1	31125300	0.909091
chr1	31130900	0.752066
chr1	31135800	0.652893
chr1	31164100	0.735537
chr1	31200400	0.677686
chr1	31205800	0.661157
chr1	31233300	0.785124
chr1	31256000	0.867769
chr1	31274900	0.892562
chr1	31278100	0.760331
chr1	31281200	0.685950
chr1	31283300	0.727273
chr1	31289400	0.884298
chr1	31314300	0.884298
chr1	31317700	0.884298
chr1	31320800	0.702479
chr1	31326600	0.743802
chr1	31329100	0.867769
chr1	31344900	0.900826
chr1	31353400	0.743802
chr1	31356800	0.644628
chr1	31361200	0.933884
chr1	31365400	0.644628
chr1	31368800	0.661157
chr1	31375400	0.818182

chr1	31465700	0.644628
chr1	31595300	0.801653
chr1	31627100	0.652893
chr1	31644000	0.809917
chr1	31654900	0.752066
chr1	31688700	0.768595
chr1	31840000	0.826446
chr1	31889600	0.867769
chr1	31894600	0.669421
chr1	31907300	0.727273
chr1	32030000	0.917355
chr1	32045200	0.702479
chr1	32048700	0.826446
chr1	32059900	0.801653
chr1	32132400	0.851240
chr1	32136200	0.669421
chr1	32167000	0.677686
chr1	32172000	0.859504
chr1	32211900	0.694215
chr1	32218500	0.925620
chr1	32224600	0.842975
chr1	32234700	0.735537
chr1	32246800	0.644628
chr1	32248900	0.909091
chr1	32264200	0.752066
chr1	32397300	0.826446
chr1	32401100	0.702479
chr1	32418200	0.884298
chr1	32421500	1.000000
chr1	32453100	0.719008
chr1	32473800	0.677686
chr1	32526300	0.719008
chr1	32529100	0.776860
chr1	32534800	0.785124
chr1	32593200	0.859504
chr1	32663800	0.702479
chr1	32715700	0.760331
chr1	32798200	0.909091
chr1	32803900	0.842975
chr1	32819300	0.768595
chr1	32893400	0.752066
chr1	32904200	0.702479
chr1	32913800	0.694215
chr1	33098700	0.966942
chr1	33102300	0.966942
chr1	33178800	0.652893
chr1	33182600	0.826446
chr1	33191100	0.809917
chr1	33199800	0.677686
chr1	33203000	0.826446
chr1	33209700	0.752066
chr1	33215500	0.652893
chr1	33223100	0.768595
chr1	33240900	0.752066

chr1	33338600	0.851240
chr1	33341900	0.694215
chr1	33344200	0.851240
chr1	33348100	0.702479
chr1	33446500	0.876033
chr1	33450400	0.966942
chr1	33458400	0.933884
chr1	33471900	0.685950
chr1	33586000	0.760331
chr1	33603900	0.677686
chr1	33606500	0.966942
chr1	33617300	0.834711
chr1	33619600	0.644628
chr1	33627100	0.851240
chr1	33631100	0.727273
chr1	33635200	0.801653
chr1	33700800	0.694215
chr1	33713600	0.694215
chr1	33737600	0.702479
chr1	33740200	0.719008
chr1	33766100	0.735537
chr1	33804600	0.950413
chr1	33809000	0.727273
chr1	33813000	0.685950
chr1	33827200	0.859504
chr1	33829900	0.842975
chr1	33845200	0.661157
chr1	33982500	0.760331
chr1	34101700	0.950413
chr1	34106800	0.760331
chr1	34314100	0.900826
chr1	34433000	0.752066
chr1	34436600	0.876033
chr1	34439500	0.644628
chr1	34485600	0.710744
chr1	34503800	0.917355
chr1	34523500	0.809917
chr1	34579800	0.900826
chr1	34613100	0.694215
chr1	34615800	0.727273
chr1	34645400	0.826446
chr1	34654900	0.719008
chr1	35056300	0.826446
chr1	35063800	0.809917
chr1	35120100	0.710744
chr1	35122500	0.685950
chr1	35150300	0.909091
chr1	35205900	0.719008
chr1	35215700	0.776860
chr1	35229200	0.851240
chr1	35262600	0.735537
chr1	35319100	0.975207
chr1	35384600	0.661157
chr1	35389300	0.809917

chr1	35436800	0.760331
chr1	35676600	0.842975
chr1	35687300	0.677686
chr1	35856400	0.909091
chr1	35860100	0.818182
chr1	35937300	0.702479
chr1	36002500	0.851240
chr1	36005600	0.842975
chr1	36019600	0.719008
chr1	36054200	0.735537
chr1	36088900	0.876033
chr1	36157100	0.925620
chr1	36165300	0.801653
chr1	36168500	0.735537
chr1	36192100	0.644628
chr1	36563000	0.917355
chr1	36565600	0.644628
chr1	36584700	0.702479
chr1	36617000	0.702479
chr1	36641900	0.884298
chr1	36653700	0.851240
chr1	36670000	0.719008
chr1	36817600	0.710744
chr1	36840300	0.842975
chr1	36906600	0.685950
chr1	36980000	0.677686
chr1	37042200	0.818182
chr1	37047100	0.677686
chr1	37052600	0.851240
chr1	37056800	0.925620
chr1	37090800	0.644628
chr1	37113200	0.752066
chr1	37139100	0.809917
chr1	37169700	0.793388
chr1	37177100	0.859504
chr1	37231400	0.694215
chr1	37236000	0.644628
chr1	37290300	0.991736
chr1	37308300	0.702479
chr1	37313100	0.884298
chr1	37321400	0.743802
chr1	37379500	0.710744
chr1	37408100	0.685950
chr1	37448500	0.727273
chr1	37467700	0.801653
chr1	37472400	0.776860
chr1	37495300	0.752066
chr1	37506000	0.760331
chr1	37508900	0.801653
chr1	37542100	0.768595
chr1	37701100	0.661157
chr1	37706700	0.851240
chr1	37709400	0.876033
chr1	37733000	0.685950

chr1	37740400	0.727273
chr1	37771500	0.735537
chr1	37781200	0.851240
chr1	37784300	0.801653
chr1	37829100	0.942149
chr1	37846500	0.677686
chr1	37857500	0.652893
chr1	37861200	0.867769
chr1	37876500	0.768595
chr1	37896300	0.727273
chr1	37904700	0.785124
chr1	37918100	0.727273
chr1	37937600	0.867769
chr1	37943100	0.851240
chr1	37945800	0.867769
chr1	38077600	0.991736
chr1	38126800	0.892562
chr1	38129500	0.900826
chr1	38463300	0.958678
chr1	38475100	0.785124
chr1	38492100	0.694215
chr1	38496300	0.719008
chr1	38507600	0.677686
chr1	38530500	0.685950
chr1	38534300	0.719008
chr1	38539300	0.818182
chr1	38542200	0.677686
chr1	38549300	0.892562
chr1	38559300	0.710744
chr1	38607300	0.892562
chr1	38619600	0.900826
chr1	38648800	0.851240
chr1	38685000	0.834711
chr1	38709600	0.950413
chr1	38712700	0.644628
chr1	38722600	0.719008
chr1	38736500	0.991736
chr1	38739100	0.776860
chr1	38750500	0.768595
chr1	38753000	0.876033
chr1	38758300	0.925620
chr1	38803400	0.702479
chr1	38831100	0.818182
chr1	38837100	0.958678
chr1	38890100	0.942149
chr1	38914900	0.644628
chr1	38931200	0.851240
chr1	38940900	0.702479
chr1	38951800	0.909091
chr1	38955700	0.818182
chr1	38963000	0.702479
chr1	38965400	0.834711
chr1	38974600	0.727273
chr1	39036300	0.710744

chr1	39074300	0.933884
chr1	39091300	0.644628
chr1	39193500	0.884298
chr1	39207000	0.694215
chr1	39255100	0.685950
chr1	39267200	0.760331
chr1	39280300	0.867769
chr1	39301300	0.719008
chr1	39407400	0.892562
chr1	39419300	0.867769
chr1	39425200	0.710744
chr1	39451600	0.867769
chr1	39460400	0.685950
chr1	39463200	0.760331
chr1	39467500	0.966942
chr1	39470600	0.685950
chr1	39482800	0.909091
chr1	39564100	0.917355
chr1	39574300	0.677686
chr1	39581400	0.859504
chr1	39595700	0.669421
chr1	39599000	0.760331
chr1	39608000	1.000000
chr1	39616500	0.876033
chr1	39619900	0.710744
chr1	39626500	0.826446
chr1	39630800	0.801653
chr1	39636800	0.983471
chr1	39650300	0.719008
chr1	39657100	0.743802
chr1	39670900	0.958678
chr1	39680600	0.876033
chr1	39687300	0.760331
chr1	39696400	0.909091
chr1	39736800	0.735537
chr1	39744000	0.818182
chr1	39813200	0.752066
chr1	39857900	0.644628
chr1	39864300	0.685950
chr1	39880400	0.669421
chr1	39978500	0.702479
chr1	40112300	0.776860
chr1	40149700	0.735537
chr1	40160800	0.677686
chr1	40167200	0.677686
chr1	40177900	0.727273
chr1	40344000	0.743802
chr1	40358400	0.867769
chr1	40361500	0.925620
chr1	40415700	0.785124
chr1	40446200	0.826446
chr1	40498300	0.727273
chr1	40525200	0.694215
chr1	40875100	0.818182

chr1	41078500	0.661157
chr1	41116100	0.702479
chr1	41129000	0.776860
chr1	41198300	0.909091
chr1	41239400	0.925620
chr1	41260400	0.661157
chr1	41267200	0.677686
chr1	41269500	0.876033
chr1	41290800	0.917355
chr1	41365400	0.694215
chr1	41398200	0.661157
chr1	41418500	0.917355
chr1	41431600	0.710744
chr1	41448400	0.776860
chr1	41487000	0.760331
chr1	41752200	0.801653
chr1	41780600	0.801653
chr1	41786200	0.661157
chr1	41824600	0.710744
chr1	41834500	0.933884
chr1	41837900	0.768595
chr1	41853900	0.818182
chr1	41857800	0.644628
chr1	41865200	0.876033
chr1	41870500	0.809917
chr1	41874200	0.826446
chr1	41889700	0.743802
chr1	41901700	0.909091
chr1	41940800	0.727273
chr1	41948800	0.752066
chr1	41962100	0.983471
chr1	41973600	0.661157
chr1	42085700	0.735537
chr1	42122100	0.719008
chr1	42246200	0.760331
chr1	42289500	0.983471
chr1	42308800	0.719008
chr1	42328400	0.727273
chr1	42336400	0.719008
chr1	42340200	0.685950
chr1	42420500	0.785124
chr1	42447300	0.768595
chr1	42732600	0.933884
chr1	42744800	0.677686
chr1	42806400	0.685950
chr1	43207000	0.958678
chr1	43393800	0.809917
chr1	43397900	0.760331
chr1	43400000	0.851240
chr1	43403300	0.760331
chr1	43407600	0.735537
chr1	43410300	0.768595
chr1	43412700	0.661157
chr1	43416100	0.909091

chr1	43418800	0.892562
chr1	43427200	0.719008
chr1	43429600	0.950413
chr1	43433600	0.950413
chr1	43444600	0.859504
chr1	43459400	0.644628
chr1	43472300	0.859504
chr1	43475000	0.652893
chr1	43524900	0.760331
chr1	43535200	0.752066
chr1	43557400	0.900826
chr1	43609400	0.760331
chr1	43673000	0.983471
chr1	43693200	0.884298
chr1	43696200	0.685950
chr1	43749400	0.694215
chr1	43827300	0.677686
chr1	43888300	0.685950
chr1	43960000	0.719008
chr1	44000400	0.685950
chr1	44013000	0.801653
chr1	44015900	0.851240
chr1	44018400	0.826446
chr1	44024500	0.867769
chr1	44029600	0.801653
chr1	44031800	0.644628
chr1	44119300	0.966942
chr1	44126200	0.876033
chr1	44232800	0.950413
chr1	44270400	0.702479
chr1	44301000	0.950413
chr1	44307500	0.942149
chr1	44324700	0.867769
chr1	44351100	0.867769
chr1	44354100	0.776860
chr1	44379400	0.677686
chr1	44396500	0.710744
chr1	44404600	0.743802
chr1	44408900	0.735537
chr1	44419300	0.710744
chr1	44499600	0.694215
chr1	44502600	0.702479
chr1	44509400	0.710744
chr1	44535400	0.652893
chr1	44555400	0.809917
chr1	44704200	0.826446
chr1	44720900	0.685950
chr1	44723000	0.710744
chr1	44727700	0.834711
chr1	44784600	0.876033
chr1	44793900	0.793388
chr1	44813800	0.842975
chr1	44834500	0.719008
chr1	44887500	0.859504

chr1	44890800	0.719008
chr1	44894800	0.801653
chr1	44902700	0.793388
chr1	44907000	0.768595
chr1	44926300	0.892562
chr1	44938800	0.685950
chr1	44969100	0.669421
chr1	44973900	0.884298
chr1	44977400	0.752066
chr1	44998300	0.793388
chr1	45002500	0.719008
chr1	45006500	0.685950
chr1	45042500	0.991736
chr1	45058300	0.834711
chr1	45061100	0.776860
chr1	45076800	0.809917
chr1	45115900	0.950413
chr1	45119100	0.685950
chr1	45147900	0.867769
chr1	45155100	0.735537
chr1	45172100	0.859504
chr1	45174400	0.785124
chr1	45244500	0.669421
chr1	45259200	0.826446
chr1	45272300	0.776860
chr1	45275200	0.809917
chr1	45772400	0.809917
chr1	45787100	0.900826
chr1	45826400	0.785124
chr1	46009900	0.727273
chr1	46039500	0.826446
chr1	46054900	0.702479
chr1	46144500	0.685950
chr1	46148300	0.661157
chr1	46314400	0.652893
chr1	46379200	0.818182
chr1	46388000	0.685950
chr1	46395900	0.776860
chr1	46420000	0.892562
chr1	46435000	0.710744
chr1	46485200	0.768595
chr1	46509400	0.760331
chr1	46633000	0.818182
chr1	46640100	0.694215
chr1	46646100	0.644628
chr1	46687700	0.892562
chr1	46689900	0.752066
chr1	46764500	0.785124
chr1	46773100	0.876033
chr1	46775200	0.950413
chr1	46867600	0.925620
chr1	46912400	0.685950
chr1	46916400	0.735537
chr1	46919000	0.925620

chr1	46935800	0.867769
chr1	46940200	0.677686
chr1	46942800	0.826446
chr1	46946300	0.710744
chr1	46951600	0.859504
chr1	46957200	0.669421
chr1	46960300	0.925620
chr1	46977800	0.694215
chr1	46983000	0.801653
chr1	46986200	0.909091
chr1	46988700	0.842975
chr1	46996800	0.809917
chr1	47016400	0.776860
chr1	47077500	0.917355
chr1	47079800	1.000000
chr1	47131800	0.644628
chr1	47141400	0.876033
chr1	47143600	0.743802
chr1	47190500	0.818182
chr1	47194400	0.677686
chr1	47223500	0.859504
chr1	47288700	0.859504
chr1	47341700	0.694215
chr1	47449600	0.719008
chr1	47483800	0.710744
chr1	47557700	0.719008
chr1	47573600	0.727273
chr1	47578700	0.677686
chr1	47609700	0.752066
chr1	47641300	0.752066
chr1	47656000	0.685950
chr1	47890500	0.801653
chr1	47905700	0.793388
chr1	47912300	0.900826
chr1	47915900	0.842975
chr1	47918600	0.677686
chr1	47925400	0.958678
chr1	47940600	0.644628
chr1	47961200	0.884298
chr1	47989500	0.752066
chr1	48014300	0.818182
chr1	48030700	0.876033
chr1	48038700	0.917355
chr1	48041800	0.743802
chr1	48049400	0.661157
chr1	48152600	0.900826
chr1	48154700	0.677686
chr1	48243000	0.735537
chr1	48293600	0.719008
chr1	48308700	0.867769
chr1	48360200	0.826446
chr1	48364100	0.958678
chr1	48452400	0.859504
chr1	48458800	0.727273

chr1	48471900	0.884298
chr1	48494000	0.942149
chr1	48505800	0.793388
chr1	48509400	0.793388
chr1	48526800	0.702479
chr1	48576400	0.694215
chr1	48674100	0.710744
chr1	48960400	0.685950
chr1	48965600	0.719008
chr1	49061800	0.702479
chr1	49085600	0.826446
chr1	49111900	0.710744
chr1	49147500	0.851240
chr1	49176400	1.000000
chr1	49339800	0.710744
chr1	50717900	0.760331
chr1	50741800	0.727273
chr1	50815200	0.652893
chr1	50859500	0.661157
chr1	50893500	0.842975
chr1	50937500	0.760331
chr1	51077100	0.694215
chr1	51086900	0.710744
chr1	51134200	0.644628
chr1	51171600	0.661157
chr1	51339500	0.809917
chr1	51889300	0.966942
chr1	52200600	0.702479
chr1	52324900	0.975207
chr1	52376500	0.694215
chr1	52423500	0.752066
chr1	52426800	0.768595
chr1	52429700	0.818182
chr1	52463900	0.826446
chr1	52472100	0.917355
chr1	52585500	0.694215
chr1	52682400	0.694215
chr1	52814600	0.669421
chr1	52817000	0.892562
chr1	52920700	0.652893
chr1	52927400	0.710744
chr1	53034300	0.644628
chr1	53079800	0.719008
chr1	53126100	0.950413
chr1	53128900	0.958678
chr1	53145200	0.884298
chr1	53152200	0.685950
chr1	53298700	0.710744
chr1	53341700	0.752066
chr1	53371400	0.809917
chr1	53442300	0.966942
chr1	53515300	0.867769
chr1	53547800	0.652893
chr1	53554300	0.702479

chr1	53582000	0.776860
chr1	53586300	0.702479
chr1	53753700	0.842975
chr1	53770000	0.900826
chr1	53842300	0.694215
chr1	53863600	0.661157
chr1	53882700	0.867769
chr1	53884900	0.652893
chr1	53896600	0.983471
chr1	53908000	0.826446
chr1	53914600	0.991736
chr1	53936400	0.760331
chr1	53951700	0.768595
chr1	53969700	0.834711
chr1	54018600	0.876033
chr1	54025400	0.719008
chr1	54059500	0.694215
chr1	54072400	0.768595
chr1	54100600	0.776860
chr1	54139400	0.867769
chr1	54147900	0.785124
chr1	54151600	0.694215
chr1	54160100	0.834711
chr1	54171300	1.000000
chr1	54191000	0.752066
chr1	54379500	0.958678
chr1	54415100	0.785124
chr1	54583100	0.851240
chr1	54587600	0.727273
chr1	54590900	0.752066
chr1	54601600	0.909091
chr1	54604000	0.661157
chr1	54689600	0.801653
chr1	54694300	0.685950
chr1	54723900	0.644628
chr1	54738900	0.851240
chr1	54753100	0.644628
chr1	54758300	0.694215
chr1	54761000	0.661157
chr1	54773100	0.768595
chr1	54778100	0.942149
chr1	54780900	0.876033
chr1	54786900	0.710744
chr1	54793800	0.867769
chr1	54796000	0.909091
chr1	54798400	0.892562
chr1	54801300	0.859504
chr1	54808300	0.917355
chr1	54816000	0.909091
chr1	54819100	0.669421
chr1	54827500	0.776860
chr1	54830600	0.842975
chr1	54839100	0.710744
chr1	54842500	0.958678

chr1	54845800	0.677686
chr1	54850600	0.644628
chr1	54858100	0.735537
chr1	54878200	0.652893
chr1	54925600	0.867769
chr1	54928300	0.727273
chr1	54931100	0.851240
chr1	54937600	0.859504
chr1	54943000	0.818182
chr1	54946600	0.917355
chr1	54956600	0.694215
chr1	55006600	0.710744
chr1	55014900	0.652893
chr1	55035700	0.834711
chr1	55038000	0.933884
chr1	55041200	0.793388
chr1	55045200	0.884298
chr1	55048800	0.884298
chr1	55076900	0.991736
chr1	55081200	0.677686
chr1	55085600	0.661157
chr1	55241000	0.743802
chr1	55247300	0.652893
chr1	55255000	0.876033
chr1	55261000	0.727273
chr1	55266900	0.685950
chr1	55280800	0.851240
chr1	55349800	0.776860
chr1	55404100	0.661157
chr1	55416600	0.925620
chr1	55429000	0.851240
chr1	55444200	0.867769
chr1	55460800	0.834711
chr1	55476600	0.727273
chr1	55488100	0.727273
chr1	55677600	0.652893
chr1	55743200	0.752066
chr1	55789500	0.785124
chr1	56124200	0.776860
chr1	56184700	0.818182
chr1	56655300	0.752066
chr1	56836500	0.867769
chr1	56840800	0.669421
chr1	56903100	0.975207
chr1	56919400	0.975207
chr1	56960700	0.661157
chr1	56976800	0.892562
chr1	56983100	0.884298
chr1	56994000	0.752066
chr1	56997500	0.661157
chr1	57001000	0.859504
chr1	57003500	0.826446
chr1	57010100	0.975207
chr1	57016200	0.983471

chr1	57019000	0.917355
chr1	57029200	0.809917
chr1	57032600	0.818182
chr1	57036500	0.776860
chr1	57154300	0.652893
chr1	57285400	0.867769
chr1	57400800	0.719008
chr1	57405200	0.752066
chr1	57568500	0.801653
chr1	57578400	0.727273
chr1	57623700	0.743802
chr1	57629100	0.776860
chr1	57632100	0.743802
chr1	57637100	0.818182
chr1	57661600	0.818182
chr1	57664600	0.644628
chr1	57681400	0.776860
chr1	57728000	0.776860
chr1	57803100	0.925620
chr1	57837100	0.669421
chr1	57916900	0.694215
chr1	58017500	0.719008
chr1	58066700	0.892562
chr1	58089700	0.702479
chr1	58099200	0.727273
chr1	58177300	0.710744
chr1	58190900	0.710744
chr1	58298500	0.834711
chr1	58324600	0.743802
chr1	58450000	0.809917
chr1	58453900	0.669421
chr1	58469600	0.834711
chr1	58473100	0.876033
chr1	58497500	0.991736
chr1	58504900	0.661157
chr1	58526300	0.694215
chr1	58530100	0.644628
chr1	58555100	0.694215
chr1	58625000	0.884298
chr1	58629100	0.727273
chr1	58699600	0.884298
chr1	58702600	0.727273
chr1	58774100	0.950413
chr1	58811600	0.818182
chr1	58829800	0.677686
chr1	58854400	0.685950
chr1	58857800	0.785124
chr1	58899000	0.776860
chr1	58932400	0.694215
chr1	58963800	0.793388
chr1	59039200	0.785124
chr1	59085700	0.677686
chr1	59170600	1.000000
chr1	59289700	0.661157

chr1	59319300	0.801653
chr1	59329100	0.702479
chr1	59340800	0.892562
chr1	59448100	0.702479
chr1	59479500	0.983471
chr1	59482000	0.710744
chr1	59523700	0.677686
chr1	59529600	0.743802
chr1	59538200	0.710744
chr1	59545900	0.966942
chr1	59582900	0.933884
chr1	59609100	0.876033
chr1	59617100	0.702479
chr1	59776100	0.842975
chr1	59783600	0.644628
chr1	59849800	0.710744
chr1	59859100	0.694215
chr1	59884600	0.743802
chr1	59912500	0.644628
chr1	60139500	0.669421
chr1	60144100	0.892562
chr1	60152800	0.661157
chr1	60186200	0.776860
chr1	60225300	0.752066
chr1	60463800	0.842975
chr1	61172600	0.735537
chr1	61314900	0.834711
chr1	61327600	0.834711
chr1	61337000	0.652893
chr1	61352600	0.719008
chr1	61360400	0.859504
chr1	61372200	0.710744
chr1	61377900	0.867769
chr1	61387100	0.694215
chr1	61402900	0.768595
chr1	61436100	0.743802
chr1	61440700	0.768595
chr1	61471600	0.917355
chr1	61545900	0.710744
chr1	61577400	0.685950
chr1	61625100	0.644628
chr1	61700500	0.652893
chr1	61704200	0.900826
chr1	61714200	0.793388
chr1	61716300	0.685950
chr1	61750400	0.917355
chr1	61762100	0.892562
chr1	61767700	0.727273
chr1	61786100	0.727273
chr1	61803800	0.760331
chr1	61819100	0.818182
chr1	61928400	0.768595
chr1	61947800	0.694215
chr1	62055000	0.909091

chr1	62102800	0.809917
chr1	62319700	0.743802
chr1	62416800	0.876033
chr1	62419600	0.859504
chr1	62499400	0.842975
chr1	62506600	0.702479
chr1	62525000	0.727273
chr1	62576300	0.652893
chr1	62608600	0.900826
chr1	62641000	0.834711
chr1	62643200	0.876033
chr1	62646800	0.933884
chr1	62649800	0.776860
chr1	62694300	0.892562
chr1	62729500	0.826446
chr1	62745300	0.702479
chr1	62748900	0.644628
chr1	62762100	0.884298
chr1	62793700	0.793388
chr1	62803600	0.785124
chr1	62872600	0.826446
chr1	62942600	0.801653
chr1	63051500	0.892562
chr1	63142800	0.752066
chr1	63156600	0.760331
chr1	63243900	0.669421
chr1	63384000	0.801653
chr1	63419600	0.785124
chr1	63429200	0.661157
chr1	63508000	0.694215
chr1	63570400	0.685950
chr1	63578100	0.685950
chr1	63654500	0.826446
chr1	63761000	0.776860
chr1	63765400	0.768595
chr1	63770900	0.785124
chr1	64023500	0.735537
chr1	64040200	0.851240
chr1	64067400	0.752066
chr1	64078900	0.735537
chr1	64091600	0.768595
chr1	64128500	0.727273
chr1	64149000	0.818182
chr1	64187900	0.719008
chr1	64195000	0.652893
chr1	64198400	0.652893
chr1	64212700	0.818182
chr1	64237900	0.884298
chr1	64244100	0.719008
chr1	64246800	0.826446
chr1	64249100	1.000000
chr1	64255400	0.966942
chr1	64257600	0.743802
chr1	64260500	0.876033

chr1	64263100	0.743802
chr1	64265800	0.661157
chr1	64270500	0.884298
chr1	64283300	0.776860
chr1	64295900	0.644628
chr1	64299800	0.826446
chr1	64302600	0.917355
chr1	64308400	0.876033
chr1	64313300	0.818182
chr1	64329900	0.677686
chr1	64333900	0.842975
chr1	64337300	0.669421
chr1	64343800	0.735537
chr1	64357500	0.702479
chr1	64374100	1.000000
chr1	64379200	0.809917
chr1	64382800	0.685950
chr1	64387100	0.685950
chr1	64438500	0.644628
chr1	64472400	0.677686
chr1	64504900	0.710744
chr1	64512400	0.685950
chr1	64527200	0.867769
chr1	64539000	0.743802
chr1	64568000	0.677686
chr1	64575000	0.694215
chr1	64603100	0.727273
chr1	64614200	0.669421
chr1	64711400	0.809917
chr1	64822900	0.826446
chr1	64825000	0.644628
chr1	64940600	0.685950
chr1	64945700	0.752066
chr1	64959800	0.925620
chr1	64963100	0.983471
chr1	64965300	0.719008
chr1	64973700	0.900826
chr1	64979500	0.826446
chr1	64983500	0.719008
chr1	64986300	0.785124
chr1	64993100	0.710744
chr1	65003200	0.892562
chr1	65006300	0.826446
chr1	65011300	0.677686
chr1	65030800	0.752066
chr1	65035700	0.702479
chr1	65038400	0.702479
chr1	65055100	0.644628
chr1	65108300	0.710744
chr1	65290200	0.661157
chr1	65327500	0.776860
chr1	65363900	0.867769
chr1	65373000	0.727273
chr1	65394400	0.669421

chr1	65422200	0.826446
chr1	65426600	0.661157
chr1	65619900	0.760331
chr1	65891200	0.925620
chr1	65898900	0.818182
chr1	65910600	0.900826
chr1	66153700	0.694215
chr1	66642400	0.710744
chr1	66731100	0.685950
chr1	66737700	0.859504
chr1	66801500	0.933884
chr1	66815700	0.685950
chr1	66817800	0.884298
chr1	66820100	0.859504
chr1	66822600	0.958678
chr1	66911800	0.719008
chr1	67006600	0.727273
chr1	67196600	0.801653
chr1	67485300	0.727273
chr1	67506400	0.669421
chr1	67524500	0.743802
chr1	67563600	0.826446
chr1	67603500	0.685950
chr1	67615200	0.768595
chr1	67690800	0.677686
chr1	67722800	0.752066
chr1	67728200	0.735537
chr1	67808100	0.917355
chr1	67814300	0.809917
chr1	67855900	0.743802
chr1	67898600	0.760331
chr1	67924900	0.950413
chr1	67930000	0.818182
chr1	68021500	0.785124
chr1	68067500	0.793388
chr1	68209400	0.694215
chr1	68212200	0.892562
chr1	68215300	0.966942
chr1	68219100	0.685950
chr1	68232700	0.809917
chr1	68270300	0.752066
chr1	68289400	0.661157
chr1	68305400	0.826446
chr1	68354400	0.652893
chr1	68607100	0.892562
chr1	68744100	0.851240
chr1	68758400	0.644628
chr1	68931100	0.644628
chr1	69066700	0.702479
chr1	69443100	0.983471
chr1	69446000	0.818182
chr1	69542800	0.661157
chr1	69557600	0.818182
chr1	69595100	0.702479

chr1	69648100	0.702479
chr1	69700300	0.669421
chr1	69710200	0.966942
chr1	70204200	0.669421
chr1	70892200	0.727273
chr1	70907800	0.785124
chr1	70910100	0.867769
chr1	71012900	0.719008
chr1	71022000	0.669421
chr1	71053300	0.925620
chr1	71141500	0.743802
chr1	71194000	0.669421
chr1	71354700	0.818182
chr1	71695100	0.818182
chr1	71859300	0.677686
chr1	71904400	0.652893
chr1	72059100	0.719008
chr1	72204200	0.669421
chr1	72303100	0.710744
chr1	72484800	0.652893
chr1	72500800	0.677686
chr1	73136900	0.661157
chr1	73453100	0.793388
chr1	74409700	0.785124
chr1	74990300	0.685950
chr1	75247100	0.677686
chr1	75540700	0.917355
chr1	75545100	0.644628
chr1	75549200	0.933884
chr1	75808600	0.719008
chr1	76095900	0.743802
chr1	76138500	0.966942
chr1	76186100	0.785124
chr1	76514500	0.702479
chr1	76518900	0.900826
chr1	76521600	0.685950
chr1	76620200	0.710744
chr1	76695000	0.876033
chr1	76697300	0.933884
chr1	76704800	0.867769
chr1	76713400	0.768595
chr1	76716700	0.719008
chr1	76967600	0.983471
chr1	76977100	0.867769
chr1	77049100	0.809917
chr1	77320500	0.776860
chr1	77374500	0.661157
chr1	77385100	0.644628
chr1	77399700	0.793388
chr1	77485000	0.900826
chr1	77491900	0.661157
chr1	77524400	0.793388
chr1	77547900	0.719008
chr1	77980500	1.000000

chr1	78100700	0.644628
chr1	78470600	0.743802
chr1	78587700	0.768595
chr1	78621000	0.785124
chr1	78703800	0.785124
chr1	78729700	0.752066
chr1	78903700	0.685950
chr1	79351200	0.785124
chr1	79659100	0.809917
chr1	79701800	0.719008
chr1	79728600	1.000000
chr1	80192600	0.685950
chr1	80647400	0.768595
chr1	80999800	0.768595
chr1	81003700	0.727273
chr1	81140400	0.785124
chr1	81623200	0.719008
chr1	81692300	0.644628
chr1	81704000	0.942149
chr1	81717200	0.826446
chr1	81731200	0.702479
chr1	81771800	0.661157
chr1	81777800	0.892562
chr1	81780300	0.793388
chr1	81795000	0.785124
chr1	81814600	0.909091
chr1	81816800	0.801653
chr1	81845600	0.685950
chr1	81875600	0.694215
chr1	81880200	0.892562
chr1	81897300	0.983471
chr1	81975500	0.834711
chr1	81978400	0.719008
chr1	82012500	0.876033
chr1	82014600	0.917355
chr1	82063800	0.727273
chr1	82070900	0.702479
chr1	82129800	0.958678
chr1	82147900	0.892562
chr1	82165500	0.669421
chr1	82170000	0.727273
chr1	82246400	0.735537
chr1	82263500	0.809917
chr1	82274900	0.685950
chr1	82277500	0.876033
chr1	82299600	0.818182
chr1	82340800	0.694215
chr1	82431200	0.735537
chr1	82444900	0.975207
chr1	82951900	0.975207
chr1	83137800	0.661157
chr1	83279800	0.826446
chr1	83311000	0.884298
chr1	83393700	0.644628

chr1	83648300	0.661157
chr1	83674600	0.925620
chr1	83682400	0.661157
chr1	83684600	0.652893
chr1	83755700	0.702479
chr1	83880000	0.909091
chr1	84083600	0.925620
chr1	84159000	0.917355
chr1	84207300	0.752066
chr1	84217200	0.735537
chr1	84227100	0.694215
chr1	84236100	0.694215
chr1	84254000	0.661157
chr1	84436900	0.760331
chr1	84640300	0.785124
chr1	84720700	0.859504
chr1	84737600	0.859504
chr1	84769000	0.909091
chr1	85037800	0.661157
chr1	85049500	0.859504
chr1	85141600	0.793388
chr1	85163800	0.776860
chr1	85168300	0.661157
chr1	85181400	0.710744
chr1	85206400	0.768595
chr1	85223300	0.909091
chr1	85230400	0.851240
chr1	85240900	0.719008
chr1	85373400	0.727273
chr1	85410800	0.685950
chr1	85516100	0.719008
chr1	85691200	0.851240
chr1	85705900	0.669421
chr1	85774400	0.892562
chr1	85796100	0.859504
chr1	85809200	0.685950
chr1	85859400	0.710744
chr1	85863400	0.694215
chr1	85902900	0.685950
chr1	85912700	0.793388
chr1	85940400	0.661157
chr1	85987300	0.644628
chr1	85993200	0.785124
chr1	86050100	0.975207
chr1	86073500	0.958678
chr1	86095000	0.834711
chr1	86284600	0.702479
chr1	86442200	0.727273
chr1	86566600	0.851240
chr1	87399900	0.917355
chr1	87416800	0.685950
chr1	87491300	0.694215
chr1	87614900	0.785124
chr1	87618000	0.793388

chr1	87656200	0.644628
chr1	87667200	0.909091
chr1	87692500	0.818182
chr1	87894600	0.950413
chr1	87903100	0.719008
chr1	87930400	0.685950
chr1	88109400	0.859504
chr1	88152000	0.842975
chr1	88189200	0.760331
chr1	88369100	0.752066
chr1	88407300	0.661157
chr1	88417900	0.826446
chr1	88434400	0.644628
chr1	88439900	0.644628
chr1	88602900	0.710744
chr1	88713500	0.677686
chr1	88763600	0.677686
chr1	88870500	0.867769
chr1	89114400	0.652893
chr1	89164100	0.661157
chr1	89240100	0.876033
chr1	89243300	0.876033
chr1	89248400	0.933884
chr1	89287200	0.661157
chr1	89843900	0.652893
chr1	89871400	0.644628
chr1	89993100	0.710744
chr1	89996700	0.760331
chr1	90032100	1.000000
chr1	90056400	0.685950
chr1	90064700	0.826446
chr1	90267900	0.694215
chr1	90274200	0.950413
chr1	90289800	0.661157
chr1	90299800	0.917355
chr1	90302600	0.785124
chr1	90305400	0.677686
chr1	90372100	0.785124
chr1	90376800	1.000000
chr1	90384500	0.958678
chr1	90395200	0.975207
chr1	90399100	0.917355
chr1	90405600	0.743802
chr1	90415800	0.719008
chr1	90739400	0.719008
chr1	90741600	0.809917
chr1	90967400	0.694215
chr1	90975100	0.685950
chr1	91000500	0.677686
chr1	91003900	0.677686
chr1	91026900	0.793388
chr1	91088500	0.710744
chr1	91170600	0.644628
chr1	91176900	0.842975

chr1	91180000	0.752066
chr1	91182700	0.743802
chr1	91227800	0.809917
chr1	91250200	0.694215
chr1	91275100	0.842975
chr1	91298800	0.669421
chr1	91308600	0.768595
chr1	91385300	0.776860
chr1	91389700	0.735537
chr1	91392800	0.983471
chr1	91423900	0.809917
chr1	91438000	0.644628
chr1	91440300	0.925620
chr1	91460900	0.785124
chr1	91473900	0.785124
chr1	91481900	0.933884
chr1	91502700	0.685950
chr1	91532300	0.669421
chr1	91570400	0.900826
chr1	91586600	0.909091
chr1	91590400	0.958678
chr1	91604600	0.818182
chr1	91631100	0.859504
chr1	91853100	1.000000
chr1	92012800	0.644628
chr1	92031200	0.694215
chr1	92077700	0.685950
chr1	92083500	0.685950
chr1	92111200	0.727273
chr1	92149400	0.917355
chr1	92179200	0.826446
chr1	92256700	0.702479
chr1	92907700	0.669421
chr1	92942100	0.702479
chr1	93258000	0.785124
chr1	93466400	0.942149
chr1	93482100	0.661157
chr1	93741800	0.909091
chr1	93762600	0.768595
chr1	93824500	0.785124
chr1	93877200	0.925620
chr1	94018700	0.809917
chr1	94041300	0.752066
chr1	94060100	0.760331
chr1	94064100	0.661157
chr1	94074700	0.735537
chr1	94080700	0.735537
chr1	94083600	0.743802
chr1	94101700	0.933884
chr1	94104900	0.752066
chr1	94110500	0.818182
chr1	94142100	0.727273
chr1	94188900	0.743802
chr1	94199300	0.694215

chr1	94235100	0.752066
chr1	94273300	0.677686
chr1	94280900	0.809917
chr1	94286800	0.900826
chr1	94294600	0.933884
chr1	94445300	0.793388
chr1	94479000	0.735537
chr1	94487400	0.785124
chr1	94514300	0.702479
chr1	94560100	0.842975
chr1	94571200	0.793388
chr1	94579400	0.925620
chr1	94585800	0.801653
chr1	94612300	0.669421
chr1	94861100	0.752066
chr1	95004400	0.727273
chr1	95075000	0.719008
chr1	95089400	0.776860
chr1	95123500	1.000000
chr1	95127000	0.991736
chr1	95133800	0.677686
chr1	95154700	0.909091
chr1	95179600	0.644628
chr1	95200600	0.677686
chr1	95274700	0.727273
chr1	95320700	0.851240
chr1	95328600	0.768595
chr1	95331700	0.991736
chr1	95349400	0.702479
chr1	95353600	0.859504
chr1	95360600	0.809917
chr1	95375900	0.752066
chr1	95385800	0.644628
chr1	95401800	0.710744
chr1	95419800	0.677686
chr1	95436800	0.842975
chr1	95546600	0.685950
chr1	95551100	0.859504
chr1	95663700	0.644628
chr1	95873000	0.826446
chr1	95875200	0.793388
chr1	95976700	0.710744
chr1	95984100	0.917355
chr1	96049100	0.917355
chr1	96469000	0.900826
chr1	96526800	0.768595
chr1	96569700	0.694215
chr1	96650000	0.809917
chr1	96725400	0.793388
chr1	96931600	0.760331
chr1	96948000	0.644628
chr1	96954100	0.876033
chr1	96985800	0.933884
chr1	96989100	0.719008

chr1	97010400	0.702479
chr1	97025700	0.975207
chr1	97028500	0.900826
chr1	97046700	0.900826
chr1	97048900	0.776860
chr1	97181300	0.694215
chr1	97580700	0.743802
chr1	98553100	0.661157
chr1	98671500	0.768595
chr1	98719100	0.694215
chr1	98924400	0.743802
chr1	98968400	0.644628
chr1	99457500	0.727273
chr1	99535300	0.801653
chr1	99793300	0.743802
chr1	100111700	0.867769
chr1	100514700	0.702479
chr1	100613600	0.909091
chr1	100791400	0.859504
chr1	100877300	0.892562
chr1	100900400	0.727273
chr1	100906100	0.652893
chr1	100924900	0.669421
chr1	100928200	0.933884
chr1	101416100	0.942149
chr1	101437500	0.735537
chr1	101602900	0.834711
chr1	101757400	0.644628
chr1	102098700	0.719008
chr1	102119600	0.942149
chr1	102179700	0.760331
chr1	102309500	0.776860
chr1	102573000	0.867769
chr1	103242000	0.735537
chr1	103716600	0.859504
chr1	104047000	0.694215
chr1	104600000	0.661157
chr1	104627400	0.702479
chr1	104635600	0.685950
chr1	107066400	0.801653
chr1	107289600	0.727273
chr1	107496500	0.694215
chr1	107527600	0.735537
chr1	107718400	0.694215
chr1	107738000	0.743802
chr1	107790900	0.727273
chr1	107799300	0.842975
chr1	107820000	0.719008
chr1	107963800	0.793388
chr1	108066400	0.776860
chr1	108113300	0.685950
chr1	108293000	0.760331
chr1	108309800	0.677686
chr1	108364900	0.727273

chr1	108410700	0.818182
chr1	108420300	0.735537
chr1	108474700	0.652893
chr1	108479300	0.958678
chr1	108542700	0.785124
chr1	108593500	0.768595
chr1	108596800	0.876033
chr1	108600700	0.685950
chr1	108752000	0.809917
chr1	108804000	0.818182
chr1	108975800	0.892562
chr1	109076500	0.859504
chr1	109228300	0.768595
chr1	109359500	0.942149
chr1	109491000	0.966942
chr1	109626500	0.727273
chr1	109768400	0.652893
chr1	109773700	0.842975
chr1	109780400	0.876033
chr1	109785400	0.694215
chr1	109791500	0.661157
chr1	109796600	0.859504
chr1	109805900	0.793388
chr1	109828200	0.760331
chr1	109849900	0.826446
chr1	109903900	0.768595
chr1	109914300	0.793388
chr1	109926200	0.958678
chr1	109935300	0.669421
chr1	110011600	0.652893
chr1	110041100	0.884298
chr1	110104600	0.652893
chr1	110107100	0.958678
chr1	110151900	0.685950
chr1	110160300	0.661157
chr1	110166300	0.760331
chr1	110179100	0.867769
chr1	110186600	0.727273
chr1	110326200	0.702479
chr1	110375700	0.900826
chr1	110416600	0.900826
chr1	110420100	0.867769
chr1	110455600	0.809917
chr1	110473900	0.917355
chr1	110502700	0.694215
chr1	110546400	0.966942
chr1	110550200	0.842975
chr1	110573600	0.685950
chr1	110614300	0.661157
chr1	110632500	0.768595
chr1	110673500	0.867769
chr1	110679800	0.735537
chr1	110765500	0.677686
chr1	110776800	0.685950

chr1	110784100	0.793388
chr1	110786300	0.834711
chr1	110855600	0.851240
chr1	110890100	0.909091
chr1	110933800	0.842975
chr1	110976800	0.851240
chr1	111025400	0.818182
chr1	111037800	0.876033
chr1	111056400	0.652893
chr1	111059900	0.710744
chr1	111067400	0.710744
chr1	111108500	0.677686
chr1	111292300	0.785124
chr1	111746900	0.685950
chr1	111757500	0.685950
chr1	111762500	0.743802
chr1	111907600	0.925620
chr1	112084800	0.644628
chr1	112135800	0.809917
chr1	112263400	0.876033
chr1	112272900	0.834711
chr1	112321900	0.710744
chr1	112395200	0.644628
chr1	112431900	0.644628
chr1	112447100	0.677686
chr1	112507000	0.710744
chr1	112546700	0.652893
chr1	112550100	0.975207
chr1	112636200	0.818182
chr1	112713400	0.735537
chr1	112782300	0.826446
chr1	112803900	0.710744
chr1	112903100	0.661157
chr1	112934100	0.677686
chr1	112946900	0.859504
chr1	113009200	0.884298
chr1	113067700	0.702479
chr1	113070700	0.644628
chr1	113215000	1.000000
chr1	113241200	0.793388
chr1	113243700	0.793388
chr1	113247100	0.735537
chr1	113254800	0.710744
chr1	113266100	0.719008
chr1	113313700	0.727273
chr1	113337900	0.942149
chr1	113347500	0.809917
chr1	113350000	0.743802
chr1	113352300	0.669421
chr1	113361100	0.909091
chr1	113365200	0.727273
chr1	113368900	0.760331
chr1	113448500	0.950413
chr1	113453000	0.768595

chr1	113481000	0.752066
chr1	113484800	0.743802
chr1	113564200	0.677686
chr1	113570300	0.933884
chr1	113587100	0.991736
chr1	113596800	0.809917
chr1	113600000	0.892562
chr1	113702500	0.760331
chr1	113743300	0.842975
chr1	113754700	0.685950
chr1	113763900	0.661157
chr1	113796100	0.652893
chr1	113798700	0.933884
chr1	113819300	0.644628
chr1	113864300	0.677686
chr1	114173400	0.801653
chr1	114313400	0.694215
chr1	114480500	0.644628
chr1	114519300	0.851240
chr1	114525800	0.842975
chr1	114546100	0.752066
chr1	114551100	0.859504
chr1	114567100	0.735537
chr1	114579100	0.793388
chr1	114582300	0.983471
chr1	114617400	0.917355
chr1	114619700	0.719008
chr1	114622900	0.719008
chr1	114632100	0.842975
chr1	114637000	0.669421
chr1	114648700	0.776860
chr1	114656800	0.793388
chr1	114660900	0.719008
chr1	114666200	0.859504
chr1	114688600	0.727273
chr1	114724300	0.785124
chr1	114757900	0.652893
chr1	114825800	0.702479
chr1	115007900	0.652893
chr1	115353200	0.710744
chr1	115435500	0.743802
chr1	115444700	0.826446
chr1	115599400	0.702479
chr1	115626200	0.644628
chr1	115642300	0.727273
chr1	115763300	0.685950
chr1	115843100	0.669421
chr1	116035400	0.826446
chr1	116088700	0.900826
chr1	116175500	1.000000
chr1	116193400	0.900826
chr1	116197700	0.801653
chr1	116202700	0.694215
chr1	116257400	0.752066

chr1	116280100	0.793388
chr1	116314600	0.801653
chr1	116337800	0.735537
chr1	116373300	0.710744
chr1	116467500	0.909091
chr1	116497700	0.776860
chr1	116512600	0.677686
chr1	116546000	0.661157
chr1	116682000	0.661157
chr1	116774000	0.644628
chr1	116888900	0.694215
chr1	116958900	0.719008
chr1	116997000	0.727273
chr1	117021400	0.809917
chr1	117096400	0.768595
chr1	117148500	0.719008
chr1	117159800	0.677686
chr1	117167800	0.685950
chr1	117172700	0.785124
chr1	117186200	0.884298
chr1	117189700	0.776860
chr1	117207600	0.677686
chr1	117240100	0.793388
chr1	117255700	0.991736
chr1	117339700	0.661157
chr1	117351000	0.719008
chr1	117375300	0.842975
chr1	117378600	0.694215
chr1	117396500	0.983471
chr1	117421600	0.867769
chr1	117546600	0.685950
chr1	117636100	0.909091
chr1	117678100	0.677686
chr1	117714800	0.884298
chr1	117752100	0.644628
chr1	117811500	0.818182
chr1	117814800	0.818182
chr1	117889500	0.785124
chr1	117959500	0.809917
chr1	118117100	0.851240
chr1	118175800	0.925620
chr1	118251200	0.958678
chr1	118510500	0.809917
chr1	118540300	0.760331
chr1	118617300	0.685950
chr1	118620300	0.809917
chr1	118627500	0.677686
chr1	118790600	0.702479
chr1	118793100	0.785124
chr1	119026300	0.983471
chr1	119029400	0.991736
chr1	119463400	0.917355
chr1	119503400	0.900826
chr1	119534300	0.867769

chr1	119740400	0.776860
chr1	119837400	0.752066
chr1	119846200	0.644628
chr1	119875200	0.669421
chr1	119890800	0.694215
chr1	119931100	0.743802
chr1	119993300	0.842975
chr1	120090900	0.661157
chr1	120154500	0.719008
chr1	120174700	0.818182
chr1	120202800	0.694215
chr1	120217100	0.661157
chr1	120222300	0.933884
chr1	120230000	0.884298
chr1	120258600	0.702479
chr1	120287100	0.702479
chr1	120316500	0.768595
chr1	120544200	0.958678
chr1	120557900	0.793388
chr1	120579800	0.719008
chr1	120585700	0.727273
chr1	120587800	0.876033
chr1	120602100	0.859504
chr1	120605000	0.826446
chr1	120679100	0.793388
chr1	120759500	0.760331
chr1	120765400	0.809917
chr1	120780200	0.652893
chr1	120807600	0.727273
chr1	121103800	0.942149
chr1	121370400	0.702479
chr1	121468300	0.661157
chr1	121480300	0.793388
chr1	121485500	0.834711
chr1	142967800	0.776860
chr1	143732500	0.834711
chr1	143750900	0.669421
chr1	143945400	0.900826
chr1	143963500	0.785124
chr1	143967000	0.776860
chr1	143987200	0.652893
chr1	143993200	0.776860
chr1	144008500	0.867769
chr1	144010900	0.884298
chr1	144029000	0.859504
chr1	144048200	0.710744
chr1	144062300	0.900826
chr1	144091200	0.876033
chr1	144321400	0.966942
chr1	144325100	0.669421
chr1	144328800	0.702479
chr1	144389200	0.652893
chr1	144511700	0.793388
chr1	144529100	0.809917

chr1	144538600	0.834711
chr1	144688500	0.644628
chr1	144692500	0.826446
chr1	144697300	0.785124
chr1	144700500	0.694215
chr1	144707200	0.925620
chr1	144860000	0.859504
chr1	144914000	0.644628
chr1	144937200	0.884298
chr1	144941400	0.909091
chr1	144960900	0.975207
chr1	144965400	0.809917
chr1	144967900	0.727273
chr1	144973000	0.661157
chr1	144975800	0.719008
chr1	145006500	0.652893
chr1	145014800	0.752066
chr1	145017000	0.735537
chr1	145023600	0.842975
chr1	145112800	0.702479
chr1	145144900	0.710744
chr1	145185300	0.652893
chr1	145219500	0.685950
chr1	145229300	0.661157
chr1	145233800	0.842975
chr1	145236300	0.727273
chr1	145263800	0.925620
chr1	145455500	0.842975
chr1	145457800	0.735537
chr1	145487500	0.867769
chr1	145542900	0.859504
chr1	145555300	0.694215
chr1	145701500	0.677686
chr1	145727700	0.661157
chr1	145986400	0.652893
chr1	146017200	0.809917
chr1	146124600	0.644628
chr1	146133300	0.735537
chr1	146140200	0.719008
chr1	146149000	0.785124
chr1	146592700	0.652893
chr1	146596200	0.694215
chr1	146719300	0.727273
chr1	146808600	0.917355
chr1	147016900	0.859504
chr1	147039500	0.892562
chr1	147042400	0.677686
chr1	147060800	0.760331
chr1	147063400	0.752066
chr1	147148400	0.652893
chr1	147245700	0.768595
chr1	147253400	0.644628
chr1	147306300	0.975207
chr1	147309700	0.991736

chr1	147364800	0.694215
chr1	147666500	0.710744
chr1	147690400	0.933884
chr1	147818300	0.710744
chr1	148240600	0.801653
chr1	148534500	0.785124
chr1	148606200	0.818182
chr1	148768100	0.818182
chr1	148782000	0.710744
chr1	149035600	0.892562
chr1	149291300	0.644628
chr1	149328800	0.776860
chr1	149528300	0.710744
chr1	149583600	0.867769
chr1	149588800	0.834711
chr1	149595900	0.644628
chr1	149747000	0.776860
chr1	149908800	0.768595
chr1	149911600	0.768595
chr1	149968400	0.793388
chr1	149994500	0.685950
chr1	150137000	0.793388
chr1	150275500	0.975207
chr1	150335200	0.694215
chr1	150346900	0.727273
chr1	150381400	0.809917
chr1	150445000	0.842975
chr1	150511200	0.892562
chr1	150536800	0.685950
chr1	150542400	0.760331
chr1	150546500	0.727273
chr1	150576900	0.975207
chr1	150582400	0.933884
chr1	150595100	0.834711
chr1	150608200	0.900826
chr1	150614400	0.942149
chr1	150771500	0.685950
chr1	150779800	0.842975
chr1	150941500	0.768595
chr1	150949500	0.776860
chr1	150951900	0.694215
chr1	150954100	0.950413
chr1	150956300	0.685950
chr1	151009600	0.867769
chr1	151263400	0.818182
chr1	151267800	0.958678
chr1	151343000	0.719008
chr1	151345300	0.685950
chr1	151519800	0.958678
chr1	151675200	0.652893
chr1	151677800	0.661157
chr1	151684300	0.661157
chr1	151696900	0.834711
chr1	151701400	0.768595

chr1	151707900	0.669421
chr1	151798800	0.925620
chr1	151801900	0.925620
chr1	151819200	0.859504
chr1	151833800	0.834711
chr1	151941200	0.694215
chr1	151961900	0.677686
chr1	151986400	0.785124
chr1	152016300	0.760331
chr1	152020900	0.677686
chr1	152969600	0.785124
chr1	153095800	0.842975
chr1	153265700	0.909091
chr1	153279500	0.661157
chr1	153439100	0.958678
chr1	153456900	0.801653
chr1	153468300	0.884298
chr1	153511200	0.851240
chr1	153517900	0.942149
chr1	153520600	0.867769
chr1	153538600	0.834711
chr1	153541300	0.826446
chr1	153571700	0.991736
chr1	153581800	0.851240
chr1	153585400	0.727273
chr1	153596200	0.826446
chr1	153670700	0.842975
chr1	153716000	0.685950
chr1	153719100	0.975207
chr1	153750300	0.685950
chr1	153766200	0.768595
chr1	153837100	0.834711
chr1	153846100	0.909091
chr1	153855000	0.685950
chr1	153932700	0.851240
chr1	154152400	1.000000
chr1	154160100	0.950413
chr1	154403600	0.727273
chr1	154405800	0.834711
chr1	154528500	0.809917
chr1	154583200	0.685950
chr1	154587500	0.876033
chr1	154594600	0.652893
chr1	154703600	0.818182
chr1	154715200	0.818182
chr1	154717700	0.834711
chr1	154745100	0.834711
chr1	154786800	0.818182
chr1	154795500	0.809917
chr1	154812800	0.900826
chr1	154819500	0.669421
chr1	154822900	0.958678
chr1	154835400	0.909091
chr1	154839500	0.710744

chr1	154844200	0.727273
chr1	154851200	0.710744
chr1	154913600	0.719008
chr1	154926400	0.685950
chr1	154981600	0.842975
chr1	154985700	0.809917
chr1	155046000	0.719008
chr1	155060200	0.793388
chr1	155064100	0.793388
chr1	155067100	0.818182
chr1	155103400	0.818182
chr1	155159200	0.975207
chr1	155168300	0.694215
chr1	155249500	0.661157
chr1	155267500	0.785124
chr1	155403700	0.818182
chr1	155569300	0.702479
chr1	155606200	0.661157
chr1	155911100	0.743802
chr1	155916200	0.892562
chr1	155937300	0.933884
chr1	155951300	0.884298
chr1	155954200	0.677686
chr1	156033500	0.661157
chr1	156036200	0.719008
chr1	156042600	0.826446
chr1	156045300	0.644628
chr1	156048000	0.942149
chr1	156056300	0.644628
chr1	156060500	0.677686
chr1	156067100	0.809917
chr1	156073600	0.752066
chr1	156093600	0.809917
chr1	156096800	0.842975
chr1	156099500	0.983471
chr1	156116500	0.834711
chr1	156123800	0.727273
chr1	156131100	0.727273
chr1	156142800	0.661157
chr1	156146800	0.859504
chr1	156152700	0.644628
chr1	156166700	0.735537
chr1	156186600	0.917355
chr1	156195600	0.685950
chr1	156215800	0.644628
chr1	156361900	0.834711
chr1	156369900	0.834711
chr1	156372200	0.719008
chr1	156393500	0.669421
chr1	156402600	0.719008
chr1	156406400	0.710744
chr1	156410700	0.644628
chr1	156432000	0.661157
chr1	156434100	0.710744

chr1	156446900	0.727273
chr1	156460400	0.942149
chr1	156466700	0.743802
chr1	156473300	0.983471
chr1	156475400	0.892562
chr1	156496100	0.818182
chr1	156554500	0.826446
chr1	156589500	0.892562
chr1	156613800	0.677686
chr1	156630200	0.925620
chr1	156632300	0.793388
chr1	156634400	0.809917
chr1	156640000	0.710744
chr1	156653600	0.702479
chr1	156681500	0.661157
chr1	156712300	0.900826
chr1	156730300	0.834711
chr1	156827300	0.694215
chr1	156836200	0.743802
chr1	156846500	0.793388
chr1	156855300	0.867769
chr1	156857700	0.727273
chr1	156903100	0.661157
chr1	156973300	0.884298
chr1	157064300	0.661157
chr1	157077900	0.752066
chr1	157081700	0.760331
chr1	157087300	0.727273
chr1	157092800	0.818182
chr1	157125600	0.884298
chr1	157132200	0.991736
chr1	157134400	0.719008
chr1	157200900	0.702479
chr1	157210800	0.809917
chr1	157280900	0.818182
chr1	157296200	0.768595
chr1	157509900	0.842975
chr1	157536200	0.752066
chr1	157610700	0.677686
chr1	157723800	0.644628
chr1	157949100	0.859504
chr1	157966100	0.826446
chr1	157972600	0.925620
chr1	157980400	0.867769
chr1	157984700	0.958678
chr1	157987100	0.719008
chr1	157990100	0.727273
chr1	157992400	0.644628
chr1	157995600	0.652893
chr1	158008500	0.719008
chr1	158025600	0.694215
chr1	158041400	0.818182
chr1	158044500	0.909091
chr1	158048300	0.826446

chr1	158066600	0.842975
chr1	158073700	1.000000
chr1	158088600	0.768595
chr1	158090700	0.727273
chr1	158156700	0.809917
chr1	158161300	0.710744
chr1	158393300	0.652893
chr1	158476500	0.677686
chr1	158557200	0.867769
chr1	159111800	0.867769
chr1	159151700	0.710744
chr1	159178400	0.669421
chr1	159189400	0.991736
chr1	159870600	0.694215
chr1	159880700	0.900826
chr1	159884100	0.933884
chr1	159886600	0.719008
chr1	159907400	0.842975
chr1	159911400	0.842975
chr1	160016800	0.818182
chr1	160032400	0.826446
chr1	160037600	0.809917
chr1	160064600	0.702479
chr1	160074200	0.809917
chr1	160079100	0.834711
chr1	160088000	0.694215
chr1	160094900	0.702479
chr1	160139500	0.760331
chr1	160142100	0.834711
chr1	160163300	0.793388
chr1	160177800	0.735537
chr1	160180500	0.876033
chr1	160277300	0.851240
chr1	160332400	0.942149
chr1	160344900	0.669421
chr1	160378400	0.834711
chr1	160380800	0.851240
chr1	160456700	0.677686
chr1	160490500	0.694215
chr1	160641900	0.851240
chr1	160696700	0.909091
chr1	160717200	0.702479
chr1	160780800	0.644628
chr1	160788300	0.644628
chr1	160924500	0.776860
chr1	160942200	0.694215
chr1	160951400	0.719008
chr1	160957200	0.884298
chr1	160983800	0.876033
chr1	161166500	0.760331
chr1	161170100	0.785124
chr1	161230900	0.876033
chr1	161236800	0.818182
chr1	161255500	0.719008

chr1	161338100	0.793388
chr1	161476500	0.727273
chr1	161483600	0.702479
chr1	161559500	0.752066
chr1	161641300	0.859504
chr1	161707100	0.809917
chr1	161979000	0.735537
chr1	161984800	0.702479
chr1	161991200	0.809917
chr1	162042800	0.859504
chr1	162057300	0.743802
chr1	162107400	0.900826
chr1	162114500	0.743802
chr1	162118500	0.727273
chr1	162142300	0.834711
chr1	162145600	0.958678
chr1	162168200	0.752066
chr1	162185700	0.884298
chr1	162189600	0.809917
chr1	162200100	0.785124
chr1	162202600	0.669421
chr1	162207900	0.644628
chr1	162220400	0.975207
chr1	162270100	0.735537
chr1	162303900	0.652893
chr1	162368900	0.760331
chr1	162441400	0.809917
chr1	162500600	0.752066
chr1	162603200	0.826446
chr1	162661400	0.867769
chr1	162666500	0.975207
chr1	162996400	0.702479
chr1	163102400	0.702479
chr1	163125900	0.743802
chr1	163173400	0.991736
chr1	163370100	1.000000
chr1	163380400	0.677686
chr1	163397400	0.702479
chr1	163474500	0.661157
chr1	163493300	0.727273
chr1	163507600	0.900826
chr1	163531600	0.760331
chr1	163535500	0.669421
chr1	163566800	0.834711
chr1	163580800	0.710744
chr1	163584700	0.743802
chr1	164024100	0.909091
chr1	164392400	0.735537
chr1	164544300	0.793388
chr1	164548200	0.768595
chr1	164552600	0.958678
chr1	164555100	0.743802
chr1	164558000	0.950413
chr1	164561600	0.876033

chr1	164564400	0.685950
chr1	164567300	0.644628
chr1	164574500	0.735537
chr1	164577200	0.719008
chr1	164581800	0.884298
chr1	164586300	0.809917
chr1	164593200	0.644628
chr1	164603500	0.752066
chr1	164607700	0.719008
chr1	164618300	0.933884
chr1	164620700	0.702479
chr1	164626900	0.661157
chr1	164629500	0.966942
chr1	164640000	0.842975
chr1	164693700	0.702479
chr1	164700300	0.644628
chr1	164729000	0.652893
chr1	164741100	0.942149
chr1	164749400	0.661157
chr1	164756900	0.950413
chr1	164825400	0.652893
chr1	165017500	0.760331
chr1	165040500	0.752066
chr1	165078700	0.884298
chr1	165087300	0.752066
chr1	165191000	0.900826
chr1	165219000	0.702479
chr1	165223800	0.735537
chr1	165245400	0.685950
chr1	165257500	0.776860
chr1	165314400	0.760331
chr1	165436800	0.752066
chr1	165513500	0.867769
chr1	165530300	0.727273
chr1	165586400	0.677686
chr1	165604100	0.644628
chr1	165629000	0.867769
chr1	165803000	0.793388
chr1	165812100	0.818182
chr1	165823300	0.842975
chr1	165826800	0.793388
chr1	165844500	0.710744
chr1	165853400	0.652893
chr1	165868800	0.834711
chr1	165903100	0.727273
chr1	165912700	0.735537
chr1	166002100	0.644628
chr1	166010000	0.677686
chr1	166154700	0.644628
chr1	166272400	0.677686
chr1	166352900	0.925620
chr1	166355400	0.760331
chr1	166497300	0.925620
chr1	166919500	0.661157

chr1	166921900	0.702479
chr1	166941600	0.743802
chr1	167028900	0.752066
chr1	167063800	0.669421
chr1	167073200	0.842975
chr1	167075600	0.661157
chr1	167085600	0.710744
chr1	167117900	0.826446
chr1	167127700	0.950413
chr1	167132600	0.942149
chr1	167136000	0.958678
chr1	167143700	0.768595
chr1	167195500	0.851240
chr1	167238100	0.661157
chr1	167298900	0.677686
chr1	167314500	0.933884
chr1	167331700	0.785124
chr1	167343400	0.818182
chr1	167394300	0.677686
chr1	167396700	0.867769
chr1	167400900	0.785124
chr1	167427200	0.677686
chr1	167437300	0.669421
chr1	167455200	0.685950
chr1	167459000	0.867769
chr1	167461900	0.851240
chr1	167472600	0.900826
chr1	167503200	0.669421
chr1	167605000	0.818182
chr1	167656900	0.677686
chr1	167674900	0.958678
chr1	167688500	0.793388
chr1	167765700	0.809917
chr1	167773500	0.719008
chr1	167826000	0.752066
chr1	167831600	0.710744
chr1	167874600	0.702479
chr1	167882700	0.652893
chr1	168051500	0.752066
chr1	168080700	0.702479
chr1	168219700	0.818182
chr1	168254400	0.669421
chr1	168261300	0.677686
chr1	168303700	0.669421
chr1	168397800	0.801653
chr1	168451400	0.661157
chr1	168798100	0.661157
chr1	168888700	0.991736
chr1	168940400	0.685950
chr1	169072000	0.719008
chr1	169079300	0.719008
chr1	169083000	0.652893
chr1	169448800	0.859504
chr1	169498900	0.983471

chr1	169660000	0.818182
chr1	169681100	0.818182
chr1	169715300	0.785124
chr1	169750700	0.900826
chr1	169860400	0.793388
chr1	169865900	0.677686
chr1	170096300	0.702479
chr1	170396300	0.652893
chr1	170457300	0.842975
chr1	170473200	0.685950
chr1	170532600	0.933884
chr1	170550300	0.884298
chr1	170556000	0.710744
chr1	170626900	0.719008
chr1	171002500	0.859504
chr1	171188500	0.694215
chr1	171191700	0.735537
chr1	171228300	0.842975
chr1	171268100	1.000000
chr1	171275600	0.743802
chr1	171283800	0.661157
chr1	171390700	0.727273
chr1	171398900	0.909091
chr1	171401600	0.983471
chr1	171471000	0.685950
chr1	171475900	0.652893
chr1	171610600	0.876033
chr1	171614500	0.760331
chr1	171723700	0.768595
chr1	171771500	0.892562
chr1	171787300	0.785124
chr1	171868600	0.760331
chr1	171879400	0.975207
chr1	171909300	0.768595
chr1	171936200	0.859504
chr1	172062400	0.826446
chr1	172111100	0.834711
chr1	172154900	0.776860
chr1	172248800	0.975207
chr1	172327800	0.867769
chr1	172330800	0.793388
chr1	172351800	0.685950
chr1	172360200	0.661157
chr1	172365200	0.652893
chr1	172401200	0.694215
chr1	172506800	0.958678
chr1	172608500	0.685950
chr1	172683600	0.859504
chr1	172796000	0.685950
chr1	173092400	0.983471
chr1	173172200	0.710744
chr1	173248700	0.859504
chr1	173370000	0.785124
chr1	173387500	0.743802

chr1	173400600	0.826446
chr1	173408700	0.644628
chr1	173414400	0.809917
chr1	173418200	0.785124
chr1	173476800	0.809917
chr1	173497800	0.661157
chr1	173500400	0.727273
chr1	173651900	0.644628
chr1	173822200	0.801653
chr1	173832900	0.900826
chr1	173858000	0.991736
chr1	173869100	0.677686
chr1	173872500	0.702479
chr1	173903900	0.768595
chr1	174342500	0.644628
chr1	174662300	0.818182
chr1	174841200	0.950413
chr1	174900300	0.694215
chr1	174999200	0.776860
chr1	175005900	0.727273
chr1	175041600	0.702479
chr1	175098400	0.933884
chr1	175191100	0.933884
chr1	175390500	0.685950
chr1	175533600	0.826446
chr1	175549800	0.809917
chr1	175643000	0.727273
chr1	175671300	0.851240
chr1	175753900	0.677686
chr1	175766500	0.694215
chr1	175834600	0.975207
chr1	175857600	0.752066
chr1	175888400	0.644628
chr1	175891600	0.842975
chr1	176520000	0.793388
chr1	176665000	0.785124
chr1	176928700	0.809917
chr1	176965800	0.677686
chr1	177002800	0.669421
chr1	177014000	0.884298
chr1	177322800	0.900826
chr1	177748500	0.669421
chr1	177764200	0.983471
chr1	177767500	0.677686
chr1	177929600	0.661157
chr1	177980200	0.677686
chr1	178001300	0.743802
chr1	178030600	0.933884
chr1	178115700	0.719008
chr1	178143200	0.661157
chr1	178222800	0.785124
chr1	178234100	0.867769
chr1	178236500	0.917355
chr1	178240700	0.776860

chr1	178247300	0.851240
chr1	178251500	0.760331
chr1	178254200	0.652893
chr1	178263500	0.933884
chr1	178341100	0.826446
chr1	178545400	0.900826
chr1	178550700	0.809917
chr1	178840300	0.793388
chr1	178898500	0.776860
chr1	178915600	0.867769
chr1	178998300	0.760331
chr1	179028200	0.719008
chr1	179057000	0.785124
chr1	179498100	0.834711
chr1	179757600	0.851240
chr1	179801600	0.809917
chr1	179891600	0.760331
chr1	180137500	0.900826
chr1	180208900	0.925620
chr1	180213800	0.892562
chr1	180217300	0.768595
chr1	180219500	0.669421
chr1	180224000	0.867769
chr1	180237500	0.702479
chr1	180241000	0.818182
chr1	180403100	0.851240
chr1	180511500	0.900826
chr1	180663800	0.661157
chr1	180806000	0.834711
chr1	180869000	0.793388
chr1	180892500	0.966942
chr1	180895600	0.851240
chr1	180897900	0.661157
chr1	180923300	0.644628
chr1	180925800	0.801653
chr1	180951200	0.942149
chr1	181064900	0.867769
chr1	181069000	0.669421
chr1	181079400	0.652893
chr1	181087300	0.818182
chr1	181098300	0.900826
chr1	181103900	0.958678
chr1	181111300	0.834711
chr1	181135800	0.727273
chr1	181149500	0.826446
chr1	181151800	0.752066
chr1	181159400	0.694215
chr1	181176400	0.752066
chr1	181473800	0.727273
chr1	181552600	0.743802
chr1	181561800	0.768595
chr1	181578500	1.000000
chr1	181684000	0.809917
chr1	181687500	0.677686

chr1	181939700	0.925620
chr1	181956300	0.793388
chr1	182053900	0.694215
chr1	182071000	1.000000
chr1	182113300	0.768595
chr1	182176200	0.710744
chr1	182301700	0.702479
chr1	182328300	0.884298
chr1	182414300	0.677686
chr1	182454200	0.652893
chr1	182558500	0.818182
chr1	182581600	0.661157
chr1	182587400	0.785124
chr1	182595700	0.652893
chr1	182603600	0.842975
chr1	182626900	0.785124
chr1	182631900	0.859504
chr1	182636300	0.719008
chr1	182639300	0.776860
chr1	182641500	0.809917
chr1	182654000	0.884298
chr1	182673500	0.735537
chr1	182751700	0.685950
chr1	182771900	0.661157
chr1	182775800	0.876033
chr1	182815400	0.661157
chr1	182879600	0.801653
chr1	182928100	0.884298
chr1	182996200	0.644628
chr1	183006400	0.677686
chr1	183040300	0.644628
chr1	183063100	0.669421
chr1	183068000	0.950413
chr1	183071000	0.735537
chr1	183078700	0.760331
chr1	183134000	0.859504
chr1	183164600	0.983471
chr1	183188300	0.644628
chr1	183248400	0.785124
chr1	183287800	0.702479
chr1	183290500	0.933884
chr1	183300400	0.752066
chr1	183373200	0.933884
chr1	183431200	0.752066
chr1	183468800	0.661157
chr1	183516500	0.917355
chr1	183782400	0.752066
chr1	183841200	0.677686
chr1	183851500	0.677686
chr1	183915100	0.975207
chr1	183995000	0.785124
chr1	184026500	0.669421
chr1	184123400	0.818182
chr1	184241000	0.661157

chr1	184353400	0.801653
chr1	184378100	0.785124
chr1	184386300	0.694215
chr1	184391300	0.752066
chr1	184395700	0.909091
chr1	184425100	0.760331
chr1	184429600	0.826446
chr1	184449900	0.917355
chr1	184455900	0.809917
chr1	184463900	0.867769
chr1	184467300	0.834711
chr1	184473300	0.818182
chr1	184489100	0.694215
chr1	184599400	0.685950
chr1	184634000	0.719008
chr1	184638600	0.933884
chr1	184643100	0.876033
chr1	184654600	0.685950
chr1	184855500	0.694215
chr1	185040400	0.661157
chr1	185043400	0.925620
chr1	185501300	0.950413
chr1	185615800	0.983471
chr1	185669500	0.785124
chr1	185812900	0.785124
chr1	185818400	0.826446
chr1	185882200	0.933884
chr1	186954600	0.818182
chr1	186980100	0.661157
chr1	187016900	0.785124
chr1	187378000	0.743802
chr1	187446600	0.735537
chr1	187594300	0.809917
chr1	187601000	0.966942
chr1	187608000	0.909091
chr1	187898400	0.785124
chr1	188058800	0.694215
chr1	188639400	0.752066
chr1	189283100	0.809917
chr1	191143700	0.727273
chr1	192763100	0.900826
chr1	192800800	0.727273
chr1	192808800	0.900826
chr1	192947000	0.710744
chr1	193082900	0.785124
chr1	193432600	0.652893
chr1	193512000	0.818182
chr1	193558300	0.876033
chr1	193565400	0.644628
chr1	193572500	0.727273
chr1	193697100	0.685950
chr1	193899600	0.685950
chr1	194988800	0.710744
chr1	195306400	0.958678

chr1	195818200	0.727273
chr1	196003700	0.876033
chr1	196275700	0.917355
chr1	196282000	0.760331
chr1	196314500	0.884298
chr1	196547400	0.884298
chr1	196847500	0.661157
chr1	196961400	0.644628
chr1	197099200	0.669421
chr1	197407100	0.752066
chr1	197411400	0.661157
chr1	197433000	0.752066
chr1	197440400	0.735537
chr1	197510900	0.743802
chr1	197564700	0.727273
chr1	197780100	0.933884
chr1	197784400	0.867769
chr1	197860800	0.702479
chr1	197878300	0.710744
chr1	197883900	0.669421
chr1	198203600	0.776860
chr1	198215700	0.694215
chr1	198260600	0.669421
chr1	198284800	0.735537
chr1	198287100	0.661157
chr1	198489700	0.752066
chr1	198621600	0.727273
chr1	198669000	0.669421
chr1	198671200	0.909091
chr1	198711700	0.719008
chr1	198758300	0.950413
chr1	199086200	0.685950
chr1	199280900	0.958678
chr1	199657100	0.892562
chr1	199661100	0.710744
chr1	199717500	0.958678
chr1	199752800	0.719008
chr1	199835400	0.884298
chr1	200017600	0.917355
chr1	200019700	0.851240
chr1	200045000	0.677686
chr1	200050800	0.793388
chr1	200099100	0.925620
chr1	200143800	0.719008
chr1	200195700	0.950413
chr1	200212400	0.685950
chr1	200283800	0.719008
chr1	200299800	0.661157
chr1	200306600	0.702479
chr1	200332100	0.983471
chr1	200334400	0.983471
chr1	200343100	0.834711
chr1	200358400	0.867769
chr1	200361400	0.975207

chr1	200422900	0.900826
chr1	200453300	0.917355
chr1	200531600	0.842975
chr1	200635600	0.743802
chr1	200845000	0.867769
chr1	200857200	0.719008
chr1	200864900	0.983471
chr1	200885900	0.661157
chr1	200980000	0.867769
chr1	200983500	0.702479
chr1	200986100	0.661157
chr1	200990100	0.669421
chr1	200994900	0.685950
chr1	201032300	0.851240
chr1	201096600	0.909091
chr1	201124800	0.752066
chr1	201154300	0.710744
chr1	201252900	0.809917
chr1	201259100	0.768595
chr1	201264500	0.768595
chr1	201316000	0.685950
chr1	201331500	0.710744
chr1	201351700	0.677686
chr1	201363800	0.942149
chr1	201390300	0.644628
chr1	201405500	0.694215
chr1	201417800	0.793388
chr1	201420500	0.694215
chr1	201425600	0.719008
chr1	201470000	0.876033
chr1	201472600	0.925620
chr1	201481900	0.652893
chr1	201501200	0.801653
chr1	201515500	0.809917
chr1	201552300	0.818182
chr1	201560800	0.760331
chr1	201563600	0.702479
chr1	201588300	0.793388
chr1	201597900	0.867769
chr1	201605600	0.710744
chr1	201621800	0.644628
chr1	201645400	0.975207
chr1	201648200	0.958678
chr1	201657500	0.760331
chr1	201663400	0.892562
chr1	201673200	0.644628
chr1	201689300	0.760331
chr1	201706600	0.867769
chr1	201711700	0.669421
chr1	201717600	0.760331
chr1	201721300	0.867769
chr1	201723400	0.809917
chr1	201729900	0.785124
chr1	201733000	0.694215

chr1	201811600	0.801653
chr1	201850800	0.760331
chr1	201864200	0.876033
chr1	201881500	0.735537
chr1	201911000	0.727273
chr1	201977600	0.694215
chr1	201987900	0.917355
chr1	202004100	0.727273
chr1	202054200	0.719008
chr1	202056900	0.727273
chr1	202070800	0.842975
chr1	202074600	0.958678
chr1	202076900	0.842975
chr1	202101600	0.677686
chr1	202104600	0.958678
chr1	202108000	0.652893
chr1	202123500	0.661157
chr1	202154400	0.727273
chr1	202205900	0.884298
chr1	202217500	0.702479
chr1	202250300	0.818182
chr1	202284600	0.727273
chr1	202331400	0.809917
chr1	202349300	0.900826
chr1	202380100	0.917355
chr1	202389400	0.652893
chr1	202508800	0.685950
chr1	202535100	0.818182
chr1	202537400	0.942149
chr1	202546300	0.859504
chr1	202594000	0.876033
chr1	202607400	0.793388
chr1	202650200	0.842975
chr1	202681900	0.719008
chr1	202768400	0.669421
chr1	202804800	0.685950
chr1	202855200	0.685950
chr1	202968700	0.694215
chr1	203005100	0.735537
chr1	203009100	0.859504
chr1	203018200	0.702479
chr1	203021600	0.735537
chr1	203023800	0.669421
chr1	203051400	0.661157
chr1	203053800	0.694215
chr1	203087200	0.768595
chr1	203103500	0.661157
chr1	203116700	0.826446
chr1	203119500	0.892562
chr1	203122500	0.809917
chr1	203142100	0.702479
chr1	203147900	0.644628
chr1	203155600	0.669421
chr1	203185600	0.743802

chr1	203248100	0.785124
chr1	203256600	0.793388
chr1	203259900	0.851240
chr1	203290500	0.669421
chr1	203294700	0.876033
chr1	203298600	0.743802
chr1	203412800	0.727273
chr1	203433500	0.809917
chr1	203444900	0.867769
chr1	203449900	0.809917
chr1	203461100	0.661157
chr1	203463400	0.661157
chr1	203477900	0.702479
chr1	203483300	0.826446
chr1	203486300	0.652893
chr1	203489000	0.793388
chr1	203491700	0.876033
chr1	203494500	0.867769
chr1	203514100	0.801653
chr1	203518900	0.884298
chr1	203525200	0.950413
chr1	203541600	0.892562
chr1	203558900	0.727273
chr1	203561800	0.975207
chr1	203567500	0.710744
chr1	203619600	0.669421
chr1	203626700	0.892562
chr1	203632700	0.669421
chr1	203660400	0.735537
chr1	203713000	0.694215
chr1	203954900	0.933884
chr1	203964700	0.743802
chr1	203973100	0.818182
chr1	203997900	0.685950
chr1	204034800	1.000000
chr1	204047200	0.793388
chr1	204050700	0.793388
chr1	204053900	0.958678
chr1	204057900	0.966942
chr1	204060600	0.760331
chr1	204063800	0.900826
chr1	204068500	0.859504
chr1	204072700	0.909091
chr1	204075000	0.760331
chr1	204078100	0.975207
chr1	204083200	0.809917
chr1	204096200	0.760331
chr1	204099200	0.991736
chr1	204111400	0.652893
chr1	204116900	0.892562
chr1	204160000	0.727273
chr1	204176400	0.719008
chr1	204224600	0.991736
chr1	204248600	0.685950

chr1	204256900	0.917355
chr1	204265600	0.727273
chr1	204275100	0.859504
chr1	204277300	0.685950
chr1	204279800	0.719008
chr1	204283700	0.826446
chr1	204291700	0.677686
chr1	204310600	0.685950
chr1	204312700	0.719008
chr1	204320400	0.933884
chr1	204329000	0.925620
chr1	204368000	0.694215
chr1	204416000	0.694215
chr1	204422300	0.669421
chr1	204424400	0.801653
chr1	204426500	0.892562
chr1	204451200	0.859504
chr1	204530100	0.685950
chr1	204534900	0.752066
chr1	204540900	0.685950
chr1	204543400	0.768595
chr1	204548700	0.892562
chr1	204554600	0.727273
chr1	204569500	0.809917
chr1	204571700	0.785124
chr1	204616600	0.909091
chr1	204621400	0.917355
chr1	204644000	0.702479
chr1	204662400	0.809917
chr1	204676100	0.859504
chr1	204680600	0.702479
chr1	204684300	0.735537
chr1	204721100	0.842975
chr1	204733600	0.669421
chr1	204803800	0.710744
chr1	204890500	0.694215
chr1	204894300	0.925620
chr1	204899900	0.834711
chr1	204910800	0.661157
chr1	204919900	0.669421
chr1	204961000	0.785124
chr1	204972800	0.867769
chr1	205014500	0.743802
chr1	205031000	0.669421
chr1	205107600	0.785124
chr1	205157100	0.917355
chr1	205162400	0.735537
chr1	205171400	0.834711
chr1	205217000	0.702479
chr1	205228000	0.661157
chr1	205231900	0.768595
chr1	205236600	0.785124
chr1	205254000	0.809917
chr1	205262100	0.644628

chr1	205264800	0.942149
chr1	205268000	0.942149
chr1	205273000	0.933884
chr1	205281100	0.644628
chr1	205283500	0.768595
chr1	205287400	0.677686
chr1	205294600	0.851240
chr1	205305200	0.694215
chr1	205320100	0.801653
chr1	205323900	0.785124
chr1	205326100	0.900826
chr1	205342100	0.942149
chr1	205345400	0.851240
chr1	205410700	0.925620
chr1	205414100	0.909091
chr1	205421600	0.644628
chr1	205442700	0.661157
chr1	205446700	0.752066
chr1	205450200	0.900826
chr1	205455000	0.867769
chr1	205461800	0.809917
chr1	205464800	0.727273
chr1	205470400	0.917355
chr1	205482800	0.776860
chr1	205488700	0.801653
chr1	205492800	0.867769
chr1	205498400	0.958678
chr1	205513000	0.826446
chr1	205617900	0.743802
chr1	205630900	0.735537
chr1	205634800	0.876033
chr1	205638700	0.669421
chr1	205642200	0.719008
chr1	205645500	0.652893
chr1	205670700	0.842975
chr1	205684300	0.677686
chr1	205713100	0.743802
chr1	205779400	0.710744
chr1	205796600	0.851240
chr1	205902900	0.785124
chr1	205909500	0.760331
chr1	205912900	0.644628
chr1	205922500	0.661157
chr1	206079800	0.702479
chr1	206084700	0.859504
chr1	206088300	0.661157
chr1	206107600	0.884298
chr1	206265200	0.735537
chr1	206485300	0.677686
chr1	206491300	0.884298
chr1	206499800	0.743802
chr1	206524700	0.644628
chr1	206554100	0.801653
chr1	206646600	0.752066

chr1	206685800	0.752066
chr1	206832300	0.834711
chr1	206846300	0.743802
chr1	206861900	0.776860
chr1	206868200	0.793388
chr1	206876300	0.876033
chr1	206880400	0.743802
chr1	206897400	0.776860
chr1	207156000	0.727273
chr1	207159300	0.743802
chr1	207176800	0.727273
chr1	207199100	0.925620
chr1	207203600	0.694215
chr1	207206300	0.983471
chr1	207232800	0.809917
chr1	207475000	0.677686
chr1	207480400	0.785124
chr1	207490600	0.752066
chr1	207597600	0.644628
chr1	207759000	0.652893
chr1	207793900	0.661157
chr1	207804800	0.925620
chr1	207807700	0.768595
chr1	207843100	0.652893
chr1	207922000	0.644628
chr1	207978500	0.661157
chr1	208098400	0.876033
chr1	208171900	0.644628
chr1	208250900	0.801653
chr1	208256800	0.768595
chr1	208260300	0.793388
chr1	208267300	0.743802
chr1	208272100	0.809917
chr1	208275400	0.652893
chr1	208294200	0.793388
chr1	208301600	0.801653
chr1	208305500	0.669421
chr1	208308500	0.809917
chr1	208318600	0.735537
chr1	208332900	0.809917
chr1	208400000	0.669421
chr1	208405100	0.661157
chr1	208407800	0.900826
chr1	208410100	0.652893
chr1	208412800	0.834711
chr1	208426000	0.735537
chr1	208529100	0.760331
chr1	208548100	0.677686
chr1	208603800	0.685950
chr1	208705800	0.669421
chr1	208774700	0.776860
chr1	208955200	0.900826
chr1	209008600	0.768595
chr1	209238400	0.892562

chr1	209279800	0.884298
chr1	209328900	0.909091
chr1	209331600	0.909091
chr1	209364400	0.702479
chr1	209372800	0.900826
chr1	209376800	0.727273
chr1	209401300	0.760331
chr1	209423000	0.669421
chr1	209530200	0.768595
chr1	209549400	0.669421
chr1	209557500	0.950413
chr1	209571900	0.719008
chr1	209579400	0.842975
chr1	209601800	0.876033
chr1	209604200	0.785124
chr1	209607100	0.975207
chr1	209726500	0.917355
chr1	209729900	0.892562
chr1	209738900	0.677686
chr1	209750500	0.669421
chr1	209794300	0.719008
chr1	209801300	0.719008
chr1	209803600	0.801653
chr1	209815100	0.768595
chr1	210036900	0.685950
chr1	210396400	0.727273
chr1	210411200	0.892562
chr1	210433600	0.809917
chr1	210446500	0.809917
chr1	210472400	0.669421
chr1	210532200	0.884298
chr1	210543100	0.719008
chr1	210547600	0.966942
chr1	210574300	0.710744
chr1	210581100	0.785124
chr1	210607700	0.661157
chr1	211421300	0.752066
chr1	211430400	0.793388
chr1	211503100	0.644628
chr1	211505300	0.652893
chr1	211551900	0.710744
chr1	211594200	0.727273
chr1	211643600	0.909091
chr1	211722200	0.743802
chr1	211755000	0.942149
chr1	211854500	0.669421
chr1	212102900	0.669421
chr1	212204700	0.760331
chr1	212271800	0.917355
chr1	212279600	0.942149
chr1	212462100	0.702479
chr1	212464600	0.743802
chr1	212483600	0.834711
chr1	212627500	0.661157

chr1	212680600	0.776860
chr1	212688800	0.776860
chr1	212725500	0.801653
chr1	212761500	0.818182
chr1	212769500	0.859504
chr1	212779000	0.793388
chr1	212785600	0.859504
chr1	212788000	0.793388
chr1	212808900	0.677686
chr1	212927600	0.652893
chr1	213053800	0.652893
chr1	213073900	0.685950
chr1	213079600	0.834711
chr1	213083500	0.834711
chr1	213088200	0.694215
chr1	213090800	0.826446
chr1	213102200	0.694215
chr1	213107800	0.661157
chr1	213129000	0.925620
chr1	213132900	0.644628
chr1	213142200	0.925620
chr1	213155200	0.975207
chr1	213229900	0.719008
chr1	213240100	0.644628
chr1	213405700	0.834711
chr1	213418700	0.661157
chr1	213455000	0.785124
chr1	213545700	0.661157
chr1	213626000	0.826446
chr1	213681500	0.842975
chr1	213829900	0.669421
chr1	213843900	0.859504
chr1	213935500	0.661157
chr1	214151200	0.735537
chr1	214190300	0.694215
chr1	214200800	0.909091
chr1	214204500	0.892562
chr1	214252700	0.925620
chr1	214281200	0.685950
chr1	214328600	0.809917
chr1	214366900	0.743802
chr1	214422800	0.867769
chr1	214463700	0.752066
chr1	214492600	0.884298
chr1	214502500	0.727273
chr1	214511200	0.809917
chr1	214553800	0.950413
chr1	214578200	0.933884
chr1	214593400	0.983471
chr1	214613500	0.991736
chr1	214618200	0.760331
chr1	214620600	0.983471
chr1	214649900	0.842975
chr1	214680700	0.685950

chr1	214688000	0.892562
chr1	214711800	0.702479
chr1	214721300	0.727273
chr1	214858000	0.785124
chr1	215150600	0.669421
chr1	215214000	0.685950
chr1	215374400	0.851240
chr1	215773300	0.677686
chr1	215821000	0.644628
chr1	215825400	0.884298
chr1	216269100	0.752066
chr1	216318500	0.735537
chr1	216356800	0.743802
chr1	216394900	0.785124
chr1	216420300	0.644628
chr1	216561700	0.694215
chr1	216860700	0.859504
chr1	216913200	0.702479
chr1	216945800	0.710744
chr1	216979100	0.743802
chr1	216999100	0.685950
chr1	217101200	0.727273
chr1	217118700	0.743802
chr1	217126500	0.669421
chr1	217517600	0.710744
chr1	217543100	0.776860
chr1	217546200	0.917355
chr1	218075800	0.818182
chr1	218151700	0.661157
chr1	218208300	0.917355
chr1	218275300	0.702479
chr1	218330400	0.719008
chr1	218390800	0.867769
chr1	218470100	0.966942
chr1	218474700	0.925620
chr1	218486800	0.644628
chr1	218633600	0.991736
chr1	218667800	0.768595
chr1	218787100	0.702479
chr1	218848200	0.892562
chr1	219438400	0.652893
chr1	219729000	0.669421
chr1	219999700	0.801653
chr1	220007200	0.867769
chr1	220398800	0.702479
chr1	220447900	0.727273
chr1	220510100	0.884298
chr1	220513400	0.983471
chr1	220542300	0.768595
chr1	220565000	0.677686
chr1	220583800	0.785124
chr1	220690000	0.702479
chr1	220924500	0.652893
chr1	220983700	0.685950

chr1	221056000	0.933884
chr1	221061000	0.892562
chr1	221065300	0.950413
chr1	221069700	0.867769
chr1	221095700	0.842975
chr1	221213400	0.809917
chr1	221401800	0.743802
chr1	221613600	0.710744
chr1	221804800	0.652893
chr1	221889900	0.818182
chr1	222146100	0.685950
chr1	222274200	0.669421
chr1	222421400	0.735537
chr1	222440200	0.685950
chr1	222613200	0.768595
chr1	222910300	0.867769
chr1	222998300	0.752066
chr1	223017700	0.842975
chr1	223184700	0.884298
chr1	223192100	0.677686
chr1	223195100	0.818182
chr1	223197900	0.685950
chr1	223206100	0.826446
chr1	223214900	0.685950
chr1	223248500	0.884298
chr1	223293500	0.727273
chr1	223303100	0.785124
chr1	223314800	0.652893
chr1	223354300	0.685950
chr1	223429700	0.677686
chr1	223493800	0.859504
chr1	223653700	0.652893
chr1	223740900	0.685950
chr1	223745600	0.727273
chr1	223834700	0.743802
chr1	223903100	0.661157
chr1	223906100	0.677686
chr1	223914900	0.785124
chr1	223918800	0.719008
chr1	223921900	0.752066
chr1	223940900	0.867769
chr1	224020700	0.776860
chr1	224058800	0.752066
chr1	224304500	0.652893
chr1	224352400	0.884298
chr1	224358000	0.661157
chr1	224363800	0.752066
chr1	224391600	0.958678
chr1	224401300	0.950413
chr1	224411700	0.652893
chr1	224424200	0.785124
chr1	224428100	0.661157
chr1	224444900	0.727273
chr1	224527200	0.975207

chr1	224618800	0.694215
chr1	224644900	0.677686
chr1	224681400	0.661157
chr1	224686400	0.991736
chr1	224690900	0.933884
chr1	224694500	0.652893
chr1	224782300	0.669421
chr1	224795300	0.801653
chr1	224820900	0.760331
chr1	224838700	0.677686
chr1	224891900	0.669421
chr1	224925700	0.785124
chr1	224954900	0.652893
chr1	224968000	0.719008
chr1	224988800	0.966942
chr1	225040100	0.661157
chr1	225068800	0.735537
chr1	225612800	0.735537
chr1	225634700	0.867769
chr1	225648700	0.644628
chr1	225653500	0.768595
chr1	225663100	0.710744
chr1	225672500	0.652893
chr1	225688700	0.785124
chr1	225697000	0.809917
chr1	225721300	0.652893
chr1	225726200	0.644628
chr1	225764300	0.710744
chr1	225797200	0.694215
chr1	225921200	0.677686
chr1	225934600	0.760331
chr1	225950500	0.826446
chr1	225955700	0.752066
chr1	225959000	0.685950
chr1	225968500	0.719008
chr1	226005600	0.661157
chr1	226012200	0.892562
chr1	226078200	0.917355
chr1	226087500	0.983471
chr1	226096800	0.669421
chr1	226133500	0.661157
chr1	226139100	0.876033
chr1	226190600	0.760331
chr1	226242700	0.752066
chr1	226292700	0.719008
chr1	226295800	0.909091
chr1	226312800	0.702479
chr1	226315800	0.677686
chr1	226323700	0.743802
chr1	226391500	0.694215
chr1	226398500	0.776860
chr1	226401200	0.752066
chr1	226408400	0.694215
chr1	226590600	0.818182

chr1	226616900	0.661157
chr1	226662300	0.760331
chr1	226679700	0.801653
chr1	226684100	0.735537
chr1	226708600	0.966942
chr1	226739400	0.735537
chr1	226796300	0.776860
chr1	226823900	0.652893
chr1	226842000	0.975207
chr1	226851000	0.677686
chr1	226876400	0.776860
chr1	226911500	0.975207
chr1	226919000	0.760331
chr1	226951800	0.801653
chr1	226954300	0.735537
chr1	227015800	0.669421
chr1	227064100	0.685950
chr1	227130000	0.702479
chr1	227133400	0.793388
chr1	227176400	0.859504
chr1	227182300	0.851240
chr1	227327000	0.719008
chr1	227447000	0.719008
chr1	227508300	0.661157
chr1	227545100	0.727273
chr1	227774800	0.942149
chr1	227918500	0.760331
chr1	227951500	0.694215
chr1	227982000	0.900826
chr1	228073700	0.644628
chr1	228084100	0.719008
chr1	228095200	0.694215
chr1	228103200	0.859504
chr1	228107800	0.661157
chr1	228111100	0.842975
chr1	228197600	0.669421
chr1	228233900	0.818182
chr1	228261900	0.752066
chr1	228273800	0.867769
chr1	228337600	0.851240
chr1	228350600	0.727273
chr1	228362200	0.867769
chr1	228555900	0.685950
chr1	228659700	0.669421
chr1	228663000	0.743802
chr1	228666900	0.859504
chr1	228859600	0.743802
chr1	228940900	0.677686
chr1	228947400	0.785124
chr1	228950300	0.735537
chr1	228990000	0.719008
chr1	228999800	0.644628
chr1	229002900	0.661157
chr1	229018700	0.694215

chr1	229047400	0.785124
chr1	229073900	0.842975
chr1	229115000	0.867769
chr1	229164000	0.933884
chr1	229183600	0.925620
chr1	229199100	0.644628
chr1	229225000	0.785124
chr1	229230200	0.983471
chr1	229244500	0.950413
chr1	229284100	0.768595
chr1	229286900	0.727273
chr1	229326600	0.842975
chr1	229339700	0.867769
chr1	229363600	0.735537
chr1	229367600	0.983471
chr1	229389000	0.900826
chr1	229516300	0.710744
chr1	229532800	0.710744
chr1	229699400	0.685950
chr1	229830100	0.867769
chr1	229846700	0.785124
chr1	229852100	0.644628
chr1	229998000	0.884298
chr1	230165600	0.710744
chr1	230183100	0.826446
chr1	230222400	0.776860
chr1	230237600	0.917355
chr1	230241400	0.842975
chr1	230247800	0.851240
chr1	230262800	0.743802
chr1	230278400	0.719008
chr1	230321700	0.942149
chr1	230356100	0.669421
chr1	230367000	0.834711
chr1	230387600	0.859504
chr1	230404600	0.702479
chr1	230413300	0.909091
chr1	230429400	0.694215
chr1	230468500	0.768595
chr1	230511900	0.710744
chr1	230518100	0.818182
chr1	230529600	0.925620
chr1	230537900	0.752066
chr1	230558200	0.785124
chr1	230564700	0.975207
chr1	230567000	0.727273
chr1	230652000	0.834711
chr1	230654100	0.966942
chr1	230708900	0.760331
chr1	230745600	0.719008
chr1	230849900	0.702479
chr1	230864800	0.785124
chr1	230889600	0.909091
chr1	230921600	0.727273

chr1	230987000	0.727273
chr1	230999600	0.776860
chr1	231030800	0.702479
chr1	231121300	0.735537
chr1	231320100	0.925620
chr1	231329300	0.677686
chr1	231654800	0.702479
chr1	231662400	0.719008
chr1	231686200	0.735537
chr1	231826500	0.842975
chr1	231906800	0.652893
chr1	231992400	0.809917
chr1	232036800	0.661157
chr1	232086000	0.652893
chr1	232147900	0.851240
chr1	232151800	0.644628
chr1	232181500	0.702479
chr1	232221200	0.727273
chr1	232241800	0.661157
chr1	232253400	0.826446
chr1	232264400	0.776860
chr1	232266700	0.909091
chr1	232270200	0.917355
chr1	232273100	0.727273
chr1	232279100	0.735537
chr1	232302800	0.785124
chr1	232394300	0.859504
chr1	232417200	0.900826
chr1	232442800	0.859504
chr1	232514700	0.644628
chr1	232529600	0.719008
chr1	232590400	0.710744
chr1	232608700	0.768595
chr1	232639100	0.818182
chr1	232644300	0.776860
chr1	232674400	0.793388
chr1	232691600	0.933884
chr1	232694200	0.685950
chr1	232752300	0.677686
chr1	232756400	0.876033
chr1	232763300	0.752066
chr1	232861900	0.694215
chr1	232884800	0.867769
chr1	232902100	0.652893
chr1	232906400	0.925620
chr1	233047200	0.752066
chr1	233090800	0.768595
chr1	233216000	0.785124
chr1	233244700	0.719008
chr1	233248600	0.991736
chr1	233250700	0.917355
chr1	233316300	0.818182
chr1	233413900	0.776860
chr1	233418100	0.809917

chr1	233425200	0.950413
chr1	233428300	0.768595
chr1	233436200	0.776860
chr1	233468100	0.710744
chr1	233477400	0.669421
chr1	233504500	0.966942
chr1	233596200	0.760331
chr1	233607100	0.958678
chr1	233659100	0.966942
chr1	233724300	0.851240
chr1	233772100	0.983471
chr1	233775800	0.710744
chr1	233815800	0.644628
chr1	234019700	0.760331
chr1	234362700	0.719008
chr1	234365700	0.652893
chr1	234453000	0.884298
chr1	234462800	0.743802
chr1	234611700	0.644628
chr1	234627200	0.644628
chr1	234645800	0.702479
chr1	234651500	0.942149
chr1	234659200	0.735537
chr1	234667500	0.677686
chr1	234748600	0.809917
chr1	234753900	0.900826
chr1	234791900	0.909091
chr1	234812300	0.867769
chr1	234820000	0.644628
chr1	234859000	0.719008
chr1	234862900	0.900826
chr1	234908000	0.818182
chr1	234918900	0.826446
chr1	234921400	0.809917
chr1	234945200	0.669421
chr1	234988300	0.727273
chr1	235062300	0.776860
chr1	235077900	0.652893
chr1	235093900	0.958678
chr1	235106300	0.991736
chr1	235108800	0.892562
chr1	235120700	0.661157
chr1	235123400	0.975207
chr1	235159200	0.710744
chr1	235243300	0.710744
chr1	235247300	0.793388
chr1	235253900	0.876033
chr1	235257300	0.727273
chr1	235268000	0.677686
chr1	235362000	0.917355
chr1	235423200	0.917355
chr1	235434200	0.694215
chr1	235442700	0.702479
chr1	235521400	0.644628

chr1	235710500	0.867769
chr1	235762900	0.768595
chr1	235787000	0.677686
chr1	235791800	0.644628
chr1	235800100	0.760331
chr1	235803900	0.694215
chr1	235806000	0.727273
chr1	235808200	0.685950
chr1	235827200	0.727273
chr1	235832500	0.760331
chr1	235836800	0.768595
chr1	235839500	0.983471
chr1	235939400	0.702479
chr1	236009100	0.834711
chr1	236083800	0.859504
chr1	236266700	0.677686
chr1	236292800	0.793388
chr1	236313800	0.785124
chr1	236465100	0.677686
chr1	236650700	0.710744
chr1	236666500	0.727273
chr1	236680400	0.685950
chr1	236823600	0.785124
chr1	237115500	0.884298
chr1	237180500	0.983471
chr1	237195600	0.694215
chr1	237209200	0.884298
chr1	237238400	0.851240
chr1	237347200	0.809917
chr1	237403000	0.958678
chr1	237452100	0.677686
chr1	237729500	0.661157
chr1	237996800	0.876033
chr1	238037500	0.818182
chr1	238126900	0.801653
chr1	238275900	0.752066
chr1	238340900	0.719008
chr1	238416000	0.867769
chr1	238418700	0.644628
chr1	238540900	0.991736
chr1	238544100	0.966942
chr1	238622500	0.785124
chr1	238811700	0.950413
chr1	238916500	0.859504
chr1	239271100	0.842975
chr1	239289900	0.677686
chr1	239406400	0.884298
chr1	239472900	0.694215
chr1	239918600	0.710744
chr1	239974000	0.834711
chr1	239978300	0.776860
chr1	240061300	0.876033
chr1	240078600	0.793388
chr1	240235800	0.661157

chr1	240238900	0.760331
chr1	240303300	0.661157
chr1	241051700	0.661157
chr1	241119600	0.661157
chr1	241176200	0.942149
chr1	241197900	0.768595
chr1	241209100	0.834711
chr1	241278700	0.818182
chr1	241327200	0.752066
chr1	241603800	0.809917
chr1	242234000	0.661157
chr1	242249900	0.809917
chr1	242310900	0.685950
chr1	242328100	0.727273
chr1	242341900	0.652893
chr1	242359500	0.801653
chr1	242366800	0.694215
chr1	242375400	0.743802
chr1	242388800	0.727273
chr1	242611400	0.983471
chr1	242618700	0.743802
chr1	242653600	0.752066
chr1	242683100	0.735537
chr1	242749800	0.950413
chr1	243278000	0.768595
chr1	243286100	0.752066
chr1	243368600	0.719008
chr1	243381400	0.694215
chr1	243414600	0.669421
chr1	243448200	0.710744
chr1	243479800	0.900826
chr1	243634300	0.818182
chr1	243637300	0.685950
chr1	243639400	0.859504
chr1	243655000	0.966942
chr1	243671000	0.652893
chr1	243896000	0.685950
chr1	243900400	0.694215
chr1	243903700	0.942149
chr1	243910400	0.760331
chr1	244088300	0.950413
chr1	244094700	0.892562
chr1	244110400	0.876033
chr1	244132100	0.776860
chr1	244175400	0.834711
chr1	244185100	0.702479
chr1	244215300	0.702479
chr1	244231000	0.661157
chr1	244324400	0.652893
chr1	244327800	0.867769
chr1	244339100	0.884298
chr1	244500400	0.661157
chr1	244505500	0.942149
chr1	244517100	1.000000

chr1	244525300	0.983471
chr1	244533600	0.809917
chr1	244573500	0.652893
chr1	244603200	0.702479
chr1	244609000	0.876033
chr1	244611500	0.892562
chr1	244618300	0.876033
chr1	244806300	0.743802
chr1	244819500	0.809917
chr1	244822500	0.727273
chr1	244899800	0.991736
chr1	244923800	0.661157
chr1	244997000	0.644628
chr1	245038800	0.752066
chr1	245136200	0.661157
chr1	245182200	0.661157
chr1	245209800	0.752066
chr1	245224000	0.818182
chr1	245344500	0.851240
chr1	245365200	0.933884
chr1	245398300	0.942149
chr1	245420400	0.867769
chr1	245474600	0.719008
chr1	245500000	0.694215
chr1	245509000	0.727273
chr1	245542000	0.760331
chr1	245647700	0.950413
chr1	245676400	0.727273
chr1	245875200	0.661157
chr1	245973800	0.652893
chr1	246098000	0.661157
chr1	246132500	0.702479
chr1	246171900	0.669421
chr1	246229300	0.826446
chr1	246231500	0.776860
chr1	246235600	0.752066
chr1	246271700	0.685950
chr1	246383300	0.752066
chr1	246589000	0.661157
chr1	246592100	0.925620
chr1	246599000	0.719008
chr1	246917400	0.661157
chr1	246931800	0.867769
chr1	246952900	0.743802
chr1	246959200	0.694215
chr1	246976400	0.909091
chr1	247112200	0.677686
chr1	247230100	0.661157
chr1	247398200	0.661157
chr1	247456100	0.950413
chr1	247458800	0.834711
chr1	247462900	0.793388
chr1	247498900	0.851240
chr1	247501700	0.710744

chr1	247511600	0.719008
chr1	247535100	0.735537
chr1	247538600	0.669421
chr1	247542000	0.942149
chr1	247553800	0.785124
chr1	247637700	0.652893
chr1	249113300	0.818182
chr1	249192100	0.909091
chr20	247800	0.661157
chr20	259000	0.735537
chr20	283500	0.694215
chr20	302600	0.685950
chr20	311300	0.752066
chr20	335400	0.785124
chr20	341400	0.644628
chr20	381700	0.702479
chr20	415400	0.793388
chr20	552900	0.826446
chr20	555700	0.809917
chr20	592900	0.842975
chr20	626500	0.776860
chr20	639600	0.933884
chr20	642900	0.933884
chr20	646000	0.785124
chr20	680700	0.710744
chr20	720000	0.752066
chr20	756800	0.851240
chr20	797000	0.743802
chr20	897500	0.661157
chr20	925600	0.892562
chr20	937100	0.677686
chr20	1193400	0.884298
chr20	1356700	0.719008
chr20	1370100	0.851240
chr20	1404800	0.909091
chr20	1408300	0.917355
chr20	1481300	0.991736
chr20	1604200	0.669421
chr20	1703200	0.933884
chr20	1788900	0.752066
chr20	1792400	0.644628
chr20	1797800	0.652893
chr20	1800000	0.884298
chr20	1819900	0.851240
chr20	1862100	0.785124
chr20	1871600	0.669421
chr20	1878800	0.661157
chr20	1881500	0.752066
chr20	1910900	0.760331
chr20	1926200	0.818182
chr20	1930300	0.669421
chr20	1932400	0.669421
chr20	1936500	0.909091
chr20	1946700	0.768595

chr20	1975100	0.661157
chr20	1979800	0.677686
chr20	1984900	0.735537
chr20	1987600	0.710744
chr20	2069600	0.818182
chr20	2110800	0.652893
chr20	2129300	0.752066
chr20	2178000	0.818182
chr20	2216000	0.909091
chr20	2226200	0.859504
chr20	2245500	0.768595
chr20	2278000	0.719008
chr20	2360900	0.942149
chr20	2517500	0.677686
chr20	2669100	0.851240
chr20	2675900	0.735537
chr20	2679000	0.694215
chr20	2682000	0.776860
chr20	2687000	0.727273
chr20	2690300	0.677686
chr20	2719700	0.694215
chr20	2724000	0.768595
chr20	2985800	0.768595
chr20	3023000	0.876033
chr20	3264200	0.669421
chr20	3359200	0.818182
chr20	3442300	0.702479
chr20	3611200	0.851240
chr20	3666000	0.694215
chr20	3714200	0.677686
chr20	3720500	0.785124
chr20	3782000	0.685950
chr20	3788300	0.834711
chr20	3998600	0.768595
chr20	4003500	0.834711
chr20	4007000	0.702479
chr20	4015900	0.694215
chr20	4019600	0.818182
chr20	4041400	0.702479
chr20	4045100	0.644628
chr20	4063800	0.900826
chr20	4089800	0.809917
chr20	4116200	0.801653
chr20	4143100	0.851240
chr20	4148100	0.834711
chr20	4461800	0.818182
chr20	4488900	0.735537
chr20	4527300	0.900826
chr20	4562800	0.793388
chr20	4573600	0.809917
chr20	4678300	0.743802
chr20	4685200	0.793388
chr20	4701300	0.719008
chr20	4712200	0.685950

chr20	4730200	0.685950
chr20	4816900	0.669421
chr20	4877500	0.743802
chr20	5131400	0.652893
chr20	5147300	0.702479
chr20	5150000	0.876033
chr20	5152500	0.776860
chr20	5156700	0.669421
chr20	5172900	0.933884
chr20	5177100	0.652893
chr20	5246500	0.842975
chr20	5251500	0.933884
chr20	5490900	0.776860
chr20	5785700	0.793388
chr20	5803800	0.942149
chr20	5806400	0.884298
chr20	5852200	0.719008
chr20	5863200	0.735537
chr20	6035100	0.752066
chr20	6678900	0.826446
chr20	6745500	0.900826
chr20	6795700	0.876033
chr20	6830400	0.644628
chr20	6879600	0.669421
chr20	6889700	0.950413
chr20	7094600	0.669421
chr20	7195100	0.760331
chr20	7637700	0.785124
chr20	7965100	0.809917
chr20	8109500	0.685950
chr20	8290900	0.859504
chr20	8341400	0.702479
chr20	8695600	0.735537
chr20	8790000	0.652893
chr20	8792900	0.834711
chr20	8882400	0.661157
chr20	8955100	0.685950
chr20	9003100	0.925620
chr20	9202000	0.710744
chr20	9374200	0.776860
chr20	9499300	0.826446
chr20	9501900	0.892562
chr20	9553700	0.900826
chr20	9557300	0.768595
chr20	9811400	0.727273
chr20	9943800	0.702479
chr20	10113300	0.809917
chr20	10206300	0.743802
chr20	10221800	0.644628
chr20	10230200	0.809917
chr20	10261300	0.834711
chr20	10295600	0.884298
chr20	10332900	0.925620
chr20	10445600	0.776860

chr20	10448000	0.925620
chr20	10471800	0.991736
chr20	10482800	0.884298
chr20	10492000	0.809917
chr20	10503200	0.793388
chr20	10517500	0.694215
chr20	10530900	0.818182
chr20	10533500	0.727273
chr20	10539700	0.785124
chr20	10565200	0.661157
chr20	10607600	0.652893
chr20	10610900	0.719008
chr20	10614200	0.768595
chr20	10617700	0.851240
chr20	10628900	0.727273
chr20	10631400	0.793388
chr20	10641700	0.793388
chr20	10645300	0.826446
chr20	10647800	0.867769
chr20	10673200	0.702479
chr20	10700200	0.727273
chr20	10761300	0.677686
chr20	10765000	0.834711
chr20	10775200	0.694215
chr20	11229800	0.785124
chr20	11248800	0.735537
chr20	11252300	0.661157
chr20	11332800	0.677686
chr20	11360400	0.942149
chr20	11393000	0.685950
chr20	11428200	0.694215
chr20	11433100	0.719008
chr20	11449400	0.785124
chr20	11518100	0.991736
chr20	11521600	1.000000
chr20	11524600	0.694215
chr20	11634600	0.685950
chr20	11660300	0.661157
chr20	11698000	0.685950
chr20	11747800	0.727273
chr20	11758700	0.809917
chr20	11764300	0.900826
chr20	11874700	0.719008
chr20	11897300	0.702479
chr20	11960200	0.785124
chr20	12642000	0.735537
chr20	12734000	0.983471
chr20	12741400	0.842975
chr20	12802500	0.644628
chr20	12873700	0.669421
chr20	12906200	0.884298
chr20	12990200	0.752066
chr20	13167000	0.801653
chr20	13181800	0.752066

chr20	13255300	0.677686
chr20	13337700	0.834711
chr20	13397800	0.685950
chr20	13472700	0.975207
chr20	13607700	0.909091
chr20	13630700	0.669421
chr20	13855400	0.950413
chr20	13971600	0.644628
chr20	14315000	0.768595
chr20	14452100	0.884298
chr20	14480200	0.743802
chr20	14691900	0.661157
chr20	14694600	0.743802
chr20	14697300	1.000000
chr20	14841700	0.685950
chr20	14900600	0.909091
chr20	14928700	0.760331
chr20	14932100	0.760331
chr20	14984700	0.710744
chr20	15050500	0.644628
chr20	15201900	0.776860
chr20	15271800	0.727273
chr20	15542600	0.859504
chr20	15608700	0.876033
chr20	15619400	0.818182
chr20	15639600	0.644628
chr20	15642100	0.933884
chr20	15769500	0.743802
chr20	15799600	0.710744
chr20	15869900	0.652893
chr20	15930100	0.752066
chr20	15957300	1.000000
chr20	15986800	1.000000
chr20	16030800	0.652893
chr20	16046400	0.752066
chr20	16196000	0.694215
chr20	16546800	0.677686
chr20	16560800	0.644628
chr20	16923400	0.801653
chr20	17005400	0.884298
chr20	17183500	0.809917
chr20	17411900	0.809917
chr20	17514500	0.859504
chr20	17539000	0.892562
chr20	17554000	0.793388
chr20	17575400	0.809917
chr20	17583400	0.884298
chr20	17592200	0.900826
chr20	17598100	0.760331
chr20	17627700	0.826446
chr20	17636900	0.809917
chr20	17674600	0.669421
chr20	17691600	0.752066
chr20	17733600	0.975207

chr20	17752600	0.768595
chr20	17787500	0.793388
chr20	17804900	0.669421
chr20	17817500	0.719008
chr20	17840900	0.768595
chr20	17851900	0.785124
chr20	17854100	0.809917
chr20	17856400	0.801653
chr20	17864300	0.859504
chr20	17876800	0.710744
chr20	17910200	0.710744
chr20	17915000	0.793388
chr20	17920500	0.942149
chr20	17940000	0.752066
chr20	17964600	0.950413
chr20	17986000	0.727273
chr20	18014200	0.677686
chr20	18147700	0.818182
chr20	18205800	0.942149
chr20	18304700	0.966942
chr20	18393700	0.900826
chr20	18650600	0.760331
chr20	18664200	0.785124
chr20	18679700	0.710744
chr20	18720300	0.661157
chr20	18837900	0.760331
chr20	18938300	0.694215
chr20	18965600	0.652893
chr20	18996500	0.785124
chr20	19076800	0.950413
chr20	19187300	0.859504
chr20	19384900	0.818182
chr20	19404000	0.859504
chr20	19491300	0.719008
chr20	19564600	0.702479
chr20	19601500	0.876033
chr20	19642700	0.694215
chr20	19751200	0.669421
chr20	19917000	0.661157
chr20	19940000	0.909091
chr20	19974600	0.809917
chr20	20059800	0.677686
chr20	20087700	0.743802
chr20	20353000	0.842975
chr20	20389700	0.669421
chr20	20392800	0.842975
chr20	20433400	0.884298
chr20	20442100	0.768595
chr20	20446400	0.793388
chr20	20458500	0.669421
chr20	20473300	0.892562
chr20	20540100	0.661157
chr20	20647000	0.884298
chr20	20724700	0.942149

chr20	20796000	0.859504
chr20	20802000	0.851240
chr20	20868900	0.958678
chr20	20878900	0.768595
chr20	20942000	0.644628
chr20	21001000	0.652893
chr20	21034200	0.801653
chr20	21071200	0.925620
chr20	21161500	0.892562
chr20	21168700	0.669421
chr20	21170900	0.826446
chr20	21173500	0.793388
chr20	21194600	0.661157
chr20	21216200	0.892562
chr20	21304600	0.652893
chr20	21374000	0.677686
chr20	21480000	0.776860
chr20	21491100	0.669421
chr20	21500400	0.727273
chr20	21552100	0.776860
chr20	21687100	0.809917
chr20	21690100	0.760331
chr20	21843700	0.892562
chr20	21918300	0.925620
chr20	22198500	0.727273
chr20	22320500	0.644628
chr20	22371200	0.785124
chr20	22384400	0.785124
chr20	22410900	0.793388
chr20	22437100	0.958678
chr20	22444300	0.727273
chr20	22471700	0.867769
chr20	22511100	0.842975
chr20	22537400	0.900826
chr20	22539800	0.735537
chr20	22542300	0.892562
chr20	22544500	0.842975
chr20	22552700	0.818182
chr20	22556200	0.768595
chr20	22560500	0.826446
chr20	22566100	0.702479
chr20	22571600	0.884298
chr20	22601100	0.909091
chr20	22633700	0.710744
chr20	22644400	0.644628
chr20	22672900	0.702479
chr20	22781800	0.644628
chr20	23023200	0.685950
chr20	23043400	0.851240
chr20	23106600	0.776860
chr20	23141800	0.677686
chr20	23186800	0.851240
chr20	23236700	0.685950
chr20	23306000	0.644628

chr20	23374500	0.710744
chr20	23427600	0.735537
chr20	23433800	0.661157
chr20	23454200	0.694215
chr20	23488900	0.710744
chr20	23508700	0.925620
chr20	23513700	0.776860
chr20	23566000	0.644628
chr20	23607100	0.652893
chr20	23757200	0.958678
chr20	23839100	0.735537
chr20	23848100	0.776860
chr20	23904600	0.694215
chr20	23936900	0.669421
chr20	23962800	0.685950
chr20	24040300	0.644628
chr20	24134100	0.652893
chr20	24193200	0.719008
chr20	24404400	0.900826
chr20	24406600	0.809917
chr20	24426300	0.743802
chr20	24436200	0.760331
chr20	24443600	0.809917
chr20	24472900	0.876033
chr20	24508700	0.652893
chr20	24591200	0.768595
chr20	24616300	0.900826
chr20	24724300	0.859504
chr20	24770700	0.685950
chr20	24998300	0.694215
chr20	25052800	0.694215
chr20	25179700	0.661157
chr20	25186400	0.768595
chr20	25278100	0.785124
chr20	25305000	0.842975
chr20	25331900	0.727273
chr20	25334800	0.785124
chr20	25338500	0.966942
chr20	25345700	0.652893
chr20	25418000	0.652893
chr20	25470400	0.851240
chr20	25513400	0.876033
chr20	25520800	0.942149
chr20	25525800	0.950413
chr20	25529800	0.876033
chr20	25560100	0.859504
chr20	25844200	0.644628
chr20	26051600	0.743802
chr20	26142000	0.876033
chr20	26190200	0.884298
chr20	26314500	0.677686
chr20	26319200	0.735537
chr20	29833100	0.743802
chr20	30105200	0.644628

chr20	30148200	0.884298
chr20	30154700	0.834711
chr20	30157500	0.851240
chr20	30164900	0.694215
chr20	30169300	0.942149
chr20	30173900	0.950413
chr20	30182300	0.793388
chr20	30184600	0.942149
chr20	30190700	0.702479
chr20	30199600	0.842975
chr20	30266900	0.752066
chr20	30280200	0.785124
chr20	30283200	0.785124
chr20	30287300	0.719008
chr20	30296600	0.983471
chr20	30302100	0.793388
chr20	30305400	0.644628
chr20	30372500	0.818182
chr20	30411100	0.818182
chr20	30421900	0.760331
chr20	30429500	0.727273
chr20	30433900	0.859504
chr20	30438300	0.867769
chr20	30474000	0.752066
chr20	30494000	0.991736
chr20	30499400	0.834711
chr20	30502500	0.735537
chr20	30508000	0.727273
chr20	30589500	0.644628
chr20	30595600	0.677686
chr20	30598700	0.669421
chr20	30612300	0.735537
chr20	30616800	0.661157
chr20	30628600	0.743802
chr20	30679800	0.760331
chr20	30704500	0.884298
chr20	30756500	0.752066
chr20	30792000	0.958678
chr20	30932900	0.677686
chr20	31033100	0.661157
chr20	31052200	0.801653
chr20	31058500	0.768595
chr20	31062400	0.900826
chr20	31085800	0.876033
chr20	31110300	0.768595
chr20	31116100	0.834711
chr20	31122800	0.785124
chr20	31126800	0.644628
chr20	31152100	0.719008
chr20	31158900	0.801653
chr20	31162800	0.661157
chr20	31174800	0.652893
chr20	31261400	0.743802
chr20	31264000	0.793388

chr20	31269000	0.768595
chr20	31291000	0.735537
chr20	31337700	0.867769
chr20	31341700	0.801653
chr20	31360600	0.818182
chr20	31369000	0.975207
chr20	31379300	0.942149
chr20	31391800	0.925620
chr20	31396100	0.909091
chr20	31446500	0.826446
chr20	31459900	0.652893
chr20	31562500	0.785124
chr20	31691300	0.702479
chr20	31720900	0.752066
chr20	31766600	0.719008
chr20	31865700	0.685950
chr20	31936800	0.669421
chr20	31940500	0.752066
chr20	32065400	0.809917
chr20	32143600	0.768595
chr20	32156700	0.876033
chr20	32242400	0.933884
chr20	32282100	0.818182
chr20	32298400	0.909091
chr20	32322200	0.809917
chr20	32385000	0.694215
chr20	32414300	0.809917
chr20	32430100	0.909091
chr20	32434500	0.826446
chr20	32451300	0.669421
chr20	32502900	0.727273
chr20	32588000	0.677686
chr20	32598500	0.933884
chr20	32601100	0.867769
chr20	32604200	0.702479
chr20	32612400	0.892562
chr20	32621500	0.677686
chr20	32633800	0.900826
chr20	32656100	0.958678
chr20	32888900	0.818182
chr20	32906700	0.867769
chr20	32920200	0.710744
chr20	32954300	0.694215
chr20	33111200	0.768595
chr20	33343000	0.661157
chr20	33379700	0.652893
chr20	33541100	0.809917
chr20	33568900	0.760331
chr20	33572300	0.826446
chr20	33678500	0.677686
chr20	33754400	0.909091
chr20	33762600	0.710744
chr20	33780000	0.801653
chr20	33796400	0.685950

chr20	33847600	0.669421
chr20	33850100	0.884298
chr20	33892200	0.834711
chr20	34026900	0.719008
chr20	34241200	0.702479
chr20	34357100	0.809917
chr20	34496100	0.752066
chr20	34522600	0.818182
chr20	34535200	0.669421
chr20	34686100	0.933884
chr20	34702400	0.735537
chr20	34710700	0.710744
chr20	34744900	0.702479
chr20	34761600	0.909091
chr20	34791100	0.702479
chr20	34847600	0.719008
chr20	34856400	0.661157
chr20	34891200	0.661157
chr20	34961500	0.942149
chr20	34968500	0.776860
chr20	34997000	0.876033
chr20	35006700	0.652893
chr20	35019700	0.743802
chr20	35029700	0.652893
chr20	35083700	0.752066
chr20	35089000	0.776860
chr20	35094200	0.925620
chr20	35101300	0.710744
chr20	35120700	0.752066
chr20	35172300	0.652893
chr20	35191400	0.876033
chr20	35214200	0.958678
chr20	35226400	0.818182
chr20	35252800	0.752066
chr20	35274900	0.892562
chr20	35448500	0.768595
chr20	35464600	0.900826
chr20	35471500	0.768595
chr20	35483700	0.652893
chr20	35515900	0.966942
chr20	35540400	0.809917
chr20	35575800	0.975207
chr20	35851100	0.644628
chr20	35855900	0.685950
chr20	35858500	0.867769
chr20	35871900	0.652893
chr20	35874100	0.975207
chr20	35880500	0.801653
chr20	35887300	0.785124
chr20	35896500	0.983471
chr20	35914800	0.644628
chr20	35944100	0.752066
chr20	35947500	0.975207
chr20	35965400	0.644628

chr20	36023300	0.752066
chr20	36027100	0.900826
chr20	36034900	0.776860
chr20	36037600	0.834711
chr20	36040400	0.694215
chr20	36046500	0.644628
chr20	36049400	0.719008
chr20	36134300	0.900826
chr20	36152800	0.644628
chr20	36226800	0.892562
chr20	36355800	0.785124
chr20	36379200	0.785124
chr20	36445000	0.727273
chr20	36475200	0.768595
chr20	36497900	0.933884
chr20	36537300	0.834711
chr20	36546200	0.727273
chr20	36561800	0.809917
chr20	36597800	0.834711
chr20	36601200	0.842975
chr20	36605900	0.942149
chr20	36772700	0.719008
chr20	36881400	0.652893
chr20	36928800	0.826446
chr20	36968400	0.809917
chr20	36972200	0.917355
chr20	36983600	0.917355
chr20	37037600	0.644628
chr20	37045500	0.793388
chr20	37228400	0.760331
chr20	37288300	0.702479
chr20	37295200	0.727273
chr20	37300400	0.867769
chr20	37408400	0.991736
chr20	37432700	0.710744
chr20	37447100	0.719008
chr20	37455000	0.851240
chr20	37489700	0.793388
chr20	37565300	0.743802
chr20	37639100	0.768595
chr20	37752500	0.818182
chr20	37775500	0.710744
chr20	37911700	0.818182
chr20	37939400	0.760331
chr20	38164100	0.727273
chr20	38458500	0.752066
chr20	38482300	0.661157
chr20	38528800	0.925620
chr20	38615400	0.900826
chr20	38702900	0.909091
chr20	38897500	0.818182
chr20	38904400	0.661157
chr20	38908000	0.867769
chr20	39132100	0.702479

chr20	39310600	0.760331
chr20	39321900	0.661157
chr20	39367600	0.702479
chr20	39406200	0.727273
chr20	39487500	0.677686
chr20	39597700	0.710744
chr20	39626300	0.677686
chr20	39632400	0.685950
chr20	39675800	0.677686
chr20	39764100	0.768595
chr20	39769700	0.933884
chr20	39783700	0.719008
chr20	39788000	0.702479
chr20	39874300	0.818182
chr20	39878900	0.669421
chr20	39937400	0.743802
chr20	40024800	0.933884
chr20	40129500	0.669421
chr20	40141900	0.685950
chr20	40192300	0.719008
chr20	40201000	0.685950
chr20	40203800	0.942149
chr20	40319500	0.842975
chr20	40367600	0.876033
chr20	40398600	0.793388
chr20	40406200	0.669421
chr20	40427500	0.644628
chr20	40518900	0.826446
chr20	40789400	0.652893
chr20	40938100	0.743802
chr20	41021500	0.917355
chr20	41075800	0.694215
chr20	41092400	0.644628
chr20	41465200	0.876033
chr20	41609200	0.776860
chr20	41612200	0.719008
chr20	41662100	0.669421
chr20	41725400	0.685950
chr20	41872100	0.727273
chr20	41924900	0.661157
chr20	42059600	0.735537
chr20	42213800	0.652893
chr20	42350300	0.876033
chr20	42471200	0.743802
chr20	42523100	0.917355
chr20	42528700	0.851240
chr20	42533100	0.876033
chr20	42555200	0.694215
chr20	42571900	0.652893
chr20	42577900	0.644628
chr20	42590100	0.876033
chr20	42649300	0.735537
chr20	42766200	0.727273
chr20	42860400	0.851240

chr20	42901000	0.842975
chr20	42953500	0.743802
chr20	42965100	0.900826
chr20	42999400	0.652893
chr20	43017700	0.669421
chr20	43153200	0.834711
chr20	43214300	0.785124
chr20	43220000	0.743802
chr20	43238600	0.900826
chr20	43245000	0.669421
chr20	43271600	0.892562
chr20	43275200	0.950413
chr20	43285300	0.785124
chr20	43330300	0.760331
chr20	43355900	0.752066
chr20	43358300	0.834711
chr20	43364700	0.685950
chr20	43413900	0.942149
chr20	43425800	0.735537
chr20	43434900	0.685950
chr20	43465100	0.661157
chr20	43541100	0.793388
chr20	43646400	0.900826
chr20	43670800	0.991736
chr20	43705700	0.735537
chr20	43740800	0.752066
chr20	43744000	0.785124
chr20	43835800	0.760331
chr20	43838900	0.900826
chr20	43887500	0.834711
chr20	43930100	0.760331
chr20	43935000	0.752066
chr20	43937300	0.851240
chr20	43966600	0.669421
chr20	43969400	0.743802
chr20	43973700	0.785124
chr20	43987700	0.710744
chr20	44116400	0.694215
chr20	44212400	0.785124
chr20	44394600	0.917355
chr20	44400300	0.842975
chr20	44453100	0.867769
chr20	44483300	0.958678
chr20	44567600	0.975207
chr20	44612100	0.710744
chr20	44622700	0.818182
chr20	44629600	0.892562
chr20	44633900	0.719008
chr20	44637300	0.925620
chr20	44640300	0.818182
chr20	44658400	0.661157
chr20	44666100	0.793388
chr20	44699800	0.669421
chr20	44703200	0.958678

chr20	44706000	0.826446
chr20	44761900	0.694215
chr20	44765000	0.661157
chr20	44768400	0.652893
chr20	44774800	0.677686
chr20	44828400	0.743802
chr20	44832000	0.743802
chr20	44845200	0.851240
chr20	44849100	0.669421
chr20	44852000	0.752066
chr20	44855500	0.727273
chr20	44866100	0.652893
chr20	44902900	0.719008
chr20	44906800	0.661157
chr20	44912100	0.661157
chr20	44921500	0.809917
chr20	44963000	0.719008
chr20	44977000	0.826446
chr20	44980000	0.884298
chr20	45030200	0.842975
chr20	45053000	0.950413
chr20	45108000	0.710744
chr20	45150000	0.859504
chr20	45180900	0.884298
chr20	45183900	0.785124
chr20	45187700	0.685950
chr20	45191400	1.000000
chr20	45212700	0.933884
chr20	45223800	0.669421
chr20	45251400	0.694215
chr20	45255700	0.652893
chr20	45260800	0.644628
chr20	45267000	0.702479
chr20	45269400	0.685950
chr20	45303200	0.768595
chr20	45333800	0.826446
chr20	45343800	0.669421
chr20	45436500	0.644628
chr20	45574500	0.743802
chr20	45608300	0.842975
chr20	45619400	0.752066
chr20	45621700	0.917355
chr20	45624500	0.776860
chr20	45627500	0.735537
chr20	45754800	0.694215
chr20	45835700	0.950413
chr20	45896100	0.710744
chr20	45932400	0.727273
chr20	45938500	0.702479
chr20	45944400	0.752066
chr20	45950400	0.768595
chr20	45954600	0.719008
chr20	45960400	0.818182
chr20	45965100	0.933884

chr20	45975700	0.983471
chr20	45979300	0.876033
chr20	45982200	0.876033
chr20	45984600	0.991736
chr20	45987500	0.677686
chr20	45991200	0.859504
chr20	45994300	0.661157
chr20	46000000	0.719008
chr20	46002200	0.752066
chr20	46006500	0.710744
chr20	46012800	0.652893
chr20	46137500	0.793388
chr20	46149700	0.851240
chr20	46171800	0.851240
chr20	46186700	0.842975
chr20	46202700	0.776860
chr20	46205700	0.801653
chr20	46216100	0.719008
chr20	46278300	0.958678
chr20	46283200	0.917355
chr20	46293600	0.694215
chr20	46304700	0.685950
chr20	46317700	0.900826
chr20	46395300	0.834711
chr20	46467700	0.776860
chr20	46523800	0.652893
chr20	46541300	0.661157
chr20	46602600	0.735537
chr20	46607900	0.851240
chr20	46621200	0.809917
chr20	46653700	0.776860
chr20	46755600	0.702479
chr20	46905000	0.942149
chr20	46921300	0.702479
chr20	46924600	0.983471
chr20	46938500	0.702479
chr20	46997200	0.661157
chr20	47002600	0.958678
chr20	47004700	0.900826
chr20	47024100	0.661157
chr20	47028000	0.685950
chr20	47035900	0.859504
chr20	47084700	0.702479
chr20	47089600	0.809917
chr20	47107000	0.942149
chr20	47155500	0.685950
chr20	47220500	0.669421
chr20	47237600	0.842975
chr20	47252700	0.677686
chr20	47296500	0.776860
chr20	47299600	0.933884
chr20	47364400	0.818182
chr20	47368000	0.966942
chr20	47371200	0.710744

chr20	47380400	0.719008
chr20	47385900	0.851240
chr20	47411400	1.000000
chr20	47414300	0.801653
chr20	47437200	0.694215
chr20	47441600	0.809917
chr20	47463600	0.826446
chr20	47521900	0.752066
chr20	47652500	0.685950
chr20	47668100	0.669421
chr20	47833800	0.826446
chr20	47904200	0.975207
chr20	47923600	0.652893
chr20	47937500	0.743802
chr20	47941100	0.752066
chr20	47997300	0.677686
chr20	48062000	0.677686
chr20	48064100	0.867769
chr20	48067200	0.909091
chr20	48136700	0.652893
chr20	48179000	0.983471
chr20	48189600	0.809917
chr20	48248400	0.867769
chr20	48275400	0.917355
chr20	48292900	0.694215
chr20	48301900	0.694215
chr20	48304700	0.727273
chr20	48311300	1.000000
chr20	48373300	0.644628
chr20	48375900	0.842975
chr20	48382300	0.752066
chr20	48385200	0.909091
chr20	48388700	0.768595
chr20	48448800	0.842975
chr20	48470800	0.644628
chr20	48505400	0.826446
chr20	48508200	0.760331
chr20	48587300	0.685950
chr20	48596200	0.644628
chr20	48604000	0.768595
chr20	48606800	0.826446
chr20	48629300	0.652893
chr20	48671600	0.859504
chr20	48692600	0.677686
chr20	48702500	0.925620
chr20	48716100	0.669421
chr20	48759300	0.685950
chr20	48763900	0.685950
chr20	48767500	0.818182
chr20	48796100	0.735537
chr20	48814900	0.661157
chr20	48884800	0.702479
chr20	48922800	0.834711
chr20	48925900	0.735537

chr20	48977200	0.834711
chr20	49029200	0.669421
chr20	49050500	0.694215
chr20	49067900	0.652893
chr20	49074100	0.727273
chr20	49227800	0.801653
chr20	49245300	0.644628
chr20	49253000	0.743802
chr20	49255200	0.933884
chr20	49258900	0.958678
chr20	49262400	0.942149
chr20	49354300	0.768595
chr20	49356500	0.685950
chr20	49384100	0.884298
chr20	49386400	0.735537
chr20	49407500	0.933884
chr20	49416700	0.661157
chr20	49435200	0.892562
chr20	49465500	0.760331
chr20	49468000	0.694215
chr20	49472600	0.776860
chr20	49482000	0.652893
chr20	49486500	0.793388
chr20	49531000	0.710744
chr20	49533700	0.760331
chr20	49609100	0.909091
chr20	49631300	0.933884
chr20	49637200	0.933884
chr20	49687900	0.685950
chr20	49704300	0.694215
chr20	49709000	0.809917
chr20	49738900	0.793388
chr20	49838800	0.925620
chr20	49901500	0.644628
chr20	49925600	0.809917
chr20	49929000	0.801653
chr20	49935100	0.710744
chr20	50003800	0.892562
chr20	50007100	0.818182
chr20	50014400	0.685950
chr20	50040900	0.793388
chr20	50214700	0.834711
chr20	50277500	0.743802
chr20	50313000	0.950413
chr20	50322500	0.892562
chr20	50327500	0.950413
chr20	50410700	0.652893
chr20	50428400	0.652893
chr20	50503800	0.752066
chr20	50506300	0.710744
chr20	50527400	0.933884
chr20	50575400	0.727273
chr20	50619500	0.669421
chr20	50663400	0.768595

chr20	50666400	0.727273
chr20	50677800	0.793388
chr20	50712100	0.702479
chr20	50826400	0.859504
chr20	50925800	0.760331
chr20	50928100	0.859504
chr20	51118500	0.917355
chr20	51233100	0.702479
chr20	51293200	0.694215
chr20	51521800	0.644628
chr20	51540200	0.685950
chr20	51683100	0.743802
chr20	51699300	0.661157
chr20	51779500	0.669421
chr20	51882400	0.826446
chr20	51898300	0.809917
chr20	51900900	0.661157
chr20	51948200	0.661157
chr20	52029400	0.776860
chr20	52164600	0.785124
chr20	52195700	0.760331
chr20	52197900	0.735537
chr20	52200700	0.975207
chr20	52203400	0.950413
chr20	52205700	0.859504
chr20	52212800	0.966942
chr20	52216300	0.752066
chr20	52226500	0.900826
chr20	52230100	0.702479
chr20	52239900	0.950413
chr20	52259700	0.669421
chr20	52296900	0.702479
chr20	52324200	0.694215
chr20	52329000	0.884298
chr20	52351100	0.652893
chr20	52353400	0.991736
chr20	52355700	0.975207
chr20	52357800	0.851240
chr20	52370000	0.983471
chr20	52380800	0.801653
chr20	52385400	0.950413
chr20	52426200	0.685950
chr20	52474600	0.876033
chr20	52480800	0.826446
chr20	52484200	0.950413
chr20	52489700	0.892562
chr20	52502600	0.785124
chr20	52515600	0.735537
chr20	52518700	0.719008
chr20	52551900	0.685950
chr20	52557100	0.925620
chr20	52559600	0.925620
chr20	52686600	0.834711
chr20	52740300	0.900826

chr20	52839700	0.801653
chr20	52884800	0.884298
chr20	52908600	0.785124
chr20	52963400	0.743802
chr20	53064000	0.776860
chr20	53095700	0.793388
chr20	53127300	0.669421
chr20	53131300	0.702479
chr20	53143100	0.652893
chr20	53241800	0.851240
chr20	53250400	0.793388
chr20	53256300	0.727273
chr20	53279000	0.958678
chr20	53707100	0.719008
chr20	53782900	0.702479
chr20	54039800	0.809917
chr20	54154700	0.917355
chr20	54207300	0.958678
chr20	54923500	0.876033
chr20	54998000	0.661157
chr20	55249100	0.661157
chr20	55265600	0.735537
chr20	55311200	0.983471
chr20	55314800	0.727273
chr20	55324400	0.818182
chr20	55354300	0.785124
chr20	55380100	0.851240
chr20	55383500	0.818182
chr20	55405600	0.859504
chr20	55437000	0.793388
chr20	55473100	0.694215
chr20	55502800	0.760331
chr20	55505500	0.768595
chr20	55531400	0.851240
chr20	55543500	0.685950
chr20	55589200	0.785124
chr20	55597600	0.735537
chr20	55629800	0.669421
chr20	55643300	0.727273
chr20	55667500	0.760331
chr20	55734500	0.900826
chr20	55773700	0.793388
chr20	55787500	0.859504
chr20	55807600	0.826446
chr20	55831600	0.727273
chr20	55834600	0.925620
chr20	55845600	0.900826
chr20	55949900	0.842975
chr20	55970800	0.917355
chr20	56004300	0.661157
chr20	56016200	0.942149
chr20	56043100	0.727273
chr20	56046000	0.818182
chr20	56058000	0.859504

chr20	56089500	0.644628
chr20	56099400	0.743802
chr20	56230300	0.652893
chr20	56236900	0.818182
chr20	56242600	0.933884
chr20	56252600	0.752066
chr20	56260600	0.702479
chr20	56264800	0.661157
chr20	56298300	0.768595
chr20	56348000	0.768595
chr20	56410900	0.900826
chr20	56443000	0.801653
chr20	56514400	0.727273
chr20	56574500	0.727273
chr20	56619100	0.644628
chr20	56631300	0.652893
chr20	56706500	0.851240
chr20	56785200	0.694215
chr20	56870300	0.900826
chr20	56918300	0.702479
chr20	56948300	0.710744
chr20	57010600	0.743802
chr20	57015300	0.652893
chr20	57024000	0.776860
chr20	57028100	0.785124
chr20	57042900	0.743802
chr20	57057200	0.669421
chr20	57088100	0.652893
chr20	57098800	0.685950
chr20	57153700	0.685950
chr20	57194900	0.768595
chr20	57223300	0.793388
chr20	57230200	0.826446
chr20	57232300	0.859504
chr20	57234900	0.826446
chr20	57237200	0.669421
chr20	57240600	0.801653
chr20	57243600	0.851240
chr20	57246400	0.752066
chr20	57250700	0.776860
chr20	57267800	0.884298
chr20	57270500	0.942149
chr20	57328900	0.859504
chr20	57365400	0.760331
chr20	57422400	0.752066
chr20	57450700	0.669421
chr20	57453800	0.809917
chr20	57458600	0.950413
chr20	57470600	0.727273
chr20	57473700	0.900826
chr20	57475800	0.933884
chr20	57697600	0.661157
chr20	57701700	0.925620
chr20	57733700	0.694215

chr20	57844400	0.826446
chr20	57961900	0.661157
chr20	57980500	0.661157
chr20	58028700	0.710744
chr20	58045000	0.809917
chr20	58521100	0.768595
chr20	58567000	0.826446
chr20	58633700	0.760331
chr20	58680200	0.652893
chr20	59008100	0.925620
chr20	59155300	0.818182
chr20	59238100	0.776860
chr20	59267000	0.727273
chr20	59269100	1.000000
chr20	59291000	0.652893
chr20	59304400	0.975207
chr20	59364600	0.727273
chr20	59379900	0.826446
chr20	59473500	0.752066
chr20	59480500	0.661157
chr20	59589900	0.644628
chr20	59746300	0.760331
chr20	59804400	0.644628
chr20	59812400	0.826446
chr20	59825100	0.669421
chr20	59846800	0.785124
chr20	59907900	0.710744
chr20	59925600	0.876033
chr20	59928000	0.685950
chr20	60009600	0.669421
chr20	60012000	0.900826
chr20	60015900	0.719008
chr20	60018300	0.876033
chr20	60021000	0.719008
chr20	60023600	0.793388
chr20	60036200	0.785124
chr20	60069100	0.669421
chr20	60071900	0.917355
chr20	60075800	0.776860
chr20	60090900	0.884298
chr20	60096800	0.669421
chr20	60142300	0.669421
chr20	60145700	0.776860
chr20	60157300	0.743802
chr20	60211500	0.826446
chr20	60251800	0.719008
chr20	60294700	0.876033
chr20	60509500	0.727273
chr20	60526600	0.801653
chr20	60602600	0.694215
chr20	60623200	0.768595
chr20	60679900	0.801653
chr20	60724200	0.958678
chr20	60727500	0.644628

chr20	60731500	0.909091
chr20	60803200	0.661157
chr20	60812700	0.752066
chr20	60818700	0.702479
chr20	60880900	0.859504
chr20	60929800	0.917355
chr20	60932100	0.809917
chr20	60934500	0.933884
chr20	60937200	0.851240
chr20	60939600	0.867769
chr20	60945000	0.743802
chr20	60950300	0.950413
chr20	60952800	0.950413
chr20	61006300	0.826446
chr20	61053500	0.859504
chr20	61058000	0.661157
chr20	61060600	0.685950
chr20	61062700	0.834711
chr20	61071300	0.884298
chr20	61075600	0.719008
chr20	61175000	0.661157
chr20	61179300	0.727273
chr20	61203700	0.942149
chr20	61283500	0.842975
chr20	61372400	0.818182
chr20	61384100	0.859504
chr20	61456500	0.925620
chr20	61507900	0.644628
chr20	61510900	0.743802
chr20	61517200	0.776860
chr20	61532100	0.950413
chr20	61546100	0.983471
chr20	61549100	0.768595
chr20	61571900	0.669421
chr20	61592800	0.900826
chr20	61602800	0.768595
chr20	61611200	0.677686
chr20	61618300	0.818182
chr20	61626700	0.661157
chr20	61629800	0.834711
chr20	61632900	0.669421
chr20	61640700	0.876033
chr20	61646500	0.826446
chr20	61661500	0.842975
chr20	61665200	0.834711
chr20	61731800	0.743802
chr20	61762100	0.694215
chr20	61799400	0.818182
chr20	61867500	0.652893
chr20	61879200	0.661157
chr20	61922800	0.685950
chr20	61927400	0.760331
chr20	61974800	0.743802
chr20	61984000	0.884298

chr20	62009400	0.876033
chr20	62013400	0.644628
chr20	62025800	0.925620
chr20	62036000	0.702479
chr20	62041300	0.909091
chr20	62049200	0.652893
chr20	62065600	0.752066
chr20	62086500	0.909091
chr20	62106000	0.677686
chr20	62113500	0.743802
chr20	62119900	0.727273
chr20	62128200	0.909091
chr20	62133900	0.851240
chr20	62166500	0.719008
chr20	62169600	0.743802
chr20	62179700	0.743802
chr20	62328700	0.851240
chr20	62427800	0.644628
chr20	62466900	0.760331
chr20	62479600	0.793388
chr20	62485800	0.743802
chr20	62490300	0.809917
chr20	62574500	0.867769
chr20	62579900	0.933884
chr20	62582200	0.793388
chr20	62590800	0.900826
chr20	62667900	0.826446
chr20	62677000	0.809917
chr20	62685700	0.719008
chr20	62689100	0.809917
chr20	62701300	0.677686
chr20	62735700	0.677686
chr20	62787600	0.644628
chr20	62791600	0.752066
chr20	62796200	0.669421
chr20	62808600	0.925620
chr20	62820600	0.909091
chr20	62823000	0.768595
chr21	9827500	0.859504
chr21	10493200	0.991736
chr21	10807300	0.776860
chr21	11186400	0.719008
chr21	14365500	0.834711
chr21	15475700	0.644628
chr21	15975200	0.743802
chr21	15989300	0.991736
chr21	16284700	0.644628
chr21	16289000	0.991736
chr21	16359900	0.677686
chr21	16374900	0.760331
chr21	16423600	0.900826
chr21	16430000	0.859504
chr21	16494200	0.743802
chr21	16572200	0.917355

chr21	16594800	0.768595
chr21	16614800	0.842975
chr21	16779700	0.644628
chr21	16796400	0.983471
chr21	17111700	0.826446
chr21	17125800	1.000000
chr21	17652800	0.933884
chr21	17689700	0.644628
chr21	18203300	0.702479
chr21	18423400	0.694215
chr21	18492500	0.661157
chr21	18888900	0.727273
chr21	18891300	0.859504
chr21	18898800	0.925620
chr21	19033000	0.694215
chr21	19040300	0.743802
chr21	19062900	0.644628
chr21	19201800	0.702479
chr21	19232300	0.669421
chr21	19362400	0.644628
chr21	19528200	0.983471
chr21	19784100	0.834711
chr21	19833500	0.785124
chr21	20492900	0.669421
chr21	20926800	0.842975
chr21	20977900	0.743802
chr21	22000300	0.818182
chr21	22034300	0.785124
chr21	22162100	0.950413
chr21	22195500	0.809917
chr21	22307000	0.702479
chr21	22419000	0.909091
chr21	22737600	0.785124
chr21	23224100	0.867769
chr21	24258300	0.719008
chr21	24354600	0.834711
chr21	26314600	0.710744
chr21	26705900	0.685950
chr21	26711400	0.760331
chr21	26745700	0.917355
chr21	27174200	0.760331
chr21	27333000	0.933884
chr21	27359600	0.644628
chr21	27369900	0.859504
chr21	27372800	0.752066
chr21	27380900	0.702479
chr21	27396000	0.809917
chr21	27430500	0.719008
chr21	27437300	0.793388
chr21	27441400	0.851240
chr21	27443800	0.876033
chr21	27454000	0.942149
chr21	27472000	0.652893
chr21	27479100	0.900826

chr21	27481300	0.685950
chr21	27486700	0.644628
chr21	27489500	0.801653
chr21	27495300	0.685950
chr21	27498100	0.876033
chr21	27500800	0.644628
chr21	27531000	0.677686
chr21	27654000	0.809917
chr21	27885800	0.776860
chr21	28105300	0.859504
chr21	28282400	0.719008
chr21	28319100	0.809917
chr21	28797000	0.669421
chr21	29441700	0.892562
chr21	29653200	0.677686
chr21	29655600	0.644628
chr21	29698700	0.826446
chr21	29927600	0.677686
chr21	29953500	0.876033
chr21	29963800	0.661157
chr21	30295400	0.710744
chr21	30969200	0.685950
chr21	30988900	0.743802
chr21	31048600	0.685950
chr21	31093900	0.859504
chr21	31243800	0.669421
chr21	31248100	0.826446
chr21	31444700	0.809917
chr21	31456500	0.942149
chr21	32062900	0.677686
chr21	32277000	0.669421
chr21	32531400	0.661157
chr21	32566200	0.743802
chr21	32628300	0.652893
chr21	32649300	0.652893
chr21	32683600	0.867769
chr21	32791300	0.743802
chr21	32843800	0.801653
chr21	32882000	0.818182
chr21	32887400	0.958678
chr21	32903300	0.710744
chr21	33043900	0.768595
chr21	33097800	0.677686
chr21	33217600	0.685950
chr21	33238900	0.768595
chr21	33265700	0.942149
chr21	33267900	0.842975
chr21	33284300	0.842975
chr21	33468800	0.644628
chr21	33473100	0.694215
chr21	33528700	0.793388
chr21	33605700	0.652893
chr21	33678800	0.884298
chr21	33707700	0.925620

chr21	33801200	0.669421
chr21	33854700	0.710744
chr21	33865500	0.834711
chr21	33868400	0.669421
chr21	33897500	0.983471
chr21	33934000	0.876033
chr21	33972800	0.809917
chr21	33977600	0.834711
chr21	34127400	0.752066
chr21	34178900	0.644628
chr21	34322800	0.677686
chr21	34327400	0.818182
chr21	34331300	0.793388
chr21	34349900	0.867769
chr21	34406100	0.752066
chr21	34408500	0.876033
chr21	34437000	0.685950
chr21	34445600	0.719008
chr21	34448400	0.842975
chr21	34478100	0.694215
chr21	34489500	0.768595
chr21	34494400	0.661157
chr21	34500500	0.694215
chr21	34506700	0.719008
chr21	34519200	0.677686
chr21	34521800	0.752066
chr21	34569900	0.842975
chr21	34604900	0.694215
chr21	34734400	0.677686
chr21	34740300	0.958678
chr21	35023400	0.859504
chr21	35051200	0.661157
chr21	35056300	0.950413
chr21	35064500	0.917355
chr21	35076300	0.644628
chr21	35084800	0.842975
chr21	35101300	0.694215
chr21	35166800	0.694215
chr21	35231100	0.909091
chr21	35264900	0.669421
chr21	35267800	0.818182
chr21	35320500	0.983471
chr21	35340900	0.809917
chr21	35343900	0.768595
chr21	35348800	0.644628
chr21	35358400	0.669421
chr21	35402800	0.669421
chr21	35473300	0.884298
chr21	35486600	0.809917
chr21	35502700	0.867769
chr21	35553300	0.933884
chr21	35571300	0.661157
chr21	35576700	0.652893
chr21	35724700	0.735537

chr21	35756300	0.917355
chr21	35893700	0.694215
chr21	35918000	0.669421
chr21	36003200	0.661157
chr21	36045000	0.669421
chr21	36169400	0.669421
chr21	36197200	0.685950
chr21	36238300	0.776860
chr21	36332900	0.644628
chr21	36739800	0.652893
chr21	36777100	0.884298
chr21	36787200	0.867769
chr21	36843700	0.661157
chr21	36847600	0.859504
chr21	36876500	0.719008
chr21	36880000	0.669421
chr21	36893700	0.909091
chr21	36959800	0.727273
chr21	37126000	0.669421
chr21	37255000	0.661157
chr21	37401100	0.710744
chr21	37408100	0.685950
chr21	37469900	0.991736
chr21	37473300	0.991736
chr21	37582200	0.909091
chr21	37618300	0.727273
chr21	37623200	0.760331
chr21	37626800	0.859504
chr21	37826900	0.851240
chr21	37834600	0.661157
chr21	37837900	0.727273
chr21	37915100	0.933884
chr21	38068400	0.644628
chr21	38077000	0.818182
chr21	38091300	0.752066
chr21	38122800	0.942149
chr21	38164400	0.809917
chr21	38192900	0.933884
chr21	38286800	0.793388
chr21	38290200	0.859504
chr21	38319700	0.892562
chr21	38350200	0.644628
chr21	38420000	0.661157
chr21	38518300	0.776860
chr21	38527700	0.669421
chr21	38617900	0.876033
chr21	38646500	0.818182
chr21	38654400	0.842975
chr21	38728100	0.702479
chr21	38743300	0.694215
chr21	38753500	0.834711
chr21	38756100	0.818182
chr21	38787400	0.719008
chr21	38792000	0.752066

chr21	38803200	0.942149
chr21	38808100	0.685950
chr21	38818900	0.644628
chr21	38831200	0.867769
chr21	38835100	0.710744
chr21	38870900	0.661157
chr21	38893500	0.710744
chr21	38924000	0.752066
chr21	38937200	0.818182
chr21	38963900	0.842975
chr21	38966400	0.834711
chr21	39004000	0.818182
chr21	39041300	0.677686
chr21	39048300	0.785124
chr21	39056000	0.909091
chr21	39131600	0.669421
chr21	39192600	0.644628
chr21	39216200	0.677686
chr21	39222200	0.661157
chr21	39543900	0.909091
chr21	39600300	0.900826
chr21	39668900	0.867769
chr21	39696600	0.851240
chr21	39728800	0.719008
chr21	39785500	0.719008
chr21	39820900	0.801653
chr21	39844800	0.876033
chr21	39861500	0.917355
chr21	39878700	0.710744
chr21	39968800	0.859504
chr21	39977400	0.652893
chr21	40015400	0.818182
chr21	40036400	0.735537
chr21	40182200	1.000000
chr21	40185600	0.661157
chr21	40282400	0.644628
chr21	40317600	0.859504
chr21	40351700	0.917355
chr21	40354500	0.809917
chr21	40360000	0.826446
chr21	40362500	0.685950
chr21	40379700	0.834711
chr21	40393000	0.743802
chr21	40395300	0.677686
chr21	40402800	0.942149
chr21	40454900	0.867769
chr21	40509900	0.892562
chr21	40723200	0.685950
chr21	40802700	0.710744
chr21	40823700	0.851240
chr21	40909900	0.983471
chr21	40974300	0.793388
chr21	40998900	0.735537
chr21	41009400	0.768595

chr21	41013700	0.859504
chr21	41016400	0.652893
chr21	41079300	0.942149
chr21	41223300	0.677686
chr21	41323500	0.719008
chr21	41538200	0.793388
chr21	41643300	0.743802
chr21	41663700	0.876033
chr21	41708100	0.735537
chr21	41888400	0.760331
chr21	41995400	0.785124
chr21	42040600	0.685950
chr21	42236900	0.644628
chr21	42440900	0.735537
chr21	42485500	0.933884
chr21	42520000	0.702479
chr21	42833200	0.694215
chr21	42852200	0.826446
chr21	42908600	0.685950
chr21	43014500	0.818182
chr21	43025600	0.702479
chr21	43033200	0.785124
chr21	43077600	0.793388
chr21	43093900	0.644628
chr21	43099200	0.768595
chr21	43136000	0.661157
chr21	43172200	0.917355
chr21	43175200	0.652893
chr21	43192900	0.859504
chr21	43198700	0.661157
chr21	43256400	1.000000
chr21	43282600	0.735537
chr21	43317400	0.818182
chr21	43346500	0.975207
chr21	43353700	0.661157
chr21	43371400	0.743802
chr21	43442200	0.917355
chr21	43477300	0.768595
chr21	43512400	0.743802
chr21	43543100	0.644628
chr21	43582200	0.834711
chr21	43730300	0.694215
chr21	43733500	0.884298
chr21	43737800	0.842975
chr21	43756500	0.851240
chr21	43795600	0.809917
chr21	43809800	0.776860
chr21	43835500	0.776860
chr21	43882800	0.710744
chr21	43894700	0.818182
chr21	43937100	0.892562
chr21	43943900	0.685950
chr21	43951600	0.826446
chr21	43970700	0.760331

chr21	44013700	0.818182
chr21	44053300	0.760331
chr21	44062000	0.942149
chr21	44077200	0.644628
chr21	44087200	0.793388
chr21	44089600	0.694215
chr21	44092600	0.851240
chr21	44094900	0.842975
chr21	44097200	0.801653
chr21	44100000	0.727273
chr21	44128000	0.867769
chr21	44167900	0.719008
chr21	44204100	0.876033
chr21	44214500	0.752066
chr21	44227500	0.876033
chr21	44230100	0.710744
chr21	44238700	0.719008
chr21	44253800	0.859504
chr21	44345300	1.000000
chr21	44348200	0.661157
chr21	44358200	0.933884
chr21	44376100	0.801653
chr21	44379900	0.884298
chr21	44392000	0.917355
chr21	44397400	0.685950
chr21	44401600	0.694215
chr21	44413100	0.851240
chr21	44425800	0.702479
chr21	44465600	0.925620
chr21	44490200	0.892562
chr21	44500400	0.743802
chr21	44503200	0.677686
chr21	44507300	0.677686
chr21	44530300	0.694215
chr21	44535300	0.809917
chr21	44559400	0.809917
chr21	44567000	0.776860
chr21	44580200	0.884298
chr21	44585800	0.867769
chr21	44597500	0.752066
chr21	44698200	0.867769
chr21	44733200	0.818182
chr21	44753400	0.826446
chr21	44803500	0.892562
chr21	44808100	0.694215
chr21	44814700	0.644628
chr21	44817200	0.826446
chr21	44830700	0.644628
chr21	44835600	0.925620
chr21	44860000	0.669421
chr21	44914500	0.719008
chr21	44938500	0.834711
chr21	44946200	0.760331
chr21	45123200	0.851240

chr21	45154700	0.710744
chr21	45160600	0.652893
chr21	45188200	0.694215
chr21	45198700	0.793388
chr21	45231800	0.710744
chr21	45235100	0.694215
chr21	45246700	0.768595
chr21	45304900	0.760331
chr21	45309800	0.851240
chr21	45340500	0.776860
chr21	45345300	0.719008
chr21	45377900	0.991736
chr21	45564100	0.669421
chr21	45623700	0.685950
chr21	45664900	0.859504
chr21	45683700	0.652893
chr21	45705700	0.809917
chr21	45790100	0.768595
chr21	45913100	0.719008
chr21	45917500	0.785124
chr21	45930700	0.644628
chr21	45969200	0.983471
chr21	46013300	0.867769
chr21	46018300	0.842975
chr21	46057800	0.661157
chr21	46067500	0.760331
chr21	46079500	0.884298
chr21	46107000	0.818182
chr21	46115700	0.669421
chr21	46118100	0.685950
chr21	46124600	0.669421
chr21	46143700	0.818182
chr21	46169200	0.652893
chr21	46173300	0.727273
chr21	46180600	0.809917
chr21	46187100	0.966942
chr21	46235200	0.694215
chr21	46264700	0.677686
chr21	46286600	0.867769
chr21	46341100	0.826446
chr21	46347600	0.892562
chr21	46350100	0.685950
chr21	46378600	0.776860
chr21	46390500	0.933884
chr21	46423600	0.669421
chr21	46432600	0.933884
chr21	46447200	0.735537
chr21	46471200	0.669421
chr21	46484600	0.776860
chr21	46581500	0.727273
chr21	46669100	0.776860
chr21	46714700	0.752066
chr21	46743600	0.801653
chr21	46753900	0.644628

chr21	46779600	0.760331
chr21	46781800	0.809917
chr21	46785200	0.900826
chr21	46798600	0.909091
chr21	46828600	0.776860
chr21	46831000	0.859504
chr21	46834400	0.867769
chr21	46837100	0.785124
chr21	46844300	0.661157
chr21	46851400	0.958678
chr21	46855100	0.719008
chr21	46859300	0.644628
chr21	46876900	0.677686
chr21	46909100	0.768595
chr21	46976100	0.694215
chr21	46980300	0.760331
chr21	46994600	0.851240
chr21	47321100	0.652893
chr21	47397200	0.727273
chr21	47469600	0.900826
chr21	47503800	0.694215
chr21	47548900	0.785124
chr21	47573500	1.000000
chr21	47601300	0.909091
chr21	47703700	0.685950
chr21	47738200	0.752066
chr21	47801700	0.719008
chr21	47831100	0.892562
chr21	47842100	0.719008
chr21	48025400	0.851240
chr22	16862000	0.694215
chr22	17275200	0.776860
chr22	17306400	0.793388
chr22	17332600	0.785124
chr22	17675500	0.826446
chr22	17682100	0.842975
chr22	17699200	0.826446
chr22	17716200	0.900826
chr22	17718600	0.661157
chr22	17737700	0.975207
chr22	17742100	0.909091
chr22	17745000	0.727273
chr22	17748800	0.743802
chr22	17751000	0.743802
chr22	17754000	0.950413
chr22	17788700	0.727273
chr22	17820400	0.776860
chr22	17854300	0.685950
chr22	17861000	0.884298
chr22	17865300	0.834711
chr22	17867600	0.851240
chr22	17888800	0.685950
chr22	17892200	0.966942
chr22	17898500	0.661157

chr22	17902200	0.809917
chr22	17911600	0.735537
chr22	17913800	0.776860
chr22	17916600	0.719008
chr22	17930300	0.669421
chr22	17942700	0.776860
chr22	17947100	0.719008
chr22	17955500	0.826446
chr22	17971200	0.743802
chr22	18016700	0.644628
chr22	18043200	0.900826
chr22	18050700	0.793388
chr22	18063600	0.652893
chr22	18161600	0.710744
chr22	18183200	0.752066
chr22	18225100	0.743802
chr22	18227200	0.801653
chr22	18386800	0.768595
chr22	18405400	0.710744
chr22	18426500	0.685950
chr22	18429200	0.942149
chr22	18443800	0.859504
chr22	18447500	0.727273
chr22	18455800	0.884298
chr22	18463900	0.669421
chr22	18473000	0.669421
chr22	18475400	0.768595
chr22	18640500	0.644628
chr22	18648600	0.652893
chr22	18785500	0.735537
chr22	18796000	0.768595
chr22	18808900	0.727273
chr22	18892100	0.661157
chr22	18896800	0.900826
chr22	18900000	0.867769
chr22	18913700	0.909091
chr22	18916300	0.752066
chr22	18918500	0.942149
chr22	18925600	0.652893
chr22	18936100	0.884298
chr22	19098100	0.702479
chr22	19135000	0.809917
chr22	19162200	0.966942
chr22	19164300	0.768595
chr22	19202000	0.702479
chr22	19206100	0.776860
chr22	19273900	0.991736
chr22	19411000	0.876033
chr22	19595400	0.760331
chr22	19598700	0.876033
chr22	19603300	0.644628
chr22	19605600	0.719008
chr22	19609500	0.909091
chr22	19629400	0.867769

chr22	19651600	0.776860
chr22	19667100	0.677686
chr22	19679600	0.842975
chr22	19693200	0.942149
chr22	19718800	0.818182
chr22	19742700	0.785124
chr22	19745100	0.727273
chr22	19755400	0.694215
chr22	19832700	0.652893
chr22	19856600	0.859504
chr22	19869100	0.975207
chr22	19879000	0.826446
chr22	19891200	0.826446
chr22	19947900	0.710744
chr22	19950900	0.702479
chr22	19962400	0.983471
chr22	19985400	0.652893
chr22	19988500	0.760331
chr22	20001000	0.685950
chr22	20071000	0.851240
chr22	20108400	0.851240
chr22	20146900	0.842975
chr22	20162700	0.669421
chr22	20191800	0.842975
chr22	20195000	0.652893
chr22	20226000	0.644628
chr22	20228500	0.818182
chr22	20231700	0.702479
chr22	20233900	0.719008
chr22	20257900	0.702479
chr22	20302300	0.760331
chr22	20304800	0.809917
chr22	20360600	0.694215
chr22	20403200	0.851240
chr22	20421400	0.685950
chr22	20725900	0.710744
chr22	20843600	0.652893
chr22	20886000	0.809917
chr22	20905300	0.719008
chr22	20961500	0.776860
chr22	21133700	0.752066
chr22	21138600	0.661157
chr22	21195700	0.719008
chr22	21240000	0.694215
chr22	21265600	0.776860
chr22	21280700	0.768595
chr22	21291900	0.801653
chr22	21322500	0.933884
chr22	21325700	0.809917
chr22	21334100	0.727273
chr22	21365000	0.933884
chr22	21406300	0.719008
chr22	21410400	0.644628
chr22	21457500	0.785124

chr22	21559000	0.727273
chr22	21610400	0.892562
chr22	21615200	0.694215
chr22	21778600	0.826446
chr22	21792800	0.727273
chr22	21795100	0.652893
chr22	21797300	0.991736
chr22	21803000	0.760331
chr22	21810500	0.909091
chr22	21847500	0.950413
chr22	21853400	0.652893
chr22	21865300	0.752066
chr22	21928100	0.644628
chr22	22008600	0.644628
chr22	22173100	0.801653
chr22	22272100	0.809917
chr22	22276800	0.975207
chr22	22280300	0.867769
chr22	22302100	0.685950
chr22	22385600	0.727273
chr22	22462600	0.727273
chr22	22480500	0.867769
chr22	22496900	0.719008
chr22	22703700	0.727273
chr22	22814700	1.000000
chr22	22818200	0.644628
chr22	23302300	0.652893
chr22	23377400	0.702479
chr22	23387600	0.793388
chr22	23415000	0.644628
chr22	23470400	0.677686
chr22	23484500	0.694215
chr22	23495000	0.842975
chr22	23499100	0.826446
chr22	23507300	0.652893
chr22	23509500	0.752066
chr22	23530800	0.785124
chr22	23534700	0.876033
chr22	23561300	0.958678
chr22	23565600	0.826446
chr22	23572500	0.933884
chr22	23574800	0.652893
chr22	23579500	0.760331
chr22	23587100	0.826446
chr22	23589700	0.710744
chr22	23592000	0.793388
chr22	23604000	0.884298
chr22	23610000	0.669421
chr22	23612200	0.727273
chr22	23623600	0.685950
chr22	23726400	0.909091
chr22	23775800	0.735537
chr22	23875600	0.735537
chr22	23934100	0.793388

chr22	24017100	0.785124
chr22	24020300	0.785124
chr22	24105600	0.933884
chr22	24184300	0.702479
chr22	24188700	0.727273
chr22	24191300	0.760331
chr22	24240000	0.785124
chr22	24250700	0.752066
chr22	24294000	0.785124
chr22	24297000	0.842975
chr22	24328700	0.719008
chr22	24343800	0.677686
chr22	24349000	0.661157
chr22	24542500	0.917355
chr22	24554100	0.685950
chr22	24639500	0.793388
chr22	24670000	0.719008
chr22	24681500	0.752066
chr22	24734700	0.991736
chr22	24737900	0.752066
chr22	24803600	0.677686
chr22	24815100	0.876033
chr22	24817500	0.719008
chr22	24825500	0.842975
chr22	24841900	0.685950
chr22	24903500	0.859504
chr22	24912600	0.694215
chr22	24996000	0.669421
chr22	25026100	0.661157
chr22	25028200	0.892562
chr22	25047200	0.710744
chr22	25082200	0.661157
chr22	25087000	0.859504
chr22	25092200	0.950413
chr22	25096200	0.818182
chr22	25103100	0.702479
chr22	25158400	0.809917
chr22	25199900	0.685950
chr22	25206700	0.809917
chr22	25231200	0.710744
chr22	25322200	0.884298
chr22	25362900	0.942149
chr22	25365900	0.950413
chr22	25373000	0.851240
chr22	25378700	0.669421
chr22	25381400	0.710744
chr22	25392900	0.900826
chr22	25403200	0.942149
chr22	25457900	0.752066
chr22	25465700	0.743802
chr22	25495200	0.727273
chr22	25511100	0.694215
chr22	25516700	0.818182
chr22	25545200	0.785124

chr22	25562700	0.685950
chr22	25571300	0.652893
chr22	25641500	0.834711
chr22	25779900	0.776860
chr22	25782500	0.942149
chr22	25785700	0.809917
chr22	25796000	0.661157
chr22	25803400	0.867769
chr22	25815600	0.685950
chr22	25828500	0.694215
chr22	25833900	0.818182
chr22	25904300	0.752066
chr22	25963600	0.702479
chr22	26011600	0.652893
chr22	26016400	0.925620
chr22	26309200	0.801653
chr22	26320600	0.834711
chr22	26326600	0.710744
chr22	26331700	0.851240
chr22	26338900	0.644628
chr22	26362500	0.644628
chr22	26367200	0.958678
chr22	26462900	0.933884
chr22	26527700	0.743802
chr22	26570600	0.809917
chr22	26756600	0.669421
chr22	26771500	0.760331
chr22	26813200	0.752066
chr22	26946000	0.884298
chr22	26950800	0.983471
chr22	26956000	0.727273
chr22	26960500	0.867769
chr22	26963900	0.991736
chr22	26966100	0.826446
chr22	26969100	0.793388
chr22	26972000	0.652893
chr22	26976900	0.851240
chr22	27011900	0.826446
chr22	27014300	0.719008
chr22	27039300	0.809917
chr22	27044600	0.909091
chr22	27057100	0.727273
chr22	27061800	0.743802
chr22	27065000	0.876033
chr22	27077400	0.652893
chr22	27086100	0.933884
chr22	27143000	0.752066
chr22	27145200	0.677686
chr22	27147500	0.842975
chr22	27149900	0.851240
chr22	27173900	0.834711
chr22	27194900	0.867769
chr22	27218500	0.644628
chr22	27232500	0.694215

chr22	27255100	0.727273
chr22	27283600	0.710744
chr22	27289900	0.925620
chr22	27297300	0.892562
chr22	27301500	0.752066
chr22	27306700	0.669421
chr22	27309500	0.652893
chr22	27319700	0.834711
chr22	27350600	0.793388
chr22	27355700	0.669421
chr22	27428700	0.719008
chr22	27482600	0.884298
chr22	27496700	0.884298
chr22	27571400	0.892562
chr22	27606700	0.669421
chr22	27655800	0.776860
chr22	27670600	0.661157
chr22	27695500	0.793388
chr22	27719600	0.661157
chr22	27728100	0.826446
chr22	27766700	0.685950
chr22	27769600	0.942149
chr22	27803700	0.760331
chr22	27813500	0.867769
chr22	27829900	0.719008
chr22	27832500	0.892562
chr22	27839400	0.933884
chr22	27844000	0.661157
chr22	27851100	0.685950
chr22	27858200	0.809917
chr22	27871000	0.685950
chr22	27877000	0.826446
chr22	27891400	0.925620
chr22	27897700	0.669421
chr22	27914700	0.661157
chr22	27937800	0.743802
chr22	27946900	0.694215
chr22	27969800	0.785124
chr22	28013400	0.677686
chr22	28023300	0.719008
chr22	28026900	0.801653
chr22	28032700	0.801653
chr22	28035000	0.735537
chr22	28038300	0.859504
chr22	28051000	0.768595
chr22	28067100	0.983471
chr22	28115100	0.900826
chr22	28123100	0.776860
chr22	28144600	0.669421
chr22	28154100	0.760331
chr22	28164900	0.685950
chr22	28172500	0.793388
chr22	28176000	0.685950
chr22	28180300	0.685950

chr22	28182800	0.719008
chr22	28189400	0.743802
chr22	28200700	0.743802
chr22	28206900	0.876033
chr22	28231900	0.834711
chr22	28240900	0.719008
chr22	28246600	0.876033
chr22	28274900	0.776860
chr22	28280000	0.876033
chr22	28283500	0.710744
chr22	28326800	0.809917
chr22	28376600	0.851240
chr22	28387700	0.942149
chr22	28504800	0.661157
chr22	28508700	0.818182
chr22	28525800	0.710744
chr22	28605600	0.644628
chr22	28672400	0.826446
chr22	28754700	0.752066
chr22	28821000	0.719008
chr22	28937300	0.644628
chr22	29046700	0.735537
chr22	29090400	0.652893
chr22	29132900	0.851240
chr22	29156600	0.966942
chr22	29198500	0.743802
chr22	29214800	0.694215
chr22	29293400	0.917355
chr22	29296200	0.652893
chr22	29299800	0.900826
chr22	29314200	0.809917
chr22	29321900	0.958678
chr22	29336400	0.818182
chr22	29339500	0.735537
chr22	29352200	0.685950
chr22	29365700	0.644628
chr22	29380000	0.900826
chr22	29383200	0.661157
chr22	29385400	0.958678
chr22	29396200	0.909091
chr22	29405100	0.685950
chr22	29408000	0.933884
chr22	29428600	0.834711
chr22	29451900	0.685950
chr22	29458700	0.652893
chr22	29479000	0.719008
chr22	29537800	0.892562
chr22	29548400	0.768595
chr22	29550800	0.677686
chr22	29599200	0.768595
chr22	29605800	0.900826
chr22	29611100	0.743802
chr22	29616400	0.793388
chr22	29641900	0.710744

chr22	29650300	0.859504
chr22	29765400	0.818182
chr22	29873900	0.685950
chr22	29878800	0.652893
chr22	30014400	0.809917
chr22	30023600	0.677686
chr22	30085300	0.809917
chr22	30087600	0.702479
chr22	30097300	0.752066
chr22	30123200	0.785124
chr22	30131300	0.644628
chr22	30197600	0.752066
chr22	30268200	0.752066
chr22	30313500	0.677686
chr22	30389600	0.743802
chr22	30592500	0.966942
chr22	30611200	0.834711
chr22	30625100	0.685950
chr22	30632200	0.876033
chr22	30635100	0.702479
chr22	30653500	0.785124
chr22	30759100	0.867769
chr22	30794900	0.719008
chr22	30798400	0.702479
chr22	30841200	0.859504
chr22	30930000	0.677686
chr22	30957900	0.644628
chr22	30964600	0.669421
chr22	31005700	0.834711
chr22	31051500	0.851240
chr22	31134900	0.652893
chr22	31227600	0.743802
chr22	31278000	0.917355
chr22	31290900	0.826446
chr22	31416200	0.644628
chr22	31451600	0.644628
chr22	31486500	0.785124
chr22	31494900	0.652893
chr22	31516500	0.644628
chr22	31519300	0.909091
chr22	31523800	0.859504
chr22	31537500	0.776860
chr22	31640200	0.842975
chr22	31644700	0.669421
chr22	31650500	0.867769
chr22	31666400	0.851240
chr22	31669200	0.685950
chr22	31709800	0.776860
chr22	31736600	0.826446
chr22	32012100	0.702479
chr22	32020100	0.694215
chr22	32023300	0.752066
chr22	32044000	0.876033
chr22	32263800	0.818182

chr22	32288100	0.685950
chr22	32347300	0.710744
chr22	32349500	0.644628
chr22	32603100	0.702479
chr22	32716400	0.776860
chr22	32722300	0.983471
chr22	32732500	0.909091
chr22	32752100	0.702479
chr22	33012200	0.677686
chr22	33018600	0.991736
chr22	33161200	0.644628
chr22	33200100	0.669421
chr22	33206700	0.793388
chr22	33226400	0.644628
chr22	33277300	0.685950
chr22	33382700	0.644628
chr22	33396500	0.884298
chr22	33432100	0.776860
chr22	33434700	0.694215
chr22	33438600	0.710744
chr22	33446400	0.983471
chr22	33458700	0.925620
chr22	33548300	0.735537
chr22	33833700	0.735537
chr22	33888200	0.900826
chr22	33909600	0.661157
chr22	33966000	0.851240
chr22	33981700	0.644628
chr22	34063500	0.842975
chr22	34066300	0.760331
chr22	34107000	0.661157
chr22	34136600	0.809917
chr22	34151900	0.785124
chr22	34154100	0.950413
chr22	34165300	0.644628
chr22	34200500	0.677686
chr22	34208300	0.776860
chr22	34217900	0.644628
chr22	34232100	0.785124
chr22	34237300	0.768595
chr22	34240400	0.909091
chr22	34243800	0.727273
chr22	34247000	1.000000
chr22	34250100	0.933884
chr22	34257000	0.842975
chr22	34264900	0.735537
chr22	34269600	0.768595
chr22	34279200	0.867769
chr22	34293400	0.925620
chr22	34295800	0.867769
chr22	34298500	0.727273
chr22	34308200	0.884298
chr22	34383300	0.900826
chr22	34388100	0.710744

chr22	34502200	0.685950
chr22	34557000	0.752066
chr22	34621400	0.909091
chr22	34676600	0.694215
chr22	34960700	0.975207
chr22	35180000	0.743802
chr22	35237900	0.917355
chr22	35248700	0.644628
chr22	35368500	0.752066
chr22	35420400	0.876033
chr22	35531400	0.909091
chr22	35568900	0.652893
chr22	35748800	0.661157
chr22	35772600	0.958678
chr22	35775100	0.694215
chr22	35798800	0.776860
chr22	35822800	0.884298
chr22	35827600	0.710744
chr22	35845100	0.702479
chr22	35855000	0.801653
chr22	35864300	0.685950
chr22	35907400	0.719008
chr22	35923200	0.727273
chr22	35944800	0.776860
chr22	35948700	0.809917
chr22	36018700	0.950413
chr22	36033800	0.884298
chr22	36045000	0.702479
chr22	36058100	0.752066
chr22	36071300	0.851240
chr22	36149100	0.884298
chr22	36226600	0.652893
chr22	36271600	0.727273
chr22	36279500	0.768595
chr22	36291200	0.776860
chr22	36297200	0.652893
chr22	36308200	0.677686
chr22	36358600	0.785124
chr22	36406600	0.909091
chr22	36506500	0.685950
chr22	36537200	0.702479
chr22	36541400	0.752066
chr22	36553000	0.809917
chr22	36580500	0.677686
chr22	36585800	0.661157
chr22	36593400	0.851240
chr22	36610700	0.719008
chr22	36617400	0.677686
chr22	36655600	0.942149
chr22	36674100	0.785124
chr22	36724600	0.867769
chr22	36727000	0.892562
chr22	36729900	0.950413
chr22	36732900	0.677686

chr22	36748400	0.958678
chr22	36751600	0.785124
chr22	36753900	0.710744
chr22	36758900	0.958678
chr22	36762800	0.685950
chr22	36765000	0.983471
chr22	36767400	0.859504
chr22	36770500	0.768595
chr22	36775900	0.644628
chr22	36815800	0.776860
chr22	36828800	0.842975
chr22	36831900	0.884298
chr22	36839200	0.669421
chr22	36848400	0.661157
chr22	36852300	0.644628
chr22	36855700	0.933884
chr22	36858500	0.942149
chr22	36958600	0.809917
chr22	36966400	0.768595
chr22	36981900	0.644628
chr22	37006000	0.743802
chr22	37026000	0.760331
chr22	37033300	0.818182
chr22	37178300	0.818182
chr22	37192600	0.760331
chr22	37210700	0.685950
chr22	37219400	0.818182
chr22	37297300	0.991736
chr22	37358200	0.694215
chr22	37398700	0.644628
chr22	37429800	0.669421
chr22	37458800	0.752066
chr22	37465000	0.661157
chr22	37475700	0.760331
chr22	37585900	0.710744
chr22	37608600	0.752066
chr22	37630700	0.727273
chr22	37641600	0.710744
chr22	37656000	0.867769
chr22	37663800	0.752066
chr22	37680300	0.851240
chr22	37691700	0.661157
chr22	37699100	0.776860
chr22	37701500	0.793388
chr22	37723300	0.760331
chr22	37726600	0.694215
chr22	37737100	0.760331
chr22	37765000	0.652893
chr22	37799500	0.859504
chr22	37818000	0.652893
chr22	37868900	0.768595
chr22	37882700	0.859504
chr22	37894600	0.652893
chr22	37898200	0.760331

chr22	37935300	0.702479
chr22	37942400	0.785124
chr22	37948700	0.702479
chr22	37953100	0.851240
chr22	37959900	0.867769
chr22	37966100	0.694215
chr22	37981700	0.694215
chr22	37998100	0.884298
chr22	38019900	0.702479
chr22	38030100	0.859504
chr22	38137400	0.776860
chr22	38219000	0.867769
chr22	38292200	0.958678
chr22	38295000	0.900826
chr22	38330000	0.719008
chr22	38368300	0.909091
chr22	38396000	0.760331
chr22	38426900	0.685950
chr22	38430700	0.925620
chr22	38448500	0.842975
chr22	38485700	0.867769
chr22	38507100	0.677686
chr22	38511000	0.710744
chr22	38516800	0.719008
chr22	38550400	0.818182
chr22	38616500	0.950413
chr22	38636700	0.991736
chr22	38658500	0.710744
chr22	38705400	0.685950
chr22	38709600	0.900826
chr22	38716000	0.884298
chr22	38721100	0.818182
chr22	38724200	0.925620
chr22	38730500	0.752066
chr22	38747200	0.743802
chr22	38750300	0.677686
chr22	38779100	0.719008
chr22	38784900	0.950413
chr22	38787400	0.834711
chr22	38796900	0.685950
chr22	38809200	0.942149
chr22	38819700	0.842975
chr22	38857200	0.727273
chr22	38859900	0.851240
chr22	38862200	0.710744
chr22	38882000	0.644628
chr22	38909900	0.710744
chr22	39100600	0.652893
chr22	39109000	0.917355
chr22	39112600	0.884298
chr22	39232300	0.719008
chr22	39257300	0.892562
chr22	39260600	0.975207
chr22	39272400	0.661157

chr22	39323800	0.702479
chr22	39343200	0.727273
chr22	39362400	0.677686
chr22	39377900	0.752066
chr22	39381900	0.743802
chr22	39389100	0.909091
chr22	39393400	0.702479
chr22	39410500	0.834711
chr22	39428100	0.842975
chr22	39435200	0.677686
chr22	39445800	0.661157
chr22	39448500	0.909091
chr22	39473200	0.710744
chr22	39494100	0.900826
chr22	39497200	0.652893
chr22	39507300	0.685950
chr22	39511000	0.768595
chr22	39520500	0.760331
chr22	39527400	0.859504
chr22	39545000	0.900826
chr22	39567900	0.958678
chr22	39571000	0.991736
chr22	39573400	0.768595
chr22	39576100	0.652893
chr22	39611400	0.909091
chr22	39624000	0.677686
chr22	39626400	0.933884
chr22	39630300	0.859504
chr22	39633400	0.785124
chr22	39651000	0.702479
chr22	39688000	0.752066
chr22	39693700	0.867769
chr22	39696600	0.661157
chr22	39705300	0.661157
chr22	39711100	0.710744
chr22	39740300	0.809917
chr22	39748400	0.669421
chr22	39757100	0.735537
chr22	39760700	0.884298
chr22	39780800	0.719008
chr22	39825900	0.892562
chr22	39859100	0.677686
chr22	39866600	0.892562
chr22	39868800	0.677686
chr22	39871300	0.710744
chr22	39907200	0.743802
chr22	39925600	0.859504
chr22	39930900	0.826446
chr22	39956100	0.834711
chr22	39994200	0.752066
chr22	40020500	0.785124
chr22	40028100	0.735537
chr22	40031500	0.677686
chr22	40063400	0.743802

chr22	40081800	0.768595
chr22	40087300	0.884298
chr22	40128600	0.685950
chr22	40311900	0.710744
chr22	40329800	0.793388
chr22	40337100	0.735537
chr22	40343100	0.669421
chr22	40356000	0.809917
chr22	40385900	0.685950
chr22	40394400	0.809917
chr22	40398700	0.859504
chr22	40405700	0.892562
chr22	40452000	0.834711
chr22	40454800	0.719008
chr22	40481000	0.884298
chr22	40497900	0.710744
chr22	40572300	0.694215
chr22	40577900	0.661157
chr22	40583600	0.826446
chr22	40587600	0.859504
chr22	40595400	0.876033
chr22	40600200	0.851240
chr22	40605600	0.950413
chr22	40608300	0.661157
chr22	40616100	0.809917
chr22	40618200	0.669421
chr22	40621300	0.826446
chr22	40634100	0.876033
chr22	40721500	0.743802
chr22	40723800	0.933884
chr22	40727800	0.669421
chr22	40730700	0.884298
chr22	40793500	0.793388
chr22	40874800	0.652893
chr22	40914300	0.719008
chr22	40930900	0.867769
chr22	40949900	0.669421
chr22	41048200	0.793388
chr22	41065300	0.801653
chr22	41079000	0.776860
chr22	41084500	0.685950
chr22	41110500	0.851240
chr22	41117400	0.801653
chr22	41124100	0.809917
chr22	41128900	0.809917
chr22	41144700	0.710744
chr22	41405800	0.661157
chr22	41577900	0.694215
chr22	41613400	0.677686
chr22	41634600	0.768595
chr22	41653700	0.768595
chr22	41687000	0.884298
chr22	41757000	0.942149
chr22	41760900	0.793388

chr22	41765100	0.818182
chr22	41875900	0.942149
chr22	41884600	0.768595
chr22	41887500	0.702479
chr22	41909300	0.884298
chr22	41929000	0.851240
chr22	41957400	0.743802
chr22	42063600	0.834711
chr22	42093600	0.818182
chr22	42151600	0.669421
chr22	42220300	0.735537
chr22	42240700	0.685950
chr22	42242800	0.900826
chr22	42259100	0.719008
chr22	42304400	0.661157
chr22	42306800	0.801653
chr22	42344400	0.685950
chr22	42347900	0.876033
chr22	42363000	0.685950
chr22	42461800	0.942149
chr22	42592600	0.677686
chr22	42614400	0.818182
chr22	42670100	0.760331
chr22	42672300	0.925620
chr22	42686100	0.842975
chr22	42690100	0.809917
chr22	42693500	0.776860
chr22	42698100	0.735537
chr22	42711100	0.925620
chr22	42719100	0.661157
chr22	42721800	0.719008
chr22	42740900	0.818182
chr22	42750400	0.644628
chr22	42756300	0.760331
chr22	42767400	0.900826
chr22	42773400	0.818182
chr22	42780900	0.661157
chr22	42796000	0.710744
chr22	42801200	0.785124
chr22	42808900	0.867769
chr22	42836500	0.768595
chr22	42855900	0.710744
chr22	42858600	0.793388
chr22	42875000	0.702479
chr22	42895100	0.809917
chr22	42905200	0.801653
chr22	42909500	1.000000
chr22	42951700	0.661157
chr22	42967200	0.677686
chr22	43005200	0.859504
chr22	43014000	0.826446
chr22	43039900	0.727273
chr22	43095000	0.933884
chr22	43113700	0.818182

chr22	43139300	0.677686
chr22	43154300	0.925620
chr22	43168900	0.719008
chr22	43202000	0.743802
chr22	43278600	0.677686
chr22	43291800	0.735537
chr22	43300200	0.793388
chr22	43312600	0.975207
chr22	43334200	0.801653
chr22	43336400	0.644628
chr22	43343800	0.842975
chr22	43356200	0.719008
chr22	43359000	0.710744
chr22	43361500	0.892562
chr22	43374800	0.644628
chr22	43381900	0.917355
chr22	43397000	0.809917
chr22	43401100	0.685950
chr22	43584500	0.644628
chr22	43599600	0.859504
chr22	43613900	0.743802
chr22	43623000	0.834711
chr22	43627600	0.834711
chr22	43632100	0.768595
chr22	43671900	0.793388
chr22	43676300	0.933884
chr22	43685400	0.809917
chr22	43738400	0.710744
chr22	43760100	0.760331
chr22	43877700	0.735537
chr22	44054700	0.818182
chr22	44120000	0.785124
chr22	44146500	0.702479
chr22	44218400	0.851240
chr22	44225000	0.694215
chr22	44240700	0.669421
chr22	44244500	0.752066
chr22	44255500	0.669421
chr22	44318000	0.851240
chr22	44448400	0.818182
chr22	44464200	0.809917
chr22	44504000	1.000000
chr22	44529000	0.652893
chr22	44541700	0.876033
chr22	44545500	0.743802
chr22	44554400	0.793388
chr22	44653200	0.768595
chr22	44658900	0.917355
chr22	44667900	0.727273
chr22	44672700	0.719008
chr22	44688600	0.727273
chr22	44721800	0.809917
chr22	44725300	0.669421
chr22	44733700	0.818182

chr22	44737800	0.925620
chr22	44743700	0.752066
chr22	44747000	0.776860
chr22	44753900	0.652893
chr22	44781100	0.677686
chr22	44788100	0.694215
chr22	44888100	0.809917
chr22	44943300	0.677686
chr22	44992800	0.710744
chr22	45016200	0.876033
chr22	45057200	0.909091
chr22	45065600	0.669421
chr22	45072900	0.644628
chr22	45081400	0.752066
chr22	45095200	0.884298
chr22	45101000	0.694215
chr22	45117200	0.867769
chr22	45134200	0.752066
chr22	45142700	0.933884
chr22	45163000	0.793388
chr22	45192300	0.685950
chr22	45195600	0.685950
chr22	45204200	0.809917
chr22	45216200	0.826446
chr22	45233100	0.876033
chr22	45237200	0.776860
chr22	45261700	0.710744
chr22	45284800	0.652893
chr22	45337200	0.851240
chr22	45341300	0.685950
chr22	45353600	0.942149
chr22	45362000	0.735537
chr22	45368000	0.793388
chr22	45370700	0.801653
chr22	45393300	0.735537
chr22	45395800	0.859504
chr22	45400000	0.950413
chr22	45408500	0.727273
chr22	45419700	0.702479
chr22	45428800	0.669421
chr22	45449500	0.966942
chr22	45468500	0.859504
chr22	45478500	0.719008
chr22	45486800	0.743802
chr22	45498100	0.760331
chr22	45520900	0.867769
chr22	45556000	0.917355
chr22	45577100	0.710744
chr22	45588700	0.685950
chr22	45594400	0.809917
chr22	45608700	0.917355
chr22	45621200	0.727273
chr22	45660000	0.735537
chr22	45704200	0.826446

chr22	45714100	0.710744
chr22	45865800	0.752066
chr22	45892500	0.892562
chr22	45905700	0.644628
chr22	45911700	0.677686
chr22	45962200	0.710744
chr22	46024600	0.677686
chr22	46029100	0.925620
chr22	46153500	0.710744
chr22	46159700	0.842975
chr22	46164900	0.933884
chr22	46239000	0.842975
chr22	46241700	0.867769
chr22	46274300	0.900826
chr22	46277100	0.942149
chr22	46292800	0.652893
chr22	46348800	0.785124
chr22	46351900	0.669421
chr22	46361100	0.809917
chr22	46363400	0.842975
chr22	46368800	0.677686
chr22	46403100	0.768595
chr22	46428300	0.752066
chr22	46470800	0.677686
chr22	46475000	0.859504
chr22	46477600	0.652893
chr22	46597700	0.809917
chr22	46774500	0.983471
chr22	46839400	0.842975
chr22	46880700	0.694215
chr22	46884300	0.719008
chr22	46896200	0.727273
chr22	46906300	0.991736
chr22	46911200	0.809917
chr22	46919000	0.644628
chr22	46938700	0.710744
chr22	46960200	0.884298
chr22	46980700	0.909091
chr22	46985500	0.719008
chr22	47009600	0.793388
chr22	47023700	0.950413
chr22	47048900	0.727273
chr22	47127800	0.644628
chr22	47131800	0.677686
chr22	47170600	0.975207
chr22	47199600	0.661157
chr22	47250100	0.644628
chr22	47304800	0.702479
chr22	47354500	0.768595
chr22	47409400	0.710744
chr22	47424200	0.752066
chr22	47439800	0.661157
chr22	47448200	0.644628
chr22	47480500	0.644628

chr22	47499000	1.000000
chr22	48072400	0.677686
chr22	48074800	0.942149
chr22	48094100	0.644628
chr22	48106300	0.851240
chr22	48113700	0.760331
chr22	48120500	0.710744
chr22	48124800	0.793388
chr22	48204200	0.826446
chr22	48543200	0.909091
chr22	48584200	0.884298
chr22	48635900	0.760331
chr22	48650100	0.710744
chr22	48663600	0.669421
chr22	48675000	0.669421
chr22	48737100	0.801653
chr22	48739800	0.694215
chr22	48753500	0.975207
chr22	48765600	0.859504
chr22	48773300	0.661157
chr22	48787300	0.793388
chr22	48890300	0.818182
chr22	48892900	0.785124
chr22	48895900	0.925620
chr22	48906800	0.867769
chr22	48982000	0.760331
chr22	49026000	0.842975
chr22	49113700	0.710744
chr22	49192500	0.867769
chr22	49357300	0.661157
chr22	49449700	0.652893
chr22	49458900	0.685950
chr22	49516000	0.867769
chr22	49548300	0.842975
chr22	49594000	0.818182
chr22	49701000	0.785124
chr22	49731100	0.768595
chr22	49768800	0.752066
chr22	49877600	0.644628
chr22	49881800	0.975207
chr22	49907300	0.925620
chr22	49921700	0.694215
chr22	49930500	0.983471
chr22	49942600	0.909091
chr22	49946200	0.644628
chr22	50118600	0.685950
chr22	50175400	0.644628
chr22	50232800	0.644628
chr22	50250500	0.735537
chr22	50328600	0.785124
chr22	50340700	0.694215
chr22	50357600	0.644628
chr22	50364700	0.884298
chr22	50447100	0.661157

chr22	50453400	0.859504
chr22	50468800	0.694215
chr22	50587900	0.760331
chr22	50629100	0.809917
chr22	50657400	0.702479
chr22	50701400	0.842975
chr22	50721600	0.851240
chr22	50729900	0.851240
chr22	50732200	0.958678
chr22	50740000	0.801653
chr22	50742900	0.710744
chr22	50747800	0.851240
chr22	50777600	0.685950
chr22	50835800	0.685950
chr22	50908800	0.694215
chr22	50937300	0.685950
chr22	51051500	0.752066
chr22	51059700	0.785124
chr22	51143900	0.644628
chr2	17900	0.818182
chr2	65600	0.867769
chr2	80900	0.834711
chr2	114800	0.710744
chr2	134000	0.644628
chr2	140400	0.743802
chr2	143400	0.677686
chr2	149500	0.842975
chr2	153100	0.851240
chr2	267500	0.727273
chr2	284600	0.760331
chr2	382100	0.719008
chr2	449900	0.710744
chr2	506300	0.661157
chr2	527600	0.661157
chr2	674000	0.735537
chr2	724600	0.694215
chr2	729500	0.867769
chr2	731800	0.876033
chr2	736000	0.677686
chr2	767600	0.652893
chr2	775000	0.892562
chr2	826900	0.661157
chr2	862000	0.727273
chr2	869800	0.677686
chr2	876700	0.735537
chr2	879900	0.760331
chr2	883900	0.809917
chr2	888900	0.851240
chr2	900400	0.892562
chr2	921300	0.867769
chr2	942400	0.677686
chr2	952400	0.727273
chr2	980900	0.719008
chr2	1024400	0.727273

chr2	1029200	0.900826
chr2	1050800	0.842975
chr2	1151800	0.760331
chr2	1344800	0.917355
chr2	1492600	0.768595
chr2	1586300	0.644628
chr2	1595600	0.702479
chr2	1610300	0.884298
chr2	1618300	0.719008
chr2	1622900	0.652893
chr2	1626700	0.842975
chr2	1628800	0.669421
chr2	1631300	0.785124
chr2	1690100	0.834711
chr2	1705600	0.842975
chr2	1708900	0.719008
chr2	1712200	0.925620
chr2	1726500	0.644628
chr2	1735700	0.785124
chr2	1744800	0.859504
chr2	1771200	0.801653
chr2	1775900	0.826446
chr2	1792500	0.677686
chr2	1841000	0.884298
chr2	1852400	0.694215
chr2	1869000	0.760331
chr2	1911700	0.867769
chr2	1978400	0.694215
chr2	1982900	0.760331
chr2	1990500	0.876033
chr2	2011800	1.000000
chr2	2019300	0.859504
chr2	2029600	0.661157
chr2	2049100	0.694215
chr2	2131300	0.644628
chr2	2151100	0.793388
chr2	2163400	0.743802
chr2	2179100	0.735537
chr2	2299500	0.694215
chr2	2328700	0.809917
chr2	2335700	0.867769
chr2	2703400	0.785124
chr2	2792700	0.933884
chr2	3070100	0.644628
chr2	3172800	0.768595
chr2	3202100	0.752066
chr2	3204900	0.702479
chr2	3248300	0.743802
chr2	3270200	0.851240
chr2	3307600	0.760331
chr2	3325300	0.768595
chr2	3403600	0.735537
chr2	3466800	0.694215
chr2	3497800	0.752066

chr2	3642800	0.727273
chr2	3682100	0.702479
chr2	3704900	0.826446
chr2	3707600	0.719008
chr2	3754400	0.719008
chr2	3862700	0.669421
chr2	4294600	0.958678
chr2	4428300	0.884298
chr2	4583800	0.760331
chr2	4625500	0.818182
chr2	5136100	0.752066
chr2	5265900	0.933884
chr2	5331600	0.743802
chr2	5513400	0.867769
chr2	5594100	0.661157
chr2	5620200	0.876033
chr2	5623400	0.834711
chr2	5626000	0.677686
chr2	5634800	0.677686
chr2	5647300	0.694215
chr2	5680400	0.727273
chr2	5843800	0.685950
chr2	5865200	0.818182
chr2	5886400	0.669421
chr2	6055500	0.909091
chr2	6067500	0.685950
chr2	6078700	0.661157
chr2	6086700	1.000000
chr2	6116300	0.950413
chr2	6122000	0.760331
chr2	6152400	0.644628
chr2	6194400	0.917355
chr2	6209800	0.975207
chr2	6214300	0.892562
chr2	6299700	0.661157
chr2	6478000	0.702479
chr2	6675700	0.776860
chr2	6779000	0.867769
chr2	6819600	0.768595
chr2	6821900	0.818182
chr2	6905600	0.801653
chr2	6944000	0.909091
chr2	6947200	0.801653
chr2	6953500	0.710744
chr2	7060600	0.727273
chr2	7158400	0.702479
chr2	7189600	0.677686
chr2	7201300	0.818182
chr2	7238000	0.760331
chr2	7331500	0.652893
chr2	7366800	0.735537
chr2	7464200	0.702479
chr2	7571300	0.826446
chr2	7589400	0.661157

chr2	7597200	0.661157
chr2	7844000	0.991736
chr2	7914400	0.661157
chr2	8012300	0.702479
chr2	8021800	0.801653
chr2	8029400	0.702479
chr2	8031700	0.785124
chr2	8040100	0.752066
chr2	8056000	0.685950
chr2	8102500	0.743802
chr2	8333900	0.752066
chr2	8358100	0.644628
chr2	8364500	0.785124
chr2	8380100	0.933884
chr2	8395700	0.760331
chr2	8398400	0.743802
chr2	8532500	0.909091
chr2	8628500	0.694215
chr2	8715700	0.719008
chr2	8724200	0.842975
chr2	8735500	0.644628
chr2	8779000	0.727273
chr2	8782500	0.859504
chr2	8833700	0.743802
chr2	9034300	0.768595
chr2	9136900	0.652893
chr2	9173800	0.702479
chr2	9215500	0.694215
chr2	9236500	0.851240
chr2	9279200	0.834711
chr2	9283500	0.669421
chr2	9293500	0.644628
chr2	9358600	0.785124
chr2	9375100	0.826446
chr2	9385600	0.884298
chr2	9389900	0.785124
chr2	9397000	0.735537
chr2	9416100	0.768595
chr2	9429400	0.826446
chr2	9447400	0.644628
chr2	9496600	0.677686
chr2	9533200	0.809917
chr2	9789400	0.851240
chr2	9815600	0.818182
chr2	9894300	0.793388
chr2	9898600	0.644628
chr2	9910700	0.818182
chr2	9942000	0.644628
chr2	10024500	0.669421
chr2	10141800	0.652893
chr2	10169700	0.826446
chr2	10213700	0.702479
chr2	10265700	0.685950
chr2	10272900	0.876033

chr2	10312500	0.793388
chr2	10464500	0.785124
chr2	10470800	0.719008
chr2	10473200	0.851240
chr2	10515800	0.644628
chr2	10522800	0.851240
chr2	10533500	0.884298
chr2	10688700	0.818182
chr2	10692400	0.694215
chr2	10720500	0.735537
chr2	10955000	0.719008
chr2	10972900	0.892562
chr2	10984200	0.661157
chr2	11024800	0.925620
chr2	11035200	0.652893
chr2	11050300	0.859504
chr2	11054200	0.644628
chr2	11060200	0.719008
chr2	11076500	0.661157
chr2	11270400	0.702479
chr2	11432700	0.677686
chr2	11515400	0.694215
chr2	11529300	0.876033
chr2	11531500	0.661157
chr2	11636100	0.842975
chr2	11639300	0.867769
chr2	11680300	0.669421
chr2	11682700	0.661157
chr2	11690900	0.669421
chr2	11719500	0.677686
chr2	11723400	0.776860
chr2	11810600	0.685950
chr2	11818300	0.900826
chr2	11822000	0.652893
chr2	11867000	0.842975
chr2	11884900	0.785124
chr2	11919300	0.644628
chr2	11925500	0.661157
chr2	11970600	0.917355
chr2	12004400	0.694215
chr2	12037000	0.727273
chr2	12223800	0.867769
chr2	12237400	0.661157
chr2	12256900	0.760331
chr2	12343000	0.652893
chr2	12454600	0.859504
chr2	12568900	0.719008
chr2	12765600	0.719008
chr2	12825300	0.933884
chr2	12862500	0.710744
chr2	12895300	0.735537
chr2	12914200	0.694215
chr2	13962900	0.743802
chr2	14337700	0.809917

chr2	14373600	0.892562
chr2	14471400	0.867769
chr2	15043700	0.694215
chr2	15183500	0.776860
chr2	15200300	0.644628
chr2	15209500	0.702479
chr2	15237200	0.809917
chr2	15259900	0.826446
chr2	15299000	0.851240
chr2	15400500	0.867769
chr2	15498600	0.768595
chr2	15777700	0.694215
chr2	15888200	0.818182
chr2	15949900	0.661157
chr2	15952800	0.685950
chr2	15966500	0.776860
chr2	16032000	0.661157
chr2	16086200	0.694215
chr2	16098300	0.942149
chr2	16116800	0.661157
chr2	16120400	0.776860
chr2	16153200	0.702479
chr2	16163100	0.735537
chr2	16174500	0.694215
chr2	16191300	0.669421
chr2	16243700	0.776860
chr2	16247100	0.661157
chr2	16366500	0.735537
chr2	16400700	0.669421
chr2	16408900	0.826446
chr2	16430800	0.958678
chr2	16485200	0.661157
chr2	16500100	0.793388
chr2	16511900	0.809917
chr2	16770300	0.958678
chr2	16807000	0.809917
chr2	16834900	0.685950
chr2	16845400	0.719008
chr2	16958900	0.793388
chr2	16980300	0.743802
chr2	17483700	0.735537
chr2	17516800	0.702479
chr2	17729400	0.776860
chr2	17739700	0.867769
chr2	17761300	0.677686
chr2	17766300	0.727273
chr2	17768700	0.727273
chr2	17772300	0.818182
chr2	17778700	0.702479
chr2	17788100	0.685950
chr2	17929100	0.983471
chr2	17997400	0.793388
chr2	18009000	0.661157
chr2	18063600	0.685950

chr2	18074700	0.826446
chr2	18097500	0.793388
chr2	18116600	0.851240
chr2	18637500	0.776860
chr2	18728200	0.652893
chr2	19259300	0.900826
chr2	19286100	0.776860
chr2	19428500	0.727273
chr2	19553000	0.867769
chr2	19626100	0.900826
chr2	19661700	0.900826
chr2	19974800	0.785124
chr2	19987100	0.752066
chr2	20271800	0.842975
chr2	20293800	0.768595
chr2	20385900	0.652893
chr2	20388900	0.859504
chr2	20391300	0.710744
chr2	20398900	0.685950
chr2	20411200	0.677686
chr2	20414900	0.809917
chr2	20421400	0.818182
chr2	20431700	0.702479
chr2	20515000	0.710744
chr2	20521400	0.677686
chr2	20553100	0.644628
chr2	20650200	0.752066
chr2	20687700	0.834711
chr2	20737800	0.867769
chr2	20757600	0.776860
chr2	20767600	0.909091
chr2	20784600	0.966942
chr2	20795800	0.661157
chr2	20814300	0.694215
chr2	20861900	0.677686
chr2	20864200	0.669421
chr2	20875500	0.785124
chr2	21291100	0.776860
chr2	21474400	0.685950
chr2	21511700	0.826446
chr2	21675800	0.900826
chr2	21691200	0.801653
chr2	21866600	0.867769
chr2	21891000	0.727273
chr2	22079900	0.644628
chr2	22367100	0.818182
chr2	22417400	0.867769
chr2	22917900	0.735537
chr2	23018700	0.652893
chr2	23023700	0.876033
chr2	23109000	0.859504
chr2	23169600	0.644628
chr2	23193800	0.702479
chr2	23258500	0.677686

chr2	23454100	0.719008
chr2	23508900	0.702479
chr2	23575000	0.818182
chr2	23627300	0.942149
chr2	23646300	0.743802
chr2	23659800	0.743802
chr2	23698600	0.793388
chr2	23701500	0.826446
chr2	23704500	0.644628
chr2	23720100	0.859504
chr2	23747600	0.727273
chr2	23756400	0.702479
chr2	23798100	0.768595
chr2	23819100	0.644628
chr2	23831500	0.933884
chr2	23837700	0.702479
chr2	23908200	0.669421
chr2	23922100	0.677686
chr2	23935700	0.818182
chr2	23974800	0.818182
chr2	24086100	0.876033
chr2	24139900	0.851240
chr2	24247100	0.694215
chr2	24249300	1.000000
chr2	24605900	0.710744
chr2	24634900	0.652893
chr2	24690200	0.867769
chr2	24803800	0.743802
chr2	24834700	0.892562
chr2	24837100	0.925620
chr2	24978500	0.685950
chr2	24997100	0.710744
chr2	25045200	0.842975
chr2	25050600	0.793388
chr2	25056300	0.719008
chr2	25121800	0.719008
chr2	25149900	0.768595
chr2	25312800	0.867769
chr2	25321800	0.809917
chr2	25383200	0.768595
chr2	25430500	0.867769
chr2	25471900	0.752066
chr2	25480400	0.768595
chr2	25482900	0.892562
chr2	25493200	0.859504
chr2	25500500	0.644628
chr2	25509100	0.785124
chr2	25518100	0.694215
chr2	25527300	0.669421
chr2	25534200	0.826446
chr2	25538200	0.958678
chr2	25582000	0.644628
chr2	25600000	0.933884
chr2	25717700	0.669421

chr2	25781600	0.859504
chr2	25816100	0.727273
chr2	25838300	0.719008
chr2	25887800	0.818182
chr2	25892900	0.917355
chr2	25921200	0.818182
chr2	25964400	0.958678
chr2	25994800	0.677686
chr2	26136300	0.752066
chr2	26174200	0.752066
chr2	26202800	0.677686
chr2	26235400	0.975207
chr2	26267300	0.752066
chr2	26291100	0.842975
chr2	26541900	0.776860
chr2	26563100	0.685950
chr2	26646500	0.809917
chr2	26686700	0.942149
chr2	26754100	0.710744
chr2	26764200	0.876033
chr2	26782000	0.876033
chr2	26785600	0.851240
chr2	26791300	0.859504
chr2	26832800	0.652893
chr2	26836400	0.942149
chr2	26838900	0.727273
chr2	26864200	0.685950
chr2	26878300	0.743802
chr2	26915100	0.685950
chr2	26944200	0.801653
chr2	26978400	0.884298
chr2	26981300	0.925620
chr2	27124900	0.661157
chr2	27138000	0.809917
chr2	27198500	0.851240
chr2	27247400	0.752066
chr2	27304400	0.958678
chr2	27319300	0.768595
chr2	27388800	0.644628
chr2	27432000	0.677686
chr2	27515300	0.669421
chr2	27544100	0.677686
chr2	27576800	0.669421
chr2	27727000	0.809917
chr2	27846000	0.818182
chr2	27926700	0.685950
chr2	27948100	0.867769
chr2	27975000	0.710744
chr2	28486400	0.727273
chr2	28498300	0.727273
chr2	28542200	0.652893
chr2	28545300	0.851240
chr2	28549400	0.719008
chr2	28565400	0.801653

chr2	28637200	0.900826
chr2	28816900	0.719008
chr2	28848400	0.859504
chr2	28903900	0.925620
chr2	28906700	0.793388
chr2	28919100	0.661157
chr2	28978300	0.702479
chr2	29121300	0.867769
chr2	29175300	0.710744
chr2	29234800	0.760331
chr2	29276600	0.685950
chr2	29287900	0.719008
chr2	29353400	0.644628
chr2	29671100	0.685950
chr2	29777500	0.727273
chr2	29826600	0.909091
chr2	30122000	0.826446
chr2	30124700	0.677686
chr2	30187500	0.851240
chr2	30213700	0.842975
chr2	30253900	0.867769
chr2	30338400	0.785124
chr2	30372900	0.983471
chr2	30414300	0.752066
chr2	30431700	0.842975
chr2	30441000	0.669421
chr2	30524300	0.942149
chr2	30543900	0.669421
chr2	30628000	0.818182
chr2	30644900	0.752066
chr2	30679600	0.652893
chr2	30682000	0.859504
chr2	30688400	0.752066
chr2	30836400	0.818182
chr2	30851200	0.669421
chr2	30875600	0.859504
chr2	30934800	0.677686
chr2	31022000	0.809917
chr2	31095200	0.694215
chr2	31109200	0.925620
chr2	31160900	0.677686
chr2	31298800	0.702479
chr2	31354600	0.710744
chr2	31456000	0.677686
chr2	31495500	0.644628
chr2	31522700	0.842975
chr2	31605900	0.809917
chr2	32117300	0.867769
chr2	32206400	0.652893
chr2	33141700	0.983471
chr2	33157300	0.776860
chr2	33163600	0.867769
chr2	33239200	0.685950
chr2	33246300	0.702479

chr2	33288400	0.776860
chr2	33363400	0.644628
chr2	33368500	0.694215
chr2	33701500	0.801653
chr2	33765700	0.677686
chr2	33809100	0.900826
chr2	33828400	0.743802
chr2	33830500	0.793388
chr2	33833100	0.950413
chr2	33839300	0.785124
chr2	33929900	0.809917
chr2	34591500	0.859504
chr2	34672900	0.991736
chr2	34960000	0.900826
chr2	35402000	0.776860
chr2	35454100	0.892562
chr2	35518700	0.859504
chr2	36047700	0.644628
chr2	36498400	0.900826
chr2	36524500	0.652893
chr2	36549600	0.661157
chr2	36580200	0.760331
chr2	36587500	0.735537
chr2	36594500	0.727273
chr2	36599000	0.933884
chr2	36604200	0.917355
chr2	36607700	0.644628
chr2	36620300	0.735537
chr2	36626100	0.818182
chr2	36648600	0.983471
chr2	36684000	0.685950
chr2	36688200	0.834711
chr2	36895300	0.710744
chr2	36981400	0.950413
chr2	37009000	0.818182
chr2	37012500	0.818182
chr2	37051800	0.834711
chr2	37062400	0.842975
chr2	37071400	0.727273
chr2	37153800	0.644628
chr2	37179000	0.818182
chr2	37409400	0.942149
chr2	37417200	0.702479
chr2	37419800	0.851240
chr2	37426500	0.942149
chr2	37432900	0.892562
chr2	37455400	0.785124
chr2	37576500	0.768595
chr2	37617800	0.719008
chr2	37881800	0.859504
chr2	37885000	0.909091
chr2	37895500	0.644628
chr2	37902100	0.859504
chr2	38108900	0.685950

chr2	38113000	0.909091
chr2	38265900	0.694215
chr2	38307200	0.834711
chr2	38313700	0.685950
chr2	38323600	0.826446
chr2	38327500	0.702479
chr2	38333600	0.884298
chr2	38336200	0.867769
chr2	38373900	0.842975
chr2	38387900	0.768595
chr2	38420200	0.685950
chr2	38452300	0.867769
chr2	38501000	0.876033
chr2	38505800	0.818182
chr2	38529300	0.991736
chr2	38634300	0.975207
chr2	38636900	0.958678
chr2	38655600	0.826446
chr2	38660100	0.710744
chr2	38666500	0.752066
chr2	38670400	0.793388
chr2	38674200	0.661157
chr2	38727500	0.801653
chr2	38750700	0.752066
chr2	38755500	0.685950
chr2	38770700	0.818182
chr2	38826200	0.652893
chr2	38936900	0.644628
chr2	39225700	0.768595
chr2	39246300	0.752066
chr2	39285700	0.661157
chr2	39319100	0.950413
chr2	39430600	0.933884
chr2	39447400	0.694215
chr2	39454700	0.652893
chr2	39508700	0.652893
chr2	39528300	0.694215
chr2	39593400	0.892562
chr2	39604900	0.760331
chr2	39659000	0.867769
chr2	39696800	0.966942
chr2	39706700	0.652893
chr2	39712100	0.859504
chr2	39715200	0.677686
chr2	39744000	0.950413
chr2	39843300	0.685950
chr2	39964900	0.776860
chr2	39999700	0.909091
chr2	40079000	0.727273
chr2	40377500	0.677686
chr2	40471300	0.694215
chr2	40532300	0.669421
chr2	41292100	0.727273
chr2	41461600	0.867769

chr2	41891800	0.760331
chr2	42092800	0.702479
chr2	42164700	0.768595
chr2	42175100	0.917355
chr2	42178200	0.867769
chr2	42191500	0.727273
chr2	42195200	0.834711
chr2	42252900	0.661157
chr2	42257000	0.851240
chr2	42259800	0.950413
chr2	42269100	0.677686
chr2	42279700	0.727273
chr2	42282600	0.818182
chr2	42300100	0.694215
chr2	42314500	0.652893
chr2	42329900	0.669421
chr2	42332900	0.867769
chr2	42345500	0.867769
chr2	42360500	0.859504
chr2	42364700	0.685950
chr2	42410000	0.677686
chr2	42424200	0.809917
chr2	42647600	0.644628
chr2	42686000	0.768595
chr2	42725500	0.669421
chr2	42750100	0.710744
chr2	42772400	0.975207
chr2	42774800	0.884298
chr2	42779200	0.644628
chr2	42781900	0.983471
chr2	42794600	0.694215
chr2	42850900	0.768595
chr2	42879400	0.719008
chr2	42881700	0.685950
chr2	42891300	0.685950
chr2	42950000	0.752066
chr2	43133800	0.661157
chr2	43151500	0.933884
chr2	43154300	0.859504
chr2	43157800	0.900826
chr2	43185000	0.842975
chr2	43189400	0.826446
chr2	43202400	0.652893
chr2	43249300	0.652893
chr2	43252100	0.661157
chr2	43269400	0.851240
chr2	43285600	0.876033
chr2	43295600	0.677686
chr2	43311400	0.834711
chr2	43314100	0.785124
chr2	43328600	0.644628
chr2	43354700	0.743802
chr2	43367700	0.702479
chr2	43380400	0.752066

chr2	43396200	0.661157
chr2	43406300	0.950413
chr2	43424700	0.727273
chr2	43445400	0.826446
chr2	43457500	0.661157
chr2	43495700	0.677686
chr2	43561200	0.801653
chr2	43573500	0.942149
chr2	43675800	0.710744
chr2	43717800	0.719008
chr2	44172300	0.743802
chr2	44220000	0.785124
chr2	44264900	0.851240
chr2	44279400	0.752066
chr2	44314700	0.834711
chr2	44353500	0.694215
chr2	44362600	0.958678
chr2	44365800	0.950413
chr2	44393600	0.752066
chr2	44460500	0.801653
chr2	44464200	0.685950
chr2	44625800	0.644628
chr2	44657500	0.801653
chr2	44682600	0.694215
chr2	44691000	0.950413
chr2	44707300	0.644628
chr2	44725800	0.760331
chr2	44770400	0.801653
chr2	44773100	0.876033
chr2	44866200	0.694215
chr2	44880100	0.867769
chr2	44902400	0.727273
chr2	44933500	0.851240
chr2	44936000	0.809917
chr2	44973700	0.900826
chr2	45000200	0.727273
chr2	45002500	0.818182
chr2	45007000	0.710744
chr2	45018500	0.975207
chr2	45034400	0.652893
chr2	45107000	0.752066
chr2	45113000	0.809917
chr2	45153400	0.669421
chr2	45192500	0.685950
chr2	45200400	0.950413
chr2	45208400	0.685950
chr2	45233900	0.743802
chr2	45297800	0.661157
chr2	45302800	0.735537
chr2	45484700	0.826446
chr2	45501700	0.669421
chr2	45573200	0.719008
chr2	45618000	0.933884
chr2	45622500	0.719008

chr2	45640600	0.768595
chr2	45685800	0.652893
chr2	45693900	0.884298
chr2	45697700	0.793388
chr2	45781800	0.719008
chr2	46064100	0.652893
chr2	46242900	1.000000
chr2	46362000	0.694215
chr2	46396200	0.760331
chr2	46457100	0.909091
chr2	46563000	0.909091
chr2	46717300	0.793388
chr2	46782900	0.851240
chr2	46929800	0.809917
chr2	46933600	0.876033
chr2	46944900	0.867769
chr2	47153500	0.702479
chr2	47211900	0.727273
chr2	47214200	0.669421
chr2	47236100	0.859504
chr2	47262600	0.966942
chr2	47270700	0.685950
chr2	47284000	0.809917
chr2	47299700	0.900826
chr2	47303600	0.933884
chr2	47306700	0.661157
chr2	47405800	0.760331
chr2	47414700	0.710744
chr2	47466600	0.809917
chr2	47500200	0.685950
chr2	47537700	0.702479
chr2	47550600	0.710744
chr2	47559800	0.851240
chr2	47574600	0.900826
chr2	47584800	0.694215
chr2	47615800	0.652893
chr2	47717000	0.752066
chr2	47744200	0.661157
chr2	47762400	0.785124
chr2	47779400	0.826446
chr2	47784700	0.826446
chr2	47819500	0.669421
chr2	47823600	0.710744
chr2	47828800	0.661157
chr2	47863900	0.867769
chr2	47882800	0.710744
chr2	47889900	0.942149
chr2	47922900	0.859504
chr2	47926400	0.909091
chr2	47995400	0.768595
chr2	48098300	0.685950
chr2	48125800	0.826446
chr2	48274700	0.752066
chr2	48334200	0.909091

chr2	48426700	0.702479
chr2	48434400	0.859504
chr2	48507000	0.685950
chr2	48698400	0.809917
chr2	48736200	0.983471
chr2	48739500	0.719008
chr2	48762400	0.710744
chr2	48788000	0.752066
chr2	48812700	0.900826
chr2	48816200	0.677686
chr2	48921700	0.958678
chr2	48957200	0.818182
chr2	48960800	0.694215
chr2	49018000	0.702479
chr2	49115600	0.900826
chr2	49457100	1.000000
chr2	49631400	0.876033
chr2	49868200	0.785124
chr2	50164300	0.785124
chr2	50200200	0.685950
chr2	50232500	0.958678
chr2	50344300	0.842975
chr2	50346700	0.826446
chr2	50452900	0.661157
chr2	50571200	0.661157
chr2	50842600	0.661157
chr2	50915400	0.859504
chr2	50948200	0.900826
chr2	50950900	0.702479
chr2	50957200	0.842975
chr2	51065700	0.661157
chr2	51229000	0.933884
chr2	51231500	0.909091
chr2	51234300	0.685950
chr2	51240900	0.942149
chr2	51490900	0.652893
chr2	51519400	0.884298
chr2	52044100	0.826446
chr2	52282300	0.694215
chr2	52566300	0.876033
chr2	52572500	0.677686
chr2	52655800	0.710744
chr2	52667900	0.719008
chr2	52684500	0.644628
chr2	52694800	0.644628
chr2	52785800	0.859504
chr2	52905300	0.652893
chr2	53125100	0.801653
chr2	53192500	0.644628
chr2	53331800	0.644628
chr2	53410700	0.743802
chr2	53461700	0.809917
chr2	53662200	0.950413
chr2	53669600	0.677686

chr2	54011600	0.735537
chr2	54054300	0.719008
chr2	54192100	0.851240
chr2	54331400	0.644628
chr2	54580600	0.677686
chr2	54605300	0.644628
chr2	54612500	0.785124
chr2	54725200	0.793388
chr2	54747500	0.644628
chr2	54778700	0.644628
chr2	54790100	0.661157
chr2	54797500	0.851240
chr2	54816300	0.661157
chr2	54832900	0.727273
chr2	54838100	0.652893
chr2	54984800	0.677686
chr2	55048100	0.661157
chr2	55184700	0.710744
chr2	55247400	0.644628
chr2	55259100	0.752066
chr2	55351400	0.661157
chr2	55361300	0.991736
chr2	55384300	0.644628
chr2	55389400	0.768595
chr2	55392000	0.702479
chr2	55452600	0.694215
chr2	55507400	0.933884
chr2	55946200	0.842975
chr2	56064700	0.644628
chr2	56236500	0.809917
chr2	56243000	0.768595
chr2	56329600	0.958678
chr2	56347800	0.785124
chr2	56498700	0.768595
chr2	56611500	0.702479
chr2	56701100	0.776860
chr2	56935200	0.677686
chr2	57412800	0.809917
chr2	57635700	0.809917
chr2	57929100	0.975207
chr2	57938900	0.669421
chr2	57986900	0.801653
chr2	58023800	0.909091
chr2	58130100	0.809917
chr2	58282800	0.760331
chr2	58434800	0.991736
chr2	58458100	0.826446
chr2	58462300	0.719008
chr2	58585500	0.685950
chr2	58662600	0.661157
chr2	58707000	0.702479
chr2	58722300	0.859504
chr2	58724800	0.884298
chr2	58762600	0.785124

chr2	58766700	0.867769
chr2	58771300	0.826446
chr2	58780700	0.719008
chr2	58797100	0.669421
chr2	58811000	0.727273
chr2	58862100	0.909091
chr2	58948200	0.809917
chr2	58989300	0.661157
chr2	59059200	0.661157
chr2	59080900	0.669421
chr2	59083600	0.702479
chr2	59140700	0.826446
chr2	59182200	0.685950
chr2	59201600	0.950413
chr2	59203800	0.685950
chr2	59409600	0.644628
chr2	59690900	0.677686
chr2	60014400	0.661157
chr2	60017600	0.809917
chr2	60102700	0.661157
chr2	60115600	0.900826
chr2	60229600	0.669421
chr2	60280100	0.818182
chr2	60543700	0.685950
chr2	60581200	0.851240
chr2	60593300	0.710744
chr2	60611300	0.867769
chr2	60637700	0.743802
chr2	60644300	0.752066
chr2	60650300	0.677686
chr2	60680600	1.000000
chr2	60693900	0.702479
chr2	60698600	0.644628
chr2	60703600	0.652893
chr2	60724700	0.818182
chr2	60735600	0.834711
chr2	60738200	0.925620
chr2	60740800	0.809917
chr2	60743400	0.859504
chr2	60748700	0.884298
chr2	60752700	0.818182
chr2	60759000	0.677686
chr2	60763200	0.851240
chr2	60766800	0.842975
chr2	60772100	0.826446
chr2	60830700	0.760331
chr2	60902400	0.801653
chr2	61653300	0.644628
chr2	61661700	0.710744
chr2	61667100	0.760331
chr2	61758700	0.776860
chr2	61778000	0.975207
chr2	61809800	1.000000
chr2	61825500	0.826446

chr2	61860000	0.694215
chr2	61863000	0.942149
chr2	61867500	0.652893
chr2	61899000	0.801653
chr2	62198800	0.933884
chr2	62277600	0.925620
chr2	62300000	0.933884
chr2	62338400	0.785124
chr2	62363700	0.644628
chr2	62378800	0.925620
chr2	62427200	0.669421
chr2	62429700	0.876033
chr2	62442400	1.000000
chr2	62493800	0.876033
chr2	62521200	0.884298
chr2	62532300	0.661157
chr2	62534500	0.851240
chr2	62537200	0.768595
chr2	62548800	0.925620
chr2	62553800	0.900826
chr2	62556900	0.694215
chr2	62577200	0.710744
chr2	62582200	1.000000
chr2	62590400	0.652893
chr2	62601600	0.669421
chr2	62639100	0.859504
chr2	62695300	0.867769
chr2	62703000	0.851240
chr2	62757400	0.851240
chr2	62900200	0.950413
chr2	62957900	0.818182
chr2	62964500	0.983471
chr2	62972000	0.702479
chr2	63562500	0.917355
chr2	63652300	0.685950
chr2	63668100	0.685950
chr2	63733600	0.702479
chr2	63783300	0.727273
chr2	63799800	0.925620
chr2	63818600	0.652893
chr2	63839600	0.793388
chr2	63845100	0.925620
chr2	63866600	0.867769
chr2	63895800	0.917355
chr2	63974100	0.966942
chr2	63992200	1.000000
chr2	64008300	0.925620
chr2	64012600	0.752066
chr2	64047700	0.661157
chr2	64078500	0.669421
chr2	64081300	0.983471
chr2	64100800	0.909091
chr2	64108400	0.851240
chr2	64154200	0.842975

chr2	64221200	0.851240
chr2	64262800	0.685950
chr2	64294300	0.677686
chr2	64362800	0.685950
chr2	64431800	0.991736
chr2	64447800	0.809917
chr2	64474600	0.793388
chr2	64478500	0.859504
chr2	64489900	0.669421
chr2	64495000	0.933884
chr2	64500500	0.851240
chr2	64504200	0.942149
chr2	64515800	0.876033
chr2	64558900	0.892562
chr2	64591300	0.719008
chr2	64628200	0.892562
chr2	64633100	0.661157
chr2	64677700	0.710744
chr2	64697600	0.818182
chr2	64701600	0.793388
chr2	64715800	0.702479
chr2	64720100	0.710744
chr2	64766500	0.677686
chr2	64848000	0.818182
chr2	64854900	0.661157
chr2	64867600	0.842975
chr2	64894300	0.727273
chr2	65051300	0.884298
chr2	65085300	0.652893
chr2	65103600	0.826446
chr2	65108000	0.925620
chr2	65272900	0.933884
chr2	65277100	0.652893
chr2	65303100	0.710744
chr2	65422700	0.727273
chr2	65528500	0.859504
chr2	65605300	0.826446
chr2	65632900	0.735537
chr2	65640000	0.900826
chr2	65648400	0.685950
chr2	65666200	0.727273
chr2	65807300	0.876033
chr2	65840300	0.983471
chr2	66061700	0.702479
chr2	66091600	0.685950
chr2	66185500	0.735537
chr2	66219200	0.710744
chr2	66335300	0.735537
chr2	66362300	0.876033
chr2	66664700	0.694215
chr2	66668700	0.859504
chr2	66671000	0.785124
chr2	66697200	0.760331
chr2	66723400	0.867769

chr2	66726400	0.652893
chr2	66735200	0.677686
chr2	66753200	0.710744
chr2	66764200	0.834711
chr2	66810700	0.826446
chr2	66906800	0.785124
chr2	66913900	0.851240
chr2	66927400	0.685950
chr2	67442500	0.735537
chr2	67552600	0.661157
chr2	67557400	0.900826
chr2	67616800	0.933884
chr2	67775800	0.859504
chr2	68122900	0.776860
chr2	68130400	0.644628
chr2	68148500	0.710744
chr2	68154700	0.793388
chr2	68157400	0.652893
chr2	68350000	0.669421
chr2	68452600	0.768595
chr2	68467800	0.768595
chr2	68476000	0.768595
chr2	68484200	0.760331
chr2	68585300	0.661157
chr2	68646400	0.826446
chr2	68913500	0.644628
chr2	68917700	0.661157
chr2	68924000	0.776860
chr2	68972100	0.677686
chr2	68993500	0.776860
chr2	69136000	0.760331
chr2	69305100	0.743802
chr2	69339300	0.735537
chr2	69405000	0.892562
chr2	69417100	0.719008
chr2	69437600	0.826446
chr2	69537200	0.809917
chr2	69678200	0.801653
chr2	69728100	0.669421
chr2	69752600	0.702479
chr2	69805200	0.793388
chr2	69867000	0.644628
chr2	69911000	0.719008
chr2	69936700	0.661157
chr2	69941000	0.719008
chr2	69945400	0.958678
chr2	70004800	0.975207
chr2	70009200	0.743802
chr2	70017700	0.884298
chr2	70203100	0.768595
chr2	70257400	0.983471
chr2	70311200	0.801653
chr2	70331700	0.760331
chr2	70335900	0.834711

chr2	70340000	0.793388
chr2	70343400	0.677686
chr2	70346400	0.826446
chr2	70354800	0.685950
chr2	70359900	0.809917
chr2	70367600	0.669421
chr2	70471500	0.768595
chr2	70686500	0.760331
chr2	70716900	0.669421
chr2	70733400	0.694215
chr2	70736600	0.644628
chr2	70751400	0.892562
chr2	70854100	0.917355
chr2	70914300	0.909091
chr2	70938500	0.851240
chr2	70943600	0.826446
chr2	70947900	0.710744
chr2	70955500	0.785124
chr2	70960500	0.884298
chr2	70974000	0.694215
chr2	70981600	0.735537
chr2	70991900	0.785124
chr2	70996700	0.719008
chr2	71055700	0.710744
chr2	71089300	0.694215
chr2	71116600	0.818182
chr2	71124600	0.785124
chr2	71135000	0.834711
chr2	71150200	0.719008
chr2	71163100	0.652893
chr2	71170800	0.661157
chr2	71212200	0.727273
chr2	71229600	0.859504
chr2	71310500	0.727273
chr2	71313000	0.776860
chr2	71330700	0.900826
chr2	71384300	0.851240
chr2	71411200	0.801653
chr2	71415000	0.776860
chr2	71463300	0.776860
chr2	71567200	0.702479
chr2	71645100	0.644628
chr2	71683300	0.826446
chr2	71688700	0.661157
chr2	71709600	0.752066
chr2	71724000	0.710744
chr2	71727600	0.644628
chr2	71755000	0.809917
chr2	71786500	0.809917
chr2	71859100	0.644628
chr2	71919200	0.768595
chr2	71922600	0.933884
chr2	72023400	0.669421
chr2	72358100	0.760331

chr2	72365500	0.834711
chr2	72369600	0.793388
chr2	72401100	0.917355
chr2	72464000	0.735537
chr2	72766500	0.735537
chr2	72946700	0.909091
chr2	72983200	0.661157
chr2	73122400	0.909091
chr2	73154200	0.702479
chr2	73163800	0.719008
chr2	73168400	0.801653
chr2	73178900	0.983471
chr2	73191900	0.826446
chr2	73202600	0.884298
chr2	73260000	0.652893
chr2	73263700	0.917355
chr2	73277700	0.867769
chr2	73291400	0.884298
chr2	73308800	0.826446
chr2	73419400	0.785124
chr2	73463900	0.719008
chr2	73466100	0.917355
chr2	73483000	0.851240
chr2	73488000	0.677686
chr2	74120500	0.644628
chr2	74135500	0.760331
chr2	74148700	0.851240
chr2	74216700	0.644628
chr2	74227700	0.950413
chr2	74232300	0.694215
chr2	74234500	0.710744
chr2	74237000	0.884298
chr2	74254000	0.983471
chr2	74258300	0.743802
chr2	74334200	0.809917
chr2	74415300	0.950413
chr2	74419300	0.661157
chr2	74431700	0.809917
chr2	74606500	0.702479
chr2	74643000	0.661157
chr2	74652900	0.661157
chr2	74665000	0.958678
chr2	74787800	0.760331
chr2	74815100	0.760331
chr2	74860300	0.834711
chr2	75006000	0.966942
chr2	75066000	0.851240
chr2	75073400	0.809917
chr2	75077000	0.942149
chr2	75175000	0.652893
chr2	75283600	0.942149
chr2	75381700	0.983471
chr2	75401500	0.884298
chr2	75504600	0.851240

chr2	75549000	0.768595
chr2	75571600	0.661157
chr2	75633800	0.735537
chr2	75660300	0.768595
chr2	75780800	0.785124
chr2	75783000	0.785124
chr2	76000400	0.942149
chr2	76186800	0.677686
chr2	76658900	0.694215
chr2	76674400	0.818182
chr2	76800300	0.966942
chr2	76840800	0.867769
chr2	76988100	0.644628
chr2	77181200	0.652893
chr2	77312000	0.785124
chr2	77314400	0.892562
chr2	77555900	0.768595
chr2	77746700	0.694215
chr2	77750300	0.743802
chr2	78263200	0.933884
chr2	78532100	0.752066
chr2	78760600	0.743802
chr2	78796100	0.719008
chr2	78985200	0.917355
chr2	79066500	0.743802
chr2	79206300	0.768595
chr2	79429000	0.669421
chr2	79504100	0.652893
chr2	79601500	0.818182
chr2	79665200	0.900826
chr2	79823900	0.752066
chr2	79867200	0.752066
chr2	79897900	0.652893
chr2	80010400	0.892562
chr2	80060400	0.859504
chr2	80076900	0.710744
chr2	80087500	0.652893
chr2	80117400	0.752066
chr2	80199500	0.760331
chr2	80203700	0.818182
chr2	80235900	0.892562
chr2	80281800	0.892562
chr2	80286000	0.793388
chr2	80364200	0.719008
chr2	80550200	0.710744
chr2	80552700	0.900826
chr2	80591800	0.694215
chr2	80775600	0.719008
chr2	81101800	0.851240
chr2	81276300	0.719008
chr2	81279000	0.644628
chr2	81623200	0.776860
chr2	81875200	0.950413
chr2	82139600	0.694215

chr2	82241100	0.644628
chr2	82289900	0.752066
chr2	82426600	0.727273
chr2	83182500	0.685950
chr2	83511600	0.677686
chr2	83812600	0.876033
chr2	83971700	0.719008
chr2	84171600	0.818182
chr2	84765000	0.644628
chr2	85009800	0.685950
chr2	85026800	0.859504
chr2	85083000	0.768595
chr2	85121900	0.735537
chr2	85154500	0.727273
chr2	85167500	0.776860
chr2	85181600	0.677686
chr2	85184800	1.000000
chr2	85187500	0.909091
chr2	85202600	0.702479
chr2	85205000	0.950413
chr2	85236100	0.644628
chr2	85262700	0.834711
chr2	85285300	0.785124
chr2	85292600	0.760331
chr2	85317100	0.661157
chr2	85386600	0.826446
chr2	85390600	0.867769
chr2	85402900	0.760331
chr2	85405900	0.958678
chr2	85409900	0.867769
chr2	85415900	0.694215
chr2	85439900	0.842975
chr2	85443700	0.818182
chr2	85459600	0.991736
chr2	85463200	1.000000
chr2	85467300	0.727273
chr2	85470300	0.801653
chr2	85474700	0.867769
chr2	85477000	0.826446
chr2	85481000	0.727273
chr2	85484500	0.776860
chr2	85486700	0.966942
chr2	85499000	0.694215
chr2	85505400	0.710744
chr2	85512900	0.884298
chr2	85536900	0.661157
chr2	85637000	0.834711
chr2	85648200	0.644628
chr2	85655800	0.809917
chr2	85658300	0.950413
chr2	85660700	0.702479
chr2	85665100	0.909091
chr2	85683600	0.793388
chr2	85686400	0.942149

chr2	85695700	0.785124
chr2	85752100	0.669421
chr2	85929000	0.834711
chr2	85969700	0.685950
chr2	85994000	0.694215
chr2	85996900	0.727273
chr2	85999800	0.809917
chr2	86055300	0.710744
chr2	86184400	0.685950
chr2	86342100	0.892562
chr2	86448600	0.661157
chr2	86705900	0.677686
chr2	86967200	0.685950
chr2	87001400	0.743802
chr2	87067300	0.909091
chr2	87083700	0.694215
chr2	87284500	0.785124
chr2	87373900	0.661157
chr2	87419700	0.950413
chr2	87511600	0.818182
chr2	87754800	0.661157
chr2	87768600	0.694215
chr2	87783200	0.652893
chr2	87785800	0.983471
chr2	87795000	0.884298
chr2	87798100	0.826446
chr2	87800600	0.776860
chr2	87804500	0.677686
chr2	87816100	0.859504
chr2	87831200	0.793388
chr2	87836300	0.644628
chr2	87851900	0.694215
chr2	87873400	0.851240
chr2	87929800	0.933884
chr2	87944500	0.867769
chr2	87962200	1.000000
chr2	87987600	0.793388
chr2	88124800	0.983471
chr2	88303000	0.884298
chr2	88319300	0.900826
chr2	88324400	0.801653
chr2	88382700	0.801653
chr2	88490900	0.669421
chr2	88500600	0.719008
chr2	88511700	0.892562
chr2	88620400	0.661157
chr2	88629900	0.809917
chr2	88655300	0.710744
chr2	88715800	0.942149
chr2	88741700	0.710744
chr2	88744000	0.925620
chr2	88771500	0.776860
chr2	88817600	0.826446
chr2	88837800	0.983471

chr2	88900400	0.966942
chr2	88924100	0.727273
chr2	88931100	0.958678
chr2	88959000	0.933884
chr2	88998000	0.776860
chr2	89014700	0.685950
chr2	89157300	0.909091
chr2	89846800	0.776860
chr2	89849300	0.685950
chr2	90134800	0.818182
chr2	90161700	0.801653
chr2	90229600	0.983471
chr2	90276900	0.818182
chr2	90457000	0.768595
chr2	90471700	0.776860
chr2	91687100	0.809917
chr2	92273200	0.661157
chr2	92304100	0.826446
chr2	92326100	0.677686
chr2	95475300	0.818182
chr2	95745200	0.785124
chr2	96440600	0.842975
chr2	96715100	0.669421
chr2	96755900	0.694215
chr2	96758100	0.884298
chr2	96765800	0.884298
chr2	96774700	0.884298
chr2	96804700	0.776860
chr2	96814600	0.975207
chr2	96818400	0.719008
chr2	96871600	0.785124
chr2	96926700	0.710744
chr2	97075200	0.768595
chr2	97102800	0.867769
chr2	97152200	0.884298
chr2	97206500	0.652893
chr2	97215800	0.851240
chr2	97220900	0.900826
chr2	97351400	0.743802
chr2	97430700	0.727273
chr2	97436400	0.702479
chr2	97485900	0.760331
chr2	97498800	0.958678
chr2	97525900	0.652893
chr2	97539100	0.826446
chr2	97544000	0.768595
chr2	97547900	1.000000
chr2	97572900	0.950413
chr2	97586900	0.975207
chr2	97623200	0.702479
chr2	97988500	0.785124
chr2	98308200	0.785124
chr2	98317000	0.685950
chr2	98337100	0.785124

chr2	98365200	0.661157
chr2	98369000	0.818182
chr2	98387400	0.735537
chr2	98508500	0.694215
chr2	98552700	0.892562
chr2	99011000	0.652893
chr2	99025400	0.677686
chr2	99059300	0.917355
chr2	99069400	0.942149
chr2	99071700	0.859504
chr2	99076000	0.900826
chr2	99080600	0.859504
chr2	99082800	0.867769
chr2	99092300	0.776860
chr2	99095700	0.900826
chr2	99101600	0.867769
chr2	99251600	0.702479
chr2	99391000	0.669421
chr2	99486100	0.909091
chr2	99526300	0.876033
chr2	99531200	0.793388
chr2	100056700	0.859504
chr2	100078700	0.661157
chr2	100167300	0.644628
chr2	100189500	0.727273
chr2	100327400	0.669421
chr2	100365100	0.661157
chr2	100439100	0.735537
chr2	100498600	0.710744
chr2	100507600	0.710744
chr2	100577600	0.743802
chr2	100726000	0.702479
chr2	100799300	0.826446
chr2	100898400	0.950413
chr2	100902700	0.669421
chr2	100908400	0.644628
chr2	101028600	0.909091
chr2	101031000	0.876033
chr2	101220700	0.876033
chr2	101256600	0.694215
chr2	101426500	0.685950
chr2	101504100	0.884298
chr2	101513000	0.644628
chr2	101566600	0.801653
chr2	101622100	0.669421
chr2	101624700	0.851240
chr2	101643500	0.942149
chr2	101730200	0.884298
chr2	101752200	0.851240
chr2	101762700	0.842975
chr2	101779100	0.834711
chr2	101866400	0.702479
chr2	101944800	0.867769
chr2	101947100	0.950413

chr2	101956200	0.669421
chr2	101971700	0.785124
chr2	102051900	0.760331
chr2	102084200	0.776860
chr2	102134900	0.760331
chr2	102150400	0.950413
chr2	102183600	0.652893
chr2	102321500	0.677686
chr2	102339000	0.677686
chr2	102342700	0.685950
chr2	102350700	0.727273
chr2	102358300	0.975207
chr2	102360700	0.685950
chr2	102363100	0.644628
chr2	102368400	0.644628
chr2	102372700	0.727273
chr2	102380200	0.809917
chr2	102385000	0.669421
chr2	102389500	0.818182
chr2	102394800	0.760331
chr2	102410200	0.752066
chr2	102422300	0.950413
chr2	102435300	0.834711
chr2	102439900	0.685950
chr2	102449300	0.669421
chr2	102510200	0.909091
chr2	102586300	0.661157
chr2	102844500	0.859504
chr2	103203100	0.669421
chr2	103409600	0.644628
chr2	103905700	0.801653
chr2	103909900	0.727273
chr2	104018900	0.933884
chr2	104401300	0.925620
chr2	104405700	0.652893
chr2	104426900	0.760331
chr2	104435100	0.735537
chr2	104640300	0.768595
chr2	104657700	0.694215
chr2	104830600	0.677686
chr2	104903600	0.859504
chr2	104961500	0.677686
chr2	105032300	0.826446
chr2	105051400	0.818182
chr2	105363000	0.661157
chr2	105366400	0.661157
chr2	105375200	0.958678
chr2	105383500	0.785124
chr2	105407100	0.685950
chr2	105463800	0.644628
chr2	105480800	0.719008
chr2	105586100	0.702479
chr2	105657400	0.752066
chr2	105681600	0.644628

chr2	105690100	0.834711
chr2	105697500	0.809917
chr2	105719800	0.909091
chr2	105731300	0.677686
chr2	105783800	0.867769
chr2	105794300	0.859504
chr2	105797500	0.900826
chr2	105925900	0.727273
chr2	105941800	0.785124
chr2	106020500	0.661157
chr2	106044000	0.859504
chr2	106058100	0.644628
chr2	106060700	0.685950
chr2	106120000	0.809917
chr2	106193500	0.727273
chr2	106200200	0.727273
chr2	106209800	0.826446
chr2	106214600	0.892562
chr2	106263900	0.677686
chr2	106269200	0.801653
chr2	106364300	0.644628
chr2	106370400	0.661157
chr2	106377100	0.752066
chr2	106386000	0.842975
chr2	106394100	0.818182
chr2	106397600	0.776860
chr2	106406800	0.760331
chr2	106413500	0.809917
chr2	106423800	0.694215
chr2	106426100	0.917355
chr2	106460500	0.735537
chr2	106470400	0.892562
chr2	106507500	0.694215
chr2	106568100	0.710744
chr2	106578800	0.966942
chr2	106690300	0.677686
chr2	106713900	0.661157
chr2	106735700	0.801653
chr2	106747300	0.925620
chr2	106782000	0.950413
chr2	106836600	0.694215
chr2	106886900	0.776860
chr2	106889000	0.950413
chr2	107125600	0.884298
chr2	107128200	0.702479
chr2	107192900	0.669421
chr2	107468900	0.884298
chr2	107486600	0.702479
chr2	107507200	0.900826
chr2	107535900	0.661157
chr2	108119500	0.834711
chr2	108302300	0.702479
chr2	108334500	0.842975
chr2	108453900	0.644628

chr2	108691500	0.694215
chr2	108746400	0.752066
chr2	108905300	0.884298
chr2	108933400	0.801653
chr2	108964800	0.677686
chr2	108994400	0.983471
chr2	108999500	0.900826
chr2	109020600	0.958678
chr2	109192100	0.652893
chr2	109203400	0.694215
chr2	109210500	0.735537
chr2	109237500	0.958678
chr2	109278500	0.644628
chr2	109551000	0.752066
chr2	109580700	0.776860
chr2	109636900	0.685950
chr2	109793900	0.809917
chr2	109816200	0.644628
chr2	109896300	0.719008
chr2	109906200	0.661157
chr2	109949400	0.785124
chr2	109999800	0.694215
chr2	110002900	0.768595
chr2	110026700	0.925620
chr2	110367300	0.760331
chr2	110417800	0.801653
chr2	110464000	0.669421
chr2	110526200	0.669421
chr2	110560300	0.669421
chr2	110656700	0.752066
chr2	110801100	0.694215
chr2	110813100	0.975207
chr2	110829600	0.801653
chr2	110925200	0.685950
chr2	111045500	0.710744
chr2	111057200	0.677686
chr2	111074000	0.991736
chr2	111086100	0.644628
chr2	111337500	0.669421
chr2	111432900	0.851240
chr2	111438100	0.859504
chr2	111444800	0.818182
chr2	111460600	0.661157
chr2	111493200	0.702479
chr2	111495700	0.801653
chr2	111499000	0.942149
chr2	111506500	0.859504
chr2	111510800	0.727273
chr2	111528600	0.768595
chr2	111559200	0.900826
chr2	111569400	0.644628
chr2	111592000	0.702479
chr2	111594400	0.793388
chr2	111596500	0.785124

chr2	111616700	0.884298
chr2	111623800	0.743802
chr2	111662700	0.851240
chr2	111826400	0.752066
chr2	111915600	0.785124
chr2	111985800	0.842975
chr2	112020900	0.760331
chr2	112046000	0.950413
chr2	112078900	0.793388
chr2	112135100	0.685950
chr2	112153500	0.776860
chr2	112156300	0.669421
chr2	112177500	0.760331
chr2	112192500	0.785124
chr2	112196200	0.719008
chr2	112200500	0.719008
chr2	112207300	0.785124
chr2	112210100	0.685950
chr2	112212500	0.909091
chr2	112222100	0.975207
chr2	112231300	0.834711
chr2	112251900	0.966942
chr2	112293100	0.752066
chr2	112335000	0.644628
chr2	112341600	0.677686
chr2	112355500	0.669421
chr2	112417300	0.785124
chr2	112436400	0.760331
chr2	112460200	0.776860
chr2	112463800	1.000000
chr2	112499400	0.677686
chr2	112531200	0.983471
chr2	112661500	0.867769
chr2	112789700	0.925620
chr2	112805800	0.710744
chr2	113372500	0.892562
chr2	113379900	0.727273
chr2	113450500	0.702479
chr2	113542300	0.685950
chr2	113571500	0.776860
chr2	113607000	0.809917
chr2	113610100	0.809917
chr2	113628600	0.694215
chr2	113786000	0.785124
chr2	113923500	0.710744
chr2	113934800	0.743802
chr2	113983800	0.859504
chr2	114030500	0.669421
chr2	114041500	0.933884
chr2	114049100	0.818182
chr2	114100100	0.710744
chr2	114164200	0.809917
chr2	114309900	0.710744
chr2	114328600	0.859504

chr2	114430000	0.876033
chr2	114585000	0.652893
chr2	114652600	0.776860
chr2	114662400	0.694215
chr2	114866800	0.851240
chr2	115006800	0.677686
chr2	115149900	0.710744
chr2	115274200	0.652893
chr2	115442700	0.809917
chr2	115472300	0.818182
chr2	115972400	0.809917
chr2	116118400	0.719008
chr2	116122200	0.760331
chr2	116152900	0.743802
chr2	116160100	0.685950
chr2	116201600	0.694215
chr2	116291100	0.661157
chr2	116301100	0.760331
chr2	116316600	0.669421
chr2	116354600	0.991736
chr2	116934500	0.702479
chr2	116997500	0.677686
chr2	117512200	0.925620
chr2	117738200	0.685950
chr2	117845300	0.859504
chr2	118250000	0.909091
chr2	118309200	0.710744
chr2	118311500	0.950413
chr2	118361400	0.644628
chr2	118419300	0.669421
chr2	118570300	0.702479
chr2	118599700	0.677686
chr2	118609700	0.851240
chr2	118785700	0.694215
chr2	119013800	0.950413
chr2	119076600	0.917355
chr2	119079700	0.661157
chr2	119098300	0.793388
chr2	119132900	0.727273
chr2	119208000	0.710744
chr2	119232100	0.727273
chr2	119248300	0.793388
chr2	119288000	0.909091
chr2	119401700	0.677686
chr2	119496100	0.818182
chr2	119514000	0.652893
chr2	119595400	0.644628
chr2	119602400	0.702479
chr2	119607800	0.677686
chr2	119639500	0.768595
chr2	119769500	0.793388
chr2	119826600	0.644628
chr2	119871000	0.785124
chr2	119876000	0.826446

chr2	119883900	0.768595
chr2	119888300	0.859504
chr2	119905600	0.661157
chr2	119925000	0.661157
chr2	119954000	0.818182
chr2	119976200	0.793388
chr2	119979500	0.669421
chr2	119987900	0.743802
chr2	119992100	0.727273
chr2	119998100	0.710744
chr2	120013700	1.000000
chr2	120017400	0.834711
chr2	120192200	0.892562
chr2	120233300	0.785124
chr2	120284800	0.925620
chr2	120396700	0.710744
chr2	120430100	0.884298
chr2	120441600	0.876033
chr2	120452000	0.768595
chr2	120458800	0.801653
chr2	120488500	0.776860
chr2	120505200	0.669421
chr2	120522200	0.685950
chr2	120524500	0.834711
chr2	120529000	0.719008
chr2	120549500	0.785124
chr2	120554300	0.760331
chr2	120557700	0.685950
chr2	120560300	0.785124
chr2	120581900	0.727273
chr2	120613200	0.661157
chr2	120787900	0.776860
chr2	120891600	0.818182
chr2	120964200	0.652893
chr2	120997300	0.776860
chr2	121003800	0.909091
chr2	121036500	0.785124
chr2	121041500	0.661157
chr2	121068100	0.809917
chr2	121071100	0.652893
chr2	121101400	0.677686
chr2	121131300	0.925620
chr2	121134600	0.785124
chr2	121142800	0.719008
chr2	121153600	0.661157
chr2	121187900	0.933884
chr2	121192600	0.867769
chr2	121195300	0.876033
chr2	121247100	0.710744
chr2	121284300	0.867769
chr2	121322400	0.801653
chr2	121333100	0.685950
chr2	121349700	0.752066
chr2	121367900	0.727273

chr2	121372000	0.867769
chr2	121419300	0.900826
chr2	121437800	0.727273
chr2	121453900	0.925620
chr2	121459900	0.801653
chr2	121462500	0.727273
chr2	121465300	0.710744
chr2	121467600	0.851240
chr2	121470200	0.644628
chr2	121480700	0.834711
chr2	121487700	0.793388
chr2	121508300	0.909091
chr2	121516300	0.867769
chr2	121530500	0.694215
chr2	121544200	0.966942
chr2	121549600	0.661157
chr2	121551700	0.776860
chr2	121555900	0.892562
chr2	121560400	0.793388
chr2	121579000	0.859504
chr2	121583000	0.809917
chr2	121591900	0.727273
chr2	121616600	0.834711
chr2	121619300	0.768595
chr2	121625300	0.876033
chr2	121632700	0.719008
chr2	121637600	0.710744
chr2	121645400	0.859504
chr2	121651400	0.809917
chr2	121658000	0.851240
chr2	121673000	0.685950
chr2	121692700	0.702479
chr2	121739800	0.743802
chr2	121760400	0.876033
chr2	121776800	0.942149
chr2	121829000	0.826446
chr2	121890800	0.867769
chr2	122238200	0.694215
chr2	122248100	0.652893
chr2	122271700	0.958678
chr2	122290700	0.685950
chr2	122420500	0.644628
chr2	122445100	0.727273
chr2	122454300	0.859504
chr2	122549000	1.000000
chr2	122562700	0.983471
chr2	122569100	0.859504
chr2	122728100	0.644628
chr2	122732900	0.710744
chr2	122790100	0.776860
chr2	122813800	0.727273
chr2	122839600	0.785124
chr2	123273400	0.867769
chr2	123413400	0.834711

chr2	123492700	0.644628
chr2	123684900	0.677686
chr2	123757600	0.892562
chr2	123760100	0.669421
chr2	124273000	0.735537
chr2	124921700	0.652893
chr2	124943300	0.694215
chr2	124945600	0.933884
chr2	125086500	0.909091
chr2	125184500	0.752066
chr2	125279500	0.727273
chr2	125753100	0.917355
chr2	125933500	0.818182
chr2	126595000	0.752066
chr2	127264900	0.702479
chr2	127476400	0.661157
chr2	127516800	0.710744
chr2	127589500	0.809917
chr2	127634600	0.702479
chr2	127700300	0.752066
chr2	127705400	0.685950
chr2	127729300	0.702479
chr2	127734300	0.669421
chr2	127822800	0.884298
chr2	127826000	0.785124
chr2	127839800	0.842975
chr2	127844900	0.950413
chr2	127853000	0.809917
chr2	127856200	0.719008
chr2	127861900	0.776860
chr2	127886400	0.793388
chr2	127916000	0.760331
chr2	127923600	0.752066
chr2	127934700	0.719008
chr2	127941400	0.818182
chr2	127945500	0.685950
chr2	127951700	0.950413
chr2	127955400	0.644628
chr2	127963100	0.669421
chr2	127966500	0.834711
chr2	128117400	0.809917
chr2	128183000	0.801653
chr2	128207500	0.933884
chr2	128214200	0.793388
chr2	128221400	0.983471
chr2	128238400	0.950413
chr2	128395900	0.776860
chr2	128587700	0.867769
chr2	128617900	0.793388
chr2	128800500	0.900826
chr2	129018800	0.867769
chr2	129040700	0.867769
chr2	129047300	0.743802
chr2	129049900	0.685950

chr2	129064500	0.942149
chr2	129072900	0.710744
chr2	129119900	0.851240
chr2	129122100	0.826446
chr2	129127400	0.851240
chr2	129137100	0.719008
chr2	129147600	0.809917
chr2	129154300	0.876033
chr2	129165800	0.809917
chr2	129185600	0.752066
chr2	129200300	0.876033
chr2	129247100	0.801653
chr2	129251300	0.652893
chr2	129275000	0.801653
chr2	129295100	0.966942
chr2	129307700	0.644628
chr2	129396700	0.991736
chr2	129402500	0.991736
chr2	129405000	0.727273
chr2	129440300	0.685950
chr2	129449500	0.818182
chr2	129544700	0.909091
chr2	129637100	0.892562
chr2	129834200	0.743802
chr2	129949300	0.909091
chr2	130323700	0.644628
chr2	130364900	0.876033
chr2	130369700	0.652893
chr2	130431700	0.644628
chr2	130462300	0.776860
chr2	130475300	0.818182
chr2	130480400	0.785124
chr2	130516100	0.975207
chr2	130560000	0.876033
chr2	130564700	0.859504
chr2	130645200	0.719008
chr2	130649600	0.743802
chr2	131018100	0.743802
chr2	131127100	0.900826
chr2	131145800	0.661157
chr2	131269400	0.644628
chr2	131278700	0.644628
chr2	131281500	0.694215
chr2	131367200	0.851240
chr2	131554000	0.743802
chr2	131577600	0.818182
chr2	131580000	0.884298
chr2	131582100	0.735537
chr2	131592400	0.735537
chr2	131601400	0.776860
chr2	131650300	0.834711
chr2	131654700	0.917355
chr2	131724500	0.884298
chr2	131887800	0.644628

chr2	131912000	0.834711
chr2	131993500	0.851240
chr2	132178100	0.975207
chr2	132180900	0.859504
chr2	132340000	0.719008
chr2	132360300	0.669421
chr2	132981300	0.669421
chr2	132987200	0.661157
chr2	132997700	0.768595
chr2	133000400	0.669421
chr2	133003500	0.652893
chr2	133013000	0.900826
chr2	133017200	0.677686
chr2	133023100	0.842975
chr2	133038900	0.702479
chr2	133095500	0.652893
chr2	133150100	0.661157
chr2	133280700	0.644628
chr2	133363700	0.735537
chr2	133390200	0.752066
chr2	133404100	0.661157
chr2	133519000	0.760331
chr2	133897600	0.752066
chr2	134100400	0.834711
chr2	134127700	0.685950
chr2	134135600	0.669421
chr2	134175500	0.702479
chr2	134179100	0.752066
chr2	134182800	0.661157
chr2	134399600	0.950413
chr2	134402000	0.702479
chr2	134432800	0.801653
chr2	134468800	0.661157
chr2	134644700	0.743802
chr2	134728700	0.776860
chr2	134845000	0.818182
chr2	134865400	0.702479
chr2	134891900	0.975207
chr2	134895300	0.752066
chr2	134905000	0.644628
chr2	134917900	0.933884
chr2	134927400	0.776860
chr2	134960100	0.685950
chr2	134965100	0.768595
chr2	134978600	0.834711
chr2	135019000	0.727273
chr2	135189300	0.793388
chr2	135218500	0.685950
chr2	135257600	0.925620
chr2	135290700	0.801653
chr2	135429000	0.752066
chr2	135435800	0.694215
chr2	135464100	0.776860
chr2	135531800	0.859504

chr2	135763500	0.644628
chr2	135901900	0.842975
chr2	135940200	0.776860
chr2	135970700	0.983471
chr2	136010900	0.661157
chr2	136344100	0.760331
chr2	136555400	0.735537
chr2	136718900	0.826446
chr2	136764400	0.710744
chr2	136851100	0.768595
chr2	136984100	0.702479
chr2	137085900	0.652893
chr2	137165600	0.876033
chr2	137195000	0.677686
chr2	137197400	0.669421
chr2	137268500	0.793388
chr2	137455700	0.702479
chr2	137577300	0.727273
chr2	137685000	0.694215
chr2	137951800	0.760331
chr2	137965000	0.966942
chr2	137977100	0.677686
chr2	138020200	0.851240
chr2	138113800	0.652893
chr2	138501700	0.661157
chr2	138582500	0.785124
chr2	138615500	0.834711
chr2	138722100	0.867769
chr2	138852200	0.685950
chr2	138893100	0.644628
chr2	138940300	0.884298
chr2	138946300	0.652893
chr2	138993000	0.735537
chr2	139025200	0.710744
chr2	139034800	1.000000
chr2	139272700	0.809917
chr2	139406800	0.809917
chr2	139428700	0.735537
chr2	139457600	0.644628
chr2	139526900	0.652893
chr2	139914500	0.834711
chr2	140029700	0.661157
chr2	140382600	0.710744
chr2	140443200	0.661157
chr2	140479300	0.644628
chr2	141653500	0.719008
chr2	141663000	0.710744
chr2	141744700	0.652893
chr2	141787300	0.644628
chr2	142339300	0.644628
chr2	142369100	0.958678
chr2	142486400	0.818182
chr2	142669600	0.661157
chr2	142810500	0.685950

chr2	143475300	0.884298
chr2	143732400	0.983471
chr2	143999500	0.752066
chr2	144105600	0.909091
chr2	144239300	0.909091
chr2	144366000	0.900826
chr2	144450100	0.917355
chr2	144516800	0.719008
chr2	144519800	0.983471
chr2	144554300	0.719008
chr2	144566200	0.900826
chr2	144577500	0.727273
chr2	144648900	0.735537
chr2	144665000	0.669421
chr2	144692200	0.818182
chr2	144797300	0.652893
chr2	144892200	0.677686
chr2	145025800	0.652893
chr2	145104200	0.801653
chr2	145107900	0.719008
chr2	145189300	0.685950
chr2	145202400	0.975207
chr2	145206700	0.702479
chr2	145210400	0.809917
chr2	145228900	0.876033
chr2	145238100	0.710744
chr2	145262400	0.661157
chr2	145268500	0.925620
chr2	145325800	0.925620
chr2	145339000	0.661157
chr2	145400100	0.694215
chr2	145407100	0.768595
chr2	145469700	0.892562
chr2	145653400	0.685950
chr2	146189500	0.719008
chr2	146349800	0.719008
chr2	146427100	0.760331
chr2	146445500	0.818182
chr2	146509800	0.694215
chr2	146687900	0.818182
chr2	146799000	0.735537
chr2	147296800	0.917355
chr2	147300000	0.933884
chr2	147341200	0.743802
chr2	147436300	0.644628
chr2	147445000	0.644628
chr2	147852500	0.768595
chr2	147933000	0.735537
chr2	147957800	0.876033
chr2	148207500	0.884298
chr2	148226900	0.793388
chr2	148405100	0.925620
chr2	148618100	0.950413
chr2	148620800	0.752066

chr2	148634700	0.801653
chr2	148805500	0.776860
chr2	148893300	0.793388
chr2	148959200	0.801653
chr2	149276900	0.685950
chr2	149281100	0.685950
chr2	149312500	0.809917
chr2	149335900	0.809917
chr2	149344500	0.801653
chr2	149372300	0.818182
chr2	149432600	0.776860
chr2	149571500	1.000000
chr2	149639500	0.975207
chr2	149830800	0.859504
chr2	149856300	0.743802
chr2	149884700	0.735537
chr2	149928000	0.735537
chr2	149933700	0.842975
chr2	149939800	0.652893
chr2	150009500	0.710744
chr2	150016300	0.834711
chr2	150080500	0.925620
chr2	150148100	0.942149
chr2	150171500	0.719008
chr2	150246000	0.743802
chr2	150308500	0.702479
chr2	150346100	0.685950
chr2	150454400	0.735537
chr2	150524000	0.727273
chr2	150657400	0.884298
chr2	150681700	0.727273
chr2	150903300	0.719008
chr2	150920700	0.900826
chr2	151065600	0.776860
chr2	151124500	0.743802
chr2	151268000	0.719008
chr2	151282000	0.644628
chr2	151286500	0.702479
chr2	151334700	0.677686
chr2	151386300	0.669421
chr2	151592900	0.818182
chr2	151660800	0.809917
chr2	151716600	0.719008
chr2	151726300	0.917355
chr2	151729400	0.809917
chr2	151732000	0.669421
chr2	151968800	0.867769
chr2	152032200	0.859504
chr2	152035100	0.793388
chr2	152172200	0.685950
chr2	152196000	0.644628
chr2	152214700	0.933884
chr2	152355300	0.735537
chr2	152586300	0.644628

chr2	152804300	0.826446
chr2	152858300	0.859504
chr2	152891000	0.925620
chr2	152907400	0.917355
chr2	153252900	0.685950
chr2	153264100	0.942149
chr2	153267000	0.685950
chr2	153291200	0.925620
chr2	153309800	0.942149
chr2	153323400	0.776860
chr2	153325700	0.867769
chr2	153328600	0.661157
chr2	153350700	0.991736
chr2	153385700	0.925620
chr2	153558800	0.752066
chr2	154245700	0.677686
chr2	154312700	0.719008
chr2	154331700	0.685950
chr2	154344700	0.801653
chr2	154400700	0.884298
chr2	154669000	0.661157
chr2	154747600	0.834711
chr2	155097500	0.818182
chr2	155227800	0.776860
chr2	155257700	0.768595
chr2	155341100	0.644628
chr2	155436700	0.801653
chr2	155446400	1.000000
chr2	155448500	0.719008
chr2	155511400	0.809917
chr2	155570300	0.694215
chr2	155652900	0.644628
chr2	156120600	0.652893
chr2	156261700	0.851240
chr2	156584200	0.809917
chr2	156698500	0.685950
chr2	156714500	0.752066
chr2	156878200	0.710744
chr2	156982000	0.685950
chr2	157063300	0.735537
chr2	157182900	0.710744
chr2	157418400	0.652893
chr2	157450200	0.743802
chr2	157529000	0.685950
chr2	157656900	0.917355
chr2	157659800	0.727273
chr2	157731400	0.793388
chr2	158103000	0.950413
chr2	158177900	0.677686
chr2	158182700	0.859504
chr2	158202600	0.842975
chr2	158416400	0.719008
chr2	158644500	0.752066
chr2	158693000	0.702479

chr2	158696700	0.776860
chr2	158728100	0.743802
chr2	158760300	0.644628
chr2	158781000	0.785124
chr2	158790800	0.851240
chr2	158982900	0.661157
chr2	159345900	0.834711
chr2	159350100	0.727273
chr2	159353800	0.776860
chr2	159362500	0.710744
chr2	159652300	0.719008
chr2	159702800	0.876033
chr2	159779400	0.661157
chr2	159852200	0.661157
chr2	159888600	0.834711
chr2	159921400	0.727273
chr2	160373200	0.669421
chr2	160466900	0.702479
chr2	160477300	0.669421
chr2	160644300	0.685950
chr2	160804300	0.694215
chr2	160909100	0.644628
chr2	160912400	0.859504
chr2	161166200	0.743802
chr2	161181600	0.652893
chr2	161224200	0.818182
chr2	161234100	0.884298
chr2	161245100	0.859504
chr2	161254700	0.917355
chr2	161259200	0.834711
chr2	161274500	0.727273
chr2	161329600	0.826446
chr2	161335600	0.661157
chr2	161440300	0.801653
chr2	161491900	0.669421
chr2	161561000	0.909091
chr2	161587700	0.661157
chr2	161782400	0.702479
chr2	161799200	0.776860
chr2	162046800	0.776860
chr2	162103700	0.727273
chr2	162178500	0.702479
chr2	162221300	0.652893
chr2	162603600	0.710744
chr2	162858900	0.809917
chr2	162893900	0.677686
chr2	162970000	0.652893
chr2	163568600	0.760331
chr2	163681100	0.727273
chr2	163934400	0.760331
chr2	164450800	0.917355
chr2	164458300	0.851240
chr2	164478100	0.652893
chr2	164555300	0.892562

chr2	164558500	0.818182
chr2	164925000	0.727273
chr2	165071500	0.685950
chr2	165450400	0.818182
chr2	165641300	0.685950
chr2	165754400	0.652893
chr2	165770000	0.719008
chr2	165926500	0.942149
chr2	166226200	0.793388
chr2	166263000	0.826446
chr2	166328300	0.677686
chr2	166484300	0.842975
chr2	166490500	0.867769
chr2	166554700	0.950413
chr2	166563600	0.842975
chr2	166984200	0.851240
chr2	167193800	0.685950
chr2	167229800	0.785124
chr2	167485700	0.851240
chr2	167621800	0.661157
chr2	167630100	0.884298
chr2	167698600	0.644628
chr2	167748900	0.727273
chr2	167829200	0.884298
chr2	168116000	0.710744
chr2	168153700	0.710744
chr2	168199900	0.735537
chr2	168241300	0.644628
chr2	168245100	0.760331
chr2	168263300	0.859504
chr2	168337200	0.818182
chr2	168408000	0.685950
chr2	168537000	0.644628
chr2	168554700	0.909091
chr2	168589300	0.793388
chr2	168613200	0.958678
chr2	168615500	0.760331
chr2	168640300	0.842975
chr2	168649700	0.677686
chr2	168701500	0.661157
chr2	168718500	1.000000
chr2	168803100	0.942149
chr2	168973400	0.727273
chr2	168994800	0.776860
chr2	169022300	0.743802
chr2	169029500	0.652893
chr2	169037400	0.768595
chr2	169047900	0.661157
chr2	169061000	0.818182
chr2	169097500	0.719008
chr2	169101200	0.702479
chr2	169281500	0.801653
chr2	169322800	0.669421
chr2	169328000	0.644628

chr2	169347200	0.851240
chr2	169375500	0.768595
chr2	169378100	0.752066
chr2	169382700	0.752066
chr2	169407900	0.925620
chr2	169446300	0.867769
chr2	169482700	0.760331
chr2	169496000	0.743802
chr2	169506400	0.644628
chr2	169518400	0.909091
chr2	169588000	0.793388
chr2	169637400	0.818182
chr2	169655700	0.917355
chr2	169659100	0.694215
chr2	169764400	0.752066
chr2	169769600	0.760331
chr2	169966800	0.752066
chr2	170157800	0.776860
chr2	170252600	0.685950
chr2	170264200	0.752066
chr2	170593500	0.652893
chr2	170646700	0.710744
chr2	170719900	0.735537
chr2	170932400	0.677686
chr2	170934500	0.991736
chr2	170943000	0.958678
chr2	171012400	0.834711
chr2	171069800	0.694215
chr2	171071900	0.950413
chr2	171079200	0.867769
chr2	171093500	0.685950
chr2	171140000	0.727273
chr2	171197000	0.867769
chr2	171211400	0.966942
chr2	171227000	0.809917
chr2	171293600	0.801653
chr2	171319000	0.719008
chr2	171362400	0.710744
chr2	171368900	0.652893
chr2	171382200	0.710744
chr2	171387300	0.661157
chr2	171508700	0.694215
chr2	171536900	0.694215
chr2	171551700	0.727273
chr2	171561100	0.685950
chr2	171621600	0.933884
chr2	171775500	0.785124
chr2	171941100	0.760331
chr2	171950800	0.661157
chr2	171997400	0.727273
chr2	172012600	0.644628
chr2	172117900	0.933884
chr2	172171900	0.942149
chr2	172177400	0.685950

chr2	172181300	0.768595
chr2	172183900	0.785124
chr2	172243300	0.851240
chr2	172266200	0.958678
chr2	172288200	0.950413
chr2	172339300	0.685950
chr2	172348500	0.917355
chr2	172370800	0.785124
chr2	172645100	0.933884
chr2	172652000	0.892562
chr2	172878100	0.818182
chr2	172887000	0.818182
chr2	172945500	0.661157
chr2	172955300	0.735537
chr2	172957800	0.768595
chr2	172963600	0.793388
chr2	172973100	0.842975
chr2	172997100	0.842975
chr2	172999300	0.652893
chr2	173047000	0.900826
chr2	173096900	0.685950
chr2	173116400	0.710744
chr2	173125500	0.909091
chr2	173150200	0.809917
chr2	173161000	0.727273
chr2	173244500	0.760331
chr2	173247000	0.950413
chr2	173296400	0.818182
chr2	173300300	0.933884
chr2	173303300	0.917355
chr2	173307200	0.834711
chr2	173327300	0.677686
chr2	173398200	0.859504
chr2	173400500	0.859504
chr2	173416200	0.933884
chr2	173532200	0.661157
chr2	173771400	0.735537
chr2	173796100	0.884298
chr2	173809900	0.876033
chr2	174049300	0.644628
chr2	174056900	0.876033
chr2	174060900	0.776860
chr2	174101500	0.925620
chr2	174129800	0.776860
chr2	174212400	0.710744
chr2	174259900	0.694215
chr2	174322500	0.793388
chr2	174436400	0.925620
chr2	174488700	0.818182
chr2	174505900	0.677686
chr2	174611900	0.760331
chr2	174615500	0.685950
chr2	174663100	0.834711
chr2	174835200	0.652893

chr2	174847900	0.818182
chr2	174879800	0.752066
chr2	174889300	0.892562
chr2	174916600	0.826446
chr2	174918800	0.909091
chr2	174928300	0.933884
chr2	174949400	0.867769
chr2	174971500	0.859504
chr2	174980400	0.694215
chr2	174989600	0.859504
chr2	174991900	0.743802
chr2	175023100	0.752066
chr2	175064100	0.859504
chr2	175132400	0.785124
chr2	175194000	0.760331
chr2	175360700	0.826446
chr2	175450800	0.859504
chr2	175532600	0.785124
chr2	175558800	0.661157
chr2	175636900	0.991736
chr2	175642100	0.809917
chr2	175652000	0.785124
chr2	175654100	0.892562
chr2	175661300	0.983471
chr2	175692600	0.925620
chr2	175701500	0.859504
chr2	175706900	0.661157
chr2	175709800	0.892562
chr2	175712500	0.809917
chr2	175715500	0.743802
chr2	175730400	0.859504
chr2	175738400	0.702479
chr2	175745300	0.793388
chr2	175747800	0.876033
chr2	175788900	0.933884
chr2	175791900	0.876033
chr2	175817500	0.727273
chr2	175824400	0.801653
chr2	175828900	0.958678
chr2	175831200	1.000000
chr2	175856800	0.801653
chr2	175872600	0.760331
chr2	175922700	0.809917
chr2	176238300	0.669421
chr2	176699600	0.694215
chr2	176749000	0.785124
chr2	176933500	0.735537
chr2	176937100	0.793388
chr2	176948400	0.925620
chr2	176958700	0.702479
chr2	176965200	0.892562
chr2	176969400	0.867769
chr2	176972600	0.892562
chr2	176993800	0.900826

chr2	177017200	0.818182
chr2	177025300	0.809917
chr2	177030100	0.809917
chr2	177044300	0.644628
chr2	177057300	0.644628
chr2	177190900	0.793388
chr2	177315000	0.859504
chr2	177510900	0.776860
chr2	177518900	0.834711
chr2	177540800	0.892562
chr2	177544500	0.834711
chr2	177611100	0.892562
chr2	177617000	0.644628
chr2	177620900	0.661157
chr2	177628500	0.892562
chr2	177631100	0.661157
chr2	177679000	0.652893
chr2	177682100	0.892562
chr2	177684200	0.661157
chr2	177717600	0.644628
chr2	177737700	0.917355
chr2	177941500	0.652893
chr2	178030200	0.710744
chr2	178035500	0.743802
chr2	178044200	0.768595
chr2	178069900	0.917355
chr2	178107400	0.727273
chr2	178110500	0.669421
chr2	178197500	1.000000
chr2	178331300	0.669421
chr2	178401500	0.677686
chr2	178462900	0.702479
chr2	178596600	0.694215
chr2	178628400	0.793388
chr2	178662500	0.661157
chr2	178754500	0.867769
chr2	178762400	0.644628
chr2	179077100	0.776860
chr2	179084900	0.735537
chr2	179371900	0.694215
chr2	179711300	0.727273
chr2	180031600	0.776860
chr2	180046200	0.958678
chr2	180089800	0.785124
chr2	180293800	0.884298
chr2	180308400	0.925620
chr2	180415400	0.752066
chr2	180427500	0.685950
chr2	180631000	0.644628
chr2	180666100	0.661157
chr2	180748900	0.694215
chr2	180869500	0.743802
chr2	180899200	0.958678
chr2	180921700	1.000000

chr2	180924000	0.743802
chr2	180936800	0.925620
chr2	181009900	0.652893
chr2	181012300	0.942149
chr2	181061600	0.661157
chr2	181193900	0.785124
chr2	181544600	0.768595
chr2	181770200	0.818182
chr2	181848900	0.884298
chr2	181866500	0.694215
chr2	181923300	0.669421
chr2	181931400	0.719008
chr2	181971900	0.892562
chr2	182276300	0.942149
chr2	182451900	0.834711
chr2	182543100	0.892562
chr2	182550500	0.752066
chr2	183107100	0.752066
chr2	183490800	0.809917
chr2	183684000	0.851240
chr2	183772400	0.735537
chr2	183875400	0.735537
chr2	183914900	0.867769
chr2	183945500	0.669421
chr2	184050500	0.652893
chr2	184157200	0.669421
chr2	184272900	0.652893
chr2	184398000	0.867769
chr2	184401400	0.669421
chr2	185237600	0.793388
chr2	186349700	0.867769
chr2	186989600	0.719008
chr2	186995000	0.719008
chr2	187120900	0.867769
chr2	187172700	0.719008
chr2	187409500	0.900826
chr2	187469900	0.669421
chr2	187579800	0.685950
chr2	187599900	0.834711
chr2	187645900	0.644628
chr2	188128900	0.933884
chr2	188269500	0.685950
chr2	188375900	0.991736
chr2	188384900	0.876033
chr2	188431900	0.785124
chr2	188483800	0.917355
chr2	188842300	0.793388
chr2	188858700	0.702479
chr2	188860800	0.851240
chr2	188865300	0.644628
chr2	188916500	0.644628
chr2	189046700	0.685950
chr2	189164000	0.876033
chr2	189167800	0.876033

chr2	189172200	0.669421
chr2	189184200	0.752066
chr2	189187900	0.809917
chr2	189205300	0.685950
chr2	189264000	0.719008
chr2	189285200	0.661157
chr2	189304800	0.685950
chr2	189319100	0.752066
chr2	189321500	0.909091
chr2	189329800	0.809917
chr2	189343500	0.727273
chr2	189371700	0.743802
chr2	189380700	0.768595
chr2	189558200	0.677686
chr2	190082600	0.892562
chr2	190291300	0.652893
chr2	190550600	0.710744
chr2	190690900	0.793388
chr2	190715400	0.661157
chr2	191079500	0.710744
chr2	191081700	0.834711
chr2	191278300	0.661157
chr2	191401700	0.760331
chr2	191564300	0.776860
chr2	191656000	0.776860
chr2	191666300	0.818182
chr2	191725600	0.917355
chr2	191734800	0.818182
chr2	191895500	0.652893
chr2	191952000	0.768595
chr2	192083200	0.644628
chr2	192113300	0.909091
chr2	192135100	0.719008
chr2	192139000	0.710744
chr2	192145500	0.884298
chr2	192183900	0.694215
chr2	192187300	0.859504
chr2	192696300	0.859504
chr2	192709900	0.785124
chr2	192792800	0.818182
chr2	192895100	0.685950
chr2	194449100	0.801653
chr2	194544900	0.727273
chr2	196135600	0.661157
chr2	196451200	0.851240
chr2	196477600	0.867769
chr2	196511400	0.867769
chr2	196524900	0.669421
chr2	196560000	0.892562
chr2	196640300	0.743802
chr2	196668700	0.661157
chr2	196673400	0.809917
chr2	196695900	0.727273
chr2	196924600	0.834711

chr2	196962300	0.851240
chr2	196966900	0.652893
chr2	196977400	0.892562
chr2	197069900	0.694215
chr2	197502400	0.710744
chr2	197823100	0.677686
chr2	198029400	0.834711
chr2	198442600	0.669421
chr2	198452200	0.991736
chr2	198493300	0.917355
chr2	198538400	0.793388
chr2	198540500	0.661157
chr2	198574200	0.685950
chr2	198722600	0.776860
chr2	198738100	0.818182
chr2	199654200	0.719008
chr2	200029300	0.809917
chr2	200133500	0.793388
chr2	200275000	0.669421
chr2	200289200	0.793388
chr2	200296300	0.702479
chr2	200330800	0.719008
chr2	200446700	0.644628
chr2	200474400	0.702479
chr2	200605000	0.793388
chr2	200616600	0.669421
chr2	200669900	0.884298
chr2	200708000	0.685950
chr2	200734300	0.983471
chr2	200844800	0.694215
chr2	200853000	0.752066
chr2	200868400	0.694215
chr2	200905100	0.661157
chr2	200954400	0.669421
chr2	201001200	0.942149
chr2	201023900	0.958678
chr2	201034100	0.669421
chr2	201068100	0.991736
chr2	201175700	0.785124
chr2	201178500	0.809917
chr2	201180800	0.975207
chr2	201182900	0.958678
chr2	201185100	0.702479
chr2	201188300	0.644628
chr2	201217200	0.859504
chr2	201220100	0.975207
chr2	201222700	0.677686
chr2	201225400	0.925620
chr2	201242000	0.669421
chr2	201244600	0.909091
chr2	201259300	0.710744
chr2	201281700	0.933884
chr2	201318400	0.677686
chr2	201345900	0.669421

chr2	201602000	0.942149
chr2	201606400	0.776860
chr2	201655400	0.834711
chr2	202022900	0.768595
chr2	202085600	0.834711
chr2	202123100	0.909091
chr2	202336100	0.752066
chr2	202540200	0.702479
chr2	202639200	0.685950
chr2	202672800	0.685950
chr2	202723700	0.727273
chr2	202737700	0.867769
chr2	202747900	0.719008
chr2	202753500	0.793388
chr2	202771800	0.925620
chr2	202789000	0.694215
chr2	202793900	0.644628
chr2	202799100	0.801653
chr2	202803700	0.652893
chr2	202814600	0.983471
chr2	202827900	0.867769
chr2	202831500	0.652893
chr2	202834700	0.719008
chr2	202839000	0.867769
chr2	202843400	0.752066
chr2	202850100	0.661157
chr2	202861000	0.933884
chr2	202864500	0.669421
chr2	202880300	0.644628
chr2	202884900	0.743802
chr2	202888600	0.983471
chr2	202891200	0.752066
chr2	202905000	0.669421
chr2	202916800	0.867769
chr2	202929500	0.933884
chr2	202938000	0.818182
chr2	202949000	0.884298
chr2	203009800	0.702479
chr2	203014000	0.884298
chr2	203036400	0.719008
chr2	203115000	0.966942
chr2	203134700	0.710744
chr2	203221300	0.669421
chr2	203283800	0.776860
chr2	203359800	0.743802
chr2	203666800	0.652893
chr2	204205500	0.925620
chr2	204349200	0.909091
chr2	204502500	0.818182
chr2	204584700	0.669421
chr2	204587000	1.000000
chr2	204947200	0.677686
chr2	205010700	0.694215
chr2	205070500	0.644628

chr2	205129100	0.669421
chr2	205137400	0.925620
chr2	205146800	0.851240
chr2	205176800	0.719008
chr2	205179000	0.909091
chr2	205190500	0.760331
chr2	205235200	0.768595
chr2	205384900	0.644628
chr2	205395900	0.710744
chr2	205912400	0.975207
chr2	206005000	0.752066
chr2	206062900	0.909091
chr2	206132100	0.710744
chr2	206138200	0.867769
chr2	206141200	0.884298
chr2	206162400	0.909091
chr2	206223500	0.958678
chr2	206256100	0.702479
chr2	206328500	0.735537
chr2	206346000	0.851240
chr2	206348600	0.966942
chr2	206515000	0.727273
chr2	206544600	0.685950
chr2	206568200	0.694215
chr2	206585200	0.950413
chr2	206595500	0.793388
chr2	206599500	0.785124
chr2	206612000	0.661157
chr2	206614200	0.826446
chr2	206628700	0.702479
chr2	206701200	0.851240
chr2	206822500	0.793388
chr2	206830900	0.685950
chr2	206896800	0.652893
chr2	207078000	0.884298
chr2	207083000	0.909091
chr2	207095400	0.884298
chr2	207122200	0.793388
chr2	207194000	0.677686
chr2	207303600	0.842975
chr2	207312800	0.677686
chr2	207376300	0.752066
chr2	207668500	0.851240
chr2	207671300	0.702479
chr2	207678500	0.958678
chr2	207727100	0.644628
chr2	207836100	0.702479
chr2	207847900	0.727273
chr2	207873200	0.983471
chr2	207906400	0.842975
chr2	207939400	0.669421
chr2	207958200	0.983471
chr2	207961700	0.694215
chr2	207998700	0.694215

chr2	208009000	0.842975
chr2	208014400	0.694215
chr2	208016500	0.793388
chr2	208019900	0.818182
chr2	208026400	0.909091
chr2	208090500	0.884298
chr2	208110500	0.851240
chr2	208123700	0.851240
chr2	208150000	0.719008
chr2	208154100	0.793388
chr2	208199600	0.942149
chr2	208250200	0.644628
chr2	208272900	0.809917
chr2	208286500	0.685950
chr2	208338100	0.694215
chr2	208625300	0.644628
chr2	208627500	0.652893
chr2	208637300	0.735537
chr2	208656300	0.727273
chr2	208661800	0.942149
chr2	208666500	0.677686
chr2	208675600	0.661157
chr2	208678400	0.958678
chr2	208703400	0.644628
chr2	208752800	0.752066
chr2	208757300	0.644628
chr2	208760600	0.900826
chr2	208798900	0.909091
chr2	208816100	0.917355
chr2	208833000	0.727273
chr2	208835500	1.000000
chr2	208871400	0.793388
chr2	208972800	0.694215
chr2	209044300	0.644628
chr2	209054800	0.785124
chr2	209240900	0.735537
chr2	209459900	0.884298
chr2	209537100	0.702479
chr2	209757400	0.694215
chr2	210004200	0.661157
chr2	210006400	0.677686
chr2	210010700	0.950413
chr2	210170500	0.859504
chr2	210222500	0.809917
chr2	210316600	0.917355
chr2	210322800	0.793388
chr2	210479400	0.909091
chr2	210526300	0.925620
chr2	210552400	0.727273
chr2	210656300	0.685950
chr2	210682800	0.900826
chr2	210738300	0.702479
chr2	210825200	0.727273
chr2	210937700	0.710744

chr2	211042700	0.983471
chr2	211046400	0.892562
chr2	211346600	0.818182
chr2	211400100	0.652893
chr2	211421700	0.752066
chr2	211583000	0.884298
chr2	211628100	0.851240
chr2	211724800	0.652893
chr2	211727100	0.818182
chr2	212211200	0.876033
chr2	212224200	0.793388
chr2	212226600	0.917355
chr2	212256000	0.710744
chr2	212631500	0.719008
chr2	212975500	0.966942
chr2	213102900	0.719008
chr2	213249100	0.727273
chr2	213292500	0.710744
chr2	213618100	0.834711
chr2	213781200	0.710744
chr2	213805700	0.983471
chr2	213951200	0.966942
chr2	214053000	0.892562
chr2	214155000	0.669421
chr2	214993200	0.942149
chr2	215315300	0.677686
chr2	215458800	0.752066
chr2	215670800	0.900826
chr2	215769600	0.669421
chr2	215887300	0.719008
chr2	216178700	0.669421
chr2	216253300	0.842975
chr2	216411500	0.652893
chr2	216637000	0.785124
chr2	216645200	0.644628
chr2	216783700	0.818182
chr2	216793500	0.685950
chr2	216867000	0.884298
chr2	216871600	0.867769
chr2	216949100	0.867769
chr2	216972700	0.644628
chr2	216979900	1.000000
chr2	217049900	0.685950
chr2	217052500	0.867769
chr2	217060900	0.801653
chr2	217107900	0.793388
chr2	217146700	0.826446
chr2	217149500	0.694215
chr2	217165600	0.785124
chr2	217181100	0.859504
chr2	217205300	0.694215
chr2	217210300	0.942149
chr2	217214500	0.884298
chr2	217273400	0.735537

chr2	217367000	0.644628
chr2	217415400	0.669421
chr2	217418100	0.685950
chr2	217428400	0.652893
chr2	217434500	0.727273
chr2	217449300	0.760331
chr2	217471900	0.909091
chr2	217484500	0.793388
chr2	217509200	0.801653
chr2	217514500	0.801653
chr2	217530900	0.859504
chr2	217553900	0.727273
chr2	217628500	0.710744
chr2	217631100	0.884298
chr2	217720100	0.669421
chr2	217850900	0.826446
chr2	218074200	0.958678
chr2	218135200	0.867769
chr2	218155000	0.768595
chr2	218196800	0.669421
chr2	218223300	0.644628
chr2	218299000	0.752066
chr2	218344200	0.661157
chr2	218376000	0.644628
chr2	218417200	0.644628
chr2	218420400	0.785124
chr2	218462000	0.669421
chr2	218487400	0.892562
chr2	218515500	0.826446
chr2	218578600	0.719008
chr2	218654800	0.884298
chr2	218701800	0.909091
chr2	218777600	0.719008
chr2	218783800	0.801653
chr2	218787000	0.818182
chr2	218791500	0.776860
chr2	218798700	0.760331
chr2	218801700	0.727273
chr2	218808600	0.851240
chr2	218813000	0.834711
chr2	218830900	0.785124
chr2	218853100	0.900826
chr2	218861200	0.702479
chr2	218867200	0.818182
chr2	218880200	0.694215
chr2	218891000	0.685950
chr2	218933900	0.942149
chr2	218990200	0.677686
chr2	219126800	0.867769
chr2	219191300	0.694215
chr2	219197400	0.661157
chr2	219222100	0.677686
chr2	219258800	0.644628
chr2	219284100	0.743802

chr2	219420400	0.752066
chr2	219442900	0.851240
chr2	219585600	0.760331
chr2	219597600	0.900826
chr2	219664500	0.826446
chr2	219718500	0.867769
chr2	219721200	0.727273
chr2	219736500	0.900826
chr2	219738600	0.842975
chr2	219745300	0.677686
chr2	219749100	0.669421
chr2	219763200	0.776860
chr2	219829300	0.743802
chr2	219844900	0.851240
chr2	219847200	0.752066
chr2	219864300	0.752066
chr2	219927600	0.644628
chr2	219938900	0.809917
chr2	220114200	0.752066
chr2	220142500	0.661157
chr2	220151200	0.851240
chr2	220164200	0.950413
chr2	220197200	0.760331
chr2	220267800	0.677686
chr2	220270000	0.768595
chr2	220281100	0.776860
chr2	220284200	0.834711
chr2	220326200	0.661157
chr2	220357400	0.652893
chr2	220367200	0.710744
chr2	220372100	0.876033
chr2	220376700	0.892562
chr2	220379200	0.661157
chr2	220386200	0.900826
chr2	220395100	0.892562
chr2	220428200	0.991736
chr2	220433200	0.801653
chr2	220508100	0.694215
chr2	220536600	0.892562
chr2	220550300	0.991736
chr2	220563900	0.719008
chr2	220567300	0.801653
chr2	220572400	0.793388
chr2	220717200	0.743802
chr2	220736500	0.793388
chr2	220767500	0.710744
chr2	220829900	0.719008
chr2	220863200	0.677686
chr2	221134500	0.719008
chr2	221370100	0.809917
chr2	222021400	0.652893
chr2	222054500	0.834711
chr2	222110000	0.768595
chr2	222237400	0.950413

chr2	222328700	0.661157
chr2	222343800	0.785124
chr2	222360800	0.685950
chr2	222378300	0.801653
chr2	222417700	0.793388
chr2	222420200	0.793388
chr2	222574000	0.900826
chr2	222645200	0.644628
chr2	222658100	0.958678
chr2	222891500	0.644628
chr2	222918000	0.909091
chr2	222939600	0.842975
chr2	222967700	0.685950
chr2	223073400	0.661157
chr2	223103400	0.719008
chr2	223123200	0.735537
chr2	223134000	0.876033
chr2	223148900	0.719008
chr2	223159700	0.669421
chr2	223169000	0.669421
chr2	223172000	0.768595
chr2	223233500	0.785124
chr2	223258600	0.809917
chr2	223343300	0.694215
chr2	223383700	0.702479
chr2	223574100	0.727273
chr2	223735300	0.735537
chr2	223772800	0.702479
chr2	223836800	0.818182
chr2	223847100	0.685950
chr2	223900900	0.925620
chr2	223903900	0.768595
chr2	223916500	0.801653
chr2	223966700	0.793388
chr2	224591500	1.000000
chr2	224697900	0.793388
chr2	224795400	0.867769
chr2	224800300	0.702479
chr2	224803800	0.776860
chr2	224815700	0.661157
chr2	224825700	0.801653
chr2	224868500	0.677686
chr2	224874200	0.785124
chr2	224890500	0.685950
chr2	224893000	0.719008
chr2	224897600	0.760331
chr2	225086800	0.677686
chr2	225129200	0.685950
chr2	225158600	0.669421
chr2	225298800	0.892562
chr2	225350400	0.876033
chr2	225435200	0.644628
chr2	225440100	0.727273
chr2	225452900	0.661157

chr2	225809900	0.702479
chr2	225937900	0.801653
chr2	226293900	0.644628
chr2	226897500	0.727273
chr2	227022600	0.743802
chr2	227024900	0.735537
chr2	227346700	0.694215
chr2	227348800	0.966942
chr2	227515100	0.983471
chr2	227660100	0.909091
chr2	227734100	0.876033
chr2	227842300	0.661157
chr2	227972000	0.661157
chr2	228050600	0.677686
chr2	228105100	0.768595
chr2	228288700	0.661157
chr2	228481800	0.826446
chr2	228526300	0.719008
chr2	228581300	0.652893
chr2	228622300	0.702479
chr2	228624700	0.809917
chr2	228631400	0.702479
chr2	228637800	0.809917
chr2	228728500	0.644628
chr2	228843600	0.685950
chr2	229372700	0.834711
chr2	229380200	0.710744
chr2	229960200	0.685950
chr2	230045800	0.917355
chr2	230274800	0.793388
chr2	230283000	0.818182
chr2	230351300	0.884298
chr2	230443300	0.743802
chr2	230563400	0.727273
chr2	230598700	0.917355
chr2	230639500	0.694215
chr2	230642300	0.677686
chr2	230646200	0.834711
chr2	230775900	0.768595
chr2	230817400	0.719008
chr2	230821100	0.809917
chr2	230845500	0.793388
chr2	231044300	0.694215
chr2	231176800	0.685950
chr2	231322500	0.776860
chr2	231519300	0.694215
chr2	231522100	0.702479
chr2	231531900	0.752066
chr2	231582800	0.834711
chr2	231586500	0.876033
chr2	231589000	0.727273
chr2	231595900	0.669421
chr2	231620700	0.702479
chr2	231624600	0.719008

chr2	231649800	0.867769
chr2	231732700	0.801653
chr2	231735000	0.644628
chr2	231737300	0.876033
chr2	231751300	0.785124
chr2	231767500	0.933884
chr2	231770300	0.933884
chr2	231849900	0.842975
chr2	231880700	0.834711
chr2	232028400	0.842975
chr2	232055200	0.785124
chr2	232093300	0.876033
chr2	232125500	0.719008
chr2	232162000	0.719008
chr2	232188400	0.661157
chr2	232207300	0.925620
chr2	232221000	0.710744
chr2	232237200	0.851240
chr2	232240100	0.652893
chr2	232242500	0.983471
chr2	232253900	0.776860
chr2	232257800	0.760331
chr2	232265100	0.900826
chr2	232274900	0.859504
chr2	232278900	0.867769
chr2	232317700	0.925620
chr2	232325300	0.644628
chr2	232428400	0.785124
chr2	232433700	0.892562
chr2	232436900	0.900826
chr2	232439500	0.892562
chr2	232442100	0.925620
chr2	232447900	0.867769
chr2	232455700	0.644628
chr2	232466000	0.909091
chr2	232486400	0.958678
chr2	232526900	0.826446
chr2	232531400	0.909091
chr2	232536200	0.685950
chr2	232539900	0.933884
chr2	232549900	0.859504
chr2	232554700	0.669421
chr2	232579400	0.958678
chr2	232610400	0.793388
chr2	232742800	0.809917
chr2	232766000	0.661157
chr2	232874900	0.735537
chr2	232879500	0.677686
chr2	233059900	0.743802
chr2	233065100	0.685950
chr2	233069500	0.661157
chr2	233096500	0.950413
chr2	233135200	0.652893
chr2	233186600	0.768595

chr2	233189600	0.950413
chr2	233200400	0.685950
chr2	233227900	0.669421
chr2	233230400	0.884298
chr2	233234900	0.859504
chr2	233239700	0.719008
chr2	233288200	0.900826
chr2	233297400	0.776860
chr2	233354500	0.892562
chr2	233358800	0.677686
chr2	233370800	0.735537
chr2	233387000	0.719008
chr2	233486000	0.694215
chr2	233564500	0.644628
chr2	233755800	0.867769
chr2	233766400	0.760331
chr2	233830900	0.752066
chr2	233848600	0.826446
chr2	233914000	0.661157
chr2	233927700	0.719008
chr2	233937900	0.719008
chr2	233940700	0.735537
chr2	233950500	0.644628
chr2	234077900	0.719008
chr2	234118200	0.818182
chr2	234238500	0.752066
chr2	234309200	0.743802
chr2	234328900	0.727273
chr2	234608700	0.652893
chr2	234838600	0.884298
chr2	235045000	0.661157
chr2	235167200	0.719008
chr2	235214200	0.842975
chr2	235238100	0.818182
chr2	235242900	0.685950
chr2	235272000	0.735537
chr2	235319600	0.677686
chr2	235357400	0.809917
chr2	235411500	0.768595
chr2	235413600	0.644628
chr2	235438000	0.933884
chr2	235454200	0.644628
chr2	235457200	0.834711
chr2	235459600	0.719008
chr2	235505700	0.991736
chr2	235541300	0.983471
chr2	235550200	0.685950
chr2	235579800	0.685950
chr2	235585400	0.867769
chr2	235596100	0.942149
chr2	235616800	0.694215
chr2	235619600	0.933884
chr2	235702100	0.933884
chr2	235794000	0.702479

chr2	235841800	0.933884
chr2	235849900	0.809917
chr2	235864900	0.809917
chr2	235867100	0.652893
chr2	235869600	0.685950
chr2	235878000	0.851240
chr2	235884100	0.884298
chr2	235890100	0.942149
chr2	235905600	0.983471
chr2	235909100	0.702479
chr2	235914800	0.958678
chr2	235917300	0.685950
chr2	235920700	0.933884
chr2	235924000	0.702479
chr2	235948000	0.776860
chr2	235973700	0.752066
chr2	235976300	0.950413
chr2	235979100	0.876033
chr2	236022800	0.677686
chr2	236066900	0.842975
chr2	236115900	0.867769
chr2	236171400	0.702479
chr2	236233900	0.768595
chr2	236293700	0.892562
chr2	236408100	0.735537
chr2	236410300	0.909091
chr2	236413900	0.975207
chr2	236423400	0.644628
chr2	236437200	0.809917
chr2	236482400	0.743802
chr2	236486600	0.925620
chr2	236489400	0.892562
chr2	236494700	0.925620
chr2	236504400	0.661157
chr2	236514600	0.793388
chr2	236547900	0.644628
chr2	236550000	0.809917
chr2	236575200	0.793388
chr2	236600100	0.851240
chr2	236612400	0.942149
chr2	236641300	0.933884
chr2	236656200	0.776860
chr2	236663300	0.826446
chr2	236682700	0.867769
chr2	236715900	0.727273
chr2	236745300	0.735537
chr2	236761100	0.669421
chr2	236813300	0.760331
chr2	236859300	0.818182
chr2	236897800	0.719008
chr2	236920900	0.768595
chr2	236923500	0.867769
chr2	236963000	0.752066
chr2	236970800	0.900826

chr2	236995700	0.909091
chr2	237017100	0.785124
chr2	237026600	0.876033
chr2	237039700	0.702479
chr2	237051900	0.719008
chr2	237070500	0.768595
chr2	237084000	0.694215
chr2	237089200	0.694215
chr2	237149200	0.892562
chr2	237216500	0.669421
chr2	237251100	0.818182
chr2	237394700	0.677686
chr2	237461000	0.983471
chr2	237481200	0.917355
chr2	237570000	0.809917
chr2	237593700	0.661157
chr2	237653800	0.661157
chr2	237702500	0.776860
chr2	237712500	0.702479
chr2	237732500	0.834711
chr2	237734800	0.876033
chr2	237737100	0.694215
chr2	237756700	0.983471
chr2	237762400	0.768595
chr2	237765600	0.694215
chr2	237858400	0.727273
chr2	237874700	0.719008
chr2	237881800	0.677686
chr2	237907900	0.694215
chr2	237951100	0.876033
chr2	238107900	0.991736
chr2	238117400	0.694215
chr2	238120600	0.752066
chr2	238123100	0.694215
chr2	238127000	0.735537
chr2	238266300	0.768595
chr2	238343700	0.661157
chr2	238347100	0.892562
chr2	238360900	0.694215
chr2	238399300	0.991736
chr2	238406400	0.801653
chr2	238481100	0.826446
chr2	238490800	0.735537
chr2	238494700	0.785124
chr2	238503700	1.000000
chr2	238507100	0.876033
chr2	238511400	0.727273
chr2	238522300	0.983471
chr2	238524400	0.694215
chr2	238572100	0.867769
chr2	238574600	0.710744
chr2	238583500	0.826446
chr2	238603500	0.735537
chr2	238606000	0.752066

chr2	238609500	0.710744
chr2	238611600	0.884298
chr2	238621300	0.950413
chr2	238624100	0.752066
chr2	238632800	0.694215
chr2	238653200	0.818182
chr2	238773200	0.752066
chr2	238832100	0.851240
chr2	238888400	0.909091
chr2	238936600	0.859504
chr2	238972100	0.661157
chr2	238989000	0.892562
chr2	239005800	0.876033
chr2	239017800	0.768595
chr2	239136900	0.644628
chr2	239194900	0.826446
chr2	239289600	0.809917
chr2	239324200	0.909091
chr2	239330400	0.793388
chr2	239394600	0.644628
chr2	239418600	0.975207
chr2	239566400	0.859504
chr2	239625400	0.694215
chr2	239693300	0.752066
chr2	239726600	0.661157
chr2	239773300	0.801653
chr2	240123500	0.892562
chr2	240132100	0.785124
chr2	240168500	0.933884
chr2	240186700	0.826446
chr2	240211000	0.702479
chr2	240213700	0.958678
chr2	240216400	0.793388
chr2	240218600	0.776860
chr2	240251700	0.710744
chr2	240260000	0.669421
chr2	240303200	0.851240
chr2	240441500	0.760331
chr2	240524400	0.876033
chr2	240746500	0.685950
chr2	240753400	0.710744
chr2	240842500	0.900826
chr2	241079800	0.834711
chr2	241090100	0.752066
chr2	241134500	0.735537
chr2	241145100	0.859504
chr2	241232600	0.685950
chr2	241381200	0.661157
chr2	241390600	0.661157
chr2	241398600	0.776860
chr2	241497500	0.900826
chr2	241544000	0.818182
chr2	241588500	0.727273
chr2	241625700	0.727273

chr2	241638900	0.652893
chr2	241732900	0.685950
chr2	241752100	0.694215
chr2	241755600	0.851240
chr2	241786800	0.818182
chr2	241862400	0.834711
chr2	241949800	0.694215
chr2	242003300	0.859504
chr2	242033900	0.694215
chr2	242214100	0.669421
chr2	242219600	0.710744
chr2	242260800	0.644628
chr2	242306800	0.760331
chr2	242486600	0.644628
chr2	242557000	0.975207
chr2	242717500	0.661157
chr2	242750000	0.719008
chr2	242808800	0.685950
chr2	242825300	0.694215
chr2	242833100	0.727273
chr2	242845600	0.661157
chr2	242885500	0.710744
chr2	242888600	0.719008
chr2	242893200	0.669421
chr2	242988600	0.644628
chr2	243006400	0.867769
chr3	115500	0.669421
chr3	139900	0.694215
chr3	153300	0.867769
chr3	271900	0.644628
chr3	492900	0.752066
chr3	501600	0.818182
chr3	1229000	0.834711
chr3	1548100	0.685950
chr3	1555700	0.735537
chr3	1719900	0.677686
chr3	2396800	0.710744
chr3	2536500	0.727273
chr3	2553300	0.710744
chr3	2695200	0.727273
chr3	2699700	0.776860
chr3	2728300	0.859504
chr3	2945300	0.752066
chr3	3004600	0.776860
chr3	3021100	0.735537
chr3	3058400	0.727273
chr3	3062500	0.851240
chr3	3102500	0.785124
chr3	3124700	0.710744
chr3	3152300	0.834711
chr3	3330100	0.644628
chr3	3463400	0.652893
chr3	3658500	0.735537
chr3	3750100	0.958678

chr3	3781500	0.661157
chr3	3805400	0.685950
chr3	3846300	0.669421
chr3	3849000	0.818182
chr3	3862000	0.644628
chr3	3891400	0.876033
chr3	4113400	0.876033
chr3	4351200	0.834711
chr3	4353300	0.834711
chr3	4431500	0.669421
chr3	4474300	0.760331
chr3	4490200	0.909091
chr3	4546900	0.859504
chr3	4557300	0.710744
chr3	4575500	0.735537
chr3	4638700	0.900826
chr3	4648900	0.694215
chr3	4762100	0.859504
chr3	4775000	0.809917
chr3	5055600	0.661157
chr3	5138000	0.685950
chr3	5175500	0.735537
chr3	5178800	0.702479
chr3	5203300	0.677686
chr3	5239400	0.768595
chr3	5255500	0.900826
chr3	5264800	0.702479
chr3	5506100	0.933884
chr3	5676300	0.644628
chr3	6164500	0.818182
chr3	6463700	0.719008
chr3	7693400	0.661157
chr3	7830600	0.834711
chr3	7855800	0.694215
chr3	7996000	0.909091
chr3	8095000	0.652893
chr3	8137400	0.760331
chr3	8155400	0.727273
chr3	8363000	0.867769
chr3	8486100	0.842975
chr3	8540400	0.876033
chr3	8542900	0.966942
chr3	8546000	0.743802
chr3	8561700	0.710744
chr3	8665100	0.834711
chr3	8774900	0.644628
chr3	8783700	0.834711
chr3	8826400	0.809917
chr3	8923900	0.950413
chr3	9007000	0.900826
chr3	9030200	0.809917
chr3	9035000	0.677686
chr3	9119800	0.677686
chr3	9144300	0.991736

chr3	9179200	0.727273
chr3	9196400	0.661157
chr3	9210000	0.743802
chr3	9226200	0.834711
chr3	9240700	0.809917
chr3	9244000	0.842975
chr3	9246500	0.685950
chr3	9267300	0.801653
chr3	9272700	0.776860
chr3	9322600	0.818182
chr3	9359200	0.900826
chr3	9393700	0.950413
chr3	9435500	0.768595
chr3	9447100	0.842975
chr3	9464600	0.900826
chr3	9624200	0.719008
chr3	9944700	0.776860
chr3	9997200	0.909091
chr3	10229500	0.669421
chr3	10241000	0.694215
chr3	10267800	0.942149
chr3	10277000	0.809917
chr3	10280700	0.652893
chr3	10305800	0.652893
chr3	10328800	0.801653
chr3	10436100	0.909091
chr3	10438200	0.917355
chr3	10537200	0.694215
chr3	10554000	0.661157
chr3	10595700	0.991736
chr3	10670100	0.694215
chr3	11010200	0.685950
chr3	11019900	0.867769
chr3	11025400	0.652893
chr3	11138300	0.677686
chr3	11517300	0.793388
chr3	11519700	0.727273
chr3	11522400	0.677686
chr3	11551500	0.677686
chr3	11603400	0.694215
chr3	11625600	0.743802
chr3	11630800	0.867769
chr3	11633500	0.933884
chr3	11643100	0.983471
chr3	11645600	0.685950
chr3	11655500	0.842975
chr3	11658500	0.793388
chr3	11661700	0.652893
chr3	11677500	0.735537
chr3	11681500	0.661157
chr3	11731800	0.677686
chr3	11758600	0.661157
chr3	11812300	0.950413
chr3	11837500	0.644628

chr3	11875000	0.859504
chr3	11966500	0.644628
chr3	12037200	0.826446
chr3	12050000	0.719008
chr3	12066800	0.809917
chr3	12102000	0.958678
chr3	12486700	0.933884
chr3	12604700	0.694215
chr3	12735700	0.685950
chr3	12827300	0.702479
chr3	12875300	0.801653
chr3	12878700	0.735537
chr3	12926900	0.652893
chr3	12986400	0.685950
chr3	13001700	0.727273
chr3	13019500	0.842975
chr3	13028900	0.900826
chr3	13053400	0.669421
chr3	13063800	0.669421
chr3	13085800	0.694215
chr3	13093000	0.652893
chr3	13134500	0.685950
chr3	13199800	0.760331
chr3	13202600	0.818182
chr3	13204700	0.859504
chr3	13213400	0.752066
chr3	13246000	0.735537
chr3	13277500	0.917355
chr3	13332900	0.685950
chr3	13338900	0.826446
chr3	13343100	0.942149
chr3	13386700	0.694215
chr3	13395700	0.677686
chr3	13407000	0.876033
chr3	13431300	0.867769
chr3	13446100	0.685950
chr3	13493400	0.685950
chr3	13519100	0.677686
chr3	13549600	0.702479
chr3	13568000	0.834711
chr3	13616900	0.776860
chr3	13629600	0.702479
chr3	13636700	0.727273
chr3	13690200	0.735537
chr3	13729500	0.785124
chr3	13770300	0.892562
chr3	13827800	0.859504
chr3	13915600	0.743802
chr3	13990300	0.818182
chr3	14186300	0.925620
chr3	14189900	0.669421
chr3	14298500	0.644628
chr3	14320600	0.776860
chr3	14327000	0.652893

chr3	14332100	0.859504
chr3	14359300	0.702479
chr3	14371300	0.793388
chr3	14386300	0.669421
chr3	14395400	0.719008
chr3	14438000	0.710744
chr3	14454100	0.933884
chr3	14457500	0.793388
chr3	14460300	0.652893
chr3	14464000	0.909091
chr3	14468400	0.809917
chr3	14473700	0.801653
chr3	14482500	0.925620
chr3	14499800	0.851240
chr3	14577200	0.710744
chr3	14579800	0.785124
chr3	14597700	0.933884
chr3	14603000	0.727273
chr3	14606600	0.834711
chr3	14611300	0.652893
chr3	14815900	0.818182
chr3	14833400	0.851240
chr3	14864800	0.685950
chr3	14878900	0.809917
chr3	14889600	0.925620
chr3	14903400	0.801653
chr3	14905800	0.859504
chr3	14921300	0.768595
chr3	14942600	0.826446
chr3	15032300	0.842975
chr3	15149900	0.719008
chr3	15156500	0.867769
chr3	15190700	0.644628
chr3	15356300	0.644628
chr3	15398400	0.975207
chr3	15403600	0.727273
chr3	15537700	0.793388
chr3	15563400	0.768595
chr3	15675000	0.983471
chr3	15696600	0.710744
chr3	15698800	0.652893
chr3	15798100	0.876033
chr3	15820600	0.644628
chr3	15839200	0.925620
chr3	15935600	0.735537
chr3	16343300	0.975207
chr3	16371200	0.851240
chr3	16394400	0.826446
chr3	16411800	0.884298
chr3	16497200	0.909091
chr3	16537600	0.710744
chr3	16540500	0.826446
chr3	16558700	0.768595
chr3	16575800	0.933884

chr3	16578300	0.702479
chr3	16592200	0.818182
chr3	16699200	0.892562
chr3	16701400	0.983471
chr3	16705900	0.983471
chr3	16711300	0.677686
chr3	16713400	0.958678
chr3	16728600	0.966942
chr3	16739600	0.991736
chr3	16831400	0.727273
chr3	16835100	0.694215
chr3	16852100	0.776860
chr3	16964700	0.958678
chr3	16977900	0.719008
chr3	17041200	0.669421
chr3	17136400	0.925620
chr3	17229500	0.669421
chr3	17237400	0.743802
chr3	17560400	0.760331
chr3	17882700	0.644628
chr3	18258900	0.776860
chr3	18276200	0.867769
chr3	18387300	0.677686
chr3	18390900	0.710744
chr3	18799900	0.669421
chr3	19017700	0.743802
chr3	19116500	0.958678
chr3	19268500	0.702479
chr3	19308900	1.000000
chr3	19335100	0.859504
chr3	19343600	0.685950
chr3	19513000	0.760331
chr3	19515900	0.669421
chr3	19740700	0.727273
chr3	19850500	0.826446
chr3	20264000	0.694215
chr3	20292600	0.884298
chr3	20386000	0.685950
chr3	20522500	0.661157
chr3	20899800	0.710744
chr3	21032500	0.661157
chr3	21585500	0.983471
chr3	21684100	0.719008
chr3	21707300	0.851240
chr3	22348400	0.652893
chr3	22364700	0.702479
chr3	22537000	0.760331
chr3	22733400	0.909091
chr3	23109900	0.933884
chr3	23121000	0.793388
chr3	23330900	0.859504
chr3	23431100	0.975207
chr3	23436600	0.694215
chr3	23478200	0.752066

chr3	23484900	0.710744
chr3	23496800	0.702479
chr3	23531700	0.768595
chr3	23578700	0.760331
chr3	23650600	0.760331
chr3	23687000	0.702479
chr3	23728500	0.859504
chr3	23751900	0.884298
chr3	23754600	0.752066
chr3	23770900	0.768595
chr3	23777600	0.644628
chr3	23782000	0.818182
chr3	23785200	0.669421
chr3	23788100	0.826446
chr3	23793200	0.851240
chr3	23802800	0.892562
chr3	23907400	0.760331
chr3	23934700	0.834711
chr3	23965600	0.652893
chr3	24202200	0.719008
chr3	24204500	0.644628
chr3	24347800	0.661157
chr3	24401900	0.710744
chr3	24431100	0.719008
chr3	24438500	0.785124
chr3	24452800	0.867769
chr3	24519300	0.917355
chr3	24540400	0.925620
chr3	24561100	0.975207
chr3	24726400	0.834711
chr3	24730500	0.735537
chr3	24887500	0.685950
chr3	24994300	0.743802
chr3	25198100	0.958678
chr3	25330500	0.909091
chr3	25461800	0.991736
chr3	25466300	0.760331
chr3	25468500	0.685950
chr3	25496300	0.727273
chr3	25634600	0.793388
chr3	25789800	0.785124
chr3	25862500	0.876033
chr3	25931300	0.644628
chr3	26315900	0.694215
chr3	26881500	0.685950
chr3	26963000	0.776860
chr3	27067400	0.785124
chr3	27085200	0.942149
chr3	27115000	0.776860
chr3	27124800	0.727273
chr3	27132600	0.661157
chr3	27147700	0.768595
chr3	27396500	0.818182
chr3	27575500	0.900826

chr3	27627000	0.652893
chr3	27649200	0.694215
chr3	27695900	0.917355
chr3	27708400	0.900826
chr3	27722800	0.867769
chr3	28013600	0.801653
chr3	28028000	0.933884
chr3	28119300	0.760331
chr3	28123700	0.884298
chr3	28160000	0.644628
chr3	28280800	0.760331
chr3	28653200	0.644628
chr3	28715900	0.661157
chr3	28721500	0.677686
chr3	29099300	0.909091
chr3	29198300	0.685950
chr3	29218100	0.685950
chr3	29327500	0.735537
chr3	29330300	0.661157
chr3	29482200	0.685950
chr3	29517400	0.867769
chr3	29669000	0.669421
chr3	29681500	0.702479
chr3	29735800	0.727273
chr3	29747700	0.743802
chr3	29813900	0.917355
chr3	29976300	0.743802
chr3	30288300	0.685950
chr3	30301100	0.801653
chr3	30591200	0.892562
chr3	30623500	0.785124
chr3	30670300	0.834711
chr3	30701200	0.884298
chr3	30737200	0.685950
chr3	30785100	0.727273
chr3	30789200	0.644628
chr3	30932800	0.677686
chr3	30950900	0.661157
chr3	31083200	0.752066
chr3	31207800	0.644628
chr3	31283900	0.661157
chr3	31317800	0.652893
chr3	31392600	0.677686
chr3	31433100	0.826446
chr3	31508700	0.933884
chr3	31547800	0.768595
chr3	31578500	0.900826
chr3	31604200	0.694215
chr3	31737800	0.694215
chr3	31747100	0.710744
chr3	31766100	0.925620
chr3	31884400	0.760331
chr3	31921800	0.694215
chr3	31924300	0.710744

chr3	31932400	0.834711
chr3	31942900	0.735537
chr3	31953200	0.710744
chr3	31958900	1.000000
chr3	31961100	0.834711
chr3	31975200	0.950413
chr3	31989800	0.677686
chr3	32004800	0.694215
chr3	32044700	0.669421
chr3	32087200	0.661157
chr3	32338500	0.752066
chr3	32344700	0.735537
chr3	32353200	0.661157
chr3	32355700	0.768595
chr3	32362700	0.983471
chr3	32367300	0.702479
chr3	32389300	0.826446
chr3	32391700	0.652893
chr3	32401600	0.694215
chr3	32443400	0.776860
chr3	32449000	0.884298
chr3	32452200	0.661157
chr3	32456000	0.752066
chr3	32462100	0.859504
chr3	32489800	0.694215
chr3	32501900	0.933884
chr3	32504500	0.652893
chr3	32509100	0.685950
chr3	32545800	0.669421
chr3	32634200	0.818182
chr3	32637000	0.743802
chr3	32776500	0.818182
chr3	32869800	0.685950
chr3	32879900	0.793388
chr3	32888300	0.661157
chr3	32893800	0.867769
chr3	32901200	0.735537
chr3	32903900	0.826446
chr3	32906500	0.826446
chr3	32909200	0.809917
chr3	32917400	0.702479
chr3	32923200	0.727273
chr3	32928100	0.826446
chr3	32989200	0.644628
chr3	33047100	0.859504
chr3	33173900	0.652893
chr3	33419500	0.677686
chr3	33467000	0.925620
chr3	33469200	0.760331
chr3	33472200	0.694215
chr3	33618700	0.834711
chr3	33831100	0.826446
chr3	34187400	0.702479
chr3	34225700	0.793388

chr3	34255100	0.776860
chr3	34296500	0.661157
chr3	34300200	0.785124
chr3	34302500	0.776860
chr3	34342200	0.677686
chr3	34907000	0.884298
chr3	34922000	0.685950
chr3	35638100	0.719008
chr3	35786200	0.809917
chr3	36361600	0.801653
chr3	36405500	0.776860
chr3	36424200	0.677686
chr3	36811600	0.677686
chr3	36869500	0.694215
chr3	36974800	0.958678
chr3	36979700	0.785124
chr3	37007400	0.685950
chr3	37031700	0.710744
chr3	37313000	0.785124
chr3	37499600	0.702479
chr3	37600800	0.776860
chr3	37709700	0.785124
chr3	37760500	0.694215
chr3	37808600	0.644628
chr3	37848300	0.702479
chr3	37906000	0.644628
chr3	37940800	0.909091
chr3	37947100	0.809917
chr3	38004100	0.694215
chr3	38015700	0.685950
chr3	38033000	0.900826
chr3	38045500	0.735537
chr3	38141000	0.785124
chr3	38215500	0.652893
chr3	38499600	0.859504
chr3	38561500	0.950413
chr3	38574100	0.719008
chr3	38658400	0.644628
chr3	38678600	0.743802
chr3	38682900	0.892562
chr3	38688400	0.644628
chr3	38700600	0.785124
chr3	38704400	0.884298
chr3	38762900	0.785124
chr3	38878600	0.752066
chr3	39032400	0.834711
chr3	39169100	0.669421
chr3	39188800	0.884298
chr3	39201100	0.768595
chr3	39212500	0.661157
chr3	39253000	0.768595
chr3	39389200	0.867769
chr3	39445700	0.719008
chr3	39451400	0.867769

chr3	39489700	0.727273
chr3	39547700	0.801653
chr3	39584900	0.677686
chr3	39618900	0.743802
chr3	39911700	0.735537
chr3	40004500	0.661157
chr3	40079500	0.652893
chr3	40370200	1.000000
chr3	40627100	0.677686
chr3	40654300	0.925620
chr3	40889700	0.743802
chr3	40967300	0.785124
chr3	41038400	0.785124
chr3	41117600	0.809917
chr3	41143200	0.785124
chr3	41173200	0.677686
chr3	41200600	0.694215
chr3	41246200	0.768595
chr3	41255200	0.776860
chr3	41258700	0.652893
chr3	41319700	0.760331
chr3	41363400	0.760331
chr3	41372400	0.851240
chr3	41404900	0.900826
chr3	41412600	0.867769
chr3	41492800	0.661157
chr3	41521000	0.851240
chr3	41611300	0.710744
chr3	41619100	0.826446
chr3	41722800	0.685950
chr3	41752800	0.801653
chr3	41836300	0.991736
chr3	41930700	0.644628
chr3	42067200	0.876033
chr3	42074200	0.859504
chr3	42084800	0.685950
chr3	42094300	0.909091
chr3	42099400	0.867769
chr3	42105100	0.991736
chr3	42108600	0.925620
chr3	42113500	0.884298
chr3	42122600	0.735537
chr3	42132800	0.644628
chr3	42164900	0.735537
chr3	42204100	0.793388
chr3	42221100	0.809917
chr3	42411100	0.942149
chr3	42489900	0.760331
chr3	42538900	0.752066
chr3	42549400	0.677686
chr3	42577800	0.760331
chr3	42673000	0.876033
chr3	42689500	0.851240
chr3	42697800	0.710744

chr3	42731100	0.652893
chr3	42777600	0.735537
chr3	42873000	0.834711
chr3	42932800	0.933884
chr3	43023000	0.760331
chr3	43030300	0.727273
chr3	43033600	0.966942
chr3	43066700	0.776860
chr3	43204000	0.818182
chr3	43293400	0.669421
chr3	43385700	0.793388
chr3	43403000	0.760331
chr3	43414900	0.933884
chr3	43428500	0.876033
chr3	43477700	0.809917
chr3	43520300	0.818182
chr3	43522600	0.677686
chr3	43578400	0.867769
chr3	43581900	0.677686
chr3	43627400	0.710744
chr3	43680700	0.867769
chr3	43738400	0.925620
chr3	43814900	0.702479
chr3	43897500	0.685950
chr3	43900200	0.884298
chr3	43914700	0.719008
chr3	43921000	0.809917
chr3	43934200	0.851240
chr3	43965600	0.801653
chr3	44009200	0.826446
chr3	44013000	0.661157
chr3	44020600	0.867769
chr3	44061400	0.760331
chr3	44090200	0.669421
chr3	44100000	0.892562
chr3	44103900	0.884298
chr3	44107400	0.826446
chr3	44118500	0.727273
chr3	44140400	0.768595
chr3	44170000	0.900826
chr3	44226500	0.752066
chr3	44401200	0.768595
chr3	44941000	0.851240
chr3	45035200	0.933884
chr3	45080100	0.685950
chr3	45102300	0.727273
chr3	45180600	0.884298
chr3	45225200	0.719008
chr3	45249200	0.669421
chr3	45314600	0.685950
chr3	45317300	0.719008
chr3	45355400	0.876033
chr3	45361100	0.644628
chr3	45379800	0.752066

chr3	45468300	0.694215
chr3	45483800	0.644628
chr3	45504900	0.892562
chr3	45507000	0.743802
chr3	45510600	0.760331
chr3	45551900	0.876033
chr3	45595800	0.685950
chr3	45609300	0.768595
chr3	45614600	0.909091
chr3	45617500	0.652893
chr3	45620700	0.652893
chr3	45637900	0.834711
chr3	45678300	0.793388
chr3	45714800	0.727273
chr3	45718000	0.735537
chr3	45720500	0.925620
chr3	45726500	0.991736
chr3	45735800	0.834711
chr3	45742700	0.991736
chr3	45789300	0.884298
chr3	45914200	0.677686
chr3	46034500	0.851240
chr3	46120700	0.685950
chr3	46417400	0.644628
chr3	46422700	0.661157
chr3	46547900	0.694215
chr3	46587600	1.000000
chr3	46600000	0.925620
chr3	46605600	0.702479
chr3	46615000	0.950413
chr3	46641000	0.958678
chr3	46651500	0.917355
chr3	46671300	0.917355
chr3	46698500	0.884298
chr3	46704700	0.752066
chr3	46905100	0.652893
chr3	46930400	0.900826
chr3	46940600	0.743802
chr3	46977400	0.991736
chr3	46985400	0.644628
chr3	46995300	0.743802
chr3	47002400	0.735537
chr3	47023700	0.785124
chr3	47029800	0.669421
chr3	47059000	0.793388
chr3	47194200	0.702479
chr3	47292600	0.669421
chr3	47413200	0.785124
chr3	47485600	0.876033
chr3	47514800	0.710744
chr3	47523900	0.851240
chr3	47563200	0.677686
chr3	47596100	0.644628
chr3	47883500	0.677686

chr3	47917000	0.801653
chr3	47932100	0.942149
chr3	47934700	0.809917
chr3	48003700	0.727273
chr3	48149100	0.694215
chr3	48167300	0.661157
chr3	48238700	0.727273
chr3	48244800	0.652893
chr3	48318500	0.876033
chr3	48320900	0.760331
chr3	48412000	0.958678
chr3	48467600	0.702479
chr3	48473700	0.909091
chr3	48477800	0.719008
chr3	48483600	0.669421
chr3	48507200	0.991736
chr3	48511400	0.933884
chr3	48516600	0.909091
chr3	48598600	0.809917
chr3	48601400	0.785124
chr3	48633900	0.694215
chr3	48661800	0.694215
chr3	48693900	0.661157
chr3	48703000	0.793388
chr3	48732400	0.966942
chr3	48740800	0.694215
chr3	48746900	0.685950
chr3	49000700	0.785124
chr3	49037800	0.867769
chr3	49211600	0.842975
chr3	49454900	0.859504
chr3	49492800	0.851240
chr3	49497300	0.958678
chr3	49500400	0.735537
chr3	49572800	0.966942
chr3	49608000	0.925620
chr3	49654000	0.694215
chr3	49742000	0.909091
chr3	49844600	0.661157
chr3	49902200	0.892562
chr3	49918600	0.859504
chr3	49941400	0.876033
chr3	49944100	0.859504
chr3	49946600	0.851240
chr3	49949700	0.661157
chr3	50005000	0.702479
chr3	50163900	0.752066
chr3	50178100	0.801653
chr3	50189000	0.719008
chr3	50196600	0.727273
chr3	50205500	0.966942
chr3	50232800	0.933884
chr3	50249100	0.661157
chr3	50280900	0.909091

chr3	50284900	0.785124
chr3	50302500	0.991736
chr3	50311200	0.801653
chr3	50341000	0.801653
chr3	50356000	0.842975
chr3	50361800	0.677686
chr3	50394900	0.644628
chr3	50412900	0.719008
chr3	50426100	0.652893
chr3	50456600	0.694215
chr3	50487900	0.685950
chr3	50492100	0.966942
chr3	50494200	0.710744
chr3	50510100	0.661157
chr3	50519000	0.669421
chr3	50531300	0.702479
chr3	50535000	0.876033
chr3	50553500	0.826446
chr3	50556600	0.677686
chr3	50559100	0.958678
chr3	50563200	0.735537
chr3	50568200	0.818182
chr3	50571600	0.710744
chr3	50577000	0.925620
chr3	50663000	0.867769
chr3	50674500	0.719008
chr3	50679400	0.652893
chr3	50689800	0.818182
chr3	50775500	0.983471
chr3	50787600	0.735537
chr3	50844200	0.851240
chr3	50928900	0.735537
chr3	50950700	0.685950
chr3	50962200	0.818182
chr3	50964600	0.884298
chr3	51378500	0.818182
chr3	51427000	0.884298
chr3	51431700	0.743802
chr3	51434800	0.859504
chr3	51614600	0.719008
chr3	51716000	0.661157
chr3	51737600	0.776860
chr3	51768900	0.785124
chr3	51787800	0.694215
chr3	51807700	0.925620
chr3	51833100	0.727273
chr3	51836500	0.950413
chr3	51879800	0.677686
chr3	51998400	0.752066
chr3	52032300	0.661157
chr3	52036700	0.776860
chr3	52040000	0.851240
chr3	52079800	0.719008
chr3	52201700	0.694215

chr3	52255400	0.652893
chr3	52407400	0.859504
chr3	52457500	0.776860
chr3	52478700	0.694215
chr3	52485000	0.958678
chr3	52488100	0.793388
chr3	52528300	0.768595
chr3	52573400	0.661157
chr3	52853900	0.743802
chr3	52869500	0.685950
chr3	52907600	0.752066
chr3	52909900	0.842975
chr3	53065500	0.876033
chr3	53190700	0.644628
chr3	53202200	0.727273
chr3	53206200	0.892562
chr3	53217500	0.694215
chr3	53228200	0.785124
chr3	53230800	0.842975
chr3	53254200	0.752066
chr3	53270500	0.876033
chr3	53272900	0.752066
chr3	53278200	0.793388
chr3	53282000	0.661157
chr3	53286400	0.818182
chr3	53293300	0.950413
chr3	53304400	0.661157
chr3	53322000	0.685950
chr3	53566400	0.743802
chr3	53651100	0.950413
chr3	53778500	0.785124
chr3	53783000	0.801653
chr3	53803200	0.743802
chr3	53808600	0.644628
chr3	53877400	0.909091
chr3	54043000	0.677686
chr3	54076900	0.669421
chr3	54113800	0.785124
chr3	54181000	0.685950
chr3	54203000	0.809917
chr3	54220400	0.752066
chr3	54227200	0.677686
chr3	54270700	0.735537
chr3	54379200	0.661157
chr3	54383800	0.752066
chr3	54634600	0.768595
chr3	54667900	0.909091
chr3	54675500	0.661157
chr3	54678700	0.727273
chr3	54704800	0.876033
chr3	54725700	0.677686
chr3	54732300	0.752066
chr3	54855800	0.702479
chr3	54892600	0.752066

chr3	54970900	0.669421
chr3	54988800	0.909091
chr3	55033700	0.685950
chr3	55040700	0.685950
chr3	55223800	0.661157
chr3	55317400	0.793388
chr3	55326800	0.661157
chr3	55546300	0.685950
chr3	55556400	0.950413
chr3	55566100	0.669421
chr3	55572700	0.834711
chr3	55574900	0.677686
chr3	55588900	0.859504
chr3	55591000	0.694215
chr3	55606100	0.950413
chr3	55627000	0.909091
chr3	55638700	0.644628
chr3	55650000	0.958678
chr3	55673900	0.942149
chr3	55680800	0.677686
chr3	55683500	0.859504
chr3	55692900	0.685950
chr3	55698300	0.793388
chr3	55724500	0.776860
chr3	55733500	0.719008
chr3	55790400	0.652893
chr3	55916100	0.867769
chr3	55922900	0.727273
chr3	56036300	0.661157
chr3	56086900	0.793388
chr3	56150100	0.983471
chr3	56157300	0.900826
chr3	56165400	0.793388
chr3	56230500	0.652893
chr3	56239500	0.652893
chr3	56289400	0.834711
chr3	56319500	0.661157
chr3	56337600	0.652893
chr3	56358200	0.727273
chr3	56448000	0.669421
chr3	56511400	0.694215
chr3	56560900	0.735537
chr3	56857900	0.925620
chr3	56950600	0.876033
chr3	56963400	0.793388
chr3	57023800	0.809917
chr3	57051400	0.884298
chr3	57095300	0.884298
chr3	57144400	0.851240
chr3	57150100	0.644628
chr3	57160500	0.851240
chr3	57163000	0.669421
chr3	57165200	1.000000
chr3	57177100	0.727273

chr3	57186400	0.801653
chr3	57204600	0.644628
chr3	57235400	0.685950
chr3	57530400	0.801653
chr3	57645800	0.859504
chr3	57774500	0.735537
chr3	57813100	0.752066
chr3	57938100	0.801653
chr3	57955900	0.983471
chr3	58005600	0.785124
chr3	58007700	0.867769
chr3	58013800	0.909091
chr3	58021300	0.950413
chr3	58025400	0.818182
chr3	58028000	0.909091
chr3	58030300	0.933884
chr3	58035100	0.991736
chr3	58045400	0.917355
chr3	58048800	0.694215
chr3	58051100	0.966942
chr3	58151100	0.760331
chr3	58159400	0.818182
chr3	58161500	0.661157
chr3	58175100	0.677686
chr3	58203500	0.644628
chr3	58307500	0.867769
chr3	58310100	0.768595
chr3	58380800	0.727273
chr3	58390300	0.768595
chr3	58398900	0.818182
chr3	58421200	0.743802
chr3	58427700	0.685950
chr3	58449500	0.842975
chr3	58457000	0.669421
chr3	58483300	0.867769
chr3	58508400	0.892562
chr3	58512400	0.644628
chr3	58543300	0.768595
chr3	58547700	0.669421
chr3	58567300	0.876033
chr3	58571400	0.661157
chr3	58615800	0.669421
chr3	58623400	0.793388
chr3	58851900	0.661157
chr3	59115500	0.933884
chr3	59136400	0.900826
chr3	59172100	0.710744
chr3	59391100	0.685950
chr3	59703800	0.768595
chr3	59799200	0.710744
chr3	59804000	0.801653
chr3	59954700	0.694215
chr3	59984400	0.785124
chr3	59996500	0.793388

chr3	60106000	0.677686
chr3	60302300	0.727273
chr3	60535200	0.743802
chr3	60541800	0.950413
chr3	60559200	0.702479
chr3	60595600	0.727273
chr3	60655600	0.975207
chr3	60729600	0.760331
chr3	60756900	0.842975
chr3	61067100	0.975207
chr3	61127600	0.826446
chr3	61318300	0.834711
chr3	61523400	0.983471
chr3	61557100	0.644628
chr3	61564700	0.809917
chr3	61572500	0.669421
chr3	61578900	0.719008
chr3	61607900	0.652893
chr3	61612100	0.760331
chr3	61615600	0.644628
chr3	61634100	0.652893
chr3	61649800	0.652893
chr3	61655500	0.694215
chr3	61670400	0.842975
chr3	61674500	0.925620
chr3	61678400	0.735537
chr3	61683100	0.975207
chr3	61685700	1.000000
chr3	61688000	0.991736
chr3	61691000	0.842975
chr3	61694400	0.727273
chr3	61705100	0.942149
chr3	61716500	0.809917
chr3	61754800	0.727273
chr3	61764400	0.669421
chr3	61769500	0.702479
chr3	61782300	0.826446
chr3	61798200	0.694215
chr3	61814800	0.917355
chr3	61833600	0.975207
chr3	61854300	0.669421
chr3	61857800	0.942149
chr3	61861000	0.991736
chr3	61880900	0.793388
chr3	61889800	0.652893
chr3	61897600	0.785124
chr3	61904800	0.917355
chr3	61917300	0.685950
chr3	61922800	0.710744
chr3	61930500	0.966942
chr3	61932800	0.900826
chr3	61938900	0.735537
chr3	61951800	0.752066
chr3	61958200	0.661157

chr3	62154900	0.652893
chr3	62175300	0.859504
chr3	62194300	0.661157
chr3	62215000	0.685950
chr3	62228600	0.851240
chr3	62231500	0.685950
chr3	62234600	0.694215
chr3	62246800	0.719008
chr3	62321100	0.776860
chr3	62361500	0.809917
chr3	62365800	0.710744
chr3	62401100	0.669421
chr3	62425500	0.694215
chr3	62431500	0.809917
chr3	62441300	0.735537
chr3	62525000	0.942149
chr3	62541800	0.826446
chr3	62665000	0.867769
chr3	62694700	0.743802
chr3	62697000	0.694215
chr3	62704700	0.735537
chr3	62710000	0.809917
chr3	62781900	0.644628
chr3	62785800	0.652893
chr3	62870900	0.991736
chr3	62905900	0.702479
chr3	62926800	0.685950
chr3	62981100	0.743802
chr3	63001700	0.900826
chr3	63062300	0.652893
chr3	63081000	0.702479
chr3	63116900	0.876033
chr3	63121400	0.942149
chr3	63213900	0.776860
chr3	63296100	0.710744
chr3	63330200	0.743802
chr3	63398900	0.809917
chr3	63428200	0.727273
chr3	63430300	0.950413
chr3	63509100	0.776860
chr3	63513400	0.851240
chr3	63563100	0.834711
chr3	63611700	0.727273
chr3	63631000	0.677686
chr3	63711200	0.809917
chr3	63800200	0.719008
chr3	63847300	0.727273
chr3	63864000	0.859504
chr3	63923600	0.735537
chr3	63942200	0.842975
chr3	63946200	0.818182
chr3	63954200	0.760331
chr3	63958100	0.702479
chr3	63979500	0.719008

chr3	63986000	0.785124
chr3	64015100	0.669421
chr3	64035500	0.834711
chr3	64049700	0.818182
chr3	64069300	0.677686
chr3	64072700	0.900826
chr3	64080700	0.942149
chr3	64092900	0.752066
chr3	64095700	0.793388
chr3	64143500	1.000000
chr3	64148900	0.809917
chr3	64175200	0.727273
chr3	64202000	0.702479
chr3	64211400	0.694215
chr3	64225000	0.983471
chr3	64264500	0.710744
chr3	64330700	0.685950
chr3	64403900	0.743802
chr3	64428400	0.859504
chr3	64475300	0.818182
chr3	64477600	0.867769
chr3	64519600	0.719008
chr3	64573200	0.710744
chr3	64628400	0.685950
chr3	64666800	0.669421
chr3	64669000	0.752066
chr3	64680200	0.735537
chr3	64711600	0.669421
chr3	64734500	0.917355
chr3	64782600	0.644628
chr3	64896200	0.785124
chr3	64902900	0.876033
chr3	64931200	0.801653
chr3	64978900	0.867769
chr3	64981700	0.900826
chr3	65065200	0.818182
chr3	65132300	0.942149
chr3	65182300	0.735537
chr3	65330700	0.661157
chr3	65376900	0.727273
chr3	65388600	0.669421
chr3	65394600	0.735537
chr3	65464800	0.942149
chr3	65470000	0.876033
chr3	65495000	0.892562
chr3	65526100	0.925620
chr3	65538000	0.710744
chr3	65573800	0.818182
chr3	65577200	0.834711
chr3	65605400	0.892562
chr3	65644700	0.685950
chr3	65699500	0.834711
chr3	65702300	0.776860
chr3	65707300	0.876033

chr3	65718300	0.694215
chr3	65734000	0.735537
chr3	65736700	0.760331
chr3	65748100	0.892562
chr3	65801500	0.842975
chr3	65880400	0.801653
chr3	65888100	0.768595
chr3	65910800	0.685950
chr3	65936100	0.909091
chr3	65939600	0.991736
chr3	65950500	0.933884
chr3	65996800	0.710744
chr3	66008600	0.702479
chr3	66019600	0.685950
chr3	66041300	0.743802
chr3	66119900	0.876033
chr3	66288300	0.644628
chr3	66396000	0.652893
chr3	66460400	0.826446
chr3	66479300	0.702479
chr3	66482700	0.818182
chr3	66489200	0.884298
chr3	66495300	0.702479
chr3	66504500	0.685950
chr3	66515200	0.661157
chr3	66519200	0.702479
chr3	66521700	0.884298
chr3	66527400	0.851240
chr3	66529800	0.809917
chr3	66535300	0.867769
chr3	66537700	0.917355
chr3	66543700	0.719008
chr3	66580700	0.851240
chr3	66582800	0.917355
chr3	66603600	0.876033
chr3	66623500	0.900826
chr3	66643300	0.925620
chr3	66660800	0.768595
chr3	66680100	0.694215
chr3	66763200	0.685950
chr3	66846300	0.809917
chr3	66849300	0.884298
chr3	66909600	0.752066
chr3	66981400	0.694215
chr3	66983900	0.900826
chr3	67022800	0.719008
chr3	67033500	0.661157
chr3	67039700	0.975207
chr3	67126200	0.702479
chr3	67249200	0.694215
chr3	67665300	0.652893
chr3	67680300	0.809917
chr3	67748400	0.809917
chr3	68054900	0.760331

chr3	68185300	0.710744
chr3	68316500	0.661157
chr3	68584100	0.884298
chr3	68681500	0.760331
chr3	68766400	0.661157
chr3	68814000	0.702479
chr3	68938500	0.876033
chr3	68968600	0.776860
chr3	68974600	0.669421
chr3	69059000	1.000000
chr3	69231300	0.661157
chr3	69250200	0.867769
chr3	69286500	0.735537
chr3	69290400	0.644628
chr3	69292800	0.834711
chr3	69298800	0.743802
chr3	69337700	0.876033
chr3	69342100	0.776860
chr3	69442100	0.743802
chr3	69457100	0.735537
chr3	69732500	0.735537
chr3	69768900	0.884298
chr3	69942400	0.752066
chr3	70115200	0.710744
chr3	70125800	0.644628
chr3	70232100	0.685950
chr3	70374800	0.702479
chr3	70526100	0.900826
chr3	70569700	0.661157
chr3	70582100	0.818182
chr3	70664800	0.991736
chr3	70882700	0.776860
chr3	70903800	0.760331
chr3	71012900	0.727273
chr3	71020900	0.752066
chr3	71032000	0.983471
chr3	71057300	0.752066
chr3	71072100	0.760331
chr3	71100100	0.892562
chr3	71102900	0.801653
chr3	71106700	0.768595
chr3	71111500	0.694215
chr3	71137000	0.776860
chr3	71179700	0.950413
chr3	71186700	0.900826
chr3	71191900	0.743802
chr3	71201600	0.702479
chr3	71205800	0.834711
chr3	71258500	0.776860
chr3	71293300	0.652893
chr3	71296600	0.892562
chr3	71349100	0.900826
chr3	71421900	0.735537
chr3	71434200	0.876033

chr3	71439700	0.917355
chr3	71463700	0.694215
chr3	71518000	0.983471
chr3	71545300	0.884298
chr3	71548000	0.909091
chr3	71555800	0.785124
chr3	71570000	0.719008
chr3	71592100	0.727273
chr3	71702900	0.900826
chr3	71721600	0.917355
chr3	71730600	0.661157
chr3	71740000	0.719008
chr3	71779100	0.966942
chr3	71805500	0.694215
chr3	71820900	0.710744
chr3	71829600	0.884298
chr3	71849000	0.719008
chr3	71851600	0.752066
chr3	71925500	0.735537
chr3	71959500	0.677686
chr3	71976200	0.710744
chr3	72005000	0.743802
chr3	72052000	0.884298
chr3	72078400	0.735537
chr3	72308200	0.842975
chr3	72348400	0.719008
chr3	72353600	0.661157
chr3	72384600	0.826446
chr3	72418900	0.694215
chr3	72424400	0.677686
chr3	72427100	0.661157
chr3	72430000	0.876033
chr3	72432100	0.867769
chr3	72440700	0.661157
chr3	72446500	0.694215
chr3	72453500	0.710744
chr3	72457000	0.677686
chr3	72487900	0.652893
chr3	72537300	0.727273
chr3	72585600	0.884298
chr3	72610500	0.892562
chr3	72631700	1.000000
chr3	72640700	0.719008
chr3	72677600	0.727273
chr3	72704800	0.694215
chr3	72747500	0.942149
chr3	72816200	0.925620
chr3	72859800	0.950413
chr3	73074300	0.743802
chr3	73120400	0.826446
chr3	73144400	0.719008
chr3	73225200	0.661157
chr3	73250300	0.702479
chr3	73274900	0.719008

chr3	73532700	0.677686
chr3	73535900	0.644628
chr3	73540100	0.917355
chr3	73560900	0.859504
chr3	73608500	0.776860
chr3	73671300	0.776860
chr3	73718000	0.917355
chr3	73734000	0.685950
chr3	73776600	0.644628
chr3	73812700	1.000000
chr3	73816800	0.818182
chr3	73924100	0.834711
chr3	74060500	0.909091
chr3	74104700	0.669421
chr3	74116000	0.818182
chr3	74452400	0.727273
chr3	75151200	0.966942
chr3	75304400	0.942149
chr3	75868400	0.834711
chr3	76088200	0.917355
chr3	76093200	0.991736
chr3	76247800	0.752066
chr3	76292400	0.727273
chr3	76402600	0.760331
chr3	76611500	0.652893
chr3	76711300	0.909091
chr3	76752100	0.776860
chr3	77063900	0.785124
chr3	77101900	0.719008
chr3	77129500	0.983471
chr3	77197500	0.785124
chr3	77331300	0.644628
chr3	77398400	0.685950
chr3	77525500	0.917355
chr3	77735100	0.768595
chr3	77756900	0.785124
chr3	78084900	0.776860
chr3	78372400	0.644628
chr3	78387300	0.752066
chr3	78397000	0.677686
chr3	78921700	0.661157
chr3	79028400	0.727273
chr3	79043900	0.909091
chr3	79062400	0.826446
chr3	79115900	0.644628
chr3	79137400	0.685950
chr3	79228300	0.826446
chr3	79249700	0.867769
chr3	79252600	0.752066
chr3	79295100	0.884298
chr3	79322000	0.702479
chr3	79356200	0.661157
chr3	79375500	0.983471
chr3	79721600	0.719008

chr3	81675600	0.685950
chr3	81941000	0.685950
chr3	82023800	0.694215
chr3	82055100	0.719008
chr3	82473000	0.884298
chr3	82478400	0.842975
chr3	83563700	0.892562
chr3	83617300	0.719008
chr3	84597400	0.685950
chr3	84650800	0.917355
chr3	84886200	0.909091
chr3	85017300	0.900826
chr3	85131300	0.652893
chr3	85142500	0.859504
chr3	85676800	0.652893
chr3	85939500	0.851240
chr3	85953200	0.694215
chr3	86224900	0.975207
chr3	86388700	0.760331
chr3	86457900	0.694215
chr3	86597300	0.834711
chr3	86611800	0.677686
chr3	86864400	0.793388
chr3	87008100	0.719008
chr3	87665000	0.809917
chr3	87776900	0.826446
chr3	88268800	0.694215
chr3	88961800	0.966942
chr3	89221200	0.694215
chr3	89295600	0.760331
chr3	89317400	0.661157
chr3	89327800	0.925620
chr3	89381500	0.669421
chr3	89786900	0.694215
chr3	89900800	0.644628
chr3	89939000	0.876033
chr3	90086800	0.752066
chr3	90119100	0.727273
chr3	90131100	0.760331
chr3	90448100	0.661157
chr3	90477900	0.793388
chr3	93850000	0.727273
chr3	93865800	0.719008
chr3	94093400	0.652893
chr3	95039000	0.809917
chr3	95068500	0.743802
chr3	95073800	0.743802
chr3	95434600	0.661157
chr3	95648000	0.776860
chr3	96168800	0.677686
chr3	96337000	0.909091
chr3	96523200	0.677686
chr3	96845200	0.842975
chr3	96986700	0.925620

chr3	96988900	0.793388
chr3	97152300	0.710744
chr3	97158700	0.801653
chr3	97381500	0.735537
chr3	97406700	0.661157
chr3	97712500	0.719008
chr3	97901800	0.644628
chr3	97960600	0.677686
chr3	98048100	0.834711
chr3	98343200	0.644628
chr3	98604400	0.768595
chr3	98705300	0.702479
chr3	99013900	0.727273
chr3	99249300	0.685950
chr3	99573800	0.851240
chr3	99615600	0.892562
chr3	99639900	0.785124
chr3	99647100	0.950413
chr3	99649400	0.685950
chr3	99664200	0.735537
chr3	99725100	0.851240
chr3	99766900	0.818182
chr3	99772900	0.719008
chr3	99792800	0.776860
chr3	99807500	0.710744
chr3	99930100	0.975207
chr3	100170500	0.677686
chr3	100280900	0.801653
chr3	100384600	0.661157
chr3	100452600	0.694215
chr3	100455900	0.884298
chr3	100473100	0.859504
chr3	100489000	0.685950
chr3	100967900	0.685950
chr3	100990900	0.818182
chr3	101102400	0.818182
chr3	101185900	0.652893
chr3	101249700	0.958678
chr3	101343900	0.702479
chr3	101500400	0.909091
chr3	101548600	0.793388
chr3	101560900	0.933884
chr3	101902600	0.818182
chr3	101956900	0.760331
chr3	102333400	0.669421
chr3	102406600	0.892562
chr3	102468800	0.669421
chr3	102644600	0.818182
chr3	102729000	0.652893
chr3	102831500	0.661157
chr3	103538400	0.727273
chr3	103895000	0.669421
chr3	104158400	0.867769
chr3	104233500	0.702479

chr3	104238900	0.950413
chr3	104564400	0.694215
chr3	104574800	0.826446
chr3	104604400	0.661157
chr3	104628400	0.652893
chr3	104680700	0.925620
chr3	104733100	0.710744
chr3	104875900	0.900826
chr3	104972900	0.677686
chr3	105331600	0.644628
chr3	105517400	0.727273
chr3	105520900	0.975207
chr3	105597400	0.694215
chr3	105850200	0.661157
chr3	106028500	0.801653
chr3	106115900	0.685950
chr3	106118700	0.694215
chr3	106212900	0.685950
chr3	106545000	0.685950
chr3	106547900	0.991736
chr3	106550000	0.702479
chr3	106599100	0.760331
chr3	106632500	0.834711
chr3	106783600	0.818182
chr3	107003300	0.661157
chr3	107086500	0.876033
chr3	107104300	0.900826
chr3	107298000	0.975207
chr3	107317800	0.983471
chr3	107362400	0.685950
chr3	107380900	0.694215
chr3	107384500	0.818182
chr3	107718100	0.685950
chr3	107734300	0.652893
chr3	108108300	0.702479
chr3	108204800	0.710744
chr3	108383800	0.685950
chr3	108766700	0.785124
chr3	108953600	0.702479
chr3	108994900	0.669421
chr3	109071700	0.884298
chr3	109076500	0.867769
chr3	109550600	0.743802
chr3	109884800	0.851240
chr3	109991600	0.735537
chr3	110153400	0.685950
chr3	110246900	0.710744
chr3	110296300	0.826446
chr3	110302800	0.661157
chr3	110318000	0.983471
chr3	110403100	0.966942
chr3	110405400	0.818182
chr3	110429800	0.966942
chr3	110462400	0.652893

chr3	110663500	0.702479
chr3	110683800	0.652893
chr3	110754600	0.685950
chr3	110765600	0.892562
chr3	110800000	0.735537
chr3	110804500	0.710744
chr3	110807500	0.826446
chr3	110825700	0.669421
chr3	110848000	0.710744
chr3	111372000	0.900826
chr3	111470800	0.818182
chr3	111581900	0.702479
chr3	111593800	0.661157
chr3	111635900	0.867769
chr3	111639200	0.652893
chr3	111673200	0.702479
chr3	111676800	0.884298
chr3	111811700	0.735537
chr3	111845100	0.834711
chr3	111856300	0.975207
chr3	112013000	0.801653
chr3	112033700	0.842975
chr3	112038400	0.677686
chr3	112041300	0.900826
chr3	112055300	0.983471
chr3	112070900	0.685950
chr3	112074200	0.975207
chr3	112110400	0.892562
chr3	112118900	0.900826
chr3	112137100	0.826446
chr3	112352300	0.950413
chr3	112360100	0.818182
chr3	112362800	0.768595
chr3	112366400	0.909091
chr3	112395900	0.710744
chr3	112405700	0.661157
chr3	112470000	0.644628
chr3	112822500	0.685950
chr3	112824700	0.950413
chr3	112865000	0.685950
chr3	112885900	0.884298
chr3	112894300	0.661157
chr3	112942600	0.809917
chr3	112948100	0.842975
chr3	112961000	1.000000
chr3	112966500	0.702479
chr3	112983500	0.958678
chr3	112994900	0.851240
chr3	113160600	0.677686
chr3	113304800	0.710744
chr3	113326200	0.884298
chr3	113405200	0.983471
chr3	113451100	0.818182
chr3	113472400	0.900826

chr3	113822500	0.743802
chr3	113874500	0.842975
chr3	113896200	0.735537
chr3	113933700	0.867769
chr3	114037700	0.876033
chr3	114052400	0.983471
chr3	114075300	0.793388
chr3	114096500	0.652893
chr3	114261200	0.644628
chr3	114290700	0.685950
chr3	114392400	0.743802
chr3	114420100	0.826446
chr3	114433800	0.644628
chr3	114576300	0.727273
chr3	114622100	0.867769
chr3	114931400	0.909091
chr3	115124000	0.694215
chr3	115155600	0.669421
chr3	115195000	0.760331
chr3	115273600	0.743802
chr3	115346000	0.702479
chr3	115485700	0.851240
chr3	115551000	0.735537
chr3	115565800	0.644628
chr3	115578600	0.950413
chr3	115582600	0.818182
chr3	115617700	0.644628
chr3	115705000	0.685950
chr3	115758700	0.776860
chr3	115837800	0.719008
chr3	115979100	0.752066
chr3	116005100	0.677686
chr3	116018600	0.702479
chr3	116057900	0.776860
chr3	116061900	0.719008
chr3	116108500	0.743802
chr3	116162100	0.644628
chr3	116504500	0.785124
chr3	116568000	0.942149
chr3	116572100	0.677686
chr3	116764500	0.801653
chr3	116800000	0.942149
chr3	117075200	0.785124
chr3	117077900	0.776860
chr3	117124400	0.776860
chr3	117159100	0.727273
chr3	117330100	0.661157
chr3	117407700	0.694215
chr3	117411200	0.661157
chr3	117416100	0.669421
chr3	117720400	0.669421
chr3	117829600	0.826446
chr3	117836500	0.809917
chr3	118198400	0.975207

chr3	118220600	0.677686
chr3	118347900	0.652893
chr3	118491600	0.793388
chr3	118627000	0.652893
chr3	118688700	0.719008
chr3	118707600	0.834711
chr3	118735400	0.685950
chr3	118839400	0.900826
chr3	119029500	0.710744
chr3	119068800	0.909091
chr3	119072600	0.702479
chr3	119079300	0.925620
chr3	119100300	0.702479
chr3	119115600	0.752066
chr3	119121100	0.851240
chr3	119308800	0.818182
chr3	119514400	0.743802
chr3	119723300	0.727273
chr3	119759300	0.801653
chr3	119896300	0.661157
chr3	120061200	0.669421
chr3	120136400	0.991736
chr3	120151600	0.900826
chr3	120155400	0.785124
chr3	120188900	0.991736
chr3	120194900	0.834711
chr3	120218100	0.644628
chr3	120223200	0.867769
chr3	120280300	0.719008
chr3	120286700	0.925620
chr3	120317700	0.727273
chr3	120396000	0.834711
chr3	120398200	0.752066
chr3	120450000	0.851240
chr3	120464200	0.669421
chr3	120483000	0.859504
chr3	120514100	0.743802
chr3	120550500	0.702479
chr3	120558200	0.710744
chr3	120565300	0.694215
chr3	120595500	0.785124
chr3	120782900	0.793388
chr3	121289600	0.677686
chr3	121313200	0.950413
chr3	121343700	0.719008
chr3	121362500	0.743802
chr3	121613400	0.776860
chr3	121615500	0.694215
chr3	121675600	0.644628
chr3	121715000	0.710744
chr3	122162400	0.917355
chr3	122188700	0.776860
chr3	122255400	0.685950
chr3	122279300	0.760331

chr3	122292400	0.842975
chr3	122458900	0.809917
chr3	122480000	0.958678
chr3	122496700	0.900826
chr3	122609600	0.752066
chr3	122613100	0.735537
chr3	122662400	0.719008
chr3	122666200	0.694215
chr3	122672200	0.685950
chr3	122675700	0.876033
chr3	122678200	0.859504
chr3	122693200	0.702479
chr3	122695300	0.685950
chr3	122697600	0.752066
chr3	122702900	0.925620
chr3	122707100	0.809917
chr3	122716600	0.818182
chr3	122725900	0.710744
chr3	122742400	0.752066
chr3	122765700	0.685950
chr3	122804500	0.677686
chr3	122818500	0.917355
chr3	122840600	0.743802
chr3	122862900	0.752066
chr3	122914100	0.669421
chr3	123029800	0.793388
chr3	123034900	0.900826
chr3	123039000	0.909091
chr3	123125500	0.644628
chr3	123129000	0.669421
chr3	123177000	0.743802
chr3	123354200	0.809917
chr3	123356500	0.652893
chr3	123371200	0.661157
chr3	123405600	0.702479
chr3	123414400	0.760331
chr3	123431300	0.743802
chr3	123486000	0.966942
chr3	123519100	0.719008
chr3	123600800	0.661157
chr3	123686400	0.719008
chr3	123877400	0.851240
chr3	123942800	0.710744
chr3	123949900	0.694215
chr3	123975400	0.669421
chr3	124017800	0.661157
chr3	124079800	0.900826
chr3	124232100	0.669421
chr3	124239700	0.702479
chr3	124312000	0.892562
chr3	124354300	0.768595
chr3	124357300	0.776860
chr3	124427000	0.644628
chr3	124471400	0.644628

chr3	124493500	0.958678
chr3	124499700	0.950413
chr3	124520100	0.900826
chr3	124546700	0.983471
chr3	124554300	0.785124
chr3	124578500	0.760331
chr3	124592700	0.719008
chr3	124595600	0.702479
chr3	124602500	0.876033
chr3	124608400	0.752066
chr3	124711900	0.719008
chr3	124715200	0.760331
chr3	124758400	0.743802
chr3	124762600	0.842975
chr3	124771400	0.826446
chr3	124795000	0.768595
chr3	124835500	0.917355
chr3	124863200	0.859504
chr3	124866800	0.661157
chr3	124875400	0.710744
chr3	124906600	0.752066
chr3	124911600	0.710744
chr3	124990800	0.677686
chr3	125154100	0.685950
chr3	125242800	0.685950
chr3	125310400	0.685950
chr3	125322100	0.661157
chr3	125331400	0.702479
chr3	125366900	0.685950
chr3	125555300	0.760331
chr3	125648200	0.661157
chr3	125741600	0.685950
chr3	125843300	0.876033
chr3	125852100	0.942149
chr3	126034700	0.826446
chr3	126099600	0.685950
chr3	126139100	0.685950
chr3	126159700	0.661157
chr3	126203900	0.743802
chr3	126218100	0.652893
chr3	126220700	0.710744
chr3	126268100	0.909091
chr3	126398600	0.776860
chr3	126403200	0.900826
chr3	126472200	0.925620
chr3	126503400	0.909091
chr3	126536100	0.735537
chr3	126568800	0.760331
chr3	126577600	0.677686
chr3	126614400	0.785124
chr3	126680000	0.809917
chr3	126691100	0.727273
chr3	126704900	0.752066
chr3	126718000	0.801653

chr3	126721400	0.933884
chr3	126723600	0.694215
chr3	126762100	0.801653
chr3	126764700	0.669421
chr3	126780700	0.661157
chr3	126851900	0.876033
chr3	126854800	0.892562
chr3	126878700	0.884298
chr3	127115600	0.942149
chr3	127142000	0.876033
chr3	127178300	0.727273
chr3	127186600	0.884298
chr3	127256100	0.851240
chr3	127294100	0.685950
chr3	127319600	0.685950
chr3	127363800	0.727273
chr3	127409200	0.752066
chr3	127416700	0.834711
chr3	127451200	0.768595
chr3	127725800	0.661157
chr3	127730400	0.801653
chr3	127814400	0.876033
chr3	127817300	0.975207
chr3	127819800	0.669421
chr3	127887100	0.776860
chr3	127893900	0.809917
chr3	127912400	0.834711
chr3	127915700	0.685950
chr3	127989600	0.950413
chr3	127996000	0.735537
chr3	127999900	0.826446
chr3	128002300	0.719008
chr3	128006400	0.917355
chr3	128068100	0.644628
chr3	128086600	0.669421
chr3	128132300	0.876033
chr3	128145400	0.644628
chr3	128154200	0.776860
chr3	128193200	0.818182
chr3	128198800	0.809917
chr3	128210600	0.677686
chr3	128212800	0.785124
chr3	128278800	0.644628
chr3	128287700	0.958678
chr3	128303300	0.801653
chr3	128382400	0.669421
chr3	128498600	0.694215
chr3	128507400	0.710744
chr3	128514000	0.661157
chr3	128522900	0.727273
chr3	128584000	0.867769
chr3	128594800	0.727273
chr3	128718100	0.818182
chr3	128722800	0.768595

chr3	128746200	0.710744
chr3	128826000	0.669421
chr3	128835800	0.826446
chr3	128948100	0.818182
chr3	129024400	0.818182
chr3	129071400	0.644628
chr3	129116000	0.743802
chr3	129144100	0.859504
chr3	129149000	0.661157
chr3	129167200	0.966942
chr3	129201400	0.900826
chr3	129205700	0.859504
chr3	129214500	0.652893
chr3	129245800	0.752066
chr3	129249400	0.752066
chr3	129258600	0.694215
chr3	129271900	0.685950
chr3	129277400	0.669421
chr3	129292200	0.694215
chr3	129312900	0.942149
chr3	129318100	0.752066
chr3	129331000	0.859504
chr3	129338100	0.644628
chr3	129382500	0.694215
chr3	129404000	0.760331
chr3	129408800	0.702479
chr3	129418500	0.694215
chr3	129420900	0.661157
chr3	129434300	0.809917
chr3	129438500	0.644628
chr3	129442400	0.842975
chr3	129452100	0.876033
chr3	129556400	0.768595
chr3	129609900	0.669421
chr3	129690500	0.710744
chr3	129813300	0.661157
chr3	130548400	0.793388
chr3	130550900	0.834711
chr3	130558800	0.942149
chr3	130616700	0.909091
chr3	130797000	0.818182
chr3	131071300	0.669421
chr3	131280700	0.652893
chr3	131287600	0.826446
chr3	131342600	0.743802
chr3	131388600	0.793388
chr3	131425000	0.752066
chr3	131430500	0.743802
chr3	131515900	0.884298
chr3	131632100	0.925620
chr3	131636700	0.876033
chr3	131676600	0.884298
chr3	131693800	0.694215
chr3	131748400	0.818182

chr3	131802900	0.652893
chr3	131899600	0.826446
chr3	131926200	0.710744
chr3	131945100	0.702479
chr3	132003900	0.958678
chr3	132036900	0.867769
chr3	132062200	0.694215
chr3	132070300	0.801653
chr3	132280200	0.719008
chr3	132450300	0.818182
chr3	132502600	0.735537
chr3	132563200	0.975207
chr3	132753700	0.818182
chr3	132760100	0.702479
chr3	132774200	0.760331
chr3	132911600	0.727273
chr3	132926200	0.760331
chr3	132930100	0.933884
chr3	132990300	0.669421
chr3	133048700	0.685950
chr3	133094200	0.694215
chr3	133124000	0.685950
chr3	133171800	0.743802
chr3	133199100	0.644628
chr3	133208900	0.743802
chr3	133291600	0.735537
chr3	133540800	0.801653
chr3	133661600	0.793388
chr3	133708000	0.876033
chr3	133750500	0.735537
chr3	133825300	0.685950
chr3	133955100	0.644628
chr3	133963500	0.826446
chr3	133971800	0.917355
chr3	133980300	0.958678
chr3	134027600	0.876033
chr3	134034800	0.719008
chr3	134045900	0.933884
chr3	134050500	0.661157
chr3	134077100	0.785124
chr3	134081500	0.859504
chr3	134101200	0.752066
chr3	134128200	0.809917
chr3	134139000	0.776860
chr3	134168500	0.785124
chr3	134183200	1.000000
chr3	134321200	0.818182
chr3	134337900	0.702479
chr3	134362100	0.768595
chr3	134383200	0.685950
chr3	134413500	0.644628
chr3	134517600	0.677686
chr3	134529000	0.834711
chr3	134570200	0.661157

chr3	134612000	0.752066
chr3	134624400	0.652893
chr3	134650400	0.694215
chr3	134656800	0.884298
chr3	134711000	0.677686
chr3	134769900	0.677686
chr3	134880600	0.710744
chr3	134936200	0.694215
chr3	134939400	0.661157
chr3	134950900	0.991736
chr3	134956700	0.694215
chr3	134978400	0.685950
chr3	135172000	0.801653
chr3	135222900	0.884298
chr3	135455900	0.661157
chr3	135458700	0.677686
chr3	135687400	0.652893
chr3	135716900	0.710744
chr3	135954900	0.785124
chr3	135996500	0.900826
chr3	136455900	0.702479
chr3	136468200	0.760331
chr3	136558200	0.867769
chr3	136612700	0.809917
chr3	136621200	0.685950
chr3	136626100	0.644628
chr3	136633700	0.669421
chr3	136754700	0.652893
chr3	136766300	0.785124
chr3	136785000	0.809917
chr3	136824900	0.785124
chr3	136828500	0.685950
chr3	136850300	0.685950
chr3	137301500	0.661157
chr3	137362000	0.735537
chr3	137366300	0.909091
chr3	137414400	0.743802
chr3	137481600	0.677686
chr3	137527600	0.793388
chr3	137564400	0.710744
chr3	137677600	0.702479
chr3	137766300	0.694215
chr3	137929700	0.685950
chr3	138128400	0.942149
chr3	138141700	0.644628
chr3	138186200	0.652893
chr3	138298600	0.644628
chr3	138543500	0.884298
chr3	138565000	0.983471
chr3	138580300	0.727273
chr3	138652600	0.892562
chr3	139039200	0.652893
chr3	139048500	0.818182
chr3	139174800	0.793388

chr3	139211000	0.859504
chr3	139243200	0.793388
chr3	139254000	0.884298
chr3	139373900	0.685950
chr3	139387500	0.752066
chr3	139394600	0.851240
chr3	139440700	0.776860
chr3	139493600	0.809917
chr3	139557700	0.669421
chr3	139812000	0.785124
chr3	139919800	0.669421
chr3	139923400	0.876033
chr3	139925600	0.859504
chr3	139970900	0.710744
chr3	139984700	0.719008
chr3	140018300	0.760331
chr3	140035200	0.661157
chr3	140051600	0.702479
chr3	140061000	1.000000
chr3	140071200	0.710744
chr3	140189100	0.685950
chr3	140224900	0.818182
chr3	140229400	0.867769
chr3	140241000	0.652893
chr3	140513500	0.785124
chr3	140542100	0.677686
chr3	140670700	0.669421
chr3	140764800	0.768595
chr3	140791100	0.818182
chr3	140793500	0.958678
chr3	140796500	0.719008
chr3	140802500	0.760331
chr3	140805100	0.785124
chr3	140811500	0.719008
chr3	140813900	0.644628
chr3	140822800	0.876033
chr3	140894000	0.719008
chr3	140985600	0.661157
chr3	141046000	0.727273
chr3	141053800	0.710744
chr3	141087200	0.892562
chr3	141094700	0.768595
chr3	141106200	0.735537
chr3	141112400	0.983471
chr3	141119200	0.685950
chr3	141131700	0.702479
chr3	141148600	0.776860
chr3	141209100	0.735537
chr3	141330000	0.785124
chr3	141338300	0.719008
chr3	141716300	0.677686
chr3	141784900	0.892562
chr3	141888000	0.702479
chr3	141929500	0.809917

chr3	141935600	0.702479
chr3	142572300	0.661157
chr3	142575900	0.685950
chr3	142582200	0.958678
chr3	142672400	0.743802
chr3	142701400	0.661157
chr3	142790500	0.710744
chr3	143097400	0.644628
chr3	143127700	0.809917
chr3	143149000	0.809917
chr3	143317300	0.867769
chr3	143320500	0.793388
chr3	143502000	0.644628
chr3	143514600	0.867769
chr3	143525500	0.661157
chr3	143567000	0.776860
chr3	143608000	0.909091
chr3	143750800	0.685950
chr3	143831700	0.727273
chr3	143940900	0.669421
chr3	144189000	0.677686
chr3	145275200	0.859504
chr3	145437300	0.735537
chr3	145516700	0.768595
chr3	145534400	0.752066
chr3	145852400	0.884298
chr3	145984100	0.735537
chr3	146241000	0.785124
chr3	146268300	0.652893
chr3	146499200	0.719008
chr3	147016600	0.743802
chr3	147060200	0.867769
chr3	147079400	0.735537
chr3	147090600	0.768595
chr3	147098700	0.842975
chr3	147102600	0.694215
chr3	147106800	0.685950
chr3	147119900	0.809917
chr3	147125100	0.652893
chr3	147450500	0.644628
chr3	147652900	0.892562
chr3	147657800	0.669421
chr3	147680500	0.900826
chr3	148437600	0.826446
chr3	148515900	0.652893
chr3	148674000	0.652893
chr3	148738500	0.975207
chr3	148833100	0.669421
chr3	148903900	0.694215
chr3	148911300	0.809917
chr3	148914400	0.677686
chr3	148917500	0.867769
chr3	149051300	0.950413
chr3	149058300	0.661157

chr3	149088400	1.000000
chr3	149095700	0.694215
chr3	149254800	0.867769
chr3	149281100	0.859504
chr3	149322500	0.776860
chr3	149337200	0.652893
chr3	149371800	0.652893
chr3	149388700	0.793388
chr3	149425400	0.842975
chr3	149543900	0.950413
chr3	149684700	0.644628
chr3	149740900	0.809917
chr3	149749100	0.735537
chr3	149753500	0.917355
chr3	149814900	0.942149
chr3	149825000	0.983471
chr3	149837200	0.727273
chr3	149848500	0.991736
chr3	149940700	0.801653
chr3	149945900	0.719008
chr3	149968000	0.685950
chr3	150015000	0.776860
chr3	150053700	0.834711
chr3	150063000	0.785124
chr3	150088500	0.892562
chr3	150103400	0.727273
chr3	150132400	0.702479
chr3	150190400	0.719008
chr3	150231100	0.669421
chr3	150317600	1.000000
chr3	150325700	0.768595
chr3	150328500	0.669421
chr3	150484300	0.727273
chr3	150516400	0.710744
chr3	150524300	0.851240
chr3	150689000	0.760331
chr3	150693800	0.909091
chr3	150801000	0.768595
chr3	150807100	0.694215
chr3	150825500	0.867769
chr3	150863800	0.842975
chr3	150900600	0.826446
chr3	150946400	0.818182
chr3	150984100	0.917355
chr3	151023100	0.917355
chr3	151034500	0.727273
chr3	151065600	0.719008
chr3	151067700	0.669421
chr3	151112400	0.867769
chr3	151196300	0.743802
chr3	151219400	0.652893
chr3	151310200	0.702479
chr3	151426900	0.702479
chr3	152017000	0.793388

chr3	152020300	0.752066
chr3	152026500	0.867769
chr3	152109200	0.826446
chr3	152121500	0.694215
chr3	152235700	0.917355
chr3	152314700	0.644628
chr3	152328100	0.768595
chr3	152374400	0.876033
chr3	152804900	0.661157
chr3	152812000	0.826446
chr3	152928000	0.876033
chr3	153066800	0.793388
chr3	153115200	0.760331
chr3	153358600	0.710744
chr3	153360800	0.710744
chr3	153406600	0.669421
chr3	153421100	0.677686
chr3	153486900	0.669421
chr3	153774800	0.710744
chr3	153778200	0.719008
chr3	153849400	0.719008
chr3	153862200	0.752066
chr3	153914000	0.652893
chr3	153966000	0.661157
chr3	154038100	0.702479
chr3	154187700	0.669421
chr3	154494800	0.677686
chr3	154737400	0.809917
chr3	155060500	0.644628
chr3	155079500	0.768595
chr3	155082400	0.785124
chr3	155087300	0.900826
chr3	155100800	0.719008
chr3	155139500	0.925620
chr3	155230300	0.876033
chr3	155337100	0.958678
chr3	155357800	0.685950
chr3	155379300	0.752066
chr3	155422600	0.677686
chr3	155465300	0.851240
chr3	155509100	0.818182
chr3	155667400	0.900826
chr3	155976600	0.644628
chr3	156036100	0.685950
chr3	156083900	0.743802
chr3	156091400	0.851240
chr3	156175100	1.000000
chr3	156181300	0.859504
chr3	156469300	0.900826
chr3	156553200	0.776860
chr3	156637000	0.694215
chr3	156763600	0.867769
chr3	156783300	0.933884
chr3	156810900	0.776860

chr3	156823500	0.867769
chr3	156848000	0.793388
chr3	156993300	0.652893
chr3	157053700	0.958678
chr3	157324400	0.677686
chr3	157472400	0.694215
chr3	158486600	0.702479
chr3	158679600	0.900826
chr3	159171900	0.694215
chr3	159278500	0.884298
chr3	159352800	0.735537
chr3	159358700	0.809917
chr3	159450400	0.719008
chr3	159493500	0.933884
chr3	159505900	0.702479
chr3	159532300	0.851240
chr3	159570400	0.884298
chr3	159579100	0.793388
chr3	159590200	0.661157
chr3	159601000	0.727273
chr3	159711700	0.735537
chr3	159880400	0.876033
chr3	160032900	0.917355
chr3	160098200	0.652893
chr3	160271900	0.685950
chr3	160503000	0.785124
chr3	160533600	0.644628
chr3	160603800	0.735537
chr3	160687000	0.735537
chr3	160701000	0.876033
chr3	160788300	0.867769
chr3	160902700	0.694215
chr3	161082000	0.743802
chr3	161084100	0.851240
chr3	161093400	0.661157
chr3	161138700	0.801653
chr3	161141400	0.652893
chr3	161350400	0.694215
chr3	161393100	0.710744
chr3	162436900	0.752066
chr3	162439600	0.685950
chr3	162459400	0.859504
chr3	163279000	0.702479
chr3	163547500	0.776860
chr3	164039300	0.719008
chr3	164050900	0.851240
chr3	164569200	0.859504
chr3	164582300	0.685950
chr3	164608400	0.809917
chr3	165555200	0.694215
chr3	165603500	0.652893
chr3	165610100	0.652893
chr3	166228400	0.677686
chr3	166461600	0.925620

chr3	167173500	0.743802
chr3	167371600	0.975207
chr3	167441600	0.776860
chr3	167445300	0.644628
chr3	167498300	0.677686
chr3	167613900	0.933884
chr3	167748200	0.652893
chr3	167860700	0.694215
chr3	167990100	0.752066
chr3	168125500	0.644628
chr3	168136200	0.768595
chr3	168231600	0.983471
chr3	168249600	0.826446
chr3	168300100	0.842975
chr3	168332900	0.735537
chr3	168335400	0.950413
chr3	168337600	0.826446
chr3	168343300	0.677686
chr3	168373600	0.685950
chr3	168525400	0.867769
chr3	168546600	0.760331
chr3	168558400	0.933884
chr3	168657800	0.826446
chr3	168718600	0.842975
chr3	168721200	0.917355
chr3	168891400	0.760331
chr3	168946000	0.768595
chr3	168962500	0.809917
chr3	169345900	0.685950
chr3	169758700	0.735537
chr3	169775100	0.719008
chr3	169777300	0.834711
chr3	170040400	0.669421
chr3	170072500	0.669421
chr3	170416100	0.702479
chr3	170452600	0.685950
chr3	170530900	0.834711
chr3	170548200	0.644628
chr3	170883700	0.652893
chr3	170905200	0.752066
chr3	170924800	0.677686
chr3	170983500	0.694215
chr3	171039800	0.661157
chr3	171048500	0.900826
chr3	171063600	0.694215
chr3	171102500	0.752066
chr3	171111400	0.933884
chr3	171132700	0.685950
chr3	171145500	0.793388
chr3	171147600	0.785124
chr3	171154700	0.793388
chr3	171159000	0.710744
chr3	171171600	0.975207
chr3	171437000	0.818182

chr3	171445300	0.694215
chr3	171466700	0.826446
chr3	171489200	0.776860
chr3	171509700	0.834711
chr3	171592700	0.785124
chr3	171601000	0.661157
chr3	171678000	0.752066
chr3	171791300	0.685950
chr3	171805700	0.743802
chr3	171817100	0.983471
chr3	171822800	0.876033
chr3	171844500	0.884298
chr3	171854200	0.842975
chr3	171858200	0.958678
chr3	171870900	0.933884
chr3	171874100	0.991736
chr3	171878400	0.809917
chr3	171889600	0.950413
chr3	171893100	0.933884
chr3	171927300	0.909091
chr3	171931800	0.768595
chr3	171936200	0.644628
chr3	171940500	0.685950
chr3	171966200	0.685950
chr3	171976800	0.661157
chr3	171979100	0.785124
chr3	172018800	0.727273
chr3	172083100	0.776860
chr3	172241400	0.834711
chr3	172572100	0.942149
chr3	172593600	0.785124
chr3	172623100	0.710744
chr3	172891900	0.801653
chr3	173020100	0.867769
chr3	173129100	0.710744
chr3	173538000	0.876033
chr3	173552700	0.694215
chr3	173584300	0.991736
chr3	173632300	0.933884
chr3	173651200	0.842975
chr3	173820300	0.851240
chr3	173948500	0.694215
chr3	173951500	0.892562
chr3	174131200	0.719008
chr3	174254100	0.644628
chr3	174256400	0.876033
chr3	174258700	0.983471
chr3	174771600	0.677686
chr3	175129400	0.669421
chr3	175244400	0.677686
chr3	175554600	0.677686
chr3	175704200	0.669421
chr3	175890300	0.752066
chr3	175938200	0.685950

chr3	176046400	0.834711
chr3	176051100	0.727273
chr3	176224000	0.867769
chr3	176240000	0.942149
chr3	176381800	0.760331
chr3	176415100	0.644628
chr3	176633200	0.677686
chr3	176731900	0.727273
chr3	176765000	0.760331
chr3	176792900	0.661157
chr3	176828500	0.768595
chr3	176843000	0.694215
chr3	176863700	0.710744
chr3	176873000	0.661157
chr3	176892800	0.677686
chr3	176909300	0.644628
chr3	176997400	0.826446
chr3	177000100	0.768595
chr3	177002300	0.702479
chr3	177063600	0.702479
chr3	177079300	0.661157
chr3	177178300	0.652893
chr3	177234600	0.644628
chr3	177292900	0.702479
chr3	177308100	0.719008
chr3	177325300	0.694215
chr3	177329200	0.884298
chr3	177772400	0.652893
chr3	177803100	0.991736
chr3	177877700	0.867769
chr3	177955600	0.752066
chr3	178267800	0.768595
chr3	178598400	0.842975
chr3	179157200	0.809917
chr3	179172300	0.652893
chr3	179245000	0.958678
chr3	179252600	0.644628
chr3	179379900	0.685950
chr3	179384600	0.785124
chr3	179419200	0.925620
chr3	179427700	0.983471
chr3	179490400	0.669421
chr3	179544200	0.735537
chr3	179600400	0.983471
chr3	179660600	0.694215
chr3	179668100	0.727273
chr3	179691900	0.785124
chr3	179750700	0.727273
chr3	179820300	0.694215
chr3	179832500	0.652893
chr3	179835100	0.727273
chr3	179853800	0.661157
chr3	180100800	0.685950
chr3	180375200	0.719008

chr3	180402800	0.760331
chr3	180426100	0.991736
chr3	180429100	0.743802
chr3	180523400	0.652893
chr3	180573100	0.685950
chr3	180588200	0.933884
chr3	180607200	0.842975
chr3	180641200	0.652893
chr3	180793700	0.735537
chr3	180970400	0.925620
chr3	181101800	0.909091
chr3	181136400	0.669421
chr3	181201800	0.677686
chr3	181329400	0.876033
chr3	181416500	0.719008
chr3	181426200	0.719008
chr3	181440200	0.818182
chr3	181449400	0.760331
chr3	181578100	0.785124
chr3	181605200	0.710744
chr3	181634300	0.661157
chr3	181658900	0.917355
chr3	181661400	0.727273
chr3	181797000	0.809917
chr3	181810600	0.809917
chr3	181840100	0.677686
chr3	181858600	0.834711
chr3	181861200	0.975207
chr3	181891100	0.702479
chr3	181903200	0.768595
chr3	181956300	0.652893
chr3	181976000	0.743802
chr3	182003000	0.867769
chr3	182045500	0.644628
chr3	182169400	0.710744
chr3	182218600	0.752066
chr3	182227500	0.685950
chr3	182284800	0.834711
chr3	182327500	0.652893
chr3	182339400	0.768595
chr3	182423300	0.685950
chr3	182443900	0.892562
chr3	182520500	0.719008
chr3	182663300	0.859504
chr3	182684400	0.884298
chr3	182832500	0.661157
chr3	182967400	0.776860
chr3	182981400	0.719008
chr3	182986100	0.669421
chr3	183017000	0.710744
chr3	183048900	0.876033
chr3	183102700	0.768595
chr3	183153000	0.801653
chr3	183204600	0.752066

chr3	183236900	0.727273
chr3	183251600	0.818182
chr3	183271700	0.694215
chr3	183294800	0.727273
chr3	183309800	0.925620
chr3	183673400	0.884298
chr3	183703200	0.942149
chr3	183896300	0.834711
chr3	184007500	0.785124
chr3	184035200	0.809917
chr3	184052900	0.685950
chr3	184056700	0.652893
chr3	184062900	0.719008
chr3	184069700	0.950413
chr3	184089500	0.768595
chr3	184218400	0.785124
chr3	184276900	0.752066
chr3	184281600	0.743802
chr3	184284700	0.743802
chr3	184313800	0.909091
chr3	184351400	0.735537
chr3	184374700	0.909091
chr3	184377500	0.785124
chr3	184380900	0.826446
chr3	184397100	0.801653
chr3	184403500	0.661157
chr3	184435000	0.958678
chr3	184445500	0.702479
chr3	184459700	0.644628
chr3	184466500	0.677686
chr3	184470300	0.818182
chr3	184474500	0.809917
chr3	184504000	0.776860
chr3	184513600	0.661157
chr3	184607500	0.834711
chr3	184618400	0.975207
chr3	184625800	0.719008
chr3	184630000	0.942149
chr3	184636900	0.785124
chr3	184671400	0.694215
chr3	184893600	0.735537
chr3	184934700	0.710744
chr3	185279300	0.793388
chr3	185364600	0.859504
chr3	185420400	0.900826
chr3	185427000	0.685950
chr3	185430000	0.652893
chr3	185433200	0.942149
chr3	185435800	0.719008
chr3	185444600	0.884298
chr3	185464300	0.677686
chr3	185468200	0.818182
chr3	185477900	0.652893
chr3	185483000	0.694215

chr3	185491700	0.735537
chr3	185497600	0.892562
chr3	185504300	0.983471
chr3	185508500	0.958678
chr3	185510900	0.826446
chr3	185520700	0.776860
chr3	185525700	0.661157
chr3	185531900	0.950413
chr3	185534300	0.851240
chr3	185538600	0.867769
chr3	185645100	0.892562
chr3	185649600	0.743802
chr3	185702000	0.809917
chr3	185775500	0.826446
chr3	185798500	0.900826
chr3	185814300	0.710744
chr3	185821700	0.743802
chr3	185840200	0.661157
chr3	185928200	0.644628
chr3	185960600	0.768595
chr3	185977600	0.719008
chr3	186022300	0.876033
chr3	186126800	0.727273
chr3	186230600	0.752066
chr3	186315100	0.834711
chr3	186361700	0.743802
chr3	186372700	0.801653
chr3	186381600	0.966942
chr3	186428900	0.702479
chr3	186458500	0.760331
chr3	186464000	0.735537
chr3	186491000	0.702479
chr3	186494200	0.892562
chr3	186584600	0.694215
chr3	186604600	0.834711
chr3	186614600	0.809917
chr3	186666600	0.685950
chr3	186679800	0.834711
chr3	186683400	0.975207
chr3	186694500	0.826446
chr3	186716800	0.834711
chr3	186719200	0.851240
chr3	186782000	0.884298
chr3	186790400	0.768595
chr3	186880300	0.785124
chr3	186883400	0.735537
chr3	186892400	0.685950
chr3	186899400	0.884298
chr3	186902300	1.000000
chr3	186905700	0.702479
chr3	186928100	0.752066
chr3	186937900	0.685950
chr3	186993000	0.950413
chr3	187078300	0.851240

chr3	187183400	0.991736
chr3	187237600	0.818182
chr3	187285600	0.669421
chr3	187309400	0.752066
chr3	187336800	0.884298
chr3	187346400	0.661157
chr3	187388200	0.727273
chr3	187432600	0.661157
chr3	187437800	0.884298
chr3	187465500	0.925620
chr3	187468400	0.909091
chr3	187492200	0.669421
chr3	187576700	0.652893
chr3	187619100	0.842975
chr3	187633000	0.652893
chr3	187636200	0.884298
chr3	187707900	0.785124
chr3	187825500	0.958678
chr3	187876100	0.694215
chr3	187887500	0.735537
chr3	187897600	0.694215
chr3	187903300	0.644628
chr3	187926000	0.694215
chr3	187935700	0.694215
chr3	187985800	0.727273
chr3	187988500	0.818182
chr3	187998000	0.669421
chr3	188017100	0.925620
chr3	188021000	0.942149
chr3	188390800	0.661157
chr3	188415400	0.710744
chr3	189022900	0.851240
chr3	189088000	0.669421
chr3	189353200	0.661157
chr3	189701000	0.933884
chr3	189719000	0.859504
chr3	189730100	0.975207
chr3	189732900	0.950413
chr3	189736400	0.760331
chr3	189758300	0.950413
chr3	189760600	0.950413
chr3	189762800	0.867769
chr3	189767300	0.950413
chr3	189776900	0.669421
chr3	189804400	0.801653
chr3	189811100	0.644628
chr3	189816100	0.826446
chr3	189901300	0.834711
chr3	190044000	0.909091
chr3	190093600	0.644628
chr3	190256200	0.644628
chr3	190271400	0.859504
chr3	190363300	0.710744
chr3	190573500	0.694215

chr3	190957200	0.685950
chr3	190971600	0.710744
chr3	190979100	0.727273
chr3	191087400	0.785124
chr3	191407000	0.892562
chr3	191612800	0.719008
chr3	191647000	0.900826
chr3	191676800	0.644628
chr3	191782300	0.702479
chr3	191905200	0.801653
chr3	191991600	0.727273
chr3	192050600	0.727273
chr3	192135000	0.768595
chr3	192185800	0.644628
chr3	192370900	0.809917
chr3	192465400	0.983471
chr3	192468700	0.743802
chr3	192537800	0.867769
chr3	192552200	0.661157
chr3	192585500	0.727273
chr3	192597800	0.909091
chr3	192607300	0.958678
chr3	192611100	0.933884
chr3	192640200	0.661157
chr3	192734500	0.809917
chr3	192883900	0.752066
chr3	192944600	0.702479
chr3	192948500	0.801653
chr3	193065300	0.652893
chr3	193096200	0.702479
chr3	193110100	0.661157
chr3	193125800	0.702479
chr3	193290500	0.669421
chr3	193345100	0.743802
chr3	193375800	0.917355
chr3	193379300	0.702479
chr3	193428900	0.909091
chr3	193449300	0.677686
chr3	193492900	0.661157
chr3	193499300	0.669421
chr3	193509300	0.776860
chr3	193530900	0.719008
chr3	193533400	0.809917
chr3	193537500	0.950413
chr3	193544900	0.851240
chr3	193570300	0.909091
chr3	193587800	0.950413
chr3	193593200	0.669421
chr3	193629400	0.710744
chr3	193656800	0.900826
chr3	193660100	0.694215
chr3	193693300	0.983471
chr3	193714700	0.851240
chr3	193719900	0.743802

chr3	193727600	0.776860
chr3	193772800	0.727273
chr3	193776500	0.867769
chr3	193804800	0.909091
chr3	193808300	0.677686
chr3	193830800	0.743802
chr3	193847800	0.859504
chr3	193850600	1.000000
chr3	193860200	0.809917
chr3	193879300	0.727273
chr3	193885000	0.727273
chr3	193923800	0.661157
chr3	193933800	0.669421
chr3	193943100	0.842975
chr3	193988600	0.661157
chr3	193993000	0.892562
chr3	194004000	0.793388
chr3	194006200	0.652893
chr3	194013000	0.900826
chr3	194015900	0.859504
chr3	194024200	0.809917
chr3	194033900	0.685950
chr3	194060300	0.884298
chr3	194085900	0.644628
chr3	194090700	0.644628
chr3	194097000	0.793388
chr3	194101300	0.842975
chr3	194192600	0.644628
chr3	194209200	0.685950
chr3	194469100	0.842975
chr3	194471400	0.876033
chr3	194490200	0.768595
chr3	194498900	0.685950
chr3	194522600	0.826446
chr3	194646600	0.669421
chr3	194706300	0.834711
chr3	194756600	0.917355
chr3	194763900	0.669421
chr3	194801600	0.669421
chr3	194808200	0.719008
chr3	194827500	0.818182
chr3	194838800	0.735537
chr3	194840900	0.933884
chr3	194871300	0.933884
chr3	194873900	0.859504
chr3	194877000	0.685950
chr3	194881700	0.809917
chr3	194904100	0.884298
chr3	194909100	0.677686
chr3	194916400	0.900826
chr3	194930700	0.884298
chr3	194941500	0.694215
chr3	194963300	0.685950
chr3	194971200	0.669421

chr3	194973700	0.694215
chr3	194976600	0.884298
chr3	195077700	0.677686
chr3	195102800	0.867769
chr3	195106700	0.727273
chr3	195118600	0.702479
chr3	195164900	0.661157
chr3	195167500	0.760331
chr3	195275700	0.859504
chr3	195355500	0.884298
chr3	195425200	0.743802
chr3	195432500	0.727273
chr3	195529100	0.719008
chr3	195533300	0.950413
chr3	195539100	0.677686
chr3	195566300	0.710744
chr3	195613400	0.859504
chr3	195616600	0.809917
chr3	195619800	0.900826
chr3	195642000	0.760331
chr3	195648600	0.644628
chr3	195785200	0.975207
chr3	195835400	0.719008
chr3	195838100	0.677686
chr3	195906700	0.867769
chr3	195913900	0.909091
chr3	195919700	0.842975
chr3	195954900	0.652893
chr3	195989200	0.975207
chr3	196000600	0.867769
chr3	196102700	0.719008
chr3	196336500	0.776860
chr3	196366900	0.809917
chr3	196373500	0.694215
chr3	196375800	0.884298
chr3	196393100	0.900826
chr3	196412000	0.925620
chr3	196481100	0.669421
chr3	196515200	0.975207
chr3	196625900	0.983471
chr3	196910800	0.661157
chr3	196932100	0.677686
chr3	196939800	0.652893
chr3	196967500	0.719008
chr3	196970300	0.925620
chr3	197036800	0.752066
chr3	197061300	0.661157
chr3	197091300	0.884298
chr3	197095400	0.752066
chr3	197145200	0.760331
chr3	197209500	0.859504
chr3	197236500	0.661157
chr3	197248500	0.818182
chr3	197267600	0.884298

chr3	197274600	0.793388
chr3	197277100	0.776860
chr3	197301600	0.975207
chr3	197304600	0.694215
chr3	197307500	0.694215
chr3	197326700	0.776860
chr3	197390200	0.760331
chr3	197439600	0.826446
chr3	197582200	0.991736
chr3	197586100	0.768595
chr3	197608100	0.867769
chr3	197610400	0.900826
chr3	197616500	0.859504
chr3	197627200	0.785124
chr3	197640100	0.958678
chr3	197664000	0.768595
chr4	573900	0.710744
chr4	619100	0.677686
chr4	678000	0.768595
chr4	786100	0.793388
chr4	962100	0.809917
chr4	973500	0.685950
chr4	1013100	0.694215
chr4	1033900	0.661157
chr4	1037900	0.917355
chr4	1044700	0.710744
chr4	1049800	0.809917
chr4	1288700	0.793388
chr4	1291400	0.752066
chr4	1397000	0.958678
chr4	1408900	0.669421
chr4	1568600	0.644628
chr4	1573900	0.818182
chr4	1601700	0.785124
chr4	1647100	0.966942
chr4	1750500	0.685950
chr4	1754500	0.677686
chr4	1770300	0.743802
chr4	1772600	0.892562
chr4	1779400	0.793388
chr4	1864700	0.743802
chr4	2053500	0.876033
chr4	2063700	0.677686
chr4	2074100	0.842975
chr4	2144200	0.685950
chr4	2217800	0.793388
chr4	2222300	0.793388
chr4	2225000	0.727273
chr4	2271100	0.801653
chr4	2341600	0.661157
chr4	2360200	0.702479
chr4	2393000	0.834711
chr4	2395900	0.876033
chr4	2443800	0.809917

chr4	2483300	0.826446
chr4	2572600	0.694215
chr4	2584800	0.785124
chr4	2593600	0.768595
chr4	2600000	0.966942
chr4	2620100	0.735537
chr4	2623800	0.900826
chr4	2780600	0.975207
chr4	2789800	0.669421
chr4	2802800	0.876033
chr4	2813300	0.925620
chr4	2822200	0.917355
chr4	2839400	0.826446
chr4	2864300	0.925620
chr4	2884200	0.917355
chr4	2925300	0.983471
chr4	3044000	0.884298
chr4	3168400	0.719008
chr4	3239900	0.925620
chr4	3245200	0.760331
chr4	3306900	0.917355
chr4	3311700	0.818182
chr4	3320700	0.661157
chr4	3325800	0.694215
chr4	3350800	0.644628
chr4	3386900	0.677686
chr4	3389100	0.710744
chr4	3391700	0.851240
chr4	3436900	0.735537
chr4	3480000	0.719008
chr4	3482700	0.752066
chr4	3487900	0.851240
chr4	3496100	0.735537
chr4	3626000	0.694215
chr4	3658400	0.661157
chr4	3662800	0.652893
chr4	3705700	0.809917
chr4	3747700	0.933884
chr4	3755900	0.644628
chr4	3766000	0.644628
chr4	3782500	0.710744
chr4	3804700	0.809917
chr4	3823700	0.743802
chr4	3831600	0.669421
chr4	3837500	0.876033
chr4	3853900	0.818182
chr4	3864600	0.859504
chr4	3882400	0.900826
chr4	3935100	0.801653
chr4	3975000	0.785124
chr4	4008300	0.702479
chr4	4112200	0.644628
chr4	4230000	0.661157
chr4	4325900	0.826446

chr4	4329700	0.677686
chr4	4360700	0.685950
chr4	4384500	0.702479
chr4	4425600	0.834711
chr4	4429600	0.851240
chr4	4432700	0.743802
chr4	4464700	0.685950
chr4	4483300	0.842975
chr4	4498400	0.719008
chr4	4523000	0.809917
chr4	4609900	0.776860
chr4	4644200	0.727273
chr4	4654200	0.694215
chr4	4671300	0.743802
chr4	4679900	0.834711
chr4	4760700	0.743802
chr4	4764300	0.694215
chr4	4768300	0.702479
chr4	4786300	0.768595
chr4	4793700	0.859504
chr4	4840400	0.644628
chr4	4857100	0.652893
chr4	4860300	0.801653
chr4	4868400	0.842975
chr4	4871500	0.685950
chr4	4875100	0.760331
chr4	5168000	0.801653
chr4	5201400	0.652893
chr4	5220300	0.661157
chr4	5594400	0.694215
chr4	5633900	0.884298
chr4	5754400	0.950413
chr4	5775600	0.900826
chr4	5865800	0.661157
chr4	5876500	0.801653
chr4	5904400	0.909091
chr4	5907800	0.834711
chr4	5914700	0.991736
chr4	5933200	0.785124
chr4	5956300	0.685950
chr4	5958600	0.859504
chr4	6008300	0.652893
chr4	6021600	0.801653
chr4	6073600	0.702479
chr4	6094300	0.719008
chr4	6107000	0.851240
chr4	6115200	0.727273
chr4	6118500	0.801653
chr4	6120800	0.727273
chr4	6136900	0.669421
chr4	6156800	0.652893
chr4	6197700	0.876033
chr4	6259600	0.826446
chr4	6276700	0.809917

chr4	6327100	0.743802
chr4	6329900	0.685950
chr4	6339300	0.644628
chr4	6369600	0.760331
chr4	6372400	0.735537
chr4	6381400	0.669421
chr4	6397500	0.710744
chr4	6405000	0.694215
chr4	6417100	0.776860
chr4	6465400	0.719008
chr4	6495500	0.793388
chr4	6566300	0.785124
chr4	6691400	0.735537
chr4	6781900	0.834711
chr4	6842100	0.644628
chr4	6848300	0.818182
chr4	6919800	0.842975
chr4	6940000	0.859504
chr4	6943100	0.942149
chr4	6947100	0.884298
chr4	6956600	0.909091
chr4	6961500	0.669421
chr4	6964100	0.809917
chr4	6975700	0.834711
chr4	6991500	0.661157
chr4	7009800	0.694215
chr4	7022200	0.685950
chr4	7103600	0.685950
chr4	7204300	0.669421
chr4	7299300	0.818182
chr4	7416900	0.776860
chr4	7430300	0.710744
chr4	7432400	0.694215
chr4	7444800	0.727273
chr4	7463800	0.719008
chr4	7474700	0.727273
chr4	7487500	0.685950
chr4	7518100	0.859504
chr4	7529300	0.834711
chr4	7531900	0.735537
chr4	7534200	0.727273
chr4	7598000	0.727273
chr4	7653500	0.842975
chr4	7703300	0.727273
chr4	7809000	0.834711
chr4	7830000	0.710744
chr4	7863300	0.975207
chr4	7893600	0.644628
chr4	7901700	0.793388
chr4	7903800	0.644628
chr4	7913000	0.900826
chr4	7931400	0.752066
chr4	7943700	0.793388
chr4	7962500	0.925620

chr4	7967000	0.644628
chr4	7981300	0.710744
chr4	8040900	0.735537
chr4	8051600	0.958678
chr4	8053900	0.884298
chr4	8065200	0.933884
chr4	8073900	0.661157
chr4	8082300	0.652893
chr4	8111600	0.900826
chr4	8119400	0.859504
chr4	8135800	0.884298
chr4	8146400	0.743802
chr4	8227800	0.917355
chr4	8240600	0.785124
chr4	8244600	0.694215
chr4	8308600	0.661157
chr4	8344900	0.694215
chr4	8412800	0.768595
chr4	8439400	0.851240
chr4	8495800	0.752066
chr4	8508700	0.735537
chr4	8535200	0.851240
chr4	8537400	0.909091
chr4	8544400	0.760331
chr4	8562200	0.685950
chr4	8569400	1.000000
chr4	8655300	0.652893
chr4	8666500	0.752066
chr4	8708200	0.685950
chr4	8839500	0.892562
chr4	8857500	0.793388
chr4	8869600	0.942149
chr4	8872600	0.752066
chr4	8879800	0.851240
chr4	8893700	0.694215
chr4	9531900	0.685950
chr4	9737500	0.644628
chr4	9885500	0.876033
chr4	9900800	0.702479
chr4	9922800	0.694215
chr4	9941100	0.834711
chr4	9951600	0.644628
chr4	9981200	0.900826
chr4	10052300	0.652893
chr4	10058600	0.661157
chr4	10065400	0.925620
chr4	10096300	0.950413
chr4	10102500	0.702479
chr4	10104800	0.958678
chr4	10107100	0.685950
chr4	10112400	0.933884
chr4	10115000	1.000000
chr4	10124900	0.719008
chr4	10298900	0.826446

chr4	10456400	0.694215
chr4	10498100	0.876033
chr4	10576000	0.727273
chr4	10591500	0.958678
chr4	10879500	0.669421
chr4	10960000	0.677686
chr4	10966500	0.867769
chr4	10985000	0.851240
chr4	11071200	0.677686
chr4	11152000	0.818182
chr4	11438600	0.834711
chr4	11471700	0.801653
chr4	11643600	0.652893
chr4	11712900	0.719008
chr4	11824400	0.842975
chr4	11849100	0.719008
chr4	11910800	0.776860
chr4	12052300	0.710744
chr4	12101500	0.752066
chr4	12245300	0.776860
chr4	12509500	0.644628
chr4	12607800	0.652893
chr4	12642300	0.884298
chr4	12726000	0.768595
chr4	12886600	0.892562
chr4	13271000	0.909091
chr4	13514900	0.760331
chr4	13527100	0.892562
chr4	13530900	0.809917
chr4	13537100	0.793388
chr4	13539200	0.644628
chr4	13541800	0.685950
chr4	13643000	0.719008
chr4	14086000	0.909091
chr4	14117200	0.644628
chr4	14341700	0.685950
chr4	14622900	0.892562
chr4	14746400	0.719008
chr4	15160900	0.743802
chr4	15200100	0.909091
chr4	15378000	0.809917
chr4	15471700	0.760331
chr4	15475000	0.851240
chr4	15545700	0.851240
chr4	15594000	0.644628
chr4	15607800	0.867769
chr4	15686500	0.950413
chr4	15692500	0.876033
chr4	15697200	0.818182
chr4	15908300	0.768595
chr4	15965000	0.785124
chr4	16031600	0.702479
chr4	16034200	0.809917
chr4	16036800	0.785124

chr4	16039100	0.719008
chr4	16042400	0.719008
chr4	16046200	0.652893
chr4	16077400	0.702479
chr4	16080000	0.710744
chr4	16087900	0.685950
chr4	16198900	0.735537
chr4	16208400	0.776860
chr4	16238200	0.644628
chr4	16324900	0.752066
chr4	16452000	0.776860
chr4	16600400	0.669421
chr4	16610800	0.925620
chr4	16675900	0.669421
chr4	16696000	0.826446
chr4	16731200	0.694215
chr4	16734200	0.652893
chr4	16755400	0.644628
chr4	16777800	0.925620
chr4	16792300	0.735537
chr4	16819600	0.793388
chr4	16822300	0.644628
chr4	16828700	0.983471
chr4	16844200	0.702479
chr4	16846500	0.719008
chr4	16848600	0.768595
chr4	16859800	0.818182
chr4	16896300	0.851240
chr4	16902100	0.826446
chr4	16910700	0.983471
chr4	16920000	0.818182
chr4	16944100	0.785124
chr4	16996700	0.793388
chr4	17006300	0.958678
chr4	17035100	0.694215
chr4	17040400	0.694215
chr4	17079100	0.710744
chr4	17158300	0.652893
chr4	17288900	0.793388
chr4	17326300	0.710744
chr4	17419400	0.685950
chr4	17496500	0.677686
chr4	17516900	0.752066
chr4	17519700	0.892562
chr4	17762900	0.719008
chr4	18580400	0.661157
chr4	18692500	0.876033
chr4	18694600	0.652893
chr4	18963700	0.776860
chr4	19013000	0.760331
chr4	19097700	0.776860
chr4	19196600	0.892562
chr4	19198800	0.685950
chr4	20216100	0.652893

chr4	20356100	0.685950
chr4	20433600	0.719008
chr4	20574500	0.719008
chr4	20679500	0.768595
chr4	20811700	0.776860
chr4	21120200	0.776860
chr4	21205300	0.669421
chr4	21363100	0.694215
chr4	21975400	0.710744
chr4	22193900	0.719008
chr4	22476800	0.851240
chr4	22503700	0.719008
chr4	22507200	0.851240
chr4	22510200	0.892562
chr4	23163800	0.958678
chr4	23211300	0.644628
chr4	23236000	0.909091
chr4	23336300	0.809917
chr4	23424900	0.958678
chr4	23457500	0.793388
chr4	23573700	0.677686
chr4	23585200	0.793388
chr4	23776400	0.851240
chr4	23880800	0.867769
chr4	23883300	0.933884
chr4	23888000	0.760331
chr4	23892800	0.702479
chr4	23931900	0.652893
chr4	23935400	0.818182
chr4	23981800	0.760331
chr4	23995500	0.669421
chr4	24246900	0.669421
chr4	24250900	0.685950
chr4	24258500	0.809917
chr4	24263700	0.694215
chr4	24268200	0.710744
chr4	24281800	0.685950
chr4	24323000	0.809917
chr4	24331000	0.834711
chr4	24354300	0.702479
chr4	24415300	0.876033
chr4	24479700	0.768595
chr4	24490800	0.644628
chr4	24509400	0.644628
chr4	24513100	0.966942
chr4	24548800	0.752066
chr4	24562100	0.677686
chr4	24645000	0.809917
chr4	24659900	0.809917
chr4	24662000	0.900826
chr4	24682700	0.867769
chr4	24691000	0.685950
chr4	24706700	0.876033
chr4	24722900	0.925620

chr4	24785200	0.752066
chr4	24810800	0.743802
chr4	24869800	0.842975
chr4	24919400	0.752066
chr4	24927000	0.661157
chr4	24990900	0.735537
chr4	25133500	0.900826
chr4	25212100	0.669421
chr4	25372300	0.719008
chr4	25448300	0.809917
chr4	25452500	0.652893
chr4	25483400	0.900826
chr4	25488200	0.785124
chr4	25526400	0.685950
chr4	25560600	0.760331
chr4	25579800	0.975207
chr4	25582600	0.776860
chr4	25654400	0.760331
chr4	25666200	0.884298
chr4	25798000	0.710744
chr4	25809800	0.669421
chr4	25816100	0.685950
chr4	25824600	0.710744
chr4	25844500	0.876033
chr4	25848000	0.884298
chr4	25893300	0.793388
chr4	25951900	0.892562
chr4	25955200	0.719008
chr4	25957700	0.752066
chr4	25979700	0.942149
chr4	25990100	0.768595
chr4	25995000	0.909091
chr4	26033800	0.942149
chr4	26071300	0.694215
chr4	26141700	0.760331
chr4	26175600	0.727273
chr4	26241100	0.933884
chr4	26280700	0.685950
chr4	26300300	0.933884
chr4	26302700	0.842975
chr4	26332700	0.801653
chr4	26347400	0.752066
chr4	26361900	0.867769
chr4	26370600	0.719008
chr4	26374600	0.677686
chr4	26404700	0.785124
chr4	26409200	0.892562
chr4	26424700	0.644628
chr4	26454700	0.727273
chr4	26491600	0.644628
chr4	26649800	0.884298
chr4	26789700	0.644628
chr4	26821100	0.752066
chr4	26913700	0.694215

chr4	27083500	0.702479
chr4	27089300	0.652893
chr4	27092400	0.900826
chr4	27098400	0.809917
chr4	27125400	0.983471
chr4	27129100	0.760331
chr4	27291600	0.760331
chr4	27704500	0.710744
chr4	27732200	0.710744
chr4	28293500	0.834711
chr4	28481600	0.727273
chr4	28492300	0.801653
chr4	28550600	0.661157
chr4	28685200	0.760331
chr4	28756100	0.826446
chr4	29004200	0.818182
chr4	30151500	0.925620
chr4	30838800	0.809917
chr4	30842000	0.685950
chr4	30976600	0.644628
chr4	31032600	0.768595
chr4	31172200	0.834711
chr4	31293300	0.991736
chr4	32135700	0.752066
chr4	32660000	0.702479
chr4	32932200	0.644628
chr4	33843300	0.644628
chr4	34263400	0.652893
chr4	35248700	0.719008
chr4	35303400	0.702479
chr4	35527200	0.809917
chr4	36028600	0.834711
chr4	36266700	0.685950
chr4	37391000	0.975207
chr4	37542600	0.950413
chr4	37605000	0.661157
chr4	37644200	0.917355
chr4	37653600	0.785124
chr4	37655700	0.785124
chr4	37664700	0.785124
chr4	37691200	0.694215
chr4	37731500	0.702479
chr4	37903900	0.752066
chr4	37910800	0.644628
chr4	37981200	0.719008
chr4	37984100	0.867769
chr4	37990600	0.983471
chr4	37993600	0.892562
chr4	37996800	0.669421
chr4	38004400	0.958678
chr4	38006900	0.867769
chr4	38087900	0.900826
chr4	38115600	0.942149
chr4	38130700	0.785124

chr4	38133900	0.752066
chr4	38182100	0.661157
chr4	38289100	0.702479
chr4	38320900	0.776860
chr4	38330400	0.644628
chr4	38491300	0.966942
chr4	38514900	0.909091
chr4	38607600	0.809917
chr4	38673800	0.677686
chr4	38676300	0.834711
chr4	38697400	0.677686
chr4	38730400	0.752066
chr4	38755000	0.842975
chr4	38859600	0.702479
chr4	39064200	0.818182
chr4	39168100	0.760331
chr4	39545100	0.652893
chr4	39619600	0.644628
chr4	39636800	0.909091
chr4	39891400	0.991736
chr4	39900700	0.685950
chr4	39939000	0.818182
chr4	39944100	0.702479
chr4	39949700	0.809917
chr4	40121600	0.809917
chr4	40195200	0.826446
chr4	40198400	0.884298
chr4	40214300	0.719008
chr4	40233400	0.644628
chr4	40308600	0.727273
chr4	40311600	0.983471
chr4	40319300	0.867769
chr4	40324400	0.793388
chr4	40337200	0.760331
chr4	40398200	0.677686
chr4	40457200	0.752066
chr4	40517500	0.661157
chr4	40527800	0.859504
chr4	40567500	0.909091
chr4	40589000	0.752066
chr4	40624200	0.694215
chr4	40634300	0.727273
chr4	40651000	0.884298
chr4	40653700	0.661157
chr4	40675100	0.884298
chr4	40720900	0.966942
chr4	40842900	0.652893
chr4	40850800	0.793388
chr4	40933900	0.685950
chr4	40936900	0.710744
chr4	40945500	0.719008
chr4	40965000	0.735537
chr4	41000800	0.652893
chr4	41127900	0.768595

chr4	41136400	0.942149
chr4	41149100	0.760331
chr4	41328000	0.644628
chr4	41330200	0.966942
chr4	41342500	0.752066
chr4	41435400	0.975207
chr4	41506600	0.834711
chr4	41527600	0.950413
chr4	41622500	0.752066
chr4	41625800	0.661157
chr4	41646800	0.785124
chr4	41747000	0.661157
chr4	41750900	0.867769
chr4	41760200	0.694215
chr4	41798900	0.925620
chr4	41987600	0.826446
chr4	42122900	0.685950
chr4	42139300	0.727273
chr4	42175500	0.760331
chr4	42271100	0.785124
chr4	42274100	0.644628
chr4	42296600	0.991736
chr4	42305100	0.702479
chr4	42313400	0.752066
chr4	42316400	0.925620
chr4	42342600	0.677686
chr4	42393600	0.652893
chr4	42427600	0.876033
chr4	42430500	0.826446
chr4	42487400	0.917355
chr4	42504400	0.652893
chr4	42514100	0.735537
chr4	42517400	0.942149
chr4	42788800	0.809917
chr4	43148200	0.694215
chr4	43460400	0.694215
chr4	43995000	0.942149
chr4	44057600	0.925620
chr4	44059800	0.942149
chr4	44230900	0.677686
chr4	44417400	0.942149
chr4	44543600	0.644628
chr4	44865700	0.900826
chr4	45014400	0.752066
chr4	45987400	0.669421
chr4	46494600	0.710744
chr4	47078600	0.851240
chr4	47183800	0.644628
chr4	47209900	0.727273
chr4	47533700	0.694215
chr4	47719700	0.801653
chr4	47770700	0.727273
chr4	48129700	0.983471
chr4	48372300	0.859504

chr4	48383200	0.859504
chr4	48696100	0.685950
chr4	48700900	0.644628
chr4	48706200	0.752066
chr4	48749200	0.768595
chr4	48751800	0.661157
chr4	48963800	0.760331
chr4	52716500	0.884298
chr4	52719700	0.752066
chr4	52725100	0.719008
chr4	52751200	0.909091
chr4	52865300	0.694215
chr4	53112900	0.652893
chr4	53236600	0.702479
chr4	53243700	0.768595
chr4	53421400	0.884298
chr4	53508300	0.652893
chr4	53510700	0.669421
chr4	53522700	0.785124
chr4	53701100	0.942149
chr4	53707100	0.900826
chr4	53721700	0.685950
chr4	53731700	0.752066
chr4	53740400	0.900826
chr4	53749900	0.892562
chr4	53792700	0.876033
chr4	53806600	0.785124
chr4	53986200	0.785124
chr4	54188100	0.644628
chr4	54342700	0.851240
chr4	54345300	0.809917
chr4	54363800	0.727273
chr4	54386800	0.702479
chr4	54398900	0.735537
chr4	54569300	0.719008
chr4	54719200	0.652893
chr4	54786700	0.685950
chr4	54796500	0.710744
chr4	54825900	0.677686
chr4	54880200	0.710744
chr4	54962400	0.768595
chr4	54968300	0.801653
chr4	55121100	0.644628
chr4	55144700	0.785124
chr4	55414500	0.661157
chr4	55416600	0.719008
chr4	55438300	0.826446
chr4	55542400	0.776860
chr4	55545600	0.809917
chr4	55683800	0.669421
chr4	55843400	0.735537
chr4	55852800	0.966942
chr4	55896600	0.644628
chr4	55946200	1.000000

chr4	55983700	0.669421
chr4	55999700	0.768595
chr4	56051100	0.876033
chr4	56053700	0.735537
chr4	56139100	0.644628
chr4	56145900	0.909091
chr4	56194500	0.884298
chr4	56231300	0.958678
chr4	56233500	0.652893
chr4	56253000	0.743802
chr4	56298800	0.760331
chr4	56335100	0.652893
chr4	56344500	0.867769
chr4	56386800	0.652893
chr4	56430200	0.776860
chr4	56500100	0.644628
chr4	56641200	0.652893
chr4	56686300	0.785124
chr4	56805700	0.677686
chr4	56840100	0.669421
chr4	56907700	0.719008
chr4	56952200	0.909091
chr4	56978600	0.785124
chr4	57077400	0.818182
chr4	57093100	0.966942
chr4	57112400	0.785124
chr4	57256200	0.685950
chr4	57267000	0.909091
chr4	57276100	0.669421
chr4	57278500	0.793388
chr4	57291900	0.942149
chr4	57306400	0.785124
chr4	57470200	0.719008
chr4	57529200	0.842975
chr4	57547800	0.842975
chr4	57624300	0.785124
chr4	57635000	0.719008
chr4	57711400	0.942149
chr4	57741100	0.818182
chr4	57945100	0.933884
chr4	57958000	0.702479
chr4	58035200	0.752066
chr4	58053700	0.694215
chr4	58060400	0.743802
chr4	58154900	0.892562
chr4	58562600	0.768595
chr4	58847000	0.702479
chr4	59361300	0.785124
chr4	59381600	0.677686
chr4	59466800	0.727273
chr4	59957600	0.702479
chr4	60001800	0.719008
chr4	60026000	0.818182
chr4	60104800	0.652893

chr4	60323600	0.760331
chr4	60786600	0.677686
chr4	60955500	0.859504
chr4	61270100	0.719008
chr4	61758300	0.677686
chr4	62071300	0.661157
chr4	62076200	0.851240
chr4	62135200	0.685950
chr4	62137700	0.644628
chr4	62164400	0.760331
chr4	62202800	0.975207
chr4	62210000	0.677686
chr4	62255000	0.685950
chr4	62261800	0.710744
chr4	62359400	0.809917
chr4	62387100	0.727273
chr4	62586800	0.768595
chr4	62637200	0.727273
chr4	62639400	0.966942
chr4	62703700	0.818182
chr4	62719500	0.834711
chr4	62777500	0.809917
chr4	62939400	0.685950
chr4	62946800	0.719008
chr4	64594800	0.909091
chr4	64850100	0.743802
chr4	65593600	0.859504
chr4	65595800	0.859504
chr4	65603500	0.942149
chr4	65723800	0.768595
chr4	65912600	0.702479
chr4	66425400	0.669421
chr4	66455700	0.727273
chr4	66532200	0.677686
chr4	66657800	0.752066
chr4	66810800	0.702479
chr4	66880500	0.809917
chr4	67472900	0.669421
chr4	67602600	0.685950
chr4	67917200	0.776860
chr4	67950900	0.669421
chr4	68266600	0.925620
chr4	68441900	0.661157
chr4	68578700	0.677686
chr4	68992400	0.644628
chr4	69139000	0.917355
chr4	69182700	0.702479
chr4	69238900	0.768595
chr4	69257400	0.685950
chr4	69270200	0.768595
chr4	69276100	1.000000
chr4	69312900	0.677686
chr4	69317500	0.933884
chr4	69527500	0.727273

chr4	69770500	0.743802
chr4	69886000	0.776860
chr4	69924000	0.867769
chr4	70296900	0.900826
chr4	70333600	0.685950
chr4	70389500	0.652893
chr4	70584800	0.743802
chr4	71735100	0.661157
chr4	71793800	0.702479
chr4	71870500	0.876033
chr4	72071000	0.685950
chr4	72169200	0.818182
chr4	72434100	0.702479
chr4	72459100	0.826446
chr4	72559200	0.677686
chr4	72618500	0.685950
chr4	72632300	0.719008
chr4	72694500	0.958678
chr4	72723700	0.950413
chr4	73178300	0.768595
chr4	73530500	0.702479
chr4	73753300	0.933884
chr4	73920700	0.685950
chr4	74065300	0.950413
chr4	74088400	0.826446
chr4	74091200	0.735537
chr4	74274700	0.818182
chr4	74316500	0.884298
chr4	74319000	1.000000
chr4	74353100	0.661157
chr4	74386900	0.900826
chr4	74439800	0.768595
chr4	74450100	0.818182
chr4	74465400	0.818182
chr4	74529000	0.818182
chr4	74550100	0.834711
chr4	74571000	0.909091
chr4	74719600	0.760331
chr4	74818000	0.785124
chr4	74831100	0.760331
chr4	74851600	0.925620
chr4	74922200	0.669421
chr4	75186800	0.809917
chr4	76215400	0.702479
chr4	76250700	0.776860
chr4	76437200	0.743802
chr4	76487300	0.694215
chr4	76594300	0.892562
chr4	76621600	0.677686
chr4	76628400	0.876033
chr4	76630800	0.983471
chr4	76633800	0.867769
chr4	76640800	0.685950
chr4	76753300	0.694215

chr4	76807300	0.752066
chr4	76845500	0.644628
chr4	76977500	0.942149
chr4	76994200	0.983471
chr4	77120100	0.710744
chr4	77138700	0.644628
chr4	77157100	0.768595
chr4	77299200	0.834711
chr4	77302100	0.983471
chr4	77318800	0.661157
chr4	77380700	0.900826
chr4	77407300	0.925620
chr4	77457200	0.694215
chr4	77480500	0.669421
chr4	77492000	0.685950
chr4	77509600	0.909091
chr4	77512900	0.826446
chr4	77520500	0.719008
chr4	77529000	0.983471
chr4	77532500	0.851240
chr4	77545300	0.867769
chr4	77561700	0.652893
chr4	77613400	0.719008
chr4	77617500	0.917355
chr4	77620500	0.776860
chr4	77625900	0.859504
chr4	77630700	0.661157
chr4	77637400	0.818182
chr4	77640400	0.727273
chr4	77645300	0.867769
chr4	77651200	0.925620
chr4	77654200	0.694215
chr4	77704300	0.966942
chr4	77891800	0.801653
chr4	77903000	0.710744
chr4	77908600	0.669421
chr4	77919000	0.785124
chr4	77923700	0.933884
chr4	77944300	0.809917
chr4	78096600	0.760331
chr4	78099300	0.909091
chr4	78131200	0.694215
chr4	78621500	0.702479
chr4	78799300	0.826446
chr4	78916000	0.652893
chr4	78930000	1.000000
chr4	78987400	0.826446
chr4	78993900	0.719008
chr4	78997000	0.917355
chr4	79013900	0.892562
chr4	79083900	0.793388
chr4	79092900	0.669421
chr4	79096700	0.859504
chr4	79100200	0.867769

chr4	79140000	0.727273
chr4	79144200	0.826446
chr4	79148800	0.694215
chr4	79150900	0.785124
chr4	79174100	0.900826
chr4	79229500	0.669421
chr4	79234700	0.785124
chr4	79253500	0.909091
chr4	79258100	0.834711
chr4	79281300	0.925620
chr4	79291000	0.826446
chr4	79318500	0.958678
chr4	79339000	0.661157
chr4	79483100	0.727273
chr4	79583600	0.677686
chr4	79627700	0.801653
chr4	79642700	0.818182
chr4	80004900	0.702479
chr4	80138500	0.669421
chr4	80274100	0.776860
chr4	80473800	0.818182
chr4	80971400	0.760331
chr4	81111600	0.652893
chr4	81118100	0.694215
chr4	81128700	0.644628
chr4	81190000	0.719008
chr4	81287500	0.719008
chr4	81341600	0.661157
chr4	81375800	0.719008
chr4	81381900	0.809917
chr4	81393100	0.661157
chr4	82075000	0.826446
chr4	82348800	0.892562
chr4	82407300	0.735537
chr4	82440300	0.842975
chr4	82471800	0.867769
chr4	82480000	0.652893
chr4	82522100	0.685950
chr4	82568900	0.801653
chr4	82835100	0.760331
chr4	82912000	0.785124
chr4	83189700	0.685950
chr4	83422700	0.793388
chr4	83506200	0.768595
chr4	83619300	0.727273
chr4	83631900	0.661157
chr4	83660800	0.793388
chr4	83666200	0.859504
chr4	83680700	0.644628
chr4	83706900	0.876033
chr4	83733500	0.685950
chr4	84131500	0.694215
chr4	84283300	0.966942
chr4	84311800	0.851240

chr4	84634600	0.661157
chr4	84773300	0.859504
chr4	84837200	0.826446
chr4	84844500	0.685950
chr4	84972000	0.892562
chr4	84979500	0.785124
chr4	85145700	0.719008
chr4	85207400	0.661157
chr4	85246300	0.727273
chr4	85300200	0.685950
chr4	85304000	0.776860
chr4	85306900	0.917355
chr4	85401600	0.785124
chr4	85424700	0.818182
chr4	85487300	0.958678
chr4	85490200	0.727273
chr4	85501100	0.743802
chr4	85569000	0.859504
chr4	85612300	0.702479
chr4	85739400	0.669421
chr4	85792300	0.661157
chr4	85814500	0.743802
chr4	85873700	0.694215
chr4	85876600	0.652893
chr4	85879700	0.661157
chr4	85891800	0.776860
chr4	86050000	0.818182
chr4	86410700	0.644628
chr4	86744900	0.644628
chr4	86748400	0.702479
chr4	86851700	0.743802
chr4	87145000	0.685950
chr4	87155000	0.785124
chr4	87187400	0.652893
chr4	87279700	0.818182
chr4	87281800	0.702479
chr4	87343900	0.677686
chr4	87354200	0.727273
chr4	87371800	0.917355
chr4	87394700	0.785124
chr4	87402500	0.933884
chr4	87405600	0.842975
chr4	87779200	0.900826
chr4	87804000	0.661157
chr4	87825800	0.859504
chr4	87869100	0.991736
chr4	87872000	0.743802
chr4	87880800	0.950413
chr4	87883600	0.809917
chr4	87887200	0.983471
chr4	87892300	0.694215
chr4	87923600	0.983471
chr4	87933000	0.851240
chr4	87935900	0.710744

chr4	87952200	0.867769
chr4	87958100	0.644628
chr4	87960300	0.933884
chr4	87965000	0.809917
chr4	88021400	0.851240
chr4	88038400	0.752066
chr4	88068200	0.694215
chr4	88447400	0.768595
chr4	88601200	1.000000
chr4	88655800	0.685950
chr4	88740800	0.644628
chr4	88770200	0.710744
chr4	88774700	0.727273
chr4	88777600	0.710744
chr4	88818000	0.727273
chr4	88837600	0.661157
chr4	88840000	0.917355
chr4	88842400	0.983471
chr4	88872200	0.743802
chr4	88877200	0.694215
chr4	88887000	0.661157
chr4	88894500	0.644628
chr4	89043700	0.818182
chr4	89072300	0.694215
chr4	89099800	0.966942
chr4	89241800	0.776860
chr4	89365000	0.685950
chr4	89376600	0.702479
chr4	89455200	0.834711
chr4	89519900	0.694215
chr4	89534900	0.669421
chr4	89568300	0.975207
chr4	89660300	0.900826
chr4	89680100	0.917355
chr4	89881200	0.925620
chr4	90148300	0.644628
chr4	90341200	0.727273
chr4	90413600	0.702479
chr4	90433700	0.685950
chr4	90879000	0.661157
chr4	90919200	0.652893
chr4	90963700	0.652893
chr4	90974300	0.768595
chr4	91137100	0.685950
chr4	91674400	0.661157
chr4	91755100	0.975207
chr4	91986000	0.834711
chr4	92049000	0.710744
chr4	92127700	0.776860
chr4	92189100	0.661157
chr4	92202400	0.752066
chr4	92490100	0.942149
chr4	92499300	0.809917
chr4	92544300	0.727273

chr4	92826000	0.793388
chr4	93280900	0.842975
chr4	93354300	0.809917
chr4	93359800	0.917355
chr4	93368700	0.842975
chr4	93371600	0.809917
chr4	93385900	0.735537
chr4	93402100	0.752066
chr4	93405200	0.685950
chr4	93409400	0.851240
chr4	93422300	0.801653
chr4	93441300	0.867769
chr4	93444000	0.900826
chr4	93477000	0.661157
chr4	93497700	0.685950
chr4	94006600	0.752066
chr4	94038800	0.727273
chr4	94054200	0.900826
chr4	94069300	0.719008
chr4	94074100	0.669421
chr4	94120700	0.892562
chr4	94220400	0.685950
chr4	94286600	0.760331
chr4	94330800	0.677686
chr4	94652200	0.785124
chr4	94670300	0.809917
chr4	94701100	0.685950
chr4	94707600	0.876033
chr4	94736400	0.644628
chr4	94868900	0.785124
chr4	94881900	0.710744
chr4	94990500	0.950413
chr4	95069500	0.694215
chr4	95379300	0.661157
chr4	95456100	0.933884
chr4	95458500	0.909091
chr4	95462400	0.826446
chr4	95497200	0.735537
chr4	95917500	0.867769
chr4	95966000	0.685950
chr4	96080600	0.694215
chr4	96102600	0.826446
chr4	96226500	0.818182
chr4	96561700	0.793388
chr4	96661600	0.644628
chr4	96720200	0.719008
chr4	96934600	0.702479
chr4	97181700	0.851240
chr4	97915400	0.644628
chr4	98426100	0.710744
chr4	99031900	0.876033
chr4	99097900	0.752066
chr4	99115200	0.909091
chr4	99206000	0.884298

chr4	99408400	0.818182
chr4	99427900	0.809917
chr4	99436500	0.727273
chr4	99476900	0.776860
chr4	99480000	0.785124
chr4	99487700	0.834711
chr4	99528100	0.661157
chr4	99531100	0.834711
chr4	99533700	0.702479
chr4	99548000	0.702479
chr4	99584600	0.826446
chr4	99669100	0.735537
chr4	99826900	0.661157
chr4	99830300	0.719008
chr4	99935000	0.851240
chr4	99945900	0.710744
chr4	100143900	0.752066
chr4	100326500	0.752066
chr4	100380600	0.727273
chr4	100546100	0.719008
chr4	100567600	0.661157
chr4	101008600	0.743802
chr4	101014800	0.785124
chr4	101022600	0.743802
chr4	101035000	0.842975
chr4	101230200	0.677686
chr4	101531400	0.752066
chr4	101712400	0.776860
chr4	101732900	0.669421
chr4	101835300	0.652893
chr4	101896700	0.685950
chr4	101950700	0.644628
chr4	101953800	0.966942
chr4	101966400	0.743802
chr4	102079900	0.834711
chr4	102090800	0.644628
chr4	102113000	0.851240
chr4	102127700	0.661157
chr4	102182200	0.644628
chr4	102203600	0.694215
chr4	102227500	0.677686
chr4	102875300	0.768595
chr4	103140600	0.876033
chr4	103179200	0.677686
chr4	103184400	0.685950
chr4	103240500	0.909091
chr4	103244200	0.933884
chr4	103263600	0.768595
chr4	103313400	0.719008
chr4	103538100	0.793388
chr4	103741200	0.644628
chr4	103744100	0.702479
chr4	104346500	0.925620
chr4	104350600	0.793388

chr4	104399600	0.661157
chr4	104695500	0.644628
chr4	104699800	0.809917
chr4	104869100	0.900826
chr4	104918000	0.966942
chr4	105342600	0.727273
chr4	105368500	0.909091
chr4	105513300	0.991736
chr4	105537800	0.809917
chr4	105781300	0.743802
chr4	105837300	0.752066
chr4	106080300	0.669421
chr4	106375200	0.743802
chr4	106819500	0.727273
chr4	106894800	0.942149
chr4	106920600	0.644628
chr4	106978800	0.661157
chr4	107408600	0.685950
chr4	107435200	0.669421
chr4	107902400	0.644628
chr4	108020000	0.834711
chr4	108069900	0.669421
chr4	108270900	0.876033
chr4	108422100	0.768595
chr4	108570400	0.661157
chr4	108609300	0.710744
chr4	108655500	0.917355
chr4	108784000	0.702479
chr4	108828100	0.768595
chr4	108834800	0.652893
chr4	108964900	0.719008
chr4	108972400	0.702479
chr4	109031500	0.669421
chr4	109035500	0.702479
chr4	109056900	0.694215
chr4	109065300	0.727273
chr4	109082200	0.900826
chr4	109225100	0.669421
chr4	109636800	0.669421
chr4	109861900	0.669421
chr4	109915800	0.685950
chr4	110085100	0.884298
chr4	110340800	0.834711
chr4	110510700	0.661157
chr4	110584800	0.644628
chr4	110603900	0.661157
chr4	110732100	0.752066
chr4	110739300	0.669421
chr4	110834400	0.793388
chr4	110894300	0.710744
chr4	111028400	0.859504
chr4	111106200	0.752066
chr4	111115300	0.842975
chr4	111394400	0.694215

chr4	111397800	0.727273
chr4	111401900	0.669421
chr4	111409300	0.826446
chr4	111533100	0.801653
chr4	111539600	0.661157
chr4	111548000	0.851240
chr4	111564800	0.710744
chr4	111715600	0.892562
chr4	112131200	0.768595
chr4	112188500	0.776860
chr4	112572100	0.933884
chr4	112729800	0.950413
chr4	113049200	0.719008
chr4	113052000	0.867769
chr4	113192700	0.652893
chr4	113261800	0.760331
chr4	113265100	0.909091
chr4	113282600	0.950413
chr4	113300800	0.768595
chr4	113430300	0.768595
chr4	113575800	0.983471
chr4	113669100	1.000000
chr4	113674100	0.991736
chr4	113680700	0.975207
chr4	113683800	0.677686
chr4	113782600	0.950413
chr4	113817300	0.958678
chr4	113895100	0.694215
chr4	113906700	0.793388
chr4	113948300	0.644628
chr4	113971000	0.809917
chr4	113974100	0.727273
chr4	114052900	0.719008
chr4	114066900	0.900826
chr4	114090600	0.818182
chr4	114131000	0.867769
chr4	114192700	0.942149
chr4	114215700	0.719008
chr4	114234100	0.661157
chr4	114374600	0.925620
chr4	114390100	0.975207
chr4	114481300	0.785124
chr4	114505700	0.950413
chr4	114567600	0.867769
chr4	115147500	0.669421
chr4	115369700	0.685950
chr4	115416900	0.917355
chr4	115483200	0.892562
chr4	115702600	0.752066
chr4	115843700	0.652893
chr4	115846300	0.669421
chr4	115908700	0.677686
chr4	116156100	0.925620
chr4	116518300	0.760331

chr4	116895600	0.925620
chr4	118862900	0.644628
chr4	119322000	0.685950
chr4	119487700	0.834711
chr4	119507100	0.818182
chr4	119748600	0.900826
chr4	119768200	0.710744
chr4	119770300	0.950413
chr4	119775200	0.735537
chr4	119786900	0.710744
chr4	119869900	0.801653
chr4	119889500	0.752066
chr4	119961500	0.776860
chr4	119963700	0.818182
chr4	120201600	0.892562
chr4	120317100	0.694215
chr4	120440300	0.652893
chr4	120516500	0.760331
chr4	120647300	0.719008
chr4	120707500	0.727273
chr4	120985400	0.661157
chr4	121438100	0.710744
chr4	121445800	0.776860
chr4	121476200	0.710744
chr4	121483300	1.000000
chr4	121841200	0.661157
chr4	121886600	0.702479
chr4	121916000	0.826446
chr4	122099300	0.809917
chr4	122421700	0.719008
chr4	122431300	0.743802
chr4	122633200	0.818182
chr4	122670900	0.826446
chr4	122717800	0.966942
chr4	123021900	0.851240
chr4	123525100	0.685950
chr4	123539000	0.842975
chr4	123697500	0.793388
chr4	123702900	0.702479
chr4	123705600	0.793388
chr4	123710100	0.925620
chr4	123717500	0.884298
chr4	123720000	0.942149
chr4	123753600	0.851240
chr4	123757200	0.900826
chr4	123769500	0.818182
chr4	123775900	0.702479
chr4	123778800	0.710744
chr4	123782700	0.677686
chr4	124079800	0.661157
chr4	124226400	0.851240
chr4	124234500	0.644628
chr4	124273500	0.652893
chr4	124300500	0.975207

chr4	124311500	0.809917
chr4	124340100	0.925620
chr4	124365400	0.867769
chr4	124468500	0.958678
chr4	124707600	0.983471
chr4	124874500	0.884298
chr4	125372200	0.818182
chr4	125381200	0.851240
chr4	125404600	0.768595
chr4	125556700	0.669421
chr4	125616500	0.669421
chr4	125660400	0.644628
chr4	125728800	0.719008
chr4	125732000	0.652893
chr4	125795300	0.876033
chr4	125858700	0.702479
chr4	126033100	0.669421
chr4	126047100	0.851240
chr4	126189900	0.859504
chr4	126279700	0.942149
chr4	126482400	0.669421
chr4	126560800	0.975207
chr4	126636000	0.809917
chr4	126656800	0.768595
chr4	127102200	0.685950
chr4	127967300	0.983471
chr4	127970400	0.826446
chr4	128047500	0.661157
chr4	128125700	0.727273
chr4	128424500	0.818182
chr4	128439600	0.727273
chr4	128526300	0.694215
chr4	128767300	0.644628
chr4	128774100	0.719008
chr4	128778600	0.834711
chr4	128893700	0.719008
chr4	129243300	0.685950
chr4	129276300	0.652893
chr4	129284100	0.644628
chr4	129383500	0.760331
chr4	129439300	0.900826
chr4	129447400	0.710744
chr4	129450400	0.743802
chr4	129490600	0.677686
chr4	129557800	0.876033
chr4	129561500	0.669421
chr4	129575200	0.694215
chr4	129604400	1.000000
chr4	129646600	0.702479
chr4	129724100	1.000000
chr4	129753100	0.966942
chr4	129780800	0.694215
chr4	130032300	0.900826
chr4	130035500	0.760331

chr4	130089300	0.776860
chr4	130241300	0.760331
chr4	130244200	0.727273
chr4	130784100	0.735537
chr4	130847600	0.644628
chr4	130851500	0.669421
chr4	130943600	0.809917
chr4	130946700	0.842975
chr4	130989800	0.694215
chr4	131032700	0.644628
chr4	131255500	0.644628
chr4	131643900	0.685950
chr4	131935300	0.859504
chr4	133046400	0.818182
chr4	133414100	0.818182
chr4	133418000	0.867769
chr4	134053200	0.719008
chr4	134389000	0.661157
chr4	134397500	0.702479
chr4	134513200	0.760331
chr4	134515500	0.900826
chr4	135006600	0.826446
chr4	135440000	0.685950
chr4	135450900	0.644628
chr4	135474000	0.677686
chr4	135545800	0.793388
chr4	135831400	0.644628
chr4	135857500	0.685950
chr4	136053600	0.652893
chr4	136106500	0.793388
chr4	136307200	0.842975
chr4	137195200	0.768595
chr4	137199500	0.694215
chr4	137203300	0.735537
chr4	137426700	0.760331
chr4	137466800	0.669421
chr4	137612100	0.661157
chr4	137672900	0.669421
chr4	137718900	0.884298
chr4	137820800	0.719008
chr4	138372700	0.727273
chr4	138455500	0.867769
chr4	138457900	0.818182
chr4	138463300	0.743802
chr4	138466800	0.851240
chr4	138585200	0.743802
chr4	138968600	0.809917
chr4	138972100	0.661157
chr4	139035300	0.727273
chr4	139138400	0.677686
chr4	139144100	0.925620
chr4	139238500	0.735537
chr4	139320300	0.710744
chr4	139397100	0.735537

chr4	139580000	0.958678
chr4	139582100	0.768595
chr4	139604400	0.900826
chr4	139618400	0.768595
chr4	139646300	0.892562
chr4	139681100	0.677686
chr4	139812900	0.685950
chr4	139858900	0.925620
chr4	139894900	0.975207
chr4	139899100	0.768595
chr4	139941900	0.884298
chr4	139949800	0.842975
chr4	139952100	0.793388
chr4	140066000	0.867769
chr4	140075900	0.966942
chr4	140079800	0.942149
chr4	140163100	0.950413
chr4	140228500	0.669421
chr4	140236200	0.925620
chr4	140251600	0.867769
chr4	140382100	0.917355
chr4	140472600	0.752066
chr4	140546500	0.826446
chr4	140555100	0.785124
chr4	140683800	0.884298
chr4	140696800	0.652893
chr4	140705000	0.785124
chr4	140737200	0.809917
chr4	140746600	0.975207
chr4	140774600	0.677686
chr4	140797400	0.859504
chr4	140809400	0.727273
chr4	140815100	0.719008
chr4	140823100	0.661157
chr4	140926200	0.859504
chr4	140934100	0.694215
chr4	140968200	0.876033
chr4	140989700	0.768595
chr4	140999900	0.909091
chr4	141003600	0.842975
chr4	141013700	0.834711
chr4	141016500	0.710744
chr4	141059500	0.884298
chr4	141134700	0.809917
chr4	141148800	0.710744
chr4	141161300	0.900826
chr4	141164700	0.644628
chr4	141265900	0.900826
chr4	141271100	0.719008
chr4	141539100	0.892562
chr4	141563400	0.793388
chr4	141575300	0.925620
chr4	141656900	0.669421
chr4	141659900	0.652893

chr4	141778100	0.644628
chr4	141797600	0.727273
chr4	141846700	0.867769
chr4	142546400	0.661157
chr4	142693600	0.702479
chr4	143116100	0.661157
chr4	143191100	0.652893
chr4	143327500	0.818182
chr4	143357000	0.983471
chr4	143630100	0.685950
chr4	143906300	0.652893
chr4	144184600	0.644628
chr4	144207900	0.818182
chr4	144273400	0.867769
chr4	144277100	0.669421
chr4	144299800	0.710744
chr4	144312500	0.677686
chr4	144320300	0.669421
chr4	144376300	0.743802
chr4	144635500	0.851240
chr4	145099900	0.644628
chr4	145111600	0.652893
chr4	145274800	0.685950
chr4	145278200	0.991736
chr4	145571000	0.785124
chr4	145609700	0.694215
chr4	145654800	0.793388
chr4	145986600	0.652893
chr4	146139400	0.652893
chr4	146461400	0.702479
chr4	146608500	0.719008
chr4	146625700	0.876033
chr4	146727200	0.727273
chr4	146752400	0.669421
chr4	146760300	0.818182
chr4	146773500	0.694215
chr4	146802800	0.652893
chr4	146805200	0.884298
chr4	146809200	0.776860
chr4	146816000	0.719008
chr4	146828800	0.661157
chr4	146831600	0.818182
chr4	146835900	0.669421
chr4	146851900	0.719008
chr4	146954000	0.685950
chr4	147075600	0.719008
chr4	147101400	0.644628
chr4	147302800	0.652893
chr4	147394000	0.702479
chr4	147554800	0.785124
chr4	147796000	0.719008
chr4	147865500	0.644628
chr4	147924100	0.884298
chr4	147933100	0.842975

chr4	148120100	0.685950
chr4	148300300	0.661157
chr4	148488400	0.719008
chr4	148547300	0.669421
chr4	148550200	0.818182
chr4	148675800	0.669421
chr4	148785200	0.760331
chr4	148788100	0.801653
chr4	148906100	0.801653
chr4	149019500	0.719008
chr4	149033700	0.669421
chr4	149803700	0.793388
chr4	150105200	0.669421
chr4	151166000	0.644628
chr4	151178100	0.677686
chr4	151181200	0.652893
chr4	151237100	0.760331
chr4	151289300	0.644628
chr4	151307100	0.752066
chr4	151606000	0.652893
chr4	151706000	0.727273
chr4	151898600	0.867769
chr4	152035200	0.652893
chr4	152087600	0.702479
chr4	152206100	0.933884
chr4	152216700	0.694215
chr4	152284200	0.801653
chr4	152351600	0.719008
chr4	152354300	0.834711
chr4	152364900	0.884298
chr4	152396400	0.719008
chr4	152406300	0.975207
chr4	152410700	0.826446
chr4	152446100	0.900826
chr4	152459900	0.818182
chr4	152471100	0.983471
chr4	152475500	0.809917
chr4	152526700	0.669421
chr4	152609200	0.710744
chr4	152667200	0.768595
chr4	152689800	0.644628
chr4	152707000	0.892562
chr4	152710700	0.793388
chr4	152732300	0.752066
chr4	152796200	0.975207
chr4	152808400	0.644628
chr4	152833700	0.727273
chr4	152878400	0.892562
chr4	152913900	0.752066
chr4	152931300	0.669421
chr4	152996600	0.694215
chr4	153086600	0.966942
chr4	153200100	0.876033
chr4	153274000	0.826446

chr4	153325200	0.702479
chr4	153346200	0.834711
chr4	153436100	0.727273
chr4	153461900	0.760331
chr4	153512900	0.685950
chr4	153526700	0.793388
chr4	153531400	0.661157
chr4	153561900	0.644628
chr4	153565300	0.818182
chr4	153577000	0.851240
chr4	153590200	0.644628
chr4	153622200	0.776860
chr4	153626700	0.785124
chr4	153717900	0.785124
chr4	153772100	0.958678
chr4	153848000	0.942149
chr4	153851700	0.892562
chr4	153871400	0.818182
chr4	153903100	0.909091
chr4	153918100	0.884298
chr4	153923200	0.900826
chr4	153930500	0.818182
chr4	153937600	0.719008
chr4	153960600	0.677686
chr4	153989100	0.719008
chr4	154004700	0.677686
chr4	154048600	0.661157
chr4	154077500	0.669421
chr4	154111200	0.669421
chr4	154128800	0.702479
chr4	154154200	0.735537
chr4	154176900	0.644628
chr4	154182600	0.892562
chr4	154190100	0.785124
chr4	154196900	0.710744
chr4	154373700	0.801653
chr4	154400700	0.669421
chr4	154410800	0.925620
chr4	154419700	0.685950
chr4	154444200	0.884298
chr4	154457400	0.900826
chr4	154460000	0.710744
chr4	154469100	0.702479
chr4	154540800	0.809917
chr4	154703200	0.768595
chr4	154738300	0.644628
chr4	154951900	0.801653
chr4	155059200	0.760331
chr4	155103300	0.743802
chr4	155105600	0.710744
chr4	155198300	0.776860
chr4	155642100	0.644628
chr4	155660100	0.719008
chr4	155981700	0.652893

chr4	156043800	0.768595
chr4	156170100	0.727273
chr4	156241000	0.652893
chr4	156940900	0.652893
chr4	157031900	0.793388
chr4	157427100	0.785124
chr4	157692400	0.925620
chr4	157863800	0.842975
chr4	157936100	0.661157
chr4	158117600	0.743802
chr4	158479600	0.760331
chr4	159175600	0.892562
chr4	159182600	0.842975
chr4	159493700	0.834711
chr4	159767000	0.983471
chr4	159791800	0.685950
chr4	159824100	0.867769
chr4	159884000	0.727273
chr4	159938100	0.909091
chr4	159943500	0.983471
chr4	159945800	0.966942
chr4	160019200	0.727273
chr4	160058500	0.669421
chr4	160071900	0.735537
chr4	160094900	0.735537
chr4	160097100	1.000000
chr4	160113700	0.644628
chr4	160125500	0.644628
chr4	160150700	0.867769
chr4	160154000	0.909091
chr4	160204900	0.702479
chr4	160424800	0.925620
chr4	160461300	0.661157
chr4	160480000	0.669421
chr4	160607100	0.694215
chr4	160872300	0.851240
chr4	162027000	0.652893
chr4	162111200	0.809917
chr4	162315100	0.826446
chr4	162446700	0.743802
chr4	162982700	0.669421
chr4	163342800	0.842975
chr4	163842500	0.661157
chr4	164486700	0.677686
chr4	164532900	0.669421
chr4	164909200	0.702479
chr4	165126000	0.652893
chr4	165448400	0.652893
chr4	165711000	0.652893
chr4	165720700	0.950413
chr4	165744100	0.644628
chr4	165788300	0.925620
chr4	165802100	0.710744
chr4	166578300	0.752066

chr4	166590200	0.685950
chr4	166678600	0.776860
chr4	166704900	0.644628
chr4	166959700	0.669421
chr4	167637000	0.892562
chr4	169072600	0.760331
chr4	169526300	0.661157
chr4	169536600	0.801653
chr4	169555200	0.760331
chr4	169576000	0.702479
chr4	169657200	0.834711
chr4	169706200	0.818182
chr4	169725500	0.983471
chr4	169738200	0.917355
chr4	169757600	0.677686
chr4	169762700	0.900826
chr4	169767900	0.652893
chr4	169770600	0.743802
chr4	169841500	0.644628
chr4	169843900	0.702479
chr4	169939200	0.859504
chr4	169971900	0.652893
chr4	169991600	0.975207
chr4	170025500	0.801653
chr4	170065000	0.727273
chr4	170106900	0.702479
chr4	170165700	0.760331
chr4	170173600	0.966942
chr4	170178900	0.661157
chr4	170581400	0.644628
chr4	170841100	0.925620
chr4	170856500	0.801653
chr4	170862100	0.652893
chr4	170866700	0.677686
chr4	170879600	0.661157
chr4	170909300	0.752066
chr4	170923200	0.785124
chr4	170938600	0.760331
chr4	171031900	0.900826
chr4	171150400	0.661157
chr4	171350400	0.793388
chr4	171604000	0.644628
chr4	172032400	0.694215
chr4	172933000	0.661157
chr4	173469800	0.801653
chr4	173630200	0.785124
chr4	173730800	0.677686
chr4	173935300	0.834711
chr4	173959900	0.826446
chr4	173995300	0.975207
chr4	174013700	0.925620
chr4	174042800	0.719008
chr4	174093600	0.867769
chr4	174095800	0.776860

chr4	174113100	0.776860
chr4	174133500	0.694215
chr4	174139700	0.735537
chr4	174164600	0.677686
chr4	174171900	0.867769
chr4	174177600	0.793388
chr4	174185700	0.677686
chr4	174265200	0.727273
chr4	174302800	0.793388
chr4	174411200	0.752066
chr4	174427700	0.702479
chr4	174440000	0.909091
chr4	174446100	0.652893
chr4	174448700	0.785124
chr4	174452900	0.743802
chr4	174838800	0.669421
chr4	174858100	0.785124
chr4	174963200	0.867769
chr4	175133300	0.900826
chr4	175324400	0.776860
chr4	175528400	0.925620
chr4	175635000	0.719008
chr4	175954500	0.917355
chr4	176693700	0.884298
chr4	176697700	0.900826
chr4	176708900	0.694215
chr4	176734600	0.785124
chr4	176827300	0.752066
chr4	176892900	0.834711
chr4	176920600	0.710744
chr4	177108100	0.818182
chr4	177225500	0.644628
chr4	177279100	0.735537
chr4	178150400	0.702479
chr4	178171600	0.652893
chr4	178406100	0.785124
chr4	178480100	0.669421
chr4	178560500	0.743802
chr4	179012400	0.859504
chr4	179494800	0.652893
chr4	179777600	0.702479
chr4	180047300	0.710744
chr4	180067600	0.644628
chr4	180213500	0.752066
chr4	180337300	0.661157
chr4	180386900	0.991736
chr4	180631500	0.942149
chr4	180972100	0.694215
chr4	180998800	0.652893
chr4	181055200	0.652893
chr4	181376700	0.685950
chr4	181452400	0.710744
chr4	181460200	0.727273
chr4	181463600	0.727273

chr4	181689000	0.859504
chr4	181714500	0.677686
chr4	181938700	0.760331
chr4	181983000	0.694215
chr4	182087800	0.685950
chr4	182114800	0.760331
chr4	182168200	0.669421
chr4	182272400	0.702479
chr4	182397200	0.776860
chr4	182405400	0.644628
chr4	182425600	0.669421
chr4	182609700	0.677686
chr4	182714500	0.876033
chr4	182742500	0.719008
chr4	182744600	0.842975
chr4	182871300	0.983471
chr4	182883700	0.702479
chr4	183006100	0.942149
chr4	183058400	0.818182
chr4	183062300	0.685950
chr4	183068800	0.793388
chr4	183072200	0.702479
chr4	183100500	0.876033
chr4	183128300	0.677686
chr4	183131500	0.702479
chr4	183159600	0.801653
chr4	183195200	0.909091
chr4	183215200	0.710744
chr4	183252000	0.644628
chr4	183306000	0.644628
chr4	183337800	0.644628
chr4	183376000	0.652893
chr4	183416700	0.727273
chr4	183423500	0.743802
chr4	183425900	0.818182
chr4	183451400	0.719008
chr4	183458900	0.809917
chr4	183470200	0.685950
chr4	183475100	0.710744
chr4	183482000	0.909091
chr4	183506900	0.702479
chr4	183637500	0.818182
chr4	183689800	0.644628
chr4	183692500	0.950413
chr4	183775200	0.702479
chr4	183784100	0.669421
chr4	184002000	0.685950
chr4	184023600	0.727273
chr4	184026700	0.826446
chr4	184034900	0.776860
chr4	184040200	0.966942
chr4	184066800	0.702479
chr4	184076300	0.735537
chr4	184086700	0.752066

chr4	184105600	0.892562
chr4	184108300	0.743802
chr4	184111200	0.644628
chr4	184162500	0.826446
chr4	184178500	0.719008
chr4	184557700	0.776860
chr4	184644700	0.735537
chr4	184695200	0.933884
chr4	184779600	0.669421
chr4	184857200	0.809917
chr4	184890200	0.842975
chr4	184893600	0.727273
chr4	184909100	0.826446
chr4	184961400	0.669421
chr4	185018600	0.669421
chr4	185086400	0.702479
chr4	185090100	0.917355
chr4	185092600	0.743802
chr4	185189700	0.760331
chr4	185209800	0.644628
chr4	185235000	0.644628
chr4	185268900	1.000000
chr4	185308200	0.958678
chr4	185311600	0.859504
chr4	185323200	0.991736
chr4	185329500	0.818182
chr4	185351700	0.661157
chr4	185363500	0.958678
chr4	185369900	0.859504
chr4	185375500	0.851240
chr4	185383100	0.669421
chr4	185433800	0.710744
chr4	185470200	0.876033
chr4	185538600	0.958678
chr4	185566000	0.760331
chr4	185574900	0.727273
chr4	185610000	0.842975
chr4	185657400	0.801653
chr4	185668700	0.876033
chr4	185933000	0.652893
chr4	185935500	0.727273
chr4	185976200	0.809917
chr4	186007400	0.909091
chr4	186029800	0.983471
chr4	186041600	0.661157
chr4	186070600	0.743802
chr4	186105800	0.661157
chr4	186123300	0.900826
chr4	186134700	0.809917
chr4	186138600	0.801653
chr4	186193600	0.661157
chr4	186228100	0.983471
chr4	186254000	0.652893
chr4	186495100	0.760331

chr4	186562400	0.677686
chr4	186572600	0.851240
chr4	186578400	0.933884
chr4	186623100	0.661157
chr4	186659100	0.644628
chr4	186661300	0.776860
chr4	186697100	0.644628
chr4	186727100	0.694215
chr4	186732700	0.743802
chr4	186758000	0.950413
chr4	186761800	0.900826
chr4	186769100	0.669421
chr4	186803100	0.661157
chr4	186877400	0.933884
chr4	186934700	0.933884
chr4	186936800	0.644628
chr4	186949400	0.661157
chr4	186951700	0.694215
chr4	186953800	0.694215
chr4	186990300	0.710744
chr4	187000300	0.685950
chr4	187003100	0.867769
chr4	187029900	0.669421
chr4	187162600	0.983471
chr4	187170000	0.966942
chr4	187172900	0.851240
chr4	187187200	0.892562
chr4	187219400	0.702479
chr4	187266900	0.661157
chr4	187556000	0.900826
chr4	187575100	0.752066
chr4	187590800	0.661157
chr4	187607000	0.809917
chr4	187610100	0.776860
chr4	187614900	0.851240
chr4	187624000	0.851240
chr4	187642200	0.834711
chr4	187941600	0.768595
chr4	187992500	0.768595
chr4	188180100	0.727273
chr4	188701600	0.644628
chr4	188719900	0.661157
chr4	188805000	0.768595
chr4	188914600	0.950413
chr4	188938600	0.702479
chr4	188944400	0.801653
chr4	188953700	0.727273
chr4	189028100	0.685950
chr4	189031600	0.809917
chr4	189043800	0.818182
chr4	189167800	0.644628
chr4	189189700	0.760331
chr4	189321400	0.991736
chr4	189350500	0.909091

chr4	189358400	0.867769
chr4	189373300	0.900826
chr4	189541300	0.776860
chr4	189601500	0.694215
chr4	189744300	0.743802
chr4	189898200	1.000000
chr4	189968300	1.000000
chr4	189971200	0.652893
chr4	189981400	1.000000
chr4	190083200	0.975207
chr4	190099700	0.933884
chr4	190107400	0.950413
chr4	190123700	0.768595
chr4	190575100	0.900826
chr4	190582200	0.801653
chr4	190587300	0.768595
chr4	190714200	0.752066
chr5	82600	0.917355
chr5	86200	0.727273
chr5	130500	0.851240
chr5	188500	0.702479
chr5	208000	0.661157
chr5	298200	0.950413
chr5	302700	0.694215
chr5	316900	0.702479
chr5	346400	0.942149
chr5	368200	0.677686
chr5	374200	0.842975
chr5	393300	0.818182
chr5	400300	0.983471
chr5	493000	0.785124
chr5	496400	0.785124
chr5	529300	0.760331
chr5	673100	0.958678
chr5	794200	0.851240
chr5	799000	0.818182
chr5	983500	0.661157
chr5	1010900	0.768595
chr5	1094700	0.694215
chr5	1097900	0.818182
chr5	1100200	0.942149
chr5	1102400	0.859504
chr5	1104800	0.909091
chr5	1119000	0.793388
chr5	1141500	0.735537
chr5	1168400	0.735537
chr5	1247400	0.818182
chr5	1316500	0.900826
chr5	1381200	0.809917
chr5	1480700	0.661157
chr5	1505500	0.867769
chr5	1507700	0.752066
chr5	1512200	0.710744
chr5	1517900	0.876033

chr5	1601500	0.669421
chr5	1607700	0.917355
chr5	1641200	0.793388
chr5	1751800	0.652893
chr5	1828500	0.900826
chr5	1832100	0.776860
chr5	1835100	0.809917
chr5	1841800	0.694215
chr5	1878200	0.884298
chr5	1889500	0.826446
chr5	1928300	0.652893
chr5	1953000	0.809917
chr5	1967100	0.644628
chr5	2039800	0.785124
chr5	2165200	0.702479
chr5	2204300	0.876033
chr5	2335600	0.785124
chr5	2533200	0.851240
chr5	2538000	0.752066
chr5	2741900	0.752066
chr5	2767500	0.661157
chr5	2780900	0.876033
chr5	2878300	0.677686
chr5	2915200	0.818182
chr5	3100500	0.809917
chr5	3104600	0.917355
chr5	3107800	0.818182
chr5	3119600	0.768595
chr5	3121900	0.818182
chr5	3124300	0.991736
chr5	3186900	0.710744
chr5	3194900	0.644628
chr5	3200400	0.669421
chr5	3395200	0.644628
chr5	3511600	0.719008
chr5	3531200	0.859504
chr5	3593000	0.677686
chr5	3710000	0.809917
chr5	3869400	0.760331
chr5	4197000	0.677686
chr5	4199400	0.710744
chr5	4207400	0.719008
chr5	4212800	0.925620
chr5	4231400	0.685950
chr5	4296300	0.719008
chr5	4399400	0.644628
chr5	4546100	0.661157
chr5	4554800	0.644628
chr5	5078600	0.975207
chr5	5179700	0.950413
chr5	5182900	0.925620
chr5	5266900	0.735537
chr5	5271600	0.867769
chr5	5313500	0.793388

chr5	5326600	0.933884
chr5	5331100	0.702479
chr5	5379600	0.652893
chr5	5484100	0.685950
chr5	5495500	0.685950
chr5	5522600	0.727273
chr5	5543500	0.710744
chr5	5573100	0.669421
chr5	5672300	0.735537
chr5	5695400	0.834711
chr5	5887400	0.752066
chr5	6070000	0.719008
chr5	6302700	0.694215
chr5	6383800	0.661157
chr5	6451100	0.694215
chr5	6468600	0.661157
chr5	6649600	0.710744
chr5	6701400	0.752066
chr5	6720400	0.710744
chr5	6730600	0.652893
chr5	6748100	0.644628
chr5	6766200	0.694215
chr5	7310100	0.942149
chr5	7315800	0.719008
chr5	7331900	0.776860
chr5	7348100	0.644628
chr5	7363600	0.752066
chr5	7401800	0.760331
chr5	7412900	0.752066
chr5	7423200	0.933884
chr5	7425800	0.983471
chr5	7447400	0.826446
chr5	7467300	0.760331
chr5	7473900	0.983471
chr5	7516100	0.685950
chr5	7564800	0.834711
chr5	7597100	0.867769
chr5	7607500	1.000000
chr5	7664100	0.785124
chr5	7705600	0.652893
chr5	7755500	0.652893
chr5	7785300	0.760331
chr5	7816200	0.776860
chr5	8060800	0.892562
chr5	8181300	0.652893
chr5	8848800	0.801653
chr5	8871100	0.809917
chr5	9169100	0.809917
chr5	9197700	0.644628
chr5	9222900	0.768595
chr5	9266000	0.909091
chr5	9295600	0.876033
chr5	9432400	0.735537
chr5	9456800	0.925620

chr5	9484100	0.652893
chr5	9596900	0.677686
chr5	9830100	0.801653
chr5	9834800	0.834711
chr5	10103400	0.991736
chr5	10157500	0.768595
chr5	10162300	0.669421
chr5	10202500	0.942149
chr5	10284500	0.677686
chr5	10339800	0.942149
chr5	10455600	0.652893
chr5	10461900	0.677686
chr5	10468400	0.694215
chr5	10481400	0.719008
chr5	10514400	0.735537
chr5	10525100	0.661157
chr5	10534000	0.834711
chr5	10551800	1.000000
chr5	10586000	0.710744
chr5	10589500	0.685950
chr5	10607700	0.925620
chr5	10612500	0.793388
chr5	10635600	0.826446
chr5	10637900	0.809917
chr5	10642600	0.727273
chr5	10689900	0.785124
chr5	10700800	0.834711
chr5	10710500	0.752066
chr5	10857500	0.892562
chr5	10905500	0.793388
chr5	11251800	0.685950
chr5	11588800	0.859504
chr5	11651700	0.776860
chr5	11791700	0.752066
chr5	11852200	0.834711
chr5	12231700	0.760331
chr5	12238300	0.735537
chr5	12324000	0.958678
chr5	12331400	0.983471
chr5	12413600	0.669421
chr5	12505100	0.735537
chr5	12623400	0.776860
chr5	12669200	0.983471
chr5	12672200	0.793388
chr5	14108800	0.727273
chr5	14115800	0.966942
chr5	14119100	0.694215
chr5	14147400	0.966942
chr5	14149500	0.776860
chr5	14166000	0.719008
chr5	14171900	0.785124
chr5	14174400	0.834711
chr5	14178700	0.884298
chr5	14185800	0.669421

chr5	14188500	0.677686
chr5	14197400	0.727273
chr5	14217100	0.859504
chr5	14219300	0.661157
chr5	14223100	0.801653
chr5	14237600	0.867769
chr5	14240500	0.900826
chr5	14250300	0.776860
chr5	14259500	0.983471
chr5	14262200	0.859504
chr5	14265100	0.942149
chr5	14270500	0.933884
chr5	14284900	0.661157
chr5	14342400	0.917355
chr5	14387200	0.867769
chr5	14400200	0.867769
chr5	14417900	0.776860
chr5	14446300	0.867769
chr5	14728800	0.809917
chr5	14850700	0.942149
chr5	14957100	0.884298
chr5	15009600	0.644628
chr5	15027700	0.743802
chr5	15234700	0.661157
chr5	15264300	0.652893
chr5	15539100	0.834711
chr5	15544400	0.785124
chr5	15554700	0.958678
chr5	15601700	0.669421
chr5	15645300	0.694215
chr5	15717200	0.661157
chr5	15794600	0.801653
chr5	15797000	0.677686
chr5	15834800	0.694215
chr5	15919400	0.727273
chr5	16428400	0.743802
chr5	16509100	0.719008
chr5	16575200	0.661157
chr5	16702400	0.768595
chr5	16713800	0.884298
chr5	16722000	0.876033
chr5	16728900	0.768595
chr5	16734000	0.818182
chr5	16754500	0.834711
chr5	16867000	0.909091
chr5	16895200	0.834711
chr5	16898100	0.859504
chr5	16901100	0.950413
chr5	16930900	0.694215
chr5	16953300	0.710744
chr5	17097300	0.776860
chr5	17116100	0.743802
chr5	17133500	0.793388
chr5	17145200	0.652893

chr5	17147600	0.694215
chr5	17152100	0.752066
chr5	17213900	0.768595
chr5	17223900	0.892562
chr5	17229500	0.743802
chr5	17241900	0.760331
chr5	17374700	0.669421
chr5	17404500	0.702479
chr5	17482000	0.702479
chr5	18099500	0.719008
chr5	18394300	0.652893
chr5	18544900	0.661157
chr5	18797500	0.776860
chr5	18800500	0.876033
chr5	18858900	0.842975
chr5	19314400	0.685950
chr5	19691600	0.752066
chr5	19745700	0.809917
chr5	19777800	0.677686
chr5	19881400	0.859504
chr5	19928700	0.743802
chr5	20609200	0.702479
chr5	20973000	0.694215
chr5	21615000	0.975207
chr5	22139800	0.785124
chr5	22165300	0.719008
chr5	22171700	0.661157
chr5	22188300	0.900826
chr5	22213200	0.892562
chr5	22929600	0.768595
chr5	23209400	0.752066
chr5	23234600	0.884298
chr5	23419600	0.743802
chr5	23507200	0.669421
chr5	24209000	0.727273
chr5	24382600	0.652893
chr5	24623600	0.644628
chr5	25021900	0.892562
chr5	25449000	0.909091
chr5	25451700	0.661157
chr5	27084200	0.909091
chr5	27088100	0.710744
chr5	27316600	0.933884
chr5	28391600	0.710744
chr5	29150900	0.867769
chr5	29525000	0.826446
chr5	30100900	0.900826
chr5	30596700	0.702479
chr5	30608100	0.661157
chr5	30995100	0.801653
chr5	31049500	0.685950
chr5	31107300	0.876033
chr5	31153900	0.710744
chr5	31382900	0.752066

chr5	31427700	0.851240
chr5	31614600	0.785124
chr5	31618900	0.917355
chr5	31657400	0.710744
chr5	31689000	0.702479
chr5	31731200	0.917355
chr5	31754700	0.909091
chr5	31770000	0.694215
chr5	31993400	0.719008
chr5	31995700	0.776860
chr5	32018500	0.652893
chr5	32028000	0.669421
chr5	32128500	0.669421
chr5	32232800	0.735537
chr5	32274700	0.958678
chr5	32277500	0.694215
chr5	32284400	0.933884
chr5	32286600	0.694215
chr5	32298600	0.652893
chr5	32320100	0.694215
chr5	32359700	0.809917
chr5	32439500	0.644628
chr5	32474900	0.859504
chr5	32501300	0.752066
chr5	32505600	0.876033
chr5	32583700	0.942149
chr5	32680600	0.785124
chr5	32953000	0.867769
chr5	32964700	0.694215
chr5	33340000	0.727273
chr5	33445600	0.933884
chr5	33448700	0.950413
chr5	33473200	0.694215
chr5	33489200	0.900826
chr5	33564700	0.834711
chr5	33589100	0.760331
chr5	33686200	0.669421
chr5	33764100	0.785124
chr5	33833500	0.677686
chr5	33850500	0.694215
chr5	33855000	0.719008
chr5	33859400	0.909091
chr5	33928700	0.694215
chr5	34297400	0.719008
chr5	34410100	0.851240
chr5	34413200	0.966942
chr5	34416400	0.958678
chr5	34475700	0.991736
chr5	34483400	0.933884
chr5	34486700	0.743802
chr5	34497200	0.752066
chr5	34522500	0.876033
chr5	34530300	0.652893
chr5	34538500	0.743802

chr5	34541700	0.876033
chr5	34544500	0.842975
chr5	34548800	0.991736
chr5	34564300	0.842975
chr5	34610300	0.801653
chr5	34666900	0.859504
chr5	34673600	0.900826
chr5	34679600	0.900826
chr5	34685500	1.000000
chr5	34687800	0.801653
chr5	34690600	0.917355
chr5	34693500	0.834711
chr5	34700100	0.776860
chr5	34704300	0.842975
chr5	34710200	0.710744
chr5	34715400	0.644628
chr5	34718500	0.876033
chr5	34734100	0.685950
chr5	34806100	0.710744
chr5	34864100	0.909091
chr5	35112700	0.834711
chr5	35333400	0.669421
chr5	35335900	0.793388
chr5	35338500	0.983471
chr5	35754400	0.752066
chr5	35795000	0.661157
chr5	36041700	0.760331
chr5	36079900	0.793388
chr5	36418400	0.768595
chr5	36442500	0.776860
chr5	36591200	0.826446
chr5	36596900	0.727273
chr5	36619300	0.983471
chr5	36655100	0.743802
chr5	36747300	0.727273
chr5	37661500	0.661157
chr5	37844300	0.801653
chr5	37847700	0.652893
chr5	37875800	0.727273
chr5	38099500	0.652893
chr5	38167800	0.677686
chr5	38379600	0.644628
chr5	38468100	0.760331
chr5	38533300	0.669421
chr5	38725500	0.785124
chr5	38979100	0.644628
chr5	39022400	0.933884
chr5	39064900	0.669421
chr5	39413300	0.694215
chr5	39527300	0.652893
chr5	39679700	0.652893
chr5	39800500	0.867769
chr5	40142900	0.768595
chr5	40308900	0.694215

chr5	40386100	0.743802
chr5	40497200	0.735537
chr5	40677300	0.842975
chr5	40832300	0.694215
chr5	40871500	0.694215
chr5	40915600	0.859504
chr5	40929600	0.735537
chr5	40934700	0.776860
chr5	41334900	0.644628
chr5	41419100	0.752066
chr5	41575500	0.793388
chr5	41637200	0.644628
chr5	41649600	0.834711
chr5	42361200	0.892562
chr5	42811800	0.752066
chr5	42814200	0.859504
chr5	42858900	0.702479
chr5	42950800	0.677686
chr5	43016200	0.661157
chr5	43020500	0.727273
chr5	43038200	0.644628
chr5	43303300	0.851240
chr5	43314900	0.818182
chr5	44048800	0.760331
chr5	44482900	0.661157
chr5	44697400	0.652893
chr5	44943100	0.933884
chr5	45058300	0.809917
chr5	45061300	0.917355
chr5	45110400	0.884298
chr5	45207000	0.842975
chr5	45288800	0.826446
chr5	45567900	0.909091
chr5	45678200	0.702479
chr5	45848400	0.975207
chr5	49406100	0.652893
chr5	49431600	0.677686
chr5	49450900	0.826446
chr5	49517400	0.727273
chr5	49708300	0.710744
chr5	49846300	0.760331
chr5	49970300	0.900826
chr5	49987500	0.677686
chr5	49995500	0.719008
chr5	50010900	0.760331
chr5	50018700	0.661157
chr5	50469200	0.900826
chr5	50522500	0.983471
chr5	50622900	0.685950
chr5	50691700	0.933884
chr5	50982200	0.719008
chr5	51178300	0.991736
chr5	51264900	0.801653
chr5	51339800	0.809917

chr5	51748500	0.809917
chr5	51779900	0.743802
chr5	51978600	0.884298
chr5	52046300	0.661157
chr5	52099900	0.677686
chr5	52714800	0.942149
chr5	52841800	0.917355
chr5	52929600	0.735537
chr5	52938300	0.917355
chr5	52968400	0.909091
chr5	53111700	0.867769
chr5	53179400	0.851240
chr5	53241400	0.652893
chr5	53294400	0.834711
chr5	53304200	0.785124
chr5	53312600	0.793388
chr5	53314700	0.991736
chr5	53359700	0.776860
chr5	53384800	0.710744
chr5	53511700	0.884298
chr5	53513900	0.644628
chr5	53592300	0.785124
chr5	53621100	0.652893
chr5	53746600	0.785124
chr5	53753800	0.710744
chr5	53830200	0.727273
chr5	53999500	0.768595
chr5	54040100	0.834711
chr5	54176200	0.669421
chr5	54285000	0.661157
chr5	54289700	0.834711
chr5	54333000	0.652893
chr5	54515100	0.719008
chr5	54527100	0.826446
chr5	54610400	0.776860
chr5	54734600	0.991736
chr5	54777200	0.859504
chr5	54780900	0.876033
chr5	54788600	0.719008
chr5	54794000	0.669421
chr5	54834900	0.661157
chr5	54866000	0.950413
chr5	54868400	0.909091
chr5	54899200	0.661157
chr5	54921200	1.000000
chr5	54987300	0.644628
chr5	55471800	0.818182
chr5	55531100	0.652893
chr5	55566700	0.661157
chr5	55904100	0.809917
chr5	55912000	0.801653
chr5	55934000	0.793388
chr5	55959600	0.743802
chr5	55965800	0.710744

chr5	55995600	0.867769
chr5	56115200	0.719008
chr5	56129500	0.859504
chr5	56141700	0.785124
chr5	56503600	0.809917
chr5	57112500	0.809917
chr5	57131600	0.677686
chr5	57555700	0.876033
chr5	57701600	0.652893
chr5	57758100	0.942149
chr5	57763700	0.743802
chr5	57786700	0.909091
chr5	57799900	0.669421
chr5	57925600	0.768595
chr5	58013800	0.925620
chr5	58018300	0.867769
chr5	58031500	0.702479
chr5	58096500	0.661157
chr5	58146200	0.694215
chr5	58224000	0.719008
chr5	58296200	0.785124
chr5	58299400	0.752066
chr5	58421000	0.669421
chr5	58423600	0.909091
chr5	58479200	0.834711
chr5	58613500	0.884298
chr5	58654200	0.958678
chr5	58780000	0.644628
chr5	58859100	0.694215
chr5	58866200	0.851240
chr5	58880500	0.719008
chr5	58883800	1.000000
chr5	59005700	0.983471
chr5	59096600	0.710744
chr5	59165500	0.876033
chr5	59218300	0.900826
chr5	60057500	0.867769
chr5	60132700	0.752066
chr5	60148900	0.694215
chr5	60573800	0.644628
chr5	60615700	0.694215
chr5	60682000	0.752066
chr5	60697500	0.677686
chr5	60707300	0.793388
chr5	60710300	0.694215
chr5	60955400	0.900826
chr5	61028300	0.743802
chr5	61337500	0.652893
chr5	61370900	0.991736
chr5	61435100	0.702479
chr5	61439600	0.727273
chr5	61479000	0.760331
chr5	61504700	0.661157
chr5	61819200	0.710744

chr5	61858600	0.677686
chr5	61865400	0.735537
chr5	61890700	0.752066
chr5	61923100	0.793388
chr5	62013400	0.685950
chr5	62551800	0.859504
chr5	62642200	0.661157
chr5	62983800	0.892562
chr5	63252800	0.818182
chr5	63450500	0.702479
chr5	63798500	0.694215
chr5	63982100	0.644628
chr5	64002200	0.950413
chr5	64041000	0.694215
chr5	64236100	0.661157
chr5	64399200	0.785124
chr5	64438500	0.793388
chr5	64623300	0.710744
chr5	64944200	0.685950
chr5	64992300	0.818182
chr5	65062800	0.768595
chr5	65277900	0.669421
chr5	65295900	0.776860
chr5	65529300	0.652893
chr5	65634000	0.710744
chr5	65976600	0.752066
chr5	66138700	0.760331
chr5	66187800	0.644628
chr5	66200100	0.785124
chr5	66204400	0.842975
chr5	66256200	0.760331
chr5	66338500	0.752066
chr5	66355400	0.785124
chr5	66365700	0.694215
chr5	66390900	0.834711
chr5	66462400	0.942149
chr5	66478300	0.760331
chr5	66486900	0.685950
chr5	66530000	0.669421
chr5	66542100	0.719008
chr5	66713700	0.685950
chr5	66975400	0.727273
chr5	67027700	0.760331
chr5	67102300	0.966942
chr5	67141200	0.669421
chr5	67266100	0.760331
chr5	67318400	0.685950
chr5	67489800	0.727273
chr5	67547700	0.818182
chr5	67702700	0.652893
chr5	68110900	0.661157
chr5	68114400	0.925620
chr5	68218100	0.859504
chr5	68221000	0.975207

chr5	68432100	0.661157
chr5	68584900	0.933884
chr5	68591300	0.702479
chr5	68785200	0.652893
chr5	68799200	0.876033
chr5	68824200	0.669421
chr5	68959000	0.652893
chr5	69292000	0.685950
chr5	69354800	0.710744
chr5	69387100	0.958678
chr5	69760500	0.752066
chr5	70133300	0.719008
chr5	70226100	0.719008
chr5	70260500	0.884298
chr5	70392200	0.752066
chr5	70395500	0.785124
chr5	70451800	0.644628
chr5	70538900	0.710744
chr5	70686400	0.743802
chr5	70694600	0.685950
chr5	70701100	0.652893
chr5	70717600	0.818182
chr5	70990500	0.661157
chr5	71079300	0.685950
chr5	71082500	0.644628
chr5	71147100	0.677686
chr5	71235300	0.768595
chr5	71240200	0.661157
chr5	71333000	0.925620
chr5	71336800	0.768595
chr5	71361500	0.876033
chr5	71366900	0.677686
chr5	71406800	0.710744
chr5	71417900	0.958678
chr5	71441200	0.727273
chr5	71447400	0.652893
chr5	71449700	0.743802
chr5	71462400	0.933884
chr5	71479500	0.685950
chr5	71505700	0.644628
chr5	71531600	0.884298
chr5	71535100	0.909091
chr5	71538700	0.694215
chr5	71598300	1.000000
chr5	71601900	0.776860
chr5	71665900	0.669421
chr5	71748100	0.677686
chr5	71781800	0.834711
chr5	71915600	0.917355
chr5	72115300	0.652893
chr5	72125700	0.710744
chr5	72139000	0.768595
chr5	72327100	0.826446
chr5	72335300	0.677686

chr5	72419400	0.661157
chr5	72467400	0.677686
chr5	72485800	0.685950
chr5	72497900	0.661157
chr5	72510000	0.652893
chr5	72542400	0.851240
chr5	72563300	0.702479
chr5	72578100	0.719008
chr5	72585200	0.842975
chr5	72592400	0.727273
chr5	72597300	0.834711
chr5	72708200	0.917355
chr5	72740100	0.677686
chr5	72750100	0.818182
chr5	72779500	0.719008
chr5	72781800	0.809917
chr5	72938300	0.719008
chr5	72940900	0.768595
chr5	72969300	0.727273
chr5	72989800	0.735537
chr5	73147800	0.644628
chr5	73502900	0.768595
chr5	73553700	0.776860
chr5	73556100	0.652893
chr5	73568200	0.760331
chr5	73691400	0.925620
chr5	73729800	0.859504
chr5	73772500	0.685950
chr5	73796000	0.644628
chr5	73812200	0.702479
chr5	73832500	0.669421
chr5	73839600	0.983471
chr5	73924100	0.694215
chr5	73927600	0.677686
chr5	74112200	0.942149
chr5	74118100	0.892562
chr5	74121200	0.661157
chr5	74129900	0.768595
chr5	74140000	0.743802
chr5	74145200	0.685950
chr5	74157700	0.884298
chr5	74164400	0.851240
chr5	74179800	0.900826
chr5	74231500	0.752066
chr5	74303400	0.892562
chr5	74346200	0.652893
chr5	74352100	1.000000
chr5	74439100	0.809917
chr5	74571500	0.719008
chr5	74617100	0.942149
chr5	74746400	0.652893
chr5	74762000	0.694215
chr5	74771900	0.900826
chr5	74832700	0.809917

chr5	75410300	0.661157
chr5	75441100	0.834711
chr5	75490800	0.776860
chr5	75493500	0.793388
chr5	75524400	0.768595
chr5	75634100	0.694215
chr5	75638100	0.768595
chr5	75718400	0.735537
chr5	75723200	0.760331
chr5	75779000	0.743802
chr5	75781600	0.719008
chr5	75785500	0.983471
chr5	75791900	0.793388
chr5	75795200	0.884298
chr5	75798400	0.876033
chr5	75801600	0.661157
chr5	75810700	0.710744
chr5	75817500	0.727273
chr5	75820500	0.933884
chr5	75831800	0.652893
chr5	75839700	0.801653
chr5	75903500	0.694215
chr5	75926500	0.644628
chr5	75934700	0.743802
chr5	76105000	0.892562
chr5	76127100	0.785124
chr5	76171100	0.685950
chr5	76254900	0.677686
chr5	76618400	0.917355
chr5	76630000	0.818182
chr5	76692100	0.768595
chr5	76924300	0.876033
chr5	76928900	0.760331
chr5	76932900	0.851240
chr5	76937200	0.644628
chr5	77044200	0.776860
chr5	77047600	0.867769
chr5	77069400	0.834711
chr5	77129200	0.834711
chr5	77143100	0.851240
chr5	77207200	0.685950
chr5	77220500	0.892562
chr5	77256500	0.842975
chr5	77280800	0.776860
chr5	77284200	0.768595
chr5	77286900	0.826446
chr5	77290200	0.752066
chr5	77321800	0.735537
chr5	77342200	0.669421
chr5	77637400	0.760331
chr5	77791400	0.942149
chr5	77801200	0.710744
chr5	77830700	0.892562
chr5	77839100	1.000000

chr5	77865100	0.892562
chr5	77898000	0.702479
chr5	77907400	0.950413
chr5	77909500	0.735537
chr5	77936000	0.958678
chr5	77987000	0.966942
chr5	78024400	0.661157
chr5	78027200	0.710744
chr5	78204500	0.834711
chr5	78223700	0.842975
chr5	78317800	0.719008
chr5	78427600	0.652893
chr5	78552000	0.661157
chr5	78555900	0.809917
chr5	78558900	0.900826
chr5	78771400	0.677686
chr5	78804700	0.859504
chr5	79223000	0.702479
chr5	79298100	0.892562
chr5	79352000	0.768595
chr5	79390100	0.801653
chr5	79393900	0.958678
chr5	79406500	0.752066
chr5	79435400	0.842975
chr5	79438900	0.900826
chr5	79445800	0.652893
chr5	79455500	0.719008
chr5	79458000	0.685950
chr5	79477500	0.834711
chr5	79479800	0.958678
chr5	79482000	0.983471
chr5	79484900	0.735537
chr5	79489400	0.851240
chr5	79495400	0.661157
chr5	79501100	0.867769
chr5	79506900	0.685950
chr5	79541700	0.917355
chr5	79543800	0.917355
chr5	79790400	0.727273
chr5	79850700	0.876033
chr5	79858300	0.735537
chr5	80057000	0.694215
chr5	80157300	0.685950
chr5	80259900	0.752066
chr5	80287000	0.801653
chr5	80477000	0.950413
chr5	80543100	0.826446
chr5	80886700	0.975207
chr5	80902600	0.644628
chr5	81049500	0.859504
chr5	81051900	0.876033
chr5	81083900	0.983471
chr5	81134300	0.669421
chr5	81146300	0.818182

chr5	81276600	0.900826
chr5	81320400	0.801653
chr5	81327600	0.900826
chr5	81402800	0.793388
chr5	81426100	0.809917
chr5	81461500	0.892562
chr5	81554000	0.702479
chr5	81571200	0.768595
chr5	81592700	0.710744
chr5	81603300	0.644628
chr5	81702900	0.801653
chr5	81887700	0.752066
chr5	81935800	0.727273
chr5	81958600	0.719008
chr5	82090500	0.702479
chr5	82093100	0.694215
chr5	82100200	0.685950
chr5	82118100	0.752066
chr5	82129600	0.644628
chr5	82297500	0.669421
chr5	82300100	0.685950
chr5	82303900	0.719008
chr5	82323100	1.000000
chr5	82332000	0.669421
chr5	82362900	0.661157
chr5	82367400	0.719008
chr5	82650900	0.975207
chr5	82674800	0.785124
chr5	82697200	0.685950
chr5	82789900	0.925620
chr5	83223400	0.652893
chr5	83409400	0.851240
chr5	83562400	0.760331
chr5	83836600	0.661157
chr5	83861200	0.793388
chr5	83895800	0.826446
chr5	83946400	0.685950
chr5	84158300	0.661157
chr5	84365700	0.867769
chr5	85110900	0.867769
chr5	85937300	0.735537
chr5	85941000	0.958678
chr5	86316100	0.644628
chr5	86593800	0.694215
chr5	86884800	0.818182
chr5	87050400	0.942149
chr5	87077600	0.694215
chr5	87195700	0.710744
chr5	87240800	0.801653
chr5	87247200	0.661157
chr5	87469200	0.785124
chr5	87526800	0.727273
chr5	87532000	0.834711
chr5	87537500	0.743802

chr5	87540700	0.842975
chr5	87544200	0.851240
chr5	87552700	0.933884
chr5	87660800	0.702479
chr5	87684600	0.991736
chr5	87687700	0.677686
chr5	87690600	0.661157
chr5	87693100	0.917355
chr5	87779900	0.685950
chr5	87786100	0.685950
chr5	87836200	0.669421
chr5	87961600	0.677686
chr5	87991800	0.958678
chr5	88004700	0.727273
chr5	88018700	0.851240
chr5	88081000	0.719008
chr5	88115400	0.652893
chr5	88123300	0.652893
chr5	88193600	0.644628
chr5	88245100	0.785124
chr5	88251700	0.876033
chr5	88464800	0.719008
chr5	88743800	0.958678
chr5	88877400	0.685950
chr5	89000200	0.867769
chr5	89141500	0.801653
chr5	89284800	0.694215
chr5	89311700	0.644628
chr5	89561000	0.644628
chr5	89601400	0.933884
chr5	89663200	0.859504
chr5	89717400	0.702479
chr5	89776100	0.793388
chr5	89843300	0.661157
chr5	89850400	0.710744
chr5	89858000	0.925620
chr5	89860200	0.702479
chr5	89967000	0.785124
chr5	90174800	0.685950
chr5	90208500	0.760331
chr5	90226100	0.727273
chr5	90236300	0.933884
chr5	90334300	0.917355
chr5	90392100	0.975207
chr5	90437700	0.661157
chr5	90635300	0.900826
chr5	90717500	0.661157
chr5	90721100	0.925620
chr5	90753600	0.735537
chr5	91072900	0.669421
chr5	91995500	0.685950
chr5	92246000	0.735537
chr5	92248400	0.702479
chr5	92258700	0.818182

chr5	92393400	0.793388
chr5	92582400	0.652893
chr5	92636100	0.942149
chr5	92757600	0.925620
chr5	92762600	0.702479
chr5	92914700	0.669421
chr5	92921500	0.760331
chr5	92924400	0.933884
chr5	92935700	0.710744
chr5	92938200	0.834711
chr5	92940300	0.752066
chr5	92943900	0.818182
chr5	92948400	0.793388
chr5	93013400	0.876033
chr5	93290800	0.694215
chr5	93303800	0.694215
chr5	93393700	0.768595
chr5	93494500	0.719008
chr5	93617600	0.644628
chr5	93691200	0.876033
chr5	93694200	0.685950
chr5	93699200	0.710744
chr5	93713000	0.652893
chr5	93721900	0.702479
chr5	94043500	0.661157
chr5	94185800	0.652893
chr5	94189200	0.909091
chr5	94199700	0.743802
chr5	94429300	0.760331
chr5	94511500	0.669421
chr5	94514000	0.900826
chr5	94617300	0.909091
chr5	94776400	0.652893
chr5	94868400	0.710744
chr5	95146700	0.710744
chr5	95194800	0.727273
chr5	95231700	0.933884
chr5	95277000	0.975207
chr5	95299800	0.669421
chr5	95420000	1.000000
chr5	95424100	0.661157
chr5	95622000	0.661157
chr5	95775700	0.785124
chr5	95988700	0.719008
chr5	96051000	0.900826
chr5	96070900	0.702479
chr5	96079300	0.669421
chr5	96127200	0.793388
chr5	96132100	0.719008
chr5	96152400	0.735537
chr5	96286800	0.768595
chr5	96432400	0.925620
chr5	96437900	0.760331
chr5	96701500	0.966942

chr5	96736600	0.785124
chr5	97379000	0.925620
chr5	97645900	0.801653
chr5	97768300	0.900826
chr5	97992500	0.694215
chr5	98089400	0.826446
chr5	98099400	0.752066
chr5	98127200	0.702479
chr5	98274500	0.834711
chr5	98346300	0.859504
chr5	98505100	0.735537
chr5	98714700	0.801653
chr5	100094500	0.652893
chr5	100126300	0.661157
chr5	101125000	0.842975
chr5	101553300	0.776860
chr5	102078400	0.669421
chr5	102085800	0.702479
chr5	102097500	0.768595
chr5	102634600	0.702479
chr5	102682400	0.933884
chr5	102726000	0.743802
chr5	102729700	0.942149
chr5	102902100	0.743802
chr5	102909700	0.826446
chr5	102983200	0.975207
chr5	103988400	0.727273
chr5	104837000	0.652893
chr5	105032000	0.694215
chr5	105421600	0.793388
chr5	105590600	0.876033
chr5	105643000	0.801653
chr5	106091300	0.876033
chr5	106309900	0.818182
chr5	106320400	0.876033
chr5	106353800	0.685950
chr5	106361900	0.685950
chr5	106704000	0.950413
chr5	106723800	0.694215
chr5	106732200	0.661157
chr5	106738500	0.925620
chr5	106741200	0.702479
chr5	106792400	0.694215
chr5	106800700	0.793388
chr5	106805900	0.677686
chr5	106808500	0.909091
chr5	106814500	0.719008
chr5	106823400	0.652893
chr5	106835400	0.793388
chr5	106838400	0.785124
chr5	106848400	0.669421
chr5	106855100	0.793388
chr5	106865600	0.909091
chr5	106901900	0.933884

chr5	106908000	0.925620
chr5	106913100	0.801653
chr5	106925500	0.909091
chr5	106941400	0.876033
chr5	106989300	0.735537
chr5	107032300	0.851240
chr5	107041500	0.842975
chr5	107081200	0.752066
chr5	107108500	0.917355
chr5	107135700	0.652893
chr5	107141500	0.892562
chr5	107145300	0.909091
chr5	107162000	0.710744
chr5	107204700	0.727273
chr5	107269200	0.884298
chr5	107311600	0.677686
chr5	107333300	0.694215
chr5	107416700	0.793388
chr5	107438400	0.677686
chr5	107459800	0.685950
chr5	107549000	0.785124
chr5	107575700	0.760331
chr5	107972500	0.900826
chr5	108022700	1.000000
chr5	108506700	0.685950
chr5	108528700	0.768595
chr5	108531700	0.760331
chr5	108749200	0.933884
chr5	108980200	0.644628
chr5	109033800	0.669421
chr5	109040600	0.677686
chr5	109075200	0.958678
chr5	109079700	0.925620
chr5	109141900	0.859504
chr5	109173200	0.892562
chr5	109176300	0.760331
chr5	109332800	0.900826
chr5	109392600	0.661157
chr5	109515500	0.644628
chr5	109587700	0.685950
chr5	109812300	0.710744
chr5	109853300	0.776860
chr5	109872500	0.991736
chr5	110235800	0.727273
chr5	110453100	0.776860
chr5	110467800	0.661157
chr5	110505300	0.735537
chr5	110783700	0.876033
chr5	110887900	0.909091
chr5	111011600	0.644628
chr5	111056400	0.933884
chr5	111073300	0.859504
chr5	111108300	0.652893
chr5	111110800	0.834711

chr5	111502400	0.950413
chr5	111593300	0.752066
chr5	111669500	0.785124
chr5	111684300	0.900826
chr5	111839600	0.752066
chr5	111964500	0.669421
chr5	112046200	0.677686
chr5	112132400	0.933884
chr5	112305200	0.900826
chr5	112319600	0.876033
chr5	112367300	0.958678
chr5	112394700	0.685950
chr5	112405600	0.884298
chr5	112417500	0.842975
chr5	112477400	0.661157
chr5	112538900	0.809917
chr5	112549100	0.900826
chr5	112551600	0.809917
chr5	112554200	0.752066
chr5	112582600	0.884298
chr5	112602700	0.752066
chr5	112605900	0.900826
chr5	112609400	0.685950
chr5	112611600	0.942149
chr5	112617600	0.644628
chr5	112714600	0.644628
chr5	112942300	0.710744
chr5	113015300	0.884298
chr5	113112500	0.793388
chr5	113399300	0.685950
chr5	113591400	0.834711
chr5	113644100	0.983471
chr5	113648000	0.933884
chr5	113691700	0.867769
chr5	113723600	0.743802
chr5	113744300	0.776860
chr5	113783100	0.661157
chr5	113785700	0.826446
chr5	113788900	0.685950
chr5	114364700	0.719008
chr5	114426100	0.809917
chr5	114445600	0.644628
chr5	114526300	0.652893
chr5	114664700	0.958678
chr5	114705800	1.000000
chr5	114936000	0.867769
chr5	114970600	0.818182
chr5	115021400	0.702479
chr5	115071400	0.685950
chr5	115073600	0.818182
chr5	115142900	0.652893
chr5	115290000	0.900826
chr5	115713900	0.768595
chr5	115774400	0.760331

chr5	115786100	0.702479
chr5	115843000	0.925620
chr5	115845900	0.652893
chr5	115854200	0.826446
chr5	115864500	0.917355
chr5	115890500	0.876033
chr5	115899500	0.669421
chr5	115905000	0.702479
chr5	115965400	0.694215
chr5	115988400	0.958678
chr5	115991100	0.727273
chr5	116032200	0.652893
chr5	116039800	0.677686
chr5	116070200	0.876033
chr5	116095800	0.917355
chr5	116109900	0.942149
chr5	116114300	0.925620
chr5	116146600	0.826446
chr5	116156500	0.983471
chr5	116160900	0.661157
chr5	116192500	0.851240
chr5	116200400	0.991736
chr5	116274700	0.958678
chr5	116378800	0.917355
chr5	116408300	0.752066
chr5	116432400	0.834711
chr5	116439600	0.669421
chr5	116773600	0.702479
chr5	117380300	0.661157
chr5	117630700	0.719008
chr5	117717700	0.661157
chr5	117781800	0.710744
chr5	117822400	0.760331
chr5	117861200	0.809917
chr5	117965100	0.694215
chr5	118131800	0.702479
chr5	118271400	0.702479
chr5	118288900	0.950413
chr5	118368900	0.719008
chr5	118386300	0.942149
chr5	118410000	0.859504
chr5	118415500	0.991736
chr5	118626500	0.859504
chr5	118658100	0.925620
chr5	118664200	0.727273
chr5	118684500	0.785124
chr5	118694600	0.909091
chr5	118702800	0.669421
chr5	118738700	0.917355
chr5	118754300	0.644628
chr5	118792700	0.876033
chr5	118806400	0.809917
chr5	118865700	0.685950
chr5	119285400	0.661157

chr5	119844000	0.752066
chr5	119848100	0.950413
chr5	119912200	0.719008
chr5	119952200	0.966942
chr5	120043300	0.818182
chr5	120179400	0.818182
chr5	120188300	0.793388
chr5	120191500	0.669421
chr5	120278600	0.685950
chr5	120814800	0.644628
chr5	121022500	0.710744
chr5	121027600	0.685950
chr5	121435400	0.661157
chr5	121457400	0.818182
chr5	121518800	0.818182
chr5	121627900	0.652893
chr5	121659500	0.743802
chr5	121669400	0.644628
chr5	121690400	0.950413
chr5	121704900	0.743802
chr5	121751400	0.735537
chr5	121765900	0.958678
chr5	121809800	0.760331
chr5	121907300	0.752066
chr5	122251900	0.644628
chr5	122256900	0.743802
chr5	122269500	0.694215
chr5	122296200	0.661157
chr5	122455600	0.760331
chr5	122488000	0.933884
chr5	122510500	0.818182
chr5	122895000	0.644628
chr5	122918500	0.867769
chr5	123009200	0.727273
chr5	123080000	0.694215
chr5	123146400	0.644628
chr5	123151300	0.975207
chr5	123210300	0.859504
chr5	123212400	0.801653
chr5	123215400	0.694215
chr5	123361400	0.652893
chr5	123570800	0.909091
chr5	123597400	0.719008
chr5	123600100	0.719008
chr5	123625800	0.983471
chr5	123646300	0.694215
chr5	123774900	0.991736
chr5	123802400	0.677686
chr5	123804800	0.933884
chr5	123813000	0.884298
chr5	123885900	0.652893
chr5	123890800	0.719008
chr5	123909800	0.694215
chr5	123917200	0.917355

chr5	123965300	0.776860
chr5	123975300	0.900826
chr5	123987400	0.727273
chr5	123998200	0.785124
chr5	124005000	0.892562
chr5	124012200	0.652893
chr5	124014800	0.652893
chr5	124034000	0.876033
chr5	124036600	0.900826
chr5	124040500	0.760331
chr5	124043800	0.743802
chr5	124069200	0.661157
chr5	124071400	0.991736
chr5	124073800	0.710744
chr5	124076400	0.685950
chr5	124087100	0.942149
chr5	124142500	0.809917
chr5	124203100	0.677686
chr5	124224500	0.801653
chr5	124242200	0.719008
chr5	124258800	0.661157
chr5	124299300	0.892562
chr5	124315400	0.661157
chr5	124332900	0.884298
chr5	124339200	0.884298
chr5	124342300	0.768595
chr5	124389300	0.876033
chr5	124393300	0.925620
chr5	124456100	0.719008
chr5	124487500	0.900826
chr5	124496900	0.785124
chr5	124526600	0.727273
chr5	124532200	0.710744
chr5	124536600	0.925620
chr5	124572500	0.884298
chr5	124653500	0.834711
chr5	124672800	0.834711
chr5	124685100	0.876033
chr5	124709100	0.958678
chr5	124745400	0.644628
chr5	124748700	0.983471
chr5	124821600	0.661157
chr5	124828300	0.644628
chr5	124911500	0.851240
chr5	124996100	0.644628
chr5	125005100	0.867769
chr5	125056000	0.760331
chr5	125184800	0.719008
chr5	125206400	0.694215
chr5	125345200	0.727273
chr5	125390100	0.933884
chr5	125393000	0.942149
chr5	125459900	0.826446
chr5	125474300	0.652893

chr5	125525900	0.884298
chr5	125599900	0.661157
chr5	125685100	0.942149
chr5	125704500	0.966942
chr5	125709500	0.809917
chr5	125721400	0.743802
chr5	125808100	0.859504
chr5	125935100	0.685950
chr5	126010000	0.685950
chr5	126016100	0.727273
chr5	126081700	0.685950
chr5	126084100	0.859504
chr5	126162800	0.685950
chr5	126174600	0.768595
chr5	126182900	0.892562
chr5	126253700	0.702479
chr5	126257500	0.677686
chr5	126309000	0.735537
chr5	126377100	0.801653
chr5	126412900	0.719008
chr5	126432100	0.743802
chr5	126592700	0.785124
chr5	126629600	0.867769
chr5	126634900	0.661157
chr5	126643800	0.909091
chr5	126647700	0.785124
chr5	126657800	0.867769
chr5	126661000	0.661157
chr5	126665400	0.677686
chr5	126667600	0.925620
chr5	126669800	0.760331
chr5	126672900	0.652893
chr5	126675400	0.669421
chr5	126742600	0.702479
chr5	126925800	0.661157
chr5	126985800	0.826446
chr5	127146200	0.909091
chr5	127180900	0.760331
chr5	127270900	0.909091
chr5	127536800	0.685950
chr5	127882800	0.958678
chr5	128010000	0.884298
chr5	128018200	0.702479
chr5	128151300	0.719008
chr5	128206100	0.917355
chr5	128320500	0.909091
chr5	128452900	0.719008
chr5	128735300	0.785124
chr5	128746600	0.892562
chr5	129059300	0.966942
chr5	129061800	0.851240
chr5	129107500	0.694215
chr5	129492200	0.801653
chr5	129503100	0.801653

chr5	129914600	0.685950
chr5	130660700	0.652893
chr5	130900200	0.851240
chr5	130925600	0.702479
chr5	130951100	0.661157
chr5	131311200	0.892562
chr5	131330700	0.669421
chr5	131335600	0.809917
chr5	131338900	0.909091
chr5	131453100	0.727273
chr5	131605200	0.652893
chr5	131658200	0.933884
chr5	131690900	0.892562
chr5	131723000	0.809917
chr5	131763100	0.909091
chr5	131781200	0.900826
chr5	131800800	0.768595
chr5	131811000	0.677686
chr5	131837100	0.694215
chr5	132089800	0.669421
chr5	132107500	0.727273
chr5	132143400	0.768595
chr5	132159200	0.669421
chr5	132243400	0.652893
chr5	132407700	0.743802
chr5	132529900	0.661157
chr5	132558900	0.669421
chr5	132587800	0.884298
chr5	132626300	0.743802
chr5	132655800	0.900826
chr5	132664000	0.818182
chr5	132667800	0.644628
chr5	132677800	0.661157
chr5	132680100	0.652893
chr5	132701800	0.818182
chr5	132719800	0.884298
chr5	132737400	0.752066
chr5	132760200	0.661157
chr5	132762700	0.809917
chr5	132804600	0.801653
chr5	132821000	0.793388
chr5	132860900	0.752066
chr5	132893100	0.694215
chr5	132919000	0.652893
chr5	133011800	0.694215
chr5	133205200	0.710744
chr5	133331600	0.776860
chr5	133343800	0.776860
chr5	133383000	0.917355
chr5	133386600	0.818182
chr5	133389700	0.702479
chr5	133476900	0.801653
chr5	133576600	0.702479
chr5	133763200	0.809917

chr5	133765500	0.975207
chr5	133775200	0.694215
chr5	133839300	0.710744
chr5	133842600	0.661157
chr5	133848700	0.876033
chr5	133871000	0.752066
chr5	133876200	1.000000
chr5	133892200	0.760331
chr5	133913100	0.694215
chr5	133923100	0.702479
chr5	133928300	0.983471
chr5	134264000	0.685950
chr5	134294300	0.752066
chr5	134325900	0.685950
chr5	134384500	0.652893
chr5	134405600	0.661157
chr5	134408700	0.752066
chr5	134486500	0.768595
chr5	134549100	0.702479
chr5	134643500	0.710744
chr5	134813500	0.735537
chr5	134835000	0.735537
chr5	134897100	0.652893
chr5	134901100	0.859504
chr5	134912200	0.644628
chr5	134939600	0.677686
chr5	135039800	0.661157
chr5	135051500	0.685950
chr5	135059000	0.768595
chr5	135065100	0.652893
chr5	135158000	0.685950
chr5	135216400	0.760331
chr5	135411600	0.727273
chr5	135434000	0.735537
chr5	135585700	0.685950
chr5	135717700	0.710744
chr5	136112600	0.702479
chr5	136227700	0.876033
chr5	136243100	0.661157
chr5	136289800	0.983471
chr5	136372400	0.867769
chr5	136426800	0.892562
chr5	136537000	0.760331
chr5	136664500	0.661157
chr5	136940900	0.669421
chr5	136966000	0.909091
chr5	137170700	0.818182
chr5	137414100	0.826446
chr5	137420000	0.752066
chr5	137429200	0.719008
chr5	137433000	0.743802
chr5	137446700	0.867769
chr5	137469500	0.793388
chr5	137582000	0.801653

chr5	137785700	0.892562
chr5	137791400	0.702479
chr5	137957900	0.669421
chr5	137985000	0.661157
chr5	138000600	0.685950
chr5	138038600	0.809917
chr5	138042900	0.834711
chr5	138045300	0.900826
chr5	138058100	0.983471
chr5	138061600	0.793388
chr5	138129000	0.661157
chr5	138156000	0.735537
chr5	138167100	0.727273
chr5	138170100	0.834711
chr5	138172900	0.702479
chr5	138207100	0.884298
chr5	138211800	0.867769
chr5	138214400	0.801653
chr5	138220200	0.983471
chr5	138271900	0.933884
chr5	138281900	0.851240
chr5	138284700	0.652893
chr5	138291100	0.710744
chr5	138299800	0.644628
chr5	138389000	0.694215
chr5	138441400	0.644628
chr5	138462700	0.702479
chr5	138495600	0.652893
chr5	138633800	0.652893
chr5	138640200	0.652893
chr5	138724300	0.851240
chr5	138755600	0.719008
chr5	138839200	0.785124
chr5	138853000	0.925620
chr5	138897900	0.851240
chr5	138901900	0.727273
chr5	138904200	0.867769
chr5	138906400	0.826446
chr5	138923500	0.892562
chr5	138971800	0.644628
chr5	139013700	0.685950
chr5	139019400	0.669421
chr5	139023200	0.842975
chr5	139035200	0.776860
chr5	139039700	0.776860
chr5	139043300	0.702479
chr5	139048200	0.917355
chr5	139051300	0.785124
chr5	139057500	0.826446
chr5	139063800	0.942149
chr5	139066700	0.727273
chr5	139069200	0.818182
chr5	139078200	0.652893
chr5	139081200	0.842975

chr5	139087400	0.702479
chr5	139113600	0.694215
chr5	139143300	0.727273
chr5	139155200	0.669421
chr5	139224300	0.752066
chr5	139260500	0.652893
chr5	139281600	0.727273
chr5	139295700	0.702479
chr5	139335700	0.834711
chr5	139348700	0.760331
chr5	139362100	0.809917
chr5	139491100	0.801653
chr5	139533600	0.876033
chr5	139562900	0.702479
chr5	139585800	0.859504
chr5	139621200	0.900826
chr5	139625800	0.876033
chr5	139748200	0.702479
chr5	140051400	0.752066
chr5	140308500	0.776860
chr5	140365700	0.975207
chr5	140369000	0.702479
chr5	140379400	0.818182
chr5	140384000	0.801653
chr5	140480100	0.743802
chr5	140858100	0.694215
chr5	140875800	0.909091
chr5	140888600	0.834711
chr5	140895500	0.644628
chr5	140937600	0.884298
chr5	140940100	0.958678
chr5	140944100	0.677686
chr5	140972000	0.644628
chr5	141003200	0.661157
chr5	141054600	0.801653
chr5	141063800	0.818182
chr5	141098300	0.685950
chr5	141123300	0.950413
chr5	141127400	0.743802
chr5	141133300	0.694215
chr5	141138200	0.719008
chr5	141140900	0.710744
chr5	141154100	0.652893
chr5	141159700	0.867769
chr5	141183700	0.743802
chr5	141186900	0.727273
chr5	141191700	0.933884
chr5	141194200	0.694215
chr5	141196500	1.000000
chr5	141201700	0.818182
chr5	141206500	0.652893
chr5	141215800	0.867769
chr5	141218900	0.925620
chr5	141223400	0.842975

chr5	141226500	0.768595
chr5	141231400	0.785124
chr5	141233500	0.719008
chr5	141235900	0.776860
chr5	141238100	0.983471
chr5	141252900	0.983471
chr5	141260700	0.842975
chr5	141325400	0.694215
chr5	141340100	0.644628
chr5	141428500	0.685950
chr5	141478300	0.768595
chr5	141532500	0.702479
chr5	141536900	0.892562
chr5	141565400	0.702479
chr5	141601100	0.652893
chr5	141615500	0.991736
chr5	141643200	0.694215
chr5	141648900	0.677686
chr5	141660500	0.760331
chr5	141667800	0.669421
chr5	141670700	0.818182
chr5	141675400	0.900826
chr5	141690700	0.694215
chr5	141698300	0.760331
chr5	141740300	0.942149
chr5	141743000	0.975207
chr5	141770400	0.851240
chr5	141855400	0.834711
chr5	141871300	0.644628
chr5	141879900	0.661157
chr5	141901000	0.702479
chr5	142004900	0.644628
chr5	142064100	0.867769
chr5	142068200	0.669421
chr5	142077800	0.809917
chr5	142082200	0.900826
chr5	142123500	0.776860
chr5	142163300	0.859504
chr5	142198300	0.776860
chr5	142207800	0.685950
chr5	142211700	0.826446
chr5	142225500	0.892562
chr5	142228900	0.644628
chr5	142237400	0.752066
chr5	142241700	0.768595
chr5	142244100	0.975207
chr5	142253200	0.818182
chr5	142261700	0.983471
chr5	142275400	0.900826
chr5	142281100	0.842975
chr5	142312000	0.818182
chr5	142362400	0.950413
chr5	142382200	0.727273
chr5	142399500	0.776860

chr5	142417200	0.909091
chr5	142457700	0.644628
chr5	142470100	0.851240
chr5	142473700	0.719008
chr5	142490500	0.669421
chr5	142500200	0.661157
chr5	142510500	0.702479
chr5	142553300	0.842975
chr5	142609800	0.727273
chr5	142659600	0.669421
chr5	142777700	0.669421
chr5	142780300	0.842975
chr5	143022200	0.694215
chr5	143162600	0.933884
chr5	143251500	0.876033
chr5	143473300	0.793388
chr5	143594100	0.743802
chr5	143648000	0.719008
chr5	143736500	0.727273
chr5	143863900	0.801653
chr5	143867900	0.809917
chr5	143884700	0.892562
chr5	143974400	0.727273
chr5	144492700	0.785124
chr5	144538900	0.809917
chr5	144617800	0.694215
chr5	144812600	0.652893
chr5	145167500	0.644628
chr5	145299300	0.719008
chr5	145318800	0.834711
chr5	145362100	0.694215
chr5	145405600	0.727273
chr5	145453200	0.859504
chr5	145456100	0.842975
chr5	145481400	0.644628
chr5	145688400	0.702479
chr5	145711200	0.809917
chr5	145720400	0.743802
chr5	145722800	0.900826
chr5	145745500	0.644628
chr5	145895700	0.793388
chr5	145940400	0.644628
chr5	146000800	0.785124
chr5	146051700	0.809917
chr5	146079800	0.834711
chr5	146112200	0.694215
chr5	146170100	0.917355
chr5	146176600	0.727273
chr5	146185600	0.785124
chr5	146194000	0.917355
chr5	146219700	0.669421
chr5	146232200	0.867769
chr5	146252100	0.950413
chr5	146254900	0.710744

chr5	146268100	0.685950
chr5	146298800	0.925620
chr5	146345900	0.644628
chr5	146478200	0.743802
chr5	146522200	0.752066
chr5	146571000	0.669421
chr5	146596300	0.776860
chr5	146670200	0.876033
chr5	146675900	0.669421
chr5	146716700	0.685950
chr5	146762700	0.702479
chr5	146768300	0.900826
chr5	146826000	0.876033
chr5	146836000	0.644628
chr5	146844300	0.760331
chr5	146859700	0.752066
chr5	146863300	0.892562
chr5	146867200	0.933884
chr5	146871000	0.867769
chr5	146934200	0.892562
chr5	146938100	0.801653
chr5	146942700	0.876033
chr5	146945600	0.851240
chr5	146949000	0.942149
chr5	146954000	0.760331
chr5	146957800	0.950413
chr5	147038600	0.760331
chr5	147115000	0.752066
chr5	147130300	0.785124
chr5	147139600	0.661157
chr5	147240300	0.900826
chr5	147248700	0.909091
chr5	147255900	0.719008
chr5	147773100	0.785124
chr5	147778000	0.859504
chr5	147834400	0.719008
chr5	147938700	0.677686
chr5	148293100	0.876033
chr5	148336600	0.925620
chr5	148357000	0.677686
chr5	148442500	0.809917
chr5	148644800	0.710744
chr5	148675300	0.661157
chr5	148786500	0.644628
chr5	148797500	0.661157
chr5	148804300	0.917355
chr5	148822500	0.793388
chr5	148825400	0.644628
chr5	148832500	0.851240
chr5	148877200	0.809917
chr5	148898800	0.900826
chr5	148926900	0.702479
chr5	148941600	0.917355
chr5	149012800	0.917355

chr5	149094100	0.917355
chr5	149151200	0.719008
chr5	149157200	0.909091
chr5	149159800	0.710744
chr5	149185700	0.735537
chr5	149401800	0.710744
chr5	149438000	0.785124
chr5	149449600	0.834711
chr5	149462000	0.727273
chr5	149516500	0.851240
chr5	149518700	0.867769
chr5	149524100	0.694215
chr5	149535500	0.768595
chr5	149541200	0.702479
chr5	149547100	0.834711
chr5	149565700	0.768595
chr5	149586200	0.760331
chr5	149588600	0.892562
chr5	149591200	0.884298
chr5	149607600	0.677686
chr5	149636000	0.785124
chr5	149668300	0.652893
chr5	149684800	0.752066
chr5	149730400	0.677686
chr5	149739800	0.710744
chr5	149787800	0.644628
chr5	149816000	0.809917
chr5	149821500	0.834711
chr5	149826300	0.710744
chr5	149834400	0.702479
chr5	149841000	0.809917
chr5	149845600	0.644628
chr5	149862100	0.793388
chr5	149872200	0.859504
chr5	149878300	0.900826
chr5	149887300	0.652893
chr5	149890800	0.776860
chr5	149905500	0.826446
chr5	149909800	0.685950
chr5	149937200	0.661157
chr5	149940400	0.669421
chr5	150017500	0.867769
chr5	150020000	0.669421
chr5	150024800	0.884298
chr5	150216500	0.884298
chr5	150447000	0.752066
chr5	150473800	0.669421
chr5	150521400	0.909091
chr5	150530500	0.727273
chr5	150605200	0.760331
chr5	150618400	0.669421
chr5	150620900	0.694215
chr5	150699000	0.652893
chr5	150706100	0.652893

chr5	150725100	0.851240
chr5	150737000	0.900826
chr5	150765200	0.719008
chr5	150767900	0.991736
chr5	150785600	0.752066
chr5	150805200	0.834711
chr5	150876200	0.818182
chr5	150950500	0.884298
chr5	150961700	0.776860
chr5	150977400	0.826446
chr5	150999600	0.917355
chr5	151055500	0.677686
chr5	151076300	0.851240
chr5	151078900	0.677686
chr5	151112200	0.685950
chr5	151120100	0.925620
chr5	151155500	0.694215
chr5	151205400	0.685950
chr5	151292600	0.702479
chr5	151297800	0.785124
chr5	151304700	0.685950
chr5	151432100	0.719008
chr5	151446700	0.900826
chr5	151504200	0.884298
chr5	151642700	0.677686
chr5	151891500	0.743802
chr5	151987300	0.652893
chr5	152100000	0.752066
chr5	152234400	0.669421
chr5	152502200	0.710744
chr5	152730300	0.834711
chr5	152836400	0.719008
chr5	152896800	0.876033
chr5	152931600	0.842975
chr5	153008900	0.702479
chr5	153040800	0.644628
chr5	153160800	0.809917
chr5	153163600	0.743802
chr5	153204800	0.719008
chr5	153339600	0.958678
chr5	153344500	0.842975
chr5	153438700	0.719008
chr5	153487600	0.983471
chr5	153543200	0.702479
chr5	153547600	0.909091
chr5	153574300	0.900826
chr5	153582900	0.809917
chr5	153587600	0.842975
chr5	153589900	0.760331
chr5	153599900	0.900826
chr5	153618700	0.669421
chr5	153667200	0.818182
chr5	153750100	0.727273
chr5	153775700	0.942149

chr5	153841400	0.644628
chr5	153847600	0.685950
chr5	153863700	0.892562
chr5	153988700	0.925620
chr5	154033400	0.768595
chr5	154071200	0.900826
chr5	154076900	0.991736
chr5	154101400	0.975207
chr5	154120900	0.818182
chr5	154129500	0.768595
chr5	154140600	0.710744
chr5	154146400	0.867769
chr5	154150800	0.933884
chr5	154155000	0.760331
chr5	154159700	0.851240
chr5	154162700	0.851240
chr5	154167300	0.859504
chr5	154385000	0.694215
chr5	154426000	0.867769
chr5	154429600	0.735537
chr5	154654500	0.867769
chr5	154665800	0.652893
chr5	154913300	0.743802
chr5	154919900	0.867769
chr5	155001000	0.892562
chr5	155004600	0.677686
chr5	155200100	0.752066
chr5	155351100	0.760331
chr5	155872100	0.719008
chr5	156083000	0.834711
chr5	156144300	0.752066
chr5	156225700	0.851240
chr5	156228800	0.851240
chr5	156230900	0.884298
chr5	156251800	0.785124
chr5	156341300	0.983471
chr5	156798500	0.809917
chr5	156891800	0.644628
chr5	156982000	0.727273
chr5	156989400	0.743802
chr5	156998700	0.727273
chr5	157065600	0.669421
chr5	157291400	0.644628
chr5	157341000	0.876033
chr5	157351400	0.702479
chr5	157354300	0.909091
chr5	157393200	0.975207
chr5	157398900	0.735537
chr5	157422100	0.677686
chr5	157440700	0.966942
chr5	157563000	0.867769
chr5	157700200	0.867769
chr5	157741200	0.900826
chr5	157753200	0.735537

chr5	157965400	0.851240
chr5	158018600	0.694215
chr5	158125100	0.727273
chr5	158127700	0.826446
chr5	158154800	0.867769
chr5	158251900	0.652893
chr5	158308800	0.834711
chr5	158374400	0.677686
chr5	158379100	0.925620
chr5	158385300	0.851240
chr5	158407200	0.793388
chr5	158438600	0.644628
chr5	158839600	0.694215
chr5	158869500	0.826446
chr5	159195600	0.958678
chr5	159306500	0.644628
chr5	159335600	0.694215
chr5	159472600	0.966942
chr5	159510700	0.785124
chr5	159560600	0.958678
chr5	159588400	0.975207
chr5	159592100	0.727273
chr5	159599000	0.694215
chr5	159603600	0.652893
chr5	159615500	0.917355
chr5	159626700	0.818182
chr5	159665200	0.876033
chr5	159673200	0.743802
chr5	159688000	0.793388
chr5	159693800	0.785124
chr5	159699100	0.867769
chr5	159705900	0.652893
chr5	159713000	0.652893
chr5	159715700	0.983471
chr5	159721600	0.776860
chr5	159772600	0.644628
chr5	159787200	0.752066
chr5	159895100	0.661157
chr5	159904700	0.859504
chr5	159912800	0.983471
chr5	160084800	0.685950
chr5	160172100	0.644628
chr5	160202000	0.834711
chr5	160351300	0.644628
chr5	160617200	0.801653
chr5	160656000	0.677686
chr5	160685400	0.727273
chr5	160859400	0.752066
chr5	161479800	0.727273
chr5	161555600	0.958678
chr5	161702100	0.776860
chr5	162068700	0.884298
chr5	162758100	0.966942
chr5	162843300	0.834711

chr5	162959500	0.809917
chr5	162962000	0.983471
chr5	162971100	0.768595
chr5	162991100	0.719008
chr5	162994900	0.644628
chr5	163342900	0.776860
chr5	163575700	0.661157
chr5	163611400	0.925620
chr5	163724000	0.834711
chr5	163926700	0.661157
chr5	163942500	0.694215
chr5	164105800	0.776860
chr5	164108600	0.702479
chr5	164225700	0.685950
chr5	164565900	0.719008
chr5	164633800	0.760331
chr5	164660000	0.983471
chr5	164885200	0.652893
chr5	165498300	0.719008
chr5	165502600	0.991736
chr5	165515800	0.933884
chr5	165524200	0.710744
chr5	165747700	0.727273
chr5	165752000	0.909091
chr5	165769800	0.842975
chr5	165857100	0.950413
chr5	165859200	0.942149
chr5	165957600	0.644628
chr5	166052200	0.752066
chr5	166099400	0.702479
chr5	166118500	0.801653
chr5	166209300	0.760331
chr5	166310300	0.925620
chr5	166390100	0.752066
chr5	166409700	0.735537
chr5	166420000	0.702479
chr5	166583200	0.652893
chr5	166681500	0.851240
chr5	166723200	0.950413
chr5	166731100	0.727273
chr5	166733400	0.727273
chr5	166782500	0.752066
chr5	166786200	0.942149
chr5	166944500	0.644628
chr5	166988500	0.652893
chr5	166998400	0.760331
chr5	167126200	0.892562
chr5	167219900	0.892562
chr5	167230300	0.644628
chr5	167316800	0.743802
chr5	167333500	0.776860
chr5	167343900	0.644628
chr5	167378400	0.669421
chr5	167532100	0.842975

chr5	167537800	0.793388
chr5	167545800	0.859504
chr5	167564400	0.727273
chr5	167634800	0.652893
chr5	167643400	0.727273
chr5	167646800	0.752066
chr5	167696700	0.661157
chr5	167716100	0.818182
chr5	167727500	0.925620
chr5	167730000	0.826446
chr5	167741700	0.719008
chr5	167744200	0.834711
chr5	167755800	0.834711
chr5	167758600	0.801653
chr5	167761500	0.950413
chr5	167769500	0.702479
chr5	167775500	0.793388
chr5	167778900	0.826446
chr5	167831200	0.652893
chr5	167959700	0.752066
chr5	168027400	0.702479
chr5	168133900	0.710744
chr5	168183800	0.719008
chr5	168255000	0.917355
chr5	168261000	0.752066
chr5	168360000	0.735537
chr5	168404100	0.710744
chr5	168412300	0.859504
chr5	168440800	0.942149
chr5	168490500	0.826446
chr5	168493700	0.735537
chr5	168508400	0.644628
chr5	168511200	0.652893
chr5	168515700	0.909091
chr5	168528200	0.760331
chr5	168553500	0.652893
chr5	168644800	0.652893
chr5	168706300	0.826446
chr5	168767300	0.809917
chr5	168885600	0.876033
chr5	169195600	0.892562
chr5	169235800	0.909091
chr5	169239500	0.834711
chr5	169404300	0.685950
chr5	169473500	0.669421
chr5	169497500	0.652893
chr5	169527800	0.702479
chr5	169537300	0.768595
chr5	169540600	0.809917
chr5	169578700	0.644628
chr5	169581900	0.685950
chr5	169611400	0.793388
chr5	169618100	0.669421
chr5	169680000	0.702479

chr5	169693600	0.826446
chr5	169701300	0.652893
chr5	169706900	0.785124
chr5	169760800	0.818182
chr5	169775200	0.809917
chr5	169780300	0.685950
chr5	169814200	0.793388
chr5	169820900	0.694215
chr5	169824400	0.950413
chr5	169836000	0.933884
chr5	169848100	0.677686
chr5	169901300	0.826446
chr5	170031400	0.652893
chr5	170039700	0.933884
chr5	170044900	0.826446
chr5	170052700	0.801653
chr5	170164500	0.801653
chr5	170166600	0.685950
chr5	170173400	1.000000
chr5	170188800	0.966942
chr5	170200800	0.851240
chr5	170218100	0.677686
chr5	170291500	0.652893
chr5	170521700	0.851240
chr5	170742600	0.768595
chr5	170745400	0.950413
chr5	170850000	0.818182
chr5	170861300	0.685950
chr5	170864900	0.719008
chr5	170878400	0.661157
chr5	170889900	0.727273
chr5	170897100	0.652893
chr5	170922100	0.983471
chr5	170948100	0.983471
chr5	171043000	0.702479
chr5	171050900	0.743802
chr5	171072700	0.752066
chr5	171095200	0.966942
chr5	171164400	0.644628
chr5	171167000	0.818182
chr5	171275600	0.677686
chr5	171284700	0.644628
chr5	171352300	0.760331
chr5	171381000	0.752066
chr5	171384400	0.760331
chr5	171393500	0.727273
chr5	171398400	0.669421
chr5	171405900	0.909091
chr5	171408000	0.661157
chr5	171410300	0.925620
chr5	171421200	0.727273
chr5	171424200	0.809917
chr5	171427400	0.925620
chr5	171430000	0.677686

chr5	171471700	0.669421
chr5	171476600	0.644628
chr5	171498800	0.826446
chr5	171530900	0.950413
chr5	171534300	0.876033
chr5	171538400	0.900826
chr5	171601600	0.876033
chr5	171606300	0.735537
chr5	171688600	0.694215
chr5	171830400	0.933884
chr5	171846500	0.760331
chr5	171873800	0.694215
chr5	171876200	0.685950
chr5	171878600	0.685950
chr5	171883300	0.661157
chr5	171999700	0.727273
chr5	172002300	0.834711
chr5	172013800	0.933884
chr5	172074400	0.867769
chr5	172122100	0.677686
chr5	172124900	0.685950
chr5	172144100	0.776860
chr5	172175900	0.710744
chr5	172192500	0.826446
chr5	172216500	0.727273
chr5	172260100	0.652893
chr5	172280000	0.801653
chr5	172293600	0.710744
chr5	172296100	0.876033
chr5	172300000	0.644628
chr5	172330200	0.661157
chr5	172333200	0.826446
chr5	172370800	0.818182
chr5	172443300	0.768595
chr5	172456300	0.834711
chr5	172472700	0.760331
chr5	172481500	0.793388
chr5	172566700	0.727273
chr5	172602800	0.685950
chr5	172630100	0.785124
chr5	172656300	0.727273
chr5	172663900	0.644628
chr5	172667100	0.661157
chr5	172673600	0.644628
chr5	172711300	0.669421
chr5	172719100	0.867769
chr5	172729200	0.743802
chr5	172731600	0.776860
chr5	172743500	0.809917
chr5	172751500	0.735537
chr5	172769100	0.818182
chr5	172926400	0.743802
chr5	172983900	0.884298
chr5	173055300	0.950413

chr5	173068100	0.685950
chr5	173071000	0.719008
chr5	173096500	0.950413
chr5	173098700	0.942149
chr5	173125600	0.710744
chr5	173147900	0.793388
chr5	173172700	0.892562
chr5	173180600	0.834711
chr5	173191900	0.685950
chr5	173198600	0.644628
chr5	173251400	0.727273
chr5	173343300	0.958678
chr5	173369000	0.991736
chr5	173391000	0.669421
chr5	173396100	0.809917
chr5	173447700	0.652893
chr5	173535100	0.793388
chr5	173622800	0.661157
chr5	173632100	0.809917
chr5	173728500	0.685950
chr5	173744600	0.661157
chr5	173756000	0.917355
chr5	173947700	0.867769
chr5	173959800	0.884298
chr5	174027300	0.909091
chr5	174053500	0.669421
chr5	174117200	0.752066
chr5	174161100	0.727273
chr5	174222100	0.752066
chr5	174344200	0.776860
chr5	174348500	0.851240
chr5	174487200	0.727273
chr5	174520600	0.652893
chr5	174558500	0.702479
chr5	174622700	0.842975
chr5	174779700	0.719008
chr5	174862000	0.743802
chr5	174865100	0.719008
chr5	174875600	0.669421
chr5	174961500	0.900826
chr5	174967700	0.685950
chr5	174975800	0.818182
chr5	174984800	0.677686
chr5	174988800	0.818182
chr5	175001200	0.694215
chr5	175024400	0.669421
chr5	175031600	0.966942
chr5	175065600	0.644628
chr5	175117900	0.793388
chr5	175123500	0.900826
chr5	175126200	0.743802
chr5	175158300	0.752066
chr5	175174500	0.652893
chr5	175237400	0.694215

chr5	175251200	0.876033
chr5	175264500	0.925620
chr5	175274800	0.710744
chr5	175302300	0.743802
chr5	175322900	0.892562
chr5	175335700	0.859504
chr5	175406500	0.876033
chr5	175438400	0.702479
chr5	175491900	0.884298
chr5	175579800	0.867769
chr5	175869400	0.884298
chr5	175960600	0.884298
chr5	175968000	0.661157
chr5	175980700	0.727273
chr5	176005300	0.644628
chr5	176030700	0.652893
chr5	176040400	0.743802
chr5	176061500	0.727273
chr5	176070800	0.727273
chr5	176077200	0.669421
chr5	176088200	0.826446
chr5	176092000	0.768595
chr5	176099900	0.719008
chr5	176103500	0.719008
chr5	176107400	0.842975
chr5	176115200	0.785124
chr5	176120100	0.900826
chr5	176124200	0.876033
chr5	176128200	0.702479
chr5	176139800	0.743802
chr5	176152800	0.776860
chr5	176160600	0.719008
chr5	176166600	0.917355
chr5	176173000	0.785124
chr5	176202300	0.760331
chr5	176212200	0.719008
chr5	176216000	0.694215
chr5	176220500	0.834711
chr5	176230800	0.834711
chr5	176243200	0.925620
chr5	176267800	0.884298
chr5	176274200	0.677686
chr5	176290200	0.818182
chr5	176294300	0.826446
chr5	176297000	0.677686
chr5	176327600	0.785124
chr5	176371200	0.991736
chr5	176463700	0.876033
chr5	176525700	0.694215
chr5	176544100	0.909091
chr5	176728300	0.809917
chr5	176742000	0.710744
chr5	176745300	0.669421
chr5	176753300	0.818182

chr5	176764100	0.925620
chr5	176791700	0.685950
chr5	176797100	0.851240
chr5	176801600	0.685950
chr5	176805300	0.793388
chr5	176809800	0.669421
chr5	176817700	0.644628
chr5	176867500	0.851240
chr5	176871900	0.677686
chr5	176876700	0.669421
chr5	176885000	0.966942
chr5	176897000	0.768595
chr5	176902900	0.851240
chr5	176921700	0.851240
chr5	176928800	0.710744
chr5	176937100	0.768595
chr5	177034800	0.958678
chr5	177206700	0.694215
chr5	177291400	0.892562
chr5	177386100	0.768595
chr5	177423200	0.760331
chr5	177508900	0.776860
chr5	177535500	0.719008
chr5	177545200	0.735537
chr5	177548500	0.966942
chr5	177550600	0.933884
chr5	177553200	0.909091
chr5	177556400	0.785124
chr5	177573300	0.702479
chr5	177605100	0.809917
chr5	177607500	0.793388
chr5	177609700	0.644628
chr5	177651100	0.900826
chr5	177665900	0.644628
chr5	177707300	0.735537
chr5	177756100	0.892562
chr5	177775800	0.743802
chr5	177778800	0.735537
chr5	177789300	0.743802
chr5	177798800	0.793388
chr5	177810600	0.876033
chr5	177821400	0.669421
chr5	177826400	0.694215
chr5	177847800	0.933884
chr5	177856300	0.727273
chr5	177880700	0.776860
chr5	177888500	0.743802
chr5	177913600	0.710744
chr5	177915700	0.776860
chr5	177953900	0.909091
chr5	177982700	0.933884
chr5	177988600	0.702479
chr5	177991900	0.867769
chr5	178047100	0.809917

chr5	178613300	0.752066
chr5	178615400	0.727273
chr5	178655400	0.793388
chr5	178699900	0.694215
chr5	178758200	0.644628
chr5	179132600	0.884298
chr5	179182200	0.867769
chr5	179190400	0.966942
chr5	179230500	0.768595
chr5	179240900	0.900826
chr5	179246000	0.702479
chr5	179344400	0.867769
chr5	179435100	0.652893
chr5	179454700	0.793388
chr5	179481800	0.859504
chr5	179487100	0.884298
chr5	179495300	0.900826
chr5	179509600	0.867769
chr5	179512800	0.900826
chr5	179518900	0.685950
chr5	179759200	0.933884
chr5	179769800	0.975207
chr5	179772300	0.727273
chr5	179775100	0.785124
chr5	179918900	0.809917
chr5	179950100	0.661157
chr5	179953500	0.867769
chr5	180059600	0.752066
chr5	180137600	0.925620
chr5	180212200	0.975207
chr5	180215400	0.677686
chr5	180218100	0.669421
chr5	180231600	0.859504
chr5	180271900	0.727273
chr5	180463900	0.735537
chr5	180536400	0.644628
chr5	180587900	0.933884
chr5	180625300	0.694215
chr5	180627800	0.685950
chr5	180666100	0.669421
chr6	221200	0.652893
chr6	236400	0.702479
chr6	314700	0.818182
chr6	324700	0.719008
chr6	352300	0.752066
chr6	374300	0.710744
chr6	552100	0.933884
chr6	583000	0.702479
chr6	625200	0.644628
chr6	650600	0.694215
chr6	660200	0.958678
chr6	675400	0.867769
chr6	681300	0.900826
chr6	701800	0.933884

chr6	707800	0.842975
chr6	732000	0.727273
chr6	795600	0.818182
chr6	830200	0.842975
chr6	1105500	0.876033
chr6	1203300	0.776860
chr6	1335700	0.685950
chr6	1348800	0.876033
chr6	1379700	0.677686
chr6	1385700	0.710744
chr6	1388500	0.710744
chr6	1394200	0.851240
chr6	1410400	0.785124
chr6	1438900	0.694215
chr6	1498200	0.743802
chr6	1511100	0.727273
chr6	1527500	0.826446
chr6	1575500	0.776860
chr6	1597200	0.685950
chr6	1612400	0.702479
chr6	1621900	0.644628
chr6	1644300	0.876033
chr6	1721100	0.669421
chr6	1838400	0.702479
chr6	1870500	0.743802
chr6	1907100	0.793388
chr6	1909500	0.652893
chr6	1920300	0.710744
chr6	1926400	0.644628
chr6	2022000	0.760331
chr6	2130300	0.644628
chr6	2141100	0.900826
chr6	2222900	0.752066
chr6	2297500	0.760331
chr6	2467200	0.677686
chr6	2641500	0.851240
chr6	2656700	0.900826
chr6	2665500	0.710744
chr6	2732000	0.776860
chr6	2735600	0.884298
chr6	2738500	0.694215
chr6	2791100	0.677686
chr6	2849200	0.809917
chr6	2854800	0.917355
chr6	2858800	0.685950
chr6	2864200	0.826446
chr6	2866600	1.000000
chr6	2878100	0.702479
chr6	2886500	0.685950
chr6	2908700	0.884298
chr6	2917800	0.842975
chr6	2921500	0.785124
chr6	2926500	0.644628
chr6	2929400	0.818182

chr6	2932100	0.752066
chr6	2953300	0.867769
chr6	2960600	0.752066
chr6	2985600	0.661157
chr6	3006100	0.818182
chr6	3010800	0.768595
chr6	3024300	0.909091
chr6	3056500	0.652893
chr6	3074000	0.727273
chr6	3077500	0.702479
chr6	3144400	0.652893
chr6	3160700	0.909091
chr6	3166600	0.983471
chr6	3196000	0.983471
chr6	3209300	0.867769
chr6	3282600	0.925620
chr6	3327000	0.752066
chr6	3333500	0.851240
chr6	3337800	0.842975
chr6	3340100	1.000000
chr6	3343600	0.752066
chr6	3352500	0.661157
chr6	3356600	0.983471
chr6	3371400	0.652893
chr6	3403900	0.694215
chr6	3410600	0.685950
chr6	3415200	0.909091
chr6	3418700	0.652893
chr6	3422100	0.983471
chr6	3424900	0.727273
chr6	3431400	0.694215
chr6	3443900	0.677686
chr6	3467200	0.900826
chr6	3483200	0.851240
chr6	3490000	0.793388
chr6	3502600	0.892562
chr6	3512000	0.867769
chr6	3527300	0.694215
chr6	3575500	0.702479
chr6	3625100	0.818182
chr6	3742800	0.793388
chr6	3746400	0.727273
chr6	3757200	0.735537
chr6	3771000	0.694215
chr6	3802000	0.661157
chr6	3814000	0.669421
chr6	3818200	0.727273
chr6	3821400	0.752066
chr6	3862500	0.752066
chr6	3895800	0.859504
chr6	3904700	0.818182
chr6	3906800	0.801653
chr6	3910300	0.652893
chr6	3926300	0.727273

chr6	3998000	0.776860
chr6	4169600	0.768595
chr6	4288400	0.743802
chr6	4292300	0.752066
chr6	4323500	0.661157
chr6	4359500	0.661157
chr6	4384300	0.909091
chr6	4406200	0.652893
chr6	4471200	0.669421
chr6	4473400	0.669421
chr6	4481800	0.909091
chr6	4487500	0.743802
chr6	4504900	0.884298
chr6	4648200	0.876033
chr6	4656000	0.685950
chr6	4720300	0.710744
chr6	4733900	0.710744
chr6	4752700	0.644628
chr6	4779900	0.867769
chr6	4782100	0.644628
chr6	4787000	0.710744
chr6	4790100	0.760331
chr6	4794300	0.743802
chr6	4804300	0.809917
chr6	4820200	0.644628
chr6	4884800	0.735537
chr6	4889800	0.661157
chr6	4904700	0.652893
chr6	4913800	0.983471
chr6	4921900	0.785124
chr6	4925900	0.727273
chr6	4932500	0.958678
chr6	4947900	0.677686
chr6	4974600	0.801653
chr6	5036400	0.727273
chr6	5133200	0.892562
chr6	5139600	0.710744
chr6	5192900	0.710744
chr6	5206000	0.942149
chr6	5233500	0.644628
chr6	5282800	0.768595
chr6	5293900	0.710744
chr6	5314100	0.851240
chr6	5379100	0.933884
chr6	5412700	0.876033
chr6	5439600	0.909091
chr6	5457800	0.685950
chr6	5464000	0.644628
chr6	5467000	0.818182
chr6	5469700	0.727273
chr6	5472700	0.867769
chr6	5500700	0.842975
chr6	5511900	0.710744
chr6	5564300	0.702479

chr6	5628900	0.661157
chr6	5673200	0.942149
chr6	5714000	0.826446
chr6	5724200	0.842975
chr6	5753000	0.785124
chr6	5783300	0.752066
chr6	5993300	0.760331
chr6	6169700	0.768595
chr6	6293400	0.719008
chr6	6318400	0.859504
chr6	6539000	0.752066
chr6	6679200	0.900826
chr6	6725500	0.652893
chr6	6743600	0.694215
chr6	6750400	0.661157
chr6	6759700	0.809917
chr6	6812200	0.818182
chr6	6857600	0.900826
chr6	6873300	0.727273
chr6	6891400	0.950413
chr6	6901400	0.785124
chr6	7009300	0.743802
chr6	7042500	0.661157
chr6	7087800	0.884298
chr6	7113400	0.909091
chr6	7119800	0.785124
chr6	7125300	0.826446
chr6	7143100	0.661157
chr6	7145300	0.933884
chr6	7147900	0.719008
chr6	7151500	0.661157
chr6	7158800	0.661157
chr6	7166000	0.727273
chr6	7176500	0.785124
chr6	7196000	0.743802
chr6	7225400	0.892562
chr6	7243200	0.826446
chr6	7339500	0.652893
chr6	7432500	0.685950
chr6	7455700	0.694215
chr6	7506200	1.000000
chr6	7532900	0.710744
chr6	7546100	0.917355
chr6	7548800	0.776860
chr6	7625500	0.809917
chr6	7673800	0.677686
chr6	7692500	0.685950
chr6	7768800	0.826446
chr6	7771200	0.942149
chr6	8048600	0.760331
chr6	8155800	0.669421
chr6	8180900	0.983471
chr6	8384200	0.727273
chr6	8504400	0.793388

chr6	9113200	0.694215
chr6	9456900	0.958678
chr6	9606900	0.743802
chr6	10269200	0.768595
chr6	10307700	0.677686
chr6	10311000	0.867769
chr6	10315800	0.809917
chr6	10383900	0.851240
chr6	10387500	0.818182
chr6	10396300	0.851240
chr6	10407500	0.727273
chr6	10424400	0.719008
chr6	10521400	0.801653
chr6	10523900	0.652893
chr6	10574300	0.867769
chr6	10577400	0.818182
chr6	10580900	0.727273
chr6	10585000	0.735537
chr6	10591600	0.842975
chr6	10596400	0.950413
chr6	10599400	0.876033
chr6	10603000	0.685950
chr6	10613900	0.694215
chr6	10633900	0.652893
chr6	10978700	0.719008
chr6	10995700	0.809917
chr6	11026900	0.776860
chr6	11077100	0.768595
chr6	11130600	0.652893
chr6	11146800	0.677686
chr6	11155000	0.702479
chr6	11195300	0.793388
chr6	11203800	0.851240
chr6	11211400	0.785124
chr6	11229900	0.933884
chr6	11242300	0.661157
chr6	11277900	0.752066
chr6	11405200	0.669421
chr6	11429800	0.776860
chr6	11557200	0.752066
chr6	11602800	0.818182
chr6	11650900	0.958678
chr6	11657100	0.743802
chr6	11688400	0.950413
chr6	11767200	0.652893
chr6	11771000	0.892562
chr6	11791400	0.727273
chr6	11901500	0.842975
chr6	11926400	0.702479
chr6	11938900	0.834711
chr6	11973400	0.685950
chr6	12040100	0.842975
chr6	12045100	0.702479
chr6	12061400	0.884298

chr6	12085600	0.669421
chr6	12172000	0.685950
chr6	12246300	0.661157
chr6	12325400	0.966942
chr6	12328400	0.710744
chr6	12336800	0.842975
chr6	12366100	0.661157
chr6	12603600	0.719008
chr6	12609800	0.851240
chr6	12657700	0.644628
chr6	12730000	0.677686
chr6	12826800	0.661157
chr6	12867800	0.958678
chr6	12881600	0.735537
chr6	12887500	0.983471
chr6	12911800	0.801653
chr6	12922800	0.710744
chr6	12932100	0.669421
chr6	12993600	0.950413
chr6	13103000	0.677686
chr6	13120400	0.842975
chr6	13149500	0.892562
chr6	13153100	0.933884
chr6	13175700	0.851240
chr6	13248800	0.768595
chr6	13340100	0.661157
chr6	13351500	0.826446
chr6	13358400	0.826446
chr6	13368400	0.694215
chr6	13381500	0.752066
chr6	13399200	0.735537
chr6	13411300	0.685950
chr6	13423200	0.983471
chr6	13429200	0.652893
chr6	13457300	0.661157
chr6	13463200	0.876033
chr6	13471000	0.727273
chr6	13496500	0.652893
chr6	13536800	0.685950
chr6	13545000	0.851240
chr6	13555600	0.867769
chr6	13636900	0.677686
chr6	13847800	0.776860
chr6	13909500	0.884298
chr6	13927800	0.685950
chr6	13981300	0.685950
chr6	14010100	0.900826
chr6	14025200	0.719008
chr6	14036100	0.785124
chr6	14056100	0.685950
chr6	14129400	0.743802
chr6	14162800	0.677686
chr6	14194700	0.851240
chr6	14213100	0.727273

chr6	14259600	0.834711
chr6	14273700	0.909091
chr6	14369700	0.735537
chr6	14379600	0.702479
chr6	14384200	0.909091
chr6	14410600	0.661157
chr6	14450600	0.710744
chr6	14599100	0.677686
chr6	14606900	0.785124
chr6	14622300	0.826446
chr6	14626700	0.958678
chr6	14640000	0.644628
chr6	14642400	0.752066
chr6	14665100	0.685950
chr6	14677800	0.694215
chr6	14729500	0.909091
chr6	14733400	0.851240
chr6	14778700	0.661157
chr6	14782600	0.743802
chr6	14785600	0.983471
chr6	14789700	0.694215
chr6	14825100	0.842975
chr6	14849800	0.942149
chr6	14861600	0.776860
chr6	14864700	0.768595
chr6	14875100	0.768595
chr6	14881100	0.743802
chr6	14913700	0.710744
chr6	14924900	0.900826
chr6	14933400	0.884298
chr6	14966200	0.900826
chr6	14975500	0.702479
chr6	15037700	0.884298
chr6	15039900	0.900826
chr6	15077800	0.735537
chr6	15130500	0.785124
chr6	15171200	0.842975
chr6	15207400	0.644628
chr6	15209500	0.958678
chr6	15224200	0.727273
chr6	15240100	0.710744
chr6	15252200	0.925620
chr6	15254600	0.942149
chr6	15256700	0.809917
chr6	15260000	0.735537
chr6	15276900	0.867769
chr6	15281500	0.669421
chr6	15294000	0.661157
chr6	15308900	0.801653
chr6	15314000	0.859504
chr6	15317400	0.669421
chr6	15321500	0.752066
chr6	15323600	0.842975
chr6	15327800	0.793388

chr6	15337800	0.809917
chr6	15342300	0.851240
chr6	15350100	0.900826
chr6	15352500	0.909091
chr6	15355200	0.644628
chr6	15359200	0.776860
chr6	15362200	0.884298
chr6	15367500	0.768595
chr6	15371600	0.702479
chr6	15383000	0.917355
chr6	15386000	0.818182
chr6	15401900	0.818182
chr6	15410100	0.851240
chr6	15426800	0.876033
chr6	15430400	0.702479
chr6	15434700	0.818182
chr6	15437300	0.809917
chr6	15448100	0.760331
chr6	15455200	0.644628
chr6	15460600	0.768595
chr6	15476700	0.694215
chr6	15484800	0.793388
chr6	15492700	0.859504
chr6	15508100	0.884298
chr6	15578300	0.785124
chr6	15616800	0.685950
chr6	15675800	0.727273
chr6	15895400	0.983471
chr6	15897800	0.752066
chr6	15906300	0.818182
chr6	15909700	0.735537
chr6	15913000	0.991736
chr6	15950100	0.644628
chr6	15953600	0.735537
chr6	16060500	0.917355
chr6	16065000	0.702479
chr6	16303200	0.834711
chr6	16306500	0.727273
chr6	16320600	0.727273
chr6	16325200	0.752066
chr6	16332400	0.719008
chr6	16457800	0.694215
chr6	16467700	0.867769
chr6	16497700	0.859504
chr6	16518400	0.685950
chr6	16636300	0.900826
chr6	16713400	0.735537
chr6	16724500	0.677686
chr6	16770400	0.719008
chr6	16804300	0.694215
chr6	17418600	0.851240
chr6	17432300	0.752066
chr6	17684700	0.685950
chr6	17925600	0.644628

chr6	17931500	0.752066
chr6	18016700	0.719008
chr6	18023700	0.900826
chr6	18043300	0.719008
chr6	18186500	0.983471
chr6	18261300	0.669421
chr6	18303700	0.809917
chr6	18307600	0.652893
chr6	18328400	0.727273
chr6	18389900	0.735537
chr6	18395700	0.983471
chr6	18492000	0.677686
chr6	18504600	0.652893
chr6	18753300	0.752066
chr6	18920800	0.719008
chr6	18998100	0.719008
chr6	19086900	0.818182
chr6	19299600	0.735537
chr6	19312400	0.909091
chr6	19538400	0.661157
chr6	19663500	0.917355
chr6	19673400	0.669421
chr6	19715700	0.752066
chr6	19720500	0.859504
chr6	19852100	0.842975
chr6	20161400	0.644628
chr6	20205200	0.760331
chr6	20207500	0.669421
chr6	20238800	0.661157
chr6	20329400	0.776860
chr6	20416900	0.801653
chr6	20468100	0.743802
chr6	20631500	0.710744
chr6	20728000	0.719008
chr6	20772000	0.785124
chr6	20811700	0.991736
chr6	20867500	0.983471
chr6	20869700	0.702479
chr6	20879700	0.669421
chr6	20882600	0.958678
chr6	20933000	0.644628
chr6	20956100	0.719008
chr6	20994400	0.652893
chr6	21079200	0.652893
chr6	21099000	0.760331
chr6	21110200	0.702479
chr6	21112500	0.719008
chr6	21131400	0.818182
chr6	21134300	0.917355
chr6	21186800	0.776860
chr6	21200000	0.719008
chr6	21327700	0.710744
chr6	21348900	0.677686
chr6	21368500	0.669421

chr6	21602900	0.727273
chr6	21680800	0.801653
chr6	21692400	0.842975
chr6	21695100	0.785124
chr6	21712400	0.685950
chr6	21716300	0.652893
chr6	21731900	0.760331
chr6	21734400	0.776860
chr6	21737300	0.909091
chr6	21739800	0.727273
chr6	21742900	0.925620
chr6	21745900	0.677686
chr6	21748700	0.917355
chr6	21751500	0.702479
chr6	21754600	0.809917
chr6	21776100	0.735537
chr6	21801800	0.677686
chr6	21826900	0.743802
chr6	21837200	0.909091
chr6	21843700	0.809917
chr6	21846800	0.785124
chr6	21885800	0.694215
chr6	21891500	0.851240
chr6	21908900	0.644628
chr6	21922700	0.694215
chr6	22012200	0.685950
chr6	22025900	0.983471
chr6	22036500	0.809917
chr6	22043500	0.743802
chr6	22061700	0.694215
chr6	22071600	0.776860
chr6	22079300	0.685950
chr6	22146300	0.809917
chr6	22223400	0.752066
chr6	22251100	0.975207
chr6	22364100	0.809917
chr6	22396600	0.793388
chr6	22808600	0.710744
chr6	22878400	0.975207
chr6	23003000	0.842975
chr6	23659700	0.760331
chr6	23955600	0.677686
chr6	24251700	0.702479
chr6	24283900	0.661157
chr6	24314100	0.768595
chr6	24401000	0.925620
chr6	24502600	0.652893
chr6	24659400	0.884298
chr6	24684400	0.966942
chr6	24876900	0.983471
chr6	24880200	0.917355
chr6	24919500	0.768595
chr6	24924200	0.892562
chr6	24932400	0.677686

chr6	24934600	0.876033
chr6	24937300	0.735537
chr6	24978700	0.900826
chr6	24981300	0.851240
chr6	24986600	0.793388
chr6	24997900	0.809917
chr6	25018100	0.685950
chr6	25062300	0.983471
chr6	25313100	0.743802
chr6	25316000	0.801653
chr6	25335000	0.876033
chr6	25342500	0.942149
chr6	25359800	0.669421
chr6	25370200	0.834711
chr6	25379200	0.710744
chr6	25407900	0.958678
chr6	25414100	0.809917
chr6	25449200	0.876033
chr6	25462800	0.900826
chr6	25627300	0.710744
chr6	25899900	0.917355
chr6	25966800	0.735537
chr6	25998900	0.669421
chr6	26019000	0.752066
chr6	26023900	0.826446
chr6	26029500	0.983471
chr6	26035800	0.785124
chr6	26045900	0.942149
chr6	26103100	0.644628
chr6	26202500	0.719008
chr6	26208000	0.727273
chr6	26220300	0.834711
chr6	26227800	0.669421
chr6	26243700	0.760331
chr6	26355300	0.661157
chr6	26474200	0.727273
chr6	26476900	0.752066
chr6	26529100	0.685950
chr6	26705200	0.909091
chr6	26914800	0.669421
chr6	27034700	0.851240
chr6	27129200	0.900826
chr6	27536400	0.644628
chr6	27727000	0.669421
chr6	27836900	0.785124
chr6	27842400	0.785124
chr6	27850400	0.719008
chr6	27856200	0.900826
chr6	27973200	0.710744
chr6	28324100	0.933884
chr6	28474700	0.710744
chr6	28483600	0.652893
chr6	28738800	0.785124
chr6	28746700	0.785124

chr6	28754300	0.685950
chr6	28780500	0.719008
chr6	29194200	0.818182
chr6	29525800	0.859504
chr6	29567200	0.669421
chr6	29571100	0.752066
chr6	29630000	0.933884
chr6	29632100	0.702479
chr6	29635100	0.975207
chr6	29652100	0.909091
chr6	29664000	0.743802
chr6	29727500	1.000000
chr6	29731000	0.694215
chr6	29933000	0.958678
chr6	30039700	0.702479
chr6	30113800	0.694215
chr6	30170100	0.652893
chr6	30175600	0.867769
chr6	30290900	0.859504
chr6	30359700	0.735537
chr6	30419100	0.785124
chr6	30483000	0.644628
chr6	30581200	0.809917
chr6	30647600	0.727273
chr6	30650500	0.661157
chr6	30692300	0.702479
chr6	30732200	0.818182
chr6	30737800	0.752066
chr6	30778700	0.793388
chr6	30796500	0.760331
chr6	30812100	0.933884
chr6	30817100	0.685950
chr6	30846200	0.785124
chr6	30858800	0.785124
chr6	30910400	0.702479
chr6	30950300	0.826446
chr6	31038700	0.909091
chr6	31047600	0.710744
chr6	31078900	0.669421
chr6	31082400	0.851240
chr6	31093500	0.685950
chr6	31107200	0.826446
chr6	31130000	0.925620
chr6	31142700	0.768595
chr6	31154100	0.694215
chr6	31171000	0.685950
chr6	31178900	0.677686
chr6	31214400	0.818182
chr6	31334900	0.644628
chr6	31358700	0.884298
chr6	31362200	0.652893
chr6	31373900	0.719008
chr6	31410100	0.752066
chr6	31455700	0.900826

chr6	31546600	0.677686
chr6	31583600	0.867769
chr6	31679300	0.702479
chr6	31682300	0.801653
chr6	31686300	0.694215
chr6	31706100	0.735537
chr6	31731400	0.685950
chr6	31745400	0.826446
chr6	31793100	0.909091
chr6	31867700	0.652893
chr6	31874500	0.743802
chr6	32014600	0.859504
chr6	32039400	0.743802
chr6	32076900	0.925620
chr6	32114700	0.818182
chr6	32135100	0.694215
chr6	32141500	0.917355
chr6	32160300	0.900826
chr6	32166400	0.661157
chr6	32192200	0.702479
chr6	32375300	0.661157
chr6	32547200	0.776860
chr6	32765200	0.702479
chr6	32829100	0.867769
chr6	32916200	0.685950
chr6	33116000	0.809917
chr6	33165800	0.652893
chr6	33194800	0.752066
chr6	33203300	0.859504
chr6	33248100	0.809917
chr6	33288000	0.801653
chr6	33313200	0.776860
chr6	33333100	0.842975
chr6	33340900	0.892562
chr6	33397400	0.669421
chr6	33551600	0.652893
chr6	33560900	0.776860
chr6	33574800	0.809917
chr6	33579000	0.826446
chr6	33596200	0.876033
chr6	33604400	0.661157
chr6	33609000	0.743802
chr6	33625000	0.942149
chr6	33660100	0.661157
chr6	33665500	0.727273
chr6	33692800	0.727273
chr6	33695400	0.702479
chr6	33713400	0.900826
chr6	33738000	0.834711
chr6	33740500	0.677686
chr6	33746100	0.685950
chr6	33750600	0.793388
chr6	33753700	0.884298
chr6	33758900	0.884298

chr6	33762700	0.876033
chr6	33782100	0.867769
chr6	33791300	0.809917
chr6	33796700	0.818182
chr6	33807100	0.760331
chr6	33819800	0.669421
chr6	33837700	0.851240
chr6	33872500	0.694215
chr6	33895400	0.909091
chr6	33903100	0.793388
chr6	33918500	0.702479
chr6	33942600	0.719008
chr6	33974500	0.884298
chr6	33984000	0.694215
chr6	34000700	0.644628
chr6	34051900	0.677686
chr6	34063400	0.851240
chr6	34067700	0.900826
chr6	34097200	0.760331
chr6	34100000	0.900826
chr6	34105900	0.760331
chr6	34110300	0.719008
chr6	34117200	0.735537
chr6	34119800	0.776860
chr6	34164300	0.842975
chr6	34168700	0.793388
chr6	34171100	0.785124
chr6	34174200	0.644628
chr6	34192700	0.685950
chr6	34195300	0.991736
chr6	34209100	0.768595
chr6	34212000	0.925620
chr6	34285100	0.867769
chr6	34331200	0.685950
chr6	34352200	0.694215
chr6	34425700	0.702479
chr6	34444000	0.702479
chr6	34481200	0.661157
chr6	34495400	0.685950
chr6	34528700	0.685950
chr6	34543400	0.760331
chr6	34549200	0.727273
chr6	34552700	0.661157
chr6	34558000	0.727273
chr6	34570300	0.826446
chr6	34625400	0.876033
chr6	34641600	0.710744
chr6	34820400	0.644628
chr6	34955200	0.719008
chr6	34988800	0.669421
chr6	34991700	0.793388
chr6	34995500	0.694215
chr6	35005100	0.983471
chr6	35011100	0.909091

chr6	35013900	0.884298
chr6	35017300	0.776860
chr6	35036600	0.743802
chr6	35039800	0.867769
chr6	35116700	0.900826
chr6	35150200	0.818182
chr6	35170400	0.702479
chr6	35204500	0.644628
chr6	35207400	0.785124
chr6	35266300	0.752066
chr6	35271200	0.801653
chr6	35295600	0.661157
chr6	35328100	0.719008
chr6	35366000	0.719008
chr6	35383300	0.785124
chr6	35387400	0.644628
chr6	35396100	0.991736
chr6	35422700	0.785124
chr6	35439800	0.694215
chr6	35452300	0.752066
chr6	35457900	0.694215
chr6	35462000	0.776860
chr6	35474200	0.900826
chr6	35476500	0.826446
chr6	35487700	0.727273
chr6	35490700	0.710744
chr6	35612000	0.685950
chr6	35631400	0.933884
chr6	35686200	0.958678
chr6	35688700	0.818182
chr6	35706400	0.942149
chr6	35708700	0.752066
chr6	35711000	0.793388
chr6	35723100	0.752066
chr6	35767000	0.884298
chr6	35863800	0.925620
chr6	35899100	0.785124
chr6	35952900	0.727273
chr6	36089100	0.702479
chr6	36100700	0.710744
chr6	36108300	0.917355
chr6	36213200	0.975207
chr6	36238100	0.685950
chr6	36257600	0.702479
chr6	36295000	0.876033
chr6	36628900	0.785124
chr6	36635300	0.900826
chr6	36644800	0.983471
chr6	36683700	0.669421
chr6	36699300	0.727273
chr6	36703400	0.834711
chr6	36745600	0.719008
chr6	36757300	0.900826
chr6	36761900	0.933884

chr6	36773100	0.760331
chr6	36785900	0.710744
chr6	36789700	0.661157
chr6	36803300	0.735537
chr6	36827100	0.942149
chr6	36918100	0.727273
chr6	36931100	0.760331
chr6	36949000	0.876033
chr6	36985900	0.644628
chr6	37019800	0.652893
chr6	37024200	0.809917
chr6	37027400	0.966942
chr6	37029700	0.694215
chr6	37037200	0.785124
chr6	37040100	0.966942
chr6	37045600	0.842975
chr6	37073400	0.900826
chr6	37091000	0.694215
chr6	37096000	0.942149
chr6	37099500	0.933884
chr6	37102700	0.958678
chr6	37107300	0.859504
chr6	37233600	0.694215
chr6	37293900	0.702479
chr6	37428000	0.685950
chr6	37449200	0.950413
chr6	37458000	0.652893
chr6	37460600	0.851240
chr6	37476000	0.669421
chr6	37513900	0.661157
chr6	37556300	0.859504
chr6	37562800	0.710744
chr6	37568400	0.685950
chr6	37593700	0.727273
chr6	37641900	0.719008
chr6	37660200	0.661157
chr6	37732500	0.752066
chr6	37744800	0.752066
chr6	37748000	0.710744
chr6	37759300	0.826446
chr6	37790900	0.785124
chr6	37849900	0.727273
chr6	37894900	0.735537
chr6	37962600	0.652893
chr6	37965400	0.677686
chr6	38105900	0.834711
chr6	38110100	0.652893
chr6	38112800	0.991736
chr6	38155500	0.677686
chr6	38160600	0.685950
chr6	38201600	0.900826
chr6	38230100	0.842975
chr6	38232400	0.752066
chr6	38238400	0.719008

chr6	38248900	0.785124
chr6	38359800	0.702479
chr6	38396100	0.900826
chr6	38446100	0.644628
chr6	38471400	0.809917
chr6	38477700	0.776860
chr6	38752000	0.917355
chr6	38844500	0.644628
chr6	38870200	0.752066
chr6	39022300	0.669421
chr6	39053500	0.793388
chr6	39056600	0.892562
chr6	39060100	0.710744
chr6	39173800	0.842975
chr6	39186100	0.933884
chr6	39199700	0.818182
chr6	39224400	0.702479
chr6	39231800	0.644628
chr6	39236400	0.727273
chr6	39241300	0.735537
chr6	39259400	0.801653
chr6	39307900	0.727273
chr6	39311800	0.785124
chr6	39330300	0.876033
chr6	39463400	0.859504
chr6	39619200	0.652893
chr6	39637700	0.644628
chr6	39677900	0.652893
chr6	39701700	0.909091
chr6	39745000	0.760331
chr6	39762100	0.768595
chr6	39776500	0.793388
chr6	39778800	0.669421
chr6	39790000	0.702479
chr6	39865700	0.760331
chr6	39907000	0.760331
chr6	39915900	0.809917
chr6	40008100	0.768595
chr6	40132400	0.661157
chr6	40146000	0.801653
chr6	40152400	0.966942
chr6	40196000	0.702479
chr6	40253600	0.760331
chr6	40257200	0.842975
chr6	40290700	0.760331
chr6	40297200	0.834711
chr6	40299500	0.867769
chr6	40310700	0.785124
chr6	40327000	0.991736
chr6	40331900	0.826446
chr6	40366800	0.652893
chr6	40387600	0.917355
chr6	40492400	0.842975
chr6	40497400	0.652893

chr6	40506100	0.669421
chr6	40509000	0.760331
chr6	40511100	0.760331
chr6	40514000	0.917355
chr6	40524400	0.867769
chr6	40527100	0.652893
chr6	40531100	0.677686
chr6	40616600	0.727273
chr6	40688200	0.942149
chr6	40698700	0.644628
chr6	40766400	0.826446
chr6	40800200	0.702479
chr6	40836400	0.644628
chr6	40886300	0.859504
chr6	40916200	0.958678
chr6	40919200	0.809917
chr6	40951700	0.776860
chr6	40965800	0.809917
chr6	40968200	0.884298
chr6	41007000	0.719008
chr6	41068400	0.851240
chr6	41139300	0.925620
chr6	41262600	0.826446
chr6	41321300	0.685950
chr6	41337200	0.719008
chr6	41344100	0.677686
chr6	41381000	0.826446
chr6	41384400	0.694215
chr6	41392500	0.801653
chr6	41398200	0.818182
chr6	41424700	0.818182
chr6	41431300	0.859504
chr6	41440900	0.917355
chr6	41456600	0.909091
chr6	41460200	0.867769
chr6	41463000	0.933884
chr6	41496300	0.809917
chr6	41499400	0.818182
chr6	41505200	0.669421
chr6	41520500	0.694215
chr6	41523200	0.644628
chr6	41525700	0.752066
chr6	41530700	0.727273
chr6	41536800	0.727273
chr6	41541300	0.710744
chr6	41552500	0.752066
chr6	41559400	0.735537
chr6	41565200	0.710744
chr6	41609100	0.793388
chr6	41611200	0.661157
chr6	41624100	0.669421
chr6	41631800	0.694215
chr6	41641700	0.801653
chr6	41645500	0.702479

chr6	41648100	0.652893
chr6	41651700	0.809917
chr6	41655800	0.809917
chr6	41669000	0.719008
chr6	41691700	0.917355
chr6	41699300	0.661157
chr6	41704400	0.644628
chr6	41906400	0.760331
chr6	41933000	0.983471
chr6	41935900	0.826446
chr6	41978300	0.793388
chr6	41999100	0.719008
chr6	42014000	0.793388
chr6	42101800	0.694215
chr6	42104100	0.925620
chr6	42106700	0.768595
chr6	42120400	0.876033
chr6	42124900	0.644628
chr6	42130500	0.793388
chr6	42134700	0.702479
chr6	42153900	0.768595
chr6	42161900	0.669421
chr6	42210500	0.859504
chr6	42225500	0.991736
chr6	42228800	0.735537
chr6	42238000	0.661157
chr6	42273000	0.644628
chr6	42284300	0.809917
chr6	42374500	0.983471
chr6	42385600	0.793388
chr6	42387800	0.710744
chr6	42396800	0.743802
chr6	42405200	0.752066
chr6	42407400	0.710744
chr6	42411500	0.776860
chr6	42424300	0.776860
chr6	42586500	0.661157
chr6	42672500	0.768595
chr6	42678100	0.685950
chr6	42724100	0.719008
chr6	42727100	0.710744
chr6	42739400	0.884298
chr6	42758300	0.851240
chr6	42788900	0.826446
chr6	42867600	0.652893
chr6	42907300	0.752066
chr6	42931700	0.710744
chr6	43051200	0.661157
chr6	43064700	0.884298
chr6	43071500	0.834711
chr6	43074000	0.694215
chr6	43082200	0.735537
chr6	43091500	0.917355
chr6	43094100	0.950413

chr6	43111300	0.867769
chr6	43142800	0.644628
chr6	43211600	0.743802
chr6	43236000	0.727273
chr6	43238200	0.669421
chr6	43246500	0.851240
chr6	43255200	0.685950
chr6	43286500	0.661157
chr6	43364700	0.752066
chr6	43450000	0.719008
chr6	43460500	0.661157
chr6	43463500	0.669421
chr6	43480100	0.859504
chr6	43635700	0.851240
chr6	43663800	0.983471
chr6	43667200	0.842975
chr6	43670800	0.975207
chr6	43675200	0.826446
chr6	43682800	0.752066
chr6	43714900	0.760331
chr6	43735500	0.892562
chr6	43743000	0.776860
chr6	43802400	0.694215
chr6	43837900	0.900826
chr6	43842300	0.760331
chr6	43859400	0.768595
chr6	43894900	0.818182
chr6	43901200	0.661157
chr6	43933000	0.661157
chr6	43962300	0.694215
chr6	43975700	0.694215
chr6	44009200	0.925620
chr6	44011700	0.884298
chr6	44020400	0.661157
chr6	44044400	0.652893
chr6	44070800	0.776860
chr6	44089200	0.710744
chr6	44101100	0.809917
chr6	44106800	0.743802
chr6	44151400	0.818182
chr6	44154300	0.694215
chr6	44160500	0.884298
chr6	44171400	0.859504
chr6	44174800	0.834711
chr6	44189200	0.818182
chr6	44194800	0.702479
chr6	44202000	0.867769
chr6	44218200	0.644628
chr6	44236500	0.710744
chr6	44240500	0.702479
chr6	44318300	0.743802
chr6	44440900	0.958678
chr6	44510500	0.966942
chr6	44589100	0.694215

chr6	44592600	0.834711
chr6	44594900	0.867769
chr6	44630900	0.991736
chr6	44635900	0.809917
chr6	44669800	0.694215
chr6	44740200	0.776860
chr6	44799700	0.752066
chr6	45414000	0.818182
chr6	45473200	0.851240
chr6	45481300	0.909091
chr6	45867600	0.809917
chr6	45870700	0.768595
chr6	45895900	0.760331
chr6	45898500	0.809917
chr6	45902100	0.760331
chr6	46016800	0.652893
chr6	46081500	0.818182
chr6	46259400	0.694215
chr6	46293800	0.818182
chr6	46326600	0.710744
chr6	46403700	0.669421
chr6	46433900	0.644628
chr6	46437000	0.900826
chr6	46673800	0.661157
chr6	46708400	0.776860
chr6	46717600	0.950413
chr6	46725000	0.652893
chr6	46904000	0.685950
chr6	47074100	1.000000
chr6	47114900	0.785124
chr6	47120900	0.768595
chr6	47256800	0.694215
chr6	47264300	0.735537
chr6	47274200	0.694215
chr6	47339700	0.685950
chr6	47397100	0.966942
chr6	47462500	0.793388
chr6	47638100	0.851240
chr6	47669000	0.719008
chr6	47702500	0.727273
chr6	47704600	0.702479
chr6	47757200	0.818182
chr6	48041900	0.644628
chr6	48228400	0.669421
chr6	48532500	0.760331
chr6	48732700	0.884298
chr6	48807200	0.727273
chr6	48885300	0.677686
chr6	48887600	0.785124
chr6	49111000	0.727273
chr6	49123000	0.735537
chr6	49300800	0.685950
chr6	49310300	0.983471
chr6	49495000	0.694215

chr6	49859000	0.719008
chr6	49879300	0.661157
chr6	49886800	0.768595
chr6	49934100	0.809917
chr6	50632600	0.760331
chr6	50676700	0.694215
chr6	50679900	0.677686
chr6	50692600	0.719008
chr6	50735700	0.785124
chr6	50785400	0.677686
chr6	50789800	0.884298
chr6	50795500	0.785124
chr6	50804100	0.859504
chr6	50808800	0.909091
chr6	51204900	0.900826
chr6	51360200	0.702479
chr6	51679700	0.752066
chr6	51707700	0.917355
chr6	51730700	0.826446
chr6	51773400	0.644628
chr6	51801700	0.694215
chr6	51848800	0.661157
chr6	51882200	0.644628
chr6	51886800	0.735537
chr6	51941900	0.975207
chr6	51944500	0.702479
chr6	52002000	0.958678
chr6	52017600	0.644628
chr6	52021000	0.950413
chr6	52191200	0.909091
chr6	52382800	0.909091
chr6	52409200	0.661157
chr6	52417300	0.768595
chr6	52423500	0.966942
chr6	52435200	0.669421
chr6	52438600	0.859504
chr6	52463300	0.677686
chr6	52601200	0.917355
chr6	52614700	0.867769
chr6	52721700	0.735537
chr6	52747300	0.760331
chr6	52788500	0.652893
chr6	52796400	0.760331
chr6	52825900	0.644628
chr6	53057800	0.652893
chr6	53153100	0.925620
chr6	53192200	0.768595
chr6	53209000	0.809917
chr6	53276400	0.735537
chr6	53349800	0.685950
chr6	53392300	0.876033
chr6	53395900	0.942149
chr6	53589700	0.760331
chr6	53671100	0.867769

chr6	53676300	0.776860
chr6	53680000	0.669421
chr6	53691900	0.710744
chr6	53752400	0.694215
chr6	53821200	0.669421
chr6	53827100	0.917355
chr6	53836600	0.710744
chr6	53852500	0.661157
chr6	53937900	0.652893
chr6	53940100	0.677686
chr6	55115600	0.809917
chr6	55142600	0.768595
chr6	55298900	0.834711
chr6	55647800	0.710744
chr6	55740600	0.884298
chr6	55778700	0.884298
chr6	56143400	0.677686
chr6	56387500	0.760331
chr6	56391000	0.768595
chr6	56578100	0.710744
chr6	56581200	0.842975
chr6	56590100	0.710744
chr6	56606800	0.743802
chr6	56609500	0.752066
chr6	56612100	0.785124
chr6	56622300	0.702479
chr6	56637700	0.801653
chr6	56640900	0.975207
chr6	56659500	0.975207
chr6	56671600	0.727273
chr6	56702900	0.685950
chr6	56705600	0.661157
chr6	56792900	0.727273
chr6	56890700	0.661157
chr6	56935400	0.842975
chr6	57273100	0.942149
chr6	57286300	0.876033
chr6	57288700	1.000000
chr6	57329100	0.694215
chr6	57338300	0.752066
chr6	57353900	0.661157
chr6	57358500	0.719008
chr6	57382600	0.702479
chr6	57384900	0.900826
chr6	57639500	0.842975
chr6	57732300	0.834711
chr6	61914000	0.694215
chr6	62284400	0.917355
chr6	62316400	0.859504
chr6	62393400	0.743802
chr6	62690900	0.669421
chr6	62737800	0.702479
chr6	62900300	0.644628
chr6	62976300	0.785124

chr6	63103900	0.694215
chr6	63190000	0.719008
chr6	63226800	0.826446
chr6	63534100	0.652893
chr6	63946800	0.694215
chr6	64384300	0.950413
chr6	64654100	0.719008
chr6	64674600	0.710744
chr6	64722300	0.735537
chr6	64989800	0.644628
chr6	65901200	0.942149
chr6	66277600	0.760331
chr6	66279900	0.892562
chr6	67373200	0.768595
chr6	68544900	0.768595
chr6	68691300	0.793388
chr6	68749300	0.958678
chr6	68751700	0.685950
chr6	68849500	0.685950
chr6	69269300	0.677686
chr6	69286400	0.694215
chr6	69359900	0.685950
chr6	69448100	0.735537
chr6	69718300	0.859504
chr6	69926300	1.000000
chr6	69930900	0.776860
chr6	70502100	0.735537
chr6	70512900	0.752066
chr6	70818000	0.702479
chr6	71184600	0.809917
chr6	71273600	0.661157
chr6	71435900	0.776860
chr6	71469100	0.842975
chr6	71574600	0.925620
chr6	71595200	0.793388
chr6	71738500	0.694215
chr6	71864200	0.785124
chr6	71867300	0.694215
chr6	71891500	0.975207
chr6	72114200	0.661157
chr6	72616900	0.752066
chr6	72792600	0.727273
chr6	72795700	0.719008
chr6	72861700	0.776860
chr6	73008300	0.727273
chr6	73122200	0.685950
chr6	73738200	0.859504
chr6	73795500	0.785124
chr6	74059100	0.933884
chr6	74233400	0.710744
chr6	74276100	0.983471
chr6	74290500	0.876033
chr6	74295900	0.950413
chr6	74932800	0.735537

chr6	75355800	0.925620
chr6	75459700	0.661157
chr6	75923200	0.942149
chr6	75940700	0.710744
chr6	75960200	0.842975
chr6	75978100	1.000000
chr6	75983300	0.842975
chr6	75986100	0.685950
chr6	75989900	0.958678
chr6	76000700	0.809917
chr6	76010100	0.727273
chr6	76018900	0.677686
chr6	76037100	0.785124
chr6	76042200	0.727273
chr6	76045400	0.752066
chr6	76085100	0.892562
chr6	76266000	0.644628
chr6	76656200	0.743802
chr6	76703600	1.000000
chr6	76762200	0.652893
chr6	77149200	0.785124
chr6	77268200	0.677686
chr6	77394300	0.900826
chr6	78047400	0.942149
chr6	78144300	0.652893
chr6	78426300	0.702479
chr6	79055600	0.685950
chr6	79280100	0.785124
chr6	79553700	0.702479
chr6	79792100	0.702479
chr6	79862500	0.991736
chr6	79928200	0.826446
chr6	80048400	0.851240
chr6	80204900	0.685950
chr6	80255800	0.884298
chr6	80280400	1.000000
chr6	80284400	0.776860
chr6	80417900	0.644628
chr6	80487800	0.735537
chr6	80520300	0.818182
chr6	80646000	0.851240
chr6	80653000	0.826446
chr6	80703400	0.809917
chr6	80921000	0.685950
chr6	81041600	0.884298
chr6	81044200	0.702479
chr6	81348700	0.760331
chr6	81684900	0.859504
chr6	82467300	0.652893
chr6	82500100	0.975207
chr6	82680100	0.892562
chr6	83095000	0.644628
chr6	83448800	0.661157
chr6	83452400	0.826446

chr6	83669500	1.000000
chr6	83780200	0.859504
chr6	84227600	0.876033
chr6	84622800	0.727273
chr6	84686400	0.785124
chr6	84762500	0.892562
chr6	84772400	0.876033
chr6	84799000	0.892562
chr6	85135500	0.694215
chr6	85157800	0.677686
chr6	85663500	0.661157
chr6	85716600	0.842975
chr6	85836800	0.776860
chr6	86033600	0.652893
chr6	86113700	0.909091
chr6	86168600	0.842975
chr6	86290500	0.809917
chr6	86379900	0.785124
chr6	86482400	0.752066
chr6	87187900	0.661157
chr6	87196700	0.669421
chr6	87221700	0.743802
chr6	87466700	0.983471
chr6	87873800	0.677686
chr6	87875900	0.851240
chr6	87880600	0.661157
chr6	87884400	0.826446
chr6	87890700	0.892562
chr6	87916600	0.818182
chr6	87919300	0.644628
chr6	87924100	0.719008
chr6	88039300	0.685950
chr6	88193700	0.743802
chr6	88289200	0.677686
chr6	88363700	0.958678
chr6	88381200	0.776860
chr6	88433300	0.785124
chr6	88607000	0.702479
chr6	88613900	0.735537
chr6	88629000	0.727273
chr6	88755800	0.652893
chr6	88838300	0.694215
chr6	88921700	0.876033
chr6	89091000	0.644628
chr6	89284900	0.719008
chr6	89294600	0.710744
chr6	89435400	0.942149
chr6	89623800	0.644628
chr6	89654500	0.768595
chr6	89682900	0.842975
chr6	89732000	0.975207
chr6	89745300	0.652893
chr6	89901100	0.694215
chr6	89953300	0.644628

chr6	90009600	0.743802
chr6	90085600	0.859504
chr6	90116100	0.669421
chr6	90193800	0.768595
chr6	90202200	0.785124
chr6	90212800	0.826446
chr6	90265600	0.685950
chr6	90378400	0.925620
chr6	90387500	0.851240
chr6	90390300	0.892562
chr6	90398000	0.834711
chr6	90400300	0.958678
chr6	90402400	0.793388
chr6	90503800	0.710744
chr6	90510000	0.917355
chr6	90620600	0.793388
chr6	90650500	0.652893
chr6	90688700	0.867769
chr6	90926300	0.834711
chr6	90928700	0.942149
chr6	90940800	0.644628
chr6	90995100	0.677686
chr6	91008800	0.834711
chr6	91074900	0.677686
chr6	91079200	0.900826
chr6	91095200	0.694215
chr6	91114100	0.826446
chr6	91123700	0.685950
chr6	91151500	0.677686
chr6	91160700	0.867769
chr6	91188700	0.826446
chr6	91200200	0.752066
chr6	91203700	0.933884
chr6	91205800	0.942149
chr6	91208100	0.661157
chr6	91218900	0.826446
chr6	91386000	0.768595
chr6	91388200	0.702479
chr6	91402400	0.685950
chr6	91411100	0.644628
chr6	91490200	0.702479
chr6	91518400	0.719008
chr6	91792500	0.685950
chr6	91797900	0.752066
chr6	91989900	0.900826
chr6	92009500	0.694215
chr6	92147300	0.644628
chr6	92343900	0.760331
chr6	92364400	0.677686
chr6	92498300	0.735537
chr6	93558400	0.801653
chr6	93626200	0.768595
chr6	93789100	0.677686
chr6	94047500	0.710744

chr6	94494800	0.933884
chr6	94497300	0.892562
chr6	94531000	0.727273
chr6	94546000	0.685950
chr6	94569800	0.933884
chr6	94572700	0.776860
chr6	94755500	0.834711
chr6	94760100	0.661157
chr6	94800100	0.694215
chr6	95964300	0.685950
chr6	96134200	0.644628
chr6	96604600	0.851240
chr6	96611300	0.966942
chr6	96645400	0.950413
chr6	96793700	0.669421
chr6	96840200	0.661157
chr6	96967900	0.727273
chr6	97515800	0.776860
chr6	97654100	0.685950
chr6	97891300	0.710744
chr6	97914400	0.826446
chr6	98117300	0.727273
chr6	98220100	0.776860
chr6	98226500	0.991736
chr6	98267700	0.842975
chr6	98299100	0.933884
chr6	98363500	0.685950
chr6	98365700	0.685950
chr6	98487100	0.900826
chr6	98489400	0.975207
chr6	98491600	0.859504
chr6	98495200	0.669421
chr6	98585600	0.809917
chr6	98744200	0.710744
chr6	98876200	0.694215
chr6	98934600	0.752066
chr6	98974200	0.876033
chr6	98990900	0.652893
chr6	99075400	0.900826
chr6	99082300	0.785124
chr6	99085500	0.735537
chr6	99090700	0.661157
chr6	99164900	0.710744
chr6	99223800	0.685950
chr6	99236200	0.876033
chr6	99285900	0.661157
chr6	99381200	0.785124
chr6	99668200	0.768595
chr6	99718300	0.652893
chr6	99818000	0.785124
chr6	100039100	0.752066
chr6	100053200	0.917355
chr6	100055600	0.876033
chr6	100059500	0.677686

chr6	100608900	0.909091
chr6	100611500	0.950413
chr6	100616300	0.785124
chr6	100624600	0.677686
chr6	100732000	0.661157
chr6	100820900	0.702479
chr6	100868800	0.793388
chr6	100875300	0.661157
chr6	100897800	0.859504
chr6	100902300	0.719008
chr6	100915200	0.851240
chr6	100917800	0.801653
chr6	101289700	0.785124
chr6	101340700	0.950413
chr6	101532000	0.727273
chr6	101565600	0.694215
chr6	101674800	0.727273
chr6	101918300	0.677686
chr6	102046300	0.644628
chr6	102063200	0.884298
chr6	102164200	0.834711
chr6	102179400	0.809917
chr6	102189500	0.842975
chr6	102214800	0.818182
chr6	102225200	0.661157
chr6	102272800	0.661157
chr6	102295600	0.909091
chr6	103449200	0.776860
chr6	103982300	0.702479
chr6	104232600	0.677686
chr6	104571700	0.719008
chr6	104618100	0.652893
chr6	104920900	0.917355
chr6	105148600	0.859504
chr6	105417500	0.958678
chr6	105449400	0.727273
chr6	105473100	0.950413
chr6	105486300	0.727273
chr6	105630100	0.867769
chr6	105705100	0.760331
chr6	105708700	0.719008
chr6	105721500	0.818182
chr6	105753100	0.719008
chr6	105848400	0.685950
chr6	106201900	0.867769
chr6	106317500	0.644628
chr6	106450200	0.719008
chr6	106453800	0.892562
chr6	106463200	0.826446
chr6	106499400	0.776860
chr6	106611900	0.809917
chr6	106615300	0.966942
chr6	106734700	0.685950
chr6	106902200	0.768595

chr6	106943200	0.694215
chr6	106985500	0.661157
chr6	107062000	0.933884
chr6	107065000	0.760331
chr6	107074700	0.694215
chr6	107115000	0.743802
chr6	107140700	0.933884
chr6	107162100	0.685950
chr6	107164200	0.669421
chr6	107197700	0.685950
chr6	107221700	0.644628
chr6	107247700	0.661157
chr6	107252600	0.958678
chr6	107272000	0.909091
chr6	107319200	0.917355
chr6	107382700	0.735537
chr6	107389000	0.644628
chr6	107424300	0.719008
chr6	107427300	0.942149
chr6	107439700	0.735537
chr6	107451100	0.768595
chr6	107473400	0.652893
chr6	107476000	0.884298
chr6	107531300	0.669421
chr6	107539400	0.752066
chr6	107588200	0.677686
chr6	107736600	0.818182
chr6	107787600	0.727273
chr6	107832700	0.809917
chr6	107855800	0.710744
chr6	107900600	1.000000
chr6	107907500	0.702479
chr6	107920800	0.776860
chr6	107928500	0.809917
chr6	107932300	0.652893
chr6	107937400	0.785124
chr6	107939600	0.826446
chr6	107979600	0.669421
chr6	107987600	0.752066
chr6	107992500	0.876033
chr6	107996900	0.694215
chr6	108021400	0.710744
chr6	108026200	0.685950
chr6	108058000	0.826446
chr6	108186100	0.669421
chr6	108303900	0.867769
chr6	108309900	0.933884
chr6	108313600	0.661157
chr6	108320700	0.727273
chr6	108393400	0.859504
chr6	108427900	0.909091
chr6	108437000	0.661157
chr6	108456400	0.809917
chr6	108492700	0.760331

chr6	108501000	0.834711
chr6	108886200	0.785124
chr6	108903900	0.950413
chr6	108910300	0.785124
chr6	108931500	0.826446
chr6	108947400	0.719008
chr6	108950100	0.669421
chr6	108960400	0.892562
chr6	108971900	0.644628
chr6	108989000	0.876033
chr6	109028700	0.818182
chr6	109042700	0.661157
chr6	109052600	0.867769
chr6	109055100	0.859504
chr6	109180900	0.793388
chr6	109242900	0.884298
chr6	109250100	0.727273
chr6	109328400	0.652893
chr6	109403600	0.652893
chr6	109583800	0.743802
chr6	109798900	0.851240
chr6	109813000	0.983471
chr6	109829700	0.909091
chr6	109835900	0.975207
chr6	109902200	0.809917
chr6	110000700	0.785124
chr6	110057600	0.801653
chr6	110145000	0.694215
chr6	110176600	0.644628
chr6	110191000	0.809917
chr6	110214500	0.950413
chr6	110223200	0.743802
chr6	110266100	1.000000
chr6	110361300	0.809917
chr6	110491200	0.677686
chr6	110591700	0.900826
chr6	110644300	0.743802
chr6	110674000	0.892562
chr6	110676900	0.851240
chr6	110832900	0.694215
chr6	110872600	0.768595
chr6	110934300	0.793388
chr6	110940400	0.661157
chr6	110967900	0.669421
chr6	110978000	0.809917
chr6	111010500	0.685950
chr6	111205600	0.859504
chr6	111355000	0.809917
chr6	111412000	0.909091
chr6	111547600	0.702479
chr6	111573600	0.884298
chr6	111799400	0.661157
chr6	111862700	0.834711
chr6	111873800	0.867769

chr6	111887900	0.900826
chr6	111936900	0.702479
chr6	111953400	0.851240
chr6	111975100	0.719008
chr6	111985100	0.826446
chr6	111989800	0.694215
chr6	111994600	0.719008
chr6	112007900	0.768595
chr6	112033300	0.818182
chr6	112053000	0.801653
chr6	112066600	0.652893
chr6	112069500	0.702479
chr6	112072700	0.991736
chr6	112086300	0.917355
chr6	112092600	0.793388
chr6	112097400	0.661157
chr6	112121400	0.669421
chr6	112163900	0.694215
chr6	112166600	0.669421
chr6	112179300	0.826446
chr6	112183600	0.909091
chr6	112187600	0.727273
chr6	112205500	0.917355
chr6	112242300	0.859504
chr6	112271300	0.685950
chr6	112306600	0.884298
chr6	112318700	0.867769
chr6	112422200	0.900826
chr6	112845600	0.793388
chr6	112884900	0.652893
chr6	112943600	0.677686
chr6	113101200	0.801653
chr6	113349600	0.652893
chr6	113387500	0.727273
chr6	113602400	0.942149
chr6	113860700	0.669421
chr6	113885500	0.768595
chr6	113908800	0.991736
chr6	113914000	0.925620
chr6	113994300	0.727273
chr6	114098700	0.752066
chr6	114282700	0.884298
chr6	114331900	0.834711
chr6	114443600	0.785124
chr6	114466500	0.652893
chr6	114476000	0.876033
chr6	114492400	0.661157
chr6	114498500	0.644628
chr6	114515900	0.661157
chr6	114522700	0.743802
chr6	114525000	0.909091
chr6	114599700	0.760331
chr6	114603400	0.925620
chr6	114641400	0.867769

chr6	114655600	0.661157
chr6	114666900	0.859504
chr6	114681900	0.677686
chr6	114684400	0.966942
chr6	114686500	0.702479
chr6	114700900	0.842975
chr6	114707300	0.685950
chr6	114741000	0.702479
chr6	114743200	0.669421
chr6	114752000	0.809917
chr6	114762600	0.652893
chr6	114793300	0.694215
chr6	114845100	0.892562
chr6	115736700	0.958678
chr6	115778400	0.719008
chr6	116424500	0.867769
chr6	116427300	0.768595
chr6	116550700	0.752066
chr6	116593000	0.884298
chr6	116645300	0.909091
chr6	116720700	0.644628
chr6	116726900	0.909091
chr6	116754000	0.818182
chr6	116833500	0.661157
chr6	116851200	0.661157
chr6	116855100	0.851240
chr6	117066600	0.818182
chr6	117271500	0.768595
chr6	117393900	0.669421
chr6	117732700	0.727273
chr6	117772700	0.652893
chr6	117871400	0.644628
chr6	117934800	0.818182
chr6	117938200	0.677686
chr6	118061900	0.727273
chr6	118117200	0.685950
chr6	118260400	0.652893
chr6	118273400	1.000000
chr6	118519600	0.652893
chr6	118645500	0.785124
chr6	118699300	0.735537
chr6	118887100	0.644628
chr6	118902400	0.818182
chr6	118951700	0.694215
chr6	119027400	0.818182
chr6	119030800	0.917355
chr6	119069500	0.942149
chr6	119122100	0.719008
chr6	119203700	0.677686
chr6	119221100	0.909091
chr6	119349000	0.809917
chr6	119356700	0.826446
chr6	119359600	0.677686
chr6	119374000	0.702479

chr6	119450300	0.644628
chr6	119617800	0.842975
chr6	119662600	0.826446
chr6	119849500	0.685950
chr6	120038700	0.652893
chr6	120063700	0.719008
chr6	120121200	0.727273
chr6	120278900	0.727273
chr6	120516000	0.661157
chr6	120906000	0.702479
chr6	121252700	0.809917
chr6	121256500	0.842975
chr6	121427000	0.644628
chr6	121508900	0.735537
chr6	121727100	0.727273
chr6	121752600	0.917355
chr6	121766000	0.851240
chr6	121806900	0.694215
chr6	121809600	0.760331
chr6	121813300	0.809917
chr6	121940900	0.867769
chr6	122172600	0.752066
chr6	122242200	0.652893
chr6	122254000	0.776860
chr6	122320900	0.925620
chr6	122586000	0.661157
chr6	123038700	0.677686
chr6	123048500	0.842975
chr6	123056400	0.710744
chr6	123074700	0.644628
chr6	123101200	0.892562
chr6	123150700	0.834711
chr6	123155000	0.851240
chr6	123183600	0.644628
chr6	123681800	0.652893
chr6	123738900	0.694215
chr6	123840100	0.669421
chr6	123910100	0.735537
chr6	124091500	0.719008
chr6	124110600	0.644628
chr6	124395500	0.768595
chr6	124479400	0.685950
chr6	124982100	0.933884
chr6	125341200	0.925620
chr6	125399000	0.652893
chr6	125512700	0.900826
chr6	125541000	0.685950
chr6	125678900	0.652893
chr6	125743200	0.975207
chr6	125753300	0.801653
chr6	125790500	0.644628
chr6	125824300	0.892562
chr6	125850400	0.925620
chr6	125906000	0.834711

chr6	125910100	0.677686
chr6	125933500	0.727273
chr6	125992200	0.950413
chr6	126000500	0.652893
chr6	126022700	0.909091
chr6	126029400	0.776860
chr6	126189100	0.793388
chr6	126239900	0.818182
chr6	126274700	0.842975
chr6	126310500	0.876033
chr6	126429000	0.834711
chr6	126503200	0.991736
chr6	126663600	0.719008
chr6	126848200	0.958678
chr6	127038300	0.702479
chr6	127094900	0.661157
chr6	127284800	0.743802
chr6	127476000	0.644628
chr6	127484600	0.644628
chr6	127612800	0.727273
chr6	127622100	0.685950
chr6	127780300	0.644628
chr6	127803400	0.942149
chr6	127914700	0.644628
chr6	128639500	0.743802
chr6	128813300	0.760331
chr6	128816800	0.644628
chr6	128829400	0.834711
chr6	128838200	0.760331
chr6	129046200	0.644628
chr6	129065500	1.000000
chr6	129276200	0.760331
chr6	129517300	0.776860
chr6	129639900	0.644628
chr6	129762100	0.900826
chr6	129768600	0.743802
chr6	130268300	0.975207
chr6	130524400	0.859504
chr6	130554700	0.702479
chr6	130699400	0.669421
chr6	130712400	0.685950
chr6	130728100	0.694215
chr6	130746800	0.710744
chr6	130947600	0.694215
chr6	130954500	0.685950
chr6	131034400	0.842975
chr6	131115600	0.719008
chr6	131147400	0.801653
chr6	131160400	0.727273
chr6	131175100	0.801653
chr6	131325700	0.652893
chr6	131351300	0.719008
chr6	131483500	0.966942
chr6	131504900	0.884298

chr6	131529900	0.652893
chr6	131603000	0.900826
chr6	131614200	0.925620
chr6	131643900	0.661157
chr6	131666300	0.661157
chr6	131781900	0.892562
chr6	131832100	0.785124
chr6	132066300	0.644628
chr6	132131800	0.694215
chr6	132181400	0.652893
chr6	132222300	0.752066
chr6	132229100	0.785124
chr6	132259400	0.719008
chr6	132274400	0.917355
chr6	132347300	0.727273
chr6	132410300	0.669421
chr6	132453300	0.809917
chr6	132713600	0.743802
chr6	132873000	0.743802
chr6	132892100	0.752066
chr6	133376900	0.975207
chr6	133753400	0.801653
chr6	133870200	0.710744
chr6	133967000	0.710744
chr6	134003500	0.975207
chr6	134214600	0.669421
chr6	134238400	0.743802
chr6	134245900	0.793388
chr6	134312400	0.644628
chr6	134317900	0.685950
chr6	134376300	0.859504
chr6	134379200	0.652893
chr6	134381900	0.900826
chr6	134384800	0.793388
chr6	134395900	0.933884
chr6	134482500	0.991736
chr6	134505100	0.925620
chr6	134551400	0.661157
chr6	134673700	0.727273
chr6	134726900	0.727273
chr6	134775300	0.991736
chr6	134838500	0.694215
chr6	135051100	0.809917
chr6	135079800	0.669421
chr6	135347000	0.735537
chr6	135537100	0.785124
chr6	135592600	0.925620
chr6	135644300	0.669421
chr6	135722100	0.735537
chr6	135825500	0.669421
chr6	135859200	0.826446
chr6	135902800	0.859504
chr6	136147700	0.644628
chr6	136243900	0.735537

chr6	136425600	0.809917
chr6	136607500	0.694215
chr6	136673300	0.752066
chr6	136755600	0.942149
chr6	136759300	0.760331
chr6	136761500	0.685950
chr6	136788700	0.826446
chr6	136793600	0.644628
chr6	136825200	0.892562
chr6	136849500	0.661157
chr6	136852800	0.669421
chr6	136868000	0.842975
chr6	136910300	0.669421
chr6	136913500	0.652893
chr6	136965500	0.785124
chr6	137094600	0.735537
chr6	137278400	0.991736
chr6	137281400	0.743802
chr6	137298400	0.702479
chr6	137617300	0.801653
chr6	137619600	0.776860
chr6	137687300	0.710744
chr6	137745200	0.950413
chr6	137812900	0.760331
chr6	137834600	0.892562
chr6	137840100	0.685950
chr6	137844500	0.859504
chr6	137864000	0.785124
chr6	137868400	0.818182
chr6	137872100	0.900826
chr6	137936700	0.685950
chr6	138186100	0.752066
chr6	138201200	0.760331
chr6	138247500	0.851240
chr6	138504500	0.991736
chr6	138509800	0.884298
chr6	138613200	0.785124
chr6	138653100	0.950413
chr6	138667200	0.669421
chr6	138694100	0.644628
chr6	138805000	0.743802
chr6	138807700	0.991736
chr6	138814800	0.851240
chr6	138818600	0.818182
chr6	138833900	0.735537
chr6	138848700	0.925620
chr6	138860300	0.661157
chr6	138877000	0.933884
chr6	138881800	0.793388
chr6	138885300	0.677686
chr6	138891000	0.677686
chr6	138898900	0.694215
chr6	138910800	0.710744
chr6	138915000	0.909091

chr6	139139500	0.851240
chr6	139289500	0.694215
chr6	139293400	0.933884
chr6	139315200	0.809917
chr6	139354900	0.925620
chr6	139633100	0.702479
chr6	139636100	0.958678
chr6	139666000	0.793388
chr6	139689200	0.975207
chr6	139698800	0.851240
chr6	139717300	0.661157
chr6	139789800	0.710744
chr6	139795200	0.975207
chr6	139801600	0.793388
chr6	139804100	0.851240
chr6	139933100	0.685950
chr6	139948200	0.644628
chr6	140043800	0.735537
chr6	140202600	0.752066
chr6	140205800	0.652893
chr6	140210000	0.983471
chr6	140304000	0.809917
chr6	140372100	0.735537
chr6	140417000	0.859504
chr6	140427400	0.801653
chr6	140757700	0.892562
chr6	140958100	0.669421
chr6	141427200	0.925620
chr6	141474300	0.909091
chr6	141710000	0.719008
chr6	141793500	0.752066
chr6	142151500	0.727273
chr6	142310700	0.842975
chr6	142336000	0.818182
chr6	142477300	0.933884
chr6	142722200	0.809917
chr6	142738200	0.652893
chr6	142762300	0.867769
chr6	142786600	0.652893
chr6	142891000	0.727273
chr6	142969000	0.702479
chr6	143014500	0.661157
chr6	143024100	0.776860
chr6	143133100	0.719008
chr6	143167600	0.785124
chr6	143176200	0.735537
chr6	143179200	0.661157
chr6	143204300	0.702479
chr6	143206700	0.900826
chr6	143384600	0.793388
chr6	143555800	0.768595
chr6	143586900	0.719008
chr6	143610100	0.942149
chr6	143834800	0.760331

chr6	143861200	0.809917
chr6	143891800	0.818182
chr6	143969700	0.785124
chr6	143996800	0.933884
chr6	144015900	0.834711
chr6	144064800	0.652893
chr6	144068200	1.000000
chr6	144071900	0.735537
chr6	144074900	0.677686
chr6	144125300	0.785124
chr6	144258700	0.719008
chr6	144263200	0.727273
chr6	144287000	0.785124
chr6	144294700	0.966942
chr6	144555700	0.900826
chr6	144587100	1.000000
chr6	144600300	0.826446
chr6	144632900	0.652893
chr6	144641900	0.644628
chr6	144644100	0.760331
chr6	144646400	0.768595
chr6	144648900	0.942149
chr6	144652200	0.760331
chr6	144665500	0.694215
chr6	144712000	0.793388
chr6	144904300	0.719008
chr6	144938900	0.719008
chr6	144965800	0.884298
chr6	144967900	0.958678
chr6	144979200	0.818182
chr6	144984400	0.900826
chr6	145047800	0.702479
chr6	145066200	0.719008
chr6	145168600	0.685950
chr6	145192400	0.793388
chr6	145217500	0.743802
chr6	145240800	0.710744
chr6	145299000	0.652893
chr6	145749700	0.652893
chr6	145854200	0.867769
chr6	146157400	0.702479
chr6	146387800	0.685950
chr6	146710300	0.677686
chr6	146866900	0.669421
chr6	147235600	0.727273
chr6	147374700	0.991736
chr6	147377500	0.644628
chr6	147631700	0.652893
chr6	147744500	0.644628
chr6	147760000	0.702479
chr6	147769000	0.793388
chr6	147995700	0.719008
chr6	147998900	0.809917
chr6	148521600	0.669421

chr6	148543300	0.735537
chr6	148583500	0.661157
chr6	148618200	0.677686
chr6	148666800	0.694215
chr6	148669600	0.760331
chr6	148695200	0.785124
chr6	148706900	0.851240
chr6	148734500	0.669421
chr6	148762500	0.661157
chr6	148765500	0.867769
chr6	148769400	0.818182
chr6	148830200	0.851240
chr6	148838100	0.710744
chr6	148852400	0.933884
chr6	148872300	0.644628
chr6	148920900	0.876033
chr6	149082900	0.975207
chr6	149087400	0.685950
chr6	149124900	0.735537
chr6	149137700	0.652893
chr6	149140400	0.991736
chr6	149172800	0.876033
chr6	149176400	0.867769
chr6	149186200	0.884298
chr6	149207200	0.876033
chr6	149245800	0.702479
chr6	149269200	0.644628
chr6	149289200	0.694215
chr6	149294000	1.000000
chr6	149301500	0.768595
chr6	149310600	0.950413
chr6	149336000	0.859504
chr6	149358600	0.884298
chr6	149367900	0.702479
chr6	149657200	0.702479
chr6	149753500	0.719008
chr6	150030300	0.768595
chr6	150182500	0.834711
chr6	150190100	0.760331
chr6	150225500	0.644628
chr6	150310000	0.702479
chr6	150346900	0.727273
chr6	150392200	0.669421
chr6	150471700	0.768595
chr6	150547400	0.842975
chr6	150800200	0.909091
chr6	150850200	0.752066
chr6	150868800	0.719008
chr6	150910200	0.727273
chr6	151042100	0.785124
chr6	151075300	0.884298
chr6	151197500	0.743802
chr6	151200300	0.876033
chr6	151330600	0.884298

chr6	151366500	0.975207
chr6	151412300	0.909091
chr6	151497400	0.661157
chr6	151576300	0.809917
chr6	151612600	0.719008
chr6	151634400	0.933884
chr6	151695600	0.669421
chr6	151969900	0.859504
chr6	152013500	0.685950
chr6	152038100	0.719008
chr6	152085600	0.785124
chr6	152127100	0.661157
chr6	152483100	0.685950
chr6	152964500	0.685950
chr6	153003100	0.702479
chr6	153298400	0.818182
chr6	153380900	0.851240
chr6	153739200	0.661157
chr6	153758100	0.933884
chr6	153938100	0.776860
chr6	154153600	0.702479
chr6	154449300	0.661157
chr6	154565100	0.801653
chr6	154568500	0.991736
chr6	154755100	0.710744
chr6	154825300	0.859504
chr6	154833800	0.809917
chr6	154839700	0.677686
chr6	154858700	0.826446
chr6	154863800	0.933884
chr6	155059900	0.892562
chr6	155064100	0.644628
chr6	155194300	0.727273
chr6	155230900	0.842975
chr6	155254100	0.942149
chr6	155328800	0.669421
chr6	155334600	0.677686
chr6	155337000	0.909091
chr6	155406300	0.793388
chr6	155421800	0.752066
chr6	155441500	1.000000
chr6	155464600	0.983471
chr6	155470400	0.867769
chr6	155497400	0.661157
chr6	155507300	0.925620
chr6	155583000	0.785124
chr6	155594900	0.834711
chr6	155739800	0.644628
chr6	155770600	0.768595
chr6	155945100	0.991736
chr6	156216500	0.942149
chr6	156276500	0.966942
chr6	156489300	0.685950
chr6	156497400	0.834711

chr6	156563500	0.719008
chr6	156718400	0.900826
chr6	156940400	0.719008
chr6	156976500	0.942149
chr6	156980400	0.752066
chr6	157042300	0.950413
chr6	157074000	0.677686
chr6	157096000	0.917355
chr6	157116700	0.925620
chr6	157130700	0.644628
chr6	157141100	0.768595
chr6	157148100	0.685950
chr6	157161200	0.685950
chr6	157169300	0.859504
chr6	157174400	0.983471
chr6	157187600	0.752066
chr6	157191400	0.933884
chr6	157203400	0.793388
chr6	157209000	0.834711
chr6	157241100	0.710744
chr6	157254800	0.892562
chr6	157265500	0.909091
chr6	157274400	0.834711
chr6	157286300	0.669421
chr6	157289800	0.834711
chr6	157299200	0.776860
chr6	157305200	0.727273
chr6	157310000	0.818182
chr6	157329700	0.710744
chr6	157334900	0.694215
chr6	157358500	0.851240
chr6	157372400	0.685950
chr6	157391800	0.752066
chr6	157399400	0.776860
chr6	157410000	0.892562
chr6	157479000	0.710744
chr6	157494200	0.661157
chr6	157550900	0.776860
chr6	157639500	0.652893
chr6	157796500	0.785124
chr6	157828100	0.694215
chr6	157845600	0.644628
chr6	157849800	0.793388
chr6	157853600	0.785124
chr6	157858500	0.702479
chr6	157877000	0.669421
chr6	157939400	0.785124
chr6	157956700	0.743802
chr6	157967900	0.859504
chr6	157973600	1.000000
chr6	158027400	0.851240
chr6	158050200	0.685950
chr6	158070100	0.768595
chr6	158089000	0.727273

chr6	158091300	0.760331
chr6	158129300	0.702479
chr6	158132700	0.876033
chr6	158163100	0.735537
chr6	158184400	0.900826
chr6	158252600	0.809917
chr6	158255300	0.842975
chr6	158260700	0.752066
chr6	158300000	0.710744
chr6	158326700	0.867769
chr6	158333200	0.859504
chr6	158382800	0.735537
chr6	158406800	0.842975
chr6	158411600	0.785124
chr6	158418200	0.702479
chr6	158423800	0.735537
chr6	158453200	0.958678
chr6	158460400	0.669421
chr6	158471000	0.743802
chr6	158583100	0.958678
chr6	158622300	0.669421
chr6	158626500	0.677686
chr6	158705400	0.991736
chr6	158744500	0.727273
chr6	158750000	0.727273
chr6	158760300	0.842975
chr6	158885400	0.694215
chr6	158920000	0.661157
chr6	158969700	0.917355
chr6	158986400	0.743802
chr6	158997400	0.892562
chr6	159032700	0.842975
chr6	159084700	0.661157
chr6	159125900	0.727273
chr6	159139800	0.661157
chr6	159179500	0.867769
chr6	159194700	0.801653
chr6	159198800	0.652893
chr6	159223500	0.909091
chr6	159229700	0.900826
chr6	159234600	0.884298
chr6	159271300	0.702479
chr6	159273500	0.727273
chr6	159275900	0.975207
chr6	159290600	0.669421
chr6	159300300	0.818182
chr6	159331800	0.983471
chr6	159343500	0.950413
chr6	159360600	0.785124
chr6	159490500	0.685950
chr6	159525500	0.661157
chr6	160241500	0.785124
chr6	160259400	0.677686
chr6	160296900	0.776860

chr6	160402500	0.809917
chr6	160533200	0.694215
chr6	160554200	0.702479
chr6	160559400	0.859504
chr6	160582800	0.917355
chr6	160598200	0.818182
chr6	160643600	0.760331
chr6	160647600	0.917355
chr6	160661900	0.851240
chr6	160668800	0.884298
chr6	160671900	0.867769
chr6	160680900	0.809917
chr6	160715100	0.710744
chr6	160721700	0.685950
chr6	160850900	0.867769
chr6	161298600	0.727273
chr6	161305200	0.818182
chr6	161321400	0.818182
chr6	161435200	0.702479
chr6	161456100	0.719008
chr6	161585300	0.785124
chr6	161589400	0.917355
chr6	161591800	0.785124
chr6	161650500	0.801653
chr6	161665100	0.644628
chr6	161676400	0.694215
chr6	161681100	0.652893
chr6	161713900	0.834711
chr6	161865500	0.834711
chr6	162259700	0.834711
chr6	162312400	0.867769
chr6	162368300	0.644628
chr6	162766600	0.661157
chr6	162964800	0.710744
chr6	163271900	0.661157
chr6	163278600	0.834711
chr6	163504500	0.669421
chr6	163510300	0.644628
chr6	163520900	0.917355
chr6	163607100	0.652893
chr6	163665400	0.719008
chr6	163681600	0.644628
chr6	163685600	0.661157
chr6	163818200	0.710744
chr6	163839500	0.909091
chr6	163844300	0.785124
chr6	163849700	0.809917
chr6	163858800	0.644628
chr6	163865000	0.661157
chr6	163881800	0.702479
chr6	163963400	0.719008
chr6	163965600	0.719008
chr6	164000900	0.809917
chr6	164057000	0.867769

chr6	164285000	0.652893
chr6	164362400	0.867769
chr6	164367600	0.818182
chr6	164436500	0.933884
chr6	164586400	0.652893
chr6	164767100	0.991736
chr6	165062300	0.694215
chr6	165380700	0.834711
chr6	165658700	0.752066
chr6	165966800	0.793388
chr6	166013600	0.793388
chr6	166029900	0.809917
chr6	166050600	0.644628
chr6	166190500	0.694215
chr6	166231500	0.826446
chr6	166233900	0.752066
chr6	166256000	0.909091
chr6	166261000	0.652893
chr6	166417100	0.768595
chr6	166471600	0.644628
chr6	166541800	0.760331
chr6	166587200	0.752066
chr6	166611400	0.710744
chr6	166654700	0.727273
chr6	166682200	0.826446
chr6	166744700	0.644628
chr6	166779100	1.000000
chr6	166954300	0.694215
chr6	166965800	0.942149
chr6	166978600	0.685950
chr6	166986700	0.909091
chr6	167003100	0.776860
chr6	167012200	0.694215
chr6	167029200	0.685950
chr6	167034800	0.884298
chr6	167054700	0.685950
chr6	167063700	0.818182
chr6	167085600	0.760331
chr6	167101600	0.776860
chr6	167116700	0.892562
chr6	167161400	0.842975
chr6	167171600	0.859504
chr6	167181000	0.809917
chr6	167363800	0.719008
chr6	167402500	0.851240
chr6	167554800	0.719008
chr6	167560700	0.892562
chr6	167578200	0.743802
chr6	167587600	0.669421
chr6	167624500	0.661157
chr6	167637100	0.826446
chr6	167639700	0.834711
chr6	167647300	0.735537
chr6	167650100	0.768595

chr6	167652700	0.876033
chr6	167655200	0.719008
chr6	167658600	0.834711
chr6	167662100	0.892562
chr6	167691600	0.661157
chr6	167694500	0.694215
chr6	167706300	0.760331
chr6	167715200	0.677686
chr6	167719200	0.900826
chr6	167728400	0.892562
chr6	167821200	0.685950
chr6	167852400	0.677686
chr6	167854800	0.991736
chr6	167885400	0.834711
chr6	168052300	0.801653
chr6	168090300	0.867769
chr6	168152100	0.776860
chr6	168197500	0.900826
chr6	168200400	0.760331
chr6	168218500	0.909091
chr6	168248500	0.809917
chr6	168250800	0.983471
chr6	168260000	0.983471
chr6	168262700	0.809917
chr6	168294500	0.950413
chr6	168398100	0.677686
chr6	168417800	0.661157
chr6	168489400	0.826446
chr6	168500300	0.859504
chr6	168503400	0.876033
chr6	168522300	0.677686
chr6	168533500	0.743802
chr6	168558800	0.834711
chr6	168572700	0.991736
chr6	168580000	0.809917
chr6	168583100	0.768595
chr6	168610100	0.867769
chr6	168617300	0.917355
chr6	168627000	0.752066
chr6	168633300	0.710744
chr6	168674400	0.809917
chr6	168714800	0.950413
chr6	168727400	0.809917
chr6	168829400	0.710744
chr6	168844700	0.752066
chr6	168852000	0.685950
chr6	168897600	0.851240
chr6	168925700	0.644628
chr6	168939400	0.702479
chr6	168942300	0.801653
chr6	169008900	0.702479
chr6	169032400	0.752066
chr6	169042000	0.867769
chr6	169050300	0.702479

chr6	169083700	0.809917
chr6	169093400	0.859504
chr6	169096100	0.917355
chr6	169237800	0.867769
chr6	169326400	0.710744
chr6	169357300	0.760331
chr6	169400500	0.776860
chr6	169407300	0.933884
chr6	169513300	0.991736
chr6	169650800	0.710744
chr6	169671200	0.851240
chr6	169828100	0.743802
chr6	170020300	0.818182
chr6	170023800	0.644628
chr6	170201600	0.727273
chr6	170210800	0.685950
chr6	170227700	0.818182
chr6	170351700	0.842975
chr6	170356300	0.685950
chr6	170468700	0.719008
chr6	170535000	0.702479
chr6	170545400	0.661157
chr6	170554100	0.652893
chr6	170596100	0.867769
chr6	170623400	0.801653
chr6	170654000	0.776860
chr6	170712700	0.801653
chr6	170743800	0.768595
chr6	170748100	0.669421
chr6	170752900	0.760331
chr6	170807400	0.661157
chr7	10300	0.652893
chr7	35000	0.702479
chr7	59900	0.785124
chr7	64600	0.685950
chr7	82100	0.652893
chr7	91800	0.785124
chr7	94200	0.909091
chr7	97700	0.818182
chr7	101300	0.661157
chr7	119500	0.677686
chr7	121700	0.768595
chr7	140200	0.702479
chr7	156400	0.826446
chr7	184100	0.694215
chr7	189100	0.785124
chr7	220300	0.644628
chr7	362600	0.652893
chr7	413600	0.710744
chr7	461800	0.710744
chr7	466000	0.776860
chr7	475300	0.818182
chr7	477900	0.785124
chr7	520400	0.785124

chr7	524700	0.719008
chr7	555200	0.752066
chr7	612900	0.933884
chr7	642300	0.752066
chr7	661800	0.768595
chr7	704000	0.793388
chr7	814200	0.710744
chr7	853100	0.950413
chr7	863400	0.809917
chr7	866300	0.661157
chr7	872100	0.950413
chr7	913300	0.809917
chr7	928900	0.685950
chr7	941400	0.834711
chr7	949500	0.644628
chr7	985500	0.760331
chr7	1020000	0.793388
chr7	1040400	0.785124
chr7	1092700	0.983471
chr7	1100400	0.867769
chr7	1106100	0.842975
chr7	1163100	0.776860
chr7	1216100	0.752066
chr7	1266000	0.958678
chr7	1280700	0.760331
chr7	1284400	0.768595
chr7	1288500	0.719008
chr7	1452700	0.719008
chr7	1469300	0.694215
chr7	1494900	0.876033
chr7	1563100	0.702479
chr7	1577000	0.876033
chr7	1595200	0.884298
chr7	1607400	0.702479
chr7	1616600	0.917355
chr7	1647500	0.727273
chr7	1734300	0.851240
chr7	1744700	0.768595
chr7	1753600	0.826446
chr7	1765200	0.793388
chr7	1796200	0.834711
chr7	1903600	0.776860
chr7	1915200	0.727273
chr7	1982200	0.900826
chr7	1985000	0.760331
chr7	2092600	0.735537
chr7	2121700	0.644628
chr7	2146400	0.743802
chr7	2149000	0.735537
chr7	2198800	0.950413
chr7	2309900	0.760331
chr7	2321900	0.785124
chr7	2341100	0.917355
chr7	2349800	0.900826

chr7	2465400	0.694215
chr7	2491600	0.752066
chr7	2514300	0.702479
chr7	2535800	0.867769
chr7	2540000	0.958678
chr7	2564800	0.983471
chr7	2567500	0.925620
chr7	2571000	0.743802
chr7	2625900	0.652893
chr7	2661500	0.925620
chr7	2681700	0.909091
chr7	2685500	0.884298
chr7	2770200	0.768595
chr7	2774800	0.909091
chr7	2798300	0.719008
chr7	2801500	0.900826
chr7	2818300	0.685950
chr7	2840500	0.644628
chr7	2843500	0.909091
chr7	2851000	0.752066
chr7	2854400	0.983471
chr7	2858500	0.983471
chr7	2861400	0.958678
chr7	2866900	0.900826
chr7	2870200	0.669421
chr7	2885000	0.719008
chr7	2903500	0.760331
chr7	2913900	0.785124
chr7	2916400	0.710744
chr7	2920800	0.842975
chr7	3216100	0.900826
chr7	3232800	0.743802
chr7	3234900	0.826446
chr7	3237500	0.826446
chr7	3304300	0.694215
chr7	3322800	0.694215
chr7	3417000	0.826446
chr7	3420900	0.900826
chr7	3439900	0.685950
chr7	3470700	0.735537
chr7	3550000	0.694215
chr7	3589000	0.859504
chr7	3593300	0.785124
chr7	3613300	0.793388
chr7	3625600	0.909091
chr7	3631800	0.859504
chr7	3723800	0.909091
chr7	3762500	0.743802
chr7	3794700	0.661157
chr7	3865100	0.702479
chr7	3902100	0.892562
chr7	3983600	0.834711
chr7	4012700	0.785124
chr7	4019700	0.801653

chr7	4046500	0.826446
chr7	4049700	0.644628
chr7	4079900	0.900826
chr7	4083000	0.768595
chr7	4170900	0.785124
chr7	4173800	0.702479
chr7	4176100	0.942149
chr7	4184700	0.933884
chr7	4280100	0.727273
chr7	4509900	0.652893
chr7	4653300	0.793388
chr7	4778700	0.669421
chr7	4848700	0.661157
chr7	4911900	0.669421
chr7	5231900	0.644628
chr7	5293200	0.661157
chr7	5373300	0.644628
chr7	5391800	0.685950
chr7	5422500	0.793388
chr7	5424900	0.685950
chr7	5428700	0.669421
chr7	5447300	0.884298
chr7	5488400	0.768595
chr7	5518100	0.892562
chr7	5532100	0.876033
chr7	5534900	0.809917
chr7	5566300	0.818182
chr7	5614800	0.809917
chr7	5647000	0.818182
chr7	5666400	0.818182
chr7	5674200	0.884298
chr7	5676500	0.867769
chr7	5731100	0.652893
chr7	5743800	0.652893
chr7	5751500	0.785124
chr7	5790300	0.685950
chr7	5807500	0.867769
chr7	5871100	0.950413
chr7	6211900	0.942149
chr7	6238700	0.818182
chr7	6298000	0.776860
chr7	6308900	0.793388
chr7	6357700	0.669421
chr7	6386400	0.661157
chr7	6699000	0.644628
chr7	6941900	0.785124
chr7	7042500	0.867769
chr7	7139900	0.719008
chr7	7145000	0.867769
chr7	7171100	0.727273
chr7	7216100	0.685950
chr7	7226500	0.661157
chr7	7241600	0.975207
chr7	7248100	0.900826

chr7	7251900	0.652893
chr7	7254400	0.661157
chr7	7268800	0.768595
chr7	7273800	0.933884
chr7	7277900	0.917355
chr7	7386300	0.677686
chr7	7392800	0.966942
chr7	7621700	0.694215
chr7	7747600	0.752066
chr7	7750200	0.652893
chr7	7813600	0.694215
chr7	7832700	0.909091
chr7	7861100	0.793388
chr7	7874500	0.661157
chr7	7910800	0.702479
chr7	7937500	0.710744
chr7	7958800	0.710744
chr7	7976600	0.685950
chr7	7994800	0.710744
chr7	8075600	0.694215
chr7	8109100	0.909091
chr7	8124700	0.867769
chr7	8151600	0.958678
chr7	8158800	0.685950
chr7	8186900	0.644628
chr7	8229500	0.752066
chr7	8261100	0.884298
chr7	8299200	0.677686
chr7	8336000	0.685950
chr7	8400300	0.925620
chr7	8402800	0.983471
chr7	8477200	0.760331
chr7	8600800	0.735537
chr7	8655200	0.644628
chr7	9053900	1.000000
chr7	9170100	0.710744
chr7	9271700	0.727273
chr7	9320400	0.785124
chr7	9977800	0.925620
chr7	10162600	0.644628
chr7	10948900	0.958678
chr7	11101900	0.652893
chr7	11122800	0.768595
chr7	11277300	0.719008
chr7	11477300	0.793388
chr7	11548200	0.809917
chr7	11557800	0.900826
chr7	11618100	0.661157
chr7	11653600	0.710744
chr7	11857100	0.652893
chr7	12131300	0.760331
chr7	12134800	0.958678
chr7	12239200	0.669421
chr7	12337800	0.661157

chr7	12369200	0.669421
chr7	12463800	0.702479
chr7	12529800	0.694215
chr7	12597300	0.892562
chr7	12770700	0.776860
chr7	12789300	0.719008
chr7	12810300	1.000000
chr7	12983700	0.661157
chr7	13025500	0.785124
chr7	13061800	0.719008
chr7	13840800	0.727273
chr7	13873900	0.925620
chr7	13876000	0.991736
chr7	13890600	0.892562
chr7	13949400	0.785124
chr7	13960200	0.644628
chr7	13989800	0.694215
chr7	14021400	0.909091
chr7	14032500	0.752066
chr7	14834200	0.776860
chr7	14873300	0.909091
chr7	14876500	0.925620
chr7	14886100	0.710744
chr7	15051300	0.710744
chr7	15236700	0.669421
chr7	15728700	0.661157
chr7	15764800	0.710744
chr7	15822100	0.933884
chr7	15879400	0.958678
chr7	16245000	0.735537
chr7	16502200	0.669421
chr7	16505300	0.801653
chr7	16524700	0.719008
chr7	16688700	0.892562
chr7	16708000	0.661157
chr7	16710800	0.652893
chr7	16784800	0.859504
chr7	16818100	0.644628
chr7	16945300	0.892562
chr7	17018400	0.727273
chr7	17345000	0.669421
chr7	17354200	0.735537
chr7	17537700	0.842975
chr7	17719700	0.842975
chr7	17812100	0.851240
chr7	17903800	0.702479
chr7	17922400	0.933884
chr7	17940600	0.694215
chr7	17942800	0.685950
chr7	17961500	0.876033
chr7	17964100	0.793388
chr7	17972700	0.694215
chr7	18358200	0.702479
chr7	18424800	0.876033

chr7	18437300	0.702479
chr7	18439900	0.834711
chr7	18549200	0.826446
chr7	18980700	0.851240
chr7	19152500	0.851240
chr7	19408800	0.785124
chr7	19445100	0.776860
chr7	19548500	0.719008
chr7	20023800	0.867769
chr7	20059600	0.983471
chr7	20093000	0.826446
chr7	20105000	0.851240
chr7	20116800	0.942149
chr7	20140100	0.743802
chr7	20153800	0.661157
chr7	20159200	0.752066
chr7	20166200	0.983471
chr7	20237000	0.661157
chr7	20241000	0.719008
chr7	20259600	0.752066
chr7	20347500	0.966942
chr7	20380700	0.966942
chr7	20383000	0.752066
chr7	20688600	0.876033
chr7	20905700	0.685950
chr7	20993500	0.702479
chr7	21064400	0.652893
chr7	21067800	0.793388
chr7	21070600	0.842975
chr7	21081500	0.809917
chr7	21084900	0.909091
chr7	21116200	0.925620
chr7	21165300	0.867769
chr7	21186600	0.727273
chr7	21212400	0.685950
chr7	21215700	0.685950
chr7	21295100	0.752066
chr7	21301500	0.942149
chr7	21316200	0.652893
chr7	21410900	0.859504
chr7	21453100	0.644628
chr7	21579800	0.719008
chr7	21585500	0.950413
chr7	21682200	0.652893
chr7	21805300	0.694215
chr7	21813700	0.661157
chr7	21906600	0.842975
chr7	21909800	0.776860
chr7	21962800	0.776860
chr7	21967800	0.702479
chr7	21979800	0.719008
chr7	21982400	0.743802
chr7	22012000	0.809917
chr7	22135700	0.644628

chr7	22181900	0.644628
chr7	22193900	0.942149
chr7	22224900	0.966942
chr7	22229500	0.760331
chr7	22360000	0.867769
chr7	22368200	0.884298
chr7	22370500	0.983471
chr7	22374000	0.743802
chr7	22473200	0.991736
chr7	22475300	0.727273
chr7	22492500	0.785124
chr7	22528400	0.694215
chr7	22601700	0.876033
chr7	22605500	0.669421
chr7	22629700	0.884298
chr7	22708000	0.818182
chr7	22711900	0.685950
chr7	22743000	0.685950
chr7	22749500	0.661157
chr7	22767000	0.677686
chr7	22842200	0.801653
chr7	22847500	0.917355
chr7	22850500	0.975207
chr7	22852600	0.867769
chr7	22882300	0.818182
chr7	22982500	0.652893
chr7	22989400	0.727273
chr7	22992700	0.735537
chr7	23028800	0.917355
chr7	23040500	0.752066
chr7	23149400	0.661157
chr7	23153400	0.933884
chr7	23158000	0.793388
chr7	23458100	0.669421
chr7	23471800	0.727273
chr7	23498500	0.685950
chr7	23501800	0.702479
chr7	23526000	0.950413
chr7	23573500	0.661157
chr7	23698600	0.785124
chr7	23702900	0.975207
chr7	23764000	0.785124
chr7	23996800	0.685950
chr7	24008000	0.851240
chr7	24065100	0.884298
chr7	24159700	0.694215
chr7	24268600	0.644628
chr7	24377100	0.884298
chr7	24412800	0.834711
chr7	24419200	0.785124
chr7	24421900	0.851240
chr7	24430300	0.900826
chr7	24432800	0.818182
chr7	24443900	0.950413

chr7	24446700	0.652893
chr7	24462400	0.735537
chr7	24467900	0.826446
chr7	24477200	0.892562
chr7	24495800	0.727273
chr7	24517800	0.867769
chr7	24546000	0.760331
chr7	24562000	0.859504
chr7	24576100	0.743802
chr7	24626500	0.743802
chr7	24644500	0.900826
chr7	24649300	0.983471
chr7	24705400	0.677686
chr7	24762200	0.685950
chr7	24766200	1.000000
chr7	24894200	0.669421
chr7	24918500	0.801653
chr7	24957200	0.735537
chr7	24972000	0.826446
chr7	24976000	0.719008
chr7	25006300	0.644628
chr7	25011600	0.685950
chr7	25045900	0.809917
chr7	25189600	0.727273
chr7	25211400	0.801653
chr7	25336400	0.801653
chr7	25343600	0.851240
chr7	25608700	0.677686
chr7	25668200	0.694215
chr7	25736400	0.809917
chr7	25766500	0.677686
chr7	25772300	0.909091
chr7	25783500	0.702479
chr7	25799800	0.884298
chr7	25871300	0.719008
chr7	25900500	0.677686
chr7	25913000	0.727273
chr7	25947200	0.801653
chr7	25966800	0.652893
chr7	25993300	0.661157
chr7	26062800	0.644628
chr7	26113000	0.702479
chr7	26119000	0.710744
chr7	26121800	0.694215
chr7	26130700	0.652893
chr7	26141400	0.867769
chr7	26156100	0.710744
chr7	26199200	0.677686
chr7	26206300	0.776860
chr7	26233900	0.644628
chr7	26342800	0.710744
chr7	26460500	0.652893
chr7	26478000	0.685950
chr7	26481300	0.818182

chr7	26491800	0.727273
chr7	26516300	0.677686
chr7	26548500	0.842975
chr7	26593500	0.768595
chr7	26627100	0.785124
chr7	26643500	0.652893
chr7	26660300	0.760331
chr7	26698400	0.743802
chr7	26707600	0.644628
chr7	26776100	0.917355
chr7	26824200	0.768595
chr7	26960000	0.793388
chr7	26984300	0.793388
chr7	26988700	1.000000
chr7	27070100	0.702479
chr7	27128200	0.685950
chr7	27131900	0.851240
chr7	27138200	0.900826
chr7	27150700	0.818182
chr7	27153600	0.669421
chr7	27179300	0.760331
chr7	27187800	0.892562
chr7	27191100	0.727273
chr7	27196100	0.743802
chr7	27204700	0.809917
chr7	27215300	0.644628
chr7	27224400	0.818182
chr7	27228700	0.867769
chr7	27232300	0.842975
chr7	27239300	0.826446
chr7	27245100	0.677686
chr7	27260400	0.702479
chr7	27265300	0.760331
chr7	27279500	0.966942
chr7	27292100	0.785124
chr7	27416000	0.652893
chr7	27473600	0.917355
chr7	27556400	0.851240
chr7	27604900	0.644628
chr7	27615900	0.768595
chr7	27626900	0.702479
chr7	27629500	0.661157
chr7	27642700	0.851240
chr7	27646300	0.760331
chr7	27675900	0.685950
chr7	27729600	0.785124
chr7	27882900	0.677686
chr7	27908800	0.818182
chr7	27936900	0.917355
chr7	27946500	0.652893
chr7	27950200	0.983471
chr7	27960900	0.652893
chr7	27963100	0.727273
chr7	27973700	0.826446

chr7	28060100	0.743802
chr7	28073100	0.760331
chr7	28111300	0.669421
chr7	28128400	0.900826
chr7	28145900	0.752066
chr7	28165300	0.735537
chr7	28212900	0.851240
chr7	28230800	0.661157
chr7	28270400	0.644628
chr7	28276500	0.818182
chr7	28331100	0.917355
chr7	28333400	0.975207
chr7	28401600	0.801653
chr7	28557000	0.685950
chr7	28570600	0.892562
chr7	28598400	0.743802
chr7	28612200	0.685950
chr7	28667400	0.652893
chr7	28683600	0.694215
chr7	28725900	0.644628
chr7	28748300	0.743802
chr7	28780000	0.768595
chr7	28787500	0.719008
chr7	28834000	0.685950
chr7	28862300	0.702479
chr7	28929300	0.884298
chr7	28931400	0.834711
chr7	28962200	0.652893
chr7	28973600	0.727273
chr7	29002200	0.727273
chr7	29020000	0.644628
chr7	29163100	0.661157
chr7	29196800	0.735537
chr7	29229800	0.719008
chr7	29236900	0.727273
chr7	29257000	0.760331
chr7	29283600	0.793388
chr7	29316200	0.661157
chr7	29328700	0.793388
chr7	29334900	0.685950
chr7	29337800	0.818182
chr7	29348100	0.710744
chr7	29359900	0.677686
chr7	29409800	0.677686
chr7	29415800	0.694215
chr7	29447900	0.818182
chr7	29482300	0.710744
chr7	29535600	0.818182
chr7	29541900	0.752066
chr7	29578400	0.661157
chr7	29597700	0.719008
chr7	29609500	0.834711
chr7	29648300	0.669421
chr7	29652400	0.743802

chr7	29660300	0.719008
chr7	29763100	0.768595
chr7	29833600	0.925620
chr7	29865900	0.776860
chr7	29922000	0.652893
chr7	30202800	0.702479
chr7	30208900	0.793388
chr7	30321100	0.719008
chr7	30330000	0.710744
chr7	30361200	0.975207
chr7	30395500	0.735537
chr7	30407800	0.685950
chr7	30430800	0.867769
chr7	30453400	0.685950
chr7	30473100	0.727273
chr7	30509200	0.892562
chr7	30589100	0.652893
chr7	30596700	0.702479
chr7	30608600	0.760331
chr7	30624300	0.900826
chr7	30724300	0.644628
chr7	30749500	0.644628
chr7	30767000	0.983471
chr7	30771200	0.694215
chr7	30920300	0.702479
chr7	30927600	0.743802
chr7	30953500	0.685950
chr7	31029000	0.735537
chr7	31060500	0.842975
chr7	31063000	0.677686
chr7	31067000	0.801653
chr7	31082900	0.884298
chr7	31088300	0.719008
chr7	31174400	0.752066
chr7	31293300	0.735537
chr7	31411400	0.661157
chr7	31505800	0.818182
chr7	31533700	0.826446
chr7	31653100	0.719008
chr7	31691300	0.884298
chr7	31727400	0.727273
chr7	31729500	0.884298
chr7	31750400	0.776860
chr7	31865600	0.652893
chr7	32084700	0.644628
chr7	32099300	0.768595
chr7	32156500	0.652893
chr7	32159600	0.652893
chr7	32298500	0.735537
chr7	32333900	0.644628
chr7	32552800	0.669421
chr7	32566100	0.884298
chr7	32571600	0.644628
chr7	32627200	0.859504

chr7	32643700	0.900826
chr7	32651100	0.975207
chr7	32658200	0.785124
chr7	32720700	0.983471
chr7	32770700	0.644628
chr7	32828100	0.652893
chr7	32904100	0.702479
chr7	32988200	0.768595
chr7	33004500	0.661157
chr7	33038300	0.876033
chr7	33116500	0.760331
chr7	33154500	0.735537
chr7	33301900	0.760331
chr7	33331800	0.652893
chr7	33458400	0.793388
chr7	33499100	0.752066
chr7	33527000	0.735537
chr7	33532300	0.743802
chr7	33574700	0.735537
chr7	33714800	0.661157
chr7	33744800	0.785124
chr7	33747000	0.851240
chr7	33751500	0.685950
chr7	33767100	0.776860
chr7	33802700	0.652893
chr7	33830500	0.925620
chr7	33913400	0.801653
chr7	33937500	0.842975
chr7	34173800	0.685950
chr7	34178700	0.818182
chr7	34308700	0.685950
chr7	34434700	0.694215
chr7	34529300	0.743802
chr7	34607600	0.677686
chr7	34817400	0.694215
chr7	34941200	0.702479
chr7	34979600	0.809917
chr7	34992800	0.669421
chr7	35055000	0.983471
chr7	35096800	0.685950
chr7	35290600	0.644628
chr7	35320800	0.884298
chr7	35406900	0.652893
chr7	35545000	0.917355
chr7	35710900	0.925620
chr7	35720400	0.793388
chr7	35731800	0.719008
chr7	35754500	0.644628
chr7	35948600	0.884298
chr7	36018800	0.793388
chr7	36051400	0.842975
chr7	36205900	0.694215
chr7	36247800	0.652893
chr7	36255000	0.867769

chr7	36298600	0.702479
chr7	36314300	0.661157
chr7	36318800	0.859504
chr7	36321800	0.983471
chr7	36324100	0.644628
chr7	36331600	0.702479
chr7	36344100	0.719008
chr7	36433000	0.669421
chr7	36445700	0.694215
chr7	36481400	0.892562
chr7	36556300	0.793388
chr7	36603900	0.644628
chr7	36608000	0.975207
chr7	36693700	0.826446
chr7	36698200	0.677686
chr7	36803000	0.842975
chr7	36814700	0.685950
chr7	36916100	0.809917
chr7	37026500	0.958678
chr7	37071900	0.950413
chr7	37086700	0.809917
chr7	37157200	0.851240
chr7	37191000	0.644628
chr7	37222300	0.694215
chr7	37265300	0.685950
chr7	37286000	0.652893
chr7	37288400	0.991736
chr7	37305400	0.661157
chr7	37324800	0.661157
chr7	37382900	0.727273
chr7	37394200	0.842975
chr7	37429800	0.768595
chr7	37474300	0.900826
chr7	37528900	0.950413
chr7	37592100	0.752066
chr7	37684200	0.669421
chr7	37734900	0.768595
chr7	37744400	0.661157
chr7	38228900	0.685950
chr7	38371400	0.975207
chr7	38792200	0.867769
chr7	38822000	0.669421
chr7	38838400	0.719008
chr7	38943700	0.669421
chr7	38972500	0.677686
chr7	39018500	0.661157
chr7	39125500	0.661157
chr7	39268900	0.925620
chr7	39311600	0.677686
chr7	39350600	0.818182
chr7	39373800	0.785124
chr7	39393700	0.925620
chr7	39473100	0.809917
chr7	39502500	0.710744

chr7	39579900	0.727273
chr7	39706800	0.785124
chr7	39922300	0.876033
chr7	40018500	0.884298
chr7	40025200	0.917355
chr7	40135400	0.669421
chr7	40458200	0.644628
chr7	40609900	0.768595
chr7	40740800	0.694215
chr7	40797100	0.710744
chr7	40867000	0.809917
chr7	40873000	0.710744
chr7	41012300	0.677686
chr7	41099200	0.892562
chr7	41236700	0.685950
chr7	41352400	0.661157
chr7	41579000	0.950413
chr7	41611600	0.661157
chr7	41632500	0.809917
chr7	41670700	1.000000
chr7	41848700	0.801653
chr7	41872800	0.644628
chr7	41980300	0.818182
chr7	42017300	0.727273
chr7	42131500	0.876033
chr7	42178000	0.752066
chr7	42190200	0.677686
chr7	42253400	0.760331
chr7	42262300	1.000000
chr7	42265800	0.727273
chr7	42270000	0.735537
chr7	42273000	0.727273
chr7	42377800	0.669421
chr7	42385400	0.785124
chr7	42387700	0.785124
chr7	42725200	0.760331
chr7	42727500	0.719008
chr7	42933300	0.768595
chr7	42953100	0.644628
chr7	43032500	0.719008
chr7	43148200	0.834711
chr7	43155700	0.710744
chr7	43159600	0.727273
chr7	43172000	0.669421
chr7	43175800	0.785124
chr7	43178800	0.776860
chr7	43181000	0.975207
chr7	43210500	0.694215
chr7	43248800	0.917355
chr7	43251300	0.719008
chr7	43254800	0.677686
chr7	43301200	0.801653
chr7	43329100	0.876033
chr7	43331500	0.892562

chr7	43334600	0.991736
chr7	43342400	0.677686
chr7	43436100	0.876033
chr7	43442200	0.669421
chr7	43479600	0.710744
chr7	43503200	0.851240
chr7	43595700	0.685950
chr7	43687100	0.983471
chr7	43691300	0.950413
chr7	43699900	0.818182
chr7	43709300	0.892562
chr7	43735400	0.801653
chr7	43790100	0.809917
chr7	43803700	0.661157
chr7	43878600	0.793388
chr7	43955800	0.752066
chr7	44026300	0.851240
chr7	44030600	0.677686
chr7	44076300	0.752066
chr7	44103300	0.892562
chr7	44105900	0.768595
chr7	44172200	0.826446
chr7	44186900	0.925620
chr7	44224300	0.669421
chr7	44254100	0.983471
chr7	44303800	0.652893
chr7	44316100	0.867769
chr7	44448800	0.826446
chr7	44486200	0.652893
chr7	44670900	0.760331
chr7	44679400	0.933884
chr7	44791300	0.702479
chr7	44794900	0.661157
chr7	45164000	0.917355
chr7	45291900	0.884298
chr7	45536200	0.752066
chr7	45538800	0.644628
chr7	45552100	0.652893
chr7	45600300	0.851240
chr7	45604200	0.685950
chr7	45617400	0.818182
chr7	45623000	0.685950
chr7	45922300	0.652893
chr7	45929900	0.661157
chr7	45964000	0.842975
chr7	46055400	0.867769
chr7	46184800	0.760331
chr7	46321600	0.876033
chr7	46392300	0.644628
chr7	46400300	0.685950
chr7	46574800	0.826446
chr7	46710900	0.867769
chr7	47078800	0.768595
chr7	47354700	0.776860

chr7	47371400	0.776860
chr7	47411700	0.909091
chr7	47420300	0.884298
chr7	47443000	0.710744
chr7	47494900	0.694215
chr7	47510700	0.925620
chr7	47515000	0.677686
chr7	47520900	0.826446
chr7	47528300	0.768595
chr7	47532500	0.677686
chr7	47560600	0.727273
chr7	47579000	0.760331
chr7	47584000	0.801653
chr7	47613000	0.719008
chr7	47619000	0.801653
chr7	47623300	0.644628
chr7	47632000	0.694215
chr7	47666000	0.892562
chr7	47675300	0.983471
chr7	47687100	0.735537
chr7	47694300	0.702479
chr7	47764600	0.892562
chr7	47784000	0.851240
chr7	47936900	0.809917
chr7	47945000	0.768595
chr7	48031700	0.727273
chr7	48078300	0.702479
chr7	48080600	0.785124
chr7	48082700	0.644628
chr7	48126500	0.942149
chr7	48131800	0.669421
chr7	48222900	0.867769
chr7	48293400	0.661157
chr7	48650800	0.785124
chr7	48748800	0.685950
chr7	49041500	0.801653
chr7	49063500	0.966942
chr7	49077900	0.867769
chr7	49140200	0.842975
chr7	49142500	0.958678
chr7	49268900	0.859504
chr7	49285200	0.966942
chr7	49524900	0.694215
chr7	49527500	0.652893
chr7	49672300	0.677686
chr7	49830900	0.785124
chr7	49892600	0.743802
chr7	49895600	0.876033
chr7	50109000	0.950413
chr7	50237500	0.760331
chr7	50355900	0.834711
chr7	50446700	0.884298
chr7	50450900	0.685950
chr7	50489300	0.752066

chr7	50535900	0.669421
chr7	50599600	0.785124
chr7	50624700	0.694215
chr7	50671500	0.892562
chr7	50696200	0.644628
chr7	50701000	0.983471
chr7	50749900	0.652893
chr7	50766300	0.710744
chr7	50835200	0.801653
chr7	50837600	0.958678
chr7	50843500	0.677686
chr7	50880500	0.884298
chr7	50935100	0.760331
chr7	51050600	0.652893
chr7	51102500	0.826446
chr7	51148400	0.834711
chr7	51214800	0.685950
chr7	51230900	0.661157
chr7	51243600	0.950413
chr7	51281300	0.702479
chr7	51297700	0.826446
chr7	51306900	0.661157
chr7	51360800	0.669421
chr7	51374100	0.694215
chr7	51378900	0.735537
chr7	51381900	0.768595
chr7	51393100	0.809917
chr7	51397400	0.685950
chr7	51434400	0.826446
chr7	51514900	0.859504
chr7	51520300	0.710744
chr7	51524800	0.669421
chr7	51798900	0.702479
chr7	51842100	0.710744
chr7	51946700	0.719008
chr7	51988900	0.677686
chr7	52018100	1.000000
chr7	52129500	0.867769
chr7	52132500	0.669421
chr7	52153800	0.975207
chr7	52186100	0.661157
chr7	52188200	0.826446
chr7	52197400	0.661157
chr7	52240600	0.727273
chr7	52615600	0.677686
chr7	52697300	0.669421
chr7	52703300	0.694215
chr7	52953200	0.710744
chr7	52985000	0.710744
chr7	53306300	0.652893
chr7	53711500	0.727273
chr7	53812200	0.685950
chr7	54213900	0.652893
chr7	54256700	0.867769

chr7	54346700	0.818182
chr7	54400500	0.785124
chr7	54487100	0.760331
chr7	54705900	0.925620
chr7	54748400	0.727273
chr7	54926100	0.702479
chr7	55134300	0.760331
chr7	55167200	0.702479
chr7	55177000	0.735537
chr7	55257200	0.768595
chr7	55260000	0.867769
chr7	55367200	0.834711
chr7	55589900	0.677686
chr7	55595000	0.685950
chr7	55604800	0.809917
chr7	55608000	0.966942
chr7	55615000	0.834711
chr7	55621100	0.652893
chr7	55625800	0.826446
chr7	55637100	0.719008
chr7	55757400	0.900826
chr7	55842800	0.710744
chr7	56039200	0.661157
chr7	57546400	0.694215
chr7	57552900	0.719008
chr7	58008800	0.669421
chr7	61079300	0.702479
chr7	61085500	0.876033
chr7	61111500	0.702479
chr7	61264600	0.669421
chr7	61391400	0.851240
chr7	61519600	0.644628
chr7	61802800	0.727273
chr7	61986400	0.867769
chr7	64458900	0.909091
chr7	64711000	0.768595
chr7	64847300	0.685950
chr7	65090000	0.867769
chr7	65372800	0.652893
chr7	65554200	0.719008
chr7	65565200	0.842975
chr7	65617600	0.743802
chr7	65767500	0.809917
chr7	65780700	0.702479
chr7	65817600	0.735537
chr7	65956300	0.867769
chr7	66084600	0.950413
chr7	66165500	0.669421
chr7	66313000	0.983471
chr7	66332600	0.710744
chr7	66395700	0.892562
chr7	66554900	0.785124
chr7	66584300	0.669421
chr7	66654800	0.826446

chr7	66657200	0.851240
chr7	66722800	0.793388
chr7	66800800	0.826446
chr7	66805200	0.710744
chr7	66857900	0.933884
chr7	67084200	0.776860
chr7	67090600	0.677686
chr7	67382700	0.702479
chr7	67523600	0.809917
chr7	67703000	0.669421
chr7	67711300	0.785124
chr7	67721900	0.735537
chr7	67741700	0.710744
chr7	67875300	0.760331
chr7	68153900	0.652893
chr7	68437500	0.809917
chr7	68446700	0.818182
chr7	68570000	0.719008
chr7	68573300	0.842975
chr7	68698100	0.727273
chr7	68814700	0.694215
chr7	68895800	0.752066
chr7	68915800	0.867769
chr7	68923300	0.942149
chr7	68983700	0.942149
chr7	68999600	0.727273
chr7	69066600	0.694215
chr7	69075300	0.909091
chr7	69111200	0.876033
chr7	69124700	0.776860
chr7	69176300	0.818182
chr7	69197800	0.834711
chr7	69203200	0.884298
chr7	69215200	0.685950
chr7	69265700	0.809917
chr7	69283200	0.677686
chr7	69297100	0.859504
chr7	69302900	0.867769
chr7	69323500	0.809917
chr7	69427700	0.966942
chr7	69432900	0.768595
chr7	69486800	0.669421
chr7	69513200	0.876033
chr7	69525900	0.694215
chr7	69559800	0.644628
chr7	69597800	0.652893
chr7	69626400	0.669421
chr7	69629300	0.702479
chr7	69648700	0.735537
chr7	69779000	0.719008
chr7	69821200	0.677686
chr7	69858600	0.826446
chr7	69882400	0.760331
chr7	69914500	0.694215

chr7	70006200	0.735537
chr7	70048300	0.950413
chr7	70050400	0.719008
chr7	70079800	0.793388
chr7	70084800	0.867769
chr7	70097000	0.793388
chr7	70103400	0.702479
chr7	70123300	0.809917
chr7	70139800	0.644628
chr7	70165300	0.859504
chr7	70168500	0.644628
chr7	70172600	0.669421
chr7	70175900	0.644628
chr7	70190400	0.809917
chr7	70194300	0.834711
chr7	70200400	0.859504
chr7	70202500	0.859504
chr7	70217600	0.867769
chr7	70228000	0.785124
chr7	70237300	0.925620
chr7	70295100	0.735537
chr7	70314300	0.917355
chr7	70322300	0.950413
chr7	70349900	0.942149
chr7	70359600	0.991736
chr7	70372800	0.793388
chr7	70385000	0.793388
chr7	70417400	0.768595
chr7	70532500	0.677686
chr7	70611500	0.710744
chr7	70641800	0.801653
chr7	70701300	0.867769
chr7	70703600	0.859504
chr7	70720800	0.884298
chr7	70733000	0.826446
chr7	70751300	0.661157
chr7	70790500	0.851240
chr7	70793800	0.900826
chr7	70813800	0.793388
chr7	70849800	0.842975
chr7	70899300	0.727273
chr7	70908800	0.752066
chr7	70922600	0.975207
chr7	70926800	0.727273
chr7	70930200	0.834711
chr7	70932400	0.818182
chr7	70935800	0.909091
chr7	70939400	0.884298
chr7	70973500	0.785124
chr7	71024100	0.685950
chr7	71086100	0.727273
chr7	71098900	0.818182
chr7	71101800	0.991736
chr7	71148100	0.958678

chr7	71205300	0.727273
chr7	71249600	0.859504
chr7	71530900	0.719008
chr7	71693300	0.685950
chr7	71911900	0.719008
chr7	71993900	0.991736
chr7	72087700	0.809917
chr7	72149200	0.768595
chr7	72154200	0.842975
chr7	72179000	0.842975
chr7	72187700	0.727273
chr7	72201500	0.876033
chr7	72321000	0.818182
chr7	72380500	0.727273
chr7	72436900	0.818182
chr7	72733700	0.652893
chr7	72745300	0.727273
chr7	72838600	0.768595
chr7	72843600	0.685950
chr7	73021800	0.760331
chr7	73085600	0.719008
chr7	73118500	0.809917
chr7	73140400	0.785124
chr7	73150200	0.710744
chr7	73155700	0.867769
chr7	73158700	0.677686
chr7	73176700	0.768595
chr7	73242400	0.958678
chr7	73248500	0.991736
chr7	73284200	0.719008
chr7	73316400	0.966942
chr7	73334000	0.884298
chr7	73441200	0.719008
chr7	73443600	0.834711
chr7	73448000	0.677686
chr7	73458100	0.909091
chr7	73466400	0.727273
chr7	73507400	0.727273
chr7	73509600	0.719008
chr7	73553700	0.900826
chr7	73598100	0.702479
chr7	73634800	0.677686
chr7	73687200	0.826446
chr7	73694300	0.884298
chr7	73699200	0.900826
chr7	73708600	0.677686
chr7	73809100	0.785124
chr7	73820100	0.661157
chr7	73843600	0.826446
chr7	73846100	0.826446
chr7	73895100	0.991736
chr7	73940700	0.809917
chr7	73983700	0.685950
chr7	74002500	0.909091

chr7	74032000	0.818182
chr7	74042500	0.942149
chr7	74058200	0.702479
chr7	74069700	0.710744
chr7	74076900	0.859504
chr7	74347200	0.652893
chr7	74382100	0.677686
chr7	74409000	0.942149
chr7	74685500	0.661157
chr7	74837700	0.933884
chr7	75028600	0.818182
chr7	75228300	0.909091
chr7	75241400	0.884298
chr7	75266000	0.677686
chr7	75358100	0.661157
chr7	75409500	0.661157
chr7	75418600	0.859504
chr7	75496300	0.677686
chr7	75535800	0.851240
chr7	75547900	0.710744
chr7	75586700	0.826446
chr7	75683700	0.776860
chr7	75698600	0.785124
chr7	75793000	0.917355
chr7	75815400	0.859504
chr7	75823200	0.652893
chr7	75847300	0.809917
chr7	75850100	0.859504
chr7	75861200	0.842975
chr7	75880400	0.727273
chr7	75891500	0.752066
chr7	75899000	0.842975
chr7	75902800	0.900826
chr7	75920700	0.710744
chr7	75924100	0.710744
chr7	75936500	0.644628
chr7	75946700	0.702479
chr7	75989800	0.735537
chr7	75994400	0.743802
chr7	76053300	0.950413
chr7	76088600	0.851240
chr7	76110300	0.818182
chr7	76115500	0.900826
chr7	76139900	0.892562
chr7	76230000	0.892562
chr7	76303200	0.975207
chr7	76384800	0.702479
chr7	76586000	0.785124
chr7	76613900	0.884298
chr7	76871800	0.876033
chr7	76938800	0.776860
chr7	76957300	0.661157
chr7	76978400	0.743802
chr7	77017200	0.710744

chr7	77025200	0.760331
chr7	77043100	0.834711
chr7	77055200	0.900826
chr7	77058200	0.867769
chr7	77185400	0.702479
chr7	77358000	0.900826
chr7	77620000	0.685950
chr7	77624600	0.661157
chr7	77647200	0.677686
chr7	77667000	0.884298
chr7	77669400	0.669421
chr7	77682100	0.842975
chr7	77703600	0.694215
chr7	77710000	0.785124
chr7	77740600	0.876033
chr7	77760000	0.859504
chr7	77789400	0.859504
chr7	77802000	0.710744
chr7	77845100	0.644628
chr7	78028200	0.669421
chr7	78030900	0.801653
chr7	78095900	0.834711
chr7	78119400	0.685950
chr7	78188700	0.776860
chr7	78193300	0.975207
chr7	78197800	0.801653
chr7	78206200	0.884298
chr7	78209200	0.991736
chr7	78346500	0.694215
chr7	78372500	0.694215
chr7	78377000	0.661157
chr7	78856700	0.694215
chr7	78947500	0.727273
chr7	79597800	0.652893
chr7	79738800	0.644628
chr7	79757500	0.818182
chr7	79812500	0.793388
chr7	79816700	0.702479
chr7	79854400	0.785124
chr7	80352400	0.884298
chr7	80389700	0.677686
chr7	80393900	0.793388
chr7	80785500	0.644628
chr7	80963200	0.876033
chr7	81179000	0.958678
chr7	81290300	0.710744
chr7	81302700	0.735537
chr7	81320100	0.867769
chr7	81381800	0.892562
chr7	81392000	0.652893
chr7	81399500	0.760331
chr7	81579400	0.917355
chr7	81739700	0.669421
chr7	81752200	0.760331

chr7	81791800	0.652893
chr7	81809700	0.669421
chr7	81860800	0.652893
chr7	81916100	0.793388
chr7	81919000	0.719008
chr7	81935100	0.685950
chr7	81943100	0.793388
chr7	82004400	0.776860
chr7	82012000	0.661157
chr7	82101000	0.752066
chr7	82201900	0.719008
chr7	82224500	0.809917
chr7	82352900	0.727273
chr7	82389900	0.950413
chr7	82434900	0.884298
chr7	82446000	0.785124
chr7	82469600	0.876033
chr7	83041700	0.826446
chr7	83045600	0.859504
chr7	83054300	0.785124
chr7	83058800	0.818182
chr7	83075600	0.661157
chr7	83081700	0.851240
chr7	83085500	0.710744
chr7	83087700	0.859504
chr7	83259300	0.735537
chr7	83270600	0.842975
chr7	83301300	0.702479
chr7	83314700	0.669421
chr7	83322300	0.710744
chr7	83325200	0.818182
chr7	83565800	0.776860
chr7	83715000	0.809917
chr7	83718600	0.743802
chr7	83740000	0.842975
chr7	83743400	0.685950
chr7	83753600	0.768595
chr7	83774900	0.975207
chr7	83786600	0.669421
chr7	83830300	0.743802
chr7	83857900	0.727273
chr7	83881300	0.685950
chr7	83907300	0.983471
chr7	84178200	0.793388
chr7	84542600	0.702479
chr7	84630000	0.702479
chr7	84649600	0.644628
chr7	85379900	0.834711
chr7	85931200	0.768595
chr7	85958800	0.652893
chr7	85968800	0.685950
chr7	86277800	0.768595
chr7	86297900	0.933884
chr7	86390000	0.909091

chr7	86455600	0.752066
chr7	86577300	0.760331
chr7	86586500	0.735537
chr7	86594600	0.859504
chr7	86602000	0.768595
chr7	86605700	0.677686
chr7	86629900	0.669421
chr7	86759000	0.900826
chr7	87094700	0.818182
chr7	87377200	0.917355
chr7	87439900	0.694215
chr7	87556200	0.818182
chr7	87731400	0.669421
chr7	87783100	0.776860
chr7	87830600	0.859504
chr7	87844800	0.685950
chr7	88124000	0.644628
chr7	88196500	0.644628
chr7	88264500	0.760331
chr7	88294600	0.694215
chr7	88594900	0.826446
chr7	88720900	0.652893
chr7	89845900	0.685950
chr7	89853800	1.000000
chr7	90251400	0.876033
chr7	90272800	0.801653
chr7	90292300	0.851240
chr7	90307500	0.867769
chr7	90318100	0.652893
chr7	90339400	0.652893
chr7	90356100	0.809917
chr7	90582000	0.983471
chr7	90599900	0.735537
chr7	90660500	0.752066
chr7	90663200	0.809917
chr7	90673900	0.933884
chr7	90696500	0.685950
chr7	90765200	0.694215
chr7	90985400	0.933884
chr7	90997900	0.900826
chr7	91050900	0.669421
chr7	91184500	0.842975
chr7	91322900	0.694215
chr7	91909000	0.719008
chr7	91923100	0.768595
chr7	92066600	0.702479
chr7	92313800	0.818182
chr7	92353800	0.702479
chr7	92380500	0.652893
chr7	92385600	0.677686
chr7	92393500	0.942149
chr7	92395900	0.809917
chr7	92398000	0.933884
chr7	92439500	0.694215

chr7	92443400	0.727273
chr7	92459400	0.661157
chr7	92546400	0.818182
chr7	92556000	0.909091
chr7	92669600	0.809917
chr7	92676100	0.752066
chr7	92777000	0.735537
chr7	92968000	0.768595
chr7	93023600	0.776860
chr7	93106400	0.842975
chr7	93116500	0.991736
chr7	93188600	0.694215
chr7	93252300	0.735537
chr7	93268600	0.685950
chr7	93446800	0.652893
chr7	93611200	0.925620
chr7	93683100	0.661157
chr7	93784800	0.652893
chr7	93820100	0.702479
chr7	93858300	0.719008
chr7	93930800	0.900826
chr7	93960600	0.694215
chr7	94004800	0.966942
chr7	94021700	0.851240
chr7	94144900	0.685950
chr7	94331400	0.793388
chr7	94510900	0.719008
chr7	94539900	0.710744
chr7	94542700	0.809917
chr7	94573900	0.752066
chr7	94599500	0.842975
chr7	94892200	0.644628
chr7	94972500	0.884298
chr7	95057400	0.735537
chr7	95100500	0.892562
chr7	95131000	0.768595
chr7	95133600	0.826446
chr7	95141300	0.652893
chr7	95457900	0.727273
chr7	95773800	0.818182
chr7	95827700	0.702479
chr7	95841600	0.760331
chr7	95938800	0.727273
chr7	95944200	0.644628
chr7	96045300	0.719008
chr7	96221300	0.652893
chr7	96287600	0.785124
chr7	96369400	0.818182
chr7	96416200	0.950413
chr7	96615100	0.694215
chr7	96619000	0.851240
chr7	96625800	0.727273
chr7	96628100	0.859504
chr7	96642000	0.677686

chr7	96648000	0.760331
chr7	96756100	0.867769
chr7	96797400	0.917355
chr7	97503400	0.809917
chr7	97546700	0.652893
chr7	97681000	0.685950
chr7	97740000	0.826446
chr7	97808400	0.661157
chr7	97841400	0.867769
chr7	97913600	0.900826
chr7	97916700	0.933884
chr7	97920600	0.694215
chr7	97953200	0.925620
chr7	97990100	0.677686
chr7	97992700	0.818182
chr7	98258900	0.867769
chr7	98424700	0.818182
chr7	98435300	0.809917
chr7	98452000	0.785124
chr7	98465500	0.685950
chr7	98696900	0.809917
chr7	98735700	0.842975
chr7	98760500	0.652893
chr7	98964500	0.694215
chr7	98978200	0.776860
chr7	99227000	0.884298
chr7	99291500	0.826446
chr7	99437700	0.925620
chr7	99527200	0.818182
chr7	99531900	0.710744
chr7	99542300	0.966942
chr7	99574000	0.785124
chr7	99578400	0.768595
chr7	99731000	0.842975
chr7	99800500	0.661157
chr7	99811800	0.644628
chr7	99824800	0.677686
chr7	99841700	0.760331
chr7	99845600	0.983471
chr7	99885400	0.719008
chr7	99967600	0.809917
chr7	100042500	0.933884
chr7	100053900	0.876033
chr7	100067700	0.867769
chr7	100084400	0.776860
chr7	100087900	0.958678
chr7	100139800	0.975207
chr7	100143700	0.917355
chr7	100165500	0.727273
chr7	100170200	0.685950
chr7	100180800	0.669421
chr7	100187600	0.900826
chr7	100224600	0.677686
chr7	100238500	0.652893

chr7	100240900	0.867769
chr7	100245500	0.793388
chr7	100277400	0.727273
chr7	100287600	0.694215
chr7	100293300	0.801653
chr7	100392300	0.776860
chr7	100397200	0.677686
chr7	100401600	0.785124
chr7	100433200	0.652893
chr7	100528000	0.768595
chr7	100544700	0.892562
chr7	100547500	0.867769
chr7	100662500	0.900826
chr7	100703000	0.710744
chr7	100710200	0.652893
chr7	100721200	0.719008
chr7	100743600	0.966942
chr7	100765800	0.884298
chr7	100770700	0.867769
chr7	100777800	0.776860
chr7	100803900	0.685950
chr7	100826100	0.669421
chr7	100844000	0.727273
chr7	100877600	0.785124
chr7	100880400	0.652893
chr7	100930600	0.661157
chr7	100935200	0.925620
chr7	100938600	0.884298
chr7	101022500	0.942149
chr7	101030400	0.801653
chr7	101035100	0.735537
chr7	101039200	0.809917
chr7	101042300	0.826446
chr7	101045600	0.917355
chr7	101055400	0.925620
chr7	101063700	0.752066
chr7	101085900	0.752066
chr7	101114400	0.661157
chr7	101130200	0.950413
chr7	101138200	0.909091
chr7	101146800	0.933884
chr7	101160600	0.727273
chr7	101186500	0.685950
chr7	101208000	0.727273
chr7	101218600	0.876033
chr7	101242100	0.702479
chr7	101246100	0.925620
chr7	101252600	0.776860
chr7	101258200	0.942149
chr7	101267900	0.950413
chr7	101270100	0.661157
chr7	101288400	0.719008
chr7	101308500	0.793388
chr7	101375000	0.661157

chr7	101377700	0.768595
chr7	101463200	0.917355
chr7	101475800	0.834711
chr7	101482800	0.644628
chr7	101487500	0.809917
chr7	101500200	0.859504
chr7	101505700	0.884298
chr7	101512100	0.677686
chr7	101514200	0.933884
chr7	101522000	0.694215
chr7	101533600	0.752066
chr7	101535900	0.669421
chr7	101575400	0.909091
chr7	101581000	0.677686
chr7	101584700	0.975207
chr7	101588300	0.669421
chr7	101590900	0.661157
chr7	101629200	0.950413
chr7	101707900	0.859504
chr7	101720500	0.669421
chr7	101723400	0.966942
chr7	101732900	0.776860
chr7	101736900	0.785124
chr7	101754800	0.900826
chr7	101765400	0.768595
chr7	101768500	0.950413
chr7	101796700	0.809917
chr7	101833800	0.909091
chr7	101892100	0.876033
chr7	101895500	0.644628
chr7	101944600	0.710744
chr7	101950800	0.677686
chr7	102065600	0.669421
chr7	102081200	0.661157
chr7	102092200	0.743802
chr7	102132000	0.892562
chr7	102148300	0.661157
chr7	102154600	0.818182
chr7	102180400	0.776860
chr7	102184500	0.652893
chr7	102222700	0.793388
chr7	102231100	0.884298
chr7	102245100	0.859504
chr7	102253600	0.818182
chr7	102279700	0.826446
chr7	102283700	0.776860
chr7	102321700	0.644628
chr7	102500600	0.851240
chr7	102553800	0.661157
chr7	102567600	0.818182
chr7	102574400	0.983471
chr7	102576600	0.785124
chr7	102579500	0.644628
chr7	102582900	0.851240

chr7	102585300	0.768595
chr7	102782500	0.727273
chr7	102812200	0.719008
chr7	102967600	0.719008
chr7	103088000	0.752066
chr7	103498100	0.702479
chr7	103816000	0.991736
chr7	103958900	0.768595
chr7	104004200	0.727273
chr7	104413500	0.942149
chr7	104461300	0.876033
chr7	104585700	0.950413
chr7	104620000	0.900826
chr7	104641900	0.710744
chr7	104851400	0.652893
chr7	104869400	0.809917
chr7	104891500	0.793388
chr7	104897800	0.702479
chr7	104902700	0.727273
chr7	104909700	0.669421
chr7	104939600	0.719008
chr7	104995000	0.719008
chr7	104999800	0.719008
chr7	105018100	0.727273
chr7	105237600	0.776860
chr7	105276000	0.834711
chr7	105281600	0.644628
chr7	105284700	0.818182
chr7	105289500	0.785124
chr7	105296500	0.876033
chr7	105306600	0.933884
chr7	105313900	0.752066
chr7	105330100	0.909091
chr7	105333100	0.694215
chr7	105336800	0.884298
chr7	105338900	0.826446
chr7	105359400	0.826446
chr7	105362200	0.867769
chr7	105389500	0.884298
chr7	105410000	0.710744
chr7	105413800	0.942149
chr7	105417300	0.727273
chr7	105429600	0.669421
chr7	105447100	0.801653
chr7	105471400	0.694215
chr7	105480400	0.735537
chr7	105490000	0.809917
chr7	105492700	0.768595
chr7	105494900	0.752066
chr7	105502200	0.785124
chr7	105546000	0.884298
chr7	105574100	0.768595
chr7	105603600	0.727273
chr7	105606600	0.677686

chr7	105616200	0.652893
chr7	105621900	0.834711
chr7	105632300	0.677686
chr7	105638600	0.851240
chr7	105650600	0.909091
chr7	105656500	0.801653
chr7	105661800	0.694215
chr7	105672300	0.652893
chr7	105691100	0.685950
chr7	105750300	0.867769
chr7	105817300	0.644628
chr7	106041800	0.644628
chr7	106209600	0.776860
chr7	106359000	0.685950
chr7	106377500	0.793388
chr7	106546200	0.685950
chr7	106588900	0.669421
chr7	106606900	0.702479
chr7	106609100	0.768595
chr7	106688500	0.685950
chr7	106708800	0.834711
chr7	106814700	0.942149
chr7	107006600	0.644628
chr7	107031800	0.760331
chr7	107428400	0.752066
chr7	107576300	0.900826
chr7	107578400	0.727273
chr7	107593500	0.818182
chr7	107600200	0.876033
chr7	107607700	0.719008
chr7	107616300	0.644628
chr7	107704400	0.752066
chr7	107877300	0.809917
chr7	107880700	0.826446
chr7	107964700	0.661157
chr7	107967800	0.661157
chr7	107969900	0.694215
chr7	108089900	0.834711
chr7	108163600	0.818182
chr7	108222900	0.933884
chr7	108233800	0.760331
chr7	108258900	0.793388
chr7	108767200	0.685950
chr7	108769400	0.677686
chr7	109231800	0.793388
chr7	109922000	0.818182
chr7	110029200	0.644628
chr7	110290800	0.652893
chr7	110333300	0.661157
chr7	110359200	0.818182
chr7	110512200	0.760331
chr7	110585300	0.826446
chr7	110659700	0.851240
chr7	110690600	0.776860

chr7	110692900	0.661157
chr7	110723000	0.644628
chr7	110731600	0.801653
chr7	110734600	0.702479
chr7	110737700	0.933884
chr7	111397600	0.677686
chr7	111411500	0.702479
chr7	111425300	0.685950
chr7	111495600	0.900826
chr7	111514500	0.793388
chr7	111567800	0.942149
chr7	111571600	0.644628
chr7	111721500	0.685950
chr7	111779000	0.719008
chr7	111800700	0.809917
chr7	111803500	0.669421
chr7	111855600	0.793388
chr7	111889600	0.694215
chr7	111893300	0.834711
chr7	111895700	0.644628
chr7	111926600	0.644628
chr7	111971600	0.826446
chr7	112063000	0.942149
chr7	112287100	0.818182
chr7	112573400	0.834711
chr7	112739800	0.727273
chr7	112971900	0.842975
chr7	113793000	0.719008
chr7	113795700	0.892562
chr7	114004800	0.644628
chr7	114270500	0.884298
chr7	114302000	0.801653
chr7	114419700	0.801653
chr7	114483300	0.785124
chr7	114497100	0.876033
chr7	114570200	0.958678
chr7	114585200	0.867769
chr7	114646300	0.826446
chr7	114649700	0.818182
chr7	114674600	0.644628
chr7	114866500	0.694215
chr7	114940300	0.644628
chr7	115264000	0.652893
chr7	115347700	0.727273
chr7	115453000	0.661157
chr7	115456900	0.884298
chr7	116007800	0.661157
chr7	116162900	0.925620
chr7	116233400	0.867769
chr7	116256700	0.818182
chr7	116347600	0.785124
chr7	116423300	0.776860
chr7	116497300	0.710744
chr7	116545700	0.710744

chr7	116610100	0.991736
chr7	116660600	0.809917
chr7	116751100	0.661157
chr7	116779200	0.867769
chr7	116789000	0.793388
chr7	117125800	0.702479
chr7	117227200	0.685950
chr7	117271500	0.859504
chr7	117293800	0.917355
chr7	117313400	0.702479
chr7	117316100	0.776860
chr7	117323900	0.809917
chr7	117453900	0.809917
chr7	117459900	0.743802
chr7	117475100	0.809917
chr7	117510500	0.760331
chr7	117517200	0.933884
chr7	117528900	0.867769
chr7	117603400	0.917355
chr7	117792800	0.925620
chr7	118270600	0.768595
chr7	118428500	0.661157
chr7	118466100	0.661157
chr7	118572500	0.644628
chr7	118818900	0.743802
chr7	119235300	0.652893
chr7	119261500	0.727273
chr7	119673300	0.677686
chr7	119830800	0.867769
chr7	119918900	0.801653
chr7	120101300	0.834711
chr7	120142800	0.809917
chr7	120162300	0.859504
chr7	120190700	0.809917
chr7	120224700	0.719008
chr7	120324900	0.743802
chr7	120369700	0.801653
chr7	120379300	0.710744
chr7	120382900	0.785124
chr7	120425800	0.702479
chr7	120478500	0.776860
chr7	120482500	0.876033
chr7	120562700	0.644628
chr7	120567500	0.727273
chr7	120690000	0.768595
chr7	120743200	0.983471
chr7	120918600	0.669421
chr7	121052000	0.710744
chr7	121097000	0.735537
chr7	121120900	0.950413
chr7	121129400	0.727273
chr7	121145300	0.900826
chr7	121151600	0.942149
chr7	121261600	0.752066

chr7	121317500	0.685950
chr7	121437600	0.942149
chr7	121440200	0.644628
chr7	121451300	0.876033
chr7	121517400	0.735537
chr7	121519500	0.719008
chr7	121525300	0.694215
chr7	121527500	0.727273
chr7	121565600	0.743802
chr7	121574900	0.743802
chr7	121577400	0.909091
chr7	121579500	0.942149
chr7	121581900	0.975207
chr7	121606200	0.669421
chr7	121723000	0.793388
chr7	121731100	0.826446
chr7	121776800	0.842975
chr7	121786700	0.710744
chr7	121789000	0.900826
chr7	121791600	0.917355
chr7	121895800	0.859504
chr7	121901800	0.793388
chr7	121909700	0.702479
chr7	121969600	0.842975
chr7	121977300	0.702479
chr7	122027200	0.669421
chr7	122053300	0.661157
chr7	122070700	0.851240
chr7	122078500	0.892562
chr7	122082800	0.727273
chr7	122089800	0.842975
chr7	122109300	1.000000
chr7	122111400	0.661157
chr7	122140600	0.644628
chr7	122159300	0.892562
chr7	122218900	0.785124
chr7	122317300	0.834711
chr7	122319800	0.983471
chr7	122326800	0.892562
chr7	122359500	0.785124
chr7	122376500	0.859504
chr7	122387300	0.752066
chr7	122407800	0.834711
chr7	122411600	0.719008
chr7	122443100	0.942149
chr7	122474800	0.702479
chr7	122488900	0.677686
chr7	122496100	0.644628
chr7	122757700	0.644628
chr7	122925600	0.694215
chr7	123074000	0.834711
chr7	123080500	0.892562
chr7	123215100	0.809917
chr7	123235300	0.834711

chr7	123284500	0.991736
chr7	123384900	0.818182
chr7	123438500	0.685950
chr7	123569900	0.851240
chr7	123586100	0.900826
chr7	123902900	0.900826
chr7	123916000	0.702479
chr7	126165400	0.652893
chr7	126331700	0.942149
chr7	127117900	0.900826
chr7	127177100	0.644628
chr7	127241300	0.884298
chr7	127269500	0.801653
chr7	127295500	0.661157
chr7	127301000	0.760331
chr7	127307600	0.834711
chr7	127314300	0.669421
chr7	127318800	0.677686
chr7	127326000	0.727273
chr7	127366700	0.917355
chr7	127371200	0.710744
chr7	127386300	0.768595
chr7	127422800	0.834711
chr7	127439400	0.719008
chr7	127453100	0.768595
chr7	127456100	0.702479
chr7	127463400	0.859504
chr7	127488500	0.867769
chr7	127507100	0.661157
chr7	127528400	0.735537
chr7	127536400	0.702479
chr7	127541400	0.735537
chr7	127544600	0.925620
chr7	127567400	0.743802
chr7	127577500	0.735537
chr7	127594200	0.677686
chr7	127616500	0.942149
chr7	127636100	0.644628
chr7	127652400	0.727273
chr7	127659800	0.909091
chr7	127668000	0.876033
chr7	127756600	0.702479
chr7	127763900	0.818182
chr7	127795300	0.768595
chr7	127804500	0.876033
chr7	127832700	0.793388
chr7	127850300	0.743802
chr7	127852500	0.652893
chr7	127870300	0.652893
chr7	127900000	0.801653
chr7	127907400	0.909091
chr7	127912400	0.842975
chr7	127939400	0.966942
chr7	127942000	0.760331

chr7	128071800	0.702479
chr7	128075500	0.776860
chr7	128239200	0.752066
chr7	128340000	0.685950
chr7	128345200	0.801653
chr7	128348800	0.917355
chr7	128452800	0.677686
chr7	128468200	0.710744
chr7	128481900	0.785124
chr7	128520200	0.851240
chr7	128529300	0.892562
chr7	128552300	0.652893
chr7	128594700	0.710744
chr7	128626800	0.694215
chr7	128681800	0.752066
chr7	128707000	0.867769
chr7	128750900	0.884298
chr7	128790700	0.694215
chr7	128811300	0.851240
chr7	128858800	0.661157
chr7	128899100	0.933884
chr7	128919500	0.652893
chr7	128922900	0.694215
chr7	128981600	0.752066
chr7	129008000	0.933884
chr7	129015600	0.917355
chr7	129110200	0.702479
chr7	129154400	0.793388
chr7	129163000	0.735537
chr7	129174100	0.727273
chr7	129198300	0.727273
chr7	129257700	0.661157
chr7	129266600	0.669421
chr7	129270400	0.809917
chr7	129286700	0.826446
chr7	129305000	0.702479
chr7	129338800	0.719008
chr7	129382100	0.892562
chr7	129411900	0.942149
chr7	129427100	0.710744
chr7	129442300	0.661157
chr7	129469700	0.768595
chr7	129472300	0.685950
chr7	129475700	0.727273
chr7	129483800	0.991736
chr7	129503100	0.760331
chr7	129516600	0.669421
chr7	129520500	0.768595
chr7	129548700	0.702479
chr7	129913400	0.966942
chr7	129915800	0.809917
chr7	129978600	0.925620
chr7	129985600	0.859504
chr7	129996400	0.942149

chr7	130013700	0.801653
chr7	130129200	0.851240
chr7	130563400	0.743802
chr7	130588800	0.702479
chr7	130595000	0.685950
chr7	130606700	0.644628
chr7	130692900	0.892562
chr7	130725500	0.652893
chr7	130727700	0.785124
chr7	130761700	0.677686
chr7	130776000	1.000000
chr7	130869000	0.661157
chr7	130875500	0.983471
chr7	130904600	0.983471
chr7	131032700	0.685950
chr7	131055800	0.661157
chr7	131094600	0.801653
chr7	131196600	0.809917
chr7	131198700	0.876033
chr7	131204900	0.768595
chr7	131207500	0.900826
chr7	131214600	0.644628
chr7	131218700	0.694215
chr7	131223600	0.933884
chr7	131225700	0.900826
chr7	131230600	0.801653
chr7	131233500	0.785124
chr7	131235700	0.975207
chr7	131253500	0.677686
chr7	131271900	0.884298
chr7	131287700	0.975207
chr7	131292200	1.000000
chr7	131305600	0.991736
chr7	131311200	0.917355
chr7	131323200	0.694215
chr7	131347700	0.842975
chr7	131352400	0.652893
chr7	131377300	0.644628
chr7	131419200	0.669421
chr7	131505100	0.669421
chr7	131515100	0.735537
chr7	131581300	0.851240
chr7	131630500	0.851240
chr7	131793300	0.685950
chr7	131827700	0.785124
chr7	131842300	0.652893
chr7	131873100	0.950413
chr7	131935100	0.892562
chr7	131960900	0.685950
chr7	132003300	0.768595
chr7	132032700	0.727273
chr7	132064800	0.834711
chr7	132071400	0.776860
chr7	132088100	0.842975

chr7	132110300	0.743802
chr7	132160600	0.933884
chr7	132171700	0.644628
chr7	132194300	0.652893
chr7	132226700	0.793388
chr7	132230600	0.809917
chr7	132250100	0.694215
chr7	132318700	0.669421
chr7	132373200	0.975207
chr7	132436200	0.760331
chr7	132464800	0.669421
chr7	132562100	0.710744
chr7	132564500	0.818182
chr7	132681400	0.760331
chr7	132684000	0.917355
chr7	132686600	0.727273
chr7	132730200	0.785124
chr7	132736700	0.644628
chr7	132756100	0.851240
chr7	132764000	0.859504
chr7	132872800	0.768595
chr7	132882200	0.834711
chr7	132893300	0.859504
chr7	132978500	0.719008
chr7	133015400	0.719008
chr7	133031000	0.809917
chr7	133040000	0.702479
chr7	133071300	0.900826
chr7	133105600	0.785124
chr7	133112900	0.834711
chr7	133116400	0.917355
chr7	133128500	0.694215
chr7	133427200	0.661157
chr7	133455400	0.735537
chr7	133619900	0.677686
chr7	133713400	0.876033
chr7	133807300	0.652893
chr7	133815200	0.991736
chr7	133819500	0.743802
chr7	133825800	0.685950
chr7	133839500	0.776860
chr7	133882300	0.727273
chr7	133962400	0.677686
chr7	134092700	0.735537
chr7	134194600	0.644628
chr7	134212900	0.727273
chr7	134354900	0.727273
chr7	134384300	0.669421
chr7	134467300	0.685950
chr7	134475400	0.727273
chr7	134550800	0.685950
chr7	134760700	0.743802
chr7	134763000	0.859504
chr7	135071300	0.694215

chr7	135246200	0.958678
chr7	135248300	0.785124
chr7	135357900	0.652893
chr7	135439100	0.727273
chr7	135443600	0.702479
chr7	135458700	0.900826
chr7	135615900	0.859504
chr7	136085800	0.719008
chr7	136091300	0.702479
chr7	136322200	0.793388
chr7	136563900	0.884298
chr7	136945100	0.702479
chr7	136974300	0.884298
chr7	137010500	0.925620
chr7	137341400	0.644628
chr7	137343600	0.933884
chr7	137403400	0.727273
chr7	137431900	0.884298
chr7	137439200	0.933884
chr7	137505700	0.809917
chr7	137524800	0.685950
chr7	137529700	0.859504
chr7	137559200	0.760331
chr7	137564600	0.975207
chr7	137571100	0.809917
chr7	137584500	0.900826
chr7	137602600	0.702479
chr7	137618500	0.743802
chr7	137621800	0.652893
chr7	137628400	0.743802
chr7	137638900	0.975207
chr7	137700200	0.818182
chr7	137711900	0.859504
chr7	137846800	0.644628
chr7	137851800	0.925620
chr7	137854500	0.942149
chr7	137856600	0.925620
chr7	137872600	0.694215
chr7	137889000	0.933884
chr7	137892200	0.851240
chr7	137894400	0.950413
chr7	137928400	0.694215
chr7	137932500	0.826446
chr7	138119400	0.735537
chr7	138157400	0.785124
chr7	138186900	0.685950
chr7	138347600	0.652893
chr7	138350900	0.719008
chr7	138353700	0.876033
chr7	138423500	0.851240
chr7	138437800	0.909091
chr7	138440600	0.884298
chr7	138458800	0.834711
chr7	138483200	0.842975

chr7	138538600	0.925620
chr7	138646100	0.661157
chr7	138661100	0.785124
chr7	138703500	0.644628
chr7	138723000	0.669421
chr7	138779300	0.876033
chr7	138803500	0.719008
chr7	138867700	0.892562
chr7	139006500	0.644628
chr7	139227000	0.826446
chr7	139298900	0.942149
chr7	139337300	0.793388
chr7	139343700	0.719008
chr7	139346000	0.867769
chr7	139367900	0.694215
chr7	139412100	0.859504
chr7	139414400	0.876033
chr7	139445500	0.818182
chr7	139673400	0.752066
chr7	139676300	0.760331
chr7	139787400	0.752066
chr7	140022800	0.719008
chr7	140090500	0.884298
chr7	140149000	0.834711
chr7	140155800	0.966942
chr7	140158600	0.677686
chr7	140185500	0.652893
chr7	140207100	0.785124
chr7	140219000	0.785124
chr7	140241500	0.768595
chr7	140326500	0.694215
chr7	140335900	0.950413
chr7	140338300	0.909091
chr7	140347100	0.644628
chr7	140776600	0.917355
chr7	140803800	0.760331
chr7	140826400	0.677686
chr7	140913900	0.694215
chr7	140939100	0.884298
chr7	140995200	0.727273
chr7	141025100	0.785124
chr7	141066400	0.892562
chr7	141108800	0.834711
chr7	141112100	0.661157
chr7	141130400	0.900826
chr7	141133500	0.661157
chr7	141190200	0.933884
chr7	141245100	0.752066
chr7	141277400	0.785124
chr7	141285200	1.000000
chr7	141288100	0.644628
chr7	141352700	0.710744
chr7	141391100	0.710744
chr7	141398300	0.785124

chr7	141594400	0.694215
chr7	141654500	0.801653
chr7	141676400	0.793388
chr7	141812300	0.661157
chr7	142186700	0.661157
chr7	142252300	0.752066
chr7	142255600	0.760331
chr7	142266500	1.000000
chr7	142273400	0.652893
chr7	142375300	0.669421
chr7	142404700	0.983471
chr7	142412600	0.661157
chr7	142417900	0.644628
chr7	142507700	0.644628
chr7	142511300	0.991736
chr7	142560700	0.818182
chr7	142563700	0.652893
chr7	142583800	0.867769
chr7	142659900	0.652893
chr7	142981100	0.710744
chr7	143067300	0.818182
chr7	143081300	0.768595
chr7	143084100	0.917355
chr7	143107700	0.644628
chr7	143110300	0.644628
chr7	143215100	0.768595
chr7	143398600	0.876033
chr7	143604100	0.809917
chr7	143895100	0.826446
chr7	144050000	0.892562
chr7	144172800	0.644628
chr7	144185200	0.925620
chr7	144263100	0.892562
chr7	144555300	0.685950
chr7	144582700	0.727273
chr7	144606300	0.685950
chr7	145415600	0.776860
chr7	145600400	0.793388
chr7	145635400	0.809917
chr7	145694600	1.000000
chr7	145947300	0.900826
chr7	146111600	0.809917
chr7	146332800	0.719008
chr7	146489300	0.983471
chr7	147134100	0.760331
chr7	147281900	0.735537
chr7	147284300	0.743802
chr7	147298300	0.652893
chr7	147303400	0.768595
chr7	147697400	0.892562
chr7	147718700	0.793388
chr7	147729100	0.694215
chr7	147804900	0.851240
chr7	148015600	0.719008

chr7	148032300	1.000000
chr7	148034700	0.876033
chr7	148082300	0.768595
chr7	148084600	0.677686
chr7	148093900	0.867769
chr7	148199500	0.950413
chr7	148265100	0.851240
chr7	148286600	0.892562
chr7	148305200	0.785124
chr7	148402800	0.834711
chr7	148405900	0.793388
chr7	148409400	0.661157
chr7	148417600	0.652893
chr7	148420200	0.702479
chr7	148444100	0.900826
chr7	148470800	0.735537
chr7	148497500	0.834711
chr7	148606400	0.884298
chr7	148661500	0.735537
chr7	148673700	0.900826
chr7	148676000	0.669421
chr7	148703200	0.677686
chr7	148721300	0.983471
chr7	148842100	0.809917
chr7	148848100	0.793388
chr7	148903000	0.685950
chr7	149019000	0.727273
chr7	149083900	0.851240
chr7	149175300	0.925620
chr7	149359900	0.727273
chr7	149379300	0.760331
chr7	149383800	0.661157
chr7	149472600	0.702479
chr7	149480700	0.710744
chr7	149485000	0.727273
chr7	149500800	0.644628
chr7	149509200	0.644628
chr7	149513600	0.669421
chr7	149529000	0.818182
chr7	149552400	0.859504
chr7	149583100	0.818182
chr7	149672500	0.685950
chr7	149676500	0.669421
chr7	149729000	0.644628
chr7	149973900	0.801653
chr7	149977200	0.661157
chr7	150009500	0.752066
chr7	150022700	0.801653
chr7	150027100	1.000000
chr7	150032200	0.975207
chr7	150055100	0.677686
chr7	150063300	0.752066
chr7	150079700	0.867769
chr7	150083600	0.793388

chr7	150092800	0.702479
chr7	150096300	0.776860
chr7	150104800	0.834711
chr7	150109200	0.710744
chr7	150146000	0.818182
chr7	150148800	0.661157
chr7	150263900	0.652893
chr7	150433600	0.727273
chr7	150462400	0.644628
chr7	150518300	0.735537
chr7	150574400	0.884298
chr7	150598800	0.801653
chr7	150652700	0.760331
chr7	150662500	0.702479
chr7	150666700	0.925620
chr7	150668800	0.826446
chr7	150678200	0.785124
chr7	150686400	0.834711
chr7	150690600	0.735537
chr7	150706000	0.842975
chr7	150711000	0.719008
chr7	150748300	0.669421
chr7	150764900	0.851240
chr7	150801800	0.826446
chr7	150807700	0.834711
chr7	150813100	0.743802
chr7	150821300	0.669421
chr7	150824600	0.677686
chr7	150908200	0.818182
chr7	150944300	0.743802
chr7	150979100	0.768595
chr7	151000900	0.644628
chr7	151070600	0.917355
chr7	151113700	0.685950
chr7	151150300	0.735537
chr7	151154300	0.942149
chr7	151157700	0.694215
chr7	151166000	0.752066
chr7	151169300	0.942149
chr7	151191900	0.834711
chr7	151202300	0.644628
chr7	151206900	0.644628
chr7	151236600	0.801653
chr7	151326400	0.867769
chr7	151385000	0.743802
chr7	151393300	0.768595
chr7	151402900	0.801653
chr7	151433100	0.735537
chr7	151437700	0.719008
chr7	151440500	0.760331
chr7	151443900	0.677686
chr7	151480200	0.652893
chr7	151491700	0.818182
chr7	151511200	0.768595

chr7	151534800	0.809917
chr7	151594600	0.859504
chr7	151733000	0.727273
chr7	151747800	0.644628
chr7	151774200	0.933884
chr7	151779400	0.661157
chr7	151956700	0.652893
chr7	152067300	0.785124
chr7	152096800	0.735537
chr7	152129400	0.702479
chr7	152220200	0.727273
chr7	152241600	0.975207
chr7	152251000	0.719008
chr7	152403100	0.719008
chr7	152431400	0.768595
chr7	152433500	0.685950
chr7	152530500	0.834711
chr7	152608100	0.694215
chr7	152630800	0.719008
chr7	152666800	0.900826
chr7	152683300	0.793388
chr7	152687300	0.966942
chr7	152720100	0.652893
chr7	152855400	0.785124
chr7	153707000	0.818182
chr7	153762600	0.685950
chr7	153769400	0.661157
chr7	153818500	0.818182
chr7	153868200	0.768595
chr7	153881200	0.743802
chr7	153952500	0.752066
chr7	154006000	0.818182
chr7	154016100	0.652893
chr7	154114500	0.743802
chr7	154535700	0.735537
chr7	154557400	0.950413
chr7	154660800	0.776860
chr7	154706100	0.702479
chr7	154722600	0.743802
chr7	154788800	0.975207
chr7	154997700	0.760331
chr7	155002000	0.644628
chr7	155020000	0.818182
chr7	155142500	0.652893
chr7	155168100	0.867769
chr7	155172200	0.652893
chr7	155202200	0.669421
chr7	155250100	0.727273
chr7	155253100	0.884298
chr7	155276300	0.859504
chr7	155289300	0.793388
chr7	155455400	0.694215
chr7	155477700	0.727273
chr7	155542100	0.661157

chr7	155556600	0.644628
chr7	155607400	0.727273
chr7	155629200	0.694215
chr7	155889300	0.694215
chr7	156372200	0.933884
chr7	156379400	0.710744
chr7	156412900	0.966942
chr7	156459000	0.950413
chr7	156546300	0.644628
chr7	156702000	0.710744
chr7	156795200	0.702479
chr7	156798600	0.644628
chr7	156802100	0.768595
chr7	156807100	0.942149
chr7	156811600	0.661157
chr7	156815200	0.842975
chr7	156934600	0.677686
chr7	157086000	0.818182
chr7	157092800	0.793388
chr7	157104100	0.933884
chr7	157113300	0.826446
chr7	157138200	0.685950
chr7	157141900	0.793388
chr7	157148600	0.950413
chr7	157168400	0.685950
chr7	157180200	0.727273
chr7	157190000	0.743802
chr7	157198700	0.685950
chr7	157201200	0.818182
chr7	157203500	0.785124
chr7	157206300	0.851240
chr7	157225700	0.752066
chr7	157257800	0.702479
chr7	157293600	0.677686
chr7	157378600	0.644628
chr7	157463500	0.768595
chr7	157478900	0.768595
chr7	157530700	0.644628
chr7	157588100	0.727273
chr7	157599900	0.669421
chr7	157628100	0.694215
chr7	157633600	0.851240
chr7	157644500	0.809917
chr7	157648600	0.685950
chr7	157668400	0.702479
chr7	157692600	0.710744
chr7	157697600	0.867769
chr7	157724200	0.760331
chr7	157747200	0.793388
chr7	157755300	0.884298
chr7	157776600	0.661157
chr7	157801300	0.785124
chr7	157813800	0.809917
chr7	158068100	0.677686

chr7	158097200	0.768595
chr7	158110700	0.975207
chr7	158118600	0.785124
chr7	158175600	0.710744
chr7	158180100	0.826446
chr7	158231100	0.677686
chr7	158243700	0.644628
chr7	158261100	0.925620
chr7	158288400	0.768595
chr7	158294000	0.818182
chr7	158343100	0.809917
chr7	158355600	0.743802
chr7	158360100	0.644628
chr7	158363100	0.958678
chr7	158379300	0.644628
chr7	158388200	0.644628
chr7	158410200	0.776860
chr7	158522700	0.727273
chr7	158526000	0.859504
chr7	158604900	0.710744
chr7	158608100	0.933884
chr7	158611400	1.000000
chr7	158614600	0.677686
chr7	158636900	0.933884
chr7	158651700	0.950413
chr7	158654500	0.809917
chr7	158769600	0.685950
chr7	158808900	0.669421
chr7	158825200	0.652893
chr7	158854800	0.776860
chr7	158899400	0.801653
chr7	158906400	0.826446
chr7	158934600	0.793388
chr8	430500	0.809917
chr8	438200	0.743802
chr8	457300	0.727273
chr8	467500	0.776860
chr8	483900	0.652893
chr8	486500	0.909091
chr8	514600	0.710744
chr8	521900	0.884298
chr8	674800	0.892562
chr8	744100	0.793388
chr8	810500	0.743802
chr8	839700	0.801653
chr8	843400	0.652893
chr8	851000	0.892562
chr8	858800	0.909091
chr8	876600	0.719008
chr8	890900	0.735537
chr8	897200	0.793388
chr8	992400	0.677686
chr8	1012900	0.900826
chr8	1019100	0.933884

chr8	1030500	0.818182
chr8	1067500	0.743802
chr8	1076600	0.851240
chr8	1082600	0.851240
chr8	1114200	0.743802
chr8	1212200	0.694215
chr8	1304400	0.752066
chr8	1325000	0.859504
chr8	1427900	0.768595
chr8	1527000	0.925620
chr8	1555600	0.735537
chr8	1600100	0.867769
chr8	1606700	0.685950
chr8	1653700	0.801653
chr8	1693900	0.727273
chr8	1717800	0.851240
chr8	1736100	0.669421
chr8	1748500	0.801653
chr8	1751900	0.661157
chr8	1759400	0.669421
chr8	1762700	0.677686
chr8	1765700	0.909091
chr8	1777300	0.842975
chr8	1788900	0.785124
chr8	1845600	0.950413
chr8	1959700	0.752066
chr8	2012500	0.776860
chr8	2034700	0.661157
chr8	2075600	0.685950
chr8	2104400	0.719008
chr8	2187500	0.776860
chr8	2232700	0.710744
chr8	2384000	0.933884
chr8	2743700	0.719008
chr8	2774300	0.818182
chr8	2916900	0.710744
chr8	3102100	0.842975
chr8	3105400	0.685950
chr8	3141900	0.694215
chr8	3412700	0.694215
chr8	3576500	0.710744
chr8	3822100	0.702479
chr8	3897600	0.768595
chr8	3923800	0.991736
chr8	3983800	0.842975
chr8	4050200	0.702479
chr8	4354300	0.677686
chr8	4510300	0.727273
chr8	4573700	0.752066
chr8	5023000	0.801653
chr8	5025100	0.925620
chr8	5028000	1.000000
chr8	5527500	0.644628
chr8	5533300	0.900826

chr8	5664800	0.702479
chr8	5908400	0.793388
chr8	5992400	0.694215
chr8	6398700	0.785124
chr8	6405300	0.867769
chr8	6408400	0.818182
chr8	6419200	0.876033
chr8	6434900	0.719008
chr8	6451900	0.892562
chr8	6474000	0.933884
chr8	6584000	0.719008
chr8	6590200	0.719008
chr8	6607700	0.661157
chr8	6658500	0.851240
chr8	6698800	0.826446
chr8	6768300	0.661157
chr8	6843400	0.710744
chr8	6862300	0.694215
chr8	6967000	0.677686
chr8	7071300	0.702479
chr8	7252100	0.801653
chr8	7306000	0.826446
chr8	7326400	0.735537
chr8	7367100	0.809917
chr8	7394800	0.685950
chr8	7473100	0.702479
chr8	7641500	0.735537
chr8	7656900	0.677686
chr8	7700400	0.834711
chr8	7721300	0.909091
chr8	7760600	0.694215
chr8	7920700	0.661157
chr8	7988700	0.760331
chr8	8000300	0.785124
chr8	8025400	0.958678
chr8	8034600	0.694215
chr8	8038100	0.793388
chr8	8041800	0.760331
chr8	8057000	0.685950
chr8	8068500	0.735537
chr8	8182300	0.950413
chr8	8239800	0.900826
chr8	8256400	0.958678
chr8	8319500	0.661157
chr8	8342100	0.669421
chr8	8361300	0.652893
chr8	8402900	0.909091
chr8	8406800	0.760331
chr8	8422300	0.710744
chr8	8432500	0.644628
chr8	8440900	0.834711
chr8	8523100	0.768595
chr8	8562400	0.933884
chr8	8601200	0.735537

chr8	8640700	0.644628
chr8	8647900	0.975207
chr8	8686900	0.727273
chr8	8702500	0.776860
chr8	8727000	0.735537
chr8	8742500	0.876033
chr8	8771100	0.876033
chr8	8799600	0.694215
chr8	8898200	0.925620
chr8	8915900	0.966942
chr8	8990700	0.685950
chr8	9005900	0.818182
chr8	9018800	0.710744
chr8	9030000	0.752066
chr8	9095100	0.834711
chr8	9097200	0.644628
chr8	9122000	1.000000
chr8	9127400	0.966942
chr8	9161000	0.752066
chr8	9195600	0.677686
chr8	9237800	0.661157
chr8	9241300	0.785124
chr8	9244500	0.719008
chr8	9260200	0.652893
chr8	9754600	0.702479
chr8	9768300	0.652893
chr8	9821400	0.991736
chr8	9905700	0.652893
chr8	9914800	0.677686
chr8	9947900	0.752066
chr8	9963500	0.809917
chr8	9988100	0.776860
chr8	10002300	0.834711
chr8	10010400	0.702479
chr8	10125100	0.867769
chr8	10192900	0.892562
chr8	10195000	1.000000
chr8	10200900	0.842975
chr8	10223100	0.975207
chr8	10268900	0.876033
chr8	10277400	0.925620
chr8	10283000	0.851240
chr8	10390100	0.694215
chr8	10412900	0.735537
chr8	10421800	0.826446
chr8	10483900	0.702479
chr8	10494600	0.694215
chr8	10530200	0.644628
chr8	10574100	0.752066
chr8	10642400	0.702479
chr8	10786400	0.842975
chr8	10818800	0.727273
chr8	10855700	0.752066
chr8	10859800	0.727273

chr8	10865700	0.776860
chr8	10879400	0.644628
chr8	10906500	0.892562
chr8	10911000	0.776860
chr8	10924800	0.652893
chr8	10930000	0.801653
chr8	10982300	0.727273
chr8	10997400	0.652893
chr8	11002400	0.867769
chr8	11055100	0.652893
chr8	11085900	0.644628
chr8	11116500	0.818182
chr8	11144800	0.652893
chr8	11150600	0.644628
chr8	11273100	0.727273
chr8	11281100	0.809917
chr8	11283300	0.909091
chr8	11287500	0.661157
chr8	11296600	0.760331
chr8	11312800	0.694215
chr8	11332700	0.752066
chr8	11471000	0.743802
chr8	11475600	0.694215
chr8	11489400	0.793388
chr8	11499600	0.694215
chr8	11534300	0.661157
chr8	11538100	0.685950
chr8	11562900	0.727273
chr8	11566000	0.851240
chr8	11575300	0.677686
chr8	11578700	0.661157
chr8	11617200	0.760331
chr8	11644600	0.661157
chr8	11655300	0.727273
chr8	11670300	0.809917
chr8	11742800	0.661157
chr8	11813600	0.826446
chr8	12081000	0.752066
chr8	12339100	0.752066
chr8	12343400	0.892562
chr8	12355700	0.975207
chr8	12383800	0.743802
chr8	12445800	0.975207
chr8	12504900	0.760331
chr8	12632900	0.801653
chr8	12652800	0.644628
chr8	12727100	0.685950
chr8	12749000	0.933884
chr8	12758600	0.652893
chr8	12830600	0.685950
chr8	12837300	0.752066
chr8	12891400	0.661157
chr8	12938500	0.809917
chr8	12980300	0.900826

chr8	12987100	0.677686
chr8	13078500	0.801653
chr8	13544800	0.743802
chr8	13575300	0.876033
chr8	13626200	0.677686
chr8	13735100	0.735537
chr8	13925800	0.735537
chr8	14422800	0.760331
chr8	14702700	0.760331
chr8	15431600	0.652893
chr8	15443700	0.966942
chr8	15446500	0.644628
chr8	15452800	0.644628
chr8	15622100	0.652893
chr8	15885400	0.719008
chr8	16092200	0.884298
chr8	16142100	1.000000
chr8	16611500	0.818182
chr8	16638200	0.917355
chr8	16652200	0.884298
chr8	16909900	0.644628
chr8	17026000	0.925620
chr8	17223300	0.710744
chr8	17225800	0.826446
chr8	17442100	0.727273
chr8	17533800	0.702479
chr8	17579700	0.958678
chr8	17768400	0.677686
chr8	18218400	0.942149
chr8	18238800	0.710744
chr8	18396600	0.702479
chr8	18414100	0.942149
chr8	18459700	0.776860
chr8	18471400	0.710744
chr8	18624700	0.644628
chr8	18628100	0.991736
chr8	18708900	0.719008
chr8	18746000	0.859504
chr8	18831800	0.834711
chr8	18836200	0.685950
chr8	18866500	0.669421
chr8	18905500	0.702479
chr8	18926900	0.652893
chr8	19009600	0.892562
chr8	19035200	0.851240
chr8	19082900	0.900826
chr8	19086600	0.785124
chr8	19103000	0.661157
chr8	19296000	0.909091
chr8	19309900	0.694215
chr8	19354700	0.842975
chr8	19496200	0.950413
chr8	19536600	0.958678
chr8	19712200	0.826446

chr8	19715400	0.694215
chr8	19719600	0.719008
chr8	19731700	0.925620
chr8	19820000	0.661157
chr8	19865800	0.752066
chr8	19961400	0.785124
chr8	20090200	0.876033
chr8	20093900	0.644628
chr8	20112900	0.834711
chr8	20126000	0.933884
chr8	20129200	0.834711
chr8	20131600	0.727273
chr8	20134000	0.925620
chr8	20138300	0.925620
chr8	20146300	0.834711
chr8	20152600	0.785124
chr8	20171000	0.727273
chr8	20187000	0.818182
chr8	20207800	0.801653
chr8	20211600	0.809917
chr8	20233900	0.801653
chr8	20236600	0.702479
chr8	20293300	0.942149
chr8	20313700	0.768595
chr8	20316900	0.983471
chr8	20325200	0.644628
chr8	20359700	0.719008
chr8	20367400	0.752066
chr8	20557400	0.677686
chr8	20676700	0.966942
chr8	20725200	0.685950
chr8	20899100	0.826446
chr8	21180100	0.685950
chr8	21188900	0.677686
chr8	21203000	0.685950
chr8	21399300	0.842975
chr8	21495700	0.842975
chr8	21538300	0.727273
chr8	21593500	0.644628
chr8	21614400	0.710744
chr8	21625900	0.793388
chr8	21669600	0.867769
chr8	21744000	0.900826
chr8	21759600	0.859504
chr8	21770600	0.694215
chr8	21773600	0.793388
chr8	21780300	0.743802
chr8	21900300	0.785124
chr8	21906400	0.743802
chr8	21913600	0.851240
chr8	21927500	0.694215
chr8	21938900	0.735537
chr8	21972600	0.752066
chr8	21975700	0.669421

chr8	21991300	0.644628
chr8	22027700	0.661157
chr8	22037000	0.834711
chr8	22046600	0.735537
chr8	22213700	0.933884
chr8	22228000	0.661157
chr8	22241000	0.776860
chr8	22245500	0.694215
chr8	22250300	0.743802
chr8	22255300	0.727273
chr8	22282100	0.768595
chr8	22407500	0.685950
chr8	22414400	0.785124
chr8	22419300	0.669421
chr8	22432600	0.710744
chr8	22443100	0.719008
chr8	22445200	0.768595
chr8	22453000	0.661157
chr8	22486200	0.900826
chr8	22516700	0.933884
chr8	22597300	0.743802
chr8	22613900	0.785124
chr8	22642200	0.826446
chr8	22670700	0.661157
chr8	22696600	0.834711
chr8	22705300	0.685950
chr8	22725200	0.900826
chr8	22731800	0.685950
chr8	22735800	0.892562
chr8	22773800	0.950413
chr8	22779300	0.677686
chr8	22811200	0.719008
chr8	22832100	0.644628
chr8	22839200	0.884298
chr8	22861500	0.661157
chr8	22866800	0.859504
chr8	22872500	0.694215
chr8	22876200	0.900826
chr8	22907600	0.859504
chr8	22932400	0.677686
chr8	22942000	0.950413
chr8	23018400	0.735537
chr8	23034800	0.727273
chr8	23098700	0.785124
chr8	23151000	0.719008
chr8	23161700	0.661157
chr8	23194100	0.900826
chr8	23211700	0.966942
chr8	23273900	0.727273
chr8	23285500	0.702479
chr8	23310100	0.900826
chr8	23313000	0.710744
chr8	23390300	0.702479
chr8	23398700	0.834711

chr8	23415700	0.818182
chr8	23433400	0.644628
chr8	23554200	0.842975
chr8	23634700	0.867769
chr8	23643500	0.958678
chr8	23645700	0.785124
chr8	23689000	0.685950
chr8	23719700	0.933884
chr8	23776000	0.842975
chr8	23821000	1.000000
chr8	23833000	0.644628
chr8	23837100	0.991736
chr8	23849200	0.768595
chr8	24050200	0.776860
chr8	24052400	0.669421
chr8	24151900	0.801653
chr8	24272800	0.752066
chr8	24367300	0.859504
chr8	24623700	0.652893
chr8	24625900	0.752066
chr8	24760700	0.694215
chr8	24769000	0.669421
chr8	24931400	0.826446
chr8	24956600	0.925620
chr8	25055800	0.909091
chr8	25240200	0.834711
chr8	25364400	0.826446
chr8	25367700	0.644628
chr8	25373300	0.760331
chr8	25457600	0.842975
chr8	25489400	0.826446
chr8	25539500	0.760331
chr8	25656600	0.644628
chr8	25688900	0.661157
chr8	25765900	0.776860
chr8	25793600	0.933884
chr8	25800400	0.669421
chr8	25816400	1.000000
chr8	25827000	0.694215
chr8	25841200	0.933884
chr8	25847700	0.677686
chr8	25865000	0.677686
chr8	26041400	0.851240
chr8	26049100	0.644628
chr8	26124000	0.702479
chr8	26172100	0.719008
chr8	26179100	0.702479
chr8	26184900	0.661157
chr8	26194900	0.727273
chr8	26197000	0.652893
chr8	26214200	0.834711
chr8	26319800	0.752066
chr8	26323500	0.809917
chr8	26429400	0.685950

chr8	26440100	0.644628
chr8	26449300	0.809917
chr8	26453600	0.958678
chr8	26459100	0.661157
chr8	26467700	0.785124
chr8	26474100	0.719008
chr8	26493700	0.768595
chr8	26496800	0.909091
chr8	26499600	0.884298
chr8	26682300	0.892562
chr8	26762600	0.685950
chr8	27175200	0.826446
chr8	27207100	0.900826
chr8	27224600	0.644628
chr8	27268800	0.892562
chr8	27303900	0.818182
chr8	27373200	0.702479
chr8	27450400	0.793388
chr8	27465800	0.884298
chr8	27469600	0.909091
chr8	27522900	0.702479
chr8	27549200	0.677686
chr8	27560500	0.834711
chr8	27588000	0.826446
chr8	27766100	0.727273
chr8	28044900	0.661157
chr8	28166300	0.677686
chr8	28206100	0.719008
chr8	28227100	0.826446
chr8	28277400	0.702479
chr8	28325900	0.892562
chr8	28355300	0.685950
chr8	28363900	0.727273
chr8	28373100	0.983471
chr8	28447600	0.826446
chr8	28451800	0.710744
chr8	28459300	0.685950
chr8	28498700	0.644628
chr8	28516100	0.727273
chr8	28565500	0.983471
chr8	28571100	0.826446
chr8	28585600	0.727273
chr8	28609700	0.950413
chr8	28728400	0.842975
chr8	28800400	0.694215
chr8	28869000	0.685950
chr8	28902100	0.644628
chr8	28922300	0.900826
chr8	28929000	0.727273
chr8	28938700	0.652893
chr8	29004600	0.694215
chr8	29053600	0.652893
chr8	29087500	0.760331
chr8	29099100	0.818182

chr8	29176400	0.694215
chr8	29203300	0.892562
chr8	29317000	0.685950
chr8	29443100	0.884298
chr8	29595300	0.884298
chr8	29636000	0.677686
chr8	29662100	0.768595
chr8	29707000	0.685950
chr8	29741700	0.851240
chr8	29744000	0.942149
chr8	29891900	0.776860
chr8	30137600	0.834711
chr8	30140700	0.735537
chr8	30197500	0.917355
chr8	30247500	0.809917
chr8	30250700	0.966942
chr8	30252900	0.900826
chr8	30257500	0.851240
chr8	30262400	0.975207
chr8	30265600	0.719008
chr8	30273100	0.892562
chr8	30280800	0.867769
chr8	30283600	0.752066
chr8	30286500	0.694215
chr8	30290700	0.867769
chr8	30297300	0.801653
chr8	30300300	0.983471
chr8	30306600	0.925620
chr8	30311100	0.776860
chr8	30318700	0.768595
chr8	30324800	0.768595
chr8	30340600	0.677686
chr8	30351400	0.818182
chr8	30358400	0.710744
chr8	30360600	1.000000
chr8	30389900	0.859504
chr8	30396800	0.710744
chr8	30399100	0.991736
chr8	30402300	0.975207
chr8	30406400	0.851240
chr8	30408500	0.669421
chr8	30414500	0.867769
chr8	30506400	0.785124
chr8	30573600	0.842975
chr8	30720900	0.892562
chr8	30776300	0.710744
chr8	30926800	0.760331
chr8	30933000	0.826446
chr8	30964400	0.834711
chr8	31097000	0.925620
chr8	31336600	0.809917
chr8	31354900	0.669421
chr8	31881300	0.652893
chr8	32024200	0.677686

chr8	32076500	0.669421
chr8	32345400	0.743802
chr8	32716000	0.719008
chr8	32858800	0.735537
chr8	32985300	0.975207
chr8	32987500	0.760331
chr8	33037600	0.768595
chr8	33057400	0.702479
chr8	33132600	0.842975
chr8	33202100	0.917355
chr8	33262400	0.719008
chr8	33272000	0.900826
chr8	33282200	0.644628
chr8	33455500	0.776860
chr8	33462500	0.768595
chr8	33484000	0.991736
chr8	33576100	0.834711
chr8	33670500	0.859504
chr8	33773900	0.694215
chr8	33848200	0.669421
chr8	33896800	0.685950
chr8	33969500	0.867769
chr8	34184500	0.685950
chr8	34704200	0.677686
chr8	34764800	0.991736
chr8	34843700	0.991736
chr8	34864500	0.859504
chr8	34938600	0.727273
chr8	35005000	0.867769
chr8	35098200	0.719008
chr8	35151300	0.685950
chr8	35161400	0.966942
chr8	35179200	0.743802
chr8	35185400	0.760331
chr8	35202500	0.785124
chr8	35231900	0.752066
chr8	35235100	0.909091
chr8	35246800	0.685950
chr8	35270300	0.785124
chr8	35273500	0.644628
chr8	35276200	0.752066
chr8	35318000	0.685950
chr8	35345400	0.776860
chr8	35350500	0.933884
chr8	35358900	0.776860
chr8	35397300	0.727273
chr8	35402300	0.991736
chr8	35418600	0.702479
chr8	35440000	0.677686
chr8	35452900	0.793388
chr8	35483300	0.677686
chr8	35500300	0.859504
chr8	35574800	0.851240
chr8	35577300	0.801653

chr8	35720400	0.719008
chr8	35731500	0.661157
chr8	35762900	0.785124
chr8	35803200	0.776860
chr8	35985500	0.809917
chr8	36583800	0.694215
chr8	36603200	0.661157
chr8	36781700	0.917355
chr8	36808200	0.801653
chr8	36924700	0.685950
chr8	36937700	0.719008
chr8	36943000	0.933884
chr8	36955000	0.719008
chr8	36957900	0.652893
chr8	36964900	0.710744
chr8	37132700	0.685950
chr8	37176400	0.652893
chr8	37184000	0.669421
chr8	37186400	0.719008
chr8	37375200	0.702479
chr8	37418300	0.826446
chr8	37439400	0.909091
chr8	37459700	0.719008
chr8	37533200	0.702479
chr8	37585100	0.942149
chr8	37652700	0.752066
chr8	37661800	0.867769
chr8	37672700	0.900826
chr8	37679300	0.900826
chr8	37684600	0.925620
chr8	37687700	0.818182
chr8	37701200	0.652893
chr8	37738400	0.876033
chr8	37740900	0.933884
chr8	37744200	0.669421
chr8	37747900	0.859504
chr8	37761500	0.884298
chr8	37773700	0.942149
chr8	37797300	0.851240
chr8	37891400	0.768595
chr8	37924500	0.842975
chr8	37939600	0.644628
chr8	37947900	0.867769
chr8	38011300	0.644628
chr8	38146700	0.768595
chr8	38269400	0.842975
chr8	38286700	0.661157
chr8	38288800	0.776860
chr8	38293900	0.975207
chr8	38302200	0.768595
chr8	38307600	0.859504
chr8	38309700	0.710744
chr8	38317000	0.818182
chr8	38320900	0.768595

chr8	38329900	0.867769
chr8	38332400	0.727273
chr8	38342200	0.669421
chr8	38367800	0.909091
chr8	38371800	0.884298
chr8	38385500	0.644628
chr8	38387900	0.950413
chr8	38398600	0.727273
chr8	38415700	0.760331
chr8	38417900	0.727273
chr8	38420200	0.760331
chr8	38422500	0.793388
chr8	38424900	0.917355
chr8	38484000	0.975207
chr8	38508600	0.768595
chr8	38570600	0.752066
chr8	38579200	0.942149
chr8	38592700	0.644628
chr8	38691400	0.760331
chr8	38693500	0.702479
chr8	38723700	0.801653
chr8	38794400	0.966942
chr8	38835200	0.644628
chr8	38857000	0.702479
chr8	39336800	0.727273
chr8	39585100	0.842975
chr8	39749900	0.785124
chr8	39792600	0.917355
chr8	39803000	0.735537
chr8	39806700	0.983471
chr8	39809200	0.809917
chr8	39951100	0.909091
chr8	40011100	0.809917
chr8	40056100	0.719008
chr8	40096100	0.958678
chr8	40177900	0.743802
chr8	40181100	0.752066
chr8	40271300	0.909091
chr8	40315400	0.685950
chr8	40351800	0.925620
chr8	40404700	0.876033
chr8	40490300	0.768595
chr8	40594300	0.644628
chr8	40614300	0.644628
chr8	40627600	0.793388
chr8	40637500	0.694215
chr8	40705300	0.727273
chr8	40726200	0.801653
chr8	40732200	0.743802
chr8	40746100	0.768595
chr8	40851100	0.776860
chr8	40935500	0.892562
chr8	40937800	0.851240
chr8	40957700	0.644628

chr8	40963400	0.652893
chr8	40984500	0.793388
chr8	41054000	0.702479
chr8	41073200	0.785124
chr8	41127300	0.752066
chr8	41147100	0.719008
chr8	41152100	0.760331
chr8	41158800	0.785124
chr8	41161300	0.776860
chr8	41176400	0.900826
chr8	41179100	0.760331
chr8	41188800	0.752066
chr8	41200500	0.677686
chr8	41206400	0.677686
chr8	41250100	0.851240
chr8	41272500	0.809917
chr8	41400800	0.809917
chr8	41463900	0.867769
chr8	41467900	0.710744
chr8	41569500	0.743802
chr8	41572000	0.669421
chr8	41585400	0.826446
chr8	41594300	0.876033
chr8	41615200	0.685950
chr8	41639000	0.702479
chr8	41649500	0.669421
chr8	41676900	0.900826
chr8	41740100	0.669421
chr8	41788700	0.652893
chr8	41981700	0.826446
chr8	42053800	0.892562
chr8	42063500	0.859504
chr8	42067000	0.842975
chr8	42235000	0.818182
chr8	42326500	0.826446
chr8	42331100	0.752066
chr8	42342500	0.834711
chr8	42350600	0.966942
chr8	42354600	0.884298
chr8	42358900	0.851240
chr8	42361000	0.694215
chr8	42592300	0.809917
chr8	42596600	0.694215
chr8	42619900	0.685950
chr8	43097300	0.727273
chr8	43768500	0.842975
chr8	43806200	0.743802
chr8	43827200	0.644628
chr8	43833800	0.768595
chr8	43838800	0.661157
chr8	46841900	0.694215
chr8	46853500	0.743802
chr8	46880100	0.735537
chr8	46906700	0.793388

chr8	48425700	0.842975
chr8	48437500	1.000000
chr8	48486700	0.661157
chr8	48523200	0.685950
chr8	48572800	0.776860
chr8	48680000	0.710744
chr8	48734700	0.933884
chr8	48905600	0.876033
chr8	48975800	0.710744
chr8	49036700	0.669421
chr8	49049900	0.950413
chr8	49166800	0.942149
chr8	49173900	0.933884
chr8	49231700	0.694215
chr8	49343200	0.834711
chr8	49435900	0.793388
chr8	49438300	0.793388
chr8	49442600	0.793388
chr8	49455200	0.950413
chr8	49458300	0.752066
chr8	49462700	0.694215
chr8	49587500	0.644628
chr8	49614200	0.785124
chr8	49677100	0.876033
chr8	49701500	0.950413
chr8	49704900	0.694215
chr8	49778100	0.760331
chr8	49816500	0.851240
chr8	49978200	0.719008
chr8	50004900	0.925620
chr8	50146600	0.719008
chr8	50157400	0.644628
chr8	50255900	0.776860
chr8	50294800	0.867769
chr8	50535400	0.661157
chr8	50629800	0.900826
chr8	50711300	0.917355
chr8	50735400	0.752066
chr8	50947100	0.809917
chr8	50969500	0.644628
chr8	50981100	0.851240
chr8	51751800	0.652893
chr8	51768300	0.669421
chr8	52414900	0.652893
chr8	52432900	0.785124
chr8	52531100	0.958678
chr8	52783300	0.793388
chr8	52802100	0.933884
chr8	52875200	0.644628
chr8	52921400	0.685950
chr8	53064000	0.867769
chr8	53082100	0.801653
chr8	53135100	0.776860
chr8	53162500	0.876033

chr8	53167400	0.760331
chr8	53243700	0.694215
chr8	53319200	0.942149
chr8	53326700	0.785124
chr8	53366100	0.727273
chr8	53471900	0.909091
chr8	53475000	0.661157
chr8	53502700	0.809917
chr8	53512900	0.876033
chr8	53541200	0.884298
chr8	53544500	0.859504
chr8	53567100	0.809917
chr8	53596600	0.644628
chr8	53607000	0.702479
chr8	53884700	0.876033
chr8	53931900	0.851240
chr8	54131700	0.710744
chr8	54136800	0.818182
chr8	54230200	0.942149
chr8	54416400	0.669421
chr8	54435600	0.809917
chr8	54462500	0.867769
chr8	54522100	0.768595
chr8	54544700	0.644628
chr8	54605400	0.752066
chr8	54647500	0.694215
chr8	54676700	0.876033
chr8	54785300	0.809917
chr8	54816500	0.685950
chr8	54819100	0.809917
chr8	54857600	0.834711
chr8	54931400	0.710744
chr8	54944300	0.867769
chr8	55019100	0.735537
chr8	55078700	0.727273
chr8	55091800	0.801653
chr8	55105200	0.669421
chr8	55181400	0.644628
chr8	55467100	0.793388
chr8	55625000	0.652893
chr8	55795200	0.752066
chr8	55918700	0.909091
chr8	56032200	0.925620
chr8	56036700	0.785124
chr8	56041400	0.702479
chr8	56043600	0.884298
chr8	56131900	0.892562
chr8	56226900	0.933884
chr8	56285600	0.958678
chr8	56322600	0.661157
chr8	56328400	0.710744
chr8	56494900	0.834711
chr8	56544900	0.652893
chr8	56761100	0.851240

chr8	56774200	0.702479
chr8	56805500	0.793388
chr8	56814100	0.760331
chr8	56826300	0.826446
chr8	56830400	0.785124
chr8	56832700	0.983471
chr8	56843100	0.900826
chr8	56851700	0.842975
chr8	56875900	0.702479
chr8	56895300	0.776860
chr8	56897900	0.818182
chr8	56961100	0.661157
chr8	57085400	0.776860
chr8	57517900	0.743802
chr8	58477300	0.768595
chr8	58833200	0.842975
chr8	58926800	0.669421
chr8	58952000	0.876033
chr8	58961000	0.760331
chr8	59004000	0.677686
chr8	59035800	0.685950
chr8	59231200	0.694215
chr8	59341900	0.694215
chr8	59366700	0.876033
chr8	59576700	0.652893
chr8	59584300	0.702479
chr8	59746300	0.710744
chr8	59840400	0.752066
chr8	59900100	0.801653
chr8	59919400	0.834711
chr8	59951900	0.702479
chr8	59955700	0.735537
chr8	59968200	0.900826
chr8	59971900	0.752066
chr8	59977700	0.950413
chr8	59988500	0.661157
chr8	60022600	0.925620
chr8	60252500	0.892562
chr8	60858100	0.661157
chr8	60872600	0.644628
chr8	61049100	0.743802
chr8	61227400	0.719008
chr8	61241600	0.719008
chr8	61589100	0.842975
chr8	61597500	0.950413
chr8	61606600	0.735537
chr8	61621100	0.710744
chr8	61648700	0.702479
chr8	61651800	0.652893
chr8	61677500	0.710744
chr8	61680200	0.702479
chr8	61683300	0.785124
chr8	61686600	0.694215
chr8	61785300	0.842975

chr8	61825500	0.719008
chr8	61861200	0.818182
chr8	61884600	0.909091
chr8	61896700	0.752066
chr8	61910700	0.809917
chr8	61916900	0.768595
chr8	61925500	0.652893
chr8	61989200	0.644628
chr8	61991500	0.785124
chr8	62034500	0.710744
chr8	62084400	0.809917
chr8	62176100	0.644628
chr8	62736500	0.975207
chr8	62749400	0.776860
chr8	62753300	0.942149
chr8	62825400	0.752066
chr8	63186600	0.644628
chr8	63209100	0.826446
chr8	63475600	0.892562
chr8	63507000	0.768595
chr8	63516800	0.694215
chr8	63539500	0.760331
chr8	63582400	0.694215
chr8	63699400	0.842975
chr8	63922800	0.859504
chr8	64109500	0.652893
chr8	64113000	0.760331
chr8	64122400	0.801653
chr8	64276100	0.727273
chr8	64278700	0.752066
chr8	64518500	0.834711
chr8	64583200	0.752066
chr8	64992800	0.661157
chr8	65070200	0.851240
chr8	65074400	0.694215
chr8	65196200	0.702479
chr8	65515200	0.652893
chr8	65591000	0.719008
chr8	65604400	0.710744
chr8	65843600	0.834711
chr8	65936700	0.702479
chr8	66078400	0.785124
chr8	66131900	0.760331
chr8	66187500	0.867769
chr8	66196800	0.685950
chr8	66571600	0.867769
chr8	66630300	0.991736
chr8	66702600	0.694215
chr8	66707500	0.760331
chr8	66712600	0.876033
chr8	66738300	0.752066
chr8	66747600	0.677686
chr8	66976900	0.917355
chr8	67037100	0.735537

chr8	67099100	0.743802
chr8	67165000	0.685950
chr8	67261000	0.892562
chr8	67265000	0.752066
chr8	67319900	0.760331
chr8	67330400	0.735537
chr8	67358600	0.942149
chr8	67371200	0.727273
chr8	67384800	0.876033
chr8	67425200	0.760331
chr8	67434900	0.661157
chr8	67446400	0.669421
chr8	67813800	0.644628
chr8	68142200	0.785124
chr8	68155100	0.735537
chr8	68251800	0.752066
chr8	68282100	0.851240
chr8	68293700	0.793388
chr8	68348200	0.892562
chr8	68392000	0.793388
chr8	68402700	0.694215
chr8	68424700	0.743802
chr8	68434900	0.743802
chr8	68498000	0.735537
chr8	68605300	0.652893
chr8	68631400	0.884298
chr8	68633500	0.983471
chr8	68639700	0.669421
chr8	68642600	0.867769
chr8	68663600	0.743802
chr8	68767200	0.669421
chr8	68824700	0.669421
chr8	68862500	0.793388
chr8	68867700	0.735537
chr8	68873100	0.760331
chr8	68877000	0.975207
chr8	68881000	0.933884
chr8	68944800	0.942149
chr8	68970100	0.669421
chr8	69013900	0.710744
chr8	69137600	0.661157
chr8	69142100	0.925620
chr8	69553400	0.975207
chr8	69561600	0.884298
chr8	69672400	0.884298
chr8	69712400	0.842975
chr8	69753900	0.793388
chr8	69757200	0.834711
chr8	69781400	0.669421
chr8	69796800	0.710744
chr8	69883500	0.694215
chr8	70281000	0.809917
chr8	70284000	0.669421
chr8	70360300	0.661157

chr8	70372400	0.834711
chr8	70381300	0.743802
chr8	70401000	0.752066
chr8	70465400	0.652893
chr8	70476900	0.710744
chr8	70501700	0.694215
chr8	70509700	0.809917
chr8	70532400	0.735537
chr8	70602600	1.000000
chr8	70703600	0.917355
chr8	70837500	0.669421
chr8	70859000	0.652893
chr8	70866600	0.702479
chr8	70890000	0.876033
chr8	70892700	0.743802
chr8	70920200	0.768595
chr8	70974100	0.644628
chr8	70987600	0.677686
chr8	70995500	0.760331
chr8	71001200	0.933884
chr8	71005500	0.851240
chr8	71061100	0.826446
chr8	71069300	0.760331
chr8	71097700	0.917355
chr8	71129500	0.958678
chr8	71142400	0.661157
chr8	71157900	0.710744
chr8	71228800	0.702479
chr8	71240100	0.694215
chr8	71245500	0.842975
chr8	71285200	0.694215
chr8	71291500	0.677686
chr8	71354900	0.793388
chr8	71717500	0.793388
chr8	72018900	0.809917
chr8	72115000	0.661157
chr8	72131200	0.702479
chr8	72269300	0.851240
chr8	72473300	0.809917
chr8	72504200	0.859504
chr8	72548900	0.917355
chr8	72554200	0.677686
chr8	72566200	0.801653
chr8	72586400	0.966942
chr8	72600500	0.768595
chr8	72605800	0.694215
chr8	72608500	0.876033
chr8	72628900	0.991736
chr8	72650600	0.727273
chr8	72746000	0.801653
chr8	72806800	0.735537
chr8	72844300	0.644628
chr8	72928600	0.867769
chr8	73040400	0.900826

chr8	73606100	0.752066
chr8	73647300	0.801653
chr8	73659300	0.900826
chr8	73670600	0.702479
chr8	73674800	0.752066
chr8	73687600	0.735537
chr8	73736300	0.909091
chr8	73798800	0.677686
chr8	73906500	0.685950
chr8	73913600	0.884298
chr8	73917000	0.801653
chr8	73992000	0.876033
chr8	74058700	0.942149
chr8	74202900	0.644628
chr8	74217000	0.760331
chr8	74219700	0.776860
chr8	74269300	0.661157
chr8	74332400	0.661157
chr8	74396600	0.685950
chr8	74403200	0.801653
chr8	74545200	0.702479
chr8	74711400	0.727273
chr8	74897100	0.752066
chr8	74989700	0.768595
chr8	75055900	0.760331
chr8	75076600	0.760331
chr8	75079700	0.661157
chr8	75134000	0.776860
chr8	75188100	0.669421
chr8	75214200	0.661157
chr8	75243400	0.809917
chr8	75250600	0.719008
chr8	75348000	0.652893
chr8	75480300	0.801653
chr8	75654300	0.950413
chr8	75913000	0.884298
chr8	75917000	0.710744
chr8	75989200	0.685950
chr8	76129600	0.909091
chr8	76132200	0.975207
chr8	76221600	0.809917
chr8	76314900	0.735537
chr8	76888700	0.694215
chr8	77147900	0.719008
chr8	77599700	0.752066
chr8	77677300	0.933884
chr8	77680100	0.677686
chr8	77688500	0.983471
chr8	77712900	0.925620
chr8	78996200	0.644628
chr8	79064500	0.842975
chr8	79144400	0.710744
chr8	79424100	0.743802
chr8	79467900	0.661157

chr8	79514300	0.925620
chr8	79675100	0.694215
chr8	79885300	0.809917
chr8	80031700	0.867769
chr8	80034300	0.933884
chr8	80037700	0.876033
chr8	80049400	0.644628
chr8	80533300	0.752066
chr8	80549900	0.694215
chr8	80578600	0.719008
chr8	80590600	0.644628
chr8	80599800	0.719008
chr8	80673300	0.685950
chr8	80845200	0.719008
chr8	80872800	0.859504
chr8	80879800	0.876033
chr8	80997000	0.876033
chr8	81020300	0.727273
chr8	81028000	0.677686
chr8	81080800	0.735537
chr8	81124400	1.000000
chr8	81143000	0.719008
chr8	81153200	0.834711
chr8	81192100	0.925620
chr8	81290100	0.826446
chr8	81359000	0.727273
chr8	81421400	0.851240
chr8	81441200	0.644628
chr8	81476100	0.685950
chr8	81488100	0.876033
chr8	81503000	0.826446
chr8	81515600	0.768595
chr8	81523900	0.760331
chr8	81550700	0.809917
chr8	81828400	0.710744
chr8	81846800	0.669421
chr8	81873200	0.685950
chr8	81947800	0.661157
chr8	82213600	0.735537
chr8	82400700	0.710744
chr8	82513200	0.661157
chr8	82809100	0.801653
chr8	82811400	0.727273
chr8	82825400	0.669421
chr8	82958500	0.933884
chr8	83433500	0.644628
chr8	84243100	0.694215
chr8	85032600	0.702479
chr8	85166000	0.735537
chr8	85202800	0.909091
chr8	85266600	0.760331
chr8	85361300	0.826446
chr8	86110200	0.743802
chr8	86230100	0.719008

chr8	86374300	0.669421
chr8	86463500	0.727273
chr8	86494400	0.719008
chr8	86504100	0.842975
chr8	86934600	0.809917
chr8	87072600	0.859504
chr8	87078000	0.785124
chr8	87404200	0.983471
chr8	87426600	0.925620
chr8	87716600	0.685950
chr8	87722200	0.826446
chr8	87905500	0.694215
chr8	88025600	0.809917
chr8	88844900	0.892562
chr8	89124600	0.809917
chr8	89140800	0.652893
chr8	89220200	0.677686
chr8	89231300	0.719008
chr8	89246200	0.669421
chr8	89256400	0.966942
chr8	89310600	0.818182
chr8	89315400	0.719008
chr8	89427100	0.719008
chr8	89578000	0.702479
chr8	89603100	0.842975
chr8	89718800	0.710744
chr8	89895600	0.661157
chr8	90248500	0.644628
chr8	90421600	0.727273
chr8	90428000	0.851240
chr8	90637200	0.909091
chr8	90737800	0.826446
chr8	90750000	0.760331
chr8	90773200	0.694215
chr8	90837200	0.719008
chr8	90895200	0.727273
chr8	91099100	0.752066
chr8	91131200	0.669421
chr8	91173700	0.818182
chr8	91227300	0.884298
chr8	91294000	0.900826
chr8	91581600	0.966942
chr8	91584200	0.743802
chr8	91591500	0.851240
chr8	91593700	0.743802
chr8	91640600	0.694215
chr8	91650700	0.785124
chr8	91683700	0.818182
chr8	91689000	0.727273
chr8	91692300	0.710744
chr8	91694900	0.884298
chr8	91698900	0.793388
chr8	91816300	0.851240
chr8	91833900	0.719008

chr8	91848100	0.685950
chr8	92041600	0.735537
chr8	92047600	0.867769
chr8	92054900	0.710744
chr8	92072900	0.694215
chr8	92087800	0.661157
chr8	92404900	0.917355
chr8	92646300	0.661157
chr8	93064000	0.768595
chr8	93074400	0.710744
chr8	93078700	0.826446
chr8	93080900	0.867769
chr8	93095100	0.727273
chr8	93103000	0.677686
chr8	93117900	0.685950
chr8	93144100	0.851240
chr8	93161500	0.735537
chr8	93259600	0.867769
chr8	93477400	0.735537
chr8	93479700	0.685950
chr8	93517100	0.644628
chr8	93698300	0.702479
chr8	93724600	0.685950
chr8	93770900	0.892562
chr8	93921000	0.735537
chr8	94229000	0.942149
chr8	94373600	0.909091
chr8	94508400	0.859504
chr8	94859300	0.793388
chr8	94892000	0.669421
chr8	94902700	0.694215
chr8	94956400	1.000000
chr8	95047300	0.851240
chr8	95092400	0.694215
chr8	95284800	0.826446
chr8	95369800	0.644628
chr8	95430300	0.669421
chr8	95445100	0.644628
chr8	95450200	0.793388
chr8	95452500	0.842975
chr8	95457600	0.892562
chr8	95987600	0.669421
chr8	96000300	0.669421
chr8	96180000	0.842975
chr8	96205900	0.669421
chr8	96443200	0.958678
chr8	96504800	0.669421
chr8	96695000	0.942149
chr8	96705700	0.702479
chr8	97294400	0.694215
chr8	97302900	0.702479
chr8	97340000	0.834711
chr8	97352200	0.652893
chr8	97389700	0.727273

chr8	97469500	0.719008
chr8	97519900	0.735537
chr8	97526900	0.735537
chr8	97530900	0.752066
chr8	97533500	0.876033
chr8	97536200	0.958678
chr8	97549200	0.892562
chr8	97557400	0.669421
chr8	97564000	0.785124
chr8	97567200	0.644628
chr8	97585400	0.644628
chr8	97592100	0.661157
chr8	98019200	0.719008
chr8	98099300	0.719008
chr8	98193700	0.851240
chr8	98272300	0.694215
chr8	98337600	0.685950
chr8	98504900	0.694215
chr8	98768500	0.851240
chr8	98774200	0.991736
chr8	98791600	0.826446
chr8	98795900	0.958678
chr8	98798800	0.991736
chr8	98844000	0.867769
chr8	98861800	0.743802
chr8	98930600	0.768595
chr8	98945000	0.842975
chr8	98950100	0.685950
chr8	98956500	0.793388
chr8	98963800	0.652893
chr8	99018100	0.884298
chr8	99027900	0.785124
chr8	99046100	0.644628
chr8	99082200	0.727273
chr8	99177600	0.760331
chr8	99182800	0.752066
chr8	99259800	0.652893
chr8	99380300	0.760331
chr8	99394900	0.851240
chr8	99671100	0.669421
chr8	99738800	0.719008
chr8	99914100	0.652893
chr8	99964100	0.917355
chr8	100241600	0.661157
chr8	100298000	0.826446
chr8	100508300	1.000000
chr8	100649600	0.677686
chr8	100655200	0.752066
chr8	101228900	0.735537
chr8	101388000	0.842975
chr8	101428300	0.867769
chr8	101446800	0.768595
chr8	101456600	0.661157
chr8	101471300	0.900826

chr8	101474100	0.685950
chr8	101507700	0.677686
chr8	101522600	0.966942
chr8	101586600	0.677686
chr8	101590200	0.950413
chr8	101676100	0.669421
chr8	101680000	0.975207
chr8	101693400	0.694215
chr8	101801500	0.867769
chr8	101822300	0.834711
chr8	101912800	0.950413
chr8	101971400	0.702479
chr8	102006900	0.933884
chr8	102016100	0.892562
chr8	102039500	0.834711
chr8	102054200	0.801653
chr8	102057000	0.652893
chr8	102064000	0.719008
chr8	102076000	0.702479
chr8	102084000	0.694215
chr8	102109400	0.669421
chr8	102116000	0.727273
chr8	102121800	0.859504
chr8	102131700	0.727273
chr8	102149800	0.867769
chr8	102168100	0.867769
chr8	102179200	0.859504
chr8	102257800	0.685950
chr8	102300100	0.801653
chr8	102307400	0.710744
chr8	102373600	0.900826
chr8	102450400	0.966942
chr8	102473200	0.925620
chr8	102513900	0.661157
chr8	102517700	0.933884
chr8	102527100	0.909091
chr8	102542100	0.859504
chr8	102551100	0.950413
chr8	102553200	0.702479
chr8	102669000	0.743802
chr8	102730100	0.652893
chr8	102772900	0.752066
chr8	102800200	0.743802
chr8	102862400	0.809917
chr8	102902100	0.818182
chr8	103036500	0.710744
chr8	103073000	0.644628
chr8	103092800	0.702479
chr8	103120900	0.694215
chr8	103125100	0.884298
chr8	103203800	0.669421
chr8	103248600	0.950413
chr8	103264000	0.669421
chr8	103379500	0.892562

chr8	103388400	0.661157
chr8	103395800	0.925620
chr8	103397900	0.710744
chr8	103413500	0.950413
chr8	103420300	0.727273
chr8	103439700	0.851240
chr8	103448800	0.900826
chr8	103517400	0.892562
chr8	103524400	0.768595
chr8	103530300	0.768595
chr8	103541200	0.760331
chr8	103547500	0.966942
chr8	103551100	0.669421
chr8	103556700	0.785124
chr8	103589400	0.826446
chr8	103598000	0.950413
chr8	103679500	0.752066
chr8	103711300	0.685950
chr8	103742500	0.900826
chr8	103795100	0.818182
chr8	103801400	0.909091
chr8	103807600	0.785124
chr8	103821500	0.884298
chr8	103826800	0.702479
chr8	103871300	0.710744
chr8	103899500	0.727273
chr8	103907100	0.842975
chr8	103911600	0.702479
chr8	103916000	0.826446
chr8	103919000	0.892562
chr8	103922500	0.884298
chr8	103940000	0.950413
chr8	103943000	0.966942
chr8	103952600	0.785124
chr8	103974000	0.950413
chr8	104012400	1.000000
chr8	104177700	0.818182
chr8	104229700	0.702479
chr8	104240800	0.727273
chr8	104268200	0.702479
chr8	104317600	0.727273
chr8	104424200	0.809917
chr8	104457900	0.752066
chr8	104492000	0.652893
chr8	104609600	0.702479
chr8	104637100	0.669421
chr8	104656300	0.826446
chr8	104663100	0.933884
chr8	104665400	0.925620
chr8	105041800	0.826446
chr8	105306600	0.793388
chr8	105338100	0.719008
chr8	105354900	0.942149
chr8	105392200	0.652893

chr8	105520000	0.793388
chr8	106596100	0.851240
chr8	106605700	0.694215
chr8	106666600	0.809917
chr8	106674300	0.793388
chr8	106762100	0.892562
chr8	106765300	0.859504
chr8	106879300	0.694215
chr8	107002400	0.842975
chr8	107099000	0.867769
chr8	107115900	0.793388
chr8	107384400	0.710744
chr8	107553900	0.710744
chr8	107672500	0.702479
chr8	107676500	0.801653
chr8	108352500	0.735537
chr8	108393600	0.760331
chr8	108442300	0.785124
chr8	108456900	0.702479
chr8	108499200	0.851240
chr8	108502500	0.685950
chr8	108507500	0.727273
chr8	108648000	0.661157
chr8	109299300	0.900826
chr8	109756600	0.752066
chr8	110146900	0.900826
chr8	110151100	0.710744
chr8	110275200	0.966942
chr8	110294300	0.818182
chr8	110296600	0.694215
chr8	110455900	0.669421
chr8	110607900	0.644628
chr8	110909200	0.694215
chr8	111848400	0.933884
chr8	112163200	0.942149
chr8	112241900	0.752066
chr8	112340000	0.793388
chr8	112692300	0.925620
chr8	112757800	0.644628
chr8	113230700	0.785124
chr8	113266500	0.884298
chr8	113686800	0.760331
chr8	113818600	0.842975
chr8	113821700	0.702479
chr8	113894200	0.925620
chr8	114993500	1.000000
chr8	115292200	0.793388
chr8	115465900	0.685950
chr8	115565500	0.842975
chr8	115582800	0.685950
chr8	116073600	0.685950
chr8	116128000	0.727273
chr8	116170000	0.892562
chr8	116221300	0.719008

chr8	116231000	0.760331
chr8	116480700	0.950413
chr8	116507200	0.842975
chr8	117102200	0.818182
chr8	117140900	0.818182
chr8	117171900	0.719008
chr8	117366500	0.743802
chr8	117487800	0.644628
chr8	117703600	0.702479
chr8	117743500	0.909091
chr8	117792400	0.694215
chr8	117796300	0.661157
chr8	118032200	0.719008
chr8	118056900	0.743802
chr8	118241600	0.727273
chr8	118316800	0.752066
chr8	118342300	0.727273
chr8	118370500	0.942149
chr8	118473400	0.661157
chr8	118615900	0.719008
chr8	118725800	0.867769
chr8	118765100	0.752066
chr8	118825300	0.859504
chr8	118897000	0.669421
chr8	118930700	0.834711
chr8	118942800	0.851240
chr8	118946600	0.801653
chr8	118960000	0.669421
chr8	118971500	0.933884
chr8	118974700	0.694215
chr8	119059500	0.694215
chr8	119065700	0.768595
chr8	119084400	0.752066
chr8	119089200	0.694215
chr8	119092000	1.000000
chr8	119117200	0.793388
chr8	119119500	0.710744
chr8	119139400	0.801653
chr8	119158300	0.669421
chr8	119222600	0.719008
chr8	119262400	0.661157
chr8	119269800	0.884298
chr8	119320600	0.735537
chr8	119517800	0.702479
chr8	119558900	0.719008
chr8	119608800	0.719008
chr8	119879700	0.834711
chr8	119960500	0.727273
chr8	120578800	0.752066
chr8	120609600	0.892562
chr8	120615500	0.685950
chr8	120668200	0.661157
chr8	120870000	0.818182
chr8	120991000	0.735537

chr8	121060800	0.942149
chr8	121278900	0.768595
chr8	121752000	0.652893
chr8	121769800	0.768595
chr8	121798900	0.900826
chr8	121808900	0.884298
chr8	121816400	0.884298
chr8	121819200	0.702479
chr8	122142400	0.694215
chr8	122167800	0.966942
chr8	122199400	0.760331
chr8	122599100	0.876033
chr8	122831100	0.801653
chr8	123651400	0.702479
chr8	123801500	0.710744
chr8	123833100	0.702479
chr8	123850300	0.950413
chr8	123853600	0.685950
chr8	123912800	0.702479
chr8	123928100	0.727273
chr8	123954400	0.652893
chr8	124043200	0.785124
chr8	124087400	0.677686
chr8	124209600	0.809917
chr8	124248300	0.710744
chr8	124492200	1.000000
chr8	124500000	0.925620
chr8	124502300	0.933884
chr8	124767800	0.669421
chr8	124890600	0.752066
chr8	124955900	0.818182
chr8	125213000	0.669421
chr8	125275400	0.677686
chr8	125367800	0.669421
chr8	125449500	0.900826
chr8	125639800	0.652893
chr8	125700100	0.925620
chr8	125733100	0.867769
chr8	125861100	0.652893
chr8	125976800	0.983471
chr8	126132800	0.652893
chr8	126160500	0.818182
chr8	126189700	0.719008
chr8	126238100	0.917355
chr8	126240900	0.644628
chr8	126250000	0.900826
chr8	126266900	0.735537
chr8	126290600	0.801653
chr8	126399200	0.958678
chr8	126405000	0.785124
chr8	126428100	0.876033
chr8	126439400	0.892562
chr8	126447600	0.669421
chr8	126459000	0.900826

chr8	126618000	0.694215
chr8	126688200	0.652893
chr8	126713600	0.743802
chr8	126959500	0.793388
chr8	127385300	0.834711
chr8	127423000	0.900826
chr8	127435600	0.991736
chr8	127438800	0.942149
chr8	127461900	0.760331
chr8	127478900	0.859504
chr8	127535700	0.719008
chr8	127559700	0.785124
chr8	127584000	0.834711
chr8	127592500	0.892562
chr8	127623800	0.900826
chr8	127647800	0.900826
chr8	127833400	0.826446
chr8	127857300	0.809917
chr8	127908800	0.710744
chr8	127964300	0.776860
chr8	128047600	0.661157
chr8	128264400	0.735537
chr8	128493500	0.677686
chr8	128655900	0.851240
chr8	128753200	0.743802
chr8	128810600	0.925620
chr8	128818300	0.652893
chr8	128821400	0.834711
chr8	128824700	0.958678
chr8	128830800	0.983471
chr8	128834300	0.710744
chr8	128837600	0.710744
chr8	128840500	0.809917
chr8	128852600	0.644628
chr8	128855400	0.661157
chr8	128858300	0.942149
chr8	128861900	0.917355
chr8	128864200	0.818182
chr8	128867500	0.942149
chr8	128871600	0.752066
chr8	128873700	0.661157
chr8	128875800	0.900826
chr8	128880000	0.809917
chr8	128882700	0.669421
chr8	128886100	0.719008
chr8	128889100	0.752066
chr8	128897100	0.652893
chr8	128903300	0.809917
chr8	128906800	0.669421
chr8	128911600	0.917355
chr8	128917500	0.826446
chr8	128919800	0.694215
chr8	128922000	0.966942
chr8	128926200	0.867769

chr8	128931900	0.851240
chr8	128939000	0.768595
chr8	128954800	0.809917
chr8	128981900	0.975207
chr8	128999500	0.768595
chr8	129014300	0.644628
chr8	129035500	0.818182
chr8	129045900	0.743802
chr8	129050900	0.752066
chr8	129059300	0.743802
chr8	129075000	0.710744
chr8	129079500	0.735537
chr8	129089400	0.752066
chr8	129098700	0.743802
chr8	129103400	0.694215
chr8	129121700	0.867769
chr8	129326300	1.000000
chr8	129329500	0.661157
chr8	129374500	0.652893
chr8	129526800	0.677686
chr8	129697100	0.719008
chr8	129780900	0.644628
chr8	129867200	0.876033
chr8	129873600	0.793388
chr8	129886900	0.752066
chr8	129911100	0.677686
chr8	129988200	0.793388
chr8	129995900	0.892562
chr8	130090100	0.900826
chr8	130259000	0.644628
chr8	130316000	0.710744
chr8	130526100	0.834711
chr8	130528300	0.991736
chr8	130548300	0.768595
chr8	130694700	0.685950
chr8	130830800	0.768595
chr8	130839000	0.669421
chr8	130883300	0.933884
chr8	130946500	0.677686
chr8	130985600	0.710744
chr8	130998000	0.884298
chr8	131000800	0.702479
chr8	131009800	0.776860
chr8	131023100	0.801653
chr8	131041100	0.958678
chr8	131207500	0.768595
chr8	131216600	0.834711
chr8	131300100	0.710744
chr8	131310200	0.851240
chr8	131319000	0.801653
chr8	131326000	0.652893
chr8	131334700	0.719008
chr8	131339200	0.884298
chr8	131349400	0.942149

chr8	131357500	0.710744
chr8	131360100	0.851240
chr8	131380700	0.719008
chr8	131401900	0.826446
chr8	131429500	0.677686
chr8	131439100	0.685950
chr8	131457400	0.669421
chr8	131461300	0.950413
chr8	131516100	0.859504
chr8	131519100	0.685950
chr8	131531400	0.933884
chr8	131540900	0.735537
chr8	131662400	0.743802
chr8	131815300	0.785124
chr8	132045300	0.884298
chr8	132201200	0.661157
chr8	132404700	0.851240
chr8	132638100	0.735537
chr8	132790200	0.719008
chr8	132919000	0.768595
chr8	132948700	0.719008
chr8	132965300	0.975207
chr8	132968100	0.867769
chr8	132986600	1.000000
chr8	133455800	0.925620
chr8	133528500	0.661157
chr8	133700300	0.909091
chr8	133715800	0.900826
chr8	133724800	0.743802
chr8	133781200	0.727273
chr8	133976900	0.710744
chr8	134053100	0.801653
chr8	134060300	0.768595
chr8	134069100	0.644628
chr8	134084300	0.677686
chr8	134124900	0.818182
chr8	134246800	0.669421
chr8	134353900	0.677686
chr8	134446400	0.785124
chr8	134468100	0.834711
chr8	134515100	0.644628
chr8	134545600	0.925620
chr8	134570000	0.685950
chr8	134572400	0.694215
chr8	134581200	0.785124
chr8	134636800	0.702479
chr8	134646900	0.760331
chr8	134781900	0.975207
chr8	134863900	0.776860
chr8	134910200	0.677686
chr8	135533900	0.702479
chr8	135586500	0.669421
chr8	135698200	0.677686
chr8	135747400	0.752066

chr8	136181400	0.942149
chr8	136410000	0.652893
chr8	136475500	0.719008
chr8	136803100	0.785124
chr8	136955000	0.669421
chr8	137349400	0.785124
chr8	138040000	0.710744
chr8	138136000	0.842975
chr8	138332900	0.685950
chr8	139089200	0.826446
chr8	139093900	0.884298
chr8	139145000	0.644628
chr8	139207600	0.892562
chr8	139240800	0.768595
chr8	139357700	0.702479
chr8	139441900	0.644628
chr8	139594600	0.884298
chr8	139750000	0.735537
chr8	139977600	0.892562
chr8	140277800	0.933884
chr8	140325800	0.644628
chr8	140379500	0.694215
chr8	140418300	0.785124
chr8	140440900	0.859504
chr8	140583000	0.727273
chr8	140622000	0.652893
chr8	140689600	0.685950
chr8	140785400	0.661157
chr8	140834300	0.719008
chr8	140854300	0.867769
chr8	140970000	0.644628
chr8	141024600	0.785124
chr8	141062400	0.793388
chr8	141068700	0.752066
chr8	141072300	0.735537
chr8	141186700	0.859504
chr8	141207800	0.966942
chr8	141249200	0.785124
chr8	141272700	0.661157
chr8	141274900	0.776860
chr8	141324100	0.710744
chr8	141342200	0.966942
chr8	141358800	0.884298
chr8	141368500	0.793388
chr8	141435700	0.694215
chr8	141536700	0.644628
chr8	141600700	0.950413
chr8	141609900	0.942149
chr8	141627600	0.685950
chr8	141630200	0.851240
chr8	141637400	0.677686
chr8	141641000	0.818182
chr8	141648300	0.851240
chr8	141780900	0.694215

chr8	141784500	0.727273
chr8	141801400	0.735537
chr8	141855700	0.958678
chr8	141873500	0.975207
chr8	141891600	0.842975
chr8	141908800	0.826446
chr8	141973700	0.818182
chr8	141986300	0.644628
chr8	142002000	0.892562
chr8	142037100	0.933884
chr8	142045700	0.785124
chr8	142048600	0.809917
chr8	142052100	0.710744
chr8	142066000	0.867769
chr8	142083500	0.702479
chr8	142085700	0.867769
chr8	142094900	0.727273
chr8	142126200	0.867769
chr8	142230900	0.900826
chr8	142233000	0.743802
chr8	142236900	0.785124
chr8	142239000	0.735537
chr8	142245500	0.644628
chr8	142249400	0.661157
chr8	142292900	0.702479
chr8	142309800	0.760331
chr8	142362300	0.867769
chr8	142369400	0.917355
chr8	142385500	0.776860
chr8	142394700	0.842975
chr8	142412200	0.909091
chr8	142416500	0.752066
chr8	142425300	0.884298
chr8	142431100	0.892562
chr8	142441200	0.917355
chr8	142444100	0.917355
chr8	142446200	0.842975
chr8	142457000	0.694215
chr8	142461700	0.776860
chr8	142483000	0.826446
chr8	142518500	0.925620
chr8	142606600	0.892562
chr8	142626800	0.826446
chr8	142651900	0.669421
chr8	142672800	0.735537
chr8	142677000	0.719008
chr8	142692200	0.809917
chr8	142710800	0.735537
chr8	142734200	0.694215
chr8	142877700	0.925620
chr8	142927500	0.851240
chr8	142986800	0.694215
chr8	143058600	0.702479
chr8	143101400	0.933884

chr8	143112300	0.760331
chr8	143177900	0.776860
chr8	143210400	0.834711
chr8	143294900	0.776860
chr8	143409800	0.727273
chr8	143437900	0.793388
chr8	143450200	0.876033
chr8	143459100	0.735537
chr8	143477200	0.710744
chr8	143498600	0.933884
chr8	143537300	0.719008
chr8	143551700	0.719008
chr8	143554000	0.776860
chr8	143581100	0.661157
chr8	143627200	0.867769
chr8	143678900	0.776860
chr8	143681300	0.909091
chr8	143690500	0.991736
chr8	143694100	0.892562
chr8	143697900	0.818182
chr8	143947300	0.818182
chr8	144050000	0.677686
chr8	144055900	0.661157
chr8	144073400	0.884298
chr8	144106100	0.892562
chr8	144176300	0.727273
chr8	144368200	0.702479
chr8	144414500	0.661157
chr8	144421500	0.818182
chr8	144453900	0.818182
chr8	144466100	0.776860
chr8	144484300	0.760331
chr8	144487600	0.669421
chr8	144502800	0.644628
chr8	144505700	0.834711
chr8	144517700	0.842975
chr8	144549400	0.966942
chr8	144558400	0.727273
chr8	144576600	0.727273
chr8	144614600	0.727273
chr8	144632500	0.661157
chr8	144649600	0.644628
chr8	144764600	0.834711
chr8	144846200	0.735537
chr8	144905700	0.900826
chr8	144907800	0.925620
chr8	144935700	0.793388
chr8	144939100	0.702479
chr8	144948400	0.760331
chr8	145020100	0.900826
chr8	145024200	0.793388
chr8	145033300	0.809917
chr8	145069400	0.727273
chr8	145083100	0.677686

chr8	145234800	0.876033
chr8	145329500	0.818182
chr8	145539400	0.661157
chr8	145559000	0.719008
chr8	145567100	0.710744
chr8	145575100	0.793388
chr8	145596800	0.669421
chr8	145639100	0.776860
chr8	145648900	0.785124
chr8	145655500	0.677686
chr8	145709900	0.768595
chr8	145721800	0.760331
chr8	145865700	0.710744
chr8	145896900	0.702479
chr8	145917400	0.768595
chr8	145987100	0.892562
chr8	145989500	0.727273
chr8	146014500	0.975207
chr8	146022300	0.917355
chr9	36700	0.867769
chr9	43500	0.644628
chr9	331400	0.776860
chr9	371300	0.677686
chr9	390100	0.719008
chr9	472800	0.661157
chr9	558500	0.859504
chr9	614400	0.710744
chr9	650400	0.933884
chr9	653700	0.859504
chr9	656200	0.793388
chr9	673900	0.677686
chr9	684500	0.925620
chr9	698800	0.661157
chr9	708600	0.719008
chr9	729400	0.834711
chr9	847700	0.809917
chr9	899700	0.669421
chr9	909000	0.826446
chr9	967900	0.710744
chr9	981800	0.644628
chr9	1775100	0.950413
chr9	1951500	0.768595
chr9	1995400	0.760331
chr9	2019900	0.685950
chr9	2157400	0.710744
chr9	2159700	0.958678
chr9	2180600	0.991736
chr9	2182700	0.702479
chr9	2195300	0.710744
chr9	2275600	0.752066
chr9	2281300	0.958678
chr9	2284400	0.859504
chr9	2360200	0.694215
chr9	2418900	0.644628

chr9	2613800	0.925620
chr9	2627000	0.685950
chr9	2632400	0.677686
chr9	2701000	0.909091
chr9	2760500	0.702479
chr9	2765700	0.702479
chr9	2809400	0.801653
chr9	2813400	0.975207
chr9	2816400	0.859504
chr9	2818600	0.710744
chr9	2857700	0.677686
chr9	3211500	0.661157
chr9	3241700	0.752066
chr9	3367500	0.760331
chr9	3402100	0.727273
chr9	3509200	0.752066
chr9	3742500	0.727273
chr9	3751500	0.694215
chr9	3874900	0.942149
chr9	3955000	0.809917
chr9	4035400	0.719008
chr9	4238300	0.735537
chr9	4514900	0.909091
chr9	4617800	0.760331
chr9	4621000	0.768595
chr9	4664900	0.661157
chr9	4671000	0.694215
chr9	4763500	0.834711
chr9	4798200	0.834711
chr9	4864800	0.661157
chr9	4902100	0.719008
chr9	4927500	0.818182
chr9	5096500	0.652893
chr9	5269400	0.768595
chr9	5327300	0.710744
chr9	5417600	0.685950
chr9	5519400	0.859504
chr9	5544700	0.652893
chr9	6079500	0.760331
chr9	6107100	0.685950
chr9	6172700	0.809917
chr9	6419000	0.685950
chr9	6595200	0.652893
chr9	6633400	0.818182
chr9	6648100	0.842975
chr9	6693100	0.983471
chr9	6765300	0.743802
chr9	6777500	0.975207
chr9	6785400	0.826446
chr9	6788900	0.867769
chr9	6819300	0.867769
chr9	6870000	0.785124
chr9	6902400	0.826446
chr9	6908600	0.768595

chr9	6955500	0.785124
chr9	7053400	0.809917
chr9	7103800	0.677686
chr9	7129800	0.702479
chr9	7212000	0.942149
chr9	7235200	0.652893
chr9	7240900	0.991736
chr9	7284800	0.669421
chr9	7543000	0.859504
chr9	7880200	0.727273
chr9	7967900	0.694215
chr9	8140200	0.867769
chr9	8395500	0.644628
chr9	8504200	0.702479
chr9	8678600	0.661157
chr9	8693700	0.867769
chr9	8716400	0.685950
chr9	8719200	0.842975
chr9	8734000	0.950413
chr9	8751500	0.842975
chr9	8843000	0.842975
chr9	9521200	0.735537
chr9	9557700	0.983471
chr9	10134400	0.809917
chr9	10339200	0.809917
chr9	10372600	0.694215
chr9	10446800	0.694215
chr9	10922100	0.826446
chr9	11915900	0.702479
chr9	12054800	0.867769
chr9	12718900	0.842975
chr9	13034600	0.785124
chr9	13143800	0.669421
chr9	13163900	0.727273
chr9	13428700	0.776860
chr9	13432000	0.727273
chr9	13448400	0.801653
chr9	13490700	0.818182
chr9	13514100	0.925620
chr9	13578000	0.760331
chr9	13587500	0.669421
chr9	13596800	0.694215
chr9	13608400	0.644628
chr9	13688900	0.925620
chr9	13860900	0.818182
chr9	13900600	0.685950
chr9	14096300	0.793388
chr9	14107700	0.652893
chr9	14192900	0.785124
chr9	14232100	0.884298
chr9	14238400	0.892562
chr9	14271300	0.644628
chr9	14274500	0.909091
chr9	14319100	0.801653

chr9	14341600	0.661157
chr9	14354000	0.677686
chr9	14373300	0.685950
chr9	14422200	0.917355
chr9	14424600	0.950413
chr9	14466800	0.975207
chr9	14479500	0.661157
chr9	14515900	0.702479
chr9	14532100	0.727273
chr9	14542400	0.785124
chr9	14547600	1.000000
chr9	14562100	0.793388
chr9	14722600	0.826446
chr9	14750300	0.727273
chr9	14847100	0.710744
chr9	14889600	0.752066
chr9	14907400	0.876033
chr9	14939700	0.652893
chr9	15116300	0.793388
chr9	15135500	0.644628
chr9	15138500	1.000000
chr9	15244600	0.644628
chr9	15247100	0.719008
chr9	15250000	0.818182
chr9	15274500	0.677686
chr9	15347000	0.727273
chr9	15514000	0.884298
chr9	16031900	0.793388
chr9	16058300	0.661157
chr9	16061000	0.818182
chr9	16155000	0.710744
chr9	16193000	0.652893
chr9	16243800	0.661157
chr9	16253900	0.677686
chr9	16310700	0.785124
chr9	16432700	0.801653
chr9	16459500	0.851240
chr9	16486300	0.942149
chr9	16488600	0.884298
chr9	16491400	0.685950
chr9	16505800	0.694215
chr9	16516000	0.983471
chr9	16519800	0.743802
chr9	16569500	0.669421
chr9	16594800	0.768595
chr9	16602600	0.677686
chr9	16609400	0.644628
chr9	16653800	0.727273
chr9	16656600	0.826446
chr9	16679500	0.669421
chr9	16705300	0.892562
chr9	16711000	0.876033
chr9	16713900	0.991736
chr9	16718500	0.859504

chr9	16733500	0.661157
chr9	16769500	0.884298
chr9	16787400	0.710744
chr9	16795900	0.826446
chr9	16804100	0.669421
chr9	16807300	0.669421
chr9	16809500	0.909091
chr9	16812600	0.884298
chr9	16817400	0.719008
chr9	16825200	0.801653
chr9	16829300	0.760331
chr9	16848700	0.661157
chr9	16865000	0.661157
chr9	16867900	0.661157
chr9	16874000	0.710744
chr9	17082400	0.727273
chr9	17204900	0.793388
chr9	17230500	0.925620
chr9	17395100	0.917355
chr9	17397700	0.735537
chr9	18036900	0.809917
chr9	18106200	0.719008
chr9	18130200	0.727273
chr9	18212900	0.694215
chr9	18305700	0.809917
chr9	18379600	0.727273
chr9	18605900	0.752066
chr9	18669300	0.719008
chr9	18806000	0.652893
chr9	18911900	0.867769
chr9	19414200	0.818182
chr9	19424500	0.644628
chr9	19446900	0.991736
chr9	19457300	0.768595
chr9	19472400	0.719008
chr9	19656800	0.652893
chr9	19700000	0.743802
chr9	19741800	0.933884
chr9	19800700	0.933884
chr9	19805100	0.826446
chr9	19808000	1.000000
chr9	19926800	0.644628
chr9	19968400	0.950413
chr9	20111700	0.884298
chr9	20190100	0.925620
chr9	20301500	0.884298
chr9	20308300	0.727273
chr9	20340300	0.694215
chr9	20356900	0.809917
chr9	20397700	0.644628
chr9	20408800	0.851240
chr9	20635100	0.669421
chr9	20665300	0.644628
chr9	20782900	0.685950

chr9	20873400	0.735537
chr9	20949000	0.909091
chr9	21251600	0.776860
chr9	21382100	0.727273
chr9	21549300	0.644628
chr9	21922000	0.669421
chr9	21971300	0.710744
chr9	21990300	0.727273
chr9	22102800	0.752066
chr9	22118000	0.743802
chr9	22431000	0.760331
chr9	22484000	0.818182
chr9	22970000	0.892562
chr9	23089300	0.685950
chr9	23230300	0.677686
chr9	23556400	0.818182
chr9	23572300	0.644628
chr9	23660100	0.859504
chr9	23683600	0.710744
chr9	23686000	0.851240
chr9	23725600	0.727273
chr9	23737900	0.652893
chr9	23741000	0.950413
chr9	23743100	0.727273
chr9	23764000	0.735537
chr9	23769600	0.900826
chr9	23773500	0.776860
chr9	23776300	0.768595
chr9	23779700	0.776860
chr9	23809300	0.652893
chr9	23813900	0.809917
chr9	23818400	0.867769
chr9	23842400	0.710744
chr9	23934400	0.661157
chr9	24073600	0.785124
chr9	24293500	0.867769
chr9	24463000	0.669421
chr9	24487900	0.793388
chr9	24498100	0.958678
chr9	24500200	0.702479
chr9	24503000	0.785124
chr9	24597300	0.677686
chr9	25205700	0.661157
chr9	25226900	0.661157
chr9	25397100	0.958678
chr9	25602900	0.760331
chr9	25608600	0.818182
chr9	25716600	0.793388
chr9	26026900	0.983471
chr9	26604000	0.801653
chr9	26746900	0.983471
chr9	26755100	0.809917
chr9	26936800	0.859504
chr9	26974700	0.768595

chr9	26977600	0.793388
chr9	27111000	0.719008
chr9	27309100	0.950413
chr9	27334000	0.909091
chr9	27341600	0.743802
chr9	27354100	0.652893
chr9	27366900	0.752066
chr9	27369800	0.859504
chr9	27386800	0.768595
chr9	27395700	0.809917
chr9	27399800	0.809917
chr9	27421900	0.661157
chr9	27431200	0.826446
chr9	27457600	0.900826
chr9	27489500	0.983471
chr9	27496000	0.644628
chr9	27499000	0.710744
chr9	27536600	0.809917
chr9	27645500	0.793388
chr9	27665600	0.743802
chr9	27671700	0.917355
chr9	27750100	0.975207
chr9	27772200	0.991736
chr9	27944000	0.785124
chr9	28032300	0.719008
chr9	28365700	0.859504
chr9	28650700	0.685950
chr9	28688700	0.809917
chr9	28762800	0.644628
chr9	28804800	0.661157
chr9	28820600	0.793388
chr9	28957500	0.719008
chr9	28972100	0.950413
chr9	29161400	0.752066
chr9	29180600	0.702479
chr9	29326100	0.694215
chr9	29514600	0.652893
chr9	29715300	0.694215
chr9	29807600	0.685950
chr9	30362700	0.727273
chr9	30762200	0.652893
chr9	30816200	0.669421
chr9	30913600	0.917355
chr9	31030900	0.818182
chr9	31034800	0.752066
chr9	31737100	0.876033
chr9	31917000	0.644628
chr9	32297300	0.719008
chr9	32343800	0.966942
chr9	32346400	0.966942
chr9	32352200	0.644628
chr9	32509200	0.661157
chr9	33014600	0.950413
chr9	33373300	0.851240

chr9	33375700	0.900826
chr9	33381400	0.867769
chr9	33391400	0.685950
chr9	33443900	0.859504
chr9	33453400	0.685950
chr9	33580200	0.727273
chr9	33658900	0.760331
chr9	33722500	0.867769
chr9	33796500	0.884298
chr9	33850400	0.735537
chr9	33881200	0.719008
chr9	33925900	0.752066
chr9	33934900	0.859504
chr9	34072800	0.876033
chr9	34151900	0.644628
chr9	34198600	0.925620
chr9	34334700	0.884298
chr9	34364200	0.760331
chr9	34443900	0.685950
chr9	34451800	0.735537
chr9	34513800	0.785124
chr9	34555200	0.768595
chr9	34560300	0.909091
chr9	34566100	0.669421
chr9	34568800	0.727273
chr9	34580500	0.809917
chr9	34586200	0.851240
chr9	34593300	0.694215
chr9	34624800	0.685950
chr9	34645200	0.900826
chr9	34653900	0.752066
chr9	34687600	0.727273
chr9	34985400	0.669421
chr9	34993600	0.884298
chr9	35027300	0.702479
chr9	35074000	0.669421
chr9	35105200	0.752066
chr9	35156100	0.652893
chr9	35518600	0.776860
chr9	35599900	0.760331
chr9	35651000	0.925620
chr9	35729800	0.752066
chr9	35775700	0.892562
chr9	35841200	0.677686
chr9	35844300	0.892562
chr9	35869200	0.677686
chr9	35882700	0.719008
chr9	35908200	0.900826
chr9	35916100	0.694215
chr9	36092800	0.776860
chr9	36100300	0.768595
chr9	36163100	0.975207
chr9	36194300	0.719008
chr9	36292700	0.867769

chr9	36381700	0.727273
chr9	36445900	0.669421
chr9	36490100	0.661157
chr9	36560100	0.694215
chr9	36726300	0.793388
chr9	36773200	0.801653
chr9	36820600	0.652893
chr9	36865000	0.818182
chr9	36913900	0.661157
chr9	36939200	0.818182
chr9	36950500	0.669421
chr9	36980800	0.900826
chr9	36996600	0.768595
chr9	37000300	0.644628
chr9	37005200	0.702479
chr9	37040900	0.768595
chr9	37054600	0.876033
chr9	37100800	0.727273
chr9	37191700	0.842975
chr9	37251600	0.991736
chr9	37260600	0.702479
chr9	37337500	0.785124
chr9	37340500	0.727273
chr9	37415400	1.000000
chr9	37425200	0.727273
chr9	37434200	0.884298
chr9	37472400	0.958678
chr9	37559400	0.661157
chr9	37566300	0.851240
chr9	37573900	0.834711
chr9	37624400	0.983471
chr9	37668800	0.743802
chr9	37731500	0.867769
chr9	37920200	0.644628
chr9	37928800	0.719008
chr9	37944200	0.834711
chr9	37946400	0.652893
chr9	37998100	0.727273
chr9	38007400	0.892562
chr9	38014800	0.876033
chr9	38018400	0.834711
chr9	38020700	0.685950
chr9	38024400	0.768595
chr9	38027800	0.876033
chr9	38037600	0.644628
chr9	38040000	0.727273
chr9	38043000	0.710744
chr9	38048000	0.867769
chr9	38050600	0.743802
chr9	38058100	0.776860
chr9	38063200	0.892562
chr9	38065600	0.727273
chr9	38083300	0.702479
chr9	38172700	0.776860

chr9	38187000	0.644628
chr9	38203200	0.743802
chr9	38227800	0.809917
chr9	38278300	0.677686
chr9	38284800	0.826446
chr9	38310600	0.801653
chr9	38347000	0.801653
chr9	38380300	0.661157
chr9	38415000	0.727273
chr9	38421800	0.702479
chr9	38484300	0.975207
chr9	38487000	0.768595
chr9	38555900	0.876033
chr9	38564400	0.876033
chr9	38672500	0.809917
chr9	38685600	0.818182
chr9	38687800	0.942149
chr9	38690500	0.884298
chr9	38761800	0.818182
chr9	39171000	0.694215
chr9	39552500	0.900826
chr9	39567400	0.801653
chr9	39571200	0.768595
chr9	39573700	1.000000
chr9	39604700	0.752066
chr9	39900500	0.735537
chr9	40192100	0.694215
chr9	40556500	0.842975
chr9	40880600	0.884298
chr9	40895400	0.702479
chr9	40898500	0.719008
chr9	40901500	0.950413
chr9	41110300	0.677686
chr9	41131700	0.859504
chr9	41203600	0.909091
chr9	41312200	0.669421
chr9	41697400	0.818182
chr9	41712100	0.719008
chr9	41715500	0.719008
chr9	41718700	0.983471
chr9	41749800	0.760331
chr9	41919600	0.702479
chr9	42081200	0.644628
chr9	42281400	0.694215
chr9	42416300	0.809917
chr9	42819300	0.950413
chr9	43054700	0.710744
chr9	43456500	0.958678
chr9	43484000	0.760331
chr9	43487800	0.776860
chr9	43491000	0.644628
chr9	43506600	0.876033
chr9	43823300	0.801653
chr9	44070900	0.768595

chr9	44177100	0.719008
chr9	44210300	0.669421
chr9	44420100	0.669421
chr9	44463100	0.785124
chr9	44615100	0.710744
chr9	44640600	0.809917
chr9	44644000	0.644628
chr9	44648200	0.735537
chr9	44662300	0.909091
chr9	44859400	0.710744
chr9	45004500	0.785124
chr9	45352700	0.793388
chr9	45362600	0.694215
chr9	45737700	0.727273
chr9	46123600	0.942149
chr9	46272900	0.818182
chr9	47049300	0.694215
chr9	47168200	0.776860
chr9	47304800	0.727273
chr9	65494600	0.644628
chr9	65657000	0.644628
chr9	66129100	0.933884
chr9	66150100	0.917355
chr9	66500900	0.669421
chr9	66518900	0.685950
chr9	66530900	0.958678
chr9	66800000	0.785124
chr9	66812700	0.702479
chr9	66822600	0.661157
chr9	66971400	0.876033
chr9	67018900	0.801653
chr9	67334600	0.851240
chr9	67780400	0.851240
chr9	67974600	0.644628
chr9	69711300	0.942149
chr9	70002400	0.743802
chr9	70010300	0.727273
chr9	70077000	0.685950
chr9	70717700	0.702479
chr9	71178200	0.735537
chr9	71283300	0.727273
chr9	71371000	0.958678
chr9	71487500	0.975207
chr9	71490400	0.710744
chr9	71514200	0.768595
chr9	71562100	0.991736
chr9	71565600	0.818182
chr9	71597100	0.735537
chr9	71614300	0.652893
chr9	71639100	0.743802
chr9	71660700	0.793388
chr9	71815500	0.719008
chr9	71819700	0.661157
chr9	71842900	0.694215

chr9	71847300	0.900826
chr9	71923300	0.933884
chr9	71947300	0.768595
chr9	72028000	0.851240
chr9	72091700	0.652893
chr9	72282900	0.776860
chr9	72359700	0.661157
chr9	72361900	0.826446
chr9	72377300	0.842975
chr9	72379800	0.917355
chr9	72437700	0.884298
chr9	72441000	0.942149
chr9	72579700	0.743802
chr9	72653400	0.661157
chr9	72675900	0.867769
chr9	72725800	0.851240
chr9	72730700	0.677686
chr9	73021900	0.727273
chr9	73095200	0.884298
chr9	73148400	0.669421
chr9	73161700	0.652893
chr9	73169400	0.958678
chr9	73182300	0.661157
chr9	73217800	0.685950
chr9	73345800	0.760331
chr9	73547400	0.702479
chr9	73556100	0.644628
chr9	73731000	0.661157
chr9	73733700	0.661157
chr9	73739100	0.719008
chr9	73783900	0.661157
chr9	73859500	0.942149
chr9	73861700	0.644628
chr9	73865600	0.892562
chr9	73948700	0.694215
chr9	73979000	0.842975
chr9	73983100	0.727273
chr9	73998800	0.719008
chr9	74134200	0.942149
chr9	74167900	0.710744
chr9	74223800	0.785124
chr9	74278700	0.933884
chr9	74299900	0.727273
chr9	74360400	0.644628
chr9	74373600	0.661157
chr9	74376600	0.768595
chr9	74422100	0.793388
chr9	74516800	0.925620
chr9	74519400	0.851240
chr9	74535900	0.975207
chr9	74540400	0.834711
chr9	74568500	0.933884
chr9	74582000	0.933884
chr9	74584200	0.652893

chr9	74600300	0.710744
chr9	74640200	0.801653
chr9	74930800	0.867769
chr9	75070400	0.983471
chr9	75142600	0.876033
chr9	75295600	0.909091
chr9	75297900	0.859504
chr9	75666200	0.851240
chr9	75767400	0.925620
chr9	75830400	0.983471
chr9	76057400	0.859504
chr9	76461600	0.991736
chr9	76694900	0.826446
chr9	76699100	0.917355
chr9	76706800	0.851240
chr9	76995100	0.644628
chr9	77102500	0.719008
chr9	77107400	0.644628
chr9	77109500	0.719008
chr9	77139000	0.661157
chr9	77245300	0.719008
chr9	77288900	0.669421
chr9	77539100	0.818182
chr9	77699000	0.826446
chr9	77974400	1.000000
chr9	77999300	0.652893
chr9	78029700	0.652893
chr9	78075900	0.876033
chr9	78086400	0.909091
chr9	78455900	0.727273
chr9	78510100	0.669421
chr9	78541600	0.809917
chr9	78589500	0.834711
chr9	78653200	0.809917
chr9	78660000	0.776860
chr9	78751900	0.652893
chr9	78913200	0.752066
chr9	79048200	0.685950
chr9	79207200	0.719008
chr9	79210700	0.851240
chr9	79258600	0.859504
chr9	79306900	0.785124
chr9	79418700	0.867769
chr9	79488300	0.752066
chr9	79502900	0.834711
chr9	79517100	0.644628
chr9	79643600	0.694215
chr9	79707700	0.859504
chr9	79714400	0.685950
chr9	79743200	0.892562
chr9	79758600	0.702479
chr9	79781800	0.785124
chr9	79795500	0.677686
chr9	80055400	0.851240

chr9	80076000	0.652893
chr9	80149500	0.925620
chr9	80158800	0.776860
chr9	80200200	0.983471
chr9	80217200	0.677686
chr9	80332900	1.000000
chr9	80335900	0.669421
chr9	80350900	0.702479
chr9	80355200	0.677686
chr9	80499700	0.785124
chr9	80572200	0.884298
chr9	80576600	0.710744
chr9	80636600	0.669421
chr9	80639000	0.900826
chr9	80641500	0.685950
chr9	80939600	0.743802
chr9	80980100	0.719008
chr9	81009000	0.752066
chr9	81012600	0.834711
chr9	81021800	0.776860
chr9	81053700	0.818182
chr9	81117700	0.752066
chr9	81158500	0.876033
chr9	81186000	0.685950
chr9	81256000	0.702479
chr9	81585400	0.661157
chr9	81587700	0.983471
chr9	81600200	0.892562
chr9	81823800	0.719008
chr9	81855900	0.776860
chr9	81917600	0.652893
chr9	81967600	0.834711
chr9	81975400	0.793388
chr9	81983000	0.818182
chr9	82011500	0.776860
chr9	82024800	0.735537
chr9	82199100	0.842975
chr9	82225600	0.669421
chr9	82232100	0.644628
chr9	82266900	0.661157
chr9	82276900	0.867769
chr9	82279100	0.685950
chr9	82286700	0.685950
chr9	82307200	0.702479
chr9	82309800	0.768595
chr9	82433400	0.685950
chr9	82449800	0.768595
chr9	82552200	0.652893
chr9	82578000	0.867769
chr9	83016100	0.983471
chr9	83025200	0.884298
chr9	83064700	0.727273
chr9	83497100	0.933884
chr9	83727700	0.834711

chr9	83987100	0.677686
chr9	83990200	0.652893
chr9	84162200	0.785124
chr9	84164900	0.917355
chr9	84207900	0.652893
chr9	84211600	0.859504
chr9	84214100	0.719008
chr9	84216700	0.842975
chr9	84237100	0.950413
chr9	84255600	0.694215
chr9	84262300	0.884298
chr9	84265600	0.652893
chr9	84274900	0.719008
chr9	84280400	0.983471
chr9	84296000	0.669421
chr9	84309600	0.785124
chr9	84333300	0.768595
chr9	84362600	0.685950
chr9	84435100	0.867769
chr9	84697900	0.826446
chr9	84786100	0.818182
chr9	84881500	0.785124
chr9	85345400	0.710744
chr9	85350000	0.669421
chr9	85365400	0.652893
chr9	85497600	0.884298
chr9	85563200	0.652893
chr9	85814000	0.661157
chr9	85851100	0.669421
chr9	86047100	0.752066
chr9	86236200	0.694215
chr9	86246500	0.685950
chr9	86262700	0.652893
chr9	86766700	0.752066
chr9	86892400	0.785124
chr9	86946600	0.644628
chr9	86955400	0.752066
chr9	87188600	0.644628
chr9	87323800	0.735537
chr9	87344100	0.644628
chr9	87426600	0.892562
chr9	87434500	0.776860
chr9	87467100	0.966942
chr9	87478400	0.710744
chr9	87552400	0.760331
chr9	87560500	0.661157
chr9	87617500	0.851240
chr9	87620400	0.652893
chr9	87772100	0.685950
chr9	87782400	0.727273
chr9	87828800	0.710744
chr9	87883200	0.677686
chr9	88036300	0.768595
chr9	88082400	0.644628

chr9	88216100	0.859504
chr9	88338400	0.826446
chr9	88359300	0.867769
chr9	88493700	0.785124
chr9	88651000	0.710744
chr9	88678600	0.793388
chr9	88738000	0.834711
chr9	88742000	0.818182
chr9	88796500	0.925620
chr9	88801900	0.818182
chr9	88902600	0.983471
chr9	88918700	0.685950
chr9	88942900	0.809917
chr9	89050000	0.644628
chr9	89193500	0.710744
chr9	89410600	0.842975
chr9	89416300	0.652893
chr9	89509100	0.909091
chr9	89555900	0.661157
chr9	89558400	0.677686
chr9	89763500	0.768595
chr9	89814800	0.661157
chr9	90005900	0.669421
chr9	90008500	0.809917
chr9	90012700	0.768595
chr9	90018800	0.669421
chr9	90021300	0.958678
chr9	90024700	0.867769
chr9	90050200	0.694215
chr9	90106500	0.677686
chr9	90131700	0.785124
chr9	90134600	0.818182
chr9	90140200	0.710744
chr9	90145900	0.743802
chr9	90167100	0.867769
chr9	90183300	0.752066
chr9	90187100	0.826446
chr9	90211200	0.876033
chr9	90216100	0.900826
chr9	90224200	0.809917
chr9	90230700	0.760331
chr9	90259400	0.859504
chr9	90282200	0.826446
chr9	90300700	0.685950
chr9	90380000	0.719008
chr9	90470800	0.685950
chr9	90490000	0.661157
chr9	90498300	0.727273
chr9	90622200	0.669421
chr9	90640700	0.752066
chr9	90660700	0.851240
chr9	90665600	0.677686
chr9	90693200	0.793388
chr9	90776300	0.842975

chr9	90889600	0.677686
chr9	90893300	0.752066
chr9	90896300	0.685950
chr9	90928700	0.958678
chr9	90994300	0.727273
chr9	91014800	0.743802
chr9	91033500	1.000000
chr9	91036500	0.917355
chr9	91058800	0.892562
chr9	91076100	0.743802
chr9	91127200	0.661157
chr9	91166700	0.818182
chr9	91276000	0.710744
chr9	91362100	0.776860
chr9	91434000	0.727273
chr9	91623700	0.685950
chr9	91627600	0.694215
chr9	92028200	0.942149
chr9	92045000	0.826446
chr9	92048600	0.876033
chr9	92052700	0.859504
chr9	92055600	0.933884
chr9	92074800	0.760331
chr9	92080400	0.760331
chr9	92096900	0.727273
chr9	92110200	0.809917
chr9	92114300	0.702479
chr9	92159500	0.809917
chr9	92208300	0.826446
chr9	92234600	0.727273
chr9	92290200	0.752066
chr9	92293400	0.966942
chr9	92310400	0.942149
chr9	92316600	0.727273
chr9	92445600	0.661157
chr9	92450900	0.694215
chr9	92455700	0.735537
chr9	92506600	0.752066
chr9	92509900	0.644628
chr9	92756800	0.834711
chr9	92789400	0.685950
chr9	92796900	0.685950
chr9	92824400	0.719008
chr9	92929300	0.768595
chr9	93167800	0.677686
chr9	93546300	0.925620
chr9	93652400	0.735537
chr9	93658500	0.818182
chr9	93727400	0.760331
chr9	93730100	0.818182
chr9	93852200	0.743802
chr9	93880900	0.842975
chr9	93922600	0.727273
chr9	93925200	0.710744

chr9	93961200	0.694215
chr9	94079000	0.710744
chr9	94106700	0.834711
chr9	94109300	0.677686
chr9	94321700	0.818182
chr9	94327000	0.719008
chr9	94335800	0.661157
chr9	94456300	0.743802
chr9	94533200	0.677686
chr9	94538100	0.677686
chr9	94620200	0.652893
chr9	94647500	0.661157
chr9	94662000	0.876033
chr9	94665000	0.884298
chr9	94683600	0.702479
chr9	94691100	0.644628
chr9	94702600	0.842975
chr9	94708000	0.785124
chr9	94811800	0.933884
chr9	94818200	0.991736
chr9	94896900	0.793388
chr9	94901000	0.702479
chr9	94909800	0.785124
chr9	95141700	0.644628
chr9	95243400	0.685950
chr9	95352700	0.669421
chr9	95422700	0.809917
chr9	95427200	0.884298
chr9	95442500	0.735537
chr9	95518800	0.661157
chr9	95523600	0.727273
chr9	95533100	0.710744
chr9	95645200	0.727273
chr9	95675000	0.727273
chr9	95732900	0.983471
chr9	95751100	0.876033
chr9	95755300	0.876033
chr9	95815900	0.669421
chr9	95819600	0.661157
chr9	95825100	0.661157
chr9	95857200	0.735537
chr9	95879000	0.743802
chr9	95906700	0.876033
chr9	95922600	0.702479
chr9	95961300	0.776860
chr9	95966500	0.694215
chr9	96024400	0.677686
chr9	96106100	0.727273
chr9	96116300	0.842975
chr9	96223400	0.677686
chr9	96226700	0.768595
chr9	96229500	0.892562
chr9	96362400	0.909091
chr9	96381100	0.719008

chr9	96521400	0.644628
chr9	96572700	0.685950
chr9	96576500	0.851240
chr9	96626900	0.735537
chr9	96632100	0.933884
chr9	96675100	0.942149
chr9	96705400	0.785124
chr9	96709900	0.677686
chr9	96715600	0.760331
chr9	96720700	0.669421
chr9	96723200	0.942149
chr9	96765100	0.702479
chr9	96847600	0.669421
chr9	96857900	0.694215
chr9	96865700	0.710744
chr9	96911200	0.661157
chr9	96913600	0.694215
chr9	96975200	0.776860
chr9	97091400	0.669421
chr9	97149600	0.975207
chr9	97332200	0.661157
chr9	97366000	0.842975
chr9	97370400	0.900826
chr9	97393600	0.867769
chr9	97587600	0.793388
chr9	97664300	0.793388
chr9	97676800	0.859504
chr9	97679800	0.652893
chr9	97710900	0.702479
chr9	97743400	0.644628
chr9	97820200	0.842975
chr9	97825300	0.834711
chr9	97840100	0.661157
chr9	97843200	0.727273
chr9	97854800	0.818182
chr9	97893800	0.834711
chr9	97933700	0.760331
chr9	97945900	0.892562
chr9	97977900	0.785124
chr9	98197000	0.735537
chr9	98210700	0.818182
chr9	98213300	0.752066
chr9	98225500	0.892562
chr9	98243700	0.760331
chr9	98260200	0.727273
chr9	98276500	0.801653
chr9	98291500	0.776860
chr9	98333400	0.710744
chr9	98344100	0.876033
chr9	98346900	0.694215
chr9	98368200	0.826446
chr9	98429000	0.776860
chr9	98517500	0.933884
chr9	98526900	0.884298

chr9	98529300	0.776860
chr9	98534900	0.677686
chr9	98582000	0.842975
chr9	98687900	0.719008
chr9	98699200	0.719008
chr9	98810100	0.669421
chr9	98850100	0.909091
chr9	98888700	0.735537
chr9	98892000	0.793388
chr9	98899900	0.727273
chr9	98903100	0.834711
chr9	98932100	0.975207
chr9	98949500	0.900826
chr9	98963900	0.760331
chr9	98977700	0.661157
chr9	98980700	0.677686
chr9	99018900	0.776860
chr9	99021600	0.677686
chr9	99083800	0.884298
chr9	99106200	0.983471
chr9	99157900	0.842975
chr9	99161500	0.760331
chr9	99183400	0.727273
chr9	99222800	0.892562
chr9	99264800	0.809917
chr9	99318000	0.652893
chr9	99367700	0.719008
chr9	99375300	0.793388
chr9	99622100	0.785124
chr9	99691400	0.677686
chr9	99693900	0.735537
chr9	99772200	0.735537
chr9	99808700	0.826446
chr9	99813400	0.735537
chr9	99816900	0.867769
chr9	99836200	0.991736
chr9	99850700	0.644628
chr9	99909000	0.719008
chr9	99931100	0.710744
chr9	99976000	0.669421
chr9	100034400	0.876033
chr9	100039400	0.884298
chr9	100069700	0.867769
chr9	100125800	0.652893
chr9	100150300	0.785124
chr9	100161500	0.644628
chr9	100202600	0.669421
chr9	100290800	0.892562
chr9	100306100	0.661157
chr9	100327300	0.776860
chr9	100361000	0.644628
chr9	100474000	0.966942
chr9	100534800	0.925620
chr9	100572400	0.669421

chr9	100594100	0.867769
chr9	100617800	0.727273
chr9	100631700	0.727273
chr9	100653000	0.818182
chr9	100849200	0.842975
chr9	100854700	0.702479
chr9	100895900	0.876033
chr9	100908800	0.644628
chr9	100931300	0.652893
chr9	100934400	0.677686
chr9	100937700	0.752066
chr9	100962500	0.834711
chr9	100981500	1.000000
chr9	100983600	0.652893
chr9	101077600	0.727273
chr9	101143000	0.661157
chr9	101172900	0.644628
chr9	101175900	0.752066
chr9	101266400	0.851240
chr9	101359700	0.702479
chr9	101441000	0.900826
chr9	101554100	0.752066
chr9	101565000	0.793388
chr9	101572700	0.776860
chr9	101601200	0.669421
chr9	101725500	0.925620
chr9	101733700	0.785124
chr9	101737600	0.776860
chr9	101741200	0.801653
chr9	101859300	0.727273
chr9	101875000	0.644628
chr9	101932500	0.809917
chr9	101935600	0.710744
chr9	102021800	0.661157
chr9	102060200	0.710744
chr9	102250800	0.966942
chr9	102368600	0.702479
chr9	102485800	0.900826
chr9	102547600	0.809917
chr9	102573900	0.818182
chr9	102657600	0.669421
chr9	102683000	0.644628
chr9	102730100	0.809917
chr9	102826200	0.727273
chr9	102846400	0.669421
chr9	102902800	0.685950
chr9	102913100	0.702479
chr9	103255400	0.727273
chr9	103358100	0.669421
chr9	103401900	0.867769
chr9	103433800	0.644628
chr9	103464400	0.694215
chr9	103817400	0.685950
chr9	103835800	0.661157

chr9	103850600	0.743802
chr9	103859200	0.644628
chr9	103863200	0.735537
chr9	103877100	0.876033
chr9	103881900	0.710744
chr9	103893800	0.685950
chr9	103910500	0.768595
chr9	103993500	0.652893
chr9	103997400	0.801653
chr9	104032600	0.818182
chr9	104088800	0.685950
chr9	104225000	0.892562
chr9	104276700	0.892562
chr9	104286800	0.826446
chr9	104346000	0.776860
chr9	105405400	0.685950
chr9	105800800	0.661157
chr9	106008000	0.743802
chr9	106463200	0.685950
chr9	106477500	0.752066
chr9	106753600	0.694215
chr9	107017600	0.694215
chr9	107626600	0.719008
chr9	107631700	0.933884
chr9	107648800	0.925620
chr9	107652400	0.793388
chr9	107666100	0.719008
chr9	107696800	0.702479
chr9	107724000	0.958678
chr9	107826800	0.735537
chr9	107878800	0.652893
chr9	107901200	0.900826
chr9	107903400	0.793388
chr9	107936300	0.818182
chr9	108004500	0.867769
chr9	108050700	0.677686
chr9	108054400	0.826446
chr9	108057200	0.867769
chr9	108059300	0.900826
chr9	108062300	0.842975
chr9	108070800	0.661157
chr9	108091400	0.735537
chr9	108152500	0.909091
chr9	108155000	0.644628
chr9	108157700	0.694215
chr9	108165100	0.925620
chr9	108167600	0.867769
chr9	108175800	0.768595
chr9	108178000	0.677686
chr9	108221000	0.669421
chr9	108409900	0.694215
chr9	108414400	0.925620
chr9	108674200	0.991736
chr9	108720500	0.760331

chr9	108731700	0.685950
chr9	108754800	0.826446
chr9	108782400	0.958678
chr9	108801100	0.909091
chr9	109230000	0.834711
chr9	109384000	0.694215
chr9	109440700	0.884298
chr9	109447800	0.710744
chr9	109521200	0.842975
chr9	109633300	0.719008
chr9	109646400	0.702479
chr9	109651300	0.867769
chr9	109655700	0.669421
chr9	109683600	0.801653
chr9	109697500	0.925620
chr9	109722400	0.867769
chr9	109733000	0.925620
chr9	109811900	0.752066
chr9	109893600	0.851240
chr9	109991200	0.834711
chr9	110011800	0.826446
chr9	110052000	0.685950
chr9	110184800	0.743802
chr9	110201100	0.975207
chr9	110302300	0.900826
chr9	110436800	0.983471
chr9	110514400	0.644628
chr9	110582900	0.842975
chr9	110635600	0.942149
chr9	110761600	0.776860
chr9	110815300	0.842975
chr9	110849600	0.785124
chr9	110856700	0.876033
chr9	111034800	0.735537
chr9	111096200	0.826446
chr9	111305800	0.669421
chr9	111439400	0.851240
chr9	111861100	0.818182
chr9	112021300	0.884298
chr9	112033300	0.694215
chr9	112073500	0.776860
chr9	112076500	0.776860
chr9	112078600	0.743802
chr9	112108000	0.867769
chr9	112172500	0.892562
chr9	112203500	0.801653
chr9	112283100	0.727273
chr9	112444300	0.652893
chr9	112529400	0.719008
chr9	112533900	0.752066
chr9	112631300	0.644628
chr9	112650500	0.694215
chr9	112790200	0.768595
chr9	112862200	0.644628

chr9	112866200	0.661157
chr9	112868600	0.644628
chr9	112873900	0.685950
chr9	112877300	0.975207
chr9	112884100	0.661157
chr9	112888300	0.900826
chr9	112890500	0.809917
chr9	112893600	0.859504
chr9	112920800	0.851240
chr9	113306000	0.727273
chr9	113344400	0.834711
chr9	113476900	0.685950
chr9	113748400	0.752066
chr9	113766100	0.669421
chr9	113861400	0.842975
chr9	113995500	0.884298
chr9	114219500	0.743802
chr9	114290100	0.884298
chr9	114292600	0.884298
chr9	114426200	0.661157
chr9	114800100	0.644628
chr9	114804500	0.851240
chr9	114829000	0.644628
chr9	114854500	0.793388
chr9	114861100	0.892562
chr9	114908300	0.743802
chr9	114933100	0.702479
chr9	114943600	0.661157
chr9	115073100	0.818182
chr9	115111300	0.876033
chr9	115117300	0.652893
chr9	115235100	0.768595
chr9	115252600	0.942149
chr9	115309800	0.859504
chr9	115314800	0.851240
chr9	115344600	0.793388
chr9	115380700	0.768595
chr9	115417300	0.834711
chr9	115443400	0.719008
chr9	115546800	0.644628
chr9	115573300	0.719008
chr9	115608900	0.867769
chr9	115611100	0.818182
chr9	115630900	0.652893
chr9	115822600	0.809917
chr9	115828200	0.727273
chr9	115833300	0.743802
chr9	115838900	0.834711
chr9	115844100	0.917355
chr9	115850100	0.809917
chr9	115923700	0.785124
chr9	116263800	0.867769
chr9	116269000	0.834711
chr9	116298900	0.867769

chr9	116303400	0.925620
chr9	116337600	0.909091
chr9	116362000	0.859504
chr9	116365400	0.867769
chr9	116372500	0.925620
chr9	116379700	0.768595
chr9	116383300	0.859504
chr9	116386300	0.925620
chr9	116417500	0.694215
chr9	116464700	0.884298
chr9	116468500	0.768595
chr9	116471700	0.685950
chr9	116556800	0.661157
chr9	116620600	0.694215
chr9	116636400	0.644628
chr9	116648300	0.867769
chr9	116674900	0.752066
chr9	116800800	0.760331
chr9	116840700	0.685950
chr9	116862700	0.677686
chr9	116871000	0.809917
chr9	116926000	0.685950
chr9	116967800	0.743802
chr9	116992000	0.760331
chr9	117048000	0.685950
chr9	117050600	0.876033
chr9	117111600	0.842975
chr9	117130900	0.752066
chr9	117133100	0.933884
chr9	117145800	0.818182
chr9	117152000	0.702479
chr9	117158000	0.710744
chr9	117168200	0.867769
chr9	117176100	0.644628
chr9	117197400	0.842975
chr9	117264300	0.966942
chr9	117270800	0.719008
chr9	117346000	0.975207
chr9	117376000	0.669421
chr9	117415900	0.793388
chr9	117424900	0.727273
chr9	117568700	0.867769
chr9	117632500	0.859504
chr9	117651200	0.826446
chr9	117945400	0.685950
chr9	118059600	0.727273
chr9	118223400	0.677686
chr9	118232500	0.917355
chr9	118234800	0.768595
chr9	118242000	0.768595
chr9	118276100	0.867769
chr9	118287100	0.727273
chr9	118358900	0.652893
chr9	118608300	0.661157

chr9	119034600	0.925620
chr9	119041900	0.983471
chr9	119046100	0.785124
chr9	119049900	0.727273
chr9	119085400	0.727273
chr9	119094500	0.776860
chr9	119097400	0.842975
chr9	119100200	0.677686
chr9	119104800	0.801653
chr9	119233900	0.991736
chr9	119239400	0.677686
chr9	119253800	0.876033
chr9	119386300	0.652893
chr9	119504100	0.735537
chr9	119562900	0.776860
chr9	119599400	0.735537
chr9	119605100	0.735537
chr9	119691400	1.000000
chr9	119709300	0.851240
chr9	119807100	0.685950
chr9	119835200	0.818182
chr9	119843800	0.859504
chr9	119854300	0.942149
chr9	120002100	0.752066
chr9	120031900	0.752066
chr9	120049300	0.669421
chr9	120107700	0.760331
chr9	120139400	0.652893
chr9	120163000	0.851240
chr9	120311600	0.785124
chr9	120358600	0.909091
chr9	120772100	0.851240
chr9	121285300	0.752066
chr9	121406000	0.818182
chr9	121447300	0.743802
chr9	121840700	0.677686
chr9	121849100	0.942149
chr9	121912200	0.710744
chr9	122035600	0.826446
chr9	122047200	0.644628
chr9	122060800	1.000000
chr9	122066900	0.694215
chr9	122096700	0.900826
chr9	122112300	0.900826
chr9	122123100	0.876033
chr9	122128000	0.710744
chr9	122133800	0.685950
chr9	122138000	0.710744
chr9	122203300	0.776860
chr9	122250000	0.702479
chr9	122296000	0.694215
chr9	122438500	0.760331
chr9	122521800	0.884298
chr9	122536800	0.702479

chr9	122647900	0.834711
chr9	122726600	0.991736
chr9	122733900	0.644628
chr9	122803500	0.702479
chr9	122840800	0.826446
chr9	122843400	0.743802
chr9	122846100	0.719008
chr9	123070600	0.669421
chr9	123087700	0.801653
chr9	123111500	0.851240
chr9	123174600	0.925620
chr9	123246000	0.876033
chr9	123253200	0.644628
chr9	123287300	0.694215
chr9	123291600	0.925620
chr9	123614700	1.000000
chr9	123636200	0.991736
chr9	123657100	0.776860
chr9	123683000	0.752066
chr9	123728300	0.958678
chr9	123969100	0.644628
chr9	123976000	0.876033
chr9	123983700	0.983471
chr9	124016600	0.752066
chr9	124023300	0.909091
chr9	124033700	0.661157
chr9	124041200	0.661157
chr9	124052200	0.652893
chr9	124060700	0.727273
chr9	124130200	0.793388
chr9	124149200	0.917355
chr9	124232300	0.710744
chr9	124234800	0.710744
chr9	124241700	0.793388
chr9	124268600	0.851240
[truncated: 358,619 more chars]
