# Supplementary material for: RFECS: A Random-Forest Based Algorithm for Enhancer Identification from Chromatin State
Source: PLoS Comput Biol. 2013 Mar 14;9(3):e1002968. doi: 10.1371/journal.pcbi.1002968 (PMC3597546; doi:10.1371/journal.pcbi.1002968)
Supplement: Table S15 — Enhancer predictions in the ENCODE cell-types Huvec, K562, Nha, Nhdfad, Nhek and Nhlf (hg18). (PDF) [file pcbi.1002968.s022.pdf]

Huvec enhancers

Chromosome	Location	Voting_cutoff
chr10	174000	0.677686
chr10	183800	0.727273
chr10	228800	0.958678
chr10	234800	1.000000
chr10	247500	0.983471
chr10	313600	0.826446
chr10	343900	0.966942
chr10	370200	1.000000
chr10	376000	0.933884
chr10	467300	0.859504
chr10	547300	0.809917
chr10	600500	0.851240
chr10	706200	0.900826
chr10	990800	0.909091
chr10	1090100	0.925620
chr10	1092500	0.661157
chr10	1108000	0.859504
chr10	1111100	0.859504
chr10	1156600	0.933884
chr10	2940300	1.000000
chr10	2958400	0.727273
chr10	2968200	0.900826
chr10	3028500	0.876033
chr10	3088100	0.727273
chr10	3097200	0.834711
chr10	3113600	0.900826
chr10	3116100	0.950413
chr10	3129600	0.826446
chr10	3132400	0.851240
chr10	3134900	0.975207
chr10	3138000	0.818182
chr10	3140100	0.900826
chr10	3145700	0.975207
chr10	3235800	0.942149
chr10	3240000	0.876033
chr10	3282800	0.900826
chr10	3291300	0.867769
chr10	3305500	0.785124
chr10	3307700	0.760331
chr10	3321200	0.710744
chr10	3442600	0.933884
chr10	3466900	0.818182
chr10	3469400	0.950413
chr10	3478100	0.727273
chr10	3494600	0.727273
chr10	3498200	0.743802
chr10	3507800	0.933884
chr10	3511300	0.983471
chr10	3514800	0.818182
chr10	3524800	0.776860
chr10	3568400	0.710744

chr10	3581000	0.958678
chr10	3790200	0.933884
chr10	3797300	0.958678
chr10	3818200	0.950413
chr10	3820500	0.760331
chr10	3829500	0.900826
chr10	3847500	0.983471
chr10	3851100	0.983471
chr10	3853700	0.793388
chr10	3871600	1.000000
chr10	3878100	0.727273
chr10	3894800	0.900826
chr10	3903600	0.983471
chr10	3919700	0.900826
chr10	3928600	0.950413
chr10	3931400	0.884298
chr10	3935300	0.826446
chr10	3937700	0.983471
chr10	3939800	0.942149
chr10	3962300	0.966942
chr10	3966900	0.983471
chr10	3972900	0.950413
chr10	3978400	0.958678
chr10	3985300	0.727273
chr10	3991400	0.677686
chr10	4038100	0.710744
chr10	4086700	0.991736
chr10	4109900	0.958678
chr10	4112500	0.917355
chr10	4116400	0.876033
chr10	4122600	0.966942
chr10	4124800	0.809917
chr10	4170700	0.933884
chr10	4174200	1.000000
chr10	4283300	0.958678
chr10	4287100	0.933884
chr10	4289500	0.859504
chr10	4302900	0.826446
chr10	4323200	0.752066
chr10	4394300	0.933884
chr10	4397500	0.975207
chr10	4415000	0.867769
chr10	4426800	0.661157
chr10	4493700	0.983471
chr10	4543700	0.694215
chr10	4583800	0.867769
chr10	4676100	0.884298
chr10	4692900	0.876033
chr10	4705700	0.983471
chr10	4718400	0.909091
chr10	4721600	0.826446
chr10	4743700	0.925620
chr10	4747400	0.900826
chr10	4768400	0.966942

chr10	4806500	0.975207
chr10	4808600	0.983471
chr10	4811200	0.925620
chr10	4814800	1.000000
chr10	4820700	0.892562
chr10	4824000	0.801653
chr10	4847200	1.000000
chr10	4849800	0.859504
chr10	4851900	0.900826
chr10	4857600	0.958678
chr10	4890200	0.892562
chr10	4910300	0.942149
chr10	4984600	0.966942
chr10	5062400	0.966942
chr10	5136900	0.719008
chr10	5327000	0.975207
chr10	5334000	0.991736
chr10	5336400	0.975207
chr10	5469800	0.661157
chr10	5488700	0.685950
chr10	5492800	0.867769
chr10	5588100	0.727273
chr10	5591000	0.677686
chr10	5596600	0.842975
chr10	5602100	0.884298
chr10	5629500	0.710744
chr10	5632400	0.768595
chr10	5638500	0.826446
chr10	5903700	0.801653
chr10	5934900	0.966942
chr10	5957700	0.884298
chr10	5984500	0.752066
chr10	5989000	0.842975
chr10	6000700	0.900826
chr10	6012100	0.842975
chr10	6014400	0.942149
chr10	6017900	0.909091
chr10	6125800	0.909091
chr10	6134100	0.933884
chr10	6156900	0.867769
chr10	6193700	1.000000
chr10	6199900	0.925620
chr10	6205000	0.727273
chr10	6212500	0.867769
chr10	6214700	0.958678
chr10	6230600	0.925620
chr10	6237400	0.669421
chr10	6270300	0.727273
chr10	6281800	0.966942
chr10	6317600	0.925620
chr10	6443900	0.677686
chr10	6714100	0.942149
chr10	6763600	0.950413
chr10	6772200	0.991736

chr10	6780300	0.743802
chr10	6783400	0.677686
chr10	6791200	0.958678
chr10	7306900	0.867769
chr10	7929700	0.694215
chr10	7974600	0.917355
chr10	7990900	0.991736
chr10	7998900	0.909091
chr10	8011800	1.000000
chr10	8014600	0.785124
chr10	8041400	0.685950
chr10	8069000	0.727273
chr10	8081900	0.876033
chr10	8085400	0.661157
chr10	8090100	0.950413
chr10	8103600	0.735537
chr10	8108100	1.000000
chr10	8114200	0.743802
chr10	8116400	0.917355
chr10	8365400	0.669421
chr10	8374700	0.776860
chr10	8412100	0.917355
chr10	8431800	0.694215
chr10	9054500	0.966942
chr10	9060300	0.983471
chr10	9828500	0.966942
chr10	10542400	0.677686
chr10	10655700	0.685950
chr10	10828000	0.966942
chr10	10830100	0.876033
chr10	10834900	0.975207
chr10	10838400	0.752066
chr10	10840600	0.867769
chr10	10847800	0.842975
chr10	10867300	0.975207
chr10	10873000	1.000000
chr10	10912200	0.892562
chr10	10915300	0.933884
chr10	11077700	0.991736
chr10	11187700	0.694215
chr10	11252300	0.983471
chr10	11254500	0.702479
chr10	11510800	0.851240
chr10	11581900	0.785124
chr10	11589400	0.925620
chr10	11594500	1.000000
chr10	11657900	0.661157
chr10	11690000	0.685950
chr10	11692200	0.983471
chr10	11716200	0.991736
chr10	11722500	0.933884
chr10	11727900	0.950413
chr10	11799900	0.793388
chr10	11841200	0.917355

chr10	11856500	0.900826
chr10	11913600	0.826446
chr10	11917800	0.793388
chr10	12036400	0.933884
chr10	12049100	0.760331
chr10	12114000	0.876033
chr10	12228900	0.958678
chr10	12233900	0.760331
chr10	12256900	0.760331
chr10	12277600	0.876033
chr10	12657000	0.966942
chr10	12746300	0.851240
chr10	12749700	0.975207
chr10	12752000	0.826446
chr10	12754600	0.892562
chr10	12762700	0.966942
chr10	12887500	0.966942
chr10	12891800	0.958678
chr10	13344200	0.785124
chr10	13420500	0.685950
chr10	13425300	0.743802
chr10	13482500	0.785124
chr10	13571900	0.669421
chr10	13650700	0.702479
chr10	13680100	0.909091
chr10	13702400	0.925620
chr10	13717300	0.867769
chr10	13725300	0.826446
chr10	13729700	0.677686
chr10	13744100	0.867769
chr10	13747000	0.892562
chr10	13750800	0.793388
chr10	13755400	0.958678
chr10	13758600	0.884298
chr10	13799200	0.942149
chr10	13816400	0.950413
chr10	13818800	0.950413
chr10	13826200	0.859504
chr10	13829300	0.966942
chr10	13832100	1.000000
chr10	13851600	0.958678
chr10	13856600	0.884298
chr10	13858700	0.983471
chr10	13863100	0.867769
chr10	13874000	0.834711
chr10	13877300	0.917355
chr10	13882000	0.909091
chr10	13887100	0.826446
chr10	13890200	0.685950
chr10	13896600	0.942149
chr10	13900500	1.000000
chr10	13902700	0.884298
chr10	13905100	0.958678
chr10	13907600	0.925620

chr10	13909700	0.925620
chr10	13915200	0.719008
chr10	13919800	0.669421
chr10	13922000	0.661157
chr10	13924600	0.842975
chr10	13926700	0.950413
chr10	13934800	0.834711
chr10	13936900	0.842975
chr10	13966800	0.933884
chr10	13981700	0.826446
chr10	13987800	0.950413
chr10	13992700	0.685950
chr10	14002500	0.975207
chr10	14015000	0.925620
chr10	14026800	0.983471
chr10	14030200	0.991736
chr10	14032800	0.884298
chr10	14035000	0.983471
chr10	14039700	0.661157
chr10	14058200	0.776860
chr10	14075000	0.685950
chr10	14078600	0.933884
chr10	14114300	0.785124
chr10	14116800	0.983471
chr10	14216200	0.727273
chr10	14370100	0.735537
chr10	14549500	0.834711
chr10	14570400	0.966942
chr10	14588200	0.785124
chr10	14596100	0.851240
chr10	14598600	0.851240
chr10	14602300	0.851240
chr10	14607700	0.760331
chr10	14613600	0.933884
chr10	14615900	0.991736
chr10	14618300	0.909091
chr10	14620600	0.958678
chr10	14623800	0.925620
chr10	14627500	0.793388
chr10	14631300	0.983471
chr10	14643100	0.661157
chr10	14652200	0.983471
chr10	14659300	0.983471
chr10	14672600	0.752066
chr10	14791400	0.884298
chr10	14821100	0.892562
chr10	14823800	0.851240
chr10	14825900	0.958678
chr10	14840900	0.950413
chr10	14862600	0.752066
chr10	15077900	0.900826
chr10	15149500	0.950413
chr10	15166300	0.735537
chr10	15188500	0.925620

chr10	15212200	0.950413
chr10	15238100	0.876033
chr10	15240300	0.719008
chr10	15243000	0.991736
chr10	15245200	0.859504
chr10	15259100	0.991736
chr10	15261900	0.933884
chr10	15281300	0.884298
chr10	15290300	0.892562
chr10	15299300	0.826446
chr10	15356200	0.925620
chr10	15368100	0.991736
chr10	15387200	0.900826
chr10	15391300	0.917355
chr10	15428300	0.975207
chr10	15481900	0.942149
chr10	15621900	0.925620
chr10	15631300	0.884298
chr10	15672500	0.867769
chr10	15714900	0.859504
chr10	15746300	0.826446
chr10	15939600	0.900826
chr10	16479300	0.727273
chr10	16686600	0.950413
chr10	16820000	0.884298
chr10	16870000	0.801653
chr10	16872200	0.917355
chr10	16875000	0.975207
chr10	16899100	0.785124
chr10	16929700	0.661157
chr10	16933900	0.842975
chr10	16952900	0.958678
chr10	16962000	0.793388
chr10	16987100	0.983471
chr10	16993000	0.966942
chr10	16996400	0.933884
chr10	17008200	0.719008
chr10	17011600	0.983471
chr10	17029500	0.966942
chr10	17035400	0.900826
chr10	17038100	0.694215
chr10	17040200	0.859504
chr10	17044800	0.851240
chr10	17048200	0.834711
chr10	17051400	0.925620
chr10	17066100	1.000000
chr10	17069700	0.966942
chr10	17073100	0.917355
chr10	17091600	0.933884
chr10	17099100	0.685950
chr10	17104100	0.710744
chr10	17109600	0.942149
chr10	17116900	0.966942
chr10	17119100	0.694215

chr10	17127100	0.719008
chr10	17143800	0.834711
chr10	17158200	0.991736
chr10	17191000	0.677686
chr10	17241200	0.818182
chr10	17248500	0.809917
chr10	17257000	0.925620
chr10	17259300	0.752066
chr10	17269300	0.727273
chr10	17277100	0.677686
chr10	17299700	0.768595
chr10	17468400	0.694215
chr10	17487800	0.694215
chr10	17518100	0.661157
chr10	17552300	0.909091
chr10	17555400	0.917355
chr10	17569800	0.983471
chr10	17697800	0.661157
chr10	17708100	0.983471
chr10	17777700	0.958678
chr10	17787000	0.876033
chr10	17811400	0.743802
chr10	17826000	0.983471
chr10	17860100	0.867769
chr10	17879200	0.826446
chr10	18033800	0.991736
chr10	18058400	0.917355
chr10	18072800	0.983471
chr10	18106900	0.752066
chr10	18126100	0.826446
chr10	18294800	0.710744
chr10	18897100	0.942149
chr10	18900200	0.950413
chr10	20211900	0.966942
chr10	20221100	0.925620
chr10	21291500	1.000000
chr10	21562200	0.702479
chr10	21582000	0.900826
chr10	21604000	0.983471
chr10	21619700	0.983471
chr10	21624200	0.859504
chr10	21626300	0.983471
chr10	21652500	0.900826
chr10	21655000	0.900826
chr10	21658900	0.950413
chr10	21696200	0.809917
chr10	21817900	0.809917
chr10	21822100	0.818182
chr10	22016900	0.909091
chr10	22267900	0.884298
chr10	22536000	0.710744
chr10	22717500	0.793388
chr10	22723100	0.818182
chr10	22765700	0.669421

chr10	22888600	0.710744
chr10	22905300	0.760331
chr10	22909500	0.950413
chr10	22911600	0.776860
chr10	22913700	0.966942
chr10	22921200	0.966942
chr10	22937500	0.966942
chr10	22962300	0.710744
chr10	22971500	0.909091
chr10	23106100	0.851240
chr10	23111100	0.859504
chr10	23114000	0.884298
chr10	23217100	0.719008
chr10	23389200	0.933884
chr10	24738200	0.743802
chr10	24846200	0.661157
chr10	24935200	0.900826
chr10	24970200	0.694215
chr10	24995900	0.950413
chr10	25138600	0.925620
chr10	25141100	0.950413
chr10	25147400	0.966942
chr10	25155500	0.809917
chr10	25186900	0.752066
chr10	25191100	0.867769
chr10	25210400	0.768595
chr10	25241300	0.809917
chr10	25247600	0.975207
chr10	25312500	0.975207
chr10	25349600	0.900826
chr10	25352000	0.925620
chr10	25357400	0.842975
chr10	25782900	0.975207
chr10	25845800	0.942149
chr10	26911400	0.694215
chr10	27312900	1.000000
chr10	27609200	0.694215
chr10	28297600	0.966942
chr10	28343600	0.760331
chr10	28377600	0.909091
chr10	28381500	0.925620
chr10	28384900	0.958678
chr10	28400700	0.818182
chr10	28419800	0.942149
chr10	28427400	0.892562
chr10	28430600	0.933884
chr10	28444900	0.661157
chr10	28467800	0.752066
chr10	28839900	0.809917
chr10	28958200	0.685950
chr10	29008700	0.743802
chr10	29011400	0.694215
chr10	29196100	0.793388
chr10	29259600	0.719008

chr10	29273600	0.826446
chr10	29402500	0.727273
chr10	29421600	0.900826
chr10	29439300	0.909091
chr10	29449200	0.859504
chr10	29606900	0.818182
chr10	29633700	0.694215
chr10	29772100	0.892562
chr10	29824700	0.859504
chr10	29845400	0.966942
chr10	29864300	0.925620
chr10	29872200	0.826446
chr10	29875600	0.876033
chr10	29879000	0.892562
chr10	29925500	0.983471
chr10	29935400	0.801653
chr10	29937800	0.950413
chr10	29948000	0.793388
chr10	29951000	0.975207
chr10	29958400	0.809917
chr10	29977900	0.991736
chr10	29981800	0.983471
chr10	30014600	0.892562
chr10	30035300	0.983471
chr10	30046900	0.925620
chr10	30061800	0.727273
chr10	30073900	0.925620
chr10	30084500	0.925620
chr10	30088300	0.876033
chr10	30092200	0.735537
chr10	30115900	0.694215
chr10	30139000	0.991736
chr10	30147500	0.710744
chr10	30152000	0.776860
chr10	30185000	0.983471
chr10	30236600	0.702479
chr10	30238700	0.966942
chr10	30241200	0.859504
chr10	30257600	0.851240
chr10	30332000	0.826446
chr10	30338500	0.983471
chr10	30345300	0.685950
chr10	30352200	0.917355
chr10	30400700	0.826446
chr10	30404200	0.809917
chr10	30413900	0.933884
chr10	30444800	0.900826
chr10	30472200	0.661157
chr10	30475200	0.983471
chr10	30478200	0.975207
chr10	30488600	0.859504
chr10	30497300	0.776860
chr10	30505000	0.942149
chr10	30509300	0.809917

chr10	30743800	0.768595
chr10	30827100	0.694215
chr10	30843500	0.826446
chr10	30872900	0.958678
chr10	30894800	0.694215
chr10	30927200	0.735537
chr10	30930100	0.776860
chr10	30933700	0.991736
chr10	30937400	0.942149
chr10	30940700	0.950413
chr10	30958500	0.677686
chr10	31009200	0.694215
chr10	31026000	0.859504
chr10	31073900	0.966942
chr10	31077700	0.834711
chr10	31080800	0.826446
chr10	31103300	0.983471
chr10	31106600	0.884298
chr10	31110300	0.966942
chr10	31144600	0.933884
chr10	31148700	0.933884
chr10	31172500	0.925620
chr10	31233900	0.694215
chr10	31253900	0.966942
chr10	31257400	0.900826
chr10	31270500	0.727273
chr10	31273200	0.933884
chr10	31275900	0.933884
chr10	31278600	0.958678
chr10	31286000	0.826446
chr10	31289000	0.983471
chr10	31292300	0.925620
chr10	31300700	0.892562
chr10	31303000	0.925620
chr10	31319500	0.776860
chr10	31323000	0.942149
chr10	31340900	1.000000
chr10	31348700	0.760331
chr10	31390500	0.826446
chr10	31423300	0.942149
chr10	31436300	0.983471
chr10	31532300	0.958678
chr10	31567800	0.966942
chr10	31604400	0.983471
chr10	31673800	0.991736
chr10	31712600	0.669421
chr10	31715900	0.925620
chr10	31816500	0.983471
chr10	31872000	0.685950
chr10	31939100	0.942149
chr10	31986900	0.975207
chr10	31990400	0.867769
chr10	32034700	0.942149
chr10	32039200	0.842975

chr10	32097800	1.000000
chr10	32153900	0.925620
chr10	32186500	0.975207
chr10	32194900	0.818182
chr10	32229800	0.975207
chr10	32233700	0.983471
chr10	32237000	0.966942
chr10	32267000	0.966942
chr10	32332800	0.685950
chr10	32341100	0.719008
chr10	32420700	1.000000
chr10	32428500	0.900826
chr10	32430700	0.933884
chr10	32438900	0.966942
chr10	32444500	0.983471
chr10	32486100	0.768595
chr10	32495100	0.983471
chr10	32552300	0.694215
chr10	32587600	0.909091
chr10	32702000	0.983471
chr10	33227600	0.991736
chr10	33240200	0.892562
chr10	33250300	0.793388
chr10	33252400	0.752066
chr10	33272000	0.950413
chr10	33275200	0.991736
chr10	33294800	0.917355
chr10	33321800	0.983471
chr10	33342500	0.950413
chr10	33415300	0.925620
chr10	33446400	0.925620
chr10	33468000	0.975207
chr10	33483600	0.892562
chr10	33487200	0.975207
chr10	33503300	0.752066
chr10	33507000	0.867769
chr10	33516500	0.925620
chr10	33519500	0.983471
chr10	33521900	0.983471
chr10	33525900	0.661157
chr10	33537400	0.719008
chr10	33542100	0.933884
chr10	33544500	0.966942
chr10	33549100	0.669421
chr10	33553200	0.983471
chr10	33555300	0.909091
chr10	33557500	0.776860
chr10	33561500	0.933884
chr10	33563600	0.785124
chr10	33566500	0.793388
chr10	33575200	0.958678
chr10	33591700	0.867769
chr10	33604200	0.942149
chr10	33606800	0.685950

chr10	33653100	0.727273
chr10	33655700	0.876033
chr10	33666400	0.793388
chr10	33798400	0.826446
chr10	33950800	0.809917
chr10	34011400	0.925620
chr10	34190100	0.851240
chr10	34402600	0.694215
chr10	34411100	0.991736
chr10	34435100	0.801653
chr10	34470900	0.702479
chr10	34474600	0.801653
chr10	34569900	1.000000
chr10	34588500	0.743802
chr10	34627200	0.743802
chr10	34695500	0.933884
chr10	34809300	0.669421
chr10	34815300	0.859504
chr10	34817500	0.942149
chr10	34869800	0.842975
chr10	34872100	0.917355
chr10	34988400	0.991736
chr10	34997200	0.892562
chr10	35022500	0.966942
chr10	35029200	0.685950
chr10	35047900	0.983471
chr10	35090400	0.876033
chr10	35283800	0.785124
chr10	35330700	0.776860
chr10	35347500	0.983471
chr10	35461900	1.000000
chr10	35484600	0.694215
chr10	35514500	0.909091
chr10	35621700	1.000000
chr10	35630300	0.743802
chr10	35658000	0.975207
chr10	35661800	0.983471
chr10	35667400	0.876033
chr10	35669700	0.834711
chr10	35684000	0.735537
chr10	35698600	0.834711
chr10	35702400	0.884298
chr10	35708800	0.677686
chr10	35711300	0.975207
chr10	35714700	0.917355
chr10	35735600	0.876033
chr10	35739500	0.776860
chr10	35743200	0.909091
chr10	35752500	0.991736
chr10	35775700	0.702479
chr10	35784600	0.900826
chr10	35853800	0.834711
chr10	35864300	0.925620
chr10	35867500	0.685950

chr10	35897000	0.727273
chr10	35916200	0.735537
chr10	36144800	0.752066
chr10	36179700	1.000000
chr10	36199200	0.661157
chr10	36202300	0.818182
chr10	36276400	0.719008
chr10	36461000	0.834711
chr10	36463200	0.917355
chr10	36471300	0.801653
chr10	36543600	0.727273
chr10	36614300	0.785124
chr10	36778500	0.859504
chr10	36780600	0.851240
chr10	36782700	0.909091
chr10	36789100	0.735537
chr10	36824100	0.884298
chr10	36828200	1.000000
chr10	36862500	0.785124
chr10	36895200	0.743802
chr10	36897800	0.991736
chr10	36924400	0.694215
chr10	38806100	0.719008
chr10	38872300	0.834711
chr10	38877800	0.735537
chr10	38881500	0.933884
chr10	42364900	0.818182
chr10	42400300	0.702479
chr10	42404400	0.710744
chr10	42546200	0.702479
chr10	42799500	0.661157
chr10	43362100	0.702479
chr10	43573000	0.752066
chr10	43597800	0.685950
chr10	43617300	0.719008
chr10	43636400	0.867769
chr10	43656400	0.933884
chr10	43783300	0.702479
chr10	43858000	0.669421
chr10	43894800	0.975207
chr10	43898300	0.735537
chr10	44189800	0.760331
chr10	44269700	0.975207
chr10	44342400	0.933884
chr10	44352500	0.909091
chr10	44354600	0.694215
chr10	44358000	0.859504
chr10	44360600	0.909091
chr10	44363700	0.933884
chr10	44504200	0.884298
chr10	45065400	1.000000
chr10	45248700	0.768595
chr10	45297000	0.669421
chr10	45374700	0.818182

chr10	45470500	0.842975
chr10	45475600	0.925620
chr10	45478500	0.694215
chr10	45481300	0.768595
chr10	45491900	0.983471
chr10	45554400	0.983471
chr10	45640400	0.760331
chr10	45731400	0.983471
chr10	45735100	0.892562
chr10	45869900	0.760331
chr10	45966500	1.000000
chr10	46052800	0.966942
chr10	46767600	0.917355
chr10	46908400	0.661157
chr10	46971300	0.826446
chr10	46975200	0.661157
chr10	47129700	0.892562
chr10	47151500	0.727273
chr10	47248500	0.867769
chr10	47391700	0.785124
chr10	47395900	0.719008
chr10	48181300	0.942149
chr10	48326300	1.000000
chr10	48336700	0.809917
chr10	49462400	0.859504
chr10	49517600	0.809917
chr10	49573500	0.892562
chr10	49599400	1.000000
chr10	49608400	0.983471
chr10	49639000	0.677686
chr10	49657600	0.785124
chr10	49664900	0.661157
chr10	49678700	0.909091
chr10	49684600	0.727273
chr10	49705800	0.842975
chr10	49735000	1.000000
chr10	49758600	0.950413
chr10	49761100	0.909091
chr10	49797900	0.950413
chr10	49817400	0.851240
chr10	49832900	0.950413
chr10	49848200	0.793388
chr10	50153600	0.867769
chr10	50385900	0.876033
chr10	50392300	0.842975
chr10	50396700	0.710744
chr10	50402900	0.909091
chr10	50606700	0.752066
chr10	50742700	0.942149
chr10	50744800	0.884298
chr10	50761400	0.842975
chr10	50887700	0.661157
chr10	51039900	0.950413
chr10	51050400	0.958678

chr10	51053300	0.719008
chr10	51373500	0.702479
chr10	51498800	0.809917
chr10	51559700	0.752066
chr10	51567700	0.677686
chr10	51572300	0.752066
chr10	51575300	0.958678
chr10	51577500	0.983471
chr10	52086200	0.809917
chr10	52153500	0.933884
chr10	52191600	0.876033
chr10	52299200	0.925620
chr10	52364700	0.925620
chr10	52413200	0.909091
chr10	52420200	0.702479
chr10	52462000	0.950413
chr10	53011100	0.669421
chr10	53101300	0.760331
chr10	53228600	0.661157
chr10	53403700	0.950413
chr10	53599400	0.669421
chr10	53679300	0.958678
chr10	53790600	0.735537
chr10	54068400	0.826446
chr10	54078100	0.760331
chr10	54092900	0.925620
chr10	54095900	0.892562
chr10	54098700	0.743802
chr10	54111700	0.851240
chr10	54147700	0.743802
chr10	54203800	0.950413
chr10	54240000	0.727273
chr10	54247300	0.909091
chr10	54251300	0.884298
chr10	54299900	0.694215
chr10	54339000	0.983471
chr10	54357800	0.884298
chr10	54392800	0.958678
chr10	54428000	0.942149
chr10	54436700	0.719008
chr10	54464100	0.983471
chr10	54538700	0.702479
chr10	54790300	0.991736
chr10	55164000	0.884298
chr10	55339900	0.983471
chr10	56310200	0.727273
chr10	56348100	0.900826
chr10	56373400	0.983471
chr10	56381900	0.892562
chr10	56505100	0.669421
chr10	56518900	0.702479
chr10	56557800	0.933884
chr10	56904800	0.685950
chr10	57170700	0.710744

chr10	60000800	0.851240
chr10	60015000	0.702479
chr10	60025000	0.669421
chr10	60034300	0.942149
chr10	60040700	0.966942
chr10	60043400	0.884298
chr10	60235800	0.925620
chr10	60350300	0.710744
chr10	61296800	0.884298
chr10	61321300	0.834711
chr10	61668400	0.900826
chr10	61814700	0.685950
chr10	61883500	0.768595
chr10	61935800	0.710744
chr10	61942900	0.661157
chr10	61970500	0.719008
chr10	62042000	0.826446
chr10	62044400	0.917355
chr10	62060400	0.735537
chr10	62065500	0.966942
chr10	62074700	0.933884
chr10	62145000	0.892562
chr10	62148600	0.876033
chr10	62175000	0.917355
chr10	62184500	0.950413
chr10	62186700	0.719008
chr10	62189300	0.942149
chr10	62207100	0.677686
chr10	62542400	0.809917
chr10	62571000	0.735537
chr10	62581000	0.809917
chr10	62589000	0.892562
chr10	62606000	0.900826
chr10	62716500	0.760331
chr10	62724400	0.958678
chr10	62739800	0.892562
chr10	62789000	0.685950
chr10	62823900	0.743802
chr10	62896300	0.801653
chr10	63235600	0.925620
chr10	63247500	0.743802
chr10	63349600	0.975207
chr10	63381400	0.991736
chr10	63393900	0.859504
chr10	63396200	0.694215
chr10	63402900	0.933884
chr10	63452800	0.710744
chr10	63512300	0.685950
chr10	63521300	0.809917
chr10	63546600	0.677686
chr10	63553800	0.950413
chr10	63595800	0.983471
chr10	63598000	0.809917
chr10	63657500	0.859504

chr10	63699300	0.991736
chr10	63703100	0.991736
chr10	63753500	0.950413
chr10	63806100	0.909091
chr10	63808700	0.909091
chr10	63811000	0.702479
chr10	63842100	0.801653
chr10	63844500	0.851240
chr10	63881000	0.975207
chr10	63890900	1.000000
chr10	63991400	0.983471
chr10	64000600	0.702479
chr10	64003800	0.983471
chr10	64137700	0.776860
chr10	64140000	0.950413
chr10	64144600	0.826446
chr10	64193500	0.983471
chr10	64203800	0.909091
chr10	64447500	0.677686
chr10	64449800	0.975207
chr10	64520500	0.991736
chr10	64967100	0.892562
chr10	64970800	0.884298
chr10	64996700	0.991736
chr10	65001900	0.991736
chr10	65004000	0.801653
chr10	65008100	0.859504
chr10	65030400	0.793388
chr10	65033800	0.991736
chr10	65175800	0.892562
chr10	65192300	0.942149
chr10	65195000	0.892562
chr10	65198900	0.933884
chr10	65314000	0.966942
chr10	65333200	1.000000
chr10	65388100	0.884298
chr10	65403800	0.876033
chr10	65406000	0.900826
chr10	65430300	0.900826
chr10	65432900	0.826446
chr10	65460600	0.917355
chr10	65467600	0.851240
chr10	65473700	0.975207
chr10	65482800	0.950413
chr10	65533400	0.826446
chr10	65559200	0.950413
chr10	65801700	0.719008
chr10	66627800	0.702479
chr10	66633200	0.975207
chr10	67088600	0.975207
chr10	67101900	0.983471
chr10	67115400	0.884298
chr10	67330300	0.694215
chr10	69362600	0.975207

chr10	69367400	0.991736
chr10	69426500	0.809917
chr10	69430300	0.991736
chr10	69535500	0.958678
chr10	69854200	0.909091
chr10	69857500	0.710744
chr10	69893700	0.876033
chr10	69896900	0.958678
chr10	69913900	0.826446
chr10	69944900	0.768595
chr10	70097100	0.677686
chr10	70772600	0.925620
chr10	70775000	0.958678
chr10	70817500	0.950413
chr10	70820900	0.760331
chr10	70823200	0.867769
chr10	70826100	0.975207
chr10	70846300	0.884298
chr10	70851000	0.702479
chr10	70853700	0.760331
chr10	70862300	0.867769
chr10	70976200	0.834711
chr10	70982600	0.685950
chr10	70985500	0.818182
chr10	70989500	0.826446
chr10	70997900	0.859504
chr10	71032100	0.702479
chr10	71038400	0.884298
chr10	71076400	0.661157
chr10	71081900	0.867769
chr10	71085900	0.694215
chr10	71088000	0.851240
chr10	71094900	0.917355
chr10	71099200	0.851240
chr10	71109700	0.727273
chr10	71121000	0.950413
chr10	71123100	0.900826
chr10	71136100	0.710744
chr10	71138200	0.975207
chr10	71168900	0.958678
chr10	71210100	0.669421
chr10	71213500	0.768595
chr10	71221000	0.677686
chr10	71224700	0.900826
chr10	71228700	0.958678
chr10	71234800	0.768595
chr10	71239300	0.694215
chr10	71241400	0.876033
chr10	71258100	0.851240
chr10	71390300	0.710744
chr10	71482100	0.727273
chr10	71488400	0.983471
chr10	71529400	0.677686
chr10	71564600	0.661157

chr10	71568000	1.000000
chr10	71579900	1.000000
chr10	71590100	0.942149
chr10	71599100	0.942149
chr10	71605100	0.851240
chr10	71608200	0.933884
chr10	71620900	0.925620
chr10	71624400	0.991736
chr10	71626900	0.950413
chr10	71629900	0.760331
chr10	71634400	0.776860
chr10	71662000	0.801653
chr10	71672300	0.702479
chr10	71907400	0.776860
chr10	71934000	0.743802
chr10	72056800	0.842975
chr10	72138200	0.909091
chr10	72201300	0.859504
chr10	72218400	0.851240
chr10	72241800	0.785124
chr10	72258900	0.694215
chr10	72261800	0.958678
chr10	72269200	0.818182
chr10	72279200	0.933884
chr10	72590100	0.793388
chr10	72593300	1.000000
chr10	72651700	0.694215
chr10	72775800	0.950413
chr10	72872600	0.983471
chr10	72977500	0.702479
chr10	73004800	0.884298
chr10	73015200	0.809917
chr10	73031700	0.719008
chr10	73107900	0.694215
chr10	73115400	0.809917
chr10	73157300	0.776860
chr10	73398200	0.677686
chr10	73453600	0.925620
chr10	73506700	0.735537
chr10	73526900	0.694215
chr10	73529400	0.867769
chr10	73596500	0.958678
chr10	73608800	0.876033
chr10	73620700	0.942149
chr10	73631500	0.760331
chr10	73716700	0.892562
chr10	73734800	0.925620
chr10	73740900	0.892562
chr10	73769600	0.884298
chr10	73819200	0.826446
chr10	73857300	0.925620
chr10	73931200	0.867769
chr10	73946800	0.991736
chr10	73983500	0.983471

chr10	74003300	0.991736
chr10	74008500	0.826446
chr10	74015200	0.892562
chr10	74056800	0.867769
chr10	74058900	0.933884
chr10	74069400	0.900826
chr10	74077500	0.818182
chr10	74080400	0.785124
chr10	74083100	0.933884
chr10	74085300	0.785124
chr10	74088400	0.917355
chr10	74091800	0.702479
chr10	74097400	0.842975
chr10	74124400	0.727273
chr10	74448200	0.685950
chr10	74469400	0.917355
chr10	74496200	0.809917
chr10	74552300	0.925620
chr10	74560600	0.809917
chr10	74562800	0.966942
chr10	74647000	1.000000
chr10	74804400	0.991736
chr10	74836900	1.000000
chr10	74878200	1.000000
chr10	74934500	0.942149
chr10	74943500	0.900826
chr10	75261600	0.694215
chr10	75346500	0.942149
chr10	75351600	0.867769
chr10	75407900	0.661157
chr10	75480700	0.851240
chr10	75535400	0.694215
chr10	75547800	0.661157
chr10	75575700	0.983471
chr10	75579900	0.876033
chr10	75597100	0.818182
chr10	75603600	0.818182
chr10	75626000	0.801653
chr10	75646800	0.752066
chr10	75648900	0.669421
chr10	75654600	0.958678
chr10	75659300	0.826446
chr10	75665800	0.785124
chr10	75668900	0.966942
chr10	75697800	0.826446
chr10	75700900	0.859504
chr10	75717000	0.719008
chr10	75725900	0.710744
chr10	75728400	0.842975
chr10	75761300	0.867769
chr10	75768400	0.719008
chr10	75771800	0.752066
chr10	75781100	0.719008
chr10	75816200	0.966942

chr10	75818300	0.685950
chr10	75831300	0.983471
chr10	75840800	0.851240
chr10	75905500	0.776860
chr10	75925400	0.950413
chr10	75950000	1.000000
chr10	75984200	0.933884
chr10	75988200	0.966942
chr10	75994000	0.884298
chr10	75998700	0.983471
chr10	76199100	0.909091
chr10	76205600	0.685950
chr10	76220900	0.975207
chr10	76260500	0.983471
chr10	76263600	0.983471
chr10	76349500	0.909091
chr10	76499100	0.710744
chr10	76560300	0.958678
chr10	76684000	0.661157
chr10	76887300	0.950413
chr10	76904100	0.950413
chr10	76940800	0.876033
chr10	76952100	0.851240
chr10	76990500	0.966942
chr10	77055200	0.752066
chr10	77229200	0.661157
chr10	77414500	0.892562
chr10	77483900	0.983471
chr10	77548700	0.933884
chr10	77606000	0.669421
chr10	78069400	0.785124
chr10	78089900	0.892562
chr10	78097800	0.760331
chr10	78112400	0.958678
chr10	78252000	0.867769
chr10	78303900	0.900826
chr10	78872400	0.685950
chr10	78916600	0.942149
chr10	78920700	0.917355
chr10	78923000	0.925620
chr10	78925200	0.867769
chr10	78965100	0.909091
chr10	79110900	0.719008
chr10	79146800	0.991736
chr10	79150800	0.677686
chr10	79153400	0.702479
chr10	79497900	0.867769
chr10	79596500	0.826446
chr10	79605200	0.958678
chr10	79633900	0.859504
chr10	79638200	0.743802
chr10	79658900	0.661157
chr10	79709100	0.685950
chr10	79744500	0.677686

chr10	79786000	0.834711
chr10	79796400	0.743802
chr10	80016000	0.785124
chr10	80062800	0.842975
chr10	80095700	0.942149
chr10	80122400	0.768595
chr10	80216300	0.826446
chr10	80219000	0.991736
chr10	80262300	0.917355
chr10	80460700	0.710744
chr10	80515100	0.702479
chr10	80517700	0.867769
chr10	80692100	0.768595
chr10	80698300	0.900826
chr10	80725300	0.661157
chr10	80732000	0.727273
chr10	80735300	0.661157
chr10	80831900	0.776860
chr10	80844200	0.859504
chr10	80854100	0.851240
chr10	80873100	0.842975
chr10	80887500	0.917355
chr10	80890100	0.735537
chr10	80896100	0.917355
chr10	80898200	0.768595
chr10	80904600	0.793388
chr10	80910100	0.785124
chr10	80912600	0.900826
chr10	80916200	0.950413
chr10	80918300	0.727273
chr10	80920500	0.760331
chr10	80939600	0.942149
chr10	80943900	0.776860
chr10	80981900	0.950413
chr10	80984000	0.743802
chr10	80997600	0.826446
chr10	81000900	0.710744
chr10	81003500	0.768595
chr10	81007000	0.793388
chr10	81025900	0.661157
chr10	81046900	0.900826
chr10	81054300	0.917355
chr10	81059100	0.743802
chr10	81077800	0.925620
chr10	81159400	0.900826
chr10	81163500	0.727273
chr10	81173500	0.735537
chr10	81195000	0.661157
chr10	81410500	0.818182
chr10	81538200	0.942149
chr10	81540600	0.950413
chr10	81588600	0.900826
chr10	81627000	0.909091
chr10	81694100	0.991736

chr10	81905900	0.785124
chr10	81918700	0.842975
chr10	81926200	0.884298
chr10	81959400	0.876033
chr10	81962900	0.942149
chr10	81967500	0.958678
chr10	82117000	0.677686
chr10	82172300	0.669421
chr10	82174500	0.677686
chr10	82176800	0.966942
chr10	82212900	0.727273
chr10	82217600	0.677686
chr10	82219900	0.900826
chr10	82222400	0.983471
chr10	82228300	0.793388
chr10	82233500	0.950413
chr10	82237400	0.983471
chr10	82245600	0.743802
chr10	82251400	0.719008
chr10	82255700	0.884298
chr10	82260300	0.966942
chr10	82263400	0.851240
chr10	82266200	0.876033
chr10	82271700	0.942149
chr10	82423000	0.669421
chr10	83624100	0.801653
chr10	83626800	0.983471
chr10	83695000	0.793388
chr10	83718600	0.983471
chr10	83721600	0.867769
chr10	83723900	0.958678
chr10	84420600	0.760331
chr10	84502600	0.752066
chr10	84742500	0.942149
chr10	84756100	0.702479
chr10	84835900	0.752066
chr10	85306000	0.983471
chr10	85363900	0.760331
chr10	85368500	0.900826
chr10	85372100	0.768595
chr10	85408000	0.892562
chr10	85485100	0.867769
chr10	85743800	0.677686
chr10	86056600	0.983471
chr10	86067800	0.793388
chr10	86070100	0.942149
chr10	86073900	0.991736
chr10	86184600	0.991736
chr10	86238200	0.752066
chr10	86254500	0.933884
chr10	86269400	0.900826
chr10	86271600	0.702479
chr10	87402800	1.000000
chr10	88171200	0.859504

chr10	88239300	0.801653
chr10	88471600	0.727273
chr10	88507900	0.752066
chr10	88622000	0.719008
chr10	88628600	0.719008
chr10	88633400	0.809917
chr10	88720000	0.694215
chr10	88726800	0.884298
chr10	88728900	0.809917
chr10	88842200	0.900826
chr10	88844300	0.991736
chr10	88971900	0.752066
chr10	89060200	0.909091
chr10	89062700	0.933884
chr10	89143200	0.892562
chr10	89195300	0.983471
chr10	89197800	0.760331
chr10	89292700	0.975207
chr10	89395400	0.983471
chr10	89417700	0.892562
chr10	89422500	0.966942
chr10	89435400	0.958678
chr10	89473300	0.809917
chr10	89476300	0.958678
chr10	89481300	0.702479
chr10	89483600	0.983471
chr10	89520400	0.983471
chr10	89535900	0.966942
chr10	89635000	0.834711
chr10	89675900	0.983471
chr10	89678500	0.933884
chr10	89687100	0.958678
chr10	89721000	0.975207
chr10	89823100	0.727273
chr10	89912900	0.900826
chr10	89918000	1.000000
chr10	89923000	0.677686
chr10	90004000	0.950413
chr10	90014100	0.669421
chr10	90147000	0.677686
chr10	90407300	0.826446
chr10	90411800	0.942149
chr10	90441100	0.685950
chr10	90463100	0.785124
chr10	90467600	0.867769
chr10	90514700	0.892562
chr10	90642600	1.000000
chr10	90695300	0.677686
chr10	90792600	0.975207
chr10	90834100	0.958678
chr10	90875100	0.991736
chr10	90878300	0.685950
chr10	90882700	0.876033
chr10	90905200	0.933884

chr10	90984900	0.876033
chr10	91037800	0.834711
chr10	91040100	0.719008
chr10	91062200	0.669421
chr10	91087400	0.793388
chr10	91111900	0.743802
chr10	91218500	0.867769
chr10	91543500	0.785124
chr10	91958800	0.727273
chr10	91987400	0.958678
chr10	92066400	0.876033
chr10	92074000	0.752066
chr10	92076300	0.694215
chr10	92079200	0.925620
chr10	92179100	0.975207
chr10	92347100	0.677686
chr10	92672500	0.917355
chr10	92675400	0.933884
chr10	92682300	0.917355
chr10	92688100	0.727273
chr10	92691100	0.917355
chr10	92693200	0.859504
chr10	92698000	0.842975
chr10	93101500	0.991736
chr10	93109300	0.867769
chr10	93348400	0.809917
chr10	93351300	0.958678
chr10	93357300	0.859504
chr10	93364700	0.925620
chr10	93374000	0.950413
chr10	93469500	0.768595
chr10	93661900	0.958678
chr10	93884300	0.925620
chr10	93902500	0.851240
chr10	93906200	0.834711
chr10	93970900	0.735537
chr10	94053300	0.851240
chr10	94351000	0.809917
chr10	94364500	0.677686
chr10	94400300	0.685950
chr10	94455000	0.859504
chr10	94457100	0.933884
chr10	94459700	0.942149
chr10	94510900	0.752066
chr10	94515300	0.710744
chr10	94517500	0.710744
chr10	94520700	0.925620
chr10	94572100	0.917355
chr10	94576100	0.958678
chr10	94579700	0.710744
chr10	94582300	0.925620
chr10	94586400	0.677686
chr10	94596600	0.743802
chr10	94599900	0.966942

chr10	94603200	1.000000
chr10	94631700	0.966942
chr10	94633900	0.991736
chr10	94636900	0.851240
chr10	94652600	0.834711
chr10	94732200	0.867769
chr10	94828800	0.867769
chr10	94959100	0.834711
chr10	95106900	0.876033
chr10	95124600	0.793388
chr10	95136600	0.983471
chr10	95140400	0.884298
chr10	95144400	0.975207
chr10	95164000	0.975207
chr10	95167100	0.842975
chr10	95172500	0.917355
chr10	95178300	0.785124
chr10	95182000	1.000000
chr10	95194800	0.702479
chr10	95197600	0.677686
chr10	95199800	0.876033
chr10	95202500	0.876033
chr10	95205900	0.834711
chr10	95217800	0.917355
chr10	95220600	0.950413
chr10	95222800	0.983471
chr10	95225800	0.909091
chr10	95228100	0.702479
chr10	95231400	0.884298
chr10	95233700	0.743802
chr10	95236200	0.983471
chr10	95239000	0.884298
chr10	95258700	0.842975
chr10	95302400	0.809917
chr10	95308600	0.925620
chr10	95315100	0.661157
chr10	95326700	0.685950
chr10	95751900	0.719008
chr10	96362700	0.975207
chr10	96990100	0.917355
chr10	96996600	0.851240
chr10	97021200	0.793388
chr10	97026800	0.933884
chr10	97029300	0.859504
chr10	97033800	0.900826
chr10	97055600	0.719008
chr10	97068600	0.925620
chr10	97091000	0.669421
chr10	97094600	0.900826
chr10	97205800	0.933884
chr10	97239900	0.958678
chr10	97245500	0.818182
chr10	97253400	0.760331
chr10	97259200	0.677686

chr10	97268900	0.942149
chr10	97273900	0.834711
chr10	97277800	0.876033
chr10	97293200	0.876033
chr10	97296100	1.000000
chr10	97489800	0.727273
chr10	97502400	0.950413
chr10	97516300	0.702479
chr10	97522300	0.958678
chr10	97553300	0.842975
chr10	97574900	1.000000
chr10	97578800	0.991736
chr10	97799000	0.983471
chr10	97807200	0.900826
chr10	97811700	0.752066
chr10	97814000	0.966942
chr10	97825900	0.966942
chr10	97833600	0.950413
chr10	98126300	0.677686
chr10	98348500	0.768595
chr10	98480500	0.735537
chr10	98561100	0.958678
chr10	98624100	0.991736
chr10	98784400	0.958678
chr10	98797800	0.702479
chr10	98978000	0.958678
chr10	99016400	0.743802
chr10	99115800	0.785124
chr10	99183100	0.785124
chr10	99189000	0.842975
chr10	99276800	0.694215
chr10	99279100	0.867769
chr10	99301800	0.900826
chr10	99304400	0.818182
chr10	99307000	0.710744
chr10	99334700	0.719008
chr10	99356800	0.991736
chr10	99408900	0.942149
chr10	99448200	0.752066
chr10	99486700	0.900826
chr10	99642600	0.859504
chr10	99649300	0.900826
chr10	99655700	0.917355
chr10	99790400	0.685950
chr10	99985700	0.867769
chr10	100076600	0.752066
chr10	100083000	0.694215
chr10	100104400	0.685950
chr10	100113300	0.826446
chr10	100194500	1.000000
chr10	100220900	1.000000
chr10	100227700	0.685950
chr10	100288100	0.867769
chr10	100316200	0.818182

chr10	100994400	0.685950
chr10	101134900	0.867769
chr10	101152200	0.925620
chr10	101156000	0.942149
chr10	101163900	0.867769
chr10	101226500	0.925620
chr10	101228900	0.867769
chr10	101295600	0.818182
chr10	101421700	0.958678
chr10	101427100	0.727273
chr10	101536100	0.917355
chr10	101539100	0.900826
chr10	101542000	0.966942
chr10	101604900	0.950413
chr10	101623800	0.677686
chr10	101641100	0.884298
chr10	101666700	0.942149
chr10	101669000	0.809917
chr10	101673500	0.966942
chr10	101677900	0.933884
chr10	101682500	0.950413
chr10	101686100	0.834711
chr10	101689100	0.867769
chr10	101691500	0.859504
chr10	101693700	0.933884
chr10	101698900	0.801653
chr10	101704000	0.983471
chr10	101706100	0.950413
chr10	101709200	0.702479
chr10	101718500	0.991736
chr10	101722400	0.925620
chr10	101726200	0.991736
chr10	101728300	0.801653
chr10	101736200	0.842975
chr10	101738600	0.933884
chr10	101741700	0.702479
chr10	101745100	0.851240
chr10	101754300	1.000000
chr10	101757100	0.966942
chr10	101761900	0.727273
chr10	101765800	0.933884
chr10	101804000	0.942149
chr10	101861100	0.867769
chr10	102119300	0.942149
chr10	102124800	0.983471
chr10	102131400	0.867769
chr10	102297700	0.694215
chr10	102322300	0.793388
chr10	102642600	0.884298
chr10	102650600	0.966942
chr10	102762300	0.925620
chr10	102773200	0.867769
chr10	102802700	0.752066
chr10	102822800	0.876033

chr10	102881200	0.752066
chr10	102985300	0.669421
chr10	103124700	0.694215
chr10	103127800	0.851240
chr10	103296200	0.818182
chr10	103330400	0.876033
chr10	103369600	0.685950
chr10	103539500	0.710744
chr10	103608500	0.876033
chr10	103616600	0.702479
chr10	103698200	0.933884
chr10	103701900	0.975207
chr10	103733800	0.818182
chr10	103746800	0.760331
chr10	103776700	0.685950
chr10	103875700	0.727273
chr10	103877900	0.801653
chr10	103910800	0.834711
chr10	103985900	0.892562
chr10	104040900	0.917355
chr10	104060000	0.876033
chr10	104070400	0.867769
chr10	104103300	0.809917
chr10	104159500	0.768595
chr10	104269400	0.768595
chr10	104339900	0.834711
chr10	104353400	0.735537
chr10	104356400	0.776860
chr10	104360800	0.661157
chr10	104365200	0.958678
chr10	104371100	0.735537
chr10	104387000	0.900826
chr10	104390500	0.909091
chr10	104393100	0.818182
chr10	104401300	0.669421
chr10	104409100	0.776860
chr10	104418000	0.975207
chr10	104434200	0.768595
chr10	104513300	0.809917
chr10	104534000	0.710744
chr10	104542400	0.975207
chr10	104556600	0.867769
chr10	104562000	0.917355
chr10	104564100	0.826446
chr10	104573100	0.752066
chr10	104576500	0.677686
chr10	104913300	0.925620
chr10	104916100	0.909091
chr10	104928600	0.727273
chr10	104941500	0.925620
chr10	104953900	0.677686
chr10	104992400	0.801653
chr10	105001300	0.752066
chr10	105033300	0.661157

chr10	105037600	0.710744
chr10	105158200	0.661157
chr10	105188800	0.752066
chr10	105206300	1.000000
chr10	105209700	0.685950
chr10	105264500	0.933884
chr10	105358200	0.735537
chr10	105428700	0.851240
chr10	105453300	0.818182
chr10	105456700	0.900826
chr10	105519800	0.933884
chr10	105522800	0.702479
chr10	105525400	0.752066
chr10	105532700	0.859504
chr10	105570500	0.677686
chr10	105572800	0.933884
chr10	105610400	0.991736
chr10	105648400	0.917355
chr10	105654000	0.834711
chr10	105656100	0.900826
chr10	105668500	0.983471
chr10	105675700	0.933884
chr10	105679000	0.851240
chr10	105740200	0.834711
chr10	105743500	0.975207
chr10	105781600	1.000000
chr10	105809800	0.909091
chr10	105814900	0.884298
chr10	105836900	1.000000
chr10	105859600	0.727273
chr10	105869800	0.743802
chr10	106021400	0.719008
chr10	106053300	0.942149
chr10	106059200	0.809917
chr10	106061500	0.702479
chr10	106084900	0.801653
chr10	106089300	0.694215
chr10	106092300	0.925620
chr10	106094800	0.661157
chr10	106103300	0.917355
chr10	106109800	0.685950
chr10	106113500	0.719008
chr10	109501900	0.917355
chr10	111644500	0.752066
chr10	111649900	0.950413
chr10	111659500	0.950413
chr10	111675100	0.933884
chr10	111695400	0.991736
chr10	111725900	0.834711
chr10	111728200	0.892562
chr10	111818200	0.884298
chr10	111834400	0.925620
chr10	111837700	0.793388
chr10	111841700	0.859504

chr10	111936400	0.900826
chr10	111982000	0.743802
chr10	112066100	0.818182
chr10	112070900	1.000000
chr10	112086600	0.983471
chr10	112113800	0.983471
chr10	112136400	0.842975
chr10	112140000	0.975207
chr10	112148400	0.876033
chr10	112152400	0.710744
chr10	112156000	0.809917
chr10	112159500	0.950413
chr10	112161800	0.776860
chr10	112168100	0.669421
chr10	112171400	0.900826
chr10	112175000	0.950413
chr10	112180600	0.917355
chr10	112207600	0.909091
chr10	112216000	0.719008
chr10	112219800	0.677686
chr10	112248300	0.867769
chr10	112252200	0.661157
chr10	112265600	0.793388
chr10	112268800	0.892562
chr10	112272900	0.867769
chr10	112285800	0.818182
chr10	112287900	0.842975
chr10	112290000	0.983471
chr10	112296500	0.743802
chr10	112508000	1.000000
chr10	112519000	0.983471
chr10	112631100	0.685950
chr10	114073900	0.818182
chr10	114121900	0.958678
chr10	114136000	0.818182
chr10	114149400	0.950413
chr10	114165100	0.685950
chr10	114410700	0.677686
chr10	114432000	0.752066
chr10	114502400	0.809917
chr10	114539200	0.727273
chr10	114575600	0.793388
chr10	114672700	0.983471
chr10	114716000	0.685950
chr10	114788000	0.776860
chr10	114795900	0.685950
chr10	114805700	0.966942
chr10	114813900	0.991736
chr10	114817200	0.900826
chr10	114820200	0.958678
chr10	114831200	0.942149
chr10	114847400	0.991736
chr10	114853600	0.942149
chr10	115142200	0.826446

chr10	115310400	1.000000
chr10	115314200	0.975207
chr10	115317600	0.876033
chr10	115323300	0.719008
chr10	115442200	0.752066
chr10	115469100	0.917355
chr10	115617500	0.719008
chr10	115634700	0.826446
chr10	115640300	0.991736
chr10	115676200	0.776860
chr10	115690700	0.991736
chr10	115705000	0.809917
chr10	115786200	0.884298
chr10	115788300	0.776860
chr10	115898600	0.818182
chr10	116064400	0.917355
chr10	116132500	0.768595
chr10	116135500	0.760331
chr10	116149400	0.694215
chr10	116153400	0.752066
chr10	116164100	0.661157
chr10	116173100	0.702479
chr10	116186700	0.785124
chr10	116206500	0.834711
chr10	116249800	0.958678
chr10	116253200	0.702479
chr10	116262000	0.661157
chr10	116265200	0.983471
chr10	116267700	0.719008
chr10	116311300	0.694215
chr10	116383900	1.000000
chr10	116394300	0.958678
chr10	116405700	0.925620
chr10	116408200	0.884298
chr10	116414200	1.000000
chr10	116426000	0.669421
chr10	116441900	0.809917
chr10	116445300	0.785124
chr10	116457300	0.925620
chr10	116473800	0.975207
chr10	116475900	0.925620
chr10	116540000	0.892562
chr10	116585400	0.834711
chr10	116616400	1.000000
chr10	116629500	0.975207
chr10	116685600	1.000000
chr10	117697400	0.785124
chr10	117701600	0.801653
chr10	118648300	0.859504
chr10	118651300	0.925620
chr10	118707600	0.942149
chr10	118734200	0.966942
chr10	118897700	0.867769
chr10	119124900	0.818182

chr10	119215200	0.669421
chr10	119218000	0.867769
chr10	119221700	0.669421
chr10	119801300	0.752066
chr10	120458700	0.694215
chr10	120465800	0.760331
chr10	120489700	0.983471
chr10	120630700	0.776860
chr10	120790900	0.785124
chr10	120860900	0.925620
chr10	120896300	0.884298
chr10	120926900	0.950413
chr10	120958200	0.851240
chr10	120963000	0.950413
chr10	120971100	0.966942
chr10	120992400	0.966942
chr10	120994500	0.933884
chr10	120997600	0.851240
chr10	121007100	0.925620
chr10	121010300	0.925620
chr10	121013700	0.826446
chr10	121024200	0.694215
chr10	121028900	0.826446
chr10	121032100	0.925620
chr10	121061700	1.000000
chr10	121065000	0.702479
chr10	121068000	0.826446
chr10	121075000	0.727273
chr10	121077500	0.809917
chr10	121095900	0.842975
chr10	121098000	0.950413
chr10	121128400	0.752066
chr10	121131600	0.851240
chr10	121155100	0.876033
chr10	121161600	0.983471
chr10	121165000	0.950413
chr10	121168400	0.785124
chr10	121170800	0.859504
chr10	121277300	0.842975
chr10	121281500	0.909091
chr10	121415200	0.859504
chr10	121419200	0.950413
chr10	121427700	0.892562
chr10	121440900	0.768595
chr10	121446700	1.000000
chr10	121484400	0.801653
chr10	121497800	1.000000
chr10	121510600	0.859504
chr10	121532200	0.925620
chr10	121543400	0.983471
chr10	122154200	0.942149
chr10	122176000	0.785124
chr10	122182600	0.661157
chr10	122186300	0.983471

chr10	122188700	0.892562
chr10	122220900	0.900826
chr10	122237200	0.859504
chr10	122367600	0.669421
chr10	122371600	0.867769
chr10	122373700	1.000000
chr10	122388200	0.809917
chr10	122392400	0.958678
chr10	122396400	0.851240
chr10	122401500	1.000000
chr10	122410900	0.743802
chr10	122443500	0.991736
chr10	122486900	0.760331
chr10	122492000	0.900826
chr10	122495500	0.983471
chr10	122515500	0.776860
chr10	122520200	0.669421
chr10	123243200	0.760331
chr10	123247500	0.793388
chr10	123277300	0.768595
chr10	123279400	0.735537
chr10	123325000	0.842975
chr10	123496600	0.950413
chr10	123513900	0.942149
chr10	123551800	0.867769
chr10	123702900	0.842975
chr10	123707800	0.735537
chr10	123716600	0.991736
chr10	123823000	0.710744
chr10	123871400	0.727273
chr10	123875700	0.958678
chr10	123887700	0.991736
chr10	123898800	0.685950
chr10	123902600	0.851240
chr10	123906700	0.818182
chr10	123909300	0.950413
chr10	123943000	0.950413
chr10	123955900	0.743802
chr10	124024400	0.669421
chr10	124027700	0.834711
chr10	124067000	0.950413
chr10	124069300	0.834711
chr10	124071600	0.793388
chr10	124210300	0.966942
chr10	124220400	0.710744
chr10	124228900	0.909091
chr10	124232400	0.842975
chr10	124235400	0.785124
chr10	124253400	0.702479
chr10	124256000	0.867769
chr10	124264200	0.743802
chr10	124290100	0.950413
chr10	124301300	0.702479
chr10	124612900	0.966942

chr10	124615000	0.909091
chr10	124621300	0.958678
chr10	124624400	0.818182
chr10	124699600	0.809917
chr10	124892700	0.785124
chr10	124917000	0.826446
chr10	125560500	0.867769
chr10	125568900	0.991736
chr10	125866200	0.900826
chr10	126043800	0.818182
chr10	126301600	0.694215
chr10	126334800	0.710744
chr10	126337500	0.958678
chr10	126402800	0.702479
chr10	126407900	0.818182
chr10	126417600	0.743802
chr10	126429900	0.942149
chr10	126433900	0.760331
chr10	126454700	0.685950
chr10	126613500	0.966942
chr10	126664800	0.900826
chr10	126724300	0.909091
chr10	126734400	0.892562
chr10	126737800	0.975207
chr10	126744700	0.776860
chr10	126748200	0.983471
chr10	126750700	0.884298
chr10	126765700	0.884298
chr10	126770100	0.942149
chr10	126772800	0.834711
chr10	126774900	0.867769
chr10	126826400	0.975207
chr10	126840400	0.884298
chr10	126845600	0.727273
chr10	127239300	0.991736
chr10	127295300	1.000000
chr10	127315500	0.950413
chr10	127368600	0.933884
chr10	127530100	0.727273
chr10	127688700	0.694215
chr10	127730900	0.900826
chr10	127738800	0.991736
chr10	127748000	0.826446
chr10	127936300	0.950413
chr10	128771500	0.991736
chr10	128880300	0.876033
chr10	128947000	0.983471
chr10	129052800	0.669421
chr10	129112800	0.727273
chr10	129675000	0.752066
chr10	129679200	0.677686
chr10	129686100	0.752066
chr10	129707800	0.859504
chr10	129717000	0.867769

chr10	129719200	0.958678
chr10	129721900	0.958678
chr10	129724600	0.983471
chr10	129726800	0.859504
chr10	129738400	0.727273
chr10	129741300	0.876033
chr10	129745100	0.743802
chr10	129747900	0.975207
chr10	129767400	1.000000
chr10	129775000	0.842975
chr10	129778100	0.892562
chr10	129782200	0.768595
chr10	129784700	0.694215
chr10	129788700	0.752066
chr10	129791800	0.727273
chr10	129795400	0.727273
chr10	129798900	0.793388
chr10	129805000	0.760331
chr10	129809100	0.991736
chr10	129816600	0.793388
chr10	129835100	0.950413
chr10	129846000	0.983471
chr10	129862000	0.933884
chr10	130007200	0.834711
chr10	130019600	0.743802
chr10	130053400	0.710744
chr10	130833900	0.983471
chr10	130837200	0.785124
chr10	130839400	0.867769
chr10	130841700	0.900826
chr10	130845100	0.966942
chr10	130847400	0.884298
chr10	130895000	0.719008
chr10	130904100	0.752066
chr10	131057200	0.975207
chr10	131103800	0.983471
chr10	131216400	0.983471
chr10	131265500	0.735537
chr10	131937300	0.983471
chr10	133737000	0.719008
chr10	133742200	0.809917
chr10	133745200	0.966942
chr10	133748900	0.768595
chr10	133759000	0.793388
chr10	133762900	0.851240
chr10	133786600	0.685950
chr10	133811400	0.661157
chr10	134150700	0.876033
chr10	134215400	0.842975
chr10	134221800	0.818182
chr10	134225300	0.909091
chr10	134232000	0.818182
chr10	134331200	0.933884
chr10	134350500	0.719008

chr10	134353900	0.876033
chr10	134385900	0.735537
chr10	134389100	0.900826
chr10	134402100	0.975207
chr10	134404800	0.933884
chr10	134407200	0.950413
chr10	134442600	0.801653
chr10	134479800	0.942149
chr10	134482200	0.842975
chr10	134498600	0.942149
chr10	134924100	0.809917
chr10	135071700	0.752066
chr10	135106900	0.834711
chr10	135171000	0.752066
chr10	135188100	0.727273
chr11	211000	0.851240
chr11	307700	0.661157
chr11	327400	0.884298
chr11	335800	0.975207
chr11	355900	0.735537
chr11	407100	0.826446
chr11	415200	0.892562
chr11	448800	0.776860
chr11	469900	0.842975
chr11	519100	0.809917
chr11	537200	0.801653
chr11	588200	0.818182
chr11	637700	0.785124
chr11	690500	0.991736
chr11	706900	0.793388
chr11	721200	0.809917
chr11	796100	0.793388
chr11	812200	0.801653
chr11	822000	0.867769
chr11	827400	0.842975
chr11	836000	0.942149
chr11	931000	0.661157
chr11	933300	0.991736
chr11	940900	0.859504
chr11	1284000	0.842975
chr11	1325900	0.669421
chr11	1357700	0.859504
chr11	1410700	0.826446
chr11	1568700	0.694215
chr11	1660900	0.834711
chr11	1715200	0.925620
chr11	1718400	0.669421
chr11	1781900	0.727273
chr11	1794100	0.958678
chr11	1848800	0.818182
chr11	1898600	0.900826
chr11	1927700	0.983471
chr11	1930200	0.975207
chr11	1970900	0.669421

chr11	2020200	0.677686
chr11	2023900	0.702479
chr11	2098900	0.966942
chr11	2118700	0.909091
chr11	2121900	0.735537
chr11	2130800	0.818182
chr11	2144000	0.752066
chr11	2402400	0.677686
chr11	2405100	0.776860
chr11	2408600	0.818182
chr11	2420800	0.685950
chr11	2554800	0.727273
chr11	2801800	0.760331
chr11	2913800	0.884298
chr11	2920400	0.669421
chr11	2954900	0.760331
chr11	2967200	0.785124
chr11	2972700	0.975207
chr11	2975300	0.859504
chr11	3010000	0.983471
chr11	3042500	0.702479
chr11	3071500	0.892562
chr11	3117500	0.884298
chr11	3145800	0.752066
chr11	3182000	0.677686
chr11	3191000	0.818182
chr11	3312100	0.942149
chr11	3357900	0.900826
chr11	3442800	0.710744
chr11	3744900	0.768595
chr11	3808500	0.760331
chr11	3820000	0.867769
chr11	3856700	0.710744
chr11	3887200	0.975207
chr11	3889300	0.975207
chr11	3901900	0.933884
chr11	3907500	0.727273
chr11	4017700	0.842975
chr11	4047600	0.991736
chr11	4079700	0.942149
chr11	4156900	0.958678
chr11	4502500	0.975207
chr11	5302100	0.876033
chr11	5321800	0.867769
chr11	5385600	0.975207
chr11	5388700	0.958678
chr11	5618300	0.743802
chr11	5642700	0.983471
chr11	6115800	0.785124
chr11	6241300	0.776860
chr11	6343800	0.933884
chr11	6348300	1.000000
chr11	6399800	0.900826
chr11	6409700	0.925620

chr11	6490900	0.661157
chr11	6496300	0.727273
chr11	6598000	0.958678
chr11	6628700	0.966942
chr11	6631100	0.859504
chr11	6634900	0.752066
chr11	6638100	0.785124
chr11	6656200	0.950413
chr11	6665100	0.818182
chr11	6669100	0.776860
chr11	6696300	0.685950
chr11	6698800	0.818182
chr11	6717000	0.991736
chr11	6740500	0.760331
chr11	6745900	0.710744
chr11	6749900	0.776860
chr11	6768000	0.793388
chr11	7026400	0.809917
chr11	7078800	0.983471
chr11	7226800	0.950413
chr11	7599300	0.876033
chr11	7622000	0.975207
chr11	7627100	0.991736
chr11	7659100	0.859504
chr11	7695300	0.677686
chr11	7999000	1.000000
chr11	8007800	0.876033
chr11	8090700	0.768595
chr11	8228800	0.884298
chr11	8290300	0.727273
chr11	8295100	0.702479
chr11	8693200	0.950413
chr11	8709400	0.785124
chr11	8721800	0.710744
chr11	8736500	0.760331
chr11	8742000	0.991736
chr11	8754500	0.950413
chr11	8856200	0.760331
chr11	8861900	0.950413
chr11	8886800	0.867769
chr11	8935900	0.818182
chr11	9023700	1.000000
chr11	9038800	0.727273
chr11	9162100	0.867769
chr11	9172400	0.958678
chr11	9181900	0.975207
chr11	9191400	0.876033
chr11	9203100	0.909091
chr11	9272300	0.909091
chr11	9275000	0.983471
chr11	9282800	0.677686
chr11	9337400	0.909091
chr11	9383900	0.950413
chr11	9386500	0.876033

chr11	9549500	0.983471
chr11	9573900	0.685950
chr11	9588000	0.975207
chr11	9591100	0.933884
chr11	9594100	0.677686
chr11	9634400	0.867769
chr11	9637100	0.743802
chr11	9646700	0.917355
chr11	9649200	0.925620
chr11	9661000	0.834711
chr11	9673900	0.917355
chr11	9684900	0.785124
chr11	9708200	0.752066
chr11	9710700	0.966942
chr11	9713800	0.958678
chr11	9725000	0.975207
chr11	9728800	0.768595
chr11	9730900	0.884298
chr11	9733600	0.950413
chr11	9738000	0.702479
chr11	9741600	0.958678
chr11	9743700	0.785124
chr11	9748300	0.966942
chr11	9772400	0.801653
chr11	9777100	0.826446
chr11	9780000	0.851240
chr11	9782400	0.942149
chr11	9787500	0.876033
chr11	9796900	0.727273
chr11	9874200	0.983471
chr11	9880400	0.958678
chr11	9884700	0.983471
chr11	9927700	0.991736
chr11	9999700	0.892562
chr11	10004100	0.925620
chr11	10043400	0.909091
chr11	10048100	0.826446
chr11	10140400	0.826446
chr11	10148400	0.776860
chr11	10283800	0.727273
chr11	10331200	0.677686
chr11	10344800	0.958678
chr11	10347200	0.826446
chr11	10349400	0.942149
chr11	10351600	0.925620
chr11	10374300	0.983471
chr11	10422700	0.694215
chr11	10428700	0.900826
chr11	10472500	0.768595
chr11	10477900	0.661157
chr11	10527500	1.000000
chr11	10531400	0.685950
chr11	10546300	0.966942
chr11	10551400	0.876033

chr11	10595500	0.834711
chr11	10602700	0.933884
chr11	10606400	0.876033
chr11	10628400	0.884298
chr11	10679300	0.842975
chr11	10681800	0.818182
chr11	10696800	0.735537
chr11	10757600	0.950413
chr11	10806500	0.909091
chr11	10826600	0.867769
chr11	10922800	0.884298
chr11	10933700	0.818182
chr11	11043800	0.768595
chr11	11413800	0.834711
chr11	11882200	0.876033
chr11	11997400	0.719008
chr11	12002400	0.909091
chr11	12007700	0.909091
chr11	12010900	0.983471
chr11	12020500	0.966942
chr11	12027500	1.000000
chr11	12041600	0.743802
chr11	12065900	1.000000
chr11	12069000	0.818182
chr11	12102000	0.966942
chr11	12112600	0.859504
chr11	12131500	0.719008
chr11	12134700	0.702479
chr11	12136900	0.925620
chr11	12144900	0.851240
chr11	12147400	0.950413
chr11	12159800	0.983471
chr11	12184400	0.677686
chr11	12186500	0.917355
chr11	12190200	0.743802
chr11	12198100	0.900826
chr11	12203300	0.768595
chr11	12205800	0.942149
chr11	12208100	0.983471
chr11	12223600	0.900826
chr11	12235800	0.859504
chr11	12238700	0.942149
chr11	12241400	0.950413
chr11	12244100	0.958678
chr11	12246500	0.958678
chr11	12254400	0.876033
chr11	12257800	0.900826
chr11	12260500	0.768595
chr11	12262600	0.917355
chr11	12290600	0.884298
chr11	12292700	0.710744
chr11	12303200	0.801653
chr11	12306800	0.851240
chr11	12310700	0.909091

chr11	12433900	0.975207
chr11	12452500	0.867769
chr11	12476300	0.801653
chr11	12485300	0.785124
chr11	12491300	0.785124
chr11	12534800	0.801653
chr11	12561500	0.925620
chr11	12708100	0.991736
chr11	12713800	0.760331
chr11	12716400	0.892562
chr11	12725800	0.710744
chr11	12729200	0.661157
chr11	12737700	0.727273
chr11	12747700	0.900826
chr11	12791800	0.975207
chr11	12809700	0.702479
chr11	12821800	0.991736
chr11	12824900	0.950413
chr11	12836600	0.933884
chr11	12846100	0.958678
chr11	12864200	0.975207
chr11	12922500	0.743802
chr11	13031500	0.735537
chr11	13123700	0.669421
chr11	13126900	0.760331
chr11	13133000	0.876033
chr11	13139900	1.000000
chr11	13142200	0.884298
chr11	13157800	0.826446
chr11	13167800	0.727273
chr11	13175000	0.702479
chr11	13227000	0.719008
chr11	13230100	0.983471
chr11	13238100	0.818182
chr11	13283600	0.991736
chr11	13359800	0.933884
chr11	13398000	0.884298
chr11	13462200	0.983471
chr11	13468300	0.991736
chr11	13471100	0.975207
chr11	13482100	0.735537
chr11	13707400	0.900826
chr11	13739300	0.991736
chr11	13742800	0.677686
chr11	13767100	0.785124
chr11	13895300	0.842975
chr11	13945800	0.842975
chr11	13951000	0.776860
chr11	13965000	0.818182
chr11	13984800	0.851240
chr11	14340600	0.884298
chr11	14344700	0.983471
chr11	14359100	0.966942
chr11	14398800	0.710744

chr11	14403200	0.661157
chr11	14446400	0.917355
chr11	14468100	0.958678
chr11	14600300	0.975207
chr11	14924600	0.710744
chr11	15096000	0.677686
chr11	15764500	0.719008
chr11	15767000	0.801653
chr11	15990700	0.859504
chr11	16023600	0.884298
chr11	16247900	0.735537
chr11	16635500	0.842975
chr11	16781500	1.000000
chr11	16809400	1.000000
chr11	16811600	0.735537
chr11	16814000	0.851240
chr11	16919800	0.834711
chr11	16924700	0.892562
chr11	17113600	0.809917
chr11	17168100	0.851240
chr11	17179400	0.958678
chr11	17183800	0.818182
chr11	17193500	0.892562
chr11	17232100	0.876033
chr11	17246900	0.760331
chr11	17252200	0.661157
chr11	17297500	0.702479
chr11	17672000	0.818182
chr11	17756500	0.867769
chr11	18103200	0.859504
chr11	18346700	0.760331
chr11	18406100	0.818182
chr11	18415200	0.661157
chr11	18771800	0.809917
chr11	18786000	0.867769
chr11	19047800	0.661157
chr11	19099500	0.768595
chr11	19102700	0.909091
chr11	19109500	0.950413
chr11	19126900	0.702479
chr11	19221300	0.826446
chr11	19254600	0.925620
chr11	19386200	0.702479
chr11	19389600	0.933884
chr11	19393100	0.826446
chr11	19396000	0.917355
chr11	19402300	0.884298
chr11	19450500	0.752066
chr11	19459600	0.661157
chr11	19464000	0.876033
chr11	19468900	0.727273
chr11	19533500	0.892562
chr11	19582700	0.950413
chr11	19586200	0.900826

chr11	19599500	0.966942
chr11	19616700	0.752066
chr11	19620000	0.909091
chr11	19636900	0.966942
chr11	19649900	0.925620
chr11	19682300	0.776860
chr11	19686900	0.685950
chr11	19698200	0.884298
chr11	19702200	0.975207
chr11	19727700	0.917355
chr11	19733100	0.818182
chr11	19738200	0.909091
chr11	19745800	0.900826
chr11	19762000	0.842975
chr11	19793100	0.702479
chr11	19883300	0.859504
chr11	19886300	0.950413
chr11	19890300	0.785124
chr11	19940900	0.892562
chr11	19957000	0.768595
chr11	19973600	0.958678
chr11	19976300	0.710744
chr11	19979900	0.842975
chr11	20000600	0.991736
chr11	20003400	0.909091
chr11	20026700	0.942149
chr11	20044300	0.991736
chr11	20063900	0.884298
chr11	20115700	0.760331
chr11	20119200	0.983471
chr11	20121600	0.991736
chr11	20132100	0.909091
chr11	20134700	0.661157
chr11	20185200	0.809917
chr11	20376400	0.752066
chr11	20384200	0.925620
chr11	20389300	0.958678
chr11	22454200	0.694215
chr11	22481300	0.900826
chr11	22688900	0.694215
chr11	22724700	0.950413
chr11	22807100	0.991736
chr11	22814400	0.859504
chr11	22830200	0.892562
chr11	22854400	0.826446
chr11	23146200	0.669421
chr11	23227600	0.925620
chr11	23300100	0.694215
chr11	23333700	0.801653
chr11	23336100	0.719008
chr11	23576900	0.958678
chr11	23592800	0.942149
chr11	23925100	0.983471
chr11	23954300	0.983471

chr11	24311000	0.661157
chr11	24334400	1.000000
chr11	24597200	0.925620
chr11	24609800	0.801653
chr11	24614100	0.768595
chr11	24719500	0.702479
chr11	24851100	0.727273
chr11	24855400	0.876033
chr11	25878700	0.793388
chr11	25912700	0.661157
chr11	26249100	0.950413
chr11	26251200	0.991736
chr11	26253400	0.966942
chr11	26256500	0.842975
chr11	27235200	0.859504
chr11	27237800	0.809917
chr11	27304300	0.991736
chr11	27478100	0.719008
chr11	27956400	0.950413
chr11	28112100	0.933884
chr11	28144900	0.702479
chr11	28167800	0.950413
chr11	28249900	0.958678
chr11	28325600	0.702479
chr11	28365000	0.900826
chr11	28483200	0.719008
chr11	28588600	0.900826
chr11	28643900	0.685950
chr11	28674200	0.685950
chr11	28723400	0.950413
chr11	28726400	0.760331
chr11	28761300	0.694215
chr11	28764700	0.925620
chr11	28778600	0.818182
chr11	28781400	0.925620
chr11	28789600	0.983471
chr11	28820300	0.983471
chr11	28841800	0.834711
chr11	28853700	0.900826
chr11	28856100	0.818182
chr11	28858300	0.975207
chr11	28860500	0.892562
chr11	28864400	0.884298
chr11	28882800	0.958678
chr11	28933900	0.950413
chr11	29024400	0.950413
chr11	29028200	0.917355
chr11	29030400	0.801653
chr11	29040500	0.975207
chr11	29139700	0.859504
chr11	29142900	0.834711
chr11	29304000	0.809917
chr11	29315800	0.917355
chr11	29328400	0.851240

chr11	29474200	0.876033
chr11	29503000	0.785124
chr11	30901200	0.950413
chr11	31668000	0.677686
chr11	31834900	0.818182
chr11	32109900	0.958678
chr11	32125900	0.834711
chr11	32159200	0.983471
chr11	32913800	0.694215
chr11	33169800	0.809917
chr11	33180400	0.768595
chr11	33256800	0.785124
chr11	33282000	0.719008
chr11	33394400	0.983471
chr11	33400500	0.876033
chr11	33467100	0.768595
chr11	33470800	0.694215
chr11	33474800	0.661157
chr11	33485200	0.785124
chr11	33497900	0.669421
chr11	33711200	0.760331
chr11	33714200	0.991736
chr11	33716600	0.859504
chr11	33722400	0.900826
chr11	33724500	0.851240
chr11	33726700	0.752066
chr11	33730000	0.966942
chr11	33738900	0.867769
chr11	33742100	0.909091
chr11	33744700	0.743802
chr11	33747600	0.900826
chr11	33751600	0.900826
chr11	33754300	0.900826
chr11	33759600	0.710744
chr11	33824200	0.719008
chr11	33833700	0.710744
chr11	33921300	0.991736
chr11	33951800	0.677686
chr11	33954000	0.966942
chr11	33956900	0.925620
chr11	33959900	0.785124
chr11	33964300	0.884298
chr11	34066900	0.661157
chr11	34090200	0.983471
chr11	34097200	0.909091
chr11	34273200	0.809917
chr11	34621200	0.727273
chr11	34703500	0.793388
chr11	34814500	0.727273
chr11	34831500	0.991736
chr11	34997700	0.892562
chr11	34999900	0.842975
chr11	35052600	0.892562
chr11	35057800	0.942149

chr11	35099600	0.966942
chr11	35109600	0.892562
chr11	35133300	0.884298
chr11	35163500	0.677686
chr11	35180000	0.694215
chr11	35188700	0.991736
chr11	35601800	0.719008
chr11	35638900	0.669421
chr11	35643700	1.000000
chr11	35645900	0.876033
chr11	35651700	0.975207
chr11	35654300	0.826446
chr11	35663200	0.884298
chr11	35710500	0.661157
chr11	35757400	0.958678
chr11	35773600	0.909091
chr11	35817500	0.917355
chr11	35820300	0.991736
chr11	35843900	0.900826
chr11	35930300	0.983471
chr11	35945400	0.727273
chr11	35975600	0.884298
chr11	36000700	0.826446
chr11	36024600	0.900826
chr11	36031600	0.966942
chr11	36034300	0.760331
chr11	36079500	0.966942
chr11	36094800	0.727273
chr11	36097100	0.958678
chr11	36113300	0.719008
chr11	36126800	0.735537
chr11	36138100	0.958678
chr11	36172300	0.892562
chr11	36179200	0.991736
chr11	36187100	0.809917
chr11	36253000	0.702479
chr11	36371800	0.818182
chr11	36385200	0.669421
chr11	36426500	0.768595
chr11	36431200	0.991736
chr11	36433500	0.785124
chr11	36453900	0.859504
chr11	36509700	0.975207
chr11	36522500	0.942149
chr11	36533900	0.677686
chr11	37743500	0.950413
chr11	37808300	0.785124
chr11	37810400	0.925620
chr11	37994800	0.677686
chr11	38012500	0.950413
chr11	38027700	0.975207
chr11	38031700	0.785124
chr11	39563800	0.983471
chr11	40351100	1.000000

chr11	40358600	0.925620
chr11	40360700	0.925620
chr11	42043900	0.727273
chr11	42271700	0.809917
chr11	42337400	0.909091
chr11	43350000	0.859504
chr11	43398100	0.752066
chr11	43654200	0.768595
chr11	43705200	0.719008
chr11	43708700	0.925620
chr11	43852700	0.991736
chr11	43920500	0.950413
chr11	43927500	0.983471
chr11	43942600	0.942149
chr11	43945800	0.900826
chr11	43948300	0.925620
chr11	43958400	0.983471
chr11	44112500	0.702479
chr11	44266600	0.842975
chr11	44291600	0.942149
chr11	44331400	0.710744
chr11	44338400	0.826446
chr11	44591000	0.942149
chr11	44593300	0.735537
chr11	44645700	0.876033
chr11	44656100	0.727273
chr11	44703400	0.818182
chr11	44723100	0.801653
chr11	44731300	0.785124
chr11	44747900	0.669421
chr11	44751400	0.892562
chr11	44782900	0.809917
chr11	44785700	0.991736
chr11	44788400	0.966942
chr11	44796600	0.975207
chr11	44811300	0.975207
chr11	44839600	0.917355
chr11	44846000	0.834711
chr11	44884100	0.892562
chr11	44899900	0.859504
chr11	44977000	0.975207
chr11	44999600	0.867769
chr11	45047900	0.884298
chr11	45202500	0.727273
chr11	45231900	0.975207
chr11	45286800	0.710744
chr11	45422400	0.942149
chr11	45640200	0.925620
chr11	45663900	0.727273
chr11	45668600	0.735537
chr11	45677100	0.859504
chr11	45680400	0.950413
chr11	45880700	0.950413
chr11	45921800	0.818182

chr11	46121800	0.768595
chr11	46258800	0.776860
chr11	46264900	0.752066
chr11	46364100	0.867769
chr11	46371200	0.685950
chr11	46382700	0.760331
chr11	46385900	0.760331
chr11	46402200	0.776860
chr11	46473600	0.702479
chr11	46513400	0.942149
chr11	46535500	0.851240
chr11	46542700	0.933884
chr11	46575900	0.983471
chr11	46582600	0.876033
chr11	46613000	0.818182
chr11	46619600	0.727273
chr11	46670300	0.958678
chr11	46686300	0.859504
chr11	46736600	0.826446
chr11	46822100	0.859504
chr11	46928600	0.859504
chr11	46941200	0.760331
chr11	47199500	0.669421
chr11	47239900	0.661157
chr11	47242400	0.925620
chr11	47276500	0.834711
chr11	47279800	0.834711
chr11	47287000	0.942149
chr11	47396300	0.867769
chr11	47415300	0.735537
chr11	47417700	0.851240
chr11	47421800	0.818182
chr11	47429000	0.793388
chr11	47432500	0.760331
chr11	47438300	0.818182
chr11	47483000	0.793388
chr11	47488300	0.983471
chr11	47499600	0.809917
chr11	47536700	0.925620
chr11	47540500	0.727273
chr11	47546000	0.925620
chr11	47562000	0.950413
chr11	47611500	0.785124
chr11	47790600	0.710744
chr11	47907500	0.685950
chr11	47912200	0.793388
chr11	47927500	0.942149
chr11	47936700	0.933884
chr11	47940500	1.000000
chr11	47968900	0.842975
chr11	48035300	0.694215
chr11	48041200	0.958678
chr11	48085700	0.735537
chr11	48088900	0.710744

chr11	48103100	0.909091
chr11	48131600	0.900826
chr11	48145000	0.685950
chr11	48172200	0.859504
chr11	48176300	0.942149
chr11	48192100	0.917355
chr11	48893200	0.752066
chr11	49255500	0.950413
chr11	49347900	0.942149
chr11	49350000	0.702479
chr11	49389600	0.892562
chr11	50522200	0.727273
chr11	51566900	0.752066
chr11	51569800	0.752066
chr11	51575800	0.677686
chr11	51580300	0.710744
chr11	51582500	0.933884
chr11	51588600	0.834711
chr11	51593200	0.958678
chr11	55015300	0.735537
chr11	55026000	0.950413
chr11	56840100	0.909091
chr11	56981400	0.917355
chr11	57045600	0.983471
chr11	57056800	0.818182
chr11	57079000	0.933884
chr11	57083200	0.719008
chr11	57090100	0.983471
chr11	57175200	0.834711
chr11	57181000	0.867769
chr11	57184300	0.917355
chr11	57194800	0.669421
chr11	57244100	0.752066
chr11	57250500	0.809917
chr11	57417500	0.900826
chr11	57539900	0.776860
chr11	57542600	0.818182
chr11	57546200	0.702479
chr11	57549000	0.727273
chr11	57560500	0.917355
chr11	57566700	0.876033
chr11	57569100	1.000000
chr11	57770100	0.719008
chr11	57772400	0.991736
chr11	57774600	0.694215
chr11	57792200	0.851240
chr11	57826700	0.925620
chr11	58102800	0.909091
chr11	58161700	0.900826
chr11	58341500	0.983471
chr11	58343700	0.834711
chr11	58395500	0.950413
chr11	58869800	0.669421
chr11	58994400	0.694215

chr11	59278900	0.942149
chr11	59313300	0.669421
chr11	59324100	0.760331
chr11	59384600	0.719008
chr11	59428500	0.719008
chr11	59474700	0.991736
chr11	59524900	0.719008
chr11	59540200	0.942149
chr11	59544200	0.983471
chr11	59553900	0.809917
chr11	59556300	0.859504
chr11	59569600	0.867769
chr11	59625400	0.933884
chr11	59632900	0.925620
chr11	59669200	0.966942
chr11	59866300	0.991736
chr11	59869300	0.983471
chr11	59892600	0.983471
chr11	59927500	0.975207
chr11	59929800	0.909091
chr11	59933700	0.958678
chr11	59937300	0.966942
chr11	59961200	0.917355
chr11	59964000	0.942149
chr11	59972700	0.851240
chr11	59982000	0.685950
chr11	60074100	0.917355
chr11	60083000	0.909091
chr11	60138600	0.925620
chr11	60546000	0.859504
chr11	60555600	0.983471
chr11	60578400	0.909091
chr11	60603400	0.925620
chr11	60608000	0.685950
chr11	60612800	0.925620
chr11	60659000	0.818182
chr11	60678900	0.719008
chr11	60686900	0.975207
chr11	60704900	0.719008
chr11	60897900	0.702479
chr11	60917500	0.950413
chr11	60921400	0.826446
chr11	60926600	0.933884
chr11	61065600	0.900826
chr11	61115900	0.809917
chr11	61182300	0.694215
chr11	61203000	0.661157
chr11	61284400	0.785124
chr11	61363200	0.867769
chr11	61410300	0.661157
chr11	61449900	0.983471
chr11	61463800	0.917355
chr11	61486400	0.975207
chr11	61545300	0.867769

chr11	61572500	0.809917
chr11	61582100	0.685950
chr11	61594300	0.669421
chr11	61603300	0.975207
chr11	61649200	0.876033
chr11	61655900	0.933884
chr11	61737600	0.900826
chr11	61740600	0.859504
chr11	61746400	0.851240
chr11	61793200	1.000000
chr11	61920800	0.975207
chr11	62031800	0.983471
chr11	62107700	0.983471
chr11	62128600	0.925620
chr11	62208000	0.752066
chr11	62265100	0.958678
chr11	62267400	0.793388
chr11	62270000	0.793388
chr11	62276400	0.793388
chr11	62306700	0.727273
chr11	62309100	0.884298
chr11	62316200	0.966942
chr11	62319900	0.859504
chr11	62322100	0.743802
chr11	62328100	0.900826
chr11	62346100	0.942149
chr11	62361000	0.735537
chr11	62370400	0.677686
chr11	62381300	0.710744
chr11	62390400	0.925620
chr11	62455600	0.834711
chr11	62472800	0.669421
chr11	62558000	0.702479
chr11	62652700	0.743802
chr11	62657500	0.925620
chr11	62691400	0.785124
chr11	62785400	0.793388
chr11	62913200	0.966942
chr11	62933500	0.950413
chr11	63304700	0.752066
chr11	63350200	0.884298
chr11	63377400	0.925620
chr11	63435800	0.991736
chr11	63477500	0.685950
chr11	63530700	0.834711
chr11	63605000	0.685950
chr11	63626400	0.727273
chr11	63636200	0.776860
chr11	63639800	0.702479
chr11	63656000	0.776860
chr11	63685700	0.826446
chr11	63705600	0.669421
chr11	63767500	0.694215
chr11	63771600	0.768595

chr11	63775400	0.917355
chr11	63828700	0.801653
chr11	63862600	0.876033
chr11	63949200	0.958678
chr11	63973200	0.727273
chr11	63976500	0.867769
chr11	63995700	0.661157
chr11	64007500	0.669421
chr11	64030900	0.760331
chr11	64034600	0.752066
chr11	64059800	0.702479
chr11	64071900	0.859504
chr11	64122900	0.942149
chr11	64125600	0.719008
chr11	64217000	0.760331
chr11	64321800	0.694215
chr11	64510500	0.859504
chr11	64513100	0.685950
chr11	64531700	0.785124
chr11	64540200	0.983471
chr11	64547300	0.859504
chr11	64568500	0.694215
chr11	64624800	0.876033
chr11	64626900	0.909091
chr11	64632300	0.809917
chr11	64639800	0.702479
chr11	64653400	0.735537
chr11	64685800	0.669421
chr11	64739400	0.867769
chr11	64757800	0.867769
chr11	64764700	0.892562
chr11	64815400	0.685950
chr11	64936900	0.809917
chr11	64993300	0.867769
chr11	65043000	1.000000
chr11	65046200	0.884298
chr11	65048300	0.768595
chr11	65058200	0.826446
chr11	65085800	0.818182
chr11	65097700	0.842975
chr11	65138900	0.950413
chr11	65149100	0.975207
chr11	65152700	0.785124
chr11	65156300	0.818182
chr11	65158400	0.950413
chr11	65184200	0.892562
chr11	65188300	0.727273
chr11	65199100	0.719008
chr11	65239800	0.958678
chr11	65246800	0.719008
chr11	65258700	0.909091
chr11	65261200	0.933884
chr11	65275800	0.942149
chr11	65308300	0.768595

chr11	65323200	0.710744
chr11	65329100	0.768595
chr11	65333700	0.942149
chr11	65342300	0.859504
chr11	65344600	0.710744
chr11	65359900	0.925620
chr11	65374900	0.925620
chr11	65408100	0.760331
chr11	65410800	0.768595
chr11	65415200	0.900826
chr11	65671000	0.909091
chr11	65678800	0.801653
chr11	65681200	0.942149
chr11	65709200	0.917355
chr11	65783400	0.859504
chr11	65788700	0.950413
chr11	65836800	0.917355
chr11	65848800	0.876033
chr11	65880400	0.975207
chr11	65883600	0.917355
chr11	65887100	0.909091
chr11	65894900	0.975207
chr11	65904900	0.694215
chr11	66046000	0.661157
chr11	66096100	0.727273
chr11	66105400	0.892562
chr11	66311400	0.958678
chr11	66336000	0.809917
chr11	66387400	0.752066
chr11	66389500	0.661157
chr11	66508000	0.909091
chr11	66515600	0.859504
chr11	66583400	0.851240
chr11	66622900	0.669421
chr11	66660100	0.900826
chr11	66736000	0.983471
chr11	66738800	0.793388
chr11	66839300	0.768595
chr11	66885600	0.859504
chr11	66913600	0.958678
chr11	66935800	0.661157
chr11	67006600	0.859504
chr11	67037700	0.818182
chr11	67045300	0.859504
chr11	67055200	0.892562
chr11	67119200	0.826446
chr11	67124500	0.950413
chr11	67142400	0.677686
chr11	67164800	0.818182
chr11	67170400	0.785124
chr11	67188700	0.785124
chr11	67220200	0.876033
chr11	67232400	0.966942
chr11	67273800	0.809917

chr11	67331500	0.991736
chr11	67341300	1.000000
chr11	67398800	0.818182
chr11	67782200	0.752066
chr11	67805400	0.966942
chr11	67867900	0.975207
chr11	68083100	0.909091
chr11	68096100	0.966942
chr11	68099200	0.851240
chr11	68145400	0.743802
chr11	68147500	0.677686
chr11	68149600	0.867769
chr11	68205400	0.793388
chr11	68270800	1.000000
chr11	68291800	0.991736
chr11	68317900	0.909091
chr11	68350500	0.851240
chr11	68427100	0.793388
chr11	68445300	0.685950
chr11	68452600	0.677686
chr11	68517200	0.991736
chr11	68606500	0.685950
chr11	68611700	0.677686
chr11	68637900	0.809917
chr11	68643300	0.818182
chr11	68658800	0.925620
chr11	68723100	0.892562
chr11	68891200	0.719008
chr11	69035300	0.809917
chr11	69062300	0.925620
chr11	69067600	0.793388
chr11	69071100	0.933884
chr11	69080100	0.975207
chr11	69085600	1.000000
chr11	69089700	0.768595
chr11	69165200	0.876033
chr11	69168700	0.752066
chr11	69171900	0.785124
chr11	69180000	0.925620
chr11	69231600	0.785124
chr11	69259000	0.710744
chr11	69265600	0.661157
chr11	69286600	0.917355
chr11	69308700	0.818182
chr11	69312200	0.983471
chr11	69324400	0.694215
chr11	69403000	0.867769
chr11	69451700	0.942149
chr11	69459400	0.867769
chr11	69516000	0.743802
chr11	69716100	0.851240
chr11	69795800	0.859504
chr11	69816700	0.809917
chr11	69920000	0.884298

chr11	70052000	0.710744
chr11	70127700	0.760331
chr11	70153800	0.958678
chr11	70266400	0.884298
chr11	70634800	0.958678
chr11	71201600	0.925620
chr11	71524500	0.760331
chr11	71643000	0.752066
chr11	71646300	0.991736
chr11	71649100	0.909091
chr11	71655400	0.991736
chr11	71657500	0.909091
chr11	71659700	0.842975
chr11	71674100	0.909091
chr11	71710400	0.917355
chr11	71736900	0.694215
chr11	71754000	0.669421
chr11	71760300	0.958678
chr11	71778400	0.933884
chr11	71781100	0.809917
chr11	71784300	0.710744
chr11	71789600	0.851240
chr11	71793100	0.818182
chr11	71951000	0.752066
chr11	72195000	0.892562
chr11	72336200	0.710744
chr11	72342900	0.933884
chr11	72345200	0.884298
chr11	72348800	0.727273
chr11	72354500	0.801653
chr11	72447400	0.966942
chr11	72527500	0.842975
chr11	72547600	0.876033
chr11	72568000	0.975207
chr11	72576400	0.991736
chr11	72581100	0.785124
chr11	72646900	0.727273
chr11	72677200	0.950413
chr11	72759700	0.818182
chr11	72784200	0.694215
chr11	72888900	0.917355
chr11	72897400	0.867769
chr11	72910200	0.842975
chr11	72954100	0.851240
chr11	72969000	0.900826
chr11	73000900	0.958678
chr11	73025500	0.727273
chr11	73031800	0.851240
chr11	73035700	0.867769
chr11	73042000	0.661157
chr11	73046700	0.892562
chr11	73077500	0.884298
chr11	73094300	0.925620
chr11	73097000	0.950413

chr11	73104400	0.669421
chr11	73110600	0.694215
chr11	73278200	0.785124
chr11	73372500	0.702479
chr11	73695500	0.859504
chr11	73736800	0.743802
chr11	73850200	0.818182
chr11	73866400	0.710744
chr11	73999500	0.975207
chr11	74030200	0.900826
chr11	74033400	0.983471
chr11	74037900	0.768595
chr11	74041500	0.983471
chr11	74044000	0.677686
chr11	74130000	0.983471
chr11	74138500	0.884298
chr11	74160900	0.900826
chr11	74271000	0.958678
chr11	74348200	0.752066
chr11	74408800	0.710744
chr11	74442700	0.743802
chr11	74465400	0.950413
chr11	74467500	0.900826
chr11	74481900	1.000000
chr11	74494600	0.966942
chr11	74530100	0.933884
chr11	74533900	0.925620
chr11	74551400	0.942149
chr11	74616800	0.818182
chr11	74640900	0.950413
chr11	74644100	0.900826
chr11	74649200	0.983471
chr11	74671600	0.661157
chr11	74742800	0.685950
chr11	74854700	0.760331
chr11	74872700	0.933884
chr11	74987100	0.925620
chr11	74992100	0.826446
chr11	75004700	0.661157
chr11	75017700	0.950413
chr11	75023800	0.884298
chr11	75026000	0.909091
chr11	75037400	0.892562
chr11	75040400	0.925620
chr11	75045400	0.776860
chr11	75048600	0.702479
chr11	75052300	0.942149
chr11	75055100	0.694215
chr11	75059200	0.925620
chr11	75068200	0.867769
chr11	75081100	0.760331
chr11	75097100	0.909091
chr11	75099900	0.776860
chr11	75114300	0.917355

chr11	75117700	0.983471
chr11	75130400	0.933884
chr11	75141700	0.719008
chr11	75177500	0.925620
chr11	75180900	0.884298
chr11	75201800	0.785124
chr11	75204000	0.760331
chr11	75207600	0.801653
chr11	75234400	0.768595
chr11	75245600	0.752066
chr11	75265900	0.958678
chr11	75269700	0.752066
chr11	75277300	0.677686
chr11	75315400	0.950413
chr11	75471900	0.842975
chr11	75480000	0.867769
chr11	75488500	0.727273
chr11	75523800	0.842975
chr11	75538600	0.909091
chr11	75636700	0.809917
chr11	75649900	0.859504
chr11	75657600	0.776860
chr11	75678300	0.983471
chr11	75741000	0.851240
chr11	75743300	0.793388
chr11	75785900	0.991736
chr11	75794700	0.966942
chr11	75831600	0.933884
chr11	75863700	0.851240
chr11	75952700	0.677686
chr11	76082600	0.752066
chr11	76188900	0.727273
chr11	76262200	0.900826
chr11	76271500	0.909091
chr11	76291400	0.793388
chr11	76299600	0.966942
chr11	76326600	0.867769
chr11	76335000	0.685950
chr11	76340100	0.826446
chr11	76343100	0.942149
chr11	76375500	0.702479
chr11	76379000	0.710744
chr11	76383300	0.942149
chr11	76406200	0.892562
chr11	76412800	0.818182
chr11	76475000	1.000000
chr11	76479900	0.859504
chr11	76492500	0.685950
chr11	76510600	0.884298
chr11	76534600	0.867769
chr11	76537000	0.818182
chr11	76550900	0.950413
chr11	76591500	0.768595
chr11	76596700	0.983471

chr11	76600200	0.776860
chr11	76731700	0.859504
chr11	76751100	0.809917
chr11	76785500	0.851240
chr11	76794000	0.826446
chr11	76839000	0.752066
chr11	76851500	0.768595
chr11	76879100	0.685950
chr11	76884600	0.884298
chr11	76955900	0.975207
chr11	76961900	0.851240
chr11	77119000	0.842975
chr11	77123100	0.900826
chr11	77218200	0.809917
chr11	77313700	0.983471
chr11	77407300	0.694215
chr11	77554000	1.000000
chr11	77597700	0.966942
chr11	77601900	0.975207
chr11	77625700	1.000000
chr11	77641500	0.975207
chr11	77706800	0.760331
chr11	77734400	0.702479
chr11	77741800	0.900826
chr11	77751800	0.975207
chr11	77880400	0.859504
chr11	77968300	0.834711
chr11	78010700	0.669421
chr11	78029900	0.884298
chr11	78055600	0.917355
chr11	78061900	0.950413
chr11	78126400	0.752066
chr11	78131200	0.950413
chr11	78171900	0.950413
chr11	78181200	0.900826
chr11	78272200	0.950413
chr11	78274300	0.909091
chr11	78278600	0.719008
chr11	78509000	0.983471
chr11	78724800	0.793388
chr11	78729800	0.991736
chr11	78798500	0.991736
chr11	79149200	0.677686
chr11	80009500	0.892562
chr11	80061900	0.909091
chr11	80154000	0.925620
chr11	80305100	0.983471
chr11	80307200	0.826446
chr11	80411000	1.000000
chr11	80437700	0.760331
chr11	80527500	0.925620
chr11	80558400	0.818182
chr11	80567600	0.735537
chr11	80572300	0.735537

chr11	82505800	0.983471
chr11	82565700	0.983471
chr11	82569000	0.661157
chr11	82571100	0.950413
chr11	82595100	1.000000
chr11	82601000	0.991736
chr11	82618000	0.818182
chr11	82634400	0.933884
chr11	82691400	1.000000
chr11	82719300	0.933884
chr11	82724200	0.785124
chr11	82750800	0.694215
chr11	82756300	0.702479
chr11	82758700	0.991736
chr11	82763600	0.925620
chr11	82767800	0.669421
chr11	82780100	0.876033
chr11	82969200	0.884298
chr11	85195200	1.000000
chr11	85370600	0.677686
chr11	85436900	0.991736
chr11	85447600	0.942149
chr11	85565900	0.710744
chr11	85659100	0.991736
chr11	85673100	0.958678
chr11	85680300	0.991736
chr11	85753500	0.975207
chr11	85773900	0.809917
chr11	85776500	0.925620
chr11	85863100	0.710744
chr11	85884500	0.743802
chr11	85887600	0.809917
chr11	85904800	0.917355
chr11	86102000	0.768595
chr11	86107800	0.909091
chr11	86167700	0.677686
chr11	86169800	0.677686
chr11	86172300	0.785124
chr11	86184600	0.727273
chr11	86186700	0.842975
chr11	86189800	0.834711
chr11	86193400	0.834711
chr11	86195800	0.991736
chr11	86215000	0.950413
chr11	86222700	0.743802
chr11	86226300	0.942149
chr11	86229400	0.942149
chr11	86231700	0.876033
chr11	86236000	0.983471
chr11	86259600	0.983471
chr11	86263500	0.826446
chr11	86270800	0.909091
chr11	86278900	0.900826
chr11	86286600	0.975207

chr11	86290400	0.892562
chr11	86303200	0.702479
chr11	86305300	0.809917
chr11	86308100	0.958678
chr11	86317000	0.892562
chr11	86319500	0.958678
chr11	86332500	0.859504
chr11	86344300	0.801653
chr11	86386400	0.685950
chr11	86407100	0.876033
chr11	86431800	0.933884
chr11	86446700	0.776860
chr11	86449300	1.000000
chr11	86452300	0.727273
chr11	86455400	0.793388
chr11	86515400	0.933884
chr11	86517500	0.876033
chr11	86520600	0.776860
chr11	86526800	0.950413
chr11	86529700	0.958678
chr11	86619200	0.735537
chr11	86621800	0.694215
chr11	86627000	0.933884
chr11	86630800	0.933884
chr11	86636500	0.685950
chr11	86642600	0.933884
chr11	86646300	0.760331
chr11	86649100	0.851240
chr11	86653300	0.851240
chr11	86658800	0.958678
chr11	86661900	0.677686
chr11	86680800	0.975207
chr11	86731300	0.801653
chr11	86859500	0.785124
chr11	86885000	1.000000
chr11	86950400	0.925620
chr11	86972300	0.933884
chr11	86992800	0.768595
chr11	87064600	0.842975
chr11	87077700	0.669421
chr11	87081300	0.727273
chr11	87102800	0.785124
chr11	87142500	0.685950
chr11	88046100	0.710744
chr11	88058500	0.991736
chr11	88097200	0.685950
chr11	88132100	0.909091
chr11	88134400	0.735537
chr11	88136500	0.909091
chr11	88148400	0.917355
chr11	88269300	0.942149
chr11	88271400	0.760331
chr11	88288600	0.983471
chr11	88309100	0.702479

chr11	88993900	0.752066
chr11	89065800	0.785124
chr11	89070200	0.818182
chr11	89072500	0.917355
chr11	89116100	0.826446
chr11	89139100	0.669421
chr11	89225600	0.785124
chr11	89229800	0.942149
chr11	89339500	0.966942
chr11	89341600	0.842975
chr11	89597600	0.884298
chr11	90434600	0.743802
chr11	90537500	0.809917
chr11	90653000	0.727273
chr11	91342400	0.677686
chr11	91358900	0.818182
chr11	91605200	1.000000
chr11	93262700	0.809917
chr11	93451700	0.975207
chr11	93467100	0.669421
chr11	93865200	0.867769
chr11	93867800	0.933884
chr11	93870000	0.975207
chr11	93874000	0.892562
chr11	93876100	0.933884
chr11	93882900	0.900826
chr11	93885800	0.826446
chr11	94134800	0.760331
chr11	94297100	0.719008
chr11	94312500	0.752066
chr11	94450600	0.776860
chr11	94466100	0.950413
chr11	94470900	0.785124
chr11	94484200	0.892562
chr11	94510000	0.735537
chr11	94514000	0.752066
chr11	94526300	0.834711
chr11	94529500	0.950413
chr11	94610200	0.950413
chr11	94629500	0.876033
chr11	94659100	0.950413
chr11	94661800	0.859504
chr11	94666500	0.942149
chr11	94669000	0.900826
chr11	94671900	0.975207
chr11	94841400	0.719008
chr11	94877800	0.909091
chr11	94881000	0.842975
chr11	95166600	0.735537
chr11	95647000	0.776860
chr11	95650800	0.966942
chr11	95654600	0.743802
chr11	95710700	0.991736
chr11	95745700	0.859504

chr11	95795700	0.917355
chr11	95836100	0.991736
chr11	95846300	0.884298
chr11	95870600	0.661157
chr11	95880900	0.942149
chr11	95888600	0.669421
chr11	95891300	0.933884
chr11	95896500	0.933884
chr11	95902300	0.950413
chr11	95941400	0.966942
chr11	95946100	0.768595
chr11	95949600	0.859504
chr11	95960900	0.851240
chr11	95964400	0.975207
chr11	95976600	0.942149
chr11	95987800	0.991736
chr11	95993100	0.768595
chr11	95997100	0.685950
chr11	96027600	0.661157
chr11	96036700	0.966942
chr11	96040200	0.917355
chr11	96042400	0.983471
chr11	96044900	0.966942
chr11	96048400	0.925620
chr11	96072400	0.933884
chr11	96620100	1.000000
chr11	97486100	0.842975
chr11	97491300	0.727273
chr11	97611200	0.975207
chr11	97836800	0.900826
chr11	100433200	0.942149
chr11	100490600	0.975207
chr11	100503900	0.760331
chr11	100510300	0.685950
chr11	100590800	0.661157
chr11	100688100	0.743802
chr11	100820200	0.793388
chr11	100834800	0.661157
chr11	101734300	0.966942
chr11	101741600	0.892562
chr11	101783800	0.991736
chr11	101787900	0.702479
chr11	101878700	0.685950
chr11	101882000	0.694215
chr11	102042800	0.768595
chr11	102049100	0.867769
chr11	102097200	0.991736
chr11	102139800	0.983471
chr11	102147700	0.669421
chr11	102184200	0.685950
chr11	102215600	0.892562
chr11	102254000	0.735537
chr11	102256100	0.958678
chr11	102317600	0.958678

chr11	102320700	0.925620
chr11	102329000	0.719008
chr11	102340300	0.842975
chr11	102401700	0.950413
chr11	102435300	0.669421
chr11	102470500	0.991736
chr11	102486700	0.876033
chr11	102492600	0.958678
chr11	102599000	1.000000
chr11	102601500	0.950413
chr11	102629200	0.884298
chr11	102631500	0.909091
chr11	102633700	0.933884
chr11	102651500	0.966942
chr11	102671700	0.884298
chr11	102846000	0.991736
chr11	102866500	0.950413
chr11	102868700	0.710744
chr11	102907300	0.909091
chr11	102957400	0.892562
chr11	102986100	0.776860
chr11	103304900	0.867769
chr11	103396200	0.925620
chr11	103399700	0.752066
chr11	103401900	0.925620
chr11	103404300	0.983471
chr11	103457700	0.876033
chr11	103459900	0.818182
chr11	103463300	0.735537
chr11	103472500	0.867769
chr11	103479700	0.917355
chr11	103482100	0.785124
chr11	103495200	0.752066
chr11	103497900	0.826446
chr11	103502200	0.958678
chr11	103504700	0.942149
chr11	103669300	0.925620
chr11	103671700	0.661157
chr11	103677100	0.793388
chr11	103778700	0.983471
chr11	104480800	0.661157
chr11	104576300	0.859504
chr11	104607200	0.842975
chr11	104643700	1.000000
chr11	104762600	0.776860
chr11	104769600	0.900826
chr11	104837400	0.818182
chr11	104876200	0.933884
chr11	104918300	0.942149
chr11	104944900	0.942149
chr11	104951300	0.818182
chr11	104971300	0.876033
chr11	104981400	0.942149
chr11	105013200	0.975207

chr11	105027300	0.768595
chr11	105115700	0.991736
chr11	105168800	0.752066
chr11	105337700	0.694215
chr11	105806800	0.801653
chr11	105884100	0.876033
chr11	105902600	1.000000
chr11	106343300	0.925620
chr11	106354000	0.719008
chr11	106889900	0.752066
chr11	107416000	0.983471
chr11	107433800	0.958678
chr11	107484000	0.727273
chr11	107490600	0.867769
chr11	107502500	0.966942
chr11	107567300	0.818182
chr11	107689900	0.785124
chr11	107693800	0.933884
chr11	107702000	0.760331
chr11	107710600	0.884298
chr11	107713100	0.975207
chr11	108039000	0.801653
chr11	108107700	0.975207
chr11	108487800	0.933884
chr11	108557000	0.942149
chr11	108605200	0.975207
chr11	108725200	0.942149
chr11	108728700	0.975207
chr11	108740300	0.834711
chr11	108742400	0.702479
chr11	108745000	0.975207
chr11	108751600	0.685950
chr11	108758000	0.876033
chr11	108766400	0.776860
chr11	108768500	0.909091
chr11	108777500	1.000000
chr11	108800200	0.842975
chr11	108844600	0.884298
chr11	108857100	0.867769
chr11	108905100	0.925620
chr11	108909500	0.867769
chr11	108940500	0.661157
chr11	109294300	0.710744
chr11	109736300	0.909091
chr11	109786000	0.958678
chr11	109817600	0.876033
chr11	109842400	0.909091
chr11	109940700	0.983471
chr11	109948900	0.942149
chr11	109962800	0.694215
chr11	109992300	0.834711
chr11	110019500	0.958678
chr11	110030900	0.661157
chr11	110065600	0.917355

chr11	110067900	0.925620
chr11	110071400	0.818182
chr11	110123500	0.851240
chr11	110151300	0.842975
chr11	110183100	0.975207
chr11	110188600	1.000000
chr11	110190800	0.975207
chr11	110199800	0.884298
chr11	110208900	0.892562
chr11	110228400	0.834711
chr11	110234100	0.950413
chr11	110244300	0.752066
chr11	110247400	0.661157
chr11	110268900	0.859504
chr11	110393400	0.776860
chr11	110649000	0.958678
chr11	110665900	0.809917
chr11	110672200	0.909091
chr11	110701000	0.834711
chr11	110703900	0.983471
chr11	111101800	0.661157
chr11	111504700	0.925620
chr11	111578200	0.983471
chr11	111601100	0.685950
chr11	111609300	0.884298
chr11	111616500	0.677686
chr11	111690400	0.966942
chr11	111752900	0.727273
chr11	111794300	0.966942
chr11	111797800	0.801653
chr11	111810500	0.975207
chr11	111814900	0.925620
chr11	111817100	0.966942
chr11	111820700	0.768595
chr11	111846500	0.991736
chr11	111920000	0.933884
chr11	111929000	0.785124
chr11	111991000	0.975207
chr11	111999700	0.752066
chr11	112028200	0.826446
chr11	112036000	0.669421
chr11	112046300	0.818182
chr11	112092000	0.710744
chr11	112126500	0.950413
chr11	112164400	0.958678
chr11	112188700	0.760331
chr11	112482100	0.933884
chr11	112484300	0.834711
chr11	113255600	0.933884
chr11	113744300	0.685950
chr11	113748000	0.669421
chr11	113907600	0.925620
chr11	113911900	0.694215
chr11	113915000	0.991736

chr11	113950500	0.950413
chr11	113952700	0.719008
chr11	113962500	0.975207
chr11	113982300	0.842975
chr11	114010400	0.876033
chr11	114031100	0.958678
chr11	114034000	0.983471
chr11	114038300	0.900826
chr11	114042500	0.809917
chr11	114049100	0.859504
chr11	114051900	0.826446
chr11	114069300	0.983471
chr11	114073300	0.842975
chr11	114091100	0.925620
chr11	114151800	0.876033
chr11	114162100	0.884298
chr11	114165600	0.867769
chr11	114178600	1.000000
chr11	114186600	0.859504
chr11	114211400	0.760331
chr11	114230400	0.776860
chr11	114237900	0.867769
chr11	114312800	0.669421
chr11	114433900	0.942149
chr11	114667800	0.702479
chr11	115093500	0.933884
chr11	115161900	0.975207
chr11	116165900	0.702479
chr11	116184000	0.966942
chr11	116229400	0.826446
chr11	116232600	0.958678
chr11	116238000	0.876033
chr11	116244700	0.884298
chr11	116267500	0.958678
chr11	116342800	0.975207
chr11	116429500	0.933884
chr11	116434300	0.710744
chr11	116451400	0.685950
chr11	116473000	0.983471
chr11	116621800	0.801653
chr11	116706700	0.826446
chr11	116724800	0.942149
chr11	116817500	0.900826
chr11	116915400	0.694215
chr11	116930000	0.966942
chr11	116943300	0.685950
chr11	117043700	0.735537
chr11	117051600	0.669421
chr11	117070200	0.942149
chr11	117088700	0.702479
chr11	117110100	0.851240
chr11	117152200	0.991736
chr11	117250400	0.661157
chr11	117260300	0.661157

chr11	117689300	0.900826
chr11	117694100	0.933884
chr11	117696200	0.818182
chr11	117778500	0.892562
chr11	117843700	0.859504
chr11	118077700	0.834711
chr11	118099800	0.983471
chr11	118112700	0.818182
chr11	118136900	0.826446
chr11	118145800	0.694215
chr11	118148600	0.785124
chr11	118452000	0.925620
chr11	118482600	0.809917
chr11	118485100	0.793388
chr11	118487900	0.785124
chr11	118490500	0.942149
chr11	118494600	0.694215
chr11	118529800	0.809917
chr11	118560800	0.884298
chr11	118586900	0.752066
chr11	118780100	0.933884
chr11	118784900	0.710744
chr11	118788400	0.958678
chr11	118895800	0.785124
chr11	118977400	0.719008
chr11	119046600	0.661157
chr11	119049100	0.925620
chr11	119089500	0.685950
chr11	119100700	0.983471
chr11	119134100	1.000000
chr11	119192900	0.983471
chr11	119208700	0.876033
chr11	119212700	0.719008
chr11	119227500	0.677686
chr11	119465900	0.917355
chr11	119486500	0.950413
chr11	119489400	0.950413
chr11	119542500	0.710744
chr11	119547700	0.859504
chr11	119555500	0.876033
chr11	120096400	0.818182
chr11	120200500	0.859504
chr11	120213000	0.909091
chr11	120233500	0.661157
chr11	120241400	0.727273
chr11	120262000	0.677686
chr11	120271800	0.991736
chr11	120275600	0.859504
chr11	120303000	1.000000
chr11	120435700	0.933884
chr11	120437800	0.851240
chr11	120586200	0.785124
chr11	120644800	0.917355
chr11	120651000	0.876033

chr11	120988900	0.983471
chr11	121108200	0.834711
chr11	121182800	0.719008
chr11	121335600	0.818182
chr11	121337700	0.942149
chr11	121353500	0.876033
chr11	121486000	0.834711
chr11	121497300	0.719008
chr11	121700100	0.950413
chr11	121705000	0.743802
chr11	121916300	0.933884
chr11	121921100	0.925620
chr11	121941000	0.876033
chr11	121956700	0.826446
chr11	121961900	0.752066
chr11	121967700	0.826446
chr11	121973100	0.933884
chr11	122007800	0.892562
chr11	122027900	0.735537
chr11	122030200	0.793388
chr11	122037100	0.727273
chr11	122045900	0.884298
chr11	122052700	0.884298
chr11	122057200	0.694215
chr11	122061100	0.933884
chr11	122064100	0.851240
chr11	122068000	0.991736
chr11	122081000	0.685950
chr11	122100600	0.876033
chr11	122208700	0.710744
chr11	122362700	0.851240
chr11	122513900	0.917355
chr11	122518500	0.694215
chr11	122574500	0.975207
chr11	122582900	1.000000
chr11	122586900	0.685950
chr11	122595500	0.776860
chr11	122598300	0.842975
chr11	122603600	0.975207
chr11	122606000	0.702479
chr11	122609000	0.925620
chr11	122629800	0.727273
chr11	122644600	0.842975
chr11	122648600	0.983471
chr11	122655600	0.884298
chr11	122662700	0.809917
chr11	122753900	0.677686
chr11	122768900	1.000000
chr11	122935400	0.719008
chr11	122954400	0.933884
chr11	122989600	0.867769
chr11	122993700	0.983471
chr11	123008600	0.842975
chr11	123059300	0.727273

chr11	123172700	0.785124
chr11	123278400	0.892562
chr11	123325200	0.909091
chr11	123345400	0.991736
chr11	123461600	0.925620
chr11	123712800	0.900826
chr11	123830600	0.677686
chr11	123844700	0.975207
chr11	124045100	0.793388
chr11	124146800	0.900826
chr11	124156200	0.867769
chr11	124352400	1.000000
chr11	124514400	0.991736
chr11	124544700	0.826446
chr11	124587800	0.867769
chr11	124595800	0.909091
chr11	124620100	0.900826
chr11	124627200	0.917355
chr11	124640200	0.991736
chr11	124706800	1.000000
chr11	124733600	0.694215
chr11	124736200	0.785124
chr11	124752800	0.884298
chr11	124756200	0.710744
chr11	124759200	0.776860
chr11	124762200	0.958678
chr11	124769200	0.661157
chr11	124771900	0.909091
chr11	124785600	1.000000
chr11	124791300	0.867769
chr11	124907300	0.809917
chr11	124953300	0.884298
chr11	124961800	0.942149
chr11	125062500	0.661157
chr11	125164200	0.809917
chr11	125167700	0.867769
chr11	125170900	0.917355
chr11	125184300	0.719008
chr11	125261000	0.958678
chr11	125541000	0.950413
chr11	125544500	0.991736
chr11	125647800	0.884298
chr11	125760500	0.958678
chr11	125932800	0.677686
chr11	126025000	0.818182
chr11	126100300	0.991736
chr11	126116900	0.975207
chr11	126151100	0.702479
chr11	126206000	0.933884
chr11	126208100	0.719008
chr11	126210300	0.710744
chr11	126212400	0.669421
chr11	126216100	0.933884
chr11	126219600	0.859504

chr11	126223300	0.942149
chr11	126227000	0.719008
chr11	126286800	0.867769
chr11	126301800	0.892562
chr11	126456000	0.983471
chr11	127511400	0.983471
chr11	127521800	0.867769
chr11	127897000	0.983471
chr11	127917200	0.851240
chr11	127923200	1.000000
chr11	127966000	0.900826
chr11	128023700	1.000000
chr11	128027900	0.933884
chr11	128042300	0.958678
chr11	128050800	0.966942
chr11	128065400	0.909091
chr11	128081100	0.768595
chr11	128110700	0.983471
chr11	128199400	0.950413
chr11	128204500	0.859504
chr11	128206800	0.702479
chr11	128213000	0.983471
chr11	128329000	0.884298
chr11	128363000	0.876033
chr11	128369700	0.925620
chr11	128371900	0.950413
chr11	128374000	0.867769
chr11	128377200	0.867769
chr11	128379900	0.983471
chr11	128385000	0.760331
chr11	128388100	0.752066
chr11	128394000	0.719008
chr11	128419100	1.000000
chr11	128446300	0.966942
chr11	128450500	0.669421
chr11	128457400	0.661157
chr11	128500900	0.768595
chr11	128548800	0.950413
chr11	128555200	0.694215
chr11	128557600	0.752066
chr11	128568500	0.694215
chr11	128571500	0.933884
chr11	128575600	0.958678
chr11	128579600	0.991736
chr11	128582000	0.884298
chr11	128585800	0.966942
chr11	128590600	0.760331
chr11	128600300	0.925620
chr11	128603900	0.768595
chr11	128615300	0.702479
chr11	128628400	0.859504
chr11	128644600	0.942149
chr11	128695400	0.867769
chr11	128698300	0.859504

chr11	128701300	0.983471
chr11	128706300	1.000000
chr11	128713300	0.661157
chr11	128730000	0.685950
chr11	128760900	0.752066
chr11	128778900	0.975207
chr11	128834800	0.743802
chr11	128894700	0.834711
chr11	129013300	0.892562
chr11	129148800	0.661157
chr11	129243400	0.900826
chr11	129508600	0.975207
chr11	129513400	0.702479
chr11	129565800	0.677686
chr11	129686400	0.760331
chr11	129695700	0.851240
chr11	129699000	0.768595
chr11	129732100	0.917355
chr11	129781700	0.942149
chr11	129821200	0.983471
chr11	129830800	0.719008
chr11	129893900	0.834711
chr11	129912800	0.851240
chr11	129957500	0.661157
chr11	129969300	0.677686
chr11	129973300	0.942149
chr11	129992500	0.966942
chr11	129994900	0.975207
chr11	130350900	0.719008
chr11	130450700	0.884298
chr11	130592200	0.760331
chr11	130596400	0.867769
chr11	130608200	1.000000
chr11	130652700	0.925620
chr11	130657300	0.735537
chr11	130742500	0.677686
chr11	130744600	0.859504
chr11	130752700	0.801653
chr11	130757100	0.966942
chr11	130761000	0.975207
chr11	130763800	0.669421
chr11	130767500	0.909091
chr11	130776000	0.917355
chr11	130779800	0.851240
chr11	130870200	0.975207
chr11	131583500	0.933884
chr11	131614300	0.768595
chr11	131616900	0.859504
chr11	131620400	0.900826
chr11	131625500	0.958678
chr11	131638100	0.933884
chr11	131681800	0.884298
chr11	131709800	0.760331
chr11	131725500	1.000000

chr11	131730300	0.702479
chr11	131734600	0.752066
chr11	131739300	0.942149
chr11	131759000	0.983471
chr11	131845800	0.702479
chr11	131935300	0.942149
chr11	131940300	0.834711
chr11	131942400	0.991736
chr11	131944600	0.925620
chr11	131996700	0.834711
chr11	132160100	0.900826
chr11	132182700	0.685950
chr11	132934700	0.719008
chr11	133920900	0.859504
chr11	133930900	0.826446
chr11	133944700	0.752066
chr11	133988700	0.710744
chr11	133992000	0.752066
chr11	133997100	0.851240
chr11	134100100	0.809917
chr11	134146700	0.669421
chr11	134374500	0.909091
chr11	134380600	0.768595
chr11	134612600	0.842975
chr11	134722200	0.677686
chr11	134819200	0.991736
chr12	82200	0.685950
chr12	92100	0.702479
chr12	95300	0.785124
chr12	313200	0.752066
chr12	619100	0.884298
chr12	648300	0.735537
chr12	654900	0.702479
chr12	681500	0.958678
chr12	685600	0.719008
chr12	707600	0.834711
chr12	717100	0.801653
chr12	719900	0.900826
chr12	725200	0.917355
chr12	732300	0.900826
chr12	741800	0.909091
chr12	752100	0.942149
chr12	756300	0.735537
chr12	784200	0.669421
chr12	860500	0.942149
chr12	889200	0.917355
chr12	915800	0.727273
chr12	961400	0.842975
chr12	1020900	0.694215
chr12	1024700	0.785124
chr12	1068300	0.933884
chr12	1154200	0.867769
chr12	1156900	0.842975
chr12	1187400	0.702479

chr12	1281800	0.661157
chr12	1293900	0.983471
chr12	1313900	0.892562
chr12	1326700	0.966942
chr12	1399000	0.991736
chr12	1573400	0.851240
chr12	1583700	0.983471
chr12	1690800	0.752066
chr12	1715200	0.710744
chr12	1739700	0.876033
chr12	1790700	0.975207
chr12	1870300	0.809917
chr12	1921600	0.743802
chr12	2365300	0.809917
chr12	2445900	0.983471
chr12	2800800	0.685950
chr12	2955200	0.909091
chr12	3029000	0.826446
chr12	3067300	0.743802
chr12	3071400	0.694215
chr12	3073800	0.826446
chr12	3084100	0.925620
chr12	3096200	0.826446
chr12	3124300	0.933884
chr12	3142400	0.818182
chr12	3185800	0.785124
chr12	3193000	0.859504
chr12	3206200	0.966942
chr12	3226600	0.917355
chr12	3232600	0.842975
chr12	3239500	0.710744
chr12	3262700	0.917355
chr12	3274500	0.694215
chr12	3315300	0.694215
chr12	3325900	0.685950
chr12	3329100	0.958678
chr12	3335200	0.818182
chr12	3358900	0.909091
chr12	3409700	0.752066
chr12	3414000	0.892562
chr12	3807700	0.958678
chr12	3814200	0.933884
chr12	4140700	0.752066
chr12	4274200	0.867769
chr12	4374700	0.991736
chr12	4381000	0.809917
chr12	4384300	0.685950
chr12	4671700	0.842975
chr12	4681800	0.702479
chr12	4933900	0.950413
chr12	5566500	0.867769
chr12	5695200	1.000000
chr12	5697500	0.677686
chr12	5735100	0.884298

chr12	5855000	0.983471
chr12	6136800	0.859504
chr12	6139600	0.661157
chr12	6162200	0.809917
chr12	6176100	0.818182
chr12	6179600	0.950413
chr12	6181800	0.966942
chr12	6185100	0.785124
chr12	6187500	0.892562
chr12	6190600	0.851240
chr12	6195900	0.801653
chr12	6199300	1.000000
chr12	6203800	0.851240
chr12	6207600	0.958678
chr12	6211000	0.669421
chr12	6217200	0.917355
chr12	6220300	0.950413
chr12	6222700	1.000000
chr12	6227700	0.735537
chr12	6235100	0.809917
chr12	6244500	0.917355
chr12	6249100	0.677686
chr12	6251300	0.702479
chr12	6254900	0.975207
chr12	6264300	0.809917
chr12	6267700	0.735537
chr12	6271200	0.735537
chr12	6280400	0.975207
chr12	6283300	0.917355
chr12	6287700	0.859504
chr12	6293100	0.950413
chr12	6305500	0.892562
chr12	6308500	0.768595
chr12	6313700	0.867769
chr12	6316000	0.735537
chr12	6323700	0.694215
chr12	6326300	0.942149
chr12	6330300	0.991736
chr12	6335000	0.942149
chr12	6339300	0.958678
chr12	6343100	0.876033
chr12	6371900	0.801653
chr12	6383500	0.900826
chr12	6387500	0.768595
chr12	6389700	0.925620
chr12	6395100	0.958678
chr12	6405000	0.884298
chr12	6420200	0.661157
chr12	6432700	0.983471
chr12	6442000	0.826446
chr12	6454500	0.785124
chr12	6456600	0.826446
chr12	6472800	0.867769
chr12	6481800	0.793388

chr12	6486300	0.710744
chr12	6540100	0.966942
chr12	6572700	0.710744
chr12	6641100	0.909091
chr12	6647200	0.685950
chr12	6651600	0.851240
chr12	6657800	0.719008
chr12	6662100	0.809917
chr12	6723100	0.991736
chr12	6748800	0.958678
chr12	6756600	0.768595
chr12	6873800	0.991736
chr12	6888200	0.785124
chr12	6930900	0.685950
chr12	6934600	0.702479
chr12	7000400	0.884298
chr12	7043300	0.867769
chr12	7047300	0.842975
chr12	7052300	0.768595
chr12	7062800	0.694215
chr12	7071400	0.983471
chr12	7078100	0.661157
chr12	7155100	0.785124
chr12	7245200	0.793388
chr12	7276700	0.834711
chr12	7303200	0.909091
chr12	7502600	0.909091
chr12	7585700	0.917355
chr12	7588300	0.892562
chr12	7830600	0.776860
chr12	7848200	0.909091
chr12	7995900	0.842975
chr12	8000500	0.958678
chr12	8058800	0.743802
chr12	8086400	0.677686
chr12	8091000	0.950413
chr12	8113600	0.776860
chr12	8123100	0.842975
chr12	8125300	0.950413
chr12	8130100	0.892562
chr12	8142700	0.793388
chr12	8171700	0.760331
chr12	8188300	0.685950
chr12	8191600	0.851240
chr12	8219200	0.958678
chr12	8230300	0.685950
chr12	8310100	0.851240
chr12	8707600	0.727273
chr12	8771700	0.900826
chr12	8828600	0.950413
chr12	8830700	0.760331
chr12	8833900	0.826446
chr12	8840900	0.818182
chr12	8845700	0.900826

chr12	8862000	0.702479
chr12	8865600	0.867769
chr12	9144100	0.809917
chr12	9148600	0.694215
chr12	9316600	0.942149
chr12	9320600	0.975207
chr12	9322900	0.909091
chr12	9608500	0.942149
chr12	9803900	0.884298
chr12	9826800	0.900826
chr12	9852900	0.958678
chr12	9874200	0.991736
chr12	9890300	0.785124
chr12	9903600	0.876033
chr12	9928300	0.925620
chr12	9945900	0.991736
chr12	9950400	0.983471
chr12	10163300	0.785124
chr12	10170700	0.933884
chr12	10183700	0.818182
chr12	10187000	0.925620
chr12	10195200	0.669421
chr12	10218800	0.776860
chr12	10222800	0.917355
chr12	10251200	0.809917
chr12	10373400	0.975207
chr12	10463900	0.876033
chr12	10573100	0.719008
chr12	10771100	0.917355
chr12	10796100	0.917355
chr12	10820500	0.842975
chr12	10822700	0.933884
chr12	10869400	0.900826
chr12	10902700	0.809917
chr12	11121000	0.950413
chr12	11168900	0.694215
chr12	11322100	0.702479
chr12	11419200	0.710744
chr12	11505200	0.983471
chr12	11510900	0.727273
chr12	11513600	0.909091
chr12	11545000	0.826446
chr12	11636300	0.983471
chr12	11698800	0.917355
chr12	11810400	0.909091
chr12	11826900	0.975207
chr12	11851000	0.834711
chr12	11870400	0.818182
chr12	11873200	0.867769
chr12	11878300	0.983471
chr12	11884600	0.991736
chr12	11899400	0.950413
chr12	11912200	0.892562
chr12	11919400	0.743802

chr12	11923700	0.867769
chr12	11947900	0.851240
chr12	11952000	0.975207
chr12	11955300	0.892562
chr12	11965100	0.917355
chr12	11967500	0.933884
chr12	11975700	0.826446
chr12	11987400	0.685950
chr12	11991100	0.685950
chr12	12017500	0.884298
chr12	12032200	0.991736
chr12	12035700	0.867769
chr12	12039200	0.933884
chr12	12045400	0.892562
chr12	12112200	0.966942
chr12	12186200	0.884298
chr12	12248300	0.694215
chr12	12252200	0.859504
chr12	12300600	0.727273
chr12	12313600	0.776860
chr12	12335600	0.975207
chr12	12368000	0.809917
chr12	12392700	0.809917
chr12	12447900	0.900826
chr12	12473400	0.669421
chr12	12480900	0.669421
chr12	12487400	0.702479
chr12	12489600	0.983471
chr12	12551500	0.818182
chr12	12579100	0.743802
chr12	12593000	0.983471
chr12	12596900	0.900826
chr12	12628300	0.950413
chr12	12659600	0.884298
chr12	12682300	0.669421
chr12	12716600	0.801653
chr12	12843500	0.900826
chr12	12867400	0.859504
chr12	12912200	0.958678
chr12	12929400	0.925620
chr12	12932700	1.000000
chr12	12938200	0.785124
chr12	12941800	0.933884
chr12	12957000	0.983471
chr12	12962100	0.975207
chr12	12972600	0.809917
chr12	12974700	0.892562
chr12	12977000	0.876033
chr12	12989100	0.933884
chr12	12993400	0.867769
chr12	12998600	0.661157
chr12	13024000	0.917355
chr12	13026600	0.826446
chr12	13035400	0.942149

chr12	13052300	0.826446
chr12	13062800	0.966942
chr12	13065400	0.933884
chr12	13068100	0.818182
chr12	13070200	0.859504
chr12	13080700	0.785124
chr12	13129200	1.000000
chr12	13133200	0.867769
chr12	13187100	0.760331
chr12	13233300	0.768595
chr12	13247300	0.991736
chr12	13251300	0.958678
chr12	13255000	0.694215
chr12	13269300	0.867769
chr12	13278400	0.991736
chr12	13289300	0.842975
chr12	13318100	0.942149
chr12	13320600	0.809917
chr12	13332700	0.727273
chr12	13348500	0.727273
chr12	13354700	0.818182
chr12	13358600	0.950413
chr12	13364700	0.933884
chr12	13410000	0.661157
chr12	14404500	0.851240
chr12	14409800	0.983471
chr12	14413700	0.884298
chr12	14526600	0.983471
chr12	14538400	0.917355
chr12	14543500	0.669421
chr12	14552900	0.842975
chr12	14563900	0.776860
chr12	14570400	0.661157
chr12	14574400	0.966942
chr12	14987800	0.950413
chr12	14990400	0.983471
chr12	14997100	0.661157
chr12	15002700	0.809917
chr12	15058300	0.983471
chr12	15063600	0.958678
chr12	15100100	0.826446
chr12	15103700	0.958678
chr12	15106800	0.966942
chr12	15306200	0.669421
chr12	15490500	0.884298
chr12	15749900	0.859504
chr12	15752800	0.909091
chr12	15781800	0.900826
chr12	15784300	0.975207
chr12	15787100	0.867769
chr12	15791100	0.975207
chr12	15816000	0.942149
chr12	15843400	0.842975
chr12	15854600	0.909091

chr12	15872000	0.727273
chr12	15881600	0.925620
chr12	15934100	0.727273
chr12	16081600	0.942149
chr12	16116500	0.867769
chr12	16119600	0.991736
chr12	16121800	0.661157
chr12	16130400	0.710744
chr12	16132700	0.991736
chr12	16717200	0.661157
chr12	16721900	0.801653
chr12	18081900	0.917355
chr12	19152100	0.818182
chr12	19309500	0.760331
chr12	19427100	1.000000
chr12	19522800	0.702479
chr12	19595600	0.694215
chr12	19683100	0.958678
chr12	19700200	0.942149
chr12	19726400	0.719008
chr12	19729300	0.983471
chr12	19836900	0.909091
chr12	19848500	1.000000
chr12	19865400	0.900826
chr12	19890300	1.000000
chr12	19897600	0.975207
chr12	19899700	0.834711
chr12	19953500	0.909091
chr12	20099300	0.842975
chr12	20110200	0.991736
chr12	20113300	0.966942
chr12	20121400	0.991736
chr12	20123600	0.809917
chr12	20153100	0.975207
chr12	20174800	0.685950
chr12	20177700	0.735537
chr12	20184100	0.966942
chr12	20193100	0.694215
chr12	20304000	0.884298
chr12	20365900	0.925620
chr12	20434000	0.702479
chr12	20503800	0.892562
chr12	20527600	0.793388
chr12	20589200	0.900826
chr12	20625200	0.677686
chr12	20645900	0.876033
chr12	20704200	0.975207
chr12	20755400	0.702479
chr12	21570200	0.975207
chr12	21581400	0.983471
chr12	21657200	0.677686
chr12	21768700	0.900826
chr12	21871100	0.900826
chr12	22094900	0.743802

chr12	22436000	0.917355
chr12	22441300	0.876033
chr12	22678400	0.685950
chr12	22682300	0.743802
chr12	22781300	0.710744
chr12	22840800	0.975207
chr12	23128200	0.925620
chr12	23133000	0.876033
chr12	23237500	1.000000
chr12	23406500	0.719008
chr12	23447800	0.933884
chr12	23882900	0.661157
chr12	24192800	0.760331
chr12	24199200	0.900826
chr12	24264800	0.801653
chr12	24380100	0.983471
chr12	24502600	0.909091
chr12	24754200	0.991736
chr12	24762000	0.966942
chr12	24770700	0.685950
chr12	25004800	0.975207
chr12	25007100	0.983471
chr12	25015100	0.669421
chr12	25042900	0.859504
chr12	25050100	0.983471
chr12	25053200	0.925620
chr12	25058200	0.685950
chr12	25063100	0.884298
chr12	25074900	0.983471
chr12	25085400	0.818182
chr12	25097300	0.867769
chr12	25099500	0.834711
chr12	25305200	0.719008
chr12	25611500	0.958678
chr12	25801800	0.694215
chr12	25847200	0.991736
chr12	25853200	0.661157
chr12	25872700	0.809917
chr12	25898600	0.727273
chr12	25922600	0.867769
chr12	25929200	0.826446
chr12	25938000	0.694215
chr12	25941900	0.892562
chr12	25947800	0.966942
chr12	25959600	0.727273
chr12	25973100	0.669421
chr12	25982600	0.900826
chr12	25996900	0.925620
chr12	26008900	0.834711
chr12	26078200	0.892562
chr12	26114700	0.677686
chr12	26123400	0.975207
chr12	26141800	1.000000
chr12	26151700	0.950413

chr12	26163800	0.892562
chr12	26206900	0.785124
chr12	26267300	0.669421
chr12	26306600	1.000000
chr12	26425100	0.867769
chr12	26428400	0.958678
chr12	26452200	0.760331
chr12	26471500	0.983471
chr12	26531800	0.983471
chr12	26783500	0.958678
chr12	26801700	0.975207
chr12	26830800	0.909091
chr12	26834500	0.793388
chr12	26842200	0.966942
chr12	26845900	0.933884
chr12	26849200	0.752066
chr12	26853100	0.950413
chr12	26857900	0.966942
chr12	26875100	0.727273
chr12	26877400	0.991736
chr12	26884200	1.000000
chr12	26902400	1.000000
chr12	26904500	0.917355
chr12	26940400	0.983471
chr12	26944000	0.768595
chr12	26953100	0.793388
chr12	26974700	0.975207
chr12	27010400	0.892562
chr12	27023800	0.966942
chr12	27031800	0.818182
chr12	27094000	0.900826
chr12	27097600	0.760331
chr12	27263000	0.661157
chr12	27315300	0.983471
chr12	27330200	1.000000
chr12	27333200	0.991736
chr12	27339000	0.900826
chr12	27425700	0.719008
chr12	27427900	0.727273
chr12	27438000	0.991736
chr12	27445600	0.983471
chr12	27484300	0.925620
chr12	27496700	0.917355
chr12	27519800	0.710744
chr12	27613500	0.933884
chr12	27616400	0.909091
chr12	27674500	0.818182
chr12	27679600	0.727273
chr12	27701600	0.884298
chr12	27703800	0.933884
chr12	27707700	0.917355
chr12	27710400	0.859504
chr12	27714200	0.859504
chr12	27718000	0.942149

chr12	27724600	0.867769
chr12	27727100	0.983471
chr12	27729300	0.983471
chr12	27732200	0.867769
chr12	27736900	0.867769
chr12	27769100	0.958678
chr12	27772600	1.000000
chr12	27776600	0.933884
chr12	27780800	0.983471
chr12	27784200	0.834711
chr12	27797700	0.719008
chr12	27925800	1.000000
chr12	27939000	0.925620
chr12	27975200	0.958678
chr12	28076000	0.776860
chr12	28119800	0.958678
chr12	28176800	0.950413
chr12	28188700	0.710744
chr12	28218300	0.760331
chr12	28242300	0.876033
chr12	28284100	1.000000
chr12	28578200	0.966942
chr12	28704200	0.917355
chr12	28764300	0.826446
chr12	28924100	0.909091
chr12	28935200	0.867769
chr12	29086600	0.933884
chr12	29092500	0.975207
chr12	29112300	0.958678
chr12	29115400	0.760331
chr12	29119800	0.710744
chr12	29310600	0.975207
chr12	29317900	0.958678
chr12	29381800	0.876033
chr12	29384700	0.702479
chr12	29388200	1.000000
chr12	29392000	0.925620
chr12	29394600	0.801653
chr12	29396900	0.991736
chr12	29473300	0.876033
chr12	29579000	0.826446
chr12	29581700	0.694215
chr12	29715300	0.966942
chr12	29722700	0.743802
chr12	29879100	0.909091
chr12	29894900	0.983471
chr12	30074600	0.876033
chr12	30123900	0.991736
chr12	30179800	1.000000
chr12	30200400	1.000000
chr12	30264300	0.900826
chr12	30354200	0.727273
chr12	30766700	0.950413
chr12	30860300	0.710744

chr12	30946800	0.818182
chr12	31004700	0.710744
chr12	31068600	0.702479
chr12	31079300	0.661157
chr12	31082000	0.859504
chr12	31084100	0.950413
chr12	31149500	0.809917
chr12	31153800	0.719008
chr12	31171400	0.719008
chr12	31176000	0.933884
chr12	31231500	0.933884
chr12	31256900	0.793388
chr12	31270800	0.925620
chr12	31470800	0.884298
chr12	31523500	0.892562
chr12	31644900	1.000000
chr12	31652800	0.735537
chr12	31660300	0.983471
chr12	31662500	0.661157
chr12	31670900	0.867769
chr12	31674200	0.809917
chr12	31728200	0.933884
chr12	31868800	0.933884
chr12	31884400	0.834711
chr12	31886700	0.966942
chr12	31899900	0.925620
chr12	31903700	0.917355
chr12	32028400	0.851240
chr12	32050000	0.917355
chr12	32268900	0.950413
chr12	32351100	0.776860
chr12	32393800	0.966942
chr12	32418600	0.809917
chr12	32424700	0.983471
chr12	32485800	1.000000
chr12	32584200	0.785124
chr12	32588900	0.793388
chr12	32591000	0.942149
chr12	32597700	0.942149
chr12	32600500	0.884298
chr12	32604900	0.983471
chr12	32607700	0.991736
chr12	32610400	0.991736
chr12	32621400	0.809917
chr12	32635400	0.900826
chr12	32639500	0.942149
chr12	32658900	0.991736
chr12	32702000	0.685950
chr12	32721100	0.909091
chr12	32739700	0.867769
chr12	32765900	1.000000
chr12	32906900	0.685950
chr12	33073300	0.925620
chr12	34372800	0.933884

chr12	34841700	0.661157
chr12	34846100	0.669421
chr12	34848300	0.760331
chr12	37991800	0.851240
chr12	38029200	0.818182
chr12	38038300	0.793388
chr12	38448400	0.719008
chr12	39130900	0.909091
chr12	39518100	0.768595
chr12	39593400	0.768595
chr12	39703800	0.719008
chr12	40277800	0.677686
chr12	40758500	0.685950
chr12	41086900	0.727273
chr12	41408300	0.983471
chr12	41583700	0.702479
chr12	41757200	0.991736
chr12	41880000	0.942149
chr12	41916200	0.983471
chr12	42188800	0.958678
chr12	42244400	0.669421
chr12	42324700	0.685950
chr12	42326900	0.818182
chr12	42415500	0.966942
chr12	42418100	0.876033
chr12	42485200	0.859504
chr12	42492200	0.801653
chr12	42525200	0.966942
chr12	42803800	0.950413
chr12	42814200	0.793388
chr12	42816600	0.991736
chr12	42820900	0.950413
chr12	42841600	1.000000
chr12	42863300	0.710744
chr12	42866400	0.809917
chr12	42869400	0.975207
chr12	42875000	0.867769
chr12	42959700	0.834711
chr12	42969600	0.983471
chr12	42976900	0.801653
chr12	42980100	0.661157
chr12	43032300	0.851240
chr12	43184700	0.991736
chr12	43256100	0.702479
chr12	43352300	0.859504
chr12	43354500	0.917355
chr12	43356700	0.925620
chr12	43360000	0.768595
chr12	43364000	0.983471
chr12	43424500	0.909091
chr12	43473300	0.966942
chr12	43632500	0.942149
chr12	43661800	0.983471
chr12	43671900	0.983471

chr12	43675700	0.743802
chr12	43679300	1.000000
chr12	43687000	1.000000
chr12	43727500	0.661157
chr12	43732600	0.842975
chr12	44363800	0.743802
chr12	45445200	0.727273
chr12	45585800	0.958678
chr12	45625700	0.727273
chr12	45629400	0.900826
chr12	45654000	0.826446
chr12	45661700	0.950413
chr12	45667900	0.983471
chr12	45677900	0.950413
chr12	45693100	0.975207
chr12	45698600	1.000000
chr12	45744100	0.685950
chr12	45773100	0.719008
chr12	45830000	0.785124
chr12	46006400	0.900826
chr12	46009300	0.694215
chr12	46032100	0.917355
chr12	46166000	0.735537
chr12	46356900	0.859504
chr12	46367500	0.942149
chr12	46375200	0.752066
chr12	46548000	0.851240
chr12	46604000	0.966942
chr12	46612900	0.826446
chr12	46616500	0.942149
chr12	46758900	0.809917
chr12	46761000	0.694215
chr12	46776400	0.776860
chr12	46780400	0.942149
chr12	46784800	0.991736
chr12	46797200	0.867769
chr12	46823600	0.760331
chr12	46827000	0.983471
chr12	46832500	0.933884
chr12	46834600	0.942149
chr12	46837000	0.892562
chr12	46840300	0.991736
chr12	46844000	0.975207
chr12	46846100	0.892562
chr12	46864600	0.694215
chr12	46867900	0.983471
chr12	46874600	0.900826
chr12	46877400	0.950413
chr12	46885700	0.942149
chr12	46888100	0.834711
chr12	46917800	0.950413
chr12	46928000	0.983471
chr12	46943700	0.900826
chr12	46950100	0.677686

chr12	46957100	0.991736
chr12	46980000	0.768595
chr12	47044800	0.727273
chr12	47055300	0.991736
chr12	47059200	0.958678
chr12	47061600	0.834711
chr12	47180900	0.900826
chr12	47183100	0.851240
chr12	47219700	0.793388
chr12	47225600	0.735537
chr12	47358600	0.950413
chr12	47408700	0.925620
chr12	47416300	0.958678
chr12	47479900	0.727273
chr12	47482600	0.884298
chr12	47672000	0.669421
chr12	47711000	0.900826
chr12	47755100	0.859504
chr12	47771600	0.842975
chr12	47773700	0.859504
chr12	47787200	0.892562
chr12	47899200	0.851240
chr12	47998500	0.785124
chr12	48135400	0.694215
chr12	48171200	0.851240
chr12	48177000	0.694215
chr12	48195400	0.669421
chr12	48197500	0.661157
chr12	48199700	0.925620
chr12	48203300	0.793388
chr12	48215900	0.702479
chr12	48223000	0.834711
chr12	48261800	0.661157
chr12	48291000	0.760331
chr12	48816400	0.933884
chr12	48840400	0.892562
chr12	49073400	0.710744
chr12	49247400	0.735537
chr12	49275800	0.818182
chr12	49278200	0.900826
chr12	49320400	0.677686
chr12	49355800	0.909091
chr12	49375100	0.933884
chr12	49389300	0.719008
chr12	49443800	0.925620
chr12	49482300	0.735537
chr12	49488300	0.669421
chr12	49499500	0.942149
chr12	49584100	0.785124
chr12	49592600	0.818182
chr12	49595600	0.983471
chr12	49654400	1.000000
chr12	49663200	0.975207
chr12	49690900	0.842975

chr12	49727200	0.710744
chr12	49730900	0.776860
chr12	49742000	0.702479
chr12	49764200	0.776860
chr12	49855900	0.983471
chr12	49876800	0.958678
chr12	49883800	1.000000
chr12	49895600	0.834711
chr12	49897800	0.834711
chr12	49901600	0.933884
chr12	49905500	0.760331
chr12	49909400	0.958678
chr12	49932200	0.669421
chr12	49943100	0.892562
chr12	50031500	0.776860
chr12	50039100	0.867769
chr12	50042500	0.760331
chr12	50049400	0.793388
chr12	50059300	0.884298
chr12	50068700	0.785124
chr12	50092800	0.942149
chr12	50097800	0.950413
chr12	50157500	0.710744
chr12	50179200	0.818182
chr12	50215300	0.942149
chr12	50219300	0.909091
chr12	50263200	0.950413
chr12	50288600	0.685950
chr12	50314500	0.900826
chr12	50339600	0.867769
chr12	50361400	0.694215
chr12	50378100	0.925620
chr12	50451600	0.669421
chr12	50464800	0.661157
chr12	50482400	0.892562
chr12	50578900	0.801653
chr12	50581800	0.859504
chr12	50586900	0.909091
chr12	50591200	0.876033
chr12	50595900	0.942149
chr12	50598200	0.785124
chr12	50605800	0.909091
chr12	50618400	0.950413
chr12	50627200	0.694215
chr12	50652100	0.752066
chr12	50665900	0.760331
chr12	50669900	0.694215
chr12	50784500	0.966942
chr12	50809900	0.900826
chr12	50819700	0.925620
chr12	50911600	0.942149
chr12	50927000	0.958678
chr12	50940600	0.669421
chr12	50944600	0.793388

chr12	50958900	0.685950
chr12	50962800	0.719008
chr12	50983900	0.942149
chr12	50991800	0.966942
chr12	50994400	0.793388
chr12	51004000	0.735537
chr12	51036200	0.702479
chr12	51180800	0.975207
chr12	51317800	0.966942
chr12	51379400	0.991736
chr12	51418100	0.785124
chr12	51640700	0.884298
chr12	51768500	0.859504
chr12	51787000	0.809917
chr12	51789200	0.975207
chr12	52077700	0.983471
chr12	52086100	0.826446
chr12	52099900	0.876033
chr12	52138200	0.958678
chr12	52148300	1.000000
chr12	52208500	0.776860
chr12	52227000	0.859504
chr12	52245300	0.776860
chr12	52250100	0.785124
chr12	52279000	0.991736
chr12	52281800	0.859504
chr12	52289000	0.727273
chr12	52292100	0.917355
chr12	52296000	0.892562
chr12	52308300	0.818182
chr12	52312000	0.884298
chr12	52316000	0.834711
chr12	52318200	0.793388
chr12	52321000	0.925620
chr12	52367200	0.917355
chr12	52370600	0.702479
chr12	52374800	0.809917
chr12	52398600	0.685950
chr12	52415000	0.876033
chr12	52419500	0.785124
chr12	52427100	0.991736
chr12	52438600	0.851240
chr12	52458000	0.719008
chr12	52499500	0.677686
chr12	52522300	0.826446
chr12	52540500	0.900826
chr12	52542600	0.867769
chr12	52557800	0.743802
chr12	52577900	0.942149
chr12	52582300	0.859504
chr12	52584400	0.743802
chr12	52596700	0.892562
chr12	52608600	0.851240
chr12	52611100	0.669421

chr12	52624200	0.752066
chr12	52638100	0.752066
chr12	52652700	0.669421
chr12	53268300	0.975207
chr12	53273700	0.776860
chr12	53297700	0.925620
chr12	53304800	0.760331
chr12	53318600	0.702479
chr12	53321700	0.876033
chr12	53340200	0.983471
chr12	53345600	0.719008
chr12	53432800	0.983471
chr12	53434900	0.892562
chr12	53460700	0.719008
chr12	53466300	0.694215
chr12	53576700	0.694215
chr12	53592000	0.702479
chr12	53608700	0.900826
chr12	53664400	0.669421
chr12	53684000	0.710744
chr12	53739100	0.818182
chr12	53765300	0.818182
chr12	53772900	0.876033
chr12	53806600	0.884298
chr12	53815400	0.743802
chr12	53887100	0.983471
chr12	53955700	1.000000
chr12	53985900	0.710744
chr12	53989400	0.917355
chr12	53991700	0.966942
chr12	53997800	0.900826
chr12	54012400	0.991736
chr12	54053200	0.975207
chr12	54071500	0.669421
chr12	54389900	0.702479
chr12	54624300	0.950413
chr12	54656700	0.900826
chr12	54723600	0.876033
chr12	54754300	0.966942
chr12	54763000	0.685950
chr12	54765700	0.842975
chr12	54802000	0.933884
chr12	54805300	0.719008
chr12	54809000	0.793388
chr12	54815100	0.900826
chr12	54817600	0.966942
chr12	54820300	0.842975
chr12	54824600	0.776860
chr12	54827700	0.710744
chr12	55329500	0.975207
chr12	56040500	0.818182
chr12	56112000	0.834711
chr12	56115200	0.900826
chr12	56132600	0.735537

chr12	56241800	0.942149
chr12	56308900	0.685950
chr12	56371500	0.719008
chr12	56423300	0.909091
chr12	56482300	0.818182
chr12	56488200	0.694215
chr12	56509100	0.694215
chr12	56514200	0.892562
chr12	56520100	0.900826
chr12	56537700	0.818182
chr12	56541000	0.727273
chr12	56551100	0.760331
chr12	56556100	0.851240
chr12	56563800	0.859504
chr12	56592500	0.983471
chr12	56841600	0.669421
chr12	56912700	0.983471
chr12	56915000	0.752066
chr12	56918800	0.876033
chr12	56922300	0.876033
chr12	56959900	0.909091
chr12	56968100	0.851240
chr12	56975900	0.991736
chr12	57026300	0.760331
chr12	57078800	0.876033
chr12	57115000	0.727273
chr12	57382400	0.892562
chr12	57385500	0.933884
chr12	57485100	0.735537
chr12	57541600	0.752066
chr12	57610900	0.785124
chr12	57619500	0.735537
chr12	57637400	0.793388
chr12	57674500	0.892562
chr12	57703500	0.768595
chr12	57783100	0.983471
chr12	57870000	0.727273
chr12	57911100	0.752066
chr12	57915700	0.793388
chr12	58015600	0.752066
chr12	58027000	0.702479
chr12	58135400	0.702479
chr12	58225800	0.793388
chr12	58230600	0.768595
chr12	58233800	0.801653
chr12	58237000	0.859504
chr12	58258900	0.826446
chr12	58282500	0.925620
chr12	58290600	0.834711
chr12	58597500	0.785124
chr12	58662600	0.991736
chr12	58704200	0.925620
chr12	58708200	0.785124
chr12	58731400	0.727273

chr12	58736100	1.000000
chr12	58808200	0.842975
chr12	58819400	0.975207
chr12	58903100	0.892562
chr12	58951300	0.966942
chr12	59151600	1.000000
chr12	59307900	0.867769
chr12	59315600	0.677686
chr12	59319400	1.000000
chr12	59336000	0.710744
chr12	59356000	1.000000
chr12	59435500	0.958678
chr12	59449600	0.966942
chr12	59464600	0.950413
chr12	59469100	0.933884
chr12	59471200	0.983471
chr12	59473600	0.884298
chr12	59479000	0.768595
chr12	59516600	0.818182
chr12	60233700	0.933884
chr12	60244700	0.661157
chr12	60367300	0.909091
chr12	60430200	0.942149
chr12	60466300	0.768595
chr12	60475900	0.975207
chr12	60542300	0.958678
chr12	60566600	0.851240
chr12	60673300	0.942149
chr12	60729900	0.876033
chr12	60789100	0.694215
chr12	60831700	0.884298
chr12	62522500	0.826446
chr12	62931800	0.677686
chr12	63054200	0.966942
chr12	63097200	0.900826
chr12	63118900	1.000000
chr12	63125700	0.958678
chr12	63151500	0.826446
chr12	63178800	0.966942
chr12	63181400	0.735537
chr12	63183900	0.942149
chr12	63257200	0.818182
chr12	63260800	0.917355
chr12	63297200	0.950413
chr12	63688500	1.000000
chr12	63794900	0.876033
chr12	63804200	0.975207
chr12	64240400	0.719008
chr12	64291900	0.909091
chr12	64309500	0.876033
chr12	64312700	0.900826
chr12	64334500	0.834711
chr12	64361500	0.966942
chr12	64364600	0.900826

chr12	64433300	0.727273
chr12	64482700	0.983471
chr12	64554600	0.950413
chr12	64571800	0.958678
chr12	64575400	0.975207
chr12	64581300	0.900826
chr12	64784500	0.669421
chr12	64790000	0.818182
chr12	64813600	0.867769
chr12	64851000	0.859504
chr12	64853600	0.776860
chr12	64955000	0.776860
chr12	64978100	0.942149
chr12	65007200	0.719008
chr12	65010200	0.859504
chr12	65013800	1.000000
chr12	65017100	0.900826
chr12	65019300	0.966942
chr12	65021400	0.917355
chr12	65027800	0.933884
chr12	65060200	1.000000
chr12	65063200	0.867769
chr12	65066000	0.867769
chr12	65070200	0.983471
chr12	65074000	0.958678
chr12	65138900	0.983471
chr12	65141500	1.000000
chr12	65147500	1.000000
chr12	65283400	0.710744
chr12	65515300	0.776860
chr12	65603500	0.933884
chr12	65663900	0.950413
chr12	65700700	0.958678
chr12	65702900	0.876033
chr12	65824200	0.900826
chr12	65826500	0.892562
chr12	65927300	0.983471
chr12	65929400	0.859504
chr12	65931500	0.809917
chr12	65951000	0.785124
chr12	65956100	0.925620
chr12	65960000	0.925620
chr12	65962100	0.917355
chr12	65991500	0.710744
chr12	65996200	0.719008
chr12	65998900	0.983471
chr12	66003100	0.933884
chr12	66006600	0.966942
chr12	66025100	0.826446
chr12	66027200	0.966942
chr12	66029600	0.933884
chr12	66032100	0.991736
chr12	66042000	0.801653
chr12	66044100	0.942149

chr12	66048700	0.966942
chr12	66051200	1.000000
chr12	66053500	0.942149
chr12	66089800	1.000000
chr12	66172700	0.975207
chr12	66222100	0.743802
chr12	66225900	0.661157
chr12	66235200	0.991736
chr12	66247500	0.958678
chr12	66252600	0.809917
chr12	66262000	0.925620
chr12	66271200	0.983471
chr12	66274600	0.942149
chr12	66276900	0.710744
chr12	66280500	0.991736
chr12	66284200	0.884298
chr12	66287400	0.793388
chr12	66321900	0.752066
chr12	66324700	0.958678
chr12	66330200	0.991736
chr12	66334900	0.892562
chr12	66342900	0.950413
chr12	66347100	0.950413
chr12	66350700	0.884298
chr12	66354800	0.983471
chr12	66357700	0.933884
chr12	66455700	0.818182
chr12	66539200	0.983471
chr12	66547100	0.801653
chr12	66570700	0.876033
chr12	66591700	0.834711
chr12	66611400	0.685950
chr12	66910500	0.925620
chr12	67027900	0.785124
chr12	68100300	0.710744
chr12	68634100	0.669421
chr12	68704300	0.884298
chr12	68759000	0.785124
chr12	68800100	0.859504
chr12	68849500	0.892562
chr12	68915700	0.991736
chr12	68933500	0.677686
chr12	69003900	0.661157
chr12	69025300	0.826446
chr12	69166800	0.760331
chr12	69181500	0.933884
chr12	69327200	0.752066
chr12	69421700	0.818182
chr12	69447200	0.768595
chr12	69453400	0.694215
chr12	69491000	0.983471
chr12	69648100	0.760331
chr12	69660300	0.958678
chr12	69725700	0.710744

chr12	69799400	0.975207
chr12	69837100	0.925620
chr12	69903400	0.661157
chr12	69919100	0.752066
chr12	70084100	0.851240
chr12	70216000	0.760331
chr12	70267600	0.983471
chr12	70295300	0.900826
chr12	70302400	0.991736
chr12	70382300	0.842975
chr12	70434000	0.677686
chr12	70584800	0.752066
chr12	70639700	0.892562
chr12	70642300	0.867769
chr12	70901800	0.950413
chr12	70937900	0.983471
chr12	70942200	0.909091
chr12	70944800	0.876033
chr12	70970500	0.933884
chr12	70990900	0.661157
chr12	70993800	0.966942
chr12	70997200	0.719008
chr12	71005500	0.892562
chr12	71009100	0.661157
chr12	71032900	0.917355
chr12	71038300	0.884298
chr12	71040800	0.958678
chr12	71043000	0.983471
chr12	71046800	0.884298
chr12	71055800	0.958678
chr12	71058800	0.958678
chr12	71065100	0.958678
chr12	71085400	0.958678
chr12	71113700	0.950413
chr12	71116700	0.983471
chr12	71552300	0.925620
chr12	72021000	0.669421
chr12	72104500	0.809917
chr12	72177700	0.826446
chr12	75175600	0.925620
chr12	75283800	0.966942
chr12	75728700	0.702479
chr12	75783400	0.876033
chr12	75883700	0.834711
chr12	75944600	0.760331
chr12	75958100	0.752066
chr12	75979500	0.942149
chr12	76017700	0.685950
chr12	76026300	0.743802
chr12	76029000	0.801653
chr12	76051100	0.942149
chr12	76053300	0.909091
chr12	76094200	0.942149
chr12	76101300	0.900826

chr12	76115900	0.950413
chr12	76119500	0.966942
chr12	76160900	0.661157
chr12	76175200	0.834711
chr12	76209600	0.975207
chr12	76242400	0.809917
chr12	76257200	0.867769
chr12	76259800	0.702479
chr12	76272200	0.694215
chr12	76303300	0.950413
chr12	76328500	0.983471
chr12	76333300	0.917355
chr12	76338500	0.933884
chr12	76345700	0.760331
chr12	76347800	0.826446
chr12	76371200	0.735537
chr12	76375900	0.983471
chr12	76391700	0.950413
chr12	76397400	0.669421
chr12	76414900	0.966942
chr12	76419300	0.677686
chr12	76421400	0.917355
chr12	76431800	0.942149
chr12	76480400	0.661157
chr12	76517900	1.000000
chr12	76577500	0.892562
chr12	76618100	0.735537
chr12	76620700	1.000000
chr12	76630800	0.983471
chr12	76652600	0.909091
chr12	76660600	0.983471
chr12	76669100	0.983471
chr12	76672200	0.669421
chr12	76702800	0.867769
chr12	76723200	0.884298
chr12	76731800	0.950413
chr12	76735100	0.950413
chr12	76844600	0.876033
chr12	76914300	0.925620
chr12	76950400	0.694215
chr12	77030300	0.677686
chr12	77043100	0.669421
chr12	77457000	0.966942
chr12	77496700	0.876033
chr12	77499000	0.842975
chr12	77508100	0.793388
chr12	77510500	0.917355
chr12	77513400	0.933884
chr12	77516700	0.925620
chr12	77607600	0.785124
chr12	77625100	0.727273
chr12	78146400	0.900826
chr12	78224800	0.859504
chr12	78240100	0.925620

chr12	78242400	0.991736
chr12	78282800	0.958678
chr12	78288000	0.694215
chr12	78333300	0.752066
chr12	78337100	0.677686
chr12	78341300	0.884298
chr12	78374100	0.809917
chr12	78376300	0.975207
chr12	78588600	0.719008
chr12	79092500	0.677686
chr12	79098200	0.983471
chr12	79116000	0.735537
chr12	79281200	0.991736
chr12	79448400	0.669421
chr12	79450500	1.000000
chr12	79452700	0.909091
chr12	79457300	0.983471
chr12	79577900	0.975207
chr12	79696100	0.950413
chr12	79814300	0.975207
chr12	79818400	0.983471
chr12	79833200	0.975207
chr12	79838600	0.752066
chr12	79845900	0.975207
chr12	79848800	0.851240
chr12	79909100	0.876033
chr12	79916300	1.000000
chr12	79940200	0.842975
chr12	79943400	0.842975
chr12	79950900	0.694215
chr12	79953500	0.694215
chr12	79960600	0.983471
chr12	80079200	0.760331
chr12	80235600	1.000000
chr12	80239000	0.991736
chr12	80281200	0.719008
chr12	80450800	0.834711
chr12	80459300	0.826446
chr12	80494800	0.809917
chr12	80581300	0.884298
chr12	80616200	0.983471
chr12	80620500	0.917355
chr12	80636200	0.950413
chr12	80663000	0.991736
chr12	80747600	0.900826
chr12	80750400	0.958678
chr12	80836400	0.661157
chr12	81172100	0.834711
chr12	81472900	0.975207
chr12	81602900	0.685950
chr12	81801800	0.710744
chr12	82153200	0.793388
chr12	82801300	0.900826
chr12	82868900	0.983471

chr12	82881300	0.694215
chr12	82949100	0.801653
chr12	83044600	0.818182
chr12	83229500	0.826446
chr12	83251800	0.991736
chr12	83256200	0.719008
chr12	83410000	0.752066
chr12	83478500	0.826446
chr12	85574000	0.925620
chr12	85750700	0.933884
chr12	85757600	0.661157
chr12	86020500	0.983471
chr12	86114300	0.876033
chr12	86215400	0.826446
chr12	86225500	0.768595
chr12	86390100	0.925620
chr12	86588200	0.694215
chr12	86624600	0.950413
chr12	86627300	0.735537
chr12	87106400	1.000000
chr12	87653700	0.669421
chr12	87711800	0.925620
chr12	87717200	0.950413
chr12	87727200	0.909091
chr12	88221900	0.917355
chr12	88636600	0.867769
chr12	88641400	0.801653
chr12	88693400	0.991736
chr12	88702600	0.685950
chr12	88704700	0.933884
chr12	88719600	0.983471
chr12	88948500	0.702479
chr12	89032800	0.983471
chr12	89289900	0.851240
chr12	89292700	0.975207
chr12	89320200	0.983471
chr12	89338300	0.958678
chr12	89341100	0.983471
chr12	89346800	0.752066
chr12	89354700	0.950413
chr12	89413700	0.826446
chr12	89475300	0.983471
chr12	89479600	0.818182
chr12	89511800	0.685950
chr12	89528700	0.760331
chr12	89550600	0.966942
chr12	89554700	0.942149
chr12	89557700	0.867769
chr12	89560400	0.884298
chr12	89575700	0.735537
chr12	89606700	0.776860
chr12	89619800	0.876033
chr12	89630000	0.966942
chr12	89633200	1.000000

chr12	89639000	0.752066
chr12	89653900	0.694215
chr12	89720900	0.793388
chr12	89740500	0.991736
chr12	89762300	0.900826
chr12	89766100	0.966942
chr12	89768200	0.975207
chr12	89770500	0.669421
chr12	89784200	0.950413
chr12	89834200	0.909091
chr12	89844400	0.925620
chr12	89846500	0.958678
chr12	89848700	0.942149
chr12	89855100	0.991736
chr12	89863600	0.851240
chr12	89901000	0.719008
chr12	89939000	0.719008
chr12	90257400	0.900826
chr12	90341600	0.950413
chr12	90344000	0.925620
chr12	90593400	0.983471
chr12	90605200	0.983471
chr12	90623500	1.000000
chr12	90637900	0.719008
chr12	90647800	0.925620
chr12	90685600	0.983471
chr12	90715900	0.760331
chr12	90721500	0.909091
chr12	90725600	0.867769
chr12	90909400	0.975207
chr12	90914700	0.694215
chr12	90937800	0.842975
chr12	91152300	0.900826
chr12	91316200	0.892562
chr12	91332700	0.900826
chr12	91364300	0.900826
chr12	91533200	0.702479
chr12	91762500	0.661157
chr12	91794800	0.669421
chr12	91826200	0.727273
chr12	91971800	0.685950
chr12	92172700	0.834711
chr12	92307800	0.776860
chr12	92321200	0.793388
chr12	92384800	0.983471
chr12	92388100	0.809917
chr12	92429100	0.975207
chr12	92488400	0.900826
chr12	92602800	0.933884
chr12	92618800	0.727273
chr12	92684400	0.818182
chr12	92735500	0.818182
chr12	92806000	0.719008
chr12	92873000	0.669421

chr12	92933400	1.000000
chr12	92938100	0.793388
chr12	92988100	0.925620
chr12	93140200	0.975207
chr12	93148500	0.983471
chr12	93163600	0.983471
chr12	93293700	0.900826
chr12	93362700	0.950413
chr12	93365600	0.900826
chr12	93396900	0.983471
chr12	93401700	0.685950
chr12	93404700	0.818182
chr12	93410400	0.983471
chr12	93414500	0.909091
chr12	93427000	0.991736
chr12	93444800	0.661157
chr12	93448100	0.768595
chr12	93475000	0.975207
chr12	93484800	0.983471
chr12	93507300	0.917355
chr12	93524500	0.735537
chr12	93529600	0.975207
chr12	93571800	0.867769
chr12	93592100	0.694215
chr12	93597500	0.818182
chr12	93623700	0.842975
chr12	93759900	0.851240
chr12	94172000	0.900826
chr12	94180900	0.743802
chr12	94199500	0.752066
chr12	94676800	0.826446
chr12	94703000	0.677686
chr12	94966000	0.942149
chr12	94981700	0.958678
chr12	94984100	0.975207
chr12	94986300	0.958678
chr12	94989000	0.933884
chr12	95009800	0.966942
chr12	95013400	0.925620
chr12	95016400	0.917355
chr12	95038200	0.892562
chr12	95041700	0.768595
chr12	95067900	0.958678
chr12	95070400	0.950413
chr12	95084400	1.000000
chr12	95098600	0.793388
chr12	95108400	0.983471
chr12	95111900	0.727273
chr12	95156000	0.900826
chr12	95162900	0.719008
chr12	95172600	0.909091
chr12	95199700	0.884298
chr12	95435100	0.694215
chr12	95478000	0.991736

chr12	95497200	0.975207
chr12	95512500	0.983471
chr12	95558400	0.842975
chr12	95563300	1.000000
chr12	95567700	0.677686
chr12	95571100	0.851240
chr12	95597100	0.942149
chr12	95600100	0.793388
chr12	95606600	0.909091
chr12	95622000	0.842975
chr12	95625000	0.842975
chr12	95653100	0.776860
chr12	95656900	1.000000
chr12	95671200	0.900826
chr12	95698700	0.983471
chr12	95710400	0.876033
chr12	95730200	0.933884
chr12	95752300	0.983471
chr12	95869900	0.661157
chr12	95875000	0.752066
chr12	95939700	0.826446
chr12	95998700	0.933884
chr12	96000800	0.933884
chr12	96003300	0.917355
chr12	96006200	0.942149
chr12	96018500	0.851240
chr12	96027600	0.892562
chr12	96030300	0.933884
chr12	96032800	0.942149
chr12	96057600	0.966942
chr12	96112300	0.760331
chr12	96136300	0.661157
chr12	96197200	0.966942
chr12	96200400	0.851240
chr12	96203200	0.950413
chr12	96223600	0.900826
chr12	96230400	0.991736
chr12	96232500	0.719008
chr12	96442300	0.983471
chr12	96448300	0.958678
chr12	96465100	0.752066
chr12	96472400	0.950413
chr12	96477200	0.694215
chr12	96488400	0.933884
chr12	96497000	0.884298
chr12	96502000	0.909091
chr12	96515300	0.809917
chr12	96524800	0.719008
chr12	96530400	0.892562
chr12	96535500	0.942149
chr12	96538700	0.818182
chr12	96557900	0.851240
chr12	96560900	0.942149
chr12	96564500	0.785124

chr12	96571100	0.975207
chr12	96580700	0.669421
chr12	96587400	0.768595
chr12	96593200	0.834711
chr12	96596000	0.793388
chr12	96598400	0.983471
chr12	96601000	0.735537
chr12	96603700	0.925620
chr12	96605800	0.958678
chr12	96608200	0.950413
chr12	96611500	0.909091
chr12	96613800	0.884298
chr12	96616100	0.900826
chr12	96619100	0.925620
chr12	96622800	0.826446
chr12	96627700	1.000000
chr12	96629800	0.966942
chr12	96632100	0.909091
chr12	96636300	0.933884
chr12	96644000	0.958678
chr12	96650000	0.768595
chr12	96652100	0.933884
chr12	96655500	0.801653
chr12	96658200	0.942149
chr12	96729100	0.942149
chr12	96733100	0.851240
chr12	96743700	0.735537
chr12	96746300	0.892562
chr12	96755400	0.983471
chr12	96758800	0.826446
chr12	96762300	0.983471
chr12	96764400	0.859504
chr12	96766500	0.966942
chr12	96769200	0.892562
chr12	96771500	0.752066
chr12	96775500	0.942149
chr12	96778000	0.892562
chr12	96780600	0.917355
chr12	96786400	0.727273
chr12	96796300	0.719008
chr12	96810400	0.685950
chr12	96813200	0.909091
chr12	96816800	0.909091
chr12	96819300	0.809917
chr12	96821500	0.991736
chr12	96823600	0.966942
chr12	96833400	0.702479
chr12	96836400	0.909091
chr12	96838600	0.851240
chr12	96840700	0.834711
chr12	96843000	0.785124
chr12	96860800	0.793388
chr12	96878900	0.917355
chr12	96882200	0.958678

chr12	96887400	0.809917
chr12	96894000	0.917355
chr12	96897300	0.975207
chr12	96904500	0.900826
chr12	96915100	0.834711
chr12	97081500	0.834711
chr12	97114000	0.752066
chr12	97204200	0.958678
chr12	97206500	0.859504
chr12	97283700	0.917355
chr12	97305700	0.727273
chr12	97469700	0.950413
chr12	97534100	0.768595
chr12	97536800	0.958678
chr12	97950400	0.950413
chr12	98521200	0.983471
chr12	98582700	0.727273
chr12	98746700	0.925620
chr12	98791400	0.760331
chr12	98794000	0.933884
chr12	98796100	0.710744
chr12	98818900	0.991736
chr12	98833900	0.933884
chr12	98858200	0.801653
chr12	98885400	0.867769
chr12	99030300	0.793388
chr12	100558000	0.793388
chr12	100958300	0.966942
chr12	100970700	0.966942
chr12	100978000	0.809917
chr12	101239400	0.702479
chr12	101412600	1.000000
chr12	101416000	0.743802
chr12	102032500	0.752066
chr12	102269500	0.892562
chr12	102285400	1.000000
chr12	102330200	0.933884
chr12	102335200	0.727273
chr12	102566600	0.917355
chr12	102568800	0.991736
chr12	102576500	0.983471
chr12	102592100	0.909091
chr12	102816000	0.809917
chr12	102830900	0.950413
chr12	102833300	0.752066
chr12	103352100	0.776860
chr12	104191700	0.834711
chr12	104194300	0.983471
chr12	104199800	0.933884
chr12	104405200	0.933884
chr12	104515500	0.702479
chr12	104611800	0.669421
chr12	104614600	0.900826
chr12	104671200	0.925620

chr12	104674800	1.000000
chr12	104679400	0.933884
chr12	104696400	0.694215
chr12	104765300	0.867769
chr12	104803100	0.909091
chr12	104857000	0.818182
chr12	104872300	1.000000
chr12	104874500	0.958678
chr12	104899500	0.669421
chr12	104982500	0.867769
chr12	104996900	0.735537
chr12	104999300	0.975207
chr12	105015100	0.669421
chr12	105066000	1.000000
chr12	105080100	0.752066
chr12	105089500	0.892562
chr12	105114600	0.975207
chr12	105116700	0.694215
chr12	105196400	0.760331
chr12	105347200	0.719008
chr12	105388300	0.661157
chr12	105608700	0.933884
chr12	105620100	0.809917
chr12	105623200	0.909091
chr12	105648300	0.975207
chr12	105651600	0.694215
chr12	105683200	0.669421
chr12	105686700	0.768595
chr12	105701700	0.710744
chr12	105720900	0.983471
chr12	105776900	0.727273
chr12	105787700	0.735537
chr12	105803900	0.892562
chr12	105822100	0.892562
chr12	105827800	0.900826
chr12	105838100	0.793388
chr12	105840700	0.917355
chr12	105855900	0.917355
chr12	105860400	0.677686
chr12	105869100	0.983471
chr12	105871700	0.727273
chr12	105874600	0.958678
chr12	105877300	0.867769
chr12	105879500	0.727273
chr12	105882400	0.892562
chr12	105884500	0.685950
chr12	105948900	0.991736
chr12	105974700	0.661157
chr12	105981400	0.983471
chr12	105984400	0.677686
chr12	106071000	0.661157
chr12	106132000	1.000000
chr12	106135300	0.793388
chr12	106155400	0.900826

chr12	106244800	0.776860
chr12	106247200	0.735537
chr12	106327600	0.842975
chr12	106329700	0.752066
chr12	106361600	0.834711
chr12	106415700	0.991736
chr12	106424700	0.933884
chr12	106456600	0.735537
chr12	106459000	0.900826
chr12	106469000	0.826446
chr12	106472500	1.000000
chr12	106485100	0.975207
chr12	106490900	0.818182
chr12	106501300	0.743802
chr12	106507700	0.801653
chr12	106528900	0.768595
chr12	106678900	0.677686
chr12	106764000	0.966942
chr12	106812800	1.000000
chr12	106977500	0.694215
chr12	106980500	0.661157
chr12	107235200	0.842975
chr12	107421700	0.966942
chr12	107444900	0.834711
chr12	107451400	0.975207
chr12	107485300	0.793388
chr12	107630800	0.694215
chr12	107671900	0.958678
chr12	107714900	0.661157
chr12	107909400	0.859504
chr12	108139400	0.776860
chr12	108143400	0.983471
chr12	108297900	0.669421
chr12	108690700	0.900826
chr12	108696700	0.793388
chr12	108700300	0.942149
chr12	108707300	0.917355
chr12	108727900	0.859504
chr12	109006100	0.710744
chr12	109016800	0.809917
chr12	109023600	0.975207
chr12	109038300	0.917355
chr12	109040900	0.983471
chr12	109043800	0.966942
chr12	109057900	0.942149
chr12	109060000	0.776860
chr12	109062600	0.710744
chr12	109079500	0.958678
chr12	109083000	0.991736
chr12	109086700	0.842975
chr12	109116000	0.884298
chr12	109118200	0.727273
chr12	109128500	0.900826
chr12	109195900	0.818182

chr12	109198000	0.983471
chr12	109209500	0.842975
chr12	109216800	0.975207
chr12	109219000	0.933884
chr12	109223100	0.975207
chr12	109226700	0.677686
chr12	109230100	0.942149
chr12	109232400	0.975207
chr12	109235300	0.950413
chr12	109243100	0.842975
chr12	109248600	0.942149
chr12	109252600	0.685950
chr12	109531000	0.917355
chr12	109549200	0.851240
chr12	109748100	0.685950
chr12	109886900	0.884298
chr12	109897100	0.975207
chr12	109957700	0.900826
chr12	109977600	0.892562
chr12	109983100	0.851240
chr12	110152600	0.669421
chr12	110266400	0.859504
chr12	110276000	0.942149
chr12	110326800	0.942149
chr12	110375800	0.983471
chr12	110383000	0.950413
chr12	110385200	0.801653
chr12	110427200	0.950413
chr12	110453600	0.760331
chr12	110462400	0.925620
chr12	110465300	0.776860
chr12	110477300	0.991736
chr12	110481200	0.801653
chr12	110493500	0.760331
chr12	110637900	0.950413
chr12	110662400	0.661157
chr12	110707700	0.818182
chr12	110739200	0.909091
chr12	110742300	0.975207
chr12	110751200	0.975207
chr12	110791500	0.975207
chr12	110977700	0.743802
chr12	110981400	0.958678
chr12	110984800	0.933884
chr12	111136800	0.933884
chr12	111182500	0.694215
chr12	111355500	0.983471
chr12	111753100	0.867769
chr12	111801700	0.834711
chr12	111835200	0.991736
chr12	111837700	0.975207
chr12	111841800	0.933884
chr12	111851400	0.884298
chr12	111862900	0.677686

chr12	111865000	0.933884
chr12	111867400	0.900826
chr12	111870000	0.834711
chr12	111872800	0.842975
chr12	111875900	0.826446
chr12	111878000	0.851240
chr12	111881400	0.917355
chr12	111884900	0.818182
chr12	111889500	0.768595
chr12	112008300	1.000000
chr12	112016300	0.958678
chr12	112074500	0.694215
chr12	112082000	0.760331
chr12	112179500	0.983471
chr12	112186900	0.991736
chr12	112213800	1.000000
chr12	112223600	0.900826
chr12	112363400	0.958678
chr12	112446800	0.661157
chr12	112456900	0.867769
chr12	112486000	0.859504
chr12	112534600	0.859504
chr12	112554900	0.958678
chr12	112568500	0.867769
chr12	112741300	0.900826
chr12	112891400	0.702479
chr12	112915800	0.685950
chr12	113274900	0.884298
chr12	113277100	0.884298
chr12	113279500	0.867769
chr12	113340200	0.966942
chr12	113344800	0.793388
chr12	113409500	0.735537
chr12	113445200	0.909091
chr12	113448900	0.925620
chr12	113457300	0.983471
chr12	113469100	0.669421
chr12	113479000	0.834711
chr12	113632100	1.000000
chr12	113661700	0.884298
chr12	113667600	0.983471
chr12	113670000	0.685950
chr12	113676000	0.867769
chr12	113679800	0.661157
chr12	113682500	0.801653
chr12	113684800	0.669421
chr12	113737800	0.826446
chr12	113767400	0.719008
chr12	113905600	0.818182
chr12	114219900	0.760331
chr12	114269300	0.942149
chr12	114301500	0.661157
chr12	114373700	0.942149
chr12	116411800	0.876033

chr12	116550400	0.702479
chr12	116559400	0.859504
chr12	116562300	0.826446
chr12	116574100	0.966942
chr12	116581600	0.983471
chr12	116587300	0.876033
chr12	116603300	0.669421
chr12	116610200	0.991736
chr12	116625100	0.917355
chr12	116682100	0.735537
chr12	116797300	0.983471
chr12	116814700	0.933884
chr12	116819200	0.818182
chr12	116821900	0.867769
chr12	116894100	0.983471
chr12	116946600	0.710744
chr12	116963700	0.933884
chr12	116966400	0.677686
chr12	117037600	0.859504
chr12	117114000	0.702479
chr12	117147400	0.809917
chr12	117156700	1.000000
chr12	117165200	0.925620
chr12	117210800	0.818182
chr12	117451900	0.983471
chr12	117586400	0.760331
chr12	117925400	0.917355
chr12	118100800	0.983471
chr12	118112800	0.851240
chr12	118118300	0.925620
chr12	118121900	0.685950
chr12	118124100	0.851240
chr12	118126200	0.966942
chr12	118200100	0.785124
chr12	118207100	0.933884
chr12	118314400	0.685950
chr12	118471700	0.884298
chr12	118490700	0.884298
chr12	118627700	0.950413
chr12	118740400	0.702479
chr12	118745300	0.942149
chr12	118768400	0.950413
chr12	118795900	0.851240
chr12	118799300	0.917355
chr12	118856700	0.685950
chr12	119614400	0.669421
chr12	119619000	0.743802
chr12	120112000	0.793388
chr12	120130900	0.975207
chr12	120142700	0.710744
chr12	120154400	0.942149
chr12	120307800	0.842975
chr12	120525200	0.884298
chr12	120557300	0.776860

chr12	120564700	0.942149
chr12	120605700	0.892562
chr12	120663400	0.851240
chr12	120666600	0.677686
chr12	120668800	0.735537
chr12	120671400	0.867769
chr12	120674700	0.884298
chr12	120677400	0.917355
chr12	120681400	0.966942
chr12	120683600	0.991736
chr12	120686100	0.752066
chr12	120689100	0.859504
chr12	120692900	1.000000
chr12	120697300	0.983471
chr12	120699600	0.818182
chr12	120705800	0.669421
chr12	120729400	0.735537
chr12	120740900	0.743802
chr12	120926300	0.842975
chr12	120976600	0.851240
chr12	120988100	0.851240
chr12	121079200	0.809917
chr12	121087400	0.859504
chr12	121115700	0.809917
chr12	121118300	0.768595
chr12	121136800	0.892562
chr12	121212400	0.834711
chr12	121221500	1.000000
chr12	121252700	0.743802
chr12	121288100	0.892562
chr12	121498500	0.801653
chr12	121533800	0.801653
chr12	121562500	0.925620
chr12	121567200	0.818182
chr12	121571100	0.677686
chr12	121583100	0.694215
chr12	121609400	0.909091
chr12	121625500	0.975207
chr12	121628700	1.000000
chr12	121631000	0.909091
chr12	121635000	0.950413
chr12	121643100	0.818182
chr12	121652800	0.933884
chr12	121715300	0.785124
chr12	121717600	0.677686
chr12	121826400	0.685950
chr12	121840200	0.702479
chr12	121890800	0.702479
chr12	122067800	0.677686
chr12	122097400	0.851240
chr12	122102200	0.776860
chr12	122110500	0.685950
chr12	122187100	0.900826
chr12	122222800	0.818182

chr12	122228100	0.933884
chr12	122234000	0.677686
chr12	122332200	0.859504
chr12	122356400	0.884298
chr12	122363200	0.917355
chr12	122430600	0.983471
chr12	122500700	0.834711
chr12	122503100	0.743802
chr12	122510500	0.661157
chr12	122515900	0.776860
chr12	122521000	0.752066
chr12	122527800	0.776860
chr12	122582400	0.876033
chr12	122593300	0.785124
chr12	122665200	0.793388
chr12	122667900	0.776860
chr12	122711800	0.669421
chr12	122803900	0.793388
chr12	122812600	0.950413
chr12	122851200	0.933884
chr12	122853400	0.876033
chr12	122898100	0.818182
chr12	122902900	0.983471
chr12	122949100	0.867769
chr12	123135400	0.719008
chr12	123163900	1.000000
chr12	123210500	0.842975
chr12	123319100	0.752066
chr12	123326000	0.876033
chr12	123335900	0.661157
chr12	123352800	0.710744
chr12	123355300	0.661157
chr12	123371300	0.900826
chr12	123377000	0.900826
chr12	123427900	0.793388
chr12	123430200	0.743802
chr12	123432700	0.917355
chr12	123436300	0.851240
chr12	123443100	0.867769
chr12	123469400	0.892562
chr12	123561800	0.702479
chr12	123570700	0.735537
chr12	123574900	0.884298
chr12	123579600	0.785124
chr12	123590100	0.966942
chr12	123592300	0.842975
chr12	123599400	0.842975
chr12	123611600	0.975207
chr12	123624700	0.719008
chr12	123626800	0.958678
chr12	123727400	0.983471
chr12	123752600	0.702479
chr12	123762900	0.975207
chr12	123872800	0.694215

chr12	123883700	0.933884
chr12	123918700	0.727273
chr12	123930700	0.909091
chr12	123963000	0.785124
chr12	123965100	0.826446
chr12	124004300	0.752066
chr12	124076600	0.884298
chr12	124427300	0.785124
chr12	124436800	0.842975
chr12	124525200	0.685950
chr12	124538700	0.842975
chr12	124557500	0.785124
chr12	124595600	0.900826
chr12	124673500	0.768595
chr12	124718300	1.000000
chr12	124722300	0.867769
chr12	124740800	0.694215
chr12	124768400	0.710744
chr12	124837900	0.809917
chr12	124855000	0.669421
chr12	124865100	0.925620
chr12	124868500	0.818182
chr12	124871100	0.859504
chr12	124875100	0.818182
chr12	124878000	0.826446
chr12	124908800	0.768595
chr12	124913200	0.975207
chr12	124926400	0.991736
chr12	124951100	0.842975
chr12	124983800	0.958678
chr12	124991300	0.917355
chr12	124998000	0.884298
chr12	125006400	0.867769
chr12	125009000	0.760331
chr12	125023900	0.702479
chr12	125033600	0.859504
chr12	125035900	0.933884
chr12	125052900	0.710744
chr12	125066800	0.809917
chr12	125073800	0.809917
chr12	125076900	0.892562
chr12	125093900	0.809917
chr12	125106700	0.719008
chr12	125111400	0.950413
chr12	125136600	0.710744
chr12	125139000	0.900826
chr12	125141400	0.876033
chr12	125148400	0.661157
chr12	125153900	0.818182
chr12	125165100	0.892562
chr12	125167600	0.793388
chr12	125169900	0.892562
chr12	125173300	0.892562
chr12	125180000	0.826446

chr12	125185900	0.710744
chr12	125198600	0.917355
chr12	125205600	0.966942
chr12	125212200	0.925620
chr12	125222400	0.710744
chr12	125225900	0.925620
chr12	125230500	0.933884
chr12	125323100	0.826446
chr12	125366000	0.661157
chr12	125389700	0.950413
chr12	125402200	0.702479
chr12	125405100	1.000000
chr12	125414200	0.859504
chr12	125422700	0.859504
chr12	125425900	0.900826
chr12	125582200	0.677686
chr12	127650600	0.958678
chr12	129279100	0.710744
chr12	129282500	0.942149
chr12	129286700	0.958678
chr12	129303200	0.760331
chr12	131261300	0.876033
chr12	131355800	0.694215
chr12	132222200	0.966942
chr12	132269200	0.958678
chr12	132289000	0.785124
chr12	132397400	0.694215
chr12	132488400	0.867769
chr12	132515200	0.983471
chr12	132563000	0.909091
chr12	132904500	0.760331
chr12	133083700	0.925620
chr12	133177800	0.768595
chr12	133180400	0.710744
chr12	133230900	0.900826
chr13	20161500	0.735537
chr13	20202000	0.859504
chr13	20251900	0.958678
chr13	20473900	0.727273
chr13	20531900	0.818182
chr13	21080700	0.661157
chr13	21183500	0.958678
chr13	21228900	0.892562
chr13	21284000	0.900826
chr13	21288500	0.826446
chr13	21296300	0.834711
chr13	21303400	0.909091
chr13	21362800	0.942149
chr13	21365200	0.867769
chr13	21371700	0.975207
chr13	21376500	0.991736
chr13	21387500	0.867769
chr13	21391300	0.793388
chr13	21403000	0.710744

chr13	21448000	0.851240
chr13	21457200	0.925620
chr13	21520600	0.851240
chr13	21572500	0.859504
chr13	21588300	0.917355
chr13	21592100	0.966942
chr13	21609500	0.942149
chr13	21613600	1.000000
chr13	21616300	0.884298
chr13	21620600	0.900826
chr13	21628600	0.884298
chr13	21649500	0.842975
chr13	21652700	0.702479
chr13	21655000	0.867769
chr13	21900700	0.776860
chr13	21974200	0.876033
chr13	22010200	0.925620
chr13	22158000	0.942149
chr13	22162900	0.851240
chr13	23885600	0.991736
chr13	23893900	0.876033
chr13	23896000	0.966942
chr13	23898800	0.809917
chr13	23924500	0.942149
chr13	23946900	0.958678
chr13	23954200	0.975207
chr13	24001100	0.793388
chr13	24003200	0.768595
chr13	24041000	0.752066
chr13	24133600	0.752066
chr13	24183300	0.991736
chr13	24192700	0.983471
chr13	24325100	0.983471
chr13	24369100	0.983471
chr13	24433100	0.983471
chr13	24441900	0.685950
chr13	24475400	0.851240
chr13	24477700	0.727273
chr13	24496900	0.933884
chr13	24503100	0.900826
chr13	24506600	0.801653
chr13	24638200	0.892562
chr13	24649600	0.991736
chr13	24699600	0.677686
chr13	24710200	0.669421
chr13	24717200	0.933884
chr13	24723300	0.966942
chr13	24790300	0.776860
chr13	24846000	0.892562
chr13	25075300	0.925620
chr13	25077900	0.809917
chr13	25084800	0.768595
chr13	25485400	0.917355
chr13	25946500	0.694215

chr13	26480700	0.669421
chr13	26540300	0.735537
chr13	26551800	0.966942
chr13	26582000	0.884298
chr13	26589700	0.983471
chr13	26592600	0.950413
chr13	26595800	0.818182
chr13	27050700	0.909091
chr13	27072700	0.793388
chr13	27075700	0.677686
chr13	27157800	0.942149
chr13	27161600	0.867769
chr13	27165200	0.909091
chr13	27176400	0.785124
chr13	27642100	0.694215
chr13	27706200	0.909091
chr13	27747700	0.685950
chr13	27757400	0.917355
chr13	27769600	0.991736
chr13	27833800	0.719008
chr13	27847200	0.719008
chr13	27936800	0.743802
chr13	28021800	0.735537
chr13	28033700	0.991736
chr13	28101100	0.735537
chr13	28147400	0.933884
chr13	28158100	0.942149
chr13	28174500	0.909091
chr13	28233100	0.809917
chr13	28365800	0.826446
chr13	28490200	0.669421
chr13	28495300	0.727273
chr13	28674900	0.801653
chr13	28886200	0.685950
chr13	28896700	0.966942
chr13	28899300	0.975207
chr13	28902100	0.768595
chr13	28904800	0.933884
chr13	28908000	0.818182
chr13	28910800	0.917355
chr13	28914400	0.958678
chr13	28922700	0.760331
chr13	28925300	0.768595
chr13	28928000	1.000000
chr13	28930900	0.958678
chr13	28933100	0.867769
chr13	28954000	0.876033
chr13	28959400	0.702479
chr13	28962700	0.950413
chr13	28966800	1.000000
chr13	28969000	0.785124
chr13	28972200	0.694215
chr13	28974700	0.876033
chr13	28976800	0.991736

chr13	28982100	0.933884
chr13	28984400	0.909091
chr13	28987800	0.884298
chr13	29001800	0.793388
chr13	29015500	0.809917
chr13	29025000	0.867769
chr13	29027100	0.776860
chr13	29037900	0.900826
chr13	29041600	1.000000
chr13	29050000	0.975207
chr13	29053800	0.958678
chr13	29065600	0.966942
chr13	29132800	0.793388
chr13	29149300	0.900826
chr13	29215300	1.000000
chr13	29291200	0.867769
chr13	29318600	0.975207
chr13	29324900	0.958678
chr13	30089900	0.909091
chr13	30095100	0.983471
chr13	30098500	0.958678
chr13	30112600	0.851240
chr13	30120900	0.966942
chr13	30123100	0.991736
chr13	30126100	0.826446
chr13	30138600	0.933884
chr13	30277400	0.950413
chr13	30420400	0.677686
chr13	30498400	0.842975
chr13	30712700	0.809917
chr13	30717300	0.958678
chr13	30875200	0.867769
chr13	30877300	0.809917
chr13	30887000	0.785124
chr13	30939400	0.991736
chr13	30995400	0.719008
chr13	31004600	0.818182
chr13	31020300	0.933884
chr13	31037100	0.661157
chr13	31041200	0.859504
chr13	31147200	0.958678
chr13	31198000	0.900826
chr13	31240200	0.669421
chr13	31248100	0.793388
chr13	31272300	0.958678
chr13	31283100	0.958678
chr13	31287300	0.884298
chr13	31314700	0.826446
chr13	31318100	0.983471
chr13	31338200	0.900826
chr13	31367100	0.966942
chr13	31370900	0.702479
chr13	31374100	0.958678
chr13	31377500	0.752066

chr13	31381300	0.859504
chr13	31391200	0.958678
chr13	31407500	0.694215
chr13	31439200	0.876033
chr13	31444300	0.842975
chr13	31448300	0.785124
chr13	31470300	0.859504
chr13	31474500	0.727273
chr13	31483100	0.842975
chr13	31620300	0.950413
chr13	31665200	0.752066
chr13	32112900	0.669421
chr13	32205900	0.958678
chr13	32213100	0.942149
chr13	32306900	0.661157
chr13	32368000	0.991736
chr13	32717300	0.809917
chr13	32828600	0.917355
chr13	32835400	0.760331
chr13	32837600	0.950413
chr13	32842500	0.669421
chr13	32846400	1.000000
chr13	32859500	0.933884
chr13	32874900	0.793388
chr13	32888800	0.669421
chr13	32984300	0.760331
chr13	33216900	0.685950
chr13	33250100	0.859504
chr13	33305900	0.768595
chr13	33492000	0.818182
chr13	33544000	0.958678
chr13	33579000	0.685950
chr13	33662300	0.834711
chr13	33665000	0.900826
chr13	33667200	0.983471
chr13	33685400	0.966942
chr13	33701100	0.975207
chr13	33726900	0.702479
chr13	33729000	0.983471
chr13	33743800	1.000000
chr13	33759800	0.842975
chr13	33763600	0.950413
chr13	33767700	0.826446
chr13	33771600	0.818182
chr13	33778700	1.000000
chr13	33782300	0.933884
chr13	33803100	0.743802
chr13	33805200	0.991736
chr13	33810600	0.983471
chr13	33823200	0.966942
chr13	33826300	0.884298
chr13	33851100	0.892562
chr13	33854100	0.876033
chr13	33857300	0.917355

chr13	34065400	0.991736
chr13	34069200	0.983471
chr13	34071700	0.958678
chr13	34084100	0.933884
chr13	34116100	0.909091
chr13	34118200	0.752066
chr13	34130500	0.933884
chr13	34185300	0.818182
chr13	34253800	0.966942
chr13	34271800	0.892562
chr13	34407400	0.818182
chr13	34453300	0.991736
chr13	34493200	0.809917
chr13	34600200	0.975207
chr13	34619300	0.809917
chr13	34651000	0.752066
chr13	34728000	0.958678
chr13	34732500	0.983471
chr13	34736500	0.991736
chr13	34923600	0.702479
chr13	34931600	0.950413
chr13	35034500	0.685950
chr13	35037400	0.785124
chr13	35714600	0.768595
chr13	35924000	0.826446
chr13	35963400	0.867769
chr13	35967800	0.694215
chr13	36045500	0.801653
chr13	36412900	1.000000
chr13	36708600	0.710744
chr13	36721300	0.966942
chr13	36850600	0.702479
chr13	36907400	0.983471
chr13	36992300	0.966942
chr13	36996100	0.785124
chr13	37020400	0.826446
chr13	37105800	0.727273
chr13	37108900	0.933884
chr13	37248700	0.776860
chr13	37397200	0.867769
chr13	37436800	0.942149
chr13	37463300	0.917355
chr13	37519600	0.867769
chr13	37523600	0.760331
chr13	37529500	0.884298
chr13	37540400	1.000000
chr13	37601400	0.933884
chr13	37701400	0.793388
chr13	37703600	0.900826
chr13	37709500	0.909091
chr13	37862300	0.867769
chr13	37935100	0.991736
chr13	37987400	0.661157
chr13	38191700	0.876033

chr13	38221400	0.776860
chr13	38231200	0.958678
chr13	38239900	0.702479
chr13	38256200	0.950413
chr13	38276100	0.950413
chr13	38283400	0.900826
chr13	38288800	0.760331
chr13	38290900	0.950413
chr13	38321700	0.933884
chr13	39526300	0.867769
chr13	39989500	0.991736
chr13	40030300	0.801653
chr13	40102600	0.942149
chr13	40140000	0.966942
chr13	40172500	0.760331
chr13	40348300	0.801653
chr13	40350700	0.983471
chr13	40358900	0.983471
chr13	40361000	0.975207
chr13	40393400	0.942149
chr13	40438200	0.900826
chr13	40449500	0.727273
chr13	40453200	0.909091
chr13	40456600	0.925620
chr13	40466800	0.859504
chr13	40469700	1.000000
chr13	40474700	0.900826
chr13	40526600	0.991736
chr13	40605400	0.966942
chr13	40638900	0.677686
chr13	40657300	0.818182
chr13	40690300	0.942149
chr13	40705800	0.933884
chr13	40724000	0.776860
chr13	40732400	0.694215
chr13	40765000	0.884298
chr13	40768800	0.983471
chr13	40781300	0.867769
chr13	40805200	0.851240
chr13	40829900	0.752066
chr13	40888000	0.950413
chr13	40900200	0.710744
chr13	40920700	0.793388
chr13	40977200	0.925620
chr13	40981000	0.694215
chr13	41013300	0.991736
chr13	41016100	0.991736
chr13	41020300	0.983471
chr13	41051700	0.942149
chr13	41077700	0.884298
chr13	41080000	0.917355
chr13	41082500	0.909091
chr13	41090300	0.661157
chr13	41115000	0.991736

chr13	41131000	0.818182
chr13	41133400	0.950413
chr13	41138700	0.991736
chr13	41143200	0.909091
chr13	41151400	0.685950
chr13	41155500	0.983471
chr13	41159400	0.966942
chr13	41161500	0.958678
chr13	41165400	0.851240
chr13	41184000	0.834711
chr13	41187900	0.966942
chr13	41214300	0.975207
chr13	41223500	0.900826
chr13	41247300	0.809917
chr13	41256400	0.958678
chr13	41351400	0.991736
chr13	41558000	0.793388
chr13	41560200	0.900826
chr13	41609400	0.983471
chr13	41614500	0.925620
chr13	41639100	0.950413
chr13	41649900	0.793388
chr13	41859600	0.867769
chr13	41881200	0.694215
chr13	41902300	0.768595
chr13	41920300	0.991736
chr13	42037900	0.900826
chr13	42040100	0.867769
chr13	42139000	0.950413
chr13	42170700	0.909091
chr13	42185500	0.892562
chr13	42189600	0.859504
chr13	42221000	0.933884
chr13	42223700	0.950413
chr13	42226700	0.958678
chr13	42268500	0.892562
chr13	42270900	0.909091
chr13	42363200	0.867769
chr13	42402300	0.991736
chr13	42408300	0.669421
chr13	42491500	0.991736
chr13	42586500	0.892562
chr13	42601200	0.735537
chr13	42640600	0.818182
chr13	42708400	0.702479
chr13	42871700	0.669421
chr13	43049600	0.752066
chr13	43061400	1.000000
chr13	43068900	0.685950
chr13	43113700	0.702479
chr13	43123600	0.900826
chr13	43128200	0.801653
chr13	43469200	0.785124
chr13	43534200	0.760331

chr13	43629400	0.785124
chr13	43670300	0.975207
chr13	43843600	0.909091
chr13	43858800	0.768595
chr13	43875300	0.942149
chr13	43879800	0.925620
chr13	43891300	0.859504
chr13	43922900	0.677686
chr13	43938700	0.983471
chr13	43952000	0.933884
chr13	44422900	0.950413
chr13	44588300	0.892562
chr13	44692000	0.702479
chr13	44707300	0.892562
chr13	44805700	0.933884
chr13	44812000	1.000000
chr13	44817500	0.942149
chr13	44833500	0.925620
chr13	44881700	0.950413
chr13	44887700	0.991736
chr13	44935500	0.834711
chr13	44939600	0.958678
chr13	45046000	0.892562
chr13	45048900	0.834711
chr13	45285200	0.950413
chr13	45288800	0.917355
chr13	45330400	0.925620
chr13	45338400	0.735537
chr13	45376800	0.710744
chr13	45393500	0.719008
chr13	45411900	0.942149
chr13	45417000	0.884298
chr13	45458700	0.876033
chr13	45491900	0.694215
chr13	45496700	0.859504
chr13	45504900	0.768595
chr13	45507300	0.933884
chr13	45509400	0.991736
chr13	45542600	0.760331
chr13	45548500	0.983471
chr13	45589200	0.735537
chr13	45717200	0.685950
chr13	45737100	0.983471
chr13	45781400	0.966942
chr13	45794100	0.900826
chr13	45885600	0.867769
chr13	45900300	0.685950
chr13	45903600	0.661157
chr13	45906400	0.909091
chr13	45911800	0.785124
chr13	45942200	0.685950
chr13	45971000	1.000000
chr13	45988900	0.925620
chr13	46057100	0.834711

chr13	46075100	0.958678
chr13	46086600	0.669421
chr13	46101900	0.760331
chr13	46293600	0.925620
chr13	46422400	0.859504
chr13	46425300	0.694215
chr13	46435500	0.710744
chr13	46688300	0.727273
chr13	46694800	0.752066
chr13	46699900	0.975207
chr13	46806900	0.917355
chr13	46871900	1.000000
chr13	47133600	1.000000
chr13	47170000	0.727273
chr13	47174900	0.776860
chr13	47179600	0.719008
chr13	47216000	0.966942
chr13	47229700	0.950413
chr13	47237500	0.958678
chr13	47240300	0.958678
chr13	48618400	0.933884
chr13	48684600	0.942149
chr13	48700500	0.942149
chr13	48719200	0.933884
chr13	48730400	0.966942
chr13	48735900	0.867769
chr13	48942400	0.776860
chr13	48976100	0.950413
chr13	49006500	0.958678
chr13	49011500	0.925620
chr13	49020800	0.851240
chr13	49023500	0.876033
chr13	49039400	0.809917
chr13	49066800	0.876033
chr13	49069500	0.925620
chr13	49147900	0.694215
chr13	49175300	0.818182
chr13	49466700	0.966942
chr13	49661600	0.809917
chr13	49672900	0.983471
chr13	49687500	0.867769
chr13	49721400	0.925620
chr13	49756000	0.793388
chr13	49883100	0.859504
chr13	49888400	1.000000
chr13	50190800	0.991736
chr13	50204400	0.966942
chr13	50208000	0.760331
chr13	50223700	0.661157
chr13	50245100	0.685950
chr13	50267500	0.900826
chr13	50422300	0.685950
chr13	50574100	0.950413
chr13	50652000	0.793388

chr13	50819200	0.909091
chr13	50894700	0.933884
chr13	50909400	0.983471
chr13	50940500	0.966942
chr13	50961200	0.851240
chr13	50964100	0.958678
chr13	50985700	0.826446
chr13	50996200	1.000000
chr13	51023000	0.966942
chr13	51103900	0.694215
chr13	51191800	0.975207
chr13	51253900	0.983471
chr13	51258000	0.851240
chr13	51260200	0.991736
chr13	51266100	0.933884
chr13	51272400	0.983471
chr13	51641000	0.752066
chr13	51722800	0.719008
chr13	51728800	0.818182
chr13	51777000	0.942149
chr13	51804500	0.859504
chr13	51810100	0.867769
chr13	51850900	0.909091
chr13	51889900	0.983471
chr13	51938900	0.859504
chr13	51989100	0.785124
chr13	51993800	0.669421
chr13	52218600	0.983471
chr13	52247900	0.661157
chr13	52271800	0.694215
chr13	52375900	0.876033
chr13	52636300	0.884298
chr13	52737000	0.925620
chr13	52755900	0.933884
chr13	52762200	0.685950
chr13	52804500	1.000000
chr13	52919700	0.743802
chr13	52946200	0.669421
chr13	52948900	0.702479
chr13	52989400	0.925620
chr13	53002100	0.818182
chr13	53005500	0.760331
chr13	53150700	0.958678
chr13	53176500	0.727273
chr13	53182900	0.876033
chr13	53197700	0.876033
chr13	53205600	0.917355
chr13	53276500	0.776860
chr13	53425600	0.760331
chr13	57078800	0.669421
chr13	57281100	0.793388
chr13	57293900	0.892562
chr13	57435900	0.942149
chr13	57439100	0.983471

chr13	58066700	0.793388
chr13	58544700	0.867769
chr13	58875000	0.818182
chr13	58917900	0.661157
chr13	59660700	0.743802
chr13	59663400	0.834711
chr13	59840900	0.867769
chr13	59894200	0.768595
chr13	59907500	0.760331
chr13	59911600	0.785124
chr13	59916600	0.826446
chr13	59931200	0.950413
chr13	59934600	0.661157
chr13	59969000	0.942149
chr13	60021300	0.859504
chr13	60024300	0.685950
chr13	60033300	0.884298
chr13	60035800	0.975207
chr13	60051700	0.661157
chr13	60054900	0.892562
chr13	60058400	0.685950
chr13	60066600	0.834711
chr13	60069000	1.000000
chr13	60100800	0.793388
chr13	60105600	0.917355
chr13	60108900	0.975207
chr13	60208300	0.975207
chr13	60241000	0.983471
chr13	60246300	0.710744
chr13	60330200	0.677686
chr13	60351700	0.859504
chr13	60398200	0.793388
chr13	60709900	0.760331
chr13	60723800	0.677686
chr13	60909100	0.661157
chr13	61008000	0.694215
chr13	61084500	0.958678
chr13	61334100	0.975207
chr13	61506500	0.834711
chr13	64008200	0.909091
chr13	64557900	0.694215
chr13	64808600	0.917355
chr13	64814100	0.925620
chr13	65214600	0.876033
chr13	65297900	0.925620
chr13	65344000	0.818182
chr13	65352500	0.909091
chr13	65385500	0.942149
chr13	65486600	0.925620
chr13	66467500	0.991736
chr13	66470500	0.685950
chr13	66486900	0.892562
chr13	66489900	0.801653
chr13	66517900	0.917355

chr13	67210100	0.909091
chr13	67231400	0.842975
chr13	67234500	0.933884
chr13	67490000	0.661157
chr13	67568500	0.776860
chr13	67720700	0.826446
chr13	67722900	0.958678
chr13	68638600	0.727273
chr13	69311600	0.702479
chr13	69651900	0.892562
chr13	70545000	0.958678
chr13	71209200	0.776860
chr13	71283300	0.859504
chr13	71479600	0.694215
chr13	71818700	0.842975
chr13	71823500	0.909091
chr13	71826700	0.950413
chr13	71834800	0.842975
chr13	72069300	0.892562
chr13	72073900	0.884298
chr13	72090200	0.685950
chr13	72195500	0.719008
chr13	72198700	0.958678
chr13	72201200	0.925620
chr13	72207300	0.983471
chr13	72236600	0.719008
chr13	72364100	0.859504
chr13	72542300	0.958678
chr13	72684300	0.884298
chr13	72700900	0.760331
chr13	72716400	0.752066
chr13	72741000	0.685950
chr13	72869800	0.669421
chr13	73226900	0.677686
chr13	73291300	0.735537
chr13	73630400	0.950413
chr13	73665000	0.917355
chr13	73900200	0.719008
chr13	74252000	0.842975
chr13	74340800	0.991736
chr13	74403500	0.950413
chr13	74454800	0.685950
chr13	74509000	0.900826
chr13	74512900	0.842975
chr13	74649100	0.966942
chr13	74678500	0.702479
chr13	74689500	0.909091
chr13	74692800	1.000000
chr13	74702900	0.818182
chr13	74767900	0.876033
chr13	74818700	0.942149
chr13	74862600	0.743802
chr13	75150100	0.694215
chr13	75262500	0.867769

chr13	75305400	0.958678
chr13	75399800	0.917355
chr13	75445000	0.892562
chr13	75466300	0.975207
chr13	75482100	0.842975
chr13	75504800	0.669421
chr13	75710300	0.851240
chr13	75756300	0.933884
chr13	75893600	0.884298
chr13	75898000	0.801653
chr13	75901900	0.752066
chr13	75919300	0.958678
chr13	75938500	0.925620
chr13	75942600	0.966942
chr13	76279700	1.000000
chr13	76299700	0.743802
chr13	76350700	0.925620
chr13	76363300	0.909091
chr13	76372500	0.884298
chr13	76867800	0.917355
chr13	77087200	0.983471
chr13	77116700	0.694215
chr13	77127100	0.991736
chr13	77185600	0.669421
chr13	77286200	0.950413
chr13	77297300	0.760331
chr13	77299600	0.983471
chr13	77304500	0.925620
chr13	77329100	0.735537
chr13	77332800	0.710744
chr13	77338800	0.867769
chr13	77365300	0.909091
chr13	77427600	0.983471
chr13	77440300	0.950413
chr13	77443100	0.677686
chr13	77453400	0.942149
chr13	77456300	0.776860
chr13	77758700	0.677686
chr13	77874600	0.842975
chr13	77879800	1.000000
chr13	77885700	0.685950
chr13	77890900	0.983471
chr13	78082300	0.876033
chr13	78084400	0.983471
chr13	78090800	0.917355
chr13	78096200	0.942149
chr13	78119100	0.727273
chr13	78354300	0.760331
chr13	78471100	0.867769
chr13	78482600	0.735537
chr13	78555000	0.727273
chr13	78596600	0.966942
chr13	78606600	0.909091
chr13	78695700	0.710744

chr13	78722500	0.933884
chr13	79224700	0.818182
chr13	79230200	0.867769
chr13	79239200	0.801653
chr13	79283500	0.793388
chr13	79928200	0.991736
chr13	79960800	0.702479
chr13	79968300	0.925620
chr13	80023000	0.694215
chr13	80032600	0.776860
chr13	80099900	1.000000
chr13	80222100	0.834711
chr13	80341300	0.942149
chr13	80357100	0.966942
chr13	80385800	0.900826
chr13	80487700	0.917355
chr13	80507200	1.000000
chr13	80510600	0.801653
chr13	80513200	0.859504
chr13	80612000	0.950413
chr13	80615100	0.892562
chr13	80623500	0.991736
chr13	80658100	0.884298
chr13	80684000	0.793388
chr13	80707600	0.950413
chr13	80792400	0.942149
chr13	80809900	0.958678
chr13	80851000	0.892562
chr13	81044200	0.983471
chr13	81104000	0.876033
chr13	81122200	0.991736
chr13	81126400	0.793388
chr13	81205000	0.867769
chr13	81239300	0.768595
chr13	81415400	0.809917
chr13	81446100	0.950413
chr13	81496100	0.818182
chr13	81499100	0.785124
chr13	81529500	0.752066
chr13	81531800	0.892562
chr13	81536800	0.851240
chr13	81547800	0.900826
chr13	82585400	1.000000
chr13	88111900	1.000000
chr13	90053900	0.727273
chr13	90109400	0.685950
chr13	90166300	0.933884
chr13	90168400	0.900826
chr13	90203700	0.900826
chr13	90379900	0.710744
chr13	90465600	0.909091
chr13	90543900	0.892562
chr13	91100800	0.768595
chr13	91922600	0.983471

chr13	91933400	0.859504
chr13	91979000	0.768595
chr13	92051300	0.669421
chr13	92549300	0.884298
chr13	92556800	0.876033
chr13	94410600	0.760331
chr13	94962100	0.776860
chr13	94964700	1.000000
chr13	95236600	0.900826
chr13	95294100	0.966942
chr13	95312200	0.851240
chr13	95511700	0.677686
chr13	95620600	0.776860
chr13	95652500	0.727273
chr13	95655500	0.809917
chr13	95719900	0.710744
chr13	95736400	0.933884
chr13	95740700	0.975207
chr13	95753200	0.991736
chr13	95765500	0.925620
chr13	95768500	0.991736
chr13	95781000	0.776860
chr13	95789900	0.801653
chr13	95806700	0.933884
chr13	95857000	0.950413
chr13	95866100	0.801653
chr13	95888500	0.685950
chr13	95900100	0.669421
chr13	95907500	0.950413
chr13	95920300	0.975207
chr13	95924400	0.876033
chr13	95933100	0.892562
chr13	95941800	0.884298
chr13	95996500	0.876033
chr13	96033400	0.735537
chr13	96056500	0.768595
chr13	96138000	0.925620
chr13	96182800	0.669421
chr13	96187800	0.991736
chr13	96217400	0.983471
chr13	96253800	0.851240
chr13	96257000	0.909091
chr13	96365500	0.966942
chr13	96436900	0.925620
chr13	96486000	0.801653
chr13	96511200	0.983471
chr13	96549000	0.735537
chr13	96677900	0.842975
chr13	96745300	0.768595
chr13	97302900	0.834711
chr13	97679400	0.975207
chr13	97762300	0.958678
chr13	97767900	0.942149
chr13	97775000	0.710744

chr13	97794700	0.983471
chr13	97847100	0.785124
chr13	97879500	0.710744
chr13	97881800	0.793388
chr13	97887600	0.975207
chr13	97893100	0.958678
chr13	97896500	0.975207
chr13	97899400	0.983471
chr13	97902800	1.000000
chr13	97906800	0.801653
chr13	97914700	0.867769
chr13	97917700	0.983471
chr13	97921500	0.867769
chr13	97926300	0.991736
chr13	97928800	0.909091
chr13	97932600	0.975207
chr13	97948400	0.983471
chr13	97954600	0.892562
chr13	97958800	0.834711
chr13	97961000	1.000000
chr13	97980700	0.942149
chr13	97988100	0.925620
chr13	97997100	0.851240
chr13	98021400	0.793388
chr13	98089500	0.669421
chr13	98301100	0.983471
chr13	98313200	0.859504
chr13	98501500	0.983471
chr13	98532500	0.942149
chr13	98631400	0.694215
chr13	98667400	0.909091
chr13	98825100	0.991736
chr13	98867000	0.661157
chr13	98869100	0.719008
chr13	98905700	0.983471
chr13	98908500	0.917355
chr13	98960100	0.942149
chr13	99128400	0.958678
chr13	99131000	0.884298
chr13	99134800	0.669421
chr13	99137900	0.867769
chr13	99142900	0.991736
chr13	99157000	0.752066
chr13	99159900	0.884298
chr13	99162400	0.719008
chr13	99218900	0.975207
chr13	99223600	0.917355
chr13	99404600	0.685950
chr13	99474800	0.809917
chr13	99478800	0.776860
chr13	99509500	0.925620
chr13	99533600	0.694215
chr13	99542500	0.975207
chr13	99546200	0.826446

chr13	99560700	0.694215
chr13	99562800	0.991736
chr13	99566300	1.000000
chr13	99571500	0.917355
chr13	99581200	0.958678
chr13	99600700	0.975207
chr13	99612900	0.925620
chr13	99616100	0.925620
chr13	99626100	0.975207
chr13	99631900	0.793388
chr13	99635500	0.966942
chr13	99652400	0.719008
chr13	99657200	1.000000
chr13	99661500	0.677686
chr13	99699100	0.942149
chr13	99714900	0.933884
chr13	99717000	0.867769
chr13	99777700	0.983471
chr13	99916500	0.793388
chr13	99960200	1.000000
chr13	99965700	0.966942
chr13	99968100	0.909091
chr13	99973900	0.958678
chr13	100008300	0.801653
chr13	100085500	0.909091
chr13	100158400	0.710744
chr13	100195600	0.876033
chr13	100243100	0.925620
chr13	100310800	0.752066
chr13	100608800	0.809917
chr13	100612400	0.768595
chr13	100752800	0.983471
chr13	100782300	0.867769
chr13	100785000	0.826446
chr13	101046600	0.925620
chr13	101086800	0.809917
chr13	101093600	0.842975
chr13	101116700	0.719008
chr13	101136400	0.785124
chr13	101143800	0.669421
chr13	101196700	1.000000
chr13	101241100	0.859504
chr13	101245400	0.809917
chr13	101261500	0.900826
chr13	101321000	0.925620
chr13	101528200	0.942149
chr13	101540300	0.950413
chr13	101864000	0.975207
chr13	101911000	0.818182
chr13	101996000	0.909091
chr13	102043200	0.917355
chr13	102051200	0.942149
chr13	102102900	0.991736
chr13	102105400	0.818182

chr13	102108700	0.983471
chr13	102140000	0.710744
chr13	102142400	0.917355
chr13	102145400	0.867769
chr13	102164900	0.958678
chr13	102173100	0.966942
chr13	102188900	0.876033
chr13	102228800	0.752066
chr13	102231900	0.876033
chr13	102276600	0.900826
chr13	102292900	0.983471
chr13	102314600	0.983471
chr13	102848600	0.950413
chr13	103304300	0.727273
chr13	103356400	0.983471
chr13	103360500	0.900826
chr13	103382500	0.933884
chr13	103478900	0.950413
chr13	104536200	0.933884
chr13	104601100	0.925620
chr13	104605300	0.991736
chr13	104661500	0.966942
chr13	104668300	0.925620
chr13	104677200	0.867769
chr13	104758400	0.909091
chr13	104760600	0.859504
chr13	104932200	0.661157
chr13	104968700	0.826446
chr13	104979800	0.859504
chr13	105061800	0.884298
chr13	105067500	0.685950
chr13	105637100	0.958678
chr13	105688300	0.867769
chr13	105771100	0.958678
chr13	105848900	0.909091
chr13	106089400	0.917355
chr13	106095100	0.834711
chr13	106151000	0.685950
chr13	106163600	0.950413
chr13	106166100	0.677686
chr13	106530700	0.809917
chr13	106572100	0.768595
chr13	106756800	0.966942
chr13	106762100	1.000000
chr13	106769900	0.917355
chr13	106773900	0.958678
chr13	106785800	0.743802
chr13	106792000	0.752066
chr13	106804100	0.834711
chr13	106808900	0.826446
chr13	106937600	0.694215
chr13	106971700	0.983471
chr13	107000800	0.752066
chr13	107020200	0.867769

chr13	107025600	0.991736
chr13	107029400	0.818182
chr13	107125000	0.876033
chr13	107143900	0.925620
chr13	107148500	0.983471
chr13	107156500	0.892562
chr13	107175800	0.826446
chr13	107183500	0.685950
chr13	107190400	0.685950
chr13	107270400	0.776860
chr13	107304000	0.818182
chr13	107333500	0.917355
chr13	107405800	1.000000
chr13	107569900	0.793388
chr13	107572500	0.983471
chr13	107838600	0.917355
chr13	107855300	0.917355
chr13	107863100	0.710744
chr13	107911500	0.685950
chr13	107929200	0.983471
chr13	107936900	0.933884
chr13	108013500	0.719008
chr13	108019800	0.834711
chr13	108022800	0.983471
chr13	108026500	0.694215
chr13	108059700	0.892562
chr13	108103600	0.983471
chr13	108229300	0.743802
chr13	108233600	0.892562
chr13	108238100	0.735537
chr13	108270700	0.743802
chr13	108419000	0.958678
chr13	108460100	0.669421
chr13	109550500	0.842975
chr13	109568200	0.983471
chr13	109610400	0.752066
chr13	109799500	0.925620
chr13	109804100	0.851240
chr13	109807200	0.950413
chr13	109809700	0.917355
chr13	109852100	0.760331
chr13	109895900	0.950413
chr13	109898800	0.851240
chr13	109904400	0.842975
chr13	109925900	0.933884
chr13	109928000	0.826446
chr13	109997600	0.958678
chr13	110001600	0.966942
chr13	110007800	0.876033
chr13	110076700	1.000000
chr13	110438000	0.661157
chr13	110776100	0.710744
chr13	110781100	0.950413
chr13	110869100	0.966942

chr13	110878700	0.991736
chr13	110884700	0.942149
chr13	110886800	0.809917
chr13	110892400	0.975207
chr13	110900100	0.966942
chr13	110903600	0.925620
chr13	110911300	0.884298
chr13	110913400	0.933884
chr13	110916000	0.958678
chr13	110919100	0.983471
chr13	110946700	0.694215
chr13	110951000	0.900826
chr13	110965000	0.958678
chr13	110975100	0.975207
chr13	110990600	0.983471
chr13	110994500	0.809917
chr13	111001000	0.867769
chr13	111024800	0.983471
chr13	111032600	0.842975
chr13	111034800	0.975207
chr13	111043300	0.958678
chr13	111045400	0.933884
chr13	111055300	0.785124
chr13	111059200	0.809917
chr13	111061500	0.752066
chr13	111065000	0.966942
chr13	111071500	0.818182
chr13	111074300	0.983471
chr13	111078100	0.983471
chr13	111085600	0.785124
chr13	111095800	0.702479
chr13	111123500	0.743802
chr13	111130300	0.966942
chr13	111199800	0.867769
chr13	111270300	0.809917
chr13	111452400	0.809917
chr13	111522800	0.834711
chr13	111527300	0.900826
chr13	111596700	0.991736
chr13	111607900	0.966942
chr13	111659200	0.900826
chr13	111768500	0.677686
chr13	111827200	0.826446
chr13	111830600	0.834711
chr13	111847300	0.727273
chr13	111863400	0.909091
chr13	111904100	0.801653
chr13	113180200	0.876033
chr13	113213500	0.966942
chr13	113227900	0.685950
chr13	113230000	0.933884
chr13	113258300	0.826446
chr13	113364200	0.661157
chr13	113366700	0.694215

chr13	113377300	0.909091
chr13	113415100	0.933884
chr13	113469500	0.867769
chr13	113514600	0.966942
chr13	113540700	0.925620
chr13	113677700	0.801653
chr13	113784600	0.710744
chr13	113825100	0.942149
chr13	113833100	0.851240
chr13	113838700	0.983471
chr13	113842300	0.884298
chr13	113845500	0.867769
chr13	114103800	0.661157
chr13	114148000	0.917355
chr13	114150300	0.942149
chr13	114202000	0.826446
chr13	114207700	0.991736
chr13	114301700	0.768595
chr13	114465000	0.768595
chr13	114474800	0.975207
chr13	114478500	0.900826
chr13	114489200	0.892562
chr13	114520800	0.892562
chr13	114541100	0.834711
chr13	114585000	0.925620
chr13	114600300	0.909091
chr13	114770700	0.983471
chr13	114793100	0.966942
chr13	114797900	0.892562
chr13	114802000	0.842975
chr13	114829600	0.809917
chr13	114832200	0.669421
chr13	114834500	0.859504
chr13	114873200	0.917355
chr13	114876600	0.892562
chr13	114880100	0.702479
chr13	114882200	0.884298
chr13	114884700	0.966942
chr13	114888100	0.826446
chr13	114891800	0.661157
chr13	114894800	0.776860
chr13	114900200	0.867769
chr13	114927400	0.933884
chr13	114940300	0.867769
chr13	114978000	0.958678
chr13	114985500	0.719008
chr13	114988300	0.950413
chr14	19019300	0.909091
chr14	19038200	0.710744
chr14	20869700	0.884298
chr14	20896200	0.867769
chr14	20940400	0.710744
chr14	20945500	0.909091
chr14	20967500	0.925620

chr14	20980900	0.975207
chr14	20985400	0.942149
chr14	21132100	0.727273
chr14	21166000	0.694215
chr14	21175600	0.917355
chr14	21250400	0.851240
chr14	21257600	0.867769
chr14	21268200	0.958678
chr14	21294800	0.851240
chr14	21443500	0.776860
chr14	21539900	0.694215
chr14	21686900	0.909091
chr14	21732900	0.776860
chr14	21873700	0.694215
chr14	21906600	0.694215
chr14	21936100	0.983471
chr14	22137200	0.958678
chr14	22140500	0.702479
chr14	22148600	0.785124
chr14	22336200	0.991736
chr14	22342700	0.991736
chr14	22355800	0.793388
chr14	22360600	0.752066
chr14	22382100	0.917355
chr14	22385000	0.975207
chr14	22387500	0.966942
chr14	22392400	0.933884
chr14	22538900	0.719008
chr14	22694100	0.983471
chr14	22736800	0.685950
chr14	22765300	0.743802
chr14	22898700	0.801653
chr14	22902000	0.900826
chr14	23005800	0.925620
chr14	23014800	0.752066
chr14	23018000	0.909091
chr14	23020400	0.975207
chr14	23024900	0.776860
chr14	23027000	0.966942
chr14	23029100	0.876033
chr14	23031200	0.842975
chr14	23036000	0.793388
chr14	23041600	0.876033
chr14	23056100	0.842975
chr14	23083700	0.867769
chr14	23128000	0.966942
chr14	23158700	0.851240
chr14	23161300	0.876033
chr14	23169600	0.975207
chr14	23182600	0.942149
chr14	23186600	0.785124
chr14	23209100	0.983471
chr14	23287700	0.884298
chr14	23289800	0.694215

chr14	23292200	1.000000
chr14	23294400	0.867769
chr14	23303800	0.942149
chr14	23309600	0.661157
chr14	23312500	0.925620
chr14	23318900	0.702479
chr14	23322100	0.892562
chr14	23444200	0.884298
chr14	23447400	0.793388
chr14	23479600	0.809917
chr14	23516600	0.900826
chr14	23531300	0.991736
chr14	23538600	0.925620
chr14	23548200	0.900826
chr14	23584600	0.834711
chr14	23591100	0.909091
chr14	23599300	0.661157
chr14	23607700	0.685950
chr14	23624700	0.719008
chr14	23707300	0.710744
chr14	23769600	0.669421
chr14	23789800	0.834711
chr14	24550800	0.685950
chr14	24575900	0.867769
chr14	24630300	0.702479
chr14	24641700	0.685950
chr14	24683600	0.694215
chr14	24693400	0.925620
chr14	24695500	0.793388
chr14	24766300	0.917355
chr14	24780300	0.760331
chr14	24782900	0.958678
chr14	24809100	0.925620
chr14	24835400	0.752066
chr14	24859000	0.801653
chr14	24861800	0.925620
chr14	24872900	0.834711
chr14	24888300	0.900826
chr14	24893800	0.909091
chr14	24896000	0.991736
chr14	24901900	0.727273
chr14	24905400	0.719008
chr14	24907500	0.834711
chr14	25243700	0.950413
chr14	25404100	0.677686
chr14	25455000	0.685950
chr14	25476800	0.818182
chr14	25859300	0.958678
chr14	25864700	0.975207
chr14	26423100	0.933884
chr14	26537300	0.826446
chr14	26599000	0.966942
chr14	26608400	0.892562
chr14	27178500	0.942149

chr14	27243900	1.000000
chr14	27278000	0.809917
chr14	27290700	0.809917
chr14	27305500	0.719008
chr14	27311400	0.900826
chr14	27836100	0.983471
chr14	27861700	0.991736
chr14	28141700	0.933884
chr14	28156600	0.727273
chr14	28852200	0.909091
chr14	29141100	0.958678
chr14	29146000	0.859504
chr14	29228800	0.694215
chr14	29254800	0.661157
chr14	29705700	0.950413
chr14	29707800	0.735537
chr14	29709900	0.950413
chr14	29712700	0.925620
chr14	29741300	0.719008
chr14	29912200	0.867769
chr14	30071400	0.892562
chr14	30124600	0.743802
chr14	30195900	0.884298
chr14	30211700	0.884298
chr14	30218900	0.710744
chr14	30692200	0.909091
chr14	30694500	0.950413
chr14	30697700	0.710744
chr14	31160800	0.760331
chr14	31409700	0.909091
chr14	31464700	0.933884
chr14	31508000	0.851240
chr14	31670000	0.727273
chr14	31733100	0.867769
chr14	31743200	0.867769
chr14	31753500	0.851240
chr14	31760800	0.842975
chr14	31763000	0.925620
chr14	31765100	0.983471
chr14	31826500	0.867769
chr14	31830500	0.809917
chr14	31883800	0.752066
chr14	32037600	0.752066
chr14	32288500	0.710744
chr14	32346800	0.933884
chr14	32362700	0.818182
chr14	32396900	0.983471
chr14	32403300	0.909091
chr14	32407100	0.694215
chr14	32611600	0.743802
chr14	32883900	0.966942
chr14	32887900	0.966942
chr14	32954200	0.991736
chr14	32979200	0.876033

chr14	32994600	0.942149
chr14	33404200	0.760331
chr14	33596900	0.760331
chr14	33992500	0.834711
chr14	34002200	0.958678
chr14	34009600	0.917355
chr14	34025900	0.719008
chr14	34033300	0.900826
chr14	34037500	0.975207
chr14	34039900	0.909091
chr14	34116900	0.942149
chr14	34124800	0.991736
chr14	34127300	0.677686
chr14	34149000	0.983471
chr14	34155900	0.950413
chr14	34232000	0.966942
chr14	34235000	0.818182
chr14	34237900	0.958678
chr14	34386300	0.950413
chr14	34408000	0.661157
chr14	34920500	0.909091
chr14	34966600	0.727273
chr14	34972300	0.842975
chr14	35093300	0.702479
chr14	35119000	0.900826
chr14	35135400	0.991736
chr14	35147700	0.950413
chr14	35169600	0.983471
chr14	35250700	0.983471
chr14	35312700	0.702479
chr14	35396000	0.942149
chr14	35400400	0.942149
chr14	35441300	0.818182
chr14	35520000	0.851240
chr14	35530800	0.661157
chr14	35625500	0.793388
chr14	35629300	0.975207
chr14	35683800	0.842975
chr14	35693800	0.661157
chr14	35764100	0.694215
chr14	35806500	0.859504
chr14	35811100	0.917355
chr14	35832400	0.801653
chr14	35835500	0.834711
chr14	35855100	0.917355
chr14	35867600	0.867769
chr14	35883700	1.000000
chr14	35889500	0.950413
chr14	35976500	0.892562
chr14	36004900	0.669421
chr14	36064300	0.702479
chr14	36158900	0.661157
chr14	36348500	0.942149
chr14	37051100	0.760331

chr14	37505500	1.000000
chr14	37517500	0.966942
chr14	37586600	0.991736
chr14	37665100	0.942149
chr14	37672200	0.685950
chr14	37683500	0.867769
chr14	37722300	0.793388
chr14	38037000	0.851240
chr14	38053700	0.677686
chr14	38057800	0.719008
chr14	38065400	0.760331
chr14	38613200	0.966942
chr14	38628600	0.669421
chr14	38727000	0.867769
chr14	38748700	0.727273
chr14	38759000	0.892562
chr14	38761600	0.876033
chr14	38779300	0.818182
chr14	38781400	0.785124
chr14	38819800	0.859504
chr14	38827100	0.983471
chr14	38835900	0.991736
chr14	38853200	0.710744
chr14	38856100	0.661157
chr14	38861100	0.991736
chr14	38873400	0.966942
chr14	38875700	0.958678
chr14	38889100	0.760331
chr14	39308700	0.958678
chr14	39311300	0.719008
chr14	39461800	0.851240
chr14	39464400	0.826446
chr14	39567000	0.760331
chr14	39569500	0.809917
chr14	39647100	0.801653
chr14	39688100	0.975207
chr14	39844400	0.925620
chr14	39846500	0.966942
chr14	39896100	0.793388
chr14	42662600	0.892562
chr14	42684500	0.876033
chr14	44786200	0.909091
chr14	45651000	0.917355
chr14	45719800	0.950413
chr14	46149300	0.892562
chr14	46606200	0.834711
chr14	46609900	0.752066
chr14	47041500	0.859504
chr14	47045000	0.917355
chr14	47656100	1.000000
chr14	47663600	0.876033
chr14	47677300	0.669421
chr14	47696800	0.958678
chr14	47704400	0.859504

chr14	48429100	0.867769
chr14	49979300	0.950413
chr14	50091100	0.925620
chr14	50374800	0.917355
chr14	50379100	0.818182
chr14	50413600	0.950413
chr14	50437900	0.909091
chr14	50443100	0.851240
chr14	50449400	0.983471
chr14	50465900	0.834711
chr14	50468100	0.917355
chr14	50470500	0.694215
chr14	50507700	0.719008
chr14	50527400	0.710744
chr14	50529600	0.818182
chr14	50584500	0.661157
chr14	50692700	0.809917
chr14	50788500	0.677686
chr14	50803200	0.958678
chr14	50809800	0.776860
chr14	50815000	0.735537
chr14	50818200	0.776860
chr14	50830900	0.909091
chr14	50870600	0.950413
chr14	50914900	0.958678
chr14	50969200	0.950413
chr14	50988100	0.950413
chr14	50996200	0.834711
chr14	51072800	0.710744
chr14	51125200	0.958678
chr14	51144800	0.983471
chr14	51208400	0.785124
chr14	51242300	0.983471
chr14	51249700	0.710744
chr14	51253400	0.702479
chr14	51263200	0.867769
chr14	51268000	0.933884
chr14	51271700	0.942149
chr14	51284100	0.933884
chr14	51326900	0.785124
chr14	51422100	0.900826
chr14	51428600	0.793388
chr14	51588700	0.719008
chr14	51827900	0.842975
chr14	51861300	0.685950
chr14	51865900	0.900826
chr14	51909900	0.983471
chr14	51913900	0.793388
chr14	51916000	0.768595
chr14	51925900	0.776860
chr14	51958700	0.727273
chr14	51987200	0.975207
chr14	52001500	0.975207
chr14	52006600	0.942149

chr14	52013300	0.942149
chr14	52015500	0.909091
chr14	52021200	0.975207
chr14	52048800	0.884298
chr14	52084100	0.942149
chr14	52125300	0.768595
chr14	52133200	0.710744
chr14	52314000	0.876033
chr14	52327700	0.909091
chr14	52351600	0.752066
chr14	52373000	0.867769
chr14	52470200	0.677686
chr14	52487800	0.975207
chr14	52491200	0.661157
chr14	52493400	0.983471
chr14	52496900	0.925620
chr14	52543500	0.909091
chr14	52545600	0.942149
chr14	52592300	0.876033
chr14	53167900	0.983471
chr14	53208600	0.859504
chr14	53236900	0.933884
chr14	53302000	0.950413
chr14	53370000	0.942149
chr14	53373400	0.966942
chr14	53384500	0.809917
chr14	53386600	0.950413
chr14	53393500	0.719008
chr14	53427800	0.760331
chr14	53704700	0.851240
chr14	53746000	0.785124
chr14	53833400	0.933884
chr14	53836300	0.966942
chr14	54009700	0.859504
chr14	54017200	0.975207
chr14	54125900	0.809917
chr14	54182200	0.719008
chr14	54310100	0.983471
chr14	54318300	0.752066
chr14	54346300	0.760331
chr14	54349200	0.983471
chr14	54352400	0.942149
chr14	54357700	0.966942
chr14	54361500	0.884298
chr14	54391700	0.991736
chr14	54411000	0.685950
chr14	54415200	0.669421
chr14	54430500	0.752066
chr14	54467200	0.958678
chr14	54560000	0.661157
chr14	54574000	0.793388
chr14	54576600	0.950413
chr14	54584000	0.983471
chr14	54614000	0.900826

chr14	54633400	0.710744
chr14	54636600	0.925620
chr14	54641100	0.727273
chr14	54649900	0.867769
chr14	54656800	0.826446
chr14	54661500	0.793388
chr14	54678700	0.933884
chr14	54777200	0.752066
chr14	54779300	0.661157
chr14	54793200	0.793388
chr14	54798600	0.983471
chr14	54810800	0.892562
chr14	54832500	0.966942
chr14	54902700	0.760331
chr14	54956600	0.842975
chr14	54996900	0.685950
chr14	55031100	0.752066
chr14	55037800	0.983471
chr14	55044400	0.710744
chr14	55061000	0.950413
chr14	55067000	0.966942
chr14	55090700	0.983471
chr14	55094600	0.867769
chr14	55105000	0.966942
chr14	55116100	0.909091
chr14	55118500	0.818182
chr14	55121800	0.743802
chr14	55126000	0.983471
chr14	55132500	0.958678
chr14	55144400	0.966942
chr14	55150800	0.966942
chr14	55154300	0.884298
chr14	55158200	0.933884
chr14	55179300	0.876033
chr14	55223700	0.809917
chr14	55238900	0.851240
chr14	55241100	0.867769
chr14	55344100	0.851240
chr14	55443600	0.975207
chr14	55538900	0.752066
chr14	55568800	0.743802
chr14	55571000	0.719008
chr14	55574700	0.925620
chr14	55604300	0.975207
chr14	55634600	0.851240
chr14	55647700	0.925620
chr14	55655300	0.735537
chr14	55745200	0.876033
chr14	55866100	0.958678
chr14	55885100	0.818182
chr14	55915100	0.975207
chr14	55918900	0.851240
chr14	55921600	0.950413
chr14	55925800	0.909091

chr14	55982200	0.884298
chr14	56008500	0.719008
chr14	56053100	0.677686
chr14	56067900	0.975207
chr14	56157200	0.991736
chr14	56247300	0.942149
chr14	56258200	0.950413
chr14	56270500	0.801653
chr14	56274300	1.000000
chr14	56281500	0.842975
chr14	56380900	0.892562
chr14	56639600	0.966942
chr14	56666500	0.950413
chr14	56738200	0.925620
chr14	56740500	0.975207
chr14	56800900	0.983471
chr14	56806900	0.793388
chr14	56810000	0.917355
chr14	56845600	1.000000
chr14	56879800	0.917355
chr14	57690400	0.669421
chr14	57733000	0.785124
chr14	57830400	0.884298
chr14	57849600	0.876033
chr14	57860900	0.702479
chr14	58188900	0.752066
chr14	58192700	0.950413
chr14	58549500	0.925620
chr14	58565300	0.801653
chr14	58748500	0.933884
chr14	58798100	0.859504
chr14	58820400	0.818182
chr14	58836000	0.661157
chr14	58863000	0.694215
chr14	59001600	0.818182
chr14	59198800	0.958678
chr14	59219800	0.818182
chr14	59629700	0.727273
chr14	59659000	0.727273
chr14	59683800	0.818182
chr14	59722000	0.958678
chr14	59742600	0.991736
chr14	59745700	0.909091
chr14	59768200	0.702479
chr14	59773100	0.983471
chr14	59776200	0.884298
chr14	59788200	0.950413
chr14	59799200	0.785124
chr14	59802000	0.876033
chr14	59809400	0.933884
chr14	59828100	0.776860
chr14	59831300	0.950413
chr14	59873900	0.975207
chr14	59895600	0.851240

chr14	59905400	0.991736
chr14	59921400	0.776860
chr14	59948700	0.942149
chr14	60239200	0.942149
chr14	60386500	0.818182
chr14	60561400	0.851240
chr14	60619000	0.917355
chr14	60761600	0.809917
chr14	60952800	0.801653
chr14	61092100	0.900826
chr14	61104900	0.809917
chr14	61109500	0.685950
chr14	61203700	0.834711
chr14	61224600	0.892562
chr14	61241800	0.702479
chr14	61274800	0.661157
chr14	61392400	0.818182
chr14	61445500	0.842975
chr14	61492800	0.966942
chr14	61565900	0.975207
chr14	61571600	0.892562
chr14	61593300	1.000000
chr14	61595600	0.768595
chr14	61613100	0.710744
chr14	61615200	0.958678
chr14	61644300	0.760331
chr14	61650400	0.925620
chr14	61655700	0.917355
chr14	61724000	0.834711
chr14	61747800	0.661157
chr14	61752300	0.710744
chr14	61767200	0.760331
chr14	61775300	1.000000
chr14	61787300	0.727273
chr14	61794200	0.942149
chr14	61805300	0.859504
chr14	61809100	0.900826
chr14	61811400	0.867769
chr14	61815300	0.760331
chr14	61817800	0.719008
chr14	61822600	0.950413
chr14	61826000	0.859504
chr14	61829200	0.876033
chr14	61833200	0.950413
chr14	61855700	0.983471
chr14	61858000	0.785124
chr14	61884000	0.983471
chr14	61886600	0.925620
chr14	61900000	0.975207
chr14	61905900	0.983471
chr14	61922700	0.661157
chr14	61929600	0.818182
chr14	61933000	0.933884
chr14	61939800	0.694215

chr14	61943900	0.983471
chr14	61946500	0.719008
chr14	61959700	0.694215
chr14	61965700	0.785124
chr14	61970200	0.975207
chr14	61983600	0.876033
chr14	61994700	0.735537
chr14	61998100	0.727273
chr14	62000200	0.851240
chr14	62004300	0.809917
chr14	62027700	0.826446
chr14	62030700	0.876033
chr14	62032800	0.793388
chr14	62052900	0.768595
chr14	62061600	0.669421
chr14	62066300	0.991736
chr14	62076000	0.917355
chr14	62120600	0.975207
chr14	62128900	0.991736
chr14	62135500	0.702479
chr14	62138000	0.958678
chr14	62140400	0.942149
chr14	62178900	0.685950
chr14	62219200	0.669421
chr14	62222200	0.685950
chr14	62305800	0.983471
chr14	62469600	0.925620
chr14	62564500	0.694215
chr14	62676400	0.950413
chr14	63622600	0.892562
chr14	63641900	0.942149
chr14	63646100	0.851240
chr14	63650100	0.983471
chr14	63653400	0.917355
chr14	63659100	0.983471
chr14	63670100	0.909091
chr14	63678200	0.892562
chr14	63682000	0.983471
chr14	63696300	0.719008
chr14	63715200	0.892562
chr14	63905000	0.752066
chr14	64081900	1.000000
chr14	64144900	0.917355
chr14	64331900	0.851240
chr14	64337200	0.859504
chr14	64358600	0.867769
chr14	64363600	0.933884
chr14	64366800	0.735537
chr14	64395500	0.958678
chr14	64419700	0.867769
chr14	64463300	0.661157
chr14	64541900	0.991736
chr14	64699600	0.983471
chr14	64721000	0.818182

chr14	64744800	0.776860
chr14	64746900	0.776860
chr14	64763600	0.942149
chr14	64905400	0.966942
chr14	64912400	0.884298
chr14	64968500	0.785124
chr14	64974600	0.809917
chr14	64978200	0.710744
chr14	65033700	0.900826
chr14	65121600	0.719008
chr14	65187400	0.851240
chr14	65229200	0.776860
chr14	65231300	1.000000
chr14	65347300	0.776860
chr14	65450600	0.702479
chr14	65541300	0.975207
chr14	65546700	0.760331
chr14	65565800	0.876033
chr14	65638200	0.677686
chr14	65677900	0.785124
chr14	65705700	1.000000
chr14	65709900	0.719008
chr14	65712000	0.876033
chr14	65717200	0.917355
chr14	65721900	0.685950
chr14	65725300	0.917355
chr14	65882000	0.669421
chr14	66106100	0.884298
chr14	67044900	0.661157
chr14	67112600	0.809917
chr14	67117700	0.958678
chr14	67511300	0.760331
chr14	67743200	0.702479
chr14	67829900	0.793388
chr14	67838500	0.925620
chr14	67863000	0.991736
chr14	67878900	0.760331
chr14	67881500	0.933884
chr14	68008900	0.785124
chr14	68090100	0.809917
chr14	68147400	0.983471
chr14	68202200	0.752066
chr14	68257500	0.842975
chr14	68326400	0.983471
chr14	68607300	0.950413
chr14	68611800	1.000000
chr14	68666800	1.000000
chr14	68677800	0.942149
chr14	68698800	0.983471
chr14	68711000	0.727273
chr14	68714400	0.801653
chr14	68717300	0.694215
chr14	68728200	0.710744
chr14	68731100	0.793388

chr14	68741100	0.900826
chr14	68760300	0.776860
chr14	68828200	0.909091
chr14	68832100	0.876033
chr14	68844800	0.694215
chr14	68925700	0.892562
chr14	68931900	0.942149
chr14	68943600	0.719008
chr14	68962500	0.909091
chr14	68968100	0.809917
chr14	68994200	0.958678
chr14	69014900	0.760331
chr14	69017500	0.925620
chr14	69020700	0.975207
chr14	69032500	0.818182
chr14	69037000	0.801653
chr14	69042600	0.917355
chr14	69045100	0.958678
chr14	69065800	0.966942
chr14	69142200	0.975207
chr14	69149600	0.966942
chr14	69152000	0.743802
chr14	69162300	0.958678
chr14	69170200	0.809917
chr14	69195300	0.876033
chr14	69201400	0.842975
chr14	69238900	0.950413
chr14	69247000	0.966942
chr14	69252100	0.958678
chr14	69254900	0.859504
chr14	69281400	0.776860
chr14	69283500	0.958678
chr14	69308600	0.884298
chr14	69362800	0.876033
chr14	69402800	0.677686
chr14	69405800	0.661157
chr14	69407900	0.768595
chr14	69417200	0.884298
chr14	69420400	0.950413
chr14	69422800	0.851240
chr14	69425900	0.917355
chr14	69431800	0.661157
chr14	69434200	0.661157
chr14	69438100	0.735537
chr14	69442000	0.727273
chr14	69448900	0.958678
chr14	69472800	0.876033
chr14	69481400	0.859504
chr14	69501900	0.966942
chr14	69504000	0.776860
chr14	69506200	0.743802
chr14	69510400	0.677686
chr14	69513800	0.859504
chr14	69516800	0.900826

chr14	69519500	0.991736
chr14	69522500	0.809917
chr14	69527100	0.743802
chr14	69544200	0.826446
chr14	69547800	0.958678
chr14	69561100	0.760331
chr14	69563900	0.669421
chr14	69600300	0.727273
chr14	69693300	0.793388
chr14	69722100	0.727273
chr14	69868500	0.677686
chr14	69927200	0.752066
chr14	69931800	0.966942
chr14	69951400	0.735537
chr14	70039400	0.909091
chr14	70053500	0.892562
chr14	70077500	0.702479
chr14	70082700	0.975207
chr14	70092500	0.826446
chr14	70115100	0.884298
chr14	70118500	0.793388
chr14	70169700	0.685950
chr14	70180000	0.925620
chr14	70186600	0.876033
chr14	70194000	0.925620
chr14	70228000	0.917355
chr14	70237100	0.900826
chr14	70466100	0.719008
chr14	70831900	0.842975
chr14	71350200	0.818182
chr14	71386900	0.900826
chr14	71448700	0.966942
chr14	71489200	0.909091
chr14	71597800	0.950413
chr14	71610000	0.867769
chr14	71654700	0.685950
chr14	71758200	0.892562
chr14	71839100	0.991736
chr14	72006600	0.867769
chr14	72096800	0.702479
chr14	72118200	0.983471
chr14	72126400	0.818182
chr14	72220000	0.694215
chr14	72244000	0.776860
chr14	72247100	0.983471
chr14	72270800	1.000000
chr14	72971100	0.694215
chr14	73075500	0.826446
chr14	73085100	0.991736
chr14	73105300	0.694215
chr14	73110200	0.933884
chr14	73114100	0.710744
chr14	73118200	0.991736
chr14	73131900	0.958678

chr14	73144700	0.710744
chr14	73146800	0.842975
chr14	73248700	0.942149
chr14	73268500	0.892562
chr14	73351200	0.966942
chr14	73677100	0.983471
chr14	73688800	0.776860
chr14	73695300	0.867769
chr14	73707300	0.661157
chr14	73737100	0.743802
chr14	73778600	0.818182
chr14	73891500	0.975207
chr14	73894900	0.975207
chr14	73919900	0.760331
chr14	73922500	0.685950
chr14	73927300	0.892562
chr14	73930100	0.768595
chr14	73933200	0.983471
chr14	74129400	0.834711
chr14	74193600	0.727273
chr14	74195900	0.917355
chr14	74210600	0.801653
chr14	74212800	0.983471
chr14	74216500	0.950413
chr14	74220300	0.851240
chr14	74223400	0.727273
chr14	74228500	0.776860
chr14	74232100	0.760331
chr14	74235600	0.735537
chr14	74239000	0.809917
chr14	74257800	0.842975
chr14	74269500	0.768595
chr14	74296900	0.909091
chr14	74317800	0.694215
chr14	74355600	0.735537
chr14	74363800	0.818182
chr14	74476100	0.909091
chr14	74532500	0.909091
chr14	74548500	0.925620
chr14	74707900	0.818182
chr14	74766300	0.743802
chr14	74953600	0.950413
chr14	75020500	0.909091
chr14	75036800	0.892562
chr14	75039100	0.884298
chr14	75041400	0.942149
chr14	75046900	0.991736
chr14	75050500	0.669421
chr14	75054000	0.876033
chr14	75073500	0.727273
chr14	75080800	0.950413
chr14	75087500	0.917355
chr14	75101800	0.719008
chr14	75105000	0.859504

chr14	75116500	0.958678
chr14	75126000	0.958678
chr14	75134800	0.975207
chr14	75138300	0.942149
chr14	75258000	0.768595
chr14	75260100	0.909091
chr14	75291500	0.851240
chr14	75301700	0.958678
chr14	75351200	0.694215
chr14	75375900	0.685950
chr14	75399200	1.000000
chr14	75406800	0.842975
chr14	75409800	0.710744
chr14	75413400	0.867769
chr14	75418600	0.768595
chr14	75427100	0.685950
chr14	75432000	0.975207
chr14	75440500	0.793388
chr14	75450900	0.983471
chr14	75455900	0.809917
chr14	75478600	0.909091
chr14	75492300	1.000000
chr14	75509000	0.900826
chr14	75517800	0.768595
chr14	75526700	0.991736
chr14	75535200	0.719008
chr14	75591100	1.000000
chr14	75595500	0.909091
chr14	75614600	0.942149
chr14	75663400	0.719008
chr14	75673000	0.950413
chr14	75689200	0.900826
chr14	75697300	0.785124
chr14	75701400	0.900826
chr14	75704900	0.694215
chr14	75721700	0.743802
chr14	75724100	0.842975
chr14	75726200	0.760331
chr14	75743100	0.702479
chr14	75763300	0.876033
chr14	75769000	0.785124
chr14	75990600	0.892562
chr14	76002400	1.000000
chr14	76072200	0.710744
chr14	76113000	0.685950
chr14	76130300	0.909091
chr14	76156500	0.859504
chr14	76334600	0.983471
chr14	76338300	0.958678
chr14	76345800	0.983471
chr14	76362300	0.727273
chr14	76454300	0.925620
chr14	76456800	0.884298
chr14	76624100	0.685950

chr14	76815800	0.768595
chr14	76819300	0.661157
chr14	76843500	0.743802
chr14	77182400	0.818182
chr14	77186800	0.892562
chr14	77235100	0.884298
chr14	77238600	0.958678
chr14	77240700	0.785124
chr14	77331100	0.776860
chr14	77384000	0.752066
chr14	77386500	0.884298
chr14	77416300	0.818182
chr14	77418400	0.900826
chr14	77420700	0.975207
chr14	77423100	0.884298
chr14	77426300	0.933884
chr14	77467200	0.892562
chr14	77496100	0.809917
chr14	77504500	0.826446
chr14	77506900	0.942149
chr14	77509300	0.983471
chr14	77519400	0.809917
chr14	77522100	0.884298
chr14	77526000	0.917355
chr14	77542900	1.000000
chr14	77578000	0.859504
chr14	77606500	0.785124
chr14	77770500	0.966942
chr14	78003900	0.867769
chr14	78009500	0.933884
chr14	78047400	0.917355
chr14	78049600	0.925620
chr14	78055700	0.958678
chr14	78057900	0.801653
chr14	78060400	0.983471
chr14	78070100	0.884298
chr14	78074800	0.925620
chr14	78108500	0.867769
chr14	78205500	0.900826
chr14	79033400	0.859504
chr14	79072700	0.834711
chr14	79387500	0.892562
chr14	79389800	0.809917
chr14	79417300	0.818182
chr14	79433700	0.925620
chr14	79436200	0.942149
chr14	79441200	0.958678
chr14	79478100	0.925620
chr14	79512000	1.000000
chr14	79835900	0.826446
chr14	79878200	0.752066
chr14	81088200	0.851240
chr14	81105300	0.966942
chr14	81131000	0.768595

chr14	81237000	0.983471
chr14	81421900	0.710744
chr14	81902900	0.735537
chr14	81986400	0.859504
chr14	84173800	0.818182
chr14	84442900	0.685950
chr14	84473600	0.768595
chr14	84595200	0.785124
chr14	84717500	1.000000
chr14	84720900	0.991736
chr14	84739900	0.735537
chr14	84790700	0.950413
chr14	84810600	0.710744
chr14	84841900	0.685950
chr14	84909000	0.677686
chr14	84949100	0.983471
chr14	84951200	0.826446
chr14	85216300	0.669421
chr14	85273100	0.768595
chr14	85426400	0.842975
chr14	85430100	0.966942
chr14	85433400	0.735537
chr14	85442800	0.727273
chr14	85632300	0.743802
chr14	85717500	0.851240
chr14	85777200	0.859504
chr14	85786800	0.950413
chr14	85803400	0.793388
chr14	85826100	0.685950
chr14	85883600	0.966942
chr14	85937700	0.867769
chr14	85939800	0.801653
chr14	85942400	0.818182
chr14	85983500	1.000000
chr14	85993600	0.818182
chr14	86033300	1.000000
chr14	86039100	0.842975
chr14	86047400	0.743802
chr14	86053100	0.669421
chr14	86218800	0.719008
chr14	88237600	0.884298
chr14	88340600	0.942149
chr14	88790000	0.685950
chr14	89022300	0.694215
chr14	89080000	0.991736
chr14	89345700	0.975207
chr14	89507700	0.760331
chr14	89604100	0.793388
chr14	89632100	0.991736
chr14	89707300	0.826446
chr14	89768900	0.942149
chr14	89771500	0.760331
chr14	89818300	0.900826
chr14	89874000	0.892562

chr14	89884600	0.702479
chr14	89892400	0.818182
chr14	89896700	0.884298
chr14	89910400	0.991736
chr14	89974300	0.909091
chr14	90007300	0.661157
chr14	90070900	0.702479
chr14	90082400	0.694215
chr14	90237700	0.933884
chr14	90341200	0.661157
chr14	90388800	0.917355
chr14	90394200	0.975207
chr14	90420200	0.719008
chr14	90425200	0.793388
chr14	90604000	0.909091
chr14	90607400	0.900826
chr14	90742800	0.958678
chr14	90851500	0.694215
chr14	90867400	1.000000
chr14	90876000	0.966942
chr14	90883500	0.793388
chr14	90886700	0.933884
chr14	90911000	0.785124
chr14	90920600	0.900826
chr14	90953700	0.719008
chr14	91078500	0.958678
chr14	91080800	0.909091
chr14	91083800	0.942149
chr14	91091300	0.768595
chr14	91113000	0.842975
chr14	91144500	0.991736
chr14	91225000	0.991736
chr14	91261600	0.727273
chr14	91263800	0.917355
chr14	91267800	0.752066
chr14	91272300	0.884298
chr14	91294800	0.925620
chr14	91317300	0.925620
chr14	91427800	0.867769
chr14	91436900	0.818182
chr14	91524800	0.950413
chr14	91528200	0.760331
chr14	91574800	0.669421
chr14	91576900	0.859504
chr14	91606600	0.685950
chr14	91611800	0.801653
chr14	91621600	0.669421
chr14	91669100	0.917355
chr14	91694900	0.760331
chr14	91710200	0.983471
chr14	91720100	0.785124
chr14	91725100	0.760331
chr14	91731200	0.727273
chr14	91774800	0.793388

chr14	91789500	0.892562
chr14	91793700	0.776860
chr14	91799000	0.727273
chr14	91813100	0.743802
chr14	91826200	0.702479
chr14	91836500	0.793388
chr14	91840100	0.851240
chr14	91844400	0.793388
chr14	91867100	0.900826
chr14	91869400	0.950413
chr14	91885100	0.710744
chr14	92446900	0.702479
chr14	92450000	0.909091
chr14	92463900	0.884298
chr14	92925500	0.694215
chr14	92928000	0.991736
chr14	92978300	0.876033
chr14	93007300	0.975207
chr14	93013000	0.851240
chr14	93077400	0.925620
chr14	93154300	0.727273
chr14	93159600	0.743802
chr14	93192900	0.859504
chr14	93316100	0.694215
chr14	93503500	0.685950
chr14	93506100	0.851240
chr14	93510300	0.859504
chr14	93516800	0.834711
chr14	93582800	0.727273
chr14	93653300	0.950413
chr14	93698200	0.685950
chr14	93775700	0.950413
chr14	93784700	0.991736
chr14	93797000	0.834711
chr14	94413100	0.694215
chr14	94426000	0.909091
chr14	94529400	0.818182
chr14	94577300	0.909091
chr14	94581000	0.727273
chr14	95009300	0.933884
chr14	95065700	0.818182
chr14	95067900	0.983471
chr14	95242400	0.933884
chr14	95640900	0.776860
chr14	95650400	0.983471
chr14	95652800	0.892562
chr14	95694200	0.760331
chr14	95715600	0.975207
chr14	95720000	0.942149
chr14	95727000	0.884298
chr14	95732300	0.958678
chr14	95758200	0.859504
chr14	95778700	0.859504
chr14	95828100	0.677686

chr14	95927700	0.876033
chr14	95930300	0.694215
chr14	95949900	1.000000
chr14	95962500	0.710744
chr14	95974200	0.752066
chr14	95983000	0.694215
chr14	95986700	0.842975
chr14	95990300	0.983471
chr14	95993800	0.925620
chr14	95998200	0.966942
chr14	96012500	0.958678
chr14	96766100	0.702479
chr14	97026100	0.834711
chr14	98268900	0.760331
chr14	99858700	0.958678
chr14	99903500	0.925620
chr14	99935800	0.975207
chr14	99972500	0.851240
chr14	99998200	0.991736
chr14	100046400	0.867769
chr14	100057500	0.859504
chr14	100062600	0.933884
chr14	100082800	0.958678
chr14	100086700	0.909091
chr14	100112000	0.694215
chr14	100165500	0.661157
chr14	100197100	0.859504
chr14	100200400	0.801653
chr14	100213500	0.925620
chr14	100225400	0.876033
chr14	100258900	0.876033
chr14	100261100	0.727273
chr14	100498000	0.983471
chr14	100523200	0.685950
chr14	100535300	0.859504
chr14	100573300	0.719008
chr14	100609900	0.925620
chr14	100626200	0.719008
chr14	100659500	0.933884
chr14	100680800	0.776860
chr14	100741700	0.685950
chr14	100751800	0.834711
chr14	100775800	0.785124
chr14	100808500	0.950413
chr14	100826500	0.950413
chr14	100828900	0.925620
chr14	100836300	0.884298
chr14	100839200	0.661157
chr14	100851900	0.991736
chr14	100857000	0.685950
chr14	101013700	0.785124
chr14	101053400	0.752066
chr14	101111700	0.719008
chr14	101143700	0.727273

chr14	101155500	0.702479
chr14	101159500	0.884298
chr14	101228500	0.867769
chr14	101282700	0.818182
chr14	101366400	0.776860
chr14	101416700	0.942149
chr14	101525300	0.983471
chr14	101529300	0.983471
chr14	101534400	0.933884
chr14	101538800	0.842975
chr14	101541100	0.776860
chr14	101544100	0.925620
chr14	101557600	0.983471
chr14	101563600	0.884298
chr14	101565700	0.909091
chr14	101587200	0.669421
chr14	101601400	0.958678
chr14	101614500	1.000000
chr14	102172800	0.752066
chr14	102188500	0.727273
chr14	102198200	0.884298
chr14	102302600	0.661157
chr14	102343500	0.909091
chr14	102439200	0.991736
chr14	102447300	0.752066
chr14	102538200	0.752066
chr14	102550200	0.834711
chr14	102687800	0.884298
chr14	102817900	0.809917
chr14	102856800	0.876033
chr14	102905000	0.685950
chr14	102967100	0.975207
chr14	102970600	0.917355
chr14	102994700	0.694215
chr14	103051900	0.743802
chr14	103094400	0.851240
chr14	103096700	0.991736
chr14	103121700	0.991736
chr14	103163600	0.694215
chr14	103243000	0.719008
chr14	103261800	0.818182
chr14	103389800	0.842975
chr14	103396300	0.710744
chr14	103460400	0.727273
chr14	103477500	0.958678
chr14	103481600	0.909091
chr14	103489100	0.859504
chr14	103492200	0.892562
chr14	103533700	0.966942
chr14	103542100	0.842975
chr14	103558000	0.851240
chr14	103576400	0.826446
chr14	103585100	0.752066
chr14	103588900	0.793388

chr14	103674200	0.669421
chr14	103702400	0.859504
chr14	103780800	0.785124
chr14	103839300	0.933884
chr14	103865300	0.818182
chr14	103868300	0.909091
chr14	103870900	0.727273
chr14	103923100	0.942149
chr14	103989000	0.669421
chr14	104035700	0.975207
chr14	104043500	0.917355
chr14	104073900	0.842975
chr14	104091200	0.867769
chr14	104094700	0.752066
chr14	104163300	0.677686
chr14	104178400	0.892562
chr14	104181400	0.710744
chr14	104208000	0.950413
chr14	104221800	0.735537
chr14	104322000	0.876033
chr14	104326900	0.793388
chr14	104339100	0.842975
chr14	104346900	0.842975
chr14	104357300	0.702479
chr14	104390400	0.661157
chr14	104622200	0.925620
chr14	104652000	0.983471
chr14	104767000	0.677686
chr14	104903300	0.834711
chr14	105116900	0.785124
chr14	105132400	0.685950
chr14	105144200	0.818182
chr14	105148500	0.933884
chr14	105154500	0.677686
chr14	105158500	0.685950
chr14	105161400	0.710744
chr14	105203000	0.859504
chr14	105236700	0.776860
chr14	105239800	0.834711
chr14	105242000	0.785124
chr14	105287400	0.768595
chr14	105294000	0.702479
chr14	105399300	0.785124
chr14	105438100	0.884298
chr14	105440300	0.685950
chr14	105444100	0.801653
chr14	105452900	0.727273
chr14	105482300	0.983471
chr14	105490000	0.842975
chr14	105493400	0.892562
chr14	105527500	0.785124
chr14	105635900	1.000000
chr14	105643200	0.735537
chr14	105647300	0.719008

chr14	105659000	0.867769
chr14	105663700	0.917355
chr14	105666400	0.760331
chr14	105668900	0.727273
chr14	105750800	0.842975
chr14	105758900	0.768595
chr14	105761400	0.727273
chr14	105797100	0.966942
chr14	105804500	0.826446
chr14	105864000	0.809917
chr14	105877900	0.925620
chr14	105881500	0.842975
chr14	105896200	0.851240
chr14	105898300	0.694215
chr14	105902700	0.867769
chr14	105947200	0.900826
chr14	105953700	0.768595
chr14	105956400	0.727273
chr15	20668300	0.785124
chr15	20674900	0.884298
chr15	22904600	0.900826
chr15	22911400	0.942149
chr15	22923900	0.975207
chr15	23127700	1.000000
chr15	23158500	0.702479
chr15	23195000	0.950413
chr15	23335600	0.859504
chr15	23342200	0.942149
chr15	25104200	0.909091
chr15	28378700	0.752066
chr15	28445100	0.661157
chr15	28878200	0.867769
chr15	28974000	0.818182
chr15	28996100	0.942149
chr15	29034600	0.752066
chr15	29065200	0.702479
chr15	29261700	0.760331
chr15	29269400	0.826446
chr15	29425100	0.661157
chr15	29745600	0.867769
chr15	29973100	0.694215
chr15	29978300	0.785124
chr15	29990800	0.925620
chr15	30076800	0.661157
chr15	30092600	0.851240
chr15	30094900	0.884298
chr15	30118100	0.785124
chr15	30227000	0.983471
chr15	30403000	0.917355
chr15	30704800	0.809917
chr15	30721500	0.793388
chr15	30775700	0.958678
chr15	30829300	0.892562
chr15	30846200	0.785124

chr15	30872000	0.818182
chr15	30962700	0.702479
chr15	31215200	0.785124
chr15	31276700	0.702479
chr15	31296900	0.925620
chr15	31315300	0.752066
chr15	31317600	0.677686
chr15	31320800	0.768595
chr15	31324500	0.983471
chr15	31508400	0.768595
chr15	31516400	0.768595
chr15	31635200	0.925620
chr15	31645900	0.983471
chr15	31654000	0.661157
chr15	31689700	0.991736
chr15	31698300	0.727273
chr15	31733300	0.933884
chr15	31741300	0.966942
chr15	31846500	0.842975
chr15	32746200	0.876033
chr15	32763000	0.892562
chr15	32815500	0.991736
chr15	32870900	0.785124
chr15	32887700	0.785124
chr15	32910800	0.685950
chr15	32940500	0.768595
chr15	32952200	0.685950
chr15	33011100	0.826446
chr15	33085300	0.809917
chr15	33117300	0.933884
chr15	33144000	0.900826
chr15	33148800	0.801653
chr15	33170300	0.809917
chr15	33315100	0.917355
chr15	33419100	0.983471
chr15	33424500	0.826446
chr15	33487300	0.793388
chr15	34013300	0.942149
chr15	34015500	0.900826
chr15	34017900	0.719008
chr15	34294100	0.818182
chr15	34348800	0.719008
chr15	34415400	0.983471
chr15	34440100	0.669421
chr15	34531900	0.966942
chr15	34610900	0.801653
chr15	34627600	0.900826
chr15	34690500	0.809917
chr15	34787500	0.685950
chr15	34836600	0.884298
chr15	34902400	0.909091
chr15	34913300	1.000000
chr15	34921400	0.826446
chr15	34956800	0.851240

chr15	34975000	0.884298
chr15	34992500	0.966942
chr15	35014700	0.859504
chr15	35087600	0.727273
chr15	35145400	0.661157
chr15	35148300	0.768595
chr15	35278000	0.933884
chr15	35461000	0.983471
chr15	35597500	0.892562
chr15	35599600	0.950413
chr15	35602200	0.958678
chr15	35622900	0.677686
chr15	35719800	0.818182
chr15	35770500	0.809917
chr15	35965200	0.975207
chr15	35968000	0.685950
chr15	35973500	0.884298
chr15	35982800	0.884298
chr15	35985000	0.917355
chr15	35989800	0.900826
chr15	35998300	0.917355
chr15	36018100	0.909091
chr15	36030300	0.793388
chr15	36298000	0.975207
chr15	36479300	0.694215
chr15	36663100	0.884298
chr15	36720800	0.958678
chr15	36905000	0.892562
chr15	36981500	0.983471
chr15	37085800	0.677686
chr15	37100900	0.677686
chr15	37236400	0.694215
chr15	37351000	0.909091
chr15	37377200	0.909091
chr15	37387800	0.818182
chr15	38058000	0.685950
chr15	38193600	0.958678
chr15	38335200	0.727273
chr15	38343100	0.867769
chr15	38361700	0.966942
chr15	38371700	0.752066
chr15	38420800	0.694215
chr15	38536200	0.685950
chr15	38540100	0.876033
chr15	38667500	0.685950
chr15	38690500	0.826446
chr15	38772400	0.942149
chr15	38782900	0.785124
chr15	39006800	0.661157
chr15	39050500	0.842975
chr15	39110900	0.925620
chr15	39115400	0.950413
chr15	39117500	0.694215
chr15	39119600	0.958678

chr15	39121700	0.876033
chr15	39137400	0.876033
chr15	39161000	0.933884
chr15	39176700	0.983471
chr15	39185700	0.876033
chr15	39188900	0.884298
chr15	39238500	0.685950
chr15	39287100	0.694215
chr15	39289700	0.950413
chr15	39377900	0.876033
chr15	39395300	0.892562
chr15	39402900	0.966942
chr15	39410900	0.900826
chr15	39417000	0.867769
chr15	39420400	0.983471
chr15	39425300	0.867769
chr15	39431400	0.834711
chr15	39434000	0.958678
chr15	39438400	0.694215
chr15	39464500	0.933884
chr15	39481400	0.669421
chr15	39488000	0.859504
chr15	39521900	0.909091
chr15	39524900	0.900826
chr15	39539600	0.950413
chr15	39541700	0.958678
chr15	39545100	0.933884
chr15	39553300	0.917355
chr15	39557600	0.826446
chr15	39560000	0.884298
chr15	39562800	0.900826
chr15	39566100	0.909091
chr15	39580200	0.950413
chr15	39593100	0.991736
chr15	39650400	0.859504
chr15	39652500	0.884298
chr15	39655500	0.983471
chr15	39658700	0.909091
chr15	39696500	0.677686
chr15	39720900	0.743802
chr15	39724500	0.760331
chr15	39727300	0.859504
chr15	39755400	0.834711
chr15	39767300	0.900826
chr15	39781500	0.661157
chr15	39870700	0.776860
chr15	39882000	0.702479
chr15	39916300	0.677686
chr15	39918600	0.859504
chr15	39922000	0.933884
chr15	39934500	0.991736
chr15	39950100	0.735537
chr15	40077000	0.661157
chr15	40079100	0.669421

chr15	40085600	0.661157
chr15	40144100	0.925620
chr15	40200800	0.859504
chr15	40229300	0.661157
chr15	40234000	0.677686
chr15	40269200	0.768595
chr15	40321500	0.966942
chr15	40340700	0.876033
chr15	40356800	0.991736
chr15	40361600	0.892562
chr15	40368100	0.743802
chr15	40373500	0.909091
chr15	40387800	0.685950
chr15	40392000	0.975207
chr15	40395900	0.818182
chr15	40483000	0.867769
chr15	40531600	0.933884
chr15	40559200	0.909091
chr15	40575000	0.900826
chr15	40583500	0.768595
chr15	40607000	0.834711
chr15	40618600	0.785124
chr15	40620700	0.983471
chr15	40625900	0.677686
chr15	40630400	0.851240
chr15	40633700	0.661157
chr15	40637200	0.809917
chr15	40660500	0.710744
chr15	40665300	0.694215
chr15	40799800	0.685950
chr15	41023300	0.867769
chr15	41062100	0.743802
chr15	41079900	0.785124
chr15	41085600	0.793388
chr15	41088700	1.000000
chr15	41136800	0.661157
chr15	41197700	0.966942
chr15	41232000	0.818182
chr15	41235000	0.694215
chr15	41277300	0.785124
chr15	41294000	0.785124
chr15	41310200	0.950413
chr15	41312400	0.983471
chr15	41549400	0.685950
chr15	41780000	0.785124
chr15	41783200	0.884298
chr15	41787800	0.892562
chr15	41794700	0.925620
chr15	41847400	0.760331
chr15	41952300	0.793388
chr15	41959200	0.743802
chr15	41988200	0.975207
chr15	42039200	0.685950
chr15	42070400	0.809917

chr15	42072900	0.983471
chr15	42081400	1.000000
chr15	42084400	0.991736
chr15	42089300	0.826446
chr15	42097300	0.900826
chr15	42102000	0.727273
chr15	42187600	0.760331
chr15	42210200	0.752066
chr15	42212500	0.950413
chr15	42218700	0.925620
chr15	42222400	0.991736
chr15	42225800	0.661157
chr15	42229200	0.909091
chr15	42233900	0.933884
chr15	42240500	0.966942
chr15	42243500	0.900826
chr15	42252000	0.909091
chr15	42254200	0.958678
chr15	42260600	0.900826
chr15	42349700	0.851240
chr15	42392800	0.859504
chr15	42468300	0.694215
chr15	42678900	0.768595
chr15	42750200	0.975207
chr15	42785600	0.859504
chr15	42793500	0.884298
chr15	42873800	0.743802
chr15	42876800	0.752066
chr15	42897700	0.933884
chr15	43183200	0.909091
chr15	43442400	0.834711
chr15	43462300	0.793388
chr15	43482900	0.909091
chr15	43806300	0.727273
chr15	44087300	0.975207
chr15	44203400	0.925620
chr15	44206000	0.942149
chr15	44291600	0.958678
chr15	44316200	0.966942
chr15	44320500	0.859504
chr15	44322600	1.000000
chr15	44326800	0.958678
chr15	44337900	0.809917
chr15	44373900	0.942149
chr15	44380600	0.933884
chr15	44395300	0.925620
chr15	44430500	0.892562
chr15	44450200	0.958678
chr15	44500400	0.925620
chr15	44508300	0.694215
chr15	44811700	0.859504
chr15	44834400	0.925620
chr15	44847600	0.727273
chr15	44891300	0.933884

chr15	45019300	0.975207
chr15	45088100	0.735537
chr15	45096700	0.925620
chr15	45115700	0.950413
chr15	45123600	0.966942
chr15	45131500	1.000000
chr15	45319300	0.876033
chr15	45333000	1.000000
chr15	45354000	0.950413
chr15	45362100	0.933884
chr15	45369700	0.851240
chr15	45410000	0.727273
chr15	45414600	0.710744
chr15	45571800	0.677686
chr15	45723000	0.719008
chr15	45750000	0.884298
chr15	45791600	0.719008
chr15	45881900	0.702479
chr15	45930000	0.851240
chr15	45933700	0.925620
chr15	45963000	0.892562
chr15	46007400	0.834711
chr15	46195800	0.917355
chr15	46198900	0.735537
chr15	46211100	0.834711
chr15	46213300	0.909091
chr15	46380500	0.826446
chr15	46383300	0.818182
chr15	47539100	0.933884
chr15	47676800	0.925620
chr15	47698400	0.669421
chr15	47708100	0.975207
chr15	47715500	1.000000
chr15	47732100	0.933884
chr15	47753500	0.785124
chr15	47758700	0.950413
chr15	47761100	0.710744
chr15	47767800	0.661157
chr15	47776800	0.851240
chr15	47806900	0.975207
chr15	47809900	0.958678
chr15	47816100	0.818182
chr15	47958600	0.991736
chr15	47965800	0.768595
chr15	48042700	0.867769
chr15	48104900	0.867769
chr15	48442600	0.975207
chr15	48666600	0.818182
chr15	48675000	0.735537
chr15	48684900	0.925620
chr15	48695400	0.876033
chr15	48716900	0.760331
chr15	48740400	0.785124
chr15	48744000	0.975207

chr15	48786000	0.917355
chr15	48829200	1.000000
chr15	48840200	0.876033
chr15	48847600	0.702479
chr15	48858600	0.925620
chr15	48895400	1.000000
chr15	48934300	0.834711
chr15	48988900	0.752066
chr15	49065700	0.933884
chr15	49068400	0.694215
chr15	49353100	0.942149
chr15	49393900	0.661157
chr15	49415400	0.950413
chr15	49427100	0.958678
chr15	49430700	0.702479
chr15	49438200	0.909091
chr15	49919500	0.859504
chr15	50124100	0.851240
chr15	50427200	0.702479
chr15	50458600	0.834711
chr15	50926000	0.975207
chr15	51177900	0.966942
chr15	51203200	0.694215
chr15	51232600	0.859504
chr15	51369900	0.966942
chr15	51379400	0.975207
chr15	51382400	0.991736
chr15	51389300	0.867769
chr15	51391500	0.826446
chr15	51413600	0.991736
chr15	51425000	0.983471
chr15	51483500	0.867769
chr15	51516600	0.785124
chr15	51520800	0.785124
chr15	51546100	0.752066
chr15	51559900	0.694215
chr15	51786900	0.950413
chr15	52014100	0.983471
chr15	52130300	0.975207
chr15	52134100	1.000000
chr15	52136200	0.991736
chr15	52139000	1.000000
chr15	52154300	0.851240
chr15	52161100	0.818182
chr15	52167700	0.983471
chr15	52171600	0.983471
chr15	52231700	0.710744
chr15	52274400	0.876033
chr15	52285600	0.966942
chr15	52299100	0.991736
chr15	52301200	0.851240
chr15	52310900	0.884298
chr15	52385800	0.933884
chr15	52390100	0.719008

chr15	52393200	0.785124
chr15	52395800	0.966942
chr15	52404800	0.818182
chr15	52408300	0.727273
chr15	52417100	0.975207
chr15	52436900	0.851240
chr15	52500100	0.685950
chr15	52545100	0.826446
chr15	52548000	0.801653
chr15	52554500	0.983471
chr15	52676500	0.966942
chr15	52701400	0.685950
chr15	52707700	0.942149
chr15	52710200	0.975207
chr15	52712400	0.900826
chr15	52756500	0.909091
chr15	52774400	0.966942
chr15	52777400	0.925620
chr15	52780700	0.785124
chr15	52784000	0.958678
chr15	52786600	0.975207
chr15	52790000	0.785124
chr15	52850600	0.785124
chr15	52945900	1.000000
chr15	52969100	0.702479
chr15	53021100	0.743802
chr15	53023600	0.958678
chr15	53097700	0.785124
chr15	53903700	0.966942
chr15	55522500	0.942149
chr15	55533200	0.694215
chr15	55560100	0.942149
chr15	55571900	0.818182
chr15	55574800	0.942149
chr15	55665600	0.859504
chr15	55696400	0.950413
chr15	55920300	0.809917
chr15	56030000	0.743802
chr15	56149100	0.826446
chr15	56161500	0.694215
chr15	56165300	0.958678
chr15	56187000	0.975207
chr15	56190400	0.975207
chr15	56200300	0.933884
chr15	56210400	0.694215
chr15	56235000	0.991736
chr15	56332800	0.859504
chr15	56340000	0.685950
chr15	56912600	0.661157
chr15	57023000	0.669421
chr15	57056100	0.900826
chr15	57059400	0.884298
chr15	57066800	0.851240
chr15	57098400	0.983471

chr15	57107300	0.917355
chr15	57110000	0.917355
chr15	57163700	0.702479
chr15	57179100	0.685950
chr15	57206000	0.809917
chr15	57278000	0.966942
chr15	57304200	0.702479
chr15	57323800	0.735537
chr15	57350300	0.983471
chr15	57352400	0.884298
chr15	57354500	0.966942
chr15	57374600	0.983471
chr15	57410200	0.876033
chr15	57414500	0.884298
chr15	57427500	0.925620
chr15	57439000	0.735537
chr15	57442500	0.818182
chr15	57445800	0.991736
chr15	57599000	0.818182
chr15	57613600	0.892562
chr15	57616200	0.710744
chr15	57626300	0.826446
chr15	57628500	0.917355
chr15	57630900	1.000000
chr15	57677300	0.975207
chr15	57685300	0.958678
chr15	57688100	0.892562
chr15	57695800	0.743802
chr15	57750900	0.727273
chr15	57778100	0.752066
chr15	57787600	0.685950
chr15	57798800	0.900826
chr15	57803100	0.876033
chr15	57818300	0.677686
chr15	57843000	0.809917
chr15	57845500	0.760331
chr15	57848100	0.917355
chr15	57854000	0.991736
chr15	57870900	1.000000
chr15	57900000	0.834711
chr15	57902700	0.933884
chr15	57919300	0.851240
chr15	57985200	0.719008
chr15	57988700	0.685950
chr15	58025300	0.909091
chr15	58081700	0.942149
chr15	58117200	0.785124
chr15	58120000	0.809917
chr15	58166500	0.966942
chr15	58171200	0.677686
chr15	58201700	0.826446
chr15	58215900	0.983471
chr15	58287100	0.710744
chr15	58312100	0.884298

chr15	58331200	0.933884
chr15	58454300	0.818182
chr15	58472300	0.925620
chr15	58495800	0.834711
chr15	58624500	0.884298
chr15	58673100	0.917355
chr15	58732400	0.925620
chr15	58734600	0.776860
chr15	58750500	0.950413
chr15	58765900	0.942149
chr15	58768700	0.876033
chr15	58803300	0.702479
chr15	58844400	0.950413
chr15	58920800	0.900826
chr15	58974500	0.702479
chr15	59035100	0.991736
chr15	59132300	0.768595
chr15	59244600	0.685950
chr15	59268500	0.876033
chr15	59288000	0.826446
chr15	59458400	0.925620
chr15	59460700	0.859504
chr15	59462800	0.727273
chr15	59467500	0.991736
chr15	59484000	0.958678
chr15	59493000	0.677686
chr15	59510800	0.991736
chr15	59514600	0.851240
chr15	59517400	0.785124
chr15	59525000	0.983471
chr15	59529800	1.000000
chr15	59560900	0.892562
chr15	59585200	0.917355
chr15	59589400	0.942149
chr15	59593100	0.983471
chr15	59598300	0.925620
chr15	59642400	0.950413
chr15	59659000	0.776860
chr15	59971100	1.000000
chr15	59978200	1.000000
chr15	59992400	0.801653
chr15	60298800	0.892562
chr15	60363600	0.710744
chr15	60652000	0.809917
chr15	60654900	0.966942
chr15	60657000	0.760331
chr15	60661500	0.768595
chr15	60664200	0.785124
chr15	60667500	0.917355
chr15	60669600	0.942149
chr15	60671800	0.669421
chr15	60674000	0.983471
chr15	60676500	0.925620
chr15	60681400	0.768595

chr15	60684700	0.826446
chr15	60692600	0.900826
chr15	60700800	0.983471
chr15	60714100	0.991736
chr15	60757800	0.966942
chr15	60762800	0.685950
chr15	60897900	0.842975
chr15	60906200	0.909091
chr15	60928000	0.983471
chr15	60937200	0.809917
chr15	61001300	0.917355
chr15	61011100	0.818182
chr15	61044900	0.917355
chr15	61060400	0.694215
chr15	61067300	0.826446
chr15	61254500	0.851240
chr15	61263300	1.000000
chr15	61267000	0.768595
chr15	61287600	0.859504
chr15	61327100	0.983471
chr15	61330500	0.867769
chr15	61497900	0.925620
chr15	61816500	0.983471
chr15	61821100	0.983471
chr15	62150000	0.917355
chr15	62171200	0.942149
chr15	62328200	0.834711
chr15	62356300	0.991736
chr15	62361100	0.702479
chr15	62391800	0.760331
chr15	62400700	0.925620
chr15	62403000	1.000000
chr15	62405600	0.917355
chr15	62412000	0.983471
chr15	62416700	1.000000
chr15	62418800	0.702479
chr15	62428100	0.909091
chr15	62430700	0.966942
chr15	62459000	0.801653
chr15	62464600	0.809917
chr15	63137700	0.768595
chr15	63141400	0.975207
chr15	63143800	0.933884
chr15	63151000	0.685950
chr15	63172300	0.983471
chr15	63179100	0.760331
chr15	63182200	0.785124
chr15	63187100	0.884298
chr15	63190400	0.950413
chr15	63221200	0.917355
chr15	63249600	0.917355
chr15	63306000	0.900826
chr15	63308300	0.685950
chr15	63343800	1.000000

chr15	63347100	0.892562
chr15	63389800	0.950413
chr15	63426200	0.991736
chr15	63428500	0.975207
chr15	63486700	0.884298
chr15	63505000	0.900826
chr15	63510500	0.818182
chr15	63513900	0.909091
chr15	63524900	0.983471
chr15	63528600	0.900826
chr15	63537000	0.975207
chr15	63549200	0.950413
chr15	63553200	0.925620
chr15	63580100	0.958678
chr15	63583800	0.983471
chr15	63601500	0.933884
chr15	63641000	0.900826
chr15	63667700	0.975207
chr15	63776600	0.768595
chr15	63794200	0.851240
chr15	63802500	0.942149
chr15	63832100	0.661157
chr15	63845800	0.909091
chr15	63892200	0.719008
chr15	63898600	0.710744
chr15	63903000	0.851240
chr15	63925900	0.958678
chr15	64000700	0.710744
chr15	64110900	0.834711
chr15	64119900	0.685950
chr15	64128300	0.867769
chr15	64134500	0.983471
chr15	64151700	0.834711
chr15	64189200	0.892562
chr15	64198500	0.851240
chr15	64202000	0.909091
chr15	64230800	0.818182
chr15	64233100	0.917355
chr15	64284600	0.694215
chr15	64297000	0.710744
chr15	64364500	0.950413
chr15	64433800	0.776860
chr15	64456500	0.685950
chr15	64540500	0.966942
chr15	64630300	0.966942
chr15	64636500	0.867769
chr15	64640800	0.661157
chr15	64643800	0.735537
chr15	64735600	0.809917
chr15	64737700	0.842975
chr15	64795500	0.851240
chr15	64813800	0.958678
chr15	64819600	0.834711
chr15	64823500	0.752066

chr15	64828600	0.991736
chr15	64879400	0.818182
chr15	64992200	0.719008
chr15	65010900	0.909091
chr15	65137500	0.826446
chr15	65149400	0.826446
chr15	65151700	0.719008
chr15	65176500	0.809917
chr15	65185900	0.842975
chr15	65188400	0.809917
chr15	65190600	0.917355
chr15	65197900	0.727273
chr15	65223600	0.851240
chr15	65278000	0.925620
chr15	65342400	0.702479
chr15	65403000	0.859504
chr15	65618900	0.752066
chr15	65632600	0.685950
chr15	65677900	0.876033
chr15	65805900	0.710744
chr15	65818500	0.669421
chr15	65869300	0.727273
chr15	65876900	0.983471
chr15	65935000	0.776860
chr15	65947000	0.942149
chr15	66076500	0.710744
chr15	66079900	0.966942
chr15	66089300	1.000000
chr15	66112000	0.950413
chr15	66125400	0.975207
chr15	66130600	0.710744
chr15	66133500	0.925620
chr15	66165200	0.834711
chr15	66188700	0.818182
chr15	66232600	0.892562
chr15	66662500	0.942149
chr15	66683200	0.983471
chr15	66698000	0.859504
chr15	66710400	0.818182
chr15	66753100	0.818182
chr15	66808300	1.000000
chr15	66870800	0.851240
chr15	66873900	0.851240
chr15	66905600	0.826446
chr15	66908900	0.851240
chr15	66911800	0.834711
chr15	66914800	0.966942
chr15	66932000	0.950413
chr15	66936300	0.760331
chr15	66940300	0.818182
chr15	66943300	0.851240
chr15	66947500	0.942149
chr15	66950400	0.727273
chr15	66954600	0.900826

chr15	66978200	0.743802
chr15	66992700	0.669421
chr15	66999800	1.000000
chr15	67007200	0.884298
chr15	67013300	0.867769
chr15	67016200	0.842975
chr15	67022600	0.942149
chr15	67027200	1.000000
chr15	67037100	0.958678
chr15	67041000	0.991736
chr15	67044600	0.876033
chr15	67050100	0.991736
chr15	67054000	0.785124
chr15	67056800	0.942149
chr15	67059500	0.867769
chr15	67065800	0.917355
chr15	67094700	0.677686
chr15	67108000	0.669421
chr15	67122900	0.677686
chr15	67135600	0.685950
chr15	67143500	0.892562
chr15	67176000	0.958678
chr15	67224600	0.826446
chr15	67250400	0.975207
chr15	67306500	0.851240
chr15	67318900	0.876033
chr15	67326500	0.933884
chr15	67334800	0.677686
chr15	67337200	0.958678
chr15	67342200	0.876033
chr15	67357300	0.768595
chr15	67362400	0.669421
chr15	67367400	0.958678
chr15	67370800	0.752066
chr15	67373700	0.983471
chr15	67378100	0.950413
chr15	67381100	0.809917
chr15	67384000	0.983471
chr15	67386100	0.743802
chr15	67389300	0.809917
chr15	67394000	0.950413
chr15	67399700	0.892562
chr15	67402700	0.752066
chr15	67408800	0.892562
chr15	67411500	0.768595
chr15	67414200	0.851240
chr15	67417400	0.727273
chr15	67420400	0.785124
chr15	67426000	0.884298
chr15	67428500	0.942149
chr15	67432500	0.958678
chr15	67434600	0.917355
chr15	67436800	0.983471
chr15	67444100	0.975207

chr15	67450500	0.859504
chr15	67454300	0.867769
chr15	67456800	0.851240
chr15	67466300	0.743802
chr15	67469800	0.892562
chr15	67472100	0.925620
chr15	67479000	0.669421
chr15	67491500	0.991736
chr15	67706700	0.776860
chr15	67794500	0.719008
chr15	68136400	0.842975
chr15	68392500	0.661157
chr15	68498100	0.917355
chr15	68573600	0.677686
chr15	68633400	0.826446
chr15	68640100	0.876033
chr15	68645000	0.966942
chr15	68658200	0.818182
chr15	68661400	0.950413
chr15	68672400	0.933884
chr15	68677100	0.793388
chr15	68683100	0.942149
chr15	68724300	0.719008
chr15	68836100	0.719008
chr15	68852100	0.661157
chr15	68878800	0.760331
chr15	68911700	0.818182
chr15	68993400	0.909091
chr15	68997200	0.867769
chr15	69047500	0.859504
chr15	69054800	0.925620
chr15	69058200	0.801653
chr15	69074700	0.826446
chr15	69082700	0.884298
chr15	69087900	0.958678
chr15	69338000	0.933884
chr15	69368000	0.661157
chr15	69372900	0.826446
chr15	69375300	0.958678
chr15	69385100	0.933884
chr15	69447800	0.892562
chr15	69457800	0.958678
chr15	69486900	0.826446
chr15	69545900	0.966942
chr15	69633400	0.818182
chr15	69687000	0.884298
chr15	69755500	0.876033
chr15	69767100	0.694215
chr15	69850100	0.958678
chr15	70051400	0.966942
chr15	70053600	0.983471
chr15	70091600	0.950413
chr15	70385800	0.710744
chr15	70393900	0.826446

chr15	70404700	0.661157
chr15	70419900	0.859504
chr15	70563100	0.975207
chr15	70767800	0.925620
chr15	70797700	0.752066
chr15	70801200	0.785124
chr15	70821000	0.694215
chr15	70851800	0.694215
chr15	70897800	0.801653
chr15	70943300	0.975207
chr15	70983400	0.991736
chr15	70995900	0.966942
chr15	71001600	0.933884
chr15	71005700	0.958678
chr15	71008600	0.867769
chr15	71034700	0.991736
chr15	71061000	0.826446
chr15	71063600	0.991736
chr15	71068100	0.793388
chr15	71070600	0.942149
chr15	71084700	0.983471
chr15	71087800	0.975207
chr15	71092900	0.900826
chr15	71100100	0.801653
chr15	71126600	0.702479
chr15	71149600	0.983471
chr15	71194700	0.950413
chr15	71216600	0.735537
chr15	71422100	0.760331
chr15	71424800	1.000000
chr15	71427100	0.851240
chr15	71429800	0.752066
chr15	71530300	0.851240
chr15	71544300	0.975207
chr15	71567600	0.983471
chr15	71571800	0.859504
chr15	71581200	0.950413
chr15	71588500	0.983471
chr15	71618300	0.851240
chr15	71659200	0.685950
chr15	71667500	0.991736
chr15	71677900	0.677686
chr15	71740500	0.950413
chr15	71750800	0.958678
chr15	71789300	0.760331
chr15	71865200	0.677686
chr15	71972700	0.743802
chr15	72040800	0.909091
chr15	72045700	0.933884
chr15	72054600	0.851240
chr15	72072800	0.991736
chr15	72081200	0.859504
chr15	72084600	0.776860
chr15	72099600	0.867769

chr15	72530000	0.917355
chr15	72847300	0.933884
chr15	73050800	0.909091
chr15	73866500	0.925620
chr15	73895200	0.834711
chr15	73927100	0.685950
chr15	73979300	1.000000
chr15	73981700	0.933884
chr15	73989700	1.000000
chr15	74163900	0.694215
chr15	74216800	0.826446
chr15	74219900	0.727273
chr15	74229100	0.834711
chr15	74244100	0.785124
chr15	74262000	0.876033
chr15	74265600	0.851240
chr15	74275400	0.851240
chr15	74280300	0.818182
chr15	74282600	0.834711
chr15	74304500	0.950413
chr15	74318100	0.851240
chr15	74333000	0.719008
chr15	74429000	0.719008
chr15	74472600	0.776860
chr15	74533000	0.776860
chr15	74584400	0.710744
chr15	74638000	0.694215
chr15	74658500	0.809917
chr15	74671500	0.768595
chr15	74678200	0.950413
chr15	74737000	0.809917
chr15	74804700	0.909091
chr15	74818500	0.793388
chr15	74838700	0.859504
chr15	74867100	0.917355
chr15	74881700	1.000000
chr15	74935700	0.925620
chr15	74943500	0.917355
chr15	74946500	0.958678
chr15	74956400	0.958678
chr15	75055200	0.867769
chr15	75069900	0.958678
chr15	75082600	0.752066
chr15	75158500	0.752066
chr15	75160800	0.925620
chr15	75163100	1.000000
chr15	75254000	0.966942
chr15	75257300	0.917355
chr15	75289300	0.752066
chr15	75321800	0.950413
chr15	75324700	0.867769
chr15	75382800	0.851240
chr15	75397200	0.900826
chr15	75399500	0.801653

chr15	75401800	0.694215
chr15	75464100	0.661157
chr15	75497400	0.735537
chr15	75776500	0.834711
chr15	75825400	0.958678
chr15	75913600	0.909091
chr15	75944100	0.727273
chr15	75953600	0.677686
chr15	75959400	0.925620
chr15	76051000	0.685950
chr15	76116600	0.958678
chr15	76184400	0.884298
chr15	76215300	0.876033
chr15	76222700	0.702479
chr15	76432600	0.793388
chr15	76489400	0.826446
chr15	76510200	0.702479
chr15	76633000	0.743802
chr15	76672900	0.727273
chr15	77259900	0.743802
chr15	77265600	0.801653
chr15	77321100	0.669421
chr15	77346800	0.677686
chr15	77356900	0.966942
chr15	77360300	1.000000
chr15	77411900	0.785124
chr15	77436700	0.867769
chr15	77452900	0.719008
chr15	77478500	0.776860
chr15	77480800	0.933884
chr15	77501700	0.760331
chr15	77517100	0.842975
chr15	77519500	0.677686
chr15	77575300	0.669421
chr15	77640300	0.826446
chr15	77747900	0.776860
chr15	77751600	0.991736
chr15	77771700	0.909091
chr15	77774300	0.975207
chr15	77776400	0.909091
chr15	77780000	0.818182
chr15	77786300	0.917355
chr15	77790100	0.727273
chr15	77801400	0.909091
chr15	77835800	0.801653
chr15	77897400	0.677686
chr15	77926000	0.752066
chr15	78237100	0.892562
chr15	78284200	0.983471
chr15	78288100	0.867769
chr15	78328200	0.867769
chr15	78344200	0.991736
chr15	78349600	0.900826
chr15	78352100	0.685950

chr15	78357800	0.958678
chr15	78360600	0.859504
chr15	78363000	0.809917
chr15	78367700	0.809917
chr15	78398600	0.743802
chr15	78505300	0.743802
chr15	78615200	0.661157
chr15	78622300	0.884298
chr15	78703800	0.768595
chr15	78933700	0.776860
chr15	79048300	0.826446
chr15	79052900	0.834711
chr15	79055100	0.900826
chr15	79072500	0.694215
chr15	79104900	0.892562
chr15	79164600	0.719008
chr15	79230000	0.719008
chr15	79237400	0.735537
chr15	79281200	0.826446
chr15	79285400	0.760331
chr15	79297100	0.933884
chr15	79615600	0.966942
chr15	79621700	0.925620
chr15	79932900	0.867769
chr15	79936900	0.933884
chr15	79973500	0.942149
chr15	80325600	0.966942
chr15	80365300	0.702479
chr15	80398000	0.867769
chr15	80453400	0.876033
chr15	80467100	0.677686
chr15	80471800	0.933884
chr15	80881400	0.991736
chr15	80883900	0.685950
chr15	80902400	0.925620
chr15	80908700	0.925620
chr15	80913700	0.966942
chr15	81071900	0.826446
chr15	81227500	0.859504
chr15	81271600	0.661157
chr15	81297200	0.743802
chr15	81316500	0.876033
chr15	81322400	0.909091
chr15	81332200	0.966942
chr15	81378300	0.760331
chr15	81385000	0.909091
chr15	81391600	0.710744
chr15	81406000	0.661157
chr15	81426700	0.826446
chr15	81558700	0.702479
chr15	81589300	0.727273
chr15	81797000	0.983471
chr15	81809200	0.900826
chr15	81824100	0.950413

chr15	81868200	0.685950
chr15	82386700	0.933884
chr15	82433000	0.958678
chr15	82439700	0.867769
chr15	82628800	0.859504
chr15	82702800	0.842975
chr15	82713400	0.917355
chr15	82926400	0.950413
chr15	82989600	0.809917
chr15	83089700	0.851240
chr15	83127500	0.768595
chr15	83316900	0.669421
chr15	83491100	0.785124
chr15	83501800	0.851240
chr15	83715900	0.694215
chr15	83722300	0.892562
chr15	83737200	0.876033
chr15	83761700	0.933884
chr15	83779000	0.818182
chr15	83782800	0.710744
chr15	83832400	0.917355
chr15	83834800	0.876033
chr15	83870900	0.826446
chr15	83956500	0.909091
chr15	83979600	0.785124
chr15	84517300	0.719008
chr15	84749500	0.809917
chr15	84841700	0.661157
chr15	84854900	0.900826
chr15	84963300	0.793388
chr15	85069200	0.933884
chr15	85099100	0.975207
chr15	85123100	0.818182
chr15	85131600	0.793388
chr15	85159900	0.661157
chr15	85201700	0.818182
chr15	85294400	0.826446
chr15	85308500	0.958678
chr15	85315900	0.966942
chr15	85360500	0.669421
chr15	85363000	0.975207
chr15	85365400	0.661157
chr15	85376600	0.694215
chr15	85454900	0.694215
chr15	85457500	0.933884
chr15	85491800	0.669421
chr15	85499300	0.793388
chr15	85503100	0.991736
chr15	85554400	0.933884
chr15	85569800	0.685950
chr15	85573900	0.917355
chr15	85589200	0.842975
chr15	85591500	0.966942
chr15	85668800	0.685950

chr15	85673300	0.842975
chr15	85685700	0.785124
chr15	85712500	0.735537
chr15	85720600	0.975207
chr15	85734000	0.909091
chr15	85796800	0.768595
chr15	85801300	0.669421
chr15	85840300	0.685950
chr15	85861900	0.942149
chr15	85875500	0.710744
chr15	85880900	0.768595
chr15	85887300	0.892562
chr15	85938400	1.000000
chr15	85948500	0.909091
chr15	85951800	0.859504
chr15	85957000	0.958678
chr15	85964900	0.743802
chr15	86022400	1.000000
chr15	86038000	0.900826
chr15	86071700	0.983471
chr15	86122800	0.917355
chr15	86128000	1.000000
chr15	86135800	0.892562
chr15	86142300	0.975207
chr15	86150500	0.768595
chr15	86152600	0.867769
chr15	86162600	0.768595
chr15	86193000	0.925620
chr15	86211500	0.917355
chr15	86232900	0.950413
chr15	86235000	0.876033
chr15	86245500	0.917355
chr15	86250800	0.884298
chr15	86315000	0.876033
chr15	86384000	0.685950
chr15	88134200	0.685950
chr15	88153400	0.859504
chr15	88360400	0.735537
chr15	88670500	0.661157
chr15	88679200	0.710744
chr15	88726900	0.702479
chr15	88998100	0.694215
chr15	89008400	0.966942
chr15	89167900	0.785124
chr15	89174300	0.892562
chr15	89182400	0.983471
chr15	89441000	0.900826
chr15	89561000	0.685950
chr15	89630600	0.776860
chr15	89634500	0.950413
chr15	89644700	0.809917
chr15	89648800	1.000000
chr15	89656100	0.867769
chr15	89663400	0.909091

chr15	89666600	0.884298
chr15	89673400	0.966942
chr15	89676600	0.975207
chr15	89681200	0.685950
chr15	89687200	0.818182
chr15	89709800	0.710744
chr15	89712200	0.925620
chr15	89736200	0.909091
chr15	89859200	0.727273
chr15	89950200	0.768595
chr15	89974300	0.851240
chr15	90039800	0.793388
chr15	90122000	0.768595
chr15	90131400	0.975207
chr15	90206400	0.925620
chr15	90209400	0.702479
chr15	90320100	0.842975
chr15	90338600	0.950413
chr15	90352500	0.735537
chr15	90361700	0.694215
chr15	90364300	0.710744
chr15	90367000	0.851240
chr15	90380700	0.809917
chr15	90389100	0.809917
chr15	90392100	0.892562
chr15	90395100	0.876033
chr15	90543100	0.735537
chr15	90547700	0.859504
chr15	90563500	0.867769
chr15	90577500	0.809917
chr15	90581200	0.743802
chr15	90584400	0.776860
chr15	90608800	0.892562
chr15	90701700	0.776860
chr15	90709300	1.000000
chr15	90718100	0.719008
chr15	90728300	0.743802
chr15	90755400	0.851240
chr15	90793000	0.818182
chr15	90802900	0.892562
chr15	90805100	0.975207
chr15	90807500	0.694215
chr15	90811900	0.702479
chr15	90886700	0.975207
chr15	90905900	0.925620
chr15	90908100	0.917355
chr15	90920700	0.983471
chr15	90934800	0.900826
chr15	90946800	0.859504
chr15	90949100	0.818182
chr15	90951200	0.925620
chr15	90956800	0.966942
chr15	90963100	0.991736
chr15	90979200	0.950413

chr15	90983600	0.809917
chr15	91002800	1.000000
chr15	91006800	0.933884
chr15	91063200	0.842975
chr15	91108300	0.892562
chr15	91138100	0.876033
chr15	91191500	0.917355
chr15	91194300	0.892562
chr15	91224900	0.842975
chr15	91318700	0.768595
chr15	91412500	0.768595
chr15	91426900	0.851240
chr15	91429800	0.925620
chr15	91473300	0.892562
chr15	91493100	0.851240
chr15	91498300	0.735537
chr15	91531800	0.867769
chr15	93077100	0.966942
chr15	93107400	0.702479
chr15	93167100	0.752066
chr15	93169600	0.983471
chr15	93173500	0.859504
chr15	93187600	0.892562
chr15	93189700	0.851240
chr15	93192300	0.727273
chr15	93196800	0.859504
chr15	93250500	0.685950
chr15	93252600	0.851240
chr15	93375700	1.000000
chr15	93450000	0.950413
chr15	93453500	0.942149
chr15	93458200	0.933884
chr15	93461400	0.975207
chr15	93465000	0.950413
chr15	93524900	0.991736
chr15	93572700	0.735537
chr15	93632300	0.727273
chr15	95128500	0.991736
chr15	95150500	0.752066
chr15	95152600	0.851240
chr15	95214400	0.876033
chr15	95217700	0.933884
chr15	95227500	0.942149
chr15	95244300	0.925620
chr15	95248300	0.925620
chr15	95335400	0.702479
chr15	95383800	0.975207
chr15	95386200	0.776860
chr15	95389400	0.785124
chr15	95509200	0.694215
chr15	95560700	0.793388
chr15	95562800	0.867769
chr15	95630400	0.793388
chr15	95674400	0.958678

chr15	95718600	0.950413
chr15	95720800	0.669421
chr15	95752500	0.752066
chr15	95790400	0.892562
chr15	95795700	0.909091
chr15	95798100	0.669421
chr15	95803100	0.950413
chr15	95806800	1.000000
chr15	95828800	0.859504
chr15	95833100	0.991736
chr15	95838500	0.950413
chr15	95840700	0.942149
chr15	95844100	0.900826
chr15	95849000	0.719008
chr15	95868000	0.743802
chr15	95873100	0.677686
chr15	95909000	0.909091
chr15	95911700	0.876033
chr15	95938100	0.892562
chr15	96001000	0.842975
chr15	96003500	0.966942
chr15	96007900	0.942149
chr15	96012900	0.793388
chr15	96015000	0.818182
chr15	96100300	0.991736
chr15	96103600	0.917355
chr15	96147700	0.975207
chr15	96228200	0.975207
chr15	96259600	0.727273
chr15	96278000	0.983471
chr15	96306600	0.818182
chr15	96313800	0.801653
chr15	96355900	0.661157
chr15	96391400	0.793388
chr15	96431400	0.942149
chr15	96476000	0.694215
chr15	96591000	0.925620
chr15	96596700	0.677686
chr15	96702500	0.685950
chr15	96774200	0.966942
chr15	96817000	0.942149
chr15	96819900	0.842975
chr15	96853400	0.685950
chr15	96860800	0.842975
chr15	96863500	0.735537
chr15	96891400	0.702479
chr15	96893900	0.991736
chr15	96902400	0.719008
chr15	96975400	0.925620
chr15	97393100	0.776860
chr15	97433900	0.694215
chr15	97536200	0.933884
chr15	97580100	0.694215
chr15	98045400	0.925620

chr15	98047600	0.975207
chr15	98110700	0.950413
chr15	98311700	0.909091
chr15	98522200	0.677686
chr15	98536800	0.958678
chr15	98539100	0.727273
chr15	98568900	0.933884
chr15	98971900	0.826446
chr15	99198500	0.776860
chr15	99212300	0.983471
chr15	99215300	1.000000
chr15	99270300	0.900826
chr15	99273600	0.966942
chr15	99279400	0.933884
chr15	99297700	0.851240
chr15	99343700	0.867769
chr15	99356300	0.942149
chr15	99394400	0.694215
chr15	99402200	0.669421
chr15	99425800	0.785124
chr15	99433200	0.892562
chr15	99439000	0.752066
chr15	99441400	0.942149
chr15	99446600	0.842975
chr15	99448800	0.942149
chr15	99451000	0.818182
chr15	99457300	0.867769
chr15	99459700	0.834711
chr15	99468000	0.942149
chr15	99602800	0.768595
chr15	99670600	0.958678
chr15	99675900	1.000000
chr15	99706900	0.727273
chr15	99750000	0.719008
chr15	99780000	0.677686
chr15	99846600	0.785124
chr15	99874500	0.818182
chr15	99884700	0.983471
chr15	99965800	0.892562
chr15	99982800	1.000000
chr15	99991700	0.677686
chr15	99995700	0.942149
chr15	100017500	0.991736
chr15	100027000	0.950413
chr15	100048400	0.909091
chr15	100077400	0.966942
chr15	100171800	0.876033
chr15	100223200	0.933884
chr15	100244000	0.975207
chr15	100249300	0.991736
chr15	100583700	0.991736
chr15	100651700	0.933884
chr15	100654700	0.983471
chr15	101228000	0.917355

chr15	101243900	0.876033
chr15	101253800	0.867769
chr15	101262600	0.933884
chr15	101429900	0.834711
chr15	101432000	0.842975
chr15	101439800	0.702479
chr15	101442900	0.818182
chr15	101499300	0.884298
chr15	101548300	0.776860
chr15	101611300	0.876033
chr15	101632200	0.859504
chr15	101668400	0.842975
chr15	101707500	0.876033
chr15	101709600	0.826446
chr15	101717400	0.991736
chr15	101723700	0.991736
chr15	101729300	0.752066
chr15	101740500	0.851240
chr15	101783600	0.752066
chr15	101787100	0.942149
chr15	101805400	0.719008
chr15	101833400	0.727273
chr15	101986900	0.975207
chr15	101995800	0.677686
chr15	102109000	0.735537
chr15	102120200	0.958678
chr15	102182500	0.950413
chr15	102242700	0.925620
chr15	102509600	0.768595
chr16	63300	0.702479
chr16	123400	0.892562
chr16	130100	0.793388
chr16	136200	0.719008
chr16	152800	0.859504
chr16	163900	0.884298
chr16	231200	0.900826
chr16	292100	0.743802
chr16	303100	0.900826
chr16	330800	0.743802
chr16	377900	0.917355
chr16	402000	0.661157
chr16	420600	0.793388
chr16	434100	0.702479
chr16	447400	0.768595
chr16	474000	0.661157
chr16	508000	0.991736
chr16	527900	0.933884
chr16	559600	0.892562
chr16	575100	0.818182
chr16	616900	0.867769
chr16	676900	0.876033
chr16	725700	0.776860
chr16	765700	0.719008
chr16	1014400	0.776860

chr16	1047800	0.942149
chr16	1053800	0.892562
chr16	1128700	0.892562
chr16	1133200	0.818182
chr16	1140300	0.818182
chr16	1382200	0.661157
chr16	1428200	0.702479
chr16	1458900	0.702479
chr16	1562700	0.909091
chr16	1577900	0.876033
chr16	1594400	0.991736
chr16	1599800	0.909091
chr16	1690500	0.900826
chr16	1709300	0.702479
chr16	1772400	0.851240
chr16	2073500	0.958678
chr16	2086100	0.900826
chr16	2094400	0.876033
chr16	2141900	0.727273
chr16	2174000	0.727273
chr16	2176800	0.818182
chr16	2198900	0.851240
chr16	2208000	0.900826
chr16	2210500	0.851240
chr16	2219200	0.826446
chr16	2287200	0.818182
chr16	2302700	0.933884
chr16	2421800	0.834711
chr16	2521900	0.719008
chr16	2567100	0.826446
chr16	2570400	0.743802
chr16	2633100	0.975207
chr16	2656600	0.760331
chr16	2667700	0.842975
chr16	2886000	0.909091
chr16	2892500	0.685950
chr16	2918100	0.776860
chr16	2966400	0.983471
chr16	3014600	0.925620
chr16	3058500	0.727273
chr16	3079800	0.702479
chr16	3136500	0.818182
chr16	3150600	0.867769
chr16	3152700	0.942149
chr16	3179700	0.694215
chr16	3506800	0.776860
chr16	3627400	0.884298
chr16	3696300	0.677686
chr16	3727200	0.975207
chr16	3849700	0.669421
chr16	3870400	0.867769
chr16	3996500	0.694215
chr16	4011000	0.760331
chr16	4061500	0.760331

chr16	4065900	0.685950
chr16	4103900	0.685950
chr16	4163000	0.743802
chr16	4289700	0.900826
chr16	4349500	0.933884
chr16	4357500	0.867769
chr16	4365400	0.933884
chr16	4368500	0.909091
chr16	4370600	0.909091
chr16	4378600	0.768595
chr16	4422300	0.925620
chr16	4434300	0.702479
chr16	4458700	0.801653
chr16	4461600	0.735537
chr16	4528800	0.950413
chr16	4539000	0.958678
chr16	4551300	0.983471
chr16	4566300	0.991736
chr16	4584400	0.801653
chr16	4666600	1.000000
chr16	4818800	0.760331
chr16	4900300	0.768595
chr16	4902700	0.983471
chr16	4910000	0.958678
chr16	4987100	0.793388
chr16	4995400	0.892562
chr16	8922700	0.892562
chr16	8944900	0.925620
chr16	8950900	0.735537
chr16	8954600	0.677686
chr16	8964000	0.661157
chr16	8980300	0.884298
chr16	9051700	0.892562
chr16	9054200	0.851240
chr16	9142100	0.925620
chr16	9172900	0.809917
chr16	9204100	0.983471
chr16	9209200	0.851240
chr16	9221500	0.884298
chr16	9472600	0.793388
chr16	9706700	0.702479
chr16	9891600	1.000000
chr16	9931400	0.735537
chr16	9940200	0.933884
chr16	10499300	0.966942
chr16	10653100	0.917355
chr16	10923100	0.859504
chr16	11032700	0.685950
chr16	11048600	0.867769
chr16	11052000	0.975207
chr16	11076700	0.900826
chr16	11191000	0.669421
chr16	11195500	0.859504
chr16	11215900	0.743802

chr16	11227800	0.876033
chr16	11234600	0.677686
chr16	11296100	0.876033
chr16	11422800	0.793388
chr16	11456400	0.735537
chr16	11517400	0.661157
chr16	11579200	0.950413
chr16	11589400	0.826446
chr16	11592000	0.917355
chr16	11655200	0.983471
chr16	11768900	0.710744
chr16	11875800	0.834711
chr16	11930700	0.900826
chr16	11991100	0.942149
chr16	12088600	0.851240
chr16	12183600	0.867769
chr16	12186500	0.892562
chr16	12435500	0.842975
chr16	12498400	0.917355
chr16	12502200	0.743802
chr16	12511600	0.760331
chr16	12556900	0.925620
chr16	12564700	0.809917
chr16	12738200	0.925620
chr16	12741300	0.966942
chr16	12769900	0.991736
chr16	12774000	0.727273
chr16	12823600	0.983471
chr16	12852200	1.000000
chr16	12894700	0.685950
chr16	12968700	0.743802
chr16	14051600	0.900826
chr16	14093300	0.677686
chr16	14109800	0.760331
chr16	14136900	0.983471
chr16	14144900	0.983471
chr16	14152100	0.933884
chr16	14203400	0.867769
chr16	14280300	0.735537
chr16	14290600	0.950413
chr16	14374800	0.876033
chr16	14493900	0.710744
chr16	14581300	0.743802
chr16	14586300	0.826446
chr16	14593000	0.991736
chr16	14595900	0.933884
chr16	14659300	0.975207
chr16	14725300	0.776860
chr16	14729000	0.727273
chr16	14780400	0.892562
chr16	14797600	0.851240
chr16	14836400	0.925620
chr16	14838700	0.801653
chr16	15108500	0.842975

chr16	15111600	0.900826
chr16	15119800	0.818182
chr16	15127800	0.809917
chr16	15130000	0.892562
chr16	15144000	0.892562
chr16	15155500	0.884298
chr16	15237400	0.842975
chr16	15240500	0.710744
chr16	15548100	0.661157
chr16	15567600	0.776860
chr16	15576700	0.760331
chr16	15583700	0.925620
chr16	15596400	0.983471
chr16	15675900	0.933884
%s	%d	%f
85000	0.975207	
chr16	15766900	0.884298
chr16	15796800	0.859504
chr16	15802000	0.884298
chr16	15973900	0.867769
chr16	16046400	0.752066
chr16	16055200	0.933884
chr16	16058300	0.685950
chr16	16077700	0.983471
chr16	16081000	0.966942
chr16	16084100	0.933884
chr16	16088200	0.958678
chr16	16092900	0.942149
chr16	16099000	0.925620
chr16	16116300	0.909091
chr16	16119700	0.710744
chr16	16122900	0.909091
chr16	16139600	0.752066
chr16	16194800	0.760331
chr16	16215200	0.892562
chr16	16403400	0.669421
chr16	17463900	0.991736
chr16	17474700	0.793388
chr16	17491000	0.983471
chr16	18530000	0.867769
chr16	18804500	0.842975
chr16	18808500	0.727273
chr16	18815700	1.000000
chr16	18873000	0.669421
chr16	18878200	1.000000
chr16	18881100	0.942149
chr16	19000700	0.917355
chr16	19010100	0.884298
chr16	19017400	0.677686
chr16	19074100	0.975207
chr16	19098200	0.851240
chr16	19103900	0.950413
chr16	19119600	0.983471
chr16	19124700	0.760331

chr16	19128200	0.768595
chr16	19131100	0.801653
chr16	19142500	0.818182
chr16	19202200	0.859504
chr16	19601600	0.892562
chr16	19607000	0.933884
chr16	19708100	0.892562
chr16	19712000	0.743802
chr16	19716400	0.859504
chr16	19722100	0.909091
chr16	19766200	0.975207
chr16	19777700	0.876033
chr16	19816400	0.909091
chr16	19823100	0.735537
chr16	20489100	0.719008
chr16	20756300	0.710744
chr16	20826300	0.735537
chr16	20862200	0.735537
chr16	20865300	1.000000
chr16	20877100	0.867769
chr16	20880200	0.942149
chr16	20890900	0.958678
chr16	20919000	0.834711
chr16	20922400	0.983471
chr16	20925100	0.851240
chr16	21088100	0.884298
chr16	21115700	0.735537
chr16	21200100	0.776860
chr16	21359600	0.876033
chr16	21439600	0.950413
chr16	21452300	0.776860
chr16	21462000	0.834711
chr16	21465600	0.958678
chr16	21468300	0.826446
chr16	21514600	0.661157
chr16	21523400	0.768595
chr16	21638300	0.950413
chr16	21650600	0.760331
chr16	21657500	0.785124
chr16	21781200	0.950413
chr16	21802300	0.966942
chr16	21872100	0.991736
chr16	21885100	0.801653
chr16	21897900	0.942149
chr16	21900400	0.925620
chr16	22013100	0.876033
chr16	22034900	0.719008
chr16	22037900	0.950413
chr16	22040900	0.743802
chr16	22057500	0.933884
chr16	22082500	0.983471
chr16	22086400	0.859504
chr16	22204700	0.851240
chr16	22207500	0.719008

chr16	22332100	0.942149
chr16	22380400	0.900826
chr16	22382700	0.793388
chr16	22399000	0.776860
chr16	22414000	0.958678
chr16	22493500	0.925620
chr16	22496200	0.867769
chr16	22509100	0.909091
chr16	22523000	0.958678
chr16	22554600	0.950413
chr16	22597100	0.933884
chr16	22618300	0.917355
chr16	22945200	0.818182
chr16	23083700	0.867769
chr16	23091000	0.950413
chr16	23093400	0.975207
chr16	23136200	1.000000
chr16	23143200	0.776860
chr16	23146400	0.942149
chr16	23194400	0.867769
chr16	23405500	0.900826
chr16	23435600	0.991736
chr16	23503700	0.925620
chr16	23509000	0.933884
chr16	23516500	0.702479
chr16	23619700	0.983471
chr16	23681400	0.785124
chr16	23692900	0.743802
chr16	24163100	0.983471
chr16	24556400	0.760331
chr16	24588700	0.876033
chr16	24621900	0.834711
chr16	24657800	0.884298
chr16	24680700	0.958678
chr16	24704600	0.710744
chr16	24713000	1.000000
chr16	24749100	0.950413
chr16	24760000	0.776860
chr16	24832100	0.785124
chr16	24850600	0.909091
chr16	24856300	0.669421
chr16	24859900	0.669421
chr16	24865400	0.710744
chr16	24991300	0.991736
chr16	24995200	0.933884
chr16	24997300	0.694215
chr16	24999500	0.966942
chr16	25002700	0.950413
chr16	25006100	0.834711
chr16	25018700	0.834711
chr16	25021500	0.776860
chr16	25024300	0.768595
chr16	25060200	0.793388
chr16	25062300	0.785124

chr16	25121400	0.702479
chr16	27189500	0.826446
chr16	27209000	0.735537
chr16	27246800	0.933884
chr16	27251400	1.000000
chr16	27253500	0.842975
chr16	27255800	0.925620
chr16	27265400	0.917355
chr16	27269600	0.900826
chr16	27298000	0.909091
chr16	27328600	0.983471
chr16	27338700	0.983471
chr16	27341200	0.785124
chr16	27344500	0.950413
chr16	27346700	0.876033
chr16	27376800	0.785124
chr16	27379400	0.900826
chr16	27461700	0.942149
chr16	27473400	0.933884
chr16	27482200	0.834711
chr16	27487600	0.768595
chr16	27503700	0.702479
chr16	27532800	0.867769
chr16	27688700	0.884298
chr16	27715900	1.000000
chr16	27771100	0.859504
chr16	28153900	0.834711
chr16	28157300	0.983471
chr16	28183800	0.776860
chr16	28193000	1.000000
chr16	28205400	0.884298
chr16	28220300	0.983471
chr16	28393500	0.950413
chr16	28396400	0.842975
chr16	28433100	0.958678
chr16	28437800	0.735537
chr16	28647200	0.785124
chr16	28705000	0.760331
chr16	28741900	0.785124
chr16	28744600	0.942149
chr16	28957400	0.851240
chr16	29127400	0.884298
chr16	29164400	0.834711
chr16	29167900	0.677686
chr16	29185500	0.826446
chr16	29190400	0.991736
chr16	29211900	0.760331
chr16	29232600	0.950413
chr16	29274600	0.785124
chr16	29277400	0.917355
chr16	29281500	0.859504
chr16	29284100	0.768595
chr16	29302600	0.942149
chr16	29330200	0.917355

chr16	29338300	0.933884
chr16	29340400	0.760331
chr16	29417900	0.710744
chr16	29431500	0.925620
chr16	29440500	0.727273
chr16	29445700	0.933884
chr16	29448000	0.917355
chr16	29519400	0.950413
chr16	29533000	0.842975
chr16	29542300	0.752066
chr16	29547500	0.950413
chr16	29549700	0.727273
chr16	29616800	0.785124
chr16	29821600	0.826446
chr16	29834000	0.710744
chr16	29927400	0.892562
chr16	29990100	0.851240
chr16	30022600	0.760331
chr16	30063000	1.000000
chr16	30066500	0.710744
chr16	30075200	0.735537
chr16	30079600	0.760331
chr16	30086500	0.752066
chr16	30089800	0.851240
chr16	30107400	0.669421
chr16	30132600	0.719008
chr16	30210900	0.785124
chr16	30220800	0.719008
chr16	30259600	0.867769
chr16	30272900	0.776860
chr16	30282300	0.669421
chr16	30287500	0.793388
chr16	30364200	0.719008
chr16	30368300	0.743802
chr16	30383000	0.719008
chr16	30411800	0.859504
chr16	30421700	0.694215
chr16	30437400	0.884298
chr16	30503500	0.752066
chr16	30553400	0.958678
chr16	30566500	0.785124
chr16	30573100	0.743802
chr16	30661300	0.809917
chr16	30668700	0.900826
chr16	30673300	0.685950
chr16	30725100	0.917355
chr16	30752900	0.867769
chr16	30759300	0.760331
chr16	30825900	0.867769
chr16	30908200	0.942149
chr16	30913800	0.892562
chr16	30916200	0.842975
chr16	30970600	0.685950
chr16	31076500	0.818182

chr16	31150500	0.884298
chr16	31153700	0.859504
chr16	31227300	0.785124
chr16	31366800	0.685950
chr16	31464500	0.966942
chr16	31482500	0.785124
chr16	31492400	0.727273
chr16	31881000	0.983471
chr16	31888600	0.991736
chr16	31892100	0.925620
chr16	32931300	0.669421
chr16	33736900	0.884298
chr16	33871900	0.669421
chr16	33894200	0.743802
chr16	33952900	0.809917
chr16	33955400	0.851240
chr16	33961000	0.727273
chr16	33975200	0.909091
chr16	34022500	0.966942
chr16	35239800	0.809917
chr16	46430900	0.685950
chr16	46435200	0.826446
chr16	46439000	0.785124
chr16	46657500	0.859504
chr16	46726100	0.735537
chr16	46844300	0.702479
chr16	46860800	0.818182
chr16	46894500	0.776860
chr16	47042700	0.685950
chr16	47170800	0.950413
chr16	47409200	0.760331
chr16	47558100	0.933884
chr16	47730200	0.900826
chr16	47747400	0.677686
chr16	47766900	0.719008
chr16	47814000	0.826446
chr16	48310500	0.851240
chr16	48352500	0.975207
chr16	48400200	0.669421
chr16	48479200	0.917355
chr16	48632200	0.925620
chr16	48657300	0.710744
chr16	49486500	0.752066
chr16	49644400	0.677686
chr16	49654600	0.859504
chr16	49674000	0.768595
chr16	49677800	0.942149
chr16	49698100	0.867769
chr16	49789200	0.884298
chr16	49810500	0.834711
chr16	49859200	0.966942
chr16	49861700	0.752066
chr16	49885500	0.694215
chr16	50102300	0.661157

chr16	50201000	0.801653
chr16	50229500	0.867769
chr16	50237300	0.991736
chr16	50242200	0.942149
chr16	50301200	0.966942
chr16	50347900	0.859504
chr16	50582300	0.702479
chr16	50645800	0.892562
chr16	51573300	0.991736
chr16	51603900	0.867769
chr16	51612000	0.776860
chr16	52148600	0.785124
chr16	52176400	0.884298
chr16	52288900	0.876033
chr16	53086800	0.991736
chr16	53090600	0.710744
chr16	53105700	0.892562
chr16	53114700	0.991736
chr16	53124000	0.842975
chr16	53128700	0.834711
chr16	53132100	0.743802
chr16	53134600	0.776860
chr16	53252800	0.776860
chr16	53255400	0.842975
chr16	53516500	0.743802
chr16	53538900	0.876033
chr16	53544200	0.892562
chr16	53549500	0.876033
chr16	53551600	0.760331
chr16	53553800	0.942149
chr16	53660700	0.958678
chr16	53857100	0.859504
chr16	53899600	0.983471
chr16	53927900	0.752066
chr16	54111600	0.975207
chr16	54170300	0.942149
chr16	54971700	0.892562
chr16	55505600	0.991736
chr16	55521200	0.752066
chr16	55523300	0.933884
chr16	55529100	0.818182
chr16	55531900	0.702479
chr16	55534000	0.933884
chr16	55537800	0.834711
chr16	55545900	0.727273
chr16	55558900	0.719008
chr16	55563500	0.966942
chr16	55576500	0.958678
chr16	55586900	0.669421
chr16	55592700	0.818182
chr16	55598200	0.975207
chr16	55602700	0.801653
chr16	55648100	0.983471
chr16	55735500	0.685950

chr16	56391000	0.793388
chr16	56541300	0.983471
chr16	56559600	0.909091
chr16	56649300	0.851240
chr16	56652200	0.685950
chr16	56677700	0.884298
chr16	56697100	0.719008
chr16	56710000	0.826446
chr16	56768700	0.826446
chr16	56776300	0.752066
chr16	56841500	0.991736
chr16	56846700	0.892562
chr16	56873700	0.776860
chr16	56949900	0.677686
chr16	56969800	0.743802
chr16	56995800	0.702479
chr16	57035100	0.818182
chr16	57048800	0.727273
chr16	57174400	0.958678
chr16	57179000	0.900826
chr16	57213000	0.752066
chr16	57225600	0.909091
chr16	57228900	0.991736
chr16	57294900	0.743802
chr16	57300400	0.958678
chr16	57314200	0.818182
chr16	57349400	0.669421
chr16	57351600	0.735537
chr16	57369500	0.719008
chr16	57415000	0.669421
chr16	57451600	0.752066
chr16	57504700	0.702479
chr16	57548900	0.661157
chr16	57564000	0.661157
chr16	57567100	0.826446
chr16	57625300	0.892562
chr16	57661600	0.933884
chr16	57665000	0.876033
chr16	57668400	0.826446
chr16	57671800	0.818182
chr16	57678000	0.876033
chr16	57680600	0.900826
chr16	57688400	0.975207
chr16	57712000	0.818182
chr16	57771600	0.826446
chr16	57798600	0.801653
chr16	57844600	0.917355
chr16	57850600	0.727273
chr16	57928200	0.933884
chr16	58115700	0.867769
chr16	58155500	0.942149
chr16	58221800	0.768595
chr16	58351800	0.917355
chr16	58412900	0.917355

chr16	58443600	0.876033
chr16	58485900	0.826446
chr16	58490400	1.000000
chr16	58493600	0.710744
chr16	58509000	0.768595
chr16	58534600	0.925620
chr16	58769700	0.950413
chr16	59080100	0.719008
chr16	59083200	0.933884
chr16	59101500	0.991736
chr16	59214200	0.719008
chr16	59232900	0.950413
chr16	59245500	0.958678
chr16	59498100	0.669421
chr16	59513200	0.909091
chr16	59579500	0.661157
chr16	59666600	0.942149
chr16	59669900	0.818182
chr16	60005500	0.685950
chr16	60112300	0.818182
chr16	60126000	0.966942
chr16	60216100	0.809917
chr16	63501900	0.966942
chr16	63647900	0.933884
chr16	64196900	0.983471
chr16	64425700	0.966942
chr16	64432300	0.966942
chr16	64450700	0.826446
chr16	64512600	0.892562
chr16	64997200	0.991736
chr16	65058900	0.958678
chr16	65104600	0.719008
chr16	65152900	0.785124
chr16	65693000	0.975207
chr16	66398300	0.752066
chr16	66406700	0.983471
chr16	66414100	0.950413
chr16	66417200	0.975207
chr16	66421400	0.776860
chr16	66440800	0.826446
chr16	66443100	0.867769
chr16	66547600	0.884298
chr16	66550000	0.669421
chr16	66553300	0.983471
chr16	66558400	0.958678
chr16	66598800	0.917355
chr16	66603300	0.983471
chr16	66612900	0.661157
chr16	66620000	0.809917
chr16	66642600	0.884298
chr16	66685400	0.661157
chr16	66695600	0.983471
chr16	66776600	0.776860
chr16	66878900	0.809917

chr16	66907500	0.818182
chr16	66934400	0.727273
chr16	66959300	0.818182
chr16	67062300	0.743802
chr16	67066200	0.669421
chr16	67198800	0.735537
chr16	67204700	0.785124
chr16	67212500	0.892562
chr16	67215700	0.834711
chr16	67219600	0.727273
chr16	67224400	0.975207
chr16	67228600	0.834711
chr16	67247200	0.793388
chr16	67251600	0.793388
chr16	67275200	0.776860
chr16	67292500	0.851240
chr16	67303300	1.000000
chr16	67311100	0.892562
chr16	67346100	0.842975
chr16	67363900	0.785124
chr16	67380800	0.991736
chr16	67399400	0.702479
chr16	67427800	0.834711
chr16	67441700	0.958678
chr16	67465600	0.702479
chr16	67516900	0.727273
chr16	67520900	0.760331
chr16	67550700	0.933884
chr16	67555600	0.884298
chr16	67570900	0.669421
chr16	67582900	0.892562
chr16	67590100	0.958678
chr16	67595400	0.669421
chr16	67605900	0.958678
chr16	67708000	0.975207
chr16	67711100	0.694215
chr16	67804000	0.710744
chr16	67806100	0.892562
chr16	67808200	0.842975
chr16	67878900	0.818182
chr16	67892100	0.826446
chr16	67931000	0.785124
chr16	67937100	0.925620
chr16	67960400	0.925620
chr16	68000000	0.933884
chr16	68018500	0.975207
chr16	68034500	0.669421
chr16	68099100	0.950413
chr16	68113800	0.933884
chr16	68118300	0.760331
chr16	68271800	0.942149
chr16	68292200	0.867769
chr16	68301900	0.677686
chr16	68319000	0.743802

chr16	68323100	0.950413
chr16	68325500	0.834711
chr16	68359100	0.735537
chr16	68366900	0.950413
chr16	68964200	0.942149
chr16	68996100	0.950413
chr16	69007300	0.892562
chr16	69026300	0.975207
chr16	69028500	0.859504
chr16	69051300	0.933884
chr16	69054200	0.801653
chr16	69074000	0.909091
chr16	69151500	0.702479
chr16	69200200	0.743802
chr16	69203400	0.876033
chr16	69207900	0.942149
chr16	69224400	0.669421
chr16	69251600	0.991736
chr16	69253900	0.685950
chr16	69256300	0.933884
chr16	69260200	1.000000
chr16	69335500	0.966942
chr16	69402400	0.892562
chr16	69413000	0.991736
chr16	69415900	0.867769
chr16	69430600	0.933884
chr16	69432900	0.983471
chr16	69438600	0.892562
chr16	69444400	0.975207
chr16	69451800	0.876033
chr16	69477900	0.809917
chr16	69511500	0.975207
chr16	69516900	0.966942
chr16	69565000	0.867769
chr16	69569700	0.793388
chr16	69598100	0.842975
chr16	69603000	0.685950
chr16	69654100	0.892562
chr16	69697500	0.851240
chr16	69762100	0.809917
chr16	69771700	0.867769
chr16	69882900	0.942149
chr16	69889800	0.867769
chr16	69952200	0.966942
chr16	69959100	0.842975
chr16	70032800	0.752066
chr16	70049500	0.884298
chr16	70057600	0.801653
chr16	70060800	0.884298
chr16	70111600	0.942149
chr16	70230900	0.818182
chr16	70248000	0.834711
chr16	70263700	0.884298
chr16	70266500	0.842975

chr16	70288800	0.685950
chr16	70438600	0.809917
chr16	70457400	0.859504
chr16	70461500	0.942149
chr16	70465100	0.942149
chr16	70468300	0.942149
chr16	70470800	0.694215
chr16	70510500	0.801653
chr16	70513300	0.669421
chr16	70542900	0.867769
chr16	70555300	0.793388
chr16	70561100	0.859504
chr16	70563300	0.851240
chr16	70608100	1.000000
chr16	70611400	0.677686
chr16	70622800	0.933884
chr16	70669800	0.925620
chr16	70688100	0.793388
chr16	70722500	0.752066
chr16	70728200	0.752066
chr16	70730400	0.966942
chr16	70741100	0.892562
chr16	70744100	0.710744
chr16	70780500	0.694215
chr16	70785400	0.776860
chr16	70839600	0.892562
chr16	70857800	0.925620
chr16	71317300	0.950413
chr16	71392400	0.710744
chr16	71660600	0.785124
chr16	71796700	0.842975
chr16	71915800	0.694215
chr16	71934800	0.685950
chr16	71949400	0.859504
chr16	71961300	0.834711
chr16	71963600	0.909091
chr16	72237700	0.966942
chr16	72298200	0.735537
chr16	72324800	0.958678
chr16	72396800	0.900826
chr16	72405700	0.867769
chr16	72516000	0.950413
chr16	72564700	0.917355
chr16	72571900	0.710744
chr16	72821600	0.818182
chr16	72882700	0.909091
chr16	72911700	0.983471
chr16	72928900	0.925620
chr16	72973000	0.983471
chr16	73024200	0.752066
chr16	73054200	0.834711
chr16	73086500	0.867769
chr16	73089500	0.909091
chr16	73100300	0.950413

chr16	73102900	0.867769
chr16	73105100	0.958678
chr16	73127800	0.983471
chr16	73188400	0.859504
chr16	73197800	0.776860
chr16	73204700	0.892562
chr16	73207000	0.942149
chr16	74432700	0.719008
chr16	74473000	0.719008
chr16	74483100	0.933884
chr16	74486400	0.867769
chr16	74489600	0.867769
chr16	74588200	0.818182
chr16	74629100	0.958678
chr16	74635700	0.719008
chr16	74638100	0.719008
chr16	74730200	0.719008
chr16	74808900	0.818182
chr16	74902400	0.942149
chr16	75045900	0.958678
chr16	75059900	0.966942
chr16	75063100	0.785124
chr16	75109600	0.842975
chr16	75121600	0.991736
chr16	75268600	0.900826
chr16	75273300	0.991736
chr16	75276700	0.851240
chr16	75279700	0.710744
chr16	75282200	0.710744
chr16	75289900	0.966942
chr16	75292300	0.685950
chr16	75307200	0.776860
chr16	75323600	0.900826
chr16	75330900	0.900826
chr16	75338500	0.867769
chr16	75351600	0.752066
chr16	75354400	0.950413
chr16	75374000	0.842975
chr16	75377200	0.743802
chr16	75385400	0.958678
chr16	75412100	0.677686
chr16	75415200	0.975207
chr16	75436500	0.917355
chr16	75446700	0.876033
chr16	75628900	0.909091
chr16	75662400	0.851240
chr16	75685500	0.661157
chr16	75695100	0.776860
chr16	77206700	0.834711
chr16	77248200	0.917355
chr16	77322700	0.975207
chr16	77332000	0.991736
chr16	77335500	0.809917
chr16	77342300	0.991736

chr16	77436800	0.743802
chr16	77491300	0.909091
chr16	77533900	0.925620
chr16	77599100	0.950413
chr16	77603300	0.991736
chr16	77609200	0.966942
chr16	77613700	0.876033
chr16	77620600	0.966942
chr16	77625300	0.942149
chr16	77627500	0.851240
chr16	77630600	1.000000
chr16	77652300	0.859504
chr16	77694400	0.909091
chr16	77701700	0.818182
chr16	77814700	0.809917
chr16	77868100	0.975207
chr16	77911600	0.669421
chr16	78177100	0.942149
chr16	78182200	0.966942
chr16	78352200	0.702479
chr16	80061600	0.917355
chr16	80232300	0.958678
chr16	80295100	0.983471
chr16	80333600	0.768595
chr16	80633800	0.851240
chr16	80649200	0.793388
chr16	80657800	0.719008
chr16	80691300	0.743802
chr16	80752800	0.818182
chr16	80785600	0.809917
chr16	80793800	0.933884
chr16	80803800	0.685950
chr16	80813100	0.818182
chr16	80826000	0.975207
chr16	80841500	0.933884
chr16	80970100	0.859504
chr16	80973600	0.876033
chr16	80982700	0.851240
chr16	81027500	0.917355
chr16	81031300	0.842975
chr16	81215400	0.752066
chr16	81314300	0.983471
chr16	81402000	0.826446
chr16	81417200	0.925620
chr16	81420000	0.727273
chr16	81429400	0.661157
chr16	81435100	0.958678
chr16	81438000	0.909091
chr16	81440700	0.719008
chr16	81491600	0.851240
chr16	81507400	1.000000
chr16	81513000	0.884298
chr16	81527600	0.818182
chr16	81530200	0.909091

chr16	81534400	0.975207
chr16	81536700	0.876033
chr16	81544400	0.743802
chr16	81546500	0.933884
chr16	81549100	0.834711
chr16	81553600	0.925620
chr16	81560300	0.991736
chr16	81576600	0.669421
chr16	81587900	0.983471
chr16	81602100	0.661157
chr16	81638900	0.834711
chr16	81644000	0.925620
chr16	81647300	0.933884
chr16	81650600	0.801653
chr16	81658000	0.818182
chr16	81661000	0.809917
chr16	81666500	0.842975
chr16	81668600	0.859504
chr16	81712200	0.950413
chr16	81719200	0.859504
chr16	81722300	0.842975
chr16	81725900	0.958678
chr16	81729400	0.809917
chr16	81731900	0.983471
chr16	81737200	0.677686
chr16	81741700	0.818182
chr16	81745500	0.933884
chr16	81752100	0.933884
chr16	81764200	0.867769
chr16	81780200	0.851240
chr16	81895800	0.801653
chr16	81900900	0.884298
chr16	81931000	0.685950
chr16	81936300	0.884298
chr16	82028800	0.702479
chr16	82031800	0.760331
chr16	82071700	0.669421
chr16	82226700	0.942149
chr16	82230100	0.991736
chr16	82664500	0.909091
chr16	82668700	0.801653
chr16	82672400	0.958678
chr16	82675000	0.785124
chr16	82691500	0.801653
chr16	82732700	0.942149
chr16	82735800	0.760331
chr16	82781700	0.983471
chr16	82784600	0.685950
chr16	82789400	0.966942
chr16	82795500	0.892562
chr16	82847900	0.983471
chr16	82971000	0.966942
chr16	82979600	0.991736
chr16	83013700	1.000000

chr16	83029700	0.892562
chr16	83035800	0.694215
chr16	83057300	0.727273
chr16	83080700	0.818182
chr16	83103900	0.975207
chr16	83209100	0.694215
chr16	83339000	0.933884
chr16	83341200	0.735537
chr16	83370600	0.925620
chr16	83373700	0.975207
chr16	83378000	0.983471
chr16	83437300	0.793388
chr16	83637400	0.760331
chr16	83813500	0.694215
chr16	83836500	0.834711
chr16	83869700	0.983471
chr16	83988900	0.925620
chr16	84097100	0.768595
chr16	84105500	0.991736
chr16	84129000	0.735537
chr16	84211100	0.958678
chr16	84545200	0.892562
chr16	84561200	0.876033
chr16	84565100	0.809917
chr16	84567400	0.975207
chr16	84575900	0.950413
chr16	84583300	0.834711
chr16	84631500	0.818182
chr16	84634100	0.966942
chr16	84637500	0.752066
chr16	84653000	0.950413
chr16	84694900	0.685950
chr16	84722000	0.785124
chr16	84726200	0.909091
chr16	84757200	0.727273
chr16	84760400	0.909091
chr16	84763300	0.966942
chr16	84767100	0.966942
chr16	84792800	0.933884
chr16	84808900	0.892562
chr16	84840300	0.958678
chr16	84853900	0.801653
chr16	84919200	0.900826
chr16	85011200	0.710744
chr16	85029000	0.876033
chr16	85033200	0.925620
chr16	85089300	0.958678
chr16	85157800	0.842975
chr16	85160700	0.661157
chr16	85164600	0.661157
chr16	85171100	0.876033
chr16	85178400	0.942149
chr16	85185600	0.826446
chr16	85209200	0.818182

chr16	85254200	0.876033
chr16	85325400	0.776860
chr16	85340100	0.719008
chr16	85342700	0.768595
chr16	85368600	0.710744
chr16	85384700	0.702479
chr16	85429800	0.768595
chr16	85597600	0.752066
chr16	85608500	0.719008
chr16	85647300	0.809917
chr16	85750100	0.735537
chr16	85801700	0.892562
chr16	85812600	1.000000
chr16	85824100	0.735537
chr16	85922600	0.900826
chr16	86374900	0.958678
chr16	86378600	0.859504
chr16	86421000	0.842975
chr16	86424200	0.975207
chr16	86426700	0.867769
chr16	86430200	0.933884
chr16	86432400	0.867769
chr16	86439200	0.950413
chr16	86449300	0.917355
chr16	86511400	0.702479
chr16	86554100	0.694215
chr16	86597700	0.859504
chr16	86607500	0.834711
chr16	86612100	0.950413
chr16	86619400	0.975207
chr16	86624200	0.859504
chr16	86627300	0.694215
chr16	86650900	0.710744
chr16	86696000	1.000000
chr16	86699500	0.975207
chr16	86702400	0.685950
chr16	86706300	0.760331
chr16	86710400	0.851240
chr16	86713900	0.900826
chr16	86716300	0.834711
chr16	86768300	0.834711
chr16	86775200	0.834711
chr16	86785100	0.925620
chr16	86791100	0.752066
chr16	86822300	0.661157
chr16	86831300	0.859504
chr16	86852800	0.892562
chr16	86857600	0.710744
chr16	86861400	0.950413
chr16	86870600	0.975207
chr16	86885700	0.793388
chr16	86933800	0.892562
chr16	86935900	0.851240
chr16	86949000	0.884298

chr16	86959000	0.867769
chr16	86965600	0.900826
chr16	86968800	0.900826
chr16	86980100	1.000000
chr16	86986100	0.826446
chr16	87051000	0.842975
chr16	87077500	0.826446
chr16	87087100	0.983471
chr16	87094800	0.925620
chr16	87100300	0.768595
chr16	87110800	0.876033
chr16	87152000	0.851240
chr16	87207700	0.669421
chr16	87212600	0.983471
chr16	87220300	0.818182
chr16	87379000	0.867769
chr16	87398000	0.834711
chr16	87409900	0.950413
chr16	87422500	0.983471
chr16	87425000	0.768595
chr16	87441400	0.975207
chr16	87470000	0.851240
chr16	87491500	0.975207
chr16	87495700	0.884298
chr16	87498500	0.876033
chr16	87500600	0.785124
chr16	87505700	0.719008
chr16	87519100	0.933884
chr16	87542100	0.975207
chr16	87552400	0.743802
chr16	87572400	0.719008
chr16	87593800	0.793388
chr16	87616200	0.793388
chr16	87620200	0.685950
chr16	87736500	0.983471
chr16	87757100	0.760331
chr16	87812200	0.826446
chr16	87814500	0.892562
chr16	87840500	0.950413
chr16	87874300	0.942149
chr16	87887700	0.925620
chr16	87894600	0.793388
chr16	87900500	0.752066
chr16	87979200	0.818182
chr16	87988000	0.867769
chr16	87990700	0.892562
chr16	87997400	0.983471
chr16	88000800	0.925620
chr16	88058800	0.942149
chr16	88152800	0.975207
chr16	88178800	0.867769
chr16	88220700	0.834711
chr16	88270500	1.000000
chr16	88272900	0.958678

chr16	88275400	0.834711
chr16	88277500	0.958678
chr16	88280000	0.892562
chr16	88298900	0.909091
chr16	88333000	0.710744
chr16	88350400	0.933884
chr16	88365400	0.661157
chr16	88367500	0.801653
chr16	88497100	0.933884
chr16	88519200	0.834711
chr16	88528900	0.694215
chr16	88531700	0.900826
chr16	88540400	0.826446
chr16	88566300	0.735537
chr16	88570400	0.867769
chr16	88593300	0.909091
chr16	88731900	0.785124
chr16	88830500	0.669421
chr16	88834900	0.785124
chr16	88837900	0.768595
chr16	88841000	0.950413
chr16	88988200	0.876033
chr16	88992000	0.768595
chr16	89043800	0.685950
chr16	89323900	0.876033
chr16	89364600	0.917355
chr16	89368400	0.917355
chr16	89381000	0.851240
chr16	89388100	0.966942
chr16	89390400	0.900826
chr16	89393200	0.884298
chr16	89396600	0.966942
chr16	89400100	0.851240
chr16	89402700	0.933884
chr16	89426200	0.991736
chr16	89431100	0.942149
chr16	89440300	0.867769
chr16	89450000	0.842975
chr16	89462300	0.809917
chr16	89476500	0.991736
chr16	89480400	0.694215
chr16	89488200	0.925620
chr16	89492800	0.834711
chr16	89511700	0.685950
chr16	89517000	0.983471
chr16	89520900	0.851240
chr16	89569000	0.768595
chr16	89572200	0.735537
chr16	89590300	0.950413
chr16	89598600	0.884298
chr16	89630300	0.809917
chr16	89632500	0.785124
chr16	89641600	0.685950
chr16	89647800	0.876033

chr16	89669200	0.950413
chr16	89690100	0.933884
chr16	89735300	0.760331
chr16	89766600	0.776860
chr16	89785300	0.942149
chr16	89791000	0.793388
chr16	89930800	0.801653
chr16	89984800	0.685950
chr16	89992900	0.694215
chr16	90082800	0.876033
chr16	90114000	0.809917
chr17	50300	0.958678
chr17	79100	0.826446
chr17	84100	0.983471
chr17	97900	0.768595
chr17	117400	0.859504
chr17	130000	0.942149
chr17	184100	0.867769
chr17	199300	0.727273
chr17	202800	0.702479
chr17	235500	0.818182
chr17	398200	0.685950
chr17	422200	0.983471
chr17	438000	0.735537
chr17	453100	0.991736
chr17	460900	1.000000
chr17	464200	0.983471
chr17	466700	0.942149
chr17	500300	0.917355
chr17	505000	0.892562
chr17	537200	0.909091
chr17	597800	0.966942
chr17	603000	0.826446
chr17	698500	0.867769
chr17	762800	0.917355
chr17	783900	0.983471
chr17	800900	0.710744
chr17	807800	0.768595
chr17	814600	0.950413
chr17	819300	1.000000
chr17	821500	0.933884
chr17	831400	0.983471
chr17	837100	0.983471
chr17	858300	0.884298
chr17	906500	0.743802
chr17	909600	0.842975
chr17	943400	0.727273
chr17	947200	0.983471
chr17	964700	0.859504
chr17	984200	0.809917
chr17	987300	0.694215
chr17	991300	0.776860
chr17	1015400	0.710744
chr17	1018800	0.859504

chr17	1026600	0.991736
chr17	1042900	0.702479
chr17	1068800	0.818182
chr17	1107300	0.727273
chr17	1133000	0.669421
chr17	1252000	0.818182
chr17	1263800	0.719008
chr17	1270700	0.933884
chr17	1281900	0.661157
chr17	1294500	0.677686
chr17	1317200	0.768595
chr17	1325700	0.958678
chr17	1328400	0.719008
chr17	1350400	0.867769
chr17	1360700	0.661157
chr17	1388500	0.735537
chr17	1391000	0.917355
chr17	1397500	0.694215
chr17	1415400	0.867769
chr17	1461400	0.917355
chr17	1478200	0.892562
chr17	1481200	0.942149
chr17	1493700	0.809917
chr17	1516200	0.900826
chr17	1521200	0.727273
chr17	1628300	0.809917
chr17	1656400	0.669421
chr17	1667900	0.669421
chr17	1683500	0.834711
chr17	1691100	0.859504
chr17	1811900	0.900826
chr17	1924500	0.727273
chr17	1929300	0.809917
chr17	1966000	0.892562
chr17	1969300	0.983471
chr17	1973000	0.991736
chr17	1976100	0.900826
chr17	1987200	0.710744
chr17	1990300	0.950413
chr17	1992400	0.884298
chr17	1994500	0.983471
chr17	2010400	1.000000
chr17	2026900	0.942149
chr17	2042200	0.727273
chr17	2075600	0.867769
chr17	2078400	0.983471
chr17	2083300	0.958678
chr17	2085800	0.793388
chr17	2096200	0.958678
chr17	2108300	0.900826
chr17	2116500	0.892562
chr17	2119400	0.958678
chr17	2135600	0.834711
chr17	2141500	0.859504

chr17	2144000	0.909091
chr17	2148100	0.876033
chr17	2263400	0.983471
chr17	2298300	0.892562
chr17	2301300	0.876033
chr17	2308700	0.818182
chr17	2310800	0.859504
chr17	2394800	0.876033
chr17	2496300	0.661157
chr17	2715800	0.892562
chr17	2757800	0.685950
chr17	2858900	0.925620
chr17	2863900	0.826446
chr17	2907800	0.975207
chr17	2932800	0.760331
chr17	2935200	0.851240
chr17	3507100	0.851240
chr17	3544000	0.752066
chr17	3563300	0.809917
chr17	3599500	0.776860
chr17	3623700	0.925620
chr17	3699300	0.669421
chr17	3722500	0.760331
chr17	3774600	0.719008
chr17	3788900	0.752066
chr17	3791400	0.933884
chr17	3793900	0.909091
chr17	3798300	0.842975
chr17	3820600	0.735537
chr17	3848400	0.834711
chr17	3868400	0.727273
chr17	3880500	0.900826
chr17	3887600	0.884298
chr17	3906000	0.727273
chr17	4002400	0.851240
chr17	4005400	0.933884
chr17	4034400	0.958678
chr17	4065200	0.793388
chr17	4092800	0.950413
chr17	4134000	0.933884
chr17	4217200	0.685950
chr17	4333400	0.909091
chr17	4337200	0.719008
chr17	4387300	0.776860
chr17	4405200	0.727273
chr17	4408500	0.669421
chr17	4413500	0.661157
chr17	4423100	0.851240
chr17	4426100	0.776860
chr17	4435300	0.859504
chr17	4438900	0.735537
chr17	4441200	0.925620
chr17	4444100	0.735537
chr17	4487900	0.669421

chr17	4544100	0.661157
chr17	4579300	0.677686
chr17	4582900	0.925620
chr17	4625200	0.694215
chr17	4640100	0.677686
chr17	4689700	0.793388
chr17	4805200	0.975207
chr17	4812400	0.776860
chr17	4848400	0.818182
chr17	4853700	0.909091
chr17	4857000	0.925620
chr17	4869500	0.727273
chr17	4873000	0.785124
chr17	4915300	0.727273
chr17	4935200	0.685950
chr17	5187500	0.685950
chr17	5420100	0.760331
chr17	5437700	0.719008
chr17	5489100	0.909091
chr17	5534100	0.958678
chr17	5663400	0.834711
chr17	5684100	0.834711
chr17	5689100	0.876033
chr17	5730400	0.909091
chr17	5733000	0.933884
chr17	5736500	0.991736
chr17	5783400	0.834711
chr17	5801200	0.719008
chr17	5932000	0.685950
chr17	5994000	0.719008
chr17	6002000	0.867769
chr17	6006100	0.925620
chr17	6014500	0.809917
chr17	6082800	0.958678
chr17	6091400	0.760331
chr17	6094800	0.809917
chr17	6135700	0.917355
chr17	6143100	0.909091
chr17	6157700	0.859504
chr17	6161300	0.867769
chr17	6230900	0.925620
chr17	6346800	0.685950
chr17	6355300	0.743802
chr17	6360600	0.752066
chr17	6409900	0.966942
chr17	6414100	0.876033
chr17	6416300	1.000000
chr17	6478800	0.925620
chr17	6559400	0.752066
chr17	6655300	0.834711
chr17	6659100	0.958678
chr17	6690700	0.991736
chr17	6817600	0.661157
chr17	6923000	0.801653

chr17	6934300	0.933884
chr17	6941700	0.685950
chr17	6946900	0.793388
chr17	6961800	0.677686
chr17	6964200	0.933884
chr17	7036800	0.975207
chr17	7078100	0.785124
chr17	7108600	0.933884
chr17	7117900	0.842975
chr17	7140900	0.727273
chr17	7167900	0.859504
chr17	7348800	0.694215
chr17	7379800	0.826446
chr17	7384000	0.834711
chr17	7390700	0.677686
chr17	7401000	0.785124
chr17	7461700	0.785124
chr17	7479600	0.818182
chr17	7483100	0.892562
chr17	7530800	0.685950
chr17	7608800	0.685950
chr17	7649500	0.669421
chr17	7741600	0.909091
chr17	7744300	0.900826
chr17	7792800	0.743802
chr17	7819500	0.702479
chr17	7833400	0.710744
chr17	7838000	0.702479
chr17	7959700	0.958678
chr17	7966500	0.818182
chr17	7983400	0.752066
chr17	8042500	0.785124
chr17	8057400	0.760331
chr17	8060700	0.892562
chr17	8091300	0.834711
chr17	8095900	0.942149
chr17	8130300	0.694215
chr17	8184700	0.826446
chr17	8216700	0.710744
chr17	8262600	0.826446
chr17	8265200	0.760331
chr17	8270700	0.884298
chr17	8281200	0.859504
chr17	8288800	0.842975
chr17	8349200	1.000000
chr17	8578000	1.000000
chr17	8584700	0.892562
chr17	8822600	0.694215
chr17	8826300	0.867769
chr17	9143500	0.826446
chr17	9162800	0.826446
chr17	9274500	0.851240
chr17	9290200	0.884298
chr17	9373800	0.776860

chr17	9377100	0.727273
chr17	9416200	0.933884
chr17	9548700	0.809917
chr17	11944900	0.743802
chr17	11993600	0.925620
chr17	12011700	0.826446
chr17	12185900	0.694215
chr17	13965600	0.966942
chr17	13995500	0.685950
chr17	14096700	0.727273
chr17	14213100	0.661157
chr17	15161000	0.876033
chr17	15302400	0.933884
chr17	15310100	0.702479
chr17	15340100	0.661157
chr17	15345200	0.867769
chr17	15348500	0.983471
chr17	15352000	0.842975
chr17	15361200	0.768595
chr17	15373400	0.776860
chr17	15383200	0.958678
chr17	15394500	0.983471
chr17	15405600	1.000000
chr17	15414100	0.867769
chr17	15420000	0.950413
chr17	15425700	0.809917
chr17	15493500	0.942149
chr17	15546200	0.991736
chr17	15549200	0.925620
chr17	15553100	0.809917
chr17	15563600	0.909091
chr17	15818900	0.768595
chr17	15846400	0.859504
chr17	15850800	0.743802
chr17	15858100	0.975207
chr17	15860600	0.677686
chr17	15862900	0.950413
chr17	15867200	0.876033
chr17	15870500	0.752066
chr17	15885400	0.867769
chr17	15888100	0.834711
chr17	15917700	0.925620
chr17	15931600	0.966942
chr17	15989100	0.826446
chr17	16054200	0.958678
chr17	16095100	0.950413
chr17	16102900	0.685950
chr17	16191400	0.776860
chr17	16233200	0.752066
chr17	16322700	0.859504
chr17	16345800	0.884298
chr17	16367200	0.768595
chr17	16373700	0.950413
chr17	16380900	0.892562

chr17	16391800	0.727273
chr17	16420400	0.834711
chr17	16570900	0.702479
chr17	16768500	0.826446
chr17	16813800	0.909091
chr17	16914800	0.735537
chr17	16935100	0.991736
chr17	16948900	0.917355
chr17	16955600	0.826446
chr17	16959300	0.834711
chr17	16978200	0.917355
chr17	17044200	0.785124
chr17	17055700	0.892562
chr17	17088600	0.702479
chr17	17094800	0.983471
chr17	17255800	0.842975
chr17	17307400	0.958678
chr17	17316200	0.727273
chr17	17319100	0.752066
chr17	17321300	0.966942
chr17	17323900	0.768595
chr17	17339000	0.909091
chr17	17348400	0.735537
chr17	17350800	0.719008
chr17	17465400	0.950413
chr17	17580800	0.826446
chr17	17588100	0.793388
chr17	17590400	0.710744
chr17	17604000	0.975207
chr17	17613700	0.776860
chr17	17616500	0.884298
chr17	17631000	0.768595
chr17	17642400	0.851240
chr17	17656800	0.909091
chr17	17677600	0.694215
chr17	17687600	0.694215
chr17	17723100	0.710744
chr17	17727300	0.958678
chr17	17745100	0.752066
chr17	17772800	0.677686
chr17	17781200	0.966942
chr17	17804300	0.876033
chr17	17810600	0.859504
chr17	17817700	0.710744
chr17	17825200	1.000000
chr17	17846400	0.991736
chr17	17851600	0.702479
chr17	17861300	0.867769
chr17	17863500	0.909091
chr17	17935900	0.876033
chr17	18057100	0.735537
chr17	18062200	0.719008
chr17	18091700	0.752066
chr17	18095000	0.933884

chr17	18129100	0.661157
chr17	18139300	0.719008
chr17	18152200	0.900826
chr17	18204800	0.942149
chr17	18221300	0.818182
chr17	18245800	0.958678
chr17	18362600	0.859504
chr17	18529200	0.768595
chr17	18622800	0.859504
chr17	18625600	0.958678
chr17	18725200	0.818182
chr17	18804700	0.892562
chr17	18825000	0.818182
chr17	18872500	0.793388
chr17	18885600	0.917355
chr17	18905700	0.685950
chr17	18933600	0.892562
chr17	18936500	0.851240
chr17	18941000	0.685950
chr17	18945400	0.818182
chr17	18947700	0.950413
chr17	18951500	0.793388
chr17	18956700	0.785124
chr17	18965100	0.834711
chr17	18968300	0.694215
chr17	18974200	0.760331
chr17	18982000	0.884298
chr17	18992000	0.867769
chr17	18995000	0.694215
chr17	18997100	0.884298
chr17	19012100	0.851240
chr17	19024400	0.859504
chr17	19033800	0.942149
chr17	19035900	0.743802
chr17	19040800	0.809917
chr17	19045200	0.983471
chr17	19047700	0.826446
chr17	19062000	0.892562
chr17	19064400	0.694215
chr17	19066800	0.760331
chr17	19090900	0.826446
chr17	19094300	0.884298
chr17	19118100	0.892562
chr17	19120600	0.702479
chr17	19123000	0.909091
chr17	19138100	0.859504
chr17	19165700	0.818182
chr17	19168400	0.710744
chr17	19173000	0.776860
chr17	19207400	0.983471
chr17	19215500	0.876033
chr17	19220800	0.768595
chr17	19302300	0.752066
chr17	19352600	0.983471

chr17	19628300	0.760331
chr17	19648600	0.768595
chr17	19656700	0.760331
chr17	19860300	0.950413
chr17	19862400	0.900826
chr17	19873500	0.694215
chr17	20077900	0.727273
chr17	20110000	0.842975
chr17	20166200	0.909091
chr17	20354400	0.719008
chr17	20840000	0.991736
chr17	20938200	0.677686
chr17	20947500	0.809917
chr17	21065100	0.669421
chr17	21090100	0.842975
chr17	21186600	0.719008
chr17	21192300	0.752066
chr17	21220500	0.685950
chr17	22020700	0.859504
chr17	25267600	0.809917
chr17	25534300	0.950413
chr17	25572100	0.942149
chr17	25574900	0.950413
chr17	25583700	0.966942
chr17	25590700	1.000000
chr17	25592900	0.851240
chr17	25612500	0.958678
chr17	25625300	0.719008
chr17	25650900	0.752066
chr17	25660400	0.867769
chr17	25670900	0.892562
chr17	25673200	0.809917
chr17	25676200	0.950413
chr17	25679700	0.809917
chr17	25682000	0.925620
chr17	25704100	0.793388
chr17	25707300	0.867769
chr17	25709400	0.809917
chr17	25711500	0.991736
chr17	25774200	1.000000
chr17	25794000	0.743802
chr17	25801100	0.909091
chr17	25862100	0.983471
chr17	25879200	0.826446
chr17	25891400	1.000000
chr17	25894100	0.917355
chr17	25905100	0.933884
chr17	25929900	0.785124
chr17	25948300	0.826446
chr17	25958900	0.669421
chr17	26145100	0.991736
chr17	26306100	0.933884
chr17	26309500	1.000000
chr17	26316300	0.942149

chr17	26361400	0.900826
chr17	26439800	0.942149
chr17	26458800	0.785124
chr17	26461000	0.983471
chr17	26472400	0.768595
chr17	26498200	0.958678
chr17	26501200	0.983471
chr17	26516600	0.876033
chr17	26555300	0.702479
chr17	26578800	0.909091
chr17	26666000	0.859504
chr17	26673000	0.826446
chr17	26675300	0.785124
chr17	26708400	0.834711
chr17	26722100	0.776860
chr17	26973900	0.892562
chr17	27038500	0.702479
chr17	27045900	0.785124
chr17	27050000	0.826446
chr17	27052400	0.909091
chr17	27088300	0.909091
chr17	27134900	0.933884
chr17	27137300	0.983471
chr17	27193000	0.876033
chr17	27217500	0.867769
chr17	27220600	0.892562
chr17	27236100	0.818182
chr17	27239600	0.909091
chr17	27253500	0.966942
chr17	27272200	0.900826
chr17	27274400	0.925620
chr17	27309900	0.884298
chr17	27370300	0.801653
chr17	27467800	0.925620
chr17	27476000	0.925620
chr17	27483400	0.958678
chr17	27503900	0.801653
chr17	27568200	0.900826
chr17	27583100	0.661157
chr17	27697300	0.685950
chr17	27740300	0.983471
chr17	27838300	0.892562
chr17	27913500	0.834711
chr17	27922500	0.917355
chr17	27940700	0.933884
chr17	27942800	0.685950
chr17	27966500	0.892562
chr17	27970900	0.917355
chr17	27992800	0.975207
chr17	28010600	0.884298
chr17	28019700	0.900826
chr17	28050000	0.842975
chr17	28052900	0.884298
chr17	28055000	0.892562

chr17	28060000	0.776860
chr17	28068200	0.958678
chr17	28071900	0.983471
chr17	28106300	0.752066
chr17	28246600	0.876033
chr17	28254800	0.900826
chr17	28591500	1.000000
chr17	28667900	0.975207
chr17	28761000	0.809917
chr17	28847100	0.975207
chr17	28974100	0.661157
chr17	28983200	0.892562
chr17	28995100	0.785124
chr17	29014100	0.743802
chr17	29249900	0.702479
chr17	29395200	0.975207
chr17	29517500	0.743802
chr17	29596800	0.917355
chr17	29601700	0.710744
chr17	29641500	0.975207
chr17	29648700	0.933884
chr17	29889800	0.851240
chr17	29892400	0.719008
chr17	29925000	0.917355
chr17	30052500	0.876033
chr17	30081600	0.900826
chr17	30088200	0.809917
chr17	30148400	0.876033
chr17	30152400	0.793388
chr17	30170200	0.909091
chr17	30390100	0.752066
chr17	30396200	0.760331
chr17	30399300	0.909091
chr17	30407100	0.743802
chr17	30489000	0.760331
chr17	30519000	0.950413
chr17	30533200	0.801653
chr17	30683200	0.809917
chr17	30830600	0.702479
chr17	30846900	0.950413
chr17	30864200	0.983471
chr17	30878900	0.958678
chr17	31070200	0.876033
chr17	31098800	1.000000
chr17	31120900	0.859504
chr17	31123200	0.867769
chr17	31126300	0.983471
chr17	31129900	0.884298
chr17	31132600	0.958678
chr17	31135200	0.925620
chr17	31146500	0.900826
chr17	31149400	0.917355
chr17	31181500	0.933884
chr17	31197700	0.983471

chr17	31254400	0.710744
chr17	31258500	0.818182
chr17	31264200	0.842975
chr17	32505000	1.000000
chr17	32562100	0.851240
chr17	32564200	0.809917
chr17	32567000	0.933884
chr17	32570900	0.702479
chr17	32580300	0.950413
chr17	32586600	0.975207
chr17	32628100	0.719008
chr17	32710200	0.950413
chr17	32714500	0.694215
chr17	32740400	0.958678
chr17	33350100	0.958678
chr17	33355900	0.917355
chr17	33390700	0.909091
chr17	33402800	0.661157
chr17	33409600	0.661157
chr17	33417400	0.793388
chr17	33478300	0.991736
chr17	33572700	0.909091
chr17	33581500	0.983471
chr17	33584700	0.983471
chr17	33623200	0.876033
chr17	33673500	0.925620
chr17	33703400	0.735537
chr17	33761500	0.768595
chr17	33787900	0.776860
chr17	33815000	0.702479
chr17	33865400	0.834711
chr17	33868100	0.909091
chr17	33870900	0.867769
chr17	33916600	0.842975
chr17	33944600	0.735537
chr17	33957800	0.909091
chr17	34040600	0.925620
chr17	34129000	0.933884
chr17	34142000	0.785124
chr17	34202900	0.909091
chr17	34258100	0.685950
chr17	34301700	0.917355
chr17	34305800	0.793388
chr17	34316000	0.834711
chr17	34345100	0.851240
chr17	34839100	0.826446
chr17	34885500	0.752066
chr17	34998700	0.851240
chr17	35078900	0.809917
chr17	35205000	0.933884
chr17	35207200	0.826446
chr17	35209300	0.859504
chr17	35294100	0.801653
chr17	35337300	0.975207

chr17	35447400	0.950413
chr17	35504900	0.966942
chr17	35537300	0.958678
chr17	35540600	0.884298
chr17	35618800	0.694215
chr17	35649900	0.859504
chr17	35656600	0.859504
chr17	35717400	0.966942
chr17	35825600	0.966942
chr17	35837100	0.991736
chr17	35970500	0.760331
chr17	36008900	0.991736
chr17	36070800	0.826446
chr17	36184700	0.826446
chr17	36204600	0.752066
chr17	36442700	0.942149
chr17	36483100	0.760331
chr17	36583100	0.785124
chr17	36587500	0.702479
chr17	36604300	0.719008
chr17	36612900	0.785124
chr17	36621600	0.950413
chr17	36627000	0.909091
chr17	36629200	0.661157
chr17	36666300	0.809917
chr17	36715000	0.661157
chr17	36844300	0.702479
chr17	36881100	0.685950
chr17	36888400	0.785124
chr17	36899100	0.892562
chr17	36907000	0.776860
chr17	36946800	0.809917
chr17	37020100	0.958678
chr17	37036500	0.958678
chr17	37050200	0.743802
chr17	37052900	0.776860
chr17	37057500	0.785124
chr17	37063000	0.677686
chr17	37065200	0.785124
chr17	37115100	0.942149
chr17	37159500	0.776860
chr17	37332200	0.694215
chr17	37335100	0.719008
chr17	37366500	0.867769
chr17	37508300	0.661157
chr17	37673800	1.000000
chr17	37697800	0.760331
chr17	37816200	1.000000
chr17	37863800	0.710744
chr17	37868600	0.710744
chr17	37872600	0.867769
chr17	37880300	0.735537
chr17	37896500	0.884298
chr17	38081000	0.727273

chr17	38086100	0.859504
chr17	38170600	0.851240
chr17	38172800	0.909091
chr17	38175800	0.892562
chr17	38178000	0.801653
chr17	38181100	0.925620
chr17	38183700	0.867769
chr17	38221900	0.727273
chr17	38225500	0.917355
chr17	38228500	0.818182
chr17	38232100	0.661157
chr17	38254800	0.685950
chr17	38258400	0.793388
chr17	38265100	0.975207
chr17	38270300	0.859504
chr17	38290700	0.917355
chr17	38333800	0.752066
chr17	38348000	0.834711
chr17	38359400	0.752066
chr17	38440600	0.925620
chr17	38460100	0.677686
chr17	38464600	0.702479
chr17	38468300	0.859504
chr17	38471200	0.917355
chr17	38477100	0.933884
chr17	38483000	0.876033
chr17	38487300	0.685950
chr17	38491800	0.900826
chr17	38497100	0.677686
chr17	38504100	0.958678
chr17	38507000	0.983471
chr17	38511900	0.661157
chr17	38604700	0.735537
chr17	38656200	0.785124
chr17	38689900	0.975207
chr17	38692900	0.694215
chr17	38695000	0.727273
chr17	38705600	0.685950
chr17	38713700	0.768595
chr17	38739000	0.867769
chr17	38801400	0.661157
chr17	38864400	0.826446
chr17	38966500	0.785124
chr17	39069400	0.669421
chr17	39256200	0.677686
chr17	39276800	0.719008
chr17	39560200	0.942149
chr17	39649300	0.818182
chr17	39679400	0.925620
chr17	39687100	0.876033
chr17	39705300	0.909091
chr17	39804800	0.876033
chr17	39835400	0.933884
chr17	39929900	0.983471

chr17	39934300	0.950413
chr17	39950100	0.785124
chr17	40072600	0.702479
chr17	40109800	0.727273
chr17	40155400	0.752066
chr17	40177000	1.000000
chr17	40192500	0.710744
chr17	40224900	0.859504
chr17	40250500	0.702479
chr17	40264800	0.942149
chr17	40284700	0.925620
chr17	40288300	0.851240
chr17	40333400	0.834711
chr17	40336300	0.851240
chr17	40380400	0.851240
chr17	40401000	0.925620
chr17	40405000	0.983471
chr17	40465400	0.842975
chr17	40471500	0.950413
chr17	40473600	0.933884
chr17	40481700	0.917355
chr17	40500000	0.842975
chr17	40531400	0.900826
chr17	40541700	0.900826
chr17	40563300	0.710744
chr17	40567200	0.942149
chr17	40570600	0.975207
chr17	40609600	0.991736
chr17	40661200	0.900826
chr17	40672600	0.867769
chr17	40674800	0.752066
chr17	40679000	0.966942
chr17	40684300	0.661157
chr17	40693700	0.801653
chr17	40806000	0.760331
chr17	40832300	0.867769
chr17	40836200	0.768595
chr17	40915700	0.942149
chr17	40927700	0.876033
chr17	40967400	0.991736
chr17	41047600	0.900826
chr17	41077200	0.785124
chr17	41137200	1.000000
chr17	41168000	0.842975
chr17	41171500	0.801653
chr17	41281700	0.983471
chr17	41381800	0.942149
chr17	41400400	0.743802
chr17	41419500	0.743802
chr17	41445500	0.851240
chr17	41509600	0.685950
chr17	41595100	0.983471
chr17	41608300	0.925620
chr17	41640700	0.727273

chr17	41656700	0.702479
chr17	41668500	0.950413
chr17	41670700	0.859504
chr17	41687300	0.859504
chr17	41710300	0.735537
chr17	41723700	0.876033
chr17	41791500	0.743802
chr17	41833000	0.719008
chr17	41892200	0.942149
chr17	41985000	0.677686
chr17	41995300	0.702479
chr17	42030600	0.694215
chr17	42072300	0.661157
chr17	42080300	0.958678
chr17	42111800	0.760331
chr17	42147200	0.900826
chr17	42188800	0.785124
chr17	42215900	0.942149
chr17	42288100	0.826446
chr17	42300900	0.809917
chr17	42357600	0.727273
chr17	42403900	0.685950
chr17	42624700	0.983471
chr17	42665100	0.909091
chr17	42677200	0.867769
chr17	42680000	0.768595
chr17	42754900	0.900826
chr17	42872900	1.000000
chr17	42898400	0.917355
chr17	42940900	0.685950
chr17	42950400	0.909091
chr17	42979500	0.909091
chr17	42983800	0.776860
chr17	42989200	0.768595
chr17	43037600	0.801653
chr17	43046100	0.727273
chr17	43058800	0.917355
chr17	43065300	0.694215
chr17	43096800	0.859504
chr17	43099200	0.776860
chr17	43130100	0.818182
chr17	43171100	0.710744
chr17	43201800	0.727273
chr17	43205700	0.760331
chr17	43222000	0.842975
chr17	43224200	0.752066
chr17	43229300	0.669421
chr17	43236100	0.851240
chr17	43249100	0.909091
chr17	43351400	0.768595
chr17	43355300	0.884298
chr17	43374000	0.842975
chr17	43385100	0.950413
chr17	43391000	0.818182

chr17	43395500	0.785124
chr17	43398700	0.818182
chr17	43416100	0.859504
chr17	43419400	0.917355
chr17	43442300	0.793388
chr17	43449300	0.975207
chr17	43472700	0.859504
chr17	43487900	0.958678
chr17	43525000	0.669421
chr17	43539800	0.842975
chr17	43549900	0.834711
chr17	43565400	1.000000
chr17	44026600	0.669421
chr17	44134700	0.991736
chr17	44178300	0.892562
chr17	44186300	0.851240
chr17	44227900	0.677686
chr17	44232800	0.950413
chr17	44241200	0.669421
chr17	44265400	0.694215
chr17	44272700	0.719008
chr17	44364300	0.661157
chr17	44586500	0.669421
chr17	44820400	0.743802
chr17	44825800	0.818182
chr17	44842400	0.892562
chr17	44896800	0.677686
chr17	44929500	0.702479
chr17	45007700	0.942149
chr17	45082800	0.859504
chr17	45148700	0.768595
chr17	45213400	0.719008
chr17	45215900	0.768595
chr17	45219600	0.677686
chr17	45234100	0.958678
chr17	45249200	0.826446
chr17	45257500	0.719008
chr17	45260600	0.851240
chr17	45307500	0.900826
chr17	45316900	0.834711
chr17	45335400	0.933884
chr17	45340400	0.867769
chr17	45344200	0.983471
chr17	45356800	0.900826
chr17	45371800	0.925620
chr17	45383300	1.000000
chr17	45393300	1.000000
chr17	45413000	0.983471
chr17	45580400	0.958678
chr17	45681900	0.818182
chr17	45700200	0.975207
chr17	45714100	0.710744
chr17	45730700	0.752066
chr17	45764600	0.942149

chr17	45811200	0.685950
chr17	45867600	0.719008
chr17	45888200	0.785124
chr17	45900400	0.685950
chr17	45912700	0.768595
chr17	45928600	0.909091
chr17	45933600	0.842975
chr17	46027300	0.776860
chr17	46080300	0.942149
chr17	46129200	0.669421
chr17	46131600	0.702479
chr17	46144000	0.694215
chr17	46205400	0.834711
chr17	46272200	0.859504
chr17	46302100	0.842975
chr17	46314300	1.000000
chr17	46316800	0.694215
chr17	46319400	0.917355
chr17	46323000	0.950413
chr17	46338900	0.719008
chr17	46373100	0.991736
chr17	46484800	0.909091
chr17	46504000	0.710744
chr17	46570300	0.884298
chr17	46623800	0.909091
chr17	46626200	0.876033
chr17	46634000	0.760331
chr17	46642100	0.925620
chr17	46651700	0.925620
chr17	46666100	0.991736
chr17	46671700	0.900826
chr17	46679100	0.760331
chr17	46683700	0.694215
chr17	46711400	0.719008
chr17	46918900	0.859504
chr17	46950900	0.669421
chr17	47039100	0.859504
chr17	47050600	0.900826
chr17	47081100	0.677686
chr17	47084900	0.694215
chr17	47091700	0.991736
chr17	47094300	0.942149
chr17	47099300	0.801653
chr17	47107400	0.991736
chr17	47113400	0.942149
chr17	47154600	0.900826
chr17	47210300	0.661157
chr17	47279100	1.000000
chr17	47285400	0.743802
chr17	47290900	0.892562
chr17	47468100	0.917355
chr17	47574700	0.694215
chr17	47675900	0.793388
chr17	47699600	0.983471

chr17	47714600	0.876033
chr17	47747900	0.933884
chr17	47766000	0.983471
chr17	47789400	0.776860
chr17	47842400	0.685950
chr17	47853500	0.826446
chr17	47893800	1.000000
chr17	47897800	0.909091
chr17	47906100	0.917355
chr17	47909300	0.950413
chr17	47911800	0.710744
chr17	47914800	0.768595
chr17	47928800	0.933884
chr17	48030300	0.909091
chr17	48046300	0.859504
chr17	48071600	0.809917
chr17	48105400	0.776860
chr17	48121400	0.785124
chr17	48125300	0.942149
chr17	48128800	0.727273
chr17	48131500	0.966942
chr17	48137500	0.966942
chr17	48140900	0.677686
chr17	48143000	0.876033
chr17	48201500	0.727273
chr17	48207900	0.752066
chr17	48217600	0.925620
chr17	48220000	0.909091
chr17	48230100	0.735537
chr17	48255900	0.942149
chr17	48277800	0.818182
chr17	48344400	0.743802
chr17	48367700	0.991736
chr17	48426300	0.719008
chr17	48501700	0.958678
chr17	48625100	0.719008
chr17	48682500	0.818182
chr17	48696900	0.677686
chr17	48718800	0.933884
chr17	48739800	0.809917
chr17	48746000	0.851240
chr17	48930100	0.685950
chr17	49008900	0.818182
chr17	49031900	0.710744
chr17	49054700	0.876033
chr17	49134700	0.801653
chr17	49142000	0.661157
chr17	49163500	0.991736
chr17	49200000	0.785124
chr17	49210700	0.991736
chr17	49261700	0.727273
chr17	49292200	0.809917
chr17	49302300	0.966942
chr17	49321200	0.867769

chr17	49328700	0.677686
chr17	50682100	0.793388
chr17	50867800	0.925620
chr17	51117600	1.000000
chr17	51183500	0.958678
chr17	53194500	0.991736
chr17	53236500	0.735537
chr17	53240100	0.892562
chr17	53254800	0.859504
chr17	53265000	0.685950
chr17	53342400	0.727273
chr17	53530100	0.900826
chr17	53654300	0.909091
chr17	53800500	0.661157
chr17	53809000	0.669421
chr17	53841600	0.983471
chr17	54263700	0.917355
chr17	54369800	0.983471
chr17	54395400	0.859504
chr17	54674900	0.702479
chr17	54756600	0.900826
chr17	54759500	0.983471
chr17	54777100	0.942149
chr17	54829100	0.842975
chr17	54832700	0.983471
chr17	54857700	0.710744
chr17	54864700	0.983471
chr17	54900300	0.966942
chr17	54914100	0.768595
chr17	54916500	0.950413
chr17	54945800	0.933884
chr17	54976900	0.876033
chr17	54980000	0.892562
chr17	54988300	0.752066
chr17	54994100	0.909091
chr17	54999800	0.727273
chr17	55015800	0.933884
chr17	55027900	0.809917
chr17	55123600	0.801653
chr17	55289100	0.859504
chr17	55293300	0.983471
chr17	55304400	0.793388
chr17	55373400	0.991736
chr17	55434700	0.867769
chr17	55442500	0.685950
chr17	55457200	0.661157
chr17	55502900	0.900826
chr17	55521000	0.892562
chr17	55615400	0.661157
chr17	55740700	1.000000
chr17	55851400	0.900826
chr17	55945800	0.909091
chr17	55952900	0.900826
chr17	55957000	0.752066

chr17	55967700	0.809917
chr17	55970900	0.900826
chr17	55980800	0.694215
chr17	56009000	0.826446
chr17	56013300	0.685950
chr17	56024700	0.826446
chr17	56027000	0.917355
chr17	56029800	0.818182
chr17	56048500	0.793388
chr17	56063000	0.694215
chr17	56067600	0.917355
chr17	56071600	0.933884
chr17	56469300	0.892562
chr17	56594400	0.710744
chr17	56602900	0.942149
chr17	56606000	0.735537
chr17	56709500	0.867769
chr17	56720100	0.942149
chr17	56780800	1.000000
chr17	57034800	0.892562
chr17	57079400	1.000000
chr17	57152000	0.884298
chr17	57171200	0.900826
chr17	57173300	0.793388
chr17	57229800	0.776860
chr17	57235500	0.859504
chr17	57237800	0.900826
chr17	57245200	0.818182
chr17	57365300	0.884298
chr17	57405300	0.933884
chr17	57440000	0.925620
chr17	57443400	0.677686
chr17	57450600	0.925620
chr17	57454300	0.818182
chr17	57456500	0.851240
chr17	57459500	0.933884
chr17	57501000	0.892562
chr17	57503100	0.834711
chr17	57505200	0.958678
chr17	57512700	0.909091
chr17	57515500	0.809917
chr17	57519100	0.983471
chr17	57684700	0.917355
chr17	57700700	0.785124
chr17	57704000	0.685950
chr17	57711700	0.785124
chr17	57748800	0.900826
chr17	57775800	0.685950
chr17	57787100	0.669421
chr17	57831600	0.876033
chr17	57834100	0.958678
chr17	57836600	0.809917
chr17	57840000	0.950413
chr17	57842300	0.950413

chr17	57844900	1.000000
chr17	57848400	0.884298
chr17	57860400	0.851240
chr17	57863100	0.917355
chr17	57865200	0.942149
chr17	57870100	0.983471
chr17	57874400	0.958678
chr17	57906100	0.818182
chr17	57908200	0.900826
chr17	57910400	0.892562
chr17	57914200	0.743802
chr17	57920400	0.809917
chr17	57923900	0.933884
chr17	57930600	0.768595
chr17	57933500	0.958678
chr17	58143600	0.884298
chr17	58179700	0.719008
chr17	58220200	0.752066
chr17	58247800	0.933884
chr17	58327000	0.917355
chr17	58347400	0.760331
chr17	58499000	0.694215
chr17	58546100	0.983471
chr17	58648000	0.760331
chr17	58652100	0.975207
chr17	58708500	0.876033
chr17	59135100	0.809917
chr17	59329700	0.694215
chr17	59532000	0.685950
chr17	59573500	0.851240
chr17	59737100	0.950413
chr17	60139800	0.694215
chr17	60256900	0.933884
chr17	60568700	0.801653
chr17	60643100	0.983471
chr17	60650900	0.876033
chr17	60730300	0.694215
chr17	60772400	0.958678
chr17	60897500	0.975207
chr17	60899900	0.900826
chr17	60970300	0.917355
chr17	60994800	0.677686
chr17	61045800	0.900826
chr17	61240700	0.925620
chr17	61352900	0.818182
chr17	61357500	0.876033
chr17	61368400	0.752066
chr17	61371200	0.966942
chr17	61386800	0.834711
chr17	61435500	0.818182
chr17	61460400	0.677686
chr17	61477100	0.884298
chr17	61498200	1.000000
chr17	61501200	0.876033

chr17	61510100	0.851240
chr17	61512200	0.933884
chr17	61525900	0.760331
chr17	61528700	0.909091
chr17	61539000	0.661157
chr17	61552200	0.867769
chr17	61555800	0.760331
chr17	61657500	0.983471
chr17	61674600	0.975207
chr17	61704400	0.702479
chr17	61711600	0.991736
chr17	61743200	0.876033
chr17	61758300	0.785124
chr17	61778800	0.710744
chr17	61784000	0.776860
chr17	61921400	0.677686
chr17	62009000	0.669421
chr17	62052200	0.933884
chr17	62063000	0.801653
chr17	62072600	0.727273
chr17	62080400	0.983471
chr17	62085800	0.793388
chr17	62087900	0.966942
chr17	62092700	0.876033
chr17	62095800	0.933884
chr17	62099400	0.892562
chr17	62103000	0.917355
chr17	62138900	0.991736
chr17	62159900	0.925620
chr17	62162100	0.818182
chr17	62175100	0.991736
chr17	62230300	0.867769
chr17	62233900	0.793388
chr17	62248200	0.900826
chr17	62250400	0.991736
chr17	62252500	0.694215
chr17	62258000	0.942149
chr17	62293900	0.950413
chr17	62360200	0.735537
chr17	62393000	0.925620
chr17	62395800	0.966942
chr17	62407900	0.818182
chr17	62410600	0.933884
chr17	62499200	0.743802
chr17	62569500	0.991736
chr17	62595900	0.677686
chr17	62604900	0.842975
chr17	62611900	0.743802
chr17	62616600	0.975207
chr17	62619900	0.983471
chr17	62628700	0.768595
chr17	62632200	0.958678
chr17	62637900	0.842975
chr17	62650200	0.851240

chr17	62654100	0.669421
chr17	62660700	0.966942
chr17	62664400	0.950413
chr17	62676000	0.917355
chr17	62690600	0.917355
chr17	62702300	0.966942
chr17	62706400	0.892562
chr17	62709800	0.991736
chr17	62716700	0.867769
chr17	62720600	0.950413
chr17	62722800	0.801653
chr17	62724900	0.892562
chr17	62727000	0.942149
chr17	62729500	0.892562
chr17	62735800	0.801653
chr17	62739000	0.900826
chr17	62744700	0.933884
chr17	62773400	0.958678
chr17	62778800	0.768595
chr17	62805100	0.735537
chr17	62815500	0.867769
chr17	62826900	0.876033
chr17	62830600	0.950413
chr17	62897300	0.685950
chr17	62924000	0.950413
chr17	62947300	0.917355
chr17	62962200	0.793388
chr17	63023800	0.801653
chr17	63031800	0.933884
chr17	63119600	0.793388
chr17	63133700	0.793388
chr17	63167400	0.785124
chr17	63169600	0.760331
chr17	63174700	0.760331
chr17	63180300	0.933884
chr17	63188400	0.958678
chr17	63225200	0.834711
chr17	63258500	0.892562
chr17	63286700	0.785124
chr17	63600500	0.834711
chr17	63657600	0.851240
chr17	63739200	0.842975
chr17	63781500	0.958678
chr17	63911400	0.694215
chr17	63915400	0.975207
chr17	64237000	0.793388
chr17	64269500	0.991736
chr17	64272800	0.752066
chr17	64301200	0.694215
chr17	64344900	0.925620
chr17	64428200	0.909091
chr17	64456300	0.975207
chr17	64458800	0.661157
chr17	64462200	0.909091

chr17	64465500	0.950413
chr17	64490300	0.950413
chr17	64504400	0.809917
chr17	64506500	0.801653
chr17	64539400	0.950413
chr17	64544300	0.983471
chr17	64546500	0.776860
chr17	64564100	0.876033
chr17	64575400	0.884298
chr17	64577500	0.925620
chr17	64658200	0.694215
chr17	64688300	0.983471
chr17	64901500	0.892562
chr17	64961900	0.669421
chr17	65213100	0.735537
chr17	65254300	0.917355
chr17	65286100	0.694215
chr17	65375900	0.752066
chr17	65387400	0.958678
chr17	65418300	0.991736
chr17	65428000	0.925620
chr17	65437400	0.983471
chr17	65440200	0.702479
chr17	65478100	0.966942
chr17	65525300	0.958678
chr17	65546200	0.983471
chr17	65603000	0.710744
chr17	65630300	0.950413
chr17	65852600	0.785124
chr17	65959600	0.809917
chr17	65970800	0.818182
chr17	66016700	0.818182
chr17	66048900	0.785124
chr17	66051100	0.785124
chr17	66058400	0.842975
chr17	66067000	0.950413
chr17	66086500	0.677686
chr17	66192600	0.785124
chr17	66197600	0.818182
chr17	66210300	0.702479
chr17	66255500	0.925620
chr17	66297400	0.776860
chr17	66301700	0.876033
chr17	66309200	0.925620
chr17	66351000	0.933884
chr17	66374300	0.735537
chr17	66378200	0.867769
chr17	66380800	0.909091
chr17	66433300	0.942149
chr17	66438600	0.983471
chr17	66506300	0.983471
chr17	66597200	0.677686
chr17	66755900	0.909091
chr17	66771500	0.983471

chr17	66774100	0.950413
chr17	66790800	0.950413
chr17	66818500	0.991736
chr17	66889600	0.669421
chr17	66905600	0.917355
chr17	66923100	0.801653
chr17	66977800	0.785124
chr17	67010000	0.867769
chr17	67042100	0.661157
chr17	67044500	0.966942
chr17	67089600	0.735537
chr17	67266100	0.925620
chr17	67413500	0.826446
chr17	67420100	1.000000
chr17	67426800	0.900826
chr17	67430700	0.702479
chr17	67441500	0.776860
chr17	67447300	1.000000
chr17	67464100	0.942149
chr17	67498500	0.884298
chr17	67515100	0.950413
chr17	67536300	0.975207
chr17	67548100	0.760331
chr17	67571300	0.818182
chr17	67577400	0.867769
chr17	67590300	0.975207
chr17	67594700	0.950413
chr17	67603800	0.842975
chr17	67605900	0.909091
chr17	67669700	0.983471
chr17	67758400	0.991736
chr17	67824500	0.942149
chr17	67853900	0.950413
chr17	67867000	0.909091
chr17	67870900	0.991736
chr17	67875200	0.966942
chr17	67993700	0.702479
chr17	68002700	0.702479
chr17	68030700	0.983471
chr17	68036100	0.917355
chr17	68038300	0.966942
chr17	68096300	0.702479
chr17	68106400	0.917355
chr17	68117700	0.991736
chr17	68133900	0.950413
chr17	68154000	0.694215
chr17	68156900	0.900826
chr17	68163100	0.925620
chr17	68169100	0.826446
chr17	68252600	0.958678
chr17	68341000	0.702479
chr17	68555300	0.942149
chr17	68560100	0.991736
chr17	70390500	0.925620

chr17	70417800	0.925620
chr17	70712700	0.834711
chr17	70897300	0.966942
chr17	70903300	0.958678
chr17	70964600	0.735537
chr17	70973900	0.925620
chr17	70981100	0.925620
chr17	71007600	0.900826
chr17	71200200	0.884298
chr17	71220900	0.685950
chr17	71225100	0.925620
chr17	71288900	0.933884
chr17	71300700	0.925620
chr17	71304900	0.785124
chr17	71339000	0.727273
chr17	72322900	0.735537
chr17	72734000	0.661157
chr17	72741300	0.735537
chr17	72754300	0.719008
chr17	72776200	0.809917
chr17	72794800	0.859504
chr17	72822200	0.884298
chr17	72858100	0.752066
chr17	72865300	0.719008
chr17	72966900	0.851240
chr17	73032600	0.876033
chr17	73062100	0.768595
chr17	73074200	0.735537
chr17	73080600	0.867769
chr17	73087900	0.842975
chr17	73092600	0.859504
chr17	73102000	0.900826
chr17	73110700	0.851240
chr17	73120700	0.834711
chr17	73155500	0.966942
chr17	73246600	0.867769
chr17	73262300	0.900826
chr17	73322500	0.975207
chr17	73362600	0.702479
chr17	73483800	0.735537
chr17	73505500	0.826446
chr17	73567800	0.743802
chr17	73630000	0.884298
chr17	73642400	0.785124
chr17	73650200	0.710744
chr17	73676200	0.760331
chr17	73680400	0.710744
chr17	73682700	1.000000
chr17	73684900	0.876033
chr17	73687800	0.867769
chr17	73695500	0.793388
chr17	73697700	0.785124
chr17	73699900	0.909091
chr17	73703500	0.834711

chr17	73717600	0.842975
chr17	73779800	0.892562
chr17	73789500	0.743802
chr17	73824000	0.983471
chr17	73840800	0.867769
chr17	73843200	0.793388
chr17	73845400	0.818182
chr17	73849400	0.834711
chr17	73854900	0.983471
chr17	73866200	0.950413
chr17	73869000	0.768595
chr17	73871400	0.867769
chr17	73966300	0.958678
chr17	74025900	0.735537
chr17	74050600	0.867769
chr17	74088100	0.867769
chr17	74118100	0.760331
chr17	74141900	0.859504
chr17	74176500	0.859504
chr17	74248700	0.743802
chr17	74257700	0.958678
chr17	74260500	0.818182
chr17	74264800	0.983471
chr17	74311200	0.900826
chr17	74333900	0.661157
chr17	74369500	0.950413
chr17	74424300	0.942149
chr17	74439200	0.793388
chr17	74441900	0.719008
chr17	74481700	0.942149
chr17	74489800	0.809917
chr17	74524200	0.867769
chr17	74526400	0.710744
chr17	74534200	0.677686
chr17	74537200	0.710744
chr17	74543400	0.801653
chr17	74556800	0.661157
chr17	74559000	0.768595
chr17	74619900	0.917355
chr17	74685300	0.942149
chr17	74692400	0.950413
chr17	74694600	0.727273
chr17	74697000	0.942149
chr17	74708000	0.876033
chr17	74712500	0.851240
chr17	74769400	0.917355
chr17	74787700	0.892562
chr17	74864700	0.983471
chr17	74868900	0.900826
chr17	74898000	0.710744
chr17	75083000	0.876033
chr17	75089200	0.942149
chr17	75091600	0.917355
chr17	75093900	0.892562

chr17	75104000	0.867769
chr17	75106300	0.975207
chr17	75108500	0.933884
chr17	75112100	0.842975
chr17	75115600	0.710744
chr17	75117700	0.966942
chr17	75121200	0.876033
chr17	75125300	0.917355
chr17	75135800	0.958678
chr17	75143700	0.900826
chr17	75147000	0.975207
chr17	75150600	0.661157
chr17	75159100	0.793388
chr17	75162100	0.884298
chr17	75168900	0.818182
chr17	75171300	0.900826
chr17	75173800	0.884298
chr17	75177700	0.925620
chr17	75196700	0.876033
chr17	75199800	0.975207
chr17	75202900	0.942149
chr17	75205200	1.000000
chr17	75230200	0.917355
chr17	75232600	0.876033
chr17	75251100	0.950413
chr17	75283200	0.983471
chr17	75312400	0.851240
chr17	75315800	0.743802
chr17	75319500	0.793388
chr17	75338800	0.950413
chr17	75386000	0.950413
chr17	75389100	0.719008
chr17	75397000	0.710744
chr17	75417200	0.801653
chr17	75419900	0.917355
chr17	75426600	0.892562
chr17	75432600	0.743802
chr17	75462200	0.702479
chr17	75828000	0.900826
chr17	75996000	0.743802
chr17	76031200	0.950413
chr17	76055500	0.677686
chr17	76126900	0.727273
chr17	76137500	0.859504
chr17	76170900	0.727273
chr17	76219800	1.000000
chr17	76223200	0.876033
chr17	76254800	0.900826
chr17	76259600	0.677686
chr17	76310500	0.876033
chr17	76313200	0.884298
chr17	76320000	0.933884
chr17	76322100	0.809917
chr17	76338400	0.942149

chr17	76349400	1.000000
chr17	76353200	0.776860
chr17	76357100	0.834711
chr17	76362100	0.727273
chr17	76370200	0.818182
chr17	76372700	0.909091
chr17	76377100	0.983471
chr17	76379500	0.942149
chr17	76381700	0.975207
chr17	76384200	0.809917
chr17	76387900	0.793388
chr17	76391500	0.752066
chr17	76410800	0.719008
chr17	76414000	0.834711
chr17	76481300	0.677686
chr17	76588900	0.942149
chr17	76662400	0.950413
chr17	76677200	0.983471
chr17	76703700	0.975207
chr17	76711800	0.966942
chr17	76758500	0.933884
chr17	76768500	0.826446
chr17	76801400	0.685950
chr17	76815400	0.983471
chr17	76822900	0.884298
chr17	76858600	0.933884
chr17	76869900	0.884298
chr17	76872500	0.727273
chr17	76876100	0.950413
chr17	76912500	0.785124
chr17	76922100	0.958678
chr17	76929300	0.702479
chr17	76932400	0.909091
chr17	76936200	0.793388
chr17	76983300	0.818182
chr17	76989700	0.685950
chr17	77008100	0.975207
chr17	77023700	0.677686
chr17	77032600	0.685950
chr17	77157700	0.842975
chr17	77637000	0.677686
chr17	77670300	0.661157
chr17	77707200	0.834711
chr17	77767200	0.793388
chr17	77772400	0.842975
chr17	77785500	0.776860
chr17	77788900	0.685950
chr17	77803400	0.677686
chr17	77893200	0.867769
chr17	77907900	0.785124
chr17	77935100	0.925620
chr17	77962400	0.900826
chr17	77965900	0.694215
chr17	78003000	0.776860

chr17	78008600	0.685950
chr17	78043000	0.867769
chr17	78045200	0.809917
chr17	78152300	0.785124
chr17	78229100	0.958678
chr17	78233800	0.834711
chr17	78237600	0.710744
chr17	78252000	0.768595
chr17	78493300	0.760331
chr17	78623900	0.917355
chr17	78645000	0.677686
chr17	78653200	0.975207
chr17	78669600	0.834711
chr17	78724300	0.826446
chr17	78733100	0.942149
chr17	78736500	0.760331
chr17	78756900	0.677686
chr17	78763200	0.768595
chr17	78767100	0.966942
chr17	78800200	0.909091
chr17	78802300	0.834711
chr17	78807100	0.933884
chr17	78833200	0.785124
chr17	78864900	0.735537
chr17	78867500	0.752066
chr17	78875600	0.933884
chr17	78902000	0.991736
chr17	78992300	0.925620
chr17	79019200	0.768595
chr17	79026400	0.694215
chr17	79069000	0.826446
chr17	79093300	0.702479
chr17	79124700	0.702479
chr17	79134400	0.917355
chr17	79259500	0.669421
chr17	79283500	0.776860
chr17	79303600	0.669421
chr17	79339700	0.867769
chr17	79386800	0.826446
chr17	79393800	0.884298
chr17	79397400	0.867769
chr17	79421200	0.685950
chr17	79424100	0.867769
chr17	79448800	0.776860
chr17	79451300	0.677686
chr17	79453800	0.867769
chr17	79456100	0.710744
chr17	79521700	0.942149
chr17	79524400	0.933884
chr17	79528700	0.983471
chr17	79531700	0.818182
chr17	79534200	0.909091
chr17	79536500	0.859504
chr17	79544000	0.768595

chr17	79550500	0.884298
chr17	79624300	0.661157
chr17	79635500	0.685950
chr17	79791900	0.760331
chr17	79881800	0.900826
chr17	79918100	0.685950
chr17	79962000	0.925620
chr17	80064600	0.801653
chr17	80164100	0.892562
chr17	80196600	0.661157
chr17	80199800	0.785124
chr17	80204900	0.818182
chr17	80225500	0.884298
chr17	80232000	0.661157
chr17	80246500	0.950413
chr17	80252500	0.925620
chr17	80257400	0.818182
chr17	80293200	0.900826
chr17	80297500	0.892562
chr17	80455500	0.669421
chr17	80489600	0.975207
chr17	80493900	0.793388
chr17	80496100	0.942149
chr17	80551300	0.983471
chr17	80561200	0.909091
chr17	80693400	0.694215
chr17	80749700	0.867769
chr17	80765400	0.925620
chr17	80808200	0.925620
chr17	80818300	0.892562
chr17	80825400	0.727273
chr17	80829400	0.933884
chr17	80834300	0.884298
chr17	80843800	0.991736
chr17	80921500	0.859504
chr17	81006500	0.859504
chr17	81041900	0.884298
chr17	81057600	0.735537
chr17	81061300	0.768595
chr17	81067500	0.809917
chr17	81131900	0.752066
chr18	111000	0.710744
chr18	316700	0.958678
chr18	319600	0.801653
chr18	323300	1.000000
chr18	395900	0.983471
chr18	413300	0.727273
chr18	421600	0.942149
chr18	424600	0.983471
chr18	447600	0.768595
chr18	457700	0.735537
chr18	475700	0.909091
chr18	516000	0.975207
chr18	519400	0.785124

chr18	525200	0.975207
chr18	635000	0.710744
chr18	703500	0.834711
chr18	777700	0.950413
chr18	786600	0.768595
chr18	806100	0.809917
chr18	809300	0.735537
chr18	830400	0.933884
chr18	845900	0.966942
chr18	908600	0.685950
chr18	988100	0.925620
chr18	991700	0.702479
chr18	996000	0.933884
chr18	1002800	0.917355
chr18	2198400	0.743802
chr18	2302800	0.925620
chr18	2305100	0.991736
chr18	2515700	0.983471
chr18	2637200	0.925620
chr18	2658600	0.991736
chr18	2777000	0.826446
chr18	2888600	0.776860
chr18	2917400	0.867769
chr18	2923900	0.760331
chr18	2978900	0.884298
chr18	2981100	0.710744
chr18	2983300	0.909091
chr18	3008600	0.975207
chr18	3011200	0.669421
chr18	3015100	1.000000
chr18	3017200	0.793388
chr18	3029800	0.776860
chr18	3060800	0.710744
chr18	3081900	0.958678
chr18	3084500	0.752066
chr18	3104500	0.991736
chr18	3153500	0.809917
chr18	3251800	0.909091
chr18	3265800	0.842975
chr18	3269600	0.768595
chr18	3272100	0.669421
chr18	3277700	0.826446
chr18	3282800	0.933884
chr18	3319600	0.694215
chr18	3321800	0.785124
chr18	3412400	0.768595
chr18	3455000	0.768595
chr18	3459300	0.966942
chr18	3466700	0.991736
chr18	3478900	0.801653
chr18	3500000	0.809917
chr18	3580300	0.727273
chr18	3592900	0.933884
chr18	3597200	0.760331

chr18	3602000	0.727273
chr18	3623100	0.801653
chr18	3625800	0.743802
chr18	3650400	0.942149
chr18	3653500	0.991736
chr18	3666100	0.925620
chr18	3711300	0.983471
chr18	3748000	0.991736
chr18	3771500	0.925620
chr18	3774600	0.727273
chr18	3795100	0.983471
chr18	3887100	0.694215
chr18	4004700	0.942149
chr18	5432400	0.702479
chr18	5453500	0.900826
chr18	5455700	0.834711
chr18	5457800	0.826446
chr18	5464200	0.801653
chr18	5466800	0.834711
chr18	5510700	0.710744
chr18	5516400	0.900826
chr18	5519100	0.876033
chr18	5521200	0.991736
chr18	5576500	0.909091
chr18	5583500	0.958678
chr18	5891100	0.834711
chr18	6056500	0.842975
chr18	6728600	0.661157
chr18	6731800	0.752066
chr18	6741500	0.925620
chr18	6747600	0.727273
chr18	6782000	0.867769
chr18	6807600	0.933884
chr18	7267900	0.793388
chr18	7324300	0.950413
chr18	7326400	0.752066
chr18	7368600	0.975207
chr18	7558400	0.900826
chr18	7566400	0.661157
chr18	7591200	0.768595
chr18	7627600	0.694215
chr18	7749600	0.809917
chr18	7761300	0.694215
chr18	7878900	0.884298
chr18	7922400	0.925620
chr18	7946700	0.851240
chr18	7954600	0.867769
chr18	7958900	0.801653
chr18	8142800	0.933884
chr18	8329400	0.702479
chr18	8626900	0.909091
chr18	8635400	0.925620
chr18	8661900	0.842975
chr18	8665800	0.966942

chr18	8730700	0.975207
chr18	8733100	0.776860
chr18	8741100	0.677686
chr18	8743500	0.966942
chr18	8747900	0.950413
chr18	8751300	0.942149
chr18	8777500	0.694215
chr18	8787500	0.950413
chr18	8791400	0.826446
chr18	8794700	0.793388
chr18	8797900	0.917355
chr18	8801900	0.826446
chr18	8814200	0.785124
chr18	8891200	1.000000
chr18	9017400	0.958678
chr18	9023100	0.685950
chr18	9038600	0.859504
chr18	9093200	0.917355
chr18	9095500	0.776860
chr18	9106300	0.818182
chr18	9115900	0.760331
chr18	9119300	0.983471
chr18	9143900	0.719008
chr18	9147900	0.809917
chr18	9177800	0.702479
chr18	9185400	0.942149
chr18	9321300	0.975207
chr18	9348800	0.958678
chr18	9401100	0.983471
chr18	9403700	0.876033
chr18	9405800	0.975207
chr18	9539800	0.669421
chr18	9572100	0.958678
chr18	9595900	0.661157
chr18	9609600	0.710744
chr18	9616700	0.685950
chr18	9619900	0.950413
chr18	9632500	0.859504
chr18	9639200	0.661157
chr18	9643900	0.975207
chr18	9706300	0.785124
chr18	9720000	0.950413
chr18	9744100	0.917355
chr18	9756200	0.966942
chr18	9825800	0.991736
chr18	9828600	0.983471
chr18	9877300	0.991736
chr18	9917600	0.909091
chr18	9968600	0.909091
chr18	10016700	0.958678
chr18	10033100	0.661157
chr18	10054800	0.710744
chr18	10057100	0.842975
chr18	10059200	0.743802

chr18	10132200	0.826446
chr18	10142200	0.685950
chr18	10170500	0.958678
chr18	10270200	0.917355
chr18	10290400	0.818182
chr18	10292500	0.859504
chr18	10455400	0.661157
chr18	10617500	0.677686
chr18	10863700	0.818182
chr18	10913300	0.942149
chr18	10917900	0.735537
chr18	10925800	0.818182
chr18	10928100	0.909091
chr18	10974300	0.719008
chr18	10989800	0.818182
chr18	11003700	0.694215
chr18	11005800	0.876033
chr18	11096800	0.818182
chr18	11383700	0.752066
chr18	11500800	1.000000
chr18	11523400	0.983471
chr18	11642100	0.752066
chr18	11857300	0.975207
chr18	11860300	0.776860
chr18	11947700	0.743802
chr18	11980600	0.818182
chr18	12071700	0.809917
chr18	12080200	1.000000
chr18	12196000	0.851240
chr18	12219000	0.719008
chr18	12254400	0.826446
chr18	12279100	0.677686
chr18	12282600	0.752066
chr18	12296400	0.925620
chr18	12416100	0.834711
chr18	12420500	0.719008
chr18	12461100	0.867769
chr18	12463700	0.983471
chr18	12516000	0.867769
chr18	12596400	0.826446
chr18	12599500	0.991736
chr18	12602600	0.702479
chr18	12607500	0.958678
chr18	12630200	0.966942
chr18	12641000	0.876033
chr18	12653200	0.900826
chr18	12660500	1.000000
chr18	12679600	0.966942
chr18	12828700	1.000000
chr18	12907700	0.793388
chr18	13052300	0.793388
chr18	13260800	0.884298
chr18	13268400	0.685950
chr18	13375600	0.958678

chr18	13380500	0.768595
chr18	13382700	0.975207
chr18	13433800	0.942149
chr18	13436100	0.958678
chr18	13452600	0.685950
chr18	13457700	0.958678
chr18	13465100	0.983471
chr18	13501200	0.859504
chr18	13611400	0.785124
chr18	13659100	0.818182
chr18	13797500	0.966942
chr18	13801400	0.760331
chr18	14972100	1.000000
chr18	18520000	0.851240
chr18	18881100	0.669421
chr18	18910800	0.719008
chr18	19031500	0.710744
chr18	19065100	0.925620
chr18	19236900	0.909091
chr18	19250100	0.842975
chr18	19259200	0.826446
chr18	19344500	0.925620
chr18	19406300	0.809917
chr18	19414400	0.942149
chr18	19477300	0.768595
chr18	19510300	0.669421
chr18	19522500	0.867769
chr18	19683600	1.000000
chr18	19759000	0.818182
chr18	19766900	0.892562
chr18	19769600	0.851240
chr18	19771900	0.818182
chr18	19774200	0.867769
chr18	19797800	0.694215
chr18	19833900	0.727273
chr18	19856200	0.694215
chr18	19862900	0.983471
chr18	19916200	0.909091
chr18	19926500	0.834711
chr18	19930500	0.834711
chr18	19966400	0.950413
chr18	19971900	0.966942
chr18	19979000	0.958678
chr18	20013000	0.950413
chr18	20015600	0.685950
chr18	20032700	0.950413
chr18	20046400	0.884298
chr18	20048900	0.876033
chr18	20052000	0.752066
chr18	20085700	0.727273
chr18	20132200	1.000000
chr18	20137600	0.884298
chr18	20141600	0.694215
chr18	20143700	0.752066

chr18	20185000	0.933884
chr18	20189700	0.950413
chr18	20248300	0.983471
chr18	20254600	0.933884
chr18	20270400	0.685950
chr18	20291400	0.743802
chr18	20515700	0.669421
chr18	20558400	0.966942
chr18	20612500	0.876033
chr18	20671600	0.834711
chr18	20687300	0.983471
chr18	20694300	0.942149
chr18	20696400	0.842975
chr18	20707300	0.776860
chr18	20714500	0.702479
chr18	20747200	0.719008
chr18	20760400	0.983471
chr18	20772400	0.768595
chr18	20774900	0.975207
chr18	20805500	0.867769
chr18	20809500	0.950413
chr18	20811700	0.966942
chr18	20818100	0.809917
chr18	20837800	0.826446
chr18	20840700	0.975207
chr18	20848900	0.983471
chr18	20860000	0.925620
chr18	20863300	0.884298
chr18	20883700	0.950413
chr18	20888200	0.743802
chr18	20892000	0.876033
chr18	20916700	0.760331
chr18	20938800	0.669421
chr18	20958400	0.909091
chr18	20960800	0.834711
chr18	20966800	0.933884
chr18	20981500	0.917355
chr18	21039100	0.776860
chr18	21068000	0.917355
chr18	21100300	0.892562
chr18	21154500	0.669421
chr18	21174000	0.983471
chr18	21199800	0.669421
chr18	21207100	0.983471
chr18	21229400	0.785124
chr18	21298700	0.884298
chr18	21365200	0.884298
chr18	21418200	0.867769
chr18	21433800	0.727273
chr18	21481200	0.950413
chr18	21492900	0.719008
chr18	21669600	0.735537
chr18	21812100	0.776860
chr18	21949200	0.743802

chr18	22133700	0.933884
chr18	22365700	0.776860
chr18	22412500	0.975207
chr18	22441200	0.851240
chr18	22443300	0.900826
chr18	22449200	0.818182
chr18	22493600	0.867769
chr18	22502700	0.694215
chr18	22567000	0.859504
chr18	22570900	0.991736
chr18	22586600	0.710744
chr18	22622600	0.669421
chr18	22635400	0.785124
chr18	22657700	0.677686
chr18	22677600	0.925620
chr18	22680900	0.917355
chr18	22703800	0.710744
chr18	22716600	0.694215
chr18	22745600	0.727273
chr18	22766200	0.851240
chr18	22795500	0.669421
chr18	22808600	0.950413
chr18	22812400	0.702479
chr18	22815800	0.884298
chr18	22819800	0.983471
chr18	22824700	0.768595
chr18	22828100	0.958678
chr18	22831400	0.909091
chr18	22833700	0.950413
chr18	22855800	0.983471
chr18	22859600	0.851240
chr18	22862800	0.958678
chr18	22865000	0.851240
chr18	22869600	0.966942
chr18	22879500	0.991736
chr18	22902800	0.727273
chr18	22911900	0.685950
chr18	22935000	0.661157
chr18	23083900	0.685950
chr18	23097400	0.842975
chr18	23966200	0.752066
chr18	24039100	0.842975
chr18	24061100	1.000000
chr18	25419400	0.950413
chr18	25423800	0.933884
chr18	25577400	0.685950
chr18	25583900	0.975207
chr18	25620000	0.752066
chr18	25651900	0.710744
chr18	25692300	0.991736
chr18	25695700	0.950413
chr18	25716300	0.776860
chr18	25728100	0.702479
chr18	25784500	0.975207

chr18	26110000	0.933884
chr18	26238500	0.710744
chr18	26271700	0.793388
chr18	26316200	0.743802
chr18	26347300	0.768595
chr18	26349700	0.975207
chr18	26373600	1.000000
chr18	26376000	0.958678
chr18	26382900	1.000000
chr18	26485800	0.851240
chr18	26565100	0.661157
chr18	26656700	0.661157
chr18	26690200	0.719008
chr18	26734300	0.925620
chr18	26738400	0.884298
chr18	26742000	0.917355
chr18	26745700	0.983471
chr18	26761800	0.867769
chr18	27674700	0.917355
chr18	28824300	0.933884
chr18	28855600	0.677686
chr18	28883400	0.710744
chr18	29212400	0.925620
chr18	29255900	0.776860
chr18	29258600	0.743802
chr18	29261400	0.917355
chr18	29304500	0.760331
chr18	29438000	0.859504
chr18	29665500	0.950413
chr18	29671400	0.801653
chr18	29930500	0.991736
chr18	29963300	0.983471
chr18	30318100	0.809917
chr18	30501800	0.933884
chr18	30590700	0.900826
chr18	30594100	0.942149
chr18	30615200	0.867769
chr18	30716300	0.991736
chr18	30764300	0.933884
chr18	31176700	0.991736
chr18	31739500	0.826446
chr18	31923100	0.661157
chr18	32139600	0.743802
chr18	32240000	0.983471
chr18	32287800	0.950413
chr18	32348100	0.958678
chr18	32353600	0.892562
chr18	32537700	0.925620
chr18	32600500	0.826446
chr18	32610200	0.776860
chr18	32619800	0.991736
chr18	32624600	0.892562
chr18	32630000	0.743802
chr18	32652400	0.909091

chr18	32655900	0.983471
chr18	32658200	0.983471
chr18	32668100	0.694215
chr18	32691000	0.685950
chr18	32820200	0.661157
chr18	32858500	0.735537
chr18	32862600	0.983471
chr18	33010800	0.851240
chr18	33016200	0.768595
chr18	33037000	0.983471
chr18	33048900	0.818182
chr18	33051000	0.694215
chr18	33059100	0.826446
chr18	33061300	0.909091
chr18	33071100	0.966942
chr18	33186200	0.983471
chr18	33194200	0.983471
chr18	33197000	0.917355
chr18	33201000	0.991736
chr18	33215000	1.000000
chr18	33283100	1.000000
chr18	33287300	0.917355
chr18	33289400	0.942149
chr18	33291600	0.933884
chr18	33294000	0.966942
chr18	33738700	0.975207
chr18	33743300	0.983471
chr18	33757000	0.900826
chr18	33760100	0.884298
chr18	33890000	0.859504
chr18	34422600	0.785124
chr18	35068000	0.859504
chr18	36638300	0.975207
chr18	36994800	0.702479
chr18	37029000	0.942149
chr18	37035300	0.983471
chr18	37043200	0.950413
chr18	37141600	0.834711
chr18	37248700	0.710744
chr18	37282300	0.752066
chr18	37420400	0.851240
chr18	37526900	0.917355
chr18	37549200	0.842975
chr18	37945900	0.900826
chr18	37948600	0.900826
chr18	37952600	0.900826
chr18	39240900	0.793388
chr18	39246900	0.669421
chr18	39284100	0.669421
chr18	39300500	0.892562
chr18	39312100	0.958678
chr18	39571900	0.702479
chr18	39578100	0.694215
chr18	40225000	0.917355

chr18	40229700	0.818182
chr18	40511700	0.975207
chr18	40741800	0.826446
chr18	40774600	0.859504
chr18	41670800	0.950413
chr18	41708800	0.826446
chr18	41870000	0.785124
chr18	42088300	0.917355
chr18	42306300	0.677686
chr18	43326200	0.727273
chr18	43336000	0.859504
chr18	43344500	0.760331
chr18	43383300	0.983471
chr18	43418700	0.710744
chr18	43531800	0.719008
chr18	43574200	0.702479
chr18	43785700	0.925620
chr18	43830600	0.975207
chr18	44126300	0.785124
chr18	44203900	0.685950
chr18	44299000	0.975207
chr18	45275600	0.677686
chr18	45379700	0.975207
chr18	45420800	0.702479
chr18	45425300	0.867769
chr18	45454900	0.752066
chr18	45530400	0.851240
chr18	45968000	0.768595
chr18	46015300	0.950413
chr18	46020500	0.785124
chr18	46072400	0.983471
chr18	46089200	0.867769
chr18	46092300	0.826446
chr18	46184200	0.685950
chr18	46207700	0.950413
chr18	46222300	0.983471
chr18	46236200	0.884298
chr18	46246700	0.760331
chr18	46262200	0.677686
chr18	46308000	0.966942
chr18	46317100	0.826446
chr18	46351900	0.694215
chr18	46354500	0.925620
chr18	46361700	0.719008
chr18	46366100	0.685950
chr18	46369400	0.983471
chr18	46371600	0.818182
chr18	46375300	0.785124
chr18	46378400	0.859504
chr18	46399800	0.793388
chr18	46428500	0.884298
chr18	46430600	0.685950
chr18	46449500	0.809917
chr18	46451600	0.842975

chr18	46454700	0.768595
chr18	46457500	0.975207
chr18	46461600	0.785124
chr18	46466000	0.958678
chr18	46468200	0.842975
chr18	46470500	0.685950
chr18	46473500	0.694215
chr18	46479500	0.710744
chr18	46482700	0.942149
chr18	46487100	0.909091
chr18	46524800	0.925620
chr18	46582600	0.776860
chr18	46703900	0.809917
chr18	46734000	0.892562
chr18	46776200	0.991736
chr18	46848400	0.950413
chr18	46925800	0.884298
chr18	46939900	0.950413
chr18	47003600	0.942149
chr18	47023600	0.669421
chr18	47174500	0.909091
chr18	47186600	0.983471
chr18	47191200	1.000000
chr18	47204500	0.950413
chr18	47212000	0.900826
chr18	47214100	0.768595
chr18	47217600	0.966942
chr18	47228000	0.834711
chr18	47249500	0.867769
chr18	47254300	0.727273
chr18	47274900	0.909091
chr18	47376500	0.950413
chr18	47385100	0.851240
chr18	47419200	0.793388
chr18	47565100	0.677686
chr18	48363400	0.735537
chr18	48609500	0.991736
chr18	48634600	0.685950
chr18	50208800	0.958678
chr18	50319300	0.851240
chr18	51675900	1.000000
chr18	51685200	0.900826
chr18	51695900	0.669421
chr18	51709200	0.809917
chr18	51736600	1.000000
chr18	51759900	0.867769
chr18	51762500	0.884298
chr18	51765600	0.958678
chr18	51778000	0.958678
chr18	51915000	0.917355
chr18	52357900	0.735537
chr18	52380000	0.834711
chr18	52443700	0.884298
chr18	52635500	0.884298

chr18	52765200	0.958678
chr18	52768300	0.958678
chr18	52777800	0.909091
chr18	52804600	0.694215
chr18	52902500	0.966942
chr18	52912100	0.942149
chr18	52919300	0.991736
chr18	52924400	0.685950
chr18	52932000	0.983471
chr18	52943100	0.719008
chr18	52984400	0.710744
chr18	52987000	0.685950
chr18	52990800	0.752066
chr18	52995800	0.975207
chr18	53000000	0.661157
chr18	53005500	0.801653
chr18	53007800	0.876033
chr18	53018800	0.859504
chr18	53054000	0.859504
chr18	53057200	0.785124
chr18	53070500	0.694215
chr18	53073600	0.950413
chr18	53081500	0.983471
chr18	53088000	0.826446
chr18	53090100	0.892562
chr18	53144600	0.719008
chr18	53147500	0.975207
chr18	53152900	0.851240
chr18	53163200	0.892562
chr18	53167400	0.975207
chr18	53170600	0.809917
chr18	53176200	0.743802
chr18	53180300	0.958678
chr18	53260300	0.768595
chr18	53399800	0.933884
chr18	53435900	0.752066
chr18	53448400	0.809917
chr18	53457900	0.942149
chr18	53496400	0.776860
chr18	53650500	0.719008
chr18	53656000	0.925620
chr18	53687500	0.834711
chr18	53689800	0.950413
chr18	53752200	0.851240
chr18	53775800	0.752066
chr18	53864500	0.942149
chr18	53867800	0.933884
chr18	53870100	0.892562
chr18	53913300	0.760331
chr18	53958800	0.975207
chr18	53998100	0.694215
chr18	54070500	0.892562
chr18	54111000	0.925620
chr18	54127000	0.950413

chr18	54169500	0.867769
chr18	54242600	0.933884
chr18	54456500	0.958678
chr18	54467400	0.842975
chr18	54558100	0.834711
chr18	54586800	0.694215
chr18	54672200	0.892562
chr18	54878000	0.785124
chr18	55105700	0.842975
chr18	55295400	0.933884
chr18	55325400	0.719008
chr18	55335200	0.983471
chr18	55342500	0.900826
chr18	55345200	0.975207
chr18	55361500	0.818182
chr18	55395800	1.000000
chr18	55411800	0.933884
chr18	55436200	0.876033
chr18	55439100	0.917355
chr18	55444100	0.793388
chr18	55447800	0.892562
chr18	55450200	0.859504
chr18	55452300	0.685950
chr18	55456400	0.975207
chr18	55460500	0.983471
chr18	55474000	0.842975
chr18	55477000	0.892562
chr18	55480700	0.785124
chr18	55487400	0.950413
chr18	55495400	0.785124
chr18	55501000	0.826446
chr18	55505800	0.925620
chr18	55507900	0.900826
chr18	55513600	0.958678
chr18	55556100	0.752066
chr18	55583800	0.801653
chr18	55593300	0.933884
chr18	55764800	0.785124
chr18	55785400	0.735537
chr18	55844500	0.925620
chr18	55854100	0.867769
chr18	55863400	0.694215
chr18	55881500	0.834711
chr18	55888600	0.661157
chr18	55891900	0.983471
chr18	55907700	0.933884
chr18	55910600	0.669421
chr18	55914900	0.677686
chr18	55930600	0.958678
chr18	55936500	0.727273
chr18	55940200	0.669421
chr18	55958000	0.983471
chr18	55981500	0.933884
chr18	55984200	0.950413

chr18	55989200	0.876033
chr18	56006500	0.925620
chr18	56029800	0.950413
chr18	56064600	0.793388
chr18	56085100	0.933884
chr18	56209400	0.710744
chr18	56456800	0.983471
chr18	56530800	0.975207
chr18	56534100	0.867769
chr18	56555400	1.000000
chr18	56579500	0.851240
chr18	56581600	0.942149
chr18	56604700	1.000000
chr18	56660700	0.917355
chr18	56663400	0.884298
chr18	56940800	0.719008
chr18	57541500	1.000000
chr18	57549800	0.983471
chr18	57553500	0.983471
chr18	57572300	0.950413
chr18	57576800	0.743802
chr18	58361100	0.933884
chr18	58421900	0.917355
chr18	58506400	0.694215
chr18	58533300	0.661157
chr18	58559900	1.000000
chr18	58603700	0.752066
chr18	58618200	0.785124
chr18	58646000	0.966942
chr18	58649700	0.975207
chr18	59067900	0.884298
chr18	59247100	0.752066
chr18	59249700	0.727273
chr18	59263600	0.743802
chr18	59269400	0.991736
chr18	59272100	0.785124
chr18	59275800	0.809917
chr18	59295400	0.859504
chr18	59361800	1.000000
chr18	59364900	0.760331
chr18	59369900	0.884298
chr18	59387300	0.991736
chr18	59416000	0.991736
chr18	59498800	0.983471
chr18	59501500	0.892562
chr18	59564400	0.809917
chr18	59617300	0.933884
chr18	59625600	0.900826
chr18	59645500	0.917355
chr18	59659800	1.000000
chr18	59666700	0.966942
chr18	59729300	0.859504
chr18	59759300	0.859504
chr18	59770900	0.719008

chr18	59773700	0.884298
chr18	59949600	0.991736
chr18	59991600	0.809917
chr18	59995900	0.793388
chr18	60002200	0.900826
chr18	60006800	0.760331
chr18	60014600	0.983471
chr18	60088300	0.925620
chr18	60093100	0.925620
chr18	60105500	0.876033
chr18	60118500	1.000000
chr18	60122900	0.677686
chr18	60153400	0.950413
chr18	60198300	0.925620
chr18	60221600	0.950413
chr18	60251800	0.909091
chr18	60418800	0.975207
chr18	60427200	0.694215
chr18	60545500	0.851240
chr18	60548900	0.950413
chr18	60564800	0.925620
chr18	60607000	0.834711
chr18	60805900	0.876033
chr18	60905800	0.909091
chr18	61547500	0.834711
chr18	61550800	0.925620
chr18	62001100	1.000000
chr18	62032600	0.809917
chr18	62072500	0.909091
chr18	64179300	0.942149
chr18	64286800	0.694215
chr18	64300600	0.743802
chr18	64493800	0.793388
chr18	64900300	0.933884
chr18	64911700	0.752066
chr18	64933800	0.975207
chr18	64978400	0.727273
chr18	65027700	0.975207
chr18	65053300	0.892562
chr18	65193100	0.966942
chr18	65399000	0.851240
chr18	65827900	0.719008
chr18	66096500	0.669421
chr18	66508300	0.925620
chr18	67152400	0.669421
chr18	67455800	0.925620
chr18	67712400	0.842975
chr18	67849200	0.991736
chr18	67858000	0.809917
chr18	67963800	0.933884
chr18	67996700	0.983471
chr18	68089000	0.950413
chr18	68132200	0.702479
chr18	68649100	0.917355

chr18	68659300	0.851240
chr18	68664000	0.909091
chr18	68668900	0.867769
chr18	68680400	0.727273
chr18	68686700	0.793388
chr18	68703400	0.809917
chr18	68705500	0.719008
chr18	68712900	0.867769
chr18	68716300	0.925620
chr18	68783500	0.876033
chr18	68877800	0.859504
chr18	68917500	0.669421
chr18	68989500	0.785124
chr18	69099300	0.760331
chr18	71923200	0.818182
chr18	72344200	0.851240
chr18	72519000	0.917355
chr18	72547000	0.859504
chr18	72549100	0.793388
chr18	72552800	0.950413
chr18	72663100	0.958678
chr18	72707000	0.983471
chr18	72761200	0.710744
chr18	72780900	0.966942
chr18	72784400	0.752066
chr18	72913500	0.909091
chr18	72916800	0.801653
chr18	72926000	0.867769
chr18	72934100	0.884298
chr18	72942400	0.983471
chr18	72951100	0.719008
chr18	72964000	0.735537
chr18	72980100	0.859504
chr18	72989300	0.809917
chr18	72993400	0.983471
chr18	74172100	0.826446
chr18	74189300	0.842975
chr18	74409900	0.925620
chr18	74540100	0.851240
chr18	74767200	0.917355
chr18	74777600	0.917355
chr18	74780900	0.851240
chr18	74784500	0.909091
chr18	74799100	0.768595
chr18	74812800	0.892562
chr18	74816700	0.933884
chr18	74826900	0.917355
chr18	76878100	0.669421
chr18	76948200	0.884298
chr18	77006300	0.785124
chr18	77129100	0.719008
chr18	77153800	0.793388
chr18	77159300	0.942149
chr18	77162700	0.685950

chr18	77166300	0.933884
chr18	77172500	0.958678
chr18	77214200	0.859504
chr18	77220800	0.760331
chr18	77225500	0.842975
chr18	77244600	0.876033
chr18	77246700	0.834711
chr18	77257800	0.933884
chr18	77268300	0.826446
chr18	77286600	0.743802
chr18	77328100	0.900826
chr19	63300	0.719008
chr19	267500	0.727273
chr19	290900	0.727273
chr19	312700	0.909091
chr19	316000	0.859504
chr19	348600	0.694215
chr19	409500	0.867769
chr19	511400	0.710744
chr19	576000	0.752066
chr19	676800	0.859504
chr19	679100	0.809917
chr19	728400	0.950413
chr19	779500	0.975207
chr19	781900	0.801653
chr19	784500	0.942149
chr19	789600	0.785124
chr19	869700	0.925620
chr19	909400	0.892562
chr19	913400	0.669421
chr19	918000	0.719008
chr19	935000	0.900826
chr19	941200	0.975207
chr19	996700	0.785124
chr19	1016800	0.884298
chr19	1018900	0.942149
chr19	1029600	0.809917
chr19	1038300	0.867769
chr19	1065500	0.809917
chr19	1071200	0.809917
chr19	1101000	0.842975
chr19	1103300	0.719008
chr19	1113800	0.785124
chr19	1126600	0.818182
chr19	1133600	0.793388
chr19	1177400	0.892562
chr19	1180900	0.925620
chr19	1184100	0.892562
chr19	1203800	0.867769
chr19	1213400	0.818182
chr19	1215700	0.685950
chr19	1233100	0.694215
chr19	1240600	0.859504
chr19	1244700	0.842975

chr19	1247800	0.677686
chr19	1254200	0.876033
chr19	1258900	0.760331
chr19	1265100	0.809917
chr19	1267300	0.694215
chr19	1295800	0.661157
chr19	1313700	0.950413
chr19	1316100	0.677686
chr19	1325400	0.917355
chr19	1354400	0.710744
chr19	1361600	0.966942
chr19	1411300	0.884298
chr19	1415800	0.917355
chr19	1455500	0.727273
chr19	1470800	0.694215
chr19	1726200	0.710744
chr19	1747800	0.776860
chr19	1812600	0.719008
chr19	1841600	0.776860
chr19	1855300	0.694215
chr19	1857800	0.950413
chr19	1865000	0.677686
chr19	1882400	0.958678
chr19	1930100	0.834711
chr19	1944000	0.743802
chr19	2028900	0.818182
chr19	2032600	0.859504
chr19	2042300	0.925620
chr19	2048300	0.909091
chr19	2056700	0.842975
chr19	2060300	0.669421
chr19	2085700	0.958678
chr19	2091200	0.892562
chr19	2094400	0.752066
chr19	2167100	0.818182
chr19	2169300	0.975207
chr19	2186600	0.933884
chr19	2208700	0.702479
chr19	2267100	0.719008
chr19	2274100	0.958678
chr19	2282200	0.719008
chr19	2290900	0.809917
chr19	2425400	0.785124
chr19	2469800	0.867769
chr19	2489100	0.859504
chr19	2491700	0.768595
chr19	2496200	0.694215
chr19	2507400	0.876033
chr19	2525200	0.685950
chr19	2529500	0.991736
chr19	2533100	0.892562
chr19	2540200	0.925620
chr19	2543800	0.867769
chr19	2547100	0.958678

chr19	2553000	0.975207
chr19	2555900	0.892562
chr19	2577800	0.760331
chr19	2603100	0.876033
chr19	2611300	0.661157
chr19	2621200	0.669421
chr19	2628400	0.867769
chr19	2695800	0.735537
chr19	2775900	0.983471
chr19	2948000	0.983471
chr19	2969100	0.917355
chr19	2972400	0.917355
chr19	2974900	0.694215
chr19	2983900	0.892562
chr19	3048000	0.785124
chr19	3099100	0.760331
chr19	3140700	0.727273
chr19	3142800	0.900826
chr19	3155300	0.942149
chr19	3161400	0.834711
chr19	3347100	0.743802
chr19	3369700	0.933884
chr19	3375800	0.752066
chr19	3388300	0.983471
chr19	3404200	0.983471
chr19	3423800	0.876033
chr19	3428700	0.925620
chr19	3433700	0.801653
chr19	3435800	0.776860
chr19	3441900	0.909091
chr19	3444900	0.867769
chr19	3451300	0.925620
chr19	3458200	0.809917
chr19	3509500	0.909091
chr19	3553300	0.933884
chr19	3572200	0.785124
chr19	3576800	0.661157
chr19	3656100	0.842975
chr19	3670100	0.859504
chr19	3684700	0.826446
chr19	3967900	0.760331
chr19	4053800	0.719008
chr19	4058900	0.727273
chr19	4062300	0.950413
chr19	4078400	0.760331
chr19	4146200	0.727273
chr19	4338700	0.909091
chr19	4369200	0.900826
chr19	4374000	0.818182
chr19	4377000	0.983471
chr19	4380500	0.727273
chr19	4386300	0.867769
chr19	4392100	0.785124
chr19	4394700	0.942149

chr19	4397900	0.851240
chr19	4401700	0.909091
chr19	4471000	0.818182
chr19	4506900	0.917355
chr19	4535300	0.776860
chr19	4540600	0.727273
chr19	4558200	0.917355
chr19	4560700	0.793388
chr19	4565700	0.892562
chr19	4575600	0.892562
chr19	4634100	0.793388
chr19	4636200	0.950413
chr19	4638700	0.719008
chr19	4647900	0.876033
chr19	4650000	0.710744
chr19	4694700	0.785124
chr19	4714000	0.983471
chr19	4813200	0.867769
chr19	4816000	0.727273
chr19	4912600	0.785124
chr19	4916500	0.900826
chr19	4972600	0.694215
chr19	4984000	0.661157
chr19	5037500	0.867769
chr19	5049300	0.677686
chr19	5173600	0.694215
chr19	5293300	0.661157
chr19	5626000	0.685950
chr19	5692900	0.743802
chr19	5804000	0.826446
chr19	5895000	0.859504
chr19	5934600	0.842975
chr19	5951800	0.727273
chr19	5954400	0.983471
chr19	5962600	0.892562
chr19	5968900	0.801653
chr19	5980100	0.752066
chr19	5985900	0.752066
chr19	5994500	0.925620
chr19	6013900	0.842975
chr19	6023600	0.942149
chr19	6056600	0.892562
chr19	6059000	0.867769
chr19	6062900	0.884298
chr19	6065600	0.958678
chr19	6068000	0.876033
chr19	6086500	0.785124
chr19	6089000	0.983471
chr19	6092800	0.785124
chr19	6100700	0.909091
chr19	6104500	0.933884
chr19	6220500	0.677686
chr19	6234200	0.917355
chr19	6237700	0.867769

chr19	6241000	0.925620
chr19	6244800	0.942149
chr19	6250000	0.768595
chr19	6270200	0.669421
chr19	6276900	0.991736
chr19	6374400	0.669421
chr19	6531200	0.826446
chr19	6611400	0.859504
chr19	6675300	0.793388
chr19	6734800	0.950413
chr19	6745200	0.818182
chr19	6753800	0.776860
chr19	6767600	0.826446
chr19	6802200	0.958678
chr19	6863600	0.685950
chr19	6948500	0.942149
chr19	7129300	0.694215
chr19	7168000	0.776860
chr19	7183200	0.975207
chr19	7189100	0.933884
chr19	7197600	0.785124
chr19	7310200	0.727273
chr19	7456500	0.942149
chr19	7515500	0.917355
chr19	7548100	0.859504
chr19	7557600	0.884298
chr19	7566200	0.768595
chr19	7702000	0.694215
chr19	7717400	0.809917
chr19	7720800	0.752066
chr19	7853800	0.793388
chr19	7860700	0.966942
chr19	7864600	0.909091
chr19	7927300	0.785124
chr19	7934300	0.694215
chr19	7939800	0.661157
chr19	7994100	0.909091
chr19	8052200	0.801653
chr19	8067300	0.900826
chr19	8305700	0.661157
chr19	8334300	0.752066
chr19	8389400	0.818182
chr19	8400600	0.669421
chr19	8406100	0.909091
chr19	8419900	0.958678
chr19	8422000	0.925620
chr19	8426900	0.950413
chr19	8432700	0.950413
chr19	8457100	0.859504
chr19	8459800	0.958678
chr19	8461900	0.917355
chr19	8488800	0.966942
chr19	8512700	0.851240
chr19	8525000	0.942149

chr19	8657500	0.859504
chr19	9239400	0.876033
chr19	9969300	0.661157
chr19	10207300	0.851240
chr19	10347200	0.966942
chr19	10379400	0.685950
chr19	10382600	0.809917
chr19	10397700	0.727273
chr19	10400300	0.710744
chr19	10406600	0.809917
chr19	10511800	0.793388
chr19	10527500	0.661157
chr19	10530900	0.677686
chr19	10535300	0.842975
chr19	10539000	0.900826
chr19	10543100	0.743802
chr19	10590400	0.809917
chr19	10707600	0.685950
chr19	10716000	0.942149
chr19	10736400	0.834711
chr19	10853400	0.851240
chr19	10859900	0.669421
chr19	10862600	0.892562
chr19	10891400	0.966942
chr19	10924000	0.735537
chr19	10948100	0.669421
chr19	11087000	0.809917
chr19	11185500	0.925620
chr19	11196400	0.818182
chr19	11204100	0.983471
chr19	11207500	0.760331
chr19	11210500	0.900826
chr19	11242200	0.892562
chr19	11248300	1.000000
chr19	11250400	0.760331
chr19	11254700	0.776860
chr19	11263800	0.950413
chr19	11276500	0.785124
chr19	11278900	0.743802
chr19	11281000	0.834711
chr19	11290200	1.000000
chr19	11306200	0.892562
chr19	11309200	0.694215
chr19	11314800	0.975207
chr19	11354400	0.661157
chr19	11363900	0.958678
chr19	11374900	0.867769
chr19	11377100	0.818182
chr19	11380200	0.925620
chr19	11404900	0.818182
chr19	11455000	0.942149
chr19	11489100	0.892562
chr19	11493600	0.826446
chr19	11530000	0.752066

chr19	11593800	0.785124
chr19	11616100	0.677686
chr19	11647000	0.942149
chr19	11851000	0.685950
chr19	12606500	0.727273
chr19	12655600	0.735537
chr19	12886300	0.752066
chr19	12890600	0.727273
chr19	12895600	0.884298
chr19	12900100	0.669421
chr19	12905500	0.719008
chr19	12917400	0.669421
chr19	12952200	0.669421
chr19	12958800	0.851240
chr19	12978700	0.719008
chr19	12996700	0.727273
chr19	13030300	0.785124
chr19	13063100	0.917355
chr19	13065500	0.702479
chr19	13076500	0.925620
chr19	13130100	0.677686
chr19	13134200	0.719008
chr19	13137500	0.719008
chr19	13148000	0.768595
chr19	13163000	0.685950
chr19	13167300	0.818182
chr19	13172100	0.776860
chr19	13190700	0.694215
chr19	13202600	0.917355
chr19	13205700	0.752066
chr19	13208100	0.867769
chr19	13210500	0.925620
chr19	13215600	0.925620
chr19	13267700	0.942149
chr19	13274100	0.942149
chr19	13277000	0.900826
chr19	13279400	0.958678
chr19	13283700	0.942149
chr19	13345800	0.669421
chr19	13388700	0.677686
chr19	13510300	0.975207
chr19	13540300	0.950413
chr19	13841200	0.752066
chr19	13855100	0.842975
chr19	13892800	0.826446
chr19	13899000	0.950413
chr19	13908800	0.826446
chr19	13945400	0.942149
chr19	13948900	0.867769
chr19	13954900	0.743802
chr19	13957000	0.884298
chr19	13959200	0.702479
chr19	13961600	0.785124
chr19	13963900	0.917355

chr19	13972700	0.760331
chr19	13976900	0.776860
chr19	13991600	0.909091
chr19	14090200	0.909091
chr19	14098800	0.793388
chr19	14115800	0.710744
chr19	14169200	0.661157
chr19	14218900	0.801653
chr19	14224100	0.727273
chr19	14229800	0.710744
chr19	14259100	0.842975
chr19	14261200	0.661157
chr19	14339700	0.752066
chr19	14359900	0.785124
chr19	14395200	0.884298
chr19	14474500	0.752066
chr19	14495600	0.702479
chr19	14547200	0.677686
chr19	14551400	0.917355
chr19	14586500	0.942149
chr19	14608300	0.760331
chr19	14618700	0.867769
chr19	14630900	0.752066
chr19	14633900	0.966942
chr19	14671700	0.867769
chr19	15334500	0.752066
chr19	15363600	0.661157
chr19	15486000	0.900826
chr19	15544300	0.669421
chr19	15560300	0.685950
chr19	15568500	0.752066
chr19	15575000	0.892562
chr19	15578100	0.942149
chr19	15580900	0.694215
chr19	15662400	0.677686
chr19	16152100	0.966942
chr19	16175000	0.694215
chr19	16191700	0.975207
chr19	16196000	0.669421
chr19	16199200	0.876033
chr19	16207400	0.719008
chr19	16369700	0.743802
chr19	16373600	0.685950
chr19	16390400	0.933884
chr19	16394800	0.975207
chr19	16401300	0.900826
chr19	16404800	0.834711
chr19	16427500	0.975207
chr19	16440000	0.752066
chr19	16450700	0.958678
chr19	16458900	0.975207
chr19	16462600	1.000000
chr19	16494200	0.826446
chr19	16531300	0.735537

chr19	16547600	0.942149
chr19	16558500	0.900826
chr19	16568900	0.867769
chr19	16574100	0.710744
chr19	16677400	0.710744
chr19	16695300	0.991736
chr19	16699400	0.942149
chr19	16845100	0.942149
chr19	16936400	0.752066
chr19	16945600	0.752066
chr19	16949300	0.991736
chr19	16983000	0.966942
chr19	16988000	0.859504
chr19	16999600	0.991736
chr19	17190900	0.867769
chr19	17201100	0.917355
chr19	17208700	0.685950
chr19	17220000	0.975207
chr19	17247900	0.925620
chr19	17357700	0.892562
chr19	17375300	0.900826
chr19	17407400	0.719008
chr19	17414200	0.785124
chr19	17439800	0.793388
chr19	17458100	0.719008
chr19	17460200	0.983471
chr19	17463900	0.702479
chr19	17490400	0.719008
chr19	17494000	0.818182
chr19	17516200	0.752066
chr19	17523800	1.000000
chr19	17529800	0.760331
chr19	17559400	0.752066
chr19	17600200	0.950413
chr19	17611000	0.760331
chr19	17634400	0.842975
chr19	17650500	0.851240
chr19	17664500	0.942149
chr19	17671500	0.975207
chr19	17717200	0.760331
chr19	17791400	0.933884
chr19	17797900	0.793388
chr19	17828700	0.818182
chr19	17833900	0.752066
chr19	17858800	0.785124
chr19	17890100	0.677686
chr19	17906000	0.702479
chr19	17953300	0.859504
chr19	17959200	0.661157
chr19	18058400	0.661157
chr19	18111900	0.710744
chr19	18131500	0.677686
chr19	18135300	0.743802
chr19	18209300	0.925620

chr19	18215700	0.702479
chr19	18268400	0.768595
chr19	18272200	0.694215
chr19	18303000	0.752066
chr19	18386100	0.702479
chr19	18393700	0.752066
chr19	18415900	0.942149
chr19	18431700	0.966942
chr19	18435100	0.884298
chr19	18475300	0.933884
chr19	18477700	0.809917
chr19	18485600	0.966942
chr19	18503900	0.801653
chr19	18526400	0.851240
chr19	18528800	0.942149
chr19	18533400	0.677686
chr19	18540300	0.933884
chr19	18571200	0.694215
chr19	18590800	0.909091
chr19	18618500	0.685950
chr19	18626200	0.867769
chr19	18630500	0.735537
chr19	18653700	0.719008
chr19	18671200	0.867769
chr19	18673400	0.892562
chr19	18723800	0.826446
chr19	18761800	0.834711
chr19	18789100	0.859504
chr19	18812100	0.834711
chr19	18950200	0.876033
chr19	18959800	0.867769
chr19	18962500	0.851240
chr19	18971000	0.743802
chr19	19078900	0.950413
chr19	19081100	0.842975
chr19	19083700	0.933884
chr19	19096700	0.826446
chr19	19110300	0.818182
chr19	19173900	0.677686
chr19	19221700	0.834711
chr19	19281500	0.735537
chr19	19336400	0.809917
chr19	19440300	0.909091
chr19	19476100	0.760331
chr19	19484100	0.925620
chr19	19495600	0.842975
chr19	19502700	0.735537
chr19	19506000	0.685950
chr19	19555600	0.669421
chr19	19569500	0.776860
chr19	19573300	0.776860
chr19	19575500	0.876033
chr19	19601100	0.727273
chr19	24526300	0.892562

chr19	27740400	0.917355
chr19	27764800	0.735537
chr19	27967300	0.793388
chr19	28781300	0.710744
chr19	28804800	0.876033
chr19	28822300	0.942149
chr19	30153800	0.892562
chr19	30207300	0.702479
chr19	30364500	0.776860
chr19	31821100	0.925620
chr19	32715800	0.685950
chr19	32843100	0.809917
chr19	32864900	0.950413
chr19	32913200	0.975207
chr19	32944800	0.834711
chr19	32949700	0.826446
chr19	33153300	0.900826
chr19	33185600	0.661157
chr19	33555500	0.677686
chr19	33601300	0.776860
chr19	33867800	0.983471
chr19	33888500	0.867769
chr19	33926400	0.694215
chr19	33964800	0.958678
chr19	33998800	0.768595
chr19	34113200	0.809917
chr19	34273100	0.702479
chr19	34311800	0.785124
chr19	34359900	0.785124
chr19	34375500	0.876033
chr19	34397200	1.000000
chr19	34662600	0.685950
chr19	34668200	0.867769
chr19	34724100	0.975207
chr19	34759900	0.917355
chr19	34762400	0.909091
chr19	34765800	0.975207
chr19	34768600	0.826446
chr19	34773900	0.685950
chr19	34804300	0.876033
chr19	34807700	0.826446
chr19	34811200	0.917355
chr19	34819200	0.966942
chr19	34823700	0.991736
chr19	34922100	0.743802
chr19	34927900	0.859504
chr19	34966100	0.661157
chr19	34971900	0.842975
chr19	35184100	0.776860
chr19	35329200	0.809917
chr19	35476700	0.950413
chr19	35486000	0.752066
chr19	35489000	1.000000
chr19	35494500	0.735537

chr19	35497500	0.958678
chr19	35529000	0.975207
chr19	35532200	0.851240
chr19	35615600	0.669421
chr19	35705200	0.793388
chr19	35810200	0.900826
chr19	35950500	0.669421
chr19	36049200	0.743802
chr19	36066700	1.000000
chr19	36133100	0.859504
chr19	36164500	0.743802
chr19	36247600	0.950413
chr19	36360000	0.685950
chr19	36422500	0.809917
chr19	36500800	0.743802
chr19	36561900	0.694215
chr19	36619300	0.917355
chr19	36628100	0.694215
chr19	36642800	0.727273
chr19	36660700	0.950413
chr19	36716400	0.909091
chr19	37853400	0.900826
chr19	37998000	0.719008
chr19	38423200	0.975207
chr19	38453000	0.958678
chr19	38489000	0.719008
chr19	38491500	0.942149
chr19	38496700	0.876033
chr19	38510900	0.966942
chr19	38514400	0.917355
chr19	38516500	0.793388
chr19	38522200	0.942149
chr19	38551700	1.000000
chr19	38571000	0.842975
chr19	38700800	0.925620
chr19	38719900	0.876033
chr19	38742500	0.909091
chr19	38757800	0.735537
chr19	38765600	0.677686
chr19	38768800	0.942149
chr19	38881400	0.900826
chr19	38908800	0.727273
chr19	39142600	1.000000
chr19	39146400	0.966942
chr19	39150800	0.983471
chr19	39154200	0.743802
chr19	39158400	0.867769
chr19	39162200	0.867769
chr19	39166800	0.925620
chr19	39170200	0.958678
chr19	39175700	0.826446
chr19	39178000	0.884298
chr19	39182400	0.702479
chr19	39185300	0.958678

chr19	39197500	0.727273
chr19	39199600	0.809917
chr19	39225100	0.768595
chr19	39269500	0.727273
chr19	39283700	0.685950
chr19	39296200	0.900826
chr19	39353600	0.942149
chr19	39369200	0.892562
chr19	39431000	0.867769
chr19	39441100	0.677686
chr19	39483800	0.793388
chr19	39564900	0.983471
chr19	39569500	0.884298
chr19	39574600	0.743802
chr19	39623000	0.851240
chr19	39635500	0.876033
chr19	39651600	0.768595
chr19	39826700	0.867769
chr19	39863700	0.702479
chr19	39889000	0.793388
chr19	39895700	0.719008
chr19	39901400	0.900826
chr19	39919300	0.942149
chr19	39922400	0.909091
chr19	39924600	0.909091
chr19	40033300	0.966942
chr19	40059300	0.669421
chr19	40448200	0.818182
chr19	40512400	0.867769
chr19	40653800	0.776860
chr19	40726000	0.942149
chr19	40737600	0.710744
chr19	40741100	0.809917
chr19	40748400	0.661157
chr19	40792200	0.760331
chr19	40863800	0.842975
chr19	40872100	0.793388
chr19	40923400	0.925620
chr19	40939700	0.743802
chr19	41091800	0.966942
chr19	41094700	0.942149
chr19	41106000	0.768595
chr19	41109700	0.917355
chr19	41116300	0.867769
chr19	41121900	0.685950
chr19	41140500	0.760331
chr19	41147200	0.760331
chr19	41152500	0.983471
chr19	41169400	0.768595
chr19	41172800	0.867769
chr19	41228000	0.966942
chr19	41255700	0.809917
chr19	41267800	0.867769
chr19	41304300	0.743802

chr19	41310500	0.685950
chr19	41313100	0.942149
chr19	41316900	0.826446
chr19	41427400	0.685950
chr19	41448500	0.983471
chr19	41641900	0.661157
chr19	41728300	0.867769
chr19	41733500	0.818182
chr19	41773300	0.867769
chr19	41779100	0.842975
chr19	41813300	0.809917
chr19	41835500	0.818182
chr19	41855500	0.867769
chr19	41874000	0.966942
chr19	41882700	0.727273
chr19	41908300	1.000000
chr19	41913000	0.834711
chr19	41934900	0.685950
chr19	41962800	0.983471
chr19	41982800	0.685950
chr19	41986900	0.702479
chr19	42007600	0.900826
chr19	42028700	0.760331
chr19	42038300	0.983471
chr19	42056100	0.867769
chr19	42061800	0.710744
chr19	42391400	0.768595
chr19	42433100	0.793388
chr19	42593100	0.876033
chr19	42596500	0.942149
chr19	42616900	0.818182
chr19	42619100	0.793388
chr19	42626300	0.966942
chr19	42637700	0.661157
chr19	42712000	0.884298
chr19	42723300	0.752066
chr19	42750200	0.685950
chr19	42754500	0.702479
chr19	42760800	0.694215
chr19	42782700	0.900826
chr19	42787300	0.909091
chr19	42790900	0.958678
chr19	42818000	0.669421
chr19	42891700	0.677686
chr19	42900400	0.702479
chr19	42937900	0.851240
chr19	42954600	0.793388
chr19	43033200	0.834711
chr19	43972400	0.851240
chr19	43979800	0.933884
chr19	44102900	0.702479
chr19	44168500	0.909091
chr19	44257000	0.760331
chr19	44264300	0.661157

chr19	44269700	0.942149
chr19	44279100	0.859504
chr19	44282500	0.760331
chr19	44289200	0.818182
chr19	44303400	0.669421
chr19	44324800	0.685950
chr19	45089700	0.892562
chr19	45092400	0.909091
chr19	45096400	0.917355
chr19	45106100	0.900826
chr19	45108500	0.942149
chr19	45110700	0.991736
chr19	45126800	0.842975
chr19	45129800	0.950413
chr19	45138100	0.793388
chr19	45152200	1.000000
chr19	45181000	0.917355
chr19	45188800	0.933884
chr19	45195900	0.933884
chr19	45223100	0.768595
chr19	45225800	0.826446
chr19	45227900	0.776860
chr19	45245400	0.983471
chr19	45250900	0.735537
chr19	45253700	0.669421
chr19	45257200	0.991736
chr19	45261400	0.950413
chr19	45264300	0.661157
chr19	45277900	0.942149
chr19	45300500	0.752066
chr19	45312500	0.818182
chr19	45324100	0.727273
chr19	45348100	0.710744
chr19	45354600	0.884298
chr19	45360100	0.752066
chr19	45372200	0.991736
chr19	45374400	0.950413
chr19	45379500	0.785124
chr19	45409400	0.776860
chr19	45418400	0.752066
chr19	45602100	0.768595
chr19	45657300	0.909091
chr19	45668000	0.710744
chr19	45674700	0.975207
chr19	45715300	0.876033
chr19	45719300	0.950413
chr19	45739200	0.727273
chr19	45748700	0.867769
chr19	45776200	0.876033
chr19	45784200	0.975207
chr19	45844100	0.785124
chr19	45880000	0.909091
chr19	45889400	0.793388
chr19	45902100	0.925620

chr19	45905700	0.876033
chr19	45929400	0.884298
chr19	45932200	0.793388
chr19	45947800	1.000000
chr19	45959500	0.950413
chr19	46002500	0.859504
chr19	46009200	0.801653
chr19	46018500	0.851240
chr19	46072400	0.809917
chr19	46084700	0.942149
chr19	46096100	0.909091
chr19	46103000	0.727273
chr19	46110800	0.917355
chr19	46118800	0.917355
chr19	46134300	0.950413
chr19	46146100	0.925620
chr19	46172400	0.942149
chr19	46181200	0.719008
chr19	46185100	0.768595
chr19	46222000	0.768595
chr19	46225600	0.727273
chr19	46313600	0.933884
chr19	46319500	0.818182
chr19	46324200	0.950413
chr19	46380400	0.760331
chr19	46431500	0.917355
chr19	46457100	0.859504
chr19	46459900	0.909091
chr19	46463400	0.818182
chr19	46503900	0.661157
chr19	46518600	0.685950
chr19	46525800	0.727273
chr19	46573000	1.000000
chr19	46625900	0.851240
chr19	46637000	0.983471
chr19	46766600	0.760331
chr19	46787400	0.925620
chr19	46792600	0.900826
chr19	46801900	0.793388
chr19	46804800	0.892562
chr19	46855800	0.743802
chr19	46874700	0.702479
chr19	46878100	0.785124
chr19	46920100	0.809917
chr19	46991000	0.768595
chr19	47221600	0.752066
chr19	47225900	0.727273
chr19	47230700	0.743802
chr19	47234700	0.900826
chr19	47237100	0.752066
chr19	47258700	0.950413
chr19	47317400	0.950413
chr19	47323700	0.719008
chr19	47333400	0.710744

chr19	47359900	0.933884
chr19	47367200	0.785124
chr19	47370400	1.000000
chr19	47377700	0.892562
chr19	47382200	0.826446
chr19	47408300	0.818182
chr19	47417300	0.702479
chr19	47428600	0.834711
chr19	47435900	0.900826
chr19	47439100	0.818182
chr19	47459000	0.942149
chr19	47466500	0.776860
chr19	47478600	0.942149
chr19	47494900	0.809917
chr19	47507400	0.776860
chr19	47611100	0.958678
chr19	47638600	0.991736
chr19	47655900	0.892562
chr19	47660300	0.867769
chr19	47662800	0.694215
chr19	47678300	0.876033
chr19	47680400	0.975207
chr19	47683400	0.942149
chr19	47692700	0.818182
chr19	47694900	0.859504
chr19	47706700	0.851240
chr19	47714500	0.884298
chr19	47729600	0.942149
chr19	47736400	0.685950
chr19	47743700	0.842975
chr19	47747700	0.950413
chr19	47828100	0.743802
chr19	47833900	0.752066
chr19	47867700	0.876033
chr19	47931800	0.909091
chr19	48013600	0.793388
chr19	48027000	0.776860
chr19	48076700	0.702479
chr19	48099500	0.727273
chr19	48102100	0.793388
chr19	48116900	0.842975
chr19	48222700	0.809917
chr19	48233700	0.851240
chr19	48245900	0.933884
chr19	48266400	0.702479
chr19	48280200	0.842975
chr19	48611300	0.892562
chr19	48614600	0.834711
chr19	48625000	0.892562
chr19	48627400	0.925620
chr19	48629500	0.809917
chr19	48656700	0.925620
chr19	48700800	0.826446
chr19	48713600	0.991736

chr19	48722800	0.818182
chr19	48747200	0.793388
chr19	48750100	0.925620
chr19	48754800	0.950413
chr19	48761000	0.710744
chr19	48763300	0.925620
chr19	48814200	0.950413
chr19	48820800	0.892562
chr19	48824700	0.851240
chr19	48836500	0.917355
chr19	48868200	0.685950
chr19	48897600	0.826446
chr19	48919200	0.685950
chr19	48928500	0.950413
chr19	48984200	0.900826
chr19	48991600	0.818182
chr19	49046700	0.975207
chr19	49057400	0.661157
chr19	49072200	0.677686
chr19	49127600	0.867769
chr19	49134000	0.884298
chr19	49141700	0.818182
chr19	49149600	0.685950
chr19	49178900	0.900826
chr19	49200100	0.776860
chr19	49218500	0.809917
chr19	49228700	0.958678
chr19	49230800	0.809917
chr19	49245100	0.710744
chr19	49250100	0.669421
chr19	49284500	0.768595
chr19	49298800	0.809917
chr19	49315600	0.752066
chr19	49339200	0.685950
chr19	49380500	0.826446
chr19	49399200	0.809917
chr19	49412300	0.900826
chr19	49437200	0.677686
chr19	49466500	0.909091
chr19	49631700	0.702479
chr19	49637300	0.710744
chr19	49646500	0.768595
chr19	49649300	0.710744
chr19	49668800	0.842975
chr19	49693100	0.685950
chr19	49702400	0.859504
chr19	49860400	0.991736
chr19	50015800	0.727273
chr19	50031500	0.859504
chr19	50058600	0.983471
chr19	50093500	0.768595
chr19	50117600	0.942149
chr19	50134800	0.719008
chr19	50183500	0.661157

chr19	50191700	0.958678
chr19	50335800	0.735537
chr19	50362800	0.983471
chr19	50373000	0.661157
chr19	50400800	0.661157
chr19	50430800	0.785124
chr19	50458600	0.818182
chr19	50508000	0.851240
chr19	50804500	0.966942
chr19	50852900	0.933884
chr19	50878400	0.727273
chr19	50890900	0.702479
chr19	51161800	0.876033
chr19	51206500	0.933884
chr19	51226500	0.685950
chr19	51231600	0.818182
chr19	51600600	0.702479
chr19	51866500	0.834711
chr19	51897800	0.834711
chr19	52184700	0.685950
chr19	52191000	0.677686
chr19	52196000	0.702479
chr19	52207600	0.760331
chr19	53046600	0.876033
chr19	53219400	0.958678
chr19	53226400	0.702479
chr19	53239600	0.876033
chr19	53494800	0.867769
chr19	53590900	0.966942
chr19	54050800	0.809917
chr19	54313400	0.818182
chr19	54346200	0.950413
chr19	54352700	0.933884
chr19	54369500	0.809917
chr19	54385300	0.694215
chr19	54493400	0.900826
chr19	54495600	0.876033
chr19	54497700	0.752066
chr19	54511900	0.942149
chr19	54523500	0.925620
chr19	54527100	0.809917
chr19	54539200	0.900826
chr19	54652800	0.867769
chr19	54665500	0.892562
chr19	54707700	0.892562
chr19	54714100	0.950413
chr19	54752000	0.859504
chr19	54900700	0.735537
chr19	54963000	0.876033
chr19	54972500	0.776860
chr19	54980600	0.876033
chr19	54984800	0.785124
chr19	55030200	1.000000
chr19	55032800	0.966942

chr19	55035100	0.925620
chr19	55526500	0.892562
chr19	55550000	0.876033
chr19	55553400	0.991736
chr19	55572200	0.677686
chr19	55581100	0.842975
chr19	55626000	0.752066
chr19	55691000	0.710744
chr19	55700900	0.925620
chr19	55718800	0.900826
chr19	55721700	0.983471
chr19	55728400	0.818182
chr19	55763300	0.958678
chr19	55766600	0.851240
chr19	55771500	0.809917
chr19	55850400	0.851240
chr19	55865700	0.752066
chr19	55999900	0.851240
chr19	56028800	0.900826
chr19	56058600	0.743802
chr19	56061900	0.876033
chr19	56098600	0.694215
chr19	56116000	0.785124
chr19	56151700	0.719008
chr19	56169800	0.851240
chr19	56189300	0.859504
chr19	56191500	0.661157
chr19	56578300	0.859504
chr19	56592600	0.743802
chr19	56622800	0.876033
chr19	56633400	0.710744
chr19	56665000	0.842975
chr19	56674000	0.735537
chr19	56785100	0.900826
chr19	56813400	0.933884
chr19	58341800	0.942149
chr19	58862000	0.793388
chr19	58868400	0.776860
chr19	59050000	0.859504
chr19	59067800	0.768595
chr19	59072000	0.661157
chr1	13700	0.685950
chr1	21700	0.801653
chr1	569800	0.694215
chr1	743300	0.892562
chr1	752900	0.727273
chr1	856800	0.677686
chr1	877000	0.785124
chr1	976200	0.809917
chr1	999600	0.867769
chr1	1004800	0.694215
chr1	1015500	0.884298
chr1	1093800	0.826446
chr1	1148500	0.801653

chr1	1150900	0.851240
chr1	1196700	0.859504
chr1	1200700	0.859504
chr1	1206600	0.950413
chr1	1218100	0.702479
chr1	1366000	0.925620
chr1	1503300	0.925620
chr1	1618000	0.950413
chr1	1714400	0.909091
chr1	1779300	0.842975
chr1	1790700	1.000000
chr1	2064900	0.818182
chr1	2080500	0.942149
chr1	2126700	0.710744
chr1	2131000	0.818182
chr1	2136400	0.859504
chr1	2144100	0.826446
chr1	2146700	0.884298
chr1	2166500	0.752066
chr1	2178500	0.983471
chr1	2188700	0.884298
chr1	2207200	0.884298
chr1	2211100	0.966942
chr1	2222700	0.809917
chr1	2226400	0.702479
chr1	2231000	0.876033
chr1	2233100	0.834711
chr1	2245800	0.900826
chr1	2253900	0.950413
chr1	2325500	0.809917
chr1	2337400	0.925620
chr1	2345900	0.661157
chr1	2383600	0.983471
chr1	2477800	0.776860
chr1	2480300	0.933884
chr1	2486200	0.942149
chr1	2488400	0.727273
chr1	2510300	0.966942
chr1	2513500	0.975207
chr1	2525400	0.826446
chr1	3230600	0.884298
chr1	3421000	0.809917
chr1	3457000	0.743802
chr1	3459800	0.859504
chr1	3507700	0.950413
chr1	3510700	0.760331
chr1	3514800	0.694215
chr1	3536100	0.826446
chr1	6118900	0.826446
chr1	6265800	0.768595
chr1	6377300	0.859504
chr1	6409000	0.884298
chr1	6419800	1.000000
chr1	6446200	0.884298

chr1	6480200	0.909091
chr1	6485000	0.801653
chr1	6515300	0.694215
chr1	6546700	0.809917
chr1	6553500	0.826446
chr1	6562200	0.669421
chr1	6605100	0.876033
chr1	6607500	0.818182
chr1	6664800	0.900826
chr1	6803900	0.842975
chr1	6855900	0.983471
chr1	6865400	0.826446
chr1	6920200	0.966942
chr1	6996000	0.876033
chr1	7359900	0.942149
chr1	7439800	0.975207
chr1	7449600	0.669421
chr1	7462400	0.768595
chr1	7531600	0.760331
chr1	7609800	0.991736
chr1	7613000	0.851240
chr1	7821000	0.867769
chr1	8065400	0.925620
chr1	8075700	0.917355
chr1	8080200	0.983471
chr1	8082800	0.735537
chr1	8088400	0.983471
chr1	8093300	0.785124
chr1	8101200	0.867769
chr1	8108200	0.983471
chr1	8122400	0.958678
chr1	8138400	0.933884
chr1	8144200	0.818182
chr1	8150500	0.991736
chr1	8155200	0.702479
chr1	8177500	0.909091
chr1	8183000	0.958678
chr1	8194100	0.991736
chr1	8198100	0.966942
chr1	8230100	0.925620
chr1	8242800	0.958678
chr1	8255000	0.917355
chr1	8258400	0.925620
chr1	8272200	0.900826
chr1	8277500	0.917355
chr1	8285500	0.710744
chr1	8298700	0.909091
chr1	8302000	0.702479
chr1	8319500	0.966942
chr1	8413800	0.760331
chr1	8434000	0.834711
chr1	8444900	0.975207
chr1	8454900	0.900826
chr1	8458100	1.000000

chr1	8468700	0.966942
chr1	8472700	0.991736
chr1	8484900	0.743802
chr1	8499600	0.867769
chr1	8516900	0.793388
chr1	8523800	0.950413
chr1	8577500	0.694215
chr1	8586000	0.876033
chr1	8598700	0.743802
chr1	8601400	0.933884
chr1	8632000	1.000000
chr1	8658700	0.743802
chr1	8661400	0.818182
chr1	8663500	0.975207
chr1	8708000	0.991736
chr1	8712300	0.826446
chr1	8721300	0.809917
chr1	8728700	0.809917
chr1	8747500	0.950413
chr1	8771500	0.876033
chr1	8774000	0.892562
chr1	8797600	0.719008
chr1	8873500	1.000000
chr1	8875700	0.702479
chr1	8883000	0.760331
chr1	8918100	0.851240
chr1	8920400	0.677686
chr1	8935700	0.892562
chr1	8940300	0.727273
chr1	8960400	0.834711
chr1	8964200	0.884298
chr1	9002400	0.669421
chr1	9007600	0.950413
chr1	9036500	0.818182
chr1	9046600	0.917355
chr1	9078900	0.900826
chr1	9120200	0.685950
chr1	9207300	0.867769
chr1	9222400	0.719008
chr1	9224700	0.975207
chr1	9226900	0.925620
chr1	9258900	0.776860
chr1	9271600	0.892562
chr1	9300200	0.975207
chr1	9335900	0.892562
chr1	9356200	0.801653
chr1	9361000	0.760331
chr1	9363800	0.942149
chr1	9378100	0.975207
chr1	9398700	0.826446
chr1	9401800	0.842975
chr1	9422000	0.752066
chr1	9431900	0.933884
chr1	9434100	0.958678

chr1	9437400	0.933884
chr1	9458500	0.983471
chr1	9462800	0.851240
chr1	9471200	0.760331
chr1	9474400	0.966942
chr1	9496000	0.859504
chr1	9499000	0.818182
chr1	9502400	0.991736
chr1	9602500	0.669421
chr1	9622100	0.727273
chr1	9653400	0.900826
chr1	9712300	0.801653
chr1	9816400	0.694215
chr1	9885700	0.661157
chr1	9896200	0.752066
chr1	9926300	0.826446
chr1	9935600	0.727273
chr1	9950300	0.685950
chr1	9953800	0.966942
chr1	10051100	0.909091
chr1	10057800	0.834711
chr1	10092200	0.694215
chr1	10127300	0.851240
chr1	10168900	0.966942
chr1	10400300	0.925620
chr1	10448400	0.925620
chr1	10463800	0.958678
chr1	10475900	0.917355
chr1	10511800	0.785124
chr1	10518700	0.933884
chr1	10555500	0.669421
chr1	10570000	0.719008
chr1	10572100	0.669421
chr1	10588600	0.950413
chr1	10601200	0.925620
chr1	10636000	0.983471
chr1	10699000	0.694215
chr1	11208000	0.851240
chr1	11297300	0.917355
chr1	11328000	0.892562
chr1	11342100	0.983471
chr1	11519200	0.983471
chr1	11539400	0.842975
chr1	11753600	0.884298
chr1	11780000	0.677686
chr1	11791100	0.801653
chr1	11845400	0.801653
chr1	11851200	0.966942
chr1	11858200	0.991736
chr1	11881900	0.942149
chr1	11991200	0.851240
chr1	12045800	0.975207
chr1	12193500	0.760331
chr1	12204100	0.867769

chr1	12208000	0.933884
chr1	12213700	0.892562
chr1	12222100	0.809917
chr1	12237300	0.842975
chr1	12240200	0.983471
chr1	12244000	0.776860
chr1	12356800	0.917355
chr1	12466200	0.950413
chr1	12484200	0.834711
chr1	12493000	0.834711
chr1	12495200	0.900826
chr1	12497500	0.685950
chr1	12514300	0.719008
chr1	12518000	0.884298
chr1	12521500	0.966942
chr1	12538700	0.851240
chr1	12541900	0.975207
chr1	12570600	0.768595
chr1	12600500	0.867769
chr1	12602700	0.735537
chr1	12610100	0.876033
chr1	12656100	0.983471
chr1	12659900	0.826446
chr1	12665100	0.834711
chr1	12680200	0.785124
chr1	12696200	0.983471
chr1	14078200	0.809917
chr1	15333500	0.669421
chr1	15347900	0.785124
chr1	15417400	0.677686
chr1	15485700	0.752066
chr1	15504000	0.669421
chr1	15506100	0.983471
chr1	15515400	0.950413
chr1	15527400	0.909091
chr1	15536700	0.776860
chr1	15545200	1.000000
chr1	15573900	0.809917
chr1	15588100	0.950413
chr1	15655800	0.884298
chr1	15668700	0.958678
chr1	15679400	0.950413
chr1	15739700	0.842975
chr1	15742700	0.669421
chr1	15744800	0.876033
chr1	15820400	0.884298
chr1	15842100	0.842975
chr1	15851700	0.785124
chr1	15911400	0.702479
chr1	15943200	0.702479
chr1	15947000	0.991736
chr1	16010300	0.669421
chr1	16018800	0.884298
chr1	16077500	0.867769

chr1	16083000	0.834711
chr1	16092000	0.859504
chr1	16121400	0.818182
chr1	16124100	0.950413
chr1	16213000	0.909091
chr1	16263900	0.719008
chr1	16266100	0.776860
chr1	16276000	0.752066
chr1	16285900	0.958678
chr1	16293200	0.909091
chr1	16339700	0.818182
chr1	16375600	0.702479
chr1	16403000	0.851240
chr1	16433700	0.727273
chr1	16461100	0.826446
chr1	16469700	0.909091
chr1	16474500	0.900826
chr1	16484100	0.834711
chr1	16491100	0.876033
chr1	16494600	0.776860
chr1	16499500	0.942149
chr1	16501800	0.859504
chr1	16509400	0.793388
chr1	16533500	0.966942
chr1	16576700	0.917355
chr1	16593500	0.909091
chr1	16620400	0.677686
chr1	16700900	0.677686
chr1	16703400	1.000000
chr1	16713600	0.966942
chr1	16723700	0.900826
chr1	16840900	0.752066
chr1	16847700	0.851240
chr1	16859000	0.768595
chr1	16974500	0.710744
chr1	17020400	0.702479
chr1	17034600	0.851240
chr1	17066800	0.768595
chr1	17086500	0.719008
chr1	17222800	0.677686
chr1	17248600	0.760331
chr1	17287800	0.719008
chr1	17294400	0.809917
chr1	17311600	0.925620
chr1	17343100	0.735537
chr1	17446100	0.785124
chr1	17449500	0.892562
chr1	17455100	0.991736
chr1	17578000	0.785124
chr1	17872200	0.677686
chr1	19234800	0.818182
chr1	19240500	0.933884
chr1	19251100	0.950413
chr1	19253300	0.917355

chr1	19255700	0.933884
chr1	19268800	0.925620
chr1	19272000	0.867769
chr1	19275800	0.702479
chr1	19299700	0.942149
chr1	19336100	0.727273
chr1	19338800	0.768595
chr1	19407500	0.933884
chr1	19411100	0.669421
chr1	19531300	0.876033
chr1	19601000	0.818182
chr1	19642500	0.983471
chr1	19713200	0.685950
chr1	19718000	0.925620
chr1	19756900	0.983471
chr1	19763200	0.768595
chr1	19776500	0.983471
chr1	19784400	0.785124
chr1	19796000	0.933884
chr1	19802500	0.801653
chr1	19804600	0.933884
chr1	19829400	0.867769
chr1	19832100	0.859504
chr1	19922700	0.661157
chr1	19973000	0.694215
chr1	19978500	0.909091
chr1	19991800	0.785124
chr1	20092400	0.900826
chr1	20219300	0.776860
chr1	20407500	0.760331
chr1	20437500	0.818182
chr1	20820600	0.834711
chr1	20822700	0.876033
chr1	20916200	0.942149
chr1	21012100	0.727273
chr1	21023700	0.694215
chr1	21060700	0.925620
chr1	21174600	0.752066
chr1	21309000	0.768595
chr1	21349100	0.958678
chr1	21417200	0.752066
chr1	21424900	0.719008
chr1	21427800	0.818182
chr1	21476400	0.876033
chr1	21519300	0.892562
chr1	21578800	0.867769
chr1	21582000	0.942149
chr1	21592400	0.884298
chr1	21596300	0.867769
chr1	21601400	0.892562
chr1	21607800	0.933884
chr1	21621900	0.983471
chr1	21626500	0.892562
chr1	21629000	0.785124

chr1	21631100	0.818182
chr1	21640100	0.785124
chr1	21648400	0.826446
chr1	21651500	0.950413
chr1	21653800	0.867769
chr1	21657200	0.909091
chr1	21660300	0.826446
chr1	21662900	0.851240
chr1	21665700	0.661157
chr1	21901200	0.694215
chr1	21913000	0.958678
chr1	21996200	0.752066
chr1	22000300	0.793388
chr1	22030700	0.752066
chr1	22048300	0.966942
chr1	22091900	0.966942
chr1	22100300	0.876033
chr1	22102700	0.776860
chr1	22106200	0.859504
chr1	22143900	0.876033
chr1	22148200	0.727273
chr1	22185200	0.842975
chr1	22188700	0.677686
chr1	22197100	0.793388
chr1	22200700	0.677686
chr1	22222600	0.958678
chr1	22227300	0.710744
chr1	22230100	0.917355
chr1	22232500	0.917355
chr1	22235000	0.991736
chr1	22238500	0.966942
chr1	22242800	0.892562
chr1	22244900	0.942149
chr1	22247100	0.917355
chr1	22250000	0.776860
chr1	22264900	0.884298
chr1	22267700	0.909091
chr1	22271300	0.710744
chr1	22274000	0.760331
chr1	22285400	0.933884
chr1	22288900	0.818182
chr1	22291000	0.710744
chr1	22297600	0.826446
chr1	22299700	0.966942
chr1	22301800	0.925620
chr1	22323900	0.809917
chr1	22326900	0.942149
chr1	22346000	0.933884
chr1	22350600	0.719008
chr1	22366700	0.925620
chr1	22382600	0.719008
chr1	22416500	0.933884
chr1	22442100	0.809917
chr1	22455200	0.785124

chr1	22600600	0.851240
chr1	22675200	0.760331
chr1	22814800	0.925620
chr1	22826700	0.884298
chr1	23004800	0.669421
chr1	23029700	0.842975
chr1	23069300	0.867769
chr1	23072200	0.884298
chr1	23075000	0.942149
chr1	23077900	0.669421
chr1	23082700	0.851240
chr1	23148000	0.975207
chr1	23153200	0.834711
chr1	23156900	0.876033
chr1	23159500	0.801653
chr1	23203600	0.809917
chr1	23206700	0.776860
chr1	23215500	0.983471
chr1	23280200	0.669421
chr1	23303000	0.900826
chr1	23376900	0.966942
chr1	23396100	0.909091
chr1	23425900	0.842975
chr1	23428000	0.983471
chr1	23430500	0.975207
chr1	23441400	0.859504
chr1	23446500	0.694215
chr1	23449800	0.752066
chr1	23456700	0.991736
chr1	23459300	0.966942
chr1	23496900	0.702479
chr1	23505500	0.826446
chr1	23515600	0.859504
chr1	23521400	0.859504
chr1	23524900	0.900826
chr1	23551900	0.966942
chr1	23609900	1.000000
chr1	23615700	0.743802
chr1	23619200	0.991736
chr1	23630500	1.000000
chr1	23667600	0.851240
chr1	23672100	0.702479
chr1	23698100	0.768595
chr1	23704200	0.826446
chr1	23711300	0.727273
chr1	23720700	0.876033
chr1	23730200	0.933884
chr1	23734100	0.677686
chr1	23749500	0.768595
chr1	23855300	0.694215
chr1	23875200	0.818182
chr1	23881600	0.892562
chr1	23887000	0.768595
chr1	23889100	1.000000

chr1	23895200	0.867769
chr1	23904400	0.917355
chr1	24106900	0.735537
chr1	24125500	0.727273
chr1	24281900	0.950413
chr1	24292000	0.710744
chr1	24303700	0.735537
chr1	24308000	0.925620
chr1	24311300	0.958678
chr1	24335800	0.991736
chr1	24345700	0.884298
chr1	24435300	0.925620
chr1	24438600	0.876033
chr1	24477100	0.809917
chr1	24610700	0.685950
chr1	24711300	0.958678
chr1	24718600	0.900826
chr1	24720900	0.793388
chr1	24967900	0.661157
chr1	25010500	0.702479
chr1	25022400	0.884298
chr1	25032200	0.983471
chr1	25047100	0.917355
chr1	25051800	0.975207
chr1	25054000	0.925620
chr1	25057400	0.991736
chr1	25078900	0.950413
chr1	25081500	0.991736
chr1	25092200	0.942149
chr1	25095900	0.892562
chr1	25129400	0.768595
chr1	25133200	0.867769
chr1	25136400	0.892562
chr1	25155400	0.876033
chr1	25174800	0.950413
chr1	25586800	0.975207
chr1	25647300	0.785124
chr1	25653500	0.867769
chr1	25657100	0.991736
chr1	25689000	0.991736
chr1	25691500	0.694215
chr1	25788900	0.677686
chr1	25795600	0.809917
chr1	25859300	0.694215
chr1	25876200	0.743802
chr1	25886000	0.851240
chr1	25888300	0.785124
chr1	26022400	0.727273
chr1	26132900	0.818182
chr1	26188000	0.925620
chr1	26222900	0.867769
chr1	26421300	0.685950
chr1	26429400	0.991736
chr1	26450200	0.892562

chr1	26483200	0.801653
chr1	26488700	0.661157
chr1	26552400	0.768595
chr1	26602300	0.851240
chr1	26604800	0.669421
chr1	26611800	0.669421
chr1	26624900	0.702479
chr1	26677700	0.900826
chr1	27004200	0.677686
chr1	27018600	0.685950
chr1	27027100	0.793388
chr1	27033000	0.785124
chr1	27050300	0.975207
chr1	27071200	0.917355
chr1	27079400	0.966942
chr1	27095600	0.785124
chr1	27099200	0.776860
chr1	27112400	0.785124
chr1	27127100	0.966942
chr1	27175800	0.752066
chr1	27192700	0.719008
chr1	27251000	0.851240
chr1	27276800	0.752066
chr1	27318200	0.983471
chr1	27322000	0.694215
chr1	27327300	0.950413
chr1	27337900	0.685950
chr1	27358500	0.909091
chr1	27362900	0.834711
chr1	27371800	0.727273
chr1	27437000	0.760331
chr1	27439400	0.933884
chr1	27449000	0.933884
chr1	27676600	0.818182
chr1	27719300	0.694215
chr1	27754300	0.694215
chr1	27758200	0.991736
chr1	27763600	0.876033
chr1	27787200	0.975207
chr1	27849100	0.793388
chr1	27855100	0.958678
chr1	27867300	0.661157
chr1	27869800	0.776860
chr1	27883600	0.719008
chr1	27895400	0.892562
chr1	27902500	0.983471
chr1	27939700	0.760331
chr1	27943300	0.801653
chr1	27958200	0.884298
chr1	27961100	0.842975
chr1	27986700	0.818182
chr1	27989600	0.785124
chr1	28116200	0.983471
chr1	28160000	0.768595

chr1	28288200	0.719008
chr1	28319400	0.983471
chr1	28355800	1.000000
chr1	28372700	0.983471
chr1	28389800	0.909091
chr1	28393400	0.859504
chr1	28439400	0.851240
chr1	28487700	0.933884
chr1	28499600	0.776860
chr1	28570600	0.917355
chr1	28575800	0.884298
chr1	28743700	0.818182
chr1	28749100	0.975207
chr1	28764600	0.719008
chr1	28776900	0.867769
chr1	28782800	0.991736
chr1	28786300	0.876033
chr1	28928900	0.785124
chr1	28975100	0.842975
chr1	28994700	0.851240
chr1	29066600	0.884298
chr1	29072900	0.867769
chr1	29075600	0.826446
chr1	29078200	0.925620
chr1	29211700	0.801653
chr1	29225300	0.661157
chr1	29254200	0.991736
chr1	29449000	0.727273
chr1	29451100	0.669421
chr1	29529300	0.719008
chr1	29586800	0.669421
chr1	29710700	0.859504
chr1	30162000	0.809917
chr1	30180800	0.826446
chr1	30185400	0.958678
chr1	30447500	0.876033
chr1	30557100	0.925620
chr1	31214600	0.785124
chr1	31255800	0.719008
chr1	31297400	0.917355
chr1	31314100	0.884298
chr1	31320300	0.677686
chr1	31375200	0.702479
chr1	31380900	0.768595
chr1	31440000	0.884298
chr1	31447900	0.768595
chr1	31480600	0.909091
chr1	31513700	0.677686
chr1	31745800	0.793388
chr1	31867900	0.694215
chr1	32015500	0.826446
chr1	32029400	0.975207
chr1	32039700	0.983471
chr1	32047000	0.760331

chr1	32050300	0.867769
chr1	32053200	0.743802
chr1	32055600	0.942149
chr1	32062800	0.975207
chr1	32136100	0.768595
chr1	32146700	0.710744
chr1	32155700	0.752066
chr1	32166900	0.669421
chr1	32178700	0.966942
chr1	32181000	0.942149
chr1	32227100	0.793388
chr1	32230500	0.685950
chr1	32238400	0.876033
chr1	32262400	0.669421
chr1	32265400	0.694215
chr1	32269000	0.876033
chr1	32355300	0.958678
chr1	32396900	0.785124
chr1	32399100	0.900826
chr1	32401200	0.834711
chr1	32405000	0.925620
chr1	32410400	0.942149
chr1	32421600	0.983471
chr1	32448000	0.677686
chr1	32451100	0.727273
chr1	32529000	0.942149
chr1	32532600	0.942149
chr1	32567100	0.966942
chr1	32605000	0.925620
chr1	32638600	0.702479
chr1	32647900	0.785124
chr1	32690100	0.710744
chr1	32701900	0.884298
chr1	32714500	0.702479
chr1	32716800	0.900826
chr1	32732800	0.876033
chr1	32742000	0.776860
chr1	32815100	0.900826
chr1	32827900	0.834711
chr1	32962200	0.793388
chr1	32966300	0.867769
chr1	32968400	0.900826
chr1	33000000	0.694215
chr1	33191200	0.884298
chr1	33241400	0.793388
chr1	33280700	0.661157
chr1	33363300	0.661157
chr1	33399900	0.917355
chr1	33405200	0.785124
chr1	33407700	0.983471
chr1	33415200	0.661157
chr1	33419400	0.975207
chr1	33438800	0.719008
chr1	33447100	0.991736

chr1	33449200	0.669421
chr1	33455200	0.925620
chr1	33458500	0.884298
chr1	33462300	0.950413
chr1	33465400	0.834711
chr1	33470000	0.818182
chr1	33472700	0.983471
chr1	33627000	0.661157
chr1	33629800	0.867769
chr1	33772900	0.818182
chr1	33800600	0.719008
chr1	33804800	0.942149
chr1	33808200	0.818182
chr1	33812100	0.809917
chr1	33816300	0.818182
chr1	33887200	0.966942
chr1	33899800	0.991736
chr1	33909200	0.950413
chr1	33924600	0.760331
chr1	35308900	0.785124
chr1	35311100	0.876033
chr1	35332100	0.719008
chr1	35351500	0.818182
chr1	35395200	0.743802
chr1	35513400	0.909091
chr1	35528800	0.983471
chr1	35654200	0.661157
chr1	35754500	0.933884
chr1	35972100	0.851240
chr1	36043200	0.702479
chr1	36102900	0.752066
chr1	36181700	0.685950
chr1	36228000	0.991736
chr1	36474500	0.958678
chr1	36591100	0.694215
chr1	36599900	0.793388
chr1	36603300	0.892562
chr1	36617000	0.884298
chr1	36625200	0.752066
chr1	36628500	0.975207
chr1	36638100	0.950413
chr1	36640200	0.752066
chr1	36654000	0.942149
chr1	36714300	0.975207
chr1	36766200	0.851240
chr1	36804900	0.669421
chr1	36815200	0.760331
chr1	36832800	0.975207
chr1	36835000	0.859504
chr1	36840900	0.694215
chr1	36844000	0.669421
chr1	37218600	0.801653
chr1	37226700	0.768595
chr1	37272400	0.710744

chr1	37937200	0.710744
chr1	37945700	0.818182
chr1	38000300	0.991736
chr1	38022800	0.785124
chr1	38040900	0.776860
chr1	38043100	0.884298
chr1	38077200	0.917355
chr1	38411200	0.752066
chr1	38461900	0.950413
chr1	38468100	0.851240
chr1	38474900	0.702479
chr1	38482300	0.942149
chr1	38491700	0.710744
chr1	38496100	0.909091
chr1	38510600	0.743802
chr1	38580800	0.975207
chr1	38713000	0.942149
chr1	38921800	0.818182
chr1	39194200	1.000000
chr1	39201500	0.752066
chr1	39306400	0.768595
chr1	39407600	0.752066
chr1	39513600	0.909091
chr1	39568700	0.876033
chr1	39575900	0.950413
chr1	39581300	0.685950
chr1	39583600	0.958678
chr1	39590700	0.743802
chr1	39593400	0.991736
chr1	39596200	0.925620
chr1	39598900	0.735537
chr1	39614000	0.809917
chr1	39616900	1.000000
chr1	39621200	0.933884
chr1	39624300	0.818182
chr1	39626400	0.966942
chr1	39631500	0.909091
chr1	39638200	0.685950
chr1	39644800	0.966942
chr1	39652200	0.950413
chr1	39655600	0.991736
chr1	39658100	0.983471
chr1	39660400	0.900826
chr1	39662500	0.876033
chr1	39679300	0.925620
chr1	39704300	0.950413
chr1	39734100	0.983471
chr1	39738300	0.685950
chr1	39784400	0.950413
chr1	39848300	0.917355
chr1	39853500	0.925620
chr1	39858900	0.933884
chr1	39861300	0.867769
chr1	39865300	0.991736

chr1	39882700	0.785124
chr1	39897800	0.884298
chr1	39917400	0.950413
chr1	39957400	0.842975
chr1	39981200	0.727273
chr1	40236500	0.768595
chr1	40254800	0.752066
chr1	40311600	0.702479
chr1	40364900	0.925620
chr1	40480300	0.950413
chr1	40503000	0.752066
chr1	40508900	0.752066
chr1	40522900	0.710744
chr1	40525800	0.958678
chr1	40557100	0.892562
chr1	40598500	0.694215
chr1	40714700	0.743802
chr1	41178900	0.851240
chr1	41267900	0.776860
chr1	41307300	0.826446
chr1	41334400	0.768595
chr1	41341700	0.975207
chr1	41344800	0.933884
chr1	41364100	0.925620
chr1	41398000	0.925620
chr1	41414800	0.942149
chr1	41434000	0.958678
chr1	41469500	0.900826
chr1	41481500	0.793388
chr1	41487600	0.752066
chr1	41490600	0.851240
chr1	41510200	0.884298
chr1	41568300	0.933884
chr1	41575300	0.933884
chr1	41704600	0.743802
chr1	41807900	0.900826
chr1	41827300	0.818182
chr1	41884900	0.876033
chr1	41898700	0.900826
chr1	41962100	0.925620
chr1	41982400	0.694215
chr1	42179800	0.983471
chr1	42190000	0.851240
chr1	42193400	0.884298
chr1	42205100	0.859504
chr1	42218400	0.760331
chr1	42278500	0.900826
chr1	42306900	0.818182
chr1	42309600	0.685950
chr1	42312700	0.876033
chr1	42318100	0.661157
chr1	42328400	0.826446
chr1	42336400	0.752066
chr1	42357300	0.909091

chr1	42364000	0.933884
chr1	42368800	0.793388
chr1	42402100	0.702479
chr1	42421400	0.991736
chr1	42643400	0.851240
chr1	42650200	0.661157
chr1	42652300	0.867769
chr1	42677100	0.867769
chr1	42680600	0.950413
chr1	42707000	0.950413
chr1	42785600	0.983471
chr1	42789000	0.867769
chr1	42893400	0.909091
chr1	42903100	0.677686
chr1	42906100	0.752066
chr1	43154700	0.685950
chr1	43251300	0.834711
chr1	43405900	0.842975
chr1	43409800	0.884298
chr1	43444500	0.867769
chr1	43505400	0.727273
chr1	43673800	0.694215
chr1	43698300	0.900826
chr1	43740400	0.760331
chr1	43761000	0.983471
chr1	43765400	0.760331
chr1	43839000	0.933884
chr1	43932300	0.760331
chr1	43937400	0.900826
chr1	43939500	0.991736
chr1	43943000	0.826446
chr1	43996100	0.966942
chr1	44004700	0.702479
chr1	44006900	0.826446
chr1	44011400	0.710744
chr1	44031500	0.851240
chr1	44154500	0.884298
chr1	44176000	0.867769
chr1	44178100	0.710744
chr1	44249700	0.950413
chr1	44288000	1.000000
chr1	44319200	0.842975
chr1	44402100	0.710744
chr1	44514200	0.925620
chr1	44793900	0.876033
chr1	44868000	0.876033
chr1	44884100	0.702479
chr1	44889600	0.892562
chr1	44951700	0.966942
chr1	45138200	0.925620
chr1	45142200	0.818182
chr1	45196700	0.892562
chr1	45223800	0.719008
chr1	45244400	0.677686

chr1	45252300	0.702479
chr1	45265300	0.710744
chr1	45269600	0.859504
chr1	45277100	0.752066
chr1	45279500	0.975207
chr1	45285400	0.892562
chr1	45390700	0.950413
chr1	45413400	1.000000
chr1	45434400	0.826446
chr1	45436600	0.851240
chr1	45712500	0.950413
chr1	45732400	0.991736
chr1	45739000	0.793388
chr1	45769600	0.685950
chr1	45772700	0.702479
chr1	45800500	0.834711
chr1	45808000	0.859504
chr1	45859700	0.991736
chr1	46009800	0.991736
chr1	46052600	0.661157
chr1	46074700	0.983471
chr1	46083300	0.702479
chr1	46113800	0.677686
chr1	46145000	0.925620
chr1	46233100	0.685950
chr1	46268100	0.669421
chr1	46330100	0.669421
chr1	46358200	0.966942
chr1	46395000	0.661157
chr1	46411300	0.677686
chr1	46547500	0.917355
chr1	46603700	0.933884
chr1	46633000	0.809917
chr1	46637300	0.710744
chr1	46647600	0.842975
chr1	46649800	0.892562
chr1	46860400	0.842975
chr1	46999400	0.768595
chr1	47147900	0.925620
chr1	47152100	0.876033
chr1	47190000	0.983471
chr1	47323400	0.694215
chr1	47489800	0.826446
chr1	47496700	0.776860
chr1	47574000	0.975207
chr1	47648200	0.917355
chr1	47656400	0.793388
chr1	47679500	0.677686
chr1	47682900	0.933884
chr1	47691700	0.677686
chr1	47701300	0.859504
chr1	47707500	0.942149
chr1	47710100	0.892562
chr1	47798600	0.694215

chr1	47898600	0.834711
chr1	48035500	0.975207
chr1	48116500	0.776860
chr1	48123000	0.809917
chr1	48175500	0.900826
chr1	48360900	0.768595
chr1	48372000	0.991736
chr1	48463100	0.694215
chr1	48936000	0.975207
chr1	50890600	0.669421
chr1	51069600	1.000000
chr1	51133300	0.991736
chr1	51190800	0.677686
chr1	51528800	0.876033
chr1	51730300	0.983471
chr1	51734200	0.983471
chr1	51762900	0.884298
chr1	51769900	0.917355
chr1	51776900	0.702479
chr1	51780300	0.809917
chr1	51899800	0.991736
chr1	52015100	0.876033
chr1	52024700	0.950413
chr1	52035500	0.925620
chr1	52041700	0.818182
chr1	52102800	0.669421
chr1	52127100	0.950413
chr1	52137000	0.867769
chr1	52139600	0.884298
chr1	52203000	0.851240
chr1	52206700	0.826446
chr1	52216200	0.942149
chr1	52363700	0.991736
chr1	52411700	0.859504
chr1	52429700	0.818182
chr1	52605800	0.867769
chr1	52704200	0.958678
chr1	52706700	0.851240
chr1	52747700	0.933884
chr1	53035600	0.677686
chr1	53140700	0.752066
chr1	53169200	0.818182
chr1	53217700	0.785124
chr1	53257900	0.818182
chr1	53512700	0.694215
chr1	53516200	0.842975
chr1	53683000	0.727273
chr1	53759900	0.834711
chr1	53790900	0.768595
chr1	54231600	0.991736
chr1	54448600	0.768595
chr1	54513100	0.834711
chr1	54587700	0.801653
chr1	54603600	0.867769

chr1	54639000	0.727273
chr1	54673800	0.925620
chr1	54715900	0.859504
chr1	54729200	0.768595
chr1	54739300	0.776860
chr1	54753800	0.909091
chr1	54764200	0.776860
chr1	54779100	0.818182
chr1	54793000	0.983471
chr1	54801000	0.859504
chr1	54804100	0.768595
chr1	54851200	0.785124
chr1	54874500	0.958678
chr1	54879300	0.975207
chr1	54928100	0.661157
chr1	54931300	0.834711
chr1	54940800	0.826446
chr1	54965200	0.776860
chr1	55008000	0.818182
chr1	55036500	0.752066
chr1	55350300	0.809917
chr1	55416400	0.710744
chr1	55421500	0.909091
chr1	55437200	0.661157
chr1	55462700	0.727273
chr1	55505900	0.685950
chr1	55594800	0.826446
chr1	55669400	0.834711
chr1	55742400	0.933884
chr1	55776800	0.909091
chr1	55779900	0.867769
chr1	55784200	0.950413
chr1	55788800	0.661157
chr1	55798900	0.917355
chr1	55887100	0.719008
chr1	55896100	0.892562
chr1	55904800	0.760331
chr1	55914400	0.925620
chr1	55922900	0.669421
chr1	55940700	0.685950
chr1	56534900	0.983471
chr1	56539700	0.933884
chr1	56546100	0.950413
chr1	56603700	0.975207
chr1	56614800	0.884298
chr1	56768100	0.661157
chr1	56877400	0.925620
chr1	56884000	0.719008
chr1	56923300	0.933884
chr1	56936500	0.917355
chr1	56943800	0.900826
chr1	56949200	0.991736
chr1	56957100	0.900826
chr1	56962700	0.809917

chr1	56968500	0.834711
chr1	56972800	0.900826
chr1	56979300	0.826446
chr1	56982500	0.677686
chr1	57039100	0.677686
chr1	57298600	0.900826
chr1	58082400	0.768595
chr1	58861400	0.909091
chr1	58900200	0.760331
chr1	59049800	0.950413
chr1	59052600	0.900826
chr1	59058800	0.933884
chr1	59085000	1.000000
chr1	59144600	0.661157
chr1	59184500	0.983471
chr1	59193700	0.867769
chr1	59215200	0.743802
chr1	59219700	0.876033
chr1	59222800	0.768595
chr1	59228100	0.694215
chr1	59231200	0.975207
chr1	59239500	0.942149
chr1	59279300	0.876033
chr1	59285800	0.818182
chr1	59290100	0.752066
chr1	59315000	0.983471
chr1	59321400	0.983471
chr1	59347300	0.826446
chr1	59350000	0.942149
chr1	59355100	0.917355
chr1	59359700	0.991736
chr1	59363000	0.842975
chr1	59365700	0.942149
chr1	59369600	0.925620
chr1	59375300	1.000000
chr1	59479400	0.925620
chr1	59482000	0.826446
chr1	59486200	0.933884
chr1	59491400	0.859504
chr1	59495800	0.809917
chr1	59528500	0.983471
chr1	59538100	0.958678
chr1	59549900	0.859504
chr1	59583100	0.727273
chr1	59594600	0.991736
chr1	59598400	0.785124
chr1	59602500	0.710744
chr1	59609200	0.991736
chr1	59612600	0.859504
chr1	59616900	0.950413
chr1	59622700	0.685950
chr1	59639600	0.809917
chr1	59742400	0.900826
chr1	59758700	0.975207

chr1	59763600	0.685950
chr1	59782600	0.867769
chr1	59799000	0.876033
chr1	59801500	0.785124
chr1	59949900	0.834711
chr1	60036600	0.966942
chr1	60062300	0.975207
chr1	60144400	0.818182
chr1	60173200	0.702479
chr1	60197100	0.752066
chr1	60209400	0.975207
chr1	60216400	0.983471
chr1	60225300	0.661157
chr1	60238300	0.710744
chr1	60243300	0.900826
chr1	60280600	0.661157
chr1	60345900	0.859504
chr1	60567500	0.842975
chr1	60693900	0.892562
chr1	60746500	0.768595
chr1	60748600	0.801653
chr1	60889300	0.752066
chr1	60936300	0.975207
chr1	60970700	0.785124
chr1	60974400	0.661157
chr1	61039700	0.983471
chr1	61055300	0.917355
chr1	61062900	0.983471
chr1	61086800	0.950413
chr1	61149100	0.661157
chr1	61341000	0.942149
chr1	61377900	0.909091
chr1	61390200	0.867769
chr1	61414900	0.983471
chr1	61423600	0.966942
chr1	61426000	0.884298
chr1	61430000	0.892562
chr1	61448800	0.760331
chr1	61456200	0.884298
chr1	61472300	0.677686
chr1	61520000	0.685950
chr1	61545200	0.842975
chr1	61598400	0.917355
chr1	61605500	0.991736
chr1	61607800	0.851240
chr1	61647700	0.669421
chr1	61650200	0.768595
chr1	61667100	0.867769
chr1	61720900	0.867769
chr1	61737400	0.851240
chr1	61753600	0.983471
chr1	61755800	0.760331
chr1	61784500	0.925620
chr1	61804600	0.958678

chr1	61818600	0.719008
chr1	61824600	0.768595
chr1	61854500	0.826446
chr1	61869900	0.966942
chr1	61885400	0.768595
chr1	61900000	0.760331
chr1	61903200	0.685950
chr1	61916400	0.834711
chr1	61919900	0.909091
chr1	62072500	0.942149
chr1	62102600	0.818182
chr1	62107800	0.702479
chr1	62188800	0.685950
chr1	62209000	0.694215
chr1	62294900	0.752066
chr1	62318900	0.752066
chr1	63156200	0.942149
chr1	63293100	0.818182
chr1	63590900	0.950413
chr1	63783500	0.876033
chr1	63790000	0.677686
chr1	63929500	0.975207
chr1	64128500	0.785124
chr1	64164300	0.876033
chr1	64198000	0.876033
chr1	64212800	0.966942
chr1	64218400	0.958678
chr1	64340200	0.694215
chr1	64386500	0.760331
chr1	64472400	0.851240
chr1	64638300	0.966942
chr1	64697200	0.677686
chr1	64711900	0.694215
chr1	64729800	0.710744
chr1	64758200	0.933884
chr1	64786200	0.710744
chr1	64809600	0.685950
chr1	64942500	1.000000
chr1	64944800	0.760331
chr1	64947300	0.859504
chr1	64980200	0.719008
chr1	64983300	0.702479
chr1	65006400	0.991736
chr1	65090400	0.760331
chr1	65153700	0.983471
chr1	65157700	0.826446
chr1	65301500	0.735537
chr1	65304200	0.966942
chr1	65329600	0.900826
chr1	65331700	0.876033
chr1	65337300	1.000000
chr1	65340600	0.842975
chr1	65344300	0.909091
chr1	65347500	0.776860

chr1	65350800	0.942149
chr1	65357700	0.966942
chr1	65364100	0.991736
chr1	65382300	0.942149
chr1	65384600	0.768595
chr1	65387100	0.909091
chr1	65399800	0.975207
chr1	65415300	0.925620
chr1	65428900	0.942149
chr1	65467100	0.900826
chr1	65494000	0.661157
chr1	65885300	0.661157
chr1	65889500	0.909091
chr1	65892700	0.933884
chr1	65897200	0.983471
chr1	65935900	0.876033
chr1	65960400	0.669421
chr1	65979000	0.768595
chr1	65993400	0.760331
chr1	66004400	0.768595
chr1	66072200	0.743802
chr1	66157500	0.661157
chr1	66278100	0.925620
chr1	66419300	0.900826
chr1	66536500	0.702479
chr1	66709800	0.966942
chr1	66713200	0.909091
chr1	66728500	0.818182
chr1	66730600	0.909091
chr1	66733700	0.909091
chr1	66737700	0.876033
chr1	66767200	0.851240
chr1	66774100	0.677686
chr1	66797300	0.694215
chr1	66802000	0.851240
chr1	66805500	0.892562
chr1	66809600	0.983471
chr1	66813500	0.983471
chr1	66816000	0.983471
chr1	66818500	0.801653
chr1	66821300	0.859504
chr1	66836800	0.975207
chr1	66839900	0.818182
chr1	66904800	0.966942
chr1	66911500	0.925620
chr1	66956300	0.892562
chr1	66970200	0.669421
chr1	66993800	0.975207
chr1	66998400	0.925620
chr1	67008800	1.000000
chr1	67030600	0.909091
chr1	67064700	0.710744
chr1	67090100	0.958678
chr1	67142800	0.983471

chr1	67192200	0.661157
chr1	67195800	0.983471
chr1	67198800	0.942149
chr1	67218300	0.760331
chr1	67399600	0.826446
chr1	67404400	0.694215
chr1	67411100	0.876033
chr1	67420600	0.925620
chr1	67485900	0.958678
chr1	67730900	1.000000
chr1	67752100	0.842975
chr1	67913100	0.719008
chr1	67934600	0.785124
chr1	67991400	0.694215
chr1	68030200	0.826446
chr1	68036600	0.710744
chr1	68056400	0.900826
chr1	68078500	0.735537
chr1	68084600	0.975207
chr1	68092300	0.900826
chr1	68096300	0.884298
chr1	68140900	0.925620
chr1	68149100	0.818182
chr1	68166900	0.884298
chr1	68173500	0.685950
chr1	68176400	0.867769
chr1	68179600	0.917355
chr1	68186400	0.909091
chr1	68189400	0.900826
chr1	68192900	0.892562
chr1	68201400	0.909091
chr1	68203900	0.809917
chr1	68214600	0.809917
chr1	68217200	0.909091
chr1	68220600	0.785124
chr1	68226100	0.677686
chr1	68232700	0.925620
chr1	68237300	0.909091
chr1	68251900	0.991736
chr1	68255700	0.950413
chr1	68258100	0.983471
chr1	68276400	0.834711
chr1	68280600	0.834711
chr1	68296200	0.900826
chr1	68304200	0.818182
chr1	68307100	0.942149
chr1	68313400	0.876033
chr1	68316700	0.867769
chr1	68322000	0.975207
chr1	68349200	0.785124
chr1	68355800	0.991736
chr1	68362100	0.851240
chr1	68364200	0.752066
chr1	68374000	0.727273

chr1	68436100	0.900826
chr1	68641700	0.983471
chr1	68644200	0.884298
chr1	68646400	0.892562
chr1	68665000	0.933884
chr1	68671200	0.669421
chr1	68673300	0.975207
chr1	68688200	0.719008
chr1	68759400	0.677686
chr1	68779800	0.752066
chr1	68797300	0.785124
chr1	68845200	0.793388
chr1	68850600	0.991736
chr1	68943000	1.000000
chr1	70035700	0.785124
chr1	70325000	0.826446
chr1	70381200	0.661157
chr1	70443100	0.818182
chr1	70460300	0.826446
chr1	70510900	0.975207
chr1	70676800	0.950413
chr1	70689700	0.727273
chr1	70737800	0.669421
chr1	70756500	0.909091
chr1	70786200	0.694215
chr1	71770000	0.710744
chr1	71831700	0.760331
chr1	72577700	0.677686
chr1	72632400	0.768595
chr1	72752600	0.685950
chr1	72904700	0.900826
chr1	73045600	0.826446
chr1	74059400	0.966942
chr1	74070800	0.933884
chr1	75183400	0.719008
chr1	75812000	0.950413
chr1	75939000	0.950413
chr1	75969200	0.818182
chr1	75988200	0.983471
chr1	76005600	0.793388
chr1	76015700	0.768595
chr1	76045200	0.909091
chr1	76049200	0.710744
chr1	76092400	0.909091
chr1	76231700	0.900826
chr1	76256500	0.776860
chr1	76534800	0.991736
chr1	76542900	0.785124
chr1	76552400	0.702479
chr1	76620700	0.719008
chr1	76695100	0.735537
chr1	76720300	0.958678
chr1	76735300	0.925620
chr1	76891800	1.000000

chr1	77252400	0.710744
chr1	77737900	0.727273
chr1	77858500	0.719008
chr1	77926200	0.809917
chr1	77970900	0.925620
chr1	77977600	0.876033
chr1	77980900	0.793388
chr1	78003300	0.917355
chr1	78022500	0.727273
chr1	78090300	0.809917
chr1	78105400	0.694215
chr1	78263000	0.900826
chr1	78297300	0.917355
chr1	78300000	0.950413
chr1	78414500	0.785124
chr1	78441500	0.669421
chr1	78504400	0.892562
chr1	78518900	0.735537
chr1	78536900	0.983471
chr1	78539300	0.884298
chr1	78541700	0.983471
chr1	78545900	0.983471
chr1	78561500	0.983471
chr1	78563600	0.694215
chr1	78567600	0.991736
chr1	78638100	0.677686
chr1	78741700	0.818182
chr1	78752600	0.925620
chr1	78806000	0.942149
chr1	78963400	0.743802
chr1	79116100	0.727273
chr1	79297900	0.983471
chr1	79300000	0.842975
chr1	79473700	0.719008
chr1	79491100	0.826446
chr1	79506800	0.851240
chr1	79509000	0.842975
chr1	79545800	0.876033
chr1	79617300	0.719008
chr1	79627100	1.000000
chr1	79654200	0.975207
chr1	79659200	0.925620
chr1	79701400	0.958678
chr1	80041300	0.975207
chr1	80172500	0.669421
chr1	80277200	0.983471
chr1	80325700	1.000000
chr1	80858600	0.685950
chr1	81186900	0.661157
chr1	81348600	0.677686
chr1	81617000	0.727273
chr1	81645300	0.933884
chr1	81772100	0.669421
chr1	82051400	0.694215

chr1	82136500	0.710744
chr1	82147800	0.991736
chr1	82168800	0.950413
chr1	82170900	0.892562
chr1	82173300	0.925620
chr1	82197800	0.859504
chr1	82278300	0.743802
chr1	82343500	0.958678
chr1	82353600	0.983471
chr1	82383700	0.669421
chr1	82867000	0.975207
chr1	83031500	0.975207
chr1	83161500	0.933884
chr1	83170600	0.859504
chr1	83258300	0.958678
chr1	83303800	0.793388
chr1	83386500	1.000000
chr1	83397200	0.917355
chr1	83611900	0.950413
chr1	83617100	0.884298
chr1	83620700	0.884298
chr1	83624800	0.694215
chr1	83631600	0.793388
chr1	84009700	0.694215
chr1	84355900	0.785124
chr1	84359700	0.809917
chr1	84569400	0.685950
chr1	84609700	0.710744
chr1	84612800	0.694215
chr1	84622300	0.966942
chr1	84637200	0.818182
chr1	84641000	0.991736
chr1	84711000	0.892562
chr1	84714000	0.900826
chr1	84719600	0.950413
chr1	84766800	0.958678
chr1	84831600	0.933884
chr1	84899500	0.950413
chr1	85065400	0.991736
chr1	85069500	0.867769
chr1	85098900	0.776860
chr1	85164400	0.727273
chr1	85320400	1.000000
chr1	85411200	0.809917
chr1	85739600	0.702479
chr1	85744500	0.892562
chr1	85765100	0.743802
chr1	85774400	0.950413
chr1	85778800	0.851240
chr1	85782000	0.909091
chr1	85786000	1.000000
chr1	85789000	0.991736
chr1	85791800	0.785124
chr1	85794900	0.975207

chr1	85797300	0.991736
chr1	85800400	0.958678
chr1	85804900	0.958678
chr1	85807500	0.983471
chr1	85810900	0.842975
chr1	85826500	0.909091
chr1	85832500	0.950413
chr1	85864600	0.826446
chr1	85873100	0.933884
chr1	85884400	0.975207
chr1	85906700	0.991736
chr1	85913400	0.950413
chr1	85915500	0.867769
chr1	85927700	0.685950
chr1	86036300	0.884298
chr1	86038400	0.867769
chr1	86040500	0.950413
chr1	86072500	0.925620
chr1	86095000	0.917355
chr1	86156100	0.669421
chr1	86534000	0.752066
chr1	86849100	0.793388
chr1	86855200	0.727273
chr1	86896100	0.801653
chr1	86993300	0.958678
chr1	87162300	0.975207
chr1	87192400	0.983471
chr1	87240300	1.000000
chr1	87245200	0.826446
chr1	87254400	0.909091
chr1	87259200	0.917355
chr1	87494500	0.983471
chr1	87598000	0.801653
chr1	87618100	0.710744
chr1	87678400	0.694215
chr1	87692400	0.983471
chr1	87801000	0.809917
chr1	87939400	0.933884
chr1	88151500	0.876033
chr1	88319900	0.958678
chr1	88346900	0.685950
chr1	88434200	0.983471
chr1	88452900	0.851240
chr1	88479200	0.694215
chr1	88518000	0.677686
chr1	88676400	0.818182
chr1	88793700	0.983471
chr1	89153200	0.719008
chr1	89164200	0.925620
chr1	89450900	0.942149
chr1	89459500	0.834711
chr1	89591600	0.760331
chr1	89993100	0.661157
chr1	90004300	0.925620

chr1	90013600	0.842975
chr1	90071600	0.727273
chr1	90102900	0.975207
chr1	90109500	0.785124
chr1	90138000	0.851240
chr1	90158600	1.000000
chr1	90162200	0.834711
chr1	90164500	0.991736
chr1	90174600	0.884298
chr1	90176700	0.942149
chr1	90187500	0.867769
chr1	90190500	0.983471
chr1	90223400	0.925620
chr1	90227200	0.809917
chr1	90321600	0.702479
chr1	90331800	0.876033
chr1	90351300	1.000000
chr1	90360200	1.000000
chr1	90364200	0.975207
chr1	90370200	0.925620
chr1	90373100	0.991736
chr1	90376000	0.826446
chr1	90428100	0.694215
chr1	90441500	0.867769
chr1	90531600	0.669421
chr1	90862900	0.966942
chr1	90869000	0.884298
chr1	91191500	0.661157
chr1	91429900	0.876033
chr1	91739900	0.809917
chr1	91852800	0.933884
chr1	91968700	0.785124
chr1	92022100	0.983471
chr1	92035700	0.826446
chr1	92048700	0.694215
chr1	92103400	0.975207
chr1	92151600	0.719008
chr1	92155600	0.983471
chr1	92167000	1.000000
chr1	92191900	0.942149
chr1	92197800	0.818182
chr1	92247500	0.983471
chr1	92249700	0.933884
chr1	92254700	0.735537
chr1	92273000	0.925620
chr1	92275500	0.735537
chr1	92280200	0.884298
chr1	92317000	0.933884
chr1	92357800	0.958678
chr1	92362600	0.851240
chr1	92378800	0.892562
chr1	92497100	0.702479
chr1	92515400	0.760331
chr1	92583400	0.702479

chr1	92910000	0.719008
chr1	93003900	0.925620
chr1	93050300	0.809917
chr1	93171600	0.966942
chr1	93258200	0.966942
chr1	93270900	0.809917
chr1	93481400	0.785124
chr1	93547800	0.925620
chr1	93589200	0.884298
chr1	93621300	0.851240
chr1	93804300	0.958678
chr1	93806900	0.677686
chr1	93919900	0.851240
chr1	93937500	0.900826
chr1	93991100	0.685950
chr1	94017500	0.677686
chr1	94021200	0.942149
chr1	94032600	0.991736
chr1	94055100	0.958678
chr1	94072000	0.842975
chr1	94075000	0.809917
chr1	94083900	0.876033
chr1	94088600	0.661157
chr1	94091700	0.991736
chr1	94095100	0.842975
chr1	94102300	0.958678
chr1	94128100	0.950413
chr1	94130200	0.752066
chr1	94133300	0.867769
chr1	94135800	0.991736
chr1	94139100	0.826446
chr1	94141700	0.785124
chr1	94159900	0.719008
chr1	94167800	0.892562
chr1	94188600	0.859504
chr1	94220300	0.983471
chr1	94245400	0.876033
chr1	94264200	1.000000
chr1	94270300	0.983471
chr1	94282600	0.909091
chr1	94310500	0.793388
chr1	94371800	0.752066
chr1	94487100	0.950413
chr1	94492200	0.826446
chr1	94503300	0.859504
chr1	94506400	0.842975
chr1	94509400	0.735537
chr1	94512000	0.958678
chr1	94516400	0.942149
chr1	94527100	0.909091
chr1	94529400	0.966942
chr1	94532300	0.685950
chr1	94534900	0.842975
chr1	94538200	0.983471

chr1	94557200	0.867769
chr1	94561400	0.892562
chr1	94576000	0.677686
chr1	94608200	0.950413
chr1	94647100	0.801653
chr1	94657300	0.685950
chr1	94659700	0.909091
chr1	94674300	0.991736
chr1	94683100	0.966942
chr1	94685200	0.909091
chr1	94688000	0.685950
chr1	94694900	0.776860
chr1	94698100	0.826446
chr1	94705300	0.909091
chr1	94721100	0.958678
chr1	94725200	0.826446
chr1	94732100	0.983471
chr1	94747400	0.942149
chr1	94750800	0.917355
chr1	94761800	0.975207
chr1	94764000	0.842975
chr1	94766100	0.917355
chr1	94770600	0.892562
chr1	94773000	0.719008
chr1	94776500	0.834711
chr1	94782700	0.925620
chr1	94785500	0.884298
chr1	94792000	0.950413
chr1	94795800	0.669421
chr1	95119600	0.661157
chr1	95201700	0.975207
chr1	95249800	0.991736
chr1	95258700	1.000000
chr1	95262000	0.867769
chr1	95267600	0.818182
chr1	95272700	0.661157
chr1	95313200	0.677686
chr1	95320600	0.983471
chr1	95329300	0.950413
chr1	95331400	0.876033
chr1	95342400	0.710744
chr1	95401500	0.942149
chr1	95420400	0.909091
chr1	95437000	0.975207
chr1	95448400	0.809917
chr1	95460200	0.900826
chr1	95479700	0.900826
chr1	95488400	0.809917
chr1	95493300	0.752066
chr1	95553100	0.991736
chr1	96396800	0.892562
chr1	96828900	0.983471
chr1	96836400	0.991736
chr1	96840200	0.909091

chr1	96854300	0.909091
chr1	96882200	0.867769
chr1	97270100	0.702479
chr1	97331700	0.876033
chr1	97479900	1.000000
chr1	97560000	0.925620
chr1	97604100	0.950413
chr1	97610800	0.925620
chr1	97658600	0.958678
chr1	97744000	0.942149
chr1	97862400	0.735537
chr1	97917700	0.884298
chr1	97955700	0.685950
chr1	98069500	0.933884
chr1	98263400	0.975207
chr1	98315300	0.942149
chr1	98366700	0.925620
chr1	98369700	0.818182
chr1	98372600	0.900826
chr1	98403700	0.958678
chr1	98452100	0.975207
chr1	98474600	0.768595
chr1	98485500	0.917355
chr1	98496500	0.983471
chr1	98521300	0.768595
chr1	98552400	0.900826
chr1	98729900	0.958678
chr1	98781500	0.876033
chr1	98791800	0.966942
chr1	98794500	0.958678
chr1	98802300	0.983471
chr1	98811500	0.942149
chr1	98849500	0.892562
chr1	98851700	0.818182
chr1	98854400	1.000000
chr1	98871500	0.710744
chr1	98886500	0.669421
chr1	98933700	0.909091
chr1	99369100	0.983471
chr1	99372500	1.000000
chr1	99470500	0.801653
chr1	99907500	0.867769
chr1	100001000	0.859504
chr1	100010100	0.966942
chr1	100013000	0.925620
chr1	100055200	0.677686
chr1	100057300	0.958678
chr1	100060400	0.958678
chr1	100064500	0.900826
chr1	100105000	0.950413
chr1	100110200	0.743802
chr1	100115700	0.966942
chr1	100118000	0.925620
chr1	100120200	0.917355

chr1	100122800	0.983471
chr1	100126000	0.950413
chr1	100138600	0.925620
chr1	100146000	0.942149
chr1	100148400	0.867769
chr1	100152000	0.900826
chr1	100155300	0.859504
chr1	100158400	0.834711
chr1	100194100	0.818182
chr1	100422700	0.983471
chr1	100429000	0.834711
chr1	100506400	0.727273
chr1	100556300	0.933884
chr1	100755800	0.966942
chr1	100759300	0.950413
chr1	100853500	0.826446
chr1	100866400	0.768595
chr1	100881500	0.933884
chr1	100909800	0.991736
chr1	101050500	0.735537
chr1	101062700	0.942149
chr1	101088100	0.809917
chr1	101092700	0.966942
chr1	101174400	0.900826
chr1	101179700	0.933884
chr1	101232600	0.942149
chr1	101236200	1.000000
chr1	101265700	0.983471
chr1	101298500	0.719008
chr1	101302100	0.958678
chr1	101332100	0.925620
chr1	101421300	0.933884
chr1	101425700	0.975207
chr1	101455700	0.958678
chr1	101461300	0.892562
chr1	101468500	0.768595
chr1	101472200	0.892562
chr1	101577800	0.818182
chr1	101602900	1.000000
chr1	101608500	0.752066
chr1	101616000	1.000000
chr1	101628100	0.958678
chr1	101641500	0.933884
chr1	101648600	0.991736
chr1	101653100	0.975207
chr1	101708600	0.925620
chr1	101716500	0.966942
chr1	101725400	0.933884
chr1	101728000	1.000000
chr1	101731000	0.942149
chr1	101748200	0.760331
chr1	101757600	0.950413
chr1	101764400	0.942149
chr1	101766500	0.950413

chr1	101768800	1.000000
chr1	101775600	0.834711
chr1	101832500	0.685950
chr1	101875900	0.942149
chr1	103345700	0.776860
chr1	103347800	0.702479
chr1	103358100	0.801653
chr1	103716300	0.900826
chr1	104052100	0.685950
chr1	107562200	0.867769
chr1	108361200	0.793388
chr1	108404700	0.892562
chr1	108479600	0.958678
chr1	108815700	0.785124
chr1	108964400	0.818182
chr1	109128700	0.958678
chr1	109133100	0.942149
chr1	109135200	0.876033
chr1	109224800	0.834711
chr1	109263100	0.958678
chr1	109309900	0.793388
chr1	109343700	0.892562
chr1	109359400	0.909091
chr1	109373800	0.818182
chr1	109433000	0.933884
chr1	109435600	0.685950
chr1	109491100	0.917355
chr1	109623100	0.909091
chr1	109656400	0.842975
chr1	109713600	0.785124
chr1	109735000	0.966942
chr1	109740100	0.900826
chr1	109792600	0.785124
chr1	109935700	0.768595
chr1	110009600	0.826446
chr1	110035400	0.776860
chr1	110075400	0.685950
chr1	110089300	0.760331
chr1	110111800	0.801653
chr1	110124400	0.776860
chr1	110138500	0.983471
chr1	110156200	0.950413
chr1	110158900	0.900826
chr1	110161200	0.859504
chr1	110202000	0.710744
chr1	110262400	0.776860
chr1	110326700	0.785124
chr1	110333600	0.842975
chr1	110336700	0.876033
chr1	110420200	0.727273
chr1	110435400	0.842975
chr1	110438700	0.859504
chr1	110442100	0.727273
chr1	110460800	0.727273

chr1	110476900	0.826446
chr1	110522700	0.958678
chr1	110546800	0.884298
chr1	110627400	0.752066
chr1	110693700	0.851240
chr1	110779100	0.975207
chr1	111691300	0.694215
chr1	112017000	0.884298
chr1	112050900	0.818182
chr1	112135700	0.743802
chr1	112263000	0.677686
chr1	112276000	0.818182
chr1	112305000	0.884298
chr1	112713200	0.785124
chr1	112930700	0.867769
chr1	112932800	0.719008
chr1	112942400	0.768595
chr1	112947200	0.859504
chr1	112974100	0.950413
chr1	112976300	0.793388
chr1	112978600	1.000000
chr1	112982700	0.991736
chr1	112987200	0.950413
chr1	113009500	0.958678
chr1	113087500	0.909091
chr1	113119300	0.735537
chr1	113134900	0.991736
chr1	113164900	0.892562
chr1	113180000	0.958678
chr1	113245900	0.710744
chr1	113261800	0.826446
chr1	113362600	1.000000
chr1	113409000	0.917355
chr1	113412700	0.867769
chr1	113449200	0.776860
chr1	113478500	0.942149
chr1	113574100	0.776860
chr1	113586400	0.818182
chr1	113602000	0.876033
chr1	113685000	0.909091
chr1	113943900	0.727273
chr1	114000200	0.793388
chr1	114094800	0.776860
chr1	114202000	0.950413
chr1	114226800	1.000000
chr1	114356100	0.892562
chr1	114403800	0.950413
chr1	114411000	0.752066
chr1	114474900	0.702479
chr1	114480100	0.975207
chr1	114490200	0.917355
chr1	114519200	1.000000
chr1	114522300	0.933884
chr1	114525400	0.669421

chr1	114991600	1.000000
chr1	115001100	0.942149
chr1	115397900	0.702479
chr1	115605500	0.966942
chr1	115628200	0.669421
chr1	115677300	0.661157
chr1	115697300	0.710744
chr1	115708800	0.768595
chr1	115711900	0.826446
chr1	115723000	0.661157
chr1	115733000	0.834711
chr1	115794700	0.991736
chr1	115969200	0.933884
chr1	116089400	0.842975
chr1	116198000	0.950413
chr1	116354500	0.867769
chr1	116382100	0.677686
chr1	116694900	0.933884
chr1	116899300	0.884298
chr1	116901600	0.677686
chr1	116919700	0.859504
chr1	116924000	0.991736
chr1	116982700	0.983471
chr1	117029300	0.669421
chr1	117032300	0.975207
chr1	117051800	0.991736
chr1	117131500	0.694215
chr1	117390600	0.710744
chr1	117393200	0.900826
chr1	117396600	0.991736
chr1	117421600	0.933884
chr1	117429500	0.859504
chr1	117569400	1.000000
chr1	117572800	0.727273
chr1	117620200	0.950413
chr1	117636300	0.983471
chr1	117640300	0.900826
chr1	118009600	0.925620
chr1	118214600	0.809917
chr1	118229900	0.942149
chr1	118301500	0.694215
chr1	118364800	0.983471
chr1	118369800	0.942149
chr1	118381300	0.685950
chr1	119530400	0.752066
chr1	119532900	0.809917
chr1	119544700	0.702479
chr1	119551400	0.685950
chr1	119703900	0.801653
chr1	119729900	0.669421
chr1	119822700	0.950413
chr1	120113000	0.669421
chr1	120146600	0.925620
chr1	120170100	0.818182

chr1	120176000	0.925620
chr1	120179800	0.942149
chr1	120188300	0.760331
chr1	120215900	0.859504
chr1	120218800	0.710744
chr1	120256700	0.727273
chr1	120271100	0.909091
chr1	120402400	0.933884
chr1	120538500	0.752066
chr1	120543800	0.851240
chr1	120553000	0.727273
chr1	120558400	0.900826
chr1	120595400	0.942149
chr1	120605000	0.842975
chr1	120685200	0.884298
chr1	120688600	0.933884
chr1	120753700	0.735537
chr1	120762000	0.760331
chr1	120766700	0.826446
chr1	120779600	0.975207
chr1	120789200	0.958678
chr1	120795400	0.917355
chr1	120808800	0.950413
chr1	120821700	0.933884
chr1	120859100	0.900826
chr1	121098400	0.983471
chr1	121105500	0.909091
chr1	121116400	0.842975
chr1	121126900	0.884298
chr1	121329700	0.942149
chr1	121485200	0.710744
chr1	143709100	0.719008
chr1	143893200	0.909091
chr1	143930600	0.975207
chr1	143944100	0.818182
chr1	143956900	1.000000
chr1	143963100	0.719008
chr1	143973100	0.991736
chr1	143985900	0.876033
chr1	143991000	0.942149
chr1	143998700	0.884298
chr1	144008300	0.735537
chr1	144011500	0.867769
chr1	144015700	0.842975
chr1	144028300	0.991736
chr1	144032000	0.925620
chr1	144060900	0.925620
chr1	144077300	0.752066
chr1	144085300	0.768595
chr1	144092500	0.942149
chr1	144533500	0.677686
chr1	144535900	0.834711
chr1	144538800	0.801653
chr1	144541600	0.884298

chr1	144577900	0.909091
chr1	144584100	0.735537
chr1	144679800	0.776860
chr1	144692200	0.818182
chr1	144892600	0.900826
chr1	144907500	0.892562
chr1	144924300	0.933884
chr1	144941500	0.702479
chr1	144955400	0.818182
chr1	144967700	0.842975
chr1	145009800	0.942149
chr1	145032800	0.727273
chr1	145090700	0.909091
chr1	145128100	0.809917
chr1	145130700	0.975207
chr1	145216500	0.942149
chr1	145219500	0.801653
chr1	145222000	0.776860
chr1	145224200	0.685950
chr1	145226600	0.966942
chr1	145263300	0.661157
chr1	145277200	0.975207
chr1	145283100	0.834711
chr1	145455400	0.867769
chr1	145457500	0.867769
chr1	145480000	0.793388
chr1	145525200	0.809917
chr1	145527300	0.950413
chr1	145544100	0.892562
chr1	145563300	0.685950
chr1	145585300	0.892562
chr1	145649400	0.950413
chr1	145652700	0.958678
chr1	145683500	0.933884
chr1	145714000	0.809917
chr1	145945200	0.710744
chr1	146518600	1.000000
chr1	146745300	0.876033
chr1	147012300	0.793388
chr1	147148700	0.925620
chr1	147235200	0.694215
chr1	147253500	1.000000
chr1	147267000	0.966942
chr1	147273400	0.694215
chr1	147322200	0.884298
chr1	147324400	0.826446
chr1	147350400	0.842975
chr1	147355200	0.818182
chr1	147486900	0.743802
chr1	147505700	0.727273
chr1	147809000	0.776860
chr1	147860700	0.851240
chr1	147874900	0.735537
chr1	147994200	0.677686

chr1	148236600	0.719008
chr1	148532700	0.826446
chr1	148537300	0.950413
chr1	148543200	0.842975
chr1	148604800	0.743802
chr1	148610800	0.768595
chr1	148767100	0.909091
chr1	148931300	0.900826
chr1	149035200	0.842975
chr1	149398500	0.661157
chr1	149523400	0.776860
chr1	149672600	0.727273
chr1	149764600	0.661157
chr1	149825900	0.710744
chr1	149903600	0.876033
chr1	149911000	0.983471
chr1	149968600	0.719008
chr1	150082500	0.925620
chr1	150114500	1.000000
chr1	150117400	0.826446
chr1	150125800	0.975207
chr1	150128400	0.677686
chr1	150131600	0.768595
chr1	150135800	0.942149
chr1	150228800	0.900826
chr1	150245600	0.760331
chr1	150480900	0.909091
chr1	150525500	0.702479
chr1	150531400	0.900826
chr1	150536200	0.909091
chr1	150542100	0.991736
chr1	150547100	0.694215
chr1	150553200	0.933884
chr1	150577300	0.909091
chr1	150583100	0.826446
chr1	150585200	0.942149
chr1	150587700	0.966942
chr1	150592900	0.942149
chr1	150596300	0.933884
chr1	150661300	0.925620
chr1	150665300	0.991736
chr1	150737700	0.983471
chr1	150949700	0.859504
chr1	150952400	0.793388
chr1	150972000	0.900826
chr1	151029300	0.768595
chr1	151035400	0.892562
chr1	151037800	0.710744
chr1	151079700	0.743802
chr1	151104400	0.917355
chr1	151128000	0.760331
chr1	151284200	0.867769
chr1	151286800	0.975207
chr1	151344900	0.884298

chr1	151467700	0.958678
chr1	151520300	0.743802
chr1	151634300	0.702479
chr1	151653400	0.975207
chr1	151676400	0.834711
chr1	151801900	0.677686
chr1	151813700	0.694215
chr1	151819300	0.785124
chr1	151821400	0.876033
chr1	151841000	0.917355
chr1	151860000	0.925620
chr1	151871500	0.842975
chr1	151877900	0.966942
chr1	151907700	0.661157
chr1	151919000	0.991736
chr1	151933600	0.727273
chr1	151936500	0.743802
chr1	151942000	0.685950
chr1	151945700	0.876033
chr1	151953500	0.983471
chr1	151960400	0.933884
chr1	151963400	0.685950
chr1	151967800	0.727273
chr1	152020800	1.000000
chr1	153517900	0.892562
chr1	153522000	0.876033
chr1	153537900	0.776860
chr1	153541500	0.884298
chr1	153560200	0.677686
chr1	153571900	0.950413
chr1	153722500	0.776860
chr1	153725600	0.826446
chr1	153736500	0.942149
chr1	153743400	0.842975
chr1	153746400	0.685950
chr1	153752800	0.760331
chr1	153763100	0.966942
chr1	153853900	0.991736
chr1	153856100	0.669421
chr1	153901500	0.942149
chr1	154128000	0.818182
chr1	154149700	0.933884
chr1	154152600	0.768595
chr1	154301200	0.719008
chr1	154320100	0.669421
chr1	154325800	0.809917
chr1	154393100	0.892562
chr1	154396700	0.859504
chr1	154404300	0.760331
chr1	154436200	0.900826
chr1	154453400	0.975207
chr1	154457300	0.859504
chr1	154459600	0.851240
chr1	154462800	0.933884

chr1	154466300	0.752066
chr1	154468400	0.917355
chr1	154471000	0.793388
chr1	154494500	0.851240
chr1	154532300	0.669421
chr1	154540700	0.752066
chr1	154556800	0.966942
chr1	154699400	0.735537
chr1	154703100	0.933884
chr1	154783100	0.694215
chr1	154813100	0.884298
chr1	154850300	0.793388
chr1	154857200	0.859504
chr1	154939700	0.719008
chr1	154972400	0.694215
chr1	154977800	0.867769
chr1	154990100	1.000000
chr1	155022300	0.818182
chr1	155026500	0.752066
chr1	155096700	0.892562
chr1	155139300	0.942149
chr1	155163700	0.884298
chr1	155195200	1.000000
chr1	155210900	0.785124
chr1	155215500	0.661157
chr1	155220700	0.760331
chr1	155281300	0.719008
chr1	155291500	0.735537
chr1	155506100	0.975207
chr1	155618600	0.859504
chr1	155733600	0.867769
chr1	155819700	0.669421
chr1	155859900	0.983471
chr1	155863900	0.892562
chr1	155900100	0.694215
chr1	155908600	0.818182
chr1	155911200	0.677686
chr1	155931300	0.933884
chr1	155933400	0.925620
chr1	155941300	0.958678
chr1	155950800	0.768595
chr1	155959300	0.719008
chr1	156039900	0.661157
chr1	156047400	0.685950
chr1	156056500	0.909091
chr1	156067200	0.727273
chr1	156071100	0.694215
chr1	156076500	0.991736
chr1	156088200	0.876033
chr1	156090800	0.818182
chr1	156094400	0.851240
chr1	156097800	0.826446
chr1	156100400	0.966942
chr1	156116600	0.933884

chr1	156118700	0.909091
chr1	156131500	0.867769
chr1	156186000	0.851240
chr1	156188400	0.958678
chr1	156203600	0.876033
chr1	156215800	0.776860
chr1	156261300	0.760331
chr1	156339400	0.793388
chr1	156391300	0.677686
chr1	156406000	0.776860
chr1	156426400	0.669421
chr1	156436200	0.884298
chr1	156447300	0.884298
chr1	156460800	0.859504
chr1	156467100	0.743802
chr1	156472200	0.661157
chr1	156475600	0.892562
chr1	156563500	0.785124
chr1	156595000	0.719008
chr1	156627500	0.892562
chr1	156634300	0.818182
chr1	156643900	0.900826
chr1	156674900	0.669421
chr1	156716200	0.760331
chr1	156751700	0.669421
chr1	156776600	0.760331
chr1	156783700	0.727273
chr1	156786400	0.768595
chr1	156799100	0.719008
chr1	156811500	0.694215
chr1	156815900	0.785124
chr1	156819300	0.685950
chr1	156830300	0.876033
chr1	156838200	0.694215
chr1	156841300	0.727273
chr1	156845900	0.876033
chr1	156849600	0.760331
chr1	156862400	0.834711
chr1	156872700	0.909091
chr1	156875100	0.834711
chr1	156877500	0.834711
chr1	156890400	0.677686
chr1	156897700	0.685950
chr1	157013000	0.710744
chr1	157076500	0.867769
chr1	157124900	0.859504
chr1	157360900	0.983471
chr1	157382000	0.842975
chr1	157939300	0.892562
chr1	157949000	0.735537
chr1	157981900	0.768595
chr1	157987500	0.958678
chr1	157990600	0.933884
chr1	158008400	0.900826

chr1	158072000	0.950413
chr1	158083600	0.661157
chr1	158985600	0.942149
chr1	159058700	0.694215
chr1	159062100	1.000000
chr1	159131500	0.685950
chr1	159158400	0.685950
chr1	159181300	0.719008
chr1	159252700	0.743802
chr1	159289300	0.867769
chr1	159379100	0.950413
chr1	159418200	0.975207
chr1	159439800	0.933884
chr1	159443200	0.983471
chr1	159462900	0.818182
chr1	159565000	0.925620
chr1	159568300	1.000000
chr1	159623200	0.942149
chr1	159625300	0.677686
chr1	159744700	0.900826
chr1	159749200	0.917355
chr1	159756500	0.900826
chr1	159852300	0.834711
chr1	159860100	1.000000
chr1	159879900	0.694215
chr1	159882100	0.942149
chr1	159885900	0.793388
chr1	159888100	0.793388
chr1	159891100	0.909091
chr1	159915500	0.727273
chr1	160054200	0.768595
chr1	160178200	0.991736
chr1	160180600	0.760331
chr1	160189100	0.876033
chr1	160230100	0.669421
chr1	160309700	0.884298
chr1	160373300	0.752066
chr1	160965600	0.884298
chr1	160967800	0.958678
chr1	160982600	0.884298
chr1	160984900	0.859504
chr1	161085300	0.975207
chr1	161089700	0.809917
chr1	161135300	0.727273
chr1	161155700	0.933884
chr1	161176900	0.867769
chr1	161187300	0.661157
chr1	161198100	0.909091
chr1	161228800	0.677686
chr1	161277100	0.719008
chr1	161352400	0.983471
chr1	161442300	0.735537
chr1	161469900	0.909091
chr1	161476700	0.818182

chr1	161503300	0.776860
chr1	161576100	0.694215
chr1	161583400	0.768595
chr1	161697700	0.834711
chr1	161700200	0.925620
chr1	161739500	0.900826
chr1	161742300	0.818182
chr1	161762700	0.892562
chr1	161813400	0.958678
chr1	161880300	0.884298
chr1	161900100	0.752066
chr1	161932100	0.966942
chr1	162005600	0.710744
chr1	162025300	0.892562
chr1	162051000	0.867769
chr1	162058700	0.884298
chr1	162161400	0.900826
chr1	162181700	0.785124
chr1	162183800	0.661157
chr1	162272800	0.727273
chr1	162275800	0.950413
chr1	162280500	0.958678
chr1	162319800	0.950413
chr1	162459700	0.917355
chr1	162508700	0.958678
chr1	162512000	1.000000
chr1	162530500	0.735537
chr1	162534500	0.710744
chr1	162538800	0.702479
chr1	162575600	0.983471
chr1	162596900	0.842975
chr1	162600500	0.975207
chr1	162641200	0.942149
chr1	162643900	0.776860
chr1	162646600	0.917355
chr1	162651800	0.719008
chr1	162660600	0.991736
chr1	162666400	0.925620
chr1	162679900	0.933884
chr1	162727200	0.694215
chr1	162824300	0.867769
chr1	162826600	0.958678
chr1	162828700	0.950413
chr1	162844100	0.900826
chr1	162863600	0.694215
chr1	162878800	0.842975
chr1	162892500	0.752066
chr1	162943500	0.834711
chr1	162954600	0.950413
chr1	162958000	0.661157
chr1	163005900	0.958678
chr1	163039400	0.694215
chr1	163042400	0.942149
chr1	163053000	0.818182

chr1	163055700	0.876033
chr1	163086500	0.719008
chr1	163117300	0.876033
chr1	163132400	0.958678
chr1	163135900	0.735537
chr1	163138000	0.942149
chr1	163140800	0.876033
chr1	163143600	0.826446
chr1	163166500	0.966942
chr1	163169500	0.793388
chr1	163172900	0.702479
chr1	163175900	0.752066
chr1	163677000	0.991736
chr1	163679600	0.842975
chr1	163766000	0.661157
chr1	163777100	0.685950
chr1	163845800	0.958678
chr1	164124400	0.859504
chr1	164530800	0.702479
chr1	164676600	0.710744
chr1	164681900	0.983471
chr1	164690400	0.752066
chr1	165367500	0.958678
chr1	165812100	0.793388
chr1	165840800	0.710744
chr1	165854200	0.917355
chr1	165864600	0.719008
chr1	165867800	0.950413
chr1	165870500	0.685950
chr1	165873300	0.983471
chr1	165880500	0.909091
chr1	166135600	0.661157
chr1	167166000	0.752066
chr1	167267300	0.710744
chr1	167373600	0.793388
chr1	167599800	0.694215
chr1	167624600	0.933884
chr1	167744600	0.966942
chr1	167766000	0.917355
chr1	167789700	0.983471
chr1	167832300	0.768595
chr1	167934000	0.677686
chr1	168051300	0.710744
chr1	168198100	0.768595
chr1	168231000	0.991736
chr1	168256800	0.669421
chr1	168289700	0.991736
chr1	168889200	0.851240
chr1	168891300	0.958678
chr1	168893400	0.966942
chr1	169078900	0.909091
chr1	169081100	0.842975
chr1	169083700	0.950413
chr1	169257500	0.867769

chr1	169296500	0.925620
chr1	169332600	0.975207
chr1	169429900	0.661157
chr1	169453200	0.694215
chr1	169478300	0.669421
chr1	169481000	0.661157
chr1	169535600	0.694215
chr1	169579800	0.826446
chr1	169588100	0.851240
chr1	169593900	0.768595
chr1	169640600	0.884298
chr1	169645400	0.983471
chr1	169666000	0.776860
chr1	169709500	0.983471
chr1	169749300	0.900826
chr1	169872200	0.735537
chr1	169884000	0.735537
chr1	169904200	0.735537
chr1	170463800	0.801653
chr1	170532500	0.933884
chr1	170604200	0.818182
chr1	170658900	0.785124
chr1	170662600	0.900826
chr1	170670400	0.785124
chr1	170677600	0.942149
chr1	171404400	0.900826
chr1	171457700	0.933884
chr1	171624800	0.884298
chr1	171709100	0.942149
chr1	171763500	0.900826
chr1	171882800	0.801653
chr1	171936100	0.661157
chr1	171973800	0.719008
chr1	172010100	0.851240
chr1	172074800	0.752066
chr1	172078500	0.867769
chr1	172082700	0.975207
chr1	172105400	0.892562
chr1	172111500	0.809917
chr1	172115200	0.677686
chr1	172117800	0.818182
chr1	172124300	1.000000
chr1	172224500	0.942149
chr1	172290100	0.818182
chr1	172292500	0.785124
chr1	172313000	0.958678
chr1	172321100	0.900826
chr1	172326500	0.727273
chr1	172329500	0.867769
chr1	172350200	1.000000
chr1	172353000	0.983471
chr1	172368500	0.917355
chr1	172517800	0.685950
chr1	172551200	0.735537

chr1	172554800	0.834711
chr1	172557900	0.892562
chr1	172612300	0.743802
chr1	172624500	0.669421
chr1	172668700	0.851240
chr1	172715800	0.950413
chr1	172730900	0.867769
chr1	172795800	0.826446
chr1	172829600	0.966942
chr1	172852900	0.851240
chr1	172862400	0.785124
chr1	172864500	0.983471
chr1	172868000	0.942149
chr1	172872900	0.975207
chr1	172876900	0.925620
chr1	172879900	0.917355
chr1	172883700	0.991736
chr1	172886200	0.942149
chr1	172888500	0.876033
chr1	172898800	0.900826
chr1	172902000	0.900826
chr1	172905600	0.925620
chr1	172916400	0.975207
chr1	172918800	0.909091
chr1	172923700	0.975207
chr1	172927100	0.933884
chr1	172929700	0.851240
chr1	172936800	0.900826
chr1	172945800	0.950413
chr1	172960300	0.900826
chr1	172979200	0.768595
chr1	173013200	0.975207
chr1	173018000	0.950413
chr1	173021300	0.801653
chr1	173033400	0.785124
chr1	173081700	0.975207
chr1	173092200	0.876033
chr1	173131200	0.909091
chr1	173159000	0.785124
chr1	173161600	0.859504
chr1	173165400	0.933884
chr1	173170500	0.768595
chr1	173238800	0.884298
chr1	173300300	0.694215
chr1	173399300	0.909091
chr1	173443500	0.958678
chr1	173519000	0.735537
chr1	173524300	0.867769
chr1	173639100	0.702479
chr1	173822700	0.900826
chr1	173839500	0.966942
chr1	173930400	0.884298
chr1	174937800	0.958678
chr1	174960100	0.826446

chr1	174965900	0.809917
chr1	174978800	0.710744
chr1	175138000	0.719008
chr1	175141100	0.694215
chr1	175167200	1.000000
chr1	175170500	0.983471
chr1	175287400	0.975207
chr1	175332800	0.776860
chr1	175475500	0.900826
chr1	175478500	0.991736
chr1	175481600	0.950413
chr1	175484300	0.933884
chr1	175490700	0.809917
chr1	175506000	0.917355
chr1	175513000	0.966942
chr1	175567500	0.958678
chr1	175584500	0.661157
chr1	175642900	1.000000
chr1	175752900	0.942149
chr1	175946800	0.966942
chr1	176003200	0.942149
chr1	176147600	0.818182
chr1	177150900	0.702479
chr1	177903300	0.727273
chr1	177981000	0.975207
chr1	178022600	0.983471
chr1	178096300	0.958678
chr1	178162200	0.900826
chr1	178208300	1.000000
chr1	178210900	0.867769
chr1	178224400	0.727273
chr1	178237400	0.958678
chr1	178310800	0.942149
chr1	178314300	0.966942
chr1	178378200	0.900826
chr1	178387500	0.760331
chr1	178454700	0.892562
chr1	178631700	0.694215
chr1	178651800	0.917355
chr1	178655100	0.900826
chr1	178753600	0.942149
chr1	178840900	0.851240
chr1	179057300	0.867769
chr1	179095300	0.966942
chr1	179099300	0.958678
chr1	179107600	0.909091
chr1	179113400	0.727273
chr1	179116000	0.975207
chr1	179160100	0.876033
chr1	179203700	0.743802
chr1	179633200	0.975207
chr1	179679600	0.975207
chr1	179786100	0.694215
chr1	179854000	0.702479

chr1	179995700	0.851240
chr1	180009300	0.818182
chr1	180105900	0.958678
chr1	180121100	0.966942
chr1	180204800	0.694215
chr1	180219500	0.702479
chr1	180352000	0.867769
chr1	180475000	0.917355
chr1	180477400	0.917355
chr1	180485700	0.834711
chr1	180489200	0.925620
chr1	180500000	0.859504
chr1	180535800	0.752066
chr1	180823500	0.958678
chr1	181003000	0.983471
chr1	181069400	0.867769
chr1	181071500	0.876033
chr1	181076300	0.867769
chr1	181105000	0.983471
chr1	181108900	0.776860
chr1	181111500	0.793388
chr1	181128800	0.818182
chr1	181221000	0.760331
chr1	182362000	0.694215
chr1	182573900	0.768595
chr1	182922400	0.793388
chr1	182932300	0.859504
chr1	182960000	0.826446
chr1	182972400	0.785124
chr1	182989000	0.942149
chr1	182999300	0.917355
chr1	183009500	0.958678
chr1	183022000	0.702479
chr1	183028600	0.900826
chr1	183030700	0.933884
chr1	183040400	0.925620
chr1	183049900	0.685950
chr1	183109200	0.991736
chr1	183133000	0.933884
chr1	183150100	0.867769
chr1	183155500	0.884298
chr1	183164600	0.867769
chr1	183188200	0.669421
chr1	183199800	0.818182
chr1	183204200	0.892562
chr1	183240200	0.909091
chr1	183242500	0.776860
chr1	183248600	0.859504
chr1	183261300	0.669421
chr1	183413800	0.694215
chr1	183450300	0.760331
chr1	183484300	0.834711
chr1	183621100	0.809917
chr1	183624500	0.776860

chr1	183726500	0.950413
chr1	183753500	0.834711
chr1	183773500	0.677686
chr1	183777200	0.661157
chr1	183782400	0.719008
chr1	183786800	1.000000
chr1	183791500	0.661157
chr1	183807700	0.966942
chr1	183812300	1.000000
chr1	183817600	0.909091
chr1	183820600	0.876033
chr1	183839200	0.826446
chr1	183841600	0.991736
chr1	183844200	0.859504
chr1	183847000	0.942149
chr1	183851200	0.991736
chr1	183857400	0.966942
chr1	183885500	0.983471
chr1	183972000	0.685950
chr1	184212100	0.735537
chr1	184229200	0.851240
chr1	184266000	0.958678
chr1	184269800	0.752066
chr1	184299400	0.661157
chr1	184461000	0.867769
chr1	184477000	0.793388
chr1	184567300	0.710744
chr1	184762100	0.933884
chr1	184771100	0.710744
chr1	184824000	0.950413
chr1	185336900	0.950413
chr1	185342400	0.859504
chr1	185346100	0.975207
chr1	185396400	0.933884
chr1	185489700	0.818182
chr1	185536600	0.933884
chr1	185615800	0.917355
chr1	185643800	0.966942
chr1	185663500	0.958678
chr1	185789500	0.851240
chr1	185831200	0.760331
chr1	185864200	0.818182
chr1	185978400	0.909091
chr1	186063300	0.975207
chr1	186072100	0.958678
chr1	186157800	0.991736
chr1	186256400	0.735537
chr1	186374400	0.933884
chr1	186410000	0.776860
chr1	186412100	0.950413
chr1	186445800	0.966942
chr1	186452400	0.677686
chr1	186468500	0.917355
chr1	186505300	1.000000

chr1	186544700	0.925620
chr1	186596000	0.966942
chr1	186625200	0.933884
chr1	186632300	0.876033
chr1	186652100	0.669421
chr1	186732500	0.983471
chr1	186823800	0.685950
chr1	186886800	1.000000
chr1	186897600	0.760331
chr1	186913100	0.975207
chr1	186915300	0.909091
chr1	186917800	0.900826
chr1	186920600	0.909091
chr1	186943400	0.760331
chr1	186945500	0.933884
chr1	186947700	0.900826
chr1	186954700	0.917355
chr1	187003000	0.735537
chr1	187624300	0.892562
chr1	187660700	0.809917
chr1	187817800	0.876033
chr1	187997000	0.958678
chr1	188012100	0.760331
chr1	188014200	0.776860
chr1	188059600	0.933884
chr1	188207000	0.950413
chr1	188341200	0.661157
chr1	188622300	0.983471
chr1	188669200	0.975207
chr1	188675200	0.851240
chr1	188693100	0.669421
chr1	189566200	0.735537
chr1	189775400	0.801653
chr1	190992000	0.966942
chr1	191104500	0.909091
chr1	191793200	0.809917
chr1	192074200	0.958678
chr1	192088300	0.842975
chr1	192129300	0.677686
chr1	192139900	0.983471
chr1	192195500	0.801653
chr1	192229800	0.793388
chr1	192233300	0.966942
chr1	192409000	0.859504
chr1	192413000	0.950413
chr1	192415100	0.876033
chr1	192422800	0.942149
chr1	192459200	0.760331
chr1	192482300	0.801653
chr1	192491000	0.900826
chr1	192499700	0.942149
chr1	192509100	0.917355
chr1	192514000	0.909091
chr1	192516100	0.966942

chr1	192529000	0.983471
chr1	192670400	0.983471
chr1	192776200	0.793388
chr1	192780700	0.727273
chr1	192796300	0.958678
chr1	192906300	0.892562
chr1	192921400	0.958678
chr1	192936900	0.983471
chr1	193084700	0.892562
chr1	193291700	0.983471
chr1	193387900	0.669421
chr1	193404000	0.685950
chr1	193528100	0.743802
chr1	193584300	0.917355
chr1	193599200	0.966942
chr1	193994300	0.719008
chr1	194032400	0.958678
chr1	196150700	0.991736
chr1	196336500	0.909091
chr1	196370100	0.991736
chr1	196527900	0.776860
chr1	197110200	0.710744
chr1	197634000	0.760331
chr1	197807900	0.925620
chr1	197882100	0.719008
chr1	197885400	0.752066
chr1	197888800	0.677686
chr1	198090300	0.752066
chr1	198092500	0.975207
chr1	198095700	0.859504
chr1	198121200	0.991736
chr1	198124900	0.851240
chr1	198139000	0.983471
chr1	198189900	1.000000
chr1	198204100	0.776860
chr1	198237600	0.909091
chr1	198239700	0.958678
chr1	198242900	0.834711
chr1	198413000	0.917355
chr1	198415100	0.900826
chr1	198419100	0.909091
chr1	198461300	0.950413
chr1	198638600	0.925620
chr1	198651300	0.727273
chr1	198745300	0.809917
chr1	198747700	0.892562
chr1	198749900	0.958678
chr1	198850500	0.917355
chr1	198859800	0.867769
chr1	198862800	0.842975
chr1	198879100	0.892562
chr1	198882400	0.694215
chr1	198885300	0.966942
chr1	198888600	0.826446

chr1	198899400	0.735537
chr1	198902200	0.801653
chr1	199196200	0.917355
chr1	199240800	0.768595
chr1	199243000	0.991736
chr1	199260000	0.785124
chr1	199271400	0.933884
chr1	199403800	0.793388
chr1	199542600	0.685950
chr1	199654800	0.942149
chr1	199657800	0.966942
chr1	199661200	0.991736
chr1	199703200	0.776860
chr1	199712300	0.950413
chr1	199728600	0.818182
chr1	199750300	0.917355
chr1	199753600	0.917355
chr1	199824600	0.966942
chr1	199829200	0.900826
chr1	199837000	0.743802
chr1	200031800	0.884298
chr1	200041600	0.867769
chr1	200045400	0.925620
chr1	200123600	0.818182
chr1	200148700	1.000000
chr1	200201700	0.826446
chr1	200211800	0.768595
chr1	200306000	0.834711
chr1	200332800	0.752066
chr1	200385400	0.966942
chr1	200422700	0.826446
chr1	200452900	0.909091
chr1	200609900	0.925620
chr1	200635500	0.966942
chr1	200669300	0.719008
chr1	200699500	0.892562
chr1	200743700	0.760331
chr1	200842800	0.685950
chr1	200979200	0.735537
chr1	200983700	0.884298
chr1	200992400	0.834711
chr1	200997800	0.752066
chr1	201083700	0.743802
chr1	201431000	0.859504
chr1	201455400	0.983471
chr1	201460500	0.975207
chr1	201465700	0.942149
chr1	201470000	0.859504
chr1	201481700	0.818182
chr1	201504700	0.809917
chr1	201512000	0.966942
chr1	201521200	0.933884
chr1	201524300	0.942149
chr1	201526500	0.735537

chr1	201532100	0.876033
chr1	201540300	0.851240
chr1	201547000	0.694215
chr1	201562700	0.933884
chr1	201566200	0.702479
chr1	201568300	0.776860
chr1	201572400	0.760331
chr1	201579100	0.942149
chr1	201603500	0.867769
chr1	201606800	0.735537
chr1	201617700	0.892562
chr1	201645400	0.867769
chr1	201681600	0.760331
chr1	201684500	0.958678
chr1	201704500	0.669421
chr1	201716300	0.661157
chr1	201720600	0.983471
chr1	201723000	0.958678
chr1	201742900	0.983471
chr1	201750900	0.958678
chr1	201758600	0.809917
chr1	201762500	0.818182
chr1	201815000	0.818182
chr1	201819500	0.983471
chr1	201828800	0.909091
chr1	201874100	0.710744
chr1	201886600	0.710744
chr1	201915900	0.710744
chr1	202129600	0.702479
chr1	202154500	0.851240
chr1	202163000	0.900826
chr1	202183700	0.834711
chr1	202205400	0.851240
chr1	202248900	0.909091
chr1	202251800	0.933884
chr1	202253900	0.876033
chr1	202256100	0.785124
chr1	202281500	0.702479
chr1	202426000	0.925620
chr1	202435500	0.975207
chr1	202449400	0.669421
chr1	202555500	0.685950
chr1	202559400	0.867769
chr1	202562300	0.719008
chr1	202585200	0.950413
chr1	202679500	0.752066
chr1	202682500	0.719008
chr1	202828300	0.669421
chr1	202922300	0.991736
chr1	202996100	0.669421
chr1	203123000	0.925620
chr1	203242800	0.801653
chr1	203256700	0.677686
chr1	203290200	0.966942

chr1	203294500	0.743802
chr1	203372100	0.719008
chr1	203481000	0.743802
chr1	203483200	0.743802
chr1	203489900	0.661157
chr1	203576200	0.991736
chr1	203595300	0.669421
chr1	203600900	0.776860
chr1	203608300	0.925620
chr1	203614700	0.702479
chr1	203620000	0.892562
chr1	203632400	0.776860
chr1	203641400	0.768595
chr1	203643800	0.892562
chr1	203662200	0.917355
chr1	203768000	0.694215
chr1	203798700	0.983471
chr1	203819700	0.851240
chr1	203837300	0.909091
chr1	203858500	0.958678
chr1	203876500	0.884298
chr1	204014900	0.785124
chr1	204027000	0.884298
chr1	204034800	0.801653
chr1	204048100	0.917355
chr1	204057900	0.867769
chr1	204063900	0.685950
chr1	204071600	0.735537
chr1	204074200	0.752066
chr1	204078000	0.809917
chr1	204097000	0.958678
chr1	204100500	0.826446
chr1	204141400	0.867769
chr1	204160400	0.851240
chr1	204166800	0.727273
chr1	204169000	0.884298
chr1	204256800	0.892562
chr1	204283300	0.752066
chr1	204304800	0.785124
chr1	204327300	0.842975
chr1	204329600	0.760331
chr1	204332700	0.760331
chr1	204337600	0.735537
chr1	204354500	0.727273
chr1	204386900	0.818182
chr1	204401400	0.793388
chr1	204408000	0.909091
chr1	204411300	0.752066
chr1	204422600	0.900826
chr1	204433700	0.809917
chr1	204436100	0.942149
chr1	204447000	0.900826
chr1	204473400	0.917355
chr1	204941600	0.983471

chr1	205114700	0.958678
chr1	205145100	0.884298
chr1	205236900	0.719008
chr1	205240500	0.859504
chr1	205254300	0.818182
chr1	205257400	0.892562
chr1	205263700	0.719008
chr1	205267800	0.851240
chr1	205274200	0.809917
chr1	205283300	0.942149
chr1	205474200	0.818182
chr1	205498700	0.727273
chr1	205513000	0.933884
chr1	205551900	0.752066
chr1	205561900	0.735537
chr1	205566500	0.768595
chr1	205580200	0.983471
chr1	205634400	0.826446
chr1	205638800	0.752066
chr1	205670300	0.842975
chr1	205903200	0.991736
chr1	205921400	0.752066
chr1	206078800	0.925620
chr1	206094800	1.000000
chr1	206108300	0.892562
chr1	206151400	0.776860
chr1	206158700	0.859504
chr1	206308500	1.000000
chr1	206319700	0.983471
chr1	206491000	0.966942
chr1	206494700	0.785124
chr1	206522900	0.933884
chr1	206525100	0.818182
chr1	206532600	0.842975
chr1	206540200	0.694215
chr1	206548600	0.983471
chr1	206554900	0.859504
chr1	206566600	0.669421
chr1	206576800	0.752066
chr1	206578900	0.884298
chr1	206603900	0.950413
chr1	206623400	0.942149
chr1	206630000	0.818182
chr1	206634400	0.859504
chr1	206645700	0.884298
chr1	206660800	0.677686
chr1	206909200	0.694215
chr1	207379400	0.991736
chr1	207392400	0.917355
chr1	207414800	0.710744
chr1	207458300	0.942149
chr1	207472200	0.719008
chr1	207476400	0.669421
chr1	207478500	0.933884

chr1	207483700	0.892562
chr1	207562900	0.752066
chr1	207567200	0.735537
chr1	207669700	0.694215
chr1	207928300	0.694215
chr1	207979500	0.768595
chr1	207989300	0.760331
chr1	207992800	0.785124
chr1	207997600	0.900826
chr1	208001700	0.793388
chr1	208020400	0.917355
chr1	208022600	0.892562
chr1	208027200	0.975207
chr1	208032600	0.719008
chr1	208037700	0.719008
chr1	208063900	0.991736
chr1	208066800	0.685950
chr1	208092000	0.685950
chr1	208096600	0.851240
chr1	208111600	0.942149
chr1	208115500	0.900826
chr1	208118400	0.950413
chr1	208122800	0.925620
chr1	208132900	0.900826
chr1	208137100	0.975207
chr1	208202800	0.809917
chr1	208205300	0.661157
chr1	208281600	0.776860
chr1	208306300	0.710744
chr1	208329500	0.776860
chr1	208332700	0.826446
chr1	208349000	0.900826
chr1	208352500	0.892562
chr1	208355800	0.933884
chr1	208358700	0.867769
chr1	208374300	0.942149
chr1	208377300	0.809917
chr1	208381000	0.950413
chr1	208383700	0.942149
chr1	208386900	0.991736
chr1	208389000	0.884298
chr1	208391300	0.884298
chr1	208396800	0.760331
chr1	208400000	0.975207
chr1	208405200	0.851240
chr1	208855800	0.909091
chr1	209008100	0.719008
chr1	209058500	0.661157
chr1	209092900	0.809917
chr1	209116400	0.727273
chr1	209118800	0.991736
chr1	209125400	0.966942
chr1	209342100	0.677686
chr1	209750800	0.735537

chr1	209815800	0.661157
chr1	209822400	0.900826
chr1	209824800	0.809917
chr1	209831000	0.801653
chr1	209921500	0.760331
chr1	209943200	0.909091
chr1	209975300	0.884298
chr1	209981500	0.950413
chr1	209996900	0.950413
chr1	209999000	0.859504
chr1	210005000	0.851240
chr1	210009300	0.983471
chr1	210012000	0.867769
chr1	210014800	0.752066
chr1	210046900	0.834711
chr1	210057600	0.950413
chr1	210069500	0.975207
chr1	210298000	0.834711
chr1	210408500	0.710744
chr1	210410900	0.867769
chr1	210413600	0.892562
chr1	210420200	0.669421
chr1	210466100	0.727273
chr1	210484900	0.933884
chr1	210547600	0.842975
chr1	210612700	0.900826
chr1	211307300	0.727273
chr1	211509400	0.694215
chr1	211545300	0.661157
chr1	211643000	0.826446
chr1	211688900	0.950413
chr1	211715700	0.776860
chr1	211725600	0.892562
chr1	211748500	0.975207
chr1	211813700	0.900826
chr1	211822000	0.933884
chr1	211827400	0.884298
chr1	212091800	0.685950
chr1	212125600	0.851240
chr1	212204700	0.933884
chr1	212241100	0.942149
chr1	212340300	1.000000
chr1	212375200	0.694215
chr1	212415800	0.983471
chr1	212464400	0.867769
chr1	212659500	0.842975
chr1	212687800	0.793388
chr1	212735300	0.867769
chr1	212740400	0.669421
chr1	212769800	0.991736
chr1	212795400	0.909091
chr1	212811100	0.876033
chr1	212839100	0.876033
chr1	212841800	0.991736

chr1	212886400	0.917355
chr1	212895700	0.719008
chr1	212956300	0.991736
chr1	212968100	0.669421
chr1	213296900	0.694215
chr1	213299500	0.710744
chr1	213309200	0.685950
chr1	213438400	0.851240
chr1	213935500	0.702479
chr1	214146900	0.768595
chr1	214151200	1.000000
chr1	214176500	0.884298
chr1	214276200	0.859504
chr1	214281100	0.925620
chr1	214289800	0.809917
chr1	214298300	1.000000
chr1	214361000	0.702479
chr1	214388100	0.785124
chr1	214433500	0.917355
chr1	214469600	0.785124
chr1	214576000	0.884298
chr1	214579300	0.842975
chr1	214590200	0.884298
chr1	214601200	0.876033
chr1	214603700	0.818182
chr1	214610700	0.661157
chr1	214613100	0.735537
chr1	214619200	0.958678
chr1	214621500	0.851240
chr1	214626500	0.900826
chr1	214629000	0.867769
chr1	214634500	0.677686
chr1	214650500	0.991736
chr1	214716300	0.958678
chr1	214740600	0.685950
chr1	214744200	0.991736
chr1	214788700	0.983471
chr1	214797200	0.685950
chr1	214927500	0.801653
chr1	215075800	0.966942
chr1	215288100	0.834711
chr1	215447500	0.975207
chr1	215456200	0.991736
chr1	217699800	0.950413
chr1	218486400	0.859504
chr1	218522300	0.735537
chr1	218524900	0.876033
chr1	218548200	0.925620
chr1	218550800	0.826446
chr1	218554300	0.685950
chr1	218557600	0.991736
chr1	218564500	0.702479
chr1	218633800	0.785124
chr1	218637300	0.909091

chr1	218647300	0.983471
chr1	218653200	0.942149
chr1	218655300	0.958678
chr1	218661600	0.933884
chr1	218670200	0.942149
chr1	218672800	0.925620
chr1	218731700	0.942149
chr1	218759700	0.950413
chr1	218765900	0.851240
chr1	218794600	0.776860
chr1	218834100	0.966942
chr1	218879800	0.958678
chr1	218939500	0.685950
chr1	219047800	0.983471
chr1	219078000	0.900826
chr1	219104700	0.710744
chr1	219116100	0.669421
chr1	219362600	1.000000
chr1	219445700	0.776860
chr1	219453100	0.892562
chr1	219470800	0.661157
chr1	219475700	0.842975
chr1	219478500	0.900826
chr1	219484200	0.867769
chr1	219486500	0.801653
chr1	219496500	0.661157
chr1	219498700	0.942149
chr1	219503300	0.991736
chr1	219536700	0.710744
chr1	220174700	0.950413
chr1	220311000	0.975207
chr1	220372300	0.661157
chr1	220474100	0.975207
chr1	220905700	0.942149
chr1	220908100	0.983471
chr1	220923800	0.743802
chr1	221017100	0.892562
chr1	221050400	1.000000
chr1	221061300	0.809917
chr1	221065100	0.776860
chr1	221206100	1.000000
chr1	221209600	0.991736
chr1	221274400	0.760331
chr1	221335300	0.760331
chr1	221343200	0.958678
chr1	221391200	0.685950
chr1	221438000	0.933884
chr1	221567800	0.991736
chr1	221584800	0.950413
chr1	221589100	0.975207
chr1	221613000	0.933884
chr1	221615100	0.801653
chr1	221933200	0.909091
chr1	221935600	0.917355

chr1	221983700	0.900826
chr1	221997800	0.909091
chr1	222000600	0.900826
chr1	222003300	0.983471
chr1	222006300	0.950413
chr1	222013500	0.900826
chr1	222042800	0.661157
chr1	222638900	0.710744
chr1	222756000	0.900826
chr1	222775000	0.900826
chr1	222800700	0.694215
chr1	223063200	0.677686
chr1	223065700	0.760331
chr1	223168200	0.991736
chr1	223250900	0.776860
chr1	223276800	0.983471
chr1	223283600	0.834711
chr1	223307700	0.727273
chr1	223327200	0.760331
chr1	223407300	0.892562
chr1	223743900	0.892562
chr1	223746000	0.842975
chr1	223889000	0.859504
chr1	223893800	0.917355
chr1	223896300	0.859504
chr1	223903900	0.884298
chr1	223906300	0.892562
chr1	223915000	0.785124
chr1	223918200	0.719008
chr1	223922000	0.933884
chr1	223924800	0.991736
chr1	223929200	0.884298
chr1	223931800	0.685950
chr1	223937100	0.958678
chr1	223961500	0.975207
chr1	223965000	0.933884
chr1	223995000	0.677686
chr1	224011200	0.785124
chr1	224020000	0.991736
chr1	224025400	0.719008
chr1	224220500	0.933884
chr1	224230400	0.719008
chr1	224239900	0.842975
chr1	224242000	0.842975
chr1	224246400	0.942149
chr1	224357100	0.975207
chr1	224393200	1.000000
chr1	224411900	0.950413
chr1	224433300	0.925620
chr1	224440000	0.809917
chr1	224447800	0.983471
chr1	224450600	0.859504
chr1	224488300	0.867769
chr1	224615600	0.826446

chr1	224618400	0.917355
chr1	224635300	0.776860
chr1	224645100	0.950413
chr1	224647300	0.826446
chr1	224686400	0.942149
chr1	224691300	0.851240
chr1	224717400	0.760331
chr1	224729800	0.876033
chr1	224733100	0.958678
chr1	224753100	0.958678
chr1	224811900	0.933884
chr1	224820600	0.925620
chr1	224840700	0.900826
chr1	225344100	0.752066
chr1	225610600	0.826446
chr1	225613000	0.785124
chr1	225633100	0.892562
chr1	225648400	0.727273
chr1	225655600	0.884298
chr1	225696700	0.925620
chr1	225790900	0.752066
chr1	225843500	1.000000
chr1	225845800	0.950413
chr1	225882300	0.851240
chr1	225888100	0.900826
chr1	225925300	0.702479
chr1	225934800	0.933884
chr1	225943600	0.785124
chr1	225951500	0.925620
chr1	225955200	0.917355
chr1	225999400	0.743802
chr1	226005400	0.793388
chr1	226068700	0.752066
chr1	226107900	0.958678
chr1	226184900	0.975207
chr1	226190700	0.818182
chr1	226315900	0.702479
chr1	226554200	0.884298
chr1	226838400	0.933884
chr1	226844300	0.925620
chr1	226848900	0.933884
chr1	226851200	0.719008
chr1	226853300	0.677686
chr1	226863500	0.900826
chr1	226868200	0.785124
chr1	226878800	0.958678
chr1	226885100	0.752066
chr1	226890900	0.743802
chr1	226898700	0.876033
chr1	226912100	0.826446
chr1	226966800	0.818182
chr1	226972000	0.776860
chr1	226987300	0.793388
chr1	227001900	0.727273

chr1	227004400	0.917355
chr1	227007200	0.983471
chr1	227009900	0.826446
chr1	227016100	0.933884
chr1	227060100	0.702479
chr1	227080200	0.702479
chr1	227178100	0.958678
chr1	227250700	0.677686
chr1	227322700	1.000000
chr1	227324800	0.859504
chr1	227413600	0.983471
chr1	227486500	0.834711
chr1	227497700	0.702479
chr1	227500600	0.801653
chr1	227539300	0.983471
chr1	227544100	0.950413
chr1	227546200	0.900826
chr1	227558600	0.933884
chr1	227586600	0.950413
chr1	227616100	0.909091
chr1	227620000	1.000000
chr1	227625500	0.719008
chr1	227635700	0.743802
chr1	227916500	0.818182
chr1	227964200	0.818182
chr1	228248400	0.752066
chr1	228279000	0.851240
chr1	228336900	0.735537
chr1	228351500	0.851240
chr1	228458300	0.975207
chr1	228566800	0.752066
chr1	228660800	0.760331
chr1	228667100	0.661157
chr1	228962200	0.859504
chr1	228974400	0.983471
chr1	228978300	0.785124
chr1	229039300	0.909091
chr1	229074600	0.768595
chr1	229163800	0.809917
chr1	229168500	0.743802
chr1	229195300	0.793388
chr1	229394600	0.685950
chr1	229416800	0.710744
chr1	229715300	0.884298
chr1	229800000	0.942149
chr1	230220700	0.892562
chr1	230226000	0.884298
chr1	230240700	0.859504
chr1	230249900	0.942149
chr1	230322400	0.958678
chr1	230327300	0.909091
chr1	230341800	0.917355
chr1	230346500	0.983471
chr1	230350900	0.966942

chr1	230406800	0.958678
chr1	230410300	0.900826
chr1	230444000	0.801653
chr1	230985700	0.950413
chr1	231113200	0.719008
chr1	231157000	0.776860
chr1	231164800	1.000000
chr1	231206900	0.809917
chr1	231209000	0.669421
chr1	231275600	0.884298
chr1	231408700	1.000000
chr1	231415200	0.909091
chr1	231460000	0.768595
chr1	231469600	0.859504
chr1	231487800	0.950413
chr1	231526800	0.966942
chr1	231532000	0.876033
chr1	231544300	0.975207
chr1	231561600	0.685950
chr1	231629700	0.917355
chr1	231681100	0.983471
chr1	231708100	0.917355
chr1	231720100	0.867769
chr1	231739600	0.677686
chr1	231754900	0.826446
chr1	231764900	0.661157
chr1	231826400	0.859504
chr1	231918200	0.983471
chr1	231950300	0.966942
chr1	231963200	0.991736
chr1	232021200	0.925620
chr1	232054900	0.842975
chr1	232065700	0.809917
chr1	232107700	0.983471
chr1	232126500	0.834711
chr1	232149100	0.975207
chr1	232260500	0.842975
chr1	232286600	0.958678
chr1	232607700	0.727273
chr1	232659900	0.727273
chr1	232695300	0.818182
chr1	232714800	0.933884
chr1	232726000	0.801653
chr1	232803300	0.727273
chr1	232820500	0.966942
chr1	232822600	0.884298
chr1	232826300	0.917355
chr1	232840900	0.983471
chr1	232845600	0.793388
chr1	233099200	0.933884
chr1	233248400	0.950413
chr1	233250700	0.925620
chr1	234566900	0.677686
chr1	234609400	0.752066

chr1	234635600	0.900826
chr1	234651600	0.933884
chr1	234659600	1.000000
chr1	234667500	0.760331
chr1	234680500	1.000000
chr1	234686600	0.719008
chr1	234735100	0.950413
chr1	234738700	0.727273
chr1	234746200	0.669421
chr1	234749500	0.876033
chr1	234754900	0.859504
chr1	234767600	0.991736
chr1	234793200	1.000000
chr1	234795500	0.851240
chr1	234812200	0.685950
chr1	234854100	0.983471
chr1	234857600	0.685950
chr1	234860300	0.900826
chr1	234864100	0.950413
chr1	234867700	0.867769
chr1	234880000	0.983471
chr1	234908500	0.942149
chr1	234980700	0.958678
chr1	234987700	0.867769
chr1	234998800	0.661157
chr1	235002800	0.909091
chr1	235030200	0.983471
chr1	235035400	0.884298
chr1	235048000	0.685950
chr1	235053200	0.785124
chr1	235056200	0.818182
chr1	235058300	0.917355
chr1	235060900	0.933884
chr1	235063100	0.851240
chr1	235069500	0.818182
chr1	235093200	0.685950
chr1	235096600	0.818182
chr1	235098700	0.776860
chr1	235106400	0.909091
chr1	235111100	0.966942
chr1	235114700	0.702479
chr1	235117600	0.694215
chr1	235120100	0.727273
chr1	235122200	0.909091
chr1	235124500	0.909091
chr1	235132500	0.859504
chr1	235135100	0.925620
chr1	235144400	0.909091
chr1	235148000	0.677686
chr1	235184100	0.851240
chr1	235243200	0.809917
chr1	235247100	0.933884
chr1	235409900	0.917355
chr1	235481400	0.669421

chr1	235606600	0.826446
chr1	235633100	0.685950
chr1	235796400	0.702479
chr1	235805800	0.669421
chr1	236073500	0.892562
chr1	236081200	0.991736
chr1	236097400	0.719008
chr1	236135500	0.975207
chr1	236147600	0.991736
chr1	236150600	1.000000
chr1	236162900	0.917355
chr1	236165600	0.925620
chr1	236173500	0.925620
chr1	236666700	0.983471
chr1	236695300	0.702479
chr1	236698000	0.768595
chr1	236787300	0.917355
chr1	236897800	0.834711
chr1	237766300	0.694215
chr1	240194800	0.983471
chr1	240929200	0.966942
chr1	240950600	0.851240
chr1	240967100	0.876033
chr1	240994900	0.917355
chr1	241042000	0.702479
chr1	241111200	0.719008
chr1	241639500	0.958678
chr1	241646700	1.000000
chr1	241670500	0.925620
chr1	241776200	0.983471
chr1	241805100	0.669421
chr1	242089900	0.925620
chr1	243335200	0.859504
chr1	243337700	0.983471
chr1	243398200	0.719008
chr1	243400400	0.851240
chr1	243421400	0.677686
chr1	243424600	0.661157
chr1	243428200	0.975207
chr1	243431800	1.000000
chr1	243434100	0.867769
chr1	243449600	0.867769
chr1	243451900	0.884298
chr1	243457600	1.000000
chr1	243554400	0.925620
chr1	243636600	0.909091
chr1	243646600	0.719008
chr1	243688600	0.892562
chr1	243801000	0.677686
chr1	243806900	0.892562
chr1	243809200	0.818182
chr1	243882800	0.966942
chr1	243955500	0.983471
chr1	244006600	0.793388

chr1	244079500	0.909091
chr1	244090500	1.000000
chr1	244102800	0.975207
chr1	244175800	0.702479
chr1	244215500	0.727273
chr1	244302200	0.909091
chr1	244326000	0.809917
chr1	244328400	0.983471
chr1	244334400	0.702479
chr1	244343900	0.818182
chr1	244377800	0.933884
chr1	244399800	0.909091
chr1	244412100	0.975207
chr1	244436900	0.991736
chr1	244463400	0.925620
chr1	244467300	0.677686
chr1	244478600	0.950413
chr1	244486300	0.925620
chr1	244489100	0.876033
chr1	244505300	0.933884
chr1	244510600	0.842975
chr1	244513800	0.909091
chr1	244517600	0.958678
chr1	244599000	0.958678
chr1	244601400	0.950413
chr1	244609000	0.958678
chr1	244612300	0.702479
chr1	244763600	0.942149
chr1	244849400	0.661157
chr1	244996900	0.776860
chr1	245001600	0.685950
chr1	245128100	0.983471
chr1	245132000	0.735537
chr1	245152600	0.909091
chr1	245255600	0.975207
chr1	246038700	0.966942
chr1	246041900	0.942149
chr1	246120600	0.842975
chr1	246150400	0.942149
chr1	246303300	0.925620
chr1	246305900	0.768595
chr1	246319900	0.685950
chr1	246456300	0.867769
chr1	246459600	0.942149
chr1	246520100	0.933884
chr1	246547400	0.727273
chr1	246667000	0.702479
chr1	246732500	0.909091
chr1	246735400	0.892562
chr1	246751500	0.983471
chr1	246772600	0.983471
chr1	246898500	0.991736
chr1	246948500	0.925620
chr1	247014200	0.975207

chr1	247111200	0.867769
chr1	247123500	0.867769
chr1	247218800	0.661157
chr1	247367900	0.809917
chr1	248571700	0.702479
chr1	249118200	0.834711
chr1	249123000	0.834711
chr20	273100	0.710744
chr20	281700	0.884298
chr20	295000	0.925620
chr20	310100	0.719008
chr20	350500	0.876033
chr20	355000	0.958678
chr20	419700	0.768595
chr20	426100	0.776860
chr20	440100	0.925620
chr20	517400	0.892562
chr20	520900	0.702479
chr20	555500	0.917355
chr20	592800	0.809917
chr20	816900	0.793388
chr20	825800	0.909091
chr20	831500	0.760331
chr20	1103500	0.669421
chr20	1147800	0.834711
chr20	1166000	0.776860
chr20	1206800	0.661157
chr20	1223500	0.942149
chr20	1294600	0.983471
chr20	1302800	0.760331
chr20	1308700	0.884298
chr20	1310800	0.966942
chr20	1314000	0.876033
chr20	1325400	0.958678
chr20	1349800	0.851240
chr20	1352600	0.661157
chr20	1355000	0.925620
chr20	1357400	0.752066
chr20	1361300	0.958678
chr20	1363500	0.694215
chr20	1375400	0.867769
chr20	1377600	0.975207
chr20	1381100	0.991736
chr20	1397100	0.768595
chr20	1399500	0.975207
chr20	1416100	0.966942
chr20	1451000	0.975207
chr20	1473800	0.876033
chr20	1542200	0.793388
chr20	1548400	0.991736
chr20	1550500	0.925620
chr20	1598000	0.983471
chr20	1605500	0.752066
chr20	1767700	0.892562

chr20	1790000	0.677686
chr20	1794000	1.000000
chr20	1797200	0.966942
chr20	1801700	0.677686
chr20	1805800	0.950413
chr20	2199100	0.950413
chr20	2265900	0.694215
chr20	2270100	0.975207
chr20	2458100	0.793388
chr20	2673300	0.677686
chr20	2752700	0.834711
chr20	2851800	0.685950
chr20	2856800	0.719008
chr20	3052800	0.669421
chr20	3063700	0.719008
chr20	3074100	0.743802
chr20	3102600	0.661157
chr20	3146200	0.727273
chr20	3202500	0.710744
chr20	3229900	0.801653
chr20	3359200	1.000000
chr20	3485000	0.710744
chr20	3720600	0.834711
chr20	3762200	0.727273
chr20	3788800	0.785124
chr20	3792600	0.966942
chr20	3880800	0.884298
chr20	3913600	0.818182
chr20	3952200	0.818182
chr20	3961700	0.876033
chr20	4048500	0.801653
chr20	4066600	0.925620
chr20	4101900	0.743802
chr20	4111900	0.900826
chr20	4116800	0.983471
chr20	4133500	0.826446
chr20	4153400	0.876033
chr20	4206900	0.834711
chr20	4230400	0.785124
chr20	4488300	0.950413
chr20	4490400	0.884298
chr20	4494800	0.975207
chr20	4527700	0.983471
chr20	4531800	0.966942
chr20	4536500	0.950413
chr20	4573300	0.826446
chr20	4607600	0.876033
chr20	4614600	0.983471
chr20	4646800	0.991736
chr20	4684700	0.942149
chr20	4708100	0.818182
chr20	4776400	0.785124
chr20	4793200	0.702479
chr20	4795400	0.702479

chr20	4811600	0.867769
chr20	4907200	0.983471
chr20	4932300	0.925620
chr20	4954400	0.867769
chr20	5046600	0.727273
chr20	5059300	0.909091
chr20	5071200	0.842975
chr20	5138100	0.975207
chr20	5150100	0.842975
chr20	5161400	0.991736
chr20	5196600	0.876033
chr20	5334900	0.991736
chr20	5589600	0.669421
chr20	5623500	0.966942
chr20	5627900	0.942149
chr20	5635200	0.776860
chr20	5638500	0.892562
chr20	5695600	0.876033
chr20	5705700	0.892562
chr20	5768500	0.694215
chr20	5785800	0.966942
chr20	5803800	0.669421
chr20	5816600	0.884298
chr20	5843900	0.966942
chr20	5846000	0.859504
chr20	5892400	0.677686
chr20	5900100	0.900826
chr20	6015500	0.867769
chr20	6449900	0.925620
chr20	6481900	0.900826
chr20	6500600	0.942149
chr20	6543000	1.000000
chr20	6556800	0.925620
chr20	6572200	0.942149
chr20	6644300	0.975207
chr20	6654100	0.958678
chr20	6666200	0.876033
chr20	6680700	0.851240
chr20	6685400	0.876033
chr20	6689100	0.884298
chr20	6691200	0.975207
chr20	6728200	0.867769
chr20	6937700	0.685950
chr20	7140900	0.958678
chr20	7380400	0.942149
chr20	7440200	0.917355
chr20	7479400	0.983471
chr20	7609200	0.983471
chr20	7618000	0.884298
chr20	7703800	0.661157
chr20	7822800	0.851240
chr20	7837900	0.958678
chr20	7840200	0.776860
chr20	7893200	0.950413

chr20	7905700	0.991736
chr20	8028400	0.677686
chr20	8031200	0.834711
chr20	8075900	0.975207
chr20	8097500	0.710744
chr20	8125600	0.826446
chr20	8129400	0.975207
chr20	8132400	0.909091
chr20	8134800	1.000000
chr20	8137500	1.000000
chr20	8142500	0.958678
chr20	8490900	0.685950
chr20	8523000	0.983471
chr20	8528100	0.809917
chr20	8568300	0.983471
chr20	8571400	0.900826
chr20	8627500	0.942149
chr20	8637500	0.966942
chr20	8793700	0.925620
chr20	8835500	0.859504
chr20	9032400	0.909091
chr20	9216700	0.776860
chr20	9222400	0.834711
chr20	9329200	0.677686
chr20	9381400	0.900826
chr20	9441700	0.859504
chr20	9575600	0.669421
chr20	10016100	0.702479
chr20	10021300	0.917355
chr20	10055600	0.710744
chr20	10058400	0.818182
chr20	10287900	1.000000
chr20	10290300	0.966942
chr20	10331700	0.958678
chr20	10334400	0.950413
chr20	10338200	0.768595
chr20	10341000	0.975207
chr20	10348000	0.677686
chr20	10350700	0.826446
chr20	10357300	0.834711
chr20	10396400	0.900826
chr20	10447300	0.942149
chr20	10450100	0.950413
chr20	10492600	0.991736
chr20	10495400	0.727273
chr20	10502200	0.867769
chr20	10522100	0.983471
chr20	10537300	0.925620
chr20	10559900	0.752066
chr20	10573000	0.900826
chr20	10579300	0.826446
chr20	10585700	0.966942
chr20	10603900	0.925620
chr20	10617900	0.917355

chr20	10635100	0.925620
chr20	10645900	0.958678
chr20	10739400	0.900826
chr20	10742600	1.000000
chr20	10838200	0.661157
chr20	10844900	0.958678
chr20	10891700	0.752066
chr20	10906400	0.826446
chr20	10909500	0.694215
chr20	10916500	0.851240
chr20	10944700	0.950413
chr20	10951300	0.826446
chr20	10982900	0.801653
chr20	11061700	0.859504
chr20	11082300	0.785124
chr20	11099300	0.752066
chr20	11139100	0.942149
chr20	11151100	0.958678
chr20	11159900	0.958678
chr20	11167300	0.884298
chr20	11198300	0.950413
chr20	11210900	0.966942
chr20	11216000	0.851240
chr20	11247900	0.900826
chr20	11428800	0.892562
chr20	11436700	0.958678
chr20	11673700	0.710744
chr20	11722200	0.785124
chr20	11758900	0.876033
chr20	12000800	0.727273
chr20	12033700	0.809917
chr20	12035800	0.925620
chr20	12072200	0.909091
chr20	12910000	0.983471
chr20	13228300	0.801653
chr20	13243700	0.842975
chr20	13252100	1.000000
chr20	13312500	0.661157
chr20	13506000	0.892562
chr20	13746500	0.834711
chr20	13758200	0.942149
chr20	13781700	0.743802
chr20	14426300	0.933884
chr20	14470100	0.818182
chr20	14478000	0.958678
chr20	14761500	0.785124
chr20	14894900	0.752066
chr20	14906100	0.925620
chr20	14932700	0.983471
chr20	14944300	0.917355
chr20	14966500	0.991736
chr20	15202300	0.760331
chr20	15654400	0.892562
chr20	15685000	0.958678

chr20	17501300	0.867769
chr20	17539800	0.975207
chr20	17543900	0.743802
chr20	17549300	0.818182
chr20	17575300	0.842975
chr20	17593000	0.876033
chr20	17625400	0.702479
chr20	17629600	0.685950
chr20	17636100	0.719008
chr20	17646700	0.983471
chr20	17728300	0.859504
chr20	17731400	0.859504
chr20	17767300	0.892562
chr20	17824500	0.826446
chr20	17830800	0.925620
chr20	17845300	0.694215
chr20	17877300	0.950413
chr20	17918000	0.892562
chr20	17939200	0.768595
chr20	17943400	0.834711
chr20	17953000	0.719008
chr20	17974000	0.933884
chr20	18037900	0.710744
chr20	18206300	0.801653
chr20	19680600	0.809917
chr20	19716300	0.917355
chr20	19765000	0.785124
chr20	19767400	0.966942
chr20	19783300	0.752066
chr20	19793600	0.958678
chr20	19796300	0.958678
chr20	19798800	0.818182
chr20	19808900	0.785124
chr20	19821200	0.785124
chr20	19865500	0.818182
chr20	19887900	0.727273
chr20	19895200	0.892562
chr20	19912400	0.892562
chr20	19916600	0.958678
chr20	19918900	0.793388
chr20	19926200	0.694215
chr20	19933000	0.793388
chr20	19955500	0.876033
chr20	19958000	0.917355
chr20	19966200	1.000000
chr20	19972300	0.760331
chr20	19976100	0.776860
chr20	19984300	0.760331
chr20	20018800	0.983471
chr20	20115800	0.702479
chr20	20349800	0.661157
chr20	20433400	0.834711
chr20	20474100	0.834711
chr20	20713000	1.000000

chr20	20715400	0.942149
chr20	20724800	0.694215
chr20	20733700	1.000000
chr20	20740000	0.826446
chr20	20742300	0.983471
chr20	20746800	0.917355
chr20	20783300	0.842975
chr20	21081900	0.892562
chr20	21290900	0.892562
chr20	21304600	0.975207
chr20	21318900	0.785124
chr20	21342100	1.000000
chr20	21377700	0.859504
chr20	21417200	0.933884
chr20	21486900	0.801653
chr20	22493500	0.719008
chr20	22665900	0.809917
chr20	22673000	0.826446
chr20	22679800	0.702479
chr20	22698200	0.958678
chr20	22744300	0.793388
chr20	22754300	0.677686
chr20	22775800	0.851240
chr20	22778000	0.975207
chr20	22843000	0.859504
chr20	22847900	0.925620
chr20	22902900	0.983471
chr20	23042700	0.933884
chr20	23053900	1.000000
chr20	23061200	0.884298
chr20	23068300	0.760331
chr20	23073600	0.975207
chr20	23083000	0.801653
chr20	23088000	0.776860
chr20	23100700	0.661157
chr20	23113900	0.801653
chr20	23124000	0.991736
chr20	23127200	0.975207
chr20	23129800	0.884298
chr20	23134000	0.909091
chr20	23137200	0.867769
chr20	23140200	0.884298
chr20	23143100	0.909091
chr20	23159400	0.950413
chr20	23208000	0.677686
chr20	23237100	0.685950
chr20	23239700	0.785124
chr20	23251000	0.809917
chr20	23298200	0.842975
chr20	23312800	0.710744
chr20	23341900	0.776860
chr20	23344900	0.661157
chr20	23347200	0.942149
chr20	23355200	0.958678

chr20	23367800	0.785124
chr20	23378400	0.975207
chr20	23603800	0.933884
chr20	23664300	0.975207
chr20	23674800	0.958678
chr20	23736100	0.933884
chr20	23812900	0.776860
chr20	23998500	0.958678
chr20	24008100	0.958678
chr20	24083000	0.884298
chr20	24085100	0.942149
chr20	24087200	0.867769
chr20	24096200	0.752066
chr20	24100100	0.900826
chr20	24144400	0.743802
chr20	24955900	0.719008
chr20	25017200	0.942149
chr20	25129600	0.752066
chr20	25204300	0.892562
chr20	25214500	0.834711
chr20	25218800	0.909091
chr20	25230800	0.685950
chr20	25290600	0.950413
chr20	25342000	0.876033
chr20	25465800	0.909091
chr20	25489100	0.809917
chr20	25514300	0.867769
chr20	25720600	0.735537
chr20	25849300	0.768595
chr20	26319200	0.719008
chr20	30105200	0.760331
chr20	30111700	0.876033
chr20	30137000	0.743802
chr20	30147900	0.826446
chr20	30150700	0.834711
chr20	30158300	0.950413
chr20	30161300	0.892562
chr20	30175700	0.826446
chr20	30182200	0.892562
chr20	30184400	0.892562
chr20	30196900	0.933884
chr20	30199600	0.900826
chr20	30249700	0.942149
chr20	30262000	0.809917
chr20	30266500	0.859504
chr20	30271000	0.719008
chr20	30282900	0.793388
chr20	30287000	0.719008
chr20	30289200	0.851240
chr20	30293000	0.966942
chr20	30299200	0.702479
chr20	30302000	0.768595
chr20	30305100	0.900826
chr20	30400500	0.727273

chr20	30411900	0.727273
chr20	30426700	0.760331
chr20	30430500	0.694215
chr20	30595700	0.892562
chr20	30640600	0.719008
chr20	30662300	0.702479
chr20	30672300	0.669421
chr20	30676200	0.826446
chr20	30741000	0.892562
chr20	30743800	0.694215
chr20	30914900	0.958678
chr20	30920700	0.793388
chr20	30922800	0.925620
chr20	30966700	0.991736
chr20	31020600	0.942149
chr20	31022900	0.743802
chr20	31045800	0.793388
chr20	31054900	0.743802
chr20	31057600	0.760331
chr20	31060000	0.842975
chr20	31067300	0.892562
chr20	31073400	0.743802
chr20	31086000	0.884298
chr20	31089800	0.991736
chr20	31097900	0.851240
chr20	31105200	0.867769
chr20	31269600	0.685950
chr20	31296100	0.966942
chr20	31325900	0.958678
chr20	31334600	0.876033
chr20	31381500	0.909091
chr20	31416300	0.950413
chr20	31467600	0.694215
chr20	31560300	0.719008
chr20	31951300	0.694215
chr20	32106100	0.966942
chr20	32127000	0.983471
chr20	32274600	0.727273
chr20	32322000	0.958678
chr20	32375100	1.000000
chr20	32377500	0.842975
chr20	32381100	0.925620
chr20	32402500	0.925620
chr20	32420200	0.933884
chr20	32431800	0.834711
chr20	32446100	0.867769
chr20	32498100	0.851240
chr20	32503100	0.752066
chr20	32597800	0.719008
chr20	32808900	0.661157
chr20	32900000	0.933884
chr20	32978700	0.785124
chr20	32993700	0.785124
chr20	33003100	0.752066

chr20	33047000	0.867769
chr20	33114000	0.917355
chr20	33159100	0.950413
chr20	33170000	0.851240
chr20	33277700	0.727273
chr20	33301400	0.958678
chr20	33356300	0.876033
chr20	33461000	0.793388
chr20	33496600	0.983471
chr20	33529500	0.900826
chr20	33609200	0.983471
chr20	33640800	0.983471
chr20	33643900	0.851240
chr20	33676500	0.768595
chr20	33681900	0.851240
chr20	33699500	0.892562
chr20	33704900	0.719008
chr20	33708100	0.958678
chr20	33711400	0.991736
chr20	33717900	0.900826
chr20	33722200	0.851240
chr20	33724500	0.942149
chr20	33754700	0.983471
chr20	33796300	0.876033
chr20	33813800	0.859504
chr20	33891600	0.942149
chr20	33894300	0.661157
chr20	33896600	0.834711
chr20	33900400	0.975207
chr20	33907200	0.851240
chr20	33915300	0.958678
chr20	33932500	0.867769
chr20	33984400	0.710744
chr20	33986800	1.000000
chr20	34026800	0.710744
chr20	34035000	0.702479
chr20	34049900	0.925620
chr20	34079000	0.842975
chr20	34204000	0.719008
chr20	34332200	0.884298
chr20	34356200	0.942149
chr20	34459700	0.884298
chr20	34463900	0.925620
chr20	34472200	0.975207
chr20	34490200	0.958678
chr20	34605800	1.000000
chr20	34653000	0.702479
chr20	34686000	0.760331
chr20	34742800	0.768595
chr20	34770800	0.809917
chr20	34891600	0.834711
chr20	35093600	0.909091
chr20	35095900	0.851240
chr20	35098400	0.752066

chr20	35101500	0.661157
chr20	35120700	0.876033
chr20	35128600	0.884298
chr20	35172500	0.694215
chr20	35386600	0.975207
chr20	35397900	0.966942
chr20	35461900	0.859504
chr20	35464600	0.917355
chr20	35472100	0.785124
chr20	35477100	0.900826
chr20	35486400	1.000000
chr20	35575900	0.694215
chr20	35617200	0.826446
chr20	35899200	0.818182
chr20	35905800	0.876033
chr20	35923700	0.942149
chr20	36007500	0.884298
chr20	36012500	0.900826
chr20	36031300	0.826446
chr20	36035200	0.842975
chr20	36040700	0.867769
chr20	36147800	1.000000
chr20	36162700	0.892562
chr20	36171100	0.727273
chr20	36339700	0.859504
chr20	36618900	1.000000
chr20	36630600	0.818182
chr20	36652700	0.983471
chr20	36678500	0.876033
chr20	36685900	0.884298
chr20	36702100	0.966942
chr20	36713100	0.876033
chr20	36723300	0.842975
chr20	36727800	0.909091
chr20	36737500	0.793388
chr20	36739700	0.950413
chr20	36742600	0.743802
chr20	36746300	0.867769
chr20	36752000	0.884298
chr20	36754500	0.776860
chr20	36758400	0.892562
chr20	36765700	0.710744
chr20	36768200	0.785124
chr20	36771000	0.950413
chr20	36774000	0.900826
chr20	36777400	0.925620
chr20	36779800	0.958678
chr20	36783300	0.909091
chr20	36789300	0.669421
chr20	36795300	0.719008
chr20	36799000	0.834711
chr20	36802100	0.942149
chr20	36846400	0.917355
chr20	36860400	0.859504

chr20	36889500	0.719008
chr20	36899900	0.876033
chr20	36902300	0.983471
chr20	36904700	0.975207
chr20	37079700	0.876033
chr20	37082700	0.752066
chr20	37113800	0.719008
chr20	37230800	0.842975
chr20	37275300	0.785124
chr20	37356400	0.801653
chr20	37360000	0.793388
chr20	37386300	0.958678
chr20	37428300	0.925620
chr20	37432900	0.719008
chr20	37437700	0.818182
chr20	37441300	0.991736
chr20	37454400	0.925620
chr20	37456800	0.983471
chr20	37459500	0.925620
chr20	37495500	0.702479
chr20	37557700	0.677686
chr20	37561500	0.842975
chr20	37614500	0.851240
chr20	37638100	0.710744
chr20	37737600	0.983471
chr20	38202800	0.983471
chr20	38205800	0.760331
chr20	38217100	1.000000
chr20	38289200	0.983471
chr20	38451700	0.785124
chr20	38923900	1.000000
chr20	38983100	0.776860
chr20	39272900	0.884298
chr20	39275300	0.900826
chr20	39386000	0.892562
chr20	39620400	0.917355
chr20	39631600	0.735537
chr20	39635300	0.909091
chr20	39638800	0.685950
chr20	39687500	0.950413
chr20	39701000	0.942149
chr20	39704700	0.966942
chr20	39727000	0.801653
chr20	39769700	0.735537
chr20	39771800	0.752066
chr20	39774400	0.719008
chr20	39777200	0.818182
chr20	39780600	0.826446
chr20	39829500	0.909091
chr20	39861800	0.760331
chr20	39874600	0.983471
chr20	39878100	0.933884
chr20	39930100	0.859504
chr20	39947400	0.685950

chr20	39958400	0.983471
chr20	39968900	0.760331
chr20	39995300	0.702479
chr20	40205300	0.818182
chr20	40619100	0.983471
chr20	42472700	0.909091
chr20	42478700	0.867769
chr20	42539800	0.876033
chr20	42568700	0.834711
chr20	42571500	1.000000
chr20	42585200	0.942149
chr20	42588200	0.760331
chr20	42613300	1.000000
chr20	42648300	0.661157
chr20	42833200	0.975207
chr20	43155100	0.909091
chr20	43169800	0.834711
chr20	43177800	0.975207
chr20	43182100	0.975207
chr20	43202000	0.818182
chr20	43205500	0.867769
chr20	43209300	0.983471
chr20	43214700	0.809917
chr20	43218800	0.975207
chr20	43223000	0.719008
chr20	43228100	0.884298
chr20	43230700	0.917355
chr20	43238600	0.735537
chr20	43244700	0.809917
chr20	43270700	0.801653
chr20	43275200	0.809917
chr20	43321600	0.950413
chr20	43331800	0.859504
chr20	43358200	0.669421
chr20	43375000	0.760331
chr20	43567700	0.933884
chr20	43590500	0.793388
chr20	43599300	0.719008
chr20	43622100	0.975207
chr20	43670400	0.876033
chr20	43679000	0.735537
chr20	43700300	0.776860
chr20	43727000	0.818182
chr20	43966700	0.950413
chr20	43971200	0.925620
chr20	43974400	0.661157
chr20	43989500	0.917355
chr20	44047700	0.884298
chr20	44407900	0.983471
chr20	44461800	0.776860
chr20	44464500	0.793388
chr20	44548500	0.727273
chr20	44637200	0.661157
chr20	44686900	0.760331

chr20	44689800	0.958678
chr20	44750900	0.950413
chr20	44834700	0.768595
chr20	44979300	0.842975
chr20	45029900	0.942149
chr20	45039700	0.809917
chr20	45187000	0.735537
chr20	45202400	0.669421
chr20	45887900	0.950413
chr20	45902700	0.818182
chr20	45932600	0.925620
chr20	45940000	0.694215
chr20	45949800	0.727273
chr20	45956200	0.991736
chr20	45960300	0.826446
chr20	45963400	0.991736
chr20	45965900	0.719008
chr20	45977000	0.809917
chr20	45979300	0.983471
chr20	45990800	0.776860
chr20	46013200	0.892562
chr20	46021100	0.884298
chr20	46152900	0.867769
chr20	46169900	0.661157
chr20	46175300	0.719008
chr20	46197000	0.909091
chr20	46205600	0.950413
chr20	46208300	0.909091
chr20	46239700	1.000000
chr20	46278900	0.661157
chr20	46311500	0.677686
chr20	46395200	0.925620
chr20	46402900	0.983471
chr20	46415200	0.710744
chr20	46428100	0.892562
chr20	46440800	0.900826
chr20	46507900	0.809917
chr20	46523800	0.727273
chr20	46579400	0.917355
chr20	46602000	0.884298
chr20	46908200	0.966942
chr20	46913000	0.991736
chr20	46916100	0.909091
chr20	46922800	0.702479
chr20	46925100	0.685950
chr20	46928300	0.966942
chr20	47003500	0.950413
chr20	47238000	0.909091
chr20	47255900	0.818182
chr20	47284100	0.785124
chr20	47287000	0.900826
chr20	47299200	0.694215
chr20	47303400	0.900826
chr20	47338200	0.685950

chr20	47342800	0.809917
chr20	47367900	0.917355
chr20	47377000	0.661157
chr20	47380900	0.776860
chr20	47408400	0.950413
chr20	47434600	0.983471
chr20	47439900	0.818182
chr20	47448600	0.851240
chr20	47459000	0.942149
chr20	47463100	0.950413
chr20	47496700	0.694215
chr20	47540800	0.900826
chr20	47543800	0.867769
chr20	47546000	0.859504
chr20	47548200	0.867769
chr20	47589700	0.966942
chr20	47600500	0.727273
chr20	47797000	0.760331
chr20	47864600	0.958678
chr20	47884700	0.760331
chr20	47902900	0.776860
chr20	47905000	0.933884
chr20	47935700	0.867769
chr20	47940500	0.834711
chr20	48101000	0.719008
chr20	48168700	0.768595
chr20	48179100	0.958678
chr20	48211400	0.834711
chr20	48228000	0.793388
chr20	48279200	0.818182
chr20	48293400	0.809917
chr20	48308100	0.867769
chr20	48313300	0.966942
chr20	48315600	0.809917
chr20	48318400	0.975207
chr20	48341900	0.900826
chr20	48385500	0.859504
chr20	48388800	0.925620
chr20	48393500	0.925620
chr20	48407200	0.884298
chr20	48428400	0.669421
chr20	48506300	0.669421
chr20	48555600	0.719008
chr20	48598600	0.867769
chr20	48603900	0.975207
chr20	48637300	0.785124
chr20	48643900	0.694215
chr20	48646800	0.892562
chr20	48650000	0.983471
chr20	48652200	0.842975
chr20	48661000	0.702479
chr20	48663700	0.801653
chr20	48688200	0.801653
chr20	48703000	0.909091

chr20	48720400	0.958678
chr20	48755100	0.785124
chr20	48758800	0.909091
chr20	48761600	0.958678
chr20	48764600	0.834711
chr20	48766800	0.900826
chr20	48771400	0.677686
chr20	48782900	0.958678
chr20	48789200	0.834711
chr20	48810100	0.818182
chr20	48902400	0.975207
chr20	48909400	0.925620
chr20	48925400	0.983471
chr20	48962800	0.809917
chr20	48996000	0.925620
chr20	48998700	0.752066
chr20	49004800	0.669421
chr20	49017100	0.818182
chr20	49032700	0.917355
chr20	49057900	0.917355
chr20	49063600	0.983471
chr20	49087800	0.884298
chr20	49092500	0.793388
chr20	49100400	0.867769
chr20	49108100	0.719008
chr20	49114600	0.859504
chr20	49131800	0.876033
chr20	49137800	0.834711
chr20	49140600	0.809917
chr20	49143000	0.933884
chr20	49146000	0.892562
chr20	49155700	0.859504
chr20	49159900	0.834711
chr20	49163400	0.694215
chr20	49175100	0.991736
chr20	49177500	0.785124
chr20	49179600	0.859504
chr20	49187600	0.950413
chr20	49190300	0.785124
chr20	49209100	0.785124
chr20	49262500	0.735537
chr20	49407800	0.702479
chr20	49461800	0.669421
chr20	49530900	0.884298
chr20	49533000	0.933884
chr20	49639500	0.661157
chr20	49810100	0.661157
chr20	49972000	0.966942
chr20	49976400	0.975207
chr20	49978500	0.983471
chr20	49982400	0.809917
chr20	49984500	0.884298
chr20	49991000	0.958678
chr20	49999100	0.809917

chr20	50001300	0.933884
chr20	50005300	0.975207
chr20	50010300	0.818182
chr20	50027000	0.917355
chr20	50029300	0.925620
chr20	50036900	0.809917
chr20	50045400	0.942149
chr20	50064100	0.958678
chr20	50073700	0.793388
chr20	50109100	0.925620
chr20	50114700	0.900826
chr20	50142800	0.719008
chr20	50159300	0.950413
chr20	50171400	0.925620
chr20	50180900	0.892562
chr20	50211700	0.842975
chr20	50217400	0.933884
chr20	50240900	0.842975
chr20	50245800	0.966942
chr20	50249300	0.760331
chr20	50268100	0.917355
chr20	50279100	0.925620
chr20	50312100	0.851240
chr20	50314200	0.768595
chr20	50359800	0.694215
chr20	50366900	0.826446
chr20	50369500	0.859504
chr20	50504000	0.685950
chr20	50527200	0.925620
chr20	50543700	0.867769
chr20	50547600	0.876033
chr20	50551300	0.991736
chr20	50575700	0.876033
chr20	50601900	1.000000
chr20	50612200	0.983471
chr20	50618600	0.917355
chr20	50648900	0.950413
chr20	50678000	0.892562
chr20	51107100	0.801653
chr20	51109200	0.702479
chr20	51111300	0.925620
chr20	51114600	0.826446
chr20	51697200	0.826446
chr20	52196300	1.000000
chr20	52206700	0.768595
chr20	52223100	0.752066
chr20	52226700	0.867769
chr20	52238400	0.933884
chr20	52240500	0.917355
chr20	52259600	0.917355
chr20	52277200	0.785124
chr20	52294700	0.685950
chr20	52337900	0.809917
chr20	52340600	0.661157

chr20	52391200	0.867769
chr20	52407700	0.867769
chr20	52444500	0.834711
chr20	52447200	0.909091
chr20	52483000	0.909091
chr20	52489200	0.702479
chr20	52495700	0.702479
chr20	52501800	0.752066
chr20	52507900	0.900826
chr20	52514900	0.950413
chr20	52518200	0.991736
chr20	52532100	0.859504
chr20	52534500	0.834711
chr20	52543300	0.966942
chr20	52551600	1.000000
chr20	52557600	0.983471
chr20	52566200	0.925620
chr20	52578500	0.719008
chr20	52957400	0.859504
chr20	52962200	0.966942
chr20	53174800	0.983471
chr20	54987200	0.752066
chr20	54994200	0.975207
chr20	54998000	0.859504
chr20	55081400	0.876033
chr20	55204900	0.793388
chr20	55363700	0.991736
chr20	55936900	0.760331
chr20	55965000	0.892562
chr20	55967400	0.661157
chr20	55971100	0.917355
chr20	56070400	0.768595
chr20	56203400	0.950413
chr20	56247700	0.950413
chr20	56261500	0.768595
chr20	56273900	0.900826
chr20	56294100	0.727273
chr20	56325200	0.727273
chr20	56343000	0.958678
chr20	56345200	0.859504
chr20	56348100	1.000000
chr20	56649600	0.917355
chr20	56654500	0.876033
chr20	56755100	0.694215
chr20	56812600	0.694215
chr20	56827400	0.950413
chr20	56844700	0.760331
chr20	56917400	0.983471
chr20	56982900	0.958678
chr20	57014900	0.892562
chr20	57033800	0.991736
chr20	57123100	0.669421
chr20	57125800	0.826446
chr20	57200200	0.958678

chr20	57205000	0.950413
chr20	57220700	0.867769
chr20	57234900	0.785124
chr20	57237400	0.975207
chr20	57268100	0.966942
chr20	57451000	0.909091
chr20	57470200	0.809917
chr20	57472800	0.942149
chr20	57483700	0.958678
chr20	57586800	0.768595
chr20	57604600	0.719008
chr20	57639000	0.669421
chr20	58411200	0.719008
chr20	58509300	0.719008
chr20	60601600	0.669421
chr20	60699100	0.661157
chr20	60762600	0.975207
chr20	60778700	0.892562
chr20	60795200	0.735537
chr20	60861400	0.975207
chr20	60930400	0.884298
chr20	60932500	0.851240
chr20	60935300	0.909091
chr20	60939800	0.727273
chr20	60944100	0.760331
chr20	60956400	0.900826
chr20	60996700	0.900826
chr20	61077300	0.851240
chr20	61187500	0.719008
chr20	61201200	1.000000
chr20	61283900	0.966942
chr20	61299000	0.685950
chr20	61336100	0.785124
chr20	61340600	0.669421
chr20	61372800	0.809917
chr20	61404700	0.776860
chr20	61423300	0.826446
chr20	61425800	0.743802
chr20	61448100	0.694215
chr20	61450800	0.727273
chr20	61554900	0.975207
chr20	61560100	0.842975
chr20	61583600	0.785124
chr20	61762000	0.942149
chr20	62169600	0.735537
chr20	62313800	0.727273
chr20	62317500	0.809917
chr20	62320400	0.867769
chr20	62328400	0.909091
chr20	62331700	0.917355
chr20	62406400	0.917355
chr20	62479200	0.884298
chr20	62486400	0.809917
chr20	62490400	0.793388

chr20	62495000	0.727273
chr20	62504900	0.983471
chr20	62573800	0.942149
chr20	62580700	0.909091
chr20	62604700	0.950413
chr20	62673900	0.809917
chr20	62677500	0.677686
chr20	62681700	0.809917
chr20	62683900	0.933884
chr20	62686600	0.884298
chr20	62689100	0.710744
chr20	62693700	0.760331
chr20	62715300	0.793388
chr20	62751900	0.677686
chr20	62820700	0.859504
chr21	9827300	0.900826
chr21	10492800	0.958678
chr21	10702300	0.975207
chr21	10720700	0.685950
chr21	11186400	0.983471
chr21	15679200	0.768595
chr21	15906600	0.975207
chr21	15914700	0.884298
chr21	15918500	0.694215
chr21	15975800	0.942149
chr21	16055700	0.694215
chr21	16084300	0.942149
chr21	16250500	0.900826
chr21	16253600	0.809917
chr21	16426800	0.760331
chr21	17054600	0.917355
chr21	17108600	0.975207
chr21	17114300	0.785124
chr21	17127300	0.876033
chr21	17140300	0.710744
chr21	17144200	0.685950
chr21	17161700	0.958678
chr21	17214600	0.760331
chr21	17220700	0.975207
chr21	17222800	0.859504
chr21	17225500	0.842975
chr21	17359000	0.991736
chr21	17454700	0.743802
chr21	17488800	0.842975
chr21	17491000	0.719008
chr21	17553300	0.909091
chr21	17569900	0.801653
chr21	17588000	0.752066
chr21	17614200	0.818182
chr21	17653000	0.925620
chr21	17675100	0.983471
chr21	17678200	0.851240
chr21	17790900	0.776860
chr21	17797700	0.842975

chr21	17800000	0.851240
chr21	17839700	0.727273
chr21	17869300	0.884298
chr21	17903100	0.991736
chr21	17909300	0.942149
chr21	17923400	0.933884
chr21	17927200	0.859504
chr21	17936300	0.669421
chr21	17946300	0.983471
chr21	17960300	0.917355
chr21	18284600	0.669421
chr21	18744200	0.991736
chr21	18824200	0.933884
chr21	18827500	0.793388
chr21	18861400	0.752066
chr21	18864800	1.000000
chr21	18900100	0.958678
chr21	18909500	0.983471
chr21	19528200	0.933884
chr21	19537200	0.727273
chr21	19918600	0.743802
chr21	20205500	0.702479
chr21	21179200	0.834711
chr21	21211100	0.826446
chr21	22573100	0.958678
chr21	24084300	0.966942
chr21	24264800	0.975207
chr21	24271000	0.909091
chr21	24273800	0.884298
chr21	24357500	0.702479
chr21	24386400	0.834711
chr21	24390000	0.991736
chr21	24534600	0.975207
chr21	24729000	0.760331
chr21	25419200	0.942149
chr21	26742700	0.884298
chr21	26940900	0.991736
chr21	26946700	0.975207
chr21	27061200	0.677686
chr21	27257300	0.677686
chr21	27259900	0.917355
chr21	27268900	0.983471
chr21	27271500	0.867769
chr21	27274800	0.950413
chr21	27285400	0.892562
chr21	27300700	0.909091
chr21	27303100	0.925620
chr21	27305200	0.776860
chr21	27307400	0.983471
chr21	27309800	0.942149
chr21	27311900	0.842975
chr21	27324400	0.801653
chr21	27327700	0.917355
chr21	27330200	0.909091

chr21	27333500	0.768595
chr21	27337000	0.876033
chr21	27340200	0.975207
chr21	27352900	0.966942
chr21	27356800	0.991736
chr21	27359200	0.785124
chr21	27425000	0.785124
chr21	27439000	0.702479
chr21	27443600	0.809917
chr21	27455500	0.909091
chr21	27463400	0.975207
chr21	27479100	0.900826
chr21	27488700	0.950413
chr21	27495700	0.669421
chr21	27498100	0.834711
chr21	27510600	0.727273
chr21	27513200	0.983471
chr21	27520200	0.661157
chr21	27522300	0.958678
chr21	27539000	0.950413
chr21	27762000	0.975207
chr21	27879800	0.727273
chr21	27919400	0.958678
chr21	27937000	0.917355
chr21	27943300	0.818182
chr21	27960500	0.925620
chr21	28214200	0.727273
chr21	29020100	0.900826
chr21	29022200	0.842975
chr21	29301400	0.793388
chr21	29352100	0.892562
chr21	29484000	0.719008
chr21	29602600	0.743802
chr21	29656200	0.867769
chr21	29666500	0.983471
chr21	29684700	0.694215
chr21	29711700	0.991736
chr21	29809200	0.727273
chr21	29891200	0.950413
chr21	29895500	1.000000
chr21	29911600	0.925620
chr21	29913700	0.876033
chr21	30005300	0.917355
chr21	30007500	0.933884
chr21	30009900	0.884298
chr21	30117900	0.801653
chr21	30167800	0.834711
chr21	30195600	0.933884
chr21	30316500	0.801653
chr21	30450700	0.834711
chr21	30452900	0.785124
chr21	30464400	0.925620
chr21	30517700	0.933884
chr21	30519800	0.669421

chr21	30536200	0.867769
chr21	30547900	0.958678
chr21	30554900	0.892562
chr21	30569000	1.000000
chr21	30581200	0.975207
chr21	30590200	0.925620
chr21	30631500	0.958678
chr21	30660100	0.702479
chr21	30686500	0.702479
chr21	30713900	0.694215
chr21	30716100	0.892562
chr21	31029300	0.966942
chr21	31037100	0.834711
chr21	32546500	0.669421
chr21	32955000	0.942149
chr21	33041000	0.917355
chr21	33576400	0.743802
chr21	33579400	0.685950
chr21	33621600	0.933884
chr21	33701600	1.000000
chr21	33712200	0.892562
chr21	33744300	1.000000
chr21	33776000	0.909091
chr21	33787800	0.900826
chr21	33800000	0.966942
chr21	33810300	0.933884
chr21	33815200	0.975207
chr21	33823700	0.925620
chr21	33829400	0.958678
chr21	33836400	0.983471
chr21	33848100	0.991736
chr21	33851700	0.975207
chr21	33868800	0.884298
chr21	33870900	0.743802
chr21	33873000	0.900826
chr21	33888700	0.966942
chr21	33892100	0.785124
chr21	33894200	0.892562
chr21	33898200	0.776860
chr21	33916800	0.826446
chr21	33969900	0.909091
chr21	34071800	0.776860
chr21	34075300	0.991736
chr21	34080600	0.983471
chr21	34409300	0.785124
chr21	34415500	0.909091
chr21	34437300	0.694215
chr21	34605600	0.826446
chr21	34659800	0.752066
chr21	34673500	0.884298
chr21	34721300	0.809917
chr21	34733600	0.793388
chr21	34752400	0.677686
chr21	34756300	0.900826

chr21	34792100	0.776860
chr21	34801800	0.892562
chr21	34807400	0.983471
chr21	34918300	0.669421
chr21	34981500	0.975207
chr21	35027500	0.785124
chr21	35030600	0.925620
chr21	35034800	0.661157
chr21	35055900	0.925620
chr21	35058600	0.950413
chr21	35076400	0.694215
chr21	35132400	0.818182
chr21	35136200	0.876033
chr21	35141500	0.859504
chr21	35267400	0.876033
chr21	35270800	0.785124
chr21	35320400	0.991736
chr21	35341200	0.933884
chr21	35465000	0.983471
chr21	35574600	0.669421
chr21	35709500	0.677686
chr21	35856100	0.760331
chr21	35897500	0.760331
chr21	35925900	0.917355
chr21	36179800	0.842975
chr21	36181900	0.826446
chr21	36184200	0.735537
chr21	36209700	0.818182
chr21	36247900	0.694215
chr21	36254000	0.785124
chr21	36256400	0.826446
chr21	36337900	0.785124
chr21	36355900	0.966942
chr21	36399200	0.826446
chr21	36419800	0.966942
chr21	36423900	0.677686
chr21	36458000	0.900826
chr21	36575400	0.694215
chr21	36578400	0.975207
chr21	36595300	0.793388
chr21	36605200	0.983471
chr21	36629400	0.925620
chr21	36747400	0.991736
chr21	36751200	0.892562
chr21	36762500	0.669421
chr21	36768400	0.867769
chr21	36849300	0.958678
chr21	36855900	0.760331
chr21	37016000	0.909091
chr21	37021500	0.867769
chr21	37277900	0.719008
chr21	37298200	0.867769
chr21	37305400	0.983471
chr21	37438200	0.801653

chr21	37522900	0.793388
chr21	37579900	0.685950
chr21	37700900	0.892562
chr21	37801100	0.702479
chr21	37805500	0.975207
chr21	37810200	0.966942
chr21	37822600	0.743802
chr21	37826800	0.727273
chr21	37829500	0.876033
chr21	37832200	0.942149
chr21	37838400	0.917355
chr21	37849800	0.760331
chr21	37851900	0.942149
chr21	38012800	0.900826
chr21	38020100	0.909091
chr21	38071400	0.760331
chr21	38172500	0.776860
chr21	38219800	0.950413
chr21	38233300	0.958678
chr21	38282600	0.933884
chr21	38319800	0.909091
chr21	38339200	0.768595
chr21	38353300	0.768595
chr21	38362300	0.768595
chr21	38382400	0.809917
chr21	38525800	0.958678
chr21	38571900	0.818182
chr21	38610400	0.933884
chr21	38667400	0.694215
chr21	38711300	0.900826
chr21	38714400	0.975207
chr21	38731200	0.983471
chr21	38743300	0.785124
chr21	38745700	0.818182
chr21	38747800	0.900826
chr21	38797300	0.752066
chr21	38823200	0.983471
chr21	38835900	0.917355
chr21	38876000	0.776860
chr21	38920200	0.933884
chr21	38937700	0.694215
chr21	38953200	0.917355
chr21	38995700	0.694215
chr21	39009100	0.867769
chr21	39012300	0.727273
chr21	39017200	0.942149
chr21	39020500	0.834711
chr21	39031600	0.991736
chr21	39038000	0.917355
chr21	39159700	0.842975
chr21	39288700	0.702479
chr21	39410000	0.917355
chr21	39542600	0.735537
chr21	39544700	0.768595

chr21	39613100	0.958678
chr21	39617200	0.925620
chr21	39640600	0.710744
chr21	39649700	0.702479
chr21	39668900	0.892562
chr21	39673400	0.884298
chr21	39678600	1.000000
chr21	39716000	0.884298
chr21	39724300	0.950413
chr21	39748800	0.867769
chr21	39752000	0.743802
chr21	39761500	1.000000
chr21	39807600	0.851240
chr21	39812700	0.917355
chr21	39815100	0.975207
chr21	39820300	0.975207
chr21	39827600	0.975207
chr21	39831600	0.884298
chr21	39834200	0.859504
chr21	39841900	0.809917
chr21	39845800	0.983471
chr21	39849300	0.909091
chr21	39852000	0.966942
chr21	39855800	0.983471
chr21	39858100	0.933884
chr21	39860900	0.785124
chr21	39864400	0.950413
chr21	39872000	0.876033
chr21	39874200	0.818182
chr21	40033300	0.768595
chr21	40051700	0.809917
chr21	40106500	0.834711
chr21	40116400	0.925620
chr21	40145700	1.000000
chr21	40181000	0.685950
chr21	40183600	0.991736
chr21	40196000	0.793388
chr21	40212000	0.892562
chr21	40216300	0.677686
chr21	40223000	0.942149
chr21	40225900	1.000000
chr21	40343200	0.950413
chr21	40362100	0.727273
chr21	40365600	0.983471
chr21	40372700	0.776860
chr21	40376200	0.776860
chr21	40378900	0.983471
chr21	40393000	0.727273
chr21	40410400	0.884298
chr21	40412900	0.917355
chr21	40438900	0.892562
chr21	40548100	0.826446
chr21	40568600	0.917355
chr21	40757400	0.859504

chr21	40760500	0.818182
chr21	41032400	0.925620
chr21	42537800	0.851240
chr21	42543400	0.859504
chr21	42620000	0.983471
chr21	42623600	0.958678
chr21	42626600	0.983471
chr21	43062400	0.826446
chr21	43085200	0.793388
chr21	43189000	0.669421
chr21	43316000	0.826446
chr21	43346700	0.842975
chr21	43352200	0.826446
chr21	43354300	0.933884
chr21	43368900	0.851240
chr21	43374900	0.818182
chr21	43380200	0.876033
chr21	43382500	0.834711
chr21	43461100	0.933884
chr21	43468600	0.809917
chr21	43472400	0.719008
chr21	43477600	1.000000
chr21	43518000	0.983471
chr21	43613600	0.743802
chr21	43619900	0.966942
chr21	43630400	0.983471
chr21	43642300	0.950413
chr21	43648700	0.776860
chr21	43683800	0.685950
chr21	43698100	0.917355
chr21	43894400	0.735537
chr21	44098600	0.859504
chr21	44144300	0.743802
chr21	44201800	0.669421
chr21	44204800	0.925620
chr21	44282600	0.702479
chr21	44394100	0.785124
chr21	44412800	0.975207
chr21	44454300	0.900826
chr21	44466200	0.768595
chr21	44525000	0.710744
chr21	44529600	0.933884
chr21	44583500	0.966942
chr21	44597200	0.793388
chr21	44753700	0.809917
chr21	44758400	0.900826
chr21	44761100	0.743802
chr21	44766100	0.851240
chr21	44769100	0.842975
chr21	44782500	0.834711
chr21	44848200	0.735537
chr21	44867600	0.983471
chr21	44876900	0.950413
chr21	44887700	0.719008

chr21	44900000	0.702479
chr21	44914800	0.826446
chr21	44922900	0.719008
chr21	44936100	0.694215
chr21	44940700	0.950413
chr21	45006400	0.983471
chr21	45011200	0.884298
chr21	45026200	0.776860
chr21	45029800	0.818182
chr21	45065600	0.917355
chr21	45075300	0.909091
chr21	45078300	0.859504
chr21	45146300	0.950413
chr21	45149100	0.942149
chr21	45188500	0.818182
chr21	45232100	0.760331
chr21	45304600	0.818182
chr21	45359100	0.710744
chr21	45376700	0.710744
chr21	45426600	0.842975
chr21	45503700	0.991736
chr21	45506600	0.752066
chr21	45542500	0.752066
chr21	45555500	0.950413
chr21	45558100	0.818182
chr21	45562900	0.735537
chr21	45580300	0.851240
chr21	45592800	0.685950
chr21	45604800	0.925620
chr21	45622900	0.834711
chr21	45628000	0.785124
chr21	45674700	0.851240
chr21	45725600	0.859504
chr21	46175300	0.925620
chr21	46266600	0.917355
chr21	46286800	0.793388
chr21	46289800	0.727273
chr21	46298800	0.669421
chr21	46302400	0.793388
chr21	46331600	0.661157
chr21	46335100	0.942149
chr21	46347900	0.743802
chr21	46362000	0.661157
chr21	46378900	0.727273
chr21	46383000	0.801653
chr21	46389900	0.785124
chr21	46403200	0.826446
chr21	46421100	0.727273
chr21	46425400	0.867769
chr21	46432600	0.768595
chr21	46476700	0.933884
chr21	46499900	0.661157
chr21	46512800	0.925620
chr21	46516300	0.768595

chr21	46519400	0.785124
chr21	46522400	0.917355
chr21	46524800	0.776860
chr21	46530200	0.983471
chr21	46537200	0.735537
chr21	46549000	0.793388
chr21	46553700	0.851240
chr21	46603100	0.900826
chr21	46620600	0.677686
chr21	46712500	0.842975
chr21	46715900	0.966942
chr21	46722500	0.909091
chr21	46729500	0.834711
chr21	46737200	0.685950
chr21	46740600	0.677686
chr21	46783500	0.900826
chr21	46788500	0.818182
chr21	46828000	0.942149
chr21	46847900	0.793388
chr21	46853100	0.958678
chr21	46859300	0.975207
chr21	46880800	0.867769
chr21	46891700	0.735537
chr21	46938000	0.942149
chr21	46954000	0.884298
chr21	46971600	1.000000
chr21	47564100	0.892562
chr21	47573700	0.752066
chr21	47608600	0.983471
chr21	47623200	0.785124
chr21	47704000	0.950413
chr21	47718000	0.801653
chr21	47875700	1.000000
chr21	47914700	0.867769
chr21	47920600	0.925620
chr21	47935500	0.892562
chr21	48019500	0.867769
chr22	16861700	0.661157
chr22	17382900	0.867769
chr22	17544800	1.000000
chr22	17599300	0.669421
chr22	17680100	0.752066
chr22	17887300	0.966942
chr22	18189700	0.818182
chr22	18217100	0.900826
chr22	18270000	0.785124
chr22	18393900	0.834711
chr22	18403300	0.818182
chr22	18408200	0.735537
chr22	18434400	0.950413
chr22	18452900	0.826446
chr22	18455800	0.942149
chr22	18463600	0.818182
chr22	18574900	0.867769

chr22	18597500	0.719008
chr22	18622700	0.958678
chr22	18640500	0.884298
chr22	18878200	0.867769
chr22	19050500	0.834711
chr22	19073700	0.917355
chr22	19411000	0.719008
chr22	19443900	0.884298
chr22	19470100	0.867769
chr22	19472500	0.909091
chr22	19521700	0.801653
chr22	19553800	0.859504
chr22	19556500	0.925620
chr22	19564200	0.685950
chr22	19566300	0.942149
chr22	19568500	0.727273
chr22	19582100	0.743802
chr22	19609200	0.942149
chr22	19733800	0.752066
chr22	19760900	0.876033
chr22	19794100	0.809917
chr22	19833100	0.793388
chr22	19844500	0.702479
chr22	19870800	0.884298
chr22	19873900	0.826446
chr22	19877300	0.975207
chr22	19881100	0.933884
chr22	19897500	0.818182
chr22	19925900	0.958678
chr22	19932000	0.909091
chr22	19934700	0.876033
chr22	19937900	0.966942
chr22	19947100	0.685950
chr22	19951000	0.925620
chr22	19954900	0.743802
chr22	19964100	0.834711
chr22	19972100	0.900826
chr22	19986300	0.900826
chr22	19990300	0.942149
chr22	20010600	0.685950
chr22	20049900	0.966942
chr22	20071100	0.826446
chr22	20142800	0.909091
chr22	20191300	0.851240
chr22	20193700	0.859504
chr22	20231400	0.859504
chr22	20287800	0.677686
chr22	20409600	0.826446
chr22	20425000	0.867769
chr22	20785800	0.793388
chr22	20865500	0.909091
chr22	20871700	0.859504
chr22	20875000	0.876033
chr22	20877900	0.958678

chr22	20881500	0.809917
chr22	20887000	0.842975
chr22	20903700	0.752066
chr22	20905800	0.793388
chr22	20907900	0.818182
chr22	20911600	0.809917
chr22	21006000	0.661157
chr22	21093300	0.735537
chr22	21105200	0.884298
chr22	21132400	0.975207
chr22	21134500	0.991736
chr22	21136700	0.859504
chr22	21138900	0.859504
chr22	21300700	0.983471
chr22	21334500	0.818182
chr22	21400100	0.867769
chr22	21774800	0.925620
chr22	21790500	0.975207
chr22	21823800	0.710744
chr22	21853300	0.900826
chr22	21869000	0.867769
chr22	21925000	0.809917
chr22	22068000	0.669421
chr22	22152200	0.942149
chr22	22170500	0.801653
chr22	22172800	0.900826
chr22	22191900	0.983471
chr22	22291500	0.760331
chr22	22294700	0.768595
chr22	22297100	0.966942
chr22	22300100	0.752066
chr22	22304900	0.752066
chr22	22484600	0.661157
chr22	22565100	0.727273
chr22	22974000	0.942149
chr22	23484500	0.942149
chr22	23488000	0.727273
chr22	23528200	1.000000
chr22	23530800	0.826446
chr22	23533600	0.785124
chr22	23541500	0.991736
chr22	23543900	0.785124
chr22	23555300	0.942149
chr22	23558600	0.933884
chr22	23561300	0.876033
chr22	23563900	0.859504
chr22	23569400	0.826446
chr22	23582800	0.950413
chr22	23585000	0.661157
chr22	23619900	0.776860
chr22	23625400	0.892562
chr22	23632800	0.710744
chr22	23644400	0.909091
chr22	23646500	0.909091

chr22	23649600	0.801653
chr22	23653100	0.942149
chr22	23775900	0.752066
chr22	23864900	0.917355
chr22	23908500	0.735537
chr22	24105800	0.768595
chr22	24115400	0.793388
chr22	24181300	0.669421
chr22	24255900	0.719008
chr22	24282900	0.818182
chr22	24343400	0.752066
chr22	24384400	0.669421
chr22	24397700	0.752066
chr22	24455400	0.809917
chr22	24458200	0.966942
chr22	24542600	0.842975
chr22	24597400	0.950413
chr22	24602400	0.818182
chr22	24605100	0.983471
chr22	24636400	0.975207
chr22	24638600	0.933884
chr22	24643200	0.785124
chr22	24660900	0.892562
chr22	24669400	0.661157
chr22	24685600	0.991736
chr22	24699000	0.842975
chr22	24701700	0.958678
chr22	24711500	0.917355
chr22	24715700	0.966942
chr22	24756300	0.950413
chr22	24776100	0.933884
chr22	24787200	0.950413
chr22	24793400	0.685950
chr22	24797100	0.892562
chr22	24799600	0.900826
chr22	24803600	0.900826
chr22	24816500	0.801653
chr22	24825600	0.727273
chr22	24830500	0.892562
chr22	24836600	0.661157
chr22	24852300	0.752066
chr22	24856400	0.983471
chr22	24861800	0.900826
chr22	24870600	0.710744
chr22	24878800	0.694215
chr22	24890900	0.661157
chr22	24903400	0.900826
chr22	24920700	0.950413
chr22	24941900	0.702479
chr22	24997800	0.669421
chr22	25031200	0.694215
chr22	25042100	0.834711
chr22	25082300	0.669421
chr22	25123700	0.743802

chr22	25125800	0.801653
chr22	25157600	0.719008
chr22	25209300	0.776860
chr22	25290600	0.876033
chr22	25322500	0.991736
chr22	25359400	0.942149
chr22	25362800	0.793388
chr22	25366400	0.793388
chr22	25374100	0.735537
chr22	25379400	0.801653
chr22	25382200	0.917355
chr22	25386100	0.735537
chr22	25388700	0.909091
chr22	25392500	0.933884
chr22	25398200	0.991736
chr22	25410200	0.966942
chr22	25422500	0.991736
chr22	25449500	0.752066
chr22	25457900	0.925620
chr22	25466300	0.727273
chr22	25482700	0.983471
chr22	25487400	0.925620
chr22	25511100	0.917355
chr22	25517700	0.917355
chr22	25530400	0.975207
chr22	25535400	0.710744
chr22	25539400	0.925620
chr22	25542800	0.925620
chr22	25744100	0.958678
chr22	25761700	0.669421
chr22	25797100	0.892562
chr22	25871800	0.958678
chr22	26143100	0.966942
chr22	26156200	0.925620
chr22	26827200	0.743802
chr22	26860800	0.942149
chr22	26867700	0.925620
chr22	26896500	0.966942
chr22	26900000	0.933884
chr22	26949800	0.983471
chr22	26960700	0.809917
chr22	26969800	0.983471
chr22	26981400	0.933884
chr22	26983800	0.826446
chr22	27041400	0.876033
chr22	27054700	0.768595
chr22	27077800	0.834711
chr22	27087600	0.785124
chr22	27105400	0.867769
chr22	27108000	0.900826
chr22	27110600	0.892562
chr22	27115100	0.925620
chr22	27130600	0.975207
chr22	27147500	0.975207

chr22	27291300	0.950413
chr22	27504400	0.958678
chr22	27508100	0.933884
chr22	27510700	0.892562
chr22	27512800	0.867769
chr22	27526800	0.785124
chr22	27530600	0.727273
chr22	27536100	0.909091
chr22	27550900	0.842975
chr22	27569800	0.958678
chr22	27599100	0.925620
chr22	27608900	0.966942
chr22	27620700	0.677686
chr22	27634800	0.710744
chr22	27637600	0.925620
chr22	27643900	0.826446
chr22	27695300	0.950413
chr22	27815300	0.925620
chr22	27817400	0.859504
chr22	27853500	0.876033
chr22	27871400	0.991736
chr22	27885400	0.834711
chr22	27903400	0.942149
chr22	27908400	0.942149
chr22	27973500	0.991736
chr22	28114500	0.909091
chr22	28123000	0.925620
chr22	28176300	0.694215
chr22	28196000	0.702479
chr22	28246500	0.983471
chr22	28277400	0.735537
chr22	28282600	1.000000
chr22	28411600	0.851240
chr22	28426200	0.702479
chr22	28429100	0.719008
chr22	28431300	0.867769
chr22	28447500	0.909091
chr22	28536200	0.694215
chr22	28679700	0.851240
chr22	28771800	0.685950
chr22	28807000	0.685950
chr22	28956400	0.694215
chr22	29106100	0.933884
chr22	29108200	0.851240
chr22	29146900	0.933884
chr22	29334600	0.793388
chr22	29373000	0.867769
chr22	29419900	0.900826
chr22	29426600	0.727273
chr22	29429300	0.942149
chr22	29437400	0.710744
chr22	29440700	0.785124
chr22	29598600	0.785124
chr22	29629400	0.685950

chr22	29707400	0.950413
chr22	29711200	0.851240
chr22	29760400	0.900826
chr22	29776700	0.942149
chr22	29779400	0.801653
chr22	29866600	0.694215
chr22	29877200	0.933884
chr22	29917300	0.917355
chr22	29935500	0.867769
chr22	30003100	0.942149
chr22	30014800	0.818182
chr22	30017700	0.909091
chr22	30022100	0.809917
chr22	30025800	0.892562
chr22	30031100	0.958678
chr22	30057600	0.694215
chr22	30097700	0.669421
chr22	30195400	0.768595
chr22	30217600	0.983471
chr22	30232400	0.710744
chr22	30304100	0.859504
chr22	30369800	0.685950
chr22	30390900	0.925620
chr22	30440600	0.669421
chr22	30588700	0.900826
chr22	30591200	0.900826
chr22	30593400	0.826446
chr22	30605300	0.826446
chr22	30659500	0.834711
chr22	30674100	0.909091
chr22	30687400	0.892562
chr22	30698700	0.685950
chr22	30702000	0.785124
chr22	30783300	0.727273
chr22	30805100	0.809917
chr22	30816900	0.876033
chr22	30823500	0.694215
chr22	30831800	0.884298
chr22	30979100	0.975207
chr22	31005600	0.900826
chr22	31023600	0.983471
chr22	31030600	0.760331
chr22	31045700	0.892562
chr22	31113600	0.884298
chr22	31129400	0.834711
chr22	31140100	0.909091
chr22	31218700	0.867769
chr22	31227000	0.950413
chr22	31263000	0.834711
chr22	31265100	0.809917
chr22	31318100	0.851240
chr22	31442700	0.892562
chr22	31446300	0.826446
chr22	31451700	0.719008

chr22	31469800	0.785124
chr22	31482300	0.743802
chr22	31544900	0.768595
chr22	31552000	0.818182
chr22	31569500	0.958678
chr22	31606500	0.752066
chr22	31614600	0.909091
chr22	31627700	0.851240
chr22	31631600	0.966942
chr22	31635000	0.933884
chr22	31638600	0.991736
chr22	31641400	0.892562
chr22	31650300	0.909091
chr22	31681200	0.661157
chr22	31686500	0.842975
chr22	31692300	0.743802
chr22	31927000	0.942149
chr22	31980500	0.942149
chr22	31998600	0.818182
chr22	32021900	0.900826
chr22	32024200	0.892562
chr22	32034500	0.975207
chr22	32042600	0.809917
chr22	32044900	0.859504
chr22	32056900	0.661157
chr22	32171900	0.950413
chr22	32226100	0.876033
chr22	32266600	0.776860
chr22	32268800	0.760331
chr22	32294300	0.694215
chr22	32298000	0.710744
chr22	32319400	0.752066
chr22	32343700	0.694215
chr22	32348700	0.991736
chr22	32351300	0.909091
chr22	32366900	0.776860
chr22	32727800	0.809917
chr22	32750600	0.892562
chr22	35702900	0.983471
chr22	35708000	0.719008
chr22	35710700	0.785124
chr22	35726500	0.892562
chr22	35760500	0.917355
chr22	35768300	0.983471
chr22	35773300	0.900826
chr22	35776100	0.752066
chr22	35793100	0.760331
chr22	35795400	0.661157
chr22	35843700	0.991736
chr22	35864100	0.826446
chr22	35867600	0.925620
chr22	35874200	0.760331
chr22	36044800	0.685950
chr22	36138700	0.917355

chr22	36163500	0.975207
chr22	36173800	0.975207
chr22	36233900	0.669421
chr22	36279400	0.892562
chr22	36296700	0.909091
chr22	36300100	0.842975
chr22	36306600	0.983471
chr22	36311200	0.834711
chr22	36318100	0.884298
chr22	36357900	0.975207
chr22	36368000	0.834711
chr22	36370500	0.710744
chr22	36407200	0.694215
chr22	36551200	0.685950
chr22	36554100	0.842975
chr22	36557000	0.785124
chr22	36593500	0.818182
chr22	36655600	0.950413
chr22	36701000	0.793388
chr22	36711500	0.801653
chr22	36713900	0.735537
chr22	36717500	0.760331
chr22	36719600	0.909091
chr22	36723700	0.909091
chr22	36728100	0.694215
chr22	36730300	0.661157
chr22	36732400	0.661157
chr22	36736700	0.975207
chr22	36739700	0.942149
chr22	36742400	0.892562
chr22	36745700	0.958678
chr22	36749100	0.743802
chr22	36751900	0.719008
chr22	36754500	0.991736
chr22	36758000	0.975207
chr22	36761800	0.942149
chr22	36764900	0.669421
chr22	36767800	0.884298
chr22	36770200	0.834711
chr22	36773200	0.950413
chr22	36775400	0.983471
chr22	36785200	0.776860
chr22	36792300	0.752066
chr22	36795200	0.942149
chr22	36806400	0.785124
chr22	36822800	0.702479
chr22	36827400	0.768595
chr22	36836300	0.834711
chr22	36848300	0.892562
chr22	36851400	0.950413
chr22	36858000	0.933884
chr22	36860700	0.760331
chr22	36862900	0.669421
chr22	36866000	0.983471

chr22	36903300	0.958678
chr22	36938300	0.768595
chr22	36958800	0.719008
chr22	37178000	0.752066
chr22	37219700	0.909091
chr22	37249700	0.892562
chr22	37270600	0.702479
chr22	37277200	0.933884
chr22	37281000	0.975207
chr22	37284600	0.661157
chr22	37404700	0.752066
chr22	37426800	0.760331
chr22	37437200	0.752066
chr22	37476000	0.809917
chr22	37575300	0.892562
chr22	37582700	0.776860
chr22	37586200	0.975207
chr22	37595800	0.809917
chr22	37626000	0.685950
chr22	37633600	0.677686
chr22	37651300	1.000000
chr22	37656100	0.867769
chr22	37663500	0.842975
chr22	37678800	0.818182
chr22	37701100	0.834711
chr22	37705000	0.818182
chr22	37724400	0.685950
chr22	37727800	0.743802
chr22	37731100	0.743802
chr22	37828600	0.900826
chr22	37850600	0.760331
chr22	37852700	0.842975
chr22	37860500	0.859504
chr22	37893700	0.991736
chr22	37900300	0.702479
chr22	37905300	0.768595
chr22	37908800	0.950413
chr22	37917100	0.669421
chr22	37922000	0.966942
chr22	37942500	0.966942
chr22	37948600	0.776860
chr22	37953500	0.925620
chr22	37963700	0.818182
chr22	38003800	0.694215
chr22	38030000	0.859504
chr22	38036100	0.900826
chr22	38074900	0.793388
chr22	38141200	0.677686
chr22	38149600	0.983471
chr22	38155100	0.933884
chr22	38178300	0.677686
chr22	38214700	0.933884
chr22	38277100	0.884298
chr22	38284100	0.785124

chr22	38292700	0.917355
chr22	38301100	0.727273
chr22	38326900	0.859504
chr22	38330100	0.702479
chr22	38430700	0.743802
chr22	38494600	0.776860
chr22	38531900	0.677686
chr22	38568000	0.702479
chr22	38570200	0.942149
chr22	38572500	0.867769
chr22	38574800	0.966942
chr22	38578100	0.966942
chr22	38601500	0.842975
chr22	38610300	0.851240
chr22	38614000	0.876033
chr22	38617000	0.859504
chr22	38619400	0.685950
chr22	38623600	0.719008
chr22	38627700	0.859504
chr22	38629900	0.677686
chr22	38637100	0.942149
chr22	38653700	0.975207
chr22	38655900	0.892562
chr22	38702100	0.909091
chr22	38708900	0.909091
chr22	38715100	0.884298
chr22	38724200	0.933884
chr22	38732400	0.776860
chr22	38851600	0.727273
chr22	39090200	0.859504
chr22	39093400	0.793388
chr22	39101000	0.702479
chr22	39114600	0.917355
chr22	39184800	0.909091
chr22	39261400	0.743802
chr22	39320400	0.859504
chr22	39378500	0.702479
chr22	39473200	0.735537
chr22	39568000	0.892562
chr22	39571100	0.859504
chr22	39574300	0.851240
chr22	39576400	0.900826
chr22	39581000	0.834711
chr22	39601800	0.793388
chr22	39611600	0.975207
chr22	39626800	0.884298
chr22	39630200	0.942149
chr22	39635200	0.735537
chr22	39652000	0.900826
chr22	39663800	0.735537
chr22	39676300	0.958678
chr22	39688100	0.975207
chr22	39694300	0.694215
chr22	39704400	0.950413

chr22	39706700	0.892562
chr22	39828100	0.966942
chr22	39867900	0.661157
chr22	40437600	0.793388
chr22	40454300	0.743802
chr22	40517000	0.661157
chr22	40579200	0.917355
chr22	40609900	0.743802
chr22	40616900	0.867769
chr22	40635200	1.000000
chr22	40655000	0.694215
chr22	40659700	0.917355
chr22	40662200	0.752066
chr22	40669400	0.933884
chr22	40673000	0.933884
chr22	40723600	0.966942
chr22	40760800	0.966942
chr22	40769000	0.685950
chr22	40793300	0.933884
chr22	40895300	0.983471
chr22	40930600	0.743802
chr22	41048000	0.694215
chr22	41069400	0.826446
chr22	41076300	0.677686
chr22	41107800	0.851240
chr22	41136200	0.760331
chr22	41187800	0.867769
chr22	41250200	0.694215
chr22	41260300	0.669421
chr22	41325000	0.909091
chr22	41445300	0.818182
chr22	41577300	0.933884
chr22	41581000	0.975207
chr22	41613900	0.867769
chr22	41626000	0.826446
chr22	41629400	0.958678
chr22	41634300	0.917355
chr22	41653300	0.793388
chr22	41687400	0.892562
chr22	41702200	0.834711
chr22	41723400	0.851240
chr22	41736700	0.842975
chr22	41763600	0.834711
chr22	41994400	0.834711
chr22	42014600	0.694215
chr22	42063600	0.892562
chr22	42093300	0.702479
chr22	42177400	0.801653
chr22	42221900	0.694215
chr22	42228300	0.801653
chr22	42242300	0.702479
chr22	42253800	0.702479
chr22	42306900	0.826446
chr22	42314300	0.809917

chr22	42322500	0.776860
chr22	42340300	0.900826
chr22	42373200	0.818182
chr22	42474800	0.677686
chr22	42569300	0.768595
chr22	42834000	0.727273
chr22	42840600	0.884298
chr22	42896900	0.743802
chr22	42933300	0.851240
chr22	42935800	0.876033
chr22	43005300	0.867769
chr22	43012100	0.793388
chr22	43031900	0.809917
chr22	43040500	0.710744
chr22	43042600	0.983471
chr22	43058700	0.884298
chr22	43067600	0.801653
chr22	43072900	0.958678
chr22	43077100	0.917355
chr22	43123900	0.991736
chr22	43144600	0.884298
chr22	43202300	0.809917
chr22	43216400	0.793388
chr22	43241200	0.677686
chr22	43303100	0.752066
chr22	43318200	0.909091
chr22	43322400	0.859504
chr22	43330100	0.983471
chr22	43334100	0.842975
chr22	43343100	0.826446
chr22	43354500	0.818182
chr22	43356900	0.710744
chr22	43375100	0.933884
chr22	43387200	1.000000
chr22	43391500	0.909091
chr22	43398800	0.983471
chr22	43580000	0.942149
chr22	43660200	0.842975
chr22	44242700	0.677686
chr22	44353200	0.669421
chr22	44399400	0.801653
chr22	44417000	0.900826
chr22	44423700	0.677686
chr22	44428100	0.851240
chr22	44433700	0.842975
chr22	44445300	0.710744
chr22	44447700	0.768595
chr22	44455200	0.917355
chr22	44463900	0.793388
chr22	44523300	0.933884
chr22	44525900	0.842975
chr22	44530500	0.851240
chr22	44757000	0.735537
chr22	44759600	0.975207

chr22	45016300	0.851240
chr22	45081000	0.834711
chr22	45551100	0.793388
chr22	45555900	0.950413
chr22	45608600	1.000000
chr22	45617400	0.991736
chr22	45642700	0.909091
chr22	45676000	0.958678
chr22	45681000	0.694215
chr22	45710000	0.752066
chr22	45714400	0.917355
chr22	45731400	0.727273
chr22	45733500	0.942149
chr22	45831700	0.900826
chr22	45899100	0.818182
chr22	46035300	0.710744
chr22	46077500	0.900826
chr22	46105700	0.793388
chr22	46201300	0.719008
chr22	46227400	0.809917
chr22	46365400	0.876033
chr22	46367800	0.677686
chr22	46375300	0.760331
chr22	46403700	0.743802
chr22	46423500	0.876033
chr22	46453200	0.983471
chr22	46457100	0.933884
chr22	46462300	0.752066
chr22	46472600	0.983471
chr22	46474700	0.851240
chr22	46476800	0.950413
chr22	46479200	0.719008
chr22	46481700	0.743802
chr22	46484700	0.851240
chr22	46486900	0.925620
chr22	46665900	0.851240
chr22	46684300	0.876033
chr22	46691800	0.661157
chr22	46699800	0.851240
chr22	46913700	0.958678
chr22	46944000	0.834711
chr22	47009900	0.785124
chr22	47048700	0.752066
chr22	47082100	0.735537
chr22	47128100	0.727273
chr22	47147800	0.851240
chr22	47150800	0.942149
chr22	47154800	0.785124
chr22	47166900	0.801653
chr22	47187500	0.669421
chr22	47189700	0.859504
chr22	47211900	0.842975
chr22	47242200	0.809917
chr22	47250400	0.925620

chr22	47272500	0.983471
chr22	47330900	0.785124
chr22	47338900	0.958678
chr22	47367400	0.975207
chr22	47381100	0.917355
chr22	47426700	0.760331
chr22	47462300	0.818182
chr22	47560500	0.876033
chr22	47714300	0.735537
chr22	47725500	0.785124
chr22	47736700	0.710744
chr22	50026400	0.867769
chr22	50061100	0.966942
chr22	50118400	0.818182
chr22	50182500	0.983471
chr22	50222100	0.677686
chr22	50250000	0.710744
chr22	50274400	0.958678
chr22	50324600	0.743802
chr22	50326800	0.685950
chr22	50329200	0.991736
chr22	50333000	0.727273
chr22	50346200	0.768595
chr22	50353100	0.719008
chr22	50360000	0.933884
chr22	50364600	0.933884
chr22	50528600	0.694215
chr22	50550200	0.950413
chr22	50555700	0.975207
chr22	50558800	0.884298
chr22	50623500	0.917355
chr22	50629300	0.826446
chr22	50645900	0.818182
chr22	50686400	0.859504
chr22	50701100	0.669421
chr22	50732200	0.983471
chr22	50738200	0.983471
chr22	50743100	0.702479
chr22	50753500	0.768595
chr22	50781000	0.677686
chr22	50838900	0.809917
chr22	50842400	0.876033
chr22	50970900	0.801653
chr22	51001900	0.702479
chr22	51080200	0.917355
chr22	51095300	0.809917
chr22	51116300	0.735537
chr22	51123700	0.677686
chr22	51136600	0.752066
chr2	290200	0.785124
chr2	675400	0.975207
chr2	1595000	0.933884
chr2	1608800	0.826446
chr2	1610900	0.917355

chr2	1630100	0.933884
chr2	1633100	0.842975
chr2	1657800	0.752066
chr2	1690000	0.801653
chr2	1712100	0.917355
chr2	1719700	0.884298
chr2	1727700	0.867769
chr2	1734100	0.975207
chr2	1743200	0.958678
chr2	2275400	0.884298
chr2	3390900	0.851240
chr2	3451900	0.851240
chr2	3527400	0.917355
chr2	3583600	0.710744
chr2	3626300	0.685950
chr2	3633500	0.702479
chr2	3775700	0.768595
chr2	3961300	0.876033
chr2	4096200	0.975207
chr2	4388700	0.702479
chr2	4402200	0.983471
chr2	4407500	0.685950
chr2	4468600	0.925620
chr2	4547400	0.917355
chr2	4550100	0.966942
chr2	4604000	0.900826
chr2	4710500	0.884298
chr2	4716500	0.859504
chr2	4732000	0.669421
chr2	5771700	0.793388
chr2	6921500	0.900826
chr2	6967600	0.909091
chr2	7116700	0.900826
chr2	7133500	0.917355
chr2	7148600	0.727273
chr2	7167500	0.735537
chr2	8288800	0.933884
chr2	8294900	0.785124
chr2	8311300	0.677686
chr2	8821000	0.710744
chr2	8825800	0.776860
chr2	8944500	0.892562
chr2	9124500	0.768595
chr2	9247000	0.925620
chr2	9283400	0.735537
chr2	9319600	0.826446
chr2	9341700	0.925620
chr2	9346100	0.752066
chr2	9352900	0.917355
chr2	9365600	0.892562
chr2	9368500	0.991736
chr2	9373500	0.884298
chr2	9375600	0.669421
chr2	9377900	0.975207

chr2	9390200	0.760331
chr2	9392900	0.900826
chr2	9397500	0.793388
chr2	9399600	0.694215
chr2	9401800	0.809917
chr2	9404300	0.867769
chr2	9415300	0.966942
chr2	9426400	0.826446
chr2	9429200	0.958678
chr2	9450800	0.975207
chr2	9459000	0.785124
chr2	9468100	0.975207
chr2	9487600	0.743802
chr2	9505700	0.867769
chr2	9571200	0.809917
chr2	9680300	0.884298
chr2	9693200	0.958678
chr2	9706000	0.933884
chr2	9748600	0.991736
chr2	9778500	0.809917
chr2	9796500	1.000000
chr2	9816000	0.892562
chr2	9821700	0.851240
chr2	9844200	0.801653
chr2	9855800	0.809917
chr2	9893800	0.677686
chr2	9986400	0.760331
chr2	10114100	0.727273
chr2	10132100	0.867769
chr2	10134600	0.991736
chr2	10169900	0.884298
chr2	10177300	0.958678
chr2	10183600	0.818182
chr2	10266000	0.809917
chr2	10300100	0.661157
chr2	10304600	0.867769
chr2	10310800	0.752066
chr2	10423900	0.793388
chr2	10426600	0.818182
chr2	10438400	0.801653
chr2	10464500	0.966942
chr2	10469100	0.892562
chr2	10472200	0.826446
chr2	10487600	0.966942
chr2	10522900	0.867769
chr2	10526500	0.768595
chr2	10533600	0.793388
chr2	10543100	0.958678
chr2	10549100	0.801653
chr2	10690500	0.760331
chr2	10696000	0.867769
chr2	10716400	0.933884
chr2	10852800	0.727273
chr2	10858200	0.966942

chr2	10954900	0.892562
chr2	11041700	0.710744
chr2	11052300	0.752066
chr2	11073300	0.743802
chr2	11076700	0.991736
chr2	11214000	0.743802
chr2	11269700	0.900826
chr2	11273400	0.776860
chr2	11294800	0.727273
chr2	11327900	0.809917
chr2	11344100	0.983471
chr2	11391500	0.793388
chr2	11421600	0.661157
chr2	11428700	0.710744
chr2	11433300	0.809917
chr2	11467200	0.785124
chr2	11508400	0.917355
chr2	11515000	0.950413
chr2	11526700	0.842975
chr2	11532300	0.933884
chr2	11544200	0.933884
chr2	11604200	0.702479
chr2	11743200	1.000000
chr2	11828900	0.776860
chr2	11837800	0.743802
chr2	11885100	0.900826
chr2	11894500	0.702479
chr2	11903400	0.983471
chr2	11915600	0.983471
chr2	11919000	0.950413
chr2	11970700	0.909091
chr2	11974400	0.710744
chr2	12004300	0.685950
chr2	12138500	0.851240
chr2	12296100	1.000000
chr2	12310000	0.942149
chr2	12317300	0.966942
chr2	12333100	0.983471
chr2	12427300	0.801653
chr2	12466200	0.793388
chr2	12619600	0.917355
chr2	12696600	0.892562
chr2	12803500	0.925620
chr2	12805700	0.892562
chr2	12811600	0.909091
chr2	12814100	0.876033
chr2	12816400	0.917355
chr2	12843100	0.975207
chr2	12855400	0.826446
chr2	12928500	1.000000
chr2	12936200	0.867769
chr2	12938400	0.983471
chr2	12959700	0.958678
chr2	12965100	0.677686

chr2	12997700	0.983471
chr2	13025100	0.917355
chr2	13092000	0.991736
chr2	13106000	0.983471
chr2	13122500	0.950413
chr2	13128000	0.958678
chr2	13141700	0.710744
chr2	15310200	0.991736
chr2	15364400	0.966942
chr2	15384300	0.661157
chr2	15500200	0.909091
chr2	15540600	0.694215
chr2	15698600	0.743802
chr2	15720100	1.000000
chr2	15775100	0.900826
chr2	15778200	0.842975
chr2	15780900	0.966942
chr2	15790200	0.685950
chr2	15808600	0.966942
chr2	15844800	0.867769
chr2	15849600	0.859504
chr2	15870700	0.892562
chr2	15874200	0.752066
chr2	15895300	0.867769
chr2	15901200	0.991736
chr2	16790400	0.826446
chr2	16806400	0.958678
chr2	16815300	0.876033
chr2	16832400	0.958678
chr2	16845100	0.677686
chr2	17918600	0.983471
chr2	17938500	0.851240
chr2	18498600	0.801653
chr2	18521200	0.818182
chr2	18538300	0.710744
chr2	18551400	0.694215
chr2	18595100	0.950413
chr2	18718000	0.809917
chr2	18733900	0.942149
chr2	18739700	0.661157
chr2	19330700	0.809917
chr2	19341500	0.925620
chr2	19558200	0.876033
chr2	19911500	0.975207
chr2	20001700	0.818182
chr2	20068900	0.867769
chr2	20131700	0.900826
chr2	20134100	0.876033
chr2	20172100	0.925620
chr2	20180100	0.975207
chr2	20212800	0.727273
chr2	20227800	0.925620
chr2	20234900	0.776860
chr2	20237400	0.958678

chr2	20240900	1.000000
chr2	20307000	0.768595
chr2	20314000	0.661157
chr2	20341500	0.876033
chr2	20349200	0.975207
chr2	20514600	1.000000
chr2	20516900	0.735537
chr2	20523200	0.942149
chr2	20624900	0.834711
chr2	20638900	0.900826
chr2	20644300	0.991736
chr2	20651900	0.867769
chr2	20688400	0.710744
chr2	20714300	0.727273
chr2	20716400	0.727273
chr2	20725100	0.661157
chr2	20739500	0.752066
chr2	20766700	0.950413
chr2	20769300	0.950413
chr2	20775000	0.776860
chr2	20777100	0.818182
chr2	20779200	0.925620
chr2	20782400	0.793388
chr2	20785600	0.917355
chr2	20793100	0.793388
chr2	20795200	0.776860
chr2	20797300	0.719008
chr2	20800400	0.958678
chr2	20813400	0.785124
chr2	20815900	0.685950
chr2	20818600	0.809917
chr2	20835100	0.991736
chr2	20874900	0.685950
chr2	20882700	0.685950
chr2	20899200	0.876033
chr2	20928200	0.950413
chr2	21268000	0.702479
chr2	21293500	0.768595
chr2	23604300	0.917355
chr2	23720200	0.834711
chr2	23773400	0.909091
chr2	23860400	0.727273
chr2	23980800	0.966942
chr2	23985300	0.735537
chr2	24238900	0.752066
chr2	24342300	0.685950
chr2	24485400	0.727273
chr2	24492400	0.925620
chr2	24495400	0.834711
chr2	24509200	0.677686
chr2	24556700	0.975207
chr2	24578000	0.760331
chr2	24588600	0.892562
chr2	24659200	0.694215

chr2	24713300	0.661157
chr2	24926100	0.958678
chr2	24954400	0.719008
chr2	25040200	0.975207
chr2	25049600	0.917355
chr2	25110200	0.991736
chr2	25149900	0.719008
chr2	25301700	0.669421
chr2	25354800	0.743802
chr2	25384500	0.669421
chr2	25391500	0.760331
chr2	25476300	0.884298
chr2	25491400	0.975207
chr2	25499900	0.958678
chr2	25509300	0.694215
chr2	25514300	0.876033
chr2	25518500	0.933884
chr2	25585400	0.719008
chr2	25600100	0.900826
chr2	25714900	0.925620
chr2	25781700	0.859504
chr2	25793400	0.876033
chr2	25810500	0.719008
chr2	25838800	0.809917
chr2	25841800	0.917355
chr2	25874400	0.983471
chr2	25887900	0.677686
chr2	25922100	0.776860
chr2	26018000	0.867769
chr2	26044700	0.834711
chr2	26074900	0.917355
chr2	26162600	0.661157
chr2	26184200	1.000000
chr2	26200500	0.925620
chr2	26202900	0.826446
chr2	26214700	0.694215
chr2	26225400	0.702479
chr2	26232200	0.702479
chr2	26235400	0.809917
chr2	26250000	0.677686
chr2	26291800	0.950413
chr2	26408000	0.719008
chr2	26452600	0.685950
chr2	26497100	0.892562
chr2	26678900	0.727273
chr2	26715000	0.950413
chr2	26719400	0.900826
chr2	26919800	0.983471
chr2	26980200	0.966942
chr2	26982400	0.851240
chr2	26989000	0.685950
chr2	27071000	0.859504
chr2	27214500	0.710744
chr2	27227200	0.694215

chr2	27239200	0.834711
chr2	27286300	0.958678
chr2	27304600	0.942149
chr2	27319600	0.752066
chr2	27472900	0.801653
chr2	27488000	0.685950
chr2	27511800	1.000000
chr2	27516300	0.958678
chr2	27544000	0.760331
chr2	27609300	0.735537
chr2	27654200	0.925620
chr2	27789800	1.000000
chr2	27822300	0.834711
chr2	27850000	0.950413
chr2	27890100	0.727273
chr2	27905500	0.983471
chr2	27929600	0.859504
chr2	27938600	0.900826
chr2	27943400	0.983471
chr2	27958600	0.942149
chr2	27961100	0.925620
chr2	27989200	0.884298
chr2	27997200	0.669421
chr2	28002800	0.983471
chr2	28019000	0.942149
chr2	28023300	0.917355
chr2	28032700	0.975207
chr2	28055500	0.958678
chr2	28078900	0.702479
chr2	28108900	0.975207
chr2	28143700	0.966942
chr2	28169700	0.975207
chr2	28179000	0.933884
chr2	28182900	0.958678
chr2	28200800	0.933884
chr2	28259000	0.933884
chr2	28271200	0.876033
chr2	28276100	0.966942
chr2	28303900	0.983471
chr2	28325600	0.950413
chr2	28433000	0.826446
chr2	28497400	0.785124
chr2	28499900	0.801653
chr2	28561700	0.727273
chr2	28565500	0.983471
chr2	28572000	0.809917
chr2	28579000	0.685950
chr2	28583100	0.859504
chr2	28589800	0.851240
chr2	28610500	0.991736
chr2	28622000	0.983471
chr2	28626100	0.933884
chr2	28629200	0.925620
chr2	28631400	0.752066

chr2	28633700	0.867769
chr2	28647100	0.892562
chr2	28673100	0.818182
chr2	28807700	0.760331
chr2	28817800	0.776860
chr2	28832400	0.983471
chr2	28834600	0.793388
chr2	28844800	0.983471
chr2	28872700	0.785124
chr2	28884900	0.975207
chr2	28887400	0.933884
chr2	28904500	0.950413
chr2	28915300	0.900826
chr2	28940100	0.975207
chr2	28973600	0.710744
chr2	29033900	0.694215
chr2	29150500	0.818182
chr2	29341200	0.975207
chr2	29347000	0.983471
chr2	29353500	0.958678
chr2	29483100	0.983471
chr2	30372900	0.876033
chr2	30378500	0.983471
chr2	30448600	0.727273
chr2	30476200	0.826446
chr2	30489400	0.735537
chr2	30497300	1.000000
chr2	30631100	0.876033
chr2	30633800	0.859504
chr2	30660100	0.900826
chr2	31510400	0.842975
chr2	32158000	0.950413
chr2	32509800	0.743802
chr2	32514700	0.983471
chr2	32661300	0.669421
chr2	32714400	0.826446
chr2	32817400	0.834711
chr2	32904300	0.900826
chr2	32908800	0.735537
chr2	33017700	0.900826
chr2	33024200	0.702479
chr2	33030600	0.917355
chr2	33050700	0.785124
chr2	33141100	0.884298
chr2	33152300	0.834711
chr2	33195100	0.661157
chr2	33239100	0.900826
chr2	33245900	0.727273
chr2	33250000	0.900826
chr2	33289900	0.851240
chr2	33294100	0.826446
chr2	33296400	0.942149
chr2	33298800	0.909091
chr2	33311900	0.958678

chr2	33329700	0.975207
chr2	33361800	0.776860
chr2	33383900	0.909091
chr2	33391600	0.942149
chr2	33434900	0.917355
chr2	33438500	0.785124
chr2	33441100	0.917355
chr2	33445300	0.950413
chr2	33462000	0.917355
chr2	33466100	0.991736
chr2	33468300	0.842975
chr2	33501100	0.933884
chr2	33514000	0.776860
chr2	33517500	0.966942
chr2	33565500	0.694215
chr2	33567800	0.991736
chr2	33591600	0.892562
chr2	33646200	0.991736
chr2	33651200	0.851240
chr2	33661400	0.917355
chr2	33668000	0.950413
chr2	33703400	0.809917
chr2	33719600	0.685950
chr2	33722200	0.809917
chr2	33780600	1.000000
chr2	33929700	0.859504
chr2	33944500	0.661157
chr2	34330500	0.801653
chr2	34333800	0.793388
chr2	35729200	0.950413
chr2	35736100	0.933884
chr2	36189200	0.685950
chr2	36199300	0.925620
chr2	36566300	0.719008
chr2	36580200	0.925620
chr2	36588100	0.867769
chr2	36592500	0.842975
chr2	36594900	0.958678
chr2	36597000	0.900826
chr2	36600400	0.917355
chr2	36604100	0.760331
chr2	36607200	0.702479
chr2	36609600	0.950413
chr2	36613300	0.818182
chr2	36616600	1.000000
chr2	36620700	0.991736
chr2	36625300	0.983471
chr2	36628300	0.776860
chr2	36631500	0.958678
chr2	36634700	1.000000
chr2	36637500	0.925620
chr2	36641300	0.917355
chr2	36645500	0.702479
chr2	36647900	0.867769

chr2	36658700	0.876033
chr2	36662500	0.859504
chr2	36666000	0.909091
chr2	36669400	0.991736
chr2	36671800	0.818182
chr2	36674900	0.950413
chr2	36678100	0.958678
chr2	36680500	0.859504
chr2	36683700	0.933884
chr2	36686500	0.909091
chr2	36688900	0.958678
chr2	36691300	0.975207
chr2	36693900	0.933884
chr2	36696600	0.975207
chr2	36703100	0.785124
chr2	36706000	0.925620
chr2	36708200	0.677686
chr2	36710400	0.917355
chr2	36714200	1.000000
chr2	36716900	0.975207
chr2	36719300	0.950413
chr2	36770500	0.958678
chr2	36772900	0.760331
chr2	36777000	0.975207
chr2	36786200	0.950413
chr2	36790100	0.909091
chr2	36792500	0.909091
chr2	36795700	0.933884
chr2	36798100	0.933884
chr2	36877800	0.809917
chr2	36882700	0.909091
chr2	36891600	0.727273
chr2	36905100	0.859504
chr2	36924500	0.834711
chr2	36981700	0.752066
chr2	37063000	0.661157
chr2	37074900	0.661157
chr2	37117200	0.975207
chr2	37140600	0.677686
chr2	37154100	0.942149
chr2	37158200	0.909091
chr2	37178900	0.966942
chr2	37307800	0.793388
chr2	37331400	0.842975
chr2	37377400	0.975207
chr2	37380300	0.694215
chr2	37385100	0.661157
chr2	37409200	0.917355
chr2	37471300	0.933884
chr2	37510800	0.942149
chr2	37607400	0.958678
chr2	37618200	0.925620
chr2	37677300	0.826446
chr2	37681600	0.834711

chr2	37684700	0.801653
chr2	37687300	0.867769
chr2	37789200	0.958678
chr2	37794200	0.867769
chr2	37811400	0.983471
chr2	37814700	0.933884
chr2	37832400	0.710744
chr2	37870600	0.842975
chr2	37872800	1.000000
chr2	37874900	0.809917
chr2	37877200	0.768595
chr2	37879500	0.925620
chr2	37882200	0.851240
chr2	37884400	0.983471
chr2	37886800	0.950413
chr2	37903700	0.859504
chr2	38003700	0.743802
chr2	38008200	0.892562
chr2	38050400	0.942149
chr2	38056700	0.991736
chr2	38083000	0.752066
chr2	38334300	0.809917
chr2	38374100	0.876033
chr2	38554200	0.685950
chr2	38736900	0.743802
chr2	38770200	0.983471
chr2	38826600	0.727273
chr2	38918400	0.884298
chr2	38975000	0.702479
chr2	39187800	0.719008
chr2	39225200	0.867769
chr2	39456400	0.661157
chr2	39607600	0.876033
chr2	39629800	0.859504
chr2	39635300	0.776860
chr2	39638800	0.983471
chr2	39667200	0.917355
chr2	39695600	0.892562
chr2	39697700	0.933884
chr2	39712000	0.942149
chr2	39715800	0.909091
chr2	39719700	0.884298
chr2	39722600	0.958678
chr2	39740800	0.859504
chr2	39744500	0.842975
chr2	39751100	0.975207
chr2	39757800	0.801653
chr2	39778400	0.983471
chr2	39795800	0.983471
chr2	39847700	0.727273
chr2	40557500	0.867769
chr2	40580100	0.760331
chr2	40628700	0.900826
chr2	40662300	0.925620

chr2	40739200	0.966942
chr2	41185000	0.950413
chr2	41189600	0.876033
chr2	41293700	0.842975
chr2	41379100	0.876033
chr2	41421800	0.710744
chr2	41470100	0.669421
chr2	42194000	0.966942
chr2	42252800	0.685950
chr2	42329000	0.867769
chr2	42360500	0.925620
chr2	42369400	0.892562
chr2	42451200	0.867769
chr2	42477400	0.719008
chr2	42484000	0.867769
chr2	42569900	0.876033
chr2	42648400	0.925620
chr2	42738000	0.776860
chr2	42846400	0.950413
chr2	43063400	0.900826
chr2	43087600	0.834711
chr2	43106100	0.685950
chr2	43109200	0.966942
chr2	43124300	0.743802
chr2	43134100	0.719008
chr2	43141000	0.958678
chr2	43145900	0.958678
chr2	43150900	0.776860
chr2	43154100	0.834711
chr2	43156400	0.933884
chr2	43178700	0.818182
chr2	43182100	0.975207
chr2	43188900	1.000000
chr2	43202900	0.768595
chr2	43269100	0.727273
chr2	43271800	0.834711
chr2	43274200	0.975207
chr2	43279100	1.000000
chr2	43301500	0.933884
chr2	43303700	0.834711
chr2	43310200	0.785124
chr2	43312800	0.933884
chr2	43314900	0.958678
chr2	43341700	0.958678
chr2	43358600	0.826446
chr2	43386100	0.752066
chr2	43395000	0.884298
chr2	43399000	0.909091
chr2	43402900	0.900826
chr2	43406400	0.950413
chr2	43413100	0.669421
chr2	43496400	0.909091
chr2	43521400	0.909091
chr2	43560700	1.000000

chr2	43568500	0.859504
chr2	43572300	0.983471
chr2	43639600	0.677686
chr2	43653900	0.983471
chr2	43672800	0.917355
chr2	43679600	1.000000
chr2	43683200	0.991736
chr2	43686400	0.768595
chr2	43689000	0.669421
chr2	43695500	0.975207
chr2	43941700	0.743802
chr2	44314700	0.818182
chr2	44461000	0.884298
chr2	44586300	0.966942
chr2	44776600	1.000000
chr2	44781100	0.917355
chr2	44936000	0.867769
chr2	45179900	0.743802
chr2	45182900	0.876033
chr2	45200600	0.669421
chr2	45228600	0.694215
chr2	45231900	0.801653
chr2	45252600	0.677686
chr2	45298100	0.727273
chr2	45356100	0.818182
chr2	45359500	0.752066
chr2	45465800	0.900826
chr2	45468900	0.876033
chr2	45483000	0.884298
chr2	45485400	0.785124
chr2	45488200	0.669421
chr2	45491200	0.669421
chr2	45496600	0.661157
chr2	45503100	0.834711
chr2	45505700	0.925620
chr2	45515900	0.876033
chr2	45518900	0.842975
chr2	45558000	0.685950
chr2	45581500	0.942149
chr2	45608000	0.983471
chr2	45738500	0.909091
chr2	45795300	0.983471
chr2	45797500	0.702479
chr2	45834500	0.743802
chr2	45839700	0.859504
chr2	45848000	0.694215
chr2	45896500	0.983471
chr2	45998500	0.743802
chr2	46017900	0.950413
chr2	46021800	0.909091
chr2	46040500	0.966942
chr2	46088200	0.842975
chr2	46146700	0.685950
chr2	46163300	0.834711

chr2	46166400	0.942149
chr2	46169700	0.826446
chr2	46192600	1.000000
chr2	46242700	0.983471
chr2	46257000	0.975207
chr2	46273300	0.892562
chr2	46285700	0.768595
chr2	46290600	0.752066
chr2	46293300	0.710744
chr2	46295400	0.966942
chr2	46304600	0.917355
chr2	46314100	0.942149
chr2	46325600	0.958678
chr2	46344400	0.892562
chr2	46346700	0.991736
chr2	46363000	0.752066
chr2	46392400	0.983471
chr2	46406600	0.752066
chr2	46414900	0.809917
chr2	46424500	0.719008
chr2	46427900	0.942149
chr2	46452600	0.983471
chr2	46485000	1.000000
chr2	46487200	0.859504
chr2	46495300	1.000000
chr2	46497700	0.859504
chr2	46501000	0.818182
chr2	46505800	0.950413
chr2	46523600	0.694215
chr2	46531100	0.842975
chr2	46533400	0.884298
chr2	46543600	0.760331
chr2	46551600	0.983471
chr2	46561400	0.851240
chr2	46563600	0.925620
chr2	46580300	0.793388
chr2	46590600	0.851240
chr2	46594200	0.768595
chr2	46708800	0.966942
chr2	46727100	0.719008
chr2	46883600	0.842975
chr2	46912200	0.677686
chr2	46918400	0.950413
chr2	46925500	0.719008
chr2	46933800	0.925620
chr2	46976200	0.809917
chr2	47041600	0.975207
chr2	47074600	0.950413
chr2	47078400	0.793388
chr2	47081100	0.661157
chr2	47083400	0.917355
chr2	47094400	0.909091
chr2	47100800	0.785124
chr2	47105900	0.859504

chr2	47109500	0.884298
chr2	47137500	0.884298
chr2	47140500	0.909091
chr2	47149700	0.966942
chr2	47167600	0.801653
chr2	47182600	0.801653
chr2	47192400	0.884298
chr2	47195400	0.991736
chr2	47209500	0.669421
chr2	47214300	0.719008
chr2	47263100	0.727273
chr2	47271600	0.735537
chr2	47293500	0.776860
chr2	47299500	0.983471
chr2	47303300	0.900826
chr2	47311800	0.801653
chr2	47314800	0.677686
chr2	47317200	0.876033
chr2	47346200	0.892562
chr2	47417300	0.917355
chr2	47419600	0.752066
chr2	47422600	0.785124
chr2	47433100	0.727273
chr2	47436700	0.809917
chr2	47452600	0.752066
chr2	47497100	0.884298
chr2	47499900	1.000000
chr2	47572000	0.785124
chr2	47582100	0.983471
chr2	47650000	0.719008
chr2	47688100	0.685950
chr2	47729100	0.867769
chr2	47736600	0.876033
chr2	47761700	0.917355
chr2	47916800	0.966942
chr2	48009500	0.661157
chr2	48094900	0.950413
chr2	48130000	0.661157
chr2	48334300	0.867769
chr2	48436900	0.975207
chr2	48476500	0.975207
chr2	48540900	0.752066
chr2	48571100	0.818182
chr2	48573800	0.851240
chr2	48580400	0.975207
chr2	48584400	0.983471
chr2	48613500	0.966942
chr2	48648000	0.917355
chr2	48736500	0.942149
chr2	48759800	0.719008
chr2	48939200	0.785124
chr2	49456800	0.925620
chr2	49492900	0.834711
chr2	49534700	0.694215

chr2	50877600	0.876033
chr2	51231100	0.793388
chr2	51735500	0.925620
chr2	51744100	0.950413
chr2	52040800	0.983471
chr2	52223400	0.876033
chr2	54167200	1.000000
chr2	54177300	0.776860
chr2	54189300	0.991736
chr2	54199100	0.669421
chr2	54331500	0.826446
chr2	54588000	0.933884
chr2	54643800	0.983471
chr2	54647200	0.776860
chr2	54657300	0.950413
chr2	54692500	0.876033
chr2	54697700	1.000000
chr2	54707000	0.719008
chr2	54710700	0.801653
chr2	54725300	0.785124
chr2	54752100	0.884298
chr2	54759100	0.743802
chr2	54773200	0.818182
chr2	54789200	0.677686
chr2	54798100	0.975207
chr2	54800400	0.892562
chr2	54802700	0.950413
chr2	54805400	0.900826
chr2	54807500	0.942149
chr2	54814000	0.892562
chr2	54818200	0.719008
chr2	54823600	0.884298
chr2	54831200	0.859504
chr2	54833700	0.818182
chr2	54841200	0.826446
chr2	54846200	0.710744
chr2	54859800	0.900826
chr2	54863100	0.876033
chr2	54900800	0.851240
chr2	54995500	0.942149
chr2	54999700	0.991736
chr2	55131500	0.942149
chr2	55180600	0.892562
chr2	55193800	0.719008
chr2	55270300	0.702479
chr2	55279800	0.710744
chr2	55310900	0.909091
chr2	55320700	1.000000
chr2	55324600	0.966942
chr2	55327800	0.900826
chr2	55330100	0.933884
chr2	55336800	0.876033
chr2	55339900	0.859504
chr2	55343400	0.702479

chr2	55375900	0.842975
chr2	55385200	0.727273
chr2	55388900	0.975207
chr2	55392200	0.826446
chr2	55537800	0.884298
chr2	55592100	0.727273
chr2	55777600	0.900826
chr2	55813300	0.950413
chr2	55993600	0.867769
chr2	55999500	0.991736
chr2	56006400	0.925620
chr2	56008600	0.983471
chr2	56089300	0.801653
chr2	56093100	0.925620
chr2	56097500	0.677686
chr2	56099700	0.661157
chr2	56101900	0.685950
chr2	56104000	0.884298
chr2	56107800	0.925620
chr2	56111100	0.826446
chr2	56113500	0.958678
chr2	56115800	1.000000
chr2	56119400	0.975207
chr2	56122200	0.834711
chr2	56127200	0.900826
chr2	56129500	0.966942
chr2	56131800	0.818182
chr2	56135000	0.752066
chr2	56142000	0.760331
chr2	56169500	0.677686
chr2	56188900	0.909091
chr2	56192000	0.876033
chr2	56194700	0.950413
chr2	56212100	0.867769
chr2	56214300	0.958678
chr2	56217400	0.785124
chr2	56220000	0.991736
chr2	56222500	0.933884
chr2	56235700	0.933884
chr2	56238100	0.958678
chr2	56241000	0.991736
chr2	56243600	0.966942
chr2	56246000	0.950413
chr2	56361300	0.876033
chr2	56455000	0.702479
chr2	56457400	0.942149
chr2	56816000	0.719008
chr2	56908900	0.975207
chr2	57407000	0.785124
chr2	57409500	0.975207
chr2	57600800	0.925620
chr2	58269900	0.851240
chr2	58335300	0.909091
chr2	58340300	0.826446

chr2	58365400	0.826446
chr2	58847900	0.867769
chr2	58861600	1.000000
chr2	58866000	0.950413
chr2	60809100	0.735537
chr2	61024100	1.000000
chr2	61089300	0.661157
chr2	61095000	0.661157
chr2	61112000	0.925620
chr2	61114600	0.735537
chr2	61186100	0.950413
chr2	61407100	0.818182
chr2	61483000	0.950413
chr2	61500600	0.942149
chr2	61567500	0.876033
chr2	61653600	0.884298
chr2	61687700	0.966942
chr2	61898600	0.909091
chr2	62309300	0.818182
chr2	62345800	0.909091
chr2	62444100	0.826446
chr2	62447600	0.801653
chr2	62531400	0.975207
chr2	62534600	0.768595
chr2	62609700	0.719008
chr2	62639100	0.851240
chr2	62703000	0.975207
chr2	62705600	0.958678
chr2	62805900	0.900826
chr2	63053600	0.752066
chr2	63262600	0.975207
chr2	63926600	0.876033
chr2	63962800	0.917355
chr2	63965000	0.884298
chr2	63969100	0.685950
chr2	64212700	0.842975
chr2	64248600	0.702479
chr2	64280100	0.950413
chr2	64291000	0.933884
chr2	64329000	0.925620
chr2	64332900	0.719008
chr2	64342500	0.983471
chr2	64365400	0.661157
chr2	64455800	0.661157
chr2	64488000	0.958678
chr2	64491500	0.702479
chr2	64494200	0.842975
chr2	64496500	0.909091
chr2	64500800	0.966942
chr2	64504900	0.900826
chr2	64558100	0.925620
chr2	64631500	0.991736
chr2	64716300	0.801653
chr2	64719800	0.834711

chr2	64836800	0.900826
chr2	64871500	0.719008
chr2	64923300	0.884298
chr2	64926200	0.942149
chr2	64938900	0.950413
chr2	64958100	0.818182
chr2	65006700	0.983471
chr2	65009400	0.776860
chr2	65020600	1.000000
chr2	65023000	0.966942
chr2	65025400	1.000000
chr2	65039600	0.884298
chr2	65059100	0.859504
chr2	65086800	0.900826
chr2	65092500	0.958678
chr2	65103800	0.851240
chr2	65270600	0.909091
chr2	65286600	0.975207
chr2	65292500	0.859504
chr2	65296000	0.669421
chr2	65298100	0.966942
chr2	65303600	0.752066
chr2	65309200	0.917355
chr2	65414100	0.950413
chr2	65459200	0.793388
chr2	65577500	0.727273
chr2	65594400	0.966942
chr2	65605200	0.867769
chr2	65611100	0.760331
chr2	65648000	0.661157
chr2	65773200	0.735537
chr2	65802500	0.677686
chr2	65804700	0.983471
chr2	66705000	0.743802
chr2	66708400	0.834711
chr2	66718200	0.991736
chr2	66736700	0.958678
chr2	66783400	0.925620
chr2	66789800	0.958678
chr2	67182700	0.719008
chr2	67487300	0.909091
chr2	67616600	0.975207
chr2	67622000	0.983471
chr2	67626600	0.785124
chr2	67628700	0.950413
chr2	67730400	0.983471
chr2	67788900	0.958678
chr2	68053000	0.975207
chr2	68073800	0.892562
chr2	68099200	0.983471
chr2	68145100	0.958678
chr2	68149700	0.818182
chr2	68212500	0.661157
chr2	68225000	0.958678

chr2	68378600	0.752066
chr2	68458200	0.661157
chr2	68466500	0.966942
chr2	68787300	0.685950
chr2	68881100	0.867769
chr2	69002200	0.966942
chr2	69023800	0.785124
chr2	69038700	0.876033
chr2	69056900	0.909091
chr2	69060200	0.900826
chr2	69077400	0.859504
chr2	69097500	0.661157
chr2	69106500	0.975207
chr2	69273700	0.909091
chr2	69310300	0.925620
chr2	69324600	0.669421
chr2	69366000	0.909091
chr2	69389200	0.884298
chr2	69392000	0.719008
chr2	69404900	0.925620
chr2	69441900	0.793388
chr2	69450600	0.809917
chr2	69479300	0.801653
chr2	69481500	0.900826
chr2	69503100	0.933884
chr2	69777500	0.925620
chr2	69782300	0.975207
chr2	69844300	0.752066
chr2	69863100	0.719008
chr2	69895600	1.000000
chr2	69902100	0.768595
chr2	69910400	0.900826
chr2	70025900	0.859504
chr2	70045300	0.867769
chr2	70141000	0.694215
chr2	70145400	0.917355
chr2	70161500	0.958678
chr2	70164600	0.809917
chr2	70173600	0.752066
chr2	70317900	0.776860
chr2	70322700	0.950413
chr2	70351300	0.719008
chr2	70359600	0.826446
chr2	70363600	0.975207
chr2	70370600	0.776860
chr2	70440200	0.685950
chr2	70471400	0.735537
chr2	70580100	0.702479
chr2	70585100	0.867769
chr2	70599200	0.975207
chr2	70995000	0.776860
chr2	71056400	0.809917
chr2	71115200	0.661157
chr2	71149400	0.975207

chr2	71175500	0.752066
chr2	71204200	0.785124
chr2	71637900	0.801653
chr2	71645200	0.892562
chr2	71651400	0.975207
chr2	71688100	0.958678
chr2	71696200	0.685950
chr2	71718900	0.975207
chr2	71721800	0.851240
chr2	71725200	0.909091
chr2	71728800	0.884298
chr2	71732200	0.975207
chr2	71772500	0.851240
chr2	71784400	0.776860
chr2	71832600	0.933884
chr2	71836800	0.909091
chr2	71842500	0.909091
chr2	71944500	0.892562
chr2	71970500	0.859504
chr2	71975000	0.909091
chr2	72108300	0.727273
chr2	72343200	0.884298
chr2	72401400	0.752066
chr2	72788900	0.942149
chr2	72947100	1.000000
chr2	72984500	0.876033
chr2	73005800	0.669421
chr2	73008300	0.925620
chr2	73021800	0.710744
chr2	73090000	0.760331
chr2	73123500	0.809917
chr2	73143700	0.677686
chr2	73148100	0.826446
chr2	73234600	0.925620
chr2	73241300	0.991736
chr2	73248800	0.685950
chr2	73291200	0.958678
chr2	73312000	0.942149
chr2	73317000	0.909091
chr2	73329900	0.851240
chr2	73334800	0.694215
chr2	73361500	0.851240
chr2	73394300	0.909091
chr2	73404300	0.809917
chr2	73467300	0.975207
chr2	73519000	0.685950
chr2	73521100	0.719008
chr2	73974200	0.991736
chr2	73987200	0.752066
chr2	74079600	0.776860
chr2	74198500	0.661157
chr2	74208700	0.851240
chr2	74214700	0.876033
chr2	74230000	0.801653

chr2	74433500	0.983471
chr2	74671800	0.801653
chr2	74743600	0.743802
chr2	74787900	0.677686
chr2	74968200	0.719008
chr2	74992000	0.685950
chr2	75070500	0.719008
chr2	75073000	0.851240
chr2	75120400	0.983471
chr2	75147300	0.685950
chr2	75374100	0.677686
chr2	75728400	0.834711
chr2	75731000	0.892562
chr2	75734300	0.719008
chr2	75775200	0.917355
chr2	75785000	0.661157
chr2	75789600	0.876033
chr2	75823300	0.842975
chr2	75868300	0.818182
chr2	76696600	0.793388
chr2	77095500	0.991736
chr2	77212300	0.867769
chr2	78536500	0.966942
chr2	79073300	0.685950
chr2	79168700	0.958678
chr2	80717400	0.694215
chr2	82829700	0.661157
chr2	82902900	0.793388
chr2	82918300	0.917355
chr2	83023300	0.867769
chr2	83083700	0.661157
chr2	83279200	0.867769
chr2	83282700	0.958678
chr2	83309200	1.000000
chr2	83331500	0.991736
chr2	83343200	0.801653
chr2	83396100	0.991736
chr2	83413800	0.942149
chr2	83893100	0.785124
chr2	84721700	0.991736
chr2	85122300	0.694215
chr2	85147400	0.917355
chr2	85151500	0.859504
chr2	85173800	1.000000
chr2	85186100	0.975207
chr2	85245000	0.925620
chr2	85269800	0.925620
chr2	85378600	0.760331
chr2	85383700	0.785124
chr2	85403000	0.966942
chr2	85405600	1.000000
chr2	85408000	0.809917
chr2	85476400	0.942149
chr2	85478500	0.867769

chr2	85483500	0.727273
chr2	85485800	0.826446
chr2	85492200	0.975207
chr2	85497100	0.917355
chr2	85505200	0.768595
chr2	85648100	0.834711
chr2	85665300	1.000000
chr2	85726900	0.834711
chr2	85732300	0.876033
chr2	85744200	0.793388
chr2	85777200	0.909091
chr2	85805600	0.867769
chr2	85814000	0.851240
chr2	85816100	0.859504
chr2	85833200	0.909091
chr2	85835300	0.909091
chr2	85876800	0.809917
chr2	85890200	0.892562
chr2	85906400	0.884298
chr2	85929900	0.826446
chr2	85938000	0.851240
chr2	85962700	0.975207
chr2	85970900	0.752066
chr2	85984300	0.694215
chr2	85992100	0.719008
chr2	85994700	0.909091
chr2	85999800	0.991736
chr2	86002100	0.826446
chr2	86020100	0.842975
chr2	86081400	0.702479
chr2	86089000	0.950413
chr2	86106400	0.785124
chr2	86113900	0.826446
chr2	86121000	0.719008
chr2	86165900	0.793388
chr2	86227500	0.694215
chr2	86256000	0.719008
chr2	86266300	0.735537
chr2	86273100	0.842975
chr2	86289500	0.991736
chr2	86300000	0.867769
chr2	86441100	0.669421
chr2	86452800	0.876033
chr2	86811600	0.966942
chr2	86859400	0.727273
chr2	86899100	0.842975
chr2	86932000	0.867769
chr2	87018400	0.710744
chr2	87242600	0.851240
chr2	87321100	0.933884
chr2	87416800	0.958678
chr2	87489700	0.909091
chr2	87522500	0.975207
chr2	87544600	0.809917

chr2	87546700	0.909091
chr2	87552700	0.900826
chr2	87745800	0.966942
chr2	87753200	0.892562
chr2	87760800	0.859504
chr2	87782500	0.760331
chr2	87785700	0.876033
chr2	87795700	0.966942
chr2	87798200	0.958678
chr2	87800300	0.925620
chr2	87825600	0.892562
chr2	87893400	0.909091
chr2	87901700	0.917355
chr2	87903900	0.776860
chr2	87906000	0.966942
chr2	87912500	0.909091
chr2	87935100	0.710744
chr2	87950400	0.859504
chr2	87975100	0.933884
chr2	88054500	0.793388
chr2	88124500	0.909091
chr2	88152500	0.776860
chr2	88899900	0.925620
chr2	88903100	0.677686
chr2	88958900	0.876033
chr2	89029000	0.925620
chr2	89036300	0.685950
chr2	89039800	0.760331
chr2	89056100	0.768595
chr2	89065200	0.677686
chr2	89097000	0.752066
chr2	89117700	0.727273
chr2	89127700	0.892562
chr2	89545900	0.809917
chr2	89865300	0.842975
chr2	89875200	0.826446
chr2	89879700	0.768595
chr2	91845400	0.966942
chr2	91956500	0.743802
chr2	92220100	0.768595
chr2	92269300	0.867769
chr2	92272800	0.785124
chr2	92286400	0.768595
chr2	92300600	0.719008
chr2	92325600	0.809917
chr2	95475300	0.942149
chr2	96192500	0.768595
chr2	96348600	0.826446
chr2	96533500	0.685950
chr2	96556800	0.752066
chr2	96559500	0.834711
chr2	96561900	0.685950
chr2	96566400	0.991736
chr2	96569800	0.702479

chr2	96571900	0.975207
chr2	96575700	0.983471
chr2	96578000	0.950413
chr2	96580300	0.966942
chr2	96584300	0.776860
chr2	96587400	0.900826
chr2	96811100	0.834711
chr2	96871500	0.991736
chr2	96908400	0.900826
chr2	96911800	0.809917
chr2	96917700	0.991736
chr2	96920800	0.892562
chr2	96927400	0.933884
chr2	96981800	0.793388
chr2	97152000	0.834711
chr2	97193500	0.983471
chr2	97198100	0.983471
chr2	97205700	0.809917
chr2	97292000	0.983471
chr2	97404000	0.677686
chr2	97426700	0.661157
chr2	97429400	0.809917
chr2	97532700	0.826446
chr2	97853800	0.917355
chr2	97857900	0.735537
chr2	97861500	0.983471
chr2	97863600	0.900826
chr2	97867100	0.966942
chr2	97872900	0.834711
chr2	97875900	0.917355
chr2	97899300	0.768595
chr2	98163900	0.876033
chr2	98167500	0.983471
chr2	98261600	0.735537
chr2	98294200	0.933884
chr2	98486700	0.933884
chr2	98911400	0.975207
chr2	98915500	0.842975
chr2	99011400	0.991736
chr2	99036900	0.991736
chr2	99074600	0.851240
chr2	99082200	0.876033
chr2	99096600	0.677686
chr2	99115600	0.942149
chr2	99119400	0.694215
chr2	99139700	0.818182
chr2	99215900	0.677686
chr2	99266400	0.826446
chr2	99300400	0.719008
chr2	99308600	0.958678
chr2	99318000	0.942149
chr2	99327600	0.966942
chr2	99330700	0.694215
chr2	99337800	0.661157

chr2	99342700	0.909091
chr2	99344900	0.752066
chr2	99374800	0.950413
chr2	99385500	0.884298
chr2	99411200	0.950413
chr2	99416100	0.727273
chr2	99423300	0.892562
chr2	99437700	0.760331
chr2	99440100	0.669421
chr2	99450500	0.950413
chr2	99482300	0.991736
chr2	99526200	0.958678
chr2	99529200	0.727273
chr2	99533500	0.785124
chr2	100041900	0.851240
chr2	100065700	0.925620
chr2	100092000	0.966942
chr2	100478300	0.991736
chr2	100484500	0.950413
chr2	100494400	0.702479
chr2	100498000	0.975207
chr2	100592100	0.876033
chr2	100725700	0.694215
chr2	100810000	0.942149
chr2	100815100	0.975207
chr2	100817900	0.867769
chr2	100902600	0.909091
chr2	101197800	0.900826
chr2	101256500	0.727273
chr2	101384100	0.983471
chr2	101398100	0.776860
chr2	101402900	0.950413
chr2	101430200	0.991736
chr2	101439500	0.661157
chr2	101474700	0.958678
chr2	101492400	0.950413
chr2	101495900	0.958678
chr2	101498200	0.983471
chr2	101500900	0.859504
chr2	101503400	0.752066
chr2	101508700	0.669421
chr2	101511000	0.991736
chr2	101519500	0.876033
chr2	101524200	0.942149
chr2	101540200	0.727273
chr2	101542500	0.942149
chr2	101576400	0.983471
chr2	101595200	0.933884
chr2	101601100	0.719008
chr2	101664300	0.752066
chr2	101726700	0.991736
chr2	101737700	0.975207
chr2	101742000	0.983471
chr2	101911900	0.900826

chr2	101914600	0.677686
chr2	101921600	0.900826
chr2	101944900	0.752066
chr2	101947700	0.785124
chr2	101954600	0.785124
chr2	101999400	0.876033
chr2	102058900	0.818182
chr2	102079700	0.727273
chr2	102082100	0.942149
chr2	102090800	0.859504
chr2	102103100	0.950413
chr2	102132200	0.876033
chr2	102137100	0.917355
chr2	102163100	0.818182
chr2	102176700	0.958678
chr2	102193800	0.842975
chr2	102236000	0.719008
chr2	102313600	0.752066
chr2	102318300	0.702479
chr2	102326000	0.727273
chr2	102353400	0.983471
chr2	102359100	0.933884
chr2	102363400	0.909091
chr2	102375300	0.760331
chr2	102384300	0.768595
chr2	102387500	0.785124
chr2	102413700	0.851240
chr2	102423100	1.000000
chr2	102432800	0.785124
chr2	102435400	0.785124
chr2	102440300	0.867769
chr2	102443700	0.975207
chr2	102448300	0.801653
chr2	102460700	0.694215
chr2	102481100	0.991736
chr2	102497900	0.925620
chr2	102627600	0.892562
chr2	102663100	0.966942
chr2	102667700	0.752066
chr2	102681000	0.842975
chr2	102699500	0.942149
chr2	102712000	0.702479
chr2	102721500	0.760331
chr2	102744600	0.842975
chr2	102766700	0.900826
chr2	102844900	0.867769
chr2	102857500	0.760331
chr2	102868200	0.760331
chr2	102875000	0.818182
chr2	102890400	0.710744
chr2	102918800	0.776860
chr2	102920900	0.942149
chr2	102923100	0.942149
chr2	102929800	1.000000

chr2	102932200	0.950413
chr2	102952200	0.933884
chr2	102956000	0.694215
chr2	102968100	0.685950
chr2	103053200	0.859504
chr2	103126200	0.950413
chr2	103130800	0.966942
chr2	105890900	0.752066
chr2	105934600	0.743802
chr2	105940300	0.818182
chr2	105943400	0.917355
chr2	105947200	0.702479
chr2	105987300	0.900826
chr2	105991000	0.884298
chr2	105994100	0.975207
chr2	105997300	0.958678
chr2	106003200	0.801653
chr2	106005500	0.909091
chr2	106009600	0.727273
chr2	106019700	0.727273
chr2	106021900	0.917355
chr2	106027200	0.966942
chr2	106072200	0.876033
chr2	106127600	0.677686
chr2	106162900	0.859504
chr2	106170300	0.958678
chr2	106364500	0.710744
chr2	106406800	0.975207
chr2	106412300	0.727273
chr2	106757700	0.727273
chr2	108984300	0.851240
chr2	108994300	0.933884
chr2	109158700	0.991736
chr2	109170300	0.925620
chr2	109176500	0.719008
chr2	109196700	0.892562
chr2	109201200	0.768595
chr2	109205700	0.958678
chr2	109213500	0.876033
chr2	109233600	1.000000
chr2	109235700	0.694215
chr2	109237800	0.876033
chr2	109251100	0.834711
chr2	109254200	0.958678
chr2	109257500	0.768595
chr2	109270400	0.876033
chr2	109274100	0.834711
chr2	109279000	0.975207
chr2	109288700	1.000000
chr2	109295000	0.983471
chr2	109297100	0.867769
chr2	109303500	0.776860
chr2	109306200	0.933884
chr2	109450000	0.702479

chr2	109706300	0.925620
chr2	109715300	0.983471
chr2	109781300	0.933884
chr2	109783800	0.867769
chr2	109787900	0.776860
chr2	109791700	0.719008
chr2	109802500	0.876033
chr2	109826700	0.925620
chr2	109830700	0.950413
chr2	109855200	0.809917
chr2	109857300	0.826446
chr2	109859900	0.702479
chr2	109878600	0.933884
chr2	109884600	0.735537
chr2	109892100	0.909091
chr2	109896600	0.752066
chr2	109916400	0.983471
chr2	109938500	0.933884
chr2	109942000	0.983471
chr2	109949400	0.900826
chr2	109952600	0.958678
chr2	109956400	0.892562
chr2	109966000	0.826446
chr2	109968900	0.859504
chr2	110089400	0.950413
chr2	110099600	1.000000
chr2	110104600	0.909091
chr2	110255500	0.966942
chr2	110316300	0.950413
chr2	110318600	0.900826
chr2	110322500	0.834711
chr2	110367400	0.768595
chr2	110458000	0.760331
chr2	110646600	0.867769
chr2	110653700	0.933884
chr2	110659000	0.760331
chr2	110663900	0.975207
chr2	110674000	0.958678
chr2	110678400	0.933884
chr2	110682200	0.851240
chr2	110757100	0.694215
chr2	110771200	0.677686
chr2	110774400	0.983471
chr2	110809900	0.851240
chr2	110812900	0.983471
chr2	110820100	0.743802
chr2	110849300	0.801653
chr2	110852100	0.826446
chr2	110859400	0.975207
chr2	110861600	0.785124
chr2	110864900	0.851240
chr2	110867800	0.661157
chr2	110878800	0.809917
chr2	110907600	0.991736

chr2	110911000	0.991736
chr2	110914500	0.950413
chr2	111003400	0.958678
chr2	111037500	0.851240
chr2	111057200	0.727273
chr2	111074500	0.958678
chr2	111077100	0.925620
chr2	111112400	1.000000
chr2	111115500	0.702479
chr2	111129900	0.702479
chr2	111181700	0.826446
chr2	111204200	0.975207
chr2	111207900	0.851240
chr2	111213600	0.958678
chr2	111223200	0.991736
chr2	111228300	0.719008
chr2	111231800	0.859504
chr2	111233900	0.809917
chr2	111240500	0.909091
chr2	111369100	0.818182
chr2	111433000	0.958678
chr2	111453800	0.661157
chr2	111829800	0.966942
chr2	111906800	0.983471
chr2	111920900	0.884298
chr2	112033300	0.884298
chr2	112057800	0.867769
chr2	112060800	0.760331
chr2	112073200	0.859504
chr2	112095800	0.900826
chr2	112102300	0.983471
chr2	112106000	0.661157
chr2	112114600	0.933884
chr2	112124600	0.685950
chr2	112182700	0.917355
chr2	112208100	0.909091
chr2	112210200	0.975207
chr2	112212500	0.983471
chr2	112220500	0.909091
chr2	112222700	0.917355
chr2	112225400	0.727273
chr2	112238600	0.925620
chr2	112246900	0.710744
chr2	112254800	0.917355
chr2	112291200	0.950413
chr2	112300000	0.950413
chr2	112324200	0.925620
chr2	112357000	0.834711
chr2	112375200	0.826446
chr2	112452700	0.975207
chr2	112456700	0.933884
chr2	112460200	0.867769
chr2	112463700	0.900826
chr2	112534400	0.933884

chr2	112585300	0.685950
chr2	112598100	0.743802
chr2	112658600	0.826446
chr2	112664200	0.950413
chr2	112678300	0.966942
chr2	112688800	0.975207
chr2	112694800	0.760331
chr2	112697200	0.917355
chr2	112712900	0.867769
chr2	112723300	0.834711
chr2	112725400	0.743802
chr2	112734500	0.950413
chr2	112759400	0.760331
chr2	112773300	0.793388
chr2	112784100	0.917355
chr2	112790400	0.991736
chr2	112819400	0.983471
chr2	112821800	0.818182
chr2	112823900	0.900826
chr2	112831700	0.876033
chr2	112833900	0.950413
chr2	112847200	0.925620
chr2	112850500	0.958678
chr2	112895600	0.719008
chr2	112931400	0.719008
chr2	112945900	0.892562
chr2	112956700	0.876033
chr2	112979100	0.735537
chr2	113059200	0.983471
chr2	113246000	0.694215
chr2	113251900	0.760331
chr2	113382900	0.975207
chr2	113385200	0.793388
chr2	113389300	0.917355
chr2	113398600	0.752066
chr2	113401100	0.776860
chr2	113407100	0.710744
chr2	113417000	0.785124
chr2	113419200	0.776860
chr2	113428200	0.876033
chr2	113432200	0.975207
chr2	113441500	0.884298
chr2	113446100	0.785124
chr2	113449600	0.950413
chr2	113464300	0.669421
chr2	113526200	0.851240
chr2	113541800	0.991736
chr2	113545300	0.966942
chr2	113551700	0.991736
chr2	113569000	0.785124
chr2	113571500	0.900826
chr2	113582100	0.677686
chr2	113589400	0.950413
chr2	113627600	0.735537

chr2	113638900	0.859504
chr2	113998800	0.933884
chr2	114082800	0.735537
chr2	114349500	0.809917
chr2	114520400	0.801653
chr2	114576400	0.677686
chr2	114632000	0.719008
chr2	114653400	0.669421
chr2	114656200	0.743802
chr2	114724600	0.818182
chr2	114735900	0.933884
chr2	117427300	0.694215
chr2	118594300	0.719008
chr2	118614500	0.983471
chr2	118691700	0.818182
chr2	118715900	1.000000
chr2	118739100	0.752066
chr2	118805300	0.752066
chr2	118870300	0.859504
chr2	118876800	0.752066
chr2	118881800	0.917355
chr2	119496700	0.917355
chr2	120013800	0.983471
chr2	120213500	0.983471
chr2	120402200	0.809917
chr2	120405100	0.818182
chr2	120407800	1.000000
chr2	120415700	0.752066
chr2	120561100	0.950413
chr2	120566700	0.851240
chr2	120578200	1.000000
chr2	120596200	0.785124
chr2	120668400	0.735537
chr2	120775200	0.809917
chr2	120866900	0.942149
chr2	120890100	1.000000
chr2	120892800	0.958678
chr2	120981200	0.702479
chr2	120997600	0.991736
chr2	121005800	0.826446
chr2	121013600	0.842975
chr2	121015700	0.876033
chr2	121022600	0.975207
chr2	121027200	0.669421
chr2	121266800	0.826446
chr2	121310500	0.669421
chr2	121348000	0.876033
chr2	121427800	0.677686
chr2	121480800	0.950413
chr2	121494000	0.719008
chr2	121523700	0.950413
chr2	121625400	0.661157
chr2	121670000	0.685950
chr2	121673000	0.900826

chr2	122118400	0.685950
chr2	122123800	0.752066
chr2	122290000	0.958678
chr2	122354400	0.917355
chr2	122367000	0.727273
chr2	122371400	0.851240
chr2	122457800	0.950413
chr2	122656300	0.859504
chr2	122936700	0.809917
chr2	122938800	0.950413
chr2	122942100	0.900826
chr2	123507000	0.867769
chr2	127416800	0.991736
chr2	127534900	0.801653
chr2	127819000	0.826446
chr2	127821400	0.842975
chr2	127823500	0.867769
chr2	127841800	0.760331
chr2	127845000	0.950413
chr2	127851800	0.900826
chr2	127902300	0.925620
chr2	127911400	0.925620
chr2	127984400	0.727273
chr2	128139700	0.809917
chr2	128156000	0.793388
chr2	128166000	0.933884
chr2	128169100	0.991736
chr2	128171500	0.867769
chr2	128174200	0.776860
chr2	128266500	0.975207
chr2	128327300	0.735537
chr2	128357200	0.669421
chr2	128396300	0.867769
chr2	128399700	0.909091
chr2	128405100	0.826446
chr2	128416600	0.743802
chr2	128419100	0.826446
chr2	128422200	0.983471
chr2	128439300	0.834711
chr2	128453500	0.710744
chr2	128529000	0.925620
chr2	128927500	1.000000
chr2	128937900	0.991736
chr2	128944900	0.975207
chr2	128954300	0.958678
chr2	129121800	0.710744
chr2	129153700	0.942149
chr2	129166500	0.719008
chr2	129282100	0.735537
chr2	129342500	0.909091
chr2	130738000	0.694215
chr2	130873100	0.991736
chr2	131095300	0.694215
chr2	131261900	0.917355

chr2	131285500	0.752066
chr2	131374700	0.892562
chr2	131582400	0.958678
chr2	131798000	0.710744
chr2	131981400	1.000000
chr2	132354500	0.950413
chr2	132427000	0.933884
chr2	132443600	0.768595
chr2	132560300	0.727273
chr2	132640300	0.694215
chr2	132981200	0.900826
chr2	133003200	0.966942
chr2	133012600	0.917355
chr2	133016900	0.975207
chr2	133026600	0.685950
chr2	133034400	0.752066
chr2	133196500	0.710744
chr2	133958600	0.983471
chr2	133967100	0.702479
chr2	133971300	0.917355
chr2	133990300	0.942149
chr2	134338700	0.991736
chr2	134424900	0.925620
chr2	134432900	0.876033
chr2	134548900	0.818182
chr2	134571000	0.942149
chr2	134574600	0.876033
chr2	134576900	0.975207
chr2	134580200	0.719008
chr2	134599900	1.000000
chr2	134869600	0.669421
chr2	134876600	0.685950
chr2	134890000	0.661157
chr2	134895400	0.818182
chr2	134900900	0.991736
chr2	134919000	0.958678
chr2	134933100	0.933884
chr2	134935800	0.933884
chr2	134957000	0.842975
chr2	135008800	0.677686
chr2	135048000	0.752066
chr2	135059000	0.958678
chr2	135061200	0.776860
chr2	135065100	0.991736
chr2	135068700	0.950413
chr2	135117400	0.983471
chr2	135155400	0.892562
chr2	135157500	0.884298
chr2	135159900	0.900826
chr2	135165700	0.925620
chr2	135169200	0.983471
chr2	135184300	1.000000
chr2	135190100	1.000000
chr2	135194900	0.867769

chr2	135201800	0.694215
chr2	135204400	0.983471
chr2	135208700	0.818182
chr2	135440500	0.933884
chr2	135442600	0.933884
chr2	135464800	0.942149
chr2	135494200	0.975207
chr2	135812800	0.900826
chr2	136003000	0.851240
chr2	136279900	0.661157
chr2	136424900	0.694215
chr2	136503000	0.884298
chr2	136520100	0.867769
chr2	136531700	0.975207
chr2	136544700	0.785124
chr2	136667900	0.710744
chr2	136706800	1.000000
chr2	136718900	0.793388
chr2	136993800	0.966942
chr2	136997500	0.975207
chr2	137000800	0.834711
chr2	137134600	0.710744
chr2	137181400	0.669421
chr2	138451700	0.826446
chr2	138717900	0.809917
chr2	138721100	0.859504
chr2	138727300	1.000000
chr2	139025300	0.966942
chr2	139030300	0.743802
chr2	139174100	0.661157
chr2	139179700	0.925620
chr2	139232600	0.942149
chr2	139706000	0.958678
chr2	140697700	1.000000
chr2	141414100	0.727273
chr2	141778300	0.925620
chr2	141780400	0.727273
chr2	141785200	0.851240
chr2	141799400	0.801653
chr2	141813200	0.900826
chr2	142151000	0.958678
chr2	142369100	0.917355
chr2	142943600	0.991736
chr2	142948600	0.892562
chr2	143580200	0.694215
chr2	143583300	0.851240
chr2	143599700	0.983471
chr2	143602100	0.909091
chr2	143626400	0.909091
chr2	143631900	0.884298
chr2	143702100	0.702479
chr2	143704900	0.859504
chr2	143712400	0.942149
chr2	143732200	0.958678

chr2	143841900	0.884298
chr2	143868600	0.702479
chr2	144189100	0.702479
chr2	144659100	0.925620
chr2	144857600	0.694215
chr2	144956000	0.851240
chr2	145032100	0.809917
chr2	145078500	0.909091
chr2	145091000	0.768595
chr2	145100200	0.785124
chr2	145107200	0.933884
chr2	145109400	0.851240
chr2	145116200	0.917355
chr2	145185400	0.933884
chr2	145201500	0.884298
chr2	145209800	0.710744
chr2	145211900	0.933884
chr2	145214200	0.892562
chr2	145217900	0.983471
chr2	145228700	0.669421
chr2	145271400	0.727273
chr2	145340800	0.983471
chr2	145354200	0.975207
chr2	145560000	0.702479
chr2	145594200	0.983471
chr2	145696400	1.000000
chr2	145700500	0.991736
chr2	145702700	0.950413
chr2	145754200	0.685950
chr2	145765100	0.958678
chr2	145770300	0.991736
chr2	145774900	0.842975
chr2	145868400	0.719008
chr2	145896700	0.719008
chr2	146349500	0.727273
chr2	146351900	0.983471
chr2	146356200	0.958678
chr2	146502500	0.710744
chr2	146509600	0.958678
chr2	146518600	0.917355
chr2	146569700	0.793388
chr2	146742900	0.950413
chr2	147070900	0.909091
chr2	147337300	0.909091
chr2	147424500	0.752066
chr2	147579500	0.925620
chr2	147631500	0.942149
chr2	147744100	0.851240
chr2	147789400	0.752066
chr2	148587300	0.694215
chr2	148604500	0.669421
chr2	148671700	0.719008
chr2	149096500	0.958678
chr2	149149200	0.925620

chr2	149400600	0.966942
chr2	149418500	0.818182
chr2	149639200	0.991736
chr2	149921800	0.661157
chr2	150223000	0.966942
chr2	150454700	0.942149
chr2	150505200	0.950413
chr2	150518700	0.917355
chr2	150560100	0.884298
chr2	150589600	0.752066
chr2	150591700	0.925620
chr2	150598300	0.884298
chr2	150624600	0.892562
chr2	150627300	0.909091
chr2	150715700	0.975207
chr2	150846300	0.991736
chr2	150932800	0.884298
chr2	150939000	0.900826
chr2	150945600	0.909091
chr2	150954900	0.991736
chr2	150984400	0.983471
chr2	150990500	0.983471
chr2	150997500	0.851240
chr2	151001300	0.950413
chr2	151019200	0.925620
chr2	151023600	0.983471
chr2	151091500	0.677686
chr2	151104300	0.809917
chr2	151153700	0.876033
chr2	151260400	0.933884
chr2	151265900	0.842975
chr2	151268600	0.859504
chr2	151282200	0.818182
chr2	151320500	0.900826
chr2	151324600	0.917355
chr2	151328800	0.892562
chr2	151332600	0.991736
chr2	151335000	0.727273
chr2	151339000	0.669421
chr2	151348900	0.925620
chr2	151477200	0.702479
chr2	151481200	0.966942
chr2	152154700	0.942149
chr2	152191100	0.892562
chr2	152195700	0.776860
chr2	152207600	0.900826
chr2	152231200	0.801653
chr2	152471700	0.958678
chr2	152476300	0.727273
chr2	152494500	0.983471
chr2	152622300	0.826446
chr2	152681800	0.677686
chr2	152701300	0.909091
chr2	152735600	0.983471

chr2	152743000	0.702479
chr2	153057700	0.942149
chr2	153260000	0.917355
chr2	153262600	0.975207
chr2	153272300	0.818182
chr2	153278400	1.000000
chr2	153303200	0.892562
chr2	153326300	0.685950
chr2	153350700	0.685950
chr2	153354500	0.702479
chr2	153362600	0.892562
chr2	153380000	0.752066
chr2	153382500	0.991736
chr2	153399800	1.000000
chr2	153469100	0.735537
chr2	154977600	0.983471
chr2	155085600	0.851240
chr2	155820500	0.900826
chr2	156234900	0.900826
chr2	156520400	0.694215
chr2	156553500	0.785124
chr2	157291700	0.702479
chr2	157320900	0.842975
chr2	157323900	0.685950
chr2	157889100	0.685950
chr2	157913100	0.950413
chr2	158030500	0.966942
chr2	158039600	0.975207
chr2	158047100	0.909091
chr2	158326300	0.785124
chr2	158641200	0.933884
chr2	158670500	0.694215
chr2	158676800	0.809917
chr2	158688000	0.958678
chr2	158694900	0.925620
chr2	158712600	0.851240
chr2	158868100	0.785124
chr2	159048100	0.702479
chr2	159059300	0.925620
chr2	159071400	0.834711
chr2	159317200	0.677686
chr2	159396200	0.785124
chr2	159457100	1.000000
chr2	159476200	0.752066
chr2	159492800	0.867769
chr2	159524500	0.917355
chr2	159573100	0.983471
chr2	159575500	0.735537
chr2	159783300	0.809917
chr2	159788600	0.669421
chr2	159793800	0.933884
chr2	159810800	0.917355
chr2	159824200	0.727273
chr2	159837600	0.735537

chr2	159854300	0.719008
chr2	159856400	0.900826
chr2	159882500	0.793388
chr2	159887200	0.983471
chr2	159903300	0.958678
chr2	159910300	0.917355
chr2	159921700	0.958678
chr2	159926700	0.884298
chr2	159930200	0.983471
chr2	159934200	0.859504
chr2	159941000	0.933884
chr2	159943300	0.958678
chr2	159945800	0.768595
chr2	159949000	0.884298
chr2	159951400	0.942149
chr2	159954300	0.859504
chr2	159956900	0.884298
chr2	159967700	0.842975
chr2	159975700	0.851240
chr2	159982400	0.966942
chr2	159986900	0.727273
chr2	159990300	0.876033
chr2	159993300	0.917355
chr2	159997300	0.983471
chr2	160004200	0.892562
chr2	160007100	0.785124
chr2	160014400	0.991736
chr2	160017700	0.752066
chr2	160026000	0.950413
chr2	160042100	0.661157
chr2	160045400	0.859504
chr2	160082900	0.809917
chr2	160106500	0.991736
chr2	160219800	0.966942
chr2	160354200	0.975207
chr2	160372800	0.900826
chr2	160406600	0.834711
chr2	160462800	0.719008
chr2	160542000	0.669421
chr2	160567400	0.809917
chr2	160571900	0.950413
chr2	160580200	0.925620
chr2	160593200	0.942149
chr2	160601200	0.909091
chr2	160648700	0.776860
chr2	160652800	1.000000
chr2	160798900	0.933884
chr2	160804200	0.933884
chr2	160817600	1.000000
chr2	160839000	0.661157
chr2	160890800	0.867769
chr2	160898800	0.809917
chr2	160909100	0.818182
chr2	160912200	0.801653

chr2	160932400	0.818182
chr2	161013500	0.958678
chr2	161038000	0.834711
chr2	161061300	0.892562
chr2	161064600	0.702479
chr2	161067700	0.991736
chr2	161078000	0.917355
chr2	161081200	0.917355
chr2	161083400	0.768595
chr2	161086700	1.000000
chr2	161098800	0.942149
chr2	161126800	0.793388
chr2	161136000	0.933884
chr2	161138800	0.677686
chr2	161147000	0.793388
chr2	161151600	0.975207
chr2	161156600	0.925620
chr2	161165800	0.900826
chr2	161190800	0.743802
chr2	161195500	0.694215
chr2	161227800	0.809917
chr2	161230000	0.950413
chr2	161233900	0.685950
chr2	161236500	0.925620
chr2	161238800	0.925620
chr2	161244900	0.966942
chr2	161249700	0.719008
chr2	161254300	0.809917
chr2	161260400	0.909091
chr2	161265800	0.752066
chr2	161268400	0.884298
chr2	161273100	0.933884
chr2	161276500	0.983471
chr2	161284900	0.925620
chr2	161290000	1.000000
chr2	161311200	0.727273
chr2	161508700	0.867769
chr2	161523400	0.950413
chr2	161594100	0.958678
chr2	161609400	0.909091
chr2	161933200	0.958678
chr2	161935300	0.842975
chr2	161942100	0.793388
chr2	161947300	0.834711
chr2	161959900	0.760331
chr2	161980000	0.942149
chr2	162065100	0.842975
chr2	162256700	0.793388
chr2	162271300	0.867769
chr2	162275700	0.851240
chr2	162280500	0.876033
chr2	162359200	0.950413
chr2	162762500	0.966942
chr2	162770500	0.785124

chr2	162893400	0.702479
chr2	162947300	0.867769
chr2	162950100	0.884298
chr2	162970800	0.991736
chr2	163101300	0.975207
chr2	163118200	0.851240
chr2	164015600	0.876033
chr2	164045000	0.900826
chr2	164198100	0.958678
chr2	164207100	0.950413
chr2	164509900	0.719008
chr2	164513500	0.735537
chr2	164524100	0.768595
chr2	164539600	0.809917
chr2	164631900	0.768595
chr2	164635400	0.925620
chr2	165245400	0.768595
chr2	165353900	0.702479
chr2	165420200	0.975207
chr2	165455300	0.801653
chr2	165474200	0.925620
chr2	165481300	0.842975
chr2	165597000	0.719008
chr2	165602300	0.785124
chr2	165627200	0.991736
chr2	165640200	0.809917
chr2	165643300	0.760331
chr2	165651600	0.975207
chr2	165755500	0.727273
chr2	166233700	0.826446
chr2	166467400	0.727273
chr2	166478100	0.958678
chr2	166766600	0.743802
chr2	166914700	0.809917
chr2	166924200	0.876033
chr2	166945600	0.950413
chr2	166949700	0.834711
chr2	167091200	0.933884
chr2	167096300	0.942149
chr2	167245700	0.859504
chr2	167992200	0.826446
chr2	167997300	0.719008
chr2	168366600	0.983471
chr2	168971000	0.917355
chr2	169042400	0.710744
chr2	169046700	0.752066
chr2	169050100	0.743802
chr2	169067800	0.958678
chr2	169073200	0.809917
chr2	169077800	0.933884
chr2	169347200	0.925620
chr2	169394500	0.661157
chr2	169489500	0.900826
chr2	169543300	0.917355

chr2	169579400	0.842975
chr2	169614100	0.851240
chr2	169655500	0.677686
chr2	169676400	0.727273
chr2	169849300	0.876033
chr2	169853000	0.942149
chr2	169855400	0.867769
chr2	169858800	0.958678
chr2	169864600	0.685950
chr2	169868300	0.917355
chr2	169870400	0.785124
chr2	170040800	0.909091
chr2	170334700	0.809917
chr2	170364500	0.925620
chr2	170568600	0.867769
chr2	170573600	0.859504
chr2	170646500	0.677686
chr2	170672500	0.826446
chr2	170909300	0.809917
chr2	170930400	0.719008
chr2	171012200	0.983471
chr2	171078600	0.958678
chr2	171186800	0.685950
chr2	171360100	0.942149
chr2	171362200	0.975207
chr2	171382300	0.983471
chr2	171387400	0.909091
chr2	171411300	0.719008
chr2	171679700	0.760331
chr2	171700600	0.801653
chr2	172003700	0.933884
chr2	172011600	0.818182
chr2	172172800	0.661157
chr2	172203800	0.900826
chr2	172248100	0.851240
chr2	172260100	1.000000
chr2	172268700	0.925620
chr2	172335500	0.785124
chr2	172388400	0.909091
chr2	172569800	0.983471
chr2	172655100	0.983471
chr2	172948100	0.735537
chr2	172961900	0.702479
chr2	173074300	0.925620
chr2	173096900	0.975207
chr2	173105600	0.719008
chr2	173107900	0.677686
chr2	173111900	0.661157
chr2	173114000	0.942149
chr2	173116100	0.867769
chr2	173118300	0.933884
chr2	173147500	0.925620
chr2	173149900	0.801653
chr2	173153300	0.925620

chr2	173176900	0.942149
chr2	173273800	0.677686
chr2	173296200	0.719008
chr2	173303200	0.677686
chr2	173307400	0.834711
chr2	173327600	0.785124
chr2	173345300	0.842975
chr2	173418100	0.851240
chr2	173512500	0.867769
chr2	173537700	0.793388
chr2	173608400	0.752066
chr2	173648200	0.661157
chr2	173686700	0.933884
chr2	173701500	0.983471
chr2	173725100	0.842975
chr2	173793200	0.917355
chr2	173796900	0.958678
chr2	173827900	1.000000
chr2	173832200	0.776860
chr2	173834800	0.942149
chr2	173850900	0.884298
chr2	173914300	0.933884
chr2	174016800	0.727273
chr2	174235300	0.925620
chr2	174436500	0.719008
chr2	174439800	0.851240
chr2	174455200	0.950413
chr2	174657300	0.768595
chr2	174808100	0.768595
chr2	174878000	0.793388
chr2	174901600	0.909091
chr2	174904800	0.859504
chr2	174924200	0.958678
chr2	174983500	1.000000
chr2	174986800	0.842975
chr2	175191900	0.710744
chr2	175198600	0.669421
chr2	175202100	0.710744
chr2	175208800	0.776860
chr2	175458700	0.933884
chr2	175462600	0.950413
chr2	175628600	0.991736
chr2	175651000	0.859504
chr2	175693100	0.917355
chr2	175831300	0.909091
chr2	175837000	0.834711
chr2	175839100	0.826446
chr2	175863200	0.933884
chr2	175880500	0.942149
chr2	176238400	0.801653
chr2	176953200	0.727273
chr2	176962200	0.719008
chr2	176981500	0.776860
chr2	177005600	0.933884

chr2	177044000	0.793388
chr2	177057400	0.793388
chr2	177077100	0.694215
chr2	177099300	0.975207
chr2	177109900	0.694215
chr2	177147900	0.785124
chr2	177266600	0.785124
chr2	177279600	0.991736
chr2	177285800	0.917355
chr2	177311400	0.991736
chr2	177338000	0.719008
chr2	177380600	0.735537
chr2	177384200	0.958678
chr2	177430900	0.950413
chr2	177448800	0.991736
chr2	177476100	0.669421
chr2	177478800	0.917355
chr2	177518700	0.826446
chr2	177534400	0.677686
chr2	177574600	0.710744
chr2	178008400	0.702479
chr2	178019100	0.876033
chr2	178026800	0.685950
chr2	178029600	0.950413
chr2	178033300	0.900826
chr2	178037200	0.975207
chr2	178076500	0.661157
chr2	178104800	0.884298
chr2	178107100	0.859504
chr2	178110700	1.000000
chr2	178116000	0.917355
chr2	178119700	0.991736
chr2	178139000	0.752066
chr2	178193400	0.768595
chr2	178197300	0.677686
chr2	178201000	0.909091
chr2	178203500	0.909091
chr2	178259900	0.975207
chr2	178263500	0.975207
chr2	178270300	0.966942
chr2	178283500	0.702479
chr2	178310000	0.826446
chr2	178332600	0.826446
chr2	178339800	0.958678
chr2	178413400	0.710744
chr2	178415500	0.950413
chr2	178471600	0.785124
chr2	178488300	0.702479
chr2	178589400	0.966942
chr2	178611500	0.983471
chr2	178623800	0.809917
chr2	178939600	0.793388
chr2	179107300	0.785124
chr2	179152800	0.942149

chr2	179163800	0.942149
chr2	179166200	0.694215
chr2	179317100	0.760331
chr2	179685200	0.991736
chr2	179695000	0.966942
chr2	179729000	0.983471
chr2	179735900	0.727273
chr2	179747800	0.991736
chr2	179971700	1.000000
chr2	179984200	0.859504
chr2	180095700	0.892562
chr2	180104300	0.760331
chr2	180137400	0.991736
chr2	180319900	0.909091
chr2	180325700	0.933884
chr2	180332300	0.826446
chr2	180875800	0.942149
chr2	181276800	0.966942
chr2	181389200	0.809917
chr2	181844900	0.760331
chr2	181902200	0.677686
chr2	181935400	0.677686
chr2	181937600	0.975207
chr2	181972100	0.892562
chr2	181989200	0.892562
chr2	182521900	0.752066
chr2	182608300	0.842975
chr2	182644800	0.950413
chr2	182654100	0.710744
chr2	182656600	0.991736
chr2	182670500	0.983471
chr2	182676000	0.950413
chr2	182684500	0.719008
chr2	182755700	0.677686
chr2	182764000	0.685950
chr2	182768300	0.685950
chr2	182781700	0.892562
chr2	182819400	0.909091
chr2	182942900	0.669421
chr2	182998600	1.000000
chr2	183009600	0.900826
chr2	183148700	0.983471
chr2	183237800	0.884298
chr2	183269900	0.776860
chr2	183387600	0.942149
chr2	183410100	0.801653
chr2	183413400	0.702479
chr2	183482700	0.851240
chr2	183619500	0.702479
chr2	183812100	0.859504
chr2	183814900	0.743802
chr2	183991600	0.842975
chr2	184114100	0.710744
chr2	184180800	0.991736

chr2	185632100	0.958678
chr2	185634200	0.851240
chr2	185646100	0.966942
chr2	186652500	0.801653
chr2	186995000	0.966942
chr2	187305500	0.694215
chr2	187398000	0.958678
chr2	187408200	0.851240
chr2	187410500	0.760331
chr2	187427100	0.826446
chr2	187435300	0.669421
chr2	187440200	0.909091
chr2	187460000	0.661157
chr2	187470400	0.983471
chr2	187719200	0.735537
chr2	187727900	0.826446
chr2	187748700	0.925620
chr2	187751800	0.768595
chr2	187756400	0.685950
chr2	187857800	0.677686
chr2	188001800	0.991736
chr2	188071000	0.892562
chr2	188113400	0.685950
chr2	188128900	0.983471
chr2	188131100	0.900826
chr2	188165100	0.975207
chr2	188168100	0.760331
chr2	188175600	0.694215
chr2	188182200	0.950413
chr2	188185200	0.983471
chr2	188220400	0.752066
chr2	188225400	0.876033
chr2	188237300	0.710744
chr2	188245400	0.776860
chr2	188256700	0.685950
chr2	188259500	0.677686
chr2	188263400	0.834711
chr2	188267900	0.760331
chr2	188270400	0.958678
chr2	188273100	0.719008
chr2	188280800	0.876033
chr2	188287100	0.983471
chr2	188290000	0.859504
chr2	188292600	0.727273
chr2	188295200	0.983471
chr2	188298400	0.876033
chr2	188301700	0.975207
chr2	188304900	0.950413
chr2	188313900	0.809917
chr2	188365400	0.950413
chr2	188369300	0.933884
chr2	188392000	0.983471
chr2	188406800	0.991736
chr2	188410100	0.909091

chr2	188413900	0.801653
chr2	188434300	0.966942
chr2	188440200	0.925620
chr2	188446000	0.842975
chr2	188451100	0.760331
chr2	188509900	0.884298
chr2	188573900	0.694215
chr2	188741100	0.867769
chr2	188956100	0.950413
chr2	189160600	0.685950
chr2	189168500	0.942149
chr2	189172600	0.966942
chr2	189179600	0.917355
chr2	189183600	0.950413
chr2	189185700	0.966942
chr2	189188000	0.900826
chr2	189244800	0.719008
chr2	189246900	0.950413
chr2	189653500	0.834711
chr2	189714000	0.661157
chr2	189843900	0.727273
chr2	189866700	0.743802
chr2	189962000	0.917355
chr2	190022900	0.661157
chr2	190069400	0.776860
chr2	190075400	1.000000
chr2	190087300	0.793388
chr2	190126300	0.818182
chr2	190141400	0.785124
chr2	190151400	0.818182
chr2	190193800	0.900826
chr2	190212700	0.768595
chr2	190235300	0.933884
chr2	190273100	0.975207
chr2	190434700	0.677686
chr2	190440500	0.917355
chr2	190478100	0.867769
chr2	190817100	0.809917
chr2	191199300	0.975207
chr2	191278600	0.809917
chr2	191334700	0.900826
chr2	191472700	0.719008
chr2	191492600	0.719008
chr2	191501800	0.727273
chr2	191543600	0.950413
chr2	191546900	0.884298
chr2	191549300	0.884298
chr2	191583800	0.975207
chr2	191588300	0.942149
chr2	191624500	0.917355
chr2	191654400	0.809917
chr2	191657300	0.942149
chr2	191712600	0.661157
chr2	191752400	0.834711

chr2	191813200	0.975207
chr2	191835800	0.842975
chr2	191839000	0.884298
chr2	191842900	0.884298
chr2	191846600	0.719008
chr2	191849800	0.851240
chr2	191888100	0.702479
chr2	191893200	0.677686
chr2	191896200	0.983471
chr2	191903500	0.876033
chr2	192112900	0.975207
chr2	192130600	0.975207
chr2	192187000	0.884298
chr2	192242200	0.983471
chr2	192249400	0.909091
chr2	192251600	1.000000
chr2	192331100	0.842975
chr2	192436500	0.768595
chr2	192477200	0.859504
chr2	192490300	0.876033
chr2	192503100	1.000000
chr2	192523300	0.884298
chr2	192545400	0.793388
chr2	192548300	0.710744
chr2	192554400	0.884298
chr2	192696600	0.760331
chr2	192702100	0.925620
chr2	192706200	0.727273
chr2	192713300	0.983471
chr2	192718900	0.942149
chr2	192721000	0.966942
chr2	192723100	0.785124
chr2	192729700	0.710744
chr2	192732000	0.991736
chr2	192743800	0.991736
chr2	192764400	0.991736
chr2	192772600	0.867769
chr2	192782800	0.785124
chr2	192792100	0.933884
chr2	192795200	0.966942
chr2	192815200	0.842975
chr2	192826600	0.884298
chr2	192840900	0.876033
chr2	192843500	0.727273
chr2	192848700	0.859504
chr2	193026900	0.776860
chr2	193132200	0.942149
chr2	194238400	0.834711
chr2	194689200	0.719008
chr2	194702700	0.966942
chr2	194712200	0.768595
chr2	195562900	0.727273
chr2	195565600	0.809917
chr2	196229000	0.900826

chr2	196478200	0.867769
chr2	196602700	0.735537
chr2	197025300	0.909091
chr2	197032700	0.900826
chr2	197066800	0.892562
chr2	197097500	0.884298
chr2	197099700	0.801653
chr2	197103900	0.884298
chr2	197106100	0.933884
chr2	197124900	0.983471
chr2	197128400	0.892562
chr2	197131700	0.892562
chr2	197134400	0.983471
chr2	197141100	0.818182
chr2	197144000	0.884298
chr2	197149800	1.000000
chr2	197159900	0.884298
chr2	197190400	0.925620
chr2	197195700	0.966942
chr2	197197900	0.818182
chr2	197228100	0.677686
chr2	197230500	0.991736
chr2	197238200	0.991736
chr2	197265200	0.950413
chr2	197268800	0.991736
chr2	197285200	0.917355
chr2	197293300	0.876033
chr2	197296400	0.685950
chr2	197299300	0.983471
chr2	197367200	0.735537
chr2	197466400	0.966942
chr2	197471700	0.958678
chr2	197692500	0.669421
chr2	197784000	0.900826
chr2	197831700	0.818182
chr2	197852900	0.991736
chr2	197893400	0.776860
chr2	197897000	0.834711
chr2	197944800	0.768595
chr2	198002300	0.983471
chr2	198024300	0.983471
chr2	198026700	0.694215
chr2	198029800	0.694215
chr2	198045400	0.942149
chr2	198060800	0.933884
chr2	198308700	0.677686
chr2	198361100	0.694215
chr2	198367300	0.859504
chr2	198379900	0.669421
chr2	198421200	0.933884
chr2	198692400	0.983471
chr2	198754200	0.842975
chr2	198794300	0.950413
chr2	198837100	0.710744

chr2	198930400	0.685950
chr2	198982700	0.669421
chr2	200168800	0.958678
chr2	200247300	0.727273
chr2	200297000	0.785124
chr2	200328500	0.876033
chr2	200778800	0.818182
chr2	200844700	0.851240
chr2	200868500	0.768595
chr2	200890700	0.677686
chr2	201166500	0.685950
chr2	201185200	0.801653
chr2	201205600	0.661157
chr2	201232200	0.735537
chr2	201241500	0.991736
chr2	201245600	0.983471
chr2	201248600	0.818182
chr2	201262700	0.859504
chr2	201273400	0.983471
chr2	201474500	0.710744
chr2	201578300	0.917355
chr2	201619800	0.793388
chr2	201622300	0.661157
chr2	201626500	0.991736
chr2	201628700	0.966942
chr2	201630800	0.876033
chr2	201643200	0.876033
chr2	201647200	0.669421
chr2	201651300	0.958678
chr2	201654400	0.892562
chr2	201675000	0.826446
chr2	201752700	0.661157
chr2	201810000	0.851240
chr2	201917600	0.776860
chr2	201990000	0.958678
chr2	201993400	0.785124
chr2	201999800	0.942149
chr2	202013400	0.892562
chr2	202017100	0.909091
chr2	202022900	0.826446
chr2	202050200	0.685950
chr2	202056500	0.917355
chr2	202077000	0.677686
chr2	202085000	0.975207
chr2	202125200	0.818182
chr2	202279400	0.942149
chr2	202284600	0.958678
chr2	202297700	0.991736
chr2	202331400	0.809917
chr2	202487100	0.793388
chr2	202517200	0.859504
chr2	202520500	0.719008
chr2	202560300	0.743802
chr2	202563400	0.892562

chr2	202565500	0.925620
chr2	202569400	0.867769
chr2	202572600	0.942149
chr2	202598400	0.983471
chr2	202600800	0.884298
chr2	202723900	0.710744
chr2	202726000	0.884298
chr2	202731300	0.958678
chr2	203051000	0.710744
chr2	203114600	0.768595
chr2	203137900	0.991736
chr2	203143500	0.983471
chr2	203159800	0.975207
chr2	203178800	0.760331
chr2	203182600	1.000000
chr2	203200900	0.900826
chr2	203238700	0.925620
chr2	203246500	0.826446
chr2	203273600	0.983471
chr2	203276700	0.719008
chr2	203283300	0.818182
chr2	203294100	0.966942
chr2	203359300	1.000000
chr2	203394600	0.859504
chr2	203673000	0.760331
chr2	203971600	0.933884
chr2	204145800	0.983471
chr2	204205900	0.925620
chr2	204265700	0.851240
chr2	204268800	0.958678
chr2	204306900	0.818182
chr2	204311100	0.983471
chr2	204363200	0.991736
chr2	204366200	0.884298
chr2	204401000	0.677686
chr2	204547600	0.851240
chr2	204551300	0.834711
chr2	204557500	0.909091
chr2	204624500	0.760331
chr2	204647900	0.694215
chr2	204651900	0.900826
chr2	204654800	0.867769
chr2	204673900	1.000000
chr2	204676900	0.950413
chr2	204774500	0.727273
chr2	204891500	0.826446
chr2	204899100	0.826446
chr2	204973800	0.851240
chr2	204976300	0.942149
chr2	204979200	0.983471
chr2	204981600	0.719008
chr2	205187100	0.991736
chr2	205317400	0.925620
chr2	205319500	0.842975

chr2	205344500	0.801653
chr2	205403900	0.942149
chr2	205478000	0.867769
chr2	205510200	0.900826
chr2	205530100	0.793388
chr2	205533500	0.983471
chr2	205536400	0.917355
chr2	205539400	0.975207
chr2	205557100	0.818182
chr2	205660200	0.942149
chr2	205708100	0.876033
chr2	205736000	0.793388
chr2	205890300	0.950413
chr2	206005200	0.768595
chr2	206063300	1.000000
chr2	206110600	0.677686
chr2	206135800	0.702479
chr2	206232200	0.677686
chr2	206270300	0.983471
chr2	206289700	0.851240
chr2	206292300	0.677686
chr2	206301000	0.933884
chr2	206546300	0.661157
chr2	206553500	0.983471
chr2	206564500	0.727273
chr2	206566600	0.876033
chr2	206569700	0.933884
chr2	206571900	0.933884
chr2	206574200	0.876033
chr2	206577300	0.942149
chr2	206599600	0.809917
chr2	206603100	0.892562
chr2	206622900	1.000000
chr2	206628800	0.933884
chr2	206634500	0.983471
chr2	206640100	0.677686
chr2	206665800	0.793388
chr2	206790800	0.834711
chr2	206795300	0.975207
chr2	206829400	0.818182
chr2	207161300	0.917355
chr2	207166100	0.950413
chr2	207287700	0.876033
chr2	207449700	0.743802
chr2	207662900	0.842975
chr2	207912200	0.669421
chr2	207929500	0.851240
chr2	207932400	0.785124
chr2	207939400	0.818182
chr2	207947900	0.925620
chr2	207981500	0.892562
chr2	207987000	0.867769
chr2	207996600	0.991736
chr2	207999200	0.900826

chr2	208001500	0.925620
chr2	208008200	0.991736
chr2	208015200	0.776860
chr2	208019300	0.876033
chr2	208022000	0.933884
chr2	208026200	0.809917
chr2	208041500	0.694215
chr2	208091500	0.851240
chr2	208104400	0.743802
chr2	208119100	0.719008
chr2	208123400	0.785124
chr2	208148600	0.669421
chr2	208176500	0.785124
chr2	208195800	0.900826
chr2	208201700	0.975207
chr2	208250900	0.702479
chr2	208259900	0.983471
chr2	208337800	0.942149
chr2	208415100	0.983471
chr2	208426500	0.950413
chr2	208482200	0.958678
chr2	208541900	0.925620
chr2	208548800	0.942149
chr2	208575400	0.826446
chr2	208657200	0.925620
chr2	208675800	0.818182
chr2	208693800	0.900826
chr2	208712700	0.983471
chr2	208762900	0.801653
chr2	208879000	0.942149
chr2	209272000	0.735537
chr2	210234200	0.884298
chr2	210248400	0.752066
chr2	210338400	0.851240
chr2	210359600	0.966942
chr2	210362200	0.661157
chr2	210372700	0.743802
chr2	210389800	0.851240
chr2	210391900	0.809917
chr2	210503400	0.752066
chr2	210507600	0.785124
chr2	210869700	0.677686
chr2	211063500	0.719008
chr2	211856300	0.834711
chr2	211862700	0.785124
chr2	211997800	0.933884
chr2	212251900	0.661157
chr2	212273200	0.917355
chr2	212285800	0.719008
chr2	212296000	0.702479
chr2	212602500	0.702479
chr2	212733300	0.983471
chr2	212847300	0.859504
chr2	212893000	0.776860

chr2	212897600	0.892562
chr2	212900500	0.909091
chr2	212917400	0.892562
chr2	212975300	0.801653
chr2	212989700	0.669421
chr2	213038500	0.909091
chr2	213708800	0.942149
chr2	213777600	0.966942
chr2	214103900	0.801653
chr2	214860400	0.966942
chr2	214884200	0.876033
chr2	215595500	0.809917
chr2	216178600	0.694215
chr2	216263400	0.768595
chr2	216275500	0.925620
chr2	216282700	0.834711
chr2	216286400	0.917355
chr2	216296900	0.867769
chr2	216303400	0.760331
chr2	216325200	0.884298
chr2	216330300	0.867769
chr2	216370100	0.925620
chr2	216393500	0.966942
chr2	216396300	0.925620
chr2	216401600	0.685950
chr2	216405600	0.859504
chr2	216467400	0.983471
chr2	216484500	0.917355
chr2	216492000	0.975207
chr2	216523200	0.917355
chr2	216529700	0.925620
chr2	216558700	1.000000
chr2	216565900	0.942149
chr2	216574900	0.925620
chr2	216578800	0.826446
chr2	216582500	0.975207
chr2	216584600	0.818182
chr2	216594000	0.685950
chr2	216624200	0.983471
chr2	216637400	0.933884
chr2	216659000	0.933884
chr2	216692200	0.809917
chr2	216694900	0.991736
chr2	216698000	0.892562
chr2	216701200	0.958678
chr2	216706300	0.925620
chr2	216709500	0.991736
chr2	216716500	0.917355
chr2	216771500	0.694215
chr2	216871500	0.809917
chr2	216922100	0.719008
chr2	216980000	0.694215
chr2	217014800	0.925620
chr2	217082200	0.958678

chr2	217087500	0.950413
chr2	217151900	0.694215
chr2	217163900	0.793388
chr2	217172400	0.735537
chr2	217181000	0.900826
chr2	217183800	0.818182
chr2	217212000	0.909091
chr2	217233800	0.900826
chr2	217239000	0.743802
chr2	217343300	0.950413
chr2	217351700	0.685950
chr2	217355000	0.760331
chr2	217357700	0.933884
chr2	217361100	0.909091
chr2	217366600	0.694215
chr2	217400700	0.917355
chr2	217404900	0.826446
chr2	217420200	0.669421
chr2	217501700	0.793388
chr2	217509500	0.719008
chr2	217529900	0.752066
chr2	217559300	0.851240
chr2	217920000	0.669421
chr2	217970800	0.809917
chr2	217985500	0.694215
chr2	218026300	0.735537
chr2	218088500	0.966942
chr2	218126700	0.743802
chr2	218142700	0.991736
chr2	218144800	0.727273
chr2	218681400	0.776860
chr2	218685100	0.900826
chr2	218710700	0.677686
chr2	218716200	0.760331
chr2	218753100	0.925620
chr2	218768300	0.966942
chr2	218776900	0.801653
chr2	218785000	0.892562
chr2	218793400	0.909091
chr2	218798000	0.809917
chr2	218800200	0.900826
chr2	218802600	0.950413
chr2	218809000	0.743802
chr2	218829500	0.669421
chr2	218848700	0.826446
chr2	218858000	0.925620
chr2	218860500	0.884298
chr2	218872200	0.909091
chr2	218875400	0.694215
chr2	218880200	0.752066
chr2	218899900	0.710744
chr2	219085100	0.760331
chr2	219088200	0.966942
chr2	219094200	1.000000

chr2	219124400	0.818182
chr2	219147700	0.876033
chr2	219150100	0.942149
chr2	219153400	0.867769
chr2	219158700	0.950413
chr2	219162900	0.727273
chr2	219181400	0.975207
chr2	219186700	0.892562
chr2	219232800	0.661157
chr2	219252500	0.801653
chr2	219263500	0.966942
chr2	219271400	1.000000
chr2	219366800	0.842975
chr2	219436400	0.677686
chr2	219472600	0.710744
chr2	219488100	0.768595
chr2	219496800	0.991736
chr2	219510200	0.925620
chr2	219544000	0.677686
chr2	219596700	0.942149
chr2	219697600	0.950413
chr2	219757900	0.851240
chr2	219774100	0.719008
chr2	219849400	0.958678
chr2	219858600	0.785124
chr2	220084800	0.834711
chr2	220114600	0.834711
chr2	220159800	0.661157
chr2	221954100	0.669421
chr2	221963100	0.694215
chr2	221969500	0.661157
chr2	222100900	0.950413
chr2	222285400	0.950413
chr2	222288400	0.991736
chr2	222299400	0.991736
chr2	222309000	1.000000
chr2	222313800	0.702479
chr2	222332900	0.991736
chr2	222343000	0.768595
chr2	222366400	0.917355
chr2	222368700	0.710744
chr2	222383400	0.991736
chr2	222392600	0.719008
chr2	222395700	0.809917
chr2	222506500	0.917355
chr2	222632400	0.991736
chr2	222645200	0.975207
chr2	222808100	0.950413
chr2	223163500	0.727273
chr2	223229700	0.909091
chr2	223518200	0.958678
chr2	223551400	0.702479
chr2	223560100	0.842975
chr2	223563400	0.975207

chr2	223568000	0.760331
chr2	223574500	0.975207
chr2	223608500	0.991736
chr2	223657000	0.727273
chr2	223707700	1.000000
chr2	223729000	0.785124
chr2	223754800	0.933884
chr2	223757200	0.851240
chr2	223784800	0.966942
chr2	223906500	0.694215
chr2	223979500	0.942149
chr2	223982600	0.743802
chr2	223987500	0.859504
chr2	224087800	0.685950
chr2	224125700	0.859504
chr2	224428700	0.702479
chr2	224478100	0.900826
chr2	224561800	0.743802
chr2	224564900	0.917355
chr2	224590400	0.942149
chr2	224600500	0.727273
chr2	224625600	0.892562
chr2	224630400	0.768595
chr2	224680400	0.950413
chr2	224685300	0.685950
chr2	224774800	0.719008
chr2	224784400	0.975207
chr2	224789300	0.925620
chr2	224802300	0.933884
chr2	224807000	0.801653
chr2	225007500	0.785124
chr2	225011000	0.909091
chr2	225086700	0.991736
chr2	225268300	0.892562
chr2	225271100	0.917355
chr2	225273200	0.752066
chr2	225282400	1.000000
chr2	225299500	0.669421
chr2	225307300	0.892562
chr2	225314400	0.867769
chr2	225350400	0.950413
chr2	225668100	0.950413
chr2	225682200	0.950413
chr2	225684300	0.785124
chr2	225702600	0.991736
chr2	225747600	0.966942
chr2	225775600	0.834711
chr2	225780600	0.818182
chr2	225791100	0.884298
chr2	225793300	0.900826
chr2	225803900	1.000000
chr2	225807800	0.768595
chr2	225810400	0.942149
chr2	225813800	0.983471

chr2	225875900	0.933884
chr2	225965800	0.884298
chr2	226602200	0.966942
chr2	227343300	0.933884
chr2	228271500	0.809917
chr2	228280700	0.909091
chr2	228363700	0.661157
chr2	228684100	0.719008
chr2	228720800	0.661157
chr2	228727600	0.975207
chr2	228733400	0.710744
chr2	228736800	0.851240
chr2	230045700	0.975207
chr2	230310200	0.942149
chr2	230319900	0.983471
chr2	230646400	0.809917
chr2	230681100	0.818182
chr2	230713900	1.000000
chr2	230717800	0.983471
chr2	230738300	0.669421
chr2	230775800	0.661157
chr2	230846000	0.942149
chr2	230995700	0.958678
chr2	231035500	0.752066
chr2	231191300	0.685950
chr2	231265500	0.661157
chr2	231271000	0.950413
chr2	231274600	0.975207
chr2	231283900	0.876033
chr2	231306300	0.942149
chr2	231312900	0.909091
chr2	231580500	0.719008
chr2	231622600	0.884298
chr2	231631000	0.991736
chr2	231639600	0.925620
chr2	231641700	0.685950
chr2	231643800	0.966942
chr2	231658200	0.925620
chr2	231660800	0.950413
chr2	231852100	0.669421
chr2	231924300	0.752066
chr2	231933900	0.909091
chr2	231988100	0.909091
chr2	231997300	0.834711
chr2	232025600	0.900826
chr2	232027800	1.000000
chr2	232033500	0.942149
chr2	232039200	0.859504
chr2	232045400	0.942149
chr2	232047600	0.809917
chr2	232050200	0.752066
chr2	232054700	0.694215
chr2	232056800	0.900826
chr2	232165200	0.925620

chr2	232230400	0.793388
chr2	232321600	0.694215
chr2	232348200	0.685950
chr2	232395300	0.809917
chr2	232419800	0.785124
chr2	232469600	0.876033
chr2	232479300	1.000000
chr2	232505400	0.851240
chr2	232508000	0.884298
chr2	232527200	0.851240
chr2	232531500	0.818182
chr2	232536100	0.702479
chr2	232546000	0.942149
chr2	232577900	0.851240
chr2	232581100	0.842975
chr2	232903300	0.776860
chr2	232938000	0.933884
chr2	233035300	0.669421
chr2	233125100	0.966942
chr2	233166100	0.983471
chr2	233179800	0.669421
chr2	233182000	0.768595
chr2	233457800	0.702479
chr2	233700600	0.900826
chr2	233704000	0.884298
chr2	233766900	0.950413
chr2	233793000	0.661157
chr2	233916900	0.677686
chr2	233927900	0.694215
chr2	233941500	0.925620
chr2	233948200	0.727273
chr2	233951400	0.942149
chr2	234118500	0.991736
chr2	234296900	0.809917
chr2	234323400	0.669421
chr2	234327100	0.909091
chr2	234329200	0.809917
chr2	234338800	0.909091
chr2	234396800	0.818182
chr2	234466200	0.892562
chr2	234598600	0.909091
chr2	234760500	0.925620
chr2	234777000	0.694215
chr2	234883500	0.884298
chr2	234941500	0.933884
chr2	235000900	0.776860
chr2	235046200	0.950413
chr2	235055700	0.900826
chr2	235112300	0.958678
chr2	235152600	0.917355
chr2	235155100	1.000000
chr2	235158700	0.710744
chr2	235160900	0.975207
chr2	235167200	0.966942

chr2	235200900	0.991736
chr2	235331100	0.933884
chr2	235336500	1.000000
chr2	235344400	0.991736
chr2	235354500	0.909091
chr2	235358100	0.900826
chr2	235529500	0.917355
chr2	235578700	0.669421
chr2	235589000	1.000000
chr2	235595500	0.685950
chr2	235600000	0.991736
chr2	235603200	0.727273
chr2	235733000	0.876033
chr2	235749300	1.000000
chr2	235789200	0.983471
chr2	235859800	0.776860
chr2	235869600	0.983471
chr2	235879900	0.933884
chr2	235889100	0.892562
chr2	235892500	0.876033
chr2	235895800	0.958678
chr2	235898600	0.958678
chr2	235902800	0.735537
chr2	235906500	0.694215
chr2	235909400	0.933884
chr2	235914500	0.942149
chr2	235929800	0.793388
chr2	235931900	0.859504
chr2	235934400	0.702479
chr2	235948100	0.702479
chr2	235958800	0.752066
chr2	235963600	0.818182
chr2	235997000	0.793388
chr2	236172500	0.892562
chr2	236177000	0.735537
chr2	236410300	0.925620
chr2	236427500	0.768595
chr2	236575200	0.768595
chr2	236736000	0.818182
chr2	236768300	0.958678
chr2	236770400	0.793388
chr2	236773400	0.950413
chr2	236807500	0.685950
chr2	236809900	0.842975
chr2	236910600	0.925620
chr2	237014500	0.727273
chr2	237017400	0.876033
chr2	237078300	0.760331
chr2	237084000	0.818182
chr2	237087000	0.942149
chr2	237089300	0.760331
chr2	237115400	0.876033
chr2	237146000	0.661157
chr2	237444100	0.900826

chr2	237476900	0.867769
chr2	237481200	0.719008
chr2	237483900	0.983471
chr2	237486000	0.942149
chr2	237493500	0.851240
chr2	237551400	0.702479
chr2	237570400	0.669421
chr2	237657500	0.884298
chr2	237685900	0.966942
chr2	237736900	0.991736
chr2	237742700	0.950413
chr2	237761300	0.900826
chr2	237780500	0.983471
chr2	237796100	0.867769
chr2	237799000	0.909091
chr2	237837600	0.735537
chr2	237856100	0.909091
chr2	237907200	1.000000
chr2	237921500	1.000000
chr2	237934400	0.677686
chr2	237944700	0.991736
chr2	237969400	0.867769
chr2	237982200	0.685950
chr2	237984300	0.859504
chr2	237996800	0.900826
chr2	238001400	1.000000
chr2	238004600	0.661157
chr2	238006800	1.000000
chr2	238037300	0.842975
chr2	238065700	0.735537
chr2	238166400	0.669421
chr2	238200300	0.876033
chr2	238341600	0.785124
chr2	238358600	0.818182
chr2	238374400	0.991736
chr2	238410600	0.785124
chr2	238418900	0.760331
chr2	238509400	0.685950
chr2	238512800	0.925620
chr2	238522400	0.818182
chr2	238524700	0.661157
chr2	238526800	0.876033
chr2	238581800	0.892562
chr2	238584200	0.743802
chr2	238586300	1.000000
chr2	238595400	0.900826
chr2	238598700	0.818182
chr2	238604700	0.867769
chr2	238607500	0.793388
chr2	238610300	0.851240
chr2	238614000	0.867769
chr2	238623900	0.892562
chr2	238635800	0.801653
chr2	238640400	0.842975

chr2	238642600	0.884298
chr2	238653400	0.727273
chr2	238851300	0.826446
chr2	239072700	0.892562
chr2	239080000	0.942149
chr2	239172600	0.818182
chr2	239286100	0.925620
chr2	239289600	0.900826
chr2	239348800	0.900826
chr2	239506000	0.851240
chr2	239553500	0.966942
chr2	239773200	0.983471
chr2	239878000	0.694215
chr2	239896400	0.909091
chr2	240139000	0.685950
chr2	240171700	0.785124
chr2	240189700	0.677686
chr2	240240900	0.793388
chr2	240250500	0.859504
chr2	240303000	0.975207
chr2	240752700	0.834711
chr2	240780200	0.900826
chr2	240821200	0.892562
chr2	241260000	0.966942
chr2	241327400	0.892562
chr2	241392500	0.719008
chr2	241497300	0.859504
chr2	241838300	0.768595
chr2	241853000	0.685950
chr2	241927000	0.834711
chr2	241931600	0.958678
chr2	241949600	0.975207
chr2	241976200	0.793388
chr2	242048600	0.661157
chr2	242097500	0.801653
chr2	242163600	0.851240
chr2	242175600	0.735537
chr2	242213100	0.685950
chr2	242252100	0.685950
chr2	242261500	0.933884
chr2	242294700	0.760331
chr2	242321500	0.661157
chr2	242449100	0.661157
chr2	242484900	0.809917
chr2	242487100	0.933884
chr2	242495200	0.710744
chr2	242501200	0.685950
chr2	242539600	0.677686
chr2	242549800	0.909091
chr2	242557700	0.785124
chr2	242606300	0.950413
chr2	242620900	0.958678
chr2	242670800	0.826446
chr2	242744000	0.694215

chr2	242786500	0.876033
chr2	242806500	0.661157
chr2	242949800	0.685950
chr3	898400	0.669421
chr3	2009700	0.826446
chr3	2011800	0.917355
chr3	2013900	0.900826
chr3	3215400	0.983471
chr3	4436300	0.966942
chr3	4441100	0.669421
chr3	4517400	0.975207
chr3	4537400	0.793388
chr3	4575500	0.826446
chr3	4698700	0.925620
chr3	4750400	0.958678
chr3	4778700	0.768595
chr3	4782300	0.743802
chr3	4786400	0.884298
chr3	4792600	0.958678
chr3	4856500	0.983471
chr3	4871100	0.661157
chr3	4873200	0.983471
chr3	4895200	0.892562
chr3	4898700	1.000000
chr3	4909200	0.958678
chr3	5029100	0.933884
chr3	5036800	0.677686
chr3	5054800	0.991736
chr3	5058700	0.909091
chr3	5064100	0.900826
chr3	5066400	0.925620
chr3	5068600	0.909091
chr3	5181300	0.776860
chr3	5223700	0.776860
chr3	5236600	0.925620
chr3	5239300	0.933884
chr3	6492000	0.909091
chr3	7161800	0.677686
chr3	7314100	0.950413
chr3	7330100	0.966942
chr3	7347100	0.859504
chr3	7349200	0.991736
chr3	7352200	0.958678
chr3	7388400	1.000000
chr3	7424700	0.909091
chr3	7487200	0.702479
chr3	7546600	0.925620
chr3	7548700	0.884298
chr3	7673200	0.983471
chr3	7725200	0.975207
chr3	7748400	0.851240
chr3	7752100	0.958678
chr3	7774800	0.826446
chr3	7777100	0.966942

chr3	7919000	0.983471
chr3	8152900	0.884298
chr3	8487300	0.933884
chr3	8525000	0.867769
chr3	8542100	0.702479
chr3	8548300	0.842975
chr3	8552100	0.966942
chr3	8571700	0.809917
chr3	8582400	0.735537
chr3	8887900	0.669421
chr3	8890800	0.785124
chr3	8911500	0.876033
chr3	8979300	0.950413
chr3	9359100	0.942149
chr3	9435800	0.685950
chr3	9443400	0.925620
chr3	9462200	0.966942
chr3	9465400	0.983471
chr3	9542200	0.752066
chr3	9719000	0.925620
chr3	9746200	0.809917
chr3	9788700	1.000000
chr3	9790800	0.760331
chr3	9796800	0.900826
chr3	9839400	0.677686
chr3	9851700	0.768595
chr3	9880300	0.867769
chr3	9936000	0.884298
chr3	9945100	0.884298
chr3	9958900	0.785124
chr3	10012500	0.776860
chr3	10019000	1.000000
chr3	10050900	0.768595
chr3	10057300	0.900826
chr3	10191900	0.685950
chr3	10215800	0.942149
chr3	10236100	0.834711
chr3	10240900	0.809917
chr3	10245400	0.809917
chr3	10248500	0.975207
chr3	10263100	0.958678
chr3	10270500	0.950413
chr3	10292800	0.719008
chr3	10310300	0.727273
chr3	10351600	1.000000
chr3	10355100	0.842975
chr3	10395400	0.966942
chr3	10975900	0.677686
chr3	11138700	0.942149
chr3	11178700	0.933884
chr3	11211400	0.925620
chr3	11222700	0.917355
chr3	11226000	0.966942
chr3	11228700	0.867769

chr3	11237200	0.694215
chr3	11244300	0.892562
chr3	11247100	0.884298
chr3	11250100	1.000000
chr3	11253100	0.826446
chr3	11255200	0.975207
chr3	11262200	0.859504
chr3	11265400	0.842975
chr3	11269500	0.917355
chr3	11280100	0.950413
chr3	11321100	0.884298
chr3	11328700	0.834711
chr3	11332700	0.950413
chr3	11348100	0.909091
chr3	11370100	0.983471
chr3	11373000	0.975207
chr3	11408900	0.958678
chr3	11421500	0.884298
chr3	11427800	0.983471
chr3	11434100	0.842975
chr3	11495100	0.851240
chr3	11518100	0.793388
chr3	11521000	0.818182
chr3	11536600	0.933884
chr3	11540800	0.892562
chr3	11551300	1.000000
chr3	11556100	0.958678
chr3	11565400	0.809917
chr3	11590500	0.917355
chr3	11598700	0.809917
chr3	11602600	0.966942
chr3	11613100	0.884298
chr3	11625000	0.983471
chr3	11632800	0.809917
chr3	11635100	1.000000
chr3	11642000	0.859504
chr3	11645500	0.892562
chr3	11666200	0.834711
chr3	11675600	0.933884
chr3	11678700	0.685950
chr3	11703400	0.876033
chr3	11713400	0.677686
chr3	11759100	0.727273
chr3	11806800	0.900826
chr3	11812400	0.809917
chr3	11862600	0.661157
chr3	12045900	0.661157
chr3	12220200	1.000000
chr3	12235400	0.991736
chr3	12361100	0.685950
chr3	12370600	0.752066
chr3	12447900	0.900826
chr3	12504100	0.760331
chr3	12518200	0.826446

chr3	12550100	0.909091
chr3	12626800	0.702479
chr3	12641800	0.785124
chr3	12800200	0.685950
chr3	12964800	0.851240
chr3	12990900	0.661157
chr3	13001900	0.801653
chr3	13005900	0.768595
chr3	13050900	0.752066
chr3	14184100	0.900826
chr3	14187800	0.851240
chr3	14196100	0.719008
chr3	14212500	0.950413
chr3	14365300	0.719008
chr3	14478100	0.917355
chr3	14481200	0.826446
chr3	14483600	0.809917
chr3	14493200	0.685950
chr3	14495300	0.694215
chr3	14503100	0.661157
chr3	14514600	0.917355
chr3	14522000	0.909091
chr3	14524500	0.826446
chr3	14676700	0.975207
chr3	14845400	0.942149
chr3	14848600	0.818182
chr3	14851400	0.793388
chr3	14855500	0.694215
chr3	14865400	0.933884
chr3	14871300	0.917355
chr3	14904300	0.776860
chr3	14918600	0.752066
chr3	14932100	0.669421
chr3	14992200	1.000000
chr3	15018200	0.793388
chr3	15032400	0.661157
chr3	15084000	0.809917
chr3	15086600	0.900826
chr3	15311900	0.727273
chr3	15333300	0.876033
chr3	15335600	0.991736
chr3	15338300	0.983471
chr3	15340800	0.735537
chr3	15342900	0.892562
chr3	15347500	0.752066
chr3	15351900	0.909091
chr3	15362100	0.966942
chr3	15366600	0.785124
chr3	15368700	0.867769
chr3	15370800	0.958678
chr3	15398400	0.933884
chr3	15408600	0.867769
chr3	15410900	0.900826
chr3	15462800	0.876033

chr3	15482800	0.950413
chr3	15649600	0.950413
chr3	15672300	0.867769
chr3	15675600	0.966942
chr3	15678200	0.950413
chr3	15680600	0.925620
chr3	15686400	0.793388
chr3	15690800	0.834711
chr3	15727700	0.735537
chr3	15810900	0.983471
chr3	15825400	0.975207
chr3	15840600	0.702479
chr3	15847600	0.966942
chr3	16000900	0.702479
chr3	16006500	0.933884
chr3	16093000	0.983471
chr3	16097400	1.000000
chr3	16100700	0.785124
chr3	16103300	0.867769
chr3	16106700	0.842975
chr3	16109400	0.900826
chr3	16111800	0.917355
chr3	16116300	0.917355
chr3	16127400	0.942149
chr3	16182400	0.983471
chr3	16190700	0.851240
chr3	16216300	0.801653
chr3	16219500	0.801653
chr3	16319400	0.925620
chr3	16322400	0.966942
chr3	16510200	1.000000
chr3	16515700	0.661157
chr3	16518800	0.892562
chr3	16523100	0.975207
chr3	16526700	0.983471
chr3	16536900	0.933884
chr3	16592400	0.677686
chr3	16881300	1.000000
chr3	16891700	0.983471
chr3	16906600	1.000000
chr3	16949800	1.000000
chr3	16958500	0.867769
chr3	17081400	1.000000
chr3	17627800	0.694215
chr3	17677400	0.892562
chr3	17785200	0.677686
chr3	19930900	0.933884
chr3	20065600	0.892562
chr3	20097000	0.925620
chr3	20099400	0.727273
chr3	20298100	0.975207
chr3	20504500	0.818182
chr3	20521500	0.900826
chr3	20523700	0.743802

chr3	20532900	0.785124
chr3	20690200	0.909091
chr3	21315500	0.975207
chr3	21714000	0.966942
chr3	22326300	0.909091
chr3	22965400	0.942149
chr3	23049200	0.851240
chr3	23141600	0.991736
chr3	23147100	0.694215
chr3	23201800	0.909091
chr3	23305800	0.727273
chr3	23366700	0.925620
chr3	23383200	0.710744
chr3	23481100	0.719008
chr3	23532300	0.942149
chr3	23559600	0.685950
chr3	23654100	0.925620
chr3	23671300	0.677686
chr3	23710200	0.801653
chr3	23713600	0.801653
chr3	23727800	0.702479
chr3	23754500	0.867769
chr3	23777400	1.000000
chr3	23782400	0.942149
chr3	23788600	0.719008
chr3	23793200	0.727273
chr3	23857700	0.867769
chr3	23904800	0.925620
chr3	23908200	0.991736
chr3	23916800	0.958678
chr3	23922700	0.809917
chr3	23989800	0.851240
chr3	24001000	0.950413
chr3	24033800	0.900826
chr3	24263200	0.719008
chr3	24284000	0.991736
chr3	24406900	0.809917
chr3	24630700	0.991736
chr3	24640600	0.834711
chr3	24871400	0.876033
chr3	24879700	0.752066
chr3	25193300	0.975207
chr3	25198400	0.867769
chr3	25475700	0.975207
chr3	25495800	0.983471
chr3	25497900	0.991736
chr3	25500000	0.975207
chr3	25502800	0.917355
chr3	25513200	0.685950
chr3	25521100	0.735537
chr3	25525400	0.842975
chr3	25534600	0.975207
chr3	25550000	0.876033
chr3	25552100	0.950413

chr3	25623500	0.859504
chr3	25634900	0.760331
chr3	25896400	0.966942
chr3	27009700	0.727273
chr3	27030200	0.892562
chr3	27189300	0.983471
chr3	27353400	0.933884
chr3	27368500	0.834711
chr3	27401700	0.983471
chr3	27473300	0.768595
chr3	27522600	0.677686
chr3	27549300	0.677686
chr3	27566000	0.859504
chr3	27574700	0.884298
chr3	27577400	0.975207
chr3	27586500	0.892562
chr3	27601600	0.991736
chr3	27632000	0.909091
chr3	27634200	0.776860
chr3	27638400	0.950413
chr3	27648900	0.933884
chr3	27665000	0.933884
chr3	27670900	0.991736
chr3	27699200	0.867769
chr3	27723000	0.719008
chr3	27756700	0.793388
chr3	27765500	0.702479
chr3	28760300	0.677686
chr3	28787600	0.909091
chr3	28958600	0.818182
chr3	29031200	0.826446
chr3	29107200	0.950413
chr3	29133600	0.900826
chr3	29169700	0.966942
chr3	29213200	0.950413
chr3	29228600	0.851240
chr3	29244100	0.876033
chr3	29281700	0.909091
chr3	29365700	0.925620
chr3	29373700	0.950413
chr3	29377400	0.966942
chr3	29423000	0.809917
chr3	29554300	0.826446
chr3	29557200	0.991736
chr3	29567100	0.975207
chr3	29577900	0.900826
chr3	29580300	0.851240
chr3	29644000	0.958678
chr3	29725200	0.909091
chr3	29728700	0.933884
chr3	29735200	0.826446
chr3	29737500	0.950413
chr3	29787900	0.909091
chr3	29798500	0.677686

chr3	29802400	0.809917
chr3	29965100	0.851240
chr3	30018900	0.851240
chr3	30086400	0.743802
chr3	30198800	1.000000
chr3	30204100	0.694215
chr3	30269600	0.851240
chr3	30324100	0.966942
chr3	30327200	0.710744
chr3	30329300	0.925620
chr3	30331800	0.991736
chr3	30335400	0.851240
chr3	30383900	0.933884
chr3	30386900	0.991736
chr3	30389800	0.975207
chr3	30391900	0.942149
chr3	30412500	0.958678
chr3	30434200	0.760331
chr3	30553300	0.876033
chr3	30558300	0.710744
chr3	30560400	0.900826
chr3	30562900	0.925620
chr3	30569700	1.000000
chr3	30580500	0.933884
chr3	30586100	0.966942
chr3	30588200	0.743802
chr3	30652100	0.719008
chr3	30654400	0.966942
chr3	30658300	0.669421
chr3	30663700	0.991736
chr3	30668000	0.884298
chr3	30670100	0.710744
chr3	30672300	0.933884
chr3	30677400	0.768595
chr3	30683000	0.851240
chr3	30685800	0.801653
chr3	30696800	0.892562
chr3	30700900	0.983471
chr3	30703300	0.900826
chr3	30706300	0.975207
chr3	30708900	0.900826
chr3	30714700	0.834711
chr3	30718700	0.685950
chr3	30721100	0.933884
chr3	30725000	0.925620
chr3	30728500	0.975207
chr3	30731500	0.966942
chr3	30734000	0.892562
chr3	30747200	0.876033
chr3	30750400	0.793388
chr3	30754800	0.669421
chr3	30762900	0.991736
chr3	31045900	0.958678
chr3	31051900	0.933884

chr3	31056000	0.900826
chr3	31083300	0.685950
chr3	31118900	0.942149
chr3	31135100	0.942149
chr3	31208000	1.000000
chr3	31307500	0.966942
chr3	31316700	0.958678
chr3	31319600	0.983471
chr3	31330500	0.785124
chr3	31425900	0.710744
chr3	31578200	0.702479
chr3	31617000	0.694215
chr3	31712000	0.826446
chr3	31741500	0.677686
chr3	31746200	0.958678
chr3	32005100	0.983471
chr3	32010100	0.884298
chr3	32087300	0.669421
chr3	32182600	0.925620
chr3	32185900	0.661157
chr3	32219400	0.834711
chr3	32242700	0.859504
chr3	32268700	0.900826
chr3	32287600	0.818182
chr3	32318200	0.727273
chr3	32327700	0.735537
chr3	32338400	0.727273
chr3	32355600	0.694215
chr3	32378200	0.694215
chr3	32444400	0.925620
chr3	32456400	0.950413
chr3	32460200	0.727273
chr3	32464400	0.950413
chr3	32485300	0.958678
chr3	32499500	0.867769
chr3	32504100	0.702479
chr3	32509600	0.685950
chr3	32511900	1.000000
chr3	32514800	0.909091
chr3	32542100	0.760331
chr3	33034700	0.884298
chr3	33176000	0.719008
chr3	33228900	0.900826
chr3	33253500	0.884298
chr3	33354700	0.826446
chr3	33357700	1.000000
chr3	33360800	0.925620
chr3	33401900	0.942149
chr3	33430400	0.991736
chr3	33434700	0.975207
chr3	33585500	0.958678
chr3	33620300	0.958678
chr3	33701100	0.818182
chr3	33848600	0.983471

chr3	33897200	0.917355
chr3	33925400	0.661157
chr3	34225000	0.991736
chr3	34248600	0.958678
chr3	34250700	0.851240
chr3	34254300	0.975207
chr3	34260500	0.727273
chr3	34463600	0.760331
chr3	35425600	0.975207
chr3	35464700	0.950413
chr3	35565500	0.925620
chr3	35966100	0.735537
chr3	35970900	0.834711
chr3	35974500	0.983471
chr3	36235200	0.892562
chr3	36934800	0.834711
chr3	37011500	0.735537
chr3	37031200	0.892562
chr3	37103200	0.702479
chr3	37114900	0.776860
chr3	37138800	0.975207
chr3	37156800	0.884298
chr3	37173300	0.991736
chr3	37205300	0.851240
chr3	37215400	0.727273
chr3	37225000	0.876033
chr3	37321400	0.909091
chr3	37324500	0.677686
chr3	37755200	0.809917
chr3	37902200	0.760331
chr3	37934700	0.768595
chr3	37941500	0.851240
chr3	37948200	0.909091
chr3	37951400	0.809917
chr3	37978100	0.966942
chr3	37983700	0.685950
chr3	37987900	0.842975
chr3	37998900	0.892562
chr3	38003500	0.842975
chr3	38040700	0.975207
chr3	38080900	0.760331
chr3	38676000	0.892562
chr3	38763500	0.834711
chr3	38765800	0.677686
chr3	38772100	1.000000
chr3	38781500	0.785124
chr3	38815000	0.900826
chr3	38870900	0.752066
chr3	38876300	0.975207
chr3	39032500	0.710744
chr3	39167700	0.876033
chr3	39176900	0.694215
chr3	39183600	0.702479
chr3	39187600	0.743802

chr3	39208100	0.925620
chr3	39219300	0.876033
chr3	39221700	0.958678
chr3	39419200	0.842975
chr3	39470800	0.710744
chr3	39475600	0.876033
chr3	39542500	0.677686
chr3	39548200	0.983471
chr3	39573700	1.000000
chr3	39584000	0.785124
chr3	39635100	0.760331
chr3	39679600	0.793388
chr3	39681700	0.900826
chr3	39747300	0.685950
chr3	39789800	0.991736
chr3	39792400	0.719008
chr3	39794500	0.983471
chr3	39798800	0.867769
chr3	39812600	0.677686
chr3	39855900	0.727273
chr3	39912000	0.685950
chr3	39949800	0.876033
chr3	39953200	0.867769
chr3	39958800	0.809917
chr3	39976800	0.768595
chr3	40002500	0.933884
chr3	40258300	0.669421
chr3	40341900	0.685950
chr3	40546100	0.892562
chr3	40919700	0.983471
chr3	41012400	0.851240
chr3	41015300	0.933884
chr3	41018700	0.876033
chr3	41159400	0.925620
chr3	41255700	0.661157
chr3	42062800	1.000000
chr3	42067200	0.958678
chr3	42079000	0.793388
chr3	42084300	0.925620
chr3	42086900	0.991736
chr3	42094500	0.983471
chr3	42104200	0.975207
chr3	42108500	0.925620
chr3	42114200	0.958678
chr3	42139500	0.867769
chr3	42148800	0.909091
chr3	42159100	0.917355
chr3	42162300	0.966942
chr3	42165300	0.917355
chr3	42203900	0.760331
chr3	42220800	0.942149
chr3	42225000	0.950413
chr3	42227300	0.735537
chr3	42231100	0.677686

chr3	42493400	0.917355
chr3	42514200	0.776860
chr3	42516300	0.950413
chr3	42532000	0.876033
chr3	42561900	0.661157
chr3	42652600	0.983471
chr3	42697600	0.752066
chr3	43021400	0.677686
chr3	43204600	0.851240
chr3	43223000	0.743802
chr3	43228600	0.793388
chr3	43231600	0.983471
chr3	43235500	0.867769
chr3	43240800	0.900826
chr3	43290700	0.661157
chr3	43327100	0.702479
chr3	43334700	0.694215
chr3	43339100	0.818182
chr3	43356700	0.661157
chr3	43359400	0.925620
chr3	43429100	0.702479
chr3	43431500	0.975207
chr3	43433900	0.942149
chr3	43436800	0.842975
chr3	43441100	0.900826
chr3	43455800	0.958678
chr3	43460700	0.884298
chr3	43507000	0.842975
chr3	43519700	0.950413
chr3	43538300	0.892562
chr3	43541400	0.925620
chr3	43544600	0.925620
chr3	43626900	0.760331
chr3	43664700	0.760331
chr3	43674100	0.900826
chr3	43749800	0.859504
chr3	43784800	0.900826
chr3	43790000	0.942149
chr3	43795800	0.743802
chr3	43801200	0.900826
chr3	43808700	0.991736
chr3	43812000	0.842975
chr3	43911800	0.859504
chr3	43915300	0.900826
chr3	43935900	0.702479
chr3	44037200	0.702479
chr3	44277900	0.983471
chr3	44481600	0.933884
chr3	44925100	0.892562
chr3	44927600	0.933884
chr3	44974800	0.991736
chr3	44997000	0.809917
chr3	45044300	0.876033
chr3	45053900	0.958678

chr3	45106600	0.975207
chr3	45165700	0.884298
chr3	45187500	0.685950
chr3	45209400	0.958678
chr3	45477700	1.000000
chr3	45553800	0.917355
chr3	45580300	0.983471
chr3	45586800	0.892562
chr3	45595900	0.933884
chr3	45640000	0.669421
chr3	45673000	0.966942
chr3	45682000	0.685950
chr3	45686600	0.760331
chr3	45689800	0.983471
chr3	45697900	0.859504
chr3	45706900	0.677686
chr3	45710100	0.942149
chr3	45718500	0.669421
chr3	45735900	0.727273
chr3	45838000	0.760331
chr3	45859900	0.983471
chr3	46028000	0.867769
chr3	46030700	0.876033
chr3	46428200	0.661157
chr3	46447200	0.727273
chr3	46450400	0.801653
chr3	46454700	0.917355
chr3	46558900	0.975207
chr3	46562800	0.925620
chr3	46580800	0.900826
chr3	46584300	0.760331
chr3	46598200	0.743802
chr3	46600600	0.735537
chr3	46671600	0.752066
chr3	46737400	0.876033
chr3	46743000	0.752066
chr3	46748000	0.752066
chr3	46854200	0.694215
chr3	46887600	0.785124
chr3	46934000	0.818182
chr3	46940700	0.760331
chr3	46945300	0.710744
chr3	46955600	0.677686
chr3	46968200	0.752066
chr3	46971600	0.942149
chr3	46974700	0.933884
chr3	46978300	0.818182
chr3	46984600	0.826446
chr3	46992500	0.818182
chr3	47001000	0.727273
chr3	47023200	0.785124
chr3	47030200	0.983471
chr3	47051300	0.834711
chr3	47118500	0.685950

chr3	47308900	0.842975
chr3	47356900	0.917355
chr3	47404500	0.826446
chr3	47435800	0.768595
chr3	47919500	0.925620
chr3	47952800	0.983471
chr3	48017200	0.768595
chr3	48055700	0.669421
chr3	48058300	0.876033
chr3	48061000	0.685950
chr3	48081100	0.876033
chr3	48127100	0.694215
chr3	48172400	0.785124
chr3	48182200	0.909091
chr3	48193100	0.876033
chr3	48196900	0.727273
chr3	48289700	0.859504
chr3	48443900	0.818182
chr3	48509600	0.851240
chr3	48542800	0.685950
chr3	48545400	0.743802
chr3	48598300	0.669421
chr3	48633000	0.752066
chr3	48694200	0.834711
chr3	48699200	0.809917
chr3	48732600	0.826446
chr3	48858500	0.685950
chr3	48965000	0.768595
chr3	48967300	0.942149
chr3	49256100	0.867769
chr3	49314800	0.710744
chr3	49332100	0.785124
chr3	49352000	0.884298
chr3	49379100	0.876033
chr3	49382700	0.991736
chr3	49384800	0.851240
chr3	49389900	0.950413
chr3	49397300	0.685950
chr3	49454000	0.702479
chr3	49506600	0.735537
chr3	49538600	0.909091
chr3	49578100	0.776860
chr3	49724200	0.710744
chr3	49741600	0.685950
chr3	49762600	0.669421
chr3	49785900	0.851240
chr3	49822300	0.719008
chr3	49825300	0.661157
chr3	49828200	0.685950
chr3	49844100	0.867769
chr3	49878500	0.702479
chr3	49941500	0.900826
chr3	49943800	0.727273
chr3	50120300	0.983471

chr3	50175800	0.785124
chr3	50196300	0.727273
chr3	50198600	0.809917
chr3	50205200	0.694215
chr3	50207500	0.669421
chr3	50209700	0.785124
chr3	50232300	0.719008
chr3	50243000	0.669421
chr3	50278000	0.685950
chr3	50283500	0.685950
chr3	50286300	0.768595
chr3	50290300	1.000000
chr3	50293500	0.942149
chr3	50296400	0.983471
chr3	50298600	0.876033
chr3	50302200	0.685950
chr3	50330900	0.842975
chr3	50338400	0.776860
chr3	50355100	0.702479
chr3	50362400	0.760331
chr3	50371300	0.876033
chr3	50376100	0.685950
chr3	50378300	0.743802
chr3	50383500	0.793388
chr3	50553800	0.909091
chr3	50639200	0.917355
chr3	50712600	0.785124
chr3	51378200	0.983471
chr3	51433900	0.768595
chr3	51715700	0.851240
chr3	51741500	0.677686
chr3	51977300	0.826446
chr3	51990300	0.776860
chr3	52007500	0.743802
chr3	52018300	0.719008
chr3	52039900	0.826446
chr3	52047200	0.876033
chr3	52082400	0.793388
chr3	52087100	0.884298
chr3	52121500	0.760331
chr3	52124800	0.702479
chr3	52128200	0.867769
chr3	52156100	0.826446
chr3	52162600	0.851240
chr3	52250600	0.752066
chr3	52253900	0.892562
chr3	52271300	0.950413
chr3	52293200	0.917355
chr3	52305000	0.991736
chr3	52314500	0.933884
chr3	52334800	0.859504
chr3	52348200	0.909091
chr3	52351900	0.752066
chr3	52402900	0.752066

chr3	52462800	0.834711
chr3	52465000	0.966942
chr3	52478700	0.669421
chr3	52523500	0.677686
chr3	52527900	0.743802
chr3	52532400	0.950413
chr3	52559200	0.933884
chr3	52564800	0.942149
chr3	52569100	0.760331
chr3	52573400	0.760331
chr3	52699000	0.661157
chr3	52705100	0.983471
chr3	52797800	0.925620
chr3	52956100	0.694215
chr3	53201300	0.892562
chr3	53228900	0.727273
chr3	53269700	0.809917
chr3	53272100	0.685950
chr3	53277700	0.809917
chr3	53286600	0.859504
chr3	53304500	0.785124
chr3	53385100	0.991736
chr3	53389600	0.925620
chr3	53394300	0.776860
chr3	55635800	0.942149
chr3	55654800	0.776860
chr3	55920500	0.677686
chr3	56136200	0.876033
chr3	56166600	0.867769
chr3	56316800	0.958678
chr3	56325500	1.000000
chr3	56337900	0.719008
chr3	56358600	0.694215
chr3	56793000	0.975207
chr3	56818000	1.000000
chr3	56847700	0.752066
chr3	56896800	0.809917
chr3	56945800	0.702479
chr3	56952700	0.983471
chr3	56957300	0.876033
chr3	56959700	0.950413
chr3	56963800	0.991736
chr3	57009000	0.958678
chr3	57015300	0.900826
chr3	57042100	0.768595
chr3	57095400	0.983471
chr3	57103000	0.702479
chr3	57115500	0.834711
chr3	57185100	0.776860
chr3	57193800	0.743802
chr3	57204400	0.719008
chr3	57436900	0.950413
chr3	57450200	0.859504
chr3	57504600	0.983471

chr3	57530400	0.752066
chr3	57547000	0.685950
chr3	57585200	0.719008
chr3	57616300	0.966942
chr3	57738500	0.925620
chr3	57782300	0.710744
chr3	57789700	0.975207
chr3	57936200	0.661157
chr3	57938800	0.752066
chr3	57942500	0.768595
chr3	57945600	0.975207
chr3	57955900	0.991736
chr3	57959000	0.892562
chr3	57961200	0.793388
chr3	57970100	0.818182
chr3	57985300	0.677686
chr3	57999700	0.925620
chr3	58006700	0.900826
chr3	58012100	0.917355
chr3	58026600	0.950413
chr3	58030700	0.892562
chr3	58033700	0.859504
chr3	58035800	0.661157
chr3	58039400	0.983471
chr3	58042700	0.661157
chr3	58046400	0.975207
chr3	58049000	0.776860
chr3	58052300	0.842975
chr3	58058800	0.768595
chr3	58066200	0.933884
chr3	58070300	0.950413
chr3	58072500	0.942149
chr3	58079300	0.719008
chr3	58085600	0.876033
chr3	58101100	0.661157
chr3	58103500	0.760331
chr3	58107000	0.818182
chr3	58147200	0.685950
chr3	58150800	0.942149
chr3	58158400	0.694215
chr3	58161000	0.859504
chr3	58184700	0.991736
chr3	58187100	0.661157
chr3	58258600	0.983471
chr3	58311000	0.942149
chr3	58372200	0.809917
chr3	58374400	0.950413
chr3	58449400	0.702479
chr3	58514800	0.752066
chr3	58522500	0.685950
chr3	58547300	0.842975
chr3	58551100	0.876033
chr3	58561800	0.818182
chr3	58615500	0.719008

chr3	58633500	0.851240
chr3	58637200	0.933884
chr3	58652900	0.735537
chr3	58860800	1.000000
chr3	58975300	0.768595
chr3	58984500	0.892562
chr3	60351400	0.719008
chr3	61239300	0.859504
chr3	61550700	0.669421
chr3	61632900	0.925620
chr3	61834800	0.966942
chr3	61881200	0.867769
chr3	61910400	0.719008
chr3	61964400	0.719008
chr3	61966600	0.991736
chr3	61970900	0.859504
chr3	62006100	0.983471
chr3	62120100	0.983471
chr3	62130700	0.966942
chr3	62145800	0.975207
chr3	62156000	0.975207
chr3	62548100	0.900826
chr3	62561200	0.743802
chr3	62571800	0.942149
chr3	62574200	0.925620
chr3	62587300	0.950413
chr3	62590700	0.966942
chr3	62593700	0.917355
chr3	63886400	0.685950
chr3	63920300	0.669421
chr3	63926200	0.867769
chr3	63946100	0.801653
chr3	64175900	0.958678
chr3	64196100	0.975207
chr3	64200300	0.950413
chr3	64212000	0.669421
chr3	64277300	0.694215
chr3	64337300	0.884298
chr3	64341600	0.950413
chr3	64602700	0.991736
chr3	64613600	0.752066
chr3	64636200	0.966942
chr3	64652400	0.975207
chr3	64655500	0.884298
chr3	64679400	0.719008
chr3	64782600	1.000000
chr3	64788100	0.685950
chr3	64864500	0.785124
chr3	64866900	0.694215
chr3	64876000	0.942149
chr3	64884300	0.694215
chr3	64894700	0.983471
chr3	64902800	0.983471
chr3	64908300	0.909091

chr3	64914600	0.892562
chr3	64931000	0.694215
chr3	64940800	0.685950
chr3	64974000	0.975207
chr3	64978600	0.735537
chr3	64982000	1.000000
chr3	65093200	0.991736
chr3	65120000	0.710744
chr3	65154900	0.809917
chr3	65346900	0.818182
chr3	65384900	0.925620
chr3	65394600	0.933884
chr3	65605400	0.752066
chr3	65841100	0.719008
chr3	65869800	0.661157
chr3	65875500	0.801653
chr3	65885200	0.983471
chr3	65901400	0.975207
chr3	65923600	0.942149
chr3	65932400	0.818182
chr3	65935200	1.000000
chr3	65937700	0.983471
chr3	65939800	0.842975
chr3	65942100	0.983471
chr3	65947600	0.942149
chr3	65950500	0.851240
chr3	65981100	0.826446
chr3	66093100	0.859504
chr3	66101900	0.975207
chr3	66119900	1.000000
chr3	66387100	1.000000
chr3	66404600	0.933884
chr3	66445500	0.975207
chr3	66451800	0.876033
chr3	66479400	0.983471
chr3	66491900	0.768595
chr3	66494000	0.950413
chr3	66501400	0.801653
chr3	66521800	0.909091
chr3	66525600	0.909091
chr3	66531200	0.933884
chr3	66534400	0.727273
chr3	66543500	0.909091
chr3	66774000	0.958678
chr3	66854500	0.818182
chr3	67022800	0.743802
chr3	67114900	0.867769
chr3	67134000	0.851240
chr3	67512100	0.966942
chr3	67581700	0.826446
chr3	67584500	0.809917
chr3	67661300	0.950413
chr3	67770100	0.942149
chr3	69058000	0.793388

chr3	69060100	0.925620
chr3	69081700	0.884298
chr3	69083900	0.900826
chr3	69132000	0.983471
chr3	69137200	0.900826
chr3	69139600	0.983471
chr3	69171300	0.677686
chr3	69224300	0.925620
chr3	69229100	0.991736
chr3	69232300	0.842975
chr3	69244300	0.892562
chr3	69248200	0.661157
chr3	69250600	0.900826
chr3	69253700	0.933884
chr3	69261600	0.958678
chr3	69266500	0.710744
chr3	69286200	0.809917
chr3	69289800	0.966942
chr3	69293200	0.975207
chr3	69338900	0.966942
chr3	69349400	0.884298
chr3	69377000	0.702479
chr3	69404100	0.966942
chr3	69409500	0.727273
chr3	69419600	0.983471
chr3	69459000	0.694215
chr3	69461100	0.983471
chr3	69470400	0.925620
chr3	69508600	0.818182
chr3	69512000	0.917355
chr3	69698900	0.876033
chr3	69714400	0.958678
chr3	69813200	0.909091
chr3	69820000	0.942149
chr3	69826500	0.785124
chr3	69836900	0.942149
chr3	69841600	0.801653
chr3	69853100	0.785124
chr3	69856400	0.925620
chr3	69905400	0.719008
chr3	69939800	0.785124
chr3	70161100	0.661157
chr3	70297700	0.966942
chr3	70299900	0.933884
chr3	70310500	0.727273
chr3	70766300	0.735537
chr3	70787700	0.975207
chr3	70821200	0.859504
chr3	70825600	0.685950
chr3	70878800	0.735537
chr3	70899600	0.900826
chr3	70903600	0.909091
chr3	70982600	0.752066
chr3	71030800	0.669421

chr3	71033200	0.900826
chr3	71039100	0.826446
chr3	71049100	0.785124
chr3	71055100	0.958678
chr3	71057900	0.925620
chr3	71061000	0.809917
chr3	71085200	1.000000
chr3	71096900	0.859504
chr3	71099600	0.685950
chr3	71101700	0.884298
chr3	71105100	0.752066
chr3	71107700	0.975207
chr3	71110400	0.867769
chr3	71116100	0.826446
chr3	71137300	0.694215
chr3	71150000	0.900826
chr3	71153900	0.958678
chr3	71157300	0.801653
chr3	71159700	0.867769
chr3	71161800	0.884298
chr3	71177200	0.851240
chr3	71276600	0.900826
chr3	71294400	0.925620
chr3	71298700	0.933884
chr3	71301400	0.785124
chr3	71308100	0.925620
chr3	71439000	0.950413
chr3	71441500	0.768595
chr3	71445800	0.942149
chr3	71463100	1.000000
chr3	71472700	0.702479
chr3	71478200	0.966942
chr3	71481700	0.702479
chr3	71493800	0.975207
chr3	71516000	0.876033
chr3	71518500	0.867769
chr3	71553800	0.917355
chr3	71565200	0.942149
chr3	71571800	0.975207
chr3	71582500	1.000000
chr3	71586800	0.958678
chr3	71591800	0.859504
chr3	71600800	0.991736
chr3	71635500	0.752066
chr3	71707500	0.983471
chr3	71744000	0.785124
chr3	71752100	1.000000
chr3	72235700	0.826446
chr3	72320400	0.818182
chr3	72360000	0.991736
chr3	72388200	0.834711
chr3	72462700	0.768595
chr3	72477000	0.661157
chr3	72532300	0.909091

chr3	72536500	0.694215
chr3	72776800	0.743802
chr3	72788300	0.842975
chr3	72806500	0.661157
chr3	72859700	0.677686
chr3	72870400	0.966942
chr3	72957800	0.702479
chr3	72995000	0.884298
chr3	73078700	0.694215
chr3	73085500	0.743802
chr3	73108000	0.694215
chr3	73143900	0.694215
chr3	75482900	0.694215
chr3	77717600	0.834711
chr3	77955300	0.950413
chr3	78188400	0.818182
chr3	78190500	0.991736
chr3	78192700	0.768595
chr3	78254500	0.892562
chr3	78418300	0.991736
chr3	78691400	0.933884
chr3	78787900	0.669421
chr3	78889400	0.661157
chr3	78905000	0.727273
chr3	79023700	0.727273
chr3	79026900	0.710744
chr3	79318700	0.768595
chr3	79893100	0.859504
chr3	79921800	0.933884
chr3	80068000	0.917355
chr3	80137500	0.785124
chr3	80239500	0.966942
chr3	80242500	0.933884
chr3	80668200	0.661157
chr3	80782300	0.942149
chr3	80785100	0.942149
chr3	80939200	0.867769
chr3	81155300	0.983471
chr3	81265800	0.793388
chr3	81298900	0.851240
chr3	81328500	0.818182
chr3	81357900	0.966942
chr3	81413700	0.851240
chr3	81474900	0.917355
chr3	81515300	0.983471
chr3	81522500	0.933884
chr3	81535100	0.685950
chr3	81580100	0.694215
chr3	81773200	0.909091
chr3	81776200	0.909091
chr3	81778700	0.851240
chr3	81791000	0.661157
chr3	81794700	0.966942
chr3	81808800	0.809917

chr3	82129900	0.950413
chr3	82134200	0.958678
chr3	82137700	0.966942
chr3	82244400	0.867769
chr3	83273800	0.933884
chr3	87043200	0.983471
chr3	87240200	0.694215
chr3	87321000	0.958678
chr3	87382600	0.884298
chr3	87385700	0.743802
chr3	87440400	0.710744
chr3	87446400	0.966942
chr3	87859800	0.826446
chr3	88081200	0.834711
chr3	88094100	0.909091
chr3	90367200	0.793388
chr3	90377400	0.669421
chr3	90447700	0.933884
chr3	90463900	0.710744
chr3	93707300	0.735537
chr3	93786200	0.925620
chr3	94160400	0.942149
chr3	95642600	0.925620
chr3	95985000	0.950413
chr3	95987200	0.991736
chr3	96070800	0.809917
chr3	96073900	0.685950
chr3	96169000	0.867769
chr3	96336700	0.942149
chr3	97498000	0.900826
chr3	97605200	0.702479
chr3	98375000	0.834711
chr3	98411800	0.933884
chr3	98415100	0.950413
chr3	98429000	0.677686
chr3	98449400	0.950413
chr3	98460200	0.983471
chr3	98466100	0.818182
chr3	98482100	0.752066
chr3	98484900	0.975207
chr3	98488000	0.735537
chr3	98499400	0.933884
chr3	98507600	0.661157
chr3	98510700	0.760331
chr3	98526200	0.702479
chr3	98603700	0.785124
chr3	98605800	0.975207
chr3	98621600	0.702479
chr3	98642100	0.892562
chr3	98681500	0.876033
chr3	98686300	0.983471
chr3	98688400	0.867769
chr3	98691300	0.983471
chr3	98695900	0.884298

chr3	98698200	0.975207
chr3	98705200	0.942149
chr3	98827500	0.933884
chr3	98847900	0.809917
chr3	98851100	0.727273
chr3	98965500	0.710744
chr3	99032900	0.991736
chr3	99039100	0.991736
chr3	99049700	0.669421
chr3	99074500	0.942149
chr3	99130100	0.876033
chr3	99132800	0.958678
chr3	99137400	0.768595
chr3	99201100	0.892562
chr3	99222700	0.859504
chr3	99261600	0.925620
chr3	99263900	0.983471
chr3	99266000	0.958678
chr3	99268600	0.991736
chr3	99297400	0.983471
chr3	99346300	0.710744
chr3	99353800	0.958678
chr3	99361600	0.809917
chr3	99364300	0.917355
chr3	99367800	0.933884
chr3	99370600	0.950413
chr3	99379200	0.966942
chr3	99386400	0.752066
chr3	99389500	0.793388
chr3	99418400	0.785124
chr3	99428800	0.942149
chr3	99431400	0.685950
chr3	99440800	0.925620
chr3	99471200	0.991736
chr3	99544100	0.950413
chr3	99581200	0.876033
chr3	99584900	0.892562
chr3	99592100	0.727273
chr3	99600300	0.966942
chr3	99603700	0.958678
chr3	99615400	0.983471
chr3	99620400	0.966942
chr3	99629400	0.884298
chr3	99637600	0.694215
chr3	99640100	0.950413
chr3	99645000	0.933884
chr3	99654300	0.743802
chr3	99679900	0.669421
chr3	99728200	0.950413
chr3	99759200	0.933884
chr3	99761400	0.983471
chr3	99766500	0.876033
chr3	99788600	0.942149
chr3	99844700	0.876033

chr3	99877800	0.958678
chr3	99879900	0.867769
chr3	100008400	0.661157
chr3	100104700	0.801653
chr3	100348700	0.933884
chr3	100369900	0.925620
chr3	100618300	0.966942
chr3	100620500	0.867769
chr3	100635100	0.917355
chr3	100736300	0.851240
chr3	100755300	0.917355
chr3	100785300	0.983471
chr3	100791400	1.000000
chr3	100847600	0.685950
chr3	100860700	0.892562
chr3	100915700	0.876033
chr3	101240900	0.983471
chr3	101251000	0.991736
chr3	101446400	0.694215
chr3	101481000	0.884298
chr3	101500300	0.876033
chr3	101529100	0.892562
chr3	101567600	0.685950
chr3	101592900	0.727273
chr3	101645500	0.826446
chr3	101657000	0.768595
chr3	101660500	0.942149
chr3	101674300	0.661157
chr3	101726200	0.958678
chr3	101798300	0.735537
chr3	101801200	0.809917
chr3	101812500	0.719008
chr3	101825900	0.876033
chr3	101849400	0.950413
chr3	101861200	0.966942
chr3	101876200	0.942149
chr3	101882200	0.933884
chr3	101892300	0.793388
chr3	101894900	0.925620
chr3	101897300	0.793388
chr3	101900300	0.933884
chr3	103431500	0.768595
chr3	103851900	0.966942
chr3	104323700	0.768595
chr3	104348200	1.000000
chr3	104587700	0.669421
chr3	104599200	0.842975
chr3	104620700	0.834711
chr3	104625400	0.677686
chr3	104629100	0.834711
chr3	104636000	0.760331
chr3	104914900	0.983471
chr3	105012700	0.727273
chr3	105071600	0.661157

chr3	105078300	0.933884
chr3	105081000	0.826446
chr3	105083100	0.958678
chr3	105113500	0.991736
chr3	105178700	0.669421
chr3	105549500	0.942149
chr3	105579800	0.685950
chr3	105584300	0.826446
chr3	105925800	0.933884
chr3	106285800	0.727273
chr3	106289900	0.702479
chr3	106329300	0.694215
chr3	106446500	0.826446
chr3	106449600	0.900826
chr3	106464500	0.983471
chr3	106581300	0.785124
chr3	106927300	1.000000
chr3	107310200	0.743802
chr3	107377300	0.785124
chr3	107381100	0.958678
chr3	107430400	0.752066
chr3	107661300	0.793388
chr3	107676500	0.785124
chr3	107679200	0.900826
chr3	107689300	0.983471
chr3	107701100	0.743802
chr3	107706300	0.983471
chr3	107725700	0.727273
chr3	107743900	0.785124
chr3	107755100	0.884298
chr3	107846300	0.710744
chr3	107851100	0.950413
chr3	108062200	0.958678
chr3	108191400	0.950413
chr3	108257300	0.768595
chr3	108268100	0.917355
chr3	110037300	0.826446
chr3	110063100	0.768595
chr3	110132100	0.876033
chr3	110134600	0.900826
chr3	110139200	0.958678
chr3	110240400	0.958678
chr3	110246500	0.942149
chr3	110296100	0.925620
chr3	110383300	0.933884
chr3	110529700	0.710744
chr3	110533400	0.950413
chr3	111388300	0.793388
chr3	111395800	0.950413
chr3	111414700	0.867769
chr3	111454400	0.884298
chr3	111458500	0.859504
chr3	111467100	0.925620
chr3	111566000	0.991736

chr3	111571400	0.991736
chr3	111581800	0.991736
chr3	111584800	0.884298
chr3	111593300	0.950413
chr3	111621100	0.900826
chr3	111624100	0.925620
chr3	111629800	0.661157
chr3	111634400	0.942149
chr3	111636800	0.842975
chr3	111642800	0.983471
chr3	111650700	0.867769
chr3	111652800	0.958678
chr3	111656500	0.809917
chr3	111662900	0.900826
chr3	111666300	1.000000
chr3	111676400	0.975207
chr3	111682600	0.859504
chr3	111685900	0.983471
chr3	111794100	0.950413
chr3	111809700	0.851240
chr3	111816500	0.950413
chr3	111821000	0.950413
chr3	111836100	0.818182
chr3	112012800	0.834711
chr3	112069600	0.867769
chr3	112072600	0.892562
chr3	112110200	0.876033
chr3	112114500	0.950413
chr3	112219600	0.694215
chr3	112265800	0.975207
chr3	112274700	0.925620
chr3	112311800	0.661157
chr3	112323100	0.851240
chr3	112325400	0.876033
chr3	112347300	0.975207
chr3	112352200	0.735537
chr3	112361900	0.694215
chr3	112396000	0.925620
chr3	112480300	0.760331
chr3	112505400	0.826446
chr3	113006600	0.743802
chr3	113160700	0.809917
chr3	113217600	0.975207
chr3	113276900	0.669421
chr3	113347600	0.677686
chr3	113503100	0.966942
chr3	113508200	0.900826
chr3	113523800	0.966942
chr3	113528900	0.793388
chr3	113548600	0.925620
chr3	113550700	0.917355
chr3	113673300	0.884298
chr3	113799200	0.966942
chr3	113812000	0.983471

chr3	113841700	0.975207
chr3	113871900	0.867769
chr3	113932800	0.925620
chr3	113936900	0.809917
chr3	113942200	0.933884
chr3	114103300	0.842975
chr3	114161700	0.669421
chr3	114271700	0.842975
chr3	114277200	0.719008
chr3	114285700	0.793388
chr3	114288700	0.677686
chr3	114368900	0.900826
chr3	114385800	0.702479
chr3	114791300	0.950413
chr3	114931200	0.867769
chr3	114937100	0.851240
chr3	114959000	0.983471
chr3	114964800	0.776860
chr3	114967500	0.983471
chr3	115040400	0.809917
chr3	115060500	0.826446
chr3	115070100	0.768595
chr3	115101100	0.975207
chr3	115155700	0.925620
chr3	115220300	0.859504
chr3	116648900	0.983471
chr3	116755700	0.975207
chr3	116971200	0.925620
chr3	116997900	0.752066
chr3	117341000	0.933884
chr3	117347800	0.909091
chr3	117604200	0.793388
chr3	117716400	0.809917
chr3	118268500	0.942149
chr3	118998600	1.000000
chr3	119020300	0.892562
chr3	119024400	0.702479
chr3	119030500	0.826446
chr3	119043200	0.809917
chr3	119069200	0.991736
chr3	119072000	0.677686
chr3	119152000	0.966942
chr3	119219800	0.818182
chr3	119316600	0.909091
chr3	119346600	0.834711
chr3	119355500	0.925620
chr3	119358000	0.661157
chr3	119422000	0.842975
chr3	119451200	0.983471
chr3	119470700	0.884298
chr3	119489100	0.884298
chr3	119529100	0.760331
chr3	119733400	0.661157
chr3	119872300	0.909091

chr3	119896500	0.983471
chr3	120136500	1.000000
chr3	120140500	0.900826
chr3	120148400	0.793388
chr3	120154800	0.983471
chr3	120158100	0.983471
chr3	120160400	0.966942
chr3	120215900	0.925620
chr3	120226300	0.975207
chr3	120361600	0.942149
chr3	120397700	0.950413
chr3	120412600	0.917355
chr3	120500800	0.983471
chr3	120511000	0.942149
chr3	120513300	0.867769
chr3	121015000	0.685950
chr3	121505800	0.677686
chr3	121714400	0.818182
chr3	121718200	0.925620
chr3	122202100	0.950413
chr3	122660100	0.834711
chr3	122784300	0.752066
chr3	122795800	0.942149
chr3	122798400	1.000000
chr3	122801700	0.942149
chr3	122804100	0.677686
chr3	122877000	0.727273
chr3	122950400	0.809917
chr3	123313800	0.719008
chr3	123372300	0.661157
chr3	123376700	0.884298
chr3	123380300	0.702479
chr3	123387100	0.801653
chr3	123407600	0.851240
chr3	123411000	0.826446
chr3	123414600	0.991736
chr3	123469800	1.000000
chr3	123503100	0.925620
chr3	123529000	0.867769
chr3	123554200	0.661157
chr3	123742700	0.661157
chr3	123816900	0.834711
chr3	123838300	0.867769
chr3	123876000	0.752066
chr3	123967700	0.983471
chr3	123996000	0.702479
chr3	124109300	0.801653
chr3	124128800	0.884298
chr3	124133300	0.826446
chr3	124147900	0.942149
chr3	124150900	0.735537
chr3	124216900	0.710744
chr3	124269000	0.826446
chr3	124277700	0.818182

chr3	124304000	0.694215
chr3	124340100	0.735537
chr3	124491300	0.958678
chr3	124494300	0.851240
chr3	124497300	0.785124
chr3	124551800	0.884298
chr3	124554200	0.900826
chr3	124557100	0.933884
chr3	124564500	0.834711
chr3	124577700	0.933884
chr3	124579800	0.991736
chr3	124598700	0.909091
chr3	124702200	0.669421
chr3	124704900	0.983471
chr3	124743600	0.942149
chr3	124751700	0.752066
chr3	124759200	0.793388
chr3	124762500	0.818182
chr3	124771200	0.768595
chr3	124776300	0.793388
chr3	124782200	0.942149
chr3	124827100	0.685950
chr3	124860800	0.884298
chr3	124951200	0.909091
chr3	124976700	0.892562
chr3	125037100	0.834711
chr3	125054200	0.867769
chr3	125230900	0.685950
chr3	125259600	0.950413
chr3	125322200	0.685950
chr3	125848900	0.826446
chr3	125920500	0.677686
chr3	125986600	0.958678
chr3	125994800	0.710744
chr3	126008400	0.826446
chr3	126075000	0.685950
chr3	126108300	0.727273
chr3	126191600	0.950413
chr3	126206200	0.743802
chr3	126326400	0.752066
chr3	126336200	0.752066
chr3	126630700	0.917355
chr3	126640600	0.793388
chr3	126645000	0.958678
chr3	126651300	0.760331
chr3	126701600	0.702479
chr3	126721400	0.900826
chr3	126892300	0.735537
chr3	127175400	0.793388
chr3	127319700	0.826446
chr3	127332300	0.942149
chr3	127415600	0.958678
chr3	127433100	0.925620
chr3	127455200	0.909091

chr3	127466900	0.793388
chr3	127487200	0.867769
chr3	127495000	0.826446
chr3	127499000	0.867769
chr3	127520500	0.884298
chr3	127523700	0.942149
chr3	127539700	0.975207
chr3	127634200	0.743802
chr3	127774800	0.669421
chr3	127793400	0.768595
chr3	127796100	0.892562
chr3	127813800	0.925620
chr3	127831800	0.842975
chr3	127962000	0.809917
chr3	127994000	0.900826
chr3	127998100	0.818182
chr3	128062700	0.851240
chr3	128073100	0.785124
chr3	128079500	0.818182
chr3	128085600	0.669421
chr3	128114600	0.735537
chr3	128151800	0.677686
chr3	128199900	0.834711
chr3	128216600	0.727273
chr3	128268500	0.991736
chr3	128272300	0.892562
chr3	128274700	0.975207
chr3	128278600	0.669421
chr3	128287900	0.793388
chr3	128339000	1.000000
chr3	128344000	0.685950
chr3	128513500	0.752066
chr3	128523200	0.851240
chr3	128525800	0.950413
chr3	128532300	0.958678
chr3	128724400	0.801653
chr3	128733800	0.685950
chr3	128746300	0.801653
chr3	128765100	0.834711
chr3	128773300	0.752066
chr3	128806900	0.991736
chr3	128835900	0.867769
chr3	128898900	0.851240
chr3	128915600	1.000000
chr3	128944500	0.925620
chr3	128948700	0.768595
chr3	129024900	0.909091
chr3	129072100	0.884298
chr3	129098400	0.834711
chr3	129109600	0.702479
chr3	129111900	0.793388
chr3	129116300	0.710744
chr3	129147800	0.760331
chr3	129161500	0.983471

chr3	129169600	0.933884
chr3	129201100	0.842975
chr3	129210100	0.925620
chr3	129213300	0.818182
chr3	129216800	0.694215
chr3	129219600	0.834711
chr3	129277700	0.818182
chr3	129279800	0.975207
chr3	129295600	0.933884
chr3	129306600	0.710744
chr3	129309400	0.842975
chr3	129313400	0.752066
chr3	129329200	0.743802
chr3	129331700	0.892562
chr3	129335800	0.702479
chr3	129339700	0.859504
chr3	129342700	0.876033
chr3	129347300	0.694215
chr3	129371000	0.991736
chr3	129377000	0.975207
chr3	129574900	0.867769
chr3	129609400	1.000000
chr3	129653800	0.669421
chr3	129981700	0.785124
chr3	130411900	0.950413
chr3	130419700	0.925620
chr3	130446300	0.694215
chr3	130462800	0.710744
chr3	130646100	0.727273
chr3	130683000	0.851240
chr3	130687400	0.975207
chr3	131068500	0.966942
chr3	131073300	0.983471
chr3	131105100	0.909091
chr3	131107700	0.917355
chr3	131121800	0.851240
chr3	131853400	0.950413
chr3	131855500	0.917355
chr3	132124300	0.950413
chr3	132151500	0.917355
chr3	132154600	0.909091
chr3	132164900	0.909091
chr3	132262300	0.925620
chr3	132269300	0.851240
chr3	132274600	0.884298
chr3	132297000	0.735537
chr3	132313100	0.975207
chr3	132316600	0.694215
chr3	132341500	1.000000
chr3	132349200	0.942149
chr3	132356700	0.892562
chr3	132364300	0.818182
chr3	132374800	0.710744
chr3	132387100	0.876033

chr3	132439000	0.702479
chr3	132997300	0.867769
chr3	133013200	0.933884
chr3	133020500	1.000000
chr3	133022900	0.925620
chr3	133218600	0.793388
chr3	133225400	0.966942
chr3	133251400	0.859504
chr3	133255400	0.942149
chr3	133260200	0.818182
chr3	133291500	0.983471
chr3	133295900	0.685950
chr3	133310300	0.900826
chr3	133363600	0.909091
chr3	133366000	0.752066
chr3	133375200	0.702479
chr3	133594100	0.661157
chr3	133687700	0.818182
chr3	133698800	0.909091
chr3	133728000	0.991736
chr3	133786000	0.925620
chr3	133863300	0.694215
chr3	133905900	0.826446
chr3	133920600	0.942149
chr3	133962000	0.867769
chr3	134032200	0.966942
chr3	134035400	0.925620
chr3	134044800	0.727273
chr3	134046900	0.892562
chr3	134077100	0.768595
chr3	134082100	0.859504
chr3	134084400	0.727273
chr3	134088400	0.909091
chr3	134125800	0.785124
chr3	134146700	1.000000
chr3	134165300	0.694215
chr3	134190800	0.909091
chr3	134201400	0.900826
chr3	134427900	0.909091
chr3	134516600	0.760331
chr3	134566000	0.991736
chr3	135711300	1.000000
chr3	135825300	0.867769
chr3	135861700	0.917355
chr3	135890400	0.876033
chr3	136063100	0.685950
chr3	136110000	0.752066
chr3	136262700	0.933884
chr3	136266500	0.685950
chr3	136453800	0.950413
chr3	136506000	0.917355
chr3	136558100	0.991736
chr3	136563900	0.900826
chr3	136604900	0.710744

chr3	136611600	0.958678
chr3	136613900	0.983471
chr3	136617700	0.719008
chr3	136620900	1.000000
chr3	136626600	0.719008
chr3	136639100	0.942149
chr3	136657200	0.991736
chr3	136662500	0.958678
chr3	136674700	0.834711
chr3	136714100	0.909091
chr3	136733200	0.768595
chr3	137490600	0.768595
chr3	137938500	0.925620
chr3	138043500	0.685950
chr3	138494000	0.991736
chr3	138543100	0.925620
chr3	138581900	0.925620
chr3	138594900	0.991736
chr3	138655500	0.785124
chr3	138658200	0.677686
chr3	138669200	0.743802
chr3	139977400	0.884298
chr3	140349100	0.760331
chr3	140355900	0.661157
chr3	141040300	0.710744
chr3	141045800	0.685950
chr3	141051000	0.958678
chr3	141056500	0.942149
chr3	141065600	0.694215
chr3	141080200	0.975207
chr3	141085300	0.867769
chr3	141108300	0.876033
chr3	141124400	0.917355
chr3	141134700	0.942149
chr3	141139600	0.719008
chr3	141145300	0.867769
chr3	141160600	0.677686
chr3	141201800	0.785124
chr3	141243900	0.851240
chr3	141249200	0.909091
chr3	141257700	0.809917
chr3	141271800	0.933884
chr3	141276200	0.975207
chr3	141400300	0.958678
chr3	141405900	0.884298
chr3	141427000	0.983471
chr3	141430400	0.983471
chr3	141459800	0.702479
chr3	141516600	0.884298
chr3	141560000	0.983471
chr3	141570500	0.694215
chr3	141580100	0.909091
chr3	141589400	0.851240
chr3	141659800	0.933884

chr3	141772600	0.909091
chr3	141929100	0.785124
chr3	142138100	1.000000
chr3	142220200	0.892562
chr3	142338400	0.685950
chr3	142696100	0.900826
chr3	142804300	0.801653
chr3	142854300	0.851240
chr3	142859100	0.851240
chr3	142887600	0.661157
chr3	142958200	0.933884
chr3	142960900	0.933884
chr3	143022100	0.975207
chr3	143026400	0.809917
chr3	143050000	0.809917
chr3	143063600	0.958678
chr3	143162900	0.966942
chr3	143287100	0.892562
chr3	143295900	0.975207
chr3	143455700	0.793388
chr3	143550200	0.958678
chr3	143594700	0.677686
chr3	143695100	0.677686
chr3	143948900	0.752066
chr3	144218700	0.950413
chr3	144310100	0.983471
chr3	144314700	0.917355
chr3	144490500	0.983471
chr3	144495000	0.685950
chr3	144559800	0.826446
chr3	144909600	0.876033
chr3	145811800	0.966942
chr3	145819100	0.983471
chr3	145844300	0.760331
chr3	145861300	0.768595
chr3	145864300	0.876033
chr3	145867100	0.735537
chr3	145875800	0.876033
chr3	145881700	0.909091
chr3	145885100	0.925620
chr3	145895300	0.983471
chr3	145902700	0.776860
chr3	145908200	0.909091
chr3	145913100	0.942149
chr3	145915400	0.925620
chr3	145936300	0.818182
chr3	145941100	0.909091
chr3	145960200	0.859504
chr3	145965800	0.818182
chr3	145970300	0.793388
chr3	145991900	0.917355
chr3	145994000	0.909091
chr3	145997100	0.669421
chr3	146027100	0.966942

chr3	146032300	0.983471
chr3	146114800	0.785124
chr3	146221900	0.834711
chr3	146224900	0.975207
chr3	146343300	0.884298
chr3	146464100	0.950413
chr3	146686500	0.884298
chr3	146859600	0.694215
chr3	147016700	0.702479
chr3	147045800	0.768595
chr3	147053000	0.966942
chr3	147055700	0.867769
chr3	147137400	0.760331
chr3	147530600	0.991736
chr3	147534900	0.752066
chr3	147648700	0.776860
chr3	147661400	0.909091
chr3	147742000	0.917355
chr3	147760900	0.851240
chr3	148182200	0.917355
chr3	148184400	0.950413
chr3	148231500	0.983471
chr3	148592200	0.966942
chr3	148597600	0.942149
chr3	148599800	0.867769
chr3	148735200	0.834711
chr3	148761200	0.661157
chr3	148773800	0.743802
chr3	148870100	0.785124
chr3	148897500	0.991736
chr3	148943200	0.859504
chr3	148966100	0.842975
chr3	148995600	0.818182
chr3	149018400	0.752066
chr3	149052300	0.826446
chr3	149057500	0.685950
chr3	149061600	0.983471
chr3	149071500	0.685950
chr3	149085000	0.983471
chr3	149089100	0.966942
chr3	149091300	0.719008
chr3	149100400	0.727273
chr3	149105000	0.975207
chr3	149111400	0.884298
chr3	149113800	0.983471
chr3	149119900	0.933884
chr3	149133900	0.933884
chr3	149161200	0.677686
chr3	149198200	0.925620
chr3	149224100	0.768595
chr3	149226600	0.859504
chr3	149245500	0.900826
chr3	149248100	0.991736
chr3	149251300	0.975207

chr3	149254500	0.867769
chr3	149257800	0.719008
chr3	149260800	0.942149
chr3	149263000	0.776860
chr3	149266400	0.942149
chr3	149271100	0.801653
chr3	149273600	0.842975
chr3	149277400	0.776860
chr3	149285500	0.834711
chr3	149290700	0.876033
chr3	149294400	0.975207
chr3	149297800	0.983471
chr3	149300300	0.983471
chr3	149302900	0.942149
chr3	149305600	0.975207
chr3	149307800	0.975207
chr3	149309900	0.966942
chr3	149312200	0.933884
chr3	149314900	0.925620
chr3	149317600	0.909091
chr3	149321000	0.933884
chr3	149323400	0.950413
chr3	149325600	0.818182
chr3	149328100	1.000000
chr3	149330300	0.983471
chr3	149333700	0.925620
chr3	149336800	0.809917
chr3	149339800	0.909091
chr3	149342200	0.677686
chr3	149344500	0.942149
chr3	149348200	0.719008
chr3	149353300	0.950413
chr3	149357200	0.942149
chr3	149362100	0.933884
chr3	149364200	0.925620
chr3	149366900	0.851240
chr3	149371000	0.809917
chr3	149378200	0.876033
chr3	149408100	0.760331
chr3	149673200	0.694215
chr3	149902300	0.785124
chr3	149939400	0.851240
chr3	149944200	0.933884
chr3	149951200	0.859504
chr3	149955800	0.892562
chr3	149970000	0.991736
chr3	149995300	0.818182
chr3	150019200	0.975207
chr3	150066600	0.991736
chr3	150088100	0.966942
chr3	150090300	0.826446
chr3	150115200	0.719008
chr3	150124900	0.694215
chr3	150136900	0.785124

chr3	150165200	0.809917
chr3	150168100	0.851240
chr3	150187800	0.958678
chr3	150198800	0.768595
chr3	150223100	0.768595
chr3	150274600	0.983471
chr3	150317600	0.892562
chr3	150332000	0.826446
chr3	150946500	0.900826
chr3	151484100	0.809917
chr3	151488600	0.942149
chr3	151493700	0.743802
chr3	151511800	0.702479
chr3	151582500	0.950413
chr3	151590100	0.669421
chr3	151619100	0.950413
chr3	151621900	0.966942
chr3	151627900	0.950413
chr3	151641800	0.876033
chr3	151644100	0.876033
chr3	151694400	0.975207
chr3	151737800	0.760331
chr3	151740100	0.876033
chr3	151744600	0.975207
chr3	151765500	0.876033
chr3	151839100	0.900826
chr3	151959100	0.818182
chr3	151984600	0.975207
chr3	151990600	0.685950
chr3	152006100	1.000000
chr3	152016700	0.867769
chr3	152022900	0.842975
chr3	152026700	0.867769
chr3	152033400	1.000000
chr3	152035600	0.900826
chr3	152038200	0.983471
chr3	152042000	0.826446
chr3	152045400	0.876033
chr3	152048800	0.975207
chr3	152051600	0.966942
chr3	152065600	0.892562
chr3	152070400	0.661157
chr3	152075900	0.752066
chr3	152094500	0.876033
chr3	152097300	0.661157
chr3	152100300	0.867769
chr3	152117200	0.884298
chr3	152154400	0.966942
chr3	152161500	0.801653
chr3	152218100	0.785124
chr3	152314400	0.958678
chr3	152516300	0.727273
chr3	152662100	0.859504
chr3	152761500	0.950413

chr3	152770000	0.991736
chr3	152776100	0.842975
chr3	152802500	0.677686
chr3	152848600	0.793388
chr3	152856800	0.958678
chr3	152859100	0.950413
chr3	152862900	0.991736
chr3	152927500	0.834711
chr3	152934800	0.785124
chr3	152939900	1.000000
chr3	153002500	0.876033
chr3	153317900	0.909091
chr3	153320200	0.884298
chr3	153342700	0.818182
chr3	153345200	0.925620
chr3	153347500	1.000000
chr3	153547900	0.966942
chr3	153610400	0.909091
chr3	153942700	0.768595
chr3	153948500	0.694215
chr3	154741900	0.661157
chr3	154745400	0.768595
chr3	154787900	0.983471
chr3	154790100	0.735537
chr3	155608400	0.942149
chr3	155867300	0.909091
chr3	155929700	0.867769
chr3	155978200	0.958678
chr3	156022500	0.917355
chr3	156026100	0.900826
chr3	156035800	0.958678
chr3	156151300	0.950413
chr3	156202300	0.975207
chr3	156207200	0.933884
chr3	156215000	0.909091
chr3	156253800	0.809917
chr3	156260200	0.702479
chr3	156262300	0.983471
chr3	156265900	0.677686
chr3	156270300	0.818182
chr3	156324200	0.677686
chr3	156333800	0.892562
chr3	156383300	0.785124
chr3	156396000	0.801653
chr3	156398800	0.917355
chr3	156404700	0.661157
chr3	156408500	0.958678
chr3	156412000	0.942149
chr3	156490800	0.743802
chr3	156499800	0.851240
chr3	156502900	0.876033
chr3	156506300	0.801653
chr3	156510200	0.694215
chr3	156529100	0.826446

chr3	156531200	0.900826
chr3	156534300	0.710744
chr3	156538000	0.735537
chr3	156543200	0.669421
chr3	156746500	0.834711
chr3	156808400	0.859504
chr3	156846700	0.842975
chr3	156849000	0.933884
chr3	156879800	0.760331
chr3	156892100	0.702479
chr3	156966600	1.000000
chr3	156981400	0.950413
chr3	156999700	0.851240
chr3	157003800	0.884298
chr3	157052400	0.950413
chr3	157054700	0.876033
chr3	157074200	0.677686
chr3	157077200	0.975207
chr3	157079400	0.900826
chr3	157083600	0.702479
chr3	157088000	0.669421
chr3	157094100	0.834711
chr3	157107200	0.966942
chr3	157124800	0.925620
chr3	157135500	0.818182
chr3	157161200	0.760331
chr3	157165100	0.768595
chr3	157178200	0.900826
chr3	157185700	0.851240
chr3	157205400	0.834711
chr3	157217900	0.942149
chr3	157245700	0.719008
chr3	157247900	0.735537
chr3	157253300	0.942149
chr3	157821500	0.694215
chr3	157824000	0.702479
chr3	158367900	0.917355
chr3	158377200	0.867769
chr3	158379700	0.909091
chr3	158386500	0.859504
chr3	158403200	0.826446
chr3	158417800	0.826446
chr3	158421100	1.000000
chr3	158427900	0.859504
chr3	158430500	0.983471
chr3	158436700	0.876033
chr3	158441000	0.834711
chr3	158443400	0.975207
chr3	158487100	0.975207
chr3	158636300	0.809917
chr3	158796500	0.900826
chr3	158800500	0.809917
chr3	158847100	0.818182
chr3	158876400	0.834711

chr3	158884400	0.785124
chr3	158888200	0.975207
chr3	158909400	0.950413
chr3	158970300	0.859504
chr3	159312200	0.991736
chr3	159429700	0.983471
chr3	159445800	0.942149
chr3	159450600	0.876033
chr3	159479500	0.785124
chr3	159493900	1.000000
chr3	159503500	0.760331
chr3	159506100	0.900826
chr3	159510400	0.702479
chr3	159515000	0.942149
chr3	159522700	0.719008
chr3	159542000	0.752066
chr3	159547400	0.851240
chr3	159554900	0.975207
chr3	159562300	0.727273
chr3	159570200	0.776860
chr3	159573700	0.966942
chr3	159575900	0.859504
chr3	159579900	0.958678
chr3	159590600	1.000000
chr3	159599800	0.818182
chr3	159601900	0.950413
chr3	159647400	0.900826
chr3	159843500	0.834711
chr3	159847600	0.966942
chr3	160122400	0.933884
chr3	160134400	0.776860
chr3	160165700	0.719008
chr3	160169000	0.719008
chr3	160285000	0.702479
chr3	160287900	0.785124
chr3	160359800	0.942149
chr3	160395700	0.892562
chr3	160416300	0.818182
chr3	160691400	0.677686
chr3	160753800	0.950413
chr3	160788300	0.727273
chr3	160835800	0.694215
chr3	160857100	0.669421
chr3	160889600	0.958678
chr3	160984800	0.958678
chr3	161034500	0.975207
chr3	161134100	0.983471
chr3	165361000	0.958678
chr3	167742900	0.975207
chr3	167762100	0.719008
chr3	168512600	0.983471
chr3	168520000	0.933884
chr3	168594600	0.900826
chr3	168744900	1.000000

chr3	168760100	0.818182
chr3	168762700	0.950413
chr3	168828900	0.743802
chr3	168831700	0.884298
chr3	168836000	0.958678
chr3	168851900	0.760331
chr3	168856000	0.694215
chr3	168858400	0.809917
chr3	168867500	0.719008
chr3	168870200	0.991736
chr3	168925700	0.661157
chr3	168933500	0.958678
chr3	168936500	0.942149
chr3	168946400	1.000000
chr3	168951100	0.925620
chr3	168956500	0.768595
chr3	168958600	0.859504
chr3	168962800	0.760331
chr3	168983100	0.677686
chr3	169004800	0.876033
chr3	169027100	1.000000
chr3	169029500	0.991736
chr3	169042100	0.826446
chr3	169055800	0.768595
chr3	169067500	0.950413
chr3	169072900	0.867769
chr3	169106500	0.785124
chr3	169108900	0.917355
chr3	169129100	0.702479
chr3	169149100	1.000000
chr3	169172300	0.842975
chr3	169178400	0.859504
chr3	169197400	0.760331
chr3	169213900	0.942149
chr3	169216900	0.966942
chr3	169228100	0.933884
chr3	169243900	0.950413
chr3	169247500	0.892562
chr3	169260700	0.702479
chr3	169275900	0.834711
chr3	169293800	0.975207
chr3	169300800	0.983471
chr3	169310500	0.983471
chr3	169331400	0.917355
chr3	169338000	0.752066
chr3	169375800	0.809917
chr3	169547000	0.735537
chr3	169663400	0.694215
chr3	169721500	0.785124
chr3	170028700	0.710744
chr3	170038500	0.917355
chr3	170049200	0.900826
chr3	170062700	0.851240
chr3	170072300	0.950413

chr3	170074400	0.826446
chr3	170082800	0.776860
chr3	170140900	0.851240
chr3	170143900	0.942149
chr3	170156800	0.752066
chr3	170241700	0.933884
chr3	170282300	0.909091
chr3	170407600	0.793388
chr3	170445200	0.975207
chr3	170459500	0.942149
chr3	170463000	0.950413
chr3	170465500	0.884298
chr3	170468700	0.942149
chr3	170472500	0.942149
chr3	170477600	0.925620
chr3	170521000	1.000000
chr3	170527900	0.983471
chr3	170530000	0.826446
chr3	170716200	0.975207
chr3	170746600	0.735537
chr3	170774700	0.950413
chr3	170834900	0.694215
chr3	170912100	0.991736
chr3	170914200	0.834711
chr3	170918600	0.942149
chr3	170921600	0.884298
chr3	170924300	0.975207
chr3	170979800	0.752066
chr3	171003700	0.933884
chr3	171009200	0.859504
chr3	171024800	0.867769
chr3	171029900	0.859504
chr3	171065600	0.975207
chr3	171070100	0.785124
chr3	171073600	0.958678
chr3	171076100	0.958678
chr3	171093200	0.983471
chr3	171111400	0.859504
chr3	171114400	0.826446
chr3	171135500	0.942149
chr3	171154600	0.933884
chr3	171161300	0.958678
chr3	171242600	0.892562
chr3	171394800	0.884298
chr3	171430400	1.000000
chr3	171475700	0.785124
chr3	171489500	0.809917
chr3	171495800	0.909091
chr3	171499800	1.000000
chr3	171502400	0.760331
chr3	171505900	0.851240
chr3	171509800	0.958678
chr3	171512100	0.719008
chr3	171516100	0.966942

chr3	171519800	0.909091
chr3	171521900	0.909091
chr3	171525600	0.966942
chr3	171530000	0.727273
chr3	171543100	0.909091
chr3	171547700	1.000000
chr3	171552500	0.909091
chr3	171556200	0.933884
chr3	171559600	0.966942
chr3	171561700	0.727273
chr3	171568000	0.677686
chr3	171570200	0.925620
chr3	171578600	0.900826
chr3	171581500	0.884298
chr3	171585100	0.694215
chr3	171590900	0.909091
chr3	171594900	1.000000
chr3	171672500	0.909091
chr3	171685500	0.975207
chr3	171763700	0.892562
chr3	171773200	0.950413
chr3	171775600	0.793388
chr3	171779300	0.958678
chr3	171782200	0.942149
chr3	171784900	0.851240
chr3	171807100	0.892562
chr3	171813100	0.950413
chr3	171822800	0.677686
chr3	171845400	0.719008
chr3	171849000	0.991736
chr3	171852500	0.983471
chr3	171856200	0.942149
chr3	171859000	0.991736
chr3	171862800	0.892562
chr3	171865100	0.991736
chr3	171869100	0.851240
chr3	171871200	0.785124
chr3	171874800	0.933884
chr3	171887300	0.760331
chr3	171894200	0.669421
chr3	171898300	0.801653
chr3	171900800	0.950413
chr3	171919100	0.884298
chr3	171962000	0.702479
chr3	171964600	0.983471
chr3	171971300	0.884298
chr3	172007600	0.950413
chr3	172023800	0.727273
chr3	172033800	0.661157
chr3	172037000	0.884298
chr3	172185400	0.743802
chr3	172195100	0.983471
chr3	172230900	1.000000
chr3	172234800	0.685950

chr3	172238300	1.000000
chr3	172281100	0.925620
chr3	172313200	0.925620
chr3	172325300	0.975207
chr3	172341100	0.950413
chr3	172353600	0.719008
chr3	172365000	0.669421
chr3	172377300	0.958678
chr3	172395500	0.892562
chr3	172403700	0.694215
chr3	172430200	0.694215
chr3	172572200	0.900826
chr3	172575600	0.933884
chr3	172588100	0.752066
chr3	172594500	0.826446
chr3	172623400	0.727273
chr3	172632900	0.867769
chr3	172636400	1.000000
chr3	172657000	0.991736
chr3	173183500	0.991736
chr3	173456700	0.768595
chr3	173460300	0.933884
chr3	173462500	1.000000
chr3	173465600	0.958678
chr3	173468500	0.991736
chr3	173488900	0.694215
chr3	173610600	0.933884
chr3	173657000	0.735537
chr3	173874400	0.950413
chr3	173909900	0.867769
chr3	173915400	0.826446
chr3	174156900	0.694215
chr3	174486300	0.735537
chr3	174668300	0.933884
chr3	174897500	0.719008
chr3	176381900	0.966942
chr3	176638900	0.884298
chr3	176680400	0.909091
chr3	176824700	0.727273
chr3	176844200	0.669421
chr3	176916700	0.859504
chr3	177039200	0.942149
chr3	177063800	0.983471
chr3	177068700	0.719008
chr3	177075400	0.917355
chr3	177078800	0.958678
chr3	177170200	0.958678
chr3	177234600	0.776860
chr3	177315500	0.909091
chr3	177320400	0.826446
chr3	177397700	0.958678
chr3	177412200	0.933884
chr3	177442100	0.809917
chr3	177496600	0.826446

chr3	177528600	0.917355
chr3	177553600	0.702479
chr3	177612400	0.950413
chr3	177650700	0.991736
chr3	177797400	0.950413
chr3	177802600	0.933884
chr3	177858600	0.958678
chr3	177877900	0.991736
chr3	177889000	0.991736
chr3	177920400	0.909091
chr3	177973400	1.000000
chr3	177981500	0.900826
chr3	177993000	0.876033
chr3	178038900	0.842975
chr3	178070800	0.950413
chr3	178078200	0.867769
chr3	178100400	0.909091
chr3	178111600	0.727273
chr3	178250500	0.975207
chr3	178280600	0.867769
chr3	178668900	0.867769
chr3	178685100	0.909091
chr3	178833800	0.702479
chr3	178899100	0.876033
chr3	178965800	0.710744
chr3	178986800	0.826446
chr3	179043200	0.719008
chr3	179155200	0.661157
chr3	179191800	0.933884
chr3	179201100	0.966942
chr3	179234500	0.677686
chr3	179245000	1.000000
chr3	179331600	0.958678
chr3	179333700	0.958678
chr3	179384100	0.702479
chr3	179389900	0.917355
chr3	179400400	0.975207
chr3	179457600	0.876033
chr3	179487500	0.661157
chr3	179755100	0.727273
chr3	180151500	0.966942
chr3	180348500	0.826446
chr3	181340400	0.975207
chr3	181342600	0.859504
chr3	181346300	0.809917
chr3	181349900	0.991736
chr3	181500800	0.677686
chr3	181598600	0.694215
chr3	182083100	0.950413
chr3	182453700	0.876033
chr3	182473200	0.867769
chr3	182505800	0.801653
chr3	182525300	0.661157
chr3	182528900	0.702479

chr3	182699300	0.694215
chr3	182841400	0.933884
chr3	182847000	0.735537
chr3	182850100	0.991736
chr3	182878800	0.809917
chr3	182903900	0.694215
chr3	182963300	0.785124
chr3	182979300	0.776860
chr3	182983900	0.942149
chr3	182993300	0.892562
chr3	183002900	0.917355
chr3	183005000	0.884298
chr3	183046400	0.983471
chr3	183089100	0.884298
chr3	183167600	0.834711
chr3	183173000	0.958678
chr3	183187300	0.694215
chr3	183242500	0.933884
chr3	183245300	0.892562
chr3	183266600	0.909091
chr3	183270900	0.851240
chr3	183273800	0.809917
chr3	183276100	0.793388
chr3	183281500	0.933884
chr3	183298500	0.809917
chr3	183302700	0.876033
chr3	183305400	0.685950
chr3	183453000	1.000000
chr3	183463500	0.900826
chr3	183483600	0.942149
chr3	183724200	0.818182
chr3	183881900	0.743802
chr3	183898100	0.669421
chr3	183903000	0.867769
chr3	183948000	0.809917
chr3	183979700	0.735537
chr3	184035900	0.768595
chr3	184038200	0.892562
chr3	184056800	0.834711
chr3	184086700	0.925620
chr3	184099100	0.851240
chr3	184159500	0.702479
chr3	184167000	0.818182
chr3	184177900	1.000000
chr3	184184400	0.719008
chr3	184190900	0.842975
chr3	184243000	0.942149
chr3	184249800	0.859504
chr3	184279600	0.851240
chr3	184338100	0.975207
chr3	184456400	0.867769
chr3	184535600	0.768595
chr3	184764000	0.884298
chr3	185035300	0.727273

chr3	185348200	0.867769
chr3	185376000	0.768595
chr3	185378400	0.793388
chr3	185406800	0.983471
chr3	185420400	0.694215
chr3	185423300	0.925620
chr3	185430000	0.950413
chr3	185433300	0.933884
chr3	185435700	0.900826
chr3	185447500	1.000000
chr3	185450900	0.991736
chr3	185453000	0.892562
chr3	185456100	1.000000
chr3	185462700	0.983471
chr3	185464800	0.892562
chr3	185479000	0.991736
chr3	185481400	0.851240
chr3	185485400	0.950413
chr3	185492000	0.884298
chr3	185515700	0.983471
chr3	185533900	0.942149
chr3	185647600	0.958678
chr3	185763400	0.876033
chr3	185815800	0.842975
chr3	186259300	0.942149
chr3	186335300	0.661157
chr3	186464300	0.760331
chr3	186490700	0.760331
chr3	186516500	0.694215
chr3	186539800	0.818182
chr3	186589500	0.991736
chr3	186600900	0.950413
chr3	186606600	0.685950
chr3	186619300	0.768595
chr3	186671200	0.975207
chr3	186689800	0.933884
chr3	186730300	0.950413
chr3	186732600	0.661157
chr3	186735500	0.909091
chr3	186759400	0.933884
chr3	186762800	1.000000
chr3	186820000	0.743802
chr3	186982700	0.950413
chr3	187037300	0.702479
chr3	187122000	0.661157
chr3	187483500	0.776860
chr3	187704700	0.809917
chr3	187724900	0.768595
chr3	187905400	0.702479
chr3	187947100	0.975207
chr3	187975700	0.991736
chr3	187978800	1.000000
chr3	187980900	0.991736
chr3	187983500	0.975207

chr3	187985800	0.851240
chr3	187988500	0.966942
chr3	187991500	0.892562
chr3	188004200	0.925620
chr3	188012700	0.818182
chr3	188015400	0.975207
chr3	188063400	0.975207
chr3	188066800	0.942149
chr3	188080300	0.917355
chr3	188084000	1.000000
chr3	188104300	0.694215
chr3	188149900	0.958678
chr3	188157300	0.958678
chr3	188170500	1.000000
chr3	188186300	0.834711
chr3	188214700	0.661157
chr3	188225600	0.917355
chr3	188249300	0.966942
chr3	188265300	0.983471
chr3	188276600	0.983471
chr3	188302500	0.991736
chr3	188312300	0.950413
chr3	188425800	0.983471
chr3	188494400	0.735537
chr3	188506800	0.933884
chr3	188514600	0.793388
chr3	188599400	0.867769
chr3	188602900	0.909091
chr3	188617800	0.900826
chr3	188620100	0.702479
chr3	188632600	0.933884
chr3	188635600	0.677686
chr3	188655400	0.892562
chr3	188665600	0.768595
chr3	188669700	0.876033
chr3	188850300	0.876033
chr3	189403700	0.966942
chr3	189509200	0.685950
chr3	189621800	0.876033
chr3	189626800	0.975207
chr3	189655900	0.876033
chr3	189722000	0.950413
chr3	189730000	0.793388
chr3	189742800	0.925620
chr3	189777100	0.925620
chr3	189791200	0.809917
chr3	189795000	0.942149
chr3	189812300	0.818182
chr3	189826900	0.950413
chr3	190057600	0.991736
chr3	190063400	0.859504
chr3	190104400	0.958678
chr3	190120300	0.851240
chr3	190251900	0.900826

chr3	190284500	0.975207
chr3	190298900	0.685950
chr3	190304200	0.892562
chr3	190860400	0.975207
chr3	190956200	0.950413
chr3	190958600	0.752066
chr3	191046000	0.677686
chr3	191050400	0.983471
chr3	191053600	0.950413
chr3	191056100	0.983471
chr3	191067800	0.826446
chr3	191070400	0.760331
chr3	191074900	1.000000
chr3	191084600	1.000000
chr3	191095000	0.958678
chr3	191130900	0.950413
chr3	191480700	0.801653
chr3	191510700	0.768595
chr3	191588100	0.776860
chr3	191684300	0.842975
chr3	191773100	0.892562
chr3	191781600	0.991736
chr3	191904500	0.809917
chr3	192010100	0.768595
chr3	192200700	0.909091
chr3	192536800	0.909091
chr3	192569700	0.950413
chr3	192577000	0.710744
chr3	192587700	0.892562
chr3	192598000	0.958678
chr3	192602400	0.983471
chr3	192605600	0.983471
chr3	192608200	0.975207
chr3	192675900	0.975207
chr3	192711700	0.719008
chr3	193178700	0.958678
chr3	193499200	0.776860
chr3	193571500	0.909091
chr3	193588000	1.000000
chr3	193590800	0.694215
chr3	193603600	0.669421
chr3	193689000	0.900826
chr3	193692100	0.867769
chr3	193698400	0.933884
chr3	193705500	0.694215
chr3	193720900	0.851240
chr3	193723700	0.892562
chr3	193758700	0.842975
chr3	193761200	0.867769
chr3	193786900	0.694215
chr3	193804600	0.851240
chr3	193807600	0.950413
chr3	193839900	0.669421
chr3	193852000	0.809917

chr3	193857500	0.867769
chr3	193885000	0.776860
chr3	193910100	0.876033
chr3	193924100	0.975207
chr3	193973400	0.859504
chr3	193982700	0.752066
chr3	193984800	0.925620
chr3	193987500	0.826446
chr3	193990000	0.925620
chr3	193993000	0.826446
chr3	193995500	0.669421
chr3	194014800	0.909091
chr3	194024400	0.925620
chr3	194033300	0.933884
chr3	194143300	0.884298
chr3	194146500	1.000000
chr3	194210700	0.975207
chr3	194243300	0.966942
chr3	194247800	0.975207
chr3	194258700	0.925620
chr3	194269900	0.909091
chr3	194277600	0.933884
chr3	194282400	0.884298
chr3	194299700	0.958678
chr3	194307000	0.801653
chr3	194309200	0.892562
chr3	194314600	0.735537
chr3	194355300	0.834711
chr3	194362300	0.768595
chr3	194379300	0.983471
chr3	194382600	1.000000
chr3	194385800	0.909091
chr3	194404400	0.991736
chr3	194411900	0.917355
chr3	194432700	0.859504
chr3	194436500	0.900826
chr3	194462600	0.942149
chr3	194494200	0.760331
chr3	194553600	0.983471
chr3	194562000	0.867769
chr3	194588700	0.966942
chr3	194591200	0.809917
chr3	194593400	0.859504
chr3	194600900	1.000000
chr3	194605800	0.884298
chr3	194627400	0.900826
chr3	194648900	0.776860
chr3	194651500	0.909091
chr3	194653600	0.809917
chr3	194735700	0.719008
chr3	194742200	0.760331
chr3	194750000	0.859504
chr3	194752600	0.752066
chr3	194754900	0.685950

chr3	194780000	0.793388
chr3	194801200	0.950413
chr3	194819000	0.876033
chr3	194822300	0.942149
chr3	194824500	0.801653
chr3	194827100	0.925620
chr3	194837300	0.859504
chr3	194840300	0.785124
chr3	194842400	0.876033
chr3	194845900	0.909091
chr3	194849800	0.983471
chr3	194862200	0.966942
chr3	194868400	0.933884
chr3	194872400	0.933884
chr3	194876300	0.958678
chr3	194878800	0.851240
chr3	194883100	0.942149
chr3	194885800	0.966942
chr3	194894300	0.950413
chr3	194899900	0.760331
chr3	194904100	0.958678
chr3	194909100	0.661157
chr3	194915700	0.975207
chr3	194919100	0.983471
chr3	194926100	0.991736
chr3	194931800	0.727273
chr3	194943700	0.900826
chr3	194945800	0.876033
chr3	194983800	0.892562
chr3	195007400	0.950413
chr3	195034100	0.826446
chr3	195273200	0.710744
chr3	195311300	0.942149
chr3	195352200	0.760331
chr3	195388500	0.876033
chr3	195484800	0.685950
chr3	195614200	0.851240
chr3	195620500	0.884298
chr3	195625000	0.900826
chr3	195681900	0.768595
chr3	195714100	0.661157
chr3	196260900	0.892562
chr3	196282500	0.975207
chr3	196290800	1.000000
chr3	196339200	0.983471
chr3	196378600	0.991736
chr3	196397700	0.743802
chr3	196501500	0.909091
chr3	196520400	0.942149
chr3	196625600	0.785124
chr3	196758900	0.884298
chr3	197036800	0.851240
chr3	197120100	1.000000
chr3	197122400	0.884298

chr3	197133300	0.760331
chr3	197144600	0.735537
chr3	197155600	0.661157
chr3	197193800	0.818182
chr3	197203500	0.661157
chr3	197217600	0.933884
chr3	197237200	0.719008
chr3	197244000	0.752066
chr3	197392200	0.776860
chr3	197439900	0.975207
chr3	197520700	0.702479
chr3	197680500	0.909091
chr3	197807700	0.867769
chr4	472900	0.661157
chr4	675100	0.727273
chr4	682600	0.925620
chr4	703100	0.702479
chr4	720700	0.975207
chr4	744300	0.818182
chr4	776800	0.909091
chr4	973400	0.909091
chr4	986600	0.983471
chr4	1006700	0.768595
chr4	1038200	0.743802
chr4	1041300	0.884298
chr4	1186300	0.892562
chr4	1227100	0.884298
chr4	1303400	0.900826
chr4	1550400	0.867769
chr4	1716800	0.669421
chr4	1731100	0.727273
chr4	1754400	0.669421
chr4	1758100	0.685950
chr4	1765800	0.685950
chr4	1876100	0.677686
chr4	1942300	0.958678
chr4	2258900	0.909091
chr4	2264900	0.735537
chr4	2287400	0.925620
chr4	2332200	0.809917
chr4	2404500	0.917355
chr4	2417200	0.892562
chr4	2464700	0.685950
chr4	2484500	0.710744
chr4	2487200	0.975207
chr4	2491900	0.892562
chr4	2600800	0.809917
chr4	2715900	0.950413
chr4	2748200	0.900826
chr4	2766100	0.727273
chr4	2795500	0.834711
chr4	2799500	0.892562
chr4	2803500	0.785124
chr4	2807900	0.809917

chr4	2840100	0.876033
chr4	2847900	0.677686
chr4	2866800	0.735537
chr4	2870000	0.892562
chr4	2883500	0.785124
chr4	2934400	0.702479
chr4	2960600	0.933884
chr4	3043800	0.876033
chr4	3108500	0.867769
chr4	3110800	0.876033
chr4	3113200	0.983471
chr4	3115400	0.909091
chr4	3123500	0.933884
chr4	3125800	0.983471
chr4	3302900	0.859504
chr4	3305300	0.950413
chr4	3309000	0.867769
chr4	3325100	0.867769
chr4	3333400	1.000000
chr4	3344300	0.983471
chr4	3356900	0.942149
chr4	3373700	0.809917
chr4	3378000	0.719008
chr4	3387200	0.776860
chr4	3390700	0.950413
chr4	3438900	0.834711
chr4	3986100	0.793388
chr4	4272000	0.933884
chr4	4325800	0.859504
chr4	4475800	0.752066
chr4	4494400	0.966942
chr4	4662100	0.991736
chr4	4855300	0.710744
chr4	4858900	0.776860
chr4	5021400	0.677686
chr4	5032300	0.958678
chr4	5036000	0.966942
chr4	5040000	0.950413
chr4	5046600	0.925620
chr4	5167900	0.933884
chr4	5556100	0.785124
chr4	5749500	0.818182
chr4	5753300	0.909091
chr4	5756600	0.950413
chr4	5760700	0.876033
chr4	5826100	0.677686
chr4	5829200	0.809917
chr4	6202400	0.685950
chr4	6259500	0.760331
chr4	6275400	0.851240
chr4	6280800	0.884298
chr4	6326900	0.942149
chr4	6661100	0.801653
chr4	6691000	0.719008

chr4	6696500	0.710744
chr4	6751500	0.900826
chr4	6809300	0.950413
chr4	6858000	0.719008
chr4	6944100	0.768595
chr4	6985600	0.983471
chr4	6993600	0.925620
chr4	7057300	0.884298
chr4	7209000	0.768595
chr4	7632800	0.958678
chr4	7650500	0.884298
chr4	7652700	0.818182
chr4	7760400	0.710744
chr4	7782500	0.785124
chr4	7784700	0.892562
chr4	7788200	0.785124
chr4	7794700	0.925620
chr4	7805700	0.735537
chr4	7810300	0.958678
chr4	7816200	0.851240
chr4	7825200	0.909091
chr4	7827400	0.743802
chr4	7830100	0.826446
chr4	7834100	0.950413
chr4	7843800	0.900826
chr4	7846200	0.834711
chr4	7848300	0.966942
chr4	7857200	0.735537
chr4	7859700	0.661157
chr4	7863500	0.983471
chr4	7878800	0.975207
chr4	7889100	0.719008
chr4	7892700	0.991736
chr4	7895000	0.669421
chr4	7902700	0.983471
chr4	7906100	0.966942
chr4	7909000	1.000000
chr4	7912400	1.000000
chr4	7921200	0.867769
chr4	7926400	0.942149
chr4	7936200	0.942149
chr4	7942700	0.661157
chr4	7958100	0.950413
chr4	7960200	0.801653
chr4	7962900	0.826446
chr4	7970300	0.768595
chr4	7974000	0.801653
chr4	7988400	0.818182
chr4	8084300	0.735537
chr4	8185000	0.685950
chr4	8190400	0.694215
chr4	8193800	0.900826
chr4	8197700	0.694215
chr4	8207400	1.000000

chr4	8230600	0.925620
chr4	8271900	0.719008
chr4	8280500	0.834711
chr4	8305600	0.917355
chr4	8318200	0.710744
chr4	8393800	0.892562
chr4	8396300	0.735537
chr4	8404700	0.702479
chr4	8429000	0.669421
chr4	8438100	0.727273
chr4	8440200	0.834711
chr4	10008600	0.842975
chr4	10020800	0.933884
chr4	10080200	0.776860
chr4	10091100	0.958678
chr4	10098600	0.669421
chr4	10101900	0.809917
chr4	10107700	0.966942
chr4	10110400	0.842975
chr4	10125600	0.818182
chr4	10130800	0.710744
chr4	10174900	0.818182
chr4	10183200	0.983471
chr4	10460000	0.661157
chr4	10532200	0.991736
chr4	10554800	0.694215
chr4	10608200	0.859504
chr4	10613600	0.834711
chr4	10658900	0.991736
chr4	10666300	0.793388
chr4	11473500	0.851240
chr4	11477700	0.900826
chr4	11482800	0.719008
chr4	11507400	1.000000
chr4	11516800	0.958678
chr4	11531600	0.900826
chr4	11544700	0.694215
chr4	11547700	0.909091
chr4	11550800	0.983471
chr4	11616100	0.876033
chr4	11693500	0.966942
chr4	11701000	0.983471
chr4	11742100	0.834711
chr4	11745900	0.942149
chr4	11750100	0.983471
chr4	11776400	0.876033
chr4	11807500	0.685950
chr4	11905800	0.842975
chr4	11908000	0.818182
chr4	12272600	0.661157
chr4	13550900	0.826446
chr4	13586700	0.702479
chr4	13656400	0.818182
chr4	13890400	0.818182

chr4	13894100	0.900826
chr4	13909500	0.966942
chr4	13922600	0.876033
chr4	14170900	0.685950
chr4	14180300	1.000000
chr4	14183100	0.966942
chr4	15034100	0.867769
chr4	15044900	0.942149
chr4	15151600	0.809917
chr4	15667800	0.925620
chr4	15692900	0.933884
chr4	15756000	0.892562
chr4	15923100	0.909091
chr4	16142600	0.876033
chr4	16162500	0.785124
chr4	16194800	0.677686
chr4	16198600	0.867769
chr4	16234000	0.735537
chr4	16293000	0.752066
chr4	16534100	0.685950
chr4	16600000	0.818182
chr4	16686000	0.884298
chr4	16688700	0.975207
chr4	16723400	0.743802
chr4	16726900	0.735537
chr4	16774700	0.983471
chr4	16791500	0.760331
chr4	16797100	0.694215
chr4	16814300	0.735537
chr4	16822200	0.942149
chr4	16829900	0.983471
chr4	16854400	0.842975
chr4	16859700	0.933884
chr4	16862800	0.942149
chr4	16865000	0.900826
chr4	16868100	0.950413
chr4	16877400	0.743802
chr4	16892100	0.752066
chr4	16896000	0.909091
chr4	17015100	0.710744
chr4	17020100	0.933884
chr4	17029500	0.876033
chr4	17032500	0.983471
chr4	17144300	0.735537
chr4	17325900	0.685950
chr4	17339100	1.000000
chr4	17450600	0.900826
chr4	17495900	0.752066
chr4	17582700	0.983471
chr4	17783400	0.719008
chr4	17815800	0.925620
chr4	18107300	0.743802
chr4	18111700	0.867769
chr4	18321200	0.818182

chr4	18535600	0.966942
chr4	18538700	0.661157
chr4	18542500	0.842975
chr4	18569300	0.768595
chr4	19215600	0.752066
chr4	19510600	0.776860
chr4	19724400	0.991736
chr4	19727300	0.859504
chr4	19732000	0.925620
chr4	19741200	0.925620
chr4	19778300	0.933884
chr4	19793600	0.793388
chr4	19948100	0.975207
chr4	19984500	0.983471
chr4	19989500	0.809917
chr4	20044900	0.991736
chr4	20196000	0.991736
chr4	20289300	0.975207
chr4	20420800	0.809917
chr4	20433700	0.925620
chr4	20513900	0.991736
chr4	20664900	0.768595
chr4	20780400	0.983471
chr4	20842000	0.991736
chr4	20985900	0.768595
chr4	21013300	0.785124
chr4	21015400	0.966942
chr4	21115800	0.983471
chr4	21207900	0.809917
chr4	21658800	0.925620
chr4	21771100	0.958678
chr4	21804500	0.917355
chr4	22387500	0.859504
chr4	22418800	0.809917
chr4	22476100	0.785124
chr4	24606600	0.950413
chr4	24645300	0.859504
chr4	24845200	0.826446
chr4	24853400	0.685950
chr4	24981300	0.892562
chr4	24993000	0.983471
chr4	25004600	0.909091
chr4	25175400	0.983471
chr4	25261300	0.900826
chr4	25459300	0.933884
chr4	25469400	0.900826
chr4	25487700	0.892562
chr4	25735500	0.669421
chr4	25816400	0.925620
chr4	25862000	0.975207
chr4	25886300	0.917355
chr4	25910400	0.768595
chr4	25921500	0.876033
chr4	26024200	0.851240

chr4	26325700	0.834711
chr4	26571000	0.710744
chr4	26789800	0.793388
chr4	26814300	0.966942
chr4	26824100	0.735537
chr4	26898500	0.735537
chr4	26902400	0.925620
chr4	27007600	0.950413
chr4	27086800	0.669421
chr4	28290200	0.710744
chr4	28356700	0.900826
chr4	28447000	0.661157
chr4	29095800	0.809917
chr4	29155600	0.975207
chr4	29280600	0.958678
chr4	29310600	0.991736
chr4	29313700	0.933884
chr4	29328900	0.942149
chr4	30105200	0.983471
chr4	30399000	0.975207
chr4	30432200	0.933884
chr4	30539500	0.842975
chr4	30726900	0.661157
chr4	30729900	0.909091
chr4	30746800	0.694215
chr4	30755700	0.991736
chr4	30758700	0.900826
chr4	30766700	0.950413
chr4	30769900	0.743802
chr4	30772300	0.942149
chr4	30827200	0.785124
chr4	30865300	0.793388
chr4	30947600	0.842975
chr4	31070800	0.809917
chr4	31088000	0.842975
chr4	31091700	0.917355
chr4	31127400	0.669421
chr4	31538000	0.719008
chr4	32142900	0.983471
chr4	32199200	0.975207
chr4	33783100	0.677686
chr4	37010100	0.900826
chr4	37551600	0.851240
chr4	37606600	0.900826
chr4	37650100	0.876033
chr4	37655300	0.677686
chr4	37664600	0.933884
chr4	37669400	0.785124
chr4	37676200	0.900826
chr4	37685000	0.842975
chr4	37717800	0.743802
chr4	37838600	0.942149
chr4	37840900	0.818182
chr4	37959700	0.752066

chr4	37991900	0.719008
chr4	38025700	0.661157
chr4	38053000	0.991736
chr4	38057900	0.867769
chr4	38064000	0.702479
chr4	38070300	0.809917
chr4	38080600	0.958678
chr4	38084500	0.727273
chr4	38095000	0.768595
chr4	38101600	0.942149
chr4	38134100	0.925620
chr4	38163000	0.991736
chr4	38192500	0.867769
chr4	38212800	0.851240
chr4	38238800	0.859504
chr4	38321100	0.983471
chr4	38531400	0.925620
chr4	38664900	0.793388
chr4	38670700	0.942149
chr4	38674000	0.925620
chr4	38676900	0.760331
chr4	38680000	0.958678
chr4	38690100	1.000000
chr4	38699700	0.768595
chr4	38719800	0.975207
chr4	38807300	0.942149
chr4	38859300	0.933884
chr4	38871900	0.702479
chr4	38891100	1.000000
chr4	39027600	0.925620
chr4	39034300	0.925620
chr4	39038900	0.950413
chr4	39256600	0.925620
chr4	39258700	0.991736
chr4	39306700	0.942149
chr4	39310900	0.661157
chr4	39355500	0.892562
chr4	39392600	0.768595
chr4	39523500	0.950413
chr4	39618400	0.900826
chr4	39623400	0.950413
chr4	39698800	0.661157
chr4	39822100	0.958678
chr4	39935800	0.876033
chr4	39972000	0.661157
chr4	40123700	0.991736
chr4	40168000	0.933884
chr4	40182200	0.983471
chr4	40257700	0.966942
chr4	40314400	0.892562
chr4	40318900	0.760331
chr4	40553500	0.760331
chr4	40631900	0.727273
chr4	40675200	0.743802

chr4	40752500	0.826446
chr4	40843500	0.867769
chr4	40852000	0.727273
chr4	40911500	0.669421
chr4	40913700	0.809917
chr4	40992300	0.925620
chr4	40994500	0.942149
chr4	40996700	0.785124
chr4	41001100	1.000000
chr4	41024800	0.983471
chr4	41031300	0.991736
chr4	41053700	0.917355
chr4	41063900	0.892562
chr4	41123100	0.661157
chr4	41136000	0.768595
chr4	41144100	0.768595
chr4	41146400	0.983471
chr4	41150000	0.925620
chr4	41156500	0.793388
chr4	41159200	0.983471
chr4	41162200	0.991736
chr4	41164400	0.983471
chr4	41171800	0.727273
chr4	41178900	0.942149
chr4	41182600	0.842975
chr4	41192000	0.685950
chr4	41194600	0.933884
chr4	41201700	0.925620
chr4	41204800	0.743802
chr4	41207900	0.942149
chr4	41211600	0.892562
chr4	41310000	0.892562
chr4	41321700	0.727273
chr4	41388000	0.785124
chr4	41429900	0.925620
chr4	41434800	0.768595
chr4	41445300	0.743802
chr4	41527700	1.000000
chr4	41538000	0.925620
chr4	41552900	0.710744
chr4	42154600	0.677686
chr4	42334600	0.942149
chr4	42342200	0.991736
chr4	42414600	1.000000
chr4	42418000	0.677686
chr4	43459000	0.950413
chr4	44027700	0.925620
chr4	44123600	0.900826
chr4	44148000	0.966942
chr4	44153700	0.966942
chr4	44160400	1.000000
chr4	44163400	0.942149
chr4	47074100	0.909091
chr4	47077800	0.975207

chr4	47512900	0.933884
chr4	47527800	0.983471
chr4	47533200	0.859504
chr4	47536000	0.942149
chr4	47580200	0.975207
chr4	47704000	0.743802
chr4	48082200	0.933884
chr4	48096500	0.685950
chr4	48130300	0.975207
chr4	48378400	0.818182
chr4	48591400	0.975207
chr4	48596000	0.966942
chr4	48598900	0.801653
chr4	48637600	0.909091
chr4	48643100	0.900826
chr4	48700200	0.793388
chr4	48702300	1.000000
chr4	48724900	1.000000
chr4	48729500	0.785124
chr4	48754700	1.000000
chr4	48804500	0.983471
chr4	48807200	0.661157
chr4	48901500	0.983471
chr4	48912100	0.884298
chr4	48915800	0.958678
chr4	48942200	0.834711
chr4	49132400	0.735537
chr4	49149300	0.710744
chr4	49516700	0.669421
chr4	49659800	0.966942
chr4	52772900	0.925620
chr4	53455300	0.950413
chr4	53507800	0.785124
chr4	53581800	0.677686
chr4	53886300	0.793388
chr4	53965500	0.933884
chr4	53968000	1.000000
chr4	53970300	0.958678
chr4	54173300	0.677686
chr4	54187900	0.991736
chr4	54281700	0.719008
chr4	54408500	0.966942
chr4	54440000	0.867769
chr4	54443200	1.000000
chr4	54459200	0.933884
chr4	54513400	0.677686
chr4	54569500	0.851240
chr4	54781100	0.867769
chr4	54783700	0.727273
chr4	54810300	1.000000
chr4	54845200	0.694215
chr4	54861100	0.983471
chr4	54926800	0.685950
chr4	54933300	0.752066

chr4	54943700	0.991736
chr4	54948700	0.991736
chr4	54951800	0.685950
chr4	55084200	0.702479
chr4	55096500	0.685950
chr4	55127600	0.727273
chr4	55134200	0.702479
chr4	55143900	0.867769
chr4	55147700	0.909091
chr4	55573300	0.768595
chr4	55589800	0.743802
chr4	55593300	0.727273
chr4	55603100	0.809917
chr4	55796600	0.983471
chr4	55807700	0.975207
chr4	55809900	0.975207
chr4	55812500	0.826446
chr4	55874800	0.859504
chr4	55918100	0.702479
chr4	55936000	0.983471
chr4	55941300	0.809917
chr4	55945100	0.826446
chr4	55947400	0.867769
chr4	55950900	0.702479
chr4	55956000	0.768595
chr4	55964700	0.669421
chr4	55971800	0.669421
chr4	55976600	0.876033
chr4	55983700	0.884298
chr4	55988300	0.710744
chr4	56024400	0.677686
chr4	56026700	0.933884
chr4	56038800	0.768595
chr4	56041400	0.851240
chr4	56044900	0.892562
chr4	56048100	0.966942
chr4	56050600	0.975207
chr4	56053200	0.991736
chr4	56080500	0.909091
chr4	56114300	0.917355
chr4	56118300	0.909091
chr4	56125100	0.694215
chr4	56136500	0.834711
chr4	56162300	0.842975
chr4	56170500	0.900826
chr4	56189700	0.983471
chr4	56196800	0.909091
chr4	56225000	0.892562
chr4	56231300	0.842975
chr4	56304100	0.661157
chr4	56502700	0.702479
chr4	56621200	0.818182
chr4	56623800	0.694215
chr4	56732600	0.834711

chr4	56817300	0.661157
chr4	56893100	0.859504
chr4	57167400	0.925620
chr4	57271700	0.925620
chr4	57277200	0.925620
chr4	57411200	0.785124
chr4	57485500	0.661157
chr4	57508200	0.876033
chr4	57520300	0.876033
chr4	57550800	0.677686
chr4	57804000	0.925620
chr4	57824900	0.933884
chr4	57849800	0.842975
chr4	57886400	0.958678
chr4	57890000	0.719008
chr4	57910000	0.958678
chr4	57931600	0.909091
chr4	57943200	0.809917
chr4	57945900	0.991736
chr4	57948000	0.983471
chr4	57950300	0.958678
chr4	57957000	1.000000
chr4	57962000	0.834711
chr4	57968000	0.842975
chr4	57971400	0.842975
chr4	57978000	0.719008
chr4	57981700	0.694215
chr4	57985700	0.958678
chr4	57998900	0.661157
chr4	58003300	0.900826
chr4	62325500	0.661157
chr4	62328600	0.727273
chr4	65650400	0.933884
chr4	66249900	0.892562
chr4	66368500	0.942149
chr4	66374300	0.925620
chr4	66404300	0.950413
chr4	66519700	0.933884
chr4	66521900	0.892562
chr4	68266100	0.768595
chr4	68304200	0.710744
chr4	68389100	0.925620
chr4	68422900	0.719008
chr4	68568000	0.752066
chr4	68579600	0.933884
chr4	68587800	0.809917
chr4	68752400	0.975207
chr4	69920700	0.842975
chr4	69961500	0.991736
chr4	70296800	0.876033
chr4	70574500	0.892562
chr4	70583800	0.743802
chr4	70587200	0.925620
chr4	70592700	1.000000

chr4	70595600	0.983471
chr4	70623400	0.669421
chr4	70626300	0.859504
chr4	70629800	0.933884
chr4	70632400	0.925620
chr4	70639400	0.933884
chr4	70666500	0.851240
chr4	70689800	0.801653
chr4	70715400	0.685950
chr4	71495300	0.892562
chr4	71498000	0.958678
chr4	71556700	0.719008
chr4	71600400	0.685950
chr4	71634600	0.900826
chr4	71645200	0.933884
chr4	71699900	0.677686
chr4	72426300	0.702479
chr4	72811300	0.983471
chr4	72872500	0.909091
chr4	73178300	0.884298
chr4	73185700	0.876033
chr4	73256200	0.826446
chr4	73341700	0.760331
chr4	73837500	0.975207
chr4	73841000	0.942149
chr4	73965200	0.702479
chr4	73990500	0.669421
chr4	74053000	0.950413
chr4	74080000	0.958678
chr4	74526800	0.809917
chr4	74528900	0.991736
chr4	74545900	0.991736
chr4	74564600	0.785124
chr4	74569200	0.818182
chr4	74571800	0.950413
chr4	74574900	0.983471
chr4	74591900	0.975207
chr4	74595400	0.694215
chr4	74599200	0.801653
chr4	74601300	0.975207
chr4	74606200	0.867769
chr4	74641800	0.942149
chr4	74682900	0.958678
chr4	74710500	0.892562
chr4	74738700	0.975207
chr4	74884400	0.669421
chr4	74960100	1.000000
chr4	74963300	0.793388
chr4	74974400	0.884298
chr4	74977500	0.884298
chr4	74983200	1.000000
chr4	75097000	0.983471
chr4	75193800	0.983471
chr4	75240900	0.900826

chr4	75258000	0.876033
chr4	75260900	0.727273
chr4	75284600	0.942149
chr4	75339700	0.834711
chr4	75369800	0.991736
chr4	75372200	0.661157
chr4	75411900	0.966942
chr4	75454300	0.809917
chr4	75544000	0.975207
chr4	75548000	0.694215
chr4	75550600	0.966942
chr4	75557900	0.950413
chr4	75565800	0.983471
chr4	75608400	0.818182
chr4	75694600	0.677686
chr4	75859600	0.752066
chr4	76174300	0.958678
chr4	76437200	0.818182
chr4	76584900	0.694215
chr4	76600000	0.818182
chr4	76635700	0.975207
chr4	76644100	0.694215
chr4	76671000	0.991736
chr4	76722400	0.685950
chr4	76949900	0.669421
chr4	76957500	0.892562
chr4	77011700	0.826446
chr4	77119100	0.950413
chr4	77121500	0.950413
chr4	77129900	0.991736
chr4	77342600	0.669421
chr4	77371100	0.842975
chr4	77468100	0.958678
chr4	77507500	0.727273
chr4	77532900	0.876033
chr4	77555800	0.966942
chr4	77560500	0.842975
chr4	77563000	0.809917
chr4	77579800	0.991736
chr4	77599000	0.818182
chr4	77624300	0.694215
chr4	77626800	0.743802
chr4	77675000	0.933884
chr4	77694000	0.793388
chr4	77725700	0.991736
chr4	77752500	0.917355
chr4	77804700	0.785124
chr4	77865100	0.661157
chr4	77899200	0.950413
chr4	77905800	0.975207
chr4	77923300	0.826446
chr4	77927800	0.752066
chr4	77932400	0.958678
chr4	77935800	1.000000

chr4	77943900	0.892562
chr4	78014800	1.000000
chr4	78021700	0.942149
chr4	78558300	0.917355
chr4	78617200	0.826446
chr4	78704700	0.776860
chr4	78722800	0.958678
chr4	78743300	0.917355
chr4	78775700	0.917355
chr4	78778900	0.710744
chr4	78840300	0.876033
chr4	78844100	0.933884
chr4	78848100	0.909091
chr4	78850200	0.950413
chr4	78853800	0.867769
chr4	79439800	0.710744
chr4	79451000	0.752066
chr4	79453400	0.752066
chr4	79475000	0.867769
chr4	79482300	0.975207
chr4	79486600	0.892562
chr4	79490600	1.000000
chr4	79543900	0.685950
chr4	79567600	0.975207
chr4	79573800	0.884298
chr4	79578200	0.892562
chr4	79580500	0.884298
chr4	79583300	0.983471
chr4	79588600	0.677686
chr4	79642900	0.900826
chr4	79690900	0.760331
chr4	79811700	0.760331
chr4	80131800	0.685950
chr4	80572300	0.991736
chr4	80574400	0.752066
chr4	80773600	0.685950
chr4	80904700	0.876033
chr4	80909200	0.694215
chr4	80929000	0.867769
chr4	80933200	0.909091
chr4	80938800	0.768595
chr4	80956500	0.719008
chr4	80958600	0.983471
chr4	80991400	0.925620
chr4	80999100	0.826446
chr4	81048700	1.000000
chr4	81094900	0.743802
chr4	81128400	0.710744
chr4	81157000	0.867769
chr4	81160000	0.942149
chr4	81228000	0.710744
chr4	82668600	0.900826
chr4	83346300	0.727273
chr4	83440400	0.983471

chr4	83630400	0.876033
chr4	83702200	0.966942
chr4	83705900	0.867769
chr4	83708500	0.809917
chr4	83717300	0.727273
chr4	83761200	0.958678
chr4	83787500	0.958678
chr4	83808300	0.727273
chr4	83870200	0.884298
chr4	83901800	0.925620
chr4	83958900	0.884298
chr4	83992400	0.958678
chr4	83996800	0.892562
chr4	84015400	0.842975
chr4	84071000	0.958678
chr4	84074500	0.925620
chr4	84178600	0.826446
chr4	84242000	0.661157
chr4	84250700	0.966942
chr4	84278700	1.000000
chr4	84469900	0.975207
chr4	84474300	0.884298
chr4	84540600	0.991736
chr4	84551900	1.000000
chr4	85595100	0.818182
chr4	85603400	0.801653
chr4	85612800	0.991736
chr4	85720900	0.991736
chr4	85725300	0.966942
chr4	85735800	0.917355
chr4	85773500	0.900826
chr4	85779200	0.735537
chr4	85792700	0.859504
chr4	85798800	0.876033
chr4	85801000	0.966942
chr4	85809200	0.669421
chr4	85842200	0.917355
chr4	85859600	0.983471
chr4	86330500	0.859504
chr4	86681700	0.694215
chr4	86685700	0.842975
chr4	86690600	0.760331
chr4	86695900	0.983471
chr4	86698300	0.892562
chr4	86703600	0.859504
chr4	86706400	0.975207
chr4	86708600	0.933884
chr4	86712000	0.842975
chr4	86714800	0.975207
chr4	86717900	0.909091
chr4	86729300	0.925620
chr4	86734300	0.884298
chr4	86745300	0.983471
chr4	86748200	0.950413

chr4	86764000	0.983471
chr4	86790500	0.793388
chr4	86846200	0.785124
chr4	86853800	0.752066
chr4	86874100	1.000000
chr4	86891000	0.719008
chr4	86946100	0.685950
chr4	86954700	0.966942
chr4	86958800	1.000000
chr4	87642400	0.710744
chr4	87778700	0.669421
chr4	87846500	0.876033
chr4	87860600	0.975207
chr4	87863700	0.950413
chr4	87869200	0.884298
chr4	87889800	0.966942
chr4	87892400	0.752066
chr4	87895700	0.925620
chr4	87899200	0.975207
chr4	87921100	0.752066
chr4	87931100	0.809917
chr4	87933300	0.975207
chr4	87935900	1.000000
chr4	87953500	0.876033
chr4	87960100	0.975207
chr4	87967800	0.933884
chr4	87975800	0.735537
chr4	88000600	0.925620
chr4	88011100	0.900826
chr4	88015100	0.958678
chr4	88025100	1.000000
chr4	88029200	0.983471
chr4	88040200	0.669421
chr4	88579000	0.991736
chr4	88765500	0.801653
chr4	88817800	0.958678
chr4	88871500	0.925620
chr4	88894800	0.933884
chr4	88931800	0.735537
chr4	88939400	0.991736
chr4	88991200	0.785124
chr4	89026600	0.818182
chr4	89028800	0.917355
chr4	89126200	0.776860
chr4	89146700	0.933884
chr4	89152800	0.909091
chr4	89262800	0.983471
chr4	89454000	0.818182
chr4	89520100	0.776860
chr4	89522300	0.900826
chr4	89530600	0.991736
chr4	89534300	0.983471
chr4	89700900	0.917355
chr4	89764800	0.801653

chr4	89786500	0.950413
chr4	89788600	0.966942
chr4	89882100	0.793388
chr4	89978000	0.677686
chr4	90082400	0.776860
chr4	90115500	0.719008
chr4	90216000	0.760331
chr4	90238500	0.950413
chr4	90391300	0.752066
chr4	90404400	0.933884
chr4	90410400	0.892562
chr4	90413900	0.975207
chr4	90419400	0.991736
chr4	90428600	0.677686
chr4	90456500	0.933884
chr4	90459100	0.867769
chr4	90461300	0.950413
chr4	90471300	0.917355
chr4	90630300	0.933884
chr4	90645000	0.933884
chr4	90674000	0.867769
chr4	90676300	0.884298
chr4	90710100	0.727273
chr4	90718500	0.925620
chr4	90720700	0.809917
chr4	90723000	0.876033
chr4	90742500	0.768595
chr4	90798000	0.661157
chr4	90811000	0.958678
chr4	90822100	0.727273
chr4	90851300	0.925620
chr4	91131300	0.983471
chr4	91239100	0.991736
chr4	91366100	0.685950
chr4	91542300	0.950413
chr4	91763200	0.694215
chr4	91780400	0.892562
chr4	91825600	0.752066
chr4	91872300	0.685950
chr4	94750300	0.752066
chr4	95273600	0.669421
chr4	95284400	0.966942
chr4	95286600	0.975207
chr4	95334000	0.925620
chr4	95361200	0.735537
chr4	95370000	0.710744
chr4	95385700	0.809917
chr4	95393600	0.727273
chr4	95408100	0.958678
chr4	95410200	0.801653
chr4	95412600	0.818182
chr4	95428900	0.719008
chr4	95436700	0.685950
chr4	95440000	0.809917

chr4	95450500	0.925620
chr4	95458100	0.983471
chr4	95465200	0.768595
chr4	95467700	0.950413
chr4	95470000	0.991736
chr4	95473400	0.958678
chr4	95480600	0.743802
chr4	95483100	0.991736
chr4	95503700	0.876033
chr4	95526900	0.842975
chr4	95563100	0.975207
chr4	95571100	0.719008
chr4	95749000	0.917355
chr4	96029900	0.752066
chr4	96051300	0.917355
chr4	96103200	0.702479
chr4	96177500	0.859504
chr4	96189500	0.876033
chr4	96211200	0.942149
chr4	96214500	0.776860
chr4	96284900	0.917355
chr4	96330900	0.809917
chr4	96428700	1.000000
chr4	96616000	0.933884
chr4	96622800	0.975207
chr4	96679500	0.727273
chr4	97024500	0.851240
chr4	97269200	0.933884
chr4	97273500	0.958678
chr4	97596900	1.000000
chr4	97599100	0.702479
chr4	98429200	0.842975
chr4	98446900	0.735537
chr4	98564900	0.760331
chr4	98642900	0.933884
chr4	98681200	0.983471
chr4	98683600	0.958678
chr4	98692100	0.876033
chr4	99185500	0.685950
chr4	99236900	0.842975
chr4	99283700	0.702479
chr4	99389700	0.933884
chr4	99404400	0.917355
chr4	99438000	0.752066
chr4	99443900	0.925620
chr4	99446000	0.694215
chr4	99466200	0.669421
chr4	99468600	0.818182
chr4	99539200	0.785124
chr4	99548700	0.966942
chr4	99564700	0.917355
chr4	99569900	0.834711
chr4	99576100	0.983471
chr4	99581900	0.677686

chr4	99606900	0.991736
chr4	99610600	1.000000
chr4	99650100	1.000000
chr4	99668400	0.933884
chr4	99837600	0.842975
chr4	99854100	0.942149
chr4	99981800	0.966942
chr4	101211600	0.876033
chr4	101269800	0.793388
chr4	101342800	0.851240
chr4	101350300	0.727273
chr4	101418900	0.752066
chr4	101433300	0.975207
chr4	101440500	0.677686
chr4	101570600	0.933884
chr4	101578700	1.000000
chr4	101616900	0.851240
chr4	101658500	0.983471
chr4	101941900	0.975207
chr4	101959000	0.942149
chr4	102025300	1.000000
chr4	102098700	0.925620
chr4	102113000	0.933884
chr4	102122100	0.983471
chr4	102139600	0.925620
chr4	102177400	0.859504
chr4	102181000	0.909091
chr4	102193200	0.760331
chr4	102277800	0.966942
chr4	102340600	0.661157
chr4	102875400	0.669421
chr4	103060400	0.909091
chr4	103096400	0.826446
chr4	103351700	0.760331
chr4	103387200	0.710744
chr4	103421700	0.694215
chr4	103474100	0.975207
chr4	103515600	0.669421
chr4	103534300	0.983471
chr4	103537800	0.785124
chr4	103553700	0.925620
chr4	103562200	0.892562
chr4	103731000	0.975207
chr4	103737500	0.776860
chr4	103742000	0.900826
chr4	103744800	0.694215
chr4	104020600	0.743802
chr4	104058700	0.983471
chr4	104293700	0.851240
chr4	104513700	0.950413
chr4	104641100	0.752066
chr4	104676700	0.876033
chr4	106115800	0.958678
chr4	106119000	0.933884

chr4	106124200	0.884298
chr4	106308400	0.801653
chr4	106687700	0.719008
chr4	106817500	0.735537
chr4	106900300	0.727273
chr4	106911400	0.991736
chr4	106984100	0.669421
chr4	107128600	0.694215
chr4	107195600	0.876033
chr4	107198800	0.892562
chr4	107240100	0.702479
chr4	107254600	0.925620
chr4	107287200	0.950413
chr4	107291200	0.677686
chr4	107451400	0.950413
chr4	107462900	0.884298
chr4	107487500	1.000000
chr4	107505200	1.000000
chr4	107508700	0.975207
chr4	107612100	0.950413
chr4	107661000	0.826446
chr4	107981500	0.719008
chr4	107990100	0.942149
chr4	108043700	0.776860
chr4	108051900	0.991736
chr4	108201300	0.876033
chr4	108205000	0.925620
chr4	108482700	0.851240
chr4	108611700	0.933884
chr4	108614800	0.975207
chr4	108620900	1.000000
chr4	108631600	1.000000
chr4	108775300	0.950413
chr4	108785100	0.983471
chr4	108792200	0.942149
chr4	108815000	0.818182
chr4	108817600	0.983471
chr4	109513700	0.933884
chr4	109565600	0.867769
chr4	109701500	0.876033
chr4	109800300	0.991736
chr4	109803400	0.834711
chr4	109806500	0.735537
chr4	109876600	0.818182
chr4	109883100	0.785124
chr4	109948200	0.851240
chr4	110000800	0.661157
chr4	110105800	0.942149
chr4	110136700	0.991736
chr4	110287800	0.909091
chr4	110350900	0.975207
chr4	110357800	0.752066
chr4	110470200	0.991736
chr4	110497700	0.983471

chr4	110891600	0.958678
chr4	110981600	0.685950
chr4	111012800	0.785124
chr4	111028200	0.991736
chr4	111041500	0.685950
chr4	111086900	0.900826
chr4	111115200	0.933884
chr4	111455600	0.909091
chr4	111462400	0.983471
chr4	111471800	0.925620
chr4	111553400	0.909091
chr4	111703400	0.859504
chr4	111775800	0.694215
chr4	112191100	0.892562
chr4	112433500	0.900826
chr4	112599900	0.942149
chr4	112913200	0.677686
chr4	113003800	0.752066
chr4	113007100	0.768595
chr4	113031200	0.950413
chr4	113074600	0.719008
chr4	113091500	0.867769
chr4	113194700	0.702479
chr4	113431800	0.735537
chr4	114271100	0.909091
chr4	114305200	1.000000
chr4	114358000	0.983471
chr4	114366200	0.983471
chr4	114374500	0.942149
chr4	114388900	0.900826
chr4	114391000	0.727273
chr4	114469300	0.776860
chr4	114482600	0.785124
chr4	114486600	0.785124
chr4	114706300	0.909091
chr4	114737200	0.735537
chr4	114756500	0.859504
chr4	114804100	0.950413
chr4	114880600	0.991736
chr4	114883100	0.884298
chr4	114891600	0.909091
chr4	114893900	0.900826
chr4	114927200	0.826446
chr4	114936500	0.933884
chr4	114964100	0.677686
chr4	114999000	0.950413
chr4	115009100	0.809917
chr4	115193600	0.975207
chr4	116948800	0.859504
chr4	117139500	0.818182
chr4	117453300	0.966942
chr4	118321500	0.917355
chr4	119008200	0.826446
chr4	119202600	0.694215

chr4	119426400	0.727273
chr4	119674200	0.917355
chr4	119687700	0.859504
chr4	119694300	0.933884
chr4	119696700	0.991736
chr4	119719200	0.677686
chr4	119755000	0.768595
chr4	119763600	0.909091
chr4	119772000	0.677686
chr4	119826800	0.925620
chr4	119839700	0.975207
chr4	120015100	0.842975
chr4	120024800	0.991736
chr4	120028000	0.809917
chr4	120034500	0.909091
chr4	120038900	0.966942
chr4	120045500	0.909091
chr4	120061400	0.942149
chr4	120069500	0.685950
chr4	120126300	0.900826
chr4	120129400	0.942149
chr4	120136700	0.760331
chr4	120140900	0.818182
chr4	120152700	0.859504
chr4	120155200	0.801653
chr4	120159600	0.851240
chr4	120165800	0.933884
chr4	120192800	0.826446
chr4	120202800	0.983471
chr4	120206800	0.884298
chr4	120258400	0.975207
chr4	120447800	0.942149
chr4	120651700	0.917355
chr4	121439600	0.867769
chr4	122061100	0.909091
chr4	122077700	0.859504
chr4	122607600	0.991736
chr4	122612300	1.000000
chr4	122632900	0.900826
chr4	122711500	0.983471
chr4	122742100	0.694215
chr4	123461800	0.834711
chr4	123463900	0.685950
chr4	123556800	0.669421
chr4	123588300	0.983471
chr4	123593800	0.892562
chr4	123595900	0.966942
chr4	123610100	0.909091
chr4	123686000	0.991736
chr4	123696700	0.867769
chr4	123704900	0.983471
chr4	123776600	0.677686
chr4	123811600	0.958678
chr4	123817100	0.892562

chr4	123820000	0.842975
chr4	123939400	0.859504
chr4	123947500	0.909091
chr4	123977400	0.768595
chr4	124042800	0.760331
chr4	124049400	0.917355
chr4	124171300	0.743802
chr4	124178700	0.743802
chr4	124261000	0.876033
chr4	124263100	0.710744
chr4	124265300	0.983471
chr4	124283000	0.991736
chr4	124308200	0.958678
chr4	124315200	0.669421
chr4	124337400	0.876033
chr4	124339900	0.933884
chr4	124365600	0.933884
chr4	124371300	0.818182
chr4	124476700	0.909091
chr4	124481300	0.677686
chr4	124521300	0.975207
chr4	124542700	0.892562
chr4	124752800	0.809917
chr4	124754900	0.892562
chr4	124759100	0.685950
chr4	124778500	0.917355
chr4	124813900	0.975207
chr4	124816800	0.975207
chr4	124818900	0.925620
chr4	124844400	0.991736
chr4	124857800	0.925620
chr4	124861000	0.925620
chr4	124898100	0.942149
chr4	124902500	0.958678
chr4	124906600	0.793388
chr4	124909500	0.933884
chr4	124917300	0.834711
chr4	124982000	0.933884
chr4	125032800	0.719008
chr4	125100000	0.909091
chr4	125121300	0.909091
chr4	125125700	0.685950
chr4	125255800	0.702479
chr4	125323200	0.809917
chr4	125344500	0.752066
chr4	125348700	0.950413
chr4	125351200	0.925620
chr4	125354000	0.983471
chr4	125359700	0.826446
chr4	125399800	0.892562
chr4	125448000	0.966942
chr4	125485700	0.818182
chr4	125553200	0.942149
chr4	125556900	0.793388

chr4	125561800	0.983471
chr4	125564000	0.785124
chr4	125606500	0.818182
chr4	125609300	0.785124
chr4	125625600	0.818182
chr4	125635600	0.793388
chr4	125725100	0.661157
chr4	125825700	0.685950
chr4	125860700	0.925620
chr4	125863200	0.851240
chr4	125866700	0.917355
chr4	125871100	0.925620
chr4	125895000	0.685950
chr4	126019200	0.743802
chr4	126076900	0.826446
chr4	126120300	0.834711
chr4	126165700	0.776860
chr4	126235000	0.867769
chr4	126240700	0.661157
chr4	126290000	0.966942
chr4	126311300	0.983471
chr4	126314800	0.677686
chr4	126319000	0.950413
chr4	126342800	0.669421
chr4	126442100	0.851240
chr4	126503700	0.752066
chr4	126625000	0.735537
chr4	126641700	0.818182
chr4	126656700	0.942149
chr4	126660000	0.719008
chr4	126662100	0.768595
chr4	127137500	0.925620
chr4	127392300	0.925620
chr4	127699200	0.900826
chr4	128607600	0.768595
chr4	128792500	0.818182
chr4	128981600	0.685950
chr4	129077300	0.752066
chr4	129081300	0.743802
chr4	129084900	0.983471
chr4	129178200	0.694215
chr4	129242500	0.826446
chr4	129244600	0.991736
chr4	129259800	0.966942
chr4	129275900	0.958678
chr4	129308500	0.942149
chr4	129338400	0.661157
chr4	129376100	0.975207
chr4	129382900	0.958678
chr4	129389900	0.785124
chr4	129424500	0.917355
chr4	129438700	0.743802
chr4	129443000	0.975207
chr4	129446300	0.900826

chr4	129450700	0.942149
chr4	129459300	0.743802
chr4	129494500	0.900826
chr4	129522800	0.917355
chr4	129752600	0.719008
chr4	130284900	0.818182
chr4	130298900	0.669421
chr4	130437500	0.884298
chr4	130467700	0.950413
chr4	130483300	0.694215
chr4	130744300	0.818182
chr4	131317300	0.876033
chr4	131906900	0.892562
chr4	131923100	0.727273
chr4	132531900	0.966942
chr4	132993300	0.760331
chr4	133101800	0.909091
chr4	133912800	0.909091
chr4	133915000	0.925620
chr4	133921500	0.892562
chr4	134051900	0.909091
chr4	134724200	0.677686
chr4	135465800	0.694215
chr4	135501500	0.785124
chr4	135507400	0.950413
chr4	135544200	0.950413
chr4	135643900	0.942149
chr4	136429000	0.669421
chr4	137236100	0.743802
chr4	137286200	0.719008
chr4	137288900	0.834711
chr4	137291100	0.851240
chr4	137334100	0.991736
chr4	137572400	0.950413
chr4	138309400	0.892562
chr4	138440900	0.685950
chr4	138957000	0.983471
chr4	138960700	0.661157
chr4	138971500	0.983471
chr4	138975200	0.983471
chr4	138977800	1.000000
chr4	138984200	0.859504
chr4	139033100	0.909091
chr4	139036300	1.000000
chr4	139100000	0.900826
chr4	139121500	0.991736
chr4	139143400	0.867769
chr4	139147900	0.826446
chr4	139156100	0.966942
chr4	139159200	0.909091
chr4	139197200	0.925620
chr4	139320300	0.942149
chr4	139360700	0.991736
chr4	139483800	0.942149

chr4	139657900	1.000000
chr4	139797500	0.942149
chr4	139832300	0.917355
chr4	139964100	0.867769
chr4	140187900	0.942149
chr4	140204100	0.851240
chr4	140338600	0.842975
chr4	140342000	0.917355
chr4	140408600	0.694215
chr4	140430400	0.909091
chr4	140482100	0.834711
chr4	140528200	0.661157
chr4	140579200	0.966942
chr4	140582400	0.925620
chr4	140588900	0.876033
chr4	140656600	0.900826
chr4	140662000	0.925620
chr4	140686700	0.661157
chr4	140697800	0.719008
chr4	140713300	0.876033
chr4	140730400	0.909091
chr4	140737200	0.933884
chr4	140793600	0.909091
chr4	140812300	0.950413
chr4	140823100	0.867769
chr4	140835200	0.884298
chr4	140840200	0.661157
chr4	140843500	0.743802
chr4	140878000	0.859504
chr4	140893000	0.677686
chr4	140897400	0.983471
chr4	140910900	0.859504
chr4	140926000	0.867769
chr4	140934200	1.000000
chr4	140958700	0.991736
chr4	140968800	0.975207
chr4	140971200	0.768595
chr4	140988200	0.768595
chr4	141005800	0.694215
chr4	141010700	0.975207
chr4	141014500	0.942149
chr4	141021700	1.000000
chr4	141025400	0.983471
chr4	141038200	0.933884
chr4	141058400	0.719008
chr4	141084400	0.925620
chr4	141098400	0.752066
chr4	141108100	0.694215
chr4	141144700	0.991736
chr4	141155700	0.867769
chr4	141159200	0.991736
chr4	141164600	0.702479
chr4	141173800	0.892562
chr4	141175900	0.975207

chr4	141178600	0.958678
chr4	141189100	0.925620
chr4	141198900	0.925620
chr4	141264700	0.685950
chr4	141289300	0.818182
chr4	141490300	0.768595
chr4	141602800	0.669421
chr4	141627400	0.826446
chr4	141638600	0.925620
chr4	141640700	0.942149
chr4	141648500	0.942149
chr4	141658800	0.917355
chr4	141664500	0.851240
chr4	142271600	0.892562
chr4	142274600	0.900826
chr4	142490800	0.818182
chr4	142606400	0.859504
chr4	142681300	0.933884
chr4	142684800	0.917355
chr4	142690500	0.942149
chr4	142747200	0.950413
chr4	142834400	0.801653
chr4	143013000	0.834711
chr4	143017200	0.933884
chr4	143043300	0.933884
chr4	143050200	0.975207
chr4	143273500	0.966942
chr4	143289400	0.719008
chr4	143329300	0.834711
chr4	143471300	0.966942
chr4	144273500	1.000000
chr4	144280600	0.991736
chr4	144303500	0.958678
chr4	144324500	0.966942
chr4	144328300	0.925620
chr4	144333300	0.983471
chr4	144360200	0.851240
chr4	144453300	0.983471
chr4	144507800	0.884298
chr4	144621800	0.719008
chr4	144689600	0.975207
chr4	145100500	0.818182
chr4	145277900	0.884298
chr4	145371500	0.710744
chr4	145485900	0.942149
chr4	145500400	0.975207
chr4	145504000	0.983471
chr4	145506600	0.966942
chr4	145513500	1.000000
chr4	145515900	0.933884
chr4	145548100	0.809917
chr4	145550200	0.942149
chr4	145556800	1.000000
chr4	145574000	0.719008

chr4	145580900	0.925620
chr4	145586600	0.851240
chr4	145609700	0.694215
chr4	145617500	0.669421
chr4	145621100	1.000000
chr4	145625200	0.933884
chr4	145629200	0.966942
chr4	145632600	0.826446
chr4	145635000	0.776860
chr4	145827600	0.900826
chr4	145934500	0.933884
chr4	146010200	0.710744
chr4	146028600	0.942149
chr4	146075000	0.801653
chr4	146077100	0.942149
chr4	146093300	0.892562
chr4	146200200	0.884298
chr4	146205400	0.859504
chr4	146207700	0.917355
chr4	146309400	0.983471
chr4	146373900	0.991736
chr4	146411700	0.702479
chr4	146422500	0.867769
chr4	146425500	0.851240
chr4	146429000	0.925620
chr4	146451900	0.677686
chr4	146466100	0.958678
chr4	146468600	0.685950
chr4	146545400	0.983471
chr4	146594600	0.785124
chr4	146601400	0.942149
chr4	146654200	0.867769
chr4	146657100	0.917355
chr4	146700600	0.909091
chr4	146704300	0.966942
chr4	146725500	0.842975
chr4	146727600	0.991736
chr4	146729800	0.685950
chr4	146751500	0.727273
chr4	146764500	0.975207
chr4	146769200	0.694215
chr4	146861400	0.661157
chr4	147163100	0.991736
chr4	147252000	0.900826
chr4	147257900	0.942149
chr4	147264800	0.752066
chr4	147290000	1.000000
chr4	147293100	0.933884
chr4	147329100	0.983471
chr4	147359600	0.826446
chr4	147398600	0.991736
chr4	147897800	0.735537
chr4	148119800	0.851240
chr4	148248200	0.776860

chr4	148257400	0.909091
chr4	148499200	0.950413
chr4	148636100	0.983471
chr4	148657300	0.975207
chr4	148675600	0.785124
chr4	148690100	0.983471
chr4	148693200	0.966942
chr4	148695400	0.884298
chr4	148698400	0.661157
chr4	148742900	0.702479
chr4	148751000	0.834711
chr4	148753100	0.942149
chr4	148762800	0.776860
chr4	148776600	0.719008
chr4	148787200	0.743802
chr4	148838000	0.776860
chr4	148849200	1.000000
chr4	148891300	0.859504
chr4	148916100	0.983471
chr4	148929100	0.752066
chr4	148939200	0.925620
chr4	149088100	0.702479
chr4	149176700	0.876033
chr4	149275500	0.950413
chr4	149277700	0.752066
chr4	149297600	0.909091
chr4	149355500	0.685950
chr4	149434700	0.694215
chr4	149436800	0.933884
chr4	149753400	0.925620
chr4	149756900	0.983471
chr4	149775800	0.694215
chr4	149779200	0.826446
chr4	150042400	0.975207
chr4	150628600	0.785124
chr4	150909000	0.760331
chr4	151041100	0.834711
chr4	151057500	0.925620
chr4	151062300	0.933884
chr4	151082000	0.685950
chr4	151095300	0.925620
chr4	151151200	0.677686
chr4	151153400	0.776860
chr4	151158800	0.975207
chr4	151227300	0.975207
chr4	151230200	0.991736
chr4	151269300	0.768595
chr4	151404000	0.793388
chr4	151578700	0.661157
chr4	151586600	0.752066
chr4	151872200	0.801653
chr4	151883300	0.776860
chr4	151894200	0.702479
chr4	151920600	0.735537

chr4	151953600	0.950413
chr4	152051600	0.966942
chr4	152117300	0.983471
chr4	152124800	0.760331
chr4	152128500	0.909091
chr4	152137400	0.942149
chr4	152150000	0.851240
chr4	152205900	0.991736
chr4	152213200	0.785124
chr4	152236700	0.991736
chr4	152253100	0.702479
chr4	152339700	0.909091
chr4	152580700	0.669421
chr4	152666100	0.900826
chr4	153022100	0.867769
chr4	153027700	0.983471
chr4	153031300	0.661157
chr4	153037500	0.743802
chr4	153128300	0.909091
chr4	153165500	0.975207
chr4	153176800	0.925620
chr4	153180900	0.950413
chr4	153274300	0.669421
chr4	153320900	0.661157
chr4	153434000	0.966942
chr4	153437200	0.785124
chr4	153603200	0.859504
chr4	153879100	0.834711
chr4	153883000	1.000000
chr4	154065400	0.727273
chr4	154081300	0.975207
chr4	154178800	0.710744
chr4	154247000	0.752066
chr4	154263000	0.958678
chr4	154386700	0.661157
chr4	154408100	0.793388
chr4	154411100	0.942149
chr4	154435500	0.983471
chr4	154452500	0.834711
chr4	154544800	0.900826
chr4	154598000	0.933884
chr4	154605900	0.752066
chr4	154681100	0.677686
chr4	154713600	0.842975
chr4	155665300	0.702479
chr4	155926500	0.685950
chr4	155929200	0.958678
chr4	155948200	0.975207
chr4	155969200	0.727273
chr4	156001400	1.000000
chr4	156007500	0.909091
chr4	156451800	0.942149
chr4	156464700	0.851240
chr4	156504500	0.834711

chr4	156526500	0.694215
chr4	156550700	0.958678
chr4	156727000	0.694215
chr4	157397900	0.958678
chr4	157469200	0.685950
chr4	157499700	0.900826
chr4	157519900	0.991736
chr4	157692900	0.966942
chr4	157863800	0.710744
chr4	157867400	0.933884
chr4	157871400	0.867769
chr4	157890300	0.793388
chr4	157920900	0.983471
chr4	157925500	0.842975
chr4	158037400	0.958678
chr4	158622600	0.892562
chr4	158730500	0.661157
chr4	158899700	0.917355
chr4	158904600	0.933884
chr4	158914000	0.834711
chr4	158917300	0.991736
chr4	158927700	0.859504
chr4	158929800	0.975207
chr4	158937600	0.677686
chr4	158940200	0.776860
chr4	158942400	0.859504
chr4	158954600	0.991736
chr4	158987100	0.909091
chr4	159016200	0.702479
chr4	159081100	0.991736
chr4	159083500	0.925620
chr4	159085700	0.785124
chr4	159100700	0.760331
chr4	159131700	0.867769
chr4	159236100	0.859504
chr4	159292400	0.925620
chr4	159349700	0.685950
chr4	159463700	0.917355
chr4	159574600	0.735537
chr4	159689400	0.694215
chr4	159693000	0.702479
chr4	159696800	0.966942
chr4	159704600	0.892562
chr4	159707700	0.793388
chr4	159709900	0.776860
chr4	159731500	0.950413
chr4	159739800	0.743802
chr4	159745800	0.933884
chr4	159795400	0.826446
chr4	159817600	0.958678
chr4	159918000	0.900826
chr4	159924700	0.966942
chr4	159941000	0.661157
chr4	159948700	0.776860

chr4	159969500	0.685950
chr4	159992900	0.975207
chr4	160028700	0.702479
chr4	160080700	0.900826
chr4	160096700	0.909091
chr4	160101100	0.801653
chr4	160114300	0.925620
chr4	160125600	0.768595
chr4	160128100	0.933884
chr4	160149200	0.942149
chr4	160188800	0.892562
chr4	160193200	0.818182
chr4	160205100	0.925620
chr4	160230100	0.842975
chr4	160270800	0.809917
chr4	160281900	0.694215
chr4	160304400	0.818182
chr4	160312500	0.925620
chr4	160323400	0.950413
chr4	160350700	0.776860
chr4	160357100	0.859504
chr4	160426300	0.859504
chr4	160437800	0.702479
chr4	160441800	0.768595
chr4	162476400	1.000000
chr4	162487700	0.851240
chr4	162495400	0.727273
chr4	162499400	0.983471
chr4	162601800	0.975207
chr4	162618700	0.925620
chr4	162635600	0.933884
chr4	162850900	0.677686
chr4	162857400	1.000000
chr4	162860000	0.669421
chr4	164083100	0.933884
chr4	166136200	0.917355
chr4	166144100	0.991736
chr4	166176800	0.809917
chr4	166247200	0.966942
chr4	166361600	0.735537
chr4	166436200	0.809917
chr4	166476700	0.958678
chr4	166502000	0.983471
chr4	166543800	0.900826
chr4	166547200	0.768595
chr4	166662300	0.925620
chr4	166668600	0.702479
chr4	166685000	0.685950
chr4	166687800	0.661157
chr4	166761200	0.958678
chr4	166827000	0.884298
chr4	167096500	0.884298
chr4	167214800	0.685950
chr4	167953600	0.975207

chr4	168818600	0.950413
chr4	168824900	0.942149
chr4	169017500	0.719008
chr4	169020000	0.975207
chr4	169022300	0.727273
chr4	169040900	0.727273
chr4	169140000	0.760331
chr4	169142900	0.867769
chr4	169306300	0.909091
chr4	169310500	0.966942
chr4	169330100	0.909091
chr4	169338000	0.851240
chr4	169342900	0.983471
chr4	169355000	1.000000
chr4	169362200	0.876033
chr4	169372800	0.950413
chr4	169403300	0.694215
chr4	169467400	0.768595
chr4	169475300	0.661157
chr4	169480100	1.000000
chr4	169487100	0.677686
chr4	169491100	0.867769
chr4	169526500	0.933884
chr4	169533900	0.925620
chr4	169555200	0.702479
chr4	169558300	0.867769
chr4	169615300	0.983471
chr4	169644400	0.867769
chr4	169688000	0.892562
chr4	169718500	0.859504
chr4	169738400	0.735537
chr4	169770300	0.834711
chr4	169789800	0.958678
chr4	169799500	0.719008
chr4	169971900	0.917355
chr4	169981400	0.884298
chr4	170001900	0.975207
chr4	170020000	1.000000
chr4	170037000	0.950413
chr4	170039200	0.884298
chr4	170058900	0.991736
chr4	170068800	0.950413
chr4	170079200	0.950413
chr4	170081400	0.933884
chr4	170123000	0.950413
chr4	170147500	0.983471
chr4	170151000	0.851240
chr4	170160100	0.834711
chr4	170165900	0.793388
chr4	170170900	0.909091
chr4	170174100	0.909091
chr4	170176800	0.909091
chr4	170179200	0.892562
chr4	170182200	0.900826

chr4	170236500	0.809917
chr4	170478600	0.958678
chr4	170580500	0.900826
chr4	170583600	0.842975
chr4	170585900	0.942149
chr4	170638100	0.809917
chr4	170696300	0.842975
chr4	170713400	0.760331
chr4	172799500	0.661157
chr4	172802100	0.983471
chr4	173903700	0.933884
chr4	173907000	0.834711
chr4	173979200	0.933884
chr4	174103400	0.983471
chr4	174113000	0.925620
chr4	174132100	0.818182
chr4	174139900	0.876033
chr4	174173600	0.851240
chr4	174186000	0.710744
chr4	174240300	0.669421
chr4	174288800	0.842975
chr4	174295000	0.727273
chr4	174339300	0.925620
chr4	174415800	0.752066
chr4	174422600	0.661157
chr4	174428100	0.859504
chr4	174430900	0.677686
chr4	174443700	0.743802
chr4	175139300	0.760331
chr4	177304900	0.917355
chr4	177630500	0.917355
chr4	177691200	0.694215
chr4	177700000	0.809917
chr4	177702800	0.975207
chr4	177715500	0.933884
chr4	177732000	0.958678
chr4	177760300	0.991736
chr4	177763700	0.884298
chr4	177803900	0.900826
chr4	177806100	0.975207
chr4	177824600	0.991736
chr4	177842100	0.958678
chr4	177845000	0.752066
chr4	177909500	0.685950
chr4	177927400	0.768595
chr4	178049700	0.900826
chr4	178243900	0.909091
chr4	178405800	0.917355
chr4	183513200	0.809917
chr4	183525500	0.900826
chr4	183670700	0.909091
chr4	183673700	0.983471
chr4	183897400	0.975207
chr4	184040100	0.983471

chr4	184093200	0.801653
chr4	184097000	0.760331
chr4	184104800	0.975207
chr4	184122200	0.826446
chr4	184162600	0.710744
chr4	184221800	0.942149
chr4	184250300	0.702479
chr4	184256800	0.991736
chr4	184270400	0.859504
chr4	184277100	0.892562
chr4	184319800	0.694215
chr4	184360300	0.950413
chr4	184558500	0.801653
chr4	184798000	0.694215
chr4	185187100	0.942149
chr4	185189900	1.000000
chr4	185276600	0.925620
chr4	185292300	0.677686
chr4	185304200	0.917355
chr4	185308000	0.851240
chr4	185322600	0.942149
chr4	185339100	0.826446
chr4	185363500	0.958678
chr4	185375400	0.900826
chr4	185379100	0.909091
chr4	185456000	0.983471
chr4	185470000	0.677686
chr4	185495000	0.925620
chr4	185565100	0.669421
chr4	185568200	0.760331
chr4	185589900	0.685950
chr4	185610100	0.966942
chr4	185650200	0.966942
chr4	185714300	0.892562
chr4	186033900	0.818182
chr4	186123500	0.785124
chr4	186205300	0.743802
chr4	186239900	0.842975
chr4	186266300	0.975207
chr4	186280000	0.975207
chr4	186295800	0.694215
chr4	186306500	0.793388
chr4	186321000	0.950413
chr4	186333700	0.917355
chr4	186386400	0.851240
chr4	186435000	1.000000
chr4	186441600	0.743802
chr4	186481100	0.884298
chr4	186488500	0.859504
chr4	186495300	0.917355
chr4	186603800	0.818182
chr4	186615000	0.900826
chr4	186629900	0.900826
chr4	186632500	0.851240

chr4	186634800	0.975207
chr4	186638600	0.983471
chr4	186645200	0.743802
chr4	186651100	0.909091
chr4	186659700	0.917355
chr4	186683200	0.966942
chr4	186693400	0.727273
chr4	186697700	0.719008
chr4	186700500	0.900826
chr4	186704900	0.917355
chr4	186714000	0.892562
chr4	186754700	0.925620
chr4	186759100	0.776860
chr4	186761500	0.900826
chr4	186796500	0.983471
chr4	186887600	0.884298
chr4	186951300	0.966942
chr4	187573900	0.743802
chr4	187751400	1.000000
chr4	187754600	0.950413
chr4	187761100	0.966942
chr4	187766100	0.801653
chr4	187809300	0.760331
chr4	187822300	1.000000
chr4	187858100	0.743802
chr5	221400	0.785124
chr5	223800	0.859504
chr5	244100	0.958678
chr5	252700	0.909091
chr5	264200	0.900826
chr5	274200	0.785124
chr5	311500	0.867769
chr5	344600	0.727273
chr5	372100	0.793388
chr5	374300	0.983471
chr5	395400	0.900826
chr5	400300	0.950413
chr5	415600	0.826446
chr5	658100	0.942149
chr5	873100	0.702479
chr5	912800	0.991736
chr5	927100	0.735537
chr5	957400	0.818182
chr5	1005200	0.793388
chr5	1254600	0.727273
chr5	1559600	0.834711
chr5	1564500	0.892562
chr5	1568900	0.991736
chr5	1645600	0.793388
chr5	1790400	0.983471
chr5	1793500	0.917355
chr5	3994800	1.000000
chr5	3996900	0.702479
chr5	6373100	0.925620

chr5	6420000	0.826446
chr5	6423500	0.983471
chr5	6449300	0.851240
chr5	6583500	0.876033
chr5	6585700	0.710744
chr5	6722500	0.818182
chr5	6734100	0.776860
chr5	6766900	0.975207
chr5	7079500	0.867769
chr5	8829500	0.950413
chr5	9047500	0.727273
chr5	10227700	0.685950
chr5	10422600	0.950413
chr5	10457800	0.661157
chr5	10460800	0.710744
chr5	10467900	0.958678
chr5	10514400	1.000000
chr5	10551500	0.966942
chr5	10556300	0.867769
chr5	10577800	0.942149
chr5	10591300	0.991736
chr5	10596100	0.876033
chr5	10601600	0.983471
chr5	10627900	0.900826
chr5	10634900	0.983471
chr5	10696500	0.702479
chr5	10706200	0.900826
chr5	10720500	0.991736
chr5	10725100	0.950413
chr5	10727700	0.801653
chr5	10734400	0.991736
chr5	10759300	0.760331
chr5	12910900	0.900826
chr5	13250100	0.884298
chr5	13253300	0.933884
chr5	13664700	0.909091
chr5	13986200	0.892562
chr5	14039400	0.966942
chr5	14106300	0.876033
chr5	14147500	0.991736
chr5	14149900	0.776860
chr5	14155000	0.735537
chr5	14158100	0.760331
chr5	14165200	0.743802
chr5	14167800	0.950413
chr5	14174700	0.719008
chr5	14183800	0.809917
chr5	14188400	0.958678
chr5	14191700	0.925620
chr5	14193900	0.909091
chr5	14199900	0.859504
chr5	14202900	0.966942
chr5	14205800	0.983471
chr5	14208400	1.000000

chr5	14224000	0.958678
chr5	14227500	0.983471
chr5	14241200	0.694215
chr5	14255300	0.900826
chr5	14258900	0.991736
chr5	14264900	0.991736
chr5	14267200	0.966942
chr5	14270800	1.000000
chr5	14277700	0.842975
chr5	14343000	0.900826
chr5	14357800	0.834711
chr5	14397300	0.859504
chr5	14400600	0.983471
chr5	14406200	0.743802
chr5	14408500	0.925620
chr5	14423900	0.702479
chr5	14428800	0.884298
chr5	14440200	0.892562
chr5	14443600	0.942149
chr5	14446800	0.785124
chr5	14450400	0.785124
chr5	14463700	0.859504
chr5	14476700	0.876033
chr5	14707000	0.752066
chr5	14740100	0.983471
chr5	14758300	0.983471
chr5	14810500	0.958678
chr5	14822400	0.909091
chr5	14926100	0.842975
chr5	15009700	0.983471
chr5	15365500	0.826446
chr5	15368000	0.975207
chr5	15432400	0.719008
chr5	15497800	1.000000
chr5	15606900	0.958678
chr5	15621200	0.933884
chr5	15627700	0.669421
chr5	15645100	0.842975
chr5	15695500	0.677686
chr5	15719300	0.727273
chr5	15795100	0.958678
chr5	15835100	0.826446
chr5	15851200	0.876033
chr5	15855200	0.702479
chr5	15933900	0.925620
chr5	15939500	0.966942
chr5	15993300	0.950413
chr5	16456500	0.925620
chr5	16700800	0.942149
chr5	16705500	0.876033
chr5	16713900	0.851240
chr5	16727200	0.975207
chr5	16736500	0.900826
chr5	16742400	0.925620

chr5	16744500	0.834711
chr5	16856000	0.966942
chr5	16890400	0.925620
chr5	16894800	0.876033
chr5	16897500	0.809917
chr5	16917100	0.983471
chr5	16924700	0.950413
chr5	16931700	0.743802
chr5	16958300	0.975207
chr5	16983600	0.809917
chr5	16993000	0.966942
chr5	16995900	0.942149
chr5	17001900	0.983471
chr5	17021500	1.000000
chr5	17038300	0.933884
chr5	17070600	0.661157
chr5	17077800	0.867769
chr5	17080200	0.950413
chr5	17106500	0.834711
chr5	17119100	0.975207
chr5	17128500	1.000000
chr5	17132300	0.900826
chr5	17146700	0.694215
chr5	17158700	0.669421
chr5	17169600	0.958678
chr5	17181400	0.851240
chr5	17190700	0.892562
chr5	17239500	1.000000
chr5	17249700	0.975207
chr5	17252800	0.958678
chr5	19240800	0.933884
chr5	19768700	0.842975
chr5	19772100	0.892562
chr5	19909600	0.661157
chr5	19929500	0.834711
chr5	19951400	0.760331
chr5	21017600	0.760331
chr5	21072300	0.966942
chr5	21153000	0.991736
chr5	21237500	1.000000
chr5	21679500	0.801653
chr5	21690000	0.661157
chr5	21824000	0.933884
chr5	21916000	0.834711
chr5	22597000	0.983471
chr5	27810800	0.809917
chr5	28158000	0.842975
chr5	28165300	0.909091
chr5	28314000	0.661157
chr5	31036800	0.942149
chr5	31412800	0.760331
chr5	31427900	0.768595
chr5	31471300	0.743802
chr5	31485900	0.966942

chr5	31493600	0.966942
chr5	31861800	0.834711
chr5	32028200	0.867769
chr5	32030800	0.818182
chr5	32033500	0.966942
chr5	32071900	0.669421
chr5	32098200	0.867769
chr5	32101600	0.661157
chr5	32154200	0.884298
chr5	32193900	0.909091
chr5	32234500	0.776860
chr5	32273900	0.859504
chr5	32277800	1.000000
chr5	32284100	0.859504
chr5	32414200	0.925620
chr5	32437500	0.743802
chr5	32506600	0.925620
chr5	32597200	0.991736
chr5	32600300	0.801653
chr5	33050400	0.834711
chr5	33231100	0.966942
chr5	33242400	0.661157
chr5	33272600	0.942149
chr5	33276400	0.859504
chr5	33317500	0.760331
chr5	33321400	0.975207
chr5	33331100	0.661157
chr5	33363200	0.991736
chr5	33365400	0.876033
chr5	33370400	0.983471
chr5	33402300	0.942149
chr5	33443500	0.685950
chr5	33811000	0.876033
chr5	33817400	0.933884
chr5	33828000	1.000000
chr5	33843500	0.809917
chr5	33850600	0.842975
chr5	33855400	0.958678
chr5	34042100	0.661157
chr5	34487800	0.851240
chr5	34499500	0.975207
chr5	34512200	0.900826
chr5	34517400	0.884298
chr5	34520300	0.851240
chr5	34524000	0.917355
chr5	34527300	0.702479
chr5	34539900	0.768595
chr5	34571900	0.950413
chr5	34583400	0.983471
chr5	34586000	0.950413
chr5	34588500	0.900826
chr5	34598700	0.958678
chr5	34609300	0.975207
chr5	34611400	0.685950

chr5	34621300	0.966942
chr5	34626400	0.966942
chr5	34628500	1.000000
chr5	34649100	0.719008
chr5	34660000	0.851240
chr5	34664000	0.776860
chr5	34666800	0.669421
chr5	34672900	0.809917
chr5	34676000	0.793388
chr5	34679300	0.950413
chr5	34683300	0.991736
chr5	34685800	0.933884
chr5	34690900	0.685950
chr5	34705000	0.966942
chr5	34708100	1.000000
chr5	34711300	0.826446
chr5	34713900	0.834711
chr5	34716200	0.991736
chr5	34719600	0.735537
chr5	34743300	0.834711
chr5	34789300	0.983471
chr5	34792600	1.000000
chr5	34864200	0.834711
chr5	34924200	0.735537
chr5	35803800	0.694215
chr5	35857400	0.991736
chr5	36210300	0.735537
chr5	36573800	0.727273
chr5	36575900	0.942149
chr5	36940500	0.983471
chr5	37072600	0.958678
chr5	37198400	0.851240
chr5	37369500	0.677686
chr5	37553500	0.983471
chr5	37658800	0.735537
chr5	37668900	0.900826
chr5	37704500	0.991736
chr5	37718000	1.000000
chr5	37722000	0.942149
chr5	37738400	0.925620
chr5	38400800	0.884298
chr5	38404100	0.710744
chr5	38434000	0.933884
chr5	38463000	0.785124
chr5	38465500	0.925620
chr5	38468700	0.760331
chr5	38599200	0.975207
chr5	38604200	0.727273
chr5	38651500	0.942149
chr5	38783500	1.000000
chr5	38790300	0.669421
chr5	38796700	0.892562
chr5	38807700	0.991736
chr5	38872400	0.917355

chr5	38884600	0.685950
chr5	39077200	0.834711
chr5	39080700	0.876033
chr5	39093500	0.933884
chr5	39102100	0.710744
chr5	39117600	0.933884
chr5	39139500	0.983471
chr5	39149100	0.950413
chr5	39232000	0.917355
chr5	39275100	0.677686
chr5	39355200	0.685950
chr5	39360400	0.867769
chr5	39395800	0.909091
chr5	39400100	0.925620
chr5	39411600	0.818182
chr5	39421400	0.942149
chr5	39427300	0.991736
chr5	39447800	0.991736
chr5	39476900	0.669421
chr5	39498900	0.752066
chr5	39502100	0.925620
chr5	39513500	0.983471
chr5	39517500	0.793388
chr5	39520600	0.983471
chr5	39525100	0.842975
chr5	39528100	0.876033
chr5	39534600	0.950413
chr5	39542800	0.909091
chr5	39544900	0.661157
chr5	39547600	0.768595
chr5	39661500	0.727273
chr5	39756400	0.884298
chr5	40042100	0.710744
chr5	40047000	0.975207
chr5	40109400	0.685950
chr5	40173000	0.834711
chr5	40202900	0.966942
chr5	40224700	0.892562
chr5	40292000	0.685950
chr5	40333800	0.950413
chr5	40410900	0.975207
chr5	40430100	0.950413
chr5	40522000	0.735537
chr5	40552400	0.975207
chr5	40570500	0.942149
chr5	40677600	0.801653
chr5	40687500	0.851240
chr5	40690500	0.917355
chr5	40715700	0.685950
chr5	40734300	0.867769
chr5	40736600	0.942149
chr5	41747900	0.669421
chr5	41789500	0.983471
chr5	41793500	0.925620

chr5	41796300	0.793388
chr5	41810000	0.851240
chr5	41832800	0.950413
chr5	41836300	0.892562
chr5	41922300	0.983471
chr5	42733000	0.859504
chr5	42877000	0.743802
chr5	42945600	0.925620
chr5	42949400	0.842975
chr5	42982700	0.909091
chr5	42985300	0.900826
chr5	43000800	0.776860
chr5	43011100	0.694215
chr5	43034000	0.859504
chr5	43036200	0.884298
chr5	43045000	0.925620
chr5	43064300	0.859504
chr5	43075600	0.834711
chr5	43314900	0.834711
chr5	43601600	0.826446
chr5	43608900	0.685950
chr5	44789900	0.933884
chr5	44876000	0.950413
chr5	45377000	0.702479
chr5	45386000	1.000000
chr5	46360200	0.661157
chr5	49405600	0.785124
chr5	49450800	0.785124
chr5	49887300	0.685950
chr5	50791900	0.669421
chr5	50947700	0.909091
chr5	50949800	0.768595
chr5	50964900	0.677686
chr5	52002000	0.900826
chr5	52021200	0.867769
chr5	52024000	0.966942
chr5	52039200	0.958678
chr5	52099200	0.661157
chr5	52133600	0.760331
chr5	52136400	0.669421
chr5	52218800	0.785124
chr5	52241200	0.818182
chr5	52244400	0.975207
chr5	52280300	0.900826
chr5	52283500	0.958678
chr5	52288300	0.727273
chr5	52303100	0.991736
chr5	52305200	0.842975
chr5	52315900	0.727273
chr5	52323000	0.842975
chr5	52325200	0.933884
chr5	52328000	0.950413
chr5	52333700	0.991736
chr5	52341200	0.859504

chr5	52359400	0.834711
chr5	52365700	0.950413
chr5	52387400	0.735537
chr5	52624200	0.702479
chr5	52658100	0.975207
chr5	52694200	0.719008
chr5	52714900	0.975207
chr5	53027400	0.983471
chr5	53075800	0.991736
chr5	53383800	0.826446
chr5	53820600	1.000000
chr5	53824700	0.842975
chr5	53830100	0.909091
chr5	53833600	0.752066
chr5	53852200	0.842975
chr5	53854700	0.909091
chr5	53858000	0.809917
chr5	53907000	0.818182
chr5	53940200	0.917355
chr5	54040200	0.958678
chr5	54103900	0.677686
chr5	54132400	0.727273
chr5	54180100	0.669421
chr5	54197300	0.694215
chr5	54206800	0.991736
chr5	54238700	0.809917
chr5	54266100	0.760331
chr5	54274900	0.818182
chr5	54283200	0.909091
chr5	54287000	0.950413
chr5	54289500	0.983471
chr5	54299800	0.925620
chr5	54359600	0.801653
chr5	54442400	0.760331
chr5	54448600	0.793388
chr5	54451100	1.000000
chr5	54600000	0.958678
chr5	54686800	0.892562
chr5	54734100	0.826446
chr5	54897600	0.818182
chr5	55147500	0.892562
chr5	55149800	0.983471
chr5	55152800	0.958678
chr5	55164900	0.983471
chr5	55219200	0.950413
chr5	55221400	0.719008
chr5	55286200	0.851240
chr5	55292300	0.669421
chr5	55308400	0.842975
chr5	55336200	0.933884
chr5	55346800	0.966942
chr5	55354600	0.719008
chr5	55366900	0.669421
chr5	55381400	0.991736

chr5	55419000	0.702479
chr5	55422800	0.966942
chr5	55436200	0.966942
chr5	55460700	0.735537
chr5	55464200	0.876033
chr5	55466500	0.983471
chr5	55471900	0.900826
chr5	55479500	0.983471
chr5	55482200	0.776860
chr5	55486800	0.917355
chr5	55502100	0.925620
chr5	55508100	1.000000
chr5	55575100	0.983471
chr5	55578600	0.818182
chr5	55598300	0.727273
chr5	55625200	1.000000
chr5	55638800	0.859504
chr5	55646500	0.942149
chr5	55865500	0.785124
chr5	55917200	0.776860
chr5	55933200	0.752066
chr5	56114800	0.760331
chr5	56134300	0.925620
chr5	56147900	0.793388
chr5	56196700	0.685950
chr5	56496000	0.842975
chr5	56595000	0.876033
chr5	56630900	0.842975
chr5	56679300	0.884298
chr5	56730200	0.933884
chr5	56734600	0.958678
chr5	56738100	0.975207
chr5	56747600	1.000000
chr5	56783600	0.826446
chr5	56790100	0.966942
chr5	56792200	0.851240
chr5	56795400	0.818182
chr5	56962800	0.661157
chr5	56972900	0.661157
chr5	56989300	0.900826
chr5	56994800	0.975207
chr5	57031700	0.933884
chr5	57036800	0.702479
chr5	57074300	0.950413
chr5	57076400	0.900826
chr5	57129100	0.975207
chr5	57131500	0.867769
chr5	57134600	0.867769
chr5	57138100	0.966942
chr5	57252300	0.677686
chr5	57478300	0.677686
chr5	57484500	0.809917
chr5	57535800	0.785124
chr5	57538400	0.975207

chr5	57542400	0.975207
chr5	57555800	0.991736
chr5	57562700	0.743802
chr5	57591000	1.000000
chr5	57614500	0.702479
chr5	57706800	0.702479
chr5	57750200	0.694215
chr5	57757900	0.909091
chr5	57925600	0.760331
chr5	57945300	0.851240
chr5	57962400	0.917355
chr5	57964500	0.834711
chr5	57973500	0.966942
chr5	57976900	0.892562
chr5	58376300	0.933884
chr5	58485200	0.685950
chr5	58502600	0.818182
chr5	58519200	0.925620
chr5	58533100	0.991736
chr5	58573300	0.834711
chr5	58582700	0.809917
chr5	58585100	0.933884
chr5	58588700	0.735537
chr5	58592600	0.900826
chr5	58595300	0.909091
chr5	58597700	0.768595
chr5	58651300	0.876033
chr5	58814000	0.950413
chr5	58822100	0.884298
chr5	58858600	0.983471
chr5	58884100	0.710744
chr5	58894400	0.768595
chr5	58934600	0.834711
chr5	58945300	0.991736
chr5	58958200	0.958678
chr5	59028500	0.719008
chr5	59040200	0.884298
chr5	59042300	0.851240
chr5	59047600	0.826446
chr5	59054100	0.719008
chr5	59056300	0.975207
chr5	59059900	0.884298
chr5	59082600	0.867769
chr5	59086400	0.975207
chr5	59095400	0.991736
chr5	59505300	0.991736
chr5	60347200	0.685950
chr5	60525200	0.743802
chr5	60560300	0.991736
chr5	60571900	0.975207
chr5	60574000	0.950413
chr5	60576900	0.826446
chr5	60586500	0.958678
chr5	60594700	0.983471

chr5	60608200	0.818182
chr5	60611000	0.950413
chr5	60615300	0.975207
chr5	60619100	0.876033
chr5	60638300	0.743802
chr5	60642500	0.743802
chr5	60698600	0.909091
chr5	60704800	0.909091
chr5	60728700	0.743802
chr5	60746500	0.991736
chr5	60879100	0.834711
chr5	61520400	0.677686
chr5	61552600	0.818182
chr5	61573000	0.975207
chr5	61873500	0.933884
chr5	61881700	0.785124
chr5	61889600	0.933884
chr5	61949700	0.876033
chr5	62558500	0.900826
chr5	63507200	0.950413
chr5	63806300	0.694215
chr5	63986900	0.809917
chr5	64062700	0.685950
chr5	64090500	0.867769
chr5	64114700	0.826446
chr5	64132100	0.776860
chr5	64134600	0.983471
chr5	64330800	0.694215
chr5	64333100	0.776860
chr5	64356100	0.900826
chr5	64374900	0.719008
chr5	64399000	0.735537
chr5	64433800	0.892562
chr5	64438500	0.975207
chr5	64458300	0.785124
chr5	64483200	0.991736
chr5	64493900	0.909091
chr5	64496100	0.933884
chr5	64500400	0.826446
chr5	64503100	0.661157
chr5	64505500	0.975207
chr5	64540500	0.669421
chr5	64558500	1.000000
chr5	64656100	0.958678
chr5	64710000	0.735537
chr5	64771300	0.909091
chr5	65023700	0.925620
chr5	65026900	0.661157
chr5	65065600	0.925620
chr5	65097700	0.958678
chr5	65163600	0.975207
chr5	66139600	0.983471
chr5	66144900	1.000000
chr5	66152500	0.892562

chr5	66156500	1.000000
chr5	66160300	0.768595
chr5	66178000	0.950413
chr5	66187100	0.661157
chr5	66229200	1.000000
chr5	66241900	0.925620
chr5	66254300	0.991736
chr5	66310000	0.958678
chr5	66313200	0.661157
chr5	66335000	0.966942
chr5	66354700	0.892562
chr5	66356800	0.942149
chr5	66360300	0.859504
chr5	66379100	0.892562
chr5	66391400	0.752066
chr5	66462400	0.859504
chr5	66512100	0.925620
chr5	66531000	0.975207
chr5	66540900	0.785124
chr5	66564500	0.966942
chr5	66597200	0.776860
chr5	66600100	0.826446
chr5	66602700	0.917355
chr5	66611700	0.950413
chr5	66701500	0.983471
chr5	66709900	1.000000
chr5	67030200	0.892562
chr5	67207000	0.818182
chr5	67295200	0.966942
chr5	67324000	0.818182
chr5	67489200	0.834711
chr5	67535900	0.958678
chr5	67539100	0.983471
chr5	67544400	0.900826
chr5	67547400	0.991736
chr5	67584300	0.710744
chr5	67615500	0.719008
chr5	67627700	0.917355
chr5	67630800	0.991736
chr5	67648600	0.876033
chr5	67721200	0.809917
chr5	67730600	0.694215
chr5	67825900	0.727273
chr5	68534900	0.991736
chr5	68557600	0.942149
chr5	68773500	0.983471
chr5	68781200	0.669421
chr5	68798600	0.776860
chr5	68817300	0.975207
chr5	69188300	0.710744
chr5	69591200	0.661157
chr5	70537100	0.685950
chr5	70590800	0.677686
chr5	70670000	0.677686

chr5	70767400	0.719008
chr5	70779500	1.000000
chr5	70897200	0.925620
chr5	70995400	0.983471
chr5	71065600	0.867769
chr5	71146600	0.983471
chr5	71219000	0.834711
chr5	71221400	0.942149
chr5	71228900	0.983471
chr5	71373400	0.694215
chr5	71407200	0.685950
chr5	71411200	0.966942
chr5	71425200	0.818182
chr5	71427700	0.818182
chr5	71429800	0.983471
chr5	71431900	0.925620
chr5	71434600	0.785124
chr5	71437800	0.900826
chr5	71450300	0.933884
chr5	71453200	0.809917
chr5	71456200	0.966942
chr5	71462600	0.966942
chr5	71507600	0.909091
chr5	71530300	0.958678
chr5	71532400	0.884298
chr5	71540900	0.793388
chr5	71544400	0.917355
chr5	71548600	0.991736
chr5	71552300	0.958678
chr5	71652000	0.892562
chr5	71659400	0.917355
chr5	71662800	0.768595
chr5	71674700	0.933884
chr5	71684000	0.975207
chr5	71737900	0.917355
chr5	71751100	0.801653
chr5	71760100	0.966942
chr5	71764600	0.983471
chr5	71773700	1.000000
chr5	71782500	0.884298
chr5	71795700	0.933884
chr5	71800400	0.719008
chr5	71803800	0.809917
chr5	71807300	0.983471
chr5	71848200	0.950413
chr5	71852400	0.785124
chr5	71856900	0.991736
chr5	71860600	0.925620
chr5	71883500	0.983471
chr5	71893900	0.983471
chr5	71897700	0.768595
chr5	71901600	0.876033
chr5	71920700	0.983471
chr5	72095800	0.743802

chr5	72115900	0.685950
chr5	72118600	0.760331
chr5	72133700	0.719008
chr5	72139900	0.958678
chr5	72142500	0.743802
chr5	72148300	0.760331
chr5	72153100	0.966942
chr5	72166900	0.859504
chr5	72234000	0.925620
chr5	72248300	0.669421
chr5	72254500	0.710744
chr5	72327100	0.892562
chr5	72418600	1.000000
chr5	72451200	0.719008
chr5	72529600	0.752066
chr5	72577600	0.909091
chr5	72661700	0.966942
chr5	72667500	0.958678
chr5	72678100	0.842975
chr5	72733600	0.702479
chr5	72790900	0.768595
chr5	72796900	0.818182
chr5	72803000	0.900826
chr5	72837800	1.000000
chr5	72888900	0.859504
chr5	72900200	0.867769
chr5	72903000	0.950413
chr5	72913700	0.925620
chr5	72932100	0.900826
chr5	72935400	0.975207
chr5	72938200	0.884298
chr5	72950800	0.991736
chr5	72961700	0.661157
chr5	72969200	0.942149
chr5	72988300	0.925620
chr5	72992900	0.677686
chr5	73011200	0.669421
chr5	73036600	0.950413
chr5	73041800	0.818182
chr5	73044100	0.942149
chr5	73046700	0.884298
chr5	73056100	0.685950
chr5	73060800	0.966942
chr5	73071000	0.983471
chr5	73090200	0.942149
chr5	73093000	0.975207
chr5	73119200	0.719008
chr5	73234500	0.834711
chr5	73423400	0.719008
chr5	73436900	0.900826
chr5	73462800	0.859504
chr5	73497600	0.966942
chr5	73612300	0.702479
chr5	73623400	0.735537

chr5	73704400	1.000000
chr5	73711900	0.909091
chr5	73729700	0.851240
chr5	73798600	0.834711
chr5	73839700	0.966942
chr5	73872900	0.958678
chr5	73882900	0.975207
chr5	73888400	0.793388
chr5	73891500	0.909091
chr5	73929300	0.991736
chr5	73939300	0.958678
chr5	73983600	0.760331
chr5	74018700	0.826446
chr5	74231200	0.991736
chr5	74636300	0.661157
chr5	74854700	0.917355
chr5	74861700	0.842975
chr5	74864100	0.834711
chr5	74936900	0.917355
chr5	74965300	0.950413
chr5	74974000	0.958678
chr5	75447800	0.719008
chr5	75921400	0.892562
chr5	75939400	0.983471
chr5	75953700	0.834711
chr5	75956900	0.942149
chr5	75980400	0.950413
chr5	75984900	0.950413
chr5	75987800	0.983471
chr5	75992700	1.000000
chr5	75997300	0.727273
chr5	76000300	1.000000
chr5	76016800	0.933884
chr5	76020200	0.685950
chr5	76034800	0.975207
chr5	76078700	0.842975
chr5	76081700	0.818182
chr5	76084100	0.991736
chr5	76088200	0.842975
chr5	76097600	0.900826
chr5	76101400	0.884298
chr5	76104200	0.793388
chr5	76110600	0.818182
chr5	76117400	0.694215
chr5	76127300	0.958678
chr5	76134800	0.876033
chr5	76250100	0.661157
chr5	76255200	0.917355
chr5	76435600	0.876033
chr5	76926700	0.909091
chr5	76941300	0.661157
chr5	77039400	0.702479
chr5	77178600	0.867769
chr5	77189800	0.768595

chr5	77269100	0.661157
chr5	77347100	0.834711
chr5	77353100	0.909091
chr5	77371600	0.909091
chr5	77539900	0.785124
chr5	77584300	0.768595
chr5	77702500	0.834711
chr5	77780200	0.702479
chr5	77792300	0.842975
chr5	77800900	0.752066
chr5	77806500	0.834711
chr5	77814700	0.909091
chr5	77818100	0.801653
chr5	77820700	0.983471
chr5	77823300	0.958678
chr5	77826200	0.925620
chr5	77862700	0.975207
chr5	77865300	0.826446
chr5	77882800	0.909091
chr5	77887600	0.834711
chr5	77891000	0.884298
chr5	77894200	0.966942
chr5	77897700	0.867769
chr5	77934300	0.917355
chr5	77936700	0.991736
chr5	78016900	0.876033
chr5	78067800	0.991736
chr5	78075500	0.818182
chr5	78103000	0.958678
chr5	78137900	0.925620
chr5	78204500	0.958678
chr5	78269000	0.818182
chr5	78429800	0.834711
chr5	78497900	0.966942
chr5	79947100	0.900826
chr5	80260800	0.917355
chr5	80263800	0.991736
chr5	80281700	0.884298
chr5	80364400	0.867769
chr5	80491200	0.710744
chr5	80716200	0.925620
chr5	80722700	0.876033
chr5	80725200	0.983471
chr5	80802500	0.842975
chr5	80848900	0.933884
chr5	80880700	0.793388
chr5	80898100	0.900826
chr5	80922800	0.983471
chr5	80939000	0.710744
chr5	81048000	0.694215
chr5	81065200	0.859504
chr5	81075100	0.991736
chr5	81147700	0.809917
chr5	81153900	0.859504

chr5	81461000	0.793388
chr5	81565800	0.950413
chr5	81648900	0.975207
chr5	81653100	0.966942
chr5	81682800	0.876033
chr5	81685800	0.933884
chr5	81693000	0.991736
chr5	81701200	0.917355
chr5	81703300	0.958678
chr5	81924600	0.677686
chr5	82393800	0.851240
chr5	82873000	0.909091
chr5	82929100	0.768595
chr5	83215200	0.917355
chr5	83260400	0.909091
chr5	83324100	0.917355
chr5	83328500	0.727273
chr5	83584500	0.818182
chr5	83597900	0.818182
chr5	83603100	0.694215
chr5	83658500	0.685950
chr5	83690000	0.818182
chr5	86417200	0.983471
chr5	86447700	0.991736
chr5	86491700	0.933884
chr5	86541800	0.776860
chr5	86597000	0.702479
chr5	86689400	0.958678
chr5	86696400	0.768595
chr5	86706600	0.685950
chr5	87681100	0.884298
chr5	87957000	0.719008
chr5	87976400	0.752066
chr5	87986900	0.851240
chr5	88081200	0.876033
chr5	88083300	0.809917
chr5	88110800	0.991736
chr5	88120900	0.942149
chr5	88123200	0.809917
chr5	88125600	0.909091
chr5	88129500	0.933884
chr5	88132700	0.917355
chr5	88171700	0.900826
chr5	88176200	0.876033
chr5	88542500	0.685950
chr5	88648600	0.677686
chr5	88695600	0.685950
chr5	89226500	0.925620
chr5	89229000	0.760331
chr5	89815100	0.867769
chr5	90174700	0.991736
chr5	90205000	0.958678
chr5	90252800	0.859504
chr5	90263900	0.925620

chr5	90349500	0.826446
chr5	90411000	0.867769
chr5	90441300	0.892562
chr5	90452400	0.842975
chr5	90458800	0.958678
chr5	90462600	0.900826
chr5	90575600	0.702479
chr5	90577700	0.884298
chr5	90607300	0.702479
chr5	90610400	0.925620
chr5	90674200	0.702479
chr5	91841300	0.892562
chr5	91977400	0.818182
chr5	92243900	0.950413
chr5	92275300	0.752066
chr5	92281200	0.958678
chr5	92327500	0.727273
chr5	92414700	0.933884
chr5	92454000	0.991736
chr5	92487200	1.000000
chr5	92497100	0.809917
chr5	92500200	0.942149
chr5	92525500	0.719008
chr5	92528400	0.975207
chr5	92611000	0.909091
chr5	92614800	0.867769
chr5	92685800	0.859504
chr5	92691900	0.958678
chr5	92694000	0.851240
chr5	92696100	0.909091
chr5	92700700	0.661157
chr5	92759100	0.752066
chr5	92833100	0.942149
chr5	92846700	0.917355
chr5	92849000	0.958678
chr5	92900500	0.801653
chr5	92943900	0.876033
chr5	93127700	0.900826
chr5	93261400	0.785124
chr5	93759100	0.685950
chr5	93905200	0.975207
chr5	94029300	0.917355
chr5	94082500	0.785124
chr5	94129900	0.950413
chr5	94363700	0.760331
chr5	94368700	0.752066
chr5	94412400	0.867769
chr5	94419800	1.000000
chr5	94474500	0.793388
chr5	94514300	0.966942
chr5	94551000	0.685950
chr5	94553100	0.958678
chr5	94559900	0.975207
chr5	94574300	0.933884

chr5	94577700	0.966942
chr5	94927800	0.909091
chr5	94967900	0.991736
chr5	94982700	0.900826
chr5	95005000	0.966942
chr5	95047900	0.925620
chr5	95051800	0.933884
chr5	95064800	0.884298
chr5	95074000	0.983471
chr5	95096100	0.950413
chr5	95098300	0.694215
chr5	95109800	0.785124
chr5	95115300	0.884298
chr5	95160200	0.735537
chr5	95171400	0.859504
chr5	95193200	0.942149
chr5	95195800	0.801653
chr5	95198800	0.892562
chr5	95206000	0.975207
chr5	95237800	0.851240
chr5	95385800	0.876033
chr5	95401300	0.818182
chr5	95404000	0.826446
chr5	95415100	0.900826
chr5	95417700	0.925620
chr5	95534800	0.983471
chr5	95588900	0.702479
chr5	95591900	0.933884
chr5	95595600	0.983471
chr5	95660500	0.727273
chr5	95720500	0.809917
chr5	95743600	0.925620
chr5	95770200	0.702479
chr5	95812800	0.760331
chr5	95865100	0.661157
chr5	95872700	0.727273
chr5	95874900	0.991736
chr5	95898700	0.983471
chr5	95901000	0.942149
chr5	95903200	0.942149
chr5	95905400	0.851240
chr5	95909000	0.958678
chr5	95918700	0.991736
chr5	95922800	0.719008
chr5	95924900	0.793388
chr5	95927600	0.983471
chr5	95929700	0.793388
chr5	95931800	0.983471
chr5	95934600	0.702479
chr5	95937500	0.966942
chr5	95950000	0.983471
chr5	95966000	0.685950
chr5	96079200	0.900826
chr5	96082100	0.909091

chr5	96117000	0.917355
chr5	96127200	0.900826
chr5	96157300	0.983471
chr5	96213900	0.669421
chr5	96216400	0.966942
chr5	96218700	0.719008
chr5	96274100	0.760331
chr5	96299000	0.719008
chr5	96304200	0.925620
chr5	96345400	0.661157
chr5	96463900	0.884298
chr5	96565800	0.677686
chr5	96994100	0.966942
chr5	97174500	0.958678
chr5	97177000	0.933884
chr5	97316300	0.909091
chr5	97435600	0.966942
chr5	97444300	0.925620
chr5	97447200	0.743802
chr5	97643800	0.950413
chr5	97646900	0.958678
chr5	97650400	0.958678
chr5	97791100	0.933884
chr5	97828500	0.677686
chr5	98238800	0.752066
chr5	98241200	0.991736
chr5	98356200	1.000000
chr5	98369100	0.884298
chr5	98396600	0.710744
chr5	98523800	0.760331
chr5	98527400	0.661157
chr5	98915000	0.892562
chr5	98918100	0.884298
chr5	99066000	0.867769
chr5	99069200	1.000000
chr5	99107100	0.694215
chr5	99109500	0.834711
chr5	99130100	0.710744
chr5	99236400	0.950413
chr5	99606400	0.950413
chr5	99759600	0.809917
chr5	99912300	0.950413
chr5	99931500	0.818182
chr5	99979800	0.793388
chr5	100004100	0.909091
chr5	100113500	0.925620
chr5	100164000	0.859504
chr5	100174000	0.876033
chr5	100177500	0.884298
chr5	100183000	0.991736
chr5	100190800	0.925620
chr5	100212800	0.867769
chr5	100235600	0.859504
chr5	101632400	0.859504

chr5	102051200	0.702479
chr5	102107400	1.000000
chr5	102114400	0.809917
chr5	102143500	0.694215
chr5	102148300	0.785124
chr5	102162700	0.793388
chr5	102248000	0.909091
chr5	102294900	0.917355
chr5	102309400	0.900826
chr5	102311800	1.000000
chr5	102542700	0.809917
chr5	102544800	0.991736
chr5	102553300	1.000000
chr5	102605900	0.884298
chr5	102608200	0.735537
chr5	102694300	0.950413
chr5	102805200	0.917355
chr5	103004300	0.991736
chr5	103525600	0.727273
chr5	103849200	0.950413
chr5	103996000	0.942149
chr5	104045000	0.933884
chr5	105123600	0.669421
chr5	105507800	0.826446
chr5	105734200	0.785124
chr5	105858100	0.809917
chr5	106073400	0.669421
chr5	106295900	0.752066
chr5	106471000	0.867769
chr5	106554500	0.942149
chr5	106657800	0.983471
chr5	106725400	0.991736
chr5	106759200	0.966942
chr5	106762600	0.900826
chr5	106768300	0.867769
chr5	106801900	0.727273
chr5	106804100	0.966942
chr5	106806400	0.826446
chr5	106810300	0.892562
chr5	106813700	0.942149
chr5	106839300	0.735537
chr5	106878500	0.942149
chr5	106901500	0.975207
chr5	106907400	0.892562
chr5	106912200	0.900826
chr5	107205700	0.917355
chr5	107304700	0.917355
chr5	107321400	0.669421
chr5	107333600	0.966942
chr5	107372500	0.801653
chr5	107596300	0.727273
chr5	107632000	0.991736
chr5	107978300	0.735537
chr5	107987000	0.950413

chr5	107990000	0.867769
chr5	107997500	0.991736
chr5	108071600	0.859504
chr5	108137800	0.966942
chr5	108220600	0.958678
chr5	108235400	0.851240
chr5	108374700	0.859504
chr5	108442100	0.917355
chr5	108527400	0.760331
chr5	108532600	0.950413
chr5	108791700	0.983471
chr5	108992900	0.983471
chr5	109004500	1.000000
chr5	109079800	0.859504
chr5	109095200	0.958678
chr5	109115100	0.991736
chr5	109202200	0.983471
chr5	110406500	0.768595
chr5	110435000	0.917355
chr5	110463300	0.826446
chr5	110573400	0.776860
chr5	110866200	0.950413
chr5	110886700	0.884298
chr5	110892900	0.826446
chr5	110896600	0.669421
chr5	110900300	0.950413
chr5	110910700	0.735537
chr5	110920700	0.917355
chr5	110965600	0.694215
chr5	111017000	0.966942
chr5	111033800	0.950413
chr5	111078900	0.768595
chr5	111084300	0.983471
chr5	111590200	0.685950
chr5	111665300	0.983471
chr5	111669300	0.884298
chr5	111674900	0.900826
chr5	111687300	0.834711
chr5	111743600	0.661157
chr5	111748300	0.991736
chr5	111808700	1.000000
chr5	111822100	0.958678
chr5	112071900	0.900826
chr5	112294600	0.975207
chr5	112305600	0.851240
chr5	112356400	0.991736
chr5	112359000	0.785124
chr5	112387200	0.958678
chr5	112395300	0.892562
chr5	112406500	0.710744
chr5	112417900	0.925620
chr5	112426200	0.685950
chr5	112499500	0.892562
chr5	112555100	0.702479

chr5	112557300	0.826446
chr5	112560500	0.975207
chr5	112586500	0.925620
chr5	112607000	1.000000
chr5	112609600	0.669421
chr5	112627500	0.818182
chr5	112693900	0.752066
chr5	112707900	0.867769
chr5	112714400	0.809917
chr5	112718900	0.727273
chr5	112831700	0.892562
chr5	112860900	0.727273
chr5	112942300	0.909091
chr5	113142100	0.933884
chr5	113511800	0.966942
chr5	113656000	0.917355
chr5	113661300	0.942149
chr5	114685400	0.859504
chr5	114702300	0.983471
chr5	114755800	0.867769
chr5	114759200	0.793388
chr5	114791500	0.892562
chr5	114796600	0.991736
chr5	114867800	0.809917
chr5	114874600	0.669421
chr5	114894700	0.983471
chr5	114911900	0.975207
chr5	114970500	0.694215
chr5	115146100	0.727273
chr5	115180400	0.909091
chr5	115193300	0.768595
chr5	115209500	0.685950
chr5	115314100	0.884298
chr5	115367800	0.719008
chr5	115707100	0.991736
chr5	115718500	0.661157
chr5	115725000	0.876033
chr5	118504400	0.859504
chr5	118611100	0.958678
chr5	118639200	0.958678
chr5	118660200	0.991736
chr5	118723200	0.743802
chr5	118729800	0.842975
chr5	118733500	0.900826
chr5	119488200	0.925620
chr5	119724600	0.752066
chr5	119741400	0.975207
chr5	119782000	0.991736
chr5	120209500	0.785124
chr5	120474500	0.958678
chr5	120519200	0.876033
chr5	120525100	0.975207
chr5	120588100	0.991736
chr5	120610200	0.975207

chr5	120713600	0.685950
chr5	121299700	0.685950
chr5	121305200	0.975207
chr5	121389400	0.752066
chr5	121425700	0.942149
chr5	121490800	0.768595
chr5	121517200	0.917355
chr5	121519300	0.958678
chr5	121523600	0.818182
chr5	121528300	0.785124
chr5	121694800	0.702479
chr5	122062900	0.867769
chr5	122072500	0.793388
chr5	122092300	0.834711
chr5	122435600	0.785124
chr5	122796400	0.760331
chr5	122918600	0.752066
chr5	123036500	0.958678
chr5	123044900	0.876033
chr5	123054400	0.851240
chr5	123150100	0.950413
chr5	123218100	0.933884
chr5	123401200	0.942149
chr5	123406900	0.851240
chr5	123424400	0.735537
chr5	123872600	0.983471
chr5	123874800	0.702479
chr5	123932400	0.834711
chr5	123935700	0.958678
chr5	123938800	0.818182
chr5	123941300	0.950413
chr5	123985900	0.842975
chr5	123991900	0.983471
chr5	123995700	0.661157
chr5	123998000	0.991736
chr5	124109800	0.694215
chr5	124142500	0.826446
chr5	124149100	0.884298
chr5	124225800	0.900826
chr5	124238200	0.710744
chr5	124379500	0.909091
chr5	124381900	0.851240
chr5	124429900	0.917355
chr5	124470600	0.677686
chr5	124598900	0.991736
chr5	124610500	1.000000
chr5	124613600	1.000000
chr5	124647200	0.876033
chr5	124661800	0.727273
chr5	124744900	0.719008
chr5	124748900	0.966942
chr5	124819800	0.826446
chr5	124822100	0.966942
chr5	124885800	1.000000

chr5	124895400	1.000000
chr5	124996200	1.000000
chr5	125684900	0.876033
chr5	125690100	0.966942
chr5	125751400	0.793388
chr5	125785900	0.677686
chr5	125932800	0.851240
chr5	125960500	0.702479
chr5	126147400	0.975207
chr5	126151000	0.966942
chr5	126188500	1.000000
chr5	126205200	0.842975
chr5	126213400	0.925620
chr5	126220100	0.966942
chr5	126246000	0.785124
chr5	126255600	0.925620
chr5	126307800	0.966942
chr5	126324400	0.983471
chr5	126338100	0.933884
chr5	126348800	0.966942
chr5	126351200	0.793388
chr5	126377700	0.933884
chr5	126405900	0.950413
chr5	126409100	0.867769
chr5	126412900	0.942149
chr5	126469000	0.842975
chr5	126542300	1.000000
chr5	126694500	0.719008
chr5	127138100	0.933884
chr5	127149400	0.933884
chr5	127174800	0.826446
chr5	127194400	0.925620
chr5	127198700	1.000000
chr5	127200900	0.801653
chr5	127207700	0.991736
chr5	127211500	0.752066
chr5	127228700	0.892562
chr5	127278500	0.735537
chr5	127297300	0.917355
chr5	127324100	0.983471
chr5	127374900	1.000000
chr5	127429000	0.983471
chr5	127535300	0.727273
chr5	127968300	0.851240
chr5	127980900	0.892562
chr5	127985200	0.942149
chr5	128109700	0.735537
chr5	128118500	0.942149
chr5	128149100	0.801653
chr5	128159500	0.677686
chr5	128161800	0.925620
chr5	128231000	0.933884
chr5	128256400	0.933884
chr5	128432600	0.735537

chr5	129007000	0.950413
chr5	129189600	0.710744
chr5	129359800	0.743802
chr5	129573800	0.966942
chr5	129659900	0.909091
chr5	129668900	0.710744
chr5	129673400	0.991736
chr5	129886400	0.876033
chr5	130046600	0.950413
chr5	130578800	0.776860
chr5	130601900	0.710744
chr5	130713800	0.818182
chr5	130722900	1.000000
chr5	130797400	0.752066
chr5	130841600	0.768595
chr5	130850600	0.884298
chr5	130867700	0.859504
chr5	130871100	0.851240
chr5	130884400	0.966942
chr5	130946500	0.975207
chr5	131142600	0.809917
chr5	131151100	0.892562
chr5	131332800	0.876033
chr5	131422300	0.966942
chr5	131430800	0.702479
chr5	131439600	0.834711
chr5	131515800	0.710744
chr5	131596700	0.909091
chr5	131600500	0.694215
chr5	131602900	0.942149
chr5	131632900	0.975207
chr5	131722100	0.892562
chr5	131726400	0.826446
chr5	131748400	0.685950
chr5	131751100	0.975207
chr5	131756100	0.694215
chr5	131759000	0.851240
chr5	131792400	0.760331
chr5	131800300	0.809917
chr5	131805400	0.776860
chr5	131827600	0.768595
chr5	131831000	0.677686
chr5	131834300	0.735537
chr5	131837600	0.867769
chr5	131846000	0.859504
chr5	131992100	0.818182
chr5	132041100	0.768595
chr5	132103800	0.760331
chr5	132107000	0.966942
chr5	132118100	0.818182
chr5	132175900	0.950413
chr5	132243400	0.950413
chr5	132283900	1.000000
chr5	132385300	0.917355

chr5	132390700	0.801653
chr5	132557100	0.950413
chr5	132571900	0.818182
chr5	132576900	0.867769
chr5	132795600	0.727273
chr5	132818100	0.958678
chr5	132938800	0.983471
chr5	132947500	0.768595
chr5	133071400	1.000000
chr5	133168000	0.975207
chr5	133289200	0.809917
chr5	133326900	0.950413
chr5	133329800	0.859504
chr5	133463600	0.925620
chr5	133581700	0.669421
chr5	133591400	0.768595
chr5	133839500	0.975207
chr5	133847300	0.719008
chr5	133886300	0.809917
chr5	133888400	0.917355
chr5	133890700	0.842975
chr5	133892800	0.702479
chr5	133898300	0.785124
chr5	133905500	0.826446
chr5	133913500	0.677686
chr5	133916700	0.760331
chr5	134180600	0.867769
chr5	134527100	0.694215
chr5	134530100	0.768595
chr5	134544700	0.925620
chr5	134582700	0.776860
chr5	134600800	0.661157
chr5	134605200	0.876033
chr5	134722200	0.909091
chr5	135170600	0.685950
chr5	135327500	0.669421
chr5	135330100	0.975207
chr5	135332500	0.793388
chr5	135335700	0.694215
chr5	135348000	0.900826
chr5	135373200	1.000000
chr5	135378100	0.851240
chr5	135380300	0.925620
chr5	135389300	0.669421
chr5	135394300	0.925620
chr5	135424200	0.661157
chr5	135686900	0.851240
chr5	135944200	0.661157
chr5	135950400	0.917355
chr5	135955900	0.983471
chr5	135991500	0.975207
chr5	136065900	0.991736
chr5	136369000	0.694215
chr5	136372500	0.661157

chr5	136397500	1.000000
chr5	136408400	0.925620
chr5	136451100	0.900826
chr5	136483000	0.950413
chr5	136541000	0.950413
chr5	136633900	0.909091
chr5	136637100	0.933884
chr5	136646400	1.000000
chr5	136678300	0.958678
chr5	136682300	0.933884
chr5	136698100	0.933884
chr5	136752200	0.702479
chr5	136778700	0.818182
chr5	136805700	0.909091
chr5	136807900	0.917355
chr5	136823000	0.983471
chr5	136896500	0.785124
chr5	136899700	0.958678
chr5	136912100	0.768595
chr5	137016100	0.719008
chr5	137032500	0.876033
chr5	137042800	0.950413
chr5	137073600	0.917355
chr5	137225300	0.809917
chr5	137230600	1.000000
chr5	137232700	0.760331
chr5	137290300	0.966942
chr5	137294000	0.983471
chr5	137464500	0.768595
chr5	137485100	0.801653
chr5	137577900	0.702479
chr5	137705400	0.876033
chr5	137722300	0.760331
chr5	137947500	0.900826
chr5	138032700	0.752066
chr5	138097000	0.909091
chr5	138152300	0.983471
chr5	138180600	0.991736
chr5	138189000	0.735537
chr5	138210500	0.661157
chr5	138236400	0.983471
chr5	138239500	0.942149
chr5	138272600	0.793388
chr5	138289200	0.677686
chr5	138310400	0.925620
chr5	138350900	0.851240
chr5	138397400	0.818182
chr5	138500600	0.685950
chr5	138582600	0.785124
chr5	138612600	0.826446
chr5	138724400	0.818182
chr5	138754900	0.966942
chr5	138774900	0.685950
chr5	138778800	0.876033

chr5	138781900	0.892562
chr5	138784000	0.719008
chr5	138838300	0.859504
chr5	138853500	0.735537
chr5	138876300	0.933884
chr5	138897800	0.826446
chr5	138940300	0.727273
chr5	138945400	0.876033
chr5	138951500	0.661157
chr5	139017300	0.958678
chr5	139035800	0.677686
chr5	139038400	0.752066
chr5	139040600	0.966942
chr5	139047900	0.892562
chr5	139051300	0.859504
chr5	139077100	0.950413
chr5	139091300	0.876033
chr5	139105000	0.900826
chr5	139127200	0.669421
chr5	139148400	0.884298
chr5	139365900	0.752066
chr5	139434200	0.958678
chr5	139511900	0.735537
chr5	139525900	0.752066
chr5	139537000	0.768595
chr5	139585300	0.892562
chr5	139601700	0.669421
chr5	139624400	0.760331
chr5	139627500	0.826446
chr5	139630900	0.975207
chr5	139633800	0.950413
chr5	139641200	0.983471
chr5	139647700	0.991736
chr5	139672100	0.900826
chr5	139676000	0.851240
chr5	139678200	1.000000
chr5	139689900	0.768595
chr5	139693200	0.958678
chr5	139707200	0.768595
chr5	139709300	0.983471
chr5	139721400	0.884298
chr5	139723900	0.942149
chr5	139753100	0.793388
chr5	139763200	0.900826
chr5	139860300	0.983471
chr5	139913600	0.851240
chr5	139927500	0.966942
chr5	139938000	0.694215
chr5	139969000	0.876033
chr5	139989000	0.884298
chr5	139991700	0.958678
chr5	140052600	0.801653
chr5	140090600	0.768595
chr5	140098600	0.884298

chr5	140105700	0.727273
chr5	140357500	0.727273
chr5	140750200	0.710744
chr5	140788000	0.661157
chr5	140888800	0.950413
chr5	140900900	0.942149
chr5	140904100	0.909091
chr5	140912900	0.719008
chr5	140937700	0.975207
chr5	140940300	0.966942
chr5	140985700	0.966942
chr5	140996300	0.743802
chr5	141038300	0.925620
chr5	141056200	0.842975
chr5	141063900	0.933884
chr5	141066900	0.677686
chr5	141071800	0.909091
chr5	141186000	0.917355
chr5	141196600	0.975207
chr5	141201900	0.735537
chr5	141216000	0.842975
chr5	141219300	0.942149
chr5	141223300	0.851240
chr5	141228900	0.793388
chr5	141231200	0.917355
chr5	141250900	0.735537
chr5	141254300	0.661157
chr5	141294100	0.710744
chr5	141321200	0.834711
chr5	141327400	0.661157
chr5	141333200	0.909091
chr5	141338000	0.909091
chr5	141393800	0.685950
chr5	141396000	0.942149
chr5	141404600	0.752066
chr5	141443900	0.925620
chr5	141572300	0.958678
chr5	141577200	0.991736
chr5	141580600	0.793388
chr5	141582700	0.851240
chr5	141596000	0.909091
chr5	141602600	0.752066
chr5	141615300	0.867769
chr5	141626000	0.884298
chr5	141660400	0.719008
chr5	141667400	0.925620
chr5	141670600	0.909091
chr5	141673800	0.991736
chr5	141677700	0.975207
chr5	141692800	0.925620
chr5	141696300	0.983471
chr5	141701000	0.793388
chr5	141706700	0.884298
chr5	141736900	0.991736

chr5	141742900	0.942149
chr5	141805600	0.942149
chr5	141807700	0.884298
chr5	141813600	0.776860
chr5	141826000	0.966942
chr5	141852100	0.884298
chr5	141894100	0.983471
chr5	141910100	0.884298
chr5	141922400	0.983471
chr5	141925000	0.818182
chr5	141931400	0.776860
chr5	141977900	0.867769
chr5	141980500	0.859504
chr5	142024800	0.958678
chr5	142054500	0.727273
chr5	142092900	0.859504
chr5	142123700	0.958678
chr5	142128800	0.867769
chr5	142159100	0.752066
chr5	142193900	0.719008
chr5	142201800	0.661157
chr5	142206700	0.983471
chr5	142210000	0.975207
chr5	142237500	0.818182
chr5	142252800	0.942149
chr5	142255200	0.867769
chr5	142262900	0.851240
chr5	142289500	0.818182
chr5	142302700	0.983471
chr5	142305600	0.942149
chr5	142319600	0.884298
chr5	142357700	0.950413
chr5	142362700	0.859504
chr5	142382400	0.975207
chr5	142417200	0.884298
chr5	142441000	0.942149
chr5	142506400	0.809917
chr5	142585100	0.743802
chr5	142600800	1.000000
chr5	142621700	0.694215
chr5	142624100	0.884298
chr5	142690800	0.793388
chr5	142693600	0.958678
chr5	142735600	0.909091
chr5	142780300	0.876033
chr5	142975700	0.793388
chr5	143224400	0.859504
chr5	143302700	0.859504
chr5	143332900	0.842975
chr5	143357700	0.727273
chr5	143366100	0.917355
chr5	143469400	0.710744
chr5	143525100	0.991736
chr5	143567200	0.892562

chr5	143571300	0.867769
chr5	143582000	0.760331
chr5	143585100	0.735537
chr5	144583300	0.900826
chr5	144624900	0.983471
chr5	144742400	0.917355
chr5	144859400	0.834711
chr5	144875700	0.727273
chr5	144911000	0.975207
chr5	145133000	0.884298
chr5	145156000	0.752066
chr5	145177100	0.909091
chr5	145200700	0.694215
chr5	145210300	0.942149
chr5	145235900	0.785124
chr5	145305100	0.752066
chr5	145356000	0.876033
chr5	145422600	0.809917
chr5	145663200	0.859504
chr5	145720200	0.685950
chr5	145841200	1.000000
chr5	145854500	0.958678
chr5	146111800	0.983471
chr5	146683900	0.983471
chr5	146689600	0.818182
chr5	146783300	0.876033
chr5	146785400	0.710744
chr5	146846800	0.909091
chr5	146859800	0.933884
chr5	146862100	0.975207
chr5	146864800	0.925620
chr5	146867300	0.983471
chr5	146884700	0.785124
chr5	146902200	0.909091
chr5	146911200	0.950413
chr5	146915700	0.983471
chr5	147273300	0.958678
chr5	147344000	0.809917
chr5	147471900	0.661157
chr5	147525400	0.917355
chr5	147565900	0.958678
chr5	147759200	0.950413
chr5	147766500	1.000000
chr5	147778400	0.983471
chr5	147781800	0.842975
chr5	147796700	0.917355
chr5	147848800	1.000000
chr5	147851000	0.760331
chr5	147853800	1.000000
chr5	147999800	0.958678
chr5	148003700	0.685950
chr5	148186000	1.000000
chr5	148189100	0.768595
chr5	148208100	0.826446

chr5	148288900	0.991736
chr5	148291600	0.917355
chr5	148294600	0.876033
chr5	148304900	0.958678
chr5	148310400	0.661157
chr5	148341700	0.661157
chr5	148345700	0.983471
chr5	148350500	0.727273
chr5	148354500	0.776860
chr5	148357300	0.785124
chr5	148364200	0.991736
chr5	148382600	0.925620
chr5	148417200	0.776860
chr5	148420900	0.685950
chr5	148440200	0.801653
chr5	148513400	0.884298
chr5	148515600	1.000000
chr5	148523500	0.735537
chr5	148530000	0.925620
chr5	148532900	0.942149
chr5	148551700	0.892562
chr5	148554300	0.892562
chr5	148590100	0.826446
chr5	148592200	0.909091
chr5	148608100	0.933884
chr5	148667400	0.793388
chr5	148669700	0.950413
chr5	148678900	0.785124
chr5	148690700	0.785124
chr5	148700200	0.917355
chr5	148703000	0.818182
chr5	148706200	0.975207
chr5	148713800	0.966942
chr5	148721300	0.760331
chr5	148728400	0.958678
chr5	148802700	0.743802
chr5	148810400	0.694215
chr5	148819500	0.677686
chr5	148825800	0.958678
chr5	148830200	0.694215
chr5	148833500	0.834711
chr5	148844100	0.966942
chr5	148865200	0.801653
chr5	148867300	0.735537
chr5	148899400	0.677686
chr5	148923900	0.950413
chr5	148927100	0.859504
chr5	148941200	0.950413
chr5	148943700	0.950413
chr5	148960200	0.727273
chr5	148962900	0.834711
chr5	149185900	0.826446
chr5	149219500	0.661157
chr5	149237400	0.818182

chr5	149293800	0.876033
chr5	149296900	0.983471
chr5	149317500	0.884298
chr5	149319600	0.925620
chr5	149338900	0.669421
chr5	149397400	0.867769
chr5	149403100	0.834711
chr5	149424000	0.727273
chr5	149430300	0.983471
chr5	149433300	0.867769
chr5	149447800	0.702479
chr5	149462400	0.884298
chr5	149499500	0.710744
chr5	149513200	0.785124
chr5	149518200	0.685950
chr5	149644100	0.669421
chr5	149696900	0.909091
chr5	149790100	0.834711
chr5	149822100	0.694215
chr5	149845500	0.925620
chr5	149852900	0.909091
chr5	149868300	0.917355
chr5	149883700	0.677686
chr5	149892600	0.768595
chr5	149894700	0.776860
chr5	149922000	0.975207
chr5	149975600	0.983471
chr5	149980000	0.975207
chr5	149992300	0.793388
chr5	149995700	0.818182
chr5	149999100	0.677686
chr5	150006400	0.818182
chr5	150012700	0.859504
chr5	150015900	0.752066
chr5	150018200	0.950413
chr5	150024500	0.760331
chr5	150418200	0.809917
chr5	150443300	0.842975
chr5	150447900	0.925620
chr5	150450100	0.884298
chr5	150452200	0.752066
chr5	150465600	0.793388
chr5	150469300	0.909091
chr5	150473200	0.785124
chr5	150530700	0.727273
chr5	150544600	0.933884
chr5	150582700	0.677686
chr5	151020700	0.818182
chr5	151039600	0.727273
chr5	151043400	0.735537
chr5	151045900	0.727273
chr5	151049800	0.900826
chr5	151053200	0.818182
chr5	151056300	0.892562

chr5	151060200	0.892562
chr5	151062600	0.983471
chr5	151079200	0.925620
chr5	151087900	0.727273
chr5	151121600	0.900826
chr5	151124500	0.917355
chr5	151126900	0.809917
chr5	151139200	0.776860
chr5	151150000	0.776860
chr5	151183600	0.768595
chr5	152931500	0.966942
chr5	152997100	0.685950
chr5	152999600	0.983471
chr5	153008700	0.983471
chr5	153382100	0.925620
chr5	153422700	0.925620
chr5	153556400	0.661157
chr5	153576800	0.727273
chr5	153579000	0.917355
chr5	153601100	0.818182
chr5	153618300	0.719008
chr5	153688500	0.892562
chr5	153745300	0.793388
chr5	153748900	0.975207
chr5	153770000	0.925620
chr5	153785200	0.768595
chr5	153855400	0.685950
chr5	154027200	0.867769
chr5	154034500	1.000000
chr5	154062600	0.719008
chr5	154133900	0.785124
chr5	154151300	0.834711
chr5	154174300	0.867769
chr5	154196400	0.685950
chr5	154224900	0.752066
chr5	154262100	0.677686
chr5	154324900	0.950413
chr5	154347500	0.933884
chr5	154377400	0.933884
chr5	154462300	0.900826
chr5	156778600	0.983471
chr5	156811800	0.867769
chr5	156815100	0.958678
chr5	156817900	0.859504
chr5	156934300	0.876033
chr5	156939000	0.950413
chr5	156968500	0.966942
chr5	156981600	0.983471
chr5	156991500	0.958678
chr5	157020100	0.991736
chr5	157235600	0.760331
chr5	157248000	0.876033
chr5	157254900	0.950413
chr5	157502500	0.991736

chr5	157810200	0.793388
chr5	157812500	0.909091
chr5	157817900	0.983471
chr5	157842100	0.942149
chr5	158235400	0.942149
chr5	158407800	0.991736
chr5	158430300	0.743802
chr5	158438600	0.685950
chr5	158444700	0.834711
chr5	158527900	0.685950
chr5	158602700	0.661157
chr5	158607000	0.942149
chr5	159339700	0.958678
chr5	159402800	0.818182
chr5	159448600	0.950413
chr5	159499100	0.991736
chr5	159510700	0.975207
chr5	159530800	0.760331
chr5	159593300	0.942149
chr5	159596800	0.966942
chr5	159615600	0.867769
chr5	159688400	0.925620
chr5	159690600	0.768595
chr5	159696500	0.785124
chr5	159698800	0.917355
chr5	159702200	0.942149
chr5	159709100	0.685950
chr5	159715100	0.710744
chr5	159743100	0.950413
chr5	159866400	0.727273
chr5	159877400	0.909091
chr5	159891200	1.000000
chr5	159894300	0.892562
chr5	159898200	0.975207
chr5	159904200	0.677686
chr5	159907500	0.867769
chr5	162867400	0.685950
chr5	162890100	0.942149
chr5	162906200	0.942149
chr5	162935900	0.685950
chr5	162991600	0.991736
chr5	162994800	0.909091
chr5	163132300	0.859504
chr5	163134700	0.900826
chr5	163184400	0.801653
chr5	163188000	0.942149
chr5	163194700	0.925620
chr5	163202300	0.983471
chr5	163288300	0.793388
chr5	163290900	0.900826
chr5	163293500	0.851240
chr5	163295600	0.892562
chr5	163298300	0.842975
chr5	163321800	0.834711

chr5	163371200	0.991736
chr5	163396000	0.818182
chr5	163404000	0.669421
chr5	163440100	0.909091
chr5	163631600	0.933884
chr5	163662700	0.851240
chr5	163763200	1.000000
chr5	165001300	0.876033
chr5	166478200	0.801653
chr5	166722300	1.000000
chr5	167070600	0.669421
chr5	167654300	0.991736
chr5	168007600	0.884298
chr5	168140200	0.876033
chr5	168588800	0.950413
chr5	168591900	0.867769
chr5	168774900	0.851240
chr5	168778200	1.000000
chr5	169064500	0.719008
chr5	169078200	0.925620
chr5	169115900	0.669421
chr5	169121900	0.876033
chr5	169124800	0.892562
chr5	169130500	0.925620
chr5	169134200	0.975207
chr5	169144100	0.966942
chr5	169363300	0.768595
chr5	169373700	0.884298
chr5	169399000	0.842975
chr5	169401200	0.933884
chr5	169404400	0.917355
chr5	169416400	0.975207
chr5	169523400	0.685950
chr5	169559600	0.851240
chr5	169564100	0.966942
chr5	169566700	0.677686
chr5	169578600	0.917355
chr5	169646900	0.859504
chr5	169652000	0.966942
chr5	169758500	0.958678
chr5	169816600	0.826446
chr5	169894200	0.983471
chr5	169898300	0.983471
chr5	169906800	0.892562
chr5	169918900	0.966942
chr5	170060700	0.859504
chr5	170209900	0.892562
chr5	170320100	0.809917
chr5	170651500	0.669421
chr5	170739100	0.710744
chr5	170764000	0.661157
chr5	170818300	0.702479
chr5	170846500	0.743802
chr5	170878200	0.909091

chr5	170922100	0.867769
chr5	170997300	0.661157
chr5	171023800	0.991736
chr5	171028100	0.900826
chr5	171057200	0.743802
chr5	171075200	0.942149
chr5	171095300	0.942149
chr5	171109100	0.958678
chr5	171177900	0.917355
chr5	171185500	0.917355
chr5	171269500	0.669421
chr5	171381900	0.685950
chr5	171389900	0.785124
chr5	171393600	0.818182
chr5	171412000	0.793388
chr5	171431100	0.776860
chr5	171530600	0.809917
chr5	171534400	0.793388
chr5	171538200	0.859504
chr5	171584000	0.975207
chr5	171607000	0.917355
chr5	171782500	0.685950
chr5	171823000	0.785124
chr5	171840100	0.917355
chr5	171845600	0.785124
chr5	171865100	0.702479
chr5	171870500	0.768595
chr5	171874200	0.719008
chr5	171878800	0.719008
chr5	172068400	0.710744
chr5	172140900	0.735537
chr5	172143700	0.793388
chr5	172161100	0.768595
chr5	172175800	0.975207
chr5	172185300	0.876033
chr5	172192100	0.950413
chr5	172194500	0.983471
chr5	172200300	0.818182
chr5	172205200	0.710744
chr5	172216600	0.776860
chr5	172222000	0.983471
chr5	172225700	1.000000
chr5	172234500	0.950413
chr5	172281600	0.793388
chr5	172284200	0.876033
chr5	172286600	0.917355
chr5	172289200	0.983471
chr5	172297600	0.917355
chr5	172300500	0.719008
chr5	172309500	0.909091
chr5	172318800	0.942149
chr5	172324400	0.876033
chr5	172326700	0.876033
chr5	172328800	0.958678

chr5	172331300	0.917355
chr5	172338800	0.933884
chr5	172356800	0.793388
chr5	172359800	0.925620
chr5	172371000	0.710744
chr5	172382700	0.752066
chr5	172430500	0.801653
chr5	172443700	0.966942
chr5	172456700	0.966942
chr5	172463100	0.991736
chr5	172479300	0.801653
chr5	172521100	0.818182
chr5	172665700	0.818182
chr5	172719300	0.900826
chr5	172722000	0.826446
chr5	172729500	0.661157
chr5	172751200	0.685950
chr5	172761400	0.966942
chr5	172882100	0.900826
chr5	173145600	0.685950
chr5	173191600	0.966942
chr5	173194200	0.826446
chr5	173205700	0.925620
chr5	173209200	0.975207
chr5	173282000	0.735537
chr5	173313100	0.909091
chr5	173330300	0.727273
chr5	173341100	0.752066
chr5	173347300	0.867769
chr5	173350600	0.809917
chr5	173369300	0.760331
chr5	173400400	0.694215
chr5	173960200	0.909091
chr5	175349800	0.801653
chr5	175626800	0.710744
chr5	175855100	0.859504
chr5	175866600	0.743802
chr5	175869200	0.933884
chr5	175961100	0.892562
chr5	175966200	0.694215
chr5	176024200	0.859504
chr5	176042400	0.677686
chr5	176047000	0.727273
chr5	176212000	0.859504
chr5	176245200	0.826446
chr5	176327000	0.801653
chr5	176420900	0.785124
chr5	176424400	0.867769
chr5	176514400	0.719008
chr5	176520100	0.785124
chr5	176524300	0.876033
chr5	176620600	0.900826
chr5	176622700	0.991736
chr5	176724000	0.735537

chr5	176727800	0.785124
chr5	176740400	0.876033
chr5	176755400	0.958678
chr5	176785300	0.809917
chr5	176790700	0.710744
chr5	176794100	0.917355
chr5	176799000	0.702479
chr5	176827600	0.760331
chr5	176852800	0.719008
chr5	176875300	0.818182
chr5	176880200	0.661157
chr5	176889700	0.818182
chr5	176897300	0.785124
chr5	176904400	0.735537
chr5	176907200	0.685950
chr5	176919100	0.991736
chr5	176921600	0.694215
chr5	176936700	0.925620
chr5	176946000	0.743802
chr5	176973700	0.958678
chr5	177350700	0.842975
chr5	177394600	0.950413
chr5	177543400	0.702479
chr5	177546000	1.000000
chr5	177634700	0.661157
chr5	177667300	0.727273
chr5	177923900	0.942149
chr5	178017500	0.909091
chr5	178069900	0.958678
chr5	178980900	0.661157
chr5	178996200	0.900826
chr5	179043500	0.793388
chr5	179129100	0.809917
chr5	179190000	0.942149
chr5	179194100	0.826446
chr5	179205100	0.834711
chr5	179223500	0.760331
chr5	179234900	0.727273
chr5	179244400	0.801653
chr5	179246500	0.966942
chr5	179332400	1.000000
chr5	179454000	0.975207
chr5	179484000	0.917355
chr5	179758500	0.876033
chr5	180035900	0.867769
chr5	180072900	0.983471
chr5	180212600	0.694215
chr5	180214900	0.834711
chr5	180244600	0.966942
chr5	180542500	0.826446
chr5	180588000	0.818182
chr5	180634200	0.719008
chr5	180645600	0.661157
chr6	297000	0.809917

chr6	562600	0.991736
chr6	611500	0.702479
chr6	646800	0.661157
chr6	682200	0.661157
chr6	684900	0.991736
chr6	700100	0.661157
chr6	703900	1.000000
chr6	706600	0.983471
chr6	708900	0.975207
chr6	712500	0.735537
chr6	714900	0.785124
chr6	863400	0.752066
chr6	1382500	0.801653
chr6	1605000	0.677686
chr6	1609400	0.669421
chr6	1616900	0.867769
chr6	1620100	0.983471
chr6	1631200	0.669421
chr6	1635800	0.710744
chr6	1725300	0.892562
chr6	1771400	0.925620
chr6	1781600	0.933884
chr6	1788800	0.876033
chr6	1798300	0.975207
chr6	1803200	0.867769
chr6	1817500	0.909091
chr6	1822200	0.975207
chr6	1833300	0.933884
chr6	1838600	0.867769
chr6	1909500	0.983471
chr6	1911600	0.933884
chr6	1918100	0.925620
chr6	2078300	0.685950
chr6	2104200	0.950413
chr6	2111800	1.000000
chr6	2114500	0.892562
chr6	2124700	1.000000
chr6	2128100	0.892562
chr6	2132900	0.735537
chr6	2162900	0.851240
chr6	2198400	0.809917
chr6	2203800	0.991736
chr6	2222600	0.702479
chr6	2237600	0.859504
chr6	2354100	0.776860
chr6	2380100	0.876033
chr6	2523000	0.975207
chr6	2535400	0.925620
chr6	2576500	0.983471
chr6	2579500	0.743802
chr6	2626300	0.661157
chr6	2631200	0.876033
chr6	2634800	0.958678
chr6	2719100	0.983471

chr6	2726600	0.975207
chr6	2764500	0.702479
chr6	2770400	0.826446
chr6	2784300	0.991736
chr6	2800500	0.793388
chr6	2803200	0.793388
chr6	2807700	0.991736
chr6	2818300	0.950413
chr6	2821100	0.991736
chr6	2830000	0.685950
chr6	2839500	0.677686
chr6	2841900	0.884298
chr6	2847000	0.867769
chr6	2850800	0.900826
chr6	2854900	0.727273
chr6	2857000	0.950413
chr6	2859600	0.842975
chr6	2866300	0.950413
chr6	2878000	0.925620
chr6	2905200	1.000000
chr6	2925300	0.727273
chr6	2943900	0.884298
chr6	2953100	0.826446
chr6	2961100	0.785124
chr6	2977400	0.909091
chr6	2982500	0.991736
chr6	2988000	0.760331
chr6	3056500	0.743802
chr6	3089300	0.801653
chr6	3139000	0.925620
chr6	3166400	0.958678
chr6	3227900	0.719008
chr6	3261600	0.735537
chr6	3281200	0.702479
chr6	3283300	0.719008
chr6	3292400	0.917355
chr6	3325100	0.942149
chr6	3338500	0.685950
chr6	3356200	0.768595
chr6	3422900	0.694215
chr6	3539400	0.743802
chr6	3562400	1.000000
chr6	3732800	0.950413
chr6	3737200	0.661157
chr6	3740300	0.958678
chr6	3742800	0.975207
chr6	3746800	0.925620
chr6	3754700	0.719008
chr6	3796100	0.876033
chr6	3799400	0.958678
chr6	3804800	0.735537
chr6	3807300	0.958678
chr6	3810000	0.925620
chr6	3813900	0.851240

chr6	3823300	0.727273
chr6	3825400	0.752066
chr6	3827600	0.884298
chr6	3832700	0.884298
chr6	3849800	0.925620
chr6	3884500	0.942149
chr6	3914000	0.958678
chr6	3916600	0.966942
chr6	3965900	0.925620
chr6	3987500	0.876033
chr6	4049700	0.826446
chr6	4059500	0.834711
chr6	4110100	0.942149
chr6	4126500	1.000000
chr6	4128600	0.809917
chr6	4130800	0.884298
chr6	4136900	0.760331
chr6	4251300	0.809917
chr6	4254400	0.876033
chr6	4257900	0.975207
chr6	4275600	0.859504
chr6	4313900	0.975207
chr6	4317500	0.851240
chr6	4321100	0.892562
chr6	4351900	0.925620
chr6	4359300	0.933884
chr6	4381700	0.917355
chr6	4459800	0.793388
chr6	4582600	0.942149
chr6	4600300	0.842975
chr6	4607900	0.991736
chr6	4611000	0.925620
chr6	4650300	0.842975
chr6	4659100	0.719008
chr6	4689000	0.768595
chr6	4733700	0.702479
chr6	4810600	0.925620
chr6	4813600	0.859504
chr6	4849300	0.694215
chr6	5013400	0.752066
chr6	5026800	0.826446
chr6	5054300	0.892562
chr6	5133300	0.966942
chr6	5191500	0.876033
chr6	5206000	0.991736
chr6	5231100	0.950413
chr6	5334500	0.925620
chr6	5499200	0.851240
chr6	5729800	0.752066
chr6	5852000	0.743802
chr6	6313500	0.975207
chr6	6655500	0.950413
chr6	6678100	1.000000
chr6	6687900	0.818182

chr6	6697400	0.884298
chr6	6724900	0.942149
chr6	6811900	0.842975
chr6	6827100	0.735537
chr6	7042700	0.991736
chr6	7100900	0.685950
chr6	7137900	0.719008
chr6	7147100	0.966942
chr6	7173200	0.950413
chr6	7692800	0.909091
chr6	7698600	0.975207
chr6	7723600	0.793388
chr6	7733300	0.925620
chr6	7735700	0.983471
chr6	7738900	0.818182
chr6	7741300	0.694215
chr6	7743400	0.983471
chr6	7751000	0.735537
chr6	7771000	0.942149
chr6	7774100	0.809917
chr6	7786300	0.735537
chr6	7803500	0.694215
chr6	7806000	0.876033
chr6	7819400	0.942149
chr6	7828300	0.991736
chr6	7833400	0.694215
chr6	7842500	0.694215
chr6	7847400	1.000000
chr6	7881700	0.818182
chr6	7889900	0.760331
chr6	7893100	0.834711
chr6	7895200	0.876033
chr6	7897400	0.983471
chr6	7900700	0.983471
chr6	7903500	0.793388
chr6	7912100	0.818182
chr6	7915700	0.834711
chr6	7960500	0.991736
chr6	7974600	0.752066
chr6	7977800	0.859504
chr6	7993700	0.892562
chr6	7997500	0.983471
chr6	8000800	1.000000
chr6	8003500	0.917355
chr6	8006100	0.925620
chr6	8029000	0.975207
chr6	8048500	0.933884
chr6	8058800	0.925620
chr6	8080900	0.983471
chr6	8084000	0.942149
chr6	8086300	0.925620
chr6	8109000	0.983471
chr6	9482600	0.975207
chr6	9503900	0.933884

chr6	9520400	0.776860
chr6	9524200	0.694215
chr6	9540500	0.752066
chr6	9647300	0.966942
chr6	9651000	0.834711
chr6	9767000	0.859504
chr6	9834500	0.809917
chr6	9897500	0.785124
chr6	10519900	0.884298
chr6	10522100	0.876033
chr6	10526900	0.933884
chr6	10555200	0.975207
chr6	10735500	0.834711
chr6	10760100	0.867769
chr6	10887600	0.702479
chr6	11112300	0.735537
chr6	11120900	0.719008
chr6	11131700	0.785124
chr6	11194800	0.950413
chr6	11198300	0.884298
chr6	11200900	0.859504
chr6	11203500	0.950413
chr6	11206900	0.884298
chr6	11210000	0.702479
chr6	11213100	0.801653
chr6	11215300	0.892562
chr6	11217500	0.917355
chr6	11219800	0.801653
chr6	11222400	0.925620
chr6	11226100	0.958678
chr6	11237000	0.743802
chr6	11252000	0.867769
chr6	11264100	0.950413
chr6	11278200	0.785124
chr6	11316300	0.867769
chr6	11320100	0.917355
chr6	11354500	0.991736
chr6	11357700	0.859504
chr6	11365400	0.867769
chr6	11368400	0.859504
chr6	11371000	0.785124
chr6	11390500	0.925620
chr6	11393600	0.743802
chr6	11411300	0.900826
chr6	11419100	0.900826
chr6	11424600	0.991736
chr6	11426900	0.661157
chr6	11429200	0.991736
chr6	11438900	0.975207
chr6	11564600	0.677686
chr6	11587100	0.900826
chr6	11590600	0.876033
chr6	11605600	0.719008
chr6	11610100	0.727273

chr6	11612300	0.892562
chr6	11614400	0.991736
chr6	11617200	0.900826
chr6	11649500	0.826446
chr6	11653100	0.909091
chr6	11656100	1.000000
chr6	11755500	0.876033
chr6	11766600	0.884298
chr6	11769300	0.966942
chr6	11772300	0.958678
chr6	11870600	0.752066
chr6	11939100	0.809917
chr6	12015600	0.909091
chr6	12071800	0.917355
chr6	12113500	0.768595
chr6	12137600	0.768595
chr6	12149100	0.991736
chr6	12161000	0.685950
chr6	12246700	0.752066
chr6	12296800	0.719008
chr6	12314700	0.942149
chr6	12324900	0.942149
chr6	12344300	0.719008
chr6	12393800	0.966942
chr6	12403900	0.694215
chr6	12409300	0.950413
chr6	12484500	1.000000
chr6	12489700	0.851240
chr6	12492300	0.983471
chr6	12529000	0.859504
chr6	12537200	0.909091
chr6	12540400	0.917355
chr6	12546300	1.000000
chr6	12568400	0.975207
chr6	12573500	0.983471
chr6	12577500	0.876033
chr6	12583300	0.917355
chr6	12585500	0.958678
chr6	12587900	0.826446
chr6	12595700	0.760331
chr6	12603100	0.942149
chr6	12606900	0.685950
chr6	12609100	0.892562
chr6	12962300	0.859504
chr6	12964500	0.917355
chr6	12973800	0.851240
chr6	13218000	0.942149
chr6	13226600	0.818182
chr6	13304600	0.958678
chr6	13308300	0.991736
chr6	13335800	0.851240
chr6	13354700	0.950413
chr6	13356800	0.760331
chr6	13373600	0.669421

chr6	13396000	0.942149
chr6	13404800	0.942149
chr6	13423700	0.859504
chr6	13429200	0.793388
chr6	13445700	0.793388
chr6	13449300	0.760331
chr6	13454600	0.892562
chr6	13497400	0.975207
chr6	13505600	0.818182
chr6	13523400	0.909091
chr6	13528000	0.983471
chr6	13555700	0.892562
chr6	13636900	0.859504
chr6	13654400	0.867769
chr6	13678800	0.942149
chr6	13699100	0.760331
chr6	13794900	0.768595
chr6	13914000	0.752066
chr6	13936500	0.694215
chr6	14877900	1.000000
chr6	14881300	1.000000
chr6	15067400	0.966942
chr6	15266800	0.917355
chr6	15285300	0.801653
chr6	15309000	0.867769
chr6	15313300	0.925620
chr6	15400900	0.694215
chr6	15433600	0.694215
chr6	15436000	0.950413
chr6	15474000	0.851240
chr6	15505600	0.884298
chr6	15547900	0.876033
chr6	15552500	0.991736
chr6	15609200	0.768595
chr6	15628200	0.818182
chr6	15871000	0.983471
chr6	15897800	0.991736
chr6	15923100	0.966942
chr6	15946000	0.900826
chr6	15953800	0.900826
chr6	15957300	0.776860
chr6	15963000	1.000000
chr6	16093000	0.851240
chr6	16132600	0.669421
chr6	16137700	0.752066
chr6	16164100	0.719008
chr6	16207300	0.950413
chr6	16216200	0.983471
chr6	16219500	0.694215
chr6	16229900	0.694215
chr6	16237300	0.892562
chr6	16277900	0.950413
chr6	16303100	0.719008
chr6	16320600	0.975207

chr6	16332700	0.768595
chr6	16337900	0.958678
chr6	16340000	0.859504
chr6	16346000	0.975207
chr6	16352800	0.933884
chr6	16372500	0.727273
chr6	16513700	0.702479
chr6	16669900	1.000000
chr6	16675300	0.884298
chr6	16684700	0.743802
chr6	16689900	0.975207
chr6	16713500	0.933884
chr6	16719500	0.983471
chr6	16723200	0.752066
chr6	16726700	0.966942
chr6	16732300	0.776860
chr6	16738100	0.966942
chr6	16753200	1.000000
chr6	16764300	0.834711
chr6	16766900	0.983471
chr6	16770300	0.983471
chr6	16961900	0.818182
chr6	17013400	0.925620
chr6	17028100	0.991736
chr6	17033400	0.909091
chr6	17451600	0.710744
chr6	17491100	0.842975
chr6	17495600	0.991736
chr6	17498300	0.991736
chr6	17584500	0.950413
chr6	17687900	0.752066
chr6	17703600	0.933884
chr6	17708300	0.661157
chr6	17716200	0.900826
chr6	17725600	0.851240
chr6	17831700	0.966942
chr6	17848200	0.933884
chr6	17852400	0.950413
chr6	17864200	0.752066
chr6	17867300	0.933884
chr6	17870300	0.966942
chr6	17924400	0.991736
chr6	17929200	0.966942
chr6	17933300	0.876033
chr6	17965800	0.975207
chr6	17973200	1.000000
chr6	18043800	0.851240
chr6	18122900	0.917355
chr6	18243100	0.834711
chr6	18261000	0.785124
chr6	18278000	0.669421
chr6	18344500	0.876033
chr6	18347300	0.809917
chr6	18364300	0.991736

chr6	18370200	0.743802
chr6	18372300	0.867769
chr6	18380700	0.801653
chr6	18386200	0.826446
chr6	18394100	0.735537
chr6	18398800	0.917355
chr6	18400900	0.727273
chr6	18403000	0.942149
chr6	18405400	0.851240
chr6	18407800	0.876033
chr6	18435000	0.735537
chr6	18454900	0.702479
chr6	18478100	0.876033
chr6	18497700	0.876033
chr6	18504300	0.958678
chr6	18585600	0.925620
chr6	19805200	0.809917
chr6	19838200	0.768595
chr6	20378500	0.975207
chr6	20498000	0.909091
chr6	20543700	0.710744
chr6	20565300	0.900826
chr6	20648300	0.768595
chr6	20653400	0.975207
chr6	20712000	0.809917
chr6	20811700	0.950413
chr6	20827000	0.950413
chr6	20877000	0.842975
chr6	20879800	0.933884
chr6	20907000	0.727273
chr6	20953200	0.950413
chr6	20978700	0.966942
chr6	21004500	0.958678
chr6	21006800	0.983471
chr6	21023500	0.933884
chr6	21027000	0.983471
chr6	21065400	0.685950
chr6	21068300	0.942149
chr6	21070900	0.933884
chr6	21109500	0.851240
chr6	21111600	0.966942
chr6	21187100	0.842975
chr6	21189500	1.000000
chr6	21616300	0.983471
chr6	21635000	0.958678
chr6	21679900	0.900826
chr6	21708000	0.975207
chr6	21727100	0.933884
chr6	21741000	0.826446
chr6	21743200	0.793388
chr6	21752400	0.950413
chr6	21755600	0.958678
chr6	21768400	0.909091
chr6	21771000	0.958678

chr6	21773100	0.867769
chr6	21824200	0.702479
chr6	21840900	0.917355
chr6	21844500	0.900826
chr6	21848100	0.925620
chr6	21885700	0.842975
chr6	21887800	0.719008
chr6	21915900	0.710744
chr6	21926000	0.851240
chr6	21933900	0.983471
chr6	21943300	0.942149
chr6	21948600	0.966942
chr6	21951900	0.834711
chr6	21954400	0.983471
chr6	21988000	1.000000
chr6	21999500	0.909091
chr6	22026200	0.958678
chr6	22044000	0.834711
chr6	22072100	0.909091
chr6	22113600	1.000000
chr6	22141800	0.958678
chr6	22147300	0.958678
chr6	22228800	0.752066
chr6	22445000	0.966942
chr6	22447200	0.826446
chr6	22449400	0.900826
chr6	22471700	1.000000
chr6	22614300	0.966942
chr6	22765300	0.710744
chr6	22828500	0.966942
chr6	22881400	0.975207
chr6	22884900	0.991736
chr6	22888700	0.975207
chr6	22893200	0.933884
chr6	23316900	0.727273
chr6	23350800	0.677686
chr6	24730200	0.702479
chr6	24743900	0.950413
chr6	25007400	0.876033
chr6	25051100	0.867769
chr6	25086200	0.933884
chr6	25090000	0.917355
chr6	25138700	0.752066
chr6	25174400	0.966942
chr6	25176500	0.677686
chr6	25194000	0.983471
chr6	25715400	0.983471
chr6	25971700	1.000000
chr6	25992000	0.809917
chr6	26017600	0.917355
chr6	26025800	0.702479
chr6	26035200	0.892562
chr6	26043300	0.818182
chr6	26103600	0.743802

chr6	26141200	0.743802
chr6	26203900	0.702479
chr6	26207400	0.785124
chr6	26296400	0.942149
chr6	26352000	0.991736
chr6	26413500	0.867769
chr6	26475500	0.719008
chr6	26502200	0.768595
chr6	26536700	0.743802
chr6	26553700	0.694215
chr6	26771600	0.727273
chr6	27060300	0.685950
chr6	27099100	0.702479
chr6	27125700	0.719008
chr6	27219600	0.702479
chr6	27526400	0.702479
chr6	27599000	0.842975
chr6	27774800	0.710744
chr6	27804100	0.669421
chr6	27858700	0.735537
chr6	28186700	0.727273
chr6	28227300	0.752066
chr6	28237400	0.933884
chr6	28319700	0.785124
chr6	28322000	0.933884
chr6	28325500	0.867769
chr6	28557900	0.892562
chr6	28654300	0.685950
chr6	28762500	0.685950
chr6	28778400	0.768595
chr6	28916000	0.818182
chr6	28945700	0.884298
chr6	29596100	0.685950
chr6	29618100	0.776860
chr6	29732000	0.925620
chr6	29932700	0.785124
chr6	29936200	1.000000
chr6	29974800	0.694215
chr6	30033900	0.661157
chr6	30071200	0.735537
chr6	30431900	0.801653
chr6	30449800	0.859504
chr6	30460500	0.892562
chr6	30464600	0.925620
chr6	30468900	0.950413
chr6	30477400	0.834711
chr6	30484200	0.933884
chr6	30496000	0.826446
chr6	30582100	0.933884
chr6	30686400	0.818182
chr6	30692500	0.685950
chr6	30704800	0.702479
chr6	30707900	0.859504
chr6	30713900	0.818182

chr6	30719000	0.917355
chr6	30721400	0.925620
chr6	30727500	0.991736
chr6	30732000	0.710744
chr6	30738500	0.900826
chr6	30749900	0.892562
chr6	30761800	0.900826
chr6	30769300	0.933884
chr6	30780700	0.818182
chr6	30782900	0.793388
chr6	30787200	0.752066
chr6	30790000	0.958678
chr6	30845100	1.000000
chr6	30882200	0.917355
chr6	30884300	0.752066
chr6	31054900	0.950413
chr6	31062000	0.958678
chr6	31082900	0.933884
chr6	31090600	0.983471
chr6	31093400	0.793388
chr6	31171600	0.760331
chr6	31202200	0.719008
chr6	31364700	0.975207
chr6	31374000	0.677686
chr6	31460100	0.702479
chr6	31464400	0.917355
chr6	31494600	0.983471
chr6	31506700	0.752066
chr6	31528300	0.892562
chr6	31531300	0.925620
chr6	31626100	0.710744
chr6	31658600	0.685950
chr6	31679300	0.727273
chr6	31701600	0.694215
chr6	31706100	0.677686
chr6	31760900	0.892562
chr6	31782100	0.719008
chr6	31794300	0.925620
chr6	32015300	0.669421
chr6	32026800	0.743802
chr6	32154700	0.966942
chr6	32159000	0.892562
chr6	32165900	0.867769
chr6	32191300	0.892562
chr6	32203300	0.752066
chr6	32224000	0.958678
chr6	32259100	0.785124
chr6	32337700	0.925620
chr6	32340000	0.859504
chr6	32361600	0.950413
chr6	32384100	0.669421
chr6	32909400	0.694215
chr6	32961400	0.818182
chr6	32987100	0.818182

chr6	33021300	0.785124
chr6	33129900	0.776860
chr6	33161100	0.826446
chr6	33173700	0.851240
chr6	33242900	0.677686
chr6	33285500	0.727273
chr6	33287900	0.933884
chr6	33377500	0.760331
chr6	33387200	0.900826
chr6	33399000	0.719008
chr6	33545100	0.834711
chr6	33577800	0.809917
chr6	33603200	0.694215
chr6	33641300	0.884298
chr6	33645100	0.859504
chr6	33661300	0.892562
chr6	33665400	0.942149
chr6	33667800	1.000000
chr6	33738300	0.685950
chr6	33741700	0.719008
chr6	33747500	0.809917
chr6	33753700	0.867769
chr6	33808100	0.942149
chr6	34164700	0.785124
chr6	34192100	0.983471
chr6	34203600	0.900826
chr6	34208600	0.834711
chr6	34214900	0.942149
chr6	34241100	0.966942
chr6	34274300	0.818182
chr6	34309400	0.950413
chr6	34338500	0.942149
chr6	34511600	0.752066
chr6	34578500	0.909091
chr6	34624200	0.685950
chr6	34626300	0.694215
chr6	34632500	0.958678
chr6	34641100	0.809917
chr6	34778600	0.826446
chr6	34833300	0.966942
chr6	34881100	0.925620
chr6	34887400	0.785124
chr6	34979800	0.818182
chr6	34982700	0.785124
chr6	34987300	0.768595
chr6	34992300	0.892562
chr6	34995100	0.842975
chr6	35003300	0.909091
chr6	35011700	0.950413
chr6	35015800	0.785124
chr6	35031200	0.685950
chr6	35064600	0.859504
chr6	35116500	0.933884
chr6	35149900	0.727273

chr6	35207100	0.950413
chr6	35265800	0.909091
chr6	35336800	0.818182
chr6	35339100	0.710744
chr6	35359200	0.966942
chr6	35361800	0.884298
chr6	35387500	0.702479
chr6	35393000	0.909091
chr6	35396800	0.983471
chr6	35564800	0.991736
chr6	35568700	0.991736
chr6	35571300	0.983471
chr6	35573900	0.884298
chr6	35631900	0.933884
chr6	35635600	0.958678
chr6	35638700	0.925620
chr6	35646100	0.966942
chr6	35686700	0.710744
chr6	35696800	0.785124
chr6	35779600	0.809917
chr6	35814200	0.743802
chr6	35853300	0.966942
chr6	35874400	0.958678
chr6	35920400	0.859504
chr6	35938400	0.694215
chr6	35993700	0.933884
chr6	36006600	0.818182
chr6	36067800	0.933884
chr6	36088900	0.917355
chr6	36098600	0.975207
chr6	36164100	0.694215
chr6	36192700	0.702479
chr6	36213200	0.933884
chr6	36215400	0.842975
chr6	36217500	0.942149
chr6	36258800	0.743802
chr6	36308900	0.809917
chr6	36317900	0.826446
chr6	36320300	0.768595
chr6	36326500	0.818182
chr6	36333200	0.727273
chr6	36409400	0.842975
chr6	36413100	0.933884
chr6	36567800	0.785124
chr6	36602200	0.752066
chr6	36617600	0.925620
chr6	36644800	0.975207
chr6	36650200	0.942149
chr6	36703400	0.933884
chr6	36709100	0.867769
chr6	36720500	0.743802
chr6	36723900	0.950413
chr6	36726100	0.925620
chr6	36738300	0.925620

chr6	36788900	0.876033
chr6	36792200	0.702479
chr6	36797500	0.859504
chr6	36816900	0.661157
chr6	36821400	0.925620
chr6	36882300	0.834711
chr6	36917800	0.909091
chr6	36990300	0.785124
chr6	37027700	0.867769
chr6	37070300	0.669421
chr6	37094300	0.884298
chr6	37099100	1.000000
chr6	37102800	0.909091
chr6	37106500	0.958678
chr6	37136700	0.876033
chr6	37170900	0.727273
chr6	37205100	0.958678
chr6	37208100	0.991736
chr6	37210500	0.958678
chr6	37212800	0.983471
chr6	37219200	0.826446
chr6	37228000	0.876033
chr6	37292200	0.983471
chr6	37326800	0.727273
chr6	37337900	0.727273
chr6	37484200	0.669421
chr6	37492500	1.000000
chr6	37504200	0.809917
chr6	37515700	0.925620
chr6	37521900	0.942149
chr6	37548500	0.661157
chr6	37593000	0.776860
chr6	37598500	0.983471
chr6	37612000	0.809917
chr6	37617200	0.685950
chr6	37673300	0.685950
chr6	37790800	0.818182
chr6	37836400	0.958678
chr6	37857100	0.669421
chr6	37873900	0.966942
chr6	37964500	0.694215
chr6	38124100	0.909091
chr6	38161700	0.900826
chr6	38180900	0.892562
chr6	38228000	0.966942
chr6	38230600	0.685950
chr6	38314900	0.818182
chr6	38362000	0.942149
chr6	38392000	0.685950
chr6	38499400	0.785124
chr6	38540200	0.983471
chr6	38547100	0.867769
chr6	38602400	0.785124
chr6	38641600	0.900826

chr6	38694100	0.669421
chr6	38739100	0.983471
chr6	38755000	0.983471
chr6	38757200	0.793388
chr6	38764600	0.983471
chr6	38773500	0.900826
chr6	39393900	0.958678
chr6	39850900	0.801653
chr6	39856000	0.809917
chr6	40511500	0.785124
chr6	40747400	0.925620
chr6	40875100	0.925620
chr6	40905200	0.892562
chr6	40965100	0.909091
chr6	40996300	0.793388
chr6	41065900	0.975207
chr6	41288600	0.909091
chr6	41396000	0.702479
chr6	41500000	0.867769
chr6	41528900	0.892562
chr6	41546400	0.925620
chr6	41549600	0.818182
chr6	41594900	0.909091
chr6	41623900	0.834711
chr6	41651200	0.826446
chr6	41666600	0.892562
chr6	41731700	0.752066
chr6	41793200	0.933884
chr6	41814600	0.942149
chr6	41885600	0.842975
chr6	41933700	0.867769
chr6	41936000	0.933884
chr6	41978500	0.727273
chr6	41996600	0.834711
chr6	42013800	0.983471
chr6	42096600	0.925620
chr6	42099700	0.727273
chr6	42105300	0.735537
chr6	42110600	0.859504
chr6	42165900	0.685950
chr6	42178800	0.834711
chr6	42248200	0.785124
chr6	42284200	0.867769
chr6	42307000	0.859504
chr6	42372600	0.809917
chr6	42515600	0.719008
chr6	42660500	0.735537
chr6	42682300	0.826446
chr6	42694900	0.752066
chr6	42768800	0.991736
chr6	42867800	0.685950
chr6	43143200	0.710744
chr6	43192400	0.884298
chr6	43197100	0.694215

chr6	43276800	0.785124
chr6	43338200	0.702479
chr6	43351700	0.694215
chr6	43404600	0.702479
chr6	43467400	0.933884
chr6	43546900	0.818182
chr6	43594600	0.867769
chr6	43667700	0.809917
chr6	43670900	1.000000
chr6	43674600	0.834711
chr6	43677200	0.809917
chr6	43684300	0.876033
chr6	43721700	0.933884
chr6	43742700	0.892562
chr6	43900900	0.760331
chr6	43970900	0.685950
chr6	44003700	0.702479
chr6	44009100	0.793388
chr6	44021800	0.768595
chr6	44027400	0.785124
chr6	44031900	0.669421
chr6	44034800	0.983471
chr6	44040700	0.851240
chr6	44071700	0.851240
chr6	44075400	0.966942
chr6	44083900	0.909091
chr6	44086000	0.900826
chr6	44090700	0.966942
chr6	44092800	0.917355
chr6	44148300	0.768595
chr6	44154400	0.785124
chr6	44160600	0.933884
chr6	44194400	0.884298
chr6	44201800	0.909091
chr6	44205700	0.942149
chr6	44218300	0.892562
chr6	44378800	0.752066
chr6	44384200	0.727273
chr6	44424900	0.793388
chr6	44458300	0.991736
chr6	45017500	0.900826
chr6	45041000	0.958678
chr6	45043200	0.818182
chr6	45401500	0.768595
chr6	45404900	0.768595
chr6	45413700	0.958678
chr6	45446300	0.909091
chr6	45618900	0.909091
chr6	45624100	0.842975
chr6	45645500	0.991736
chr6	45983600	0.743802
chr6	46003200	0.719008
chr6	46012800	0.966942
chr6	46258600	0.966942

chr6	46703200	0.694215
chr6	46817000	0.942149
chr6	46844300	0.867769
chr6	46856400	0.826446
chr6	46870000	0.958678
chr6	46877400	0.933884
chr6	46882400	0.933884
chr6	46886900	0.809917
chr6	46889700	0.768595
chr6	46894100	0.719008
chr6	46903500	0.909091
chr6	46914400	0.818182
chr6	46916800	0.991736
chr6	46953700	0.710744
chr6	46958600	0.958678
chr6	46962100	0.983471
chr6	47012200	0.876033
chr6	47015500	0.983471
chr6	47029800	0.735537
chr6	47103100	0.884298
chr6	47210600	0.958678
chr6	47229800	0.710744
chr6	47232800	0.983471
chr6	47382600	0.801653
chr6	47442400	0.785124
chr6	47619600	0.925620
chr6	47748900	0.685950
chr6	48398300	0.867769
chr6	48795200	0.966942
chr6	48853300	0.785124
chr6	49273400	0.760331
chr6	49293400	0.942149
chr6	49295500	0.867769
chr6	49304800	0.900826
chr6	49509400	0.818182
chr6	49521400	0.669421
chr6	49524400	1.000000
chr6	49533100	0.950413
chr6	49535300	0.950413
chr6	49538400	0.950413
chr6	50626300	0.785124
chr6	50629500	1.000000
chr6	50891600	0.958678
chr6	51928900	0.793388
chr6	52137000	1.000000
chr6	52217800	0.966942
chr6	52255100	0.933884
chr6	52281000	0.900826
chr6	52283100	0.991736
chr6	52289500	0.983471
chr6	52354600	0.950413
chr6	52357200	0.727273
chr6	52369900	0.776860
chr6	52374800	0.809917

chr6	52378100	0.925620
chr6	52383000	0.694215
chr6	52386700	0.842975
chr6	52388800	0.933884
chr6	52398000	0.727273
chr6	52400500	0.966942
chr6	52408400	0.909091
chr6	52410900	0.809917
chr6	52414900	0.743802
chr6	52418100	0.975207
chr6	52425600	0.661157
chr6	52428100	0.950413
chr6	52433700	0.942149
chr6	52435900	0.991736
chr6	52438400	0.892562
chr6	52461800	0.966942
chr6	52468400	0.867769
chr6	52477400	0.925620
chr6	52499900	0.669421
chr6	52794600	0.917355
chr6	52900800	0.694215
chr6	52907400	0.743802
chr6	52913600	0.900826
chr6	53168100	0.983471
chr6	53174700	0.925620
chr6	53180500	0.966942
chr6	53358900	0.809917
chr6	53538600	0.752066
chr6	53599700	0.933884
chr6	53618200	0.876033
chr6	53680900	0.975207
chr6	53722900	0.876033
chr6	53735200	0.710744
chr6	53764600	0.991736
chr6	53775900	0.983471
chr6	53792000	0.801653
chr6	53794200	0.991736
chr6	53849200	0.942149
chr6	53919400	0.933884
chr6	53925000	0.876033
chr6	53976400	0.933884
chr6	53978800	0.859504
chr6	54058600	0.958678
chr6	54120500	0.661157
chr6	54156900	1.000000
chr6	54200600	0.884298
chr6	54232100	0.876033
chr6	54284800	0.818182
chr6	54296600	0.702479
chr6	54512400	0.876033
chr6	55570100	0.785124
chr6	55747800	1.000000
chr6	55840000	0.719008
chr6	55889600	0.917355

chr6	55892100	0.826446
chr6	55912800	0.950413
chr6	55962000	0.677686
chr6	56194400	0.958678
chr6	56200300	0.933884
chr6	56227200	1.000000
chr6	56230100	0.900826
chr6	56233800	1.000000
chr6	56237000	0.900826
chr6	56356500	0.958678
chr6	56517900	0.950413
chr6	56521500	0.694215
chr6	56537000	0.809917
chr6	56542900	0.719008
chr6	56555800	0.975207
chr6	56558600	0.826446
chr6	56560700	0.851240
chr6	56573900	0.966942
chr6	56578000	0.785124
chr6	56580600	0.950413
chr6	56584500	0.743802
chr6	56595100	0.834711
chr6	56597600	0.884298
chr6	56637500	0.661157
chr6	56668200	0.727273
chr6	56706000	0.892562
chr6	56718000	0.669421
chr6	56728800	0.991736
chr6	56733700	1.000000
chr6	56755600	0.983471
chr6	56914000	0.727273
chr6	57130800	0.661157
chr6	57179100	0.958678
chr6	57297300	0.801653
chr6	57310900	0.958678
chr6	57471800	0.983471
chr6	58142500	0.752066
chr6	61904200	0.669421
chr6	62284200	0.925620
chr6	64058000	0.933884
chr6	64063200	0.694215
chr6	64373200	0.702479
chr6	64442300	0.793388
chr6	64514700	0.818182
chr6	64517300	0.983471
chr6	64614900	0.851240
chr6	64964700	0.776860
chr6	65207800	0.900826
chr6	65230300	0.933884
chr6	65394000	0.876033
chr6	65601100	0.917355
chr6	65615000	0.834711
chr6	65999600	0.685950
chr6	68350500	0.760331

chr6	68393900	0.793388
chr6	68447000	0.760331
chr6	68476400	0.892562
chr6	69730200	0.975207
chr6	70577300	0.694215
chr6	71108800	0.793388
chr6	71958200	0.983471
chr6	72044400	0.669421
chr6	72077200	0.983471
chr6	72096200	0.983471
chr6	72102100	0.776860
chr6	72104200	0.975207
chr6	72110600	0.884298
chr6	72112700	0.809917
chr6	72115100	0.975207
chr6	72118000	0.991736
chr6	72124500	0.685950
chr6	72187900	0.892562
chr6	72191100	0.818182
chr6	72199600	0.942149
chr6	72206100	0.925620
chr6	72971500	0.694215
chr6	73074000	0.975207
chr6	73332400	0.702479
chr6	73571500	0.942149
chr6	73663400	0.942149
chr6	73676700	0.776860
chr6	73993000	0.950413
chr6	74226100	0.702479
chr6	74289100	0.892562
chr6	74295900	0.958678
chr6	74376000	0.719008
chr6	74404200	0.842975
chr6	74409800	0.983471
chr6	74414500	0.933884
chr6	74424400	0.909091
chr6	74432600	0.942149
chr6	74435100	0.818182
chr6	74498800	0.842975
chr6	74514000	0.867769
chr6	74848000	0.966942
chr6	74959700	0.909091
chr6	75279200	0.991736
chr6	75307500	0.925620
chr6	75316800	0.991736
chr6	75319900	0.983471
chr6	75657400	0.892562
chr6	75734700	0.900826
chr6	75809000	0.719008
chr6	75812100	0.925620
chr6	75836500	0.884298
chr6	75866800	0.776860
chr6	75912100	0.719008
chr6	75930400	0.694215

chr6	75978100	0.991736
chr6	75989000	0.793388
chr6	76027800	0.785124
chr6	76044800	0.818182
chr6	76059500	0.801653
chr6	76092200	0.785124
chr6	76108300	0.735537
chr6	76123400	0.983471
chr6	76127000	0.793388
chr6	76129200	0.966942
chr6	76141400	0.661157
chr6	76189100	0.834711
chr6	76193600	0.966942
chr6	76208200	0.859504
chr6	76212200	0.892562
chr6	76234400	0.966942
chr6	76445800	0.950413
chr6	76461400	0.842975
chr6	76463800	0.975207
chr6	76502100	0.859504
chr6	76552500	0.925620
chr6	76559500	0.859504
chr6	76562700	0.900826
chr6	77755800	0.768595
chr6	77845700	0.752066
chr6	78000000	0.826446
chr6	78003000	0.991736
chr6	78157500	0.743802
chr6	78165600	0.859504
chr6	78167800	0.983471
chr6	78594000	0.719008
chr6	78649000	0.752066
chr6	78945600	0.900826
chr6	79199600	0.900826
chr6	79202700	1.000000
chr6	79244000	0.685950
chr6	79271200	0.768595
chr6	79274600	0.685950
chr6	79308000	0.760331
chr6	79311600	0.768595
chr6	79314100	0.834711
chr6	79316900	0.942149
chr6	79676800	0.925620
chr6	79679300	0.867769
chr6	79715800	0.859504
chr6	79784800	0.710744
chr6	79941400	0.710744
chr6	80085700	0.677686
chr6	80087900	0.966942
chr6	80095900	0.950413
chr6	80131900	0.909091
chr6	80255100	0.991736
chr6	80344400	0.975207
chr6	80350500	0.760331

chr6	80381300	0.851240
chr6	80415600	0.842975
chr6	80447100	0.735537
chr6	80465900	0.933884
chr6	80651700	0.966942
chr6	80732900	0.685950
chr6	80892600	0.958678
chr6	81160000	0.702479
chr6	81163500	0.752066
chr6	81167600	0.917355
chr6	81233600	0.966942
chr6	81348800	0.983471
chr6	81666900	0.933884
chr6	82317400	0.851240
chr6	82324100	0.983471
chr6	82519000	1.000000
chr6	82564100	0.867769
chr6	82576000	0.966942
chr6	82648600	0.694215
chr6	82661000	0.727273
chr6	82691900	0.884298
chr6	82725200	0.991736
chr6	82727300	0.818182
chr6	82732800	0.991736
chr6	82849200	0.685950
chr6	82854700	0.909091
chr6	82937700	0.735537
chr6	82954100	0.884298
chr6	82970700	0.685950
chr6	83586100	0.809917
chr6	83780700	0.942149
chr6	83829900	0.669421
chr6	83909400	0.966942
chr6	83936800	0.685950
chr6	83981500	0.776860
chr6	84525600	0.983471
chr6	84528600	0.719008
chr6	84534500	0.925620
chr6	84578100	0.719008
chr6	84737900	1.000000
chr6	84776300	0.677686
chr6	84848300	0.909091
chr6	84892200	0.983471
chr6	84903300	0.867769
chr6	85173700	0.661157
chr6	85176800	0.942149
chr6	85202200	0.983471
chr6	85285400	1.000000
chr6	85293900	0.925620
chr6	85311500	0.842975
chr6	85328200	0.991736
chr6	85334600	0.909091
chr6	85351300	0.826446
chr6	85353700	0.743802

chr6	85356000	0.975207
chr6	85361400	0.752066
chr6	85363500	0.900826
chr6	85377300	0.892562
chr6	85441800	1.000000
chr6	85475500	0.685950
chr6	85478700	0.735537
chr6	85501300	0.983471
chr6	85507900	0.842975
chr6	85607900	1.000000
chr6	85689900	0.991736
chr6	85759400	0.892562
chr6	85951200	0.785124
chr6	86071400	0.909091
chr6	86074300	0.694215
chr6	86092800	0.942149
chr6	86095800	0.950413
chr6	86111200	0.867769
chr6	86113900	0.900826
chr6	86116100	0.975207
chr6	86167800	0.917355
chr6	86170200	1.000000
chr6	86174000	0.958678
chr6	86176400	0.851240
chr6	86279400	0.867769
chr6	86285300	0.892562
chr6	87862200	0.743802
chr6	88197600	0.991736
chr6	88225200	0.760331
chr6	88285000	0.966942
chr6	88408200	0.677686
chr6	88424500	0.694215
chr6	88433500	0.776860
chr6	88441900	0.694215
chr6	88619200	0.818182
chr6	89086900	0.884298
chr6	89090400	0.933884
chr6	89201300	0.851240
chr6	89440800	0.942149
chr6	89525500	0.826446
chr6	89578600	0.818182
chr6	89961200	0.859504
chr6	89964500	0.793388
chr6	90007700	0.876033
chr6	90022400	0.851240
chr6	90034000	0.983471
chr6	90057900	0.685950
chr6	90060000	0.727273
chr6	90550600	0.917355
chr6	90638000	0.826446
chr6	90718900	0.975207
chr6	90754900	1.000000
chr6	90797100	0.677686
chr6	90926400	0.925620

chr6	90930600	0.876033
chr6	90941100	0.900826
chr6	90994600	0.818182
chr6	91079100	0.983471
chr6	91203500	1.000000
chr6	92144500	0.966942
chr6	92148800	0.867769
chr6	92186300	0.958678
chr6	92189600	0.884298
chr6	92192800	0.983471
chr6	93231700	0.917355
chr6	93248600	0.900826
chr6	93255700	0.909091
chr6	93260300	0.917355
chr6	93689000	0.661157
chr6	93750400	0.942149
chr6	94387200	0.933884
chr6	96020300	0.727273
chr6	96049000	0.925620
chr6	97638200	0.958678
chr6	97687900	0.826446
chr6	100676700	0.859504
chr6	100706300	0.900826
chr6	100711500	0.727273
chr6	100717200	0.950413
chr6	100725100	0.966942
chr6	100744700	0.925620
chr6	100748800	0.983471
chr6	100752800	0.661157
chr6	100791200	0.702479
chr6	100794800	0.975207
chr6	100798500	0.669421
chr6	100831100	0.685950
chr6	100902600	0.710744
chr6	100909500	0.768595
chr6	101168500	0.694215
chr6	102170900	0.900826
chr6	102189700	0.661157
chr6	102254500	0.842975
chr6	104217600	0.925620
chr6	104970600	0.933884
chr6	104973800	0.942149
chr6	105086400	0.785124
chr6	105091000	0.876033
chr6	105102000	0.917355
chr6	105151600	0.826446
chr6	105154500	0.851240
chr6	105232800	0.950413
chr6	105500300	0.950413
chr6	105633000	0.884298
chr6	105637500	0.958678
chr6	105658500	0.809917
chr6	105675300	0.702479
chr6	105808300	0.818182

chr6	105812500	0.752066
chr6	105822400	0.950413
chr6	105830800	0.917355
chr6	105876400	0.876033
chr6	105880900	0.983471
chr6	105908400	0.983471
chr6	105936800	0.933884
chr6	106348600	0.950413
chr6	106429400	0.842975
chr6	106546200	0.694215
chr6	106758300	0.917355
chr6	106809000	0.719008
chr6	106976200	0.975207
chr6	106979100	0.661157
chr6	106994900	0.933884
chr6	106997000	0.867769
chr6	107023000	0.702479
chr6	107585600	0.785124
chr6	107641800	0.933884
chr6	107644900	0.975207
chr6	107765400	0.760331
chr6	107814200	0.768595
chr6	107816900	0.958678
chr6	107821300	0.661157
chr6	107834600	0.694215
chr6	107967600	0.768595
chr6	107979300	0.950413
chr6	107987100	0.933884
chr6	107992700	0.818182
chr6	108455500	0.884298
chr6	108497200	0.735537
chr6	108768800	0.876033
chr6	108774700	0.983471
chr6	108887400	0.917355
chr6	108903000	0.925620
chr6	108909700	0.983471
chr6	108913300	0.917355
chr6	108995200	0.685950
chr6	109059100	0.983471
chr6	109061600	0.859504
chr6	109103900	0.768595
chr6	109120100	0.842975
chr6	109217900	0.909091
chr6	109223900	0.958678
chr6	109381300	0.983471
chr6	109991800	0.685950
chr6	109994300	0.975207
chr6	110065100	0.743802
chr6	110081600	0.661157
chr6	110111800	0.950413
chr6	110115200	0.991736
chr6	110416600	1.000000
chr6	110418700	0.760331
chr6	110513100	0.801653

chr6	110711300	0.958678
chr6	110721300	0.876033
chr6	110831400	0.933884
chr6	110850000	0.991736
chr6	111199600	0.710744
chr6	111582200	0.727273
chr6	111745500	0.884298
chr6	111748800	0.991736
chr6	111767500	0.950413
chr6	111778000	0.677686
chr6	111784500	0.727273
chr6	111789800	0.760331
chr6	111799800	0.925620
chr6	111816700	0.900826
chr6	111873900	0.950413
chr6	111886100	0.826446
chr6	111895500	0.966942
chr6	111910700	0.975207
chr6	111912900	0.735537
chr6	111923900	0.950413
chr6	111978800	0.925620
chr6	111985200	0.694215
chr6	111988500	0.950413
chr6	112005000	0.942149
chr6	112007900	0.867769
chr6	112016200	0.685950
chr6	112021300	0.677686
chr6	112052600	0.785124
chr6	112069300	0.942149
chr6	112072600	0.991736
chr6	112076100	0.991736
chr6	112078800	0.727273
chr6	112085000	0.909091
chr6	112106000	0.983471
chr6	112125100	0.991736
chr6	112127200	0.958678
chr6	112145100	0.983471
chr6	112151600	0.710744
chr6	112216000	0.983471
chr6	112294800	0.975207
chr6	112314700	0.950413
chr6	112329800	0.669421
chr6	112340400	0.710744
chr6	112359000	0.876033
chr6	112361200	0.975207
chr6	112363500	0.917355
chr6	112423900	0.776860
chr6	112467200	0.917355
chr6	112477300	0.884298
chr6	112504900	0.685950
chr6	112520300	0.884298
chr6	112526100	0.876033
chr6	112529200	0.909091
chr6	112535800	0.925620

chr6	112538800	0.975207
chr6	112541200	0.900826
chr6	112544300	0.991736
chr6	112546400	0.826446
chr6	112548500	0.933884
chr6	112551600	0.801653
chr6	112555200	0.909091
chr6	112558200	0.933884
chr6	112562800	0.900826
chr6	112566400	0.975207
chr6	112570300	0.834711
chr6	112590400	0.710744
chr6	112619600	0.909091
chr6	112642800	0.735537
chr6	112782300	0.727273
chr6	112810100	0.661157
chr6	112961300	0.760331
chr6	112971800	0.900826
chr6	113012600	0.884298
chr6	113123400	0.933884
chr6	113149300	0.743802
chr6	113152400	0.727273
chr6	113198900	0.917355
chr6	113234200	0.743802
chr6	113261300	0.768595
chr6	113269600	0.702479
chr6	113301500	0.884298
chr6	113340200	0.933884
chr6	113371700	0.975207
chr6	113387100	0.661157
chr6	113424400	0.950413
chr6	113442100	0.975207
chr6	113444300	0.892562
chr6	113458800	0.983471
chr6	113504200	0.983471
chr6	113598300	0.942149
chr6	113639900	0.859504
chr6	113674000	0.983471
chr6	113697700	0.834711
chr6	113771300	0.760331
chr6	113779400	0.900826
chr6	113818600	0.826446
chr6	113880400	1.000000
chr6	113882700	0.834711
chr6	113913800	0.818182
chr6	113929600	0.785124
chr6	113954400	0.933884
chr6	113983700	0.909091
chr6	113994100	0.834711
chr6	114084400	0.669421
chr6	114175700	0.685950
chr6	114200500	0.925620
chr6	114317500	0.950413
chr6	114329400	0.876033

chr6	114421900	0.719008
chr6	114664100	0.793388
chr6	116330300	0.950413
chr6	116567600	0.867769
chr6	116635900	0.826446
chr6	116649600	0.958678
chr6	116710500	0.983471
chr6	116714100	0.917355
chr6	116850200	0.710744
chr6	116879600	0.710744
chr6	116890400	0.727273
chr6	116951200	0.752066
chr6	116970000	0.809917
chr6	117063100	0.867769
chr6	117591900	0.661157
chr6	117785900	0.975207
chr6	117807100	0.727273
chr6	117812400	0.884298
chr6	117815500	0.975207
chr6	117817800	1.000000
chr6	117820100	0.966942
chr6	117823700	0.768595
chr6	117843500	0.752066
chr6	117856100	0.867769
chr6	117870800	0.892562
chr6	118610100	0.702479
chr6	118705800	0.851240
chr6	118709600	0.909091
chr6	118790600	0.768595
chr6	119027600	0.909091
chr6	119030000	0.760331
chr6	119032100	0.702479
chr6	119109500	0.669421
chr6	119192000	0.851240
chr6	119451400	0.983471
chr6	119469000	0.851240
chr6	119558800	1.000000
chr6	119656300	0.702479
chr6	121640800	0.669421
chr6	121761200	0.834711
chr6	121763300	0.950413
chr6	121765500	0.727273
chr6	121803500	0.933884
chr6	121836200	0.958678
chr6	121838400	0.892562
chr6	121841100	0.950413
chr6	121843500	0.983471
chr6	121845800	0.859504
chr6	121865200	0.950413
chr6	121952000	0.776860
chr6	121969400	0.719008
chr6	121972800	0.900826
chr6	121975200	0.710744
chr6	121979400	0.867769

chr6	122147900	0.991736
chr6	122273300	0.661157
chr6	123017600	0.983471
chr6	123412800	0.950413
chr6	123804000	0.685950
chr6	123869300	0.710744
chr6	124151500	0.719008
chr6	124329400	0.752066
chr6	124362200	0.685950
chr6	124809700	0.735537
chr6	125204100	0.933884
chr6	125287100	0.818182
chr6	125306000	0.983471
chr6	125316500	0.785124
chr6	125323600	0.867769
chr6	125335500	0.669421
chr6	125424800	0.867769
chr6	125471200	0.685950
chr6	125523500	0.669421
chr6	125622700	0.776860
chr6	125808500	0.743802
chr6	125855500	0.942149
chr6	125907400	0.966942
chr6	125909900	0.900826
chr6	125933400	0.983471
chr6	126063800	0.785124
chr6	126115700	0.925620
chr6	126125300	0.834711
chr6	126134000	0.983471
chr6	126136400	0.958678
chr6	126140000	0.801653
chr6	126143500	1.000000
chr6	126156700	0.917355
chr6	126161000	0.942149
chr6	126231800	0.942149
chr6	126240400	0.975207
chr6	126265800	0.925620
chr6	126268400	0.983471
chr6	126362200	0.983471
chr6	126588400	0.925620
chr6	126623700	0.719008
chr6	126674500	0.702479
chr6	127133300	0.958678
chr6	127153000	0.942149
chr6	127199800	0.809917
chr6	127208300	0.818182
chr6	127211500	0.925620
chr6	127237300	0.909091
chr6	127241200	0.958678
chr6	127375000	0.958678
chr6	127468600	0.975207
chr6	127483800	0.909091
chr6	127497900	0.867769
chr6	127513100	0.661157

chr6	127528900	0.867769
chr6	127532300	0.694215
chr6	127622800	0.876033
chr6	127741900	0.966942
chr6	128293600	0.859504
chr6	128530400	0.966942
chr6	128597100	0.966942
chr6	128689400	0.859504
chr6	128805000	0.900826
chr6	128809500	0.752066
chr6	128811600	0.818182
chr6	128813700	0.925620
chr6	128829400	0.917355
chr6	128833200	0.975207
chr6	128837300	0.925620
chr6	128921800	0.983471
chr6	128977400	0.942149
chr6	129018000	0.867769
chr6	129125300	0.917355
chr6	129653000	0.925620
chr6	129662400	0.958678
chr6	129674600	0.950413
chr6	129814700	0.842975
chr6	129820500	0.867769
chr6	129822900	0.892562
chr6	129897100	0.950413
chr6	129899700	0.685950
chr6	129920100	0.785124
chr6	129945100	0.842975
chr6	129960000	1.000000
chr6	129966100	0.975207
chr6	129968700	0.950413
chr6	129972600	1.000000
chr6	129974800	0.694215
chr6	129989000	0.735537
chr6	129993000	0.900826
chr6	129995300	0.975207
chr6	129999600	0.966942
chr6	130003400	0.685950
chr6	130005800	0.900826
chr6	130009000	0.884298
chr6	130023100	0.950413
chr6	130035500	0.752066
chr6	130045600	0.727273
chr6	130070900	0.975207
chr6	130211400	0.809917
chr6	130322600	0.818182
chr6	130329200	0.809917
chr6	130331500	0.743802
chr6	130355800	0.685950
chr6	130451400	0.950413
chr6	130739500	0.768595
chr6	130922300	0.884298
chr6	130925100	0.685950

chr6	131002600	0.958678
chr6	131228800	0.702479
chr6	131267500	0.966942
chr6	131335200	0.950413
chr6	131337300	0.785124
chr6	131345800	0.669421
chr6	131372200	0.669421
chr6	131423500	0.801653
chr6	131428600	0.950413
chr6	131537800	0.661157
chr6	131603000	0.661157
chr6	132185400	0.925620
chr6	132273600	0.884298
chr6	132276200	0.983471
chr6	132281900	0.818182
chr6	132301600	1.000000
chr6	132317700	0.942149
chr6	132382000	0.983471
chr6	132385500	0.942149
chr6	132388500	0.768595
chr6	132393700	0.909091
chr6	132405200	0.842975
chr6	132408700	0.991736
chr6	132437700	0.710744
chr6	132451600	0.793388
chr6	132453800	0.991736
chr6	132482500	0.900826
chr6	132520200	0.793388
chr6	132678700	0.884298
chr6	132811800	0.991736
chr6	132826300	0.991736
chr6	133112700	0.785124
chr6	133285600	0.958678
chr6	133594200	0.801653
chr6	133890700	0.752066
chr6	133931500	0.752066
chr6	134095700	0.958678
chr6	134382100	0.785124
chr6	134482400	0.710744
chr6	134499800	0.859504
chr6	134557500	0.925620
chr6	134610000	0.983471
chr6	134617500	0.991736
chr6	134621100	0.826446
chr6	134694800	0.776860
chr6	134741400	0.925620
chr6	134744900	0.933884
chr6	134747300	0.826446
chr6	134757700	0.983471
chr6	134832300	0.933884
chr6	134834400	0.867769
chr6	134838600	0.867769
chr6	134840900	0.950413
chr6	134871200	0.925620

chr6	134873300	0.694215
chr6	135036000	0.727273
chr6	135041600	0.975207
chr6	135046500	0.776860
chr6	135061700	0.983471
chr6	135204700	0.677686
chr6	135230500	0.884298
chr6	135306200	1.000000
chr6	135388000	0.818182
chr6	135680200	0.958678
chr6	135740200	0.876033
chr6	135815100	0.867769
chr6	135889600	0.677686
chr6	135952700	0.933884
chr6	135989700	0.851240
chr6	136008400	0.867769
chr6	136044100	0.892562
chr6	136173400	0.950413
chr6	136243800	0.958678
chr6	136268300	0.677686
chr6	136316700	0.983471
chr6	136333800	0.752066
chr6	136357000	1.000000
chr6	136370400	0.768595
chr6	136372800	0.950413
chr6	136380300	0.735537
chr6	136387900	0.975207
chr6	136399300	0.966942
chr6	136417200	0.867769
chr6	136425100	0.851240
chr6	136430000	0.983471
chr6	136435300	0.826446
chr6	136450800	0.818182
chr6	136472200	0.669421
chr6	136495300	0.983471
chr6	136606100	0.892562
chr6	136915300	0.991736
chr6	136929000	0.727273
chr6	136931600	0.975207
chr6	136969000	0.917355
chr6	137025100	0.685950
chr6	137038600	0.933884
chr6	137059800	0.669421
chr6	137083400	0.950413
chr6	137103900	0.958678
chr6	137106700	0.909091
chr6	137109600	0.900826
chr6	137323000	0.867769
chr6	137534700	0.743802
chr6	137544600	0.975207
chr6	137555400	0.983471
chr6	137558900	0.727273
chr6	137630800	0.685950
chr6	138019600	0.991736

chr6	138097800	0.917355
chr6	138113200	0.719008
chr6	138126900	0.859504
chr6	138133000	0.785124
chr6	138175200	0.669421
chr6	138187400	0.702479
chr6	138194600	0.966942
chr6	138204900	0.933884
chr6	138222200	0.752066
chr6	138226500	0.942149
chr6	138552700	0.933884
chr6	138693300	0.925620
chr6	138732900	0.925620
chr6	138777000	0.909091
chr6	138786900	0.834711
chr6	138808000	0.719008
chr6	138863900	0.859504
chr6	138868700	0.950413
chr6	138876900	1.000000
chr6	138893600	0.834711
chr6	138913600	0.768595
chr6	138916700	0.942149
chr6	138918800	0.966942
chr6	138922600	0.884298
chr6	138968500	0.809917
chr6	138976300	0.958678
chr6	139040500	0.752066
chr6	139044700	0.958678
chr6	139089800	1.000000
chr6	139135800	0.669421
chr6	139139900	0.785124
chr6	139279800	0.851240
chr6	139285200	0.942149
chr6	139291800	0.842975
chr6	139295000	0.933884
chr6	139468900	0.785124
chr6	139612300	0.958678
chr6	139645100	0.958678
chr6	139689800	0.776860
chr6	139692000	0.842975
chr6	139855400	0.909091
chr6	139860900	0.942149
chr6	139890400	0.975207
chr6	139909500	0.884298
chr6	140029900	0.917355
chr6	140065400	0.801653
chr6	140084200	0.809917
chr6	140087400	0.752066
chr6	140141400	0.892562
chr6	140203900	0.834711
chr6	140262500	0.793388
chr6	140265100	0.900826
chr6	140295400	0.884298
chr6	140303600	0.876033

chr6	140392800	0.867769
chr6	140399900	0.760331
chr6	140459700	0.859504
chr6	140524700	0.867769
chr6	140529600	0.694215
chr6	140547400	0.842975
chr6	140665800	0.917355
chr6	140668800	0.950413
chr6	140715300	0.900826
chr6	140766800	0.694215
chr6	140888400	0.900826
chr6	140893600	1.000000
chr6	141095200	0.710744
chr6	141168400	0.983471
chr6	141172100	0.958678
chr6	141179200	0.900826
chr6	141301200	0.900826
chr6	141429100	0.826446
chr6	141648300	0.677686
chr6	141773100	0.884298
chr6	141805100	0.842975
chr6	141874100	0.983471
chr6	141883500	0.900826
chr6	142333900	0.818182
chr6	142336100	1.000000
chr6	142477100	0.966942
chr6	142488600	0.983471
chr6	142517400	0.942149
chr6	142521600	0.983471
chr6	142523800	0.677686
chr6	142619900	0.925620
chr6	142626500	0.834711
chr6	142628600	0.876033
chr6	142631700	0.818182
chr6	142634700	0.752066
chr6	142650500	0.801653
chr6	142652800	0.975207
chr6	142655500	0.809917
chr6	142665000	0.867769
chr6	142683300	0.727273
chr6	142693600	0.892562
chr6	142697000	0.892562
chr6	142700100	0.917355
chr6	142717800	0.966942
chr6	142721900	0.917355
chr6	142727000	0.694215
chr6	142729100	0.983471
chr6	142731600	0.958678
chr6	142733800	0.975207
chr6	142737900	0.958678
chr6	143014900	0.925620
chr6	143024000	0.685950
chr6	143050300	1.000000
chr6	143084700	0.917355

chr6	143103200	0.809917
chr6	143141300	0.818182
chr6	143148700	0.983471
chr6	143154800	0.859504
chr6	143164100	0.983471
chr6	143166700	0.983471
chr6	143169100	0.958678
chr6	143189200	0.991736
chr6	143193500	0.991736
chr6	143195900	0.669421
chr6	143206800	0.975207
chr6	143212100	0.793388
chr6	143224100	0.809917
chr6	143262300	0.925620
chr6	143281500	0.859504
chr6	143511400	0.669421
chr6	143516900	0.909091
chr6	143546000	0.892562
chr6	143647000	0.925620
chr6	143650700	0.661157
chr6	143896900	0.735537
chr6	143901000	0.702479
chr6	143995000	1.000000
chr6	144008600	0.983471
chr6	144018000	1.000000
chr6	144022400	0.966942
chr6	144056000	0.851240
chr6	144076900	0.966942
chr6	144081000	0.876033
chr6	144124200	0.909091
chr6	144127300	0.768595
chr6	144137400	0.669421
chr6	144142000	0.702479
chr6	144145100	0.950413
chr6	144147700	0.685950
chr6	144452500	0.900826
chr6	144478800	0.917355
chr6	144481000	0.876033
chr6	144586700	0.983471
chr6	144615300	0.958678
chr6	144635500	0.942149
chr6	144638200	0.933884
chr6	144649000	0.950413
chr6	144667800	0.727273
chr6	144670400	0.983471
chr6	144673600	0.876033
chr6	144699300	1.000000
chr6	144737300	0.909091
chr6	144756500	0.900826
chr6	144769100	0.661157
chr6	144881600	0.933884
chr6	145031800	0.694215
chr6	145119400	0.818182
chr6	145219000	0.975207

chr6	145310700	0.735537
chr6	145315400	0.685950
chr6	145337300	0.859504
chr6	145991100	0.991736
chr6	146010300	0.809917
chr6	146020900	0.991736
chr6	146026700	0.942149
chr6	146213500	0.942149
chr6	146374100	0.958678
chr6	146611800	0.983471
chr6	146658500	0.851240
chr6	146867900	0.710744
chr6	147214000	0.685950
chr6	147235300	0.975207
chr6	147238000	0.743802
chr6	147483800	0.743802
chr6	147487700	0.983471
chr6	147516600	0.826446
chr6	147527500	0.685950
chr6	147609600	0.826446
chr6	148010800	0.760331
chr6	148127100	0.793388
chr6	148174500	0.743802
chr6	148189800	0.834711
chr6	148191900	0.958678
chr6	148195800	0.925620
chr6	148342700	0.909091
chr6	148386100	0.925620
chr6	148390000	0.900826
chr6	148411600	0.950413
chr6	148460600	0.694215
chr6	148466900	0.793388
chr6	148469000	0.942149
chr6	148589000	0.892562
chr6	148591200	0.702479
chr6	148593700	0.851240
chr6	148613600	0.661157
chr6	148626000	0.909091
chr6	148628100	0.900826
chr6	148639000	0.983471
chr6	148646000	0.842975
chr6	148651200	0.983471
chr6	148662300	0.801653
chr6	148667500	0.867769
chr6	148670600	0.661157
chr6	148684800	0.859504
chr6	148687400	0.958678
chr6	148689700	0.991736
chr6	148692800	0.685950
chr6	148696200	0.793388
chr6	148704400	0.867769
chr6	148717600	0.991736
chr6	148720700	0.917355
chr6	148728800	0.983471

chr6	148736300	0.876033
chr6	148740800	0.975207
chr6	148744200	0.900826
chr6	148746300	0.702479
chr6	148748400	0.917355
chr6	148760500	0.702479
chr6	148764900	0.950413
chr6	148767500	1.000000
chr6	148769600	0.958678
chr6	148772800	0.942149
chr6	148775000	0.900826
chr6	148778900	0.975207
chr6	148783500	0.776860
chr6	148786400	0.826446
chr6	148846300	0.975207
chr6	148852100	0.983471
chr6	148881600	0.776860
chr6	148887800	0.892562
chr6	149207200	0.760331
chr6	149370800	0.694215
chr6	149382000	0.768595
chr6	149555100	0.991736
chr6	149655700	0.933884
chr6	149658300	0.661157
chr6	149803700	0.917355
chr6	150169000	0.983471
chr6	150173100	0.661157
chr6	150175500	0.809917
chr6	150179200	0.826446
chr6	150182900	0.942149
chr6	150191300	0.842975
chr6	150218000	0.727273
chr6	150280200	0.702479
chr6	150312000	0.834711
chr6	150336200	0.867769
chr6	150347000	0.776860
chr6	150725300	0.702479
chr6	150740000	0.917355
chr6	150774300	0.768595
chr6	150814500	0.983471
chr6	150938700	0.991736
chr6	150949200	0.876033
chr6	150990000	0.702479
chr6	151003900	0.809917
chr6	151011100	0.685950
chr6	151088900	0.735537
chr6	151177700	0.884298
chr6	151180000	0.958678
chr6	151185200	0.752066
chr6	151202500	0.876033
chr6	151204700	0.760331
chr6	151213700	0.809917
chr6	151216600	0.793388
chr6	151220100	0.867769

chr6	151248100	0.826446
chr6	151280700	0.950413
chr6	151302600	0.966942
chr6	151324600	0.900826
chr6	151327700	0.826446
chr6	151333700	0.975207
chr6	151335900	0.942149
chr6	151340100	0.909091
chr6	151343600	0.950413
chr6	151356700	0.925620
chr6	151360100	0.958678
chr6	151363400	0.925620
chr6	151366200	0.925620
chr6	151368500	0.867769
chr6	151380000	0.760331
chr6	151382500	0.900826
chr6	151384700	0.884298
chr6	151389200	0.884298
chr6	151392600	1.000000
chr6	151414000	1.000000
chr6	151462900	0.867769
chr6	151576400	0.900826
chr6	151606600	0.983471
chr6	151620500	0.661157
chr6	151645200	0.818182
chr6	151679000	0.933884
chr6	151696100	0.942149
chr6	151717900	0.669421
chr6	151767100	0.876033
chr6	151803800	0.793388
chr6	151865900	0.983471
chr6	152086100	0.983471
chr6	152129600	0.826446
chr6	152213000	0.991736
chr6	152295700	0.842975
chr6	152416500	0.983471
chr6	152418900	0.884298
chr6	152422000	0.983471
chr6	152451300	0.826446
chr6	152635400	0.900826
chr6	152638500	0.785124
chr6	152701600	0.669421
chr6	152783300	0.917355
chr6	152920400	0.743802
chr6	152992600	0.950413
chr6	152994900	0.942149
chr6	153016700	0.719008
chr6	153041200	0.942149
chr6	153044500	0.983471
chr6	153227000	0.785124
chr6	153365800	0.801653
chr6	153406900	0.727273
chr6	153411500	0.892562
chr6	153414500	0.892562

chr6	153489000	0.950413
chr6	154614400	0.950413
chr6	154619700	0.842975
chr6	154709000	0.983471
chr6	154720800	0.900826
chr6	154731000	0.950413
chr6	154733100	0.776860
chr6	154754600	0.884298
chr6	154769600	0.933884
chr6	154804400	0.925620
chr6	154821700	0.884298
chr6	154837500	0.826446
chr6	154891100	0.909091
chr6	154935500	0.950413
chr6	154959900	0.859504
chr6	154975300	0.991736
chr6	154983700	0.942149
chr6	155005300	0.884298
chr6	155059900	0.983471
chr6	155154100	0.892562
chr6	155164400	0.958678
chr6	155254000	0.768595
chr6	155335200	1.000000
chr6	155347700	0.727273
chr6	155376400	0.876033
chr6	155401800	0.983471
chr6	155424700	0.727273
chr6	155448600	0.950413
chr6	155462900	0.735537
chr6	155465000	0.826446
chr6	155475600	0.892562
chr6	155492700	0.950413
chr6	155495900	0.950413
chr6	155507700	0.809917
chr6	155510100	0.834711
chr6	155512800	0.892562
chr6	155522500	0.991736
chr6	155545500	1.000000
chr6	155586600	0.710744
chr6	155594000	0.933884
chr6	155596200	0.925620
chr6	155599000	0.677686
chr6	155631700	0.958678
chr6	155739600	0.809917
chr6	155750700	0.785124
chr6	155770600	0.694215
chr6	155815400	0.975207
chr6	155845500	0.892562
chr6	155850500	0.983471
chr6	155969100	0.710744
chr6	156028000	0.776860
chr6	156087100	0.909091
chr6	156103800	0.975207
chr6	156171900	0.685950

chr6	156203000	0.950413
chr6	156205500	0.909091
chr6	156488900	0.950413
chr6	156669200	0.900826
chr6	156969400	0.710744
chr6	157020800	0.727273
chr6	157041400	0.785124
chr6	157161400	0.727273
chr6	157169300	0.677686
chr6	157188400	0.818182
chr6	157271000	0.760331
chr6	157354700	0.834711
chr6	157365500	0.743802
chr6	157381500	0.694215
chr6	157408900	0.917355
chr6	157411000	0.983471
chr6	157414100	0.966942
chr6	157421700	0.768595
chr6	157450700	0.983471
chr6	157465700	1.000000
chr6	157472600	0.983471
chr6	157542200	0.694215
chr6	157610400	0.925620
chr6	157725700	0.694215
chr6	157731800	0.760331
chr6	157738400	0.983471
chr6	157746500	0.818182
chr6	157877400	0.859504
chr6	157882400	0.942149
chr6	157918400	0.834711
chr6	157922700	0.991736
chr6	157934200	0.735537
chr6	157937200	0.909091
chr6	157951900	0.917355
chr6	157957300	0.851240
chr6	158001200	0.925620
chr6	158004400	0.677686
chr6	158034400	0.884298
chr6	158059100	0.925620
chr6	158066700	0.719008
chr6	158081000	0.735537
chr6	158178800	0.975207
chr6	158182700	0.801653
chr6	158185900	0.991736
chr6	158214000	0.776860
chr6	158235000	0.834711
chr6	158243200	0.710744
chr6	158247200	0.933884
chr6	158252700	0.834711
chr6	158255200	0.710744
chr6	158262000	0.867769
chr6	158267400	0.793388
chr6	158288700	0.942149
chr6	158291100	0.851240

chr6	158384200	0.933884
chr6	158411000	0.859504
chr6	158425500	0.809917
chr6	158429200	0.826446
chr6	158433100	0.669421
chr6	158438800	0.991736
chr6	158442700	0.677686
chr6	158447200	0.809917
chr6	158449400	0.892562
chr6	158452900	0.809917
chr6	158460500	0.834711
chr6	158464600	0.867769
chr6	158478800	0.801653
chr6	158483400	0.727273
chr6	158488500	0.834711
chr6	158491000	0.859504
chr6	158493300	0.776860
chr6	158504900	0.826446
chr6	158629200	0.942149
chr6	158633100	0.818182
chr6	158707500	1.000000
chr6	158820300	0.801653
chr6	158826900	0.966942
chr6	158829000	0.925620
chr6	158831100	1.000000
chr6	158839000	0.958678
chr6	158881800	0.950413
chr6	158896700	0.950413
chr6	158903300	0.867769
chr6	158909500	0.950413
chr6	158939600	0.933884
chr6	158976500	0.975207
chr6	158978600	0.702479
chr6	158996800	0.859504
chr6	159053900	0.735537
chr6	159063300	0.909091
chr6	159128900	0.867769
chr6	159142000	0.876033
chr6	159206100	0.950413
chr6	159220700	0.933884
chr6	159225000	0.942149
chr6	159237200	0.661157
chr6	159271400	0.801653
chr6	159275400	0.991736
chr6	160102600	0.983471
chr6	160112500	0.661157
chr6	160116000	0.752066
chr6	160400400	0.685950
chr6	160403100	0.975207
chr6	161130400	0.801653
chr6	161417500	0.884298
chr6	161453200	0.933884
chr6	161590500	0.966942
chr6	161650400	0.983471

chr6	161679300	0.958678
chr6	162344000	0.859504
chr6	162603200	0.917355
chr6	162606100	0.900826
chr6	163665600	0.702479
chr6	163681700	0.909091
chr6	163705900	0.876033
chr6	163712900	0.983471
chr6	163731100	0.966942
chr6	163757200	0.975207
chr6	163775000	0.950413
chr6	163777800	0.768595
chr6	163789300	0.983471
chr6	163808500	0.801653
chr6	163815300	0.818182
chr6	163818300	0.826446
chr6	163878800	0.859504
chr6	164007400	0.793388
chr6	164356400	0.694215
chr6	164421200	0.859504
chr6	164426500	0.685950
chr6	164506500	0.975207
chr6	164519400	0.942149
chr6	164530900	0.702479
chr6	164744000	0.892562
chr6	164793400	0.867769
chr6	164818000	0.826446
chr6	164836900	0.975207
chr6	165117000	0.760331
chr6	165988100	0.752066
chr6	166067000	0.785124
chr6	166090500	0.975207
chr6	166132100	0.785124
chr6	166180300	0.983471
chr6	166183300	0.975207
chr6	166189000	0.958678
chr6	166667400	0.685950
chr6	166721700	0.743802
chr6	166779100	0.917355
chr6	166880400	0.793388
chr6	166901800	0.983471
chr6	166905300	0.851240
chr6	166970500	0.867769
chr6	166981200	0.661157
chr6	167003100	0.900826
chr6	167010500	0.801653
chr6	167014700	0.892562
chr6	167030400	0.793388
chr6	167038000	0.727273
chr6	167043100	0.669421
chr6	167053900	0.983471
chr6	167058000	0.719008
chr6	167097200	0.942149
chr6	167105500	0.975207

chr6	167150500	0.983471
chr6	167197700	0.834711
chr6	167373500	0.983471
chr6	167637700	0.958678
chr6	167640200	0.991736
chr6	167764900	0.710744
chr6	168048300	0.975207
chr6	168077200	0.966942
chr6	168079500	0.834711
chr6	168083900	0.933884
chr6	168100700	0.834711
chr6	168197500	0.685950
chr6	168232000	0.669421
chr6	168250900	0.958678
chr6	168257500	0.975207
chr6	168335000	0.842975
chr6	168394300	0.958678
chr6	168408100	0.768595
chr6	168433300	0.842975
chr6	168436600	0.809917
chr6	168727400	0.958678
chr6	168929200	0.966942
chr6	169971900	0.768595
chr6	170069200	0.909091
chr6	170071400	0.743802
chr6	170098200	0.966942
chr6	170100300	0.966942
chr6	170190600	0.776860
chr6	170232800	0.776860
chr6	170252200	0.975207
chr6	170467800	0.876033
chr6	170494800	0.983471
chr6	170626900	0.851240
chr6	170654500	0.925620
chr6	170669900	0.892562
chr6	170687700	0.677686
chr6	170714800	0.975207
chr6	170747600	0.867769
chr6	170832800	0.719008
chr7	24200	0.727273
chr7	89700	0.851240
chr7	101700	0.719008
chr7	191900	0.719008
chr7	373300	0.719008
chr7	391600	0.809917
chr7	394100	0.818182
chr7	402600	0.991736
chr7	406600	0.876033
chr7	421700	0.793388
chr7	475700	0.809917
chr7	495800	0.884298
chr7	501500	0.760331
chr7	504800	0.801653
chr7	508900	0.867769

chr7	511800	0.809917
chr7	553800	0.958678
chr7	556300	0.752066
chr7	578200	0.966942
chr7	583000	0.876033
chr7	641600	0.950413
chr7	645400	0.735537
chr7	648300	0.859504
chr7	651300	0.727273
chr7	659700	0.710744
chr7	737100	0.851240
chr7	768200	0.818182
chr7	811000	0.876033
chr7	817300	0.793388
chr7	819500	0.801653
chr7	845300	0.702479
chr7	853100	0.983471
chr7	871900	0.917355
chr7	906900	0.776860
chr7	909000	0.677686
chr7	913300	0.809917
chr7	918500	0.661157
chr7	962600	0.710744
chr7	1069000	0.793388
chr7	1083400	0.809917
chr7	1092900	0.793388
chr7	1095800	0.950413
chr7	1100400	0.851240
chr7	1118000	0.752066
chr7	1123700	0.776860
chr7	1126800	0.818182
chr7	1140500	0.892562
chr7	1163800	0.991736
chr7	1197000	0.942149
chr7	1209600	0.776860
chr7	1216300	0.834711
chr7	1221700	0.909091
chr7	1232400	0.917355
chr7	1495900	0.942149
chr7	1500900	0.669421
chr7	1553500	0.851240
chr7	1560500	0.925620
chr7	1570800	0.661157
chr7	1574800	0.975207
chr7	1579000	0.925620
chr7	1595500	0.727273
chr7	1782400	0.776860
chr7	2059800	0.851240
chr7	2143900	0.826446
chr7	2147100	0.917355
chr7	2150200	0.933884
chr7	2274400	0.859504
chr7	2289200	0.743802
chr7	2339600	1.000000

chr7	2350300	0.900826
chr7	2357400	0.694215
chr7	2439800	0.966942
chr7	2449700	0.983471
chr7	2452700	0.900826
chr7	2491700	0.809917
chr7	2511400	0.884298
chr7	2551500	0.942149
chr7	2563800	0.826446
chr7	2570200	0.776860
chr7	2610000	0.669421
chr7	2647500	0.909091
chr7	2664600	0.760331
chr7	2667100	0.785124
chr7	2670600	0.694215
chr7	2675700	0.867769
chr7	2680200	0.876033
chr7	2682300	0.933884
chr7	2685600	0.818182
chr7	2703200	0.818182
chr7	2730100	0.702479
chr7	2757500	0.818182
chr7	2773500	0.909091
chr7	2775700	0.884298
chr7	2797800	0.950413
chr7	2803000	0.958678
chr7	2825000	0.958678
chr7	2836400	0.958678
chr7	2842500	0.719008
chr7	2860400	0.859504
chr7	2862500	0.933884
chr7	2877500	0.991736
chr7	2903600	0.933884
chr7	2910100	0.842975
chr7	2913600	0.851240
chr7	2996800	0.818182
chr7	3039300	0.950413
chr7	3045400	0.752066
chr7	3068100	0.950413
chr7	3083000	0.694215
chr7	3234700	0.925620
chr7	3476800	0.925620
chr7	3533400	0.859504
chr7	3598900	0.809917
chr7	3645900	0.776860
chr7	3956200	0.991736
chr7	3962300	0.719008
chr7	4019900	0.776860
chr7	4054500	0.867769
chr7	4653100	0.760331
chr7	4671600	0.760331
chr7	4725100	0.743802
chr7	4740700	0.859504
chr7	4745100	0.826446

chr7	4748500	0.851240
chr7	4778800	0.867769
chr7	4783700	0.842975
chr7	4786100	0.950413
chr7	4788500	0.876033
chr7	4832300	0.818182
chr7	5231700	0.793388
chr7	5258800	0.867769
chr7	5497800	0.727273
chr7	5550200	0.966942
chr7	5595800	0.776860
chr7	5603800	0.685950
chr7	5609500	0.694215
chr7	5614800	0.966942
chr7	5621000	0.851240
chr7	5638100	0.859504
chr7	5670100	0.760331
chr7	5674000	0.900826
chr7	5676800	0.760331
chr7	5683100	0.958678
chr7	5702200	0.867769
chr7	5707600	0.685950
chr7	5717400	0.983471
chr7	5731700	0.909091
chr7	5734000	0.942149
chr7	5806700	0.669421
chr7	5813800	0.900826
chr7	5860300	0.900826
chr7	5972100	0.958678
chr7	6292200	0.859504
chr7	6422200	0.966942
chr7	6425900	0.851240
chr7	6433300	0.917355
chr7	6478800	0.933884
chr7	6483800	0.909091
chr7	6570700	0.760331
chr7	6832400	0.909091
chr7	6895300	0.743802
chr7	7139700	0.776860
chr7	7216800	0.942149
chr7	7224700	0.710744
chr7	7479000	0.867769
chr7	7483800	0.950413
chr7	7704200	0.768595
chr7	7727900	0.661157
chr7	7771600	0.859504
chr7	7802000	0.677686
chr7	7835900	0.735537
chr7	7898800	0.818182
chr7	7901600	0.752066
chr7	10197800	0.933884
chr7	10280100	0.867769
chr7	11233300	0.694215
chr7	11490400	0.743802

chr7	11498900	0.983471
chr7	11506400	0.809917
chr7	11580000	0.991736
chr7	11632000	0.752066
chr7	11665700	0.958678
chr7	11689000	0.966942
chr7	11747200	0.884298
chr7	11753800	0.735537
chr7	11756900	0.958678
chr7	11898600	0.735537
chr7	12202500	0.793388
chr7	12463400	0.942149
chr7	12660300	0.991736
chr7	12732600	0.710744
chr7	12755600	1.000000
chr7	12759800	0.942149
chr7	12768000	0.966942
chr7	12771500	0.958678
chr7	12786100	0.685950
chr7	12810900	0.958678
chr7	12900800	0.876033
chr7	13068300	0.933884
chr7	13097400	0.933884
chr7	13117300	0.752066
chr7	13134000	0.719008
chr7	13840800	0.818182
chr7	13890200	0.909091
chr7	13960000	0.925620
chr7	14020100	0.975207
chr7	14788200	1.000000
chr7	14791500	0.702479
chr7	14794700	0.867769
chr7	15011200	0.685950
chr7	15018700	0.826446
chr7	15082300	0.983471
chr7	15171400	0.983471
chr7	15544200	0.933884
chr7	15548200	0.991736
chr7	15594400	0.950413
chr7	15599600	0.752066
chr7	15601700	0.760331
chr7	15688400	0.859504
chr7	15719000	0.669421
chr7	15748000	0.917355
chr7	15750500	0.983471
chr7	15841800	0.719008
chr7	15879300	0.942149
chr7	15894200	0.991736
chr7	15961300	0.966942
chr7	16003300	0.661157
chr7	16013400	0.694215
chr7	16017800	0.900826
chr7	16067900	0.867769
chr7	16102500	0.933884

chr7	16107000	0.661157
chr7	16118300	0.685950
chr7	16123600	0.867769
chr7	16126600	0.933884
chr7	16131800	0.851240
chr7	16137800	0.966942
chr7	16169700	0.867769
chr7	16242500	1.000000
chr7	16359000	0.867769
chr7	16380400	0.768595
chr7	16688900	0.900826
chr7	16691800	0.710744
chr7	16700900	0.884298
chr7	16780300	0.950413
chr7	16783400	0.950413
chr7	16805500	0.983471
chr7	16891600	0.735537
chr7	16975400	0.809917
chr7	16980700	0.983471
chr7	17018600	0.991736
chr7	17030000	0.933884
chr7	17060100	0.752066
chr7	17080200	0.909091
chr7	17083200	0.966942
chr7	17098500	0.900826
chr7	17156200	0.785124
chr7	17184400	0.719008
chr7	17225500	0.661157
chr7	17247100	0.859504
chr7	17274400	0.975207
chr7	17276600	0.743802
chr7	17314700	0.694215
chr7	17345100	1.000000
chr7	17353000	0.983471
chr7	17411600	0.983471
chr7	17446400	0.942149
chr7	17449000	0.752066
chr7	17484500	0.966942
chr7	17503800	0.760331
chr7	17561300	0.975207
chr7	17821900	0.842975
chr7	18005600	0.685950
chr7	18059800	1.000000
chr7	18429700	0.975207
chr7	18448300	0.867769
chr7	18466600	0.991736
chr7	18543200	0.735537
chr7	18548500	0.942149
chr7	18560600	0.661157
chr7	18581200	0.826446
chr7	18707200	0.809917
chr7	18889000	0.677686
chr7	18906800	0.925620
chr7	18935700	0.966942

chr7	19017900	0.892562
chr7	19147200	0.768595
chr7	19746700	0.900826
chr7	19812800	0.677686
chr7	19956100	0.925620
chr7	19959200	0.950413
chr7	19998300	0.884298
chr7	20259600	0.933884
chr7	20270000	0.958678
chr7	20346600	0.966942
chr7	20391800	0.966942
chr7	20402700	0.727273
chr7	20423600	0.917355
chr7	20447900	0.851240
chr7	20481700	0.958678
chr7	20547100	0.991736
chr7	20554300	0.933884
chr7	20557200	0.719008
chr7	20559800	0.710744
chr7	20572900	0.760331
chr7	20601400	0.991736
chr7	20638600	0.933884
chr7	20642500	0.669421
chr7	20818100	0.818182
chr7	21354400	0.950413
chr7	21358800	1.000000
chr7	21378500	0.842975
chr7	21406200	0.983471
chr7	21657100	0.991736
chr7	21891300	0.933884
chr7	21943000	0.900826
chr7	21963300	0.818182
chr7	21979400	0.834711
chr7	22000900	0.950413
chr7	22036700	1.000000
chr7	22041000	0.801653
chr7	22154700	0.677686
chr7	22161900	0.884298
chr7	22164600	0.933884
chr7	22166700	0.876033
chr7	22168800	0.942149
chr7	22196200	0.752066
chr7	22254200	0.900826
chr7	22257000	0.776860
chr7	22260800	0.702479
chr7	22335300	0.661157
chr7	22430800	0.966942
chr7	22458900	0.933884
chr7	22472200	0.661157
chr7	22474400	0.876033
chr7	22482000	0.669421
chr7	22485600	0.950413
chr7	22520800	0.677686
chr7	22523700	0.958678

chr7	22525800	0.851240
chr7	22528600	0.801653
chr7	22537400	0.975207
chr7	22601100	0.942149
chr7	22606000	0.851240
chr7	22609800	0.950413
chr7	22615600	0.818182
chr7	22618800	0.958678
chr7	22623000	0.876033
chr7	22627500	0.983471
chr7	22630400	0.950413
chr7	22637900	0.950413
chr7	22641400	0.867769
chr7	22650400	0.793388
chr7	22671800	0.876033
chr7	22687400	0.966942
chr7	22691400	0.727273
chr7	22694100	0.900826
chr7	22703700	0.933884
chr7	22707400	0.958678
chr7	22711700	0.958678
chr7	22715600	0.983471
chr7	22740500	0.752066
chr7	22758000	0.983471
chr7	22762900	0.859504
chr7	22769500	0.793388
chr7	22896400	0.876033
chr7	22898800	0.884298
chr7	22906700	0.710744
chr7	23020400	0.950413
chr7	23029700	0.818182
chr7	23049600	0.661157
chr7	23058000	0.950413
chr7	23061200	0.975207
chr7	23117000	0.809917
chr7	23208300	0.735537
chr7	23246200	0.661157
chr7	23287700	0.776860
chr7	23313300	0.826446
chr7	23375300	0.975207
chr7	23384300	0.842975
chr7	23389500	0.933884
chr7	23396500	1.000000
chr7	23398700	0.991736
chr7	23413200	0.991736
chr7	23417000	0.975207
chr7	23420600	0.818182
chr7	23458200	0.710744
chr7	23470800	0.975207
chr7	23503300	0.942149
chr7	23800000	0.876033
chr7	24693200	0.661157
chr7	24709600	0.942149
chr7	24769400	0.809917

chr7	24827100	0.900826
chr7	24861400	0.859504
chr7	24865900	0.859504
chr7	24871200	0.975207
chr7	24883200	0.851240
chr7	24923200	0.834711
chr7	24925700	0.991736
chr7	24947600	0.958678
chr7	24961600	0.859504
chr7	25011300	0.942149
chr7	25065200	0.933884
chr7	25068700	0.983471
chr7	25872100	0.685950
chr7	25889700	0.925620
chr7	25892400	0.743802
chr7	25902300	0.677686
chr7	25933000	0.859504
chr7	25935200	0.661157
chr7	26141200	0.834711
chr7	26195000	0.801653
chr7	26199100	0.851240
chr7	26235600	0.876033
chr7	26410000	0.958678
chr7	26413600	0.867769
chr7	26457700	0.983471
chr7	26489800	0.991736
chr7	26494100	0.925620
chr7	26498700	0.826446
chr7	26704800	0.702479
chr7	26715300	0.719008
chr7	26722400	0.768595
chr7	26726000	0.991736
chr7	26728500	0.933884
chr7	26745500	0.950413
chr7	26950100	0.876033
chr7	26999100	0.933884
chr7	27086900	0.677686
chr7	27128300	0.685950
chr7	27145100	0.876033
chr7	27148900	0.851240
chr7	27159600	0.909091
chr7	27164100	0.884298
chr7	27166300	0.685950
chr7	27174700	0.909091
chr7	27177600	0.925620
chr7	27180000	0.793388
chr7	27198100	0.933884
chr7	27221200	0.677686
chr7	27245300	0.826446
chr7	27250400	0.661157
chr7	27275500	0.685950
chr7	27285700	0.801653
chr7	27291500	0.768595
chr7	27645800	0.958678

chr7	27681300	0.983471
chr7	27718300	0.917355
chr7	27726400	0.694215
chr7	27754900	0.966942
chr7	27785300	0.975207
chr7	27875500	0.983471
chr7	27912800	0.950413
chr7	27923700	0.983471
chr7	27940900	1.000000
chr7	27957800	0.991736
chr7	28077300	0.983471
chr7	28094300	0.735537
chr7	28147800	0.710744
chr7	28168800	0.867769
chr7	28192000	0.950413
chr7	28219000	0.685950
chr7	28247500	0.776860
chr7	28429800	0.752066
chr7	28570400	0.966942
chr7	28613400	0.661157
chr7	28645300	0.991736
chr7	28672200	0.876033
chr7	28728300	0.809917
chr7	28738800	0.768595
chr7	28818500	0.735537
chr7	28973800	0.991736
chr7	29075200	0.991736
chr7	29077300	0.851240
chr7	29079400	0.851240
chr7	29131300	0.719008
chr7	29153500	0.776860
chr7	29156600	0.677686
chr7	29218200	0.975207
chr7	29247500	0.842975
chr7	29295900	0.834711
chr7	29305100	0.900826
chr7	29345400	0.925620
chr7	29505300	0.727273
chr7	29529000	1.000000
chr7	29540700	0.818182
chr7	29662500	0.768595
chr7	29763300	0.933884
chr7	29788100	0.661157
chr7	30027000	0.958678
chr7	30182600	0.727273
chr7	30202900	0.975207
chr7	30205000	0.735537
chr7	30330200	0.826446
chr7	30388200	0.710744
chr7	30499700	0.900826
chr7	30508500	0.900826
chr7	30513000	0.785124
chr7	30589500	0.859504
chr7	30591600	0.950413

chr7	30599500	0.735537
chr7	30603500	0.851240
chr7	30653300	0.876033
chr7	30836400	0.719008
chr7	30839800	0.685950
chr7	30844600	0.727273
chr7	30880800	0.809917
chr7	30888700	0.793388
chr7	30892300	0.702479
chr7	30910000	0.900826
chr7	30933600	0.884298
chr7	30938200	0.661157
chr7	30954800	0.809917
chr7	31025600	0.793388
chr7	31122300	0.859504
chr7	31816300	0.859504
chr7	31855700	0.702479
chr7	31910200	0.685950
chr7	31915100	0.925620
chr7	31930600	0.776860
chr7	31975300	0.685950
chr7	32026900	0.983471
chr7	32031700	1.000000
chr7	32195000	0.702479
chr7	32198500	0.942149
chr7	32202900	0.958678
chr7	32338800	0.702479
chr7	32541300	0.966942
chr7	32576900	0.983471
chr7	32625400	0.727273
chr7	32628800	0.809917
chr7	32697900	0.966942
chr7	32709600	0.900826
chr7	32815500	0.710744
chr7	32825900	0.743802
chr7	32869600	0.876033
chr7	32933600	0.867769
chr7	32945400	0.892562
chr7	33038500	0.909091
chr7	33074800	0.685950
chr7	33094200	0.900826
chr7	33631000	0.933884
chr7	33644200	0.694215
chr7	33709100	0.983471
chr7	33732900	0.892562
chr7	33762300	0.818182
chr7	33764600	0.776860
chr7	33768800	0.727273
chr7	33804900	0.793388
chr7	33829700	0.793388
chr7	33847900	0.925620
chr7	33854600	0.876033
chr7	34027200	0.826446
chr7	34076000	0.950413

chr7	34110100	0.892562
chr7	34317200	0.950413
chr7	34338400	0.776860
chr7	34492200	0.892562
chr7	34788500	0.958678
chr7	34877500	0.859504
chr7	34975800	0.842975
chr7	35031800	0.925620
chr7	35044300	0.925620
chr7	35093900	0.776860
chr7	35636000	0.776860
chr7	35737100	0.950413
chr7	35740900	0.685950
chr7	35832700	0.892562
chr7	36077200	0.983471
chr7	36085100	0.785124
chr7	36088500	0.760331
chr7	36156600	0.669421
chr7	36332000	0.818182
chr7	36343500	0.859504
chr7	36432700	0.867769
chr7	36434800	0.785124
chr7	36514600	0.876033
chr7	37005700	0.702479
chr7	37071800	0.958678
chr7	37077300	0.719008
chr7	37091000	0.702479
chr7	37337800	0.719008
chr7	37394700	0.958678
chr7	37445400	0.727273
chr7	37448600	0.933884
chr7	37470600	0.966942
chr7	37481100	0.983471
chr7	37517100	0.694215
chr7	37520600	0.950413
chr7	37956400	0.694215
chr7	38220900	0.793388
chr7	38236600	0.991736
chr7	38256000	0.900826
chr7	38351000	0.752066
chr7	38389300	0.892562
chr7	38398300	0.867769
chr7	38423000	0.958678
chr7	38426000	0.983471
chr7	38598300	0.702479
chr7	38602100	0.983471
chr7	38719400	0.727273
chr7	39620900	0.776860
chr7	39628000	0.743802
chr7	39630100	0.950413
chr7	39632700	0.942149
chr7	39635200	0.859504
chr7	39643900	0.983471
chr7	39647500	0.950413

chr7	39651600	0.958678
chr7	39655700	0.710744
chr7	39662200	0.727273
chr7	39668100	0.892562
chr7	39673900	0.925620
chr7	39682300	0.702479
chr7	39711300	0.669421
chr7	39718900	0.793388
chr7	39723300	1.000000
chr7	39728600	0.909091
chr7	39774600	0.842975
chr7	40260700	0.917355
chr7	40336900	0.933884
chr7	40339600	0.950413
chr7	40344100	0.966942
chr7	40346300	0.958678
chr7	40350400	0.983471
chr7	40477400	0.942149
chr7	40516200	0.727273
chr7	40591800	0.966942
chr7	40621600	0.809917
chr7	40773700	0.677686
chr7	40880000	0.710744
chr7	41051100	0.818182
chr7	41209400	0.851240
chr7	41226600	0.851240
chr7	41230000	0.991736
chr7	41264000	0.892562
chr7	41278600	0.694215
chr7	41548500	0.983471
chr7	41607300	0.900826
chr7	41613100	0.793388
chr7	41616400	0.958678
chr7	41619200	0.826446
chr7	41621300	0.834711
chr7	41725600	0.842975
chr7	41731900	0.776860
chr7	41735400	0.859504
chr7	41737500	0.719008
chr7	41745100	0.768595
chr7	41956300	0.966942
chr7	42031200	0.909091
chr7	42033400	0.942149
chr7	42036600	0.818182
chr7	42149600	0.677686
chr7	42229400	0.727273
chr7	42267900	0.752066
chr7	42278600	0.785124
chr7	42284600	0.983471
chr7	42359700	0.661157
chr7	42607200	0.950413
chr7	42954700	0.983471
chr7	43012600	0.677686
chr7	43239200	0.933884

chr7	43605700	0.719008
chr7	43626400	0.809917
chr7	43631300	0.743802
chr7	43655000	0.876033
chr7	43677300	0.685950
chr7	43679900	0.958678
chr7	43710000	0.859504
chr7	43714400	1.000000
chr7	43734000	0.966942
chr7	43919800	0.867769
chr7	44008700	0.702479
chr7	44018300	0.793388
chr7	44080500	0.727273
chr7	44114200	0.719008
chr7	44127900	0.859504
chr7	44185500	0.776860
chr7	44300000	0.801653
chr7	44331600	0.876033
chr7	44349800	0.859504
chr7	44365100	0.669421
chr7	44408800	0.677686
chr7	44418800	0.876033
chr7	44422100	0.752066
chr7	44424300	0.752066
chr7	44429800	0.917355
chr7	44439200	0.900826
chr7	44448500	0.851240
chr7	44501200	0.892562
chr7	44513300	0.727273
chr7	44525600	0.752066
chr7	44672000	0.702479
chr7	44675400	0.702479
chr7	44678000	0.842975
chr7	44680300	0.933884
chr7	44766900	0.983471
chr7	44775600	0.958678
chr7	44867200	0.702479
chr7	44920800	0.834711
chr7	44923000	0.677686
chr7	45002700	0.900826
chr7	45030500	0.892562
chr7	45035100	0.991736
chr7	45067300	0.958678
chr7	45280100	0.826446
chr7	45291700	0.900826
chr7	45614500	0.669421
chr7	45729600	0.818182
chr7	45802500	0.694215
chr7	45914400	0.801653
chr7	45930200	0.975207
chr7	46018200	0.942149
chr7	46361800	0.884298
chr7	46385500	0.694215
chr7	46454600	0.950413

chr7	46585400	0.950413
chr7	46608700	0.975207
chr7	46617000	0.975207
chr7	46622000	0.719008
chr7	46644000	0.768595
chr7	46785600	0.669421
chr7	46792900	0.983471
chr7	46795500	0.735537
chr7	46800100	0.760331
chr7	46804000	1.000000
chr7	46824700	0.876033
chr7	46899900	0.925620
chr7	46919600	0.785124
chr7	46921700	0.942149
chr7	46925000	0.694215
chr7	46973400	0.685950
chr7	47079800	1.000000
chr7	47092300	0.710744
chr7	47293800	0.710744
chr7	47331400	0.942149
chr7	47369000	0.876033
chr7	47468100	0.900826
chr7	47491400	0.752066
chr7	47494400	0.892562
chr7	47498000	0.892562
chr7	47515000	0.809917
chr7	47522100	0.991736
chr7	47596800	0.743802
chr7	47641200	0.876033
chr7	47764800	0.793388
chr7	47784000	0.743802
chr7	47823100	0.900826
chr7	47826700	0.991736
chr7	47846700	0.809917
chr7	47886700	1.000000
chr7	47980100	0.727273
chr7	47984200	0.950413
chr7	47986900	0.958678
chr7	47990200	0.900826
chr7	48009100	0.975207
chr7	48011600	0.933884
chr7	48031800	0.685950
chr7	48082100	0.983471
chr7	48127100	0.892562
chr7	48131400	0.669421
chr7	48135000	0.785124
chr7	48137900	0.958678
chr7	48214000	0.966942
chr7	48281300	0.710744
chr7	48284800	0.834711
chr7	48290500	0.991736
chr7	48314800	0.735537
chr7	50132800	0.727273
chr7	50344200	0.685950

chr7	50504300	0.842975
chr7	50514600	1.000000
chr7	50565700	0.917355
chr7	50570300	0.909091
chr7	50603100	0.942149
chr7	50663100	0.677686
chr7	50674400	0.685950
chr7	50678700	0.983471
chr7	50700900	0.851240
chr7	50725700	0.933884
chr7	50732600	0.958678
chr7	50735800	0.801653
chr7	50749700	0.909091
chr7	50753000	0.942149
chr7	50762000	0.743802
chr7	50787700	0.685950
chr7	50793200	0.991736
chr7	50795600	0.991736
chr7	50799100	0.851240
chr7	50808000	0.909091
chr7	50835200	0.719008
chr7	50851300	0.752066
chr7	50856100	0.661157
chr7	50862100	0.768595
chr7	50871600	0.685950
chr7	50874000	0.735537
chr7	50880800	0.793388
chr7	50886300	0.776860
chr7	50894200	0.933884
chr7	50907100	0.925620
chr7	51022100	0.776860
chr7	51025600	0.983471
chr7	51030900	0.983471
chr7	51137800	1.000000
chr7	51143700	0.991736
chr7	51190200	0.958678
chr7	51202100	0.933884
chr7	51208000	0.983471
chr7	52180300	0.702479
chr7	52336900	0.942149
chr7	52339000	0.900826
chr7	52531500	0.983471
chr7	52787800	0.818182
chr7	52843400	0.851240
chr7	52907200	0.909091
chr7	52995400	0.867769
chr7	52997500	0.851240
chr7	53024200	0.966942
chr7	53027300	0.768595
chr7	53030100	0.694215
chr7	53163400	0.958678
chr7	53168200	0.892562
chr7	53187500	0.933884
chr7	53314900	0.694215

chr7	53678900	0.702479
chr7	54328800	0.735537
chr7	54732300	0.685950
chr7	54879200	0.983471
chr7	55053400	0.768595
chr7	55063600	0.727273
chr7	55133300	0.958678
chr7	55154800	0.884298
chr7	55202500	0.694215
chr7	55210700	0.768595
chr7	55233000	0.677686
chr7	55236100	0.669421
chr7	55238800	0.785124
chr7	55242300	0.677686
chr7	55256500	0.859504
chr7	55260800	1.000000
chr7	55266800	0.677686
chr7	55281000	0.661157
chr7	55416600	0.892562
chr7	55437300	0.991736
chr7	55491400	1.000000
chr7	55507300	0.801653
chr7	55601400	0.876033
chr7	55614700	0.834711
chr7	55623000	0.958678
chr7	55636600	0.983471
chr7	55642400	0.933884
chr7	55757200	0.966942
chr7	56045800	0.785124
chr7	56115400	0.942149
chr7	56121900	0.727273
chr7	56130400	0.991736
chr7	56148100	0.983471
chr7	56160900	0.727273
chr7	58008800	0.842975
chr7	61079300	0.925620
chr7	61085000	0.933884
chr7	61264200	0.793388
chr7	61629600	0.669421
chr7	61976400	0.818182
chr7	61986000	0.801653
chr7	64459900	0.966942
chr7	64534000	0.727273
chr7	64583900	0.661157
chr7	64638200	0.958678
chr7	64654100	0.975207
chr7	64673900	1.000000
chr7	64904200	0.892562
chr7	64906300	0.884298
chr7	65149000	0.950413
chr7	65164500	0.983471
chr7	65184200	1.000000
chr7	65196900	0.677686
chr7	65211300	0.950413

chr7	65218000	0.669421
chr7	65228000	0.925620
chr7	65260000	0.801653
chr7	65263700	1.000000
chr7	65266000	0.933884
chr7	65449300	0.661157
chr7	65565400	0.719008
chr7	65572000	0.735537
chr7	65614200	0.983471
chr7	65617900	0.909091
chr7	65748400	0.983471
chr7	65769700	0.917355
chr7	65772800	0.958678
chr7	65845200	0.859504
chr7	65850400	0.694215
chr7	65861000	0.942149
chr7	65878700	0.842975
chr7	65909100	0.958678
chr7	66245300	0.917355
chr7	66313500	0.917355
chr7	66412100	0.925620
chr7	66415700	0.950413
chr7	66418300	0.900826
chr7	66490800	0.785124
chr7	66508600	0.867769
chr7	66526700	0.834711
chr7	66586000	0.809917
chr7	66593000	0.950413
chr7	66595100	0.892562
chr7	66608700	0.785124
chr7	70170200	0.702479
chr7	72147800	0.892562
chr7	72149900	0.785124
chr7	72156600	1.000000
chr7	72230300	0.727273
chr7	72247100	0.892562
chr7	72249500	0.760331
chr7	72461100	0.818182
chr7	72618300	0.851240
chr7	72626100	0.760331
chr7	72661600	0.793388
chr7	72738500	0.727273
chr7	72765700	0.768595
chr7	72772600	0.942149
chr7	72825200	0.942149
chr7	72942500	0.950413
chr7	72993900	0.694215
chr7	72996700	0.966942
chr7	73062700	0.685950
chr7	73121700	0.760331
chr7	73184900	0.661157
chr7	73188900	0.851240
chr7	73191600	0.661157
chr7	73214400	0.710744

chr7	73242400	0.834711
chr7	73256600	0.743802
chr7	73265800	0.801653
chr7	73295800	0.884298
chr7	73301300	0.917355
chr7	73356600	0.933884
chr7	73417300	0.983471
chr7	73437600	0.867769
chr7	73444100	0.735537
chr7	73476700	0.942149
chr7	73498200	0.685950
chr7	73509100	0.834711
chr7	73512800	0.950413
chr7	73587800	0.702479
chr7	73591600	0.933884
chr7	73597700	0.925620
chr7	73602500	0.685950
chr7	73671100	0.677686
chr7	73684400	0.785124
chr7	73686900	0.925620
chr7	73694300	0.942149
chr7	73721500	0.801653
chr7	73770000	0.950413
chr7	73808900	0.950413
chr7	73867800	0.702479
chr7	73893000	0.991736
chr7	74002100	0.776860
chr7	74020900	0.669421
chr7	74075000	0.867769
chr7	74132800	0.760331
chr7	74215500	0.884298
chr7	74291300	0.859504
chr7	74561100	0.834711
chr7	74596800	0.785124
chr7	74603500	0.909091
chr7	74607800	0.876033
chr7	75195000	0.884298
chr7	75226200	0.859504
chr7	75229100	0.942149
chr7	75241300	0.917355
chr7	75279900	0.900826
chr7	75300400	0.909091
chr7	75472400	0.925620
chr7	75567700	0.801653
chr7	75585700	0.719008
chr7	75592700	0.925620
chr7	75638700	0.884298
chr7	75641500	0.884298
chr7	75716800	0.900826
chr7	75731700	0.925620
chr7	75752000	0.669421
chr7	75896900	0.735537
chr7	75921400	0.983471
chr7	75924500	0.900826

chr7	75936500	0.884298
chr7	75957000	0.859504
chr7	75970600	0.925620
chr7	75976500	0.834711
chr7	75989800	0.760331
chr7	75994400	0.818182
chr7	76003000	0.768595
chr7	76009600	0.958678
chr7	76018900	0.793388
chr7	76022900	0.694215
chr7	76027200	0.752066
chr7	76068000	0.892562
chr7	76075400	0.950413
chr7	76149800	0.933884
chr7	76198300	0.925620
chr7	76209600	0.991736
chr7	76213600	0.942149
chr7	76218200	0.991736
chr7	76235700	0.958678
chr7	76242900	0.818182
chr7	76272100	0.743802
chr7	76451100	0.867769
chr7	76639600	0.892562
chr7	76705500	0.925620
chr7	76709700	0.884298
chr7	76767600	0.719008
chr7	76786700	0.785124
chr7	76807000	0.776860
chr7	76918500	0.884298
chr7	76956000	0.834711
chr7	76960400	0.950413
chr7	76978200	0.859504
chr7	76981300	0.661157
chr7	77057900	0.933884
chr7	77072200	0.801653
chr7	77104900	0.809917
chr7	77170500	0.661157
chr7	77178200	0.685950
chr7	77182700	0.933884
chr7	77199600	0.950413
chr7	77357600	0.892562
chr7	77364500	0.884298
chr7	77441600	0.966942
chr7	77464300	0.942149
chr7	77496700	0.942149
chr7	77649600	0.909091
chr7	77909300	0.958678
chr7	78720100	0.685950
chr7	78725900	0.842975
chr7	78747500	0.785124
chr7	78912700	0.933884
chr7	79016600	0.851240
chr7	79250200	0.925620
chr7	79252800	0.735537

chr7	79282800	0.685950
chr7	79612000	0.719008
chr7	79708000	0.925620
chr7	79736400	0.983471
chr7	79769000	0.702479
chr7	79870000	0.851240
chr7	79941600	1.000000
chr7	79946300	0.719008
chr7	79999100	0.818182
chr7	80015100	0.950413
chr7	80128600	0.900826
chr7	80137700	0.694215
chr7	80571700	0.983471
chr7	80929000	0.876033
chr7	80957200	0.818182
chr7	80960900	1.000000
chr7	81129100	0.719008
chr7	81248000	0.950413
chr7	81257700	0.917355
chr7	81303700	0.685950
chr7	81314500	0.685950
chr7	81399600	0.809917
chr7	81476000	0.752066
chr7	81516800	0.768595
chr7	81666300	0.983471
chr7	81669500	0.900826
chr7	81671600	0.669421
chr7	81679600	0.702479
chr7	81738700	0.917355
chr7	81921700	0.950413
chr7	82224800	0.966942
chr7	82330000	0.801653
chr7	82435200	0.950413
chr7	82489200	0.966942
chr7	82778600	0.710744
chr7	82919400	0.760331
chr7	82931000	0.942149
chr7	83098400	0.917355
chr7	83654900	0.900826
chr7	83671800	0.942149
chr7	83690300	0.958678
chr7	83703100	0.801653
chr7	83721900	0.876033
chr7	83742800	0.975207
chr7	83753800	0.958678
chr7	83769600	0.809917
chr7	83866300	0.950413
chr7	84154800	0.867769
chr7	84266200	0.859504
chr7	84268700	0.925620
chr7	84277300	0.735537
chr7	84298900	0.942149
chr7	84304500	0.776860
chr7	84318200	0.826446

chr7	84327100	1.000000
chr7	84329800	0.685950
chr7	84331900	0.876033
chr7	84534000	0.735537
chr7	84655200	0.735537
chr7	84735000	0.991736
chr7	84766200	0.834711
chr7	84790100	0.669421
chr7	85636500	0.685950
chr7	86562000	0.884298
chr7	86686000	0.933884
chr7	87238900	0.867769
chr7	87275700	0.892562
chr7	87341900	0.719008
chr7	87650900	0.793388
chr7	87856600	0.743802
chr7	88025900	0.975207
chr7	88294400	0.842975
chr7	88312900	0.950413
chr7	89400300	0.842975
chr7	89786300	0.768595
chr7	89788700	0.933884
chr7	89807400	0.669421
chr7	89809600	0.925620
chr7	89880300	0.661157
chr7	89950400	0.669421
chr7	90013700	0.933884
chr7	90104100	0.809917
chr7	90223900	0.933884
chr7	90229100	0.909091
chr7	90242800	0.793388
chr7	90255000	0.975207
chr7	90285100	0.900826
chr7	90322000	0.719008
chr7	90339400	0.809917
chr7	90350800	0.851240
chr7	90365900	0.661157
chr7	90434100	0.661157
chr7	90446500	0.991736
chr7	90454700	0.925620
chr7	90812400	0.768595
chr7	90858300	0.842975
chr7	90869100	0.743802
chr7	90956200	0.909091
chr7	90976800	0.983471
chr7	90978900	0.842975
chr7	90981100	0.876033
chr7	91095400	0.975207
chr7	91099100	0.669421
chr7	91258400	0.801653
chr7	91761200	0.826446
chr7	92193300	0.727273
chr7	92251000	0.909091
chr7	92255300	1.000000

chr7	92261700	0.958678
chr7	92265700	0.867769
chr7	92271300	0.851240
chr7	92275000	0.917355
chr7	92324700	0.892562
chr7	92327700	0.950413
chr7	92353200	1.000000
chr7	92361800	0.900826
chr7	92382200	0.958678
chr7	92396200	0.702479
chr7	92411000	0.983471
chr7	92429000	0.752066
chr7	92439300	0.942149
chr7	92504100	0.867769
chr7	92641200	0.851240
chr7	92644000	0.677686
chr7	92646100	0.842975
chr7	92673300	0.735537
chr7	92762700	0.950413
chr7	92776000	0.950413
chr7	92909300	0.942149
chr7	93039200	0.694215
chr7	93540200	0.958678
chr7	93544600	1.000000
chr7	93550100	0.785124
chr7	93569900	0.983471
chr7	93573500	0.818182
chr7	93575800	0.826446
chr7	93578900	0.958678
chr7	93586000	0.669421
chr7	93588100	0.900826
chr7	93670600	0.818182
chr7	93694300	0.793388
chr7	93698400	0.669421
chr7	93717700	0.735537
chr7	93759400	0.842975
chr7	93779800	0.834711
chr7	93787000	0.801653
chr7	93804000	0.826446
chr7	93806100	0.925620
chr7	93808500	0.942149
chr7	93811800	0.818182
chr7	93814200	0.925620
chr7	93825200	0.694215
chr7	93931000	0.752066
chr7	93943800	0.983471
chr7	93997800	0.958678
chr7	94004900	0.909091
chr7	94044200	0.677686
chr7	94250700	0.702479
chr7	94262100	0.983471
chr7	94326800	0.900826
chr7	94328900	0.785124
chr7	94331100	1.000000

chr7	95100200	0.991736
chr7	95179500	0.719008
chr7	95504700	0.925620
chr7	95516300	0.991736
chr7	95527500	0.818182
chr7	95555600	0.768595
chr7	95617100	0.719008
chr7	95714700	0.917355
chr7	95718300	0.950413
chr7	95787600	0.719008
chr7	96009800	0.694215
chr7	96018300	0.933884
chr7	96039500	0.826446
chr7	96287700	0.958678
chr7	96304500	0.719008
chr7	96656800	0.776860
chr7	97740300	0.991736
chr7	97761200	1.000000
chr7	97793400	0.801653
chr7	97875200	0.925620
chr7	97879100	0.768595
chr7	97895800	0.900826
chr7	97916100	0.925620
chr7	97984200	0.826446
chr7	98048600	0.892562
chr7	98532100	0.842975
chr7	98544200	0.801653
chr7	98627600	0.966942
chr7	98639100	0.975207
chr7	98641200	0.859504
chr7	98649000	0.925620
chr7	98701200	0.966942
chr7	98704600	0.752066
chr7	98707800	0.975207
chr7	98739400	0.702479
chr7	98865400	0.909091
chr7	98871600	0.942149
chr7	98928400	0.909091
chr7	98976700	0.909091
chr7	98979200	0.743802
chr7	98990800	0.909091
chr7	99527300	0.842975
chr7	99595700	0.677686
chr7	99633100	0.991736
chr7	99639300	0.991736
chr7	99681000	0.884298
chr7	99684500	0.942149
chr7	99691400	0.933884
chr7	99700600	0.719008
chr7	99923600	0.661157
chr7	99935800	0.983471
chr7	99942700	0.884298
chr7	100029800	0.727273
chr7	100037400	0.727273

chr7	100041700	0.768595
chr7	100043900	0.892562
chr7	100048600	0.793388
chr7	100052700	0.826446
chr7	100083200	0.661157
chr7	100091700	0.834711
chr7	100113000	0.917355
chr7	100142600	0.900826
chr7	100146400	0.900826
chr7	100224800	0.743802
chr7	100231100	0.694215
chr7	100392600	0.685950
chr7	100401300	0.702479
chr7	100427200	0.950413
chr7	100434500	0.917355
chr7	100461700	0.702479
chr7	100464000	0.884298
chr7	100472100	0.752066
chr7	100494200	0.677686
chr7	100550700	0.702479
chr7	100660800	0.719008
chr7	100729000	0.694215
chr7	100738400	0.925620
chr7	100743100	0.694215
chr7	100745200	0.694215
chr7	100748100	0.900826
chr7	100751600	0.876033
chr7	100753700	0.743802
chr7	100756700	0.958678
chr7	100758800	0.793388
chr7	100761400	0.983471
chr7	100765600	0.966942
chr7	100768800	0.909091
chr7	100775800	0.818182
chr7	100778700	0.917355
chr7	100782000	0.909091
chr7	100806800	0.793388
chr7	100809500	0.909091
chr7	100813100	0.842975
chr7	100816800	0.702479
chr7	100875700	0.867769
chr7	100881300	0.983471
chr7	100883400	0.884298
chr7	101274500	0.776860
chr7	101325000	0.826446
chr7	101332200	0.884298
chr7	101387200	0.661157
chr7	101500100	0.669421
chr7	101503200	0.669421
chr7	101524500	0.727273
chr7	101526600	0.933884
chr7	101571700	0.702479
chr7	101580100	0.785124
chr7	101594600	0.669421

chr7	101597000	0.950413
chr7	101765700	0.933884
chr7	101813300	0.942149
chr7	101864800	0.925620
chr7	101959500	0.801653
chr7	102056600	0.677686
chr7	102081100	0.876033
chr7	102146500	0.743802
chr7	102153400	0.809917
chr7	102156500	0.694215
chr7	102158600	0.702479
chr7	102172300	0.818182
chr7	102245600	0.743802
chr7	102254500	0.694215
chr7	102271700	0.719008
chr7	102330600	0.867769
chr7	102432100	0.950413
chr7	102434900	0.752066
chr7	102437000	0.950413
chr7	102454300	0.991736
chr7	102459700	0.942149
chr7	102476400	0.991736
chr7	102512500	0.900826
chr7	102515100	0.818182
chr7	102517200	0.884298
chr7	102524000	0.752066
chr7	102529000	0.958678
chr7	102532400	0.958678
chr7	102553700	0.892562
chr7	102579500	0.933884
chr7	102611800	0.710744
chr7	102614200	0.727273
chr7	102631600	0.785124
chr7	102634700	0.975207
chr7	102702400	0.983471
chr7	102783300	0.785124
chr7	102948400	0.809917
chr7	103273900	0.710744
chr7	103278300	0.900826
chr7	103359200	0.991736
chr7	103376000	0.694215
chr7	103837100	0.950413
chr7	103865200	0.958678
chr7	104431700	0.975207
chr7	104524700	0.785124
chr7	104563800	0.892562
chr7	104586700	0.991736
chr7	104602200	0.752066
chr7	104604800	0.867769
chr7	104612900	0.958678
chr7	104615200	0.719008
chr7	104620500	0.842975
chr7	104622900	0.727273
chr7	104626200	0.950413

chr7	104644200	0.867769
chr7	104738200	0.909091
chr7	104790900	0.661157
chr7	104866000	0.933884
chr7	104910100	0.859504
chr7	104992800	1.000000
chr7	105015900	0.768595
chr7	105042600	0.859504
chr7	105080200	0.950413
chr7	105119500	1.000000
chr7	105127500	0.859504
chr7	105129600	0.909091
chr7	105134600	0.851240
chr7	105137600	0.851240
chr7	105319600	0.809917
chr7	105411000	0.760331
chr7	105423400	0.752066
chr7	105450000	0.834711
chr7	105471100	0.991736
chr7	105490400	1.000000
chr7	105706000	0.760331
chr7	105712400	0.942149
chr7	105767300	0.818182
chr7	105817800	0.677686
chr7	105831500	1.000000
chr7	105846700	0.661157
chr7	105857300	0.966942
chr7	105860000	0.966942
chr7	105954400	0.991736
chr7	105986300	0.958678
chr7	106103100	0.677686
chr7	106107600	0.809917
chr7	106113000	0.958678
chr7	106125800	0.991736
chr7	106178000	0.834711
chr7	106180100	1.000000
chr7	106183500	0.958678
chr7	106230600	0.760331
chr7	106232700	0.743802
chr7	106250100	0.950413
chr7	106320000	0.760331
chr7	106322700	0.867769
chr7	106358900	0.983471
chr7	106372000	0.991736
chr7	106377600	0.859504
chr7	106410700	0.975207
chr7	106413800	0.867769
chr7	106416900	0.842975
chr7	106606100	0.669421
chr7	106680300	0.669421
chr7	106688500	0.991736
chr7	106692000	0.785124
chr7	106717500	0.917355
chr7	106721200	0.925620

chr7	106731500	0.752066
chr7	106741500	0.983471
chr7	106744100	0.925620
chr7	106788600	0.900826
chr7	106804600	0.834711
chr7	106820900	0.768595
chr7	106871200	0.975207
chr7	107005600	0.685950
chr7	107147500	0.752066
chr7	107201500	0.719008
chr7	107223400	0.719008
chr7	107236400	0.942149
chr7	107238500	0.975207
chr7	107250200	0.669421
chr7	107263100	0.785124
chr7	107387300	0.785124
chr7	107520200	0.834711
chr7	107574400	0.685950
chr7	107579300	0.801653
chr7	107582600	0.917355
chr7	107584700	0.933884
chr7	107587200	0.925620
chr7	107589900	1.000000
chr7	107593700	0.966942
chr7	107613500	0.983471
chr7	107634200	0.809917
chr7	107638000	0.768595
chr7	107645500	0.867769
chr7	107654900	0.801653
chr7	107657200	0.867769
chr7	107661400	0.809917
chr7	107671100	0.933884
chr7	107707200	0.760331
chr7	107720900	0.991736
chr7	107725100	0.834711
chr7	107735500	1.000000
chr7	107755400	0.685950
chr7	107838000	0.818182
chr7	107852300	0.942149
chr7	107863400	0.958678
chr7	107875700	0.983471
chr7	107886900	0.942149
chr7	107889000	0.900826
chr7	107922800	0.975207
chr7	107925800	0.876033
chr7	107933200	0.950413
chr7	107935300	0.900826
chr7	107938000	0.958678
chr7	107940700	0.958678
chr7	107949000	0.809917
chr7	107951100	0.785124
chr7	107953400	0.867769
chr7	108021300	0.743802
chr7	108043600	0.842975

chr7	108054800	0.826446
chr7	108057600	0.975207
chr7	108061400	0.991736
chr7	108068800	0.735537
chr7	108075700	0.942149
chr7	108082800	0.933884
chr7	108091700	0.892562
chr7	108093900	0.661157
chr7	108126800	0.768595
chr7	108130000	0.958678
chr7	108207900	0.661157
chr7	109958300	0.925620
chr7	110403300	0.760331
chr7	110563500	0.925620
chr7	110670200	0.925620
chr7	110778700	1.000000
chr7	110872600	0.983471
chr7	111033500	0.958678
chr7	111169800	0.785124
chr7	111376600	0.768595
chr7	111385400	0.892562
chr7	111468400	0.966942
chr7	111471400	0.975207
chr7	111473500	0.900826
chr7	111475600	0.859504
chr7	111495700	0.743802
chr7	111514200	0.975207
chr7	111528500	0.983471
chr7	111599700	0.966942
chr7	111617900	0.991736
chr7	111627500	0.975207
chr7	111638900	0.917355
chr7	111641600	0.966942
chr7	111649100	0.694215
chr7	111651900	0.735537
chr7	111663100	0.975207
chr7	111666500	0.685950
chr7	111676400	0.900826
chr7	111710500	0.925620
chr7	111712700	0.950413
chr7	111720700	0.884298
chr7	111725200	0.991736
chr7	111744000	0.983471
chr7	111753800	0.719008
chr7	111770200	0.727273
chr7	111772800	1.000000
chr7	111800300	0.876033
chr7	111803700	0.925620
chr7	111813600	0.991736
chr7	111816700	0.727273
chr7	111825600	0.909091
chr7	111828600	0.702479
chr7	111830900	0.867769
chr7	111839200	0.669421

chr7	112063500	0.950413
chr7	112093800	0.867769
chr7	112121600	0.983471
chr7	112125300	0.859504
chr7	112518500	0.661157
chr7	112523600	0.760331
chr7	113435500	0.719008
chr7	113437800	0.900826
chr7	113532700	0.983471
chr7	113723300	0.685950
chr7	113996800	0.876033
chr7	114129000	0.685950
chr7	114142700	0.702479
chr7	114430000	0.917355
chr7	114514700	0.743802
chr7	114570700	0.727273
chr7	114727700	0.867769
chr7	114737700	0.966942
chr7	114763000	0.719008
chr7	114871600	0.983471
chr7	115665600	0.950413
chr7	115738000	0.892562
chr7	115741200	0.752066
chr7	115854100	0.925620
chr7	115856800	0.859504
chr7	115858900	0.958678
chr7	115870200	0.669421
chr7	115883200	0.661157
chr7	115900000	0.983471
chr7	115911900	0.735537
chr7	115914000	0.900826
chr7	115921200	1.000000
chr7	115928700	0.991736
chr7	115955200	0.710744
chr7	115959400	0.876033
chr7	115963600	0.752066
chr7	115969200	0.925620
chr7	115979500	0.975207
chr7	115981900	0.735537
chr7	115993400	0.768595
chr7	115995700	0.991736
chr7	115998300	0.818182
chr7	116003500	0.966942
chr7	116018500	0.851240
chr7	116033700	0.975207
chr7	116035800	0.933884
chr7	116058000	0.801653
chr7	116066800	0.983471
chr7	116081700	0.719008
chr7	116083900	0.933884
chr7	116095600	0.818182
chr7	116106500	0.966942
chr7	116143400	0.818182
chr7	116146200	0.768595

chr7	116150000	0.809917
chr7	116153900	0.793388
chr7	116161100	0.842975
chr7	116163500	0.966942
chr7	116175500	0.983471
chr7	116184300	0.776860
chr7	116211200	0.958678
chr7	116214100	0.966942
chr7	116217600	0.917355
chr7	116222700	0.867769
chr7	116228700	0.909091
chr7	116233100	0.851240
chr7	116250400	0.900826
chr7	116255100	1.000000
chr7	116257500	0.834711
chr7	116315400	0.917355
chr7	116320000	0.966942
chr7	116328400	0.983471
chr7	116333000	0.851240
chr7	116346300	0.851240
chr7	116357200	0.983471
chr7	116367700	0.793388
chr7	116377400	0.867769
chr7	116393000	0.834711
chr7	116397900	0.661157
chr7	116412300	0.991736
chr7	116417000	0.950413
chr7	116509800	0.933884
chr7	116512500	0.966942
chr7	116515100	0.719008
chr7	116518400	0.925620
chr7	116571000	0.702479
chr7	116654900	0.735537
chr7	116660400	0.867769
chr7	116666500	0.966942
chr7	116675600	0.958678
chr7	116709000	0.735537
chr7	116713200	0.966942
chr7	116721500	0.752066
chr7	116750900	0.809917
chr7	116759200	0.925620
chr7	116765100	0.983471
chr7	116773000	0.991736
chr7	116783700	0.966942
chr7	116798300	0.859504
chr7	116810400	0.900826
chr7	116824500	0.892562
chr7	116910100	0.966942
chr7	117228800	0.776860
chr7	117241100	0.950413
chr7	117286000	0.966942
chr7	117306300	0.933884
chr7	117458400	0.727273
chr7	119185500	0.900826

chr7	119305000	0.925620
chr7	119360900	0.975207
chr7	119961300	0.719008
chr7	120042700	0.958678
chr7	120224500	0.950413
chr7	120479100	0.950413
chr7	120571500	0.760331
chr7	120582400	0.983471
chr7	120601300	0.685950
chr7	120660900	0.892562
chr7	120664400	0.702479
chr7	120681900	0.752066
chr7	120724700	0.950413
chr7	121013100	0.876033
chr7	121025000	0.743802
chr7	121076600	0.669421
chr7	121088300	0.752066
chr7	121145100	0.966942
chr7	121170400	0.942149
chr7	121185100	0.925620
chr7	121777700	0.685950
chr7	121957200	0.809917
chr7	122066200	0.719008
chr7	122293300	0.900826
chr7	122442900	0.983471
chr7	122484600	0.966942
chr7	122521300	0.719008
chr7	122665800	0.900826
chr7	122681400	1.000000
chr7	123178500	0.909091
chr7	123274100	0.966942
chr7	123284300	0.900826
chr7	123392100	0.884298
chr7	123409600	0.735537
chr7	123505400	0.826446
chr7	123724900	0.694215
chr7	123793600	0.925620
chr7	124363000	0.909091
chr7	124408400	0.719008
chr7	124423600	1.000000
chr7	124562900	0.743802
chr7	124702300	0.842975
chr7	124777500	0.942149
chr7	124987800	0.925620
chr7	124996400	0.925620
chr7	125064400	0.950413
chr7	126341900	0.892562
chr7	126368300	0.818182
chr7	126988400	0.793388
chr7	127305900	0.809917
chr7	127309000	0.685950
chr7	127324500	0.909091
chr7	127510900	0.809917
chr7	127518200	0.950413

chr7	127528600	0.694215
chr7	127538300	0.826446
chr7	127548300	0.735537
chr7	127587100	0.809917
chr7	127716100	0.925620
chr7	127772100	0.991736
chr7	127912000	0.793388
chr7	127992100	0.685950
chr7	128001800	0.809917
chr7	128094500	0.933884
chr7	128337800	0.661157
chr7	128373400	0.702479
chr7	128474100	0.942149
chr7	128509700	0.719008
chr7	128550200	0.727273
chr7	128681800	0.735537
chr7	128809500	0.661157
chr7	129017700	0.768595
chr7	129079300	1.000000
chr7	129171000	0.685950
chr7	129254700	0.694215
chr7	129284000	0.958678
chr7	129304900	0.710744
chr7	129330400	0.677686
chr7	129425600	0.727273
chr7	129470600	0.785124
chr7	129483900	0.702479
chr7	129486300	1.000000
chr7	129488600	0.785124
chr7	129494300	0.958678
chr7	129516000	0.892562
chr7	129536900	0.876033
chr7	129547000	0.966942
chr7	129556200	0.735537
chr7	129559700	0.983471
chr7	129564200	0.735537
chr7	129598900	0.818182
chr7	129610500	0.942149
chr7	129648700	0.685950
chr7	129650900	0.785124
chr7	129781500	0.892562
chr7	129826000	0.826446
chr7	129996600	0.842975
chr7	130009800	0.685950
chr7	130013800	0.933884
chr7	130071000	1.000000
chr7	130073600	0.958678
chr7	130080700	0.702479
chr7	130126600	0.867769
chr7	130129200	0.975207
chr7	130317500	0.752066
chr7	130521400	0.768595
chr7	130523900	0.950413
chr7	130563900	0.661157

chr7	130572200	0.859504
chr7	130574400	0.909091
chr7	130576900	0.975207
chr7	130579000	0.793388
chr7	130582400	0.991736
chr7	130586300	0.909091
chr7	130589400	0.801653
chr7	130592600	0.884298
chr7	130599500	0.669421
chr7	130602600	1.000000
chr7	130607000	0.859504
chr7	130626100	0.859504
chr7	130628600	0.842975
chr7	130641800	0.661157
chr7	130645400	0.958678
chr7	130681700	0.785124
chr7	130692600	0.933884
chr7	130742200	0.900826
chr7	130754500	0.776860
chr7	130757300	0.983471
chr7	130797000	0.735537
chr7	130920700	0.950413
chr7	130923800	0.917355
chr7	130939000	0.842975
chr7	131042200	0.826446
chr7	131166000	1.000000
chr7	131171600	0.975207
chr7	131197800	0.975207
chr7	131207100	0.966942
chr7	131210700	0.900826
chr7	131214200	0.851240
chr7	131218100	0.867769
chr7	131223500	0.876033
chr7	131227000	0.876033
chr7	131230600	1.000000
chr7	131305100	0.975207
chr7	131308300	0.966942
chr7	131311300	0.669421
chr7	131314900	0.818182
chr7	131318400	0.867769
chr7	131320900	0.677686
chr7	131327600	0.834711
chr7	131338700	0.958678
chr7	131343100	0.785124
chr7	131377400	0.694215
chr7	131547100	0.776860
chr7	131580700	0.991736
chr7	131750800	0.966942
chr7	131824100	0.818182
chr7	131830500	0.876033
chr7	131941200	0.719008
chr7	131977700	0.785124
chr7	131980100	0.735537
chr7	131983900	0.966942

chr7	131997900	0.710744
chr7	132003800	0.983471
chr7	132071500	0.710744
chr7	132075800	0.669421
chr7	132092200	0.669421
chr7	132095500	0.942149
chr7	132102200	0.892562
chr7	132104700	0.983471
chr7	132108000	0.826446
chr7	132110600	0.752066
chr7	132114700	0.710744
chr7	132117000	0.785124
chr7	132133700	0.677686
chr7	132148500	0.859504
chr7	132150900	0.909091
chr7	132154500	1.000000
chr7	132171400	0.933884
chr7	132185900	0.851240
chr7	132188200	0.892562
chr7	132242800	0.661157
chr7	132255800	0.661157
chr7	132257900	0.793388
chr7	132275300	0.694215
chr7	132299900	0.669421
chr7	132303700	0.983471
chr7	132483400	0.694215
chr7	132550800	0.909091
chr7	132694900	0.991736
chr7	132749700	0.809917
chr7	132920800	0.909091
chr7	132995300	0.694215
chr7	133001100	0.719008
chr7	133040200	0.859504
chr7	133050400	0.991736
chr7	133120900	0.661157
chr7	133268000	0.933884
chr7	133859900	0.669421
chr7	134107500	0.950413
chr7	134117500	0.727273
chr7	134134300	0.983471
chr7	134139500	0.834711
chr7	134165800	0.818182
chr7	134200900	0.876033
chr7	134279000	0.909091
chr7	134299000	0.991736
chr7	134304600	0.900826
chr7	134316800	1.000000
chr7	134345100	0.884298
chr7	134348400	0.950413
chr7	134352600	0.876033
chr7	134356000	0.950413
chr7	134462600	0.900826
chr7	134471500	0.809917
chr7	134484700	0.702479

chr7	134489500	0.991736
chr7	134496500	0.702479
chr7	134499000	0.991736
chr7	134519700	0.842975
chr7	134528400	0.983471
chr7	134540800	0.958678
chr7	134551200	1.000000
chr7	134555700	0.876033
chr7	134558100	0.818182
chr7	134571200	0.975207
chr7	134576100	0.719008
chr7	134626000	0.859504
chr7	134677500	0.942149
chr7	134829400	0.925620
chr7	134836000	0.809917
chr7	134845100	0.900826
chr7	134851800	0.826446
chr7	134939300	0.892562
chr7	134967400	0.685950
chr7	134975700	0.785124
chr7	134977800	0.909091
chr7	135005500	0.785124
chr7	135175900	0.801653
chr7	135345200	0.900826
chr7	135358200	0.933884
chr7	135369700	0.983471
chr7	135377100	0.909091
chr7	135392500	0.661157
chr7	135397800	0.851240
chr7	135430800	0.900826
chr7	135443400	0.958678
chr7	135447200	0.950413
chr7	135459100	0.876033
chr7	135608800	0.975207
chr7	135628600	0.917355
chr7	135664100	0.793388
chr7	136544900	0.818182
chr7	136610300	0.983471
chr7	136638500	0.975207
chr7	136641600	1.000000
chr7	136758400	0.809917
chr7	137147500	0.694215
chr7	137193700	0.842975
chr7	137264800	0.884298
chr7	137276600	0.909091
chr7	137373200	0.694215
chr7	137457800	0.661157
chr7	137486300	0.785124
chr7	137531800	0.669421
chr7	137567900	0.925620
chr7	137617900	0.884298
chr7	137650600	0.818182
chr7	137653000	0.710744
chr7	137655700	0.851240

chr7	137663500	0.925620
chr7	137666500	0.669421
chr7	137672700	0.900826
chr7	137675700	0.892562
chr7	137678800	0.867769
chr7	137682100	0.768595
chr7	137688300	0.661157
chr7	138231000	0.966942
chr7	138620000	0.933884
chr7	138622700	0.884298
chr7	138663800	0.793388
chr7	138745000	0.966942
chr7	138759800	0.752066
chr7	138776200	0.876033
chr7	138780200	0.719008
chr7	138788600	0.818182
chr7	138803400	0.776860
chr7	139168500	0.685950
chr7	139185300	0.694215
chr7	139333700	0.900826
chr7	139364800	0.975207
chr7	139368100	0.818182
chr7	139414500	0.694215
chr7	139421200	0.834711
chr7	139430800	0.669421
chr7	139434300	0.942149
chr7	139463400	0.958678
chr7	139529600	0.768595
chr7	139615700	0.900826
chr7	139722900	0.694215
chr7	139735800	0.933884
chr7	139738600	0.876033
chr7	139743000	0.892562
chr7	139750100	0.975207
chr7	140049400	0.933884
chr7	140085400	0.677686
chr7	140340500	0.743802
chr7	140356600	0.694215
chr7	140453800	0.884298
chr7	140477600	0.727273
chr7	140482800	0.685950
chr7	141054700	0.983471
chr7	141062700	0.991736
chr7	141066300	0.710744
chr7	141354500	0.710744
chr7	141360400	0.925620
chr7	141363600	0.892562
chr7	141373600	0.950413
chr7	141375900	0.876033
chr7	141488300	0.826446
chr7	142188500	0.950413
chr7	142191300	0.876033
chr7	142373800	0.669421
chr7	142422400	0.752066

chr7	142505900	0.983471
chr7	142508200	0.975207
chr7	142716600	0.851240
chr7	142942500	0.719008
chr7	142988200	0.685950
chr7	142991500	0.785124
chr7	142993600	1.000000
chr7	143043000	0.776860
chr7	143073000	0.818182
chr7	143090000	0.966942
chr7	143093100	0.859504
chr7	143115000	0.801653
chr7	143127900	0.859504
chr7	143170000	0.785124
chr7	143188800	0.793388
chr7	143191100	0.975207
chr7	143194400	0.975207
chr7	143196500	0.966942
chr7	143198700	0.719008
chr7	143219900	0.991736
chr7	143231000	0.991736
chr7	143260700	0.991736
chr7	143330800	1.000000
chr7	143333500	0.991736
chr7	143347600	0.942149
chr7	143398600	0.661157
chr7	143410100	0.983471
chr7	143412800	1.000000
chr7	143493000	0.950413
chr7	143503700	0.884298
chr7	143518800	0.975207
chr7	143521500	0.950413
chr7	143588900	0.909091
chr7	143597200	0.917355
chr7	143676800	0.876033
chr7	144522200	0.710744
chr7	144539900	0.942149
chr7	145694200	0.983471
chr7	148470200	0.909091
chr7	148517200	0.958678
chr7	148545000	0.793388
chr7	148554300	0.760331
chr7	148661000	0.685950
chr7	148858600	0.719008
chr7	148897100	0.975207
chr7	148979600	0.876033
chr7	149439700	0.661157
chr7	149473500	0.677686
chr7	149624400	0.694215
chr7	150015400	0.727273
chr7	150020800	0.661157
chr7	150065000	0.768595
chr7	150068600	0.677686
chr7	150101500	0.694215

chr7	150163500	0.958678
chr7	150181300	0.942149
chr7	150260300	0.991736
chr7	150282300	0.851240
chr7	150284600	0.826446
chr7	150326800	0.694215
chr7	150373800	0.950413
chr7	150382700	0.776860
chr7	150436000	0.685950
chr7	150453000	0.991736
chr7	150601700	0.818182
chr7	150672400	0.876033
chr7	150686300	0.909091
chr7	150690000	0.900826
chr7	150700900	0.752066
chr7	150705600	0.867769
chr7	150735600	0.966942
chr7	150782800	0.743802
chr7	150788800	0.851240
chr7	150801800	0.851240
chr7	150812200	0.983471
chr7	150818800	0.727273
chr7	150942500	0.834711
chr7	150946100	0.818182
chr7	150949800	0.702479
chr7	151157300	0.917355
chr7	151169900	0.983471
chr7	151182700	0.933884
chr7	151204800	0.661157
chr7	151283800	0.834711
chr7	151288100	0.661157
chr7	151290300	0.983471
chr7	151294900	0.669421
chr7	151298200	0.933884
chr7	151321300	0.991736
chr7	151323600	0.801653
chr7	151385200	0.975207
chr7	151425100	0.909091
chr7	151433600	0.958678
chr7	151440500	0.917355
chr7	151451700	0.909091
chr7	151453800	0.900826
chr7	151480400	0.892562
chr7	151817900	0.876033
chr7	151834900	0.710744
chr7	151843300	0.983471
chr7	151849600	0.669421
chr7	151899400	0.900826
chr7	152011200	0.909091
chr7	152362100	0.867769
chr7	152433200	0.818182
chr7	152463100	0.719008
chr7	152503900	0.743802
chr7	155000000	0.983471

chr7	155002100	0.752066
chr7	155005300	0.851240
chr7	155069600	0.719008
chr7	155079100	0.694215
chr7	155445900	0.826446
chr7	155458900	0.876033
chr7	155463500	0.950413
chr7	155557100	0.917355
chr7	155602500	0.743802
chr7	155604900	0.719008
chr7	155678700	0.950413
chr7	155712100	0.801653
chr7	155744300	0.925620
chr7	156267100	0.694215
chr7	156401000	0.694215
chr7	156696700	0.892562
chr7	156721200	0.909091
chr7	156724900	0.983471
chr7	156793300	0.752066
chr7	156951600	0.966942
chr7	157028400	0.710744
chr7	157047700	0.677686
chr7	157069800	0.942149
chr7	157072700	0.834711
chr7	157103000	0.958678
chr7	157157000	0.719008
chr7	157163700	0.727273
chr7	157216200	0.975207
chr7	157225700	0.677686
chr7	157296000	0.983471
chr7	158119300	0.735537
chr7	158442300	0.892562
chr7	158534000	1.000000
chr7	158576000	0.942149
chr7	158608200	0.851240
chr7	158612200	0.975207
chr7	158614800	0.826446
chr7	158710800	0.826446
chr8	1650100	0.685950
chr8	1694100	0.677686
chr8	1704200	0.842975
chr8	1714800	0.975207
chr8	1734800	0.975207
chr8	1737000	0.702479
chr8	1748800	0.752066
chr8	1757600	0.776860
chr8	1765500	0.942149
chr8	1768900	0.685950
chr8	1771500	0.727273
chr8	1774300	0.884298
chr8	1777700	0.942149
chr8	1803200	0.710744
chr8	1840200	0.950413
chr8	1843200	0.776860

chr8	1850800	0.818182
chr8	1942100	0.735537
chr8	2023400	0.727273
chr8	2965600	0.925620
chr8	2970800	0.876033
chr8	5876100	0.909091
chr8	6286600	1.000000
chr8	6335600	0.677686
chr8	6344100	0.958678
chr8	6386400	0.975207
chr8	6398800	0.809917
chr8	6405800	0.826446
chr8	6415900	0.925620
chr8	6428500	1.000000
chr8	6443300	0.876033
chr8	6456300	0.851240
chr8	6459700	0.933884
chr8	6502700	0.884298
chr8	6586100	0.892562
chr8	6588200	0.991736
chr8	6591300	0.983471
chr8	6645600	0.768595
chr8	6698900	0.842975
chr8	7252600	0.925620
chr8	7774400	0.975207
chr8	7923600	0.719008
chr8	8038000	0.834711
chr8	8109500	0.917355
chr8	8123000	0.677686
chr8	8128200	0.900826
chr8	8144100	0.768595
chr8	8147900	0.933884
chr8	8152200	0.760331
chr8	8154900	0.727273
chr8	8168100	0.876033
chr8	8171700	0.818182
chr8	8189300	0.975207
chr8	8192000	0.768595
chr8	8195700	0.925620
chr8	8198700	0.975207
chr8	8201500	0.752066
chr8	8205400	0.752066
chr8	8210400	1.000000
chr8	8220700	0.900826
chr8	8395500	0.975207
chr8	8402900	0.867769
chr8	8406200	0.991736
chr8	8459000	1.000000
chr8	8482700	0.793388
chr8	8486200	0.958678
chr8	8493100	0.958678
chr8	8641300	0.892562
chr8	8649400	0.661157
chr8	8655400	0.826446

chr8	8664200	0.975207
chr8	8692100	0.669421
chr8	8702400	0.801653
chr8	8718100	0.727273
chr8	8730300	0.677686
chr8	8821300	0.710744
chr8	8852900	0.743802
chr8	8864700	0.743802
chr8	8871500	0.826446
chr8	8880500	0.909091
chr8	8924100	0.933884
chr8	8928200	0.900826
chr8	8931900	0.719008
chr8	8935800	0.983471
chr8	8938500	0.776860
chr8	8942400	0.702479
chr8	8945800	0.867769
chr8	8991000	0.685950
chr8	8993600	0.917355
chr8	8996600	0.909091
chr8	8999500	0.876033
chr8	9004600	0.661157
chr8	9009500	0.677686
chr8	9375700	0.942149
chr8	9449800	0.917355
chr8	9615800	0.661157
chr8	9764100	0.801653
chr8	9991700	0.842975
chr8	10010100	1.000000
chr8	10095700	0.859504
chr8	10104400	0.909091
chr8	10443000	0.876033
chr8	10445500	0.876033
chr8	10448900	0.752066
chr8	10483600	0.892562
chr8	10486300	0.842975
chr8	10490200	0.809917
chr8	10494400	0.917355
chr8	10530400	0.826446
chr8	10538700	0.842975
chr8	10571700	0.694215
chr8	10575000	0.793388
chr8	10634000	0.933884
chr8	10636200	0.867769
chr8	10641600	1.000000
chr8	10645600	0.909091
chr8	10647800	0.727273
chr8	10655400	0.752066
chr8	10658300	0.983471
chr8	10660500	0.966942
chr8	10664000	0.685950
chr8	10671300	0.950413
chr8	10676600	0.809917
chr8	10681900	0.834711

chr8	10708700	0.694215
chr8	11026700	0.942149
chr8	11337700	0.677686
chr8	11368300	0.900826
chr8	11373800	1.000000
chr8	11382600	0.809917
chr8	11415800	0.710744
chr8	11422100	0.859504
chr8	11584800	0.702479
chr8	11620200	0.966942
chr8	11623700	0.818182
chr8	11664100	0.752066
chr8	11711800	0.909091
chr8	11720200	0.917355
chr8	11722400	0.826446
chr8	11733000	0.859504
chr8	11736000	0.917355
chr8	11749200	0.743802
chr8	11760300	0.661157
chr8	11813500	0.867769
chr8	12343100	0.669421
chr8	12458800	0.743802
chr8	12584300	0.884298
chr8	12901400	0.917355
chr8	12909500	0.851240
chr8	12934000	0.933884
chr8	12951600	0.900826
chr8	12978800	0.950413
chr8	12981200	0.983471
chr8	12984100	0.851240
chr8	12986600	0.925620
chr8	12992100	0.818182
chr8	13008500	0.983471
chr8	13013800	0.892562
chr8	13016800	0.958678
chr8	13026200	0.983471
chr8	13051200	0.950413
chr8	13061300	0.950413
chr8	13066200	0.900826
chr8	13069300	0.661157
chr8	13082500	0.966942
chr8	13089300	0.677686
chr8	13102700	0.958678
chr8	13111000	0.752066
chr8	13115900	1.000000
chr8	13122600	0.900826
chr8	13125000	0.958678
chr8	13127600	0.958678
chr8	13130200	0.801653
chr8	13135400	0.760331
chr8	13139700	0.727273
chr8	13152700	0.727273
chr8	13171100	0.702479
chr8	13194800	0.909091

chr8	13210100	0.900826
chr8	13212200	0.867769
chr8	13215300	0.925620
chr8	13220000	0.884298
chr8	13222300	0.752066
chr8	13224400	0.975207
chr8	13226700	0.867769
chr8	13230600	0.925620
chr8	13241000	0.958678
chr8	13264000	0.884298
chr8	13266400	0.661157
chr8	13272200	0.710744
chr8	13303000	0.892562
chr8	13305300	0.735537
chr8	13311200	0.917355
chr8	13317600	0.983471
chr8	13321400	0.826446
chr8	13369800	0.917355
chr8	13406900	0.851240
chr8	13422600	0.975207
chr8	13453400	0.752066
chr8	13487900	0.793388
chr8	13513700	0.859504
chr8	13515800	0.727273
chr8	15503200	0.975207
chr8	15527000	0.809917
chr8	16395500	0.991736
chr8	16405700	0.900826
chr8	16426200	0.966942
chr8	16436400	0.966942
chr8	16461900	0.942149
chr8	16554000	0.834711
chr8	16570100	0.925620
chr8	16575700	0.933884
chr8	16606300	0.719008
chr8	16611300	0.884298
chr8	16613400	0.983471
chr8	16622200	0.710744
chr8	16684100	0.942149
chr8	16696200	0.975207
chr8	16862700	0.661157
chr8	16949300	0.917355
chr8	17016800	0.917355
chr8	17020500	0.851240
chr8	17025800	0.677686
chr8	17045500	0.867769
chr8	17083800	0.719008
chr8	17111900	0.966942
chr8	17172700	0.983471
chr8	17189900	0.685950
chr8	17205700	0.760331
chr8	17208800	0.983471
chr8	17212500	0.925620
chr8	17324500	0.867769

chr8	17353400	0.760331
chr8	17460300	0.942149
chr8	17463600	0.975207
chr8	17467100	0.958678
chr8	17469500	0.909091
chr8	17471800	0.958678
chr8	17475800	0.966942
chr8	17478000	0.909091
chr8	17506200	0.950413
chr8	17508700	1.000000
chr8	17510800	0.909091
chr8	17514100	0.958678
chr8	17517000	0.834711
chr8	17520300	0.867769
chr8	17522400	0.661157
chr8	17524900	0.966942
chr8	17527500	0.809917
chr8	17529700	0.909091
chr8	17535500	0.933884
chr8	17542200	0.760331
chr8	17544400	0.942149
chr8	17547200	0.776860
chr8	17624900	0.818182
chr8	18439600	0.975207
chr8	18442600	0.809917
chr8	18576200	0.785124
chr8	18579900	0.933884
chr8	18598700	0.842975
chr8	18605600	0.834711
chr8	18672500	0.958678
chr8	18779800	0.826446
chr8	18815400	0.933884
chr8	18835100	0.743802
chr8	18839600	0.958678
chr8	18891900	0.710744
chr8	18925500	0.826446
chr8	18942600	0.933884
chr8	18992900	0.950413
chr8	18998300	0.983471
chr8	19013000	0.884298
chr8	19050800	0.735537
chr8	19058800	0.942149
chr8	19067200	0.661157
chr8	19103000	0.942149
chr8	19125500	0.801653
chr8	19205100	0.752066
chr8	19266600	0.909091
chr8	19302900	0.694215
chr8	19330300	0.876033
chr8	19436500	0.826446
chr8	19459800	0.669421
chr8	19496600	0.809917
chr8	19533800	0.685950
chr8	19536000	0.966942

chr8	19564000	0.876033
chr8	19584400	1.000000
chr8	19977500	0.809917
chr8	20045000	0.975207
chr8	20080500	0.925620
chr8	20126600	0.727273
chr8	20161800	0.876033
chr8	20186900	0.909091
chr8	21051100	0.991736
chr8	21525400	0.842975
chr8	21646600	0.785124
chr8	21776300	0.677686
chr8	21918400	0.851240
chr8	21924200	0.842975
chr8	21996700	0.768595
chr8	22014800	0.818182
chr8	22046300	0.818182
chr8	22068900	0.909091
chr8	22085400	0.942149
chr8	22241500	0.925620
chr8	22254700	0.818182
chr8	22297900	0.661157
chr8	22301800	0.917355
chr8	22342900	0.876033
chr8	22419600	0.892562
chr8	22422500	0.685950
chr8	22437400	0.669421
chr8	22453800	0.760331
chr8	22480200	0.884298
chr8	22486100	0.710744
chr8	22516800	0.867769
chr8	22531100	0.966942
chr8	22562400	0.975207
chr8	22573300	0.859504
chr8	22605700	0.661157
chr8	22722900	0.760331
chr8	22775500	0.933884
chr8	22844700	0.876033
chr8	22878200	0.983471
chr8	22907900	0.975207
chr8	22920800	0.710744
chr8	22932600	0.909091
chr8	22942000	0.925620
chr8	22948200	0.925620
chr8	22950800	0.793388
chr8	22965800	0.801653
chr8	22979100	0.950413
chr8	23022700	0.702479
chr8	23033100	0.966942
chr8	23035700	0.892562
chr8	23038900	0.975207
chr8	23076000	0.950413
chr8	23078800	0.752066
chr8	23080900	0.760331

chr8	23156600	0.793388
chr8	23178100	0.900826
chr8	23180700	0.743802
chr8	23185200	0.776860
chr8	23192700	0.809917
chr8	23195500	0.842975
chr8	23209600	0.752066
chr8	23212800	0.975207
chr8	23215000	0.942149
chr8	23241600	0.702479
chr8	23247600	0.743802
chr8	23251100	0.669421
chr8	23258400	0.900826
chr8	23262800	0.859504
chr8	23269000	0.975207
chr8	23275200	0.983471
chr8	23313100	0.834711
chr8	23365000	0.776860
chr8	23385700	0.661157
chr8	23389600	0.942149
chr8	23391700	0.661157
chr8	23393900	0.859504
chr8	23397100	0.809917
chr8	23400300	0.727273
chr8	23405700	0.900826
chr8	23409100	0.851240
chr8	23419500	0.933884
chr8	23572300	0.859504
chr8	23618600	0.925620
chr8	23621500	0.900826
chr8	23647600	0.752066
chr8	23656200	0.958678
chr8	23719100	0.966942
chr8	23739100	0.900826
chr8	23747200	0.983471
chr8	23750700	0.933884
chr8	23755300	0.776860
chr8	24014000	0.958678
chr8	24076700	0.991736
chr8	24080400	0.727273
chr8	24086900	0.966942
chr8	24207200	0.942149
chr8	24507400	0.966942
chr8	24814100	0.694215
chr8	24931100	0.851240
chr8	25001700	0.768595
chr8	25059200	0.685950
chr8	25159300	0.685950
chr8	25166600	0.719008
chr8	25202800	0.925620
chr8	25276100	0.917355
chr8	25280800	0.966942
chr8	25347300	0.694215
chr8	25901900	0.801653

chr8	26023900	0.785124
chr8	26032300	0.950413
chr8	26062300	1.000000
chr8	26115900	0.991736
chr8	26118000	0.809917
chr8	26122300	0.685950
chr8	26125600	0.685950
chr8	26161300	0.710744
chr8	26175800	0.760331
chr8	26178600	0.958678
chr8	26184800	0.694215
chr8	26209800	0.677686
chr8	26262700	0.702479
chr8	26277300	0.859504
chr8	26298500	0.842975
chr8	26304100	0.876033
chr8	26309600	0.785124
chr8	26313400	0.793388
chr8	26315500	0.991736
chr8	26320000	0.851240
chr8	26324000	0.851240
chr8	26466000	0.834711
chr8	26468600	0.884298
chr8	26472500	0.958678
chr8	26500400	0.942149
chr8	26516600	0.685950
chr8	27139200	0.983471
chr8	27144200	0.925620
chr8	27148400	0.867769
chr8	27184700	0.768595
chr8	27198700	0.685950
chr8	27219700	0.892562
chr8	27247700	0.760331
chr8	27456100	0.975207
chr8	27469100	0.933884
chr8	27475200	0.950413
chr8	27478200	0.793388
chr8	27494400	0.983471
chr8	27524600	0.760331
chr8	27533400	0.851240
chr8	27535800	0.983471
chr8	27541400	0.842975
chr8	27547100	0.818182
chr8	27560100	0.801653
chr8	27577600	0.975207
chr8	27626900	0.983471
chr8	27976200	0.983471
chr8	28257600	0.826446
chr8	28482900	0.991736
chr8	28515800	0.719008
chr8	28562500	0.793388
chr8	28566300	1.000000
chr8	28569400	0.851240
chr8	28571500	0.917355

chr8	28704400	0.785124
chr8	28734200	1.000000
chr8	28799500	0.950413
chr8	28810800	0.727273
chr8	28890700	0.917355
chr8	28894100	0.991736
chr8	28910800	0.669421
chr8	28913600	0.917355
chr8	28930700	0.842975
chr8	28967400	1.000000
chr8	28983500	0.983471
chr8	29004200	0.785124
chr8	29023300	0.685950
chr8	29027600	0.942149
chr8	29083500	0.975207
chr8	29106900	0.991736
chr8	29110000	0.942149
chr8	29147300	0.818182
chr8	29172600	0.677686
chr8	29198600	0.900826
chr8	29230900	0.710744
chr8	29317300	0.900826
chr8	29387600	0.818182
chr8	29393700	0.785124
chr8	29465000	0.966942
chr8	29485200	0.925620
chr8	29492700	0.892562
chr8	29495600	0.884298
chr8	29512100	0.975207
chr8	29514300	0.983471
chr8	29523300	0.909091
chr8	29595500	0.735537
chr8	29604800	0.991736
chr8	29608400	0.966942
chr8	29610800	0.909091
chr8	29615000	0.958678
chr8	29628100	0.966942
chr8	29631000	0.892562
chr8	29686000	0.892562
chr8	29784300	0.818182
chr8	29833700	0.842975
chr8	29852300	0.826446
chr8	29877700	0.785124
chr8	29909600	0.975207
chr8	29917100	0.933884
chr8	29934700	0.983471
chr8	29963400	0.942149
chr8	30007700	0.900826
chr8	30015800	0.694215
chr8	30027200	0.958678
chr8	30087700	0.785124
chr8	30173300	0.727273
chr8	30278800	0.851240
chr8	30281200	0.950413

chr8	30296100	0.933884
chr8	30300400	0.818182
chr8	30311300	0.925620
chr8	30348600	0.677686
chr8	30358100	0.958678
chr8	30360200	0.768595
chr8	30362400	0.950413
chr8	30375800	0.677686
chr8	30389400	0.909091
chr8	30401200	0.809917
chr8	30454500	0.933884
chr8	30462700	0.694215
chr8	30506300	0.884298
chr8	30556900	1.000000
chr8	30586300	0.743802
chr8	30656300	0.909091
chr8	30661000	0.876033
chr8	30663100	0.735537
chr8	30726400	0.776860
chr8	30945200	0.950413
chr8	30964200	0.958678
chr8	31270500	0.661157
chr8	31385400	0.768595
chr8	31613200	0.958678
chr8	32078500	0.677686
chr8	32084600	0.900826
chr8	32129000	0.735537
chr8	32146600	0.966942
chr8	32181300	0.975207
chr8	32185700	0.735537
chr8	32189300	0.884298
chr8	32191500	0.867769
chr8	32194000	0.876033
chr8	32410600	0.809917
chr8	32413900	0.925620
chr8	32421300	0.768595
chr8	32426100	0.818182
chr8	32429600	0.966942
chr8	32435800	0.867769
chr8	32440100	0.950413
chr8	32447100	0.719008
chr8	32488600	0.884298
chr8	32492100	0.933884
chr8	32500500	0.966942
chr8	32515300	0.702479
chr8	32518500	0.760331
chr8	32522300	0.933884
chr8	32559100	0.859504
chr8	32569400	0.975207
chr8	32573100	0.950413
chr8	32596800	0.892562
chr8	32726600	0.760331
chr8	32776500	0.801653
chr8	32783000	0.983471

chr8	32858000	0.917355
chr8	32874500	0.826446
chr8	32962900	0.900826
chr8	33031300	0.900826
chr8	33062800	1.000000
chr8	33071300	1.000000
chr8	33074300	0.950413
chr8	33101600	0.983471
chr8	33185800	0.966942
chr8	33202100	0.793388
chr8	33279600	0.958678
chr8	33412700	0.768595
chr8	33422200	0.669421
chr8	33872300	0.900826
chr8	34695900	1.000000
chr8	34940900	0.752066
chr8	36949400	0.859504
chr8	37003100	0.942149
chr8	37005900	0.958678
chr8	37373300	0.752066
chr8	37379000	0.958678
chr8	37402000	0.892562
chr8	37419800	0.826446
chr8	37549400	0.694215
chr8	37596400	0.710744
chr8	37605500	0.818182
chr8	37656900	0.851240
chr8	37660200	0.876033
chr8	37684300	0.719008
chr8	37701300	0.719008
chr8	37724400	1.000000
chr8	37728000	0.859504
chr8	37744200	0.694215
chr8	37783500	0.826446
chr8	37823600	0.851240
chr8	37894800	0.785124
chr8	37911400	0.768595
chr8	37916500	0.983471
chr8	37939600	0.876033
chr8	37943700	0.950413
chr8	37949400	0.958678
chr8	37986300	0.793388
chr8	38029600	0.702479
chr8	38242300	0.809917
chr8	38259600	0.859504
chr8	38262300	0.851240
chr8	38265300	0.917355
chr8	38271500	0.661157
chr8	38274800	0.677686
chr8	38277000	0.793388
chr8	38287500	0.719008
chr8	38298200	0.884298
chr8	38300600	0.842975
chr8	38303900	0.966942

chr8	38317200	0.710744
chr8	38346300	0.727273
chr8	38368800	0.975207
chr8	38385900	0.942149
chr8	38401600	0.735537
chr8	38410100	0.710744
chr8	38559400	0.834711
chr8	38586400	0.793388
chr8	38617800	1.000000
chr8	38623400	0.859504
chr8	38625500	0.958678
chr8	38636600	0.727273
chr8	38650000	0.876033
chr8	38652800	0.983471
chr8	38661100	0.900826
chr8	38665400	0.958678
chr8	38686900	0.776860
chr8	38708500	0.702479
chr8	38795300	1.000000
chr8	38858600	0.727273
chr8	38862600	1.000000
chr8	38874700	0.900826
chr8	38882100	0.834711
chr8	38885500	0.950413
chr8	38891600	0.917355
chr8	38894200	0.925620
chr8	38896400	0.900826
chr8	38900200	1.000000
chr8	39019000	0.876033
chr8	39711000	0.669421
chr8	39730200	0.809917
chr8	39771100	0.710744
chr8	39782000	0.809917
chr8	39821400	0.876033
chr8	39823800	0.785124
chr8	39917500	0.966942
chr8	39931000	0.760331
chr8	39959400	0.917355
chr8	39966900	0.884298
chr8	39977000	0.958678
chr8	39982200	0.991736
chr8	40016800	0.966942
chr8	40022800	0.917355
chr8	40029500	0.909091
chr8	40032800	0.933884
chr8	40049500	0.793388
chr8	40056900	0.909091
chr8	40068500	0.677686
chr8	40207700	0.884298
chr8	40602800	0.966942
chr8	40605500	0.752066
chr8	40626200	0.933884
chr8	41010000	0.933884
chr8	41019500	0.793388

chr8	41059400	0.983471
chr8	41219100	0.669421
chr8	41360000	0.851240
chr8	41366300	0.909091
chr8	41393700	0.851240
chr8	41399500	0.892562
chr8	41521500	0.776860
chr8	41542800	0.983471
chr8	41570700	0.876033
chr8	41593300	0.867769
chr8	41646300	0.760331
chr8	41650200	0.892562
chr8	41655800	0.950413
chr8	41670800	0.909091
chr8	41834100	0.991736
chr8	41849300	0.892562
chr8	41863100	0.991736
chr8	41906900	0.719008
chr8	41981600	0.925620
chr8	41984100	0.933884
chr8	42000200	0.909091
chr8	42012900	0.975207
chr8	42037800	0.867769
chr8	42043700	0.685950
chr8	42051800	0.892562
chr8	42055900	1.000000
chr8	42067100	0.950413
chr8	42072900	0.884298
chr8	42103500	0.818182
chr8	42110500	0.859504
chr8	42114400	0.768595
chr8	42123900	0.975207
chr8	42128000	0.876033
chr8	42135600	0.917355
chr8	42154800	0.776860
chr8	42158100	0.909091
chr8	42270700	0.933884
chr8	42326500	0.785124
chr8	42329400	0.809917
chr8	42342100	0.900826
chr8	42350400	0.933884
chr8	42355100	0.661157
chr8	42357300	0.727273
chr8	42373900	0.925620
chr8	42387900	0.983471
chr8	42390700	0.991736
chr8	42393700	0.859504
chr8	42408000	0.661157
chr8	42410700	0.809917
chr8	42474500	0.942149
chr8	42553300	0.991736
chr8	42574900	0.760331
chr8	42577000	0.958678
chr8	42592400	0.719008

chr8	42624000	0.719008
chr8	42660900	0.752066
chr8	42712300	0.983471
chr8	42746500	0.785124
chr8	42749300	0.867769
chr8	42772600	0.702479
chr8	42775900	0.925620
chr8	42820800	0.834711
chr8	42843300	0.958678
chr8	43046600	1.000000
chr8	43794500	0.818182
chr8	43829500	0.661157
chr8	46852300	0.760331
chr8	48266400	0.966942
chr8	48393000	0.719008
chr8	48424900	0.735537
chr8	48427000	0.942149
chr8	48437500	0.909091
chr8	48460800	0.851240
chr8	48513500	0.851240
chr8	48523200	0.710744
chr8	48572600	0.867769
chr8	48589000	0.991736
chr8	48624700	0.942149
chr8	48647800	0.710744
chr8	48676300	0.785124
chr8	48772700	0.735537
chr8	49193100	0.991736
chr8	49195400	0.892562
chr8	49232000	0.867769
chr8	49282900	0.851240
chr8	49320000	0.801653
chr8	49323100	0.975207
chr8	49332300	0.958678
chr8	49341200	0.933884
chr8	49345500	0.669421
chr8	49383700	0.925620
chr8	49469000	0.933884
chr8	49503200	0.719008
chr8	49537700	0.669421
chr8	49540400	0.900826
chr8	49592300	0.842975
chr8	49648100	0.876033
chr8	49701500	0.735537
chr8	49824200	0.793388
chr8	49978400	0.768595
chr8	50969400	0.950413
chr8	51529800	0.983471
chr8	51543800	1.000000
chr8	51553100	0.958678
chr8	51556300	0.991736
chr8	52757300	0.909091
chr8	52797100	0.950413
chr8	52802800	0.685950

chr8	52807900	0.669421
chr8	52813500	0.677686
chr8	52843000	0.975207
chr8	52852000	0.719008
chr8	52875200	0.851240
chr8	53379800	0.884298
chr8	53478100	0.752066
chr8	53607900	0.818182
chr8	53653100	0.900826
chr8	53656300	0.900826
chr8	53677200	0.884298
chr8	54436500	0.768595
chr8	54493100	0.958678
chr8	54531600	0.950413
chr8	54571100	0.867769
chr8	54605800	0.859504
chr8	54640600	0.991736
chr8	54645600	0.909091
chr8	54790300	0.752066
chr8	54797700	0.950413
chr8	54818200	0.867769
chr8	54857900	0.966942
chr8	54983900	0.710744
chr8	55052500	0.933884
chr8	55056200	0.909091
chr8	55062800	0.851240
chr8	55066000	0.975207
chr8	55068200	0.752066
chr8	55078500	0.884298
chr8	55083000	0.752066
chr8	55091500	0.966942
chr8	55121900	0.793388
chr8	55125900	0.909091
chr8	55157800	0.876033
chr8	55181800	0.950413
chr8	55223300	0.942149
chr8	55238400	0.818182
chr8	55247700	0.719008
chr8	55250200	0.983471
chr8	55252400	0.851240
chr8	55294200	0.801653
chr8	55296400	0.917355
chr8	55307300	0.966942
chr8	55325900	0.876033
chr8	55379600	0.768595
chr8	55383100	0.669421
chr8	55389600	0.702479
chr8	55437800	0.876033
chr8	55467300	0.975207
chr8	56757700	0.685950
chr8	56766000	0.743802
chr8	56777400	0.933884
chr8	56779500	0.950413
chr8	56814300	0.851240

chr8	56851200	0.677686
chr8	56864600	0.661157
chr8	56873600	0.983471
chr8	56911400	0.694215
chr8	57070200	0.842975
chr8	57086600	0.958678
chr8	57133400	0.661157
chr8	57631500	0.892562
chr8	57635300	0.917355
chr8	57871900	0.975207
chr8	57886300	0.776860
chr8	57912600	0.966942
chr8	57922900	0.776860
chr8	58136200	0.925620
chr8	58138600	0.983471
chr8	58943700	0.677686
chr8	59468700	0.694215
chr8	59509000	0.859504
chr8	59555300	0.892562
chr8	59636500	0.892562
chr8	59682200	0.661157
chr8	59714400	0.958678
chr8	59730300	0.826446
chr8	59740300	0.851240
chr8	59742900	0.958678
chr8	59746800	0.909091
chr8	59755000	0.900826
chr8	59758400	0.958678
chr8	59763100	0.719008
chr8	59768400	0.743802
chr8	59771500	0.851240
chr8	59778100	0.809917
chr8	59789700	0.950413
chr8	59792700	0.743802
chr8	59846900	0.768595
chr8	59858800	0.975207
chr8	59951700	0.826446
chr8	59967400	0.859504
chr8	60019500	0.760331
chr8	60028700	0.809917
chr8	60176700	0.677686
chr8	60191100	0.909091
chr8	60220500	0.694215
chr8	60530300	0.702479
chr8	60538400	0.975207
chr8	60544100	0.801653
chr8	60579500	0.867769
chr8	60956000	0.933884
chr8	61151100	0.975207
chr8	61261400	0.727273
chr8	61368300	0.867769
chr8	61621900	0.933884
chr8	61705700	0.785124
chr8	61728600	0.958678

chr8	61740800	0.975207
chr8	61764600	0.950413
chr8	61796700	0.867769
chr8	61816500	0.983471
chr8	61822100	0.859504
chr8	61862200	0.975207
chr8	61917300	0.859504
chr8	61926200	0.694215
chr8	61936600	0.785124
chr8	61949000	0.925620
chr8	61989200	0.669421
chr8	62008700	0.760331
chr8	62449400	0.834711
chr8	62504300	0.925620
chr8	62597500	0.991736
chr8	62613200	0.785124
chr8	62631500	0.719008
chr8	62672300	0.950413
chr8	63937800	0.933884
chr8	63998700	0.851240
chr8	64107500	0.933884
chr8	64110100	0.760331
chr8	64133100	0.975207
chr8	64139300	0.917355
chr8	64145000	0.925620
chr8	66630000	0.991736
chr8	67036800	0.884298
chr8	67089700	0.834711
chr8	67334200	0.966942
chr8	67384000	0.983471
chr8	67403200	0.983471
chr8	67407100	0.760331
chr8	67419900	0.793388
chr8	67436100	0.942149
chr8	67445700	0.942149
chr8	67448400	0.975207
chr8	67460000	0.752066
chr8	67468400	0.818182
chr8	67473100	0.966942
chr8	67592200	0.719008
chr8	68013400	0.925620
chr8	68147600	0.851240
chr8	68515500	0.867769
chr8	68770600	0.900826
chr8	68824100	0.669421
chr8	68829000	1.000000
chr8	68854800	0.892562
chr8	68872800	0.925620
chr8	68877200	0.991736
chr8	68881300	0.966942
chr8	68887100	0.867769
chr8	68891300	0.991736
chr8	68893600	0.727273
chr8	68897200	0.842975

chr8	68907000	0.975207
chr8	68920000	0.925620
chr8	68922100	0.933884
chr8	68924800	0.851240
chr8	68927500	0.975207
chr8	68942500	0.991736
chr8	69333700	0.661157
chr8	69643900	0.909091
chr8	69647000	0.719008
chr8	69672500	0.859504
chr8	69901600	0.909091
chr8	70310300	0.809917
chr8	70351700	0.900826
chr8	70449400	0.694215
chr8	70495200	0.793388
chr8	70501100	0.710744
chr8	70602200	0.975207
chr8	71054100	0.991736
chr8	71056300	0.826446
chr8	71065200	0.785124
chr8	72744300	0.909091
chr8	73550000	0.661157
chr8	73607100	0.669421
chr8	73609200	0.975207
chr8	73862400	0.735537
chr8	74209500	0.785124
chr8	74253600	0.876033
chr8	74283600	0.933884
chr8	74333700	0.710744
chr8	74385400	0.694215
chr8	74396500	0.958678
chr8	74431100	0.826446
chr8	74434100	0.876033
chr8	74449900	0.785124
chr8	74473600	0.851240
chr8	74590200	0.966942
chr8	74706000	0.760331
chr8	74712200	0.685950
chr8	74782800	0.685950
chr8	74847500	0.975207
chr8	74904100	0.842975
chr8	74960900	0.859504
chr8	75179600	0.925620
chr8	75466500	0.975207
chr8	75471000	0.677686
chr8	75494400	0.900826
chr8	75674200	0.851240
chr8	77586400	0.685950
chr8	77867400	0.950413
chr8	78031300	0.966942
chr8	78207400	0.975207
chr8	78270200	0.851240
chr8	79120400	0.743802
chr8	79431700	0.694215

chr8	79436700	0.851240
chr8	79439400	0.917355
chr8	79469100	0.950413
chr8	80239100	0.966942
chr8	80246400	0.991736
chr8	80261400	0.735537
chr8	80268700	0.727273
chr8	80578700	0.785124
chr8	80682000	0.669421
chr8	80706100	0.909091
chr8	80708200	0.867769
chr8	80803500	0.768595
chr8	80820200	0.776860
chr8	80856800	0.851240
chr8	80873200	0.876033
chr8	80876200	0.925620
chr8	80933000	0.677686
chr8	80939800	0.983471
chr8	81086600	0.677686
chr8	81159700	0.917355
chr8	81170200	0.975207
chr8	81416700	0.685950
chr8	81446800	0.661157
chr8	81748300	0.776860
chr8	81758100	0.752066
chr8	81787300	0.702479
chr8	81806600	0.867769
chr8	81828500	0.776860
chr8	81831400	0.958678
chr8	81838900	0.917355
chr8	81853700	0.834711
chr8	81877800	0.991736
chr8	81884900	0.975207
chr8	81892400	1.000000
chr8	81904300	0.884298
chr8	81960400	0.752066
chr8	82139700	0.884298
chr8	82146900	0.818182
chr8	82259300	0.991736
chr8	82262700	0.900826
chr8	82274900	1.000000
chr8	82277400	0.942149
chr8	82279500	0.851240
chr8	82335100	0.909091
chr8	82452400	0.942149
chr8	82645100	0.694215
chr8	82693700	0.925620
chr8	82696000	0.694215
chr8	82726500	0.669421
chr8	84046900	0.975207
chr8	84055900	0.958678
chr8	87121400	0.859504
chr8	87296100	0.867769
chr8	87332900	0.909091

chr8	87380600	0.892562
chr8	87427000	0.694215
chr8	87535500	0.975207
chr8	87678000	0.710744
chr8	87737400	0.818182
chr8	87757700	0.768595
chr8	87781300	0.694215
chr8	88708200	0.685950
chr8	88789800	0.685950
chr8	88845000	0.876033
chr8	88951400	0.958678
chr8	89013400	0.942149
chr8	89018800	0.933884
chr8	89127200	0.793388
chr8	89142300	0.661157
chr8	89154400	0.785124
chr8	89190200	0.991736
chr8	89282600	0.719008
chr8	89289000	0.669421
chr8	89310500	0.859504
chr8	89336900	0.661157
chr8	90104300	0.950413
chr8	90271200	0.702479
chr8	90326100	0.950413
chr8	90334700	0.793388
chr8	90559200	0.685950
chr8	90576100	0.809917
chr8	90624500	0.909091
chr8	90717800	0.719008
chr8	90742300	0.966942
chr8	90772700	0.909091
chr8	90817600	0.958678
chr8	90848000	0.983471
chr8	90850200	0.743802
chr8	90861100	0.884298
chr8	90895200	0.933884
chr8	90936900	0.842975
chr8	90962000	0.917355
chr8	90987400	0.975207
chr8	90994100	0.735537
chr8	91330700	0.727273
chr8	91542800	0.669421
chr8	91592600	0.909091
chr8	91659700	0.801653
chr8	91683500	0.876033
chr8	92087800	0.867769
chr8	92244200	0.727273
chr8	92331800	0.925620
chr8	92378600	0.909091
chr8	93021000	0.991736
chr8	93050800	0.669421
chr8	93055300	0.884298
chr8	93061700	0.942149
chr8	93082500	0.851240

chr8	93085300	0.958678
chr8	93097800	0.842975
chr8	93109300	0.685950
chr8	93116700	0.702479
chr8	93385100	0.942149
chr8	93461900	0.917355
chr8	93468000	0.884298
chr8	93495900	0.983471
chr8	93503400	0.917355
chr8	93521900	0.933884
chr8	93528200	0.727273
chr8	93537200	0.983471
chr8	93681000	0.991736
chr8	93795200	0.925620
chr8	93844000	0.661157
chr8	94081000	0.785124
chr8	94083800	0.776860
chr8	94750000	0.760331
chr8	94850600	0.966942
chr8	94858900	0.991736
chr8	94886400	0.884298
chr8	94893300	0.925620
chr8	94898000	0.958678
chr8	94917400	1.000000
chr8	94928300	0.834711
chr8	94945200	0.752066
chr8	94948400	0.991736
chr8	94956700	0.842975
chr8	94968300	0.983471
chr8	94971900	0.983471
chr8	95155200	0.735537
chr8	95168900	0.719008
chr8	95237100	0.900826
chr8	95246800	0.793388
chr8	95254800	0.983471
chr8	95271600	0.975207
chr8	95430300	1.000000
chr8	95449200	0.966942
chr8	95480400	0.933884
chr8	95652400	0.752066
chr8	95875300	0.975207
chr8	95980600	0.727273
chr8	96003500	0.942149
chr8	96064500	0.942149
chr8	96085800	0.735537
chr8	96102400	0.677686
chr8	96112800	0.909091
chr8	96330200	0.661157
chr8	96420600	0.958678
chr8	96454800	0.867769
chr8	96482800	0.785124
chr8	96572300	0.900826
chr8	96575700	0.851240
chr8	96681600	0.818182

chr8	96693900	0.991736
chr8	96705300	0.958678
chr8	96707600	0.950413
chr8	96715100	0.991736
chr8	96806300	0.933884
chr8	96814400	0.983471
chr8	97116900	0.851240
chr8	97145600	0.925620
chr8	97158100	0.851240
chr8	97288800	0.727273
chr8	97325400	0.950413
chr8	97336400	0.991736
chr8	97347100	0.991736
chr8	97399500	0.958678
chr8	97414000	0.983471
chr8	97417600	0.694215
chr8	97744300	0.785124
chr8	97937100	0.752066
chr8	98001900	0.958678
chr8	98610900	0.719008
chr8	98776000	0.793388
chr8	99014500	0.842975
chr8	99046000	0.851240
chr8	99159600	0.867769
chr8	99182800	0.752066
chr8	99258300	0.942149
chr8	99371300	0.876033
chr8	99545300	0.900826
chr8	99687300	0.867769
chr8	99773100	0.677686
chr8	99957100	0.768595
chr8	100431900	0.694215
chr8	100508100	0.892562
chr8	100512000	0.933884
chr8	100631300	0.950413
chr8	100718500	0.793388
chr8	101271000	1.000000
chr8	101282400	0.933884
chr8	101336700	0.975207
chr8	101388600	0.991736
chr8	101394900	0.950413
chr8	101503300	0.677686
chr8	101522400	0.884298
chr8	101555200	0.842975
chr8	101636200	0.942149
chr8	101802400	0.991736
chr8	101822300	0.975207
chr8	101860000	0.809917
chr8	101913000	0.884298
chr8	101919100	0.991736
chr8	101951300	0.661157
chr8	101960600	0.892562
chr8	102039000	0.826446
chr8	102064300	0.892562

chr8	102121400	0.884298
chr8	102149700	0.958678
chr8	102168100	0.942149
chr8	102171500	0.983471
chr8	102188800	0.719008
chr8	102323600	0.809917
chr8	102345500	0.909091
chr8	102369000	0.892562
chr8	102387200	0.834711
chr8	102389500	0.917355
chr8	102405600	0.892562
chr8	102465000	0.851240
chr8	102472500	0.793388
chr8	102620400	1.000000
chr8	102639400	0.975207
chr8	103248900	0.710744
chr8	103380700	0.975207
chr8	103416100	0.743802
chr8	103549100	0.884298
chr8	103613400	0.760331
chr8	103664900	0.727273
chr8	103671300	0.785124
chr8	103675000	0.685950
chr8	103700900	0.727273
chr8	103705200	0.884298
chr8	103742200	0.950413
chr8	103802700	0.933884
chr8	103817200	0.719008
chr8	103823200	0.983471
chr8	103826200	0.694215
chr8	103918900	0.851240
chr8	103922500	1.000000
chr8	103950100	0.677686
chr8	104082500	0.958678
chr8	104088900	0.900826
chr8	104091400	0.983471
chr8	104164700	0.991736
chr8	104197300	0.859504
chr8	104220700	0.851240
chr8	104242000	1.000000
chr8	104268300	0.983471
chr8	104281500	0.677686
chr8	104357500	0.859504
chr8	104361100	0.975207
chr8	104410100	0.917355
chr8	104532500	0.727273
chr8	104752900	0.785124
chr8	105236100	0.710744
chr8	105441100	0.983471
chr8	105655800	0.694215
chr8	105759300	0.950413
chr8	105801100	0.834711
chr8	105962400	0.776860
chr8	105987800	0.677686

chr8	106004400	0.669421
chr8	106082500	0.867769
chr8	106186100	0.950413
chr8	106221400	0.909091
chr8	106290700	0.950413
chr8	106423900	0.735537
chr8	106438400	0.752066
chr8	106441100	0.917355
chr8	106460300	0.909091
chr8	106463200	0.917355
chr8	106486600	0.958678
chr8	106511800	1.000000
chr8	106529800	0.966942
chr8	106581800	0.900826
chr8	106636900	0.983471
chr8	106643600	0.983471
chr8	106647200	0.727273
chr8	106658600	0.710744
chr8	106672200	0.966942
chr8	106729100	0.933884
chr8	106743500	0.859504
chr8	106760100	0.768595
chr8	107024100	0.818182
chr8	107058300	0.942149
chr8	107072500	0.867769
chr8	107093100	0.975207
chr8	107095900	0.950413
chr8	107221100	0.991736
chr8	107231800	0.743802
chr8	107250700	0.983471
chr8	107312800	0.760331
chr8	107405600	0.942149
chr8	107410900	0.867769
chr8	107447200	0.933884
chr8	107468500	0.983471
chr8	107474300	0.727273
chr8	107610500	0.669421
chr8	107785400	0.710744
chr8	108208500	0.694215
chr8	108344500	0.942149
chr8	108498000	1.000000
chr8	108511400	0.826446
chr8	108518400	0.743802
chr8	108545900	0.752066
chr8	108716400	0.933884
chr8	109095300	0.859504
chr8	109481000	0.900826
chr8	109487900	0.900826
chr8	109524500	0.950413
chr8	109575600	0.958678
chr8	109664200	0.900826
chr8	109961500	0.917355
chr8	110014100	0.685950
chr8	110038100	0.752066

chr8	110054800	1.000000
chr8	110274700	0.975207
chr8	110305000	1.000000
chr8	110343400	0.768595
chr8	110348900	0.694215
chr8	110375200	0.727273
chr8	110381000	0.727273
chr8	110448100	0.958678
chr8	110583800	0.909091
chr8	110589300	0.925620
chr8	110593500	0.661157
chr8	110602000	0.661157
chr8	110619400	0.917355
chr8	111521600	0.909091
chr8	111860900	0.950413
chr8	111907200	0.933884
chr8	111926700	0.702479
chr8	114114900	1.000000
chr8	114118100	0.917355
chr8	114753300	0.909091
chr8	115525000	0.958678
chr8	115552200	0.719008
chr8	116251700	0.661157
chr8	116448200	0.685950
chr8	116461600	0.702479
chr8	116464400	0.991736
chr8	116470500	0.876033
chr8	116589000	0.975207
chr8	116847600	0.743802
chr8	116963300	0.867769
chr8	116982000	0.900826
chr8	116990900	0.975207
chr8	117004300	0.677686
chr8	117049900	0.966942
chr8	117133200	0.776860
chr8	117457200	0.842975
chr8	117671000	1.000000
chr8	117699100	0.933884
chr8	117883800	0.801653
chr8	118412000	0.793388
chr8	118429000	0.793388
chr8	118615300	0.950413
chr8	118715200	1.000000
chr8	118719300	0.975207
chr8	118732000	0.867769
chr8	118744000	0.975207
chr8	118844300	0.851240
chr8	118911300	0.685950
chr8	118933700	0.983471
chr8	118950200	0.975207
chr8	118953400	0.975207
chr8	118968000	0.917355
chr8	118970300	0.983471
chr8	118990800	0.983471

chr8	118994100	0.884298
chr8	119016400	0.776860
chr8	119021600	0.702479
chr8	119024900	0.685950
chr8	119031400	0.925620
chr8	119038200	0.975207
chr8	119069000	0.925620
chr8	119075200	0.983471
chr8	119078000	0.669421
chr8	119087200	0.909091
chr8	119091500	0.975207
chr8	119098300	0.859504
chr8	119101900	0.917355
chr8	119110000	0.884298
chr8	119112200	0.942149
chr8	119119800	0.809917
chr8	119125600	0.719008
chr8	119152500	0.677686
chr8	119208400	0.892562
chr8	119214800	0.950413
chr8	119270700	0.834711
chr8	119345500	0.958678
chr8	119355700	0.818182
chr8	119359600	0.909091
chr8	119395100	0.876033
chr8	119397800	0.925620
chr8	119451200	0.892562
chr8	119453700	0.917355
chr8	119457000	0.983471
chr8	119504500	0.950413
chr8	119669700	0.975207
chr8	119891000	0.851240
chr8	119894000	0.694215
chr8	119995200	0.669421
chr8	120055700	0.983471
chr8	120058700	0.760331
chr8	120129200	0.752066
chr8	120145600	0.677686
chr8	120221000	0.710744
chr8	120711000	0.735537
chr8	120734300	0.958678
chr8	120883400	0.669421
chr8	121003200	0.826446
chr8	121256000	0.809917
chr8	121270900	0.942149
chr8	121436100	0.876033
chr8	121666900	0.942149
chr8	121669100	0.826446
chr8	121719100	0.975207
chr8	121722800	0.900826
chr8	121755700	0.727273
chr8	121790500	0.768595
chr8	121793000	0.818182
chr8	121870200	0.975207

chr8	121873900	0.661157
chr8	121945500	0.909091
chr8	122274300	0.801653
chr8	122331000	0.983471
chr8	122335500	0.958678
chr8	122351500	0.884298
chr8	122515000	0.942149
chr8	122526100	0.685950
chr8	122573200	0.975207
chr8	122612700	1.000000
chr8	122681900	0.768595
chr8	122709000	0.842975
chr8	122754900	0.900826
chr8	122824200	0.942149
chr8	122835100	0.991736
chr8	122909100	0.958678
chr8	122931700	0.983471
chr8	122954700	0.834711
chr8	123588300	0.884298
chr8	123614500	0.933884
chr8	123616800	0.925620
chr8	123618900	0.892562
chr8	124264300	0.917355
chr8	124473500	0.760331
chr8	124475800	0.950413
chr8	124479300	0.983471
chr8	124483700	0.867769
chr8	124486500	0.975207
chr8	124489000	0.892562
chr8	124491100	0.752066
chr8	124502900	0.966942
chr8	124529600	0.909091
chr8	124550300	0.983471
chr8	124683900	0.809917
chr8	124705900	0.925620
chr8	124721000	0.793388
chr8	124773500	0.768595
chr8	124790000	0.884298
chr8	124899400	0.909091
chr8	124934700	0.958678
chr8	125194500	0.801653
chr8	125210100	0.735537
chr8	125213000	0.917355
chr8	125215200	0.966942
chr8	125222100	0.966942
chr8	125225900	0.801653
chr8	125249700	0.975207
chr8	125259800	0.917355
chr8	125264200	0.900826
chr8	125267500	0.991736
chr8	125275100	0.900826
chr8	125279700	0.950413
chr8	125283900	0.942149
chr8	125333800	0.760331

chr8	125376900	0.909091
chr8	125438200	0.661157
chr8	125577400	0.966942
chr8	125606900	0.991736
chr8	125613500	0.752066
chr8	125617000	1.000000
chr8	125633900	0.685950
chr8	125650900	0.975207
chr8	125662300	0.950413
chr8	125700500	0.942149
chr8	125755800	0.826446
chr8	125763900	0.991736
chr8	125785000	0.842975
chr8	125843200	0.966942
chr8	125860800	1.000000
chr8	125879700	0.958678
chr8	125908600	0.743802
chr8	126009900	0.685950
chr8	126123200	0.900826
chr8	126131800	0.917355
chr8	126141200	0.818182
chr8	126143300	0.925620
chr8	126181300	0.892562
chr8	126186000	0.884298
chr8	126188300	0.917355
chr8	126204300	0.933884
chr8	126226400	0.950413
chr8	126232000	1.000000
chr8	126275500	0.727273
chr8	126284100	0.702479
chr8	126290000	0.876033
chr8	126293000	0.909091
chr8	126301800	0.983471
chr8	126323600	0.876033
chr8	126354900	0.892562
chr8	126373400	0.876033
chr8	126375600	0.876033
chr8	126384000	0.917355
chr8	126398700	0.702479
chr8	126401100	0.900826
chr8	126404900	0.884298
chr8	126417500	0.950413
chr8	126423100	0.950413
chr8	126438400	0.842975
chr8	126441100	0.752066
chr8	126446900	0.834711
chr8	126511400	0.801653
chr8	126522200	0.925620
chr8	126525800	0.818182
chr8	126649900	0.950413
chr8	126652200	0.842975
chr8	126656900	0.867769
chr8	126660900	0.826446
chr8	126671700	0.685950

chr8	126677000	0.702479
chr8	126843900	0.975207
chr8	126938100	0.991736
chr8	126981700	0.950413
chr8	127388800	0.933884
chr8	127447400	0.975207
chr8	127464000	1.000000
chr8	127466800	0.900826
chr8	127484000	0.983471
chr8	127509500	0.801653
chr8	127511600	0.826446
chr8	127514300	0.826446
chr8	127565500	0.785124
chr8	127576800	0.785124
chr8	127837300	0.909091
chr8	127840300	0.859504
chr8	127846400	0.950413
chr8	127910400	0.859504
chr8	127939400	0.991736
chr8	127947100	0.776860
chr8	127952400	0.842975
chr8	128004100	0.983471
chr8	128011200	0.933884
chr8	128022000	0.826446
chr8	128047900	0.685950
chr8	128118500	0.768595
chr8	128171700	0.752066
chr8	128174700	0.983471
chr8	128218600	0.991736
chr8	128221200	0.809917
chr8	128223700	0.950413
chr8	128226400	0.950413
chr8	128228800	0.884298
chr8	128235500	0.975207
chr8	128238400	0.876033
chr8	128306200	0.834711
chr8	128308300	0.859504
chr8	128343200	0.842975
chr8	128511500	0.702479
chr8	128519600	0.752066
chr8	128522000	0.867769
chr8	128589400	0.752066
chr8	128598700	0.876033
chr8	128753800	0.735537
chr8	128755900	0.851240
chr8	128811100	0.702479
chr8	128813900	1.000000
chr8	128819200	0.900826
chr8	128822300	0.966942
chr8	128825000	0.917355
chr8	128844400	0.925620
chr8	128852800	0.892562
chr8	128857200	0.859504
chr8	128860400	0.669421

chr8	128877100	0.983471
chr8	128880200	0.669421
chr8	128888300	0.859504
chr8	128902300	0.669421
chr8	128912400	0.983471
chr8	128917900	0.702479
chr8	128920500	0.975207
chr8	128924000	0.809917
chr8	128930100	0.859504
chr8	128932300	0.917355
chr8	128936900	0.785124
chr8	128941900	0.950413
chr8	128945400	0.950413
chr8	128949400	0.752066
chr8	128961900	0.834711
chr8	128964100	0.702479
chr8	128979500	0.785124
chr8	128982000	0.975207
chr8	128984200	0.867769
chr8	129030900	0.752066
chr8	129033400	0.909091
chr8	129047600	0.809917
chr8	129059200	0.892562
chr8	129066000	0.917355
chr8	129071300	0.834711
chr8	129077600	0.818182
chr8	129114900	0.867769
chr8	129121700	0.859504
chr8	129140500	0.983471
chr8	129181000	0.900826
chr8	129184800	0.917355
chr8	129188000	0.900826
chr8	129191100	0.933884
chr8	129195600	0.950413
chr8	129197900	0.950413
chr8	129239600	0.950413
chr8	129326900	0.983471
chr8	129335100	0.801653
chr8	129342500	0.892562
chr8	129603300	0.793388
chr8	129661800	0.710744
chr8	129678700	0.991736
chr8	129694900	0.669421
chr8	129800300	0.876033
chr8	129806800	0.909091
chr8	129874300	0.975207
chr8	130173600	0.933884
chr8	130192400	0.719008
chr8	130215000	0.785124
chr8	130648500	0.917355
chr8	130697300	0.892562
chr8	130699500	0.727273
chr8	130719000	0.785124
chr8	130722800	0.909091

chr8	130799500	0.950413
chr8	130861300	0.958678
chr8	130887700	0.917355
chr8	130895400	0.785124
chr8	130898400	0.942149
chr8	130984400	0.983471
chr8	130996400	0.942149
chr8	131009100	0.842975
chr8	131012500	0.950413
chr8	131074600	0.909091
chr8	131108100	0.884298
chr8	131111300	0.925620
chr8	131118400	0.933884
chr8	131138200	0.975207
chr8	131147500	0.867769
chr8	131155100	0.884298
chr8	131207200	0.983471
chr8	131213600	0.702479
chr8	131233300	0.826446
chr8	131237100	0.867769
chr8	131249600	0.892562
chr8	131257600	0.760331
chr8	131271800	0.983471
chr8	131275000	0.933884
chr8	131278700	0.917355
chr8	131281900	0.834711
chr8	131292000	0.983471
chr8	131319900	0.661157
chr8	131330300	0.842975
chr8	131336700	0.876033
chr8	131342700	0.710744
chr8	131359300	0.925620
chr8	131368700	0.768595
chr8	131420300	1.000000
chr8	131427400	0.826446
chr8	131432200	0.876033
chr8	131443400	0.776860
chr8	131450000	0.958678
chr8	131453300	0.727273
chr8	131458100	0.925620
chr8	131464700	0.933884
chr8	131490700	1.000000
chr8	131496000	0.876033
chr8	131504000	0.884298
chr8	131516100	0.809917
chr8	131527000	0.685950
chr8	131534100	0.743802
chr8	131537500	0.884298
chr8	131541200	0.950413
chr8	131617000	0.975207
chr8	131654400	0.876033
chr8	131662300	0.983471
chr8	131687600	0.752066
chr8	131740100	0.942149

chr8	131915700	0.950413
chr8	131917900	0.958678
chr8	131920000	0.818182
chr8	132870000	0.917355
chr8	132919200	0.925620
chr8	132929400	0.876033
chr8	132944900	0.719008
chr8	133028600	0.983471
chr8	133724600	1.000000
chr8	133772700	0.950413
chr8	134045900	0.900826
chr8	134230300	1.000000
chr8	134250700	0.950413
chr8	134262000	0.851240
chr8	134311400	0.719008
chr8	134315700	0.917355
chr8	134369200	0.842975
chr8	134403000	0.933884
chr8	134469200	0.983471
chr8	134499000	0.719008
chr8	134511600	0.958678
chr8	134514000	0.950413
chr8	134517000	0.677686
chr8	134533100	0.900826
chr8	134539100	0.892562
chr8	134546300	0.867769
chr8	134549900	0.727273
chr8	134553000	0.867769
chr8	134558700	1.000000
chr8	134569900	0.851240
chr8	134580600	0.851240
chr8	134588100	0.966942
chr8	134596200	0.958678
chr8	134657400	0.958678
chr8	134663800	0.942149
chr8	134666900	0.975207
chr8	134669500	0.859504
chr8	134735300	0.966942
chr8	134737800	0.892562
chr8	134742600	0.909091
chr8	134863800	0.950413
chr8	134931600	0.884298
chr8	135469900	0.685950
chr8	135593700	0.818182
chr8	135697800	0.917355
chr8	135905400	0.958678
chr8	136479200	0.933884
chr8	136556100	0.958678
chr8	136593400	0.842975
chr8	136596900	0.884298
chr8	136612100	0.719008
chr8	136641100	0.925620
chr8	138161600	0.975207
chr8	138199600	0.991736

chr8	138203800	0.685950
chr8	138313700	0.884298
chr8	138330700	0.859504
chr8	140845900	0.842975
chr8	141100900	0.735537
chr8	141109200	0.669421
chr8	141125200	0.900826
chr8	141129100	0.859504
chr8	141359800	0.842975
chr8	141423000	0.719008
chr8	141490300	0.661157
chr8	141518400	0.702479
chr8	141553700	0.859504
chr8	141555900	0.876033
chr8	141598700	0.801653
chr8	141602200	0.834711
chr8	141605300	0.917355
chr8	141609100	0.950413
chr8	141619700	0.801653
chr8	141629400	0.702479
chr8	141647300	0.983471
chr8	141656100	0.975207
chr8	141743500	0.991736
chr8	141800000	0.768595
chr8	141803400	0.909091
chr8	141807400	0.966942
chr8	141828300	0.876033
chr8	141845200	0.867769
chr8	141849800	0.942149
chr8	141864600	0.942149
chr8	141929400	0.809917
chr8	141954900	0.900826
chr8	141957700	0.735537
chr8	141962600	0.925620
chr8	141965500	0.884298
chr8	141968000	0.950413
chr8	141975000	0.669421
chr8	142008300	0.669421
chr8	142013900	0.876033
chr8	142032100	0.867769
chr8	142038100	0.925620
chr8	142045200	0.710744
chr8	142069800	0.809917
chr8	142083600	0.818182
chr8	142086000	0.925620
chr8	142090100	0.834711
chr8	142094800	0.785124
chr8	142127500	0.876033
chr8	142130300	0.826446
chr8	142137300	0.801653
chr8	142142200	0.768595
chr8	142158400	0.851240
chr8	142163800	0.958678
chr8	142167200	0.966942

chr8	142175200	0.900826
chr8	142183600	0.818182
chr8	142233500	0.966942
chr8	142237500	0.917355
chr8	142255700	0.867769
chr8	142275800	0.892562
chr8	142317800	0.743802
chr8	142339800	0.793388
chr8	142409400	0.735537
chr8	142412000	0.768595
chr8	142425300	0.917355
chr8	143534700	0.785124
chr8	143673500	0.818182
chr8	143690500	0.768595
chr8	143696000	0.710744
chr8	143749300	0.661157
chr8	143752400	0.677686
chr8	143758400	0.917355
chr8	143781400	0.991736
chr8	143807400	0.710744
chr8	143813700	0.991736
chr8	143926400	0.685950
chr8	144098100	0.661157
chr8	144105700	0.719008
chr8	144272700	1.000000
chr8	144358600	0.851240
chr8	144361200	0.867769
chr8	144393400	1.000000
chr8	144405700	0.966942
chr8	144410900	0.776860
chr8	144536400	0.909091
chr8	144576900	0.975207
chr8	144601700	0.727273
chr8	144636000	0.661157
chr8	144642200	0.694215
chr8	144654300	0.727273
chr8	144660600	0.694215
chr8	144681900	0.727273
chr8	145003200	0.933884
chr8	145008500	0.900826
chr8	145015600	0.793388
chr8	145019000	0.826446
chr8	145023000	0.826446
chr8	145025400	0.818182
chr8	145031100	0.685950
chr8	145045400	0.933884
chr8	145048000	0.735537
chr8	145060500	0.776860
chr8	145089100	0.752066
chr8	145152800	0.818182
chr8	145180700	0.785124
chr8	145192800	0.694215
chr8	145244500	0.694215
chr8	145306400	0.892562

chr8	145330100	1.000000
chr8	145475500	0.933884
chr8	145499100	1.000000
chr8	145561900	0.884298
chr8	145726600	0.859504
chr8	145870300	0.760331
chr8	145967100	0.983471
chr8	145987900	0.760331
chr8	145992800	0.958678
chr8	145996500	0.975207
chr8	146014100	0.900826
chr8	146074300	0.983471
chr8	146226700	0.752066
chr9	13900	0.876033
chr9	21900	0.719008
chr9	114300	0.867769
chr9	317100	0.752066
chr9	590300	0.719008
chr9	614700	0.719008
chr9	625500	0.727273
chr9	677700	0.776860
chr9	683000	0.793388
chr9	685400	0.826446
chr9	687900	0.917355
chr9	692700	0.768595
chr9	695400	0.884298
chr9	698000	1.000000
chr9	716100	0.793388
chr9	735300	0.917355
chr9	752400	0.983471
chr9	756200	0.942149
chr9	800700	0.776860
chr9	804900	0.710744
chr9	2022800	0.661157
chr9	2026800	1.000000
chr9	2055300	0.917355
chr9	2086200	0.776860
chr9	2094200	0.752066
chr9	2160300	0.834711
chr9	2478200	0.991736
chr9	2655800	0.925620
chr9	2691600	0.719008
chr9	2695700	0.685950
chr9	3321700	0.983471
chr9	3333200	0.950413
chr9	3337900	0.966942
chr9	3472400	0.710744
chr9	3475600	0.975207
chr9	3479500	0.958678
chr9	3497800	0.950413
chr9	3515500	0.661157
chr9	3529000	0.834711
chr9	3572100	0.768595
chr9	3863500	0.776860

chr9	3865600	0.719008
chr9	3875600	0.975207
chr9	3899400	0.876033
chr9	3906900	0.892562
chr9	3937300	0.826446
chr9	4137600	0.900826
chr9	4263100	0.801653
chr9	4293600	0.942149
chr9	4324800	0.900826
chr9	4334600	0.809917
chr9	4425200	0.950413
chr9	4431100	0.925620
chr9	4494600	1.000000
chr9	4530900	0.991736
chr9	4541900	0.859504
chr9	4548000	0.958678
chr9	4555200	0.801653
chr9	4567900	0.785124
chr9	4596700	0.983471
chr9	4763300	0.851240
chr9	4798300	0.760331
chr9	4832600	0.818182
chr9	4834800	0.900826
chr9	4988500	0.958678
chr9	5044400	0.884298
chr9	5073700	0.669421
chr9	5078400	0.702479
chr9	5081000	0.677686
chr9	5090500	0.727273
chr9	5269800	0.925620
chr9	5376100	1.000000
chr9	5436900	0.669421
chr9	5447200	0.983471
chr9	5453100	0.785124
chr9	5455300	0.900826
chr9	5460100	0.950413
chr9	5489300	0.991736
chr9	5494000	0.760331
chr9	5500000	0.785124
chr9	5512400	0.884298
chr9	5550000	0.884298
chr9	5553500	0.991736
chr9	5583300	0.942149
chr9	5586900	0.909091
chr9	5622000	0.694215
chr9	5627000	0.727273
chr9	5688400	0.809917
chr9	5719100	0.760331
chr9	5786500	0.785124
chr9	5839700	0.867769
chr9	5844600	1.000000
chr9	6098900	0.727273
chr9	6149700	0.950413
chr9	6153700	0.793388

chr9	6198400	0.859504
chr9	6217800	0.909091
chr9	6220300	0.884298
chr9	6230100	0.975207
chr9	6248300	0.892562
chr9	6331400	0.966942
chr9	6433500	0.925620
chr9	6450500	0.942149
chr9	6467300	0.909091
chr9	6551600	0.859504
chr9	6554000	0.768595
chr9	6558200	0.925620
chr9	6562700	0.867769
chr9	6567900	0.876033
chr9	6780200	0.991736
chr9	6788500	1.000000
chr9	6902900	0.809917
chr9	6944700	0.925620
chr9	7103800	0.942149
chr9	7653400	0.900826
chr9	7657100	0.793388
chr9	8365400	0.975207
chr9	8367500	0.842975
chr9	8374900	0.991736
chr9	8734200	0.851240
chr9	8908200	0.785124
chr9	9647500	0.719008
chr9	9748100	0.933884
chr9	9778200	0.966942
chr9	9783300	0.909091
chr9	9999100	1.000000
chr9	10265600	0.884298
chr9	10269000	0.884298
chr9	10876200	0.975207
chr9	10904500	0.909091
chr9	12424600	0.867769
chr9	12685300	0.958678
chr9	12815200	0.917355
chr9	12878700	0.661157
chr9	12933300	0.727273
chr9	13140200	0.942149
chr9	13209700	1.000000
chr9	13264100	0.727273
chr9	13266600	0.801653
chr9	13275400	0.917355
chr9	13522200	0.909091
chr9	13532600	0.983471
chr9	13568800	0.991736
chr9	13576000	0.983471
chr9	13587500	0.975207
chr9	13705800	0.983471
chr9	13708300	0.958678
chr9	13750600	0.917355
chr9	13897400	0.752066

chr9	13939500	0.917355
chr9	13943100	0.842975
chr9	13948200	0.900826
chr9	13976300	0.694215
chr9	13983700	0.710744
chr9	14027600	0.760331
chr9	14030700	0.942149
chr9	14056300	0.966942
chr9	14096100	0.884298
chr9	14098900	0.884298
chr9	14108000	0.809917
chr9	14155700	0.991736
chr9	14190300	0.677686
chr9	14194700	0.917355
chr9	14199700	0.991736
chr9	14203700	0.975207
chr9	14212300	1.000000
chr9	14216000	0.909091
chr9	14222200	0.991736
chr9	14236500	0.950413
chr9	14239500	0.933884
chr9	14243600	0.719008
chr9	14246300	1.000000
chr9	14252200	0.867769
chr9	14271700	0.966942
chr9	14275100	0.900826
chr9	14278500	0.966942
chr9	14299100	0.793388
chr9	14319600	0.818182
chr9	14341800	0.867769
chr9	14353200	0.826446
chr9	14372900	0.958678
chr9	14380700	0.950413
chr9	14400800	0.958678
chr9	14424000	0.909091
chr9	14433800	0.933884
chr9	14437700	0.900826
chr9	14471100	0.884298
chr9	14483100	0.818182
chr9	14555500	0.884298
chr9	14576400	0.801653
chr9	14671400	0.975207
chr9	14687500	0.702479
chr9	14691000	0.892562
chr9	14747000	1.000000
chr9	14751200	0.983471
chr9	14869700	0.793388
chr9	15069700	0.900826
chr9	15125100	0.793388
chr9	15150900	0.842975
chr9	15156200	0.983471
chr9	15247200	0.834711
chr9	15249800	0.917355
chr9	15253800	0.818182

chr9	15607000	0.859504
chr9	15640600	0.933884
chr9	15735700	0.834711
chr9	16000000	0.752066
chr9	16155000	0.793388
chr9	16338000	0.867769
chr9	16364400	0.983471
chr9	16417500	0.776860
chr9	16501700	0.958678
chr9	16505400	0.991736
chr9	16510400	0.942149
chr9	16516800	0.876033
chr9	16522700	0.917355
chr9	16526900	1.000000
chr9	16533300	0.983471
chr9	16615800	0.950413
chr9	16625600	0.925620
chr9	16629000	1.000000
chr9	16689400	0.983471
chr9	16702300	0.669421
chr9	16706400	0.842975
chr9	16709500	0.983471
chr9	16720000	0.950413
chr9	16722100	0.933884
chr9	16795500	0.685950
chr9	16829200	0.669421
chr9	16868400	0.735537
chr9	17065700	0.776860
chr9	18238700	0.975207
chr9	18268800	0.785124
chr9	18275100	0.719008
chr9	18297600	0.991736
chr9	18331400	0.925620
chr9	18334000	0.876033
chr9	18358700	0.884298
chr9	18361000	0.958678
chr9	18379800	0.669421
chr9	18393600	0.942149
chr9	18397400	0.661157
chr9	18437600	0.743802
chr9	18460400	0.876033
chr9	18466700	0.809917
chr9	18491000	0.950413
chr9	18493300	0.917355
chr9	18543500	0.975207
chr9	18566200	0.917355
chr9	18629300	0.884298
chr9	18633500	0.983471
chr9	18659100	0.950413
chr9	18782100	0.702479
chr9	18787400	1.000000
chr9	18943000	0.768595
chr9	19075200	1.000000
chr9	19115200	0.851240

chr9	19119400	0.958678
chr9	19124700	0.925620
chr9	19129900	1.000000
chr9	19162100	0.694215
chr9	19172200	0.719008
chr9	19182300	0.975207
chr9	19184800	0.983471
chr9	19192700	1.000000
chr9	19286900	0.818182
chr9	19343100	0.884298
chr9	19376200	0.900826
chr9	19387000	0.975207
chr9	19424500	0.991736
chr9	19431900	0.809917
chr9	19748900	0.975207
chr9	19926400	0.677686
chr9	19976400	0.975207
chr9	19999200	0.925620
chr9	20016000	0.768595
chr9	20029000	0.983471
chr9	20356900	0.958678
chr9	20612100	0.677686
chr9	20686900	0.710744
chr9	20701500	0.991736
chr9	20718900	0.809917
chr9	20722700	0.942149
chr9	20728900	0.966942
chr9	20732900	0.917355
chr9	20760500	0.909091
chr9	21070200	0.801653
chr9	21081900	0.719008
chr9	21091900	0.834711
chr9	21324800	0.727273
chr9	21507600	0.975207
chr9	21543500	0.867769
chr9	21550500	0.917355
chr9	21552900	0.892562
chr9	21555900	0.876033
chr9	21576600	0.851240
chr9	21591900	0.958678
chr9	21678100	0.925620
chr9	21680400	0.826446
chr9	21682500	0.983471
chr9	21685600	0.966942
chr9	21689200	1.000000
chr9	21771400	0.933884
chr9	21812700	0.900826
chr9	21832000	0.826446
chr9	21837500	1.000000
chr9	21870900	0.876033
chr9	21874300	0.966942
chr9	21904200	0.801653
chr9	21920100	0.933884
chr9	21971400	0.776860

chr9	21987300	0.867769
chr9	21990200	0.685950
chr9	22006000	0.719008
chr9	22030500	0.826446
chr9	22045300	0.876033
chr9	22053000	0.867769
chr9	22084900	0.793388
chr9	22103700	0.991736
chr9	22117700	0.958678
chr9	22129200	0.942149
chr9	22144700	0.859504
chr9	22148400	0.950413
chr9	22158600	1.000000
chr9	22238500	1.000000
chr9	22416800	0.933884
chr9	22976500	0.950413
chr9	23042500	0.677686
chr9	23044900	0.983471
chr9	23342400	0.909091
chr9	23612300	0.760331
chr9	23622100	0.991736
chr9	23683000	0.925620
chr9	25865000	0.958678
chr9	25884400	0.719008
chr9	26244100	0.991736
chr9	26260200	0.851240
chr9	26263100	0.966942
chr9	26303800	0.983471
chr9	26888800	0.768595
chr9	27054200	0.925620
chr9	27059900	0.900826
chr9	27065400	0.884298
chr9	27112000	0.950413
chr9	27119700	0.933884
chr9	27125300	0.925620
chr9	27129800	0.884298
chr9	27142600	1.000000
chr9	27145500	0.710744
chr9	27151400	0.983471
chr9	27155200	0.991736
chr9	27182100	0.950413
chr9	27185400	0.851240
chr9	27195500	0.991736
chr9	28135500	0.950413
chr9	31304900	0.867769
chr9	31336300	1.000000
chr9	31347200	0.966942
chr9	31582800	0.694215
chr9	31609000	0.743802
chr9	31612900	0.983471
chr9	32386900	0.685950
chr9	32395400	1.000000
chr9	32404300	0.727273
chr9	32423700	0.983471

chr9	32432200	0.743802
chr9	32501500	0.983471
chr9	32503700	0.842975
chr9	32581300	0.768595
chr9	32927600	0.966942
chr9	33081600	0.851240
chr9	33092900	0.702479
chr9	33107500	0.925620
chr9	33131600	0.859504
chr9	33140900	0.702479
chr9	33146300	0.702479
chr9	33151600	0.917355
chr9	33161400	0.983471
chr9	33168300	0.752066
chr9	33172500	0.867769
chr9	33296800	0.669421
chr9	33320900	0.909091
chr9	33329700	0.975207
chr9	33355300	0.933884
chr9	33375600	0.669421
chr9	33391700	0.925620
chr9	33453200	0.892562
chr9	33459300	0.776860
chr9	33506100	0.909091
chr9	33508500	0.776860
chr9	33535200	0.851240
chr9	33549500	0.975207
chr9	33719600	0.909091
chr9	33760000	0.834711
chr9	33815200	0.966942
chr9	33851700	0.727273
chr9	33885700	0.760331
chr9	33943400	0.892562
chr9	33947200	0.818182
chr9	33952900	0.793388
chr9	34024800	0.909091
chr9	34186400	1.000000
chr9	34364200	0.776860
chr9	34381000	0.842975
chr9	34420300	0.694215
chr9	34441300	0.743802
chr9	34496800	0.900826
chr9	34629200	0.900826
chr9	34991900	0.892562
chr9	34994800	0.933884
chr9	35027100	0.661157
chr9	35036800	0.851240
chr9	35113300	0.884298
chr9	35165100	0.983471
chr9	35273500	0.867769
chr9	35277700	0.942149
chr9	35335600	0.975207
chr9	35342000	0.884298
chr9	35367700	0.958678

chr9	35464800	0.661157
chr9	35499800	0.842975
chr9	35511400	0.983471
chr9	35516200	0.842975
chr9	35518500	0.859504
chr9	35521000	0.842975
chr9	35537600	0.851240
chr9	35553600	0.909091
chr9	35564000	0.826446
chr9	35617300	0.826446
chr9	35676100	0.694215
chr9	35727200	0.752066
chr9	35751100	0.909091
chr9	35754900	0.719008
chr9	35758500	0.884298
chr9	35761900	0.933884
chr9	35771700	0.942149
chr9	35775800	0.983471
chr9	35791700	0.983471
chr9	35793900	0.669421
chr9	35798600	0.958678
chr9	35812200	0.900826
chr9	35845100	0.768595
chr9	35905200	0.710744
chr9	35908300	0.760331
chr9	35911600	0.933884
chr9	35924400	0.661157
chr9	35949000	0.942149
chr9	35953200	0.867769
chr9	36041300	0.950413
chr9	36091400	0.958678
chr9	36102500	0.801653
chr9	36149400	0.925620
chr9	36152100	0.818182
chr9	36154900	0.966942
chr9	36163600	0.752066
chr9	36208200	0.900826
chr9	36352300	0.834711
chr9	36427300	0.710744
chr9	36471100	0.892562
chr9	36488800	0.851240
chr9	36625500	0.892562
chr9	36665200	0.884298
chr9	36667900	0.785124
chr9	37027600	0.785124
chr9	37034700	0.710744
chr9	37146500	0.785124
chr9	37174600	0.975207
chr9	37197500	0.793388
chr9	37200800	0.884298
chr9	37230200	0.842975
chr9	37291300	0.900826
chr9	37424700	0.710744
chr9	37472700	0.958678

chr9	37588000	0.727273
chr9	37651500	0.677686
chr9	37917200	0.867769
chr9	37930000	0.925620
chr9	37937400	0.851240
chr9	37939500	0.867769
chr9	37943900	0.950413
chr9	37954400	0.983471
chr9	37964500	0.685950
chr9	37966600	0.801653
chr9	37969800	0.685950
chr9	38007500	0.809917
chr9	38009700	0.834711
chr9	38015600	0.966942
chr9	38017800	0.859504
chr9	38020100	0.983471
chr9	38025200	0.809917
chr9	38028000	0.785124
chr9	38032800	0.776860
chr9	38036300	0.669421
chr9	38038400	0.892562
chr9	38040500	0.867769
chr9	38046800	0.702479
chr9	38048900	0.842975
chr9	38058200	0.719008
chr9	38060500	0.975207
chr9	38063800	0.735537
chr9	38074500	0.975207
chr9	38079800	0.950413
chr9	38088200	0.694215
chr9	38182200	0.776860
chr9	38186600	0.983471
chr9	38213700	0.826446
chr9	38595200	0.661157
chr9	38682600	0.925620
chr9	38687700	0.719008
chr9	39031000	0.942149
chr9	39038600	0.925620
chr9	39040700	0.975207
chr9	39079300	0.677686
chr9	39198300	0.975207
chr9	39232700	0.942149
chr9	39376500	0.867769
chr9	39392200	0.991736
chr9	39464500	0.661157
chr9	39593000	0.917355
chr9	39596600	0.859504
chr9	39727800	0.966942
chr9	39761800	0.950413
chr9	39906100	0.793388
chr9	39921400	0.991736
chr9	40063100	0.892562
chr9	40071900	0.892562
chr9	40370900	0.867769

chr9	40373100	0.933884
chr9	40380300	0.785124
chr9	40524500	0.925620
chr9	40576400	0.950413
chr9	40578900	0.809917
chr9	40594400	0.702479
chr9	40721400	0.925620
chr9	40737100	0.958678
chr9	40750100	0.710744
chr9	40758200	0.677686
chr9	40794500	0.768595
chr9	40909100	0.834711
chr9	41009700	0.983471
chr9	41093400	0.917355
chr9	41101200	0.909091
chr9	41105600	0.933884
chr9	41133400	0.677686
chr9	41271200	0.752066
chr9	41291300	0.950413
chr9	41306600	0.834711
chr9	41431300	0.760331
chr9	41521500	0.900826
chr9	41537300	0.991736
chr9	41541000	0.669421
chr9	41558300	0.752066
chr9	41696200	0.892562
chr9	41728400	0.694215
chr9	41738700	0.842975
chr9	41820000	1.000000
chr9	42151800	0.958678
chr9	42416200	0.942149
chr9	42719200	0.743802
chr9	42754400	0.909091
chr9	42757900	0.685950
chr9	42819300	0.677686
chr9	42843300	0.917355
chr9	42845400	0.694215
chr9	42847600	0.950413
chr9	42851000	0.933884
chr9	42853400	0.925620
chr9	42855500	0.842975
chr9	43380300	0.917355
chr9	43382400	0.818182
chr9	43464600	0.917355
chr9	43474400	0.685950
chr9	43478900	0.768595
chr9	43574200	0.685950
chr9	43581300	0.710744
chr9	43594600	0.933884
chr9	43609900	0.917355
chr9	43688500	0.909091
chr9	43725700	0.702479
chr9	43743500	0.933884
chr9	43796200	0.958678

chr9	44070600	0.966942
chr9	44420200	0.727273
chr9	44443600	0.867769
chr9	44539900	0.958678
chr9	44620800	0.710744
chr9	44623600	0.900826
chr9	44631500	0.876033
chr9	44991300	0.727273
chr9	44999000	0.917355
chr9	45090000	0.925620
chr9	45444300	0.752066
chr9	45446400	0.785124
chr9	45541300	0.727273
chr9	45927900	0.834711
chr9	46210800	0.851240
chr9	46293700	0.710744
chr9	46936200	0.685950
chr9	46979000	0.991736
chr9	47059800	0.834711
chr9	47196400	0.809917
chr9	47258300	0.966942
chr9	47263800	0.942149
chr9	47266000	0.933884
chr9	47305000	0.768595
chr9	65473600	0.933884
chr9	65489000	0.826446
chr9	65579500	0.702479
chr9	65696000	0.909091
chr9	65698200	0.933884
chr9	66039700	1.000000
chr9	66594800	0.933884
chr9	66812700	0.768595
chr9	66835300	0.785124
chr9	66843700	0.785124
chr9	67016600	0.867769
chr9	67020700	0.966942
chr9	67022900	0.925620
chr9	67025100	0.851240
chr9	67028300	0.991736
chr9	67291700	0.818182
chr9	67295600	0.983471
chr9	67299200	0.933884
chr9	67301300	0.776860
chr9	67304000	0.859504
chr9	67320600	0.983471
chr9	67702600	0.917355
chr9	67974200	0.834711
chr9	68377700	0.661157
chr9	68409300	0.818182
chr9	69167800	0.793388
chr9	69654200	0.917355
chr9	69656300	0.669421
chr9	69658400	0.942149
chr9	69661200	0.933884

chr9	69663600	0.966942
chr9	69666000	0.925620
chr9	69775300	0.884298
chr9	70139800	0.702479
chr9	70142300	0.958678
chr9	70327200	0.661157
chr9	70392600	0.876033
chr9	70859800	0.702479
chr9	71660200	0.685950
chr9	71698600	0.900826
chr9	71705400	0.818182
chr9	71711500	0.892562
chr9	71714600	0.818182
chr9	71744300	0.685950
chr9	71746400	0.876033
chr9	71751700	0.776860
chr9	71757800	0.917355
chr9	71765000	0.983471
chr9	71786200	0.991736
chr9	71788300	0.694215
chr9	71791300	0.867769
chr9	71794000	0.975207
chr9	71796900	0.876033
chr9	71802100	0.991736
chr9	71806800	0.867769
chr9	71867900	0.826446
chr9	71962700	0.917355
chr9	71986100	0.983471
chr9	71991800	0.735537
chr9	71995000	0.917355
chr9	72118000	0.900826
chr9	72150800	0.752066
chr9	72185800	0.933884
chr9	72189000	0.983471
chr9	72197900	0.991736
chr9	72200000	0.834711
chr9	72208400	0.743802
chr9	72216700	0.735537
chr9	72275900	0.727273
chr9	72290300	1.000000
chr9	72473200	0.685950
chr9	72500900	0.966942
chr9	72513000	0.793388
chr9	72515300	0.768595
chr9	72561800	0.958678
chr9	72591400	0.983471
chr9	72653300	0.752066
chr9	72751900	0.685950
chr9	72871600	0.892562
chr9	72906600	0.917355
chr9	73005900	1.000000
chr9	73022100	0.909091
chr9	73033100	0.917355
chr9	73036900	0.793388

chr9	73051100	0.991736
chr9	73054000	1.000000
chr9	73085600	0.826446
chr9	73096100	0.859504
chr9	73109500	0.710744
chr9	73147400	0.677686
chr9	73149600	0.983471
chr9	73169400	0.983471
chr9	73192600	0.685950
chr9	73202300	0.785124
chr9	73205100	0.975207
chr9	73216900	0.735537
chr9	73516600	0.892562
chr9	73539800	0.950413
chr9	73760400	0.975207
chr9	74062000	0.727273
chr9	74245600	0.876033
chr9	74253700	0.900826
chr9	74262500	0.842975
chr9	74293200	0.809917
chr9	74357900	0.702479
chr9	74378800	0.892562
chr9	74920400	0.826446
chr9	75056600	0.933884
chr9	75058700	0.909091
chr9	75077200	0.876033
chr9	75080500	0.991736
chr9	75088600	0.867769
chr9	75092900	0.917355
chr9	75140200	0.958678
chr9	75143400	0.950413
chr9	75173100	0.900826
chr9	75192200	0.900826
chr9	75201700	0.694215
chr9	75473300	0.933884
chr9	75540300	0.719008
chr9	75586700	0.950413
chr9	75603000	0.942149
chr9	75611300	0.983471
chr9	75622700	0.900826
chr9	75626900	0.909091
chr9	75629200	0.702479
chr9	75638600	0.991736
chr9	75689300	0.933884
chr9	75746700	0.768595
chr9	75759000	0.851240
chr9	75764700	0.983471
chr9	75771600	0.826446
chr9	75886300	0.677686
chr9	77706300	0.867769
chr9	77724100	0.909091
chr9	77763300	0.942149
chr9	77782500	0.942149
chr9	77785200	0.983471

chr9	78752500	0.669421
chr9	78936400	0.892562
chr9	78990600	0.900826
chr9	79006300	0.826446
chr9	79018600	0.851240
chr9	79030800	1.000000
chr9	79057000	0.950413
chr9	79071600	0.909091
chr9	79186600	0.867769
chr9	79266400	0.884298
chr9	79269800	0.983471
chr9	79272000	0.669421
chr9	79277800	0.991736
chr9	79282600	0.983471
chr9	79292600	0.900826
chr9	79303200	0.933884
chr9	79307000	0.925620
chr9	79318400	0.991736
chr9	79329100	0.834711
chr9	79332700	0.991736
chr9	79433400	0.933884
chr9	79627600	0.876033
chr9	79774200	0.983471
chr9	80033000	0.727273
chr9	80037200	0.983471
chr9	80040200	0.826446
chr9	80046100	0.809917
chr9	80091500	0.851240
chr9	80130700	0.933884
chr9	80203700	1.000000
chr9	80210600	0.983471
chr9	80213200	0.842975
chr9	80322100	0.909091
chr9	80324200	0.909091
chr9	80327200	0.983471
chr9	80329900	0.917355
chr9	80351100	0.851240
chr9	80366000	0.950413
chr9	80384800	0.809917
chr9	80409400	0.727273
chr9	80422800	0.975207
chr9	80435900	0.785124
chr9	80438000	0.719008
chr9	80455300	0.975207
chr9	80463900	0.661157
chr9	80467100	0.760331
chr9	80469800	0.785124
chr9	80471900	1.000000
chr9	80492000	0.727273
chr9	80512300	0.710744
chr9	80525500	0.983471
chr9	80541800	0.702479
chr9	80568300	0.942149
chr9	80576900	1.000000

chr9	80593000	0.958678
chr9	80596500	0.917355
chr9	80611600	0.942149
chr9	80614400	0.859504
chr9	80617300	0.983471
chr9	80625900	0.710744
chr9	80636800	0.909091
chr9	80654200	0.925620
chr9	80760500	0.925620
chr9	80773100	0.702479
chr9	80823100	0.834711
chr9	81220800	0.685950
chr9	81402200	0.851240
chr9	81481700	0.710744
chr9	81486700	0.925620
chr9	81548300	0.950413
chr9	81604100	0.702479
chr9	81612700	0.925620
chr9	81622900	0.702479
chr9	81629600	0.991736
chr9	81638600	0.933884
chr9	82197000	0.950413
chr9	82220000	0.669421
chr9	82239600	0.933884
chr9	82277700	0.909091
chr9	82297600	0.884298
chr9	82416800	0.876033
chr9	82556100	0.892562
chr9	82773000	0.892562
chr9	83610100	0.909091
chr9	83640400	0.710744
chr9	83643600	0.793388
chr9	83943600	0.694215
chr9	83949100	0.677686
chr9	84003800	0.942149
chr9	84006800	0.950413
chr9	84174800	0.859504
chr9	84177800	0.842975
chr9	84203500	0.925620
chr9	84210900	0.859504
chr9	84213100	0.933884
chr9	84268600	0.661157
chr9	84271500	0.958678
chr9	84298100	0.900826
chr9	84339600	0.752066
chr9	84374800	0.884298
chr9	84387100	0.809917
chr9	84430200	0.991736
chr9	84438200	0.942149
chr9	84458100	0.884298
chr9	84463700	1.000000
chr9	84487000	0.677686
chr9	84490000	0.859504
chr9	84494800	0.942149

chr9	84954600	0.809917
chr9	85497700	0.958678
chr9	85550300	0.966942
chr9	85823800	0.809917
chr9	85836300	0.950413
chr9	85883400	0.669421
chr9	85886600	0.975207
chr9	85896500	0.975207
chr9	85914700	0.942149
chr9	86047400	0.677686
chr9	86126400	0.958678
chr9	86184700	0.694215
chr9	86281100	0.991736
chr9	86314700	0.785124
chr9	86317300	0.950413
chr9	86501000	0.851240
chr9	86754500	0.909091
chr9	86762400	0.983471
chr9	86767500	0.983471
chr9	86939100	0.809917
chr9	87013100	0.694215
chr9	87322800	0.719008
chr9	87339100	0.760331
chr9	87397500	0.900826
chr9	87467100	0.909091
chr9	87476000	0.661157
chr9	87478300	0.892562
chr9	87482200	0.801653
chr9	87489800	0.966942
chr9	87636000	0.727273
chr9	87827700	0.727273
chr9	87840000	0.702479
chr9	88017600	0.900826
chr9	88036700	0.867769
chr9	88040700	0.743802
chr9	88046600	0.892562
chr9	88142700	0.983471
chr9	88709100	0.909091
chr9	88938600	0.958678
chr9	89763600	0.826446
chr9	89885700	0.785124
chr9	89952600	0.768595
chr9	90124600	0.983471
chr9	90161300	0.975207
chr9	90179200	0.958678
chr9	90184600	0.909091
chr9	90215100	0.958678
chr9	90222600	1.000000
chr9	90272300	0.933884
chr9	90301100	0.958678
chr9	90329400	0.958678
chr9	90364600	0.661157
chr9	90886300	0.950413
chr9	91079800	0.752066

chr9	91150700	0.809917
chr9	91199400	0.933884
chr9	91347300	0.933884
chr9	91349600	0.950413
chr9	91389500	0.677686
chr9	91393100	0.933884
chr9	91397400	0.834711
chr9	91409400	0.851240
chr9	91415500	0.826446
chr9	91419300	0.752066
chr9	91424500	0.760331
chr9	91468100	0.975207
chr9	91609300	0.834711
chr9	91661200	0.694215
chr9	91694300	0.710744
chr9	91753800	0.975207
chr9	91768600	0.760331
chr9	91770700	0.958678
chr9	91929800	0.752066
chr9	92040500	0.768595
chr9	92093700	0.842975
chr9	92137800	0.727273
chr9	92171600	0.867769
chr9	92218800	0.694215
chr9	92277100	0.842975
chr9	92508500	0.876033
chr9	92510800	0.950413
chr9	92516500	0.991736
chr9	92807100	0.991736
chr9	93800300	0.942149
chr9	93810500	0.933884
chr9	93819400	0.975207
chr9	93823000	0.975207
chr9	93927700	0.818182
chr9	93949600	0.950413
chr9	93956500	0.785124
chr9	94048800	0.834711
chr9	94065600	0.842975
chr9	94141000	0.785124
chr9	94158500	0.867769
chr9	94173700	0.785124
chr9	94182200	0.842975
chr9	94712600	0.892562
chr9	94860800	0.925620
chr9	94863200	0.917355
chr9	94896800	0.917355
chr9	94909900	0.975207
chr9	95075600	0.876033
chr9	95243200	0.917355
chr9	95246500	0.917355
chr9	95417000	0.950413
chr9	95434200	0.785124
chr9	95470200	0.900826
chr9	95489600	0.743802

chr9	95498800	0.776860
chr9	95502900	0.975207
chr9	95509900	0.876033
chr9	95515400	0.842975
chr9	95857500	0.867769
chr9	95861500	0.942149
chr9	95882800	0.801653
chr9	95889400	0.785124
chr9	95893600	0.727273
chr9	95946700	0.876033
chr9	96075400	0.892562
chr9	96229500	0.685950
chr9	96231700	0.818182
chr9	96263800	0.933884
chr9	96329100	0.933884
chr9	96447600	0.818182
chr9	96713900	0.752066
chr9	96819300	0.983471
chr9	96836400	0.851240
chr9	96897900	0.958678
chr9	96902300	1.000000
chr9	96919800	0.958678
chr9	96932200	0.694215
chr9	96937800	0.867769
chr9	97041200	0.834711
chr9	97496800	0.983471
chr9	97555500	0.867769
chr9	97562600	0.892562
chr9	97568200	0.983471
chr9	97584800	0.958678
chr9	97610000	0.752066
chr9	97628100	0.702479
chr9	97630300	0.983471
chr9	97635700	0.876033
chr9	97669800	0.859504
chr9	97676800	0.933884
chr9	97684800	0.925620
chr9	97692000	0.917355
chr9	97700000	0.942149
chr9	97703900	0.867769
chr9	97715400	0.958678
chr9	97758900	0.958678
chr9	97763400	0.809917
chr9	97770600	0.727273
chr9	97781300	0.892562
chr9	97788500	0.867769
chr9	97818000	0.892562
chr9	97933700	0.900826
chr9	97951900	0.991736
chr9	97955100	0.677686
chr9	98068800	0.743802
chr9	98112000	0.735537
chr9	98189100	0.768595
chr9	98225300	0.925620

chr9	98333200	0.694215
chr9	98389700	0.925620
chr9	98556900	0.983471
chr9	98564000	0.966942
chr9	98577700	0.842975
chr9	98582400	0.677686
chr9	98585600	0.909091
chr9	98615300	0.900826
chr9	98642500	1.000000
chr9	98645700	0.933884
chr9	98892100	0.950413
chr9	98921600	0.834711
chr9	98980800	0.851240
chr9	99056100	0.785124
chr9	99102300	0.983471
chr9	99106800	0.909091
chr9	99140100	0.867769
chr9	99161600	0.719008
chr9	99183200	0.876033
chr9	99437700	0.983471
chr9	99594900	0.826446
chr9	99646900	0.859504
chr9	100056300	0.842975
chr9	100070200	0.685950
chr9	100181800	0.950413
chr9	100273700	0.933884
chr9	100281600	0.917355
chr9	100413800	0.785124
chr9	100452100	0.983471
chr9	100466300	0.859504
chr9	100503800	0.859504
chr9	100565900	0.661157
chr9	100571600	0.760331
chr9	100611500	0.768595
chr9	100669000	0.669421
chr9	100770500	0.876033
chr9	100773300	0.842975
chr9	100784900	0.975207
chr9	100797900	0.694215
chr9	100843900	0.859504
chr9	100850400	0.859504
chr9	100985600	0.900826
chr9	101011200	0.983471
chr9	101080100	0.719008
chr9	101087100	0.975207
chr9	101091600	0.801653
chr9	101097000	1.000000
chr9	101104200	0.958678
chr9	101120400	0.933884
chr9	101137500	0.900826
chr9	101154600	0.719008
chr9	101172400	0.793388
chr9	101283600	0.942149
chr9	101285700	0.892562

chr9	101370300	0.983471
chr9	101373200	0.834711
chr9	101449600	0.727273
chr9	101541600	0.694215
chr9	101648900	0.677686
chr9	101947100	0.983471
chr9	101986800	0.760331
chr9	102031800	0.859504
chr9	102096900	0.933884
chr9	102102900	0.917355
chr9	102131000	0.867769
chr9	102178500	0.768595
chr9	102193500	0.785124
chr9	102196300	0.966942
chr9	102711400	0.851240
chr9	102850400	0.966942
chr9	102852700	0.677686
chr9	103174200	0.768595
chr9	103188700	0.727273
chr9	103192600	0.851240
chr9	103209100	0.661157
chr9	103265600	0.900826
chr9	103311700	0.966942
chr9	103349600	0.859504
chr9	103352500	0.760331
chr9	103855900	0.942149
chr9	103880800	0.975207
chr9	103884700	0.727273
chr9	104163400	0.702479
chr9	106995800	0.785124
chr9	107592400	1.000000
chr9	107599000	0.743802
chr9	107613300	0.785124
chr9	107631500	0.884298
chr9	107635600	0.991736
chr9	107642300	0.950413
chr9	108005600	0.818182
chr9	108059400	0.892562
chr9	108063100	0.859504
chr9	108072000	0.983471
chr9	108419100	0.818182
chr9	109561500	0.975207
chr9	109655800	0.942149
chr9	109658400	0.768595
chr9	109679500	0.991736
chr9	109723900	0.826446
chr9	109726100	0.958678
chr9	110048900	0.702479
chr9	110131100	1.000000
chr9	110253300	0.768595
chr9	110267200	0.925620
chr9	110311100	0.933884
chr9	110350000	0.900826
chr9	110400600	0.826446

chr9	110407700	0.818182
chr9	110430500	0.884298
chr9	110495700	0.958678
chr9	110509800	0.752066
chr9	110512700	0.661157
chr9	110514800	0.892562
chr9	110517900	0.975207
chr9	110803600	0.785124
chr9	110811700	0.983471
chr9	110816600	0.859504
chr9	110819400	0.909091
chr9	110905500	0.933884
chr9	110913400	0.917355
chr9	111206600	1.000000
chr9	111236200	0.677686
chr9	111241100	0.768595
chr9	111605400	0.958678
chr9	111713500	0.900826
chr9	111754000	0.975207
chr9	111757200	0.859504
chr9	111759500	0.892562
chr9	111764200	0.867769
chr9	111777000	0.735537
chr9	111798600	0.892562
chr9	111802200	0.991736
chr9	111828400	0.776860
chr9	111861000	0.991736
chr9	111864500	1.000000
chr9	111870500	0.933884
chr9	111873400	0.710744
chr9	112003700	0.661157
chr9	112013700	1.000000
chr9	112579400	0.983471
chr9	112669100	0.818182
chr9	112671200	0.876033
chr9	112673900	0.925620
chr9	112676300	0.933884
chr9	112681400	0.818182
chr9	112686000	0.942149
chr9	112730500	0.818182
chr9	112732600	0.975207
chr9	112749100	0.966942
chr9	112778400	0.983471
chr9	112789400	0.793388
chr9	112792100	0.942149
chr9	112803900	1.000000
chr9	112820800	0.966942
chr9	112829300	0.966942
chr9	112833300	0.876033
chr9	112852300	0.991736
chr9	112861500	0.760331
chr9	112872100	0.900826
chr9	112877500	0.760331
chr9	112886500	0.768595

chr9	112890700	0.950413
chr9	112894000	0.933884
chr9	112896100	0.785124
chr9	112904000	0.818182
chr9	112914800	0.851240
chr9	113318200	0.776860
chr9	113413600	0.925620
chr9	113463300	0.710744
chr9	113505200	0.933884
chr9	113507400	0.950413
chr9	113510800	0.776860
chr9	113515300	0.975207
chr9	113539700	0.842975
chr9	113560400	0.735537
chr9	114111800	0.884298
chr9	114119400	0.917355
chr9	114176500	0.834711
chr9	114220500	0.900826
chr9	114364600	1.000000
chr9	114398200	0.677686
chr9	114463200	0.983471
chr9	114491100	0.677686
chr9	114522600	0.793388
chr9	114568500	0.991736
chr9	114614100	0.661157
chr9	114629700	0.884298
chr9	114661900	0.669421
chr9	114664900	0.983471
chr9	114687400	0.925620
chr9	114689500	0.925620
chr9	114712500	0.694215
chr9	114715700	0.900826
chr9	114720600	0.950413
chr9	114723600	0.801653
chr9	114765300	0.851240
chr9	114774800	0.834711
chr9	114783500	0.991736
chr9	114792400	0.925620
chr9	114804700	0.909091
chr9	114809500	0.950413
chr9	114813100	0.958678
chr9	114821800	0.760331
chr9	114824900	0.933884
chr9	114828700	0.809917
chr9	114837300	0.933884
chr9	114846900	0.727273
chr9	114875300	0.826446
chr9	114936600	0.694215
chr9	115011200	0.834711
chr9	115013300	0.884298
chr9	115062400	0.702479
chr9	115078500	0.793388
chr9	115091100	0.743802
chr9	115111200	0.834711

chr9	115298600	0.933884
chr9	115951000	0.892562
chr9	115995800	0.942149
chr9	116160700	0.834711
chr9	116193700	0.785124
chr9	116217800	0.958678
chr9	116247400	0.933884
chr9	116262400	0.685950
chr9	116268500	0.793388
chr9	116271700	0.975207
chr9	116274800	0.900826
chr9	116280000	0.950413
chr9	116284100	0.958678
chr9	116286400	0.826446
chr9	116288500	0.867769
chr9	116311300	0.776860
chr9	116321800	0.776860
chr9	116340600	0.727273
chr9	116343600	0.991736
chr9	116349800	0.677686
chr9	116352500	0.669421
chr9	116354600	0.685950
chr9	116361900	0.975207
chr9	116364400	0.702479
chr9	116371500	0.925620
chr9	116373600	0.727273
chr9	116378700	0.694215
chr9	116385400	0.900826
chr9	116397200	0.950413
chr9	116412200	0.884298
chr9	116418500	0.942149
chr9	116437000	0.818182
chr9	116439300	0.677686
chr9	116590800	0.793388
chr9	116642100	0.818182
chr9	116649700	0.818182
chr9	116659500	0.719008
chr9	116692300	0.991736
chr9	116718500	0.942149
chr9	116721200	0.826446
chr9	116748800	0.809917
chr9	116754500	0.719008
chr9	116757000	0.876033
chr9	116766900	0.925620
chr9	116863100	0.694215
chr9	116867400	0.661157
chr9	116920400	0.900826
chr9	116940200	0.677686
chr9	116981200	0.925620
chr9	116983800	0.900826
chr9	116985900	0.876033
chr9	116989500	0.677686
chr9	117001300	0.727273
chr9	117039900	0.884298

chr9	117135200	0.867769
chr9	117148000	0.950413
chr9	117208700	0.884298
chr9	117355000	0.768595
chr9	117373900	0.760331
chr9	117384700	0.669421
chr9	117439900	0.950413
chr9	117508200	0.785124
chr9	117542000	1.000000
chr9	117568600	0.809917
chr9	117618300	0.760331
chr9	117653000	0.892562
chr9	117655200	0.793388
chr9	117658300	0.917355
chr9	117661600	0.983471
chr9	118914800	0.834711
chr9	120407400	0.793388
chr9	120441100	0.950413
chr9	120453700	0.892562
chr9	120465600	0.801653
chr9	120477800	0.785124
chr9	120525800	0.826446
chr9	121291900	0.991736
chr9	121340400	0.900826
chr9	122319100	0.818182
chr9	123128800	0.975207
chr9	123301300	0.851240
chr9	123346500	0.760331
chr9	123493100	0.958678
chr9	123504000	0.826446
chr9	123610000	0.801653
chr9	123632100	0.909091
chr9	123654900	0.702479
chr9	123657400	0.710744
chr9	123688500	0.842975
chr9	123691600	0.710744
chr9	123694300	0.909091
chr9	123699200	0.925620
chr9	123703000	0.727273
chr9	123705100	0.842975
chr9	123708800	0.892562
chr9	123713900	0.950413
chr9	123748400	0.661157
chr9	123751700	0.719008
chr9	123820300	0.727273
chr9	123824400	0.760331
chr9	124023800	0.801653
chr9	124041100	0.950413
chr9	124044500	0.776860
chr9	124051900	0.842975
chr9	124062200	0.702479
chr9	124085000	0.859504
chr9	124089600	0.859504
chr9	124114200	0.991736

chr9	124116800	1.000000
chr9	124119200	0.983471
chr9	124121300	0.942149
chr9	124123700	1.000000
chr9	124127200	0.958678
chr9	124129900	0.785124
chr9	124156500	0.694215
chr9	124161000	0.884298
chr9	124241700	0.776860
chr9	124320700	0.975207
chr9	124331200	0.917355
chr9	124362100	0.793388
chr9	124375800	0.710744
chr9	124420600	0.925620
chr9	124487500	0.826446
chr9	124498900	0.975207
chr9	124504000	0.859504
chr9	124506700	0.826446
chr9	124516600	0.801653
chr9	124527900	0.735537
chr9	124533700	0.867769
chr9	124606800	0.933884
chr9	124646300	0.917355
chr9	124650800	0.685950
chr9	124665400	0.983471
chr9	124678400	0.892562
chr9	124721200	0.859504
chr9	124724800	0.735537
chr9	124727200	0.834711
chr9	124735300	0.933884
chr9	124760500	0.793388
chr9	124779200	0.958678
chr9	124783400	0.677686
chr9	124891000	0.702479
chr9	124896700	0.892562
chr9	124906600	0.900826
chr9	124931200	0.735537
chr9	124981700	0.909091
chr9	125011200	0.983471
chr9	125015500	0.942149
chr9	125017600	0.818182
chr9	125044100	0.892562
chr9	125066000	0.925620
chr9	125068500	0.900826
chr9	125070900	0.933884
chr9	125087600	0.966942
chr9	125094600	0.859504
chr9	125102900	0.834711
chr9	125109500	1.000000
chr9	125139500	0.842975
chr9	125146600	0.917355
chr9	125152300	0.760331
chr9	125157500	0.785124
chr9	125165000	0.909091

chr9	125191400	0.818182
chr9	125208500	0.983471
chr9	125342900	0.958678
chr9	125577900	0.950413
chr9	125581800	0.876033
chr9	125584800	0.900826
chr9	125588700	0.768595
chr9	125627800	0.892562
chr9	125663600	0.867769
chr9	125686700	0.809917
chr9	125710000	0.917355
chr9	125717000	0.958678
chr9	125738000	0.991736
chr9	125762900	0.776860
chr9	125765700	0.933884
chr9	125795700	0.752066
chr9	125811700	0.719008
chr9	125913600	1.000000
chr9	125925600	0.909091
chr9	125979700	0.785124
chr9	126065300	0.793388
chr9	126577200	0.851240
chr9	126579700	0.892562
chr9	126685800	0.867769
chr9	126771100	0.710744
chr9	126774400	0.768595
chr9	126841600	0.900826
chr9	126975100	0.933884
chr9	126979200	0.925620
chr9	126983500	0.785124
chr9	127005600	0.685950
chr9	127014700	0.793388
chr9	127026900	0.966942
chr9	127029000	0.752066
chr9	127031200	0.925620
chr9	127033900	0.892562
chr9	127040700	0.909091
chr9	127042800	0.842975
chr9	127046200	0.950413
chr9	127055600	0.892562
chr9	127060700	0.752066
chr9	127063400	0.900826
chr9	127068800	0.859504
chr9	127072200	0.900826
chr9	127143800	0.900826
chr9	127153000	0.884298
chr9	127155400	0.991736
chr9	127174600	0.983471
chr9	127424200	0.851240
chr9	127428200	0.834711
chr9	127434200	0.983471
chr9	127460400	0.925620
chr9	127542100	0.900826
chr9	127558700	0.661157

chr9	127563000	0.685950
chr9	127615700	0.702479
chr9	127626000	0.677686
chr9	127634800	0.884298
chr9	127664700	0.900826
chr9	127696900	0.966942
chr9	127837700	0.826446
chr9	127935100	0.793388
chr9	127945900	0.834711
chr9	127999500	0.851240
chr9	128006300	0.892562
chr9	128026400	0.859504
chr9	128230100	0.702479
chr9	128235600	0.851240
chr9	128276200	0.892562
chr9	128284200	0.933884
chr9	128287800	0.768595
chr9	128300600	0.909091
chr9	128308100	0.727273
chr9	128313100	0.975207
chr9	128375900	0.975207
chr9	128394600	0.876033
chr9	128409300	0.983471
chr9	128413000	0.917355
chr9	128418800	0.842975
chr9	128422500	0.876033
chr9	128425600	0.966942
chr9	128438300	0.925620
chr9	128522300	0.743802
chr9	128525600	0.743802
chr9	128547700	0.966942
chr9	128694600	0.661157
chr9	129097600	0.859504
chr9	129135200	0.768595
chr9	129138300	0.851240
chr9	129149400	0.958678
chr9	129236800	1.000000
chr9	129280000	0.694215
chr9	129424800	0.702479
chr9	129569300	0.685950
chr9	129606200	0.727273
chr9	129647600	0.933884
chr9	129733800	0.909091
chr9	129811500	0.991736
chr9	129816200	0.669421
chr9	129828300	1.000000
chr9	129875700	0.834711
chr9	129917600	0.727273
chr9	129925900	1.000000
chr9	129949600	0.752066
chr9	130039900	0.834711
chr9	130100300	0.719008
chr9	130103400	0.752066
chr9	130282800	0.743802

chr9	130285900	0.876033
chr9	130293100	0.917355
chr9	130301900	0.694215
chr9	130305300	0.991736
chr9	130308700	0.785124
chr9	130313000	0.719008
chr9	130317000	0.842975
chr9	130321000	0.884298
chr9	130336400	0.809917
chr9	130341700	0.909091
chr9	130396600	0.876033
chr9	130407700	0.785124
chr9	130498800	0.933884
chr9	130517800	0.958678
chr9	130528100	0.966942
chr9	130546900	0.793388
chr9	130555900	0.917355
chr9	130579500	0.818182
chr9	130589300	0.809917
chr9	130592500	0.942149
chr9	130597500	0.917355
chr9	130603700	0.983471
chr9	130607300	0.826446
chr9	130611600	0.975207
chr9	130618600	0.735537
chr9	130621200	0.966942
chr9	130624900	0.818182
chr9	130629500	0.991736
chr9	130645600	0.867769
chr9	130659800	0.933884
chr9	130662800	0.710744
chr9	130665800	0.917355
chr9	130669200	1.000000
chr9	130680900	0.785124
chr9	130684300	0.719008
chr9	130689900	0.719008
chr9	130744200	0.809917
chr9	130764500	0.983471
chr9	130768400	0.966942
chr9	130770700	0.892562
chr9	130774100	0.958678
chr9	130785700	0.685950
chr9	130789800	0.925620
chr9	130835200	0.966942
chr9	130841200	0.710744
chr9	130856900	0.991736
chr9	130981400	0.900826
chr9	131008500	0.793388
chr9	131042200	1.000000
chr9	131045100	0.917355
chr9	131058300	0.826446
chr9	131070300	0.826446
chr9	131124300	0.867769
chr9	131127600	0.876033

chr9	131159700	0.884298
chr9	131191800	0.909091
chr9	131256700	0.818182
chr9	131285800	0.909091
chr9	131291700	0.942149
chr9	131318700	0.785124
chr9	131322300	0.966942
chr9	131327900	0.925620
chr9	131398000	0.842975
chr9	131650200	0.933884
chr9	131653500	0.785124
chr9	131655700	0.933884
chr9	131665100	0.950413
chr9	131676400	0.826446
chr9	131679600	0.785124
chr9	131889400	0.702479
chr9	131906300	0.842975
chr9	131908500	0.909091
chr9	131932500	0.851240
chr9	131936300	0.661157
chr9	131961200	0.669421
chr9	132009000	0.975207
chr9	132021200	0.685950
chr9	132083200	0.826446
chr9	132096800	0.892562
chr9	132099400	0.834711
chr9	132176000	0.966942
chr9	132199900	0.719008
chr9	132214100	0.892562
chr9	132223200	0.900826
chr9	132244400	0.876033
chr9	132253500	0.958678
chr9	132257300	0.752066
chr9	132260100	0.842975
chr9	132312900	0.768595
chr9	132339200	0.809917
chr9	132360000	0.892562
chr9	132370000	0.818182
chr9	132373000	0.719008
chr9	132382900	0.727273
chr9	132460000	0.925620
chr9	132539800	0.735537
chr9	132647400	0.661157
chr9	132654800	0.785124
chr9	132701100	0.991736
chr9	132726800	0.950413
chr9	132744200	0.975207
chr9	132798600	0.884298
chr9	132807600	0.776860
chr9	132834900	0.809917
chr9	132838300	0.859504
chr9	132873100	0.661157
chr9	132880800	0.917355
chr9	132891700	0.950413

chr9	132900900	0.983471
chr9	133034100	0.909091
chr9	133037200	0.735537
chr9	133302700	0.917355
chr9	133309100	0.818182
chr9	133320900	0.776860
chr9	133327300	0.727273
chr9	133329600	0.677686
chr9	133536600	0.818182
chr9	133604200	0.950413
chr9	133608400	0.685950
chr9	133621900	0.892562
chr9	133631700	0.983471
chr9	133638800	0.859504
chr9	133641000	1.000000
chr9	133643800	0.694215
chr9	133670400	0.933884
chr9	133672500	0.776860
chr9	133715400	0.760331
chr9	133720100	0.884298
chr9	133722600	0.867769
chr9	133724700	0.917355
chr9	133734100	0.966942
chr9	133737700	0.925620
chr9	133838200	0.917355
chr9	133993000	0.834711
chr9	134090400	0.991736
chr9	134093100	0.867769
chr9	134102200	0.983471
chr9	134104800	0.694215
chr9	134112800	0.719008
chr9	134115100	0.768595
chr9	134132200	0.809917
chr9	134145400	0.942149
chr9	134148600	0.909091
chr9	134206500	0.892562
chr9	134225600	0.694215
chr9	134268800	0.702479
chr9	134275100	0.975207
chr9	134278400	0.925620
chr9	134282600	1.000000
chr9	134294400	0.950413
chr9	134308800	0.859504
chr9	134312300	0.966942
chr9	134471400	0.793388
chr9	134484200	0.983471
chr9	134494000	0.785124
chr9	134510300	0.834711
chr9	134512600	0.876033
chr9	134516100	0.900826
chr9	134531200	0.966942
chr9	134534400	0.818182
chr9	134537400	0.983471
chr9	134545200	0.719008

chr9	134548500	0.950413
chr9	134551000	0.727273
chr9	134553100	0.958678
chr9	134555300	0.661157
chr9	134558100	0.917355
chr9	134575900	0.892562
chr9	134583500	0.983471
chr9	134585700	0.942149
chr9	134589900	0.991736
chr9	134592200	0.884298
chr9	134603800	0.892562
chr9	134608400	0.909091
chr9	134610500	0.669421
chr9	134612600	0.785124
chr9	134857000	0.917355
chr9	134896700	0.727273
chr9	134918200	0.966942
chr9	135089600	0.710744
chr9	135136700	0.933884
chr9	135195500	0.900826
chr9	135244800	0.677686
chr9	135248700	1.000000
chr9	135280200	0.768595
chr9	135422100	0.661157
chr9	135424700	0.942149
chr9	135459400	0.752066
chr9	135813300	0.892562
chr9	135989100	0.760331
chr9	136002000	0.942149
chr9	136011400	0.776860
chr9	136020100	0.867769
chr9	136109800	1.000000
chr9	136113300	0.933884
chr9	136236300	0.933884
chr9	136423700	0.909091
chr9	136549000	0.661157
chr9	136654500	0.776860
chr9	136658600	0.743802
chr9	136711000	0.876033
chr9	136721100	0.752066
chr9	136739800	0.900826
chr9	136755800	0.669421
chr9	136797700	0.942149
chr9	136801300	0.801653
chr9	136809600	0.768595
chr9	136814200	0.793388
chr9	136821000	0.975207
chr9	136825700	0.834711
chr9	136839700	0.685950
chr9	136844800	0.917355
chr9	136924500	0.727273
chr9	137028700	0.677686
chr9	137097500	0.719008
chr9	137221400	0.677686

chr9	137230900	0.743802
chr9	137233500	0.785124
chr9	137252800	0.727273
chr9	137258400	0.801653
chr9	137267900	0.950413
chr9	137270800	0.710744
chr9	137274200	0.851240
chr9	137284900	0.809917
chr9	137290000	0.950413
chr9	137296400	1.000000
chr9	137300500	0.876033
chr9	137304700	0.677686
chr9	137306900	0.925620
chr9	137327100	0.793388
chr9	137335700	0.677686
chr9	137348400	0.975207
chr9	137367700	0.950413
chr9	137444700	0.661157
chr9	137494800	1.000000
chr9	137517800	0.793388
chr9	137537200	0.884298
chr9	137562200	0.694215
chr9	137568600	0.776860
chr9	137663100	0.719008
chr9	137788900	0.851240
chr9	138408200	0.809917
chr9	138610900	0.677686
chr9	138616900	0.760331
chr9	138690100	0.727273
chr9	138742900	0.876033
chr9	138779000	0.809917
chr9	138785700	0.694215
chr9	138796700	0.842975
chr9	138801300	0.942149
chr9	138819100	0.834711
chr9	138861600	0.884298
chr9	138868700	0.842975
chr9	138885000	0.950413
chr9	138899800	0.776860
chr9	138902300	0.867769
chr9	138904700	0.892562
chr9	138924000	0.818182
chr9	138943000	0.776860
chr9	138945100	0.900826
chr9	139001800	0.900826
chr9	139025600	0.892562
chr9	139049800	0.950413
chr9	139120500	0.768595
chr9	139221100	0.661157
chr9	139237200	0.743802
chr9	139240200	0.702479
chr9	139378800	0.793388
chr9	139407500	0.925620
chr9	139422200	0.876033

chr9	139424600	0.909091
chr9	139426800	0.876033
chr9	139429200	0.834711
chr9	139432700	0.785124
chr9	139437100	0.950413
chr9	139446000	0.785124
chr9	139462800	0.809917
chr9	139482700	0.809917
chr9	139499100	0.776860
chr9	139509100	0.950413
chr9	139520000	0.776860
chr9	139537100	0.793388
chr9	139544000	0.933884
chr9	139551600	0.710744
chr9	139555100	0.859504
chr9	139566400	0.867769
chr9	139610300	0.958678
chr9	139613700	0.900826
chr9	139616200	0.826446
chr9	139695100	0.710744
chr9	139716000	0.735537
chr9	139737300	0.685950
chr9	139796700	0.842975
chr9	139822900	0.859504
chr9	139872700	0.900826
chr9	139876400	0.785124
chr9	139887100	0.743802
chr9	139890400	0.793388
chr9	139892700	0.991736
chr9	139923700	0.793388
chr9	139935800	0.752066
chr9	139938000	0.694215
chr9	139978600	0.719008
chr9	140022200	0.975207
chr9	140057700	0.818182
chr9	140063900	0.793388
chr9	140113600	0.677686
chr9	140138600	0.710744
chr9	140162900	0.900826
chr9	140189400	0.760331
chr9	140197800	0.818182
chr9	140201400	0.760331
chr9	140211000	0.942149
chr9	140215500	0.785124
chr9	140233600	0.909091
chr9	140248700	0.851240
chr9	140282200	0.752066
chr9	140301700	1.000000
chr9	140305400	0.760331
chr9	140311900	0.809917
chr9	140349400	0.925620
chr9	140507000	0.735537
chr9	140527700	0.983471
chr9	140561700	0.685950

chrX	292400	0.752066
chrX	318600	0.975207
chrX	358800	0.942149
chrX	374700	0.942149
chrX	1582000	0.909091
chrX	1601000	0.760331
chrX	1776800	0.859504
chrX	2270500	0.942149
chrX	2279400	0.727273
chrX	2530100	0.842975
chrX	2533400	0.983471
chrX	2569200	0.917355
chrX	2595800	0.785124
chrX	2612700	0.966942
chrX	2616700	0.727273
chrX	2621100	0.851240
chrX	2624200	0.909091
chrX	2626500	0.900826
chrX	2629300	0.752066
chrX	2664800	0.884298
chrX	2670800	0.983471
chrX	2807800	0.768595
chrX	2816500	0.694215
chrX	2882700	0.966942
chrX	3181100	0.933884
chrX	3189800	0.933884
chrX	3204800	0.867769
chrX	3734600	0.685950
chrX	7002300	0.958678
chrX	7026800	0.876033
chrX	7028900	0.983471
chrX	7038400	0.942149
chrX	7041100	0.685950
chrX	7051500	0.933884
chrX	7895100	0.793388
chrX	8242200	0.685950
chrX	9254100	0.793388
chrX	9286800	0.669421
chrX	9310100	0.867769
chrX	9499100	0.760331
chrX	9503400	0.966942
chrX	9517800	0.694215
chrX	9534800	0.826446
chrX	9579400	0.958678
chrX	9630500	0.933884
chrX	9633700	0.768595
chrX	9645800	0.818182
chrX	9713400	0.809917
chrX	9717000	0.867769
chrX	9733900	0.867769
chrX	9739200	0.983471
chrX	9793100	0.694215
chrX	9801000	0.917355
chrX	9815600	0.942149

chrX	9824500	0.991736
chrX	9834600	0.983471
chrX	9850800	0.909091
chrX	9852900	0.942149
chrX	9876100	0.991736
chrX	9879900	0.958678
chrX	9891600	0.818182
chrX	9908900	0.818182
chrX	9970000	0.917355
chrX	9972400	0.892562
chrX	9976900	0.785124
chrX	9979400	0.983471
chrX	9986300	0.933884
chrX	9991100	0.983471
chrX	10007700	0.793388
chrX	10013500	0.950413
chrX	10017300	0.826446
chrX	10019600	0.661157
chrX	10051500	0.966942
chrX	10057800	0.842975
chrX	10079500	0.884298
chrX	10124900	0.834711
chrX	10135100	0.925620
chrX	10143700	0.785124
chrX	10146700	0.958678
chrX	10451700	0.909091
chrX	10464900	0.925620
chrX	10474200	0.884298
chrX	10523600	0.983471
chrX	10547900	0.909091
chrX	10550700	0.925620
chrX	10724100	0.917355
chrX	10940600	0.900826
chrX	11131700	0.942149
chrX	11360500	0.975207
chrX	11386300	0.677686
chrX	11431500	0.925620
chrX	11528300	0.867769
chrX	11610200	0.727273
chrX	11905300	0.876033
chrX	11917800	0.892562
chrX	11951500	0.950413
chrX	11972000	0.842975
chrX	11984300	0.760331
chrX	11989200	0.735537
chrX	12045100	0.842975
chrX	12378900	0.983471
chrX	12389400	0.793388
chrX	12440900	0.760331
chrX	12965600	0.826446
chrX	12968500	0.966942
chrX	12974800	0.661157
chrX	12989200	0.892562
chrX	12991300	0.909091

chrX	13003400	0.950413
chrX	13048100	0.991736
chrX	13070300	0.685950
chrX	13097600	0.933884
chrX	13109000	0.900826
chrX	13113200	0.735537
chrX	13117800	0.892562
chrX	13120700	0.826446
chrX	13128500	0.884298
chrX	13138400	0.983471
chrX	13142300	1.000000
chrX	13163300	0.801653
chrX	13179400	0.958678
chrX	13183500	0.818182
chrX	13186200	0.975207
chrX	13230800	0.975207
chrX	13243300	0.826446
chrX	13301600	0.909091
chrX	13303700	0.942149
chrX	13306200	0.925620
chrX	13322400	0.867769
chrX	13359600	0.892562
chrX	13415500	0.942149
chrX	13436900	0.950413
chrX	13446700	0.991736
chrX	13451000	0.818182
chrX	13506700	0.842975
chrX	13712800	0.752066
chrX	14046000	0.975207
chrX	14909000	0.768595
chrX	14918200	0.801653
chrX	14944800	0.752066
chrX	14982300	0.892562
chrX	15288300	0.818182
chrX	15317400	0.950413
chrX	15354700	0.760331
chrX	15423500	0.768595
chrX	15493500	0.983471
chrX	15515400	0.834711
chrX	15517600	0.826446
chrX	15521400	0.942149
chrX	15523500	0.867769
chrX	15530000	0.966942
chrX	15533600	0.867769
chrX	15536000	0.900826
chrX	15539100	0.669421
chrX	15543000	0.950413
chrX	15583700	0.966942
chrX	15934800	0.909091
chrX	16007800	0.925620
chrX	16041600	0.685950
chrX	16204400	0.867769
chrX	16207300	0.859504
chrX	16502700	0.785124

chrX	16612800	0.661157
chrX	16622500	0.851240
chrX	16628500	0.694215
chrX	16677200	0.785124
chrX	16841300	0.867769
chrX	16843800	0.801653
chrX	16846300	0.950413
chrX	16856500	0.983471
chrX	16889600	0.867769
chrX	16901200	0.793388
chrX	16971900	0.925620
chrX	16975100	0.925620
chrX	17050800	0.867769
chrX	17054900	0.677686
chrX	17096700	0.966942
chrX	17572000	0.966942
chrX	17610200	0.950413
chrX	17613900	1.000000
chrX	17647400	0.892562
chrX	17651700	0.983471
chrX	17746600	0.785124
chrX	18720900	0.867769
chrX	18811600	0.917355
chrX	18891500	0.818182
chrX	18904900	0.942149
chrX	19643700	0.950413
chrX	19689000	0.909091
chrX	19697500	0.685950
chrX	19701000	0.685950
chrX	19724200	0.892562
chrX	19728600	0.917355
chrX	19777700	0.909091
chrX	19790800	0.917355
chrX	19818300	0.793388
chrX	19885700	0.917355
chrX	20134800	0.710744
chrX	20152100	0.743802
chrX	20163000	0.719008
chrX	20240600	0.677686
chrX	20282000	0.892562
chrX	20479900	0.892562
chrX	20534800	1.000000
chrX	21528500	0.917355
chrX	21548100	0.859504
chrX	21890100	0.842975
chrX	21945100	0.983471
chrX	21948500	0.743802
chrX	21951000	0.809917
chrX	21958500	0.694215
chrX	21962000	0.900826
chrX	21965500	0.975207
chrX	21971000	0.669421
chrX	21990800	0.859504
chrX	22105000	0.876033

chrX	22112000	0.661157
chrX	22154200	0.942149
chrX	22239500	0.909091
chrX	22332700	0.975207
chrX	22342100	0.859504
chrX	22392200	0.818182
chrX	22408000	1.000000
chrX	22424800	0.727273
chrX	22438900	0.867769
chrX	22717700	0.933884
chrX	22908500	0.834711
chrX	22970500	0.876033
chrX	23764200	0.867769
chrX	23792200	0.776860
chrX	23800200	0.925620
chrX	23822700	0.950413
chrX	23825800	0.942149
chrX	23829700	0.917355
chrX	23863300	0.884298
chrX	23902700	0.892562
chrX	23915800	0.694215
chrX	23917900	0.793388
chrX	23920200	0.933884
chrX	23971400	0.669421
chrX	23976300	0.727273
chrX	24164600	0.950413
chrX	24167000	0.809917
chrX	24172000	0.727273
chrX	24375400	0.884298
chrX	24446500	0.661157
chrX	25537200	0.685950
chrX	25542400	0.958678
chrX	25552700	0.892562
chrX	25888300	0.925620
chrX	25897000	0.710744
chrX	25959200	0.876033
chrX	25961300	0.859504
chrX	25974900	0.785124
chrX	26222200	0.677686
chrX	26279200	0.760331
chrX	26282300	0.917355
chrX	26802700	0.909091
chrX	26806400	0.809917
chrX	26808900	0.942149
chrX	26863600	0.925620
chrX	26932600	0.991736
chrX	26934700	0.809917
chrX	26940700	0.884298
chrX	27327200	0.925620
chrX	27458000	0.834711
chrX	27584200	0.809917
chrX	27586300	0.702479
chrX	27594200	0.768595
chrX	27616400	0.826446

chrX	27637900	0.925620
chrX	27785700	0.710744
chrX	27886000	0.727273
chrX	27908900	0.942149
chrX	27985300	0.925620
chrX	28127400	0.785124
chrX	28416500	0.818182
chrX	28422500	0.975207
chrX	28517200	0.966942
chrX	28609400	0.785124
chrX	29191200	0.909091
chrX	29336600	0.768595
chrX	29679900	0.900826
chrX	29682500	0.834711
chrX	30301900	0.892562
chrX	30327300	0.727273
chrX	30670500	0.669421
chrX	30893800	0.685950
chrX	31147300	0.950413
chrX	31227800	0.958678
chrX	31239400	0.983471
chrX	31242500	0.909091
chrX	31502200	0.776860
chrX	31517500	0.793388
chrX	31564600	0.950413
chrX	31609100	0.661157
chrX	31850600	0.702479
chrX	31866100	0.776860
chrX	33200800	0.710744
chrX	33207500	0.975207
chrX	34307200	0.809917
chrX	34390500	0.661157
chrX	34506200	0.900826
chrX	34546500	0.842975
chrX	34578400	0.867769
chrX	34581100	0.958678
chrX	34603300	0.942149
chrX	34605500	0.942149
chrX	34650100	0.842975
chrX	34671900	0.669421
chrX	34975600	0.735537
chrX	34979100	0.669421
chrX	37175600	0.983471
chrX	37211700	0.710744
chrX	37237700	0.892562
chrX	37240700	0.942149
chrX	37940600	0.933884
chrX	37955600	0.867769
chrX	37968100	0.909091
chrX	38071700	1.000000
chrX	38074500	1.000000
chrX	38164300	0.867769
chrX	38167000	0.983471
chrX	38171800	0.851240

chrX	38182900	0.950413
chrX	38185000	0.677686
chrX	38496700	0.809917
chrX	39167900	0.942149
chrX	39170400	0.900826
chrX	39278300	0.975207
chrX	39287400	0.669421
chrX	39473200	0.818182
chrX	39479000	0.727273
chrX	39548200	0.719008
chrX	39581500	0.851240
chrX	39585700	0.727273
chrX	39595900	0.983471
chrX	39612900	0.991736
chrX	39615200	0.991736
chrX	39617700	1.000000
chrX	39621000	0.743802
chrX	39668700	0.909091
chrX	39716000	0.983471
chrX	39719900	0.909091
chrX	39754700	0.801653
chrX	39765400	0.842975
chrX	39768100	0.884298
chrX	39801200	0.826446
chrX	39806800	0.900826
chrX	39829200	0.983471
chrX	39871800	0.909091
chrX	39964700	0.900826
chrX	39968600	0.859504
chrX	40008700	0.991736
chrX	40012400	0.768595
chrX	40017800	0.818182
chrX	40027300	0.933884
chrX	40030000	0.884298
chrX	40033600	0.983471
chrX	40036300	0.851240
chrX	40104900	0.793388
chrX	40121900	0.925620
chrX	40126600	0.966942
chrX	40132900	0.760331
chrX	40404000	0.950413
chrX	40504100	0.785124
chrX	40633400	0.925620
chrX	40644400	0.685950
chrX	40653900	0.925620
chrX	40726900	0.727273
chrX	40792800	0.942149
chrX	40869800	0.909091
chrX	40906100	0.867769
chrX	40943400	0.809917
chrX	41008100	0.942149
chrX	41098100	0.867769
chrX	41160600	0.975207
chrX	41190900	0.809917

chrX	41350800	0.909091
chrX	41419900	0.801653
chrX	41638800	0.958678
chrX	41879800	0.719008
chrX	42445400	0.933884
chrX	42598900	0.702479
chrX	42807600	0.793388
chrX	42843700	0.950413
chrX	42852100	0.859504
chrX	42856300	0.917355
chrX	43551400	0.876033
chrX	43553700	0.884298
chrX	43561000	0.876033
chrX	43563300	0.909091
chrX	43567400	0.966942
chrX	43832900	0.669421
chrX	43872000	0.892562
chrX	44749700	0.694215
chrX	44834400	0.809917
chrX	44942800	0.760331
chrX	45046800	0.876033
chrX	45055400	0.892562
chrX	45057700	0.876033
chrX	45061200	0.958678
chrX	45126100	0.685950
chrX	45332500	0.991736
chrX	45338900	0.917355
chrX	45364800	0.958678
chrX	45367900	0.900826
chrX	45375300	0.917355
chrX	45481600	0.942149
chrX	45556900	0.900826
chrX	45569400	0.950413
chrX	45571900	0.909091
chrX	45574000	0.793388
chrX	45576100	0.983471
chrX	45579800	0.842975
chrX	45588000	0.876033
chrX	45611100	0.925620
chrX	45613400	0.991736
chrX	45616600	1.000000
chrX	45618900	0.991736
chrX	45622100	1.000000
chrX	45625100	0.727273
chrX	45631900	0.809917
chrX	45635400	0.900826
chrX	45648100	0.925620
chrX	45662700	0.900826
chrX	45666000	0.760331
chrX	45668100	0.760331
chrX	45684100	0.685950
chrX	46139300	0.685950
chrX	46177000	0.669421
chrX	46185100	0.859504

chrX	46187900	0.727273
chrX	46407600	0.884298
chrX	46436200	0.768595
chrX	46442300	0.768595
chrX	46447200	0.958678
chrX	46486500	0.867769
chrX	46488800	0.884298
chrX	46509600	0.669421
chrX	46525100	0.975207
chrX	46530300	0.702479
chrX	46652100	0.876033
chrX	46699800	0.669421
chrX	46725100	0.834711
chrX	46764100	0.884298
chrX	46838600	0.917355
chrX	47055800	0.776860
chrX	47076700	0.925620
chrX	47442000	0.809917
chrX	47444100	0.719008
chrX	47905700	0.793388
chrX	48449200	0.851240
chrX	48452300	0.909091
chrX	48554500	0.685950
chrX	48596300	0.785124
chrX	48828900	0.809917
chrX	48897900	0.950413
chrX	48902000	0.933884
chrX	48917500	0.842975
chrX	48994200	0.677686
chrX	49012300	0.818182
chrX	49092200	0.760331
chrX	49699000	0.661157
chrX	49801400	0.752066
chrX	49946300	0.727273
chrX	49963900	0.685950
chrX	49967100	0.818182
chrX	49969300	0.851240
chrX	50338100	0.991736
chrX	50353500	0.826446
chrX	50357500	0.809917
chrX	50371000	0.900826
chrX	50373100	0.694215
chrX	50375600	0.818182
chrX	50381800	0.933884
chrX	50388100	0.925620
chrX	50390300	0.958678
chrX	50420100	0.809917
chrX	50428500	0.710744
chrX	50431600	0.743802
chrX	50441200	0.917355
chrX	50506100	0.925620
chrX	50512300	1.000000
chrX	50514500	0.925620
chrX	50542900	0.983471

chrX	50558500	0.685950
chrX	50582300	0.867769
chrX	50687300	0.710744
chrX	52005300	0.859504
chrX	52949700	0.710744
chrX	53077400	0.661157
chrX	53101200	0.719008
chrX	53118100	0.917355
chrX	53292900	0.661157
chrX	53310600	0.876033
chrX	53317300	0.966942
chrX	53343000	0.760331
chrX	53350200	0.694215
chrX	53356900	0.661157
chrX	53461100	0.719008
chrX	53622700	0.909091
chrX	53627200	0.983471
chrX	53632400	0.983471
chrX	54120200	0.876033
chrX	54204800	0.768595
chrX	54207300	0.694215
chrX	54385600	0.702479
chrX	54413000	0.661157
chrX	54509800	0.818182
chrX	54519200	0.752066
chrX	54535900	0.876033
chrX	55035000	0.867769
chrX	55083800	0.950413
chrX	55146800	0.991736
chrX	55170200	0.900826
chrX	55574100	0.760331
chrX	55737600	0.950413
chrX	55740500	0.983471
chrX	56594700	0.752066
chrX	56792000	0.785124
chrX	56820500	0.677686
chrX	57477400	0.966942
chrX	57480100	0.867769
chrX	57620900	0.900826
chrX	57927800	0.983471
chrX	57932000	0.859504
chrX	58561200	0.867769
chrX	61720900	0.661157
chrX	61758200	0.859504
chrX	61839100	0.975207
chrX	61841800	0.752066
chrX	61849700	0.768595
chrX	61881100	0.710744
chrX	62967600	0.958678
chrX	64808300	0.876033
chrX	64815900	0.876033
chrX	64821800	1.000000
chrX	64825900	0.975207
chrX	64864700	0.876033

chrX	64891200	0.735537
chrX	64893600	0.958678
chrX	64897200	0.925620
chrX	64900300	0.933884
chrX	64906400	0.942149
chrX	64910600	0.983471
chrX	64915600	0.983471
chrX	64917800	0.983471
chrX	64920000	0.661157
chrX	64924100	0.942149
chrX	64927400	0.958678
chrX	64940700	1.000000
chrX	64943700	0.694215
chrX	66778500	0.826446
chrX	66825300	0.958678
chrX	66832000	0.851240
chrX	66834400	0.909091
chrX	66954100	0.669421
chrX	67000900	1.000000
chrX	67383200	0.776860
chrX	67413300	0.867769
chrX	67636600	0.826446
chrX	67663600	0.826446
chrX	67682900	0.710744
chrX	67906600	0.942149
chrX	67912600	0.950413
chrX	67916100	0.768595
chrX	67918300	0.933884
chrX	67920400	0.867769
chrX	67923800	0.900826
chrX	68115100	0.991736
chrX	68151800	0.842975
chrX	68247800	0.925620
chrX	69246700	0.942149
chrX	69267000	0.933884
chrX	69388300	0.876033
chrX	69674900	0.876033
chrX	69697600	0.966942
chrX	69699900	0.735537
chrX	69728800	0.983471
chrX	70522900	0.917355
chrX	70598700	0.950413
chrX	70761500	0.991736
chrX	70763600	0.917355
chrX	70824900	0.983471
chrX	70827300	0.917355
chrX	70832600	0.966942
chrX	70843000	0.925620
chrX	70857600	0.950413
chrX	71224300	0.834711
chrX	71250000	0.991736
chrX	71266400	0.950413
chrX	71270200	0.710744
chrX	71273000	1.000000

chrX	71298400	0.834711
chrX	71302700	0.719008
chrX	71315300	0.933884
chrX	71321700	0.702479
chrX	71341500	0.942149
chrX	71344100	0.909091
chrX	71347200	0.950413
chrX	71351400	0.727273
chrX	71369700	0.809917
chrX	71422000	1.000000
chrX	71467800	0.760331
chrX	71489800	0.942149
chrX	71747100	0.809917
chrX	71789100	0.661157
chrX	71840400	0.876033
chrX	71843100	0.702479
chrX	71854600	0.661157
chrX	72797300	0.900826
chrX	73138800	0.917355
chrX	73143300	0.892562
chrX	73462400	0.950413
chrX	73468800	0.991736
chrX	73471700	0.867769
chrX	73756400	0.884298
chrX	75368200	0.735537
chrX	76306600	0.735537
chrX	76308700	0.917355
chrX	76648200	0.942149
chrX	76704800	0.892562
chrX	76707000	0.867769
chrX	76761300	0.669421
chrX	76765800	0.933884
chrX	76924500	0.958678
chrX	77193200	0.842975
chrX	77218200	1.000000
chrX	77364100	0.842975
chrX	77378700	0.900826
chrX	77909200	0.958678
chrX	78363600	0.933884
chrX	82013500	0.776860
chrX	84307900	0.776860
chrX	85249100	0.685950
chrX	85625800	0.760331
chrX	86647200	0.876033
chrX	86658600	0.760331
chrX	86664800	0.950413
chrX	86771600	0.884298
chrX	86780700	0.842975
chrX	86882300	0.933884
chrX	86889400	0.900826
chrX	89653300	0.958678
chrX	95222000	0.975207
chrX	95256200	0.710744
chrX	95259600	0.909091

chrX	95662700	0.892562
chrX	95686500	0.925620
chrX	95692000	0.818182
chrX	95700600	0.702479
chrX	95729900	0.760331
chrX	95959400	0.818182
chrX	96162100	0.925620
chrX	96164800	0.950413
chrX	96237600	0.859504
chrX	96276900	0.958678
chrX	96493900	0.950413
chrX	96638500	0.719008
chrX	96645300	0.933884
chrX	96780200	1.000000
chrX	96784800	0.942149
chrX	96819100	0.752066
chrX	96854300	0.950413
chrX	96872100	0.933884
chrX	97113700	0.892562
chrX	99520900	0.694215
chrX	99672700	0.677686
chrX	99705400	0.892562
chrX	99898200	0.818182
chrX	99902100	0.958678
chrX	99905300	0.966942
chrX	99909100	0.917355
chrX	99914200	0.966942
chrX	99917900	0.677686
chrX	99926900	0.975207
chrX	99930200	0.884298
chrX	99936900	0.710744
chrX	99940500	0.942149
chrX	99998600	0.801653
chrX	100001400	0.975207
chrX	100003500	0.719008
chrX	100120300	0.842975
chrX	100126200	0.702479
chrX	100167800	0.743802
chrX	100184600	0.661157
chrX	100229200	0.917355
chrX	100236200	0.966942
chrX	100250300	0.917355
chrX	100280700	0.793388
chrX	100312100	0.826446
chrX	100446800	0.694215
chrX	100474900	0.983471
chrX	100521500	0.859504
chrX	100602600	0.685950
chrX	100786200	0.876033
chrX	100938000	0.818182
chrX	100947400	0.983471
chrX	101050000	0.669421
chrX	101174300	0.677686
chrX	101242000	0.776860

chrX	101982200	0.859504
chrX	102020500	0.991736
chrX	102615700	0.900826
chrX	102651800	0.752066
chrX	102653900	0.909091
chrX	102659900	0.809917
chrX	102697700	0.991736
chrX	102708700	0.933884
chrX	102882400	0.991736
chrX	102911800	0.983471
chrX	102985900	0.925620
chrX	103357300	0.727273
chrX	103560300	0.876033
chrX	103563600	0.966942
chrX	103570000	0.909091
chrX	103580900	0.776860
chrX	103596500	0.694215
chrX	104527200	0.991736
chrX	104559400	0.834711
chrX	104573800	0.983471
chrX	104727400	0.909091
chrX	106138800	0.983471
chrX	106161500	0.942149
chrX	106449500	0.818182
chrX	106917400	0.975207
chrX	106921000	0.785124
chrX	106928100	0.776860
chrX	106930300	0.991736
chrX	106954700	0.958678
chrX	106961100	0.752066
chrX	106970000	0.776860
chrX	106975400	0.983471
chrX	106985500	1.000000
chrX	107093700	0.950413
chrX	107179300	0.719008
chrX	107265800	0.834711
chrX	107269600	0.966942
chrX	107279900	0.975207
chrX	107315700	0.909091
chrX	107341300	0.719008
chrX	107370100	0.702479
chrX	107375300	0.991736
chrX	107640900	0.826446
chrX	107643300	0.909091
chrX	108297500	0.925620
chrX	108587800	0.702479
chrX	108591100	0.892562
chrX	108716600	0.925620
chrX	108719000	0.661157
chrX	108957900	0.867769
chrX	108971600	0.752066
chrX	109092100	0.694215
chrX	109101000	0.966942
chrX	109197800	0.958678

chrX	109265000	0.834711
chrX	109269600	0.975207
chrX	109325800	0.950413
chrX	109329300	0.768595
chrX	109331900	0.975207
chrX	109404500	0.702479
chrX	109417000	0.752066
chrX	109424400	0.702479
chrX	109454000	0.909091
chrX	111625000	0.826446
chrX	112643600	0.925620
chrX	112756700	0.991736
chrX	114258200	0.933884
chrX	114265000	0.942149
chrX	114293300	0.743802
chrX	114409500	0.801653
chrX	114580000	0.834711
chrX	114602600	0.933884
chrX	114607500	0.942149
chrX	114648000	0.818182
chrX	114685200	0.710744
chrX	114695500	0.876033
chrX	114766800	0.991736
chrX	114770600	0.966942
chrX	114774500	0.884298
chrX	114794600	0.900826
chrX	114798500	0.859504
chrX	114806500	0.842975
chrX	114818300	0.876033
chrX	114826600	0.966942
chrX	114849000	0.867769
chrX	114852000	0.867769
chrX	114874100	0.892562
chrX	114924900	0.900826
chrX	117251800	0.826446
chrX	117350300	0.942149
chrX	117359800	0.909091
chrX	117362700	0.867769
chrX	117373500	0.702479
chrX	117406600	0.785124
chrX	117479200	0.727273
chrX	117484900	0.892562
chrX	117490400	0.991736
chrX	117828700	0.983471
chrX	117864200	0.991736
chrX	117886400	0.900826
chrX	118062500	0.661157
chrX	118108700	0.710744
chrX	118135200	0.942149
chrX	118150000	0.892562
chrX	118175500	0.917355
chrX	118287000	0.677686
chrX	118637400	0.933884
chrX	118675600	0.842975

chrX	118756900	0.834711
chrX	118789400	0.975207
chrX	118809900	0.752066
chrX	118819500	0.942149
chrX	118960900	0.719008
chrX	119562200	0.661157
chrX	119588000	0.727273
chrX	119620600	0.669421
chrX	119652500	0.950413
chrX	119682700	0.768595
chrX	119684800	0.975207
chrX	119696500	0.867769
chrX	119806600	1.000000
chrX	119868800	0.900826
chrX	119871700	0.884298
chrX	119883400	0.917355
chrX	119902200	0.859504
chrX	119904300	0.933884
chrX	119915700	0.917355
chrX	119925200	0.892562
chrX	119928100	1.000000
chrX	119930200	0.900826
chrX	119932500	0.966942
chrX	122862900	0.768595
chrX	122937800	0.752066
chrX	122959000	0.983471
chrX	123003400	0.818182
chrX	123093200	0.669421
chrX	123109700	0.694215
chrX	123127900	0.809917
chrX	123561100	0.925620
chrX	125606800	0.710744
chrX	125691600	0.933884
chrX	127011900	0.710744
chrX	127041300	0.760331
chrX	127077900	0.710744
chrX	127155200	0.925620
chrX	127169700	0.950413
chrX	127365400	0.710744
chrX	128628200	0.685950
chrX	128711800	0.892562
chrX	128738600	0.760331
chrX	128748500	0.818182
chrX	128757100	0.950413
chrX	128785500	0.892562
chrX	128789600	0.669421
chrX	128797500	0.917355
chrX	128800400	0.983471
chrX	128803100	0.991736
chrX	128812700	0.925620
chrX	128853500	0.933884
chrX	128856800	0.975207
chrX	128859200	0.925620
chrX	128911600	0.925620

chrX	129095800	0.809917
chrX	129194600	0.768595
chrX	129216300	0.900826
chrX	129220100	0.966942
chrX	129226500	1.000000
chrX	129232300	0.884298
chrX	129240400	1.000000
chrX	129248500	0.818182
chrX	129252600	0.966942
chrX	129255000	0.983471
chrX	129298900	0.685950
chrX	129307900	0.983471
chrX	129905300	0.776860
chrX	130116600	0.950413
chrX	130965400	0.933884
chrX	131113400	0.694215
chrX	131221500	0.702479
chrX	131321700	0.809917
chrX	131615200	0.925620
chrX	131971500	1.000000
chrX	131987300	0.727273
chrX	131992400	0.975207
chrX	132860500	0.719008
chrX	133273100	0.892562
chrX	133604000	0.950413
chrX	133646800	0.917355
chrX	133659900	0.884298
chrX	133663400	0.685950
chrX	133671700	0.925620
chrX	133706000	0.677686
chrX	133728600	0.677686
chrX	133739100	0.942149
chrX	133755800	0.834711
chrX	133759100	0.867769
chrX	133803500	0.719008
chrX	133805600	0.991736
chrX	133827000	1.000000
chrX	133852800	0.958678
chrX	133856000	0.909091
chrX	133873800	0.685950
chrX	133880100	0.983471
chrX	133955900	0.925620
chrX	133971000	0.694215
chrX	134032800	0.785124
chrX	134083600	0.958678
chrX	134125500	0.776860
chrX	134130600	0.685950
chrX	134516800	0.975207
chrX	134656800	0.719008
chrX	134659400	0.793388
chrX	135104500	0.966942
chrX	135113100	0.975207
chrX	135129600	0.760331
chrX	135138300	0.925620

chrX	135147400	0.942149
chrX	135252000	1.000000
chrX	135267800	0.917355
chrX	135273500	0.801653
chrX	135297200	0.983471
chrX	135303000	0.950413
chrX	135341900	0.975207
chrX	135344200	0.909091
chrX	135351800	0.785124
chrX	135354200	0.818182
chrX	136520300	0.900826
chrX	138821400	0.950413
chrX	138908400	0.842975
chrX	138958600	0.851240
chrX	138964400	0.983471
chrX	138973500	0.983471
chrX	138987500	0.983471
chrX	139006800	0.975207
chrX	140315100	0.950413
chrX	140430000	0.909091
chrX	140435900	0.966942
chrX	140500700	0.942149
chrX	140556200	0.892562
chrX	140558300	0.892562
chrX	141049700	0.917355
chrX	147283500	0.768595
chrX	147372800	0.991736
chrX	147960200	0.776860
chrX	148577400	0.793388
chrX	148585000	0.867769
chrX	148595500	0.950413
chrX	148607400	0.942149
chrX	149108800	0.834711
chrX	149175900	0.809917
chrX	149205000	0.851240
chrX	149231800	0.958678
chrX	149253300	0.975207
chrX	149369300	0.826446
chrX	149493800	0.983471
chrX	149615100	0.975207
chrX	149673300	0.991736
chrX	149676900	0.876033
chrX	149716500	0.669421
chrX	149750400	0.851240
chrX	149816200	0.950413
chrX	149818400	0.975207
chrX	149862800	0.752066
chrX	149872700	0.950413
chrX	150564800	0.801653
chrX	151040000	1.000000
chrX	151072000	0.950413
chrX	151151100	0.909091
chrX	151153200	0.975207
chrX	151155400	0.950413

chrX	151998700	0.752066
chrX	152065400	0.884298
chrX	152110600	0.694215
chrX	152726000	0.958678
chrX	152735700	0.743802
chrX	152740700	0.867769
chrX	152743200	0.867769
chrX	152753100	0.950413
chrX	152755200	0.793388
chrX	152757800	0.975207
chrX	152966100	0.876033
chrX	152971000	0.801653
chrX	152974400	0.719008
chrX	152992300	0.966942
chrX	153032000	0.900826
chrX	153085000	0.727273
chrX	153187800	0.942149
chrX	153191000	0.942149
chrX	153193700	0.842975
chrX	153195800	0.975207
chrX	153214100	1.000000
chrX	153233500	0.876033
chrX	153251100	0.933884
chrX	153278900	0.661157
chrX	153284400	0.776860
chrX	153286500	0.958678
chrX	153312400	0.933884
chrX	153323100	0.909091
chrX	153340400	0.876033
chrX	153348800	0.925620
chrX	153390200	0.661157
chrX	153418200	0.760331
chrX	153438400	0.917355
chrX	153475400	0.826446
chrX	153513300	0.826446
chrX	153554600	0.900826
chrX	153562900	0.925620
chrX	153572400	0.851240
chrX	153606600	0.900826
chrX	153629200	0.710744
chrX	153637800	0.785124
chrX	153655900	0.743802
chrX	153659300	0.958678
chrX	153673000	0.661157
chrX	153707400	0.983471
chrX	153718900	0.793388
chrX	153734500	0.809917
chrX	153755700	0.925620
chrX	153769400	0.892562
chrX	153771500	0.876033
chrX	153878500	0.694215
chrX	153941600	0.917355
chrX	153947800	0.983471
chrX	153956100	0.983471

chrX	153958900	0.991736
chrX	153962400	0.966942
chrX	153964500	0.975207
chrX	153967300	0.958678
chrX	154028100	0.983471
chrX	154115600	0.793388
chrX	154285600	0.842975
chrX	154612800	0.719008
chrX	154687400	0.685950
chrY	2720300	0.851240
chrY	2756200	0.917355
chrY	2811500	0.826446
chrY	2871100	0.818182
chrY	3843800	0.942149
chrY	3910300	0.735537
chrY	4212700	0.975207
chrY	7141200	0.702479
chrY	7151400	0.867769
chrY	7207700	0.950413
chrY	7212000	0.917355
chrY	7248000	0.859504
chrY	7251200	0.809917
chrY	7326900	1.000000
chrY	7330400	0.809917
chrY	7412600	0.834711
chrY	8979700	0.975207
chrY	10019300	0.876033
chrY	10021900	0.851240
chrY	10029500	0.975207
chrY	10031700	0.975207
chrY	10037700	0.859504
chrY	13194700	0.900826
chrY	13461000	0.719008
chrY	13490200	0.892562
chrY	13851800	0.694215
chrY	13869600	0.752066
chrY	14665000	0.966942
chrY	14755400	0.950413
chrY	14763600	0.859504
chrY	14830200	0.702479
chrY	15366500	0.842975
chrY	15417600	0.727273
chrY	15473900	0.826446
chrY	15587300	0.900826
chrY	16640400	0.983471
chrY	16698700	0.851240
chrY	16819800	0.752066
chrY	16906500	0.917355
chrY	16910400	0.743802
chrY	16916400	0.983471
chrY	16940400	0.917355
chrY	16946600	0.942149
chrY	18347700	0.950413
chrY	18355300	0.884298

chrY	18453900	0.727273
chrY	18461600	0.900826
chrY	18763300	0.925620
chrY	18766100	0.909091
chrY	19068100	0.892562
chrY	19157600	0.743802
chrY	19279400	0.942149
chrY	19344600	0.900826
chrY	19350400	0.917355
chrY	19384100	0.793388
chrY	19414100	0.933884
chrY	19421000	0.892562
chrY	19430700	0.859504
chrY	19432900	0.917355
chrY	21237600	0.826446
chrY	21731400	0.809917
chrY	24891200	0.776860
chrY	28784200	0.685950
chrY	58856200	0.669421

K562 enhancers

Chromosome	Location	Voting_cutoff
chr10	133800	0.727273
chr10	835400	0.619835
chr10	968000	0.851240
chr10	1055400	0.851240
chr10	1090600	0.644628
chr10	1196000	0.644628
chr10	1655500	0.644628
chr10	3028700	0.611570
chr10	3088400	0.917355
chr10	3095700	0.652893
chr10	3104200	0.735537
chr10	3113100	0.702479
chr10	3116400	1.000000
chr10	3136000	0.884298
chr10	3139300	0.925620
chr10	3141800	0.785124
chr10	3356800	0.966942
chr10	3581700	0.694215
chr10	3698100	0.595041
chr10	3708100	1.000000
chr10	3711800	0.611570
chr10	3734900	0.619835
chr10	3782700	0.628099
chr10	3797900	0.669421
chr10	3805300	0.809917
chr10	3807600	0.950413
chr10	3818200	0.603306
chr10	3822100	0.652893
chr10	3842000	0.578512
chr10	3848700	0.793388
chr10	3851200	0.578512
chr10	3859400	0.628099
chr10	3868200	0.801653
chr10	3880600	0.809917
chr10	3887400	0.619835
chr10	3895400	0.636364
chr10	3933300	0.950413
chr10	3937000	0.768595
chr10	3969500	0.958678
chr10	3976600	0.628099
chr10	4705400	0.727273
chr10	4855400	0.595041
chr10	4862500	0.735537
chr10	4864800	0.727273
chr10	4889900	0.570248
chr10	4892600	0.677686
chr10	4901600	0.578512
chr10	4908400	0.818182
chr10	4912300	0.909091
chr10	4925700	0.983471
chr10	4945600	0.801653

chr10	4983000	0.991736
chr10	4985200	0.884298
chr10	5024000	0.909091
chr10	5062900	0.925620
chr10	5113200	0.826446
chr10	5116800	0.876033
chr10	5121600	0.966942
chr10	5326500	0.958678
chr10	5334100	0.958678
chr10	5337900	0.595041
chr10	5360000	0.933884
chr10	5408100	0.669421
chr10	5446200	0.677686
chr10	5499100	0.570248
chr10	5506200	0.570248
chr10	5510300	0.942149
chr10	5512600	0.884298
chr10	5524600	0.636364
chr10	5538600	0.735537
chr10	5552700	0.735537
chr10	5571300	0.603306
chr10	5581700	0.958678
chr10	5587500	0.942149
chr10	5591200	0.884298
chr10	5593900	0.578512
chr10	5596600	0.842975
chr10	5602300	0.611570
chr10	5606000	0.743802
chr10	5699200	0.586777
chr10	5724100	0.925620
chr10	5739700	0.669421
chr10	5858900	0.636364
chr10	5871300	0.933884
chr10	5876500	0.578512
chr10	5887100	0.809917
chr10	5935400	0.586777
chr10	5941400	0.652893
chr10	5976100	0.942149
chr10	5983400	0.917355
chr10	5987100	0.578512
chr10	5990500	0.652893
chr10	6003100	0.818182
chr10	6010900	0.578512
chr10	6013200	0.892562
chr10	6023700	0.909091
chr10	6030100	0.942149
chr10	6093300	0.628099
chr10	6125700	0.570248
chr10	6134300	0.636364
chr10	6137300	0.818182
chr10	6183600	0.636364
chr10	6188400	0.636364
chr10	6193700	0.760331
chr10	6209100	0.884298

chr10	6218700	0.578512
chr10	6321700	0.611570
chr10	6443900	0.685950
chr10	6488000	0.570248
chr10	6507200	0.570248
chr10	6513500	0.826446
chr10	6517100	0.776860
chr10	6557500	0.975207
chr10	6582500	0.809917
chr10	6625700	0.884298
chr10	6964100	0.727273
chr10	6970500	0.727273
chr10	7047800	0.578512
chr10	7050200	0.818182
chr10	7057600	0.685950
chr10	7069100	0.743802
chr10	7072200	0.661157
chr10	7077500	0.735537
chr10	7085300	0.702479
chr10	7149500	0.719008
chr10	7161700	0.710744
chr10	7222000	0.933884
chr10	7229000	0.644628
chr10	7273700	0.983471
chr10	7276700	0.859504
chr10	7294400	0.950413
chr10	7299100	0.900826
chr10	7335800	0.578512
chr10	7344000	0.619835
chr10	7356900	0.652893
chr10	7414400	0.644628
chr10	7425700	0.677686
chr10	7432000	0.595041
chr10	7442500	0.983471
chr10	7449200	0.884298
chr10	7832600	0.619835
chr10	7837800	0.619835
chr10	7856800	0.586777
chr10	7929800	0.917355
chr10	7975300	0.727273
chr10	8011900	0.834711
chr10	8357100	0.768595
chr10	9867500	0.900826
chr10	9906000	0.884298
chr10	11183900	0.933884
chr10	11188100	0.628099
chr10	11191400	0.743802
chr10	11213300	0.793388
chr10	11219900	0.859504
chr10	11222300	1.000000
chr10	11273500	0.578512
chr10	11492900	0.900826
chr10	11541500	0.950413
chr10	11558600	0.669421

chr10	11581800	0.942149
chr10	11594400	0.942149
chr10	11623500	0.619835
chr10	11628600	0.818182
chr10	11631500	0.851240
chr10	11635800	0.727273
chr10	11641800	0.652893
chr10	11657000	0.892562
chr10	11682500	0.892562
chr10	11685100	0.603306
chr10	11705100	0.628099
chr10	11727100	0.760331
chr10	11729600	0.859504
chr10	11750400	0.570248
chr10	11753100	0.917355
chr10	11755400	0.909091
chr10	11780000	0.611570
chr10	11785100	0.603306
chr10	11867700	0.628099
chr10	11913900	0.851240
chr10	11916600	0.826446
chr10	11919200	0.752066
chr10	11922400	0.752066
chr10	11943500	0.752066
chr10	11955600	0.636364
chr10	12086300	0.619835
chr10	12095800	0.694215
chr10	12098500	0.958678
chr10	12113500	0.834711
chr10	12250500	0.884298
chr10	12264400	0.595041
chr10	12307400	0.677686
chr10	12527700	0.578512
chr10	12988500	0.586777
chr10	13134400	0.768595
chr10	13137700	0.793388
chr10	13144400	0.719008
chr10	13158100	0.702479
chr10	13160600	0.578512
chr10	13311100	0.867769
chr10	13314100	0.950413
chr10	13343700	0.595041
chr10	13433200	0.710744
chr10	13436300	0.595041
chr10	13445700	0.586777
chr10	13447800	0.942149
chr10	13452400	0.677686
chr10	13456900	0.809917
chr10	13460400	0.785124
chr10	13463500	0.586777
chr10	13542000	0.760331
chr10	13547400	0.619835
chr10	13755200	0.826446
chr10	13760400	0.661157

chr10	14223500	0.851240
chr10	14227700	0.950413
chr10	14915800	0.595041
chr10	14924400	0.801653
chr10	14931800	0.983471
chr10	14981500	0.694215
chr10	14993700	0.669421
chr10	15063300	0.578512
chr10	15069900	0.826446
chr10	15077900	0.933884
chr10	15141900	0.603306
chr10	15212100	0.743802
chr10	15297100	0.619835
chr10	15353300	0.661157
chr10	15356200	0.991736
chr10	15367900	0.900826
chr10	15387100	0.925620
chr10	15455800	0.636364
chr10	15484300	0.669421
chr10	15487000	0.719008
chr10	15495200	0.851240
chr10	15497500	0.859504
chr10	15503700	0.570248
chr10	15522400	0.793388
chr10	15526200	0.743802
chr10	15528400	0.652893
chr10	15631000	0.570248
chr10	15648300	0.636364
chr10	15669900	0.611570
chr10	15700300	0.892562
chr10	15767700	0.652893
chr10	15837100	0.851240
chr10	15839500	0.636364
chr10	15843900	0.867769
chr10	15847500	0.719008
chr10	15851400	0.570248
chr10	15859600	0.644628
chr10	15895500	0.595041
chr10	16512900	0.694215
chr10	16807900	0.867769
chr10	16986800	0.570248
chr10	17038600	0.983471
chr10	17065900	0.710744
chr10	17068400	0.727273
chr10	17071600	0.752066
chr10	17091700	0.644628
chr10	17238800	0.842975
chr10	17245600	0.809917
chr10	17250700	0.603306
chr10	17255400	0.851240
chr10	17258300	0.975207
chr10	17286800	0.917355
chr10	17332900	0.586777
chr10	17391000	0.776860

chr10	17457200	0.892562
chr10	17459600	0.752066
chr10	17466000	0.876033
chr10	17469000	0.859504
chr10	17472200	0.661157
chr10	17474300	0.950413
chr10	17477600	0.809917
chr10	17481400	0.884298
chr10	17486300	0.834711
chr10	17499500	0.743802
chr10	17502800	0.785124
chr10	17515000	0.685950
chr10	17531300	0.702479
chr10	17548300	0.752066
chr10	17551600	0.793388
chr10	17639900	0.578512
chr10	17698300	0.909091
chr10	17701800	0.719008
chr10	17705300	0.619835
chr10	17711000	0.652893
chr10	17716100	0.917355
chr10	17718500	0.628099
chr10	17726800	0.884298
chr10	17801200	0.652893
chr10	18048300	0.603306
chr10	18795000	0.611570
chr10	18872000	0.966942
chr10	18954000	0.677686
chr10	21331100	0.975207
chr10	21333600	0.619835
chr10	21380100	0.743802
chr10	21382200	0.586777
chr10	21387300	0.975207
chr10	21419400	0.793388
chr10	21454200	0.636364
chr10	21469600	0.578512
chr10	21474000	0.776860
chr10	21493000	0.661157
chr10	21498000	0.983471
chr10	21502300	0.652893
chr10	21562600	0.628099
chr10	21625700	0.950413
chr10	21654400	0.578512
chr10	21696100	0.644628
chr10	21809000	0.628099
chr10	21820400	0.570248
chr10	21839100	0.578512
chr10	21944900	0.636364
chr10	21951300	0.702479
chr10	21955800	0.685950
chr10	22013600	0.842975
chr10	22040700	0.727273
chr10	22087300	0.892562
chr10	22097800	0.619835

chr10	22100300	0.900826
chr10	22113800	0.669421
chr10	22184200	0.727273
chr10	22216900	0.685950
chr10	22221000	0.735537
chr10	22233900	0.975207
chr10	22239800	0.983471
chr10	22251700	0.826446
chr10	22306100	0.636364
chr10	22311900	0.909091
chr10	22376200	0.809917
chr10	22423100	0.991736
chr10	22449200	0.892562
chr10	22508700	0.685950
chr10	22536400	0.628099
chr10	22599800	0.669421
chr10	22655800	0.694215
chr10	22658800	0.851240
chr10	22664800	0.702479
chr10	22671100	0.595041
chr10	22678900	0.801653
chr10	22700100	0.884298
chr10	22716500	0.702479
chr10	22719400	0.702479
chr10	22743700	0.595041
chr10	22771400	0.578512
chr10	22778100	0.570248
chr10	22783300	0.727273
chr10	22794300	0.768595
chr10	22803200	0.851240
chr10	22840400	0.636364
chr10	22888600	0.694215
chr10	22907100	0.776860
chr10	22913100	0.950413
chr10	22917500	0.966942
chr10	22922400	0.793388
chr10	22933000	0.867769
chr10	22938200	0.669421
chr10	22951900	0.818182
chr10	22954000	0.644628
chr10	22957100	0.785124
chr10	22961700	0.958678
chr10	22965800	0.652893
chr10	22970100	0.752066
chr10	22974500	0.966942
chr10	22982800	0.809917
chr10	22987000	0.685950
chr10	22989100	0.685950
chr10	22992100	0.760331
chr10	22994900	0.785124
chr10	23009600	0.636364
chr10	23026300	0.661157
chr10	23106100	0.851240
chr10	23140900	0.818182

chr10	23144200	0.719008
chr10	23184000	0.603306
chr10	23244500	0.644628
chr10	23305700	0.760331
chr10	23335700	0.892562
chr10	23341200	0.818182
chr10	23345100	0.859504
chr10	23352800	0.859504
chr10	23801700	0.925620
chr10	24860300	0.867769
chr10	24864200	0.661157
chr10	24907400	0.834711
chr10	24932700	0.669421
chr10	24970400	0.644628
chr10	24991000	0.578512
chr10	24997300	0.702479
chr10	25005700	0.603306
chr10	25158700	0.628099
chr10	25162500	0.925620
chr10	25183700	0.595041
chr10	25185800	0.818182
chr10	25190100	0.652893
chr10	25192300	0.785124
chr10	25198000	0.991736
chr10	25205500	0.892562
chr10	25210700	0.966942
chr10	25219000	0.925620
chr10	25233800	0.752066
chr10	25243000	0.586777
chr10	25247800	0.876033
chr10	25278100	0.900826
chr10	25329000	0.793388
chr10	25337200	0.892562
chr10	25346900	0.702479
chr10	25349600	0.809917
chr10	25365900	0.909091
chr10	25369700	0.735537
chr10	25391900	0.834711
chr10	25407100	0.694215
chr10	25464100	0.619835
chr10	25781800	0.876033
chr10	25783900	0.652893
chr10	26050900	0.570248
chr10	26055800	0.619835
chr10	26955000	0.933884
chr10	27068800	0.710744
chr10	27086700	0.611570
chr10	27099500	0.950413
chr10	27133500	0.851240
chr10	27796400	0.578512
chr10	27815300	0.884298
chr10	27819400	0.752066
chr10	28675600	0.842975
chr10	28719600	0.644628

chr10	28769400	0.776860
chr10	28783600	0.876033
chr10	28785800	0.925620
chr10	28858800	0.727273
chr10	28964000	0.595041
chr10	29003700	0.611570
chr10	29009100	0.685950
chr10	29021300	0.801653
chr10	29024400	0.859504
chr10	29074300	0.826446
chr10	29087200	0.710744
chr10	29094900	0.809917
chr10	29098900	0.826446
chr10	29101800	0.942149
chr10	29113500	0.595041
chr10	29122900	0.743802
chr10	29127500	0.661157
chr10	29130000	0.661157
chr10	29139300	0.876033
chr10	29195600	0.661157
chr10	29273900	0.933884
chr10	29276200	0.752066
chr10	29298100	0.677686
chr10	29421400	0.892562
chr10	29424800	0.578512
chr10	29518800	0.818182
chr10	29543300	0.876033
chr10	29545600	0.636364
chr10	29701700	0.752066
chr10	29738400	0.669421
chr10	29760200	0.677686
chr10	30359600	0.611570
chr10	30396300	0.611570
chr10	30441400	0.991736
chr10	30450100	0.818182
chr10	30460300	0.619835
chr10	30480100	0.925620
chr10	30648700	0.743802
chr10	30685800	0.685950
chr10	30707500	0.851240
chr10	30712500	0.702479
chr10	30717000	0.867769
chr10	30720400	0.578512
chr10	30732200	0.917355
chr10	30783200	0.975207
chr10	30790100	0.578512
chr10	30792700	0.942149
chr10	30799800	0.669421
chr10	30831800	0.909091
chr10	30838300	0.603306
chr10	30847300	0.727273
chr10	30873100	0.661157
chr10	30876600	0.760331
chr10	30883300	0.570248

chr10	30901500	0.628099
chr10	31041900	0.603306
chr10	31107300	0.603306
chr10	31110300	0.595041
chr10	31423000	0.909091
chr10	31532200	0.644628
chr10	31567800	0.785124
chr10	32108800	0.776860
chr10	32152000	0.685950
chr10	32201800	0.900826
chr10	32226200	0.669421
chr10	32236600	0.966942
chr10	32241100	0.719008
chr10	32251000	0.669421
chr10	32258900	0.611570
chr10	32261700	0.983471
chr10	32264000	0.685950
chr10	32266800	0.966942
chr10	32293800	0.578512
chr10	32339800	0.603306
chr10	32348500	0.768595
chr10	32428800	0.958678
chr10	32438700	0.892562
chr10	32542700	0.859504
chr10	32622000	0.785124
chr10	32624200	0.578512
chr10	32704400	0.752066
chr10	33211600	0.818182
chr10	33227700	0.768595
chr10	33233200	0.768595
chr10	33237400	0.909091
chr10	33240200	0.586777
chr10	33251200	0.702479
chr10	33259800	0.611570
chr10	33262700	0.611570
chr10	33271700	0.611570
chr10	33275100	0.702479
chr10	33346800	0.876033
chr10	33798700	0.768595
chr10	34452100	0.785124
chr10	34917800	0.842975
chr10	35029300	0.644628
chr10	35047700	0.719008
chr10	35053500	0.851240
chr10	35070000	0.595041
chr10	35332200	0.909091
chr10	35352500	0.950413
chr10	35443000	0.818182
chr10	35481200	0.768595
chr10	35483800	0.595041
chr10	35494200	0.933884
chr10	35557900	0.809917
chr10	35622400	0.776860
chr10	35630100	0.628099

chr10	35637200	0.619835
chr10	35641200	0.859504
chr10	35643400	0.876033
chr10	35657700	0.917355
chr10	35661400	0.710744
chr10	35665800	0.876033
chr10	35670800	0.785124
chr10	35673800	0.818182
chr10	35675900	0.925620
chr10	35678000	0.966942
chr10	35680500	0.966942
chr10	35682800	0.983471
chr10	35685800	0.876033
chr10	35703700	0.876033
chr10	35710500	0.933884
chr10	35720900	0.785124
chr10	35737700	0.925620
chr10	35740500	0.942149
chr10	35747500	0.760331
chr10	35752800	0.785124
chr10	35755400	0.933884
chr10	35759200	0.685950
chr10	35782100	0.859504
chr10	35805500	0.603306
chr10	35823000	0.859504
chr10	35829800	0.876033
chr10	35894600	0.644628
chr10	38161300	0.818182
chr10	38292300	0.685950
chr10	38738900	0.644628
chr10	38759800	0.735537
chr10	39138300	0.694215
chr10	42400100	0.611570
chr10	42525700	0.586777
chr10	42876600	0.628099
chr10	43039800	0.818182
chr10	43140000	0.842975
chr10	43143700	0.760331
chr10	43151900	0.768595
chr10	43156900	0.595041
chr10	43264700	0.652893
chr10	43362800	0.611570
chr10	43783300	0.809917
chr10	43851700	0.867769
chr10	43873300	0.595041
chr10	43885900	0.768595
chr10	43889100	0.785124
chr10	43896000	0.619835
chr10	43911600	0.785124
chr10	43913900	0.966942
chr10	43916500	0.876033
chr10	43919600	0.867769
chr10	43927200	0.644628
chr10	44091400	0.727273

chr10	44095100	0.636364
chr10	44149400	0.636364
chr10	44476300	0.727273
chr10	44729300	0.826446
chr10	44733800	0.933884
chr10	44778800	0.628099
chr10	44973700	0.983471
chr10	45284700	0.942149
chr10	45409400	0.925620
chr10	45470300	0.661157
chr10	45474500	0.991736
chr10	45476900	0.652893
chr10	45482200	0.636364
chr10	45495700	0.586777
chr10	45651300	0.636364
chr10	45672300	0.842975
chr10	45737700	0.586777
chr10	45745500	0.603306
chr10	45757800	0.818182
chr10	45774800	0.983471
chr10	45798300	0.776860
chr10	45848800	0.876033
chr10	45871400	0.735537
chr10	45900300	0.942149
chr10	45903600	0.760331
chr10	45929400	0.570248
chr10	45934100	0.694215
chr10	45968200	0.801653
chr10	46044500	0.603306
chr10	46053400	0.809917
chr10	46078000	0.595041
chr10	46237100	0.876033
chr10	46353700	0.760331
chr10	46359500	0.735537
chr10	46689400	0.586777
chr10	46764800	0.760331
chr10	47008400	0.595041
chr10	47058100	0.694215
chr10	47108400	0.595041
chr10	47129600	0.768595
chr10	47224000	0.578512
chr10	47281000	0.603306
chr10	47589000	0.991736
chr10	47591900	0.652893
chr10	47745500	0.644628
chr10	48049200	0.785124
chr10	48184100	0.933884
chr10	48206100	0.611570
chr10	48332400	0.818182
chr10	48403300	0.644628
chr10	48407500	0.595041
chr10	48412700	0.950413
chr10	48416200	0.768595
chr10	48427500	0.570248

chr10	48432700	0.842975
chr10	48888900	0.595041
chr10	48954500	0.669421
chr10	48959300	0.570248
chr10	49250300	0.752066
chr10	49395500	0.768595
chr10	49498500	0.586777
chr10	49517900	0.702479
chr10	49522800	0.752066
chr10	49572700	0.917355
chr10	49592500	0.752066
chr10	49597600	0.867769
chr10	49600000	0.842975
chr10	49607800	0.884298
chr10	49614400	0.909091
chr10	49625900	0.876033
chr10	49631500	0.652893
chr10	49638700	0.809917
chr10	49664900	0.966942
chr10	49669400	0.884298
chr10	49672400	0.950413
chr10	49675800	0.719008
chr10	49680300	0.809917
chr10	49683800	0.942149
chr10	49686700	0.917355
chr10	49689000	0.809917
chr10	49692900	0.958678
chr10	49702500	0.892562
chr10	49705900	0.851240
chr10	49713900	0.933884
chr10	49716200	0.892562
chr10	49726300	0.752066
chr10	49734800	0.719008
chr10	50209500	0.595041
chr10	50217900	0.785124
chr10	50245700	0.619835
chr10	50277200	0.644628
chr10	50288000	0.628099
chr10	50326600	0.776860
chr10	50349400	0.661157
chr10	50362900	0.942149
chr10	50365200	0.611570
chr10	50367400	0.809917
chr10	50370000	1.000000
chr10	50372200	0.966942
chr10	50374300	0.950413
chr10	50377000	0.942149
chr10	50380500	0.892562
chr10	50383800	0.851240
chr10	50386200	0.950413
chr10	50388400	0.818182
chr10	50390800	0.793388
chr10	50421500	0.884298
chr10	50426200	0.694215

chr10	50433600	0.768595
chr10	50451100	0.619835
chr10	50469400	0.900826
chr10	50486600	0.801653
chr10	50510900	0.595041
chr10	50707900	0.611570
chr10	50735200	0.826446
chr10	50750800	0.628099
chr10	50801800	0.586777
chr10	51256900	0.586777
chr10	51263400	0.727273
chr10	51361400	0.586777
chr10	51397700	0.809917
chr10	51525400	0.603306
chr10	51542100	0.636364
chr10	51544500	0.702479
chr10	51560800	0.694215
chr10	51563200	0.619835
chr10	51580200	0.727273
chr10	51593400	0.958678
chr10	51597800	0.685950
chr10	51633900	0.611570
chr10	51731900	0.727273
chr10	51738300	0.826446
chr10	51994600	0.752066
chr10	52014900	0.595041
chr10	52021100	0.619835
chr10	52032300	0.603306
chr10	52035800	0.710744
chr10	52039400	0.595041
chr10	52041700	0.586777
chr10	52055400	0.900826
chr10	52069000	0.785124
chr10	52072500	0.776860
chr10	52074700	0.933884
chr10	52077800	0.983471
chr10	52080400	0.768595
chr10	52083300	0.942149
chr10	52090200	0.933884
chr10	52094000	0.950413
chr10	52096900	0.570248
chr10	52107500	0.785124
chr10	52112000	0.752066
chr10	52116800	0.925620
chr10	52120900	0.851240
chr10	52124300	0.933884
chr10	52131100	0.570248
chr10	52134800	0.710744
chr10	52140400	0.710744
chr10	52145100	0.652893
chr10	52147900	0.975207
chr10	52150700	0.595041
chr10	52153600	0.834711
chr10	52161300	0.760331

chr10	52167400	0.793388
chr10	52170700	0.867769
chr10	52180800	0.719008
chr10	52198900	0.702479
chr10	52263300	0.636364
chr10	52271900	0.842975
chr10	52287600	0.743802
chr10	52295000	0.950413
chr10	52298800	0.768595
chr10	52303800	0.917355
chr10	52306300	0.917355
chr10	52309700	0.859504
chr10	52319300	0.876033
chr10	52321500	0.991736
chr10	52324500	0.595041
chr10	52329700	0.884298
chr10	52332000	0.909091
chr10	52335100	0.851240
chr10	52340600	0.586777
chr10	52343400	0.942149
chr10	52346700	0.925620
chr10	52352200	0.685950
chr10	52355400	0.892562
chr10	52357900	0.776860
chr10	52363800	0.752066
chr10	52367700	0.652893
chr10	52370400	0.702479
chr10	52374600	0.685950
chr10	52377700	0.727273
chr10	52379900	0.685950
chr10	52389600	0.603306
chr10	52414200	0.661157
chr10	52441200	0.793388
chr10	52462000	0.925620
chr10	58130200	0.793388
chr10	59939300	0.975207
chr10	59978000	0.619835
chr10	59999800	0.710744
chr10	60082500	0.727273
chr10	60092500	0.570248
chr10	61592100	0.685950
chr10	61627000	0.694215
chr10	61633300	0.884298
chr10	61639000	0.694215
chr10	61643600	0.578512
chr10	61646400	0.644628
chr10	61653400	0.909091
chr10	61656200	0.818182
chr10	61659100	0.677686
chr10	61669900	0.661157
chr10	61726100	0.809917
chr10	61791000	0.628099
chr10	61829600	0.793388
chr10	61835200	0.570248

chr10	61857000	0.628099
chr10	61859300	0.685950
chr10	61890600	0.892562
chr10	61895100	0.958678
chr10	61908600	0.595041
chr10	61930300	0.809917
chr10	61935700	0.727273
chr10	62022900	0.884298
chr10	62042500	0.892562
chr10	62045300	0.570248
chr10	62051400	0.768595
chr10	62055600	0.636364
chr10	62065200	0.702479
chr10	62075400	0.727273
chr10	62110700	0.768595
chr10	62115100	0.876033
chr10	62423600	0.603306
chr10	62535300	0.611570
chr10	62565300	0.735537
chr10	62588200	0.851240
chr10	62698000	0.719008
chr10	62716300	0.644628
chr10	62723800	0.619835
chr10	62728300	0.735537
chr10	62736300	0.909091
chr10	62740300	0.586777
chr10	62750700	0.669421
chr10	63235200	0.652893
chr10	63349200	0.661157
chr10	63508200	0.785124
chr10	63511200	0.809917
chr10	63513700	0.603306
chr10	63517600	0.925620
chr10	63527100	0.752066
chr10	63538400	0.966942
chr10	63542100	0.586777
chr10	63546600	0.834711
chr10	63552300	0.595041
chr10	63555300	0.859504
chr10	63558500	0.570248
chr10	63572500	0.652893
chr10	63575400	0.619835
chr10	63582400	0.909091
chr10	63590400	0.851240
chr10	63595400	0.867769
chr10	63600400	0.884298
chr10	63605600	0.636364
chr10	63657700	0.809917
chr10	63741300	0.644628
chr10	63809200	0.735537
chr10	63817100	0.586777
chr10	64496100	0.818182
chr10	64862700	0.942149
chr10	64866300	0.925620

chr10	64870900	0.694215
chr10	64876200	0.983471
chr10	64986000	0.950413
chr10	64996500	0.793388
chr10	65001500	0.925620
chr10	65016600	0.644628
chr10	65019400	0.570248
chr10	65022800	0.595041
chr10	65045400	0.694215
chr10	65062800	0.578512
chr10	65083900	0.966942
chr10	65156500	0.719008
chr10	65170400	0.768595
chr10	65173800	0.694215
chr10	65183700	0.867769
chr10	65198700	0.909091
chr10	65201200	0.809917
chr10	65265100	0.900826
chr10	65283500	0.570248
chr10	65322800	0.586777
chr10	65343600	0.826446
chr10	65398000	0.619835
chr10	65467300	0.677686
chr10	66266100	0.752066
chr10	66268600	0.661157
chr10	66276300	0.942149
chr10	66280000	0.909091
chr10	66283300	0.702479
chr10	69459800	0.578512
chr10	69548500	0.743802
chr10	69550900	0.933884
chr10	69613100	0.710744
chr10	69836100	0.644628
chr10	70032100	0.685950
chr10	70088400	0.826446
chr10	70285400	0.578512
chr10	70302600	0.917355
chr10	70314100	0.669421
chr10	70316800	0.719008
chr10	70325100	0.719008
chr10	70354500	0.719008
chr10	70357300	0.595041
chr10	70570900	0.900826
chr10	70631000	0.818182
chr10	70719800	0.570248
chr10	70778900	0.768595
chr10	70805500	0.826446
chr10	70817600	0.710744
chr10	70821700	0.801653
chr10	70825300	0.636364
chr10	70829600	0.669421
chr10	70833700	0.586777
chr10	70845800	0.801653
chr10	70906700	0.809917

chr10	70970900	0.719008
chr10	70974000	0.834711
chr10	70976800	0.694215
chr10	70982100	0.685950
chr10	70989300	0.801653
chr10	70999200	0.628099
chr10	71002100	0.826446
chr10	71053200	0.752066
chr10	71068500	0.975207
chr10	71071400	0.694215
chr10	71073500	0.719008
chr10	71081500	0.702479
chr10	71092900	0.942149
chr10	71097900	0.942149
chr10	71104200	0.619835
chr10	71149700	0.867769
chr10	71168600	0.727273
chr10	71206800	0.677686
chr10	71218400	0.603306
chr10	71267700	0.570248
chr10	71494700	0.876033
chr10	71616900	0.619835
chr10	71852100	0.710744
chr10	71893700	0.628099
chr10	71897500	0.586777
chr10	71924100	0.851240
chr10	71934400	0.925620
chr10	71988300	0.652893
chr10	71995100	0.669421
chr10	72091800	0.925620
chr10	72151800	0.644628
chr10	72288800	0.760331
chr10	72318900	0.892562
chr10	72336100	0.942149
chr10	72339300	0.809917
chr10	72344900	0.586777
chr10	72349900	0.834711
chr10	72355600	0.975207
chr10	72361100	0.917355
chr10	72363500	0.842975
chr10	72378500	0.793388
chr10	72392500	0.727273
chr10	72409300	0.834711
chr10	72413400	0.884298
chr10	72417000	0.702479
chr10	72420800	0.644628
chr10	72423400	0.776860
chr10	72430200	0.743802
chr10	72435400	0.859504
chr10	72438200	0.785124
chr10	72443000	0.900826
chr10	72445700	0.942149
chr10	72454800	0.669421
chr10	72460000	0.867769

chr10	72475800	0.991736
chr10	72500900	0.909091
chr10	72504800	0.909091
chr10	72517100	0.603306
chr10	72540000	0.611570
chr10	72558300	0.694215
chr10	72574000	0.735537
chr10	72593000	0.892562
chr10	72603700	0.644628
chr10	72983500	0.809917
chr10	72989600	0.834711
chr10	72995000	0.942149
chr10	72998400	0.727273
chr10	73001400	0.719008
chr10	73015600	0.809917
chr10	73024100	0.661157
chr10	73026500	0.801653
chr10	73029700	0.611570
chr10	73073400	0.578512
chr10	73083500	0.669421
chr10	73099300	0.685950
chr10	73103500	0.727273
chr10	73105900	0.818182
chr10	73119400	0.892562
chr10	73138700	0.752066
chr10	73142800	0.785124
chr10	73307800	0.652893
chr10	73342600	0.851240
chr10	73373600	0.925620
chr10	73382500	0.735537
chr10	73390700	0.652893
chr10	73400100	0.636364
chr10	73407500	0.859504
chr10	73410000	0.900826
chr10	73419200	0.950413
chr10	73444600	0.884298
chr10	73461200	0.661157
chr10	73473400	0.876033
chr10	73488300	0.735537
chr10	73503100	0.652893
chr10	73505900	0.586777
chr10	73508000	0.909091
chr10	73513600	0.834711
chr10	73533500	0.702479
chr10	73557800	0.966942
chr10	73566000	0.752066
chr10	73597300	0.677686
chr10	73600800	0.636364
chr10	73603300	0.867769
chr10	73612600	0.669421
chr10	73615100	0.719008
chr10	73618900	0.719008
chr10	73622600	0.578512
chr10	73628200	0.950413

chr10	73634500	0.975207
chr10	73638600	0.776860
chr10	73642900	0.884298
chr10	73653900	0.752066
chr10	73680800	0.909091
chr10	73722800	0.611570
chr10	73740600	0.785124
chr10	73856300	0.842975
chr10	73858700	0.884298
chr10	73931300	0.694215
chr10	73937300	0.669421
chr10	73979200	0.661157
chr10	73983000	0.950413
chr10	74004000	0.834711
chr10	74006200	0.818182
chr10	74008900	0.685950
chr10	74014700	0.842975
chr10	74017400	0.842975
chr10	74019700	0.719008
chr10	74026300	0.917355
chr10	74031100	0.578512
chr10	74037700	0.752066
chr10	74051200	0.867769
chr10	74059400	0.735537
chr10	74065600	0.785124
chr10	74068300	0.719008
chr10	74076600	0.859504
chr10	74079600	0.859504
chr10	74083400	0.942149
chr10	74087800	0.892562
chr10	74124700	0.826446
chr10	74150600	0.578512
chr10	74226500	0.669421
chr10	74263200	0.942149
chr10	74365300	0.809917
chr10	74397300	0.933884
chr10	74402100	0.892562
chr10	74419500	0.719008
chr10	74436400	0.636364
chr10	74447600	0.876033
chr10	74450000	0.818182
chr10	74455200	0.760331
chr10	74488500	0.801653
chr10	74519600	0.570248
chr10	74554400	0.586777
chr10	74560700	0.586777
chr10	74599800	0.991736
chr10	74648400	0.809917
chr10	74664200	0.619835
chr10	74700200	0.826446
chr10	74714800	0.842975
chr10	74742000	0.826446
chr10	74840200	0.842975
chr10	74842900	0.636364

chr10	74852000	0.694215
chr10	74878400	0.892562
chr10	74942800	0.867769
chr10	75127400	0.975207
chr10	75167600	0.776860
chr10	75216900	0.900826
chr10	75220000	0.595041
chr10	75261600	0.826446
chr10	75277400	0.917355
chr10	75290700	0.644628
chr10	75346900	0.578512
chr10	75351800	0.884298
chr10	75409200	0.983471
chr10	75411500	0.818182
chr10	75414500	0.644628
chr10	75421700	0.793388
chr10	75463900	0.685950
chr10	75468000	0.801653
chr10	75474200	0.925620
chr10	75477800	0.685950
chr10	75516300	0.834711
chr10	75536700	0.636364
chr10	75569100	0.644628
chr10	75576500	1.000000
chr10	75578800	0.818182
chr10	75647000	0.694215
chr10	75654400	0.851240
chr10	75667800	0.586777
chr10	75684000	0.570248
chr10	75686400	0.578512
chr10	75692500	0.958678
chr10	75701000	0.785124
chr10	75720400	0.991736
chr10	75725900	0.876033
chr10	75739900	0.669421
chr10	75749500	0.892562
chr10	75752900	0.752066
chr10	75755300	0.909091
chr10	75763500	0.743802
chr10	75768200	0.842975
chr10	75771700	0.752066
chr10	75779200	0.950413
chr10	75783200	0.694215
chr10	75786700	0.834711
chr10	75789900	0.975207
chr10	75793200	0.619835
chr10	75800200	0.644628
chr10	75807600	0.669421
chr10	75812500	0.652893
chr10	75816300	0.785124
chr10	75827600	0.867769
chr10	75925900	0.801653
chr10	75939800	0.652893
chr10	75949800	0.760331

chr10	75998300	0.586777
chr10	76053700	0.570248
chr10	76124500	0.636364
chr10	76178600	0.801653
chr10	76194100	0.595041
chr10	76582700	0.785124
chr10	76612600	0.892562
chr10	76623500	0.685950
chr10	76681600	0.834711
chr10	76864500	0.727273
chr10	76867000	0.719008
chr10	76875500	0.636364
chr10	76881600	0.586777
chr10	76900500	0.578512
chr10	76935000	0.760331
chr10	76952100	0.677686
chr10	76957200	0.578512
chr10	76967500	0.859504
chr10	77000800	0.760331
chr10	77003600	0.727273
chr10	77036200	0.603306
chr10	79595500	0.884298
chr10	79597700	0.809917
chr10	79606900	0.743802
chr10	79649500	0.859504
chr10	79659900	0.909091
chr10	79667900	0.619835
chr10	79671700	0.909091
chr10	79680100	0.694215
chr10	79682900	0.809917
chr10	79687500	0.586777
chr10	79692500	0.595041
chr10	79699700	0.669421
chr10	79729700	0.950413
chr10	79732300	0.793388
chr10	79743800	0.859504
chr10	79746800	0.611570
chr10	79798000	0.677686
chr10	79801900	0.900826
chr10	79804200	0.834711
chr10	80015900	0.909091
chr10	80679500	0.834711
chr10	80685700	0.595041
chr10	80697900	0.677686
chr10	80725200	0.578512
chr10	80735100	0.595041
chr10	80780200	0.983471
chr10	80782700	0.818182
chr10	80787700	0.859504
chr10	80792200	0.884298
chr10	80798600	0.752066
chr10	80808300	0.917355
chr10	80839500	0.942149
chr10	80844400	0.867769

chr10	80852300	0.826446
chr10	80856300	0.636364
chr10	80874600	0.809917
chr10	80876800	0.776860
chr10	80884700	0.603306
chr10	80887500	0.884298
chr10	80889800	0.636364
chr10	80893000	0.933884
chr10	80895900	0.842975
chr10	80899400	0.644628
chr10	80905000	0.636364
chr10	80907400	0.743802
chr10	80911700	0.842975
chr10	80915300	0.570248
chr10	80918100	0.611570
chr10	80935000	0.776860
chr10	80937100	0.719008
chr10	80940200	0.917355
chr10	80943300	0.867769
chr10	80945400	0.834711
chr10	80948300	0.826446
chr10	80955700	0.669421
chr10	80999600	0.694215
chr10	81030300	0.710744
chr10	81046500	0.603306
chr10	81077900	0.776860
chr10	81103200	0.958678
chr10	81110100	0.809917
chr10	81135200	0.611570
chr10	81148200	0.859504
chr10	81152800	0.859504
chr10	81159600	0.768595
chr10	81164700	0.578512
chr10	81173300	0.900826
chr10	81175900	0.743802
chr10	81195000	0.834711
chr10	81200600	0.652893
chr10	81209700	0.710744
chr10	81212100	0.876033
chr10	81227900	0.892562
chr10	81235700	0.710744
chr10	81248900	0.611570
chr10	81251100	0.578512
chr10	81396900	0.661157
chr10	81468200	0.603306
chr10	81539100	0.719008
chr10	81542200	0.603306
chr10	81606300	0.735537
chr10	81626600	0.801653
chr10	81689500	0.801653
chr10	81818800	0.702479
chr10	81919100	1.000000
chr10	81949500	0.818182
chr10	81961200	0.793388

chr10	81971900	0.925620
chr10	82026200	0.603306
chr10	82028900	0.570248
chr10	82032800	0.900826
chr10	82174200	0.900826
chr10	82203100	0.785124
chr10	82205300	0.595041
chr10	82209900	0.570248
chr10	82217700	0.801653
chr10	82221400	0.900826
chr10	82224100	0.677686
chr10	82228400	0.586777
chr10	82245100	0.909091
chr10	82248400	0.925620
chr10	82253600	0.578512
chr10	82260300	0.975207
chr10	82265900	0.925620
chr10	82478000	0.735537
chr10	82488600	0.793388
chr10	82500100	0.768595
chr10	82512400	0.752066
chr10	82529300	0.925620
chr10	82533600	0.876033
chr10	82656900	0.793388
chr10	85743900	0.793388
chr10	85961400	0.595041
chr10	86054100	0.859504
chr10	86087000	0.842975
chr10	86094000	0.950413
chr10	86101900	0.719008
chr10	86285200	0.727273
chr10	87661800	0.661157
chr10	87784900	0.785124
chr10	87839000	0.950413
chr10	88295400	0.702479
chr10	88512800	0.818182
chr10	88522100	0.603306
chr10	88530900	0.595041
chr10	88535300	0.752066
chr10	88555800	0.702479
chr10	88648900	0.950413
chr10	88714700	0.917355
chr10	88718100	0.884298
chr10	88721300	0.636364
chr10	88726800	0.611570
chr10	88741300	0.603306
chr10	88785700	0.818182
chr10	88990400	0.834711
chr10	89061100	0.677686
chr10	89064300	0.710744
chr10	89122800	0.793388
chr10	89143100	0.685950
chr10	89158400	0.570248
chr10	89193400	0.834711

chr10	89196300	0.636364
chr10	89260800	0.900826
chr10	89306700	0.735537
chr10	89451500	0.669421
chr10	89708700	0.983471
chr10	89802000	0.595041
chr10	90846700	0.892562
chr10	91093000	0.727273
chr10	91112400	0.942149
chr10	91122900	0.933884
chr10	91131700	0.834711
chr10	91134700	0.842975
chr10	91400100	0.611570
chr10	91410200	0.570248
chr10	91467000	0.570248
chr10	92559900	0.925620
chr10	92862900	0.925620
chr10	92865400	0.851240
chr10	92951300	0.578512
chr10	92956500	0.719008
chr10	92961100	0.652893
chr10	92971600	0.619835
chr10	93008000	0.735537
chr10	93073000	0.859504
chr10	93141400	0.628099
chr10	93336300	0.925620
chr10	93348100	1.000000
chr10	93352100	0.694215
chr10	93367900	0.933884
chr10	93370200	0.768595
chr10	93373700	0.884298
chr10	93377200	0.652893
chr10	93390100	0.892562
chr10	93432300	0.661157
chr10	93450700	0.628099
chr10	93662000	0.842975
chr10	93787100	0.768595
chr10	93861400	0.842975
chr10	93870400	0.611570
chr10	93998900	0.834711
chr10	94167800	0.892562
chr10	94190400	0.595041
chr10	94247100	0.809917
chr10	94302700	0.595041
chr10	94350600	0.636364
chr10	94457300	0.636364
chr10	94460500	0.619835
chr10	94513800	0.909091
chr10	94516300	0.793388
chr10	94518700	0.628099
chr10	94521400	0.801653
chr10	94582400	0.876033
chr10	94588700	0.719008
chr10	94599300	0.809917

chr10	94607300	0.595041
chr10	94651000	0.570248
chr10	94673200	0.900826
chr10	94731600	0.884298
chr10	94733700	0.933884
chr10	94750900	0.958678
chr10	94830700	0.900826
chr10	94833500	0.636364
chr10	94838200	0.570248
chr10	95314800	0.669421
chr10	95631700	0.628099
chr10	95960400	0.719008
chr10	96137600	0.578512
chr10	96152300	0.586777
chr10	96158000	0.619835
chr10	96377500	0.644628
chr10	97026700	0.876033
chr10	97029000	0.710744
chr10	97033600	0.578512
chr10	97036600	0.743802
chr10	97038800	0.694215
chr10	97045300	0.570248
chr10	97048100	0.652893
chr10	97057500	0.975207
chr10	97064000	0.983471
chr10	97068400	0.801653
chr10	97084600	0.933884
chr10	97120800	0.603306
chr10	97154200	0.925620
chr10	97158100	0.685950
chr10	97186500	0.586777
chr10	97206100	0.644628
chr10	97227600	0.628099
chr10	97240000	0.876033
chr10	97253600	0.851240
chr10	97257100	0.859504
chr10	97261700	0.851240
chr10	97264500	0.826446
chr10	97268800	0.735537
chr10	97271400	0.595041
chr10	97278100	0.644628
chr10	97289000	0.876033
chr10	97292300	0.578512
chr10	97294400	0.644628
chr10	97297400	0.628099
chr10	97304900	0.851240
chr10	97318900	0.661157
chr10	97334700	0.611570
chr10	97406700	0.892562
chr10	97424500	0.950413
chr10	97723700	0.628099
chr10	97782700	0.768595
chr10	97832500	0.735537
chr10	97853800	0.727273

chr10	97872700	0.702479
chr10	97883400	0.801653
chr10	98243000	0.611570
chr10	98356200	0.760331
chr10	98374300	0.876033
chr10	98379300	1.000000
chr10	98383000	0.710744
chr10	98400100	0.669421
chr10	98437100	0.586777
chr10	98525400	0.628099
chr10	98561200	0.925620
chr10	98586900	0.570248
chr10	98589200	0.842975
chr10	98596100	0.685950
chr10	98600800	0.876033
chr10	98618600	0.669421
chr10	98624300	0.603306
chr10	98784100	0.578512
chr10	98982700	0.842975
chr10	99039100	0.876033
chr10	99054000	0.619835
chr10	99058100	0.950413
chr10	99067700	0.578512
chr10	99072700	0.669421
chr10	99087900	0.570248
chr10	99090300	0.611570
chr10	99107700	0.834711
chr10	99174500	0.950413
chr10	99178800	0.917355
chr10	99302900	0.975207
chr10	99357400	0.669421
chr10	99388100	0.925620
chr10	99407200	0.834711
chr10	99411200	0.933884
chr10	99413400	0.975207
chr10	99423000	0.793388
chr10	99426600	0.636364
chr10	99449800	0.917355
chr10	99478800	0.595041
chr10	99503700	1.000000
chr10	99532400	0.578512
chr10	99549000	0.842975
chr10	99551600	0.834711
chr10	99571000	0.652893
chr10	99600300	1.000000
chr10	99602900	0.652893
chr10	99649600	0.661157
chr10	99658500	0.636364
chr10	99709500	0.611570
chr10	99766500	0.876033
chr10	99996700	0.933884
chr10	100020100	0.925620
chr10	100083200	0.892562
chr10	100141000	0.702479

chr10	100164200	0.735537
chr10	100175300	0.636364
chr10	100217800	0.884298
chr10	100220600	0.942149
chr10	100223500	0.991736
chr10	100227500	0.917355
chr10	100537200	0.925620
chr10	100540300	0.942149
chr10	100668800	0.884298
chr10	100671000	0.925620
chr10	100680500	0.983471
chr10	101194100	0.619835
chr10	101196300	0.669421
chr10	101377200	0.586777
chr10	101382700	0.595041
chr10	101413600	0.595041
chr10	101427400	0.785124
chr10	101445700	0.644628
chr10	101468900	0.991736
chr10	101544900	0.917355
chr10	101744100	0.694215
chr10	101754600	0.719008
chr10	101757100	0.636364
chr10	102049800	0.719008
chr10	102111800	0.619835
chr10	102116700	0.685950
chr10	102119900	0.793388
chr10	102124800	0.776860
chr10	102130200	0.661157
chr10	102145600	0.586777
chr10	102214900	0.578512
chr10	102391400	0.710744
chr10	102396900	0.702479
chr10	102669700	0.595041
chr10	102671800	0.619835
chr10	102755400	0.644628
chr10	102761500	0.793388
chr10	102771900	0.644628
chr10	102792300	0.603306
chr10	102823800	0.851240
chr10	102831500	0.669421
chr10	103127700	0.570248
chr10	103154500	0.570248
chr10	103210300	0.719008
chr10	103214100	0.942149
chr10	103295900	0.900826
chr10	103306000	0.595041
chr10	103326700	0.611570
chr10	103336200	0.942149
chr10	103451100	0.586777
chr10	103477200	0.719008
chr10	103485500	0.636364
chr10	103509600	0.900826
chr10	103581200	0.727273

chr10	103585000	0.834711
chr10	103587900	0.619835
chr10	103591100	0.694215
chr10	103595100	0.586777
chr10	103600300	0.628099
chr10	103805500	0.595041
chr10	103817800	0.859504
chr10	103870500	0.933884
chr10	103876200	0.818182
chr10	103896100	0.760331
chr10	103911100	0.727273
chr10	103923500	0.892562
chr10	103986000	0.694215
chr10	104194100	0.603306
chr10	104202200	0.595041
chr10	104208800	0.611570
chr10	104212900	0.917355
chr10	104215900	0.983471
chr10	104221300	0.743802
chr10	104223700	0.727273
chr10	104228100	0.793388
chr10	104239300	0.694215
chr10	104353100	0.595041
chr10	104356800	0.570248
chr10	104359800	0.983471
chr10	104364500	0.595041
chr10	104399700	0.586777
chr10	104431000	0.892562
chr10	104439000	0.570248
chr10	104466300	0.586777
chr10	104470700	0.809917
chr10	104478400	0.793388
chr10	104495100	0.867769
chr10	104498400	0.983471
chr10	104518600	0.677686
chr10	104522900	0.818182
chr10	104530400	0.983471
chr10	104536600	0.917355
chr10	104543000	0.867769
chr10	104562100	0.719008
chr10	104574500	1.000000
chr10	104594300	0.842975
chr10	104616400	0.570248
chr10	104688000	0.644628
chr10	104704900	0.694215
chr10	104708300	0.595041
chr10	104723100	0.834711
chr10	104748100	0.859504
chr10	104750800	0.933884
chr10	104801100	0.867769
chr10	104814300	0.636364
chr10	104954000	0.809917
chr10	104957300	0.991736
chr10	105231500	0.785124

chr10	105264500	0.636364
chr10	105334900	0.793388
chr10	105339000	0.884298
chr10	105342400	0.702479
chr10	105369200	0.595041
chr10	105372600	0.834711
chr10	105375500	0.884298
chr10	105380600	0.950413
chr10	105451700	0.727273
chr10	105456300	0.983471
chr10	105532500	0.719008
chr10	105668500	0.950413
chr10	105670900	0.826446
chr10	105679200	0.818182
chr10	106007300	0.809917
chr10	106013100	0.776860
chr10	106020600	0.727273
chr10	106034800	0.644628
chr10	106066000	0.801653
chr10	106087700	0.735537
chr10	106091200	0.925620
chr10	106094400	0.595041
chr10	106102500	0.958678
chr10	106107300	0.950413
chr10	106110000	0.917355
chr10	106338300	0.867769
chr10	108702300	0.983471
chr10	108757000	0.851240
chr10	108773600	0.851240
chr10	108782800	0.892562
chr10	108785800	0.925620
chr10	108788500	0.735537
chr10	108793800	0.818182
chr10	108816400	0.785124
chr10	108820200	0.785124
chr10	108841800	0.818182
chr10	108844100	0.652893
chr10	111652600	0.826446
chr10	111684300	0.677686
chr10	111701400	0.611570
chr10	111738700	0.892562
chr10	111743400	0.793388
chr10	111756300	0.661157
chr10	111759000	0.603306
chr10	111765900	0.636364
chr10	111770800	0.652893
chr10	111834600	0.793388
chr10	111836700	0.644628
chr10	111841700	0.942149
chr10	111909600	0.876033
chr10	111941500	0.628099
chr10	111966100	0.611570
chr10	111973300	0.719008
chr10	111977700	0.710744

chr10	111982100	0.785124
chr10	111987900	0.628099
chr10	112011800	0.743802
chr10	112018500	0.644628
chr10	112025600	0.611570
chr10	112034600	0.603306
chr10	112117300	0.570248
chr10	112128300	0.818182
chr10	112136400	0.619835
chr10	112169800	0.801653
chr10	112175600	0.603306
chr10	112186200	0.892562
chr10	112201700	0.909091
chr10	112207200	0.867769
chr10	112243800	0.628099
chr10	112247400	0.603306
chr10	112256900	0.669421
chr10	112260200	0.644628
chr10	112264500	0.628099
chr10	112272600	0.834711
chr10	112295900	0.586777
chr10	112370000	0.570248
chr10	112373600	0.628099
chr10	112377000	0.950413
chr10	112397600	0.636364
chr10	112411800	0.743802
chr10	112429700	0.826446
chr10	112460100	0.595041
chr10	112477800	0.785124
chr10	112525600	0.991736
chr10	112532600	0.793388
chr10	112535000	0.842975
chr10	112537200	0.603306
chr10	112539400	0.578512
chr10	112546300	0.636364
chr10	112548800	0.644628
chr10	112582000	0.842975
chr10	112594500	0.933884
chr10	112598300	0.644628
chr10	112617600	0.776860
chr10	112620200	0.884298
chr10	112622800	0.611570
chr10	112625200	0.859504
chr10	112627800	0.909091
chr10	112935800	0.644628
chr10	114095500	0.611570
chr10	114136000	0.669421
chr10	114143100	0.867769
chr10	114438200	0.876033
chr10	114603700	0.983471
chr10	114672500	0.710744
chr10	114822400	0.785124
chr10	114824500	0.644628
chr10	114834000	0.636364

chr10	114847000	0.859504
chr10	114849600	0.818182
chr10	114852200	0.809917
chr10	115318100	0.652893
chr10	115580400	0.801653
chr10	115582500	0.735537
chr10	115604300	0.884298
chr10	115626200	0.570248
chr10	115707900	0.694215
chr10	115785000	0.950413
chr10	115860900	0.619835
chr10	116394900	0.652893
chr10	118707200	0.867769
chr10	118744600	0.636364
chr10	118977000	0.578512
chr10	119032300	0.900826
chr10	119038500	0.652893
chr10	119105400	0.611570
chr10	119108600	0.809917
chr10	119127100	0.578512
chr10	119140200	0.694215
chr10	119168100	0.975207
chr10	119171800	0.801653
chr10	119174700	0.735537
chr10	119184900	0.570248
chr10	119188200	0.661157
chr10	119191600	0.636364
chr10	119868100	0.933884
chr10	120229500	0.702479
chr10	120242000	0.942149
chr10	120244900	0.900826
chr10	120255600	1.000000
chr10	120458800	0.570248
chr10	120495200	0.586777
chr10	120506400	0.628099
chr10	120529100	0.983471
chr10	120836300	0.586777
chr10	120850700	0.834711
chr10	120861200	0.719008
chr10	120880400	0.884298
chr10	120891300	0.727273
chr10	120921600	0.925620
chr10	120928400	0.975207
chr10	120934900	0.785124
chr10	120945100	0.628099
chr10	120949900	0.876033
chr10	120954800	0.636364
chr10	120968100	0.611570
chr10	121010700	0.950413
chr10	121031300	0.793388
chr10	121065700	0.876033
chr10	121075500	0.702479
chr10	121226500	0.603306
chr10	121276400	0.975207

chr10	121278600	0.776860
chr10	121281000	0.950413
chr10	121296200	0.661157
chr10	121298900	0.586777
chr10	121377400	0.595041
chr10	121415600	0.727273
chr10	121418400	0.917355
chr10	121427700	0.925620
chr10	121498300	0.595041
chr10	121529800	0.570248
chr10	121546000	0.619835
chr10	121656000	0.809917
chr10	122219100	0.619835
chr10	123593800	0.603306
chr10	123695700	0.586777
chr10	123822900	0.818182
chr10	124067900	0.603306
chr10	124636500	0.719008
chr10	124654200	0.727273
chr10	124690000	0.950413
chr10	124692100	0.628099
chr10	124761500	0.644628
chr10	124764600	0.628099
chr10	124770800	0.851240
chr10	124892900	0.760331
chr10	124904900	0.958678
chr10	124917100	0.694215
chr10	124919400	0.570248
chr10	125559900	0.702479
chr10	126109500	0.892562
chr10	126113000	0.652893
chr10	126118700	0.603306
chr10	126142900	0.900826
chr10	126154100	0.900826
chr10	126170400	0.578512
chr10	126236600	0.677686
chr10	126278100	0.859504
chr10	126317100	0.950413
chr10	126330300	0.983471
chr10	126336100	0.942149
chr10	126357000	0.727273
chr10	126377900	0.892562
chr10	126382100	0.958678
chr10	126390800	0.785124
chr10	126406900	0.983471
chr10	126412500	0.595041
chr10	126429000	0.776860
chr10	126487600	0.694215
chr10	126600600	0.851240
chr10	126609500	0.603306
chr10	126636100	0.702479
chr10	126734600	0.975207
chr10	126738000	0.859504
chr10	126740800	0.603306

chr10	126746400	0.644628
chr10	126802500	0.950413
chr10	126804600	0.595041
chr10	126830600	0.925620
chr10	126834200	0.719008
chr10	126837500	0.983471
chr10	126839800	0.578512
chr10	127351100	0.768595
chr10	127506900	0.809917
chr10	127514900	0.595041
chr10	127517500	0.727273
chr10	127885900	0.983471
chr10	127888300	0.851240
chr10	127890400	0.900826
chr10	127893500	0.842975
chr10	128327100	0.570248
chr10	129861400	0.578512
chr10	130334700	0.570248
chr10	130341400	0.702479
chr10	130374700	0.611570
chr10	130396600	0.760331
chr10	130400000	0.727273
chr10	130428200	0.983471
chr10	130913200	0.884298
chr10	131937400	0.611570
chr10	131940100	0.867769
chr10	133728700	0.785124
chr10	133731300	0.966942
chr10	133733800	0.942149
chr10	133736700	0.652893
chr10	133741100	0.942149
chr10	133744400	0.595041
chr10	133747200	0.876033
chr10	133776700	0.793388
chr10	133806700	0.793388
chr10	133809600	0.570248
chr10	133812400	0.917355
chr10	133815300	0.818182
chr10	134094000	0.768595
chr10	134125100	0.892562
chr10	134134200	0.752066
chr10	134140100	0.785124
chr10	134149600	0.644628
chr10	134235400	0.933884
chr10	134261000	0.909091
chr10	134267000	0.619835
chr10	134331300	0.785124
chr10	134352200	0.603306
chr10	134358600	0.685950
chr10	134369200	0.909091
chr10	134373700	0.842975
chr10	134386000	0.983471
chr10	134442000	0.652893
chr10	134498900	1.000000

chr10	134957000	0.892562
chr10	135039100	0.702479
chr10	135059700	0.950413
chr10	135062300	0.661157
chr10	135068600	0.595041
chr10	135071600	0.851240
chr10	135089300	0.859504
chr10	135092700	0.909091
chr10	135157000	0.669421
chr10	135173800	0.884298
chr10	135195900	0.661157
chr10	135204800	0.867769
chr10	135209800	0.727273
chr10	135517000	0.859504
chr10	135519200	0.735537
chr11	87300	0.983471
chr11	106900	0.628099
chr11	129000	0.859504
chr11	183300	0.966942
chr11	185400	0.851240
chr11	204700	0.586777
chr11	210900	0.669421
chr11	216000	0.818182
chr11	218100	0.966942
chr11	220700	0.991736
chr11	223000	0.950413
chr11	257100	0.702479
chr11	278000	0.975207
chr11	290400	0.694215
chr11	298000	0.983471
chr11	306000	0.776860
chr11	323700	0.644628
chr11	326700	0.586777
chr11	334700	0.834711
chr11	337200	0.851240
chr11	342000	0.933884
chr11	345600	0.942149
chr11	349500	0.876033
chr11	353100	0.917355
chr11	361700	0.785124
chr11	389300	0.884298
chr11	393300	0.619835
chr11	422900	0.619835
chr11	444800	0.834711
chr11	447100	0.603306
chr11	457600	0.727273
chr11	460900	0.917355
chr11	469600	0.884298
chr11	503300	0.677686
chr11	519300	0.710744
chr11	537700	0.851240
chr11	552100	0.925620
chr11	554700	0.578512
chr11	574300	0.578512

chr11	580000	0.925620
chr11	613300	0.710744
chr11	666100	0.876033
chr11	691400	0.942149
chr11	698100	0.702479
chr11	724600	0.578512
chr11	727300	0.727273
chr11	738000	0.719008
chr11	742000	0.595041
chr11	745800	0.669421
chr11	752900	0.636364
chr11	806700	0.636364
chr11	838500	0.958678
chr11	844000	0.578512
chr11	859900	0.628099
chr11	862700	0.743802
chr11	876300	0.966942
chr11	878400	0.661157
chr11	908700	0.611570
chr11	915400	0.801653
chr11	922300	0.793388
chr11	938100	0.785124
chr11	943100	0.900826
chr11	1042400	0.570248
chr11	1292700	0.586777
chr11	1330300	0.636364
chr11	1357500	0.603306
chr11	1377900	0.776860
chr11	1380000	0.719008
chr11	1534000	0.578512
chr11	1542400	0.851240
chr11	1781600	0.867769
chr11	1790600	0.669421
chr11	1793900	0.892562
chr11	1866300	0.900826
chr11	1990800	0.942149
chr11	2000200	0.570248
chr11	2010200	0.975207
chr11	2012400	0.685950
chr11	2020000	0.834711
chr11	2290500	0.694215
chr11	2307400	0.867769
chr11	2315600	0.677686
chr11	2323100	0.578512
chr11	2327400	0.603306
chr11	2411200	0.595041
chr11	2555000	0.785124
chr11	2703600	0.570248
chr11	2725000	0.685950
chr11	2747200	0.595041
chr11	2761800	0.933884
chr11	2769300	0.842975
chr11	2791000	0.578512
chr11	2800100	1.000000

chr11	2802200	0.595041
chr11	2804300	0.785124
chr11	2821400	0.727273
chr11	2833500	0.752066
chr11	2858600	0.586777
chr11	2867600	0.975207
chr11	2889200	0.884298
chr11	2912200	0.966942
chr11	2920200	0.735537
chr11	2934000	0.719008
chr11	3073600	0.867769
chr11	3080600	0.933884
chr11	3170600	0.826446
chr11	3190400	0.611570
chr11	3210600	0.595041
chr11	3242600	0.702479
chr11	3253000	0.942149
chr11	3282000	0.884298
chr11	3284900	0.925620
chr11	3295700	0.611570
chr11	3306000	0.900826
chr11	3312200	0.900826
chr11	3322800	0.818182
chr11	3327500	0.917355
chr11	3330300	0.942149
chr11	3352100	0.900826
chr11	3358000	0.991736
chr11	3361700	0.975207
chr11	3367400	0.743802
chr11	3423200	0.760331
chr11	3427200	0.586777
chr11	3459000	0.867769
chr11	3506000	0.644628
chr11	3531700	0.834711
chr11	3538300	0.818182
chr11	3626400	0.785124
chr11	3636000	0.661157
chr11	3666700	0.661157
chr11	3808200	0.809917
chr11	3838700	0.652893
chr11	3847100	1.000000
chr11	3853200	0.776860
chr11	3864000	0.809917
chr11	3870200	0.628099
chr11	3874200	0.900826
chr11	3886500	0.851240
chr11	3889800	0.942149
chr11	3892500	1.000000
chr11	3899500	0.859504
chr11	3901800	0.685950
chr11	3913300	0.925620
chr11	3916100	0.958678
chr11	3925300	0.661157
chr11	3928900	0.652893

chr11	3933000	0.611570
chr11	3941900	0.578512
chr11	3951600	0.735537
chr11	3955700	0.595041
chr11	3964500	0.727273
chr11	3972100	0.694215
chr11	3976100	0.900826
chr11	3980500	0.644628
chr11	3985100	0.760331
chr11	3987700	0.727273
chr11	3998800	0.809917
chr11	4001900	0.644628
chr11	4006500	0.834711
chr11	4017900	0.801653
chr11	4038100	0.586777
chr11	4065800	0.586777
chr11	4113800	0.801653
chr11	4166700	0.892562
chr11	4183000	0.801653
chr11	4206200	0.685950
chr11	4218000	0.867769
chr11	4679400	0.570248
chr11	4903700	0.909091
chr11	4912700	0.694215
chr11	5108800	0.818182
chr11	5129000	0.694215
chr11	5145700	0.842975
chr11	5150500	0.628099
chr11	5154100	0.628099
chr11	5173200	0.942149
chr11	5176100	0.760331
chr11	5181400	0.760331
chr11	5187700	0.793388
chr11	5193700	0.752066
chr11	5217800	0.809917
chr11	5245900	0.685950
chr11	5254100	0.636364
chr11	5257300	0.694215
chr11	5278600	0.595041
chr11	5280700	0.768595
chr11	5286100	0.628099
chr11	5292800	0.834711
chr11	5295000	0.950413
chr11	5298800	0.785124
chr11	5302300	0.958678
chr11	5304400	0.909091
chr11	5307800	0.859504
chr11	5311700	0.818182
chr11	5314100	0.867769
chr11	5319200	0.884298
chr11	5321600	0.785124
chr11	5325500	0.586777
chr11	5356800	0.876033
chr11	5385400	0.975207

chr11	5433400	0.809917
chr11	5481900	0.611570
chr11	5519900	0.570248
chr11	5530100	0.909091
chr11	5552700	0.983471
chr11	5555800	0.636364
chr11	5578300	0.652893
chr11	5602300	0.809917
chr11	5613600	0.710744
chr11	5617900	0.669421
chr11	5622300	0.611570
chr11	5642400	0.842975
chr11	5686900	0.644628
chr11	5712700	0.793388
chr11	5831600	0.776860
chr11	5841800	0.603306
chr11	6246800	0.644628
chr11	6251300	0.619835
chr11	6273500	0.867769
chr11	6337600	0.636364
chr11	6378800	0.669421
chr11	6459200	0.578512
chr11	6461900	0.636364
chr11	6490900	0.636364
chr11	6560300	0.636364
chr11	6575100	0.793388
chr11	6578100	0.702479
chr11	6584700	0.636364
chr11	6590300	0.950413
chr11	6598300	0.685950
chr11	6621900	0.570248
chr11	6628400	0.867769
chr11	6657100	0.950413
chr11	6660400	0.595041
chr11	6667700	0.768595
chr11	6672500	0.867769
chr11	6689000	0.818182
chr11	6693700	0.727273
chr11	6711800	0.669421
chr11	6766500	0.752066
chr11	6768600	0.892562
chr11	7094200	0.661157
chr11	7101800	0.876033
chr11	7622000	0.694215
chr11	8224100	0.603306
chr11	8228600	0.677686
chr11	8723100	0.925620
chr11	8739700	0.685950
chr11	8752000	0.636364
chr11	8848900	0.991736
chr11	8851900	0.735537
chr11	8936000	0.793388
chr11	8963500	0.677686
chr11	8970900	0.603306

chr11	8993700	0.719008
chr11	9010400	0.644628
chr11	9016500	0.636364
chr11	9020100	0.595041
chr11	9023600	0.842975
chr11	9121800	0.760331
chr11	9146100	0.694215
chr11	9177900	0.710744
chr11	9230900	0.752066
chr11	9251700	0.842975
chr11	9266100	0.876033
chr11	9333500	0.851240
chr11	9341100	0.950413
chr11	9369300	0.719008
chr11	9383900	0.892562
chr11	9389400	0.661157
chr11	9419100	0.578512
chr11	9517900	0.925620
chr11	9552000	0.818182
chr11	9571600	0.933884
chr11	9574500	0.669421
chr11	9587500	0.661157
chr11	9591400	0.942149
chr11	9623500	0.595041
chr11	9627600	0.867769
chr11	9632900	0.884298
chr11	9648700	0.611570
chr11	9674100	0.727273
chr11	9738800	0.776860
chr11	9781500	0.603306
chr11	9806500	0.793388
chr11	9813100	0.851240
chr11	9898100	0.611570
chr11	9952700	0.768595
chr11	10004900	0.917355
chr11	10331800	0.570248
chr11	10432600	0.628099
chr11	10466700	0.842975
chr11	10476200	0.661157
chr11	10481300	0.636364
chr11	10512400	0.925620
chr11	10531100	0.743802
chr11	10604700	0.818182
chr11	10608400	0.876033
chr11	10611100	0.652893
chr11	10628500	0.834711
chr11	10663600	0.694215
chr11	10667600	0.611570
chr11	10676200	0.859504
chr11	10679100	0.661157
chr11	10681900	0.685950
chr11	10688100	0.884298
chr11	10691000	0.611570
chr11	10730100	0.710744

chr11	10757600	0.809917
chr11	10764900	0.842975
chr11	10814800	0.661157
chr11	10827100	0.628099
chr11	10845600	0.743802
chr11	10861000	0.702479
chr11	10900500	0.669421
chr11	10916700	0.917355
chr11	10919600	0.801653
chr11	10923600	0.933884
chr11	10930500	0.851240
chr11	10941800	0.834711
chr11	10953800	0.586777
chr11	11012100	0.826446
chr11	11836500	0.933884
chr11	11838600	0.685950
chr11	11869000	0.661157
chr11	11871600	0.933884
chr11	11882300	0.884298
chr11	12039800	0.677686
chr11	12050700	0.595041
chr11	12066200	0.826446
chr11	12069100	0.785124
chr11	12088500	0.619835
chr11	12111900	0.867769
chr11	12116800	0.809917
chr11	12131200	0.586777
chr11	12134500	0.776860
chr11	12138400	0.818182
chr11	12141900	0.975207
chr11	12145700	0.776860
chr11	12147900	0.884298
chr11	12150500	0.619835
chr11	12159400	0.595041
chr11	12164800	0.578512
chr11	12170300	0.661157
chr11	12173200	0.859504
chr11	12178100	0.917355
chr11	12181600	0.570248
chr11	12189600	0.735537
chr11	12194400	0.636364
chr11	12196700	0.917355
chr11	12200800	0.743802
chr11	12204700	0.975207
chr11	12209600	0.719008
chr11	12215100	0.983471
chr11	12218000	0.876033
chr11	12220700	0.619835
chr11	12223600	0.942149
chr11	12238500	0.834711
chr11	12267300	0.702479
chr11	12274100	0.586777
chr11	12280000	0.834711
chr11	12286100	0.851240

chr11	12296800	0.917355
chr11	12353800	0.636364
chr11	12388200	0.826446
chr11	12439800	0.652893
chr11	12599800	0.892562
chr11	12603800	0.619835
chr11	12624100	0.752066
chr11	12626300	0.710744
chr11	12647700	0.859504
chr11	12684200	0.595041
chr11	12702200	0.809917
chr11	12706200	0.727273
chr11	12709000	0.768595
chr11	12711200	0.892562
chr11	12713700	0.677686
chr11	12724900	0.611570
chr11	12729100	0.752066
chr11	12746100	0.950413
chr11	12748300	0.842975
chr11	12759000	0.851240
chr11	12768500	0.933884
chr11	12783400	0.578512
chr11	12786300	0.859504
chr11	12791100	0.892562
chr11	12814100	0.719008
chr11	12821900	0.867769
chr11	12825100	0.702479
chr11	12832300	0.909091
chr11	12836900	0.727273
chr11	12846000	0.917355
chr11	12848500	0.578512
chr11	12863900	0.818182
chr11	12866000	0.801653
chr11	12868300	0.867769
chr11	12874100	0.619835
chr11	12876500	0.826446
chr11	12878600	0.652893
chr11	12882100	0.942149
chr11	12889900	0.867769
chr11	12893700	0.752066
chr11	12898600	0.719008
chr11	13000700	0.603306
chr11	13012200	0.661157
chr11	13016600	0.867769
chr11	13020000	0.644628
chr11	13113500	0.975207
chr11	13124400	0.909091
chr11	13128000	0.628099
chr11	13130100	0.793388
chr11	13136700	0.595041
chr11	13157700	0.876033
chr11	13229900	0.628099
chr11	13360300	0.710744
chr11	13599500	0.611570

chr11	13707600	0.570248
chr11	13720200	0.793388
chr11	13789700	0.570248
chr11	13848600	0.636364
chr11	13898200	0.876033
chr11	13904600	0.578512
chr11	14436700	0.933884
chr11	14444300	0.933884
chr11	14559200	0.586777
chr11	14563400	0.768595
chr11	14566500	0.760331
chr11	14600700	0.677686
chr11	14671300	0.619835
chr11	14674300	0.570248
chr11	14677500	0.586777
chr11	14680400	0.793388
chr11	14690600	0.793388
chr11	14694200	0.793388
chr11	14787100	0.628099
chr11	14874200	0.578512
chr11	14916800	0.867769
chr11	15602000	0.958678
chr11	15677500	0.743802
chr11	15721900	0.644628
chr11	15906700	0.859504
chr11	15915200	0.636364
chr11	15989700	0.785124
chr11	15992700	0.859504
chr11	15998900	0.785124
chr11	16028500	0.867769
chr11	16034300	0.628099
chr11	16069100	0.950413
chr11	16115900	0.644628
chr11	16199600	0.727273
chr11	16216000	0.900826
chr11	16261400	0.636364
chr11	16299500	0.611570
chr11	16315100	0.892562
chr11	16318900	0.842975
chr11	16322400	0.685950
chr11	16348700	0.743802
chr11	16358000	0.793388
chr11	16360500	0.859504
chr11	16368600	0.826446
chr11	16372200	0.760331
chr11	16375100	0.892562
chr11	16378500	0.768595
chr11	16381300	0.628099
chr11	16386000	0.909091
chr11	16392200	0.586777
chr11	16395400	0.710744
chr11	16405000	0.719008
chr11	16414700	0.818182
chr11	16417400	0.661157

chr11	16424900	0.578512
chr11	16430100	0.595041
chr11	16446600	0.611570
chr11	16618100	0.892562
chr11	16766400	0.636364
chr11	16875700	0.867769
chr11	16879200	0.909091
chr11	16905200	0.768595
chr11	16949100	0.644628
chr11	17186600	0.776860
chr11	17208800	0.578512
chr11	17280600	0.842975
chr11	17485000	0.776860
chr11	17550600	0.785124
chr11	17721300	0.876033
chr11	17870800	0.685950
chr11	17943800	0.785124
chr11	18336200	0.991736
chr11	18348900	0.619835
chr11	18393600	0.743802
chr11	18405800	0.851240
chr11	18414500	0.752066
chr11	18420100	0.578512
chr11	18441100	0.809917
chr11	18513100	0.776860
chr11	18608100	0.595041
chr11	18638200	0.776860
chr11	18671900	0.570248
chr11	18707500	0.586777
chr11	18715400	0.851240
chr11	18965300	0.677686
chr11	19143300	0.669421
chr11	19195400	0.917355
chr11	19264900	0.636364
chr11	19463900	0.909091
chr11	19994400	0.702479
chr11	19996500	0.909091
chr11	20000400	0.966942
chr11	20002600	0.933884
chr11	20004800	0.685950
chr11	20017500	0.818182
chr11	20035400	0.776860
chr11	20090500	0.578512
chr11	20097900	0.694215
chr11	20100800	0.909091
chr11	20111600	0.876033
chr11	20123400	0.636364
chr11	20134300	1.000000
chr11	20379500	0.619835
chr11	20382200	0.570248
chr11	20384500	0.595041
chr11	20390800	0.991736
chr11	20394000	0.760331
chr11	20472700	0.578512

chr11	20478800	0.909091
chr11	20483400	0.933884
chr11	20488000	0.685950
chr11	20493500	0.727273
chr11	20496400	0.958678
chr11	20499300	0.983471
chr11	20501700	0.818182
chr11	20510100	0.760331
chr11	20515200	0.677686
chr11	20588200	0.603306
chr11	22774900	0.727273
chr11	22814000	0.735537
chr11	22817300	0.950413
chr11	27210000	0.950413
chr11	27223700	0.876033
chr11	27228800	0.867769
chr11	27231300	0.644628
chr11	27264000	0.628099
chr11	27380800	0.595041
chr11	27404600	0.661157
chr11	27414300	0.942149
chr11	27417300	0.702479
chr11	27420100	0.900826
chr11	27428800	0.760331
chr11	27434200	0.826446
chr11	27436400	0.966942
chr11	27440300	0.851240
chr11	27444000	0.917355
chr11	27448600	0.628099
chr11	27451700	0.694215
chr11	27466800	0.719008
chr11	27469500	0.983471
chr11	27484200	0.950413
chr11	28124500	0.570248
chr11	28365200	0.933884
chr11	30347700	0.644628
chr11	31441200	0.595041
chr11	31804300	0.619835
chr11	31833000	0.752066
chr11	32110200	0.669421
chr11	32401600	0.595041
chr11	32404700	0.603306
chr11	32408900	0.983471
chr11	32442000	0.611570
chr11	32444900	0.801653
chr11	32448900	0.735537
chr11	32470200	0.809917
chr11	32472300	0.942149
chr11	32475500	0.595041
chr11	32500200	0.619835
chr11	32503400	0.702479
chr11	32553400	0.727273
chr11	32592500	0.652893
chr11	33190600	0.611570

chr11	33229600	0.735537
chr11	33343000	0.785124
chr11	33364600	0.768595
chr11	33712300	0.752066
chr11	33735400	0.826446
chr11	33744200	0.719008
chr11	33746800	0.826446
chr11	33753500	0.760331
chr11	33760700	0.578512
chr11	33885600	0.595041
chr11	33897000	0.966942
chr11	33907100	0.876033
chr11	33910700	0.694215
chr11	33916800	0.760331
chr11	33920900	0.876033
chr11	33925800	0.578512
chr11	33934500	0.933884
chr11	33948700	0.950413
chr11	33954700	0.884298
chr11	33957100	0.933884
chr11	33960500	0.884298
chr11	33965100	0.917355
chr11	33968500	0.876033
chr11	33971700	0.619835
chr11	33974700	0.958678
chr11	33979500	0.619835
chr11	33992900	0.628099
chr11	34008900	0.669421
chr11	34019200	0.586777
chr11	34071400	0.619835
chr11	34077700	0.603306
chr11	34085100	0.867769
chr11	34089800	0.570248
chr11	34130400	0.752066
chr11	34256400	0.603306
chr11	34258700	0.735537
chr11	34264200	0.966942
chr11	34393500	0.966942
chr11	34449300	0.958678
chr11	34463300	0.578512
chr11	34465600	0.669421
chr11	34608000	0.950413
chr11	34621600	0.975207
chr11	34625900	0.694215
chr11	34631200	0.892562
chr11	34638500	0.900826
chr11	34641100	0.619835
chr11	34643600	0.636364
chr11	34672500	0.743802
chr11	34749300	0.752066
chr11	34780100	0.710744
chr11	34813500	0.636364
chr11	34828400	0.884298
chr11	34831500	0.991736

chr11	34847900	0.966942
chr11	34852100	0.851240
chr11	34930500	0.694215
chr11	34950500	0.644628
chr11	34958200	0.677686
chr11	35052200	0.917355
chr11	35055400	0.842975
chr11	35058600	0.884298
chr11	35062400	0.776860
chr11	35085400	0.966942
chr11	35104800	0.595041
chr11	35126300	0.595041
chr11	35364900	0.900826
chr11	35374200	0.611570
chr11	35602100	0.611570
chr11	35703300	0.776860
chr11	35807900	1.000000
chr11	36096000	0.636364
chr11	36112600	0.933884
chr11	36119500	0.983471
chr11	36123800	0.991736
chr11	36131700	0.793388
chr11	36137200	0.933884
chr11	36147700	0.619835
chr11	36171700	0.950413
chr11	36215000	0.809917
chr11	36431100	0.900826
chr11	36433300	0.661157
chr11	36475900	0.735537
chr11	36640100	0.628099
chr11	38346200	0.859504
chr11	38532100	0.785124
chr11	38539700	0.851240
chr11	38559800	0.776860
chr11	38561900	0.917355
chr11	38566000	0.884298
chr11	38606600	0.619835
chr11	38731400	0.801653
chr11	38740300	0.570248
chr11	43385500	0.611570
chr11	43388200	0.809917
chr11	43390300	0.826446
chr11	43399200	0.669421
chr11	43415800	0.636364
chr11	43710400	0.743802
chr11	43715500	0.851240
chr11	43754100	0.834711
chr11	43758400	0.801653
chr11	43762100	0.628099
chr11	43767500	0.619835
chr11	43771100	0.644628
chr11	43890900	0.983471
chr11	43942000	1.000000
chr11	43959600	0.768595

chr11	43969100	0.644628
chr11	43972700	0.834711
chr11	43984400	0.661157
chr11	44006300	0.578512
chr11	44054800	0.578512
chr11	44161300	0.834711
chr11	44265500	0.719008
chr11	44278400	0.752066
chr11	44289100	0.710744
chr11	44292000	0.793388
chr11	44304500	0.710744
chr11	44341400	0.595041
chr11	44344000	0.694215
chr11	44348700	0.793388
chr11	44355100	0.983471
chr11	44357200	0.677686
chr11	44360100	0.776860
chr11	44399600	0.611570
chr11	44419700	0.570248
chr11	44527900	0.776860
chr11	44532500	0.611570
chr11	44546200	0.826446
chr11	44550200	0.727273
chr11	44554000	0.876033
chr11	44556300	0.578512
chr11	44569600	0.942149
chr11	44572100	0.685950
chr11	44577100	0.776860
chr11	44590900	0.628099
chr11	44599400	0.785124
chr11	44629400	0.859504
chr11	44636700	0.611570
chr11	44782700	0.644628
chr11	44788300	0.611570
chr11	44791900	0.595041
chr11	44800100	0.867769
chr11	44803000	0.950413
chr11	44928600	0.636364
chr11	44933300	0.661157
chr11	44977200	0.685950
chr11	44993000	0.694215
chr11	45207300	0.991736
chr11	45670900	0.735537
chr11	45845300	0.942149
chr11	45848300	0.636364
chr11	45856300	0.851240
chr11	45899700	0.603306
chr11	45902000	1.000000
chr11	45926500	0.917355
chr11	45951900	0.603306
chr11	45967500	0.867769
chr11	45986900	0.586777
chr11	46004300	0.909091
chr11	46006700	0.628099

chr11	46013200	0.851240
chr11	46019300	0.942149
chr11	46022000	0.867769
chr11	46026600	0.570248
chr11	46031900	0.595041
chr11	46067900	0.669421
chr11	46074200	0.628099
chr11	46094200	0.793388
chr11	46097400	0.867769
chr11	46121700	0.644628
chr11	46126600	0.793388
chr11	46130400	0.867769
chr11	46136300	0.867769
chr11	46149100	0.900826
chr11	46171600	0.752066
chr11	46232200	0.578512
chr11	46241900	0.710744
chr11	46244800	0.834711
chr11	46257700	0.644628
chr11	46262600	0.876033
chr11	46269900	0.809917
chr11	46273200	0.834711
chr11	46275800	0.752066
chr11	46278600	0.933884
chr11	46287600	0.628099
chr11	46295600	0.950413
chr11	46303100	0.752066
chr11	46312200	0.619835
chr11	46315900	0.809917
chr11	46319400	0.595041
chr11	46331600	0.900826
chr11	46340400	0.966942
chr11	46352800	0.595041
chr11	46361100	0.619835
chr11	46374400	0.876033
chr11	46378700	0.570248
chr11	46383000	0.958678
chr11	46389200	0.851240
chr11	46401800	0.578512
chr11	46405000	0.586777
chr11	46443400	0.793388
chr11	46448900	0.743802
chr11	46459200	0.611570
chr11	46468100	0.834711
chr11	46479300	0.611570
chr11	46527600	0.851240
chr11	46530400	0.752066
chr11	46535200	0.586777
chr11	46555600	0.710744
chr11	46559600	0.917355
chr11	46563500	0.917355
chr11	46576200	0.966942
chr11	46580300	0.595041
chr11	46611000	0.842975

chr11	46616500	0.578512
chr11	46619600	0.809917
chr11	46634000	0.793388
chr11	46686200	0.892562
chr11	46716300	0.793388
chr11	46848200	0.595041
chr11	46850300	0.702479
chr11	46854600	0.776860
chr11	46859400	0.909091
chr11	46877000	0.603306
chr11	46908000	0.677686
chr11	46952100	0.768595
chr11	46994800	0.619835
chr11	47005500	0.611570
chr11	47027800	0.785124
chr11	47050200	0.619835
chr11	47077900	0.842975
chr11	47082900	0.942149
chr11	47279800	0.884298
chr11	47286700	0.975207
chr11	47289700	0.570248
chr11	47299100	0.851240
chr11	47359200	0.925620
chr11	47383600	0.644628
chr11	47388300	0.801653
chr11	47398000	0.603306
chr11	47401400	0.652893
chr11	47416400	0.884298
chr11	47422800	0.669421
chr11	47425600	0.876033
chr11	47511400	0.570248
chr11	47522900	0.611570
chr11	47528300	0.809917
chr11	47530900	0.925620
chr11	47534000	0.884298
chr11	47537400	0.785124
chr11	47540500	0.603306
chr11	47545900	0.958678
chr11	47627700	0.595041
chr11	47666500	0.685950
chr11	47694100	0.900826
chr11	47702700	0.570248
chr11	47797500	0.760331
chr11	47885700	0.834711
chr11	47937800	0.801653
chr11	47939900	0.793388
chr11	47961500	0.661157
chr11	47967500	0.793388
chr11	47970300	0.900826
chr11	48005500	0.900826
chr11	48008600	0.727273
chr11	48012900	0.834711
chr11	48028300	0.876033
chr11	48031400	0.834711

chr11	48077600	0.966942
chr11	48083100	1.000000
chr11	48123000	0.644628
chr11	48131500	0.694215
chr11	51582300	0.611570
chr11	51586600	0.743802
chr11	51592800	0.801653
chr11	56890000	0.809917
chr11	56892500	0.619835
chr11	56895200	0.925620
chr11	56912400	0.884298
chr11	56918300	0.818182
chr11	56923100	0.628099
chr11	57003200	0.578512
chr11	57007400	0.876033
chr11	57010700	0.966942
chr11	57019400	0.793388
chr11	57045900	0.685950
chr11	57079100	0.900826
chr11	57083900	0.595041
chr11	57089500	0.694215
chr11	57094500	0.644628
chr11	57108400	0.702479
chr11	57121200	0.834711
chr11	57126100	0.752066
chr11	57136300	0.776860
chr11	57148000	0.586777
chr11	57151100	0.776860
chr11	57154900	0.950413
chr11	57158200	0.603306
chr11	57165400	0.884298
chr11	57170600	0.851240
chr11	57175000	0.570248
chr11	57188800	0.735537
chr11	57196400	0.669421
chr11	57200400	0.942149
chr11	57232800	0.611570
chr11	57241600	0.719008
chr11	57261600	0.867769
chr11	57267700	0.909091
chr11	57280400	0.570248
chr11	57295200	0.801653
chr11	57417200	0.776860
chr11	57448700	0.892562
chr11	57499800	0.611570
chr11	57503600	0.950413
chr11	57508000	0.586777
chr11	57519100	0.950413
chr11	57540400	0.578512
chr11	57543200	0.586777
chr11	57546000	0.892562
chr11	57560400	0.909091
chr11	57567300	0.603306
chr11	57669000	0.909091

chr11	57672200	0.818182
chr11	57681600	0.776860
chr11	58373600	0.702479
chr11	58494900	0.909091
chr11	58513800	1.000000
chr11	58540200	0.719008
chr11	58545300	0.628099
chr11	58904200	0.603306
chr11	59315800	0.611570
chr11	59321200	0.595041
chr11	59415700	0.570248
chr11	59448700	0.752066
chr11	59474700	0.776860
chr11	59534800	0.933884
chr11	59554800	0.892562
chr11	59584600	0.636364
chr11	59590800	0.892562
chr11	59614200	0.826446
chr11	59617000	0.644628
chr11	59623800	0.570248
chr11	59628000	0.991736
chr11	59659700	0.851240
chr11	59706800	0.752066
chr11	59729000	0.917355
chr11	59735800	0.950413
chr11	59749000	0.859504
chr11	59824200	0.809917
chr11	59829700	0.719008
chr11	59832100	0.570248
chr11	59839200	0.809917
chr11	59846900	0.727273
chr11	59853600	0.611570
chr11	59879600	0.752066
chr11	59884400	0.859504
chr11	59888800	0.925620
chr11	59892400	0.752066
chr11	59895200	0.917355
chr11	59898300	0.619835
chr11	59953100	0.933884
chr11	59961200	0.710744
chr11	59963900	0.867769
chr11	59989600	0.884298
chr11	60010500	0.586777
chr11	60521000	0.611570
chr11	60613100	0.578512
chr11	60616700	0.768595
chr11	60621500	0.752066
chr11	60631900	0.702479
chr11	60640500	0.595041
chr11	60658900	0.958678
chr11	60667000	0.578512
chr11	60686600	0.628099
chr11	60713000	0.586777
chr11	60838900	0.809917

chr11	60897800	0.578512
chr11	60909600	0.677686
chr11	60917900	0.834711
chr11	60920300	0.859504
chr11	60932100	0.933884
chr11	60935900	0.652893
chr11	61035000	0.867769
chr11	61049600	0.776860
chr11	61054700	0.909091
chr11	61059300	0.619835
chr11	61104000	0.859504
chr11	61110100	0.983471
chr11	61112200	0.595041
chr11	61114900	0.727273
chr11	61121100	0.958678
chr11	61124900	0.685950
chr11	61135000	0.892562
chr11	61153100	0.719008
chr11	61185800	0.644628
chr11	61308900	0.603306
chr11	61356100	0.636364
chr11	61517600	0.975207
chr11	61522500	0.809917
chr11	61524600	0.793388
chr11	61563200	0.595041
chr11	61566800	0.942149
chr11	61570200	0.975207
chr11	61573000	0.702479
chr11	61577300	0.809917
chr11	61580600	0.851240
chr11	61588200	0.867769
chr11	61601200	0.801653
chr11	61604900	0.933884
chr11	61608700	0.793388
chr11	61627800	0.909091
chr11	61634000	0.991736
chr11	61639100	0.842975
chr11	61653200	0.859504
chr11	61665800	0.776860
chr11	61676800	0.727273
chr11	61690000	0.752066
chr11	61718600	0.842975
chr11	61722000	0.719008
chr11	61726900	0.818182
chr11	61730500	0.685950
chr11	61738600	0.876033
chr11	61742100	0.950413
chr11	61769200	0.644628
chr11	61780900	0.776860
chr11	61783300	0.702479
chr11	61785400	0.983471
chr11	61793100	0.595041
chr11	61801300	0.578512
chr11	61810000	0.801653

chr11	61817600	0.644628
chr11	61873100	0.719008
chr11	62136500	0.834711
chr11	62160700	0.619835
chr11	62164400	0.991736
chr11	62166600	0.809917
chr11	62169100	0.702479
chr11	62186400	0.933884
chr11	62195800	0.694215
chr11	62225800	0.958678
chr11	62230400	0.694215
chr11	62235100	0.991736
chr11	62240100	0.942149
chr11	62245800	0.694215
chr11	62255400	0.595041
chr11	62259800	0.909091
chr11	62267200	0.801653
chr11	62269300	0.933884
chr11	62273200	0.900826
chr11	62276500	0.768595
chr11	62306700	0.710744
chr11	62310700	0.942149
chr11	62316500	0.727273
chr11	62321000	0.950413
chr11	62324700	0.611570
chr11	62329100	0.876033
chr11	62337900	0.702479
chr11	62344400	0.677686
chr11	62357100	0.702479
chr11	62361200	0.942149
chr11	62370500	0.586777
chr11	62376300	0.595041
chr11	62382200	0.644628
chr11	62424900	0.661157
chr11	62448800	0.661157
chr11	62450900	0.677686
chr11	62475900	0.694215
chr11	62481200	0.694215
chr11	62557100	0.702479
chr11	62560600	0.603306
chr11	62627400	0.735537
chr11	62629900	0.983471
chr11	62645100	0.702479
chr11	62653200	0.619835
chr11	62657000	0.933884
chr11	62660500	0.578512
chr11	62673500	0.884298
chr11	62687400	0.752066
chr11	62691500	0.644628
chr11	63259700	0.776860
chr11	63270700	0.867769
chr11	63273600	0.884298
chr11	63285100	0.983471
chr11	63297100	0.628099

chr11	63299500	0.942149
chr11	63304200	0.842975
chr11	63332800	0.809917
chr11	63335400	0.884298
chr11	63342400	0.578512
chr11	63374900	0.578512
chr11	63452700	0.892562
chr11	63455100	0.809917
chr11	63457600	0.909091
chr11	63478100	0.801653
chr11	63487900	0.677686
chr11	63513800	0.710744
chr11	63570800	0.702479
chr11	63605400	0.636364
chr11	63612900	0.884298
chr11	63626300	0.595041
chr11	63639700	0.834711
chr11	63642700	0.793388
chr11	63647900	0.586777
chr11	63659400	0.628099
chr11	63686600	0.578512
chr11	63709300	0.991736
chr11	63713100	0.925620
chr11	63738900	0.644628
chr11	63775200	0.776860
chr11	63841900	0.628099
chr11	63914000	0.719008
chr11	63941700	0.603306
chr11	63949800	0.719008
chr11	63972400	0.719008
chr11	63977300	0.818182
chr11	64011500	0.694215
chr11	64016600	0.578512
chr11	64041400	0.909091
chr11	64099600	0.876033
chr11	64102900	0.752066
chr11	64109400	0.595041
chr11	64124800	0.760331
chr11	64141200	0.578512
chr11	64150300	0.628099
chr11	64153900	0.578512
chr11	64168200	0.595041
chr11	64217000	0.768595
chr11	64240700	0.851240
chr11	64269100	0.826446
chr11	64275500	0.595041
chr11	64323500	0.727273
chr11	64339700	0.809917
chr11	64397900	0.661157
chr11	64420600	0.793388
chr11	64424900	0.603306
chr11	64427800	0.694215
chr11	64433500	0.900826
chr11	64468700	0.727273

chr11	64522500	0.578512
chr11	64531000	0.768595
chr11	64543100	0.669421
chr11	64555300	0.677686
chr11	64568700	0.586777
chr11	64596000	0.925620
chr11	64610300	0.702479
chr11	64615900	0.760331
chr11	64619800	0.867769
chr11	64622900	0.900826
chr11	64632700	0.942149
chr11	64637800	0.603306
chr11	64654200	0.710744
chr11	64657800	0.966942
chr11	64662000	0.636364
chr11	64687000	0.570248
chr11	64703700	0.950413
chr11	64728300	0.570248
chr11	64757500	0.876033
chr11	64766500	0.900826
chr11	64768800	0.636364
chr11	64784200	0.603306
chr11	64811400	0.776860
chr11	64828300	0.975207
chr11	64833800	0.603306
chr11	64846200	0.785124
chr11	64866100	0.586777
chr11	64892600	0.669421
chr11	64918400	0.991736
chr11	64920800	0.900826
chr11	64939700	0.735537
chr11	64942300	0.892562
chr11	64946400	0.603306
chr11	64993200	0.586777
chr11	65043400	0.942149
chr11	65059100	0.743802
chr11	65070000	0.876033
chr11	65097400	0.760331
chr11	65106100	0.884298
chr11	65122200	0.595041
chr11	65125800	0.950413
chr11	65130300	0.669421
chr11	65134600	0.768595
chr11	65139000	0.933884
chr11	65149000	0.619835
chr11	65157100	0.735537
chr11	65171600	0.942149
chr11	65184200	0.917355
chr11	65198700	0.611570
chr11	65204200	0.595041
chr11	65211300	0.785124
chr11	65216000	0.710744
chr11	65226800	0.785124
chr11	65235700	0.595041

chr11	65241500	0.958678
chr11	65248200	0.710744
chr11	65250900	0.966942
chr11	65255500	0.793388
chr11	65258400	0.743802
chr11	65262600	0.752066
chr11	65275600	0.785124
chr11	65278800	0.603306
chr11	65290700	0.776860
chr11	65319000	0.826446
chr11	65321200	0.694215
chr11	65326100	0.768595
chr11	65328500	0.933884
chr11	65341800	0.669421
chr11	65346300	0.801653
chr11	65356000	0.859504
chr11	65363900	0.644628
chr11	65379500	0.652893
chr11	65403900	0.909091
chr11	65409800	0.595041
chr11	65460500	0.867769
chr11	65485900	0.578512
chr11	65511900	0.611570
chr11	65575100	0.570248
chr11	65635000	0.884298
chr11	65661300	0.702479
chr11	65664400	0.702479
chr11	65670400	0.991736
chr11	65674500	0.892562
chr11	65677000	0.809917
chr11	65680200	1.000000
chr11	65683700	0.851240
chr11	65696100	0.991736
chr11	65709100	0.909091
chr11	65772800	0.595041
chr11	65775300	0.636364
chr11	65781200	0.958678
chr11	65836600	0.628099
chr11	65848400	0.818182
chr11	65851700	0.652893
chr11	65883500	0.735537
chr11	65887200	0.685950
chr11	65909900	0.586777
chr11	65958700	0.578512
chr11	65990100	0.760331
chr11	66077700	0.719008
chr11	66095900	0.768595
chr11	66098200	0.652893
chr11	66119200	0.867769
chr11	66135700	0.727273
chr11	66176000	0.595041
chr11	66336300	0.652893
chr11	66363000	0.628099
chr11	66372200	0.685950

chr11	66405000	0.661157
chr11	66409900	0.578512
chr11	66420700	0.710744
chr11	66427700	0.867769
chr11	66469600	0.826446
chr11	66492400	0.710744
chr11	66496800	0.619835
chr11	66602900	0.793388
chr11	66606000	0.611570
chr11	66608100	0.958678
chr11	66636600	0.826446
chr11	66654200	0.900826
chr11	66659800	0.991736
chr11	66682800	0.611570
chr11	66686800	0.578512
chr11	66689100	0.991736
chr11	66699600	0.983471
chr11	66703600	0.834711
chr11	66718000	0.876033
chr11	66733500	0.595041
chr11	66774300	0.842975
chr11	66822900	0.752066
chr11	66845800	0.570248
chr11	66849800	0.685950
chr11	66856900	0.752066
chr11	66882500	0.661157
chr11	66936100	0.842975
chr11	67030400	0.933884
chr11	67043600	0.702479
chr11	67080200	0.991736
chr11	67083500	0.603306
chr11	67089600	0.636364
chr11	67116300	0.818182
chr11	67119300	0.611570
chr11	67123700	0.933884
chr11	67138700	0.809917
chr11	67143100	0.702479
chr11	67147200	0.983471
chr11	67149800	0.570248
chr11	67155400	0.719008
chr11	67165400	0.636364
chr11	67179000	0.694215
chr11	67181200	0.760331
chr11	67188300	0.752066
chr11	67204100	0.909091
chr11	67210200	0.578512
chr11	67230900	0.619835
chr11	67245400	0.958678
chr11	67273900	0.578512
chr11	67297300	0.578512
chr11	67312800	0.611570
chr11	67331500	0.909091
chr11	67340700	0.892562
chr11	67347500	0.892562

chr11	67349900	0.702479
chr11	67368100	0.884298
chr11	67371800	0.785124
chr11	67381900	0.917355
chr11	67385200	0.570248
chr11	67555600	0.867769
chr11	67594200	0.570248
chr11	67618500	0.619835
chr11	67652100	0.842975
chr11	67764900	0.652893
chr11	67772300	0.595041
chr11	67777800	0.719008
chr11	67811200	0.983471
chr11	67867400	0.760331
chr11	67874500	0.702479
chr11	67896500	0.619835
chr11	67906000	0.570248
chr11	67912600	0.628099
chr11	67914800	0.842975
chr11	67977100	0.859504
chr11	68025400	0.826446
chr11	68034300	0.685950
chr11	68036400	0.909091
chr11	68049900	0.793388
chr11	68065600	0.710744
chr11	68111700	0.603306
chr11	68114800	0.785124
chr11	68204700	0.933884
chr11	68237300	0.900826
chr11	68266700	0.793388
chr11	68270700	0.702479
chr11	68273100	0.876033
chr11	68281400	0.652893
chr11	68548700	0.867769
chr11	68625500	0.925620
chr11	68668800	0.628099
chr11	68673300	0.652893
chr11	68713700	0.710744
chr11	68723500	0.652893
chr11	68778800	0.652893
chr11	68841500	0.851240
chr11	68843700	0.669421
chr11	68905900	0.727273
chr11	68923800	0.925620
chr11	68928500	0.644628
chr11	68978200	0.735537
chr11	69056100	0.876033
chr11	69067300	0.933884
chr11	69069600	0.942149
chr11	69196100	0.611570
chr11	69208800	0.776860
chr11	69213000	0.966942
chr11	69215400	0.735537
chr11	69218100	0.925620

chr11	69223700	0.727273
chr11	69225900	0.909091
chr11	69235700	0.917355
chr11	69251500	0.942149
chr11	69260900	0.809917
chr11	69263200	0.983471
chr11	69265800	0.768595
chr11	69282700	0.809917
chr11	69720800	0.925620
chr11	69794700	0.842975
chr11	69796800	0.933884
chr11	69804600	0.859504
chr11	69817100	0.851240
chr11	69831100	0.652893
chr11	69846700	0.636364
chr11	69958200	0.867769
chr11	70066400	0.595041
chr11	70159600	0.991736
chr11	70181700	0.760331
chr11	70248800	0.685950
chr11	70265500	0.925620
chr11	70270500	0.859504
chr11	70302100	0.694215
chr11	70588800	0.628099
chr11	71145200	0.644628
chr11	71199600	0.636364
chr11	71242900	0.669421
chr11	71417000	0.942149
chr11	71419300	0.966942
chr11	71421400	0.611570
chr11	71424400	0.826446
chr11	71449600	0.628099
chr11	71455100	0.867769
chr11	71460400	0.851240
chr11	71476700	0.661157
chr11	71483600	1.000000
chr11	71659700	0.966942
chr11	71673700	0.628099
chr11	71706400	0.917355
chr11	71708500	0.776860
chr11	71711200	0.983471
chr11	71751700	0.727273
chr11	71781300	0.719008
chr11	71786900	0.619835
chr11	71799900	0.644628
chr11	71803100	0.752066
chr11	71811300	0.876033
chr11	71827900	0.578512
chr11	71830200	0.809917
chr11	71834200	0.851240
chr11	71863800	0.842975
chr11	71866200	0.619835
chr11	71874600	0.776860
chr11	71928900	0.619835

chr11	72020200	0.694215
chr11	72026400	0.909091
chr11	72033100	1.000000
chr11	72037700	0.826446
chr11	72044900	0.859504
chr11	72047800	0.958678
chr11	72061200	0.884298
chr11	72070500	0.611570
chr11	72123100	0.809917
chr11	72142800	0.727273
chr11	72205400	0.809917
chr11	72336700	0.611570
chr11	72344100	0.942149
chr11	72364200	0.661157
chr11	72408700	0.628099
chr11	72433400	0.950413
chr11	72446700	0.818182
chr11	72449700	0.851240
chr11	72453100	0.900826
chr11	72456100	0.859504
chr11	72460500	0.735537
chr11	72470500	0.900826
chr11	72483100	0.595041
chr11	72485400	0.867769
chr11	72489400	0.909091
chr11	72493600	0.677686
chr11	72498100	0.743802
chr11	72503800	0.578512
chr11	72506400	0.628099
chr11	72527200	0.685950
chr11	72681700	0.842975
chr11	72855600	0.884298
chr11	72871200	0.619835
chr11	72873900	0.727273
chr11	72894400	0.702479
chr11	72913500	0.876033
chr11	72953800	0.694215
chr11	73000300	0.867769
chr11	73046000	0.694215
chr11	73090000	0.661157
chr11	73096700	0.619835
chr11	73099500	0.628099
chr11	73181200	0.702479
chr11	73306100	0.570248
chr11	73358800	0.677686
chr11	73495600	0.900826
chr11	73503500	0.867769
chr11	73562600	0.933884
chr11	73591600	0.925620
chr11	73667500	0.702479
chr11	73675800	0.702479
chr11	73686000	0.628099
chr11	73690100	0.719008
chr11	73696300	0.785124

chr11	73714200	0.768595
chr11	73720700	0.801653
chr11	73723500	0.983471
chr11	73727500	0.735537
chr11	73768700	0.710744
chr11	73782400	0.900826
chr11	73801600	0.677686
chr11	73804100	0.909091
chr11	73850200	1.000000
chr11	73887200	0.595041
chr11	73897000	0.611570
chr11	73916000	0.694215
chr11	74041400	0.578512
chr11	74148800	0.661157
chr11	74170600	0.942149
chr11	74173600	0.776860
chr11	74176600	0.570248
chr11	74178900	0.661157
chr11	74191600	0.636364
chr11	74208300	0.595041
chr11	74216500	0.917355
chr11	74326500	0.595041
chr11	74345500	0.628099
chr11	74352100	0.776860
chr11	74384800	0.909091
chr11	74398400	0.595041
chr11	74408700	0.586777
chr11	74413700	0.661157
chr11	74421900	0.818182
chr11	74517800	0.892562
chr11	74529300	0.636364
chr11	74648700	0.719008
chr11	74663800	0.677686
chr11	74669300	0.710744
chr11	74675300	0.636364
chr11	74702700	0.603306
chr11	74724900	0.793388
chr11	74734700	0.586777
chr11	74740600	0.603306
chr11	74743700	0.876033
chr11	74770900	0.900826
chr11	74781500	0.900826
chr11	74798800	0.752066
chr11	74805700	0.595041
chr11	74810500	0.652893
chr11	74815400	0.661157
chr11	74818900	0.710744
chr11	74854500	1.000000
chr11	74920200	0.570248
chr11	75025300	0.983471
chr11	75040500	0.975207
chr11	75042600	0.578512
chr11	75046000	0.867769
chr11	75049100	0.983471

chr11	75051600	0.851240
chr11	75055200	0.958678
chr11	75059500	0.595041
chr11	75083600	0.917355
chr11	75099700	0.578512
chr11	75115200	0.851240
chr11	75117400	0.760331
chr11	75132000	0.909091
chr11	75179900	0.900826
chr11	75186400	0.966942
chr11	75188600	0.851240
chr11	75191400	0.991736
chr11	75193800	0.818182
chr11	75203400	0.628099
chr11	75220000	0.727273
chr11	75228400	0.669421
chr11	75244000	0.578512
chr11	75247500	0.917355
chr11	75251400	0.876033
chr11	75254800	0.694215
chr11	75259300	0.685950
chr11	75265900	0.966942
chr11	75269200	0.644628
chr11	75271300	0.859504
chr11	75276400	0.586777
chr11	75296400	0.851240
chr11	75302000	0.975207
chr11	75306300	0.809917
chr11	75308800	0.595041
chr11	75343600	0.578512
chr11	75375400	0.603306
chr11	75401100	0.834711
chr11	75425500	0.735537
chr11	75455000	0.661157
chr11	75479400	0.851240
chr11	75561900	0.793388
chr11	75587600	0.702479
chr11	75658100	0.669421
chr11	75670500	0.628099
chr11	75693400	0.809917
chr11	75736900	0.768595
chr11	75782700	0.644628
chr11	75786000	0.809917
chr11	75825900	0.917355
chr11	75896200	0.884298
chr11	75949800	0.636364
chr11	76076800	0.809917
chr11	76082600	0.776860
chr11	76088300	0.611570
chr11	76148400	0.619835
chr11	76151600	0.586777
chr11	76335800	0.611570
chr11	76356400	0.685950
chr11	76365800	0.859504

chr11	76376000	0.925620
chr11	76400900	0.661157
chr11	76414200	0.942149
chr11	76442200	0.685950
chr11	76447200	0.834711
chr11	76452200	0.801653
chr11	76456600	0.859504
chr11	76458800	0.809917
chr11	76470500	0.652893
chr11	76493500	0.611570
chr11	76500200	0.950413
chr11	76510600	0.586777
chr11	76518800	0.752066
chr11	76537200	0.909091
chr11	76549100	0.760331
chr11	76561400	0.876033
chr11	76566100	0.578512
chr11	76597100	0.909091
chr11	76600400	0.826446
chr11	76603100	0.611570
chr11	76613300	0.669421
chr11	76622500	0.628099
chr11	76628500	0.776860
chr11	76631300	0.628099
chr11	76726900	0.768595
chr11	76731700	0.677686
chr11	76742100	0.578512
chr11	76750100	0.909091
chr11	76838900	0.611570
chr11	76902700	0.603306
chr11	77011000	0.900826
chr11	77132400	0.702479
chr11	77136400	0.719008
chr11	77345700	0.719008
chr11	77355900	0.975207
chr11	77369900	0.793388
chr11	77518700	0.826446
chr11	77535100	0.694215
chr11	77595300	0.735537
chr11	77641400	0.644628
chr11	77670100	0.958678
chr11	77726600	0.619835
chr11	77739700	0.661157
chr11	77746300	0.619835
chr11	77751200	0.851240
chr11	77757500	0.586777
chr11	77762500	0.950413
chr11	77767500	0.727273
chr11	77773300	0.884298
chr11	77787600	0.834711
chr11	77805900	0.900826
chr11	77878500	0.834711
chr11	77889100	0.570248
chr11	77891400	0.669421

chr11	77903600	0.719008
chr11	77919600	0.628099
chr11	77928400	0.776860
chr11	77942500	0.578512
chr11	77962900	0.652893
chr11	77968300	0.933884
chr11	77971300	0.677686
chr11	77973400	0.909091
chr11	77979900	0.917355
chr11	77997000	0.842975
chr11	78001000	0.884298
chr11	78004800	0.876033
chr11	78007400	0.983471
chr11	78011800	0.652893
chr11	78023400	0.851240
chr11	78026500	0.834711
chr11	78030200	0.892562
chr11	78036700	0.768595
chr11	78050800	0.892562
chr11	78060200	0.851240
chr11	78063400	0.925620
chr11	78066300	0.909091
chr11	78082500	0.909091
chr11	78089300	0.669421
chr11	78092200	0.818182
chr11	78098000	0.677686
chr11	78104100	0.892562
chr11	78106700	0.636364
chr11	78111900	0.966942
chr11	78124800	0.619835
chr11	78135600	0.884298
chr11	78140600	0.876033
chr11	78215800	0.876033
chr11	78261200	0.694215
chr11	78655300	0.628099
chr11	82494200	0.842975
chr11	82501100	0.611570
chr11	82504700	0.925620
chr11	82557400	0.677686
chr11	82601300	0.628099
chr11	82615500	0.685950
chr11	82667700	0.619835
chr11	82693400	0.760331
chr11	82700900	0.644628
chr11	82703800	0.694215
chr11	82719500	0.917355
chr11	82800100	0.785124
chr11	82863100	0.900826
chr11	82865800	0.809917
chr11	82999300	0.925620
chr11	85195200	0.958678
chr11	85364600	0.842975
chr11	85719600	0.710744
chr11	85727600	0.611570

chr11	85732900	0.933884
chr11	85746200	0.719008
chr11	85749300	0.768595
chr11	85752100	0.586777
chr11	85755100	0.586777
chr11	85758300	0.595041
chr11	85764800	0.933884
chr11	85770400	0.661157
chr11	85782700	0.719008
chr11	85803200	0.694215
chr11	85820000	0.776860
chr11	85829100	0.818182
chr11	85835300	0.719008
chr11	85840600	0.801653
chr11	85844700	0.760331
chr11	85848300	0.983471
chr11	85852800	0.884298
chr11	85857000	0.859504
chr11	85861100	0.909091
chr11	85863600	0.669421
chr11	85867000	0.983471
chr11	85870900	0.892562
chr11	85874000	0.966942
chr11	85876100	0.834711
chr11	85879300	0.603306
chr11	85883200	0.636364
chr11	85888400	0.958678
chr11	85891400	0.876033
chr11	85895200	0.661157
chr11	85897300	0.950413
chr11	85904200	0.842975
chr11	85906900	0.661157
chr11	85910300	0.958678
chr11	85914700	0.628099
chr11	85923100	0.785124
chr11	85929800	0.776860
chr11	85947100	0.743802
chr11	85949200	0.760331
chr11	86134500	0.603306
chr11	86150500	0.644628
chr11	86195300	0.586777
chr11	86211500	0.801653
chr11	86222600	0.710744
chr11	86230900	0.785124
chr11	86235600	0.735537
chr11	86304000	0.677686
chr11	86417700	0.628099
chr11	86451900	0.933884
chr11	86454900	0.958678
chr11	86636100	0.661157
chr11	86638300	0.570248
chr11	86642000	0.793388
chr11	86645900	0.876033
chr11	86649000	0.859504

chr11	86651500	0.925620
chr11	86708900	0.776860
chr11	86716600	0.859504
chr11	86741800	0.603306
chr11	86746500	0.644628
chr11	86809000	0.578512
chr11	86902200	0.785124
chr11	87011700	0.727273
chr11	87064800	0.611570
chr11	88067300	0.578512
chr11	88091000	0.876033
chr11	88093100	0.851240
chr11	88148100	0.760331
chr11	88154500	0.942149
chr11	88164800	0.636364
chr11	88218800	0.834711
chr11	89968900	0.652893
chr11	91523300	0.644628
chr11	91527300	0.785124
chr11	91539200	0.652893
chr11	91638900	1.000000
chr11	91816800	0.900826
chr11	92734300	0.628099
chr11	93063300	0.619835
chr11	93259600	0.785124
chr11	93275200	0.702479
chr11	93317000	0.719008
chr11	93321500	0.809917
chr11	93447800	0.685950
chr11	93450000	0.644628
chr11	93452200	0.966942
chr11	93595400	0.578512
chr11	93603800	0.595041
chr11	93707300	0.958678
chr11	93714900	0.578512
chr11	93717300	0.975207
chr11	93722800	0.966942
chr11	93757700	0.661157
chr11	93777300	0.586777
chr11	93780300	0.818182
chr11	93811300	0.652893
chr11	93865100	0.644628
chr11	93869500	0.793388
chr11	93875500	0.727273
chr11	93885600	1.000000
chr11	94190700	0.719008
chr11	94283900	0.909091
chr11	94292100	0.826446
chr11	94297200	0.595041
chr11	94307900	0.570248
chr11	94369800	0.867769
chr11	94410700	0.909091
chr11	94415600	0.586777
chr11	94465500	0.925620

chr11	94468500	0.685950
chr11	94484200	0.595041
chr11	94494000	0.644628
chr11	94507700	0.735537
chr11	94509900	0.892562
chr11	94521500	0.876033
chr11	94524600	0.768595
chr11	94527800	0.801653
chr11	94610200	0.975207
chr11	94759400	0.760331
chr11	94783100	0.603306
chr11	94810800	0.595041
chr11	94818000	0.570248
chr11	94825600	0.958678
chr11	94833600	0.752066
chr11	94836900	0.892562
chr11	94840300	0.669421
chr11	94845800	0.619835
chr11	94855100	0.595041
chr11	94874300	0.809917
chr11	94877700	0.776860
chr11	94880000	0.925620
chr11	94882400	1.000000
chr11	94887600	0.702479
chr11	95070700	0.727273
chr11	95149300	0.826446
chr11	95184100	0.677686
chr11	95187200	0.884298
chr11	95275800	0.900826
chr11	95277900	0.851240
chr11	95280800	0.652893
chr11	95385600	0.818182
chr11	95431000	0.570248
chr11	95440100	0.636364
chr11	95451400	0.570248
chr11	95466700	0.719008
chr11	95469400	0.636364
chr11	95629800	0.578512
chr11	95718700	0.867769
chr11	95808600	0.570248
chr11	95815000	0.760331
chr11	95820100	0.619835
chr11	95836800	0.925620
chr11	95840100	0.595041
chr11	95844600	0.586777
chr11	95882500	0.958678
chr11	95888800	1.000000
chr11	95945700	0.570248
chr11	95948900	0.892562
chr11	95955500	0.636364
chr11	95980400	0.809917
chr11	95993300	0.991736
chr11	95997100	0.793388
chr11	96009800	0.818182

chr11	96012900	0.925620
chr11	96043500	0.619835
chr11	96050900	0.842975
chr11	96053400	0.834711
chr11	96058500	0.801653
chr11	96063500	0.900826
chr11	96066100	0.834711
chr11	96095500	0.644628
chr11	96100000	0.636364
chr11	96217700	0.743802
chr11	96220500	0.851240
chr11	96236100	0.818182
chr11	96242400	0.950413
chr11	96492000	0.595041
chr11	96529900	0.752066
chr11	97574600	0.628099
chr11	97611700	0.578512
chr11	97834000	0.900826
chr11	97837500	0.818182
chr11	97844000	0.900826
chr11	97846600	0.661157
chr11	97867400	0.942149
chr11	97927900	0.710744
chr11	100506300	0.942149
chr11	100518900	0.719008
chr11	100537200	0.570248
chr11	100580200	0.628099
chr11	100680400	0.793388
chr11	100732100	0.760331
chr11	100734400	0.619835
chr11	100736800	0.851240
chr11	100789900	0.578512
chr11	100825000	0.702479
chr11	100841900	0.966942
chr11	100845100	0.851240
chr11	100915200	0.867769
chr11	100920400	0.743802
chr11	100967800	0.595041
chr11	102213000	0.644628
chr11	102254900	0.876033
chr11	102257800	0.933884
chr11	102347900	0.892562
chr11	102352400	0.578512
chr11	102470700	0.628099
chr11	102492100	0.710744
chr11	107125300	0.628099
chr11	107243400	0.652893
chr11	107376700	0.628099
chr11	107433800	0.586777
chr11	107438400	0.669421
chr11	107629700	0.785124
chr11	107711800	0.958678
chr11	107799000	0.661157
chr11	107883700	0.628099

chr11	107886700	0.909091
chr11	107890800	0.859504
chr11	107896600	0.595041
chr11	107899900	0.760331
chr11	107995400	0.892562
chr11	108020200	0.619835
chr11	108071000	0.933884
chr11	108089300	0.628099
chr11	108097500	0.702479
chr11	108272400	0.760331
chr11	108372200	0.677686
chr11	108406900	0.595041
chr11	108420700	0.603306
chr11	108423900	0.909091
chr11	108427500	0.859504
chr11	108480500	0.652893
chr11	108495900	0.570248
chr11	108538500	0.586777
chr11	108564900	0.752066
chr11	108804600	0.661157
chr11	109618700	0.809917
chr11	109762400	0.925620
chr11	109765000	0.950413
chr11	109811700	0.768595
chr11	109870900	0.586777
chr11	109940700	1.000000
chr11	109945800	0.818182
chr11	109948500	0.760331
chr11	109954100	0.586777
chr11	109983500	0.586777
chr11	110045200	0.570248
chr11	110183000	0.578512
chr11	110188900	0.801653
chr11	110209200	0.619835
chr11	110307000	0.859504
chr11	110352500	0.628099
chr11	110436300	0.652893
chr11	111445800	0.652893
chr11	111472200	0.677686
chr11	111563000	0.917355
chr11	111569000	0.785124
chr11	111571100	0.685950
chr11	111600700	0.619835
chr11	111712500	0.611570
chr11	111780400	0.842975
chr11	111783600	0.743802
chr11	111789800	0.570248
chr11	111794400	0.768595
chr11	111796600	0.685950
chr11	111810100	0.578512
chr11	111820700	0.842975
chr11	111837900	0.636364
chr11	111849200	0.644628
chr11	111854100	0.958678

chr11	111859800	0.942149
chr11	111913200	0.884298
chr11	111915800	0.628099
chr11	111954200	0.619835
chr11	111960500	0.611570
chr11	111968600	0.785124
chr11	112035000	0.619835
chr11	112118100	0.595041
chr11	112125900	0.834711
chr11	112138100	0.586777
chr11	112142100	0.644628
chr11	112150200	0.578512
chr11	112157200	0.735537
chr11	112161100	0.661157
chr11	112173600	0.826446
chr11	113061800	0.793388
chr11	113319000	0.685950
chr11	113418200	0.719008
chr11	113421000	0.818182
chr11	113425900	0.892562
chr11	113450500	0.710744
chr11	113484800	0.776860
chr11	113493700	0.917355
chr11	113500200	0.628099
chr11	113506000	0.975207
chr11	113515700	0.710744
chr11	113518200	0.752066
chr11	113533900	0.661157
chr11	113537600	0.834711
chr11	113541300	0.958678
chr11	113552300	0.975207
chr11	113558900	0.619835
chr11	113561900	0.900826
chr11	113586200	0.842975
chr11	113596900	0.834711
chr11	113755800	0.586777
chr11	113950800	0.595041
chr11	113953800	0.818182
chr11	114006900	0.809917
chr11	114019000	0.710744
chr11	114021500	0.892562
chr11	114030900	0.801653
chr11	114034600	0.793388
chr11	114050400	0.570248
chr11	114054000	0.636364
chr11	114082100	0.570248
chr11	114085000	0.570248
chr11	114089200	0.636364
chr11	114091300	0.950413
chr11	114095400	0.644628
chr11	114100100	0.685950
chr11	114108300	0.652893
chr11	114111500	0.950413
chr11	114115200	0.570248

chr11	114122700	0.809917
chr11	114134700	0.785124
chr11	114144900	0.570248
chr11	114163000	0.595041
chr11	114165900	0.818182
chr11	114168500	0.909091
chr11	114178600	0.851240
chr11	114186500	0.595041
chr11	114211400	0.735537
chr11	114314800	0.892562
chr11	114318900	0.760331
chr11	116522700	0.710744
chr11	116647800	0.859504
chr11	116711800	0.793388
chr11	116723800	0.661157
chr11	116731900	0.586777
chr11	116802500	0.752066
chr11	116829500	0.619835
chr11	116842100	0.595041
chr11	116891500	0.719008
chr11	116908600	0.578512
chr11	116944500	0.826446
chr11	116958400	0.578512
chr11	116961100	0.719008
chr11	116970200	0.710744
chr11	117005900	0.727273
chr11	117009600	0.743802
chr11	117018100	0.628099
chr11	117042400	0.909091
chr11	117044900	0.785124
chr11	117052100	0.801653
chr11	117080300	0.644628
chr11	117090800	0.909091
chr11	117092900	0.809917
chr11	117095600	0.809917
chr11	117680200	0.595041
chr11	117780800	0.933884
chr11	117821600	0.719008
chr11	117824400	0.793388
chr11	117830400	0.834711
chr11	117843800	0.595041
chr11	117872100	0.793388
chr11	117875700	0.760331
chr11	117878500	0.925620
chr11	117881800	0.884298
chr11	117884000	0.644628
chr11	117889600	0.603306
chr11	117943500	0.636364
chr11	117954100	0.768595
chr11	117964200	0.578512
chr11	117973500	0.884298
chr11	118112100	0.975207
chr11	118187600	0.644628
chr11	118190500	0.851240

chr11	118210000	0.909091
chr11	118213200	0.818182
chr11	118244400	0.752066
chr11	118286400	0.892562
chr11	118311600	0.619835
chr11	118327700	0.628099
chr11	118340200	0.669421
chr11	118473400	0.909091
chr11	118476600	0.727273
chr11	118493200	0.595041
chr11	118505500	0.760331
chr11	118514300	0.958678
chr11	118561200	0.586777
chr11	118579100	0.876033
chr11	118584100	0.966942
chr11	118587000	0.834711
chr11	118591700	0.578512
chr11	118605700	0.578512
chr11	118611300	0.809917
chr11	118617000	0.644628
chr11	118657400	0.595041
chr11	118713700	0.578512
chr11	118717300	1.000000
chr11	118747600	0.570248
chr11	118756400	0.628099
chr11	118758800	0.958678
chr11	118781400	0.776860
chr11	118784500	0.867769
chr11	118790100	0.727273
chr11	118792300	0.892562
chr11	118852400	0.636364
chr11	118864300	0.661157
chr11	118872100	0.727273
chr11	118892100	0.570248
chr11	118895900	0.809917
chr11	118898500	0.719008
chr11	118902800	0.603306
chr11	118929800	0.669421
chr11	118934100	0.884298
chr11	118936300	0.611570
chr11	118975300	0.677686
chr11	118986500	0.933884
chr11	119039900	0.743802
chr11	119043500	0.991736
chr11	119046200	0.628099
chr11	119052600	0.677686
chr11	119057300	0.669421
chr11	119121800	0.892562
chr11	119130300	0.644628
chr11	119193600	0.694215
chr11	119207100	0.570248
chr11	119210200	0.636364
chr11	119223900	0.586777
chr11	119232700	0.785124

chr11	119235800	0.694215
chr11	119239200	0.966942
chr11	119242000	0.818182
chr11	119246200	0.768595
chr11	119312600	0.677686
chr11	119351300	0.735537
chr11	119455400	0.578512
chr11	119465800	0.859504
chr11	119630500	0.743802
chr11	120040100	0.619835
chr11	120152400	0.950413
chr11	120185900	0.570248
chr11	120204500	0.677686
chr11	120212600	0.628099
chr11	120215200	0.603306
chr11	120223900	0.586777
chr11	120272100	0.570248
chr11	120366000	0.685950
chr11	120469900	0.586777
chr11	122626400	0.842975
chr11	122874200	0.942149
chr11	122944700	0.809917
chr11	122954800	0.685950
chr11	123941000	0.619835
chr11	123950700	0.983471
chr11	123967000	0.958678
chr11	123971300	0.603306
chr11	123979700	0.595041
chr11	123992900	0.826446
chr11	124045000	0.776860
chr11	124061300	0.876033
chr11	124065800	0.685950
chr11	124074200	0.603306
chr11	124077800	0.694215
chr11	124079900	0.636364
chr11	124096000	0.991736
chr11	124120700	0.743802
chr11	124140600	0.619835
chr11	124146300	0.760331
chr11	124184800	0.611570
chr11	124253000	0.801653
chr11	124267000	0.925620
chr11	124311000	0.628099
chr11	124353100	0.570248
chr11	124408000	0.892562
chr11	124452100	0.702479
chr11	124495500	0.611570
chr11	124508900	0.595041
chr11	124514100	0.785124
chr11	124538300	0.818182
chr11	124617200	0.710744
chr11	124675000	0.818182
chr11	124928200	0.636364
chr11	124939400	0.925620

chr11	124941700	0.966942
chr11	124946000	0.925620
chr11	124961800	0.727273
chr11	124994500	0.685950
chr11	125143900	0.950413
chr11	125305900	0.735537
chr11	125311800	0.619835
chr11	125322200	0.636364
chr11	125324400	0.801653
chr11	125328300	0.677686
chr11	125331400	0.793388
chr11	125334500	0.702479
chr11	125353600	0.570248
chr11	125358000	0.578512
chr11	125384200	0.702479
chr11	125386800	0.694215
chr11	125403100	0.892562
chr11	125421200	0.752066
chr11	125423300	0.966942
chr11	125426500	0.818182
chr11	125458200	0.892562
chr11	125465600	0.586777
chr11	125760800	0.809917
chr11	125803400	0.636364
chr11	125840500	0.611570
chr11	126096000	0.942149
chr11	126126400	0.867769
chr11	126140800	0.595041
chr11	126211900	0.743802
chr11	126216400	0.983471
chr11	126218900	0.900826
chr11	126222400	0.644628
chr11	126229700	0.669421
chr11	126311100	0.603306
chr11	128490600	0.628099
chr11	129736500	0.859504
chr11	129800400	0.685950
chr11	129816000	0.818182
chr11	129820500	0.867769
chr11	129836600	0.628099
chr11	129876000	0.677686
chr11	129878200	0.793388
chr11	129947000	0.619835
chr11	129957600	0.892562
chr11	129959900	0.917355
chr11	129965300	0.909091
chr11	129973500	0.818182
chr11	130165300	0.760331
chr11	130179800	0.851240
chr11	130392500	0.876033
chr11	130764600	0.677686
chr11	134103700	0.876033
chr11	134107200	0.628099
chr11	134130300	0.677686

chr11	134159300	0.652893
chr11	134225700	0.834711
chr11	134353400	0.570248
chr12	61900	0.991736
chr12	64900	0.694215
chr12	67800	0.727273
chr12	77600	0.909091
chr12	90800	0.801653
chr12	148000	0.859504
chr12	150100	0.661157
chr12	153100	0.652893
chr12	161000	0.603306
chr12	166300	0.752066
chr12	186700	0.611570
chr12	208700	0.677686
chr12	213100	0.925620
chr12	216800	0.603306
chr12	220800	0.851240
chr12	224000	0.586777
chr12	242900	0.785124
chr12	281100	0.735537
chr12	309200	0.595041
chr12	516300	0.842975
chr12	671900	0.578512
chr12	685500	0.942149
chr12	688900	0.942149
chr12	697200	0.942149
chr12	700200	0.785124
chr12	702400	0.586777
chr12	719700	0.834711
chr12	755100	0.933884
chr12	772900	0.826446
chr12	776700	0.950413
chr12	784000	0.661157
chr12	803700	0.636364
chr12	826100	0.925620
chr12	829700	0.611570
chr12	859000	0.611570
chr12	868700	0.867769
chr12	899700	0.826446
chr12	904500	0.702479
chr12	920500	0.603306
chr12	1013700	0.942149
chr12	1020300	0.917355
chr12	1063000	0.859504
chr12	1314100	0.628099
chr12	1391800	0.652893
chr12	1413600	0.669421
chr12	1416300	0.851240
chr12	1419500	0.578512
chr12	1434400	0.958678
chr12	1437500	0.925620
chr12	1440100	0.925620
chr12	1453000	0.661157

chr12	1471200	0.636364
chr12	1513200	0.578512
chr12	1545800	0.842975
chr12	1549100	0.760331
chr12	1553200	0.619835
chr12	1601700	0.909091
chr12	1609600	0.925620
chr12	1628200	0.636364
chr12	1676100	0.867769
chr12	1686100	0.768595
chr12	1705500	0.595041
chr12	1711100	0.727273
chr12	1715100	0.652893
chr12	1724100	0.933884
chr12	1728400	0.925620
chr12	1732200	0.752066
chr12	1759200	0.578512
chr12	1770300	0.628099
chr12	1775300	0.809917
chr12	1778300	0.876033
chr12	1803600	0.578512
chr12	1806800	0.669421
chr12	1816000	0.735537
chr12	1821400	0.909091
chr12	1823500	0.942149
chr12	1832400	0.702479
chr12	1847400	0.727273
chr12	1919500	0.595041
chr12	1922300	0.694215
chr12	1995100	0.694215
chr12	2025100	0.586777
chr12	2027300	0.933884
chr12	2444100	0.958678
chr12	2446200	0.793388
chr12	2893600	0.586777
chr12	2902100	0.942149
chr12	3050100	0.595041
chr12	3067500	0.694215
chr12	3072500	0.818182
chr12	3086200	0.768595
chr12	3108900	0.892562
chr12	3793400	0.958678
chr12	4256100	0.644628
chr12	4262400	0.950413
chr12	4382100	0.619835
chr12	4386100	0.603306
chr12	4498300	0.909091
chr12	4502000	0.619835
chr12	4524700	0.727273
chr12	4612200	0.595041
chr12	4671600	0.809917
chr12	4676900	0.694215
chr12	4681800	0.619835
chr12	5330400	0.719008

chr12	5353800	0.685950
chr12	5722300	0.892562
chr12	5730500	0.735537
chr12	5833500	0.661157
chr12	5846200	0.586777
chr12	6264900	0.570248
chr12	6270700	0.619835
chr12	6277300	0.975207
chr12	6280900	0.586777
chr12	6362600	0.859504
chr12	6372700	0.867769
chr12	6432900	0.826446
chr12	6444700	0.727273
chr12	6448200	0.611570
chr12	6452200	0.570248
chr12	6455500	0.719008
chr12	6477600	1.000000
chr12	6480400	0.933884
chr12	6482500	0.876033
chr12	6492600	0.595041
chr12	6540100	0.892562
chr12	6561400	0.834711
chr12	6641300	0.661157
chr12	6647300	0.834711
chr12	6651500	0.727273
chr12	6654800	0.966942
chr12	6712800	0.578512
chr12	6718000	0.652893
chr12	6720800	0.826446
chr12	6724200	0.892562
chr12	6733200	0.710744
chr12	6742000	0.785124
chr12	6744600	0.702479
chr12	6747500	0.818182
chr12	6750300	0.826446
chr12	6753900	0.603306
chr12	6835200	0.578512
chr12	6856300	0.603306
chr12	6863900	0.661157
chr12	6873100	0.975207
chr12	6888200	0.702479
chr12	6920100	0.611570
chr12	6952900	0.933884
chr12	6958600	0.644628
chr12	6963600	0.677686
chr12	6975700	0.578512
chr12	6979800	0.595041
chr12	7001400	0.694215
chr12	7025300	0.652893
chr12	7029600	0.570248
chr12	7034300	0.884298
chr12	7042200	0.834711
chr12	7047800	0.636364
chr12	7052000	0.710744

chr12	7056000	0.743802
chr12	7063400	0.793388
chr12	7072700	0.909091
chr12	7104600	0.595041
chr12	7120200	0.661157
chr12	7140800	0.685950
chr12	7166100	0.760331
chr12	7168200	0.619835
chr12	7176500	0.586777
chr12	7262700	0.636364
chr12	7266500	0.586777
chr12	7271700	0.595041
chr12	7274800	0.727273
chr12	7278900	0.900826
chr12	7297700	0.892562
chr12	7347700	0.611570
chr12	7394900	0.818182
chr12	7445400	0.826446
chr12	7448400	0.975207
chr12	7450900	0.851240
chr12	7455100	0.925620
chr12	7505400	0.826446
chr12	7884300	0.710744
chr12	7996300	0.644628
chr12	8043700	0.636364
chr12	8059800	0.752066
chr12	8090900	0.859504
chr12	8102100	0.809917
chr12	8111500	0.925620
chr12	8115600	0.925620
chr12	8120500	0.743802
chr12	8139600	0.611570
chr12	8142800	0.933884
chr12	8171900	0.570248
chr12	8187900	0.768595
chr12	8191500	0.793388
chr12	8205500	0.727273
chr12	8218900	0.867769
chr12	8231700	0.628099
chr12	8309900	0.661157
chr12	8350600	0.809917
chr12	8380300	0.669421
chr12	8470900	0.694215
chr12	8473800	0.892562
chr12	8477500	0.867769
chr12	8969600	0.950413
chr12	8981200	0.743802
chr12	8994400	0.785124
chr12	9009100	0.669421
chr12	9039700	0.942149
chr12	9083900	0.611570
chr12	9608400	0.975207
chr12	9741200	0.661157
chr12	9826500	0.958678

chr12	9838500	0.900826
chr12	9895500	0.752066
chr12	9914800	0.702479
chr12	9917700	0.776860
chr12	9946100	0.685950
chr12	9950000	0.859504
chr12	10048400	0.586777
chr12	10514400	0.867769
chr12	10523200	0.669421
chr12	10770900	0.925620
chr12	10892600	0.809917
chr12	11321900	0.603306
chr12	11776600	0.578512
chr12	11949000	0.991736
chr12	11952200	0.950413
chr12	11980100	0.702479
chr12	12281200	0.636364
chr12	12344200	0.685950
chr12	12354800	0.628099
chr12	12369600	0.909091
chr12	12373800	0.595041
chr12	12377400	0.652893
chr12	12507100	0.867769
chr12	12545700	0.950413
chr12	12595300	0.752066
chr12	12628100	0.826446
chr12	12645500	0.644628
chr12	12659100	0.628099
chr12	12687100	0.677686
chr12	12716700	0.752066
chr12	12826200	0.595041
chr12	12857900	0.727273
chr12	12867600	0.760331
chr12	12887900	0.669421
chr12	12890500	0.644628
chr12	12895300	0.636364
chr12	12910800	0.876033
chr12	12941700	0.619835
chr12	12944800	0.619835
chr12	12953800	0.793388
chr12	12956700	0.677686
chr12	12960200	0.727273
chr12	12963800	0.983471
chr12	13030600	0.636364
chr12	13036400	0.586777
chr12	13063400	0.710744
chr12	13224600	0.570248
chr12	14388600	0.752066
chr12	14409500	0.818182
chr12	14411800	0.842975
chr12	14414000	0.942149
chr12	14421200	0.636364
chr12	14437400	0.942149
chr12	14461000	0.661157

chr12	14544900	0.578512
chr12	14988500	0.644628
chr12	14996300	0.685950
chr12	15114400	0.685950
chr12	15123800	0.735537
chr12	18835000	0.776860
chr12	18837100	0.644628
chr12	18905800	0.842975
chr12	18978000	0.834711
chr12	19049300	0.909091
chr12	19133500	0.636364
chr12	19297600	0.595041
chr12	19365000	0.586777
chr12	19600500	0.842975
chr12	19607400	0.603306
chr12	19636000	0.752066
chr12	19651000	0.644628
chr12	19659700	0.586777
chr12	19668200	0.983471
chr12	20704100	0.867769
chr12	21500000	0.826446
chr12	21510800	0.760331
chr12	21567500	0.685950
chr12	21803800	0.628099
chr12	22146200	0.966942
chr12	22197500	0.809917
chr12	22203600	0.884298
chr12	22504100	0.669421
chr12	23052900	0.760331
chr12	23193400	0.570248
chr12	23457100	0.611570
chr12	23569600	0.958678
chr12	23575100	0.636364
chr12	23578100	0.834711
chr12	23625400	0.595041
chr12	23655900	0.776860
chr12	23658200	0.636364
chr12	23686600	0.760331
chr12	23705600	0.570248
chr12	23710600	0.884298
chr12	23713200	0.900826
chr12	23719600	0.727273
chr12	23722700	0.603306
chr12	23787600	0.760331
chr12	23865800	0.611570
chr12	23869600	0.595041
chr12	23877100	0.595041
chr12	23884000	0.644628
chr12	23886100	0.842975
chr12	23934300	0.710744
chr12	23963900	0.793388
chr12	24041000	0.892562
chr12	24848400	0.702479
chr12	24850500	0.867769

chr12	24858800	0.578512
chr12	24862100	0.884298
chr12	24897400	0.909091
chr12	24912500	0.909091
chr12	25053400	0.595041
chr12	25086400	0.702479
chr12	25088600	0.834711
chr12	25091200	0.867769
chr12	25097500	0.818182
chr12	25105000	0.760331
chr12	25108700	0.842975
chr12	25132800	0.595041
chr12	25399200	0.628099
chr12	25429600	0.909091
chr12	25845700	1.000000
chr12	26267300	0.603306
chr12	26392300	0.595041
chr12	26424400	0.892562
chr12	26428600	0.983471
chr12	26471700	0.760331
chr12	26779600	0.909091
chr12	26841900	0.842975
chr12	26844800	0.966942
chr12	26849800	0.925620
chr12	27097500	0.644628
chr12	27169100	0.586777
chr12	27171200	0.669421
chr12	27202500	0.818182
chr12	27674300	0.892562
chr12	27703200	0.644628
chr12	27904700	0.851240
chr12	27959500	0.776860
chr12	27975100	0.785124
chr12	27997500	0.834711
chr12	28001300	0.652893
chr12	28006000	0.842975
chr12	28013100	0.884298
chr12	28017500	0.578512
chr12	28023000	0.628099
chr12	28060100	0.661157
chr12	28165600	0.884298
chr12	28180900	0.644628
chr12	28263700	0.694215
chr12	28276400	0.884298
chr12	28281200	0.586777
chr12	28283400	0.768595
chr12	28285600	0.834711
chr12	28289400	0.884298
chr12	28291700	0.603306
chr12	28302000	0.652893
chr12	28309800	0.958678
chr12	28382900	0.619835
chr12	28388100	0.859504
chr12	28394300	0.776860

chr12	28402200	0.809917
chr12	28406200	0.710744
chr12	28696900	0.586777
chr12	29317800	0.801653
chr12	30548700	0.644628
chr12	30661500	0.752066
chr12	30682700	0.702479
chr12	30904800	0.578512
chr12	30910300	0.975207
chr12	30916400	0.942149
chr12	30932400	0.661157
chr12	31219900	0.900826
chr12	31461500	0.595041
chr12	31469100	0.677686
chr12	31473100	0.661157
chr12	31515700	0.809917
chr12	31523800	0.727273
chr12	31771900	0.719008
chr12	31783600	0.710744
chr12	31867300	0.619835
chr12	31872000	0.752066
chr12	31876300	0.776860
chr12	31879700	0.578512
chr12	31885700	0.975207
chr12	31887900	0.958678
chr12	31890300	0.975207
chr12	31902000	0.760331
chr12	31904100	0.958678
chr12	31986200	0.942149
chr12	32030300	0.892562
chr12	32050500	0.892562
chr12	32106800	0.652893
chr12	32118200	0.586777
chr12	32153300	0.826446
chr12	32159200	0.867769
chr12	32165700	0.644628
chr12	32173800	0.760331
chr12	32178300	0.892562
chr12	32184500	1.000000
chr12	32187200	0.768595
chr12	32193500	0.933884
chr12	32200800	0.884298
chr12	32205000	0.752066
chr12	32207500	0.776860
chr12	32289300	0.595041
chr12	32411800	0.710744
chr12	32426800	0.876033
chr12	32443800	0.785124
chr12	32531000	0.760331
chr12	32835200	0.595041
chr12	32859600	0.809917
chr12	33136800	0.603306
chr12	33740100	0.603306
chr12	33743000	0.752066

chr12	33747400	0.719008
chr12	33750100	0.809917
chr12	33831500	0.867769
chr12	33906100	0.892562
chr12	34280900	0.892562
chr12	34446800	0.677686
chr12	34501900	0.743802
chr12	34509400	0.661157
chr12	34516600	0.752066
chr12	34833200	0.611570
chr12	34837400	0.586777
chr12	34855200	0.603306
chr12	37961300	0.900826
chr12	38449300	0.628099
chr12	39286500	0.611570
chr12	39328100	0.768595
chr12	39832700	0.702479
chr12	39853200	0.694215
chr12	40331300	0.809917
chr12	40493500	0.867769
chr12	41757200	0.950413
chr12	42471100	0.859504
chr12	42480200	0.661157
chr12	42742900	0.570248
chr12	42759000	0.719008
chr12	42811500	0.801653
chr12	42816200	0.950413
chr12	42820800	0.892562
chr12	42838400	0.628099
chr12	42840600	0.644628
chr12	42880600	0.644628
chr12	45602600	0.578512
chr12	45621800	0.867769
chr12	45626200	0.636364
chr12	45629100	0.842975
chr12	45654300	0.611570
chr12	45658100	0.677686
chr12	45668100	0.942149
chr12	45674000	0.628099
chr12	45686700	0.611570
chr12	45692900	0.950413
chr12	45709300	0.710744
chr12	45731500	0.942149
chr12	45749900	0.776860
chr12	46148300	0.694215
chr12	46156300	0.652893
chr12	46273000	0.917355
chr12	46368000	0.586777
chr12	46614200	0.785124
chr12	46616300	0.867769
chr12	46650200	0.842975
chr12	46654500	0.851240
chr12	46658300	0.586777
chr12	46760400	0.628099

chr12	46774700	0.900826
chr12	46786600	0.826446
chr12	46796000	0.570248
chr12	46800700	0.842975
chr12	46824400	1.000000
chr12	46826800	0.628099
chr12	46844500	0.818182
chr12	46850000	0.867769
chr12	46867600	0.966942
chr12	46877900	0.892562
chr12	46910400	0.661157
chr12	46918400	0.983471
chr12	46940800	0.876033
chr12	46944000	0.991736
chr12	46952300	0.867769
chr12	46980200	0.867769
chr12	47052100	0.595041
chr12	47450100	0.760331
chr12	47477200	0.818182
chr12	47479800	0.876033
chr12	47550100	0.983471
chr12	47560100	0.851240
chr12	47576400	0.702479
chr12	47583400	0.661157
chr12	47588900	0.966942
chr12	47600700	1.000000
chr12	47602900	0.710744
chr12	47609900	0.859504
chr12	47612900	0.619835
chr12	47616400	0.595041
chr12	47618500	0.702479
chr12	47639200	0.876033
chr12	47666500	0.661157
chr12	47670900	0.942149
chr12	47673200	0.776860
chr12	47675400	0.983471
chr12	47711100	0.702479
chr12	47787000	0.652893
chr12	48010100	0.892562
chr12	48031100	0.570248
chr12	48103300	0.851240
chr12	48130600	0.719008
chr12	48133800	0.892562
chr12	48163100	0.677686
chr12	48172100	0.834711
chr12	48180000	0.801653
chr12	48197300	0.619835
chr12	48202900	0.942149
chr12	48205400	0.685950
chr12	48208900	0.818182
chr12	48215600	0.727273
chr12	48222200	0.743802
chr12	48229700	0.933884
chr12	48232700	0.611570

chr12	48244900	0.983471
chr12	48261900	0.809917
chr12	48279300	0.570248
chr12	48424500	0.603306
chr12	48431600	0.628099
chr12	48461800	0.595041
chr12	48516100	0.628099
chr12	48546500	0.942149
chr12	48570100	0.834711
chr12	48631200	0.760331
chr12	48705400	0.735537
chr12	48803800	0.809917
chr12	48853800	0.628099
chr12	48933900	0.834711
chr12	48948000	0.644628
chr12	48951000	0.611570
chr12	48995000	0.966942
chr12	49034600	0.685950
chr12	49073100	0.685950
chr12	49087500	0.983471
chr12	49108600	0.586777
chr12	49142600	0.669421
chr12	49277700	0.760331
chr12	49281300	0.719008
chr12	49365800	0.603306
chr12	49393700	0.611570
chr12	49409500	0.595041
chr12	49457800	0.727273
chr12	49482200	0.818182
chr12	49521700	0.685950
chr12	49532700	0.867769
chr12	49554700	0.859504
chr12	49568100	0.570248
chr12	49595700	0.636364
chr12	49626200	0.586777
chr12	49639000	0.760331
chr12	49645800	0.652893
chr12	49650300	0.900826
chr12	49657100	0.752066
chr12	49677500	0.677686
chr12	49696000	0.727273
chr12	49700200	0.735537
chr12	49732500	0.636364
chr12	49764100	0.826446
chr12	49791100	0.677686
chr12	49794300	0.809917
chr12	49798300	0.801653
chr12	49807400	0.809917
chr12	49809900	0.809917
chr12	49816300	0.818182
chr12	50056900	0.991736
chr12	50065400	0.652893
chr12	50067800	0.892562
chr12	50093000	0.909091

chr12	50097600	0.785124
chr12	50125000	0.884298
chr12	50138700	0.636364
chr12	50144900	0.983471
chr12	50178600	0.727273
chr12	50263000	0.793388
chr12	50268800	0.752066
chr12	50286400	0.826446
chr12	50293300	0.818182
chr12	50304100	0.619835
chr12	50308300	0.595041
chr12	50339700	0.570248
chr12	50375200	0.677686
chr12	50378400	0.809917
chr12	50416600	0.611570
chr12	50436100	0.842975
chr12	50441700	0.710744
chr12	50444600	0.628099
chr12	50450100	0.785124
chr12	50465000	0.785124
chr12	50497800	0.785124
chr12	50504100	0.727273
chr12	50634500	0.760331
chr12	50636600	0.595041
chr12	50643900	0.661157
chr12	50646000	0.628099
chr12	50648600	0.991736
chr12	50665500	0.793388
chr12	50670400	0.595041
chr12	50697800	0.776860
chr12	50759800	0.677686
chr12	50798700	0.586777
chr12	50813000	0.826446
chr12	50926900	0.991736
chr12	50940000	0.570248
chr12	50944500	0.570248
chr12	50949300	0.884298
chr12	50956400	0.826446
chr12	50958900	0.570248
chr12	50969300	0.652893
chr12	50983700	0.652893
chr12	50989700	0.586777
chr12	51003300	0.694215
chr12	51005400	0.661157
chr12	51008000	0.933884
chr12	51019300	0.793388
chr12	51035900	0.917355
chr12	51143700	0.677686
chr12	51162200	0.702479
chr12	51236200	0.685950
chr12	51295900	0.851240
chr12	51317200	0.677686
chr12	51327900	0.975207
chr12	51331300	0.876033

chr12	51411400	0.876033
chr12	51413700	0.842975
chr12	51422100	0.677686
chr12	51427400	0.983471
chr12	51461700	0.710744
chr12	51555000	0.661157
chr12	51568500	0.661157
chr12	51640700	0.793388
chr12	51659900	0.578512
chr12	51713200	0.983471
chr12	51717000	0.818182
chr12	51720400	0.710744
chr12	51723200	0.942149
chr12	51735800	0.661157
chr12	51739700	0.842975
chr12	51755500	0.851240
chr12	51757600	0.578512
chr12	51763200	0.710744
chr12	51782200	0.958678
chr12	51789800	0.694215
chr12	51916800	0.595041
chr12	52222200	0.776860
chr12	52265900	0.619835
chr12	52278600	1.000000
chr12	52300600	0.826446
chr12	52303100	0.578512
chr12	52306600	0.834711
chr12	52309600	0.719008
chr12	52312200	0.628099
chr12	52317700	0.611570
chr12	52320300	0.644628
chr12	52348700	0.752066
chr12	52366700	0.867769
chr12	52374800	0.619835
chr12	52387500	0.570248
chr12	52404500	0.942149
chr12	52440000	0.611570
chr12	52446800	0.702479
chr12	52457900	0.735537
chr12	52674100	0.603306
chr12	53020700	0.644628
chr12	53031100	0.710744
chr12	53039300	0.818182
chr12	53054400	0.685950
chr12	53057300	0.578512
chr12	53184500	0.595041
chr12	53191400	1.000000
chr12	53229800	0.884298
chr12	53267300	0.702479
chr12	53269600	0.793388
chr12	53273600	0.702479
chr12	53295100	0.785124
chr12	53301300	0.909091
chr12	53308600	0.644628

chr12	53319900	0.570248
chr12	53336800	0.760331
chr12	53340800	0.694215
chr12	53346300	0.975207
chr12	53350800	0.603306
chr12	53369600	0.826446
chr12	53387100	0.892562
chr12	53405500	0.603306
chr12	53407600	0.619835
chr12	53443300	0.950413
chr12	53445500	0.876033
chr12	53451600	0.652893
chr12	53460600	0.603306
chr12	53466600	0.950413
chr12	53492900	0.595041
chr12	53509700	0.752066
chr12	53537600	0.876033
chr12	53553400	0.619835
chr12	53569200	0.669421
chr12	53577100	0.702479
chr12	53595300	0.933884
chr12	53603100	0.636364
chr12	53611800	0.661157
chr12	53638400	0.958678
chr12	53653300	0.768595
chr12	53664500	0.892562
chr12	53693000	0.942149
chr12	53703200	0.743802
chr12	53734500	0.586777
chr12	53747000	0.743802
chr12	53749300	0.892562
chr12	53760000	0.834711
chr12	53762800	0.611570
chr12	53766700	0.710744
chr12	53776800	0.611570
chr12	53782200	0.570248
chr12	53795600	0.628099
chr12	53809400	0.826446
chr12	53815300	0.818182
chr12	53817800	0.909091
chr12	53822300	0.611570
chr12	53873700	0.669421
chr12	53890300	0.694215
chr12	53963200	0.636364
chr12	53969900	0.570248
chr12	53986600	0.801653
chr12	54012200	0.785124
chr12	54054600	0.628099
chr12	54151400	0.669421
chr12	54202400	0.570248
chr12	54343400	0.809917
chr12	54358300	0.719008
chr12	54382200	0.702479
chr12	54391600	0.743802

chr12	54413700	0.710744
chr12	54475400	0.760331
chr12	54478100	0.809917
chr12	54555700	0.942149
chr12	54569600	0.909091
chr12	54578300	0.851240
chr12	54590500	0.677686
chr12	54594900	0.595041
chr12	54604200	0.743802
chr12	54608800	0.644628
chr12	54611000	0.760331
chr12	54614600	0.859504
chr12	54621600	0.818182
chr12	54624200	0.611570
chr12	54634900	0.694215
chr12	54651300	0.685950
chr12	54656000	0.884298
chr12	54659800	0.727273
chr12	54682500	0.578512
chr12	54700100	0.826446
chr12	54705800	0.925620
chr12	54717200	0.727273
chr12	54722200	0.966942
chr12	54725200	0.983471
chr12	54727600	0.760331
chr12	54733600	0.752066
chr12	54746200	0.950413
chr12	54750200	0.950413
chr12	54764300	0.677686
chr12	54768900	0.644628
chr12	54771100	0.859504
chr12	54773400	0.661157
chr12	54778600	0.876033
chr12	54781100	0.578512
chr12	54787400	0.851240
chr12	54807900	0.983471
chr12	54810000	0.851240
chr12	54815100	0.867769
chr12	54822700	1.000000
chr12	54826800	0.776860
chr12	54833200	0.652893
chr12	54880500	0.661157
chr12	55326400	0.702479
chr12	55329600	0.892562
chr12	55371800	0.933884
chr12	55374500	0.793388
chr12	55378800	0.834711
chr12	55382200	0.743802
chr12	55806500	0.752066
chr12	55894000	0.661157
chr12	56040700	0.826446
chr12	56053600	0.793388
chr12	56132200	0.586777
chr12	56140400	0.685950

chr12	56220500	0.652893
chr12	56233500	0.619835
chr12	56236800	0.991736
chr12	56242200	0.818182
chr12	56303400	0.900826
chr12	56323900	0.867769
chr12	56334300	0.685950
chr12	56370700	0.669421
chr12	56397900	0.603306
chr12	56404100	0.917355
chr12	56409800	0.578512
chr12	56440800	0.900826
chr12	56476200	0.760331
chr12	56514600	0.768595
chr12	56518800	0.892562
chr12	56525000	0.661157
chr12	56549300	0.950413
chr12	56555400	0.586777
chr12	56562000	0.719008
chr12	56596700	0.578512
chr12	56599200	0.859504
chr12	56637600	0.983471
chr12	56691000	0.917355
chr12	56696400	0.809917
chr12	56711600	0.603306
chr12	56722600	0.619835
chr12	56732600	0.884298
chr12	56769800	0.834711
chr12	56776100	0.603306
chr12	56780900	0.925620
chr12	56831300	0.801653
chr12	56853900	0.586777
chr12	56915800	0.677686
chr12	56959700	0.669421
chr12	57011300	0.619835
chr12	57018600	0.727273
chr12	57020900	0.611570
chr12	57031800	0.842975
chr12	57037400	0.702479
chr12	57062700	0.743802
chr12	57070800	0.644628
chr12	57074800	0.900826
chr12	57084700	0.776860
chr12	57089800	0.983471
chr12	57092800	0.975207
chr12	57113400	0.752066
chr12	57148000	0.892562
chr12	57179500	0.776860
chr12	57231300	0.611570
chr12	57271700	0.917355
chr12	57274800	0.710744
chr12	57292400	0.578512
chr12	57295400	0.636364
chr12	57322500	0.595041

chr12	57325300	0.884298
chr12	57335600	0.900826
chr12	57363000	0.669421
chr12	57379900	0.760331
chr12	57385600	0.719008
chr12	57395600	0.677686
chr12	57402400	0.884298
chr12	57410400	0.743802
chr12	57421500	0.917355
chr12	57433100	0.983471
chr12	57485100	0.636364
chr12	57487900	0.834711
chr12	57491000	0.900826
chr12	57496200	0.710744
chr12	57510800	0.851240
chr12	57513700	0.570248
chr12	57526900	0.851240
chr12	57532900	0.752066
chr12	57548800	0.628099
chr12	57569800	0.760331
chr12	57621600	0.595041
chr12	57634300	0.727273
chr12	57643300	0.900826
chr12	57666800	0.752066
chr12	57674600	0.884298
chr12	57706900	0.611570
chr12	57713600	0.842975
chr12	57771600	0.661157
chr12	57783000	0.603306
chr12	57811900	0.760331
chr12	57846000	0.760331
chr12	57849000	0.925620
chr12	57851200	0.834711
chr12	57860000	0.644628
chr12	57868700	0.661157
chr12	57872100	0.958678
chr12	57911000	0.925620
chr12	57932300	0.785124
chr12	57934900	0.884298
chr12	57938300	0.628099
chr12	57942500	0.743802
chr12	57983400	0.603306
chr12	58000500	0.809917
chr12	58003400	0.842975
chr12	58007700	0.900826
chr12	58011200	0.578512
chr12	58016000	0.809917
chr12	58035300	0.652893
chr12	58060900	0.644628
chr12	58161100	0.636364
chr12	58211000	0.942149
chr12	58225800	0.702479
chr12	58231000	0.859504
chr12	58255800	0.611570

chr12	58268500	0.636364
chr12	58282400	0.677686
chr12	58286200	0.586777
chr12	58292500	0.619835
chr12	58299600	0.669421
chr12	60538300	0.933884
chr12	60542200	0.842975
chr12	60624200	0.578512
chr12	60636100	0.619835
chr12	62586700	0.611570
chr12	62621300	0.694215
chr12	62626400	0.661157
chr12	62642900	1.000000
chr12	62646700	0.619835
chr12	62650300	0.768595
chr12	62660300	0.578512
chr12	62667200	0.710744
chr12	62669500	0.628099
chr12	62671700	0.595041
chr12	62674700	0.636364
chr12	62685500	0.842975
chr12	62700400	0.636364
chr12	62712600	0.595041
chr12	62866200	0.603306
chr12	63124000	0.595041
chr12	63211900	0.652893
chr12	63226700	0.685950
chr12	63254500	0.652893
chr12	63261100	0.785124
chr12	63286000	0.694215
chr12	63294400	0.933884
chr12	63301400	0.768595
chr12	63304600	1.000000
chr12	63308600	0.933884
chr12	63325700	0.603306
chr12	63338900	0.570248
chr12	63366300	0.595041
chr12	63371400	0.785124
chr12	64037800	0.685950
chr12	64794000	0.768595
chr12	64797400	0.595041
chr12	64802100	0.801653
chr12	64849500	0.942149
chr12	64993200	0.619835
chr12	65013400	0.570248
chr12	65018600	0.586777
chr12	65060500	0.933884
chr12	65063900	0.975207
chr12	65067700	0.867769
chr12	65073400	0.942149
chr12	65088400	0.727273
chr12	65115100	0.851240
chr12	65124500	0.859504
chr12	65550400	0.727273

chr12	65575900	0.702479
chr12	65586900	0.702479
chr12	65589900	0.652893
chr12	65603000	0.950413
chr12	65960100	0.661157
chr12	65997300	0.752066
chr12	66033300	0.595041
chr12	66048000	0.884298
chr12	66051000	0.925620
chr12	66060800	0.925620
chr12	66065000	0.867769
chr12	66076200	0.752066
chr12	66543900	0.636364
chr12	66558300	0.619835
chr12	66628500	0.677686
chr12	66681900	0.628099
chr12	67639600	0.644628
chr12	67672800	0.603306
chr12	68041800	0.636364
chr12	68045100	0.578512
chr12	68055300	0.727273
chr12	68746500	0.570248
chr12	68757600	0.619835
chr12	68764500	0.958678
chr12	68768300	0.636364
chr12	68772300	0.719008
chr12	68775500	1.000000
chr12	68778900	0.628099
chr12	68786200	0.867769
chr12	68841200	0.834711
chr12	68892000	0.636364
chr12	68933700	0.595041
chr12	68954300	0.793388
chr12	69002400	0.719008
chr12	69015400	0.785124
chr12	69067800	0.595041
chr12	69070500	0.603306
chr12	69165500	0.719008
chr12	69187300	0.628099
chr12	69206900	0.826446
chr12	69547300	0.636364
chr12	69637400	0.661157
chr12	69722400	0.636364
chr12	69739700	0.760331
chr12	69742400	0.669421
chr12	69748600	0.859504
chr12	69810400	0.867769
chr12	69832900	0.801653
chr12	69903300	0.694215
chr12	69911400	0.619835
chr12	70002400	0.842975
chr12	70004700	0.677686
chr12	70017600	0.834711
chr12	70021400	0.644628

chr12	70046300	0.892562
chr12	70051900	0.966942
chr12	70054200	0.884298
chr12	70066400	0.727273
chr12	70083200	0.859504
chr12	70444200	0.644628
chr12	70447500	0.876033
chr12	70544200	0.595041
chr12	70658300	0.727273
chr12	70984600	0.570248
chr12	71013900	0.628099
chr12	71039600	0.644628
chr12	71046900	0.570248
chr12	71052600	0.586777
chr12	71150300	0.900826
chr12	71561300	0.595041
chr12	71613900	0.570248
chr12	71629100	0.768595
chr12	71631700	0.818182
chr12	71634900	0.644628
chr12	71662700	0.942149
chr12	71664800	0.570248
chr12	71691600	0.884298
chr12	71693800	0.826446
chr12	71696100	0.628099
chr12	71724300	0.595041
chr12	71863600	0.768595
chr12	71961500	0.570248
chr12	71988500	0.727273
chr12	72065600	0.702479
chr12	72115900	0.760331
chr12	72125900	0.586777
chr12	72138900	0.851240
chr12	72147000	0.603306
chr12	72181100	0.652893
chr12	72272100	0.851240
chr12	72336400	0.677686
chr12	72425300	0.966942
chr12	74416100	0.611570
chr12	74629900	0.611570
chr12	74680200	0.661157
chr12	76035300	0.719008
chr12	76045400	0.570248
chr12	76078500	0.809917
chr12	76100900	0.925620
chr12	76116400	0.685950
chr12	76175200	0.834711
chr12	76231600	0.611570
chr12	76287200	0.661157
chr12	76328300	0.950413
chr12	76332000	0.917355
chr12	76338200	0.975207
chr12	76348100	0.611570
chr12	76415000	0.628099

chr12	76428600	0.652893
chr12	76469200	0.776860
chr12	76472900	0.595041
chr12	76480600	0.603306
chr12	76500000	0.727273
chr12	76507000	0.752066
chr12	76509700	0.842975
chr12	76549900	0.694215
chr12	76552900	0.669421
chr12	76574300	0.685950
chr12	76577400	0.578512
chr12	76598200	0.570248
chr12	76601200	0.677686
chr12	76610900	0.636364
chr12	76713300	0.603306
chr12	76723200	0.785124
chr12	76731500	0.595041
chr12	76796600	0.619835
chr12	76865100	0.760331
chr12	76923800	0.628099
chr12	76933900	0.809917
chr12	76938700	0.603306
chr12	76942700	0.842975
chr12	76945800	0.785124
chr12	76949900	0.826446
chr12	76956000	0.719008
chr12	77022800	0.595041
chr12	77024900	0.768595
chr12	77027600	0.785124
chr12	77030400	0.694215
chr12	77092800	0.570248
chr12	77156400	0.586777
chr12	77171800	0.768595
chr12	77174800	0.768595
chr12	77177300	0.628099
chr12	77179400	0.685950
chr12	77265400	0.669421
chr12	80178800	0.942149
chr12	80239200	0.636364
chr12	80278800	0.570248
chr12	80322600	0.892562
chr12	80331800	0.603306
chr12	80482200	0.776860
chr12	83410100	0.768595
chr12	83447200	0.851240
chr12	83496500	0.719008
chr12	83544600	0.619835
chr12	83565800	0.909091
chr12	83568900	0.710744
chr12	83590800	0.942149
chr12	83730000	0.900826
chr12	88529200	0.603306
chr12	88693400	0.570248
chr12	88828100	0.578512

chr12	89646100	0.595041
chr12	89728000	0.834711
chr12	89740600	0.876033
chr12	89764400	0.619835
chr12	89769800	0.611570
chr12	89777800	0.694215
chr12	89785400	0.917355
chr12	89787900	0.909091
chr12	89794900	0.595041
chr12	89797600	0.727273
chr12	89888400	0.859504
chr12	89922400	0.619835
chr12	89934700	0.917355
chr12	89937900	0.859504
chr12	89940900	0.578512
chr12	89959200	0.628099
chr12	89967800	0.694215
chr12	89970200	0.942149
chr12	89972600	0.942149
chr12	89987900	0.636364
chr12	89999200	0.578512
chr12	90013200	0.595041
chr12	90039900	0.661157
chr12	90042900	0.586777
chr12	90057900	0.727273
chr12	90079400	0.578512
chr12	90088500	0.752066
chr12	90150800	1.000000
chr12	90191300	0.818182
chr12	90194000	0.859504
chr12	90200300	0.710744
chr12	90219600	0.785124
chr12	90268200	0.619835
chr12	91767600	0.595041
chr12	91983200	0.694215
chr12	92012500	0.628099
chr12	92025100	0.628099
chr12	92073100	0.611570
chr12	92081300	0.818182
chr12	92155200	0.669421
chr12	92305700	0.611570
chr12	92315300	0.793388
chr12	92379500	0.727273
chr12	92382700	0.570248
chr12	92387500	0.719008
chr12	92395400	0.694215
chr12	92398800	0.909091
chr12	92469600	0.884298
chr12	92472500	0.983471
chr12	92486600	0.652893
chr12	92499900	0.636364
chr12	92502500	0.942149
chr12	92505900	0.586777
chr12	92516100	0.826446

chr12	92527300	0.702479
chr12	92543500	0.933884
chr12	92619300	0.611570
chr12	92627600	0.611570
chr12	92725000	0.785124
chr12	92735900	0.611570
chr12	92751500	0.867769
chr12	92758000	0.842975
chr12	92773500	0.867769
chr12	92783600	0.595041
chr12	92789000	0.909091
chr12	92797600	0.818182
chr12	92862400	0.900826
chr12	92935700	0.611570
chr12	93254300	0.776860
chr12	93274300	0.834711
chr12	93362600	0.727273
chr12	93396900	0.595041
chr12	93410600	0.578512
chr12	93413900	0.752066
chr12	93416600	0.578512
chr12	93437600	0.917355
chr12	93481600	0.636364
chr12	93484200	0.760331
chr12	93509300	0.942149
chr12	93534300	0.603306
chr12	93543400	0.603306
chr12	93571500	0.719008
chr12	93594000	0.677686
chr12	93623800	0.768595
chr12	93637700	0.917355
chr12	93697000	0.925620
chr12	93705400	0.570248
chr12	93707700	0.661157
chr12	93712100	0.628099
chr12	93730200	0.876033
chr12	93750500	0.570248
chr12	93758100	0.661157
chr12	93765400	0.735537
chr12	93781600	0.661157
chr12	93815700	0.661157
chr12	93822300	0.603306
chr12	93958300	0.685950
chr12	94019700	0.859504
chr12	94055500	0.636364
chr12	94074000	0.876033
chr12	94076500	0.611570
chr12	94078700	0.578512
chr12	94093600	0.603306
chr12	94100500	0.950413
chr12	94107300	0.867769
chr12	94131800	0.933884
chr12	94138600	0.785124
chr12	94143200	0.636364

chr12	94151100	0.859504
chr12	94153300	0.917355
chr12	94156700	0.966942
chr12	94163500	0.578512
chr12	94167500	0.702479
chr12	94179000	0.958678
chr12	94207800	0.603306
chr12	94241900	0.710744
chr12	94274900	0.685950
chr12	94306300	0.851240
chr12	94316800	0.776860
chr12	94332500	0.933884
chr12	94385200	0.586777
chr12	94493800	0.644628
chr12	94564400	0.636364
chr12	94598900	0.570248
chr12	94602500	0.702479
chr12	94656800	0.710744
chr12	94684600	0.677686
chr12	94780800	0.702479
chr12	94803700	0.603306
chr12	95010200	0.768595
chr12	95348400	0.876033
chr12	95494600	0.702479
chr12	95563500	0.818182
chr12	95567700	0.925620
chr12	95574500	0.876033
chr12	95577000	0.942149
chr12	95582100	0.752066
chr12	95594800	0.752066
chr12	95596900	0.702479
chr12	95599700	0.636364
chr12	95618500	0.826446
chr12	95625000	0.702479
chr12	95782500	0.661157
chr12	95857000	0.710744
chr12	95910900	0.809917
chr12	95914800	0.694215
chr12	95927800	0.669421
chr12	96442800	0.727273
chr12	96477000	0.578512
chr12	96636800	0.677686
chr12	96709200	0.694215
chr12	96723800	0.867769
chr12	96732200	0.595041
chr12	96758000	0.801653
chr12	96765500	0.826446
chr12	96768700	0.851240
chr12	96772400	0.834711
chr12	96786800	0.710744
chr12	96839900	0.669421
chr12	96895900	0.925620
chr12	97379900	0.677686
chr12	97383100	0.586777

chr12	98765500	0.595041
chr12	98793500	0.801653
chr12	98812900	0.801653
chr12	98885100	0.611570
chr12	98903200	0.644628
chr12	98927600	0.942149
chr12	98973000	0.950413
chr12	98985500	0.702479
chr12	99050500	0.884298
chr12	99065600	0.768595
chr12	99113800	0.719008
chr12	99146000	0.917355
chr12	99148500	0.818182
chr12	100438100	0.867769
chr12	100537700	0.586777
chr12	100673600	0.917355
chr12	100831500	0.801653
chr12	101804400	0.570248
chr12	102089000	0.752066
chr12	102099600	0.628099
chr12	102107400	0.570248
chr12	102187200	0.628099
chr12	102218000	0.793388
chr12	102220100	0.809917
chr12	102248600	0.867769
chr12	102250900	0.876033
chr12	102260500	0.768595
chr12	102297100	0.570248
chr12	102438300	0.586777
chr12	102711900	0.710744
chr12	102770700	0.570248
chr12	102783000	0.603306
chr12	102800400	0.595041
chr12	102815400	0.760331
chr12	102820200	0.776860
chr12	102824400	0.884298
chr12	102832100	0.958678
chr12	102835300	0.942149
chr12	102868700	0.884298
chr12	102874700	0.586777
chr12	103017200	0.578512
chr12	104194700	0.652893
chr12	104199000	0.925620
chr12	104201400	0.884298
chr12	104203700	0.842975
chr12	104253400	0.578512
chr12	104352900	0.669421
chr12	104355100	0.966942
chr12	104362900	0.586777
chr12	104368900	0.793388
chr12	104391500	0.619835
chr12	104405400	0.867769
chr12	104532800	0.669421
chr12	104549600	0.644628

chr12	104565700	0.669421
chr12	104572000	0.669421
chr12	104614000	0.760331
chr12	104647200	0.719008
chr12	104675400	0.768595
chr12	104775900	0.636364
chr12	104802900	0.950413
chr12	104806400	0.603306
chr12	104809100	0.685950
chr12	104857000	0.925620
chr12	104860000	0.586777
chr12	104863000	0.727273
chr12	104871800	0.785124
chr12	104874500	0.851240
chr12	104880400	0.710744
chr12	104884600	0.859504
chr12	104888500	0.785124
chr12	104903300	0.917355
chr12	104909900	0.876033
chr12	104917400	0.933884
chr12	104933100	0.644628
chr12	104964100	0.669421
chr12	104970400	0.768595
chr12	104973400	0.760331
chr12	104983100	0.975207
chr12	104987600	0.983471
chr12	104990800	0.900826
chr12	104993000	0.719008
chr12	104995400	0.950413
chr12	104998900	0.669421
chr12	105006400	0.818182
chr12	105062000	0.595041
chr12	105065800	0.900826
chr12	105116300	0.644628
chr12	105119400	0.925620
chr12	105122600	0.694215
chr12	105158900	0.570248
chr12	105180400	0.702479
chr12	105184100	0.694215
chr12	105353600	0.661157
chr12	105565600	0.768595
chr12	105571200	0.719008
chr12	105647900	0.669421
chr12	106234600	0.983471
chr12	106275100	0.859504
chr12	106287100	0.661157
chr12	106349100	0.909091
chr12	106351200	0.702479
chr12	106663200	0.768595
chr12	106682900	0.975207
chr12	106688100	0.702479
chr12	106693600	0.586777
chr12	107133900	0.900826
chr12	107171900	0.611570

chr12	107174300	0.752066
chr12	107225900	0.826446
chr12	107234500	0.727273
chr12	107337000	0.867769
chr12	107342100	0.652893
chr12	107369000	0.958678
chr12	107372000	0.834711
chr12	107377700	0.867769
chr12	107473300	0.917355
chr12	107504100	0.818182
chr12	108308700	0.611570
chr12	108501000	0.776860
chr12	108511000	0.942149
chr12	108514500	0.884298
chr12	108517500	0.958678
chr12	108521400	0.793388
chr12	108529400	0.685950
chr12	108707200	0.884298
chr12	108726900	0.595041
chr12	108731800	0.818182
chr12	108734400	0.644628
chr12	108871700	0.884298
chr12	108877100	0.917355
chr12	108880000	0.661157
chr12	108926500	0.909091
chr12	108947400	0.578512
chr12	108959100	0.611570
chr12	109012600	0.636364
chr12	109021100	0.619835
chr12	109025800	0.900826
chr12	109028800	0.818182
chr12	109035400	0.950413
chr12	109059300	0.694215
chr12	109065700	0.743802
chr12	109070000	0.603306
chr12	109075000	0.586777
chr12	109080200	0.933884
chr12	109083100	0.834711
chr12	109087600	0.818182
chr12	109089800	0.991736
chr12	109092500	0.884298
chr12	109096500	0.859504
chr12	109106200	0.776860
chr12	109109500	0.710744
chr12	109113500	0.876033
chr12	109115800	0.776860
chr12	109118500	0.776860
chr12	109128300	0.809917
chr12	109177500	0.818182
chr12	109214500	0.735537
chr12	109217800	0.603306
chr12	109232700	0.900826
chr12	109252400	0.611570
chr12	109549100	0.636364

chr12	109612200	0.694215
chr12	109785600	0.619835
chr12	109899200	1.000000
chr12	109901300	0.867769
chr12	110036100	0.595041
chr12	110124200	0.611570
chr12	110178200	0.719008
chr12	110291300	0.578512
chr12	110368600	0.867769
chr12	110477700	0.809917
chr12	110485000	0.586777
chr12	110494100	0.611570
chr12	110498300	1.000000
chr12	110517600	0.644628
chr12	110524100	0.743802
chr12	110527200	0.752066
chr12	110533700	0.942149
chr12	110556200	0.933884
chr12	110573100	0.677686
chr12	110695300	0.661157
chr12	110704700	0.809917
chr12	110708400	0.793388
chr12	110791900	0.933884
chr12	110838900	0.644628
chr12	110856600	0.669421
chr12	110859800	0.867769
chr12	110864500	0.652893
chr12	110894800	0.876033
chr12	110903500	0.628099
chr12	110941100	0.793388
chr12	111016400	0.752066
chr12	111023100	0.578512
chr12	111078000	0.859504
chr12	111127400	0.669421
chr12	111132400	0.669421
chr12	111137100	0.950413
chr12	111176000	0.669421
chr12	111198500	0.603306
chr12	111623700	0.760331
chr12	111831700	0.694215
chr12	111837300	0.702479
chr12	111841100	0.611570
chr12	111849600	0.958678
chr12	111852400	0.966942
chr12	111873100	0.578512
chr12	111876200	0.942149
chr12	111879900	0.628099
chr12	111883500	0.586777
chr12	111887300	0.603306
chr12	111943800	0.694215
chr12	112008100	0.925620
chr12	112016500	0.933884
chr12	112020100	0.603306
chr12	112062700	0.570248

chr12	112072400	0.793388
chr12	112076300	0.851240
chr12	112081600	0.719008
chr12	112183200	0.867769
chr12	112213300	0.735537
chr12	112215500	0.578512
chr12	112230300	0.586777
chr12	112276200	0.809917
chr12	112284000	0.776860
chr12	112342400	0.644628
chr12	112350600	0.719008
chr12	112405600	0.636364
chr12	112413200	0.909091
chr12	112417100	0.983471
chr12	112431000	0.652893
chr12	112433700	0.619835
chr12	112436800	0.603306
chr12	112446000	0.876033
chr12	112448200	0.661157
chr12	112454000	0.735537
chr12	112534700	0.958678
chr12	112543900	0.603306
chr12	112548700	0.595041
chr12	112554800	0.842975
chr12	112560400	0.694215
chr12	112566000	0.603306
chr12	112569000	0.636364
chr12	112763800	0.611570
chr12	112780400	0.586777
chr12	112791600	0.925620
chr12	112795200	0.859504
chr12	112811900	0.677686
chr12	112825100	0.859504
chr12	112852400	0.826446
chr12	112860400	0.702479
chr12	112884400	0.900826
chr12	112994000	0.809917
chr12	113022400	0.719008
chr12	113593500	0.603306
chr12	113629000	0.950413
chr12	113642800	0.884298
chr12	113645900	0.603306
chr12	113667900	0.735537
chr12	113670300	0.884298
chr12	113676300	0.975207
chr12	113683900	0.685950
chr12	113709700	0.933884
chr12	113737700	0.842975
chr12	113740800	0.710744
chr12	113761600	0.570248
chr12	113794800	0.685950
chr12	113841800	0.644628
chr12	113846400	0.867769
chr12	113854300	0.876033

chr12	113905500	0.578512
chr12	114021000	0.892562
chr12	114024800	0.793388
chr12	114050200	0.785124
chr12	114079800	0.719008
chr12	114087400	1.000000
chr12	114102100	0.785124
chr12	114123300	0.851240
chr12	114183200	0.735537
chr12	114186900	0.628099
chr12	114193400	0.834711
chr12	114196800	0.909091
chr12	114231100	0.842975
chr12	114233400	0.917355
chr12	114246900	0.619835
chr12	114258300	0.636364
chr12	114267800	0.603306
chr12	114292700	0.867769
chr12	114298600	0.578512
chr12	114301800	0.603306
chr12	114338500	0.677686
chr12	114367100	0.644628
chr12	114369700	0.760331
chr12	114372000	0.826446
chr12	114402100	0.570248
chr12	114502500	0.685950
chr12	116506600	0.644628
chr12	116533100	0.743802
chr12	116559500	0.603306
chr12	116568900	0.636364
chr12	116571700	0.760331
chr12	116581800	0.735537
chr12	116595500	0.644628
chr12	116599300	0.892562
chr12	116602800	0.785124
chr12	116612500	0.669421
chr12	116615100	0.867769
chr12	116624600	0.917355
chr12	116633500	0.809917
chr12	116639100	0.702479
chr12	116648200	0.719008
chr12	116652400	0.570248
chr12	116655700	0.652893
chr12	116674700	0.892562
chr12	116682400	0.611570
chr12	116686900	0.809917
chr12	116699400	0.570248
chr12	116703200	0.570248
chr12	116716100	0.586777
chr12	116720800	0.768595
chr12	116741300	0.603306
chr12	116759000	0.636364
chr12	116774800	0.702479
chr12	116778100	0.669421

chr12	116784500	0.942149
chr12	116793200	0.818182
chr12	116796100	0.702479
chr12	116799100	0.900826
chr12	116803200	0.628099
chr12	116811100	0.752066
chr12	116819300	0.595041
chr12	116823500	1.000000
chr12	116865000	0.842975
chr12	116872500	0.719008
chr12	116931100	0.975207
chr12	116959300	0.652893
chr12	116973100	0.619835
chr12	116984200	0.892562
chr12	116990400	0.892562
chr12	116995400	0.685950
chr12	116999200	0.900826
chr12	117015400	0.752066
chr12	117020500	0.793388
chr12	117030100	0.702479
chr12	117032400	0.636364
chr12	117037500	0.570248
chr12	117330800	0.595041
chr12	117354600	0.834711
chr12	117358100	0.826446
chr12	117362800	0.570248
chr12	117418300	0.743802
chr12	117443400	0.892562
chr12	117480400	0.727273
chr12	117483500	0.942149
chr12	117489400	0.851240
chr12	117498900	0.851240
chr12	117507800	0.685950
chr12	117529400	0.776860
chr12	117550100	0.752066
chr12	118451400	0.842975
chr12	118460500	0.826446
chr12	118502200	0.611570
chr12	118558800	0.991736
chr12	118626400	0.628099
chr12	118726200	0.818182
chr12	118768500	0.876033
chr12	118783200	0.925620
chr12	118798700	0.719008
chr12	118801800	0.578512
chr12	118848200	0.710744
chr12	119063300	0.966942
chr12	119592300	0.644628
chr12	119668300	0.694215
chr12	120040200	0.710744
chr12	120068700	0.611570
chr12	120099900	0.595041
chr12	120111600	0.818182
chr12	120663900	0.991736

chr12	120670300	0.983471
chr12	120673100	1.000000
chr12	120680600	0.785124
chr12	120683100	0.743802
chr12	120687900	0.727273
chr12	120701000	0.661157
chr12	120721200	0.628099
chr12	120728600	0.710744
chr12	120731800	0.743802
chr12	120820200	0.595041
chr12	120904800	0.578512
chr12	120910500	0.719008
chr12	120912700	0.694215
chr12	120918900	0.950413
chr12	120925800	0.983471
chr12	120964700	0.570248
chr12	120976400	0.611570
chr12	120978800	0.933884
chr12	121017300	0.586777
chr12	121036900	0.710744
chr12	121064000	0.793388
chr12	121129000	0.611570
chr12	121158600	0.991736
chr12	121269700	0.636364
chr12	121292100	0.768595
chr12	121330400	0.578512
chr12	121383000	0.603306
chr12	121397800	0.909091
chr12	121455900	0.975207
chr12	121464200	0.595041
chr12	121523000	0.570248
chr12	121567200	0.942149
chr12	121582600	0.942149
chr12	121613900	0.834711
chr12	121635600	0.669421
chr12	121641000	0.942149
chr12	121652900	0.595041
chr12	121661800	0.611570
chr12	121721200	0.719008
chr12	121821700	0.628099
chr12	121826700	0.570248
chr12	121830000	0.661157
chr12	121863000	0.859504
chr12	121952100	0.743802
chr12	122015400	0.925620
chr12	122021200	0.669421
chr12	122028700	0.710744
chr12	122049600	0.768595
chr12	122062800	0.983471
chr12	122067700	0.900826
chr12	122071300	0.942149
chr12	122087700	0.669421
chr12	122092500	0.719008
chr12	122096400	0.768595

chr12	122176300	0.966942
chr12	122180700	0.958678
chr12	122187100	0.925620
chr12	122208600	0.991736
chr12	122234000	0.950413
chr12	122281500	0.826446
chr12	122299900	0.818182
chr12	122330500	0.743802
chr12	122332600	0.619835
chr12	122365600	0.892562
chr12	122371000	0.677686
chr12	122417500	0.628099
chr12	122434900	0.694215
chr12	122442600	0.735537
chr12	122452200	0.719008
chr12	122500900	0.752066
chr12	122505100	0.719008
chr12	122510700	0.818182
chr12	122515600	0.719008
chr12	122522900	0.809917
chr12	122526200	1.000000
chr12	122530100	0.876033
chr12	122582000	0.818182
chr12	122584700	0.818182
chr12	122590800	0.578512
chr12	122596700	0.702479
chr12	122599000	0.661157
chr12	122607800	0.834711
chr12	122644600	0.586777
chr12	122680900	0.760331
chr12	122729700	0.628099
chr12	122764100	0.719008
chr12	122817900	0.983471
chr12	122833900	0.710744
chr12	122852800	0.950413
chr12	122891400	0.677686
chr12	122974100	0.859504
chr12	123129500	0.586777
chr12	123235700	0.876033
chr12	123241900	0.851240
chr12	123311100	0.570248
chr12	123328000	0.570248
chr12	123368700	0.727273
chr12	123370800	0.611570
chr12	123373600	0.917355
chr12	123431900	0.727273
chr12	123446700	0.768595
chr12	123449200	0.785124
chr12	123469300	0.991736
chr12	123485100	0.900826
chr12	123507100	0.884298
chr12	123518500	0.909091
chr12	123521300	0.892562
chr12	123547900	0.735537

chr12	123558400	0.595041
chr12	123574300	0.834711
chr12	123576900	0.644628
chr12	123579500	0.710744
chr12	123592000	0.975207
chr12	123600200	0.578512
chr12	123611600	0.900826
chr12	123625500	0.900826
chr12	123736000	0.942149
chr12	123743600	0.644628
chr12	123837400	0.595041
chr12	123842900	0.578512
chr12	123854300	0.743802
chr12	123858100	0.983471
chr12	123867000	0.586777
chr12	123874700	0.752066
chr12	123934300	0.760331
chr12	123944800	0.570248
chr12	123979700	0.776860
chr12	124200200	0.710744
chr12	124203000	0.768595
chr12	124206100	0.752066
chr12	124227900	0.669421
chr12	124338500	0.603306
chr12	124452300	0.809917
chr12	124475500	0.859504
chr12	124480300	0.595041
chr12	124484400	0.950413
chr12	124491800	0.851240
chr12	124512400	0.619835
chr12	124515900	0.826446
chr12	124655200	0.603306
chr12	124675700	0.917355
chr12	124730900	0.776860
chr12	124744600	0.867769
chr12	124758700	0.578512
chr12	124837900	0.661157
chr12	124876500	0.694215
chr12	124906800	0.570248
chr12	124909100	0.570248
chr12	124972400	0.876033
chr12	124975800	0.876033
chr12	124991300	0.909091
chr12	124994500	0.983471
chr12	124997600	0.975207
chr12	125003900	0.628099
chr12	125024700	0.586777
chr12	125034100	0.685950
chr12	125036300	0.991736
chr12	125042100	0.628099
chr12	125049200	0.785124
chr12	125058800	0.950413
chr12	125073600	0.603306
chr12	125076100	0.661157

chr12	125090500	0.900826
chr12	125093800	0.917355
chr12	125111100	0.793388
chr12	125117900	0.867769
chr12	125121200	0.958678
chr12	125162900	0.603306
chr12	125170400	0.834711
chr12	125200600	0.652893
chr12	125219500	0.652893
chr12	125230700	0.702479
chr12	125292200	0.793388
chr12	125314300	0.595041
chr12	125341000	0.876033
chr12	125349600	0.809917
chr12	125353800	0.768595
chr12	125360300	0.834711
chr12	125369400	0.785124
chr12	125372100	0.834711
chr12	125389700	0.975207
chr12	125396400	0.619835
chr12	125404500	0.586777
chr12	125414100	0.628099
chr12	125577600	0.611570
chr12	125596500	0.760331
chr12	127304600	0.644628
chr12	127354700	1.000000
chr12	127359100	0.859504
chr12	127385000	0.867769
chr12	127387900	0.735537
chr12	127390900	0.933884
chr12	127394800	0.933884
chr12	127397200	0.702479
chr12	127400000	0.710744
chr12	127433800	0.685950
chr12	127437900	0.727273
chr12	127483500	0.694215
chr12	127550100	0.636364
chr12	127562300	0.586777
chr12	127566100	0.595041
chr12	127577900	0.925620
chr12	127591800	0.702479
chr12	127600200	0.586777
chr12	127605100	0.719008
chr12	127610900	0.966942
chr12	127628000	0.636364
chr12	127645400	0.644628
chr12	127648200	0.942149
chr12	127716100	0.661157
chr12	127723800	0.603306
chr12	127780800	0.570248
chr12	130138200	0.578512
chr12	130145500	0.925620
chr12	130178600	0.685950
chr12	131311200	0.925620

chr12	131317800	0.702479
chr12	131320800	0.768595
chr12	131324700	0.677686
chr12	131338000	0.677686
chr12	131340600	0.925620
chr12	131343800	0.818182
chr12	131353700	0.752066
chr12	131362500	0.818182
chr12	131365100	0.586777
chr12	131434400	0.586777
chr12	131437400	0.958678
chr12	131443000	0.859504
chr12	131480100	0.603306
chr12	131484600	0.867769
chr12	131511100	0.760331
chr12	131702200	0.611570
chr12	131709500	0.842975
chr12	131713500	0.677686
chr12	131717000	0.619835
chr12	131719500	0.694215
chr12	131721800	0.719008
chr12	131724400	0.752066
chr12	131727200	0.818182
chr12	131732400	0.628099
chr12	131752900	0.842975
chr12	131769200	0.785124
chr12	131832900	0.685950
chr12	131836800	0.702479
chr12	131842900	0.933884
chr12	131847000	0.975207
chr12	131887100	0.859504
chr12	131892400	0.611570
chr12	131924000	0.694215
chr12	131926900	0.842975
chr12	131937200	0.685950
chr12	131941800	0.578512
chr12	132061300	0.586777
chr12	132089700	0.867769
chr12	132109300	0.950413
chr12	132112600	0.644628
chr12	132117000	0.603306
chr12	132122900	0.826446
chr12	132167100	0.975207
chr12	132317700	0.636364
chr12	132345300	0.809917
chr12	132347800	0.595041
chr12	132378400	0.619835
chr12	132412800	0.652893
chr12	132432800	0.578512
chr12	132436500	0.652893
chr12	132440500	0.793388
chr12	132443800	0.719008
chr12	132486100	0.826446
chr12	132568100	0.677686

chr12	132970800	0.925620
chr12	132999300	0.661157
chr12	133007500	0.710744
chr12	133048000	0.917355
chr12	133071100	0.859504
chr12	133079300	0.743802
chr12	133199800	0.785124
chr12	133266500	0.595041
chr12	133334400	0.785124
chr12	133342700	0.818182
chr13	19399100	0.933884
chr13	19425000	0.694215
chr13	19495000	0.834711
chr13	20288400	0.595041
chr13	20556700	0.851240
chr13	21134700	0.694215
chr13	21241300	0.578512
chr13	21376100	0.661157
chr13	21518200	0.661157
chr13	21568200	0.570248
chr13	21717300	0.735537
chr13	21952100	0.743802
chr13	22010700	0.619835
chr13	22097600	0.884298
chr13	22142400	0.867769
chr13	22146600	0.933884
chr13	22150800	0.685950
chr13	22191300	0.702479
chr13	23388500	0.983471
chr13	23423700	0.851240
chr13	23499700	0.710744
chr13	23502000	0.636364
chr13	24002500	0.752066
chr13	24144700	0.628099
chr13	24421700	0.570248
chr13	24440900	0.983471
chr13	25077400	0.892562
chr13	25080200	0.842975
chr13	25438900	0.578512
chr13	25485800	0.801653
chr13	25495100	0.570248
chr13	25506700	0.603306
chr13	25508800	0.826446
chr13	25589000	0.743802
chr13	25643300	0.669421
chr13	25655300	0.950413
chr13	25926600	1.000000
chr13	25946000	0.619835
chr13	25955600	0.900826
chr13	25966400	0.925620
chr13	25969000	0.909091
chr13	25981000	0.595041
chr13	27543800	0.776860
chr13	27550700	1.000000

chr13	27557700	0.950413
chr13	27597200	0.826446
chr13	27613700	0.636364
chr13	27619700	0.611570
chr13	27627800	0.900826
chr13	27657300	0.595041
chr13	27692600	0.603306
chr13	27701500	0.570248
chr13	27706000	0.727273
chr13	27712700	0.983471
chr13	27721000	0.884298
chr13	27742900	0.611570
chr13	27757400	0.933884
chr13	27769500	0.892562
chr13	27778000	0.685950
chr13	27817200	0.892562
chr13	27829600	0.595041
chr13	27832800	0.776860
chr13	27995900	0.983471
chr13	28033700	0.570248
chr13	28175000	0.628099
chr13	28179600	0.603306
chr13	28242500	0.685950
chr13	28254300	0.586777
chr13	28717100	0.652893
chr13	28736000	0.867769
chr13	28894300	0.644628
chr13	28924600	0.611570
chr13	28931000	0.859504
chr13	28962100	0.611570
chr13	28966900	0.619835
chr13	28976100	0.595041
chr13	28982200	0.809917
chr13	28984400	0.867769
chr13	29050700	0.876033
chr13	29103100	0.586777
chr13	29143900	0.694215
chr13	29149300	0.958678
chr13	29157500	0.727273
chr13	29165300	0.611570
chr13	29186100	0.925620
chr13	29200700	0.595041
chr13	29212400	0.578512
chr13	29282600	0.909091
chr13	29303600	0.636364
chr13	30027800	0.661157
chr13	30032200	0.917355
chr13	30034700	0.669421
chr13	30153100	0.661157
chr13	30157200	0.909091
chr13	30803400	0.950413
chr13	30885100	0.570248
chr13	30909900	0.570248
chr13	30912300	0.652893

chr13	30915100	0.958678
chr13	30938200	0.867769
chr13	30946600	0.570248
chr13	30983400	0.727273
chr13	31078600	0.685950
chr13	31240400	0.966942
chr13	31245800	0.570248
chr13	31265000	0.661157
chr13	31779100	0.942149
chr13	32836300	0.768595
chr13	32998200	0.619835
chr13	33043200	0.834711
chr13	33045800	0.702479
chr13	33071300	0.933884
chr13	33074300	0.677686
chr13	33208500	0.677686
chr13	33372800	0.685950
chr13	37388700	0.685950
chr13	38493300	0.570248
chr13	38498100	0.652893
chr13	39572900	0.818182
chr13	40690500	0.694215
chr13	41246800	0.603306
chr13	41351600	0.752066
chr13	41361600	0.702479
chr13	41548300	0.578512
chr13	41551000	0.603306
chr13	41558300	0.636364
chr13	41569700	0.611570
chr13	41573400	0.570248
chr13	41577700	0.867769
chr13	41583300	0.652893
chr13	41605000	0.578512
chr13	41627200	0.570248
chr13	41629500	0.768595
chr13	41754200	0.768595
chr13	41920500	0.619835
chr13	42037400	0.933884
chr13	42040500	0.702479
chr13	42164100	0.909091
chr13	42297600	0.826446
chr13	42326200	0.619835
chr13	42338200	0.876033
chr13	42341000	0.818182
chr13	42490900	0.694215
chr13	42515800	0.644628
chr13	42533000	0.578512
chr13	42570400	0.785124
chr13	42629500	0.685950
chr13	42749000	0.933884
chr13	42833400	0.842975
chr13	42835900	0.859504
chr13	42854600	0.851240
chr13	44833500	0.900826

chr13	44910000	0.876033
chr13	44943300	0.644628
chr13	44957000	0.619835
chr13	44961500	0.933884
chr13	44971700	0.975207
chr13	45697500	0.619835
chr13	45707900	0.702479
chr13	45717600	0.826446
chr13	45873800	0.628099
chr13	45877700	0.578512
chr13	45884000	0.677686
chr13	45899800	0.636364
chr13	45903900	0.793388
chr13	45909400	0.685950
chr13	45936800	0.818182
chr13	45947800	0.834711
chr13	45989100	0.702479
chr13	46518100	0.710744
chr13	46675100	0.586777
chr13	46688000	0.909091
chr13	46692100	0.677686
chr13	46694600	0.917355
chr13	46700000	0.942149
chr13	46750000	0.578512
chr13	46758100	0.834711
chr13	46856000	0.801653
chr13	46858700	0.851240
chr13	46871100	0.785124
chr13	47179000	0.603306
chr13	47181600	0.661157
chr13	47215300	0.669421
chr13	47217900	0.702479
chr13	47752700	0.570248
chr13	48701500	0.636364
chr13	48730600	0.570248
chr13	48736000	0.950413
chr13	48802500	0.578512
chr13	48810200	0.586777
chr13	49105600	0.636364
chr13	49108500	0.677686
chr13	49136600	0.570248
chr13	49139000	0.570248
chr13	49144700	0.603306
chr13	49200300	0.933884
chr13	49215500	0.570248
chr13	49383200	0.677686
chr13	49512900	0.702479
chr13	49636600	0.570248
chr13	49888400	0.942149
chr13	50115000	0.619835
chr13	50203200	0.586777
chr13	50210800	0.611570
chr13	50216600	0.818182
chr13	50360900	0.685950

chr13	50376900	0.628099
chr13	50422100	0.570248
chr13	50589600	0.619835
chr13	50664000	0.851240
chr13	50666100	0.776860
chr13	50696500	0.735537
chr13	50775400	0.867769
chr13	50819300	0.925620
chr13	50884900	0.669421
chr13	50887000	0.677686
chr13	50893800	0.884298
chr13	50909500	0.917355
chr13	50932000	0.661157
chr13	50964800	0.719008
chr13	50985800	0.950413
chr13	50997100	0.991736
chr13	51000600	0.644628
chr13	51034400	0.826446
chr13	51043500	0.768595
chr13	51060800	0.826446
chr13	51086900	0.867769
chr13	51187700	1.000000
chr13	51210300	0.669421
chr13	51266000	0.710744
chr13	51487300	0.619835
chr13	51491100	0.636364
chr13	51565000	0.619835
chr13	52123300	0.834711
chr13	52157300	0.619835
chr13	52162700	0.595041
chr13	52165500	0.925620
chr13	52176100	0.628099
chr13	52360700	0.570248
chr13	52368000	0.966942
chr13	52419900	0.752066
chr13	52495000	0.694215
chr13	52497300	0.942149
chr13	52500300	0.867769
chr13	52504100	0.669421
chr13	52529600	0.909091
chr13	52531700	0.768595
chr13	52534400	0.743802
chr13	52549000	0.909091
chr13	52554800	0.619835
chr13	52566500	0.694215
chr13	52570300	0.776860
chr13	52573600	0.900826
chr13	52596300	0.669421
chr13	52645800	0.809917
chr13	52692500	0.851240
chr13	52762100	0.867769
chr13	52778600	0.694215
chr13	52872500	0.702479
chr13	53088700	0.859504

chr13	59874700	0.570248
chr13	60035600	0.834711
chr13	60042800	0.595041
chr13	60069400	0.710744
chr13	60106000	0.859504
chr13	60683300	0.636364
chr13	60778900	0.570248
chr13	60789100	0.570248
chr13	60794600	0.644628
chr13	60804600	0.842975
chr13	61158300	0.900826
chr13	66651100	0.619835
chr13	67568600	0.578512
chr13	67710100	0.652893
chr13	68068000	0.619835
chr13	69272600	0.851240
chr13	69283400	0.586777
chr13	69839200	0.892562
chr13	70259300	0.603306
chr13	74039400	0.909091
chr13	75493700	0.727273
chr13	75496000	0.842975
chr13	75505300	0.603306
chr13	75900500	0.785124
chr13	76061200	0.727273
chr13	76130100	0.586777
chr13	76464200	0.570248
chr13	76521900	0.942149
chr13	76537300	0.867769
chr13	76559500	0.966942
chr13	76580400	0.603306
chr13	76597000	0.628099
chr13	76604100	0.818182
chr13	77492200	0.917355
chr13	77810400	0.644628
chr13	77875200	0.578512
chr13	77902000	0.644628
chr13	79199800	0.685950
chr13	79221800	0.661157
chr13	79224900	0.685950
chr13	79238700	0.595041
chr13	79889500	0.603306
chr13	79962200	0.628099
chr13	79967700	0.628099
chr13	79972800	0.570248
chr13	79976500	0.578512
chr13	80026000	0.752066
chr13	80059800	0.710744
chr13	80077500	0.578512
chr13	80934100	0.933884
chr13	80936400	0.884298
chr13	81173900	0.603306
chr13	92006400	0.578512
chr13	92010900	0.685950

chr13	92021900	0.644628
chr13	92191400	0.644628
chr13	93901700	0.702479
chr13	93915700	0.611570
chr13	93943000	0.727273
chr13	95309100	0.793388
chr13	95315500	0.719008
chr13	95320000	0.644628
chr13	95855200	0.603306
chr13	95860000	0.669421
chr13	95862200	0.975207
chr13	95879700	0.900826
chr13	95882700	0.834711
chr13	95896100	1.000000
chr13	95899700	0.900826
chr13	95920500	0.958678
chr13	95923900	0.867769
chr13	95927700	0.702479
chr13	95931700	0.628099
chr13	95938600	0.958678
chr13	95955700	0.603306
chr13	95969800	0.925620
chr13	96010400	0.619835
chr13	96033200	0.801653
chr13	96090400	0.702479
chr13	96130000	0.628099
chr13	96138000	0.735537
chr13	96360100	0.595041
chr13	96406700	0.900826
chr13	96409000	0.603306
chr13	96548800	0.958678
chr13	96609600	0.669421
chr13	97762500	0.619835
chr13	97774300	0.710744
chr13	97794700	0.710744
chr13	97801800	0.661157
chr13	97844100	0.842975
chr13	97847100	0.917355
chr13	97880400	0.628099
chr13	97883000	0.694215
chr13	97886900	0.570248
chr13	97892100	0.611570
chr13	97902400	0.909091
chr13	97907400	0.842975
chr13	97910500	0.933884
chr13	97914200	0.925620
chr13	97979600	0.719008
chr13	99129000	0.900826
chr13	99131400	0.942149
chr13	99134100	0.677686
chr13	99137700	1.000000
chr13	99156800	0.892562
chr13	99161500	0.735537
chr13	99194400	0.892562

chr13	99198700	0.570248
chr13	99218900	0.570248
chr13	99223500	0.818182
chr13	99449400	0.975207
chr13	99613700	0.768595
chr13	99625700	0.578512
chr13	99628300	0.752066
chr13	99630500	0.619835
chr13	99695100	0.611570
chr13	99702100	0.809917
chr13	99705400	0.884298
chr13	99708600	0.917355
chr13	99711600	0.983471
chr13	99714200	0.570248
chr13	99718000	0.760331
chr13	99730800	0.933884
chr13	99737700	0.578512
chr13	99777400	0.603306
chr13	99803800	0.603306
chr13	99805900	0.933884
chr13	99844700	0.570248
chr13	99855100	0.611570
chr13	99920200	0.867769
chr13	99960100	0.958678
chr13	99963800	0.685950
chr13	99967500	0.933884
chr13	99974800	0.933884
chr13	99977500	0.950413
chr13	99979800	0.958678
chr13	99983800	0.595041
chr13	99991600	0.884298
chr13	100010200	0.900826
chr13	100086900	0.752066
chr13	100106000	0.694215
chr13	100179100	0.578512
chr13	100752300	0.644628
chr13	101044700	0.900826
chr13	101046800	0.942149
chr13	101253700	0.983471
chr13	101261500	0.975207
chr13	103252800	0.735537
chr13	103256900	0.586777
chr13	103259300	0.900826
chr13	103264000	0.611570
chr13	103322700	0.768595
chr13	103424200	0.578512
chr13	103432300	0.884298
chr13	107222600	0.570248
chr13	108505800	0.586777
chr13	108522700	0.685950
chr13	108525900	0.603306
chr13	108529100	0.586777
chr13	108541300	0.611570
chr13	108553800	0.628099

chr13	108561300	0.735537
chr13	108568300	0.719008
chr13	108584200	0.702479
chr13	108656700	0.595041
chr13	108659500	0.842975
chr13	108878900	0.685950
chr13	109225500	0.793388
chr13	109335200	0.702479
chr13	109344900	0.909091
chr13	109364000	0.611570
chr13	109407100	0.578512
chr13	109412900	0.619835
chr13	109418900	0.570248
chr13	109422300	0.884298
chr13	109469100	0.842975
chr13	109568300	0.983471
chr13	109573900	0.876033
chr13	110076700	1.000000
chr13	110348000	0.801653
chr13	110352800	0.818182
chr13	110359400	1.000000
chr13	110365100	0.685950
chr13	110372600	0.884298
chr13	110398700	0.652893
chr13	110404300	0.586777
chr13	111108800	0.685950
chr13	111124200	0.611570
chr13	111128100	0.628099
chr13	111133800	0.859504
chr13	111156500	0.958678
chr13	111159100	0.975207
chr13	111162100	0.991736
chr13	111164300	0.809917
chr13	111177500	0.586777
chr13	111189000	0.586777
chr13	111216100	0.636364
chr13	111228000	0.842975
chr13	111254700	0.702479
chr13	111270400	0.710744
chr13	111524000	0.644628
chr13	111527200	0.876033
chr13	111562100	0.578512
chr13	111577500	0.595041
chr13	111597300	0.752066
chr13	111602500	0.727273
chr13	111605300	0.892562
chr13	111607600	0.933884
chr13	111609900	0.859504
chr13	111616600	0.909091
chr13	111619100	0.702479
chr13	111621400	0.851240
chr13	111623700	0.793388
chr13	111629400	0.950413
chr13	111634200	0.950413

chr13	111638200	0.991736
chr13	111659600	0.884298
chr13	111692400	0.677686
chr13	111696100	0.851240
chr13	111699500	0.983471
chr13	111828100	0.702479
chr13	113230400	0.619835
chr13	113238200	0.719008
chr13	113243800	0.603306
chr13	113355700	0.975207
chr13	113358900	0.743802
chr13	113363600	0.702479
chr13	113366500	0.933884
chr13	113377300	0.958678
chr13	113384000	0.570248
chr13	113395200	0.867769
chr13	113416700	0.702479
chr13	113527700	0.884298
chr13	113719400	0.603306
chr13	113793700	0.727273
chr13	113809000	0.644628
chr13	113841900	0.603306
chr13	113859900	0.710744
chr13	113868800	0.826446
chr13	113874600	0.661157
chr13	113876800	0.586777
chr13	113922400	0.619835
chr13	113928900	0.950413
chr13	114013900	0.867769
chr13	114052800	0.578512
chr13	114075100	0.785124
chr13	114160600	0.975207
chr13	114162800	0.570248
chr13	114243300	0.685950
chr13	114518800	0.752066
chr13	114556900	0.925620
chr13	114563400	0.760331
chr13	114770100	0.735537
chr13	114785300	0.867769
chr13	114813200	0.801653
chr13	114831000	0.834711
chr13	114881100	0.933884
chr13	114886300	0.636364
chr13	114894800	0.900826
chr13	114906600	0.710744
chr13	114985600	0.570248
chr13	114988200	0.859504
chr13	114992800	0.603306
chr13	115003600	0.958678
chr13	115017500	0.958678
chr13	115044600	0.917355
chr13	115050000	0.677686
chr14	19038300	0.652893
chr14	19681000	0.826446

chr14	19683500	0.735537
chr14	19894500	0.958678
chr14	19937600	0.851240
chr14	19966300	0.776860
chr14	20512800	0.570248
chr14	20580500	0.636364
chr14	20585300	0.950413
chr14	20612200	0.644628
chr14	20638900	0.595041
chr14	20644100	0.966942
chr14	20647600	0.942149
chr14	20653100	0.760331
chr14	20664100	0.661157
chr14	20731200	0.809917
chr14	20770300	0.975207
chr14	20805200	0.628099
chr14	20896100	0.694215
chr14	20898500	0.603306
chr14	20926500	0.942149
chr14	20931700	0.818182
chr14	20940600	0.570248
chr14	20945700	0.801653
chr14	20951000	0.991736
chr14	20958700	0.958678
chr14	21025400	0.768595
chr14	21027700	0.603306
chr14	21047700	0.842975
chr14	21066400	0.710744
chr14	21087000	0.595041
chr14	21125800	0.694215
chr14	21165900	0.595041
chr14	21226900	0.677686
chr14	21266600	0.909091
chr14	21270400	0.636364
chr14	21272600	0.834711
chr14	21285700	0.595041
chr14	21289300	0.958678
chr14	21317300	0.595041
chr14	21451700	0.628099
chr14	21472700	0.719008
chr14	21495300	0.842975
chr14	21512700	0.611570
chr14	21537400	0.595041
chr14	21570000	0.710744
chr14	21575300	0.933884
chr14	21581200	0.611570
chr14	21623800	0.652893
chr14	21738900	0.652893
chr14	21776100	0.685950
chr14	21786000	0.793388
chr14	21909400	0.570248
chr14	21993500	0.595041
chr14	21996900	0.884298
chr14	22803000	0.826446

chr14	22806500	0.876033
chr14	22929900	0.652893
chr14	23006500	0.966942
chr14	23025900	0.859504
chr14	23030600	0.983471
chr14	23063500	0.925620
chr14	23092300	0.719008
chr14	23140700	0.884298
chr14	23169700	0.975207
chr14	23184600	0.958678
chr14	23195400	0.900826
chr14	23218200	0.768595
chr14	23266500	0.892562
chr14	23294200	0.834711
chr14	23322800	0.669421
chr14	23338900	0.619835
chr14	23353000	0.669421
chr14	23366800	0.975207
chr14	23369500	0.595041
chr14	23421600	0.628099
chr14	23448600	0.752066
chr14	23469200	0.752066
chr14	23490300	0.884298
chr14	23494900	0.743802
chr14	23515100	0.900826
chr14	23542600	0.694215
chr14	23548500	0.867769
chr14	23567600	0.661157
chr14	23582500	0.611570
chr14	23590100	0.801653
chr14	23593200	0.867769
chr14	23596200	0.859504
chr14	23599000	0.809917
chr14	23607700	0.719008
chr14	23613700	0.735537
chr14	23616100	0.958678
chr14	23619100	0.636364
chr14	23625200	0.628099
chr14	23630000	1.000000
chr14	23633500	0.900826
chr14	23637100	0.768595
chr14	23652100	0.611570
chr14	23656400	0.661157
chr14	23665300	0.628099
chr14	23729700	0.570248
chr14	23748100	0.834711
chr14	23751400	0.603306
chr14	23764400	0.909091
chr14	23768600	0.652893
chr14	23815500	0.966942
chr14	23907600	1.000000
chr14	23910900	0.818182
chr14	23923400	0.826446
chr14	24038100	0.611570

chr14	24058900	0.677686
chr14	24115600	0.586777
chr14	24164100	0.975207
chr14	24401800	0.826446
chr14	24407300	0.570248
chr14	24410000	0.933884
chr14	24550700	0.644628
chr14	24553400	0.719008
chr14	24562700	0.652893
chr14	24578300	0.586777
chr14	24592900	0.735537
chr14	24630900	0.785124
chr14	24677200	0.743802
chr14	24725600	1.000000
chr14	24744000	0.719008
chr14	24766500	0.884298
chr14	24786200	0.900826
chr14	24802100	0.760331
chr14	24821200	0.628099
chr14	24847500	0.950413
chr14	24858300	0.752066
chr14	24861700	0.719008
chr14	24885600	0.611570
chr14	24895500	0.884298
chr14	25157700	0.975207
chr14	25435700	0.644628
chr14	25451800	0.735537
chr14	25454600	0.983471
chr14	25517600	0.776860
chr14	26750900	0.876033
chr14	26755800	0.578512
chr14	29326800	0.586777
chr14	30120300	0.966942
chr14	30159900	0.611570
chr14	30275400	0.991736
chr14	30740000	0.595041
chr14	30807500	0.578512
chr14	30843200	0.669421
chr14	31032400	0.578512
chr14	31039000	0.570248
chr14	31094100	0.570248
chr14	31291700	0.958678
chr14	31325000	0.595041
chr14	31331600	0.628099
chr14	31355800	0.578512
chr14	31378000	0.661157
chr14	31436300	0.611570
chr14	31452200	0.694215
chr14	31468000	0.595041
chr14	31498600	0.826446
chr14	31500900	0.900826
chr14	31503300	0.876033
chr14	31507700	0.834711
chr14	31512600	0.768595

chr14	31518200	0.694215
chr14	31520900	0.851240
chr14	31524400	0.644628
chr14	31528500	0.917355
chr14	31530900	0.702479
chr14	31549900	0.644628
chr14	31666800	0.867769
chr14	31669900	0.801653
chr14	31698800	0.826446
chr14	31708900	0.760331
chr14	31711700	0.950413
chr14	31875700	0.570248
chr14	32363600	0.933884
chr14	32372100	0.900826
chr14	32405000	0.743802
chr14	32414200	0.785124
chr14	32420600	0.900826
chr14	32435500	0.983471
chr14	32437700	0.603306
chr14	32476200	0.611570
chr14	32480100	0.661157
chr14	32490600	0.785124
chr14	32619900	0.652893
chr14	32800600	0.867769
chr14	32806300	0.950413
chr14	32843000	0.677686
chr14	32954100	0.966942
chr14	33828800	0.892562
chr14	33952600	0.578512
chr14	34036900	0.702479
chr14	34122500	0.867769
chr14	34278300	0.611570
chr14	34282100	0.917355
chr14	34285200	0.586777
chr14	34305900	0.710744
chr14	34321800	0.652893
chr14	34328900	0.727273
chr14	34335900	0.768595
chr14	34372500	0.760331
chr14	34381200	0.702479
chr14	34384600	0.909091
chr14	34387500	0.991736
chr14	34402600	0.768595
chr14	34407700	0.900826
chr14	34410200	0.859504
chr14	34415100	0.578512
chr14	34434000	0.917355
chr14	34438300	0.595041
chr14	34473100	0.685950
chr14	34482800	0.611570
chr14	34488700	0.578512
chr14	34499800	0.652893
chr14	34504600	0.743802
chr14	34513000	0.958678

chr14	34516600	0.966942
chr14	34522000	0.685950
chr14	34529600	0.694215
chr14	34533200	0.644628
chr14	34547500	0.909091
chr14	34551200	0.603306
chr14	34609000	0.735537
chr14	34801900	0.661157
chr14	34935000	0.942149
chr14	34966600	0.925620
chr14	34975600	0.595041
chr14	35024500	0.768595
chr14	35031300	0.578512
chr14	35103900	0.578512
chr14	35118200	0.586777
chr14	35122400	0.636364
chr14	35135900	0.570248
chr14	35162800	0.694215
chr14	35312900	0.719008
chr14	35382600	0.611570
chr14	35459700	0.694215
chr14	35520000	0.933884
chr14	35727600	0.586777
chr14	35756500	0.685950
chr14	35801500	0.644628
chr14	35806100	0.991736
chr14	35822400	0.851240
chr14	35825800	0.834711
chr14	35828200	0.685950
chr14	35834700	0.826446
chr14	35838500	0.859504
chr14	35843700	0.619835
chr14	35846000	0.793388
chr14	35856300	0.983471
chr14	35860600	0.884298
chr14	36235700	0.652893
chr14	36332300	0.578512
chr14	36348300	0.628099
chr14	36733600	0.776860
chr14	36755000	0.950413
chr14	36830700	0.570248
chr14	37243000	0.710744
chr14	37299200	0.636364
chr14	37317300	0.685950
chr14	37350000	0.669421
chr14	37445000	0.851240
chr14	37447100	0.933884
chr14	37466100	0.834711
chr14	37553200	0.900826
chr14	37559200	0.801653
chr14	37564000	0.677686
chr14	37623000	0.743802
chr14	37637300	0.876033
chr14	37672200	0.578512

chr14	38124900	0.727273
chr14	39615200	0.785124
chr14	39641900	0.719008
chr14	39673500	0.818182
chr14	39685200	0.619835
chr14	39698300	0.677686
chr14	39786200	0.685950
chr14	45600900	0.685950
chr14	50049200	0.818182
chr14	50061300	0.867769
chr14	50103600	0.644628
chr14	50155800	0.586777
chr14	50157900	0.785124
chr14	50325100	0.983471
chr14	50347300	0.975207
chr14	50349600	0.867769
chr14	50429300	0.570248
chr14	50437400	0.851240
chr14	50439600	0.644628
chr14	50444500	0.644628
chr14	50467400	0.900826
chr14	50505500	0.743802
chr14	50511900	0.842975
chr14	50523800	0.669421
chr14	50527500	0.809917
chr14	50529700	0.983471
chr14	50555100	0.603306
chr14	50809800	0.776860
chr14	50814900	0.876033
chr14	50833700	0.851240
chr14	51117600	0.884298
chr14	51130700	0.595041
chr14	51394300	1.000000
chr14	51399500	0.578512
chr14	51422700	0.727273
chr14	52488400	0.694215
chr14	52981600	0.900826
chr14	53013200	0.909091
chr14	53015700	0.611570
chr14	53202900	0.760331
chr14	53205900	0.611570
chr14	53259500	0.619835
chr14	53470700	0.694215
chr14	53576100	0.793388
chr14	53616000	0.876033
chr14	54811500	0.727273
chr14	54865500	0.578512
chr14	54902400	0.719008
chr14	54957200	0.611570
chr14	54969500	0.661157
chr14	55036000	0.603306
chr14	55140600	0.595041
chr14	55143600	0.760331
chr14	55174300	0.727273

chr14	55182200	0.603306
chr14	55188100	0.628099
chr14	55192000	0.628099
chr14	55198100	0.611570
chr14	55220900	0.818182
chr14	55223800	0.884298
chr14	55229100	0.966942
chr14	55233200	0.818182
chr14	55240300	0.917355
chr14	55272900	0.619835
chr14	55569200	0.719008
chr14	55571400	0.710744
chr14	55575000	0.975207
chr14	55583300	0.727273
chr14	55589400	0.619835
chr14	55810900	0.743802
chr14	55838100	0.859504
chr14	55849100	0.909091
chr14	55851200	0.909091
chr14	55853300	0.694215
chr14	55856100	0.570248
chr14	55890700	0.619835
chr14	55967200	0.661157
chr14	56051000	0.752066
chr14	56060700	0.892562
chr14	56248300	0.652893
chr14	56262800	0.727273
chr14	56266800	0.685950
chr14	56281500	0.942149
chr14	56666400	0.933884
chr14	56675300	0.611570
chr14	56683800	0.917355
chr14	56687300	0.983471
chr14	56800500	0.636364
chr14	56807100	0.876033
chr14	57477800	0.570248
chr14	57726600	0.628099
chr14	57728800	0.578512
chr14	57860700	0.578512
chr14	58637900	0.834711
chr14	58659500	0.652893
chr14	58663400	0.578512
chr14	58682700	0.727273
chr14	58704900	0.603306
chr14	58748500	0.826446
chr14	58798000	0.735537
chr14	59216700	0.702479
chr14	59648700	0.661157
chr14	59667400	0.892562
chr14	59809400	0.595041
chr14	59828000	0.900826
chr14	59830900	0.785124
chr14	59873300	0.760331
chr14	60286100	0.603306

chr14	60564800	0.710744
chr14	60692300	0.628099
chr14	60712900	0.611570
chr14	60720700	0.661157
chr14	60743000	0.619835
chr14	61401300	0.760331
chr14	61456500	0.900826
chr14	61568800	0.694215
chr14	61571000	0.950413
chr14	61805300	0.644628
chr14	61809900	0.826446
chr14	61823200	0.578512
chr14	61956200	0.801653
chr14	61994400	0.776860
chr14	62004600	0.644628
chr14	62019800	0.917355
chr14	62026600	0.851240
chr14	62028900	0.619835
chr14	62031000	0.867769
chr14	62178200	0.694215
chr14	63902400	0.611570
chr14	63920500	0.768595
chr14	63927800	0.578512
chr14	63938500	0.619835
chr14	63941600	0.876033
chr14	64164900	0.636364
chr14	64168800	0.842975
chr14	64180900	0.644628
chr14	64215800	0.586777
chr14	64640000	0.776860
chr14	64661900	0.586777
chr14	64733200	0.925620
chr14	64746900	0.867769
chr14	64830900	0.818182
chr14	64858700	0.735537
chr14	64861600	0.966942
chr14	64975100	0.685950
chr14	64977800	0.917355
chr14	65228900	0.652893
chr14	65295900	0.644628
chr14	65298900	0.603306
chr14	65307400	0.900826
chr14	65310000	0.669421
chr14	65313400	0.859504
chr14	65333700	0.611570
chr14	65342400	0.586777
chr14	65352900	0.603306
chr14	65446400	0.603306
chr14	65469400	0.595041
chr14	65509000	0.942149
chr14	65511600	0.958678
chr14	65525200	0.776860
chr14	65540300	0.966942
chr14	65548300	0.570248

chr14	65563700	0.652893
chr14	65574300	0.983471
chr14	65675700	0.785124
chr14	65697100	0.603306
chr14	65705600	0.661157
chr14	65711300	0.611570
chr14	65816000	0.570248
chr14	65888900	0.884298
chr14	65892600	0.611570
chr14	65895500	0.710744
chr14	65899300	0.851240
chr14	65901500	0.834711
chr14	65904300	0.900826
chr14	65906700	0.776860
chr14	65950100	0.694215
chr14	66944300	0.727273
chr14	67511200	0.644628
chr14	67654200	0.628099
chr14	67855500	0.570248
chr14	67858300	0.942149
chr14	67877300	0.909091
chr14	67881000	0.975207
chr14	67883700	0.900826
chr14	67886000	0.950413
chr14	67888600	1.000000
chr14	67892800	0.702479
chr14	67895400	0.586777
chr14	68008700	0.752066
chr14	68055900	0.876033
chr14	68089600	0.867769
chr14	68103600	1.000000
chr14	68105700	0.570248
chr14	68145700	0.768595
chr14	68148100	0.966942
chr14	68150200	0.586777
chr14	68182600	0.603306
chr14	68196400	0.661157
chr14	68553200	0.636364
chr14	68678200	0.900826
chr14	68683800	0.595041
chr14	68713300	0.876033
chr14	68729400	0.710744
chr14	68745600	0.892562
chr14	68759000	0.909091
chr14	68761100	0.603306
chr14	68763300	0.942149
chr14	68844600	0.950413
chr14	68871700	0.661157
chr14	68880600	0.685950
chr14	68950600	0.975207
chr14	68953200	0.809917
chr14	68955400	0.669421
chr14	69087200	1.000000
chr14	69097400	0.727273

chr14	69118500	0.768595
chr14	69181400	0.611570
chr14	69187100	0.851240
chr14	69192500	0.578512
chr14	69194900	0.942149
chr14	69206700	0.694215
chr14	69214100	0.578512
chr14	69216300	0.933884
chr14	69219900	0.818182
chr14	69225400	0.950413
chr14	69230200	0.950413
chr14	69246300	0.785124
chr14	69248500	0.983471
chr14	69253500	0.727273
chr14	69264400	0.793388
chr14	69273900	0.710744
chr14	69283000	0.834711
chr14	69286600	0.776860
chr14	69291800	0.727273
chr14	69321800	0.909091
chr14	69418700	0.933884
chr14	69434000	0.834711
chr14	69473200	0.636364
chr14	69485500	0.578512
chr14	69504700	0.578512
chr14	69536200	0.826446
chr14	69561700	0.603306
chr14	69599900	0.958678
chr14	69616600	0.826446
chr14	70087400	0.842975
chr14	70089800	0.611570
chr14	70092800	0.925620
chr14	70096600	0.933884
chr14	70100200	0.578512
chr14	70103800	1.000000
chr14	70106100	0.677686
chr14	70110700	0.867769
chr14	70114900	0.694215
chr14	70117900	0.628099
chr14	70120600	0.958678
chr14	70125200	0.991736
chr14	70131800	0.628099
chr14	70134100	0.586777
chr14	70151800	0.859504
chr14	70160000	0.950413
chr14	70162200	0.719008
chr14	70165100	0.991736
chr14	70167800	0.966942
chr14	70170300	0.859504
chr14	70217200	0.735537
chr14	70225700	0.900826
chr14	70704900	0.900826
chr14	71165300	0.933884
chr14	71462700	0.578512

chr14	71598000	0.933884
chr14	71610300	0.809917
chr14	71792000	0.628099
chr14	71807000	0.834711
chr14	71823000	0.876033
chr14	71827600	0.661157
chr14	71830400	0.942149
chr14	71839300	0.809917
chr14	71850900	0.743802
chr14	71885600	0.818182
chr14	71928900	0.644628
chr14	71955900	0.628099
chr14	72004300	0.628099
chr14	72006900	0.727273
chr14	72017800	0.694215
chr14	72218400	0.702479
chr14	72946200	0.760331
chr14	73066900	0.578512
chr14	73167100	0.669421
chr14	73208700	0.636364
chr14	73244000	0.595041
chr14	73290200	0.942149
chr14	73292900	0.909091
chr14	73336700	0.917355
chr14	73348300	0.636364
chr14	73374600	0.925620
chr14	73481500	0.570248
chr14	73699400	0.677686
chr14	73778600	0.909091
chr14	73791500	0.785124
chr14	73919800	0.727273
chr14	73927600	0.586777
chr14	73929700	0.619835
chr14	73934400	0.909091
chr14	73943600	0.636364
chr14	74097500	0.586777
chr14	74192700	0.909091
chr14	74195800	0.942149
chr14	74207600	0.809917
chr14	74210700	0.925620
chr14	74214100	0.809917
chr14	74217100	0.983471
chr14	74219700	0.743802
chr14	74223300	0.652893
chr14	74229600	0.958678
chr14	74232600	0.752066
chr14	74238200	0.884298
chr14	74241300	0.818182
chr14	74250900	0.677686
chr14	74256500	0.801653
chr14	74283800	0.719008
chr14	74297000	0.785124
chr14	74482700	0.975207
chr14	74639300	0.933884

chr14	74718200	0.628099
chr14	74742300	0.570248
chr14	74983500	0.578512
chr14	75083700	0.942149
chr14	75087300	0.809917
chr14	75127300	0.892562
chr14	75153800	0.586777
chr14	75162900	0.603306
chr14	75177200	0.628099
chr14	75202300	0.702479
chr14	75238300	0.578512
chr14	75314400	0.867769
chr14	75355400	0.983471
chr14	75372800	0.586777
chr14	75394200	0.809917
chr14	75408800	0.884298
chr14	75412800	0.752066
chr14	75433500	0.603306
chr14	75492300	0.975207
chr14	75512000	0.636364
chr14	75534000	0.743802
chr14	75540200	0.586777
chr14	75591200	0.925620
chr14	75638400	0.809917
chr14	75663600	0.760331
chr14	75689400	0.752066
chr14	75696900	0.933884
chr14	75701600	0.809917
chr14	75716400	0.785124
chr14	75721900	0.619835
chr14	75750700	0.900826
chr14	75763100	0.677686
chr14	75765900	0.925620
chr14	75768900	0.685950
chr14	75886300	0.644628
chr14	75909200	0.809917
chr14	75955900	0.644628
chr14	76002800	0.867769
chr14	76006800	0.900826
chr14	76009500	0.619835
chr14	76072000	0.661157
chr14	76125400	0.578512
chr14	76278100	0.752066
chr14	76297600	0.834711
chr14	76299800	0.925620
chr14	76327500	0.884298
chr14	76400000	0.950413
chr14	76402800	0.809917
chr14	76411000	0.834711
chr14	76605500	0.867769
chr14	76668600	0.603306
chr14	76722900	0.760331
chr14	76727800	0.801653
chr14	77182200	0.611570

chr14	77186900	0.578512
chr14	77239500	0.909091
chr14	77277000	0.628099
chr14	77385800	0.892562
chr14	77414400	0.727273
chr14	77422600	0.909091
chr14	77426000	0.958678
chr14	77428600	0.975207
chr14	77444600	0.685950
chr14	77490900	0.611570
chr14	77505100	0.710744
chr14	77508300	0.942149
chr14	77511100	0.900826
chr14	77513400	0.809917
chr14	77549300	0.652893
chr14	77624700	0.619835
chr14	77770100	0.966942
chr14	77799100	0.636364
chr14	77809800	0.760331
chr14	77873300	0.595041
chr14	77875900	0.826446
chr14	77881000	0.793388
chr14	78060800	0.578512
chr14	78066800	0.644628
chr14	78071100	0.909091
chr14	78074000	0.743802
chr14	78105500	0.752066
chr14	78275700	0.685950
chr14	78289400	0.909091
chr14	78292800	0.983471
chr14	78323600	0.644628
chr14	78326600	0.768595
chr14	78330500	0.842975
chr14	78351900	0.768595
chr14	78359100	0.983471
chr14	78375700	0.785124
chr14	78379000	0.661157
chr14	78383100	0.892562
chr14	78385300	0.892562
chr14	78388400	0.975207
chr14	78391000	0.925620
chr14	78396000	0.743802
chr14	78429300	0.603306
chr14	78437000	0.669421
chr14	78514600	0.685950
chr14	78781500	0.628099
chr14	78786400	0.785124
chr14	81397700	0.636364
chr14	81440200	0.661157
chr14	81472900	0.826446
chr14	81476100	0.570248
chr14	81485300	0.710744
chr14	81490000	0.851240
chr14	81630500	0.876033

chr14	81637200	0.578512
chr14	81734700	0.719008
chr14	81770100	0.958678
chr14	81820800	0.677686
chr14	81866800	0.958678
chr14	81869400	0.925620
chr14	81883400	0.892562
chr14	81889300	0.917355
chr14	81891600	0.760331
chr14	81895600	0.677686
chr14	81899100	0.818182
chr14	81986200	0.983471
chr14	88237500	0.652893
chr14	88419700	0.727273
chr14	88462100	0.942149
chr14	88465400	0.677686
chr14	88490700	0.826446
chr14	88493600	0.586777
chr14	89148700	0.595041
chr14	89282900	0.752066
chr14	89286200	0.909091
chr14	89288600	0.702479
chr14	89467400	0.636364
chr14	89625700	0.818182
chr14	89632100	0.752066
chr14	89936500	0.942149
chr14	89943400	0.760331
chr14	90803200	0.644628
chr14	90845600	0.694215
chr14	90967300	0.752066
chr14	90983400	0.752066
chr14	91000800	0.826446
chr14	91016600	0.776860
chr14	91023600	0.950413
chr14	91040900	0.735537
chr14	91044100	0.578512
chr14	91094300	0.603306
chr14	91125400	0.950413
chr14	91186300	0.636364
chr14	91228000	0.628099
chr14	91263500	0.966942
chr14	91267400	0.752066
chr14	91269900	0.785124
chr14	91273000	0.884298
chr14	91276100	0.942149
chr14	91294500	0.710744
chr14	91316100	0.685950
chr14	91496400	0.685950
chr14	91583600	0.669421
chr14	91591900	0.900826
chr14	91616100	0.644628
chr14	91669000	0.933884
chr14	91694400	0.719008
chr14	91719800	0.743802

chr14	91723000	0.710744
chr14	91732700	0.595041
chr14	91735300	0.958678
chr14	91790700	0.818182
chr14	91799100	0.595041
chr14	91831000	0.719008
chr14	91849000	0.685950
chr14	91869200	0.768595
chr14	91885200	0.768595
chr14	91892100	0.685950
chr14	91963900	0.611570
chr14	91967600	0.834711
chr14	92080600	0.933884
chr14	92241400	0.793388
chr14	92321300	0.694215
chr14	92594700	0.636364
chr14	93222400	0.710744
chr14	93226500	0.900826
chr14	93252500	0.909091
chr14	93313400	0.578512
chr14	93410800	0.652893
chr14	93460600	0.950413
chr14	93503000	0.586777
chr14	93513100	0.892562
chr14	93528000	0.710744
chr14	93532700	0.909091
chr14	93580000	0.669421
chr14	93671800	0.611570
chr14	93733200	0.611570
chr14	93736200	0.801653
chr14	93772700	0.727273
chr14	93784600	0.942149
chr14	93787400	0.611570
chr14	93790200	0.859504
chr14	94545000	0.677686
chr14	94551500	0.768595
chr14	94576900	0.603306
chr14	95652500	0.900826
chr14	95973800	0.826446
chr14	95989900	0.727273
chr14	95993400	0.867769
chr14	96007200	0.628099
chr14	96009500	0.793388
chr14	96011600	0.950413
chr14	96719200	0.752066
chr14	96816400	0.570248
chr14	96823400	0.958678
chr14	96834900	0.884298
chr14	96872500	0.578512
chr14	96874800	0.743802
chr14	97047600	0.834711
chr14	97126700	0.619835
chr14	97223500	0.842975
chr14	97269800	0.892562

chr14	97275500	0.785124
chr14	97293800	0.652893
chr14	97297400	0.760331
chr14	97354200	0.619835
chr14	97384100	0.942149
chr14	97387400	0.950413
chr14	97488200	0.661157
chr14	97544200	0.694215
chr14	99887100	0.933884
chr14	99927800	0.785124
chr14	99941800	0.859504
chr14	99944900	0.628099
chr14	100011800	0.809917
chr14	100035700	0.818182
chr14	100039100	0.842975
chr14	100073400	0.735537
chr14	100449400	0.595041
chr14	100482100	0.570248
chr14	100485700	0.867769
chr14	100532500	0.966942
chr14	100535900	0.735537
chr14	100542800	0.735537
chr14	100545800	0.752066
chr14	100598900	0.619835
chr14	100606800	0.661157
chr14	100610300	0.710744
chr14	100635000	0.595041
chr14	100659800	0.727273
chr14	100682100	0.958678
chr14	100690000	0.809917
chr14	100693400	0.669421
chr14	100710100	0.570248
chr14	100712900	0.768595
chr14	100716900	0.702479
chr14	100784700	0.578512
chr14	100855200	0.818182
chr14	100859800	0.876033
chr14	100873300	1.000000
chr14	100890500	0.809917
chr14	100906800	0.884298
chr14	100909400	0.619835
chr14	101012600	0.752066
chr14	101020600	0.619835
chr14	101072900	0.611570
chr14	101089100	0.619835
chr14	101111600	0.925620
chr14	101115300	0.661157
chr14	101135900	0.950413
chr14	101139600	0.719008
chr14	101142500	0.727273
chr14	101144800	0.975207
chr14	101147200	1.000000
chr14	101150900	0.876033
chr14	101162900	0.793388

chr14	101165600	0.983471
chr14	101228700	0.942149
chr14	101236300	0.834711
chr14	101238900	0.628099
chr14	101254800	0.735537
chr14	101258000	0.661157
chr14	101331900	1.000000
chr14	101338400	0.958678
chr14	101367000	0.859504
chr14	101827900	0.933884
chr14	102095400	0.702479
chr14	102144700	0.859504
chr14	102150700	0.975207
chr14	102155000	0.958678
chr14	102174000	0.958678
chr14	102180200	0.760331
chr14	102182600	0.900826
chr14	102193400	0.611570
chr14	102198100	0.950413
chr14	102200400	0.917355
chr14	102226100	0.785124
chr14	102232900	0.917355
chr14	102236800	0.867769
chr14	102274700	0.826446
chr14	102279300	0.900826
chr14	102281600	0.702479
chr14	102284100	0.900826
chr14	102316000	0.818182
chr14	102318300	0.611570
chr14	102320900	0.619835
chr14	102340000	0.652893
chr14	102365800	0.958678
chr14	102394000	0.834711
chr14	102426800	0.661157
chr14	102434600	0.785124
chr14	102530400	0.735537
chr14	102538500	0.809917
chr14	102555700	0.735537
chr14	102562500	1.000000
chr14	102593100	0.752066
chr14	102617300	0.595041
chr14	102621100	0.818182
chr14	102624300	0.900826
chr14	102631700	0.669421
chr14	102653900	0.570248
chr14	102657200	0.619835
chr14	102722300	0.966942
chr14	102731400	0.677686
chr14	102780800	0.702479
chr14	102904900	0.760331
chr14	102928400	0.842975
chr14	102967000	0.603306
chr14	102971000	0.917355
chr14	102977300	0.644628

chr14	102993700	0.892562
chr14	102997700	0.942149
chr14	103000100	0.603306
chr14	103005900	0.983471
chr14	103014700	0.685950
chr14	103017800	0.834711
chr14	103034900	0.719008
chr14	103037600	0.752066
chr14	103041200	0.694215
chr14	103052200	0.834711
chr14	103096700	1.000000
chr14	103105300	0.578512
chr14	103112700	0.975207
chr14	103126300	0.603306
chr14	103132400	0.809917
chr14	103150500	0.652893
chr14	103153100	0.743802
chr14	103157200	0.586777
chr14	103161500	0.619835
chr14	103237100	0.677686
chr14	103243100	0.669421
chr14	103246600	0.661157
chr14	103294700	0.677686
chr14	103466900	0.991736
chr14	103477700	0.809917
chr14	103558100	0.876033
chr14	103564800	0.611570
chr14	103566900	0.611570
chr14	103571900	0.578512
chr14	103575000	0.933884
chr14	103579800	0.595041
chr14	103582800	0.900826
chr14	103585500	0.942149
chr14	103589200	0.752066
chr14	103603400	0.942149
chr14	103671200	0.752066
chr14	103675700	0.702479
chr14	103775500	0.950413
chr14	103780200	0.793388
chr14	103796200	0.685950
chr14	103798300	0.636364
chr14	103810400	0.578512
chr14	103814300	0.603306
chr14	103822900	0.983471
chr14	103827400	0.966942
chr14	103839400	0.942149
chr14	103844700	0.983471
chr14	103860400	0.818182
chr14	103864800	0.867769
chr14	103868200	0.925620
chr14	103870900	0.595041
chr14	103875700	0.793388
chr14	103885200	0.884298
chr14	103889700	0.900826

chr14	103892900	0.702479
chr14	103895000	0.983471
chr14	103909900	0.652893
chr14	103914200	0.694215
chr14	103985700	0.909091
chr14	104026300	0.702479
chr14	104094500	0.735537
chr14	104180500	0.644628
chr14	104185300	0.595041
chr14	104196800	0.760331
chr14	104356000	0.702479
chr14	104390200	0.851240
chr14	104410500	0.966942
chr14	104440100	0.619835
chr14	105040900	0.727273
chr14	105044500	0.710744
chr14	105049500	0.760331
chr14	105144100	0.809917
chr14	105147400	0.884298
chr14	105167200	0.958678
chr14	105196300	0.884298
chr14	105235800	0.859504
chr14	105263200	0.702479
chr14	105265400	0.842975
chr14	105271500	0.876033
chr14	105294300	0.818182
chr14	105437100	0.652893
chr14	105482400	0.628099
chr14	105485400	0.603306
chr14	105542500	0.636364
chr14	105633100	0.768595
chr14	105665500	0.661157
chr14	105812500	0.776860
chr14	105844900	0.628099
chr14	105864100	0.842975
chr14	105948000	0.727273
chr14	106069600	0.826446
chr15	20301500	0.644628
chr15	20679200	0.991736
chr15	20682300	0.595041
chr15	20686100	0.719008
chr15	20689900	0.652893
chr15	20965800	0.809917
chr15	21308900	0.842975
chr15	22444000	0.892562
chr15	22446300	0.834711
chr15	22694900	0.884298
chr15	22698600	0.760331
chr15	22896300	0.710744
chr15	22924100	0.628099
chr15	22980500	0.892562
chr15	22985100	0.809917
chr15	22992400	0.677686
chr15	23037300	0.834711

chr15	23078000	0.851240
chr15	23093700	0.867769
chr15	23096600	0.752066
chr15	23128000	0.652893
chr15	23159200	0.652893
chr15	23162000	0.586777
chr15	23346700	0.975207
chr15	23353200	0.942149
chr15	23358100	0.809917
chr15	23452200	0.867769
chr15	23818300	0.578512
chr15	25661800	0.628099
chr15	25672600	0.760331
chr15	27331900	0.917355
chr15	27337900	0.677686
chr15	27364200	0.694215
chr15	27366600	0.570248
chr15	27369200	0.900826
chr15	27373400	0.958678
chr15	27379200	0.884298
chr15	27422300	0.611570
chr15	27425300	0.975207
chr15	27699800	0.942149
chr15	27713900	0.917355
chr15	27744800	0.595041
chr15	27777800	0.785124
chr15	27843800	0.628099
chr15	28538400	0.669421
chr15	28541500	0.603306
chr15	28546200	0.694215
chr15	28641200	0.876033
chr15	28646000	0.776860
chr15	28756600	0.876033
chr15	28761400	0.785124
chr15	28857100	0.801653
chr15	28864000	0.685950
chr15	28866900	0.909091
chr15	29030400	0.586777
chr15	29136600	0.661157
chr15	29142300	0.595041
chr15	29148300	0.603306
chr15	29152500	0.859504
chr15	29156400	0.859504
chr15	29159100	0.785124
chr15	29210700	0.636364
chr15	29217200	0.785124
chr15	29219900	0.661157
chr15	29226200	0.809917
chr15	29232800	0.826446
chr15	29247400	0.768595
chr15	29261200	0.933884
chr15	29265200	0.619835
chr15	29282900	0.619835
chr15	29360700	0.867769

chr15	29406200	0.595041
chr15	29419700	0.644628
chr15	29949400	0.619835
chr15	29965700	0.776860
chr15	30071500	0.611570
chr15	30108100	0.785124
chr15	30796500	0.636364
chr15	30961500	0.586777
chr15	31246700	0.578512
chr15	31576100	0.652893
chr15	31591200	0.826446
chr15	31596000	0.685950
chr15	31646100	0.586777
chr15	31654500	0.586777
chr15	31658000	0.925620
chr15	31690000	0.628099
chr15	31727900	0.603306
chr15	31741600	0.595041
chr15	32658900	0.570248
chr15	32934100	0.694215
chr15	32950200	0.669421
chr15	32952700	0.586777
chr15	33159600	0.892562
chr15	33419000	0.578512
chr15	34208300	0.809917
chr15	34339700	0.578512
chr15	34372200	0.611570
chr15	34471900	0.677686
chr15	34540800	0.727273
chr15	34545300	0.917355
chr15	34577400	0.702479
chr15	34621600	0.603306
chr15	34624200	0.876033
chr15	34723600	0.603306
chr15	34906000	0.834711
chr15	34914500	0.950413
chr15	34929300	0.578512
chr15	34937900	0.636364
chr15	34942200	0.966942
chr15	34945400	0.661157
chr15	34958300	0.735537
chr15	34960500	0.785124
chr15	35007300	0.595041
chr15	35092300	0.652893
chr15	35264800	0.685950
chr15	35286200	0.867769
chr15	35296000	0.661157
chr15	35360700	0.595041
chr15	35458200	0.958678
chr15	35461700	0.595041
chr15	35465900	0.925620
chr15	35468000	0.636364
chr15	35599100	0.991736
chr15	35601300	0.603306

chr15	35770500	0.636364
chr15	35842200	0.801653
chr15	35850000	0.636364
chr15	35887900	0.586777
chr15	35919300	0.876033
chr15	35960700	0.768595
chr15	35965200	0.884298
chr15	35967600	0.776860
chr15	35989300	0.619835
chr15	35992900	0.752066
chr15	35998100	0.652893
chr15	36606400	0.603306
chr15	36626600	0.876033
chr15	36699800	0.933884
chr15	36719200	0.570248
chr15	36721900	0.933884
chr15	36724900	0.818182
chr15	36741800	0.809917
chr15	36906500	0.570248
chr15	37224900	0.586777
chr15	37244700	0.735537
chr15	37253300	0.768595
chr15	37312700	0.809917
chr15	37349000	0.776860
chr15	37351200	0.652893
chr15	37404000	0.578512
chr15	38550400	0.595041
chr15	38616100	0.570248
chr15	38730600	0.933884
chr15	38791300	0.570248
chr15	38801400	0.619835
chr15	38881800	0.942149
chr15	38894900	0.942149
chr15	38899100	0.859504
chr15	38920300	0.595041
chr15	39420300	0.983471
chr15	39425600	0.809917
chr15	39433100	0.826446
chr15	39506100	0.809917
chr15	39645800	0.628099
chr15	39650800	0.933884
chr15	39689700	0.809917
chr15	39693200	0.669421
chr15	39697000	0.595041
chr15	39720000	0.578512
chr15	39748100	0.710744
chr15	39753800	0.570248
chr15	39804100	0.628099
chr15	39809100	0.595041
chr15	39990700	0.867769
chr15	39994200	0.677686
chr15	39997100	0.933884
chr15	40013500	0.586777
chr15	40170100	0.603306

chr15	40174400	0.694215
chr15	40185300	0.702479
chr15	40189800	0.636364
chr15	40217900	0.818182
chr15	40333100	0.661157
chr15	40337500	0.793388
chr15	40343300	0.677686
chr15	40368100	0.966942
chr15	40373800	0.793388
chr15	40377600	0.768595
chr15	40388700	0.661157
chr15	40392200	0.586777
chr15	40398100	0.785124
chr15	40430100	0.652893
chr15	40527500	0.652893
chr15	40531700	0.595041
chr15	40535700	0.859504
chr15	40559200	0.966942
chr15	40567500	0.611570
chr15	40583600	0.595041
chr15	40594000	0.859504
chr15	40601300	0.785124
chr15	40613800	0.586777
chr15	40623100	0.818182
chr15	40626700	0.876033
chr15	40630300	0.636364
chr15	40633000	0.619835
chr15	40660800	0.586777
chr15	40672200	0.826446
chr15	40719500	0.826446
chr15	40722700	0.859504
chr15	40730800	0.570248
chr15	40735800	0.652893
chr15	40742400	0.595041
chr15	40745500	0.768595
chr15	40799700	0.611570
chr15	40834500	0.793388
chr15	40846800	0.776860
chr15	40851500	0.851240
chr15	40859500	0.900826
chr15	40883600	0.595041
chr15	40915600	0.702479
chr15	41036700	0.785124
chr15	41040500	0.851240
chr15	41044500	0.595041
chr15	41049300	0.694215
chr15	41055400	0.719008
chr15	41067400	0.619835
chr15	41118800	0.900826
chr15	41139900	0.859504
chr15	41150300	0.768595
chr15	41154400	0.743802
chr15	41171800	0.611570
chr15	41199500	0.628099

chr15	41219000	0.619835
chr15	41234000	0.595041
chr15	41241900	0.743802
chr15	41526100	0.834711
chr15	41533900	0.793388
chr15	41575200	0.685950
chr15	41718800	0.661157
chr15	41730400	0.867769
chr15	41769400	0.900826
chr15	41847300	0.685950
chr15	41854600	0.876033
chr15	41858100	0.925620
chr15	41860800	0.801653
chr15	41896400	0.834711
chr15	41927100	0.661157
chr15	41951900	0.677686
chr15	41983800	0.619835
chr15	42068800	0.603306
chr15	42081800	0.636364
chr15	42209900	0.661157
chr15	42226000	0.611570
chr15	42273500	0.793388
chr15	42432700	0.603306
chr15	42451000	0.942149
chr15	42571900	0.950413
chr15	42697000	0.578512
chr15	42786100	0.644628
chr15	42790800	0.826446
chr15	42793200	0.595041
chr15	42797600	0.743802
chr15	42800900	0.900826
chr15	42838200	0.570248
chr15	42846000	0.595041
chr15	42872100	0.677686
chr15	42877200	0.809917
chr15	42881100	0.793388
chr15	42888400	0.834711
chr15	42895200	0.719008
chr15	42897900	0.942149
chr15	42900800	0.661157
chr15	42904800	0.785124
chr15	42942800	0.586777
chr15	43249400	0.826446
chr15	43386700	0.793388
chr15	43461800	0.710744
chr15	43513500	0.768595
chr15	43523100	0.942149
chr15	43531800	0.834711
chr15	43586300	0.570248
chr15	43703100	0.950413
chr15	43726400	0.950413
chr15	43733500	0.842975
chr15	43762400	0.983471
chr15	43776500	0.826446

chr15	43798400	0.743802
chr15	43801100	0.669421
chr15	43806500	0.826446
chr15	43860200	0.677686
chr15	43863400	0.603306
chr15	43960100	0.710744
chr15	44034500	0.669421
chr15	44081300	0.933884
chr15	44087500	0.842975
chr15	44106000	0.834711
chr15	44118400	0.694215
chr15	44193000	0.950413
chr15	44590600	0.743802
chr15	44677700	0.603306
chr15	44723300	0.636364
chr15	44733400	0.677686
chr15	44781700	0.793388
chr15	44834400	0.694215
chr15	44891500	0.669421
chr15	44957100	0.578512
chr15	44959400	0.768595
chr15	44975400	0.809917
chr15	45016800	0.876033
chr15	45019200	0.958678
chr15	45028200	0.785124
chr15	45073600	0.917355
chr15	45076100	0.603306
chr15	45081300	0.603306
chr15	45085300	0.900826
chr15	45089600	0.710744
chr15	45110200	0.834711
chr15	45114600	0.570248
chr15	45121500	0.892562
chr15	45124100	0.983471
chr15	45146200	0.595041
chr15	45153300	0.578512
chr15	45181100	0.702479
chr15	45290700	0.768595
chr15	45319400	0.867769
chr15	45324000	0.834711
chr15	45339800	0.694215
chr15	45342500	0.801653
chr15	45345300	0.950413
chr15	45364000	0.793388
chr15	45370400	0.818182
chr15	45375300	0.760331
chr15	45381100	0.578512
chr15	45384000	0.595041
chr15	45410600	0.628099
chr15	45461600	0.793388
chr15	45517600	0.842975
chr15	45709800	0.752066
chr15	45841100	0.876033
chr15	45876400	0.983471

chr15	48627600	0.586777
chr15	48647700	0.586777
chr15	48847600	0.619835
chr15	48872800	0.818182
chr15	48884300	0.644628
chr15	48964200	0.619835
chr15	48989300	0.809917
chr15	49012100	0.942149
chr15	49015500	0.851240
chr15	49057600	0.809917
chr15	49088700	0.619835
chr15	49144400	0.727273
chr15	49148400	0.644628
chr15	49151000	0.909091
chr15	49167500	0.818182
chr15	49176000	0.785124
chr15	49189700	0.644628
chr15	49218400	0.909091
chr15	49221400	0.636364
chr15	49238400	0.619835
chr15	49356400	0.710744
chr15	49439600	0.595041
chr15	49467600	0.785124
chr15	49518700	0.818182
chr15	49535200	0.652893
chr15	49911300	0.628099
chr15	50039900	0.942149
chr15	50044300	0.735537
chr15	50427600	0.884298
chr15	50443400	0.595041
chr15	50461500	0.603306
chr15	50482300	0.867769
chr15	50528400	0.710744
chr15	50543300	0.677686
chr15	50554300	0.884298
chr15	50580200	0.851240
chr15	50643000	0.586777
chr15	50683700	0.859504
chr15	50704200	0.595041
chr15	50711000	0.578512
chr15	50836600	0.636364
chr15	50839300	0.694215
chr15	50886900	0.818182
chr15	50933900	0.570248
chr15	50969600	0.785124
chr15	50974000	0.570248
chr15	51046000	0.933884
chr15	51055100	0.628099
chr15	51147700	0.669421
chr15	51150300	0.768595
chr15	51153700	0.752066
chr15	51169700	0.752066
chr15	51173500	0.909091
chr15	51296800	0.892562

chr15	51332000	0.636364
chr15	51378600	0.685950
chr15	51436700	0.685950
chr15	51477800	0.917355
chr15	51520700	0.859504
chr15	51861700	0.760331
chr15	51869600	0.818182
chr15	51872900	0.892562
chr15	51892900	0.867769
chr15	51910400	0.586777
chr15	51919800	0.595041
chr15	51922000	0.603306
chr15	51927100	0.859504
chr15	51939500	0.909091
chr15	51944600	0.925620
chr15	51965400	0.801653
chr15	51985000	0.611570
chr15	51989500	0.958678
chr15	51995400	0.900826
chr15	51998400	0.570248
chr15	52017600	0.619835
chr15	52019700	0.842975
chr15	52024500	0.884298
chr15	52031900	0.834711
chr15	52041900	0.603306
chr15	52048800	0.644628
chr15	52051500	0.595041
chr15	52069200	0.603306
chr15	52108900	0.818182
chr15	52135500	0.958678
chr15	52138500	0.628099
chr15	52141800	0.578512
chr15	52167300	0.933884
chr15	52171100	0.933884
chr15	52274500	0.669421
chr15	52298500	0.991736
chr15	52301100	0.983471
chr15	52303800	0.900826
chr15	52306200	0.917355
chr15	52319300	0.619835
chr15	52332200	0.752066
chr15	52334800	0.917355
chr15	52393200	0.636364
chr15	52408600	0.768595
chr15	52429800	0.586777
chr15	52436700	0.603306
chr15	52821700	0.619835
chr15	52850400	0.644628
chr15	52857800	0.826446
chr15	52945900	0.578512
chr15	55398700	0.685950
chr15	55401300	0.628099
chr15	55510500	0.702479
chr15	55514200	0.884298

chr15	55524600	0.801653
chr15	55532300	0.867769
chr15	55539800	0.801653
chr15	55543500	0.892562
chr15	55549600	0.851240
chr15	55560500	0.842975
chr15	55569900	1.000000
chr15	55572400	0.570248
chr15	55575300	0.942149
chr15	55579000	0.917355
chr15	55589600	0.578512
chr15	55637200	0.760331
chr15	55691100	0.785124
chr15	55693900	0.710744
chr15	55697900	0.586777
chr15	55805500	0.595041
chr15	56229600	0.685950
chr15	56296600	0.752066
chr15	56332500	0.603306
chr15	56345300	0.776860
chr15	56495900	0.768595
chr15	56541200	0.694215
chr15	56544000	0.892562
chr15	56586500	0.702479
chr15	56613300	0.661157
chr15	57029400	0.859504
chr15	57032400	0.603306
chr15	57051400	0.652893
chr15	57065600	0.677686
chr15	57075400	0.818182
chr15	57107900	0.628099
chr15	57175800	0.685950
chr15	57215500	0.595041
chr15	57221500	0.752066
chr15	57226900	0.570248
chr15	57263700	0.958678
chr15	57265900	0.595041
chr15	57272700	0.595041
chr15	57323900	0.719008
chr15	57328600	0.661157
chr15	57372500	0.603306
chr15	57374700	0.727273
chr15	57377600	0.677686
chr15	57436500	0.900826
chr15	57516400	0.710744
chr15	57537800	0.735537
chr15	57598700	0.644628
chr15	57604200	0.652893
chr15	57612900	0.917355
chr15	57617500	0.859504
chr15	57620600	0.661157
chr15	57627700	0.909091
chr15	57888100	0.570248
chr15	57997300	0.809917

chr15	58025400	0.710744
chr15	58242800	0.966942
chr15	58306600	0.958678
chr15	58331000	0.727273
chr15	58339200	0.619835
chr15	58342000	0.752066
chr15	58350900	0.636364
chr15	58370400	0.842975
chr15	58372600	0.917355
chr15	58400100	0.983471
chr15	58402400	0.834711
chr15	58406400	0.909091
chr15	58435100	0.909091
chr15	58459300	0.628099
chr15	58462200	0.628099
chr15	58469100	0.950413
chr15	58473100	0.876033
chr15	58481200	0.595041
chr15	58496200	0.661157
chr15	58542200	0.702479
chr15	58572000	0.644628
chr15	58574600	0.570248
chr15	58585200	0.785124
chr15	58596300	0.636364
chr15	58602400	0.628099
chr15	58605100	0.834711
chr15	58622500	0.586777
chr15	58626100	0.801653
chr15	58636900	1.000000
chr15	58644400	0.991736
chr15	58650100	0.900826
chr15	58653000	0.652893
chr15	58685300	0.867769
chr15	58693000	0.900826
chr15	58696600	0.785124
chr15	58718100	0.595041
chr15	58722400	0.867769
chr15	58732700	0.809917
chr15	58750200	0.661157
chr15	58755300	0.611570
chr15	58760000	0.685950
chr15	58778800	0.785124
chr15	58785500	0.768595
chr15	58798400	0.661157
chr15	58808200	0.595041
chr15	58810400	0.958678
chr15	58814200	0.760331
chr15	58830900	0.859504
chr15	58836900	0.661157
chr15	58839300	0.917355
chr15	58844200	0.950413
chr15	58848300	0.834711
chr15	58860700	0.909091
chr15	58877500	0.809917

chr15	58905000	0.685950
chr15	58961700	0.669421
chr15	58976800	0.628099
chr15	59024900	0.793388
chr15	59030100	0.636364
chr15	59046600	0.669421
chr15	59072000	0.818182
chr15	59122600	0.776860
chr15	59126200	0.785124
chr15	59135000	0.917355
chr15	59211200	0.727273
chr15	59217300	0.578512
chr15	59245500	0.834711
chr15	59283800	0.578512
chr15	59303700	0.867769
chr15	59403500	0.727273
chr15	59832900	0.818182
chr15	59836800	0.727273
chr15	59846500	0.636364
chr15	59978600	0.603306
chr15	59988800	0.859504
chr15	60682500	0.644628
chr15	60685300	0.900826
chr15	60687800	0.603306
chr15	60709500	0.933884
chr15	60716300	0.685950
chr15	60908000	0.909091
chr15	61914100	0.785124
chr15	62386800	0.636364
chr15	62412100	0.578512
chr15	63189700	0.644628
chr15	63344100	0.669421
chr15	63346200	0.942149
chr15	63350100	0.834711
chr15	63355500	0.942149
chr15	63368200	0.636364
chr15	63372400	0.636364
chr15	63400700	0.760331
chr15	63470400	0.570248
chr15	63488000	0.809917
chr15	63491100	1.000000
chr15	63495500	0.900826
chr15	63499300	0.818182
chr15	63503300	0.785124
chr15	63506900	0.966942
chr15	63510400	0.884298
chr15	63514300	0.669421
chr15	63519000	0.925620
chr15	63531500	0.702479
chr15	63536700	0.570248
chr15	63584800	0.900826
chr15	63634400	0.884298
chr15	63637300	0.958678
chr15	63655900	0.785124

chr15	63664500	0.809917
chr15	63666700	0.603306
chr15	63672800	0.570248
chr15	63683800	0.859504
chr15	63688200	0.950413
chr15	63691600	0.793388
chr15	63713000	0.925620
chr15	63729300	0.867769
chr15	63759200	0.595041
chr15	63800900	0.818182
chr15	63804100	0.628099
chr15	63807600	0.603306
chr15	63814500	0.892562
chr15	63817800	0.909091
chr15	63821900	0.595041
chr15	63832400	0.619835
chr15	63837400	0.735537
chr15	63903200	0.785124
chr15	64024300	0.950413
chr15	64027900	0.595041
chr15	64085300	0.785124
chr15	64099300	0.743802
chr15	64108600	0.578512
chr15	64110800	0.818182
chr15	64114400	0.801653
chr15	64119700	0.694215
chr15	64128800	0.727273
chr15	64132400	0.809917
chr15	64135000	0.933884
chr15	64142800	0.801653
chr15	64150100	0.694215
chr15	64152700	0.719008
chr15	64184500	0.942149
chr15	64190300	0.909091
chr15	64199000	0.595041
chr15	64202400	0.710744
chr15	64211500	0.917355
chr15	64231800	0.859504
chr15	64264100	0.851240
chr15	64279300	0.933884
chr15	64297100	0.842975
chr15	64306100	0.776860
chr15	64315900	0.826446
chr15	64324600	0.727273
chr15	64363900	0.925620
chr15	64398500	0.595041
chr15	64430100	0.677686
chr15	64434500	0.818182
chr15	64439100	0.826446
chr15	64443600	0.644628
chr15	64446500	0.644628
chr15	64490000	0.801653
chr15	64497600	0.991736
chr15	64509400	0.801653

chr15	64516500	0.735537
chr15	64524600	0.727273
chr15	64539600	0.950413
chr15	64722700	0.950413
chr15	64731200	0.586777
chr15	64736800	0.603306
chr15	64749400	0.909091
chr15	64756500	0.644628
chr15	64759400	0.628099
chr15	64800100	0.685950
chr15	64813700	0.710744
chr15	64819800	0.842975
chr15	64825000	0.677686
chr15	64854000	0.867769
chr15	64856400	0.909091
chr15	64859200	0.859504
chr15	64861300	0.652893
chr15	64864300	0.867769
chr15	64876300	0.933884
chr15	64889500	0.677686
chr15	64927700	0.702479
chr15	64988200	0.851240
chr15	65024100	0.611570
chr15	65042900	0.694215
chr15	65102700	0.578512
chr15	65128500	0.867769
chr15	65133100	0.619835
chr15	65137000	0.628099
chr15	65140700	0.661157
chr15	65186500	0.909091
chr15	65278300	0.644628
chr15	65336500	0.603306
chr15	65342400	0.719008
chr15	65505000	0.785124
chr15	65511200	0.884298
chr15	65514600	0.652893
chr15	65530100	0.760331
chr15	65537900	0.611570
chr15	65568300	0.876033
chr15	65582300	0.570248
chr15	65589400	0.685950
chr15	65598100	0.669421
chr15	65632500	0.702479
chr15	65642100	0.710744
chr15	65677900	0.801653
chr15	65805200	0.570248
chr15	65818200	0.595041
chr15	65825900	0.652893
chr15	65843100	0.677686
chr15	65905900	0.768595
chr15	65912300	0.685950
chr15	65931200	0.669421
chr15	66036600	0.710744
chr15	66049900	0.603306

chr15	66070400	0.677686
chr15	66074200	0.917355
chr15	66076500	0.826446
chr15	66088900	0.975207
chr15	66093800	0.958678
chr15	66097900	0.752066
chr15	66111600	0.809917
chr15	66113800	0.578512
chr15	66117000	0.595041
chr15	66125400	0.909091
chr15	66128200	0.900826
chr15	66130300	0.611570
chr15	66133000	0.884298
chr15	66155500	0.834711
chr15	66193800	0.801653
chr15	66196700	0.917355
chr15	66218300	0.636364
chr15	66263400	0.933884
chr15	66562100	0.669421
chr15	66602700	0.595041
chr15	66616200	0.685950
chr15	66662800	0.760331
chr15	66682800	0.834711
chr15	66687100	0.611570
chr15	66700600	0.793388
chr15	66727300	0.595041
chr15	66773900	0.983471
chr15	66791400	0.603306
chr15	66800500	0.619835
chr15	66879000	0.859504
chr15	66885500	0.768595
chr15	66906500	0.611570
chr15	66910200	0.685950
chr15	66914800	1.000000
chr15	66918600	0.818182
chr15	66921600	0.884298
chr15	66925200	0.851240
chr15	66932300	0.570248
chr15	66934800	0.925620
chr15	66937200	0.644628
chr15	66942900	0.809917
chr15	66945400	0.842975
chr15	66947600	0.983471
chr15	66951700	0.768595
chr15	66955200	0.834711
chr15	66963700	0.966942
chr15	66966100	0.975207
chr15	66972500	0.983471
chr15	66981300	0.768595
chr15	66993000	0.603306
chr15	67006200	0.669421
chr15	67012100	0.884298
chr15	67016200	0.966942
chr15	67059000	0.628099

chr15	67066000	0.719008
chr15	67095700	1.000000
chr15	67102800	0.652893
chr15	67143400	0.710744
chr15	67155100	0.578512
chr15	67169100	0.652893
chr15	67184700	0.578512
chr15	67187200	0.925620
chr15	67189400	0.578512
chr15	67224600	1.000000
chr15	67229000	0.636364
chr15	67305700	0.586777
chr15	67317400	0.826446
chr15	67323000	0.719008
chr15	67332100	0.834711
chr15	67334800	0.636364
chr15	67365500	0.966942
chr15	67370100	0.933884
chr15	67373500	0.925620
chr15	67375900	0.636364
chr15	67378400	0.685950
chr15	67380500	0.768595
chr15	67385200	0.702479
chr15	67388600	0.859504
chr15	67391300	0.876033
chr15	67398400	0.826446
chr15	67402200	0.570248
chr15	67411200	0.586777
chr15	67413400	0.694215
chr15	67417800	0.925620
chr15	67424700	0.661157
chr15	67430500	0.834711
chr15	67442800	0.760331
chr15	67471600	0.909091
chr15	67501000	0.727273
chr15	67561000	0.702479
chr15	67660500	0.628099
chr15	67663100	0.743802
chr15	67689000	0.652893
chr15	67692200	0.760331
chr15	67699100	0.933884
chr15	67701700	0.991736
chr15	67802900	0.768595
chr15	67811100	0.826446
chr15	67837500	0.586777
chr15	67842900	0.966942
chr15	68013400	0.710744
chr15	68064100	0.793388
chr15	68072600	0.867769
chr15	68092500	0.595041
chr15	68136800	0.685950
chr15	68145200	0.595041
chr15	68156200	0.719008
chr15	68171900	0.859504

chr15	68174800	0.768595
chr15	68192600	0.644628
chr15	68195300	0.644628
chr15	68207500	0.801653
chr15	68213400	0.611570
chr15	68216200	0.925620
chr15	68238900	0.628099
chr15	68254900	0.851240
chr15	68260900	0.727273
chr15	68343800	0.768595
chr15	68498500	0.966942
chr15	68516400	0.628099
chr15	68526900	0.851240
chr15	68565800	0.752066
chr15	68577000	0.595041
chr15	68708100	0.743802
chr15	68756800	0.595041
chr15	69090900	0.727273
chr15	69094200	0.586777
chr15	69265000	0.909091
chr15	69276900	0.876033
chr15	69283500	0.909091
chr15	69285800	0.619835
chr15	69310800	0.752066
chr15	69385900	0.809917
chr15	69406900	0.685950
chr15	69450900	0.760331
chr15	69455700	0.578512
chr15	69598100	0.595041
chr15	69749500	0.603306
chr15	69753200	0.727273
chr15	69761400	0.595041
chr15	70169000	0.826446
chr15	70379200	0.876033
chr15	70381700	0.793388
chr15	70384700	0.933884
chr15	70394800	0.685950
chr15	70404200	0.603306
chr15	70601000	0.603306
chr15	70732600	0.900826
chr15	70740500	0.900826
chr15	70745000	0.760331
chr15	70752700	0.652893
chr15	70755900	0.925620
chr15	70921100	0.834711
chr15	70925600	0.619835
chr15	72461200	0.636364
chr15	72527900	0.611570
chr15	72531400	0.793388
chr15	72574200	0.586777
chr15	72651500	0.644628
chr15	72764000	0.710744
chr15	73767400	0.933884
chr15	73774600	0.652893

chr15	73777900	0.677686
chr15	73793600	0.578512
chr15	73797400	0.909091
chr15	73857000	0.760331
chr15	73878000	0.628099
chr15	73886400	0.842975
chr15	73889000	0.752066
chr15	73894300	0.710744
chr15	74073700	0.983471
chr15	74282200	0.760331
chr15	74332000	0.578512
chr15	74495100	0.644628
chr15	74506300	0.595041
chr15	74583900	0.801653
chr15	74604600	0.942149
chr15	74607900	0.636364
chr15	74661800	0.752066
chr15	74679400	0.644628
chr15	74682100	0.867769
chr15	74685100	0.900826
chr15	74688900	0.776860
chr15	74713500	0.586777
chr15	74716300	0.735537
chr15	74718700	0.950413
chr15	74721700	0.867769
chr15	74724400	0.636364
chr15	74729400	0.702479
chr15	74735700	0.595041
chr15	74748900	0.628099
chr15	74757200	0.578512
chr15	74782700	0.677686
chr15	74828600	0.595041
chr15	74835900	0.586777
chr15	74838900	0.818182
chr15	74873600	0.818182
chr15	74877500	0.644628
chr15	74903000	1.000000
chr15	74935500	0.892562
chr15	75018600	0.628099
chr15	75072900	0.570248
chr15	75077800	0.752066
chr15	75080500	0.611570
chr15	75136800	0.586777
chr15	75149900	0.644628
chr15	75160800	0.570248
chr15	75179800	0.652893
chr15	75247200	0.752066
chr15	75314300	0.603306
chr15	75322100	0.826446
chr15	75324700	0.611570
chr15	75331600	0.752066
chr15	75336300	0.900826
chr15	75340000	0.892562
chr15	75359400	0.834711

chr15	75404900	0.958678
chr15	75417300	0.975207
chr15	75433000	0.834711
chr15	75455700	0.884298
chr15	75460000	0.628099
chr15	75471000	0.867769
chr15	75492000	0.652893
chr15	75497400	0.702479
chr15	75736900	0.628099
chr15	75835100	0.950413
chr15	75837800	0.619835
chr15	75845700	0.702479
chr15	75858500	0.570248
chr15	75883500	0.611570
chr15	75899900	0.900826
chr15	75920800	0.661157
chr15	75926500	0.851240
chr15	75928600	0.636364
chr15	75942300	0.694215
chr15	75945500	0.694215
chr15	75965500	0.603306
chr15	75987300	0.933884
chr15	76016300	0.768595
chr15	76068700	0.578512
chr15	76109300	0.603306
chr15	76119500	0.694215
chr15	76122900	0.603306
chr15	76125200	0.578512
chr15	76130000	0.595041
chr15	76141500	0.768595
chr15	76157200	0.900826
chr15	76159600	0.644628
chr15	76162500	0.793388
chr15	76192400	0.867769
chr15	76208900	0.603306
chr15	76221800	0.644628
chr15	76258100	0.619835
chr15	76491600	0.842975
chr15	76601800	0.570248
chr15	76605900	0.966942
chr15	76626700	0.570248
chr15	77281400	0.677686
chr15	77357300	0.785124
chr15	77360800	0.702479
chr15	77377900	0.867769
chr15	77479400	0.867769
chr15	77521500	0.603306
chr15	77598500	0.760331
chr15	77628400	0.603306
chr15	77707500	0.818182
chr15	77785900	0.917355
chr15	77836000	0.603306
chr15	77849800	0.677686
chr15	77862000	0.694215

chr15	77892700	0.818182
chr15	77900300	0.801653
chr15	77913700	0.991736
chr15	77916400	0.768595
chr15	77923000	0.702479
chr15	77935100	0.595041
chr15	78025400	0.826446
chr15	78028400	0.570248
chr15	78030600	0.909091
chr15	78040300	0.785124
chr15	78096500	0.669421
chr15	78252200	0.619835
chr15	78328300	0.785124
chr15	78421600	0.735537
chr15	78466300	0.752066
chr15	78504400	0.966942
chr15	78537000	0.760331
chr15	78569100	0.909091
chr15	78571200	0.570248
chr15	78594800	0.702479
chr15	78598500	0.760331
chr15	78600800	0.611570
chr15	78609200	0.578512
chr15	78748000	0.595041
chr15	79032900	0.603306
chr15	79164400	0.586777
chr15	79171700	0.586777
chr15	79194700	0.917355
chr15	79223700	0.942149
chr15	79229300	0.884298
chr15	79297500	0.842975
chr15	79462900	0.669421
chr15	79472100	0.884298
chr15	80146100	0.760331
chr15	80156600	0.619835
chr15	80178800	0.578512
chr15	80226300	0.603306
chr15	80241000	0.900826
chr15	80263700	0.859504
chr15	80270200	0.958678
chr15	80326000	0.586777
chr15	80346500	0.933884
chr15	80386400	0.628099
chr15	80397800	0.636364
chr15	80413100	0.611570
chr15	80444200	0.652893
chr15	80449200	0.809917
chr15	80453000	0.925620
chr15	81195200	0.892562
chr15	81222400	0.752066
chr15	81284400	0.644628
chr15	81290900	0.752066
chr15	81316700	0.909091
chr15	81336700	0.611570

chr15	81362200	0.694215
chr15	81378000	0.900826
chr15	81380100	0.611570
chr15	81384800	0.694215
chr15	81388700	0.983471
chr15	81394800	0.768595
chr15	81400600	0.809917
chr15	81406600	0.710744
chr15	81456900	0.975207
chr15	81459500	0.619835
chr15	81461800	0.636364
chr15	81558700	0.801653
chr15	81624000	0.595041
chr15	82143100	0.611570
chr15	82233600	0.611570
chr15	82237500	0.933884
chr15	82240700	0.958678
chr15	82243600	0.801653
chr15	82250800	0.900826
chr15	82310600	0.801653
chr15	82333400	0.586777
chr15	82368500	0.727273
chr15	82379100	0.719008
chr15	82386300	0.892562
chr15	82402700	0.628099
chr15	82436400	0.661157
chr15	82439500	0.842975
chr15	82502900	0.735537
chr15	82511900	0.595041
chr15	82524500	0.652893
chr15	82559500	0.809917
chr15	82564800	0.760331
chr15	82579800	0.685950
chr15	82655400	0.719008
chr15	82680900	0.933884
chr15	82684200	0.570248
chr15	82691100	0.570248
chr15	82733200	0.735537
chr15	82736800	0.619835
chr15	82748500	0.991736
chr15	82815800	0.884298
chr15	82951600	0.719008
chr15	82991800	0.892562
chr15	83003900	0.710744
chr15	83050500	0.595041
chr15	83057000	0.842975
chr15	83060600	0.867769
chr15	83067500	0.628099
chr15	83113200	0.619835
chr15	83124600	0.793388
chr15	83175400	0.636364
chr15	83200100	0.801653
chr15	83218500	0.917355
chr15	83240000	0.586777

chr15	83283000	0.586777
chr15	83304900	0.884298
chr15	83310200	0.950413
chr15	83312500	0.578512
chr15	83318600	0.892562
chr15	83407500	0.884298
chr15	83468000	0.661157
chr15	83471900	0.809917
chr15	83515600	0.702479
chr15	83518700	0.991736
chr15	83524900	0.801653
chr15	83561700	0.603306
chr15	83669100	0.743802
chr15	83683400	0.727273
chr15	83751900	0.669421
chr15	83755200	0.685950
chr15	83761500	0.975207
chr15	83768500	0.917355
chr15	83777900	0.685950
chr15	83848100	0.867769
chr15	83862400	0.636364
chr15	84131300	0.925620
chr15	84142200	0.727273
chr15	84166600	0.570248
chr15	84173400	0.570248
chr15	84189100	0.644628
chr15	84227900	0.578512
chr15	84781100	0.644628
chr15	84837500	0.909091
chr15	84840600	0.760331
chr15	84843400	0.578512
chr15	84852100	0.818182
chr15	84892500	0.570248
chr15	84915900	0.677686
chr15	84965900	0.884298
chr15	84975200	0.570248
chr15	85045400	0.586777
chr15	85078200	0.710744
chr15	85087600	0.685950
chr15	85091600	0.867769
chr15	85122000	0.578512
chr15	85142100	0.603306
chr15	85153100	0.611570
chr15	85160700	0.933884
chr15	85168600	0.851240
chr15	85173400	0.595041
chr15	85256600	0.611570
chr15	85268800	0.933884
chr15	85277800	0.669421
chr15	85328200	0.661157
chr15	85365300	0.661157
chr15	85373100	0.710744
chr15	85376700	0.595041
chr15	85456000	0.867769

chr15	85458600	1.000000
chr15	85473300	0.958678
chr15	85478800	0.966942
chr15	85492000	0.785124
chr15	85495900	0.925620
chr15	85504000	0.892562
chr15	85536100	0.851240
chr15	85544000	0.636364
chr15	85546600	0.619835
chr15	85551900	0.983471
chr15	85554200	0.876033
chr15	85567600	0.785124
chr15	85579900	0.595041
chr15	85591100	0.917355
chr15	85640500	0.768595
chr15	85661400	0.636364
chr15	85723000	0.570248
chr15	85731400	0.809917
chr15	85771600	0.694215
chr15	85795300	0.570248
chr15	88128400	0.570248
chr15	88152300	0.809917
chr15	88154700	0.801653
chr15	88162100	0.942149
chr15	88170400	0.818182
chr15	88175900	0.933884
chr15	88197300	0.644628
chr15	88204700	0.735537
chr15	88212700	0.892562
chr15	88215700	0.884298
chr15	88225100	0.925620
chr15	88275400	0.735537
chr15	88288300	0.876033
chr15	88291100	0.570248
chr15	88296900	0.776860
chr15	88306000	0.652893
chr15	88420900	0.636364
chr15	88769000	0.685950
chr15	89080700	0.991736
chr15	89084500	0.710744
chr15	89088400	0.595041
chr15	89107100	0.859504
chr15	89115500	0.636364
chr15	89163800	0.669421
chr15	89167600	0.909091
chr15	89171000	0.768595
chr15	89182200	0.942149
chr15	89408400	0.884298
chr15	89410500	0.669421
chr15	89420000	0.826446
chr15	89440700	0.776860
chr15	89448400	0.685950
chr15	89453600	0.727273
chr15	89461400	0.933884

chr15	89463900	0.933884
chr15	89473000	0.578512
chr15	89482600	0.933884
chr15	89516700	0.669421
chr15	89525700	0.925620
chr15	89528500	0.710744
chr15	89532300	0.834711
chr15	89545900	0.933884
chr15	89602900	0.644628
chr15	89612000	0.743802
chr15	89614700	0.983471
chr15	89617400	0.785124
chr15	89621500	0.983471
chr15	89629100	0.628099
chr15	89637100	0.785124
chr15	89640200	0.644628
chr15	89651800	0.702479
chr15	89710700	0.752066
chr15	89770200	0.892562
chr15	89774000	0.578512
chr15	89849100	0.603306
chr15	89911000	0.652893
chr15	90121900	0.644628
chr15	90124900	0.776860
chr15	90304200	0.727273
chr15	90325200	0.909091
chr15	90327300	0.652893
chr15	90364700	0.826446
chr15	90439000	0.578512
chr15	90454400	0.776860
chr15	90490500	0.950413
chr15	90544300	0.603306
chr15	90548200	0.636364
chr15	90578600	0.661157
chr15	90581800	0.867769
chr15	90584300	0.801653
chr15	90602800	0.933884
chr15	90605600	0.983471
chr15	90607800	0.595041
chr15	90642100	0.950413
chr15	90644400	0.586777
chr15	90685900	0.867769
chr15	90692500	0.876033
chr15	90696000	0.644628
chr15	90701600	0.900826
chr15	90704600	0.636364
chr15	90709400	0.884298
chr15	90719000	0.652893
chr15	90725900	0.652893
chr15	90730500	0.809917
chr15	90734800	0.752066
chr15	90742500	0.752066
chr15	90755000	0.595041
chr15	90757400	0.842975

chr15	90783000	0.818182
chr15	90785500	0.818182
chr15	90792900	0.710744
chr15	90824000	0.785124
chr15	90845000	0.719008
chr15	90850300	0.570248
chr15	90863900	0.595041
chr15	90873600	0.661157
chr15	90906200	0.809917
chr15	90908700	0.644628
chr15	90944200	0.760331
chr15	90956900	0.809917
chr15	91084200	0.735537
chr15	91101000	0.884298
chr15	91114400	0.842975
chr15	91120400	0.768595
chr15	91130500	0.603306
chr15	91141100	0.917355
chr15	91161400	0.809917
chr15	91164100	0.958678
chr15	91191900	0.859504
chr15	91194600	0.719008
chr15	91200900	0.867769
chr15	91204100	0.900826
chr15	91210300	0.644628
chr15	91227200	0.628099
chr15	91229800	0.892562
chr15	91285800	0.710744
chr15	91367900	0.595041
chr15	91378100	0.884298
chr15	91380400	0.917355
chr15	91383700	0.983471
chr15	91385800	0.966942
chr15	91389000	0.958678
chr15	91400200	0.801653
chr15	91410600	0.818182
chr15	91429700	0.685950
chr15	91439200	0.685950
chr15	91443700	0.652893
chr15	91449500	0.719008
chr15	91459500	0.909091
chr15	91466500	0.842975
chr15	91476900	0.611570
chr15	91490700	0.892562
chr15	91493600	0.966942
chr15	91532000	0.628099
chr15	91534900	0.735537
chr15	91547700	0.595041
chr15	91550700	0.595041
chr15	91561600	0.752066
chr15	91576600	0.743802
chr15	91591000	0.652893
chr15	91593300	0.644628
chr15	92013600	0.975207

chr15	92015800	0.611570
chr15	92020200	0.603306
chr15	92022600	0.768595
chr15	92080300	0.933884
chr15	92212100	0.760331
chr15	92490500	0.595041
chr15	92973100	0.628099
chr15	93161300	0.727273
chr15	93188100	0.570248
chr15	93341100	0.719008
chr15	93345000	0.851240
chr15	93348400	0.826446
chr15	93356900	0.578512
chr15	93360600	0.842975
chr15	93365900	0.570248
chr15	93392500	0.917355
chr15	93413800	0.818182
chr15	93420700	0.950413
chr15	93423500	0.694215
chr15	93452800	0.595041
chr15	93461100	0.702479
chr15	93465200	0.719008
chr15	93580200	0.595041
chr15	95294300	0.884298
chr15	95398000	0.685950
chr15	95435100	0.578512
chr15	95456400	0.727273
chr15	95712700	0.710744
chr15	95714900	0.685950
chr15	95720000	0.644628
chr15	95803700	0.603306
chr15	96101000	0.917355
chr15	96103600	0.694215
chr15	96178900	0.702479
chr15	96184900	0.619835
chr15	96189400	0.834711
chr15	96228300	0.958678
chr15	96241500	0.727273
chr15	96252600	0.752066
chr15	96254700	0.900826
chr15	96259000	0.710744
chr15	96269600	0.570248
chr15	96278000	0.586777
chr15	96386800	0.727273
chr15	96462900	0.669421
chr15	96465200	0.570248
chr15	96469800	0.611570
chr15	96472200	0.818182
chr15	96475100	0.719008
chr15	96496700	0.776860
chr15	96515900	0.818182
chr15	96586500	0.975207
chr15	96590900	0.776860
chr15	96595700	0.619835

chr15	96663100	0.636364
chr15	96666400	0.743802
chr15	96669200	0.760331
chr15	96674100	0.570248
chr15	96705800	0.950413
chr15	96808800	0.801653
chr15	96816000	0.859504
chr15	96818600	0.842975
chr15	96834100	0.644628
chr15	96860900	0.867769
chr15	96891000	0.619835
chr15	96898100	0.776860
chr15	96965700	0.735537
chr15	97068800	0.669421
chr15	98522500	0.801653
chr15	98620100	0.900826
chr15	99320500	0.669421
chr15	99526800	0.735537
chr15	99664400	0.570248
chr15	99851600	0.595041
chr15	99974100	0.661157
chr15	100119400	0.628099
chr15	100122700	0.677686
chr15	100154600	0.586777
chr15	100243900	0.586777
chr15	100249300	0.619835
chr15	100279100	0.876033
chr15	100293600	0.619835
chr15	100349000	0.727273
chr15	100526700	0.595041
chr15	100775500	0.983471
chr15	100782000	0.793388
chr15	101138900	0.867769
chr15	101648600	0.818182
chr15	101653800	0.652893
chr15	101678100	0.917355
chr15	101689800	0.876033
chr15	101708100	0.867769
chr15	101796100	0.966942
chr15	101987100	0.975207
chr15	101991400	0.727273
chr15	101996300	1.000000
chr15	102010300	0.743802
chr15	102018500	0.917355
chr15	102021700	0.586777
chr15	102154300	0.578512
chr15	102156700	0.619835
chr15	102198900	0.619835
chr15	102265500	0.586777
chr15	102270800	0.710744
chr15	102274100	0.776860
chr15	102284200	0.628099
chr15	102416800	0.983471
chr15	102446600	0.785124

chr15	102453300	0.636364
chr15	102463200	0.785124
chr15	102489000	0.694215
chr15	102491600	0.950413
chr15	102495500	0.768595
chr15	102507700	0.677686
chr16	64300	0.776860
chr16	85300	0.628099
chr16	92500	0.809917
chr16	145400	0.652893
chr16	155200	0.983471
chr16	157900	0.851240
chr16	162700	0.603306
chr16	166400	0.669421
chr16	168800	0.809917
chr16	171400	0.892562
chr16	174100	0.909091
chr16	179900	0.768595
chr16	182200	0.867769
chr16	184600	0.578512
chr16	192400	0.644628
chr16	194900	1.000000
chr16	199500	0.578512
chr16	213500	0.603306
chr16	290300	0.793388
chr16	292600	0.578512
chr16	295500	0.603306
chr16	303000	0.636364
chr16	309700	0.975207
chr16	374600	0.892562
chr16	382000	0.851240
chr16	385300	0.950413
chr16	390500	0.776860
chr16	392900	0.603306
chr16	415000	0.603306
chr16	421400	0.884298
chr16	436900	0.586777
chr16	439200	0.809917
chr16	450100	0.586777
chr16	455900	0.851240
chr16	507400	0.776860
chr16	533700	0.611570
chr16	580300	0.578512
chr16	585400	0.636364
chr16	616900	0.619835
chr16	672500	0.669421
chr16	676900	0.834711
chr16	680500	0.834711
chr16	688600	0.760331
chr16	725500	0.719008
chr16	729100	0.603306
chr16	788000	0.909091
chr16	805000	0.743802
chr16	1095000	0.595041

chr16	1243900	0.628099
chr16	1347500	0.801653
chr16	1350000	0.619835
chr16	1382000	0.851240
chr16	1521000	0.884298
chr16	1523200	0.636364
chr16	1533900	0.809917
chr16	1542300	0.578512
chr16	1593500	0.966942
chr16	1696300	0.909091
chr16	1731400	0.702479
chr16	1759400	0.661157
chr16	1773700	0.570248
chr16	1813900	0.909091
chr16	1883700	0.760331
chr16	1920800	0.735537
chr16	1928400	0.628099
chr16	1971400	0.917355
chr16	1973700	0.595041
chr16	1985800	0.743802
chr16	2054500	0.793388
chr16	2078700	0.603306
chr16	2083700	0.958678
chr16	2090400	0.595041
chr16	2177200	0.595041
chr16	2193900	0.818182
chr16	2261300	0.619835
chr16	2268000	0.719008
chr16	2276700	0.611570
chr16	2302700	0.851240
chr16	2347200	0.884298
chr16	2384000	0.595041
chr16	2393500	0.628099
chr16	2415100	0.586777
chr16	2462700	0.570248
chr16	2530000	0.942149
chr16	2535200	0.900826
chr16	2544800	0.743802
chr16	2547700	0.909091
chr16	2562200	0.983471
chr16	2567400	0.933884
chr16	2652000	0.603306
chr16	2714000	0.760331
chr16	2721500	0.702479
chr16	2726300	0.826446
chr16	2788900	0.842975
chr16	2806100	0.669421
chr16	2821000	0.677686
chr16	2862100	0.595041
chr16	2924800	0.669421
chr16	2965900	0.917355
chr16	2977400	0.661157
chr16	3018400	0.570248
chr16	3033500	0.586777

chr16	3066100	0.760331
chr16	3068500	0.842975
chr16	3120400	0.760331
chr16	3135200	0.793388
chr16	3137500	0.578512
chr16	3151100	0.570248
chr16	3213800	0.727273
chr16	3216100	0.685950
chr16	3222100	0.578512
chr16	3399100	0.917355
chr16	3438700	0.917355
chr16	3441000	0.586777
chr16	3448400	0.677686
chr16	3511400	0.669421
chr16	3524600	0.793388
chr16	3640100	0.826446
chr16	3664900	0.842975
chr16	3668600	0.884298
chr16	3693100	0.867769
chr16	3696500	0.966942
chr16	3705000	0.826446
chr16	3741300	0.603306
chr16	3746900	0.867769
chr16	3763900	0.842975
chr16	3843700	0.752066
chr16	3848400	0.603306
chr16	3854900	0.933884
chr16	3870400	0.619835
chr16	3873700	0.578512
chr16	3892700	0.603306
chr16	3896900	0.743802
chr16	3904600	0.917355
chr16	3912500	0.867769
chr16	3914900	0.570248
chr16	3921600	0.661157
chr16	3924100	0.966942
chr16	4230400	0.595041
chr16	4258600	0.809917
chr16	4294500	0.950413
chr16	4297300	0.859504
chr16	4300000	0.867769
chr16	4314100	0.760331
chr16	4338800	0.743802
chr16	4349900	0.826446
chr16	4398400	0.909091
chr16	4420800	0.818182
chr16	4424500	0.685950
chr16	4478800	0.661157
chr16	4485900	0.867769
chr16	4573400	0.876033
chr16	4577300	0.818182
chr16	4668500	1.000000
chr16	4775300	0.776860
chr16	4838600	0.710744

chr16	4855000	0.966942
chr16	4902400	0.909091
chr16	4932900	0.619835
chr16	4936700	0.661157
chr16	5030200	0.595041
chr16	5085800	0.586777
chr16	5161400	0.628099
chr16	5666200	0.570248
chr16	8042100	0.809917
chr16	8138600	0.776860
chr16	8149600	0.644628
chr16	8223600	0.603306
chr16	8230100	0.685950
chr16	8233200	0.776860
chr16	8725700	0.644628
chr16	8956500	0.760331
chr16	8958600	0.801653
chr16	8965100	0.884298
chr16	9044800	0.793388
chr16	9052000	0.611570
chr16	9054300	0.867769
chr16	9061300	0.661157
chr16	9142100	0.925620
chr16	9147200	0.685950
chr16	9164100	0.892562
chr16	9167100	0.917355
chr16	9170200	0.991736
chr16	9173000	0.801653
chr16	9175100	0.884298
chr16	9197800	0.834711
chr16	9201500	0.702479
chr16	9220300	0.752066
chr16	9691400	0.752066
chr16	9693900	0.851240
chr16	9800300	0.669421
chr16	10403400	0.859504
chr16	10407500	0.768595
chr16	10413000	0.876033
chr16	10416400	0.966942
chr16	10420000	0.760331
chr16	10434200	0.876033
chr16	10437500	0.917355
chr16	10451500	0.942149
chr16	10455500	0.834711
chr16	10457600	0.975207
chr16	10476600	0.884298
chr16	10488300	0.727273
chr16	10491300	0.809917
chr16	10499300	0.818182
chr16	10591400	0.942149
chr16	10595300	0.925620
chr16	10713700	0.859504
chr16	10716600	0.702479
chr16	10720200	0.702479

chr16	10815700	0.710744
chr16	10832400	0.917355
chr16	10835900	0.801653
chr16	10981600	0.578512
chr16	11029900	0.685950
chr16	11033500	0.619835
chr16	11041200	0.876033
chr16	11060100	0.727273
chr16	11066500	0.991736
chr16	11085700	0.743802
chr16	11087800	0.760331
chr16	11146000	0.570248
chr16	11150800	0.909091
chr16	11167100	0.636364
chr16	11178500	0.752066
chr16	11185600	0.818182
chr16	11188700	0.776860
chr16	11194100	0.876033
chr16	11196400	0.859504
chr16	11207300	0.892562
chr16	11216400	0.570248
chr16	11220500	0.628099
chr16	11223100	0.975207
chr16	11227400	0.933884
chr16	11246500	0.809917
chr16	11255200	0.809917
chr16	11259400	0.842975
chr16	11293000	0.694215
chr16	11295900	0.975207
chr16	11318800	0.892562
chr16	11326600	0.966942
chr16	11329400	0.793388
chr16	11337300	0.719008
chr16	11352600	0.677686
chr16	11391500	0.644628
chr16	11406400	0.595041
chr16	11456200	0.595041
chr16	11459300	0.603306
chr16	11473700	0.669421
chr16	11587300	0.578512
chr16	11589700	0.685950
chr16	11707500	0.851240
chr16	11710100	0.644628
chr16	11718900	0.909091
chr16	11722200	0.876033
chr16	11800200	0.851240
chr16	11828700	0.933884
chr16	11877300	0.876033
chr16	11895500	0.661157
chr16	11942800	0.578512
chr16	12005100	0.752066
chr16	12058700	0.603306
chr16	12122100	0.628099
chr16	12130900	0.801653

chr16	12142400	0.991736
chr16	12153800	0.603306
chr16	12183700	0.578512
chr16	12186200	0.636364
chr16	12192300	0.595041
chr16	12200400	0.586777
chr16	12238300	0.958678
chr16	12472000	0.586777
chr16	12481500	0.818182
chr16	12514600	0.768595
chr16	12633100	0.652893
chr16	12678800	0.719008
chr16	12704700	0.793388
chr16	12708000	0.917355
chr16	12741200	0.595041
chr16	12770300	0.661157
chr16	12776900	0.603306
chr16	12852700	0.694215
chr16	12861800	0.578512
chr16	12867600	0.611570
chr16	12887300	0.876033
chr16	12890900	0.867769
chr16	12895400	0.652893
chr16	14052100	0.570248
chr16	14093800	0.859504
chr16	14172000	0.793388
chr16	14221900	0.900826
chr16	14262900	0.578512
chr16	14401700	0.768595
chr16	14404400	0.628099
chr16	14458800	0.702479
chr16	14496700	0.768595
chr16	14579800	0.826446
chr16	14583900	0.876033
chr16	14597200	0.983471
chr16	14619200	0.578512
chr16	14622600	0.933884
chr16	14728900	0.578512
chr16	14752200	0.892562
chr16	14918900	0.677686
chr16	14942400	0.768595
chr16	14954700	0.719008
chr16	15006700	0.595041
chr16	15051200	0.611570
chr16	15065900	0.586777
chr16	15076600	0.570248
chr16	15110700	0.884298
chr16	15135700	0.785124
chr16	15241900	0.595041
chr16	15247600	0.611570
chr16	15263300	0.867769
chr16	15347500	0.570248
chr16	15391900	0.950413
chr16	15397700	0.570248

chr16	15583400	0.884298
chr16	15585800	0.586777
chr16	15596400	0.628099
chr16	15604900	0.652893
chr16	15612600	0.619835
chr16	15617700	0.933884
chr16	15640600	0.669421
chr16	15657600	0.628099
chr16	15669500	0.644628
chr16	15684400	0.884298
chr16	15687400	0.966942
chr16	15754800	0.818182
chr16	15902000	0.636364
chr16	15973400	0.966942
chr16	15975700	0.900826
chr16	15979300	0.785124
chr16	15992300	0.834711
chr16	16034100	0.760331
chr16	16037400	0.644628
chr16	16046400	0.851240
chr16	16049100	0.702479
chr16	16081600	0.685950
chr16	16085100	0.611570
chr16	16092100	0.917355
chr16	16101800	0.826446
chr16	16111800	0.785124
chr16	16114900	0.752066
chr16	16117100	0.603306
chr16	16120100	0.909091
chr16	16214900	0.983471
chr16	16284100	0.793388
chr16	16328200	0.595041
chr16	16341000	0.735537
chr16	16552900	0.975207
chr16	16597100	0.644628
chr16	16682100	0.644628
chr16	16684700	0.892562
chr16	16874900	0.578512
chr16	16923100	0.884298
chr16	17002900	0.603306
chr16	17023000	0.760331
chr16	17034500	0.586777
chr16	17147900	0.595041
chr16	17176500	0.958678
chr16	18039300	0.677686
chr16	18043800	0.619835
chr16	18312000	0.710744
chr16	18347600	0.925620
chr16	18547400	0.876033
chr16	18559500	0.876033
chr16	18772100	0.619835
chr16	18774900	0.818182
chr16	18797500	0.785124
chr16	18816200	0.942149

chr16	18881000	0.876033
chr16	18922900	0.710744
chr16	18928600	0.669421
chr16	18939700	0.603306
chr16	18996000	0.611570
chr16	19098100	0.776860
chr16	19106200	0.578512
chr16	19130000	0.685950
chr16	19142600	0.826446
chr16	19148000	0.677686
chr16	19343100	0.900826
chr16	19405500	0.719008
chr16	19407800	0.925620
chr16	19429600	0.809917
chr16	19470300	0.578512
chr16	19492800	0.851240
chr16	19497800	0.851240
chr16	19529100	0.892562
chr16	19720100	0.570248
chr16	19722300	0.991736
chr16	19732100	0.578512
chr16	19735500	0.942149
chr16	19740100	0.586777
chr16	19758000	0.636364
chr16	19854800	0.776860
chr16	20096500	0.809917
chr16	20314900	0.719008
chr16	20496200	0.859504
chr16	20684400	0.776860
chr16	20688800	0.578512
chr16	20690900	0.570248
chr16	20693500	0.983471
chr16	20756000	0.991736
chr16	20758600	0.991736
chr16	20761400	0.950413
chr16	20771500	0.859504
chr16	20779500	0.752066
chr16	20815400	0.818182
chr16	20837800	0.966942
chr16	20841300	0.603306
chr16	20877600	0.834711
chr16	20883800	0.685950
chr16	20916100	0.917355
chr16	20927600	0.876033
chr16	20930400	0.867769
chr16	20956900	0.958678
chr16	21035300	0.619835
chr16	21200400	0.685950
chr16	21246900	0.727273
chr16	21251100	0.933884
chr16	21272700	0.933884
chr16	21275600	0.826446
chr16	21289300	0.669421
chr16	21382800	0.834711

chr16	21520800	0.975207
chr16	21523100	0.710744
chr16	21525600	0.900826
chr16	21533000	0.818182
chr16	21536300	0.818182
chr16	21538600	0.818182
chr16	21546800	0.595041
chr16	21550100	0.983471
chr16	21561900	0.677686
chr16	21566400	0.595041
chr16	21620100	0.719008
chr16	21623200	0.768595
chr16	21625900	0.611570
chr16	21655600	0.702479
chr16	22006500	0.884298
chr16	22043100	0.793388
chr16	22050200	0.818182
chr16	22057900	0.669421
chr16	22066800	0.578512
chr16	22228000	0.925620
chr16	22232100	0.628099
chr16	22293800	0.950413
chr16	22298200	0.760331
chr16	22303600	0.909091
chr16	22311800	0.570248
chr16	22324300	0.694215
chr16	22365100	0.876033
chr16	22373000	0.768595
chr16	22399900	0.628099
chr16	22618300	0.776860
chr16	23092500	0.752066
chr16	23144700	0.586777
chr16	23153700	0.900826
chr16	23280800	0.743802
chr16	23448300	0.933884
chr16	23509300	0.735537
chr16	23517100	0.702479
chr16	23523500	0.595041
chr16	23530400	0.570248
chr16	23550400	0.867769
chr16	23609200	0.735537
chr16	23678800	0.950413
chr16	23712200	0.900826
chr16	23739100	0.685950
chr16	23741600	0.768595
chr16	23796600	0.933884
chr16	23814900	0.785124
chr16	23818700	0.586777
chr16	23821000	0.669421
chr16	23831000	0.661157
chr16	23834900	0.983471
chr16	23838800	0.884298
chr16	23845100	0.876033
chr16	23851700	0.760331

chr16	23854000	0.719008
chr16	23859200	0.793388
chr16	23864700	0.909091
chr16	23866900	0.586777
chr16	23871000	0.710744
chr16	23878000	0.603306
chr16	23882800	0.586777
chr16	23891600	0.760331
chr16	23911100	0.958678
chr16	23915100	0.851240
chr16	23918200	0.859504
chr16	23921300	0.975207
chr16	23954300	0.570248
chr16	23961500	0.867769
chr16	23963600	0.768595
chr16	23965800	0.661157
chr16	23971000	0.801653
chr16	23975900	0.628099
chr16	23978400	0.909091
chr16	24014000	0.933884
chr16	24020500	0.644628
chr16	24068900	0.578512
chr16	24087500	0.892562
chr16	24128000	0.917355
chr16	24147600	0.909091
chr16	24151200	0.595041
chr16	24228700	0.884298
chr16	24231400	0.685950
chr16	24475100	0.727273
chr16	24506700	0.595041
chr16	24518200	0.628099
chr16	24548700	0.685950
chr16	24749000	0.966942
chr16	24752500	0.900826
chr16	24756600	0.636364
chr16	24759500	0.892562
chr16	24840700	0.752066
chr16	24850400	0.826446
chr16	24855900	0.900826
chr16	24858400	0.768595
chr16	24895500	0.628099
chr16	24997700	0.727273
chr16	25000700	0.809917
chr16	25003500	0.694215
chr16	25006400	0.661157
chr16	25011600	0.636364
chr16	25047900	0.595041
chr16	25058000	0.975207
chr16	25061500	0.644628
chr16	25063800	0.628099
chr16	25075500	0.876033
chr16	25091800	0.909091
chr16	25093900	0.628099
chr16	25222200	0.702479

chr16	25225200	0.702479
chr16	25228700	0.809917
chr16	25272200	0.578512
chr16	25281300	0.876033
chr16	25565200	0.702479
chr16	25567600	0.735537
chr16	25782800	0.768595
chr16	25787800	0.644628
chr16	25850000	0.595041
chr16	26829100	0.595041
chr16	27210500	1.000000
chr16	27247100	0.859504
chr16	27257700	0.694215
chr16	27482500	0.950413
chr16	27550600	0.793388
chr16	27559000	0.867769
chr16	27578100	0.867769
chr16	27767800	0.909091
chr16	27771900	0.826446
chr16	28094100	0.578512
chr16	28100700	0.595041
chr16	28122100	0.851240
chr16	28136400	0.793388
chr16	28154200	0.570248
chr16	28175500	0.619835
chr16	28194900	0.735537
chr16	28219600	0.578512
chr16	28437600	0.652893
chr16	28459000	0.826446
chr16	28468200	0.595041
chr16	28505900	0.900826
chr16	28573500	0.636364
chr16	28608800	0.958678
chr16	28621200	0.909091
chr16	28678900	0.909091
chr16	28762200	0.603306
chr16	28793400	0.876033
chr16	28832000	0.719008
chr16	28949800	0.611570
chr16	28957200	0.694215
chr16	29020200	0.867769
chr16	29023200	0.909091
chr16	29040800	0.933884
chr16	29073300	0.661157
chr16	29127200	0.685950
chr16	29130300	0.801653
chr16	29164500	0.876033
chr16	29190100	0.752066
chr16	29194800	0.884298
chr16	29273700	0.983471
chr16	29277100	0.685950
chr16	29295900	0.628099
chr16	29302600	0.768595
chr16	29312000	0.570248

chr16	29361900	0.859504
chr16	29364200	0.578512
chr16	29383200	0.595041
chr16	29448900	0.801653
chr16	29471900	0.752066
chr16	29481300	0.661157
chr16	29550000	0.876033
chr16	29592200	0.809917
chr16	29607900	0.628099
chr16	29613900	0.909091
chr16	29616900	0.900826
chr16	29620800	0.644628
chr16	29626600	0.685950
chr16	29632700	0.570248
chr16	29636000	1.000000
chr16	29647800	0.950413
chr16	29657300	0.876033
chr16	29666400	0.950413
chr16	29670300	0.570248
chr16	29672900	0.809917
chr16	29685400	0.851240
chr16	29687600	0.661157
chr16	29701800	0.611570
chr16	29722100	0.752066
chr16	29724400	0.809917
chr16	29795200	0.644628
chr16	29832100	0.661157
chr16	29836800	0.595041
chr16	29955900	0.752066
chr16	29984500	0.578512
chr16	29987800	0.719008
chr16	30063000	0.719008
chr16	30066400	0.793388
chr16	30082300	0.710744
chr16	30086100	0.652893
chr16	30089700	0.719008
chr16	30102000	0.611570
chr16	30106300	0.685950
chr16	30119900	0.578512
chr16	30124800	0.958678
chr16	30151500	0.752066
chr16	30196900	0.603306
chr16	30199100	0.619835
chr16	30211100	0.917355
chr16	30290500	0.677686
chr16	30332400	0.611570
chr16	30393000	0.958678
chr16	30503300	0.966942
chr16	30536300	0.644628
chr16	30540600	0.776860
chr16	30545100	0.586777
chr16	30551100	0.719008
chr16	30553700	0.801653
chr16	30560400	0.851240

chr16	30565000	0.727273
chr16	30578000	0.570248
chr16	30586500	0.809917
chr16	30601800	0.884298
chr16	30635100	0.570248
chr16	30640600	0.727273
chr16	30703900	0.925620
chr16	30706400	0.595041
chr16	30751800	0.595041
chr16	30775600	0.661157
chr16	30790000	0.876033
chr16	30820600	0.727273
chr16	30849700	0.809917
chr16	30856600	0.909091
chr16	30866200	0.578512
chr16	30915200	0.867769
chr16	30953900	0.942149
chr16	30964900	0.785124
chr16	30985500	0.595041
chr16	31004700	0.942149
chr16	31029700	0.793388
chr16	31034200	0.578512
chr16	31107500	0.586777
chr16	31112100	0.768595
chr16	31117900	0.628099
chr16	31140500	0.966942
chr16	31146900	0.909091
chr16	31195200	0.719008
chr16	31227400	0.570248
chr16	31241500	0.917355
chr16	31260500	0.578512
chr16	31265300	1.000000
chr16	31271600	0.595041
chr16	31463600	0.818182
chr16	31467200	0.570248
chr16	31485500	0.603306
chr16	31490000	0.636364
chr16	31535400	0.834711
chr16	31538900	0.768595
chr16	31541000	0.685950
chr16	31547600	0.785124
chr16	31889000	0.818182
chr16	31893600	0.628099
chr16	32194600	0.644628
chr16	32236000	0.925620
chr16	32238500	0.933884
chr16	32252400	0.735537
chr16	32261900	0.892562
chr16	32268900	0.570248
chr16	32690500	0.983471
chr16	32694600	0.578512
chr16	32714000	0.983471
chr16	32716400	0.876033
chr16	32757500	0.801653

chr16	33136100	0.876033
chr16	33179500	0.975207
chr16	33202600	0.793388
chr16	33210400	0.710744
chr16	33259200	0.619835
chr16	33266700	0.752066
chr16	33949900	0.719008
chr16	33953000	0.661157
chr16	33955400	0.892562
chr16	33959000	0.710744
chr16	33963300	0.694215
chr16	46430900	0.677686
chr16	46617600	0.694215
chr16	46726100	0.628099
chr16	46774600	0.925620
chr16	46782400	0.884298
chr16	46792100	0.595041
chr16	46856700	0.685950
chr16	46916600	0.611570
chr16	46953500	0.752066
chr16	46977500	0.685950
chr16	47422800	0.859504
chr16	47426400	0.752066
chr16	47492100	0.859504
chr16	47503200	0.735537
chr16	47527200	0.818182
chr16	47553900	0.628099
chr16	47599600	0.570248
chr16	47612800	0.818182
chr16	47658200	0.826446
chr16	47660900	0.925620
chr16	48157400	0.900826
chr16	48334200	0.628099
chr16	48401900	0.785124
chr16	48408900	0.900826
chr16	48413400	0.752066
chr16	48428100	0.661157
chr16	48444200	0.644628
chr16	48637700	0.578512
chr16	48647300	0.809917
chr16	48651500	0.570248
chr16	49652100	0.578512
chr16	49654500	0.628099
chr16	49918400	0.785124
chr16	49920700	0.619835
chr16	50105100	0.719008
chr16	50181100	0.760331
chr16	50195100	0.603306
chr16	50199900	0.710744
chr16	50262900	0.933884
chr16	50287500	0.867769
chr16	50293600	0.677686
chr16	50295700	0.925620
chr16	50299300	0.809917

chr16	50303200	0.619835
chr16	50305400	0.884298
chr16	50308200	0.983471
chr16	50313400	0.851240
chr16	50316500	0.826446
chr16	50357900	1.000000
chr16	50368700	0.966942
chr16	50373400	0.785124
chr16	50403800	0.661157
chr16	50731400	0.570248
chr16	50875300	0.752066
chr16	53087500	0.619835
chr16	53092500	0.867769
chr16	53095800	0.685950
chr16	53100000	0.570248
chr16	53113600	0.958678
chr16	53117100	0.727273
chr16	53120000	0.859504
chr16	53128200	0.950413
chr16	53146400	0.942149
chr16	53250900	0.809917
chr16	53253900	0.859504
chr16	53258800	0.859504
chr16	53553100	0.652893
chr16	53899700	0.876033
chr16	55411000	0.685950
chr16	55469400	0.694215
chr16	55605100	0.595041
chr16	55607300	0.900826
chr16	55787300	0.925620
chr16	55870400	0.595041
chr16	56280700	0.685950
chr16	56296200	0.586777
chr16	56298900	0.950413
chr16	56309600	0.685950
chr16	56312700	0.909091
chr16	56341400	0.570248
chr16	56390700	0.834711
chr16	56448900	0.859504
chr16	56496500	0.876033
chr16	56511800	0.842975
chr16	56516400	0.793388
chr16	56640800	0.677686
chr16	56710500	0.636364
chr16	56776400	0.570248
chr16	56778900	0.685950
chr16	56869900	0.735537
chr16	56999300	0.752066
chr16	57037900	0.586777
chr16	57096400	0.950413
chr16	57099500	0.768595
chr16	57123800	0.578512
chr16	57157800	0.578512
chr16	57179900	0.966942

chr16	57190800	0.578512
chr16	57294600	0.851240
chr16	57300500	0.652893
chr16	57303100	0.933884
chr16	57324300	0.570248
chr16	57331800	0.727273
chr16	57510600	0.834711
chr16	57514300	0.752066
chr16	57578600	0.909091
chr16	57636000	0.851240
chr16	57702000	0.859504
chr16	57705600	0.578512
chr16	57710500	0.917355
chr16	57717100	0.793388
chr16	57723800	0.917355
chr16	57730000	0.719008
chr16	57735300	0.900826
chr16	57746600	0.719008
chr16	57755500	0.685950
chr16	57776800	0.685950
chr16	57799000	0.760331
chr16	57833100	0.685950
chr16	57842800	0.752066
chr16	57845700	0.570248
chr16	57850300	0.619835
chr16	57893400	0.644628
chr16	57926800	0.578512
chr16	58011400	0.983471
chr16	58016100	0.669421
chr16	58028900	0.570248
chr16	58033700	0.570248
chr16	58058500	0.595041
chr16	58067200	0.710744
chr16	58069500	0.595041
chr16	58155100	0.644628
chr16	58158800	0.636364
chr16	58213500	0.619835
chr16	58278200	0.867769
chr16	58295400	0.884298
chr16	58299000	0.652893
chr16	58318500	0.694215
chr16	58455400	0.578512
chr16	58468100	0.595041
chr16	58490700	0.570248
chr16	58493400	0.661157
chr16	58522700	0.603306
chr16	58692800	0.636364
chr16	58790400	0.826446
chr16	58826200	0.595041
chr16	58828300	0.628099
chr16	58884700	0.925620
chr16	58890200	0.603306
chr16	58894200	0.892562
chr16	59391200	0.818182

chr16	65706900	0.578512
chr16	65804100	0.900826
chr16	65844000	0.586777
chr16	65850400	0.570248
chr16	66009300	0.909091
chr16	66361300	0.595041
chr16	66546200	0.710744
chr16	66556300	0.570248
chr16	66559700	0.570248
chr16	66588700	0.570248
chr16	66608900	0.743802
chr16	66642900	0.867769
chr16	66651400	0.743802
chr16	66654300	0.942149
chr16	66657100	0.586777
chr16	66666500	0.909091
chr16	66685400	0.669421
chr16	66694700	0.933884
chr16	66722400	0.677686
chr16	66775300	0.950413
chr16	66921000	0.702479
chr16	66935000	0.628099
chr16	66939800	0.785124
chr16	66971100	0.628099
chr16	66988100	0.694215
chr16	67013100	0.661157
chr16	67048200	0.603306
chr16	67090300	0.776860
chr16	67146800	0.909091
chr16	67189600	0.578512
chr16	67199000	0.768595
chr16	67205800	0.652893
chr16	67219700	0.677686
chr16	67224600	0.735537
chr16	67229000	0.644628
chr16	67241700	0.611570
chr16	67251300	0.917355
chr16	67270700	0.636364
chr16	67274300	0.942149
chr16	67278000	0.611570
chr16	67284500	0.570248
chr16	67289100	0.743802
chr16	67302300	1.000000
chr16	67311300	0.685950
chr16	67314300	0.578512
chr16	67346400	0.636364
chr16	67427700	0.595041
chr16	67430600	0.933884
chr16	67494600	0.917355
chr16	67500000	0.578512
chr16	67509300	0.834711
chr16	67538500	0.917355
chr16	67541900	0.793388
chr16	67560700	0.909091

chr16	67629000	0.644628
chr16	67774800	0.834711
chr16	67807600	0.983471
chr16	67815200	0.958678
chr16	67817700	0.694215
chr16	67830700	0.735537
chr16	67834800	0.809917
chr16	67837100	0.826446
chr16	67848500	0.628099
chr16	67889600	0.595041
chr16	67892100	0.925620
chr16	67895300	0.611570
chr16	67919000	0.719008
chr16	67924700	0.851240
chr16	67951700	0.925620
chr16	67956400	0.752066
chr16	67993700	0.983471
chr16	68019000	0.851240
chr16	68022200	0.760331
chr16	68027800	0.702479
chr16	68033200	0.834711
chr16	68069300	0.834711
chr16	68074700	0.586777
chr16	68098800	0.826446
chr16	68106500	0.611570
chr16	68112400	0.669421
chr16	68116500	0.942149
chr16	68127300	0.859504
chr16	68148000	0.760331
chr16	68176200	0.851240
chr16	68281400	0.942149
chr16	68318100	0.661157
chr16	68324100	0.743802
chr16	68347000	0.710744
chr16	68413300	0.842975
chr16	68742100	0.578512
chr16	68758900	0.842975
chr16	68766300	0.661157
chr16	68774600	0.859504
chr16	68795100	0.611570
chr16	68805700	0.644628
chr16	69007600	0.603306
chr16	69020100	0.983471
chr16	69023500	0.636364
chr16	69147000	0.636364
chr16	69207400	0.975207
chr16	69301900	0.991736
chr16	69356500	0.611570
chr16	69361300	0.619835
chr16	69402700	0.719008
chr16	69416900	0.768595
chr16	69430600	0.570248
chr16	69433500	0.619835
chr16	69436600	0.619835

chr16	69438800	0.752066
chr16	69478400	0.702479
chr16	69511200	0.628099
chr16	69520800	0.578512
chr16	69564200	0.842975
chr16	69567500	0.694215
chr16	69624400	0.760331
chr16	69629800	0.933884
chr16	69653500	0.933884
chr16	69756500	0.752066
chr16	69758800	0.603306
chr16	69846700	0.578512
chr16	69899900	0.884298
chr16	70092600	0.570248
chr16	70118300	0.909091
chr16	70266400	0.785124
chr16	70320000	0.595041
chr16	70325200	0.809917
chr16	70366800	0.570248
chr16	70430800	0.900826
chr16	70434800	0.636364
chr16	70438700	0.818182
chr16	70442200	0.876033
chr16	70449000	0.983471
chr16	70451500	0.950413
chr16	70454000	0.884298
chr16	70456900	0.867769
chr16	70460600	0.917355
chr16	70465900	0.619835
chr16	70468600	0.892562
chr16	70475700	0.760331
chr16	70490500	0.694215
chr16	70563100	0.809917
chr16	70613600	0.685950
chr16	70688500	0.586777
chr16	70721000	0.595041
chr16	70729300	0.909091
chr16	70731400	0.719008
chr16	70760500	0.933884
chr16	70781600	0.966942
chr16	70806300	0.611570
chr16	70899000	0.925620
chr16	70929400	0.809917
chr16	71021600	0.909091
chr16	71394700	0.636364
chr16	71464900	0.942149
chr16	71526300	1.000000
chr16	71764900	0.595041
chr16	71861100	0.611570
chr16	71871400	0.859504
chr16	71925000	0.785124
chr16	71933400	0.595041
chr16	71968100	0.727273
chr16	71977800	0.578512

chr16	72047900	0.859504
chr16	72101500	0.752066
chr16	72106500	0.677686
chr16	72587600	0.760331
chr16	73071700	0.818182
chr16	74325800	0.801653
chr16	74363000	0.611570
chr16	74514700	0.743802
chr16	74542700	0.917355
chr16	74582000	0.760331
chr16	74599300	0.768595
chr16	74620800	0.776860
chr16	74626900	0.603306
chr16	74629000	0.760331
chr16	74635300	0.652893
chr16	74638000	0.644628
chr16	74694200	0.586777
chr16	74732600	0.586777
chr16	74777800	0.710744
chr16	75021100	0.834711
chr16	75023300	0.933884
chr16	75026200	0.809917
chr16	75032300	0.644628
chr16	75037200	0.834711
chr16	75045800	0.942149
chr16	75048200	0.892562
chr16	75059700	0.884298
chr16	75072500	0.677686
chr16	75075900	0.743802
chr16	75079200	0.892562
chr16	75082200	0.685950
chr16	75087900	0.925620
chr16	75090100	0.661157
chr16	75093500	0.652893
chr16	75098500	0.826446
chr16	75101200	0.851240
chr16	75103700	1.000000
chr16	75107000	0.685950
chr16	75115600	0.727273
chr16	75118100	0.644628
chr16	75121200	0.611570
chr16	75124300	0.677686
chr16	75133900	0.636364
chr16	75145400	0.900826
chr16	75164800	0.644628
chr16	75232500	0.619835
chr16	75339500	0.991736
chr16	75351600	0.909091
chr16	75358700	0.578512
chr16	75479500	0.578512
chr16	75487400	0.876033
chr16	75593900	0.710744
chr16	75603100	0.727273
chr16	75607200	0.677686

chr16	75612200	0.694215
chr16	75625300	0.933884
chr16	75628600	0.950413
chr16	75654500	0.619835
chr16	75686000	0.586777
chr16	75736200	0.851240
chr16	75815100	0.793388
chr16	77691300	0.743802
chr16	77773800	0.578512
chr16	78462600	0.958678
chr16	78477900	0.586777
chr16	78777200	0.727273
chr16	78949200	0.578512
chr16	79027700	0.628099
chr16	79434500	0.809917
chr16	79436700	0.958678
chr16	79447100	0.884298
chr16	79452200	0.925620
chr16	79802500	0.884298
chr16	79839200	0.752066
chr16	79850000	0.694215
chr16	79867500	0.727273
chr16	79898100	1.000000
chr16	79902100	0.925620
chr16	79904600	0.595041
chr16	79910000	0.983471
chr16	79924100	0.595041
chr16	79932100	0.826446
chr16	79949400	0.586777
chr16	79985500	0.611570
chr16	80000400	0.719008
chr16	80003200	0.785124
chr16	80007400	0.966942
chr16	80012000	0.595041
chr16	80022900	0.628099
chr16	80028000	0.752066
chr16	80040600	0.892562
chr16	80061600	0.752066
chr16	80063900	0.719008
chr16	81027300	0.752066
chr16	81031500	0.578512
chr16	81037300	0.578512
chr16	81099300	0.644628
chr16	81132200	0.586777
chr16	81215000	0.735537
chr16	81254400	0.892562
chr16	81257200	0.595041
chr16	81353000	0.677686
chr16	81485000	0.760331
chr16	81487400	0.892562
chr16	81490300	0.652893
chr16	81492500	0.925620
chr16	81503200	0.842975
chr16	81511900	0.611570

chr16	81515200	0.809917
chr16	81517800	0.842975
chr16	81524200	0.619835
chr16	81527000	0.570248
chr16	81530900	0.975207
chr16	81533100	0.669421
chr16	81536400	0.743802
chr16	81539800	0.867769
chr16	81547900	0.842975
chr16	81557800	0.628099
chr16	81561100	0.669421
chr16	81585600	0.793388
chr16	81617200	0.611570
chr16	81620000	0.752066
chr16	81658100	0.859504
chr16	81660600	0.892562
chr16	81663300	0.851240
chr16	81666200	0.578512
chr16	81669400	0.942149
chr16	81672900	0.727273
chr16	81686000	0.900826
chr16	81730700	0.619835
chr16	81749300	0.785124
chr16	81752600	0.661157
chr16	81764200	0.652893
chr16	81772500	0.702479
chr16	81786100	0.958678
chr16	81962900	0.652893
chr16	82187000	0.826446
chr16	83819400	0.892562
chr16	83870100	0.719008
chr16	83922600	0.826446
chr16	84045000	0.595041
chr16	84077500	0.867769
chr16	84089900	0.867769
chr16	84166600	0.768595
chr16	84171200	0.586777
chr16	84174500	0.677686
chr16	84262100	0.785124
chr16	84300800	0.752066
chr16	84558900	0.652893
chr16	84569300	0.801653
chr16	84572900	0.975207
chr16	84575700	0.636364
chr16	84583500	0.595041
chr16	84620000	0.578512
chr16	84627400	0.842975
chr16	84634100	0.834711
chr16	84637600	0.685950
chr16	84641600	0.909091
chr16	84649000	0.586777
chr16	84653300	0.578512
chr16	84711200	0.595041
chr16	84738000	0.570248

chr16	84742500	0.909091
chr16	84758000	0.760331
chr16	84761200	0.776860
chr16	84765400	0.595041
chr16	84769900	0.876033
chr16	84772200	0.578512
chr16	84785100	0.917355
chr16	84788000	0.578512
chr16	84853600	0.702479
chr16	84913300	0.950413
chr16	84915700	0.760331
chr16	84918800	0.776860
chr16	84964000	0.628099
chr16	84980900	0.619835
chr16	84987500	0.628099
chr16	85006500	0.966942
chr16	85016600	0.884298
chr16	85028300	0.760331
chr16	85034900	0.809917
chr16	85038700	0.603306
chr16	85059600	0.636364
chr16	85063400	0.876033
chr16	85076600	0.694215
chr16	85089400	0.760331
chr16	85096400	0.776860
chr16	85149000	0.628099
chr16	85160500	0.611570
chr16	85204000	0.619835
chr16	85215500	0.719008
chr16	85222800	0.743802
chr16	85234100	0.867769
chr16	85250700	0.859504
chr16	85262000	0.776860
chr16	85296300	0.950413
chr16	85309100	0.834711
chr16	85325500	0.933884
chr16	85331400	0.595041
chr16	85336000	0.603306
chr16	85339800	0.966942
chr16	85344400	0.752066
chr16	85347100	0.611570
chr16	85350600	0.851240
chr16	85353800	0.735537
chr16	85356400	0.983471
chr16	85361900	0.785124
chr16	85364800	0.809917
chr16	85383800	0.809917
chr16	85388900	0.735537
chr16	85392600	0.768595
chr16	85395400	0.743802
chr16	85404500	0.867769
chr16	85407100	0.793388
chr16	85411600	0.900826
chr16	85414500	0.578512

chr16	85417000	0.735537
chr16	85419600	0.834711
chr16	85424600	0.867769
chr16	85427700	0.768595
chr16	85430100	0.694215
chr16	85436900	0.884298
chr16	85450300	0.570248
chr16	85455500	0.652893
chr16	85470800	0.876033
chr16	85494100	0.809917
chr16	85506200	0.801653
chr16	85509000	0.611570
chr16	85525400	0.743802
chr16	85549500	0.752066
chr16	85557200	0.925620
chr16	85562800	0.842975
chr16	85568200	0.768595
chr16	85571100	0.793388
chr16	85576100	0.826446
chr16	85579000	0.892562
chr16	85585100	0.801653
chr16	85609800	0.859504
chr16	85613300	0.876033
chr16	85616300	0.685950
chr16	85619600	0.801653
chr16	85621900	0.950413
chr16	85624000	0.991736
chr16	85626600	0.595041
chr16	85635500	0.851240
chr16	85641500	0.859504
chr16	85643600	0.727273
chr16	85652600	0.925620
chr16	85655600	0.702479
chr16	85686600	0.859504
chr16	85732900	0.950413
chr16	85749700	0.636364
chr16	85767900	0.595041
chr16	85884400	0.760331
chr16	86698600	0.884298
chr16	86786700	0.578512
chr16	86935500	0.727273
chr16	86963100	0.859504
chr16	86965500	0.603306
chr16	86985900	0.595041
chr16	87087100	0.876033
chr16	87179500	0.900826
chr16	87292700	0.595041
chr16	87329500	0.719008
chr16	87337400	0.983471
chr16	87398300	0.917355
chr16	87410600	0.991736
chr16	87414900	0.719008
chr16	87423900	0.785124
chr16	87498500	0.785124

chr16	87518800	0.900826
chr16	87594900	0.793388
chr16	87616400	0.578512
chr16	87755400	0.785124
chr16	87758200	0.636364
chr16	87803200	0.619835
chr16	87808600	0.958678
chr16	87812200	0.876033
chr16	87814400	0.909091
chr16	87823900	0.694215
chr16	87840700	0.909091
chr16	87886500	0.760331
chr16	87889500	0.884298
chr16	87892200	0.801653
chr16	87894800	0.975207
chr16	87897300	0.867769
chr16	87899800	0.685950
chr16	87905200	0.859504
chr16	87913500	0.595041
chr16	87915600	0.628099
chr16	87925500	0.917355
chr16	87950100	0.603306
chr16	87990200	0.966942
chr16	88183100	0.876033
chr16	88268800	0.917355
chr16	88298700	0.909091
chr16	88320900	0.768595
chr16	88340800	0.933884
chr16	88349900	0.793388
chr16	88360000	0.892562
chr16	88365600	0.710744
chr16	88369000	0.892562
chr16	88509800	0.603306
chr16	88515400	0.685950
chr16	88525100	0.925620
chr16	88531600	0.892562
chr16	88534300	0.859504
chr16	88538600	0.628099
chr16	88540700	0.876033
chr16	88544100	0.685950
chr16	88549200	0.628099
chr16	88558100	0.876033
chr16	88560200	0.818182
chr16	88562300	0.644628
chr16	88566600	0.983471
chr16	88570900	0.702479
chr16	88575700	0.842975
chr16	88593400	0.983471
chr16	88597500	0.884298
chr16	88616300	0.644628
chr16	88619600	0.595041
chr16	88625600	0.727273
chr16	88684300	0.818182
chr16	88695000	0.776860

chr16	88701300	0.603306
chr16	88718900	0.694215
chr16	88726600	0.867769
chr16	88755200	0.702479
chr16	88767900	0.942149
chr16	88775300	0.595041
chr16	88825300	0.619835
chr16	88828000	0.578512
chr16	88830800	0.752066
chr16	88834300	0.876033
chr16	88838600	0.603306
chr16	88842200	0.677686
chr16	88857300	0.942149
chr16	88866600	0.892562
chr16	88873100	0.628099
chr16	88937800	0.826446
chr16	88949200	0.727273
chr16	88972300	0.785124
chr16	88993600	0.595041
chr16	89023500	0.909091
chr16	89046400	0.644628
chr16	89049200	0.991736
chr16	89057400	0.760331
chr16	89061900	0.966942
chr16	89066300	0.933884
chr16	89093200	0.909091
chr16	89101800	0.851240
chr16	89157100	0.876033
chr16	89165200	0.842975
chr16	89181800	0.785124
chr16	89233300	0.636364
chr16	89237900	0.603306
chr16	89287000	0.801653
chr16	89382600	0.842975
chr16	89385700	0.933884
chr16	89389200	0.925620
chr16	89394000	0.611570
chr16	89397100	0.785124
chr16	89401000	0.867769
chr16	89424100	0.834711
chr16	89431000	0.719008
chr16	89436200	0.636364
chr16	89444100	0.892562
chr16	89450100	0.826446
chr16	89455500	0.834711
chr16	89471900	0.900826
chr16	89474100	0.586777
chr16	89480700	0.578512
chr16	89506800	0.595041
chr16	89510000	0.776860
chr16	89514000	0.851240
chr16	89517200	0.644628
chr16	89520900	0.842975
chr16	89530500	0.685950

chr16	89533800	0.619835
chr16	89538200	0.578512
chr16	89541100	0.859504
chr16	89545500	0.900826
chr16	89549600	0.702479
chr16	89553000	0.851240
chr16	89569000	0.925620
chr16	89571400	0.710744
chr16	89632300	0.702479
chr16	89669700	0.595041
chr16	89674200	0.685950
chr16	89773000	0.644628
chr16	89776900	0.925620
chr16	89784700	0.876033
chr16	89790200	0.661157
chr16	89881100	0.727273
chr16	90041800	0.702479
chr16	90060700	0.743802
chr16	90113400	0.603306
chr16	90124200	0.966942
chr16	90169400	0.652893
chr16	90179300	0.595041
chr16	90183000	0.925620
chr16	90236000	0.892562
chr16	90238100	0.900826
chr16	90253300	0.611570
chr16	90256600	0.628099
chr16	90259300	0.818182
chr16	90268300	0.677686
chr16	90284300	0.842975
chr17	500300	0.719008
chr17	504000	0.859504
chr17	507500	0.983471
chr17	549700	0.652893
chr17	656700	0.570248
chr17	687900	0.570248
chr17	1041100	0.644628
chr17	1212600	0.793388
chr17	1225800	0.983471
chr17	1294700	0.851240
chr17	1392200	0.694215
chr17	1415000	0.884298
chr17	1454400	0.859504
chr17	1470300	0.677686
chr17	1493800	0.876033
chr17	1521500	0.975207
chr17	1554300	0.867769
chr17	1562300	0.603306
chr17	1608500	0.719008
chr17	1622400	0.694215
chr17	1634200	0.818182
chr17	1640500	0.900826
chr17	1696700	0.636364
chr17	1700900	0.719008

chr17	1890400	0.925620
chr17	1987600	0.578512
chr17	1992700	0.917355
chr17	2042000	0.917355
chr17	2095900	0.619835
chr17	2118000	0.801653
chr17	2199100	0.628099
chr17	2220000	0.900826
chr17	2287300	0.859504
chr17	2300300	0.809917
chr17	2310800	0.859504
chr17	2528200	0.578512
chr17	2534000	0.570248
chr17	2620300	0.619835
chr17	2689600	0.628099
chr17	3461500	0.677686
chr17	3543900	0.834711
chr17	3616400	0.595041
chr17	3642100	0.595041
chr17	3669300	0.652893
chr17	3792200	0.942149
chr17	3797600	0.628099
chr17	3817600	0.619835
chr17	3821400	0.892562
chr17	3848200	0.793388
chr17	3863600	0.925620
chr17	3872800	0.958678
chr17	3877200	0.909091
chr17	3906000	0.925620
chr17	3908200	0.702479
chr17	3910700	0.983471
chr17	3964400	0.570248
chr17	4000800	0.793388
chr17	4004400	0.983471
chr17	4065300	0.876033
chr17	4151400	0.900826
chr17	4153900	0.900826
chr17	4235300	0.735537
chr17	4245800	0.793388
chr17	4561800	0.851240
chr17	4625300	0.818182
chr17	4740400	0.644628
chr17	4744200	0.809917
chr17	4759900	0.570248
chr17	4806700	0.677686
chr17	4846300	0.611570
chr17	4848500	0.917355
chr17	4855500	0.636364
chr17	4863800	0.884298
chr17	4873100	0.925620
chr17	5343500	0.578512
chr17	5415900	0.595041
chr17	5430400	0.884298
chr17	5576500	0.578512

chr17	6354600	0.702479
chr17	6360600	0.958678
chr17	6369100	0.595041
chr17	6380000	0.619835
chr17	6479100	0.983471
chr17	6503200	0.595041
chr17	6656300	0.595041
chr17	6818900	0.834711
chr17	6822800	0.983471
chr17	6826400	0.867769
chr17	6831500	0.909091
chr17	6872900	0.743802
chr17	6897500	0.942149
chr17	6902100	0.933884
chr17	6904700	0.644628
chr17	6920800	0.942149
chr17	6925500	0.669421
chr17	6928500	0.611570
chr17	6932700	0.710744
chr17	6950100	0.578512
chr17	7118600	0.603306
chr17	7121700	0.768595
chr17	7139300	0.595041
chr17	7143800	0.619835
chr17	7152500	0.677686
chr17	7186800	0.578512
chr17	7196900	0.694215
chr17	7236700	0.669421
chr17	7341900	0.677686
chr17	7344500	0.719008
chr17	7349500	0.743802
chr17	7371100	0.702479
chr17	7379900	0.966942
chr17	7391400	0.735537
chr17	7475100	0.685950
chr17	7480300	0.578512
chr17	7482600	0.661157
chr17	7485600	0.719008
chr17	7491100	0.900826
chr17	7515400	0.702479
chr17	7550500	0.586777
chr17	7553300	0.793388
chr17	7594200	0.636364
chr17	7737400	0.652893
chr17	7743300	0.909091
chr17	7785000	0.884298
chr17	7820300	0.628099
chr17	7986500	0.619835
chr17	8009800	0.859504
chr17	8058000	0.859504
chr17	8064200	0.677686
chr17	8068000	0.900826
chr17	8094400	0.652893
chr17	8103400	0.603306

chr17	8117000	0.661157
chr17	8122200	0.818182
chr17	8230200	0.768595
chr17	8249800	0.661157
chr17	8263600	0.611570
chr17	8279900	0.661157
chr17	8282500	0.595041
chr17	8289000	0.710744
chr17	8291200	0.595041
chr17	8314300	0.595041
chr17	8321400	0.611570
chr17	8324200	0.859504
chr17	8432400	0.603306
chr17	8435800	0.900826
chr17	8515300	0.719008
chr17	10606000	0.586777
chr17	11512000	0.628099
chr17	11812300	0.909091
chr17	11950500	0.661157
chr17	11957300	0.661157
chr17	11993600	0.809917
chr17	12342600	0.735537
chr17	13250100	0.900826
chr17	13380300	0.652893
chr17	13394400	0.570248
chr17	13411600	0.851240
chr17	13414200	0.900826
chr17	13424900	0.884298
chr17	13427700	0.603306
chr17	13440100	0.842975
chr17	13444200	0.776860
chr17	13466200	0.586777
chr17	13498300	0.892562
chr17	13923400	0.719008
chr17	14065300	0.801653
chr17	14107500	0.966942
chr17	14113000	0.933884
chr17	14227100	0.661157
chr17	14235100	0.933884
chr17	14239000	0.578512
chr17	14276700	0.669421
chr17	15038300	1.000000
chr17	15088500	0.991736
chr17	15136200	0.925620
chr17	15139600	0.867769
chr17	15141700	0.752066
chr17	15144100	0.702479
chr17	15160800	0.867769
chr17	15284400	0.652893
chr17	15344900	0.785124
chr17	15394300	0.966942
chr17	15430700	0.917355
chr17	15546700	0.752066
chr17	15555200	0.867769

chr17	15789900	0.727273
chr17	15851000	0.776860
chr17	15860300	0.958678
chr17	15863100	0.942149
chr17	15870100	0.809917
chr17	15875800	0.900826
chr17	15881700	0.603306
chr17	15885200	0.983471
chr17	15887700	0.644628
chr17	15931700	0.958678
chr17	15995600	0.636364
chr17	16130000	0.727273
chr17	16147200	0.636364
chr17	16159000	0.578512
chr17	16189000	0.727273
chr17	16201400	0.884298
chr17	16217200	0.900826
chr17	16282400	0.586777
chr17	16288000	0.628099
chr17	16300200	0.983471
chr17	16302700	0.884298
chr17	16322900	0.966942
chr17	16331800	0.619835
chr17	16347400	0.710744
chr17	16424600	0.603306
chr17	16698100	0.884298
chr17	16739300	0.694215
chr17	16924500	0.611570
chr17	16931900	0.900826
chr17	16935800	0.619835
chr17	16938800	0.785124
chr17	16945300	0.628099
chr17	16948100	0.694215
chr17	16982600	0.603306
chr17	16985100	0.570248
chr17	16992100	0.842975
chr17	17075900	0.950413
chr17	17110700	0.851240
chr17	17143400	0.719008
chr17	17148200	0.586777
chr17	17156400	0.768595
chr17	17180600	0.900826
chr17	17193300	0.719008
chr17	17210000	0.842975
chr17	17319800	0.859504
chr17	17321900	0.991736
chr17	17343600	0.702479
chr17	17412900	0.925620
chr17	17442300	0.636364
chr17	17509400	0.975207
chr17	17566700	0.966942
chr17	17589300	0.876033
chr17	17597300	0.867769
chr17	17606100	0.900826

chr17	17643000	0.933884
chr17	17654700	0.694215
chr17	17684200	0.619835
chr17	17716000	0.586777
chr17	17719200	0.785124
chr17	17722700	0.892562
chr17	17724800	0.735537
chr17	17729100	0.793388
chr17	17731300	0.917355
chr17	17737400	0.752066
chr17	17745500	0.760331
chr17	17753800	1.000000
chr17	17811000	0.983471
chr17	17814700	0.628099
chr17	17817800	0.975207
chr17	17823100	0.619835
chr17	17825700	0.900826
chr17	17833400	0.595041
chr17	17846800	0.644628
chr17	17861900	0.619835
chr17	17929400	0.793388
chr17	17939700	0.677686
chr17	17946400	0.743802
chr17	17974500	0.900826
chr17	17978600	0.859504
chr17	17983100	0.644628
chr17	18067300	0.958678
chr17	18076000	0.603306
chr17	18085000	0.570248
chr17	18091600	0.570248
chr17	18150500	0.826446
chr17	18220800	0.611570
chr17	18272400	0.652893
chr17	18698100	0.595041
chr17	18720000	0.859504
chr17	18753800	0.710744
chr17	18821200	0.851240
chr17	18885700	0.760331
chr17	18901500	0.867769
chr17	18907700	0.735537
chr17	18925600	0.710744
chr17	18964400	0.661157
chr17	18968600	0.743802
chr17	18993600	0.661157
chr17	19011900	0.727273
chr17	19014400	0.702479
chr17	19016900	0.636364
chr17	19039900	0.595041
chr17	19094600	0.685950
chr17	19138300	0.876033
chr17	19152100	0.917355
chr17	19174800	0.958678
chr17	19207600	0.950413
chr17	19241400	0.578512

chr17	19250600	0.983471
chr17	19848900	0.603306
chr17	19882100	0.669421
chr17	19891600	0.785124
chr17	19917900	0.644628
chr17	19963200	0.983471
chr17	19976500	0.611570
chr17	20044800	0.677686
chr17	20093400	0.909091
chr17	20110500	0.851240
chr17	20170700	0.851240
chr17	20195000	0.595041
chr17	20326700	0.752066
chr17	20811700	0.702479
chr17	20883500	0.818182
chr17	20938200	0.909091
chr17	20940900	0.619835
chr17	21103400	0.603306
chr17	21120100	0.752066
chr17	21160700	0.818182
chr17	21176300	0.735537
chr17	21179300	0.652893
chr17	21190400	0.719008
chr17	21198200	0.619835
chr17	21220300	0.809917
chr17	21427500	0.586777
chr17	21739900	0.578512
chr17	21752900	0.809917
chr17	21755200	0.859504
chr17	21849700	0.644628
chr17	21910800	0.685950
chr17	22250800	0.636364
chr17	22253300	0.760331
chr17	25420900	0.735537
chr17	25435400	0.867769
chr17	25572500	0.785124
chr17	25620200	0.644628
chr17	25625200	0.636364
chr17	25651100	0.818182
chr17	25664400	0.785124
chr17	25668700	0.826446
chr17	25674200	0.776860
chr17	25683200	0.892562
chr17	25687900	0.735537
chr17	25690600	0.752066
chr17	25692800	0.661157
chr17	25696600	0.743802
chr17	25707500	0.876033
chr17	25731200	0.694215
chr17	25742900	0.652893
chr17	25775500	0.917355
chr17	25783000	0.595041
chr17	25786700	0.793388
chr17	25790700	0.975207

chr17	25794600	0.851240
chr17	25796700	0.694215
chr17	25798900	0.900826
chr17	25805000	0.752066
chr17	25808300	0.628099
chr17	25818900	0.793388
chr17	25824400	0.909091
chr17	25832400	0.586777
chr17	25842700	0.942149
chr17	25848400	0.611570
chr17	25853800	0.942149
chr17	25856400	0.900826
chr17	25858500	0.983471
chr17	25862600	0.677686
chr17	25872100	0.966942
chr17	25874900	0.892562
chr17	25880100	0.570248
chr17	25894700	0.983471
chr17	25899000	0.619835
chr17	25904400	0.867769
chr17	25907200	0.917355
chr17	25912300	0.950413
chr17	25925800	0.925620
chr17	25930600	0.884298
chr17	25940400	0.595041
chr17	25960800	0.950413
chr17	25962900	0.768595
chr17	25968400	0.578512
chr17	25975400	0.818182
chr17	25978900	0.652893
chr17	26217900	0.586777
chr17	26284600	0.925620
chr17	26295800	0.975207
chr17	26298100	0.859504
chr17	26301100	0.619835
chr17	26305700	0.876033
chr17	26309900	0.892562
chr17	26312600	0.735537
chr17	26314700	0.793388
chr17	26356400	0.826446
chr17	26360600	0.752066
chr17	26366400	0.867769
chr17	26380700	0.867769
chr17	26383100	0.644628
chr17	26390500	0.636364
chr17	26393100	0.595041
chr17	26402800	0.785124
chr17	26419600	0.636364
chr17	26432800	0.842975
chr17	26440000	0.958678
chr17	26444800	0.809917
chr17	26454500	0.570248
chr17	26500700	0.958678
chr17	26517000	0.884298

chr17	26553500	0.595041
chr17	26557700	0.933884
chr17	26563600	0.586777
chr17	26566500	0.661157
chr17	26595400	0.942149
chr17	26643500	0.644628
chr17	26698200	0.685950
chr17	26803100	0.611570
chr17	26852700	0.760331
chr17	26855200	0.942149
chr17	26905500	0.611570
chr17	26974600	0.595041
chr17	27062400	0.826446
chr17	27068600	0.652893
chr17	27074000	0.628099
chr17	27077800	0.851240
chr17	27090900	0.578512
chr17	27105900	0.859504
chr17	27121000	0.752066
chr17	27124500	0.694215
chr17	27129600	0.570248
chr17	27135500	0.900826
chr17	27144700	0.727273
chr17	27155300	0.851240
chr17	27161300	0.595041
chr17	27163700	0.619835
chr17	27167000	0.669421
chr17	27186100	0.636364
chr17	27195000	0.801653
chr17	27198700	0.917355
chr17	27202100	0.669421
chr17	27204200	0.611570
chr17	27208000	0.917355
chr17	27226600	0.702479
chr17	27232000	0.809917
chr17	27235800	0.776860
chr17	27239600	0.760331
chr17	27242300	0.669421
chr17	27251500	0.826446
chr17	27253800	0.842975
chr17	27263600	0.661157
chr17	27301800	0.785124
chr17	27367200	0.578512
chr17	27444000	0.619835
chr17	27452400	0.735537
chr17	27459800	0.983471
chr17	27467600	0.917355
chr17	27472600	0.603306
chr17	27478800	0.785124
chr17	27482200	0.925620
chr17	27487900	0.611570
chr17	27490200	0.950413
chr17	27496000	0.652893
chr17	27498200	0.595041

chr17	27508300	0.702479
chr17	27531900	0.595041
chr17	27546800	0.752066
chr17	27565000	0.867769
chr17	27606800	0.669421
chr17	27613900	0.694215
chr17	27720900	0.611570
chr17	27760900	0.694215
chr17	27884800	0.644628
chr17	27917400	0.677686
chr17	27942900	0.595041
chr17	27964100	0.595041
chr17	27966900	0.900826
chr17	27970200	0.818182
chr17	27977200	0.603306
chr17	27981600	0.983471
chr17	27989900	0.661157
chr17	27992300	0.867769
chr17	27995300	0.975207
chr17	28005100	0.884298
chr17	28009600	0.859504
chr17	28019700	0.793388
chr17	28032600	0.884298
chr17	28038400	0.892562
chr17	28048400	0.950413
chr17	28051300	0.793388
chr17	28054300	0.958678
chr17	28059600	0.719008
chr17	28124100	0.652893
chr17	28164000	0.685950
chr17	28172000	0.652893
chr17	28174800	0.685950
chr17	28202600	0.719008
chr17	28464300	0.710744
chr17	28531100	0.702479
chr17	28547000	0.752066
chr17	28672700	0.950413
chr17	28703800	0.760331
chr17	28732300	0.851240
chr17	28760600	0.578512
chr17	28862100	0.595041
chr17	28878200	0.595041
chr17	28948800	0.619835
chr17	28978700	0.776860
chr17	28996000	0.570248
chr17	29021400	0.603306
chr17	29028700	0.834711
chr17	29056100	0.958678
chr17	29157700	0.570248
chr17	29266700	0.578512
chr17	29379700	0.859504
chr17	29405700	0.628099
chr17	29420200	0.603306
chr17	29472500	0.595041

chr17	29475300	0.851240
chr17	29635200	0.669421
chr17	29667500	0.809917
chr17	29782600	0.876033
chr17	29785400	0.677686
chr17	29798700	0.595041
chr17	29817300	0.793388
chr17	29820000	0.917355
chr17	29826000	0.867769
chr17	29835900	0.892562
chr17	29844000	0.785124
chr17	30267900	0.611570
chr17	30396500	0.727273
chr17	30478200	0.652893
chr17	30602400	0.727273
chr17	30607300	0.925620
chr17	30611300	0.570248
chr17	30614000	0.983471
chr17	30626700	0.603306
chr17	30671100	0.669421
chr17	30673600	0.925620
chr17	30682600	0.859504
chr17	30813100	0.818182
chr17	30819300	0.644628
chr17	30864300	0.768595
chr17	31050500	0.611570
chr17	31064300	0.652893
chr17	31111200	0.603306
chr17	31113700	0.595041
chr17	31122300	0.917355
chr17	31146600	0.842975
chr17	31160800	0.595041
chr17	31187300	0.702479
chr17	31192200	0.900826
chr17	31197200	0.892562
chr17	31201300	0.611570
chr17	31210100	0.818182
chr17	31221500	0.884298
chr17	31223600	0.719008
chr17	31226900	0.884298
chr17	31231400	0.876033
chr17	31238000	0.876033
chr17	31240800	0.570248
chr17	31243100	0.727273
chr17	31274100	0.702479
chr17	31276800	0.644628
chr17	31283800	0.925620
chr17	31289400	0.702479
chr17	31298300	0.950413
chr17	31302400	0.710744
chr17	31306700	0.595041
chr17	31319800	0.933884
chr17	31325800	0.719008
chr17	31801400	0.867769

chr17	31809000	0.909091
chr17	31995400	0.752066
chr17	32106200	0.586777
chr17	32108800	0.867769
chr17	32116900	0.578512
chr17	32180300	0.578512
chr17	32186600	0.636364
chr17	32190500	0.743802
chr17	32194800	0.619835
chr17	32197600	0.644628
chr17	32201800	0.975207
chr17	32204200	0.917355
chr17	32211200	0.595041
chr17	32213400	0.793388
chr17	32216500	0.958678
chr17	32227200	0.636364
chr17	32231100	0.884298
chr17	32233400	0.859504
chr17	32236000	0.801653
chr17	32238400	0.950413
chr17	32241500	0.867769
chr17	32247200	0.570248
chr17	32249500	0.876033
chr17	32252100	0.909091
chr17	32255500	0.884298
chr17	32258600	0.950413
chr17	32262100	0.809917
chr17	32269900	0.834711
chr17	32276000	0.768595
chr17	32294200	0.719008
chr17	32351800	0.595041
chr17	32580000	0.578512
chr17	32705100	0.694215
chr17	32710400	0.619835
chr17	32714400	0.958678
chr17	32739700	0.892562
chr17	32741800	0.801653
chr17	33211700	0.578512
chr17	33214200	0.900826
chr17	33311200	0.570248
chr17	33315500	0.851240
chr17	33374200	0.685950
chr17	33378600	0.950413
chr17	33388300	0.661157
chr17	33392700	0.884298
chr17	33401400	0.595041
chr17	33410200	0.785124
chr17	33412700	0.669421
chr17	33417500	0.710744
chr17	33478300	0.842975
chr17	33640500	0.628099
chr17	33760800	0.694215
chr17	33865100	0.644628
chr17	33884600	0.776860

chr17	33888600	0.743802
chr17	33891200	0.859504
chr17	33903200	0.570248
chr17	33911700	0.677686
chr17	34077800	0.694215
chr17	34129000	0.710744
chr17	34193500	0.685950
chr17	34207500	0.925620
chr17	34226000	0.578512
chr17	34228100	0.677686
chr17	34239400	0.867769
chr17	34243000	0.950413
chr17	34247800	0.776860
chr17	34259600	0.719008
chr17	34270200	0.595041
chr17	34838800	0.776860
chr17	34885500	0.950413
chr17	34888300	0.851240
chr17	34897100	0.842975
chr17	34946900	0.842975
chr17	34951300	0.884298
chr17	34955500	0.851240
chr17	34965300	0.793388
chr17	34968500	0.975207
chr17	35062400	0.694215
chr17	35067400	0.628099
chr17	35080900	0.578512
chr17	35083300	0.652893
chr17	35086400	0.834711
chr17	35121000	0.652893
chr17	35220200	0.760331
chr17	35278700	0.611570
chr17	35285200	0.743802
chr17	35308600	0.644628
chr17	35492100	0.661157
chr17	35647200	0.669421
chr17	35658600	0.719008
chr17	35717900	0.768595
chr17	35790900	0.942149
chr17	35843600	0.743802
chr17	35879800	0.975207
chr17	35966500	0.652893
chr17	36000400	0.760331
chr17	36188700	0.900826
chr17	36193000	0.694215
chr17	36433200	0.570248
chr17	36442500	0.909091
chr17	36473600	0.909091
chr17	36510400	0.570248
chr17	36571600	0.818182
chr17	36574200	0.776860
chr17	36617300	0.842975
chr17	36741400	0.652893
chr17	36755600	0.619835

chr17	36758400	0.727273
chr17	36811600	0.661157
chr17	36831400	0.694215
chr17	36833800	0.909091
chr17	36856900	0.586777
chr17	36867700	0.909091
chr17	36875500	0.768595
chr17	36889200	0.785124
chr17	36902700	0.595041
chr17	37021200	0.570248
chr17	37038000	0.628099
chr17	37050300	0.661157
chr17	37055100	0.859504
chr17	37057700	0.809917
chr17	37083200	0.669421
chr17	37159700	0.702479
chr17	37163600	0.710744
chr17	37171100	0.983471
chr17	37173400	0.652893
chr17	37175600	0.694215
chr17	37192700	0.834711
chr17	37213500	0.570248
chr17	37331900	0.719008
chr17	37335000	0.619835
chr17	37351600	0.710744
chr17	37354300	0.603306
chr17	37401600	0.570248
chr17	37438100	0.884298
chr17	37458200	0.925620
chr17	37472700	0.603306
chr17	37482400	0.966942
chr17	37488400	0.942149
chr17	37492500	0.900826
chr17	37495400	0.851240
chr17	37509700	0.669421
chr17	37515000	0.578512
chr17	37517200	0.950413
chr17	37520400	0.570248
chr17	37533600	0.859504
chr17	37685600	0.636364
chr17	37692300	0.958678
chr17	37791800	0.603306
chr17	37796400	0.661157
chr17	37798600	0.652893
chr17	37803800	0.661157
chr17	37806400	0.876033
chr17	37810000	0.983471
chr17	37820700	0.917355
chr17	37822900	0.702479
chr17	37828400	0.694215
chr17	37831700	0.991736
chr17	37836400	0.685950
chr17	37840000	0.884298
chr17	37881500	0.636364

chr17	37896900	0.586777
chr17	38060300	0.793388
chr17	38086200	0.809917
chr17	38096400	0.933884
chr17	38115800	0.818182
chr17	38128600	0.818182
chr17	38162000	0.661157
chr17	38170800	0.694215
chr17	38225000	0.570248
chr17	38229300	0.975207
chr17	38233100	0.818182
chr17	38259100	0.677686
chr17	38261200	0.652893
chr17	38264800	0.966942
chr17	38268600	0.884298
chr17	38271900	0.595041
chr17	38309800	0.975207
chr17	38314800	0.867769
chr17	38333700	0.644628
chr17	38381000	0.702479
chr17	38422200	0.818182
chr17	38447200	0.867769
chr17	38463700	0.942149
chr17	38472600	0.603306
chr17	38476900	0.661157
chr17	38480200	0.685950
chr17	38491400	0.661157
chr17	38501400	0.867769
chr17	38504100	0.685950
chr17	38529000	0.652893
chr17	38537000	0.785124
chr17	38583100	0.644628
chr17	38589800	0.578512
chr17	38594000	0.867769
chr17	38601900	0.611570
chr17	38604200	0.586777
chr17	38628000	0.644628
chr17	38632300	0.595041
chr17	38638600	0.595041
chr17	38642500	0.785124
chr17	38647100	0.619835
chr17	38655900	0.595041
chr17	38672700	0.991736
chr17	38674900	0.710744
chr17	38690000	0.983471
chr17	38693000	0.942149
chr17	38697300	0.611570
chr17	38710700	0.950413
chr17	38713700	0.826446
chr17	38717500	0.603306
chr17	38722100	0.702479
chr17	38750600	0.859504
chr17	38942700	0.570248
chr17	38972900	0.611570

chr17	38978900	0.611570
chr17	38982700	0.892562
chr17	38986700	0.636364
chr17	38998300	0.628099
chr17	39001400	0.876033
chr17	39010700	0.677686
chr17	39223200	0.694215
chr17	39640800	0.586777
chr17	39654700	0.752066
chr17	39658100	0.719008
chr17	39662600	0.826446
chr17	39667400	0.760331
chr17	39673300	0.685950
chr17	39678400	0.942149
chr17	39689100	0.719008
chr17	39701300	0.652893
chr17	39705900	0.859504
chr17	39802600	0.851240
chr17	39805100	0.719008
chr17	39824700	0.644628
chr17	39829800	0.735537
chr17	39832400	0.661157
chr17	39836800	0.677686
chr17	39840300	0.595041
chr17	39852700	0.636364
chr17	39894500	0.702479
chr17	39938600	0.942149
chr17	39948900	0.710744
chr17	39951700	0.785124
chr17	39957500	0.702479
chr17	39968400	0.661157
chr17	40071700	0.785124
chr17	40089200	0.917355
chr17	40095000	0.991736
chr17	40109700	0.595041
chr17	40163600	0.793388
chr17	40174800	0.768595
chr17	40177000	0.950413
chr17	40224000	0.578512
chr17	40228800	0.669421
chr17	40231200	0.619835
chr17	40239500	0.785124
chr17	40243800	0.785124
chr17	40246700	0.578512
chr17	40260400	0.818182
chr17	40273100	0.586777
chr17	40288100	0.801653
chr17	40351700	0.603306
chr17	40405700	0.636364
chr17	40408600	0.677686
chr17	40418500	0.719008
chr17	40420800	0.669421
chr17	40433900	0.619835
chr17	40436600	0.611570

chr17	40446400	0.776860
chr17	40448500	0.900826
chr17	40454300	0.694215
chr17	40458100	0.644628
chr17	40462500	0.851240
chr17	40465200	0.619835
chr17	40474300	0.966942
chr17	40535000	0.619835
chr17	40542400	0.570248
chr17	40555000	0.884298
chr17	40559400	0.909091
chr17	40562100	0.884298
chr17	40567800	0.851240
chr17	40572700	0.661157
chr17	40582000	0.644628
chr17	40594300	0.933884
chr17	40605800	0.809917
chr17	40668500	0.958678
chr17	40672200	0.991736
chr17	40679400	0.719008
chr17	40693700	0.892562
chr17	40698700	0.677686
chr17	40721800	0.950413
chr17	40725600	0.793388
chr17	40758100	0.669421
chr17	40770700	0.925620
chr17	40776200	1.000000
chr17	40778800	0.644628
chr17	40781800	0.818182
chr17	40785400	0.727273
chr17	40805900	0.933884
chr17	40813400	0.892562
chr17	40826700	0.710744
chr17	40833100	0.644628
chr17	40851400	0.950413
chr17	40862100	0.595041
chr17	40903800	0.694215
chr17	40920300	0.570248
chr17	40928100	0.909091
chr17	40931200	0.834711
chr17	41080900	0.644628
chr17	41149400	0.595041
chr17	41163300	0.892562
chr17	41167500	0.652893
chr17	41171500	0.818182
chr17	41305600	0.925620
chr17	41394500	0.611570
chr17	41428800	0.727273
chr17	41445400	0.867769
chr17	41464200	0.595041
chr17	41481500	0.661157
chr17	41608300	0.925620
chr17	41613000	0.818182
chr17	41633700	0.859504

chr17	41798800	0.851240
chr17	41950100	0.595041
chr17	42030300	0.669421
chr17	42061600	0.570248
chr17	42077600	0.834711
chr17	42162200	0.834711
chr17	42164500	0.983471
chr17	42175200	0.991736
chr17	42186300	0.578512
chr17	42189100	0.710744
chr17	42210000	0.834711
chr17	42216000	0.710744
chr17	42228200	0.578512
chr17	42237700	0.818182
chr17	42245800	0.669421
chr17	42261500	0.570248
chr17	42281800	0.793388
chr17	42288500	0.603306
chr17	42300500	0.619835
chr17	42323400	0.900826
chr17	42338200	0.719008
chr17	42344500	0.809917
chr17	42346700	0.801653
chr17	42349900	0.900826
chr17	42357600	0.917355
chr17	42360700	0.925620
chr17	42366700	0.603306
chr17	42406000	0.578512
chr17	42409100	0.760331
chr17	42467400	0.867769
chr17	42471100	0.917355
chr17	42522700	0.867769
chr17	42621800	0.785124
chr17	42624800	0.950413
chr17	42639200	0.652893
chr17	42872600	0.727273
chr17	42983800	0.975207
chr17	43096500	0.628099
chr17	43099300	0.776860
chr17	43109300	0.991736
chr17	43124600	0.586777
chr17	43130400	0.570248
chr17	43201900	1.000000
chr17	43222300	0.743802
chr17	43233200	0.603306
chr17	43235700	0.752066
chr17	43240700	0.636364
chr17	43242900	0.900826
chr17	43248800	0.595041
chr17	43275400	0.851240
chr17	43302300	0.652893
chr17	43306400	0.859504
chr17	43328100	0.661157
chr17	43331800	0.925620

chr17	43334500	0.628099
chr17	43355200	0.809917
chr17	43376700	0.628099
chr17	43391600	0.677686
chr17	43396600	0.876033
chr17	43399300	0.719008
chr17	43410600	0.809917
chr17	43414000	0.884298
chr17	43417300	0.677686
chr17	43423400	0.909091
chr17	43460400	0.702479
chr17	43472500	0.950413
chr17	43503100	0.809917
chr17	43523800	0.867769
chr17	43561700	0.933884
chr17	43565600	0.595041
chr17	43570000	0.661157
chr17	43575500	0.743802
chr17	43657600	0.785124
chr17	43678900	0.727273
chr17	43691500	0.595041
chr17	43763900	0.876033
chr17	43930100	0.603306
chr17	43980500	0.603306
chr17	44026500	0.867769
chr17	44029700	0.611570
chr17	44054100	0.719008
chr17	44056300	0.884298
chr17	44088100	0.743802
chr17	44135200	0.603306
chr17	44155000	0.636364
chr17	44158000	0.933884
chr17	44160300	0.834711
chr17	44187400	0.628099
chr17	44191500	0.628099
chr17	44195900	0.735537
chr17	44200400	0.669421
chr17	44223400	0.685950
chr17	44228800	0.644628
chr17	44234700	0.760331
chr17	44237500	0.611570
chr17	44256300	0.628099
chr17	44258800	0.578512
chr17	44261000	0.818182
chr17	44263300	0.892562
chr17	44265500	0.933884
chr17	44280000	0.636364
chr17	44322400	0.867769
chr17	44364300	0.578512
chr17	44368800	0.991736
chr17	44420600	0.933884
chr17	44422900	0.768595
chr17	44518900	0.710744
chr17	44526200	0.578512

chr17	44592700	0.636364
chr17	44640700	0.892562
chr17	44676000	0.694215
chr17	44736400	0.793388
chr17	44820600	0.586777
chr17	44842300	0.842975
chr17	44847900	0.595041
chr17	44852300	0.933884
chr17	44857300	0.991736
chr17	44860400	0.603306
chr17	44863500	0.892562
chr17	44871800	0.578512
chr17	44886600	0.942149
chr17	44891700	0.785124
chr17	44914100	0.628099
chr17	44966000	0.859504
chr17	44983800	0.818182
chr17	45013600	0.950413
chr17	45021300	0.818182
chr17	45054400	0.801653
chr17	45097300	0.719008
chr17	45140400	0.752066
chr17	45149400	0.975207
chr17	45183500	0.595041
chr17	45191700	0.694215
chr17	45213700	0.669421
chr17	45234200	0.942149
chr17	45255000	0.760331
chr17	45260300	0.842975
chr17	45268500	0.570248
chr17	45275200	0.628099
chr17	45282100	0.900826
chr17	45291000	0.785124
chr17	45296200	0.876033
chr17	45298400	0.644628
chr17	45302800	0.867769
chr17	45307700	0.818182
chr17	45317600	0.859504
chr17	45321900	0.595041
chr17	45335500	0.884298
chr17	45344200	0.958678
chr17	45353200	0.826446
chr17	45357000	0.818182
chr17	45364200	0.735537
chr17	45366300	0.900826
chr17	45369400	0.966942
chr17	45372400	0.619835
chr17	45379000	0.727273
chr17	45383200	0.628099
chr17	45580300	0.975207
chr17	45589700	0.710744
chr17	45597500	0.669421
chr17	45637000	0.603306
chr17	45708600	0.586777

chr17	45730800	0.636364
chr17	45773200	0.611570
chr17	45779500	0.727273
chr17	45784400	0.925620
chr17	45823100	0.595041
chr17	45888000	0.851240
chr17	45912000	0.826446
chr17	45921800	0.669421
chr17	45943900	0.595041
chr17	45947100	0.867769
chr17	45951000	0.677686
chr17	45955700	0.619835
chr17	45979900	0.743802
chr17	46009200	0.727273
chr17	46013200	0.966942
chr17	46021300	0.603306
chr17	46024600	0.685950
chr17	46027200	0.685950
chr17	46035300	0.685950
chr17	46057300	0.702479
chr17	46061000	0.909091
chr17	46073800	0.628099
chr17	46080000	0.826446
chr17	46089300	0.677686
chr17	46104800	0.876033
chr17	46114400	0.677686
chr17	46131100	0.834711
chr17	46133600	0.925620
chr17	46226500	0.892562
chr17	46297400	0.818182
chr17	46317900	0.859504
chr17	46322100	0.710744
chr17	46324800	0.826446
chr17	46336000	0.892562
chr17	46343900	0.735537
chr17	46357400	0.900826
chr17	46389000	0.603306
chr17	46439200	0.578512
chr17	46446100	0.801653
chr17	46454000	0.768595
chr17	46462300	0.719008
chr17	46467500	0.719008
chr17	46504500	0.719008
chr17	46510500	0.595041
chr17	46517300	0.876033
chr17	46520600	0.884298
chr17	46534600	0.578512
chr17	46555700	0.983471
chr17	46562100	0.611570
chr17	46564700	0.834711
chr17	46566800	0.975207
chr17	46569200	0.925620
chr17	46572200	0.636364
chr17	46578600	0.876033

chr17	46583800	0.818182
chr17	46591100	0.834711
chr17	46597400	0.834711
chr17	46600300	0.603306
chr17	46606300	0.867769
chr17	46611900	0.636364
chr17	46614600	0.917355
chr17	46631800	0.867769
chr17	46634600	0.917355
chr17	46644800	0.694215
chr17	46648600	0.785124
chr17	46651400	0.826446
chr17	46660700	0.595041
chr17	46686700	0.595041
chr17	46699200	0.826446
chr17	46710400	0.958678
chr17	46714100	0.900826
chr17	46869200	0.669421
chr17	46903500	0.809917
chr17	47032000	0.752066
chr17	47039400	0.892562
chr17	47048600	0.669421
chr17	47050800	0.917355
chr17	47070100	0.719008
chr17	47082500	0.636364
chr17	47105900	0.801653
chr17	47154200	0.867769
chr17	47178800	0.644628
chr17	47319500	0.900826
chr17	47327000	0.677686
chr17	47330200	0.644628
chr17	47372200	0.809917
chr17	47387100	0.652893
chr17	47406200	0.719008
chr17	47416800	0.859504
chr17	47420000	0.694215
chr17	47440600	0.595041
chr17	47443100	0.570248
chr17	47465400	0.909091
chr17	47468300	0.801653
chr17	47503000	0.619835
chr17	47530200	0.595041
chr17	47592800	0.685950
chr17	47634100	0.595041
chr17	47643900	0.586777
chr17	47734000	0.586777
chr17	47787200	0.975207
chr17	47806300	0.685950
chr17	47820000	0.801653
chr17	47827600	0.644628
chr17	47832300	0.661157
chr17	47836700	0.842975
chr17	47845400	0.752066
chr17	47857500	0.958678

chr17	47910500	0.900826
chr17	47916800	0.586777
chr17	47922500	0.603306
chr17	47957600	0.851240
chr17	48020100	0.776860
chr17	48178900	0.884298
chr17	48205400	0.570248
chr17	48224300	0.636364
chr17	48232400	0.917355
chr17	48287000	0.727273
chr17	48290400	0.983471
chr17	48367600	0.892562
chr17	48385600	0.644628
chr17	48388500	0.743802
chr17	48439700	0.909091
chr17	48479300	0.644628
chr17	48501900	0.727273
chr17	48521500	0.669421
chr17	48526100	0.669421
chr17	48536700	0.628099
chr17	48619700	0.628099
chr17	48625000	0.619835
chr17	48637600	0.586777
chr17	48699100	0.636364
chr17	48704400	0.578512
chr17	48718800	0.983471
chr17	48738500	0.636364
chr17	48742700	0.909091
chr17	48788100	0.578512
chr17	48806900	0.826446
chr17	48809500	0.752066
chr17	48840900	0.611570
chr17	48895000	0.636364
chr17	48909500	0.900826
chr17	48930100	0.661157
chr17	48962000	0.793388
chr17	49013000	0.900826
chr17	49017300	0.776860
chr17	49021700	0.628099
chr17	49025500	0.826446
chr17	49028400	0.743802
chr17	49055400	0.925620
chr17	49194600	0.636364
chr17	49205700	0.834711
chr17	49210800	0.785124
chr17	49225400	0.603306
chr17	49329300	0.677686
chr17	49341300	0.611570
chr17	49348300	0.603306
chr17	49413600	0.694215
chr17	49442700	0.867769
chr17	51183500	0.966942
chr17	51696800	0.719008
chr17	51815300	0.603306

chr17	51823900	0.884298
chr17	51826100	0.735537
chr17	51842700	0.611570
chr17	52926700	0.851240
chr17	52937600	0.570248
chr17	52940600	0.677686
chr17	52943000	0.661157
chr17	52969600	0.570248
chr17	52980400	0.586777
chr17	53022800	0.636364
chr17	53049400	0.685950
chr17	53145800	0.867769
chr17	53164300	0.925620
chr17	53167900	0.958678
chr17	53380000	0.595041
chr17	53421900	0.933884
chr17	53426200	0.735537
chr17	53455400	0.801653
chr17	53526800	0.661157
chr17	53532700	0.669421
chr17	53558300	0.586777
chr17	53645000	0.917355
chr17	53654500	0.801653
chr17	53713900	0.603306
chr17	53740400	0.892562
chr17	53803100	0.586777
chr17	53809200	0.876033
chr17	53812300	0.818182
chr17	53816800	0.933884
chr17	53820500	0.611570
chr17	53838600	0.677686
chr17	53862500	0.958678
chr17	53890200	0.619835
chr17	53892900	0.586777
chr17	53895800	0.694215
chr17	53902100	0.677686
chr17	54850500	0.710744
chr17	54876900	0.586777
chr17	54954600	0.966942
chr17	54988000	0.727273
chr17	54994000	0.834711
chr17	54996500	0.801653
chr17	55015300	0.719008
chr17	55060000	0.776860
chr17	55063300	0.884298
chr17	55083100	0.570248
chr17	55095300	0.785124
chr17	55098000	0.900826
chr17	55128200	0.570248
chr17	55131800	0.826446
chr17	55145800	0.909091
chr17	55165300	0.570248
chr17	55185300	0.933884
chr17	55191100	0.595041

chr17	55333100	0.586777
chr17	55360000	0.628099
chr17	55362700	0.966942
chr17	55366000	0.752066
chr17	55407500	0.669421
chr17	55418000	0.859504
chr17	55421000	0.752066
chr17	55426700	0.636364
chr17	55433400	0.636364
chr17	55442800	0.595041
chr17	55445200	0.760331
chr17	55448100	0.669421
chr17	55453300	0.867769
chr17	55457400	0.925620
chr17	55462900	0.570248
chr17	55484600	0.834711
chr17	55498600	0.793388
chr17	55518500	0.603306
chr17	55525700	0.768595
chr17	55536800	0.966942
chr17	55558400	0.760331
chr17	55610100	0.760331
chr17	55619800	0.892562
chr17	55632900	0.636364
chr17	55910900	0.702479
chr17	55930500	0.694215
chr17	56024800	0.586777
chr17	56028700	0.876033
chr17	56062800	0.727273
chr17	56069600	0.685950
chr17	56150500	0.702479
chr17	56156300	0.867769
chr17	56158900	0.619835
chr17	56262600	0.876033
chr17	56274900	0.644628
chr17	56314600	0.603306
chr17	56322500	0.776860
chr17	56367800	0.677686
chr17	56405500	0.619835
chr17	56432500	0.628099
chr17	56438200	0.644628
chr17	56442500	0.942149
chr17	56445500	0.768595
chr17	56457200	0.851240
chr17	56469700	0.685950
chr17	56490400	0.595041
chr17	56513900	0.818182
chr17	56569900	0.842975
chr17	56653400	0.942149
chr17	56694100	0.644628
chr17	56736200	0.578512
chr17	56747700	0.735537
chr17	56757400	0.644628
chr17	56784400	0.727273

chr17	56864200	0.834711
chr17	57063200	0.851240
chr17	57172800	0.685950
chr17	57237500	0.685950
chr17	57367000	0.793388
chr17	57369300	0.752066
chr17	57374800	0.900826
chr17	57388600	0.570248
chr17	57407600	0.826446
chr17	57443100	0.710744
chr17	57450000	0.702479
chr17	57465800	0.619835
chr17	57493300	0.677686
chr17	57501100	0.793388
chr17	57599300	0.801653
chr17	57673300	0.892562
chr17	57693900	0.727273
chr17	57704800	0.694215
chr17	57708800	0.578512
chr17	57715800	0.636364
chr17	57760300	0.900826
chr17	57763000	0.851240
chr17	57774100	0.900826
chr17	57778600	0.636364
chr17	57790000	0.719008
chr17	57792600	0.628099
chr17	57807000	0.752066
chr17	57831700	0.950413
chr17	57857400	0.876033
chr17	57860600	0.925620
chr17	57866300	0.809917
chr17	57905700	0.644628
chr17	57908600	0.851240
chr17	57914500	0.611570
chr17	57920900	0.867769
chr17	57924100	0.710744
chr17	57930900	0.685950
chr17	58056700	0.925620
chr17	58114800	0.611570
chr17	58117500	0.677686
chr17	58167200	0.719008
chr17	58248300	0.694215
chr17	58264800	0.900826
chr17	58375700	0.636364
chr17	58429800	0.867769
chr17	58433500	0.884298
chr17	58440100	0.727273
chr17	58450300	0.933884
chr17	58460200	0.719008
chr17	58462400	0.669421
chr17	58547200	0.818182
chr17	59222700	0.785124
chr17	59253000	0.966942
chr17	59539900	0.578512

chr17	60163100	0.867769
chr17	60176700	0.636364
chr17	60183300	0.917355
chr17	60221300	0.942149
chr17	60234500	0.570248
chr17	60241600	0.760331
chr17	60247500	0.702479
chr17	60251000	0.595041
chr17	60256800	0.950413
chr17	60311600	0.892562
chr17	60316100	0.826446
chr17	60536700	0.966942
chr17	60548600	0.644628
chr17	60584800	0.578512
chr17	60711600	0.619835
chr17	60722500	0.685950
chr17	60731200	0.636364
chr17	60749300	0.628099
chr17	60772200	0.851240
chr17	60957800	0.636364
chr17	60994800	0.809917
chr17	61073100	0.958678
chr17	61117800	0.801653
chr17	61129400	0.595041
chr17	61393800	0.578512
chr17	61459200	0.719008
chr17	61492000	0.652893
chr17	61497900	1.000000
chr17	61509100	0.768595
chr17	61511800	0.950413
chr17	61514900	0.809917
chr17	61520400	0.826446
chr17	61526000	0.636364
chr17	61532400	0.586777
chr17	61538500	0.900826
chr17	61632500	0.570248
chr17	61649200	0.570248
chr17	61674900	0.628099
chr17	61704600	0.793388
chr17	61711800	0.917355
chr17	61752900	0.925620
chr17	61855200	0.578512
chr17	61930400	0.661157
chr17	61959600	0.595041
chr17	62008700	0.603306
chr17	62045900	0.578512
chr17	62054100	0.669421
chr17	62100900	0.884298
chr17	62103800	0.834711
chr17	62159700	0.991736
chr17	62163300	0.942149
chr17	62165900	0.578512
chr17	62188400	0.958678
chr17	62196300	0.702479

chr17	62226200	0.628099
chr17	62291000	0.933884
chr17	62294600	0.909091
chr17	62297300	0.636364
chr17	62308600	0.768595
chr17	62317600	0.851240
chr17	62406700	0.743802
chr17	62409800	0.942149
chr17	62533900	0.983471
chr17	62544200	0.644628
chr17	62569300	0.950413
chr17	62600900	0.603306
chr17	62605200	0.685950
chr17	62614700	0.760331
chr17	62632300	0.793388
chr17	62645300	0.586777
chr17	62650000	0.917355
chr17	62705800	0.884298
chr17	62789300	0.925620
chr17	62826800	0.933884
chr17	62835500	0.570248
chr17	62843000	0.851240
chr17	62891000	0.752066
chr17	62917500	0.842975
chr17	62921200	0.991736
chr17	62924100	0.958678
chr17	62935000	0.768595
chr17	62958500	0.760331
chr17	62982200	0.950413
chr17	63006600	0.619835
chr17	63054500	0.702479
chr17	63058900	0.694215
chr17	63061400	0.917355
chr17	63071000	0.595041
chr17	63127600	0.760331
chr17	63133800	0.628099
chr17	63155200	0.586777
chr17	63160500	0.867769
chr17	63168400	0.876033
chr17	63170700	0.884298
chr17	63225200	0.644628
chr17	63781600	0.619835
chr17	64247100	0.677686
chr17	64269500	0.760331
chr17	64272700	0.752066
chr17	64292700	0.644628
chr17	64327700	0.900826
chr17	64335800	0.884298
chr17	64340100	0.760331
chr17	64342700	0.933884
chr17	64346800	0.884298
chr17	64357200	0.735537
chr17	64361800	0.752066
chr17	64370800	0.743802

chr17	64397600	0.628099
chr17	64403300	0.628099
chr17	64441000	0.586777
chr17	64459500	0.636364
chr17	64463300	0.966942
chr17	64465900	0.867769
chr17	64487700	0.752066
chr17	64490600	0.991736
chr17	64499800	0.842975
chr17	64505900	0.619835
chr17	64581400	0.694215
chr17	64616700	0.694215
chr17	64652200	0.628099
chr17	64658900	0.950413
chr17	65107500	0.743802
chr17	65154200	0.958678
chr17	65214000	0.727273
chr17	65236200	0.958678
chr17	65356900	0.818182
chr17	65371200	0.818182
chr17	65428200	0.727273
chr17	65705300	0.719008
chr17	65756400	0.702479
chr17	65761900	0.677686
chr17	65775700	0.743802
chr17	65779200	0.834711
chr17	65782000	0.570248
chr17	65797300	0.644628
chr17	65825300	0.595041
chr17	65852700	0.710744
chr17	65959900	0.661157
chr17	66025900	0.809917
chr17	66082400	0.694215
chr17	66091800	0.677686
chr17	66148500	0.809917
chr17	66251900	0.702479
chr17	66433000	0.752066
chr17	66435100	0.826446
chr17	66438100	0.884298
chr17	66443200	0.785124
chr17	66457900	1.000000
chr17	66905900	0.834711
chr17	66947100	0.892562
chr17	67010200	0.768595
chr17	67045500	0.752066
chr17	67063100	0.694215
chr17	67067000	0.909091
chr17	67088100	0.702479
chr17	67091000	0.942149
chr17	67095800	0.685950
chr17	67098400	0.942149
chr17	67101200	0.933884
chr17	67138400	0.570248
chr17	67167500	0.719008

chr17	67172400	0.876033
chr17	67194900	0.644628
chr17	67235700	0.586777
chr17	67808800	0.677686
chr17	67853700	0.925620
chr17	67890600	0.685950
chr17	67904200	0.603306
chr17	67947200	0.776860
chr17	68056000	0.603306
chr17	68120900	0.611570
chr17	70563100	0.719008
chr17	70758000	0.917355
chr17	70887200	0.578512
chr17	70897600	0.909091
chr17	70903600	0.966942
chr17	70974300	0.983471
chr17	70997400	0.619835
chr17	71007300	0.743802
chr17	71197500	0.661157
chr17	71206800	0.867769
chr17	71223000	0.694215
chr17	71226000	0.818182
chr17	71285400	0.892562
chr17	71288100	0.991736
chr17	71294900	0.851240
chr17	71301500	0.719008
chr17	71304600	0.818182
chr17	71314300	0.942149
chr17	71319800	0.570248
chr17	71365600	0.809917
chr17	71470900	0.685950
chr17	72160100	0.892562
chr17	72204000	0.719008
chr17	72207600	0.595041
chr17	72216300	0.628099
chr17	72224200	0.677686
chr17	72231600	0.909091
chr17	72279400	0.785124
chr17	72291900	0.652893
chr17	72329200	0.925620
chr17	72427500	0.710744
chr17	72450500	0.768595
chr17	72460000	0.900826
chr17	72743400	0.851240
chr17	72751000	0.719008
chr17	72756700	0.975207
chr17	72775300	0.710744
chr17	72864200	0.826446
chr17	72873800	1.000000
chr17	72919800	0.611570
chr17	72927600	0.578512
chr17	72957400	0.578512
chr17	72976300	0.595041
chr17	73003100	0.677686

chr17	73015200	0.752066
chr17	73023900	0.685950
chr17	73033300	0.983471
chr17	73068200	0.983471
chr17	73070900	0.801653
chr17	73083500	0.884298
chr17	73088500	0.578512
chr17	73092900	0.983471
chr17	73110800	0.685950
chr17	73120800	0.942149
chr17	73129200	0.595041
chr17	73180600	0.719008
chr17	73185900	0.669421
chr17	73237200	0.776860
chr17	73263800	0.958678
chr17	73269700	0.652893
chr17	73314300	0.669421
chr17	73319800	0.570248
chr17	73322600	0.942149
chr17	73335100	0.909091
chr17	73338700	0.942149
chr17	73343400	0.785124
chr17	73345900	0.719008
chr17	73348800	0.859504
chr17	73357400	0.785124
chr17	73361700	0.586777
chr17	73365100	0.876033
chr17	73379600	0.661157
chr17	73393200	0.743802
chr17	73444800	0.983471
chr17	73449600	0.644628
chr17	73455700	0.595041
chr17	73483800	0.793388
chr17	73508200	0.628099
chr17	73537800	0.628099
chr17	73554000	0.809917
chr17	73567600	0.595041
chr17	73585500	0.636364
chr17	73624500	0.578512
chr17	73630000	0.909091
chr17	73638600	0.892562
chr17	73680300	0.909091
chr17	73746400	0.570248
chr17	73750300	0.760331
chr17	73784000	0.652893
chr17	73805700	0.752066
chr17	73843000	0.694215
chr17	73846100	0.900826
chr17	73855000	0.991736
chr17	73861300	0.966942
chr17	73869700	0.611570
chr17	73874600	0.595041
chr17	73947600	0.884298
chr17	73966500	0.801653

chr17	73972600	0.793388
chr17	73980000	0.867769
chr17	74026000	0.793388
chr17	74050100	0.942149
chr17	74091200	0.694215
chr17	74117500	0.867769
chr17	74137900	0.628099
chr17	74176300	0.991736
chr17	74179000	0.677686
chr17	74185500	0.983471
chr17	74193800	0.809917
chr17	74213900	0.851240
chr17	74216400	0.917355
chr17	74220400	0.867769
chr17	74229100	0.743802
chr17	74234100	0.677686
chr17	74238800	0.578512
chr17	74243200	0.702479
chr17	74252600	0.595041
chr17	74260500	0.884298
chr17	74266800	0.570248
chr17	74271300	0.834711
chr17	74273600	0.917355
chr17	74326500	0.595041
chr17	74341900	0.611570
chr17	74351900	0.570248
chr17	74366800	0.603306
chr17	74402300	0.942149
chr17	74411100	0.950413
chr17	74414300	0.785124
chr17	74438500	0.933884
chr17	74441000	0.851240
chr17	74443100	0.785124
chr17	74488000	0.603306
chr17	74502900	0.677686
chr17	74524600	0.925620
chr17	74526800	0.966942
chr17	74537200	0.966942
chr17	74559700	0.628099
chr17	74574600	0.975207
chr17	74597800	0.842975
chr17	74601800	0.760331
chr17	74608500	0.595041
chr17	74620000	0.826446
chr17	74634700	0.677686
chr17	74656400	0.909091
chr17	74664600	0.842975
chr17	74666900	0.644628
chr17	74669800	0.595041
chr17	74678400	0.851240
chr17	74686100	0.801653
chr17	74694000	0.809917
chr17	74705800	0.595041
chr17	74713000	0.900826

chr17	74728200	0.636364
chr17	74737100	0.595041
chr17	74769500	0.768595
chr17	74785900	0.933884
chr17	74788300	0.603306
chr17	74838200	0.652893
chr17	74841900	0.950413
chr17	74845300	0.834711
chr17	74864100	0.578512
chr17	75033600	0.661157
chr17	75066100	0.933884
chr17	75068400	0.966942
chr17	75072900	0.702479
chr17	75082900	0.727273
chr17	75102100	0.892562
chr17	75104700	0.826446
chr17	75117600	0.851240
chr17	75121100	0.975207
chr17	75124500	0.900826
chr17	75133700	0.809917
chr17	75136100	0.884298
chr17	75157000	0.743802
chr17	75159300	0.826446
chr17	75161700	0.735537
chr17	75176800	0.595041
chr17	75179200	0.578512
chr17	75217600	0.719008
chr17	75230100	0.933884
chr17	75272400	0.595041
chr17	75276300	0.719008
chr17	75282800	0.611570
chr17	75314800	0.586777
chr17	75321500	0.851240
chr17	75328900	0.578512
chr17	75338900	0.570248
chr17	75359800	0.975207
chr17	75362700	0.636364
chr17	75366100	0.685950
chr17	75368500	0.752066
chr17	75372100	0.834711
chr17	75382700	0.578512
chr17	75385900	0.834711
chr17	75388700	0.876033
chr17	75417100	0.776860
chr17	75423700	0.595041
chr17	75442100	0.760331
chr17	75446200	0.636364
chr17	75449800	0.876033
chr17	75456800	0.603306
chr17	75467600	0.991736
chr17	75538700	0.826446
chr17	75568200	0.710744
chr17	75767000	0.983471
chr17	75826800	0.719008

chr17	75831400	0.611570
chr17	75836700	0.909091
chr17	75860300	0.669421
chr17	75872500	0.950413
chr17	75878400	0.652893
chr17	75961700	0.619835
chr17	75997700	0.652893
chr17	76013100	0.975207
chr17	76016000	0.900826
chr17	76022800	0.966942
chr17	76026100	0.942149
chr17	76028500	0.785124
chr17	76031200	0.958678
chr17	76033700	0.776860
chr17	76043300	0.785124
chr17	76045700	0.677686
chr17	76054900	0.628099
chr17	76059100	0.611570
chr17	76078200	0.818182
chr17	76120100	0.595041
chr17	76127400	0.867769
chr17	76131000	0.768595
chr17	76133200	0.867769
chr17	76135400	0.743802
chr17	76137500	0.685950
chr17	76140400	0.842975
chr17	76158700	0.694215
chr17	76172700	0.644628
chr17	76187100	0.611570
chr17	76235900	0.644628
chr17	76243600	0.710744
chr17	76248100	0.818182
chr17	76252400	0.743802
chr17	76256100	0.950413
chr17	76259300	0.710744
chr17	76261700	0.710744
chr17	76264100	0.958678
chr17	76266300	0.909091
chr17	76269700	0.966942
chr17	76275600	0.570248
chr17	76278000	0.611570
chr17	76286300	0.719008
chr17	76297700	0.834711
chr17	76304700	0.818182
chr17	76314100	0.867769
chr17	76338600	0.661157
chr17	76352100	0.760331
chr17	76360900	0.702479
chr17	76371300	0.611570
chr17	76382500	0.644628
chr17	76407400	0.702479
chr17	76409500	0.851240
chr17	76418300	0.884298
chr17	76595600	0.595041

chr17	76662500	0.942149
chr17	76706100	0.876033
chr17	76715500	0.578512
chr17	76732300	0.702479
chr17	76744900	0.975207
chr17	76755600	0.909091
chr17	76776400	0.611570
chr17	76781300	0.826446
chr17	76784000	0.925620
chr17	76815800	0.595041
chr17	76850300	0.694215
chr17	76858600	0.884298
chr17	76879700	0.942149
chr17	76955300	0.677686
chr17	76982500	0.619835
chr17	76989300	0.735537
chr17	77008100	0.876033
chr17	77470100	0.942149
chr17	77702600	0.793388
chr17	77724200	0.661157
chr17	77774300	0.702479
chr17	77776900	0.735537
chr17	77780000	0.876033
chr17	77784200	0.702479
chr17	77787300	0.603306
chr17	77792600	0.768595
chr17	77802700	0.809917
chr17	77805900	0.694215
chr17	77809300	0.826446
chr17	77814200	0.809917
chr17	77819400	0.669421
chr17	78038200	0.842975
chr17	78043100	0.669421
chr17	78193700	0.619835
chr17	78227100	0.809917
chr17	78231300	0.991736
chr17	78236900	0.785124
chr17	78294000	0.760331
chr17	78431200	0.710744
chr17	78475700	0.628099
chr17	78574000	0.628099
chr17	78606200	0.644628
chr17	78624400	0.669421
chr17	78635000	0.644628
chr17	78638400	0.628099
chr17	78663300	0.851240
chr17	78665500	0.619835
chr17	78729700	0.793388
chr17	78765800	0.983471
chr17	78768400	0.628099
chr17	78806700	0.619835
chr17	78830400	0.760331
chr17	78866200	0.933884
chr17	78963100	0.710744

chr17	78976500	0.909091
chr17	78998200	0.619835
chr17	79014300	0.760331
chr17	79026600	0.694215
chr17	79031200	0.950413
chr17	79064600	0.661157
chr17	79069000	0.975207
chr17	79071700	0.900826
chr17	79127600	0.801653
chr17	79167900	0.834711
chr17	79175200	0.719008
chr17	79197600	0.628099
chr17	79201900	0.710744
chr17	79209200	0.876033
chr17	79218800	0.578512
chr17	79259600	0.669421
chr17	79266700	0.636364
chr17	79282700	0.603306
chr17	79285000	0.694215
chr17	79303700	0.603306
chr17	79324500	0.702479
chr17	79329000	0.859504
chr17	79339500	0.719008
chr17	79366300	0.628099
chr17	79383200	0.702479
chr17	79386700	0.942149
chr17	79439300	0.818182
chr17	79451700	0.785124
chr17	79524300	0.636364
chr17	79547300	0.859504
chr17	79611900	0.619835
chr17	79614200	0.752066
chr17	79630700	0.958678
chr17	79653200	0.570248
chr17	79668500	0.570248
chr17	79678300	0.818182
chr17	79689800	0.652893
chr17	79703300	0.611570
chr17	79708700	0.694215
chr17	79780000	0.958678
chr17	79821600	0.818182
chr17	79826000	0.942149
chr17	79851300	0.586777
chr17	79856700	0.628099
chr17	79860100	0.652893
chr17	79871200	0.892562
chr17	79878300	0.694215
chr17	79890600	0.925620
chr17	79893500	0.578512
chr17	79902800	0.628099
chr17	79926100	0.809917
chr17	79939100	0.818182
chr17	80059400	0.578512
chr17	80061900	0.677686

chr17	80074700	0.578512
chr17	80077300	0.975207
chr17	80079900	0.826446
chr17	80128800	0.884298
chr17	80158700	0.975207
chr17	80161500	0.735537
chr17	80164700	0.909091
chr17	80173000	0.884298
chr17	80176500	0.958678
chr17	80188500	0.669421
chr17	80193200	0.752066
chr17	80196700	0.677686
chr17	80198800	0.801653
chr17	80200900	0.743802
chr17	80225700	0.818182
chr17	80228600	0.669421
chr17	80245500	0.743802
chr17	80248400	0.909091
chr17	80253200	0.818182
chr17	80259700	0.628099
chr17	80267000	0.719008
chr17	80273700	0.702479
chr17	80282800	0.752066
chr17	80290500	0.719008
chr17	80293400	0.785124
chr17	80297300	0.785124
chr17	80301900	0.603306
chr17	80304300	0.983471
chr17	80307800	0.661157
chr17	80310400	0.628099
chr17	80317100	0.694215
chr17	80326700	0.768595
chr17	80339500	0.752066
chr17	80411100	0.719008
chr17	80414000	0.611570
chr17	80418800	0.586777
chr17	80422100	0.876033
chr17	80424200	0.586777
chr17	80455500	0.619835
chr17	80482400	0.595041
chr17	80489100	0.818182
chr17	80493900	0.603306
chr17	80506800	0.966942
chr17	80562000	0.677686
chr17	80569700	0.661157
chr17	80696400	1.000000
chr17	80713000	0.859504
chr17	80817700	0.851240
chr17	80821700	0.933884
chr17	80827000	0.942149
chr17	80834800	0.983471
chr17	80840500	0.619835
chr17	80842600	0.776860
chr17	81007000	0.950413

chr17	81010800	0.917355
chr17	81031300	0.884298
chr17	81040600	0.578512
chr17	81068000	0.826446
chr17	81076300	0.809917
chr17	81079900	0.892562
chr17	81104500	0.578512
chr17	81174500	0.586777
chr17	81178900	0.677686
chr18	107700	0.586777
chr18	143300	0.876033
chr18	255900	0.677686
chr18	304500	0.619835
chr18	307100	0.801653
chr18	519300	0.859504
chr18	605300	0.719008
chr18	618100	0.785124
chr18	629800	0.842975
chr18	650500	0.892562
chr18	652700	0.776860
chr18	731400	0.867769
chr18	767300	0.628099
chr18	773400	0.570248
chr18	776300	0.834711
chr18	778900	0.942149
chr18	782200	0.735537
chr18	786000	0.785124
chr18	791700	0.611570
chr18	798400	0.636364
chr18	817100	0.719008
chr18	826800	0.752066
chr18	830400	0.801653
chr18	834400	0.595041
chr18	839600	0.719008
chr18	842300	0.595041
chr18	845400	0.644628
chr18	967500	0.859504
chr18	988000	0.867769
chr18	1003100	0.578512
chr18	1050500	0.966942
chr18	1464900	0.801653
chr18	1487400	0.611570
chr18	1512000	0.859504
chr18	1529300	0.619835
chr18	2267200	0.570248
chr18	2301400	0.595041
chr18	2304800	0.876033
chr18	2317300	0.611570
chr18	2324900	0.909091
chr18	2327200	0.760331
chr18	2337500	0.669421
chr18	2525300	0.743802
chr18	2636900	0.826446
chr18	2659800	0.636364

chr18	2842300	0.644628
chr18	2849600	0.677686
chr18	2852900	0.752066
chr18	2855800	0.900826
chr18	2858000	0.752066
chr18	2875700	0.719008
chr18	2890700	0.661157
chr18	2894900	0.859504
chr18	2898400	0.685950
chr18	2970600	0.677686
chr18	2976900	0.942149
chr18	2983400	0.586777
chr18	2986700	0.727273
chr18	2994700	0.578512
chr18	2998500	0.851240
chr18	3015800	0.900826
chr18	3029500	0.694215
chr18	3039900	0.884298
chr18	3042700	0.694215
chr18	3062900	0.702479
chr18	3088600	0.611570
chr18	3112000	0.694215
chr18	3120500	0.702479
chr18	3129600	0.595041
chr18	3162500	0.578512
chr18	3178400	0.851240
chr18	3213800	0.785124
chr18	3217500	0.661157
chr18	3220000	0.760331
chr18	3259400	0.719008
chr18	3265900	0.636364
chr18	3294700	0.702479
chr18	3306400	0.752066
chr18	3321500	0.669421
chr18	3324200	0.942149
chr18	3327400	0.735537
chr18	3340900	0.917355
chr18	3381000	0.619835
chr18	3384900	0.603306
chr18	3425700	0.578512
chr18	3443200	0.826446
chr18	3445500	0.677686
chr18	3469000	0.570248
chr18	3482300	0.818182
chr18	3512600	0.909091
chr18	3521300	0.570248
chr18	3529500	0.884298
chr18	3541500	0.867769
chr18	3586100	0.768595
chr18	3599000	0.735537
chr18	3601700	0.719008
chr18	3609400	0.768595
chr18	3614300	0.851240
chr18	3616600	0.983471

chr18	3618900	0.975207
chr18	3621500	0.975207
chr18	3627300	0.900826
chr18	3632000	0.801653
chr18	3639400	0.685950
chr18	3649800	0.644628
chr18	3657300	0.801653
chr18	3659600	0.702479
chr18	3661900	0.735537
chr18	3664600	0.933884
chr18	3666700	0.842975
chr18	3675800	0.900826
chr18	3700400	0.851240
chr18	3704100	0.735537
chr18	3706900	0.851240
chr18	3710300	0.661157
chr18	3713100	0.694215
chr18	3772000	0.719008
chr18	3984100	0.694215
chr18	5298400	0.685950
chr18	6676600	0.983471
chr18	6686700	0.619835
chr18	6836500	0.661157
chr18	6885500	0.570248
chr18	6927200	0.719008
chr18	6929900	0.785124
chr18	8139800	0.975207
chr18	8450900	0.603306
chr18	8615600	0.785124
chr18	8654700	0.570248
chr18	8666100	0.595041
chr18	8704600	0.603306
chr18	8730700	0.925620
chr18	8733200	0.578512
chr18	8751100	0.578512
chr18	8835400	0.669421
chr18	8866400	0.793388
chr18	8875400	0.809917
chr18	8883300	0.859504
chr18	8891000	0.834711
chr18	8894100	0.818182
chr18	8897800	0.809917
chr18	8912100	0.669421
chr18	8918400	0.842975
chr18	8921700	0.677686
chr18	8937200	0.743802
chr18	8939800	0.669421
chr18	8944500	0.925620
chr18	8949100	0.743802
chr18	8952100	0.884298
chr18	8956700	0.801653
chr18	8963200	0.727273
chr18	8971300	0.876033
chr18	8983100	0.801653

chr18	8989900	0.909091
chr18	8994900	0.909091
chr18	8999200	0.950413
chr18	9001800	0.760331
chr18	9005600	0.884298
chr18	9007800	0.619835
chr18	9010900	0.917355
chr18	9015200	0.793388
chr18	9018800	0.842975
chr18	9022900	0.900826
chr18	9025700	0.644628
chr18	9033300	0.727273
chr18	9038600	0.619835
chr18	9049200	0.603306
chr18	9055500	0.685950
chr18	9058300	0.719008
chr18	9074000	0.628099
chr18	9095400	0.743802
chr18	9100500	0.793388
chr18	9113600	0.652893
chr18	9116700	0.975207
chr18	9119100	0.975207
chr18	9134500	0.595041
chr18	9141700	0.595041
chr18	9148000	0.776860
chr18	9157300	0.743802
chr18	9172700	0.644628
chr18	9181000	0.586777
chr18	9190000	0.892562
chr18	9338100	0.892562
chr18	9404100	0.570248
chr18	9421800	0.900826
chr18	9454500	0.595041
chr18	9506700	0.876033
chr18	9605700	0.818182
chr18	9611400	0.661157
chr18	9617200	0.867769
chr18	9674100	0.578512
chr18	9681900	0.983471
chr18	9686300	0.619835
chr18	9688800	0.826446
chr18	9700700	0.611570
chr18	9706200	0.925620
chr18	9715400	0.644628
chr18	9734400	0.595041
chr18	9758800	0.892562
chr18	9763000	0.818182
chr18	9771800	0.909091
chr18	9802300	0.694215
chr18	9805800	0.809917
chr18	9809400	0.834711
chr18	9812100	0.925620
chr18	9820900	0.743802
chr18	9824300	0.900826

chr18	9827500	0.991736
chr18	9849800	0.603306
chr18	9852900	0.636364
chr18	9868400	0.661157
chr18	9872500	0.867769
chr18	9874900	0.652893
chr18	9879300	0.958678
chr18	9883400	0.917355
chr18	9887000	0.719008
chr18	9889400	0.785124
chr18	9895600	0.900826
chr18	9901000	0.859504
chr18	9905500	0.685950
chr18	9907600	0.644628
chr18	9922500	0.628099
chr18	9925800	0.611570
chr18	9931700	0.809917
chr18	9951300	0.892562
chr18	9954900	0.570248
chr18	10081800	0.752066
chr18	10420100	0.752066
chr18	10531700	0.677686
chr18	10565400	0.876033
chr18	10578900	0.876033
chr18	10594200	0.867769
chr18	10619800	0.677686
chr18	10626900	0.826446
chr18	10629700	0.884298
chr18	10841300	0.876033
chr18	11807400	0.776860
chr18	11824000	0.685950
chr18	11846100	0.768595
chr18	11857700	0.793388
chr18	11860500	0.710744
chr18	11902800	0.867769
chr18	11917300	0.867769
chr18	11926300	0.694215
chr18	11952100	0.884298
chr18	11955700	0.760331
chr18	11963100	0.859504
chr18	12200100	0.752066
chr18	12202300	0.578512
chr18	12208900	0.809917
chr18	12212600	0.677686
chr18	12233800	0.925620
chr18	12237700	0.578512
chr18	12264700	0.644628
chr18	12284000	0.652893
chr18	12288800	0.595041
chr18	12296500	0.768595
chr18	12302700	0.983471
chr18	12306700	0.677686
chr18	12315600	0.818182
chr18	12359800	0.669421

chr18	12362000	0.636364
chr18	12373700	0.636364
chr18	12403900	0.925620
chr18	12406900	0.603306
chr18	12410500	0.570248
chr18	12468800	0.776860
chr18	12492000	0.975207
chr18	12607400	0.900826
chr18	12610800	0.685950
chr18	12632500	0.776860
chr18	12641000	0.966942
chr18	12653400	0.752066
chr18	12705500	0.595041
chr18	12741500	0.694215
chr18	12757100	0.628099
chr18	12762300	0.595041
chr18	12767400	0.752066
chr18	12838800	0.752066
chr18	12843600	0.595041
chr18	12865800	0.619835
chr18	12877000	0.760331
chr18	12880500	0.966942
chr18	12904300	0.900826
chr18	12960900	0.958678
chr18	12996600	0.603306
chr18	12998700	0.578512
chr18	13689000	0.611570
chr18	13714000	0.570248
chr18	14184000	0.793388
chr18	14238700	0.917355
chr18	14435600	0.570248
chr18	18512800	0.570248
chr18	18515700	0.719008
chr18	19138300	0.636364
chr18	19230600	0.743802
chr18	19266700	0.826446
chr18	19279400	0.884298
chr18	19315800	0.909091
chr18	19325200	0.628099
chr18	19327800	0.611570
chr18	19335100	0.884298
chr18	19646100	0.636364
chr18	19683500	0.859504
chr18	19686700	0.694215
chr18	19690600	0.950413
chr18	20032800	0.785124
chr18	20036900	0.719008
chr18	20046300	0.942149
chr18	20049000	0.958678
chr18	20052100	0.760331
chr18	20063800	0.603306
chr18	20137600	0.859504
chr18	20160300	0.884298
chr18	20247300	0.900826

chr18	20249500	0.760331
chr18	20682400	0.669421
chr18	20723800	0.694215
chr18	20840300	0.867769
chr18	20892800	0.677686
chr18	20960900	0.776860
chr18	21003100	0.958678
chr18	21013800	0.851240
chr18	21041000	0.570248
chr18	21068200	0.809917
chr18	21108000	0.867769
chr18	21110300	0.752066
chr18	21121200	0.851240
chr18	21284200	0.578512
chr18	21290200	0.595041
chr18	21299700	0.950413
chr18	21977900	0.570248
chr18	22657300	0.636364
chr18	22804500	0.603306
chr18	22811300	0.760331
chr18	22820500	0.595041
chr18	23570700	0.859504
chr18	23639700	0.917355
chr18	23665800	0.669421
chr18	23810700	0.661157
chr18	23836300	0.727273
chr18	23997600	0.586777
chr18	28148500	0.669421
chr18	28256100	0.818182
chr18	28259900	0.752066
chr18	28264300	0.661157
chr18	28392100	0.702479
chr18	28436000	0.669421
chr18	28442800	0.595041
chr18	28535700	0.619835
chr18	28679200	0.685950
chr18	28787000	0.661157
chr18	29066900	0.719008
chr18	29080800	0.694215
chr18	29084900	0.595041
chr18	29091200	0.595041
chr18	29283200	0.694215
chr18	29319100	0.892562
chr18	29402000	0.628099
chr18	29660800	0.719008
chr18	29676600	0.851240
chr18	29718600	0.801653
chr18	32560100	0.578512
chr18	32625900	0.619835
chr18	32631000	0.983471
chr18	32652600	0.727273
chr18	32676100	0.603306
chr18	32682200	0.611570
chr18	32684700	0.925620

chr18	32687100	0.975207
chr18	32689900	0.694215
chr18	32692500	0.917355
chr18	32698100	0.900826
chr18	32712000	0.966942
chr18	32715600	0.619835
chr18	32717800	0.983471
chr18	32819700	0.611570
chr18	32903000	0.776860
chr18	32942200	0.694215
chr18	32990300	0.669421
chr18	33023300	0.595041
chr18	33039700	0.975207
chr18	33043200	0.942149
chr18	33045600	0.760331
chr18	33088100	0.801653
chr18	33164800	0.611570
chr18	33260400	0.644628
chr18	33264300	0.917355
chr18	33268900	0.719008
chr18	33622000	0.619835
chr18	33643800	0.628099
chr18	33656400	0.595041
chr18	33712900	0.867769
chr18	33760200	0.892562
chr18	33780200	0.942149
chr18	33792500	0.669421
chr18	33845300	0.809917
chr18	34333800	0.793388
chr18	34379500	0.892562
chr18	34393500	0.851240
chr18	34431400	0.719008
chr18	34476700	0.644628
chr18	34530500	0.669421
chr18	34879900	0.917355
chr18	35247300	0.611570
chr18	35249900	0.710744
chr18	42305900	0.983471
chr18	42310800	0.611570
chr18	42314100	0.644628
chr18	42316900	0.983471
chr18	42419800	0.685950
chr18	42639500	0.842975
chr18	42641900	0.925620
chr18	43251000	0.933884
chr18	43267700	0.851240
chr18	43303800	0.619835
chr18	43579000	0.900826
chr18	43620400	0.966942
chr18	43632400	0.685950
chr18	43635600	0.677686
chr18	43640400	0.909091
chr18	43643900	0.801653
chr18	43729800	0.611570

chr18	43733500	0.884298
chr18	43737400	0.876033
chr18	43744500	0.826446
chr18	43749900	0.917355
chr18	43780100	0.809917
chr18	43803300	0.619835
chr18	43999400	0.685950
chr18	44002800	0.966942
chr18	44126200	0.677686
chr18	44291800	0.842975
chr18	44446500	0.752066
chr18	44448700	0.611570
chr18	44473700	0.834711
chr18	44477300	0.570248
chr18	44670800	0.917355
chr18	44699700	0.578512
chr18	44711300	0.768595
chr18	45379800	0.942149
chr18	45421000	0.685950
chr18	45428700	0.595041
chr18	45438900	0.834711
chr18	45443100	0.603306
chr18	45445700	0.570248
chr18	45452900	0.793388
chr18	45497600	0.900826
chr18	45526700	0.793388
chr18	46069700	0.966942
chr18	46072600	0.942149
chr18	46084100	0.578512
chr18	46093600	0.966942
chr18	46119400	0.743802
chr18	46132000	0.710744
chr18	46213100	0.595041
chr18	46220500	0.818182
chr18	46247000	0.876033
chr18	46259700	0.611570
chr18	46308000	0.867769
chr18	46325200	0.669421
chr18	46340900	0.983471
chr18	46355600	0.909091
chr18	46369000	0.719008
chr18	46372200	0.586777
chr18	46399600	0.677686
chr18	46423500	0.991736
chr18	46429100	0.801653
chr18	46444500	0.586777
chr18	46449300	0.702479
chr18	46451500	0.925620
chr18	46454300	0.586777
chr18	46456700	0.578512
chr18	46458900	0.743802
chr18	46461000	0.983471
chr18	46467400	0.776860
chr18	46482900	0.611570

chr18	46509400	0.570248
chr18	46524600	0.925620
chr18	46582600	0.677686
chr18	46588900	0.950413
chr18	46599100	0.900826
chr18	46602400	0.628099
chr18	46800800	0.628099
chr18	46850300	0.975207
chr18	46983600	0.603306
chr18	47003600	0.950413
chr18	47006400	0.867769
chr18	47022300	0.685950
chr18	47041400	0.586777
chr18	47051000	0.694215
chr18	47056000	0.570248
chr18	47088700	0.595041
chr18	47135000	0.752066
chr18	47252900	0.603306
chr18	47315300	0.586777
chr18	47344300	0.710744
chr18	47390800	0.611570
chr18	47406900	0.619835
chr18	47745300	0.603306
chr18	47834300	0.586777
chr18	47883100	0.917355
chr18	47886200	0.611570
chr18	47904300	0.644628
chr18	47922700	0.611570
chr18	47928300	0.578512
chr18	47951900	0.925620
chr18	47955300	0.669421
chr18	47959500	0.661157
chr18	47978600	0.677686
chr18	48332700	0.644628
chr18	48367100	0.983471
chr18	48444900	0.719008
chr18	48533600	0.966942
chr18	48535800	0.644628
chr18	48565000	0.586777
chr18	48627600	0.793388
chr18	48710700	0.661157
chr18	48718200	0.743802
chr18	51724500	0.876033
chr18	51764900	0.586777
chr18	51915300	0.983471
chr18	52379900	0.652893
chr18	52433600	0.760331
chr18	52448400	0.619835
chr18	52541100	0.983471
chr18	52549000	0.859504
chr18	52613600	0.628099
chr18	52867400	0.818182
chr18	52870100	0.570248
chr18	52969200	0.694215

chr18	53029800	0.652893
chr18	53054300	0.884298
chr18	53101000	0.628099
chr18	53126300	0.595041
chr18	53145300	0.578512
chr18	53184600	0.818182
chr18	53217200	0.867769
chr18	53258000	0.611570
chr18	53260200	0.983471
chr18	53331600	0.694215
chr18	53399000	0.826446
chr18	53405900	0.644628
chr18	53409100	0.710744
chr18	53416000	0.876033
chr18	53454200	0.644628
chr18	53617400	0.603306
chr18	53649900	0.644628
chr18	54140900	0.884298
chr18	54315800	0.727273
chr18	54328500	0.611570
chr18	54330800	0.768595
chr18	54719100	0.735537
chr18	54809100	0.752066
chr18	54811200	0.595041
chr18	55111400	0.619835
chr18	55255700	0.867769
chr18	55257800	0.735537
chr18	55261000	0.685950
chr18	55271300	0.925620
chr18	55299000	0.636364
chr18	55327300	0.851240
chr18	55334700	0.966942
chr18	55444200	0.942149
chr18	55448300	0.628099
chr18	55461200	0.652893
chr18	55487600	0.950413
chr18	55500900	0.719008
chr18	55513500	0.925620
chr18	55516000	0.636364
chr18	55577700	0.661157
chr18	55709100	0.983471
chr18	55722600	0.685950
chr18	55725700	0.578512
chr18	55737200	0.876033
chr18	55746500	0.909091
chr18	55755600	0.933884
chr18	55764900	0.719008
chr18	55785700	0.983471
chr18	55891600	0.892562
chr18	55925100	0.909091
chr18	55927500	0.834711
chr18	55930700	0.867769
chr18	55952400	0.710744
chr18	55966100	0.776860

chr18	55989200	0.677686
chr18	57214800	0.809917
chr18	57549700	0.909091
chr18	57576300	0.677686
chr18	57579000	0.652893
chr18	57586100	0.925620
chr18	57628700	0.760331
chr18	57721100	0.975207
chr18	59734000	0.578512
chr18	59838600	0.570248
chr18	59865600	0.652893
chr18	60088100	0.909091
chr18	60105600	0.719008
chr18	60153300	0.942149
chr18	60173900	0.595041
chr18	60186400	0.892562
chr18	60195300	0.669421
chr18	60222500	0.867769
chr18	60251700	0.727273
chr18	60278800	0.851240
chr18	60302400	0.735537
chr18	60327200	0.785124
chr18	60331400	0.975207
chr18	60409200	0.801653
chr18	60412400	0.876033
chr18	60446400	0.727273
chr18	60475600	0.760331
chr18	60504500	0.727273
chr18	60512900	0.752066
chr18	60518400	0.842975
chr18	60659500	0.768595
chr18	60664400	0.570248
chr18	61145300	0.743802
chr18	67615200	0.619835
chr18	67624400	0.685950
chr18	67626500	0.570248
chr18	67629400	0.892562
chr18	67648400	0.702479
chr18	67691500	0.900826
chr18	67693900	0.876033
chr18	67704900	0.966942
chr18	67819500	0.677686
chr18	67960600	0.685950
chr18	67964000	0.776860
chr18	68045300	0.933884
chr18	68052700	0.669421
chr18	68129600	0.892562
chr18	68132100	0.570248
chr18	71805500	0.768595
chr18	71893800	0.578512
chr18	71910200	0.818182
chr18	71919100	0.851240
chr18	71923600	0.603306
chr18	71936900	0.727273

chr18	71945000	0.611570
chr18	72005200	0.578512
chr18	72028500	0.595041
chr18	72147700	0.958678
chr18	72156200	0.892562
chr18	72176100	0.867769
chr18	72490500	0.702479
chr18	74189200	0.661157
chr18	74503200	0.636364
chr18	74546900	0.710744
chr18	75034900	0.669421
chr18	75697300	0.884298
chr18	75699400	0.669421
chr18	76366900	0.644628
chr18	76832000	0.611570
chr18	76916700	0.743802
chr18	76972200	0.595041
chr18	76978500	0.768595
chr18	77020600	0.909091
chr18	77173300	0.735537
chr18	77190200	0.760331
chr18	77263000	0.966942
chr18	77267900	0.909091
chr18	77276400	0.760331
chr18	77338300	0.611570
chr18	77361700	0.669421
chr18	77371400	0.710744
chr18	77703700	0.570248
chr18	77720600	0.760331
chr18	77735500	0.595041
chr18	77740500	0.611570
chr18	77743200	0.876033
chr18	77753600	0.595041
chr18	77772000	0.950413
chr18	77790000	0.809917
chr19	68000	0.611570
chr19	77400	0.925620
chr19	81600	0.760331
chr19	84400	0.991736
chr19	120800	0.636364
chr19	156800	0.702479
chr19	176300	0.677686
chr19	199200	0.892562
chr19	252700	0.834711
chr19	270700	0.818182
chr19	290800	0.760331
chr19	340600	0.710744
chr19	345600	0.677686
chr19	380800	0.628099
chr19	462600	0.702479
chr19	487600	0.611570
chr19	510900	0.785124
chr19	513500	0.595041
chr19	517000	0.818182

chr19	544600	0.752066
chr19	551900	0.776860
chr19	556400	0.900826
chr19	567500	0.694215
chr19	576500	0.636364
chr19	597700	0.570248
chr19	611300	0.933884
chr19	770900	0.727273
chr19	789600	0.900826
chr19	793600	0.586777
chr19	801900	0.595041
chr19	823900	0.752066
chr19	832000	0.685950
chr19	859600	0.760331
chr19	869700	0.892562
chr19	889300	0.876033
chr19	895600	0.785124
chr19	901000	0.793388
chr19	904100	0.950413
chr19	907000	0.768595
chr19	910300	0.776860
chr19	922500	0.735537
chr19	944600	0.892562
chr19	955000	0.826446
chr19	959700	0.851240
chr19	963400	0.966942
chr19	980400	0.694215
chr19	983000	0.842975
chr19	1001800	0.595041
chr19	1017000	0.752066
chr19	1022400	0.628099
chr19	1030600	0.876033
chr19	1045100	0.685950
chr19	1052700	0.768595
chr19	1073600	0.719008
chr19	1096100	0.661157
chr19	1099300	0.818182
chr19	1102400	0.842975
chr19	1113600	0.983471
chr19	1162000	0.603306
chr19	1165300	0.801653
chr19	1170600	0.892562
chr19	1175600	0.867769
chr19	1203400	0.595041
chr19	1205500	0.611570
chr19	1209600	0.859504
chr19	1217500	0.595041
chr19	1239900	0.677686
chr19	1246100	0.851240
chr19	1254300	0.793388
chr19	1259700	0.900826
chr19	1262700	0.818182
chr19	1265800	0.628099
chr19	1296200	0.669421

chr19	1309200	0.578512
chr19	1325100	0.578512
chr19	1332400	0.669421
chr19	1367300	0.925620
chr19	1415900	0.851240
chr19	1436200	0.760331
chr19	1444500	0.743802
chr19	1455800	0.702479
chr19	1507500	0.760331
chr19	1542500	0.636364
chr19	1600200	0.727273
chr19	1602600	0.752066
chr19	1633400	0.958678
chr19	1656100	0.975207
chr19	1666000	0.636364
chr19	1673000	0.603306
chr19	1685200	0.644628
chr19	1691500	0.669421
chr19	1767600	0.719008
chr19	1840700	0.644628
chr19	1843700	0.578512
chr19	1853500	0.818182
chr19	1858800	0.636364
chr19	1868900	0.917355
chr19	1882900	0.694215
chr19	1887500	0.892562
chr19	1907700	0.768595
chr19	1938300	0.578512
chr19	1977100	0.603306
chr19	2042100	0.892562
chr19	2052200	0.677686
chr19	2056000	0.859504
chr19	2058800	0.867769
chr19	2070000	0.752066
chr19	2087700	0.876033
chr19	2090800	0.595041
chr19	2162300	0.793388
chr19	2168000	0.892562
chr19	2170200	0.900826
chr19	2172600	0.842975
chr19	2235000	0.595041
chr19	2242000	0.685950
chr19	2263300	0.900826
chr19	2266900	0.859504
chr19	2282000	0.685950
chr19	2289900	0.785124
chr19	2346200	0.925620
chr19	2504100	0.586777
chr19	2723600	0.876033
chr19	2784600	0.752066
chr19	2865900	0.909091
chr19	2887600	0.595041
chr19	2908900	0.595041
chr19	2978200	0.636364

chr19	3023500	0.710744
chr19	3040000	0.933884
chr19	3042300	0.917355
chr19	3048800	0.611570
chr19	3063300	0.578512
chr19	3073400	0.966942
chr19	3093700	0.570248
chr19	3133700	0.958678
chr19	3155600	0.661157
chr19	3164000	0.603306
chr19	3177300	0.958678
chr19	3184000	0.636364
chr19	3189400	0.859504
chr19	3193900	0.933884
chr19	3266300	0.603306
chr19	3286800	0.595041
chr19	3307300	0.595041
chr19	3319700	0.603306
chr19	3358800	0.619835
chr19	3373200	0.570248
chr19	3375400	0.727273
chr19	3379000	0.727273
chr19	3383300	0.727273
chr19	3389700	0.942149
chr19	3451200	0.694215
chr19	3479300	0.595041
chr19	3503000	0.743802
chr19	3505300	0.611570
chr19	3520900	0.785124
chr19	3539300	0.636364
chr19	3560400	0.644628
chr19	3565800	0.603306
chr19	3569200	0.909091
chr19	3576500	0.578512
chr19	3580200	0.859504
chr19	3609900	0.925620
chr19	3613200	0.933884
chr19	3670100	0.818182
chr19	3672900	0.752066
chr19	3684100	0.644628
chr19	3721300	0.586777
chr19	3812800	0.909091
chr19	3828600	0.760331
chr19	3843400	0.876033
chr19	3850000	0.710744
chr19	3950800	0.685950
chr19	3954200	0.694215
chr19	3999600	0.611570
chr19	4005700	0.900826
chr19	4058800	0.851240
chr19	4075100	0.595041
chr19	4078100	0.975207
chr19	4089600	0.644628
chr19	4123100	0.570248

chr19	4131300	0.760331
chr19	4140900	0.570248
chr19	4145900	0.851240
chr19	4180700	0.570248
chr19	4205000	0.694215
chr19	4285100	0.586777
chr19	4288500	0.677686
chr19	4304200	0.619835
chr19	4311000	0.826446
chr19	4314500	0.595041
chr19	4369500	0.818182
chr19	4372900	0.900826
chr19	4381700	0.834711
chr19	4383900	0.578512
chr19	4391600	0.685950
chr19	4395000	0.933884
chr19	4414500	0.909091
chr19	4454900	0.793388
chr19	4470000	0.619835
chr19	4542200	0.867769
chr19	4577600	0.752066
chr19	4610400	0.578512
chr19	4623600	0.578512
chr19	4634100	0.619835
chr19	4636300	0.768595
chr19	4638400	0.785124
chr19	4655300	0.595041
chr19	4679300	0.727273
chr19	4795200	0.735537
chr19	4800000	0.578512
chr19	4813100	0.809917
chr19	4816200	0.876033
chr19	4841400	0.892562
chr19	4870500	0.892562
chr19	4914900	0.785124
chr19	4927800	0.768595
chr19	4968000	0.586777
chr19	4975600	0.644628
chr19	4988000	0.768595
chr19	4992300	0.710744
chr19	5090500	0.793388
chr19	5093600	0.966942
chr19	5128400	0.909091
chr19	5166400	0.760331
chr19	5173600	0.578512
chr19	5291300	0.892562
chr19	5346500	0.661157
chr19	5435100	0.983471
chr19	5586200	0.966942
chr19	5627000	0.900826
chr19	5629100	0.636364
chr19	5632900	0.619835
chr19	5676100	0.743802
chr19	5693700	0.735537

chr19	5799800	0.809917
chr19	5802900	0.619835
chr19	5895000	0.719008
chr19	5953300	0.975207
chr19	5957000	0.752066
chr19	5962400	0.752066
chr19	5969000	0.859504
chr19	5981100	0.586777
chr19	5983800	1.000000
chr19	5985900	0.842975
chr19	5988100	0.834711
chr19	5990700	0.702479
chr19	6048800	0.595041
chr19	6057900	0.834711
chr19	6062600	0.743802
chr19	6065000	0.694215
chr19	6067700	0.867769
chr19	6075300	0.652893
chr19	6088100	0.818182
chr19	6092600	0.900826
chr19	6100100	0.760331
chr19	6102200	0.586777
chr19	6116600	0.727273
chr19	6161600	0.710744
chr19	6239100	0.677686
chr19	6244700	0.570248
chr19	6271900	0.694215
chr19	6274500	0.685950
chr19	6276900	0.570248
chr19	6374300	0.685950
chr19	6426300	0.768595
chr19	6458100	0.685950
chr19	6504400	0.619835
chr19	6522600	0.942149
chr19	6528800	0.694215
chr19	6577600	0.785124
chr19	6580600	0.603306
chr19	6633200	0.685950
chr19	6652600	0.710744
chr19	6655100	0.702479
chr19	6667500	0.900826
chr19	6675100	0.876033
chr19	6678400	0.570248
chr19	6682900	0.876033
chr19	6718300	0.636364
chr19	6734400	0.619835
chr19	6753800	0.752066
chr19	6778900	0.876033
chr19	6802300	0.950413
chr19	6829000	0.669421
chr19	7545700	0.818182
chr19	7548700	0.851240
chr19	7559800	0.933884
chr19	7573100	0.611570

chr19	7717500	0.586777
chr19	7828900	0.752066
chr19	7834600	0.611570
chr19	7918400	0.760331
chr19	7923400	0.900826
chr19	7944300	0.702479
chr19	7973200	0.884298
chr19	7991700	0.702479
chr19	8047400	0.685950
chr19	8060200	0.735537
chr19	8066700	0.603306
chr19	8087200	0.743802
chr19	8094400	0.661157
chr19	8097400	0.652893
chr19	8101100	1.000000
chr19	8104000	0.661157
chr19	8150900	0.859504
chr19	8226000	0.570248
chr19	8259300	0.570248
chr19	8305600	0.925620
chr19	8334300	0.702479
chr19	8370400	0.586777
chr19	8394800	0.619835
chr19	8420700	0.603306
chr19	8432700	0.694215
chr19	8436400	0.851240
chr19	8453100	0.776860
chr19	8456900	0.694215
chr19	8462100	0.611570
chr19	8483500	0.933884
chr19	8489100	0.818182
chr19	8515300	0.644628
chr19	8569400	0.586777
chr19	8577200	0.752066
chr19	8580500	0.851240
chr19	8618300	0.694215
chr19	8634100	0.876033
chr19	8675200	0.719008
chr19	8816200	0.578512
chr19	9171800	0.685950
chr19	9186500	0.909091
chr19	9838800	0.834711
chr19	9898200	0.760331
chr19	9901300	0.892562
chr19	10025600	0.652893
chr19	10058700	0.694215
chr19	10203400	0.876033
chr19	10207100	0.801653
chr19	10213400	0.586777
chr19	10215500	0.991736
chr19	10310200	0.710744
chr19	10347100	0.925620
chr19	10396200	0.644628
chr19	10410200	0.752066

chr19	10412600	0.834711
chr19	10416200	0.958678
chr19	10422000	0.719008
chr19	10509600	0.884298
chr19	10511900	0.586777
chr19	10523300	0.644628
chr19	10533400	0.619835
chr19	10536900	0.595041
chr19	10562500	0.752066
chr19	10589900	0.702479
chr19	10603100	0.603306
chr19	10611500	0.578512
chr19	10695800	0.603306
chr19	10715600	0.776860
chr19	10727900	0.685950
chr19	10731800	0.628099
chr19	10759700	0.595041
chr19	10762600	0.611570
chr19	10769500	0.900826
chr19	10773500	0.661157
chr19	10806400	0.702479
chr19	10831800	0.644628
chr19	10846100	0.595041
chr19	10859700	0.917355
chr19	10862300	0.636364
chr19	10866400	0.925620
chr19	10870700	0.586777
chr19	10880300	0.702479
chr19	10920700	0.933884
chr19	10924500	0.900826
chr19	10931800	0.735537
chr19	10948300	0.727273
chr19	10950400	0.933884
chr19	10954300	0.694215
chr19	10974200	0.801653
chr19	10987600	0.958678
chr19	10992000	0.859504
chr19	11002500	0.603306
chr19	11046300	0.933884
chr19	11053300	0.702479
chr19	11074800	0.661157
chr19	11079900	0.818182
chr19	11196300	0.917355
chr19	11198700	0.595041
chr19	11207000	0.942149
chr19	11250100	0.834711
chr19	11253700	0.983471
chr19	11263600	0.776860
chr19	11268400	0.628099
chr19	11271000	0.793388
chr19	11300200	0.578512
chr19	11309400	0.586777
chr19	11454900	0.884298
chr19	11464100	0.669421

chr19	11466900	0.652893
chr19	11472700	0.603306
chr19	11488000	0.809917
chr19	11506000	0.628099
chr19	11607800	0.644628
chr19	11611400	0.619835
chr19	11647100	0.570248
chr19	11673100	0.818182
chr19	11676200	0.752066
chr19	11679700	0.942149
chr19	11689300	0.975207
chr19	11692600	1.000000
chr19	11698700	0.661157
chr19	11710700	0.595041
chr19	11752500	0.578512
chr19	11821200	0.677686
chr19	11932500	0.950413
chr19	11946400	0.727273
chr19	11972300	0.619835
chr19	12006200	0.603306
chr19	12025500	0.892562
chr19	12033400	0.677686
chr19	12444500	0.661157
chr19	12657100	0.785124
chr19	12660400	0.801653
chr19	12763500	0.578512
chr19	12890600	0.685950
chr19	12895600	0.685950
chr19	12899000	0.809917
chr19	12907300	0.826446
chr19	12936800	0.603306
chr19	12941800	0.619835
chr19	12944700	0.611570
chr19	12952600	0.628099
chr19	12959000	0.677686
chr19	12993700	0.644628
chr19	13000200	0.619835
chr19	13019900	0.826446
chr19	13024400	0.694215
chr19	13030300	0.933884
chr19	13052400	0.818182
chr19	13095900	0.991736
chr19	13102400	0.776860
chr19	13105700	0.694215
chr19	13124200	0.876033
chr19	13127000	0.768595
chr19	13129700	0.876033
chr19	13142800	0.685950
chr19	13162700	0.933884
chr19	13165100	0.752066
chr19	13172600	0.801653
chr19	13176300	0.677686
chr19	13178900	0.611570
chr19	13181400	0.628099

chr19	13190900	0.859504
chr19	13205200	0.628099
chr19	13207300	0.818182
chr19	13215600	0.933884
chr19	13237500	0.611570
chr19	13276300	0.710744
chr19	13278700	0.867769
chr19	13283600	0.735537
chr19	13346000	0.603306
chr19	13372100	0.619835
chr19	13493100	0.884298
chr19	13507800	0.652893
chr19	13510300	0.876033
chr19	13512500	0.842975
chr19	13649100	0.826446
chr19	13828200	0.578512
chr19	13841000	0.628099
chr19	13896300	0.702479
chr19	13914500	0.595041
chr19	13937200	0.652893
chr19	13944800	0.669421
chr19	13954700	0.867769
chr19	13960600	0.859504
chr19	13963000	0.917355
chr19	13967500	0.719008
chr19	13972700	0.801653
chr19	13991400	1.000000
chr19	14008300	0.702479
chr19	14016500	0.595041
chr19	14069400	0.892562
chr19	14114200	0.950413
chr19	14119500	0.933884
chr19	14126800	0.851240
chr19	14133100	0.578512
chr19	14172400	0.785124
chr19	14224800	0.768595
chr19	14229800	0.776860
chr19	14249800	0.578512
chr19	14253900	0.933884
chr19	14298800	0.719008
chr19	14331000	0.760331
chr19	14337500	0.611570
chr19	14397600	0.652893
chr19	14400000	0.785124
chr19	14406400	0.603306
chr19	14459500	0.578512
chr19	14467200	0.884298
chr19	14474200	0.760331
chr19	14478200	0.636364
chr19	14481400	0.570248
chr19	14483500	0.595041
chr19	14486700	0.909091
chr19	14496600	0.595041
chr19	14502400	0.785124

chr19	14527000	0.628099
chr19	14537000	0.752066
chr19	14551100	0.859504
chr19	14561500	0.793388
chr19	14616600	0.669421
chr19	14619100	0.661157
chr19	14621900	0.933884
chr19	14624800	0.578512
chr19	14630600	0.619835
chr19	14633600	0.685950
chr19	14638200	0.677686
chr19	14647500	0.669421
chr19	14649600	0.702479
chr19	14654000	0.793388
chr19	14656400	0.752066
chr19	14661400	0.892562
chr19	14667600	0.834711
chr19	14675100	0.661157
chr19	14685800	0.851240
chr19	14894600	0.900826
chr19	14897200	0.735537
chr19	15002400	0.842975
chr19	15237500	0.578512
chr19	15388200	0.809917
chr19	15391900	0.950413
chr19	15395200	0.851240
chr19	15405500	0.933884
chr19	15408500	0.917355
chr19	15411800	0.925620
chr19	15415400	0.859504
chr19	15432900	0.900826
chr19	15435800	0.966942
chr19	15524700	0.900826
chr19	15547600	0.611570
chr19	15561000	0.727273
chr19	15563400	0.818182
chr19	15751700	0.719008
chr19	15894100	0.570248
chr19	15945400	0.636364
chr19	15950300	0.578512
chr19	15953100	0.603306
chr19	15955700	0.669421
chr19	15970600	0.595041
chr19	15973900	0.603306
chr19	16181100	0.950413
chr19	16183600	0.603306
chr19	16189900	0.942149
chr19	16197600	0.628099
chr19	16202900	0.611570
chr19	16224600	0.578512
chr19	16264900	0.818182
chr19	16369700	0.884298
chr19	16373600	0.785124
chr19	16404800	0.694215

chr19	16427200	0.776860
chr19	16482600	0.793388
chr19	16485100	0.685950
chr19	16547300	0.884298
chr19	16553900	0.661157
chr19	16558100	0.925620
chr19	16575500	0.859504
chr19	16609200	0.570248
chr19	16673700	0.975207
chr19	16692300	1.000000
chr19	16694500	0.892562
chr19	16697400	0.702479
chr19	16701400	0.834711
chr19	16723300	0.735537
chr19	16933900	0.603306
chr19	16936600	0.619835
chr19	16998000	0.876033
chr19	17003100	0.991736
chr19	17005500	0.710744
chr19	17032500	0.801653
chr19	17108200	0.636364
chr19	17184000	0.661157
chr19	17208800	0.884298
chr19	17320800	0.950413
chr19	17331300	0.950413
chr19	17339200	0.595041
chr19	17348000	0.669421
chr19	17357700	0.958678
chr19	17360000	0.768595
chr19	17372200	0.586777
chr19	17405300	0.661157
chr19	17414600	0.685950
chr19	17419000	0.760331
chr19	17428800	0.661157
chr19	17481300	0.611570
chr19	17488500	0.851240
chr19	17510200	0.760331
chr19	17536300	0.685950
chr19	17561800	0.669421
chr19	17599800	0.752066
chr19	17619300	0.933884
chr19	17657100	0.900826
chr19	17664600	0.595041
chr19	17690900	0.727273
chr19	17705500	0.595041
chr19	17716400	0.578512
chr19	17723600	0.677686
chr19	17758900	0.685950
chr19	17791500	0.595041
chr19	17823400	0.752066
chr19	17832800	0.727273
chr19	17849500	0.876033
chr19	17855500	0.909091
chr19	17860700	0.851240

chr19	17902700	0.570248
chr19	17942500	0.619835
chr19	17974100	0.636364
chr19	17976700	0.909091
chr19	18053400	0.834711
chr19	18055800	0.652893
chr19	18058500	0.752066
chr19	18061600	0.669421
chr19	18065000	0.859504
chr19	18081100	0.917355
chr19	18110600	0.933884
chr19	18118400	0.785124
chr19	18124800	0.644628
chr19	18134900	0.628099
chr19	18176300	0.760331
chr19	18202100	0.950413
chr19	18205100	0.776860
chr19	18215400	0.760331
chr19	18268400	0.933884
chr19	18271900	0.735537
chr19	18286500	0.586777
chr19	18333000	0.677686
chr19	18374500	0.900826
chr19	18378300	0.636364
chr19	18386300	0.677686
chr19	18396300	0.661157
chr19	18401200	0.595041
chr19	18412400	0.884298
chr19	18414500	0.652893
chr19	18428500	0.876033
chr19	18431100	0.793388
chr19	18475200	0.793388
chr19	18477600	0.950413
chr19	18483100	0.876033
chr19	18487400	0.752066
chr19	18490900	0.975207
chr19	18503900	0.611570
chr19	18508700	0.595041
chr19	18513000	0.818182
chr19	18533500	0.669421
chr19	18540600	0.851240
chr19	18543700	0.743802
chr19	18551600	0.834711
chr19	18553700	0.834711
chr19	18581800	0.595041
chr19	18590300	0.900826
chr19	18592700	0.636364
chr19	18595900	0.950413
chr19	18601100	0.636364
chr19	18607500	0.685950
chr19	18618000	0.752066
chr19	18637200	0.727273
chr19	18656100	0.586777
chr19	18705000	0.685950

chr19	18708000	0.826446
chr19	18768600	0.578512
chr19	18799200	0.727273
chr19	18807400	0.785124
chr19	18842700	0.834711
chr19	18851700	0.752066
chr19	18854400	0.818182
chr19	18860800	0.603306
chr19	18875400	0.719008
chr19	18947000	0.652893
chr19	18951600	0.702479
chr19	18982800	0.570248
chr19	18995000	0.652893
chr19	19009600	0.851240
chr19	19083400	0.669421
chr19	19169500	0.628099
chr19	19177900	0.570248
chr19	19215900	0.694215
chr19	19225100	0.752066
chr19	19231900	0.735537
chr19	19281300	0.669421
chr19	19283400	0.983471
chr19	19286000	0.628099
chr19	19291000	0.578512
chr19	19318600	0.760331
chr19	19336400	0.611570
chr19	19472500	0.768595
chr19	19477600	0.619835
chr19	19483800	0.677686
chr19	19486400	0.826446
chr19	19489100	0.702479
chr19	19502500	0.925620
chr19	19506100	0.867769
chr19	19511300	0.595041
chr19	19514300	0.884298
chr19	19521700	0.710744
chr19	19555000	0.892562
chr19	19558300	0.801653
chr19	19571700	0.909091
chr19	19586400	0.677686
chr19	19588600	0.595041
chr19	19599000	0.818182
chr19	19601400	0.842975
chr19	19643700	0.768595
chr19	19709000	0.793388
chr19	20055300	0.942149
chr19	21310100	0.619835
chr19	21312300	0.809917
chr19	21341600	0.595041
chr19	21381000	0.909091
chr19	21641100	0.768595
chr19	21757100	0.760331
chr19	21776200	0.793388
chr19	21779200	0.809917

chr19	21782800	0.925620
chr19	21785200	0.900826
chr19	21788400	0.702479
chr19	21790900	0.719008
chr19	21793200	0.669421
chr19	21795400	0.983471
chr19	21801200	0.793388
chr19	21803500	0.652893
chr19	21808600	0.917355
chr19	21810900	0.826446
chr19	21815200	0.578512
chr19	21877600	0.942149
chr19	23871100	0.636364
chr19	23907500	0.809917
chr19	23953400	0.595041
chr19	27731800	0.719008
chr19	28964200	0.578512
chr19	28969700	0.727273
chr19	28973300	0.644628
chr19	28980900	0.694215
chr19	28985300	0.603306
chr19	28991100	0.677686
chr19	28994700	0.801653
chr19	28997000	0.595041
chr19	29000500	0.818182
chr19	29004000	0.925620
chr19	29007900	0.570248
chr19	29011400	0.768595
chr19	29016100	0.595041
chr19	29019600	0.685950
chr19	29038600	0.570248
chr19	29044400	0.966942
chr19	29050500	0.785124
chr19	29053500	0.719008
chr19	29055700	0.859504
chr19	29066200	0.578512
chr19	29068600	0.578512
chr19	29074200	0.628099
chr19	29077300	0.685950
chr19	29086100	0.677686
chr19	29090200	0.727273
chr19	29092500	0.661157
chr19	29103800	0.727273
chr19	29106100	0.570248
chr19	29110300	0.702479
chr19	29584200	0.586777
chr19	30118700	0.785124
chr19	30137100	0.859504
chr19	30142500	0.867769
chr19	30154300	0.826446
chr19	30162800	0.818182
chr19	30179000	0.958678
chr19	30182300	0.578512
chr19	30204000	0.586777

chr19	30297300	0.867769
chr19	30304500	0.785124
chr19	30345900	0.644628
chr19	30364700	0.628099
chr19	30372100	0.595041
chr19	30385200	0.818182
chr19	30388100	0.586777
chr19	30438600	0.719008
chr19	30443600	0.884298
chr19	30453900	0.578512
chr19	30458500	0.661157
chr19	30479900	0.900826
chr19	32860300	0.925620
chr19	32863900	0.867769
chr19	32866300	0.586777
chr19	32884200	0.760331
chr19	32909200	0.884298
chr19	32911700	0.644628
chr19	32914400	0.636364
chr19	33031600	0.586777
chr19	33040900	0.611570
chr19	33120100	0.983471
chr19	33161800	0.727273
chr19	33179000	0.719008
chr19	33199900	0.677686
chr19	33294200	0.760331
chr19	33569300	0.652893
chr19	33584800	0.950413
chr19	33592300	0.685950
chr19	33615900	0.975207
chr19	33625000	0.595041
chr19	33784800	0.793388
chr19	33867900	0.595041
chr19	34656600	0.785124
chr19	34669200	0.669421
chr19	34672600	0.925620
chr19	34680800	0.611570
chr19	34709600	0.768595
chr19	34716600	0.619835
chr19	34724000	0.983471
chr19	34752500	0.570248
chr19	34761100	0.603306
chr19	34808200	0.785124
chr19	34811000	0.719008
chr19	34819200	0.661157
chr19	34848800	0.735537
chr19	34854300	0.900826
chr19	34859300	0.570248
chr19	35222800	0.826446
chr19	35360900	0.818182
chr19	35397100	0.578512
chr19	35411500	0.975207
chr19	35466800	0.785124
chr19	35471500	0.611570

chr19	35490400	0.652893
chr19	35494400	0.950413
chr19	35532300	0.983471
chr19	35584200	0.677686
chr19	35597100	0.925620
chr19	35606100	0.685950
chr19	35652900	0.942149
chr19	35655700	0.859504
chr19	35659000	0.917355
chr19	35672800	0.752066
chr19	35676200	0.644628
chr19	35687400	0.628099
chr19	35696100	0.942149
chr19	35711500	0.975207
chr19	35714300	0.768595
chr19	35716500	0.942149
chr19	35773500	0.867769
chr19	35789500	0.818182
chr19	35793300	0.834711
chr19	35796200	0.801653
chr19	35799200	0.818182
chr19	35802200	0.809917
chr19	35807300	0.768595
chr19	35810100	1.000000
chr19	35837300	0.595041
chr19	35845000	0.719008
chr19	35871800	0.785124
chr19	35880200	0.826446
chr19	35894800	0.586777
chr19	35932600	0.727273
chr19	36066800	0.809917
chr19	36132800	0.983471
chr19	36141800	0.628099
chr19	36163500	0.619835
chr19	36233700	0.727273
chr19	36295000	0.900826
chr19	36352400	0.578512
chr19	36393200	0.595041
chr19	36398500	0.826446
chr19	36526800	0.958678
chr19	36548000	0.619835
chr19	36595500	0.809917
chr19	36632900	0.595041
chr19	38049000	0.570248
chr19	38434400	0.909091
chr19	38495800	0.603306
chr19	38583800	0.595041
chr19	38613700	0.958678
chr19	38626300	0.652893
chr19	38630500	0.586777
chr19	38644300	0.909091
chr19	38646900	0.611570
chr19	38674600	0.685950
chr19	38694900	0.710744

chr19	38708000	0.809917
chr19	38717800	0.578512
chr19	38735100	0.809917
chr19	38744600	0.611570
chr19	38758200	0.917355
chr19	38761800	0.636364
chr19	38773400	0.785124
chr19	38796000	0.760331
chr19	38802400	0.677686
chr19	38809700	0.950413
chr19	38812900	0.859504
chr19	38819400	0.619835
chr19	38861600	0.611570
chr19	38883500	0.917355
chr19	38901400	0.652893
chr19	38904100	0.933884
chr19	38908700	0.760331
chr19	38917500	0.677686
chr19	38943700	0.867769
chr19	38997500	0.743802
chr19	39006200	0.801653
chr19	39019200	0.644628
chr19	39036800	0.619835
chr19	39048000	0.900826
chr19	39095100	0.578512
chr19	39127100	0.950413
chr19	39143800	0.760331
chr19	39147200	0.710744
chr19	39150000	0.719008
chr19	39153000	0.578512
chr19	39157300	0.975207
chr19	39162100	0.702479
chr19	39169400	0.900826
chr19	39173500	0.867769
chr19	39176900	0.859504
chr19	39180400	0.983471
chr19	39219000	0.842975
chr19	39224700	0.611570
chr19	39228400	0.661157
chr19	39235300	0.652893
chr19	39241300	0.652893
chr19	39272400	0.644628
chr19	39310300	0.834711
chr19	39324000	0.570248
chr19	39350700	0.669421
chr19	39353800	0.975207
chr19	39369100	0.892562
chr19	39373700	0.661157
chr19	39376700	0.669421
chr19	39537200	0.710744
chr19	39624700	0.826446
chr19	39631000	0.752066
chr19	39641900	0.842975
chr19	39645700	0.636364

chr19	39692400	0.619835
chr19	39695500	0.793388
chr19	39743500	0.809917
chr19	39749400	0.942149
chr19	39796700	0.727273
chr19	39799900	0.925620
chr19	39802800	0.702479
chr19	39826300	0.851240
chr19	39838800	0.950413
chr19	39843000	0.909091
chr19	39847000	0.669421
chr19	39849700	0.710744
chr19	39872700	0.727273
chr19	39889200	0.942149
chr19	39892200	0.611570
chr19	39922100	0.917355
chr19	39924300	0.570248
chr19	39939300	0.611570
chr19	39970100	0.595041
chr19	39993800	0.595041
chr19	40011500	0.570248
chr19	40323300	0.669421
chr19	40325700	0.595041
chr19	40330400	0.991736
chr19	40512100	0.983471
chr19	40677400	0.578512
chr19	40715300	0.809917
chr19	40723100	0.727273
chr19	40726100	0.776860
chr19	40738400	0.743802
chr19	40773900	0.768595
chr19	40778400	0.595041
chr19	40781400	0.909091
chr19	40787500	0.876033
chr19	40793100	0.842975
chr19	40871200	0.595041
chr19	40873300	0.578512
chr19	40877700	0.950413
chr19	40905100	0.628099
chr19	40914500	0.834711
chr19	40917700	0.661157
chr19	40923100	0.917355
chr19	40941500	0.942149
chr19	40952800	0.834711
chr19	40968600	0.768595
chr19	40972900	0.603306
chr19	40994000	0.570248
chr19	41030500	0.702479
chr19	41033100	0.661157
chr19	41036100	0.669421
chr19	41101300	0.694215
chr19	41106100	0.644628
chr19	41113500	0.644628
chr19	41116200	0.685950

chr19	41121900	0.685950
chr19	41129400	0.768595
chr19	41131800	0.876033
chr19	41140500	0.677686
chr19	41146700	0.859504
chr19	41152900	0.685950
chr19	41170600	0.710744
chr19	41172800	1.000000
chr19	41193700	0.785124
chr19	41198400	0.933884
chr19	41209000	0.752066
chr19	41226800	0.702479
chr19	41260900	0.776860
chr19	41304000	0.619835
chr19	41307400	0.809917
chr19	41310800	0.925620
chr19	41315600	0.578512
chr19	41318200	0.752066
chr19	41325800	0.578512
chr19	41329400	0.966942
chr19	41332700	0.834711
chr19	41337700	0.826446
chr19	41341300	0.950413
chr19	41349900	0.842975
chr19	41368700	0.595041
chr19	41372000	0.925620
chr19	41381700	0.867769
chr19	41403200	0.942149
chr19	41407000	0.727273
chr19	41410900	0.628099
chr19	41414600	0.900826
chr19	41427700	0.752066
chr19	41435200	0.859504
chr19	41438600	0.909091
chr19	41452000	0.727273
chr19	41507800	0.669421
chr19	41628000	0.611570
chr19	41665800	0.925620
chr19	41713500	0.743802
chr19	41725400	0.867769
chr19	41730600	0.603306
chr19	41828600	0.578512
chr19	41831800	0.768595
chr19	41834200	0.867769
chr19	41855700	0.727273
chr19	41861400	0.636364
chr19	41874500	0.983471
chr19	41893100	0.677686
chr19	41901400	0.570248
chr19	41915700	0.710744
chr19	41931300	0.570248
chr19	41986000	0.826446
chr19	42347700	0.793388
chr19	42368200	0.677686

chr19	42382400	0.859504
chr19	42391100	0.801653
chr19	42431400	0.570248
chr19	42456000	0.628099
chr19	42515000	0.702479
chr19	42523100	0.909091
chr19	42704000	0.735537
chr19	42706400	0.644628
chr19	42712000	1.000000
chr19	42731000	0.768595
chr19	42734100	0.570248
chr19	42775900	0.603306
chr19	42791100	0.702479
chr19	42937900	0.694215
chr19	42954600	0.892562
chr19	43949600	0.900826
chr19	43972100	0.809917
chr19	44065600	0.776860
chr19	44111100	0.619835
chr19	44114700	0.917355
chr19	44120700	0.859504
chr19	44129200	0.834711
chr19	44139800	0.826446
chr19	44187500	0.685950
chr19	44205800	0.818182
chr19	44263600	0.768595
chr19	44269200	0.942149
chr19	44273200	0.694215
chr19	44282500	0.685950
chr19	44286200	0.933884
chr19	44288900	0.983471
chr19	44302900	0.752066
chr19	44309000	0.586777
chr19	44871000	0.586777
chr19	45056700	0.867769
chr19	45077100	0.900826
chr19	45091600	0.950413
chr19	45095200	0.694215
chr19	45142800	0.636364
chr19	45181000	0.570248
chr19	45198700	0.892562
chr19	45245500	0.917355
chr19	45256200	0.661157
chr19	45259000	0.619835
chr19	45354300	0.619835
chr19	45360000	0.876033
chr19	45407900	0.628099
chr19	45414000	0.892562
chr19	45416700	0.809917
chr19	45424100	0.611570
chr19	45430100	0.644628
chr19	45439300	0.628099
chr19	45446000	0.776860
chr19	45461200	0.644628

chr19	45506200	0.628099
chr19	45513700	0.578512
chr19	45553000	0.809917
chr19	45561800	0.619835
chr19	45581000	0.785124
chr19	45599700	0.611570
chr19	45602100	0.876033
chr19	45620500	0.661157
chr19	45630200	0.892562
chr19	45657700	0.735537
chr19	45674900	0.842975
chr19	45684900	0.735537
chr19	45696100	0.611570
chr19	45699600	0.595041
chr19	45723000	0.694215
chr19	45745500	0.826446
chr19	45749300	0.595041
chr19	45753800	0.628099
chr19	45766500	0.892562
chr19	45776300	0.991736
chr19	45803800	0.818182
chr19	45810100	0.578512
chr19	45829700	0.628099
chr19	45906300	0.669421
chr19	45919400	0.578512
chr19	45929500	0.818182
chr19	45944500	0.842975
chr19	45948700	0.636364
chr19	45959600	0.991736
chr19	45976300	0.619835
chr19	45989300	0.628099
chr19	46000500	0.619835
chr19	46002800	0.735537
chr19	46005300	0.867769
chr19	46008400	0.785124
chr19	46014800	0.942149
chr19	46017600	0.685950
chr19	46019700	0.933884
chr19	46081300	0.900826
chr19	46085900	0.603306
chr19	46091600	0.685950
chr19	46111100	0.595041
chr19	46118900	0.619835
chr19	46136200	0.636364
chr19	46170500	0.768595
chr19	46185200	0.760331
chr19	46231300	0.628099
chr19	46239500	0.785124
chr19	46284400	0.595041
chr19	46297200	0.578512
chr19	46305500	0.619835
chr19	46313300	0.900826
chr19	46377200	0.892562
chr19	46380600	0.636364

chr19	46392800	0.900826
chr19	46503400	0.652893
chr19	46697100	0.818182
chr19	46853000	0.636364
chr19	46858800	0.917355
chr19	46908500	0.743802
chr19	47034000	0.694215
chr19	47077900	0.636364
chr19	47107500	0.710744
chr19	47122700	0.677686
chr19	47125200	0.735537
chr19	47128700	0.892562
chr19	47133000	0.991736
chr19	47139400	0.685950
chr19	47143400	0.619835
chr19	47201700	0.801653
chr19	47222100	0.652893
chr19	47235300	0.917355
chr19	47240400	0.859504
chr19	47243900	0.603306
chr19	47253700	0.702479
chr19	47282000	0.570248
chr19	47285700	0.785124
chr19	47295900	0.603306
chr19	47316700	0.760331
chr19	47323800	0.628099
chr19	47344200	0.570248
chr19	47347200	0.595041
chr19	47370300	0.950413
chr19	47397300	0.760331
chr19	47407100	0.628099
chr19	47417100	0.859504
chr19	47428400	0.909091
chr19	47431000	0.710744
chr19	47462100	0.685950
chr19	47480500	0.809917
chr19	47494800	0.685950
chr19	47603400	0.884298
chr19	47609300	0.776860
chr19	47637600	0.619835
chr19	47655200	0.719008
chr19	47685900	0.809917
chr19	47692100	0.661157
chr19	47714500	0.909091
chr19	47728500	0.694215
chr19	47744900	0.685950
chr19	47748500	0.809917
chr19	47751100	0.900826
chr19	47763000	0.595041
chr19	47775600	0.636364
chr19	47781200	0.900826
chr19	47793200	0.702479
chr19	47809200	0.702479
chr19	47816900	0.851240

chr19	47819000	0.876033
chr19	47828300	0.661157
chr19	47833900	0.702479
chr19	47838100	0.727273
chr19	47840600	0.917355
chr19	47850100	0.966942
chr19	47855400	0.892562
chr19	47887000	0.909091
chr19	47972200	0.611570
chr19	47993800	0.611570
chr19	47998800	0.578512
chr19	48001100	0.966942
chr19	48003700	0.892562
chr19	48016100	0.876033
chr19	48035600	0.917355
chr19	48107600	0.644628
chr19	48151600	0.776860
chr19	48167600	0.933884
chr19	48226700	0.661157
chr19	48232100	0.595041
chr19	48234500	0.818182
chr19	48246300	0.644628
chr19	48250800	0.611570
chr19	48395500	0.983471
chr19	48422200	0.570248
chr19	48502300	0.727273
chr19	48710200	0.867769
chr19	48756000	0.752066
chr19	48813100	0.661157
chr19	48823700	0.752066
chr19	48827400	0.752066
chr19	48832700	0.760331
chr19	48835200	0.776860
chr19	48842600	0.685950
chr19	48868600	0.636364
chr19	48917800	0.611570
chr19	48922100	0.950413
chr19	48928100	0.884298
chr19	48994000	0.719008
chr19	49057300	0.694215
chr19	49067000	0.950413
chr19	49072100	0.859504
chr19	49078100	0.884298
chr19	49125500	0.842975
chr19	49128400	0.570248
chr19	49136600	0.578512
chr19	49158400	0.752066
chr19	49164400	0.909091
chr19	49178900	0.727273
chr19	49181900	0.644628
chr19	49184700	0.636364
chr19	49228900	0.876033
chr19	49243200	0.719008
chr19	49249000	0.603306

chr19	49258900	0.727273
chr19	49269500	0.719008
chr19	49284800	0.942149
chr19	49288400	0.851240
chr19	49310700	0.991736
chr19	49316000	0.636364
chr19	49334300	0.578512
chr19	49339500	0.809917
chr19	49358000	0.950413
chr19	49360900	0.933884
chr19	49371800	0.842975
chr19	49381100	0.966942
chr19	49383800	0.834711
chr19	49389800	0.760331
chr19	49395100	0.867769
chr19	49399700	0.685950
chr19	49402100	0.735537
chr19	49405900	0.677686
chr19	49422300	0.595041
chr19	49430500	0.661157
chr19	49454900	0.966942
chr19	49457200	0.727273
chr19	49464300	0.586777
chr19	49474000	0.925620
chr19	49479400	0.595041
chr19	49482500	0.743802
chr19	49524900	0.694215
chr19	49560300	0.595041
chr19	49577500	0.793388
chr19	49614200	0.760331
chr19	49636700	0.611570
chr19	49639400	0.867769
chr19	49649500	0.785124
chr19	49657100	0.710744
chr19	49729800	0.628099
chr19	49839100	0.719008
chr19	49843400	0.884298
chr19	49859800	0.677686
chr19	49867900	0.900826
chr19	49877100	0.619835
chr19	49891400	0.570248
chr19	49895000	0.719008
chr19	49907100	0.611570
chr19	49913000	0.958678
chr19	49926900	0.685950
chr19	49930000	0.727273
chr19	49939500	0.818182
chr19	49944500	0.570248
chr19	49951900	0.867769
chr19	49961600	0.570248
chr19	49973100	0.595041
chr19	49979800	0.595041
chr19	49996300	0.595041
chr19	50003400	0.652893

chr19	50006700	0.628099
chr19	50064200	1.000000
chr19	50071800	0.685950
chr19	50074400	0.876033
chr19	50117600	0.818182
chr19	50141800	0.652893
chr19	50204000	0.619835
chr19	50215600	0.900826
chr19	50231500	0.578512
chr19	50250000	0.793388
chr19	50261900	0.710744
chr19	50265300	0.958678
chr19	50273700	0.694215
chr19	50277200	0.917355
chr19	50279600	0.958678
chr19	50281800	0.694215
chr19	50309000	0.669421
chr19	50312400	0.776860
chr19	50351500	0.669421
chr19	50368100	0.570248
chr19	50375000	0.595041
chr19	50384500	0.669421
chr19	50392700	0.578512
chr19	50427300	0.834711
chr19	50458200	0.743802
chr19	50485800	0.933884
chr19	50507900	0.876033
chr19	50524500	0.925620
chr19	50526800	0.595041
chr19	50584700	0.842975
chr19	50587200	0.586777
chr19	50770300	0.595041
chr19	50773300	0.925620
chr19	50819600	0.661157
chr19	50839100	0.603306
chr19	50853900	0.628099
chr19	50864400	0.570248
chr19	50866900	0.950413
chr19	50873900	0.578512
chr19	50876300	0.966942
chr19	50886500	0.694215
chr19	50889800	0.595041
chr19	50918500	0.975207
chr19	50964900	0.768595
chr19	50970200	0.619835
chr19	51003900	0.636364
chr19	51069100	0.652893
chr19	51077300	0.727273
chr19	51085000	0.818182
chr19	51092300	0.793388
chr19	51103600	0.933884
chr19	51106800	0.942149
chr19	51109700	0.636364
chr19	51116800	0.570248

chr19	51120300	0.652893
chr19	51149700	0.694215
chr19	51153100	0.851240
chr19	51157500	0.578512
chr19	51181600	0.685950
chr19	51231200	0.818182
chr19	51239700	0.950413
chr19	51243400	0.719008
chr19	51246800	0.785124
chr19	51251800	0.975207
chr19	51264100	0.586777
chr19	51277400	0.727273
chr19	51323900	0.834711
chr19	51327300	0.834711
chr19	51382500	0.958678
chr19	51397900	0.595041
chr19	51400400	0.818182
chr19	51437400	0.603306
chr19	51506300	0.685950
chr19	51574200	0.826446
chr19	51626700	0.578512
chr19	51631700	0.809917
chr19	51640800	0.900826
chr19	51691400	0.586777
chr19	51854000	0.991736
chr19	51858200	0.867769
chr19	51866700	0.917355
chr19	51876500	0.570248
chr19	51884400	0.735537
chr19	51893500	0.570248
chr19	51896000	0.702479
chr19	51900500	0.719008
chr19	51920600	0.661157
chr19	51947000	0.776860
chr19	51952200	0.661157
chr19	51964600	0.570248
chr19	51975000	0.851240
chr19	51982200	0.619835
chr19	52004900	0.727273
chr19	52009600	0.685950
chr19	52013300	0.826446
chr19	52028800	0.958678
chr19	52030900	0.702479
chr19	52036600	0.818182
chr19	52046100	0.925620
chr19	52048900	0.842975
chr19	52068800	0.776860
chr19	52071000	0.942149
chr19	52078100	0.595041
chr19	52195600	0.578512
chr19	52202600	0.702479
chr19	52204700	0.603306
chr19	52209300	0.570248
chr19	52229900	0.636364

chr19	52237100	0.570248
chr19	52239700	0.578512
chr19	52271700	0.611570
chr19	52275300	0.661157
chr19	52281400	0.867769
chr19	52288400	0.983471
chr19	52297400	0.652893
chr19	52304900	0.628099
chr19	52308100	0.917355
chr19	52311400	0.851240
chr19	52464100	0.702479
chr19	53130700	0.578512
chr19	53150700	0.611570
chr19	53168300	0.925620
chr19	53170400	0.801653
chr19	53178800	0.768595
chr19	53183500	0.628099
chr19	53196000	0.826446
chr19	53226600	0.702479
chr19	53253400	0.570248
chr19	53339800	0.727273
chr19	53438400	0.710744
chr19	53664800	0.644628
chr19	54021300	0.619835
chr19	54272500	0.966942
chr19	54369400	0.694215
chr19	54523700	0.719008
chr19	54643800	0.661157
chr19	54666400	0.752066
chr19	54690800	0.851240
chr19	54711500	0.628099
chr19	54714800	0.950413
chr19	54718200	0.743802
chr19	54735500	0.735537
chr19	54963100	0.628099
chr19	55030300	0.578512
chr19	55084500	0.925620
chr19	55194000	0.619835
chr19	55210500	0.661157
chr19	55408100	0.611570
chr19	55416600	0.586777
chr19	55419000	0.661157
chr19	55455700	0.892562
chr19	55464600	0.619835
chr19	55510400	0.694215
chr19	55516700	0.628099
chr19	55530200	0.851240
chr19	55546500	0.925620
chr19	55551000	0.925620
chr19	55553700	0.876033
chr19	55583000	0.752066
chr19	55625000	0.801653
chr19	55656800	0.595041
chr19	55663500	0.834711

chr19	55669400	0.925620
chr19	55683100	0.595041
chr19	55690700	0.628099
chr19	55716600	0.702479
chr19	55720500	0.933884
chr19	55726700	0.809917
chr19	55759400	0.611570
chr19	55767100	0.619835
chr19	55782200	0.966942
chr19	55793600	0.793388
chr19	55808900	0.595041
chr19	55826000	0.760331
chr19	55836700	0.975207
chr19	55845700	0.702479
chr19	55887900	0.603306
chr19	55894400	0.619835
chr19	55901700	0.710744
chr19	55920600	0.636364
chr19	55929600	0.652893
chr19	55959800	0.909091
chr19	55964000	0.578512
chr19	55978700	0.983471
chr19	55999500	0.925620
chr19	56006500	0.694215
chr19	56015300	0.578512
chr19	56056600	0.958678
chr19	56059100	0.917355
chr19	56135300	0.661157
chr19	56151200	0.685950
chr19	56184800	0.917355
chr19	56189400	0.652893
chr19	56213800	0.619835
chr19	56578100	0.628099
chr19	56592800	0.694215
chr19	56629500	0.702479
chr19	56637600	1.000000
chr19	56674300	0.578512
chr19	56698500	0.892562
chr19	56761100	0.611570
chr19	56764300	0.611570
chr19	56769800	0.958678
chr19	56775300	0.826446
chr19	56818000	0.818182
chr19	56858900	0.826446
chr19	57834200	0.570248
chr19	58163000	0.702479
chr19	58167300	0.991736
chr19	58313500	0.595041
chr19	58321900	0.876033
chr19	58341400	0.669421
chr19	58842000	0.801653
chr19	58844700	0.851240
chr19	58915200	0.611570
chr19	58985200	0.628099

chr19	59000700	0.752066
chr19	59021400	0.884298
chr19	59027400	0.603306
chr19	59068000	0.669421
chr19	59080000	0.876033
chr19	59092300	0.983471
chr19	59095400	0.636364
chr19	59098900	0.851240
chr19	59101900	0.595041
chr19	59112300	0.966942
chr1	14500	0.776860
chr1	26400	0.661157
chr1	33700	0.752066
chr1	36100	0.760331
chr1	39900	0.785124
chr1	42300	0.727273
chr1	46900	0.669421
chr1	53400	0.595041
chr1	69400	0.776860
chr1	79600	0.677686
chr1	89200	0.801653
chr1	105800	0.876033
chr1	108800	0.719008
chr1	114600	0.785124
chr1	136400	0.942149
chr1	231000	0.644628
chr1	235500	0.900826
chr1	237600	0.619835
chr1	252100	0.909091
chr1	254900	0.818182
chr1	260500	0.586777
chr1	264300	0.578512
chr1	348600	0.595041
chr1	368000	0.991736
chr1	459700	0.603306
chr1	464000	0.760331
chr1	470700	0.636364
chr1	541100	0.859504
chr1	551300	0.611570
chr1	569900	0.644628
chr1	621800	0.975207
chr1	641800	0.735537
chr1	747700	0.595041
chr1	752700	0.826446
chr1	767000	0.710744
chr1	778300	0.909091
chr1	847200	0.619835
chr1	856800	0.611570
chr1	875500	0.595041
chr1	901600	0.628099
chr1	940500	0.958678
chr1	959700	0.859504
chr1	999500	0.842975
chr1	1001600	0.619835

chr1	1006300	0.611570
chr1	1044300	0.884298
chr1	1047300	0.628099
chr1	1070800	0.983471
chr1	1092100	0.644628
chr1	1121100	0.752066
chr1	1139300	0.619835
chr1	1144500	0.694215
chr1	1206800	0.578512
chr1	1251100	0.876033
chr1	1254900	0.933884
chr1	1258600	0.595041
chr1	1280700	0.710744
chr1	1293600	0.735537
chr1	1326600	0.793388
chr1	1338000	0.719008
chr1	1358900	0.785124
chr1	1369000	0.942149
chr1	1371300	0.570248
chr1	1376700	0.743802
chr1	1441000	0.818182
chr1	1491000	0.586777
chr1	1500400	0.595041
chr1	1513300	0.677686
chr1	1533100	0.925620
chr1	1583800	0.760331
chr1	1594600	0.933884
chr1	1647100	0.760331
chr1	1660200	0.694215
chr1	1690600	0.942149
chr1	1713900	0.958678
chr1	1778900	0.867769
chr1	1787700	0.685950
chr1	1790700	0.900826
chr1	1813000	0.826446
chr1	1815400	0.793388
chr1	1838200	0.727273
chr1	1841800	0.669421
chr1	1920800	0.694215
chr1	2110900	0.669421
chr1	2129000	0.867769
chr1	2132400	0.603306
chr1	2135900	0.768595
chr1	2138800	0.867769
chr1	2144600	0.884298
chr1	2158500	0.628099
chr1	2165700	0.776860
chr1	2187200	0.942149
chr1	2190200	0.702479
chr1	2193200	0.586777
chr1	2204000	0.702479
chr1	2222100	0.975207
chr1	2326100	0.851240
chr1	2455200	0.801653

chr1	2460700	0.578512
chr1	2473100	0.983471
chr1	2475700	0.983471
chr1	2480700	0.661157
chr1	2488300	0.768595
chr1	2492400	0.809917
chr1	3230300	0.958678
chr1	3448200	0.595041
chr1	3585500	0.570248
chr1	3661600	0.586777
chr1	3667800	0.818182
chr1	3676900	0.834711
chr1	3684100	0.570248
chr1	3687800	0.603306
chr1	3693700	0.859504
chr1	3710000	0.603306
chr1	3720100	0.685950
chr1	3724900	0.652893
chr1	3728300	0.719008
chr1	3739600	0.685950
chr1	3786300	0.859504
chr1	3797900	0.611570
chr1	4037200	0.677686
chr1	5946300	0.958678
chr1	5953100	0.942149
chr1	5976500	0.619835
chr1	6009800	0.661157
chr1	6041000	0.611570
chr1	6043700	0.876033
chr1	6055600	0.644628
chr1	6067600	0.942149
chr1	6070100	0.611570
chr1	6095200	0.595041
chr1	6135000	0.669421
chr1	6208800	0.694215
chr1	6261700	0.743802
chr1	6333700	0.603306
chr1	6370200	0.694215
chr1	6373800	0.719008
chr1	6377300	0.661157
chr1	6420200	0.867769
chr1	6487100	0.785124
chr1	6490300	0.876033
chr1	6531700	0.603306
chr1	6580700	0.661157
chr1	6642600	0.909091
chr1	6645400	0.842975
chr1	6670100	0.752066
chr1	6677100	0.570248
chr1	6727500	0.867769
chr1	6764500	0.578512
chr1	6818900	0.586777
chr1	6849200	0.727273
chr1	6861000	0.719008

chr1	6928900	0.694215
chr1	6972800	0.892562
chr1	7633500	0.735537
chr1	7646400	0.702479
chr1	7669300	0.669421
chr1	7690200	0.611570
chr1	7925100	0.685950
chr1	7973600	0.694215
chr1	8002200	0.743802
chr1	8011900	0.801653
chr1	8049200	0.611570
chr1	8066000	0.760331
chr1	8087000	0.652893
chr1	8167600	0.743802
chr1	8181200	0.644628
chr1	8183900	0.892562
chr1	8227700	0.586777
chr1	8230100	0.752066
chr1	8254900	0.702479
chr1	8258100	0.876033
chr1	8262700	0.595041
chr1	8272400	0.876033
chr1	8276400	0.652893
chr1	8292800	0.611570
chr1	8449400	0.942149
chr1	8452800	0.727273
chr1	8457300	0.619835
chr1	8469900	0.834711
chr1	8499500	0.900826
chr1	8524000	0.677686
chr1	8577700	0.619835
chr1	8580800	0.892562
chr1	8586700	0.586777
chr1	8602000	0.933884
chr1	8612300	0.636364
chr1	8621400	0.710744
chr1	8623900	0.578512
chr1	8632400	0.834711
chr1	8648000	0.619835
chr1	8651400	0.752066
chr1	8672500	0.644628
chr1	8686900	0.842975
chr1	8721900	0.735537
chr1	8724400	0.595041
chr1	8726500	0.727273
chr1	8728800	0.842975
chr1	8731400	0.644628
chr1	8740000	0.578512
chr1	8753900	0.768595
chr1	8773000	0.867769
chr1	8782800	0.743802
chr1	8813700	0.611570
chr1	8822400	0.636364
chr1	8825100	0.628099

chr1	8842500	0.644628
chr1	8844600	0.586777
chr1	8848500	0.892562
chr1	8853200	0.876033
chr1	8857600	0.809917
chr1	8883300	0.826446
chr1	8918700	0.801653
chr1	8932300	0.661157
chr1	8941500	0.809917
chr1	8960500	0.702479
chr1	8998700	0.636364
chr1	9224200	0.768595
chr1	9360800	0.851240
chr1	9504800	0.611570
chr1	9555800	0.619835
chr1	9589900	0.619835
chr1	9598500	0.652893
chr1	9653700	0.719008
chr1	9689900	0.586777
chr1	9718600	0.619835
chr1	9737300	0.652893
chr1	9773400	0.892562
chr1	9780000	0.669421
chr1	9813500	0.809917
chr1	9820000	0.661157
chr1	9835100	0.842975
chr1	9853800	0.586777
chr1	9865400	0.661157
chr1	9885800	0.677686
chr1	9888000	0.669421
chr1	9935500	0.628099
chr1	9961700	0.685950
chr1	9972800	0.958678
chr1	10011200	0.570248
chr1	10106000	0.917355
chr1	10125800	0.842975
chr1	10134800	0.603306
chr1	10144500	0.570248
chr1	10332600	0.685950
chr1	10380500	0.818182
chr1	10400400	0.752066
chr1	10448500	0.826446
chr1	10464100	0.636364
chr1	10475700	0.958678
chr1	10519600	0.900826
chr1	10571500	0.867769
chr1	10574400	0.677686
chr1	10577300	0.743802
chr1	10599800	0.719008
chr1	10614200	0.586777
chr1	10616800	0.983471
chr1	10777600	0.578512
chr1	11054800	0.603306
chr1	11288000	0.570248

chr1	11339800	0.669421
chr1	11509100	0.628099
chr1	11624100	0.669421
chr1	11709300	0.595041
chr1	11731600	1.000000
chr1	11750600	0.900826
chr1	11754100	0.859504
chr1	11761500	0.694215
chr1	11767700	0.809917
chr1	11780100	0.661157
chr1	11790000	0.933884
chr1	11801100	0.983471
chr1	11809200	0.702479
chr1	11813200	0.628099
chr1	11822700	0.661157
chr1	11845400	0.677686
chr1	11847700	0.760331
chr1	11850700	0.991736
chr1	11854800	0.909091
chr1	11860100	0.900826
chr1	11869900	0.867769
chr1	11872800	0.603306
chr1	11903400	0.752066
chr1	11940600	0.826446
chr1	11943300	0.975207
chr1	11953800	0.685950
chr1	11970900	0.834711
chr1	11973600	0.628099
chr1	11990700	0.900826
chr1	11999100	0.909091
chr1	12033000	0.661157
chr1	12044900	0.628099
chr1	12066600	0.942149
chr1	12074900	0.942149
chr1	12098900	0.776860
chr1	12104900	0.892562
chr1	12108100	0.760331
chr1	12112400	0.917355
chr1	12128400	0.702479
chr1	12136500	0.933884
chr1	12141900	0.925620
chr1	12145900	0.619835
chr1	12185700	0.793388
chr1	12204500	0.710744
chr1	12222400	0.851240
chr1	12233300	0.619835
chr1	12235400	0.942149
chr1	12255800	0.603306
chr1	12263800	0.669421
chr1	12278300	0.917355
chr1	12280600	0.909091
chr1	12288700	0.578512
chr1	12469800	0.933884
chr1	12488800	0.735537

chr1	12490900	0.570248
chr1	12493300	0.694215
chr1	12495800	0.917355
chr1	12498900	0.975207
chr1	12502200	0.876033
chr1	12505700	0.900826
chr1	12508000	0.636364
chr1	12515300	0.909091
chr1	12521300	0.859504
chr1	12525400	0.876033
chr1	12527800	0.710744
chr1	12529900	0.793388
chr1	12532400	0.851240
chr1	12536100	0.991736
chr1	12542100	0.942149
chr1	12554700	0.859504
chr1	12558300	0.900826
chr1	12571400	0.942149
chr1	12588100	0.900826
chr1	12591300	0.991736
chr1	12597700	0.950413
chr1	12601800	1.000000
chr1	12613400	0.768595
chr1	12633400	0.694215
chr1	12651900	0.785124
chr1	12654300	0.685950
chr1	12662000	0.685950
chr1	12664900	0.975207
chr1	12673600	0.595041
chr1	12695400	0.570248
chr1	12710700	0.677686
chr1	12805700	0.834711
chr1	12871100	0.809917
chr1	14084500	0.727273
chr1	14091500	0.595041
chr1	14135800	0.818182
chr1	14195900	0.826446
chr1	14279700	0.677686
chr1	14661400	0.876033
chr1	14745300	0.570248
chr1	14748900	0.867769
chr1	14751000	0.719008
chr1	14753200	0.578512
chr1	14766000	0.809917
chr1	14769900	0.900826
chr1	14782800	0.661157
chr1	14806600	0.801653
chr1	15094100	0.669421
chr1	15100500	0.710744
chr1	15106300	0.983471
chr1	15109200	0.851240
chr1	15112500	0.719008
chr1	15116600	0.735537
chr1	15127500	0.785124

chr1	15144300	0.909091
chr1	15153000	0.661157
chr1	15167200	0.793388
chr1	15181700	0.694215
chr1	15208800	0.859504
chr1	15228600	0.685950
chr1	15256300	0.966942
chr1	15260700	0.851240
chr1	15266700	0.925620
chr1	15297200	0.859504
chr1	15323500	0.884298
chr1	15347800	0.818182
chr1	15545400	0.884298
chr1	15551100	0.776860
chr1	15587900	0.876033
chr1	15672900	0.950413
chr1	15679200	0.834711
chr1	15685600	0.801653
chr1	15711000	0.595041
chr1	15738700	0.628099
chr1	15743200	0.619835
chr1	15750900	0.950413
chr1	15753400	0.628099
chr1	15771300	0.752066
chr1	15783400	0.884298
chr1	15791600	0.570248
chr1	15803000	0.785124
chr1	15841800	0.933884
chr1	15848300	0.826446
chr1	15868400	0.636364
chr1	15871900	0.719008
chr1	15949200	0.900826
chr1	15952200	0.892562
chr1	15955600	0.636364
chr1	16001900	0.818182
chr1	16008200	0.702479
chr1	16018700	0.917355
chr1	16107500	0.719008
chr1	16119100	0.570248
chr1	16122300	0.834711
chr1	16127100	0.851240
chr1	16171900	0.776860
chr1	16179300	0.776860
chr1	16183500	0.966942
chr1	16210300	0.867769
chr1	16220400	0.867769
chr1	16225000	0.942149
chr1	16275700	0.603306
chr1	16280800	0.900826
chr1	16284900	0.685950
chr1	16336900	0.752066
chr1	16339100	0.570248
chr1	16341500	0.801653
chr1	16358800	0.776860

chr1	16375300	0.636364
chr1	16380100	0.859504
chr1	16383000	0.966942
chr1	16403000	0.578512
chr1	16467900	0.867769
chr1	16474500	0.644628
chr1	16478000	0.876033
chr1	16488600	0.859504
chr1	16490900	0.909091
chr1	16499400	0.983471
chr1	16515600	0.636364
chr1	16522100	0.586777
chr1	16535500	0.892562
chr1	16555800	0.917355
chr1	16559500	0.933884
chr1	16570500	0.859504
chr1	16574800	0.950413
chr1	16576900	0.669421
chr1	16593900	0.892562
chr1	16669800	0.628099
chr1	16676200	0.694215
chr1	16701700	0.702479
chr1	16712900	0.578512
chr1	16718000	0.867769
chr1	16757600	0.685950
chr1	16769400	0.710744
chr1	16772200	0.801653
chr1	16846400	0.776860
chr1	16858400	0.570248
chr1	16860500	0.603306
chr1	16864300	0.950413
chr1	16866900	0.603306
chr1	16875500	0.826446
chr1	16878900	0.644628
chr1	16887400	0.661157
chr1	16892500	0.735537
chr1	16918800	0.595041
chr1	16928900	0.743802
chr1	16931600	0.578512
chr1	16942500	0.710744
chr1	16947900	0.611570
chr1	16966400	0.834711
chr1	16973400	0.694215
chr1	16981900	0.661157
chr1	16998000	0.909091
chr1	17000300	0.801653
chr1	17007600	0.677686
chr1	17030400	0.793388
chr1	17044900	0.760331
chr1	17062600	0.768595
chr1	17076600	0.595041
chr1	17088200	0.619835
chr1	17093400	0.570248
chr1	17101900	0.685950

chr1	17182100	0.636364
chr1	17197000	0.851240
chr1	17200700	0.818182
chr1	17217900	0.785124
chr1	17225000	0.677686
chr1	17229100	0.578512
chr1	17253900	0.570248
chr1	17256800	0.619835
chr1	17278400	0.619835
chr1	17336000	0.636364
chr1	17340300	0.636364
chr1	17342400	0.685950
chr1	17373100	0.636364
chr1	17383000	0.652893
chr1	17519000	0.834711
chr1	17634600	0.685950
chr1	17637000	0.867769
chr1	17675500	0.603306
chr1	17686900	0.950413
chr1	17724600	0.628099
chr1	17727700	0.586777
chr1	17731400	0.776860
chr1	17747900	0.710744
chr1	17814600	0.950413
chr1	18739300	0.933884
chr1	18742500	0.752066
chr1	18746700	0.644628
chr1	18749100	0.818182
chr1	18771200	0.570248
chr1	18857700	0.942149
chr1	18861600	0.892562
chr1	19043100	0.702479
chr1	19134200	0.876033
chr1	19181300	0.611570
chr1	19219000	0.719008
chr1	19222500	0.570248
chr1	19231200	0.603306
chr1	19236400	0.809917
chr1	19330400	0.694215
chr1	19395700	0.661157
chr1	19407600	0.975207
chr1	19440700	0.818182
chr1	19496700	0.975207
chr1	19506900	0.983471
chr1	19532500	0.760331
chr1	19627400	0.942149
chr1	19642000	0.884298
chr1	19644100	0.636364
chr1	19652500	0.818182
chr1	19668300	0.727273
chr1	19718000	0.884298
chr1	19732600	0.636364
chr1	19735300	0.760331
chr1	19748000	0.603306

chr1	19763600	0.809917
chr1	19782200	0.636364
chr1	19786200	0.966942
chr1	19792300	0.603306
chr1	19795300	0.702479
chr1	19797400	0.628099
chr1	19803800	0.851240
chr1	19807100	0.644628
chr1	19836000	0.859504
chr1	19860600	0.611570
chr1	19927100	0.694215
chr1	19930500	0.735537
chr1	19940000	0.785124
chr1	19978000	0.727273
chr1	20188400	0.710744
chr1	20205300	0.842975
chr1	20211700	0.694215
chr1	20335400	0.578512
chr1	20356200	0.768595
chr1	20498100	0.776860
chr1	20501900	0.710744
chr1	20510100	0.727273
chr1	20521800	0.727273
chr1	20524000	0.727273
chr1	20526100	0.834711
chr1	20539100	0.834711
chr1	20635200	0.578512
chr1	20803300	0.603306
chr1	20846800	0.578512
chr1	20940500	0.578512
chr1	20957700	0.834711
chr1	21060100	0.834711
chr1	21346900	0.644628
chr1	21352000	0.876033
chr1	21354800	0.884298
chr1	21385000	0.851240
chr1	21396100	0.611570
chr1	21427100	0.685950
chr1	21444500	0.776860
chr1	21492200	0.586777
chr1	21504500	0.595041
chr1	21545000	0.619835
chr1	21588300	0.900826
chr1	21592900	0.851240
chr1	21596700	0.752066
chr1	21625900	0.644628
chr1	21647100	0.859504
chr1	21651800	0.876033
chr1	21665000	0.603306
chr1	21670100	0.611570
chr1	21680300	0.876033
chr1	21716900	0.826446
chr1	21719700	0.752066
chr1	21781600	0.776860

chr1	21886300	0.719008
chr1	21901400	0.793388
chr1	21933800	0.859504
chr1	21936700	0.727273
chr1	21939800	0.842975
chr1	21943200	0.942149
chr1	21945300	0.727273
chr1	21949800	0.785124
chr1	21958000	0.859504
chr1	21961800	0.727273
chr1	21965500	0.950413
chr1	21967700	0.628099
chr1	21975100	0.702479
chr1	21994400	0.743802
chr1	21998000	0.842975
chr1	22000100	0.917355
chr1	22003200	0.661157
chr1	22012700	0.644628
chr1	22016000	0.950413
chr1	22030400	0.826446
chr1	22034000	0.652893
chr1	22076500	0.950413
chr1	22088800	0.768595
chr1	22092700	0.694215
chr1	22096100	0.793388
chr1	22102900	0.570248
chr1	22112600	0.628099
chr1	22116700	0.892562
chr1	22119000	0.603306
chr1	22145500	0.760331
chr1	22149400	0.661157
chr1	22180200	0.785124
chr1	22193400	0.818182
chr1	22213600	0.768595
chr1	22224100	0.942149
chr1	22229300	0.818182
chr1	22236000	0.710744
chr1	22238700	0.966942
chr1	22241700	0.991736
chr1	22244000	0.876033
chr1	22247500	0.818182
chr1	22251600	0.942149
chr1	22257800	0.644628
chr1	22265800	0.752066
chr1	22269300	0.694215
chr1	22283900	0.677686
chr1	22290700	0.661157
chr1	22345700	0.851240
chr1	22354300	0.603306
chr1	22364800	0.768595
chr1	22367000	0.735537
chr1	22369500	0.801653
chr1	22372300	0.801653
chr1	22375400	0.752066

chr1	22382400	0.595041
chr1	22402200	0.933884
chr1	22448600	0.685950
chr1	22490600	0.694215
chr1	22612500	0.933884
chr1	22623600	0.884298
chr1	22689800	0.975207
chr1	22774500	0.702479
chr1	22781300	0.925620
chr1	22788500	0.752066
chr1	22814800	0.669421
chr1	22864400	0.677686
chr1	23310800	0.636364
chr1	23324200	0.776860
chr1	23331000	0.611570
chr1	23430000	0.801653
chr1	23437100	0.669421
chr1	23441500	0.867769
chr1	23443900	0.801653
chr1	23452100	0.652893
chr1	23699100	0.611570
chr1	23703800	0.694215
chr1	23711600	0.958678
chr1	23730400	0.727273
chr1	23744200	0.776860
chr1	23749300	0.727273
chr1	23763000	0.867769
chr1	23771000	0.595041
chr1	23778800	0.595041
chr1	23805800	0.867769
chr1	23854700	0.603306
chr1	23858900	0.611570
chr1	23866700	0.611570
chr1	23875500	0.702479
chr1	23889200	0.776860
chr1	23903900	0.719008
chr1	23923300	0.892562
chr1	23943700	0.818182
chr1	23965000	0.669421
chr1	24043000	0.826446
chr1	24046800	0.768595
chr1	24059100	0.834711
chr1	24074400	0.826446
chr1	24107300	0.578512
chr1	24115700	0.785124
chr1	24120300	0.809917
chr1	24134100	0.677686
chr1	24157100	0.818182
chr1	24172500	0.785124
chr1	24199500	0.785124
chr1	24235800	0.867769
chr1	24241100	0.776860
chr1	24243300	0.595041
chr1	24247300	0.727273

chr1	24281800	0.710744
chr1	24289400	0.570248
chr1	24292300	0.595041
chr1	24303400	0.595041
chr1	24345600	0.768595
chr1	24426100	0.661157
chr1	24430300	0.776860
chr1	24477000	0.760331
chr1	24577100	0.570248
chr1	24621300	0.859504
chr1	24638000	0.685950
chr1	24649200	0.570248
chr1	24738100	0.834711
chr1	24745800	0.644628
chr1	24765300	0.842975
chr1	24767700	0.636364
chr1	24862800	0.570248
chr1	24913300	0.603306
chr1	24918700	0.876033
chr1	25005200	0.570248
chr1	25010800	0.619835
chr1	25021600	0.685950
chr1	25032200	0.669421
chr1	25062500	0.925620
chr1	25069700	0.991736
chr1	25075200	0.628099
chr1	25089000	0.925620
chr1	25092500	0.892562
chr1	25130400	0.619835
chr1	25279400	0.652893
chr1	25293300	0.619835
chr1	25543200	0.727273
chr1	25563900	0.809917
chr1	25587100	0.628099
chr1	25597200	0.652893
chr1	25602400	0.776860
chr1	25604600	0.859504
chr1	25607800	0.595041
chr1	25612000	0.892562
chr1	25629000	0.876033
chr1	25649900	0.859504
chr1	25667500	0.578512
chr1	25683600	0.809917
chr1	25688300	0.760331
chr1	25696900	0.801653
chr1	25735500	0.636364
chr1	25739200	0.735537
chr1	25742100	0.842975
chr1	25744500	0.752066
chr1	25748900	0.966942
chr1	25762700	0.719008
chr1	25768500	0.867769
chr1	25772600	0.809917
chr1	25788900	0.652893

chr1	25794700	0.867769
chr1	25804000	0.876033
chr1	25808700	0.884298
chr1	25812600	0.669421
chr1	25851400	0.628099
chr1	25875300	0.925620
chr1	25878000	0.834711
chr1	25880800	0.818182
chr1	25895900	0.842975
chr1	26021800	0.768595
chr1	26035100	0.743802
chr1	26087700	0.702479
chr1	26106900	0.644628
chr1	26109900	0.801653
chr1	26144600	0.636364
chr1	26149800	0.743802
chr1	26159000	0.760331
chr1	26172500	0.628099
chr1	26182800	0.661157
chr1	26189300	0.925620
chr1	26194200	0.611570
chr1	26197800	0.942149
chr1	26206600	0.644628
chr1	26213800	0.578512
chr1	26217200	0.900826
chr1	26222700	0.611570
chr1	26229700	0.652893
chr1	26247800	0.636364
chr1	26250700	0.826446
chr1	26253600	0.578512
chr1	26258800	0.611570
chr1	26261700	0.685950
chr1	26281500	0.619835
chr1	26305400	0.785124
chr1	26317300	0.578512
chr1	26320200	0.595041
chr1	26325900	0.570248
chr1	26332600	0.826446
chr1	26342100	0.578512
chr1	26348700	0.628099
chr1	26357100	0.652893
chr1	26364900	0.685950
chr1	26370600	0.983471
chr1	26376600	0.818182
chr1	26381000	0.595041
chr1	26387300	0.685950
chr1	26393800	0.917355
chr1	26396000	0.727273
chr1	26398500	0.595041
chr1	26402900	0.925620
chr1	26405700	0.925620
chr1	26408600	0.867769
chr1	26418900	0.727273
chr1	26421200	0.611570

chr1	26424700	0.859504
chr1	26431600	0.818182
chr1	26441900	0.603306
chr1	26446300	0.570248
chr1	26448400	0.876033
chr1	26456700	0.867769
chr1	26459700	0.842975
chr1	26466000	0.917355
chr1	26468800	0.727273
chr1	26471800	0.685950
chr1	26486400	0.685950
chr1	26505100	0.628099
chr1	26549200	0.669421
chr1	26602100	0.975207
chr1	26604600	0.950413
chr1	26608800	0.768595
chr1	26611500	0.966942
chr1	26613700	0.966942
chr1	26616900	0.702479
chr1	26635600	0.909091
chr1	26638800	0.702479
chr1	26642800	0.925620
chr1	26678000	0.859504
chr1	26685500	0.785124
chr1	26701700	0.752066
chr1	26706400	0.652893
chr1	26712300	0.710744
chr1	26718600	0.595041
chr1	26736400	0.851240
chr1	26739100	0.611570
chr1	26814100	0.595041
chr1	26835600	0.619835
chr1	26844400	0.586777
chr1	26858800	0.925620
chr1	26861000	0.801653
chr1	26868200	0.661157
chr1	26870300	0.851240
chr1	26882200	0.743802
chr1	26902000	0.768595
chr1	26924700	0.611570
chr1	26929800	0.727273
chr1	26939800	0.586777
chr1	26964700	0.586777
chr1	26974900	0.752066
chr1	26989900	0.702479
chr1	27004300	0.793388
chr1	27014300	0.578512
chr1	27017100	0.826446
chr1	27030300	0.586777
chr1	27033200	0.925620
chr1	27039400	0.727273
chr1	27044400	0.793388
chr1	27048300	0.669421
chr1	27070300	0.793388

chr1	27072500	0.694215
chr1	27095700	0.595041
chr1	27098500	0.892562
chr1	27106200	0.925620
chr1	27110100	0.578512
chr1	27118300	0.694215
chr1	27123800	0.851240
chr1	27126600	0.785124
chr1	27129100	0.743802
chr1	27132300	0.942149
chr1	27155600	0.884298
chr1	27158500	0.578512
chr1	27189700	0.851240
chr1	27240700	0.776860
chr1	27251200	0.611570
chr1	27277400	0.958678
chr1	27331600	0.925620
chr1	27337900	0.652893
chr1	27341400	0.603306
chr1	27344000	0.818182
chr1	27348900	0.578512
chr1	27353300	0.776860
chr1	27401000	0.983471
chr1	27426400	0.867769
chr1	27439000	0.851240
chr1	27442600	0.867769
chr1	27449400	0.793388
chr1	27464300	0.900826
chr1	27472800	0.884298
chr1	27479400	0.570248
chr1	27484100	0.842975
chr1	27492300	0.570248
chr1	27519300	0.826446
chr1	27524800	0.570248
chr1	27532900	0.776860
chr1	27547200	0.669421
chr1	27560300	0.644628
chr1	27645000	0.719008
chr1	27652300	0.595041
chr1	27669500	0.685950
chr1	27674500	0.652893
chr1	27677900	0.933884
chr1	27684100	0.619835
chr1	27691400	0.661157
chr1	27710000	0.578512
chr1	27745600	0.595041
chr1	27754100	0.636364
chr1	27758300	0.628099
chr1	27760900	0.570248
chr1	27763100	0.851240
chr1	27765500	0.636364
chr1	27776200	0.570248
chr1	27787600	0.801653
chr1	27802900	0.636364

chr1	27831700	0.983471
chr1	27835500	0.818182
chr1	27840400	0.884298
chr1	27842900	0.586777
chr1	27845100	0.942149
chr1	27850000	0.983471
chr1	27856100	0.644628
chr1	27864100	0.644628
chr1	27867200	0.818182
chr1	27869300	0.644628
chr1	27871500	0.966942
chr1	27881700	0.685950
chr1	27884100	0.966942
chr1	27887300	0.917355
chr1	27891000	0.917355
chr1	27895000	0.644628
chr1	27897700	0.685950
chr1	27901300	0.669421
chr1	27926000	0.851240
chr1	27928800	0.644628
chr1	27933100	0.942149
chr1	27945600	0.669421
chr1	27948200	0.611570
chr1	27977500	0.826446
chr1	27982300	0.859504
chr1	27993900	0.942149
chr1	27996500	0.636364
chr1	28040200	0.595041
chr1	28051600	0.785124
chr1	28106200	0.710744
chr1	28112900	0.760331
chr1	28184900	0.570248
chr1	28190800	0.958678
chr1	28193900	0.752066
chr1	28196700	0.776860
chr1	28242300	0.776860
chr1	28306100	0.851240
chr1	28329600	0.661157
chr1	28383500	0.851240
chr1	28386400	0.727273
chr1	28393100	0.818182
chr1	28402300	0.702479
chr1	28412000	0.685950
chr1	28431500	0.760331
chr1	28439500	0.909091
chr1	28442000	0.884298
chr1	28459100	0.785124
chr1	28475200	0.958678
chr1	28481600	0.776860
chr1	28499900	0.925620
chr1	28571100	0.826446
chr1	28576900	0.958678
chr1	28590000	0.768595
chr1	28594200	0.685950

chr1	28605700	0.876033
chr1	28623600	0.826446
chr1	28628600	0.834711
chr1	28659500	0.644628
chr1	28672800	0.834711
chr1	28678000	0.661157
chr1	28680900	0.743802
chr1	28700000	0.669421
chr1	28702400	0.727273
chr1	28718600	0.801653
chr1	28722300	0.876033
chr1	28730300	0.685950
chr1	28739500	0.611570
chr1	28743000	0.570248
chr1	28765000	0.859504
chr1	28768600	0.793388
chr1	28776500	0.958678
chr1	28798400	0.867769
chr1	28821600	0.603306
chr1	28828300	0.727273
chr1	28843300	0.628099
chr1	28852200	0.768595
chr1	28878600	0.710744
chr1	28982400	0.578512
chr1	28991100	0.611570
chr1	29011600	0.809917
chr1	29045800	0.892562
chr1	29066800	0.793388
chr1	29071200	0.628099
chr1	29078200	0.867769
chr1	29082000	0.603306
chr1	29108700	0.652893
chr1	29119600	0.669421
chr1	29203100	0.578512
chr1	29208700	0.876033
chr1	29211900	0.661157
chr1	29217100	0.785124
chr1	29220600	0.975207
chr1	29225300	0.917355
chr1	29229600	0.611570
chr1	29240100	0.586777
chr1	29256200	0.570248
chr1	29262600	0.851240
chr1	29267100	0.611570
chr1	29277100	0.652893
chr1	29298600	0.644628
chr1	29312300	0.586777
chr1	29360000	0.752066
chr1	29410300	0.611570
chr1	29463300	0.818182
chr1	29473100	0.652893
chr1	29485400	0.909091
chr1	29488500	0.644628
chr1	29504200	0.710744

chr1	29530000	0.735537
chr1	29535100	0.694215
chr1	29610000	0.603306
chr1	29652000	0.595041
chr1	30161700	0.859504
chr1	30593700	0.611570
chr1	30597400	0.694215
chr1	30602500	0.917355
chr1	30608100	0.917355
chr1	30632100	0.727273
chr1	30638800	0.685950
chr1	30647800	0.619835
chr1	30653100	0.727273
chr1	30655900	0.586777
chr1	30661100	0.595041
chr1	30664100	0.727273
chr1	30699800	0.595041
chr1	31190300	0.611570
chr1	31195000	0.950413
chr1	31198000	0.570248
chr1	31201100	0.900826
chr1	31204200	0.851240
chr1	31207300	0.702479
chr1	31211700	0.694215
chr1	31214100	0.958678
chr1	31216800	0.677686
chr1	31223900	0.727273
chr1	31229500	0.983471
chr1	31232700	0.834711
chr1	31236000	0.884298
chr1	31240000	0.917355
chr1	31243600	0.743802
chr1	31246700	0.851240
chr1	31249700	0.892562
chr1	31256100	1.000000
chr1	31275200	0.595041
chr1	31282600	0.925620
chr1	31290300	0.603306
chr1	31297000	0.611570
chr1	31327300	0.578512
chr1	31344600	0.776860
chr1	31347200	0.785124
chr1	31361500	0.975207
chr1	31365600	0.578512
chr1	31368800	0.867769
chr1	31372300	0.826446
chr1	31387100	0.884298
chr1	31391400	0.578512
chr1	31393500	0.586777
chr1	31486000	0.628099
chr1	31495100	0.685950
chr1	31500700	0.909091
chr1	31503000	0.909091
chr1	31543000	0.603306

chr1	31545700	0.983471
chr1	31558500	0.991736
chr1	31574200	0.917355
chr1	31584800	0.611570
chr1	31596900	0.710744
chr1	31617600	0.719008
chr1	31633000	0.933884
chr1	31636100	0.611570
chr1	31644900	0.909091
chr1	31654900	0.636364
chr1	31772700	0.661157
chr1	31844500	0.595041
chr1	31846800	0.867769
chr1	31883800	0.859504
chr1	31944700	0.900826
chr1	31956500	0.710744
chr1	32015000	0.586777
chr1	32040300	0.727273
chr1	32163600	0.719008
chr1	32167000	0.586777
chr1	32170600	0.603306
chr1	32227000	0.719008
chr1	32235600	0.842975
chr1	32247200	0.942149
chr1	32281600	0.719008
chr1	32284600	0.636364
chr1	32292400	0.867769
chr1	32332500	0.702479
chr1	32346300	0.900826
chr1	32349400	0.768595
chr1	32355100	0.719008
chr1	32371100	0.743802
chr1	32377200	0.628099
chr1	32391100	0.644628
chr1	32395100	0.619835
chr1	32407200	0.900826
chr1	32412500	0.826446
chr1	32419000	0.867769
chr1	32421600	0.785124
chr1	32423800	0.801653
chr1	32427100	0.578512
chr1	32475000	0.842975
chr1	32485400	0.652893
chr1	32488900	0.801653
chr1	32491400	0.702479
chr1	32528500	0.743802
chr1	32532600	0.586777
chr1	32540900	0.586777
chr1	32567300	0.909091
chr1	32570800	0.983471
chr1	32576500	0.677686
chr1	32580200	0.586777
chr1	32587000	0.793388
chr1	32591600	0.884298

chr1	32595200	0.719008
chr1	32609500	0.586777
chr1	32648100	0.595041
chr1	32652000	0.685950
chr1	32664800	0.586777
chr1	32701800	0.851240
chr1	32707500	0.793388
chr1	32713400	0.661157
chr1	32716500	0.669421
chr1	32728800	0.570248
chr1	32738800	0.661157
chr1	32761900	0.694215
chr1	32773200	0.760331
chr1	32777100	0.917355
chr1	32809000	0.694215
chr1	32811500	0.900826
chr1	32816300	0.595041
chr1	32819700	0.578512
chr1	32854800	0.983471
chr1	32866800	0.975207
chr1	32873000	0.570248
chr1	32879800	0.933884
chr1	32914800	0.925620
chr1	33002800	0.793388
chr1	33114000	0.586777
chr1	33161100	0.867769
chr1	33170100	0.710744
chr1	33182800	0.685950
chr1	33188100	0.909091
chr1	33206700	0.570248
chr1	33212000	0.570248
chr1	33223200	0.859504
chr1	33227500	0.859504
chr1	33232700	0.586777
chr1	33265500	0.595041
chr1	33337200	0.636364
chr1	33358800	0.595041
chr1	33386000	0.760331
chr1	33405800	0.842975
chr1	33413800	0.595041
chr1	33428200	0.603306
chr1	33432900	0.669421
chr1	33447200	0.727273
chr1	33455300	0.768595
chr1	33458500	0.793388
chr1	33470100	0.900826
chr1	33473200	0.636364
chr1	33476400	0.826446
chr1	33488500	0.900826
chr1	33490800	0.652893
chr1	33503500	0.619835
chr1	33521400	0.661157
chr1	33804700	0.834711
chr1	33812700	0.595041

chr1	33827400	0.702479
chr1	33833200	0.586777
chr1	33862500	0.917355
chr1	33902900	0.578512
chr1	34368600	0.677686
chr1	34845200	0.892562
chr1	35308700	0.685950
chr1	35312100	0.727273
chr1	35603000	0.735537
chr1	35647200	0.586777
chr1	35654100	0.578512
chr1	35688800	0.603306
chr1	35730000	0.595041
chr1	35743600	0.710744
chr1	35746100	0.867769
chr1	35752100	0.842975
chr1	35837000	0.603306
chr1	35856800	0.842975
chr1	35897300	0.809917
chr1	35965600	0.776860
chr1	35972100	0.958678
chr1	35999900	0.644628
chr1	36085200	0.652893
chr1	36095300	0.570248
chr1	36140000	0.900826
chr1	36181600	0.867769
chr1	36253800	0.867769
chr1	36276600	0.669421
chr1	36347200	0.669421
chr1	36359900	0.578512
chr1	36383500	0.586777
chr1	36401500	0.917355
chr1	36500800	0.636364
chr1	36592400	0.768595
chr1	36601300	0.859504
chr1	36628700	0.578512
chr1	36653800	0.652893
chr1	36678600	0.735537
chr1	36701900	0.570248
chr1	36714100	0.983471
chr1	36717400	0.619835
chr1	36720500	0.644628
chr1	36724100	0.628099
chr1	36738800	0.925620
chr1	36741700	0.752066
chr1	36790200	0.578512
chr1	36794900	0.603306
chr1	36814200	0.694215
chr1	36835400	0.826446
chr1	36841800	0.719008
chr1	36844100	0.793388
chr1	36848700	0.636364
chr1	36854400	0.851240
chr1	36878000	0.776860

chr1	36886900	0.685950
chr1	36914500	0.628099
chr1	36920100	0.917355
chr1	36945500	0.619835
chr1	36948000	0.983471
chr1	36955400	0.818182
chr1	36958400	0.892562
chr1	36979700	0.735537
chr1	36983300	0.917355
chr1	36990800	0.570248
chr1	37169800	0.636364
chr1	37290100	0.884298
chr1	37921000	0.694215
chr1	37933300	0.570248
chr1	37946800	0.851240
chr1	38022300	0.768595
chr1	38026000	0.809917
chr1	38030300	0.983471
chr1	38077100	0.809917
chr1	38319500	0.785124
chr1	38328300	0.636364
chr1	38331500	0.917355
chr1	38381000	0.917355
chr1	38387600	0.644628
chr1	38395000	0.933884
chr1	38463500	0.603306
chr1	38476600	0.628099
chr1	38481900	0.884298
chr1	38503200	0.900826
chr1	38519100	0.578512
chr1	38530100	0.652893
chr1	38709200	0.603306
chr1	38731400	0.958678
chr1	38775400	0.942149
chr1	38799000	0.595041
chr1	38884500	0.809917
chr1	38974100	0.685950
chr1	38978500	0.884298
chr1	38982700	0.661157
chr1	38988200	0.743802
chr1	38991600	0.975207
chr1	38993900	0.752066
chr1	38999900	0.884298
chr1	39027600	0.851240
chr1	39079300	0.710744
chr1	39123100	0.619835
chr1	39210300	0.818182
chr1	39212500	0.909091
chr1	39243700	0.900826
chr1	39249800	0.884298
chr1	39252100	0.793388
chr1	39255900	0.619835
chr1	39298100	0.917355
chr1	39381500	0.942149

chr1	39384500	0.611570
chr1	39419400	0.776860
chr1	39424900	0.925620
chr1	39435400	0.768595
chr1	39439300	0.628099
chr1	39452000	0.586777
chr1	39463000	0.578512
chr1	39513500	0.950413
chr1	39529600	0.677686
chr1	39581400	0.917355
chr1	39584100	0.727273
chr1	39596400	0.694215
chr1	39616800	0.661157
chr1	39620700	0.677686
chr1	39631400	0.917355
chr1	39636400	0.826446
chr1	39644600	0.834711
chr1	39650400	0.925620
chr1	39655500	0.851240
chr1	39658500	0.876033
chr1	39663100	0.834711
chr1	39678600	0.611570
chr1	39684000	0.636364
chr1	39687300	0.685950
chr1	39696700	0.743802
chr1	39735700	0.842975
chr1	39738600	0.661157
chr1	39742100	0.826446
chr1	39851000	0.942149
chr1	39854500	0.619835
chr1	39858400	0.619835
chr1	39861000	0.950413
chr1	39864600	0.884298
chr1	40005300	0.578512
chr1	40124000	0.619835
chr1	40168000	0.735537
chr1	40176500	0.942149
chr1	40265200	0.752066
chr1	40280400	0.611570
chr1	40282900	0.925620
chr1	40296000	0.876033
chr1	40298700	0.809917
chr1	40311900	0.595041
chr1	40317400	0.619835
chr1	40344000	0.628099
chr1	40356800	0.619835
chr1	40360000	0.710744
chr1	40364100	0.603306
chr1	40410500	0.578512
chr1	40503000	0.917355
chr1	40513000	0.603306
chr1	40515300	0.727273
chr1	40521200	0.958678
chr1	40526600	0.685950

chr1	40542900	0.966942
chr1	40558800	0.768595
chr1	40589100	0.603306
chr1	40596900	0.603306
chr1	40618800	0.768595
chr1	40642500	0.867769
chr1	40645500	0.933884
chr1	40684300	0.595041
chr1	40686600	0.752066
chr1	40689000	0.578512
chr1	40714500	0.669421
chr1	40742600	0.867769
chr1	40801500	0.603306
chr1	40805200	0.818182
chr1	40807800	0.719008
chr1	40844900	0.834711
chr1	40853400	0.636364
chr1	40855600	0.801653
chr1	40858100	0.669421
chr1	40865900	0.719008
chr1	40870500	0.677686
chr1	41099800	0.727273
chr1	41111200	0.809917
chr1	41125300	0.652893
chr1	41149600	0.710744
chr1	41165200	0.834711
chr1	41170400	0.685950
chr1	41179300	0.917355
chr1	41201000	0.611570
chr1	41240400	0.958678
chr1	41265100	0.752066
chr1	41269800	0.603306
chr1	41273400	0.669421
chr1	41281700	0.884298
chr1	41302200	0.636364
chr1	41305200	0.578512
chr1	41311200	0.867769
chr1	41331500	0.603306
chr1	41334600	0.801653
chr1	41342300	0.752066
chr1	41381200	0.685950
chr1	41405500	0.809917
chr1	41414900	0.702479
chr1	41441100	0.619835
chr1	41469500	0.900826
chr1	41514900	0.801653
chr1	41575500	0.925620
chr1	41617300	0.776860
chr1	41627300	0.694215
chr1	41667200	0.586777
chr1	41704600	0.669421
chr1	41709500	0.595041
chr1	41712300	0.834711
chr1	41799200	0.859504

chr1	41826600	0.595041
chr1	41898800	0.801653
chr1	41959400	0.983471
chr1	41962200	0.735537
chr1	42006800	0.801653
chr1	42150000	0.586777
chr1	42348100	0.884298
chr1	42357600	0.636364
chr1	42361000	0.867769
chr1	42367800	0.578512
chr1	42650400	0.603306
chr1	42688600	0.867769
chr1	42725800	0.694215
chr1	42730700	0.727273
chr1	42751900	0.578512
chr1	42786600	0.677686
chr1	42789200	0.603306
chr1	42794100	0.933884
chr1	43154800	0.628099
chr1	43222700	0.595041
chr1	43262100	0.644628
chr1	43286500	0.785124
chr1	43289000	0.917355
chr1	43295000	0.743802
chr1	43297400	0.975207
chr1	43309700	0.785124
chr1	43352900	0.578512
chr1	43392200	0.900826
chr1	43398000	0.900826
chr1	43400500	0.776860
chr1	43404600	0.859504
chr1	43408700	0.966942
chr1	43411700	0.652893
chr1	43413800	0.958678
chr1	43420400	0.785124
chr1	43427500	0.785124
chr1	43430600	0.917355
chr1	43433400	0.628099
chr1	43446200	0.710744
chr1	43448700	0.793388
chr1	43472100	0.933884
chr1	43475000	0.851240
chr1	43477500	0.851240
chr1	43488200	0.578512
chr1	43494900	0.619835
chr1	43510800	0.876033
chr1	43524800	0.933884
chr1	43527900	0.644628
chr1	43539700	0.636364
chr1	43740500	0.652893
chr1	43796200	0.851240
chr1	43810600	0.628099
chr1	43827800	0.685950
chr1	43839200	0.694215

chr1	43868200	0.661157
chr1	43871300	0.595041
chr1	43911000	0.727273
chr1	43919700	0.851240
chr1	43988300	0.760331
chr1	43990900	0.809917
chr1	44016100	0.636364
chr1	44119500	0.685950
chr1	44198500	0.752066
chr1	44216000	0.950413
chr1	44229000	0.752066
chr1	44242400	0.983471
chr1	44249500	0.785124
chr1	44301500	0.719008
chr1	44324300	0.586777
chr1	44330000	0.925620
chr1	44340500	0.694215
chr1	44400400	0.760331
chr1	44424200	0.859504
chr1	44443300	0.867769
chr1	44448300	0.842975
chr1	44460900	0.628099
chr1	44480800	0.702479
chr1	44499100	0.760331
chr1	44503200	0.636364
chr1	44510300	0.611570
chr1	44537900	0.578512
chr1	44559800	0.586777
chr1	44569500	0.677686
chr1	44613300	0.570248
chr1	44693000	0.685950
chr1	44707200	0.677686
chr1	44755400	0.719008
chr1	44758200	0.884298
chr1	44818400	0.884298
chr1	44951800	0.776860
chr1	44987700	0.793388
chr1	44990400	0.694215
chr1	44992800	0.859504
chr1	45076100	0.809917
chr1	45082600	0.595041
chr1	45090100	0.727273
chr1	45095000	0.760331
chr1	45100500	0.570248
chr1	45142700	0.818182
chr1	45174200	0.628099
chr1	45185900	0.570248
chr1	45188200	0.842975
chr1	45196600	0.842975
chr1	45208900	0.867769
chr1	45252100	0.628099
chr1	45257000	0.710744
chr1	45259600	0.694215
chr1	45264800	0.669421

chr1	45268200	0.586777
chr1	45272200	0.776860
chr1	45277600	0.809917
chr1	45297100	0.768595
chr1	45328800	0.834711
chr1	45345700	0.735537
chr1	45360600	0.586777
chr1	45362800	0.644628
chr1	45374600	0.818182
chr1	45413000	0.933884
chr1	45434400	0.809917
chr1	45436600	0.603306
chr1	45454300	0.661157
chr1	45501600	0.900826
chr1	45504800	0.809917
chr1	45638300	0.595041
chr1	45657200	0.611570
chr1	45728000	0.586777
chr1	45732300	0.884298
chr1	45736900	0.644628
chr1	45819500	0.867769
chr1	45826200	0.719008
chr1	45877400	0.603306
chr1	45895400	0.595041
chr1	45910300	0.570248
chr1	45913200	0.603306
chr1	46002600	0.892562
chr1	46040000	0.636364
chr1	46129400	0.644628
chr1	46144800	0.652893
chr1	46148800	0.636364
chr1	46189100	0.661157
chr1	46198100	0.702479
chr1	46214500	0.652893
chr1	46218200	0.570248
chr1	46235600	0.867769
chr1	46309600	0.595041
chr1	46329900	0.710744
chr1	46391000	0.578512
chr1	46395400	0.925620
chr1	46420400	0.876033
chr1	46434900	0.611570
chr1	46465100	0.826446
chr1	46519600	0.884298
chr1	46531200	0.644628
chr1	46536700	0.611570
chr1	46562600	0.801653
chr1	46580700	0.793388
chr1	46614400	0.586777
chr1	46619700	0.652893
chr1	46622500	0.958678
chr1	46625100	0.578512
chr1	46640000	0.793388
chr1	46646000	0.652893

chr1	46651400	0.644628
chr1	46671500	0.710744
chr1	46711300	0.768595
chr1	46720700	0.578512
chr1	46743800	0.768595
chr1	46751200	0.867769
chr1	46754600	0.826446
chr1	46765000	0.752066
chr1	46774700	0.652893
chr1	46804300	0.595041
chr1	46813500	0.628099
chr1	46835900	0.818182
chr1	46867600	0.652893
chr1	46872700	0.834711
chr1	46879700	0.669421
chr1	46886400	0.933884
chr1	46889100	0.760331
chr1	46903900	0.801653
chr1	46907700	0.644628
chr1	46913000	0.776860
chr1	46916000	0.834711
chr1	46927600	0.958678
chr1	46943500	0.603306
chr1	46947200	0.900826
chr1	46950800	0.801653
chr1	46975000	0.900826
chr1	46983100	0.801653
chr1	46986200	0.842975
chr1	46989300	0.983471
chr1	46993100	0.776860
chr1	47010700	0.586777
chr1	47016500	0.685950
chr1	47048600	0.752066
chr1	47061400	0.867769
chr1	47066200	0.603306
chr1	47080200	0.586777
chr1	47090200	0.652893
chr1	47135900	0.826446
chr1	47139400	0.826446
chr1	47187700	0.719008
chr1	47641300	0.611570
chr1	47649100	0.644628
chr1	47656100	0.727273
chr1	47660200	0.909091
chr1	47677100	0.818182
chr1	47680300	0.983471
chr1	47690700	0.818182
chr1	47699900	0.760331
chr1	47703800	0.925620
chr1	47707600	0.917355
chr1	47710500	0.694215
chr1	47786100	0.818182
chr1	47789500	0.942149
chr1	47817100	0.595041

chr1	47844000	0.652893
chr1	47848100	0.867769
chr1	47981300	0.768595
chr1	48473300	0.570248
chr1	48643500	0.694215
chr1	51020400	0.776860
chr1	51177900	0.859504
chr1	51186800	0.834711
chr1	51189000	0.710744
chr1	51236500	0.685950
chr1	51319100	0.702479
chr1	51339500	0.669421
chr1	51410400	0.818182
chr1	51447500	0.628099
chr1	51471300	0.661157
chr1	51580400	0.578512
chr1	51696600	0.628099
chr1	51707200	0.652893
chr1	51729400	0.752066
chr1	51747100	0.595041
chr1	51750800	0.694215
chr1	51762800	0.900826
chr1	51769700	0.611570
chr1	51774700	0.644628
chr1	51787100	0.892562
chr1	51792000	0.975207
chr1	51797200	0.760331
chr1	51884300	0.669421
chr1	51889500	0.884298
chr1	51896000	0.628099
chr1	51900100	1.000000
chr1	51915100	0.710744
chr1	51935700	0.859504
chr1	51954200	0.743802
chr1	51975700	0.677686
chr1	51979100	0.611570
chr1	51987900	0.578512
chr1	51997900	0.652893
chr1	52005800	0.603306
chr1	52024900	0.652893
chr1	52035300	0.925620
chr1	52088900	0.595041
chr1	52091100	0.719008
chr1	52096500	0.884298
chr1	52100700	0.818182
chr1	52114300	0.595041
chr1	52116400	0.842975
chr1	52127200	0.826446
chr1	52130800	0.603306
chr1	52134900	0.702479
chr1	52137600	0.917355
chr1	52156300	0.694215
chr1	52204600	0.859504
chr1	52210900	0.876033

chr1	52216300	0.933884
chr1	52316200	0.826446
chr1	52318300	0.958678
chr1	52333700	0.685950
chr1	52420800	0.892562
chr1	52423700	0.892562
chr1	52429900	0.801653
chr1	52444800	0.652893
chr1	52473500	0.727273
chr1	52511600	0.818182
chr1	52542300	0.669421
chr1	52585500	0.570248
chr1	52616400	0.727273
chr1	52648900	0.619835
chr1	52670100	0.735537
chr1	52673100	0.570248
chr1	52706400	0.628099
chr1	52754100	0.586777
chr1	52795900	0.867769
chr1	52834300	0.603306
chr1	52866300	0.652893
chr1	52890200	0.975207
chr1	52893200	0.710744
chr1	52998500	0.950413
chr1	53029200	0.677686
chr1	53078400	0.685950
chr1	53081600	0.611570
chr1	53097700	0.760331
chr1	53103300	0.760331
chr1	53140400	0.876033
chr1	53143600	0.702479
chr1	53205600	0.595041
chr1	53217800	0.842975
chr1	53299500	0.818182
chr1	53312100	0.578512
chr1	53371600	0.917355
chr1	53373800	0.752066
chr1	53376300	0.917355
chr1	53379700	0.876033
chr1	53477400	0.884298
chr1	53513500	0.917355
chr1	53554300	0.636364
chr1	53601600	0.644628
chr1	53612300	0.909091
chr1	53629000	0.694215
chr1	53648900	0.884298
chr1	53651200	0.842975
chr1	53756900	0.578512
chr1	53826300	0.636364
chr1	53833600	0.818182
chr1	53915500	0.685950
chr1	54045600	0.619835
chr1	54226800	0.570248
chr1	54277500	0.917355

chr1	54301700	0.661157
chr1	54357800	0.603306
chr1	54423000	0.776860
chr1	54428700	0.867769
chr1	54482500	0.595041
chr1	54513800	0.752066
chr1	54530400	0.570248
chr1	54539800	0.603306
chr1	54574500	0.793388
chr1	54629400	0.834711
chr1	54678600	0.818182
chr1	54681700	0.570248
chr1	54696500	0.975207
chr1	54739200	0.818182
chr1	54743300	0.578512
chr1	54749500	0.685950
chr1	54751800	0.950413
chr1	54754400	0.636364
chr1	54764400	0.719008
chr1	54772200	0.818182
chr1	54793500	0.776860
chr1	54798800	0.628099
chr1	54820300	0.702479
chr1	54823300	0.661157
chr1	54826300	0.818182
chr1	54831600	0.628099
chr1	54838100	0.595041
chr1	54841400	0.669421
chr1	54844900	0.644628
chr1	54851700	0.859504
chr1	54862800	0.966942
chr1	54866900	0.636364
chr1	54877700	0.892562
chr1	54919200	0.578512
chr1	54925400	0.570248
chr1	54930700	0.933884
chr1	54941100	0.578512
chr1	54943500	0.603306
chr1	54946900	0.933884
chr1	55030900	0.603306
chr1	55106200	0.677686
chr1	55231900	0.578512
chr1	55241200	0.628099
chr1	55327600	0.975207
chr1	55502200	0.628099
chr1	55606800	0.834711
chr1	55645500	0.925620
chr1	55656600	0.710744
chr1	55658700	0.603306
chr1	55660900	0.768595
chr1	55664600	0.677686
chr1	55669300	0.818182
chr1	55671800	0.809917
chr1	55675600	0.719008

chr1	55683500	0.677686
chr1	55711400	0.570248
chr1	55725400	0.694215
chr1	55741400	0.966942
chr1	55895500	0.570248
chr1	55910400	0.636364
chr1	55940200	0.991736
chr1	56968600	0.644628
chr1	56972000	0.768595
chr1	56976500	0.859504
chr1	56982900	0.983471
chr1	57672000	0.661157
chr1	57684400	0.942149
chr1	57693300	0.884298
chr1	57702000	0.727273
chr1	58902900	0.652893
chr1	59009500	0.570248
chr1	59015300	1.000000
chr1	59050300	0.578512
chr1	59062700	0.578512
chr1	59096200	0.809917
chr1	59221100	0.900826
chr1	59324100	0.619835
chr1	59336100	0.859504
chr1	59338600	0.867769
chr1	59341500	0.628099
chr1	59346900	0.884298
chr1	59349900	0.909091
chr1	59352200	0.685950
chr1	59359100	0.851240
chr1	59363900	0.842975
chr1	59395600	0.818182
chr1	59434500	0.917355
chr1	59442300	0.595041
chr1	59445900	0.586777
chr1	59474500	0.595041
chr1	59486000	0.842975
chr1	59509900	0.595041
chr1	59522200	0.727273
chr1	59528700	0.826446
chr1	59542800	0.776860
chr1	59550000	0.760331
chr1	59562500	0.694215
chr1	59764300	0.595041
chr1	59859300	0.975207
chr1	59971600	0.975207
chr1	60197000	0.933884
chr1	60433300	0.809917
chr1	61353000	0.760331
chr1	61361100	0.760331
chr1	61391300	0.826446
chr1	61408800	0.710744
chr1	61423900	0.958678
chr1	61427000	0.884298

chr1	61438800	0.950413
chr1	61445000	0.578512
chr1	61455800	0.735537
chr1	61460000	0.958678
chr1	61468900	0.570248
chr1	61479100	0.983471
chr1	61512800	0.768595
chr1	61514900	0.859504
chr1	61524500	0.570248
chr1	61536200	0.636364
chr1	61570600	0.876033
chr1	61581900	0.933884
chr1	61584900	0.619835
chr1	61590600	0.801653
chr1	61598200	0.710744
chr1	61605700	0.727273
chr1	61622400	0.735537
chr1	61628700	0.983471
chr1	61638200	0.809917
chr1	61641500	0.785124
chr1	61647400	0.644628
chr1	61650200	0.909091
chr1	61660000	0.603306
chr1	61669400	0.776860
chr1	61671500	0.619835
chr1	61693000	0.603306
chr1	61700000	0.892562
chr1	61704300	0.801653
chr1	61713300	0.966942
chr1	61718600	0.867769
chr1	61720700	0.966942
chr1	61734200	0.867769
chr1	61776100	0.628099
chr1	61858700	0.900826
chr1	61869900	0.586777
chr1	61912700	0.917355
chr1	61918900	0.851240
chr1	62107600	0.603306
chr1	62175000	0.892562
chr1	62247000	0.677686
chr1	63005100	0.900826
chr1	63087800	0.619835
chr1	63151500	0.669421
chr1	63156400	0.983471
chr1	63244100	0.595041
chr1	64919500	0.578512
chr1	64971500	0.578512
chr1	65223100	0.991736
chr1	65328100	0.586777
chr1	65330200	0.909091
chr1	65350500	0.702479
chr1	65356400	0.719008
chr1	65360000	0.884298
chr1	65363900	0.842975

chr1	65366600	0.834711
chr1	65379900	0.586777
chr1	65382000	0.735537
chr1	65387700	0.818182
chr1	65399900	0.851240
chr1	65411700	0.595041
chr1	65415000	0.917355
chr1	65422200	0.776860
chr1	65426900	0.636364
chr1	65487700	1.000000
chr1	65505700	0.752066
chr1	65549500	0.743802
chr1	65579300	0.636364
chr1	65624800	0.983471
chr1	65629200	0.685950
chr1	65632300	0.743802
chr1	65644800	0.636364
chr1	65675100	0.628099
chr1	65679400	0.578512
chr1	65681800	0.636364
chr1	65688300	0.917355
chr1	65691000	0.661157
chr1	65695300	0.636364
chr1	65699300	0.834711
chr1	65704200	0.619835
chr1	65724800	0.669421
chr1	65726900	0.619835
chr1	65733200	0.760331
chr1	65735400	0.628099
chr1	65746100	0.611570
chr1	65749800	0.644628
chr1	65755400	0.867769
chr1	65758200	0.851240
chr1	65761300	0.958678
chr1	65774300	0.586777
chr1	65779400	0.570248
chr1	65782200	0.694215
chr1	65786200	0.876033
chr1	65789100	0.809917
chr1	65805800	0.628099
chr1	65838500	0.983471
chr1	65844000	0.743802
chr1	65876700	0.925620
chr1	65879900	0.661157
chr1	65883200	0.595041
chr1	65890800	0.586777
chr1	65895500	0.603306
chr1	65903300	0.900826
chr1	65906800	0.570248
chr1	65934100	0.628099
chr1	65936300	0.768595
chr1	65940700	0.611570
chr1	65949200	0.694215
chr1	65957200	0.867769

chr1	65971600	0.942149
chr1	65976100	0.859504
chr1	65979500	0.611570
chr1	65984500	0.669421
chr1	65995800	0.925620
chr1	66003400	0.719008
chr1	66006000	0.851240
chr1	66020500	0.809917
chr1	66022700	0.669421
chr1	66095700	0.727273
chr1	66125200	0.595041
chr1	66153700	0.727273
chr1	66163300	0.652893
chr1	66370900	0.983471
chr1	66377800	0.785124
chr1	66999700	0.677686
chr1	67223400	0.826446
chr1	67225700	0.586777
chr1	67231200	0.603306
chr1	67401800	0.768595
chr1	67468200	0.694215
chr1	67528100	0.677686
chr1	67553500	0.628099
chr1	67567500	0.628099
chr1	67604800	0.900826
chr1	67607900	0.867769
chr1	67615300	0.760331
chr1	67656800	0.801653
chr1	67659400	0.702479
chr1	67677800	0.586777
chr1	67822100	0.925620
chr1	67842800	0.760331
chr1	67899600	0.834711
chr1	67904000	0.611570
chr1	67945200	0.966942
chr1	67948800	0.809917
chr1	67972600	0.752066
chr1	67984900	0.685950
chr1	67988100	0.628099
chr1	68001300	0.652893
chr1	68005500	0.793388
chr1	68024800	0.586777
chr1	68027700	0.669421
chr1	68036300	0.909091
chr1	68056000	0.842975
chr1	68112600	0.834711
chr1	68126500	0.661157
chr1	68140900	0.628099
chr1	68148400	0.851240
chr1	68157300	0.743802
chr1	68161000	0.942149
chr1	68166900	0.966942
chr1	68172000	0.809917
chr1	68177000	0.867769

chr1	68179800	0.892562
chr1	68182400	0.570248
chr1	68189200	0.876033
chr1	68194600	0.859504
chr1	68197800	0.884298
chr1	68201600	0.975207
chr1	68204000	0.859504
chr1	68209400	0.785124
chr1	68215600	0.818182
chr1	68220700	0.801653
chr1	68226100	0.933884
chr1	68355800	0.652893
chr1	68364500	0.925620
chr1	68382600	0.942149
chr1	68385200	0.752066
chr1	68388100	0.719008
chr1	69901800	0.834711
chr1	69946300	0.768595
chr1	69964800	0.818182
chr1	69989300	0.652893
chr1	70005700	0.884298
chr1	70097400	0.586777
chr1	70506100	0.768595
chr1	70509700	0.735537
chr1	70685500	0.578512
chr1	70690500	0.595041
chr1	70926600	0.966942
chr1	71027600	0.661157
chr1	71143400	0.611570
chr1	71217800	0.975207
chr1	71221200	0.727273
chr1	71352300	0.644628
chr1	71357000	0.785124
chr1	71363300	0.603306
chr1	71375500	0.925620
chr1	71427800	0.603306
chr1	71452100	0.603306
chr1	71481600	0.595041
chr1	71503800	0.636364
chr1	71507300	0.735537
chr1	75196000	0.661157
chr1	75739900	0.570248
chr1	75820300	0.983471
chr1	75823300	0.570248
chr1	75889000	0.702479
chr1	75906400	0.586777
chr1	75915800	0.685950
chr1	75935700	0.677686
chr1	75938400	0.966942
chr1	75987300	0.611570
chr1	76105900	0.685950
chr1	76156800	0.644628
chr1	76159700	0.900826
chr1	76173300	0.578512

chr1	76262700	0.677686
chr1	77816000	0.892562
chr1	78136200	0.636364
chr1	78171000	0.785124
chr1	78195800	0.636364
chr1	78222200	0.611570
chr1	78227800	0.826446
chr1	78299000	0.768595
chr1	78450100	0.595041
chr1	78468300	0.578512
chr1	78499600	0.793388
chr1	78518900	0.867769
chr1	78557600	0.636364
chr1	83600900	0.826446
chr1	84384100	0.644628
chr1	84386400	0.694215
chr1	84398500	0.743802
chr1	84537400	0.644628
chr1	84555900	0.570248
chr1	84569300	0.900826
chr1	84711900	0.570248
chr1	84832100	0.884298
chr1	84843800	0.636364
chr1	84893200	0.768595
chr1	85049100	0.958678
chr1	85069800	0.669421
chr1	85099000	0.884298
chr1	85109300	0.677686
chr1	85147800	0.768595
chr1	85159200	0.661157
chr1	85164400	0.933884
chr1	85171000	0.702479
chr1	85175300	0.727273
chr1	85178400	0.776860
chr1	85181700	0.793388
chr1	85187200	0.595041
chr1	85411100	0.619835
chr1	85638700	0.909091
chr1	85686400	0.933884
chr1	85691700	0.628099
chr1	85710600	0.950413
chr1	85745100	0.950413
chr1	86914600	0.743802
chr1	86926900	0.942149
chr1	87221800	0.752066
chr1	87240400	0.925620
chr1	87244500	0.636364
chr1	87254600	0.925620
chr1	87258800	0.876033
chr1	87399800	0.644628
chr1	87405200	0.842975
chr1	87408400	0.628099
chr1	87416700	0.950413
chr1	87418800	0.644628

chr1	87453500	0.644628
chr1	87456700	0.702479
chr1	87459100	0.636364
chr1	87463500	0.702479
chr1	87470900	0.595041
chr1	87569600	0.611570
chr1	87596500	0.669421
chr1	87901700	0.842975
chr1	88042600	0.834711
chr1	89144300	0.768595
chr1	89146800	0.884298
chr1	89455300	0.570248
chr1	89587800	0.785124
chr1	89592100	0.801653
chr1	89761200	0.570248
chr1	89996800	0.933884
chr1	90001000	0.702479
chr1	90003400	0.933884
chr1	90009400	0.933884
chr1	90013700	0.917355
chr1	90017000	0.925620
chr1	90032700	0.867769
chr1	90074200	0.809917
chr1	90158600	0.636364
chr1	90166200	0.785124
chr1	90169500	0.942149
chr1	90172100	0.892562
chr1	90186800	0.636364
chr1	90226500	0.636364
chr1	90252300	0.876033
chr1	90275600	0.752066
chr1	90291100	0.578512
chr1	90298500	0.644628
chr1	90302600	0.950413
chr1	90305300	0.942149
chr1	90338600	0.826446
chr1	90342400	0.760331
chr1	90344900	0.842975
chr1	90350000	0.867769
chr1	90353700	0.884298
chr1	90355800	0.834711
chr1	90358000	0.719008
chr1	90360400	0.876033
chr1	90370800	0.570248
chr1	90373900	0.933884
chr1	90377200	0.768595
chr1	90395200	0.917355
chr1	90404000	0.958678
chr1	90407200	0.851240
chr1	90412200	0.842975
chr1	90419000	0.669421
chr1	90423100	0.818182
chr1	90436400	0.628099
chr1	90441600	0.884298

chr1	90457500	0.710744
chr1	90474500	0.867769
chr1	91212700	0.611570
chr1	91278100	0.933884
chr1	91287600	0.644628
chr1	91292700	0.966942
chr1	91298100	0.636364
chr1	91389200	0.595041
chr1	91391900	0.950413
chr1	91852700	0.950413
chr1	91970700	0.727273
chr1	92022100	0.983471
chr1	92027500	0.876033
chr1	92044900	0.768595
chr1	92057700	0.851240
chr1	92062400	0.950413
chr1	92067400	0.702479
chr1	92076600	0.702479
chr1	92150700	0.785124
chr1	92163600	0.661157
chr1	92171100	0.917355
chr1	92173400	0.818182
chr1	92176000	0.652893
chr1	92178400	0.818182
chr1	92181200	0.966942
chr1	92194200	0.975207
chr1	92196300	0.793388
chr1	92199900	0.950413
chr1	92202700	0.826446
chr1	92226500	0.603306
chr1	92228900	0.975207
chr1	92231000	0.876033
chr1	92235400	0.702479
chr1	92262200	0.578512
chr1	92265200	0.694215
chr1	92269400	0.900826
chr1	92293800	0.619835
chr1	92308100	0.628099
chr1	92311800	0.578512
chr1	92338200	0.611570
chr1	92784000	0.809917
chr1	92876700	0.727273
chr1	92912700	0.909091
chr1	92926000	0.702479
chr1	92982400	0.685950
chr1	93010300	0.727273
chr1	93171100	0.570248
chr1	93174200	0.892562
chr1	93177000	0.900826
chr1	93222300	0.677686
chr1	93252500	0.702479
chr1	93270800	0.801653
chr1	93301600	0.570248
chr1	93318000	0.702479

chr1	93388300	0.842975
chr1	93390500	0.809917
chr1	93417200	0.727273
chr1	93439000	0.710744
chr1	93456300	0.818182
chr1	93459700	0.611570
chr1	93491600	0.909091
chr1	93499300	0.892562
chr1	93625600	0.743802
chr1	93768700	0.570248
chr1	93824400	0.611570
chr1	93908100	0.603306
chr1	93965200	0.851240
chr1	93967700	0.785124
chr1	93970200	0.727273
chr1	94018400	0.603306
chr1	94033900	0.644628
chr1	94081800	0.760331
chr1	94087200	0.603306
chr1	94125900	0.818182
chr1	94128100	0.942149
chr1	94130400	0.975207
chr1	94138400	0.752066
chr1	94165100	0.636364
chr1	94169300	0.694215
chr1	94176800	0.776860
chr1	94181800	0.611570
chr1	94342200	0.636364
chr1	94371200	0.644628
chr1	94466000	0.603306
chr1	94480900	0.950413
chr1	95045700	0.644628
chr1	95075300	0.578512
chr1	95087900	0.570248
chr1	95101000	0.842975
chr1	95113800	0.669421
chr1	95527300	0.727273
chr1	95531600	0.743802
chr1	95535100	0.595041
chr1	95549500	0.743802
chr1	95553300	0.975207
chr1	95559500	0.595041
chr1	95562000	0.743802
chr1	95577200	0.917355
chr1	95580400	0.950413
chr1	95588100	0.842975
chr1	95594300	0.595041
chr1	95603800	0.752066
chr1	95634000	0.619835
chr1	95636300	0.603306
chr1	95645500	0.983471
chr1	95723400	0.702479
chr1	100053000	0.636364
chr1	100066500	0.603306

chr1	100107800	0.809917
chr1	100170500	0.611570
chr1	100183300	0.851240
chr1	100211700	0.743802
chr1	100218000	0.661157
chr1	100224500	0.586777
chr1	100228600	0.570248
chr1	100233900	0.776860
chr1	100236300	0.983471
chr1	100239600	0.785124
chr1	100274600	0.933884
chr1	100313300	0.578512
chr1	100343600	0.669421
chr1	100403500	0.570248
chr1	100409200	0.842975
chr1	100494600	0.611570
chr1	100511700	0.636364
chr1	100515300	0.652893
chr1	100595500	0.619835
chr1	100608200	0.685950
chr1	100710200	0.628099
chr1	100712500	0.727273
chr1	100789400	0.892562
chr1	100791500	0.628099
chr1	100803700	0.702479
chr1	100807200	0.578512
chr1	100810200	0.743802
chr1	100812700	0.826446
chr1	100814900	0.925620
chr1	100821800	0.636364
chr1	100827000	0.785124
chr1	100861700	0.966942
chr1	100866300	0.826446
chr1	100906400	0.975207
chr1	100909700	0.595041
chr1	100922700	0.644628
chr1	101225500	0.628099
chr1	101252900	0.776860
chr1	101276400	0.818182
chr1	101415500	0.702479
chr1	101466900	0.785124
chr1	101516900	0.636364
chr1	101537500	0.834711
chr1	101552600	0.628099
chr1	101578100	0.900826
chr1	101593900	0.851240
chr1	101596600	0.727273
chr1	101665200	0.710744
chr1	101702000	0.628099
chr1	101708300	0.611570
chr1	101724300	0.917355
chr1	101728600	0.636364
chr1	101744700	0.719008
chr1	101749400	0.958678

chr1	107587400	0.586777
chr1	108622600	0.851240
chr1	108628400	0.644628
chr1	108643100	0.801653
chr1	108752500	0.719008
chr1	108778300	0.818182
chr1	109001400	0.752066
chr1	109048000	0.628099
chr1	109064900	0.900826
chr1	109072000	0.611570
chr1	109074600	0.702479
chr1	109077700	0.867769
chr1	109097600	0.876033
chr1	109100600	0.619835
chr1	109107000	0.776860
chr1	109127100	0.892562
chr1	109142300	0.586777
chr1	109189900	0.669421
chr1	109263300	0.669421
chr1	109293600	0.727273
chr1	109324300	0.958678
chr1	109359500	0.826446
chr1	109373700	0.735537
chr1	109432800	0.818182
chr1	109456100	0.983471
chr1	109500100	0.628099
chr1	109519500	0.760331
chr1	109568600	0.595041
chr1	109622700	0.727273
chr1	109627700	0.909091
chr1	109638900	0.933884
chr1	109649900	0.644628
chr1	109655600	0.785124
chr1	109669400	0.876033
chr1	109674800	0.826446
chr1	109677400	0.909091
chr1	109692100	0.661157
chr1	109694300	0.685950
chr1	109702500	0.611570
chr1	109712700	0.578512
chr1	109719000	0.917355
chr1	109724000	0.942149
chr1	109729100	0.884298
chr1	109733300	0.710744
chr1	109742100	0.685950
chr1	109744200	0.677686
chr1	109760900	0.958678
chr1	109783900	0.702479
chr1	109799100	0.636364
chr1	109817900	0.892562
chr1	109820200	0.925620
chr1	109822900	0.611570
chr1	109828400	0.859504
chr1	109834200	0.793388

chr1	109838000	0.636364
chr1	109842500	0.900826
chr1	109851800	0.578512
chr1	109900500	0.685950
chr1	109913900	0.851240
chr1	109916800	0.661157
chr1	109924900	0.685950
chr1	109929200	0.892562
chr1	109966500	0.669421
chr1	109981900	0.876033
chr1	110019700	0.595041
chr1	110046000	0.652893
chr1	110075300	0.876033
chr1	110099900	0.727273
chr1	110106100	0.694215
chr1	110165900	0.834711
chr1	110183400	0.685950
chr1	110186700	0.652893
chr1	110262000	0.933884
chr1	110268700	0.793388
chr1	110306300	0.851240
chr1	110316500	0.735537
chr1	110332800	0.859504
chr1	110337200	0.925620
chr1	110339800	0.785124
chr1	110353500	0.719008
chr1	110361300	0.809917
chr1	110365000	0.809917
chr1	110367500	0.694215
chr1	110370100	0.595041
chr1	110378300	0.917355
chr1	110395300	0.900826
chr1	110399200	0.628099
chr1	110407300	0.917355
chr1	110425100	0.966942
chr1	110428700	0.727273
chr1	110433000	0.834711
chr1	110438200	0.661157
chr1	110440400	0.818182
chr1	110444300	0.809917
chr1	110455300	0.661157
chr1	110461100	0.942149
chr1	110473900	0.818182
chr1	110477300	0.752066
chr1	110485400	0.892562
chr1	110501500	0.818182
chr1	110519600	0.636364
chr1	110523000	0.900826
chr1	110540400	0.818182
chr1	110545800	0.636364
chr1	110574100	0.652893
chr1	110602200	0.966942
chr1	110632400	0.752066
chr1	110778600	0.785124

chr1	110811700	0.611570
chr1	110871500	0.677686
chr1	110889700	0.603306
chr1	110947300	0.619835
chr1	110955100	0.636364
chr1	110957500	0.859504
chr1	110964500	0.595041
chr1	110976900	0.628099
chr1	111159500	0.851240
chr1	111162400	0.710744
chr1	111414100	0.570248
chr1	111420400	0.842975
chr1	111424200	0.801653
chr1	111431100	0.685950
chr1	111433700	0.975207
chr1	111441500	0.636364
chr1	111458800	0.586777
chr1	111461400	0.570248
chr1	111568000	0.603306
chr1	111644900	0.975207
chr1	111685900	0.636364
chr1	111704600	0.818182
chr1	111715300	0.785124
chr1	111759100	0.619835
chr1	111982800	0.925620
chr1	112006000	0.735537
chr1	112010600	0.983471
chr1	112032600	0.611570
chr1	112046700	0.834711
chr1	112056400	0.669421
chr1	112059200	0.694215
chr1	112095200	0.636364
chr1	112114000	0.809917
chr1	112130300	0.735537
chr1	112135700	0.851240
chr1	112142600	0.801653
chr1	112158600	0.619835
chr1	112168200	0.801653
chr1	112203000	0.842975
chr1	112212000	0.925620
chr1	112276400	0.859504
chr1	112286700	0.636364
chr1	112292000	0.603306
chr1	112865600	0.661157
chr1	112937100	0.809917
chr1	113006200	0.586777
chr1	113009000	0.685950
chr1	113057700	0.644628
chr1	113118400	0.785124
chr1	113126800	0.669421
chr1	113168400	0.925620
chr1	113172400	0.636364
chr1	113220800	0.644628
chr1	113231700	0.793388

chr1	113240100	0.991736
chr1	113245400	0.909091
chr1	113334400	0.677686
chr1	113337500	0.735537
chr1	113361700	0.958678
chr1	113364800	0.892562
chr1	113369200	0.818182
chr1	113375100	0.636364
chr1	113404100	0.669421
chr1	113412200	0.611570
chr1	113415700	0.917355
chr1	113421500	0.760331
chr1	113425900	0.917355
chr1	113436600	0.768595
chr1	113447100	0.983471
chr1	113478300	0.900826
chr1	113481300	0.900826
chr1	113489900	0.768595
chr1	113493500	0.644628
chr1	113564500	0.710744
chr1	113583100	0.793388
chr1	113586000	0.818182
chr1	113602300	0.917355
chr1	113631700	0.752066
chr1	113634800	0.710744
chr1	113680600	0.933884
chr1	113683400	0.900826
chr1	113685700	0.677686
chr1	113689600	0.636364
chr1	113749700	0.694215
chr1	113754500	0.900826
chr1	113760700	0.727273
chr1	113763200	0.578512
chr1	113819600	0.702479
chr1	113846000	0.619835
chr1	114202000	0.876033
chr1	114220200	0.842975
chr1	114236000	0.652893
chr1	114323400	0.842975
chr1	114329600	0.570248
chr1	114344300	0.760331
chr1	114348400	0.570248
chr1	114402600	0.677686
chr1	114414700	0.752066
chr1	114417300	0.834711
chr1	114458400	0.909091
chr1	114461100	0.925620
chr1	114476400	0.702479
chr1	114489800	0.859504
chr1	114491900	0.851240
chr1	114621100	0.966942
chr1	114624300	0.876033
chr1	114732600	0.652893
chr1	114749100	0.619835

chr1	114923200	0.694215
chr1	114929700	0.950413
chr1	114940700	0.818182
chr1	114996400	0.900826
chr1	115046900	0.661157
chr1	115060500	0.661157
chr1	115064600	0.636364
chr1	115295700	0.859504
chr1	115642200	0.603306
chr1	116187200	0.570248
chr1	116189700	0.619835
chr1	116191800	0.818182
chr1	116194500	0.603306
chr1	116198200	0.867769
chr1	116211000	0.677686
chr1	116214400	0.867769
chr1	116675100	0.876033
chr1	116699700	0.851240
chr1	116710800	0.570248
chr1	116862000	0.917355
chr1	116864800	0.884298
chr1	116879400	0.603306
chr1	116908800	0.603306
chr1	116920900	0.719008
chr1	116923700	0.785124
chr1	116926100	0.859504
chr1	116944500	0.818182
chr1	116948600	0.942149
chr1	116951000	0.669421
chr1	116954100	0.710744
chr1	116956600	0.702479
chr1	116963600	0.925620
chr1	116972400	0.636364
chr1	116983200	0.644628
chr1	117006700	0.694215
chr1	117009800	0.628099
chr1	117014400	0.867769
chr1	117019700	0.933884
chr1	117026800	0.793388
chr1	117035500	0.793388
chr1	117040700	0.652893
chr1	117048800	0.917355
chr1	117052100	0.867769
chr1	117058500	0.595041
chr1	117089300	0.652893
chr1	117099400	0.842975
chr1	117109100	0.652893
chr1	117139000	0.661157
chr1	117160600	0.611570
chr1	117164800	0.719008
chr1	117167500	0.702479
chr1	117170200	0.884298
chr1	117175000	0.719008
chr1	117215600	0.578512

chr1	117221200	0.702479
chr1	117224100	0.950413
chr1	117226200	0.719008
chr1	117228700	0.735537
chr1	117231200	0.958678
chr1	117234700	0.793388
chr1	117236800	0.842975
chr1	117240000	0.719008
chr1	117298700	0.933884
chr1	117301500	0.809917
chr1	117306400	0.694215
chr1	117366900	0.628099
chr1	117371500	0.636364
chr1	117376000	0.578512
chr1	117389500	0.884298
chr1	117424900	0.818182
chr1	117456500	0.586777
chr1	117459300	0.669421
chr1	117462300	0.834711
chr1	117465300	0.727273
chr1	117473700	0.710744
chr1	117478200	0.578512
chr1	117529300	0.735537
chr1	117564000	0.760331
chr1	117636300	0.966942
chr1	117659700	0.842975
chr1	117671000	0.892562
chr1	117842600	0.768595
chr1	117882800	0.834711
chr1	117906700	0.809917
chr1	117929700	0.578512
chr1	117944800	0.652893
chr1	117956500	0.694215
chr1	117959900	0.578512
chr1	118015900	0.785124
chr1	118034800	0.669421
chr1	118103200	0.628099
chr1	118107500	0.834711
chr1	118141600	0.702479
chr1	118144000	0.595041
chr1	118153400	0.776860
chr1	118158900	0.685950
chr1	118161300	0.760331
chr1	118170700	0.876033
chr1	118181100	0.801653
chr1	118186000	0.595041
chr1	118189000	0.586777
chr1	118193400	0.570248
chr1	118197800	0.818182
chr1	118200000	0.628099
chr1	118202700	0.785124
chr1	118207600	0.851240
chr1	118214400	0.710744
chr1	118217600	0.595041

chr1	118235400	0.685950
chr1	118240000	0.942149
chr1	118246100	0.578512
chr1	118250900	0.950413
chr1	118275400	0.867769
chr1	118297200	0.694215
chr1	118364700	0.628099
chr1	118728600	0.595041
chr1	119506500	0.776860
chr1	119627600	0.570248
chr1	119747900	0.628099
chr1	120256800	0.669421
chr1	120280100	0.826446
chr1	120358900	0.586777
chr1	120402800	0.909091
chr1	120414200	0.727273
chr1	120417000	0.719008
chr1	120463300	0.669421
chr1	120596500	0.768595
chr1	120605200	0.727273
chr1	120616700	0.652893
chr1	120643800	0.735537
chr1	120660700	0.578512
chr1	120674800	0.677686
chr1	120759800	0.900826
chr1	120768400	0.958678
chr1	120780000	0.917355
chr1	120784000	0.595041
chr1	120788400	0.876033
chr1	120795200	0.818182
chr1	120798600	0.702479
chr1	120814500	0.628099
chr1	120817600	0.677686
chr1	120822600	0.743802
chr1	120858800	0.933884
chr1	120863500	0.958678
chr1	120874300	0.595041
chr1	120909600	0.636364
chr1	120912100	0.685950
chr1	120930900	0.983471
chr1	121102700	0.586777
chr1	121105500	0.603306
chr1	121108800	0.578512
chr1	121112100	0.917355
chr1	121328900	0.867769
chr1	142623400	0.652893
chr1	142672800	0.595041
chr1	142701800	0.694215
chr1	142704000	0.578512
chr1	142723300	0.710744
chr1	142726400	0.719008
chr1	142805000	0.611570
chr1	142815000	0.727273
chr1	142822800	0.768595

chr1	142838300	0.669421
chr1	142867200	0.685950
chr1	142876800	0.834711
chr1	142885200	0.876033
chr1	143122500	0.966942
chr1	143131500	0.628099
chr1	143138000	0.578512
chr1	143141200	0.719008
chr1	143169300	0.776860
chr1	143198300	0.917355
chr1	143206300	0.867769
chr1	143220000	0.644628
chr1	143366700	0.793388
chr1	143369300	0.644628
chr1	143374700	0.578512
chr1	143388000	0.595041
chr1	143390300	0.768595
chr1	143418800	0.760331
chr1	143450200	0.859504
chr1	143454300	0.570248
chr1	143670100	0.801653
chr1	143686600	0.710744
chr1	143889100	0.867769
chr1	143893500	0.826446
chr1	143917600	0.900826
chr1	143935100	0.842975
chr1	143938400	0.570248
chr1	143942100	0.619835
chr1	143944200	0.842975
chr1	143955000	0.578512
chr1	143957700	0.785124
chr1	143964000	0.818182
chr1	143967200	0.719008
chr1	143969400	0.793388
chr1	143972200	0.859504
chr1	143984200	0.900826
chr1	143992800	0.785124
chr1	143998700	0.785124
chr1	144008000	0.818182
chr1	144021200	0.768595
chr1	144027900	0.595041
chr1	144047100	0.636364
chr1	144054700	0.595041
chr1	144090100	0.628099
chr1	144092800	0.859504
chr1	144095400	0.719008
chr1	144311700	0.652893
chr1	144314300	0.760331
chr1	144492200	0.793388
chr1	144494700	0.727273
chr1	144532100	0.570248
chr1	144536100	0.859504
chr1	144539500	0.809917
chr1	144549500	0.578512

chr1	144551700	0.636364
chr1	144564000	0.851240
chr1	144604000	0.900826
chr1	144690700	0.826446
chr1	144693300	0.603306
chr1	144700700	0.685950
chr1	144709200	0.652893
chr1	144881600	0.892562
chr1	144887500	0.719008
chr1	144894300	0.661157
chr1	144966000	0.768595
chr1	144972900	0.644628
chr1	145002500	0.900826
chr1	145006500	0.818182
chr1	145012600	0.867769
chr1	145017500	0.586777
chr1	145028500	0.677686
chr1	145032300	0.694215
chr1	145042800	0.719008
chr1	145047800	0.611570
chr1	145084100	0.735537
chr1	145087700	0.644628
chr1	145093700	0.710744
chr1	145100100	0.818182
chr1	145123100	0.595041
chr1	145135400	0.595041
chr1	145138200	0.578512
chr1	145145000	0.818182
chr1	145159400	0.669421
chr1	145165300	0.669421
chr1	145176500	0.595041
chr1	145196400	0.677686
chr1	145216500	0.743802
chr1	145224700	0.652893
chr1	145253400	0.611570
chr1	145277300	0.669421
chr1	145287900	0.595041
chr1	145351700	0.570248
chr1	145362800	0.603306
chr1	145409500	0.983471
chr1	145413300	0.942149
chr1	145422100	0.628099
chr1	145428100	0.851240
chr1	145436500	0.570248
chr1	145444500	0.669421
chr1	145449600	0.818182
chr1	145454000	0.933884
chr1	145457600	1.000000
chr1	145466900	0.570248
chr1	145502900	0.776860
chr1	145542900	0.702479
chr1	145555000	0.892562
chr1	145557300	0.834711
chr1	145606500	0.909091

chr1	145609100	0.685950
chr1	145739100	0.603306
chr1	145759500	0.942149
chr1	145829600	0.727273
chr1	145929200	0.661157
chr1	145931700	0.603306
chr1	146004000	0.578512
chr1	146085000	0.669421
chr1	146371600	0.710744
chr1	146384200	0.570248
chr1	146571200	0.628099
chr1	146629200	0.867769
chr1	146719400	0.735537
chr1	146806400	0.867769
chr1	146848500	0.677686
chr1	146851500	0.909091
chr1	146894800	0.578512
chr1	147012800	0.818182
chr1	147323800	0.603306
chr1	147394200	0.628099
chr1	147397600	0.586777
chr1	147626700	0.743802
chr1	147642400	0.644628
chr1	147730700	0.578512
chr1	147736800	0.619835
chr1	147770300	0.628099
chr1	147774700	0.694215
chr1	147809000	0.611570
chr1	147818500	0.628099
chr1	147857600	0.677686
chr1	147872300	0.752066
chr1	148242800	0.603306
chr1	148312300	0.586777
chr1	148347000	0.578512
chr1	148357200	0.694215
chr1	148523600	0.884298
chr1	148527600	0.809917
chr1	148532800	0.570248
chr1	148566200	0.595041
chr1	148604200	0.793388
chr1	148766100	0.785124
chr1	149139000	0.644628
chr1	149193100	0.710744
chr1	149205400	0.735537
chr1	149214800	0.685950
chr1	149217200	0.760331
chr1	149219500	0.603306
chr1	149291800	0.818182
chr1	149357800	0.570248
chr1	149361800	0.876033
chr1	149364300	0.760331
chr1	149374800	1.000000
chr1	149393500	0.735537
chr1	149396000	0.595041

chr1	149432700	0.628099
chr1	149435100	0.595041
chr1	149442300	0.950413
chr1	149446800	0.644628
chr1	149603500	0.710744
chr1	149605600	0.801653
chr1	149614700	0.603306
chr1	149677300	0.793388
chr1	149722100	0.595041
chr1	149742700	0.619835
chr1	149746400	0.685950
chr1	149748900	0.752066
chr1	149759800	1.000000
chr1	149768700	0.636364
chr1	149778400	0.818182
chr1	149780600	0.743802
chr1	149849700	0.628099
chr1	149854500	0.603306
chr1	149878900	0.677686
chr1	149887700	0.975207
chr1	149908900	0.809917
chr1	149911900	0.785124
chr1	149973900	0.942149
chr1	149979500	0.611570
chr1	149984400	0.611570
chr1	150049600	0.752066
chr1	150117200	0.834711
chr1	150127000	0.694215
chr1	150138000	0.925620
chr1	150159300	0.925620
chr1	150228600	0.727273
chr1	150232800	0.768595
chr1	150235600	0.619835
chr1	150245400	0.694215
chr1	150261400	0.917355
chr1	150334500	0.578512
chr1	150343900	0.685950
chr1	150346400	0.570248
chr1	150356100	0.818182
chr1	150450500	0.917355
chr1	150457900	0.818182
chr1	150479300	0.950413
chr1	150482600	0.710744
chr1	150486000	0.950413
chr1	150505300	0.685950
chr1	150510300	0.719008
chr1	150517600	0.909091
chr1	150519800	0.702479
chr1	150525800	0.942149
chr1	150528700	0.710744
chr1	150536200	0.859504
chr1	150540500	0.958678
chr1	150547200	0.669421
chr1	150577800	0.966942

chr1	150584800	0.760331
chr1	150588700	0.809917
chr1	150592300	0.851240
chr1	150595300	0.983471
chr1	150608100	0.809917
chr1	150614100	0.603306
chr1	150686000	0.818182
chr1	150722100	0.834711
chr1	150760800	0.661157
chr1	150776000	0.669421
chr1	150783700	0.628099
chr1	150943600	0.710744
chr1	150951600	0.867769
chr1	150955000	0.719008
chr1	150967000	0.809917
chr1	150971900	0.958678
chr1	150976700	0.636364
chr1	150983800	0.884298
chr1	150986400	0.983471
chr1	151025600	0.942149
chr1	151036000	0.719008
chr1	151041300	0.859504
chr1	151045100	0.636364
chr1	151115900	0.619835
chr1	151129500	0.776860
chr1	151257100	0.603306
chr1	151283100	0.900826
chr1	151286800	0.892562
chr1	151295400	0.702479
chr1	151343100	0.950413
chr1	151357500	0.834711
chr1	151360300	0.933884
chr1	151446000	0.570248
chr1	151541900	0.578512
chr1	151548100	0.867769
chr1	151554500	0.586777
chr1	151557100	0.661157
chr1	151567000	0.685950
chr1	151569300	0.942149
chr1	151611700	0.636364
chr1	151640900	0.834711
chr1	151688500	0.834711
chr1	151712700	0.793388
chr1	151741200	0.578512
chr1	151749100	0.834711
chr1	151756700	0.826446
chr1	151819100	0.669421
chr1	151871100	0.867769
chr1	151876600	0.578512
chr1	151889000	0.702479
chr1	151893800	0.842975
chr1	151895900	0.586777
chr1	151909500	0.619835
chr1	151918200	0.933884

chr1	151943200	0.661157
chr1	151945300	0.958678
chr1	151948700	0.785124
chr1	152043800	0.925620
chr1	152995300	0.578512
chr1	153013700	0.900826
chr1	153032200	0.851240
chr1	153045900	0.867769
chr1	153060400	0.595041
chr1	153069200	0.611570
chr1	153088200	0.867769
chr1	153092200	0.826446
chr1	153314400	0.628099
chr1	153316600	0.991736
chr1	153405700	0.917355
chr1	153488500	0.636364
chr1	153499300	0.834711
chr1	153505900	0.867769
chr1	153508600	0.884298
chr1	153510800	0.876033
chr1	153515400	0.669421
chr1	153518700	0.760331
chr1	153525600	0.603306
chr1	153539000	0.925620
chr1	153541200	0.867769
chr1	153582100	0.933884
chr1	153589700	0.595041
chr1	153601600	0.644628
chr1	153609000	0.595041
chr1	153621600	0.876033
chr1	153651500	0.603306
chr1	153671400	0.991736
chr1	153764200	0.752066
chr1	153778600	0.793388
chr1	153849100	0.685950
chr1	153855700	0.735537
chr1	153955500	0.578512
chr1	153997500	0.628099
chr1	154127900	0.917355
chr1	154132400	0.958678
chr1	154159900	0.760331
chr1	154247400	0.570248
chr1	154280600	0.685950
chr1	154285000	0.595041
chr1	154296100	0.619835
chr1	154327200	0.867769
chr1	154357300	0.636364
chr1	154360200	0.768595
chr1	154378200	0.628099
chr1	154414900	0.694215
chr1	154433600	0.702479
chr1	154436200	0.991736
chr1	154438900	0.685950
chr1	154463000	0.752066

chr1	154466200	0.710744
chr1	154469900	0.785124
chr1	154567500	0.570248
chr1	154572200	0.578512
chr1	154582400	0.661157
chr1	154589700	0.851240
chr1	154594900	0.578512
chr1	154687300	0.603306
chr1	154833500	0.826446
chr1	154839000	0.809917
chr1	154856800	0.834711
chr1	154859100	0.933884
chr1	154861800	0.677686
chr1	154868800	0.785124
chr1	154896500	0.619835
chr1	154898700	0.586777
chr1	154913000	0.644628
chr1	154917300	0.809917
chr1	154930600	0.685950
chr1	154936800	0.768595
chr1	154951500	0.586777
chr1	154961300	0.636364
chr1	154966200	0.942149
chr1	154971500	0.586777
chr1	154978600	0.900826
chr1	154981600	0.611570
chr1	154986200	0.975207
chr1	154990000	0.942149
chr1	155026100	0.768595
chr1	155043700	0.669421
chr1	155046000	0.677686
chr1	155055500	0.570248
chr1	155064400	0.867769
chr1	155093500	0.595041
chr1	155102600	0.818182
chr1	155111200	0.611570
chr1	155140600	0.768595
chr1	155162400	0.636364
chr1	155177000	0.694215
chr1	155189300	0.702479
chr1	155193900	0.677686
chr1	155201200	0.661157
chr1	155221100	0.743802
chr1	155233400	0.595041
chr1	155246400	0.760331
chr1	155249100	0.628099
chr1	155267400	0.628099
chr1	155273100	0.776860
chr1	155276900	0.578512
chr1	155281700	0.801653
chr1	155287200	0.570248
chr1	155290300	0.826446
chr1	155485000	0.636364
chr1	155506300	0.851240

chr1	155540300	0.884298
chr1	155569400	0.677686
chr1	155662600	0.661157
chr1	155735800	0.991736
chr1	155738500	1.000000
chr1	155806100	0.826446
chr1	155818300	0.628099
chr1	155850700	0.636364
chr1	155854600	0.958678
chr1	155863600	0.611570
chr1	155870700	0.826446
chr1	155911100	0.950413
chr1	155934700	0.644628
chr1	155940500	0.909091
chr1	155944500	0.884298
chr1	155952800	0.983471
chr1	155971600	0.933884
chr1	155973900	0.702479
chr1	156010900	0.603306
chr1	156033600	0.586777
chr1	156077700	0.752066
chr1	156083000	0.603306
chr1	156088000	0.768595
chr1	156091000	0.876033
chr1	156095000	0.710744
chr1	156097600	0.958678
chr1	156099800	0.975207
chr1	156116300	0.661157
chr1	156131600	0.636364
chr1	156143100	0.586777
chr1	156148400	0.702479
chr1	156151600	0.801653
chr1	156161600	0.603306
chr1	156185900	0.570248
chr1	156202800	0.595041
chr1	156227800	0.793388
chr1	156387700	0.727273
chr1	156391800	0.685950
chr1	156394200	0.876033
chr1	156399600	0.578512
chr1	156430900	0.942149
chr1	156459800	0.909091
chr1	156462900	1.000000
chr1	156466300	0.892562
chr1	156468400	0.644628
chr1	156472900	0.785124
chr1	156475600	0.710744
chr1	156479000	0.578512
chr1	156493900	0.942149
chr1	156586400	0.702479
chr1	156627500	0.768595
chr1	156633600	0.900826
chr1	156644000	0.603306
chr1	156647500	0.743802

chr1	156664800	0.776860
chr1	156671400	0.760331
chr1	156713100	0.842975
chr1	156715700	0.776860
chr1	156725200	0.958678
chr1	156728200	0.876033
chr1	156730300	0.917355
chr1	156741200	0.809917
chr1	156743500	0.644628
chr1	156781900	0.710744
chr1	156785700	0.818182
chr1	156818900	0.776860
chr1	156832400	0.727273
chr1	156836300	0.975207
chr1	156839800	0.776860
chr1	156853000	0.578512
chr1	156862100	0.776860
chr1	156865100	0.776860
chr1	156871200	0.570248
chr1	156877300	0.867769
chr1	156880800	0.628099
chr1	156885500	0.958678
chr1	156890400	0.619835
chr1	156894800	0.636364
chr1	156897600	0.785124
chr1	156930100	0.719008
chr1	156943900	0.586777
chr1	156963400	0.950413
chr1	156966700	0.892562
chr1	157008300	0.719008
chr1	157011300	0.652893
chr1	157013700	0.578512
chr1	157102900	0.628099
chr1	157106000	0.636364
chr1	157118000	0.851240
chr1	157121200	0.834711
chr1	157125100	0.685950
chr1	157167400	0.793388
chr1	157187500	0.867769
chr1	157197700	0.685950
chr1	157205200	0.851240
chr1	157209200	0.595041
chr1	157232700	0.801653
chr1	157237900	0.793388
chr1	157246300	0.760331
chr1	157251000	0.570248
chr1	157256600	0.677686
chr1	157259500	0.578512
chr1	157274900	0.636364
chr1	157282300	0.867769
chr1	157294600	0.760331
chr1	157327300	0.793388
chr1	157336900	0.628099
chr1	157339700	0.776860

chr1	157342400	0.710744
chr1	157347600	0.776860
chr1	157352500	0.644628
chr1	157373900	0.776860
chr1	157378200	0.743802
chr1	157457600	0.793388
chr1	158116300	0.950413
chr1	158170600	0.644628
chr1	158660000	0.785124
chr1	158664500	0.636364
chr1	158699000	0.619835
chr1	158702600	0.950413
chr1	158708500	0.652893
chr1	158742500	0.644628
chr1	158769900	0.983471
chr1	158784800	0.586777
chr1	158788900	0.595041
chr1	159047200	0.942149
chr1	159168000	0.809917
chr1	159174400	0.925620
chr1	159839400	0.892562
chr1	159850100	0.975207
chr1	159852400	0.685950
chr1	159860200	0.933884
chr1	159866200	0.719008
chr1	159870600	0.933884
chr1	159878300	0.677686
chr1	159881000	0.983471
chr1	159899700	0.669421
chr1	159924300	0.768595
chr1	159928400	0.851240
chr1	159931100	0.892562
chr1	160068100	0.628099
chr1	160071900	0.652893
chr1	160163500	0.752066
chr1	160331400	0.719008
chr1	160333500	0.826446
chr1	160338000	0.834711
chr1	160345500	0.809917
chr1	160365200	0.818182
chr1	160384800	0.743802
chr1	160391100	0.603306
chr1	160399300	0.867769
chr1	160408100	0.661157
chr1	160417400	0.644628
chr1	160430900	0.809917
chr1	160493100	0.809917
chr1	160508300	0.867769
chr1	160512600	0.933884
chr1	160516600	0.801653
chr1	160518900	0.991736
chr1	160528500	0.644628
chr1	160538300	0.752066
chr1	160545400	0.933884

chr1	160551700	0.892562
chr1	160577000	0.578512
chr1	160596900	0.876033
chr1	160600300	0.628099
chr1	160622400	0.801653
chr1	160628700	0.917355
chr1	160635700	0.652893
chr1	160645900	0.892562
chr1	160662300	0.735537
chr1	160696200	1.000000
chr1	160783500	0.586777
chr1	160915200	0.834711
chr1	160917300	0.595041
chr1	160960400	0.867769
chr1	160965000	0.818182
chr1	160967900	0.925620
chr1	160976300	0.661157
chr1	160981700	0.950413
chr1	161002900	0.636364
chr1	161053900	0.925620
chr1	161082500	0.702479
chr1	161084900	0.909091
chr1	161090800	0.752066
chr1	161133800	0.859504
chr1	161139100	0.710744
chr1	161152100	0.636364
chr1	161156700	0.950413
chr1	161163000	0.636364
chr1	161167300	0.884298
chr1	161169700	0.942149
chr1	161180300	0.611570
chr1	161190400	0.603306
chr1	161194900	0.826446
chr1	161212200	0.619835
chr1	161281800	0.776860
chr1	161286700	0.661157
chr1	161367600	0.752066
chr1	161372100	0.636364
chr1	161374600	0.685950
chr1	161377900	0.867769
chr1	161380500	0.867769
chr1	161383000	0.652893
chr1	161453100	0.628099
chr1	161457600	0.776860
chr1	161461600	0.595041
chr1	161464700	0.826446
chr1	161469500	0.694215
chr1	161472000	0.975207
chr1	161481000	0.884298
chr1	161483400	0.867769
chr1	161493700	0.644628
chr1	161504200	0.652893
chr1	161514900	0.917355
chr1	161522100	0.619835

chr1	161524600	0.719008
chr1	161528400	0.809917
chr1	161531500	0.876033
chr1	161536100	0.661157
chr1	161539200	0.876033
chr1	161542200	0.785124
chr1	161549400	0.776860
chr1	161556000	0.826446
chr1	161559500	0.867769
chr1	161565200	0.776860
chr1	161584700	0.619835
chr1	161596400	0.710744
chr1	161606000	0.611570
chr1	161609300	0.892562
chr1	161612700	0.867769
chr1	161617600	0.702479
chr1	161621000	0.694215
chr1	161623900	0.842975
chr1	161631300	0.669421
chr1	161635500	0.942149
chr1	161637900	0.735537
chr1	161641300	0.975207
chr1	161658900	0.694215
chr1	161663100	0.644628
chr1	161676700	0.876033
chr1	161682700	0.578512
chr1	161691800	0.958678
chr1	161700200	0.694215
chr1	161703000	0.669421
chr1	161707600	0.595041
chr1	161710200	0.595041
chr1	161715300	0.611570
chr1	161740200	0.619835
chr1	161742300	0.768595
chr1	161911900	0.867769
chr1	161915600	0.950413
chr1	161921300	0.710744
chr1	161923600	0.966942
chr1	162249400	0.702479
chr1	162254900	0.603306
chr1	162319800	0.925620
chr1	162323800	0.768595
chr1	162328300	0.776860
chr1	162342700	0.760331
chr1	162348200	0.685950
chr1	162351300	1.000000
chr1	162354600	0.892562
chr1	162358400	0.966942
chr1	162362300	0.570248
chr1	162366500	0.834711
chr1	162369000	0.644628
chr1	162375500	0.776860
chr1	162377700	0.669421
chr1	162380100	0.719008

chr1	162382300	0.727273
chr1	162391300	0.909091
chr1	162414900	0.842975
chr1	162417100	0.595041
chr1	162433500	0.661157
chr1	162440600	0.570248
chr1	162465800	0.595041
chr1	162520800	0.809917
chr1	162522900	0.933884
chr1	162536400	0.611570
chr1	162538600	0.892562
chr1	162601400	0.859504
chr1	162611400	0.760331
chr1	162666400	0.578512
chr1	162679800	0.570248
chr1	162682800	0.636364
chr1	164659300	0.570248
chr1	164662700	0.661157
chr1	164672700	0.876033
chr1	164677400	0.710744
chr1	164681800	0.983471
chr1	164690500	0.867769
chr1	164695100	0.727273
chr1	164705500	0.702479
chr1	164714500	0.603306
chr1	164718100	0.842975
chr1	164723800	0.677686
chr1	164739600	0.801653
chr1	164742200	0.983471
chr1	164744700	0.694215
chr1	165177600	0.661157
chr1	165362700	0.570248
chr1	165571300	0.578512
chr1	165596700	0.636364
chr1	165615600	0.909091
chr1	165717100	0.768595
chr1	165735500	0.760331
chr1	165742800	0.636364
chr1	165785900	0.677686
chr1	165802600	0.702479
chr1	165806300	0.876033
chr1	165809600	0.826446
chr1	165812200	0.859504
chr1	165816300	0.818182
chr1	165820300	0.619835
chr1	165823300	0.785124
chr1	165895000	0.636364
chr1	166440700	0.636364
chr1	166454600	0.743802
chr1	166461200	0.884298
chr1	166550000	0.570248
chr1	166554500	0.975207
chr1	166580200	0.710744
chr1	166645000	0.743802

chr1	166662100	0.603306
chr1	166664500	0.619835
chr1	166670200	0.925620
chr1	166675600	0.644628
chr1	166700000	0.768595
chr1	166842000	0.619835
chr1	166872400	0.636364
chr1	167029000	0.719008
chr1	167197000	0.710744
chr1	167199600	0.818182
chr1	167203900	0.619835
chr1	167206700	0.851240
chr1	167208800	0.603306
chr1	167218700	0.859504
chr1	167221000	0.611570
chr1	167266800	0.611570
chr1	167275300	0.619835
chr1	167298700	0.760331
chr1	167302400	0.900826
chr1	167343200	0.735537
chr1	167356600	0.735537
chr1	167390000	0.636364
chr1	167396300	0.933884
chr1	167417300	0.685950
chr1	167487100	0.975207
chr1	167496800	0.859504
chr1	167511800	0.595041
chr1	167514200	0.834711
chr1	167533700	0.867769
chr1	167570400	0.768595
chr1	167589300	0.586777
chr1	167595600	0.958678
chr1	167603100	0.586777
chr1	167606600	0.652893
chr1	167611100	0.983471
chr1	167614700	0.685950
chr1	167628300	0.785124
chr1	167640100	0.603306
chr1	167646800	0.694215
chr1	167657000	0.851240
chr1	167698300	0.727273
chr1	167716600	0.933884
chr1	167719300	0.884298
chr1	167725100	0.595041
chr1	167727300	0.595041
chr1	167731600	0.917355
chr1	167766600	0.611570
chr1	167774300	0.611570
chr1	167777900	0.595041
chr1	167791900	0.785124
chr1	167795900	0.884298
chr1	167799300	0.859504
chr1	167806400	0.644628
chr1	167812000	0.719008

chr1	167829800	0.834711
chr1	167835200	0.776860
chr1	167850100	0.933884
chr1	167874700	0.760331
chr1	167891700	0.776860
chr1	167895100	0.735537
chr1	167952900	0.578512
chr1	167955000	0.603306
chr1	168054500	0.719008
chr1	168115500	0.702479
chr1	168131200	0.917355
chr1	168187800	0.603306
chr1	168190300	0.595041
chr1	168198800	0.619835
chr1	168212900	0.636364
chr1	168251000	0.685950
chr1	168257900	0.909091
chr1	168290600	0.859504
chr1	168310600	0.818182
chr1	168335400	0.842975
chr1	168338900	0.818182
chr1	168342200	0.719008
chr1	168344600	0.884298
chr1	168348500	0.586777
chr1	168362300	0.785124
chr1	168367600	0.661157
chr1	168372100	0.983471
chr1	168415600	0.710744
chr1	168436100	0.603306
chr1	168439400	0.768595
chr1	168815100	0.669421
chr1	169030400	0.595041
chr1	169082100	0.570248
chr1	169115100	0.570248
chr1	169163100	0.578512
chr1	169240700	0.760331
chr1	169277400	0.801653
chr1	169281100	0.867769
chr1	169296900	0.975207
chr1	169308000	0.735537
chr1	169315500	0.776860
chr1	169340600	0.628099
chr1	169498500	0.611570
chr1	170011700	0.685950
chr1	170037100	0.743802
chr1	171446700	0.578512
chr1	171473800	0.570248
chr1	171584300	0.818182
chr1	171591600	0.578512
chr1	171653300	0.710744
chr1	171686700	0.760331
chr1	171723500	0.834711
chr1	171728000	0.570248
chr1	171730800	0.785124

chr1	171733200	0.735537
chr1	171749100	0.735537
chr1	171787300	0.652893
chr1	172371700	0.595041
chr1	172395100	0.809917
chr1	172419300	0.950413
chr1	172422000	0.727273
chr1	172426000	0.900826
chr1	172449200	0.710744
chr1	172456500	0.867769
chr1	172464700	0.735537
chr1	172469300	0.595041
chr1	172482800	0.685950
chr1	172484900	0.735537
chr1	172487400	0.669421
chr1	172499300	0.867769
chr1	172506900	0.570248
chr1	172514700	0.826446
chr1	172519100	0.685950
chr1	172558000	0.991736
chr1	172570000	0.867769
chr1	172607200	0.925620
chr1	172610100	0.818182
chr1	172613200	0.685950
chr1	172616500	0.603306
chr1	172628200	0.586777
chr1	172688400	0.727273
chr1	172791300	0.710744
chr1	172876700	0.834711
chr1	172903200	0.991736
chr1	173239100	0.710744
chr1	173272700	0.727273
chr1	173299700	0.983471
chr1	173324800	0.768595
chr1	173340200	0.933884
chr1	173362700	0.867769
chr1	173384100	0.743802
chr1	173392000	0.785124
chr1	173395700	0.809917
chr1	173398000	0.677686
chr1	173401100	0.785124
chr1	173405400	0.925620
chr1	173450700	0.586777
chr1	173466000	0.809917
chr1	173472900	0.636364
chr1	173523800	0.735537
chr1	173572900	0.793388
chr1	173608300	0.785124
chr1	173658100	0.900826
chr1	173661200	0.603306
chr1	173678400	0.760331
chr1	173788200	0.619835
chr1	173791200	0.570248
chr1	173978700	0.776860

chr1	173993500	0.710744
chr1	173997700	0.785124
chr1	174000500	0.867769
chr1	174037300	0.586777
chr1	174047200	0.694215
chr1	174122800	0.710744
chr1	174126100	0.628099
chr1	174132200	0.719008
chr1	174149300	0.595041
chr1	174173600	0.619835
chr1	174397600	0.826446
chr1	174715500	0.694215
chr1	174718100	0.669421
chr1	174736100	0.578512
chr1	174758300	0.809917
chr1	174761500	0.876033
chr1	174769400	0.636364
chr1	174842000	0.619835
chr1	174845100	0.595041
chr1	174859500	0.611570
chr1	174883100	0.776860
chr1	174915500	0.735537
chr1	174932900	0.818182
chr1	174935900	0.735537
chr1	174938000	0.950413
chr1	174941200	0.570248
chr1	174946200	0.710744
chr1	174966000	0.859504
chr1	174990000	0.578512
chr1	175140500	0.677686
chr1	175152000	0.719008
chr1	175156100	0.743802
chr1	175158600	0.867769
chr1	175178600	0.909091
chr1	175376100	0.661157
chr1	175379200	0.851240
chr1	175561000	0.785124
chr1	175703400	0.942149
chr1	176028400	0.628099
chr1	176816200	0.611570
chr1	176823300	0.809917
chr1	176828000	0.586777
chr1	176847100	0.752066
chr1	176850900	0.611570
chr1	178972400	0.669421
chr1	178984400	0.933884
chr1	178988700	0.578512
chr1	179000700	0.760331
chr1	179004700	0.694215
chr1	179006900	0.876033
chr1	179009200	0.966942
chr1	179012800	0.595041
chr1	179015800	0.776860
chr1	179018600	0.570248

chr1	179022400	0.950413
chr1	179028300	0.909091
chr1	179030400	0.991736
chr1	179099000	0.710744
chr1	179178800	0.752066
chr1	179187700	0.859504
chr1	179201400	0.586777
chr1	179275000	0.735537
chr1	179307500	0.793388
chr1	179332700	0.933884
chr1	179417800	0.661157
chr1	179788200	0.826446
chr1	179800700	0.669421
chr1	179849300	0.586777
chr1	179938600	0.603306
chr1	179941500	0.859504
chr1	179946000	0.834711
chr1	180027200	1.000000
chr1	180086800	0.942149
chr1	180126900	0.859504
chr1	180183300	0.578512
chr1	180258100	0.950413
chr1	180260800	0.925620
chr1	180485300	0.702479
chr1	180491700	0.743802
chr1	180496600	0.892562
chr1	180521600	0.619835
chr1	180529500	0.727273
chr1	180533800	0.595041
chr1	180541200	0.859504
chr1	180544100	0.644628
chr1	180551000	0.818182
chr1	180553800	0.586777
chr1	180564200	0.917355
chr1	180572800	0.851240
chr1	180578500	0.570248
chr1	180582400	0.768595
chr1	180585100	0.892562
chr1	180591900	0.652893
chr1	180609600	0.628099
chr1	180613300	0.719008
chr1	180823900	0.801653
chr1	180885100	0.801653
chr1	180893000	0.851240
chr1	180895800	0.900826
chr1	180902700	0.942149
chr1	180981200	0.669421
chr1	181002200	0.578512
chr1	181005600	0.743802
chr1	181012400	0.694215
chr1	181046700	0.661157
chr1	181054000	0.785124
chr1	181069300	0.900826
chr1	181071400	0.867769

chr1	181076700	0.578512
chr1	181081900	0.900826
chr1	181100700	0.859504
chr1	181105400	0.636364
chr1	181111100	0.611570
chr1	181122300	0.876033
chr1	181129100	0.595041
chr1	181143600	0.611570
chr1	181149200	0.603306
chr1	181159500	0.578512
chr1	181864100	0.752066
chr1	181875600	0.809917
chr1	182084100	0.694215
chr1	182113800	0.760331
chr1	182119000	0.925620
chr1	182140500	0.867769
chr1	182169900	0.909091
chr1	182205500	0.834711
chr1	182218500	0.818182
chr1	182225900	0.570248
chr1	182229600	0.809917
chr1	182232000	0.876033
chr1	182235700	0.595041
chr1	182238000	0.743802
chr1	182258600	0.768595
chr1	182261600	0.785124
chr1	182271300	0.950413
chr1	182279400	0.661157
chr1	182284500	0.785124
chr1	182299300	0.884298
chr1	182301800	0.851240
chr1	182305800	0.950413
chr1	182309300	0.851240
chr1	182324800	0.611570
chr1	182328900	0.876033
chr1	182332500	0.859504
chr1	182354700	0.958678
chr1	182400900	0.669421
chr1	182415500	0.702479
chr1	182545100	0.842975
chr1	182576000	0.570248
chr1	182584700	0.644628
chr1	182751400	0.892562
chr1	182754700	0.611570
chr1	182813200	0.570248
chr1	182991900	0.595041
chr1	183316000	0.809917
chr1	183444900	0.652893
chr1	183447400	0.603306
chr1	183450200	0.776860
chr1	183523000	0.801653
chr1	183537300	0.809917
chr1	183573600	0.752066
chr1	183885500	0.785124

chr1	183926700	0.851240
chr1	183952800	0.677686
chr1	183957100	0.760331
chr1	183959300	0.834711
chr1	183962800	0.834711
chr1	183966100	0.842975
chr1	183974800	0.851240
chr1	183994800	0.950413
chr1	183998300	0.669421
chr1	184002400	0.876033
chr1	184633500	0.661157
chr1	184715300	0.727273
chr1	184736700	0.636364
chr1	184740200	0.652893
chr1	184743200	0.859504
chr1	184775700	0.809917
chr1	184899500	0.933884
chr1	184924100	0.636364
chr1	184951200	0.628099
chr1	184986700	0.900826
chr1	185038300	0.595041
chr1	185049800	0.702479
chr1	185120800	0.586777
chr1	185122900	0.595041
chr1	185139900	0.570248
chr1	185210800	0.702479
chr1	185214300	0.636364
chr1	185219200	1.000000
chr1	185229500	0.842975
chr1	185233900	0.628099
chr1	185253000	0.809917
chr1	185255600	0.942149
chr1	185258000	0.809917
chr1	185261500	0.743802
chr1	185303400	0.636364
chr1	185357900	0.884298
chr1	185500700	0.735537
chr1	185503100	0.611570
chr1	185991600	0.818182
chr1	186016000	0.685950
chr1	186023900	0.578512
chr1	186047600	0.669421
chr1	186051300	0.892562
chr1	186072200	0.603306
chr1	186081800	0.760331
chr1	186092300	0.603306
chr1	186136400	0.578512
chr1	186163600	0.983471
chr1	186165700	0.818182
chr1	186257800	0.785124
chr1	187044100	0.727273
chr1	189281700	0.776860
chr1	191528200	0.652893
chr1	191545100	0.570248

chr1	191555000	0.619835
chr1	191726300	0.611570
chr1	191792200	0.694215
chr1	191821800	0.818182
chr1	191859200	0.818182
chr1	192077800	0.834711
chr1	192089000	0.702479
chr1	192093000	0.909091
chr1	192099500	0.652893
chr1	192139900	0.975207
chr1	192947400	0.619835
chr1	192955200	0.942149
chr1	193013100	0.735537
chr1	193024000	0.727273
chr1	193035100	0.603306
chr1	193040300	0.727273
chr1	193081000	0.925620
chr1	193085500	0.619835
chr1	195867600	0.652893
chr1	195870200	0.892562
chr1	195872600	0.586777
chr1	195935100	0.719008
chr1	196481100	0.719008
chr1	196486000	0.752066
chr1	196619900	0.677686
chr1	197066000	0.892562
chr1	197148400	0.933884
chr1	197411600	0.669421
chr1	197646900	0.669421
chr1	197702100	0.652893
chr1	197715300	0.801653
chr1	197718100	0.603306
chr1	197748300	0.628099
chr1	197751600	0.644628
chr1	197814700	0.677686
chr1	197913800	0.859504
chr1	198049600	0.677686
chr1	198091300	0.611570
chr1	198099000	0.801653
chr1	198131500	0.966942
chr1	198137100	0.834711
chr1	198139400	0.710744
chr1	198155000	0.628099
chr1	198157400	0.834711
chr1	198234500	0.859504
chr1	198252500	0.586777
chr1	198285100	0.595041
chr1	198450900	0.694215
chr1	198489300	0.586777
chr1	198526600	0.884298
chr1	198565300	0.719008
chr1	198569100	0.966942
chr1	198574600	0.942149
chr1	198580400	0.842975

chr1	198597700	0.702479
chr1	198601100	0.884298
chr1	198603600	0.793388
chr1	198606500	0.611570
chr1	198625500	0.867769
chr1	198638000	0.669421
chr1	200120400	0.842975
chr1	200123400	0.950413
chr1	200177400	0.677686
chr1	200195400	0.611570
chr1	200245100	0.719008
chr1	200423000	0.628099
chr1	200607800	0.876033
chr1	200611700	0.950413
chr1	200623100	0.611570
chr1	200644700	0.909091
chr1	200690000	0.619835
chr1	200746500	0.743802
chr1	200856200	0.586777
chr1	200980300	0.628099
chr1	200982400	0.834711
chr1	200985300	0.801653
chr1	200989700	0.727273
chr1	200995100	0.652893
chr1	201029200	0.727273
chr1	201120300	0.719008
chr1	201136900	0.876033
chr1	201426000	0.685950
chr1	201431200	0.859504
chr1	201476000	0.570248
chr1	201481700	0.710744
chr1	201484900	0.760331
chr1	201504900	0.900826
chr1	201521500	0.760331
chr1	201529400	0.619835
chr1	201532500	0.586777
chr1	201535500	0.876033
chr1	201539500	0.884298
chr1	201543200	0.603306
chr1	201548500	0.710744
chr1	201551600	0.867769
chr1	201555100	0.619835
chr1	201567800	0.818182
chr1	201572600	0.636364
chr1	201589300	0.652893
chr1	201596000	0.743802
chr1	201602800	0.694215
chr1	201605400	0.595041
chr1	201613300	0.685950
chr1	201616400	0.603306
chr1	201645100	0.719008
chr1	201673000	0.752066
chr1	201683700	0.826446
chr1	201701100	0.801653

chr1	201709100	0.636364
chr1	201748200	0.785124
chr1	201796300	0.669421
chr1	201801400	0.818182
chr1	201810600	0.859504
chr1	201852800	0.876033
chr1	201855500	0.909091
chr1	201857900	0.578512
chr1	201886900	0.768595
chr1	201892000	0.669421
chr1	201894300	0.842975
chr1	201899900	0.694215
chr1	201909100	0.909091
chr1	201977500	0.768595
chr1	201988000	0.611570
chr1	201992300	0.644628
chr1	201998800	0.677686
chr1	202004200	0.586777
chr1	202033900	0.809917
chr1	202045700	0.867769
chr1	202075100	0.727273
chr1	202087900	0.925620
chr1	202120000	0.628099
chr1	202125800	0.900826
chr1	202137600	0.595041
chr1	202173100	0.611570
chr1	202206000	0.719008
chr1	202308800	0.570248
chr1	202321200	0.652893
chr1	202329000	0.809917
chr1	202402500	0.636364
chr1	202429000	0.619835
chr1	202431700	0.710744
chr1	202453200	0.702479
chr1	202478700	0.702479
chr1	202485900	0.760331
chr1	202500900	0.636364
chr1	202516400	0.702479
chr1	202521200	0.628099
chr1	202523800	0.636364
chr1	202546700	0.570248
chr1	202763700	0.611570
chr1	202772000	0.685950
chr1	202791300	0.661157
chr1	202908000	0.743802
chr1	202915100	0.694215
chr1	202923800	0.752066
chr1	202929500	0.619835
chr1	202938700	0.595041
chr1	202951800	0.785124
chr1	202968500	0.661157
chr1	202995100	0.611570
chr1	202998100	0.702479
chr1	203121000	0.636364

chr1	203240400	0.644628
chr1	203247700	0.570248
chr1	203251700	0.867769
chr1	203256900	0.950413
chr1	203259800	0.826446
chr1	203269800	0.752066
chr1	203286100	0.793388
chr1	203290400	0.793388
chr1	203299000	0.636364
chr1	203334100	0.669421
chr1	203582600	0.694215
chr1	203594000	0.586777
chr1	203610800	0.925620
chr1	203615200	0.793388
chr1	203620100	0.842975
chr1	203626900	0.685950
chr1	203643300	0.900826
chr1	203648800	0.661157
chr1	203651200	0.586777
chr1	203655500	0.702479
chr1	203659100	0.776860
chr1	203662000	0.793388
chr1	203667400	0.702479
chr1	203695900	0.909091
chr1	203702600	0.578512
chr1	203768600	0.586777
chr1	203895500	0.727273
chr1	204146700	0.842975
chr1	204196800	0.925620
chr1	204256800	0.611570
chr1	204289600	0.851240
chr1	204330100	0.603306
chr1	204332900	0.677686
chr1	204347500	0.628099
chr1	204352200	0.611570
chr1	204370600	0.727273
chr1	204389300	0.743802
chr1	204392300	0.727273
chr1	204434900	0.669421
chr1	204473700	0.595041
chr1	204489000	0.752066
chr1	204493500	0.752066
chr1	204535500	0.900826
chr1	204568400	0.710744
chr1	204593200	0.909091
chr1	204595300	0.785124
chr1	204600000	0.743802
chr1	204617100	0.586777
chr1	204637800	0.570248
chr1	204639900	0.892562
chr1	204643300	0.719008
chr1	204648200	0.768595
chr1	204652500	0.818182
chr1	204657000	0.652893

chr1	204660800	1.000000
chr1	204668400	0.595041
chr1	204673900	0.644628
chr1	204676600	0.644628
chr1	204680300	0.768595
chr1	204683500	0.818182
chr1	204733300	0.619835
chr1	204829400	0.628099
chr1	204895400	0.669421
chr1	204954000	0.958678
chr1	205111300	0.785124
chr1	205126800	0.909091
chr1	205139900	0.867769
chr1	205142000	0.892562
chr1	205144900	0.909091
chr1	205165200	0.652893
chr1	205168900	0.785124
chr1	205174500	0.586777
chr1	205178100	0.661157
chr1	205184200	0.801653
chr1	205199500	0.892562
chr1	205207600	0.851240
chr1	205216700	0.694215
chr1	205218900	0.760331
chr1	205225500	0.735537
chr1	205236700	0.942149
chr1	205240200	0.727273
chr1	205254300	0.950413
chr1	205257200	0.661157
chr1	205264100	0.694215
chr1	205269900	0.570248
chr1	205296500	0.619835
chr1	205313800	0.661157
chr1	205410500	0.909091
chr1	205414300	0.603306
chr1	205417100	0.652893
chr1	205419300	0.826446
chr1	205465000	0.743802
chr1	205593700	0.570248
chr1	205606500	0.570248
chr1	205662200	0.694215
chr1	205676500	0.859504
chr1	205680700	0.776860
chr1	205704100	0.760331
chr1	205721200	0.603306
chr1	205724300	0.842975
chr1	205756800	0.768595
chr1	205771200	0.876033
chr1	205779200	0.669421
chr1	205900000	0.801653
chr1	205914900	0.719008
chr1	206079300	0.760331
chr1	206084100	0.760331
chr1	206087900	0.851240

chr1	206095600	0.834711
chr1	206098100	0.958678
chr1	206113300	0.595041
chr1	206116800	0.884298
chr1	206122100	0.859504
chr1	206132400	0.710744
chr1	206158400	0.884298
chr1	206163000	0.909091
chr1	206165500	0.652893
chr1	206266600	0.818182
chr1	206273900	0.644628
chr1	206329600	0.628099
chr1	206484000	0.644628
chr1	206490600	0.644628
chr1	206500100	0.710744
chr1	206510600	0.661157
chr1	206552800	0.570248
chr1	206555700	0.851240
chr1	206558600	0.768595
chr1	206562000	0.917355
chr1	206593000	0.809917
chr1	206613000	0.892562
chr1	206625500	0.925620
chr1	206629400	0.975207
chr1	206638300	0.595041
chr1	206661100	0.851240
chr1	206664500	0.727273
chr1	206677700	0.983471
chr1	206725100	0.842975
chr1	206729000	0.727273
chr1	206737000	0.842975
chr1	206748700	0.991736
chr1	206753500	0.702479
chr1	206764200	0.768595
chr1	206823400	0.677686
chr1	206828100	0.752066
chr1	206833300	0.793388
chr1	206837400	0.851240
chr1	206842200	0.818182
chr1	206848500	0.867769
chr1	206854000	0.752066
chr1	206862200	0.776860
chr1	206866200	0.942149
chr1	206876100	0.570248
chr1	206881700	0.619835
chr1	206884500	0.619835
chr1	206887500	0.743802
chr1	206890500	0.917355
chr1	206899900	0.603306
chr1	206908800	0.652893
chr1	206940000	0.867769
chr1	207021200	0.966942
chr1	207064300	0.685950
chr1	207080500	0.661157

chr1	207098300	0.917355
chr1	207115300	0.859504
chr1	207118500	0.859504
chr1	207129300	0.950413
chr1	207154600	0.578512
chr1	207162900	0.595041
chr1	207178600	0.727273
chr1	207181200	0.884298
chr1	207183700	0.644628
chr1	207186800	0.619835
chr1	207192100	0.644628
chr1	207196100	0.586777
chr1	207200900	0.776860
chr1	207203500	0.702479
chr1	207205900	0.661157
chr1	207208300	0.851240
chr1	207210900	0.917355
chr1	207213800	0.983471
chr1	207250200	0.570248
chr1	207258600	0.925620
chr1	207369700	0.685950
chr1	207376100	0.818182
chr1	207378800	0.900826
chr1	207392200	0.842975
chr1	207396700	0.702479
chr1	207406400	0.586777
chr1	207414900	0.752066
chr1	207417700	0.785124
chr1	207476500	0.727273
chr1	207480000	0.809917
chr1	207482500	0.760331
chr1	207492500	0.809917
chr1	207535500	0.826446
chr1	207537800	0.776860
chr1	207614300	0.842975
chr1	207631200	0.768595
chr1	207637500	0.727273
chr1	207674100	0.842975
chr1	207691400	0.735537
chr1	207695500	0.991736
chr1	207697700	0.809917
chr1	207753400	0.727273
chr1	207779400	0.900826
chr1	207793900	0.809917
chr1	207798500	0.768595
chr1	207801800	0.809917
chr1	207804400	0.909091
chr1	207808700	0.785124
chr1	207813200	0.619835
chr1	207825500	0.925620
chr1	207844500	0.909091
chr1	207854300	0.958678
chr1	207865000	0.900826
chr1	207901900	0.884298

chr1	207915600	0.636364
chr1	207919100	0.900826
chr1	207921200	0.570248
chr1	207953000	0.818182
chr1	207978200	0.677686
chr1	207980500	0.867769
chr1	207986700	0.578512
chr1	207991700	0.966942
chr1	207997100	0.876033
chr1	208001600	0.661157
chr1	208004900	0.743802
chr1	208012100	0.727273
chr1	208021800	0.636364
chr1	208024100	0.595041
chr1	208043900	0.644628
chr1	208046700	0.917355
chr1	208056700	0.595041
chr1	208059700	0.628099
chr1	208064200	0.595041
chr1	208077700	0.611570
chr1	208081800	0.752066
chr1	208091500	0.669421
chr1	208096900	0.669421
chr1	208107100	0.917355
chr1	208138700	0.685950
chr1	208192500	0.603306
chr1	208210700	0.603306
chr1	208338500	0.644628
chr1	208346000	0.892562
chr1	208352900	0.809917
chr1	208358000	0.859504
chr1	208374700	0.628099
chr1	208392600	0.586777
chr1	208395200	0.867769
chr1	208398000	0.677686
chr1	208400400	0.933884
chr1	208409600	0.950413
chr1	208412800	0.925620
chr1	208619700	0.685950
chr1	209815700	0.586777
chr1	209819100	0.966942
chr1	209821300	0.909091
chr1	209823700	0.950413
chr1	209826700	0.661157
chr1	209847300	0.933884
chr1	209862800	0.595041
chr1	209981700	0.958678
chr1	209999500	0.661157
chr1	210731500	0.727273
chr1	210771600	0.586777
chr1	211094800	0.586777
chr1	211395900	0.991736
chr1	211483100	0.694215
chr1	211495100	0.669421

chr1	211503800	0.694215
chr1	211506000	0.636364
chr1	211514600	0.636364
chr1	211517800	0.760331
chr1	211545700	0.611570
chr1	211551500	0.876033
chr1	211664400	0.933884
chr1	211677400	0.586777
chr1	211689200	0.793388
chr1	211697600	0.644628
chr1	211722300	0.876033
chr1	211730000	0.991736
chr1	211732100	0.851240
chr1	211757500	0.752066
chr1	211788400	0.694215
chr1	211791200	0.636364
chr1	211793700	0.710744
chr1	211797300	0.727273
chr1	211802900	0.570248
chr1	211806500	0.818182
chr1	211817500	0.636364
chr1	211821900	0.619835
chr1	211841400	0.595041
chr1	211869100	0.735537
chr1	211885000	0.719008
chr1	211896700	0.909091
chr1	211909200	0.727273
chr1	211974600	0.636364
chr1	212006600	0.867769
chr1	212016900	0.619835
chr1	212098500	0.595041
chr1	212101600	0.793388
chr1	212106000	0.768595
chr1	212204500	0.834711
chr1	212283400	0.694215
chr1	212291000	0.586777
chr1	212320900	0.826446
chr1	212325100	0.719008
chr1	212328200	0.644628
chr1	212333000	0.826446
chr1	212340400	0.909091
chr1	212376400	0.892562
chr1	212388300	0.809917
chr1	212393700	0.917355
chr1	212405600	0.818182
chr1	212408300	0.776860
chr1	212411900	0.743802
chr1	212417300	0.752066
chr1	212420900	0.793388
chr1	212428900	0.727273
chr1	212435500	0.735537
chr1	212464700	0.669421
chr1	212590000	0.619835
chr1	212608300	0.669421

chr1	212612700	0.884298
chr1	212651800	0.677686
chr1	212659400	0.685950
chr1	212809300	0.925620
chr1	212856900	0.611570
chr1	212870400	0.603306
chr1	212895400	0.909091
chr1	212948200	0.884298
chr1	212967900	0.785124
chr1	213090400	0.661157
chr1	213191900	0.611570
chr1	213215200	0.735537
chr1	213221500	0.702479
chr1	213231200	0.735537
chr1	213240400	0.636364
chr1	213243300	0.917355
chr1	213297300	0.785124
chr1	213301200	0.611570
chr1	213312400	0.842975
chr1	213327900	0.603306
chr1	213434200	0.636364
chr1	213470400	0.595041
chr1	213779500	0.842975
chr1	214162600	0.570248
chr1	214535600	0.842975
chr1	214593200	0.710744
chr1	215745800	0.694215
chr1	215766100	0.652893
chr1	218470700	0.760331
chr1	218476400	0.917355
chr1	218490800	0.942149
chr1	218531400	0.702479
chr1	219262700	0.983471
chr1	219293400	0.611570
chr1	219295800	0.727273
chr1	219300800	0.586777
chr1	219320400	0.851240
chr1	219323100	0.793388
chr1	219326900	0.710744
chr1	219329100	0.909091
chr1	219362600	0.611570
chr1	220012300	0.876033
chr1	220045000	0.611570
chr1	220047400	0.595041
chr1	220051700	0.909091
chr1	220057100	0.933884
chr1	220071300	0.677686
chr1	220073800	0.900826
chr1	220075900	0.702479
chr1	220078000	0.851240
chr1	220082700	0.966942
chr1	220084800	0.909091
chr1	220089100	0.735537
chr1	220093800	0.595041

chr1	220098000	0.644628
chr1	220112000	0.900826
chr1	220126500	0.578512
chr1	220133100	0.628099
chr1	220135500	0.702479
chr1	220139100	0.851240
chr1	220165600	0.636364
chr1	220212600	0.586777
chr1	220266200	0.636364
chr1	220292800	0.685950
chr1	220392900	0.619835
chr1	220448400	0.603306
chr1	220474400	0.834711
chr1	220943600	0.611570
chr1	221135500	0.586777
chr1	221140500	0.925620
chr1	221205000	0.611570
chr1	221209300	0.958678
chr1	221213700	0.628099
chr1	221220200	0.652893
chr1	221808700	0.834711
chr1	221829900	0.595041
chr1	221862200	0.570248
chr1	221880900	0.611570
chr1	221883000	0.685950
chr1	221885800	0.909091
chr1	221888000	0.925620
chr1	221890700	0.909091
chr1	221896700	0.991736
chr1	221899600	0.685950
chr1	221912600	0.595041
chr1	221933000	0.611570
chr1	221935800	0.595041
chr1	222132100	0.628099
chr1	222135100	0.909091
chr1	222161700	0.900826
chr1	222629100	0.818182
chr1	222882200	0.603306
chr1	223209700	0.727273
chr1	223318300	0.661157
chr1	223377400	0.826446
chr1	223384200	0.933884
chr1	223406900	0.958678
chr1	223417800	0.851240
chr1	223438700	0.628099
chr1	223576300	1.000000
chr1	223808400	0.595041
chr1	223886500	0.644628
chr1	223889100	0.768595
chr1	223895400	0.966942
chr1	223898700	0.925620
chr1	223904400	0.603306
chr1	223914700	0.884298
chr1	223920100	0.958678

chr1	223953300	0.760331
chr1	223958300	0.702479
chr1	223961600	0.760331
chr1	223965000	1.000000
chr1	223974600	0.867769
chr1	223978700	0.909091
chr1	223982700	0.950413
chr1	224003700	0.619835
chr1	224008100	0.611570
chr1	224015900	0.950413
chr1	224020500	0.892562
chr1	224025700	0.884298
chr1	224029500	0.586777
chr1	224036300	0.661157
chr1	224061400	0.752066
chr1	224109700	0.710744
chr1	224119200	0.900826
chr1	224124300	0.636364
chr1	224225200	0.760331
chr1	224230400	0.702479
chr1	224249300	0.892562
chr1	224256700	0.892562
chr1	224268200	0.760331
chr1	224270400	0.669421
chr1	224393100	0.876033
chr1	224401600	0.611570
chr1	224450800	0.834711
chr1	224514600	0.578512
chr1	224521900	0.925620
chr1	224581000	0.834711
chr1	224617400	0.619835
chr1	224645000	0.851240
chr1	224647800	0.719008
chr1	224687000	0.661157
chr1	224690800	0.884298
chr1	224698500	0.842975
chr1	224714800	0.570248
chr1	224717300	0.876033
chr1	224879500	0.636364
chr1	225146700	0.595041
chr1	225611700	0.578512
chr1	225623800	0.785124
chr1	225626300	0.611570
chr1	225631600	0.611570
chr1	225642800	0.867769
chr1	225655400	0.595041
chr1	225955200	0.752066
chr1	225957300	0.694215
chr1	225960800	0.578512
chr1	225999400	0.586777
chr1	226001700	0.702479
chr1	226005800	0.933884
chr1	226015400	0.611570
chr1	226087100	0.909091

chr1	226105500	0.826446
chr1	226109300	0.826446
chr1	226114200	0.983471
chr1	226117600	0.760331
chr1	226119900	0.685950
chr1	226139200	0.735537
chr1	226161100	0.818182
chr1	226192100	0.991736
chr1	226197500	0.834711
chr1	226211600	0.677686
chr1	226217200	0.785124
chr1	226222400	0.677686
chr1	226254800	0.628099
chr1	226277600	0.669421
chr1	226296000	0.652893
chr1	226300900	0.595041
chr1	226304700	0.611570
chr1	226319900	0.760331
chr1	226370200	0.768595
chr1	226404300	0.842975
chr1	226521400	0.694215
chr1	226538200	0.776860
chr1	226542200	0.702479
chr1	226589400	0.619835
chr1	226767100	0.793388
chr1	226838200	0.644628
chr1	226868000	0.619835
chr1	226911700	0.603306
chr1	226942500	0.619835
chr1	226986500	0.801653
chr1	226992900	0.884298
chr1	227016000	0.710744
chr1	227063600	0.652893
chr1	227103500	0.776860
chr1	227125700	0.586777
chr1	227130100	0.694215
chr1	227132600	0.851240
chr1	227178300	0.942149
chr1	227300300	0.578512
chr1	227323400	0.586777
chr1	227358600	0.958678
chr1	227411200	0.743802
chr1	227414300	0.694215
chr1	227429200	0.694215
chr1	227431800	0.694215
chr1	227443100	0.603306
chr1	227445900	0.603306
chr1	227448600	0.636364
chr1	227487200	0.710744
chr1	227496600	0.578512
chr1	227499200	0.586777
chr1	227523900	0.760331
chr1	227543600	0.595041
chr1	227557500	0.578512

chr1	227567300	0.867769
chr1	227576700	0.661157
chr1	227579200	0.727273
chr1	227583400	0.743802
chr1	227608500	0.628099
chr1	227617500	0.785124
chr1	227620000	0.892562
chr1	227628300	0.743802
chr1	227720300	0.925620
chr1	227760900	0.876033
chr1	227916600	0.578512
chr1	227936900	1.000000
chr1	227942300	0.867769
chr1	228035900	0.933884
chr1	228044400	0.942149
chr1	228073700	0.801653
chr1	228077200	0.900826
chr1	228107700	0.619835
chr1	228275100	0.785124
chr1	228278100	0.619835
chr1	228292200	0.603306
chr1	228298900	0.644628
chr1	228323200	0.694215
chr1	228331300	0.785124
chr1	228334700	0.727273
chr1	228337500	0.983471
chr1	228366900	0.917355
chr1	228382600	0.570248
chr1	228456000	0.652893
chr1	228478300	0.694215
chr1	228529100	0.611570
chr1	228558400	0.661157
chr1	228560800	0.818182
chr1	228579900	0.694215
chr1	228598200	0.644628
chr1	228605900	0.628099
chr1	228609800	0.834711
chr1	228615000	0.793388
chr1	228637400	0.727273
chr1	228640800	0.851240
chr1	228664100	0.776860
chr1	228677900	0.983471
chr1	228689600	0.793388
chr1	228698300	0.719008
chr1	228702300	0.677686
chr1	228974700	0.603306
chr1	229140300	0.818182
chr1	229143600	0.636364
chr1	229284200	0.834711
chr1	229335800	0.661157
chr1	229389400	0.719008
chr1	229402800	0.669421
chr1	229416500	0.628099
chr1	229441800	0.694215

chr1	229475300	0.595041
chr1	229483100	0.735537
chr1	229485700	0.876033
chr1	229488800	0.652893
chr1	229583900	0.983471
chr1	229685700	0.595041
chr1	229688700	0.619835
chr1	229757600	0.644628
chr1	229887500	0.578512
chr1	229890200	0.628099
chr1	230108600	0.727273
chr1	230165400	0.595041
chr1	230183300	0.636364
chr1	230209400	0.743802
chr1	230223100	0.950413
chr1	230237600	0.661157
chr1	230242600	0.785124
chr1	230249100	0.628099
chr1	230253400	0.752066
chr1	230256700	0.826446
chr1	230260000	0.776860
chr1	230264600	0.892562
chr1	230272200	0.570248
chr1	230276400	0.942149
chr1	230278600	0.586777
chr1	230283400	0.826446
chr1	230286500	0.760331
chr1	230289400	0.851240
chr1	230292100	0.917355
chr1	230297100	0.975207
chr1	230299500	0.570248
chr1	230302600	0.661157
chr1	230306500	0.735537
chr1	230311400	0.991736
chr1	230313700	0.586777
chr1	230321700	0.826446
chr1	230327100	0.851240
chr1	230341700	0.636364
chr1	230367400	0.669421
chr1	230404300	0.652893
chr1	230783200	0.685950
chr1	230817900	0.909091
chr1	230961200	0.636364
chr1	230992100	0.727273
chr1	230999400	0.719008
chr1	231008200	0.669421
chr1	231012000	0.785124
chr1	231052800	0.801653
chr1	231122900	0.851240
chr1	231172900	0.619835
chr1	231181900	0.785124
chr1	231184000	0.743802
chr1	231186100	0.909091
chr1	231201400	0.950413

chr1	231209200	0.677686
chr1	231228000	0.677686
chr1	231242300	0.595041
chr1	231295800	0.661157
chr1	231329800	0.628099
chr1	231335900	0.719008
chr1	231338400	0.925620
chr1	231379800	0.586777
chr1	231491000	0.768595
chr1	231544400	0.611570
chr1	231550700	0.851240
chr1	231578800	0.719008
chr1	231629800	0.818182
chr1	231667600	0.578512
chr1	231726900	0.603306
chr1	233074200	0.685950
chr1	233249300	0.942149
chr1	233577200	0.661157
chr1	233584300	0.578512
chr1	233680000	0.818182
chr1	233735200	0.644628
chr1	233743600	0.768595
chr1	233746900	0.636364
chr1	233756800	0.669421
chr1	233761800	0.950413
chr1	233767200	0.628099
chr1	233772100	0.983471
chr1	233774600	0.661157
chr1	233777900	0.785124
chr1	233792000	0.809917
chr1	233797100	0.619835
chr1	233801200	0.867769
chr1	233805100	0.735537
chr1	233809700	0.851240
chr1	233840600	0.900826
chr1	233843600	0.710744
chr1	233846400	0.851240
chr1	233866900	0.710744
chr1	234109100	0.958678
chr1	234492000	0.619835
chr1	234497500	0.917355
chr1	234505900	0.925620
chr1	234569600	0.867769
chr1	234624000	0.586777
chr1	234627100	0.710744
chr1	234629400	0.842975
chr1	234631700	0.595041
chr1	234635500	0.785124
chr1	234648600	0.752066
chr1	234657300	0.892562
chr1	234659600	0.859504
chr1	234663900	0.735537
chr1	234680400	0.975207
chr1	234683300	0.909091

chr1	234689700	0.925620
chr1	234694300	0.859504
chr1	234720000	0.876033
chr1	234747500	0.619835
chr1	234750100	0.652893
chr1	234793200	0.826446
chr1	234798200	0.578512
chr1	234807400	0.917355
chr1	234836400	0.975207
chr1	234852000	0.570248
chr1	234862800	0.710744
chr1	234866800	0.842975
chr1	234882100	0.793388
chr1	234886900	0.776860
chr1	234891700	0.785124
chr1	234908400	0.933884
chr1	234913000	0.578512
chr1	234952200	0.578512
chr1	234967100	0.661157
chr1	234979200	0.851240
chr1	234985400	0.743802
chr1	234988000	0.801653
chr1	234999000	0.586777
chr1	235001800	0.586777
chr1	235004800	0.975207
chr1	235007600	0.975207
chr1	235010100	0.900826
chr1	235012400	0.942149
chr1	235015000	0.983471
chr1	235030100	0.884298
chr1	235035500	0.809917
chr1	235054200	0.702479
chr1	235091000	0.636364
chr1	235094300	0.950413
chr1	235097200	0.743802
chr1	235100200	0.652893
chr1	235105000	0.743802
chr1	235107800	0.735537
chr1	235110200	0.652893
chr1	235113100	0.752066
chr1	235117200	0.595041
chr1	235120200	0.900826
chr1	235123100	0.652893
chr1	235148000	0.884298
chr1	235158600	0.669421
chr1	235187200	0.876033
chr1	235247100	0.603306
chr1	235254300	0.809917
chr1	235257500	0.818182
chr1	235268400	0.818182
chr1	235277000	0.983471
chr1	235293200	0.603306
chr1	235370500	0.933884
chr1	235410300	0.586777

chr1	235445700	0.752066
chr1	235453800	0.851240
chr1	235459900	0.628099
chr1	235462700	0.876033
chr1	235572500	0.677686
chr1	235575400	0.685950
chr1	235606400	0.942149
chr1	235614600	0.925620
chr1	235664800	0.702479
chr1	235752600	0.958678
chr1	235805800	0.636364
chr1	236162800	0.619835
chr1	236370500	0.628099
chr1	236404200	0.735537
chr1	236417700	0.785124
chr1	236512800	0.867769
chr1	236516100	0.991736
chr1	236518400	0.793388
chr1	236561100	0.603306
chr1	236579800	0.603306
chr1	236584900	0.727273
chr1	236673700	0.950413
chr1	236696200	0.933884
chr1	236714800	0.603306
chr1	236732000	0.661157
chr1	236917800	0.776860
chr1	237071100	0.809917
chr1	237077700	0.661157
chr1	237171300	0.644628
chr1	237176500	0.595041
chr1	241649100	0.975207
chr1	241760400	0.619835
chr1	241775100	0.694215
chr1	241834100	0.619835
chr1	241852600	0.710744
chr1	241858500	0.909091
chr1	241862300	0.842975
chr1	241865200	0.595041
chr1	241878900	0.669421
chr1	241897400	0.619835
chr1	241899600	0.685950
chr1	241912400	0.578512
chr1	241934100	0.619835
chr1	241937800	0.876033
chr1	241970700	0.851240
chr1	241974300	0.619835
chr1	241981700	0.644628
chr1	241987400	0.636364
chr1	241992700	0.842975
chr1	241997400	0.983471
chr1	242014500	0.619835
chr1	243041100	0.669421
chr1	243088500	0.628099
chr1	243197100	0.743802

chr1	243267000	0.842975
chr1	243269500	0.809917
chr1	243414900	0.628099
chr1	243427900	0.876033
chr1	243431800	0.669421
chr1	243434500	0.710744
chr1	243571100	0.603306
chr1	243585300	0.735537
chr1	243599100	0.652893
chr1	243609100	0.727273
chr1	243611500	0.892562
chr1	243636000	0.727273
chr1	244087200	0.611570
chr1	244103100	0.801653
chr1	244437400	0.966942
chr1	244463600	0.809917
chr1	244466200	0.983471
chr1	244469200	0.958678
chr1	244479200	0.586777
chr1	244486400	0.801653
chr1	244505400	0.900826
chr1	244510300	0.851240
chr1	244514100	0.652893
chr1	244517600	0.983471
chr1	244533700	0.826446
chr1	244551100	0.652893
chr1	244557200	0.900826
chr1	244603100	0.793388
chr1	244609900	0.603306
chr1	244829100	0.809917
chr1	245150400	0.603306
chr1	245159400	0.578512
chr1	245163500	0.685950
chr1	245169800	0.694215
chr1	245189700	0.570248
chr1	245205200	0.628099
chr1	245255600	0.785124
chr1	245501200	0.842975
chr1	245701600	0.669421
chr1	245955600	0.619835
chr1	246038700	0.900826
chr1	246043100	0.570248
chr1	246120400	0.636364
chr1	246154400	0.719008
chr1	246169100	0.876033
chr1	246232800	0.851240
chr1	246235500	0.760331
chr1	246241700	0.900826
chr1	246254900	0.727273
chr1	246269700	0.884298
chr1	246274500	0.776860
chr1	246276700	0.867769
chr1	246280800	0.776860
chr1	246306900	0.768595

chr1	246314800	0.776860
chr1	246317900	0.876033
chr1	246320700	0.694215
chr1	246327200	0.677686
chr1	246329600	0.900826
chr1	246332500	0.826446
chr1	246335600	0.851240
chr1	246339700	0.933884
chr1	246345400	0.851240
chr1	246351600	0.900826
chr1	246357800	0.578512
chr1	246364100	0.628099
chr1	246377100	0.752066
chr1	246379700	0.661157
chr1	246470000	0.884298
chr1	246495500	0.851240
chr1	246501500	0.884298
chr1	246514400	0.644628
chr1	246562000	0.768595
chr1	246584000	0.578512
chr1	246589500	0.710744
chr1	246592300	0.834711
chr1	246608600	0.752066
chr1	246736200	0.735537
chr1	246739200	0.785124
chr1	246748000	0.611570
chr1	246751200	0.925620
chr1	246759100	0.719008
chr1	246761200	0.694215
chr1	246862600	0.776860
chr1	247001200	0.570248
chr1	247089000	0.842975
chr1	247092400	0.586777
chr1	247123500	0.834711
chr1	247218600	0.628099
chr1	247375900	0.685950
chr1	247452600	0.842975
chr1	247455900	0.603306
chr1	247460700	0.834711
chr1	247498800	0.603306
chr1	247531800	0.809917
chr1	247535000	0.669421
chr1	247538500	0.628099
chr1	247551000	0.570248
chr1	247634100	0.578512
chr1	248009100	0.677686
chr1	248016000	0.694215
chr1	248018700	0.851240
chr1	248023000	0.619835
chr1	249122900	0.950413
chr1	249125900	0.933884
chr1	249128900	0.950413
chr1	249136200	0.851240
chr1	249146900	0.925620

chr1	249150000	0.876033
chr1	249157500	0.619835
chr1	249165600	0.685950
chr1	249176100	0.884298
chr1	249179900	0.950413
chr1	249183500	0.752066
chr1	249186700	0.694215
chr1	249195300	0.826446
chr1	249198400	0.694215
chr1	249203100	0.884298
chr1	249205200	0.661157
chr1	249208800	0.826446
chr1	249218800	0.611570
chr1	249222000	0.801653
chr1	249229100	0.694215
chr1	249233600	0.917355
chr20	185500	0.586777
chr20	239600	0.694215
chr20	247500	0.909091
chr20	335300	0.975207
chr20	342800	0.851240
chr20	345800	0.991736
chr20	347900	0.983471
chr20	350400	0.842975
chr20	352500	0.619835
chr20	354800	0.950413
chr20	356900	0.677686
chr20	360200	0.628099
chr20	365100	0.950413
chr20	380800	0.917355
chr20	383300	0.917355
chr20	387600	0.578512
chr20	397100	0.991736
chr20	426200	0.975207
chr20	440600	0.636364
chr20	510700	0.818182
chr20	517400	0.636364
chr20	526200	0.570248
chr20	530000	0.578512
chr20	544700	0.685950
chr20	549300	0.685950
chr20	558200	0.793388
chr20	606300	0.595041
chr20	623900	0.776860
chr20	626900	0.892562
chr20	636900	0.975207
chr20	641600	0.735537
chr20	648100	0.735537
chr20	651200	0.851240
chr20	654300	0.950413
chr20	665700	0.958678
chr20	667900	0.851240
chr20	684100	0.760331
chr20	755200	0.636364

chr20	793700	0.900826
chr20	802100	0.917355
chr20	810400	0.818182
chr20	816900	0.942149
chr20	819100	0.644628
chr20	955000	0.578512
chr20	1042700	0.752066
chr20	1067000	0.743802
chr20	1102200	0.768595
chr20	1106200	0.900826
chr20	1152600	0.611570
chr20	1167700	0.950413
chr20	1172100	0.876033
chr20	1196300	0.958678
chr20	1233800	0.983471
chr20	1236500	0.950413
chr20	1245100	0.785124
chr20	1249500	0.876033
chr20	1258500	0.991736
chr20	1309800	0.644628
chr20	1363400	0.735537
chr20	1415900	1.000000
chr20	1449900	0.884298
chr20	2090200	0.644628
chr20	2099800	0.694215
chr20	2294900	0.809917
chr20	2297200	0.867769
chr20	2499800	0.900826
chr20	2507500	0.727273
chr20	2510900	0.628099
chr20	2559800	0.776860
chr20	2776400	0.595041
chr20	2782200	0.661157
chr20	2784900	0.669421
chr20	2801300	0.809917
chr20	2851300	0.859504
chr20	2871800	0.933884
chr20	2922100	0.685950
chr20	2928300	0.818182
chr20	3019400	0.859504
chr20	3026000	0.570248
chr20	3058700	0.859504
chr20	3075100	0.611570
chr20	3110100	0.644628
chr20	3137900	0.652893
chr20	3401300	0.669421
chr20	3428200	0.809917
chr20	3633300	0.644628
chr20	3725900	0.611570
chr20	3765800	0.644628
chr20	3799600	0.752066
chr20	3820200	0.842975
chr20	3824200	0.991736
chr20	3826500	0.652893

chr20	3877900	0.851240
chr20	3881400	0.603306
chr20	3889500	0.611570
chr20	3891900	0.884298
chr20	3912100	0.933884
chr20	3961900	0.785124
chr20	3971400	0.570248
chr20	3976600	0.842975
chr20	3984900	0.595041
chr20	3988900	0.628099
chr20	3992500	0.603306
chr20	3998900	0.909091
chr20	4006700	0.834711
chr20	4010800	0.595041
chr20	4016000	0.917355
chr20	4019100	0.942149
chr20	4022700	0.801653
chr20	4025000	0.727273
chr20	4031600	0.727273
chr20	4041400	0.669421
chr20	4045600	0.925620
chr20	4047700	0.644628
chr20	4050100	0.909091
chr20	4053500	0.892562
chr20	4056000	0.851240
chr20	4062900	0.776860
chr20	4065200	0.884298
chr20	4067900	0.752066
chr20	4072300	0.942149
chr20	4089800	0.809917
chr20	4100700	0.694215
chr20	4111600	0.884298
chr20	4116800	0.603306
chr20	4168500	0.950413
chr20	4173400	0.975207
chr20	4198200	0.570248
chr20	4223900	0.578512
chr20	4232400	0.793388
chr20	4323700	0.578512
chr20	4350700	0.826446
chr20	4642100	0.793388
chr20	4654600	0.933884
chr20	4669700	0.595041
chr20	4954700	0.586777
chr20	5037800	0.785124
chr20	5059300	0.917355
chr20	5096600	0.603306
chr20	5103900	0.785124
chr20	5109800	0.595041
chr20	5131400	0.809917
chr20	5152200	0.677686
chr20	5470100	0.628099
chr20	5474400	0.595041
chr20	5510400	0.677686

chr20	5512800	0.842975
chr20	5516300	0.752066
chr20	5529800	0.793388
chr20	5573100	0.628099
chr20	5575900	0.652893
chr20	5578100	0.975207
chr20	5587400	0.603306
chr20	5640600	0.809917
chr20	5643800	0.619835
chr20	5652500	0.677686
chr20	5655800	0.917355
chr20	5687400	0.595041
chr20	5696000	0.611570
chr20	5700900	1.000000
chr20	5718200	0.975207
chr20	5722200	0.661157
chr20	5727400	0.611570
chr20	5734100	0.595041
chr20	5744200	0.859504
chr20	5751800	0.661157
chr20	5763100	0.983471
chr20	5766400	1.000000
chr20	5768900	0.644628
chr20	5803600	0.735537
chr20	5992900	0.685950
chr20	5995200	0.892562
chr20	6015400	0.983471
chr20	6040000	0.578512
chr20	8002700	0.644628
chr20	9117100	0.917355
chr20	9120500	0.859504
chr20	9126100	0.586777
chr20	9130500	0.925620
chr20	9191800	0.595041
chr20	10400900	0.710744
chr20	10411500	0.710744
chr20	10418700	0.694215
chr20	10429600	0.768595
chr20	10456900	0.735537
chr20	10482500	0.768595
chr20	10486500	1.000000
chr20	10489800	0.900826
chr20	10493400	0.958678
chr20	13422600	0.578512
chr20	13509900	0.595041
chr20	13606200	0.842975
chr20	13898100	0.710744
chr20	16392500	0.859504
chr20	16453000	1.000000
chr20	17418400	0.595041
chr20	17488300	0.727273
chr20	17514700	0.636364
chr20	17539200	0.859504
chr20	17543800	0.925620

chr20	17571200	0.636364
chr20	17891900	0.727273
chr20	17906600	0.884298
chr20	17916800	0.809917
chr20	17920400	0.809917
chr20	17939500	0.966942
chr20	17955100	0.595041
chr20	18237800	0.611570
chr20	18569600	0.628099
chr20	20374300	0.760331
chr20	20619200	0.636364
chr20	20927800	0.818182
chr20	21004300	0.710744
chr20	21071100	0.595041
chr20	21074400	0.611570
chr20	21099300	0.611570
chr20	21109600	0.760331
chr20	21122700	0.702479
chr20	21127500	0.760331
chr20	21304400	0.975207
chr20	21637400	0.727273
chr20	22370700	0.570248
chr20	22382900	0.685950
chr20	22393500	0.677686
chr20	22395800	0.966942
chr20	22531600	0.586777
chr20	22552300	0.636364
chr20	22598100	0.752066
chr20	22665700	0.628099
chr20	22744900	0.685950
chr20	22808000	0.685950
chr20	22814200	0.719008
chr20	22817700	0.983471
chr20	22890100	0.958678
chr20	22899600	0.586777
chr20	22902700	0.950413
chr20	22905500	0.760331
chr20	22909800	0.942149
chr20	23034000	0.842975
chr20	23041900	0.677686
chr20	23068300	0.834711
chr20	23073300	0.975207
chr20	23095600	0.818182
chr20	23097700	0.669421
chr20	23101900	0.727273
chr20	23107200	0.760331
chr20	23111300	0.652893
chr20	23115000	0.776860
chr20	23117500	0.950413
chr20	23119700	0.983471
chr20	23122000	0.628099
chr20	23124200	0.991736
chr20	23127400	0.917355
chr20	23131500	0.752066

chr20	23133600	0.859504
chr20	23136900	0.966942
chr20	23140600	0.809917
chr20	23143000	0.892562
chr20	23160500	0.776860
chr20	23174900	0.834711
chr20	23182400	0.752066
chr20	23185400	0.644628
chr20	23190700	0.603306
chr20	23292900	0.793388
chr20	23313400	0.586777
chr20	23340600	0.570248
chr20	23393300	0.661157
chr20	23474300	0.685950
chr20	23549400	0.727273
chr20	23563000	0.619835
chr20	23610000	0.611570
chr20	23641200	0.942149
chr20	24615800	0.801653
chr20	25013900	0.719008
chr20	25036600	0.570248
chr20	25204200	0.818182
chr20	25208300	0.950413
chr20	25214700	0.652893
chr20	25219100	0.975207
chr20	25291100	0.950413
chr20	25331900	0.719008
chr20	25390300	0.619835
chr20	25524400	0.603306
chr20	25529900	0.570248
chr20	25606700	0.644628
chr20	25838600	0.661157
chr20	25846500	0.710744
chr20	25991300	0.727273
chr20	26190200	0.710744
chr20	29517300	0.603306
chr20	29520800	0.702479
chr20	29528500	0.785124
chr20	29619400	0.578512
chr20	30029800	0.628099
chr20	30034600	0.694215
chr20	30061000	0.677686
chr20	30094200	0.644628
chr20	30104900	0.867769
chr20	30109900	0.752066
chr20	30130400	0.727273
chr20	30143200	0.685950
chr20	30148200	0.884298
chr20	30159800	0.611570
chr20	30179200	0.983471
chr20	30183900	0.966942
chr20	30197500	0.900826
chr20	30222100	0.818182
chr20	30232900	0.826446

chr20	30251500	0.884298
chr20	30256500	0.942149
chr20	30258800	0.628099
chr20	30261300	0.884298
chr20	30266300	0.909091
chr20	30268800	0.958678
chr20	30271700	0.983471
chr20	30279700	0.851240
chr20	30282400	0.942149
chr20	30284700	0.966942
chr20	30287800	0.942149
chr20	30292900	0.743802
chr20	30295700	0.966942
chr20	30299100	0.859504
chr20	30302300	0.669421
chr20	30334800	0.586777
chr20	30400300	0.892562
chr20	30424100	0.876033
chr20	30426800	0.694215
chr20	30506200	0.595041
chr20	30545200	0.809917
chr20	30595600	0.661157
chr20	30606300	0.578512
chr20	30624800	0.619835
chr20	30700900	0.595041
chr20	30704800	0.702479
chr20	30747900	0.876033
chr20	30792500	0.785124
chr20	30797700	0.685950
chr20	30873600	0.917355
chr20	30914300	0.867769
chr20	30922100	0.694215
chr20	30925500	0.570248
chr20	30943200	0.570248
chr20	30952400	0.669421
chr20	30979100	0.826446
chr20	30983200	0.578512
chr20	30986100	0.966942
chr20	30992200	0.942149
chr20	31065100	0.710744
chr20	31082300	0.595041
chr20	31093400	0.652893
chr20	31099200	0.743802
chr20	31112600	0.677686
chr20	31117900	0.735537
chr20	31123400	0.834711
chr20	31128100	0.925620
chr20	31142700	0.719008
chr20	31150700	0.793388
chr20	31156100	0.586777
chr20	31158300	0.925620
chr20	31160400	0.826446
chr20	31162600	0.933884
chr20	31165000	0.859504

chr20	31168200	0.909091
chr20	31174200	0.702479
chr20	31194100	0.685950
chr20	31200600	0.578512
chr20	31209500	0.727273
chr20	31212700	0.661157
chr20	31215200	0.735537
chr20	31223100	0.694215
chr20	31229100	0.933884
chr20	31231500	0.950413
chr20	31234700	0.619835
chr20	31238600	1.000000
chr20	31253100	0.867769
chr20	31261600	0.983471
chr20	31266100	0.743802
chr20	31268400	0.942149
chr20	31270900	0.818182
chr20	31274000	0.933884
chr20	31319700	0.677686
chr20	31328400	0.619835
chr20	31333000	0.801653
chr20	31341000	0.925620
chr20	31348900	0.611570
chr20	31411000	0.884298
chr20	31845100	0.809917
chr20	31927900	0.842975
chr20	32007100	0.859504
chr20	32011400	0.975207
chr20	32026900	0.611570
chr20	32029300	0.752066
chr20	32033200	0.578512
chr20	32190600	0.652893
chr20	32218400	0.636364
chr20	32254900	0.685950
chr20	32277200	0.702479
chr20	32297200	0.719008
chr20	32322200	0.710744
chr20	32331900	0.892562
chr20	32375600	0.578512
chr20	32377700	0.570248
chr20	32393900	0.603306
chr20	32417900	0.826446
chr20	32426600	0.793388
chr20	32431700	0.611570
chr20	32585800	0.876033
chr20	32592600	0.785124
chr20	32599000	0.603306
chr20	32601400	0.702479
chr20	32604700	0.685950
chr20	32611200	0.586777
chr20	32651700	0.785124
chr20	32671400	0.735537
chr20	32673900	0.917355
chr20	32676400	0.851240

chr20	32696500	0.644628
chr20	32704000	0.694215
chr20	32716300	0.677686
chr20	32724200	0.867769
chr20	32778000	0.619835
chr20	32844300	0.950413
chr20	32904300	0.743802
chr20	32910900	0.760331
chr20	32926100	0.570248
chr20	32954100	0.710744
chr20	33013400	0.595041
chr20	33049100	0.719008
chr20	33113800	0.776860
chr20	33137500	0.685950
chr20	33159300	0.834711
chr20	33277200	0.644628
chr20	33301500	0.933884
chr20	33385600	0.991736
chr20	33417300	0.661157
chr20	33462700	0.611570
chr20	33467000	0.826446
chr20	33476600	0.652893
chr20	33488100	0.570248
chr20	33492100	0.966942
chr20	33548500	0.603306
chr20	33565500	0.752066
chr20	33704200	0.966942
chr20	33706700	0.942149
chr20	33709500	0.785124
chr20	33720300	0.719008
chr20	33754400	0.727273
chr20	33762700	0.760331
chr20	33775500	0.611570
chr20	33780500	0.991736
chr20	33796300	0.628099
chr20	33859300	0.950413
chr20	33894000	0.884298
chr20	33900000	0.809917
chr20	33915000	0.900826
chr20	33917600	0.628099
chr20	33926300	0.611570
chr20	33932700	0.876033
chr20	34023100	0.570248
chr20	34049900	0.933884
chr20	34052000	0.776860
chr20	34102500	0.809917
chr20	34195100	0.611570
chr20	34199400	0.578512
chr20	34247400	0.661157
chr20	34327000	0.677686
chr20	34356400	0.611570
chr20	34365000	0.917355
chr20	34384800	0.834711
chr20	34411100	0.694215

chr20	34420100	0.685950
chr20	34459700	0.842975
chr20	34463600	0.909091
chr20	34489400	1.000000
chr20	34492200	0.842975
chr20	34495400	0.710744
chr20	34568100	0.578512
chr20	34585300	0.570248
chr20	34623000	0.867769
chr20	34630300	0.586777
chr20	34643000	0.586777
chr20	34651200	0.619835
chr20	34671100	0.628099
chr20	34673600	0.735537
chr20	34710900	0.892562
chr20	34771200	0.892562
chr20	34773400	0.636364
chr20	35050500	0.578512
chr20	35088900	0.867769
chr20	35092400	0.950413
chr20	35094500	0.760331
chr20	35101300	0.892562
chr20	35119000	0.578512
chr20	35204500	0.603306
chr20	35233300	0.752066
chr20	35329200	0.818182
chr20	35363100	0.719008
chr20	35399700	0.776860
chr20	35450800	0.842975
chr20	35465600	0.669421
chr20	35471800	0.950413
chr20	35483300	0.636364
chr20	35488100	0.595041
chr20	35502100	0.776860
chr20	35515900	0.727273
chr20	35552900	0.801653
chr20	35561500	0.735537
chr20	35583200	0.867769
chr20	35726000	0.586777
chr20	35783500	0.826446
chr20	35787500	0.570248
chr20	35791800	0.644628
chr20	35796000	0.628099
chr20	35899400	0.785124
chr20	35903600	0.685950
chr20	35924000	0.760331
chr20	35928900	0.983471
chr20	35937000	0.752066
chr20	35945000	0.900826
chr20	35965000	0.958678
chr20	36006800	0.859504
chr20	36009000	0.702479
chr20	36012700	0.793388
chr20	36025200	0.677686

chr20	36035300	0.628099
chr20	36040900	0.595041
chr20	36170800	0.595041
chr20	36331400	0.661157
chr20	36334800	0.793388
chr20	36339800	0.776860
chr20	36344200	0.710744
chr20	36462400	0.735537
chr20	36474700	0.628099
chr20	36479500	0.884298
chr20	36531300	0.603306
chr20	36587500	0.661157
chr20	36602000	0.950413
chr20	36630700	0.636364
chr20	36666600	0.578512
chr20	36713300	0.942149
chr20	36720700	0.735537
chr20	36723300	0.909091
chr20	36738400	0.710744
chr20	36742300	0.950413
chr20	36751500	0.826446
chr20	36754900	0.925620
chr20	36757700	0.793388
chr20	36763400	0.826446
chr20	36772600	0.685950
chr20	36790300	0.851240
chr20	36825400	0.867769
chr20	36971900	0.900826
chr20	36986500	0.842975
chr20	37002600	0.809917
chr20	37054600	0.595041
chr20	37058600	0.578512
chr20	37061000	0.785124
chr20	37079000	0.917355
chr20	37082200	0.735537
chr20	37108200	0.644628
chr20	37148200	0.636364
chr20	37192800	0.719008
chr20	37262800	0.595041
chr20	37295200	0.859504
chr20	37321800	0.752066
chr20	37380500	0.677686
chr20	37386300	0.661157
chr20	37455000	0.834711
chr20	37460400	0.785124
chr20	37469400	0.785124
chr20	37483600	0.867769
chr20	37487100	0.743802
chr20	37492100	0.760331
chr20	37495400	0.710744
chr20	37504000	0.603306
chr20	37518700	0.776860
chr20	37523300	0.809917
chr20	37560900	0.685950

chr20	37565200	0.595041
chr20	37567900	0.603306
chr20	37573500	0.809917
chr20	37578100	0.834711
chr20	37585900	0.884298
chr20	37588700	0.727273
chr20	37595100	0.661157
chr20	37604300	0.702479
chr20	37624600	0.884298
chr20	37627000	0.809917
chr20	37707800	0.586777
chr20	39127100	0.785124
chr20	39191800	0.677686
chr20	39194400	0.685950
chr20	39446800	0.619835
chr20	39577300	0.636364
chr20	39620100	0.975207
chr20	39632400	0.900826
chr20	39637100	0.925620
chr20	39652400	0.694215
chr20	39665000	0.793388
chr20	39674000	0.884298
chr20	39676600	0.586777
chr20	39684600	0.776860
chr20	39687500	0.950413
chr20	39700200	0.983471
chr20	39704600	0.842975
chr20	39715800	0.842975
chr20	39745300	0.966942
chr20	39763100	0.793388
chr20	39770500	0.859504
chr20	39779900	0.909091
chr20	39788300	0.702479
chr20	39842300	0.578512
chr20	39861300	0.727273
chr20	39890800	0.909091
chr20	39938000	0.834711
chr20	40054100	0.752066
chr20	40057200	0.859504
chr20	40140700	0.628099
chr20	40154300	0.760331
chr20	40164200	0.628099
chr20	40168200	0.719008
chr20	40196300	0.710744
chr20	40263900	0.743802
chr20	40272500	0.966942
chr20	40286500	0.570248
chr20	40307300	0.619835
chr20	40319900	0.900826
chr20	40324900	0.909091
chr20	40334000	0.752066
chr20	40998800	0.628099
chr20	41947900	0.719008
chr20	42059700	0.826446

chr20	42145700	0.983471
chr20	42167500	0.933884
chr20	42180400	0.677686
chr20	42185200	0.595041
chr20	42187700	0.909091
chr20	42204200	0.628099
chr20	42208800	0.925620
chr20	42213000	0.950413
chr20	42306000	0.851240
chr20	42320000	0.809917
chr20	42375600	0.669421
chr20	42588400	0.669421
chr20	42639400	0.867769
chr20	42648800	0.619835
chr20	42726900	0.867769
chr20	42833300	0.851240
chr20	42846700	0.743802
chr20	43023600	0.925620
chr20	43036600	0.785124
chr20	43085900	0.644628
chr20	43107300	0.851240
chr20	43146700	0.636364
chr20	43153000	0.818182
chr20	43170800	0.570248
chr20	43173300	0.884298
chr20	43178100	0.595041
chr20	43182000	0.983471
chr20	43205600	0.842975
chr20	43223800	0.669421
chr20	43238900	0.917355
chr20	43245400	0.909091
chr20	43256000	0.727273
chr20	43271300	0.851240
chr20	43307800	0.892562
chr20	43367800	0.710744
chr20	43486500	0.768595
chr20	43492400	0.595041
chr20	43526400	0.685950
chr20	43540900	0.801653
chr20	43590600	0.983471
chr20	43592800	0.628099
chr20	43599900	0.809917
chr20	43608200	0.958678
chr20	43622100	0.768595
chr20	43665200	0.834711
chr20	43697900	0.652893
chr20	43700400	0.652893
chr20	43952000	0.702479
chr20	43966300	0.909091
chr20	43970800	0.727273
chr20	43973000	0.966942
chr20	43987500	0.586777
chr20	44002400	0.644628
chr20	44039900	0.727273

chr20	44407800	0.628099
chr20	44410400	0.619835
chr20	44439000	0.933884
chr20	44465000	0.933884
chr20	44480300	0.983471
chr20	44483800	0.652893
chr20	44503400	0.661157
chr20	44507400	0.909091
chr20	44512000	0.595041
chr20	44514200	0.892562
chr20	44543600	0.652893
chr20	44551200	0.578512
chr20	44573200	0.826446
chr20	44598500	0.570248
chr20	44604700	0.809917
chr20	44607500	0.752066
chr20	44611900	0.636364
chr20	44711900	0.702479
chr20	44714600	0.619835
chr20	44850300	0.636364
chr20	44979500	0.983471
chr20	44997100	0.958678
chr20	45005300	0.710744
chr20	45030100	0.801653
chr20	45039600	0.628099
chr20	45192300	0.603306
chr20	45203000	0.809917
chr20	45248400	0.661157
chr20	45255900	0.727273
chr20	45260500	0.603306
chr20	45264800	0.991736
chr20	45278100	0.760331
chr20	45323700	0.611570
chr20	45568300	0.619835
chr20	45619000	0.603306
chr20	45627300	0.743802
chr20	45671600	0.644628
chr20	45817900	0.727273
chr20	45829200	0.933884
chr20	45832000	0.760331
chr20	45835100	0.586777
chr20	45883100	0.776860
chr20	45887500	0.595041
chr20	45903800	0.818182
chr20	45908800	0.958678
chr20	45933400	0.909091
chr20	45945600	0.917355
chr20	45949700	0.595041
chr20	45952000	0.809917
chr20	45955500	0.578512
chr20	45957700	0.743802
chr20	45961700	0.900826
chr20	45965400	0.719008
chr20	45969700	0.958678

chr20	45975500	0.933884
chr20	45991900	0.975207
chr20	45998000	0.834711
chr20	46001500	0.859504
chr20	46012500	0.859504
chr20	46021700	0.685950
chr20	46026200	0.727273
chr20	46061600	0.818182
chr20	46086500	0.826446
chr20	46097600	0.826446
chr20	46102500	0.776860
chr20	46108400	0.661157
chr20	46116300	0.652893
chr20	46122800	0.892562
chr20	46153000	0.842975
chr20	46169900	0.818182
chr20	46203000	0.867769
chr20	46230500	0.942149
chr20	46234600	0.801653
chr20	46237200	0.876033
chr20	46243600	0.983471
chr20	46768300	0.595041
chr20	46772100	0.595041
chr20	47238100	0.570248
chr20	47296600	0.884298
chr20	47299400	0.586777
chr20	47315600	0.652893
chr20	47339500	0.595041
chr20	47386400	0.570248
chr20	47416900	0.636364
chr20	47448700	0.595041
chr20	47458900	0.859504
chr20	47470800	0.818182
chr20	47493000	0.760331
chr20	47547200	0.867769
chr20	47669000	0.669421
chr20	47671200	0.586777
chr20	47754500	0.694215
chr20	47800800	0.652893
chr20	47833800	0.743802
chr20	47887000	0.570248
chr20	47904600	0.719008
chr20	47908100	0.719008
chr20	47936200	0.570248
chr20	47940800	0.900826
chr20	48027900	0.611570
chr20	48285800	0.595041
chr20	48288700	0.768595
chr20	48292400	0.743802
chr20	48295900	0.942149
chr20	48302600	0.942149
chr20	48305600	0.826446
chr20	48318600	0.892562
chr20	48324400	0.644628

chr20	48334600	0.611570
chr20	48364800	0.958678
chr20	48378100	0.578512
chr20	48380400	0.826446
chr20	48384100	0.867769
chr20	48392600	0.975207
chr20	48402600	0.950413
chr20	48406200	0.950413
chr20	48408300	0.950413
chr20	48411600	0.760331
chr20	48422200	0.900826
chr20	48448000	0.793388
chr20	48484900	0.677686
chr20	48509900	0.586777
chr20	48545500	0.595041
chr20	48555700	0.661157
chr20	48585900	0.578512
chr20	48595700	0.859504
chr20	48602200	0.719008
chr20	48604300	0.793388
chr20	48606400	0.917355
chr20	48629400	0.818182
chr20	48632400	0.669421
chr20	48647100	0.818182
chr20	48654500	0.578512
chr20	48672100	0.694215
chr20	48688200	0.595041
chr20	48703000	0.669421
chr20	48716000	0.818182
chr20	48718900	0.818182
chr20	48724500	0.834711
chr20	48747400	0.735537
chr20	48754700	0.851240
chr20	48763700	0.925620
chr20	48767700	0.578512
chr20	48771400	0.611570
chr20	48780200	0.975207
chr20	48796100	0.677686
chr20	48803700	0.991736
chr20	48812000	0.809917
chr20	48843700	0.578512
chr20	48864800	0.628099
chr20	48877000	0.942149
chr20	48895800	0.876033
chr20	48900300	0.661157
chr20	48909200	0.917355
chr20	48911400	0.669421
chr20	48915300	0.636364
chr20	48920600	0.719008
chr20	48925200	0.884298
chr20	48938300	0.966942
chr20	48964800	0.867769
chr20	48971700	0.760331
chr20	48986000	0.818182

chr20	48995900	0.619835
chr20	48999500	0.942149
chr20	49004300	0.917355
chr20	49044900	0.743802
chr20	49050700	1.000000
chr20	49057900	0.909091
chr20	49068000	0.694215
chr20	49072900	0.785124
chr20	49077600	0.942149
chr20	49081400	0.578512
chr20	49084800	0.694215
chr20	49092200	0.933884
chr20	49095500	0.876033
chr20	49098500	0.760331
chr20	49109000	0.801653
chr20	49116100	0.669421
chr20	49121300	0.851240
chr20	49132500	0.644628
chr20	49134700	0.809917
chr20	49136800	0.958678
chr20	49140600	0.942149
chr20	49158400	0.801653
chr20	49175200	0.859504
chr20	49187400	0.760331
chr20	49218800	0.743802
chr20	49253000	0.834711
chr20	49255700	0.842975
chr20	49258900	0.826446
chr20	49278200	0.694215
chr20	49287800	0.652893
chr20	49289900	0.983471
chr20	49292800	0.818182
chr20	49295300	0.760331
chr20	49309700	0.793388
chr20	49325100	0.661157
chr20	49327800	0.925620
chr20	49330700	0.702479
chr20	49341900	0.867769
chr20	49345300	0.925620
chr20	49352400	0.851240
chr20	49354900	0.694215
chr20	49360900	0.950413
chr20	49378600	0.743802
chr20	49381200	0.661157
chr20	49384300	0.917355
chr20	49388500	0.677686
chr20	49405100	0.876033
chr20	49407800	0.735537
chr20	49435400	0.867769
chr20	49455200	0.892562
chr20	49499000	0.834711
chr20	49519500	0.652893
chr20	49523200	0.892562
chr20	49528600	0.661157

chr20	49530900	0.851240
chr20	49533300	0.611570
chr20	49538600	0.685950
chr20	49542100	0.570248
chr20	49651000	0.933884
chr20	49935200	0.685950
chr20	49955600	0.983471
chr20	49983500	0.702479
chr20	49990900	0.900826
chr20	49996300	0.669421
chr20	50051900	0.826446
chr20	50054800	0.867769
chr20	50108700	0.776860
chr20	50111000	0.570248
chr20	50129900	0.900826
chr20	50142800	0.809917
chr20	50145700	0.776860
chr20	50153400	0.595041
chr20	50155600	0.809917
chr20	50180500	0.735537
chr20	50211900	0.966942
chr20	50216600	0.859504
chr20	50234200	0.892562
chr20	50248100	0.892562
chr20	50260200	0.685950
chr20	51041900	0.752066
chr20	51063700	0.636364
chr20	51759100	0.628099
chr20	51770800	0.669421
chr20	51779300	0.611570
chr20	51791500	0.842975
chr20	51793600	0.578512
chr20	51796900	0.826446
chr20	51803400	0.685950
chr20	51829500	0.644628
chr20	51911700	0.867769
chr20	51914200	0.785124
chr20	51916600	0.578512
chr20	51919800	0.826446
chr20	51947600	0.917355
chr20	51949800	0.892562
chr20	51987700	0.685950
chr20	52130300	0.677686
chr20	52180800	0.603306
chr20	52195900	0.702479
chr20	52201300	0.768595
chr20	52204000	0.628099
chr20	52216500	0.685950
chr20	52223000	0.752066
chr20	52228200	0.793388
chr20	52230700	0.884298
chr20	52237800	0.735537
chr20	52269200	0.578512
chr20	52272400	0.619835

chr20	52294000	0.611570
chr20	52296600	0.727273
chr20	52302400	0.743802
chr20	52361100	0.743802
chr20	52367900	0.884298
chr20	52380700	0.743802
chr20	52417700	0.867769
chr20	52464700	0.586777
chr20	52486800	0.867769
chr20	52515500	0.752066
chr20	52523600	0.942149
chr20	52529100	0.752066
chr20	52531200	0.570248
chr20	52534400	0.900826
chr20	52543300	0.652893
chr20	52550600	0.768595
chr20	52554000	0.892562
chr20	52557500	0.884298
chr20	52566700	0.603306
chr20	52660800	0.925620
chr20	52663900	0.661157
chr20	52675300	0.735537
chr20	52682400	0.892562
chr20	53711400	0.851240
chr20	53720200	1.000000
chr20	54939000	0.793388
chr20	54944100	0.628099
chr20	54949200	0.652893
chr20	54958300	0.578512
chr20	54961800	0.942149
chr20	54964800	0.603306
chr20	54982900	0.776860
chr20	54985600	0.760331
chr20	54992200	0.809917
chr20	54995800	0.966942
chr20	54998800	0.694215
chr20	55003000	0.991736
chr20	55005100	0.867769
chr20	55008600	0.909091
chr20	55023100	0.867769
chr20	55623300	0.834711
chr20	55681800	0.603306
chr20	55826700	0.661157
chr20	55964300	0.652893
chr20	55969400	0.677686
chr20	55985300	0.909091
chr20	55987900	0.975207
chr20	55990800	0.933884
chr20	55993800	0.851240
chr20	55996100	0.652893
chr20	56008300	0.991736
chr20	56010900	0.826446
chr20	56024200	0.917355
chr20	56027300	0.958678

chr20	56037000	0.727273
chr20	56039800	0.669421
chr20	56045500	0.661157
chr20	56053600	0.776860
chr20	56070200	0.727273
chr20	56102000	0.603306
chr20	56111000	0.842975
chr20	56115700	0.628099
chr20	56120600	0.644628
chr20	56136100	0.876033
chr20	56149200	0.677686
chr20	56153100	0.603306
chr20	56155700	0.677686
chr20	56158300	0.884298
chr20	56163700	0.595041
chr20	56827200	0.685950
chr20	56844100	0.933884
chr20	56870200	0.809917
chr20	56902900	0.727273
chr20	56916200	0.685950
chr20	56949600	0.942149
chr20	56967700	0.603306
chr20	56976900	0.603306
chr20	56982100	0.785124
chr20	56995900	0.661157
chr20	57116800	0.991736
chr20	57125800	0.661157
chr20	57199400	0.983471
chr20	57202700	0.595041
chr20	57210300	0.652893
chr20	57215900	0.801653
chr20	57231000	0.785124
chr20	57451300	0.611570
chr20	57483200	0.793388
chr20	57559400	0.867769
chr20	57583600	0.644628
chr20	57587300	0.710744
chr20	57590100	0.942149
chr20	57593200	0.950413
chr20	57600300	0.702479
chr20	57623800	0.595041
chr20	58523800	0.710744
chr20	60097000	0.578512
chr20	60101800	0.570248
chr20	60601400	0.975207
chr20	60606000	0.876033
chr20	60608500	0.570248
chr20	60638700	0.570248
chr20	60642300	0.595041
chr20	60663800	0.752066
chr20	60721700	0.785124
chr20	60726000	0.950413
chr20	60762300	0.735537
chr20	60810400	0.727273

chr20	60812700	0.586777
chr20	60850100	0.851240
chr20	60939600	0.785124
chr20	60944700	0.694215
chr20	60957200	0.793388
chr20	60981600	0.628099
chr20	60995300	0.595041
chr20	61023000	0.719008
chr20	61055600	0.603306
chr20	61060300	0.884298
chr20	61064900	0.785124
chr20	61070400	0.776860
chr20	61078900	0.834711
chr20	61086800	0.694215
chr20	61196600	0.842975
chr20	61283400	0.991736
chr20	61317800	0.669421
chr20	61343200	0.925620
chr20	61371900	0.909091
chr20	61407200	0.636364
chr20	61429300	0.752066
chr20	61437600	0.694215
chr20	61456200	0.652893
chr20	61464400	0.694215
chr20	61494100	0.884298
chr20	61505500	0.826446
chr20	61507600	0.842975
chr20	61549400	0.586777
chr20	61562600	0.595041
chr20	61566600	0.727273
chr20	61829000	0.636364
chr20	61844800	0.636364
chr20	61867300	0.611570
chr20	61895800	0.603306
chr20	62169700	0.809917
chr20	62180200	0.743802
chr20	62183900	0.735537
chr20	62191800	0.578512
chr20	62280100	0.603306
chr20	62288400	0.570248
chr20	62318200	0.619835
chr20	62331000	0.793388
chr20	62370400	0.661157
chr20	62382600	0.818182
chr20	62404600	0.644628
chr20	62453800	0.652893
chr20	62461500	0.636364
chr20	62479000	0.595041
chr20	62490300	0.793388
chr20	62530500	0.644628
chr20	62574200	0.702479
chr20	62581100	0.760331
chr20	62585600	0.570248
chr20	62605700	0.578512

chr20	62668500	0.818182
chr20	62677100	0.842975
chr20	62686000	0.793388
chr20	62697300	0.876033
chr20	62715300	0.570248
chr20	62752000	0.603306
chr20	62849300	0.694215
chr20	62852400	0.867769
chr20	62854900	0.669421
chr20	62858700	0.628099
chr20	62863000	0.644628
chr20	62940800	0.768595
chr20	62949700	0.603306
chr21	9662600	0.752066
chr21	9668900	0.768595
chr21	9672000	0.578512
chr21	9757100	0.586777
chr21	9759900	0.727273
chr21	9827200	0.950413
chr21	9951400	0.719008
chr21	10169800	0.727273
chr21	10185200	0.570248
chr21	10492700	0.983471
chr21	15092100	0.595041
chr21	15250000	0.652893
chr21	15296200	0.685950
chr21	15341300	0.685950
chr21	15345600	0.652893
chr21	15384100	0.652893
chr21	15441500	0.595041
chr21	15496700	0.586777
chr21	15643700	0.876033
chr21	15653500	0.685950
chr21	15663300	0.694215
chr21	15669700	0.578512
chr21	15758200	0.644628
chr21	15817900	0.603306
chr21	15823700	0.818182
chr21	15828700	0.818182
chr21	15854300	0.652893
chr21	15860700	0.652893
chr21	15863900	0.826446
chr21	15869400	0.595041
chr21	15897700	0.619835
chr21	15906700	0.669421
chr21	15920300	0.586777
chr21	15923000	0.611570
chr21	15937900	0.909091
chr21	15945100	0.619835
chr21	15952900	0.793388
chr21	15957600	0.785124
chr21	15963000	0.578512
chr21	15973100	0.851240
chr21	15975700	0.735537

chr21	16003100	0.636364
chr21	16030200	0.727273
chr21	16046300	0.793388
chr21	16051300	0.578512
chr21	16063300	0.578512
chr21	16091800	0.619835
chr21	16109400	0.851240
chr21	16117700	0.942149
chr21	16130800	0.636364
chr21	16136900	0.785124
chr21	16140600	0.636364
chr21	16144100	0.876033
chr21	17108400	0.611570
chr21	17114300	0.611570
chr21	17116400	0.966942
chr21	17121200	0.727273
chr21	17126400	0.950413
chr21	19062400	0.785124
chr21	19274300	0.702479
chr21	20834200	0.644628
chr21	20861800	0.619835
chr21	21372300	0.851240
chr21	21377200	0.586777
chr21	21459400	0.628099
chr21	21632500	0.727273
chr21	21638900	0.652893
chr21	21642700	0.603306
chr21	21645600	0.586777
chr21	22418600	0.570248
chr21	22527100	0.652893
chr21	22529400	0.661157
chr21	22531900	0.892562
chr21	22536100	0.917355
chr21	22544000	0.652893
chr21	22551700	0.834711
chr21	22553900	0.752066
chr21	22557500	0.793388
chr21	22562700	0.768595
chr21	22565700	0.636364
chr21	22568400	0.809917
chr21	22570500	0.859504
chr21	22574900	0.694215
chr21	22578100	0.636364
chr21	22582900	0.586777
chr21	23573200	0.669421
chr21	25182400	0.611570
chr21	25184600	0.884298
chr21	25262500	0.636364
chr21	25295300	0.809917
chr21	25645000	0.818182
chr21	27090300	0.851240
chr21	30207400	0.735537
chr21	30248000	0.603306
chr21	30448500	0.892562

chr21	30605500	0.636364
chr21	30662400	0.851240
chr21	30679700	0.793388
chr21	30682200	0.760331
chr21	30684400	0.793388
chr21	30687000	0.975207
chr21	30690900	0.760331
chr21	30707400	0.884298
chr21	30720400	0.768595
chr21	30806100	0.719008
chr21	30849200	0.809917
chr21	30986100	0.859504
chr21	31007400	0.876033
chr21	31010400	0.570248
chr21	31013000	0.685950
chr21	31016600	0.933884
chr21	31019600	0.842975
chr21	31024400	0.950413
chr21	31027700	0.975207
chr21	31031700	0.859504
chr21	31037500	0.619835
chr21	31042900	0.619835
chr21	31097800	0.628099
chr21	31121200	0.652893
chr21	31135800	0.892562
chr21	31312300	0.694215
chr21	33017600	0.743802
chr21	33035600	0.735537
chr21	33038300	0.595041
chr21	33085300	0.628099
chr21	33088700	0.644628
chr21	33093400	0.644628
chr21	33098100	0.826446
chr21	33107700	0.735537
chr21	33110800	0.975207
chr21	33158500	0.578512
chr21	33443400	0.851240
chr21	33504400	0.760331
chr21	33671200	0.578512
chr21	33762200	0.719008
chr21	33786000	0.619835
chr21	33800900	0.834711
chr21	33889100	0.685950
chr21	33894000	0.603306
chr21	33906200	0.628099
chr21	33910200	0.595041
chr21	33920700	0.851240
chr21	33924100	0.619835
chr21	33927700	0.851240
chr21	33940400	0.619835
chr21	33945500	0.719008
chr21	33954400	0.694215
chr21	33957500	0.595041
chr21	33959800	0.818182

chr21	33979200	0.669421
chr21	33994200	0.611570
chr21	34018200	0.719008
chr21	34055800	0.727273
chr21	34075600	0.743802
chr21	34305600	0.570248
chr21	34576500	0.669421
chr21	34584800	0.818182
chr21	34588400	0.735537
chr21	34600000	0.933884
chr21	34605100	0.826446
chr21	34674000	0.719008
chr21	34677100	0.900826
chr21	34700300	0.619835
chr21	34704500	0.826446
chr21	34737100	0.570248
chr21	34752800	0.884298
chr21	34755900	0.809917
chr21	34759100	0.809917
chr21	34779000	0.570248
chr21	34878400	0.595041
chr21	34883700	0.867769
chr21	34952300	0.586777
chr21	34957400	0.694215
chr21	35029800	0.652893
chr21	35088900	0.570248
chr21	35104700	0.628099
chr21	35134400	0.900826
chr21	35267800	0.636364
chr21	35275100	0.702479
chr21	35285300	0.644628
chr21	35297200	0.578512
chr21	35318600	0.818182
chr21	35321500	0.570248
chr21	35339800	0.842975
chr21	35347500	0.719008
chr21	35350400	0.702479
chr21	35361100	0.636364
chr21	35366100	0.826446
chr21	35395600	0.636364
chr21	35400400	0.570248
chr21	35404900	0.570248
chr21	35454000	0.710744
chr21	35459900	0.917355
chr21	35483200	0.958678
chr21	35487000	0.776860
chr21	35502000	0.595041
chr21	35532300	0.586777
chr21	35558800	0.867769
chr21	35563300	1.000000
chr21	35828600	0.793388
chr21	35856100	0.975207
chr21	35917900	0.884298
chr21	35955900	0.719008

chr21	35964600	0.793388
chr21	35984700	0.694215
chr21	36012900	0.719008
chr21	36089800	0.702479
chr21	36238200	0.785124
chr21	36254300	0.578512
chr21	36270800	0.586777
chr21	36278000	0.942149
chr21	36283300	0.917355
chr21	36292200	0.652893
chr21	36299800	0.867769
chr21	36302100	0.611570
chr21	36310700	0.867769
chr21	36315800	0.710744
chr21	36321100	0.595041
chr21	36326700	0.834711
chr21	36340200	0.925620
chr21	36346600	0.710744
chr21	36356000	0.925620
chr21	36361500	0.611570
chr21	36365800	0.702479
chr21	36368000	0.942149
chr21	36372000	0.917355
chr21	36376400	0.917355
chr21	36384200	0.611570
chr21	36387000	0.826446
chr21	36392100	0.801653
chr21	36397800	0.677686
chr21	36401300	0.900826
chr21	36407300	0.570248
chr21	36424000	0.818182
chr21	36426500	0.735537
chr21	36472800	0.685950
chr21	36477300	0.983471
chr21	36480100	0.578512
chr21	36485700	0.917355
chr21	36509300	0.818182
chr21	36511600	0.876033
chr21	36541700	0.752066
chr21	36576100	0.876033
chr21	36579500	0.619835
chr21	36599100	0.586777
chr21	36605200	0.727273
chr21	36629100	0.842975
chr21	36638400	0.603306
chr21	36655500	0.586777
chr21	36698600	0.933884
chr21	36707000	0.818182
chr21	36709500	0.884298
chr21	36715500	0.743802
chr21	36719100	0.859504
chr21	36771900	0.595041
chr21	36811300	0.619835
chr21	36834500	0.909091

chr21	36843000	0.876033
chr21	36848400	0.842975
chr21	36853600	0.677686
chr21	36855800	0.991736
chr21	36876700	0.727273
chr21	36883900	0.776860
chr21	36890600	0.603306
chr21	36920700	0.909091
chr21	36937900	0.793388
chr21	36986700	0.570248
chr21	37378000	0.603306
chr21	37403000	0.793388
chr21	37449600	0.793388
chr21	37451800	0.702479
chr21	37454900	0.595041
chr21	37486000	0.702479
chr21	37523000	0.867769
chr21	37536800	0.619835
chr21	37539500	0.776860
chr21	37670600	0.793388
chr21	37699900	0.801653
chr21	37795300	0.785124
chr21	37802400	0.975207
chr21	37810500	0.619835
chr21	38232700	0.661157
chr21	38318300	0.942149
chr21	38335600	0.611570
chr21	38347100	0.884298
chr21	38391000	0.842975
chr21	38422200	0.595041
chr21	38458900	0.826446
chr21	38530500	0.867769
chr21	38580700	0.892562
chr21	38617400	0.702479
chr21	38623900	0.570248
chr21	38642700	0.578512
chr21	38707900	0.801653
chr21	38711700	0.818182
chr21	38728700	0.776860
chr21	38748000	0.586777
chr21	38753100	0.570248
chr21	38756200	0.694215
chr21	38764400	0.727273
chr21	38780600	0.826446
chr21	38787100	0.925620
chr21	38792800	0.619835
chr21	38797900	0.719008
chr21	38808000	0.694215
chr21	38819900	0.909091
chr21	38824700	0.793388
chr21	38828000	0.851240
chr21	38831500	0.603306
chr21	38835600	0.851240
chr21	38881700	0.619835

chr21	38906100	0.603306
chr21	38909600	0.884298
chr21	38920900	0.892562
chr21	38923700	0.685950
chr21	38928400	0.826446
chr21	38931700	0.785124
chr21	38939400	0.859504
chr21	38973800	0.702479
chr21	38999400	0.619835
chr21	39014900	0.586777
chr21	39031600	0.950413
chr21	39399500	0.685950
chr21	39452800	0.801653
chr21	39464400	0.942149
chr21	39484000	0.636364
chr21	39487900	0.619835
chr21	39496000	0.694215
chr21	39551700	0.603306
chr21	39592200	0.785124
chr21	39848600	0.685950
chr21	40159700	0.628099
chr21	40162100	0.586777
chr21	40170300	0.859504
chr21	40175400	0.619835
chr21	40180700	0.776860
chr21	40204200	0.884298
chr21	40212400	0.768595
chr21	40222400	0.867769
chr21	40225000	0.859504
chr21	40229300	0.842975
chr21	40285800	0.611570
chr21	40331400	0.785124
chr21	40338000	0.578512
chr21	40358000	0.834711
chr21	40376500	0.669421
chr21	40467300	0.785124
chr21	40477900	0.801653
chr21	40546600	0.570248
chr21	40581000	0.958678
chr21	40689400	0.752066
chr21	40692400	0.768595
chr21	40704500	0.661157
chr21	40731200	0.619835
chr21	40735700	0.710744
chr21	40742700	0.925620
chr21	40749900	0.809917
chr21	40762200	0.578512
chr21	40792500	0.950413
chr21	40795600	0.743802
chr21	40802600	0.611570
chr21	40814800	0.586777
chr21	40822300	0.619835
chr21	42581200	0.719008
chr21	43256400	0.991736

chr21	43265300	0.983471
chr21	43285900	0.925620
chr21	43315900	0.975207
chr21	43322100	0.570248
chr21	43324400	0.644628
chr21	43352900	0.801653
chr21	43375600	0.801653
chr21	43425600	0.909091
chr21	43458200	0.644628
chr21	43472200	0.702479
chr21	43791100	0.595041
chr21	43809500	0.702479
chr21	43818900	0.595041
chr21	43822300	0.685950
chr21	43825500	0.570248
chr21	43835600	0.809917
chr21	43854500	0.694215
chr21	43885500	0.851240
chr21	43913400	0.619835
chr21	43960700	0.776860
chr21	43965000	0.900826
chr21	43968200	0.595041
chr21	43974400	0.826446
chr21	43982500	0.727273
chr21	44086100	0.661157
chr21	44119200	0.586777
chr21	44179900	0.760331
chr21	44311300	0.661157
chr21	44342300	0.900826
chr21	44345100	0.958678
chr21	44350200	0.801653
chr21	44352600	0.603306
chr21	44357400	0.685950
chr21	44376100	0.859504
chr21	44378300	0.694215
chr21	44391800	0.619835
chr21	44398200	0.611570
chr21	44410300	0.851240
chr21	44413100	0.768595
chr21	44457100	0.586777
chr21	44467200	0.743802
chr21	44470300	0.942149
chr21	44474100	0.694215
chr21	44492900	0.603306
chr21	44500600	0.570248
chr21	44524900	0.578512
chr21	44531000	0.685950
chr21	44551400	0.760331
chr21	44723500	0.595041
chr21	44832300	0.570248
chr21	44914700	0.925620
chr21	45032600	0.727273
chr21	45066300	0.636364
chr21	45118900	0.917355

chr21	45132700	0.652893
chr21	45147500	0.710744
chr21	45158600	0.991736
chr21	45166200	0.710744
chr21	45187400	0.652893
chr21	45192800	0.760331
chr21	45198700	0.776860
chr21	45225200	0.628099
chr21	45231100	0.776860
chr21	45235300	0.818182
chr21	45238800	0.619835
chr21	45244400	0.719008
chr21	45249900	0.586777
chr21	45274100	0.677686
chr21	45276300	0.702479
chr21	45280000	0.933884
chr21	45282100	0.628099
chr21	45284200	0.710744
chr21	45288200	0.636364
chr21	45291900	0.694215
chr21	45300500	0.752066
chr21	45303000	0.917355
chr21	45310300	0.578512
chr21	45313000	0.586777
chr21	45316700	0.826446
chr21	45322900	0.636364
chr21	45334500	0.776860
chr21	45338700	0.801653
chr21	45342400	0.983471
chr21	45355000	0.586777
chr21	45357600	0.933884
chr21	45368800	0.586777
chr21	45392900	0.694215
chr21	45439200	0.983471
chr21	45441600	0.809917
chr21	45449700	0.867769
chr21	45463900	0.884298
chr21	45556800	0.826446
chr21	45568700	0.685950
chr21	45574800	0.619835
chr21	45616800	0.867769
chr21	45622900	0.826446
chr21	45628100	0.710744
chr21	45638800	0.685950
chr21	45669800	0.760331
chr21	45758200	0.570248
chr21	46014100	0.917355
chr21	46182100	0.578512
chr21	46185800	0.785124
chr21	46198300	0.578512
chr21	46201000	0.586777
chr21	46213500	0.809917
chr21	46224500	0.842975
chr21	46255800	0.595041

chr21	46273000	0.735537
chr21	46286600	0.942149
chr21	46290000	0.694215
chr21	46298600	0.950413
chr21	46301400	0.801653
chr21	46317800	0.677686
chr21	46388500	0.710744
chr21	46425400	0.595041
chr21	46485200	0.851240
chr21	46488000	0.661157
chr21	46491200	0.611570
chr21	46507200	0.570248
chr21	46516000	0.694215
chr21	46522900	0.685950
chr21	46530800	0.727273
chr21	46534500	0.595041
chr21	46550500	0.619835
chr21	46558200	0.867769
chr21	46572500	0.909091
chr21	46588300	0.727273
chr21	46632000	0.694215
chr21	46677100	0.900826
chr21	46715400	0.727273
chr21	46719900	0.743802
chr21	46726800	0.619835
chr21	46729700	0.669421
chr21	46777100	0.743802
chr21	46780500	0.950413
chr21	46784500	0.942149
chr21	46792200	0.719008
chr21	46803100	0.603306
chr21	46830400	0.842975
chr21	46832700	0.669421
chr21	46847400	0.603306
chr21	46859200	0.735537
chr21	46890300	0.702479
chr21	46898700	0.809917
chr21	46928900	0.801653
chr21	47012600	0.669421
chr21	47369200	0.727273
chr21	47394000	0.727273
chr21	47564100	0.677686
chr21	47573300	0.884298
chr21	47593000	0.702479
chr21	47600200	0.669421
chr21	47608700	0.950413
chr21	47611700	0.694215
chr21	47621600	0.595041
chr21	47644900	0.735537
chr21	47652100	0.652893
chr21	47663800	0.595041
chr21	47668600	0.677686
chr21	47674100	0.950413
chr21	47715700	0.884298

chr21	47876100	0.859504
chr21	47904100	0.586777
chr21	47920900	0.859504
chr21	47939400	0.876033
chr21	48019300	0.735537
chr21	48100000	0.644628
chr21	48112600	1.000000
chr22	16128400	0.619835
chr22	16162300	0.917355
chr22	16205000	0.628099
chr22	17067400	0.859504
chr22	17088400	0.636364
chr22	17097500	0.636364
chr22	17105500	0.595041
chr22	17107600	0.586777
chr22	17262800	0.702479
chr22	17265600	0.652893
chr22	17568500	0.595041
chr22	17637300	0.628099
chr22	17679800	0.636364
chr22	18114700	0.661157
chr22	18118000	0.685950
chr22	18125700	0.586777
chr22	18156300	0.966942
chr22	18208000	0.644628
chr22	18243600	0.570248
chr22	18269400	0.983471
chr22	18276300	0.595041
chr22	18397700	0.603306
chr22	18401700	0.603306
chr22	18405200	0.694215
chr22	18429800	0.611570
chr22	18433500	0.603306
chr22	18448400	0.818182
chr22	18453100	0.760331
chr22	18459100	0.727273
chr22	18539500	0.719008
chr22	18833300	0.611570
chr22	18841900	0.603306
chr22	18847300	0.611570
chr22	18891700	0.677686
chr22	18895700	0.595041
chr22	18990300	0.636364
chr22	19039500	0.719008
chr22	19095500	0.743802
chr22	19111700	0.603306
chr22	19163100	0.710744
chr22	19195600	0.743802
chr22	19206200	0.578512
chr22	19228300	0.595041
chr22	19232400	0.685950
chr22	19263600	0.719008
chr22	19284800	0.619835
chr22	19288200	0.586777

chr22	19303700	0.578512
chr22	19383800	0.719008
chr22	19386800	0.776860
chr22	19391800	0.628099
chr22	19396300	0.727273
chr22	19400100	0.578512
chr22	19406100	0.685950
chr22	19409100	0.661157
chr22	19412900	0.652893
chr22	19432600	0.743802
chr22	19564900	0.851240
chr22	19570100	0.743802
chr22	19592300	0.694215
chr22	19622000	0.694215
chr22	19629300	0.933884
chr22	19655300	0.595041
chr22	19667800	0.685950
chr22	19675600	0.702479
chr22	19690700	0.669421
chr22	19715200	0.917355
chr22	19762000	0.851240
chr22	19764700	0.661157
chr22	19767200	0.570248
chr22	19794300	0.892562
chr22	19803300	0.793388
chr22	19852900	0.727273
chr22	19863900	0.636364
chr22	19879400	0.677686
chr22	19948400	0.603306
chr22	19962800	0.826446
chr22	19976700	0.603306
chr22	19987000	0.900826
chr22	19990000	0.603306
chr22	19995200	0.628099
chr22	19999000	0.719008
chr22	20001500	0.768595
chr22	20014800	0.834711
chr22	20023300	0.752066
chr22	20045000	0.570248
chr22	20057900	0.826446
chr22	20060200	0.752066
chr22	20064900	0.867769
chr22	20102500	0.644628
chr22	20108200	0.611570
chr22	20117200	0.628099
chr22	20191900	0.570248
chr22	20245400	0.628099
chr22	20310400	0.652893
chr22	20316200	0.586777
chr22	20373300	0.578512
chr22	20403400	0.644628
chr22	20407200	0.826446
chr22	20410500	0.677686
chr22	20416200	0.595041

chr22	20421600	0.578512
chr22	20441400	0.776860
chr22	20445600	0.776860
chr22	20750800	0.570248
chr22	20767100	0.652893
chr22	20810800	0.685950
chr22	20824100	0.859504
chr22	20830300	0.818182
chr22	20843200	0.603306
chr22	20866500	0.900826
chr22	20876900	0.603306
chr22	20881200	0.735537
chr22	20900700	0.677686
chr22	20904000	0.619835
chr22	20911600	0.677686
chr22	20918800	0.685950
chr22	20931800	0.851240
chr22	20969800	0.859504
chr22	20997700	0.586777
chr22	21051900	0.661157
chr22	21123600	0.669421
chr22	21131900	0.760331
chr22	21136500	0.586777
chr22	21161600	0.727273
chr22	21195400	0.925620
chr22	21199300	0.603306
chr22	21202700	0.652893
chr22	21209400	0.702479
chr22	21215600	0.586777
chr22	21218000	0.727273
chr22	21223700	0.776860
chr22	21239800	0.603306
chr22	21266700	0.661157
chr22	21281100	0.702479
chr22	21293000	0.694215
chr22	21296100	0.818182
chr22	21308000	0.586777
chr22	21323500	0.735537
chr22	21361500	0.586777
chr22	21392400	0.619835
chr22	21394700	0.677686
chr22	21398900	0.760331
chr22	21405500	0.570248
chr22	21411800	0.776860
chr22	21448100	0.851240
chr22	21457200	0.652893
chr22	21461000	0.628099
chr22	21469400	0.719008
chr22	21474800	0.685950
chr22	21635400	0.570248
chr22	21649900	0.876033
chr22	21744200	0.677686
chr22	21754300	0.652893
chr22	21759000	0.694215

chr22	21762300	0.859504
chr22	21777300	0.611570
chr22	21783200	0.652893
chr22	21788000	0.661157
chr22	21792600	0.752066
chr22	21817300	0.586777
chr22	21847600	0.677686
chr22	21851000	0.884298
chr22	21855100	0.776860
chr22	21859900	0.677686
chr22	21865200	0.595041
chr22	21887700	0.644628
chr22	21889800	0.834711
chr22	21899600	0.661157
chr22	21910500	0.595041
chr22	21927000	0.586777
chr22	21929400	0.611570
chr22	21943100	0.685950
chr22	21963100	0.685950
chr22	21974200	0.752066
chr22	21985600	0.619835
chr22	21993600	0.842975
chr22	22001400	0.727273
chr22	22041500	0.677686
chr22	22055800	0.702479
chr22	22062300	0.595041
chr22	22092700	0.735537
chr22	22098700	0.595041
chr22	22106900	0.628099
chr22	22115700	0.570248
chr22	22134000	0.611570
chr22	22139400	0.685950
chr22	22146600	0.735537
chr22	22171300	0.760331
chr22	22174500	0.859504
chr22	22178900	0.776860
chr22	22182800	0.719008
chr22	22187500	0.652893
chr22	22190700	0.661157
chr22	22198300	0.851240
chr22	22204400	0.842975
chr22	22209500	0.669421
chr22	22212800	0.603306
chr22	22217200	0.694215
chr22	22231200	0.611570
chr22	22236800	0.677686
chr22	22265200	0.842975
chr22	22303900	0.619835
chr22	22339800	0.818182
chr22	22342600	0.776860
chr22	22347300	0.768595
chr22	22357400	0.727273
chr22	22377800	0.842975
chr22	22392500	0.652893

chr22	22467900	0.611570
chr22	22477300	0.760331
chr22	22496900	0.727273
chr22	22511400	0.595041
chr22	22617800	0.694215
chr22	22624800	0.603306
chr22	22819000	0.586777
chr22	22833100	0.595041
chr22	22877000	0.677686
chr22	22885200	0.595041
chr22	22891200	0.578512
chr22	22974300	0.760331
chr22	22979500	0.942149
chr22	22994700	0.636364
chr22	22996900	0.834711
chr22	23063100	0.628099
chr22	23178900	0.644628
chr22	23271500	0.760331
chr22	23429700	0.876033
chr22	23462300	0.801653
chr22	23520000	0.743802
chr22	23529800	0.876033
chr22	23534700	0.628099
chr22	23540400	0.578512
chr22	23553500	0.570248
chr22	23556400	0.644628
chr22	23561300	0.702479
chr22	23585200	0.809917
chr22	23590900	0.760331
chr22	23594400	0.644628
chr22	23612600	0.603306
chr22	23653200	0.826446
chr22	23745400	0.752066
chr22	23773900	0.694215
chr22	23780800	0.644628
chr22	23863600	0.900826
chr22	23875900	1.000000
chr22	23878400	0.851240
chr22	23880500	0.809917
chr22	23977100	0.793388
chr22	24027300	0.892562
chr22	24105100	0.652893
chr22	24107400	0.909091
chr22	24115000	0.603306
chr22	24128700	0.619835
chr22	24231400	0.900826
chr22	24244200	0.818182
chr22	24289600	0.685950
chr22	24336700	0.644628
chr22	24360200	0.958678
chr22	24388600	0.917355
chr22	24406300	0.983471
chr22	24411200	0.578512
chr22	24464100	0.603306

chr22	24488200	0.991736
chr22	24542300	0.776860
chr22	24553600	0.619835
chr22	24640700	0.578512
chr22	24655600	0.892562
chr22	24658600	0.586777
chr22	24660900	0.809917
chr22	24681200	0.719008
chr22	24686100	0.975207
chr22	24788600	0.636364
chr22	24803300	0.876033
chr22	24841900	0.776860
chr22	24852300	0.636364
chr22	24862600	0.619835
chr22	24878800	0.595041
chr22	24907000	0.958678
chr22	24955100	0.595041
chr22	25003900	0.950413
chr22	25027100	0.809917
chr22	25029500	0.958678
chr22	25031700	0.636364
chr22	25042300	0.884298
chr22	25047800	0.818182
chr22	25099500	0.570248
chr22	25103700	0.595041
chr22	25123700	0.983471
chr22	25127200	0.917355
chr22	25130900	0.628099
chr22	25148600	0.818182
chr22	25150700	0.578512
chr22	25156200	0.727273
chr22	25497000	0.760331
chr22	25544900	0.578512
chr22	25797400	0.628099
chr22	25848700	0.793388
chr22	25942200	0.628099
chr22	25963500	0.586777
chr22	25999300	0.578512
chr22	26062700	0.570248
chr22	26122800	0.966942
chr22	26136800	0.578512
chr22	26146500	0.867769
chr22	26158700	0.950413
chr22	26249700	0.636364
chr22	26797600	0.892562
chr22	26801100	1.000000
chr22	26857300	0.661157
chr22	26859800	0.586777
chr22	26873600	0.752066
chr22	26889200	0.867769
chr22	26897400	0.859504
chr22	26951400	0.628099
chr22	26970000	0.876033
chr22	26981300	0.636364

chr22	26993400	0.652893
chr22	27003800	0.578512
chr22	27014100	1.000000
chr22	27026000	0.884298
chr22	27028200	0.818182
chr22	27030500	0.818182
chr22	27034900	0.669421
chr22	27037400	0.842975
chr22	27039500	0.727273
chr22	27054900	0.793388
chr22	27530600	0.793388
chr22	27710500	0.793388
chr22	28065400	0.892562
chr22	28067700	0.958678
chr22	28115700	0.909091
chr22	28122700	0.719008
chr22	28243200	0.826446
chr22	28308100	0.776860
chr22	28312200	0.636364
chr22	28429100	0.578512
chr22	28431400	0.793388
chr22	28640500	0.851240
chr22	28643900	0.727273
chr22	28712400	0.628099
chr22	28845100	0.884298
chr22	28940800	0.628099
chr22	28956300	0.900826
chr22	29006000	0.727273
chr22	29052700	0.652893
chr22	29175900	0.793388
chr22	29192800	0.570248
chr22	29207700	0.719008
chr22	29278700	0.727273
chr22	29282200	0.677686
chr22	29358400	0.776860
chr22	29427800	0.950413
chr22	29433900	0.595041
chr22	29446000	0.818182
chr22	29475000	0.942149
chr22	29480400	0.735537
chr22	29493600	0.917355
chr22	29499400	0.644628
chr22	29505900	0.983471
chr22	29549800	0.818182
chr22	29592200	0.950413
chr22	29598800	0.892562
chr22	29601000	0.636364
chr22	29662200	0.570248
chr22	29699900	0.752066
chr22	29705500	0.760331
chr22	29760600	0.884298
chr22	29779600	0.983471
chr22	29874600	0.685950
chr22	29878700	0.570248

chr22	29900200	0.636364
chr22	29917400	0.661157
chr22	29935900	0.586777
chr22	29960300	0.586777
chr22	29965600	0.933884
chr22	29978300	0.719008
chr22	30030100	0.661157
chr22	30097500	0.636364
chr22	30122600	0.900826
chr22	30129800	0.743802
chr22	30147400	0.760331
chr22	30177000	0.859504
chr22	30195600	0.925620
chr22	30218000	0.636364
chr22	30227900	0.760331
chr22	30236600	0.677686
chr22	30304300	0.851240
chr22	30345600	0.760331
chr22	30348400	0.950413
chr22	30350500	0.834711
chr22	30390900	0.942149
chr22	30410700	0.842975
chr22	30592300	1.000000
chr22	30602200	0.694215
chr22	30605200	1.000000
chr22	30613200	0.876033
chr22	30680100	0.685950
chr22	30683700	0.611570
chr22	30702200	0.892562
chr22	30711300	0.727273
chr22	30724600	0.694215
chr22	30789200	0.619835
chr22	30794900	0.603306
chr22	30798900	0.851240
chr22	30804900	0.578512
chr22	30831500	0.950413
chr22	30834800	0.694215
chr22	30837900	0.743802
chr22	30841400	0.900826
chr22	30903300	1.000000
chr22	30920000	0.636364
chr22	31003100	0.702479
chr22	31005500	0.975207
chr22	31031800	0.685950
chr22	31041000	0.644628
chr22	31046100	0.578512
chr22	31071300	0.958678
chr22	31094500	0.702479
chr22	31101900	0.578512
chr22	31116900	0.611570
chr22	31125100	0.925620
chr22	31268700	0.785124
chr22	31276700	0.661157
chr22	31280800	0.685950

chr22	31359200	0.603306
chr22	31387000	0.735537
chr22	31456100	0.884298
chr22	31482600	0.834711
chr22	31495000	0.636364
chr22	31498900	0.892562
chr22	31503800	0.636364
chr22	31515600	0.619835
chr22	31519200	0.619835
chr22	31524100	0.694215
chr22	31614100	0.909091
chr22	31626200	0.851240
chr22	31640200	0.661157
chr22	31688600	0.652893
chr22	31692000	0.892562
chr22	31740200	0.611570
chr22	31888500	0.628099
chr22	31914400	0.685950
chr22	31980500	0.842975
chr22	31982600	0.991736
chr22	31999400	1.000000
chr22	32004700	0.595041
chr22	32012100	0.752066
chr22	32021800	0.793388
chr22	32024700	0.909091
chr22	32074500	0.595041
chr22	32147700	0.578512
chr22	32160900	0.595041
chr22	32171300	0.603306
chr22	32263800	0.900826
chr22	32287600	0.966942
chr22	32290600	0.685950
chr22	32293700	0.619835
chr22	32297900	0.942149
chr22	32307900	0.826446
chr22	32316300	0.661157
chr22	32325600	0.578512
chr22	32333700	0.958678
chr22	32339500	0.925620
chr22	32344700	0.619835
chr22	32347400	0.867769
chr22	32366800	0.603306
chr22	32447000	0.694215
chr22	32810100	0.578512
chr22	32879200	0.809917
chr22	32928600	0.760331
chr22	32936200	0.595041
chr22	32966700	0.785124
chr22	32969600	0.942149
chr22	32973000	1.000000
chr22	33045500	0.760331
chr22	33249300	0.611570
chr22	33270100	0.975207
chr22	33276500	0.925620

chr22	33278900	0.834711
chr22	33283500	0.966942
chr22	33293800	0.644628
chr22	35461600	0.950413
chr22	35527800	0.925620
chr22	35531100	0.752066
chr22	35605400	0.735537
chr22	35608300	0.983471
chr22	35612300	0.909091
chr22	35615500	0.818182
chr22	35698200	0.628099
chr22	35773300	0.966942
chr22	35790400	0.694215
chr22	35844100	0.636364
chr22	35874100	0.595041
chr22	35877100	0.586777
chr22	35948600	0.586777
chr22	36011800	0.867769
chr22	36044900	0.801653
chr22	36118200	0.752066
chr22	36138600	0.909091
chr22	36231900	0.619835
chr22	36288500	0.652893
chr22	36300400	0.578512
chr22	36311200	0.752066
chr22	36329600	0.603306
chr22	36332900	0.570248
chr22	36391800	0.867769
chr22	36399000	0.776860
chr22	36406900	0.991736
chr22	36432400	0.809917
chr22	36445800	0.950413
chr22	36449100	0.975207
chr22	36482600	0.595041
chr22	36520500	0.694215
chr22	36552700	0.768595
chr22	36578700	0.595041
chr22	36594400	0.975207
chr22	36655300	0.942149
chr22	36659800	0.809917
chr22	36724900	0.867769
chr22	36728300	0.909091
chr22	36731400	0.933884
chr22	36751500	0.909091
chr22	36760700	0.628099
chr22	36764900	0.925620
chr22	36767300	0.752066
chr22	36779600	0.652893
chr22	36791700	0.595041
chr22	36795600	0.628099
chr22	36815900	0.785124
chr22	36819600	0.942149
chr22	36823100	0.735537
chr22	36831600	0.628099

chr22	36848100	0.975207
chr22	36851400	0.661157
chr22	36864300	0.925620
chr22	36875000	0.677686
chr22	36904300	0.842975
chr22	36940900	0.677686
chr22	37056000	0.909091
chr22	37378600	0.975207
chr22	37405000	0.603306
chr22	37411400	0.942149
chr22	37414900	0.636364
chr22	37428100	0.644628
chr22	37538600	0.818182
chr22	37626500	1.000000
chr22	37641600	0.595041
chr22	37655700	0.595041
chr22	37670000	0.834711
chr22	37700900	0.884298
chr22	37703500	0.859504
chr22	37706500	0.702479
chr22	37851800	0.710744
chr22	37905300	0.719008
chr22	37908700	0.933884
chr22	37917300	0.570248
chr22	37930300	0.719008
chr22	37935200	0.727273
chr22	37949100	0.925620
chr22	37960600	0.578512
chr22	37998200	0.809917
chr22	38030500	0.950413
chr22	38057800	0.661157
chr22	38070800	0.595041
chr22	38077800	0.727273
chr22	38158700	0.867769
chr22	38173600	0.644628
chr22	38179000	0.661157
chr22	38198200	0.578512
chr22	38200500	0.809917
chr22	38202600	0.710744
chr22	38214100	0.892562
chr22	38247400	0.595041
chr22	38288600	0.586777
chr22	38291200	0.851240
chr22	38301000	0.867769
chr22	38330200	0.859504
chr22	38396200	0.785124
chr22	38409400	0.619835
chr22	38426800	0.611570
chr22	38430300	0.991736
chr22	38523600	0.966942
chr22	38539300	0.975207
chr22	38542000	0.719008
chr22	38544800	0.628099
chr22	38559200	0.925620

chr22	38574900	0.884298
chr22	38590200	0.586777
chr22	38636900	0.842975
chr22	38662800	0.950413
chr22	38681600	0.694215
chr22	38702200	0.586777
chr22	38715200	0.851240
chr22	38784800	0.818182
chr22	38788100	0.867769
chr22	38797300	0.595041
chr22	38860000	0.619835
chr22	38906900	0.644628
chr22	38911100	0.925620
chr22	39090700	0.595041
chr22	39100400	0.636364
chr22	39108400	0.570248
chr22	39110500	0.950413
chr22	39179300	0.983471
chr22	39182100	0.933884
chr22	39184600	0.776860
chr22	39243600	0.925620
chr22	39320700	0.595041
chr22	39339700	0.702479
chr22	39362500	0.628099
chr22	39378300	0.867769
chr22	39381200	0.661157
chr22	39408700	0.636364
chr22	39415900	0.595041
chr22	39438600	0.743802
chr22	39464700	0.826446
chr22	39493500	0.619835
chr22	39527200	0.570248
chr22	39545300	0.809917
chr22	39581500	0.578512
chr22	39609300	0.685950
chr22	39632900	0.818182
chr22	39637300	0.917355
chr22	39650500	0.950413
chr22	39665300	0.595041
chr22	39670300	0.611570
chr22	39679900	1.000000
chr22	39686800	0.677686
chr22	39691100	0.719008
chr22	39696600	0.793388
chr22	39705100	0.925620
chr22	39740200	0.727273
chr22	39760300	0.925620
chr22	39792900	0.636364
chr22	39834500	0.619835
chr22	39848900	0.958678
chr22	39859100	0.983471
chr22	39861900	0.975207
chr22	39864300	0.619835
chr22	39867100	0.925620

chr22	39870500	0.586777
chr22	39873300	0.578512
chr22	39883500	0.867769
chr22	39885700	0.685950
chr22	39892100	0.652893
chr22	39896900	0.925620
chr22	39904700	0.859504
chr22	39913900	0.685950
chr22	39925400	0.636364
chr22	39930700	0.685950
chr22	39971200	0.909091
chr22	39994600	0.685950
chr22	40022100	0.975207
chr22	40031200	0.892562
chr22	40051900	0.636364
chr22	40120200	0.966942
chr22	40151800	0.644628
chr22	40161700	0.719008
chr22	40170400	0.925620
chr22	40181600	0.628099
chr22	40214400	0.966942
chr22	40217200	0.644628
chr22	40227700	0.702479
chr22	40230500	0.826446
chr22	40235900	0.851240
chr22	40241100	0.578512
chr22	40253100	0.727273
chr22	40297700	0.628099
chr22	40302700	0.710744
chr22	40310200	0.776860
chr22	40314800	0.876033
chr22	40316900	0.900826
chr22	40323300	0.842975
chr22	40326900	0.595041
chr22	40332500	0.636364
chr22	40334700	0.834711
chr22	40337600	0.586777
chr22	40341100	0.933884
chr22	40349400	0.636364
chr22	40356100	0.892562
chr22	40358400	0.900826
chr22	40362400	0.950413
chr22	40384900	0.975207
chr22	40387100	0.578512
chr22	40390200	0.826446
chr22	40395800	0.975207
chr22	40398600	0.801653
chr22	40437000	0.942149
chr22	40447900	0.595041
chr22	40451800	0.793388
chr22	40454900	0.644628
chr22	40473600	0.702479
chr22	40477800	0.834711
chr22	40480800	0.785124

chr22	40552800	0.760331
chr22	40572100	0.595041
chr22	40578900	0.661157
chr22	40582400	0.586777
chr22	40584800	0.743802
[truncated: 357,285 more chars]
